# A Quantitative Profiling Tool for Diverse Genomic Data Types Reveals Potential Associations between Chromatin and Pre-mRNA Processing

**DOI:** 10.1371/journal.pone.0132448

**Published:** 2015-07-24

**Authors:** Isaac Kremsky, Nicolás Bellora, Eduardo Eyras

**Affiliations:** 1 Computational Genomics Group, Universitat Pompeu Fabra, E08003, Barcelona, Spain; 2 Laboratorio de Microbiología Aplicada y Biotecnología, Centro Regional Universitario Bariloche, Universidad Nacional del Comahue, INIBIOMA (CONICET-UNComa), Bariloche, Argentina; 3 Catalan Institution for Research and Advanced Studies (ICREA), Barcelona, Spain; International Centre for Genetic Engineering and Biotechnology, ITALY

## Abstract

High-throughput sequencing, and genome-based datasets in general, are often represented as profiles centered at reference points to study the association of protein binding and other signals to particular regulatory mechanisms. Although these profiles often provide compelling evidence of these associations, they do not provide a quantitative assessment of the enrichment, which makes the comparison between signals and conditions difficult. In addition, a number of biases can confound profiles, but are rarely accounted for in the tools currently available. We present a novel computational method, ProfileSeq, for the quantitative assessment of biological profiles to provide an exact, nonparametric test that specific regions of the test profile have higher or lower signal densities than a control set. The method is applicable to high-throughput sequencing data (ChIP-Seq, GRO-Seq, CLIP-Seq, etc.) and to genome-based datasets (motifs, etc.). We validate ProfileSeq by recovering and providing a quantitative assessment of several results reported before in the literature using independent datasets. We show that input signal and mappability have confounding effects on the profile results, but that normalizing the signal by input reads can eliminate these biases while preserving the biological signal. Moreover, we apply ProfileSeq to ChIP-Seq data for transcription factors, as well as for motif and CLIP-Seq data for splicing factors. In all examples considered, the profiles were robust to biases in mappability of sequencing reads. Furthermore, analyses performed with ProfileSeq reveal a number of putative relationships between transcription factor binding to DNA and splicing factor binding to pre-mRNA, adding to the growing body of evidence relating chromatin and pre-mRNA processing. ProfileSeq provides a robust way to quantify genome-wide coordinate-based signal. Software and documentation are freely available for academic use at https://bitbucket.org/regulatorygenomicsupf/profileseq/.

## Introduction

Profiling is a method for data visualization that is currently widely used with high-throughput sequencing data in combination with genome annotations. This generally consists in pooling data for a set of genomic loci with similar features of interest in order to make generalized biological inferences about the feature in question. In its application to high-throughput sequencing analysis, reads are added or averaged at contiguous bins up to a specified distance from a chosen set of reference positions, e.g. transcription start sites (TSSs). By pooling data from a large number of regions, greater statistical certainty is achieved, which is desirable due to the high variability at individual loci in high-throughput sequencing data. However, profiles often simply provide a qualitative map of the genomic landscape around a feature of interest. For example, a profile was used to provide evidence that RNA Polymerase 2 (RNAPII) accumulates at sites downstream of alternatively spliced exons where CCCTC-binding factor (CTCF) is bound [1]. In a similar example, ChIP-Seq profiles were used to show qualitatively that the SR-proteins SRSF1 and SRSF2 bind to a large extent at the TSS and to a smaller extent on exons of DNA [2]. While profiles can be very useful, they generally do not provide a quantitative assessment of statistical significance, and variations of the read density could be due to experimental or data processing artifacts rather than to biology. Thus, there is a need for a profiling method that reduces biases and quantitatively assesses the statistical significance of a profile feature in order to better inform biologists of which profile results are most likely to be biologically relevant.

We present ProfileSeq, a new method for a controlled and quantitative assessment of biological profiles. In particular, ProfileSeq provides a quantitative test to assess whether specific regions of the profile have higher or lower signal densities than a control set. ProfileSeq was designed with the aim of minimizing confounding factors and for performing a proper statistical analysis of the profiles. Moreover, it is applicable for any dataset of reads or genomic ranges of any length, it can be used to generate profiles for any data type that can be reduced to a set of genomic coordinates, and can accommodate up to single nucleotide (nt) resolution, hence is also applicable to methods such as GRO-Seq [3] and iCLIP-Seq [4]. We have used ProfileSeq to reproduce previously published profiling results and to provide additional insights. We show that a number of confounding factors exists and provide novel strategies in order to eliminate or reduce those confounding factors. Finally, profiles generated with ProfileSeq reveal a number of putative relationships between transcription factor binding to DNA and splicing factor binding to pre-mRNA, adding to the growing body of evidence relating chromatin and pre-mRNA processing.

## Results and Discussion

### A quantitative method to compare genome-based profiles

**Fig 1:**
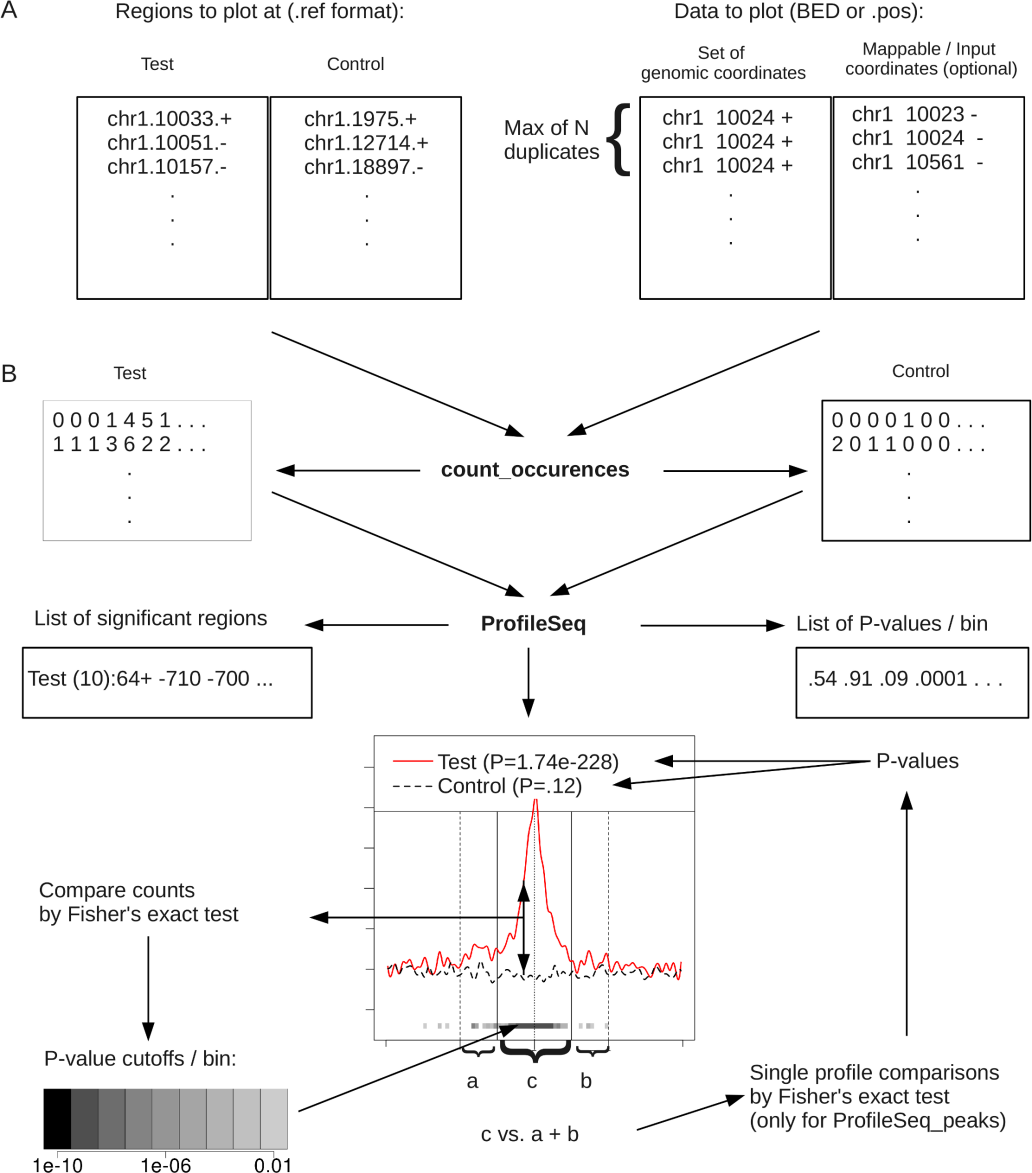
Workflow of ProfileSeq. Rectangles indicate files. Arrows leaving from files indicate outputs; arrows coming into a file indicate inputs. The user first prepares Test and Control reference files (.ref) containing the positions at which the datasets are to be plotted, as well as a BED or.pos file with the data to plot (A). The.ref and.pos formats are as displayed, except that the id field required for the.ref format is not shown. The BED or.pos file must be pre-processed such that the maximum number of times a read can be repeated at a given coordinate (chromosome position strand) is known. An optional file of genomic coordinates of mappable reads or input reads may be used for more accurate results. Each pair of.ref and BED/.pos files are then input into *count_occurences*, which will output a file of the counts of occurrences (.occ) of reads in the BED/.pos file at and around the positions in the.ref file (B). The.occ files are then used as inputs to ProfileSeq, which generates a profile like the one shown, as well as files with a list of significant regions and P-values of Test vs. Control counts in each bin. Additionally, using *ProfileSeq_peaks*, the count of occurrences in the central region c is compared to the counts in the immediately flanking regions (a and b) on the Test and Control profiles separately. Software and usage details are available at https://bitbucket.org/regulatorygenomicsupf/profileseq/.

We have developed a computational tool, ProfileSeq, to perform a quantitative comparison between profiles of genome-based signal. ProfileSeq uses as input the signal data and two sets of regions, a reference set and a control set (Fig 1A). The reference set is the list of regions of interest, and the control set contains the regions to compare with. The signal can be sequencing reads from ChIP-Seq, RNA-Seq, CLIP-Seq, etc., or any other coordinate-based measurement, like motifs, CpG islands, peak regions, etc. Optionally, a secondary signal, such as read mappability or chromatin input, can be used to correct for confounding factors. The coverage of positions by the signal is then calculated, the signal count is summarized into bins at a resolution specified as an input, and at each bin a test for enrichment is performed (Fig 1B). As output, ProfileSeq provides the corrected counts per bin, and the regions that are significantly different between reference and control, with the corresponding p-values. The novelty of ProfileSeq is that it performs a quantitative assessment of the differences in signal between the set of interest and a control set. Additionally, it performs a second quantitative test of the signal around a reference point compared to the flanking regions (Fig 1). Further details are given in the Methods section.

**Fig 2:**
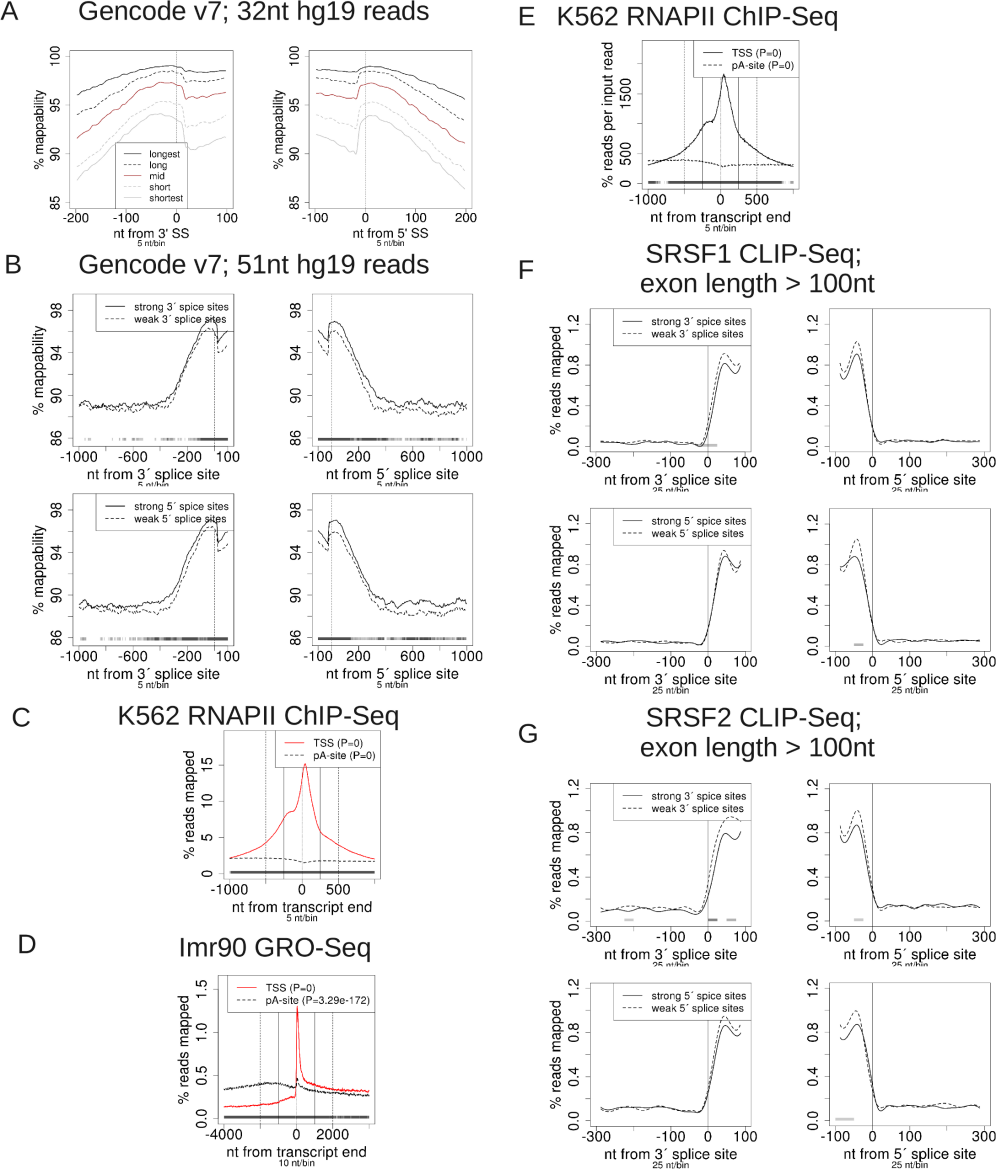
Validation Profiles. (A) Mappability according to transcript length. “Longest” gives the longest 20% of transcript lengths, “long” gives the 2^nd^ longest 20% of transcript lengths, and so on. Each class has N = 11711 reference regions. (B) Mappability at the upper vs. lower quartile of splice site strengths, “strong” and “weak”, respectively. (C)-(E) Comparisons of regions surrounding transcription start sites (TSSs) and polyadenylation sites (pA-sites). The pA profiles have been inverted, such that for all these profiles, positive values on the x-axis indicate the distance into the transcript, whereas negative values indicate the distance outside of the transcript. (F)-(G) Mouse CLIP-Seq profiles at strong vs. weak exons. Test vs. control P-values/bin are as shown in Fig 1B, with the lightest shade of grey corresponding to P-value < 0.01.

We validated ProfileSeq by reproducing prior results from the literature using multiple independent datasets, including CLIP-Seq, RNA-biding motifs, ChIP-Seq, GRO-Seq, and mappability of genomic positions. Mappability, the ability to uniquely map a read starting and ending at specific genomic coordinates, is a potentially confounding factor in profiles. We are able to reproduce and quantify the result showing that the mappability of regions flanking splice sites (SSs) of internal exons (using 32nt reads) correlates with the length of the host transcript [5], using a different annotation (Fig 2A) and for other read lengths for human and mouse (Figures A, B, and C in S1 Fig). Additionally, using exons larger than 100nt in human and mouse to account for the difference of length of the internal exons in long and short transcripts (P-value<10^−4^, Wilcoxon test) (Figure D in S1 Fig), we also find the mappability bias on the exon body (Figures E and F in S1 Fig). Thus, we recover the biases described before [5] and extend them to other regions, read lengths and species.

We also quantified an additional, relevant mappability bias, consisting of a significantly higher mappability at and around exons with strong SSs compared with exons with weak sites (Fig 2B) (Methods) with similar results for mouse (mm9 40nt reads, Figure A in S2 Fig). This bias in mappability persists in the 100nt region upstream of the 3’SS and downstream of the 5’SS when considering exons longer than 100nt in length (Figure B in S2 Fig). We observe that part of the significant difference in mappability lies on the exon. This will probably impact the calculation of exon percent spliced in (PSI) values [6, 7]. Given the potential for mappability to confound profile results, we considered it throughout our profile analyses (Methods). However, we found that in general mappability has a negligible effect on the results.

We next validated ProfileSeq for ChIP-Seq and GRO-Seq data. A number of genome-wide studies have observed a greater density of signal associated with RNAPII binding around TSSs compared to other regions, e.g. polyadenylation sites (pA-sites) [3]. We used ProfileSeq with ChIP-Seq data for RNAPII in K562 and HepG2 cells, as well as mouse embryonic fibroblasts (MEFs) (Fig 2C and Figures A and B in S3 Fig), and global run-on sequencing (GRO-Seq) for Imr90 and MEFs (Fig 2D and Figure C in S3 Fig), all centered at the same number of TSS and pA-sites from the same genes. All the obtained profiles show that the signal at the TSS is significantly greater than at pA-sites. The RNAPII profiles in HepG2 and MEF cells show a large peak just downstream of the TSS and another smaller one ~150–200nt upstream. This behavior was proposed to reflect the composite behavior of the TSSs in genes [8]. The K562 profiles behave similarly, but with a less pronounced, though still discernible, peak upstream of the TSS. We also observe significant accumulation of ChIP-Seq input reads at the TSS and depletion at the pA-site similar to the observed patterns for RNAPII. However, using ProfileSeq, we are able to show that the significant enrichment of RNAPII at the TSS persists even after accounting for the input signal in all cell lines (Fig 2E and Figures D and E in S3 Fig). Finally, stranded profiles for GRO-Seq in human (Fig 2D) and mouse (Figure C in S3 Fig) show a sharp peak just downstream of the TSS, as shown before for the human sample [3], and a smaller, but sharp peak at the pA-site as well as a second, broader peak downstream of the pA-site, which was proposed to be related to 3' cleavage before polyadenylation [3].

We also validated ProfileSeq using CLIP-Seq data. Several splicing factors are known to bind to exonic splicing enhancer (ESE) elements, thereby assisting in the inclusion of exons with weak splice-site signals [9]; various computational and experimental approaches have been used to successfully predict ESE motifs, e.g. [10,11]. The SR-proteins SRSF1 and SRSF2 are particularly well-studied splicing factors known to bind these ESEs [12]. We compared the profiles of CLIP-Seq reads for SRSF1 and SRSF2 from mouse cells [2] between the upper and lower quartiles of SS scores, for both 5' and 3' SSs separately (Fig 2F and 2G). Our profiles show a significantly greater read density in the weakest quartile compared with the strongest one, with very few reads at introns, which agrees with their main role in binding exons to enhance splicing. Additionally, the significance of the comparisons is maintained after correcting for mappability (Figures A and B in S4 Fig). Performing the same analysis for CLIP-Seq data for PTB [13] produced enrichment in the intronic region 100–125nt upstream of weak 3'SSs (Figure C in S4 Fig), consistent with previously reported patterns [14,15], whereas no difference was found when comparing weak and strong 5'SSs. Additionally, we observed significantly more PTB signal occurs on the body of exons with strong 5'SSs compared with exons with weak 5' SSs, even though this density is lower than at intronic regions (Figure C in S4 Fig). We conclude that ProfileSeq on CLIP-Seq data is consistent with the previous findings and also suggest new possible relations between PTBP1 binding and the strength of the nearby SSs.

### ChIP-Seq input signal bias as a possible confounding factor in ChIP-Seq profiles

**Fig 3:**
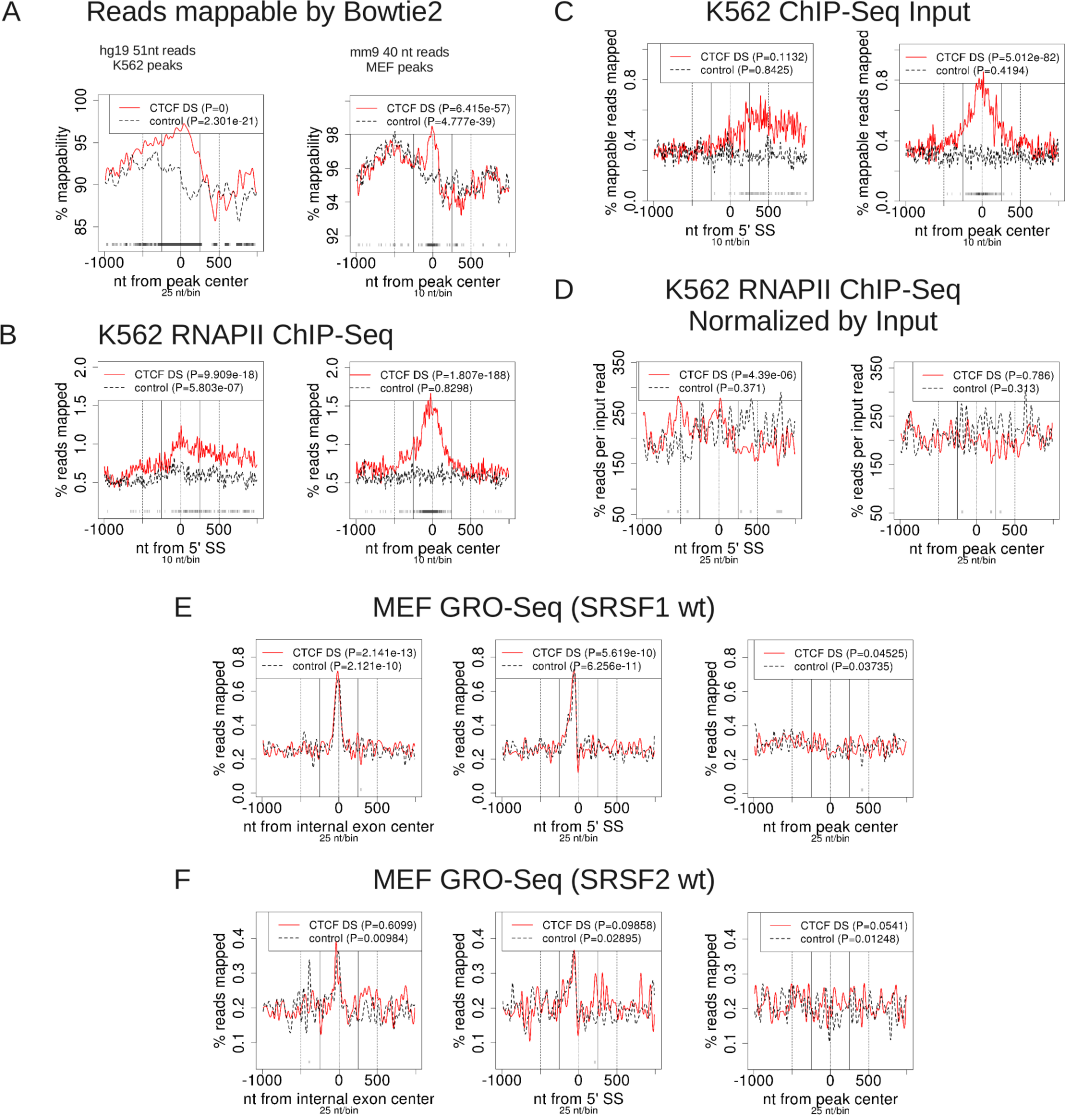
Mappability and input biases in profile results. US = peak center within 1kb upstream of 3'SS. DS = peak center within 1kb downstream of 5'SS. (A) Mappable reads at CTCF peaks vs. controls. (B)-(D) ChIP-Seq profiles from ENCODE data. (E)-(F) GRO-Seq reads at and around CTCF peaks vs. controls. SRSF1 wt is a conditional SRSF1-knockdown cell line in which SRSF1 is not knocked out; SRSF2 is defined similarly. Test vs. control P-values/bin are as shown in Fig 1B, with the lightest shade of grey corresponding to P-value < 0.01.

After validating the quantitative findings with ProfileSeq, we next investigated possible associations between chromatin and pre-mRNA splicing. It was shown before that RNAPII signal accumulates at CTCF binding sites downstream of alternatively spliced internal exons [1]. In particular, it was found that the RNAPII read density at the CTCF summits was ~3 fold greater compared with exons and with regions at > 250nt up and downstream of these summits [1]. We applied ProfileSeq to data from ENCODE to try to reproduce this result with a set of internal exons (Methods). First, we checked the mappability at the CTCF peaks in the 1kb region downstream of internal exons. We found significantly greater mappability, in both human and mouse, around the peak centers compared with control regions, defined as equivalent positions relative to the nearby SS in the 1kb region downstream of internal exons without a peak, (Fig 3A). The profiles of RNAPII ChIP-Seq reads at CTCF peaks before mappability correction in 2 human cell lines showed a similar (~2–3 fold) increase of RNAPII reads at CTCF peaks relative to the control regions, as well as relative to the 250nt upstream and downstream (Fig 3B and Figure A in S5 Fig), as observed before for alternatively spliced exons [1]. Importantly, our analysis shows that the differences are statistically significant, and that the significant difference persists after accounting for mappability (Figure B in S5 Fig), explicitly showing that the observed accumulation is not due to mappability biases.

The relationship between downstream CTCF binding and RNAPII pausing has been examined so far only in human, so we generated profiles in mouse MEF cell lines and observed the same result as in human (Figure C in S5 Fig). The profiles centered at the 5' SS looked similar to those at the exon center (Figures A and C in S5 Fig), indicating that differences between test and control sets are not due to differences in exon length distribution. Thus, we recover the significant accumulation of RNAPII ChIP-Seq reads at intragenic CTCF peaks within 1kb downstream of internal exons in both human and mouse, with similar fold-enrichment as observed just for alternatively spliced exons [1]. We also noticed a significant accumulation of ChIP-Seq input reads at CTCF peaks downstream of exons at comparable fold-enrichment as RNAPII relative to control regions in both human and mouse (Fig 3C and Figure D in S5 Fig). Moreover, when we normalized the RNAPII read profiles by input reads (Methods), we found no accumulation of RNAPII at CTCF peaks (Fig 3D and Figure E in S5 Fig); instead, there is actually a small but significant reduction in RNAPII reads per input read at CTCF peak centers relative to controls in the HepG2 profile, while there is no significant difference seen in the K562 and MEF profiles. Thus, in the three samples examined, the observed RNAPII accumulation of non-duplicate RNAPII ChIP-Seq reads at CTCF peaks is due to increased input signal at CTCF peaks. To investigate this further, we generated the profiles of sense-stranded GRO-Seq reads from the MEF cell lines (Fig 3E and 3F). This analysis shows no significant difference in reads at CTCF peaks compared with controls. Interestingly, there is a small but significant accumulation of GRO-Seq reads downstream of exons with a CTCF peak over controls in the SRSF2wt sample, but this accumulation is not related to the position of the CTCF peak (Fig 3E, right-most panel). Although these GRO-Seq profiles do not consider mappability, we showed before that the mappability bias at CTCF peaks would favor increased reads at CTCF peaks versus controls. The profiles for IgG ChIP-Seq, often used as control experiment, for MEF at CTCF peaks compared with controls show very sparse and randomly distributed signal, with none of the comparisons being statistically significant (Figure F in S5 Fig).

Finally, the original finding of RNAPII accumulation at CTCF peaks was observed downstream of exons that showed a significantly different splicing inclusion rate upon CTCF knockdown (KD) [1]. We therefore took the subset of the exons affected by CTCF KD that overlapped with our internal exon set and also had a downstream CTCF peak in HepG2. There were a total of 42 such exons. The profiles of RNAPII, the corresponding input, and RNAPII/input (Figure G in S5 Fig) show the same behavior as the profiles for the full internal exon set. Thus the accumulation of RNAPII ChIP-Seq signal that we observe is due to a bias in the input signal, regardless of whether or not the inclusion of exons is affected by CTCF KD. Our results indicate that ChIP-Seq signal recapitulates in general the ChIP input signal, suggesting that some ChIP-Seq datasets may contain unspecific binding information. We also show that normalization by dividing by input reads yields results similar to the corresponding GRO-Seq profiles, both positive (in the case of RNAPII at TSSs), and negative (in the case of RNAPII at downstream CTCF peaks). Provided that the input sample uses the same protocol and has similar sequencing depth as the samples, normalizing by input is likely to retain biological information while eliminating the unspecific signals.

These results imply that ChIP of RNAPII alone is not sufficient to estimate RNAPII elongation rates. A careful consideration of the corresponding input signal, and its appropriate normalization, are required to decouple signals due to input bias, PCR amplification bias, and biological signal. Our profiling method addresses PCR bias by only considering non-duplicate reads, and subsequently divides by non-duplicate input reads to address input bias. To our knowledge, it is the first profiling method that individually addresses both biases mentioned, and it therefore should allow for more accurate estimation of RNAPII elongation rates than previous genome-wide methods. These findings show explicitly that there is no RNAPII signal beyond input at the majority of CTCF binding sites downstream of internal exons, and casts doubt onto the hypothesis that a subset of such CTCF peaks cause RNAPII pausing at the binding site. We note, however, that the model system in which CTCF was shown explicitly to cause RNAPII pausing in vitro [1] was a CTCF peak whose summit was contained in the exon body. Our results are limited to intronic CTCF peaks and so do not contradict the result just mentioned. More work is needed to resolve whether CTCF-mediated RNAPII pausing can occur on introns.

### Increased H3K4me3 signal at internal exons with a CTCF peak downstream

**Fig 4:**
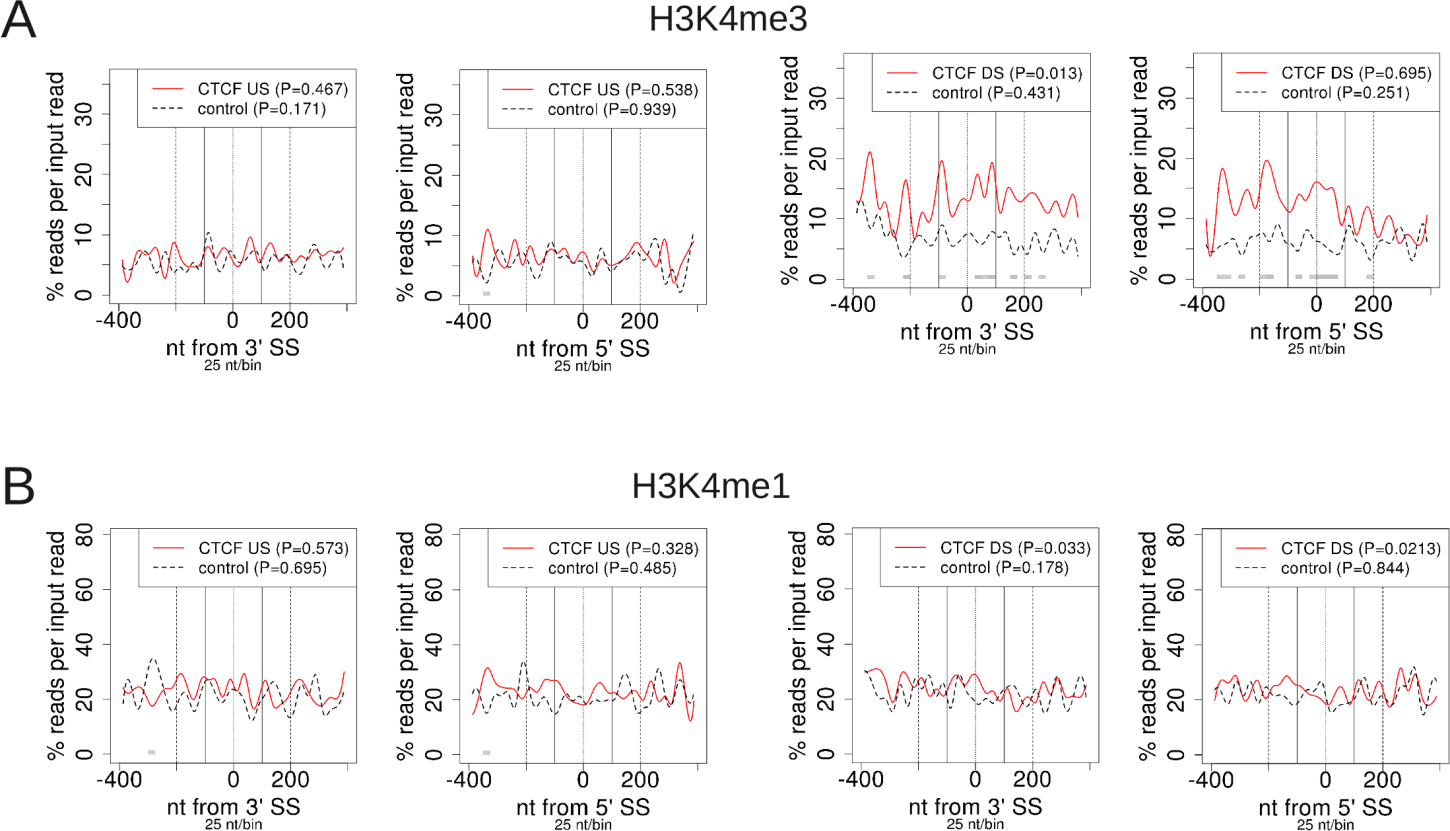
ChIP-Seq profiles of H3k4 me modifications at exons with CTCF bound either upstream or downstream. US = peak center within 1kb upstream of 3'SS. DS = peak center within 1 kb downstream of 5'SS. All exons in the profiles are longer than 100nt. Counts are normalized by the corresponding input signal for each sample. Test vs. control P-values/bin are as shown in Fig 1B, with the lightest shade of grey corresponding to P-value < 0.01.

CTCF is well known as an insulator protein, dividing regions of open and closed chromatin [16]. However, its functions have mainly been characterized in intergenic regions, and the function at intragenic regions is only beginning to be elucidated. It was shown recently that exons upstream of an intragenic CTCF peak in the STAT4 gene show higher levels of H3K4me3 occupancy than exons downstream of the peak [17]. In particular, H3K4me3 has a strong signal at the TSS, and a small, nonzero signal on exons up to the CTCF peak, at which the H3K4me3 signal drops. In contrast, on the nearby, actively transcribed STAT1 gene, there is no intragenic CTCF peak, and the H3K4me3 signal appears only at the TSS, while no signal beyond noise is evident for internal exons [17]. Yet this represents only a single example, and the signal is quite subtle. So, to provide further insights into the insulator function at intragenic regions, we used ProfileSeq to study the distribution of the signals for H3K4me1 and H3K4me3 around CTCF peaks using ENCODE data, correcting for both transcription levels and distances of profiled regions to TSS locations (Methods). Our profiles show that exons with a CTCF peak downstream, within 1kb and centered on the intron, have increased levels of H3K4me3 relative to controls, both within the exon body and at the SSs; whereas when CTCF is bound upstream, no significant difference is seen compared with the controls (Fig 4A). In contrast, no effect on H3K4me1 levels is observed when CTCF is bound either upstream or downstream (Fig 4B). This suggests that intragenic CTCF also divides genomic regions in different chromatin states. Exons downstream of CTCF peaks contain background levels of H3K4me3 signal, while exons upstream are enriched for that same mark.

H3K4me3 and H3K4me1 signals play an important role in transcription and enhancer activity [18,19]. Moreover, recently it has been shown that the activation of intragenic enhancers, which is concomitant with H3K4me3 and RNAPII signal enrichment [20], can be associated to alternative splicing of the host gene [21], which suggests a possible link between H3K4me3 and exon definition. Taken together, these results suggest that when CTCF binds intragenically, it could allow H3K4me3 to extend from the TSS up to the intragenic binding site, whereas if CTCF does not bind intragenically, H3K4me3 does not extend onto the gene body. This would also provide an alternative explanation why knocking down CTCF results in greater exclusion of internal exons, as it would result in reduced H3K4me3 on the immediately upstream exon, resulting in reduced RNAPII accumulation, and hence increased exclusion of weak exons.

### Enrichment of chromatin interactions at CTCF peaks downstream of internal exons

**Fig 5:**
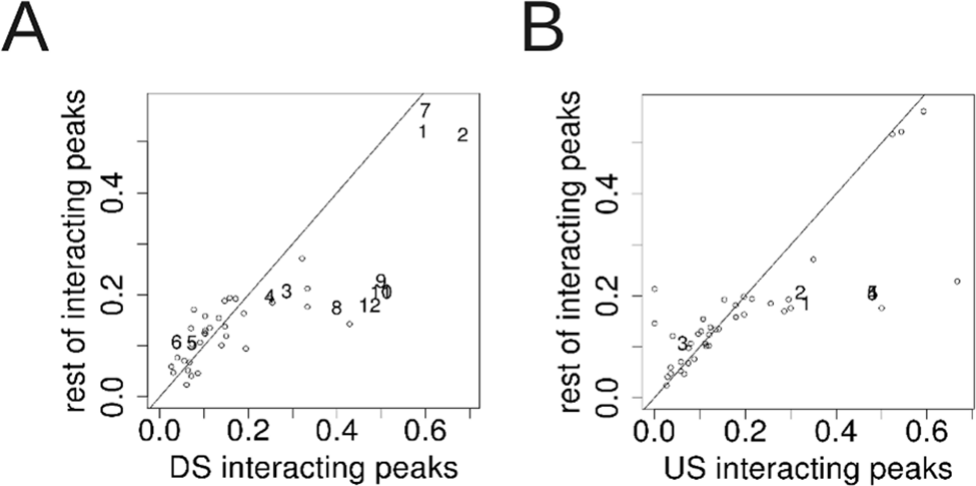
Fraction of ChIP-Seq peaks overlapping ChIA-PET interactions. Data from K562 cell line. Diagonal line is of y = x. Statistically significant data points (Fisher's Exact Test P-value < 0.05) are numbered, whereas non-significant points are shown as an empty circle. Each point represents a set of peaks. The first 5 letters of the symbol for the numbered points are listed here: (A) 1. Ctcfc 2. Ctcfl 3. Elf1s 4. MaxV0 5. Pol24 6. Pol2V 7. Rad21 8. Sp2sc 9. Thap1 10. Yy1sc 11. Yy1V0 12. Yy1V0. (B) 1. Atf3V 2. Elf1s 3. Pol2V 4. Yy1sc 5. Yy1V0. See S1 Table for the full list.

Above we have described and quantified various properties related to intragenic CTCF peaks. As CTCF plays an active role in joining distant chromatin regions to create DNA loops [16], we decided to test whether chromatin interactions might be involved in the function of intragenic CTCF. We examined the overlap of all the transcription factor (TF) peaks available from ENCODE with ChIA-PET interactions for both RNAPII and CTCF, for the cell lines K562 and MCF7. For both cell lines we compiled a list of internal exons that have an enrichment of chromatin interactions with TF peaks that are either downstream or upstream, relative to the overlap that is found genome-wide (S1 and S2 Tables). We found significantly more interacting CTCF peaks downstream of internal exons compared to the remaining CTCF peaks genome-wide (414 of 696, 59% and 16091 of 30900, 52%, respectively; Fisher's exact test P-value = 0.00011) (Fig 5A). On the other hand, upstream CTCF peaks did not have a significant difference, despite having a comparable number of peaks (Fig 5B). Similarly, there were significantly more downstream CTCF peaks that overlapped with a RNAPII interaction pair relative to the genome-wide distribution (45% versus 41%, respectively; Fisher's exact test P-value = 0.029) (Figure A in S6 Fig), while there was again no significant difference for upstream CTCF peaks (Figure B in S6 Fig). On the other hand, for MCF7 there was no significant difference in the overlap of either upstream or downstream CTCF peaks with either CTCF or RNAPII interaction pairs (Figures C-F in S6 Fig). Interestingly, in both K562 and MCF7 there were significantly greater downstream RAD21 peaks overlapping with CTCF interactions than the remaining RAD21 peaks (P-value = 0.043 and P-value = 0.005, respectively, both by Fisher's exact test). RAD21 is a cohesin subunit that has been observed to have a high overlap with CTCF binding sites and to occupy regions of enriched RNAPII ChIP signal [22]. Furthermore, we also observed significantly more upstream SPI1 (PU.1) peaks overlap with RNAPII ChIA-PET interaction pairs than the rest of SPI1 peaks genome-wide (Fisher's exact test P-value = 0.041). SPI1 has also been identified as a transcription factor with some implication in transcriptionally coupled alternative splicing regulation [23,24]. Finally, only a small fraction of downstream and upstream CTCF peaks involved in a ChIA-PET interaction pair have the other half of the pair within 1kb of any TSS for CTCF ChIA-PET in K562 (Fisher's exact test P-value < 10^−8^) (Figures G and H in S6 Fig), as well as RNAPII ChIA-PET in K562 (Figures I and J in S6 Fig) (P-value < 10^−15^); hence, the majority of transcription factor peaks flanking internal exons have significantly less interactions with the promoter-proximal region compared with interactions that occur elsewhere on the genome, as can be seen in Figures G-J in S6 Fig and S3 Table for K562, and Figures K-N in S6 Fig and S4 Table for MCF7. These results provide evidence that intragenic CTCF bound in introns downstream of internal exons are involved in DNA looping and open the possibility that chromatin-mediated modulation of alternative splicing may be mediated by long-range interactions, as suggested recently for exonic peaks [25,26]. It is as yet unclear why the enrichment of chromatin interactions at downstream CTCF peaks occurs only in K562 and not MCF7, which could be due to the difference between experiments or to cell-specific chromatin configurations.

### Quantitative tests for the enrichment of RNA-binding protein (RBP) motifs on exons

**Fig 6:**
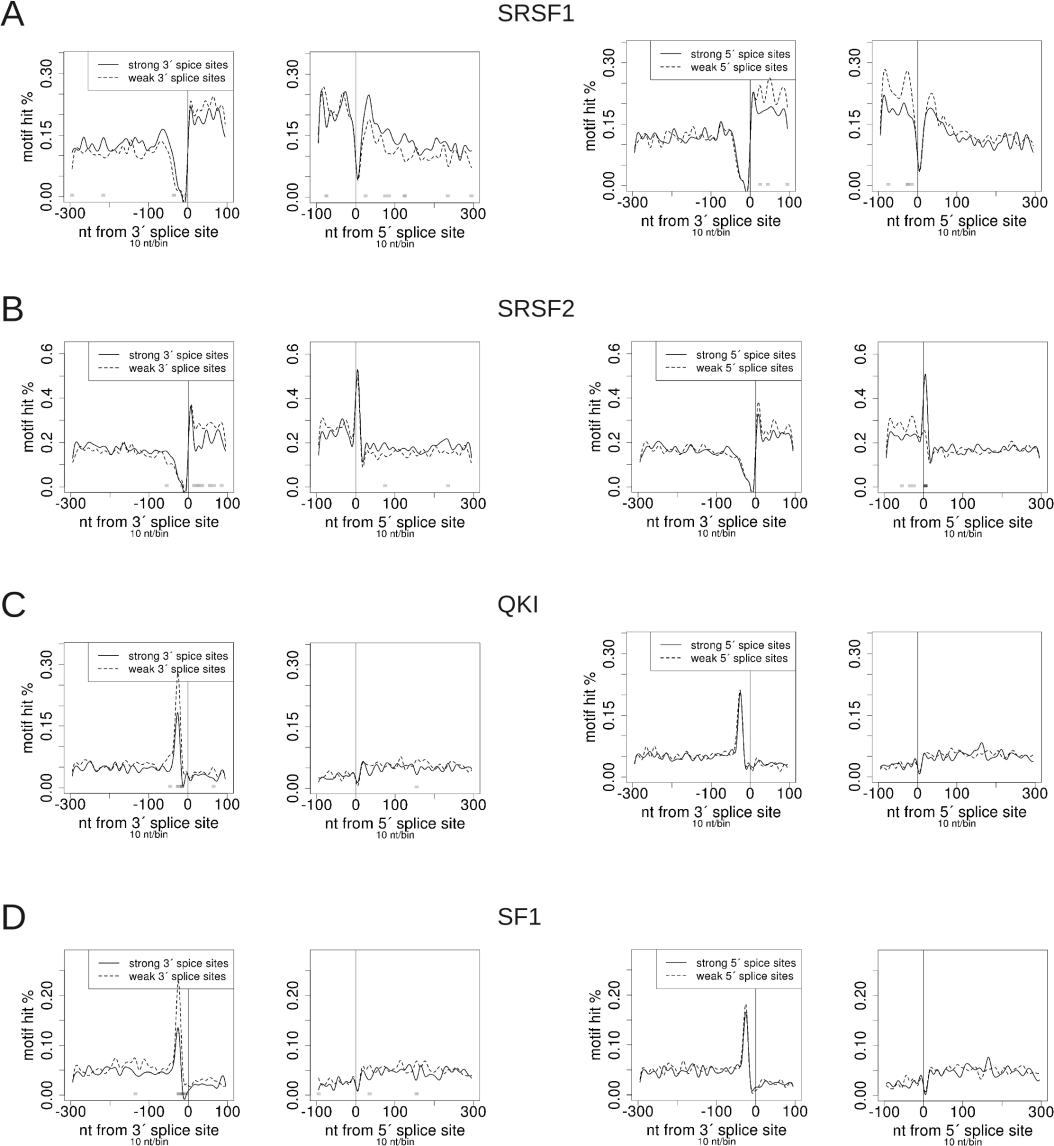
RBP motif validation profiles. “Strong” and “weak” are the upper and lower quartiles of splice site strength, respectively. Only exons longer than 100nt are considered. The corresponding profiles for the full internal exon set are in S1 File. Test vs. control P-values/bin are as shown in Fig 1B, with the lightest shade of grey corresponding to P-value < 0.01.

ProfileSeq can also be applied to motif data. We thus decided to determine quantitatively the enrichment of RBP binding motifs around exons, comparing strong and weak SSs. We first determined the occurrences of a recent compendium of motifs for RNA binding proteins [27], including SRSF1, SRSF2 and PTBP1 around the SSs. Analyzing the motifs in the upper and lower quartile of SS strengths using the full internal exon set (S1 File) as well as only internal exons longer than 100nt (S2 File), we observed an enrichment of SRSF1 and SRSF2 motifs in exons compared with introns, and on weak exons compared with strong ones (Fig 6A and 6B), consistent with their role as splicing enhancers. This behavior was also observed for SRSF9 and SRSF10, but not for SRSF7 (Figures A-C in S7 Fig). The percentage of motif hits in introns for the SR proteins is close to but generally above 0.1, consistent with the P-value cutoff of 0.001 used in determining if a sequence is a hit based on the nucleotide frequency matrix for each motif (S1 and S2 Files). Interestingly, significantly more motif hits were seen in introns flanking exons with strong 3’SSs compared with weak ones for SRSF1 and SRSF2 motifs (Fig 6A and 6B). The significant increase in mappability in introns of strong vs weak exons described above (see Fig 2) suggests greater sequence complexity in the introns flanking strong exons compared with those flanking weak ones. However, no significant difference in intronic reads was found in the CLIP-Seq profiles (see Fig 2F and Figures A and B in S4 Fig). Moreover, even though motif hit frequencies can vary in regions where there might not be actual RBP binding, e.g. in the vicinity of SSs, the motif and CLIP profiles for SRSF1 and SRSF2 all consistently show more occurrences in exons than introns, and significantly more on weak exons than strong ones, consistent with their known ESE binding activity.

For two members of the STAR family of proteins, QKI and SF1, we found a significant enrichment of motifs upstream of weak 3'SSs relative to strong ones (Fig 6C and 6D). Both factors show most of their binding affinity upstream the 3’SS, consistent with their binding to branch point (BP) and BP-like sequences [28]. In contrast to QKI and SF1, the profile of U2AF2, shows enrichment just upstream of strong 3'SSs relative to weak ones, in agreement with their function in constitutive splicing (Figure D in S7 Fig). Interestingly, HNRNPA1 and HNRNPA2B1 show a less uniform profile and, although there are more motif hits upstream of 3' SSs, both RBPs show an enrichment downstream of exons with weak 5' SSs compared with strong ones (Figures E and F in S7 Fig), consistent with their known binding activity on intronic splicing silencers [12].

### Potential coordination of chromatin factors and RBPs in the regulation of splicing

**Fig 7:**
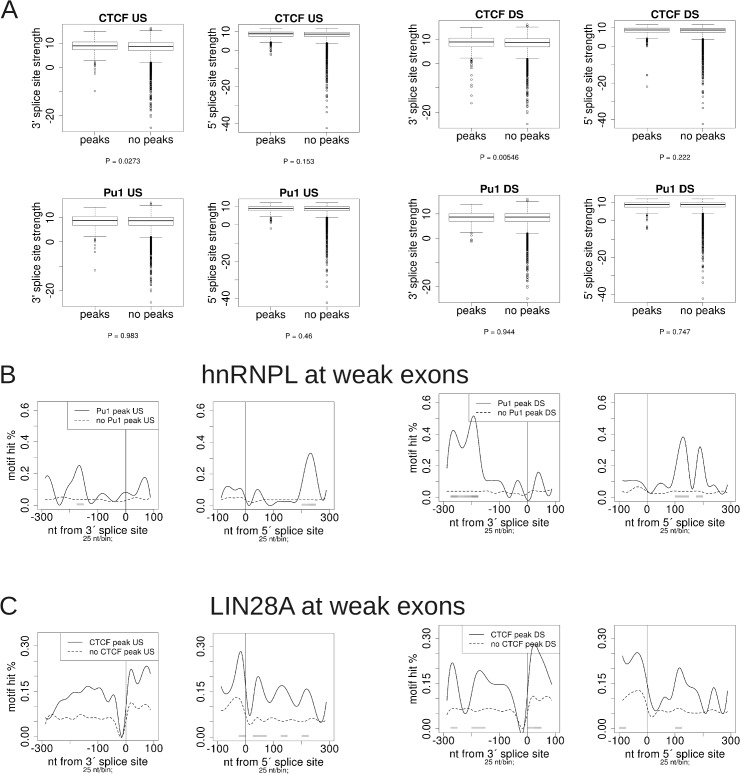
Comparison of internal exons with or without peaks nearby. US = peak center within 1kb upstream of 3'SS. DS = peak center within 1kb downstream of 5'SS. Pu1 = SPI1/PU.1 gene. (A) Splice site strengths of internal exons with or without transcription factor peaks nearby. P-values below each plot are based on the Wilcoxon test. Only exons longer than 100nt are considered. (B)-(C) RBP motif profiles at exons with or without peaks nearby. “Strong” and “weak” are the upper and lower 50% of splice site strengths, respectively. Only exons longer than 103nt are considered. Test and control exon sets for all profiles shown are matched for exon length, GC-content, and SS strength. Test vs. control P-values/bin are as shown in Fig 1B, with the lightest shade of grey corresponding to P-value < 0.01.

In order to obtain further insights into the role of protein factors binding chromatin in splicing regulation, we analyzed CTCF and SPI1 peaks near internal exons. We considered CTCF and SPI1 peaks from 8 (K562, HepG2, MCF7, A549 EtOH, A549 Dex, Ecc1, Hct, and T47D) and 2 (K562 and Gm12878) human cell lines, respectively. For CTCF, there were 944 internal exons with a reproducible peak (at least 50% overlap between 2 replicates) within 1kb upstream of an exon, and 920 exons with a reproducible CTCF peak within 1kb downstream, in at least one of the 8 samples. For SPI1, there were 424 exons with an upstream peak and 377 with a downstream peak, in at least one of the two cell lines. We compared the distribution of SS strengths of each of these exon sets to the rest of internal exons and found that the 3'SS scores of exons with a CTCF peak downstream had significantly larger SS strengths than exons without a CTCF peak downstream in all of the samples tested (Fig 7A), whereas no significant difference was observed for the other comparisons. On the other hand, there was no significant difference in either 3' or 5' SS strengths for the corresponding SPI1 comparisons.

Furthermore, exons that had a significant difference in CTCF ChIP-Seq reads between the ENCODE MCF7 and K562 cell lines in the 1kb downstream (DS) region, had significant changes in inclusion levels (PSI) in both directions, regardless of the direction of the change in DS CTCF signal (Figure A in S8 Fig). There was no detectable difference in PSI values at exons with DS CTCF vs. without in MCF7 (Figure B in S8 Fig) or K562 (data not shown) for exons that are either annotated as being skipped on at least one transcript, or had PSI < 1. We also found no significant difference in the distribution of PSI values between HepG2 and K562 ENCODE data at exons where a CTCF peak was present in K562 but not HepG2. Similar results hold for SPI1 (Figures D and E in S8 Fig). These results suggest that the presence of CTCF downstream of exons can not only enhance splicing, as originally suggested [1], but it may also repress it. A similar behavior is expected for SPI1.

The observation that the presence or absence of transcription factor binding can affect PSI values in a bidirectional manner suggests that more than a single factor determines the splicing outcome of individual exons. In particular, we hypothesized that binding of RBPs as well as chromatin and transcription factors simultaneously determine what the final splicing outcome will be. This raises the question of whether there is some coordination between transcription and splicing factors in determining the splicing outcome, or if on the contrary, they act in an independent, additive manner. To gain insights into this question, we generated profiles of the RNA compete motifs [27] around SSs, comparing exons with or without US or DS transcription factor peaks. Additionally, exons were separated according to the strength of their SSs. Profiles for the RNA binding motifs around exons with and without CTCF or SPI1 binding for both weak and strong SSs are available in S3, S4, S5 and S6 Files.

Interestingly, we found that exons with weak SSs and with a DS SPI1 peak show significant enrichment of motifs for multiple RBPs binding to AC-rich motifs, including HNRNPL, HNRNPLL, IGF2BP2 and IGF2BP3 (Fig 7B and S5 File). The location of the enrichment is ~175–275nt upstream of the 3'SS, while SPI1 binding is downstream of the 5'SS, and reduced enrichment is observed when SPI1 binds upstream (Fig 7B). This pattern is diminished or not present at strong exons (Figure A in S9 Fig and S6 File), suggesting that there might be a regulatory mechanism involving SPI1 and an RBP binding to AC-rich sequences, like HNRNPL, which is known to act both as a splicing repressor or enhancer in a position-dependent manner [12].

We acquired putative HNRNPL binding sites based on CLIP-Seq from the doRiNA database [29]. 4 samples from 2 human cell lines were used (CD4(+) and Jurkat). Only 9 of 293 exons that had a DS SPI1 peak in at least 1 of the 2 ENCODE cell lines also contained a putative HNRNPL binding event from -275 to -175nt upstream of the 3' SS. Even though these numbers are small, 8 of these 9 exons with both a DS SPI1 peak and HNRNPL binding upstream were in the weak set, while there were 143 weak exons with DS SPI1 and no HNRNPL and 150 exons with strong SSs. This difference was statistically significant (Fisher's exact test P-value = 0.036), consistent with the results of our profiles, which show that an increase in HNRNPL motif hits -275 to -175nt from the 3' SS at exons with DS SPI1 peaks vs. controls is greater in weak than strong exons. The extremely low number of exons with both HNRNPL and SPI1 binding observed may be due simply to the fact that the HNRNPL and SPI1 data are in different cell lines. In fact, there were 388 exons with a DS SPI1 peak in Gm12878, and 189 in K562, with an overlap of only 75 exons in both, suggesting a high degree of variation in SPI1 binding between cell lines. When considering all exons regardless of strength, and expanding the region to include -300 to -150nt from the 3' SS, 16 of 458 exons with a DS SPI1 peak had an HNRNPL binding site in the region just indicated, while 55 of 3092 exons without a DS SPI1 peak, from the same set of transcripts as exons with peaks, had an HNRNPL binding site. The proportion of exons with HNRNPL binding upstream was significantly larger for exons with a DS SPI1 peak (Fisher's exact test P-value = 0.018).

We also checked whether the presence or absence of a motif hit would affect the overall distribution of PSI values for alternative exons (either annotated as alternative or with PSI < 1 from RNA-Seq). We observed a significant difference in PSI values of alternative exons in Gm12878 without a DS SPI1 peak, comparing those with or without a HNRNPL motif hit (see S7 File), while the same comparison for alternative exons with a DS SPI1 peak showed no significant difference. The same trend was observed in K562 (S7 File). As only 23 alternative exons had both a DS SPI1 peak and HNRNPL motif hit (-275 to -175nt US) in Gm12878 for which PSI could be calculated, and similarly only 11 alternative exons in K562, it is not clear from these data whether the presence of a DS SPI1 peak reverses the effect on PSI values when HNRNPL binds, or whether the observed lack of significance is simply due to a small number of data points. Further work would be needed to uncover the mechanism behind the described association and the combinatorial affects of SPI1 with specific RBPs on splicing.

Although there are no reports on IGF2BP2 involvement on splicing, its motif profile is similar to HRNPL, and there is RNA-Seq data available for the knockdown of IGF2BP2 from the ENCODE project (https://www.encodeproject.org) for K562 cells. We calculated PSI values for both the knockdown (KD) and control (wt) datasets (Methods), and checked whether the distribution of changes in PSI (delta PSI) differed for exons with DS SPI1 peaks with or without an IGF2BP2 motif hit from -275 to -175nt from the 3'SS, but there was no distinguishable difference. There was also no distinguishable difference in delta PSI distribution between exons with an IGF2BP2 motif hit in the stated region with vs. without a DS SPI1 peak. These negative results involving IGF2BP2 are consistent with its characterization as functioning in the cytoplasm [30]

We also observed various RBPs that have significant differences in motif hits comparing exons with and without CTCF peaks (S3 and S4 Files). Several of these enrichments are specific to either strong or weak exons. For example, a significant enrichment of HNRNPL motif hits is observed upstream of strong exons with a DS CTCF peak, whereas no significant enrichment is observed for weak exons with DS CTCF peaks (Figures B and C in S9 Fig). No significant difference was observed in HNRNPL CLIP-Seq binding sites from -300 to -150nt from the 3'SS for all exons, regardless of strength, with DS CTCF peaks vs. no peaks. Strong exons with DS CTCF peaks had a larger proportion of cases with HNRNPL binding US from CLIP than weak exons, with marginal significance (9 of 381 vs. 2 of 381; Fisher's exact test P-value = 0.064,).

We also found LIN28A motifs significantly enriched around exons with nearby CTCF peaks (Fig 7C and Figure D in S9 Fig). The enrichment was found primarily upstream of the 3'SS and on the exon body near the 3'SS of exons with a CTCF peak DS. We confirmed an enrichment of LIN28A binding sites (doRiNA) from -300 to +25nt from the 3'SS of exons with CTCF DS (combined ENCODE peaks): 29 of 1149 exons with a DS CTCF peak had a LIN28A binding event in the region mentioned, compared with 125 of 8782 exons without a DS CTCF peak; the proportion was significantly larger for exons with DS CTCF than without (Fisher's exact test P-value = 0.007). Although LIN28A protein has been seen to bind predominantly to mRNA transcripts and be related to translation regulation [31], it can localize to the nucleus to repress the biogenesis of pre-miRNAs [32]. Our analysis brings up the possibility that LIN28A binding to intronic pre-miRNAs is related to the activity of CTCF.

We have seen some examples where the frequency of RBP motif hits depends both on the position of TF binding relative to the exon, as well as SS strength. This suggests that a possible coordination of position-specific RBP and TF binding could be involved in splicing and/or transcription regulation. Our earlier observation that CTCF divides regions of H3K4 methylation status also raises the interesting possibility of a correlation between chromatin state and the affinity for RBP binding. In light of this, our profiles suggest the possibility of coordination between CTCF binding, histone modifications, RNAPII activity, and the binding of specific RBPs.

## Conclusions

ProfileSeq provides a quantitative approach for the comparison of profiles between a reference and a control set. Additionally, it allows for straightforward determination and correction of confounding factors. We have validated ProfileSeq by recovering several qualitative results from the literature, and by providing a quantitative test for them. In addition, ProfileSeq allows taking into account mappability and input signal to perform the quantification. We found that, despite the fact that frequent observable differences in mappability between test and control profiles, correcting for mappability for reads of lengths ranging between 32–51nt has negligible effect on all cases examined. Thus ProfileSeq is robust to mappability biases over a range of scenarios.

We have also shown that ChIP input biases can have significant confounding effects on profiles. In particular, although we reproduce the accumulation of RNAPII ChIP-Seq reads at CTCF peaks downstream of internal exons in human and mouse, this RNAPII accumulation could be explained by the input signal. Although this does not contradict previous findings [1], they do suggest that the interpretation of RNAPII accumulation as pausing at the binding site of CTCF may be limited to a small number of cases. On the other had, we showed that the accumulation of RNAPII ChIP-Seq reads at the TSS persists after normalizing by input reads.

Using ProfileSeq, we have also found that SRSF1 and SRSF2 binding motif profiles agree with their corresponding CLIP-Seq profiles. Moreover, we quantified the enrichment of the CLIP and motif signal for these SR proteins on exons with weak splice-sites relative to exons with strong sites. Using a different description for the SRSF1 and SRSF2 motifs, Wang et al. [33] found the same result for SRSF1 motifs, but the opposite for SRSF2 motifs, as well as no significant difference of either SRSF1 or SRSF2 on the basis of 3' SS strength. Our quantitative analysis clarifies this result and shows that there is a significant enrichment of both proteins for motifs and CLIP signals, consistent with their role as spicing enhancers.

Finally, we found a potential coordination between SPI1 bound downstream of internal exons, and RBPs that bind to AC-rich motifs upstream of internal exons. The fact that the association is strong at weak exons, but diminished at strong exons, suggests a link with exon definition. We also found a co-occurrence of CTCF bound nearby exons and enrichment of binding sites for LIN28A, suggesting their association in pre-miRNA regulation. In summary, ProfileSeq provides a robust way to quantify genome-wide coordinate-based signals.

## Materials and Methods

### Annotation data sets

We used the human and mouse Gencode annotations (http://www.gencodegenes.org/), v7 and vM1, respectively. Entries in the GTF files were filtered out if they corresponded to “chrM” in column 1, “pseudogene” in column 3 or 6, “processed_transcript” or “TEC” in column 6, or “PUTATIVE” in column 7. From the filtered file we kept all exons (“exon” in column 3) that were not the first or last exon in the gene, and only if the 1kb region upstream and downstream of the exon did not have any overlap with any other exon, or any UTR or TSS. The set of entries whose third column was “transcript” were also extracted from the filtered file described above, from which the 5' end and 3' end of each entry was used in the file of TSSs and pA-sites, respectively, for the ChIP-Seq and GRO-Seq validation profiles. The exons for human and mouse are listed in S5–S8 Tables. S5 and S7 Tables were used in all analyses and profiles where it is mentioned that we use “internal exons” for human and mouse, respectively. The exceptions to this are the CLIP-Seq profiles at strong vs. weak exons and the analysis involving ChIA-PET interactions, in which cases exons from S6 Table were used in addition to the exons in S5 Table for human, and the exons in S8 Table were used in addition to the exons in S7 Table for mouse.

### Mappability analysis

The sequence of each region extending from 2kb upstream of the 3'SS to 2kb downstream of the 5' SS were obtained (http://genome.ucsc.edu/cgi-bin/das/) for the internal exon sets of human (hg19) and mouse (mm9). All reads of a given length were processed in FASTA format: for a read length *r* at an exon with starting at position *s* and ending position *e*, sequences of length *r* starting at *s-2000+i* were extracted and added as a single entry into the FASTA file, for all integers *i* from 0 to *e+2000-s-r+1*. A single FASTA file was used for each chromosome and each read length, which was then aligned, filtered, and reduced to a BED file of a single nucleotide position at the center of the read, using the same approach as for the profiles. This procedure was carried out for read lengths 27, 28, 30, 32, 36, 47, and 51nt for human, and 34, 36, and 40nt for mouse. These read lengths were chosen because they were the most commonly used amongst the ENCODE [34] datasets used in this study.

### Processing and filtering of high-throughput data

For publicly available ChIP-Seq data, the FASTQ files were downloaded and aligned to the human (hg19) and mouse (mm9) reference genomes using Bowtie2 [35] with flags—*no-unal-x-U-S*. SAM files thus obtained were then filtered to remove reads that were shorter than the maximum read length (thus ensuring that all reads are the same length), reads that contain one or more “N” in the sequence, and reads with MAPQ < = 30. Resulting files were converted to BAM using *samtools view* [36]. The same exact procedure was done for the CLIP-Seq data from [2]. On the other hand, for the CLIP-Seq data from [13] used in this study, *fastx_trimmer* (http://hannonlab.cshl.edu/fastx_toolkit/index.html) was first used to trim 8 nucleotides from the 3' end of each read, and only reads of length 28nt were kept, using the flags-*t 8-m 28-Q 33*, to ensure that adaptor sequences were not contained on reads, while at the same time keeping a constant read length, which allows for exact quantification according to mappability at that read length. For RNA-Seq ENCODE data, previously aligned BAM files of Poly-A+ junction reads were downloaded from (http://hgdownload.cse.ucsc.edu/goldenPath/hg19/encodeDCC/wgEncodeCaltechRnaSeq/ and http://hgdownload.cse.ucsc.edu/goldenPath/mm9/encodeDCC/wgEncodeLicrRnaSeq/).

Duplicate reads were removed from BAM files for ChIP-Seq, CLIP-Seq, and RNA-Seq using *samtools rmdup* with forced single end read mode (*-S* flag) after sorting with *samtools sort*, and the resulting BAM files were converted to SAM [36] with *samtools view*. This process was performed separately for each biological replicate. Posterior pooling of biological replicates was done using the unix command *sort-m-k3*,*3-k4n*,*4* after applying *sort-k3*,*3-k4n*,*4* to each individual replicate file. We have included as supplementary material the script used for the post-alignment processing steps just described for ChIP-Seq and CLIP-Seq (S1 Script) as well as RNA-Seq (S2 Script). Both scripts are suitable for use on a computer cluster.

### Preparation of processed SAM files for profiling

SAM files of the individual processed replicates, as well as the pooled files, were converted to BED using *pyicos (version 1*.*0*.*6c) convert* [37]. Closed coordinates were used for BED files rather than the standard half-open format. Reads were then truncated to the center position: a new BED file was created from the original BED file where the start and end position of a line in the new file were both the position halfway between the start and end position of the original file. For the cases where the halfway point was a half integer, it was rounded to the closest integer value. The script for this process is included as Supplementary Material (S3 Script). For GRO-Seq data, except for the fact that the 5' end was used instead of the read center position, the processing steps were the same as described before for the ChIP-Seq and CLIP-Seq datasets.

### Building profiles

Given a processed BED file as described above, together with a list of reference positions and a specified bin size as inputs, we count the number of reads occurring in the bed file at specified distances from the reference position with the program *count_occurrences*. For a user-defined number of bins of a specified length surrounding the set of reference regions, *count_occurrences* will count the number of reads that overlap with each bin; the source code for *count_occurrences* is available at https://bitbucket.org/regulatorygenomicsupf/profileseq/.

The resulting.*occ* file contains the count of reads at each bin for each reference region. Each.*occ* file produces a single profile. The number of replicates pooled is used to determine the maximum number of possible reads that can occur within each bin based on the filtering procedure described before. The percentage of possible reads occurring at each bin are then plotted using the R function *splines()* to generate a smooth curve that passes through most data points. Similarly, the.*occ* files of mappable reads or input reads are used to calculate and plot the percentage of mappable reads or input reads that occurred in the sample, which we have referred to as normalization. For 2-sample profiles, P-values to compare the occurrences of one sample to the other at each bin are calculated as follows: At each bin, a 2x2 contingency matrix is built as shown in Table 1:

**Table 1:** Contingency matrix for P-value calculations in ProfileSeq.

	Reads mapped	Reads not mapped
Sample	n_11_	n_12_
Control	n_21_	n_22_

where n_11_ gives the number of reads that occurred in the test set, n_21_ the number of reads occurring in control, n_12_, is computed as the maximum possible reads (or mappable reads) in that bin minus the mapped reads. If input reads are used, n_12_ is the number of input reads that occurred in the test set; n_22_ is calculated similarly for the control reads. From this table a Fisher's exact test P-value is calculated (using R). In addition, ProfileSeq counts the total number of reads in a central region of a specified length, and the total number of reads in the two flanking regions. These two flanking regions are such that they add up to the same nucleotide length as the centered region. In this case a contingency matrix analogous to Table 1 is built to determine a P-value based on Fisher's exact test to compare the centered region with the flanking regions.

### Empirical determination of False Discovery Rates

To further determine the robustness of the P-value calculation described above, a false discovery rate (FDR) is determined for every profile comparison. The test and control.*occ* files used in a profile are combined into a single file, randomly shuffled using the unix command, *shuf*, and split into two files of equal size. The two resulting.*occ* files are used to generate a profile each and quantify the differences between them. The P-value at each bin is stored into a file, and then the proportion of P-values below each cutoff, i.e, 0.01, 0.001, …1e-10 is calculated. This process is then repeated such that the file of P-values contains all P-values attained from all previous shuffling iterations as well as the current one. At the end of each iteration, the FDR for p< 0.01 is calculated as the proportion of total P-values less than 0.01. The FDR from the current iteration is then compared to the corresponding FDR from the previous iteration, and the process is continued until these two FDRs differ by less than 0.001 for 10 consecutive iterations, giving confidence of convergence to the true average.

For cases where a file of mappable reads or input reads is provided, the same procedure is performed, except that the test.*occ* file of sample counts and mappability/input counts is first combined into a single file, such that each line of the.*occ* file contains the information about both the sample and mappability/input for a single reference region. The same is done for the control set of regions. These two files are then pooled, shuffled, and split into 2 sets, as before. The file with the information for each set is then split into two.*occ* files, one for the sample count data, and the other for the mappability/input count data. Profiles and P-values are then generated for successive shuffling iterations as before until the convergence criterion stated before is met.

### Calculation of Percent Spliced In (PSI) values

RNA-Seq reads were processed as described above and *sjcount* [38] was used to count the number of junction reads at each annotated junction using Gencode version 7 for human and M1 for mouse. Given *i*, the sum of the 3' and 5' junction reads of that exon, and *e*, the skipping junction reads, i.e. one end is upstream of the 3' SS of the exon and the other end is downstream of the 5' SS, the PSI was calculated as *PSI = i/(i+2e)*. PSI was also calculated using only junction reads from either the 3' or 5' SS of internal exons, i_3_ and i_5_, respectively: 3' *PSI = i*_*3*_ */(i*_*3*_ *+e); 5' PSI = i*_*5*_ */(i*_*5*_ *+e)*.

### Splices-site scores

**SS** scores were calculated using *maxEntScan* [39] using for the 3’ SS -20 and 3nt on either side of the intron-exon boundary, and using for the 5’ SS -3 and 6nt on either side of the exon-intron boundary. The extreme values of the SS strengths obtained from *maxEntScan* were predictive of extreme values of PSI calculated by the method we have described (see S8 File).

### ChIP-Seq peaks

Previously processed broadPeak files were downloaded from ENCODE [34] for both human (http://hgdownload.cse.ucsc.edu/goldenPath/hg19/encodeDCC/wgEncodeHaibTfbs/) and mouse (http://hgdownload.cse.ucsc.edu/goldenPath/mm9/encodeDCC/wgEncodeLicrTfbs/). Files were converted from half-open to closed format. For biological replicates, only peaks that overlapped by at least 50% of the smaller peak length in both replicates were used. The overlapping peaks from the file that originated from the dataset with the larger number of reads were then used to generate a file of reference positions at the center of the peak interval. For mouse, a single BED file of peaks was provided for each biological sample since replicates were pooled before calling peaks. The reference positions for mouse were generated directly from these files. A set of US and DS peaks was constructed from the filtered peak data. US peaks were those whose center position falls within -1 and -1000nt upstream of the 3' SS of any of the exons from the internal exon set (described in “Annotation data sets”). DS peaks were similarly those peaks whose center falls within +1 and +1000nt downstream of the 5' SS.

### Profiles of RNAPII at CTCF peaks

Exons with US and DS peaks, for CTCF, SPI1, etc., attained as described above, were paired with exons on the same transcript that did not contain a US or DS peak. For example, an exon with a DS CTCF peak was paired with an exon on the same transcript that did not contain a DS CTCF peak. If no such exon was available, the exon with DS peak was discarded. If multiple exons were available, the one with the length closest to the length of the exon with DS peak was selected. A paired US and DS peak set was constructed for each sample analyzed. Furthermore, control positions were calculated in the following way: Let *d*_*i*_ be distance from the peak center to the nearest end of the exon for the *i*th DS exon (the same applies to US exons). That is, *d*_*i*_ is the distance from the 5' SS to the center of a DS peak (or the distance from the 3' SS to the center of an US peak). Then the control position for exon *i* is taken to be *d*_*i*_ nt from the nearest end of the control exon, thereby preserving the distance to the nearest SS between test and control regions.

For the profiles at peaks DS of exons affected by CTCF KD (Figure G in S5 Fig), we first took the set of all exons classified as “downstream”—that is, all exons where KD of a CTCF binding site DS had a significant effect on PSI–from Supplementary Table 4 of [1]. Since there was a small number of these exons that were contained in the internal exon set we have been using throughout this text, we expanded the exon set by ignoring the intronic behavior upstream of the exons. That is, the original exon set consists only of exons with a 1kb intronic region upstream that does not overlap with any annotated exon or UTR (and similarly for downstream). The expanded set only required that the downstream region satisfy the requirements just stated. We then took exons in the expanded set that were also in the set extracted from [1]. We required that the exon start and end coordinates matched exactly in order to be considered. We then eliminated exons from the remaining list that did not contain a DS CTCF peak in the HepG2 samples from ENCODE. The exons used to produce the profiles are listed in S9 Table.

### ChIA-PET analysis

ChIA-PET interaction BED files were downloaded from ENCODE (http://genome.ucsc.edu/cgi-bin/hgFileUi?db=hg19&g=wgEncodeGisChiaPet) and converted into closed format. For each transcription factor, peaks whose center overlapped with one half of a ChIA-PET interaction pair were obtained with the tool *fjoin* [40], using the peaks files for transcription factors and the closed BED files containing half of a ChIA-PET interaction pair per line. When 2 ChIA-PET replicates were available, the procedure described above was repeated successively for each replicate and only peaks whose centers were contained in one half of a ChIA-PET interaction from both replicates were used. For K562 CTCF ChIA-PET only one replicate was used as there was only one available from ENCODE. For some cases, the number of interaction pairs varied by more than an order of magnitude between replicates, so we removed replicates containing less than 10,000 total interaction pairs from the analysis. So even though 4 replicates were available for some samples, e.g. MCF7 RNAPII interactions, after removing small-sized replicates, each sample analyzed had only 1 or 2 replicates remaining. After obtaining the set of peaks that overlap with ChIA-PET interactions, the number of peaks whose center fell within the 1kb region upstream, and the 1kb region downstream, of our internal exon set were counted. These counts were used to construct 2x2 contingency tables (Table 2), e.g.

**Table 2:** Contingency matrix for genome-wide ChIA-PET and ChIP-Seq overlaps.

	Overlap	No overlap
US/DS	n_11_	n_12_
Genome-wide	n_21_	n_22_

The first column contains the number of peaks overlapping with ChIA-PET, whereas the 2nd column contains the number of peaks that did not overlap. The first row was for the peak in either the US or DS 1kb region, whereas in the 2^nd^ row, the remaining genome-wide counts were used. Each contingency matrix was used to calculate a P-value using Fisher's exact test.

Contingency matrices were also constructed for interactions with the TSS as follows: The count of DS peaks which overlap with half a ChIA-PET interaction pair were divided into two columns (Table 3):

**Table 3:** Contingency matrix for ChIA-PET/ChIP-Seq overlaps involving a TSS.

	Interaction with TSS	No-interaction with TSS
US/DS	n_11_	n_12_
Genome-wide	n_21_	n_22_

n_11_ gives the count of peaks where the center of the other half of the interaction pair (the one that the peak does not overlap) is contained within 1kb of any TSS, while n_12_ is the count of peaks where the center of the other half of the ChIA-PET interaction is not contained within 1kb of any TSS. The 2^nd^ row of the matrix divides the counts into the same categories by column for the remaining peaks genome-wide, i.e. peaks whose centers are not contained within 1 kb downstream of any of the exons in our internal exon set. Contingency matrices were similarly built for US peaks, and all P-values were calculated as above.

### Profiles of H3K4me1 and H3K4me3 at exons with or without CTCF peaks

CTCF peaks from the MEF cell line from ENCODE were used. Starting with the same US and DS paired CTCF peak sets described in the “Profiles of RNAPII at CTCF peaks” subsection, we considered in this analysis only exons longer than 100nt in order to avoid profile biases within the exon body. From the US and DS exon pairs longer than 100nt, we calculated the distance to the nearest TSS to each reference position (using all annotated TSSs from Gencode V7). Pairs were removed if the difference was longer than 10kb, or if either the test or control reference position was less than 1100nt from the nearest TSS. From here, the sum of all distances to the nearest TSS was calculated for both the test and control set, and the difference was taken. If the difference was greater than 800nt, the exon pair with the greatest discrepancy in distances to the TSS between test and control reference positions was removed, and the procedure was repeated until the total the difference mentioned above was smaller than 800nt. This left 327 downstream peak pairs, and 272 upstream peak pairs. In order to ensure that no biases remained between test and control sets in relation to their distribution of distances to the nearest TSS, a paired Wilcoxon rank-sum test was performed. In both cases, P-value > 0.6 was obtained, ensuring that there was no statistically relevant bias in distances to the TSS between the CTCF peak and non-peak exon pairs.

### Scanning for RBP motif hits

Human nucleotide sequences were obtained (http://genome.ucsc.edu/cgi-bin/das/hg19/) in the range -300 to +100nt from the 3'SS, and -100 to +300nt from the 5'SS, of each internal exon from the set described previously, which contains no annotated exons overlapping with the flanking intronic regions on either side within 1kb. The tool Fimo [41] was used to scan RNAcompete motifs in these sequences with a cut-off of P-value < 0.001.

### RBP motif profiles around exons

All transcription factor peaks were downloaded from ENCODE ((http://hgdownload.cse.ucsc.edu/goldenPath/hg19/encodeDCC/wgEncodeHaibTfbs/) for the cell lines described in the text. US and DS peaks were selected as described above. For a given transcription factor, all exons with US peaks from all cell lines were combined into a single file (and similarly for DS peaks). The remaining set of exons without a peak in any of the samples was then used as a pool from which to draw the control exon set. The following procedure was conducted separately for 5' and 3' SSs: The maximum and minimum maxEntScan score was determined for the set of exons with peaks and then the pool of potential control exons was reduced to only those exons whose SS score were between the minimum and maximum values for the peak set. Then, a random subset of the control pool was sampled such that it was the same size as the peak set. The distributions of SS scores, exon lengths, and GC-content for the peak set were each compared to the corresponding distributions of the random control subset. GC content distributions were matched separately for the 1kb region upstream and downstream, and the whole exon body. Distributions were compared between peak and random control subsets with a Wilcoxon rank-sum test. If the P-values for both the 3' and 5' comparisons were larger than a minimum cutoff, the random subset was kept as the control set of exons. Otherwise the procedure was repeated until the condition described in the previous sentence was met. For the comparison of SPI1 peaks vs. controls, the minimum cutoff was 0.6, and the procedure was repeated for 101 iterations, while for the CTCF comparison, the minimum cutoff was 0.5, and only 33 iterations were performed. The median count for all iterations was the value used for each bin of the control profile. Note that only exons of at least 104nt in length in this analysis were considered, as these are more useful for the generation of unbiased profiles within exon bodies. These consist of more than half the total set of internal exons.

## Supporting Information

S1 Fig Additional mappability validation profiles. (Figure A) Comparison of the mappability profiles for the upper and lower 20% of transcript lengths from Fig 1A using *ProfileSeq_ss*. (Figure B) Same as Figure A for 51nt reads. (Figure C) Same as Figure A for 36nt reads in mouse (mm9) (N = 11248). (Figure D) Cumulative distribution of exon lengths for the splice sites represented in Figures A and B. (Figure E) Same as Figure B, but limited to cases where the exon length is greater than 100nt. (Figure F) Same as Figure C, but limited to cases where the exon length is greater than 100nt. Test vs. control P-values/bin are as shown in Fig 1B, with the lightest shade of grey corresponding to P-value < 0.01.
(PDF)

S2 Fig Mappability by splice site strength. (Figure A) Mappability at the upper vs. lower quartile of splice site strengths, “strong” and “weak”, respectively. Same as Fig 2B, but for 40nt mouse (mm9) reads. (Figure B) Same as Fig 2B, but limited to exons longer than 100nt. Test vs. control P-values/bin are as shown in Fig 1B, with the lightest shade of grey corresponding to P-value < 0.01.
(PDF)

S3 Fig Additional ChIP-Seq and Gro-seq validation profiles. All profiles shown centered at the polyA-site have been inverted such that positive x-axis values indicate distance into transcript body and negative values indicate distance outside of transcript body. (Figure C) SRSF1 wt is a conditional SRSF1-knock cell line in which SRSF1 is not knocked out; SRSF2 is defined similarly. Test vs. control P-values/bin are as shown in Fig 1B, with the lightest shade of grey corresponding to P-value < 0.01.
(PDF)

S4 Fig Additional CLIP-Seq validation profiles. All profiles here are normalized by mappability. Test vs. control P-values/bin are as shown in Fig 1B, with the lightest shade of grey corresponding to P-value < 0.01.
(PDF)

S5 Fig Additional profiles at CTCF peaks vs. controls. DS = peak center within 1kb downstream of 5'ss. Test vs. control P-values/bin are as shown in Fig 1B, with the lightest shade of grey corresponding to P-value < 0.01.
(PDF)

S6 Fig Fraction of ChIP-Seq peaks overlapping ChIA-PET interactions. Diagonal line is of y = x. Statistically significant data points (Fisher's Exact Test P-value < 0.05) are shown with a number, whereas nonsignificant points are shown with an empty circle. Each point represents a set of peaks. The first 5 letters of the symbol for the numered points are listed here. (Figures A-F) Same format as in Fig 5. (Figure A) 1. Creb1 2. Ctcfc 3. Egr1V 4. Elf1s 5. Ets1V 6. GabpV 7. MaxV0 8. Pmlsc 9. Pol24 10. Pol2V 11. Six5P 12. Sp1Pc 13. SrfV0 14. Stat5 15. Tead4 16. Zbtb3 (Figure B) 1. Atf3V 2. Creb1 3. E2f6s 4. E2f6V 5. Egr1V 6. Elf1s 7. Pmlsc 8. Pol24 9. Pol2V 10. Pu1Pc 11. Sp2sc 12. SrfV0 13. Stat5 14. Taf7s 15. Usf1V 16. Yy1sc 17. Yy1V0 18. Yy1V0 (Figure C) 1. Rad21 (Figure D) 1. Sin3a (Figure E) 1. Elf1V 2. GabpV 3. MaxV0 4. Sin3a (Figure F) 1. Elf1V 2. GabpV 3. MaxV0 4. Rad21 (Figure G) 1. Cbx3s 2. Ctcfc 3. Ctcfl 4. E2f6s 5. E2f6V 6. Egr1V 7. Elf1s 8. Hdac2 9. MaxV0 10. Nr2f2 11. NrsfV 12. Pol24 13. Pol2V 14. Rad21 15. Tead4 16. Zbtb7 (Figure H) 1. Ctcfc 2. Ctcfl 3. Egr1V 4. Elf1s 5. GabpV 6. MaxV0 7. Nr2f2 8. Pol24 9. Pol2V 10. Rad21 11. Tead4 12. Zbtb7 (Figure I) 1. Atf3V 2. Atf3V 3. Cbx3s 4. Cebpb 5. Cebpd 6. Creb1 7. Ctcfc 8. Ctcfl 9. E2f6s 10. E2f6V 11. Egr1V 12. Elf1s 13. Ets1V 14. Fosl1 15. GabpV 16. Gata2 17. Hdac2 18. Hey1P 19. MaxV0 20. Mef2a 21. Nr2f2 22. NrsfV 23. Pmlsc 24. Pol24 25. Pol2V 26. Pu1Pc 27. Rad21 28. Six5P 29. Six5V 30. Sp2sc 31. Stat5 32. Taf1V 33. Tead4 34. Trim2 35. Usf1V 36. Yy1sc 37. Yy1V0 38. Zbtb3 39. Zbtb7 (Figure J) 1. Atf3V 2. Atf3V 3. Cbx3s 4. Cebpb 5. Cebpd 6. Creb1 7. Ctcfc 8. Ctcfl 9. E2f6s 10. E2f6V 11. Egr1V 12. Elf1s 13. GabpV 14. Hdac2 15. Hey1P 16. MaxV0 17. Nr2f2 18. NrsfV 19. Pmlsc 20. Pol24 21. Pol2V 22. Pu1Pc 23. Rad21 24. Sp2sc 25. Taf1V 26. Tead4 27. Usf1V 28. Yy1V0 29. Zbtb7 (Figure K) 1. MaxV0 2. Rad21 (Figure L) 1. Rad21 (Figure M) 1. Cebpb 2. Elf1V 3. Gata3 4. Hdac2 5. MaxV0 6. Nr2f2 7. Rad21 8. Sin3a (Figure N) 1. Cebpb 2. Elf1V 3. Gata3 4. Hdac2 5. MaxV0 6. Nr2f2 7. Rad21 8. Sin3a
(PDF)

S7 Fig Additional RBP motif validation profiles. Same format as Fig 6. Only considers exons longer than 100nt. Test vs. control P-values/bin are as shown in Fig 1B, with the lightest shade of grey corresponding to P-value < 0.01.
(PDF)

S8 Fig Changes in PSI and transcription factor binding. All P-values displayed below plots are based on the Wilcoxon test. (Figure A) Corr = correlation. The x-axis shows the log-fold change of CTCF ChIP-Seq reads between the indicated cell lines (M) in the 1 kb region downstream of internal exons, calculated by “pyicos enrichment” [37], while the y-axis shows the difference in PSI between the same cell lines based on RNA-Seq, calculated by MISO. Only points with a Baye's Facotr (BF) > 2 (MISO) and ||z| > 2 (pyicos) are displayed, i.e. points that have both signifcant changes in PSI and ChIP-Seq read density between the cell lines. (Figures B and D) PSI values calculated as described in the Methods for the indicated cell lines. Only exons that were either annotated as being skiped on at least one transcript, or had PSI < 1, were considered. (Figures C and E) PSI values calculated from MISO. CI: confidence interval for delta Psi (MISO). DS = peak center within 1kb downstream of 5'SS.
(PDF)

S9 Fig Additional RBP motif comparisons at exons with or without peaks nearby. Same format as Fig 7. Pu1 = SPI1/PU.1 gene. Test vs. control P-values at teach bin are as shown in Fig 1B, with the lightest shade of grey corresponding to P-value < 0.01.
(PDF)

S1 File Profiles of RNA-compete motifs at strong vs. weak splice sites. Each row consists of the profiles for a single motif, whose name is listed above each profile, and whose position-weight matrix is shown in the far-right column; “strong” is the upper quartile of splice site strengths for all internal exons, whereas “weak” is the lower quartile. The first two columns give the profiles centered at the 3' and 5' splice sites for the comparison of strong vs. weak 3' splice sites. The next two columns are the same profiles for the comparison of strong vs. weak 5' splice sites. Test vs. control P-values/bin are as shown in Fig 1B, with the lightest shade of grey corresponding to P-value < 0.01. In these profiles, only bins whose P-value cutoff had an empirical FDR < 0.05 were displayed in grey scale at the bottom, and the largest of the largest of the P-value cutoffs with FDR < 0.05 is written at the bottom of each plot.
(PDF)

S2 File Profiles of RNA-compete motifs at strong vs. weak splice sites for exons more than 100nt in length. This is exactly the same as in S1 File, except that only exons longer than 100nt were considered. So in this case “strong” is the upper quartile of splice site strengths amongst exons longer than 100nt, and similarly for “weak”.
(PDF)

S3 File RNA-compete motifs at weak exons with or without a CTCF peak nearby. US = peak center within 1kb upstream of 3' ss. DS = peak center within 1kb downstream of 5'SS, “weak” means the lowest half of 3'SS strengths for profiles centered at the 3' ss, and the lowest half of 5'SS strengths for profiles centered at the 5' ss. Test vs. control P-values/bin are as shown in Fig 1B, with the lightest shade of grey corresponding to P-value < 0.01.
(PDF)

S4 File RNA-compete motifs at strong exons with or without a CTCF peak nearby. Same format as S3 File; “strong” is defined in the same way as “weak” from S3 File, but for the largest half of splice site strengths. Test vs. control P-values/bin are as shown in Fig 1B, with the lightest shade of grey corresponding to P-value < 0.01.
(PDF)

S5 File RNA-compete motifs at weak exons with or without a SPI1 peak nearby. Same format as S3 File, Pu1 = SPI1/PU.1 gene.
(PDF)

S6 File RNA-compete motifs at strong exons with or without a SPI1 peak nearby. Same format as S5 File.
(PDF)

S7 File Boxplots of PSI distributions for exons with or without DS SPI1 peaks and/or AC-rich RBP motif hits. PSI values are from RNA-Seq from 1 of 2 cell lines–either Gm12878 or K562 – as indicated above each plot. Pu1 DS = a reproducible SPI1/PU.1 peak is found within 1 kb downstream of all internal exons considered in the given cell line. No Pu1 DS = only exons without a reproducible DS SPI1/PU.1 peak are considered. The left boxplot in each set of axes is the set of exons with an RBP motif hit from -275 to -175nt upstream of the 3' SS for the RBP indicated below the plot. The right boxplot corresponds to the exons without a motif hit in the same region (unlabeled). The P-value underneath each pair of axes is based on a Wilcoxon test. Only exons with PSI < 1 from RNA-Seq, or annotated as a skipped exon, are used in all boxplots.
(PDF)

S8 File Comparison of maxEntScan splice site score distributions at high and low PSI. “3' PSI”, was calculated using only 3' junction reads (see Methods); “5' PSI” was similarly calculated for 5' junction reads, and “PSI” uses the average of 5' and 3' junction reads. The ENCODE cell line from which RNA-Seq data was derived is listed at the top of each plot.
(PNG)

S1 Script Bash script for post-allignment filtering of SAM files. This was used to filter reads of the ChIP-Seq and CLIP-Seq data used in the paper, after alignment in Bowtie2. “Usage” shows the command used to run the script on a cluster. It may also be used with the Unix command “sh”.
(SH) Click here for additional data file.

S2 Script Bash script for post-alignment filtering of SAM files. Performs the same filtering as S1 Script. It was used to filter the pre-aligned RNA-Seq data from ENCODE that was used in the analysis of the paper. This script was needed because the SAM files used from ENCODE had a slightly different format than those obtained from alignment in Bowtie2.
(SH) Click here for additional data file.

S3 Script Commands to prepare filtered SAM files for use in ProfileSeq. This converts a SAM file into BED format, and then retains only the center coordinate. This was used throughout the paper to prepare filtered data (SAM format) for profiling. It requires Pyicoteo 1.0.6c (https://bitbucket.org/regulatorygenomicsupf/pyicoteo) to be installed. The BED file output may be directly used in ProfileSeq, or converted to a.pos file by using only columns 1, 2, and 6 of each line, for faster processing by ProfileSeq.
(SH) Click here for additional data file.

S1 Table Comparison of the overlap of K562 peaks near internal exons with ChIA-PET interactions from ENCODE to peaks genome-wide. “X peaks Y pairs” indicates the overlap of transcription factor X with Y ChIA-PET interactions, where Y = RNAPII or CTCF. DS = the peak center falls in an intronic region within 1kb downstream of an internal exon. US is similarly defined but for 1kb upstream; “more” indicates that significantly greater number of US or DS peaks of X overlapped half a Y ChIA-PET interaction pair than the remaining peaks of X genome-wide, whereas “less” indicates the opposite. Note that “more” and “less” are regardless of significance level. The last column gives the P-value for the null hypothesis that neither “more” nor “less” is true based on Fisher's exact test.
(TSV)

S2 Table Comparison of the overlap of MCF7 peaks near internal exons with ChIA-PET interactions from ENCODE to peaks genome-wide. Same as S1 Table, but for MCF7.
(TSV)

S3 Table Comparison of counts of peaks flanking internal exons that interact with a TSS to peaks not flanking internal exons that interact with a TSS based on K562 ChIP-Seq and ChIA-PET. “X peaks Y pairs” indicates that the overlap of transcription factor X with Y ChIA-PET interactions, where Y = RNAPII or CTCF. US = peak center within 1 kb upstream of 3' ss. DS = peak center within 1 kb downstream of 5'ss; “more” indicates that significantly greater US or DS peaks of X overlapped half a Y ChIA-PET interaction pair whose other half's center is within 1kb of any TSS than the remaining peaks of X overlapping Y genome-wide, whereas “less” indicates the opposite. Note that “more” and “less” are regardless of significance level. The last column gives the P-value for the null hypothesis that neither “more” nor “less” is true based on Fisher's exact test.
(TSV)

S4 Table Comparison of counts of peaks flanking internal exons that interact with a TSS to peaks not flanking internal exons that interact with a TSS based on MCF7 ChIP-seq and ChIA-PET. Same as S3 Table except for MCF7
(TSV)

S5 Table Coordinates of the human internal exon set. This is a.bed file in closed-coordinates format, i.e. with the first nucleotide at position 1. A list of all internal exons with the 1kb flanking intronic regions not overlapping with any other exon.
(BED)

S6 Table Additional. **Human internal exon coordinates**. Same format as S5 Table. Internal exons that do not contain an internal exon in the 1 kb flanking intronic regions from the same transcript, but that overlap with an internal exon from another transcript.
(BED)

S7 Table Coordinates of the mouse internal exon set. Same as S5 Table but for mouse.
(BED)

S8 Table Additional. **Human internal exon coordinates**. Same format as S6 Table but for mouse.
(BED)

S9 Table Exons affected by CTCF KD with a DS CTCF peak in HepG2. This list uses the.ref format described in the text (fields separated by “.”). The id (first field) contains the coordinates of the exon in closed format, separated by “:”, and ending with the strand. The last field of each line contains the center of the CTCF peak downstream of the exon in the HepG2 sample. DS = the peak center falls in an intronic region within 1kb downstream of an internal exon.
(REF)

S10 Table Sources of data used in this study. (PDF)

## References

[1] Shukla S, Kavak E, Gregory M, Imashimizu M, Shutinoski B, Kashlev M, et al CTCF-promoted RNA polymerase II pausing links DNA methylation to splicing. Nature. 2011;479: 74–79. 10.1038/nature10442 21964334 PMC7398428

[2] Ji X, Zhou Y, Pandit S, Huang J, Li H, Lin CY, et al SR proteins collaborate with 7SK and promoter-associated nascent RNA to release paused polymerase. Cell. 2013;153: 855–868. 10.1016/j.cell.2013.04.028 23663783 PMC4103662

[3] Core LJ, Waterfall JJ, Lis JT. Nascent RNA sequencing reveals widespread pausing and divergent initiation at human promoters. Science 2008;322: 1845–1848. 10.1126/science.1162228 19056941 PMC2833333

[4] König J, Zamack K, Rot G, Curk T, Kayiikci M, Zupan B, et al iCLIP reveals the function of hnRNP particles in splicing at individual nucleotide resolution. Nat Struct Mol Biol. 2010;17: 909–15. 10.1038/nsmb.1838 20601959 PMC3000544

[5] Schwartz S, Oren R, Ast G. Detection and removal of biases in the analysis of next generation sequencing reads. PLoS ONE. 2011;6: 1–12. 10.1371/journal.pone.0016685 PMC3031631 21304912

[6] Katz Y, Wang ET, Airoldi EM, Burge CB. Analysis and design of RNA sequencing experiments for identifying isoform regulation. Nature Methods. 2010;7 1009–1529. 10.1038/nmeth.1528 21057496 PMC3037023

[7] Kakaradov B, Xiong HY, Lee LJ, Jojic N, Frey BJ. Challenges in estimating percent inclusion of alternatively spliced junctions from RNA-seq data. BMC Bioinformatics. 2012;13: S11. 10.1186/1471-2105-13-S6-S11 PMC3330053 22537040

[8] Fenouil R, Cauchy P, Koch F, Descostes N, Cabeza JZ, Innocenti C, et al CpG islands and GC content dictate nucleosome depletion in a transcription-independent manner at mammalian promoters. Genome Research. 2012;22: 2399–2408. 10.1101/gr.138776.112 23100115 PMC3514669

[9] Black D. Mechanisms of alternative pre-messenger RNA splicing. Annu Rev Biochem. 2003;72: 291–336. 12626338 10.1146/annurev.biochem.72.121801.161720

[10] Liu HX, Zhang M, Krainer AR. Identification of functional exonic enhancer motifs recognized by individual SR proteins. Genes and Development. 1998;12: 1998–2012. 9649504 10.1101/gad.12.13.1998 PMC316967

[11] Fairbrother WG, Yeh RF, Sharp PA, Burge CB. Predictive identification of exonic splicing enhancers in human genes. Science. 2002;297: 1007–1013. 12114529 10.1126/science.1073774

[12] Fu X, Ares M Jr. Context-dependant control of alternative splicing by RNA-binding proteins. Nature Reviews Genetics. 2014;AOP: 1–13. 10.1038/nrg3778 PMC4440546 25112293

[13] Xue Y, Zhou Y, Wu T, Zhu T, Ji X, Kwon YS, et al Genome-wide analysis of PTB-RNA interactions reveals a strategy used by the general splicing repressor to modulate exon inclusion or skipping. Molecular Cell. 2009;36: 996–1006. 10.1016/j.molcel.2009.12.003 20064465 PMC2807993

[14] Sharma S, Maris C, Allain FHT, Black DL. U1 snRNA directly interacts with polypyrimidine tract-binding protein during splicing repression. Molecular Cell. 2011;41: 579–588. 10.1016/j.molcel.2011.02.012 21362553 PMC3931528

[15] Singh R, Valcarcel J, Green MR. Distinct binding specificities and functions of higher eukaryotic polypyrimidine tract-binding proteins. Science. 1995;268: 1173–1176. 7761834 10.1126/science.7761834

[16] Phillips JE, Corces VG. CTCF: master weaver of the genome. Cell. 2009;137: 1194–1211. 10.1016/j.cell.2009.06.001 19563753 PMC3040116

[17] Barski A, Cuddapah S, Cui K, Roh TY, Shones DE, Wang Z, et al High-resolution profiling of histone methylations in the human genome. Cell. 2007;129: 823–837. 17512414 10.1016/j.cell.2007.05.009

[18] Wang Z, Zang C, Cui K, Shones DE, Barski A, Peng W, et al Genome-wide mapping of HATs and HDACs reveals distinct functions in active and inactive genes. Cell. 2009;138: 1019–1034. 10.1016/j.cell.2009.06.049 19698979 PMC2750862

[19] Pekowska A, Benoukraf T, Zacarias-Cabeza J, Belhocine M, Koch F, Holota H, et al H3K4 tri-methylation provides an epigenetic signature of active enhancers. EMBO J. 2011;30: 4198–210. 10.1038/emboj.2011.295 21847099 PMC3199384

[20] Khan DH, Gonzalez C, Cooper C, Sun JM, Chen HY, Healy S, et al RNA-dependant dynamic histone acetylation regulates MCL1 alternative splicing. Nucleic Acids Research. 2014;42: 1656–1670. 10.1093/nar/gkt1134 24234443 PMC3919583

[21] Alló M, Agirre E, Bessonov S, Bertucci P, Acuña LG, Buggiano V, et al Argonaut 1 binds transcriptional enhancers and controls contitutive and alternative splicing. PNAS. 2014;111 15622–15629. 10.1073/pnas.1416858111 25313066 PMC4226100

[22] Wada Y, Ohta Y, Xu M, Tsutsumi S, Minami T, Inoue K, et al A wave of nascent transcription on activated human genes. PNAS. 2009;106: 18357–18361. 10.1073/pnas.0902573106 19826084 PMC2761237

[23] Guillouf C, Gallais I, Moreau-Gachelin F. Spi-1/PU.1 oncoprotein affects splicing decisions in a promoter binding-dependent manner. J Biol Chem. 2008;281: 19145–19155. 10.1074/jbc.M512049200 16698794

[24] Ye Z, Chen Z, Lan X, Hara S, Sunkel B, Huan THM, et al Computational analysis reveals a correlation of exon-skipping events with splicing, transcription, and epigenetic factors. Nucl Acids Res. 2013 24 12: 1–14. 24369421 10.1093/nar/gkt1338 PMC3950716

[25] Mercer TR, Edwards SL, Clark MB, Neph SJ, Wang H, Stergachis AB, et al Dnase-I hypersensitive exons colocalize with promoters and distal regulatory elements. Nature Genetics. 2013;45: 852–859. 10.1038/ng.2677 23793028 PMC4405174

[26] Acuña LIG, Kornblihtt AR. Long range chromatin organization A new layer in splicing regulation? Transcription. 2014;5: e28728. 10.4161/trns.28726 PMC4574877 25764333

[27] Ray D, Kazan H, Chan ET, Castillo LP, Chaudhry S, Talukder S, et al Rapid and systematic analysis of the RNA recognition specificities of RNA-binding proteins. Nature Biotechnology. 2009;27: 667–672. 10.1038/nbt.1550 19561594

[28] Galameau A Richard S. Target RNA motif and target mRNAs of the Quaking STAR protein. Nat Struct Mol Biol. 2005;12: 691–698. 16041388 10.1038/nsmb963

[29] Blin K, Dieterich C, Wurmus R, Rajewsk N, Landthaler M, Akalin A. DoRiNA 2.0 – upgrading the doRiNA database of RNA interactions in post-transcriptional regulation. Nucl Acids Res. 2015;43: D160–167. 10.1093/nar/gku1180 25416797 PMC4383974

[30] Nielsen J, Christiansen J, Lykke-Andersen J, Johnsen AH, Wewer UM, Nielsen FC. A family of Insulin-like Growth Factor-II mRNA-binding proteins represses translation in late development. Mol Cell Biol. 1999;19: 1262–1270. 9891060 10.1128/mcb.19.2.1262 PMC116055

[31] Cho J, Chang H, Chul Kwon S, Kim B, Kim Y, Choe J, et al LIN28A is a supressor of ER-associated translation in embryonic stem cells. Cell. 2012;151: 765–777. 10.1016/j.cell.2012.10.019 23102813

[32] Newman M, Thomsan JM, Hammond SM. Lin-28 interaction with the Let-7 precursor loop mediates regulated microRNA processing. RNA. 2008;14: 1539–1549. 10.1261/rna.1155108 18566191 PMC2491462

[33] Wang J, Smith PJ, Krainer AR, Zhang MQ. Distribution of SR protein exonic splicing enhancer motifs in human protein-coding genes. Nucleic Acids Research. 2005;33: 5053–5062. 16147989 10.1093/nar/gki810 PMC1201331

[34] The ENCODE Project Consortium. An integrated encyclopedia of DNA elements in the humna genome. Nature. 2012;489: 57–74. 10.1038/nature11247 22955616 PMC3439153

[35] Langmead B, Salzberg SL. Fast gapped-read alignment with Bowtie2. Nature Methods. 2012;9: 357–359. 10.1038/nmeth.1923 22388286 PMC3322381

[36] Li H, Handsaker B, Wysoker A, Fennell T, Ruan J, Homer N, et al The sequence alignment/map format and SAMtools. Bioinformatics. 2009;25: 2078–2079. 10.1093/bioinformatics/btp352 19505943 PMC2723002

[37] Althammer S, Gonzalez-Vallines J, Ballaré C, Beato M, Eyras E. Pyicos: a versatile toolkit for the analysis of high-throughput sequencing data. Bioinformatics. 2011;27: 3333–3340. 10.1093/bioinformatics/btr570 21994224 PMC3232367

[38] Pervouchine DD, Knowles DG, Guigo R. Intron-centric estimation of alternative splicing from RNA-seq data. Bioinformatics. 2013;23: 273–274. 10.1093/bioinformatics/bts678 PMC3546801 23172860

[39] Yeo G, Burge CB. Maximum entropy modeling of short sequence motifs with applications to RNA splicing signals. J Comp Biol. 2004;11: 377–394. 10.1089/1066527041410418 15285897

[40] Richardson JE. fjoin: simple and efficient computation of feature overlaps. J Comput Biol. 2006;13: 1457–1464. 17061921 10.1089/cmb.2006.13.1457

[41] Bailey TL, Elkan C. Fitting a mixture model by expectation maximization to discover motifs in biopolymers. Proceedings on the Second International Conference on Intelligent Systems for Molecular Biology. 1994: 28–36. 7584402

