# Supplementary material for: A Quantitative Profiling Tool for Diverse Genomic Data Types Reveals Potential Associations between Chromatin and Pre-mRNA Processing
Source: PLoS One. 2015 Jul 24;10(7):e0132448. doi: 10.1371/journal.pone.0132448 (PMC4514851; doi:10.1371/journal.pone.0132448)
Supplement: S1 Fig — (Figure A) Comparison of the mappability profiles for the upper and lower 20% of transcript lengths from Fig 1A using ProfileSeq_ss. (Figure B) Same as Figure A for 51nt reads. (Figure C) Same as Figure A for 36nt reads in mouse (mm9) (N = 11248). (Figure D) Cumulative distribution of exon lengths for the splice sites represented in Figures A and B. (Figure E) Same as Figure B, but limited to cases where the exon length is greater than 100nt. (Figure F) Same as Figure C, but limited to cases where the exon length is greater than 100nt. Test vs. control P-values/bin are as shown in Fig 1B, with the lightest shade of grey corresponding to P-value < 0.01. (PDF) [file pone.0132448.s001.pdf]

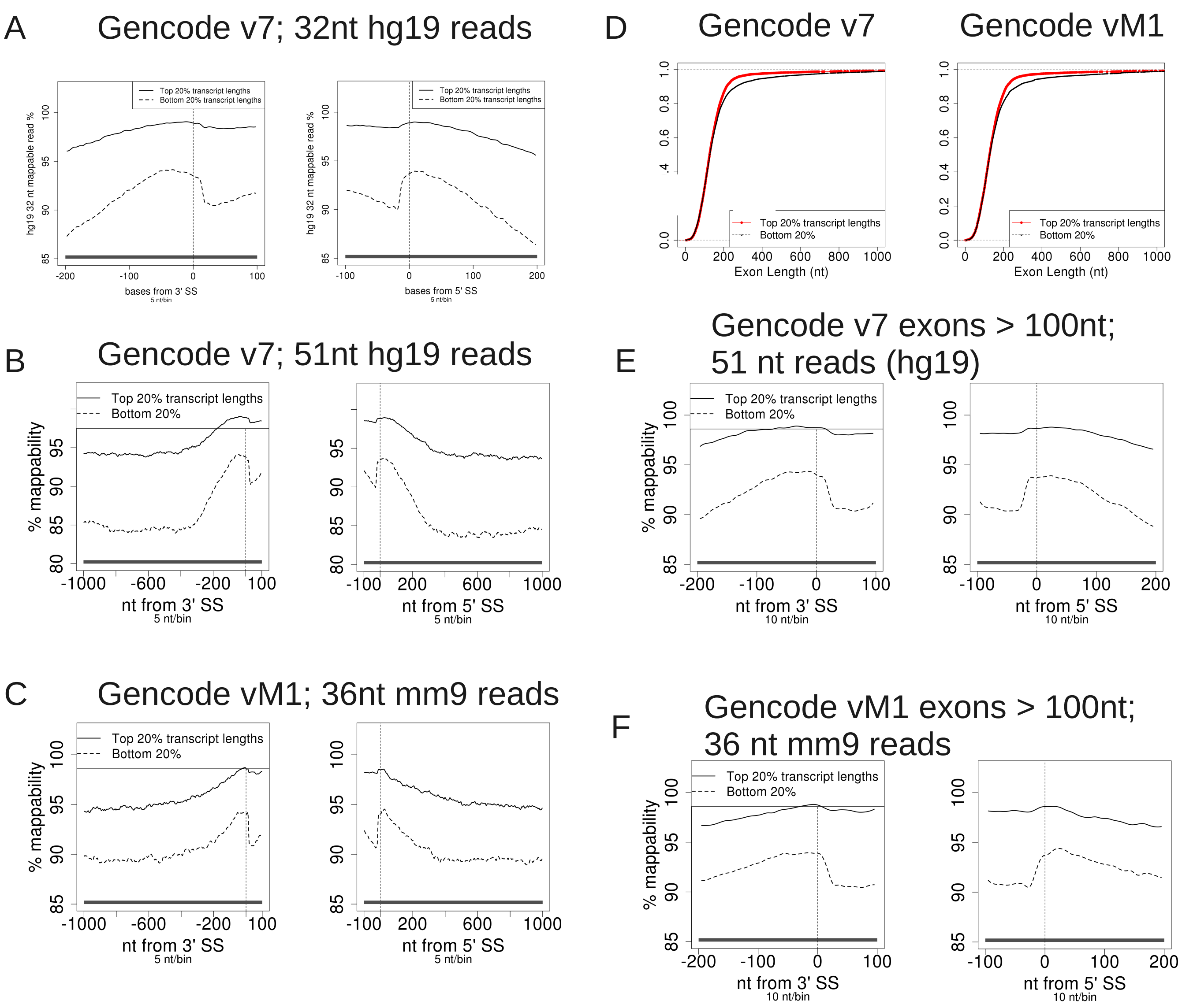
A
Gencode v7; 32nt hg19 reads
Top 20% transcript lengths
Bottom 20% transcript lengths
hg19 32 nt mappable read %
bases from 3' SS
5 nt/bin
bases from 5' SS
5 nt/bin
B
Gencode v7; 51nt hg19 reads
Top 20% transcript lengths
Bottom 20%
% mappability
nt from 3' SS
5 nt/bin
nt from 5' SS
5 nt/bin
C
Gencode vM1; 36nt mm9 reads
Top 20% transcript lengths
Bottom 20%
% mappability
nt from 3' SS
5 nt/bin
nt from 5' SS
5 nt/bin
D
Gencode v7
Gencode vM1
Top 20% transcript lengths
Bottom 20%
Exon Length (nt)
Exon Length (nt)
E
Gencode v7 exons > 100nt;
51 nt reads (hg19)
Top 20% transcript lengths
Bottom 20%
% mappability
nt from 3' SS
10 nt/bin
nt from 5' SS
10 nt/bin
F
Gencode vM1 exons > 100nt;
36 nt mm9 reads
Top 20% transcript lengths
Bottom 20%
% mappability
nt from 3' SS
10 nt/bin
nt from 5' SS
10 nt/bin
