# Supplementary figures and images for: A Quantitative Profiling Tool for Diverse Genomic Data Types Reveals Potential Associations between Chromatin and Pre-mRNA Processing
Source: PLoS One. 2015 Jul 24;10(7):e0132448. doi: 10.1371/journal.pone.0132448 (PMC4514851; doi:10.1371/journal.pone.0132448)

A

## Gencode vM1; 40 nt mm9 reads

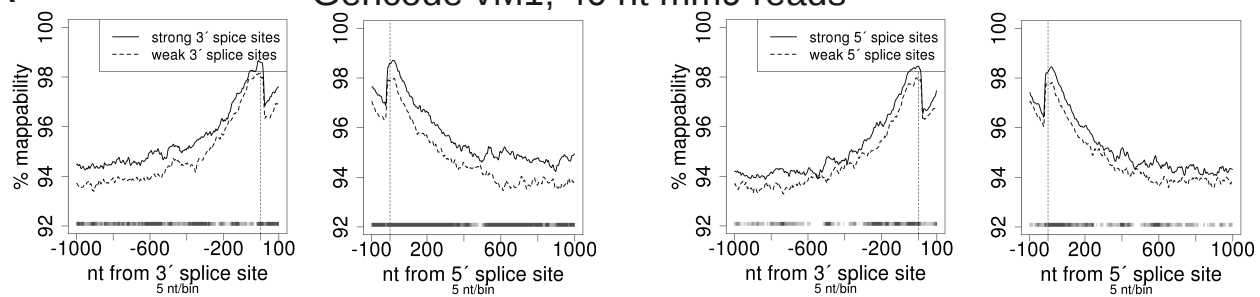

B

## Gencode v7; 51 nt hg19 reads; exon length &gt; 100nt

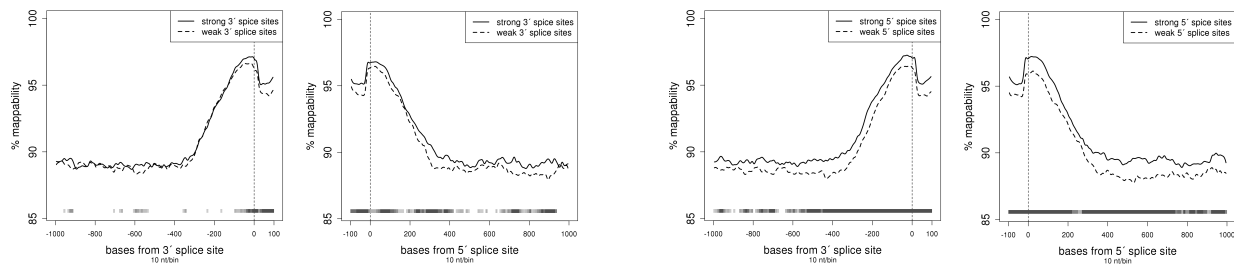

Supplement: S2 Fig — (Figure A) Mappability at the upper vs. lower quartile of splice site strengths, “strong” and “weak”, respectively. Same as Fig 2B, but for 40nt mouse (mm9) reads. (Figure B) Same as Fig 2B, but limited to exons longer than 100nt. Test vs. control P-values/bin are as shown in Fig 1B, with the lightest shade of grey corresponding to P-value < 0.01. (PDF) [file pone.0132448.s002.pdf]

# A

## SRSF1 CLIP-Seq; exon length > 100nt

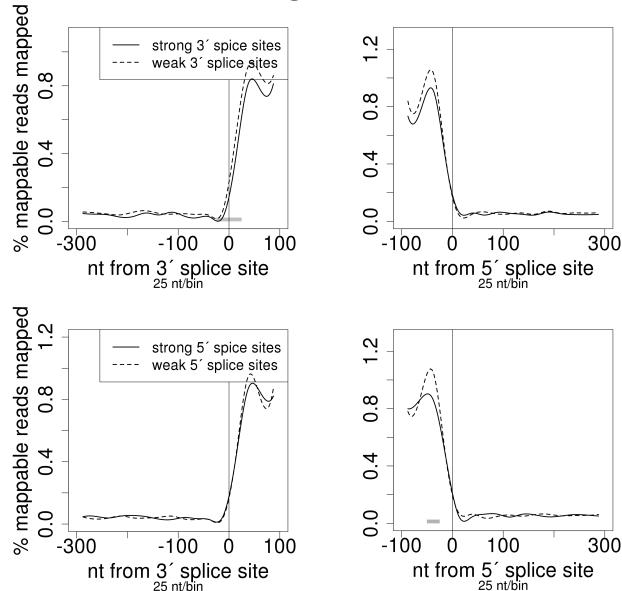

# C

## PTB CLIP-Seq; exon length > 100nt

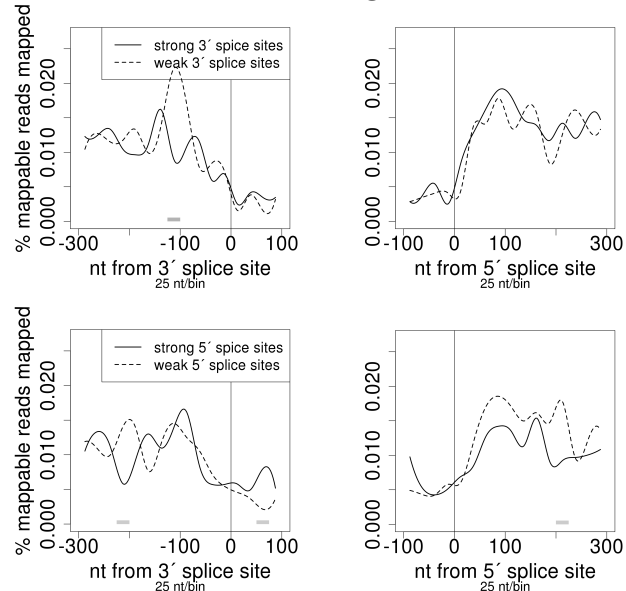

# B

## SRSF2 CLIP-Seq; exon length > 100nt

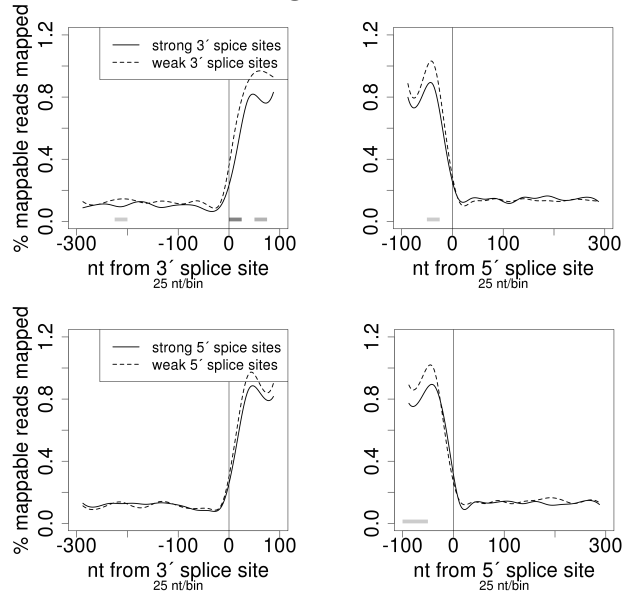

Supplement: S4 Fig — All profiles here are normalized by mappability. Test vs. control P-values/bin are as shown in Fig 1B, with the lightest shade of grey corresponding to P-value < 0.01. (PDF) [file pone.0132448.s004.pdf]

A

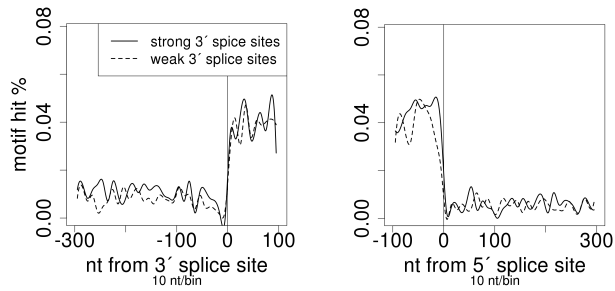

SRSF7

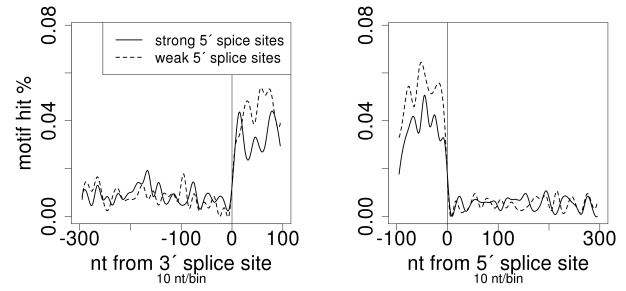

B

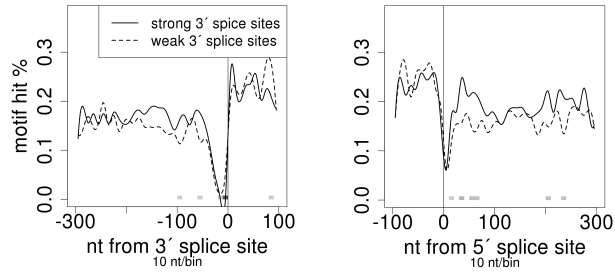

SRSF9

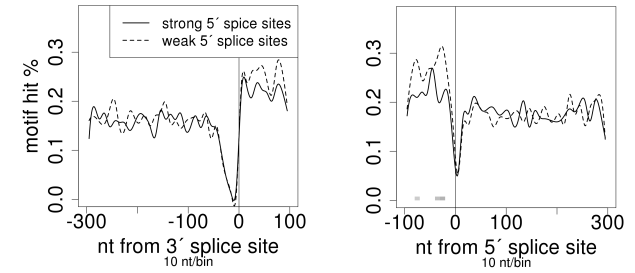

C

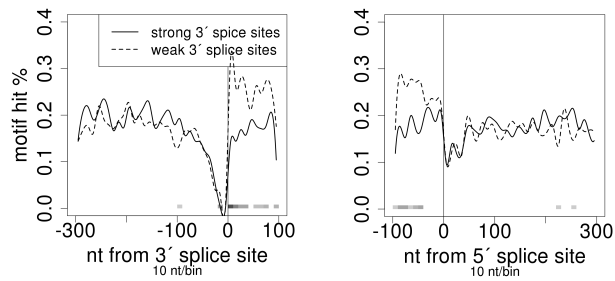

SRSF10

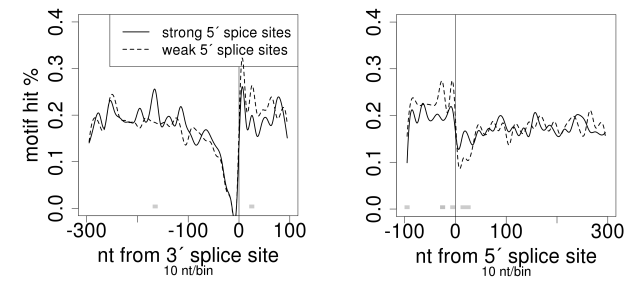

D

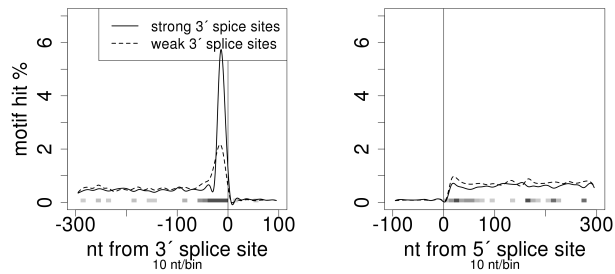

U2AF2

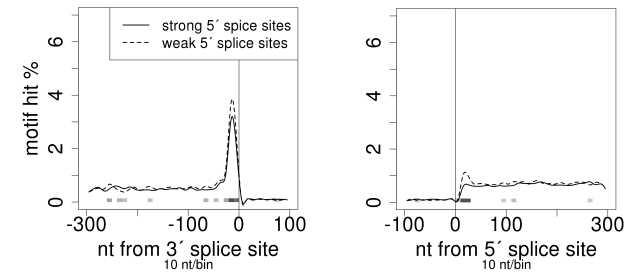

E

HNRNPA1

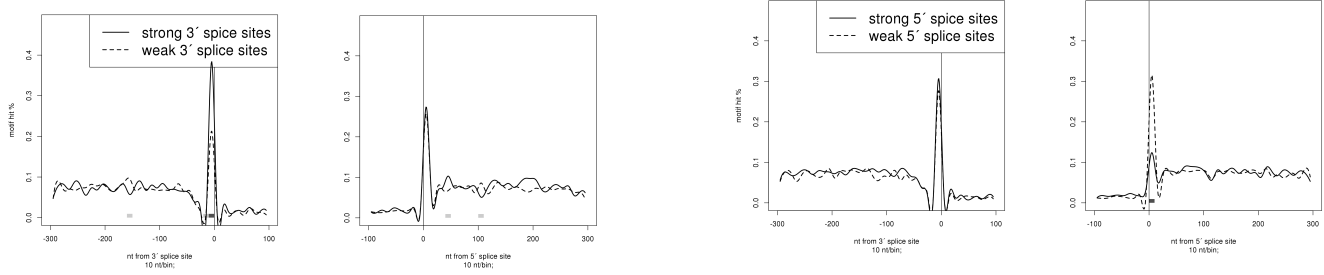

F

HNRNPA2B1

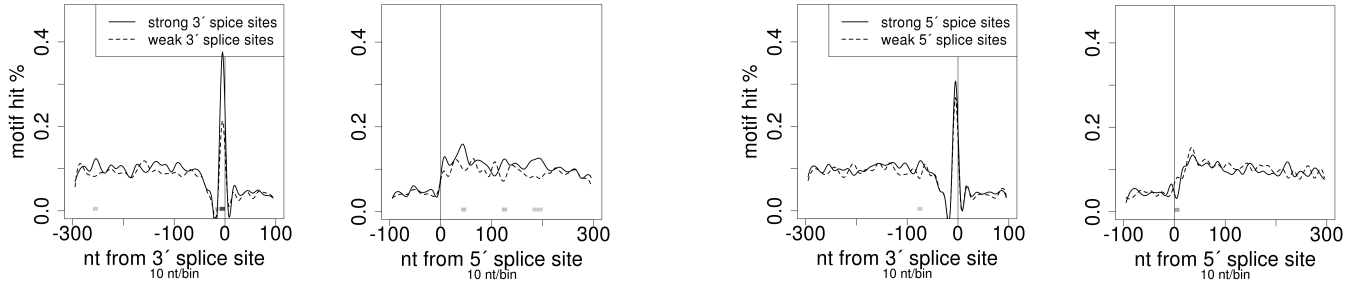

Supplement: S7 Fig — Same format as Fig 6. Only considers exons longer than 100nt. Test vs. control P-values/bin are as shown in Fig 1B, with the lightest shade of grey corresponding to P-value < 0.01. (PDF) [file pone.0132448.s007.pdf]

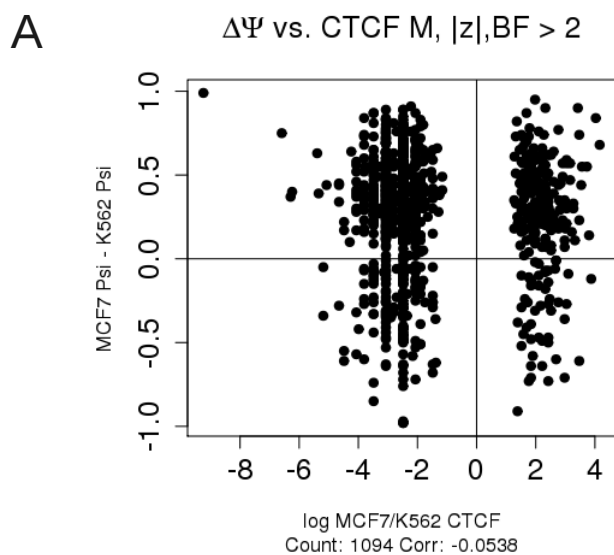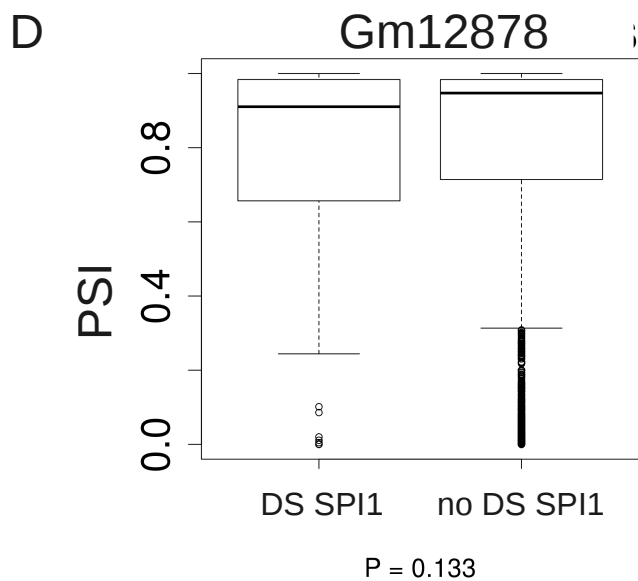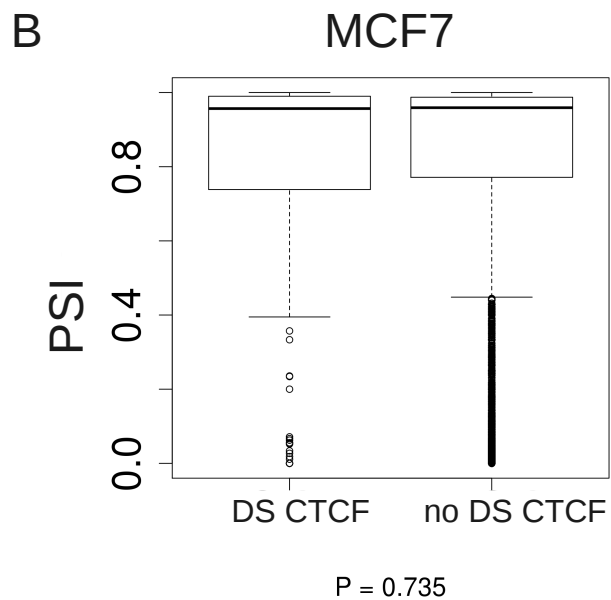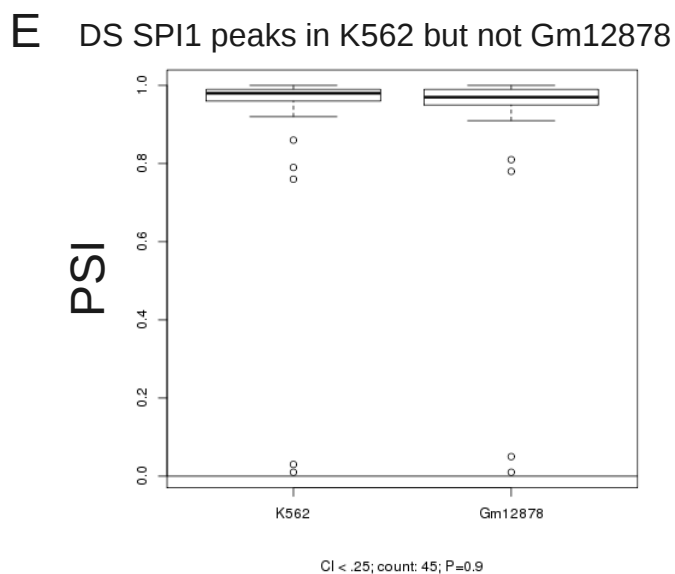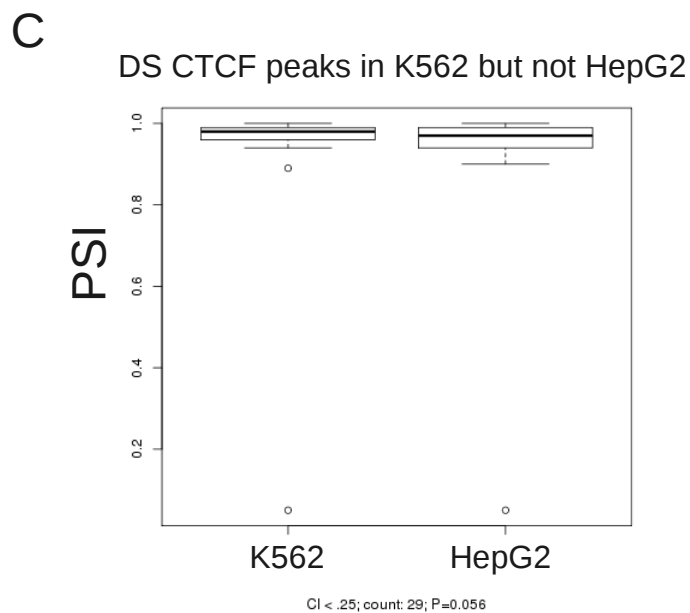

Supplement: S8 Fig — All P-values displayed below plots are based on the Wilcoxon test. (Figure A) Corr = correlation. The x-axis shows the log-fold change of CTCF ChIP-Seq reads between the indicated cell lines (M) in the 1 kb region downstream of internal exons, calculated by “pyicos enrichment” [37], while the y-axis shows the difference in PSI between the same cell lines based on RNA-Seq, calculated by MISO. Only points with a Baye's Facotr (BF) > 2 (MISO) and ||z| > 2 (pyicos) are displayed, i.e. points that have both signifcant changes in PSI and ChIP-Seq read density between the cell lines. (Figures B and D) PSI values calculated as described in the Methods for the indicated cell lines. Only exons that were either annotated as being skiped on at least one transcript, or had PSI < 1, were considered. (Figures C and E) PSI values calculated from MISO. CI: confidence interval for delta Psi (MISO). DS = peak center within 1kb downstream of 5'SS. (PDF) [file pone.0132448.s008.pdf]

A

## hnRNPL at strong exons

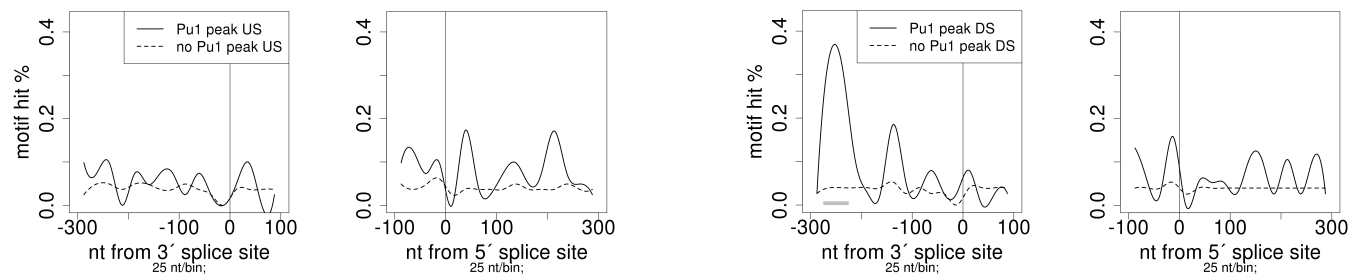

B

## hnRNPL at weak exons

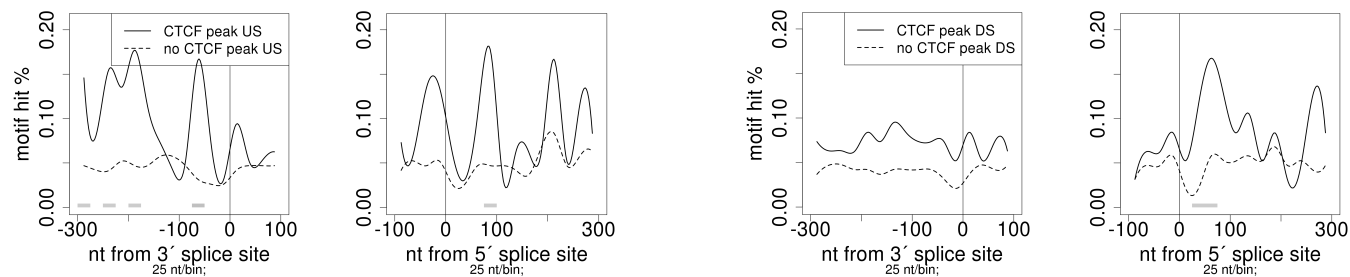

C

## hnRNPL at strong exons

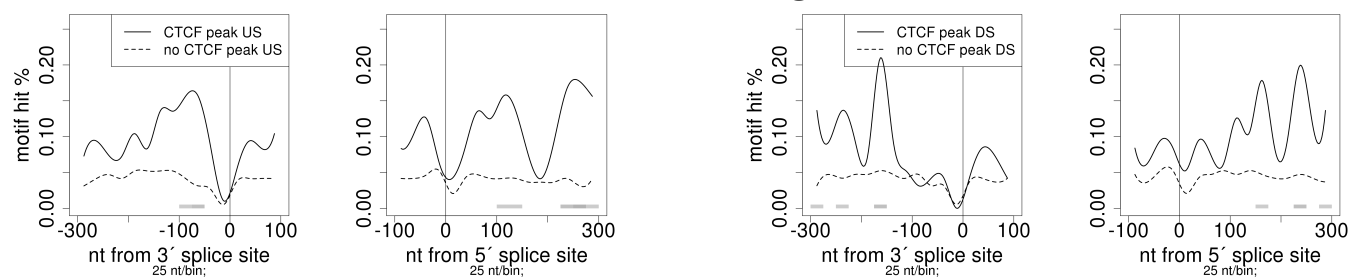

D

## LIN28A at strong exons

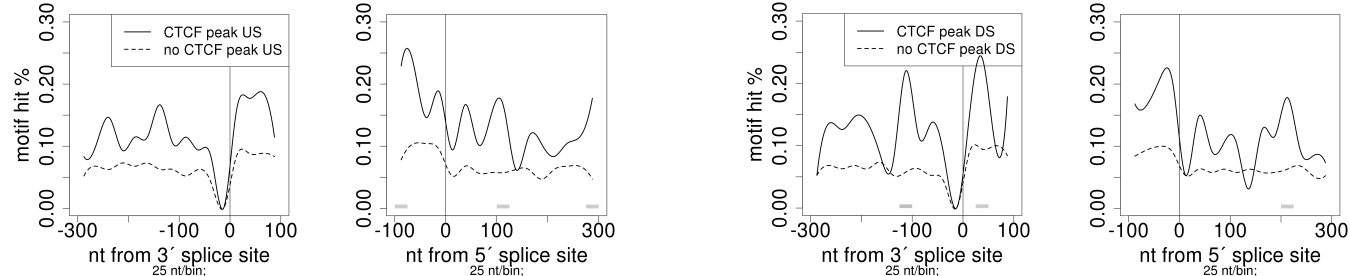

Supplement: S9 Fig — Same format as Fig 7. Pu1 = SPI1/PU.1 gene. Test vs. control P-values at teach bin are as shown in Fig 1B, with the lightest shade of grey corresponding to P-value < 0.01. (PDF) [file pone.0132448.s009.pdf]

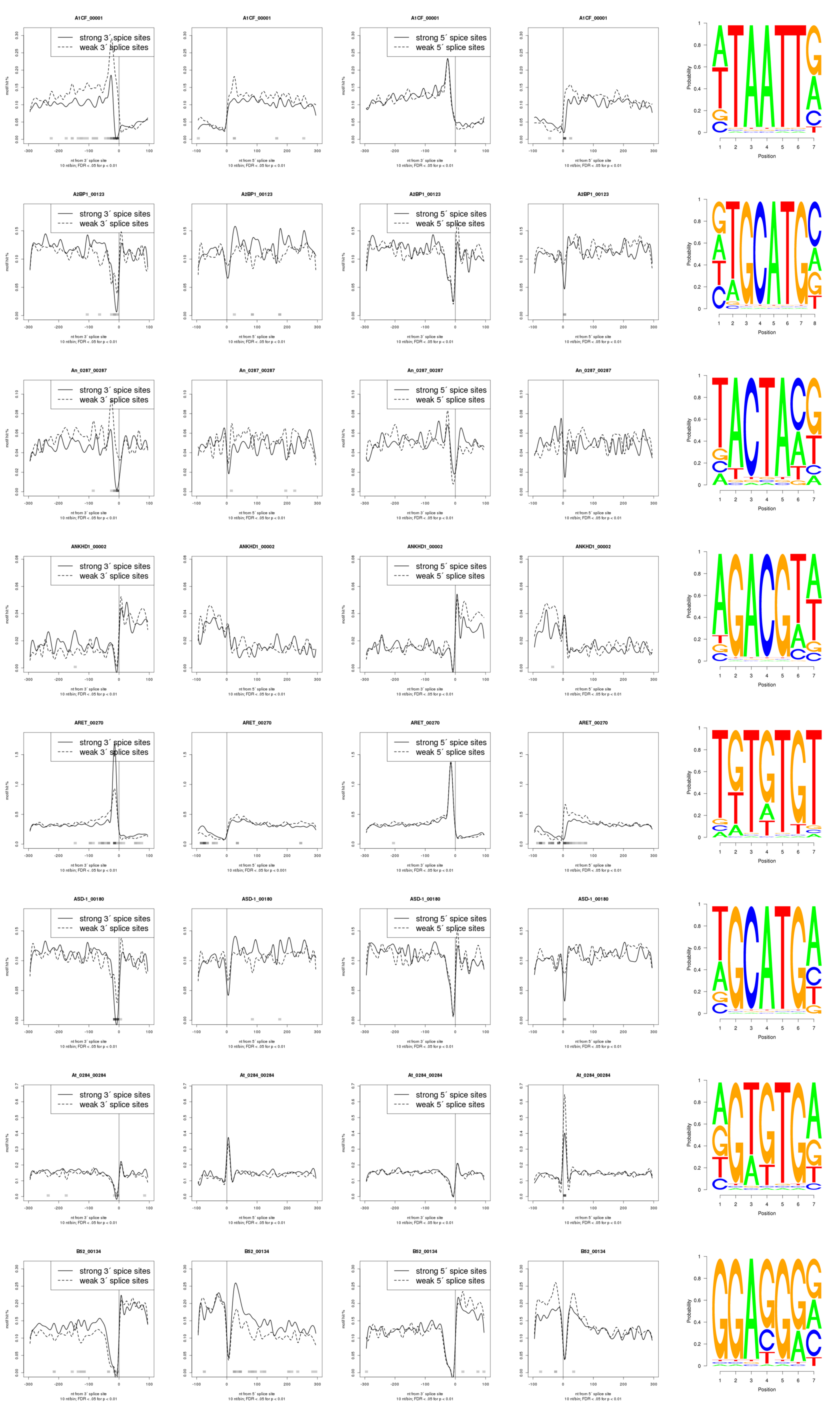

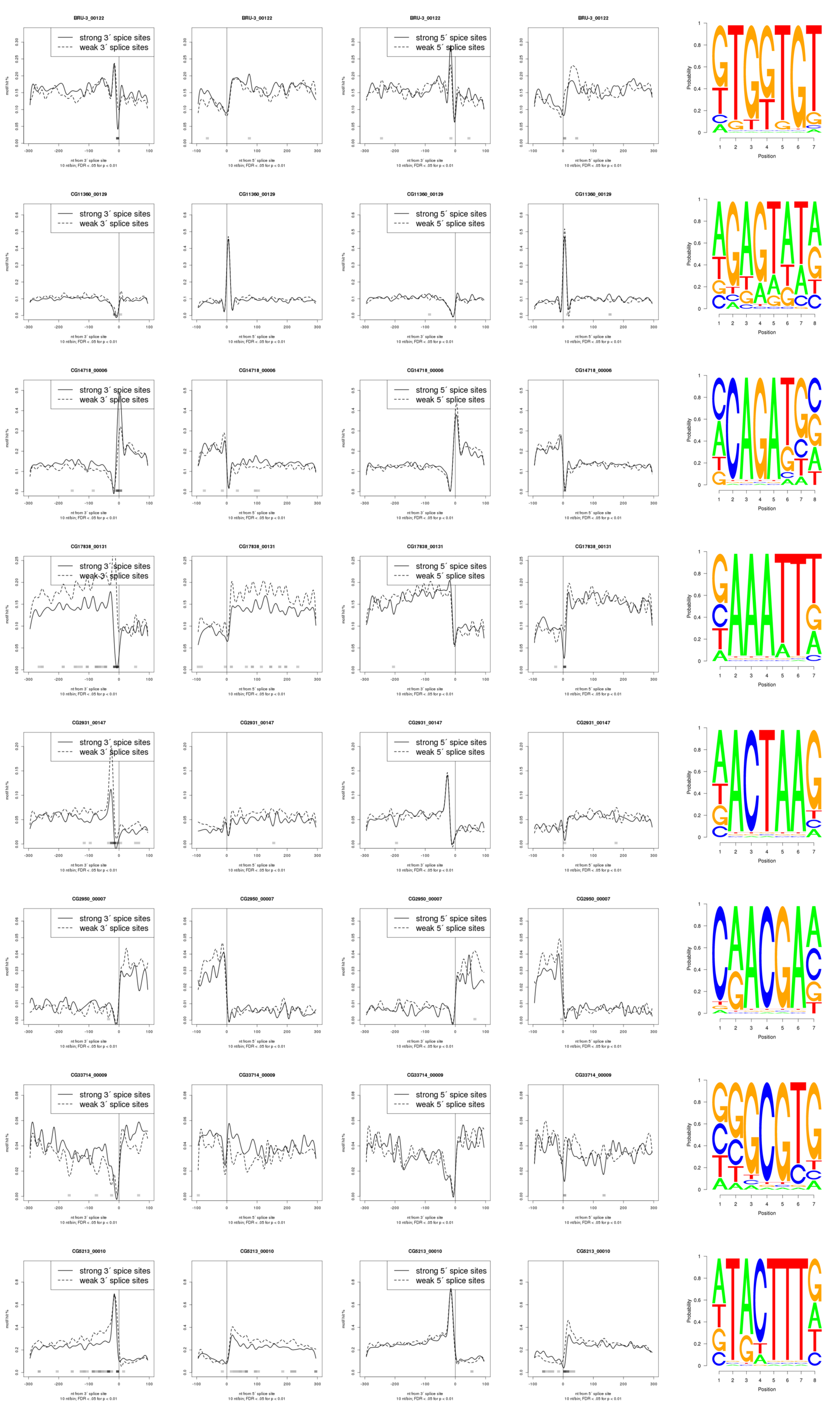

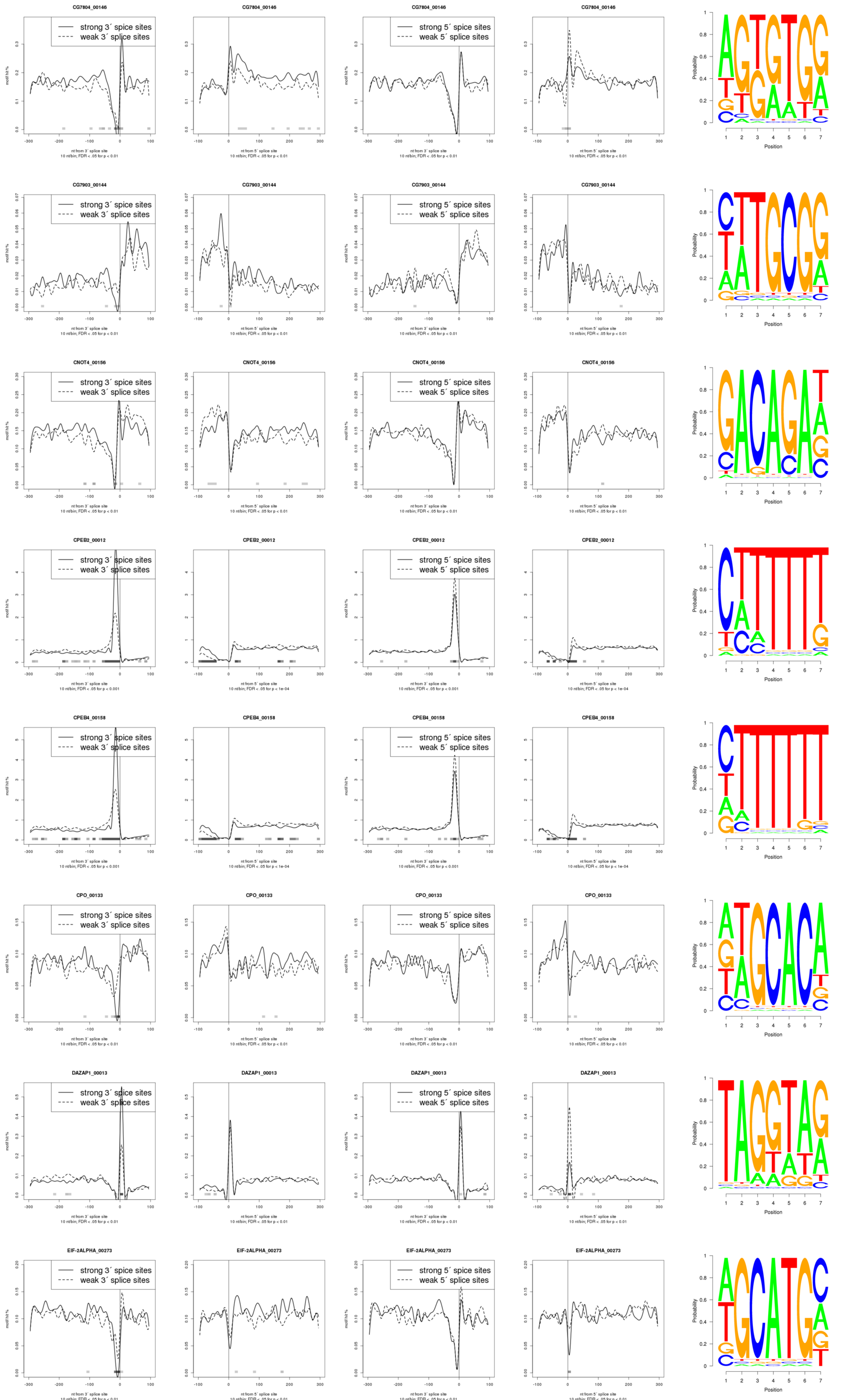

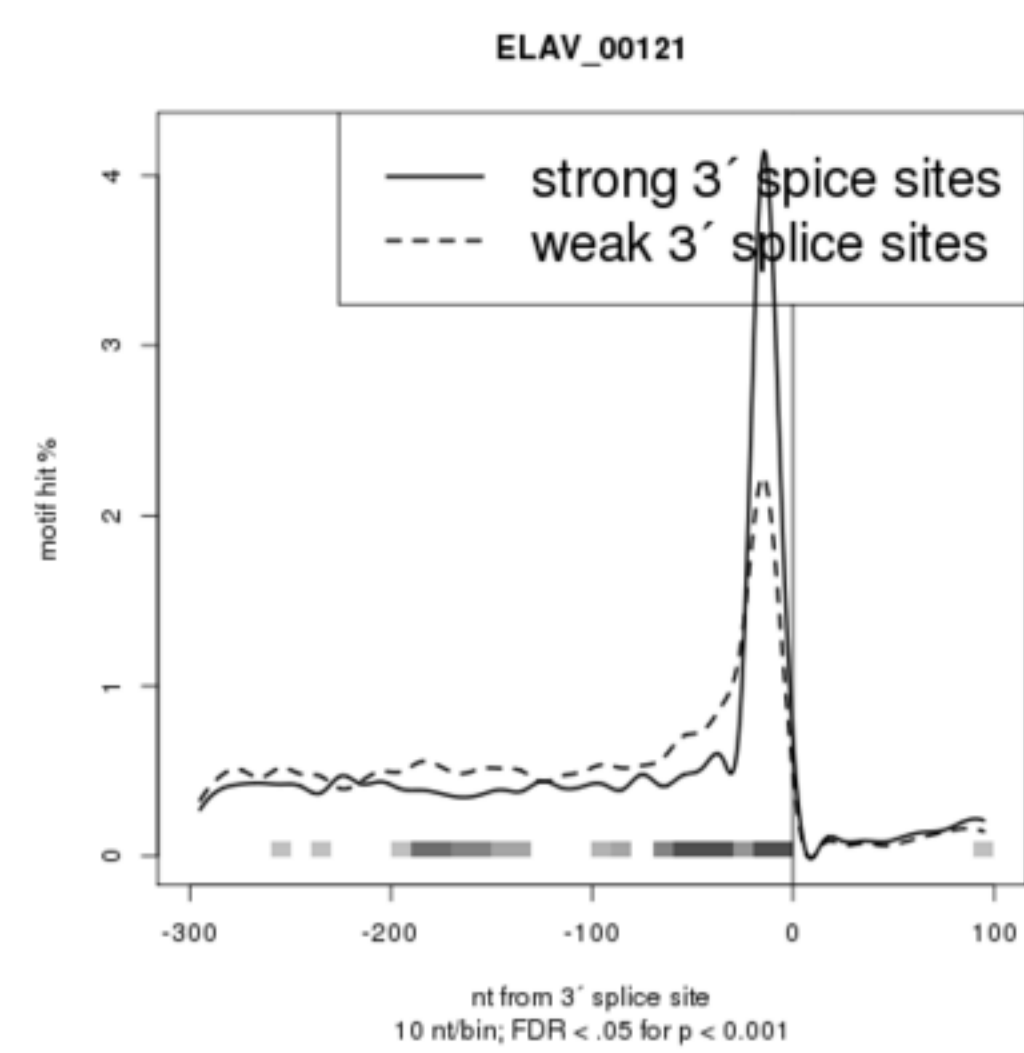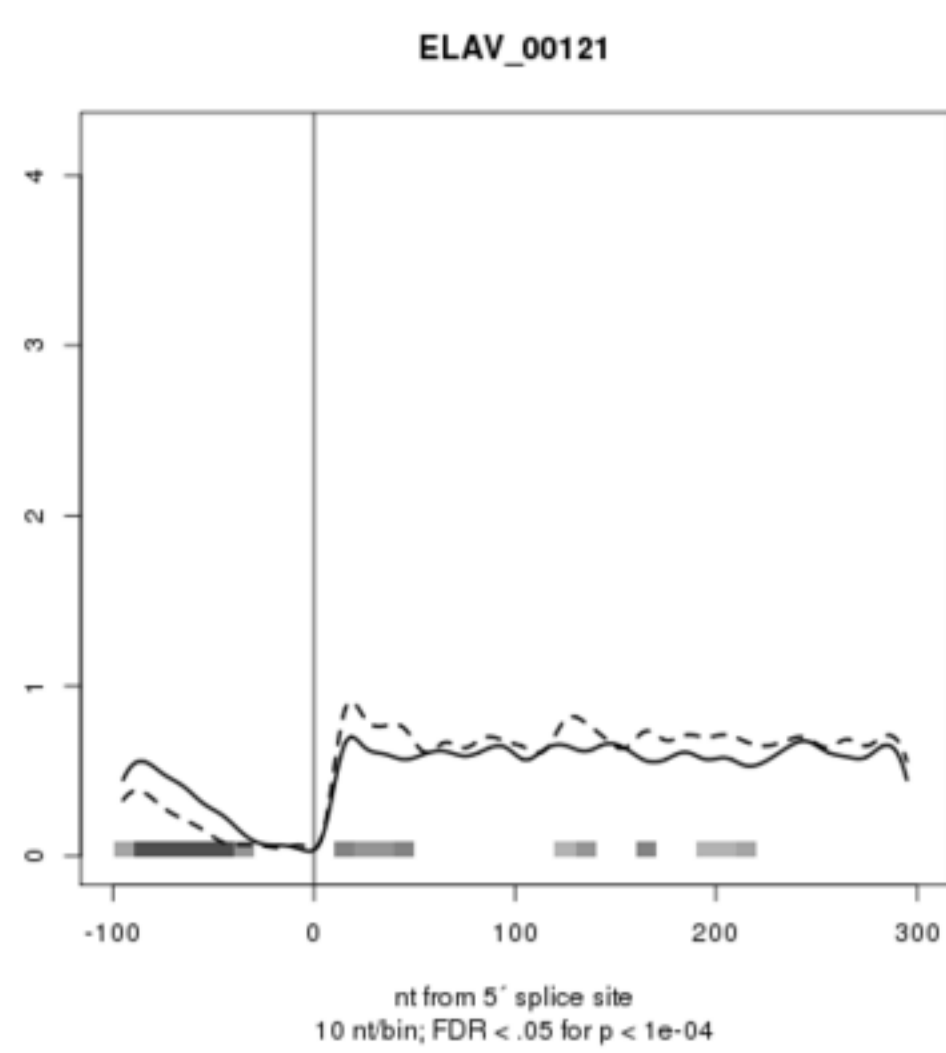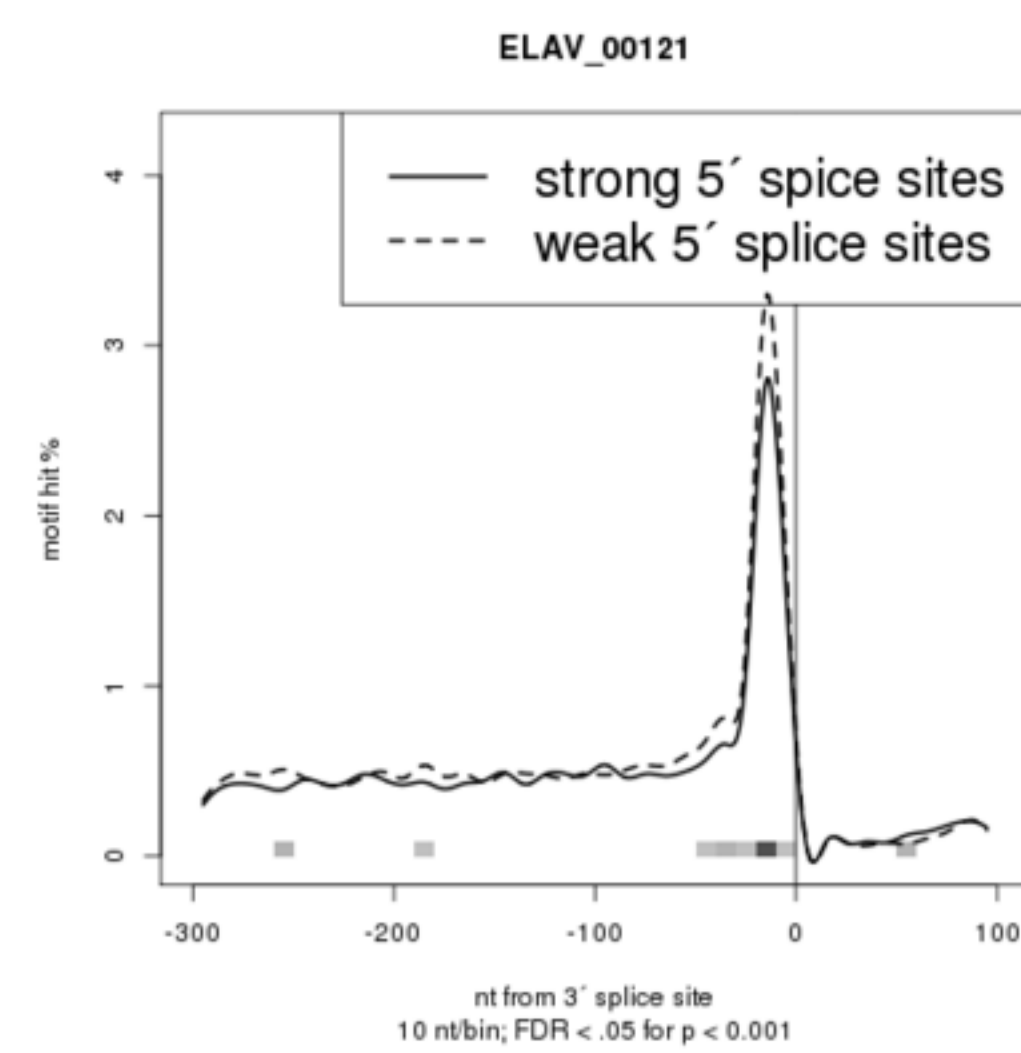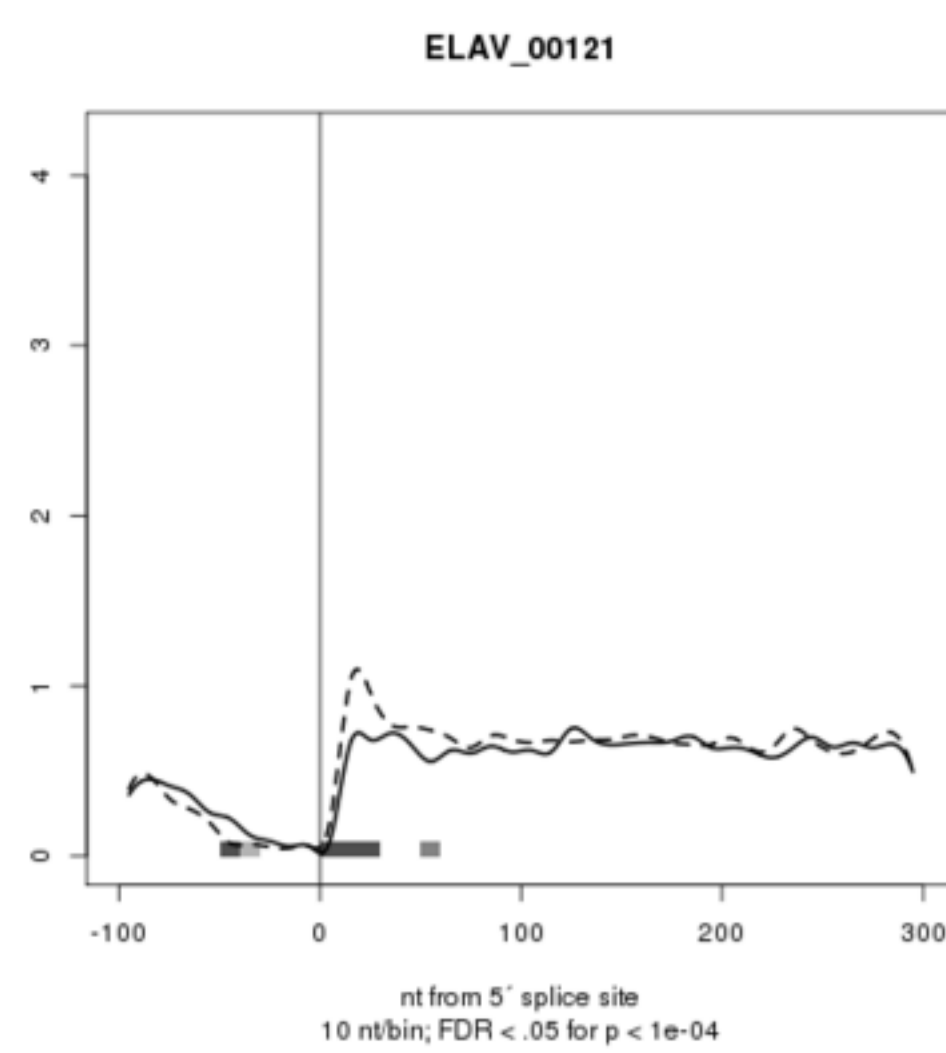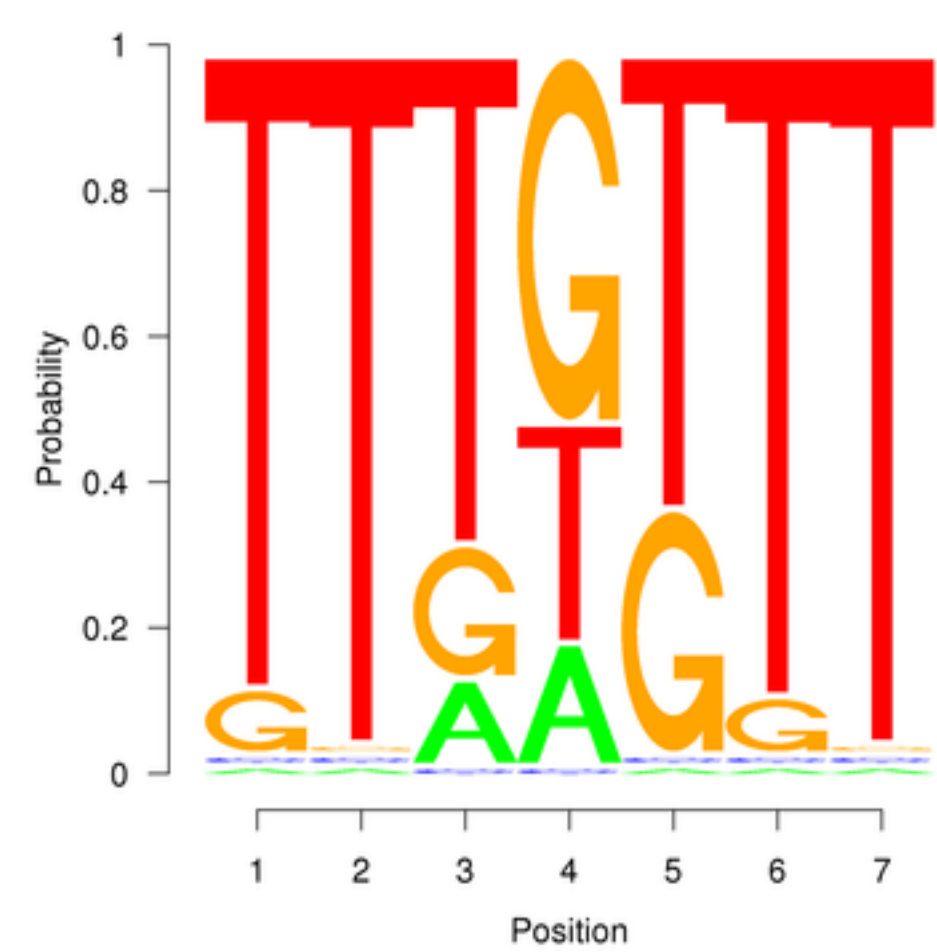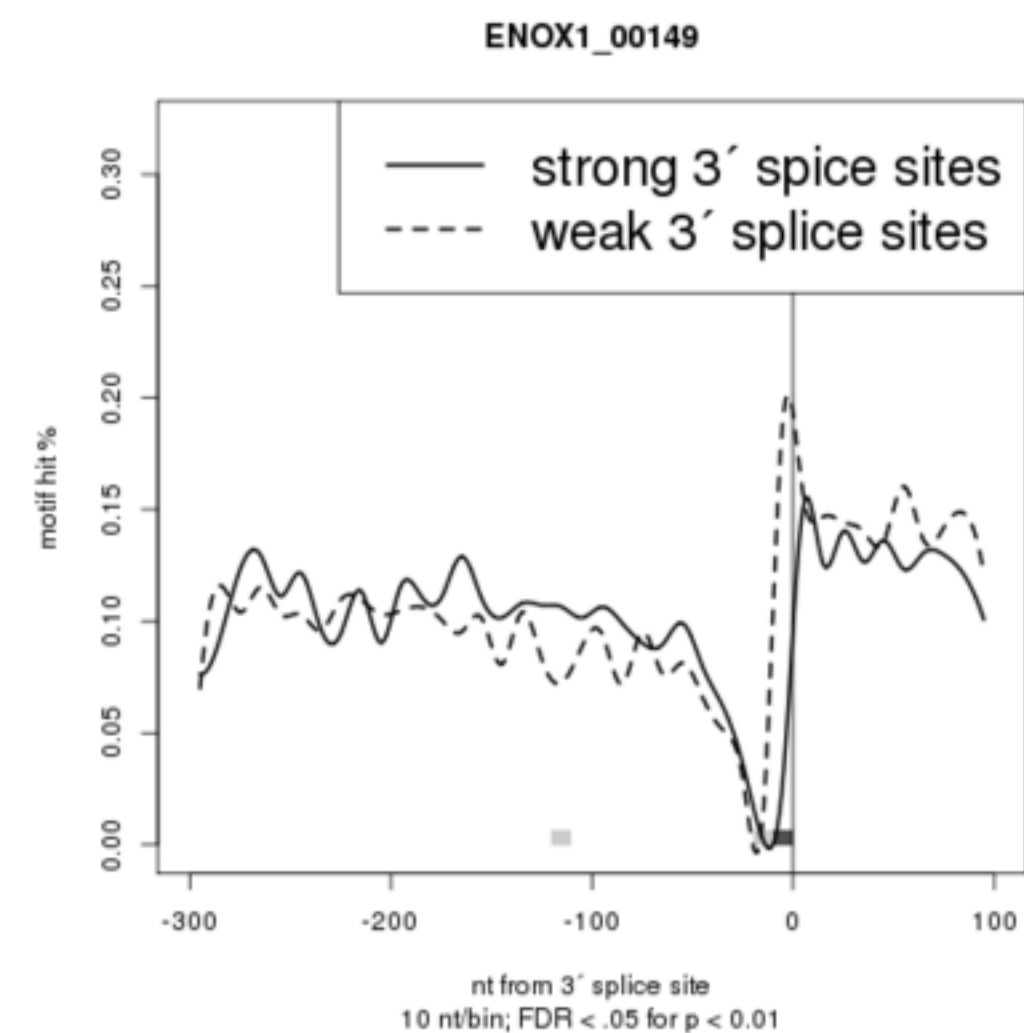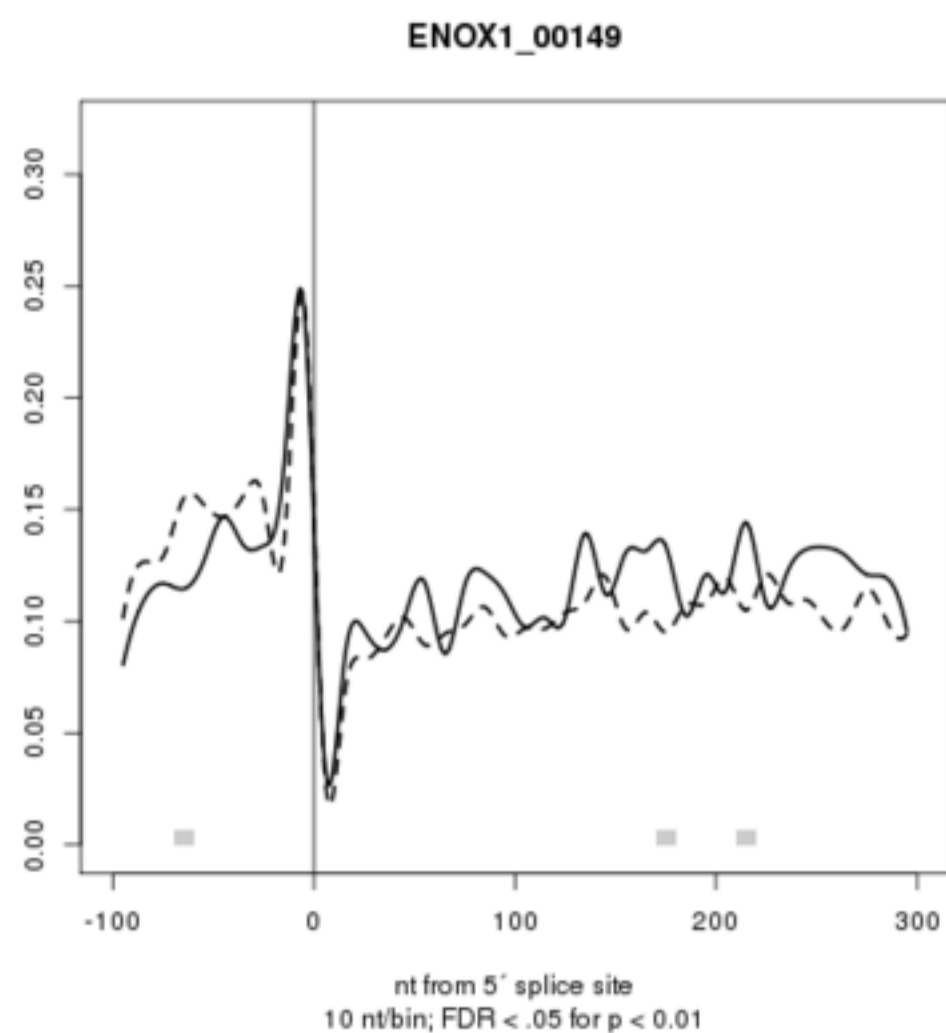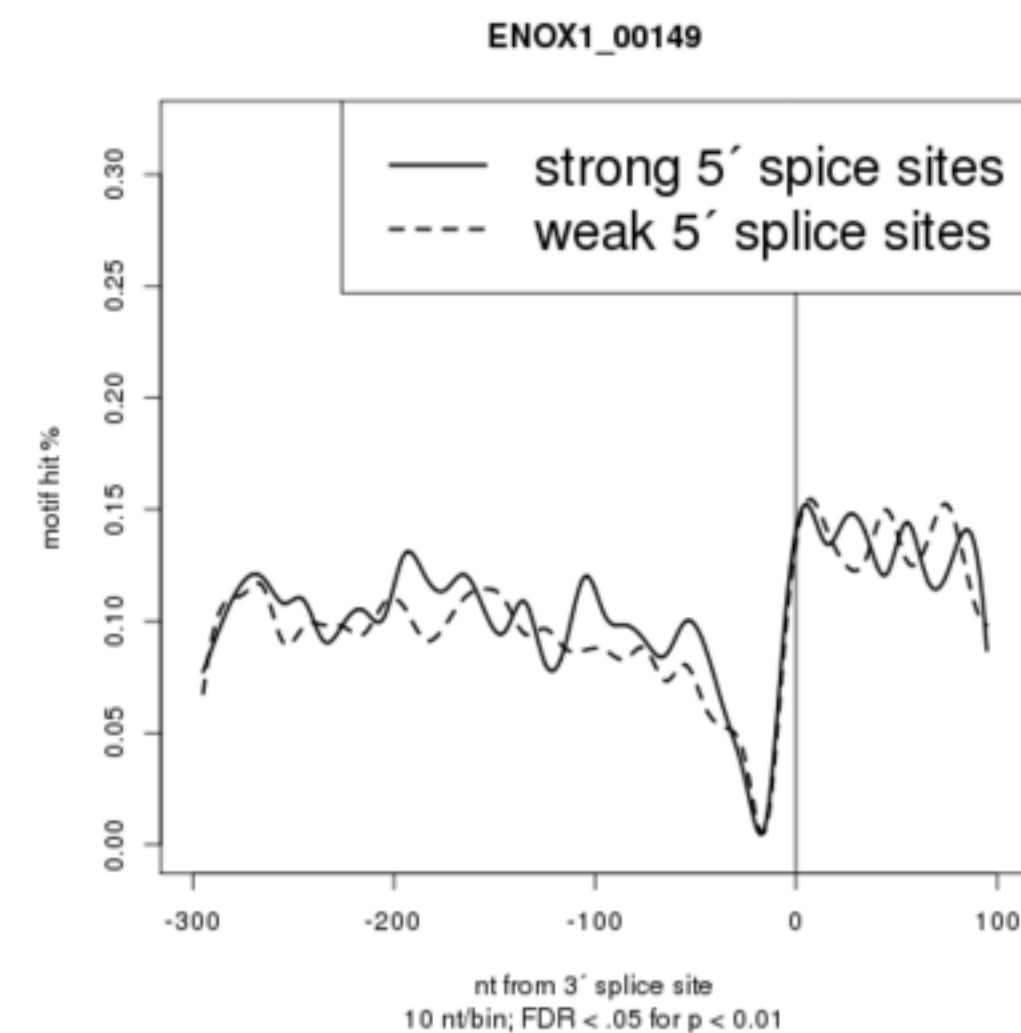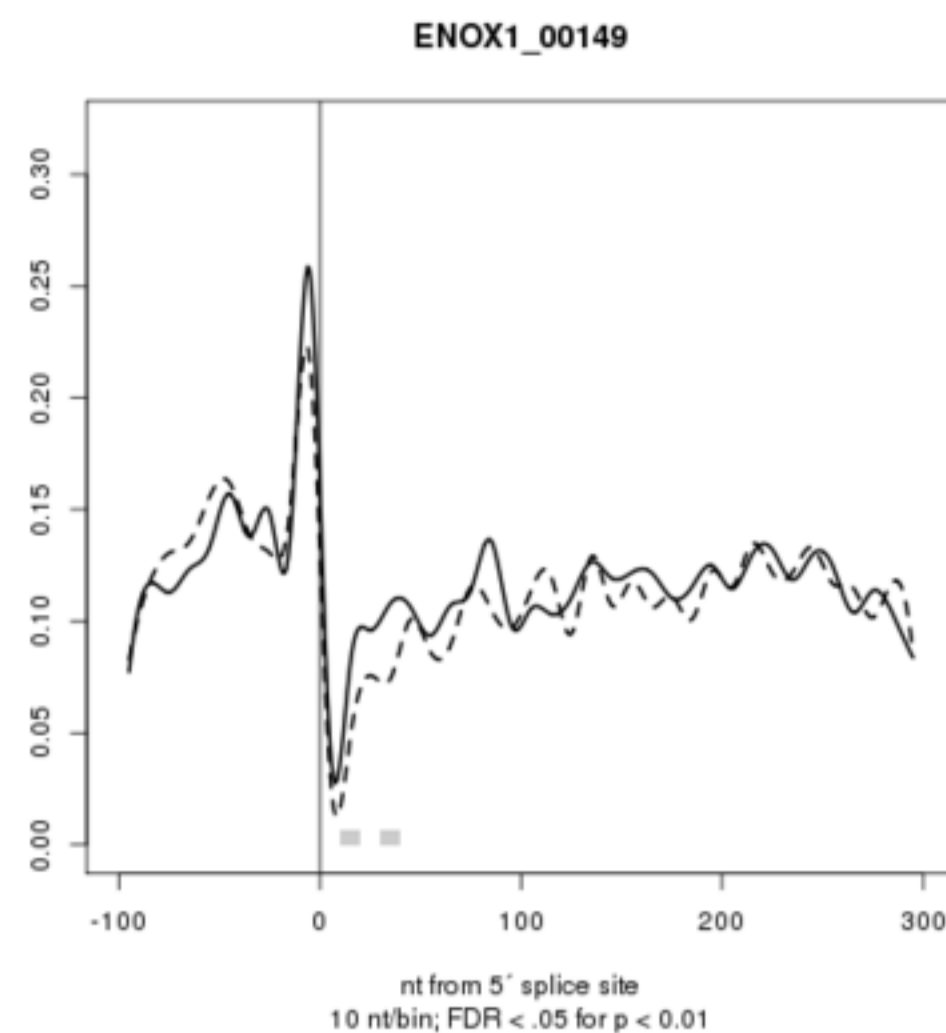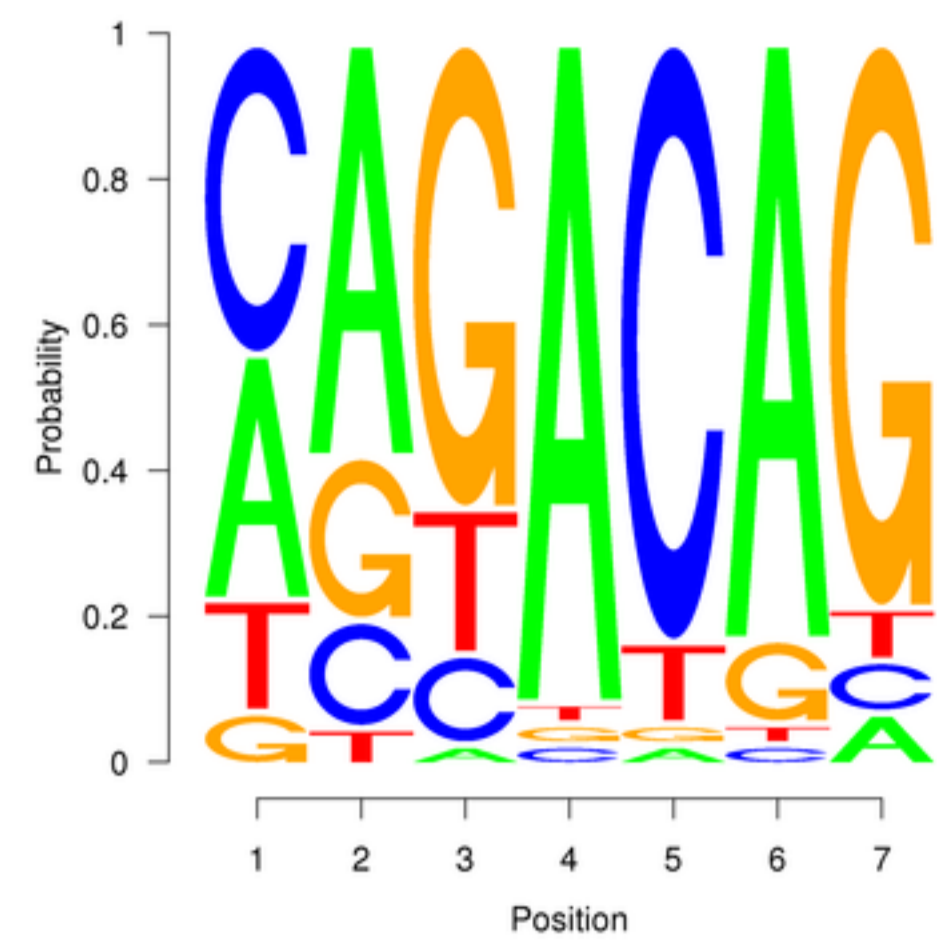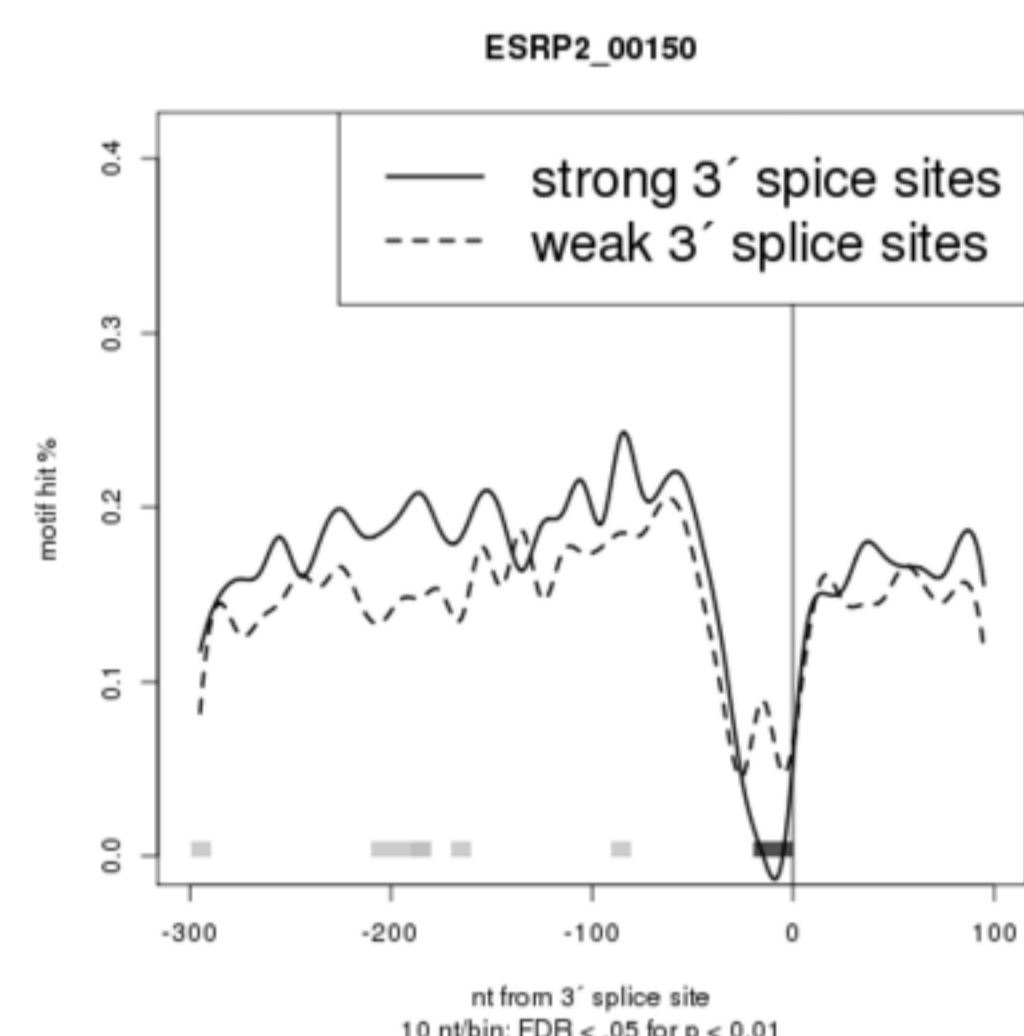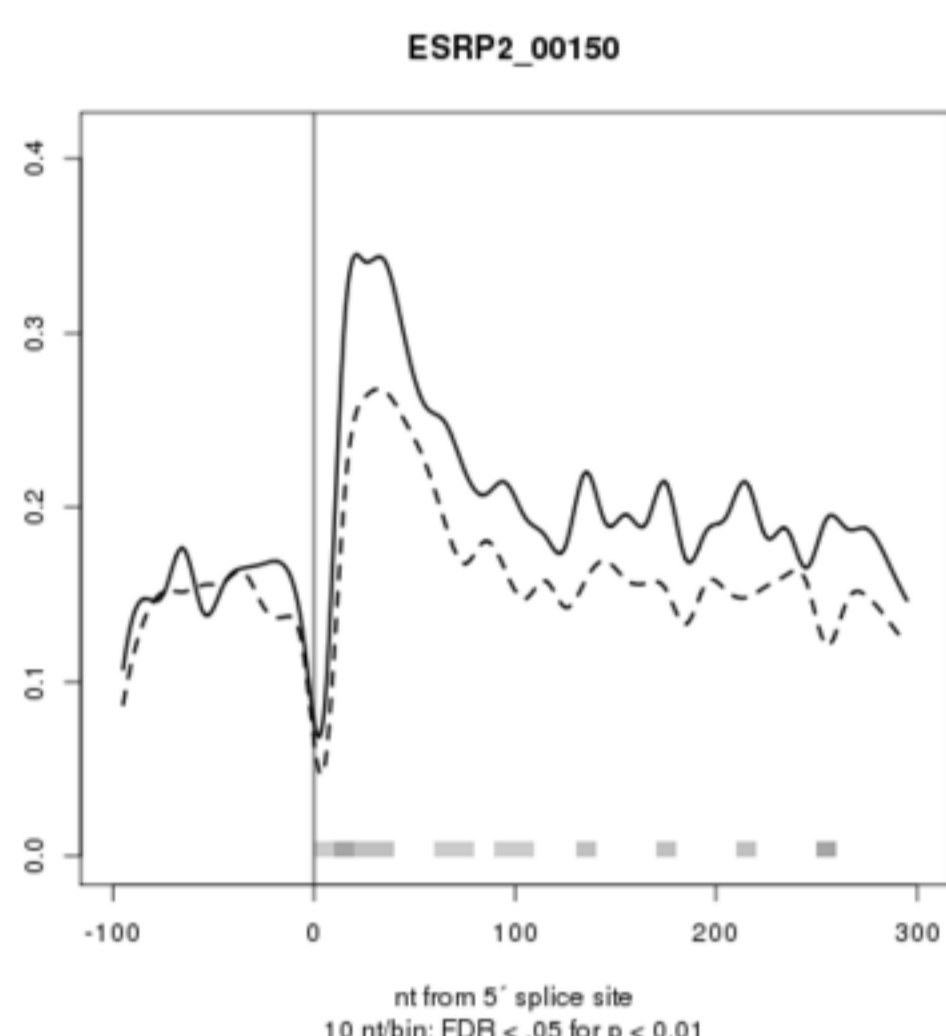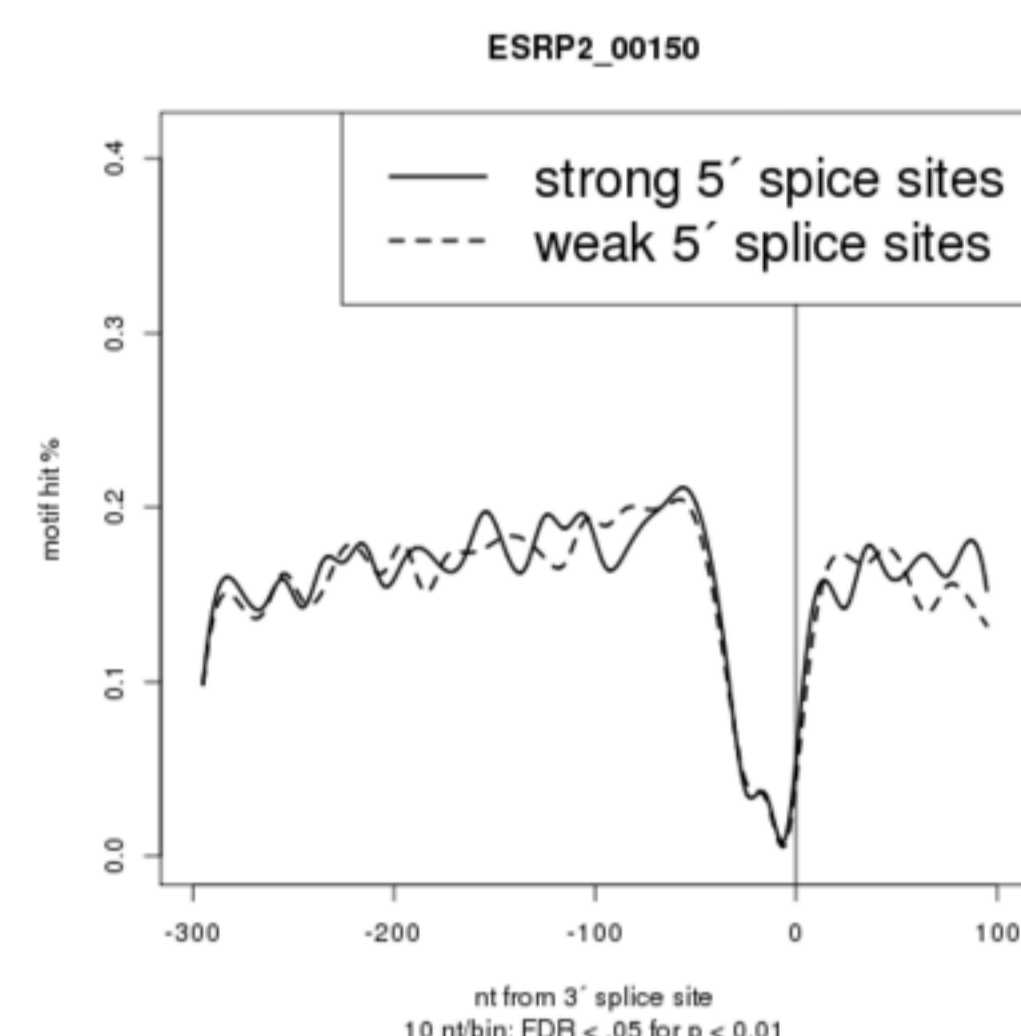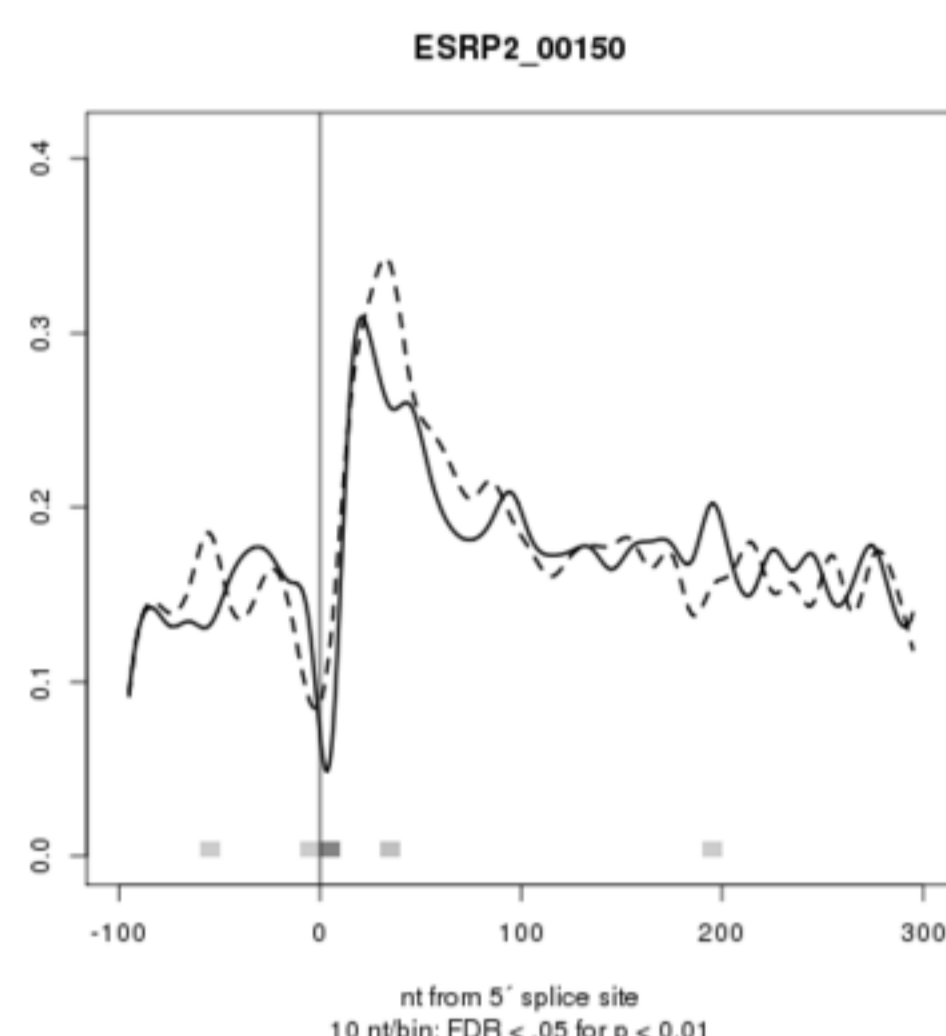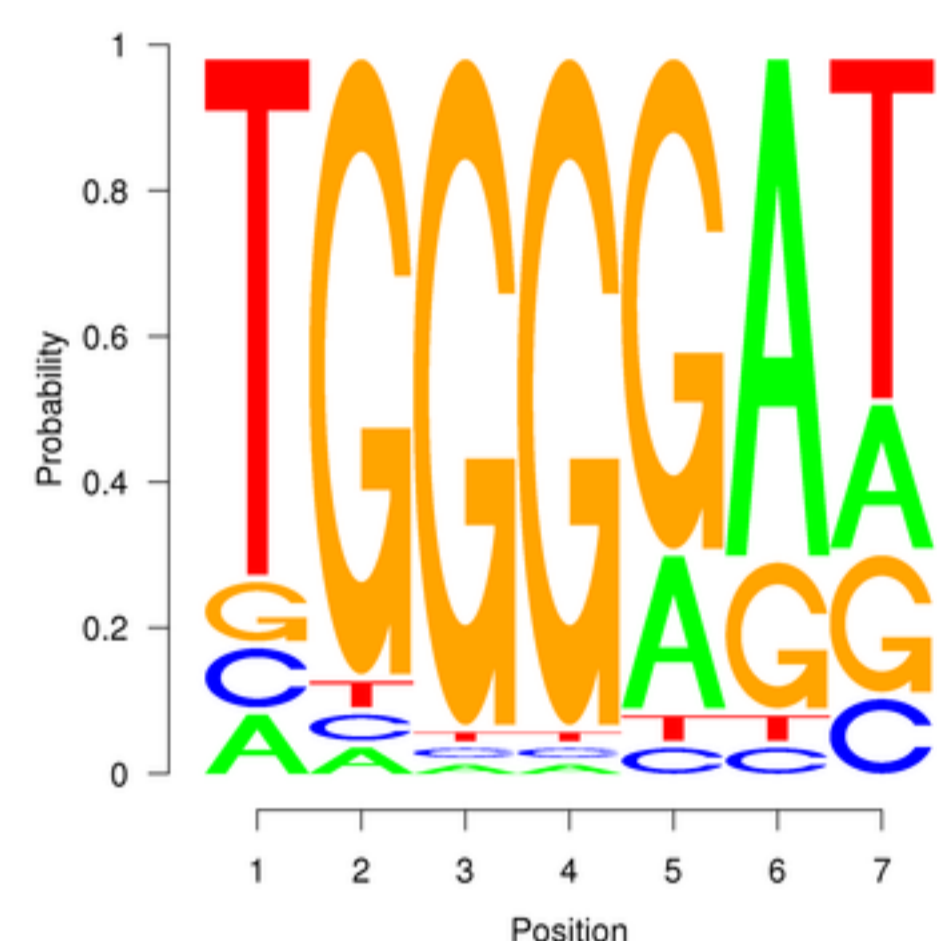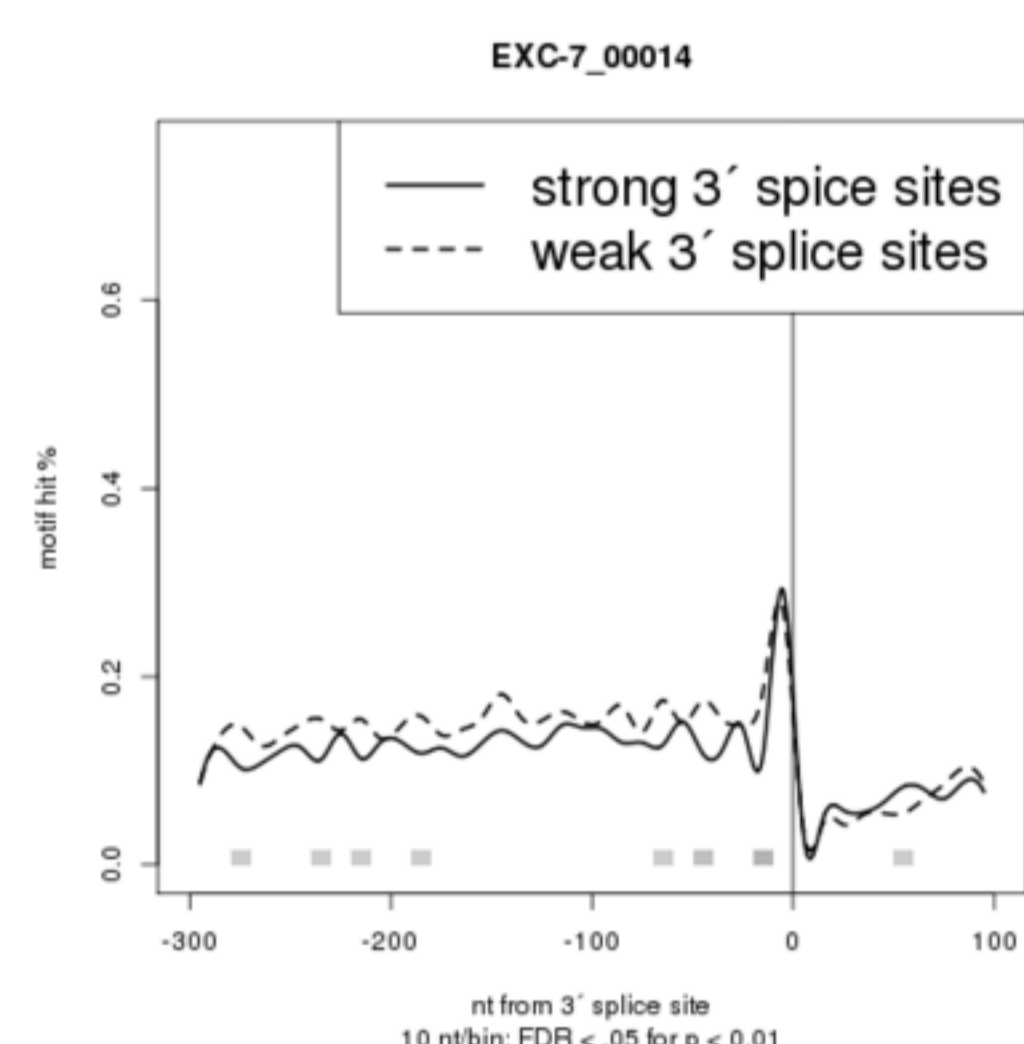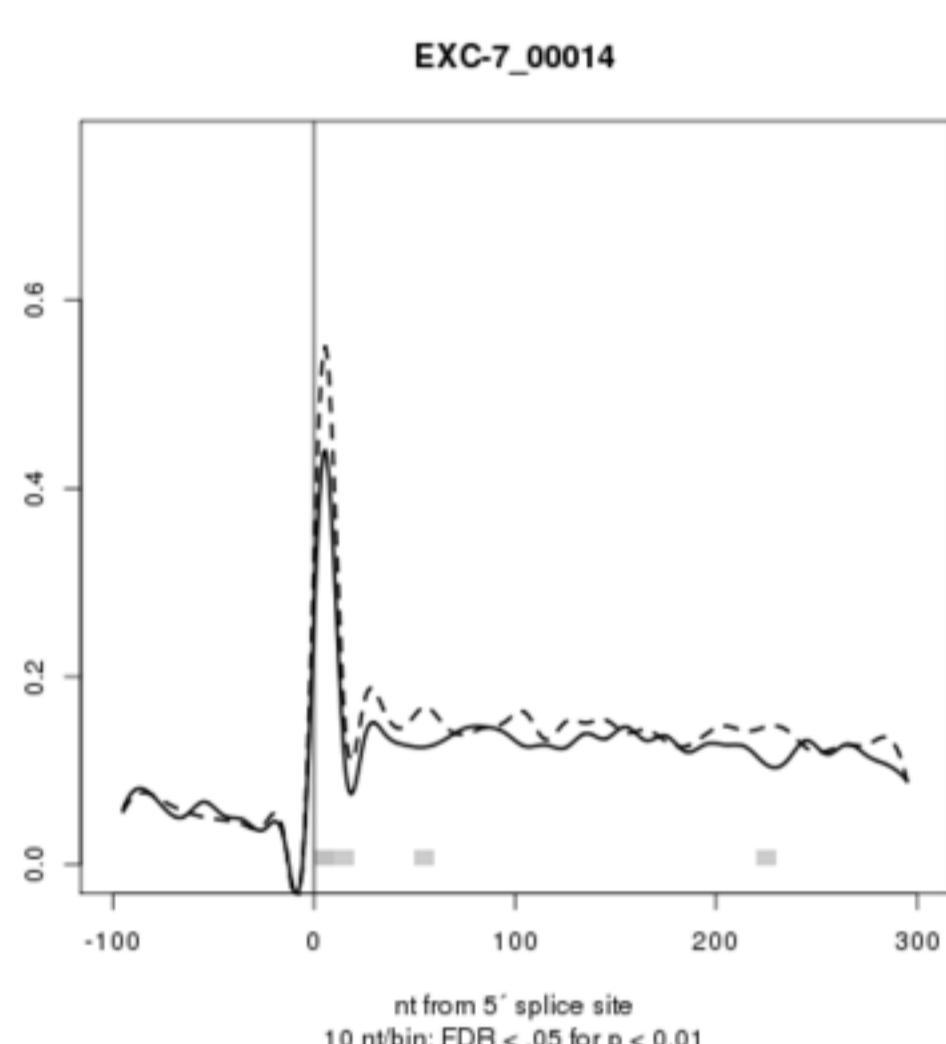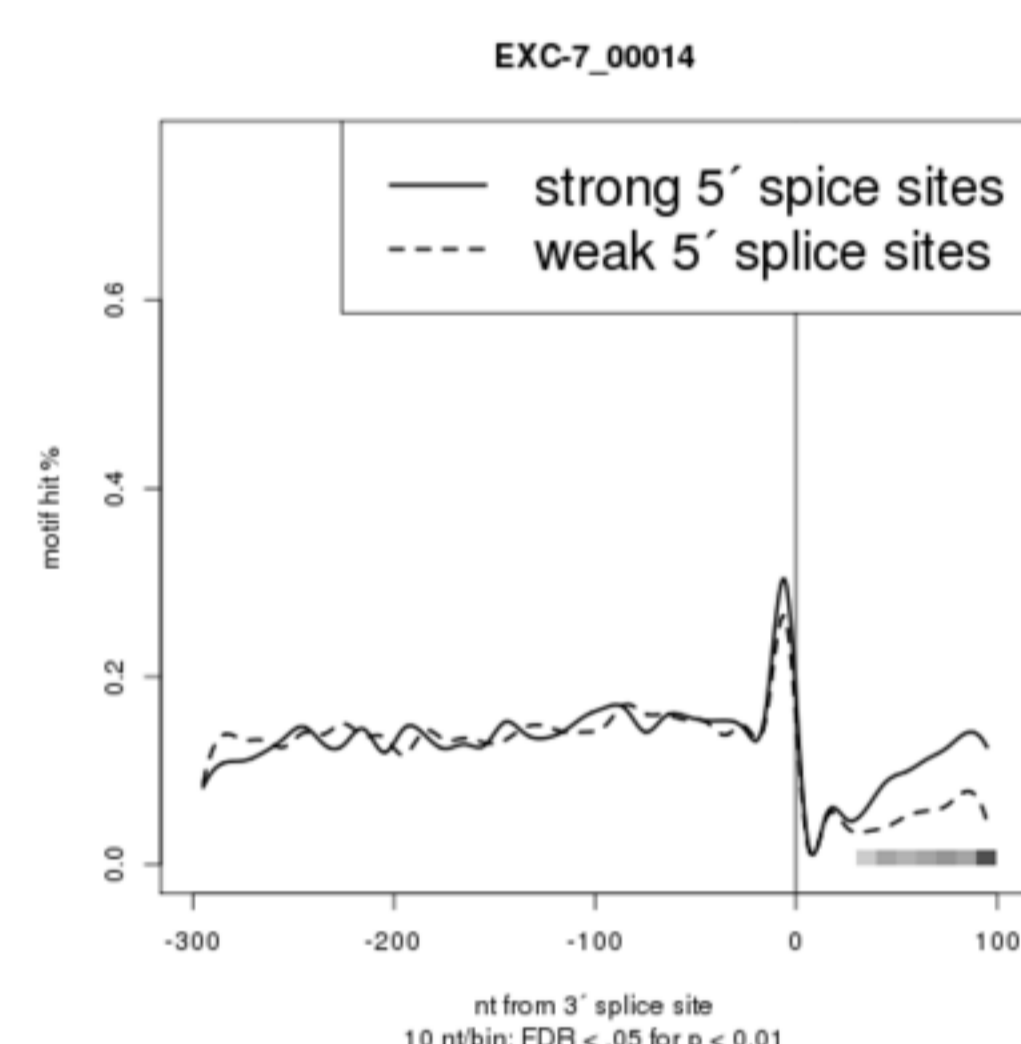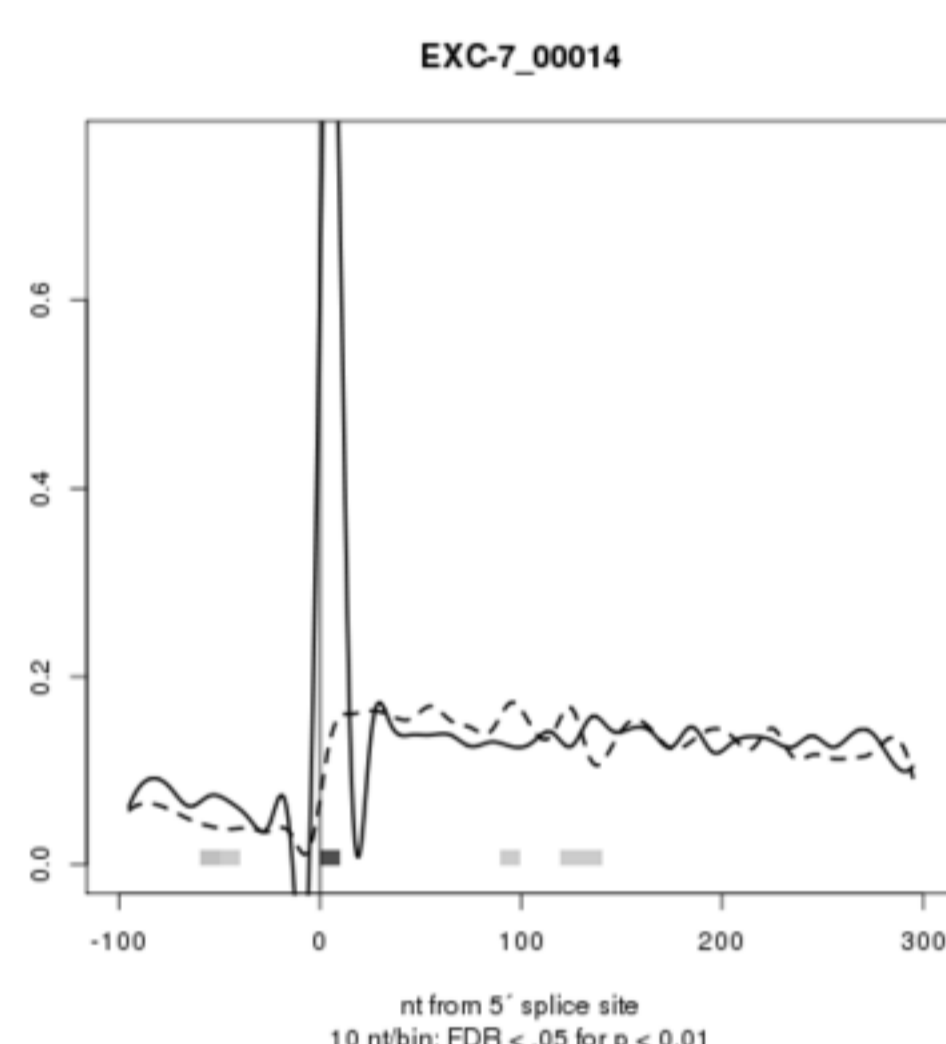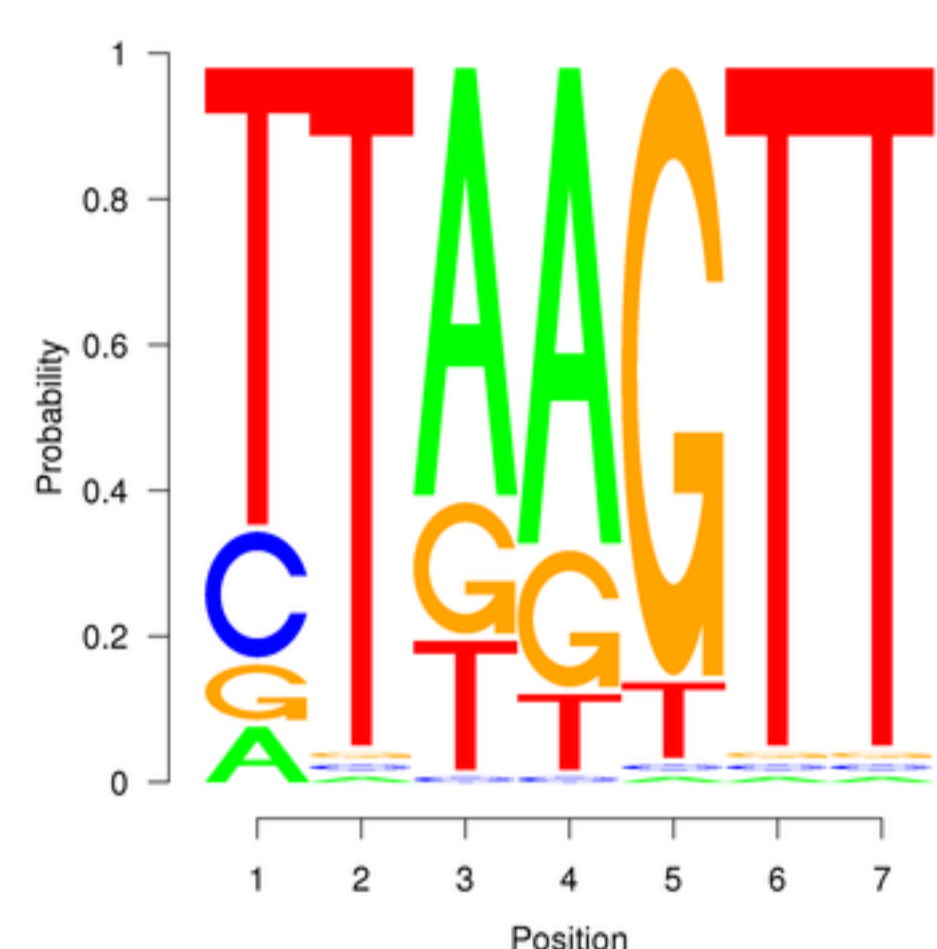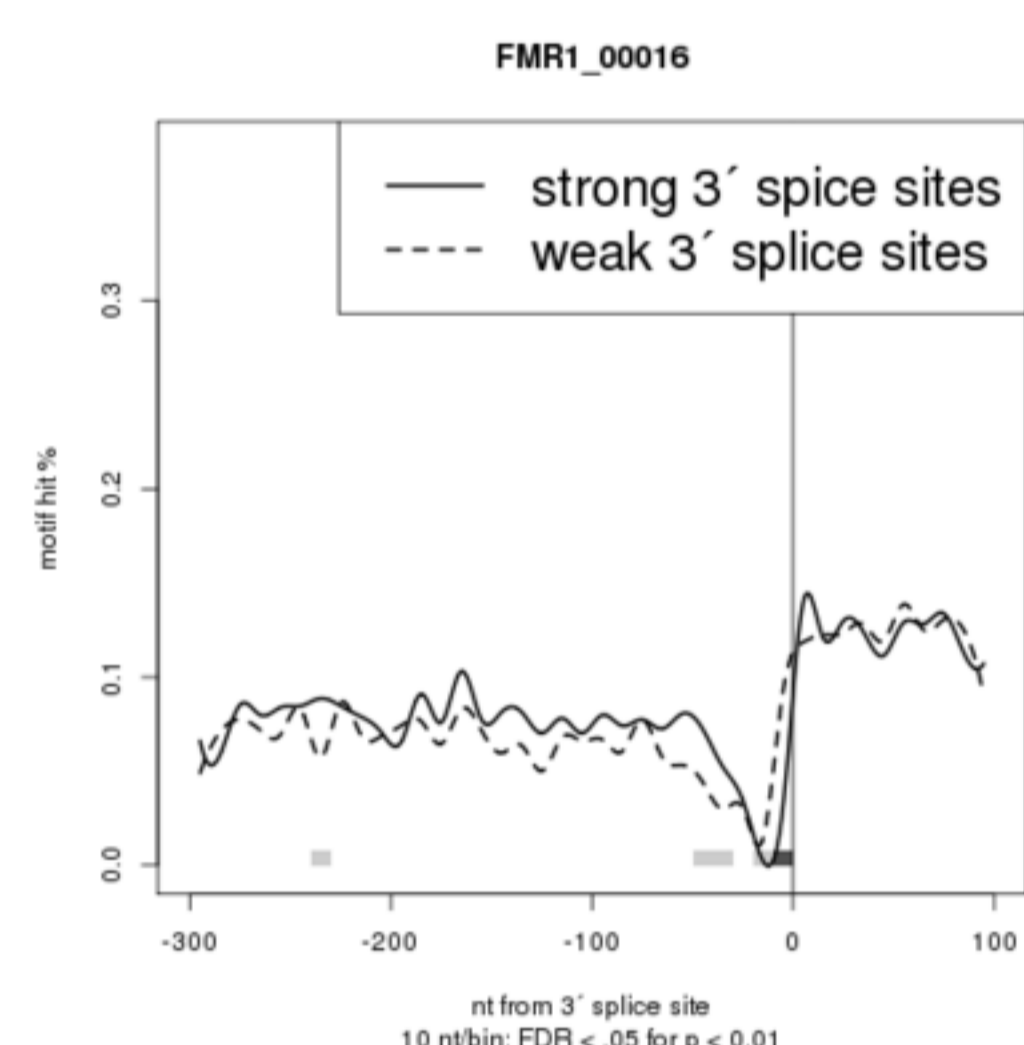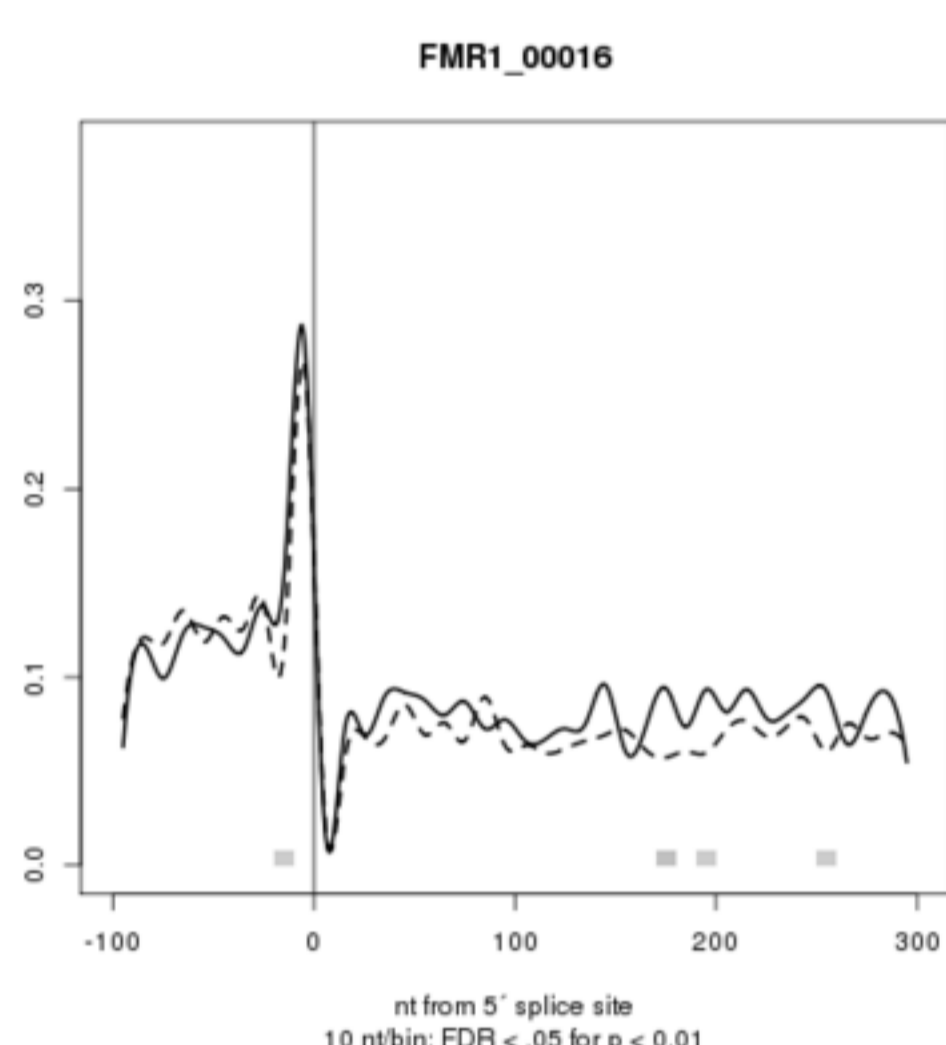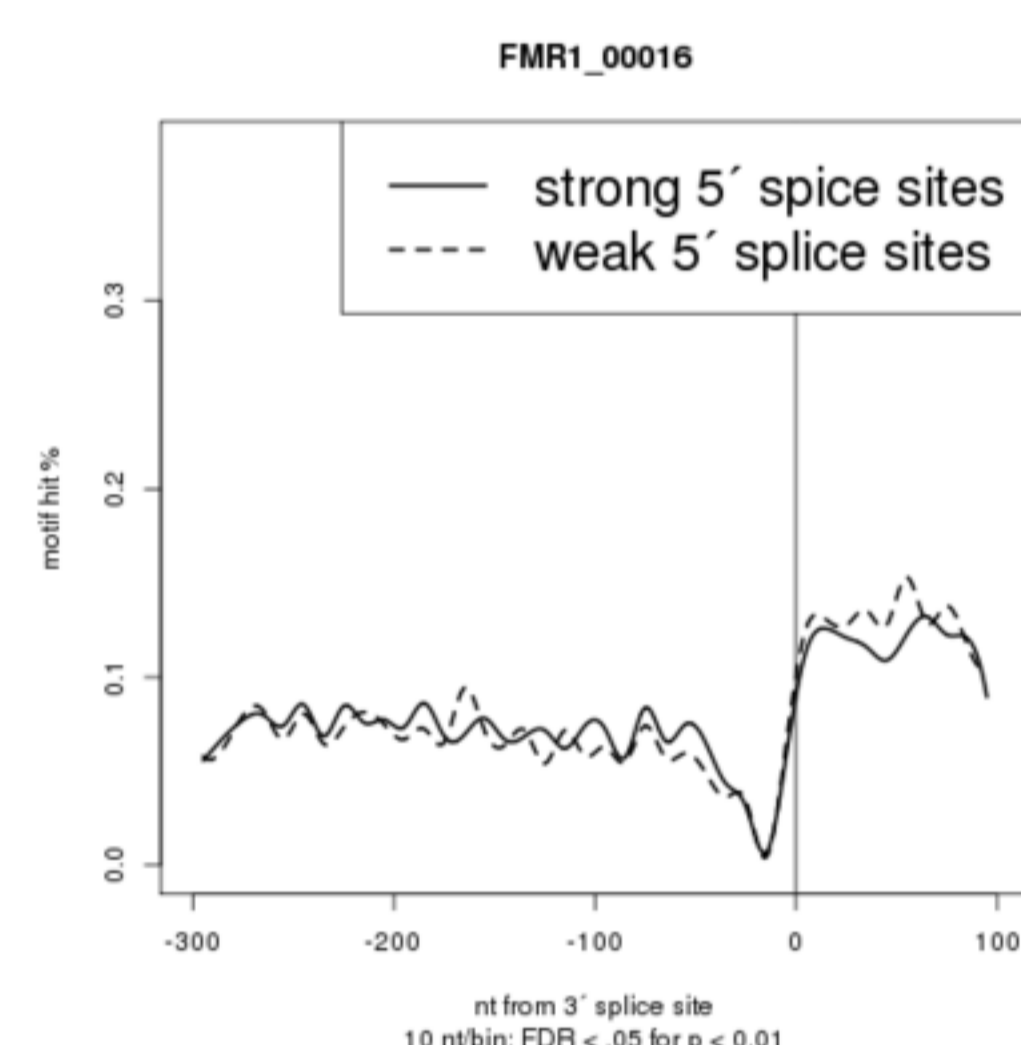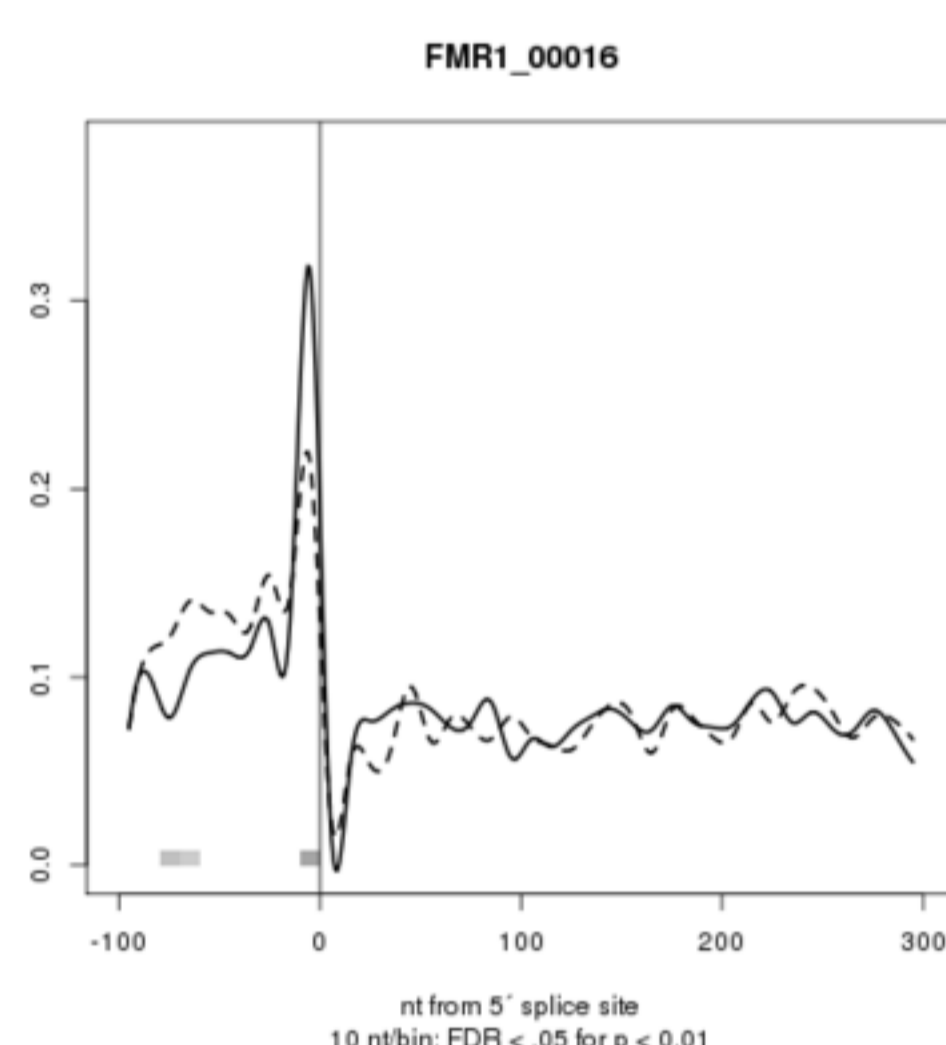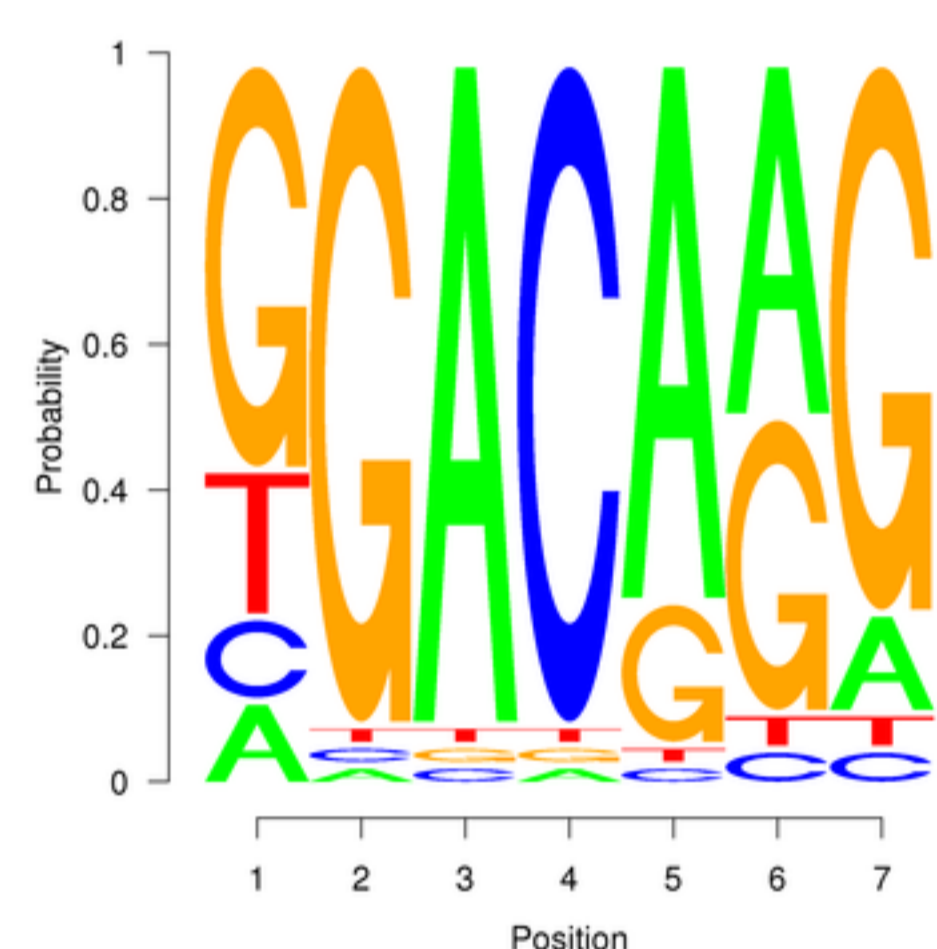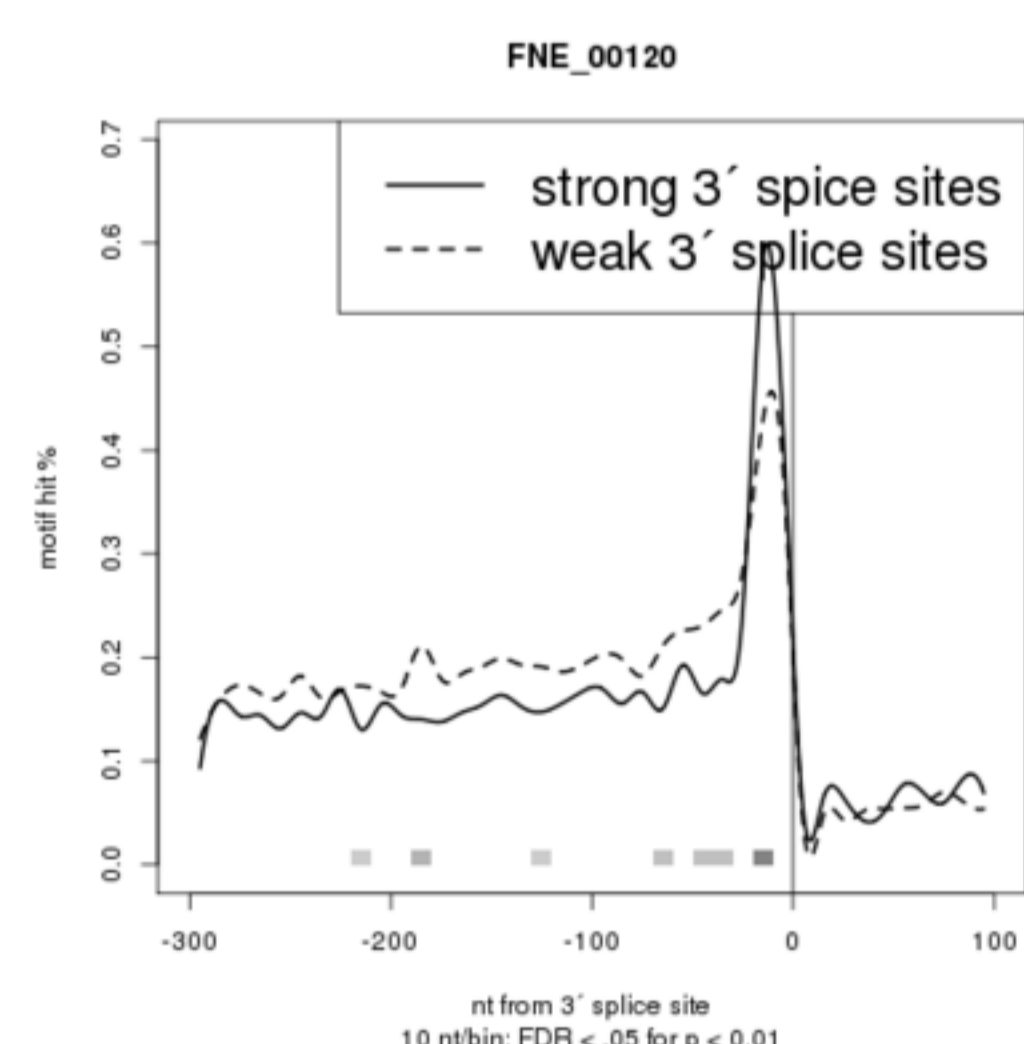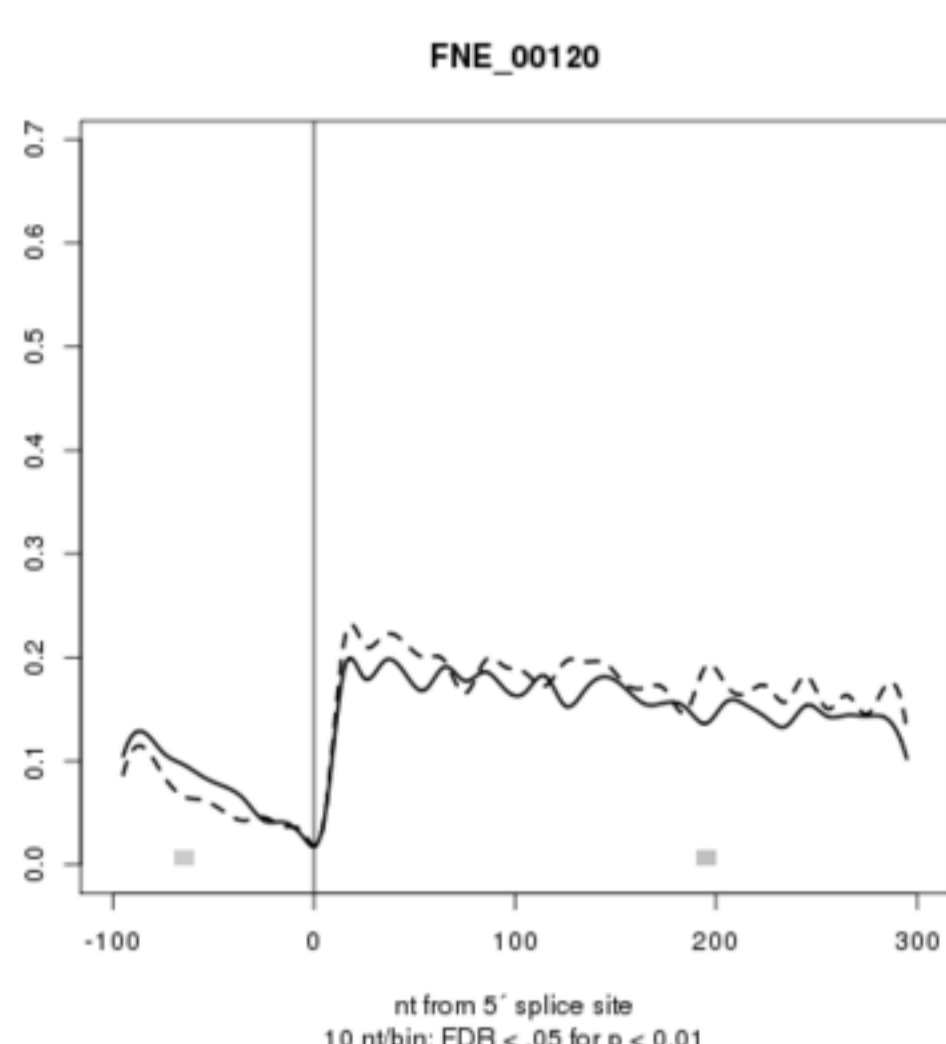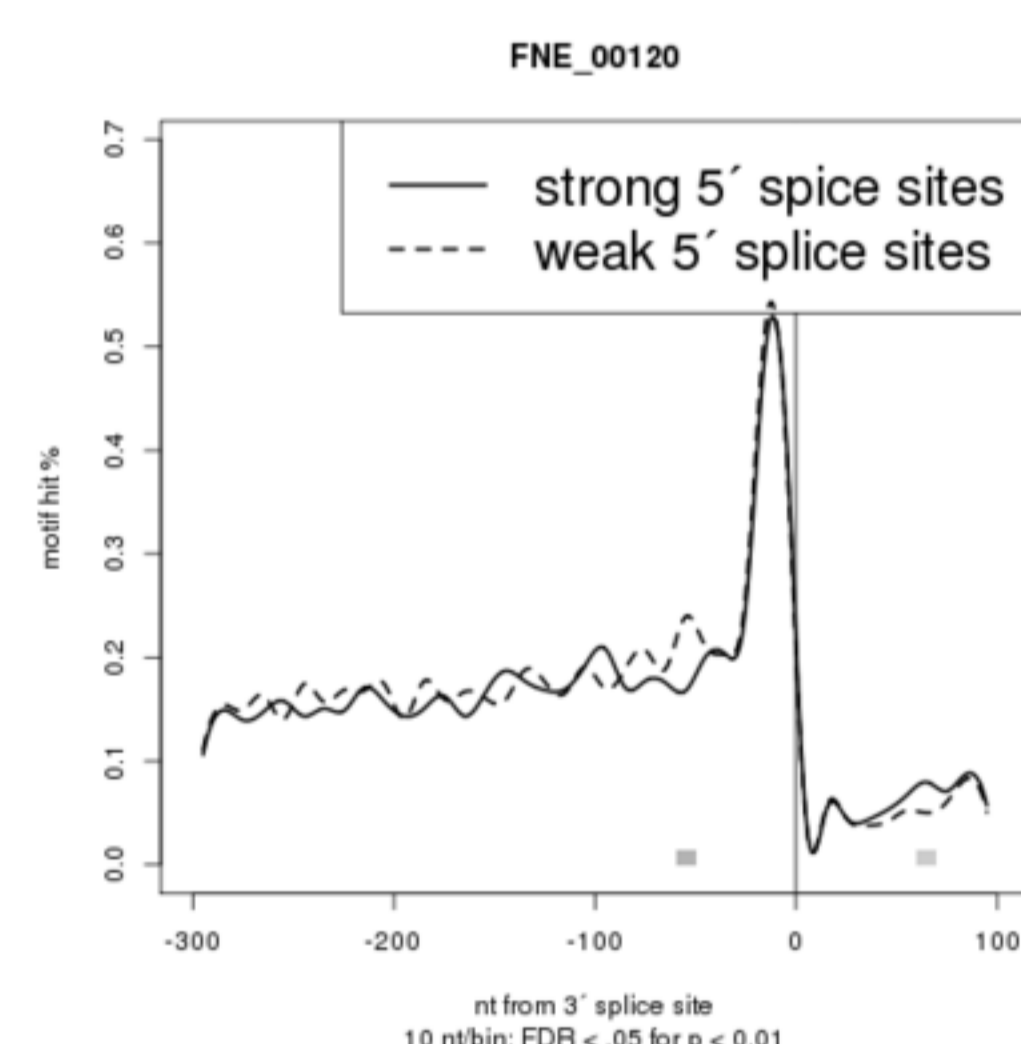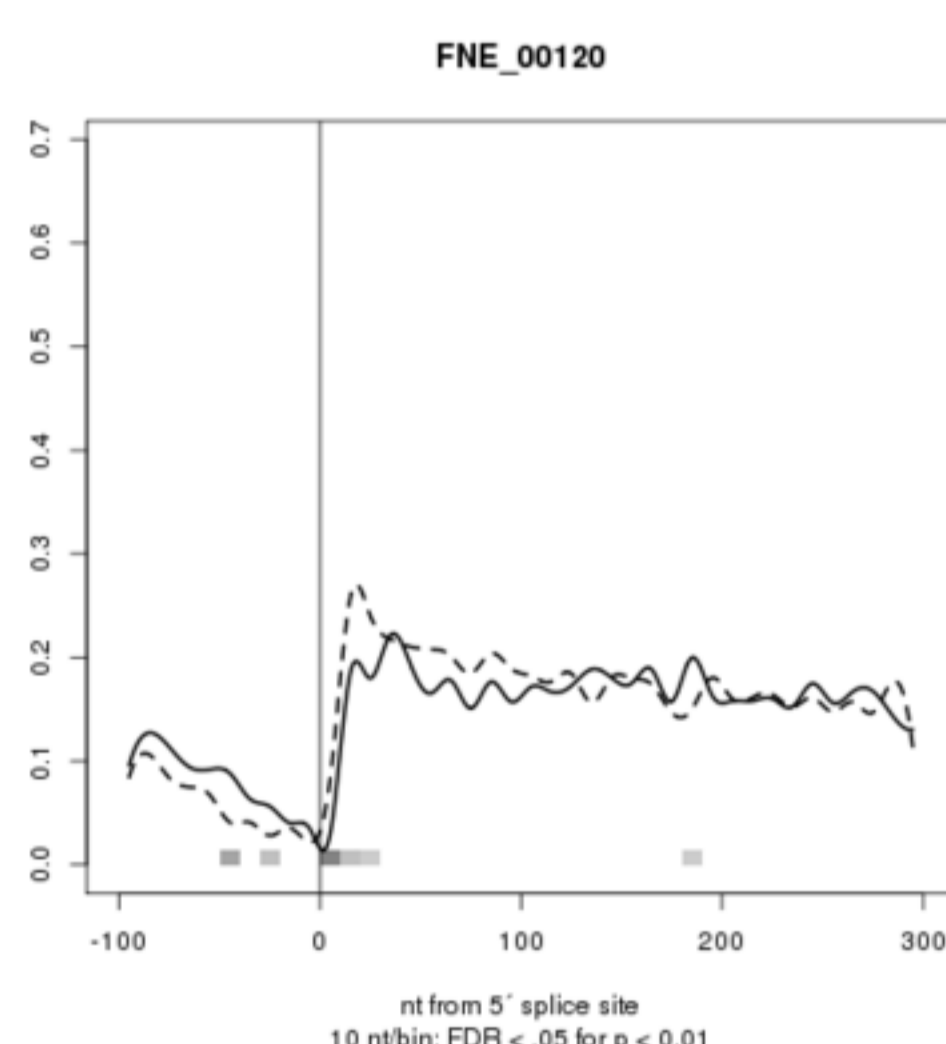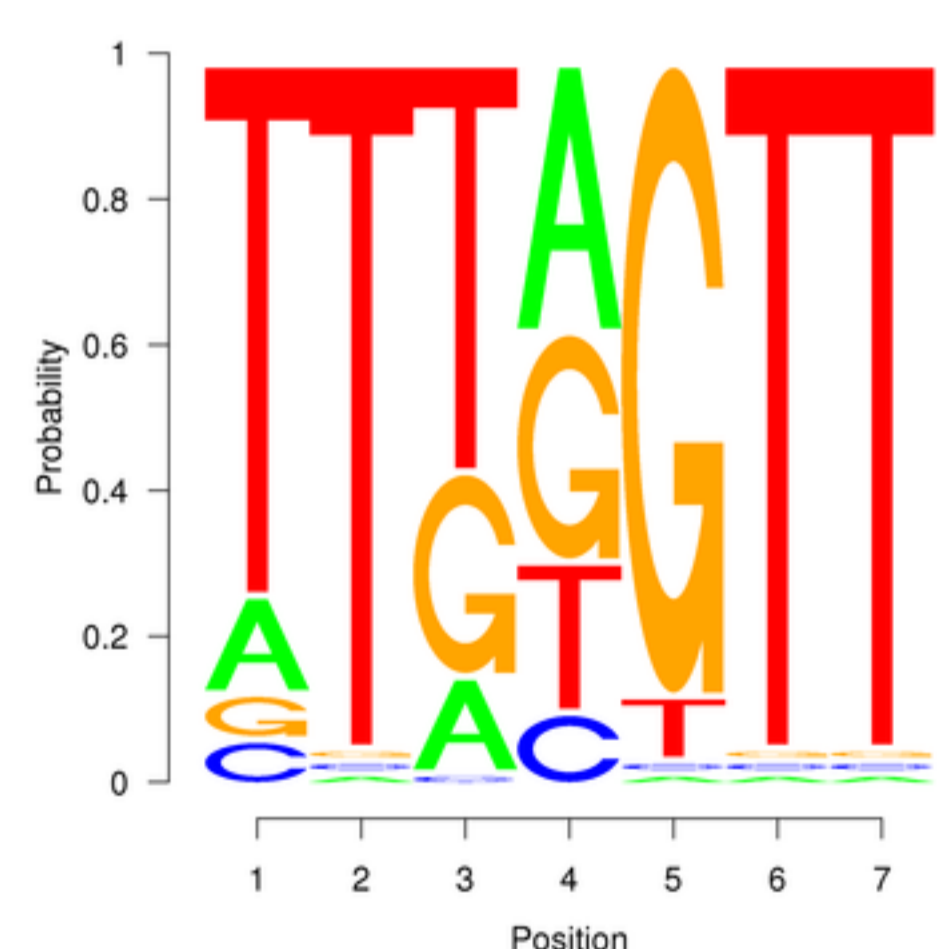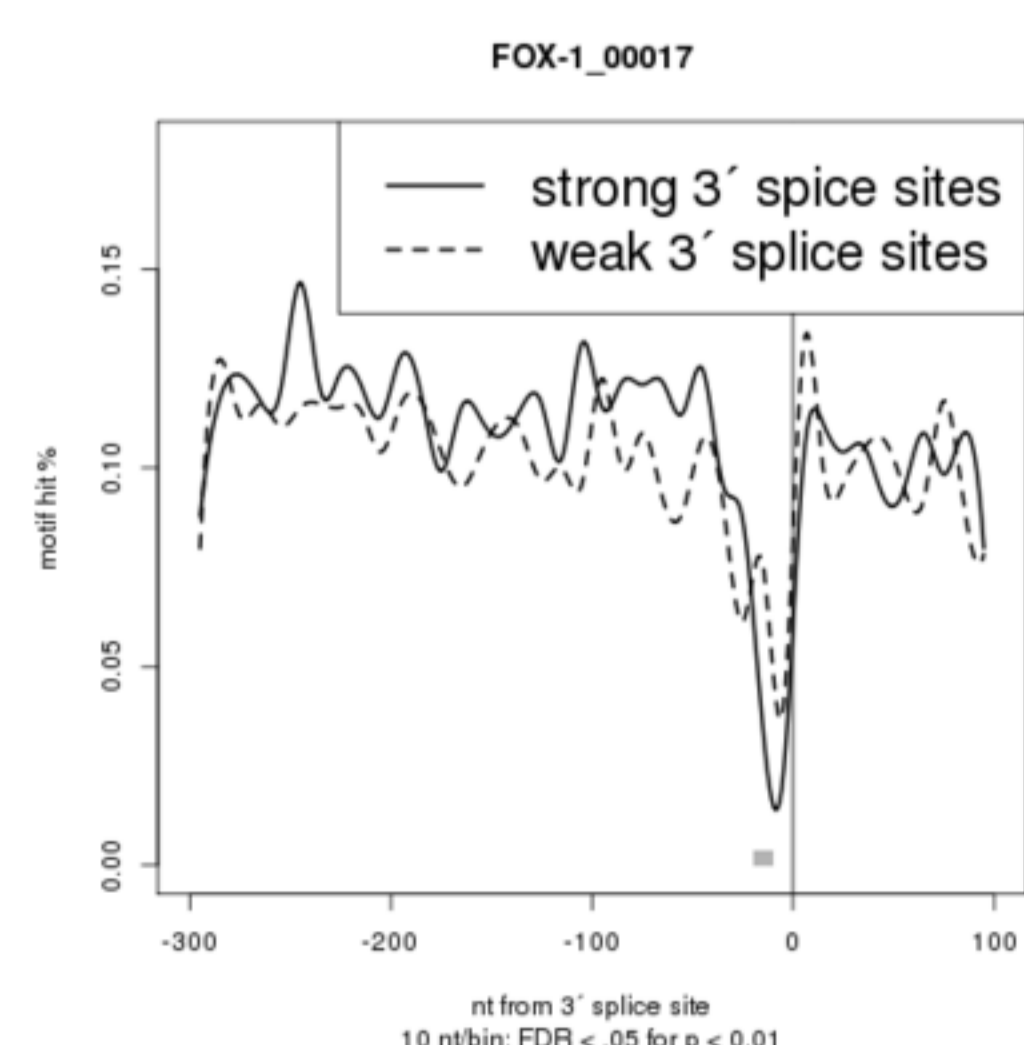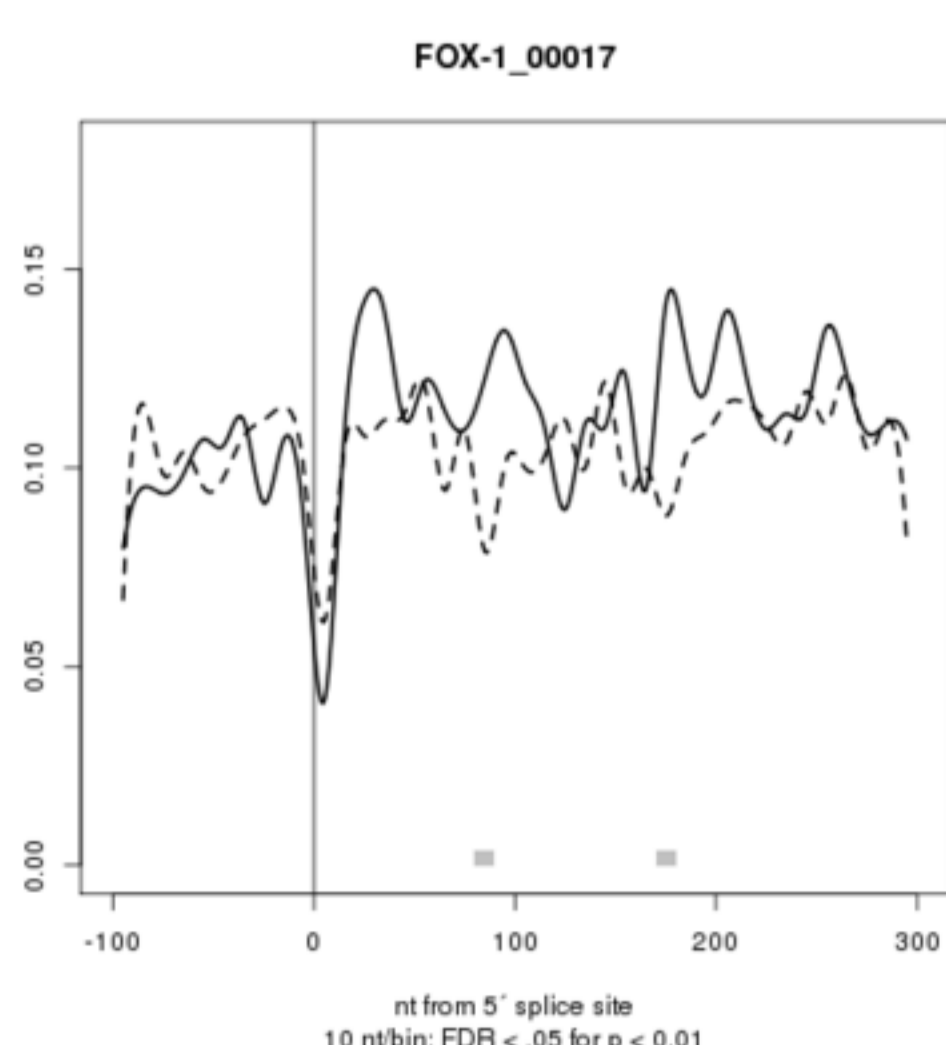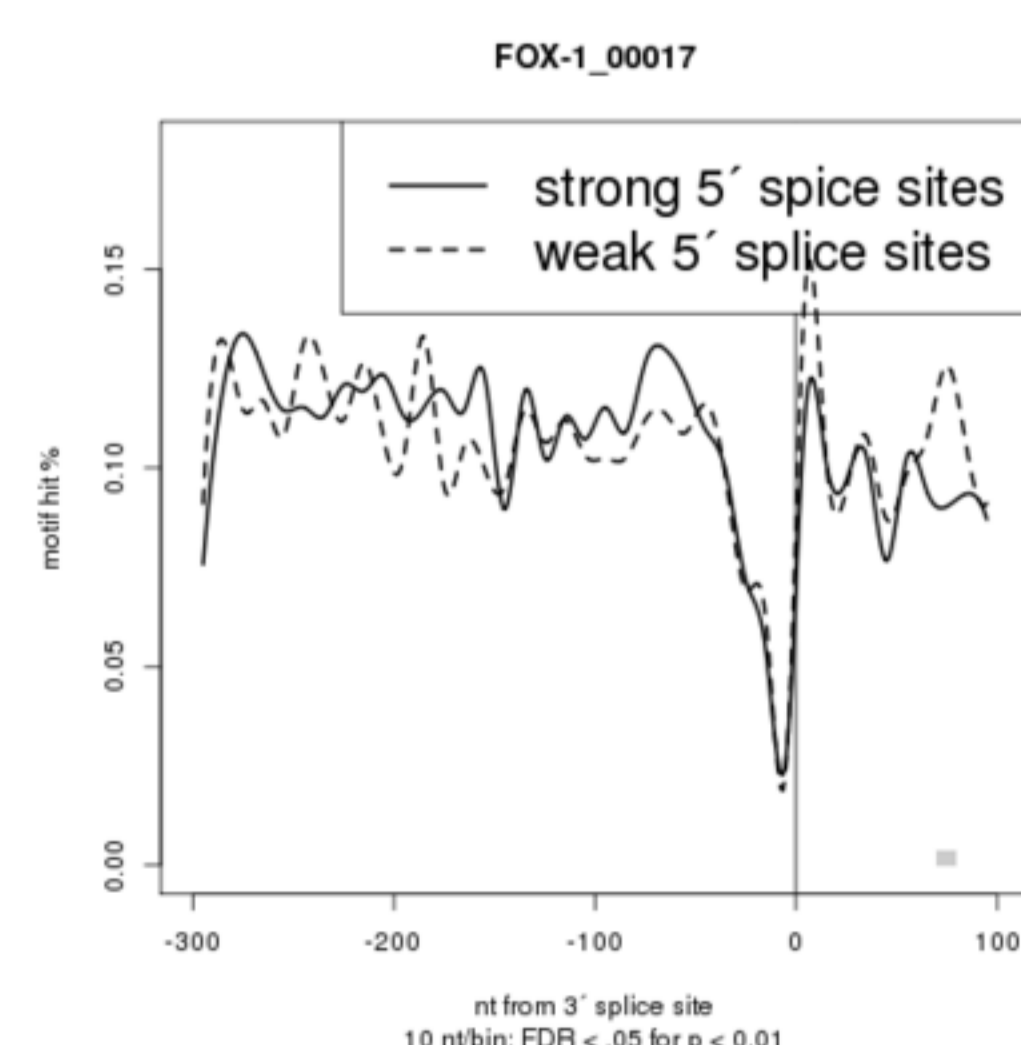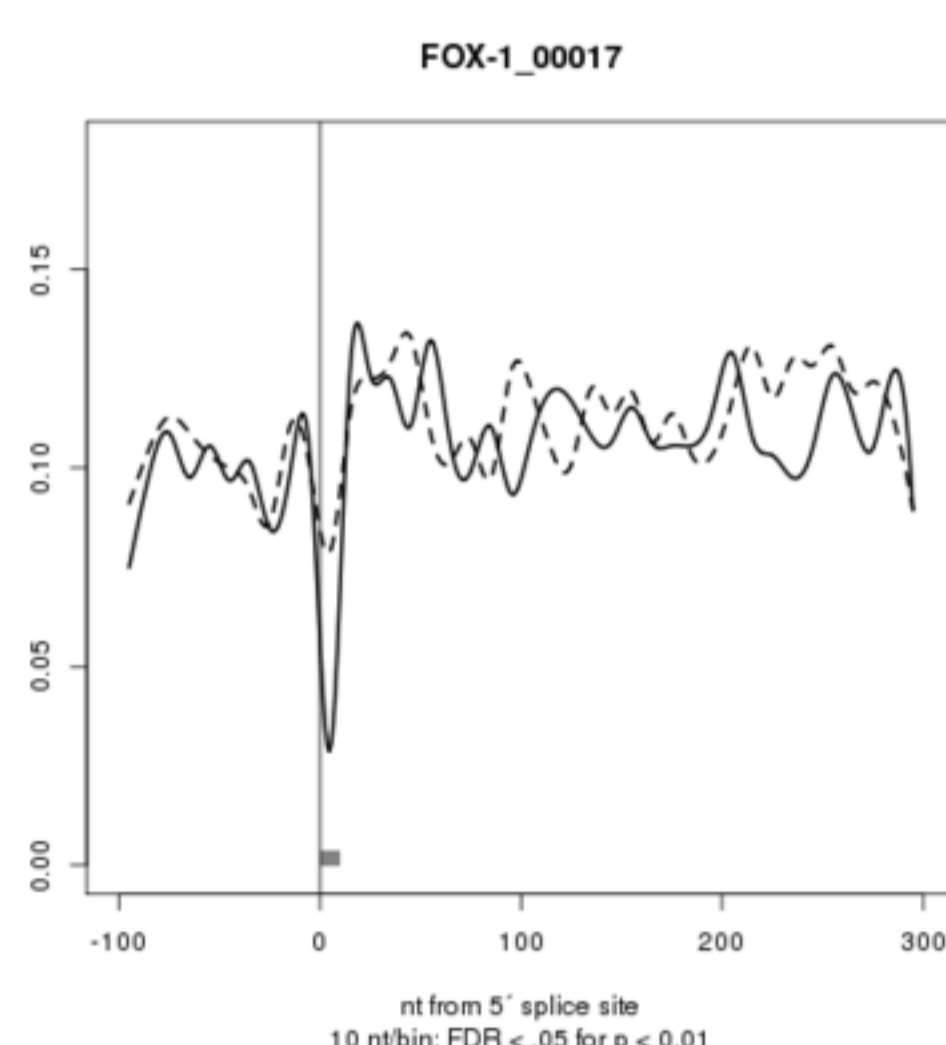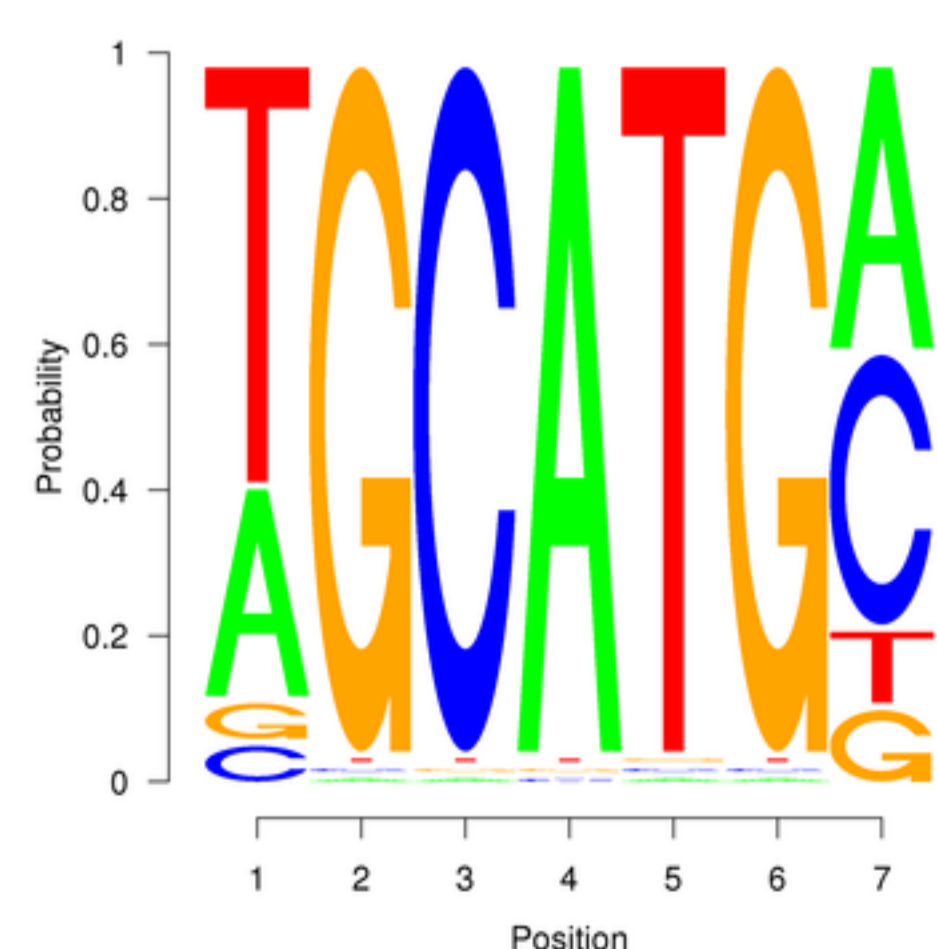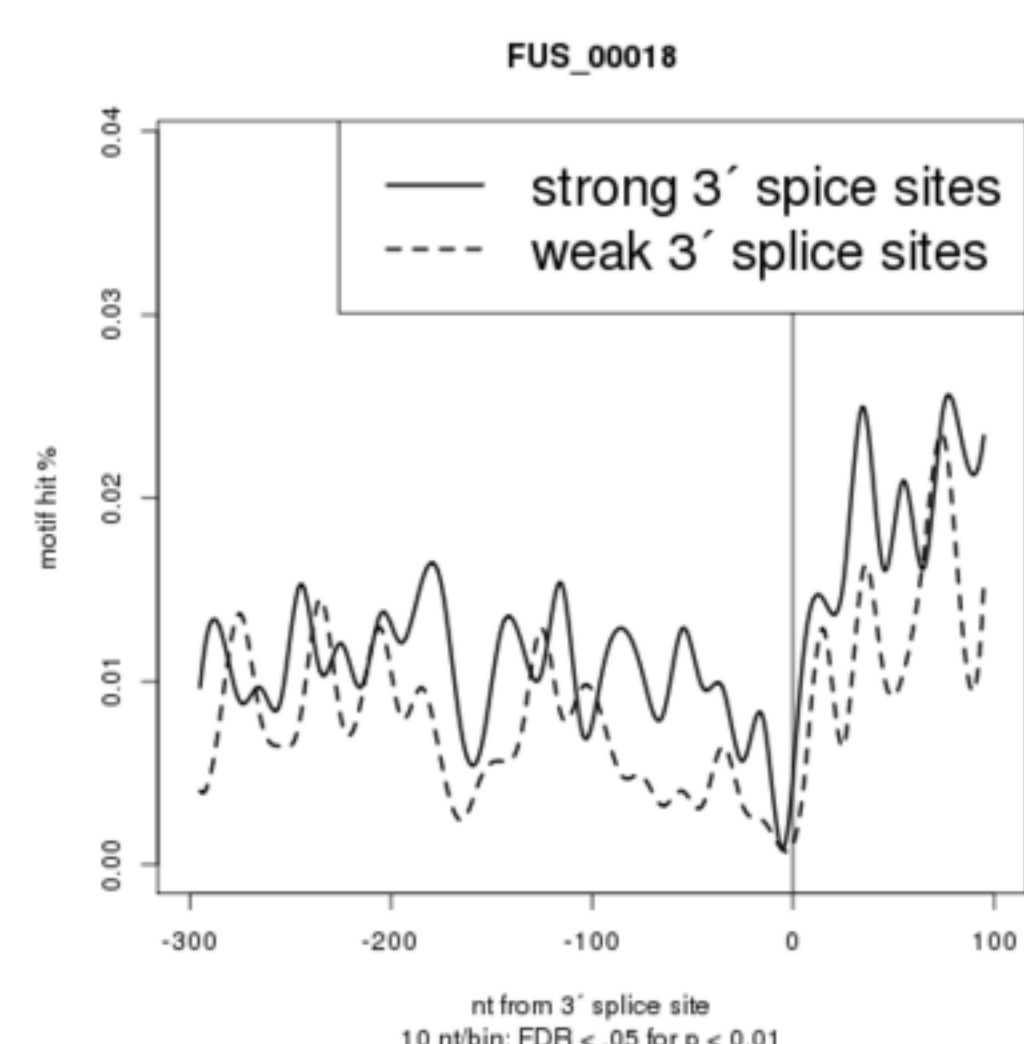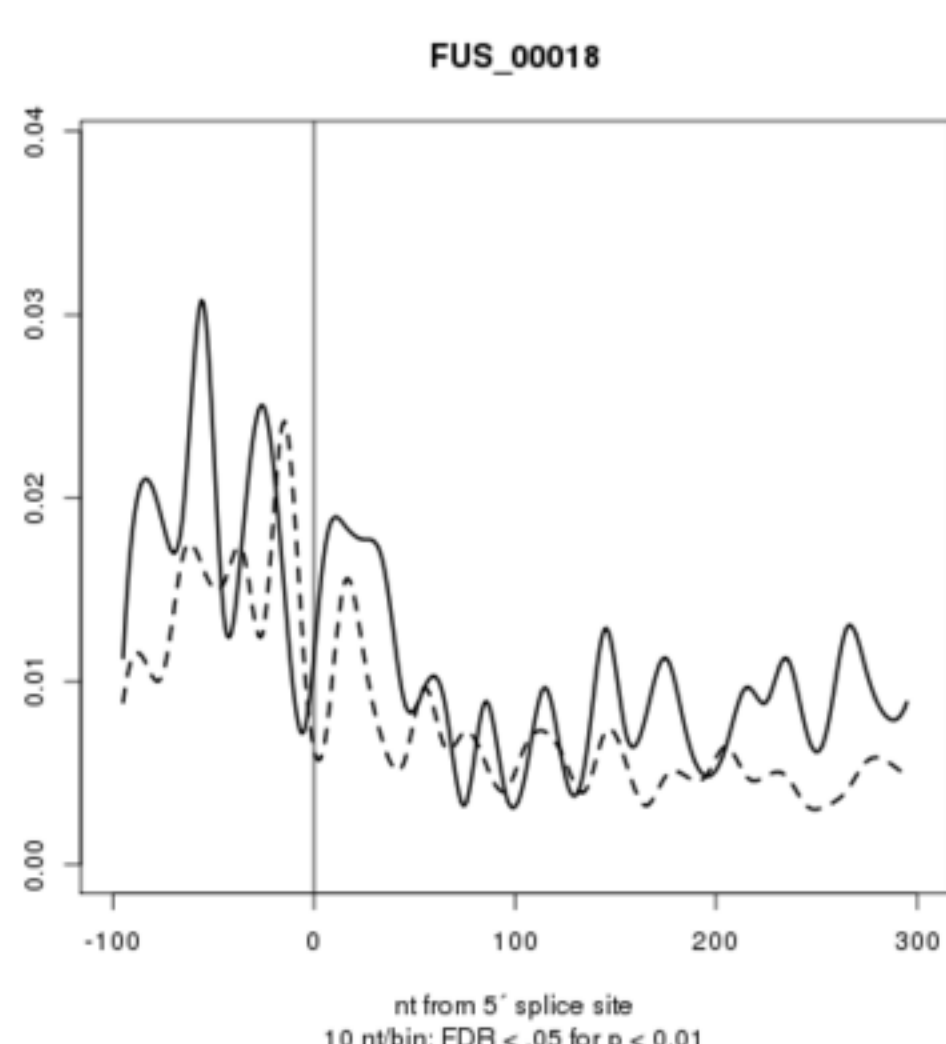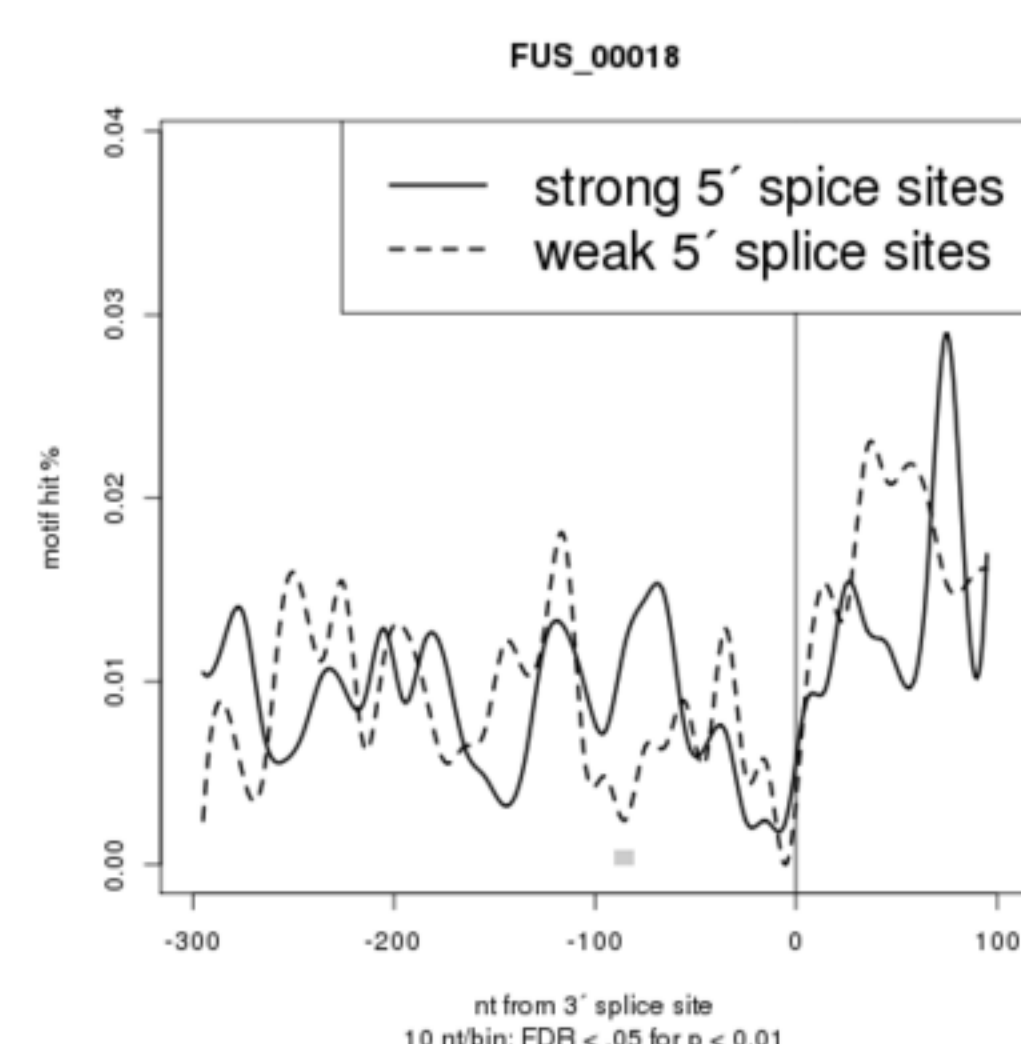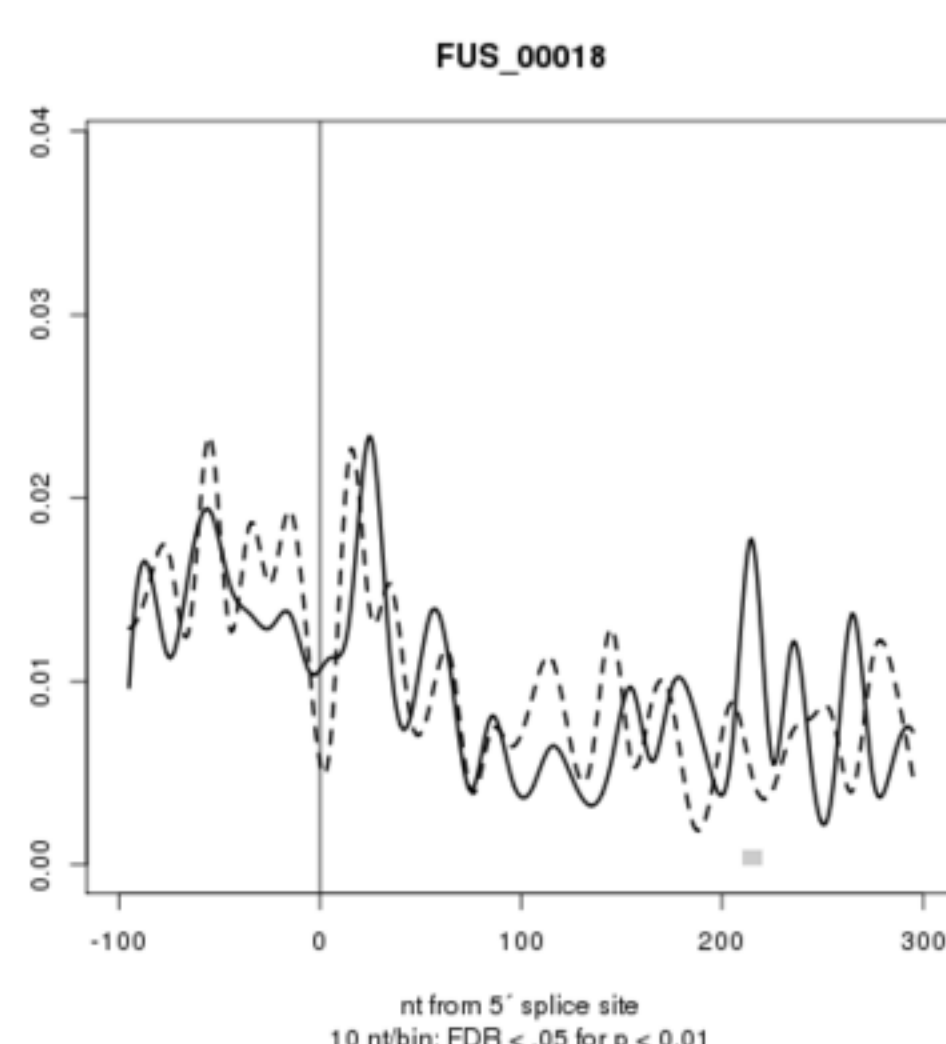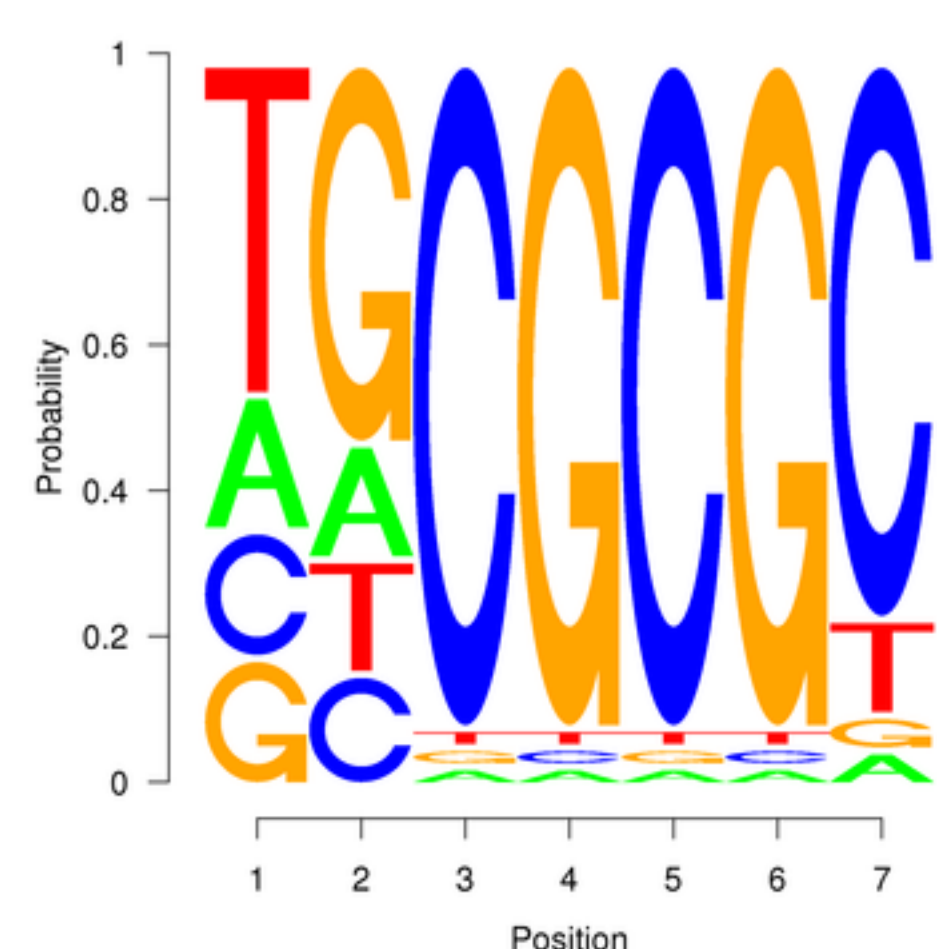

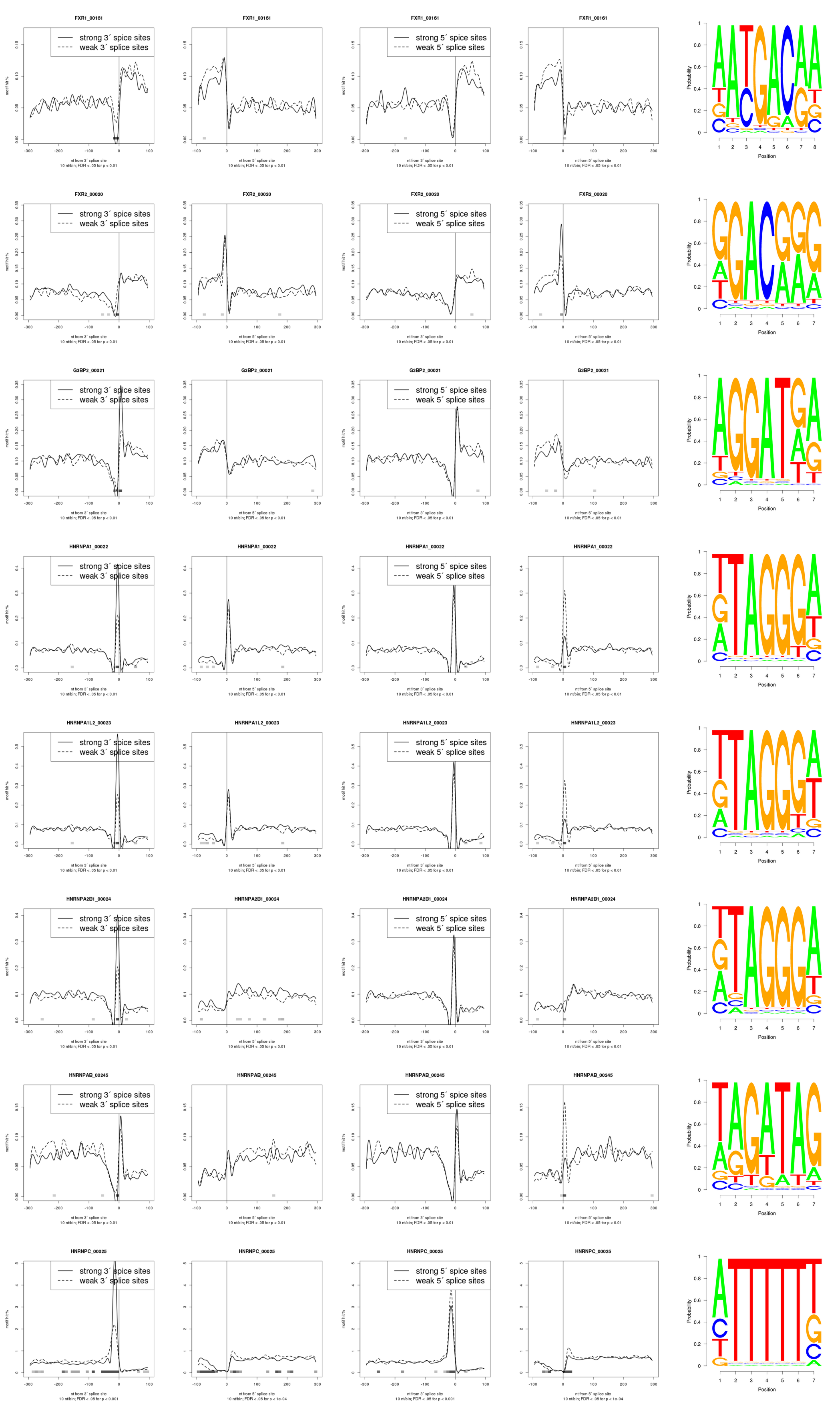

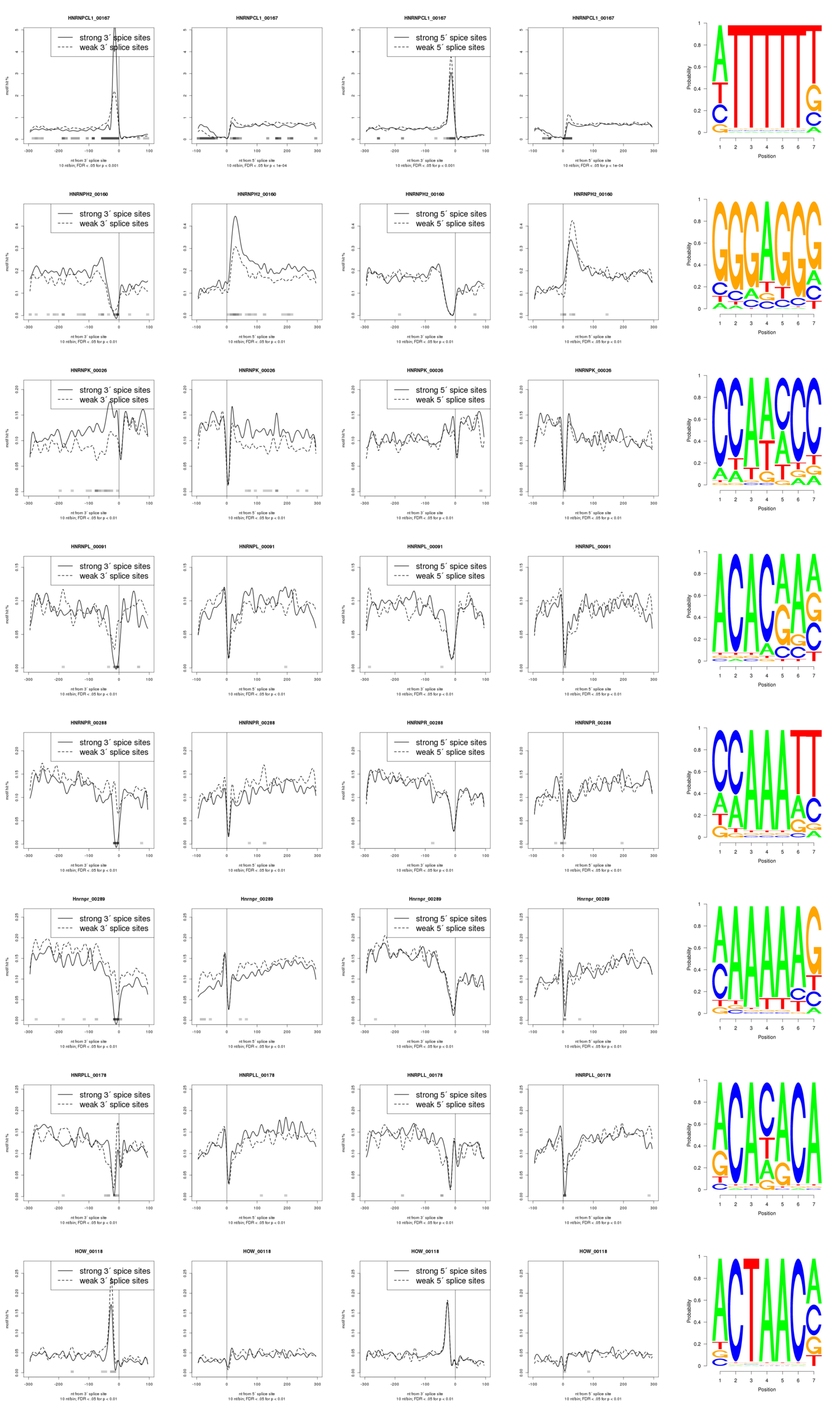

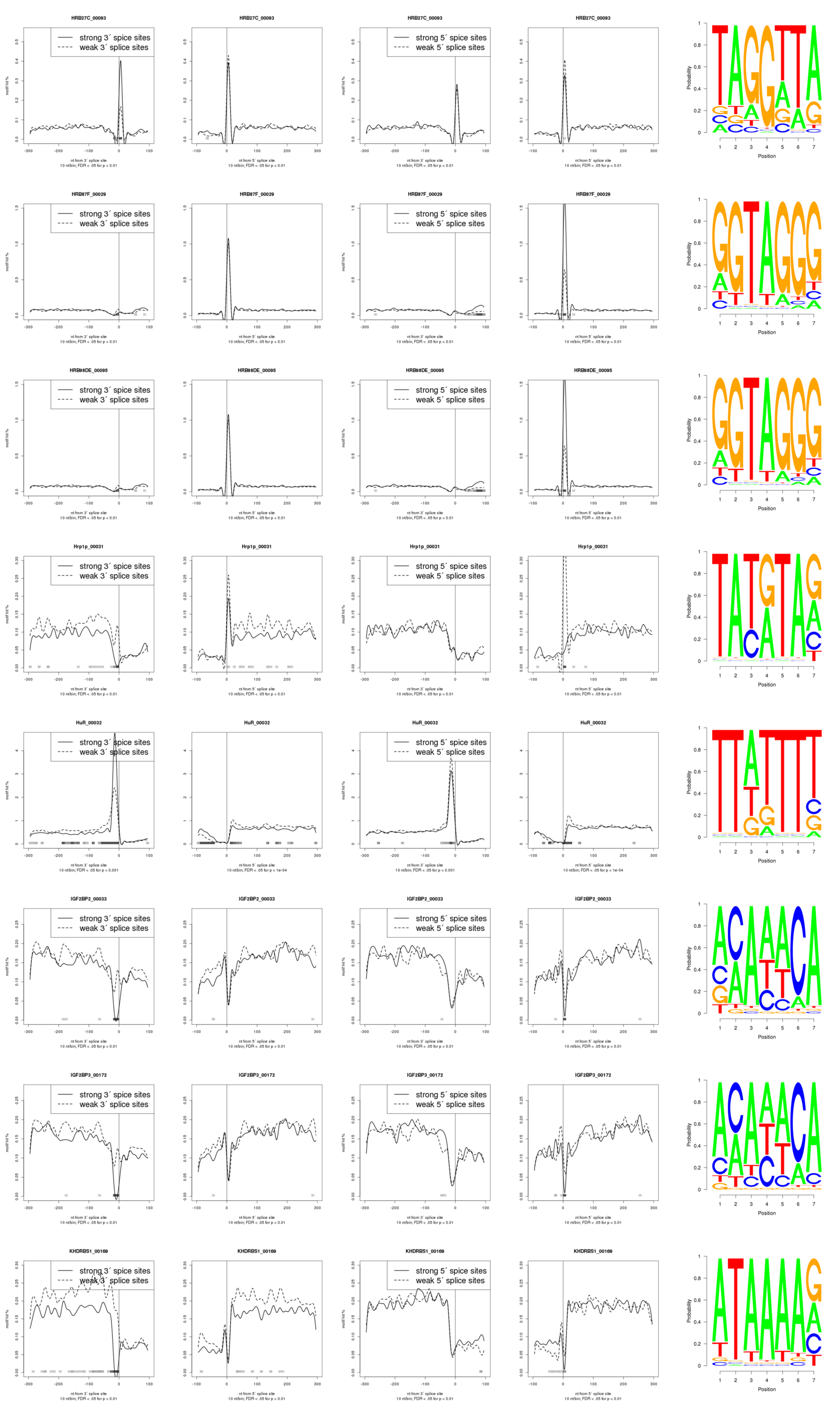

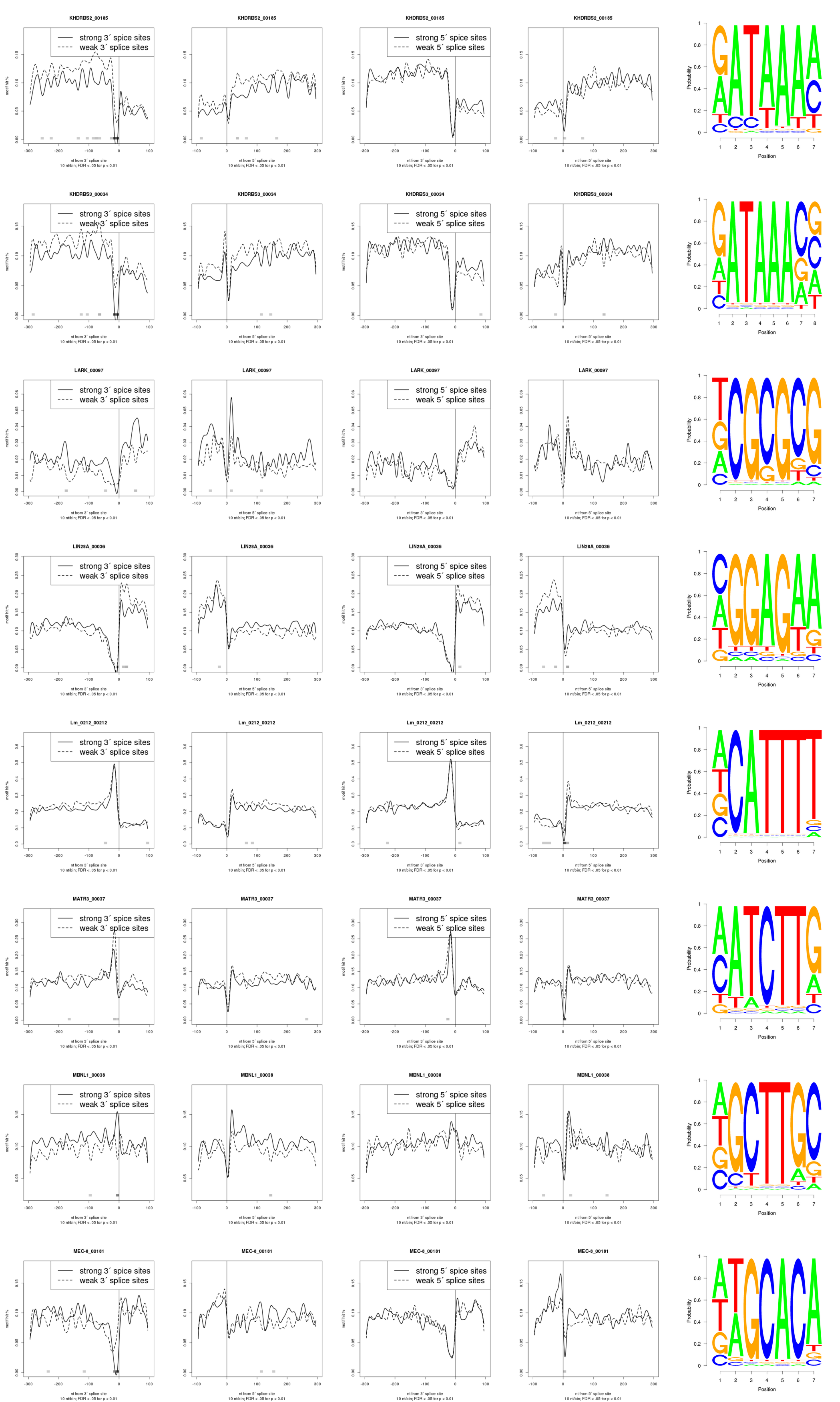

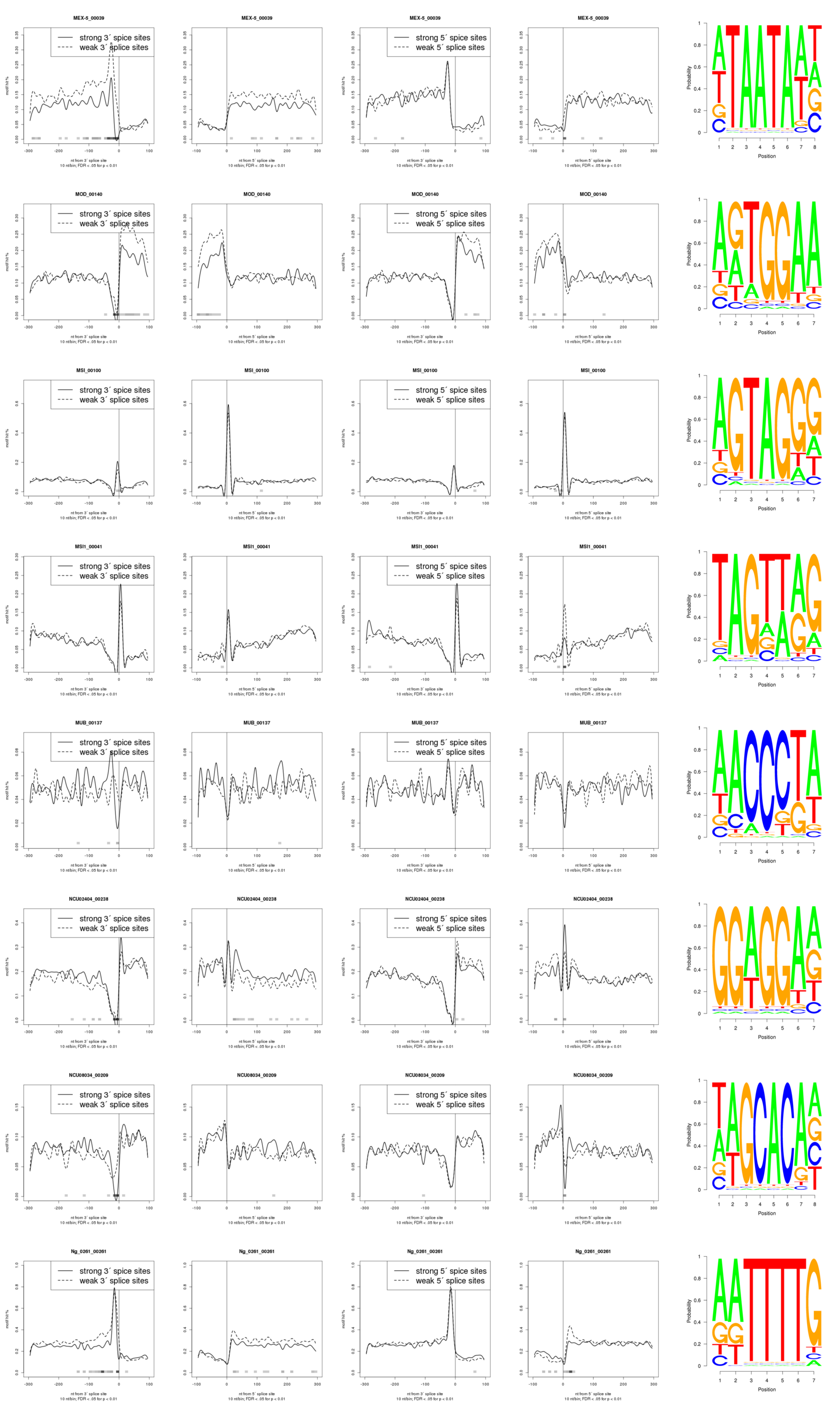

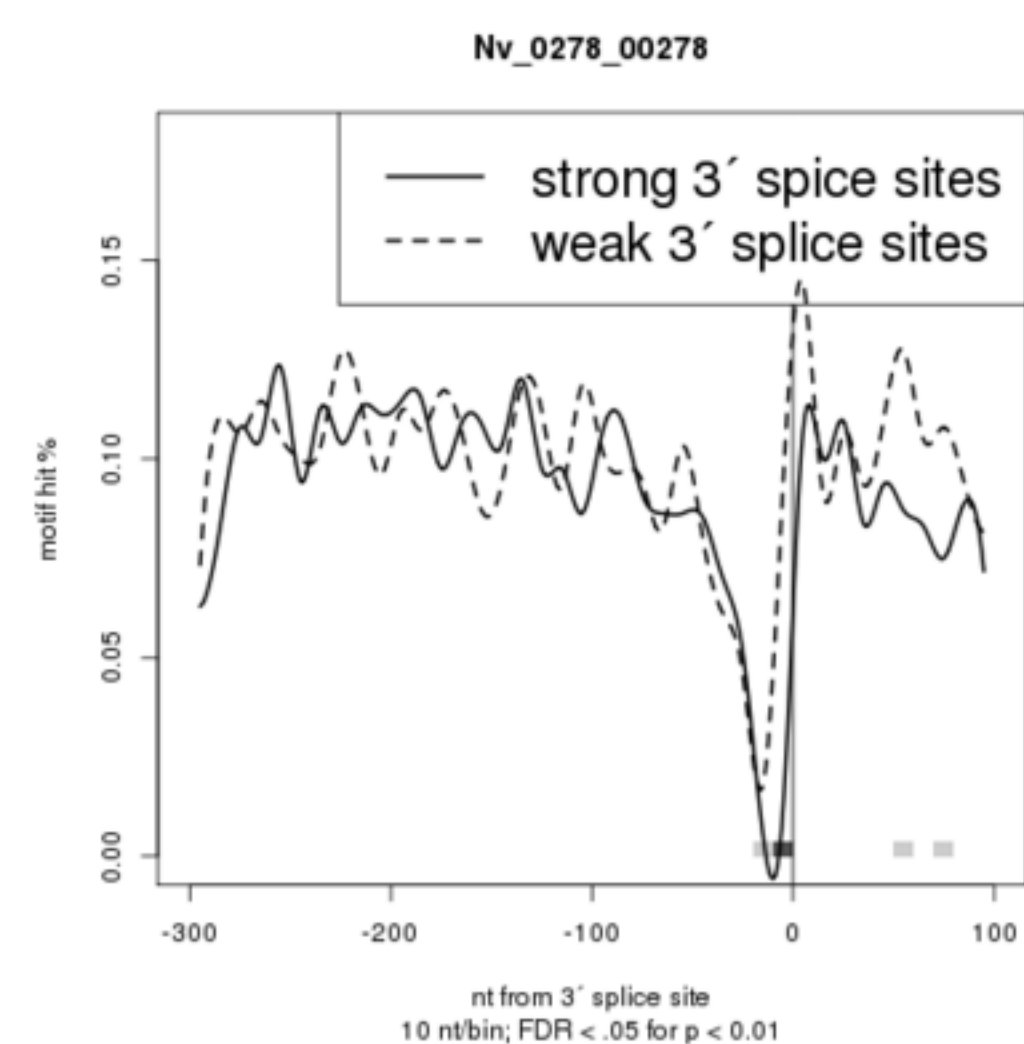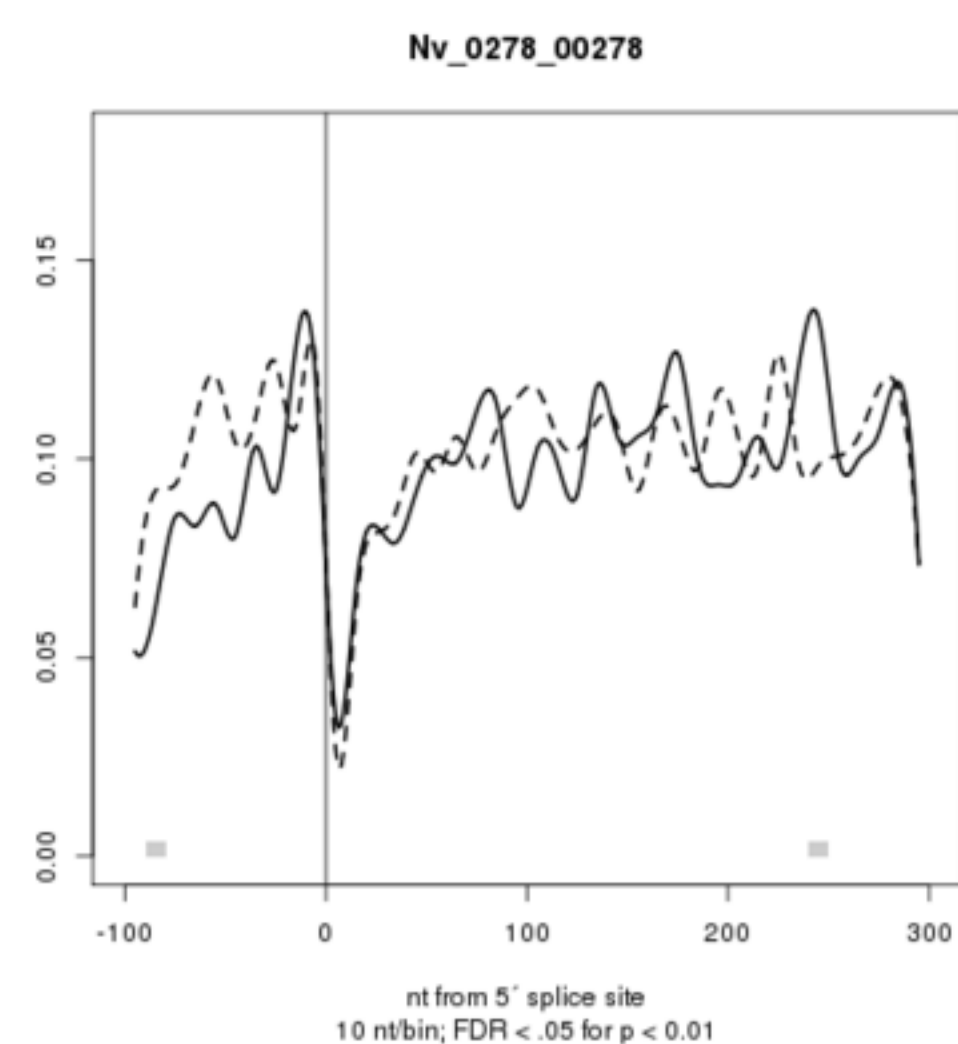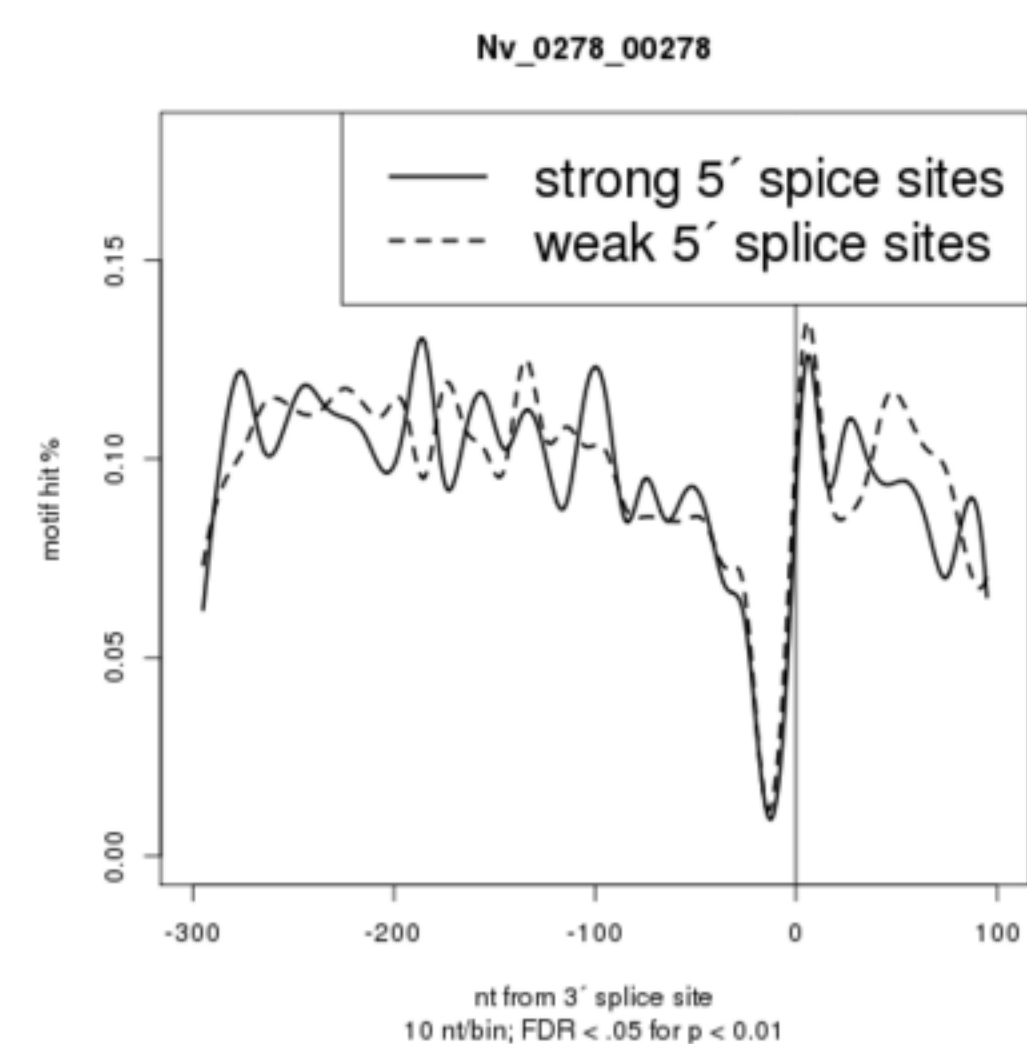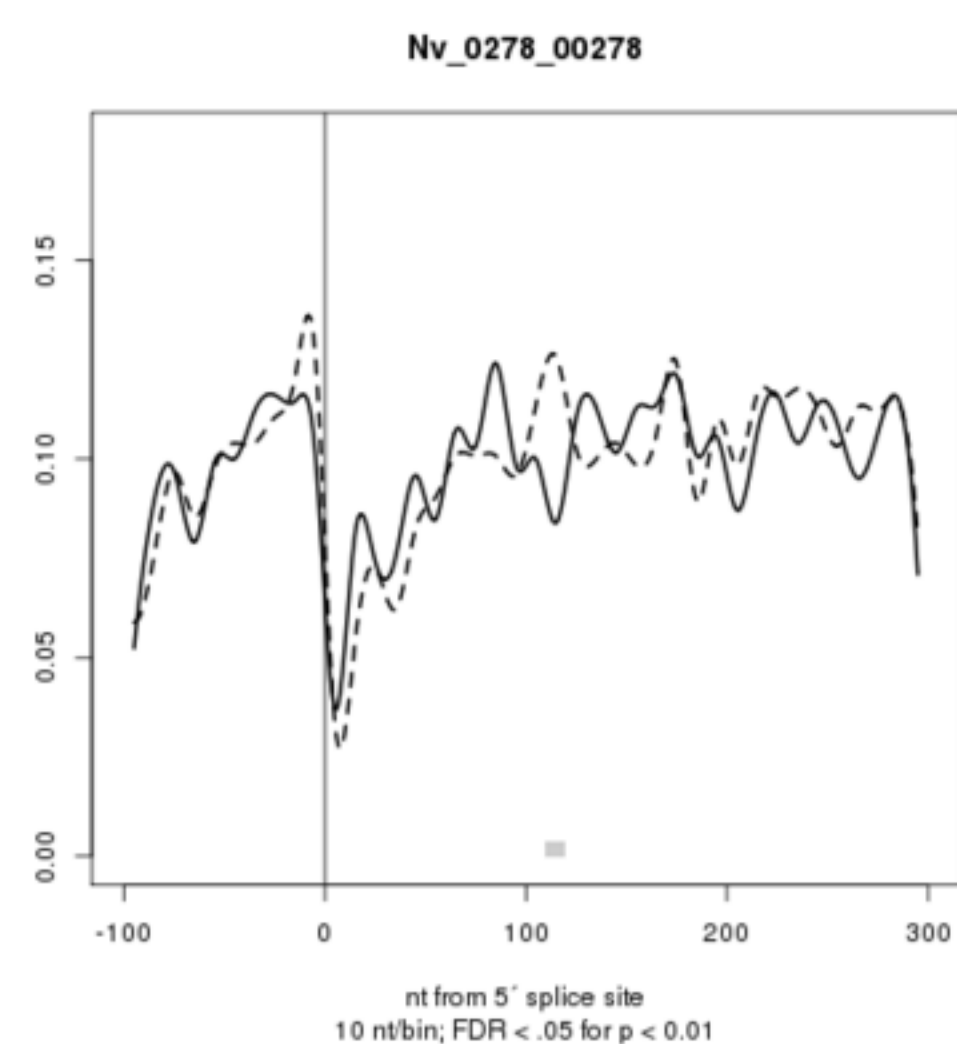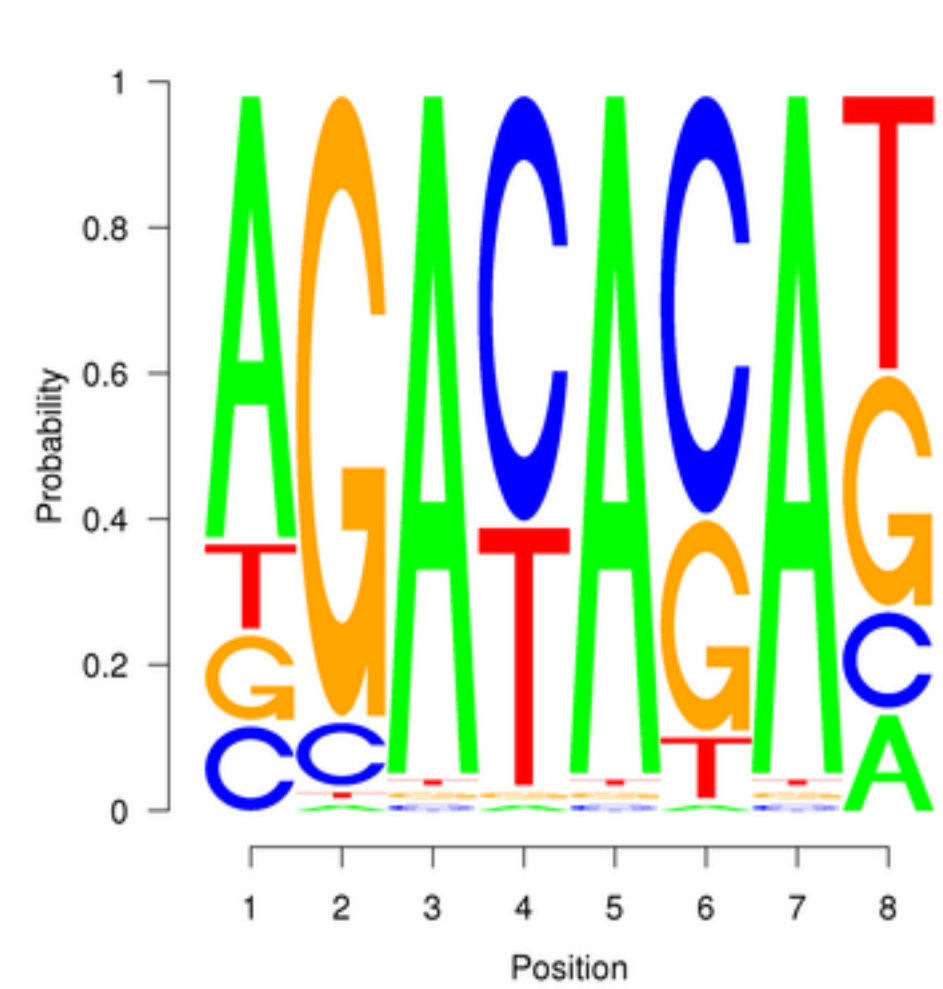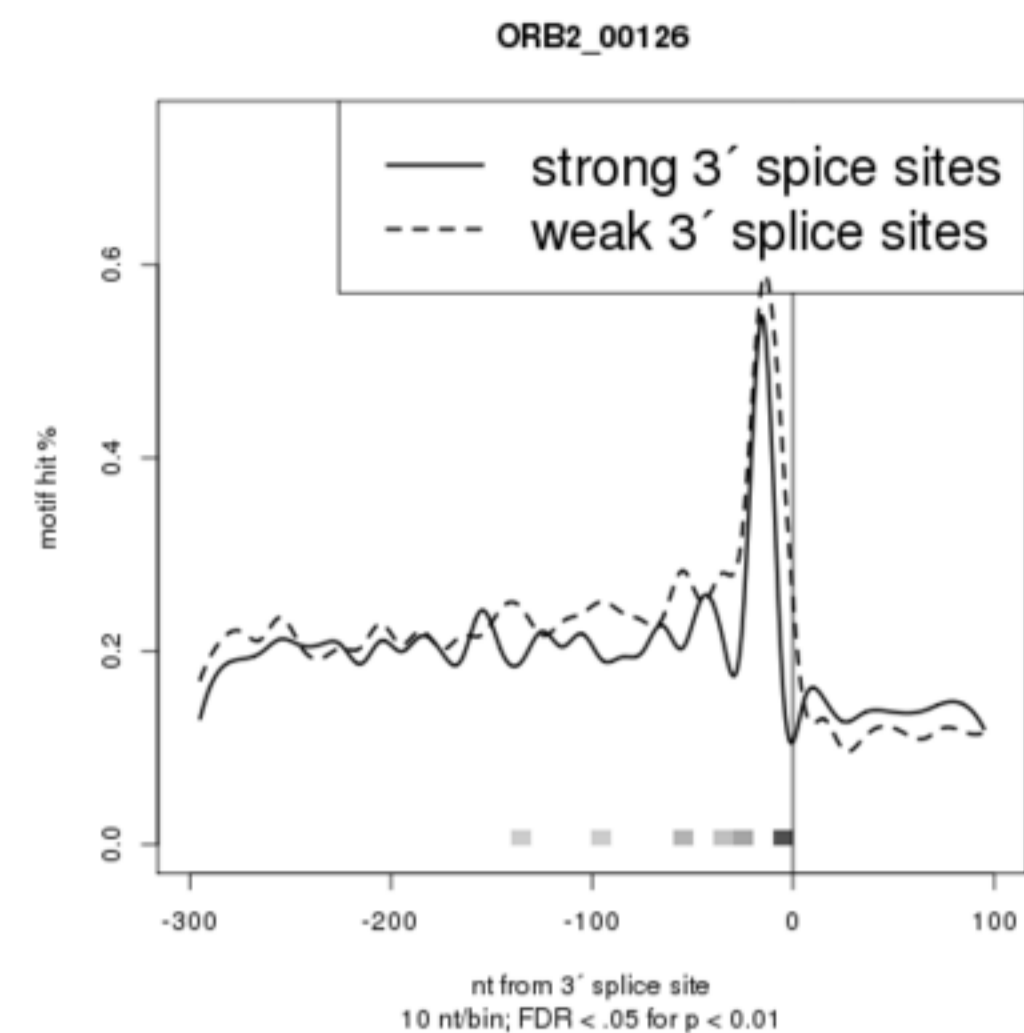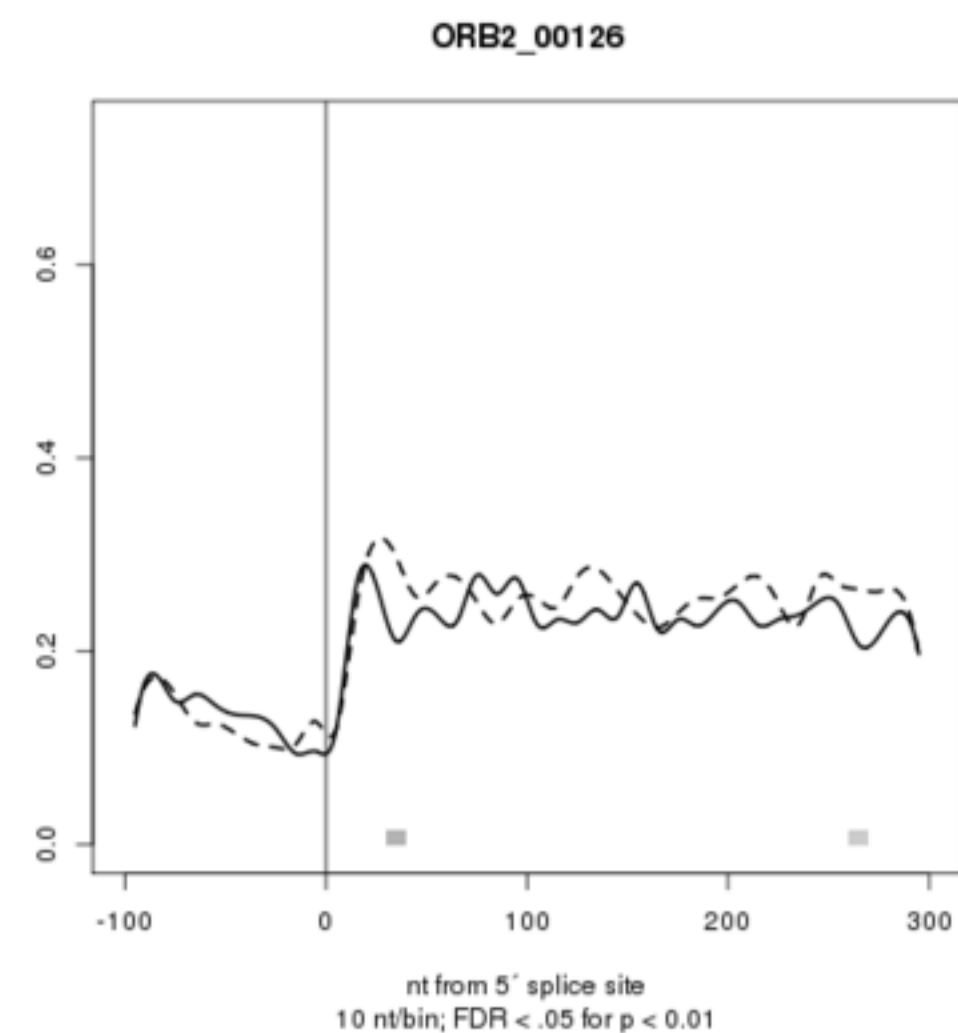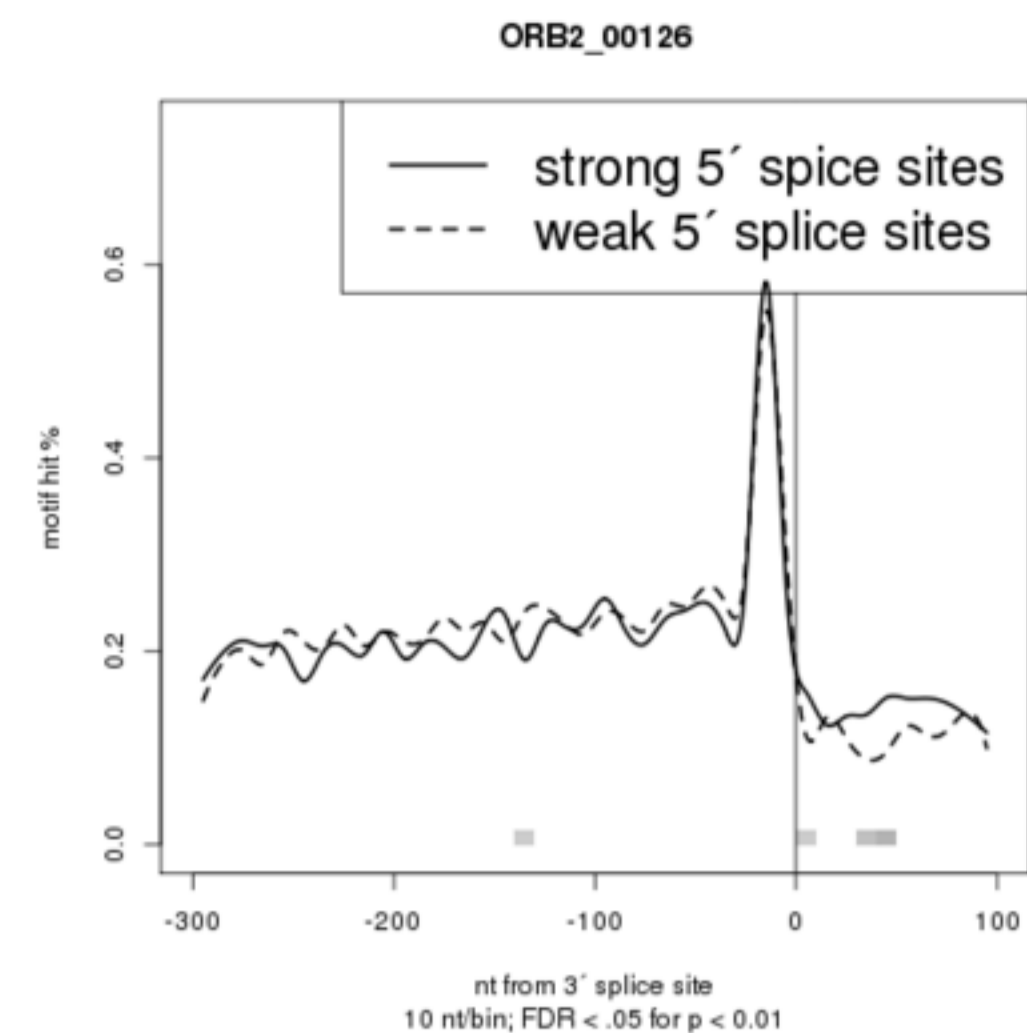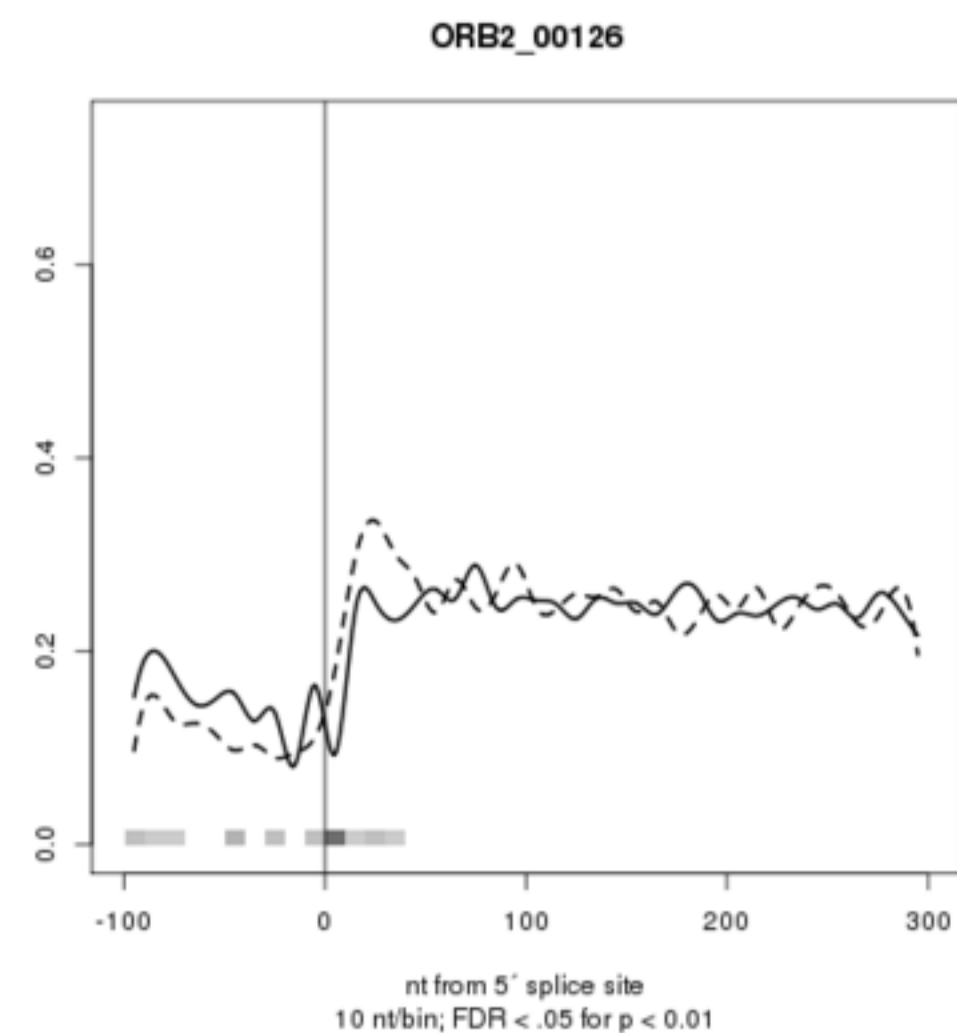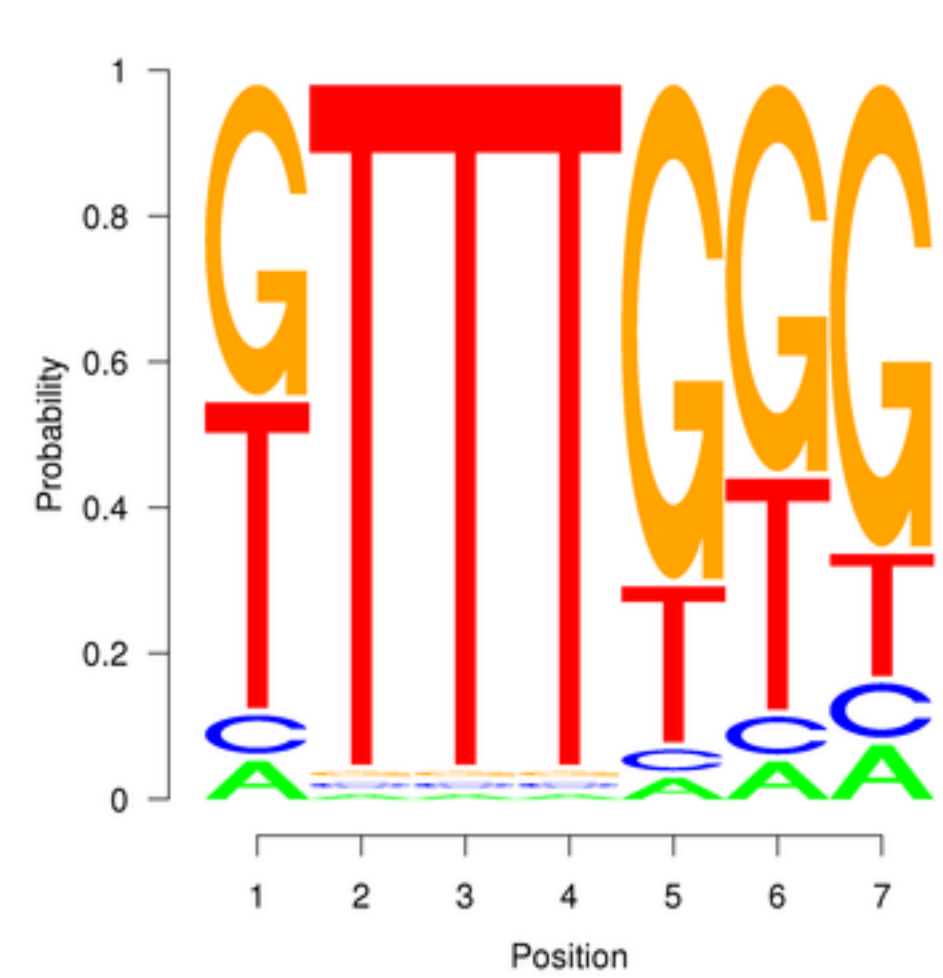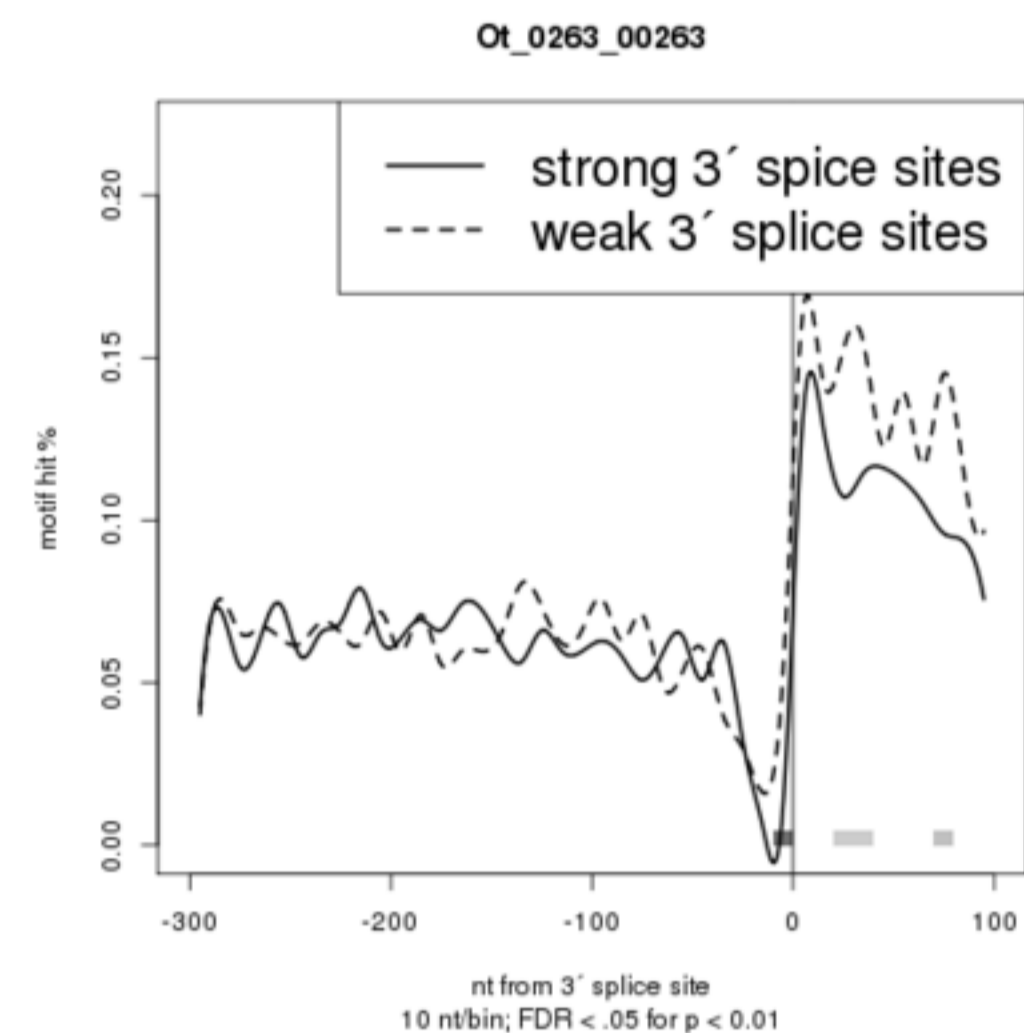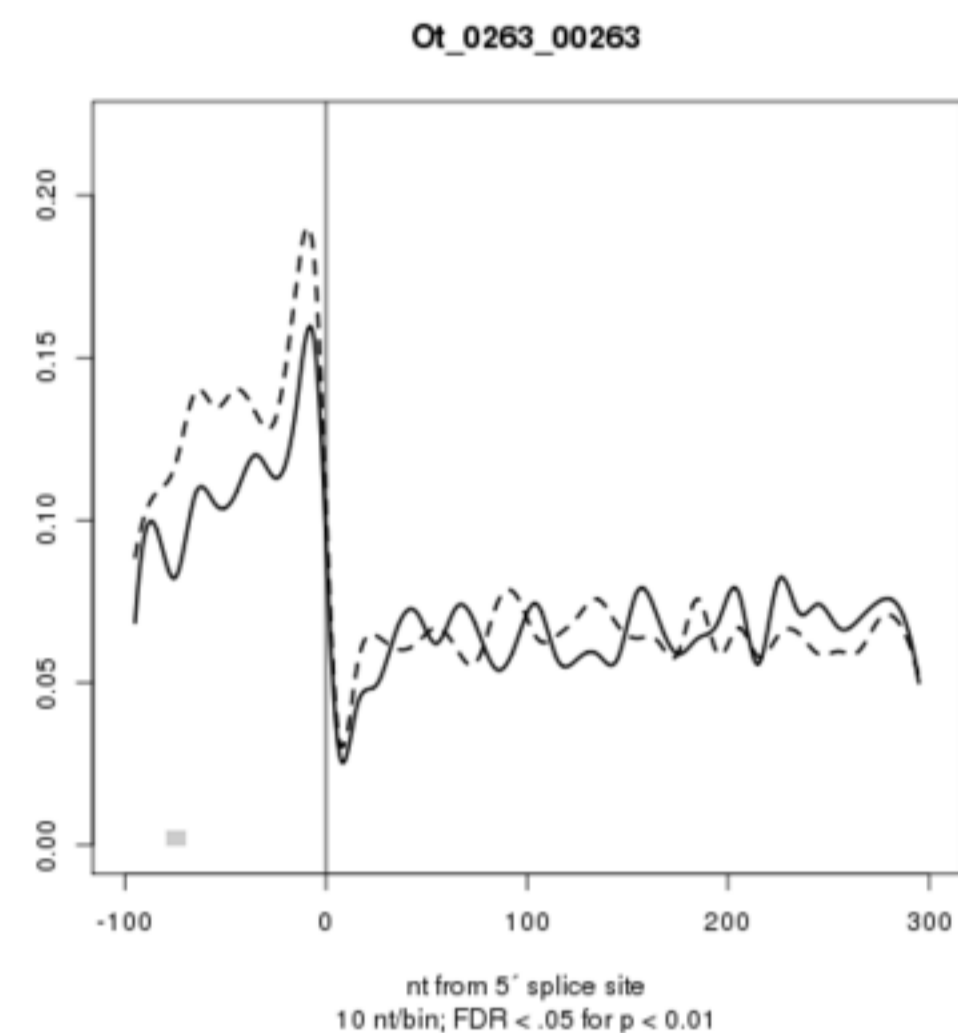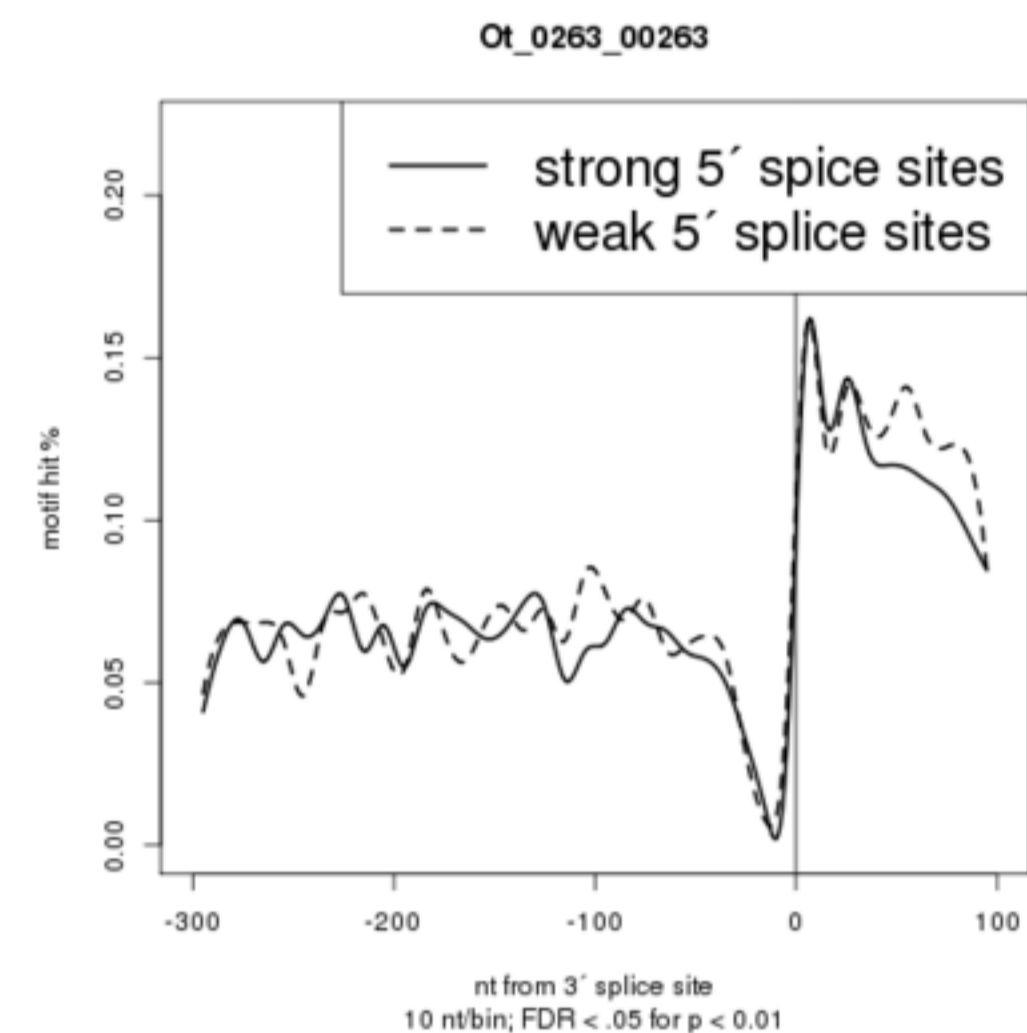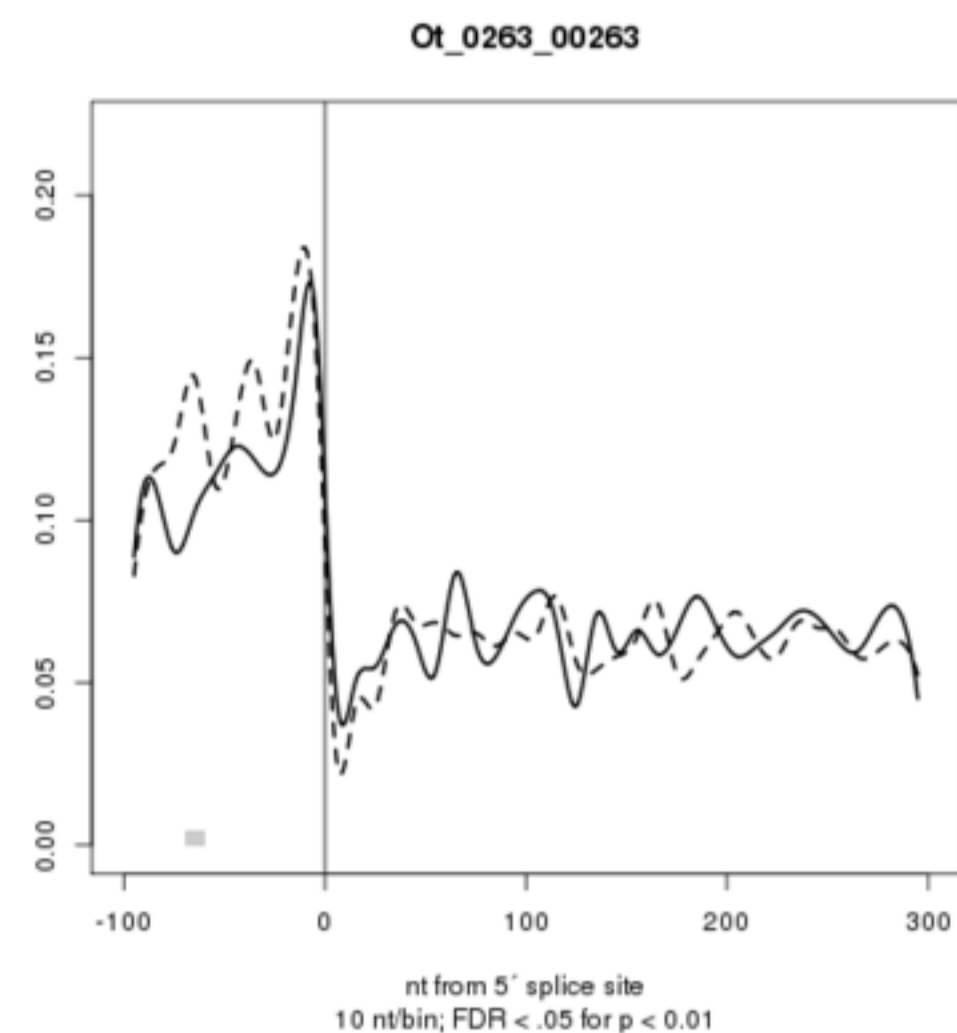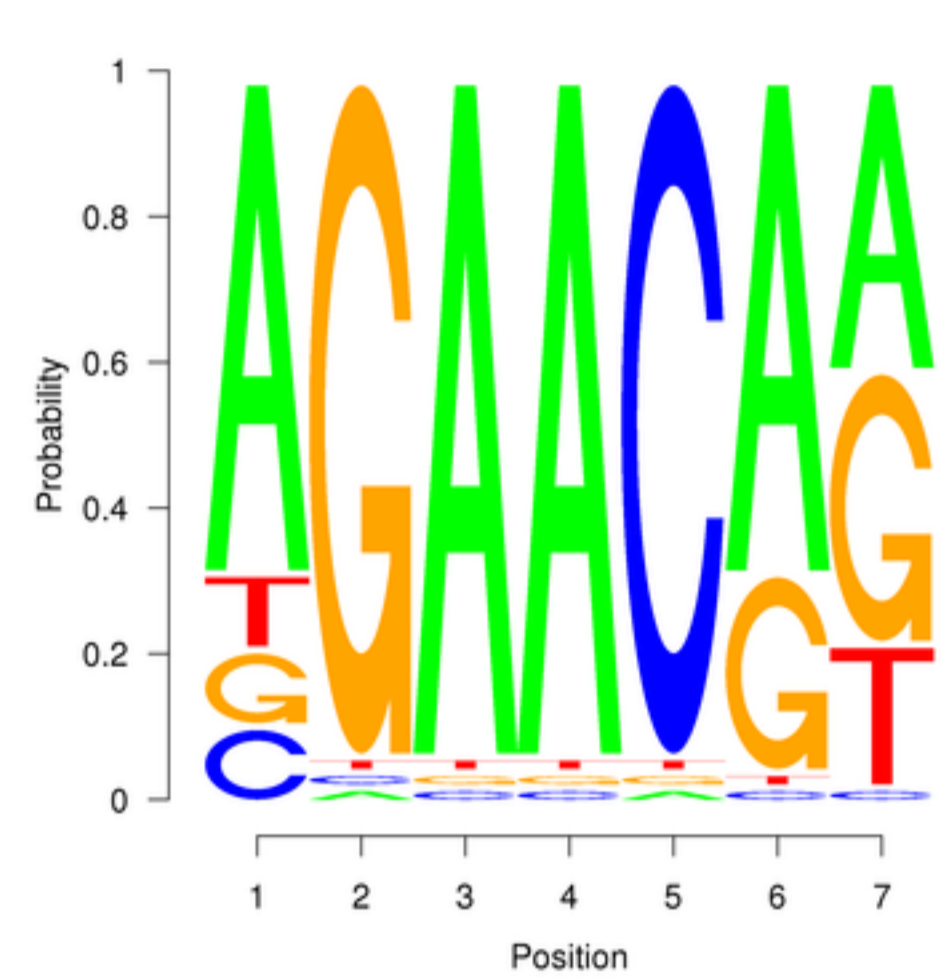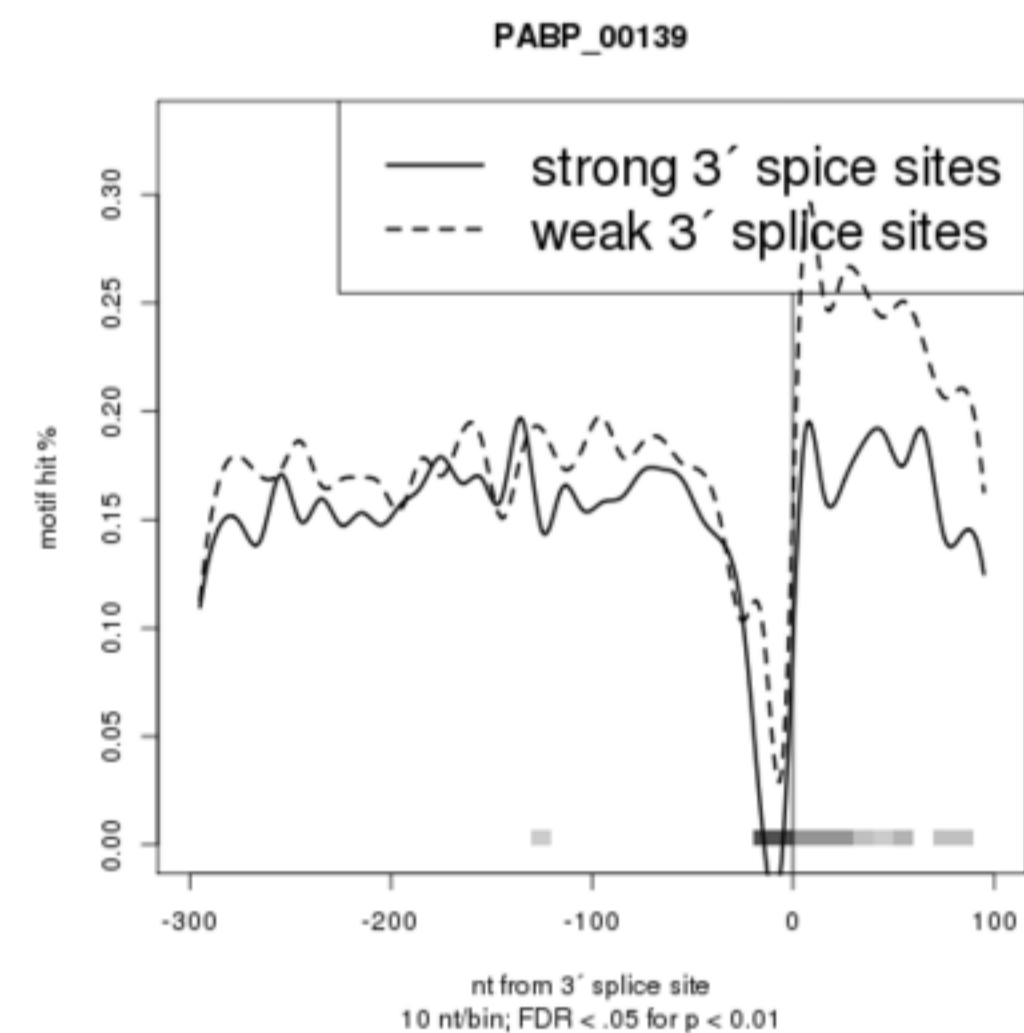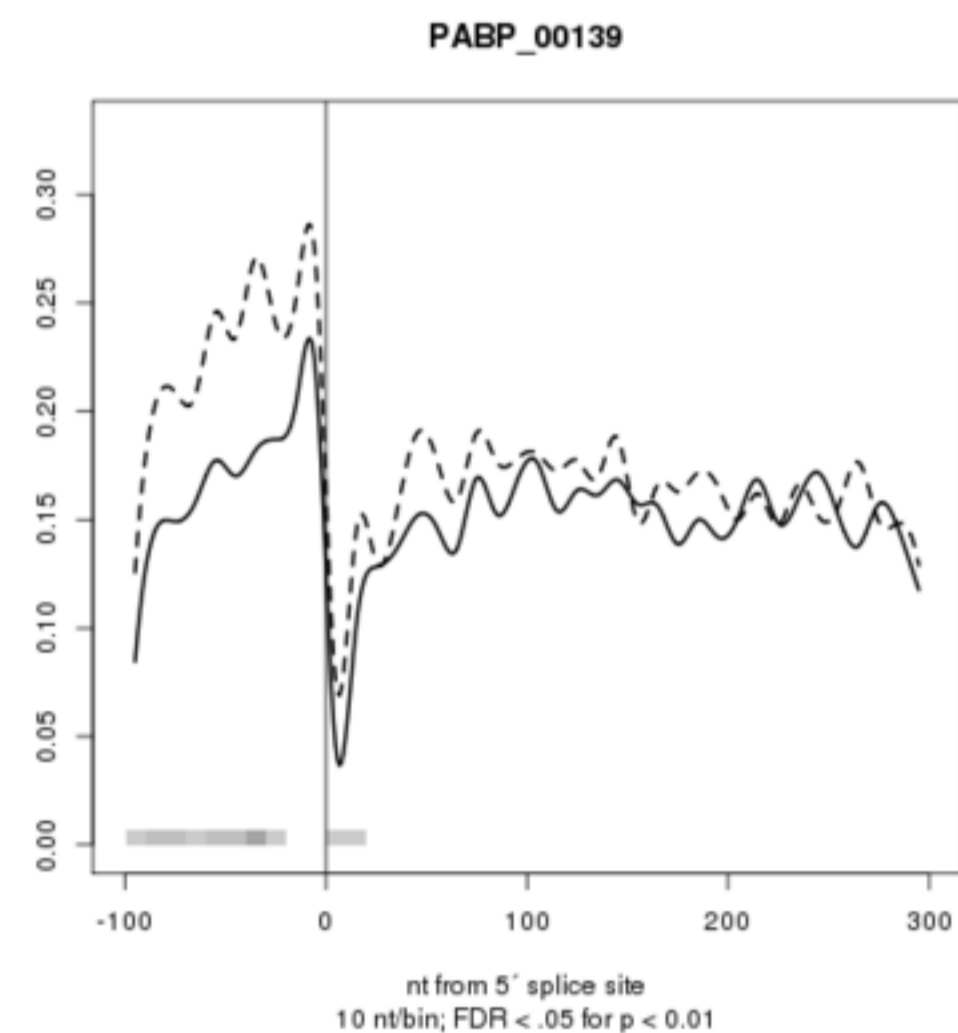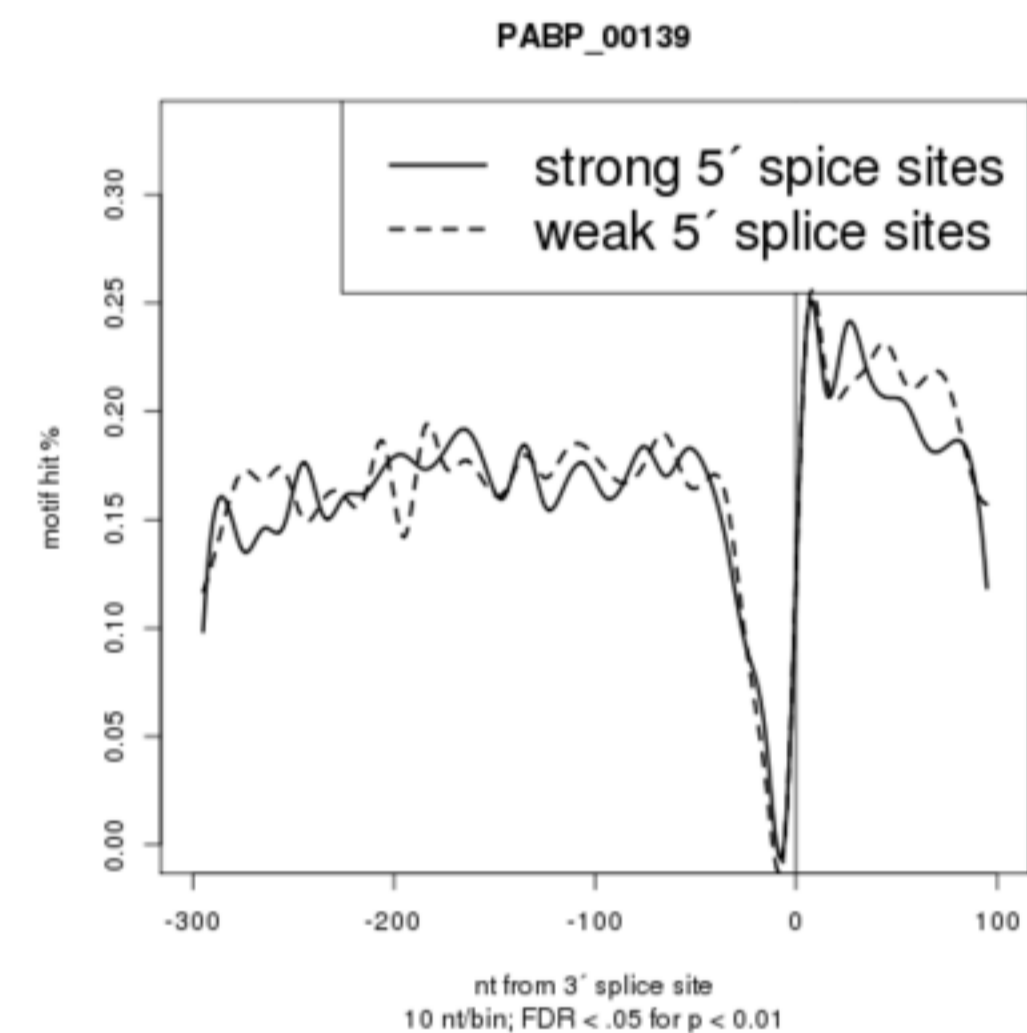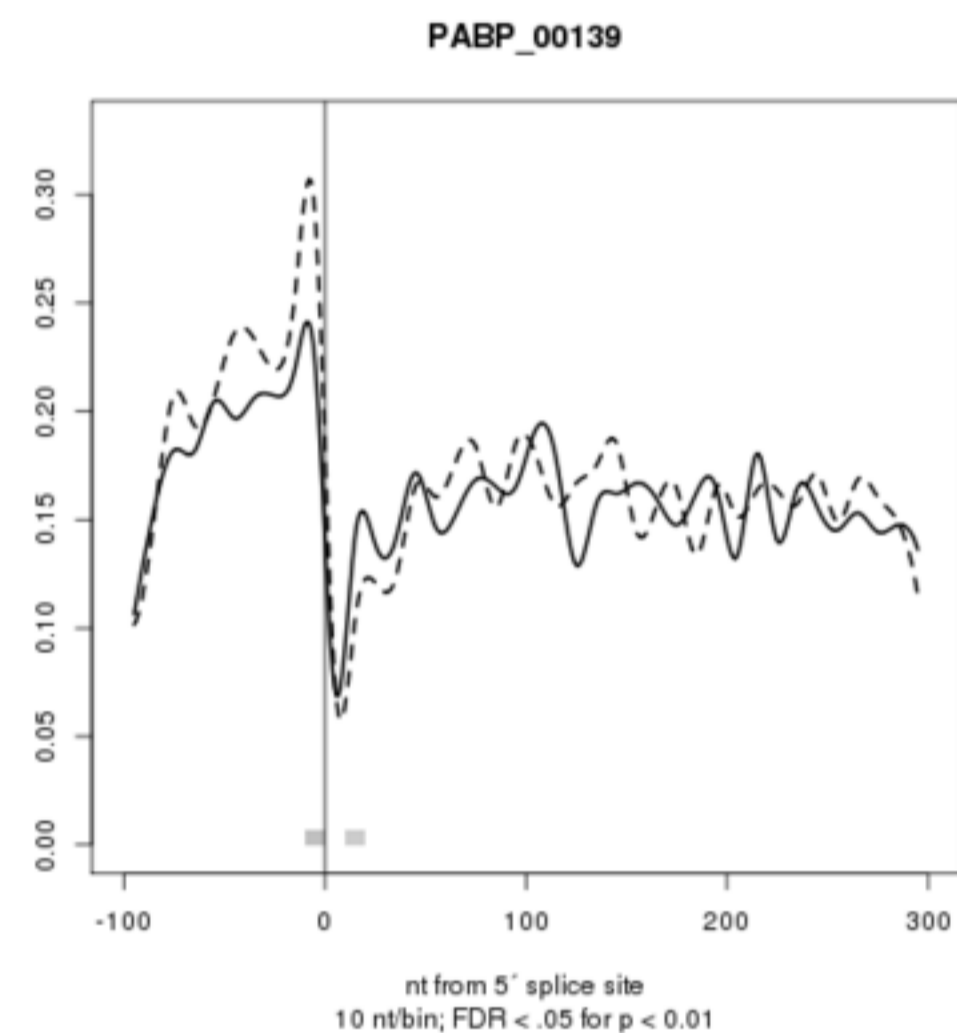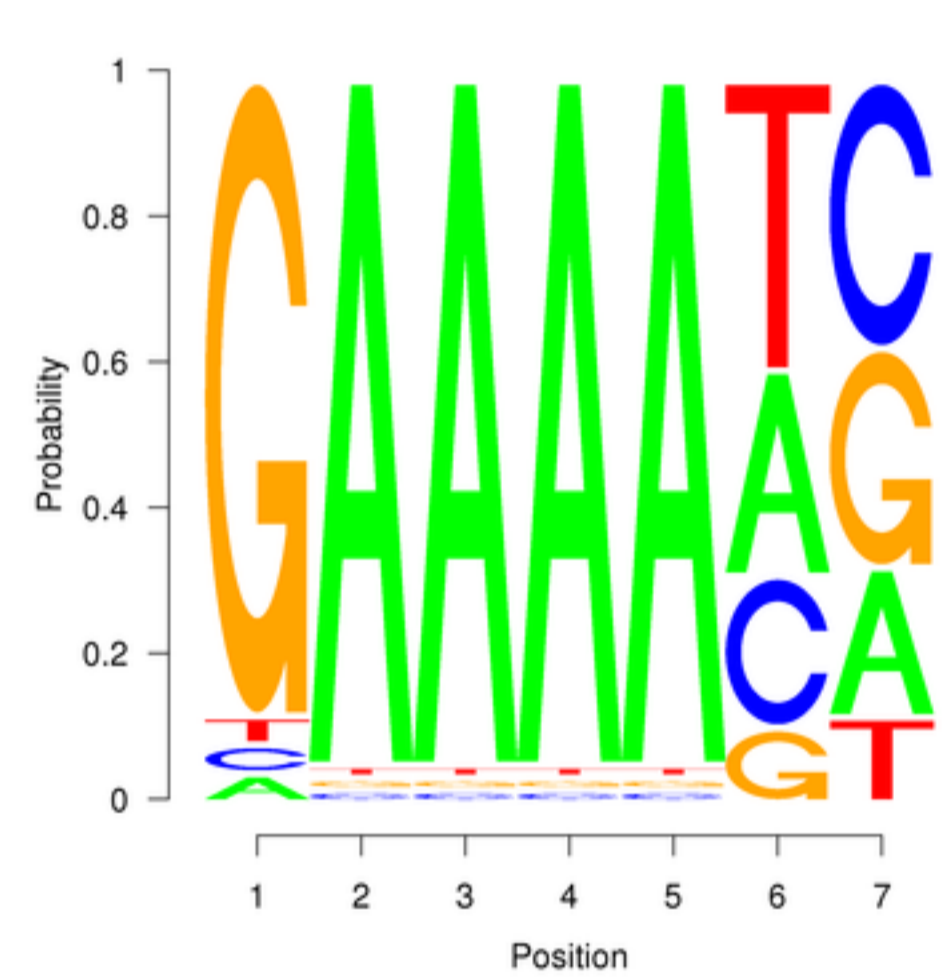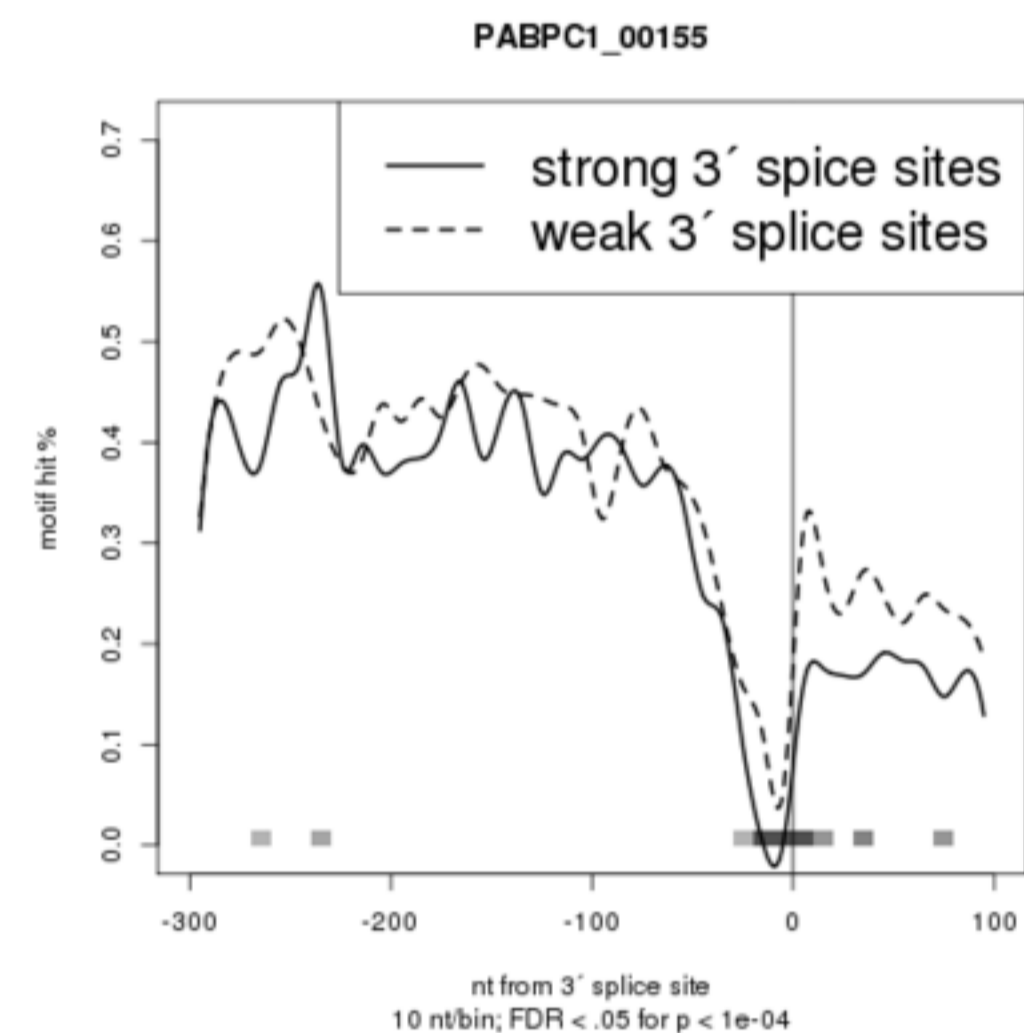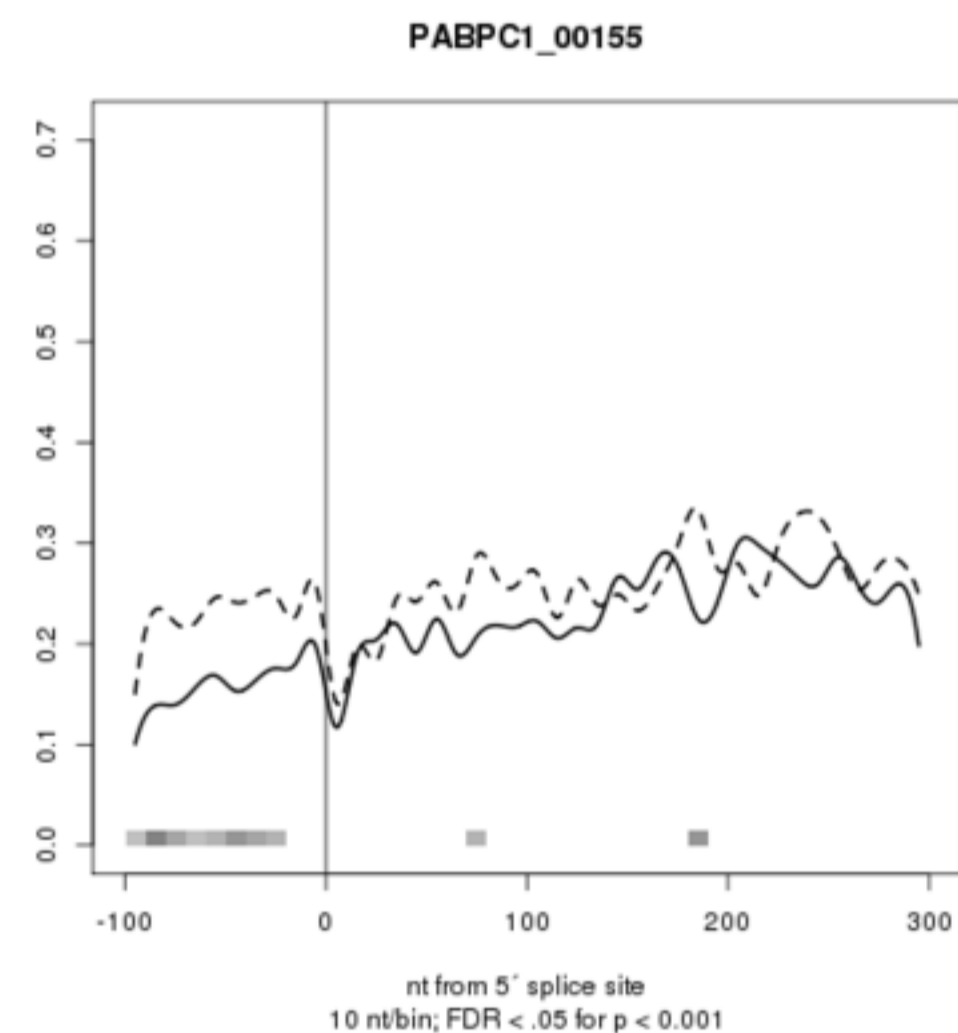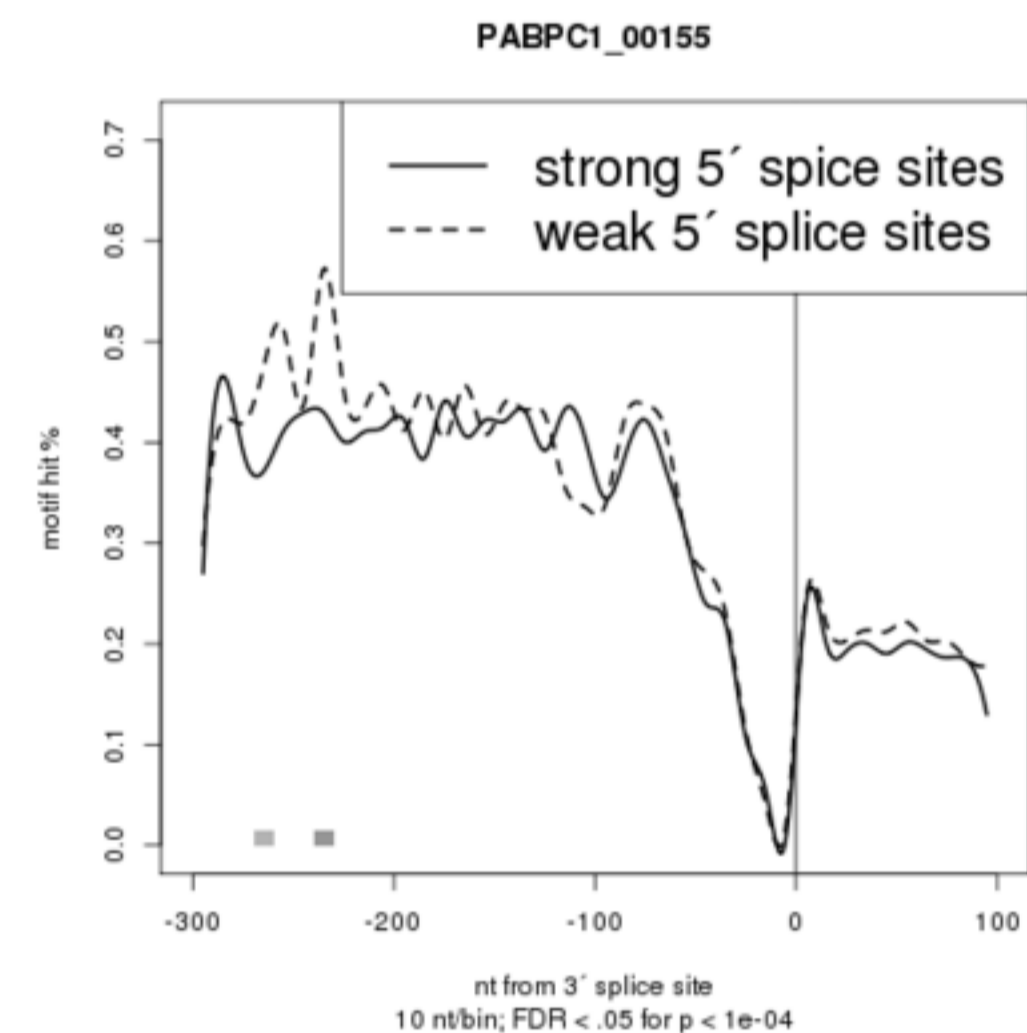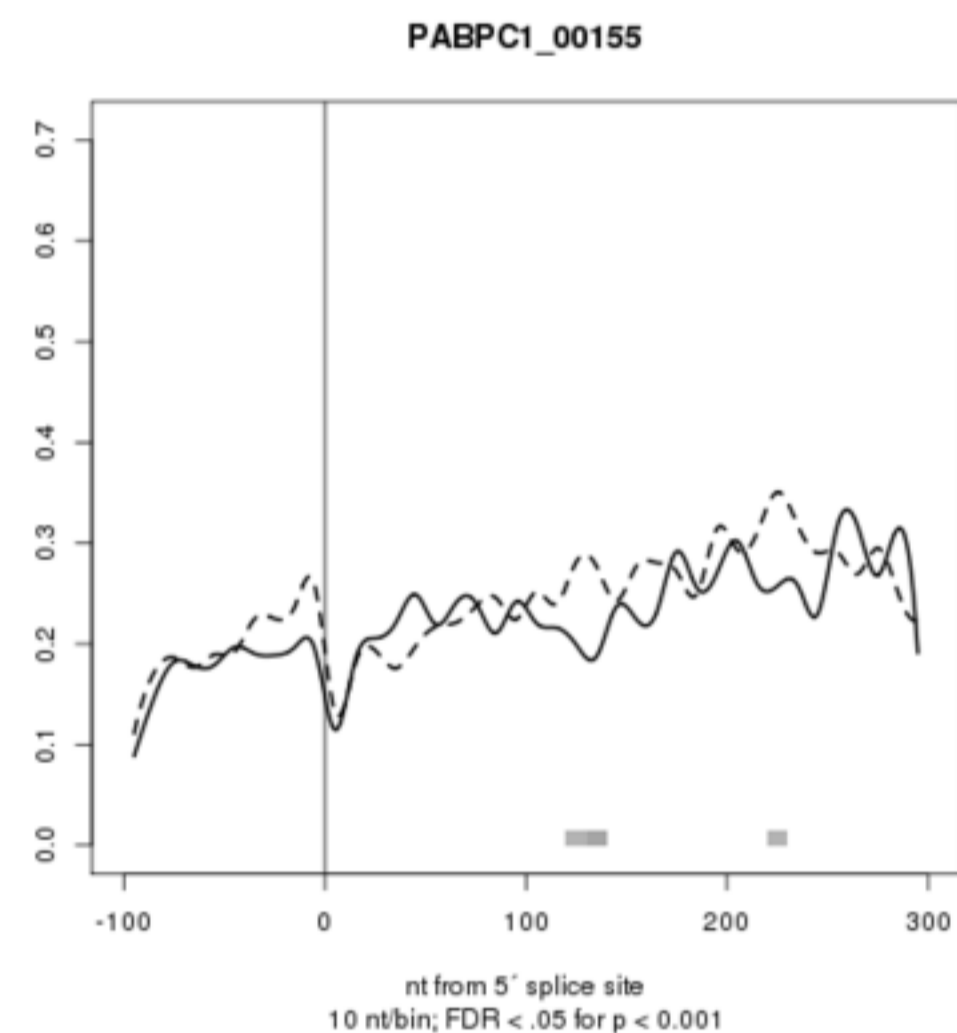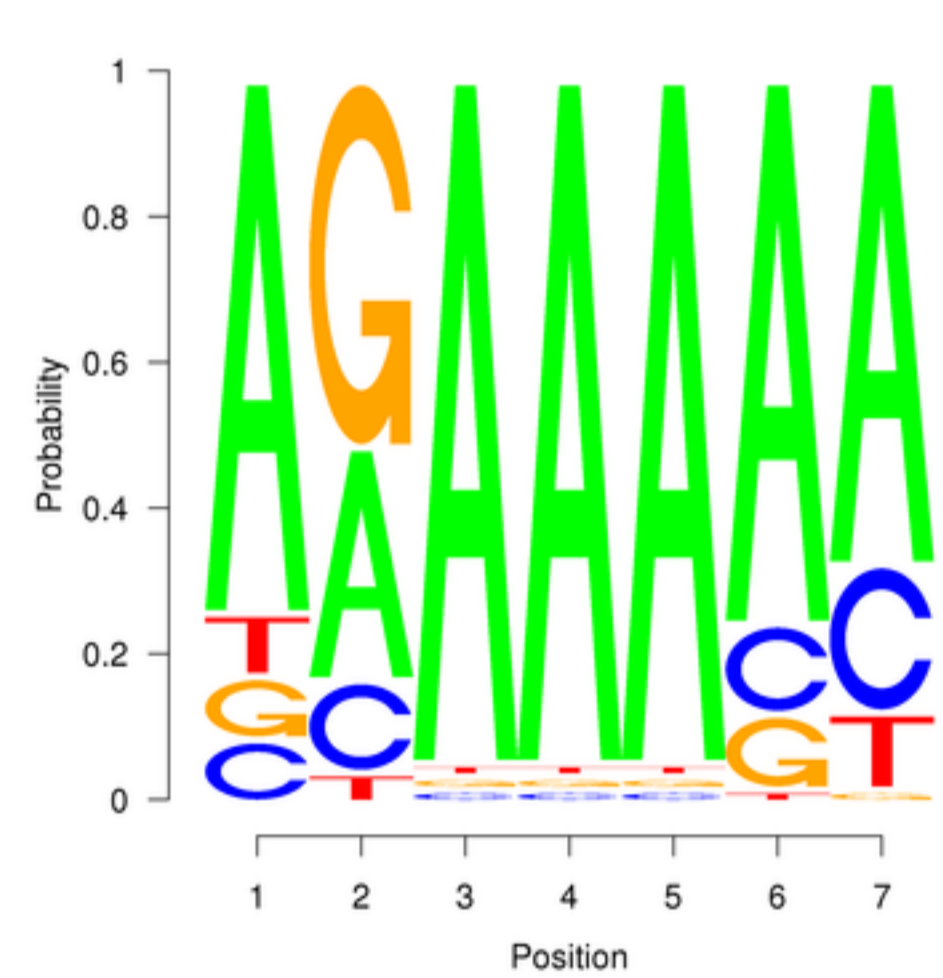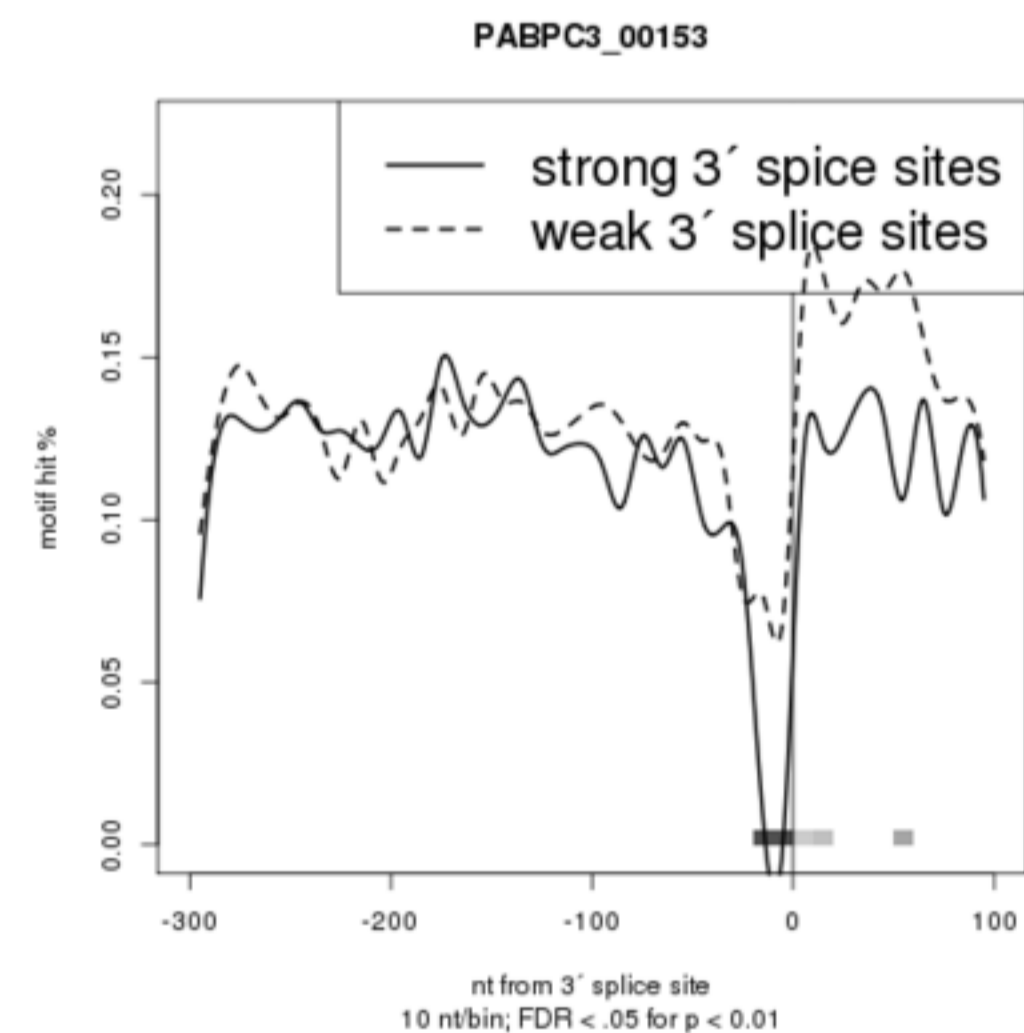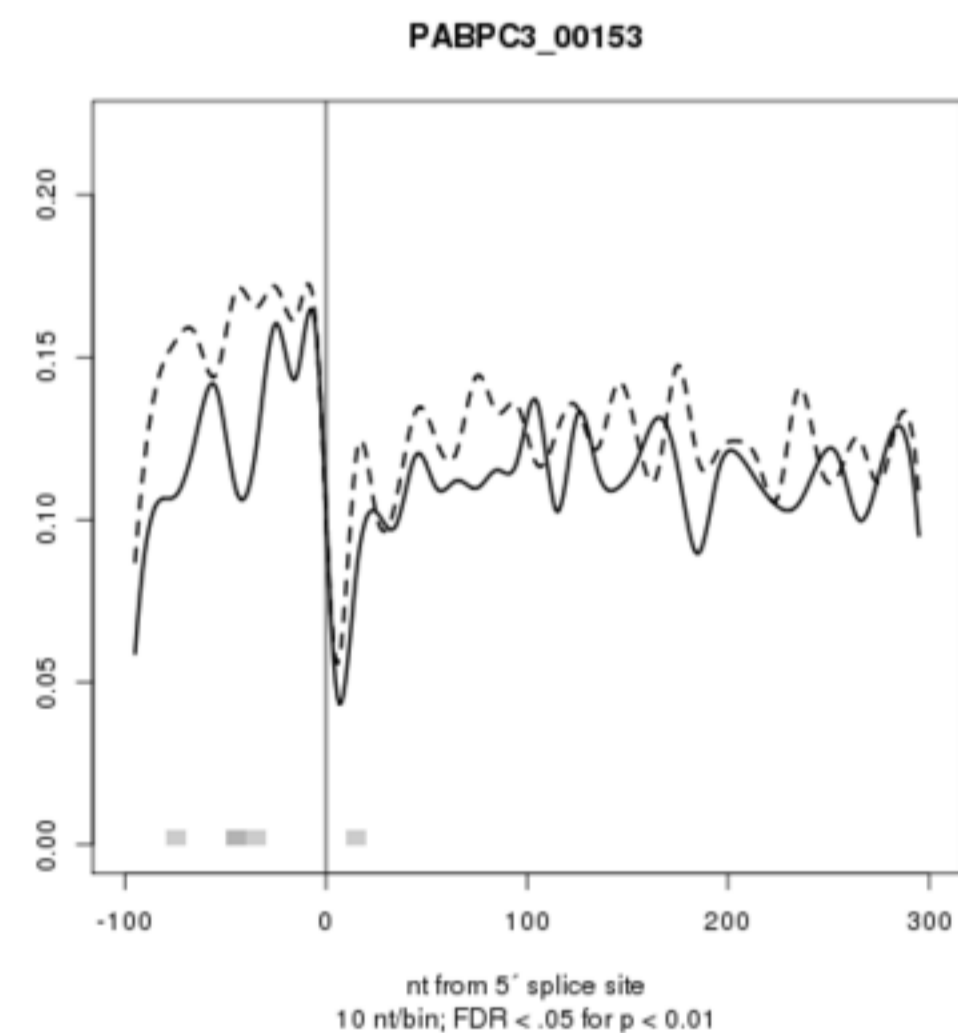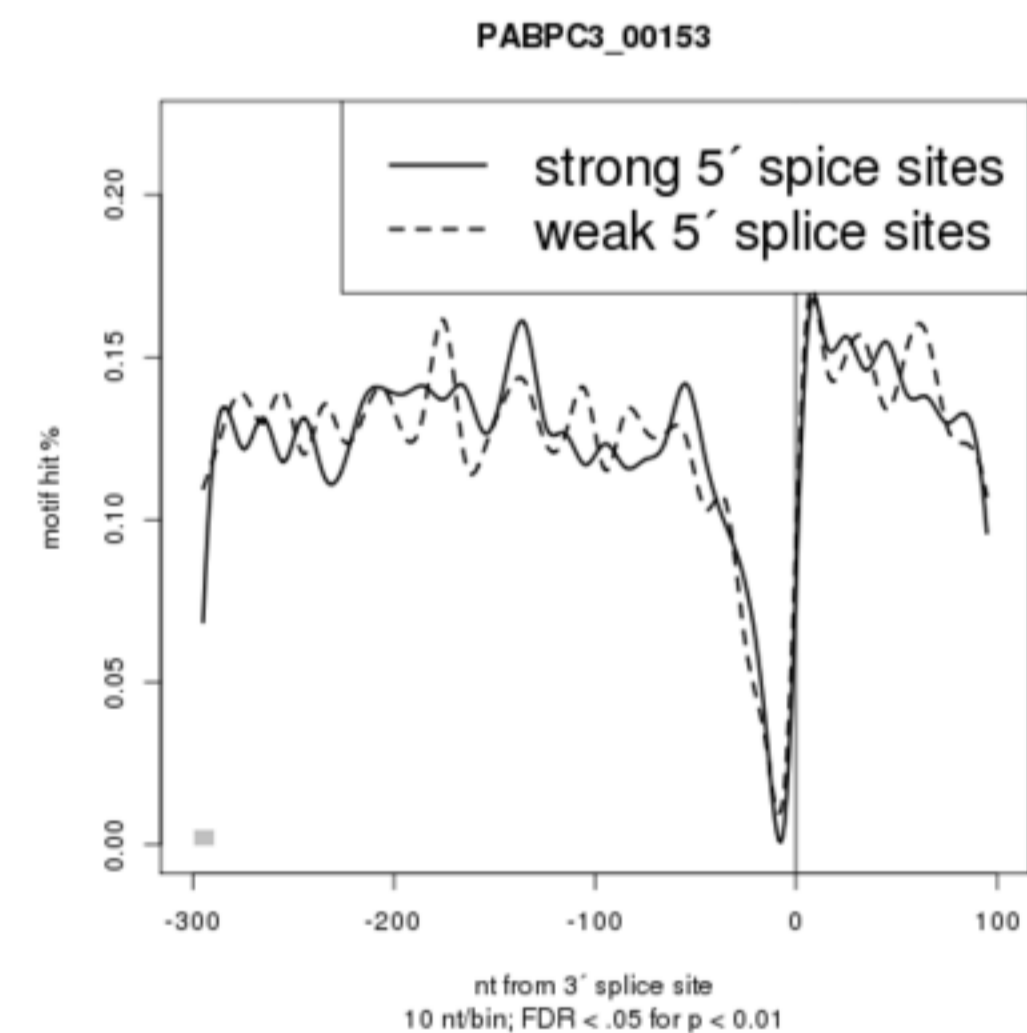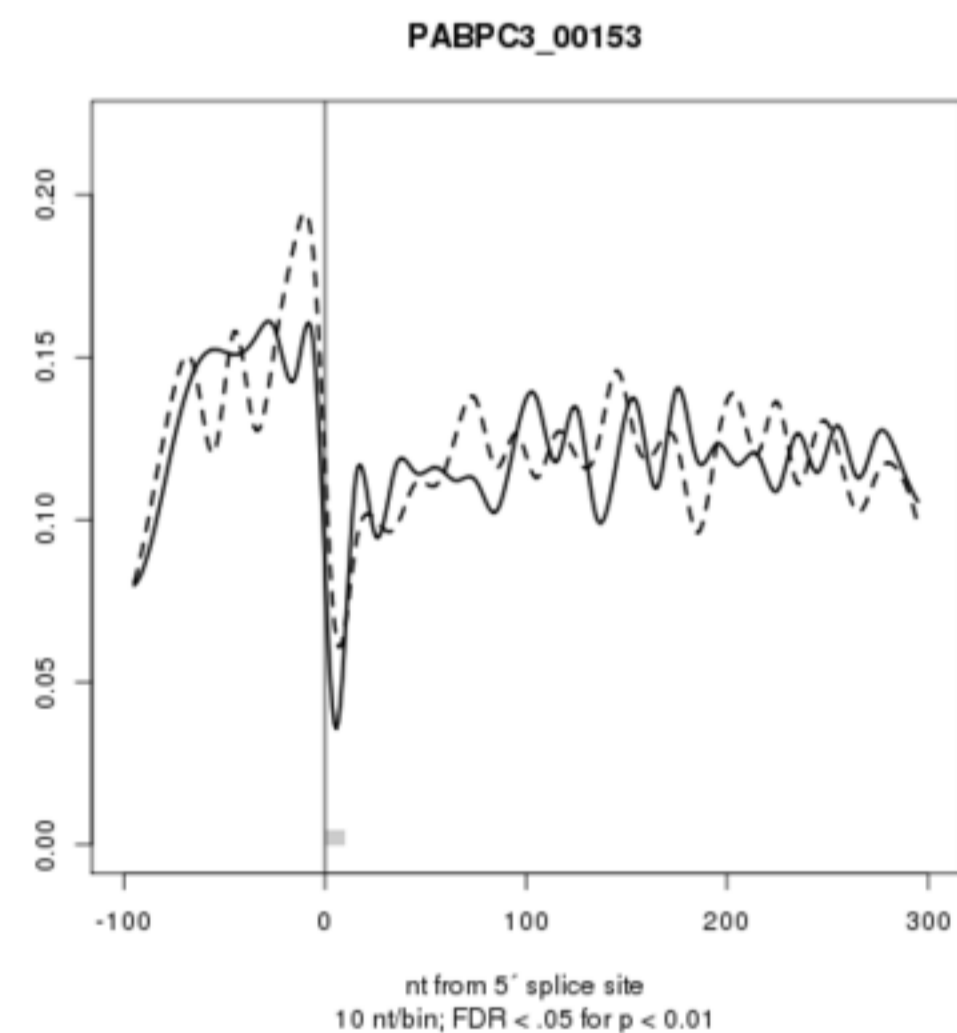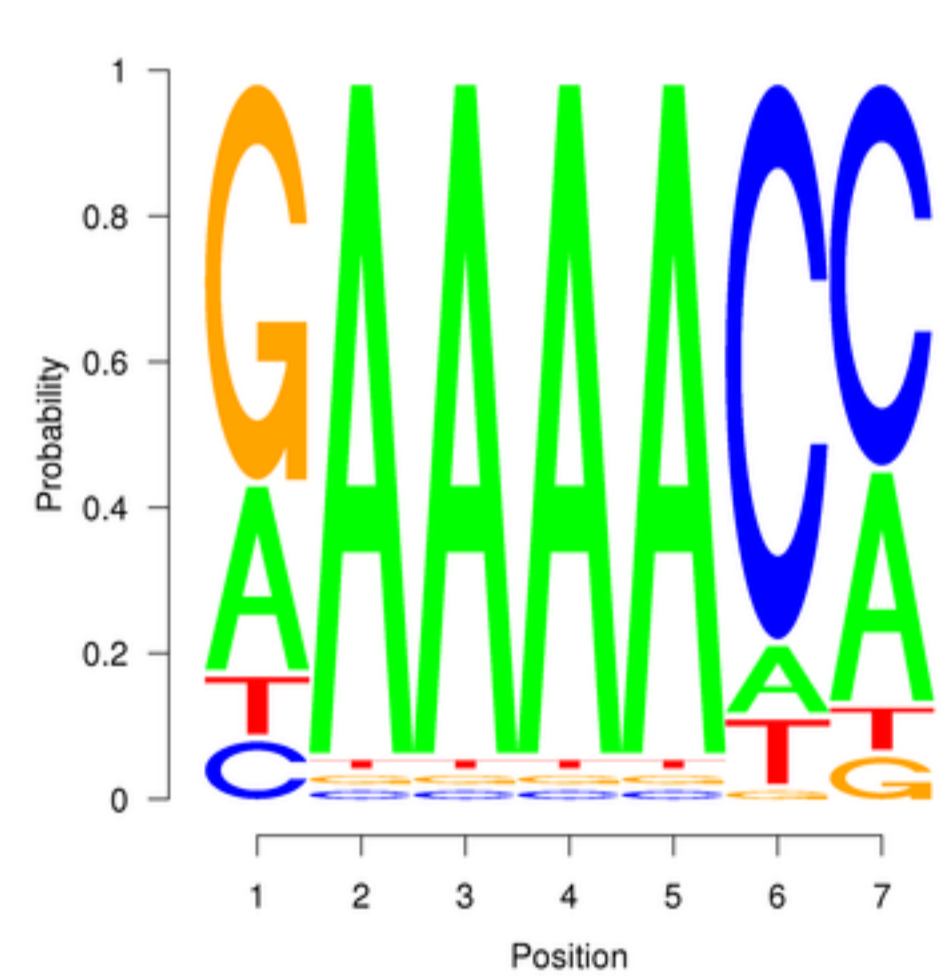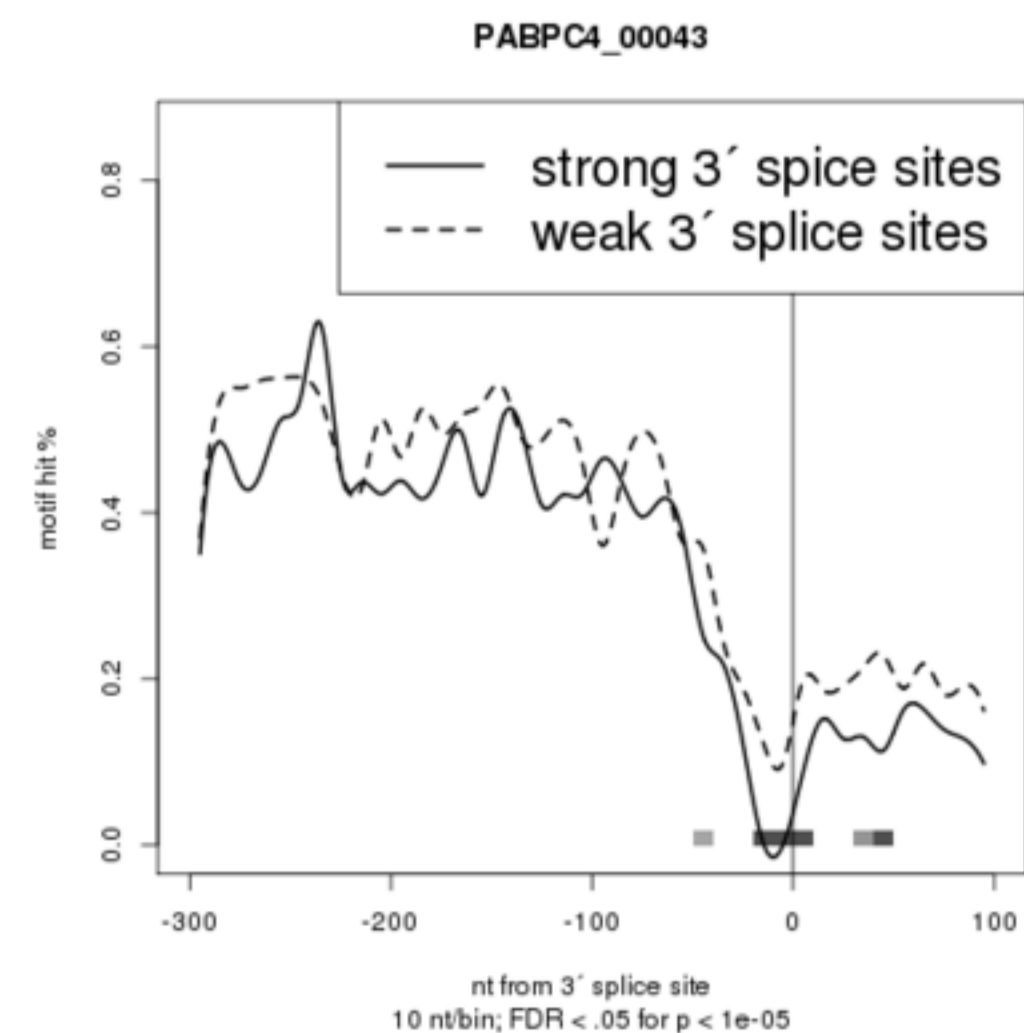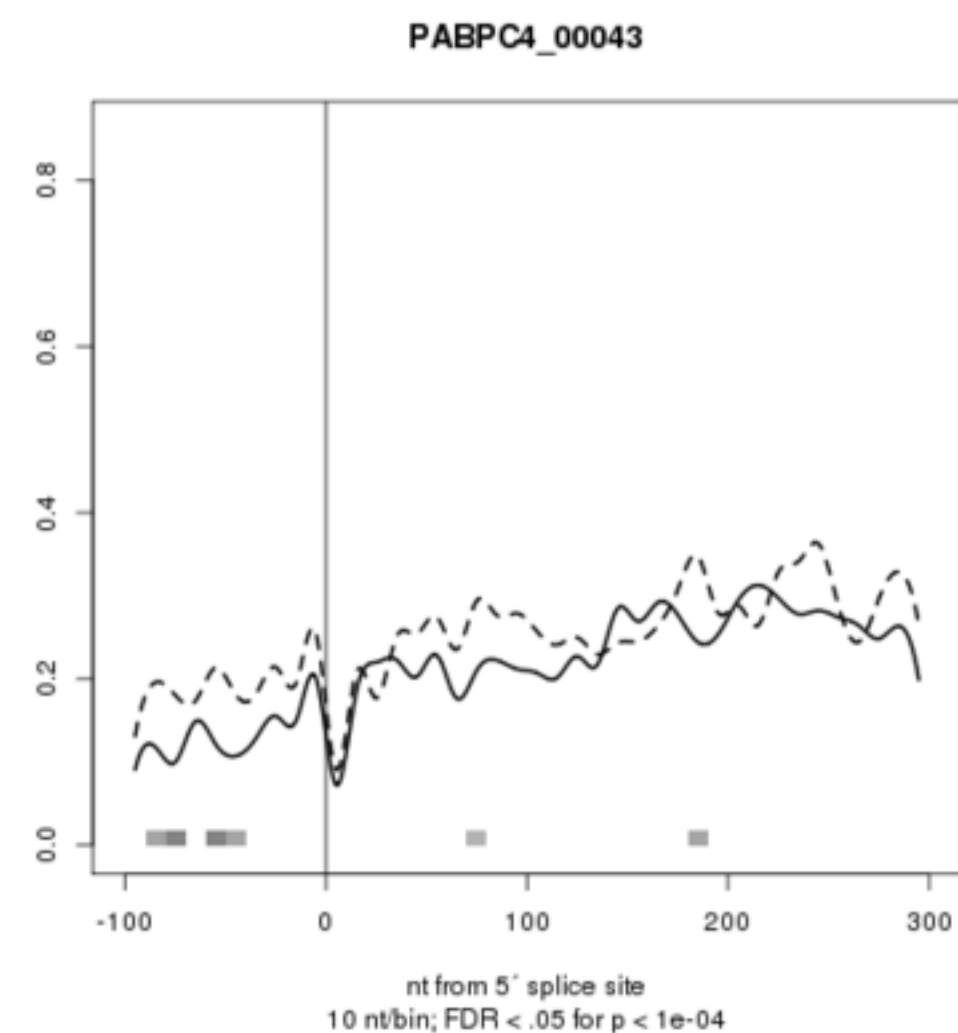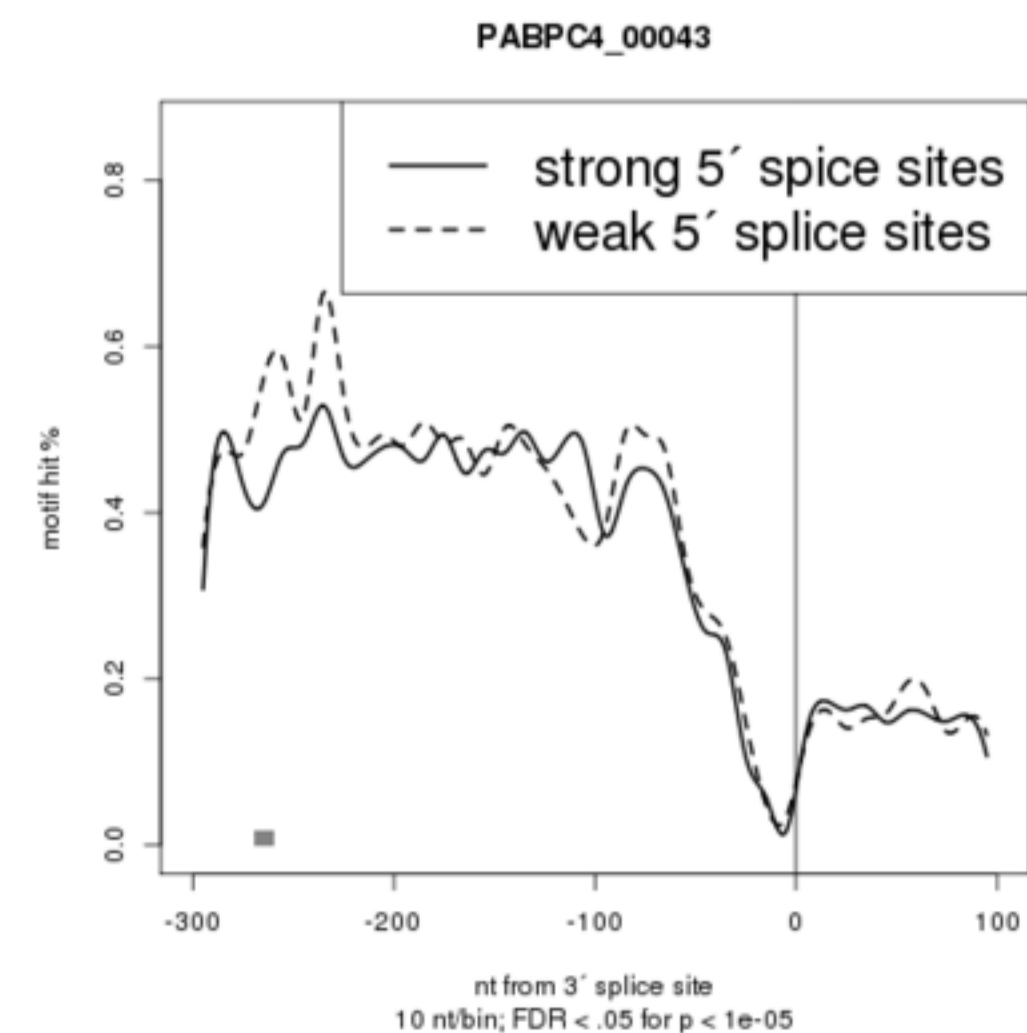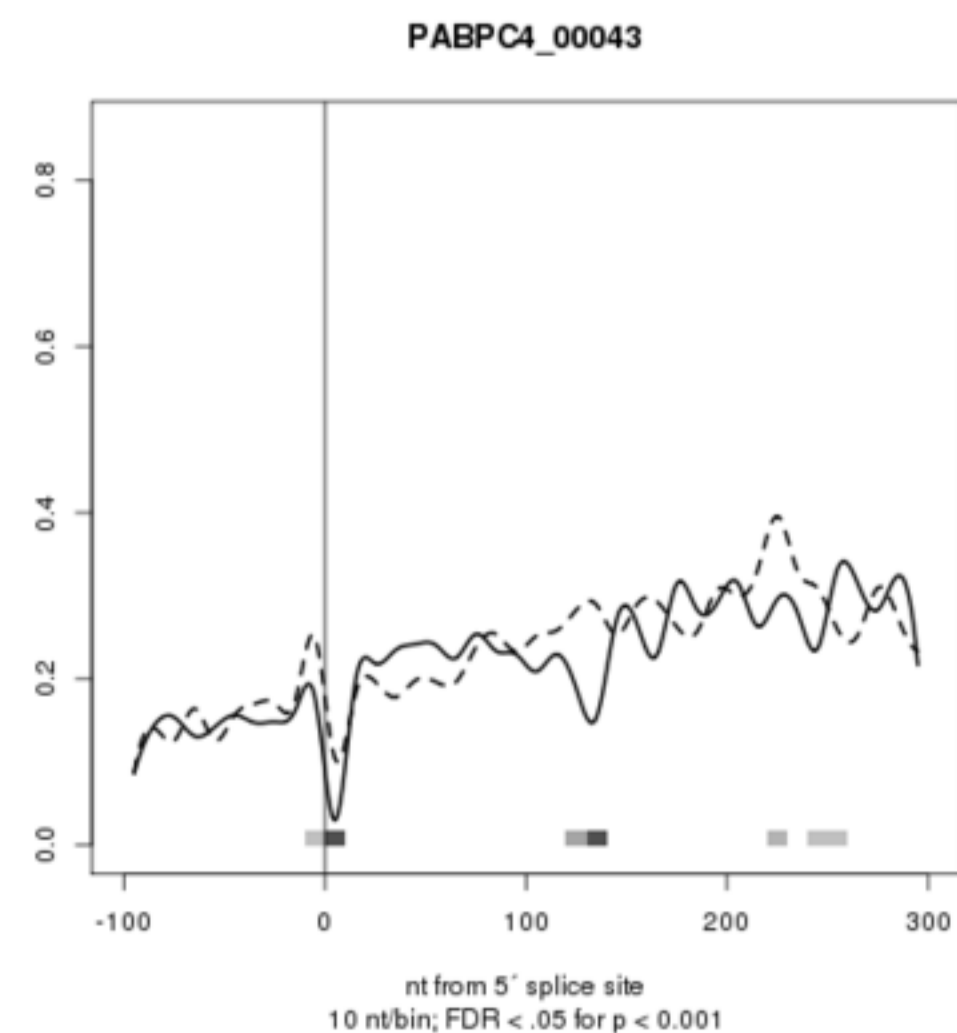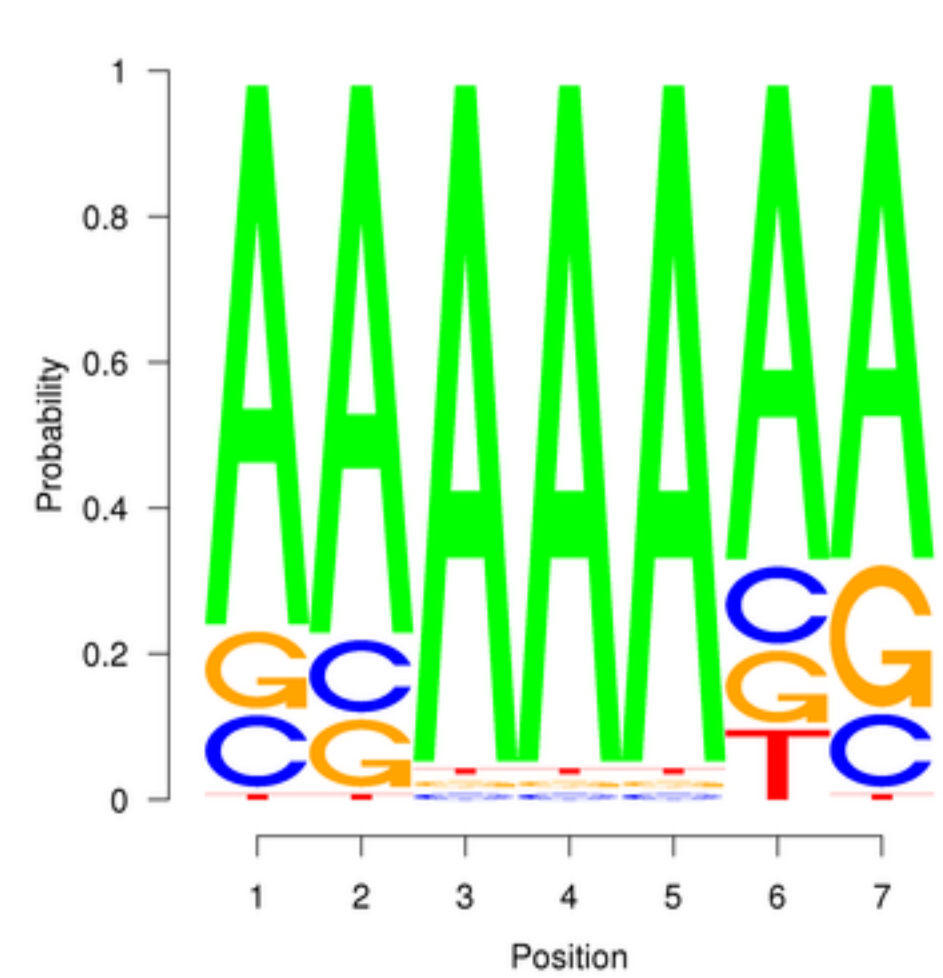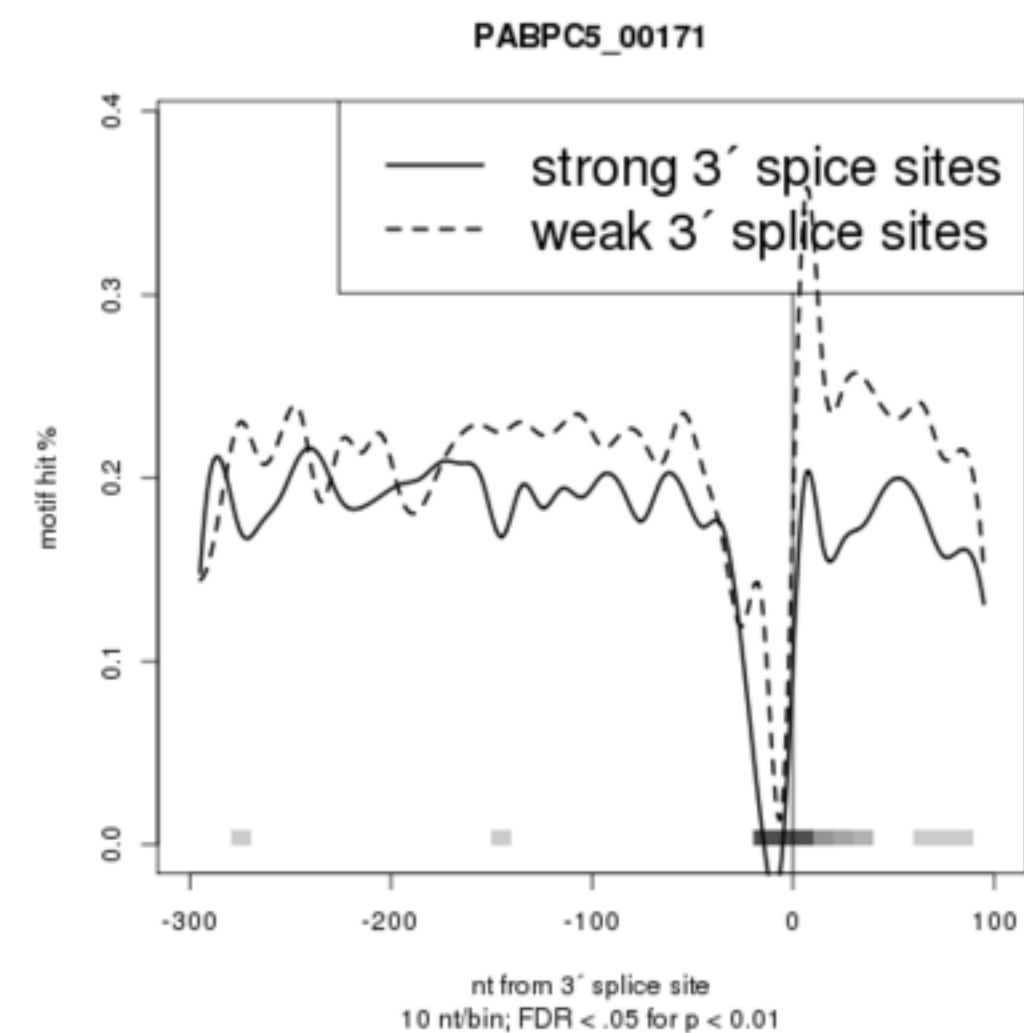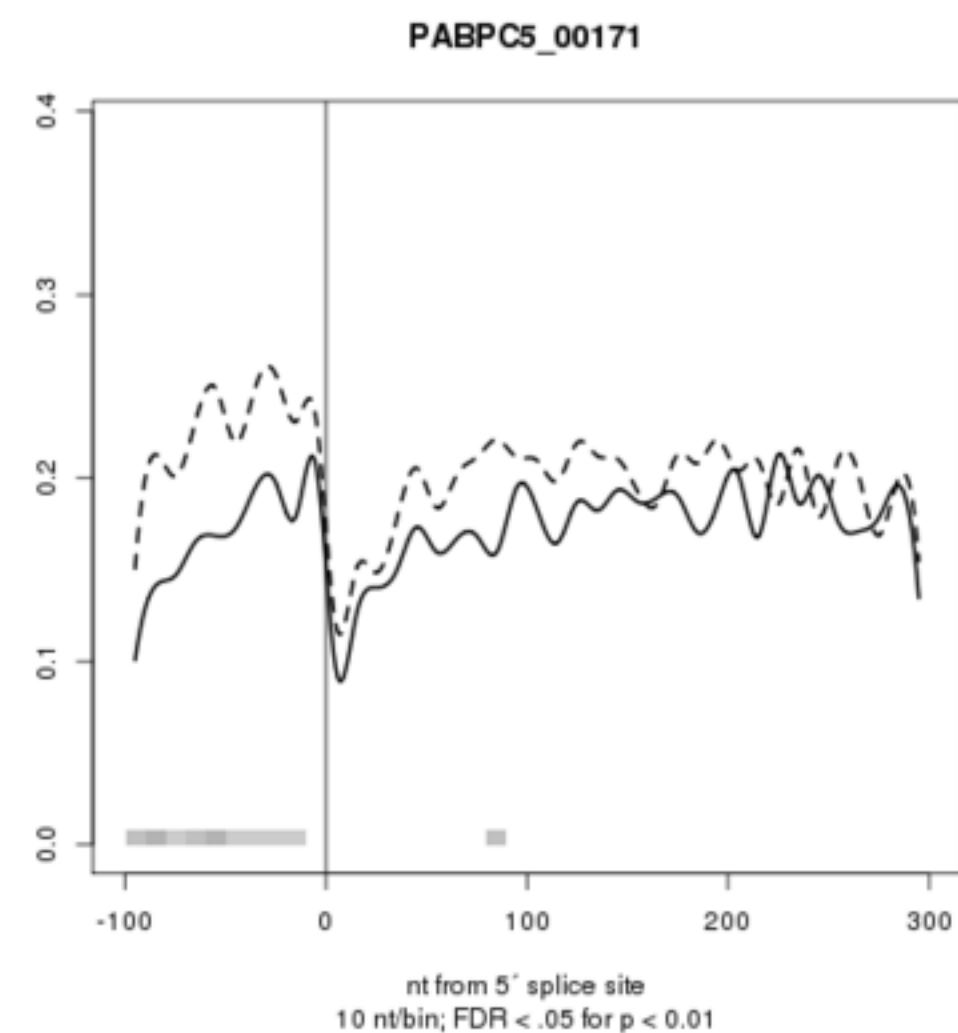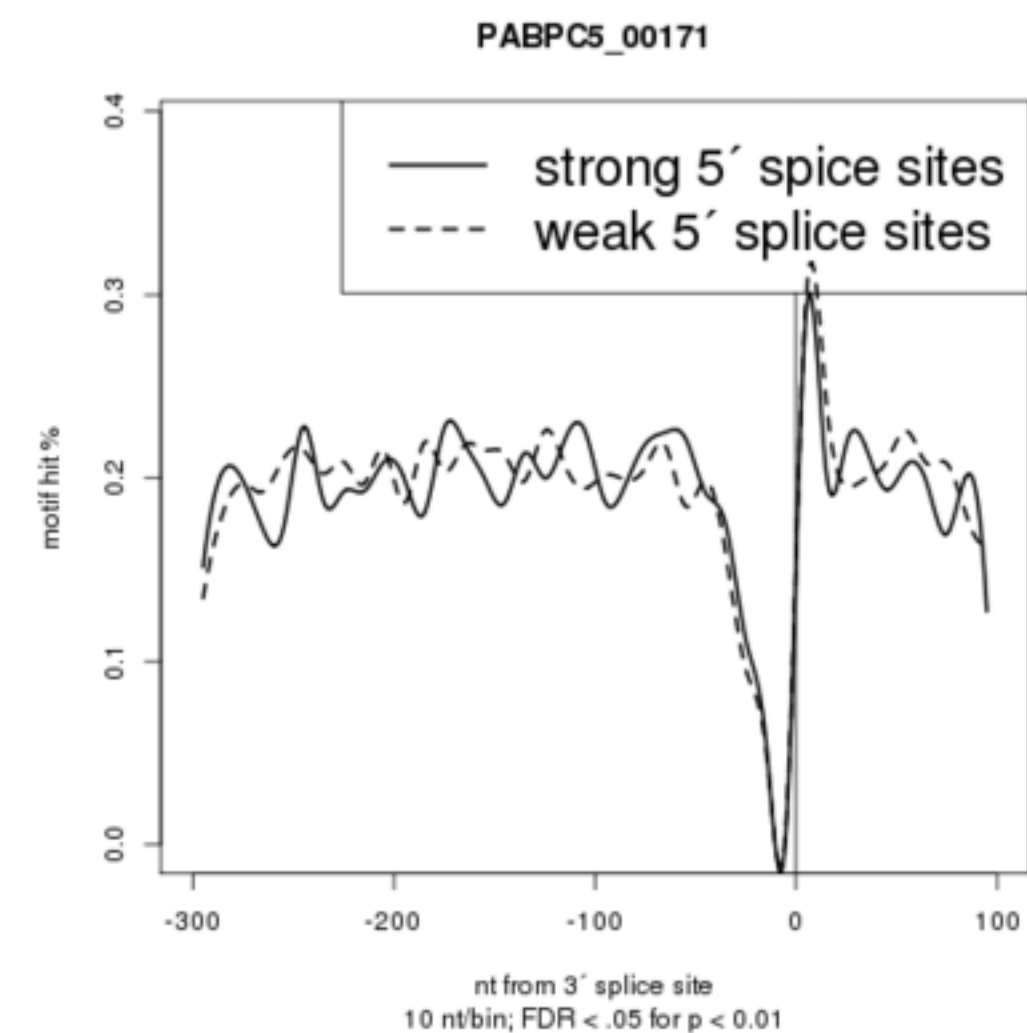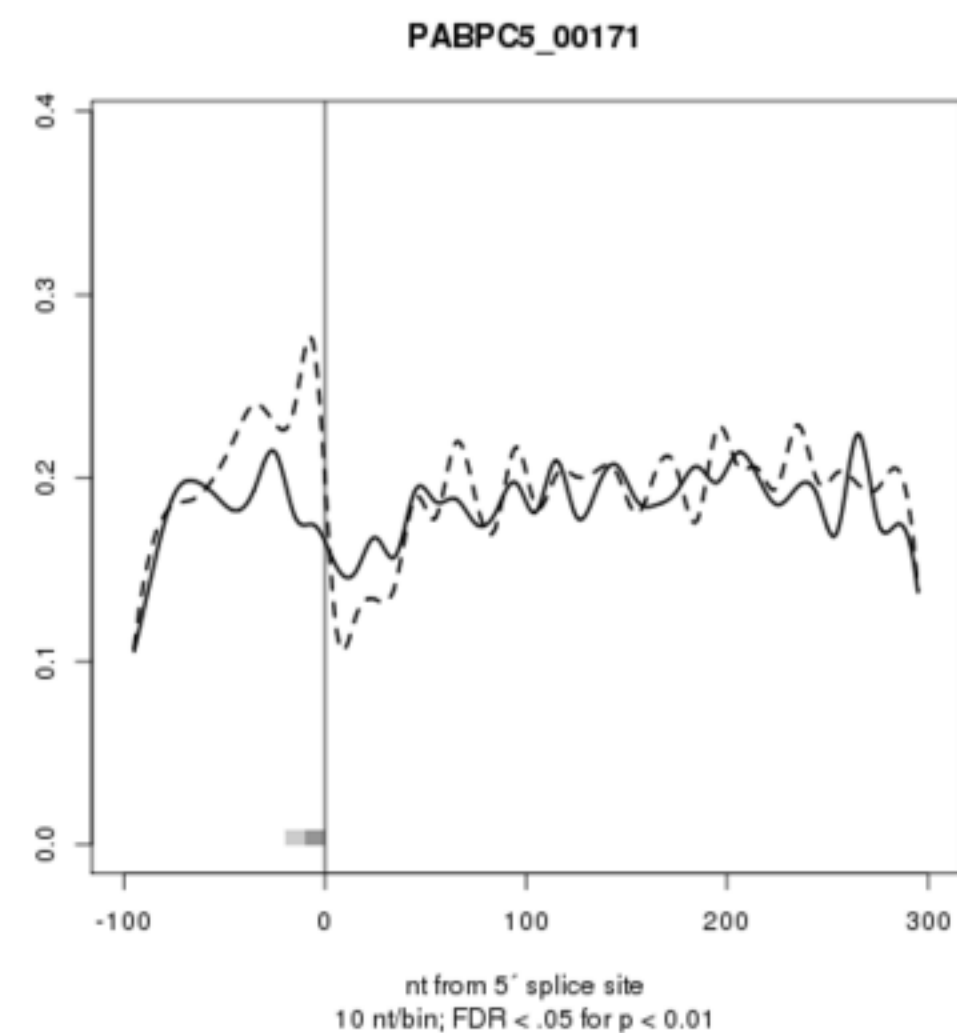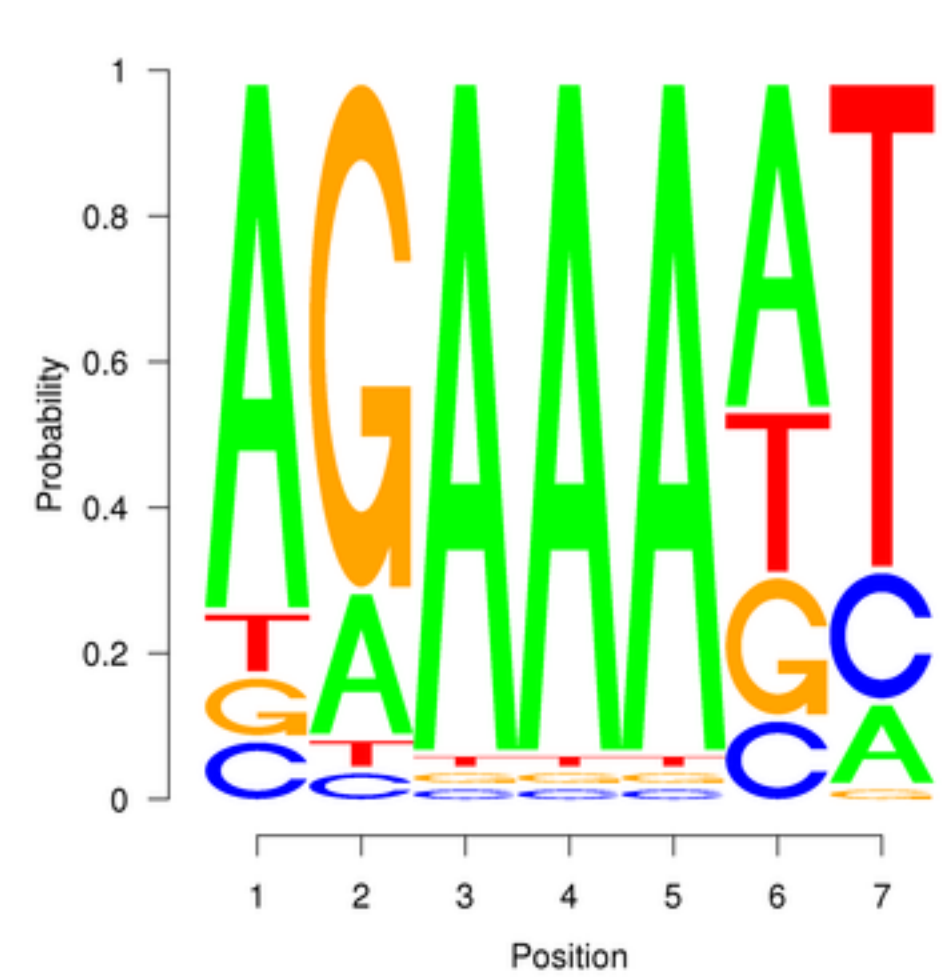

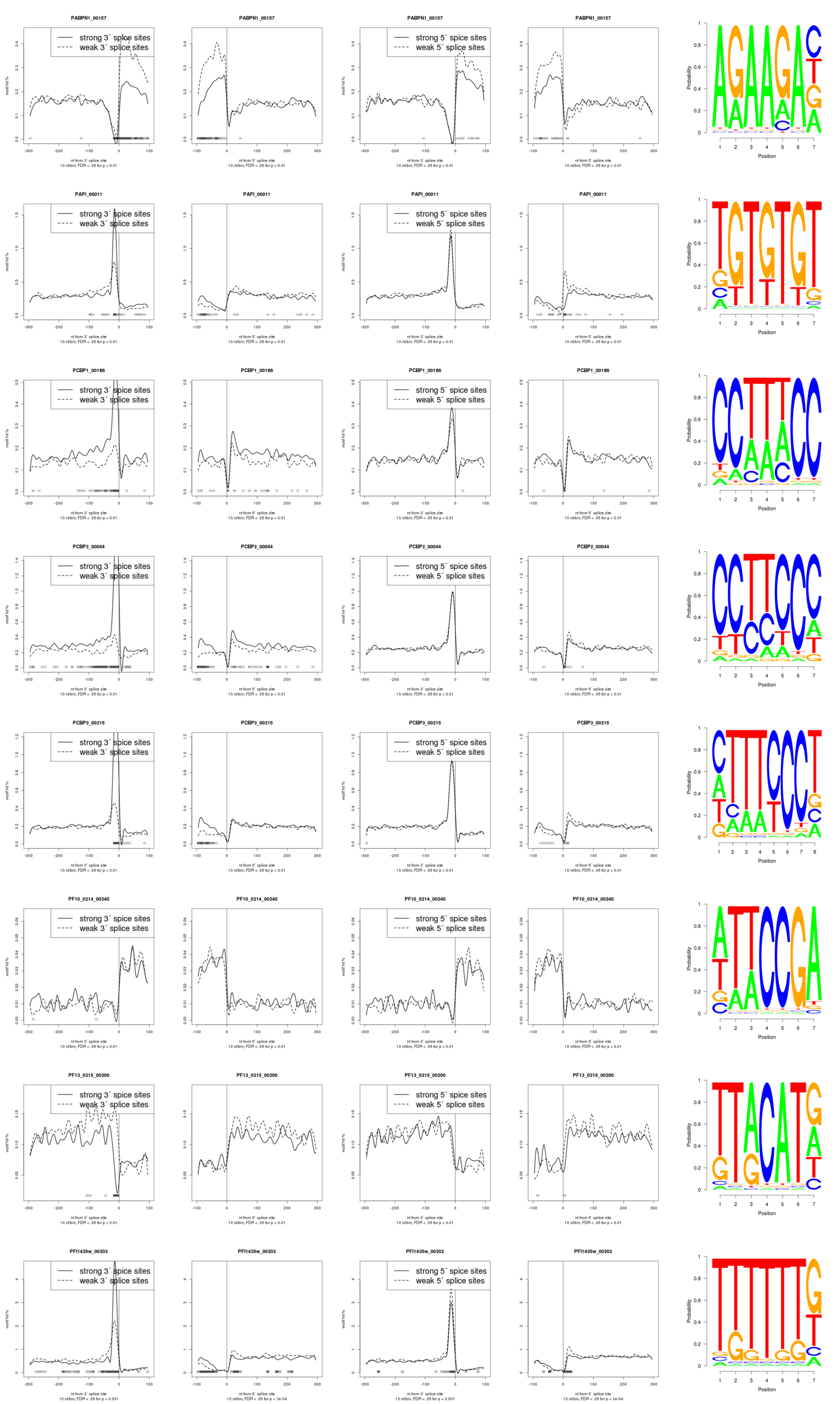

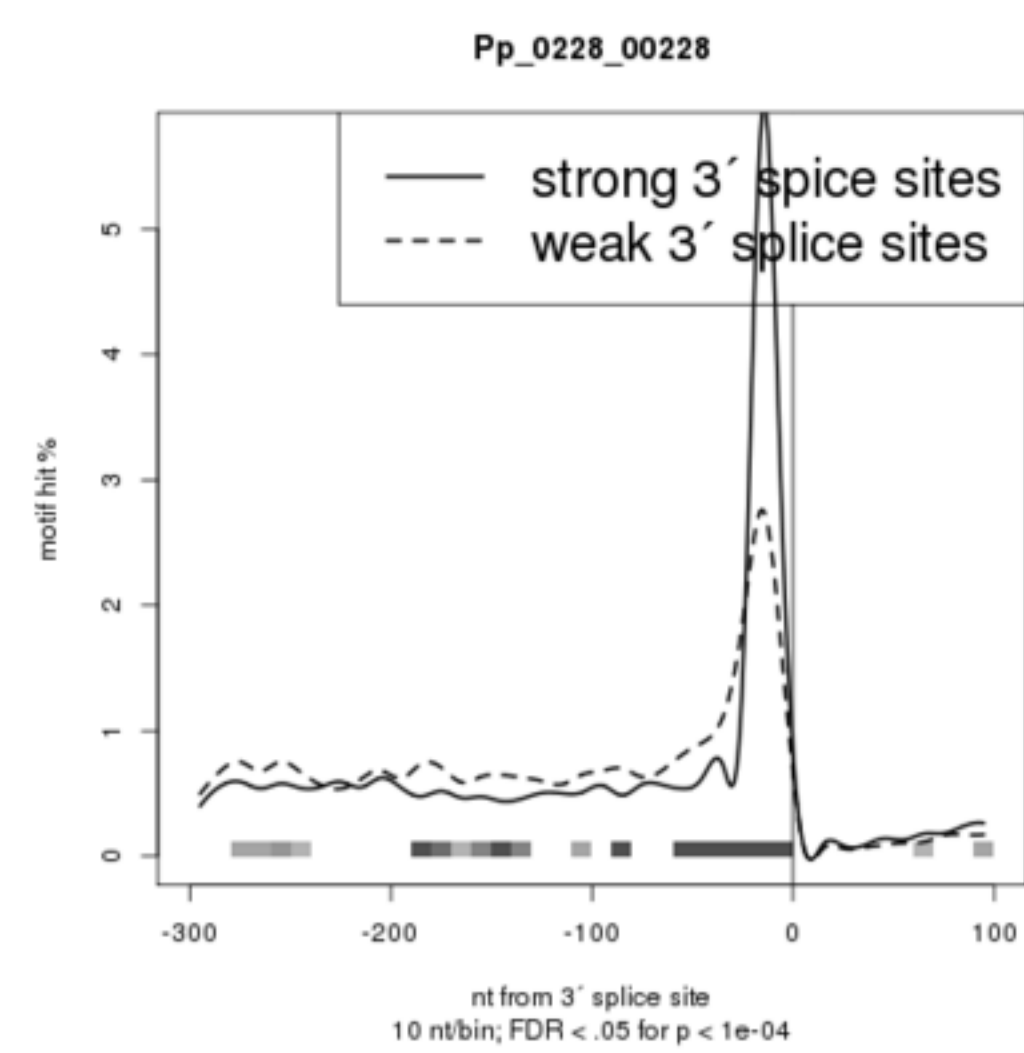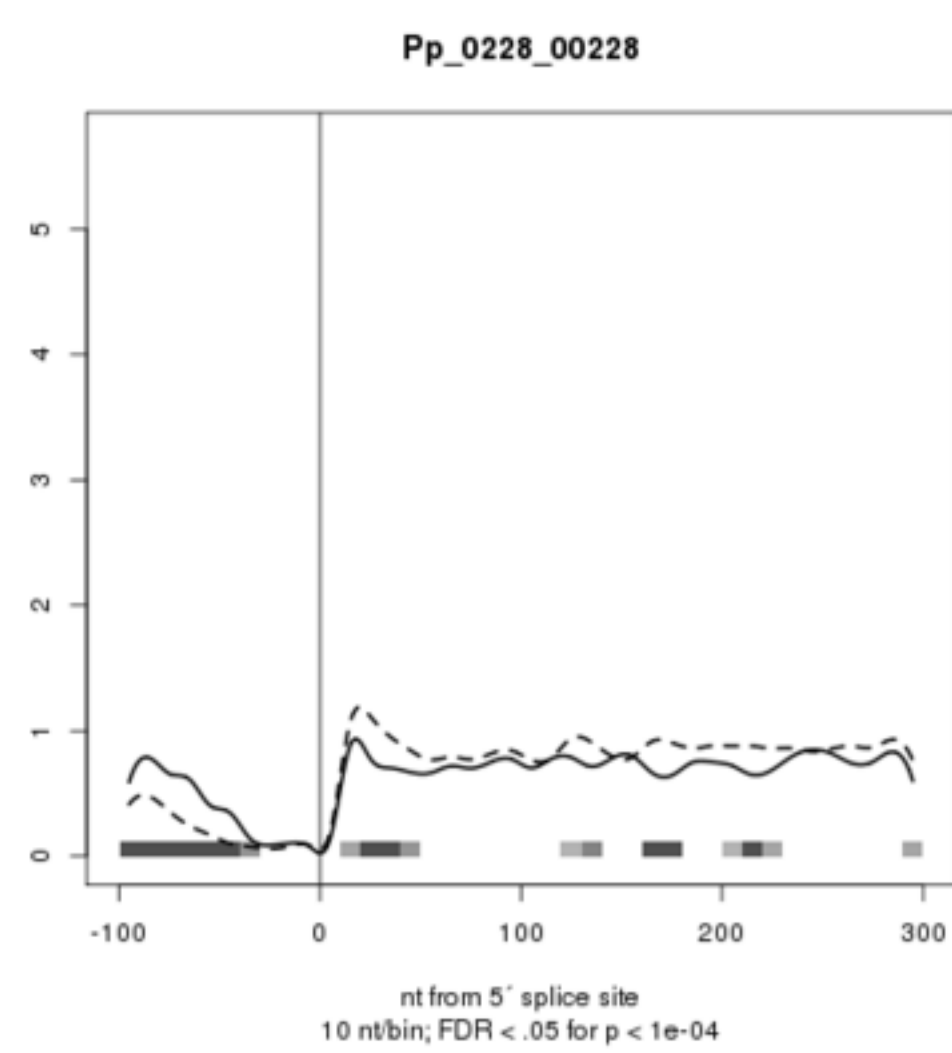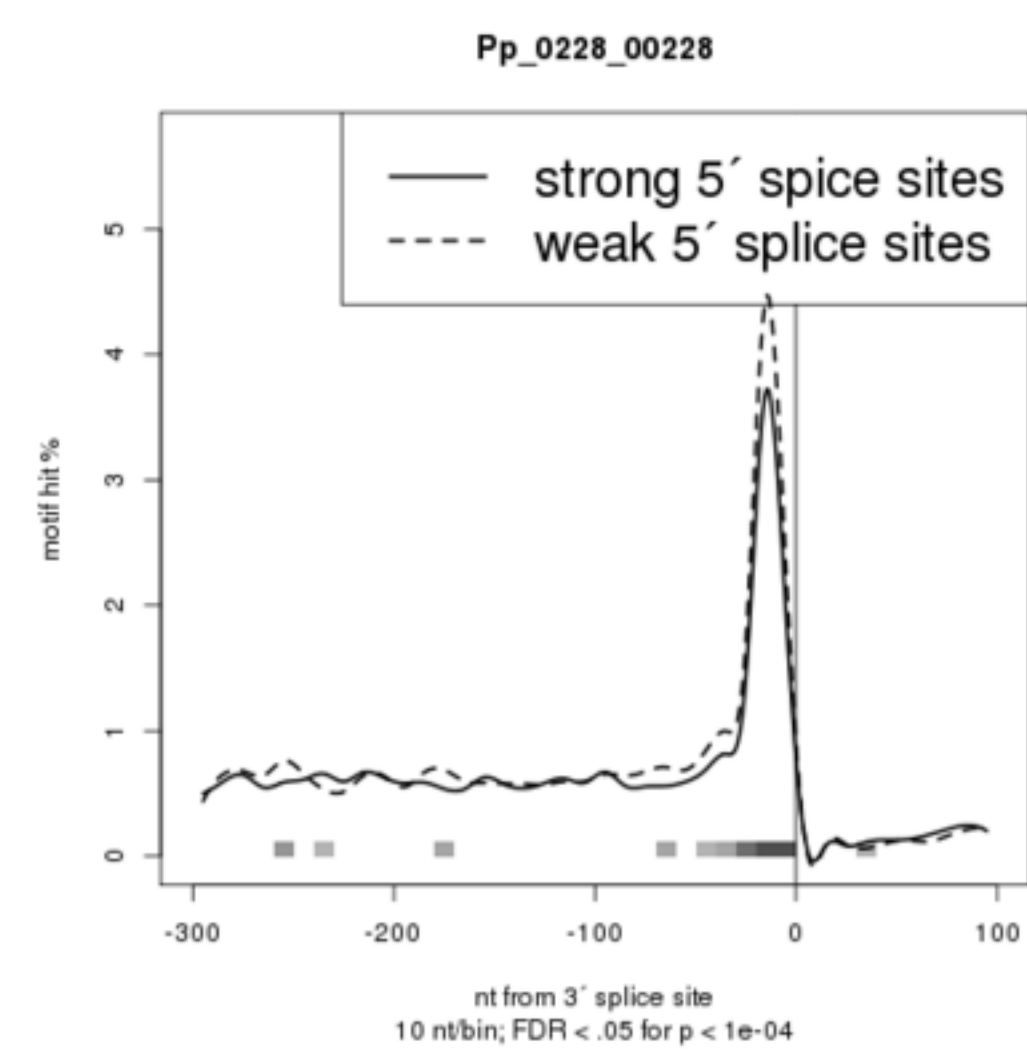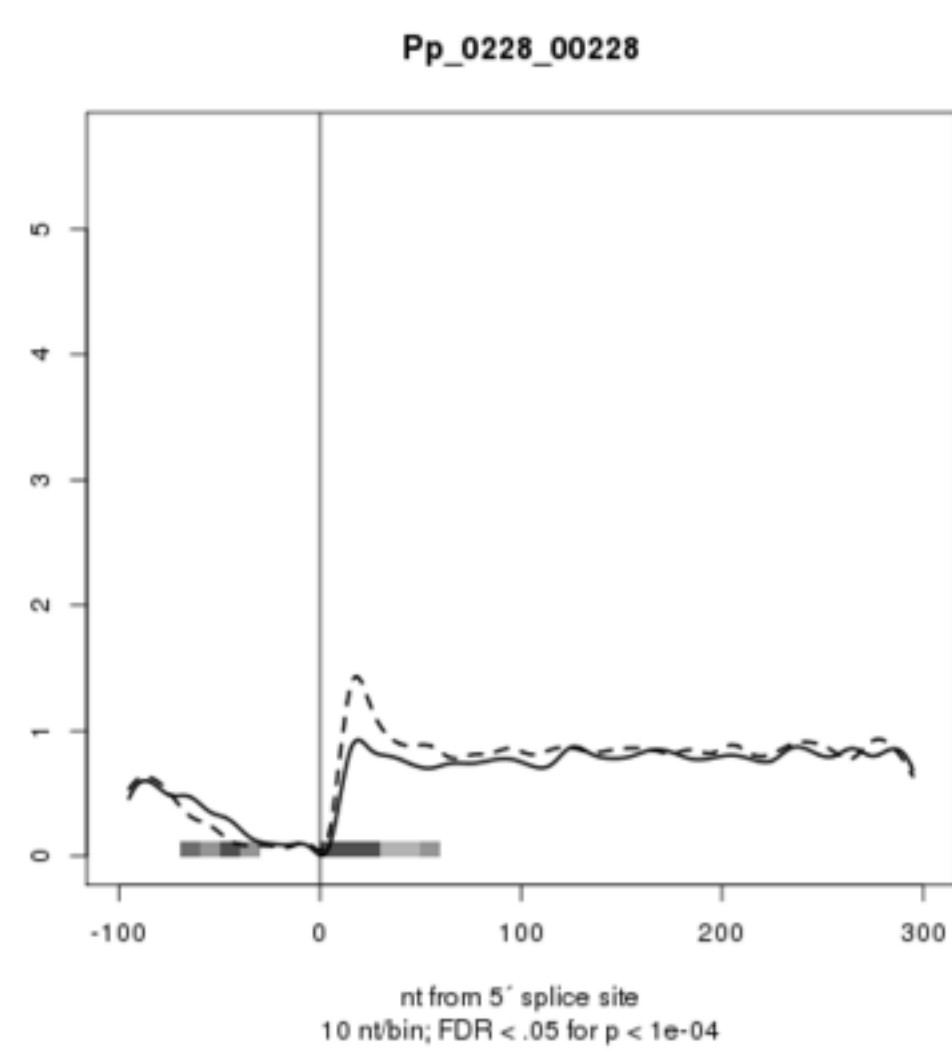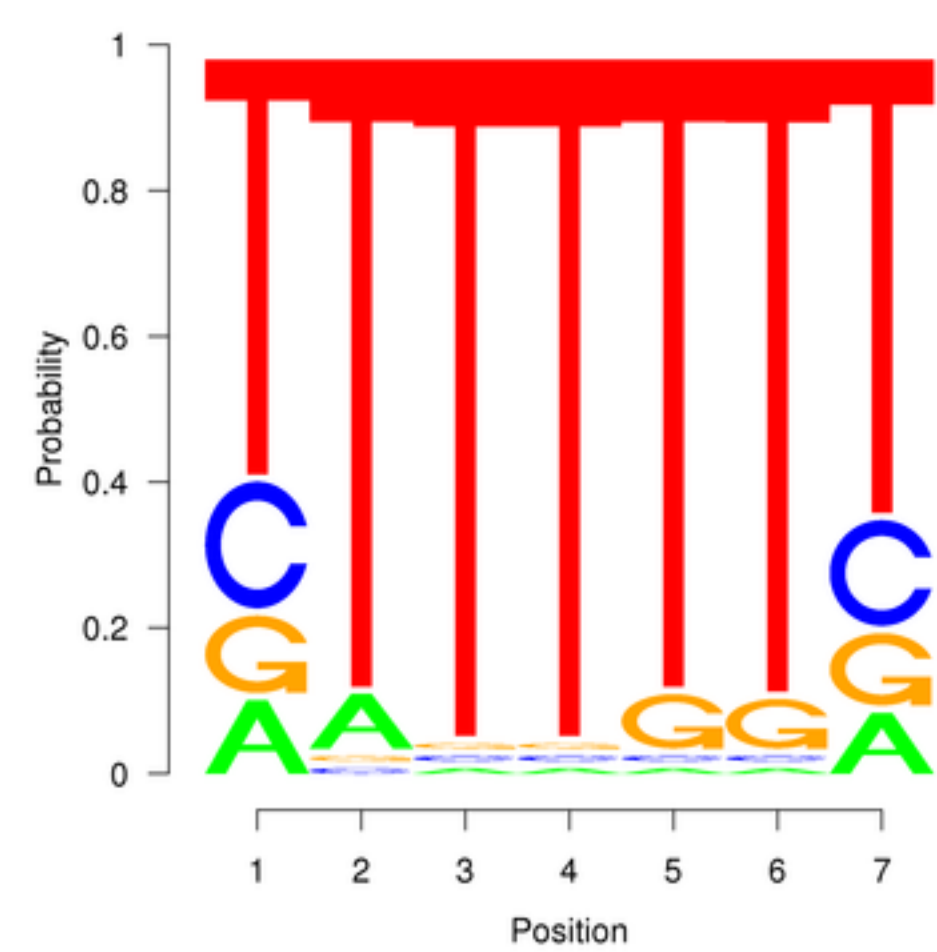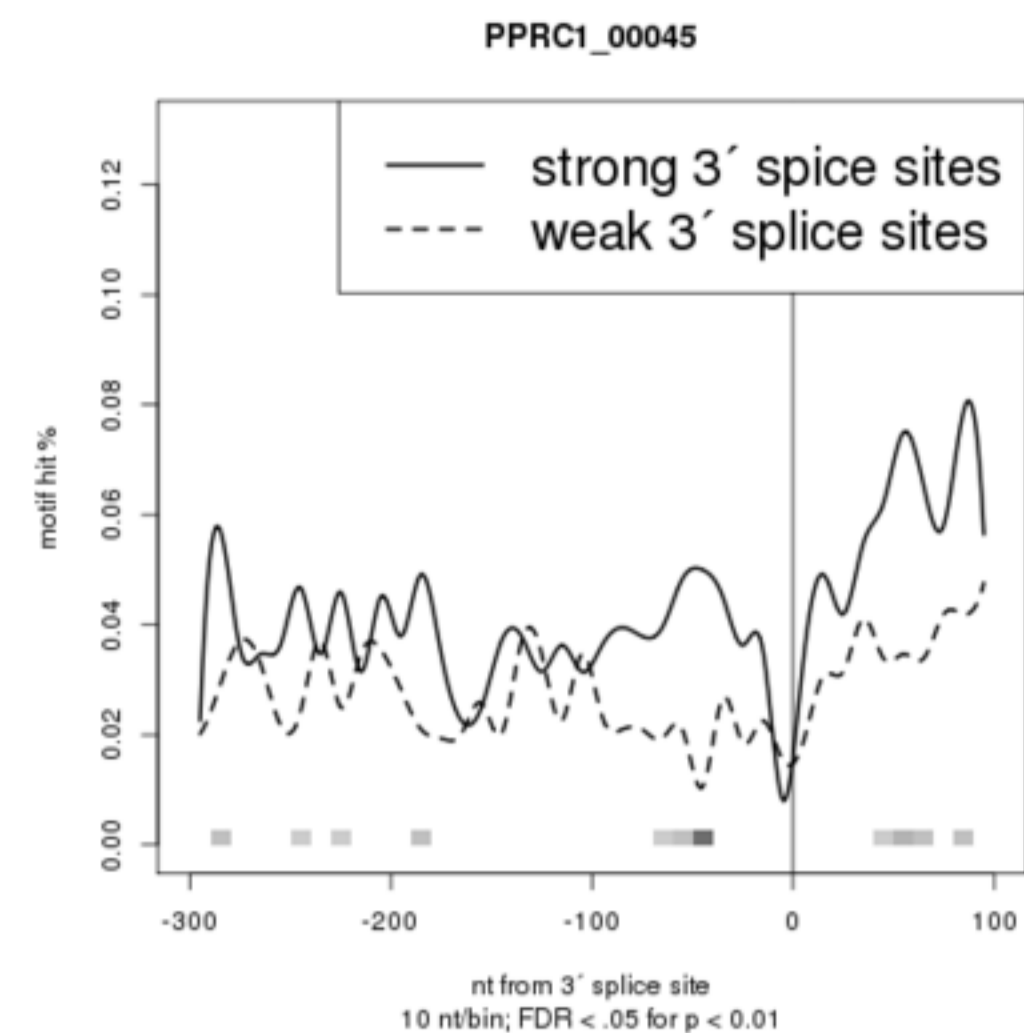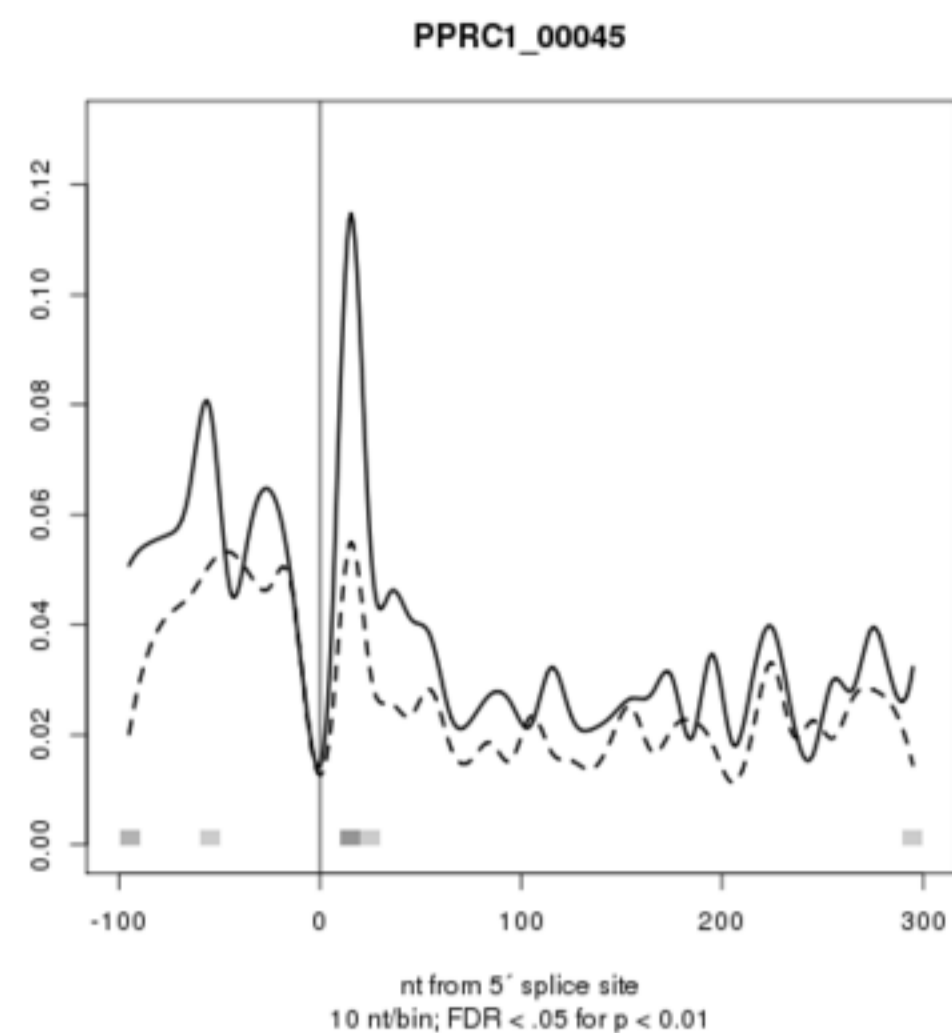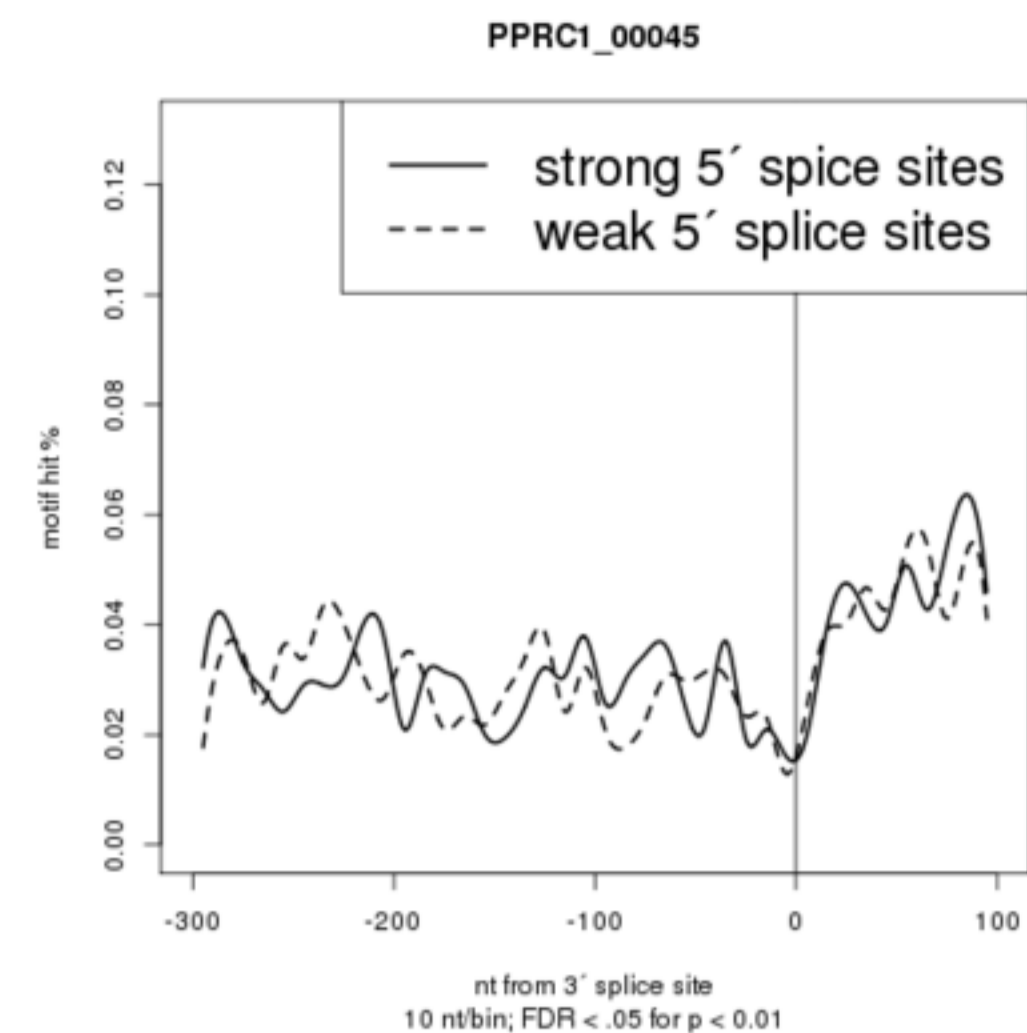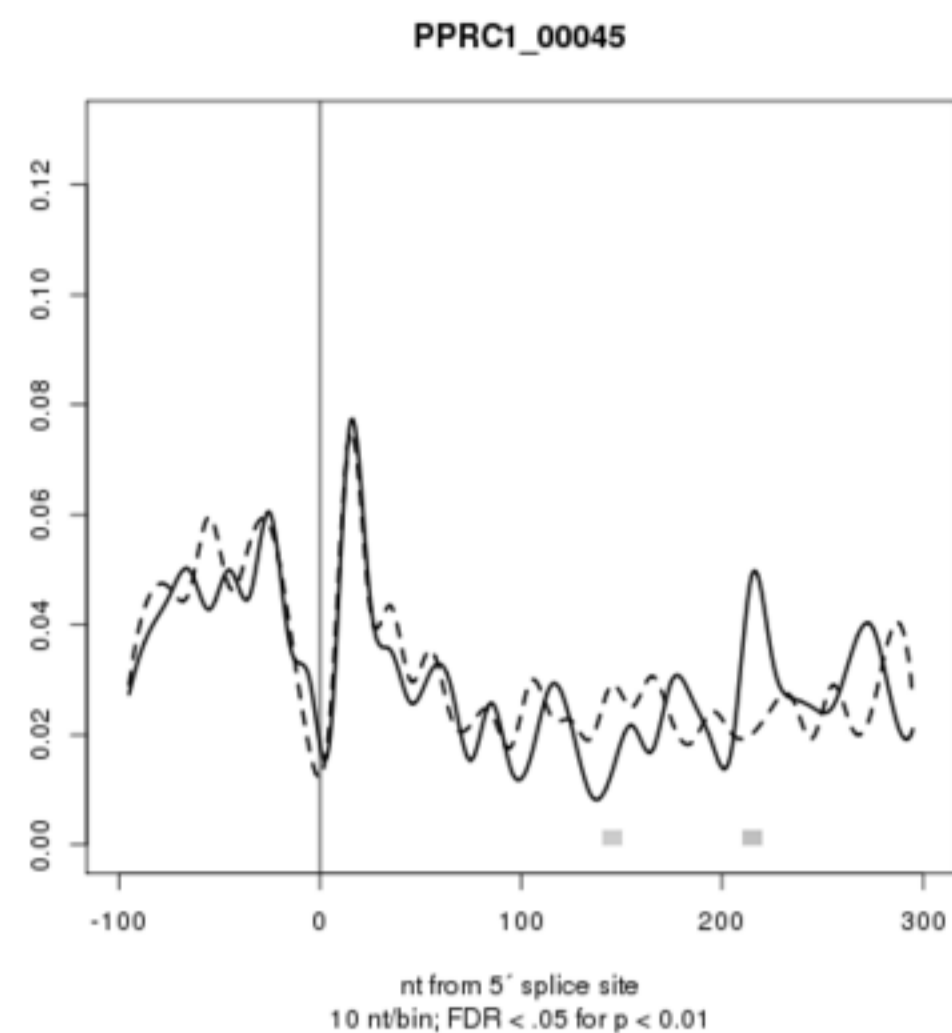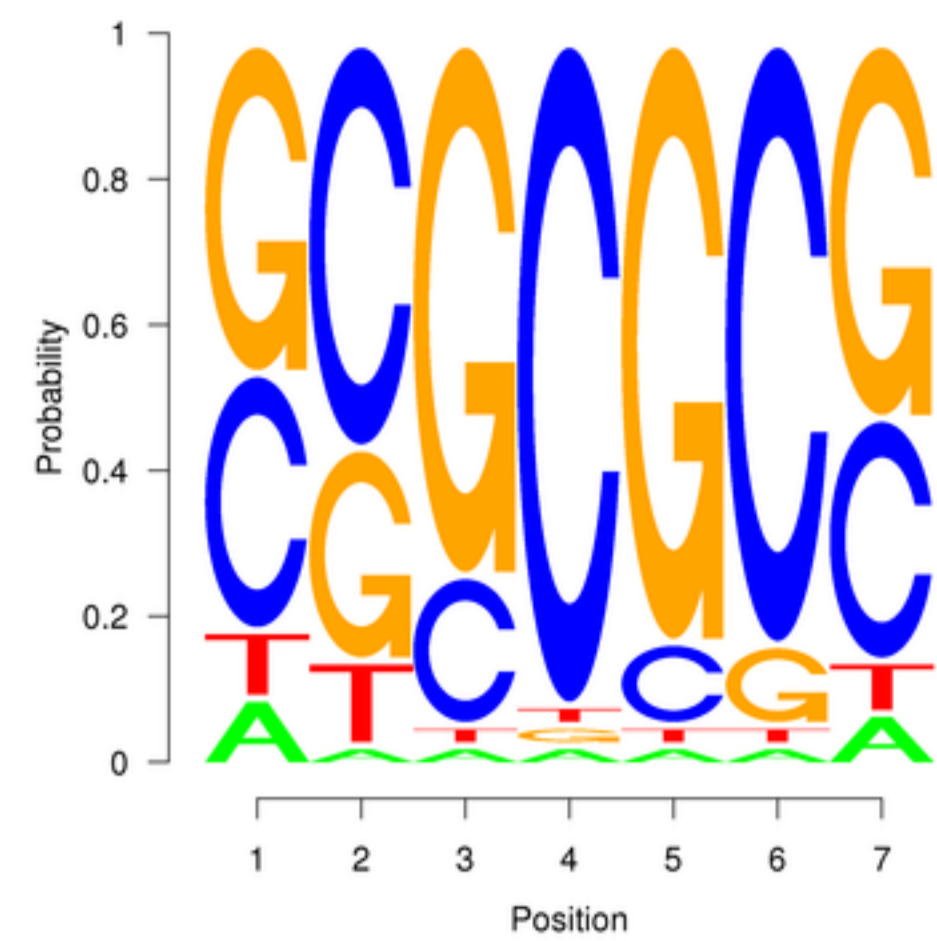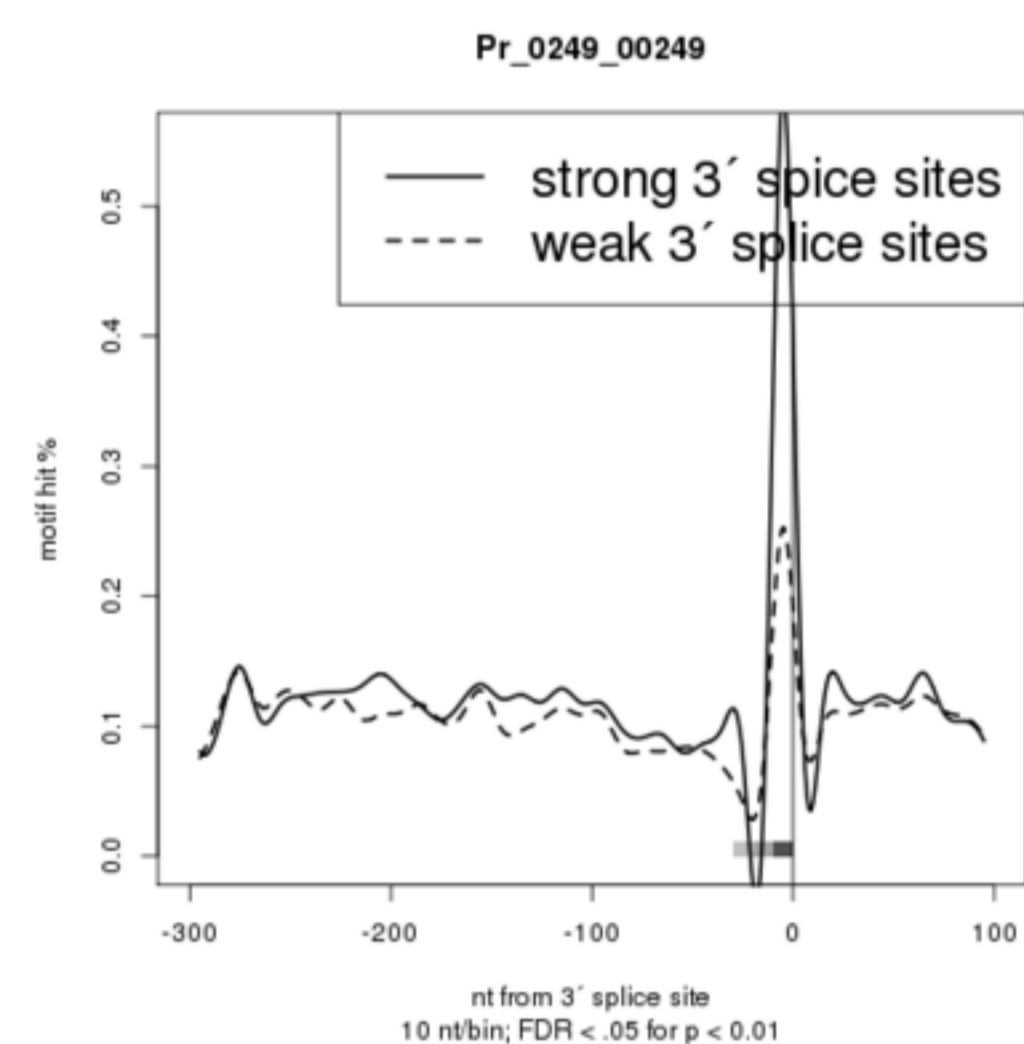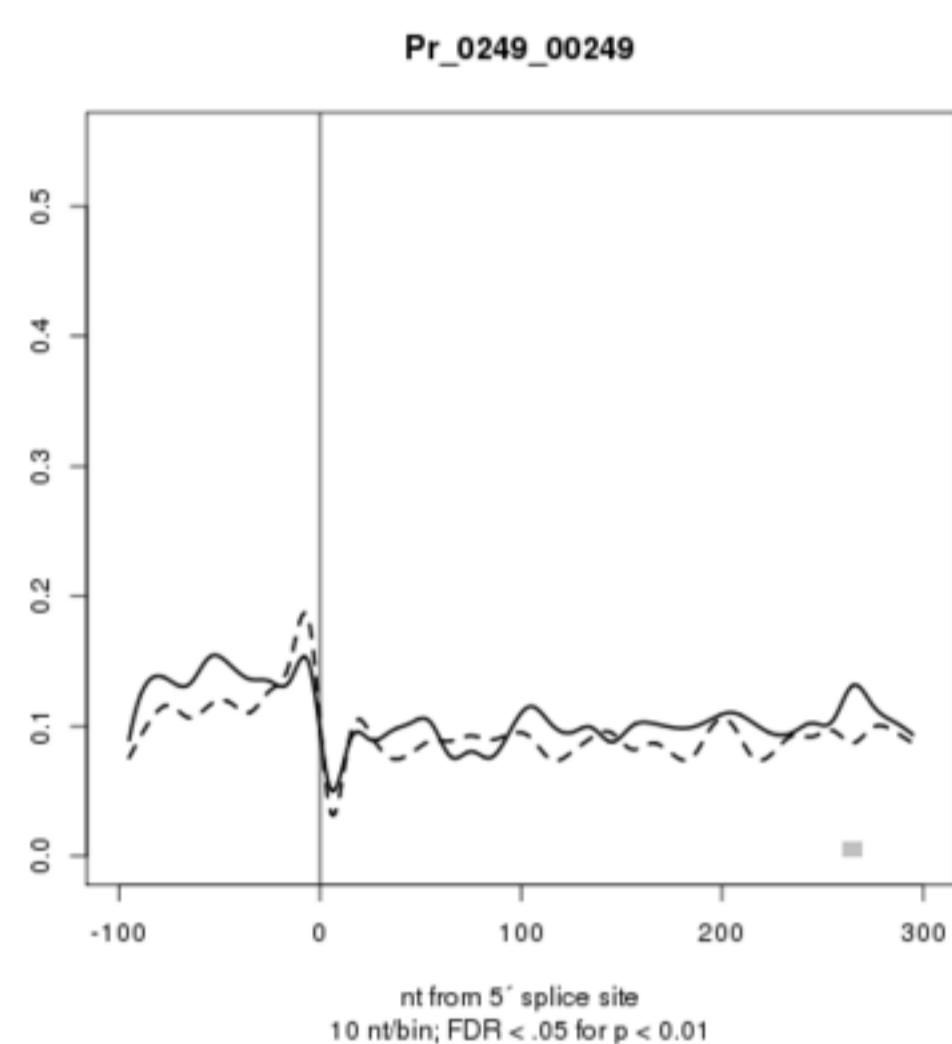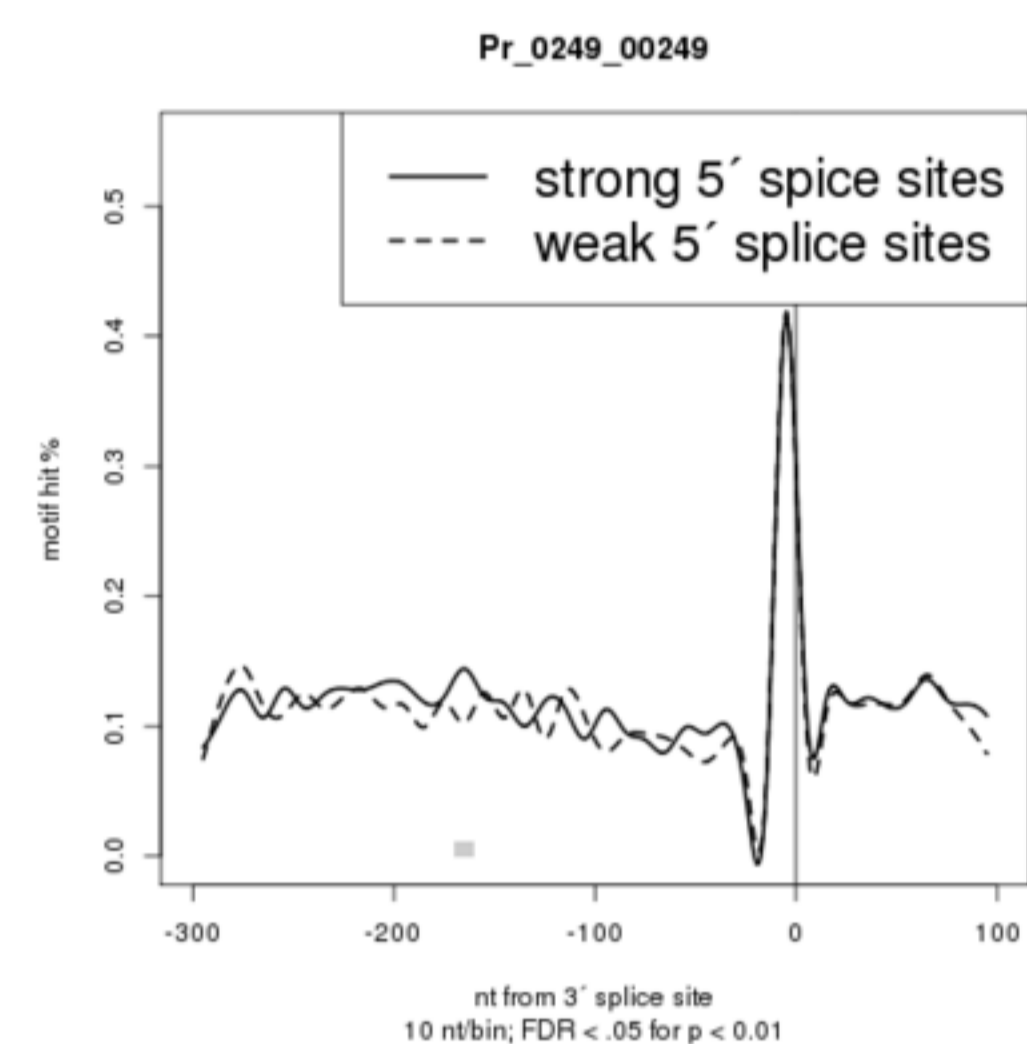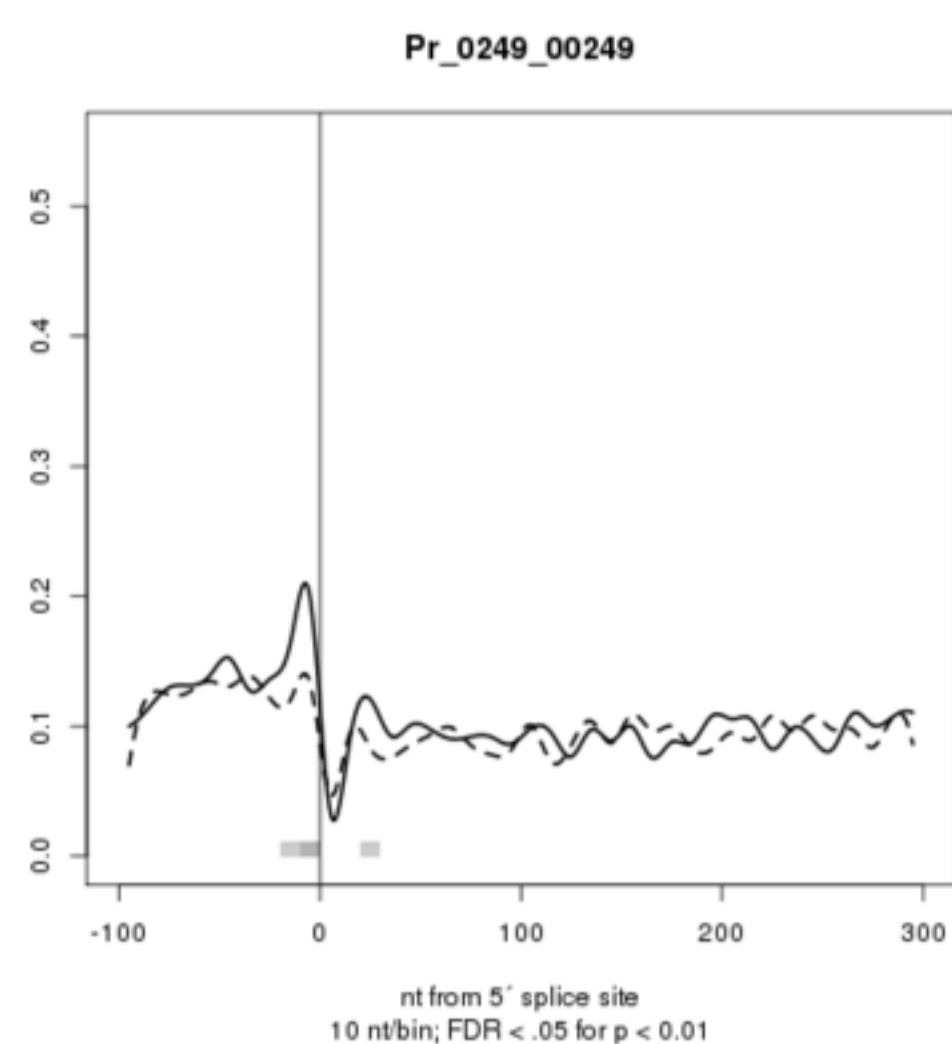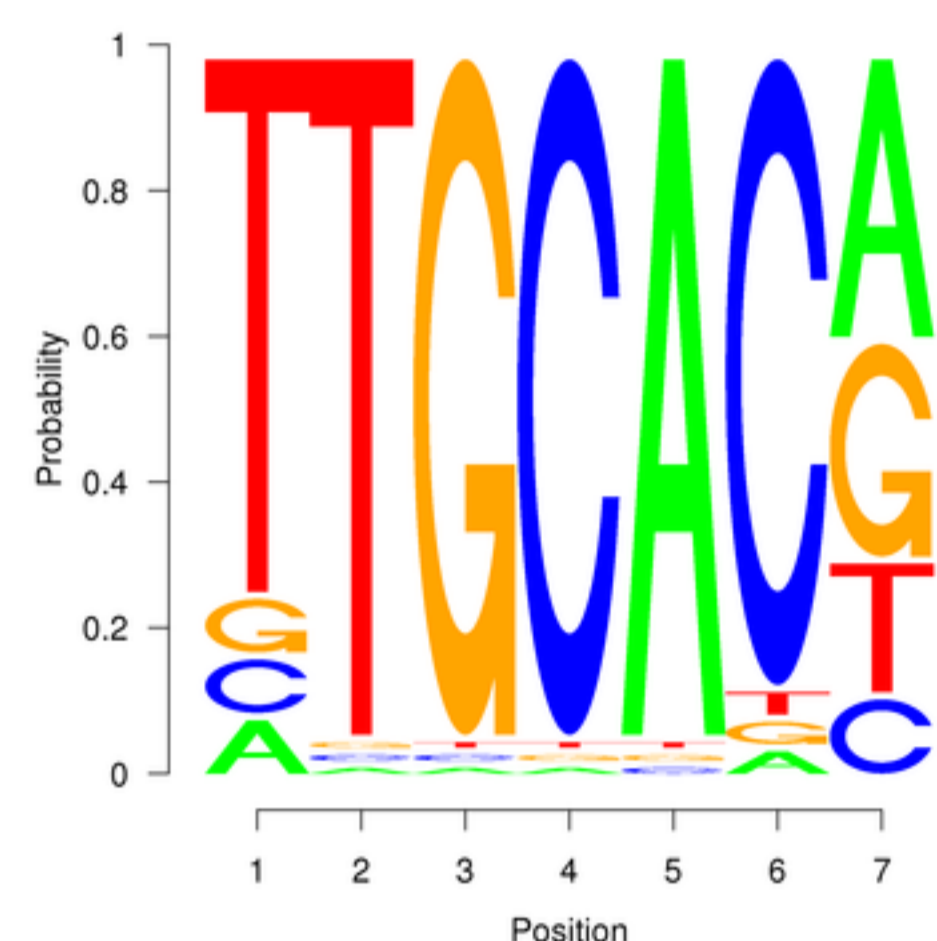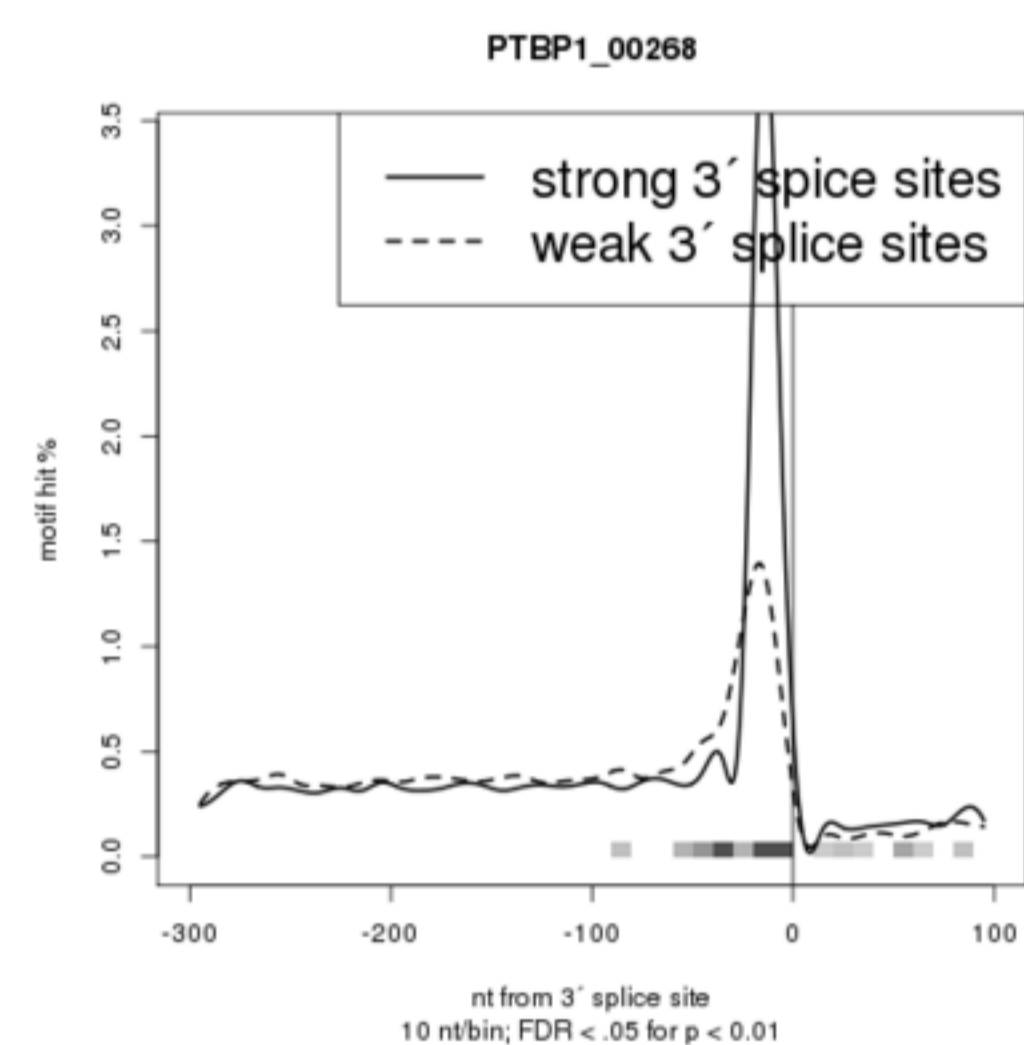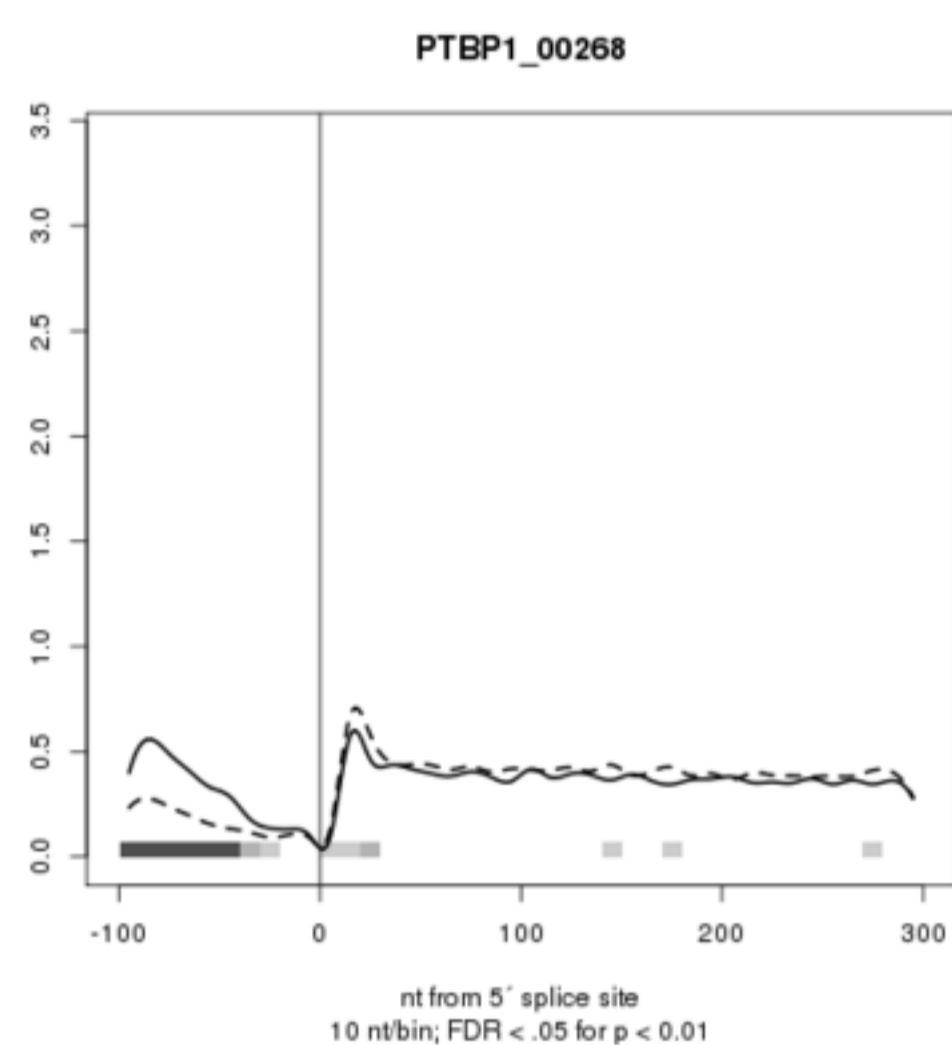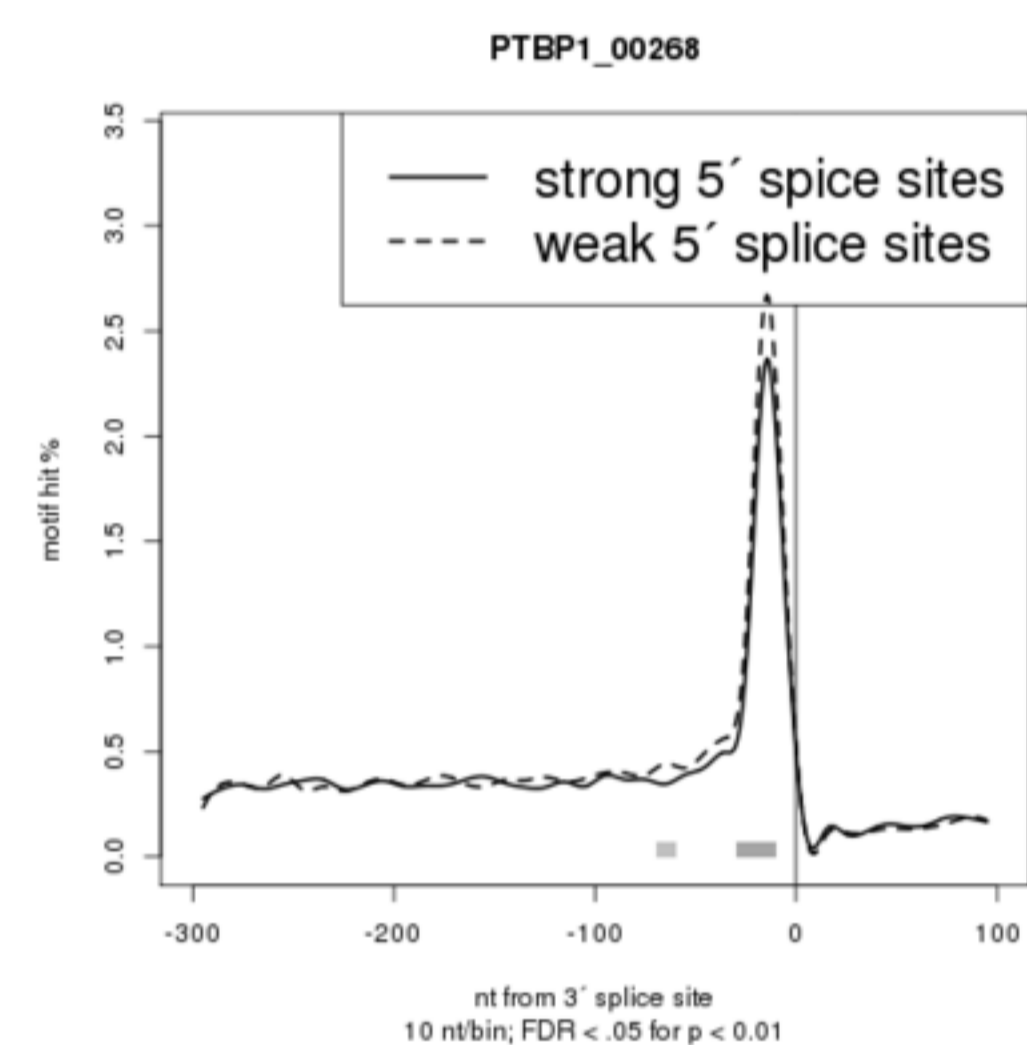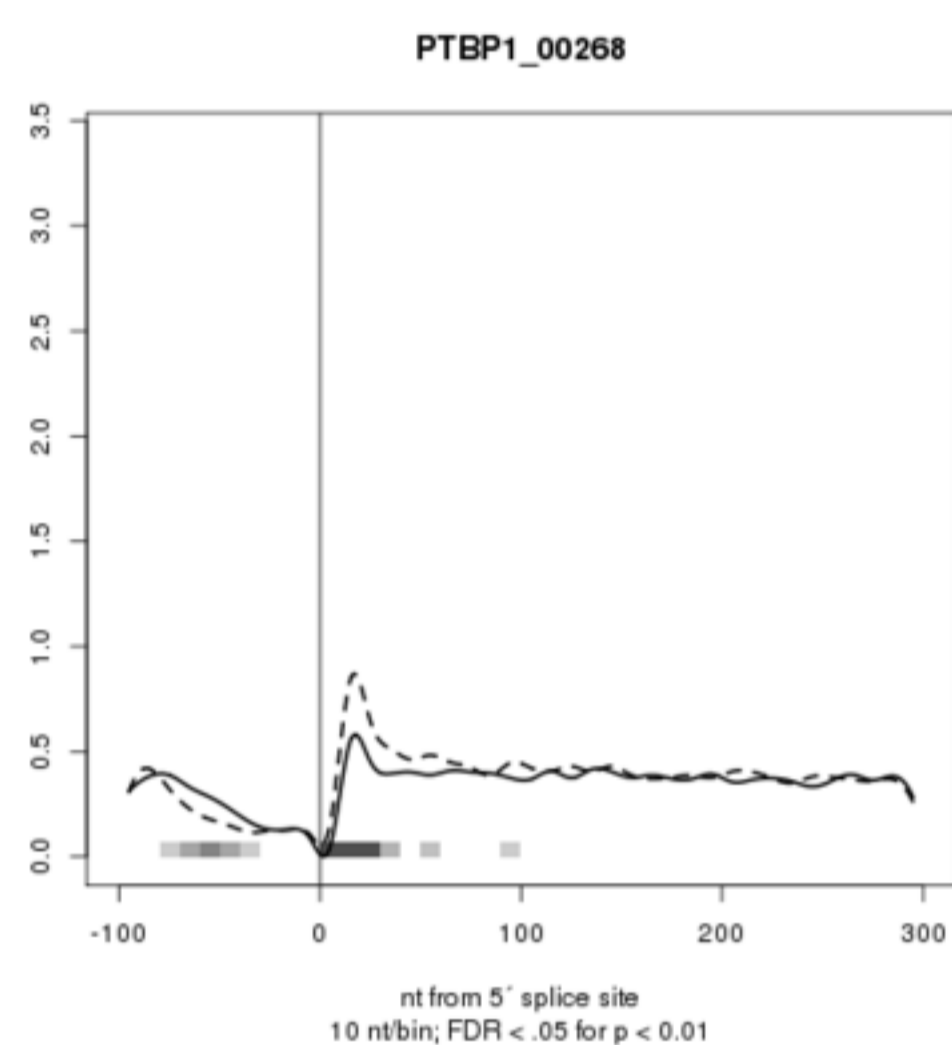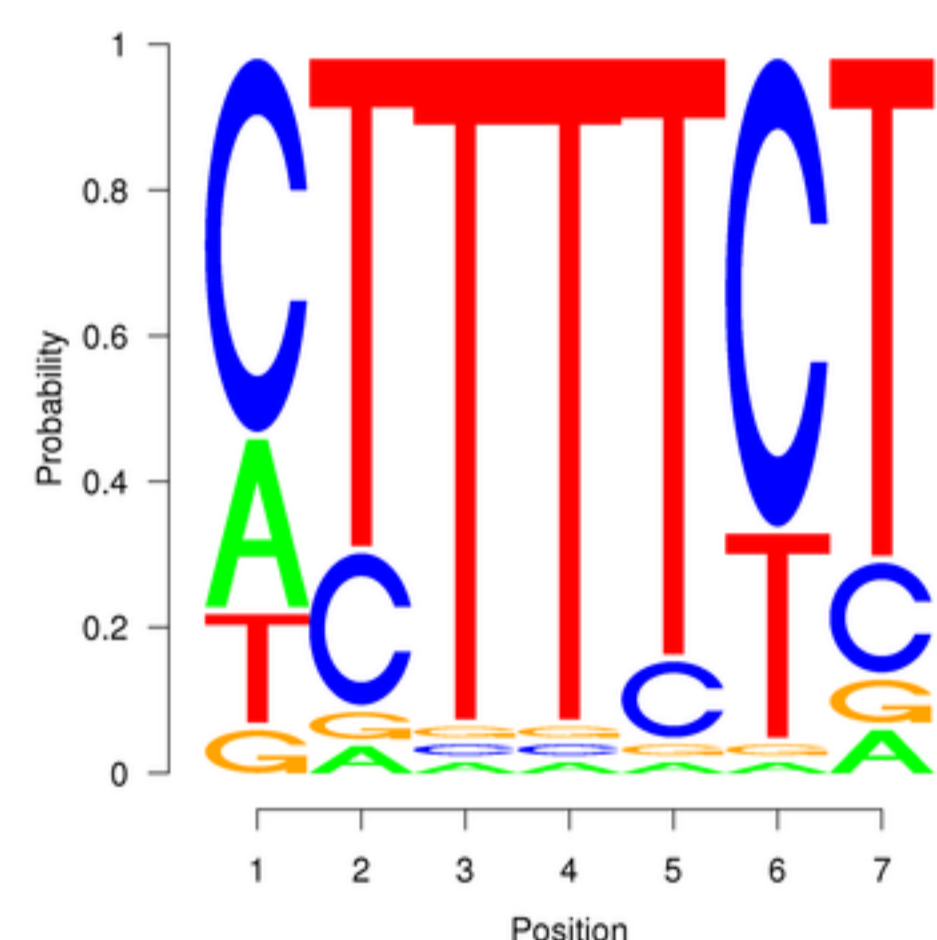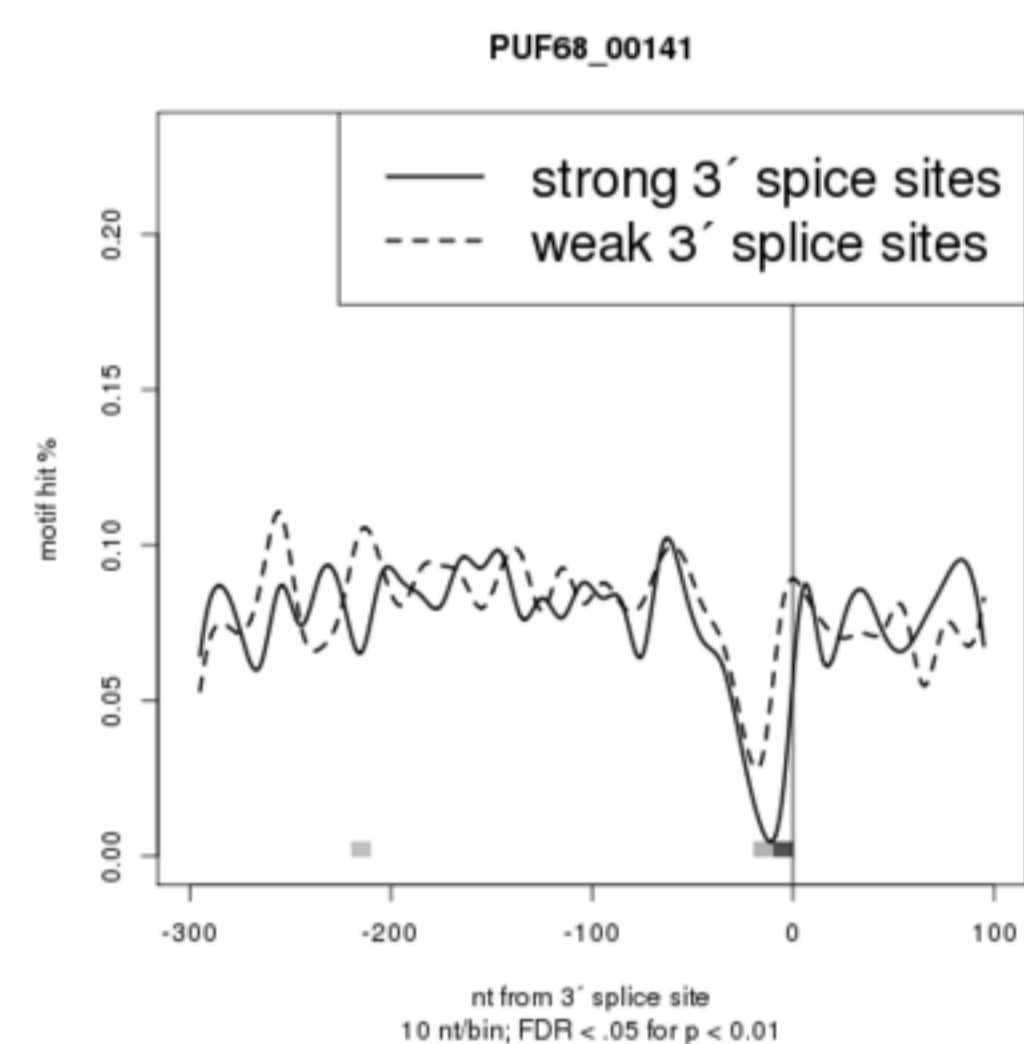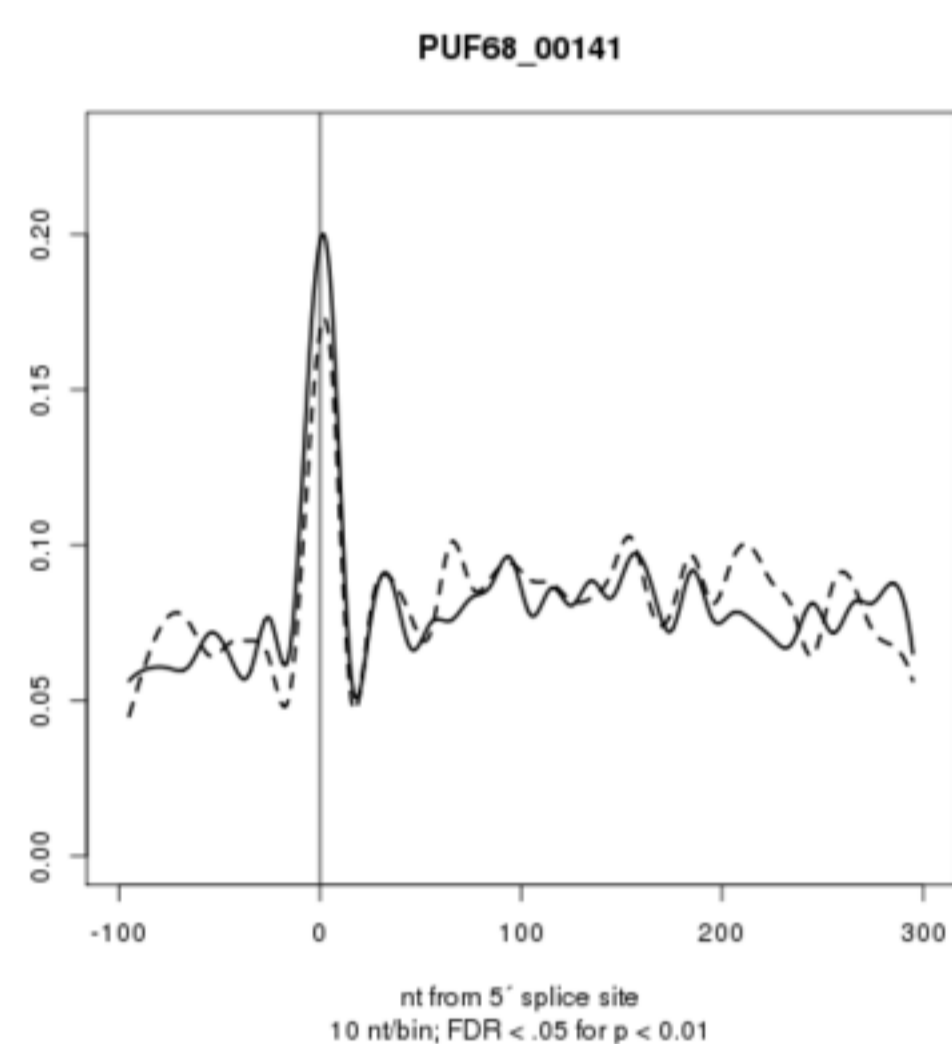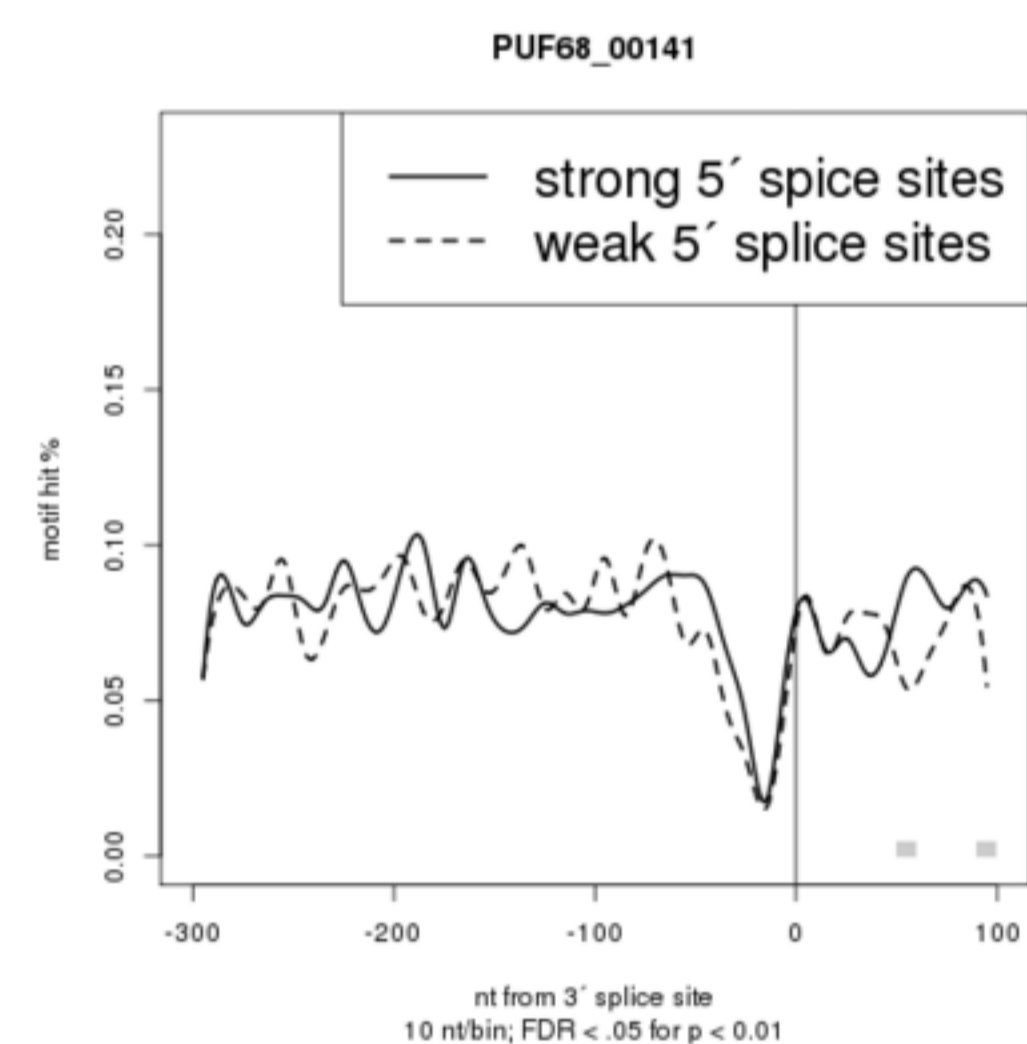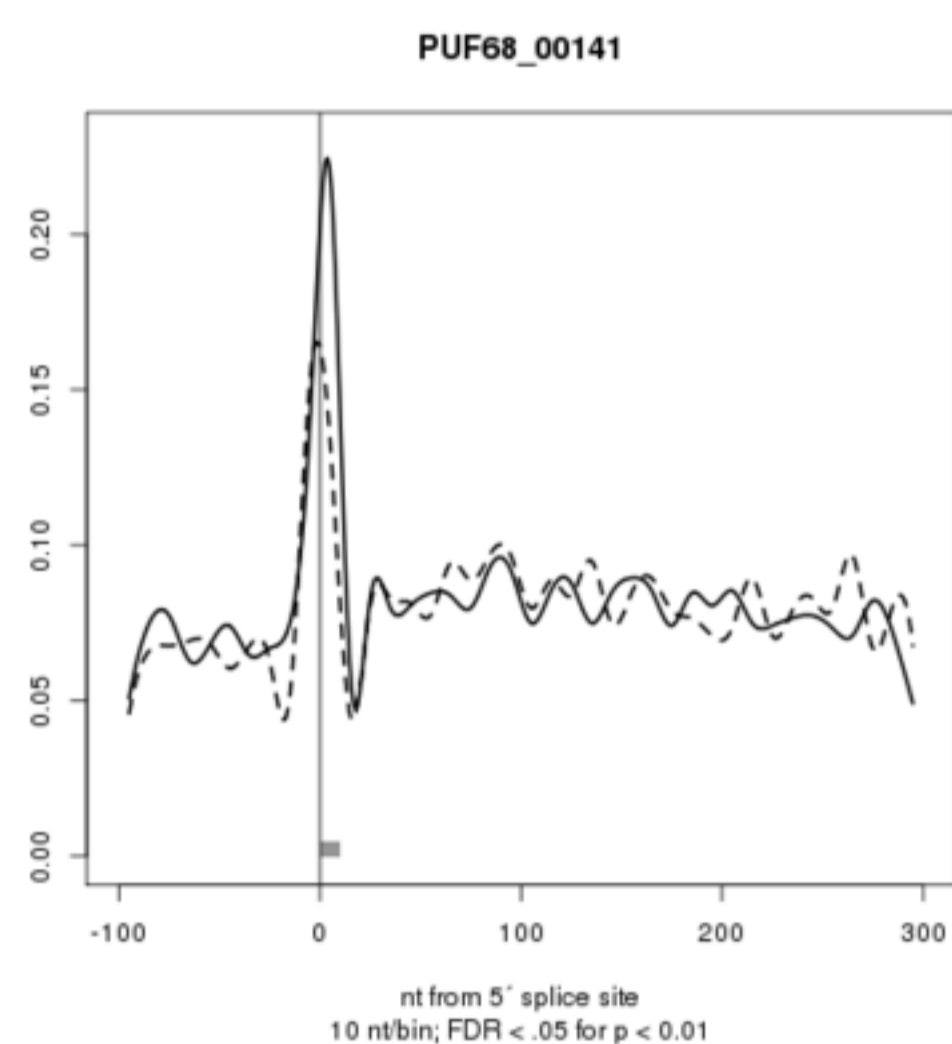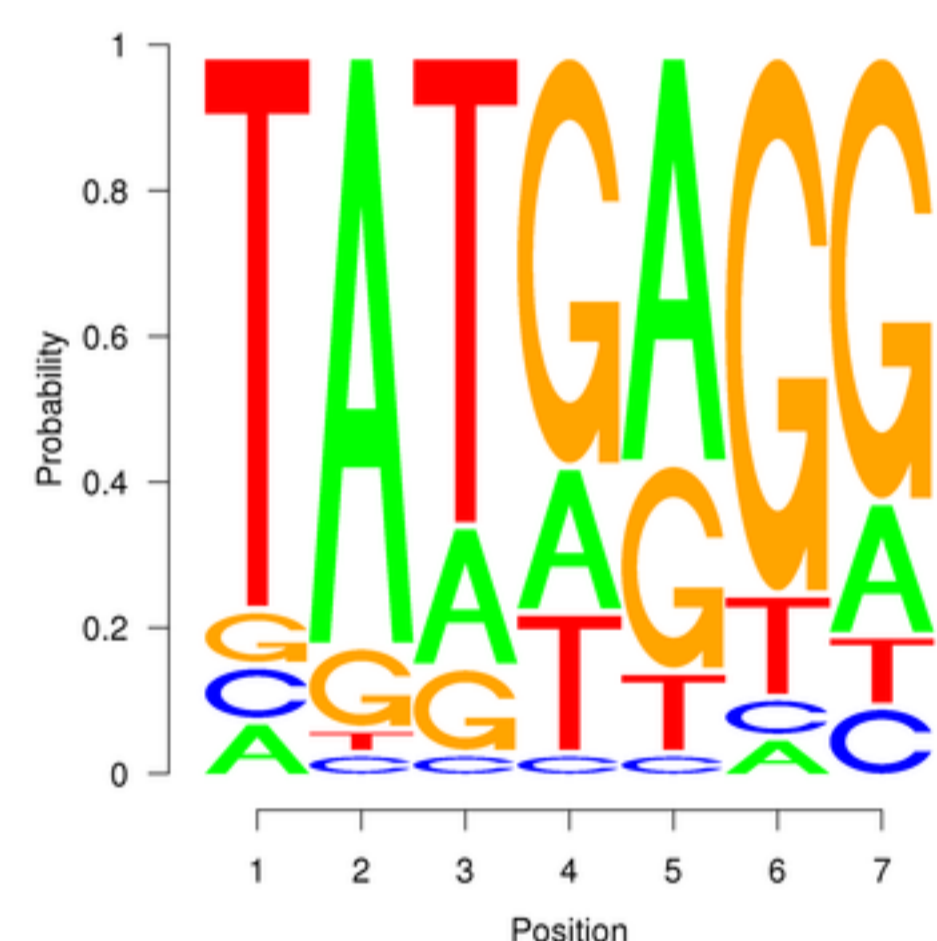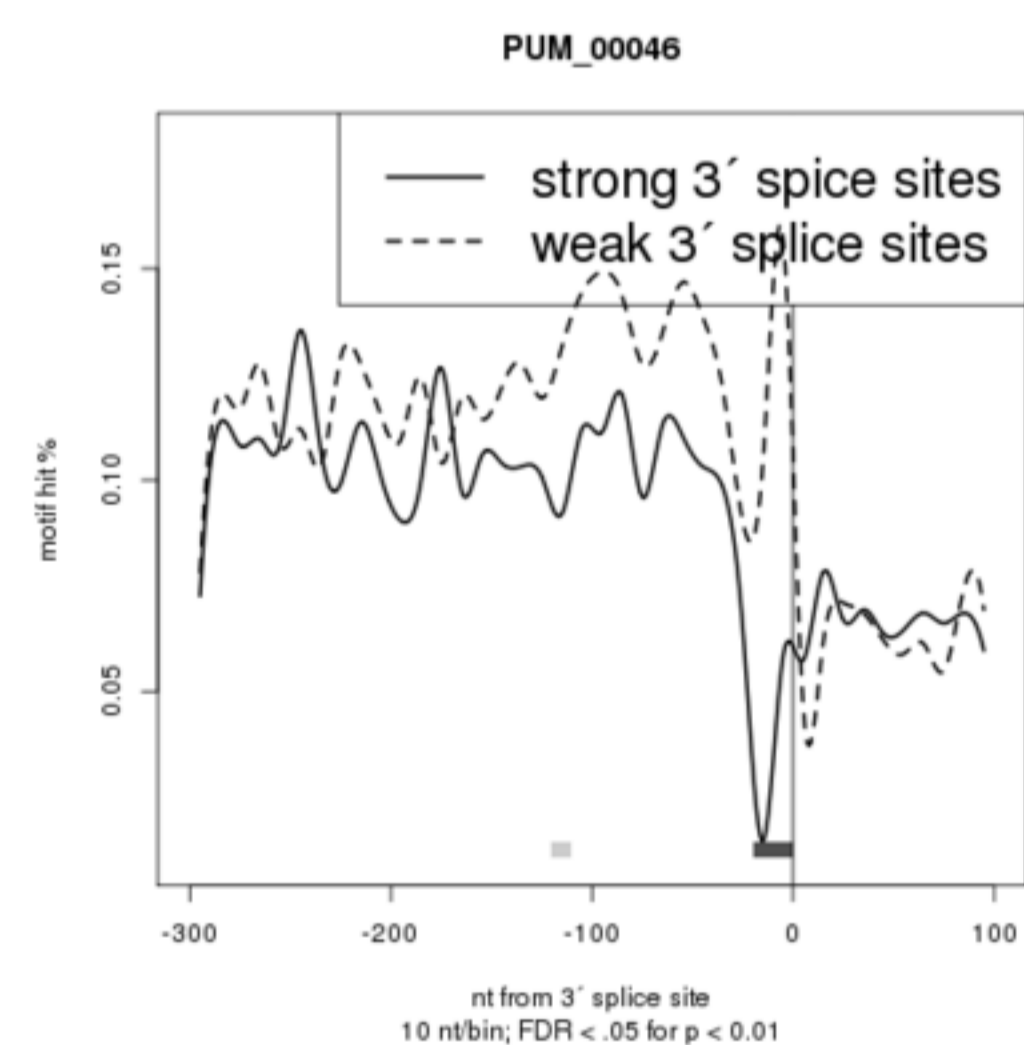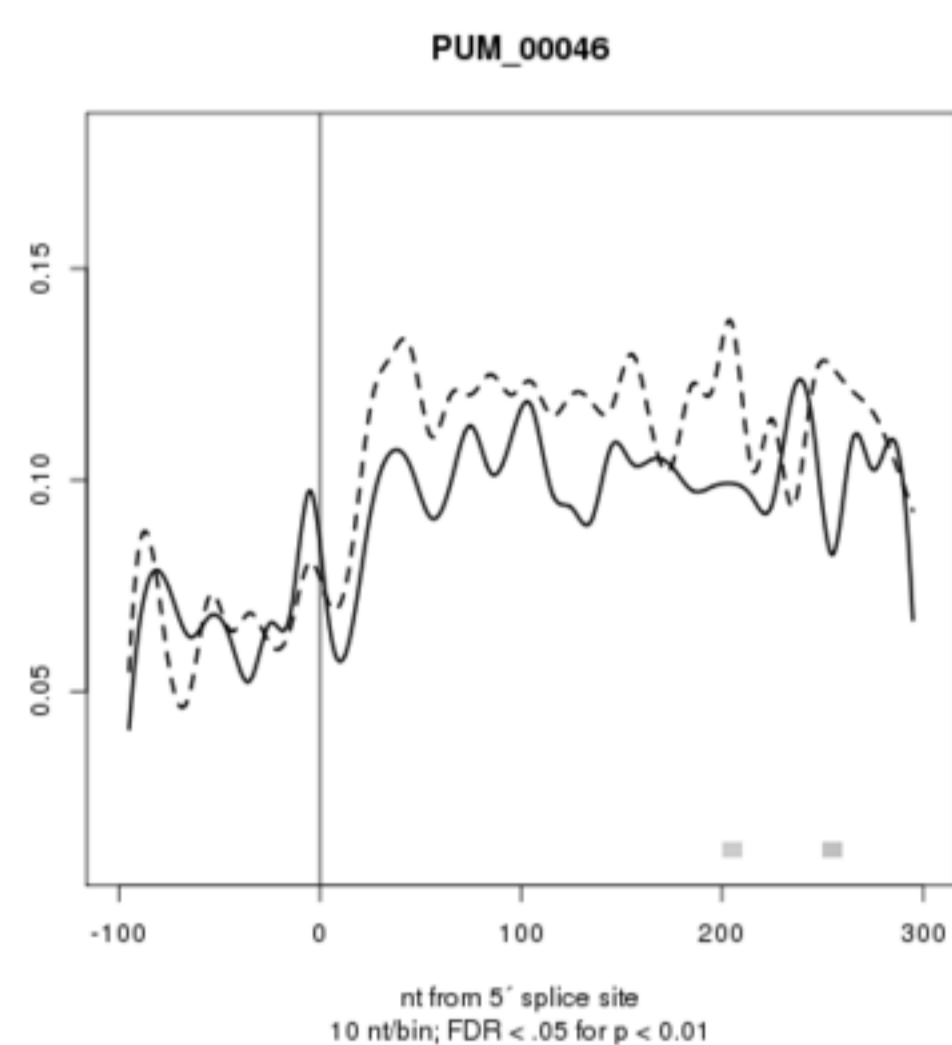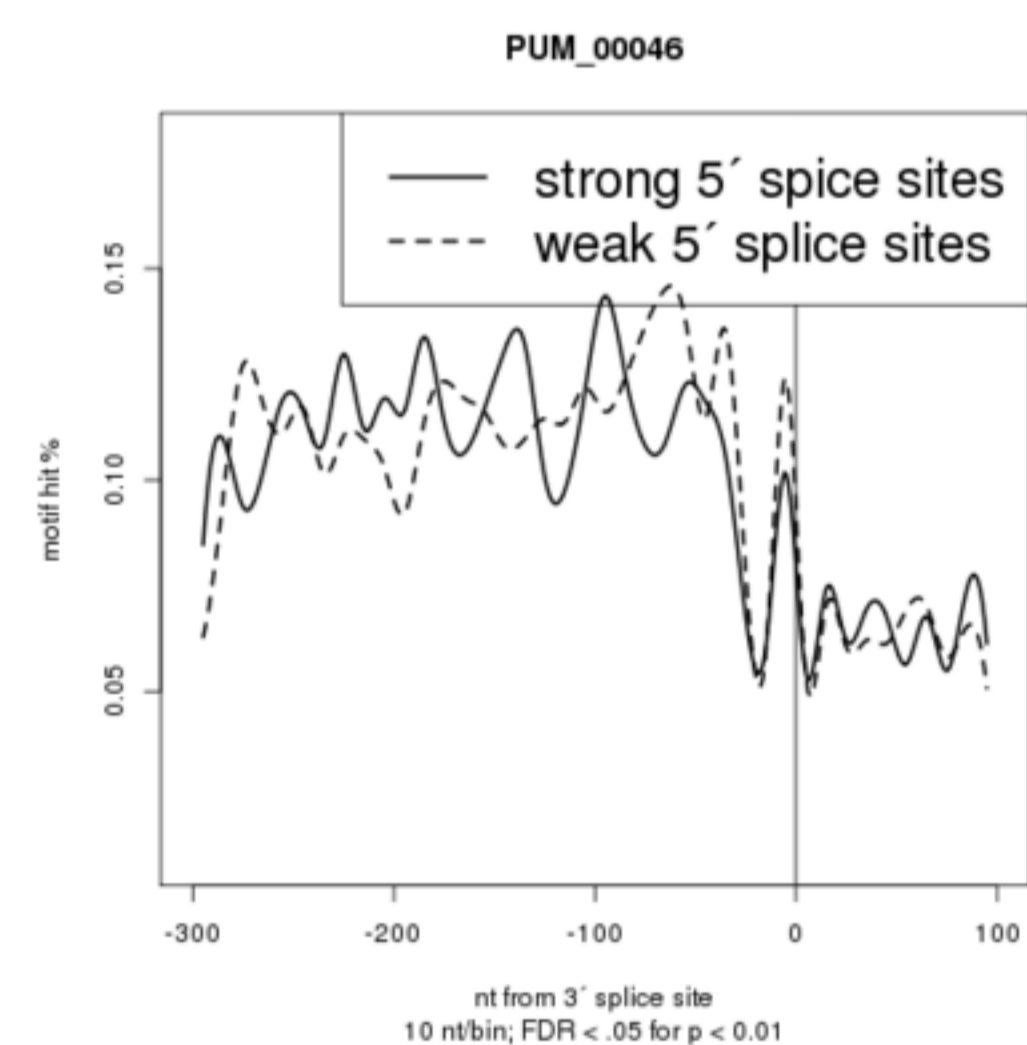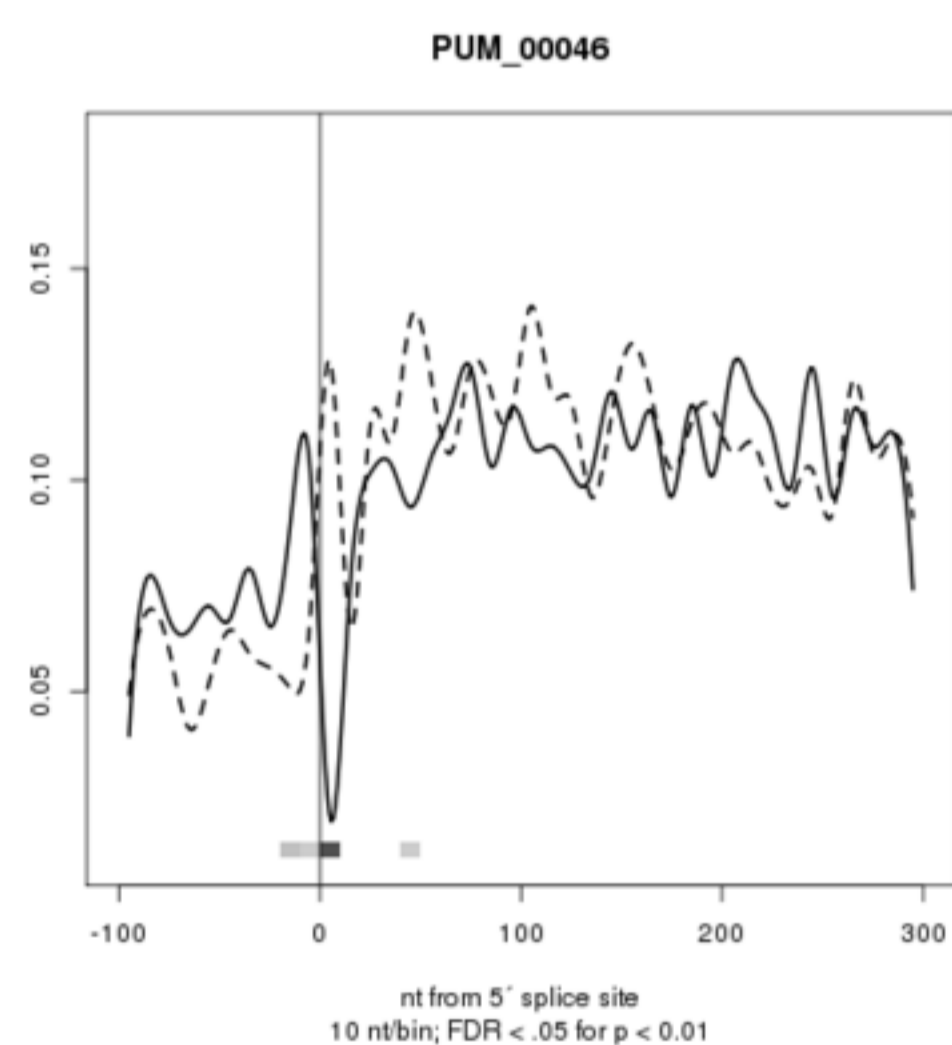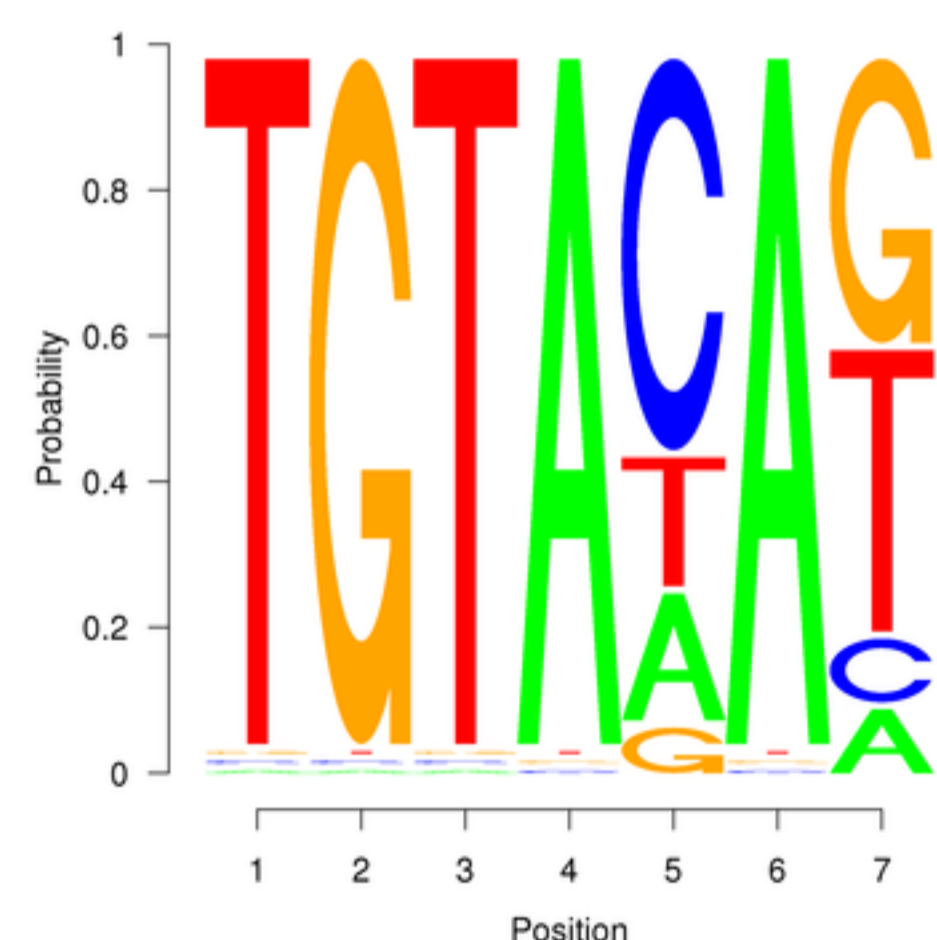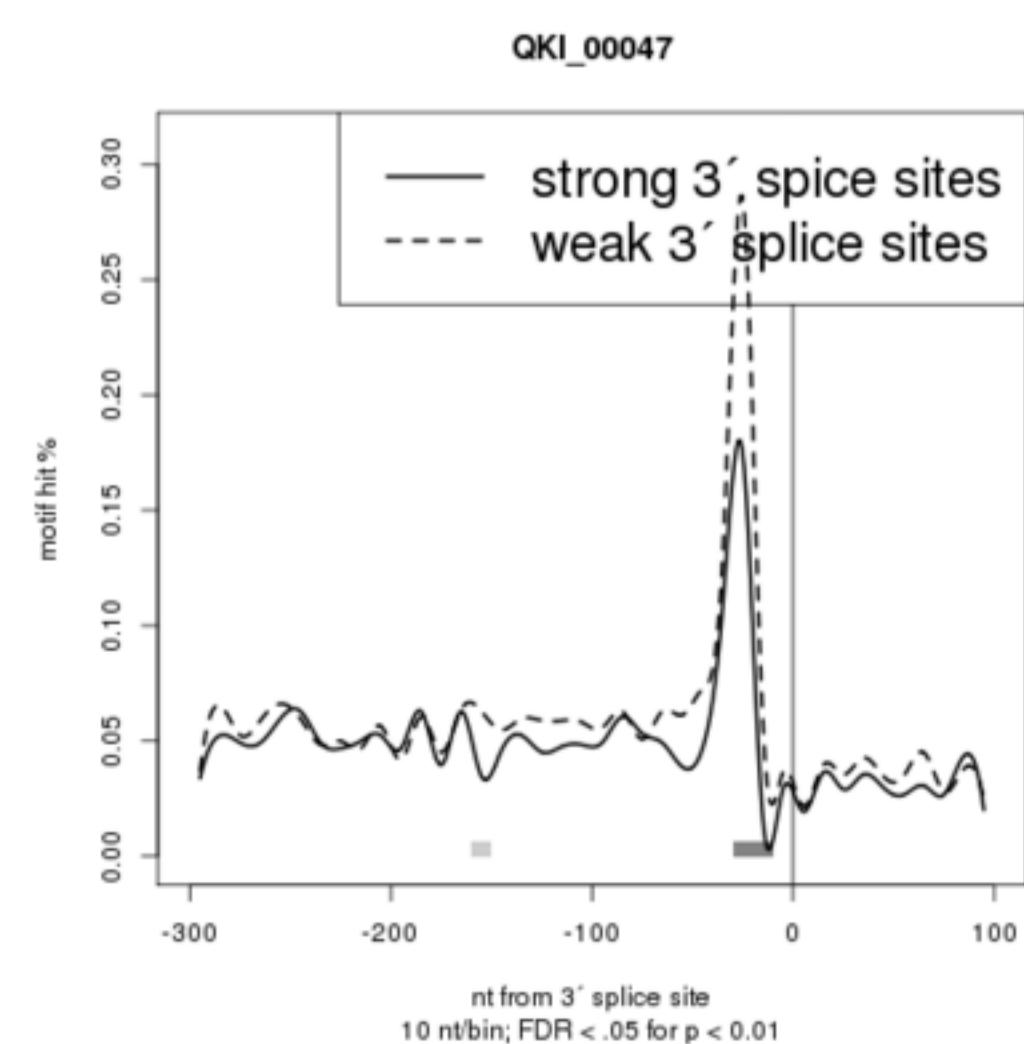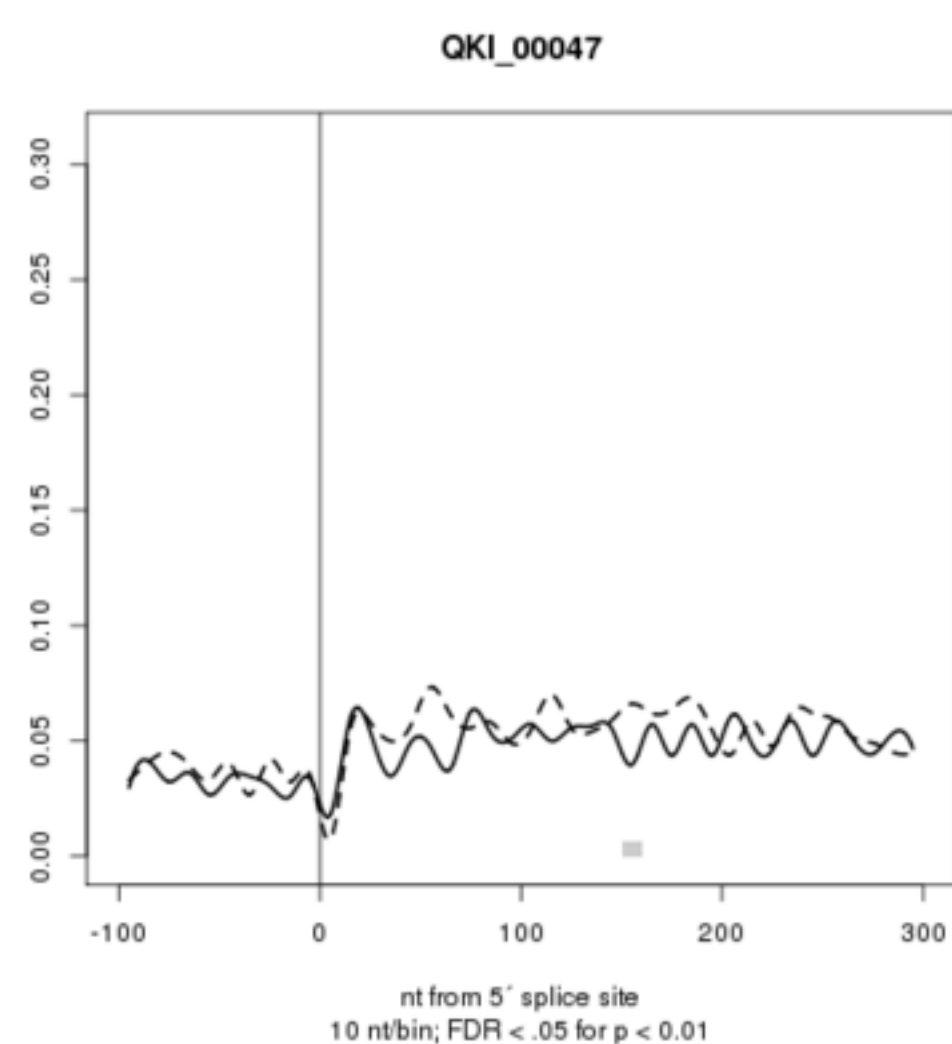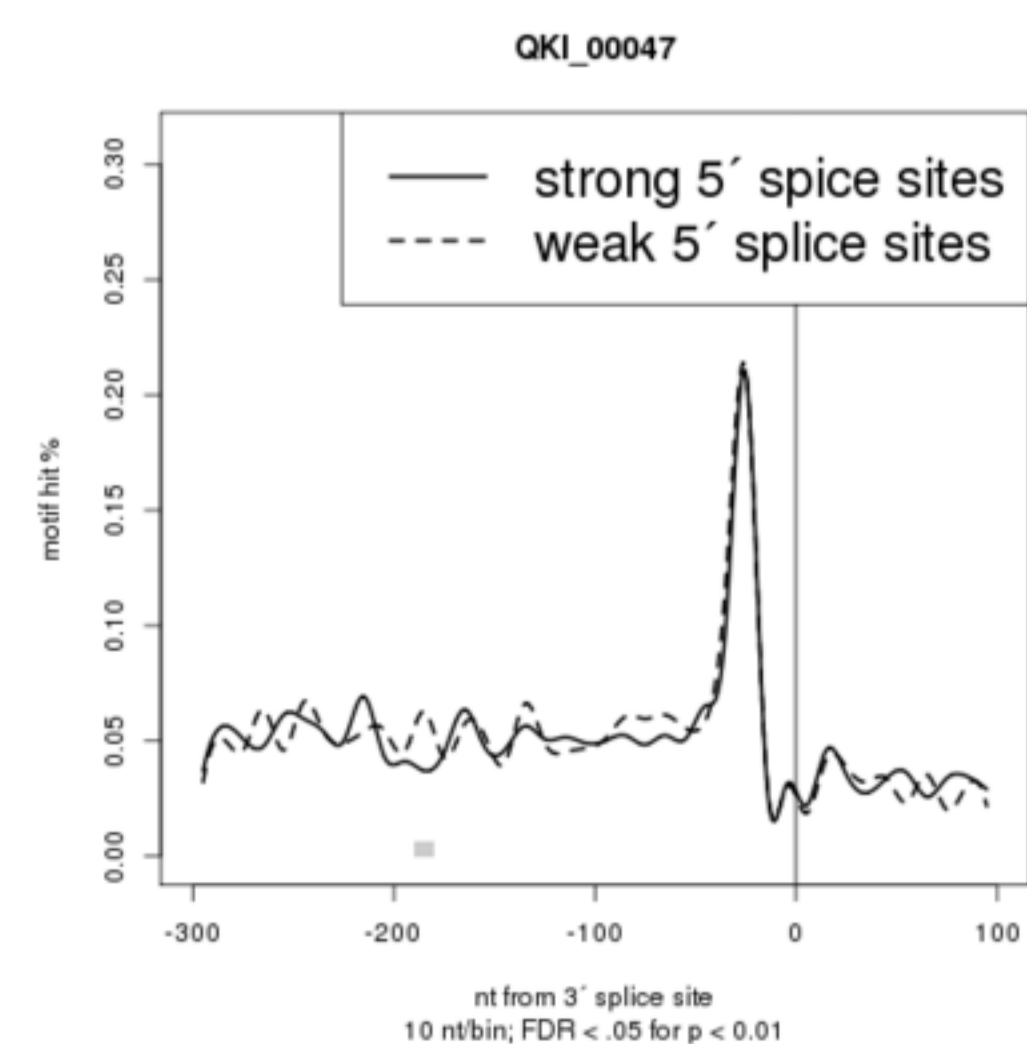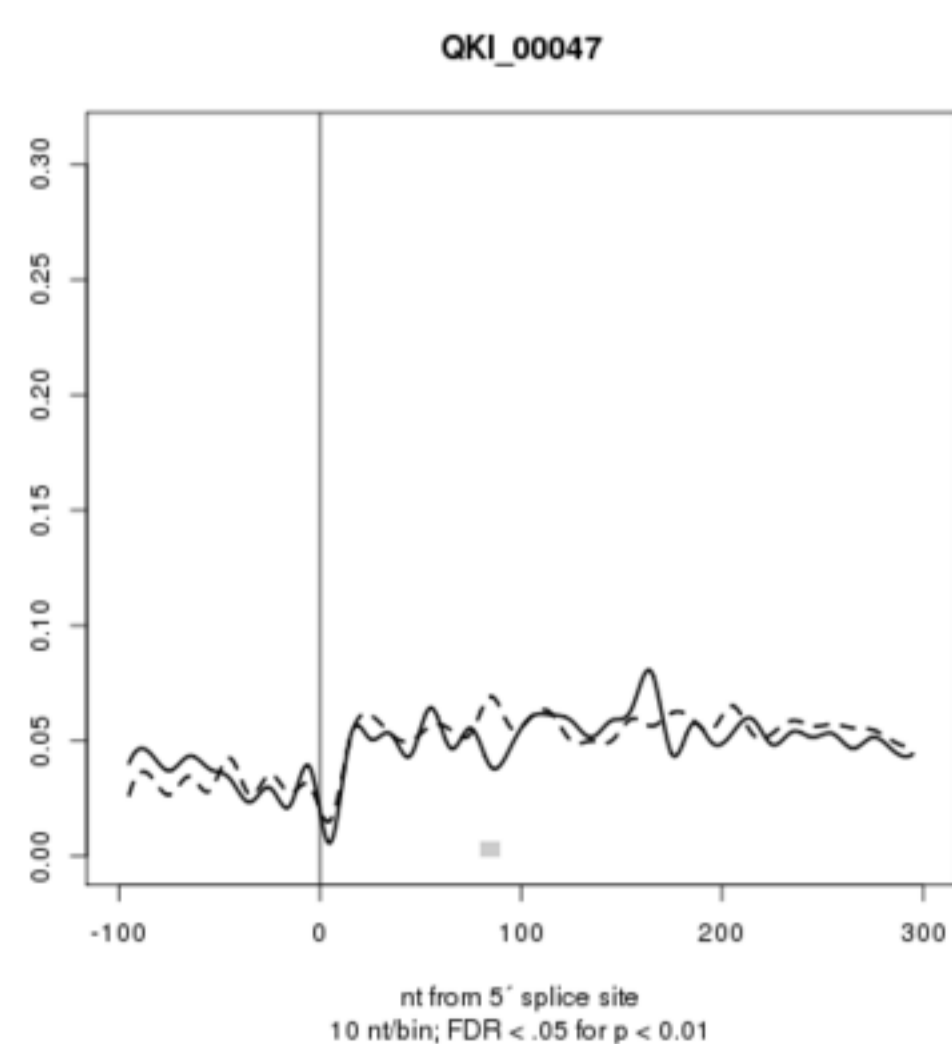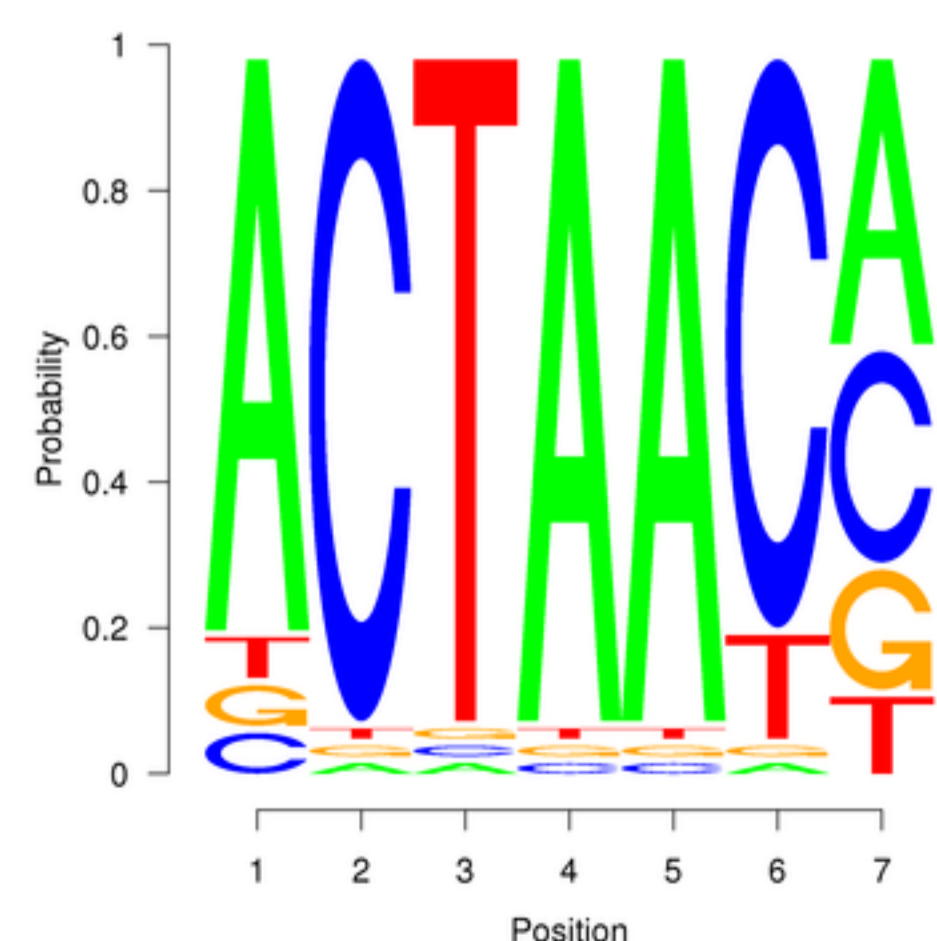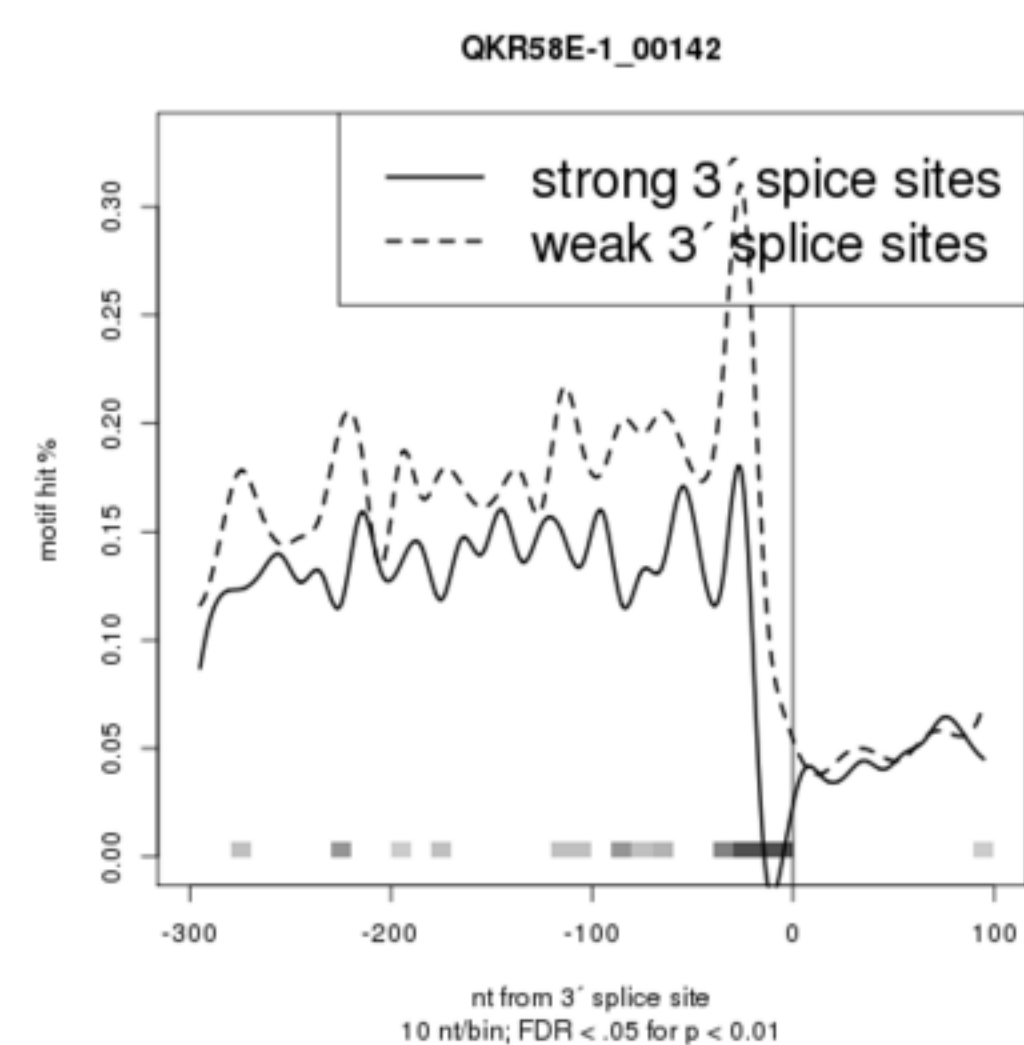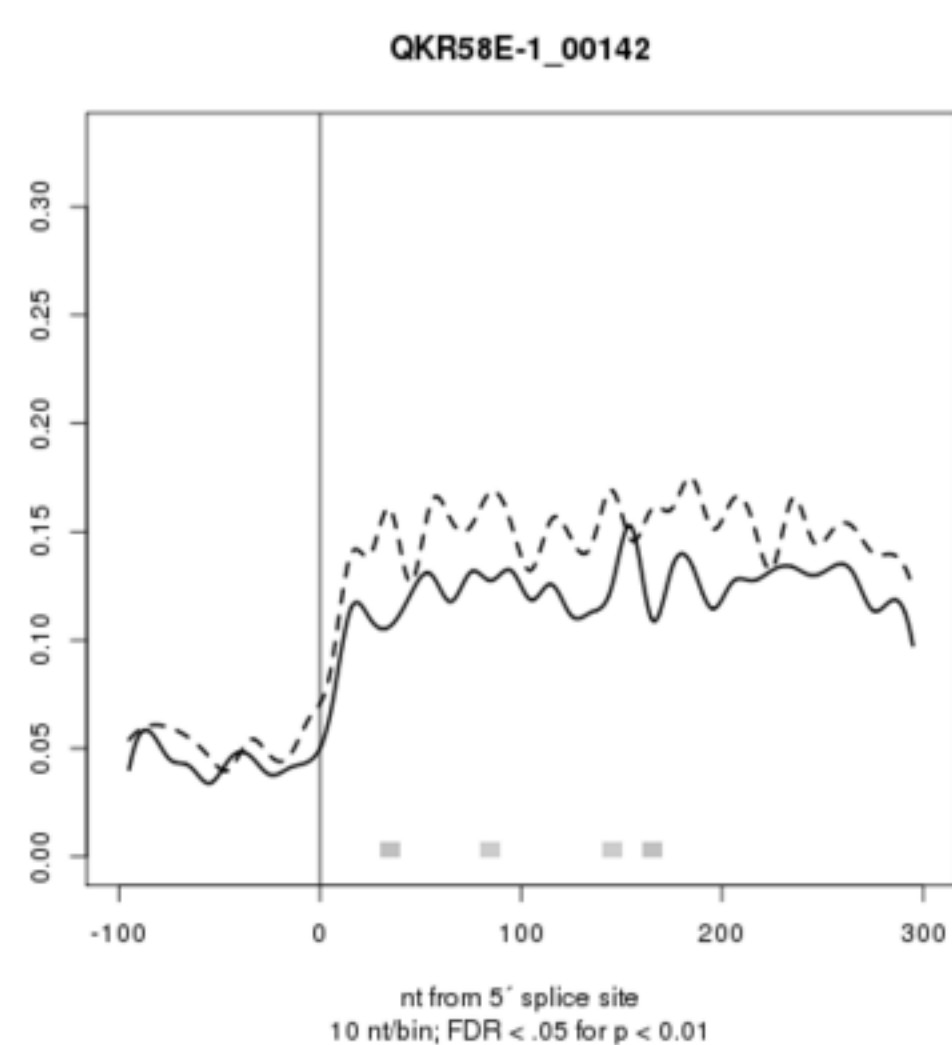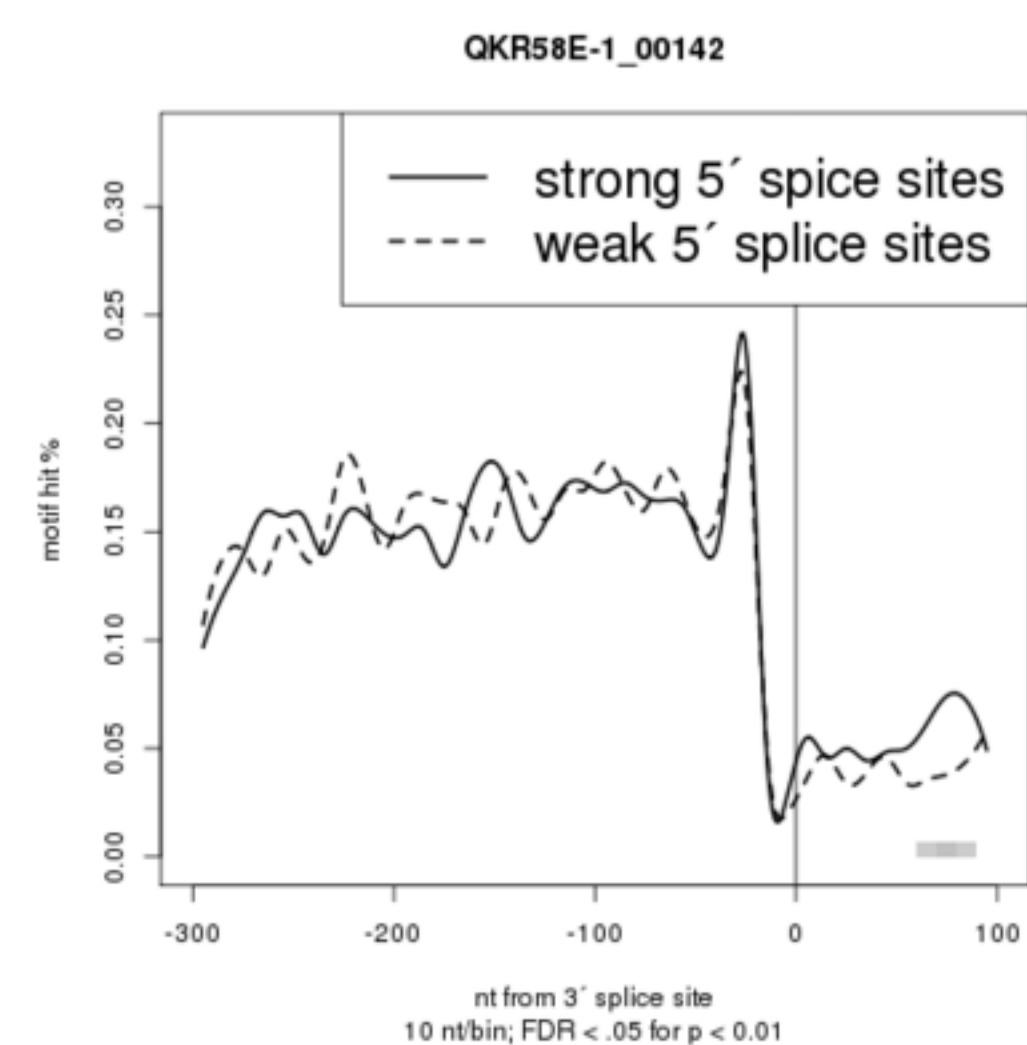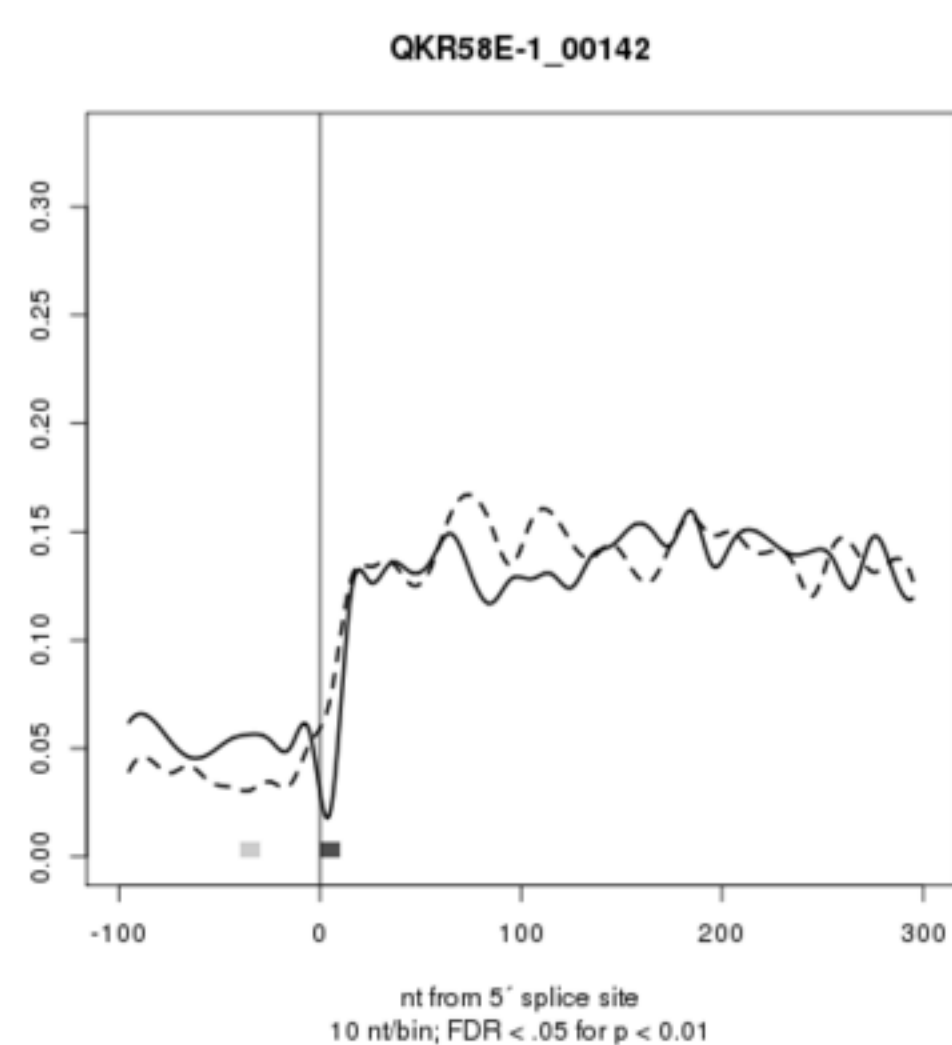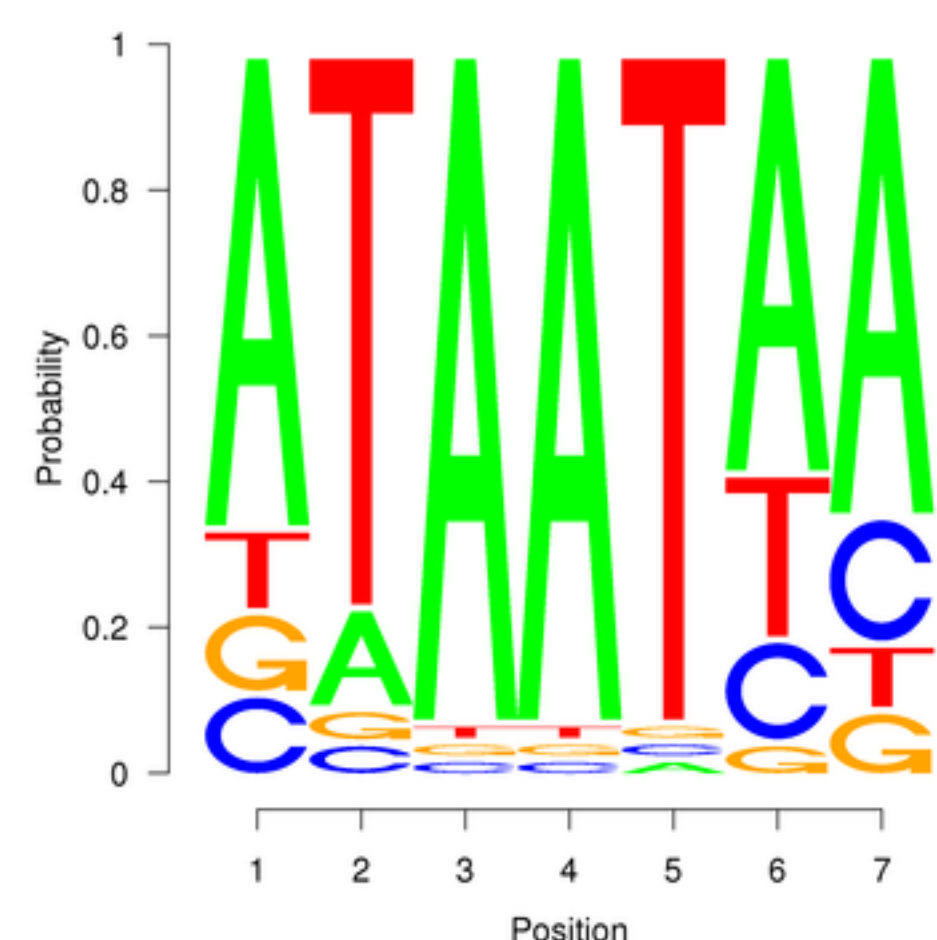

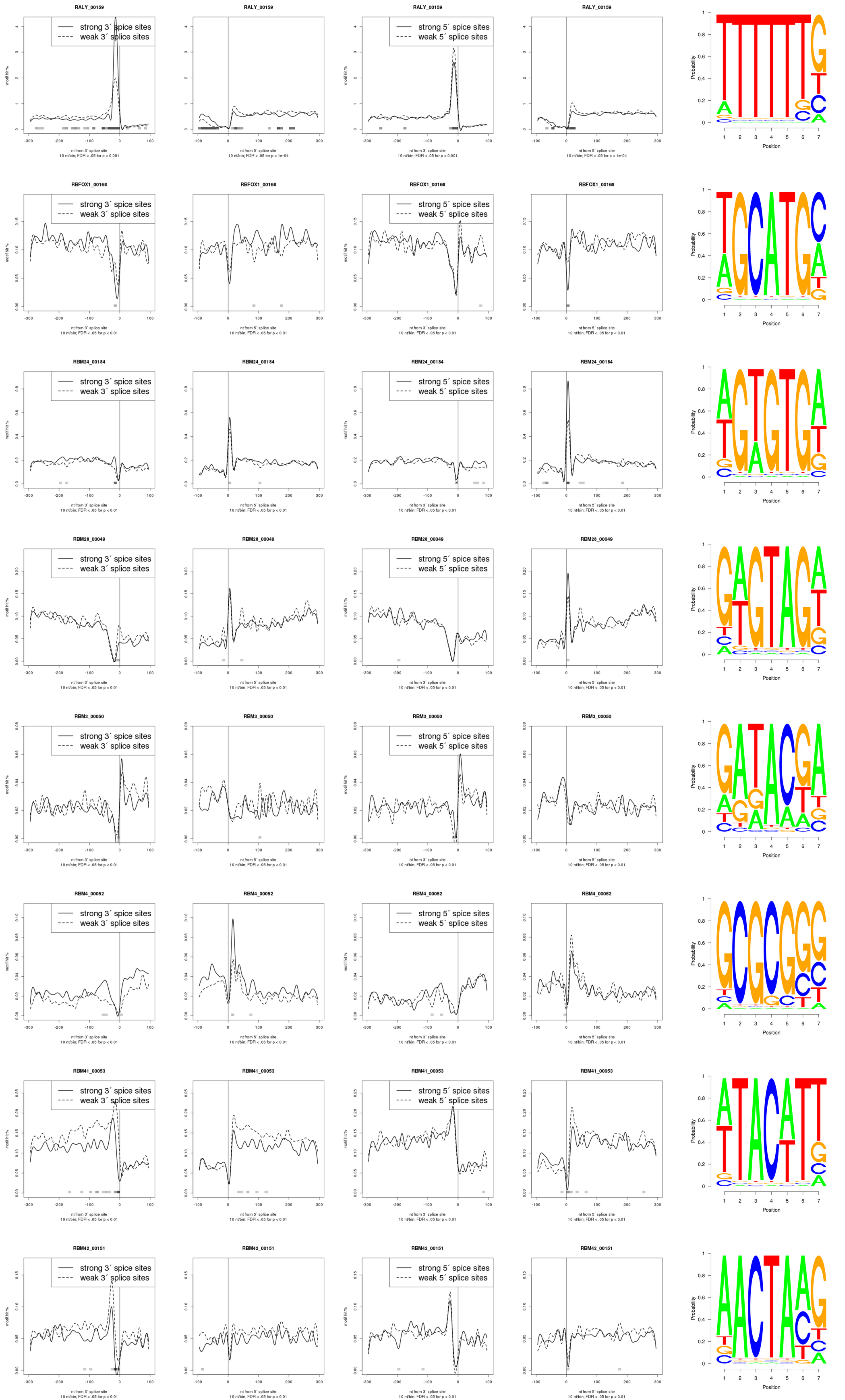

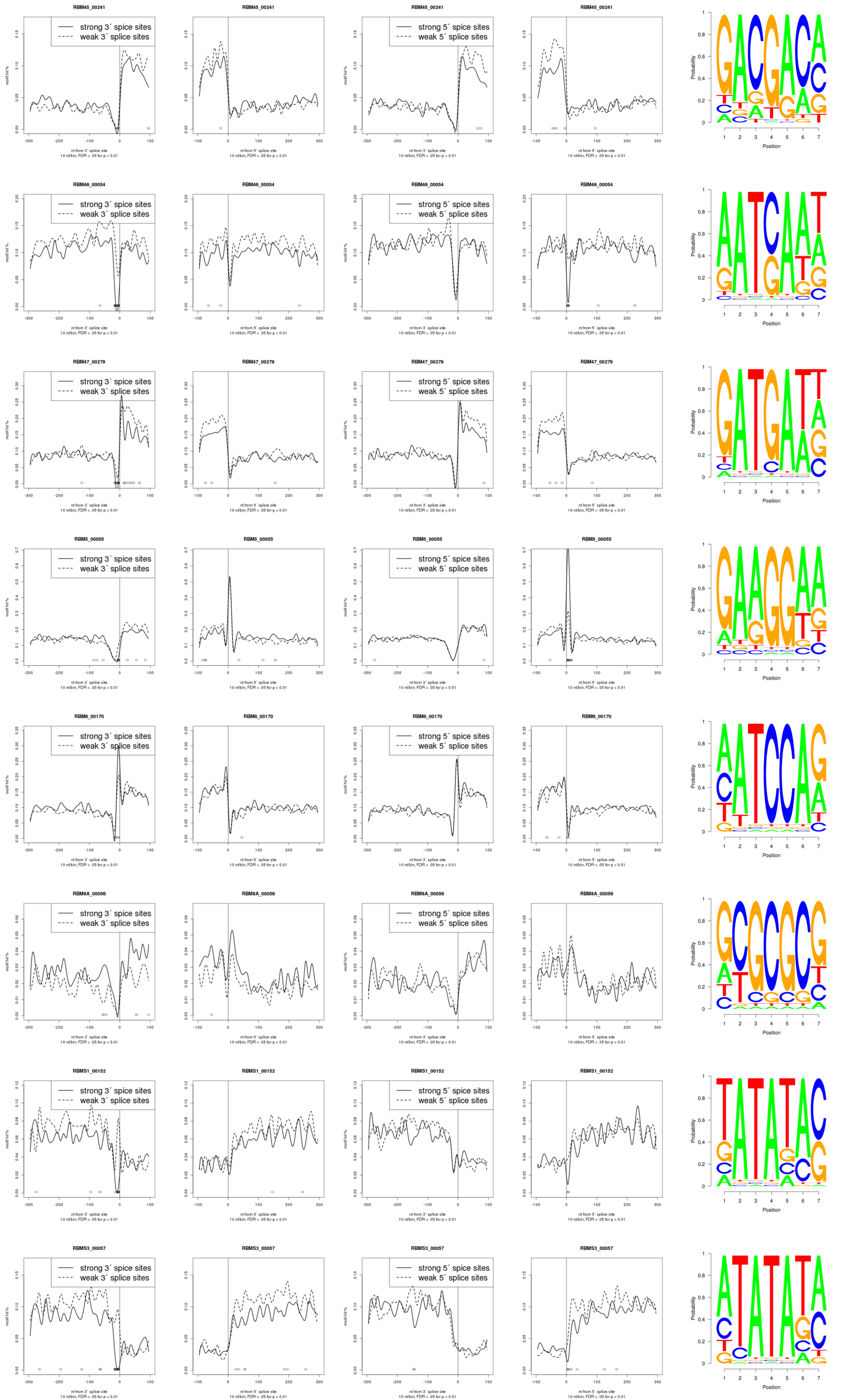

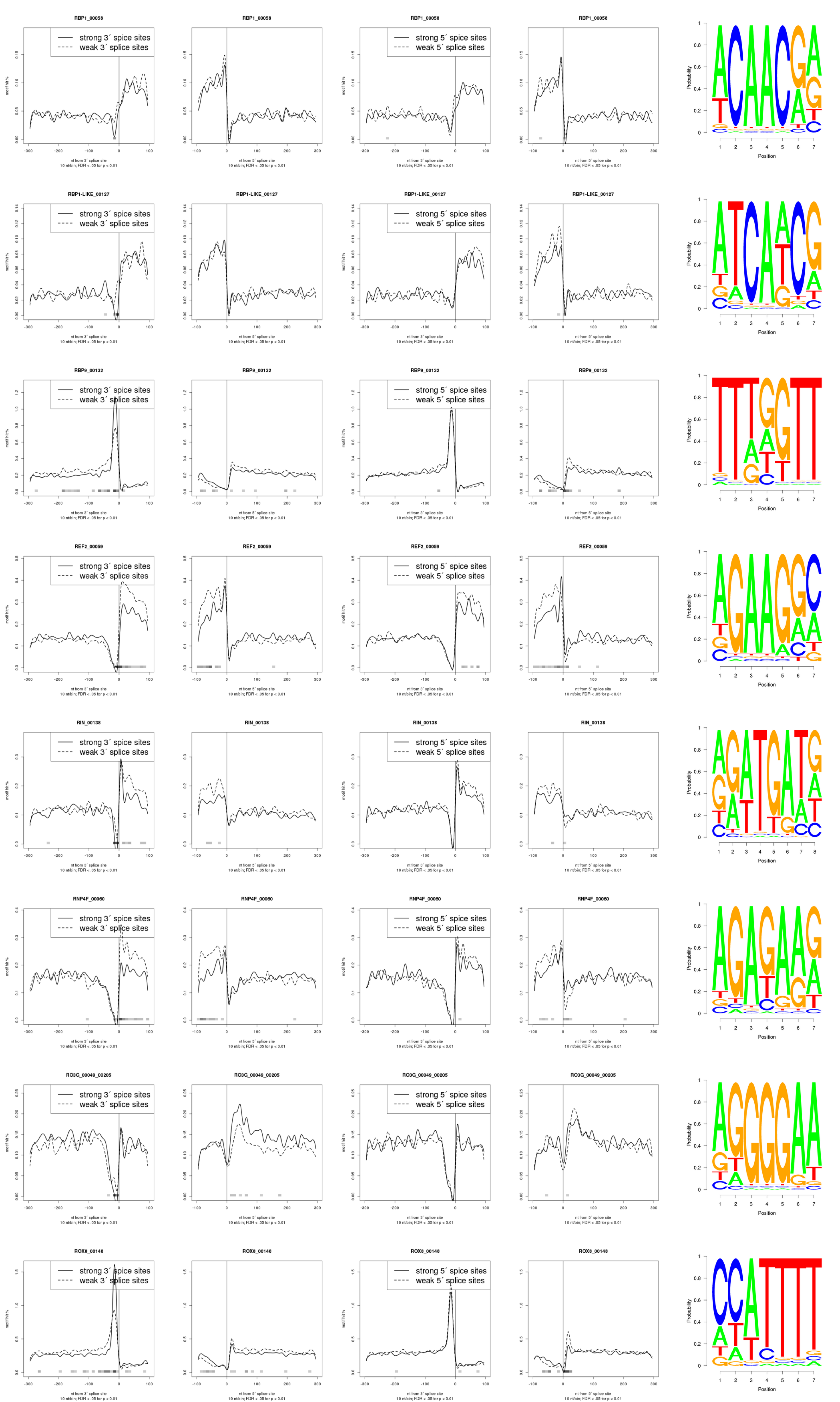

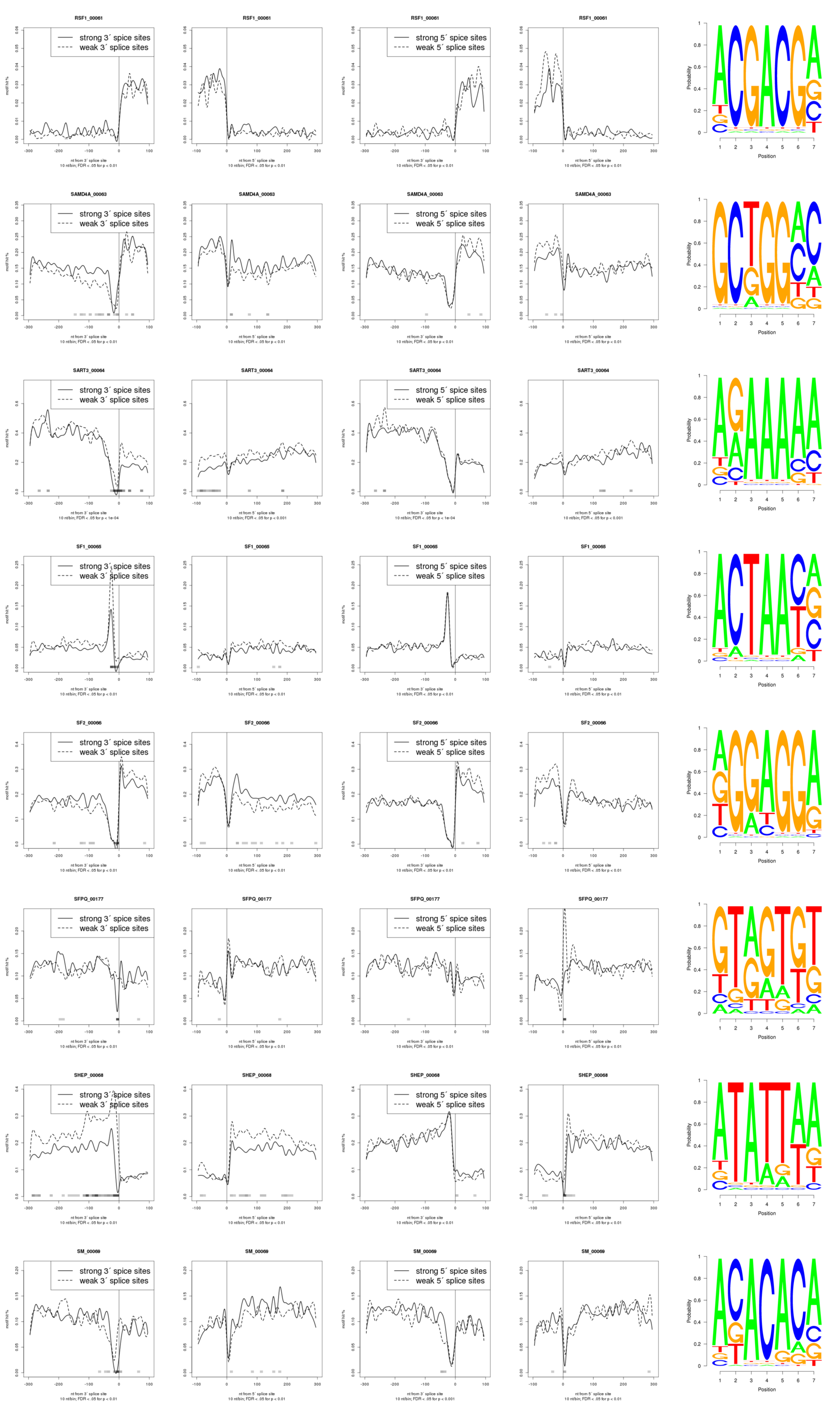

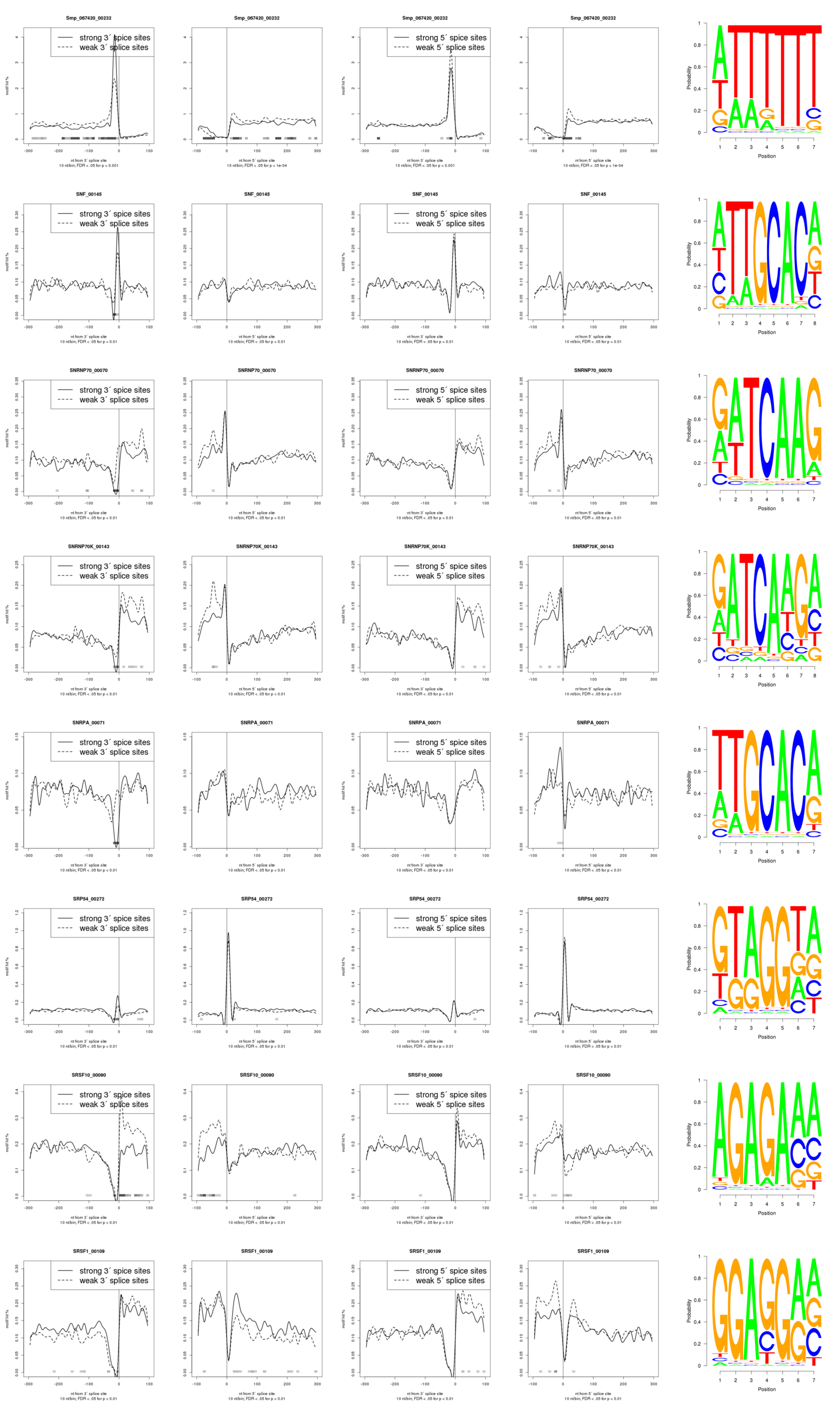

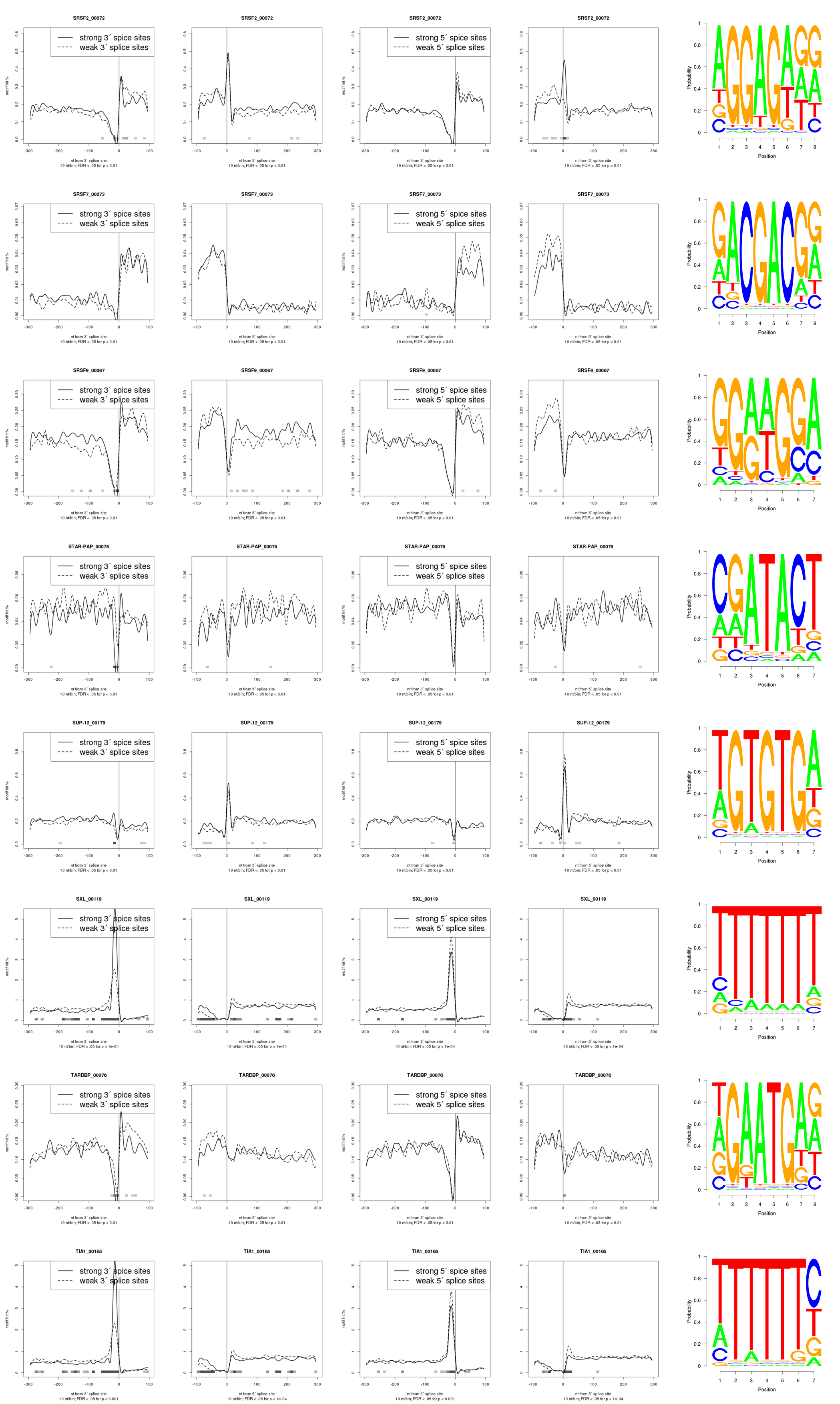

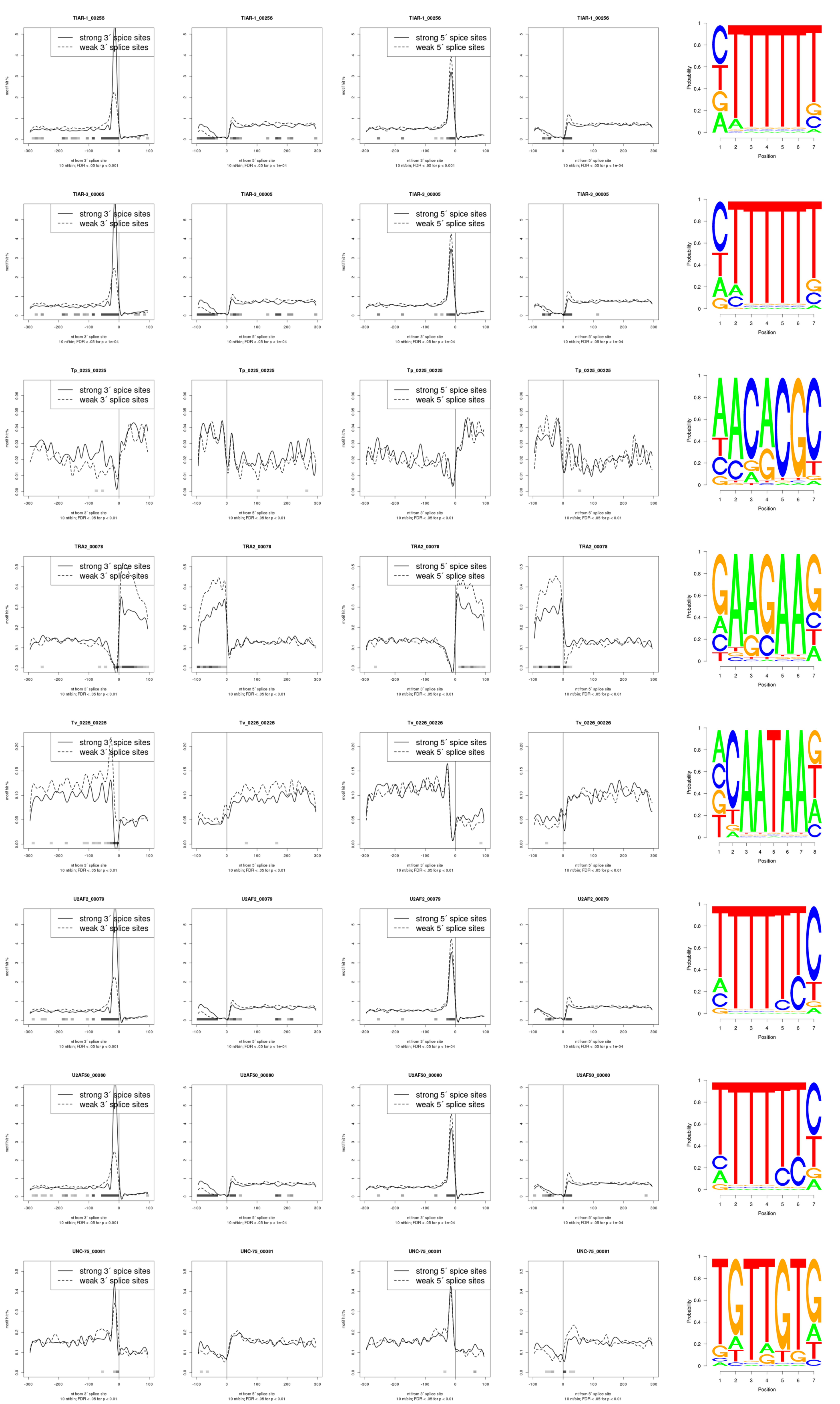

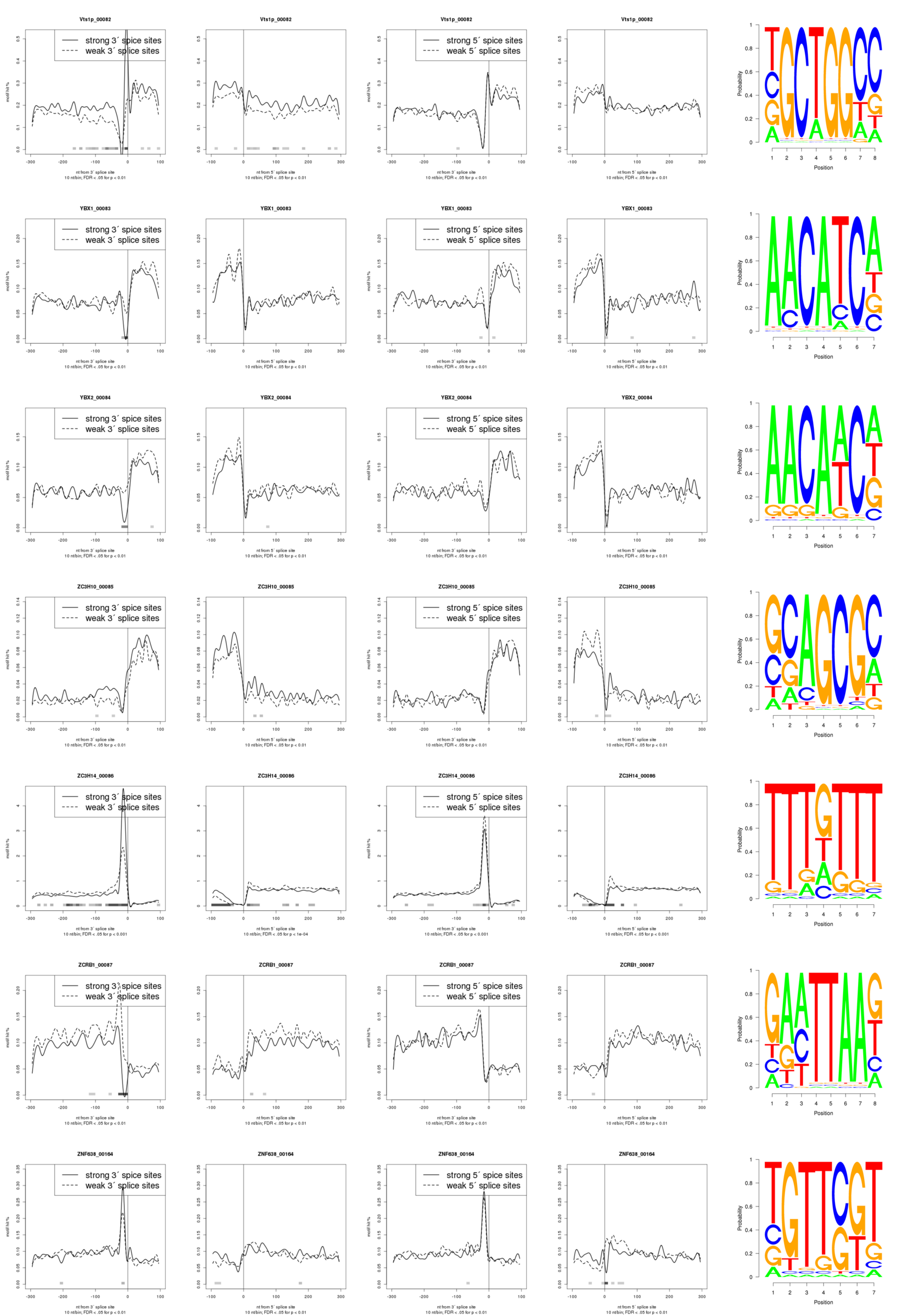

Supplement: S1 File — Each row consists of the profiles for a single motif, whose name is listed above each profile, and whose position-weight matrix is shown in the far-right column; “strong” is the upper quartile of splice site strengths for all internal exons, whereas “weak” is the lower quartile. The first two columns give the profiles centered at the 3' and 5' splice sites for the comparison of strong vs. weak 3' splice sites. The next two columns are the same profiles for the comparison of strong vs. weak 5' splice sites. Test vs. control P-values/bin are as shown in Fig 1B, with the lightest shade of grey corresponding to P-value < 0.01. In these profiles, only bins whose P-value cutoff had an empirical FDR < 0.05 were displayed in grey scale at the bottom, and the largest of the largest of the P-value cutoffs with FDR < 0.05 is written at the bottom of each plot. (PDF) [file pone.0132448.s010.pdf]

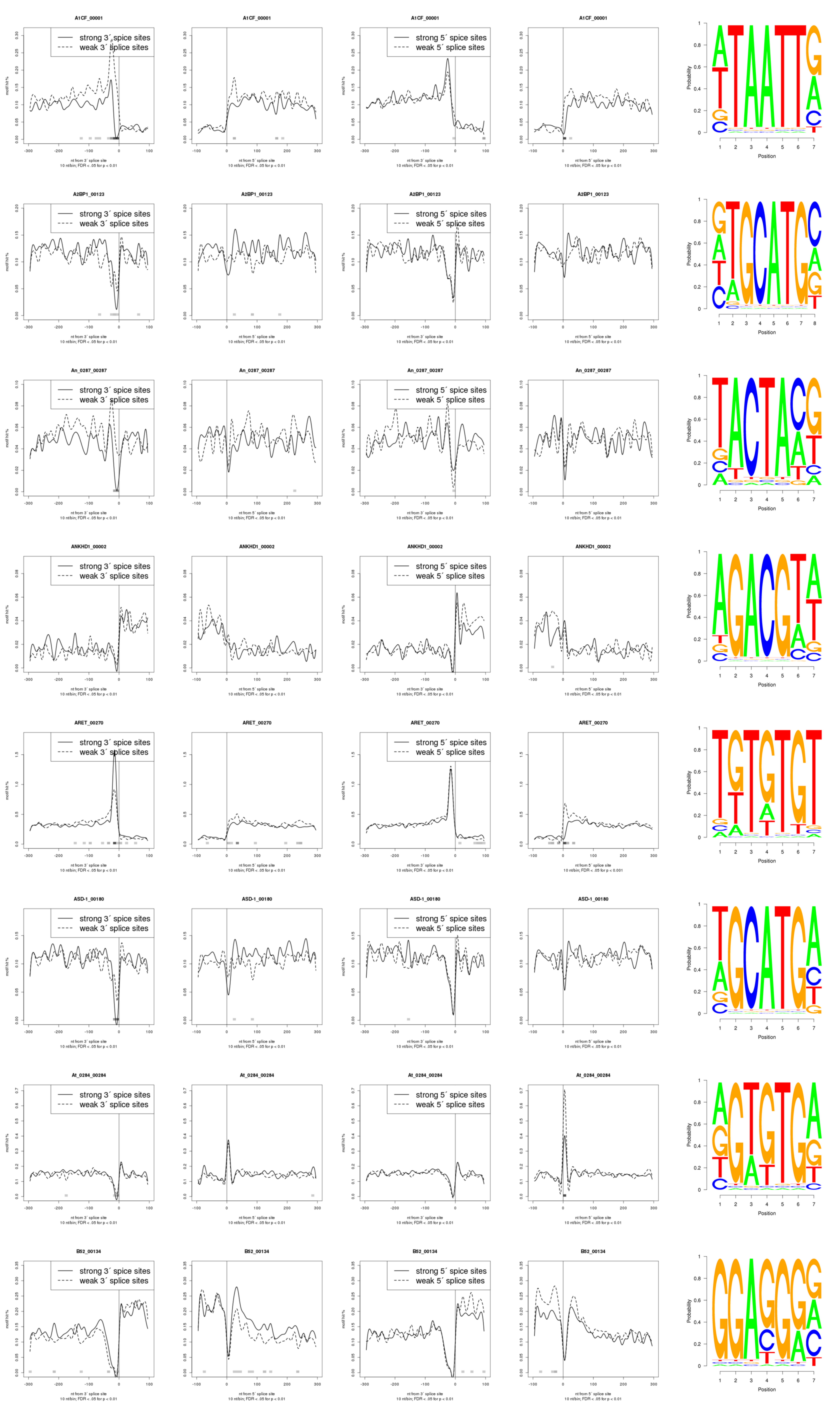

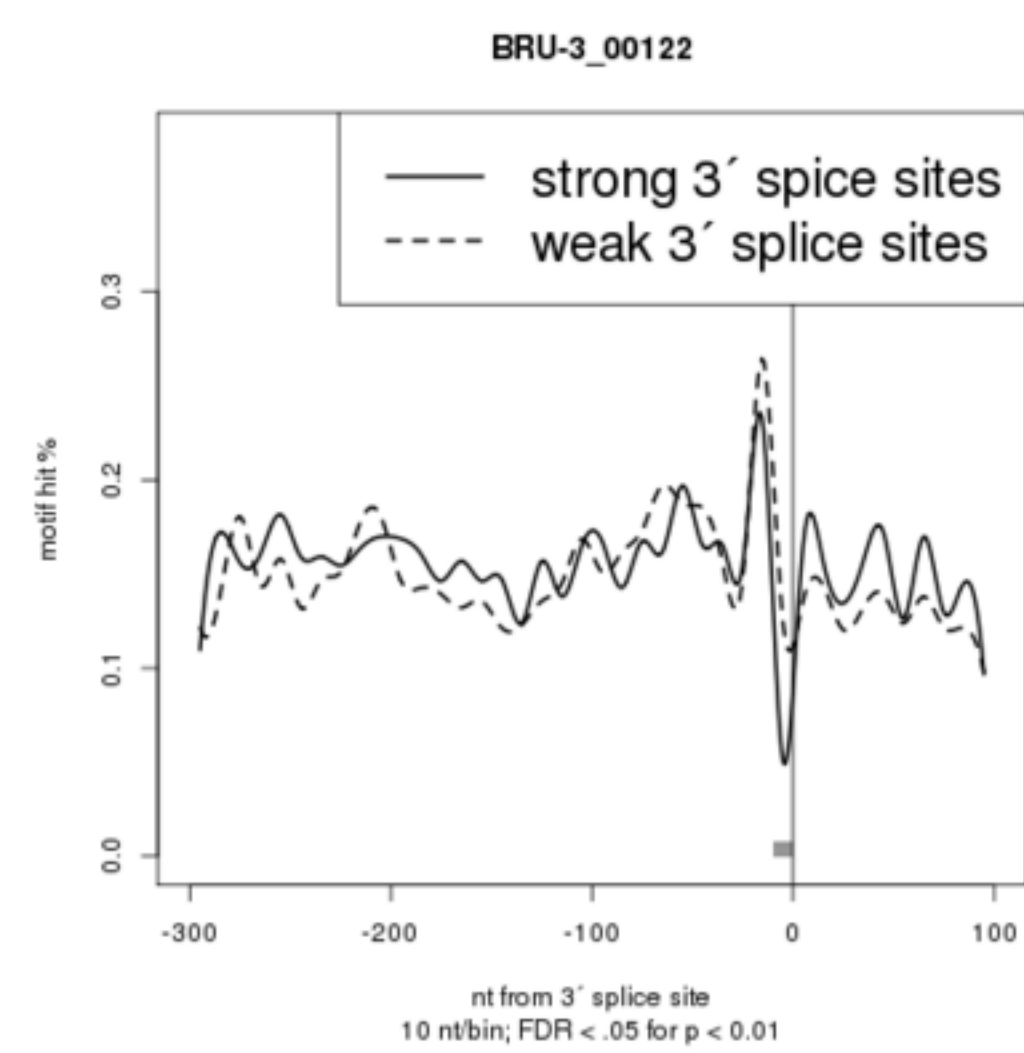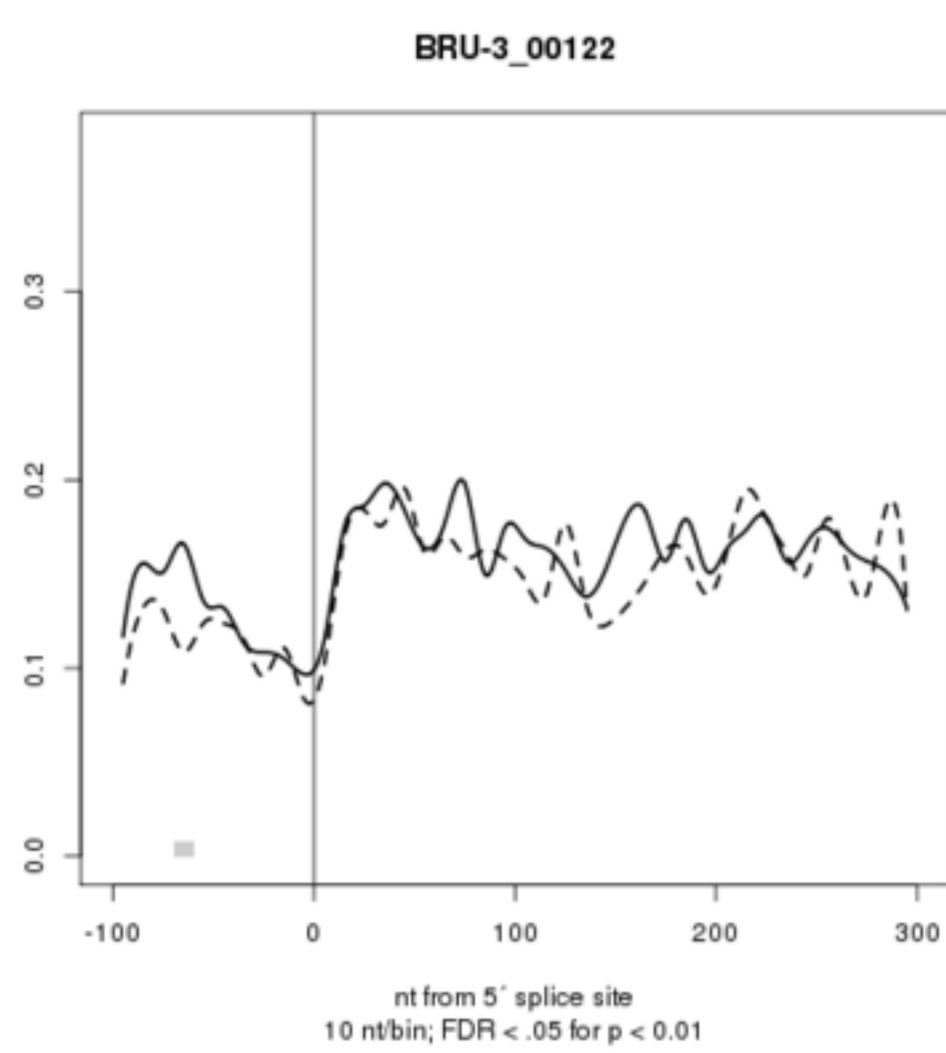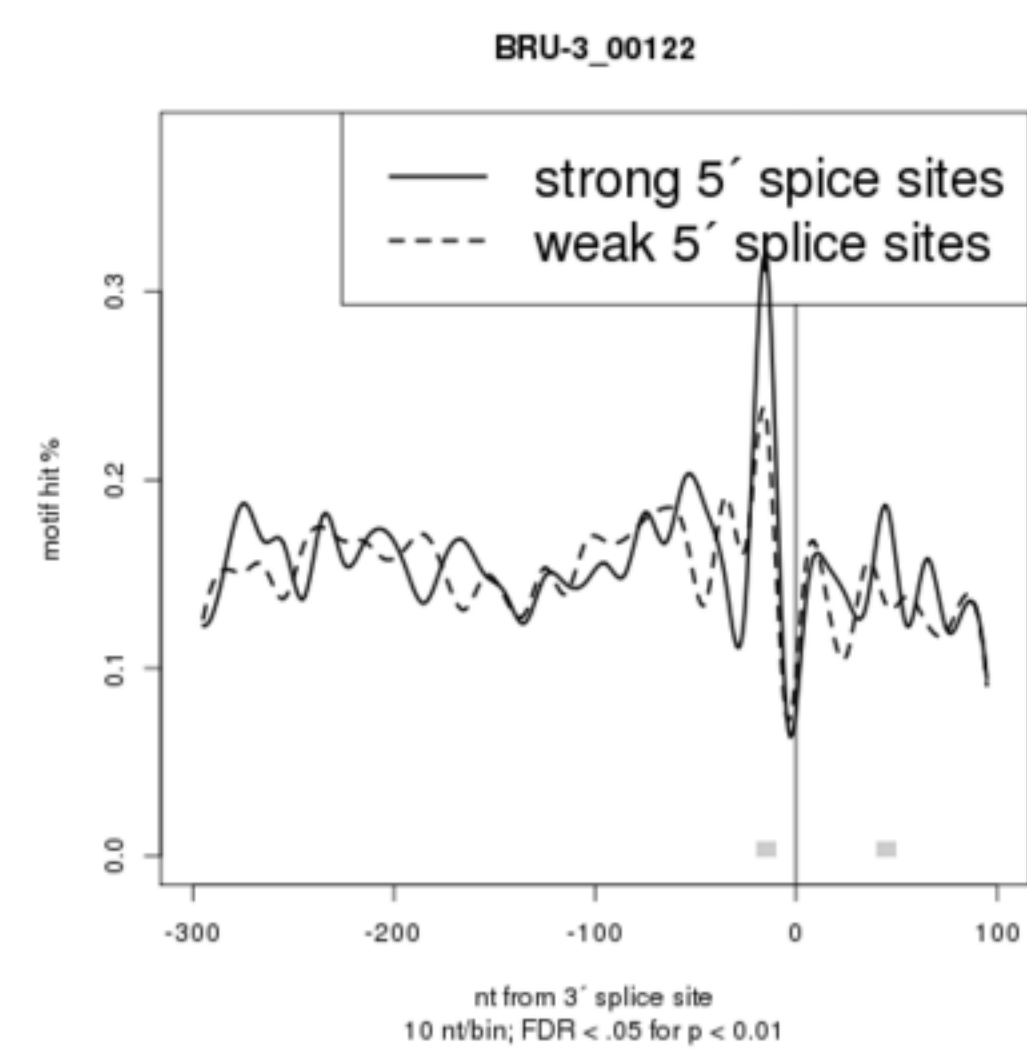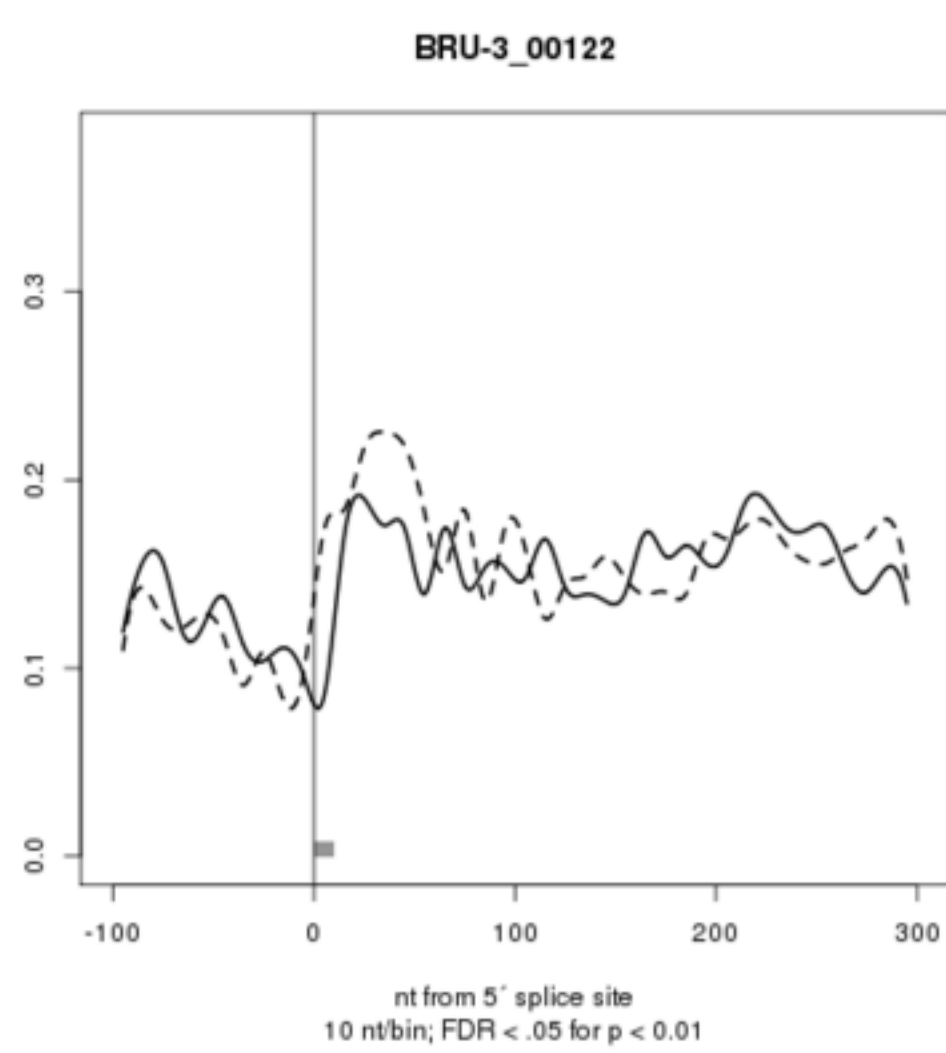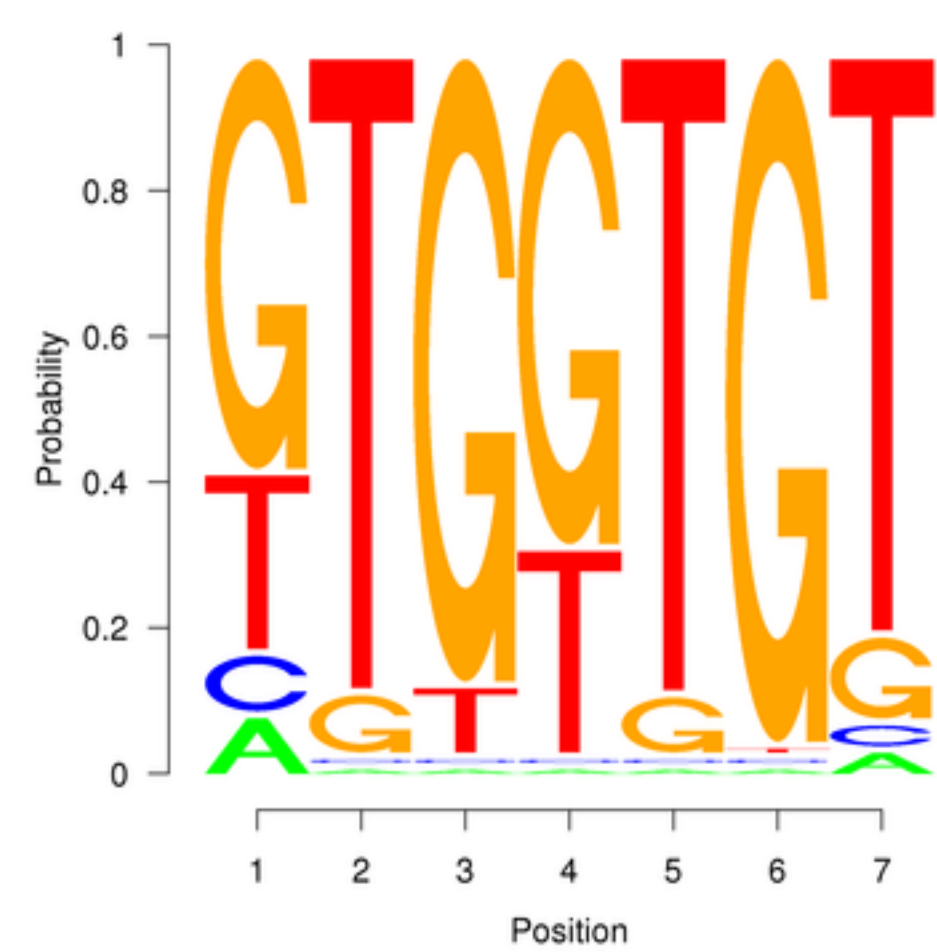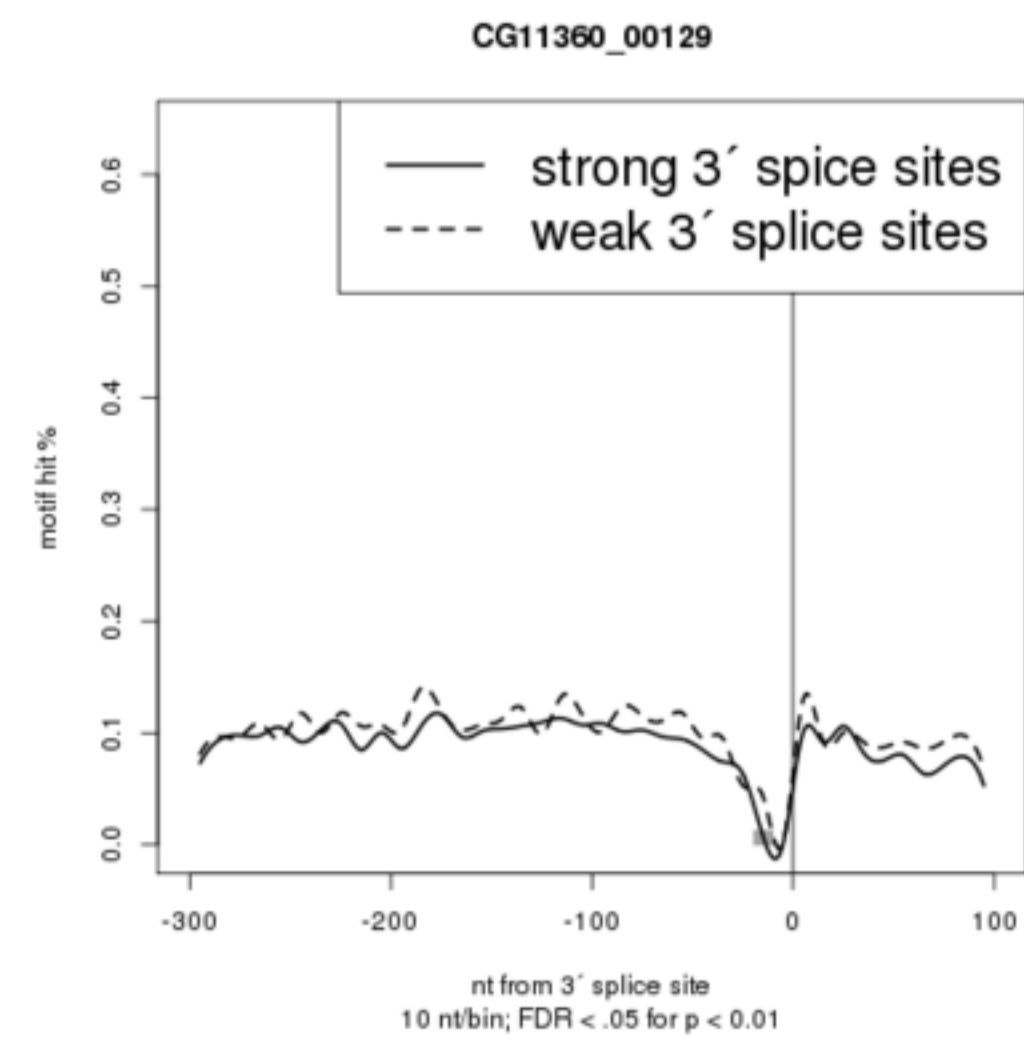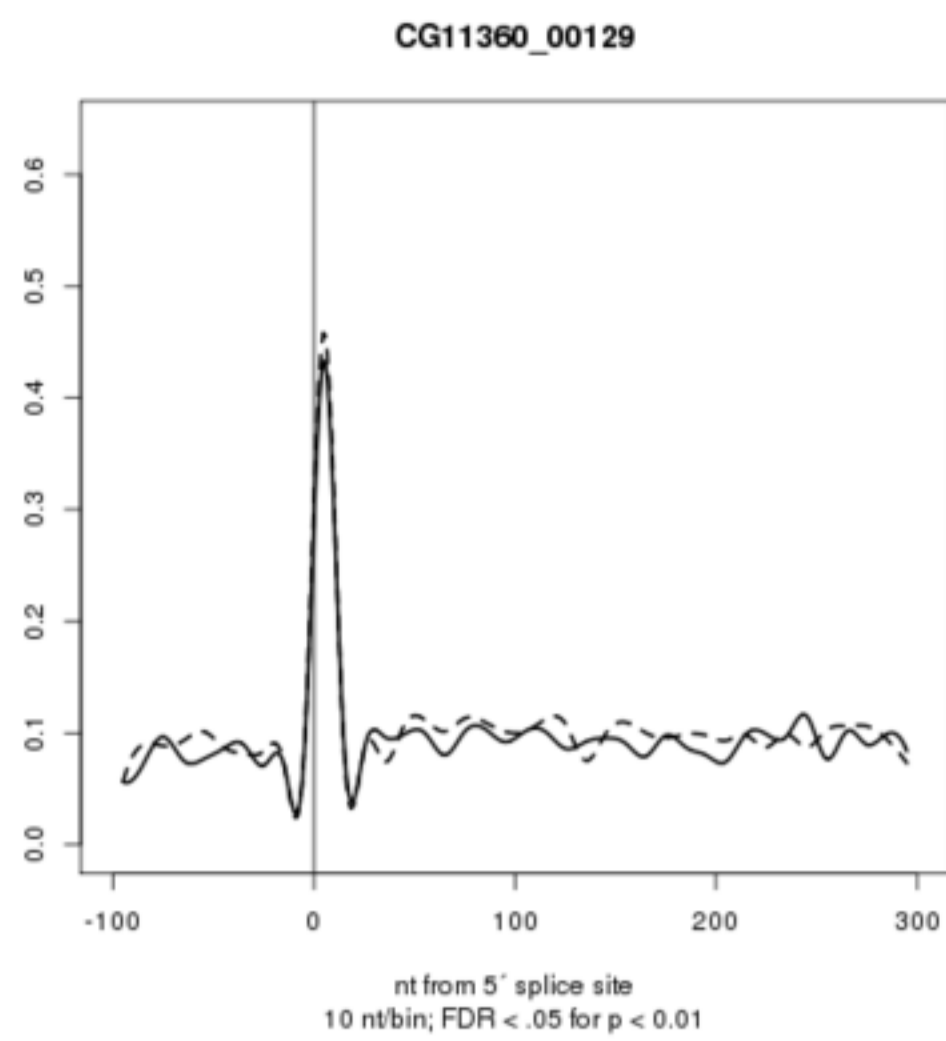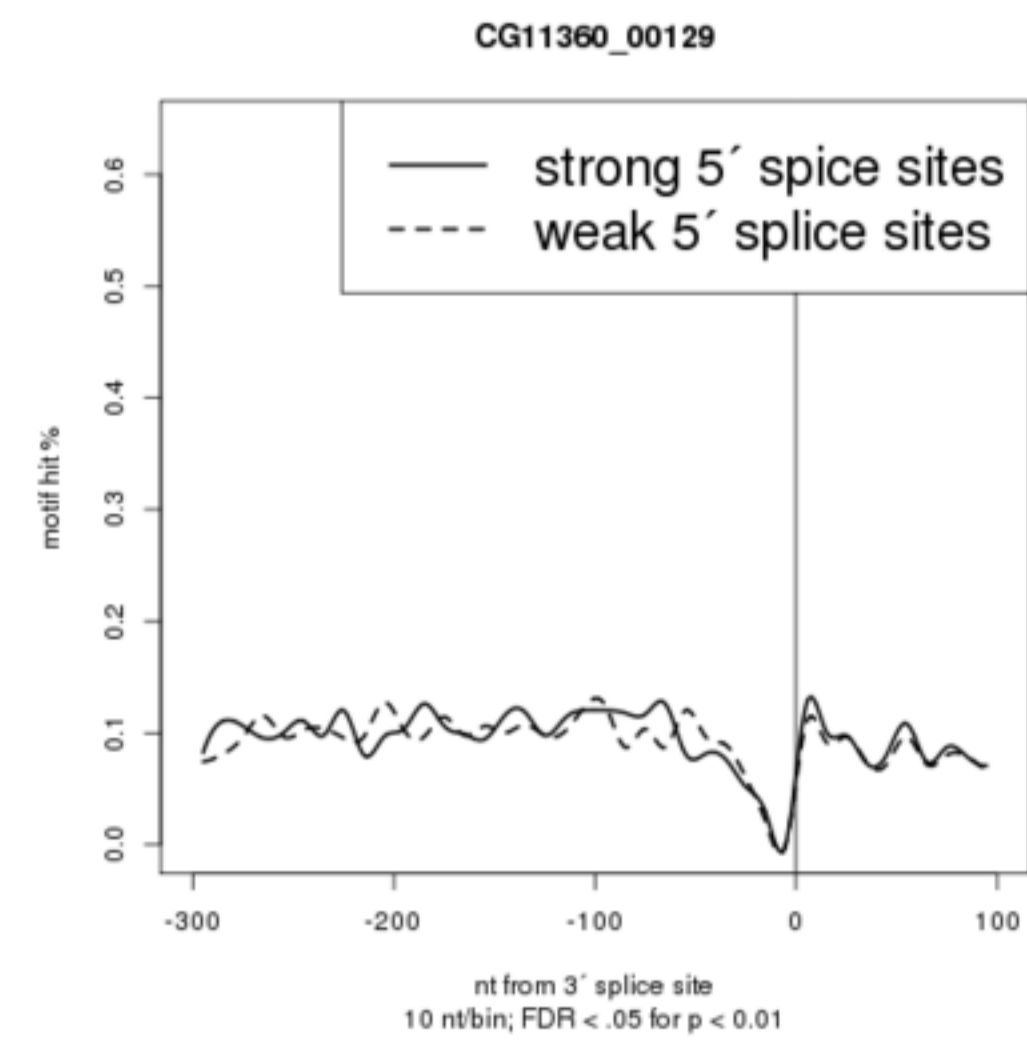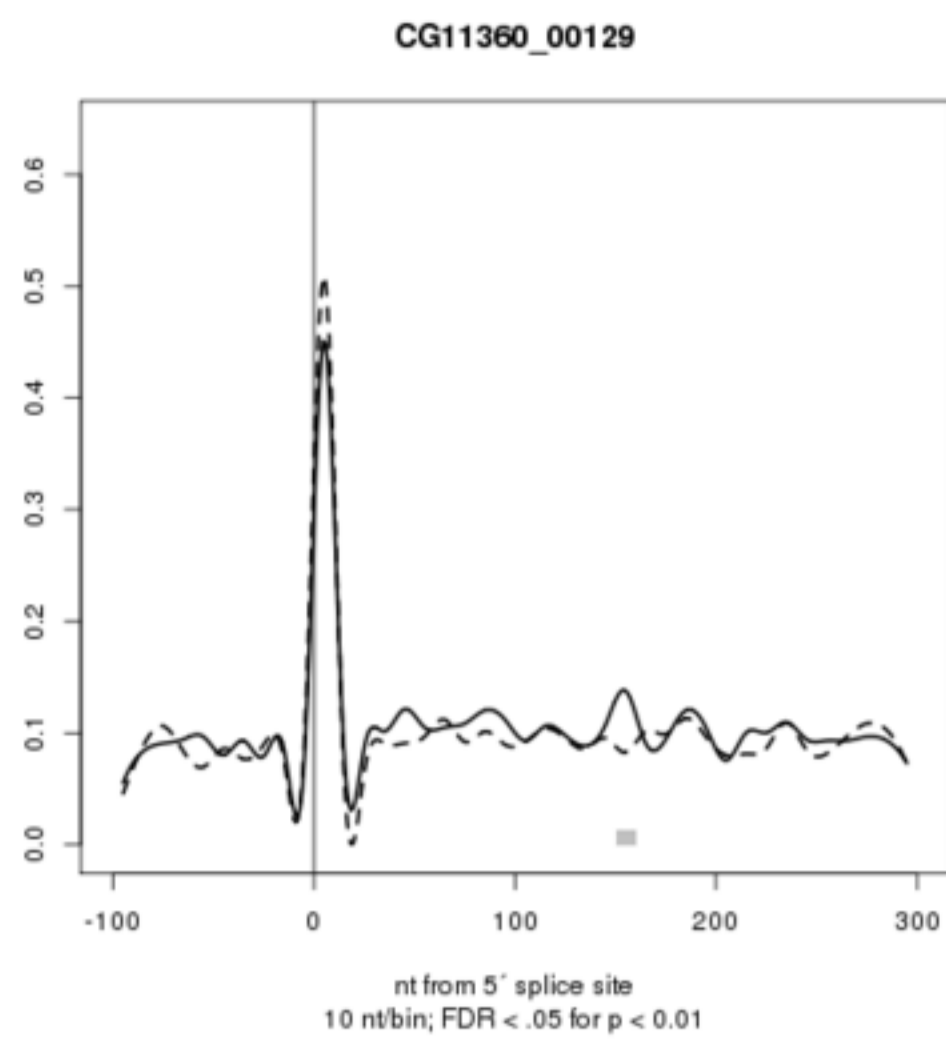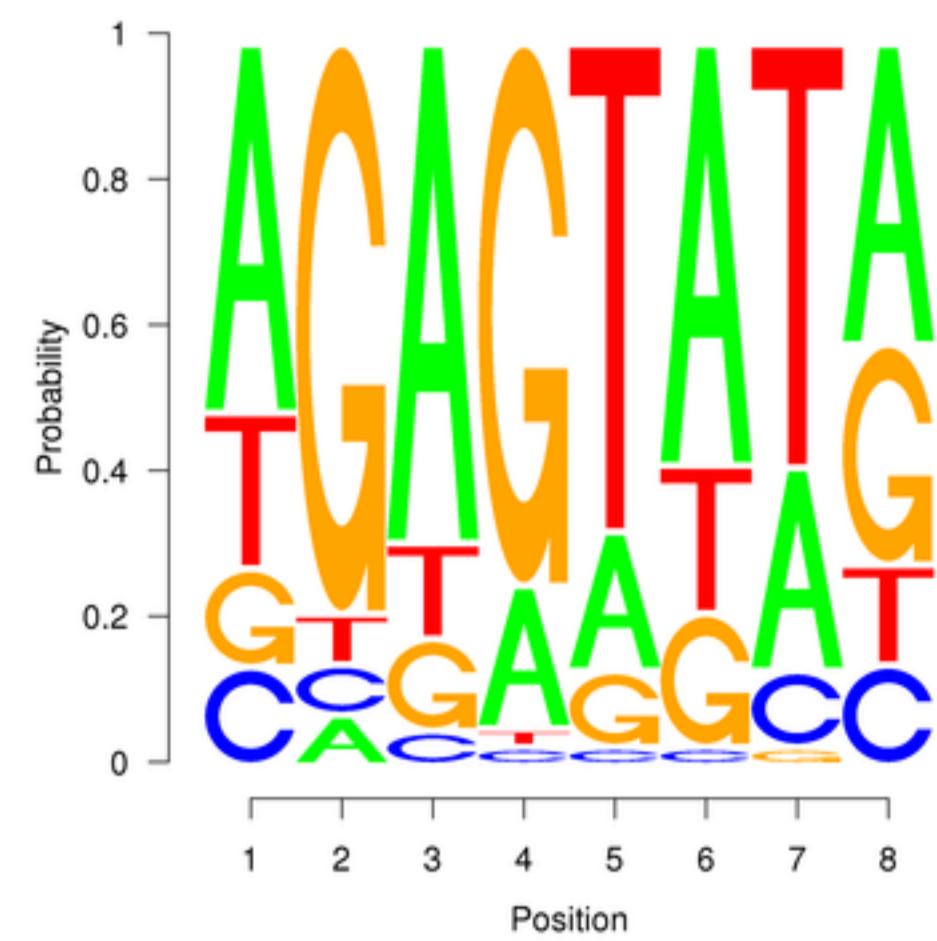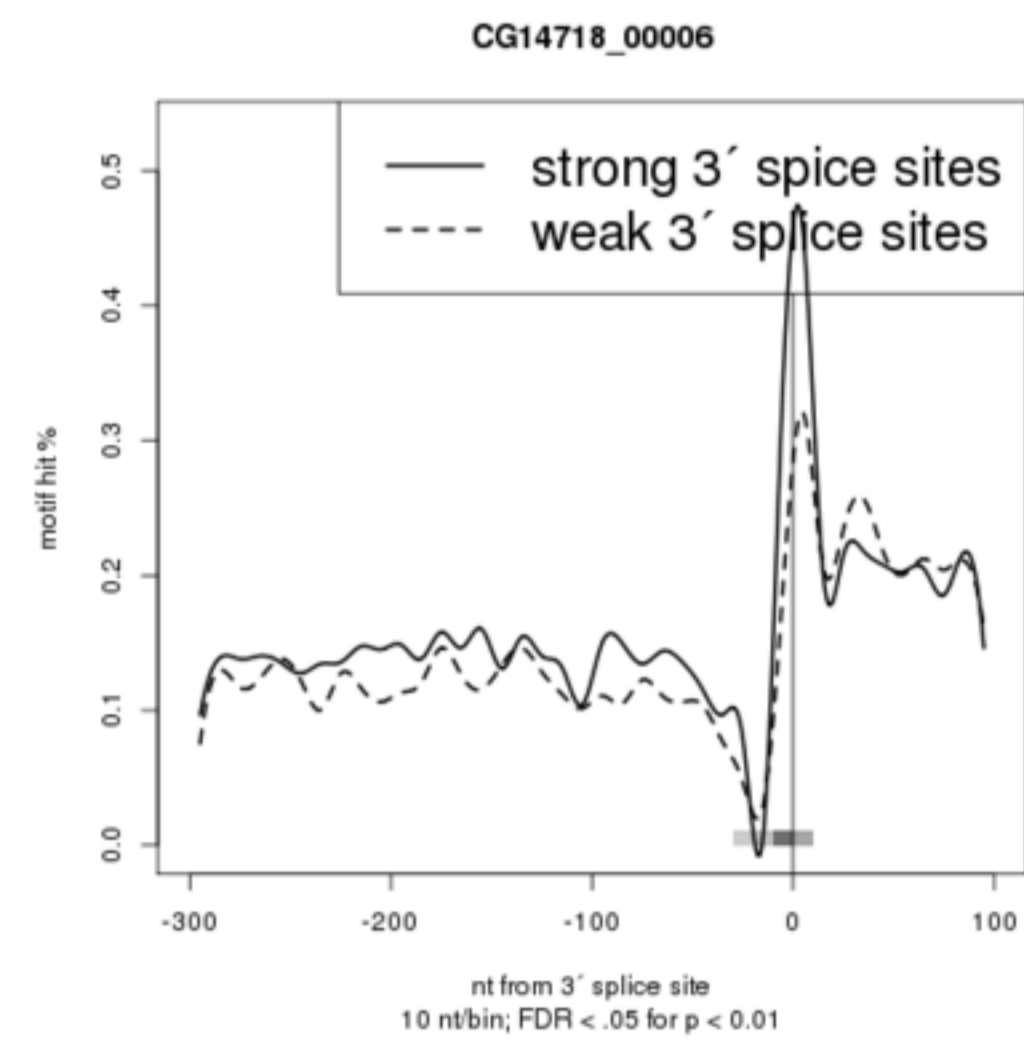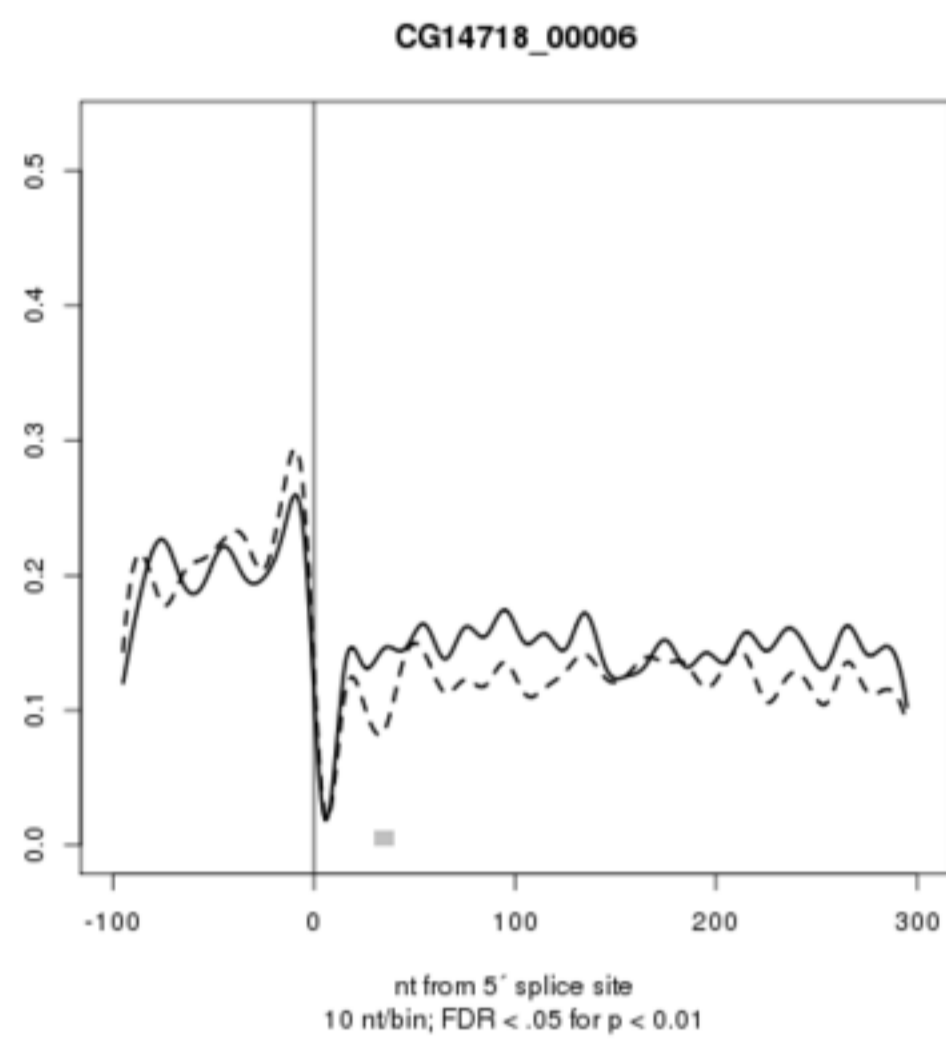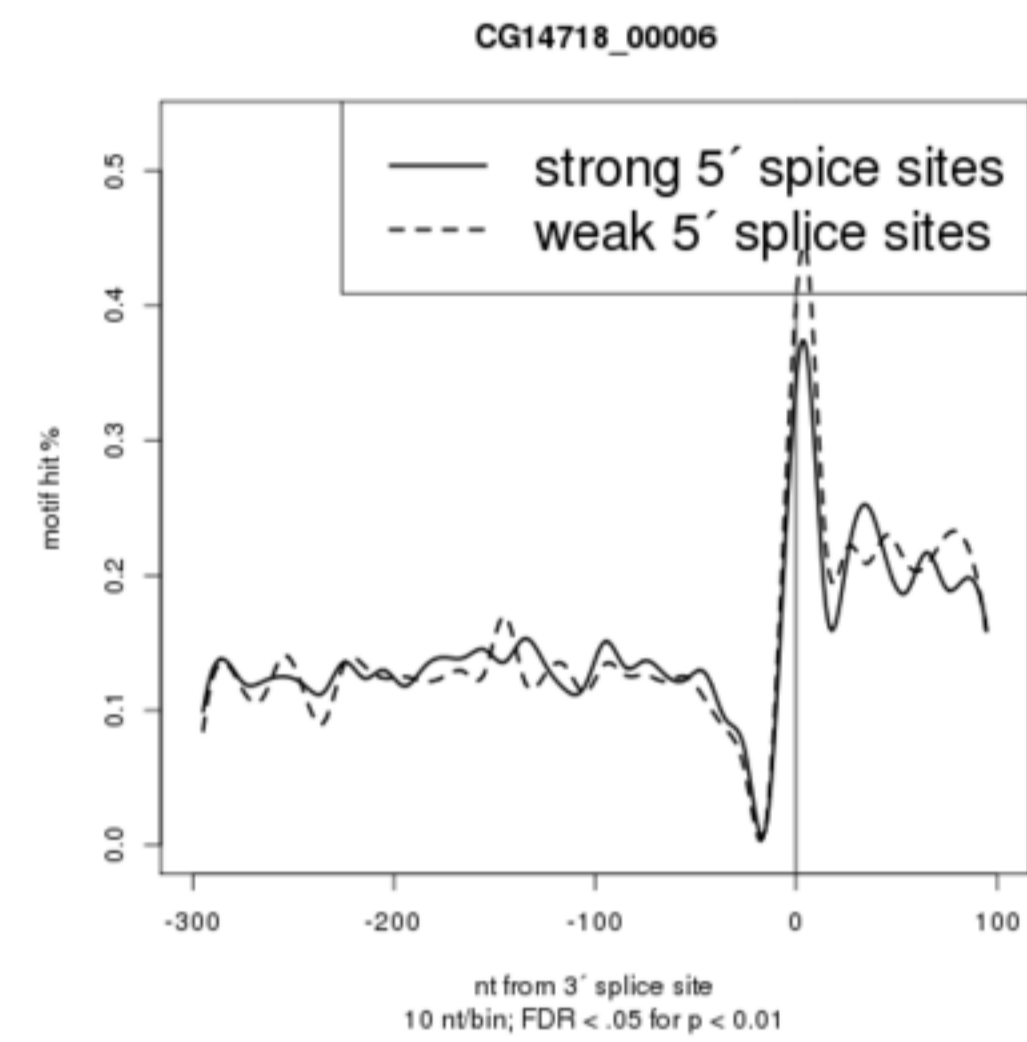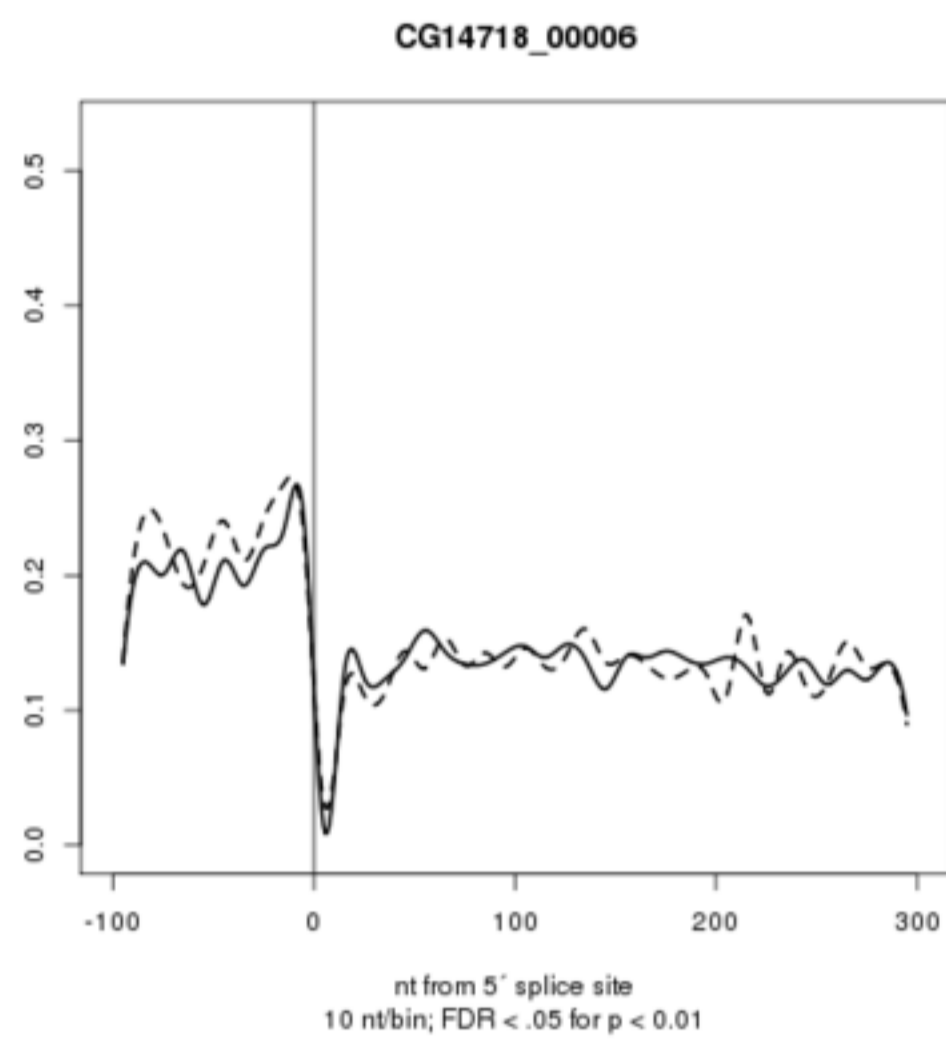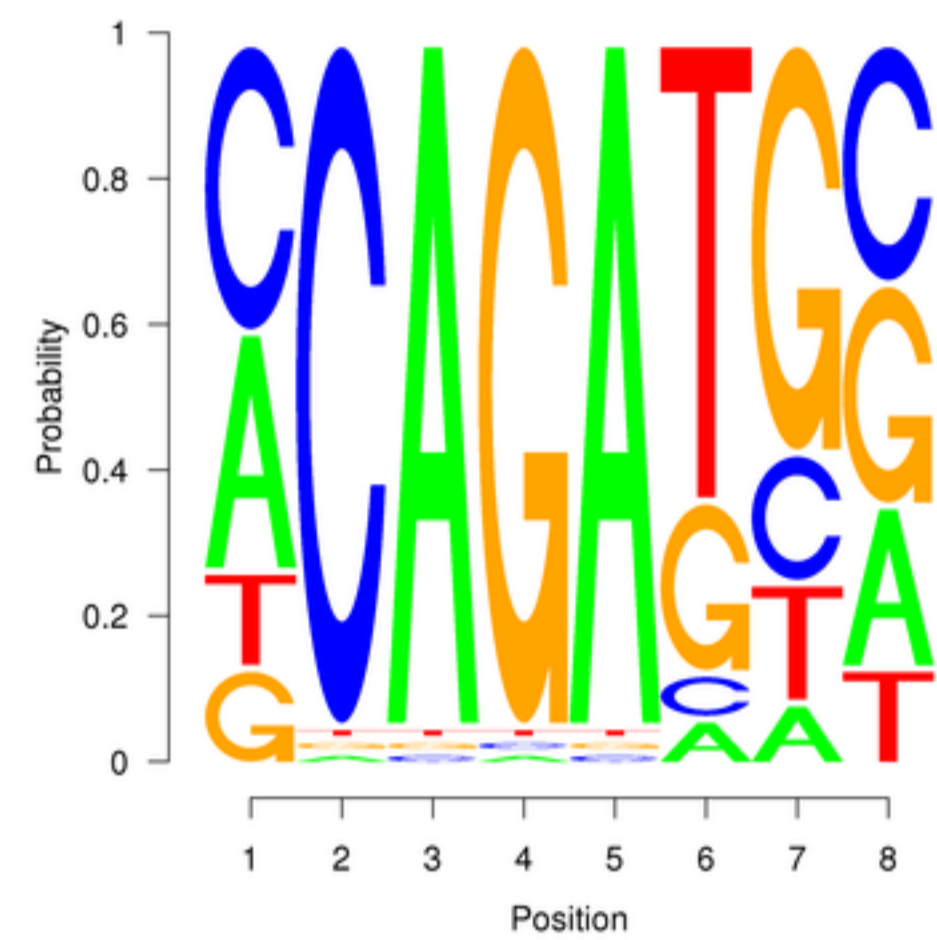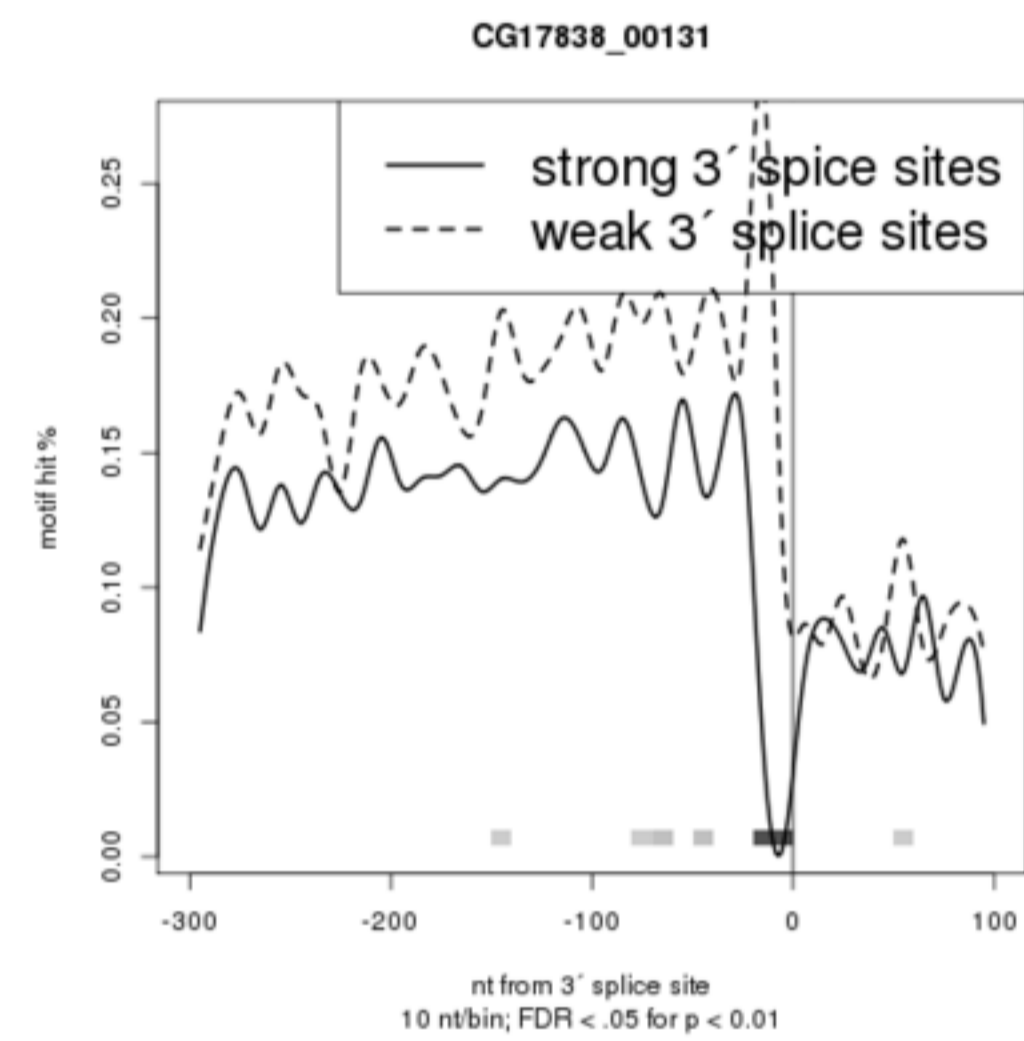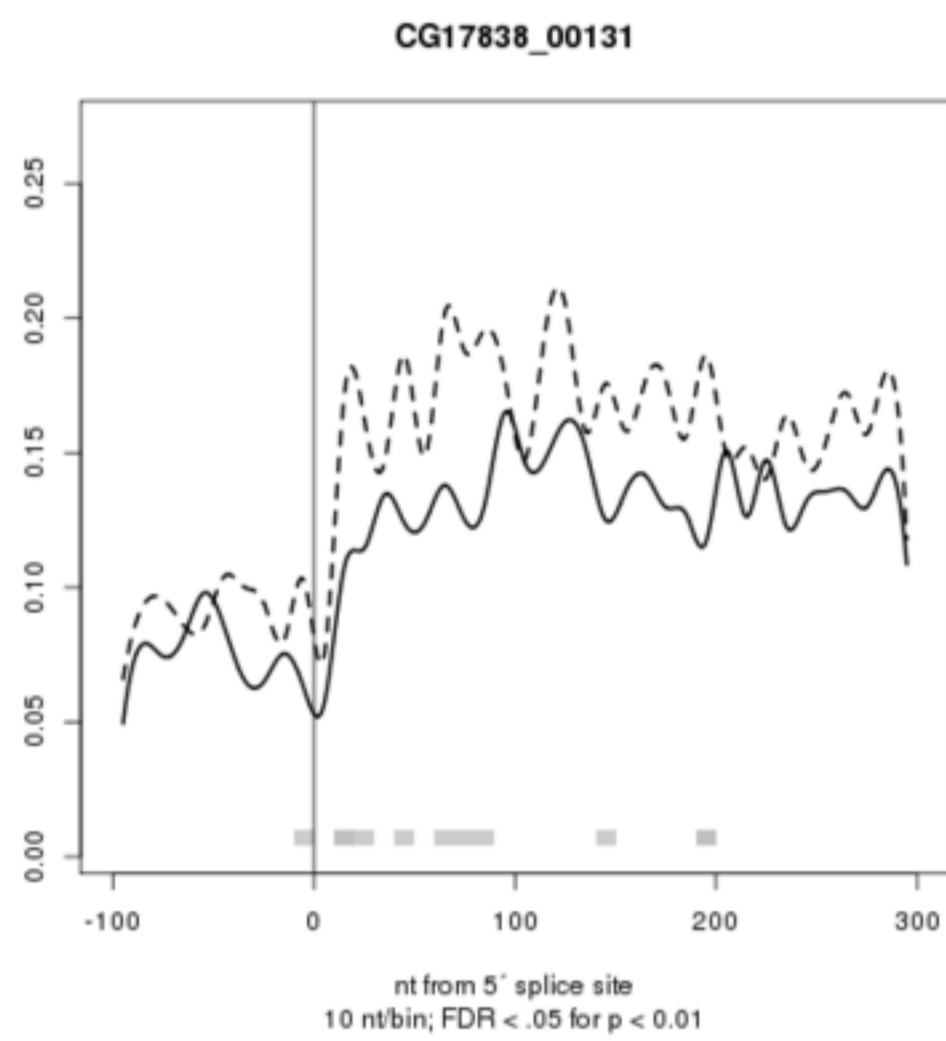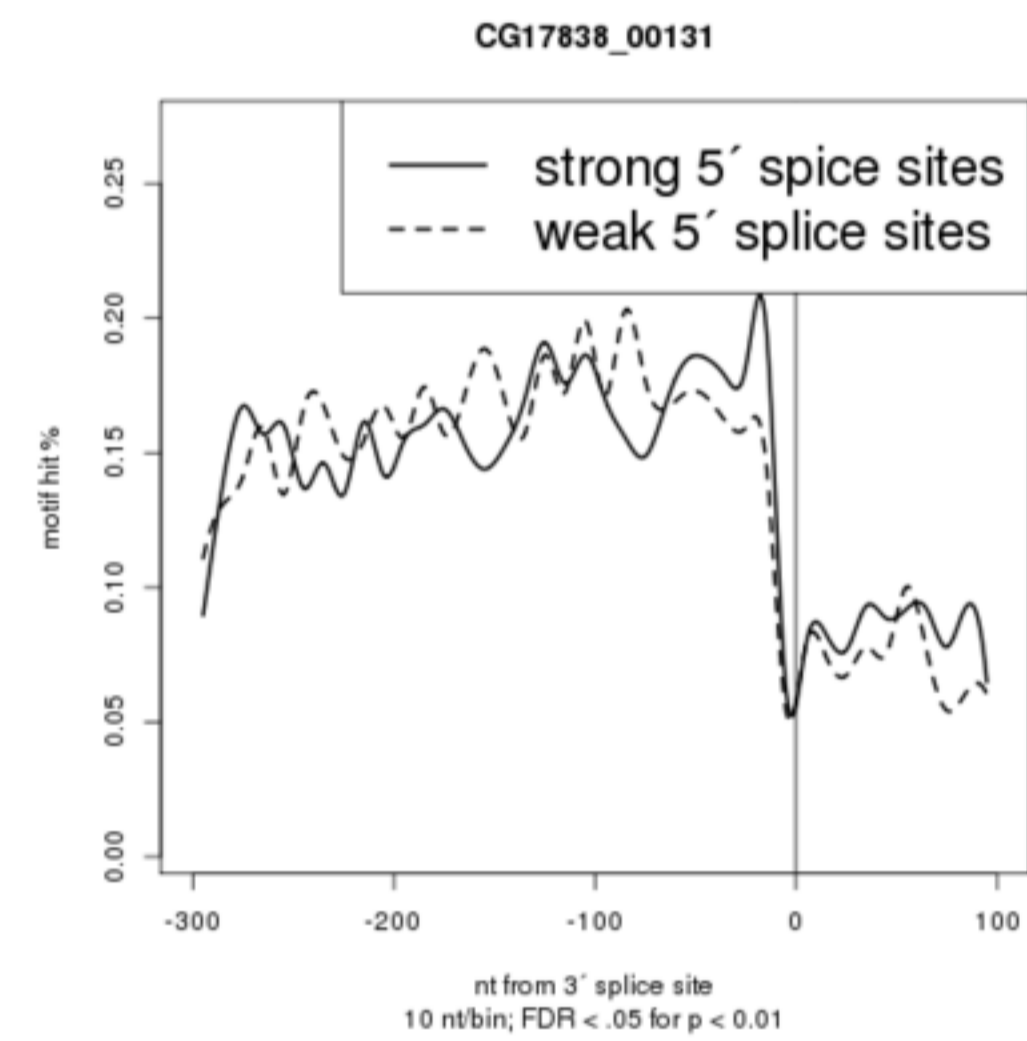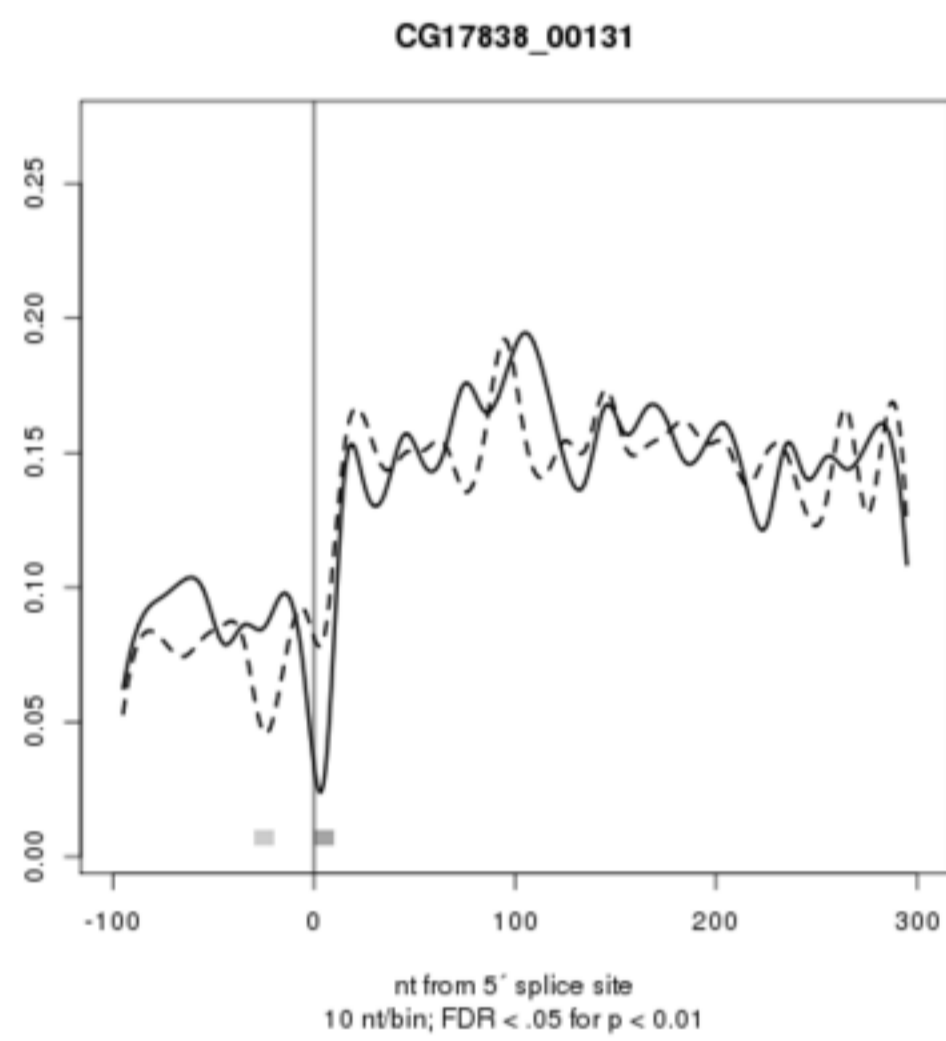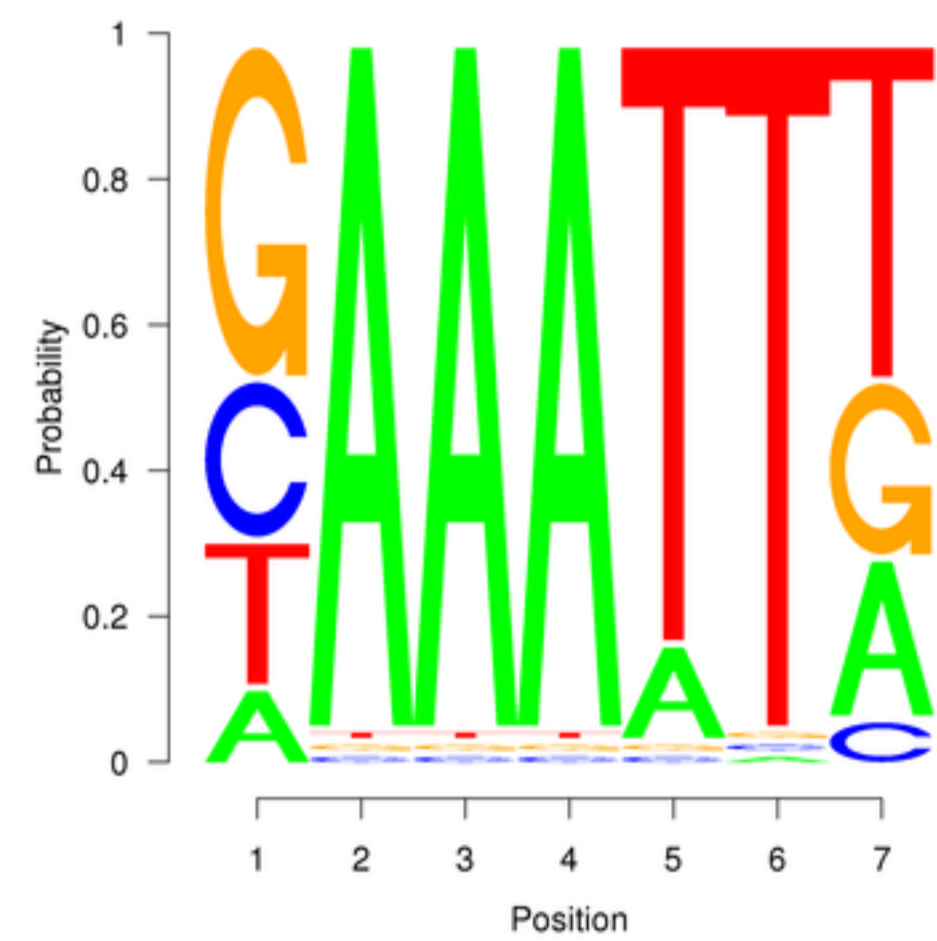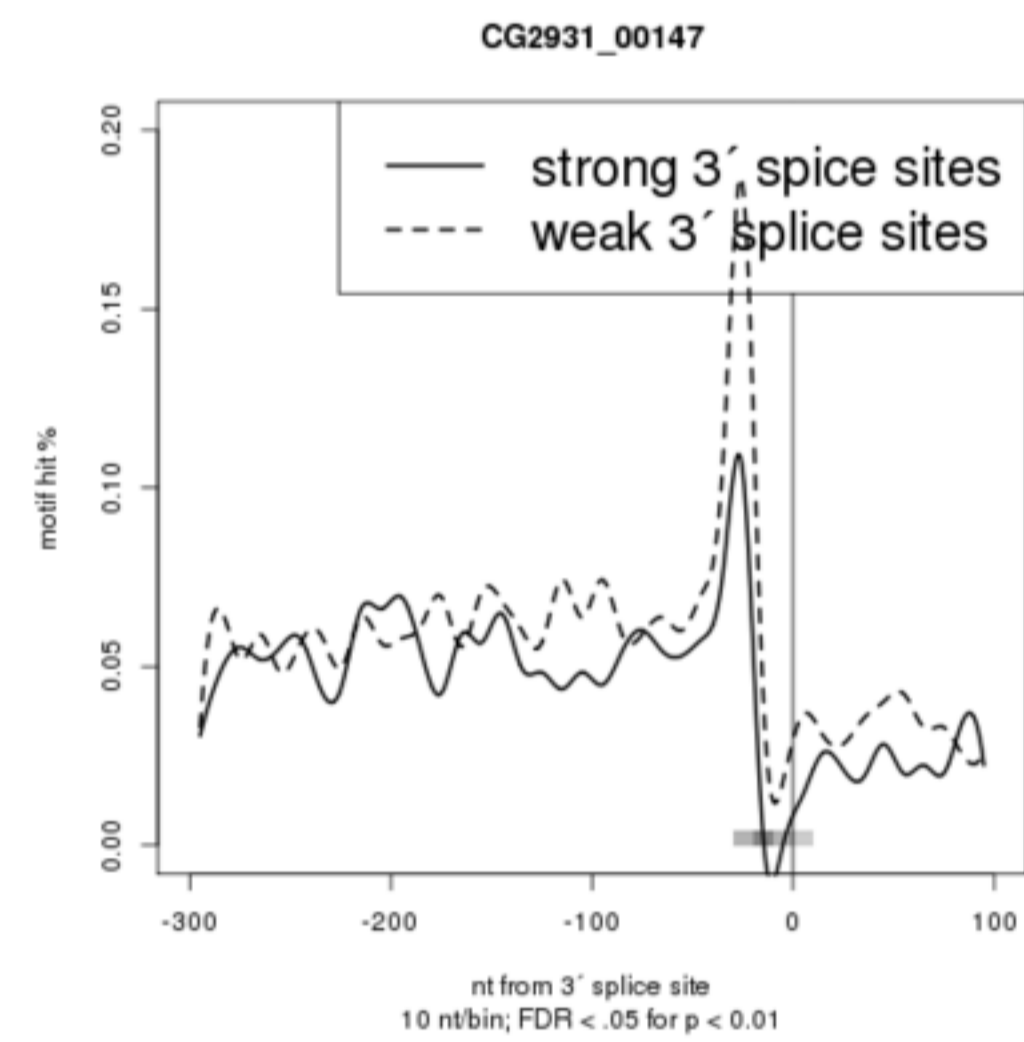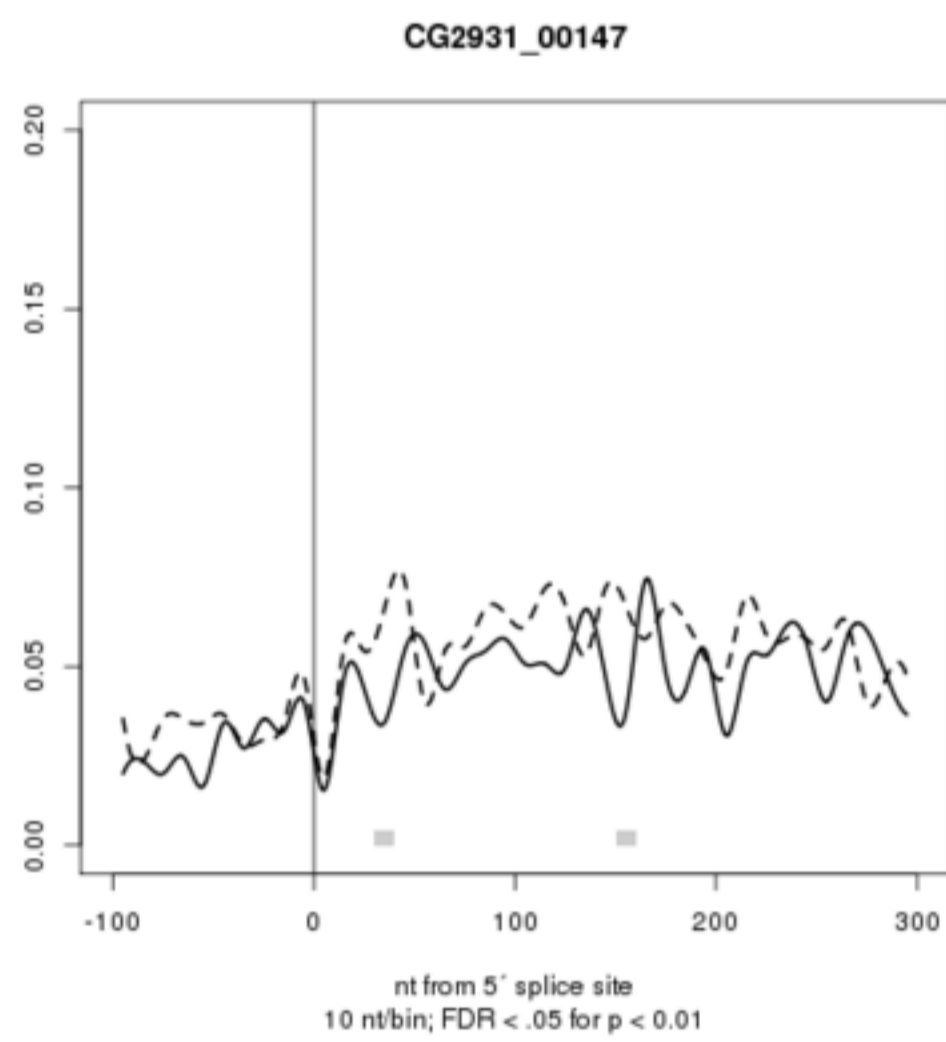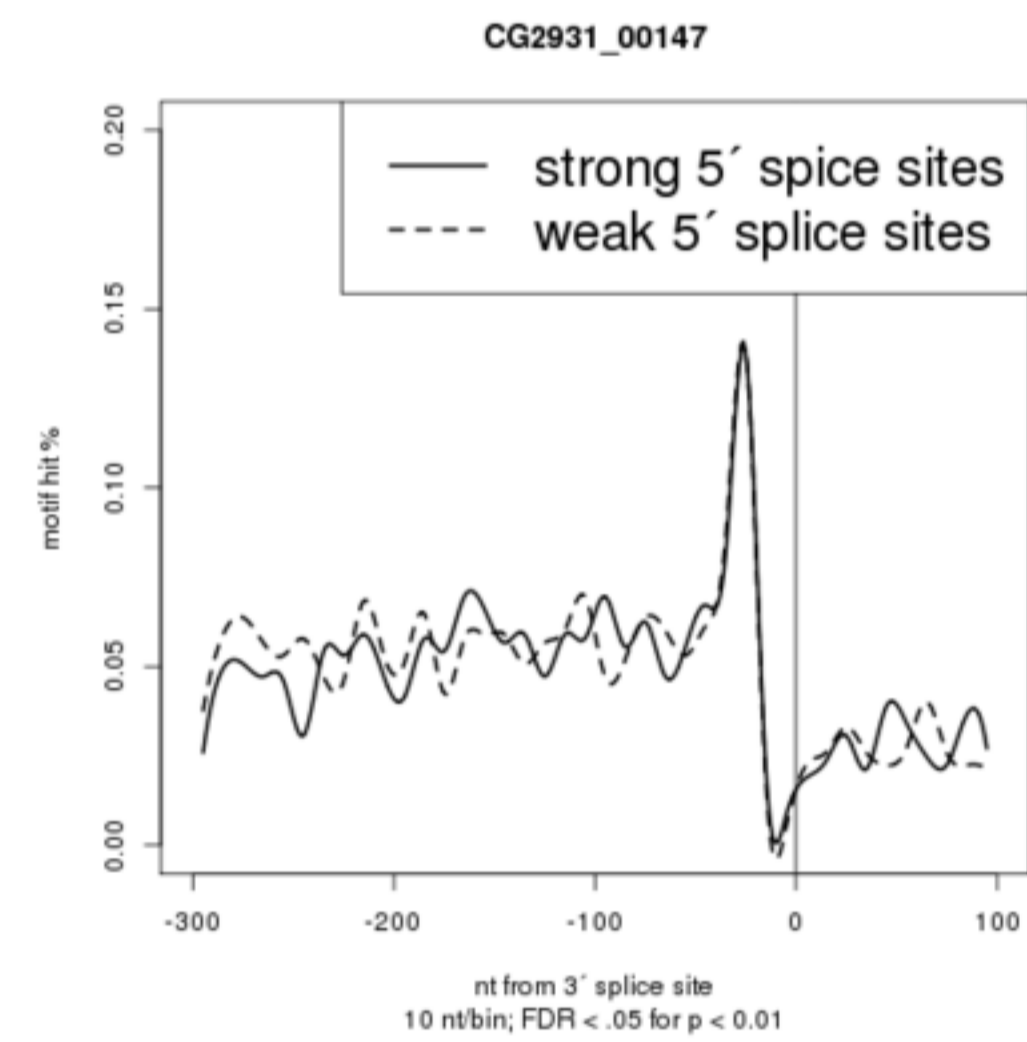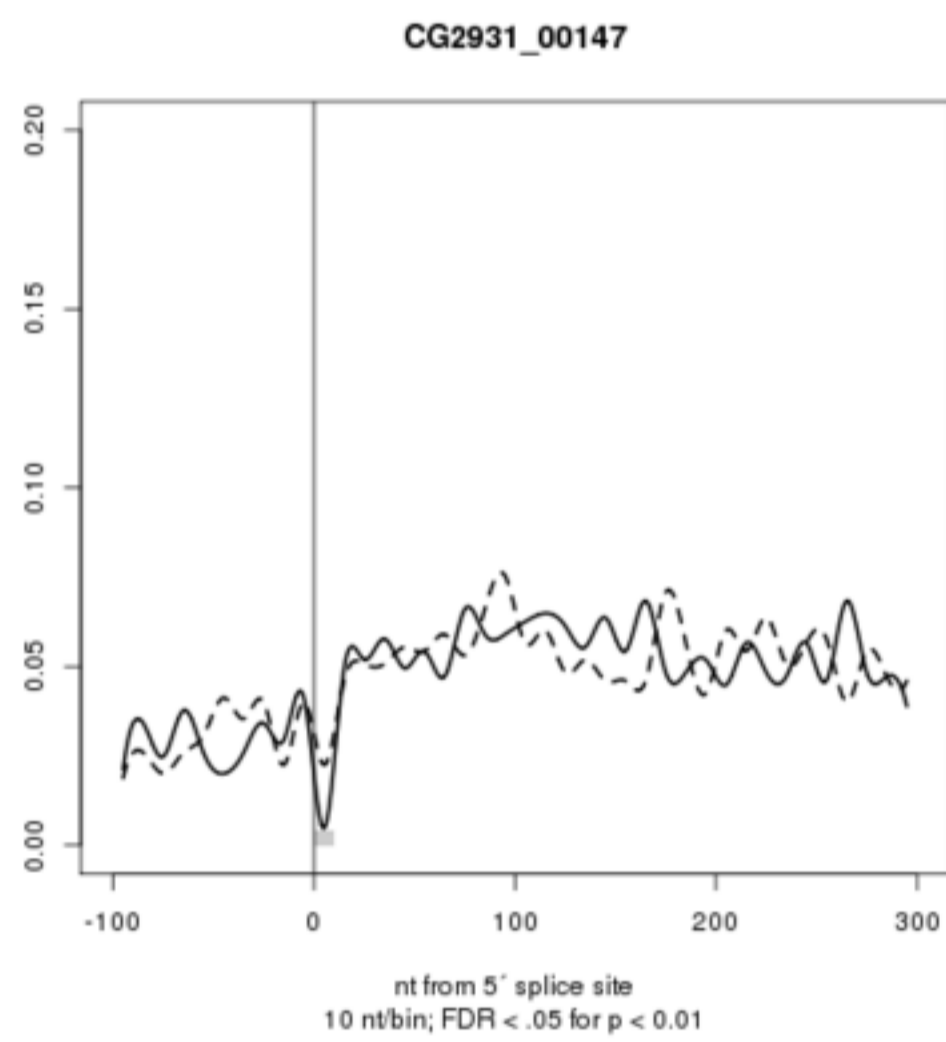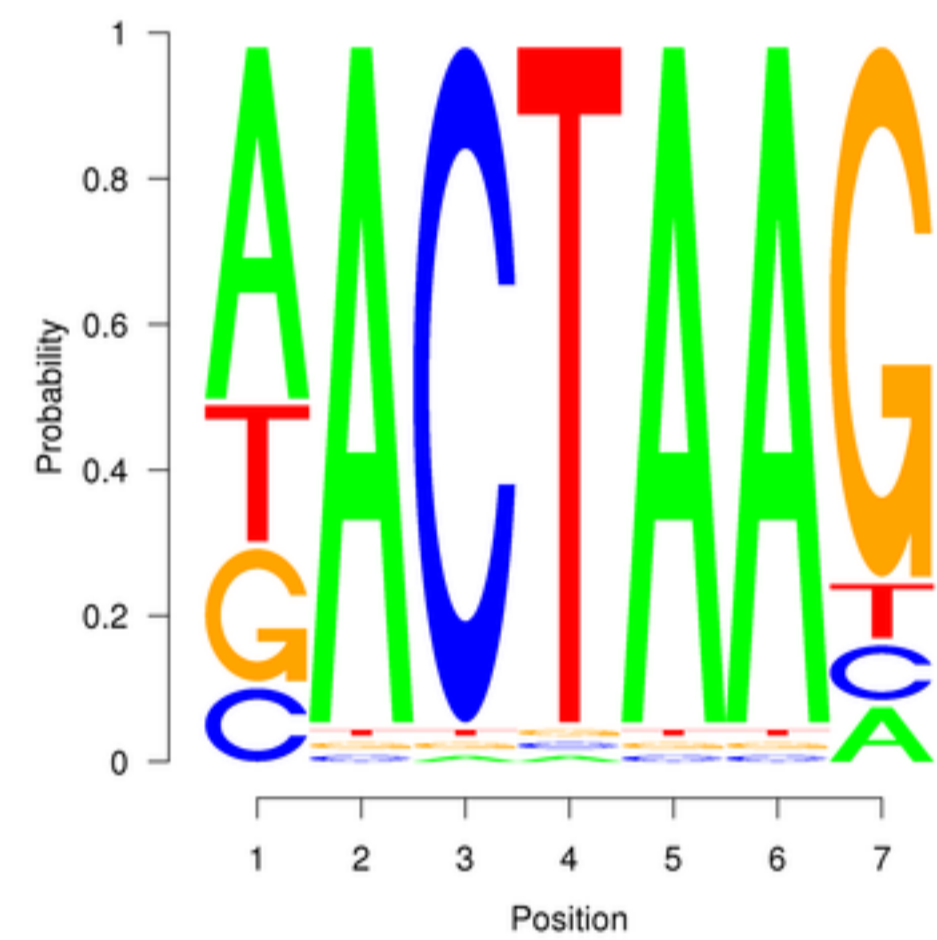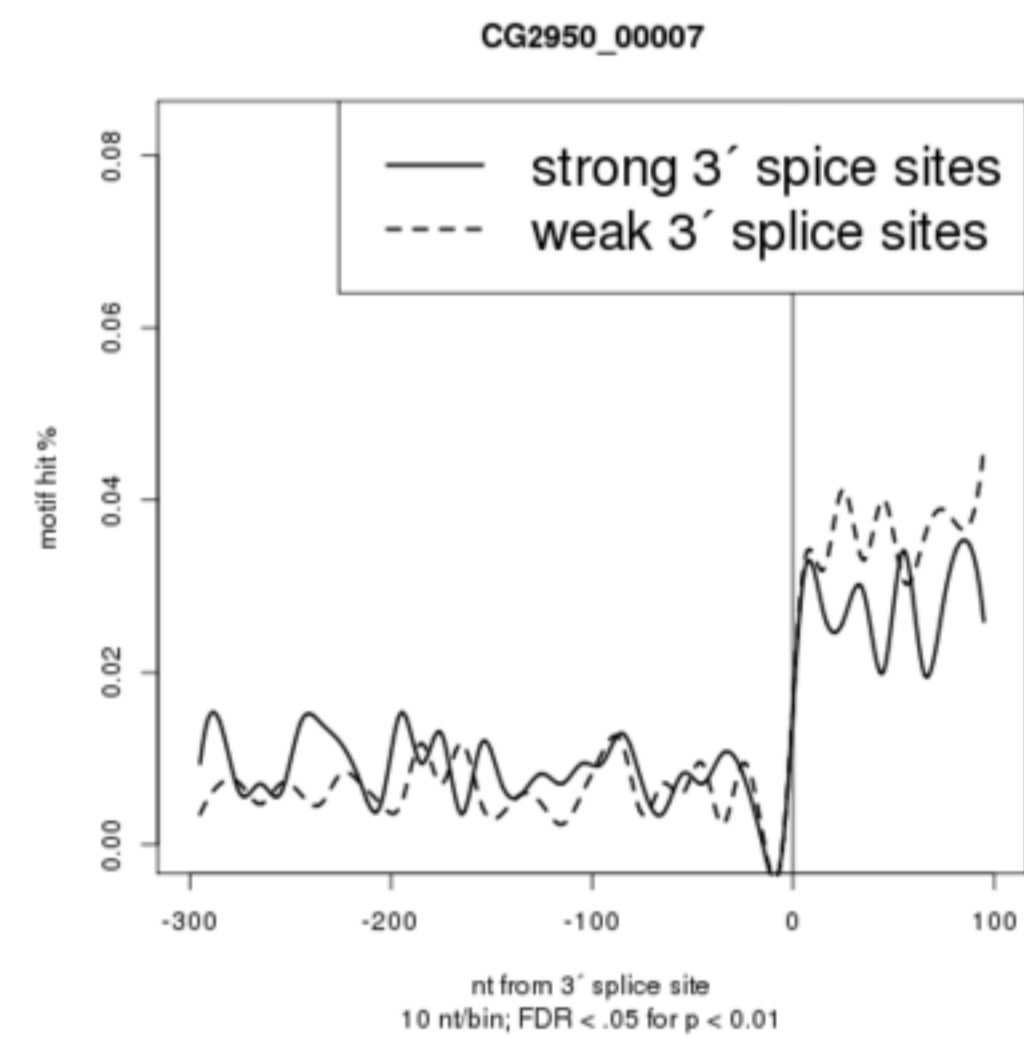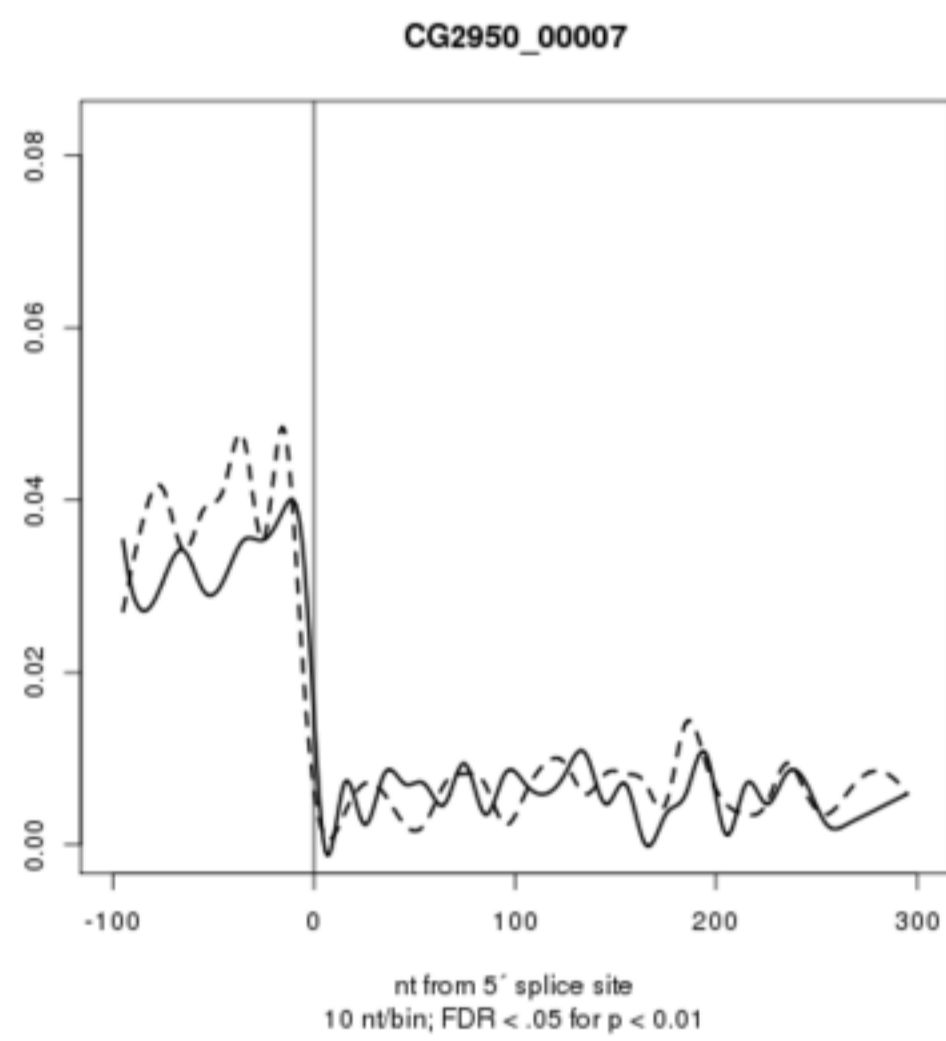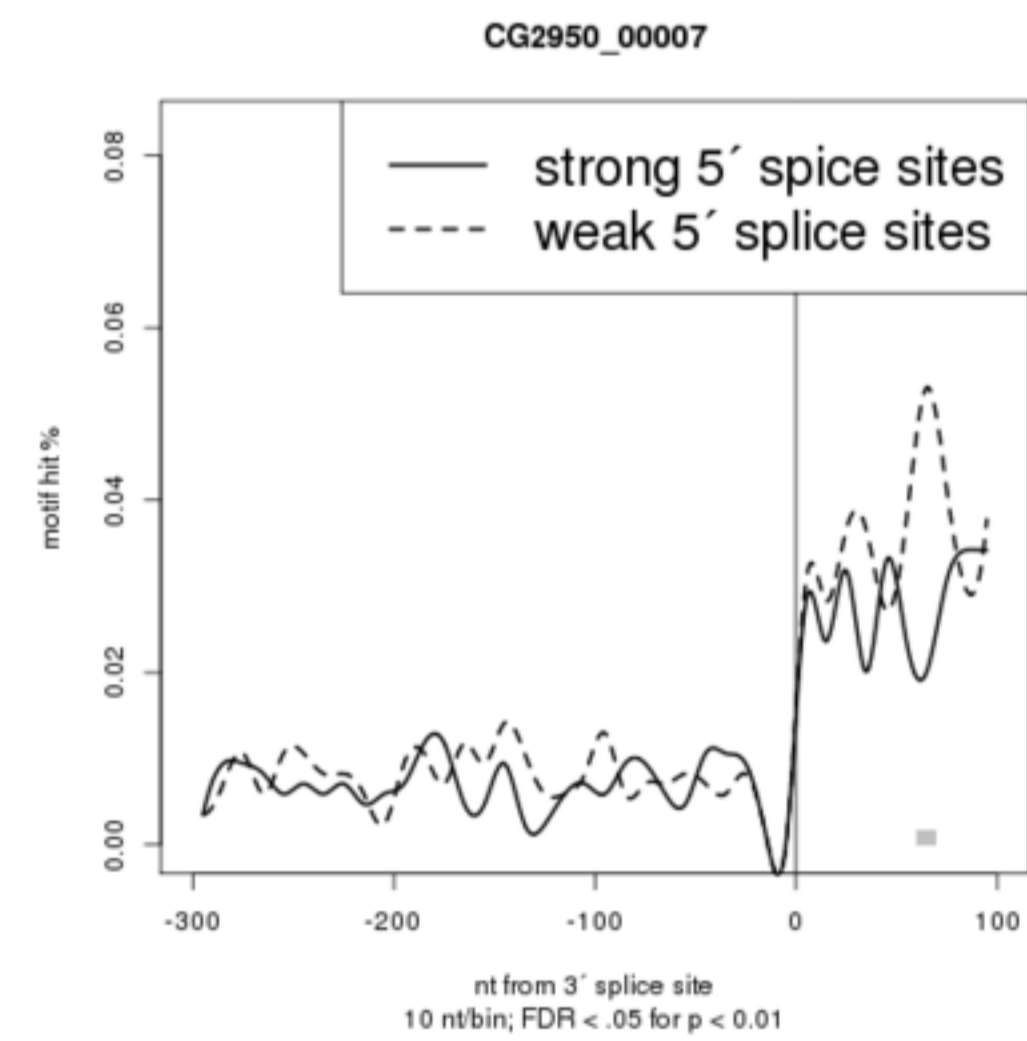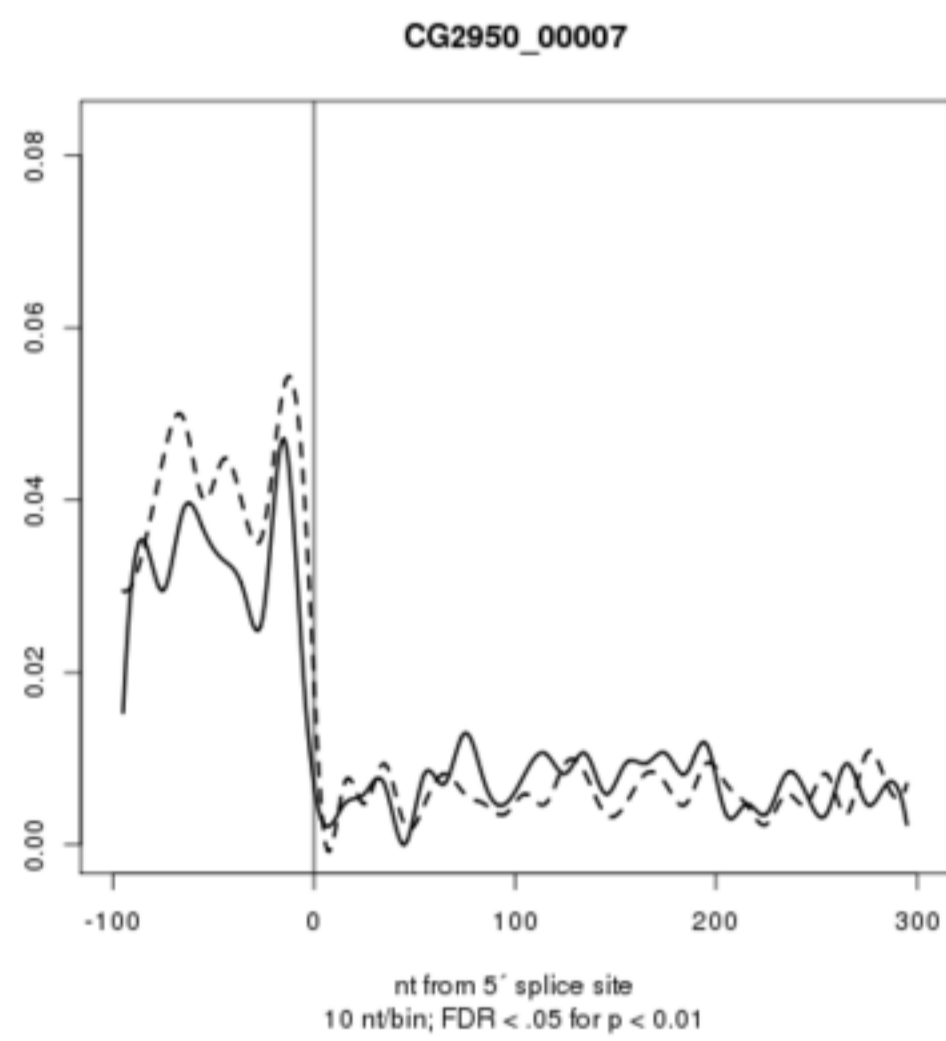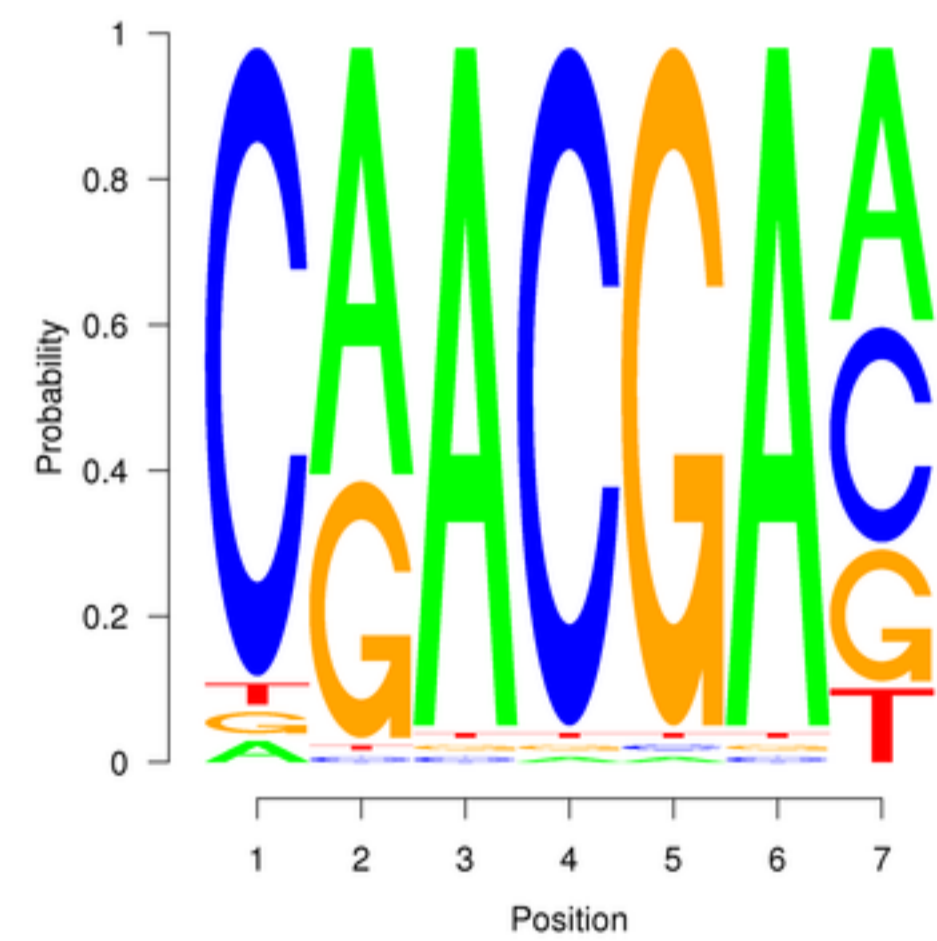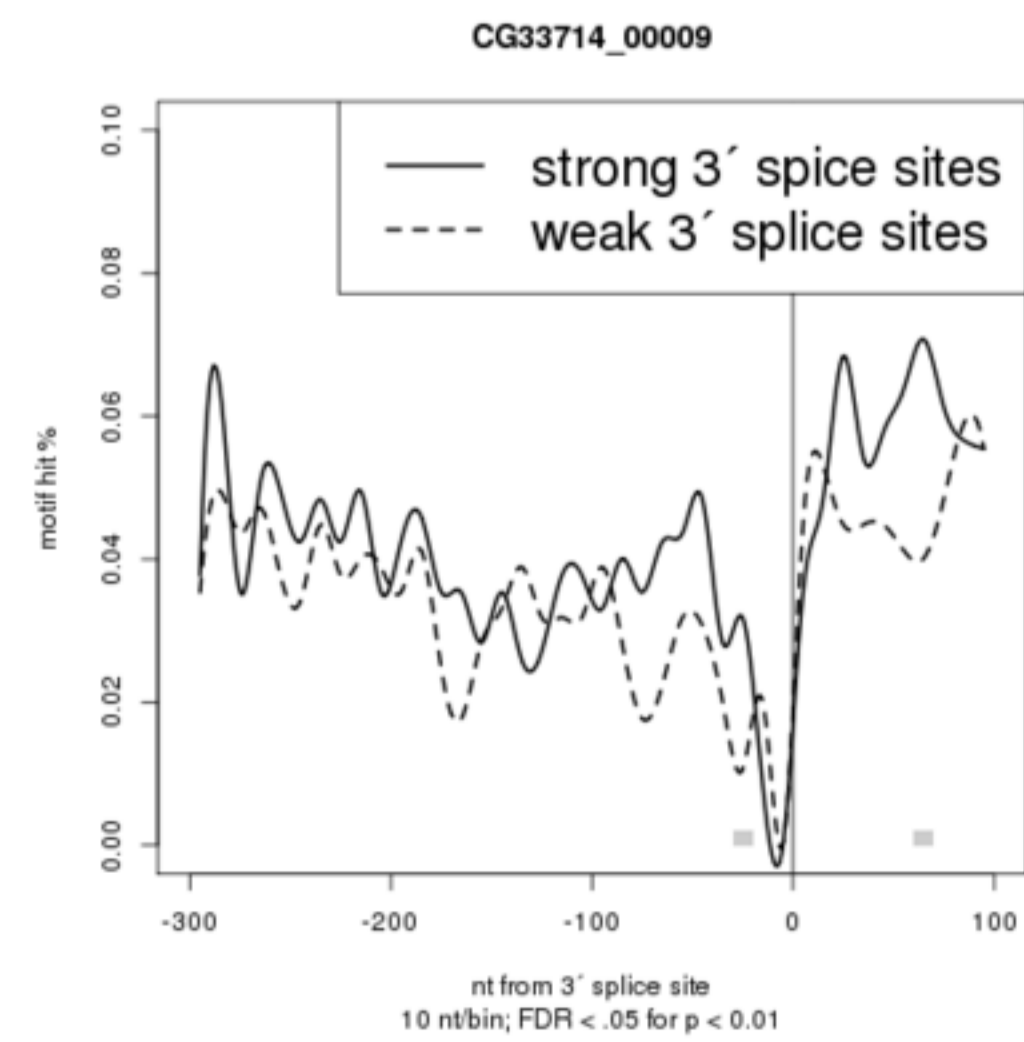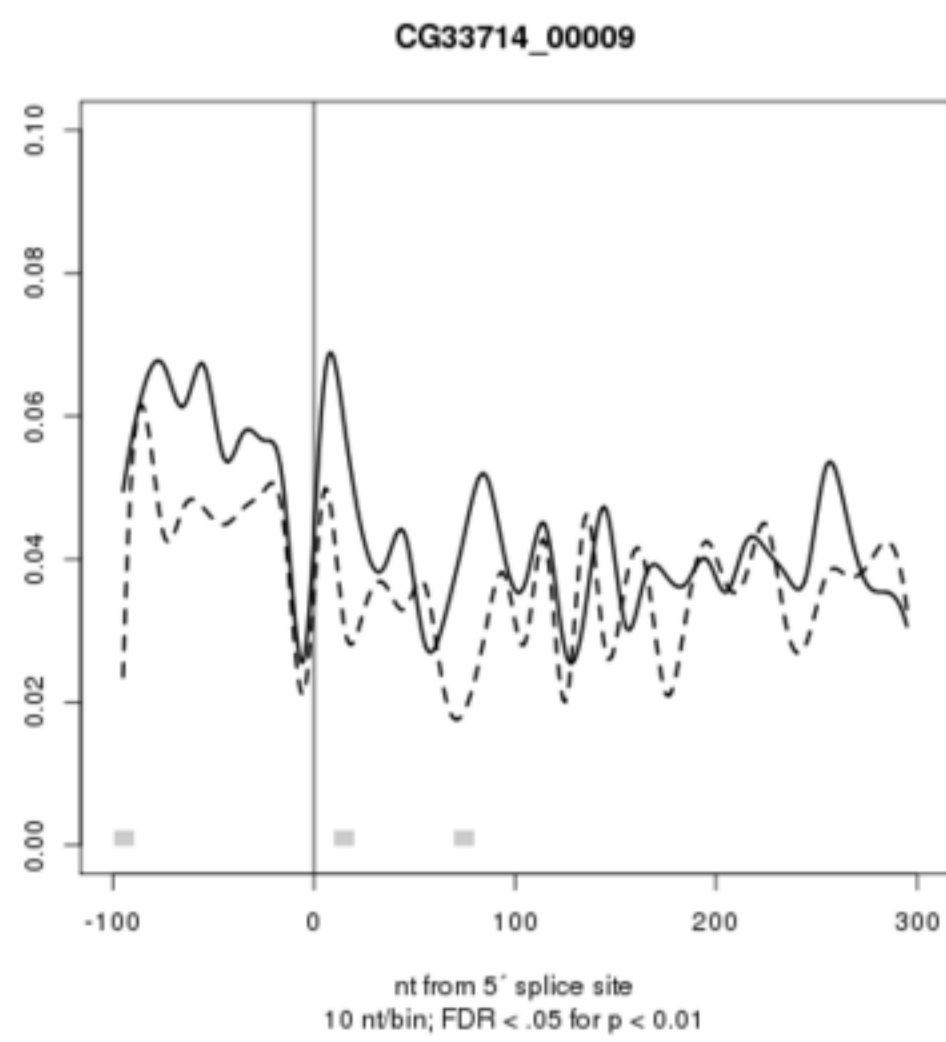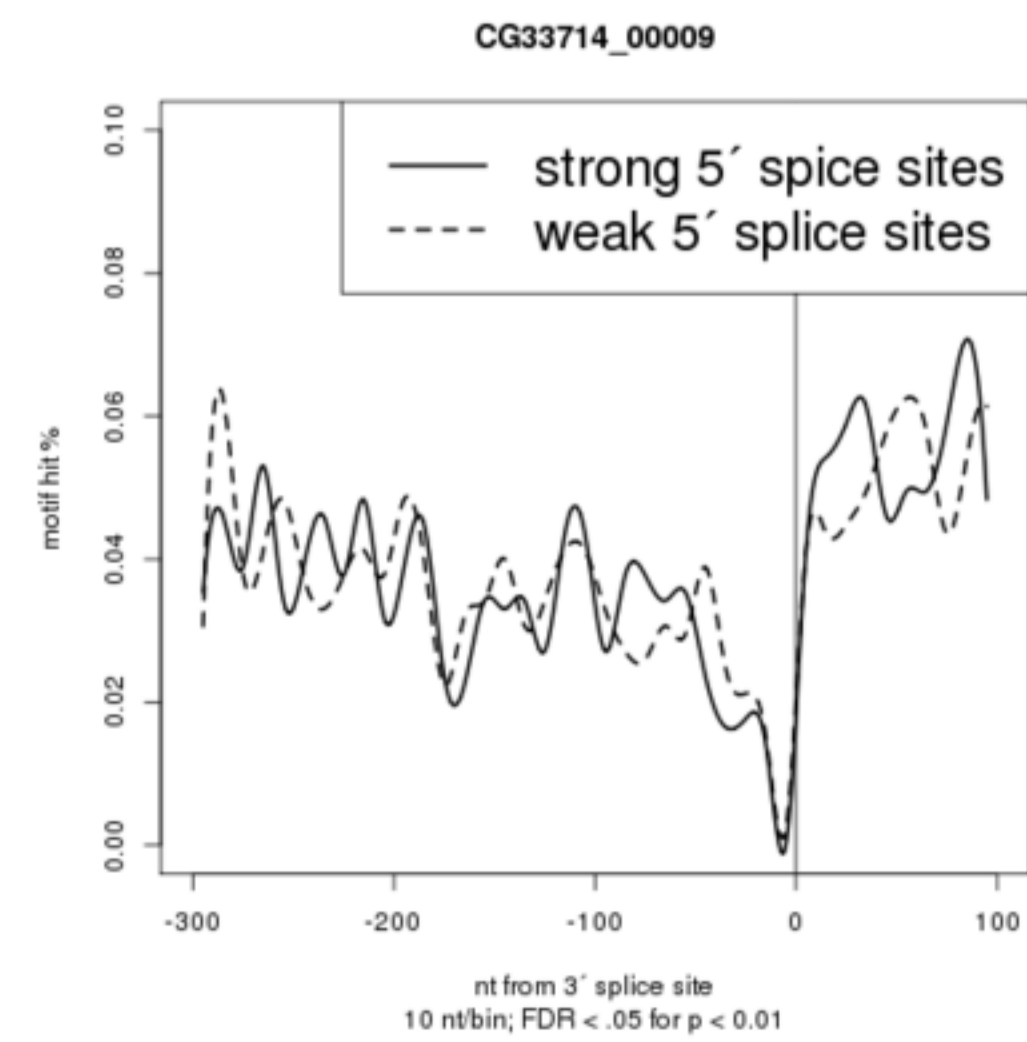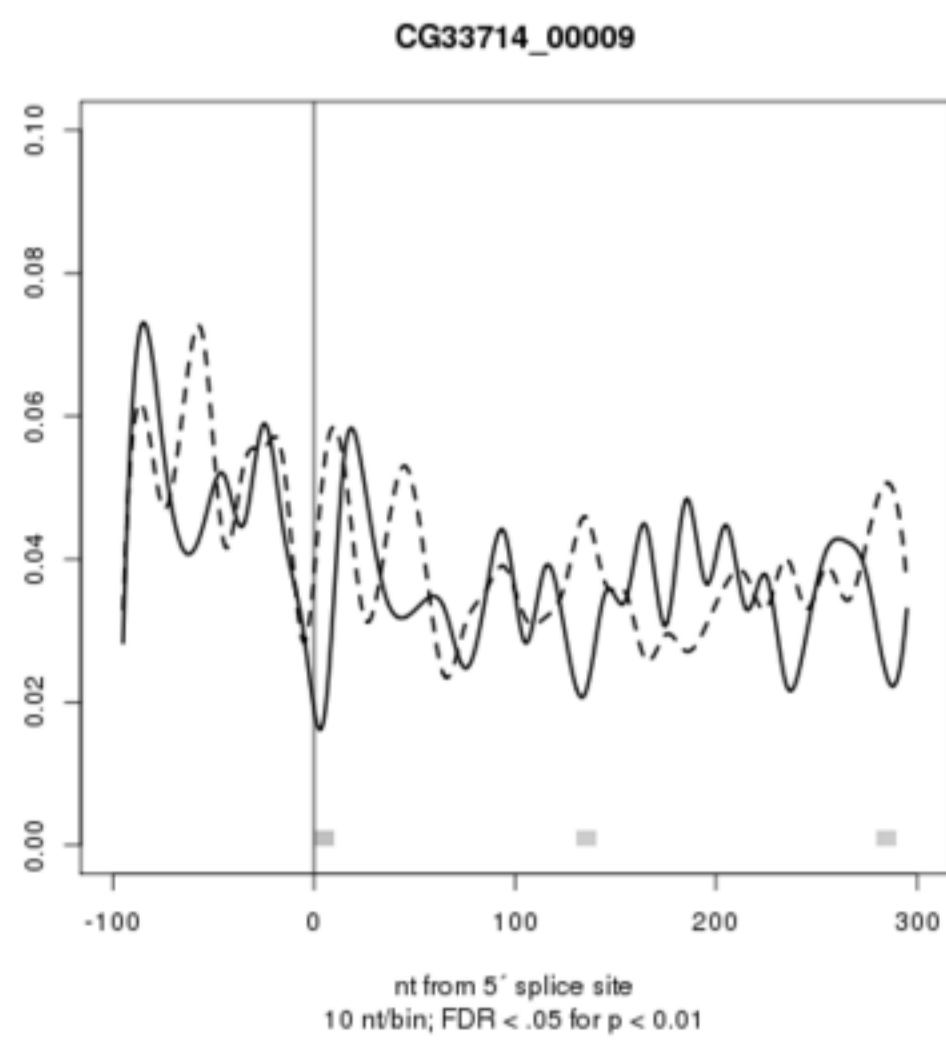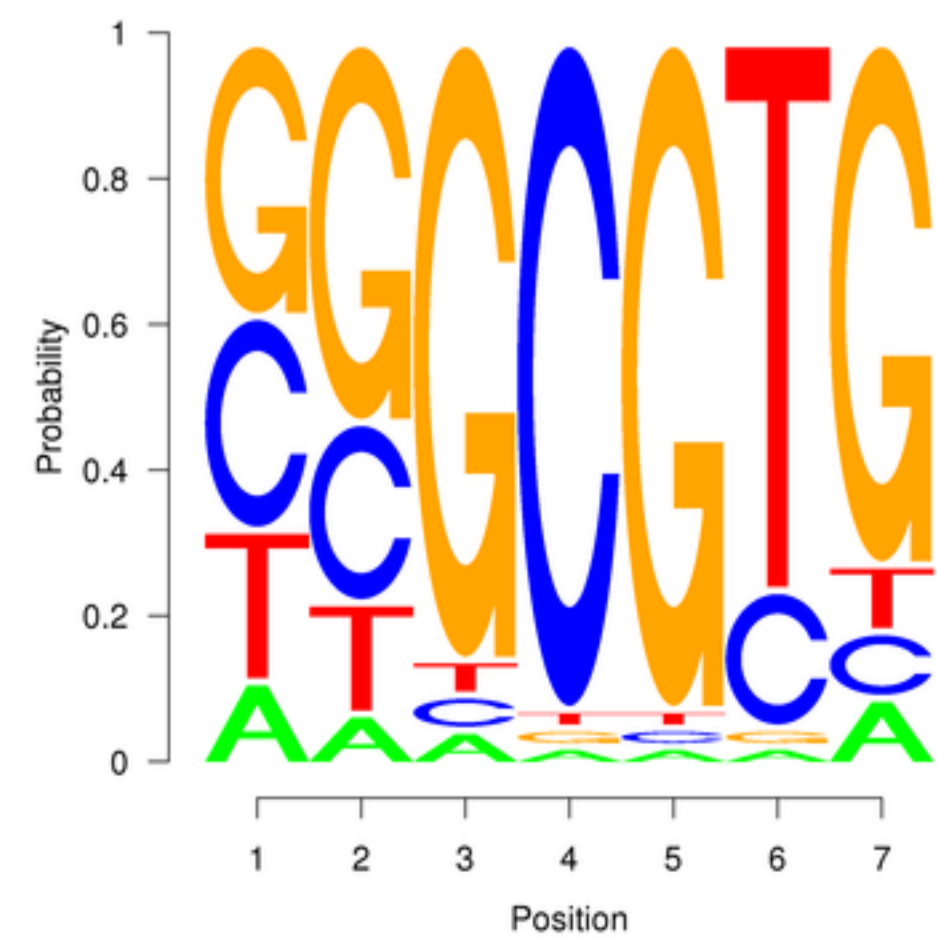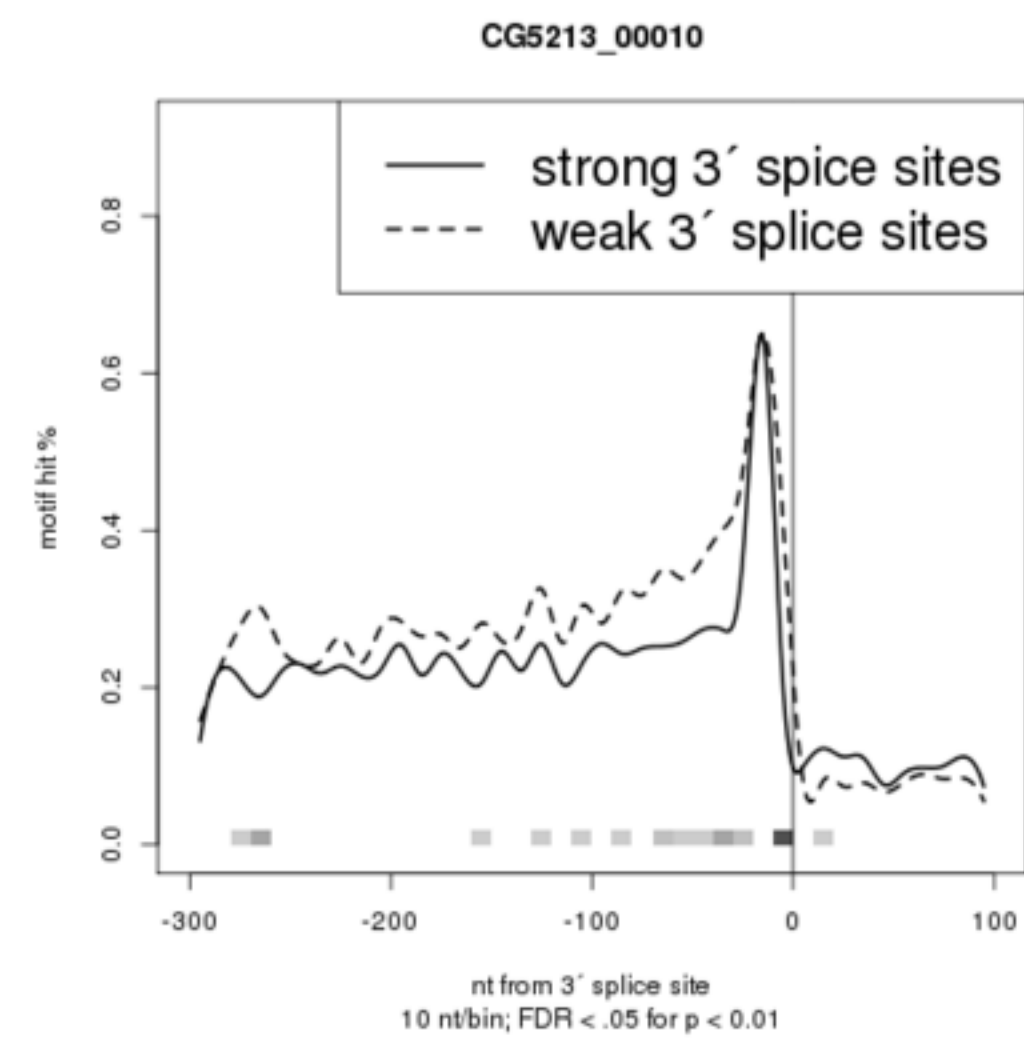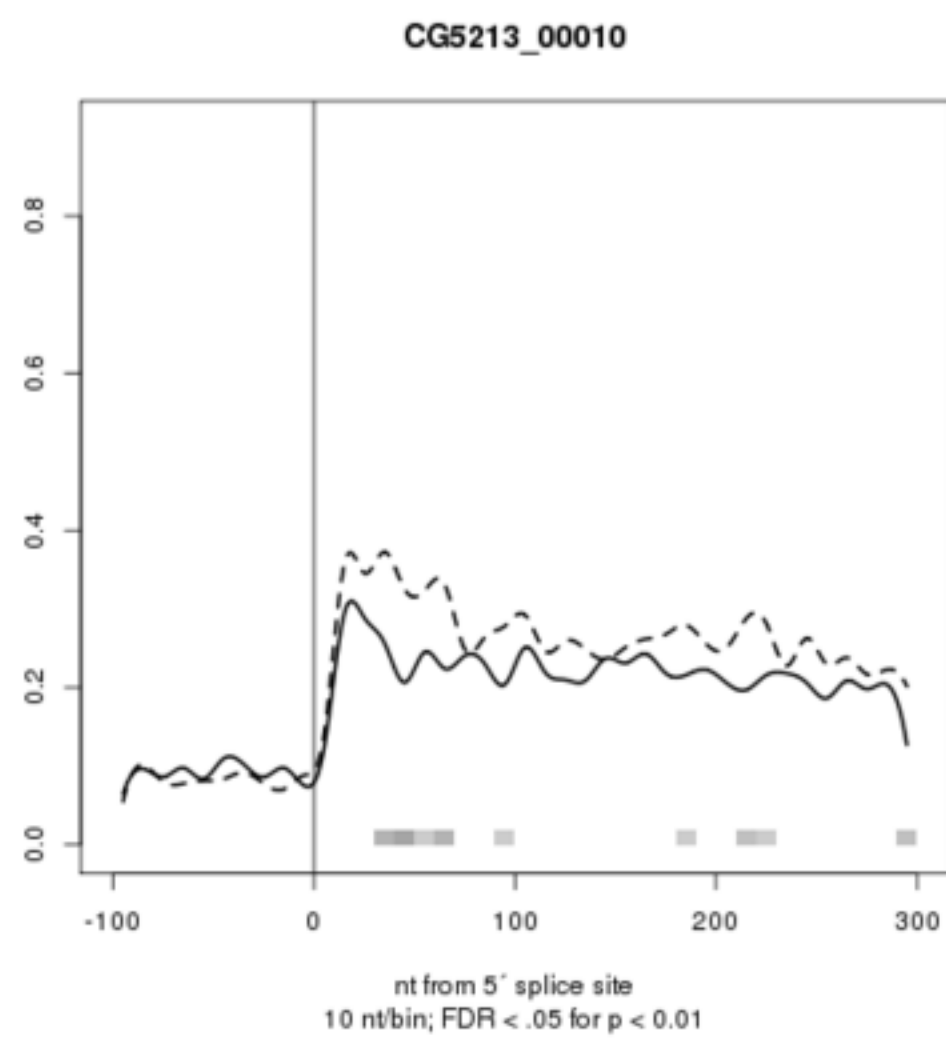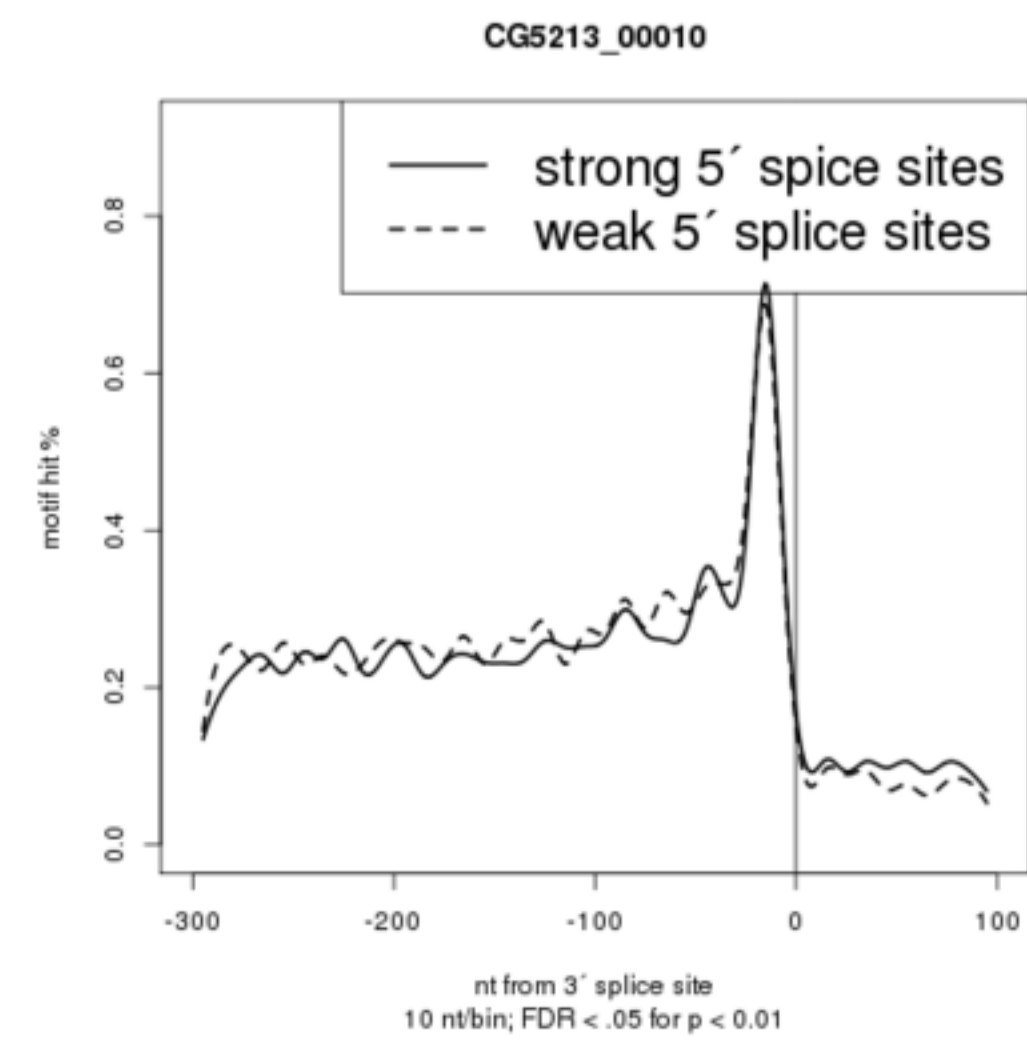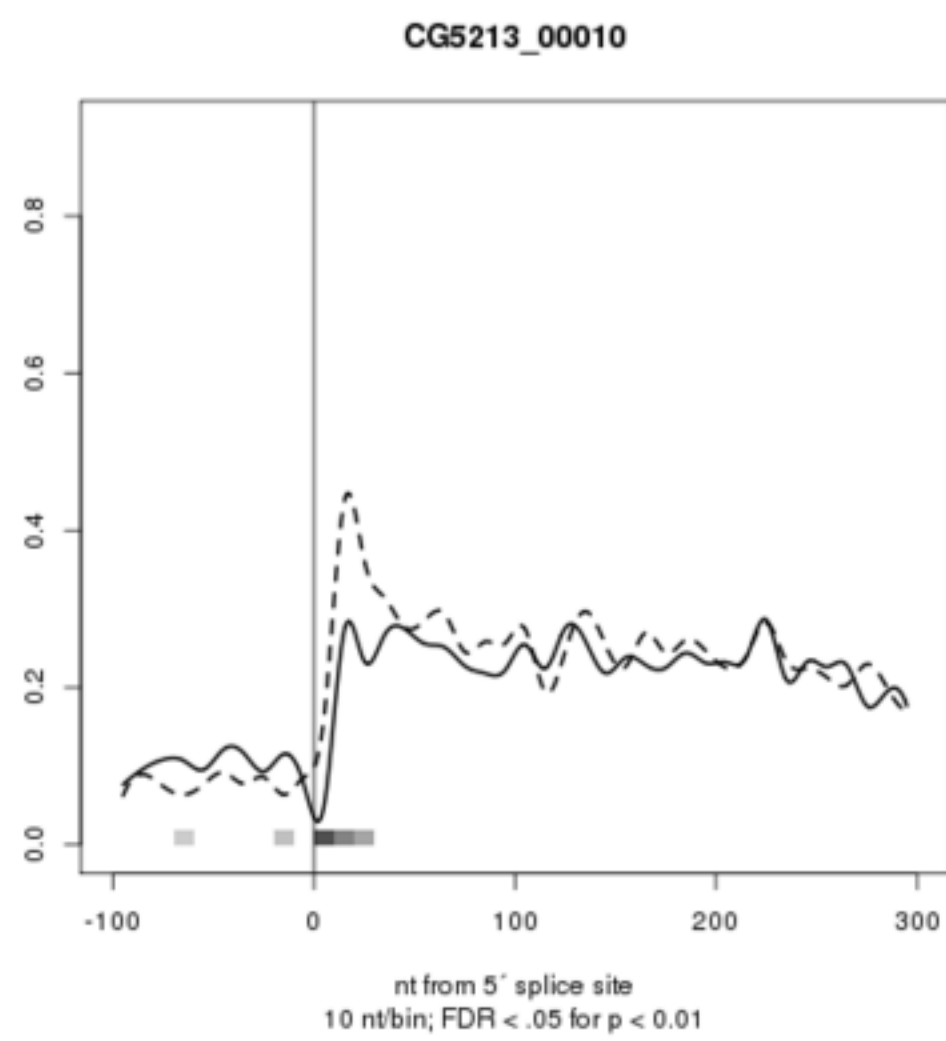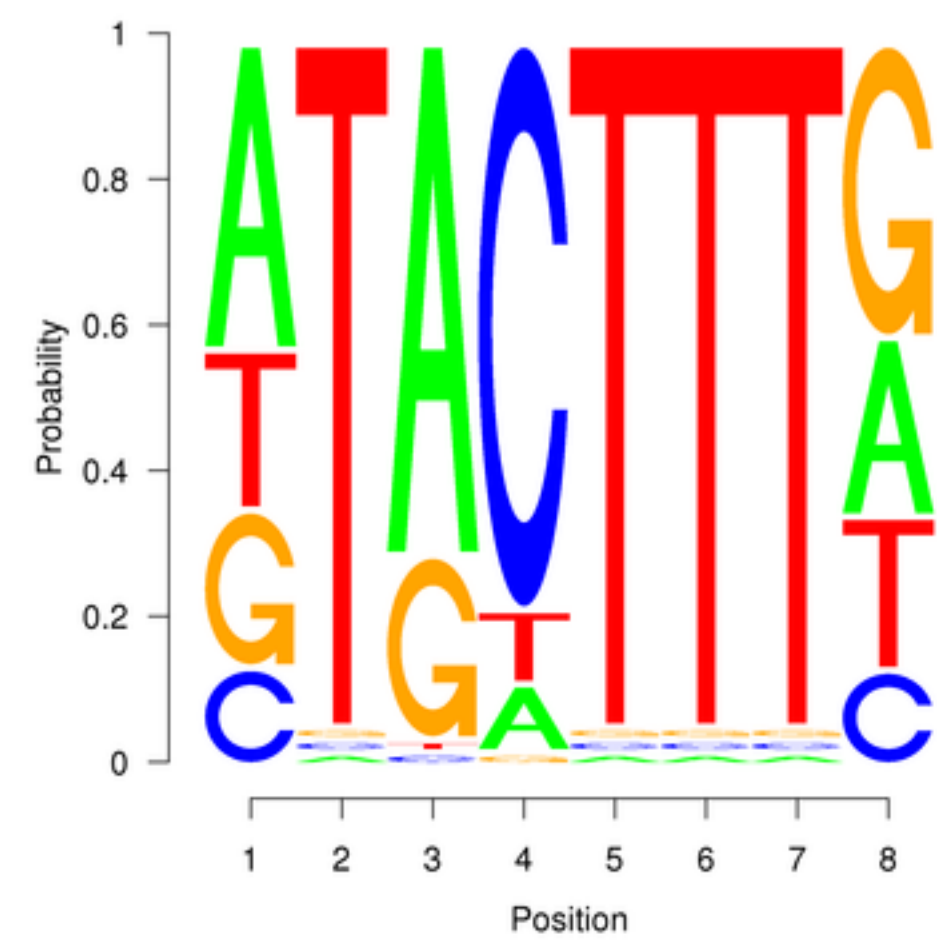

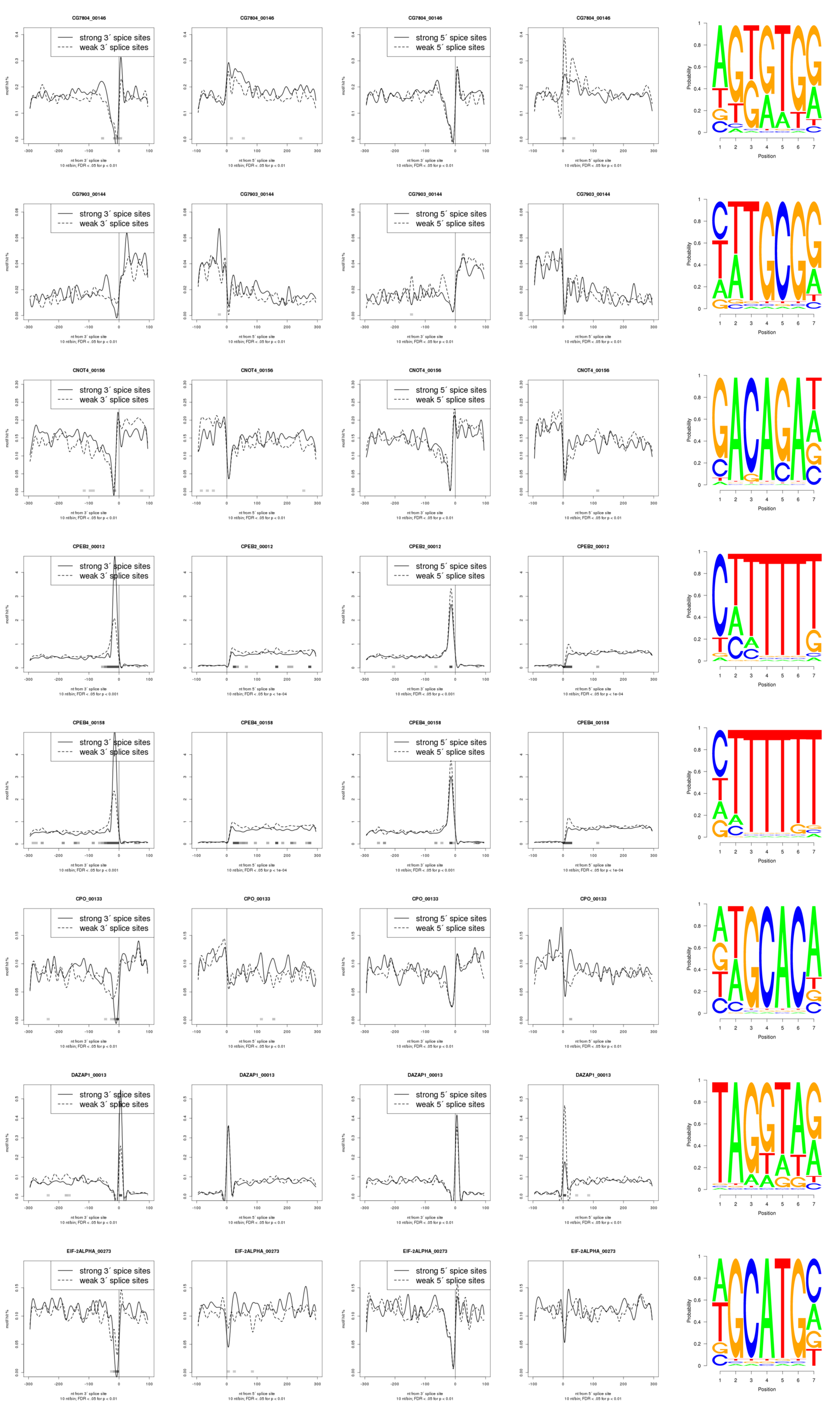

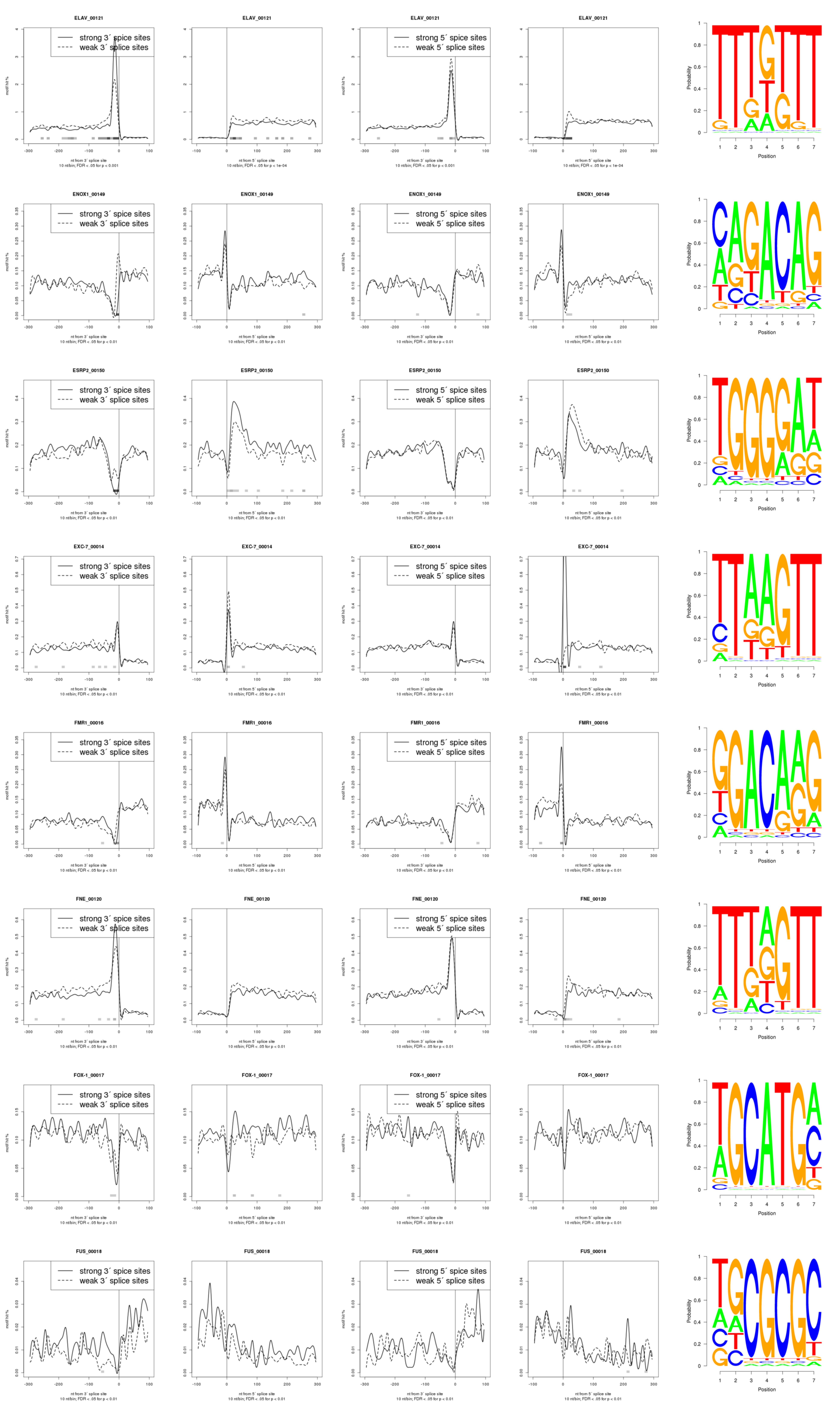

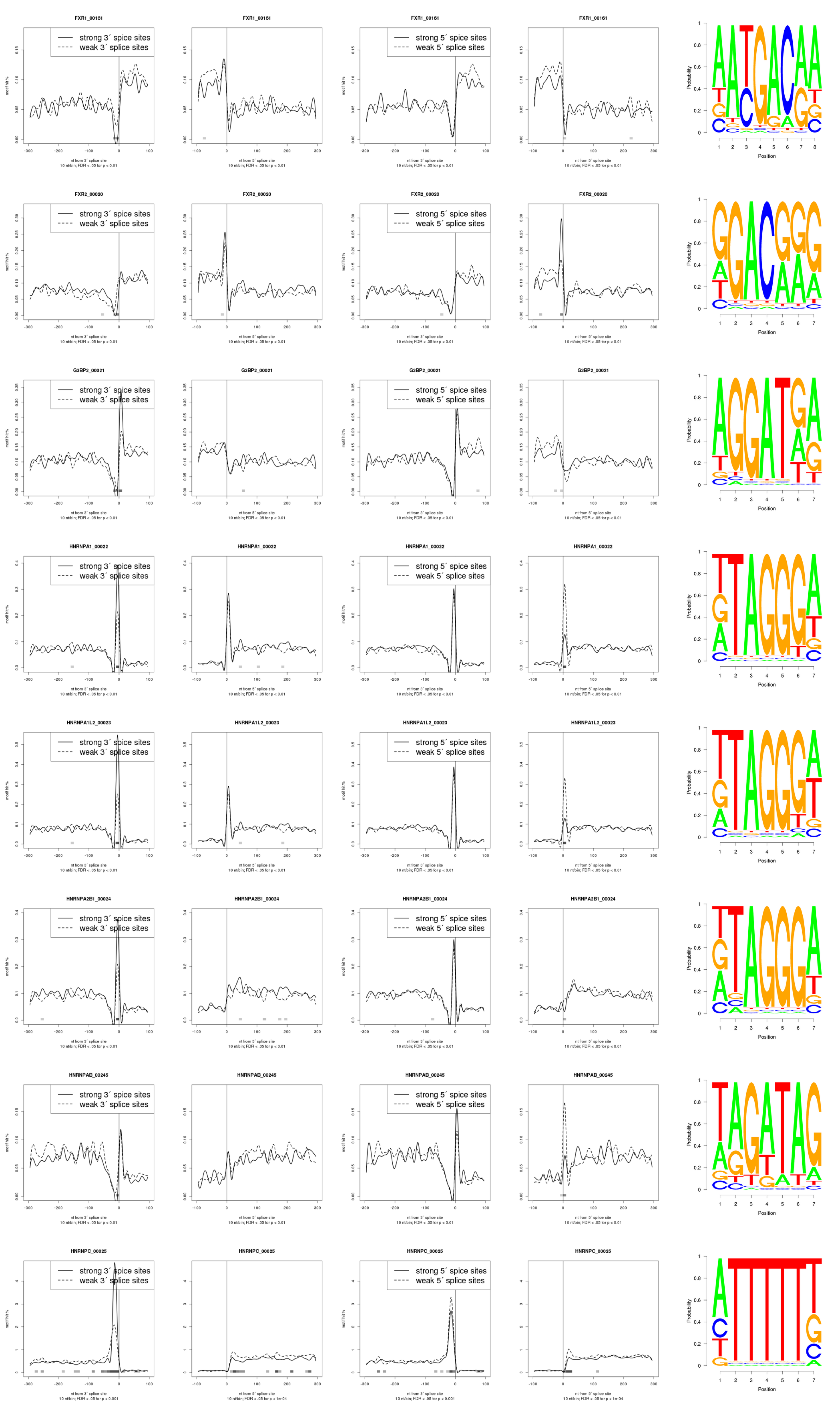

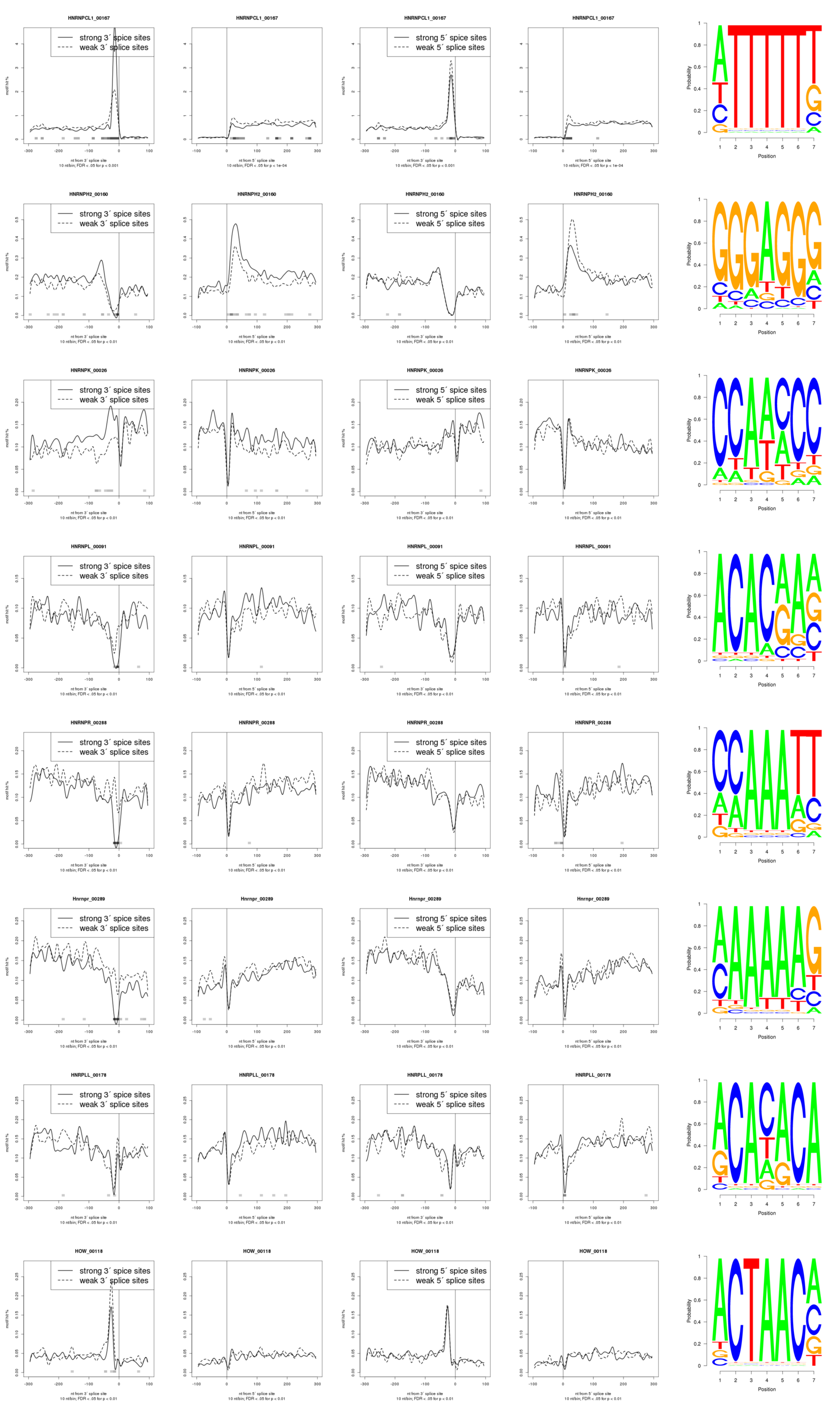

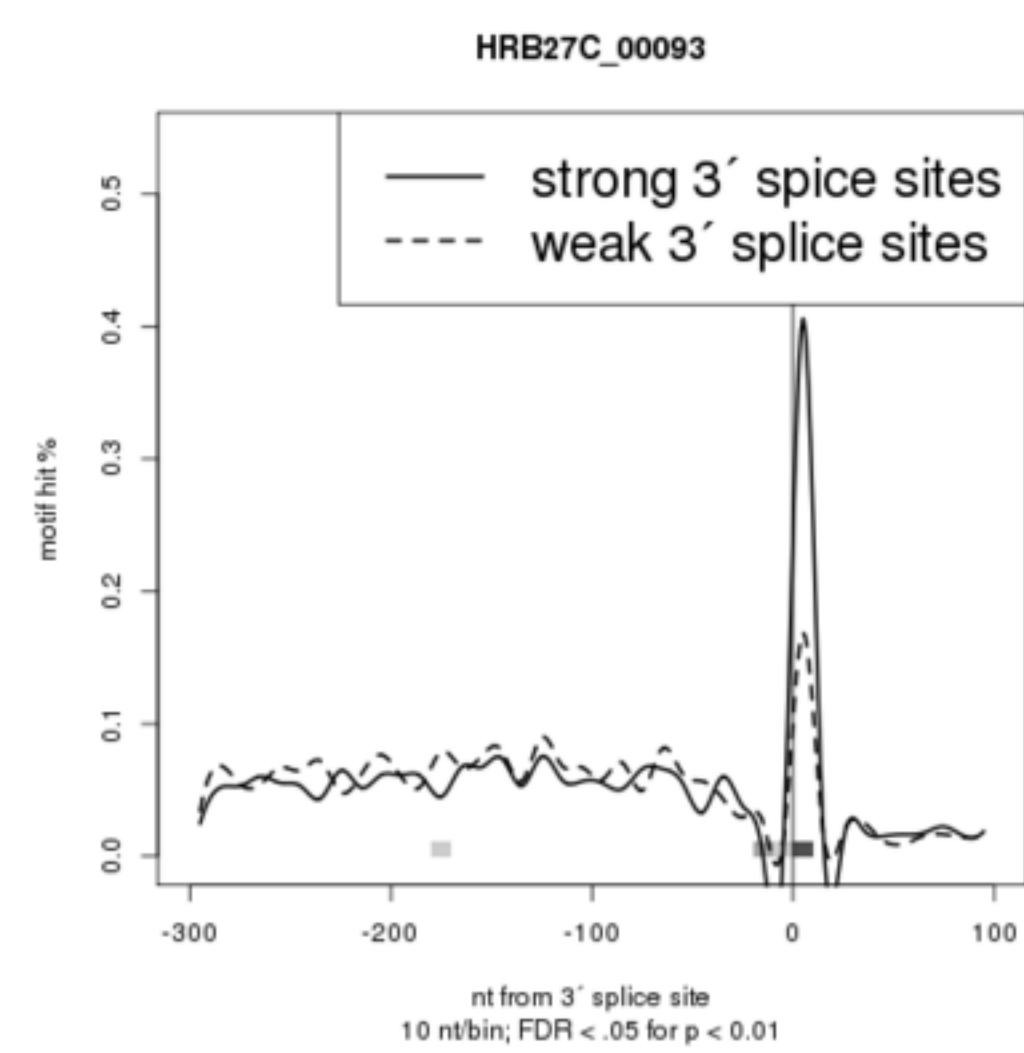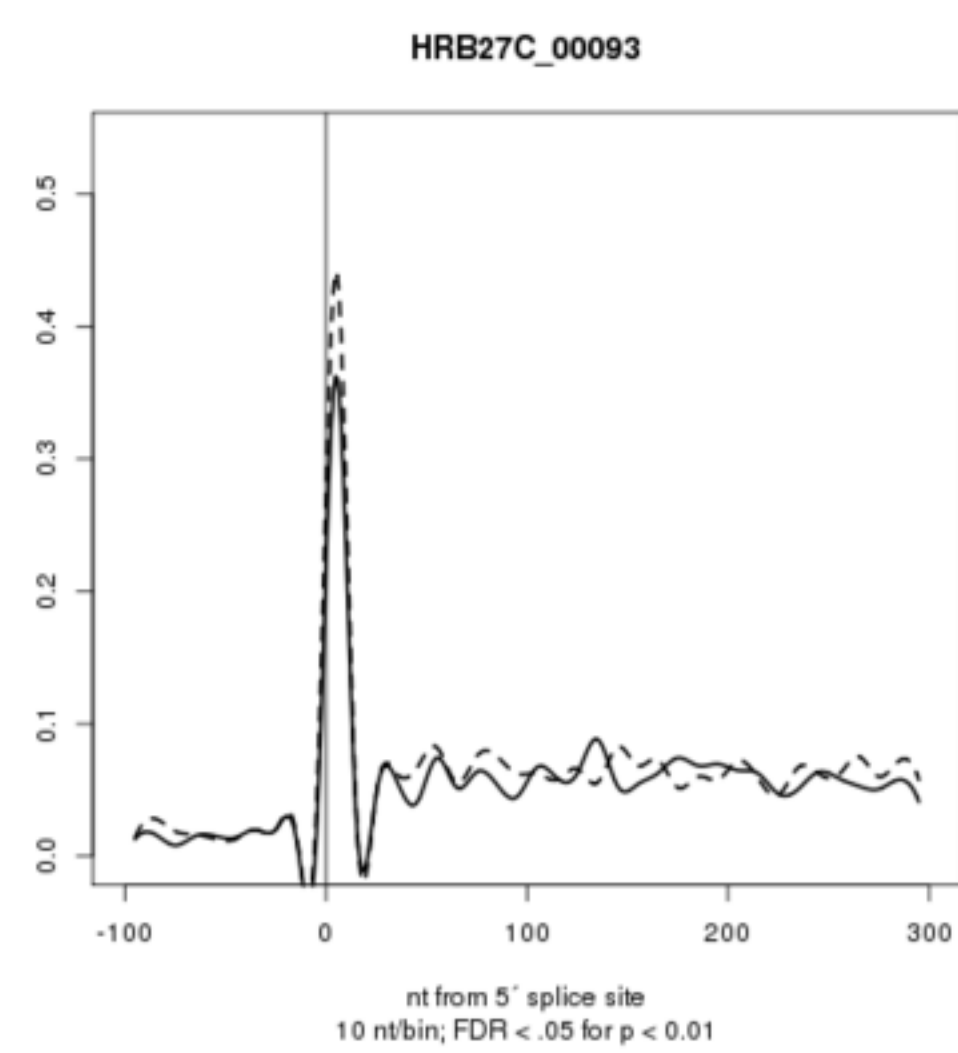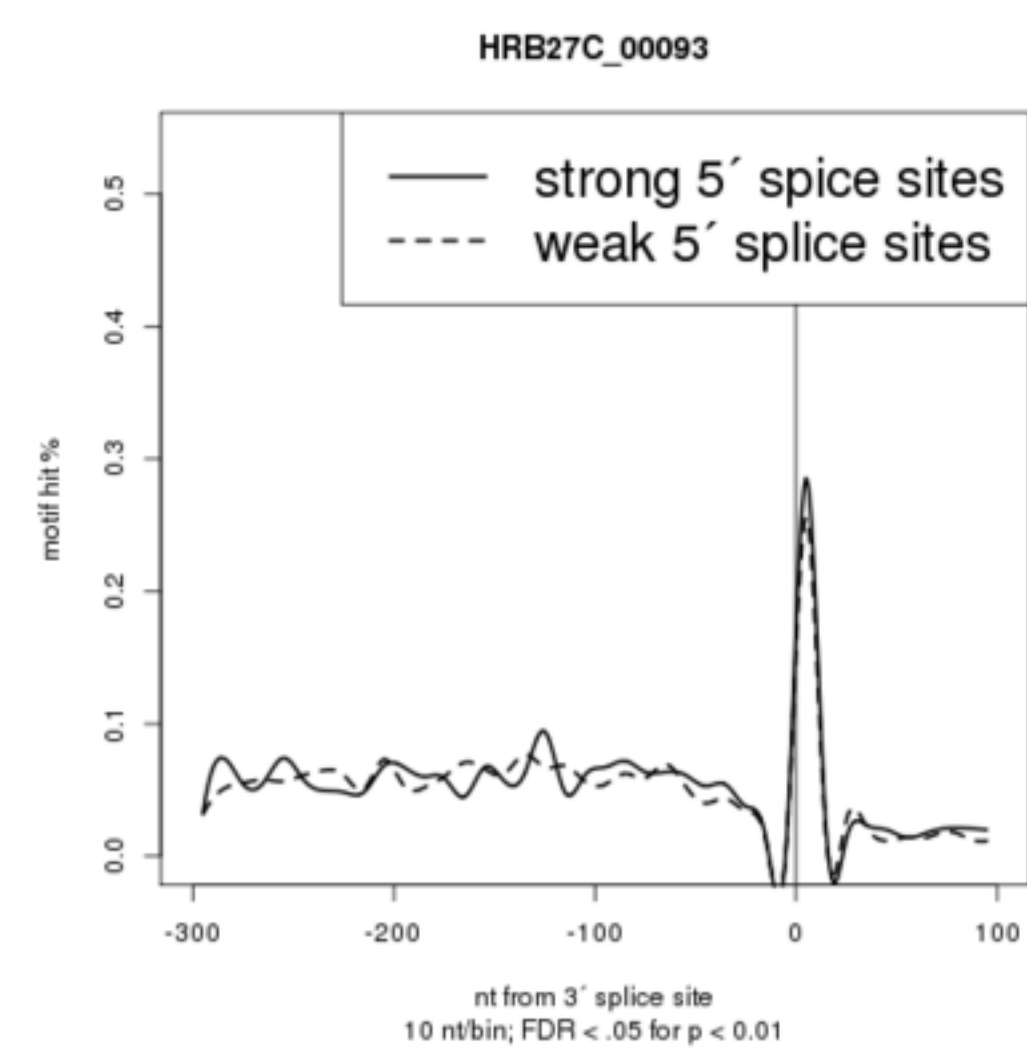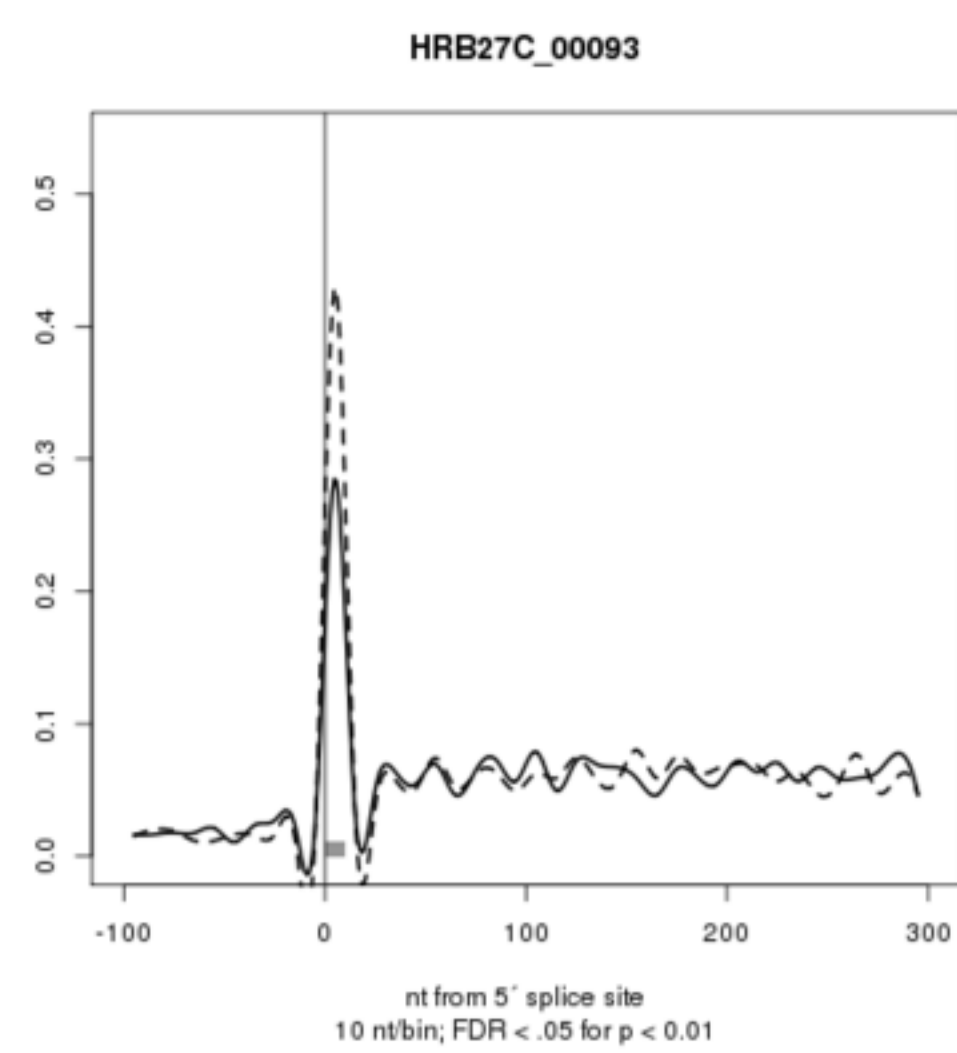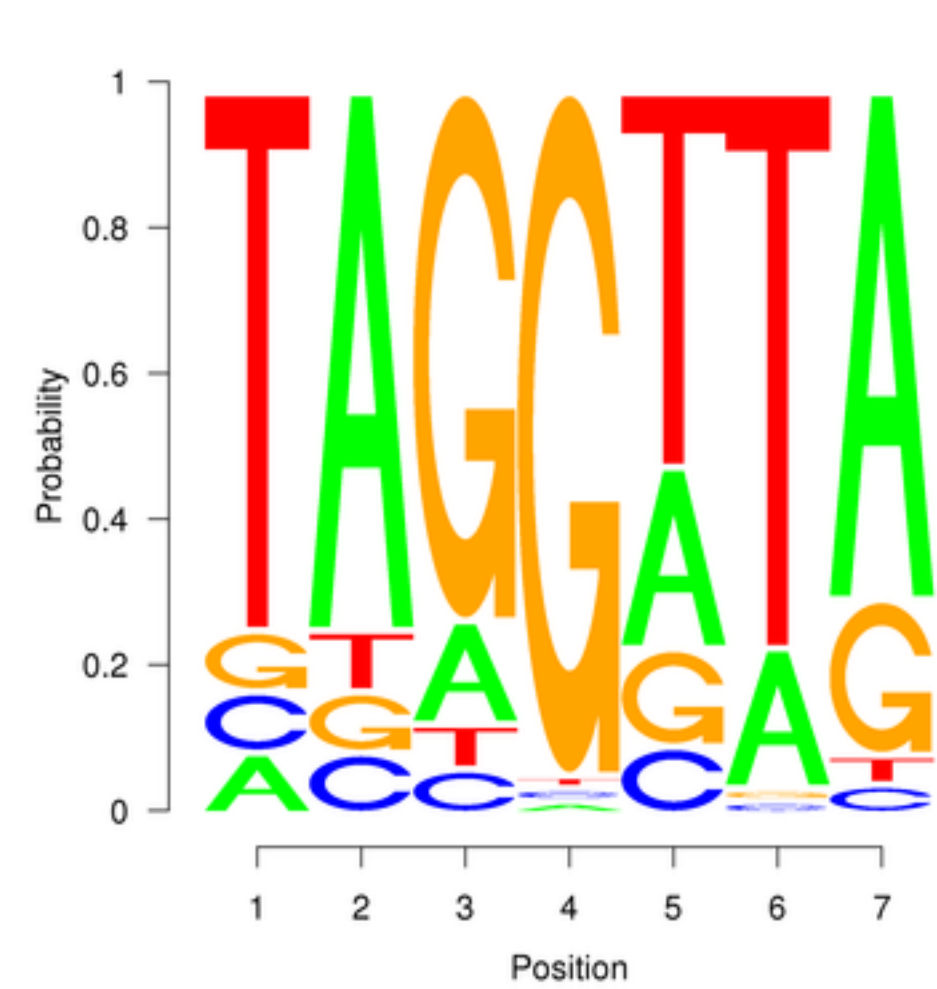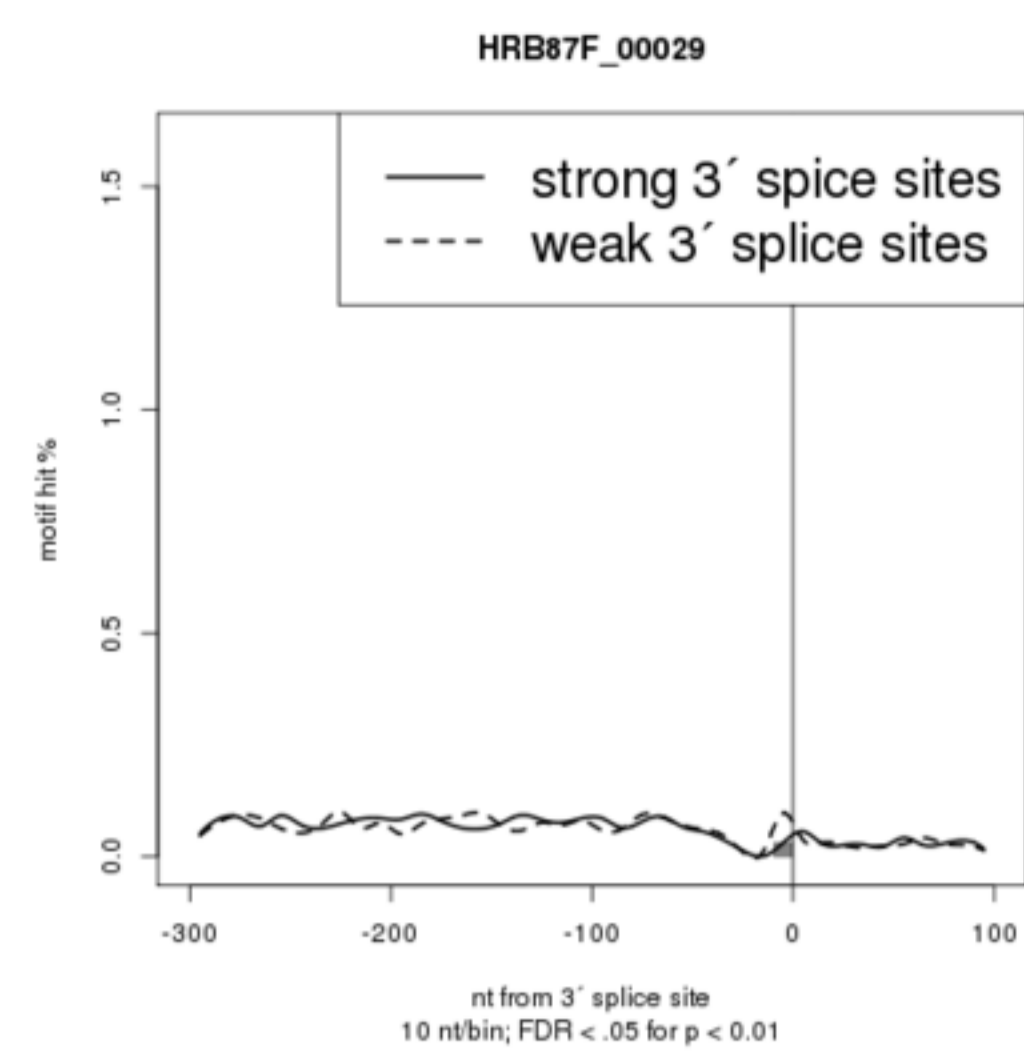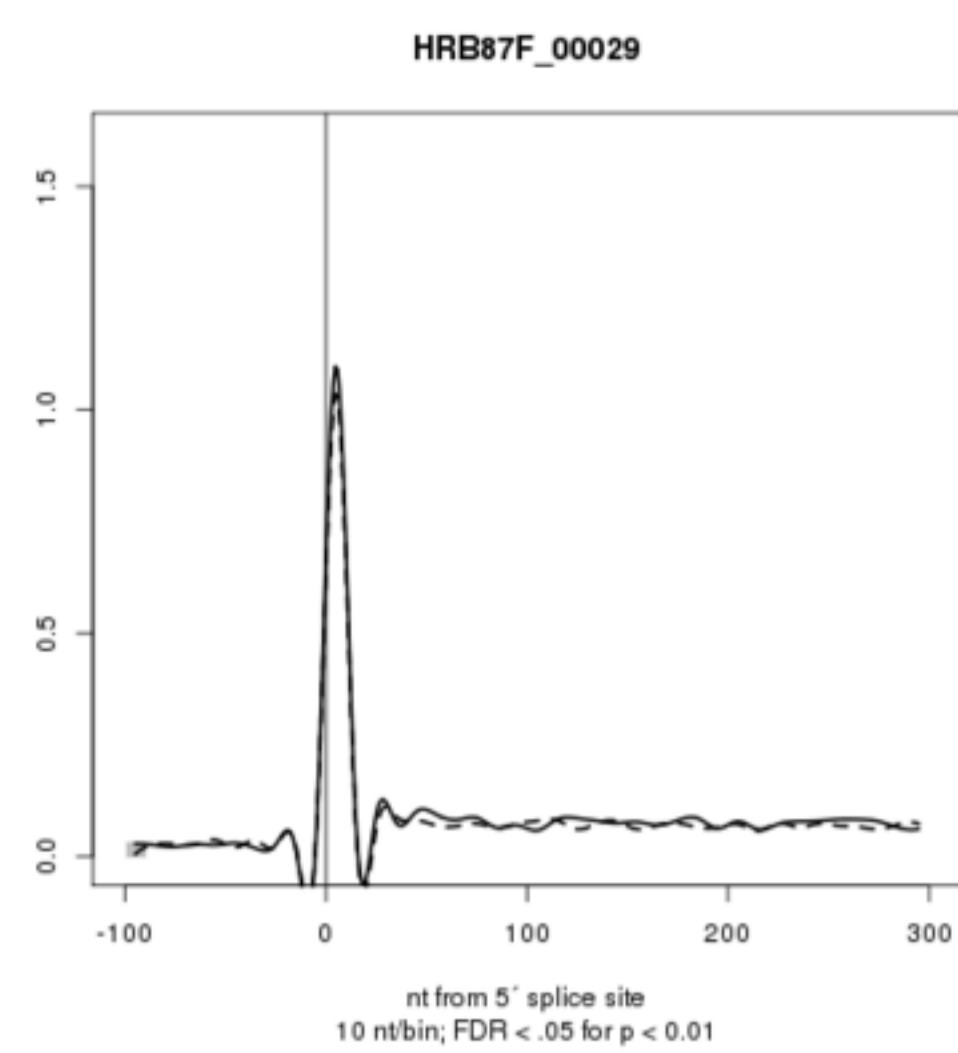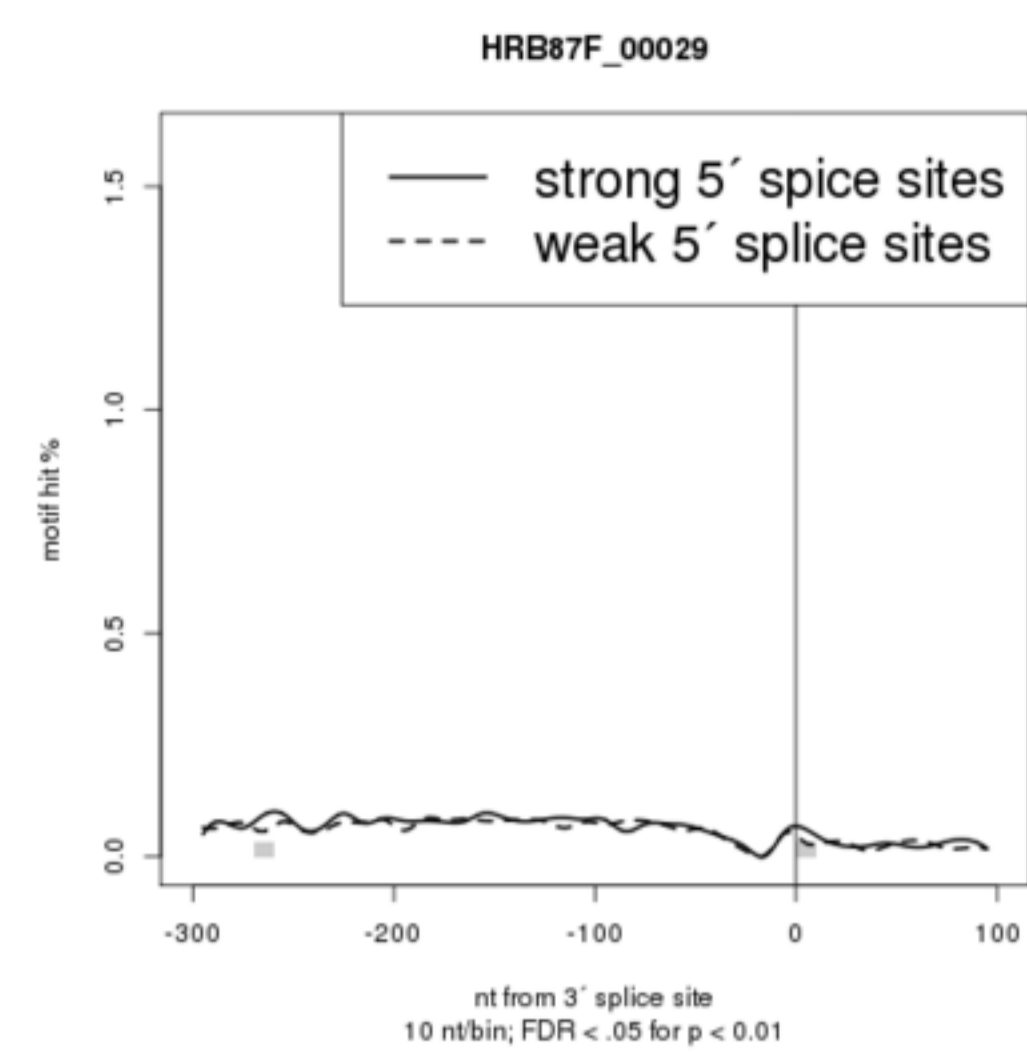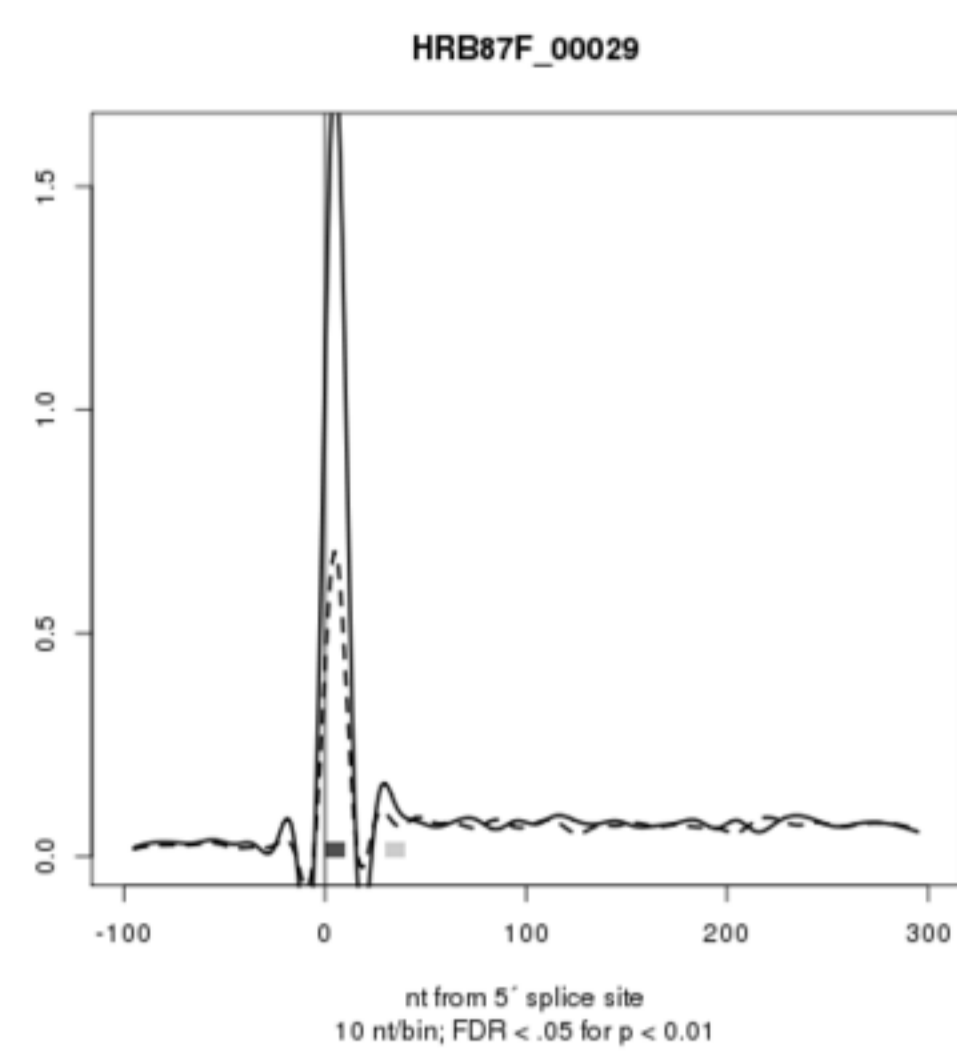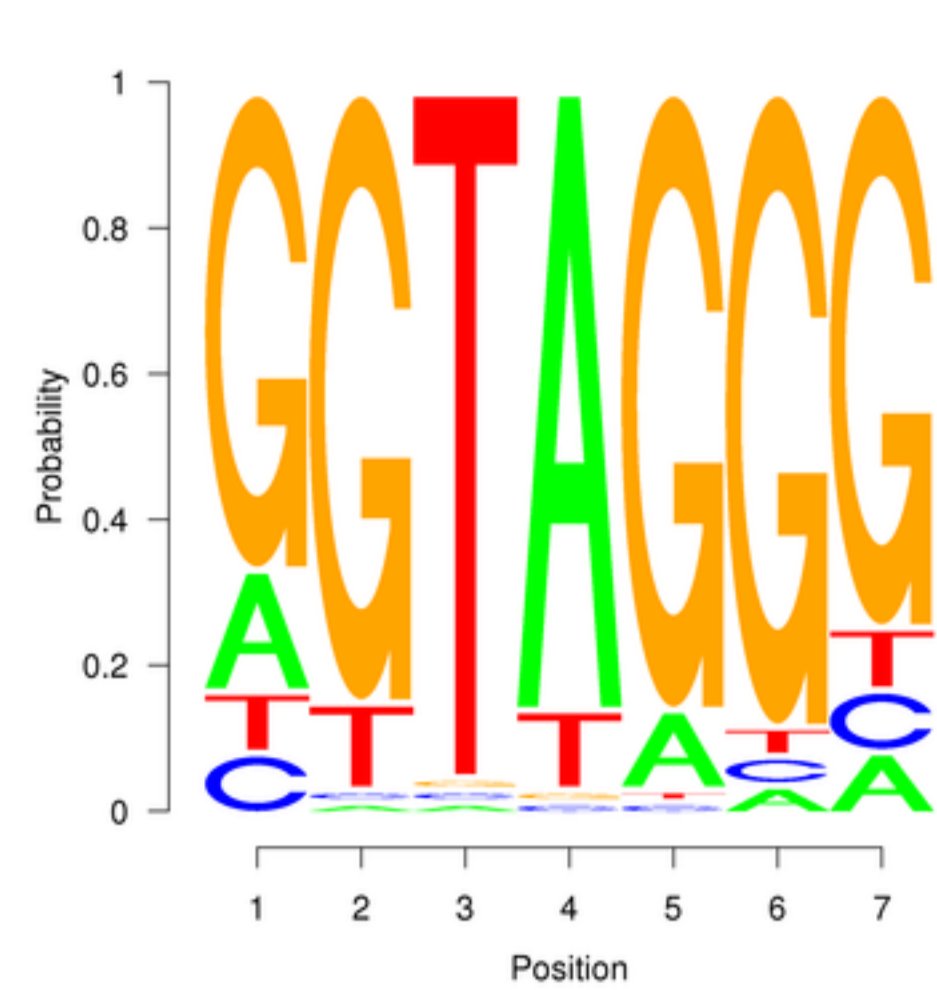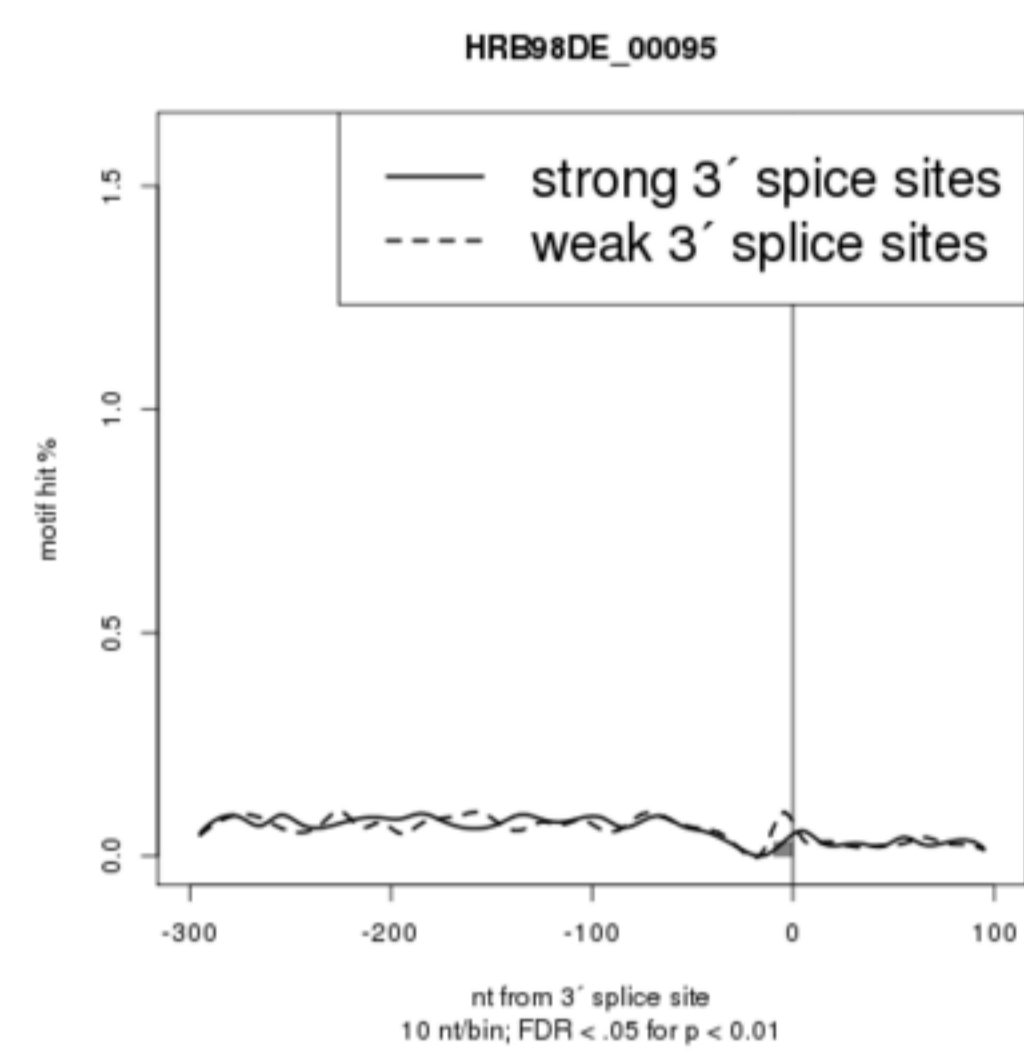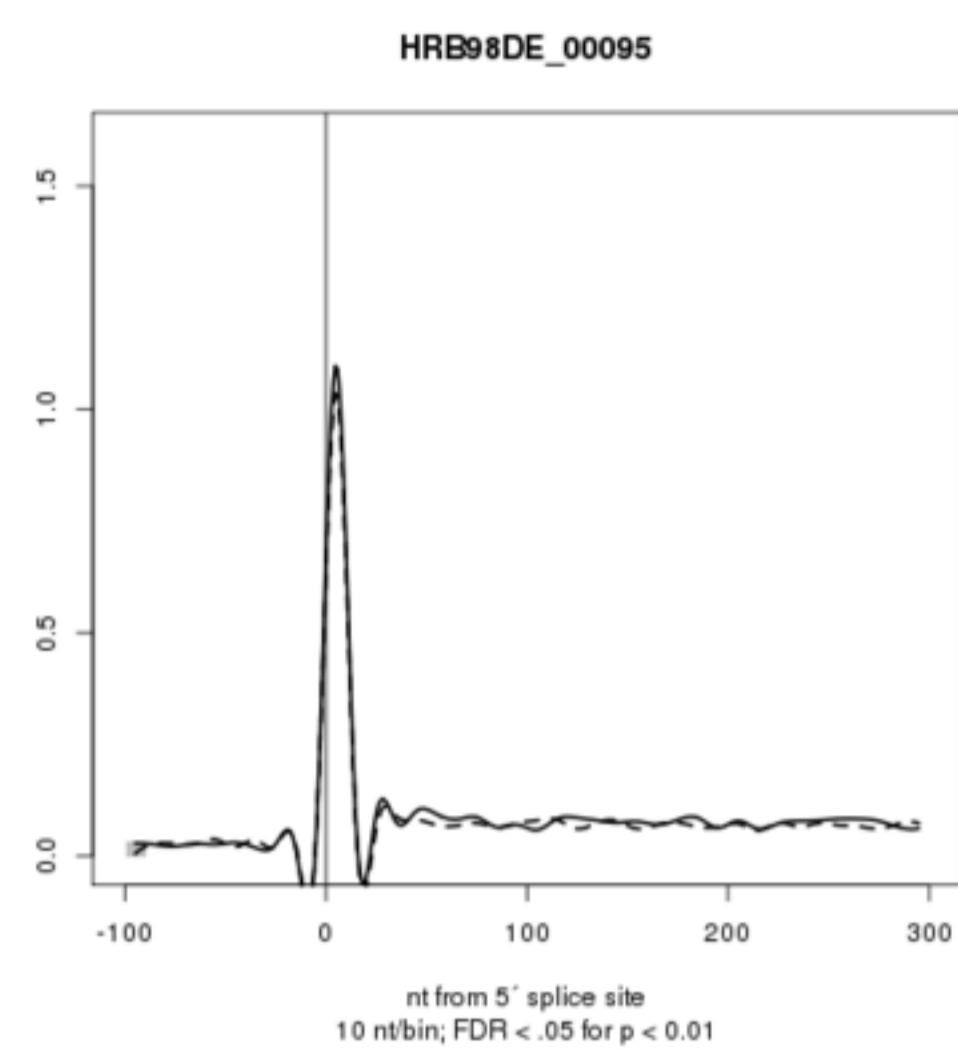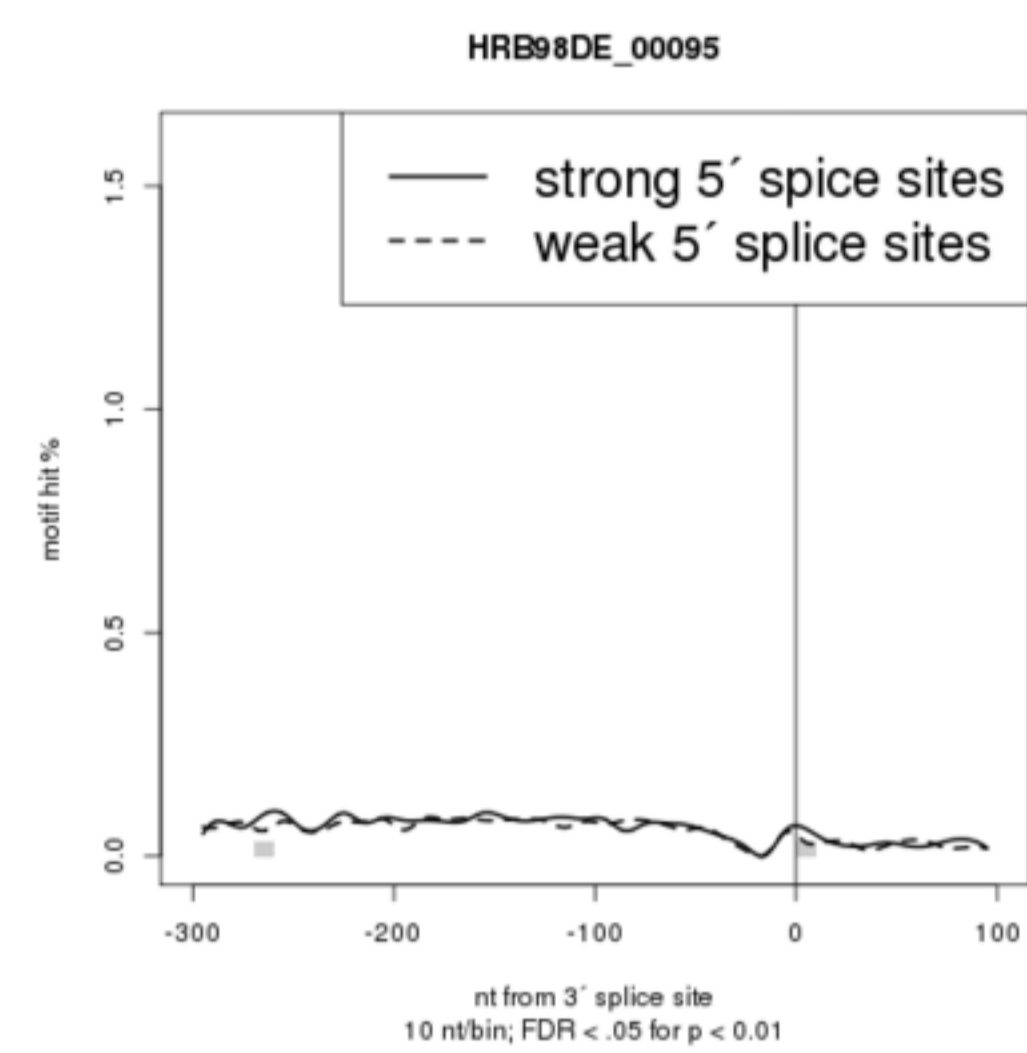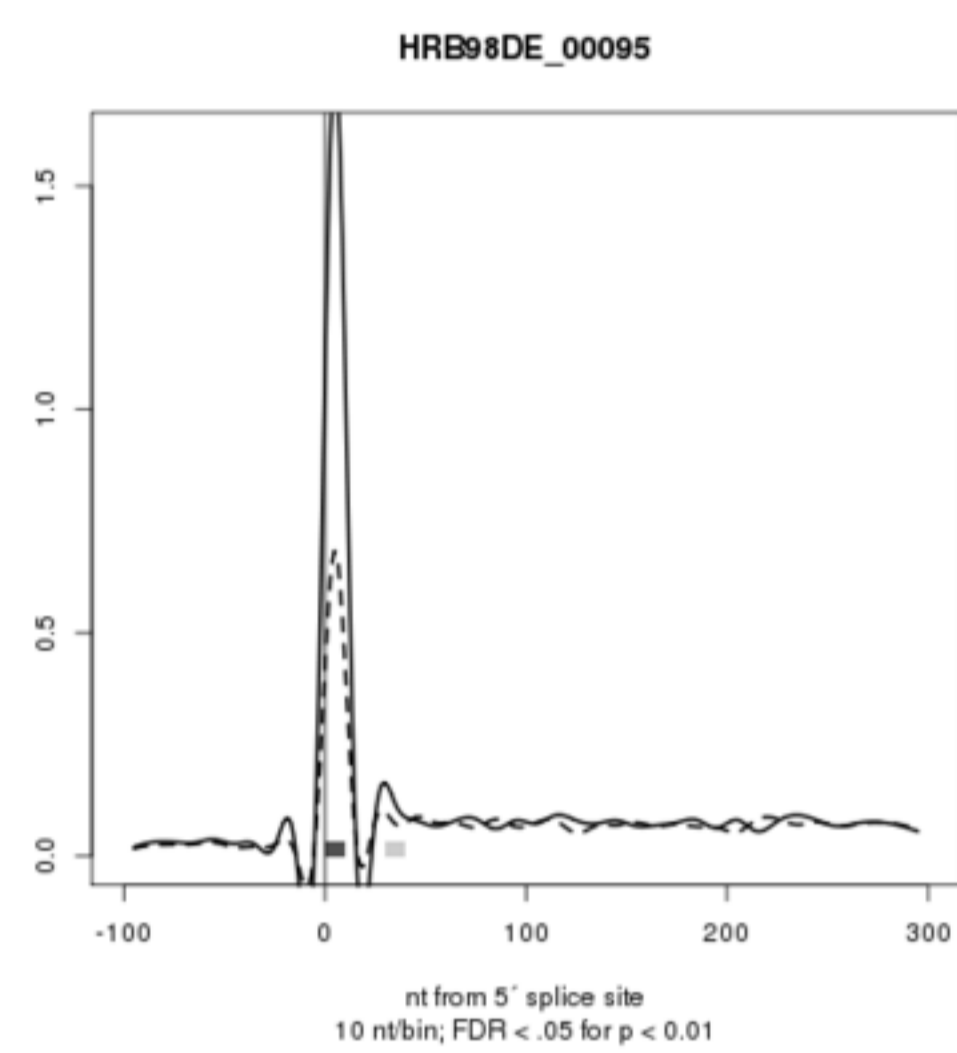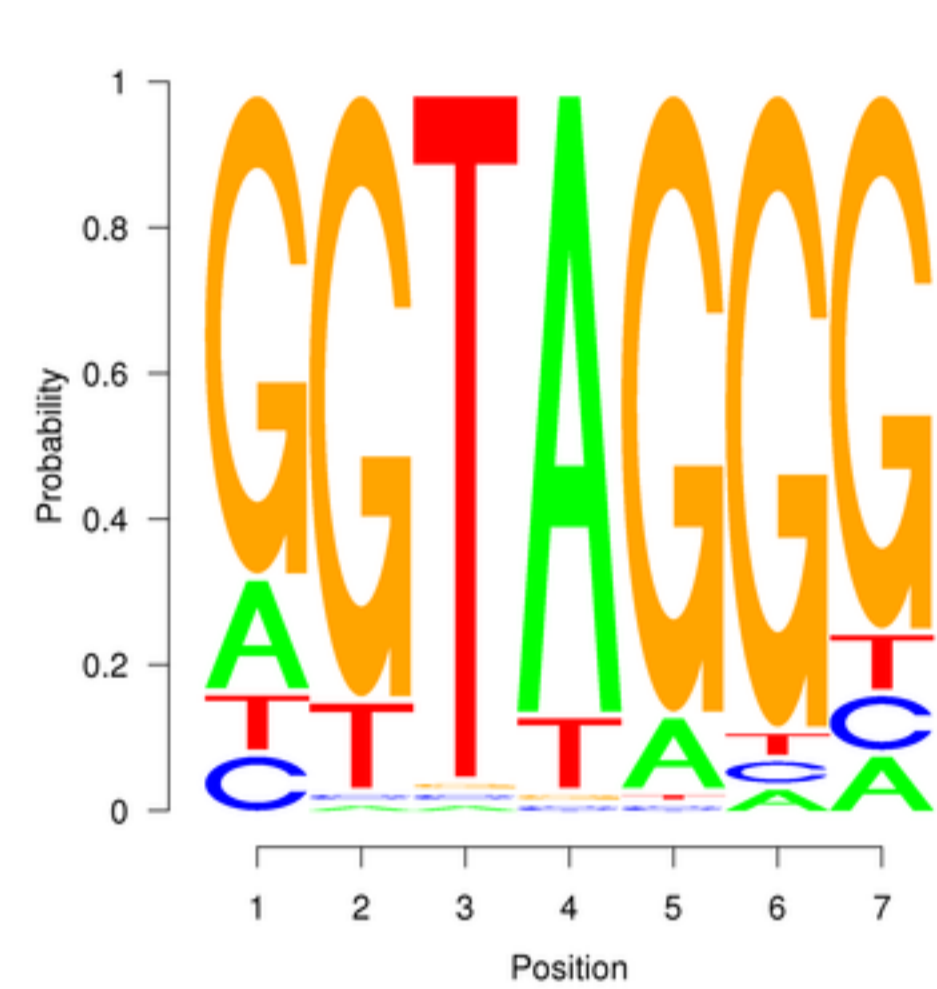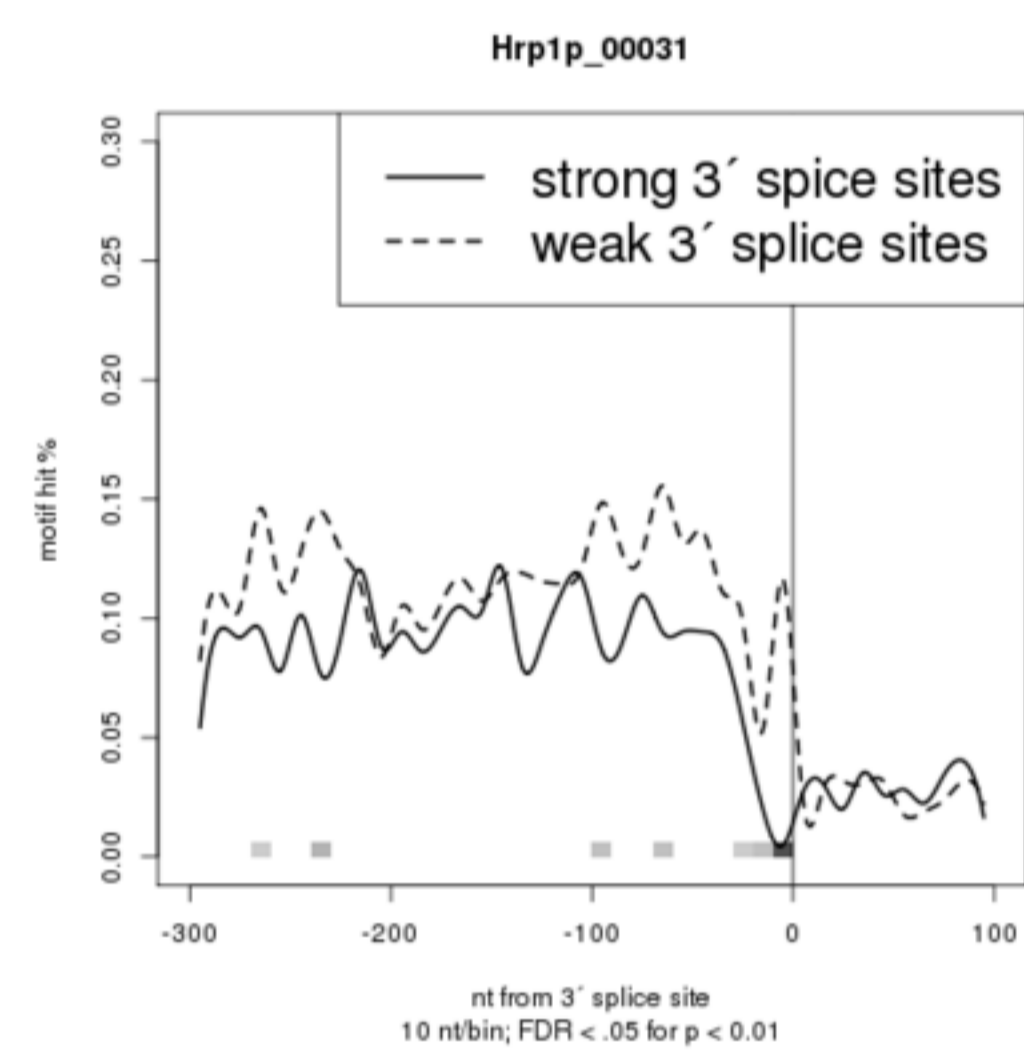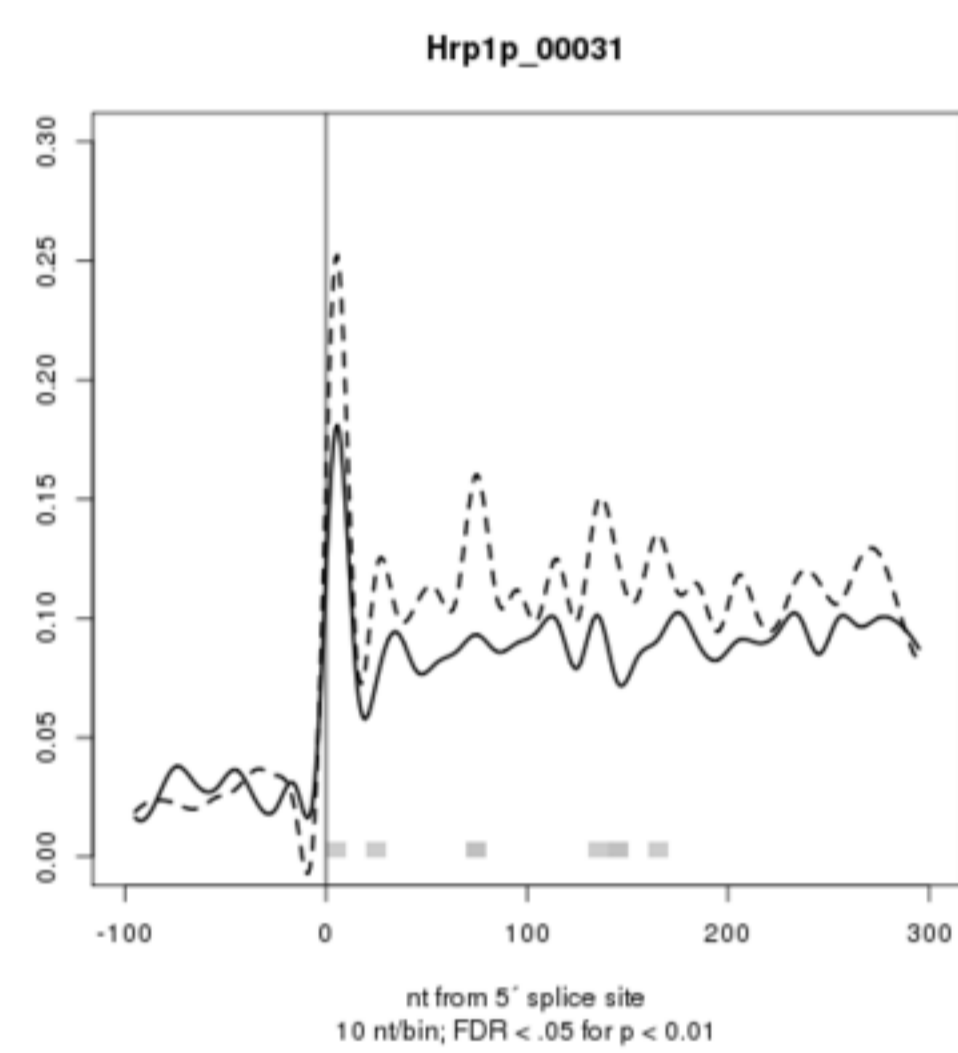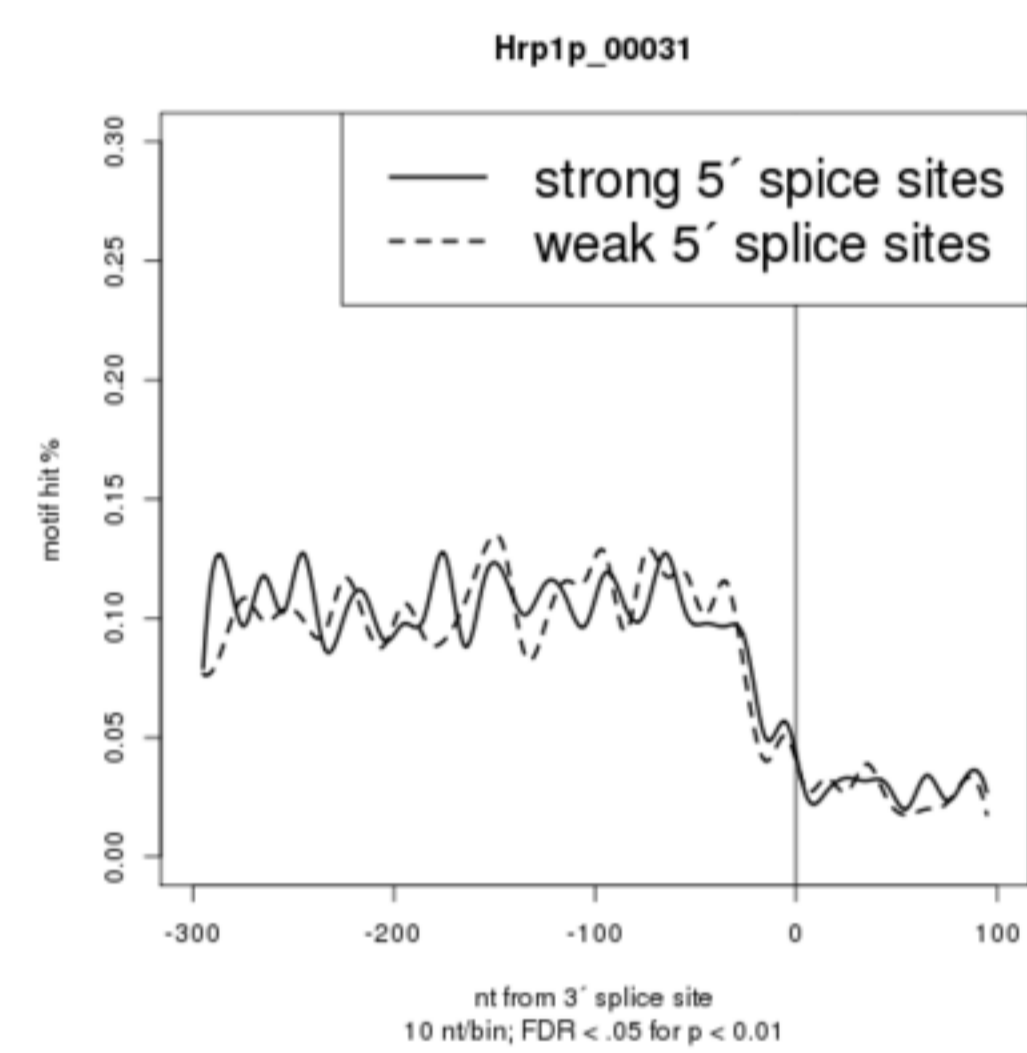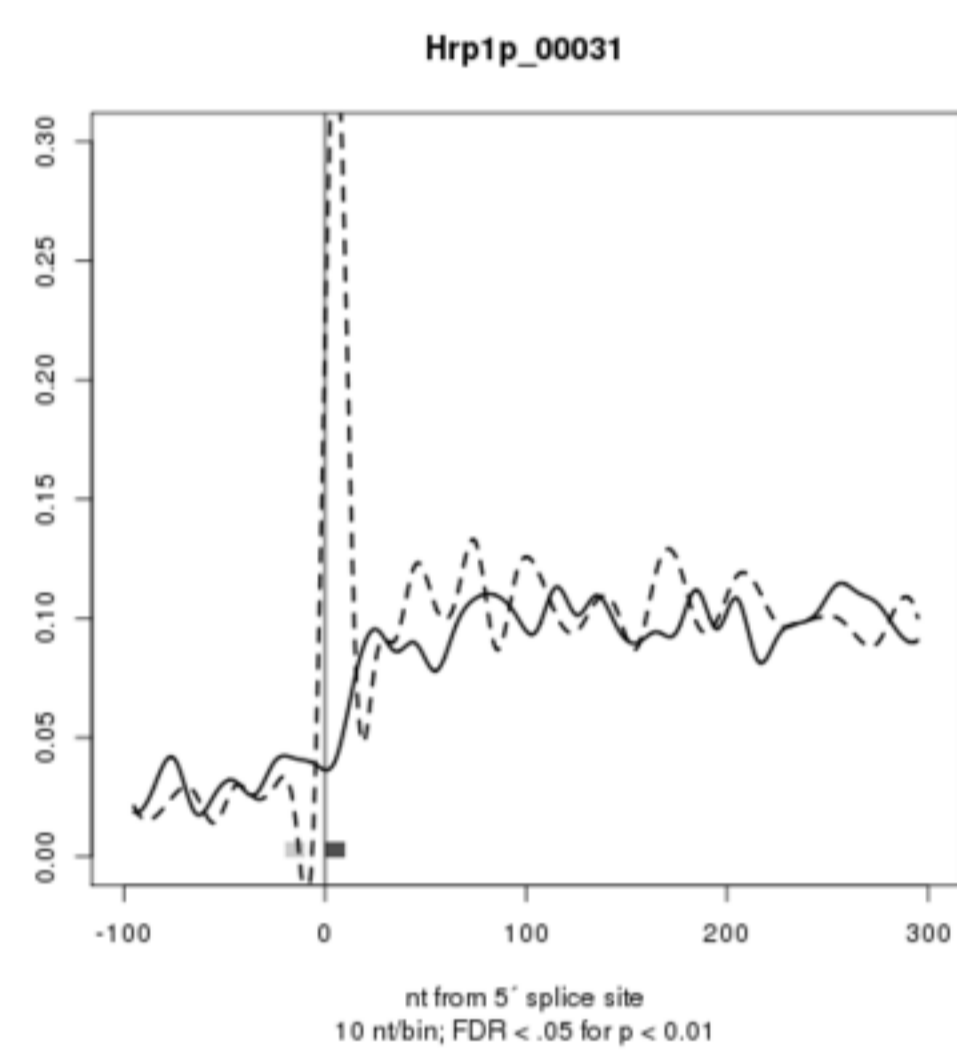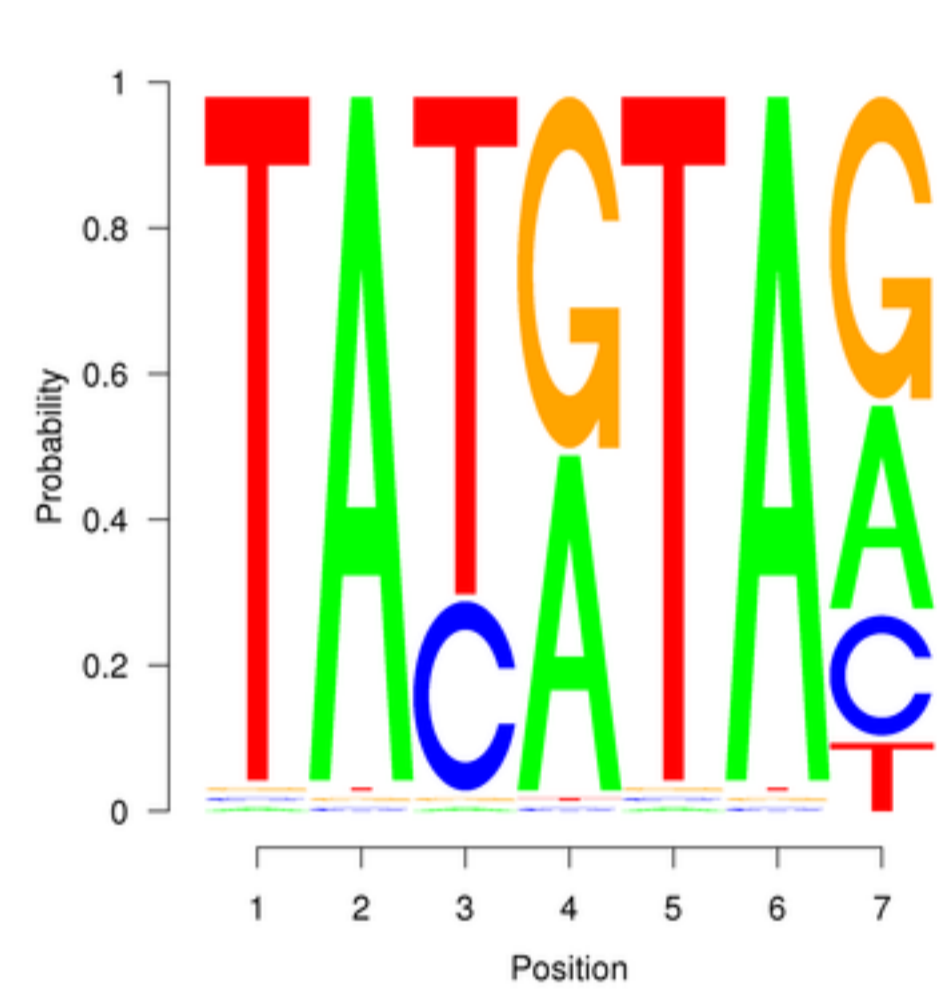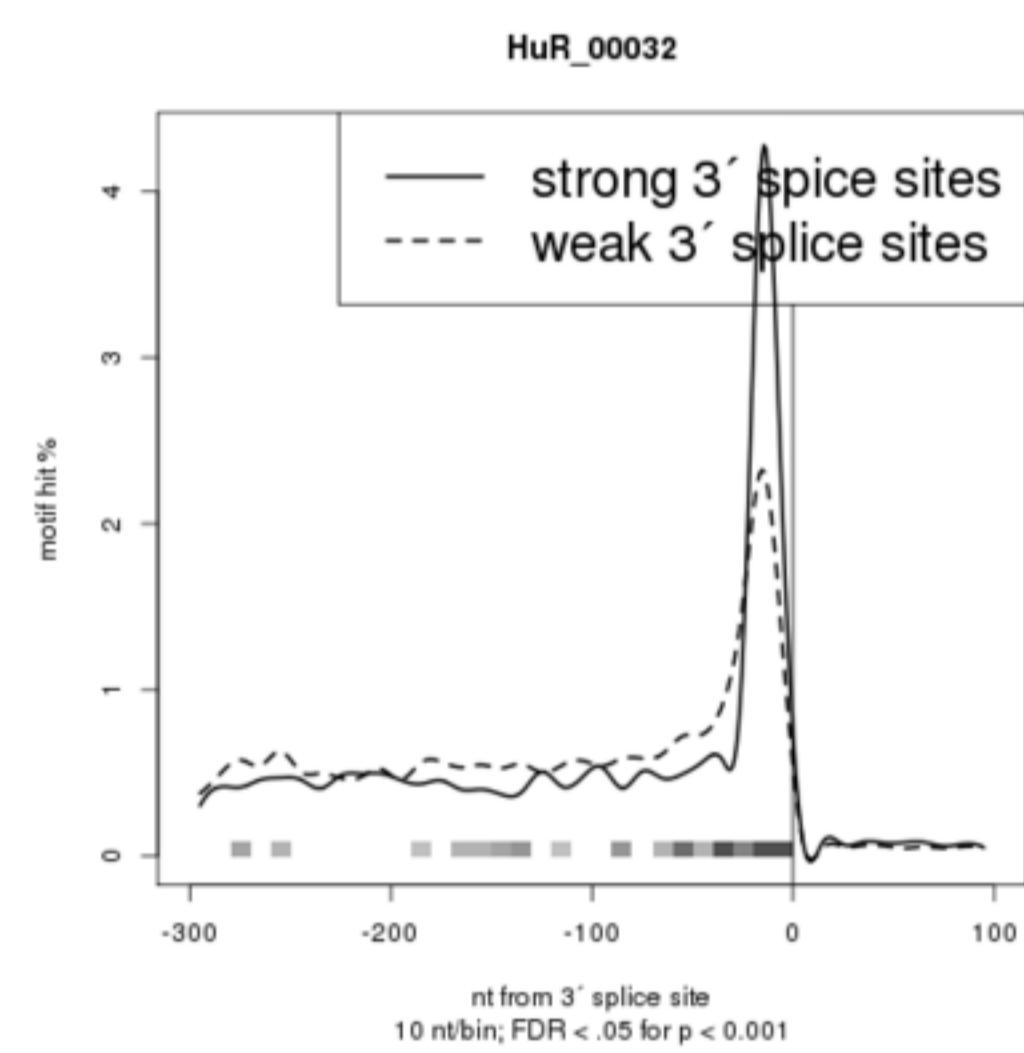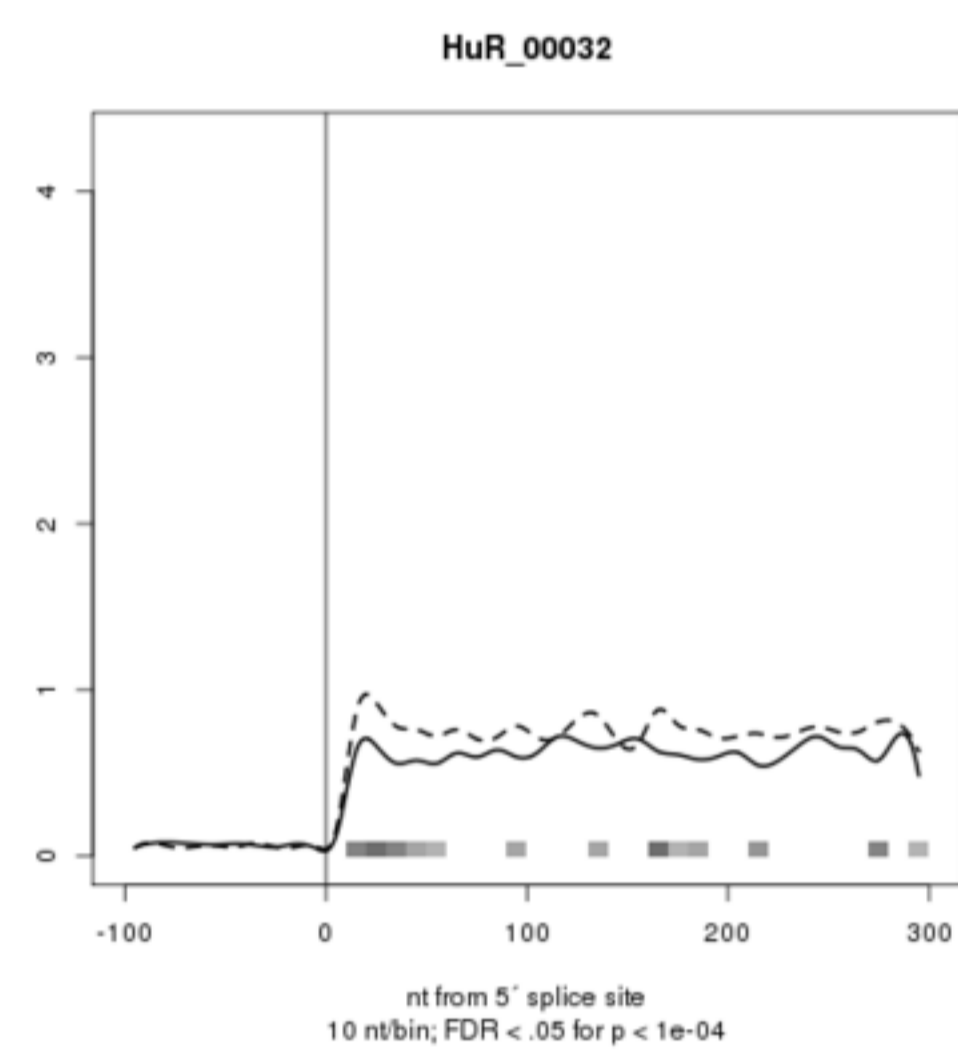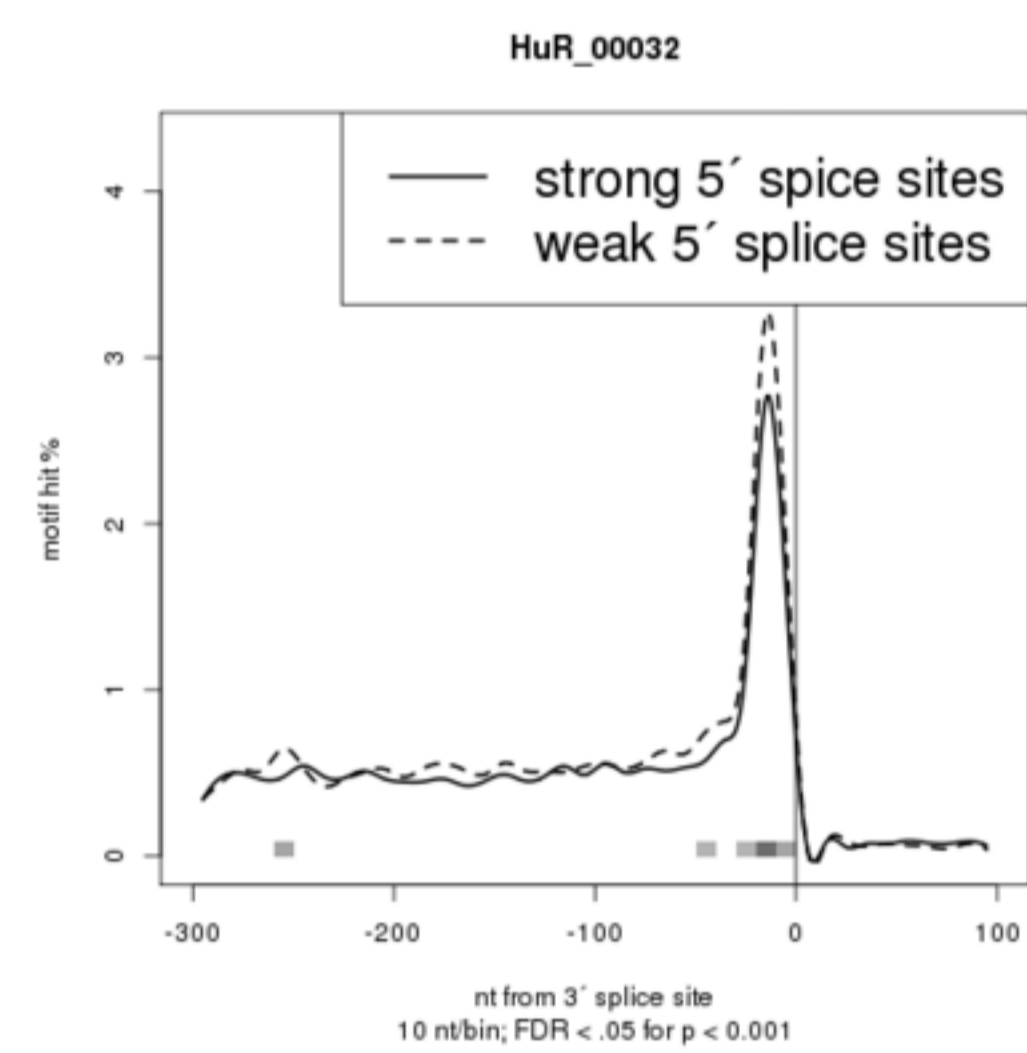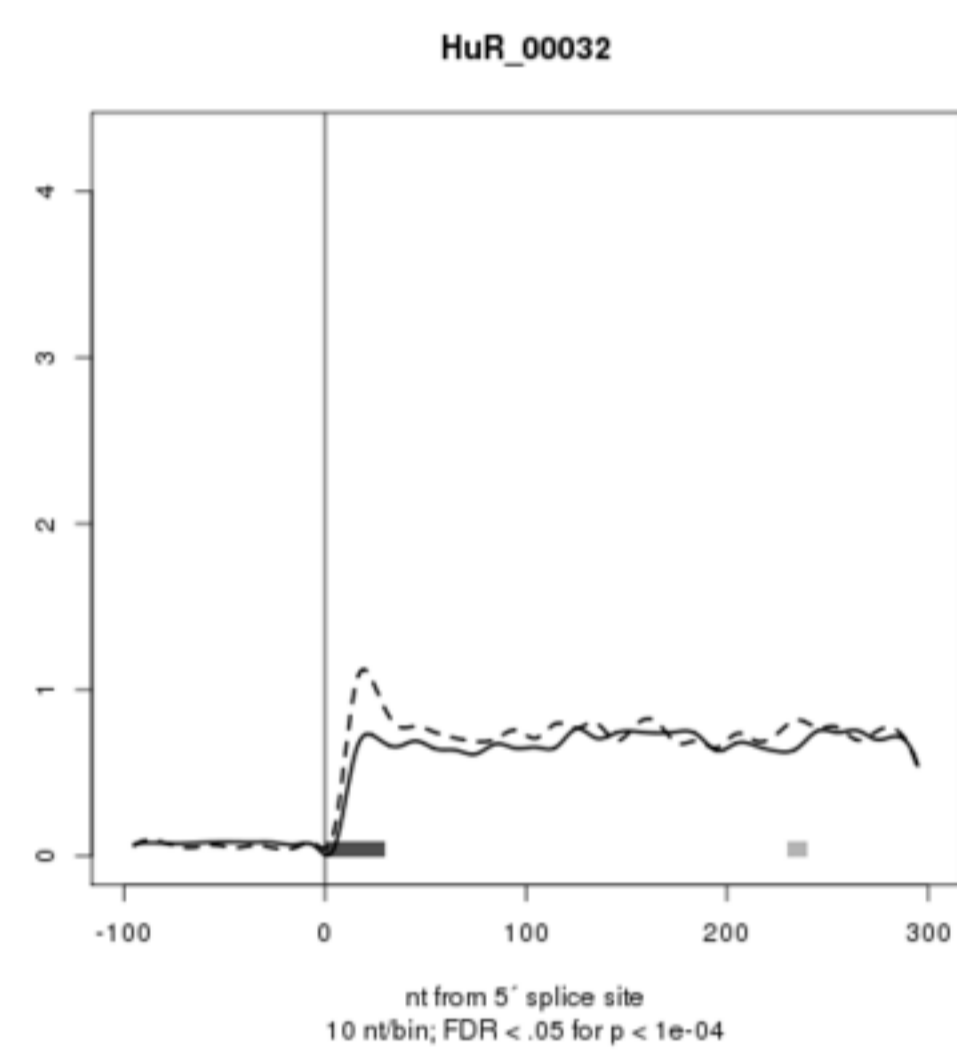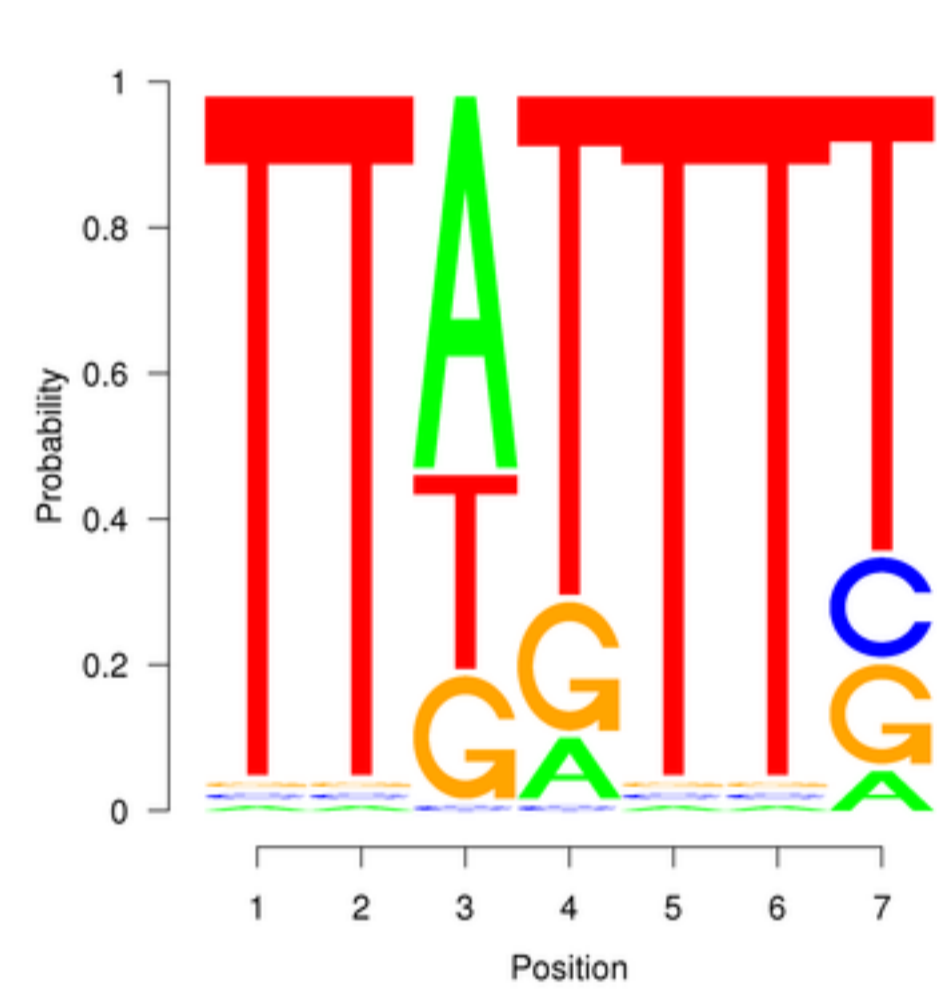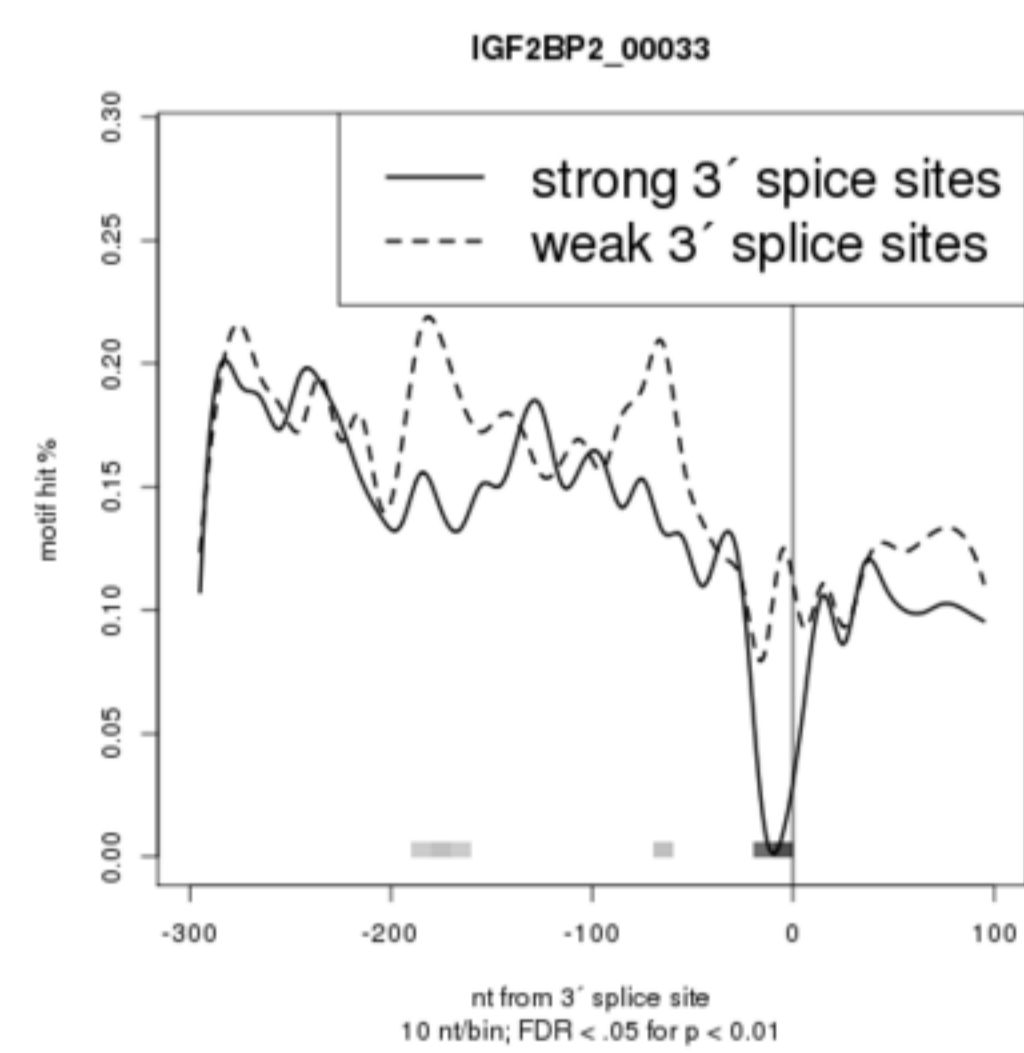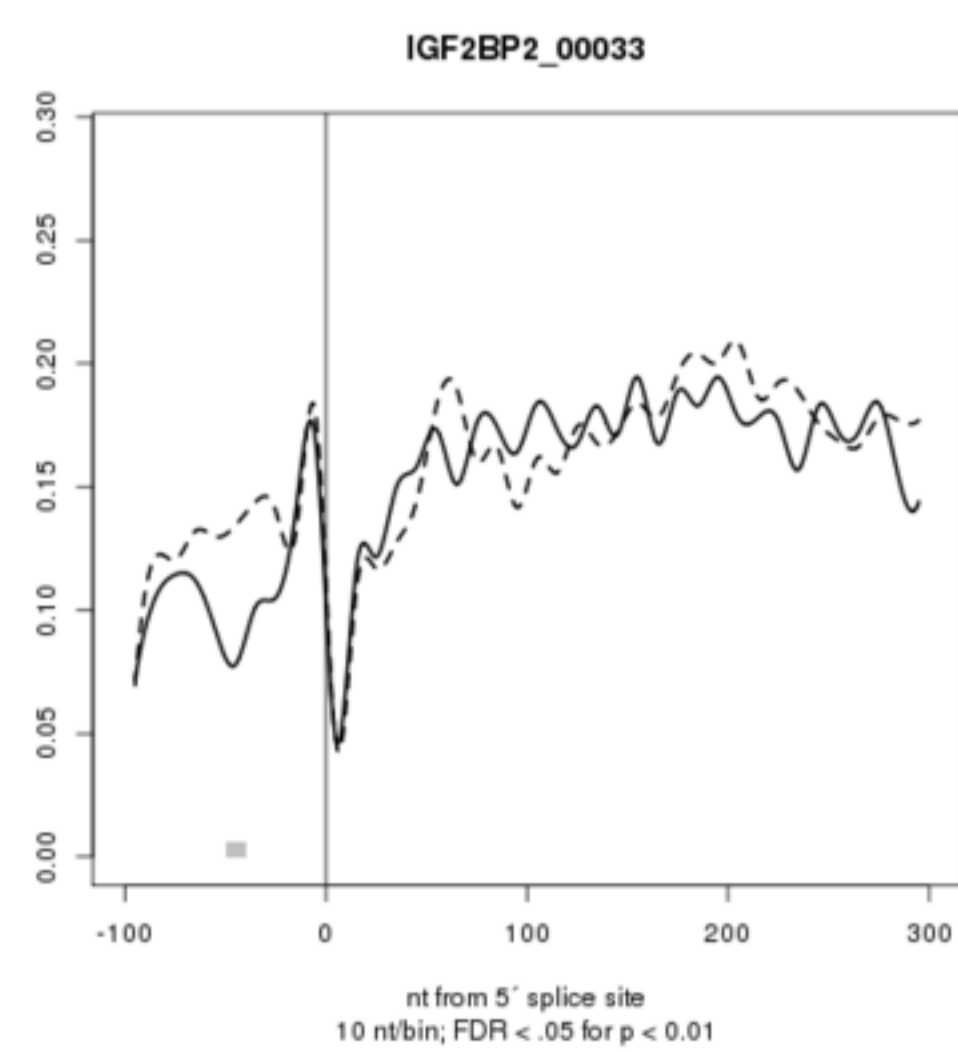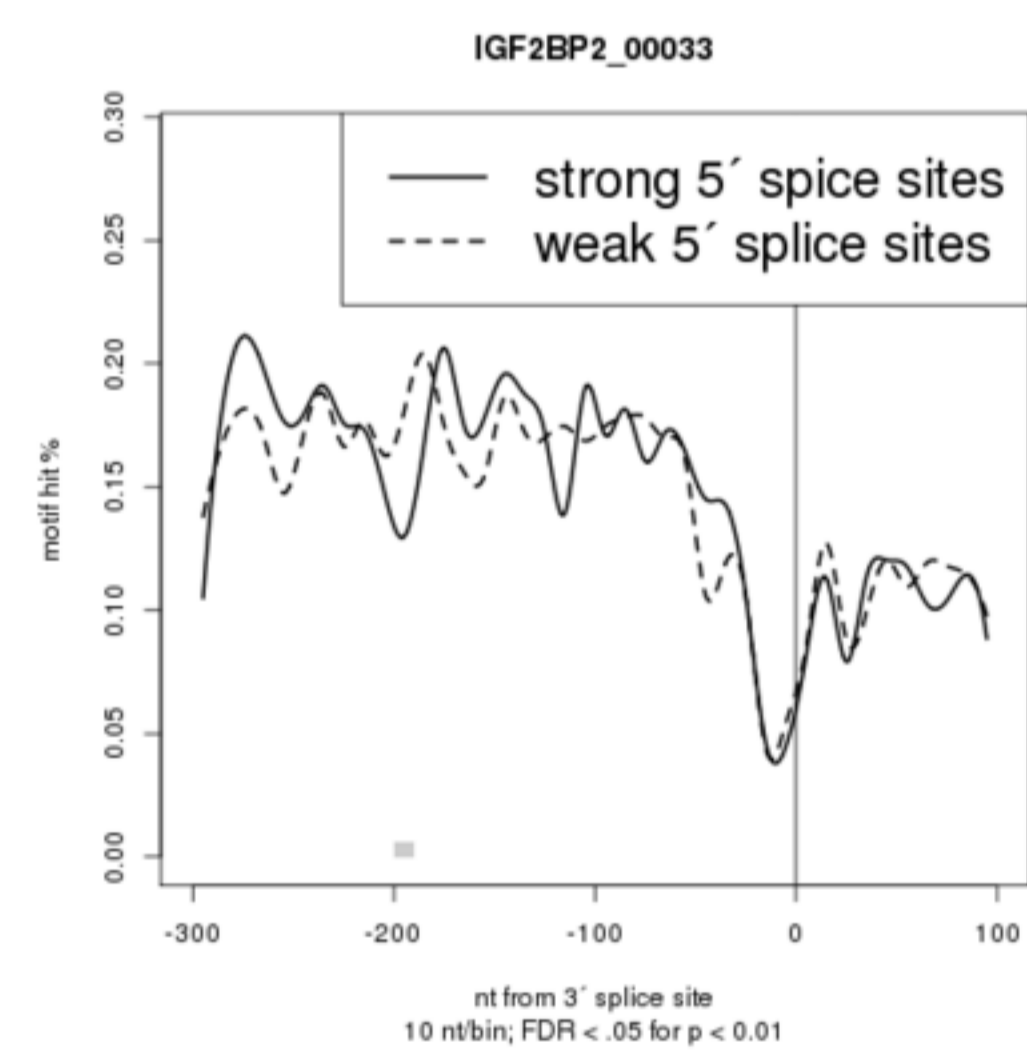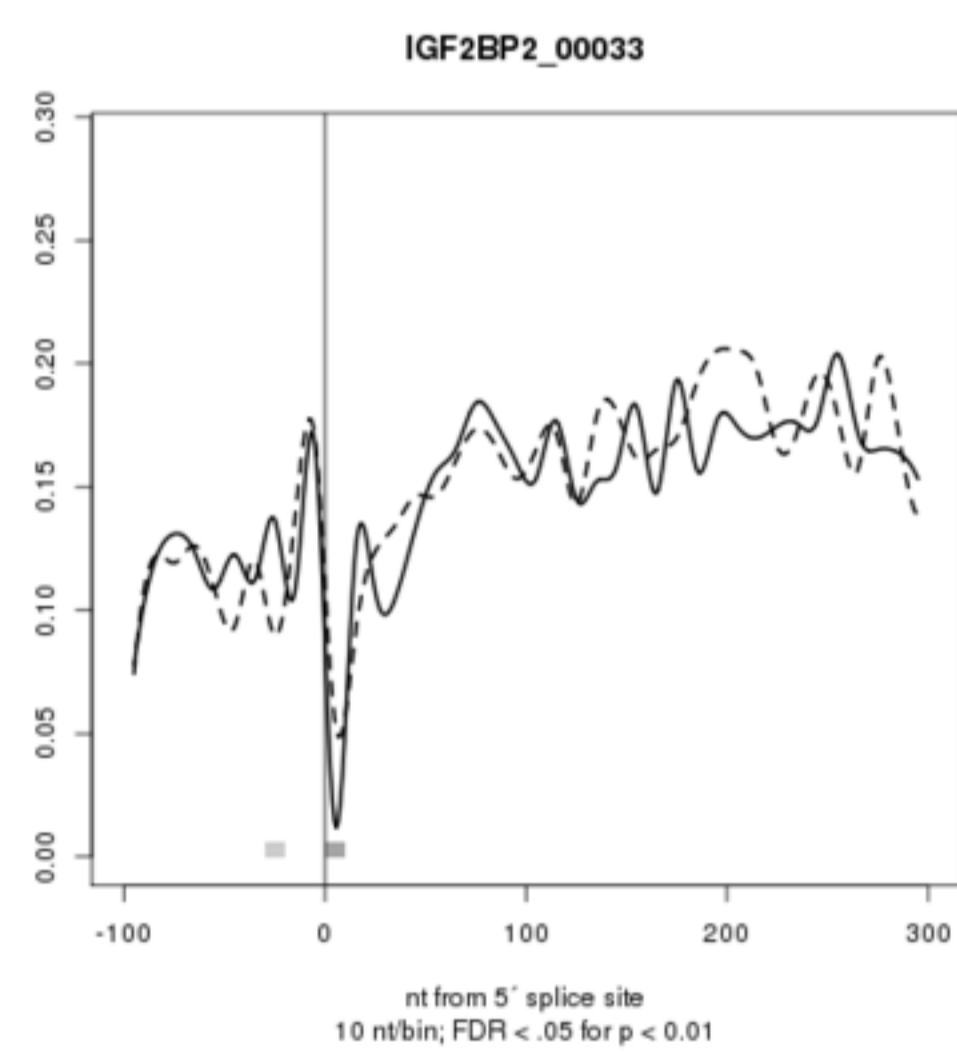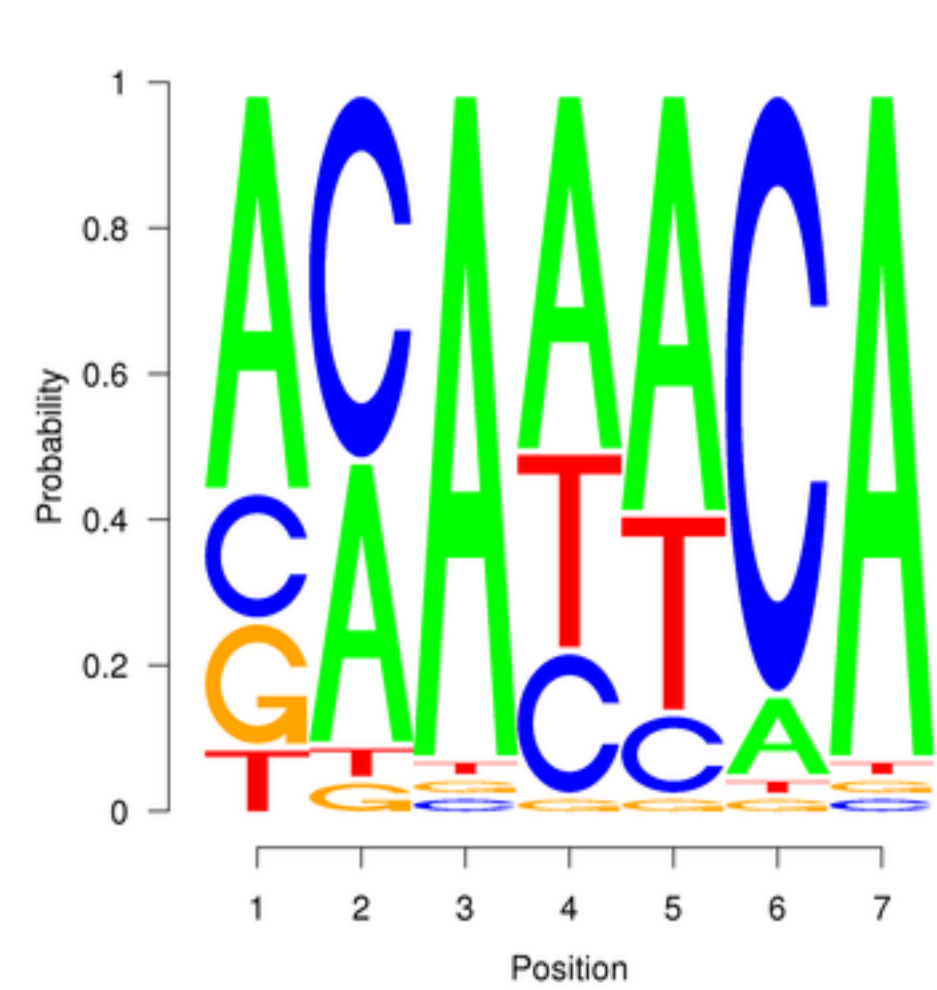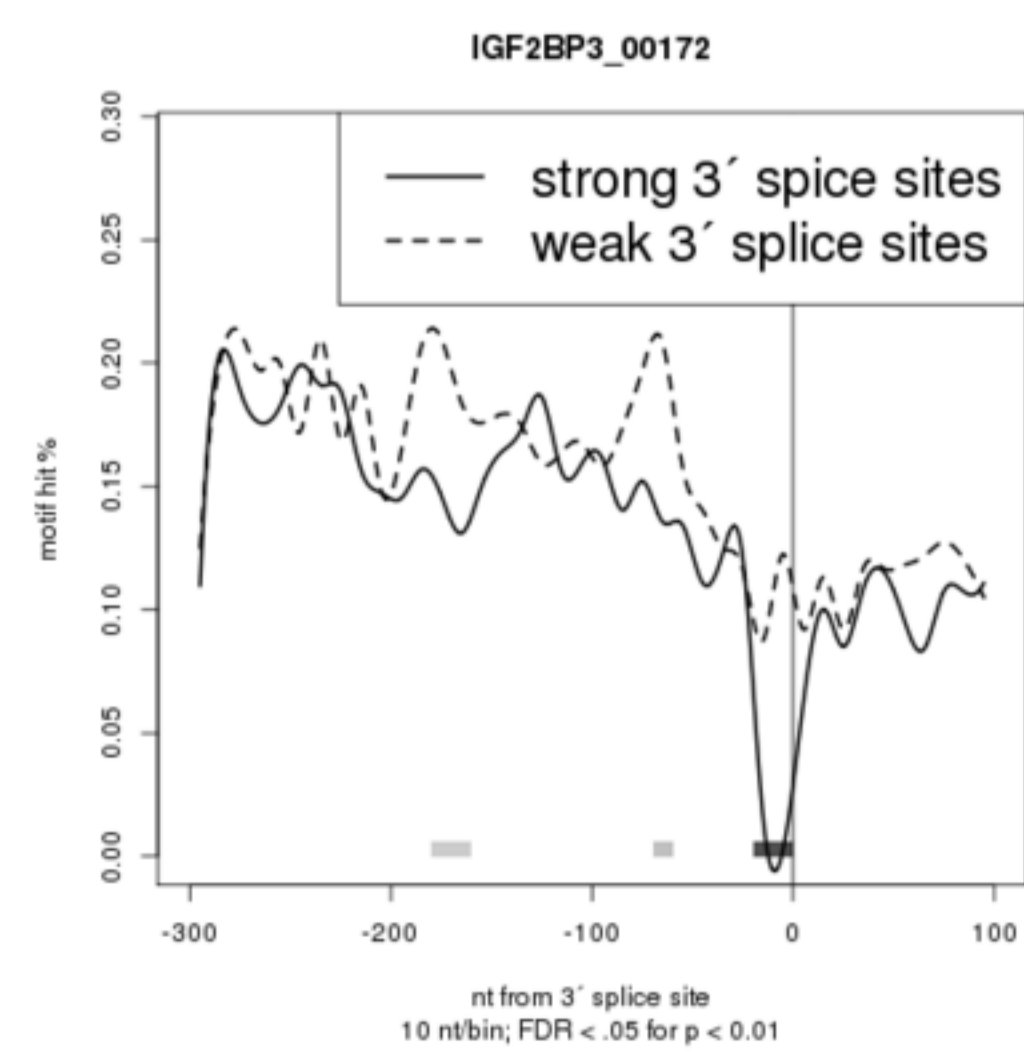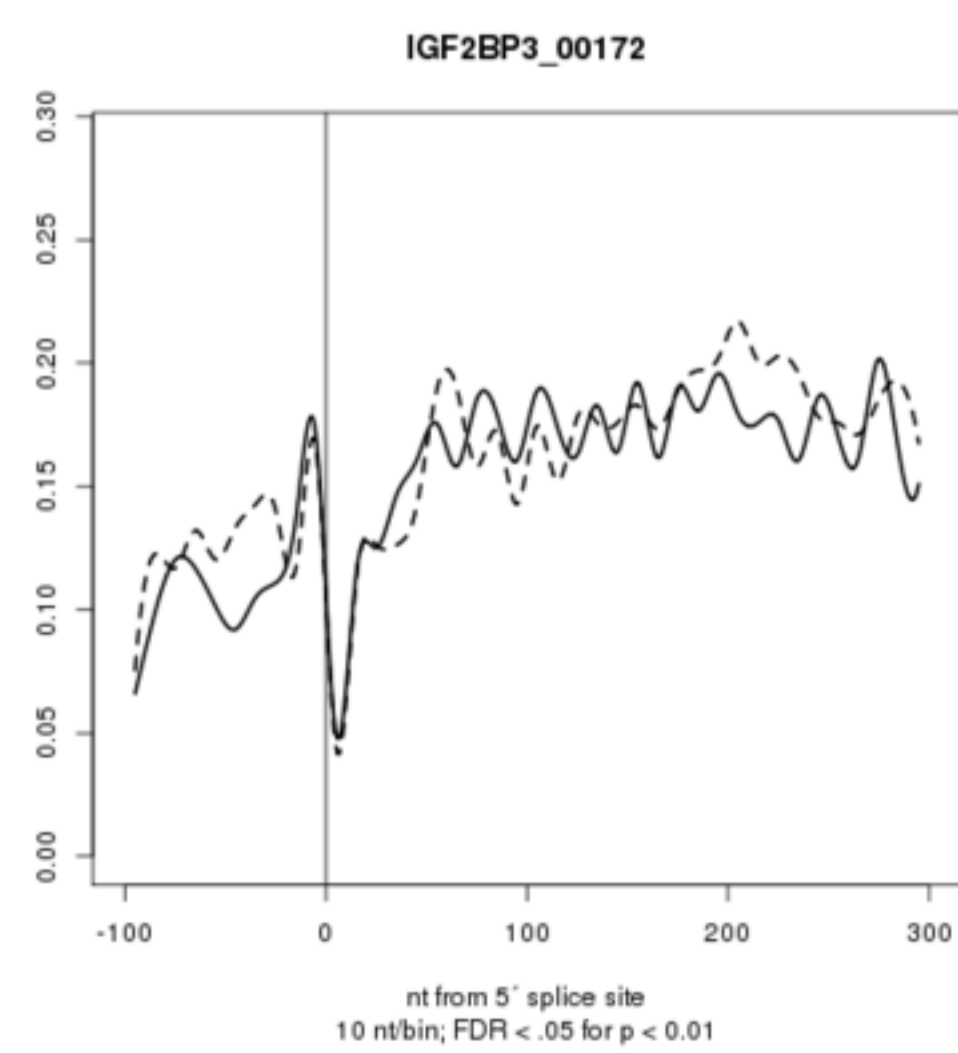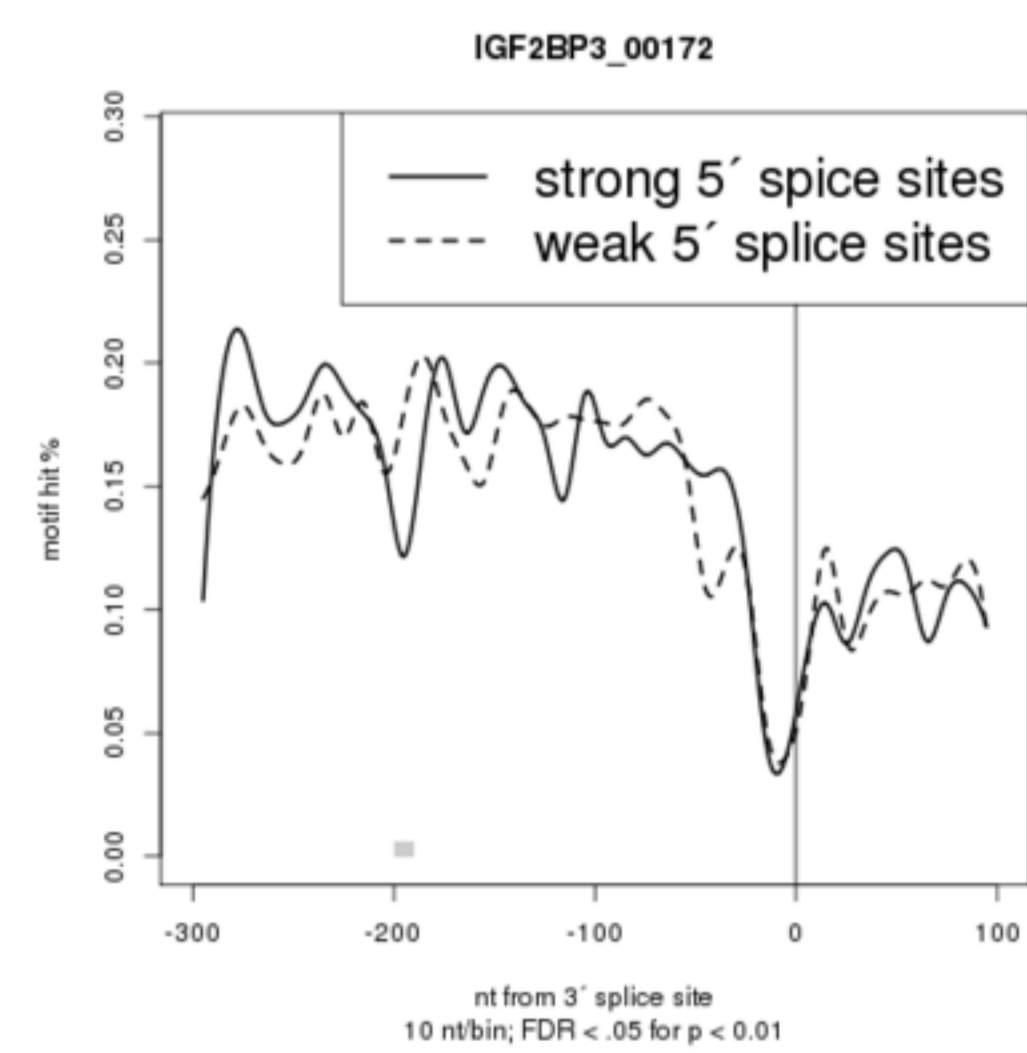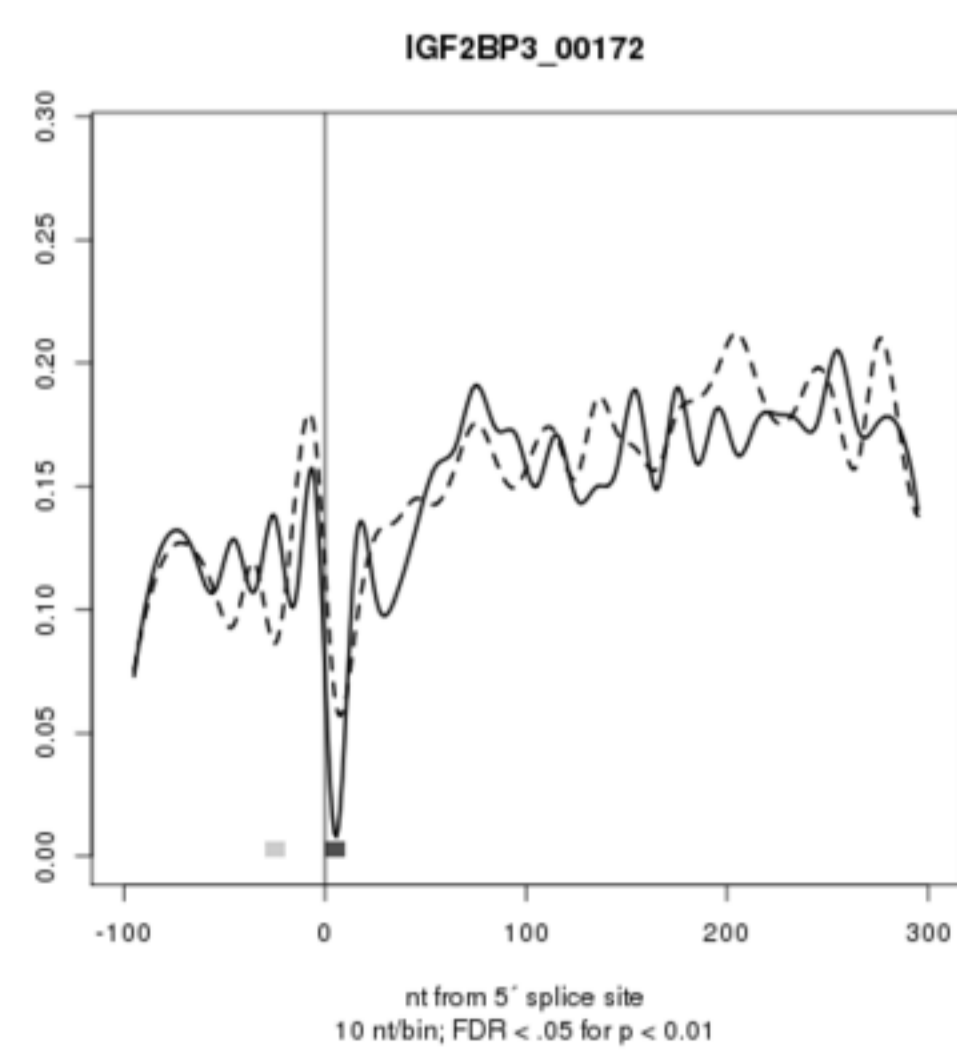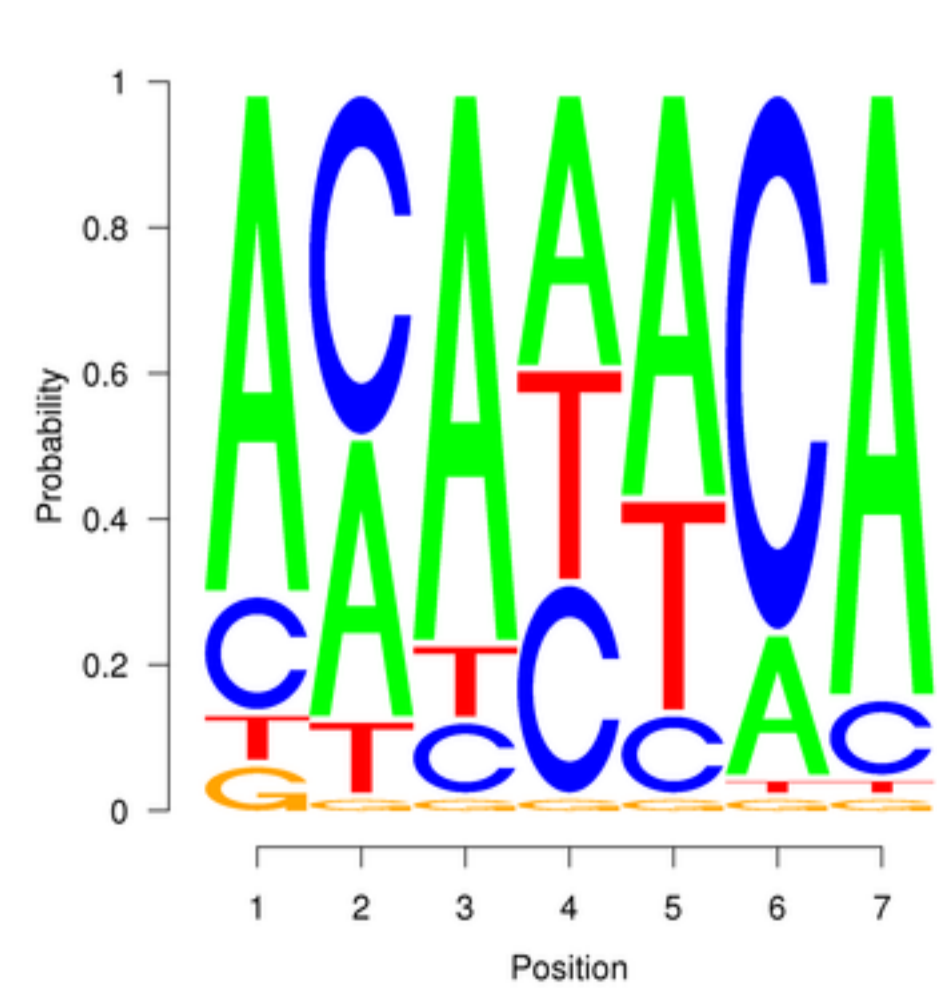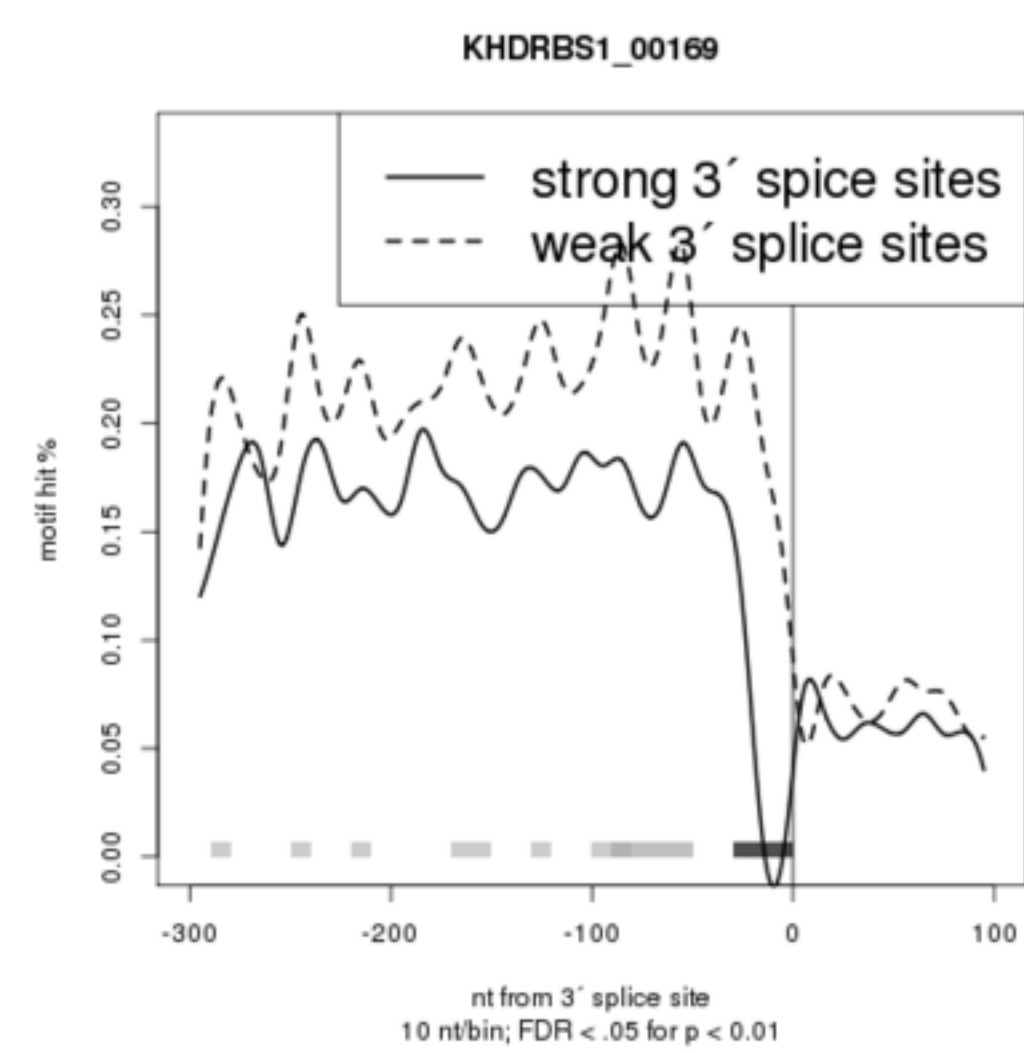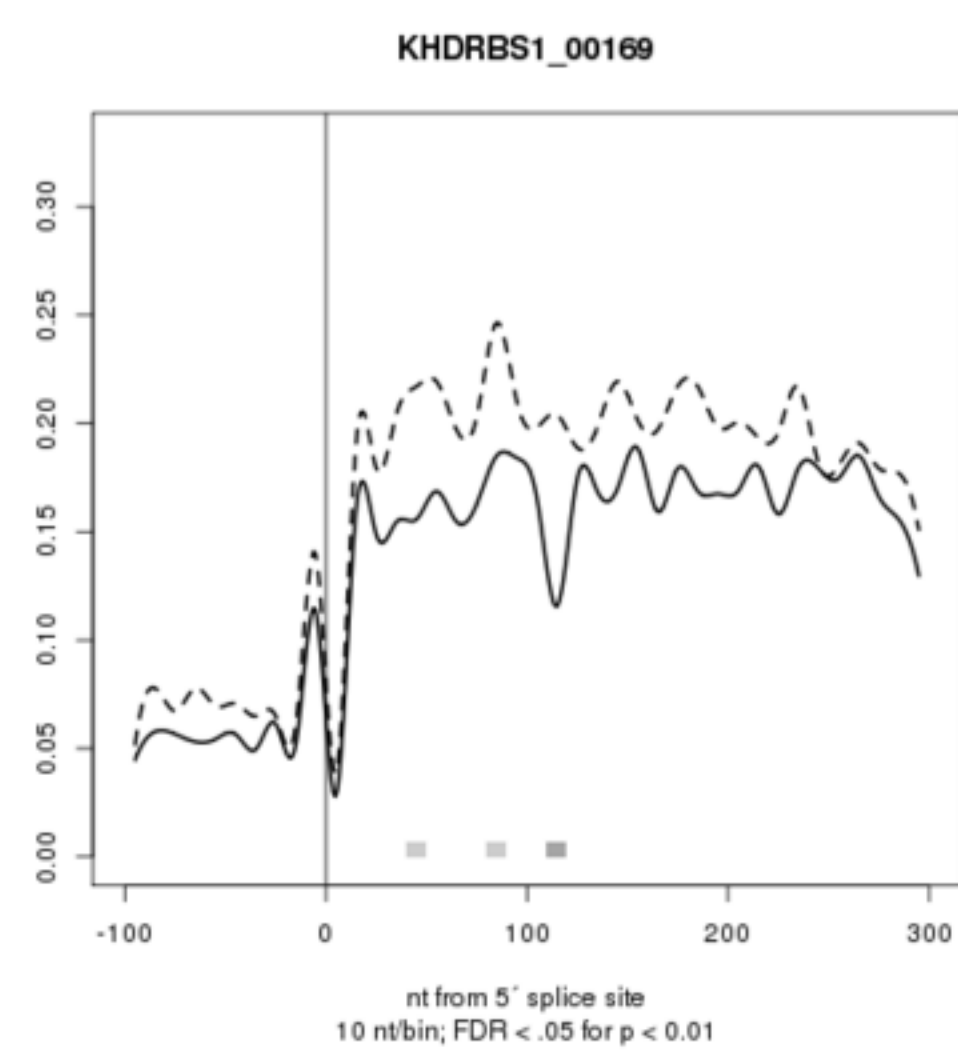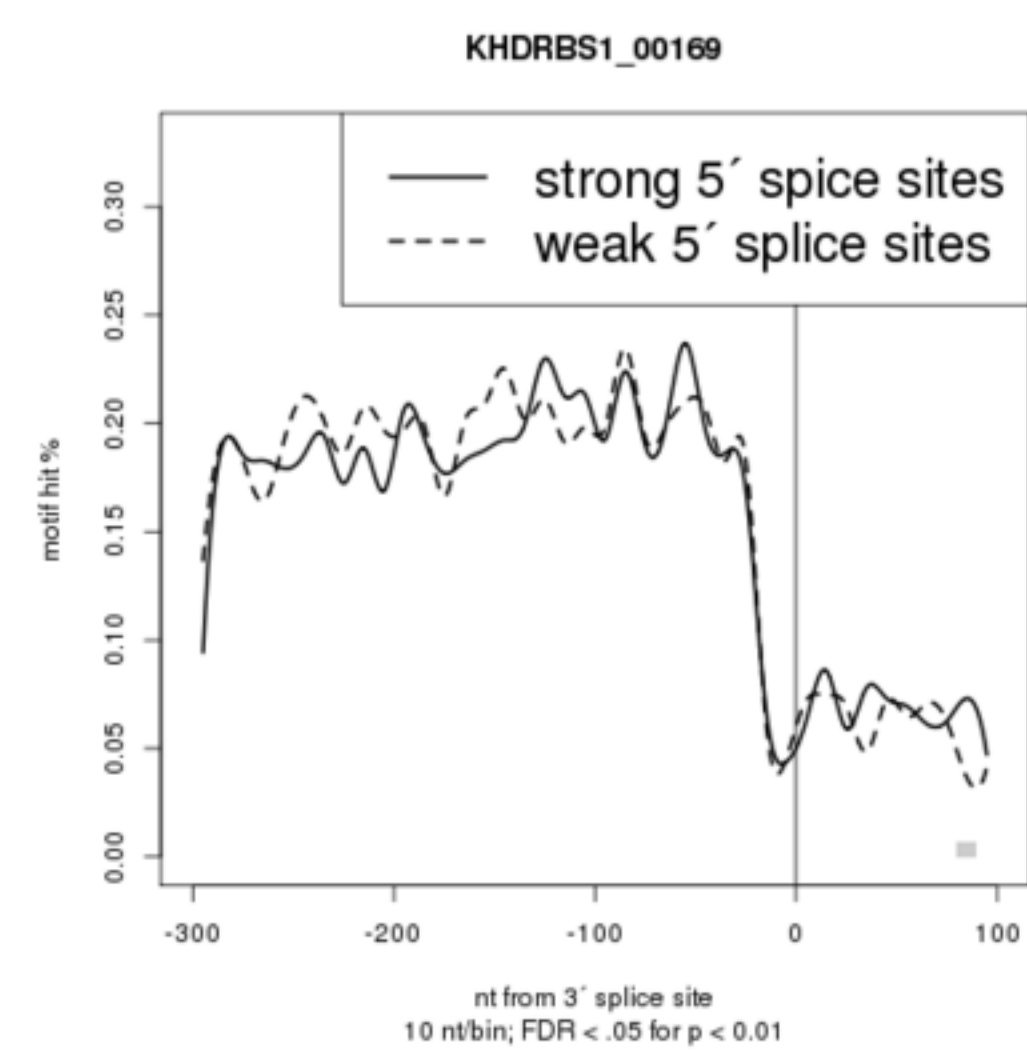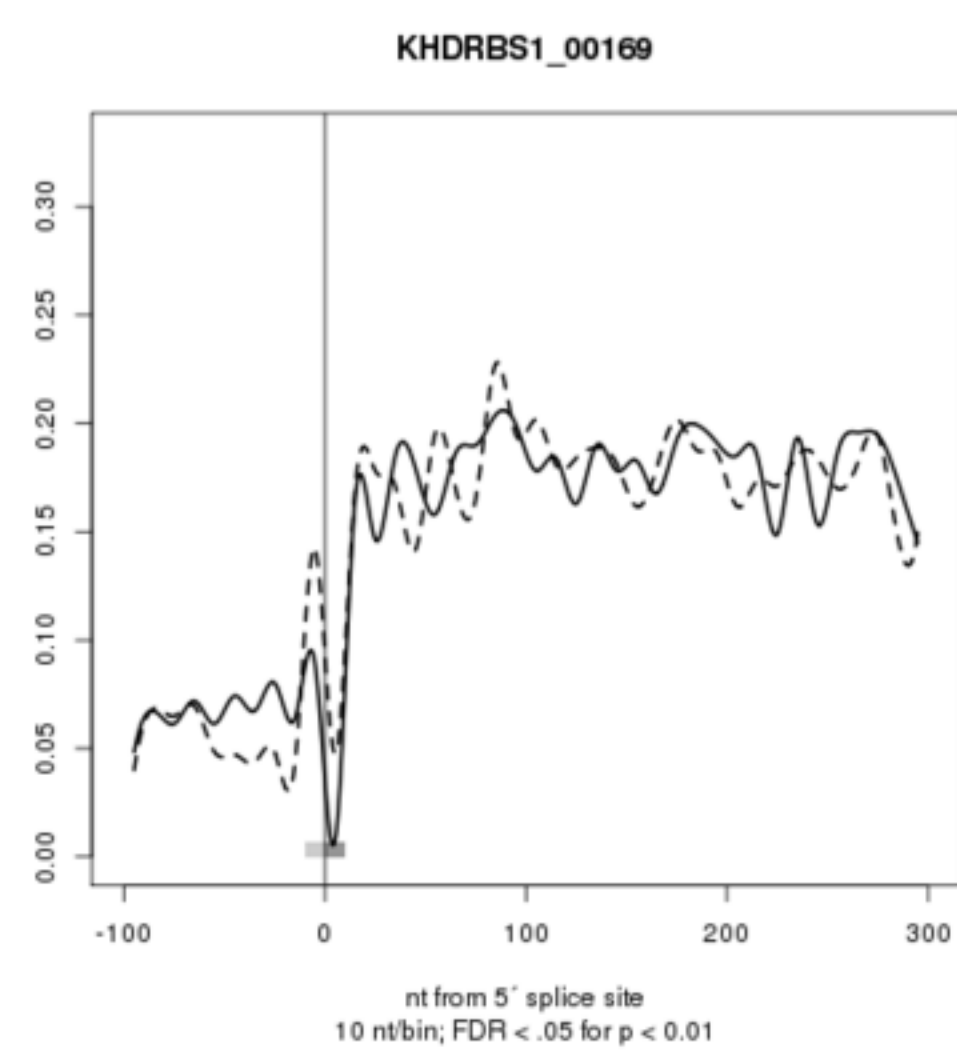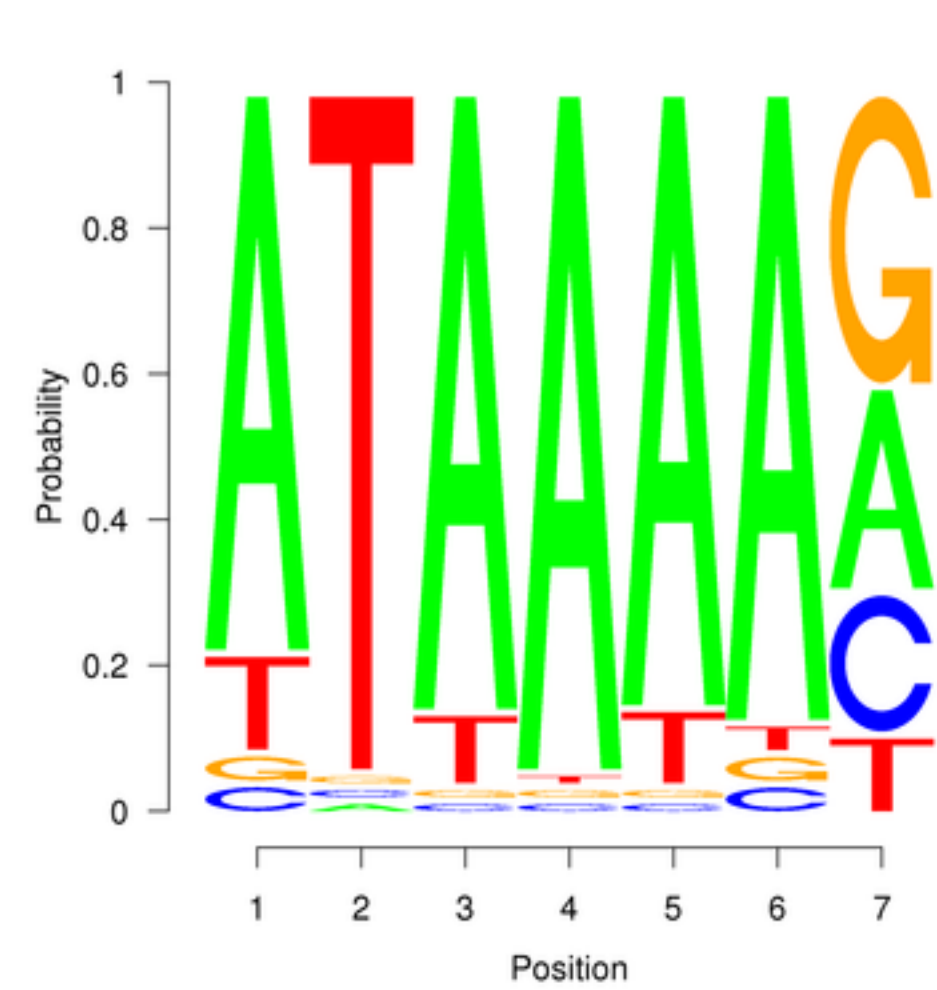

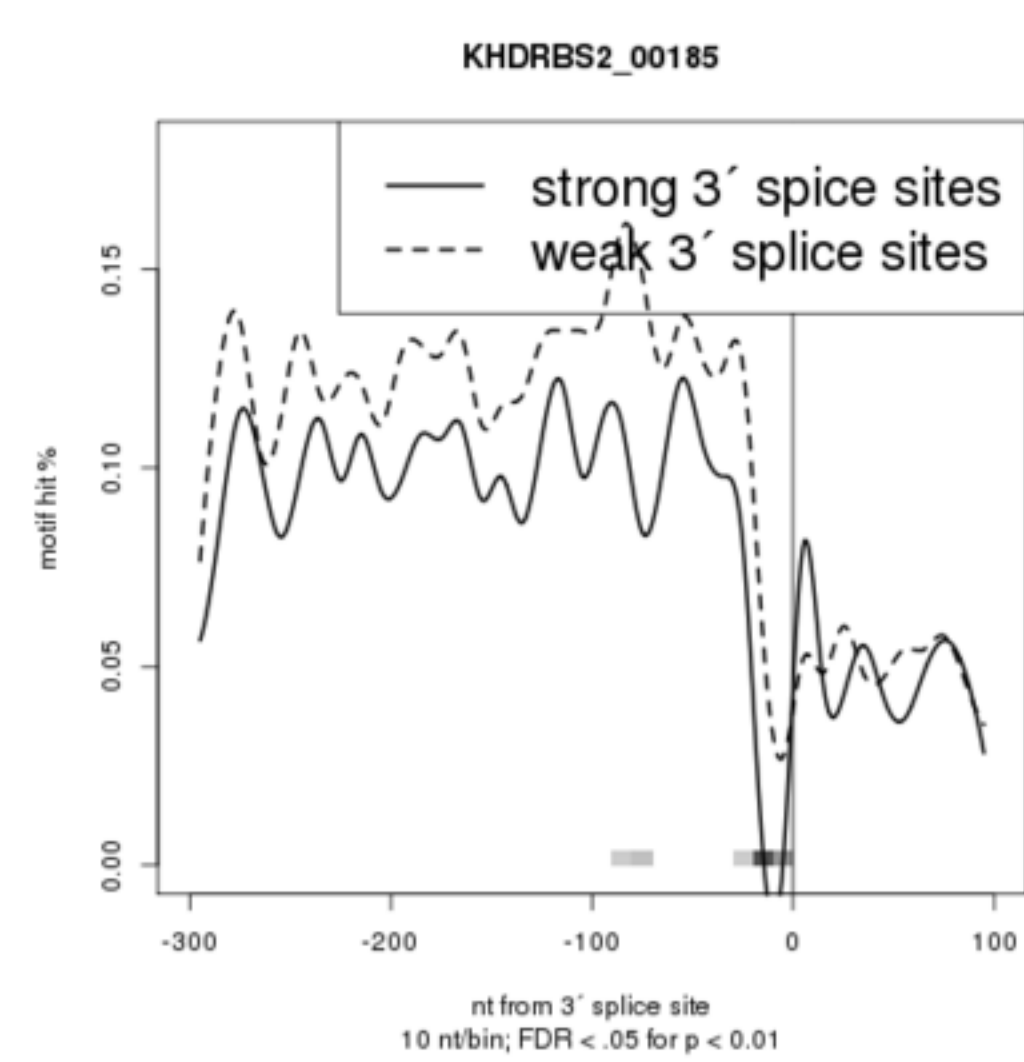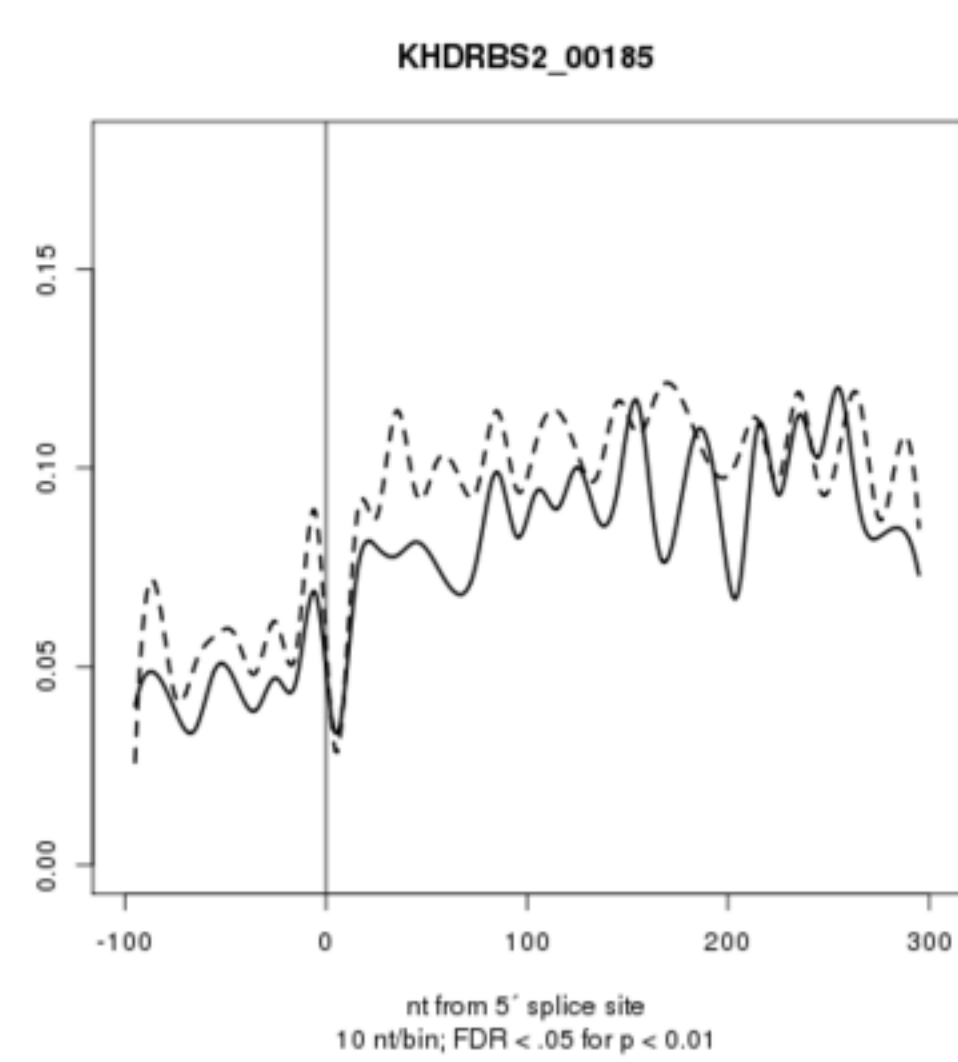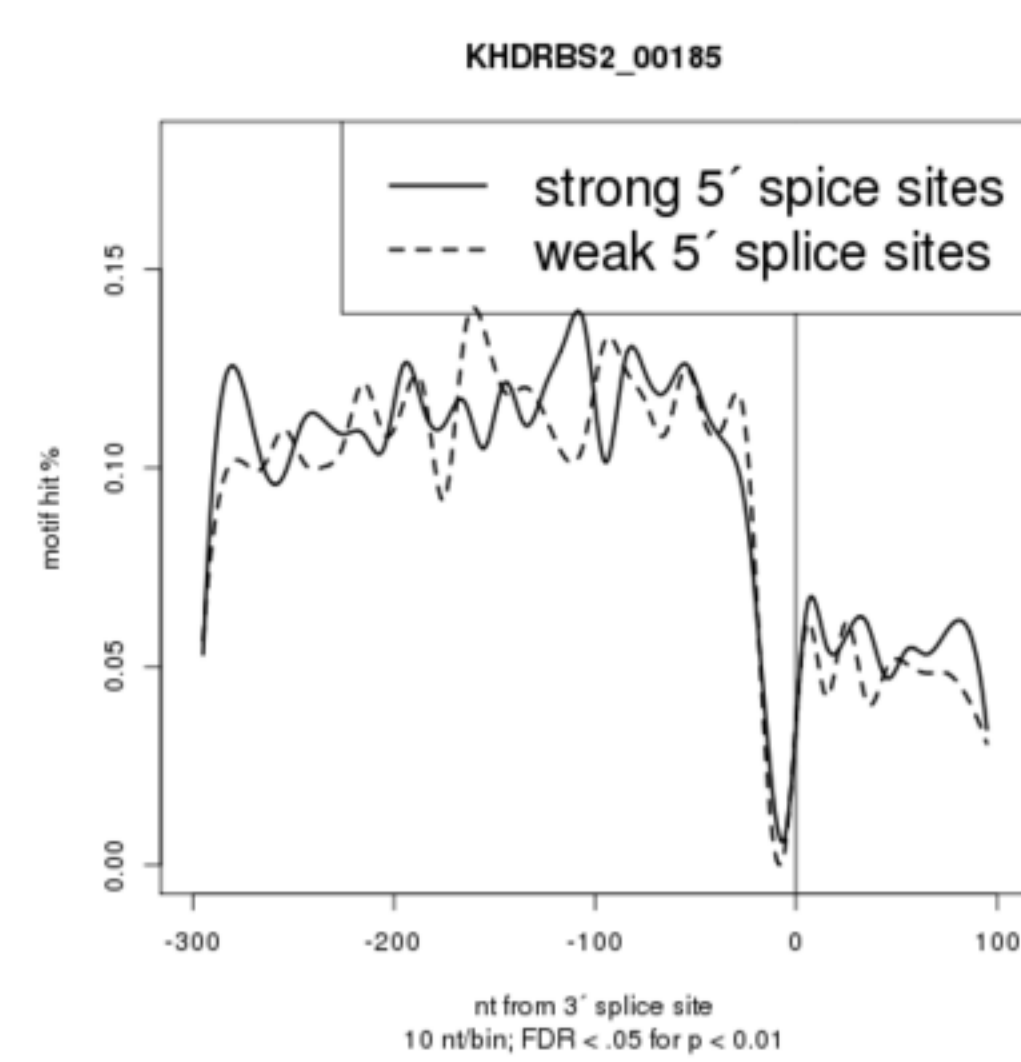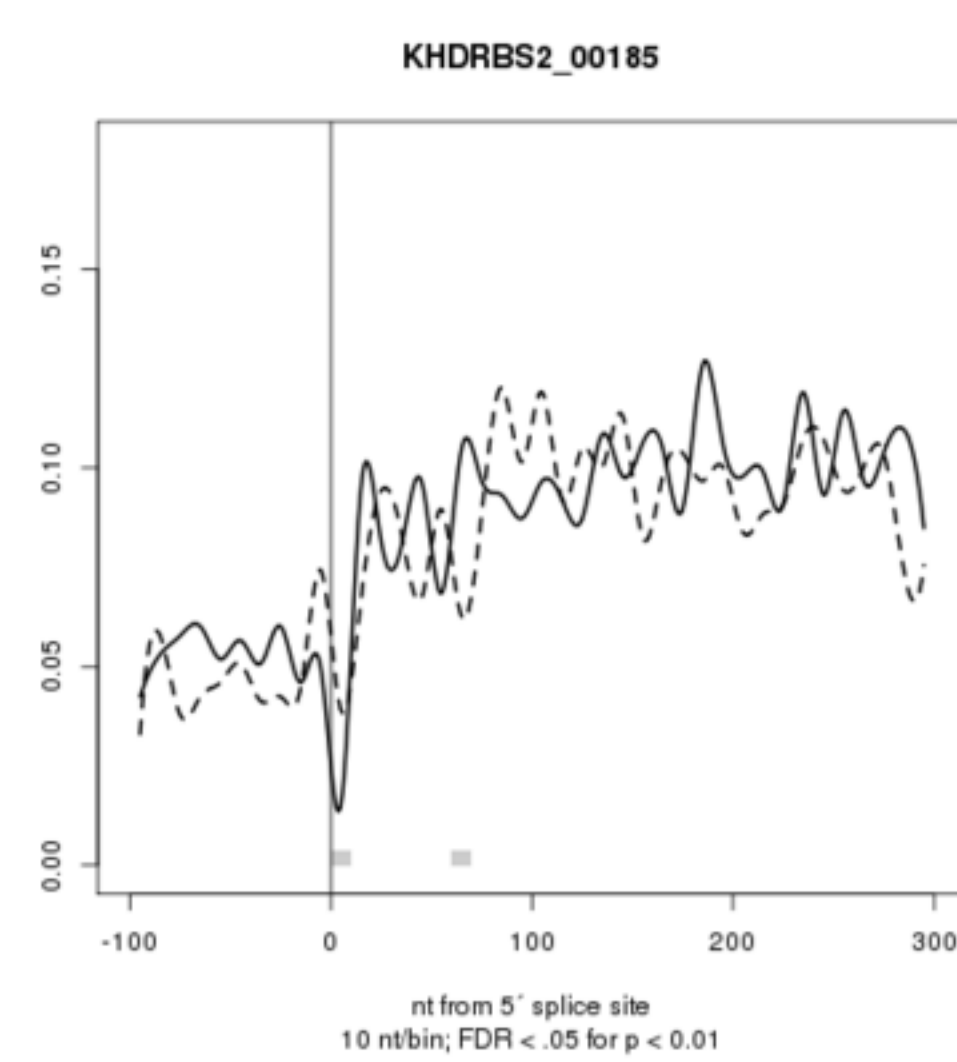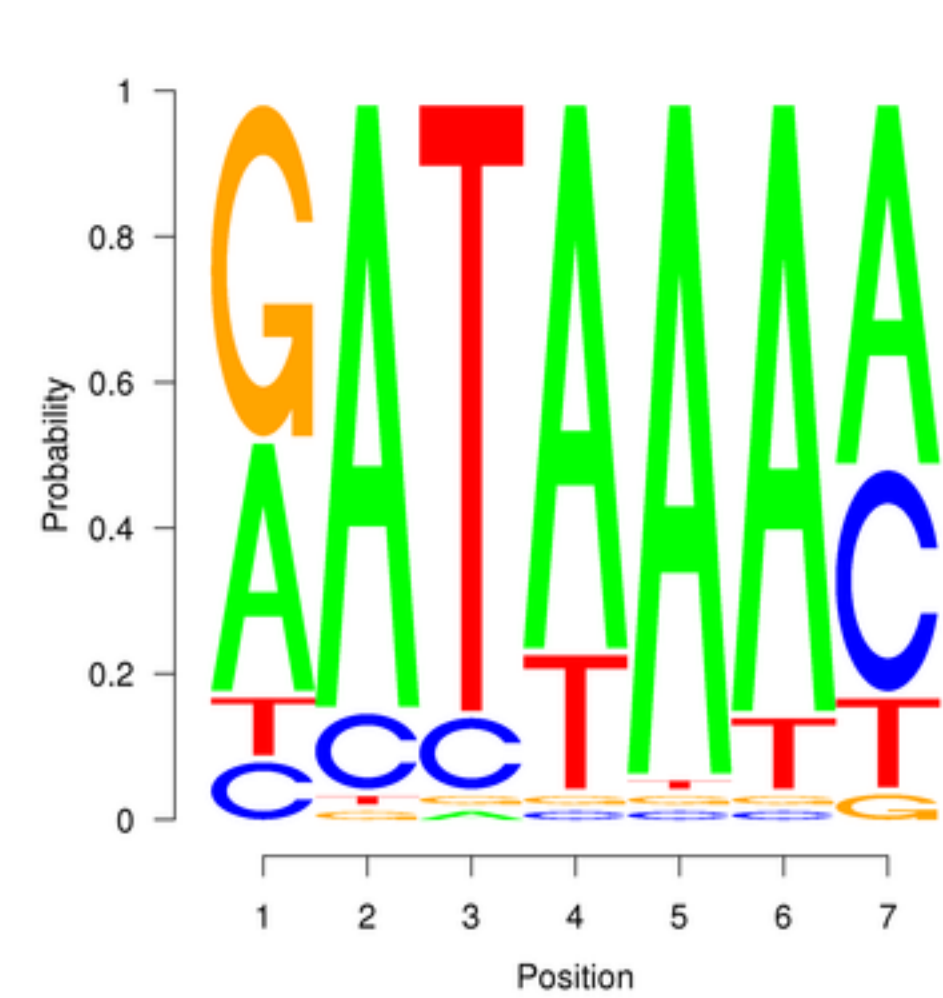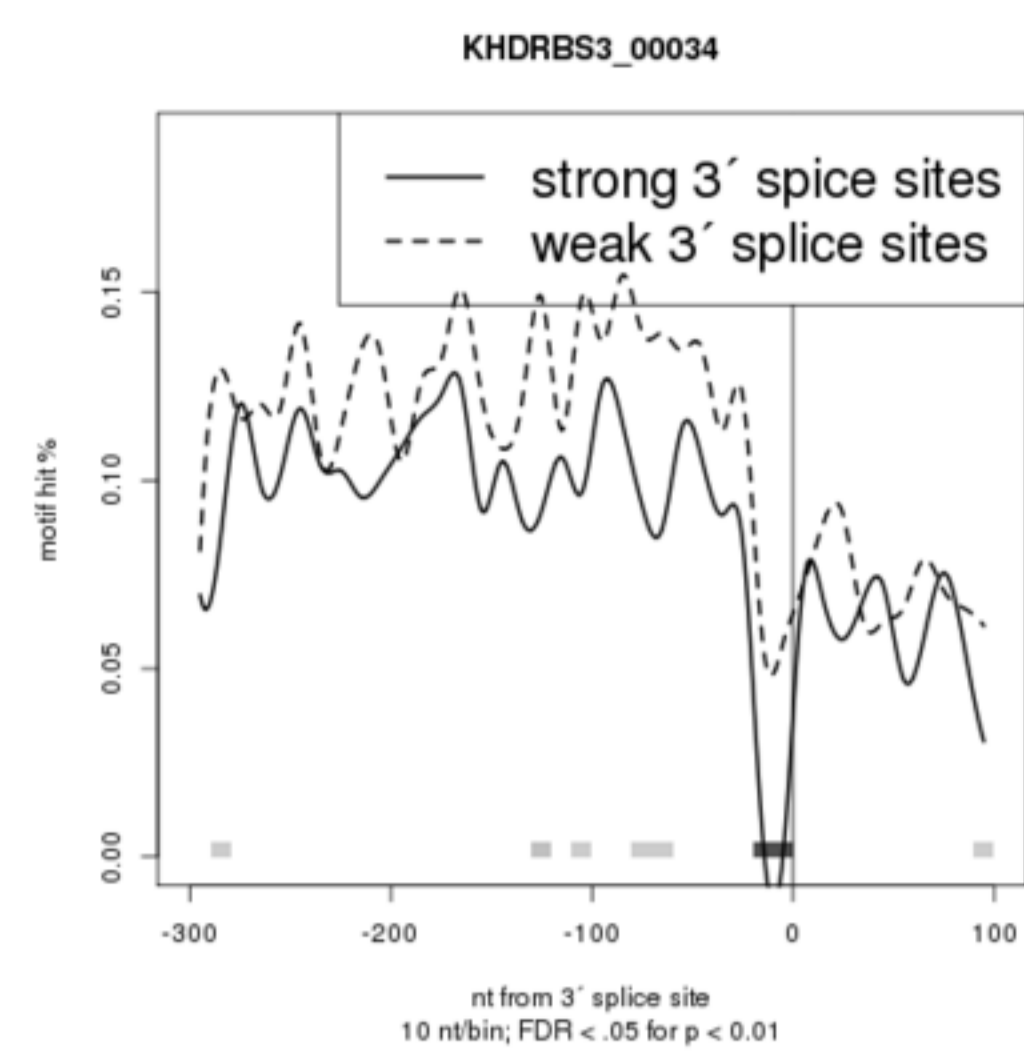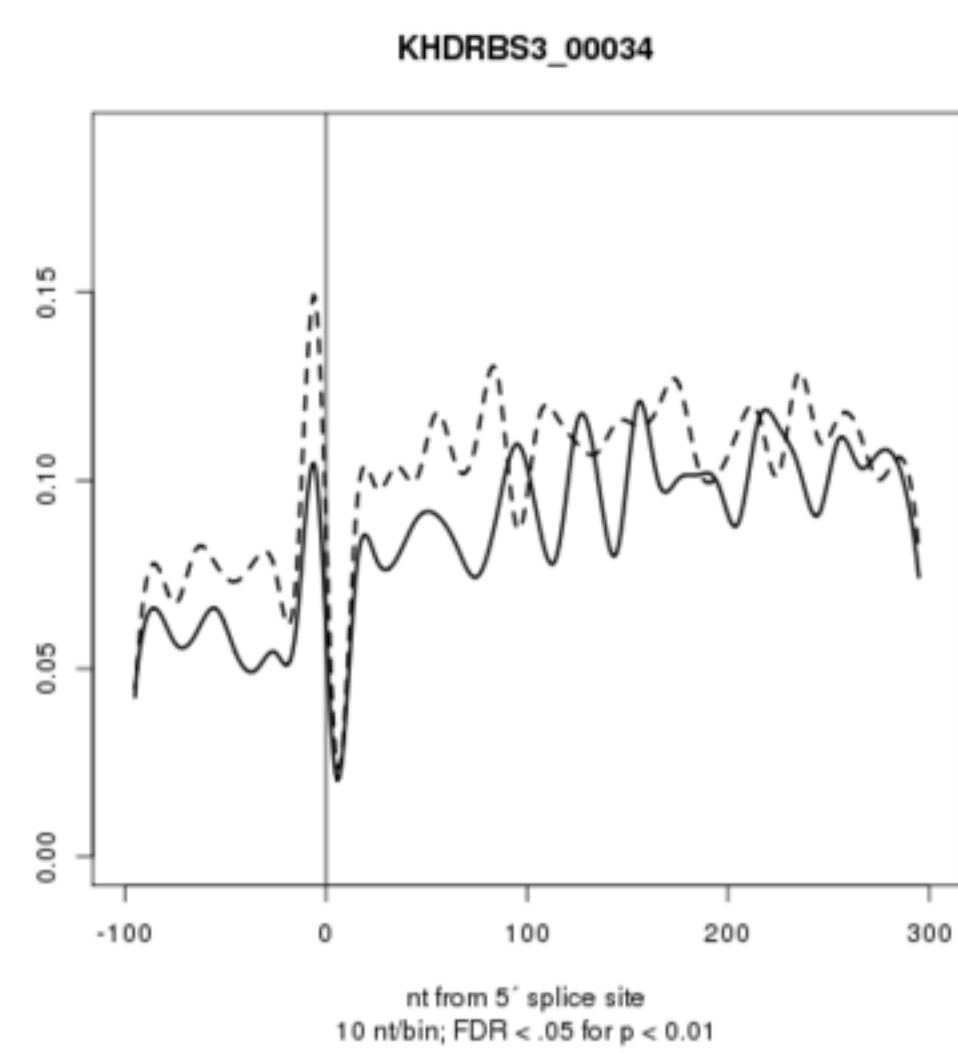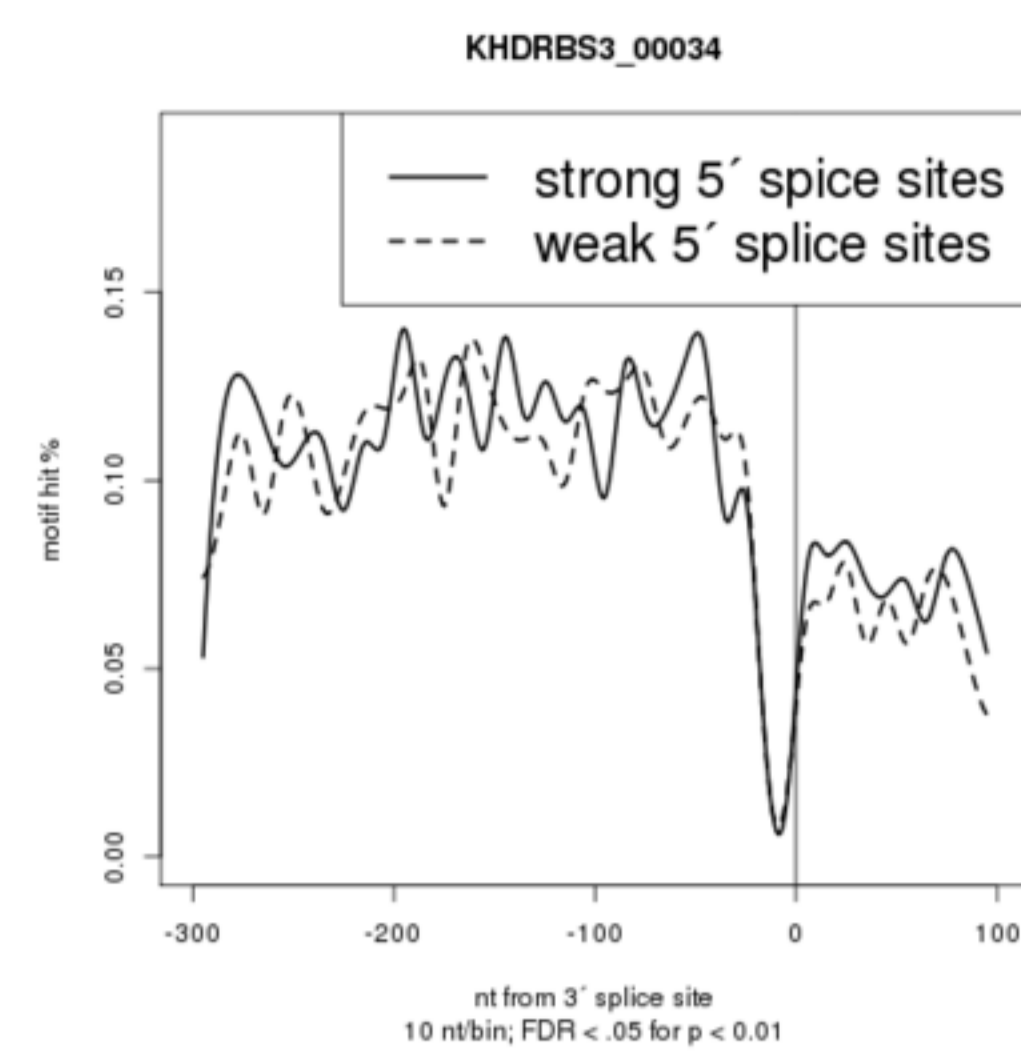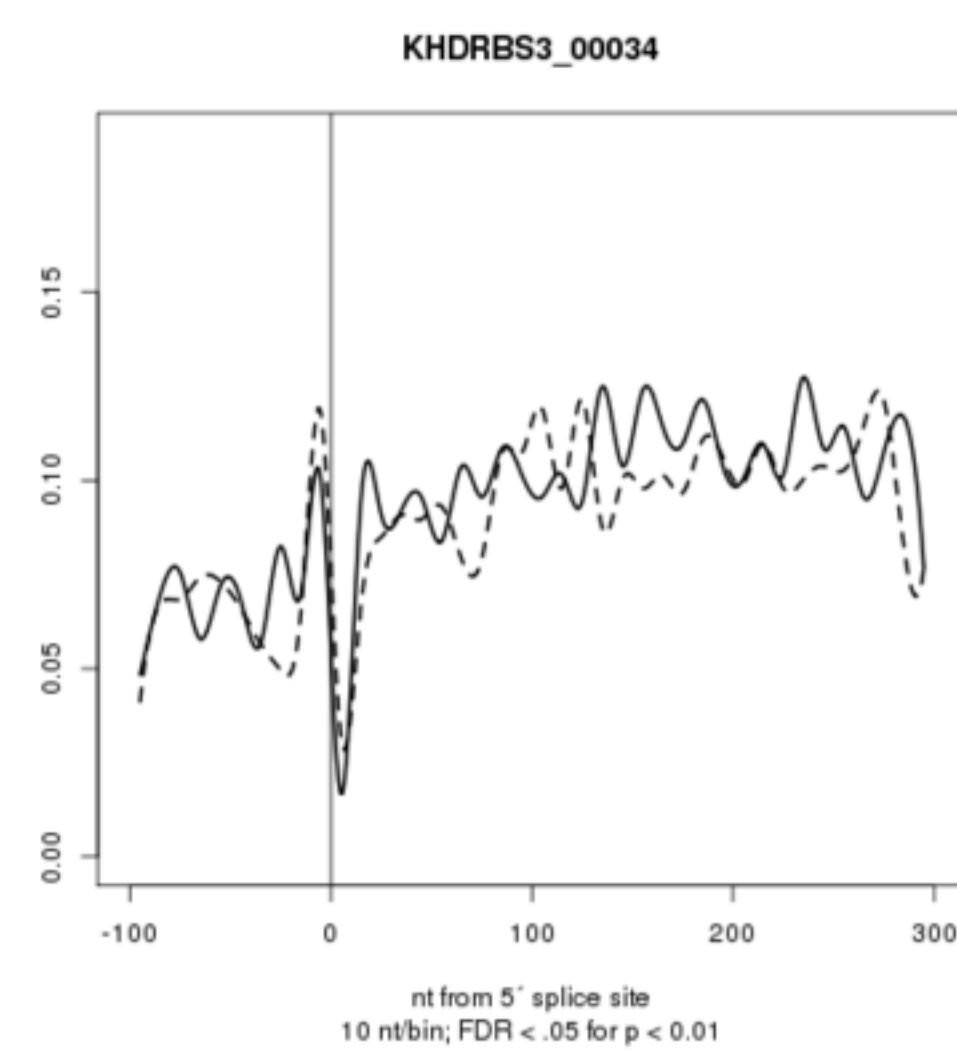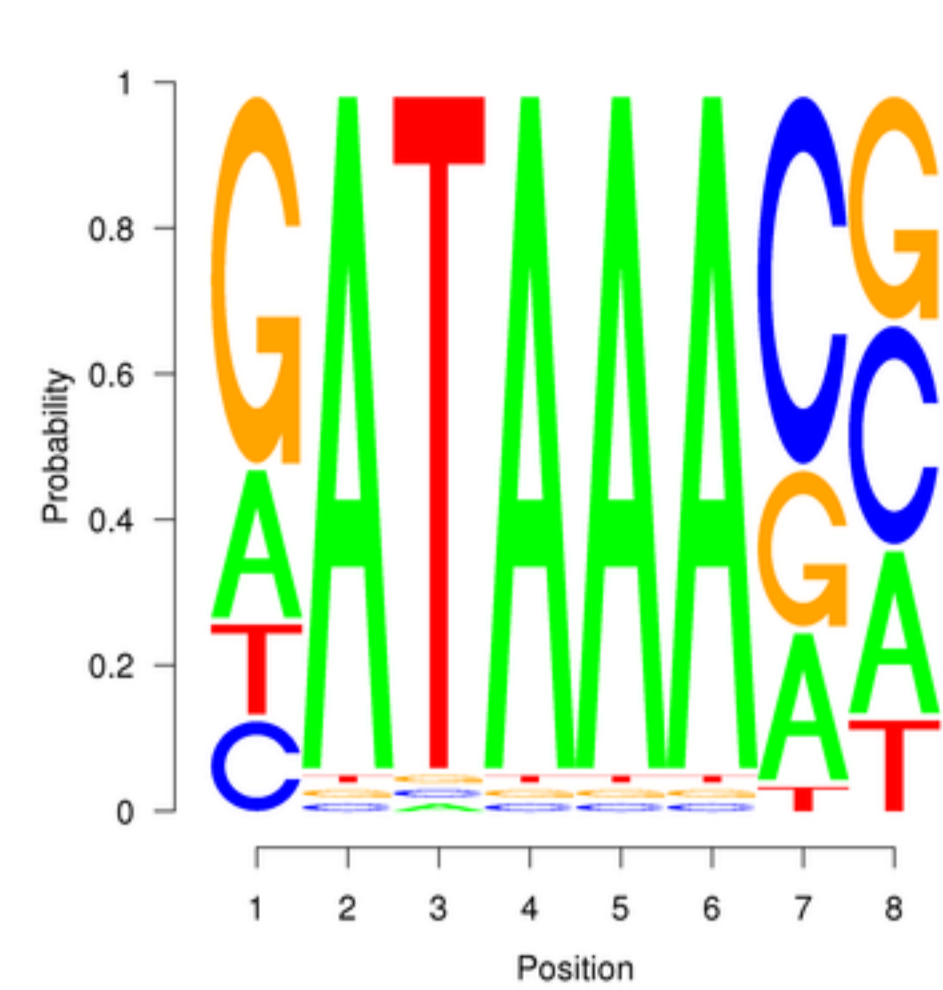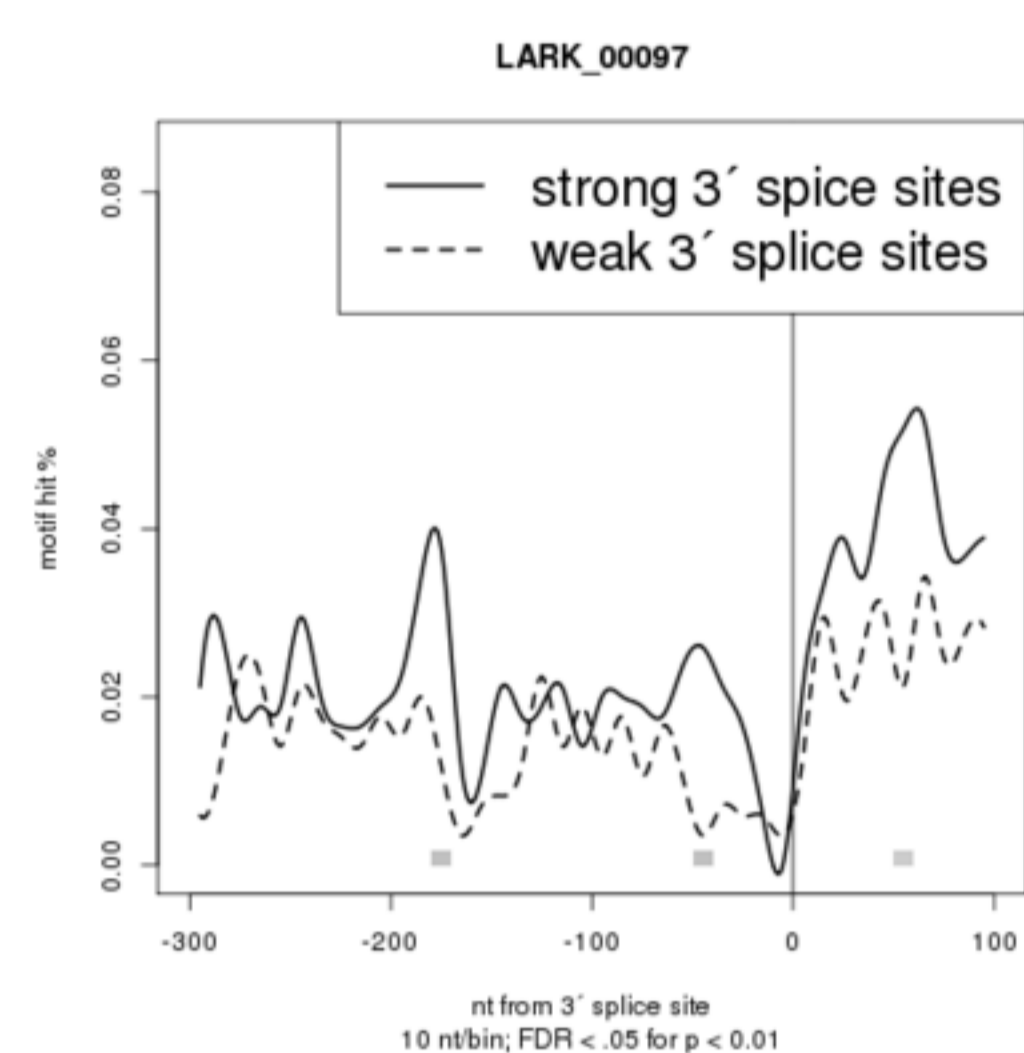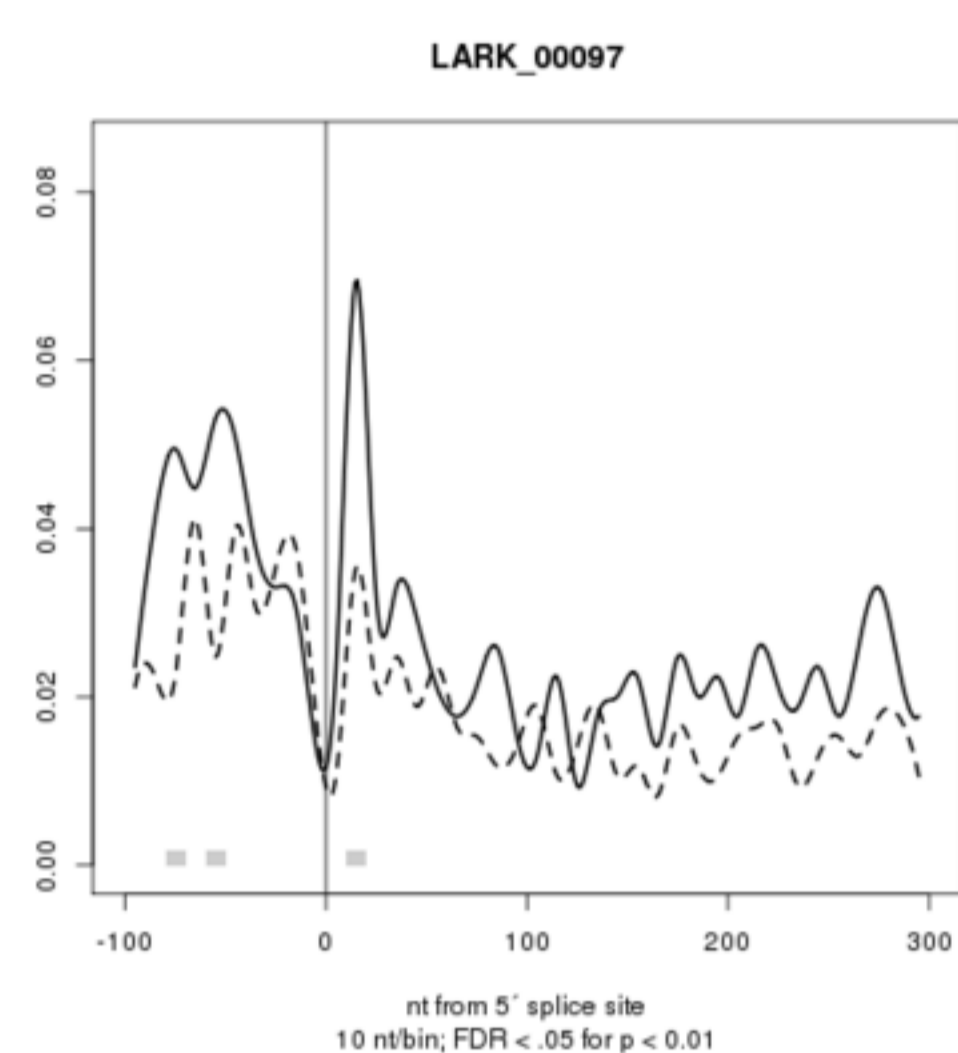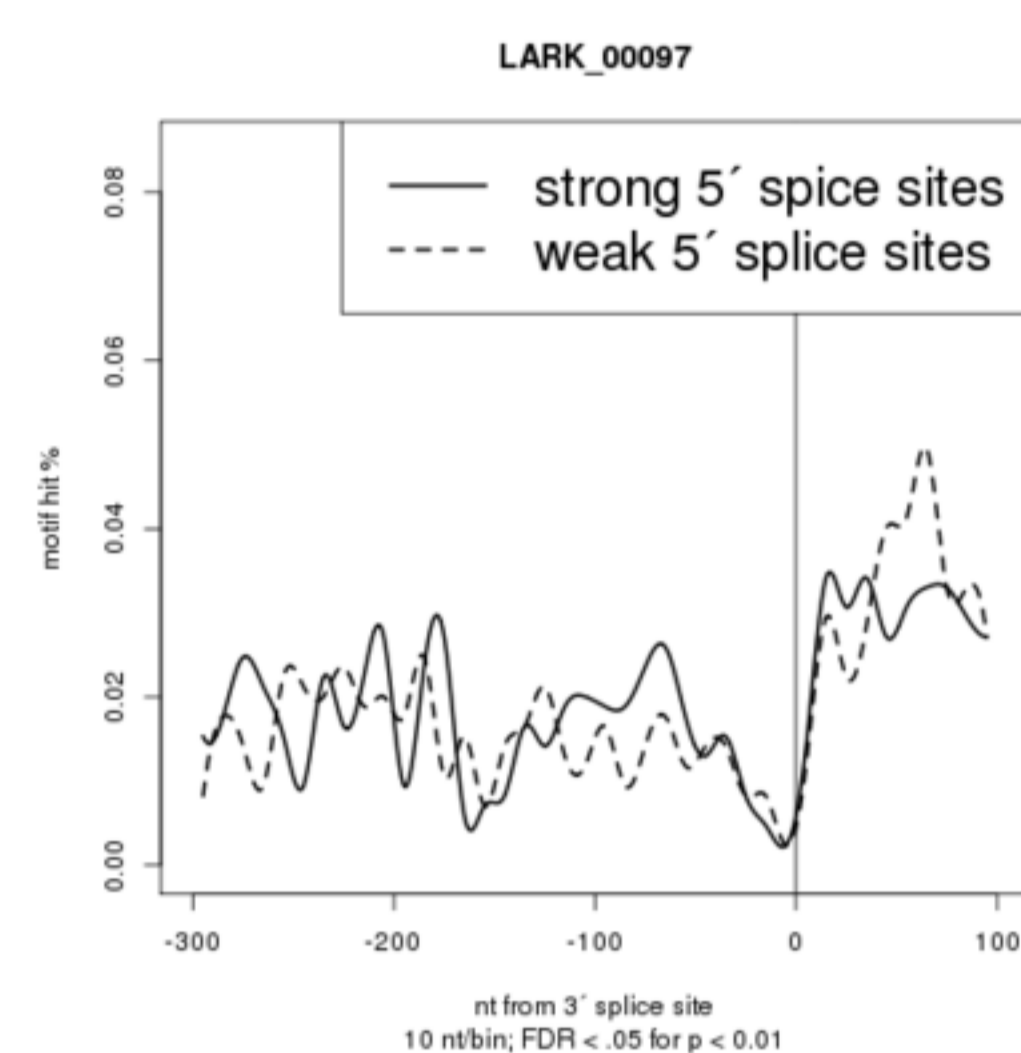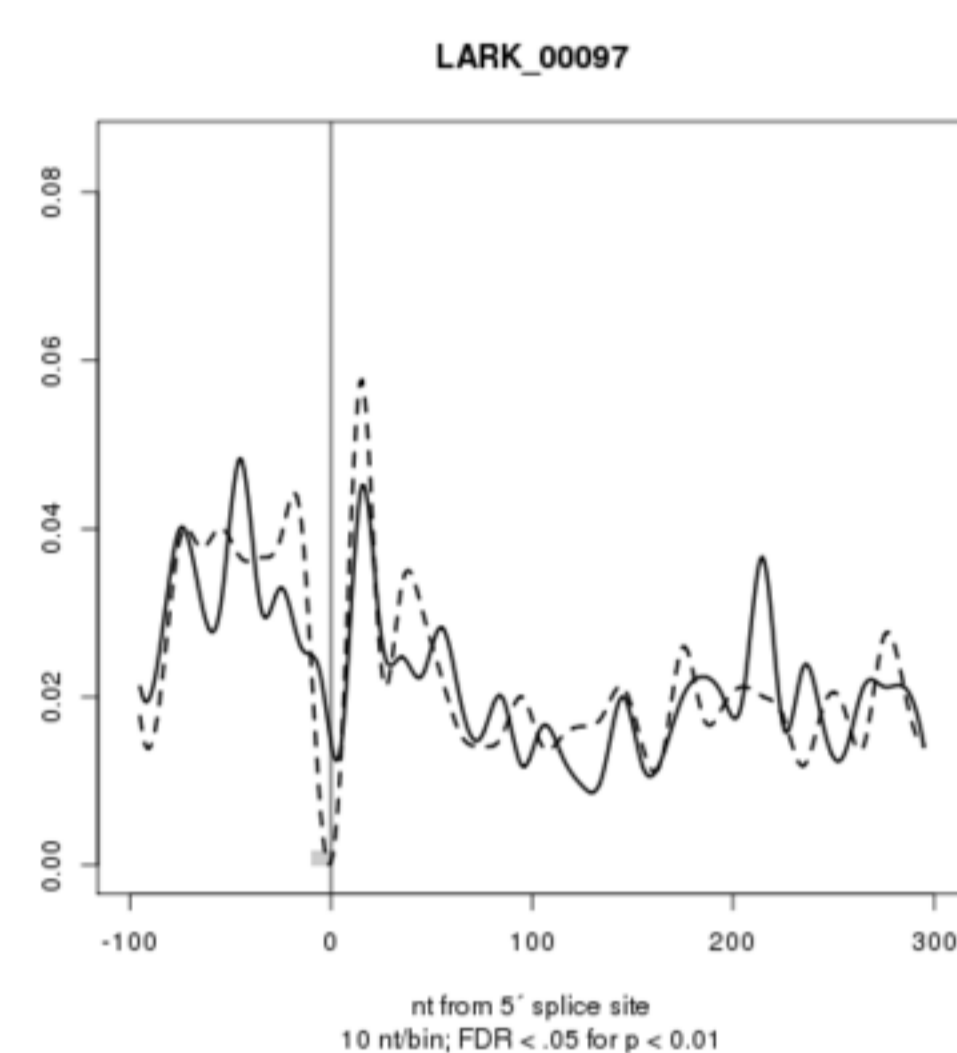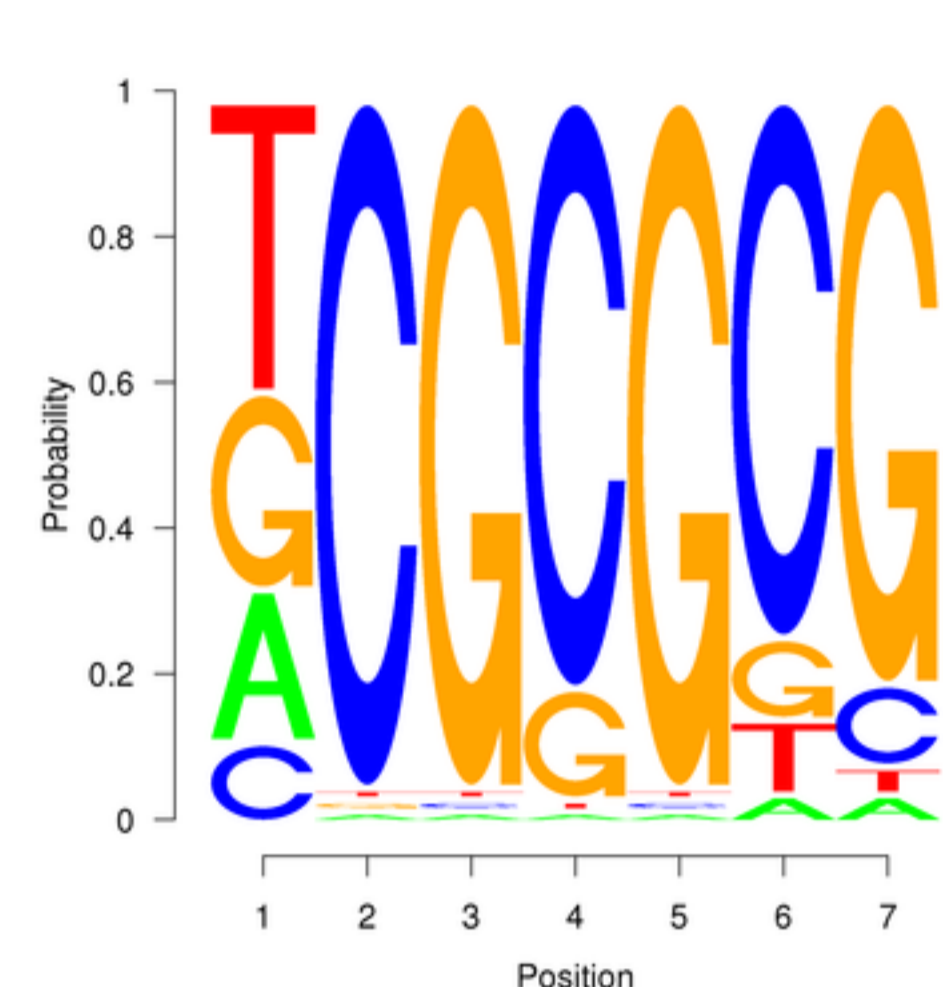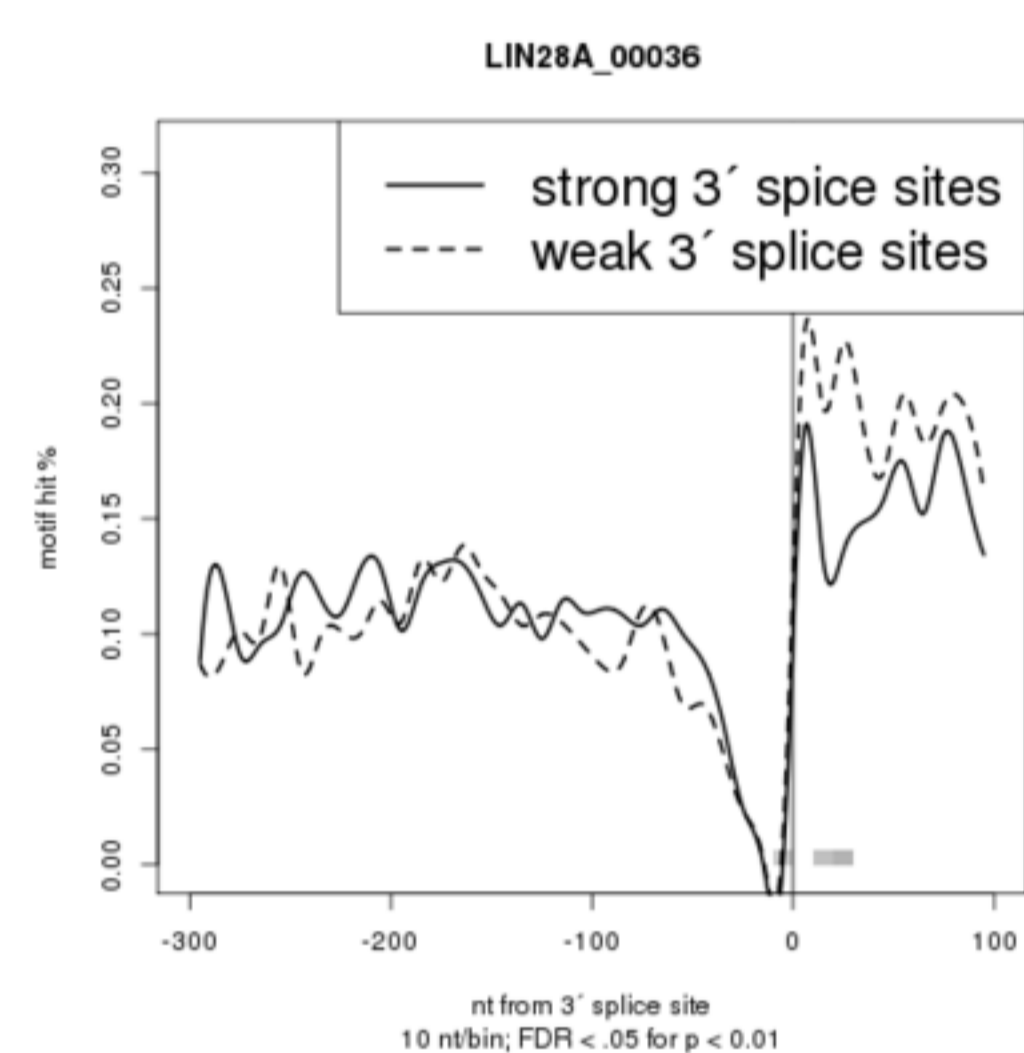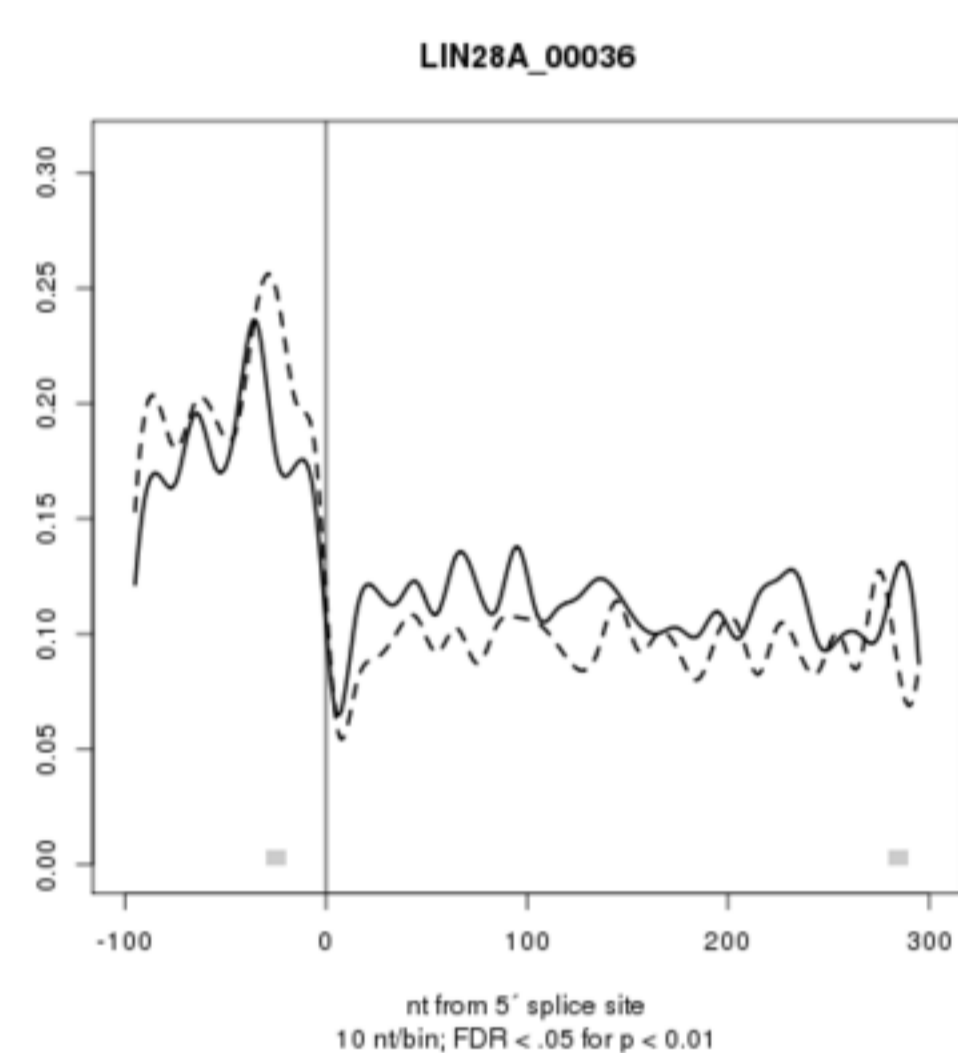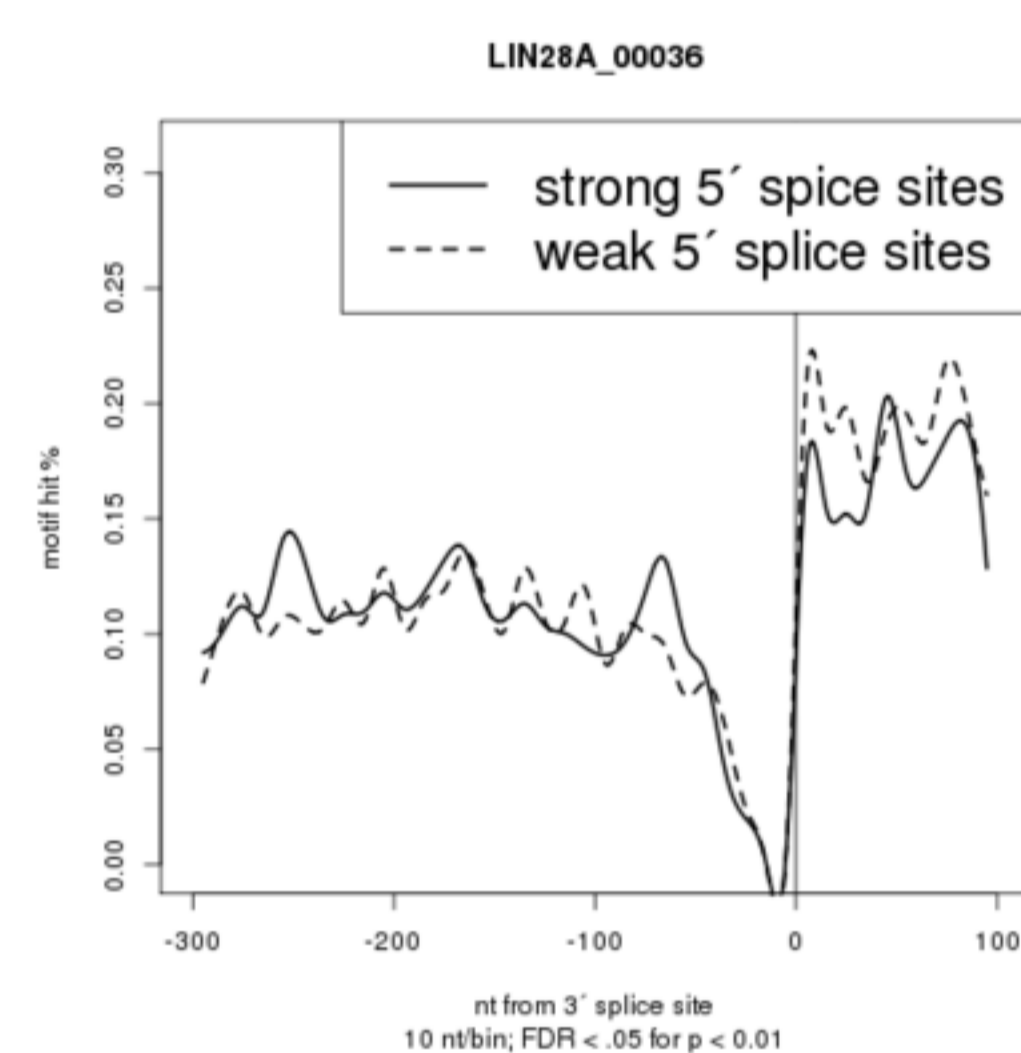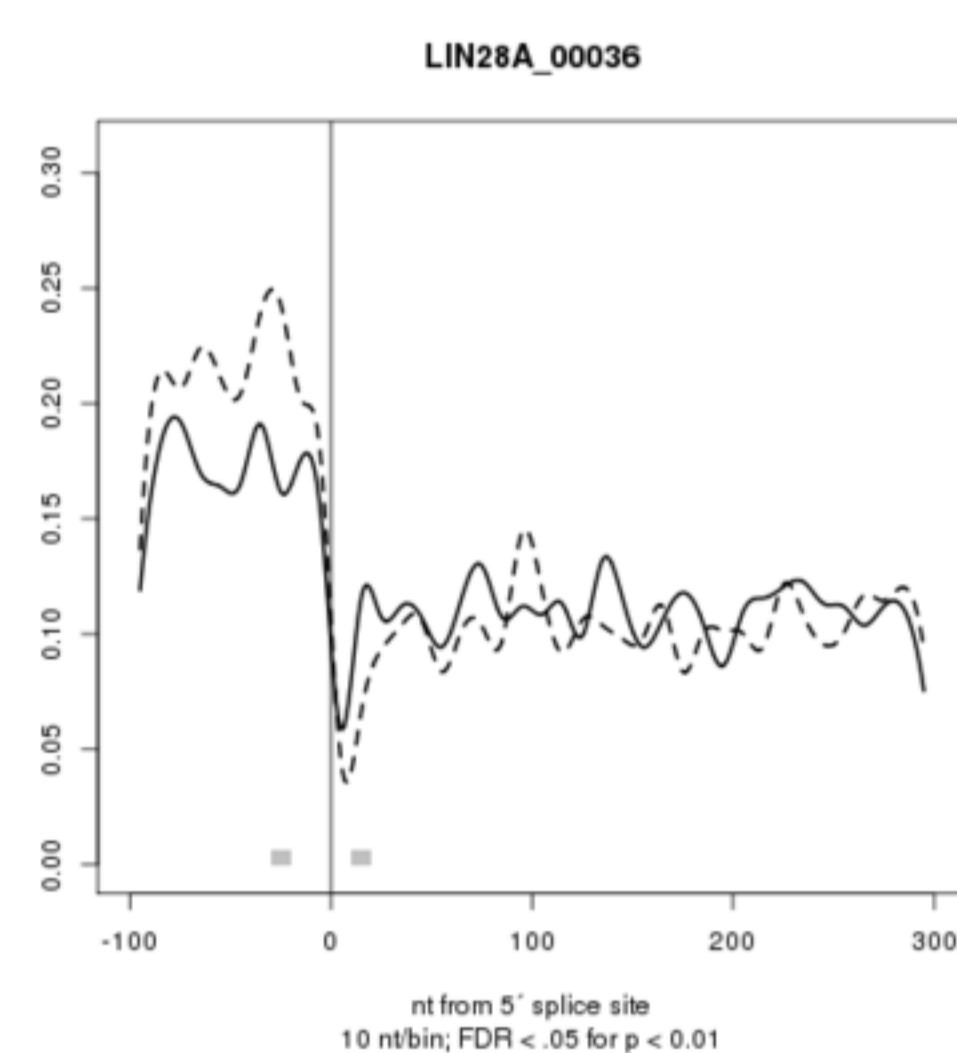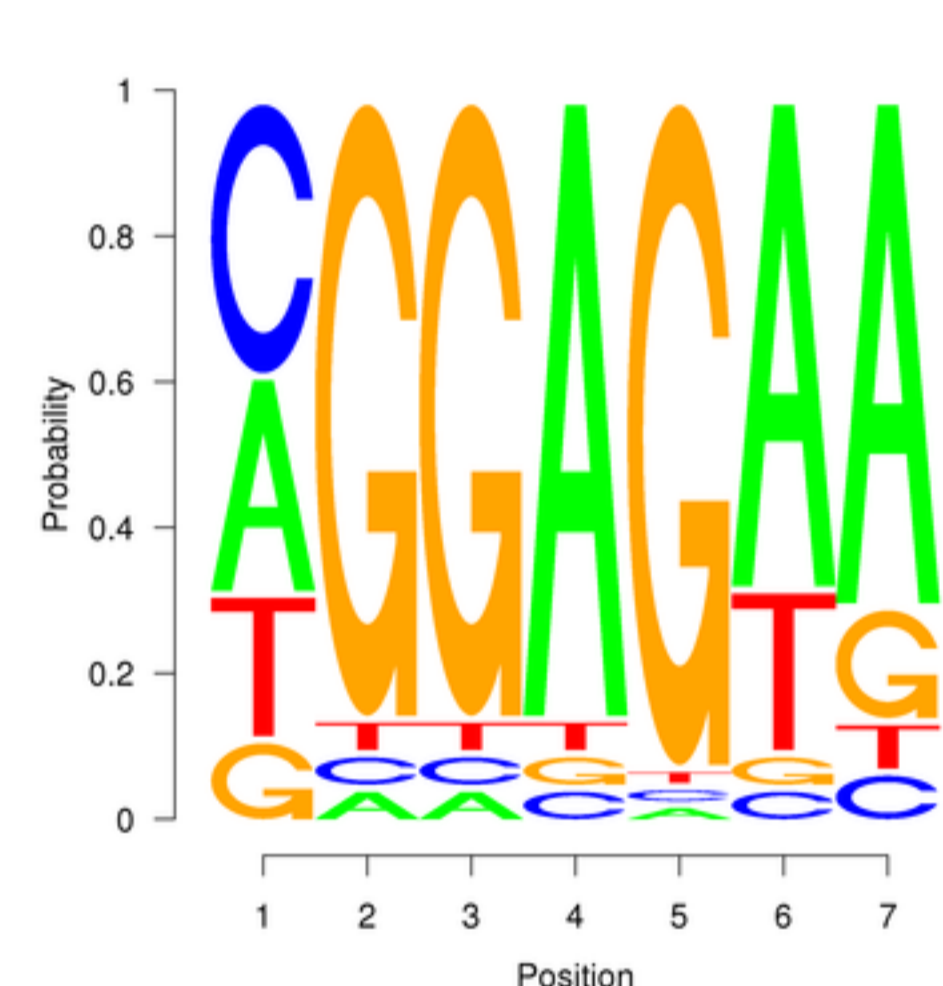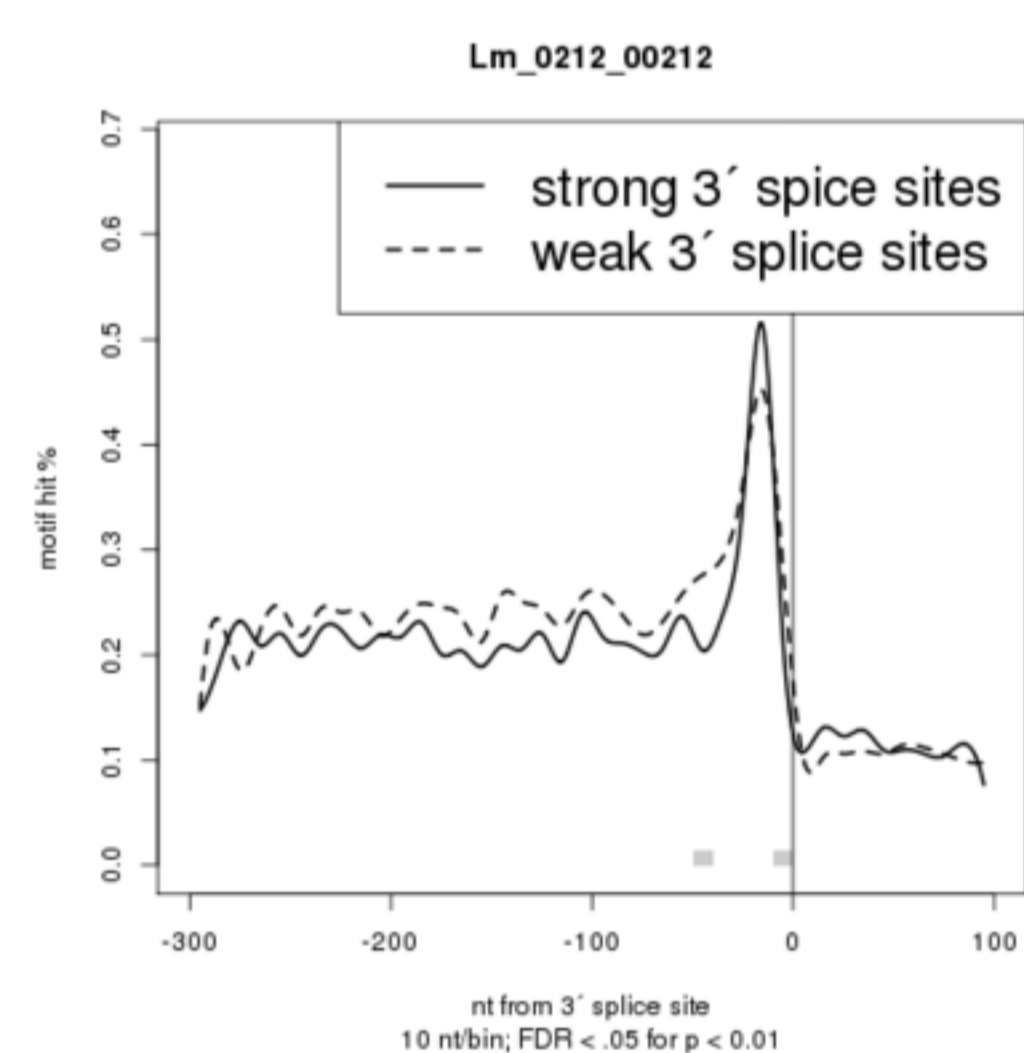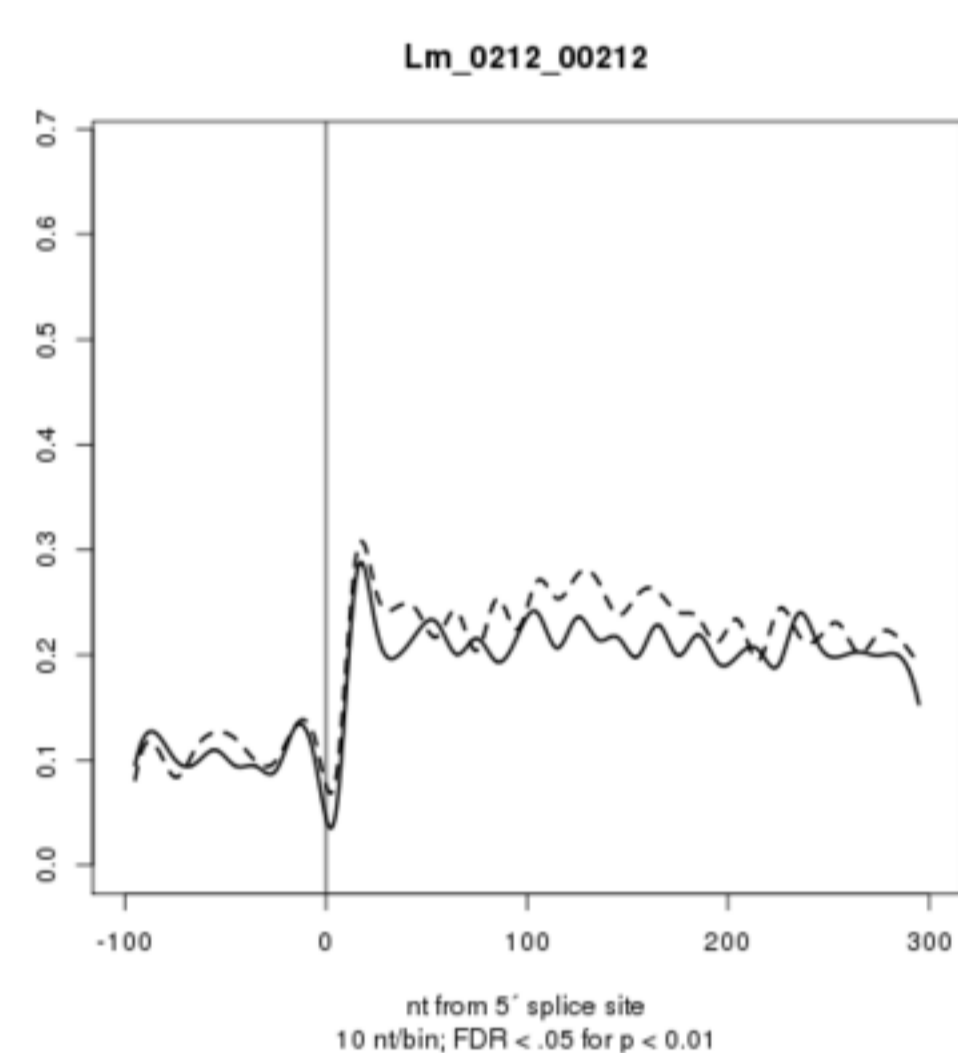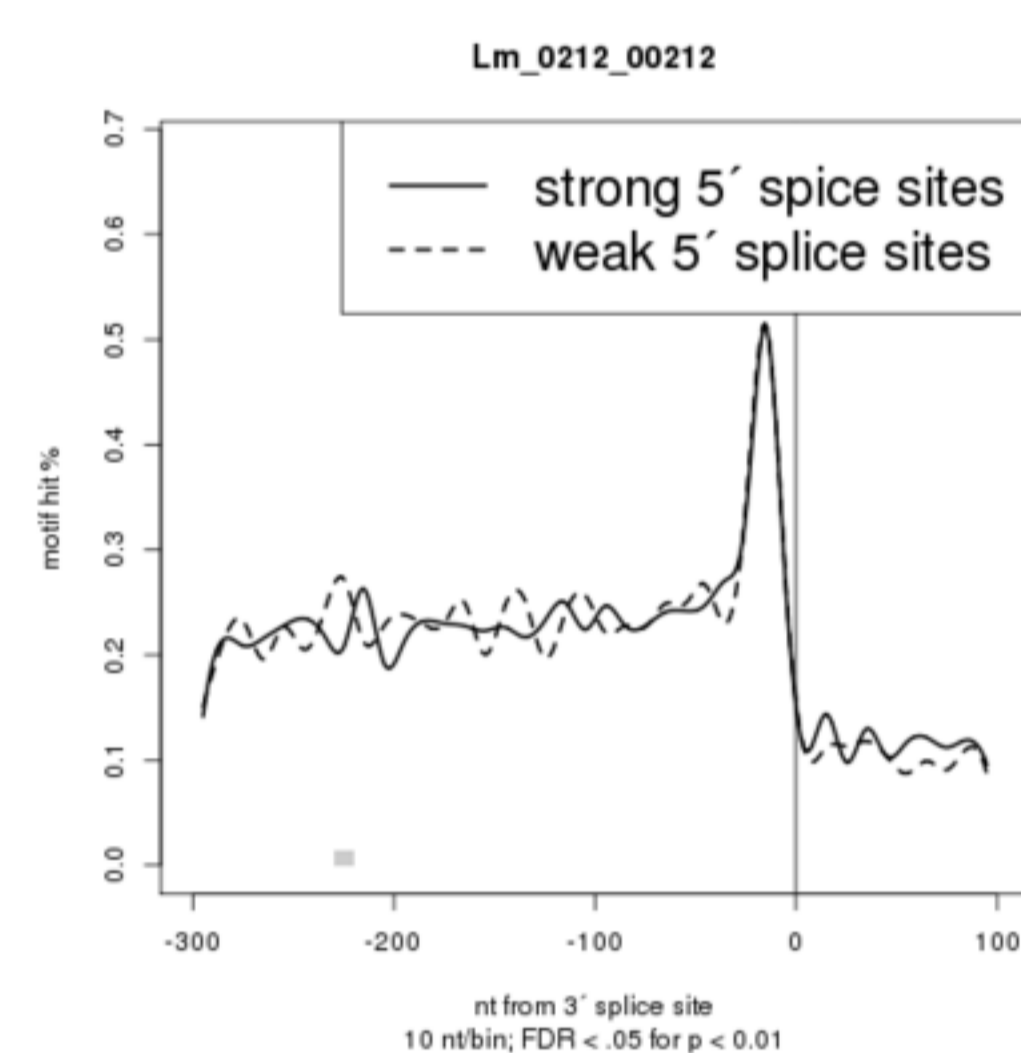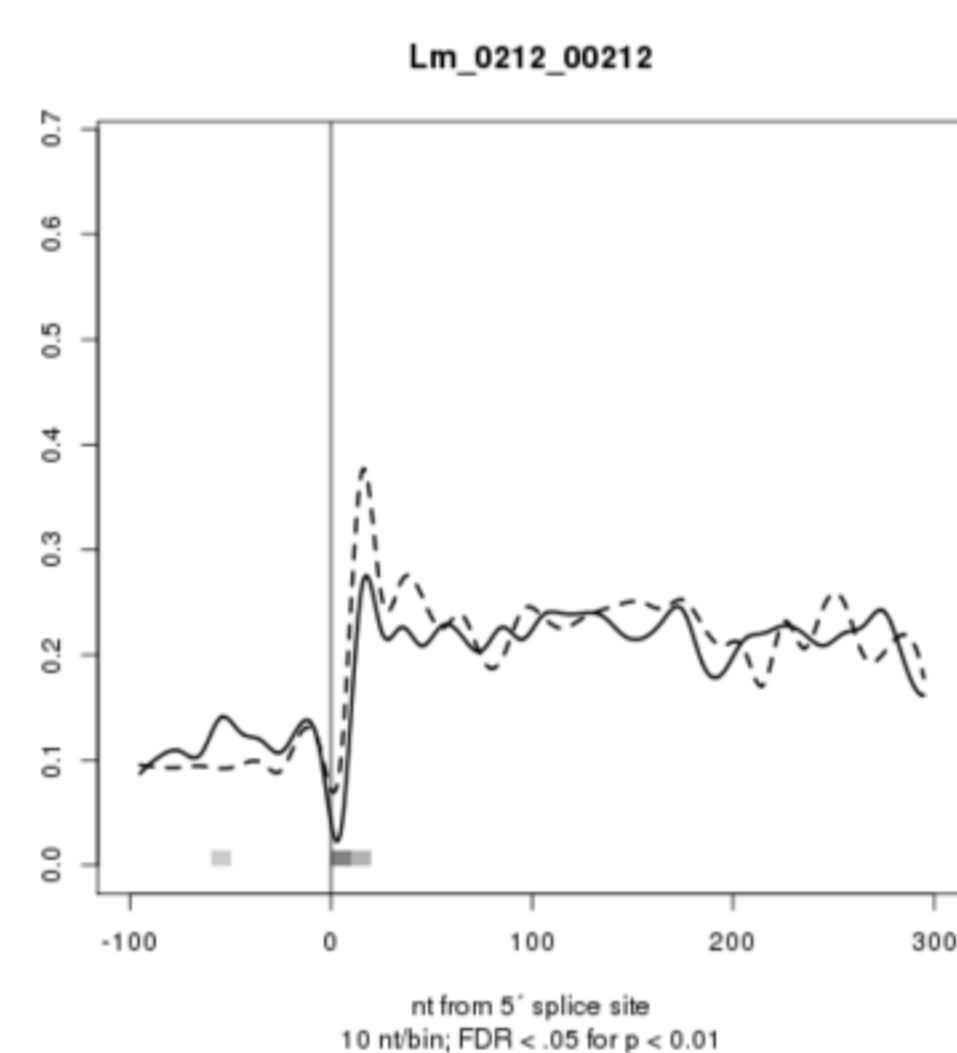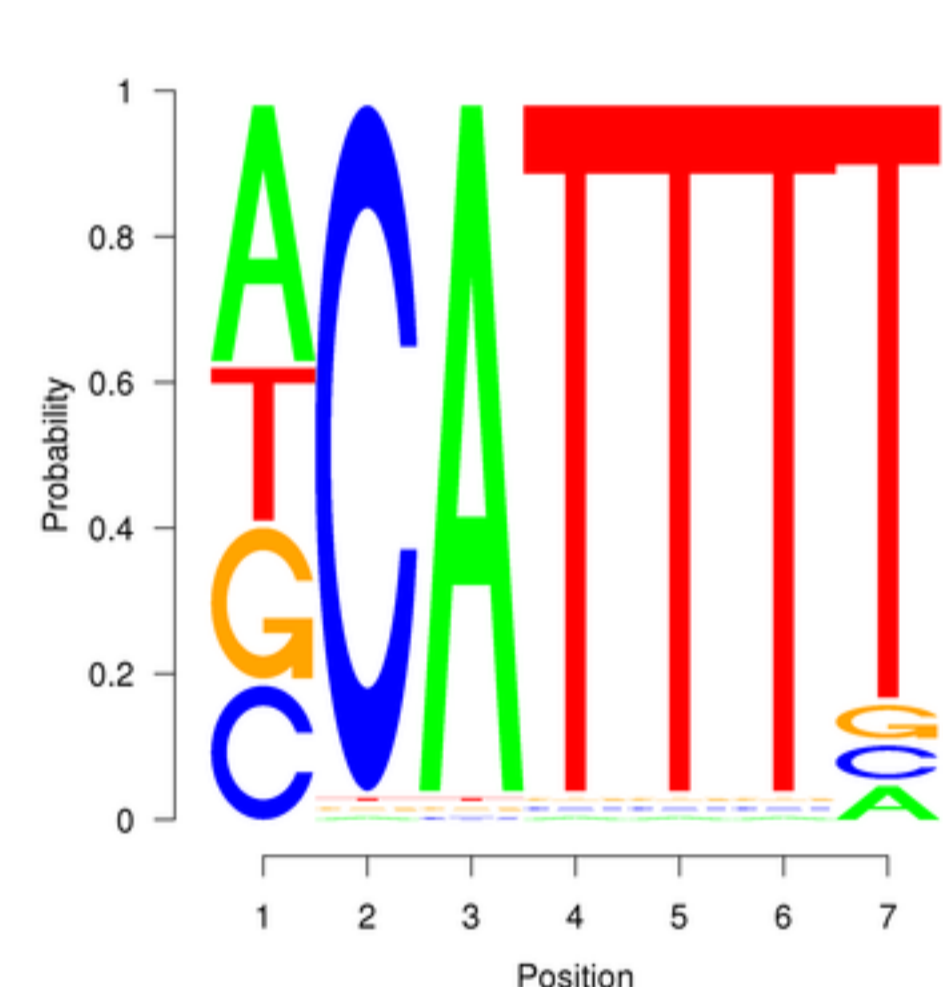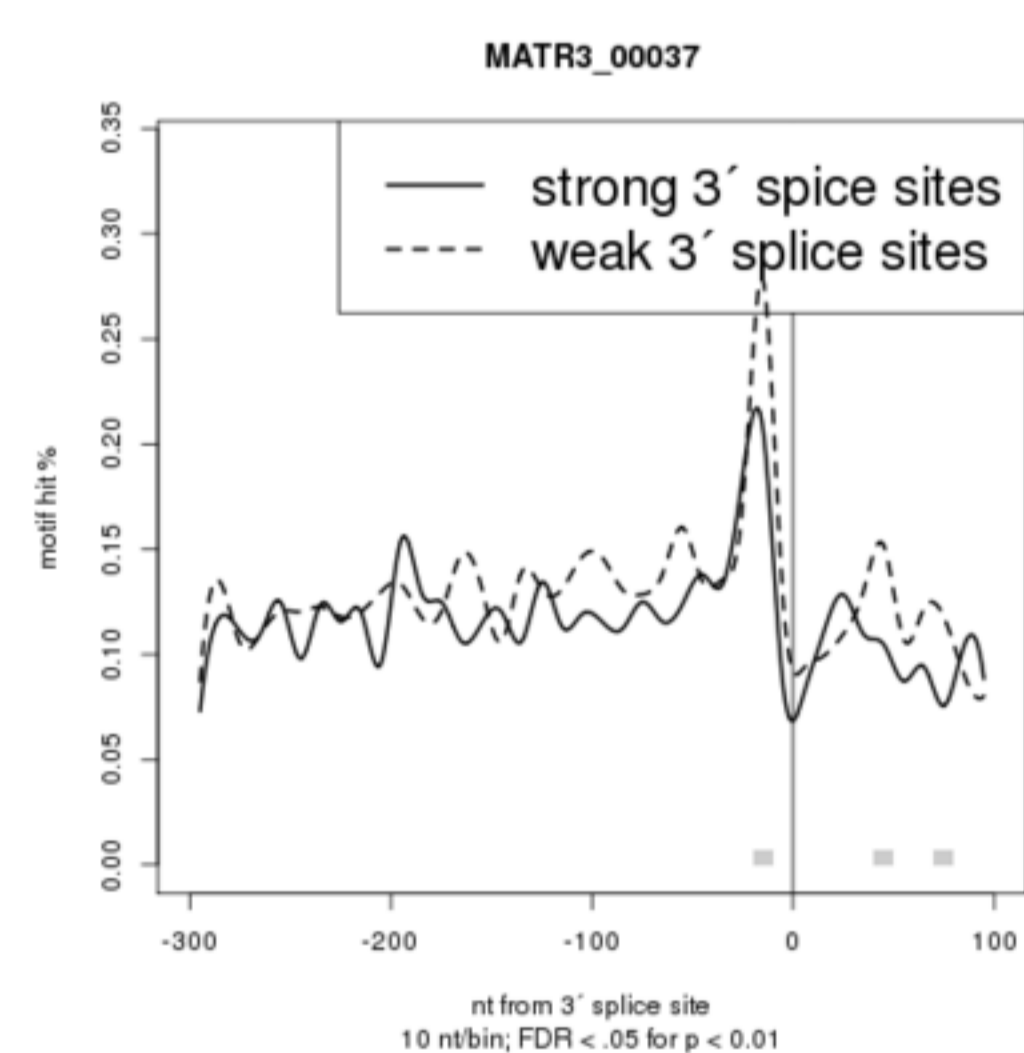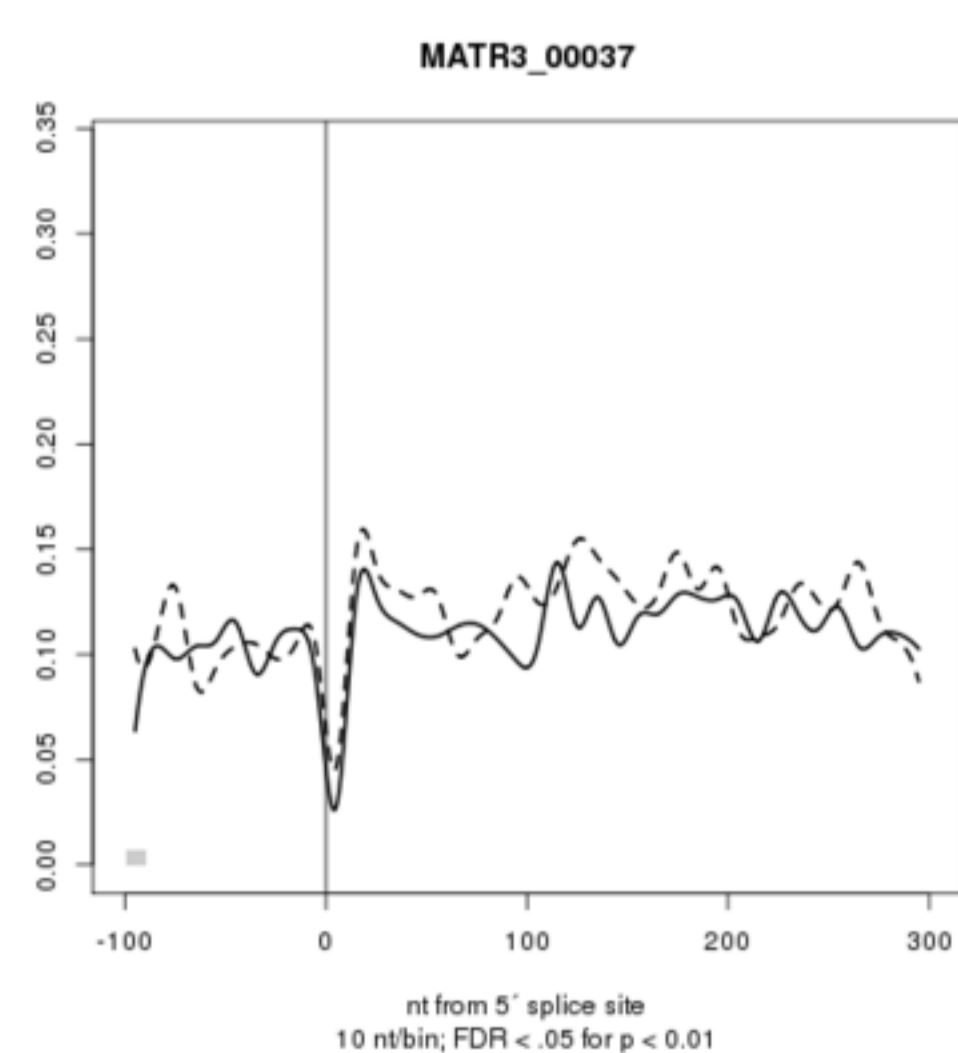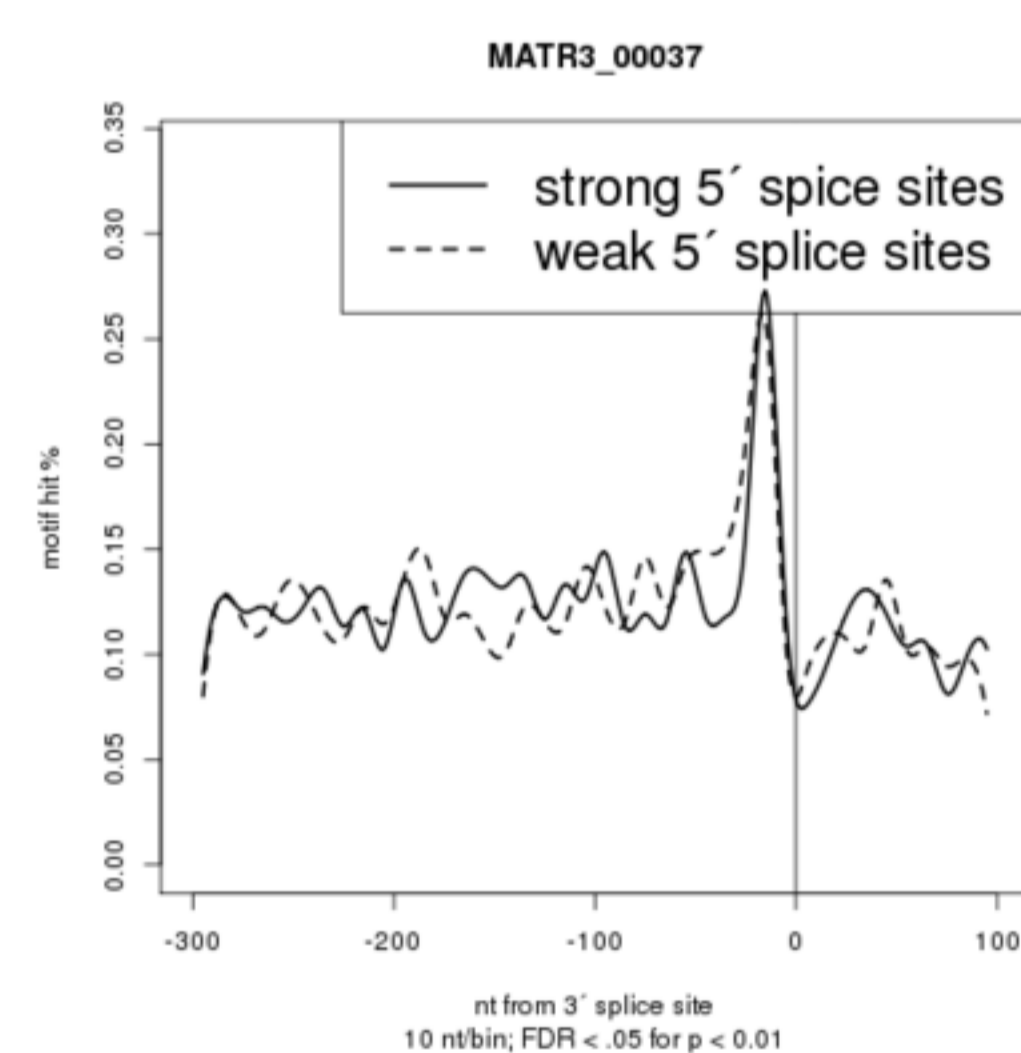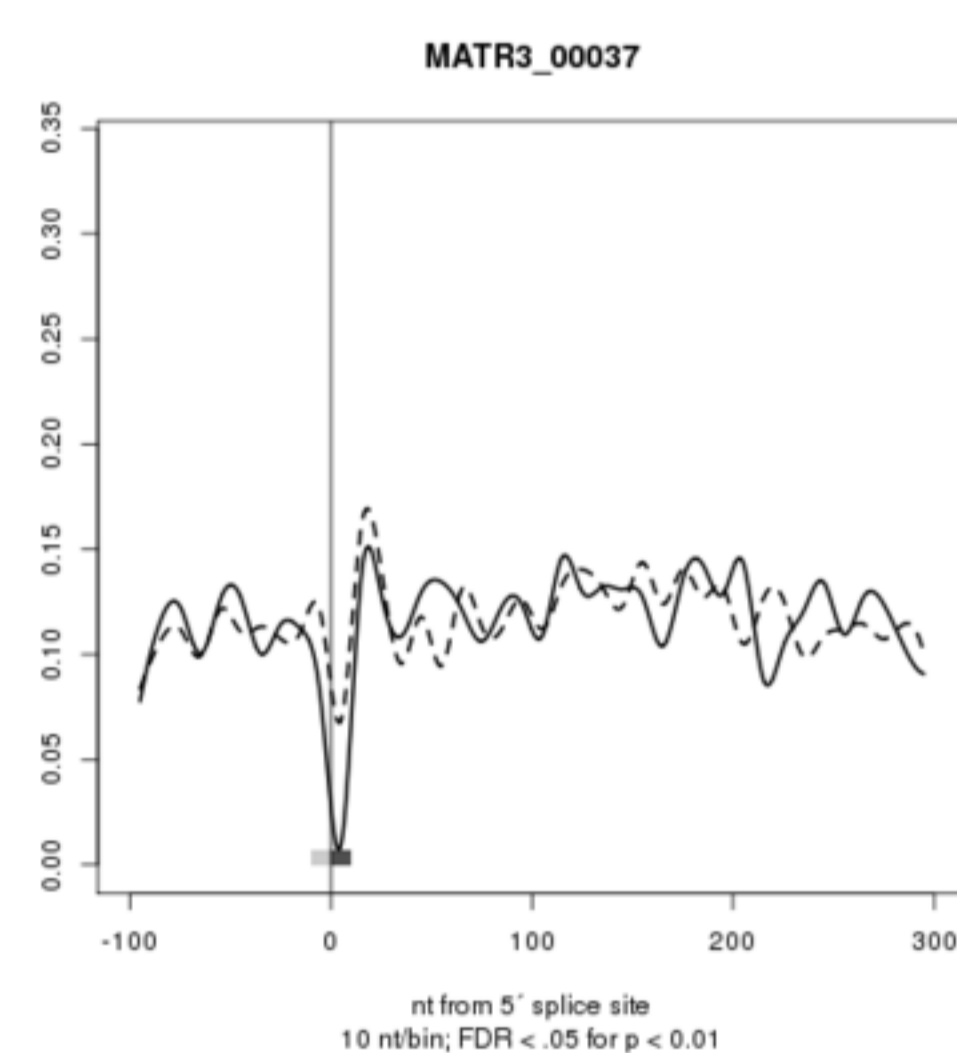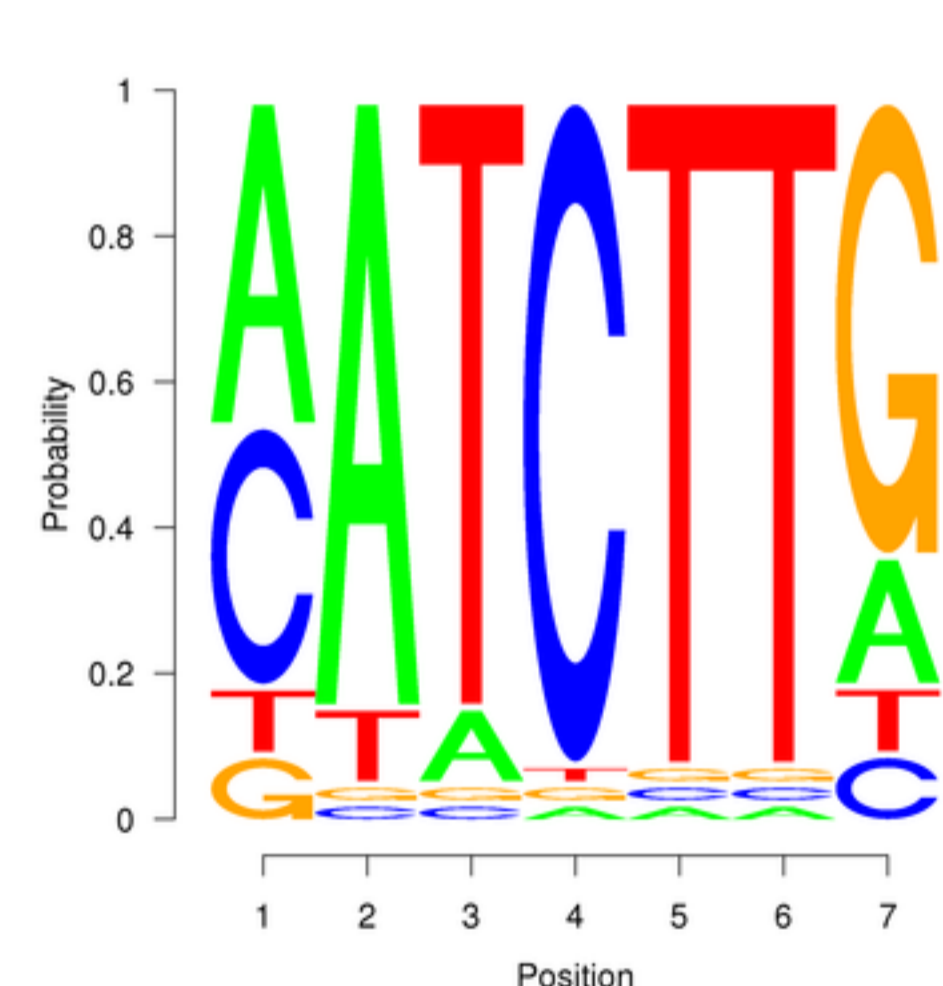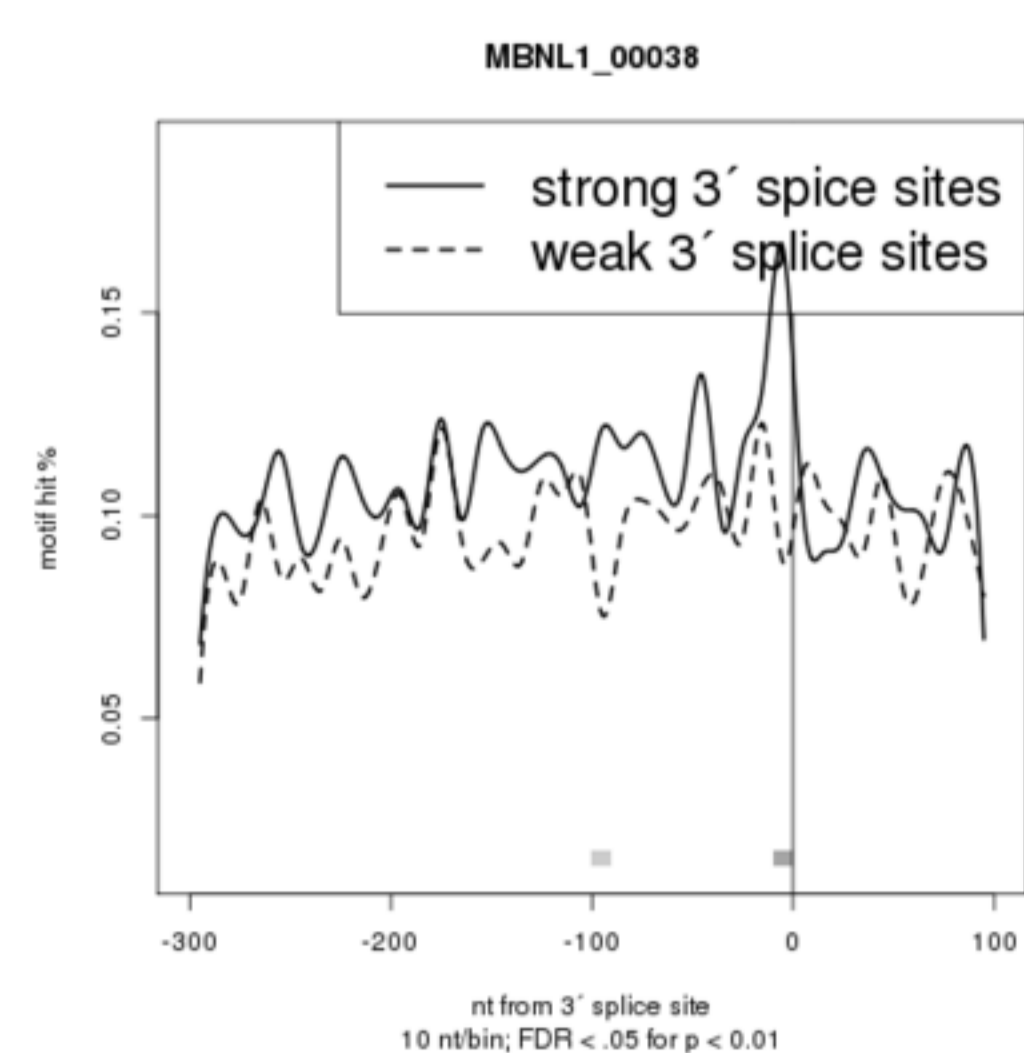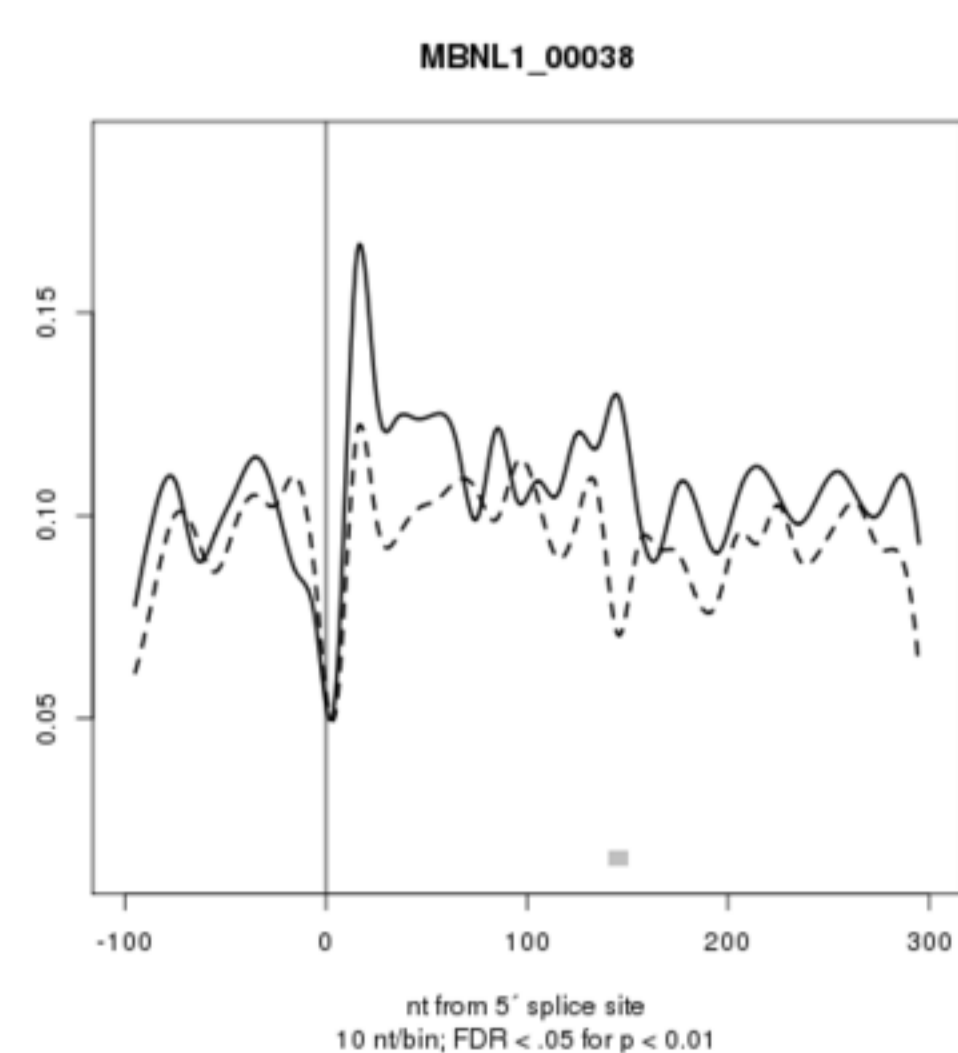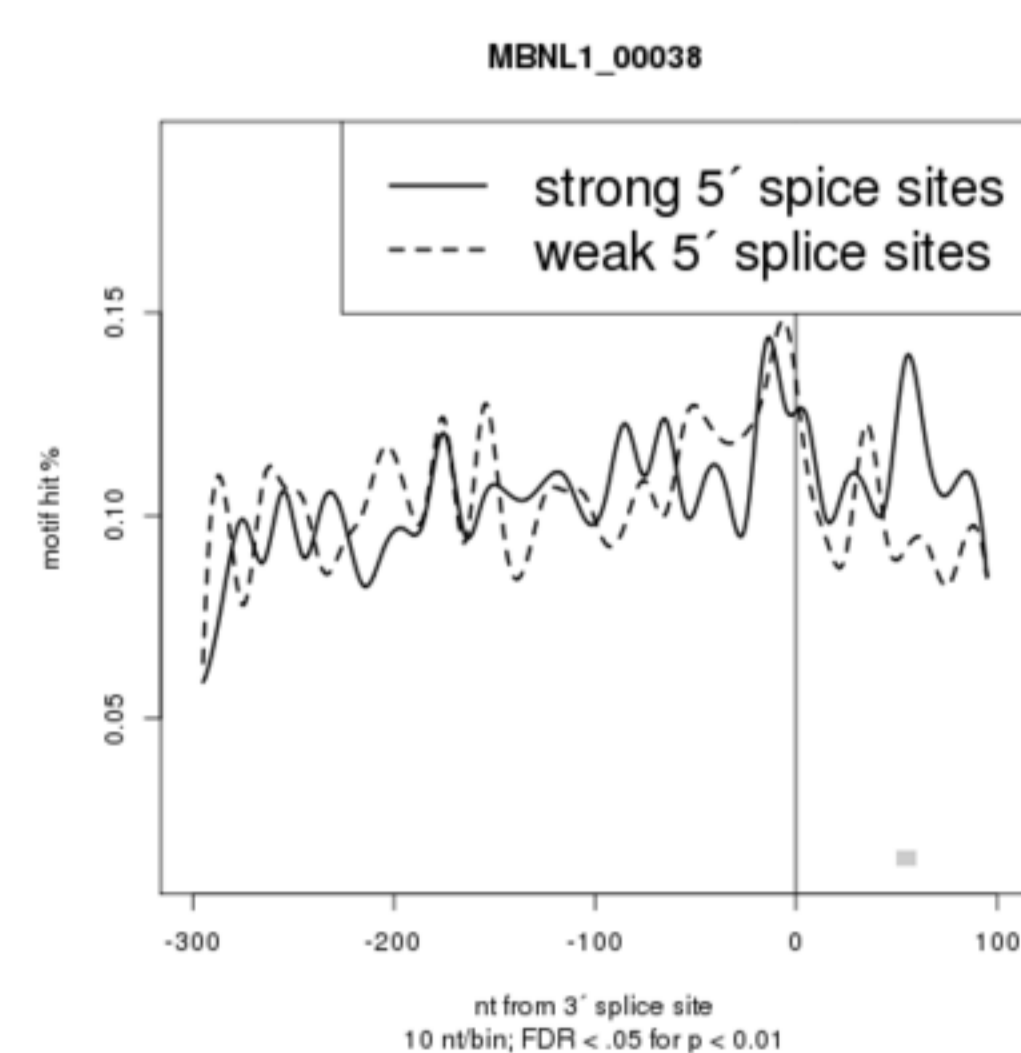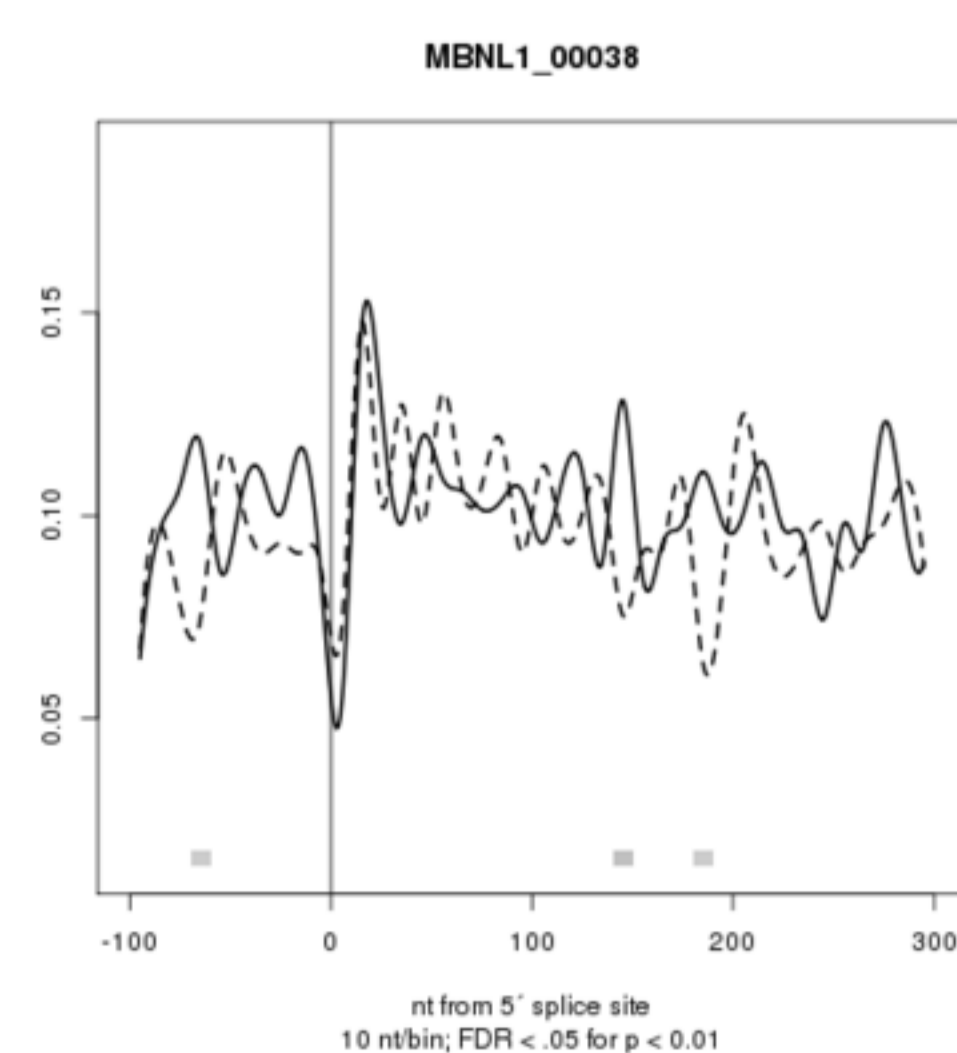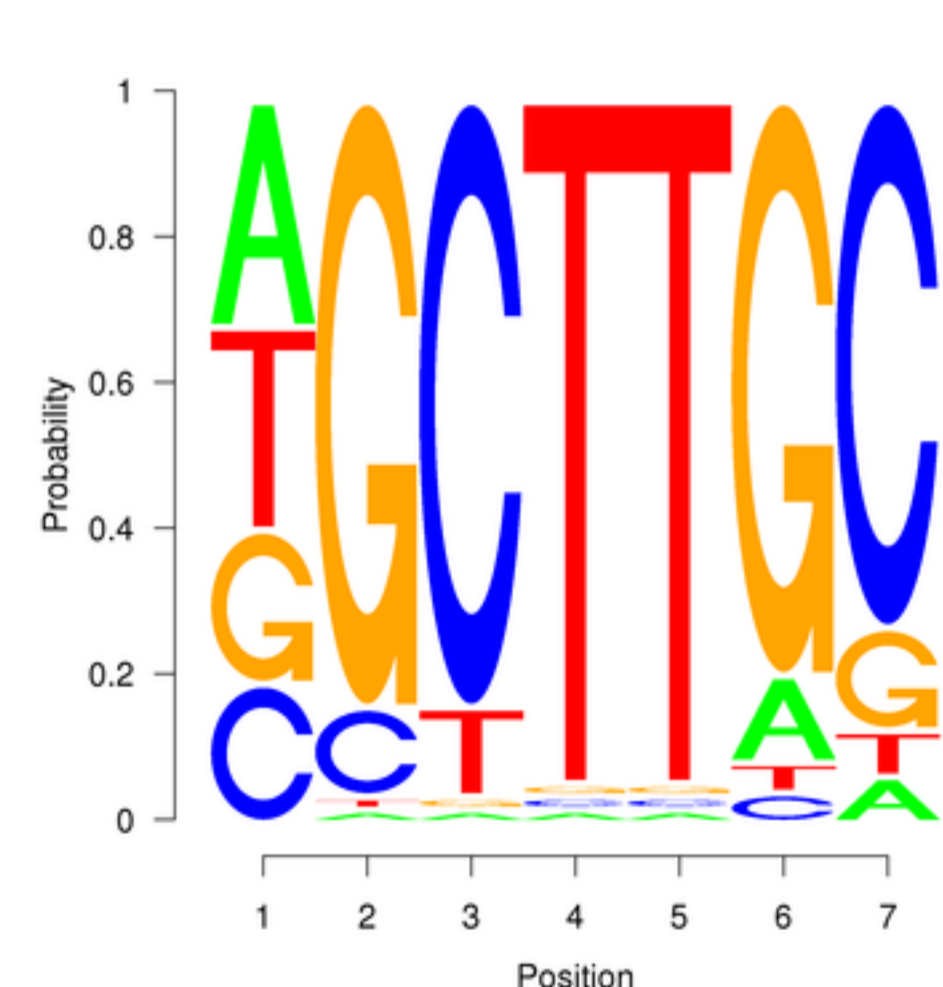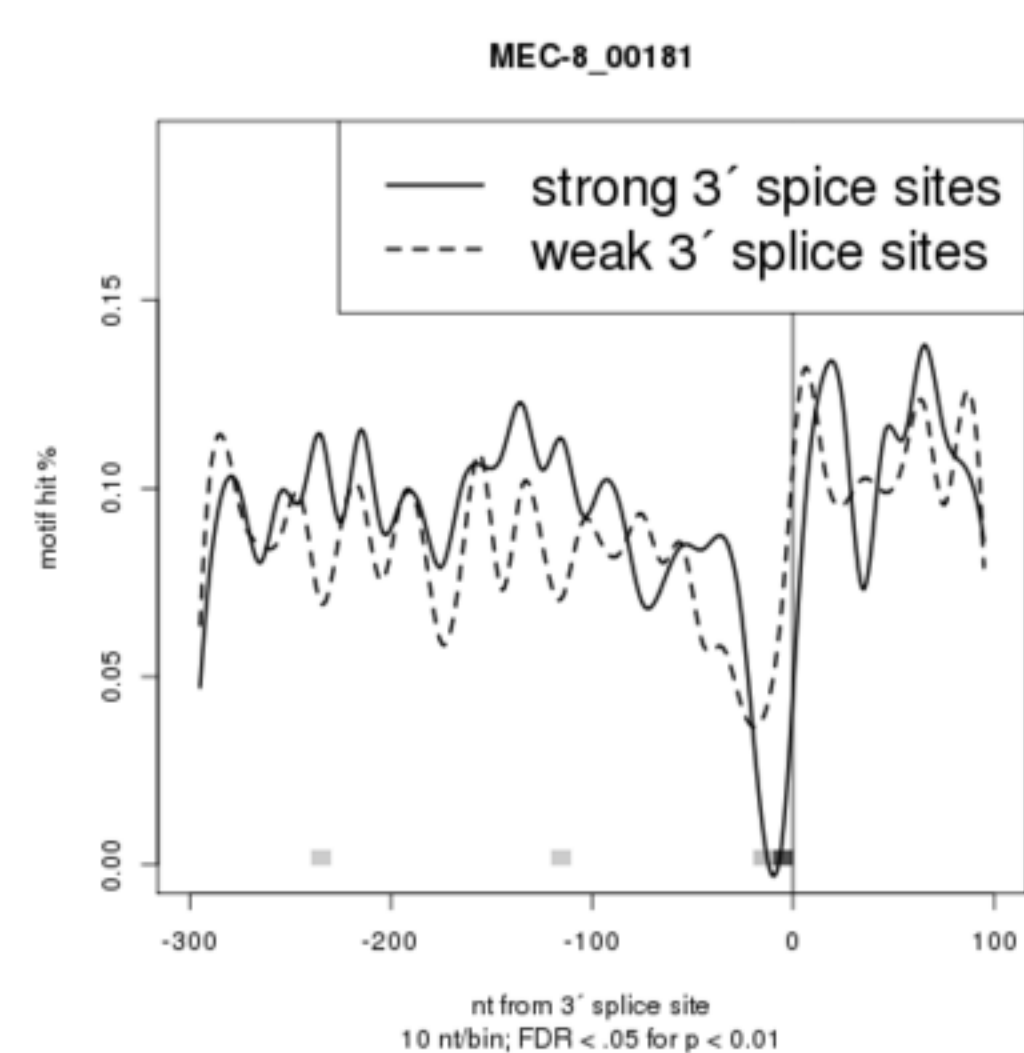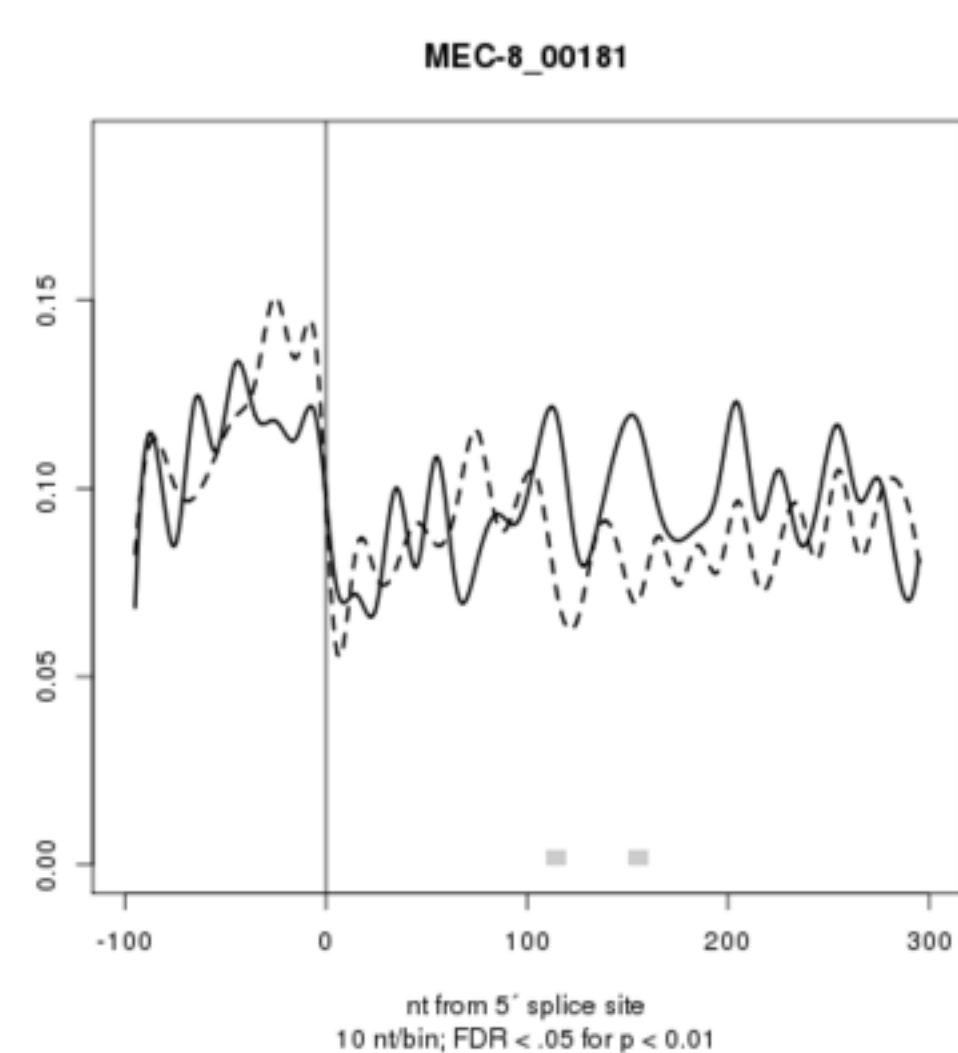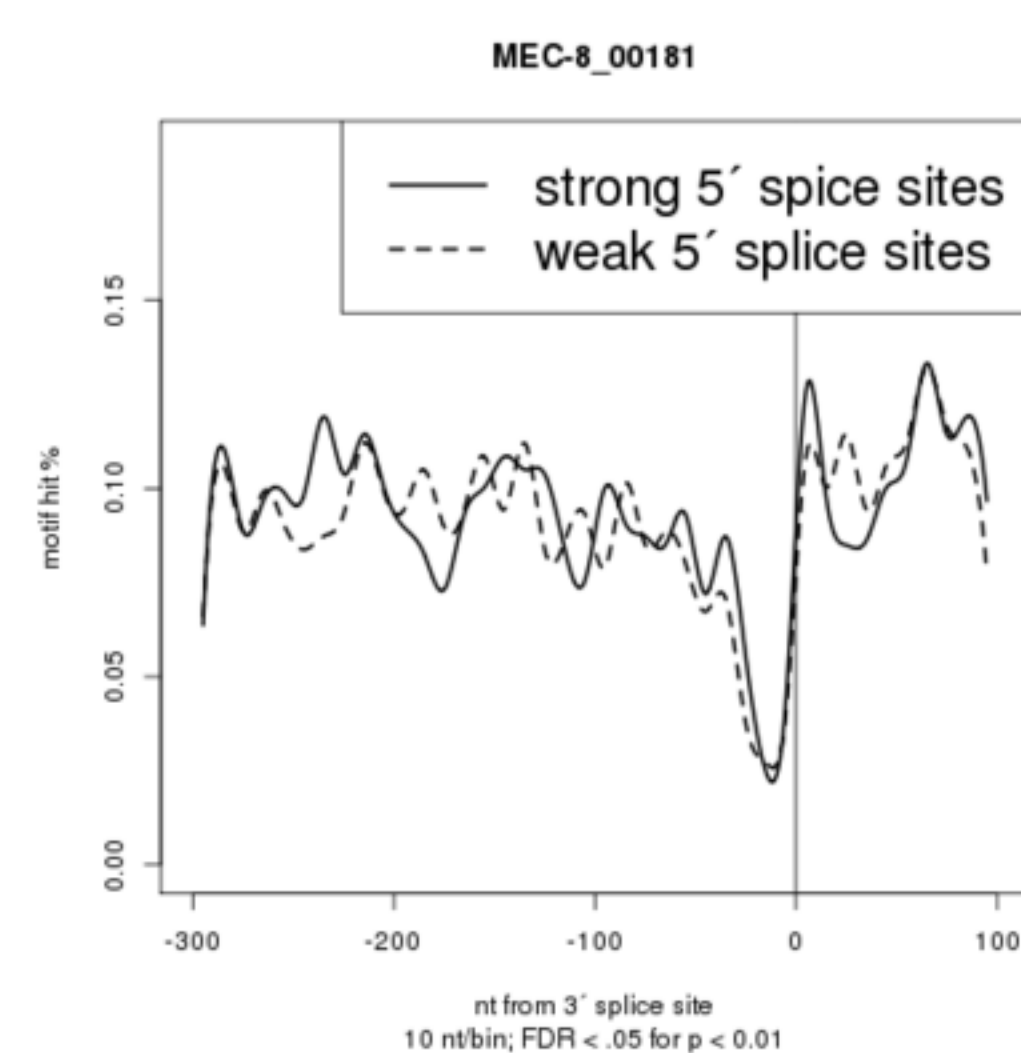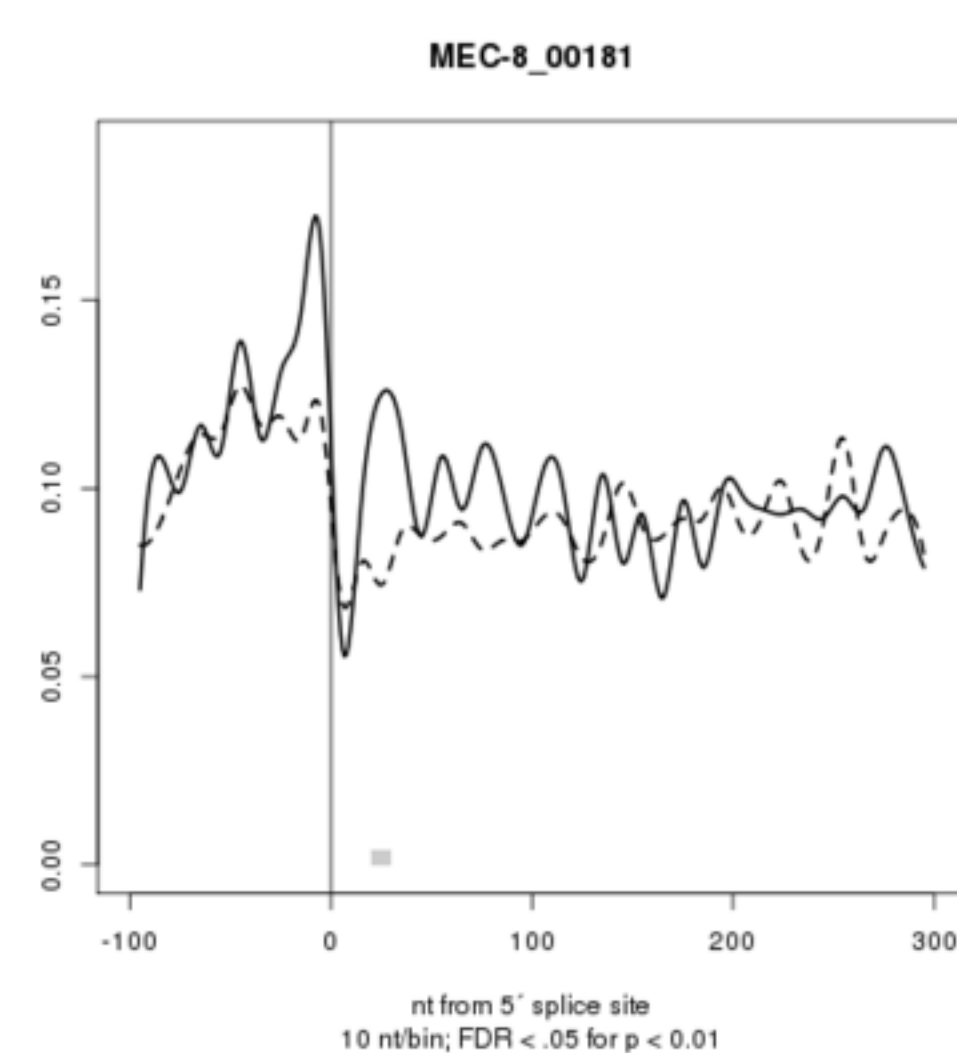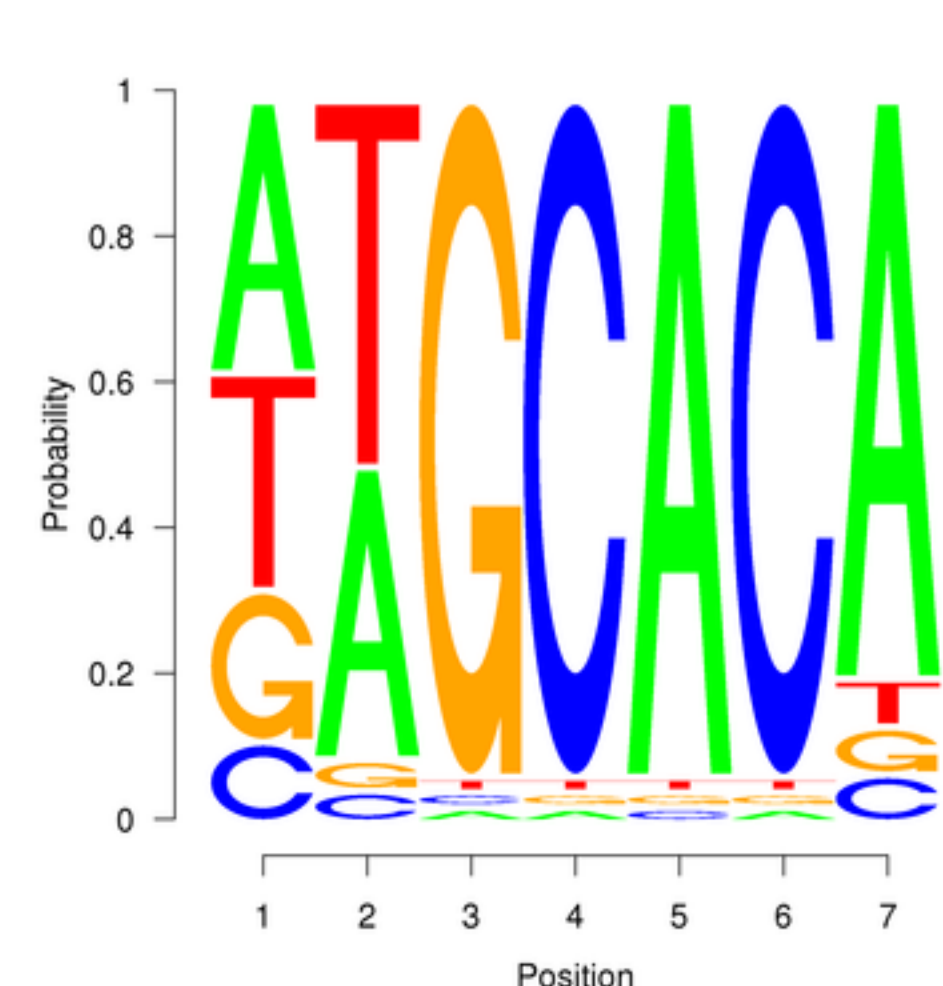

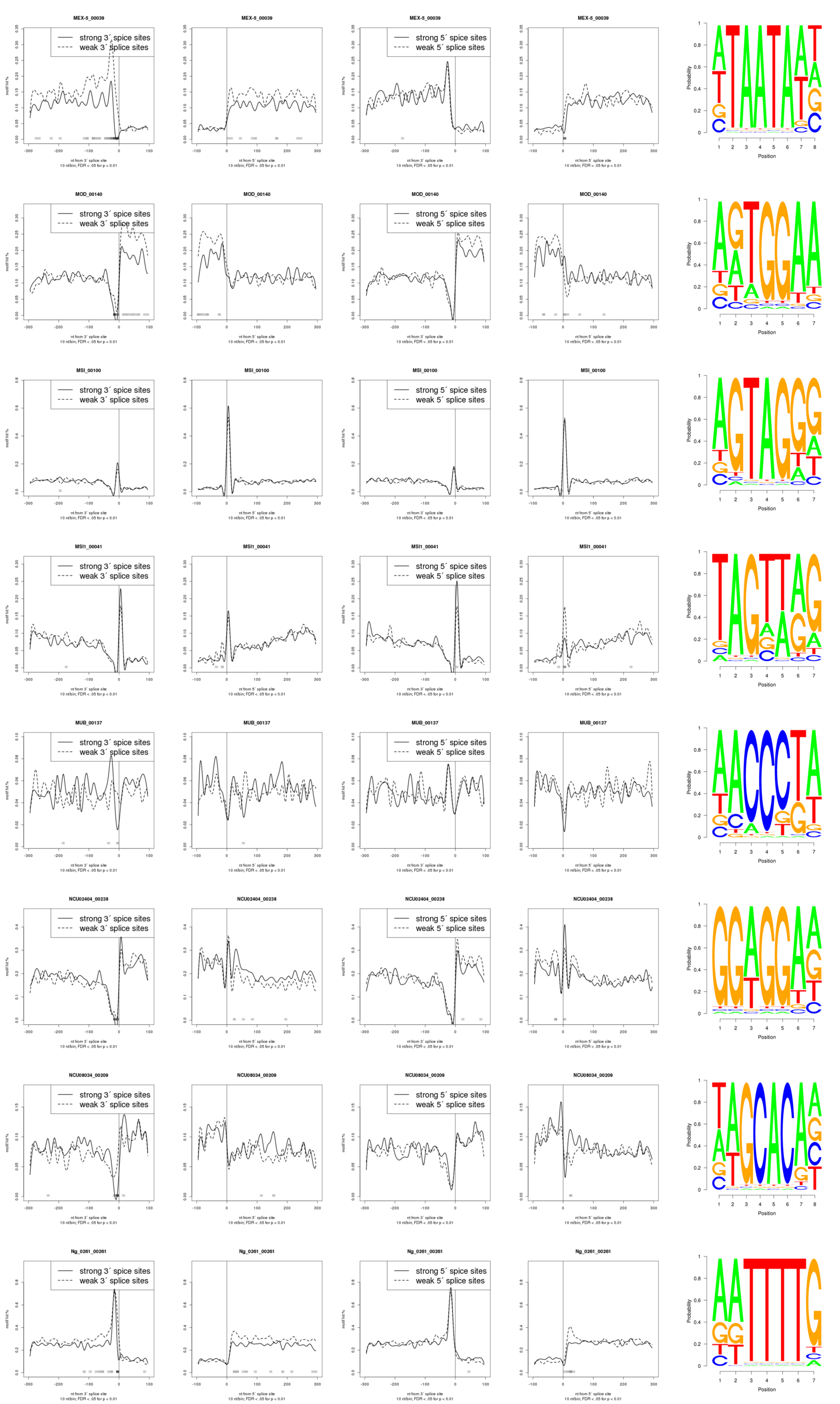

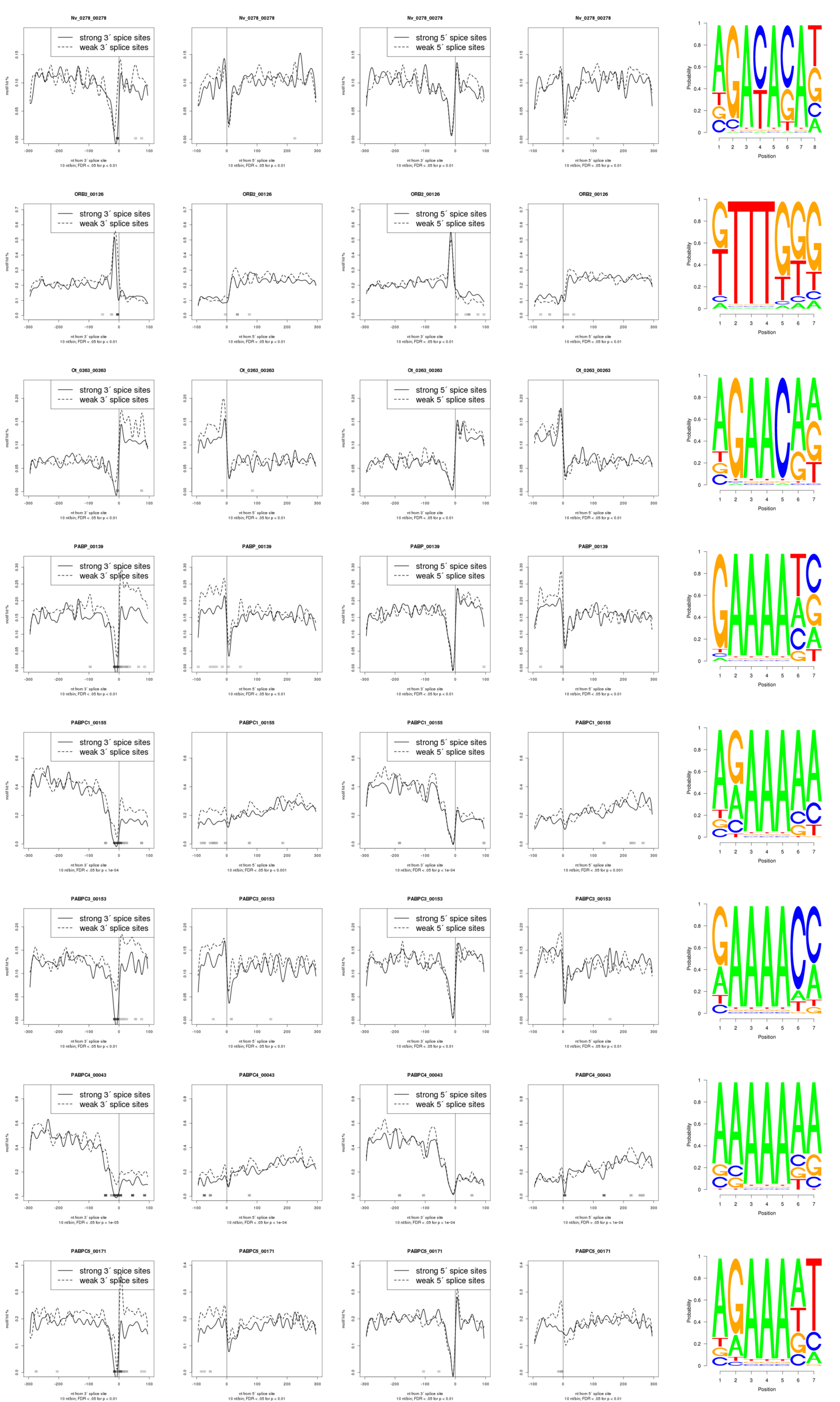

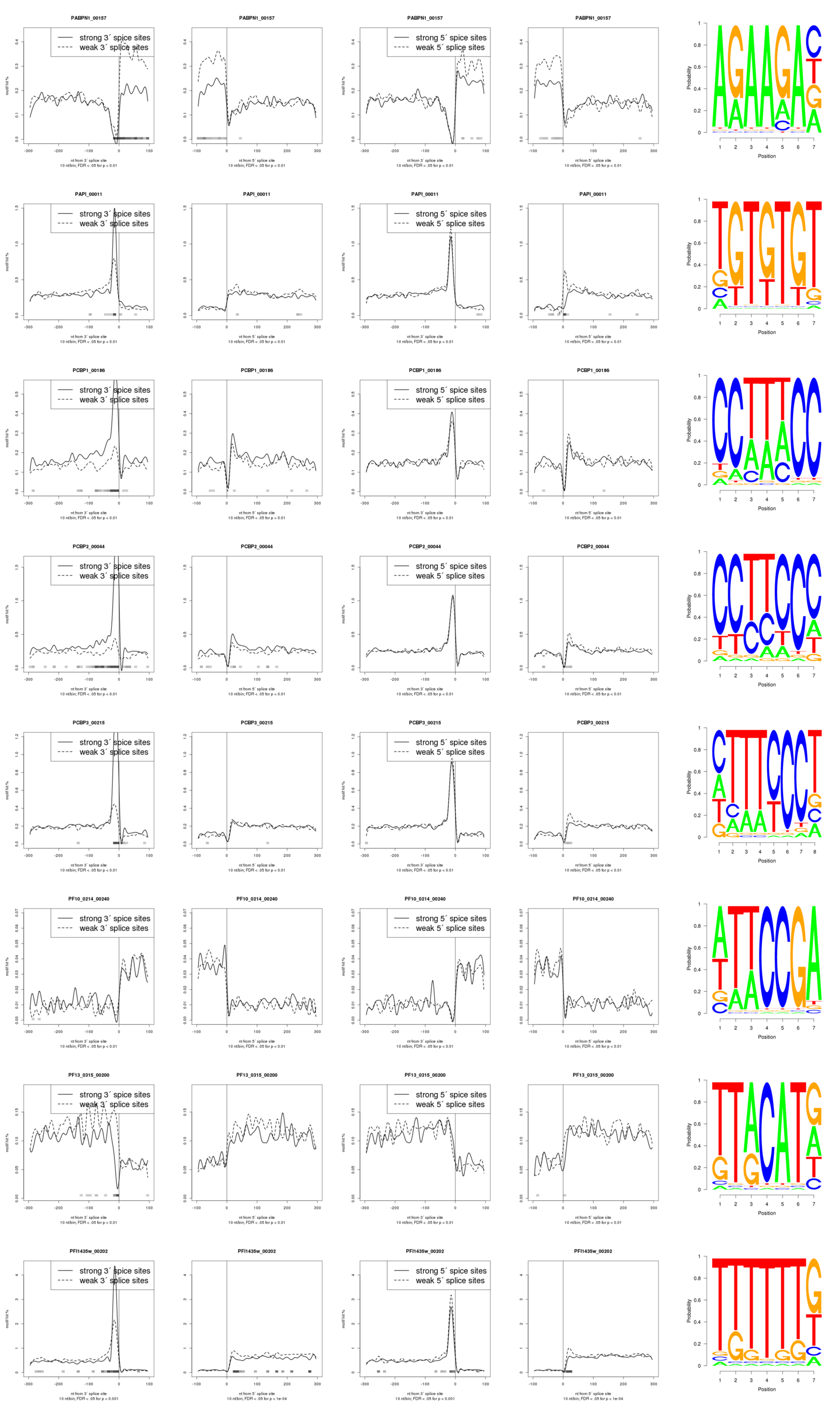

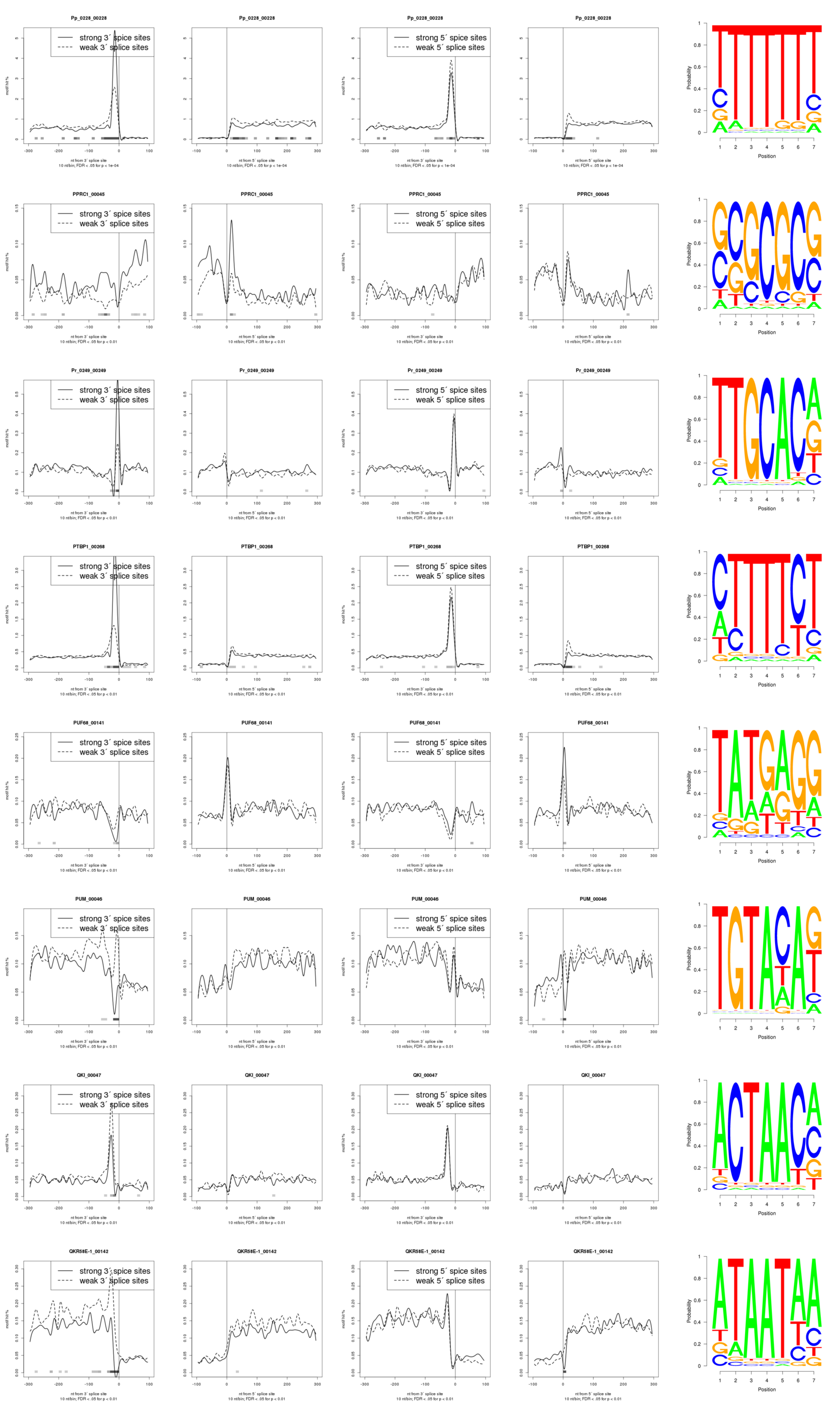

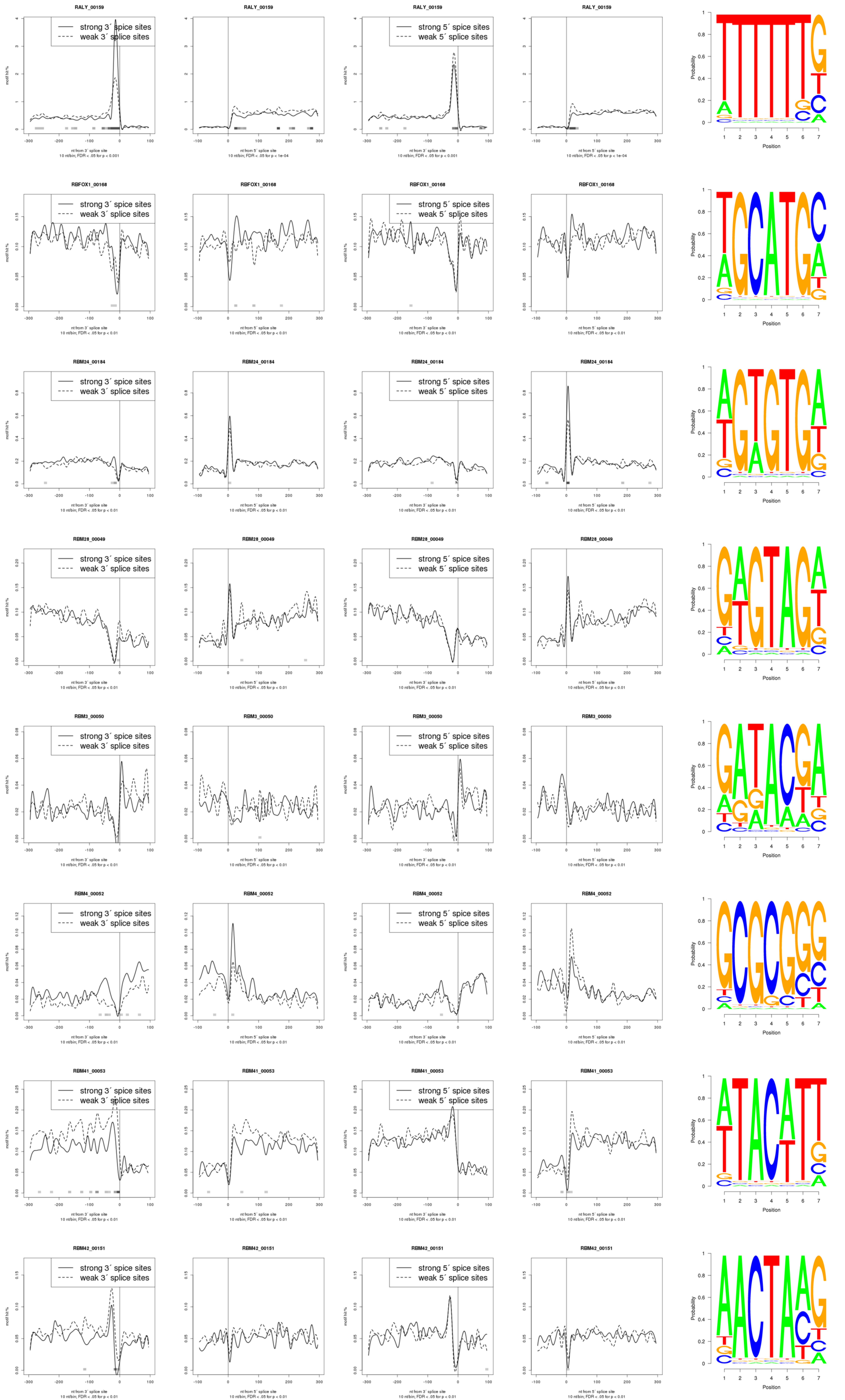

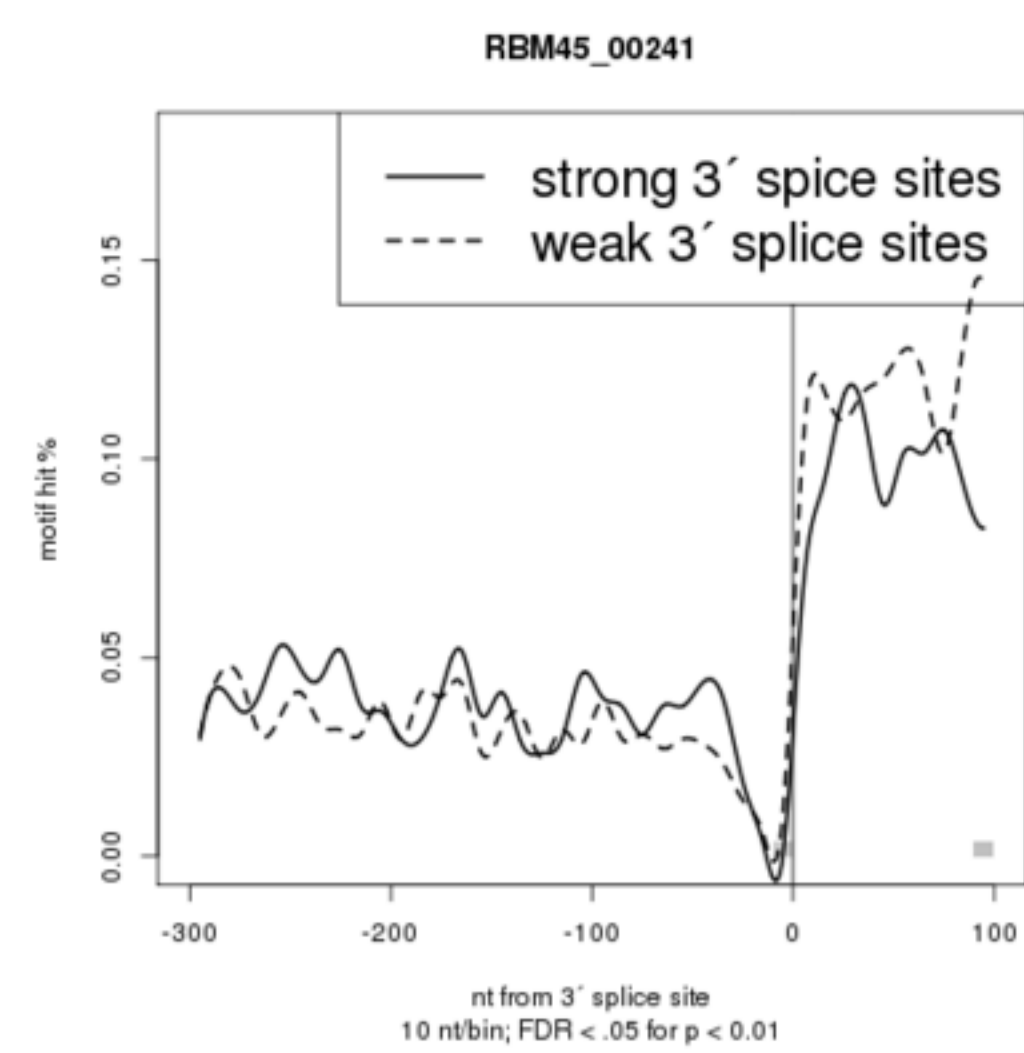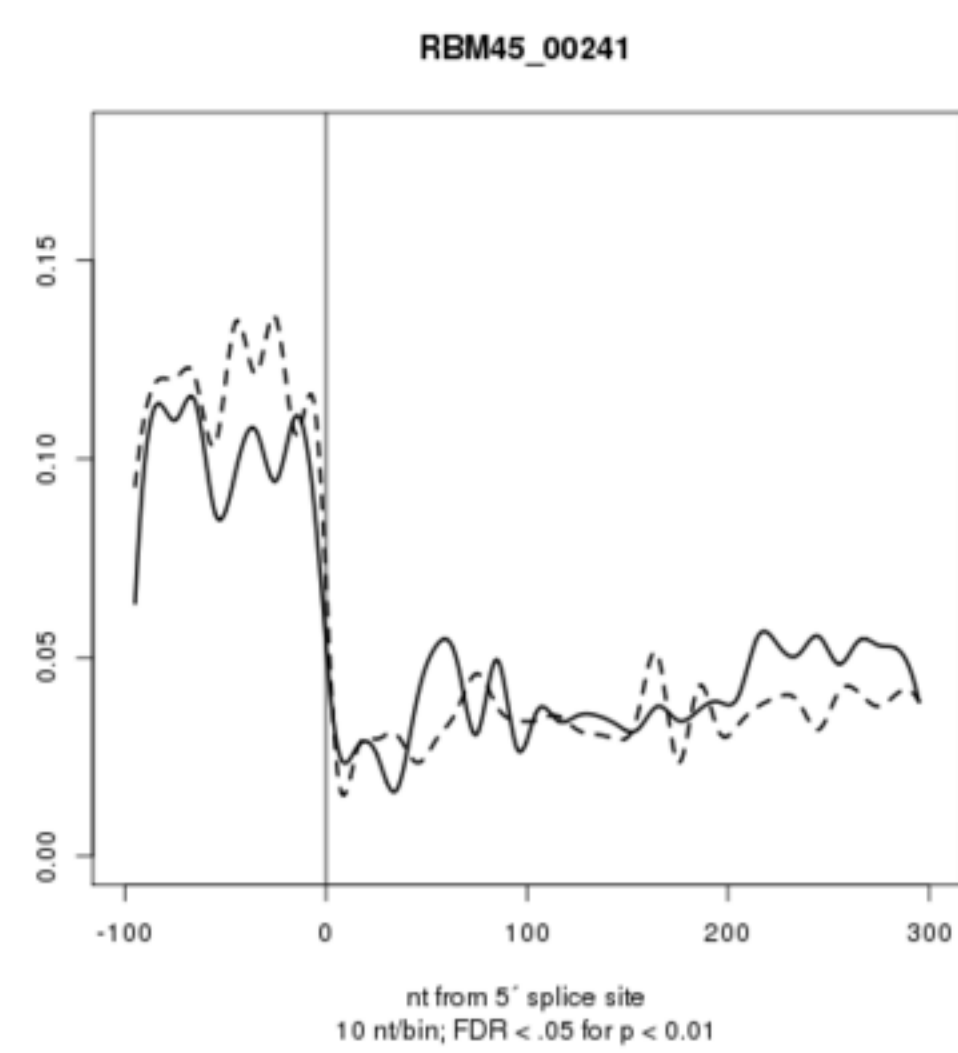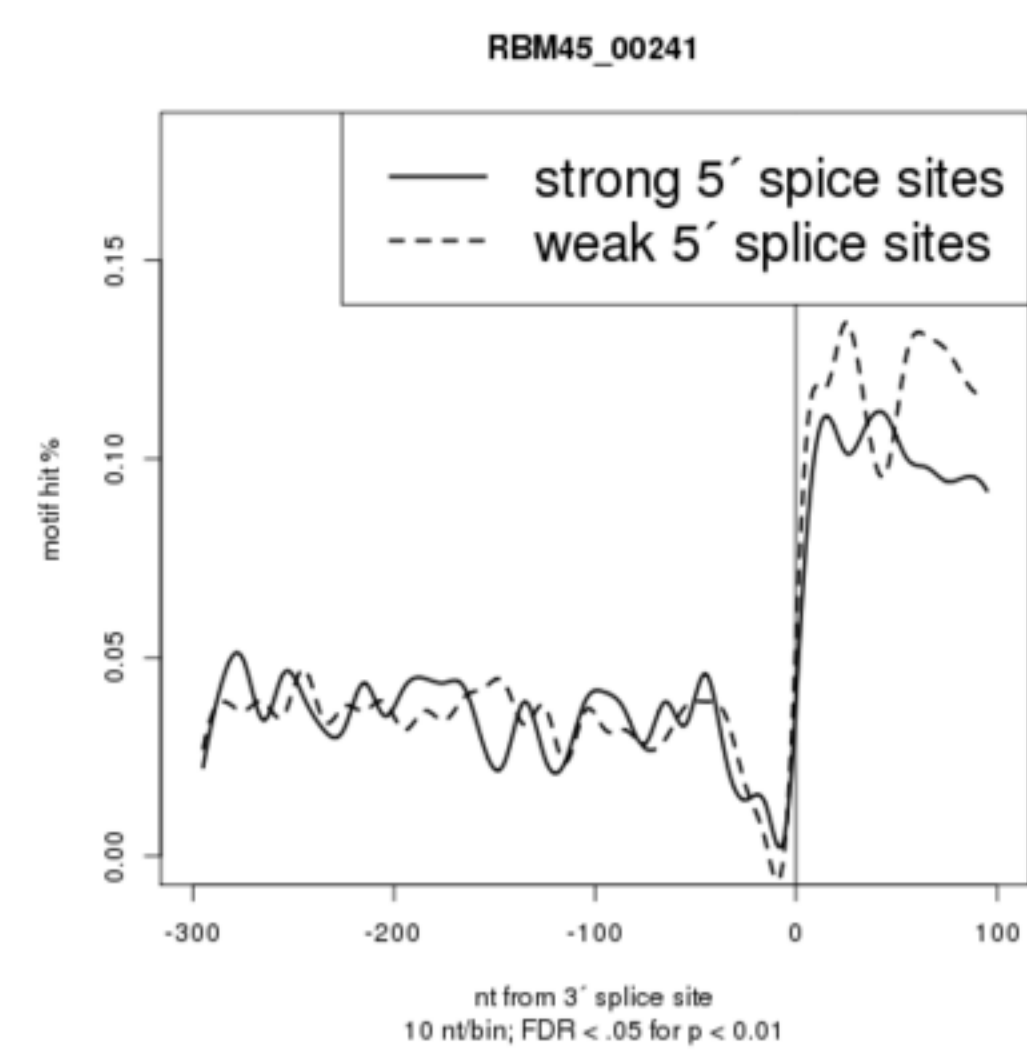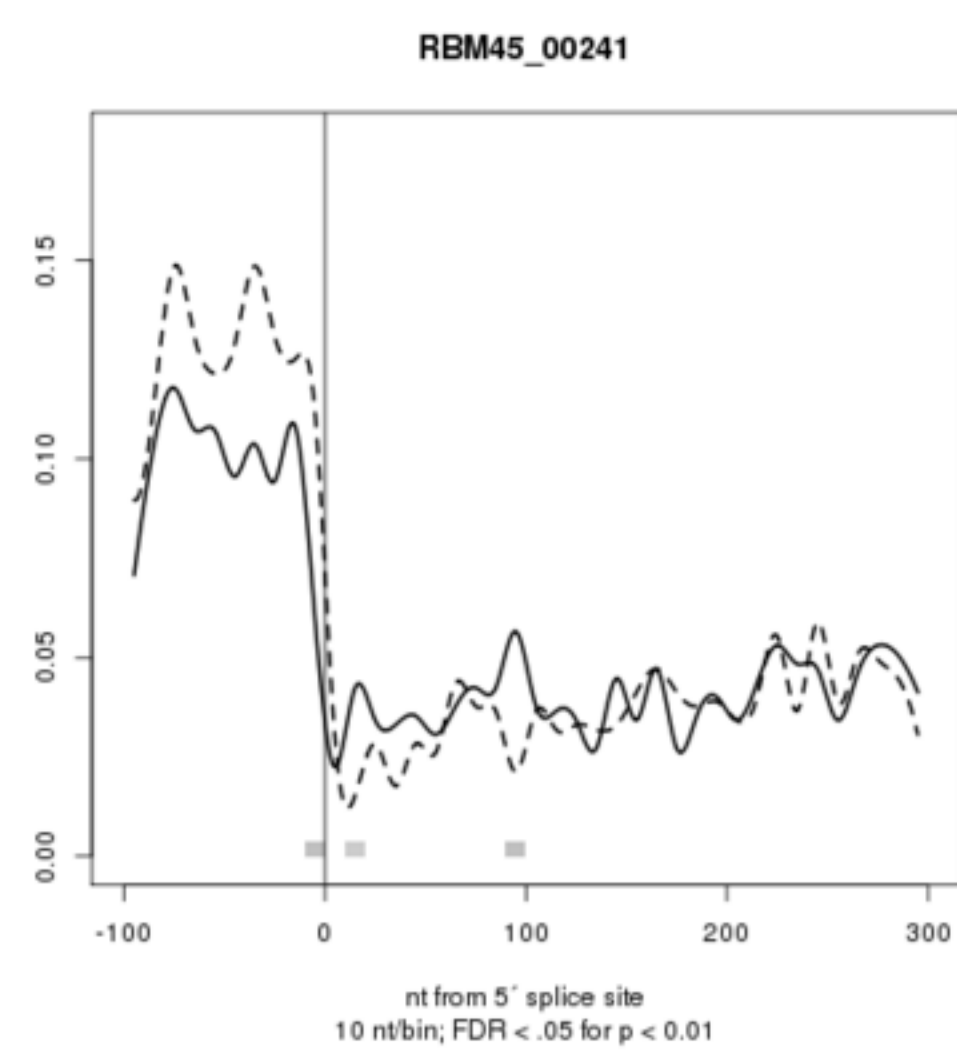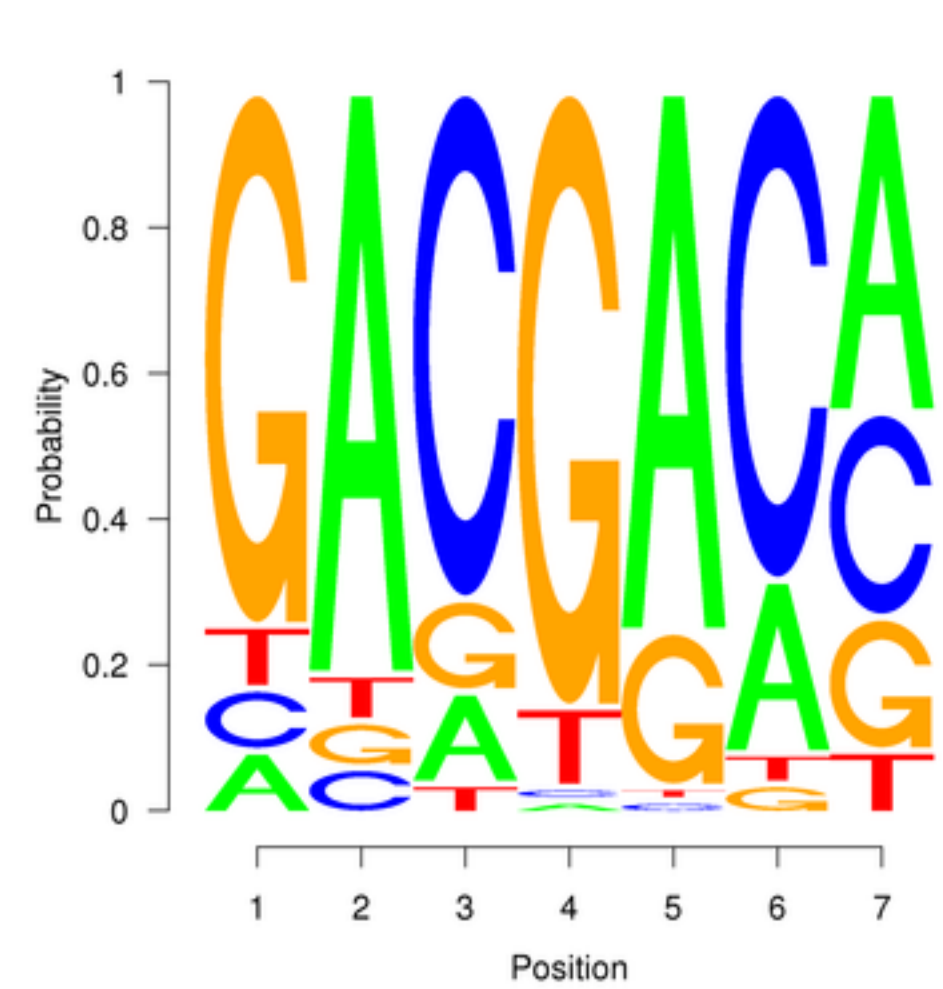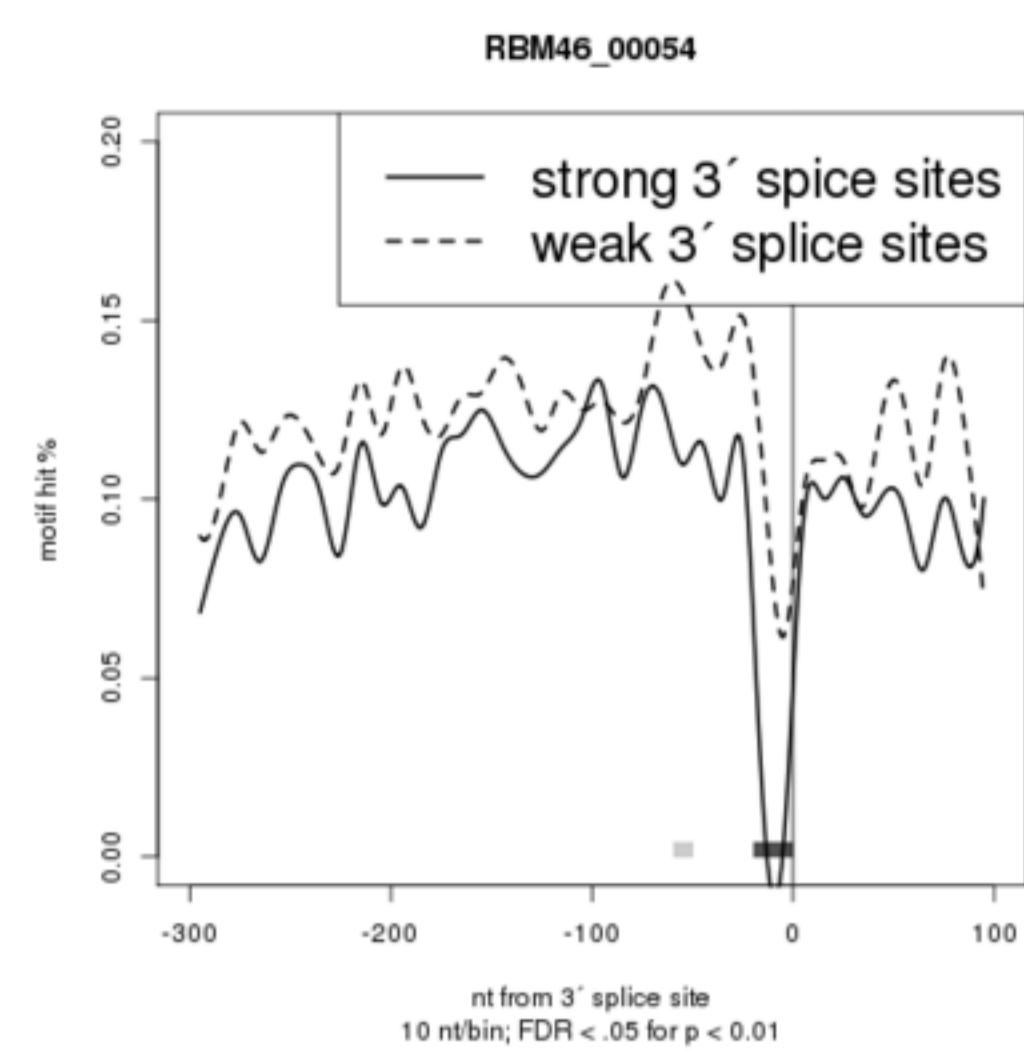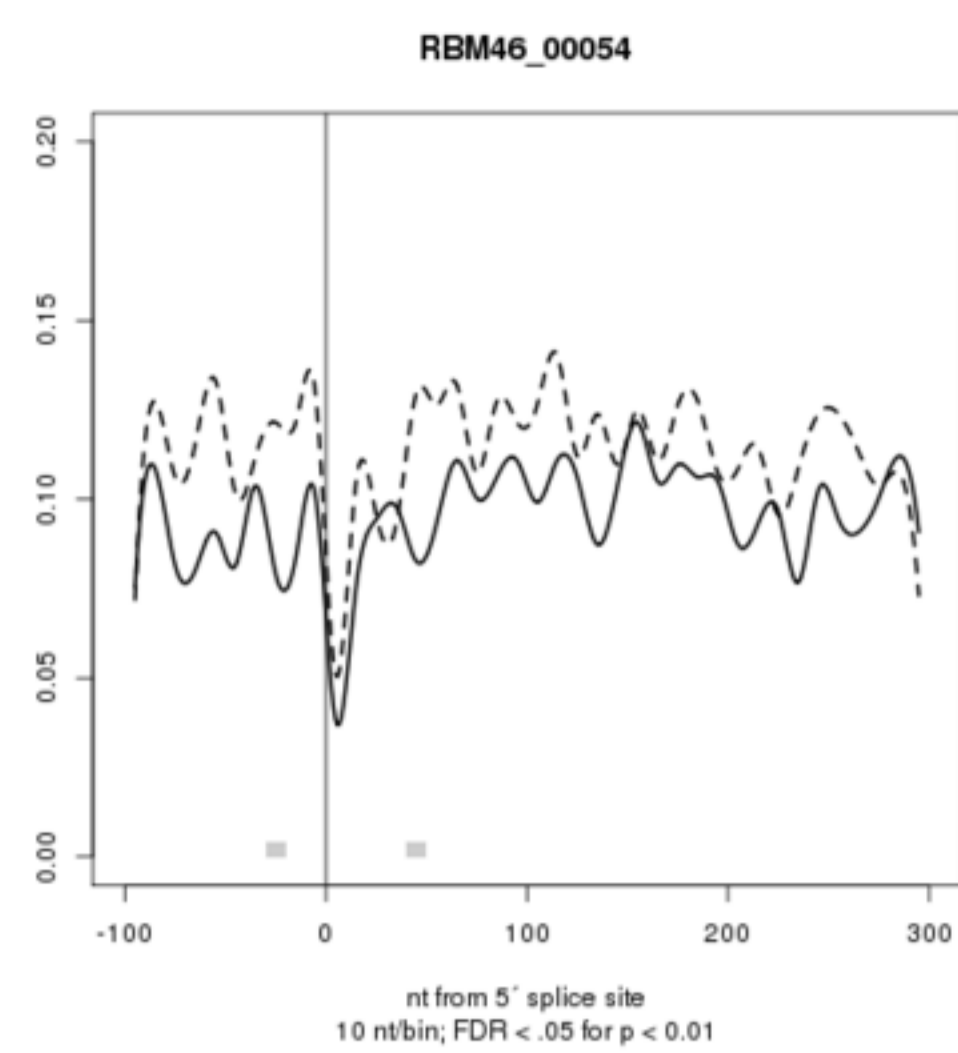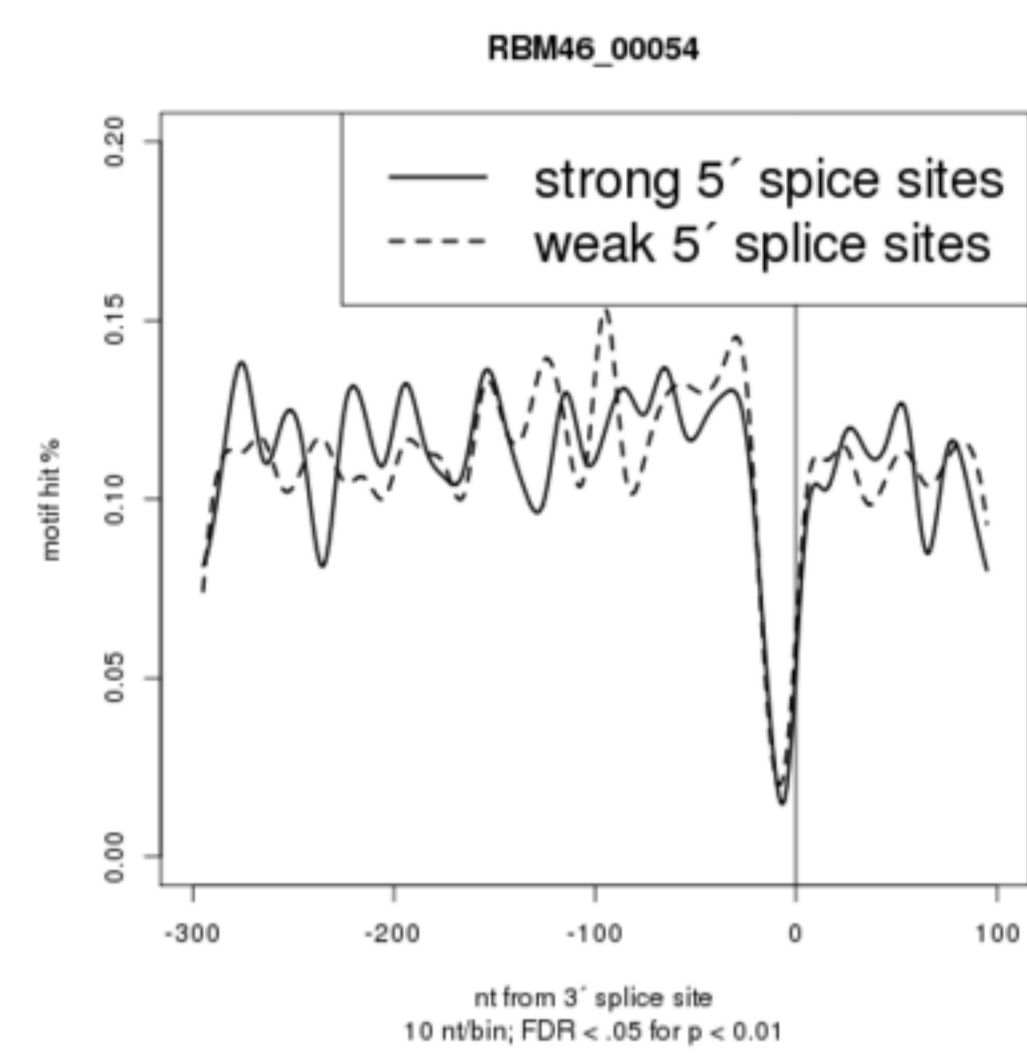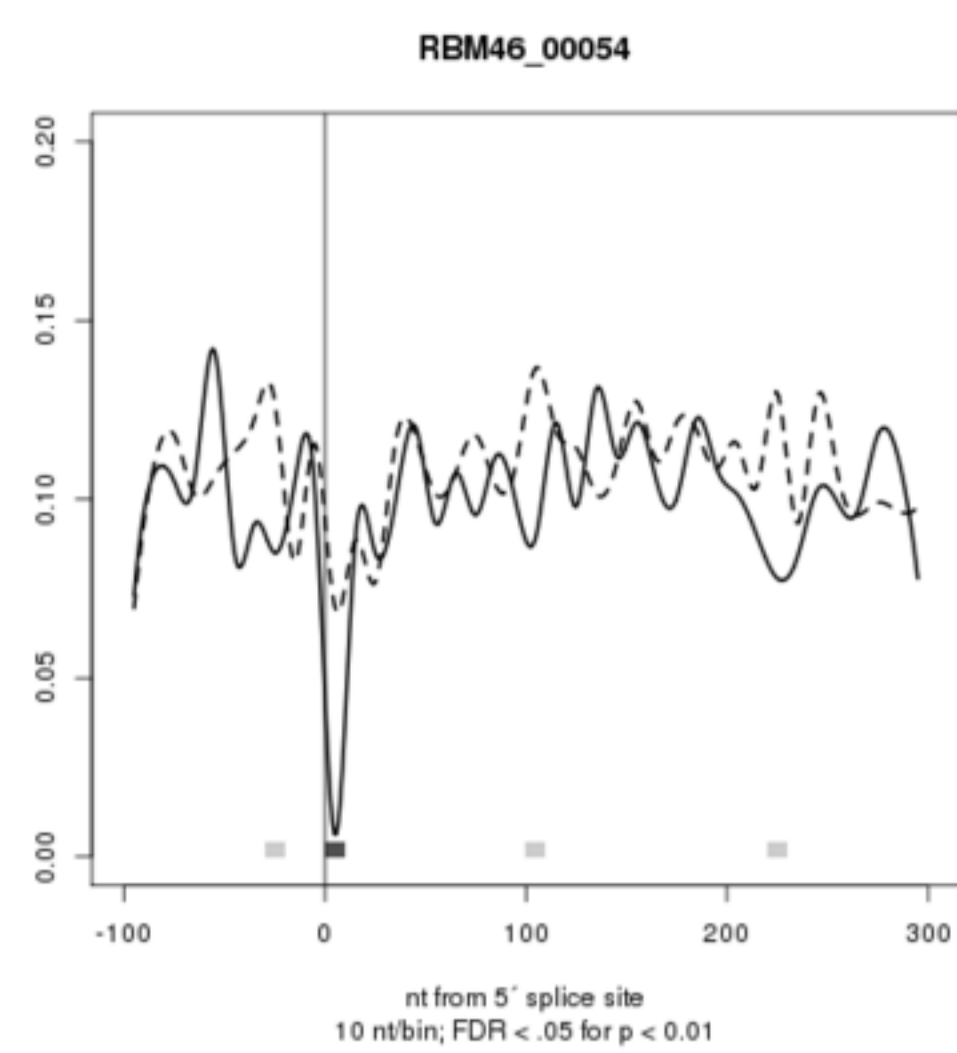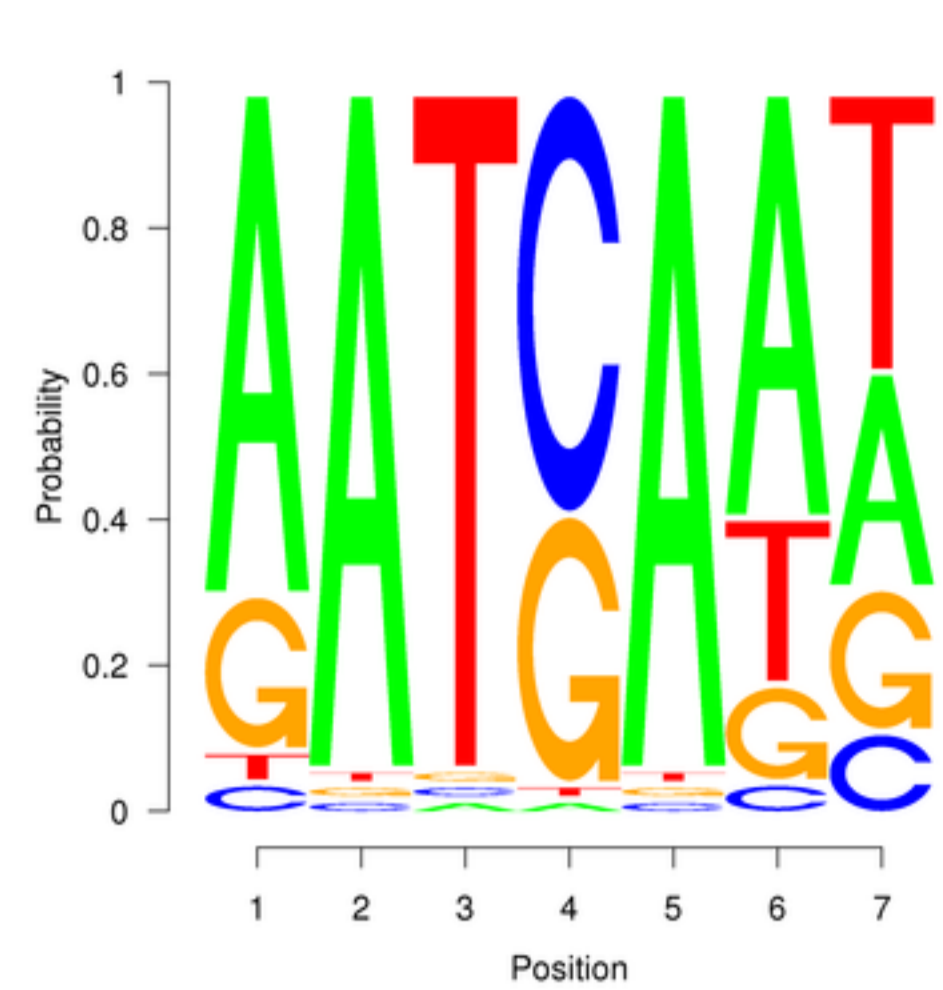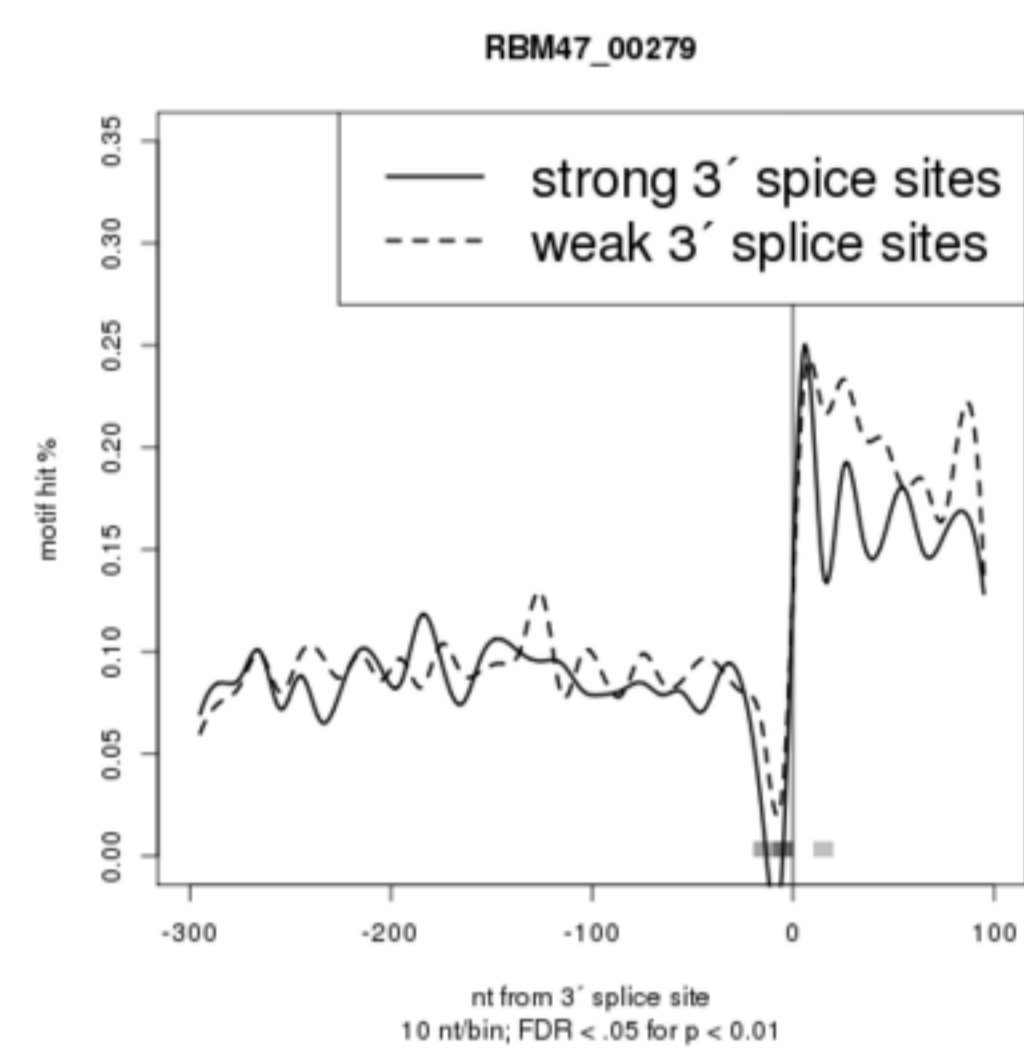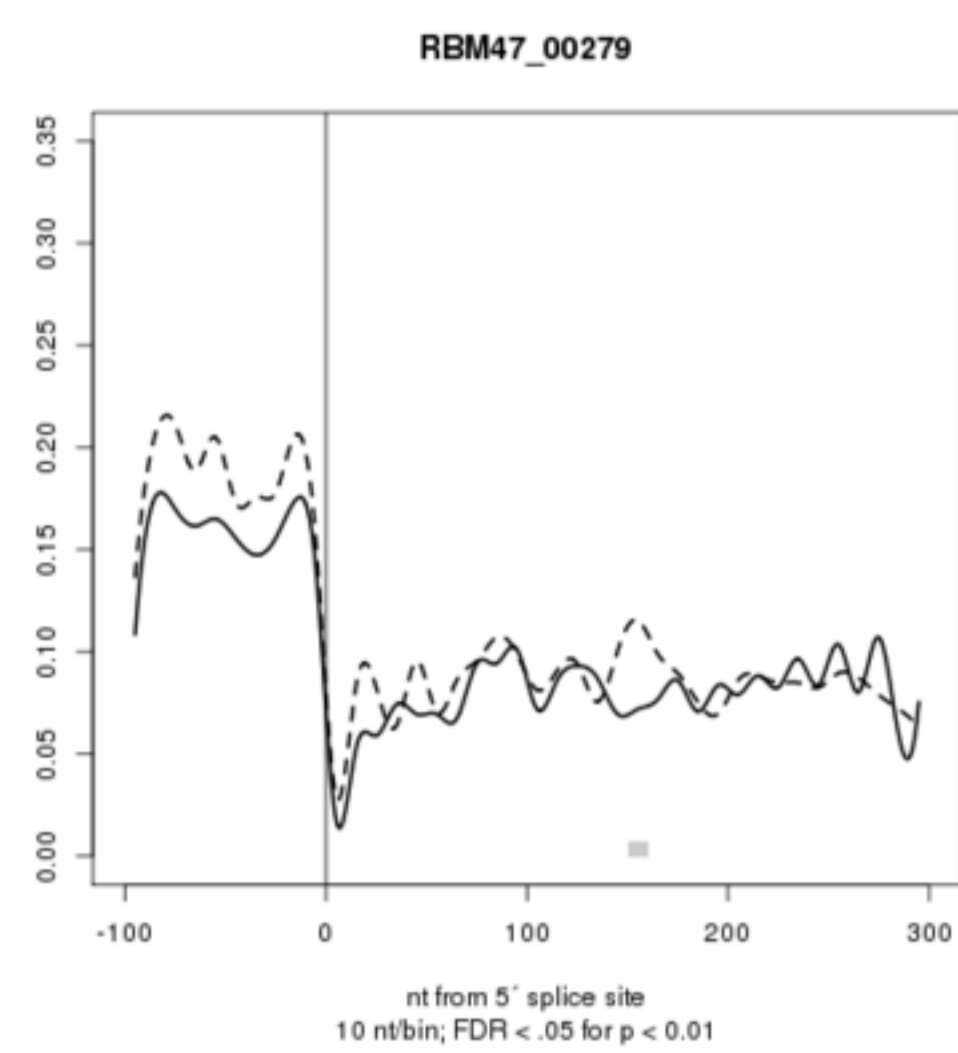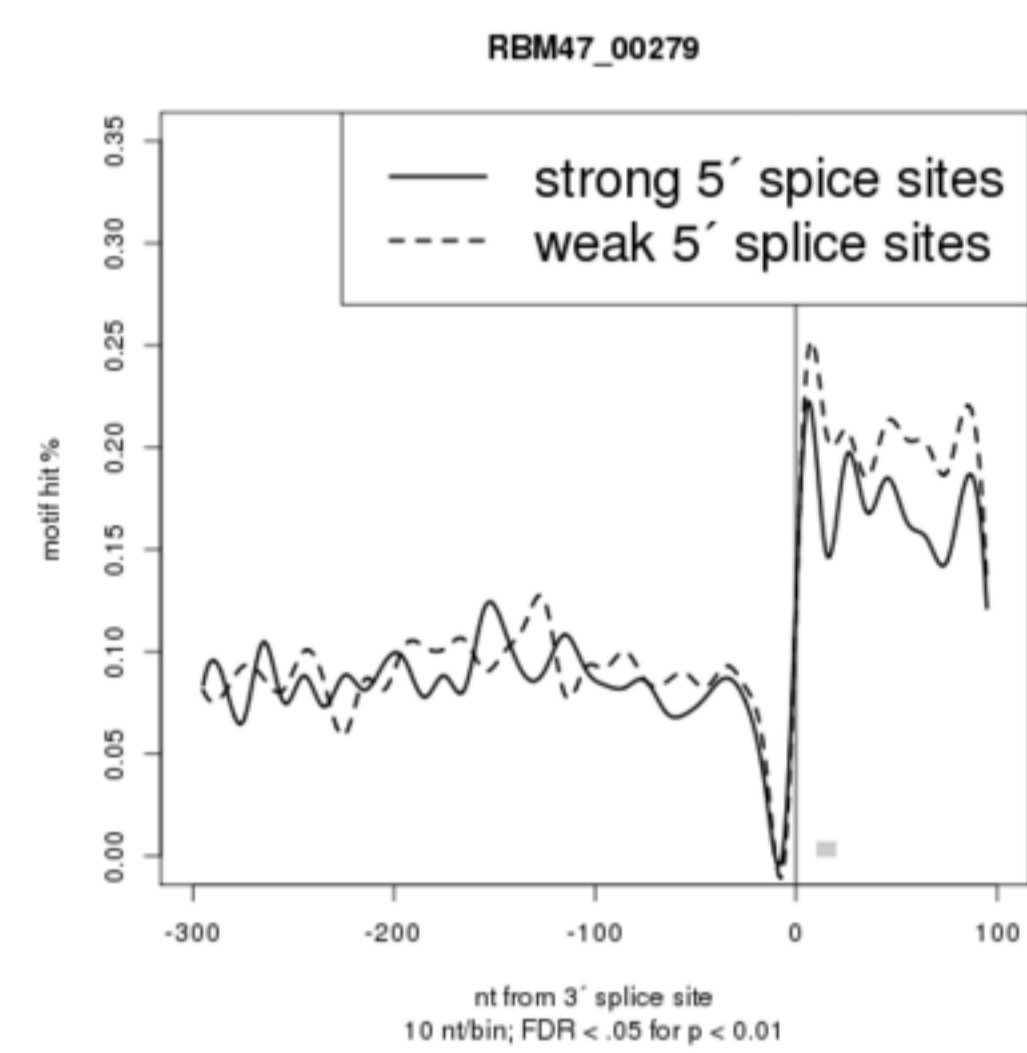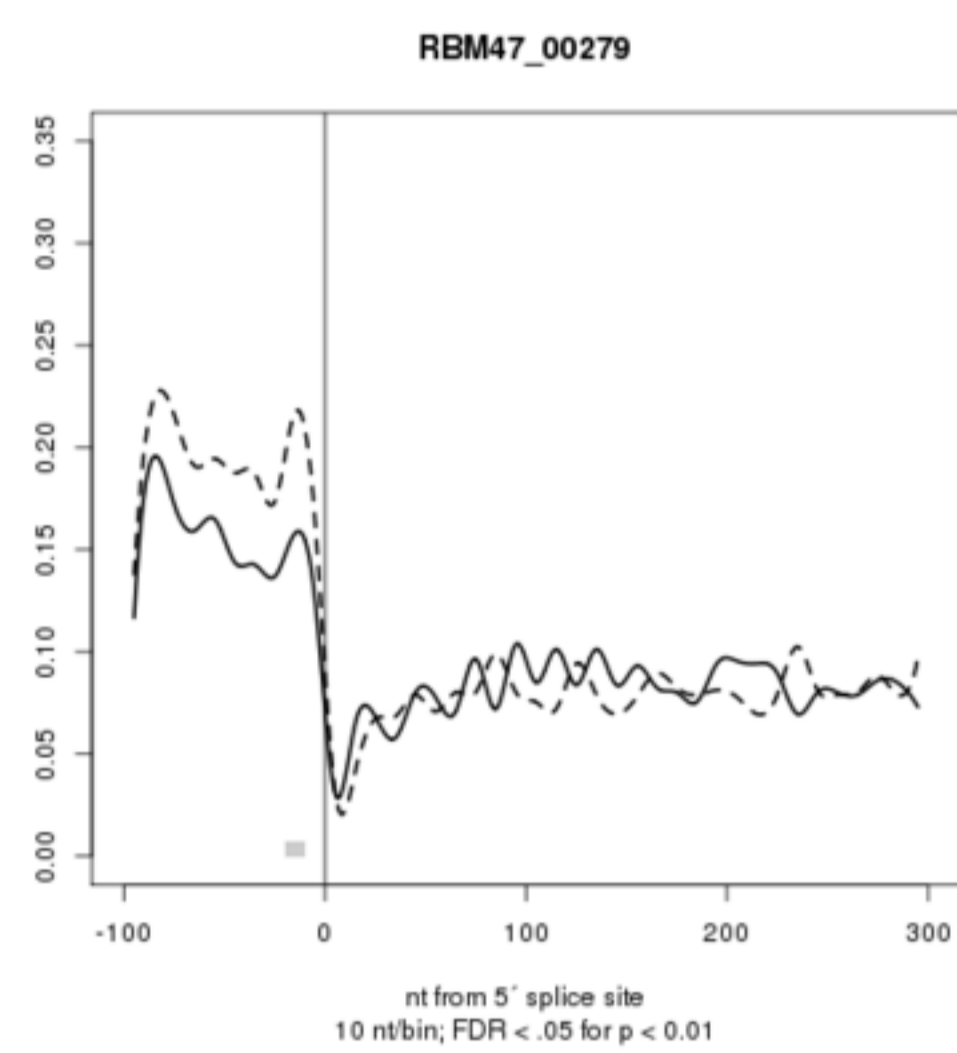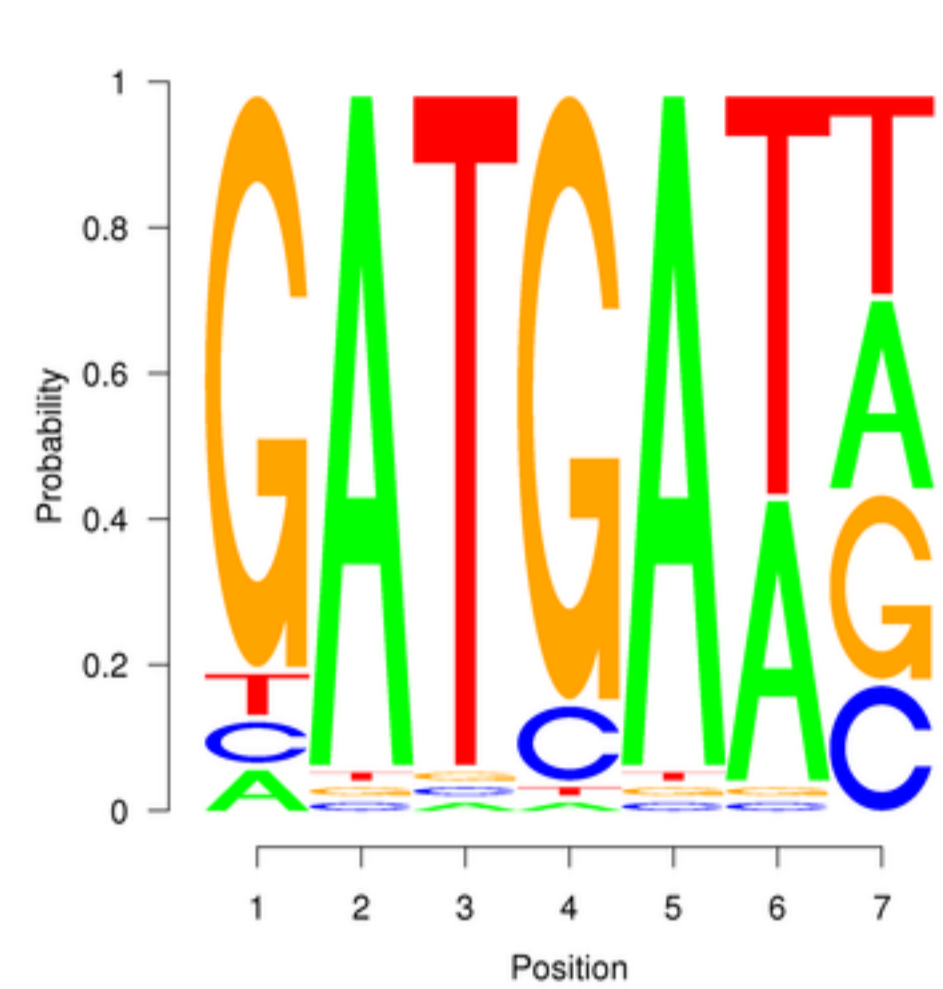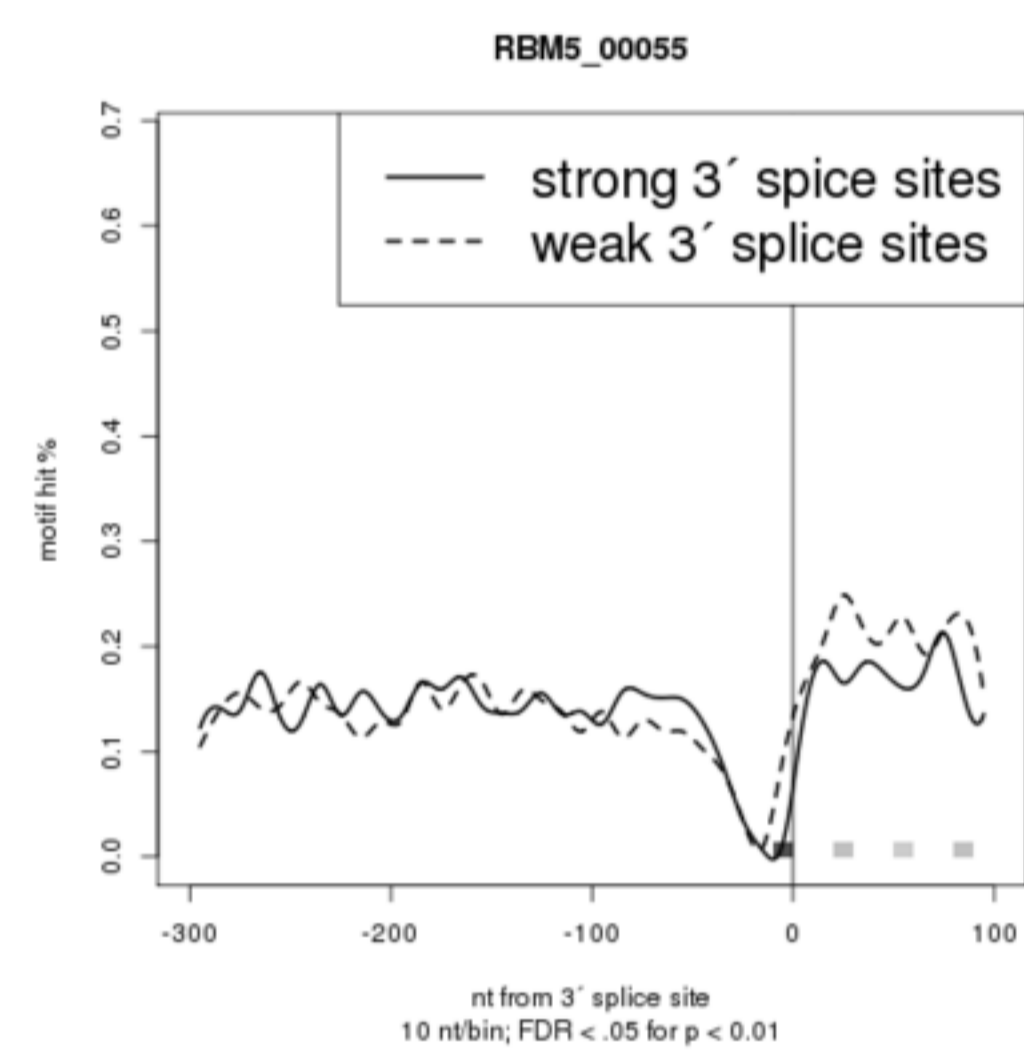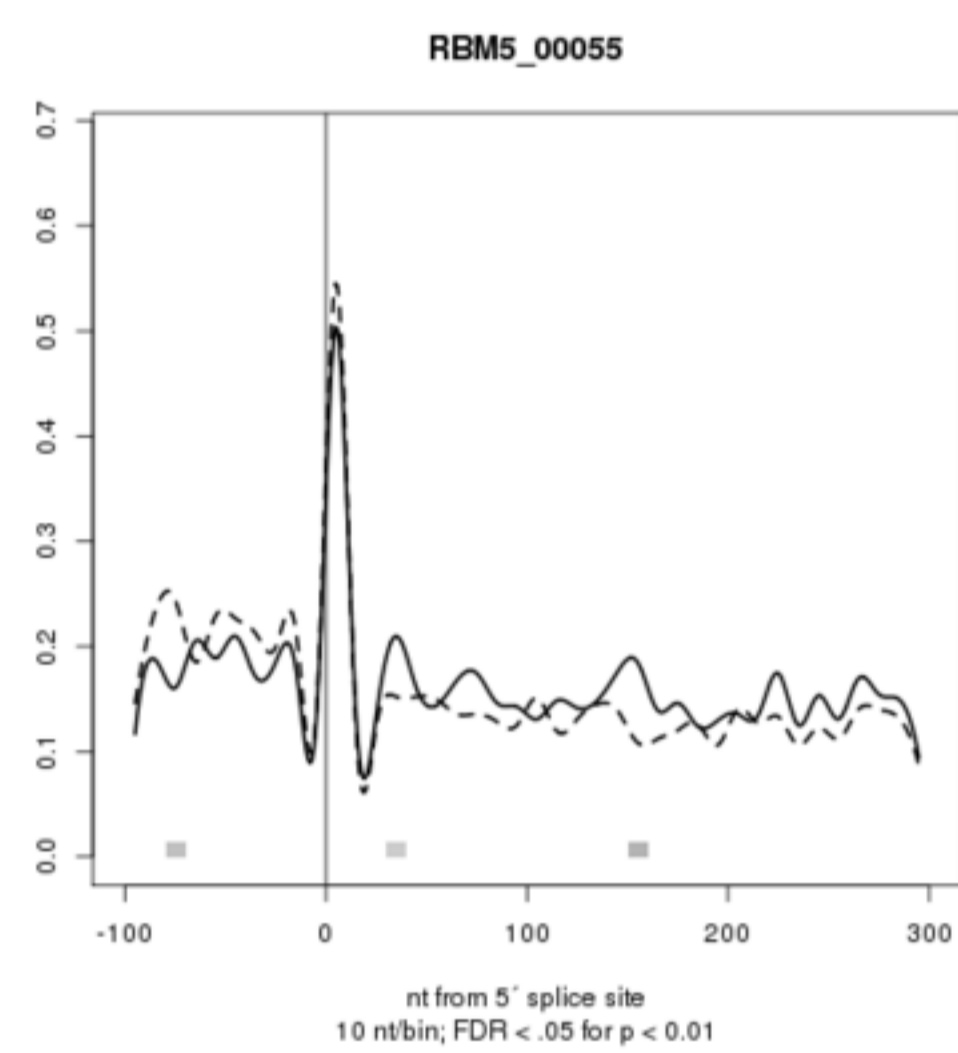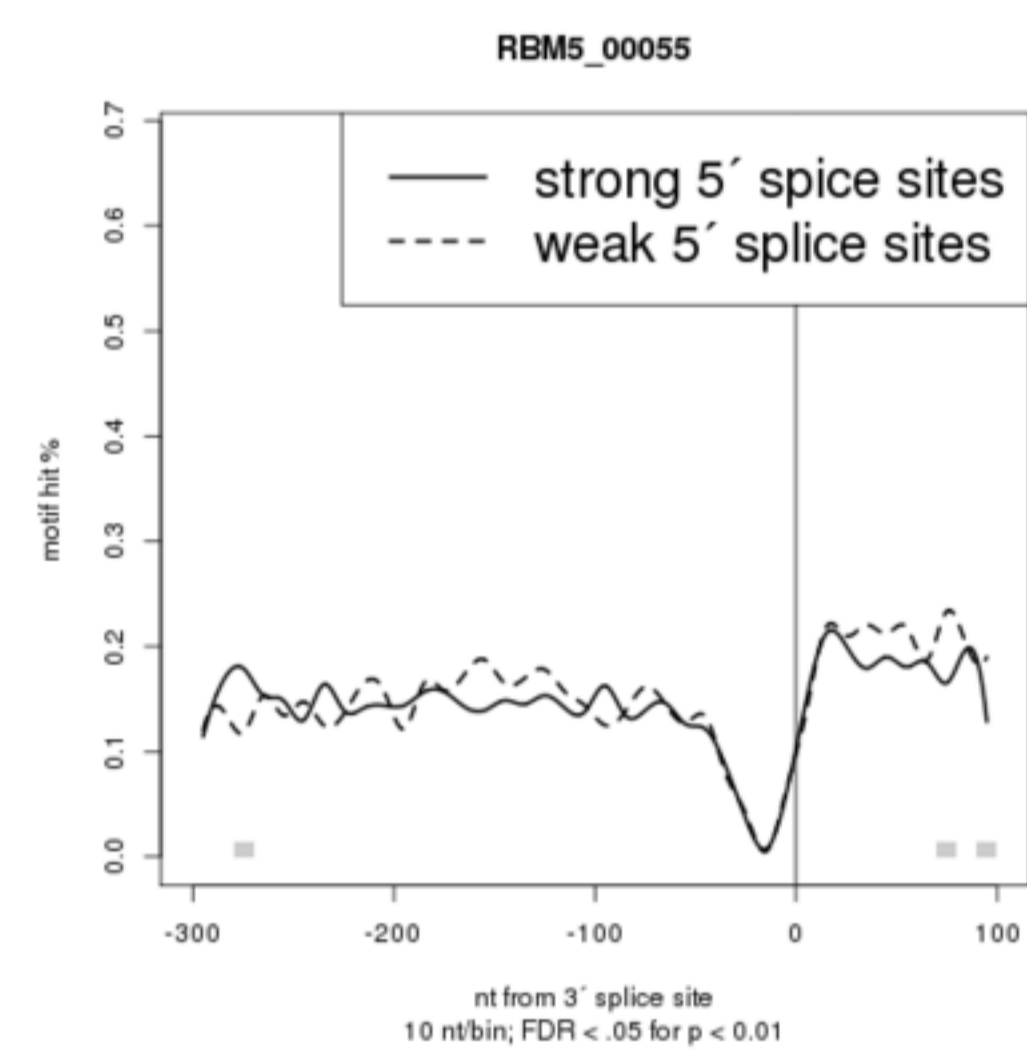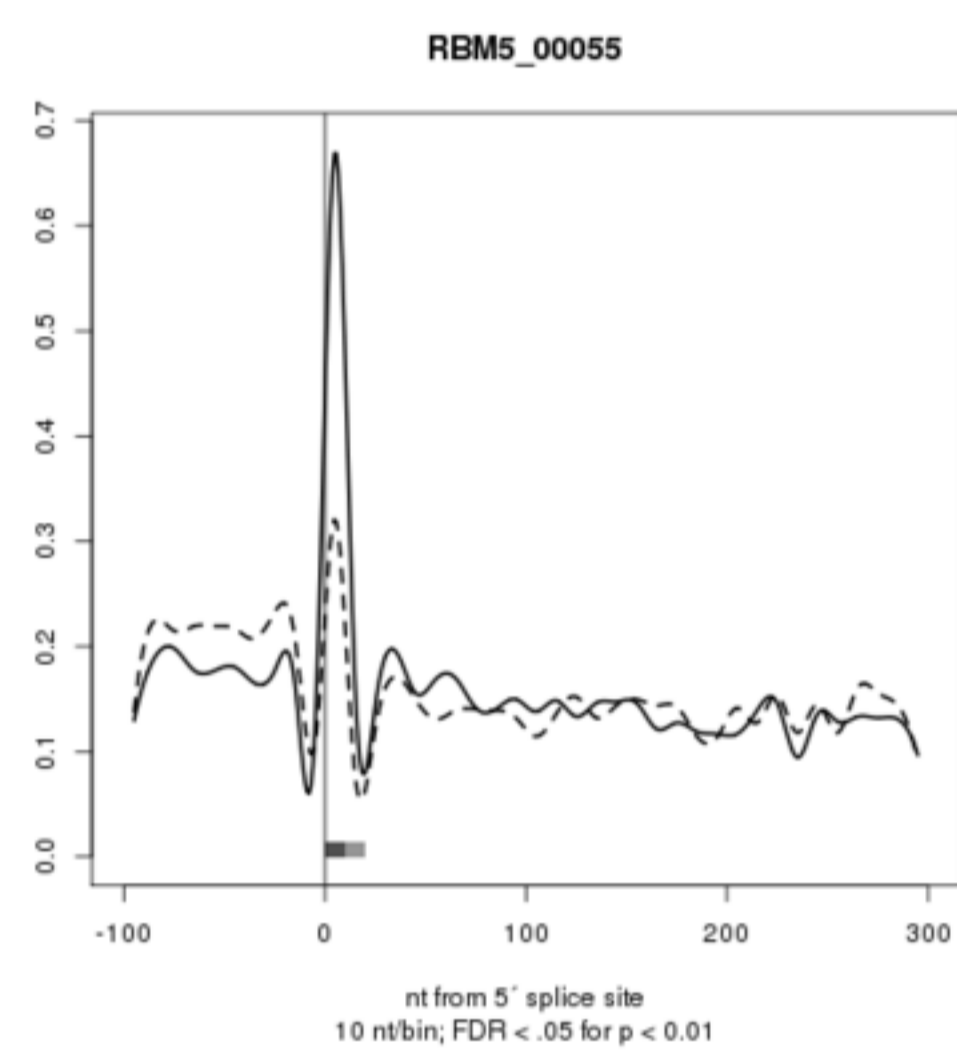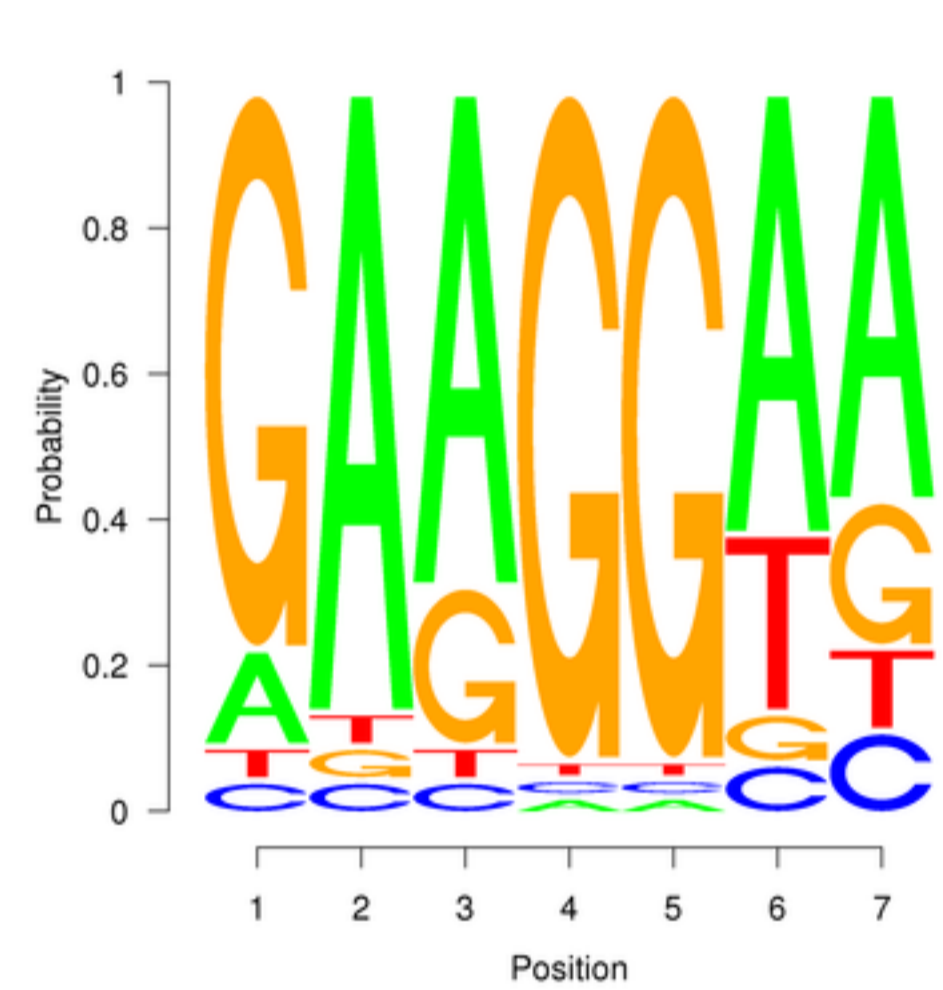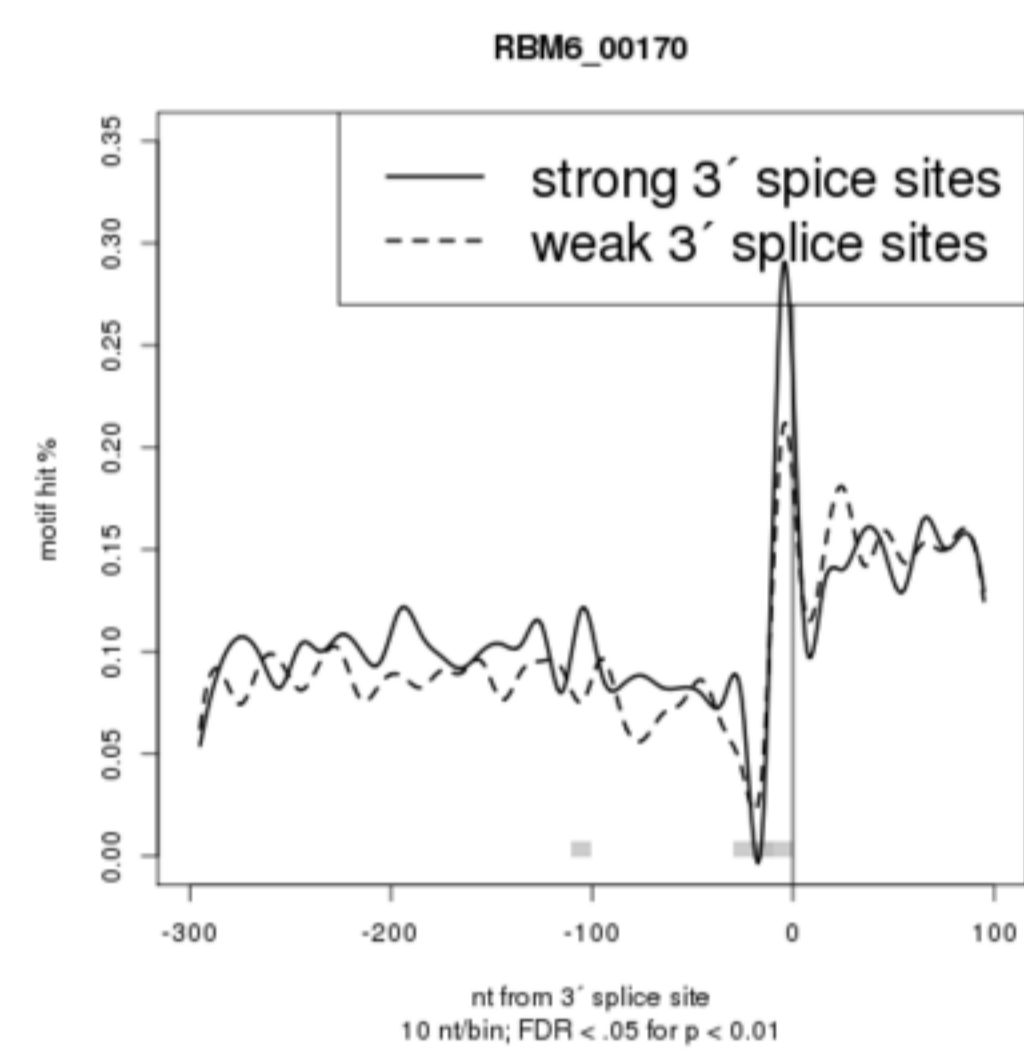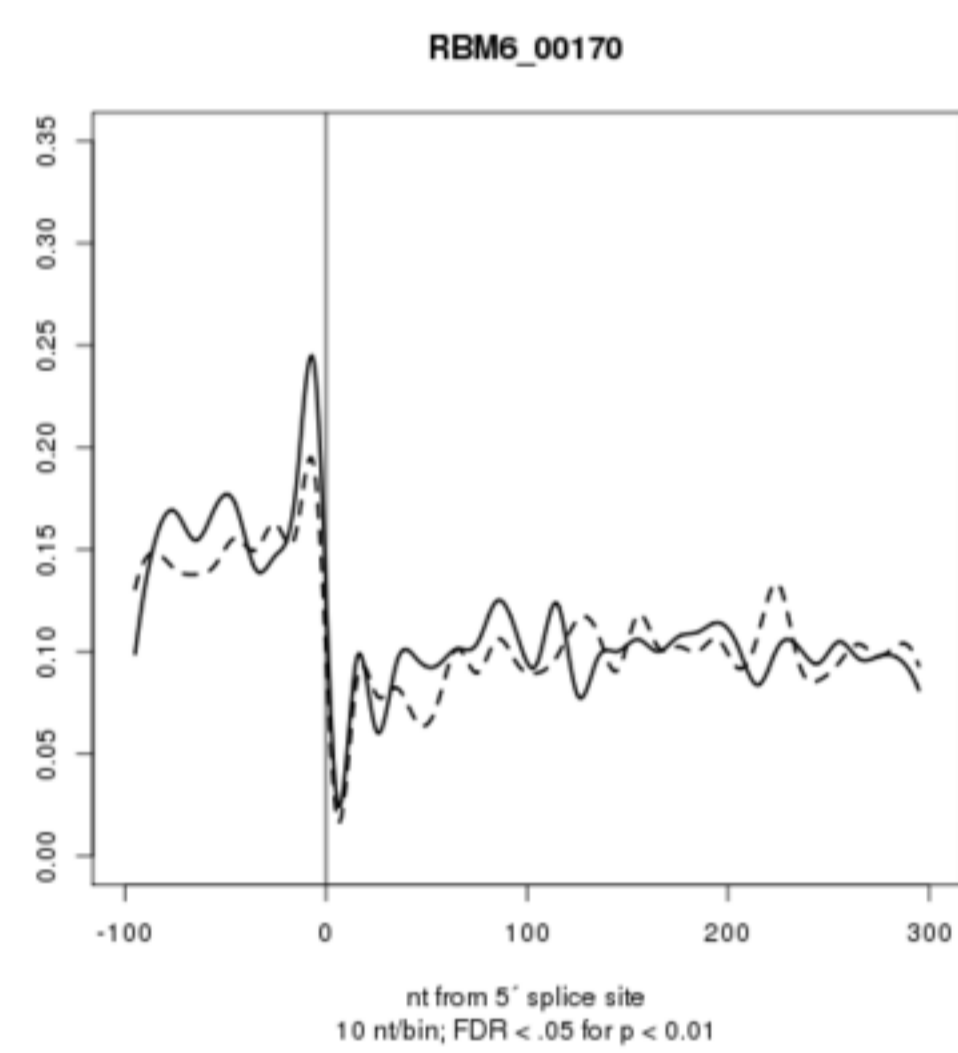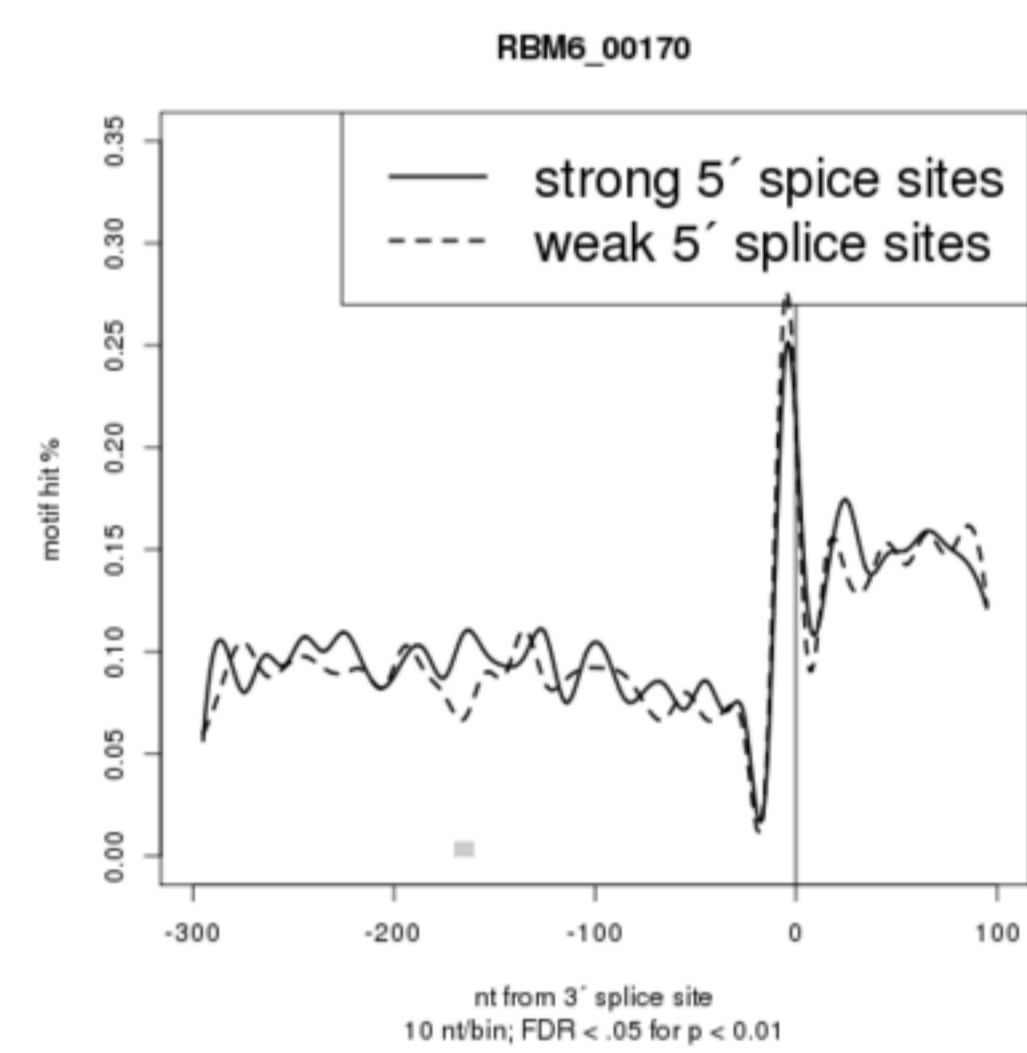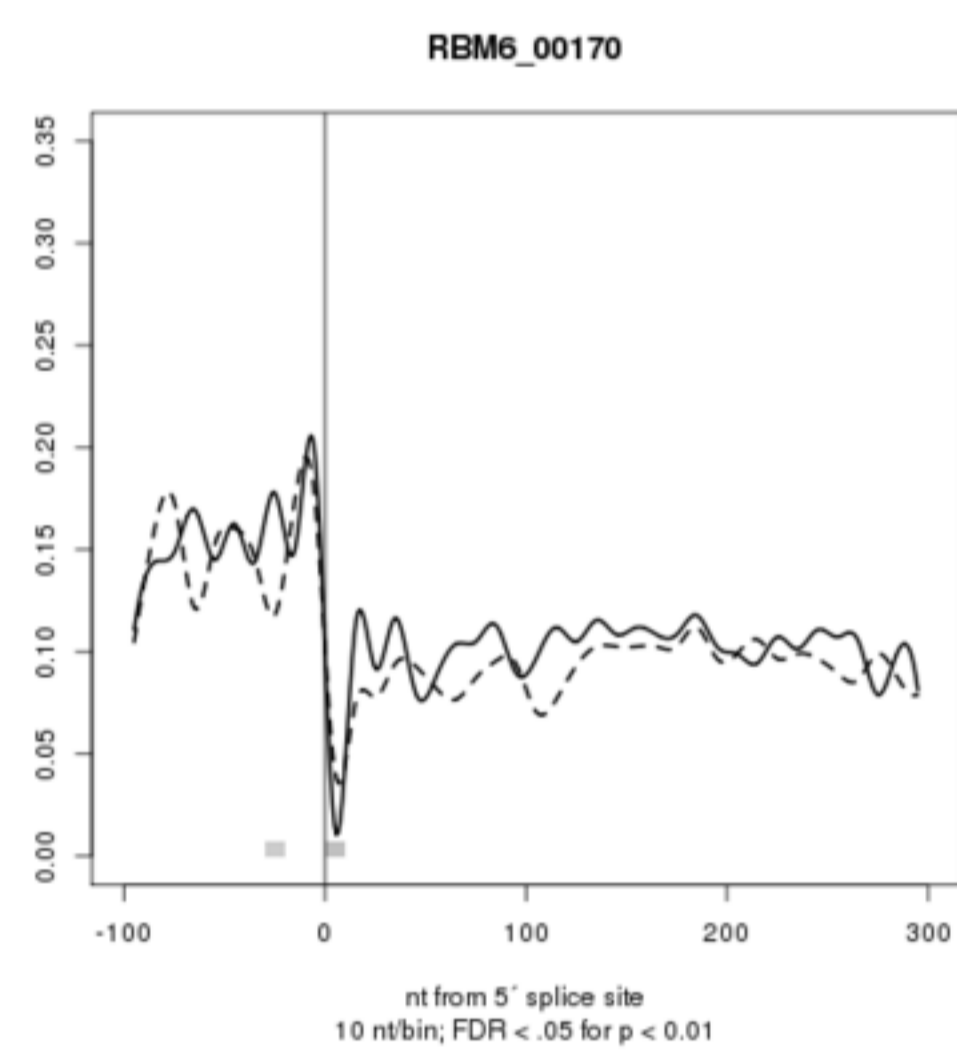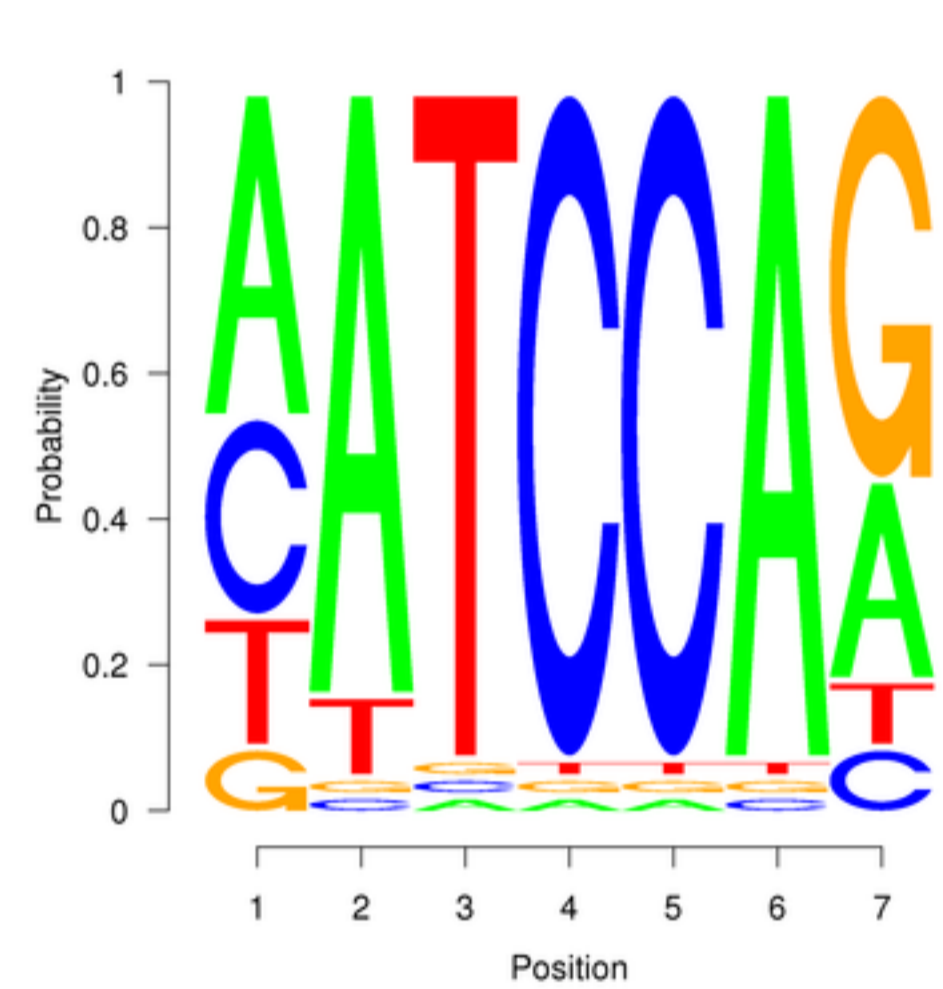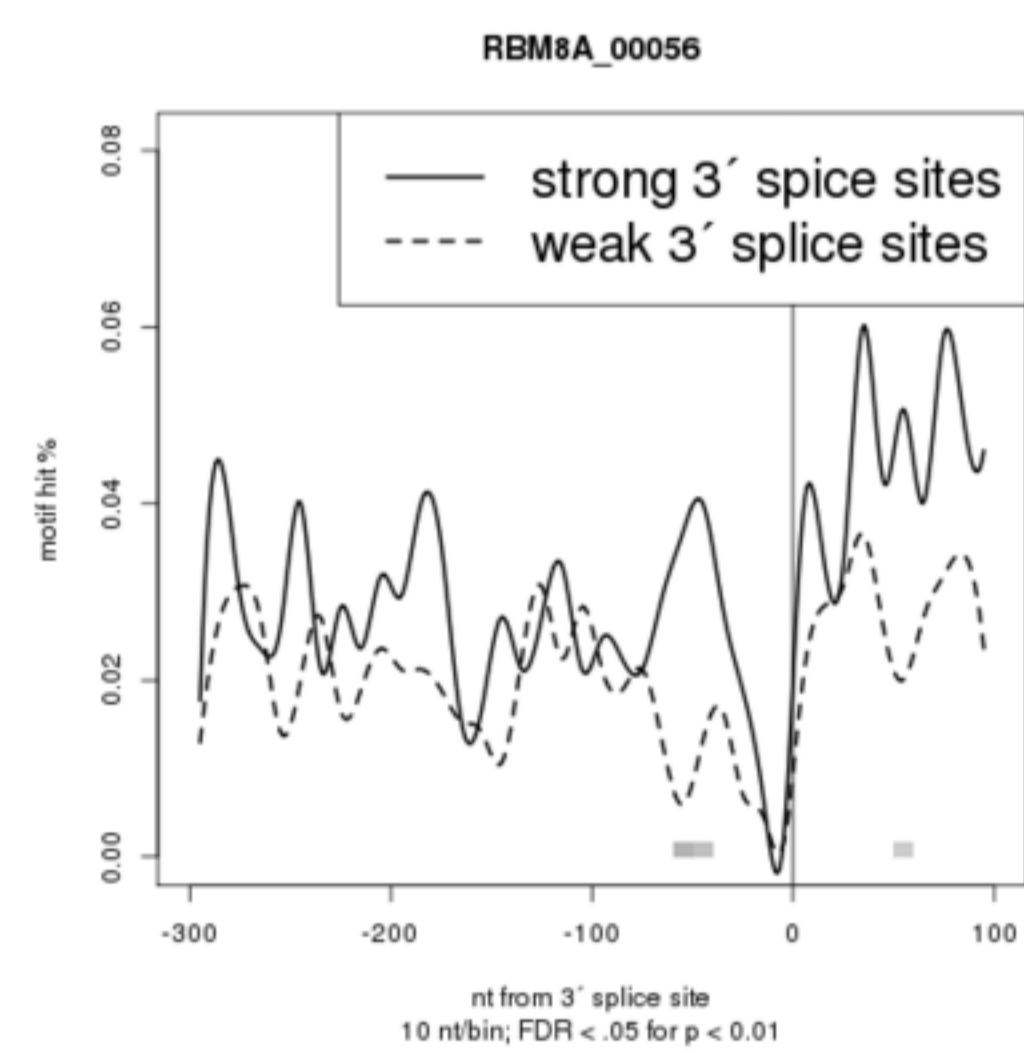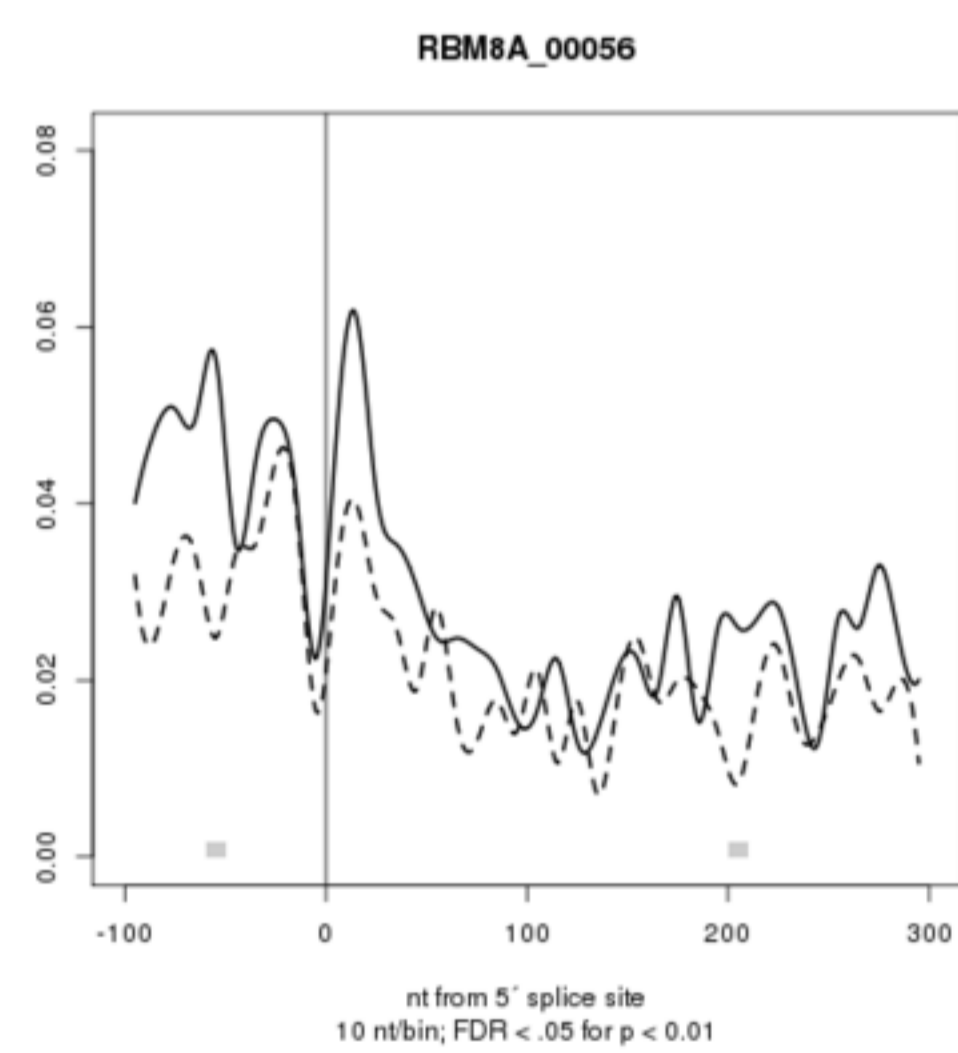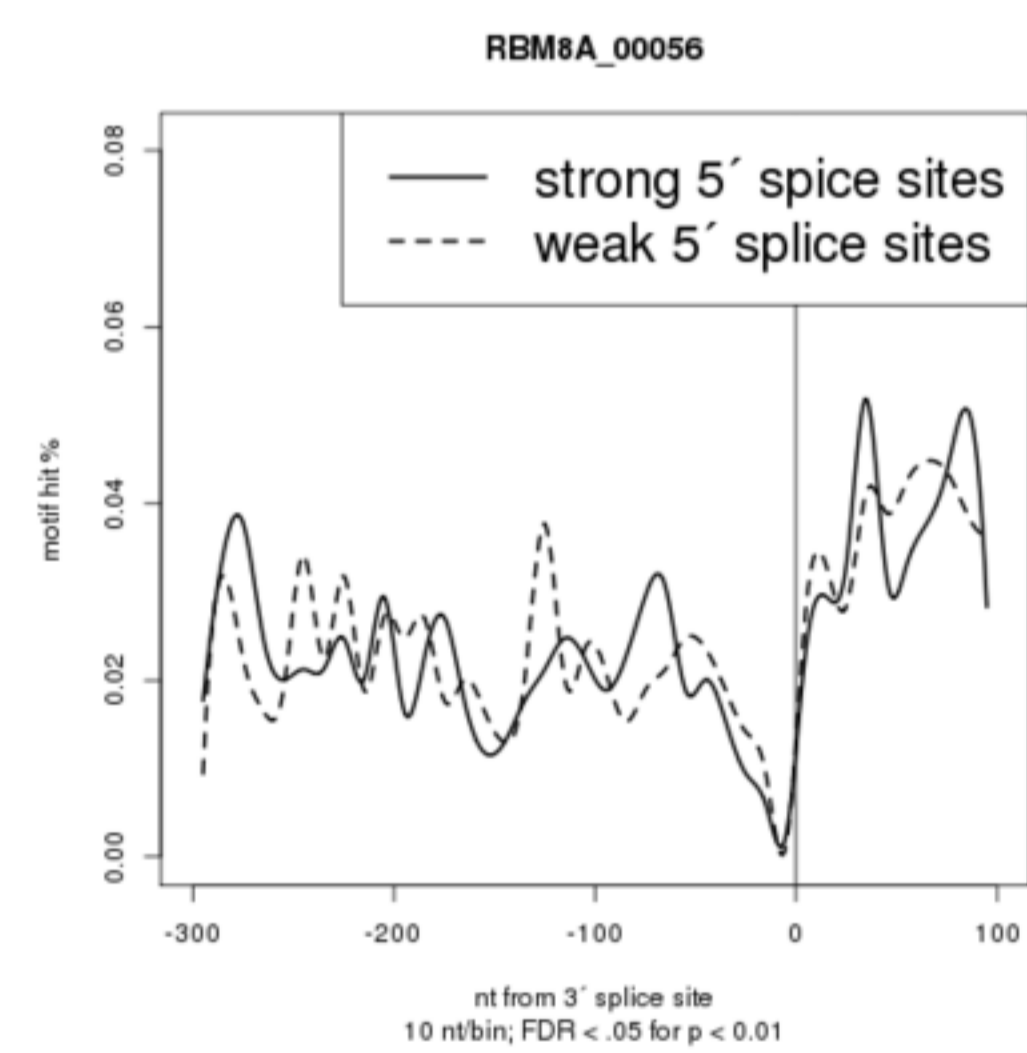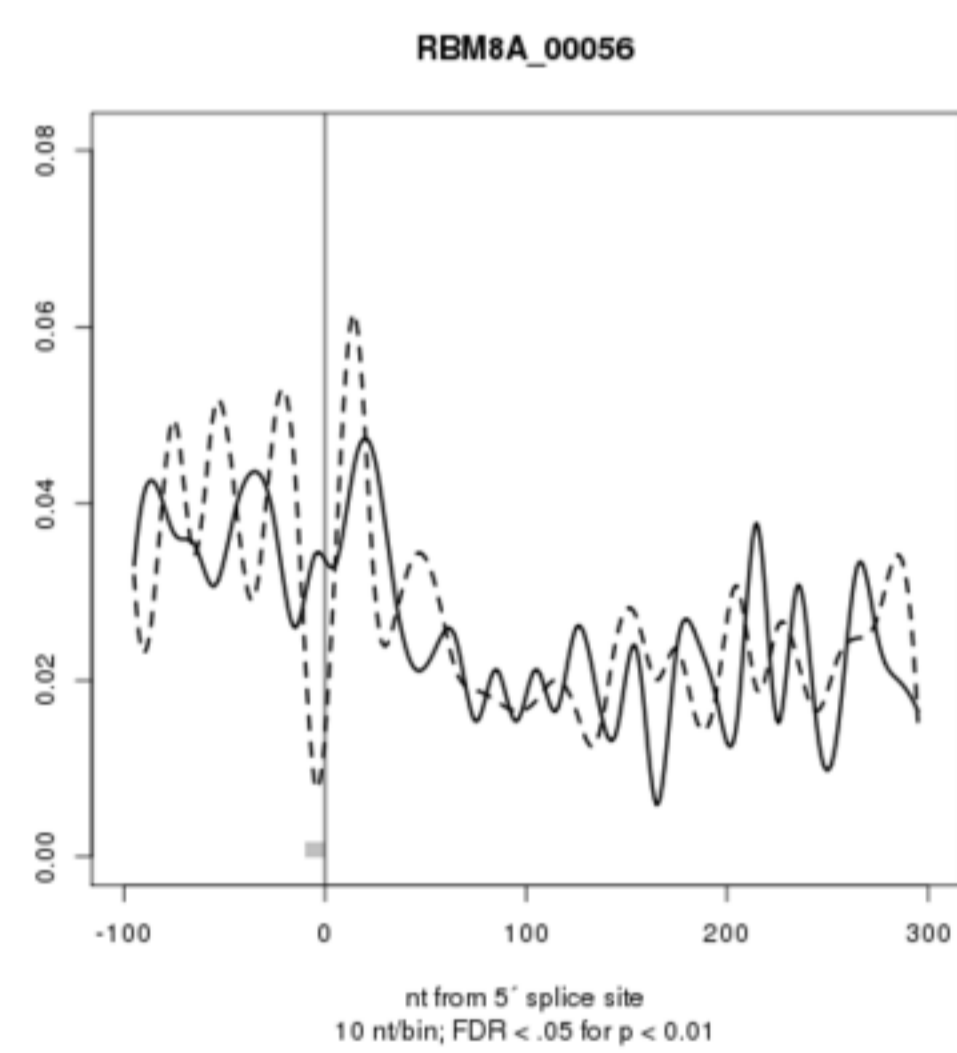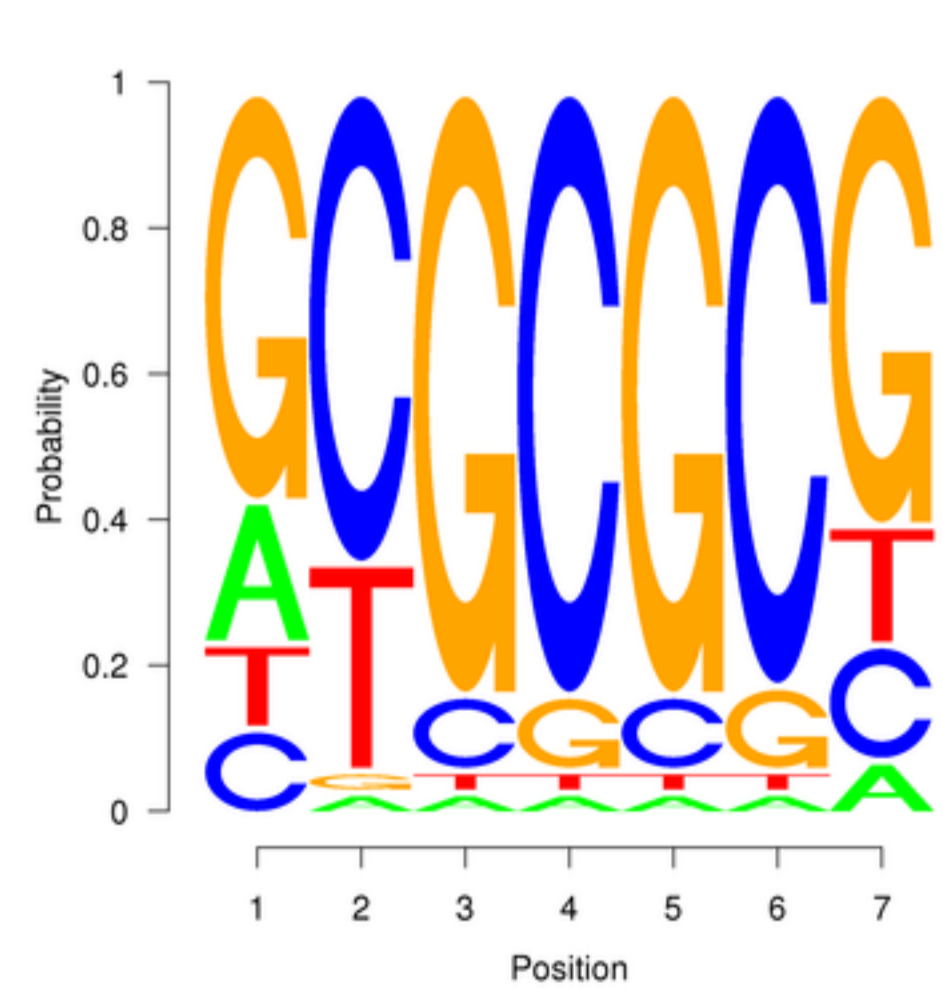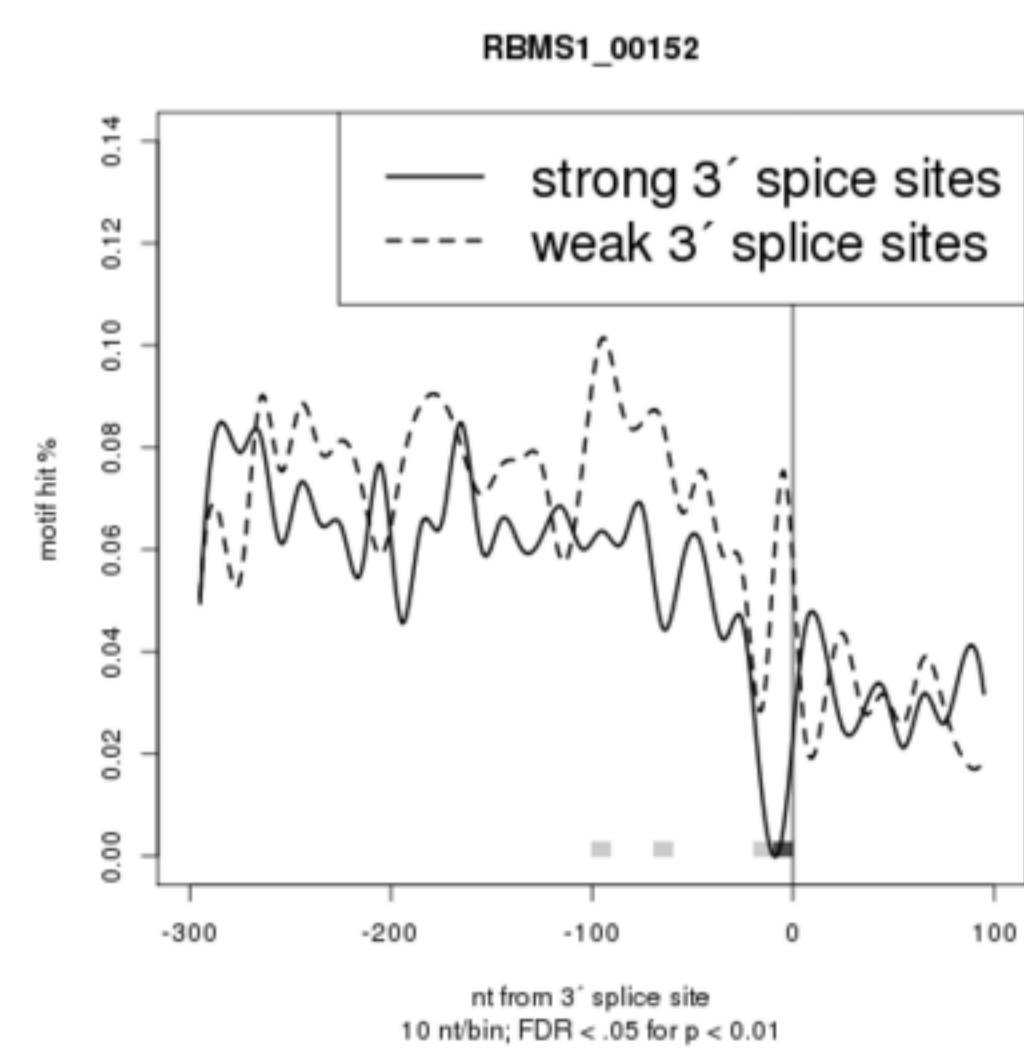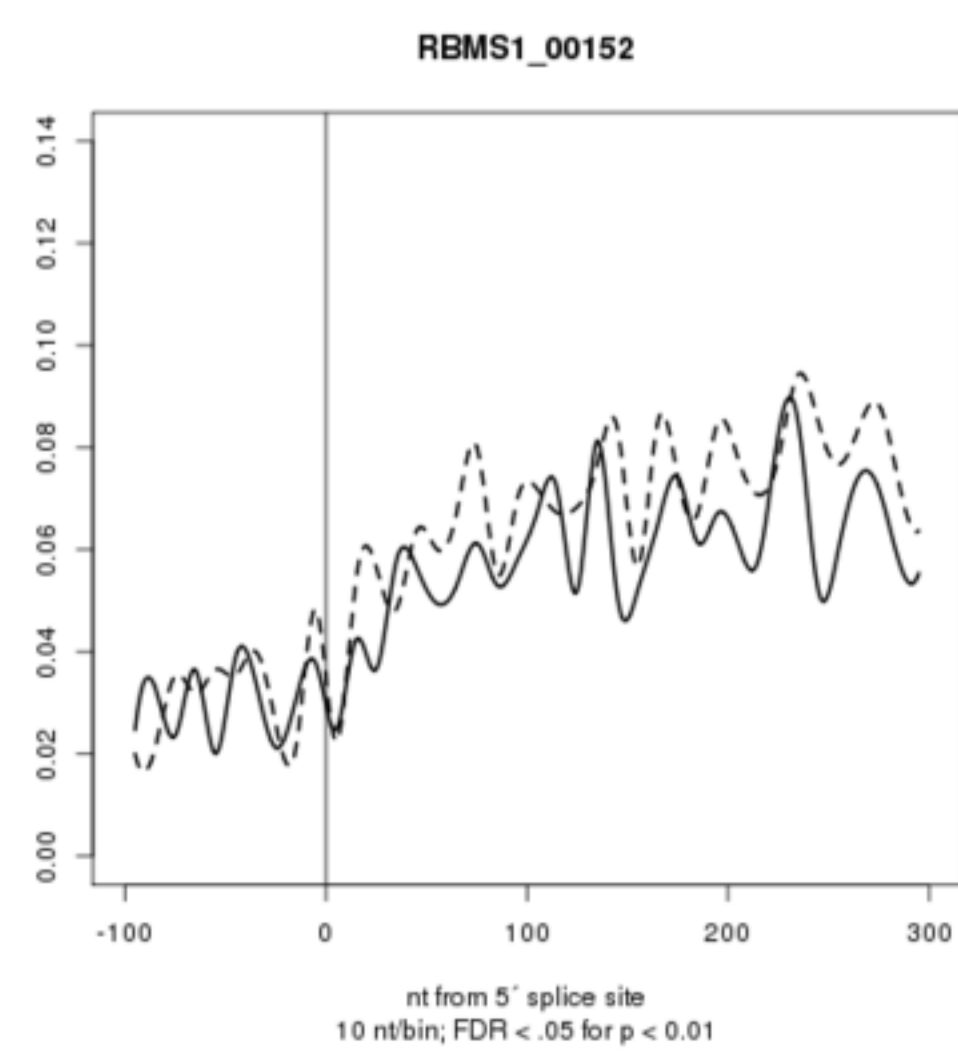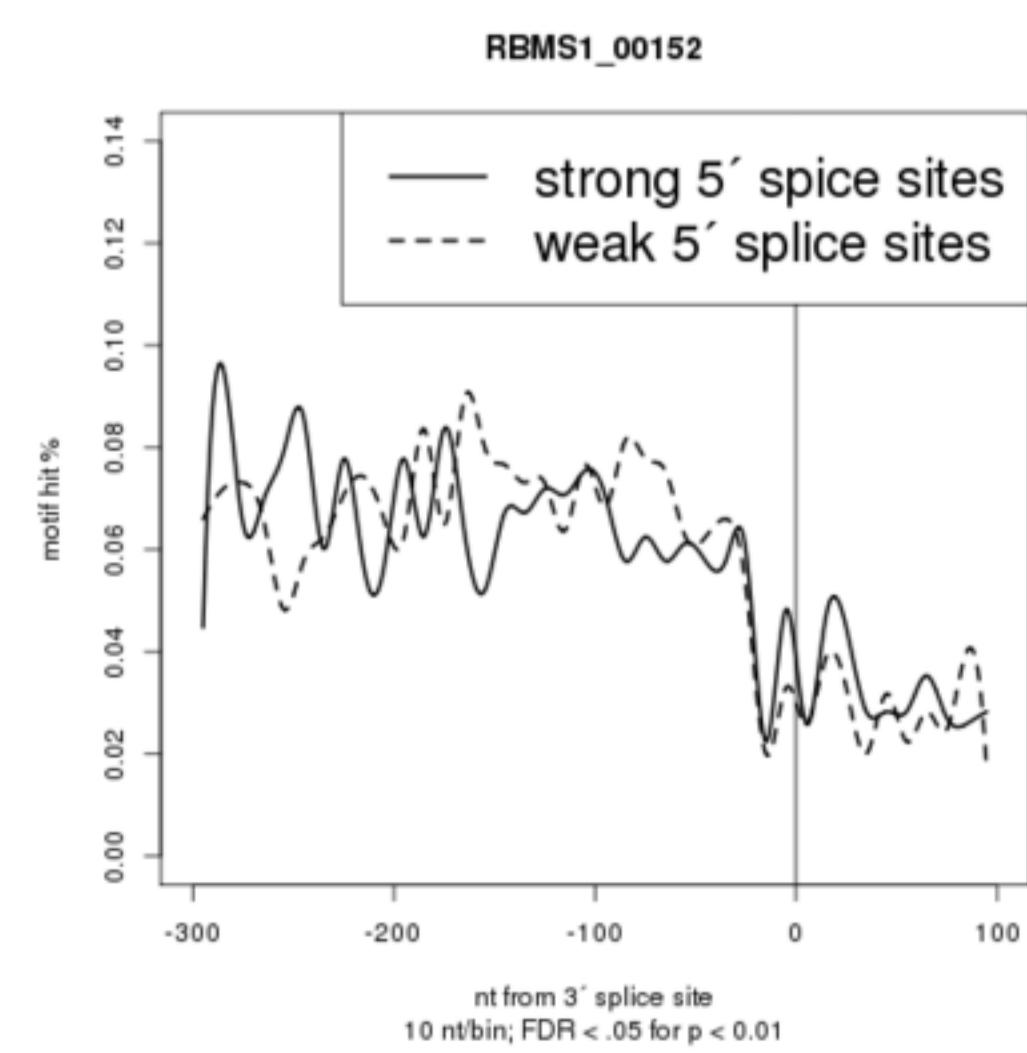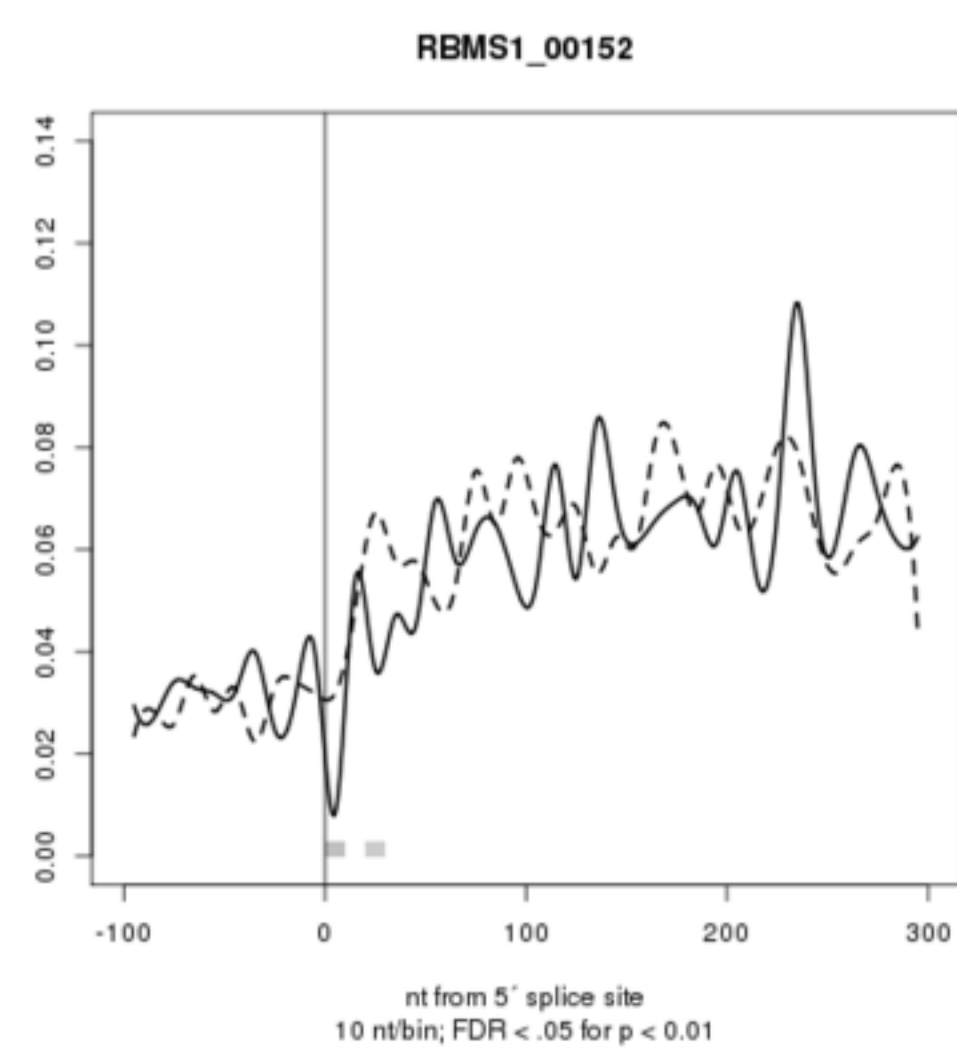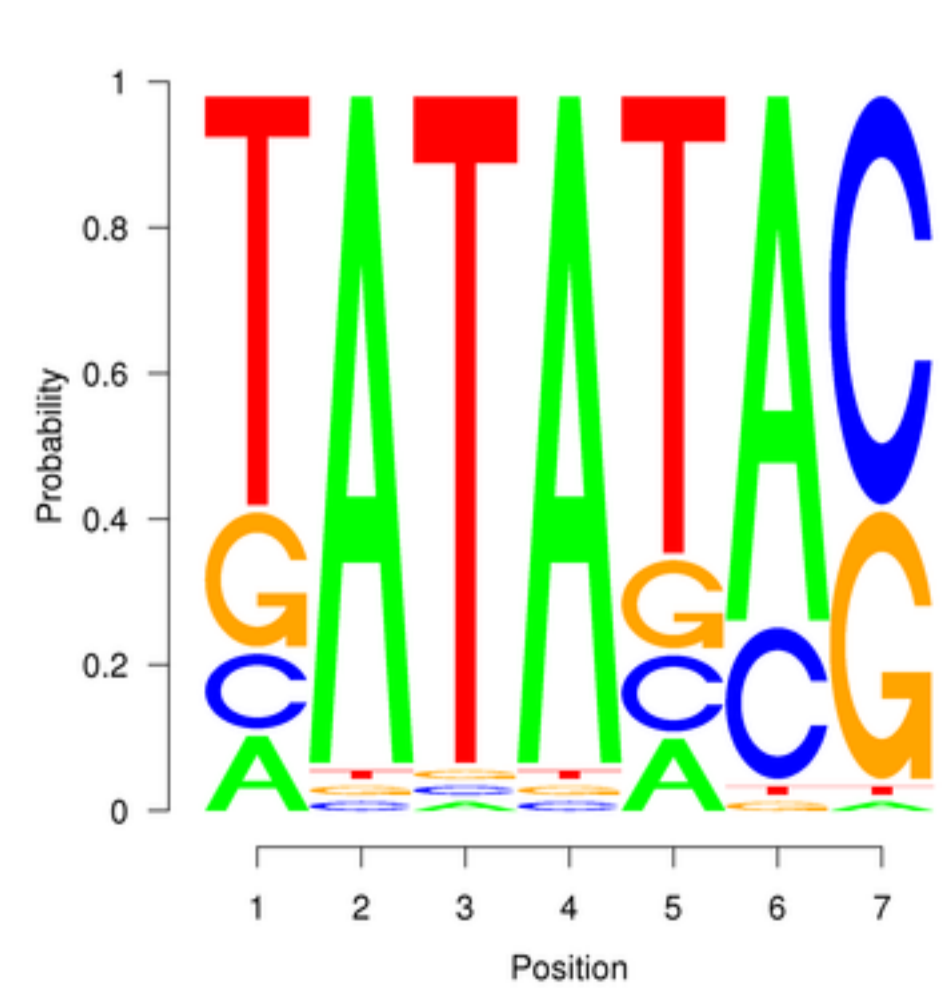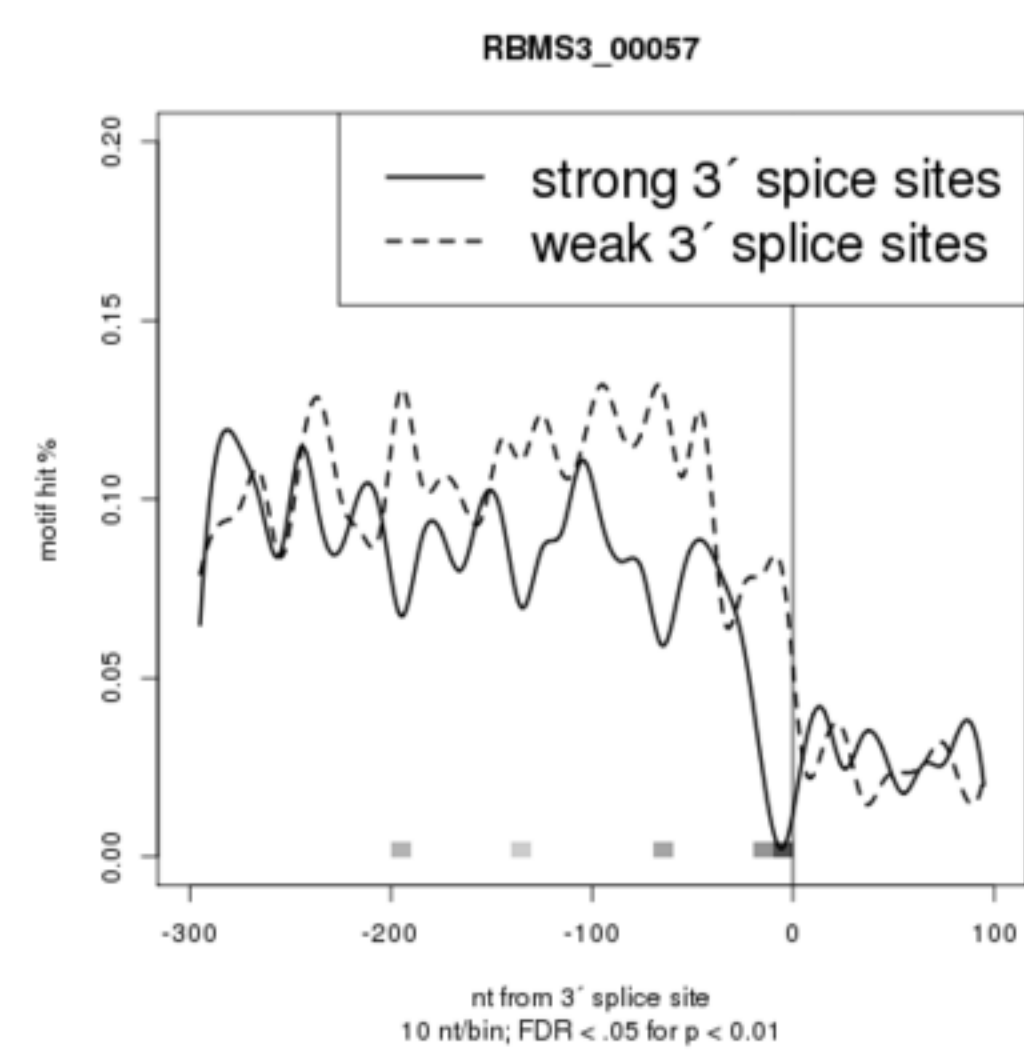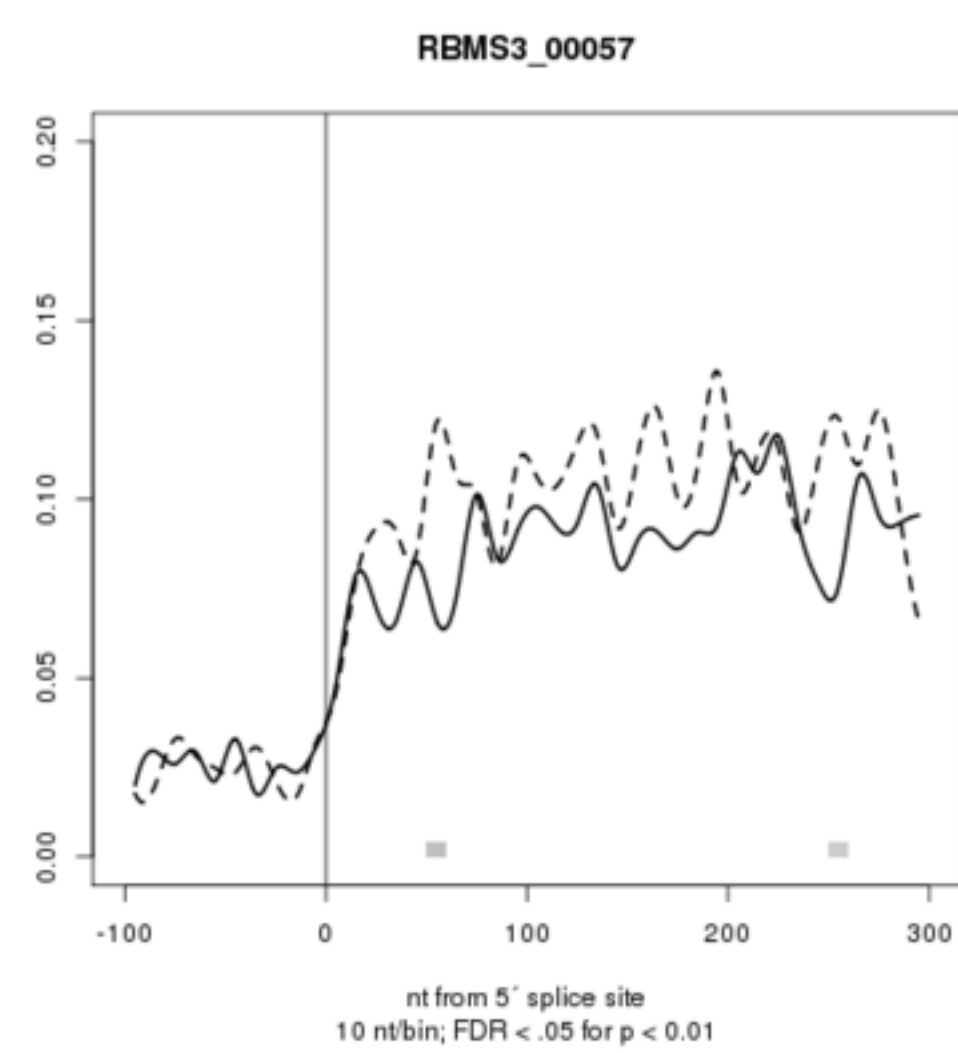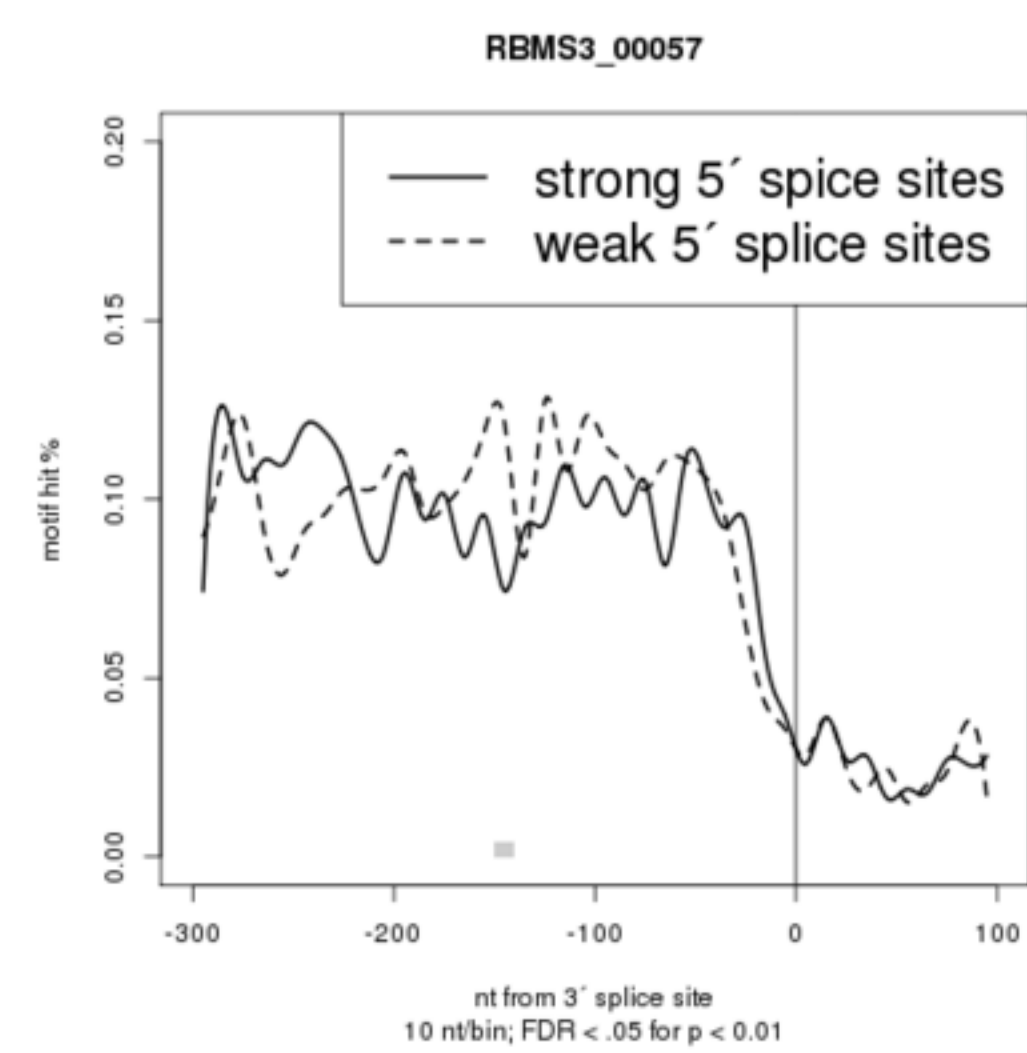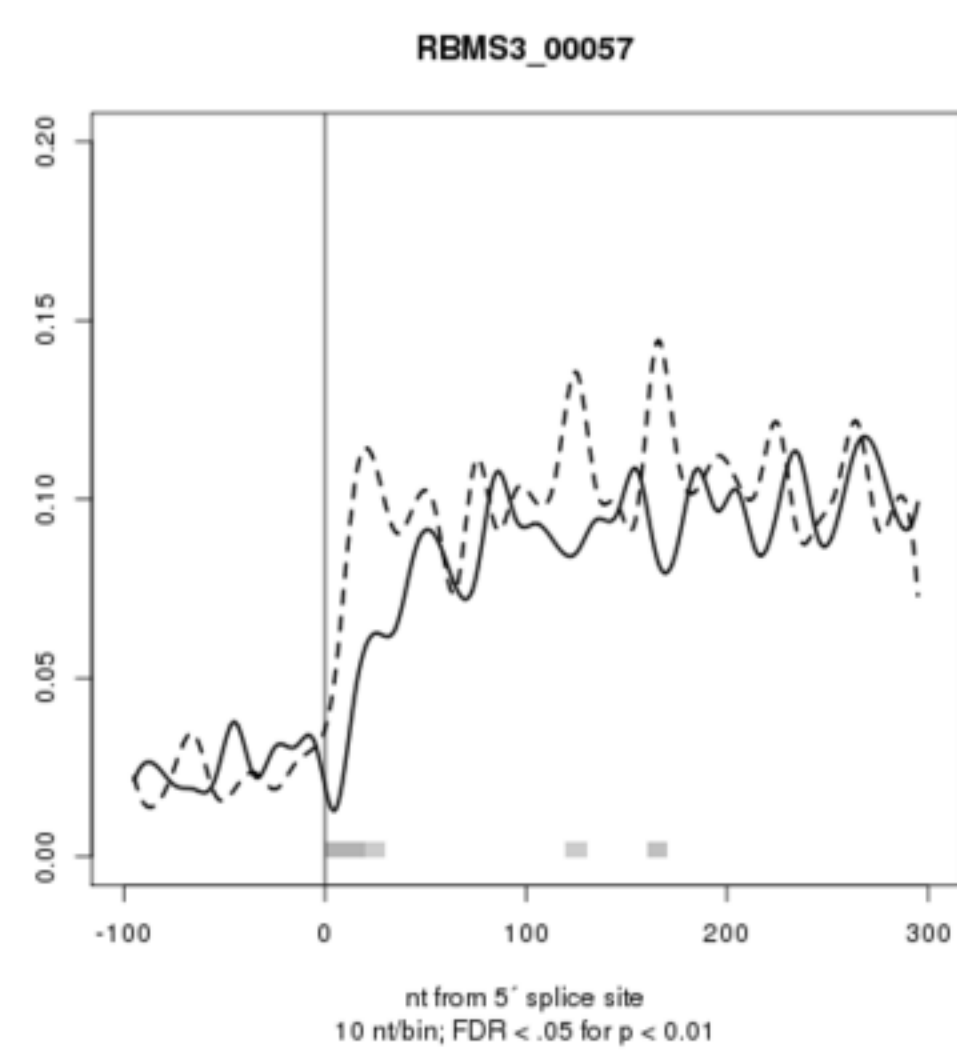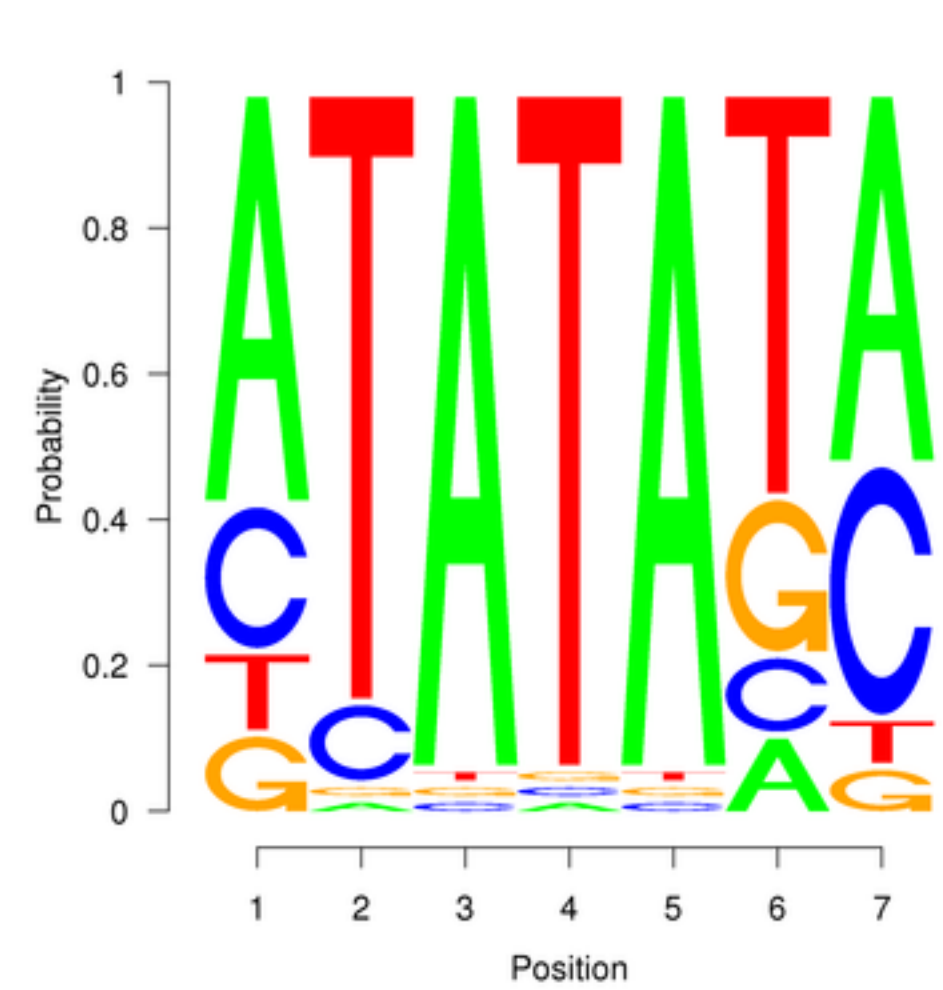

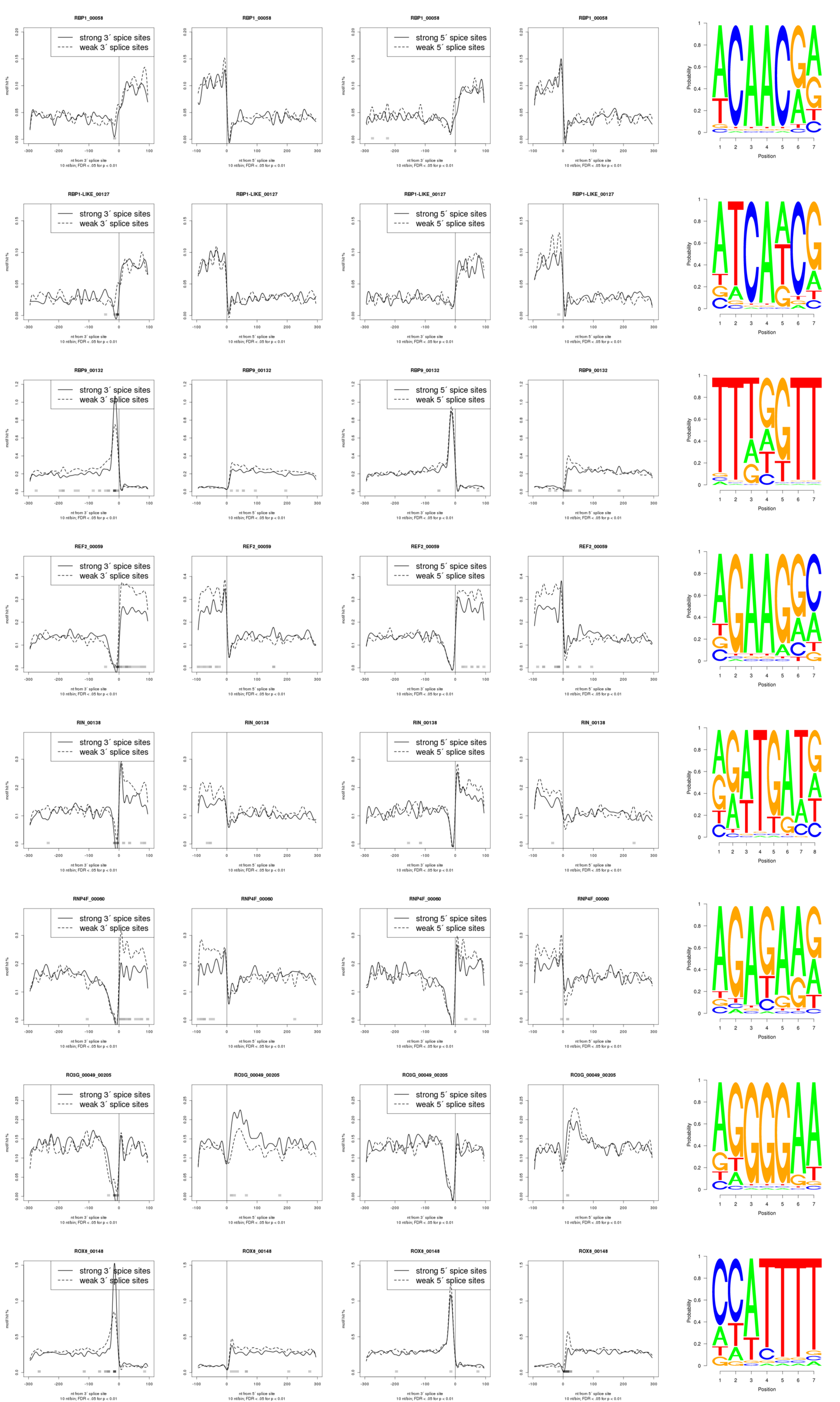

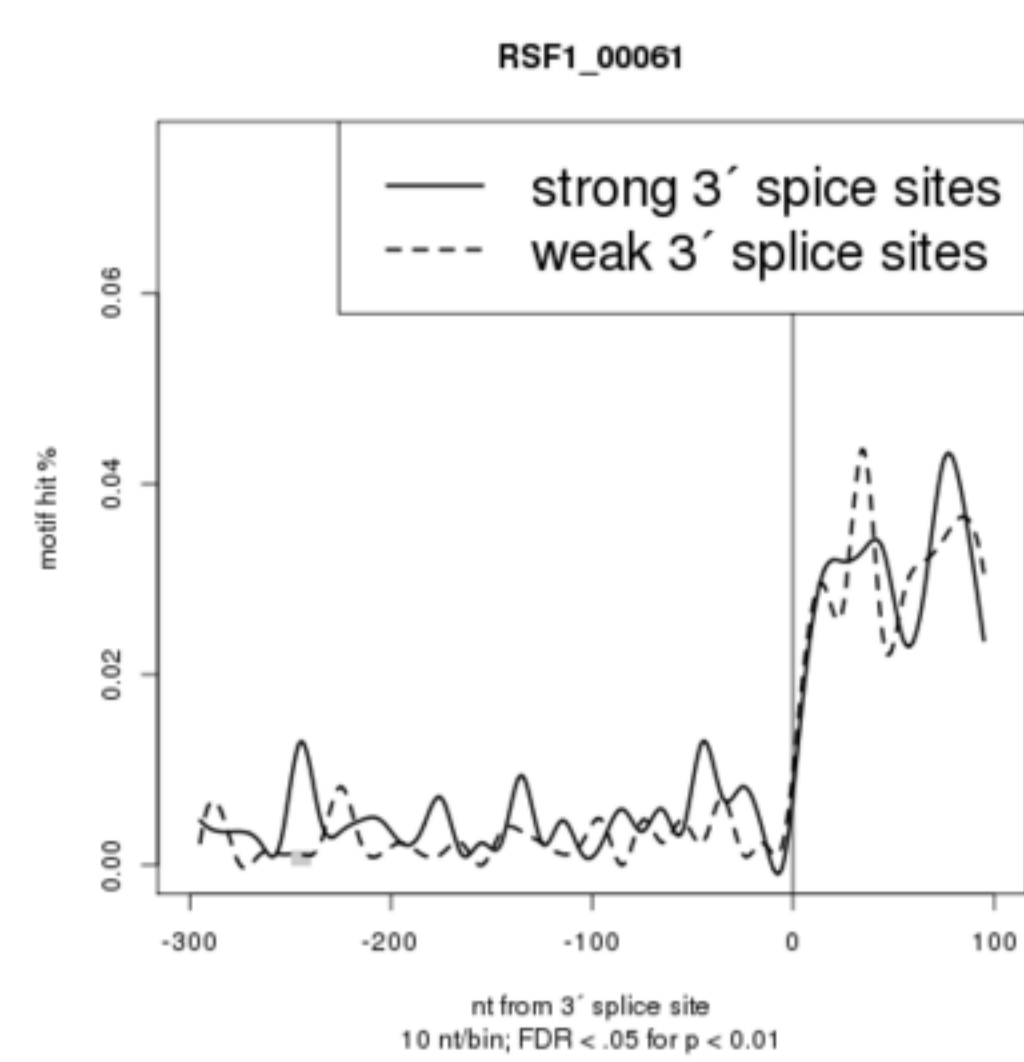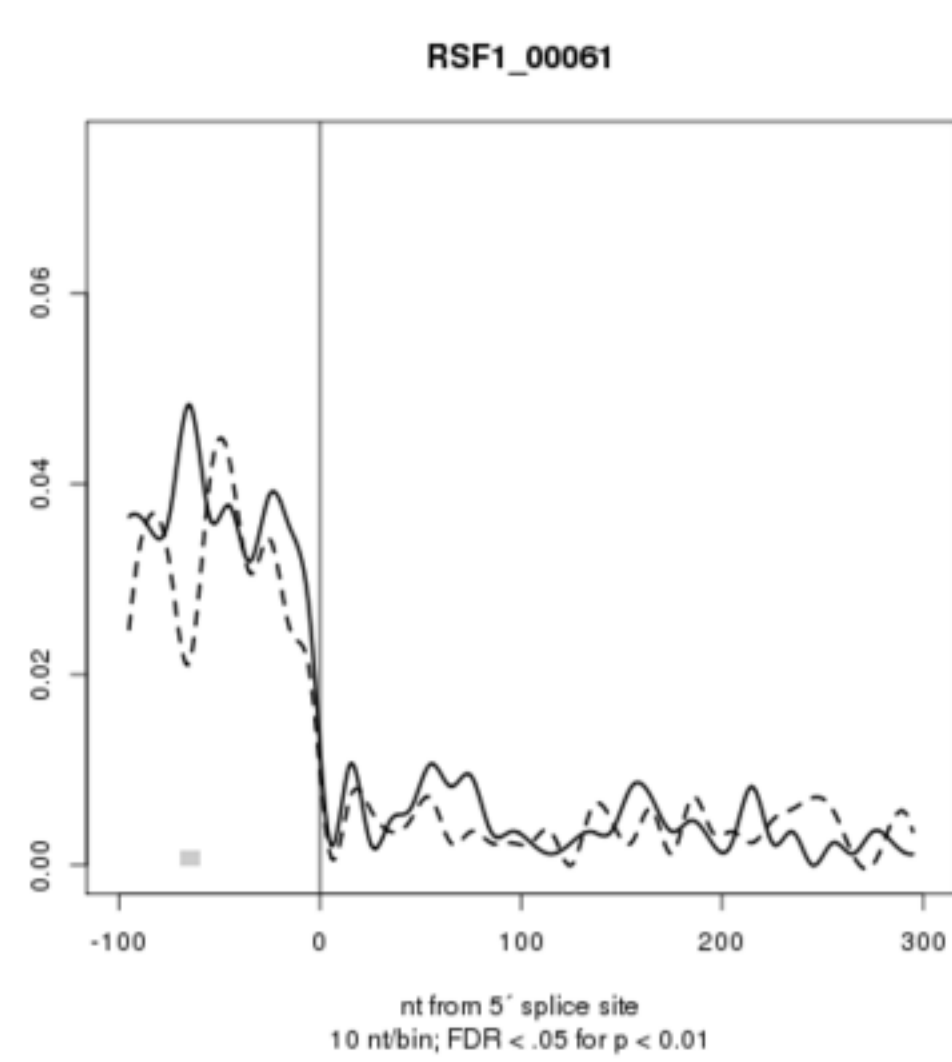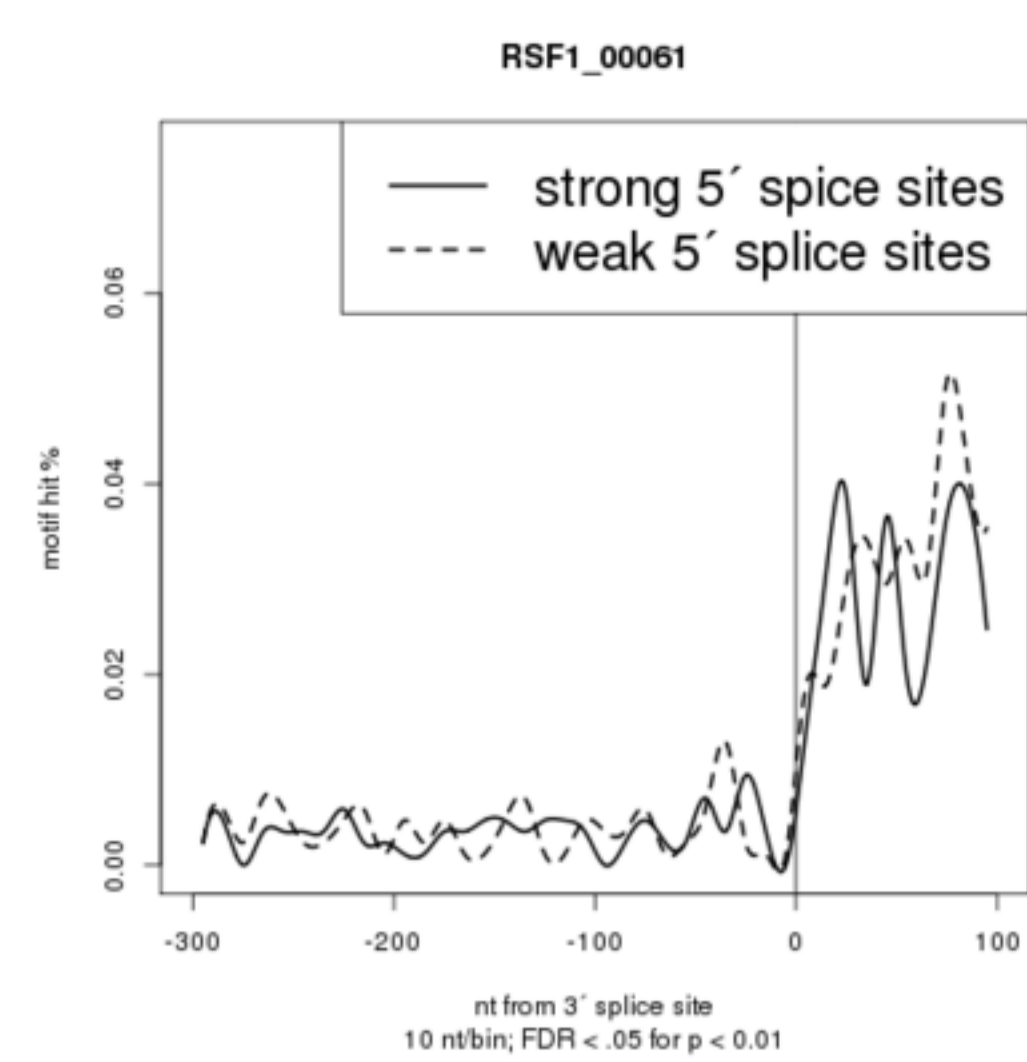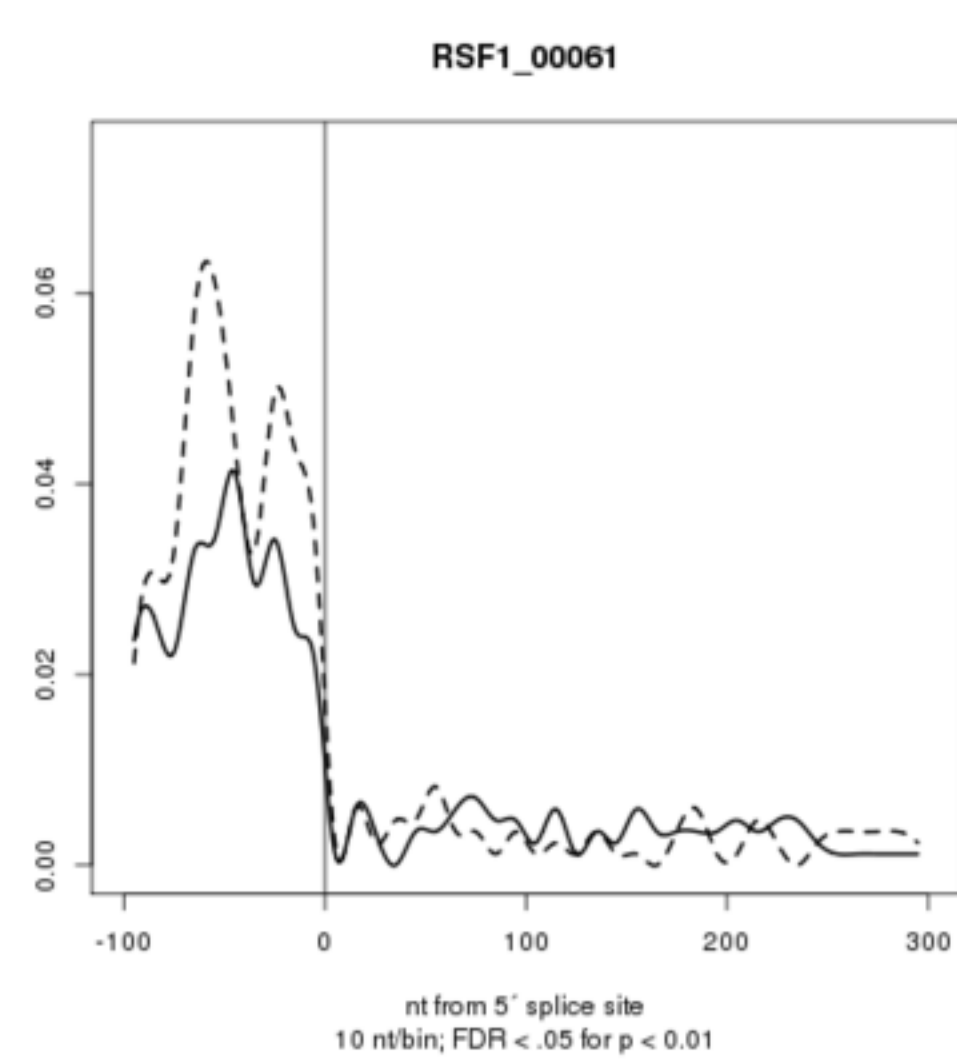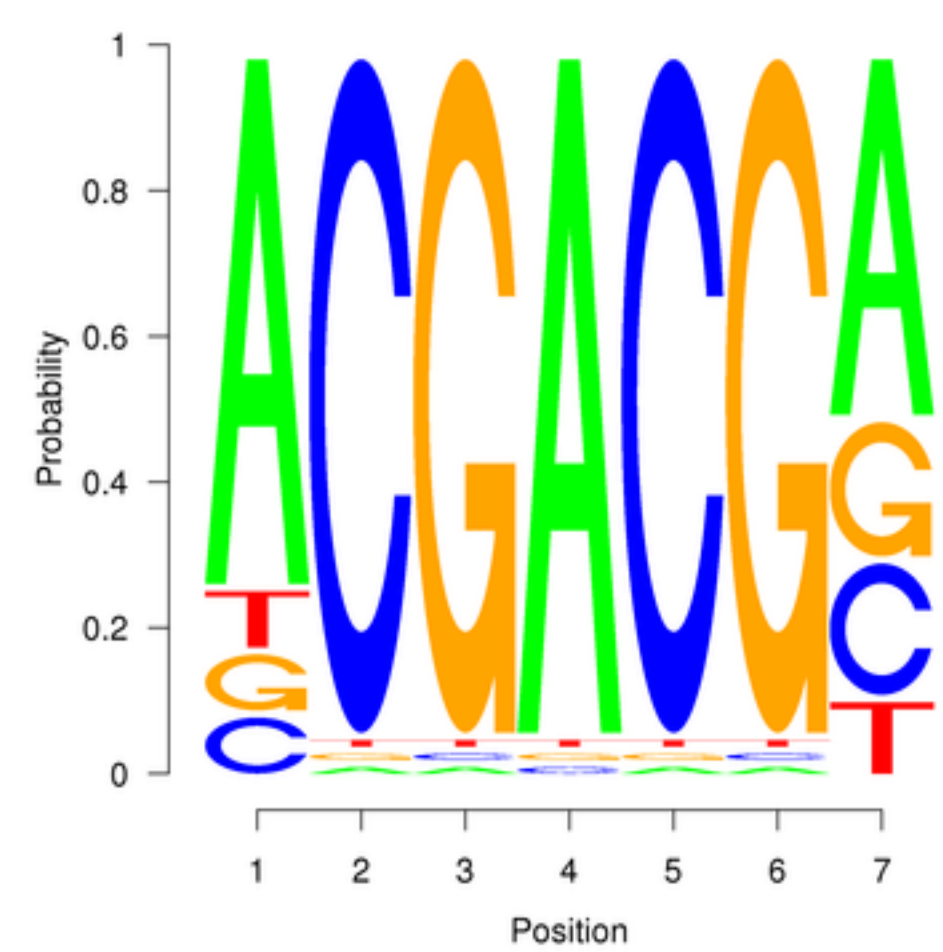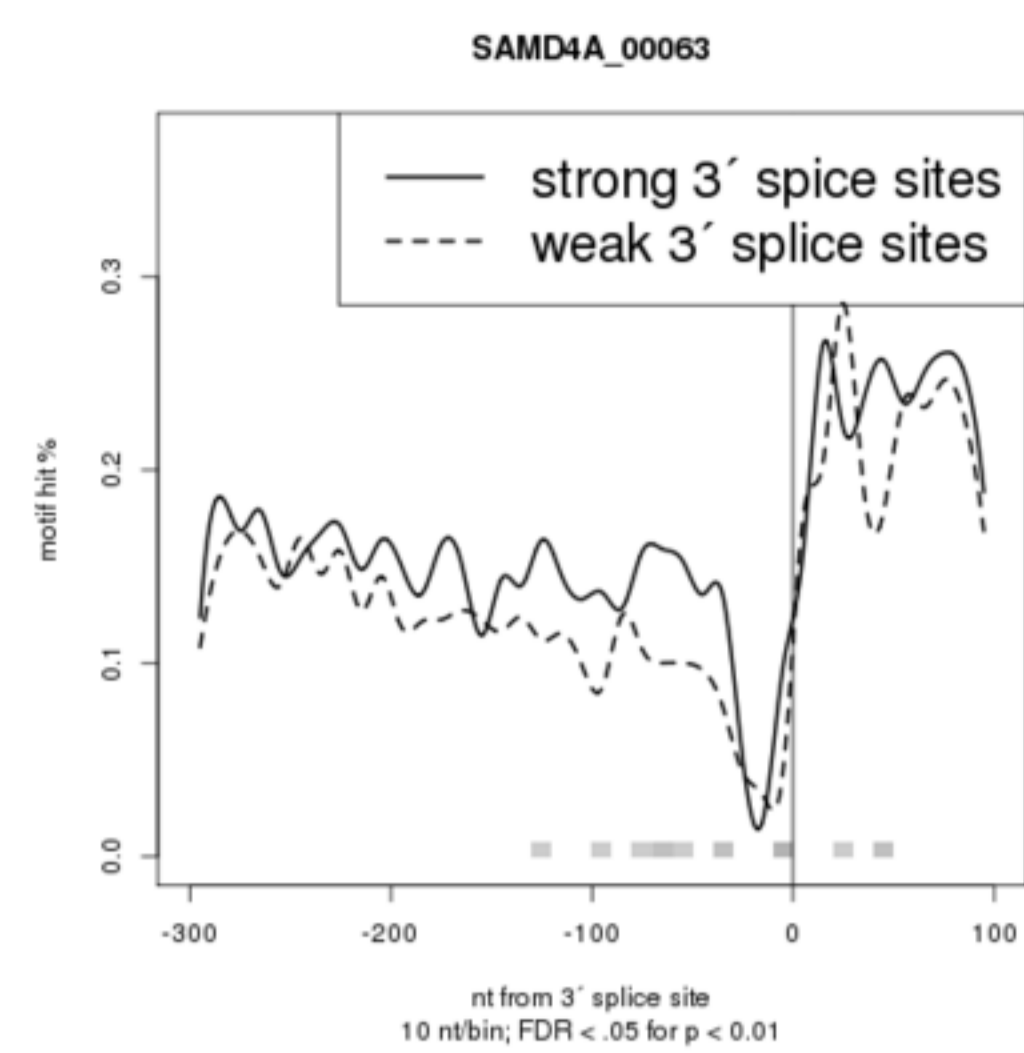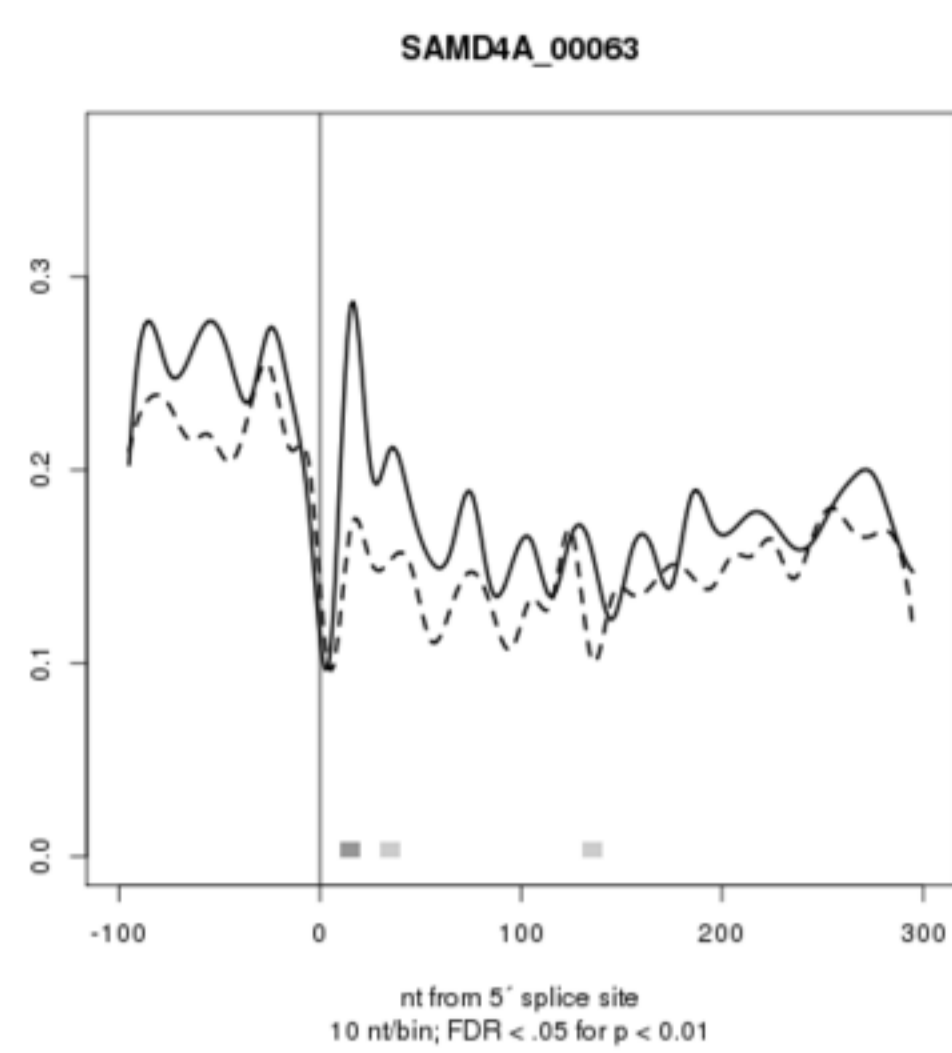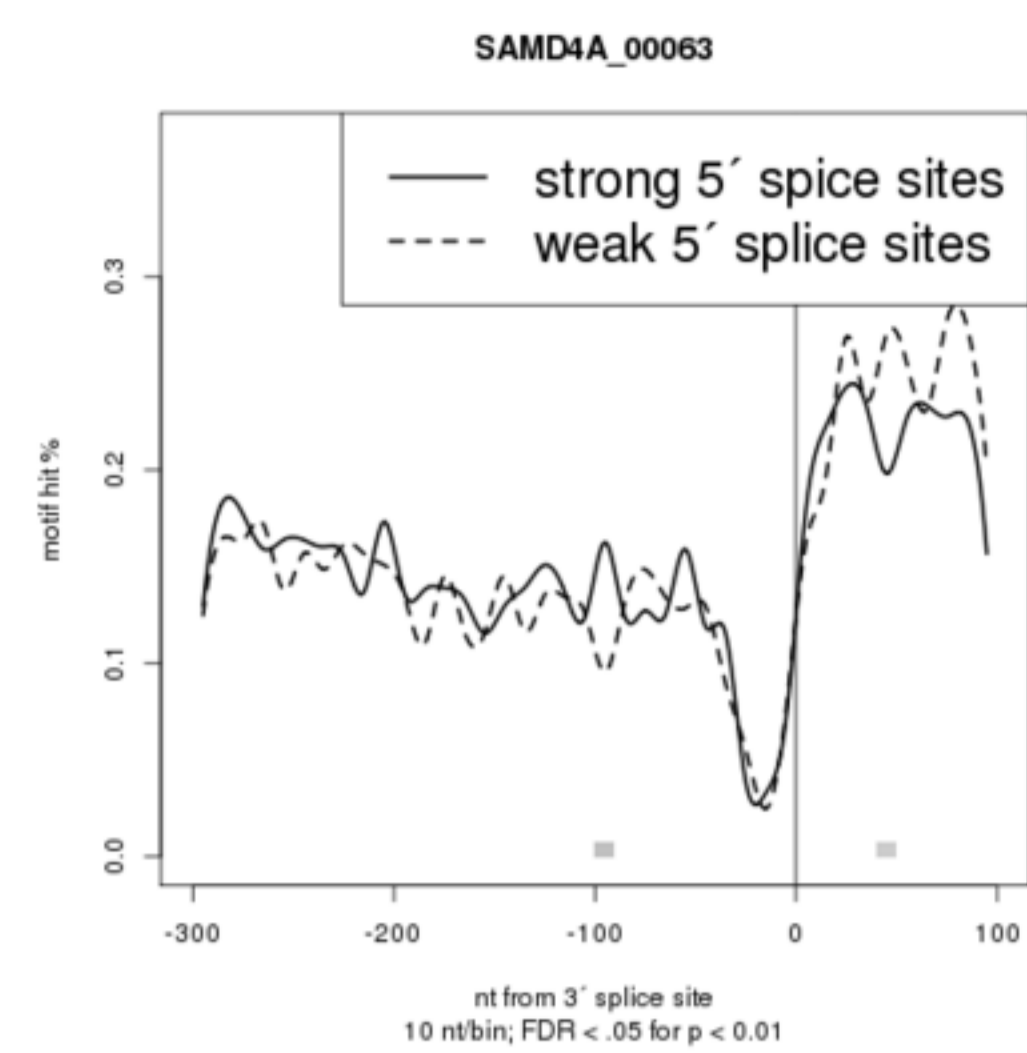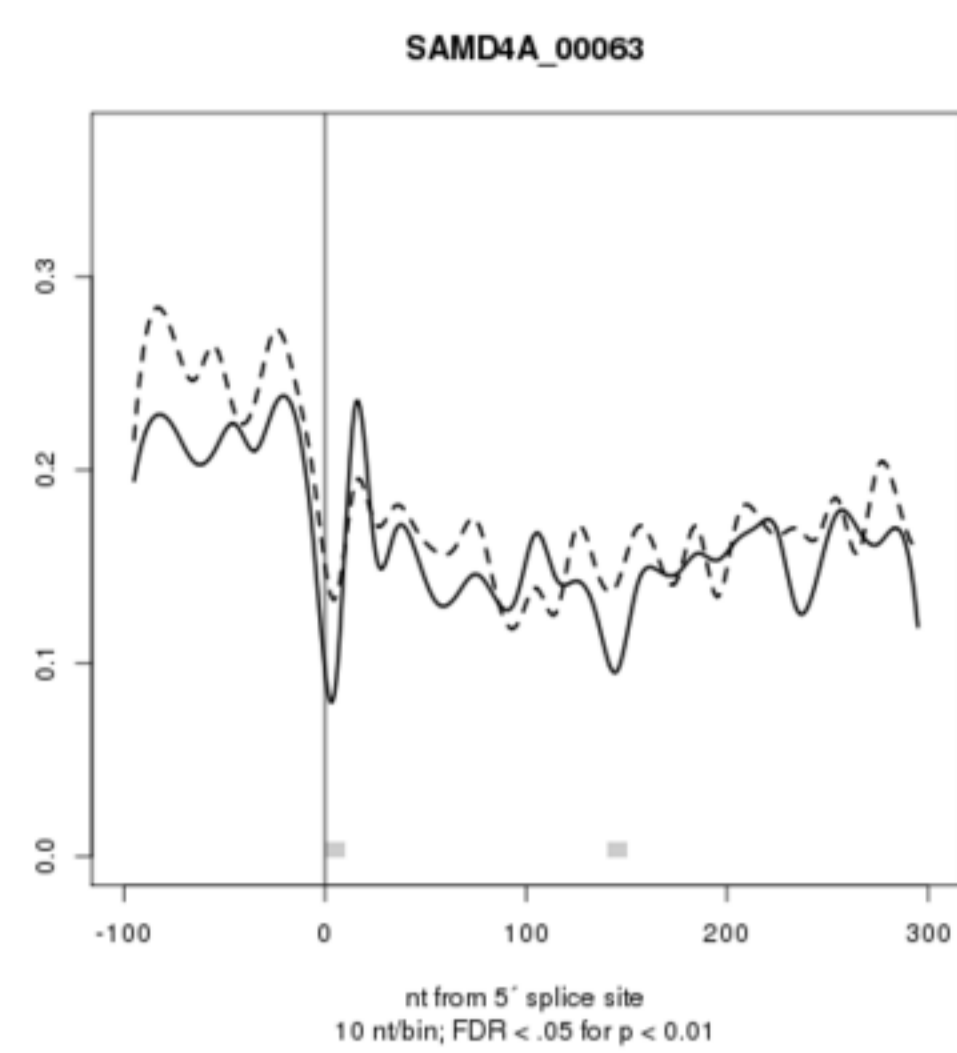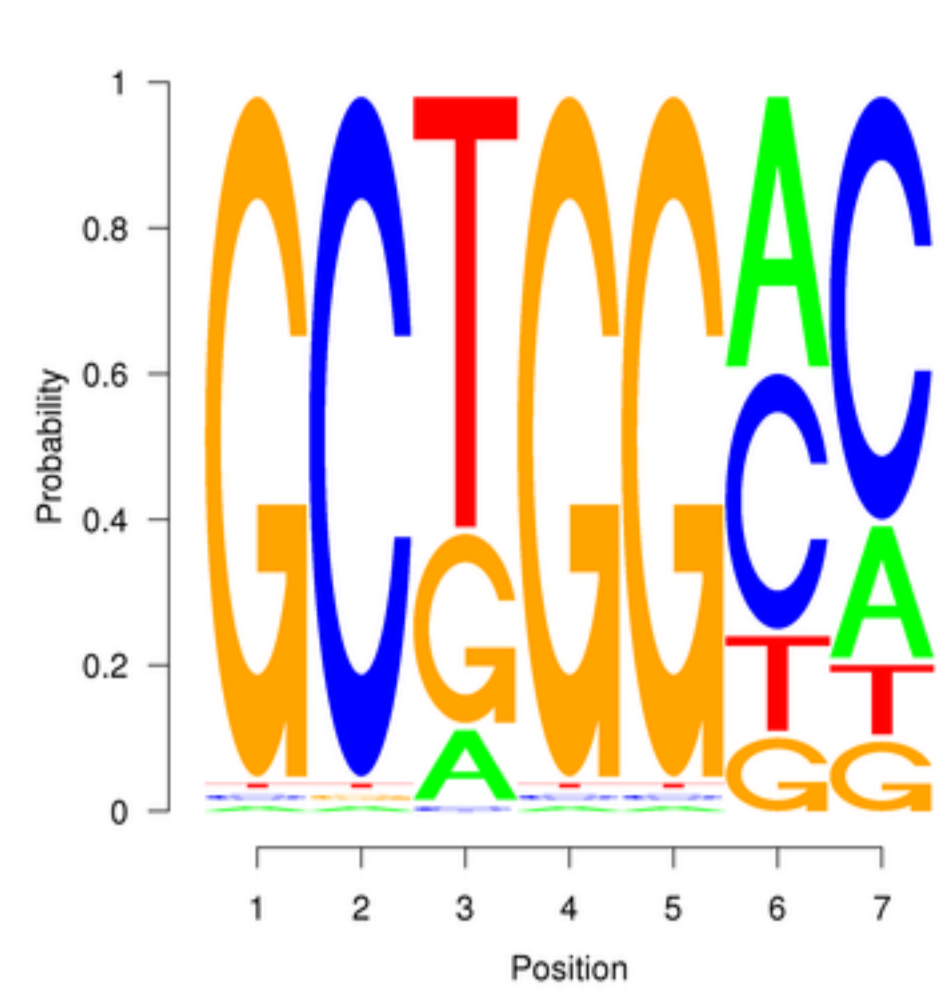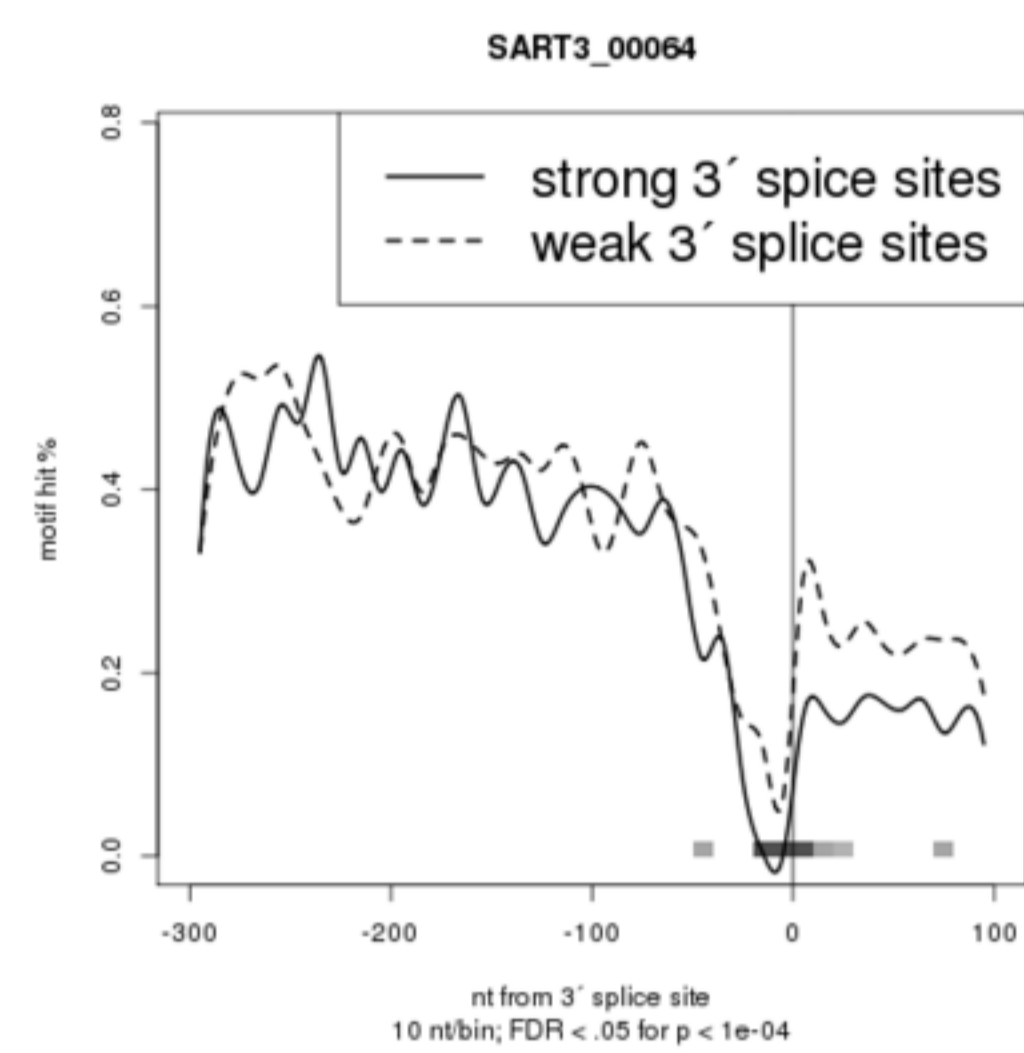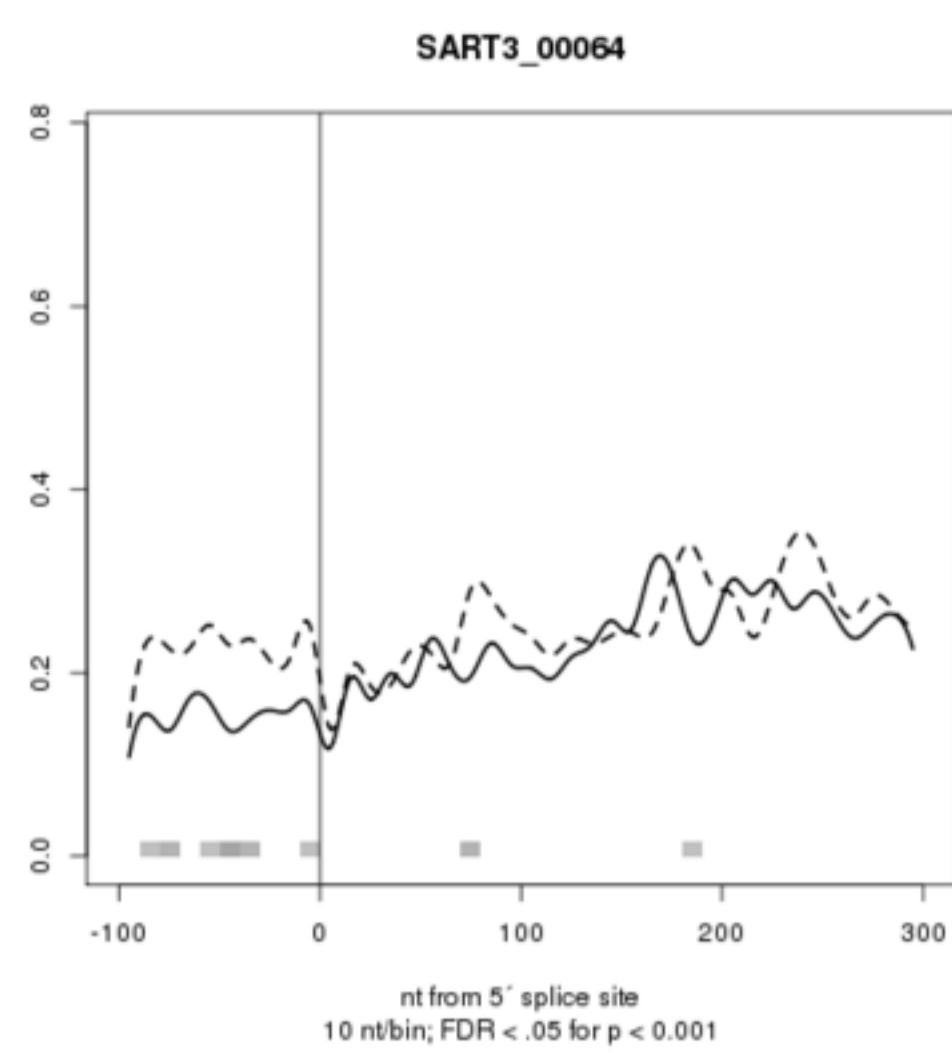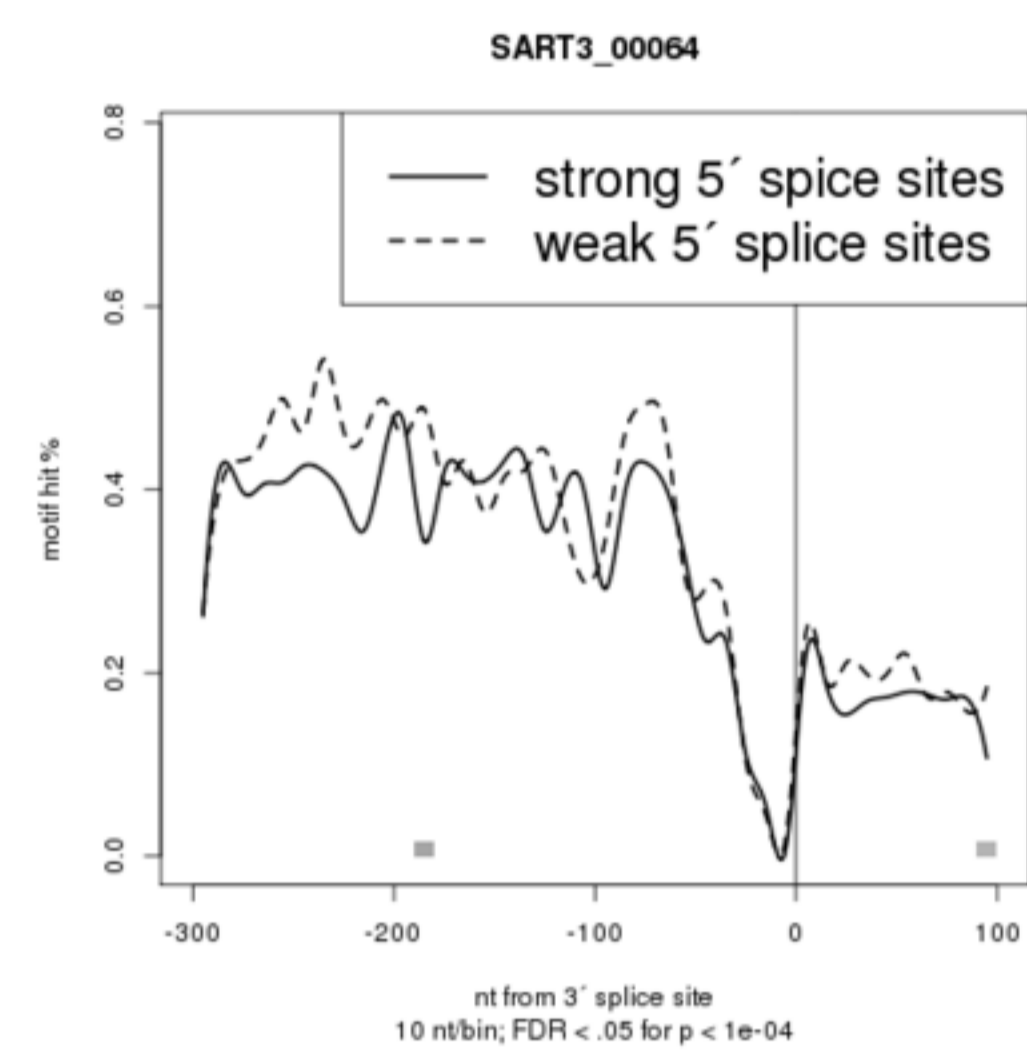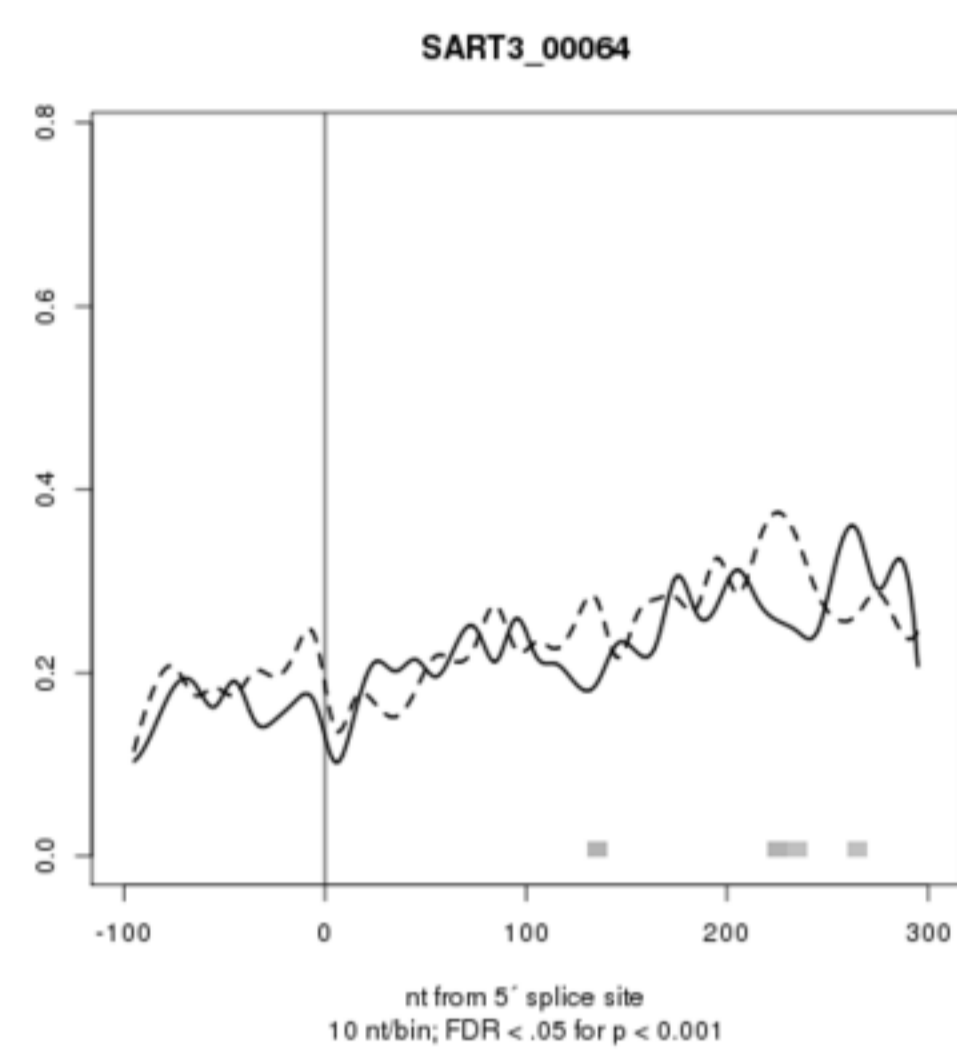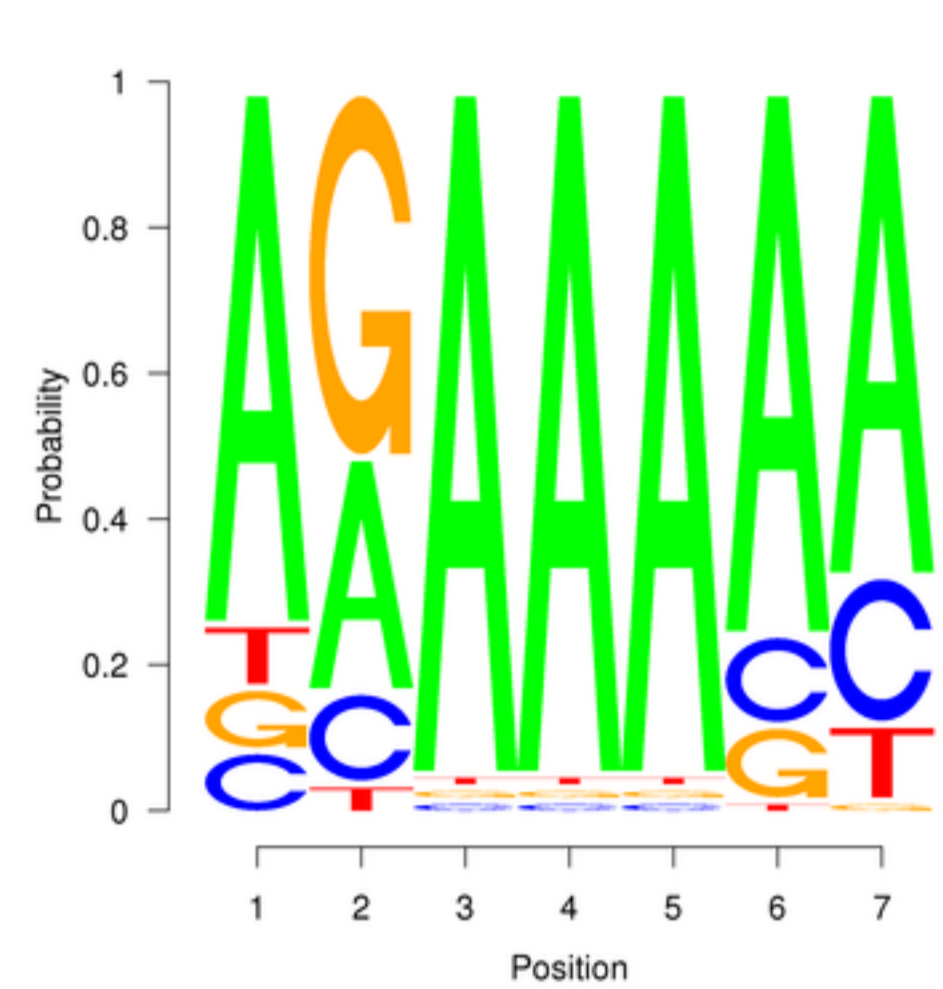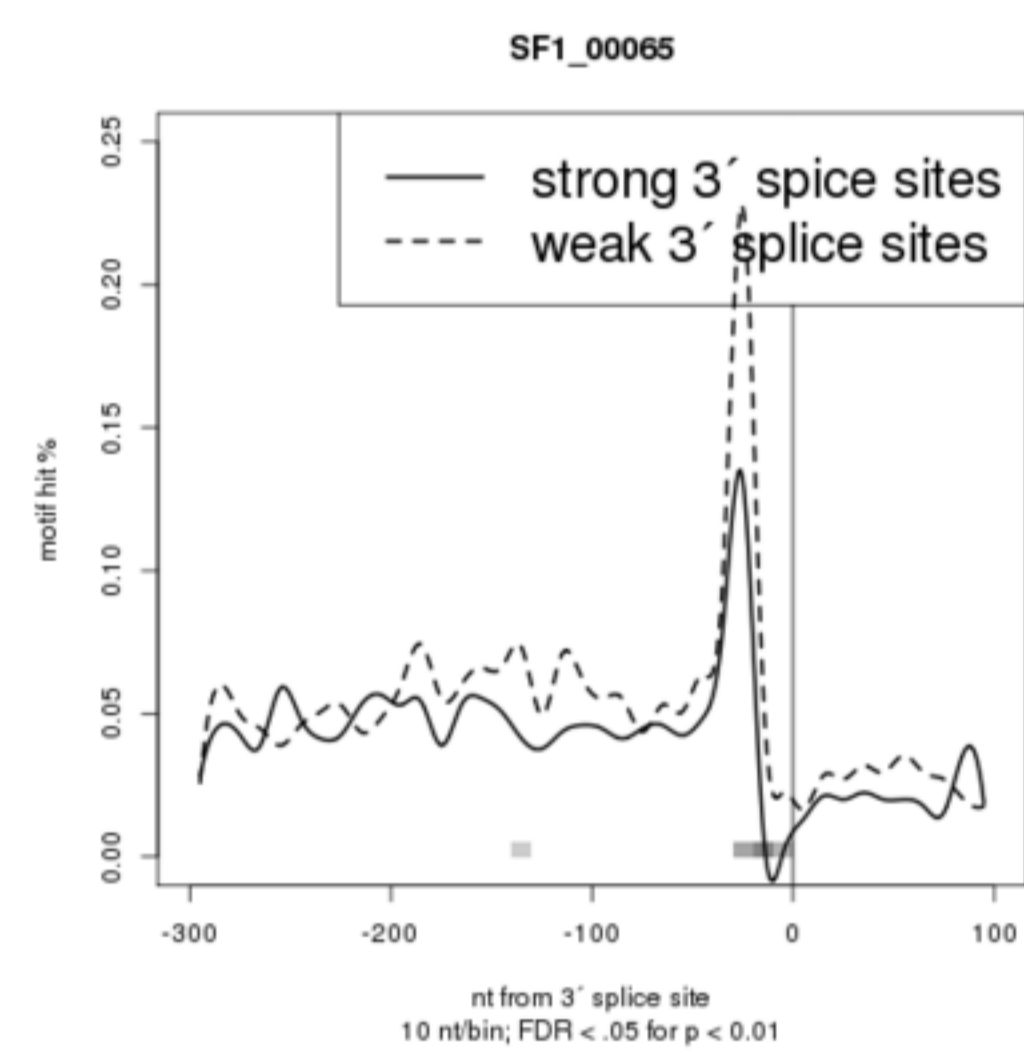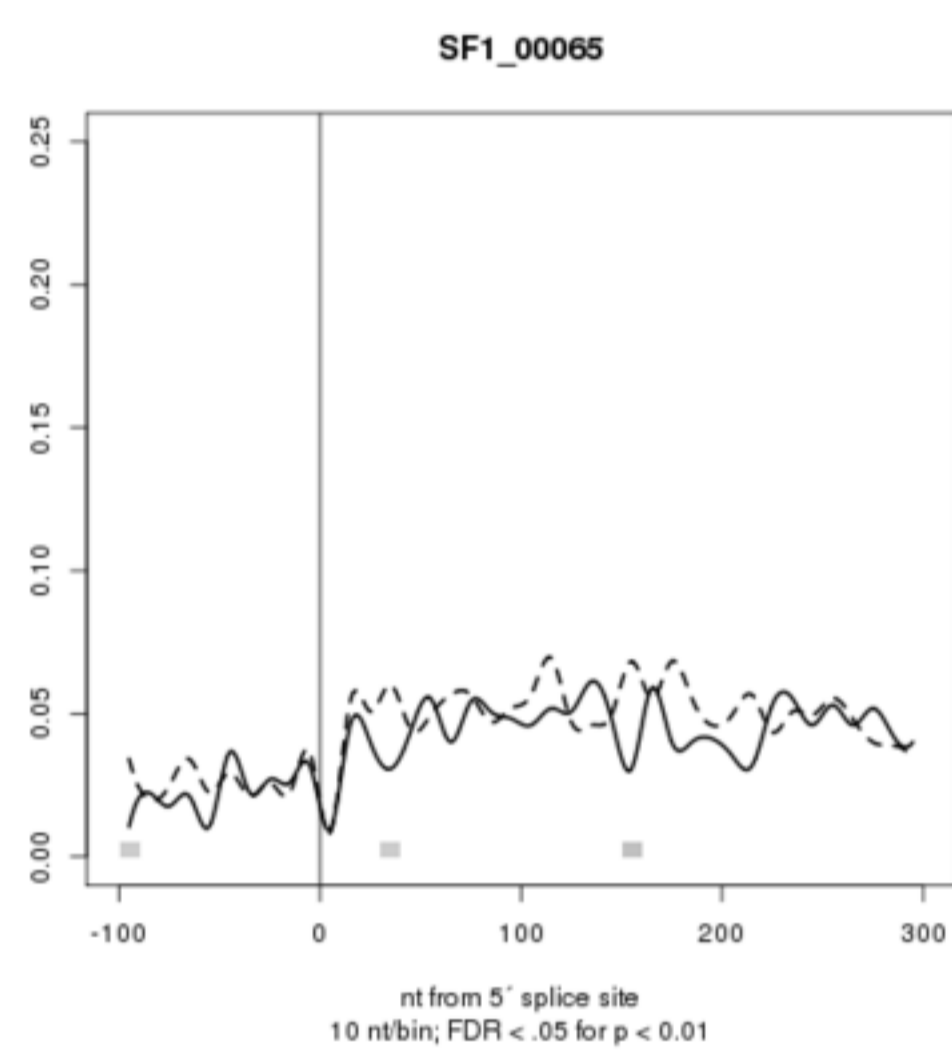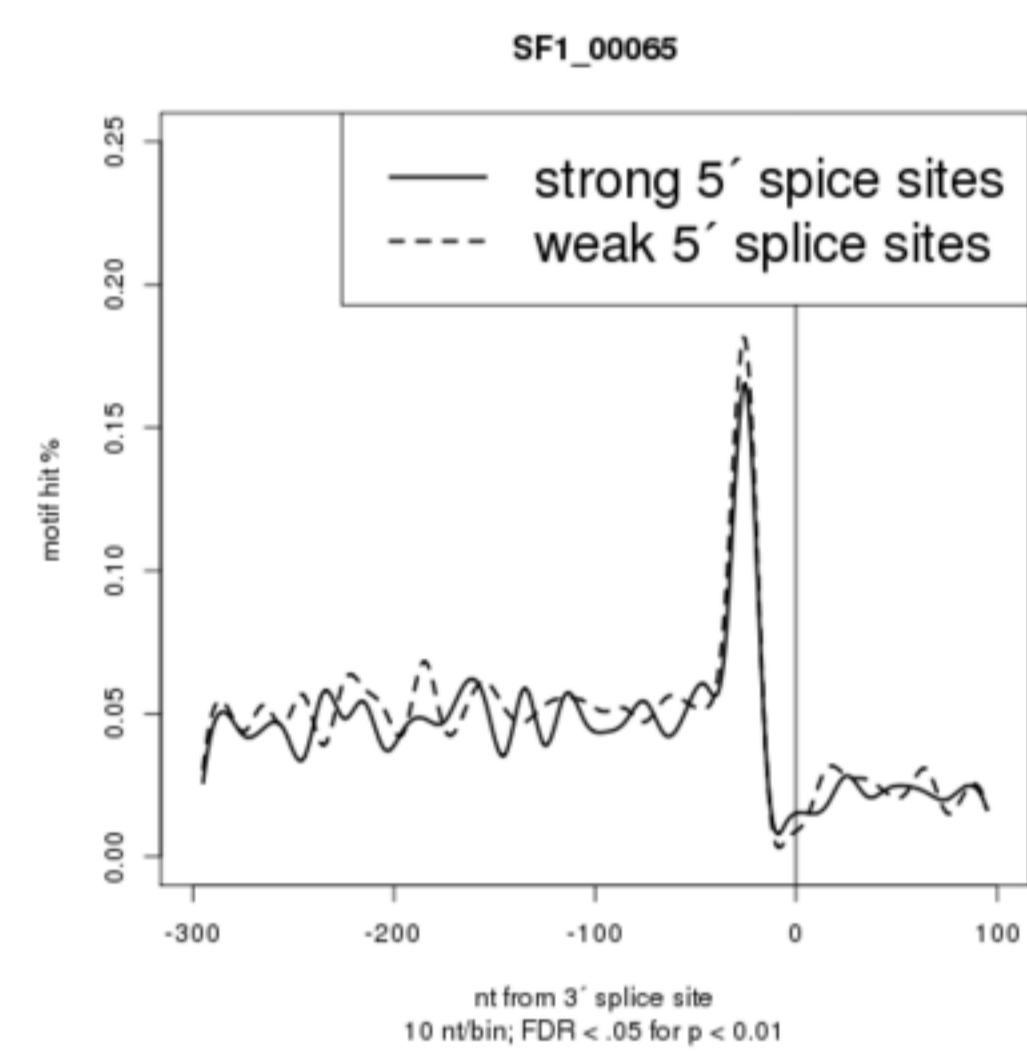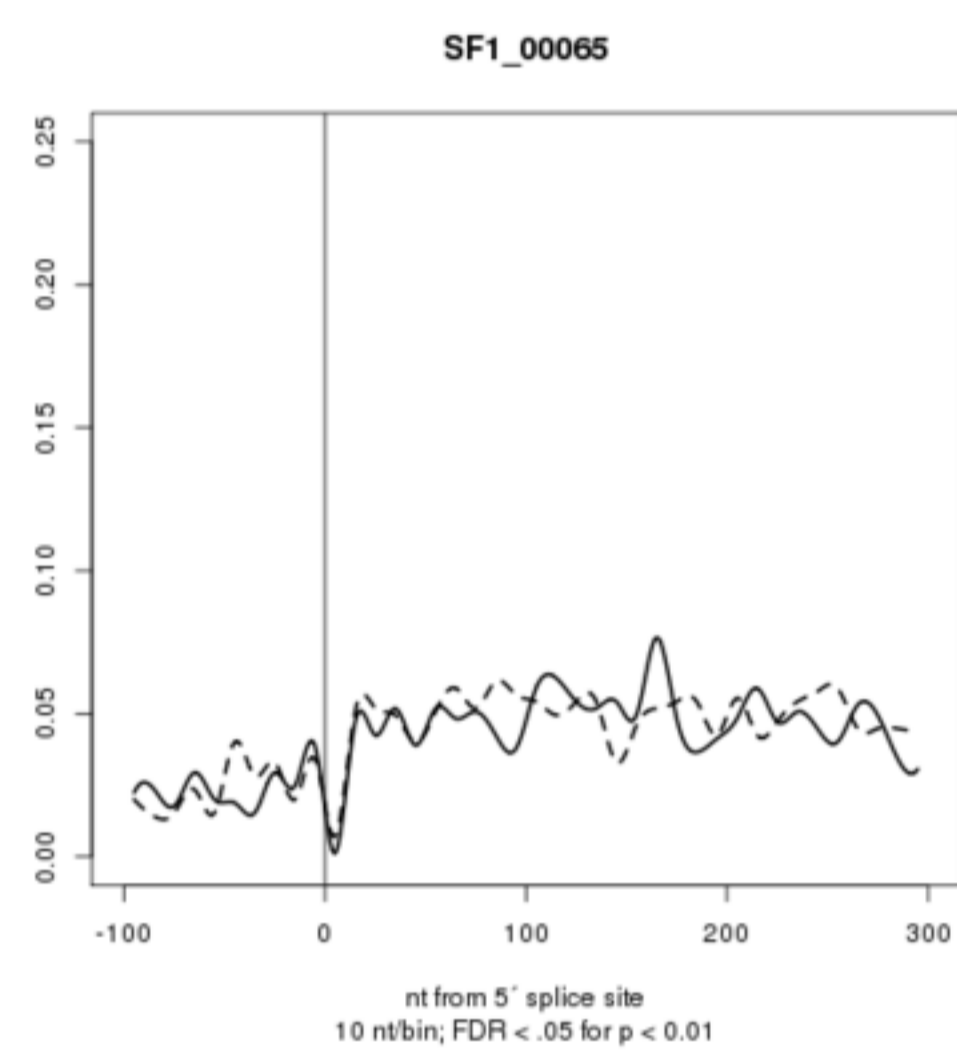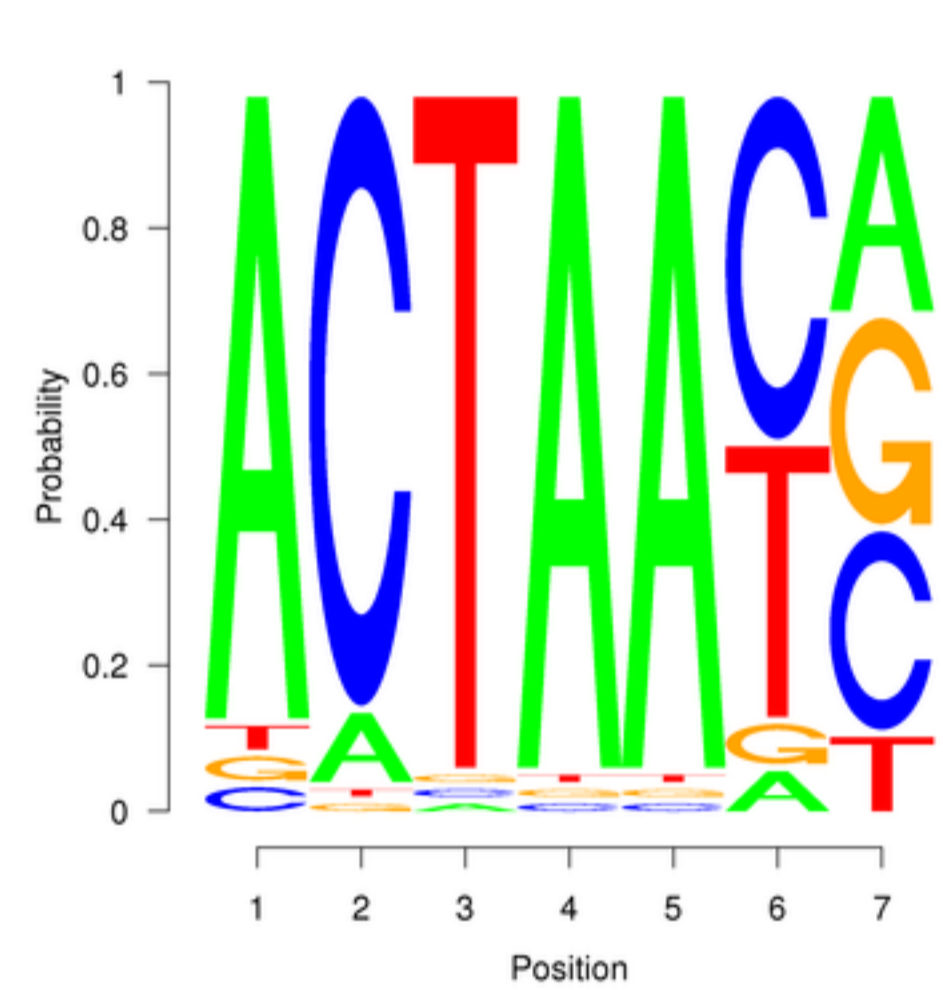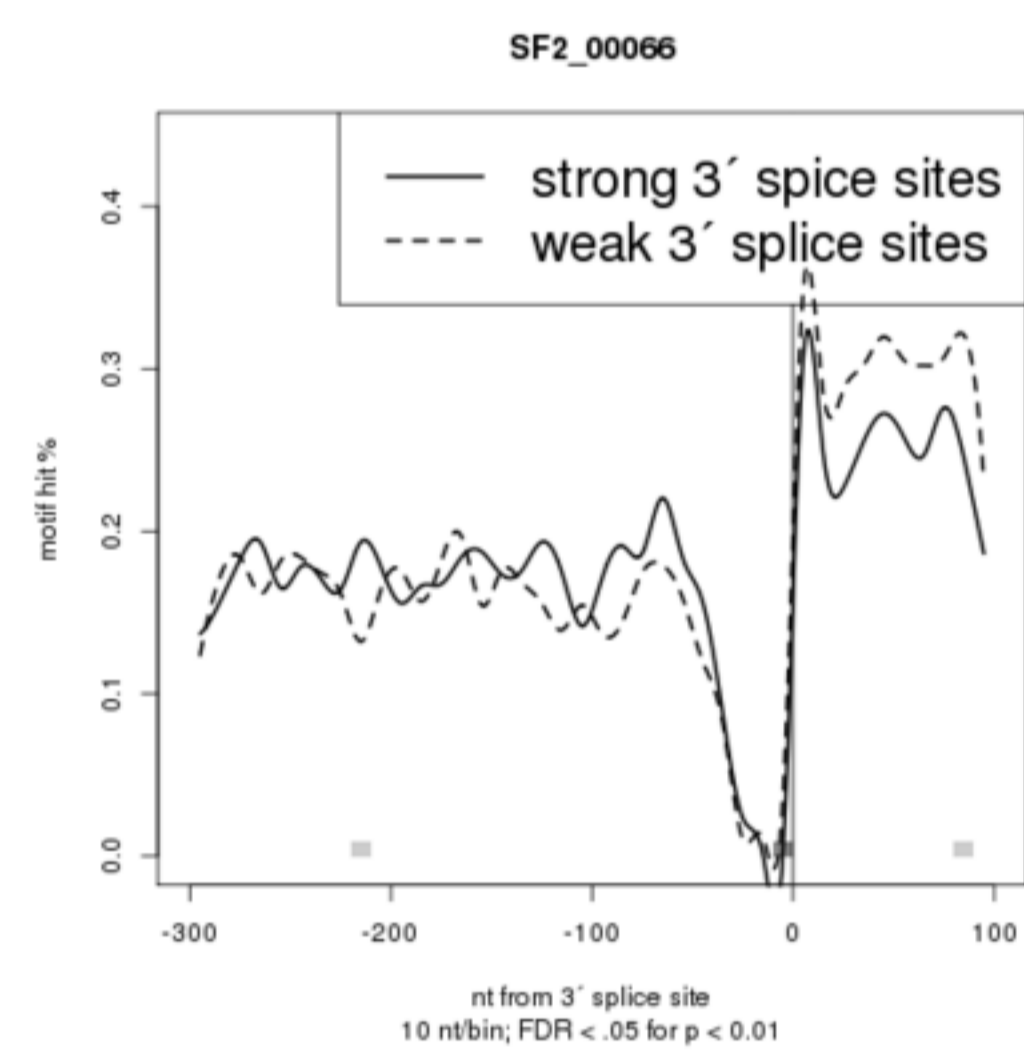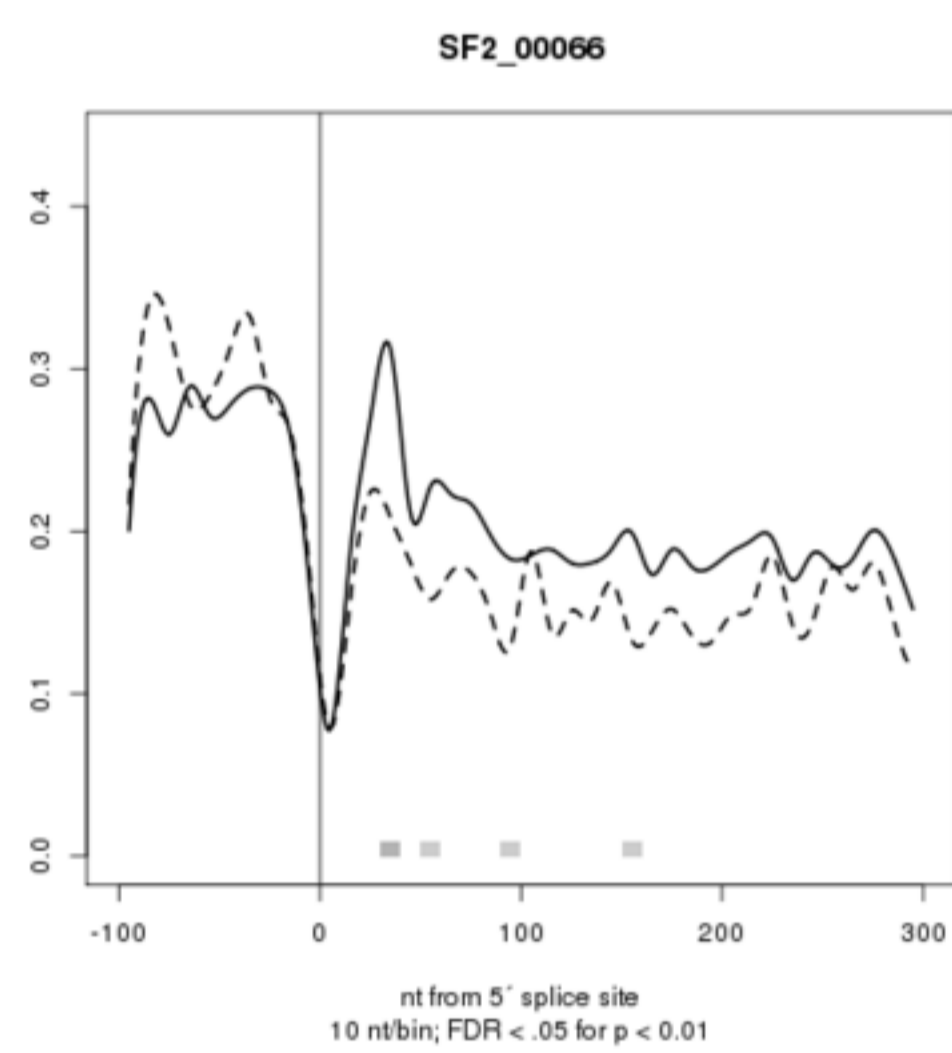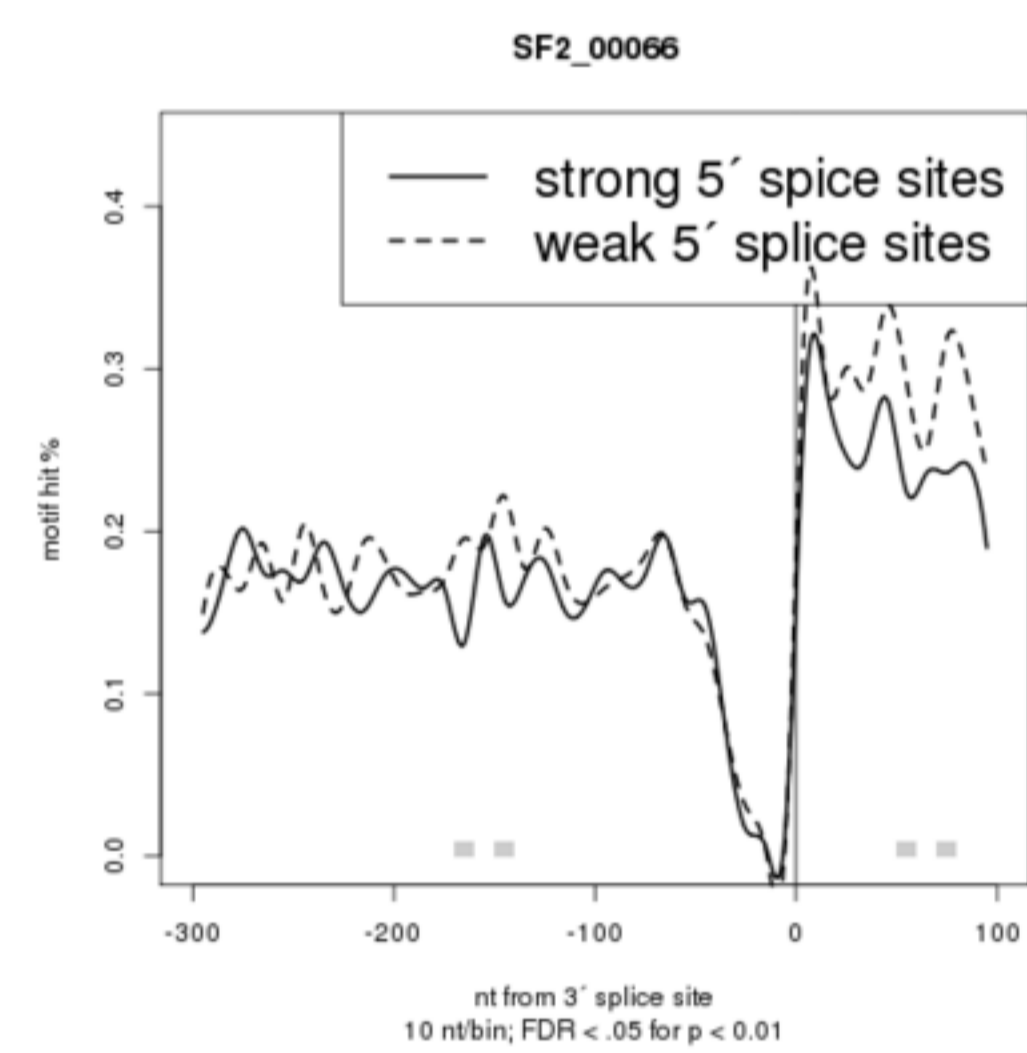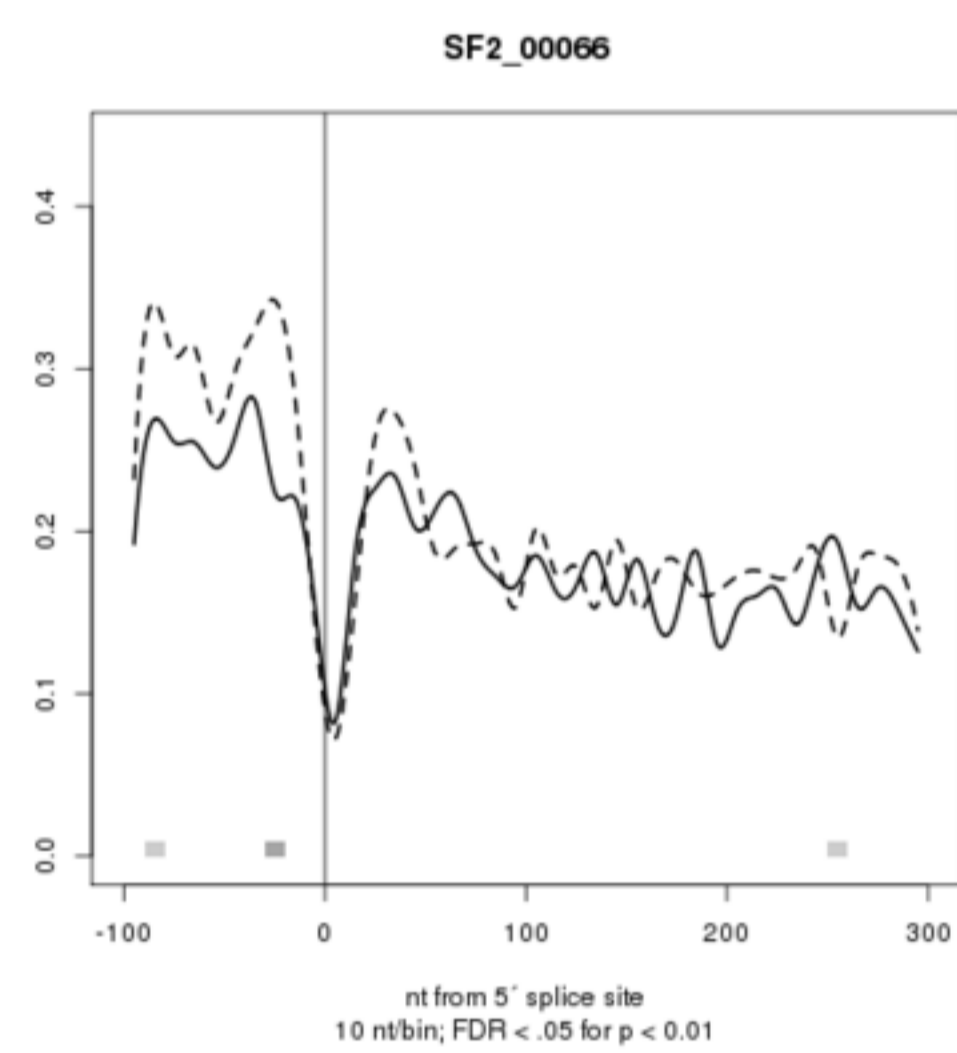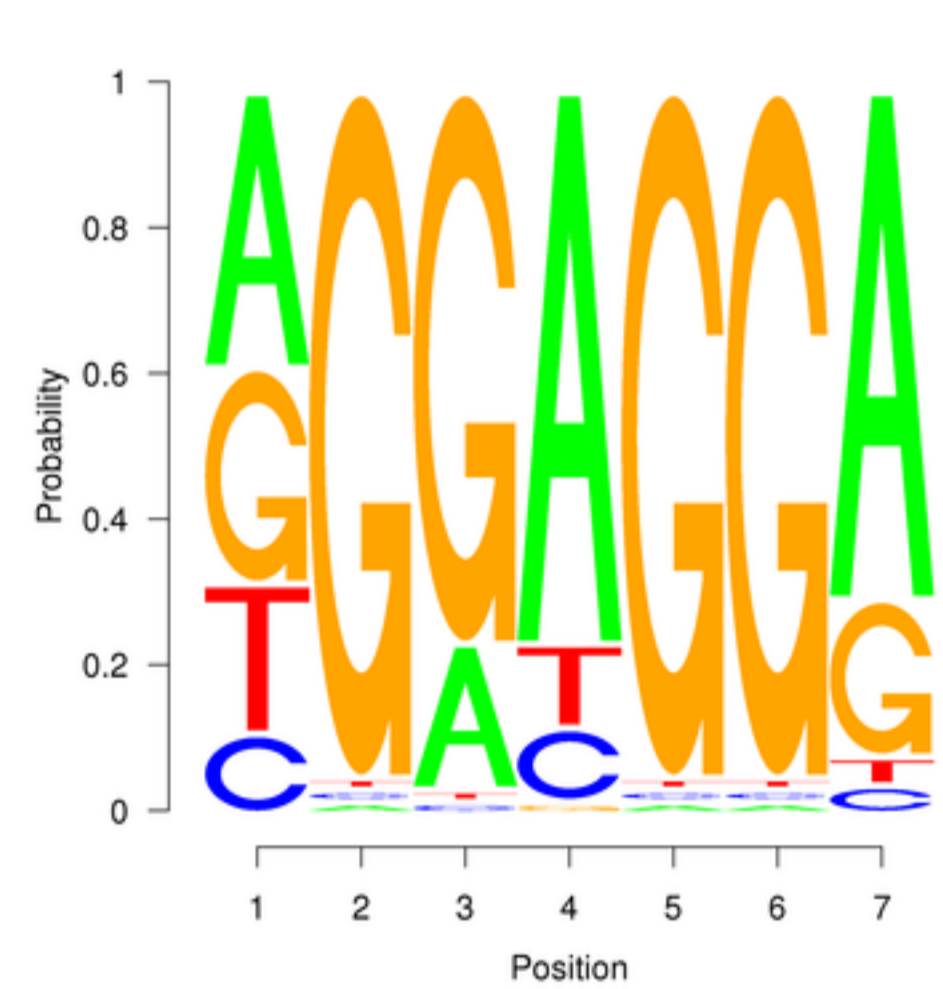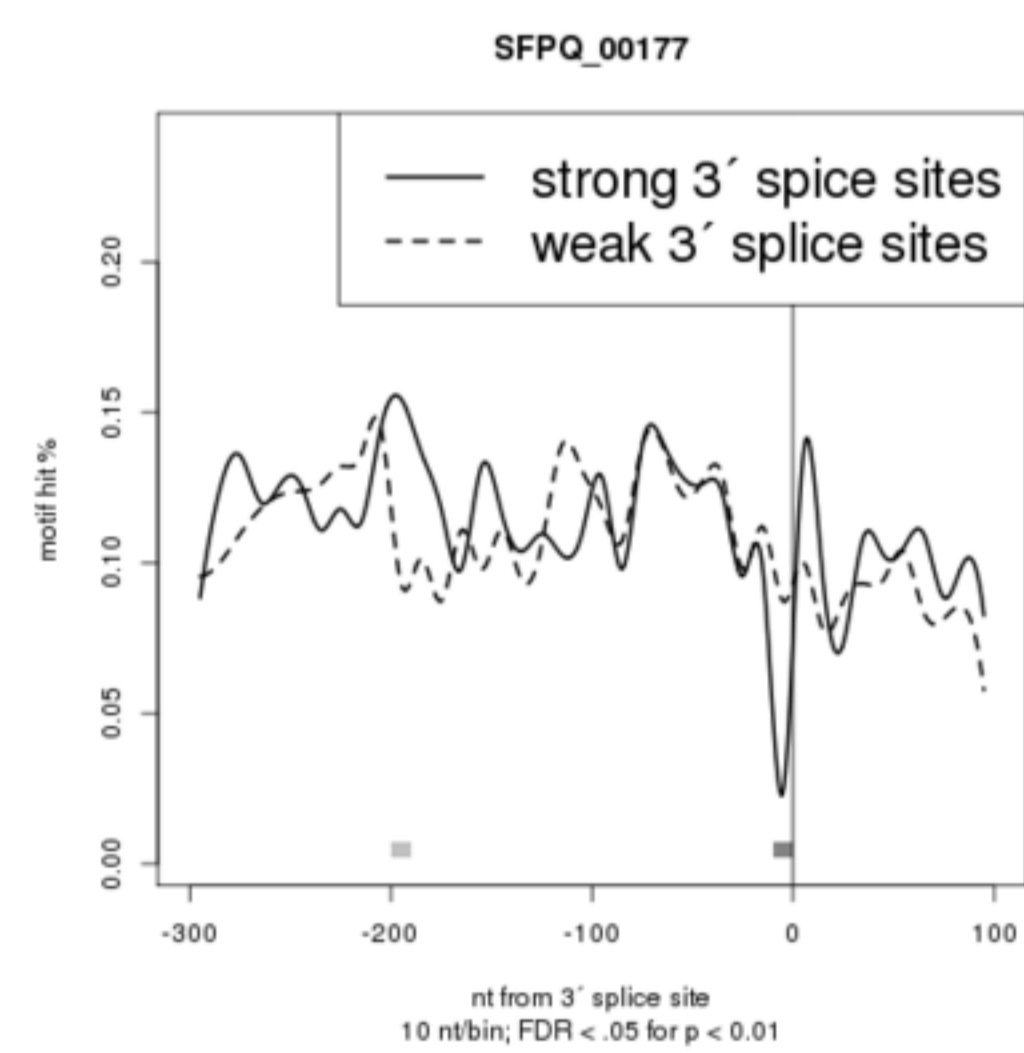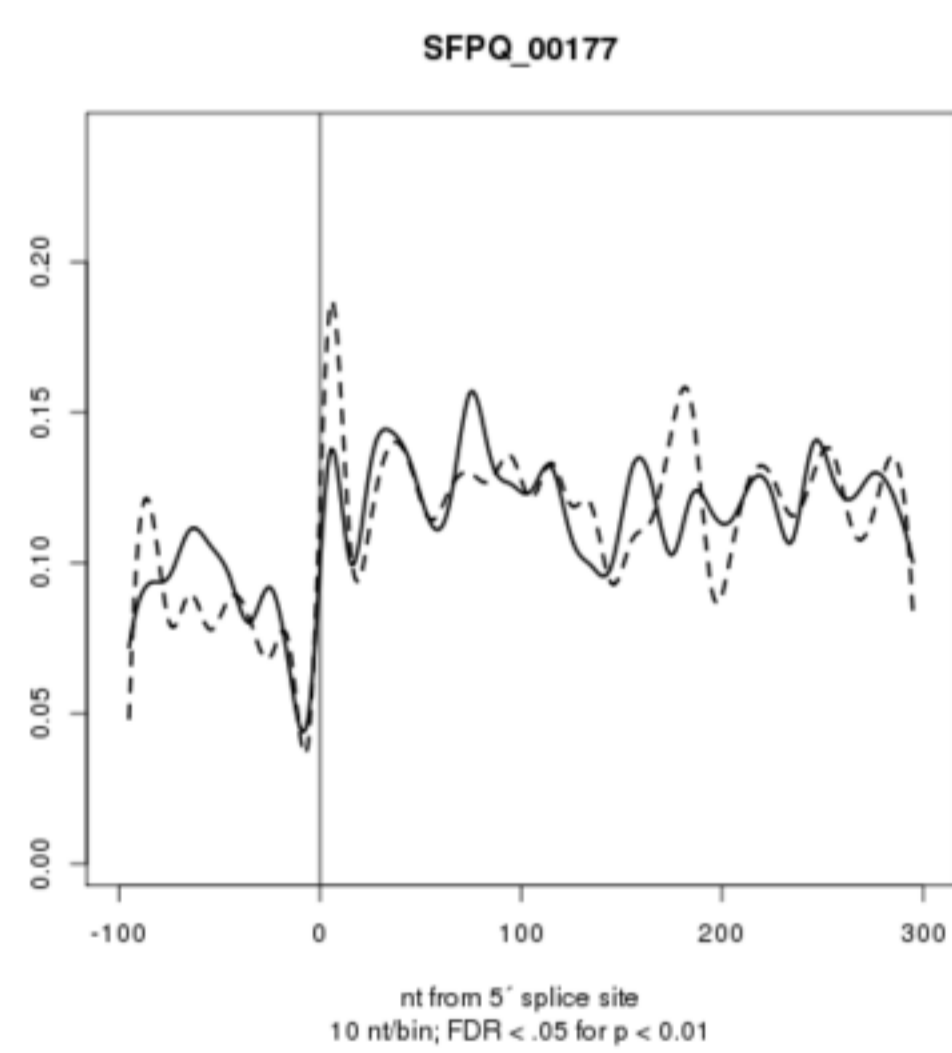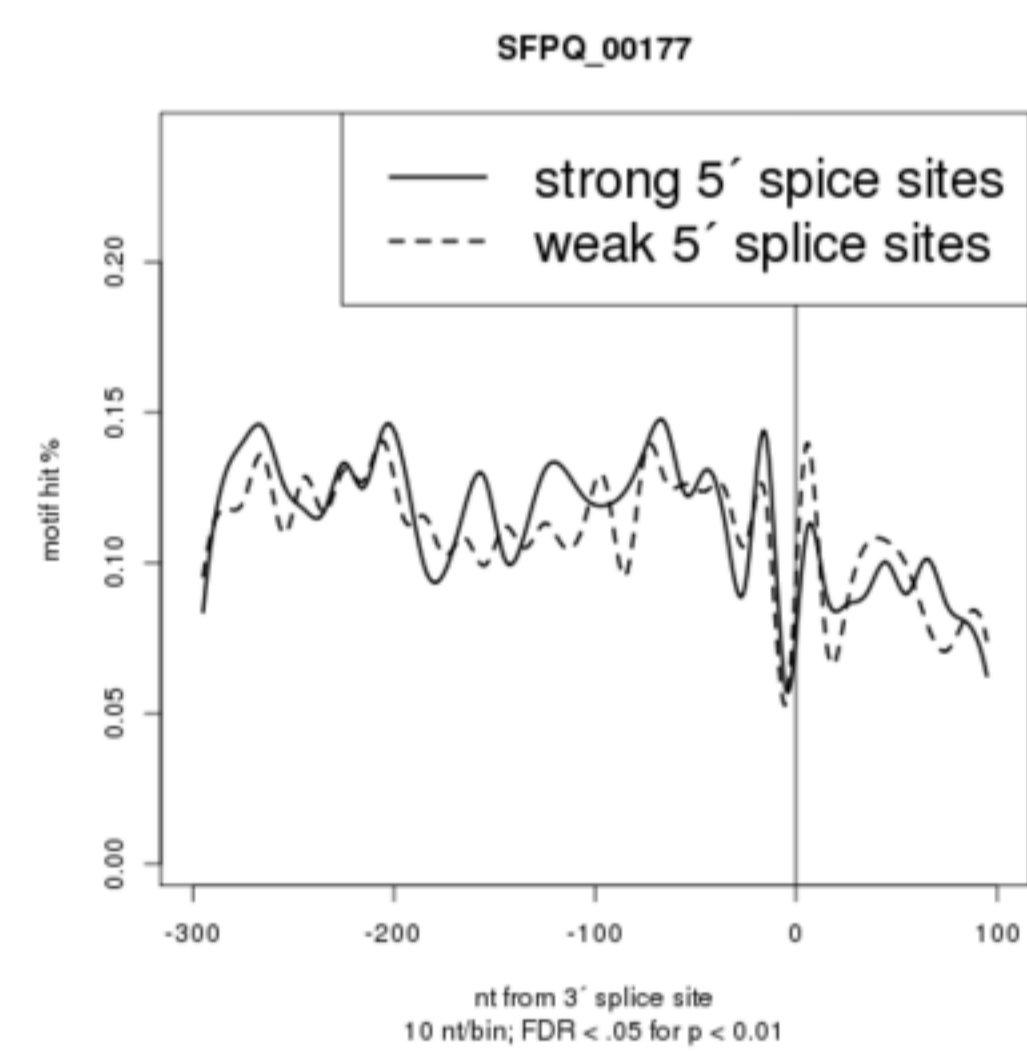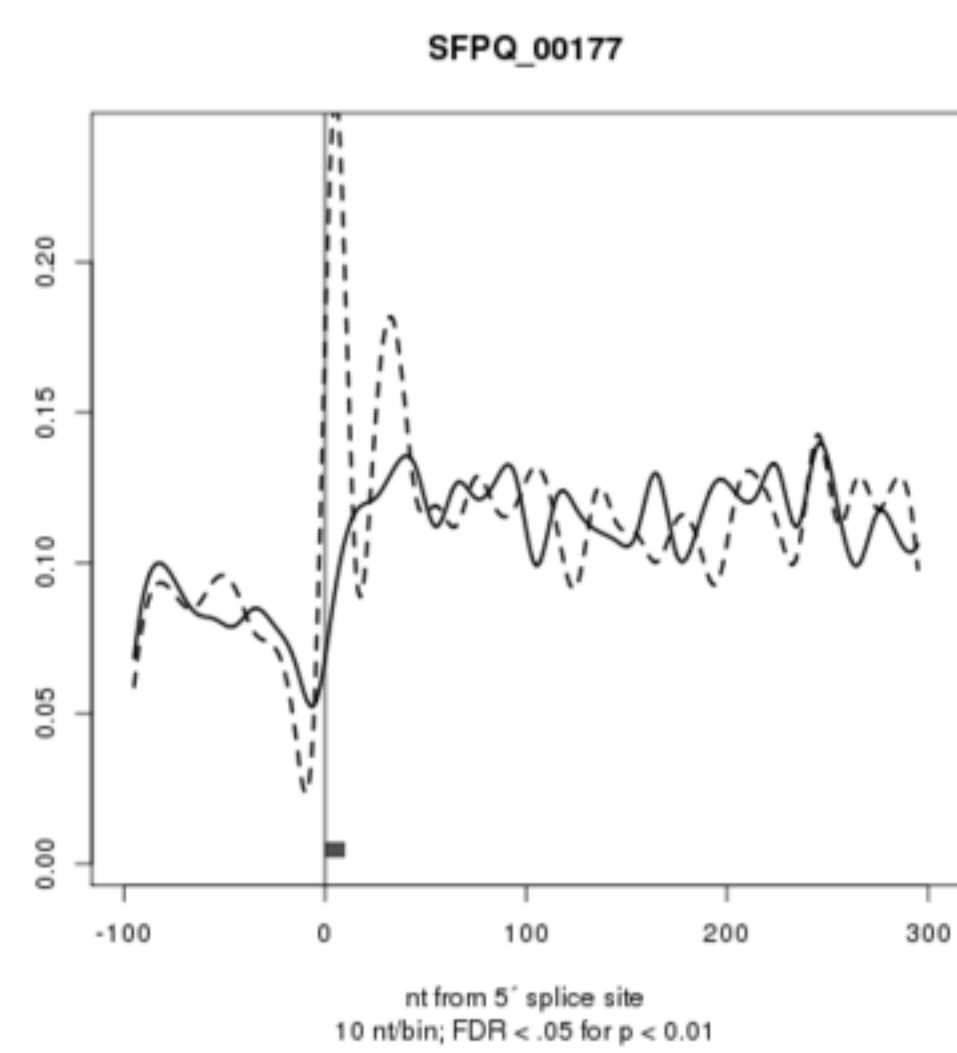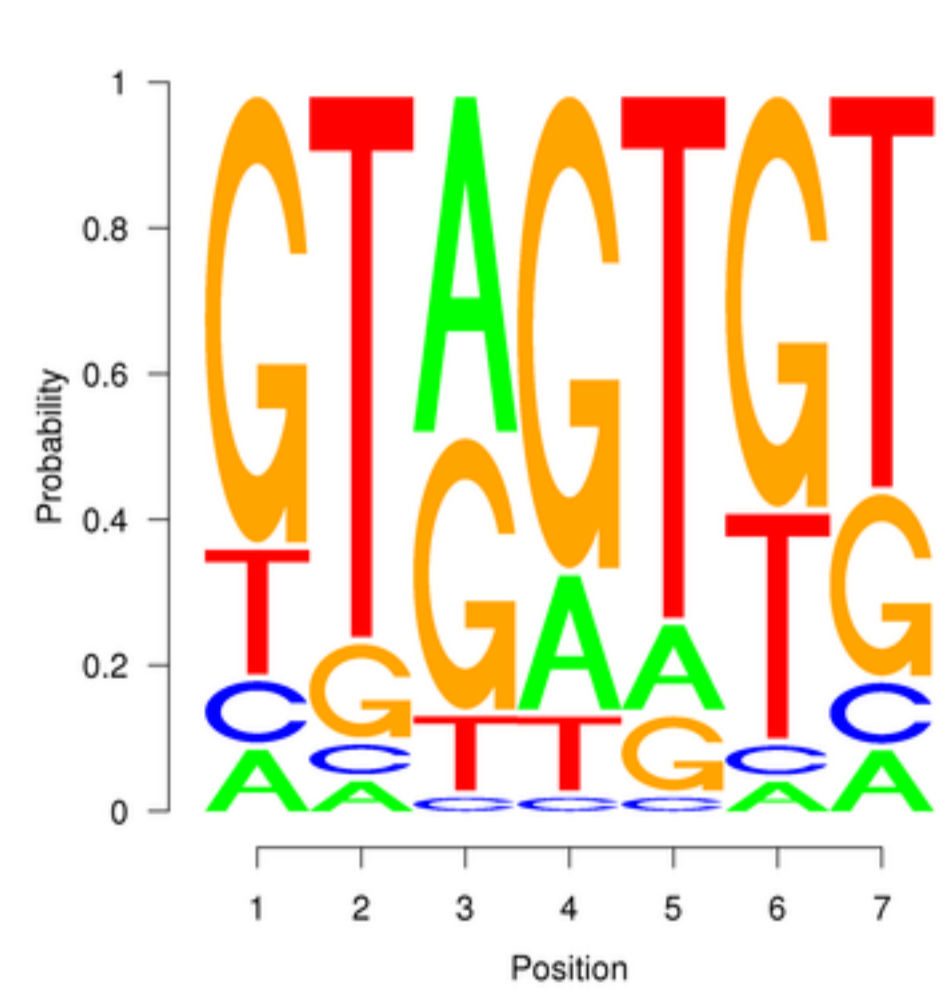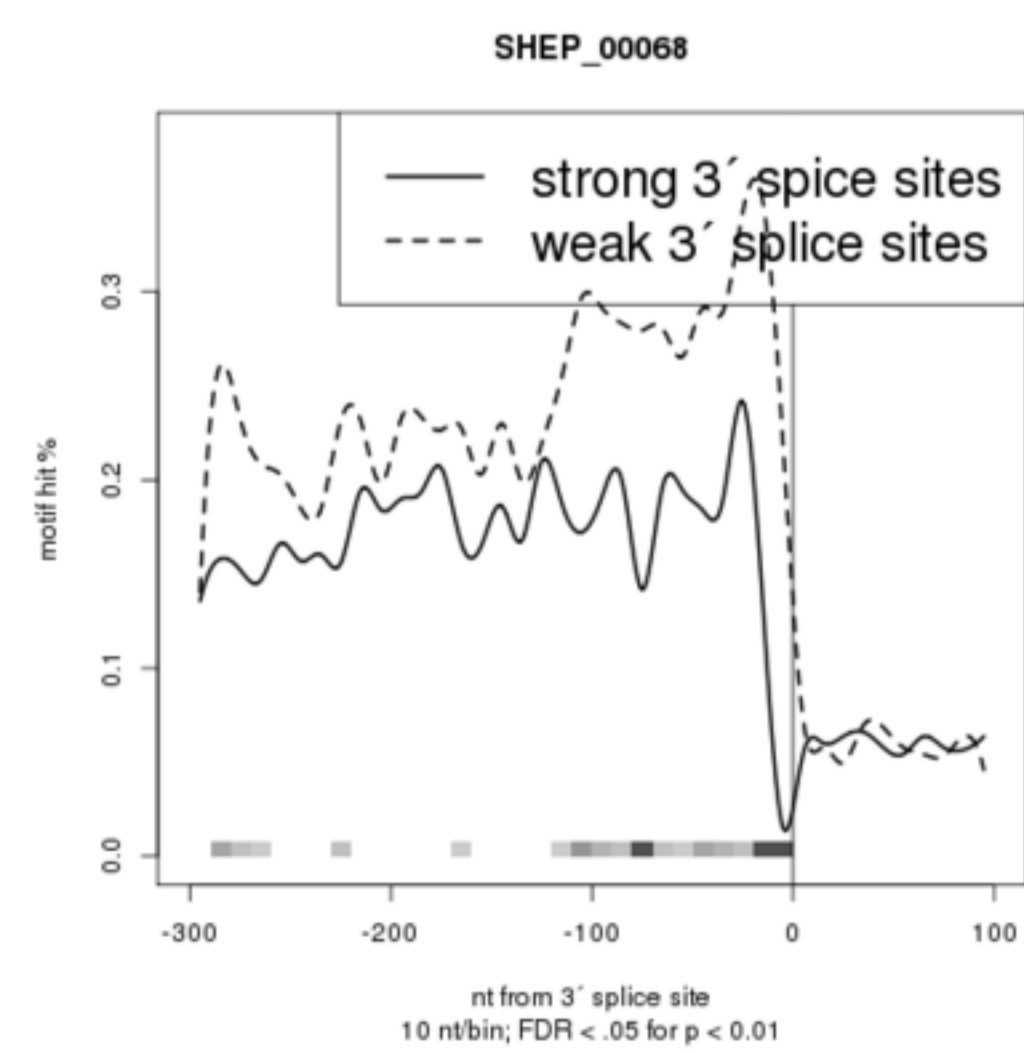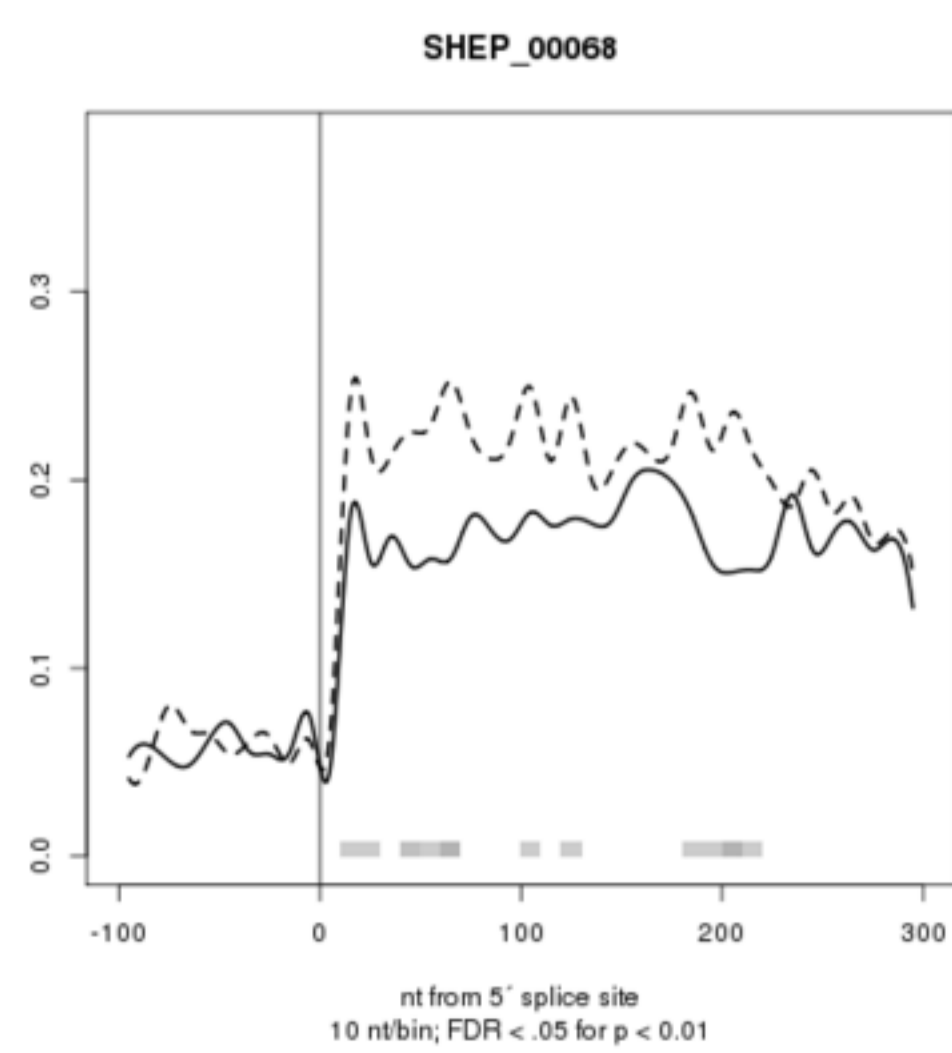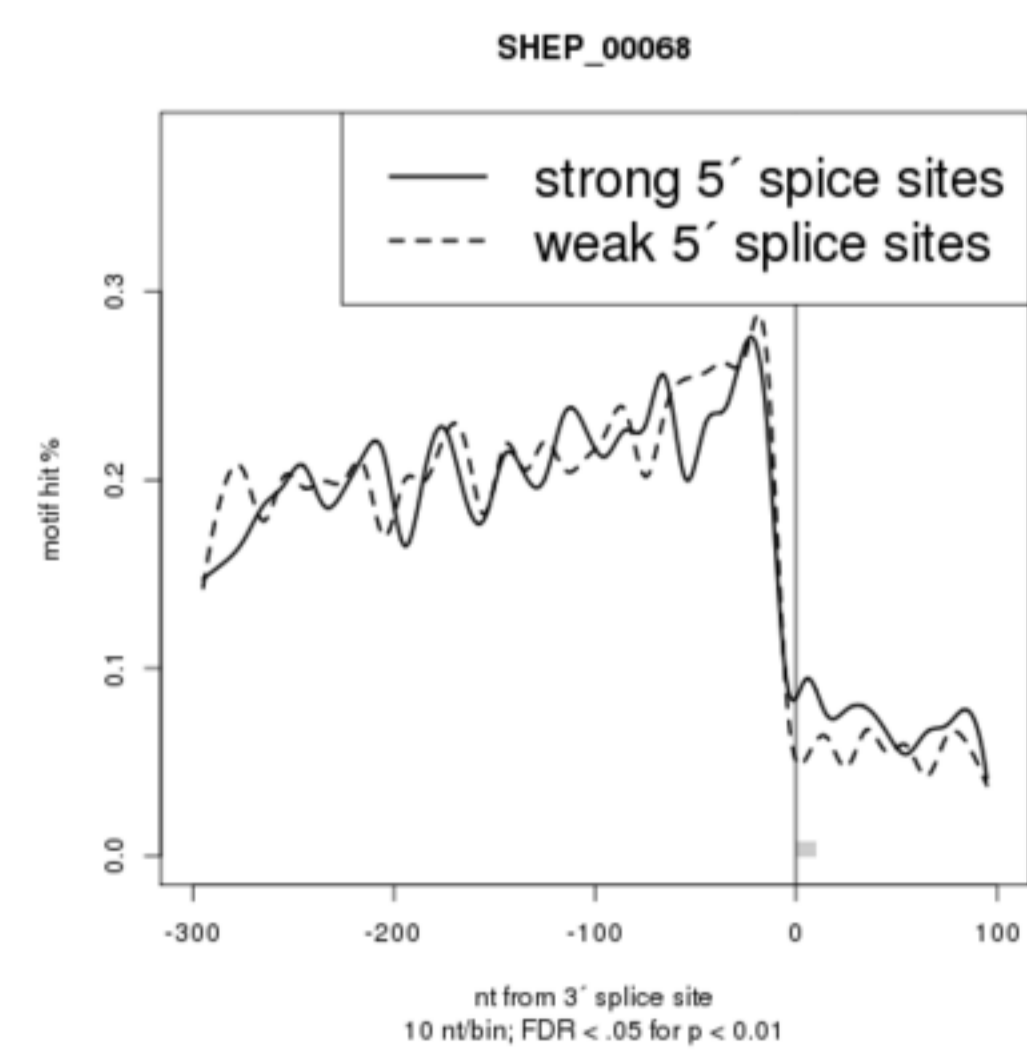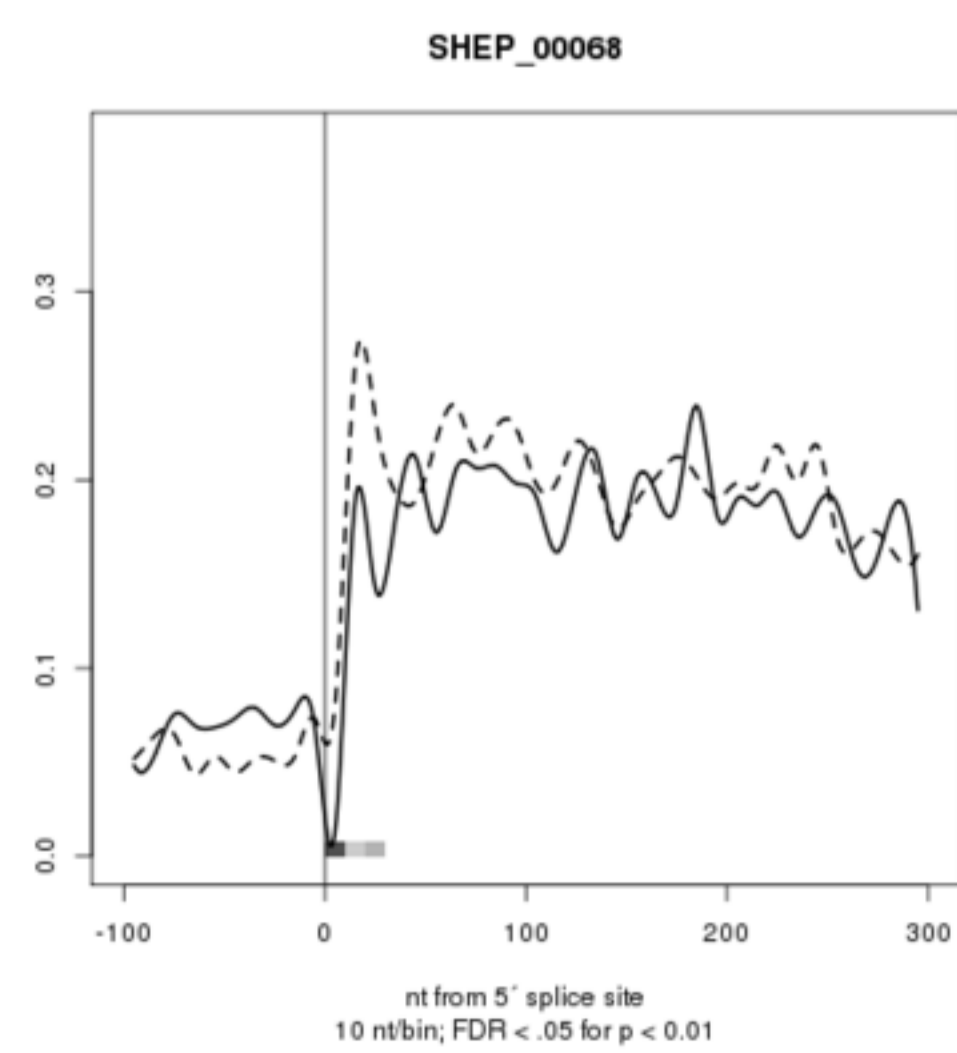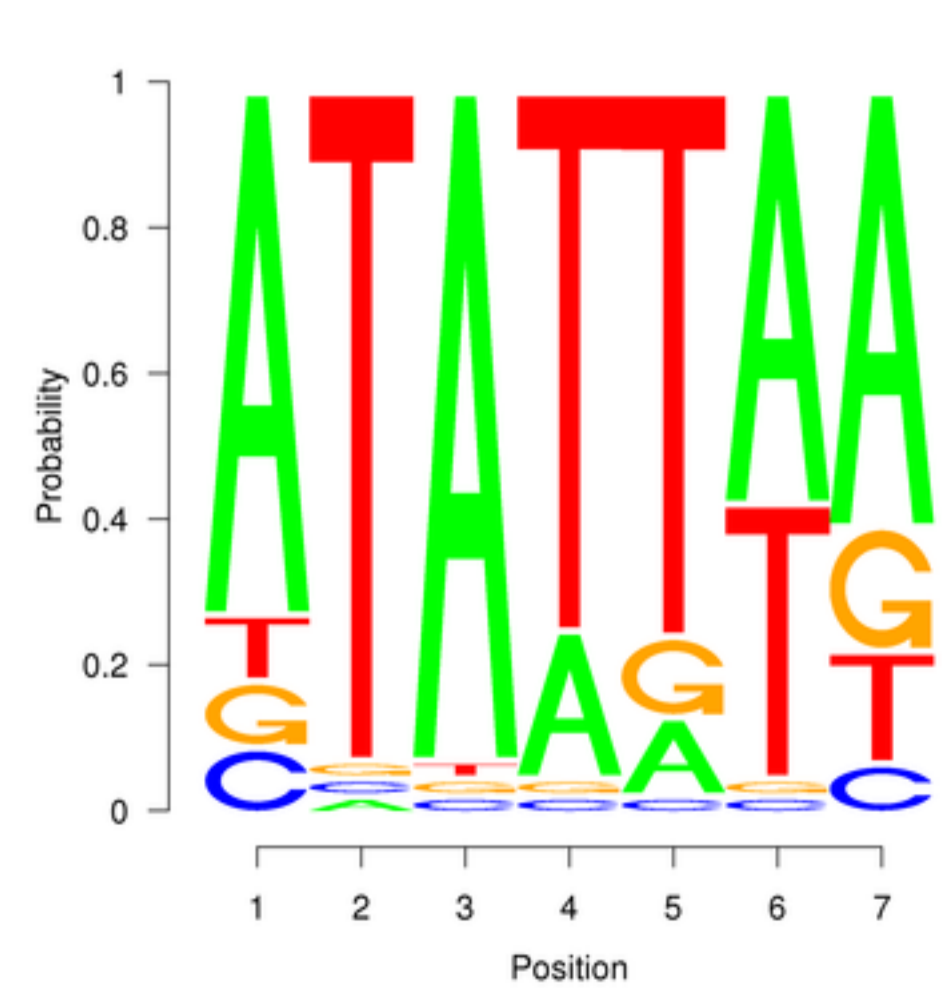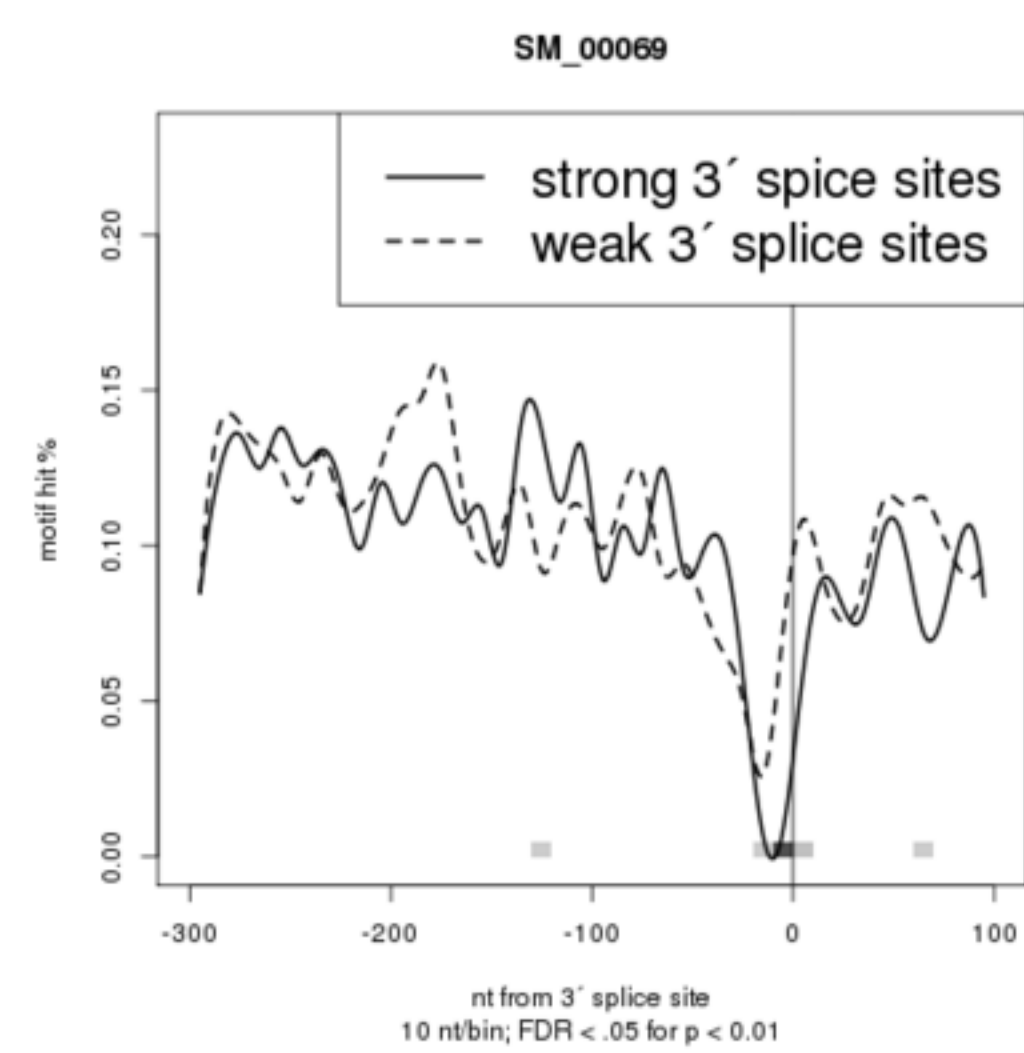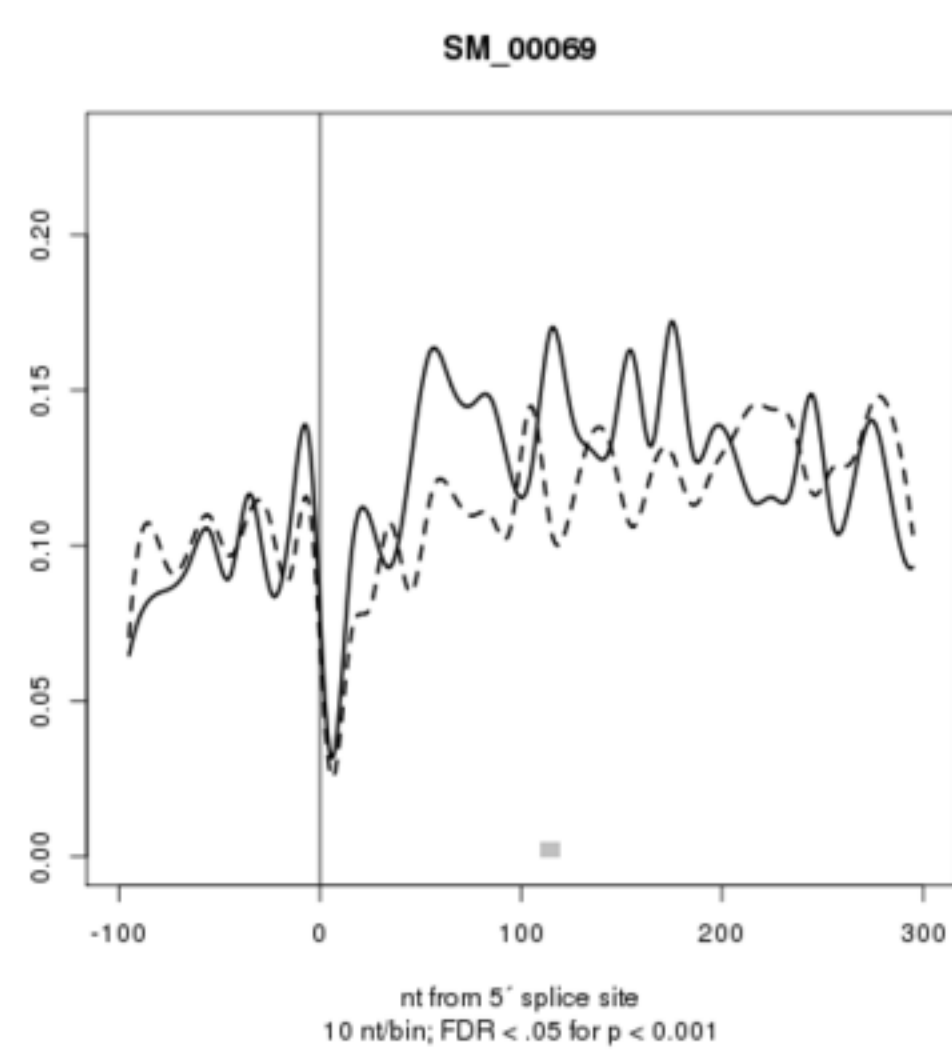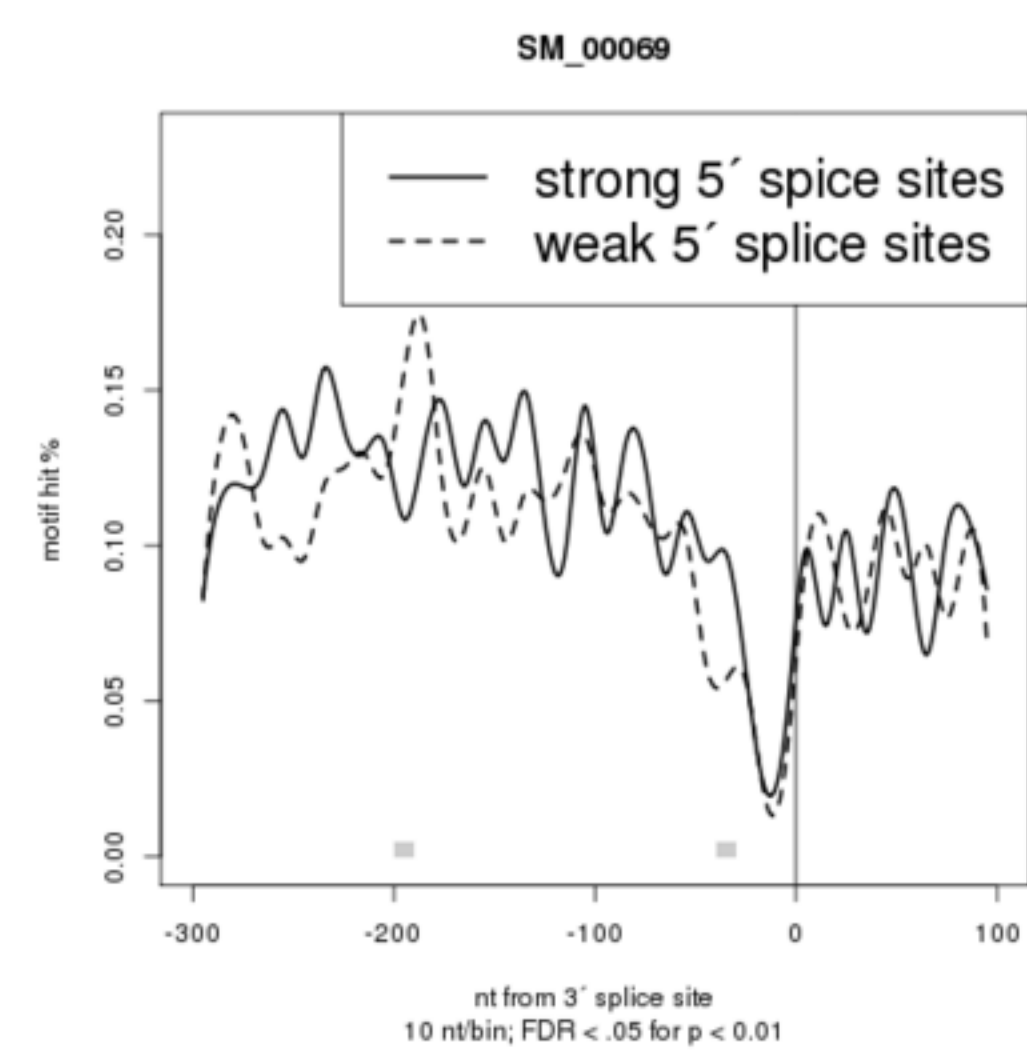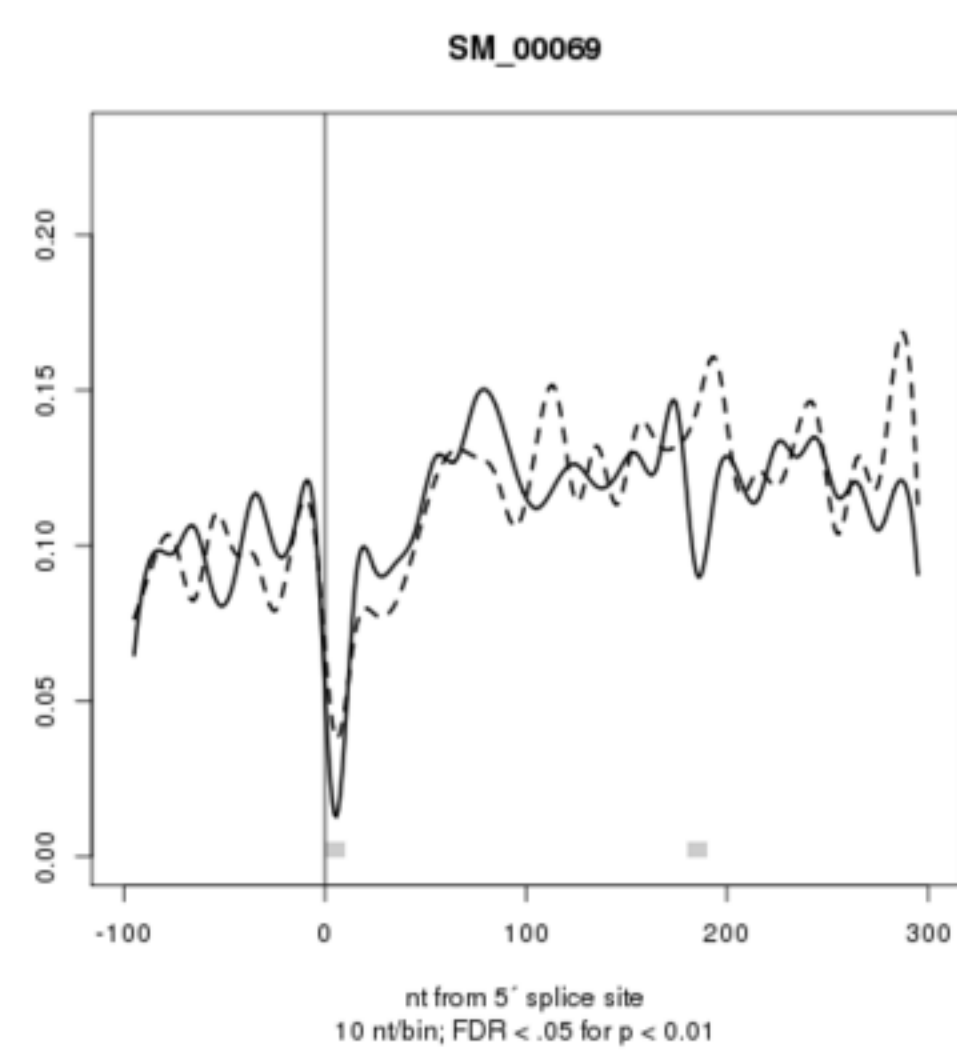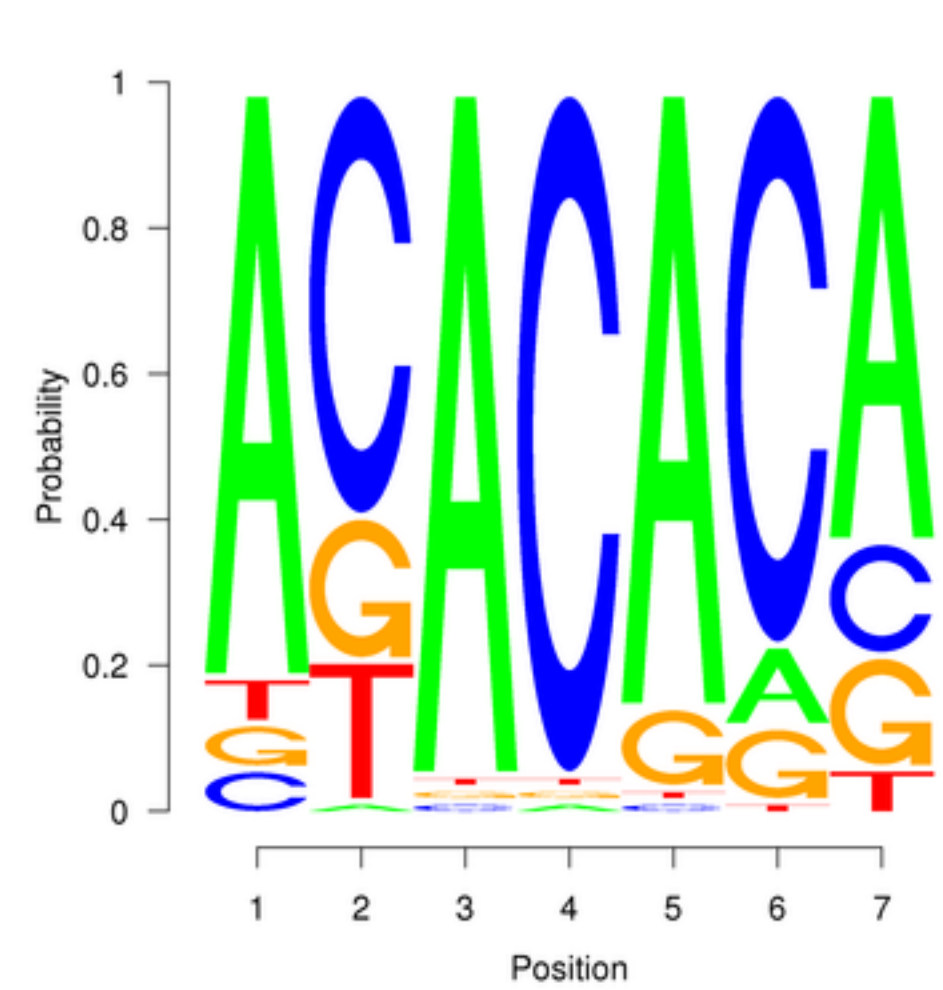

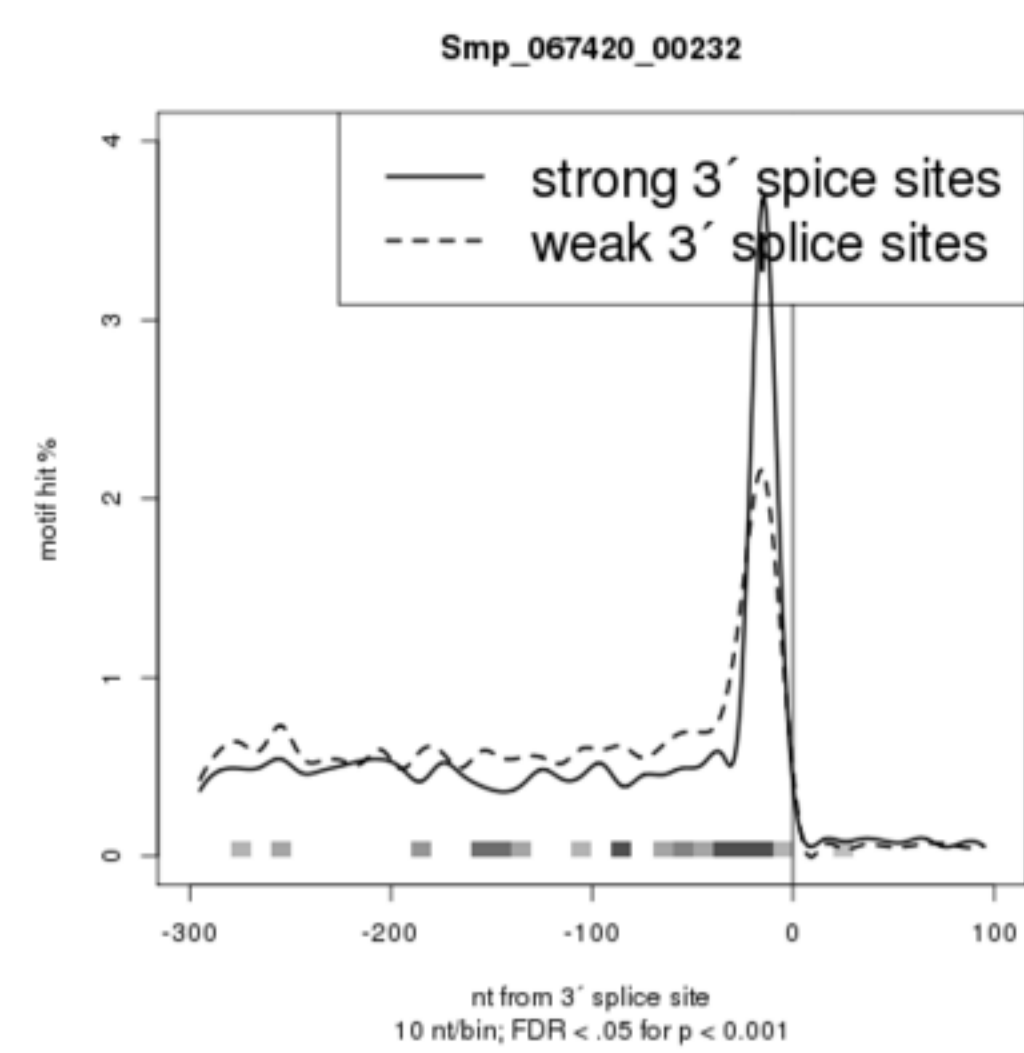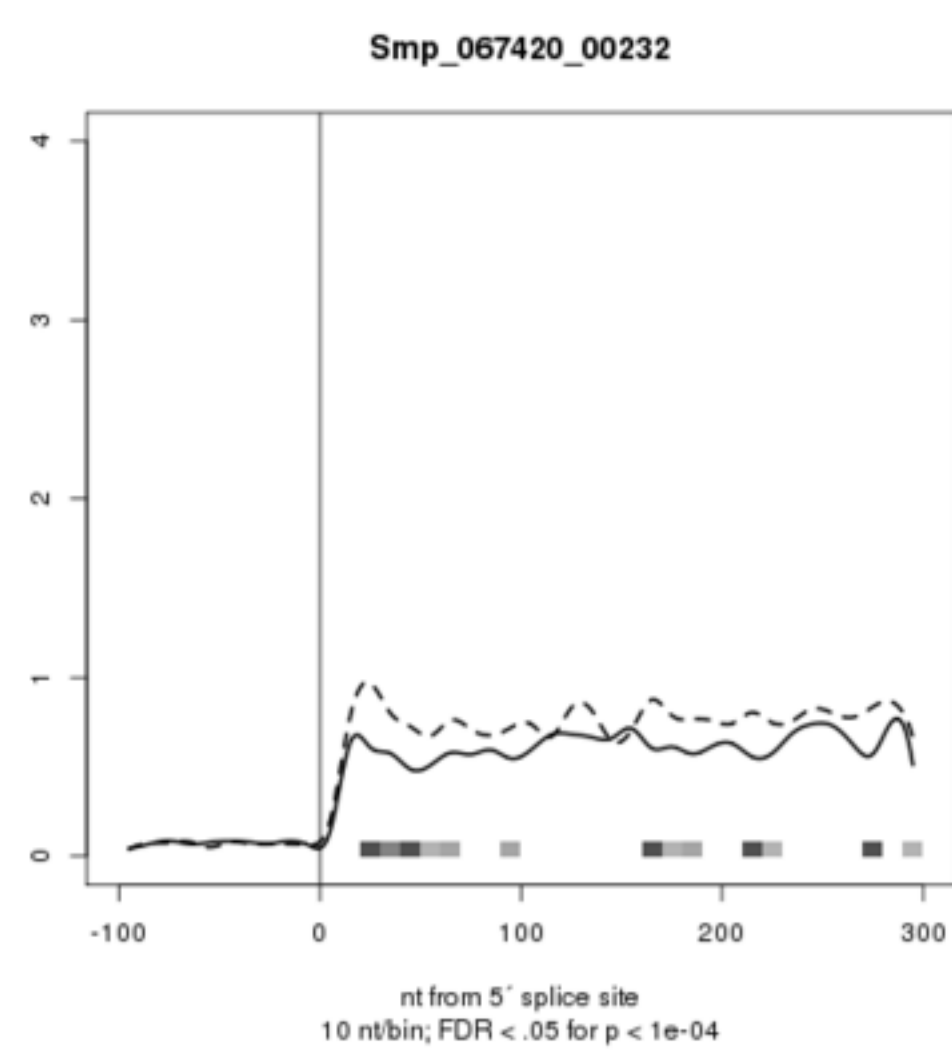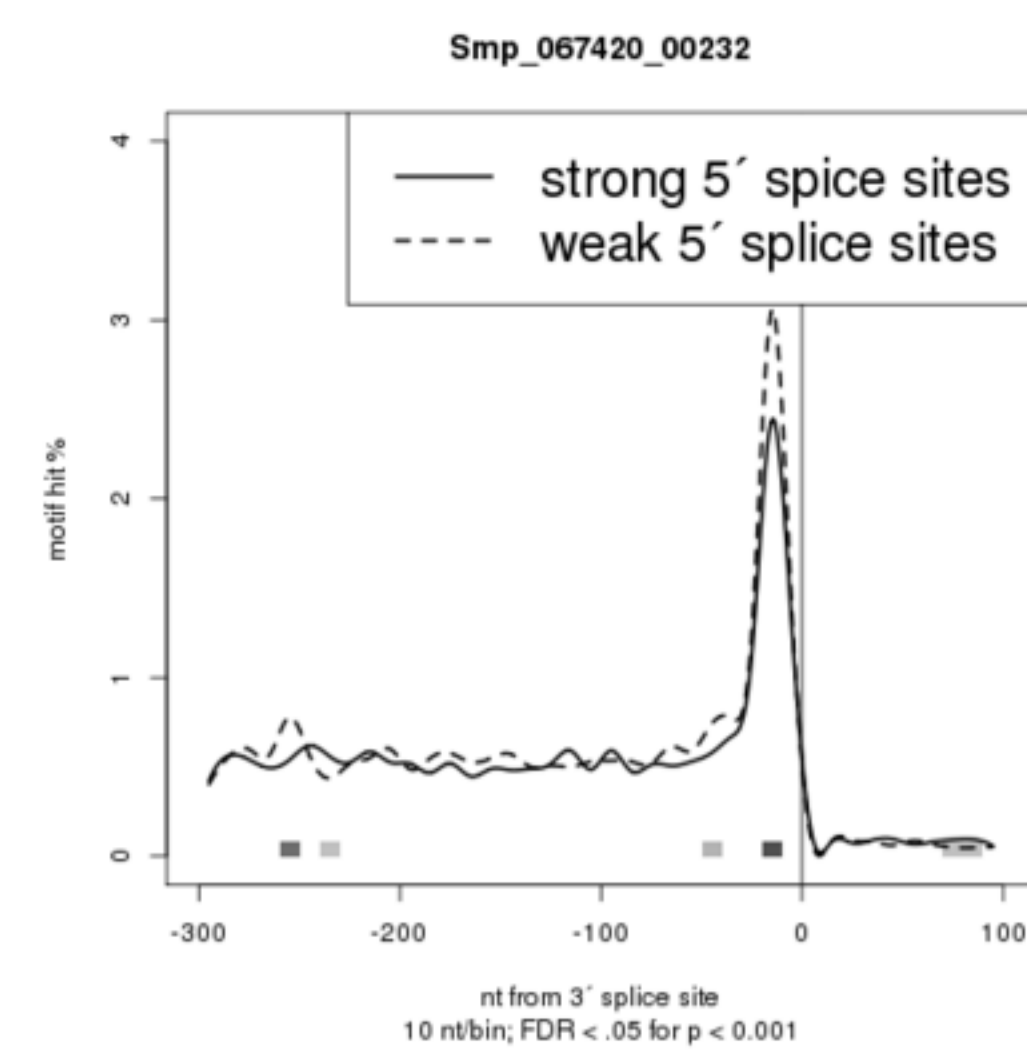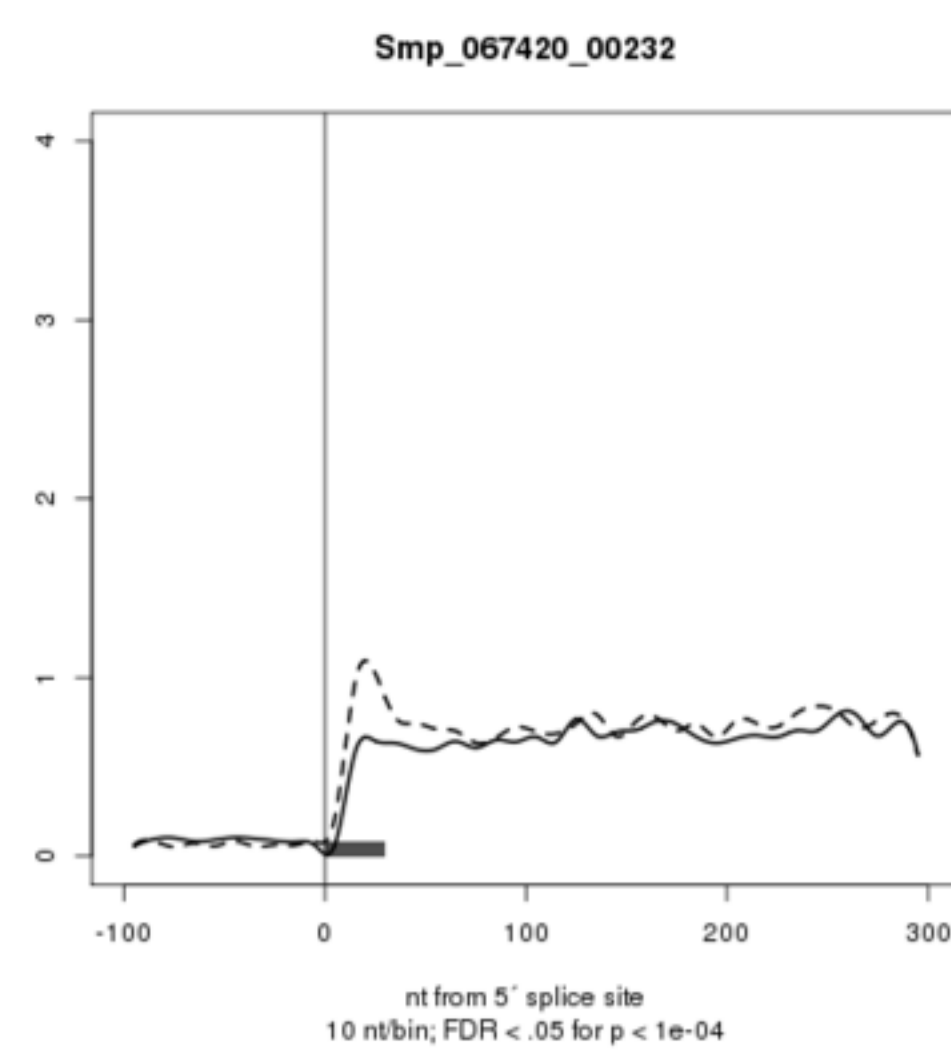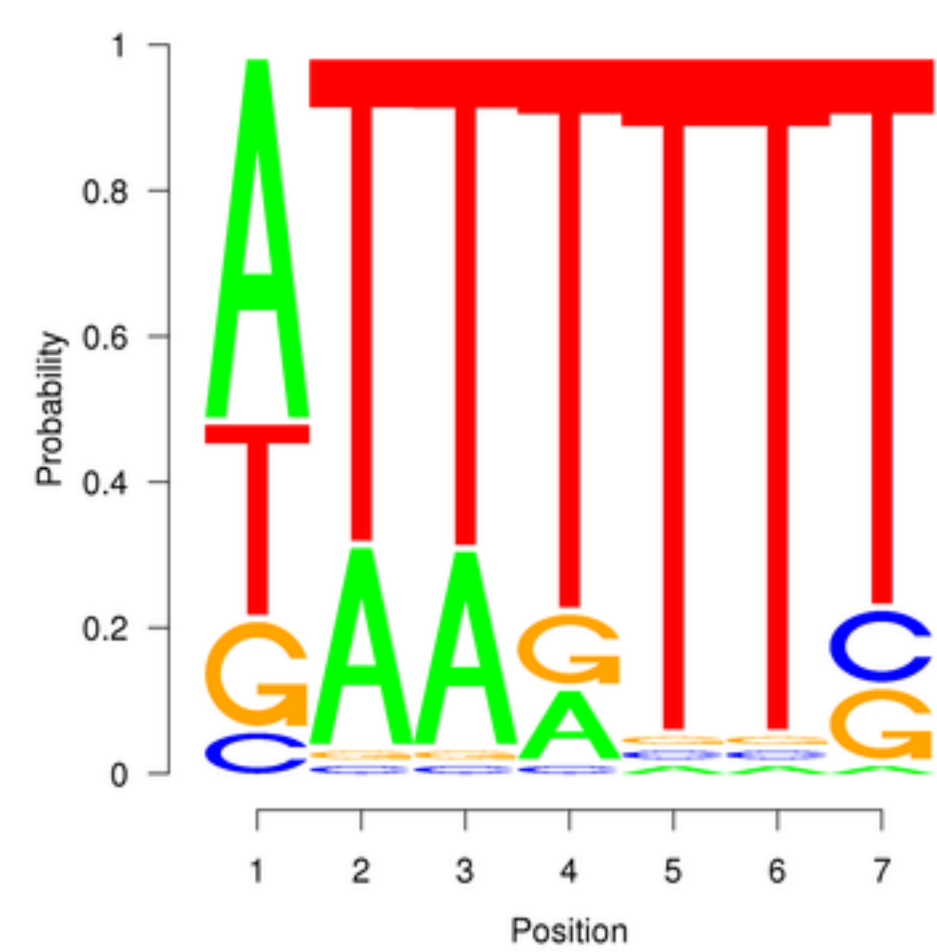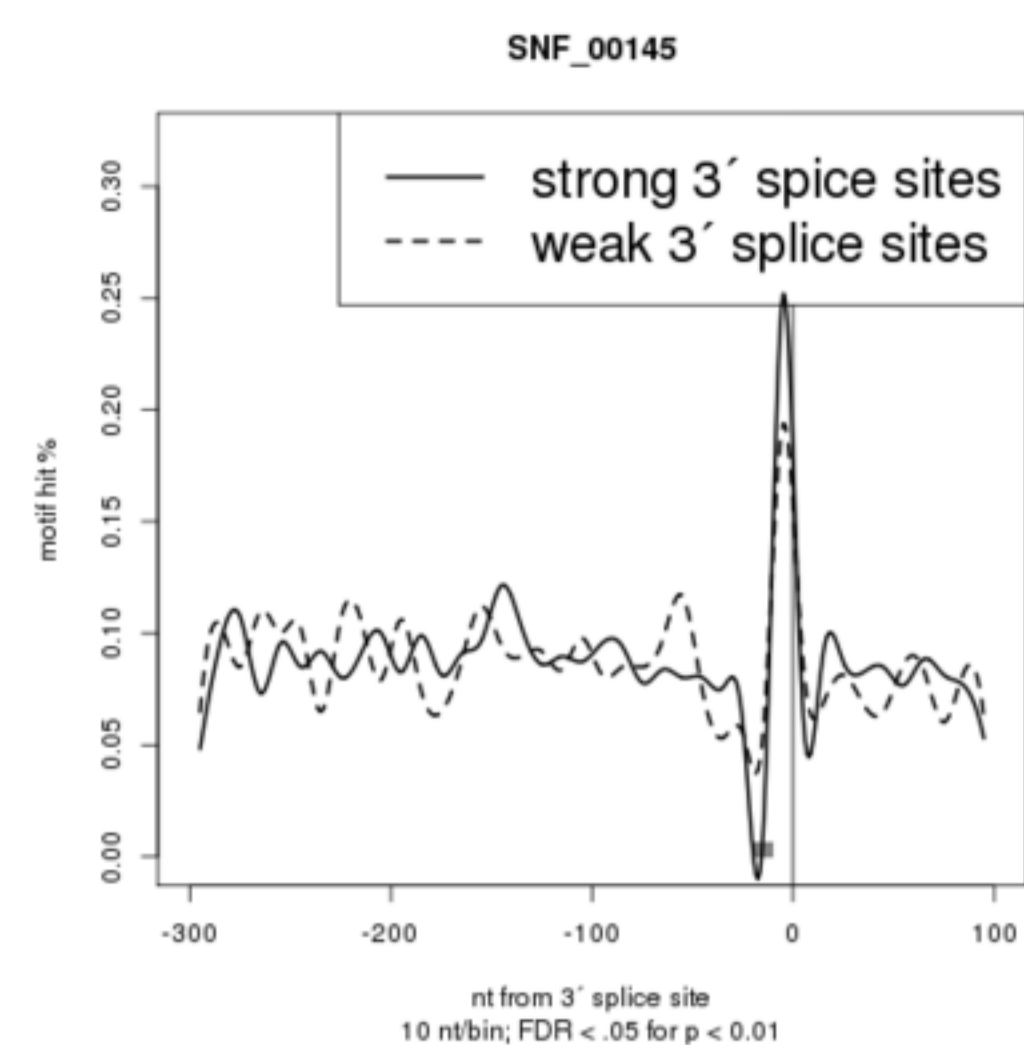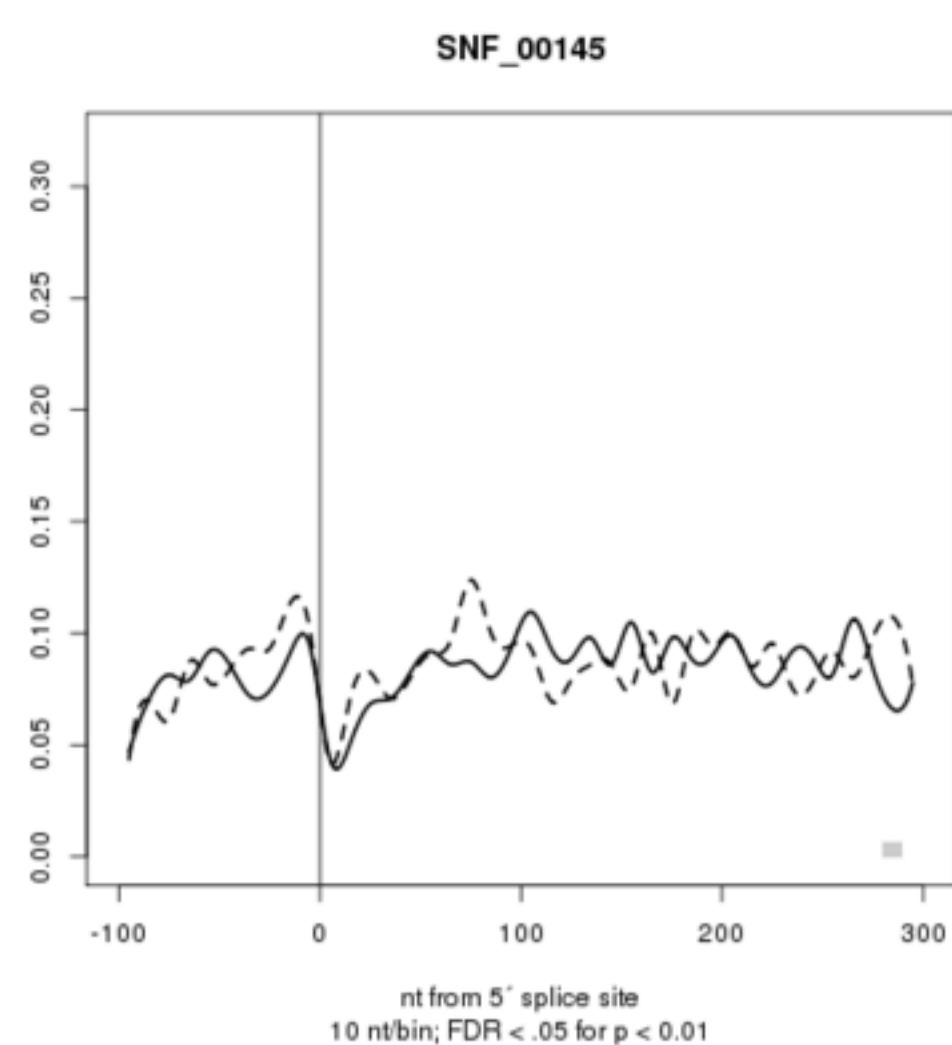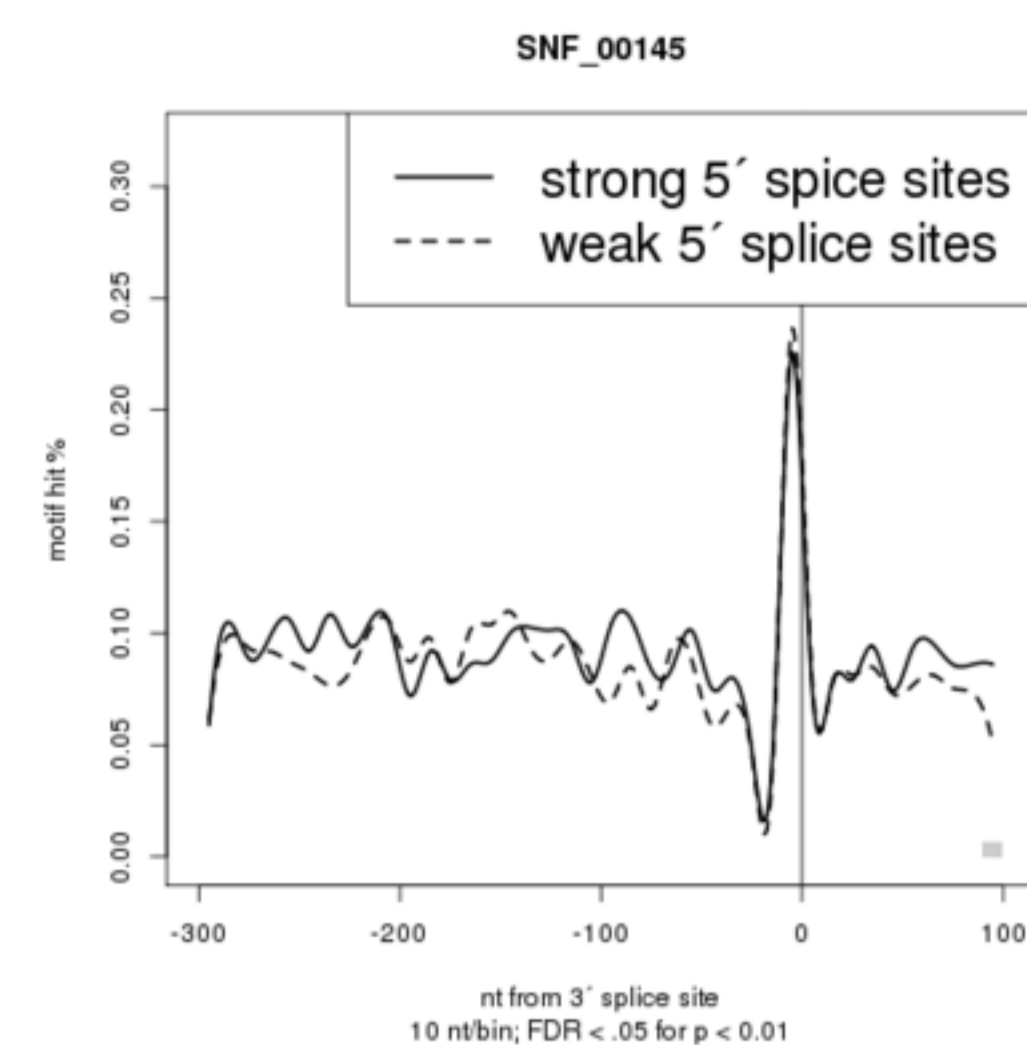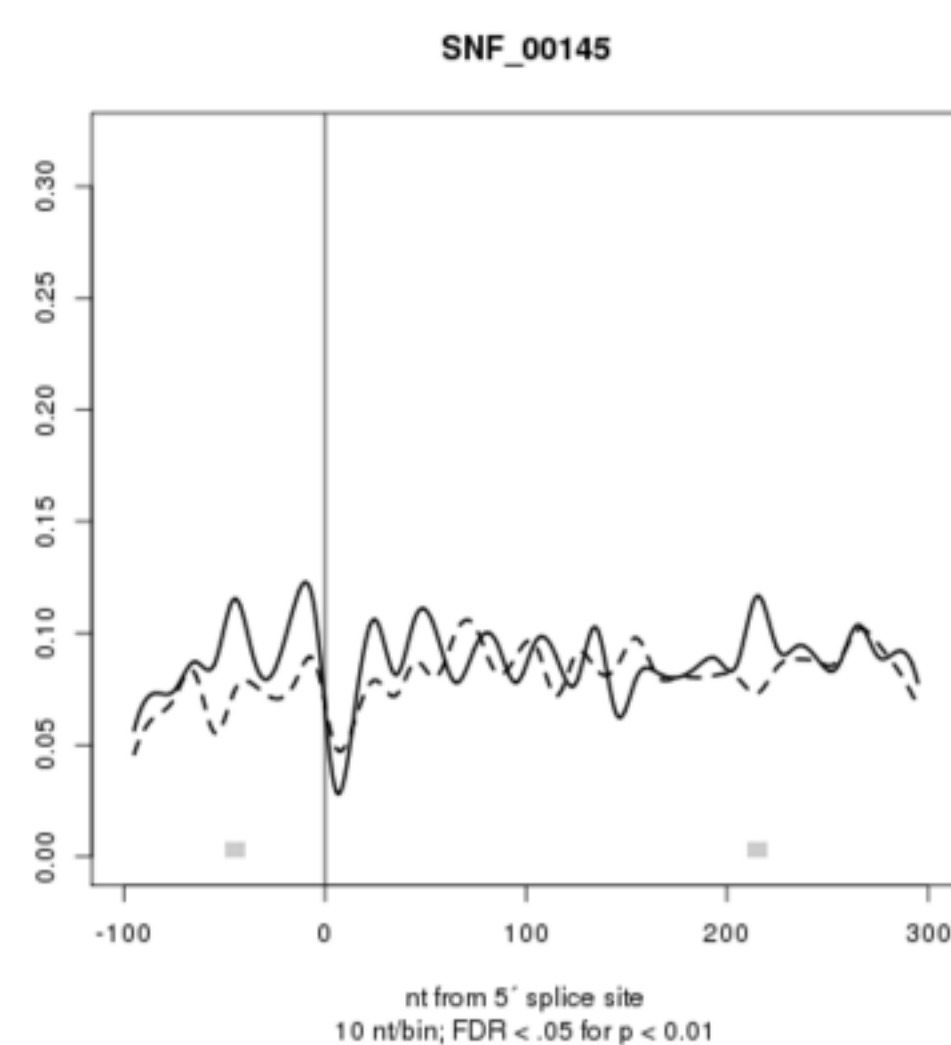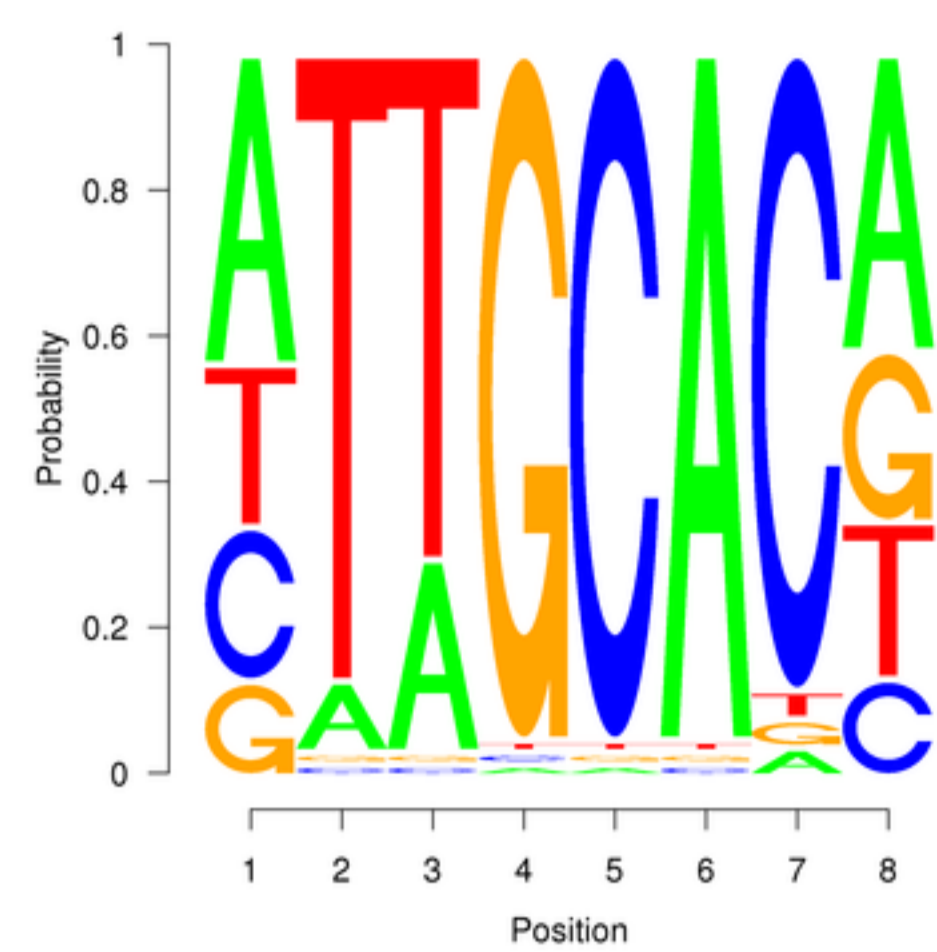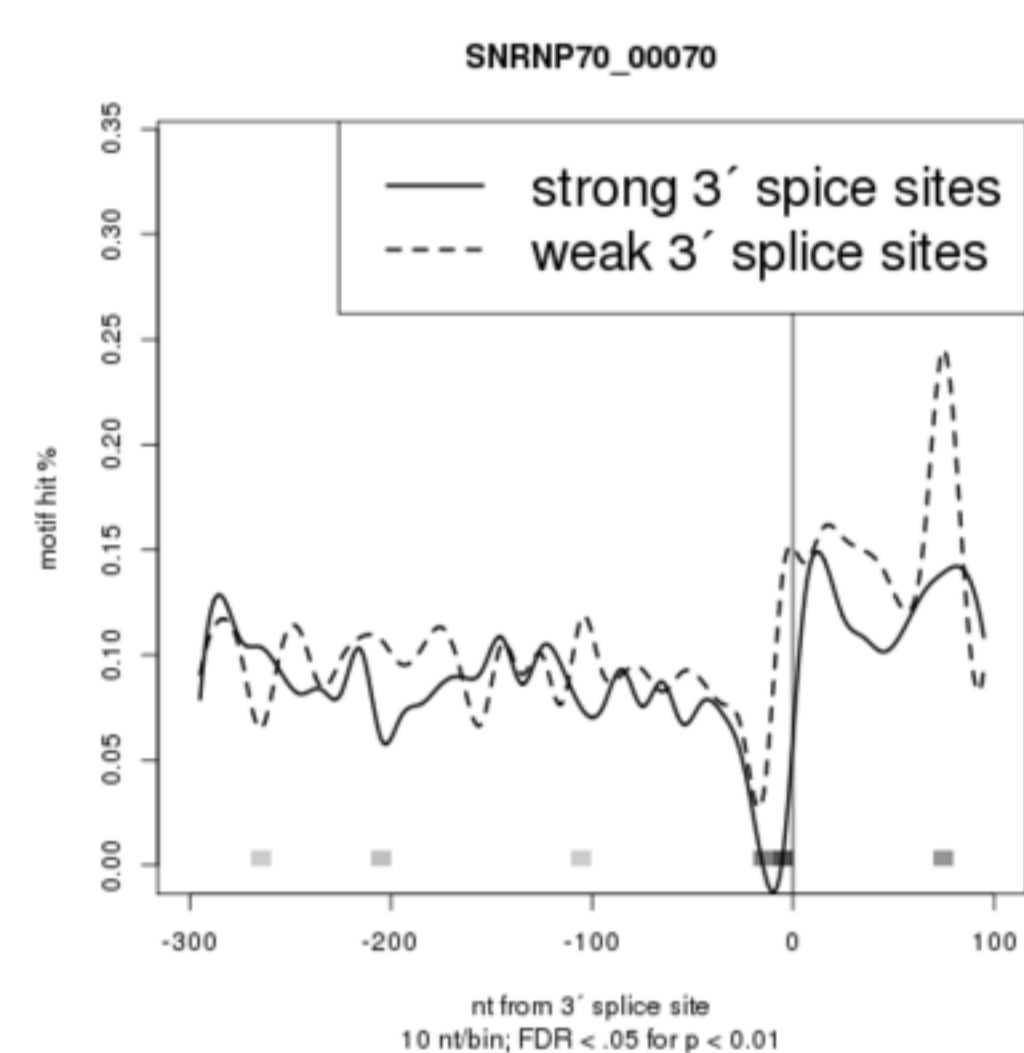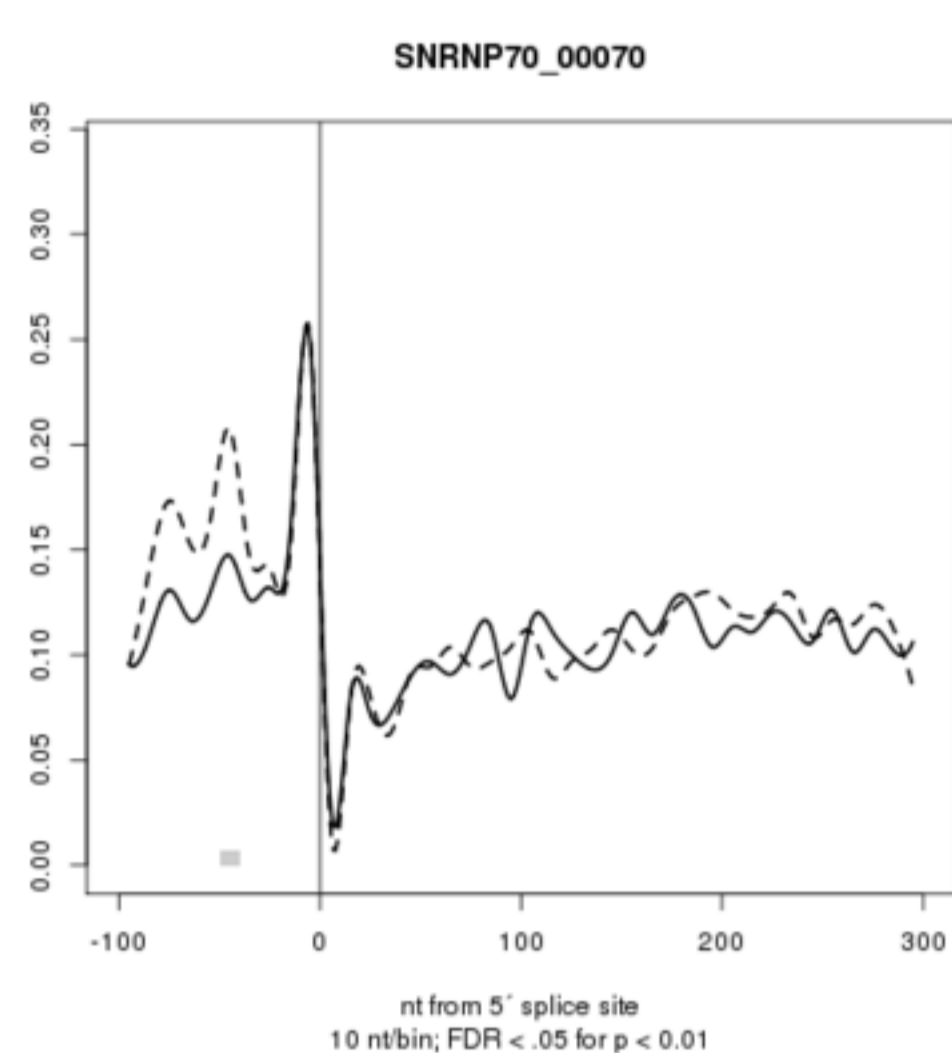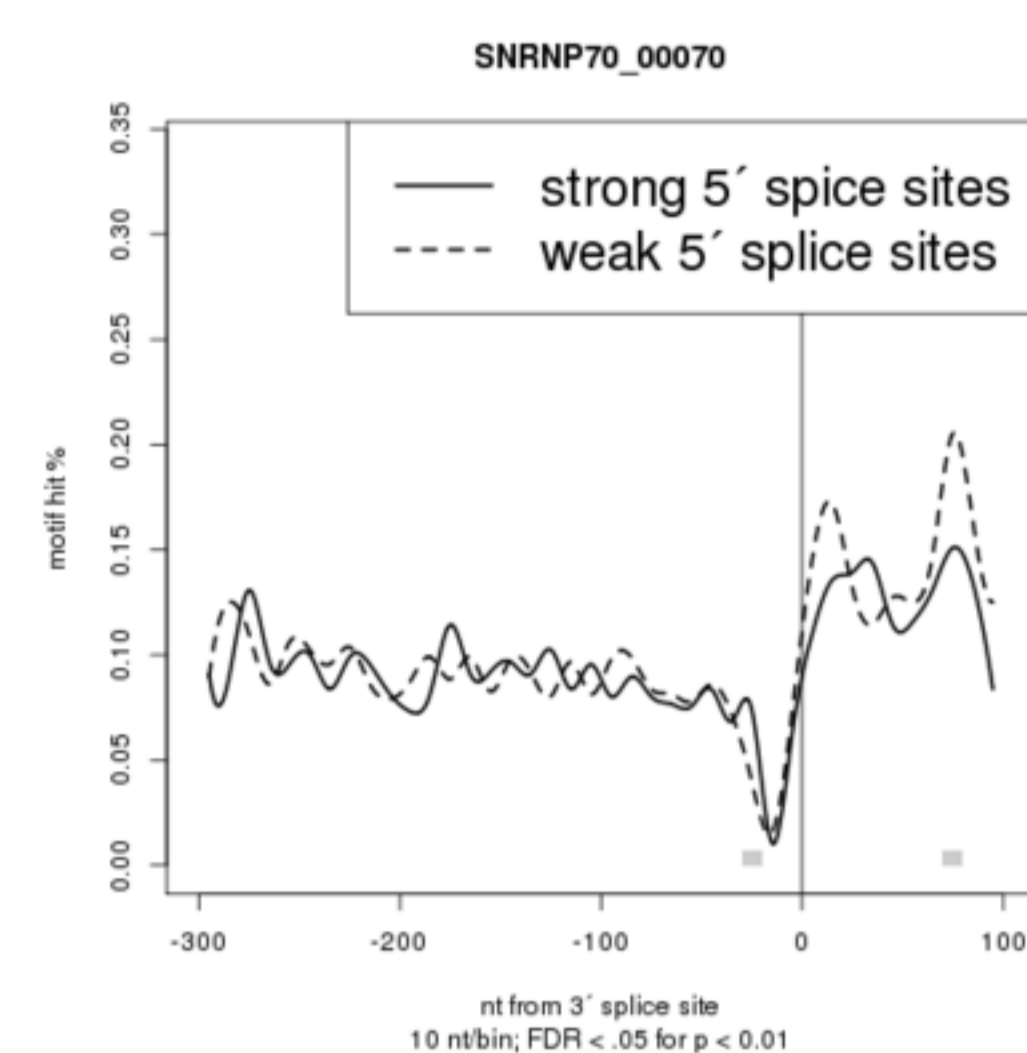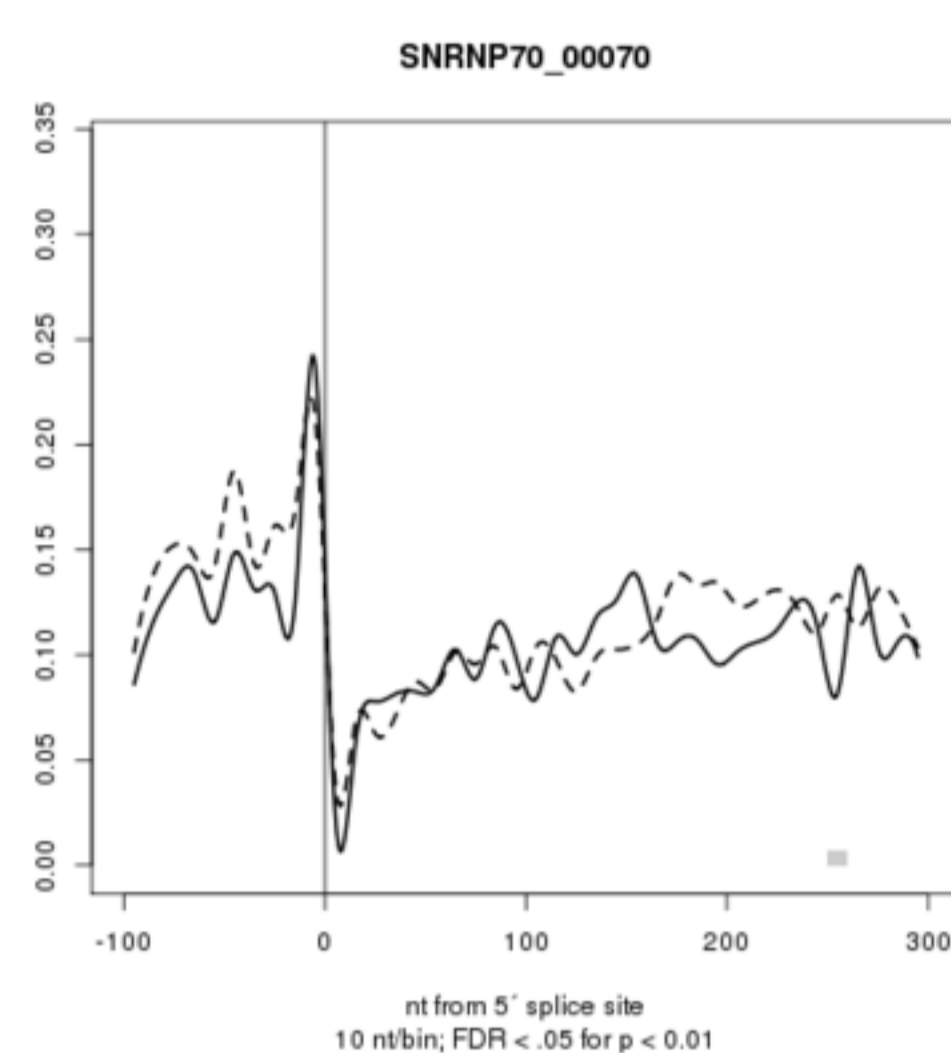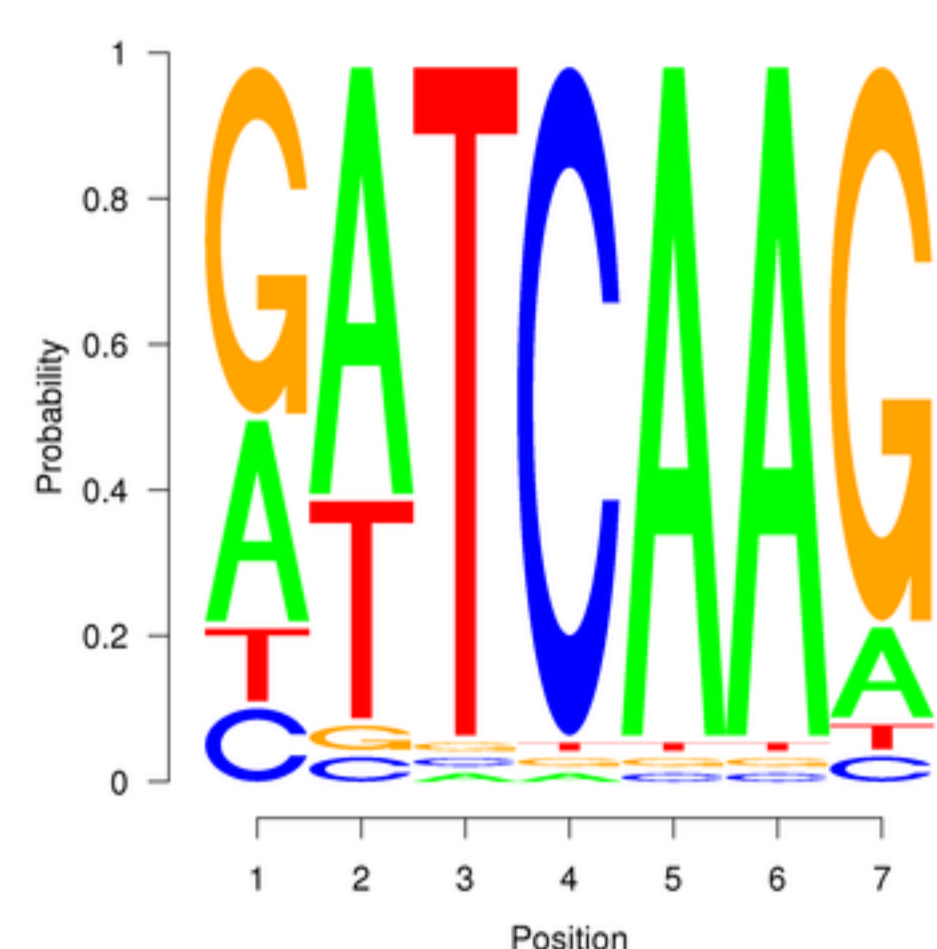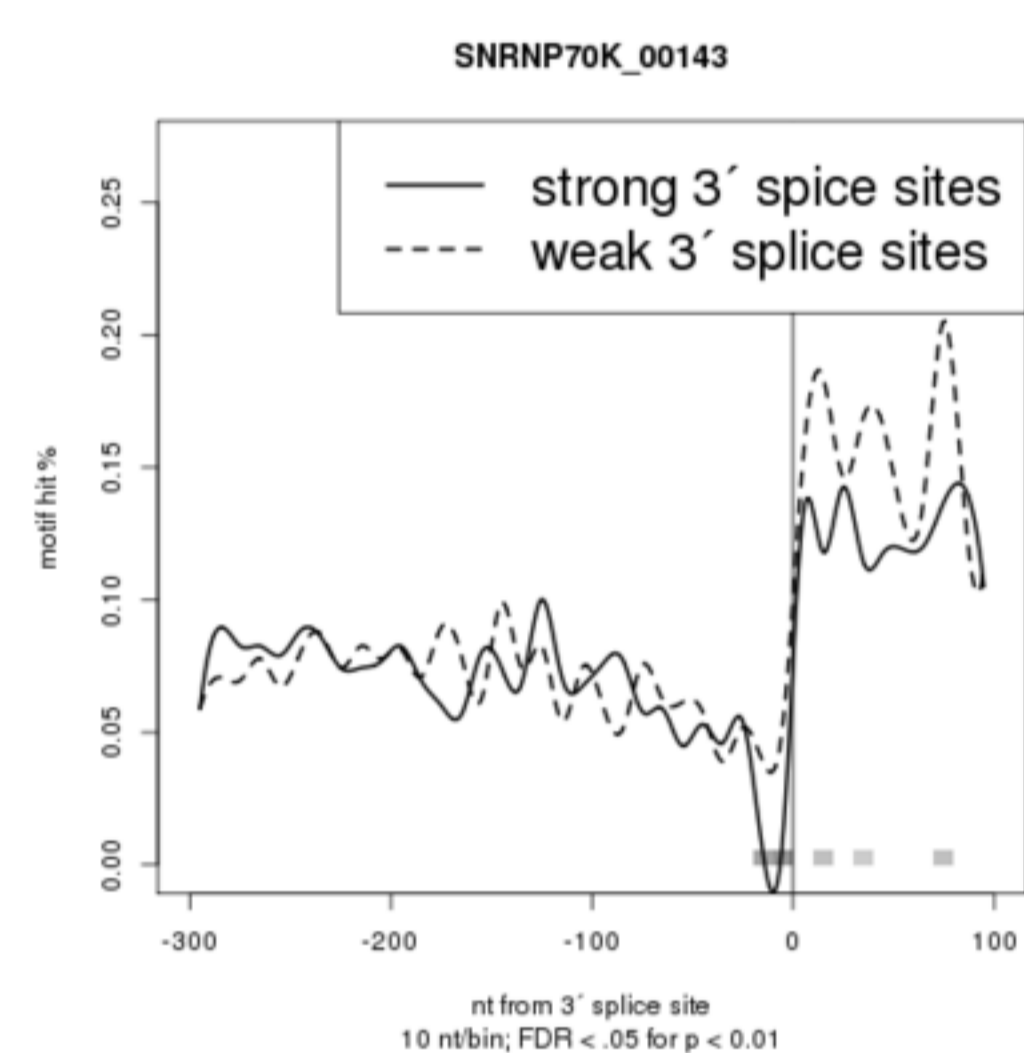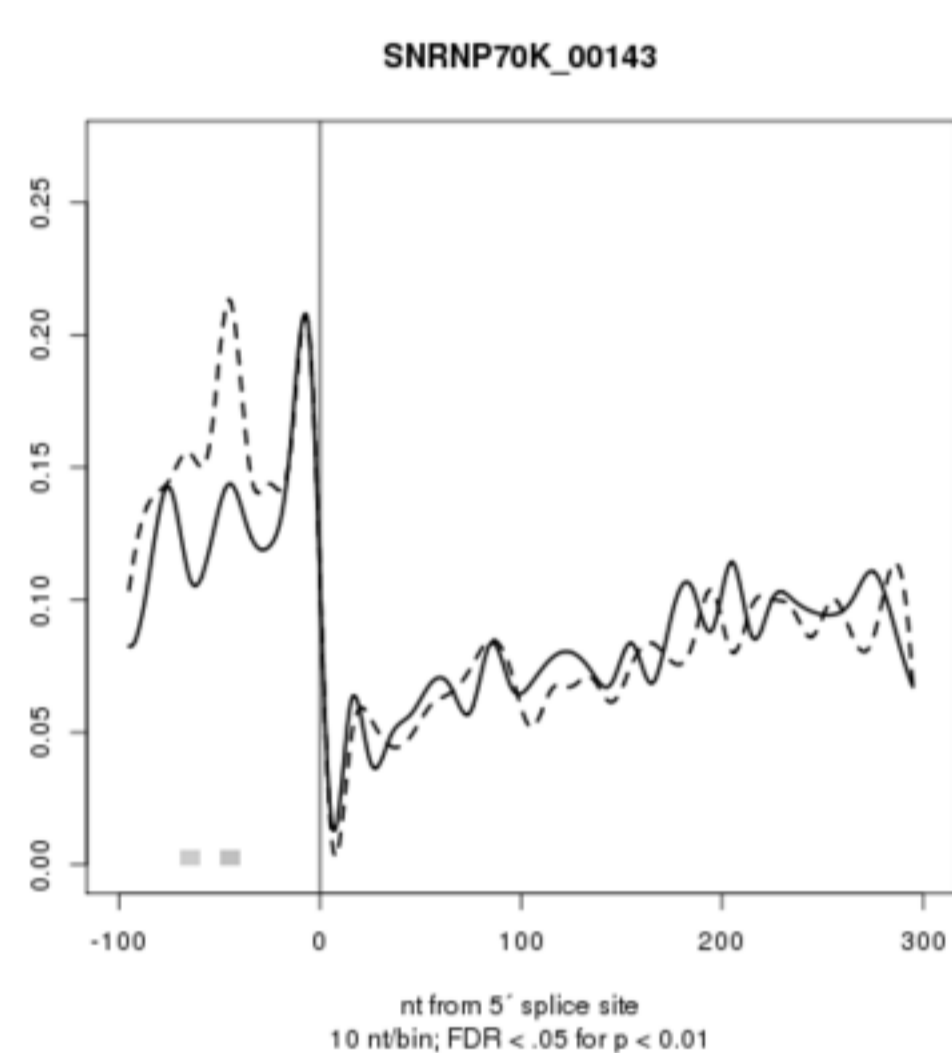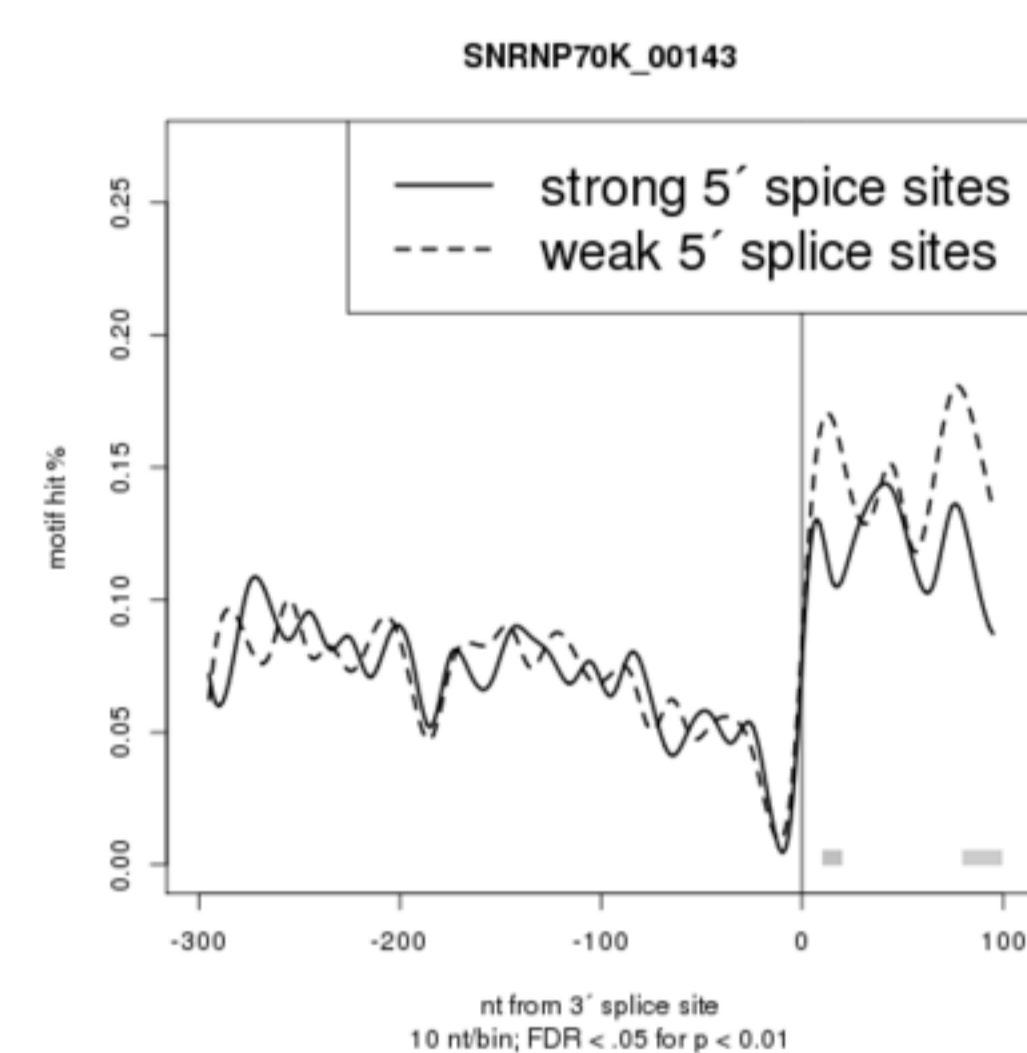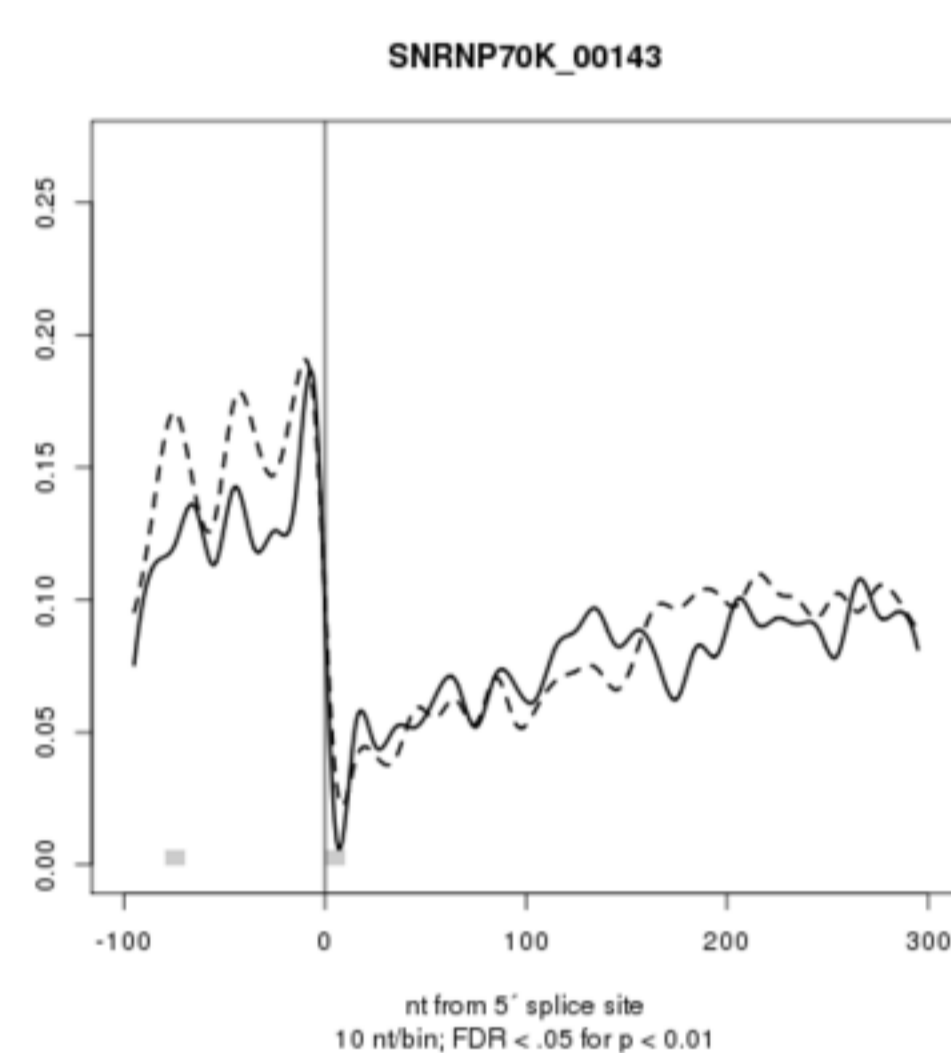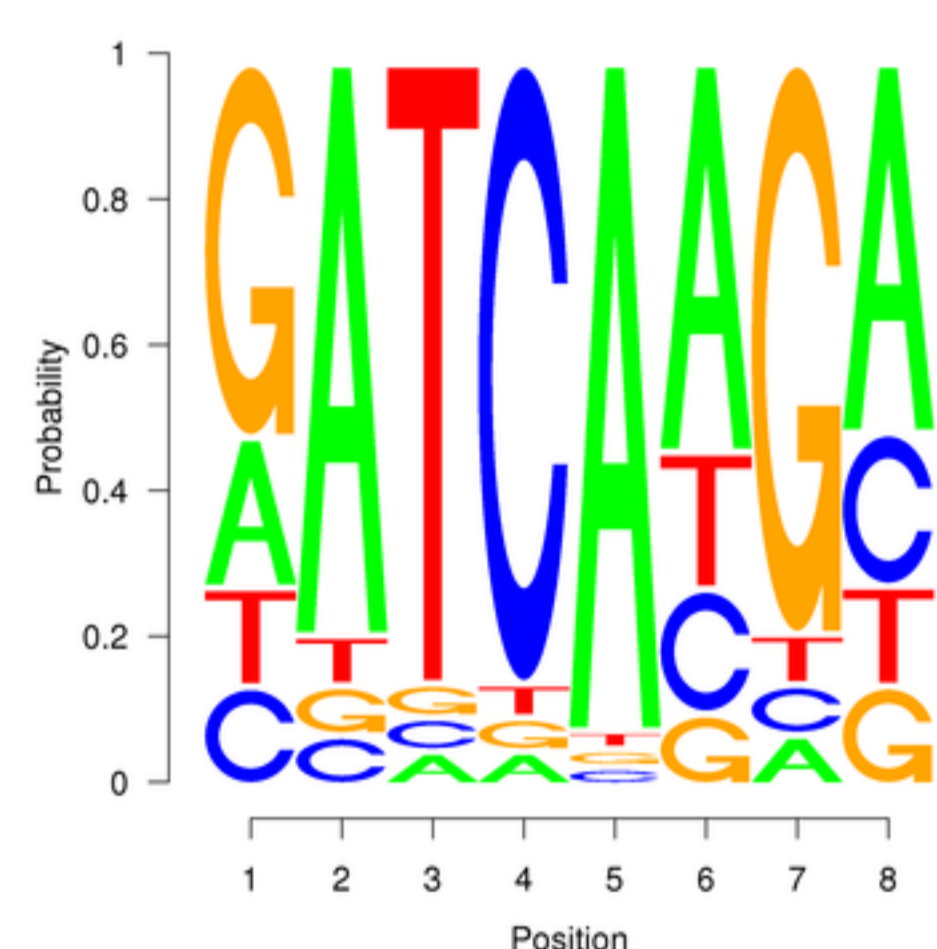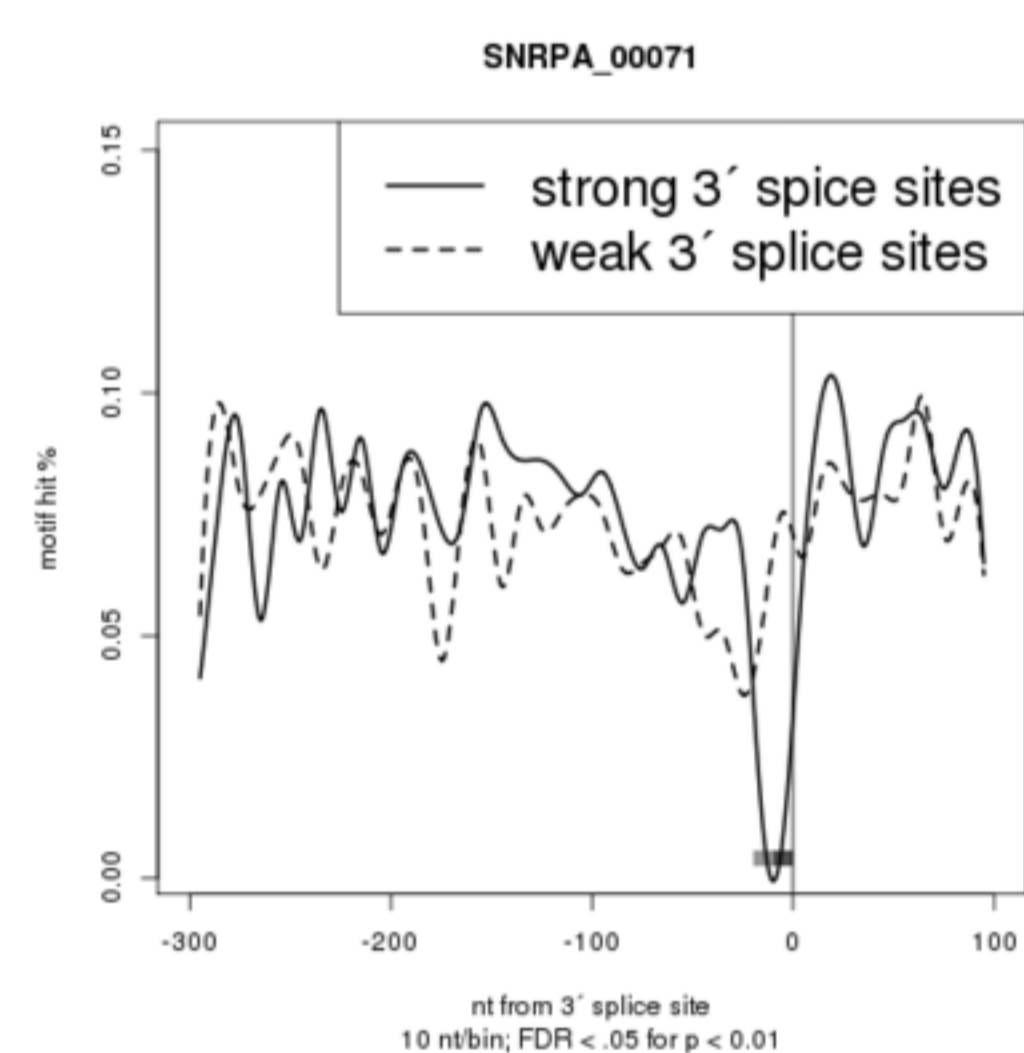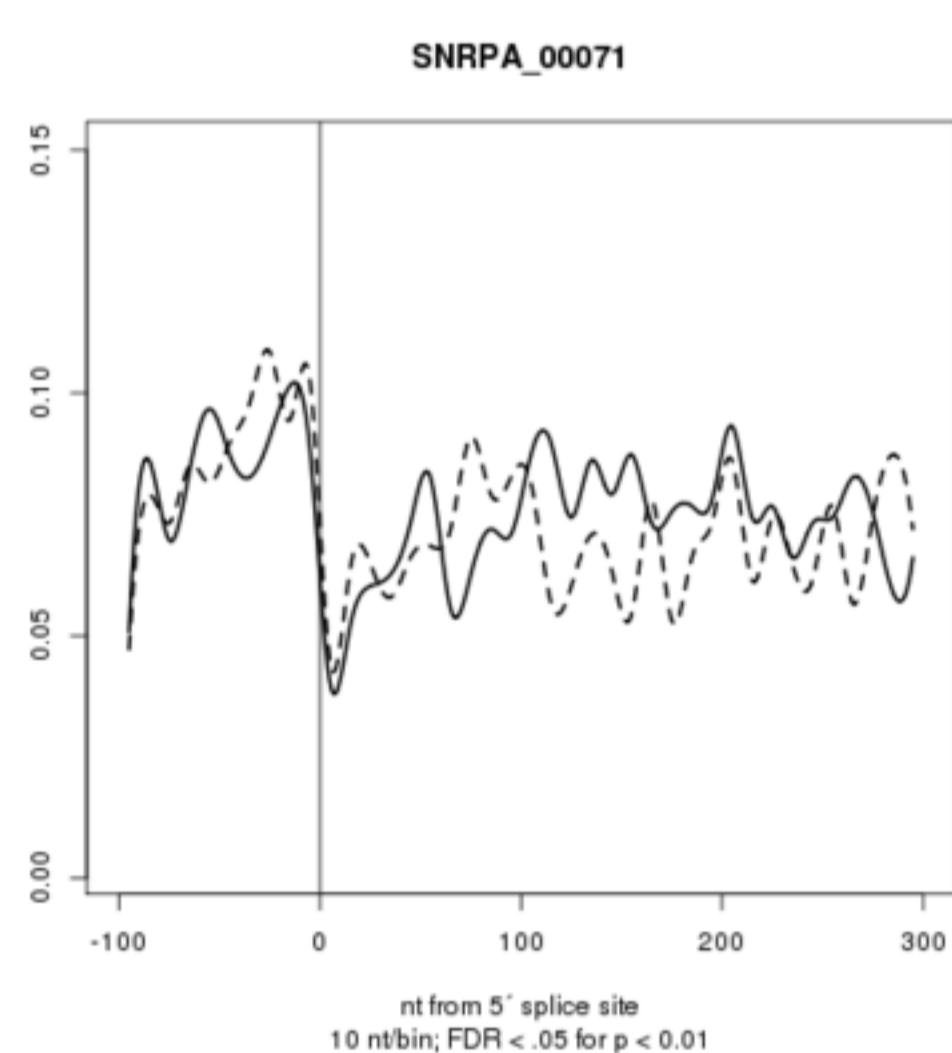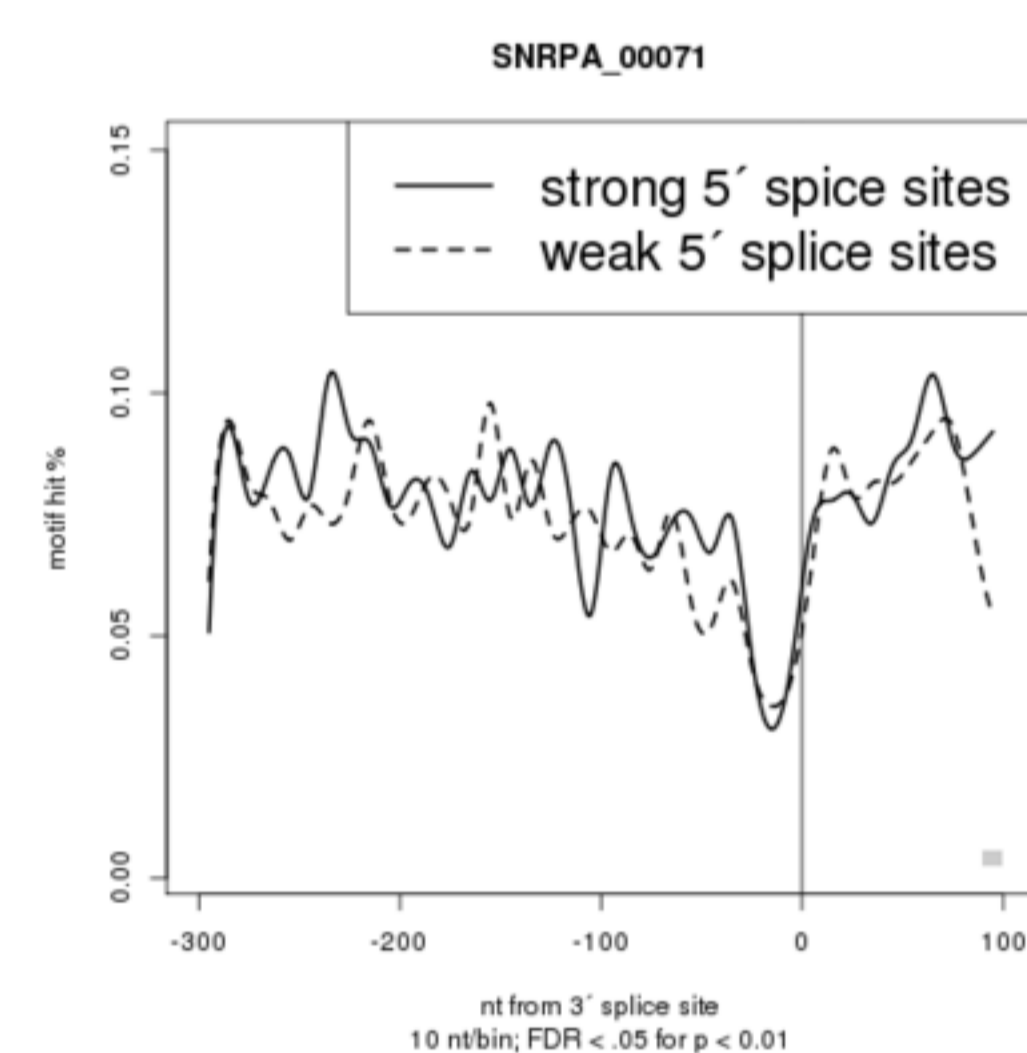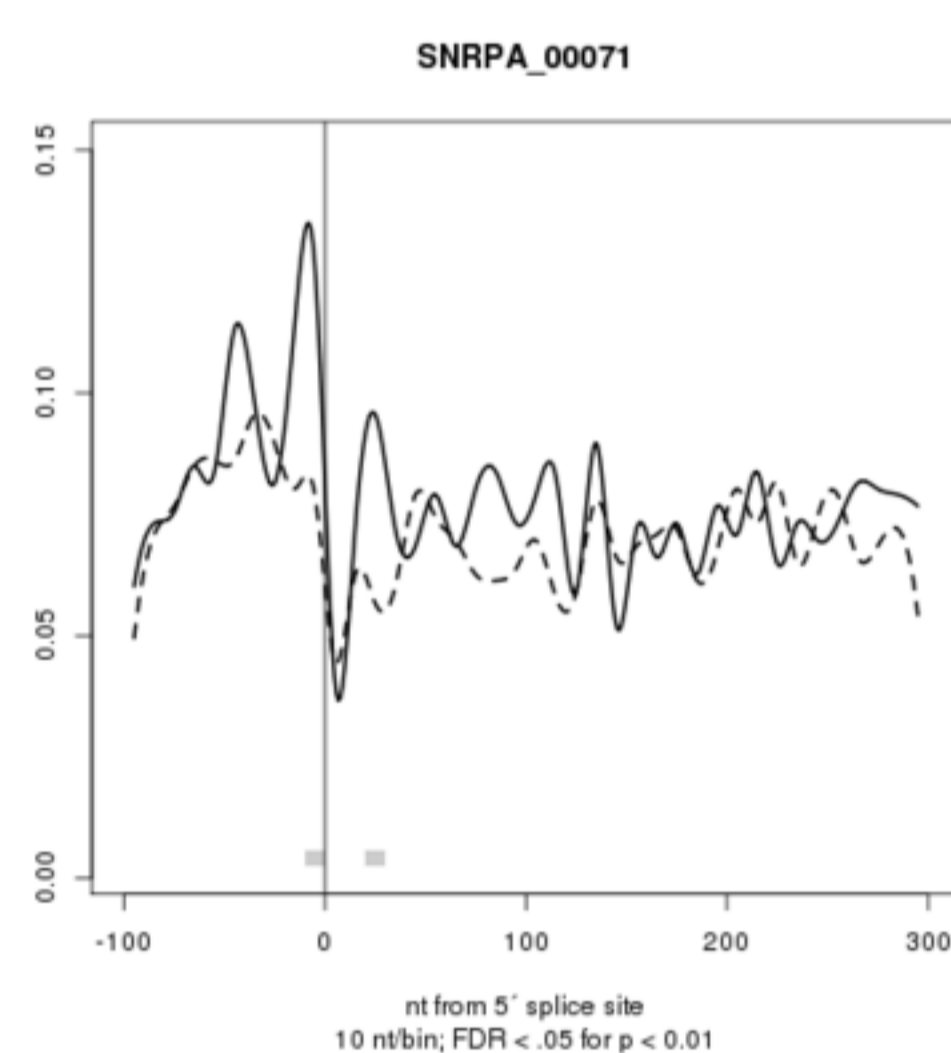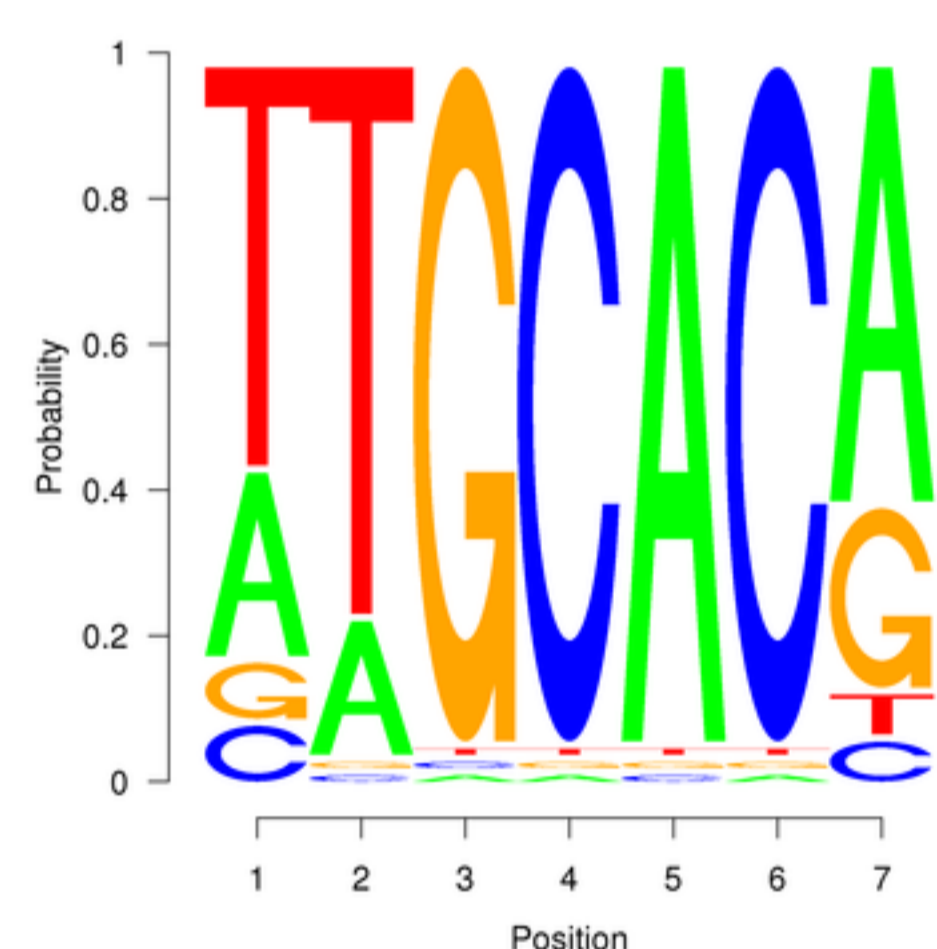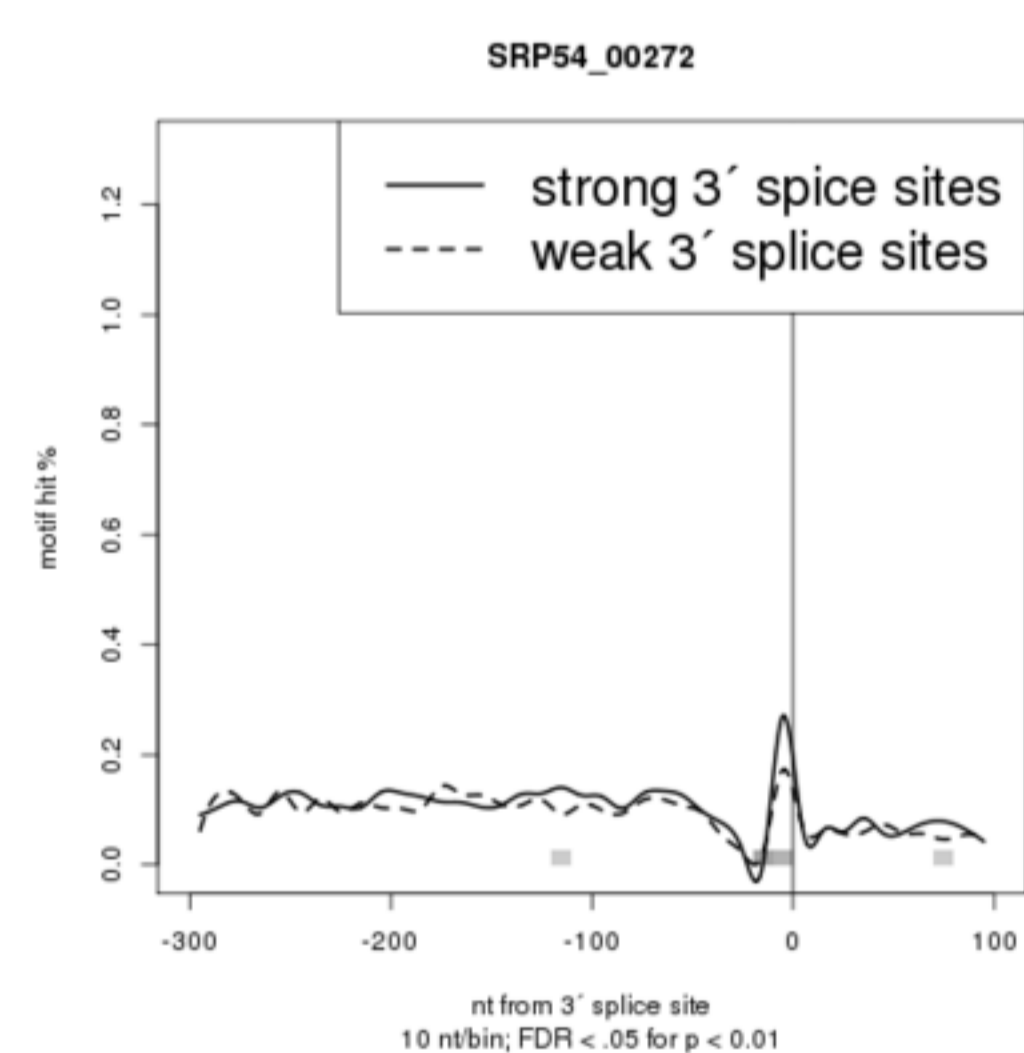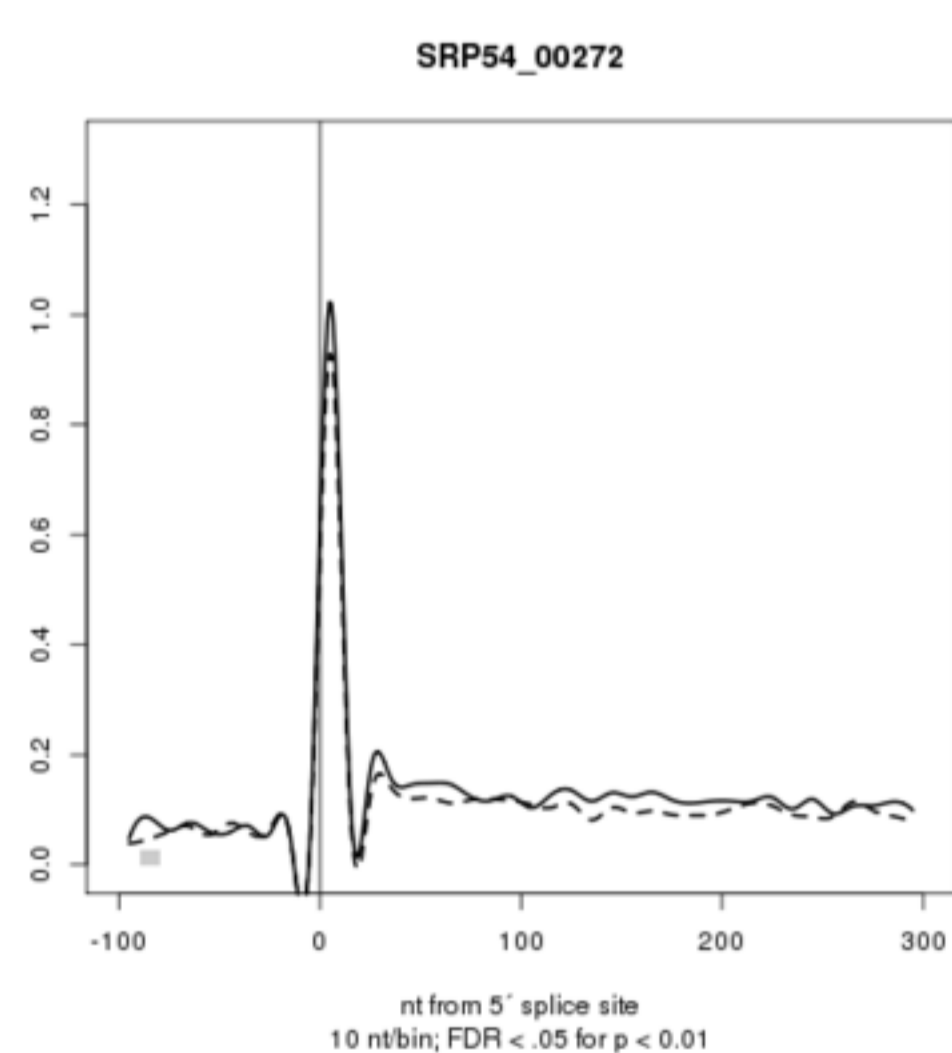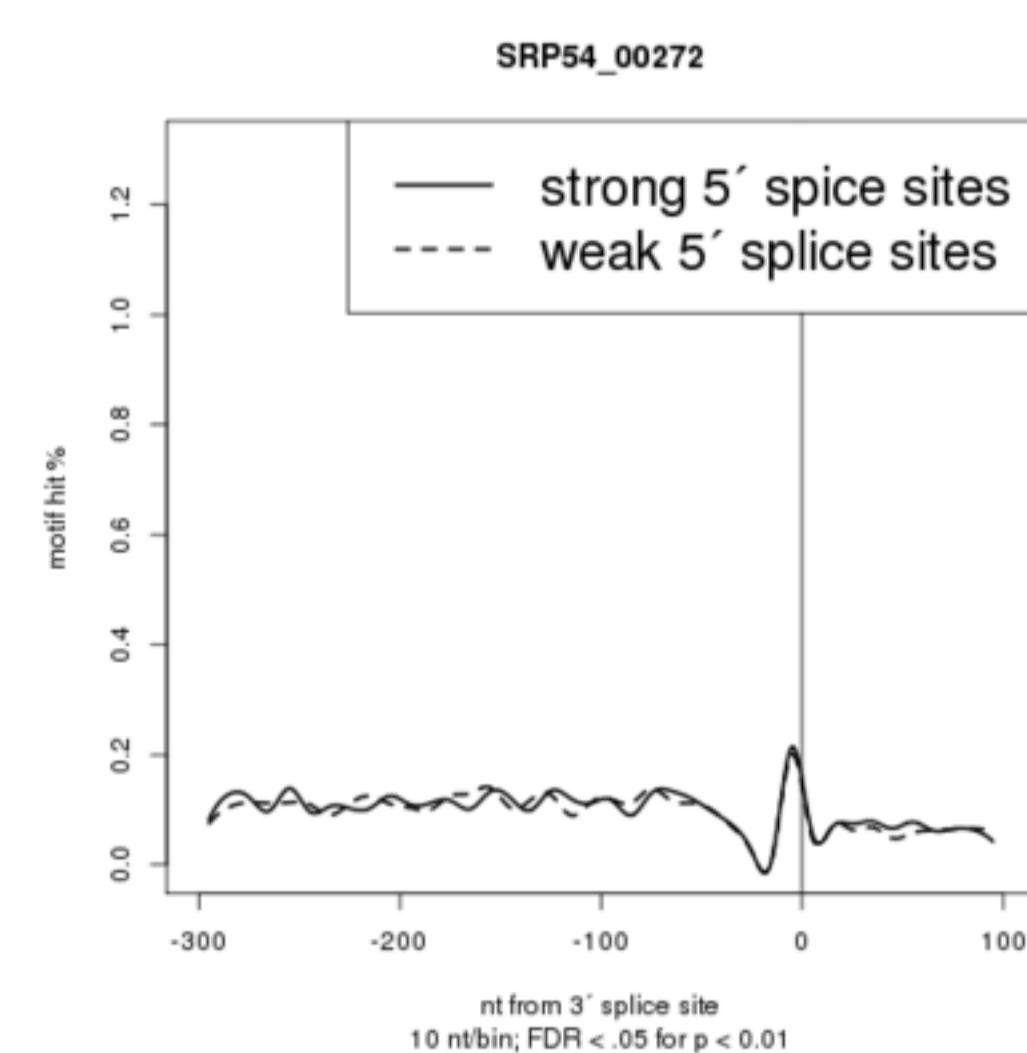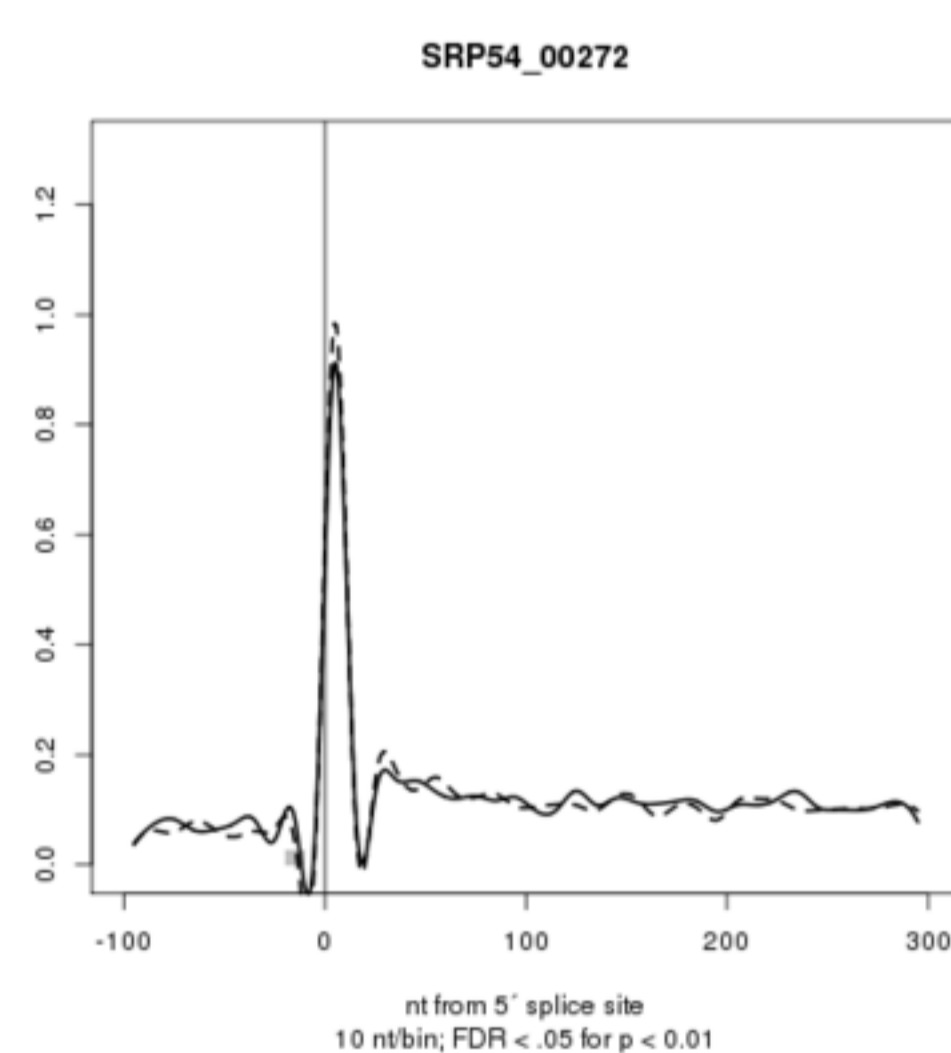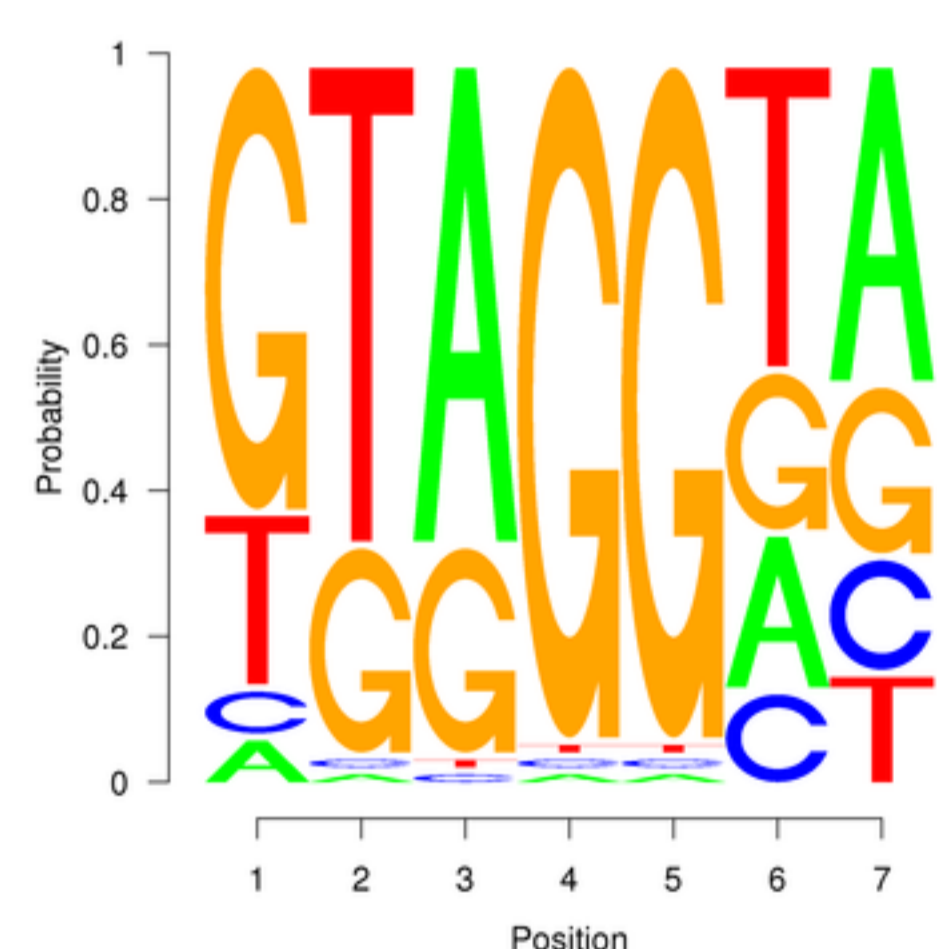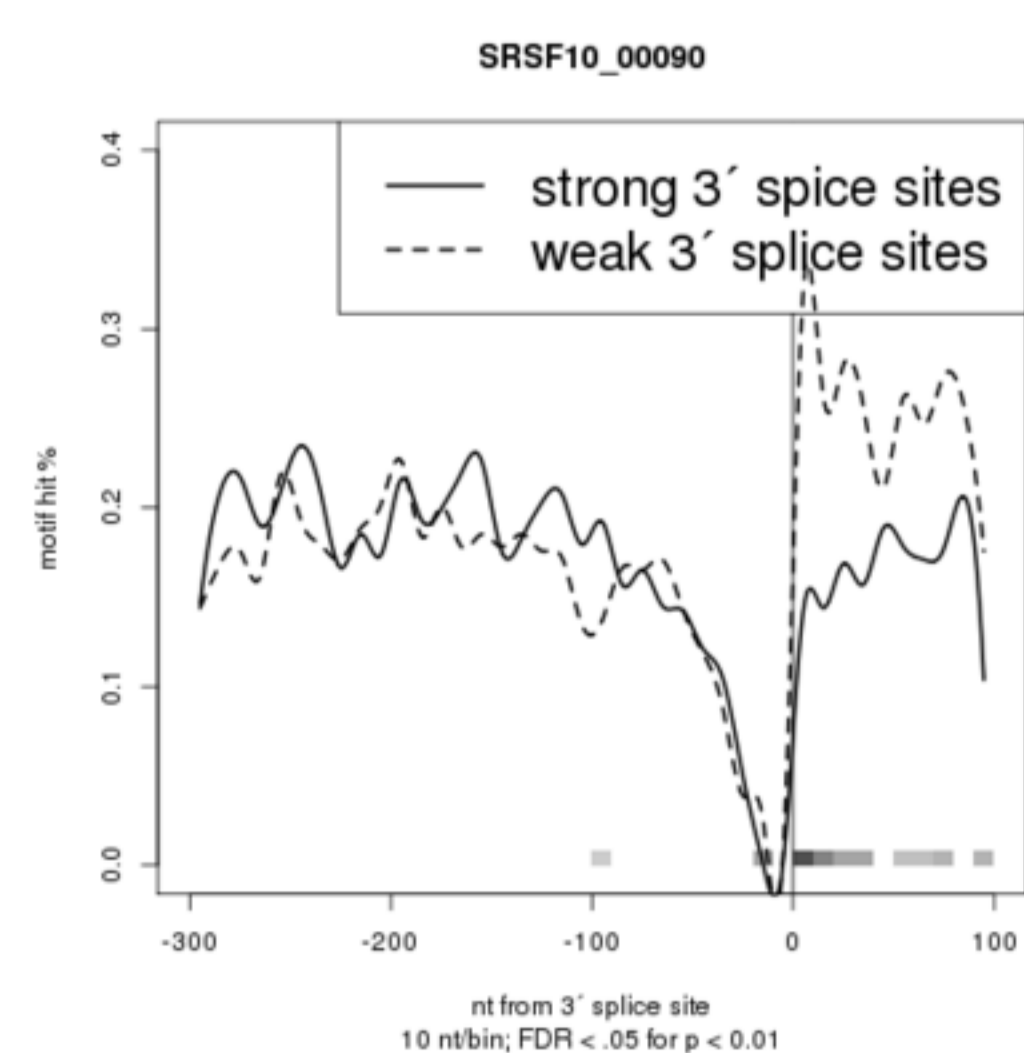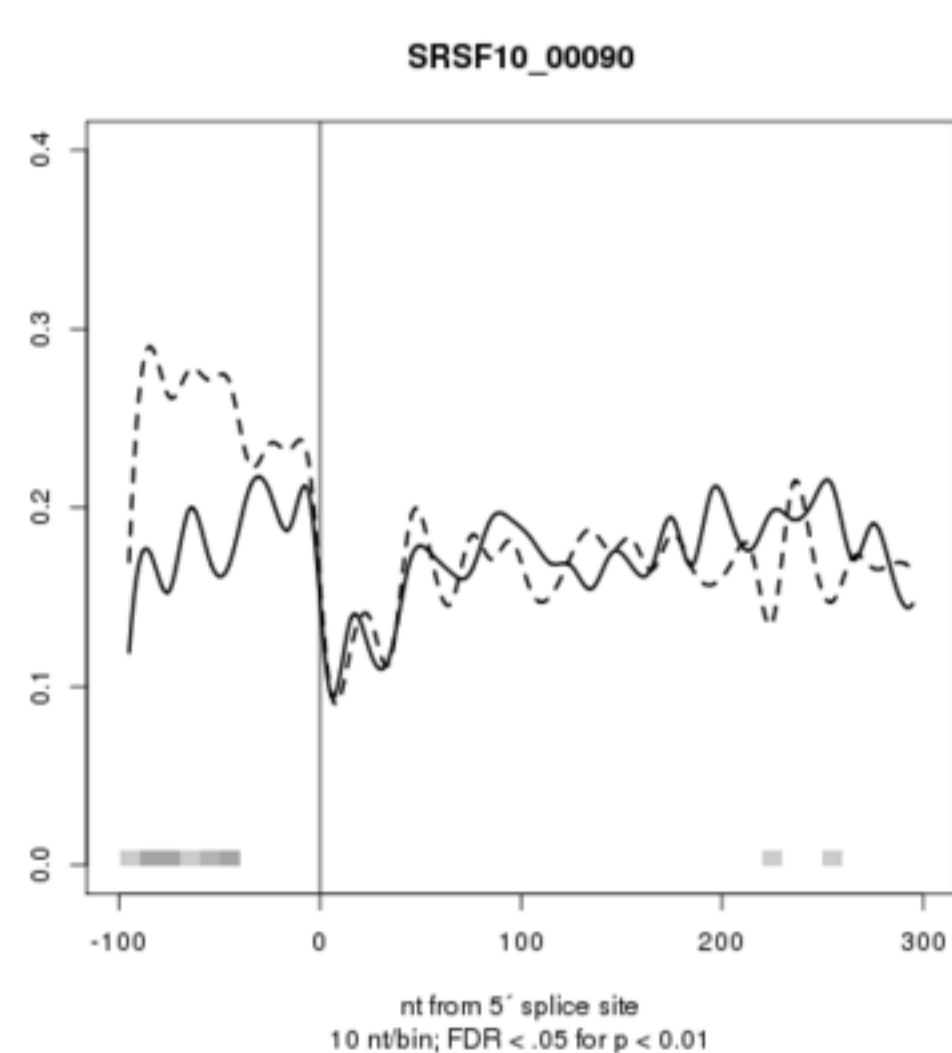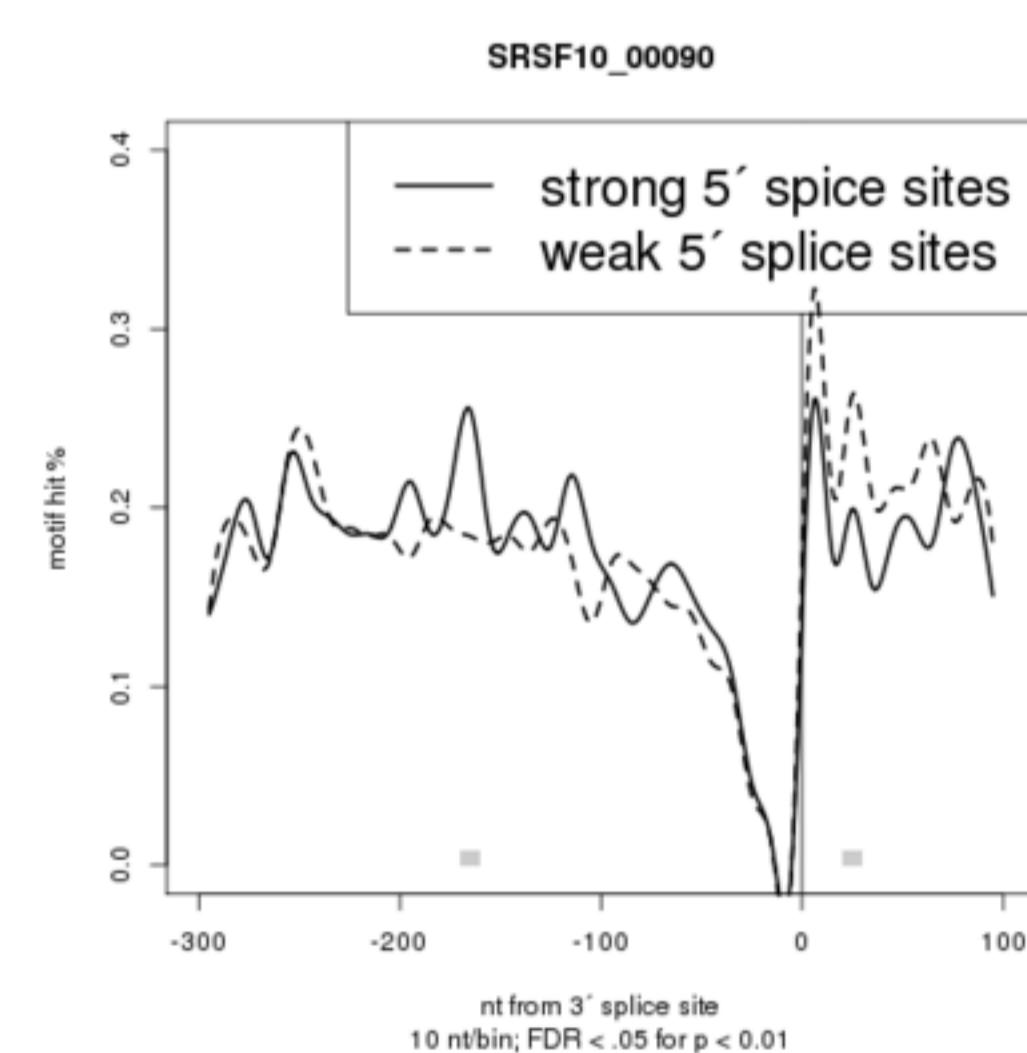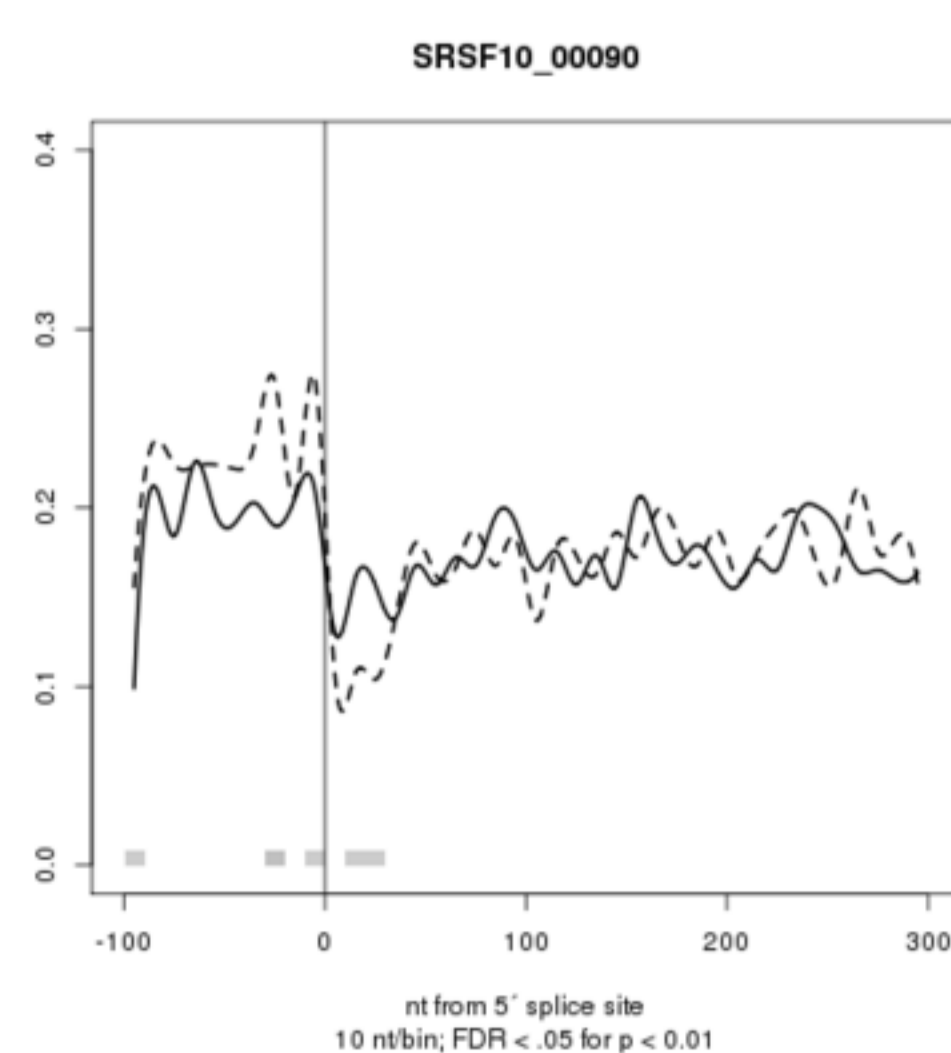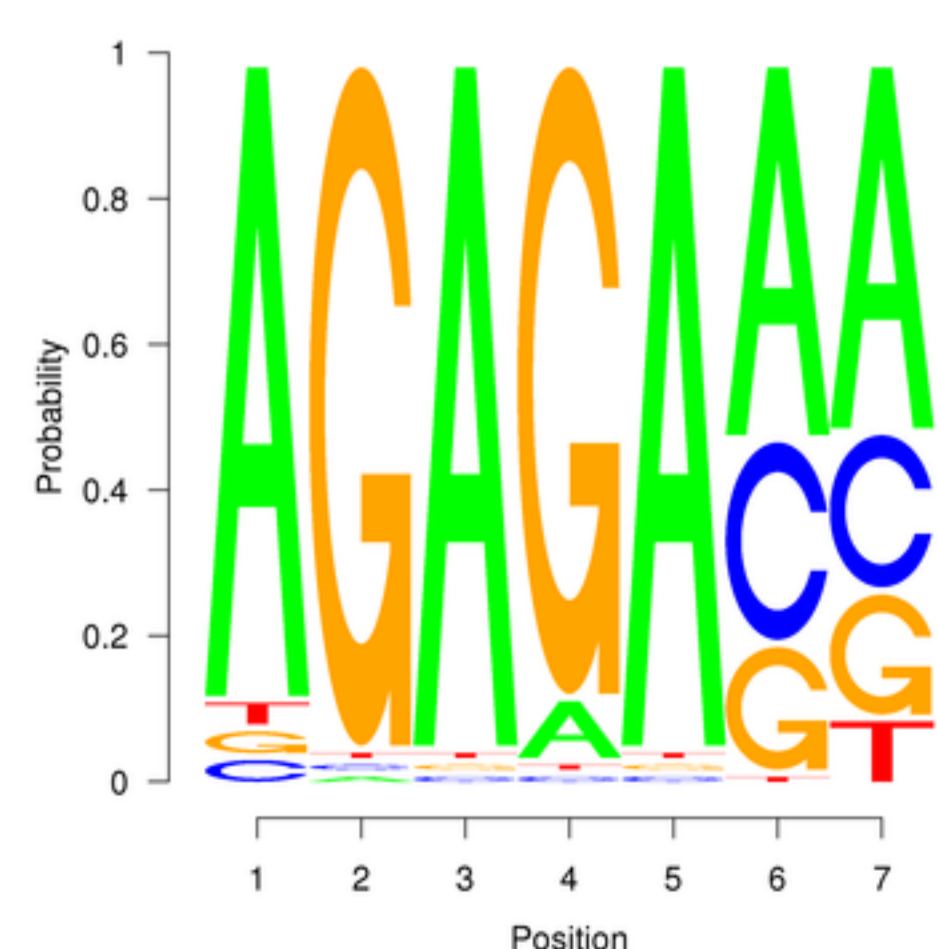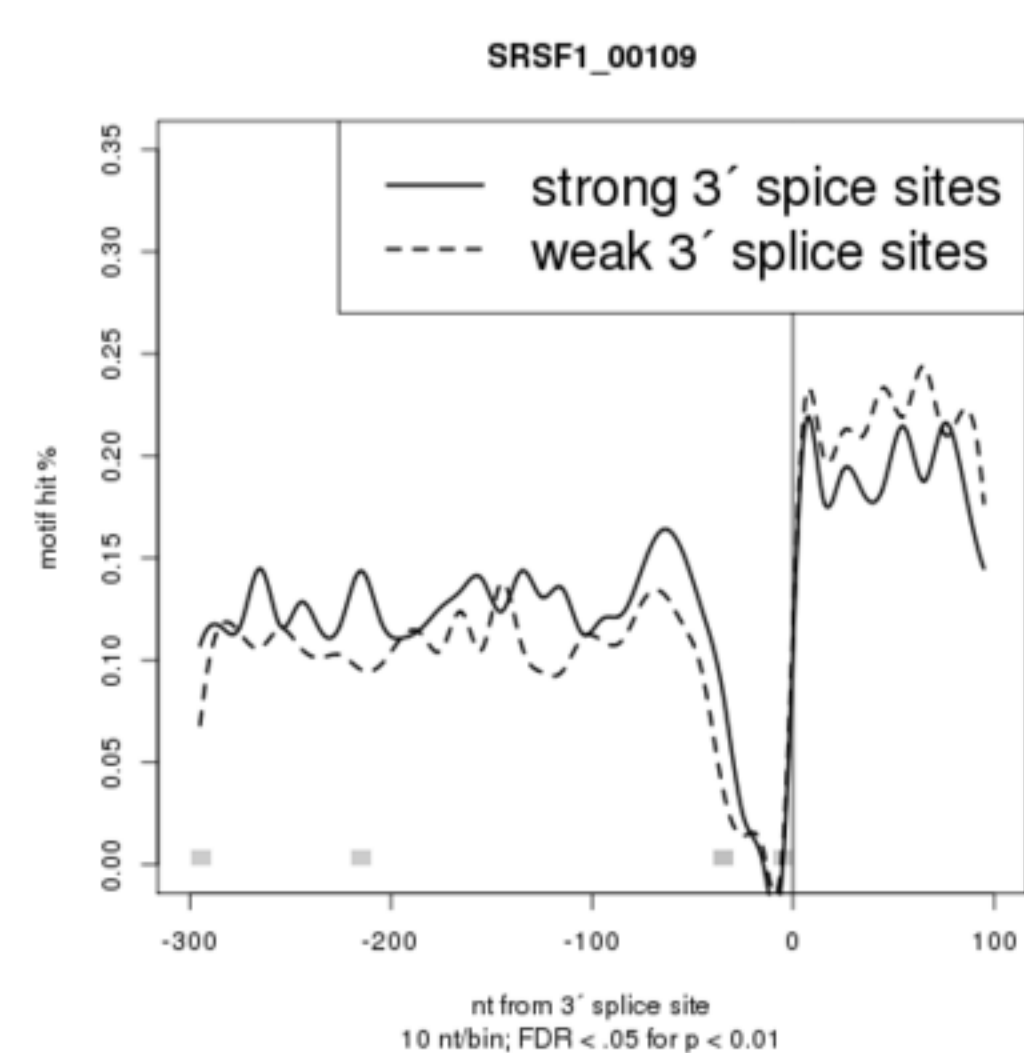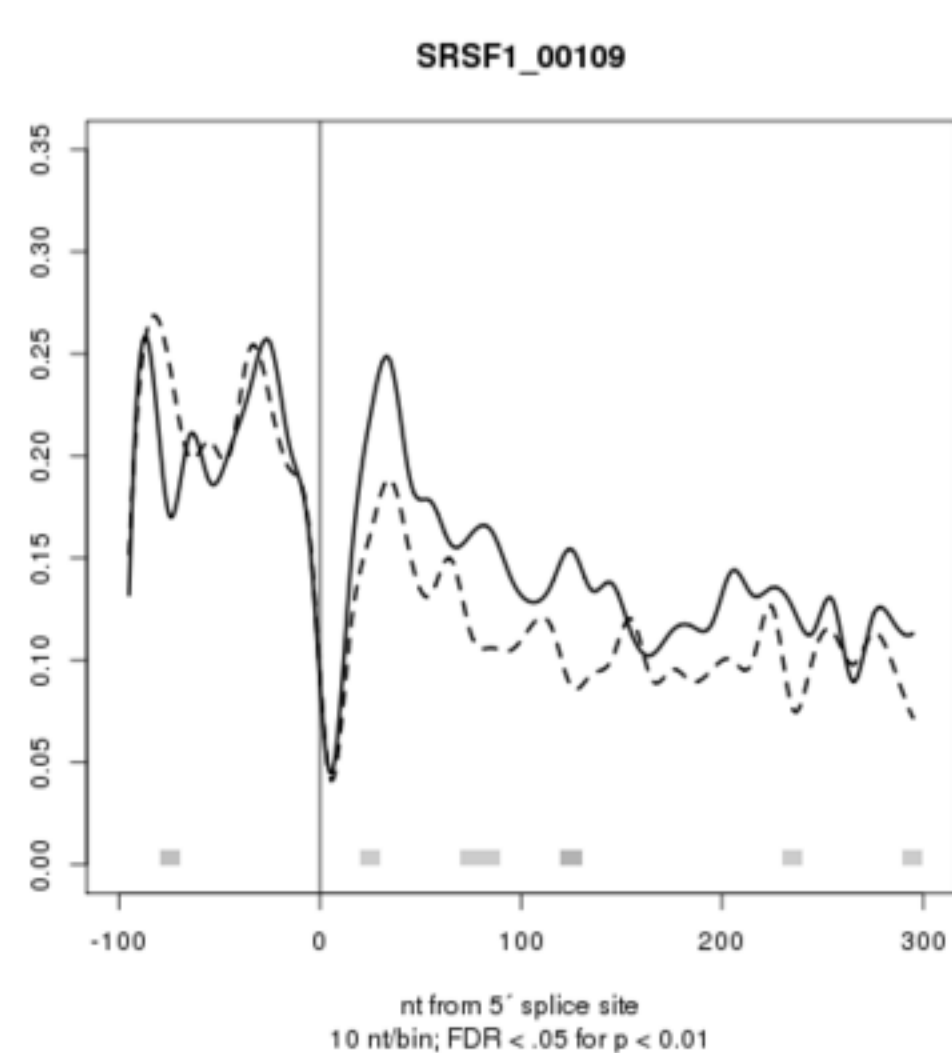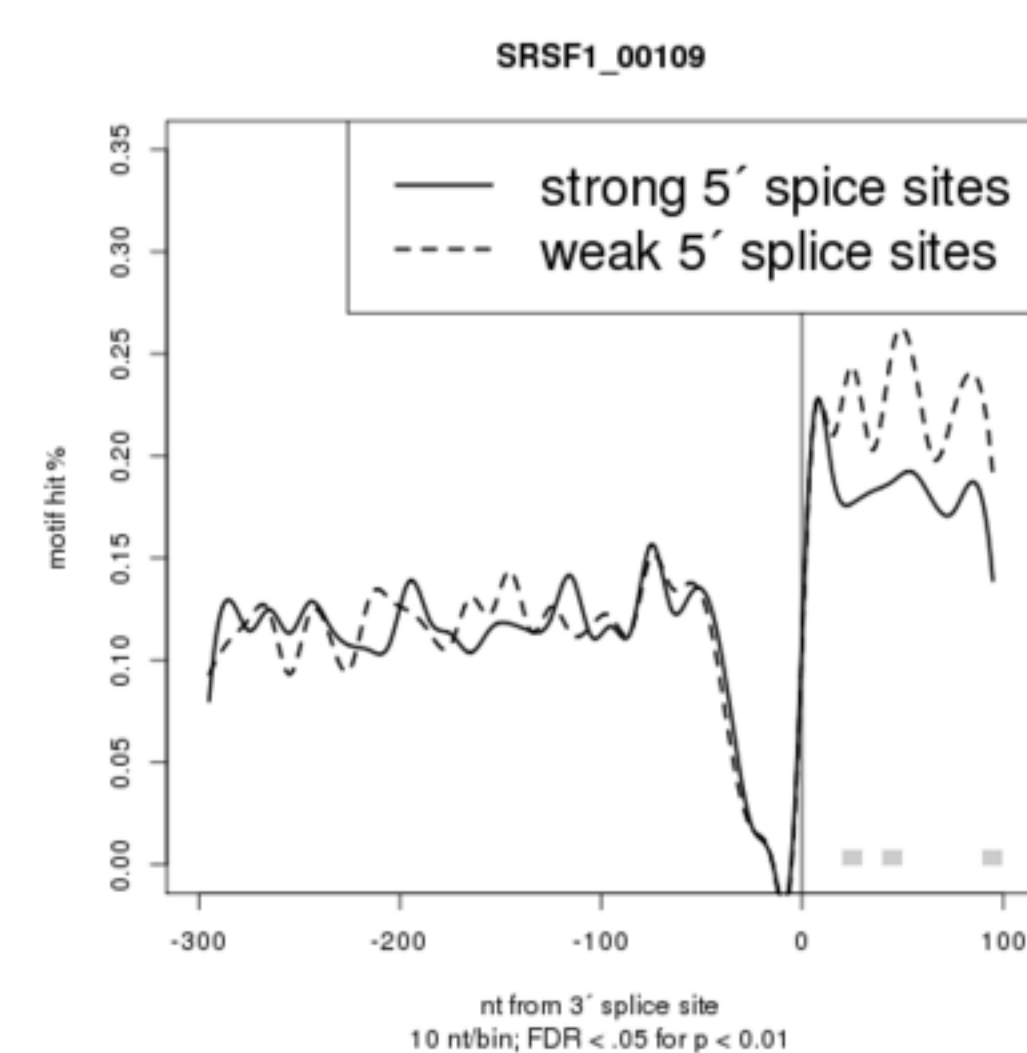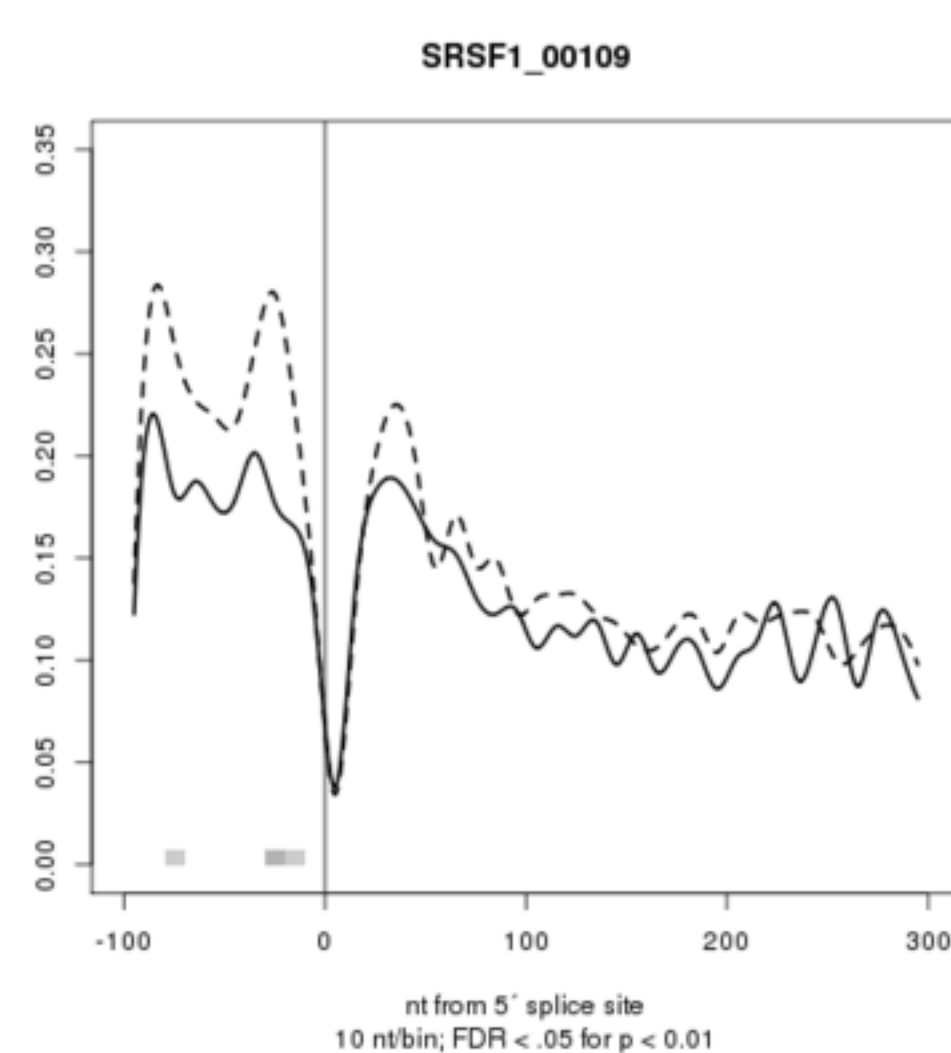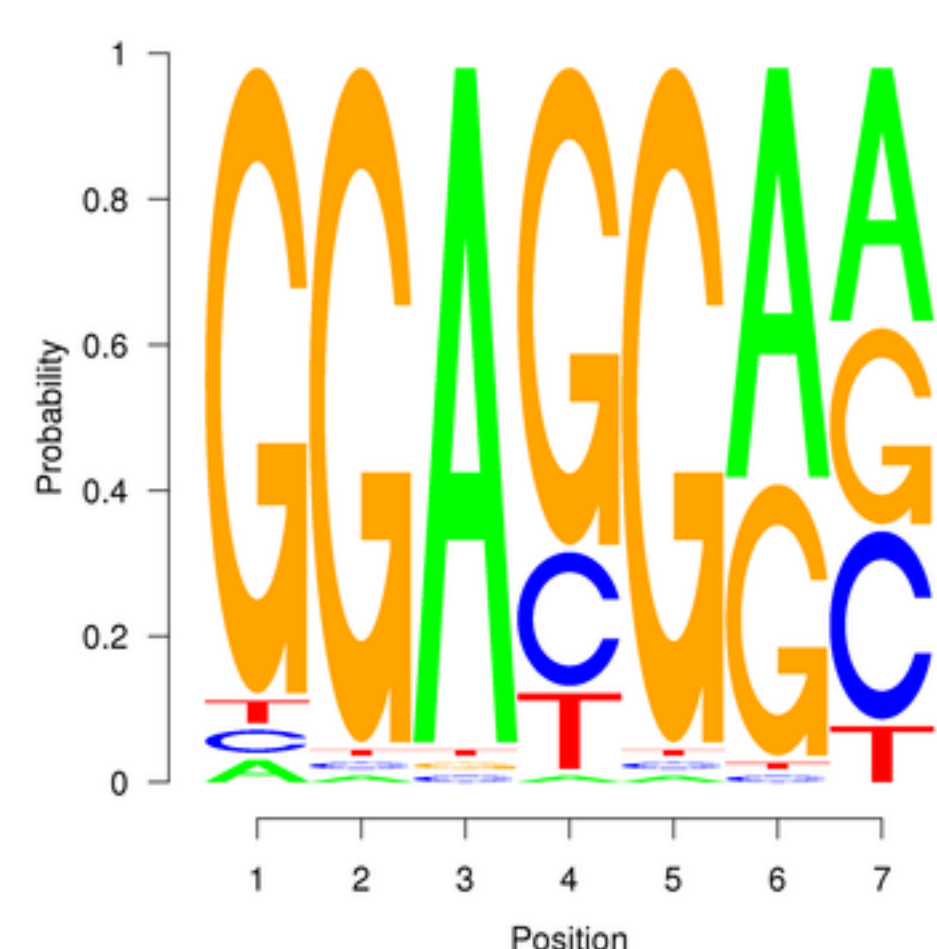

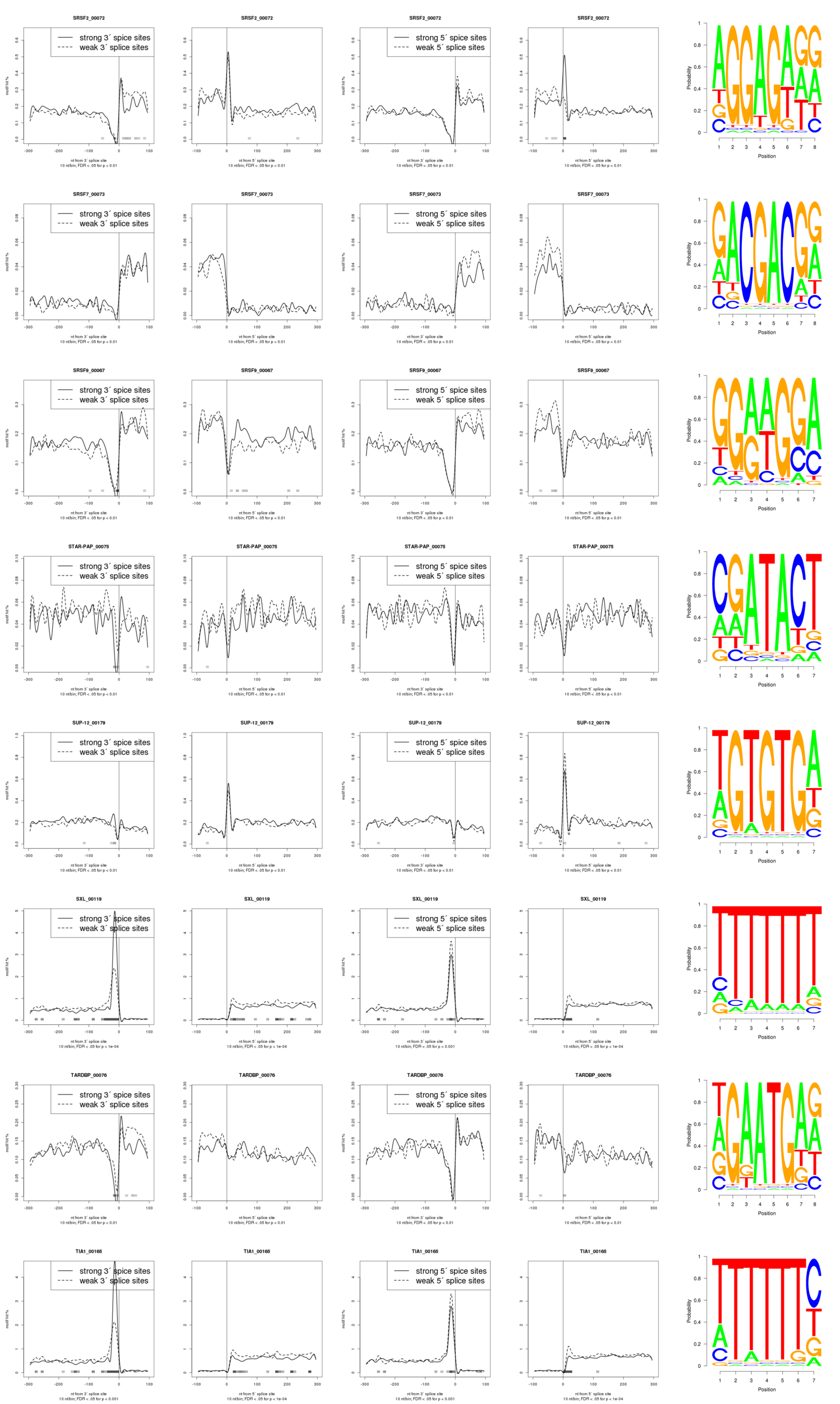

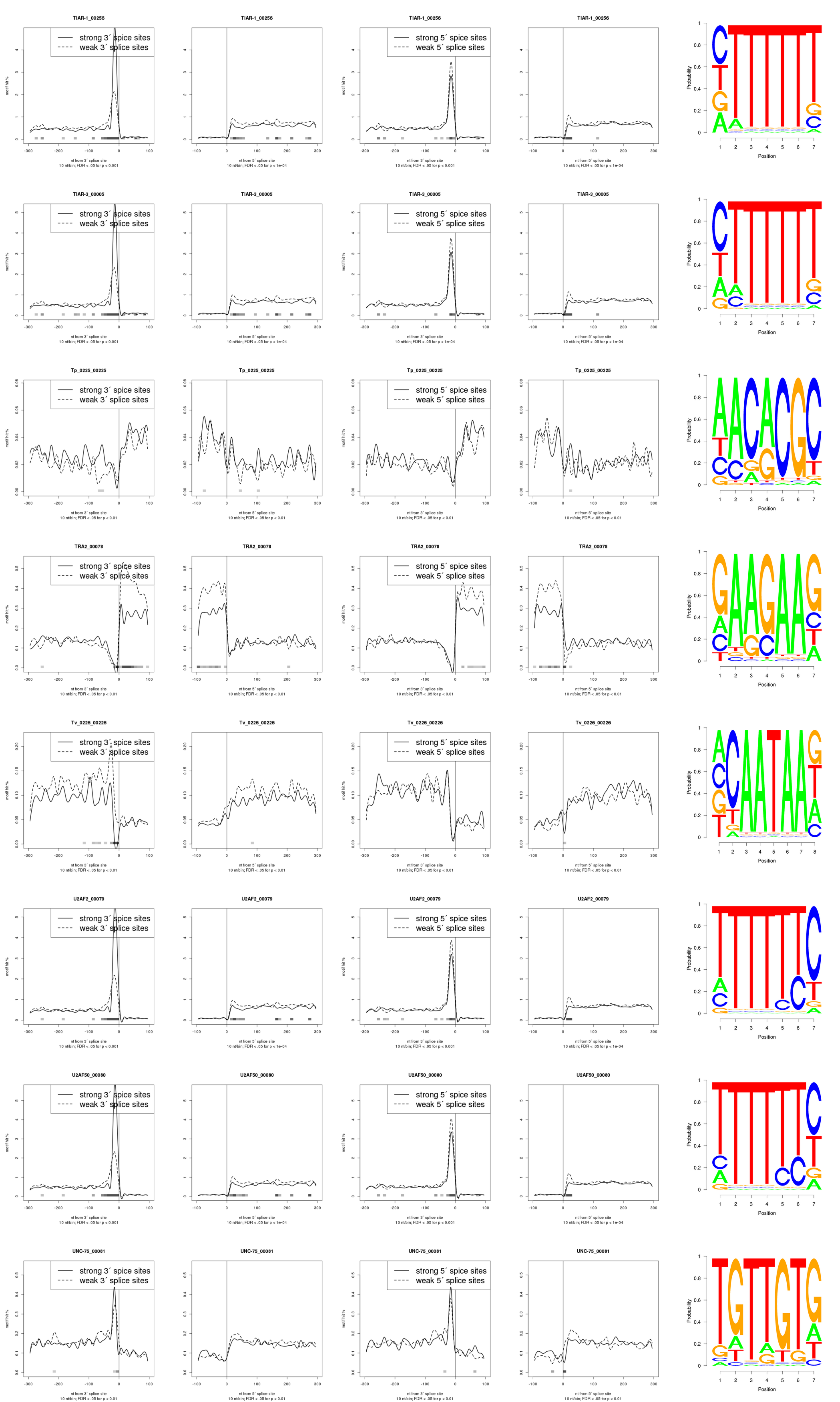

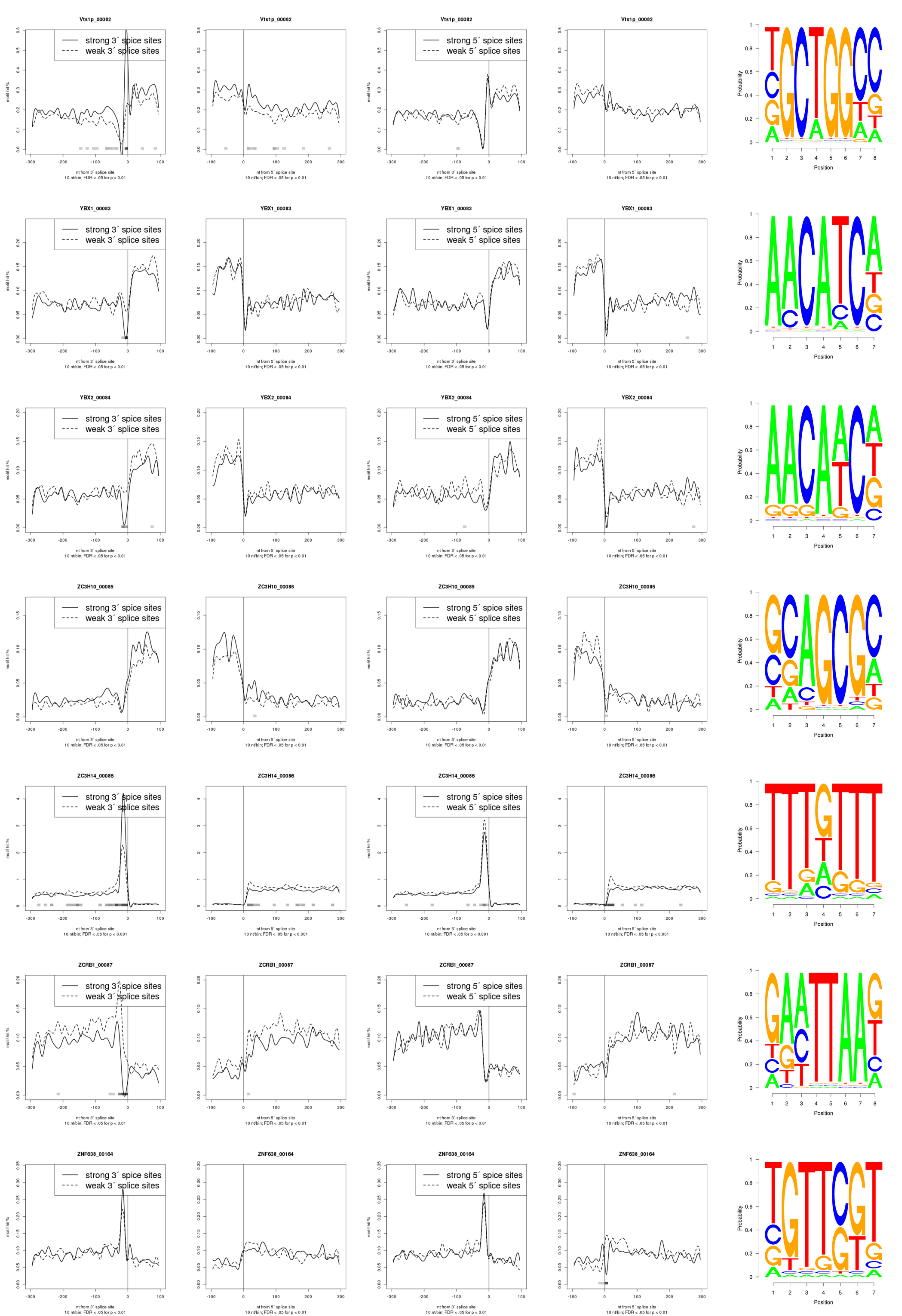

Supplement: S2 File — This is exactly the same as in S1 File, except that only exons longer than 100nt were considered. So in this case “strong” is the upper quartile of splice site strengths amongst exons longer than 100nt, and similarly for “weak”. (PDF) [file pone.0132448.s011.pdf]

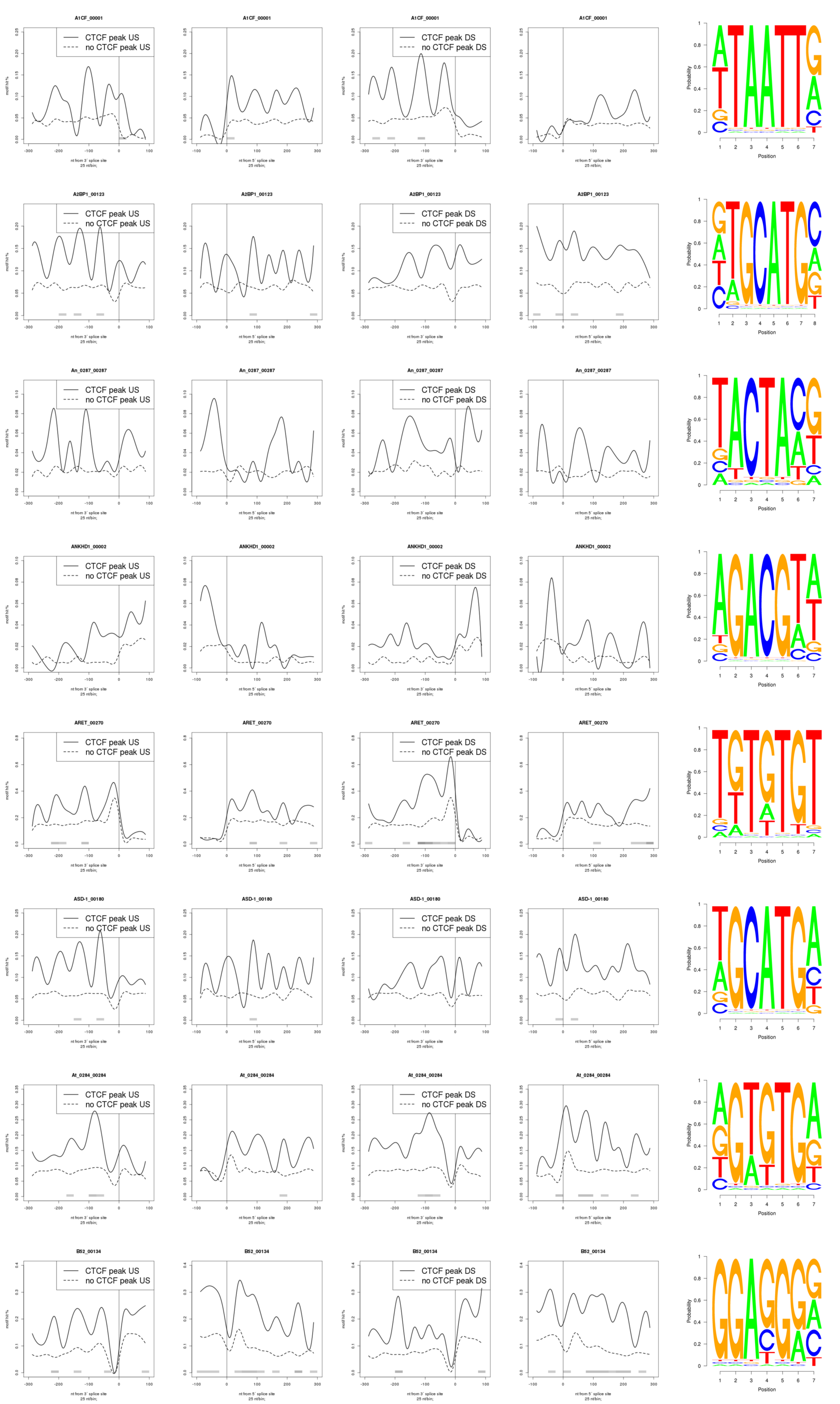

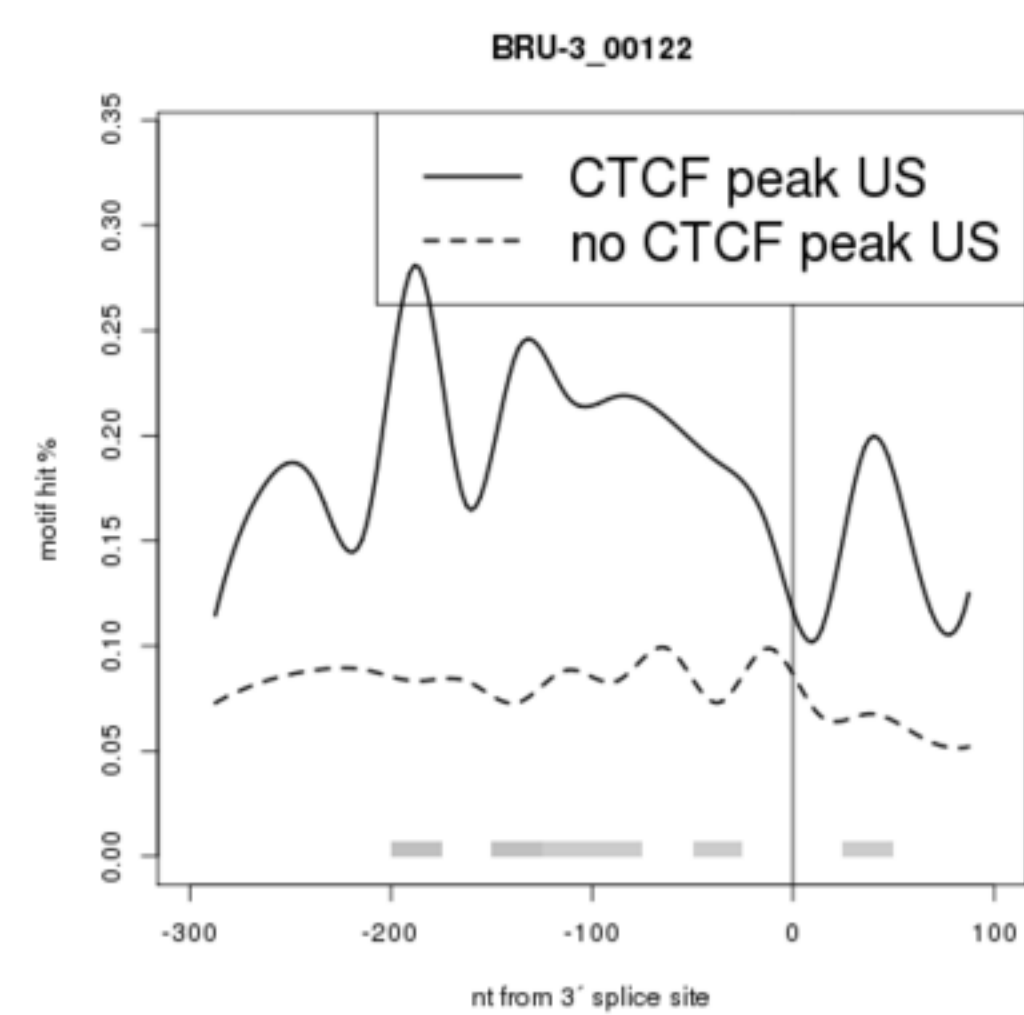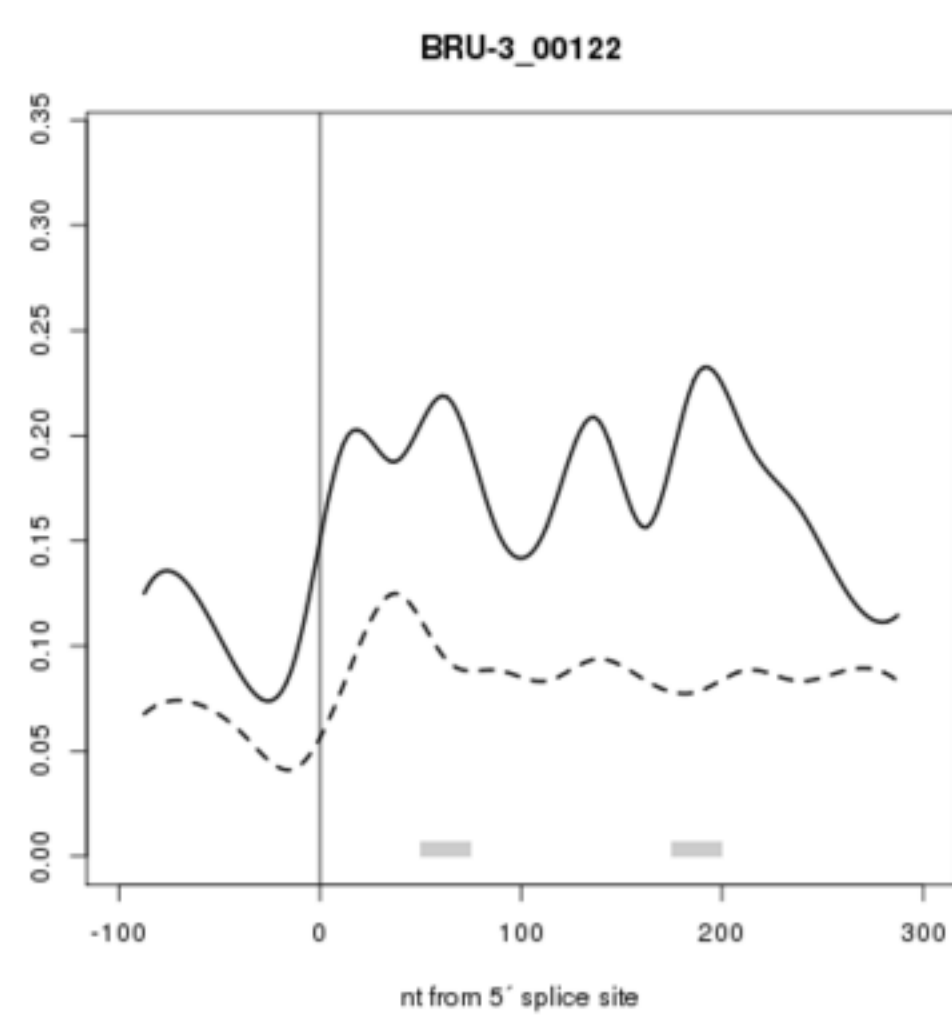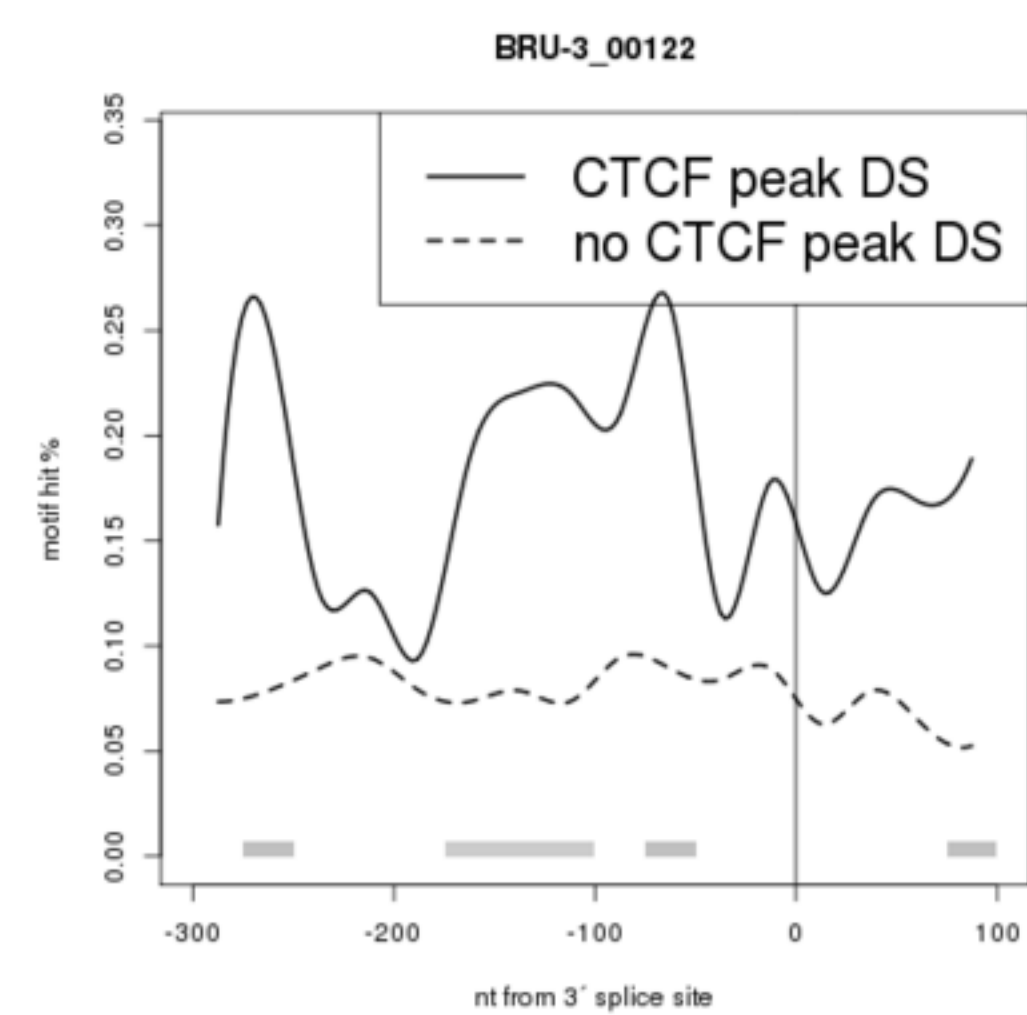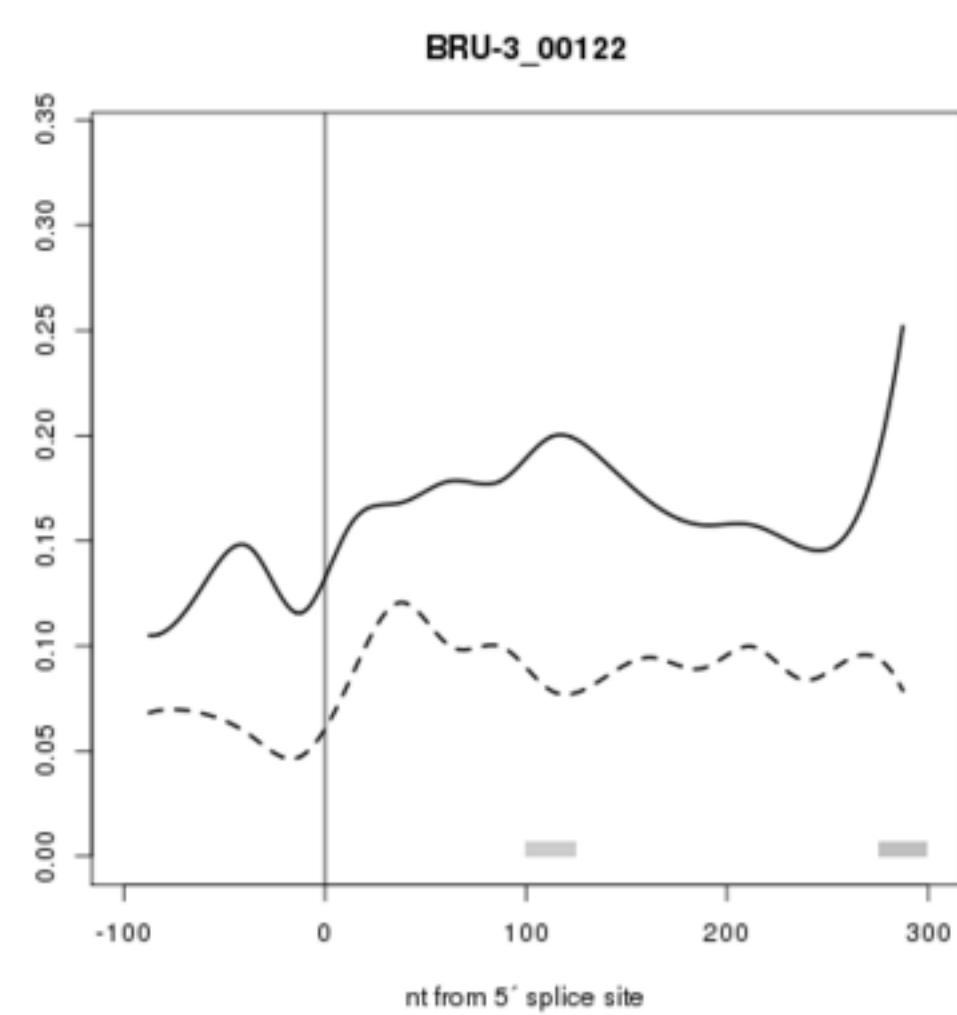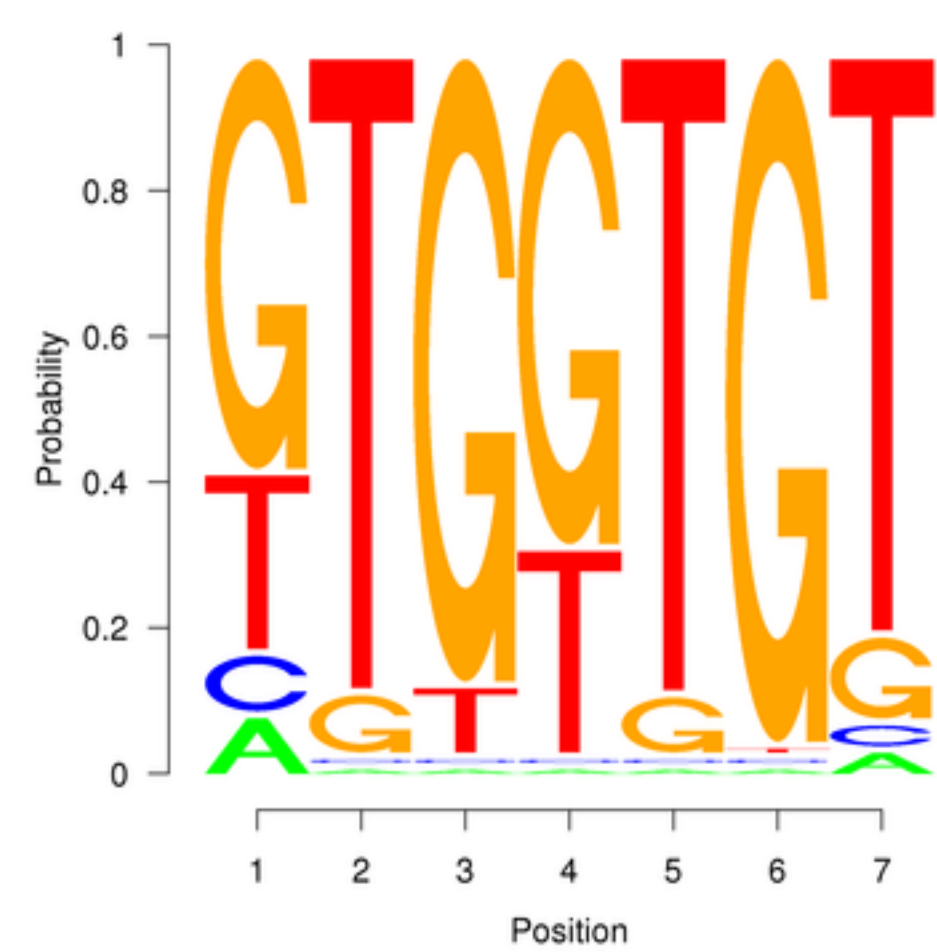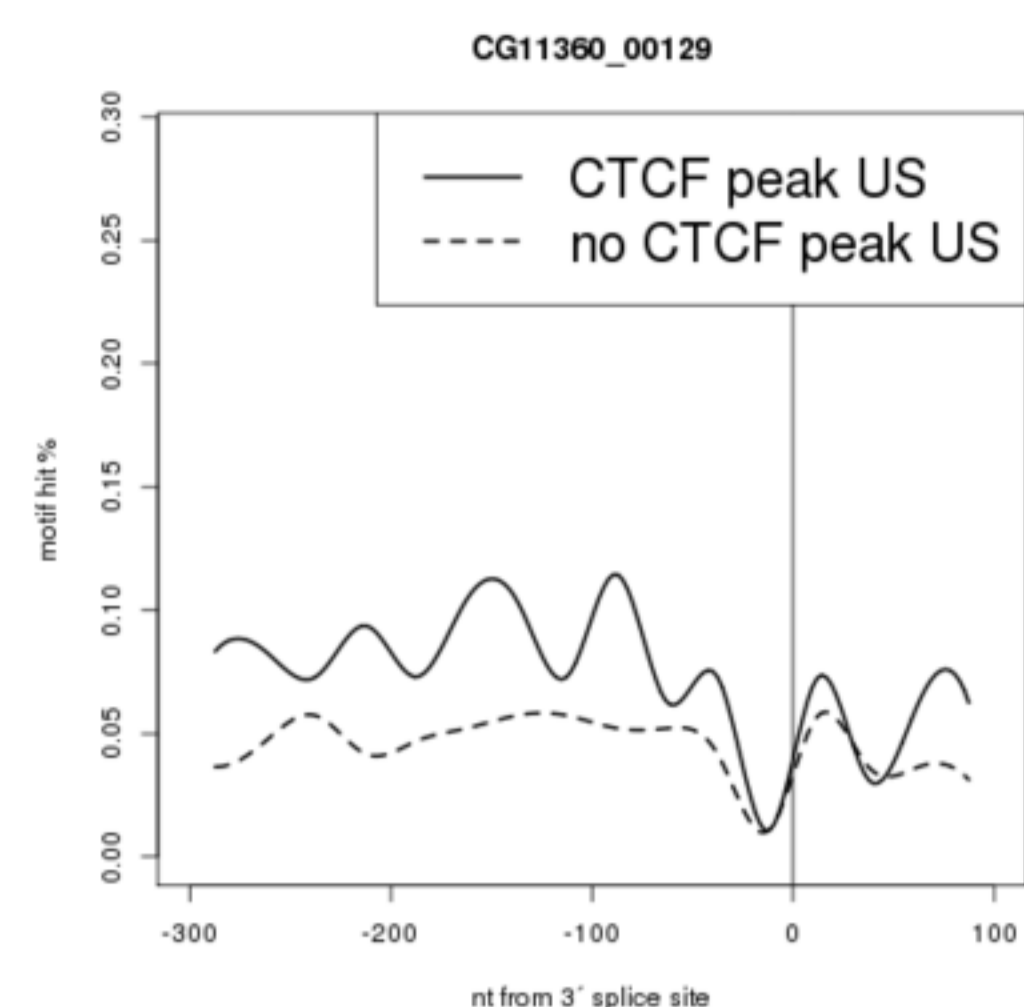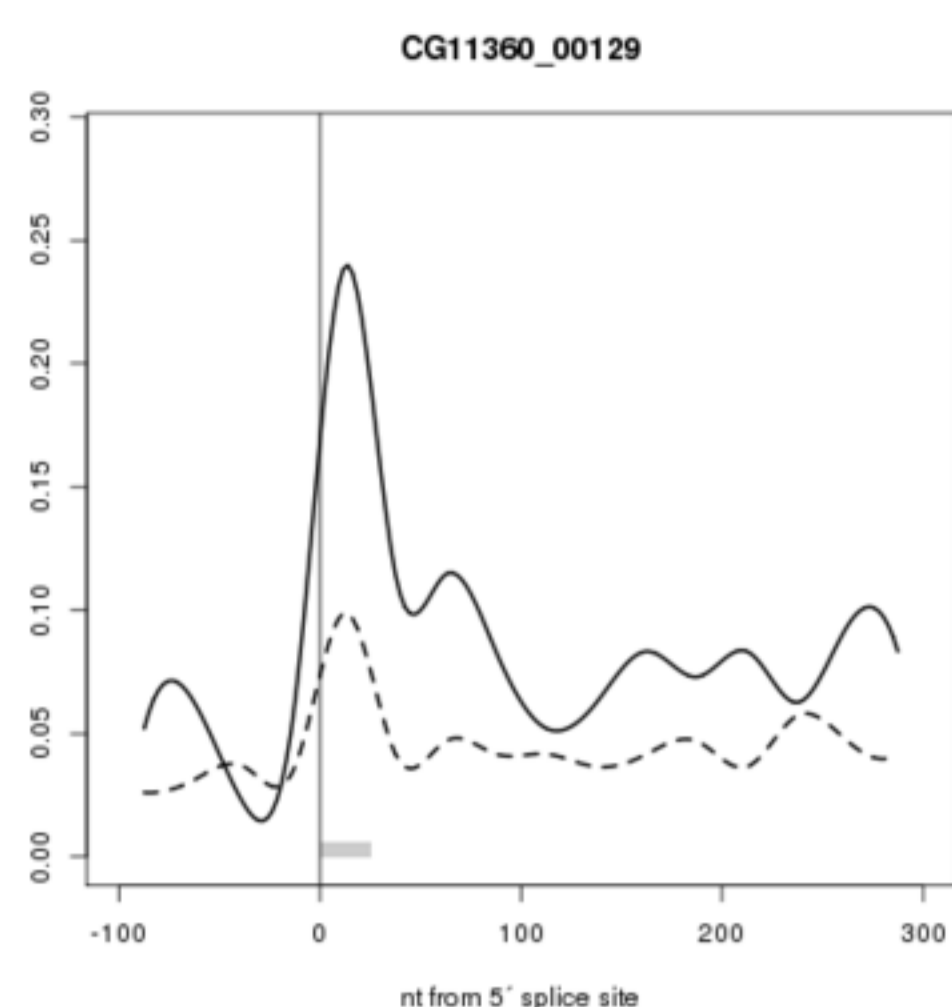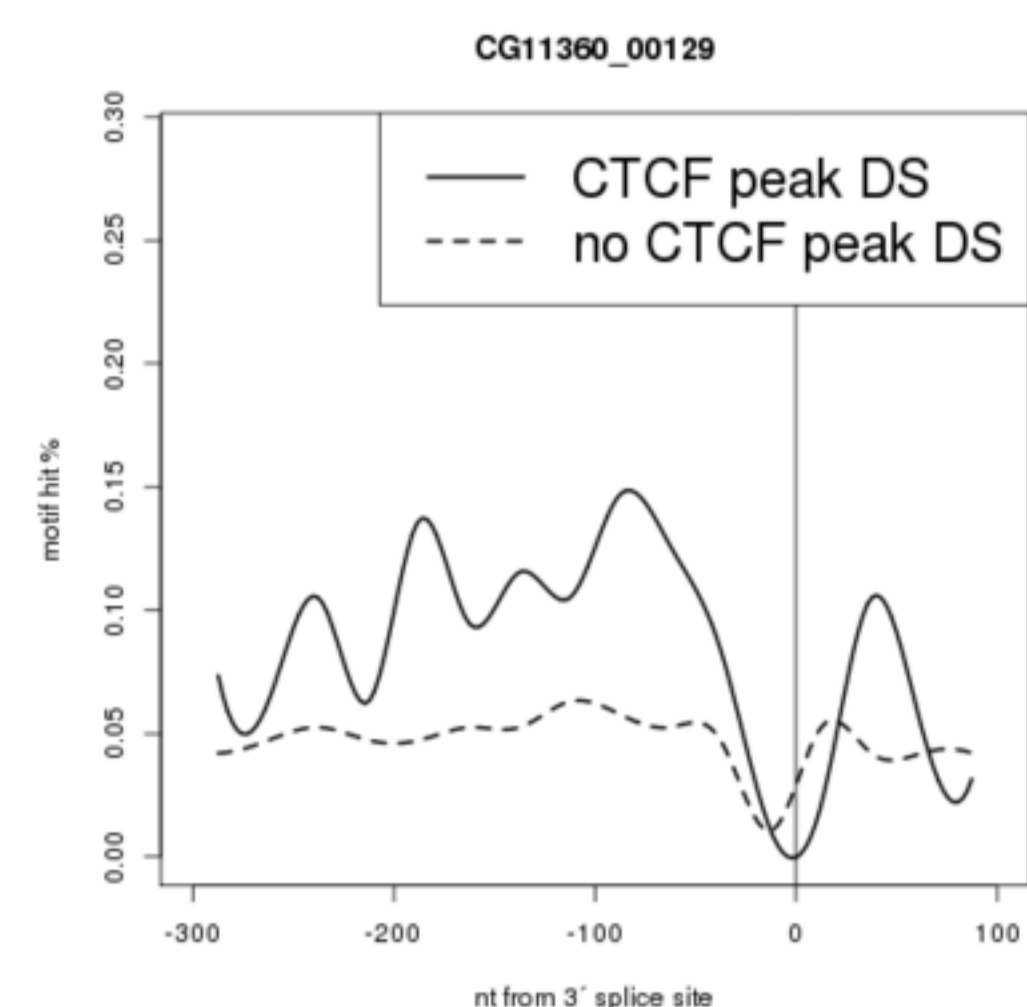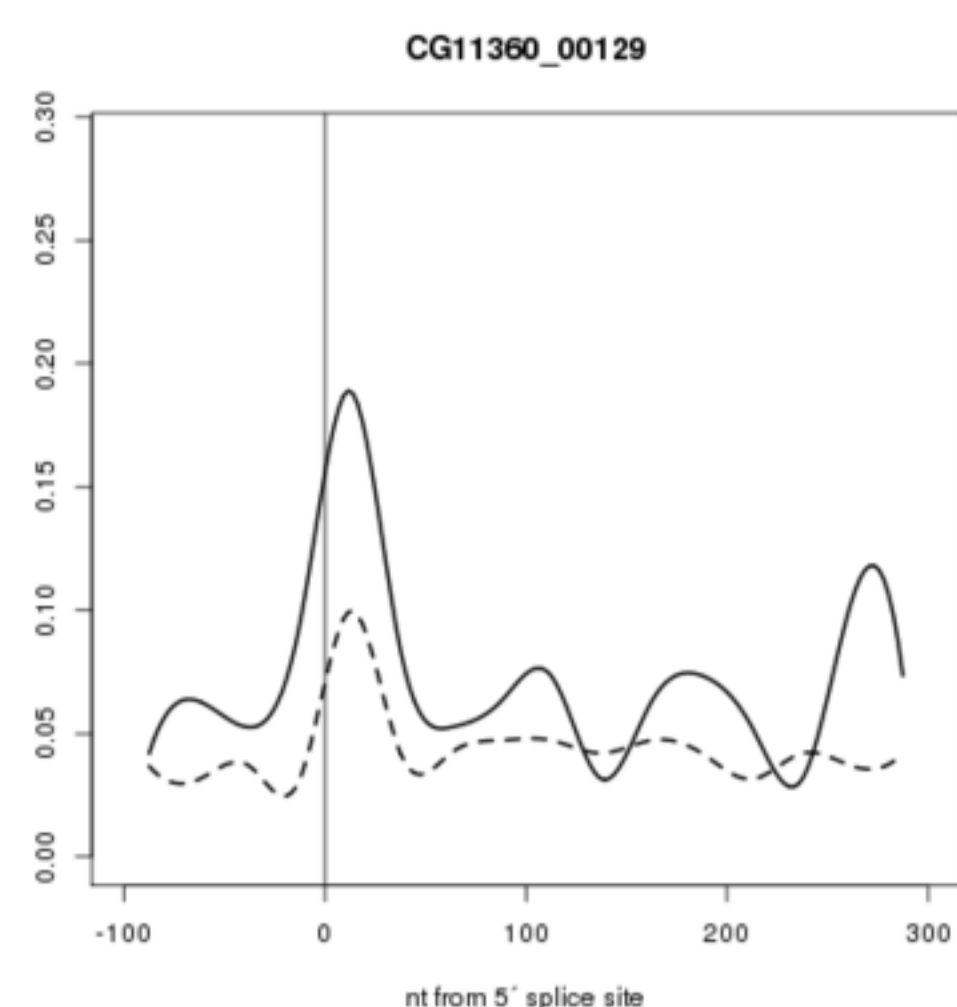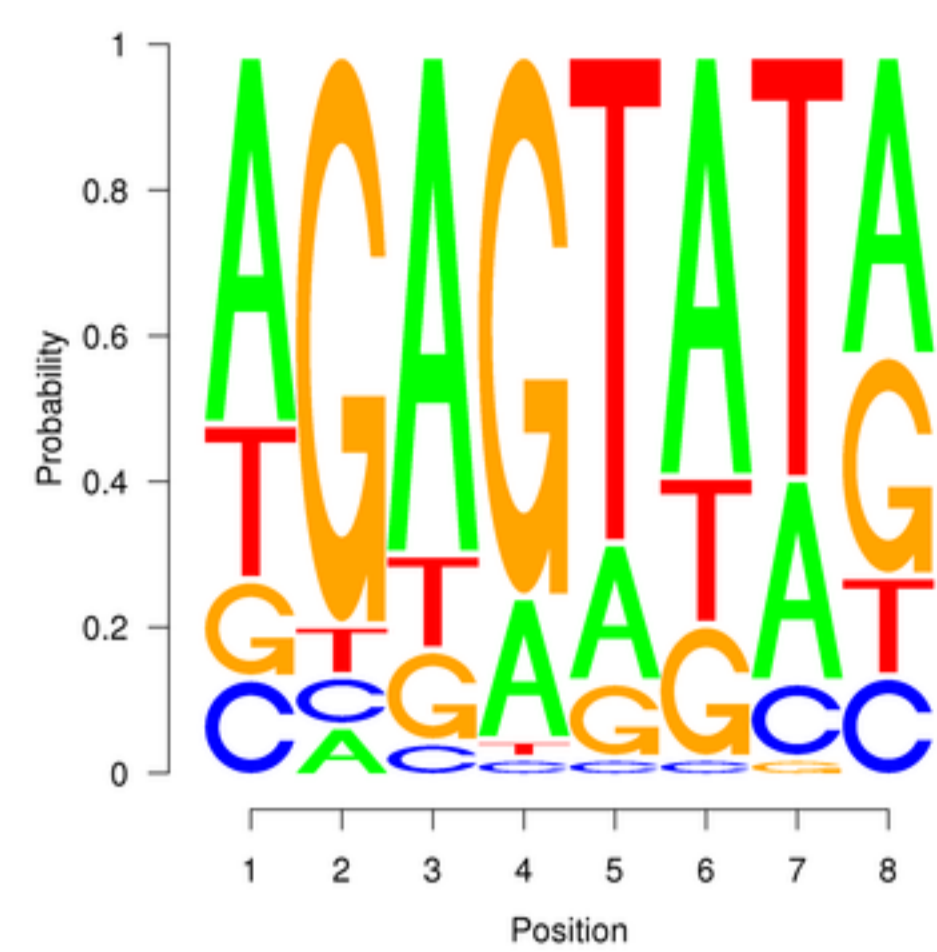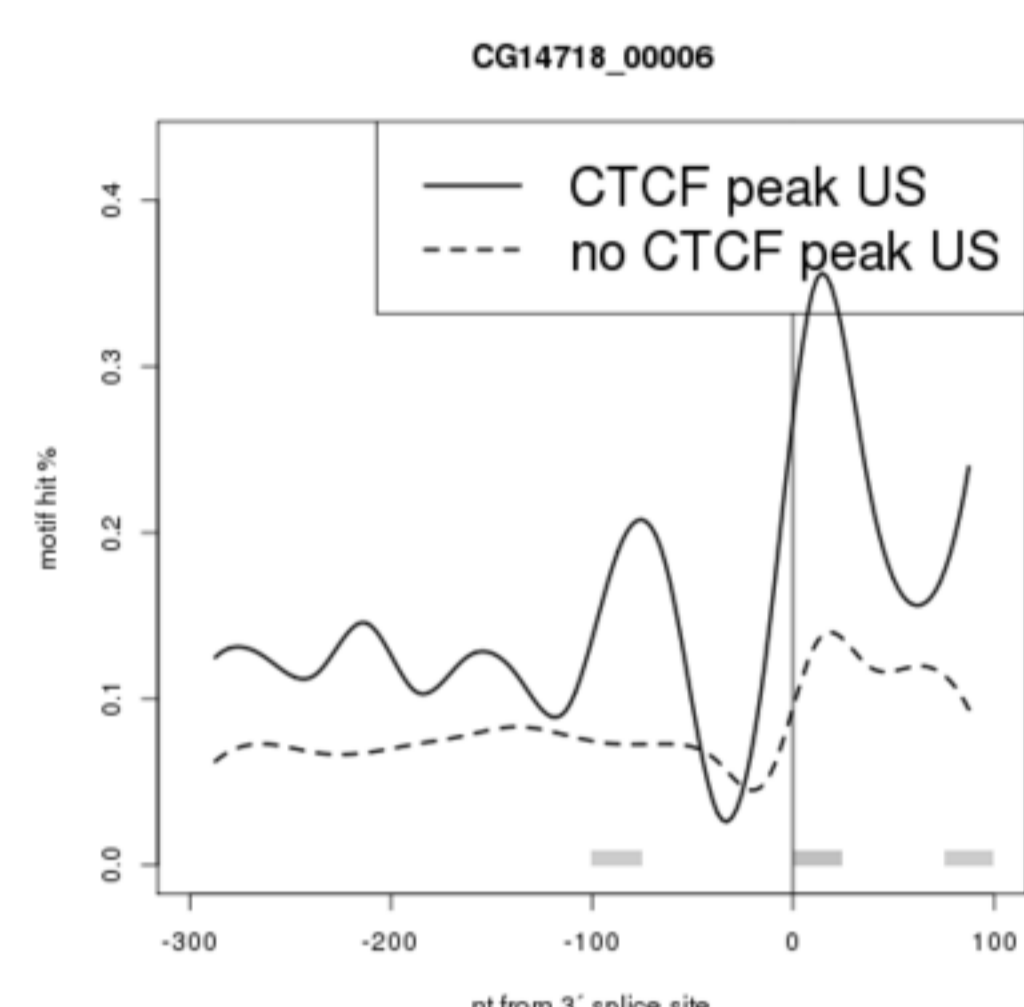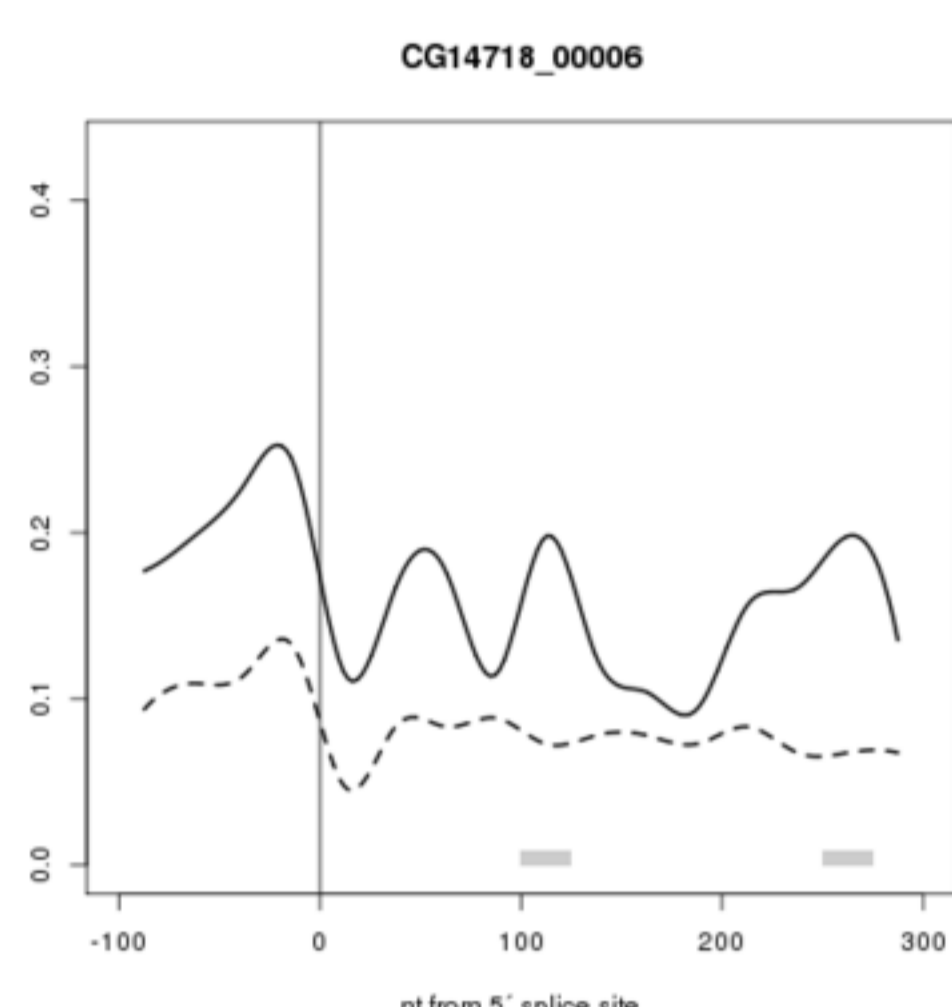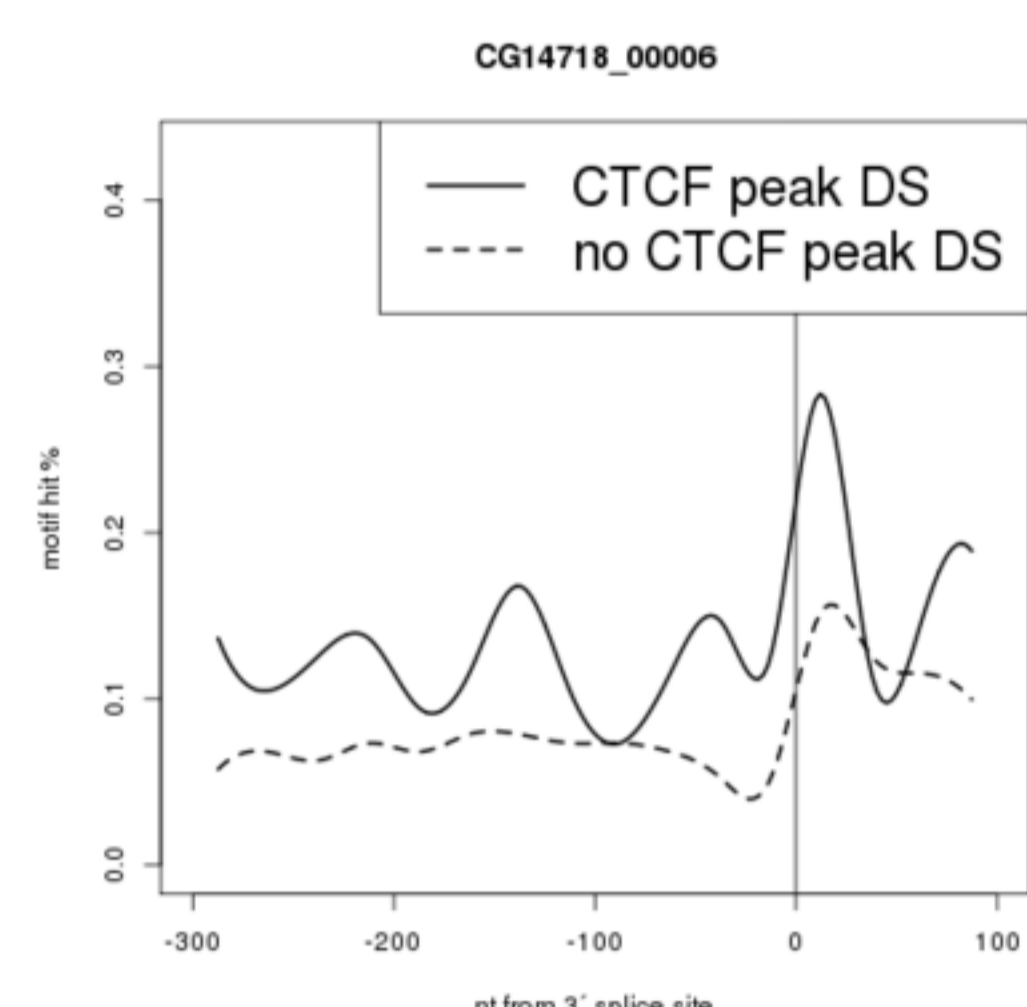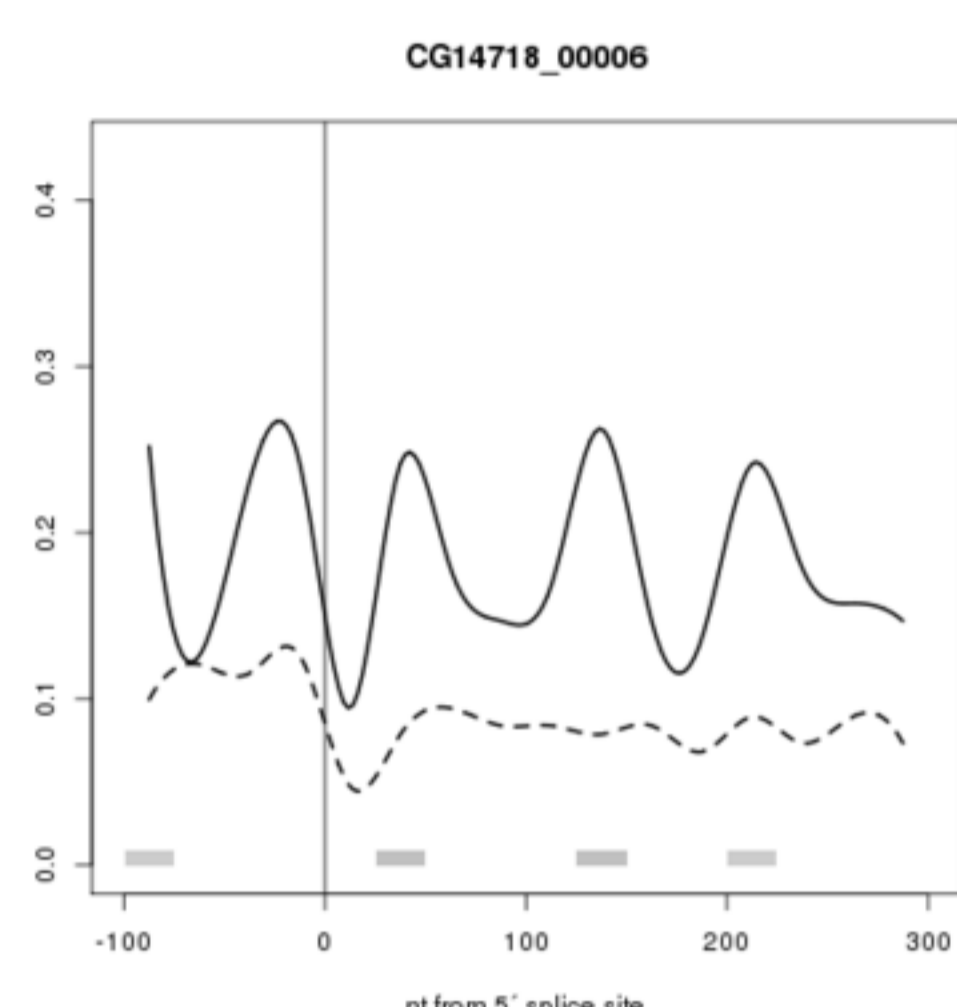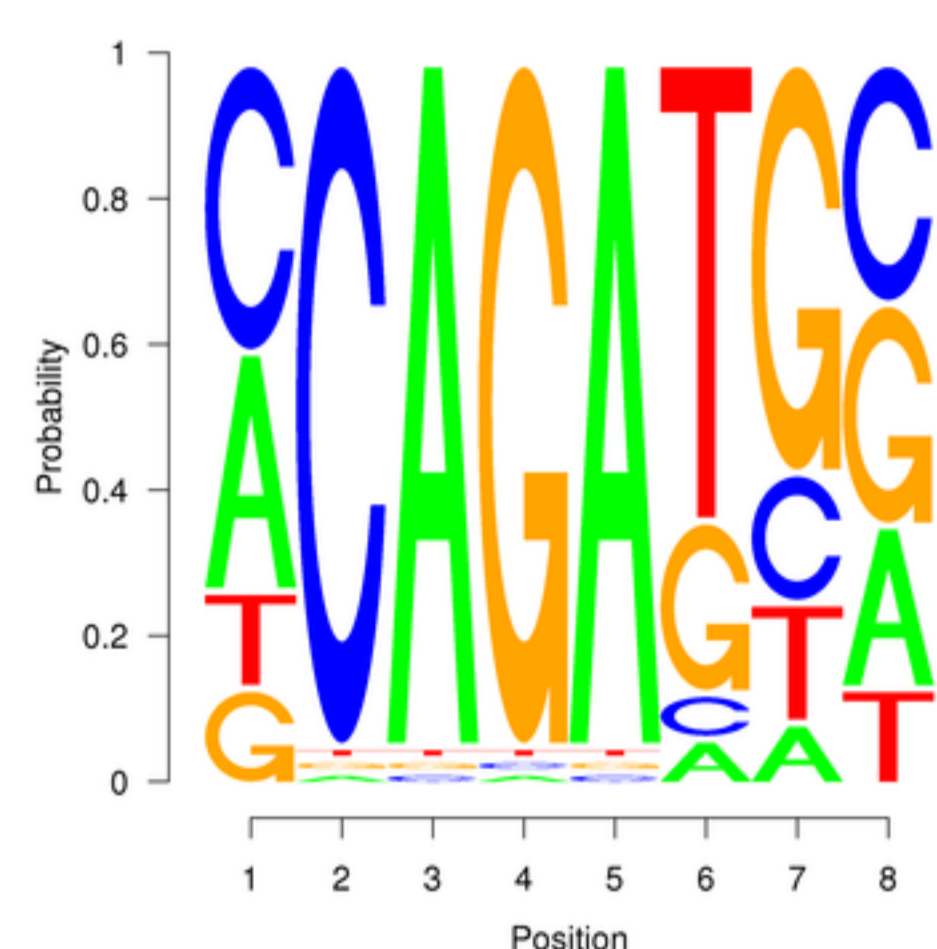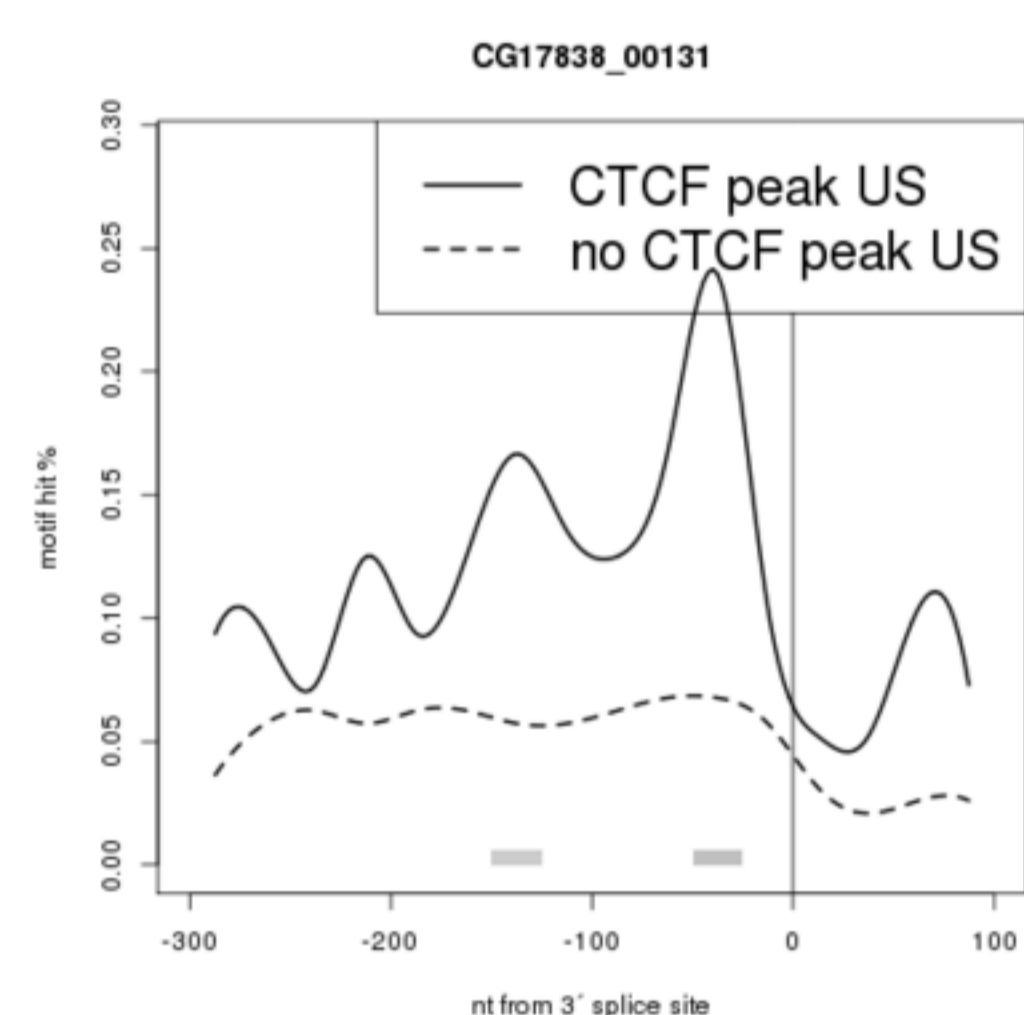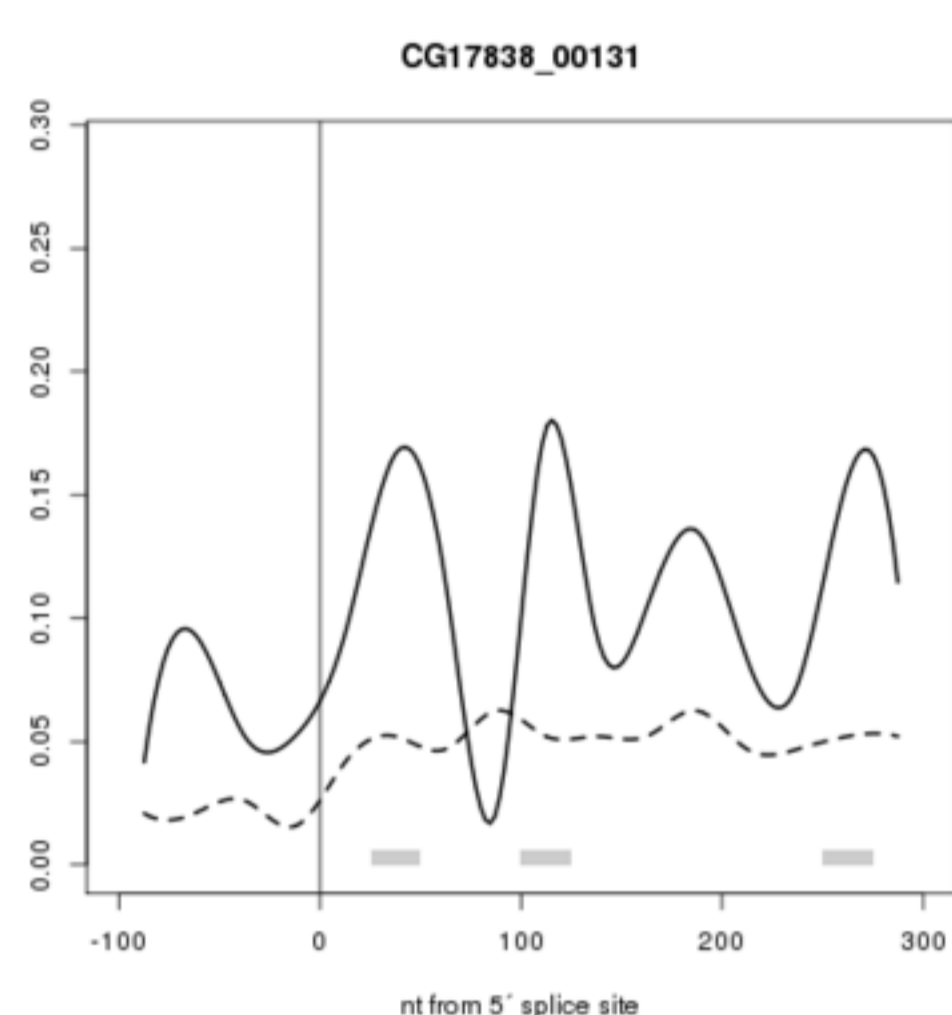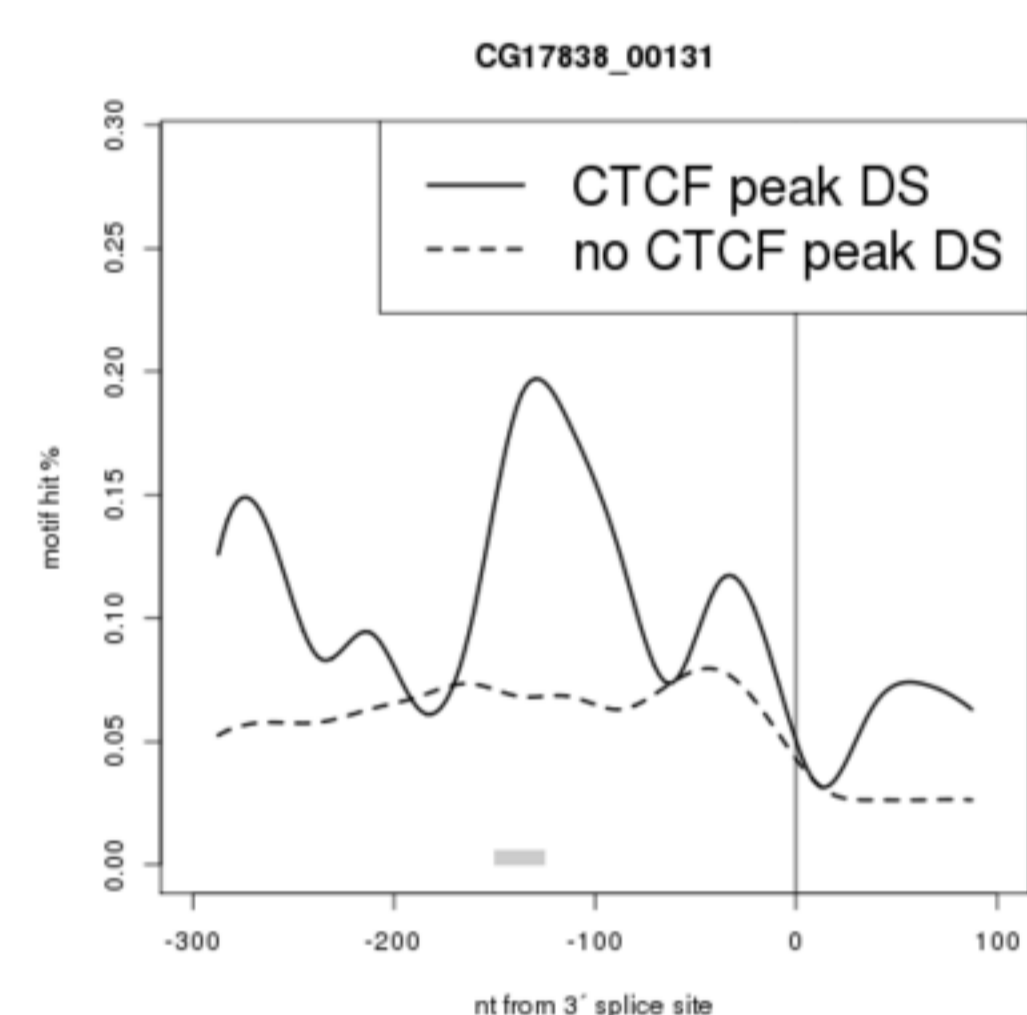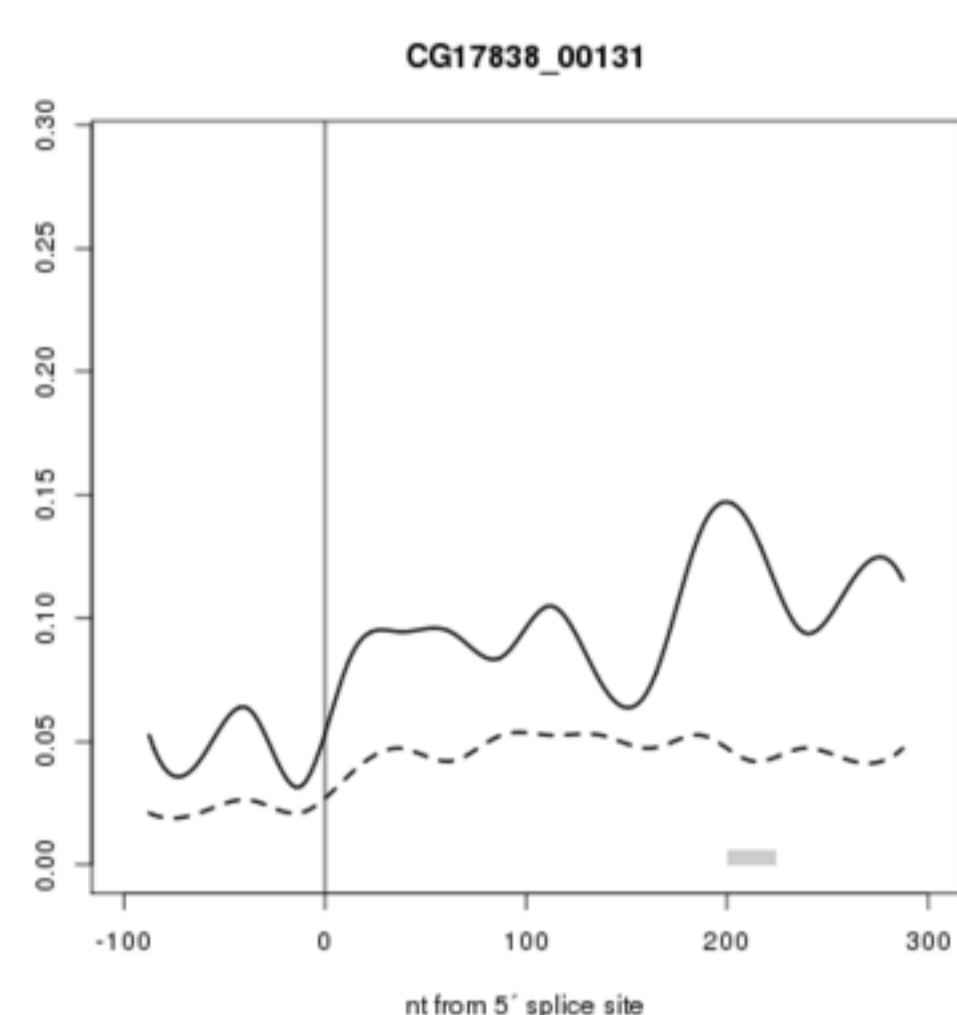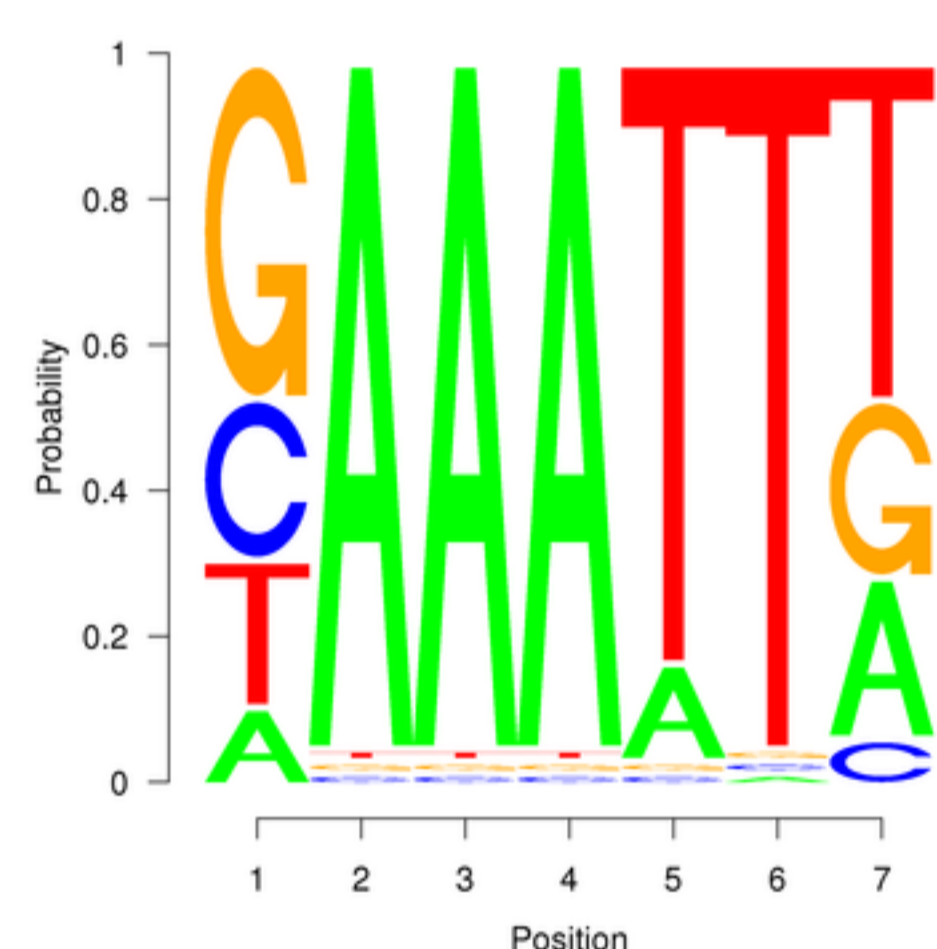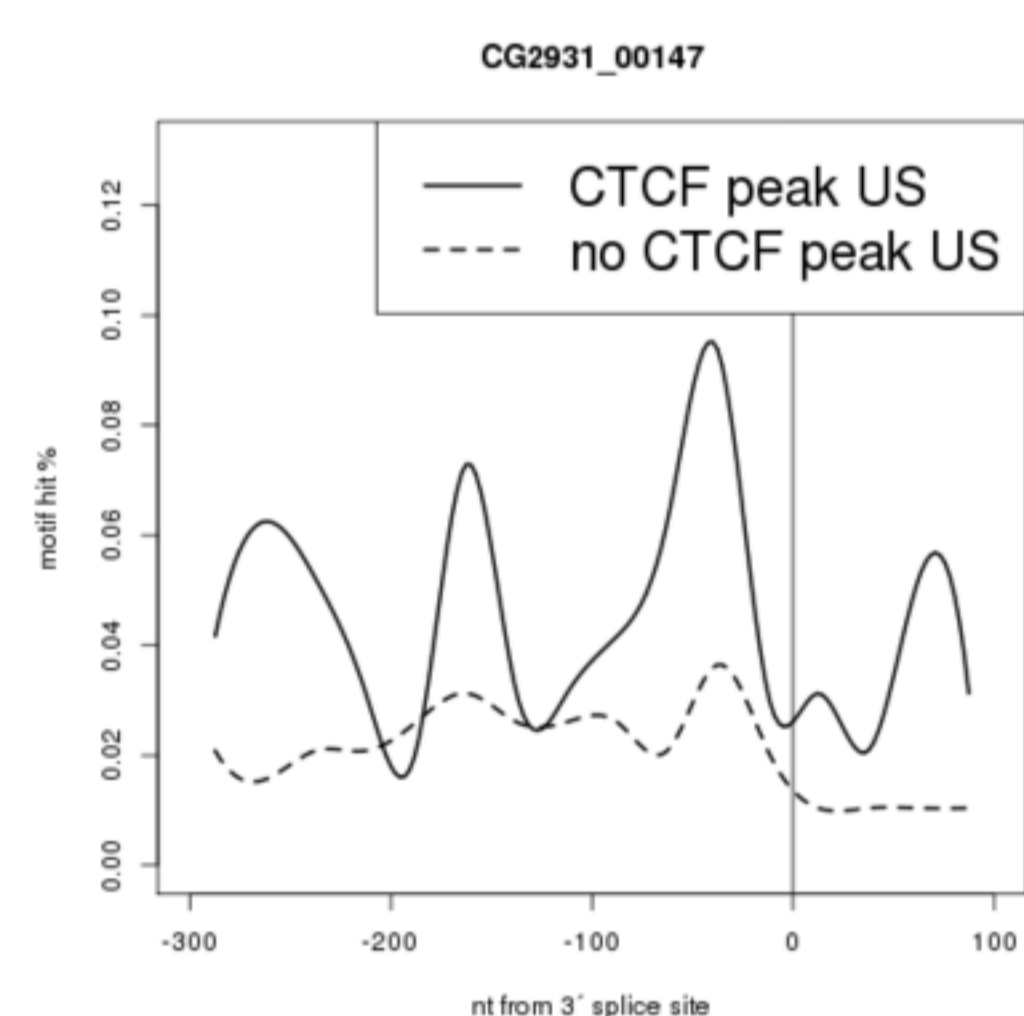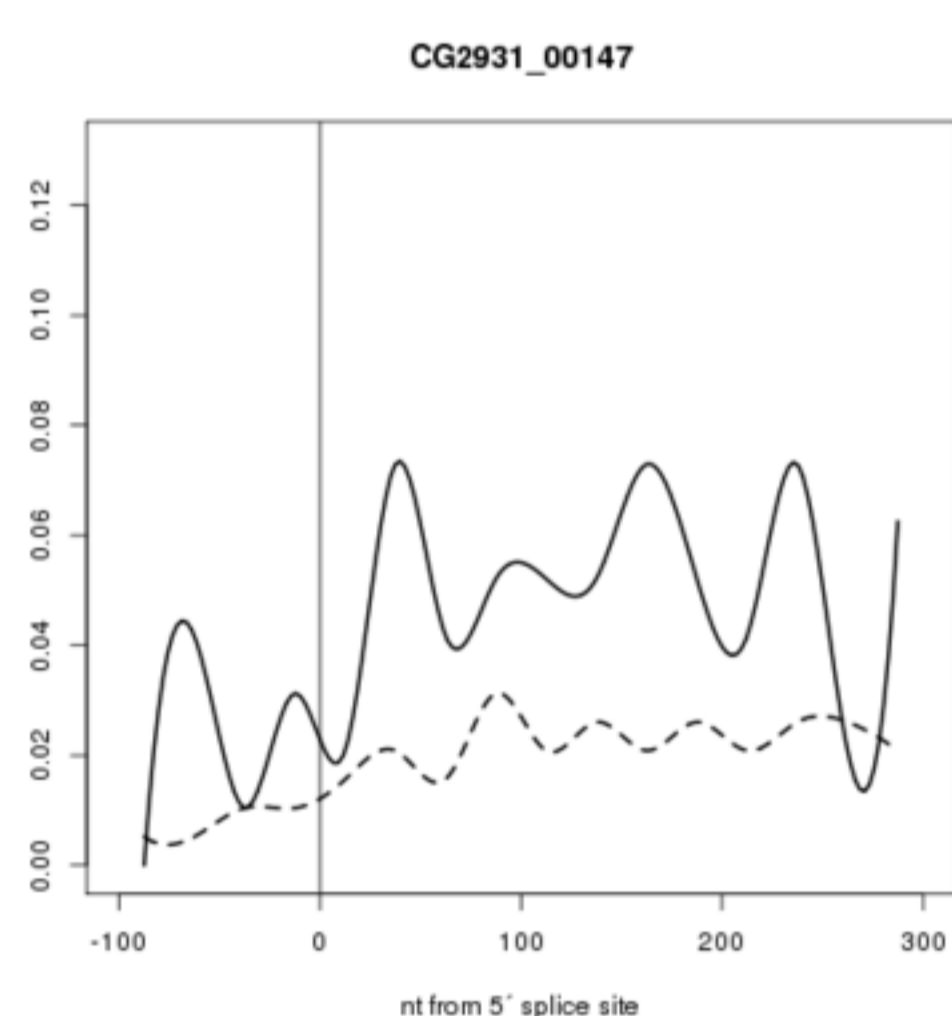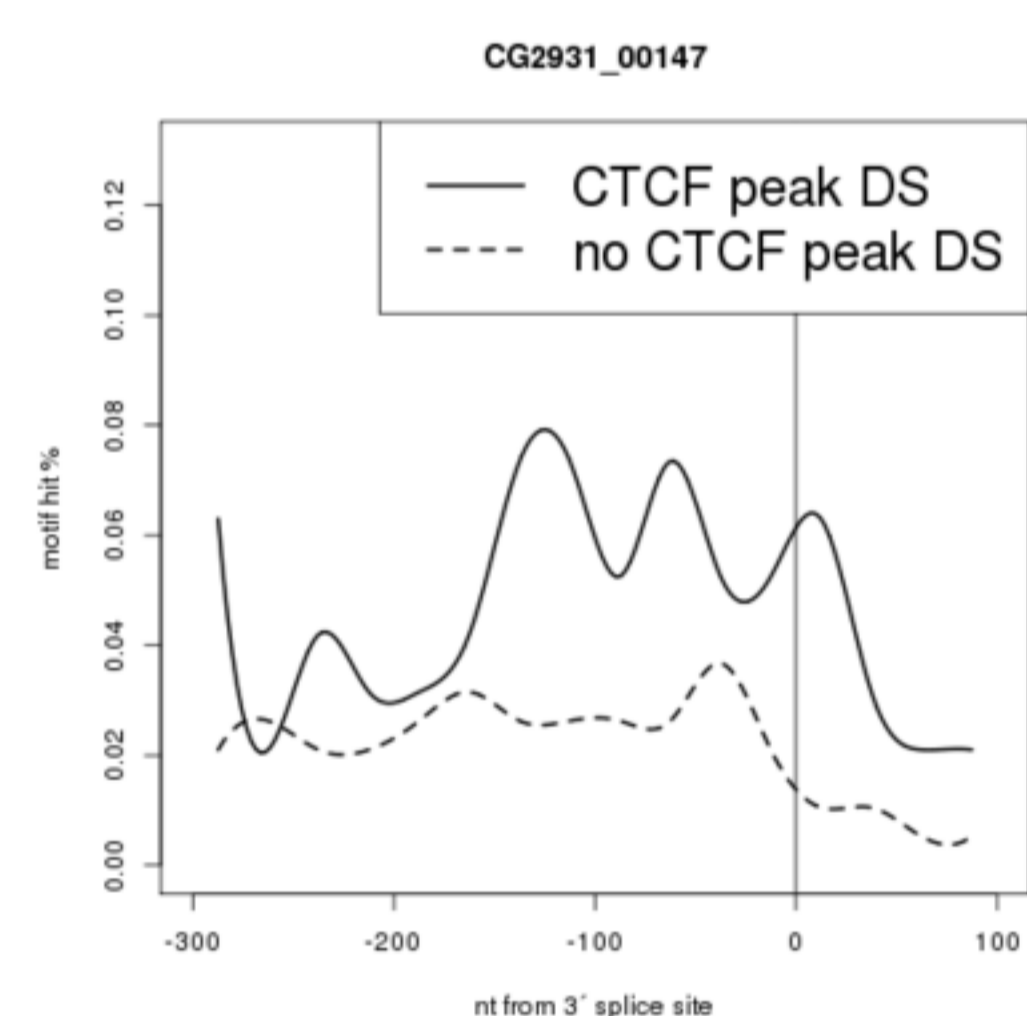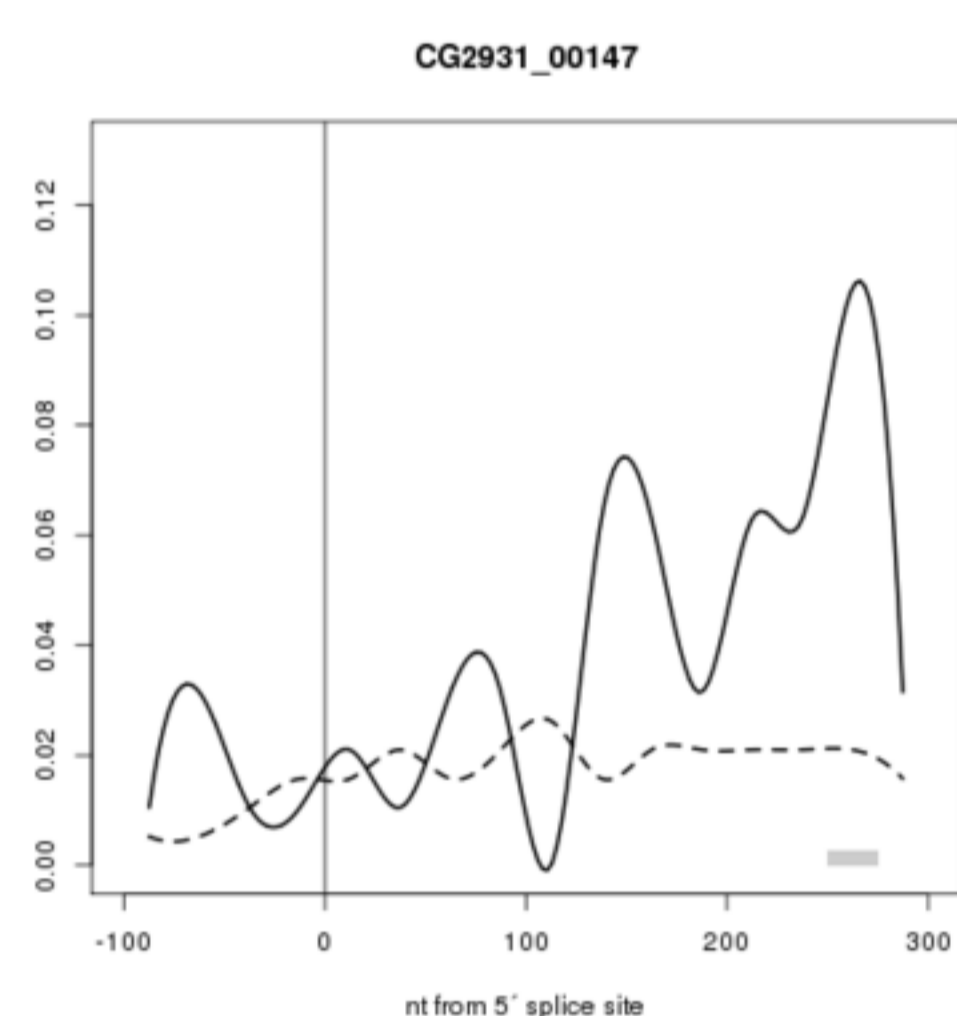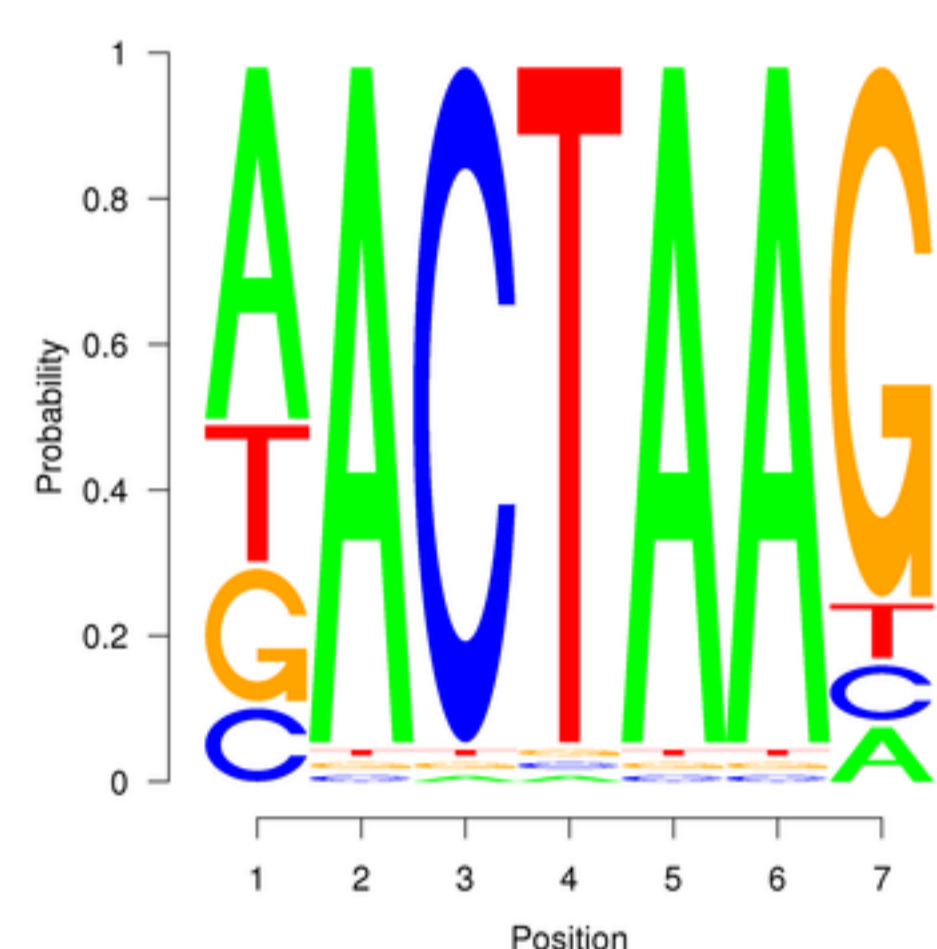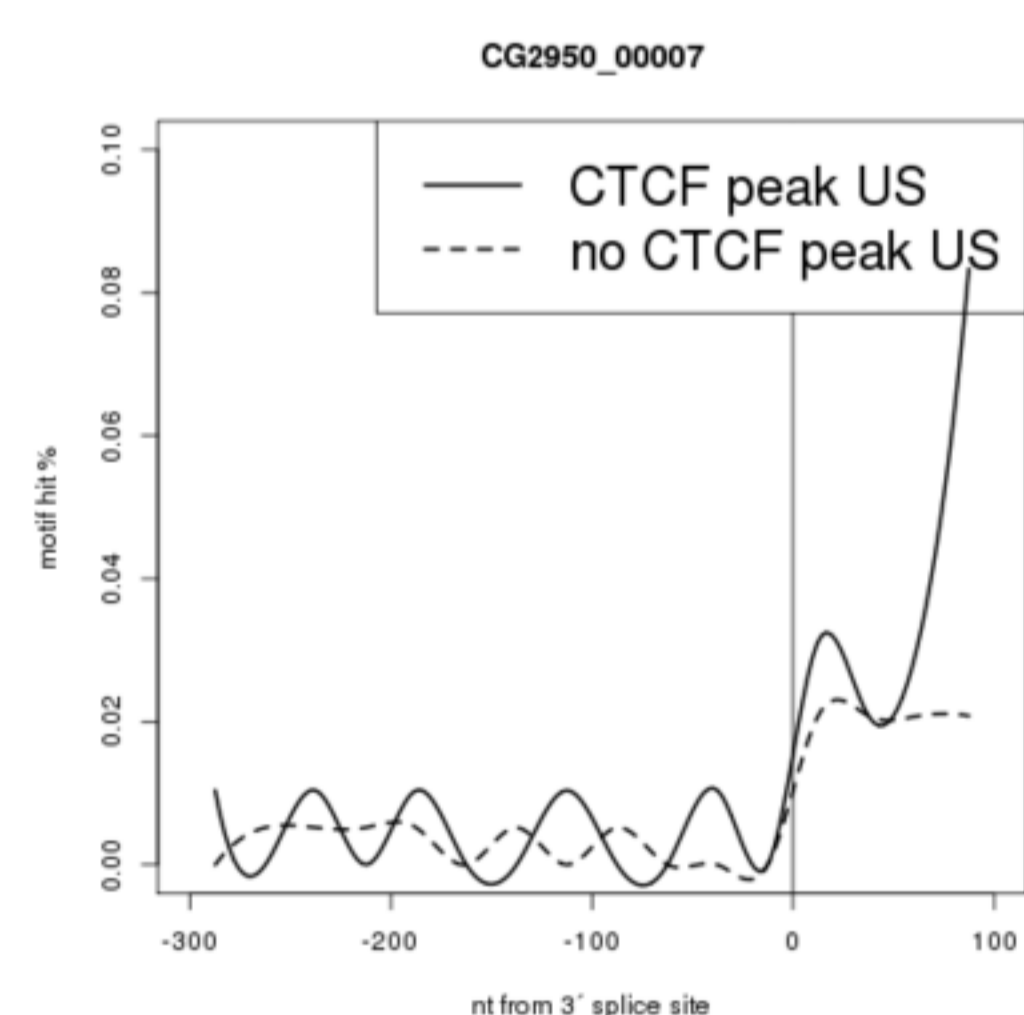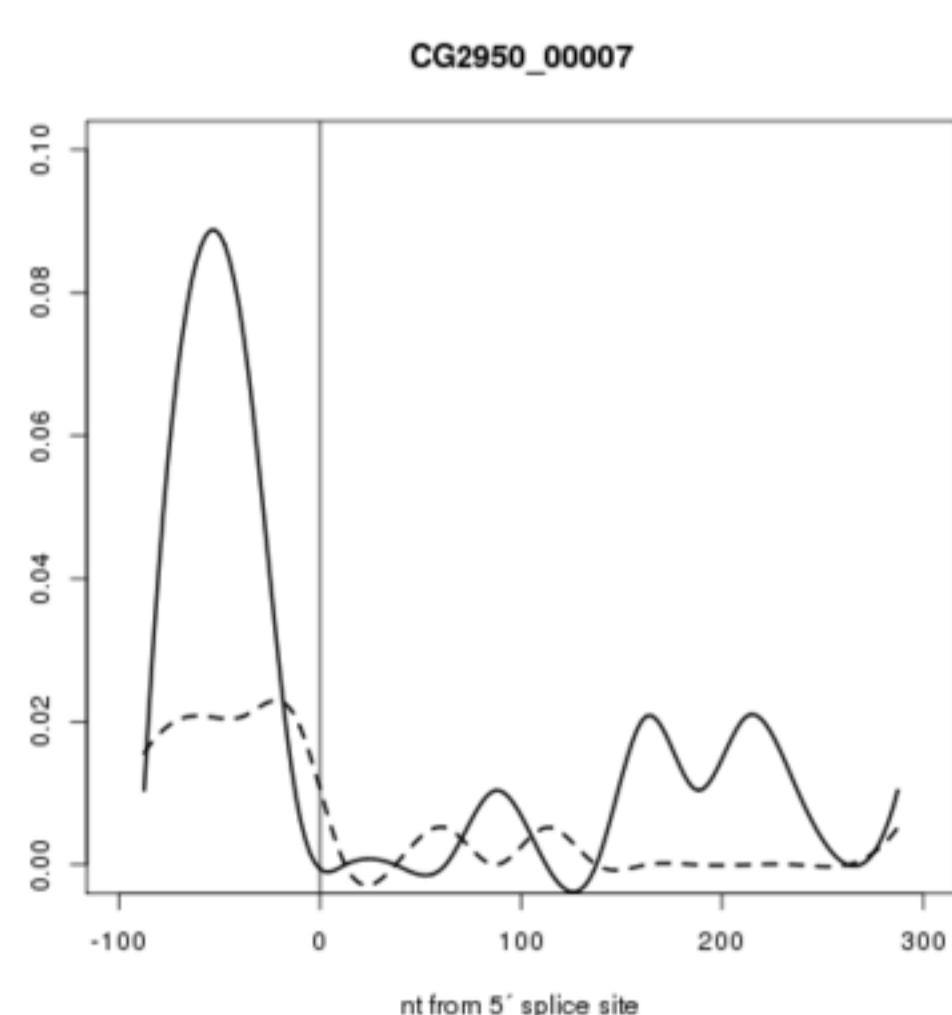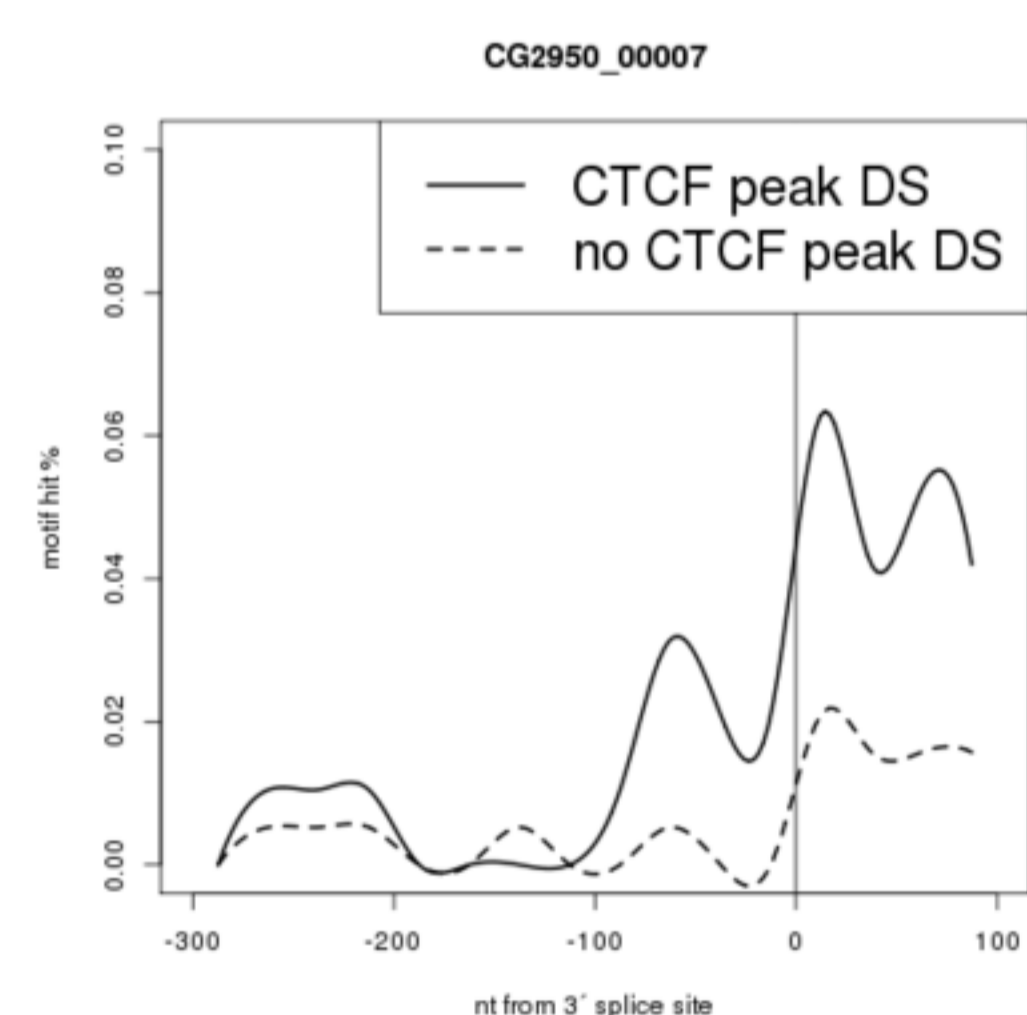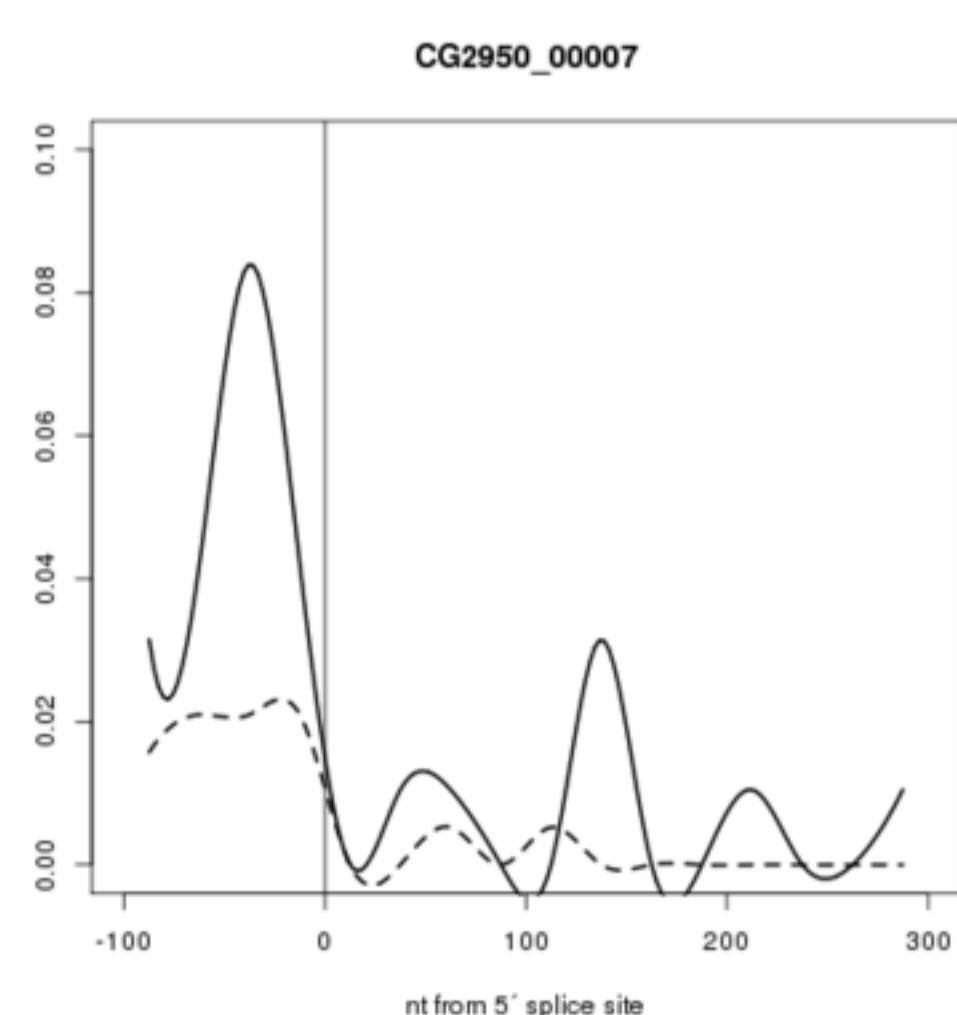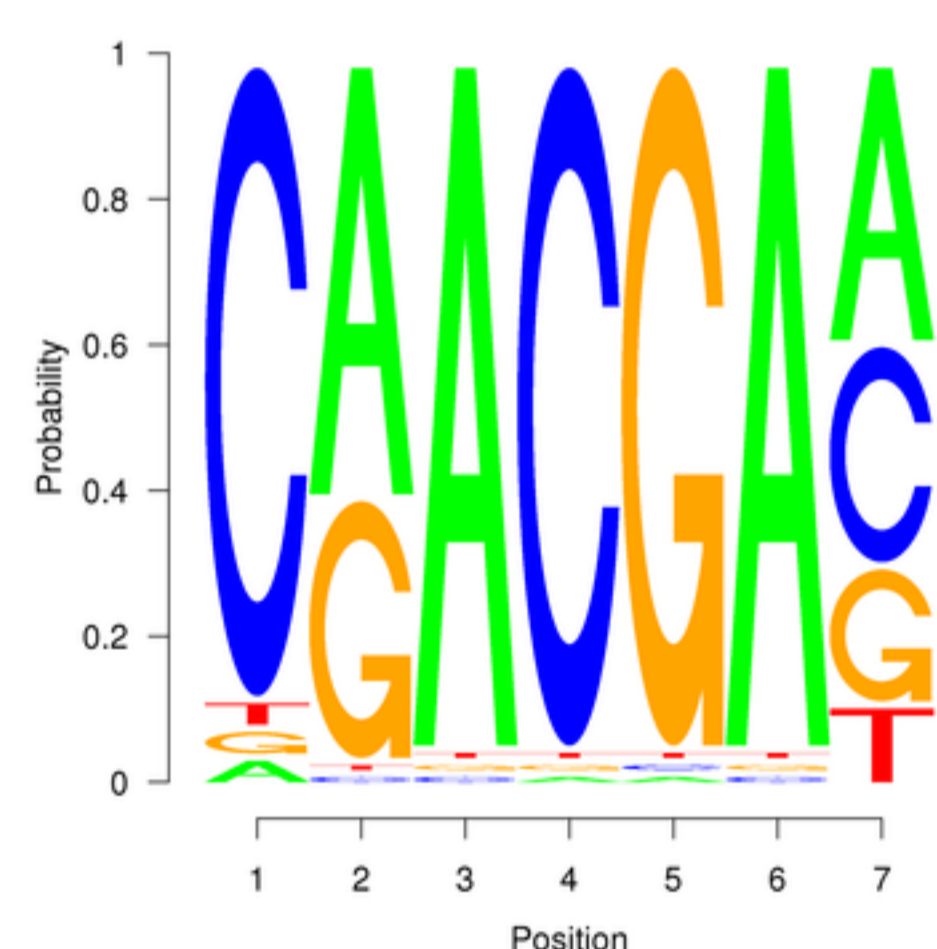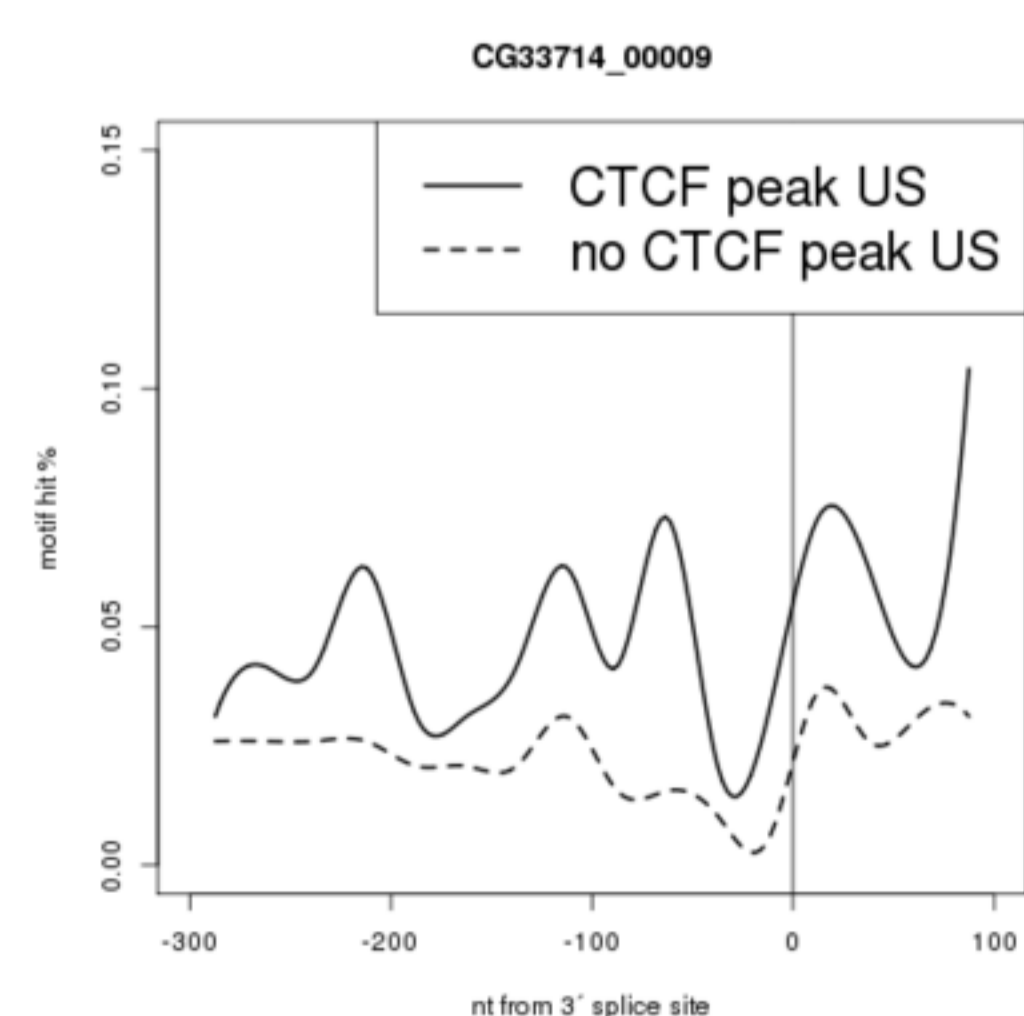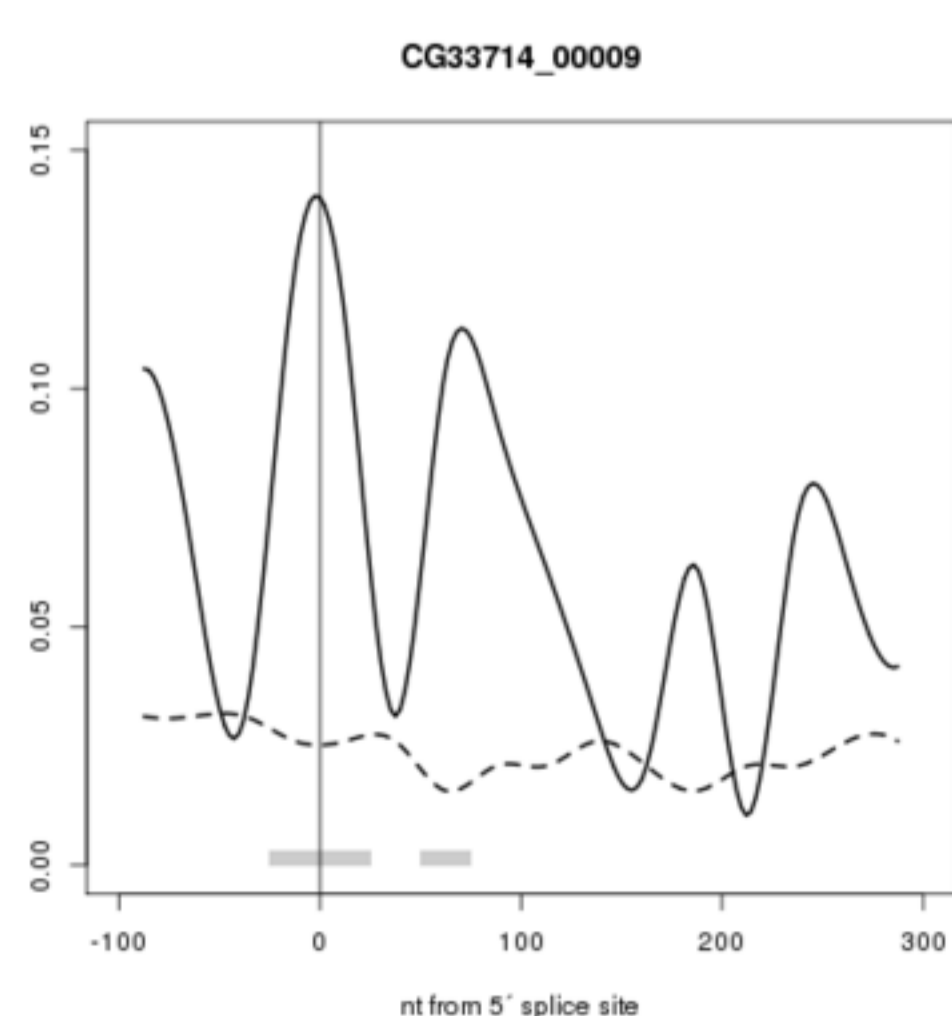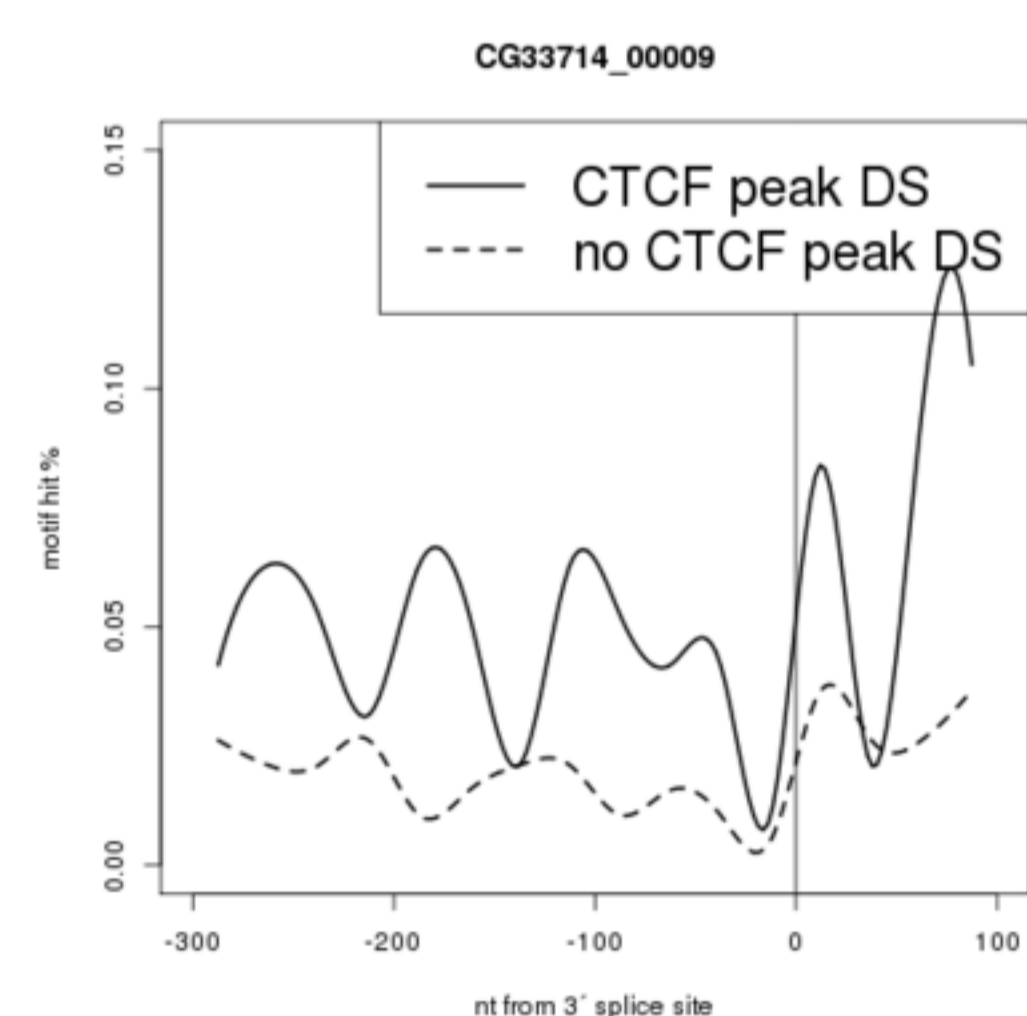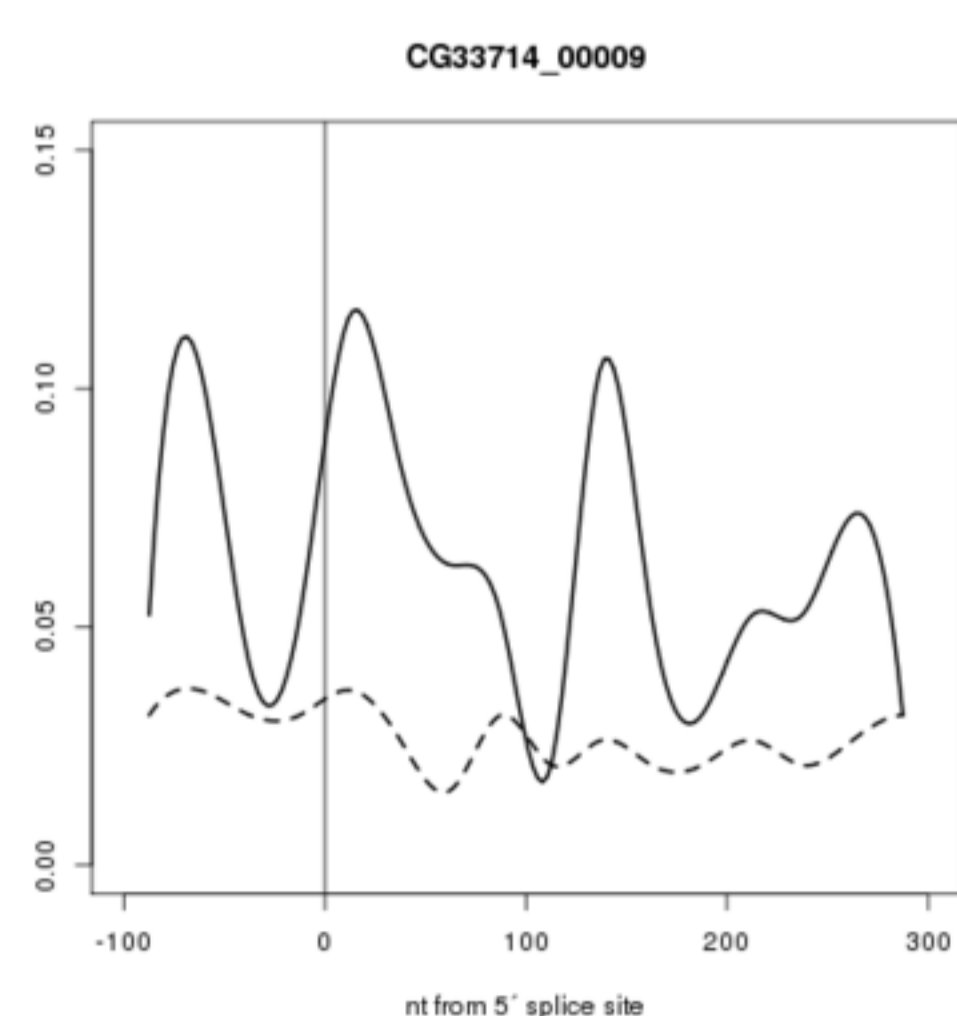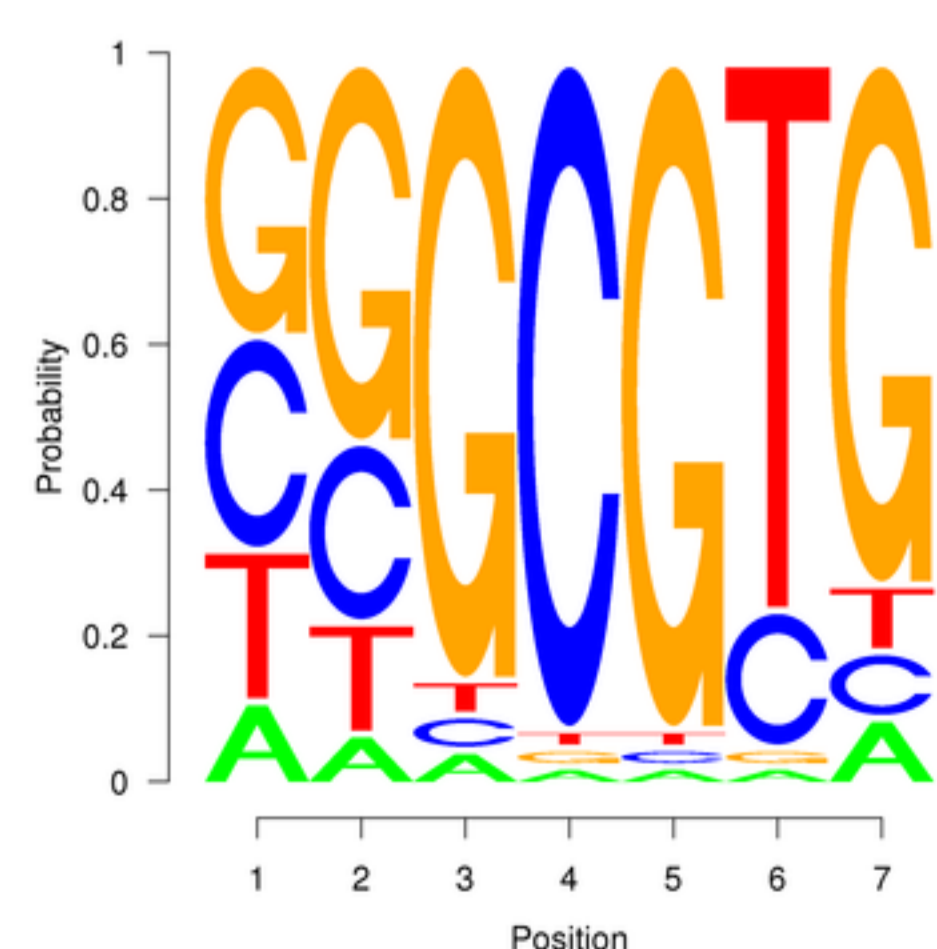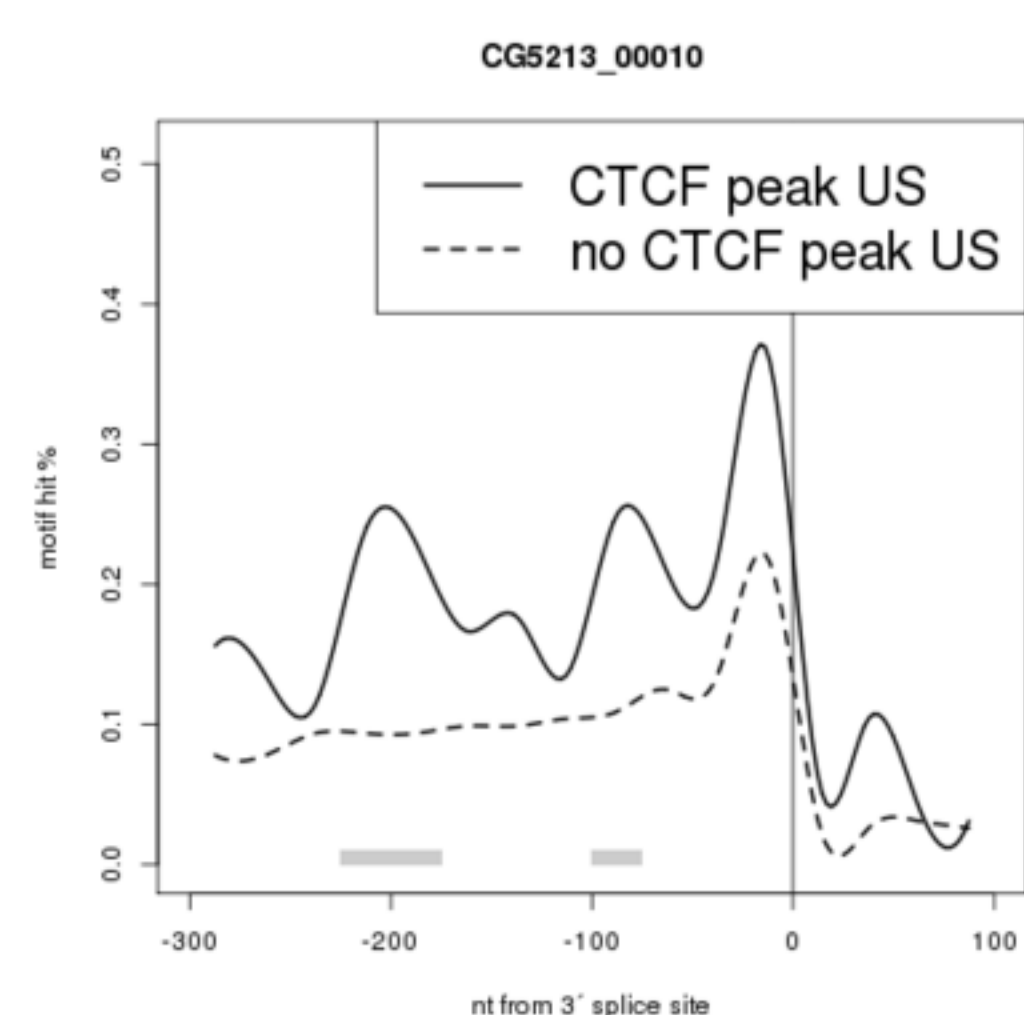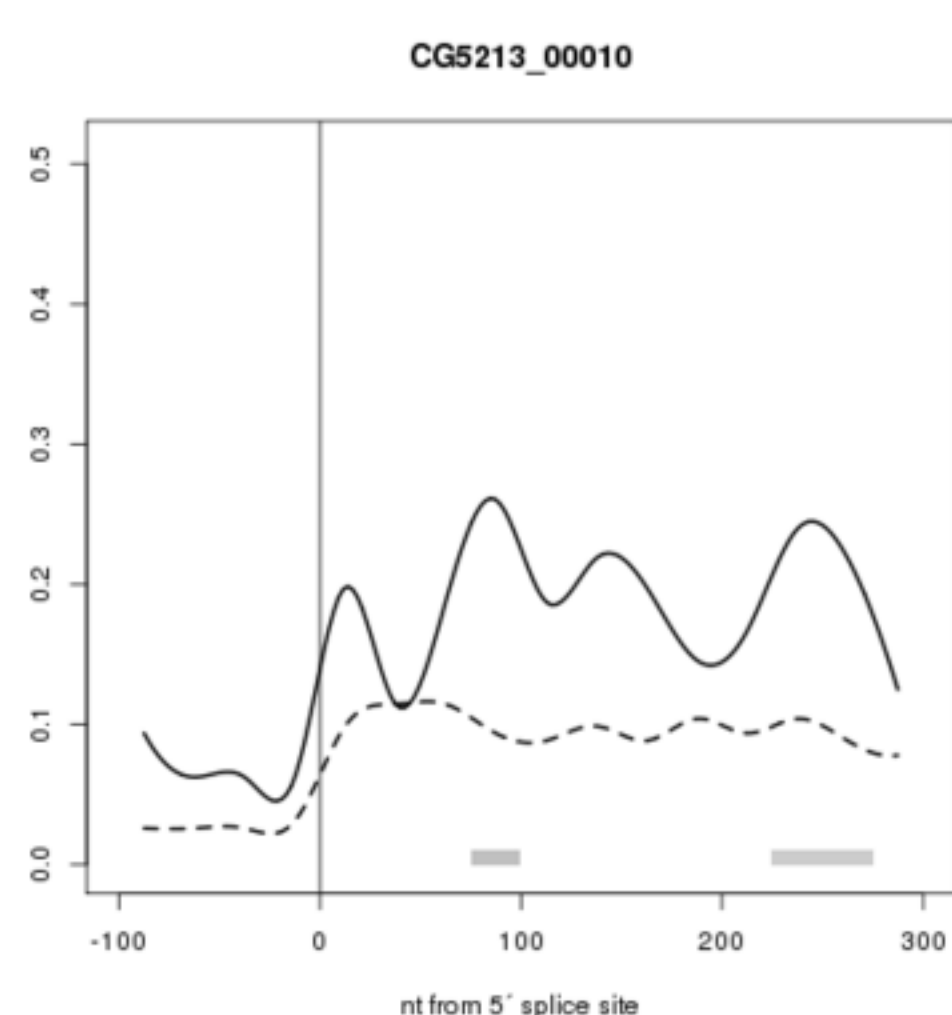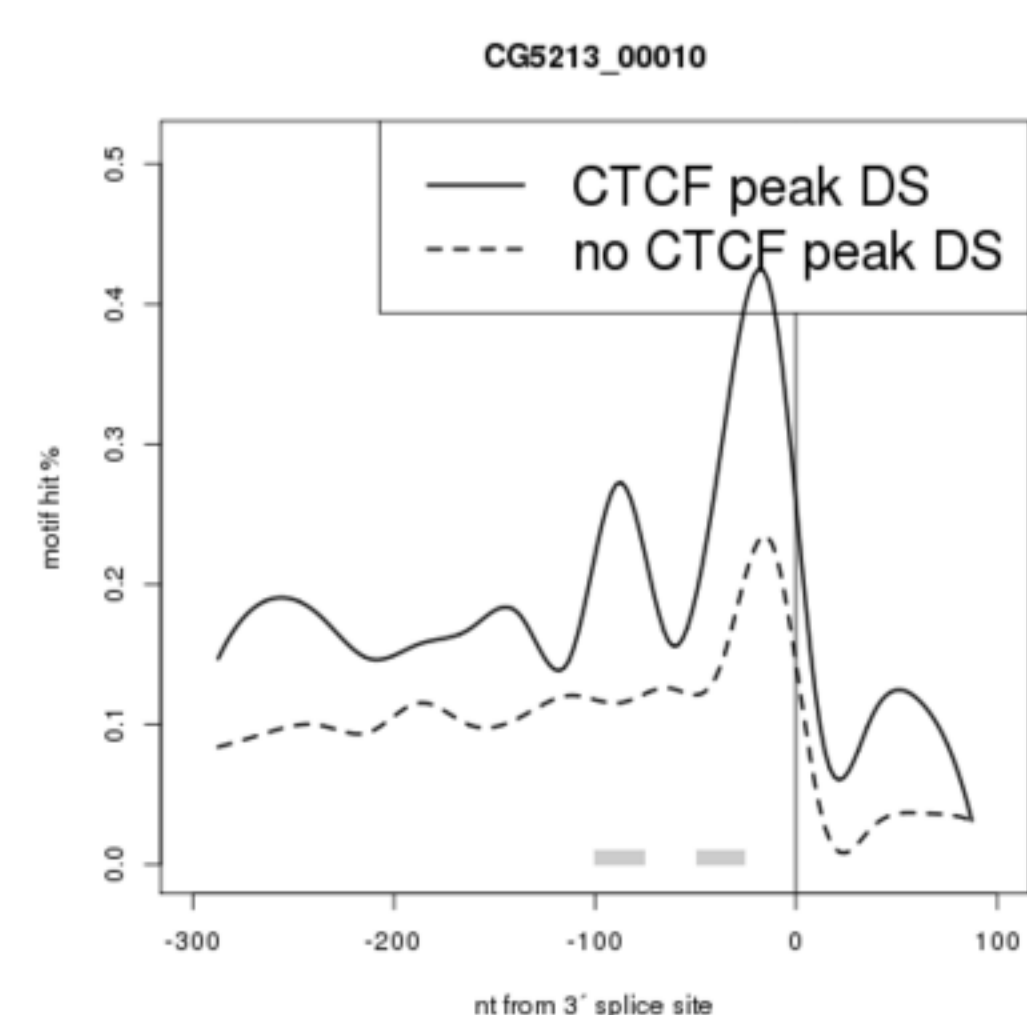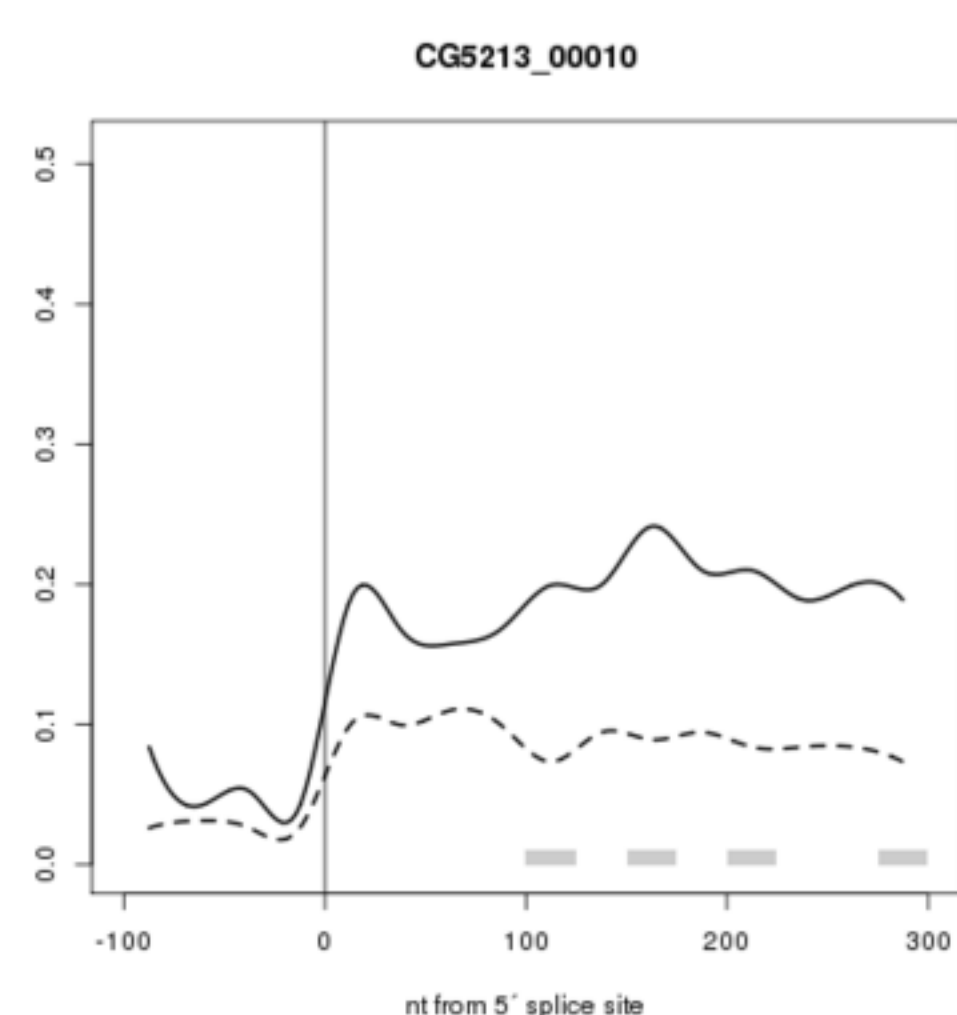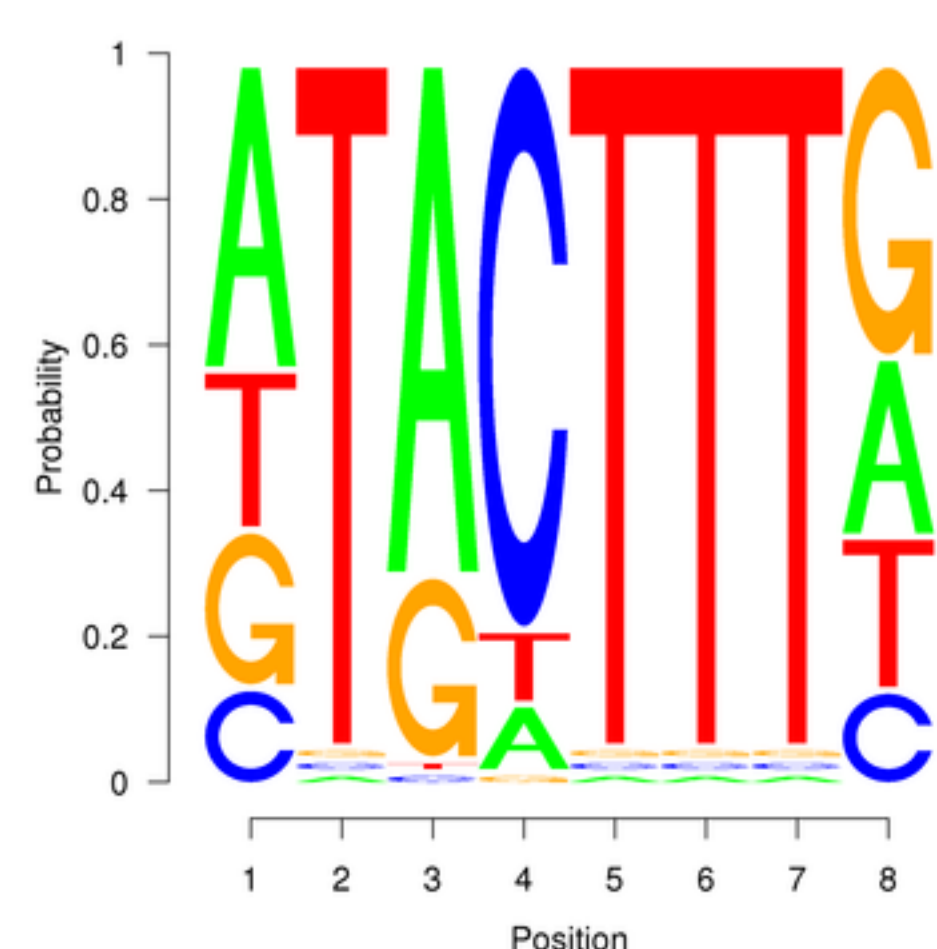

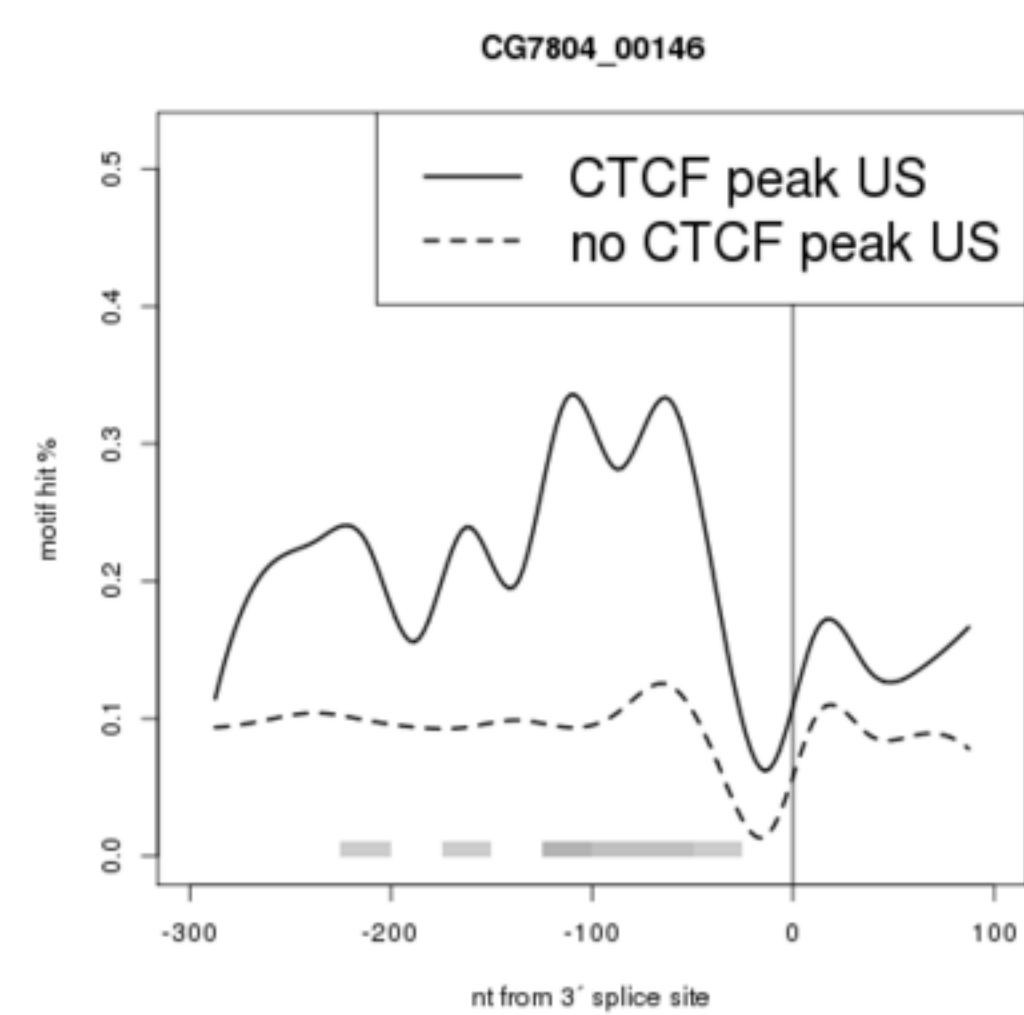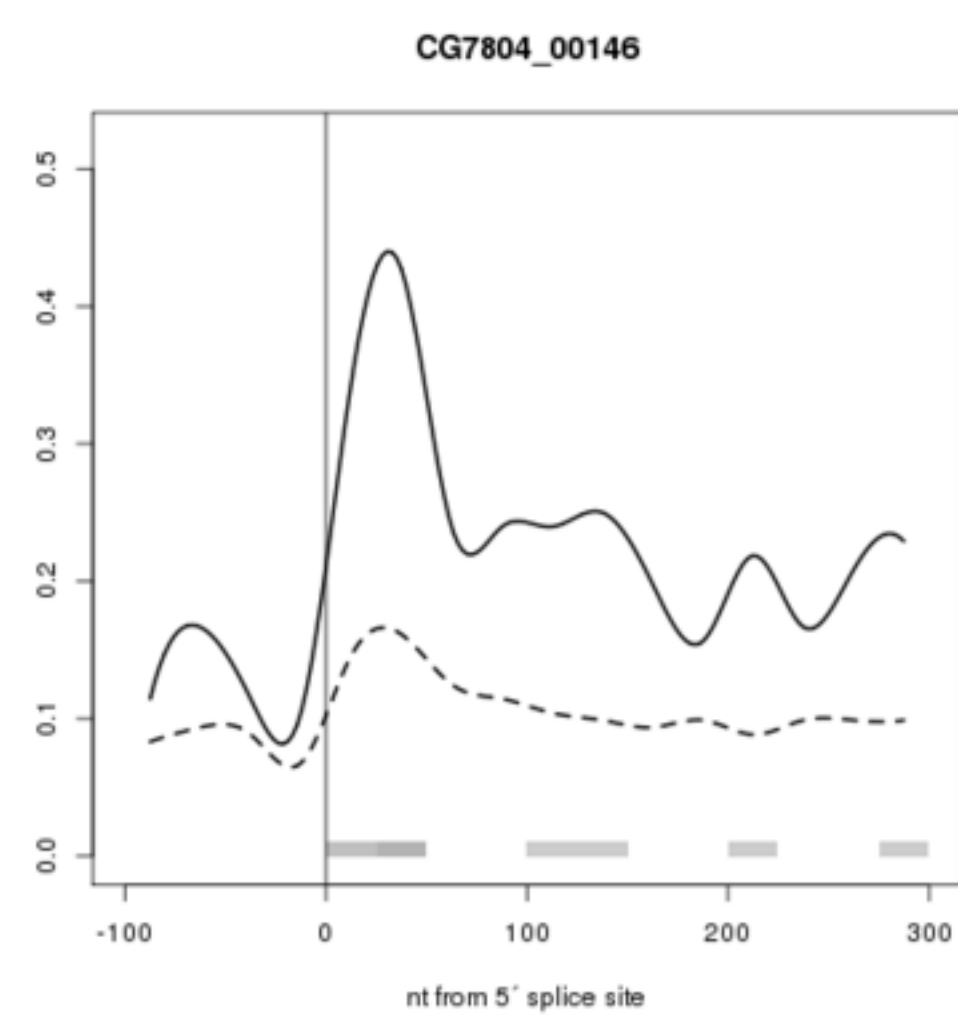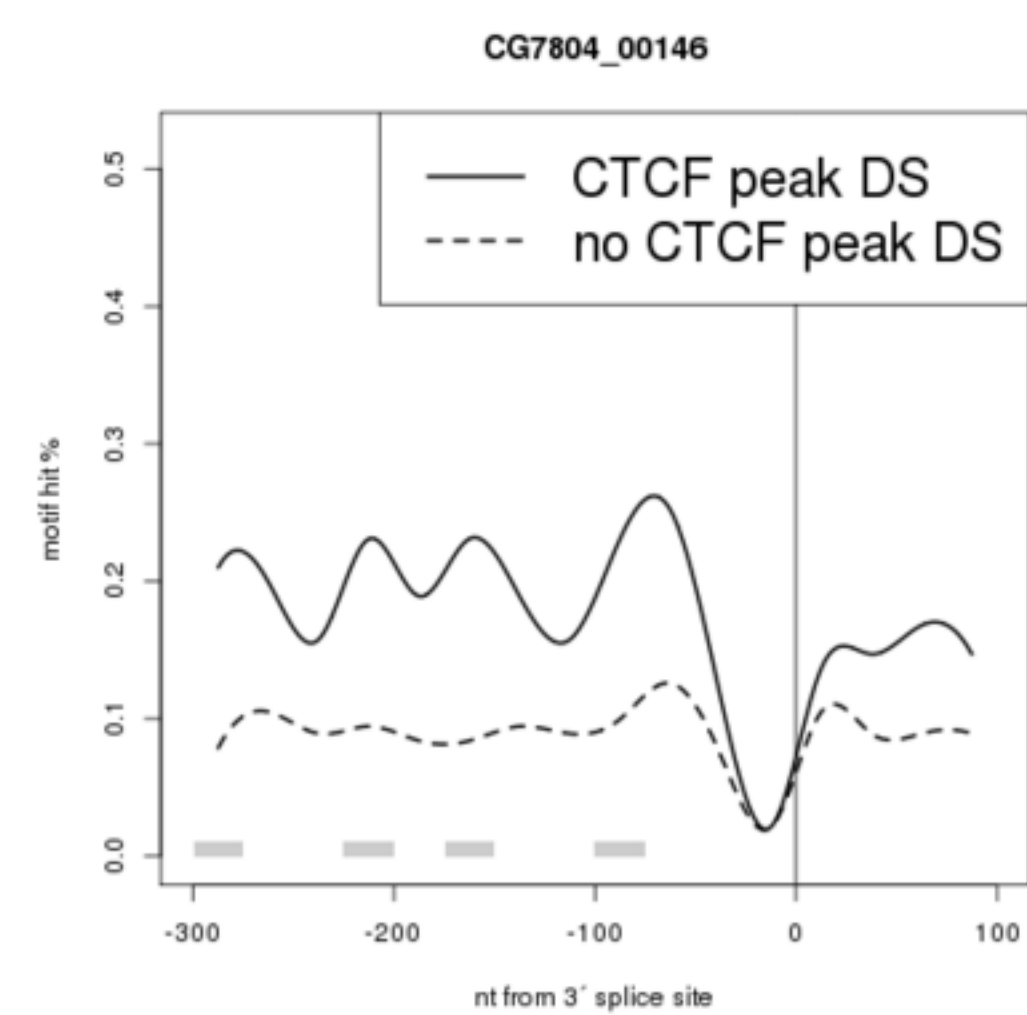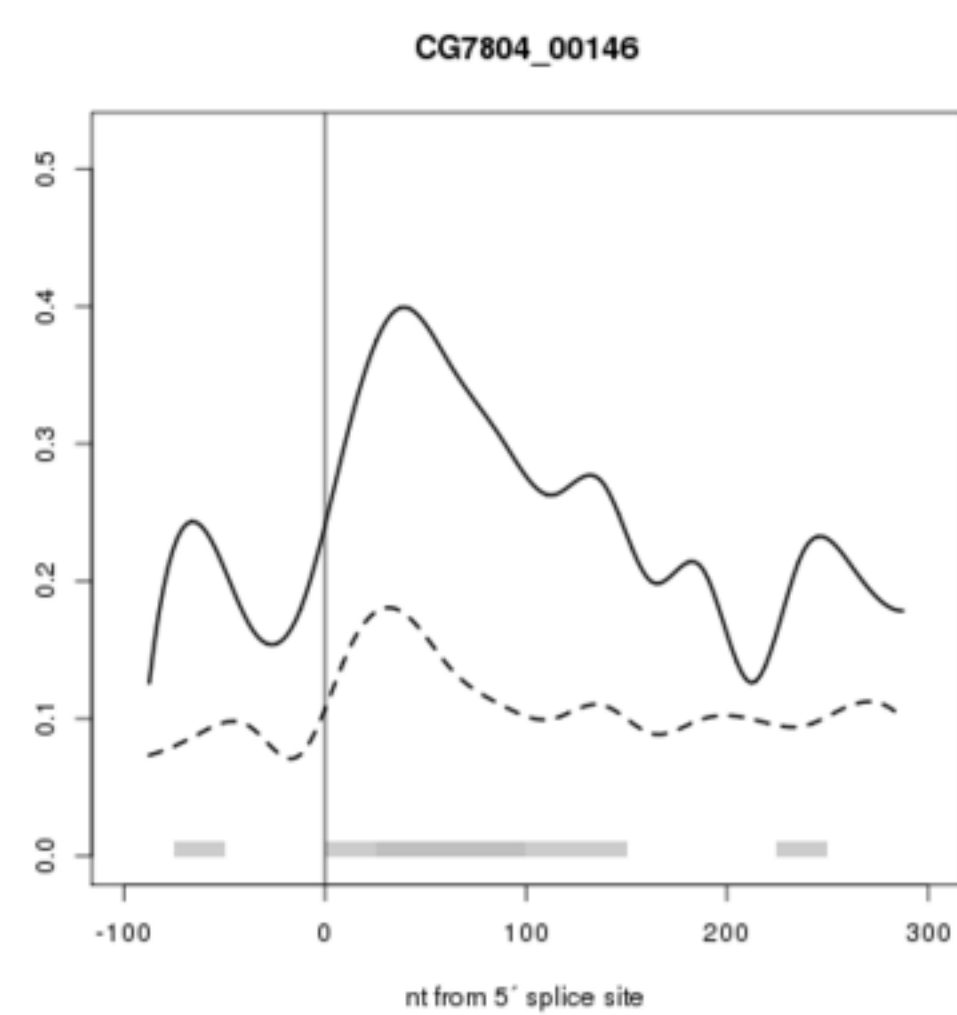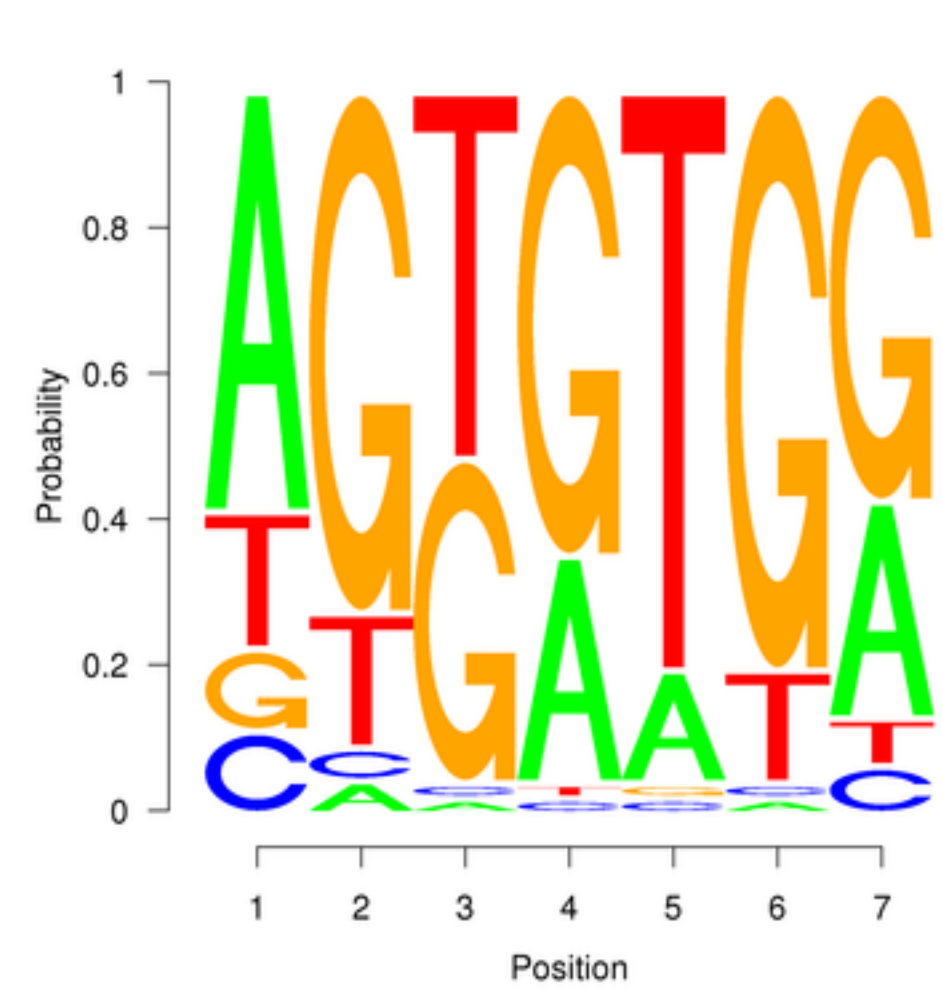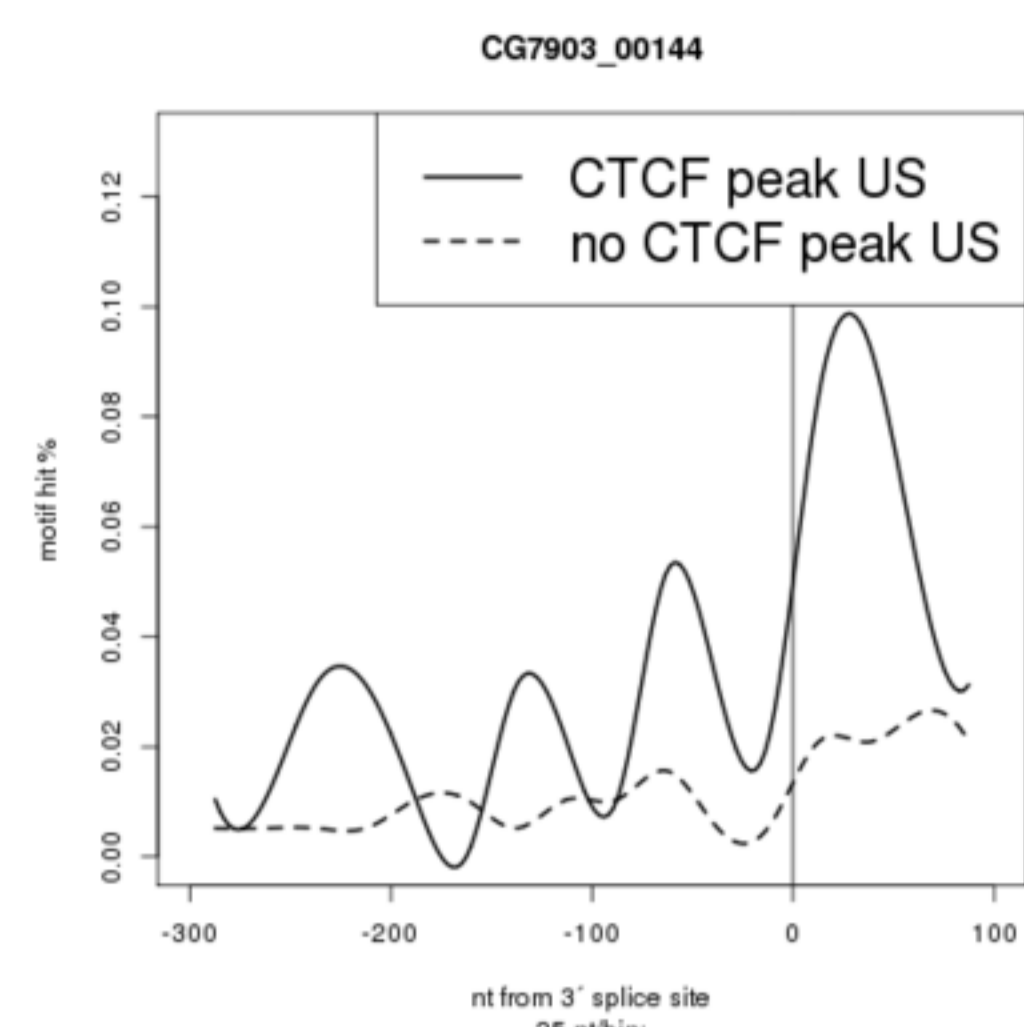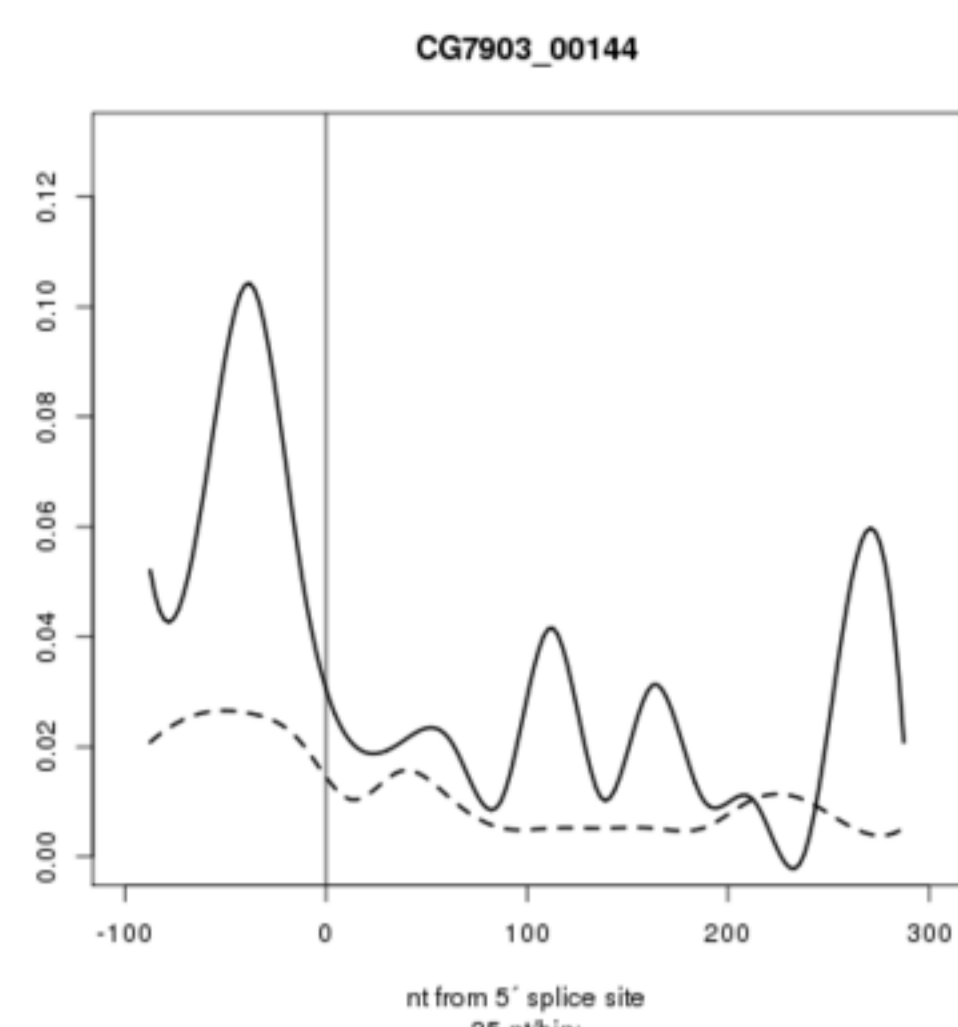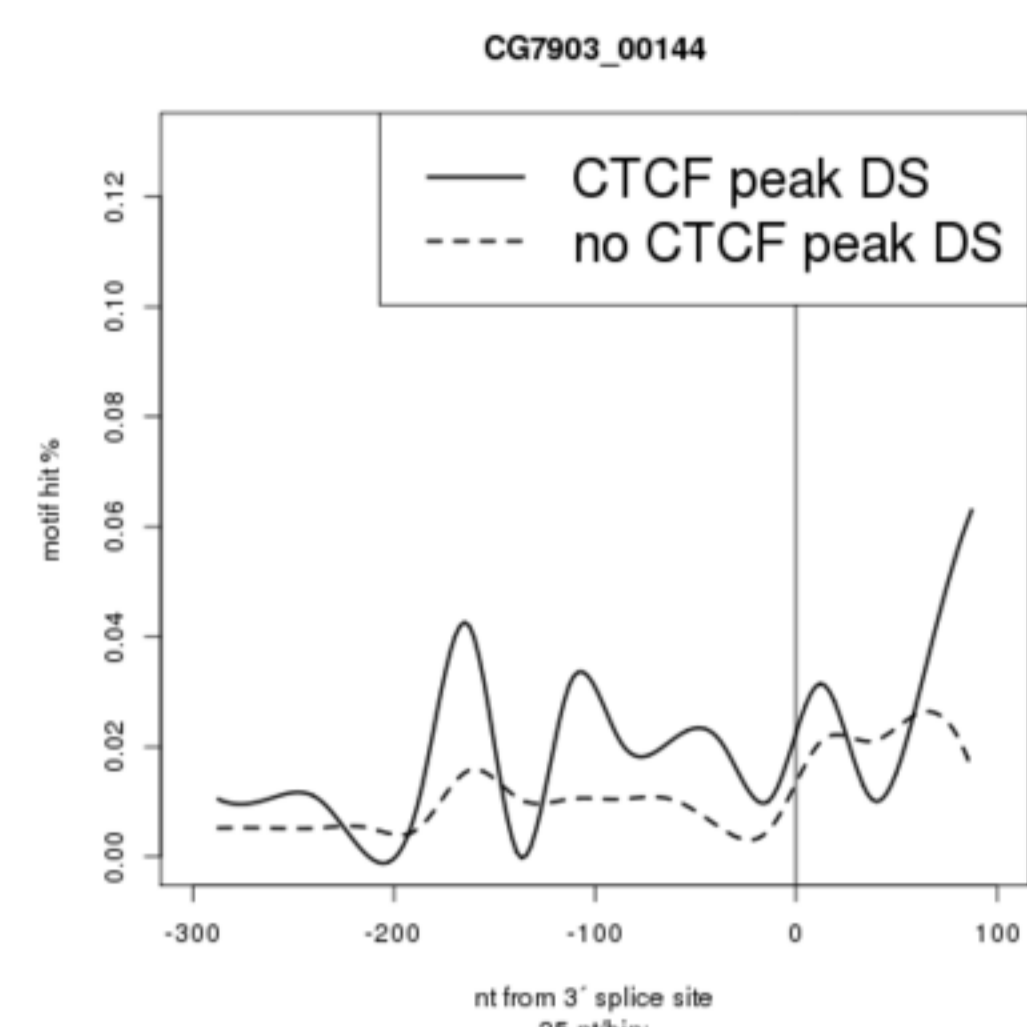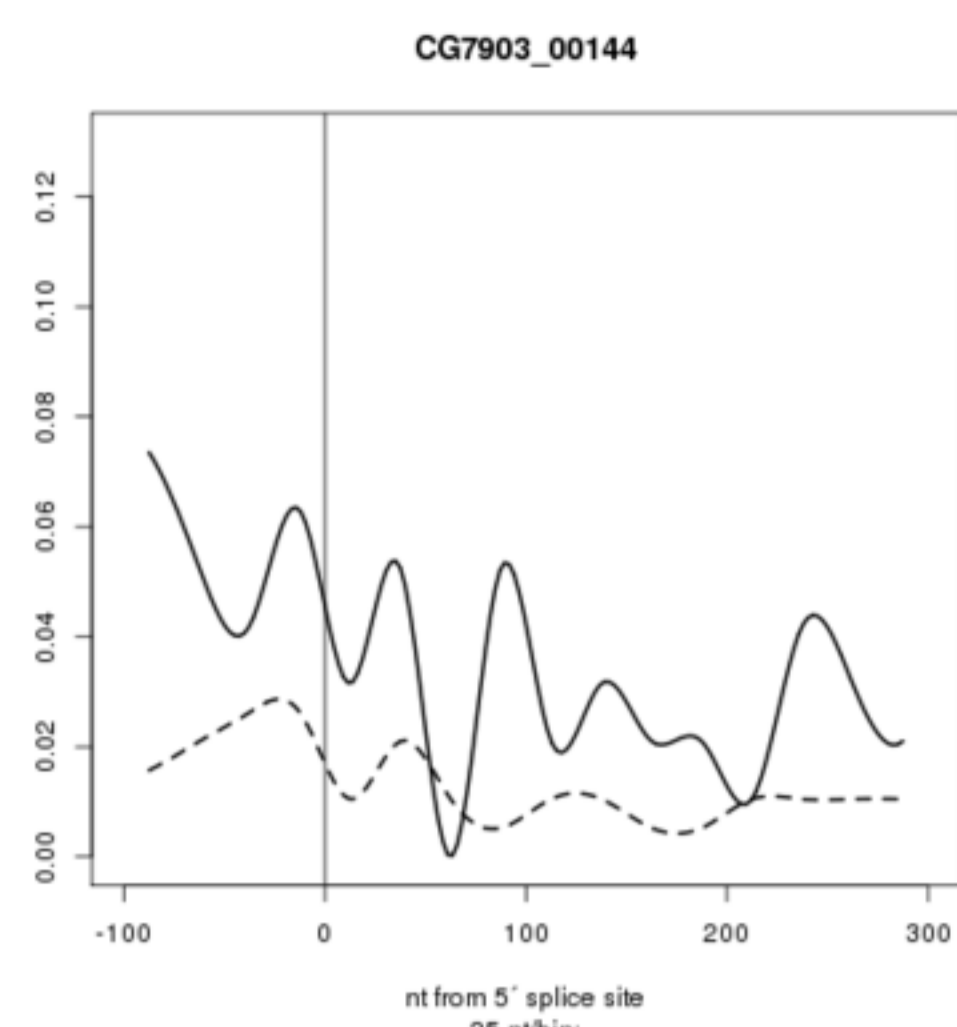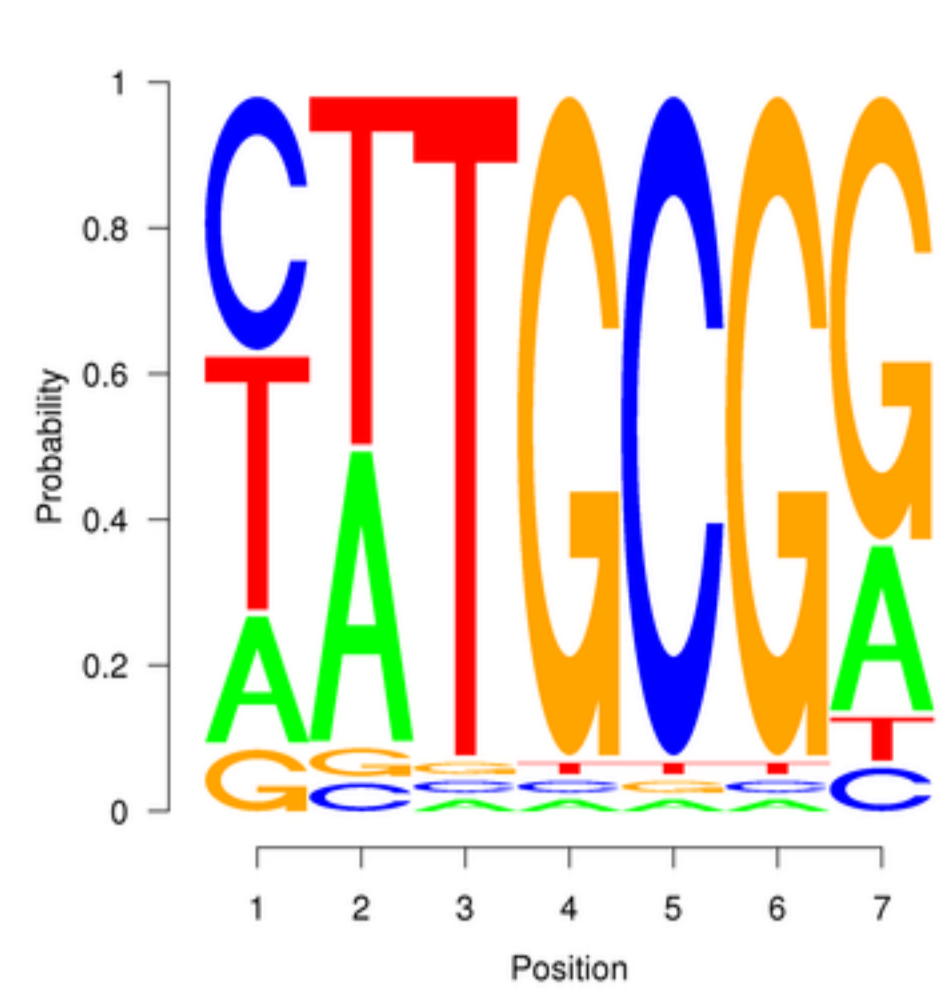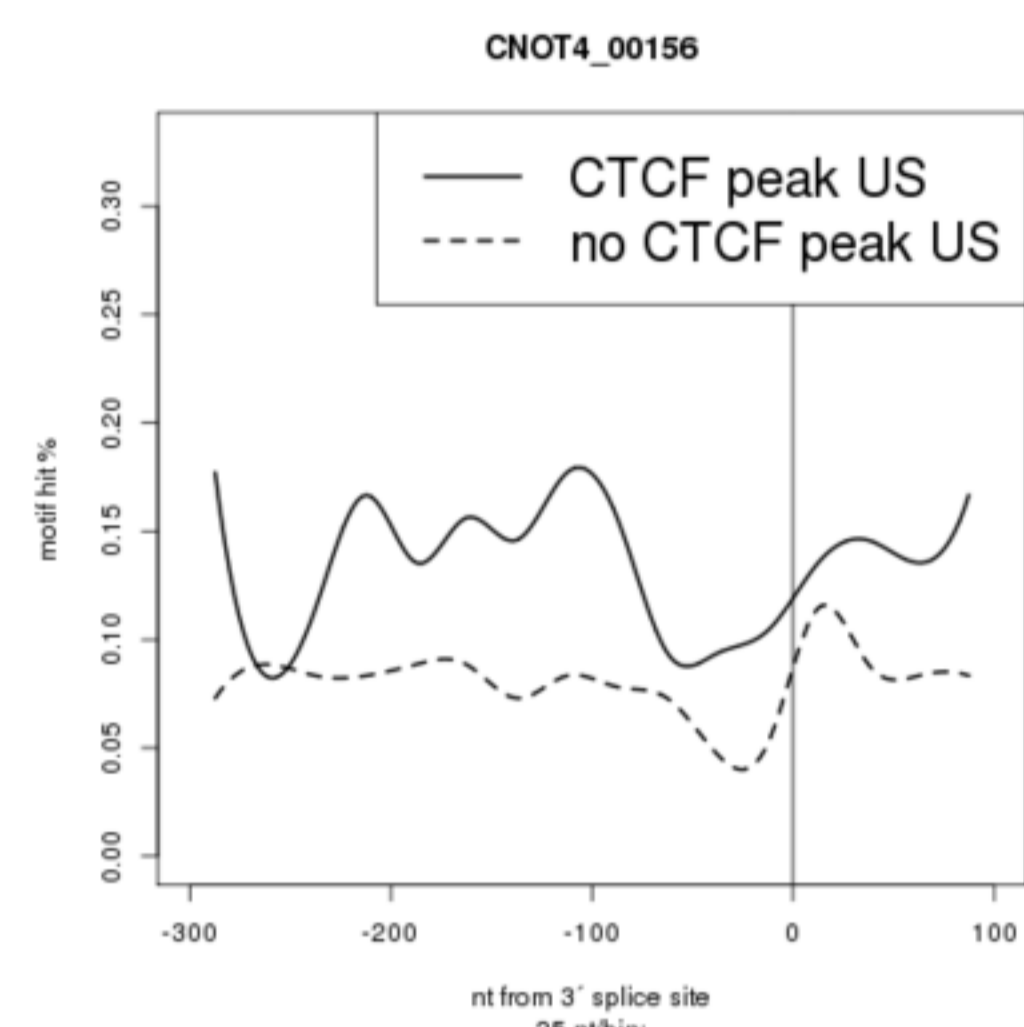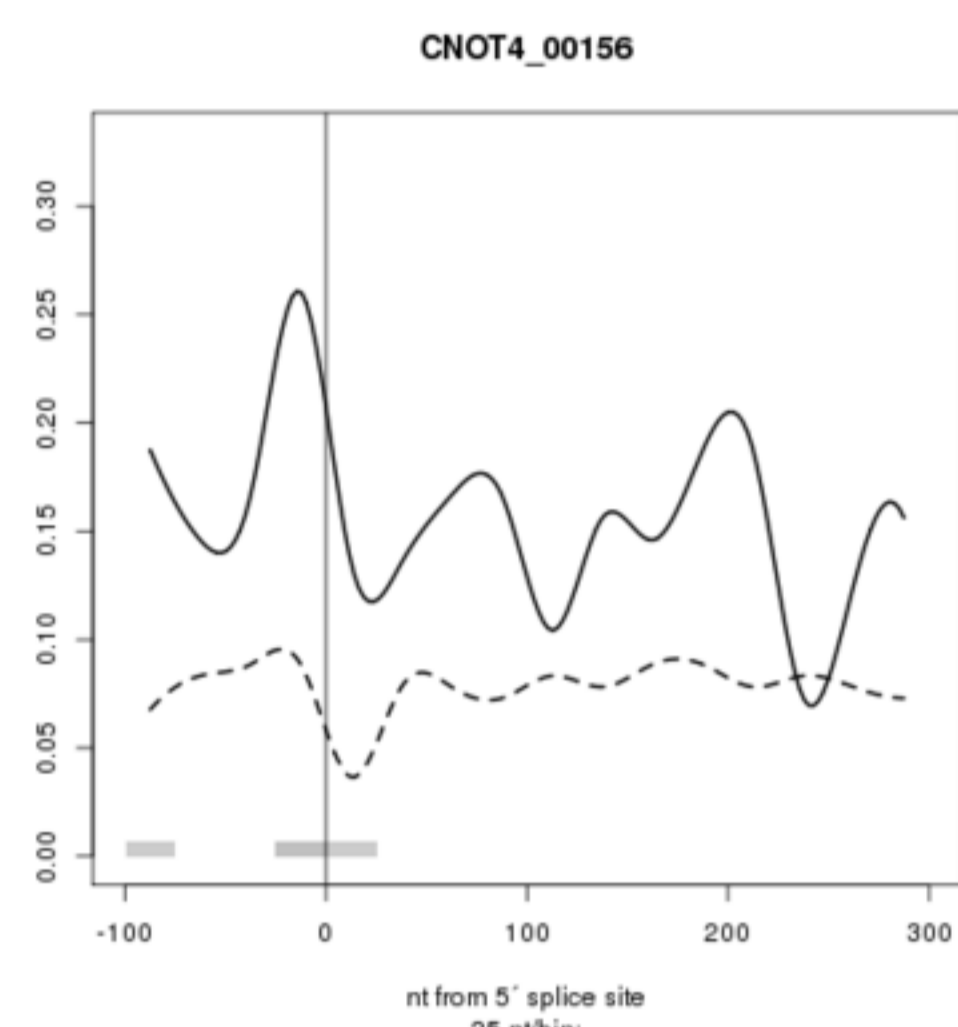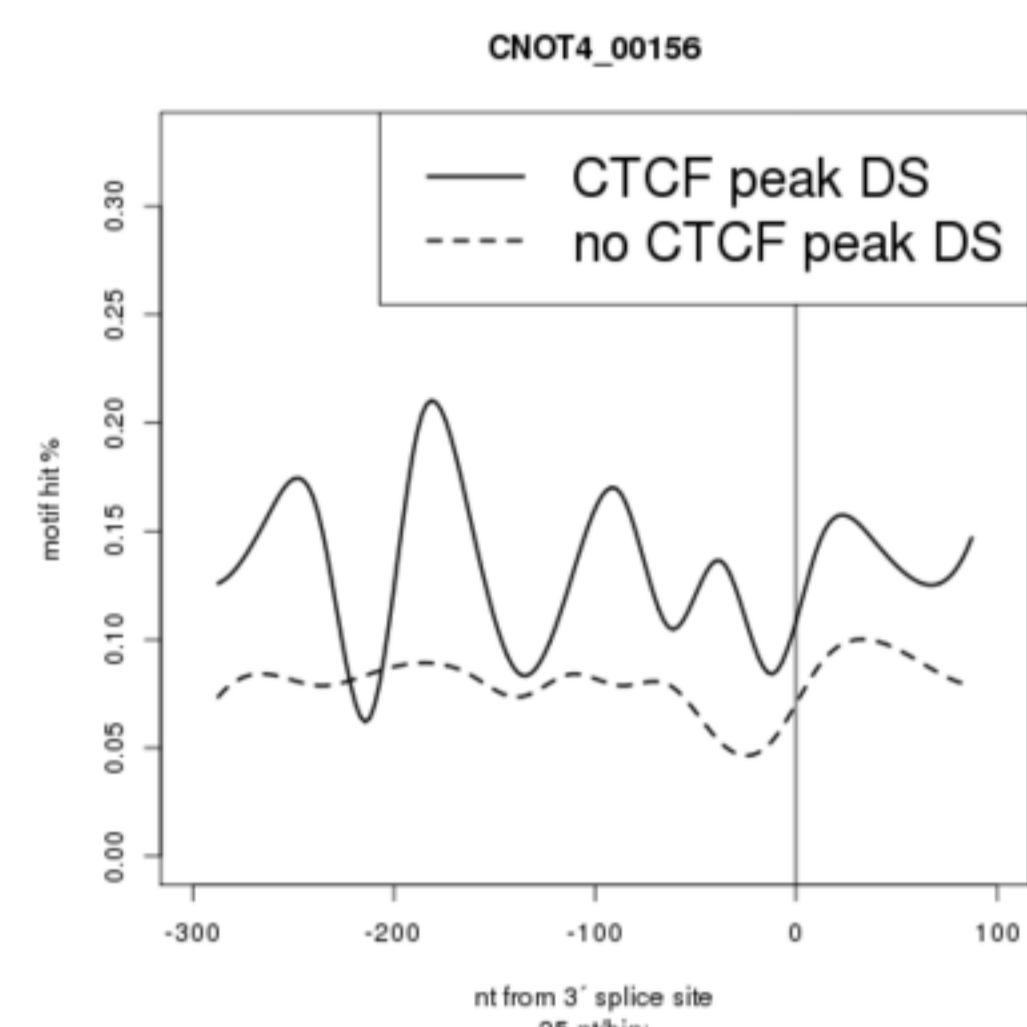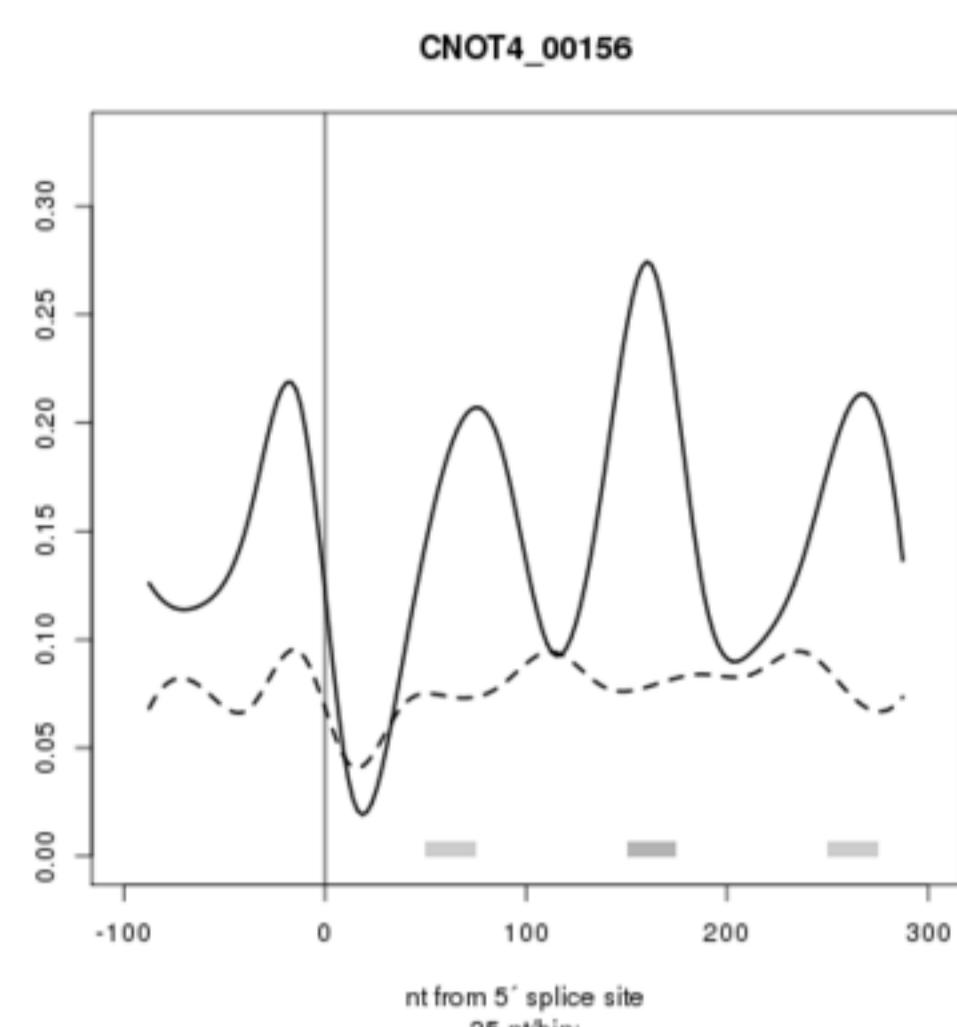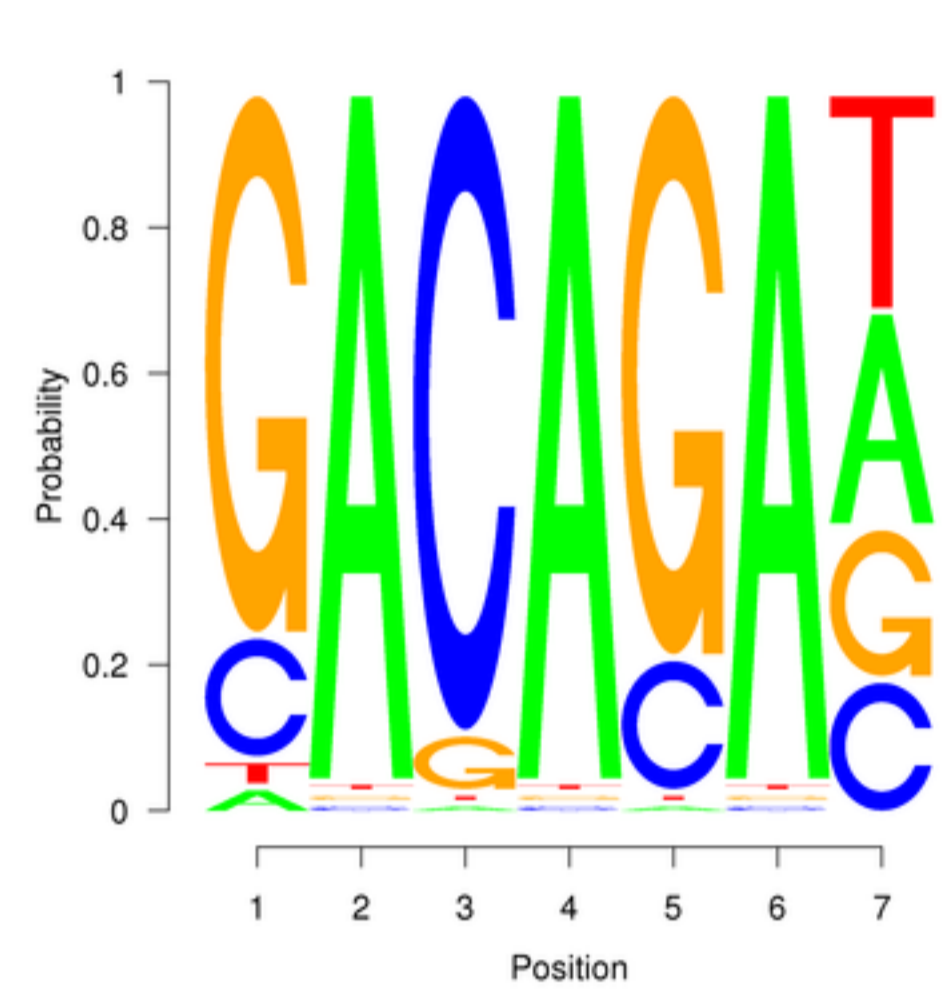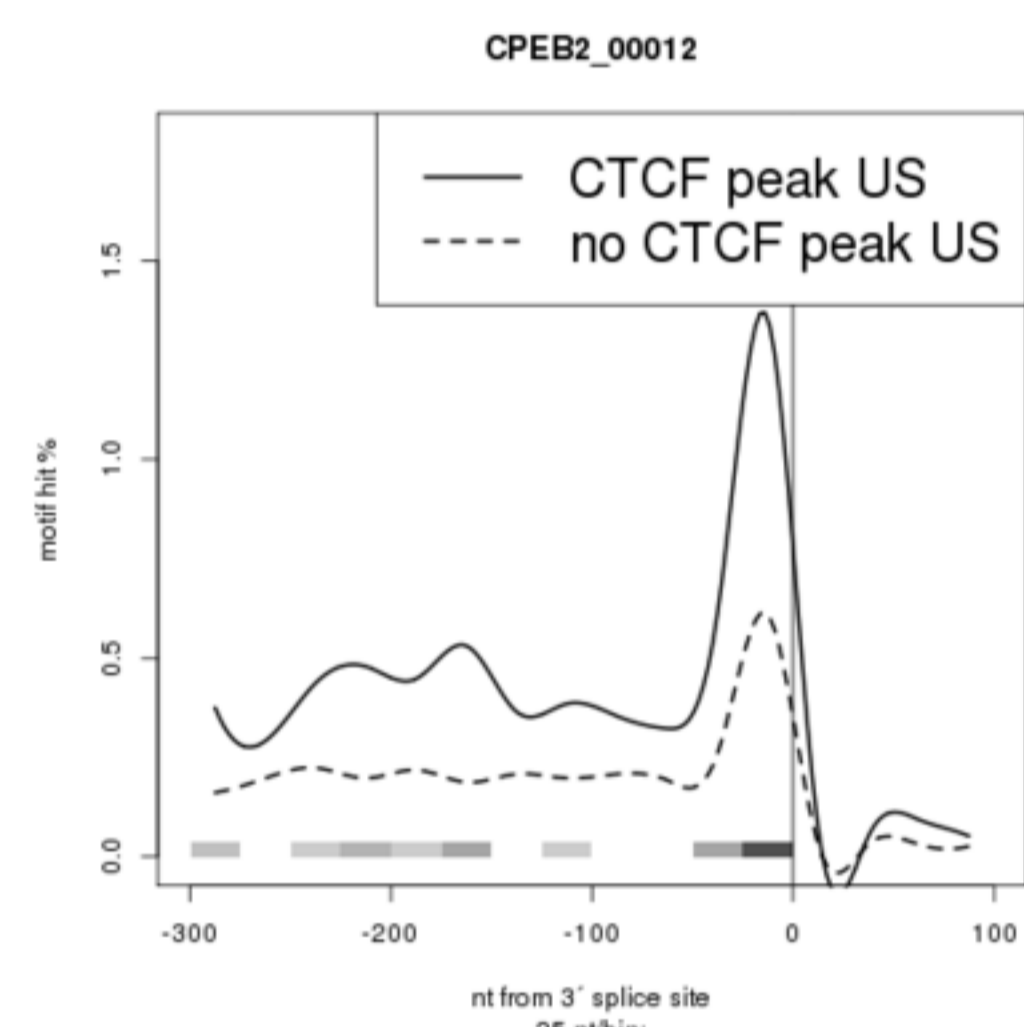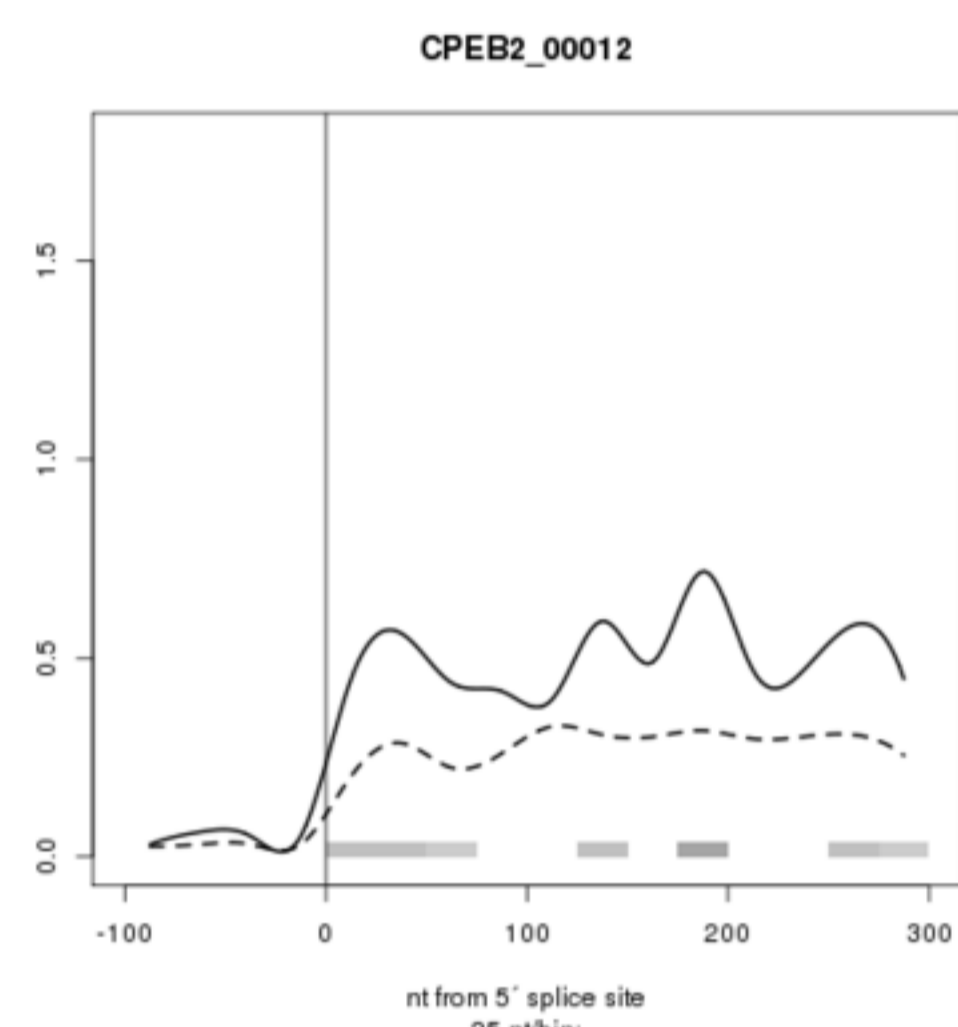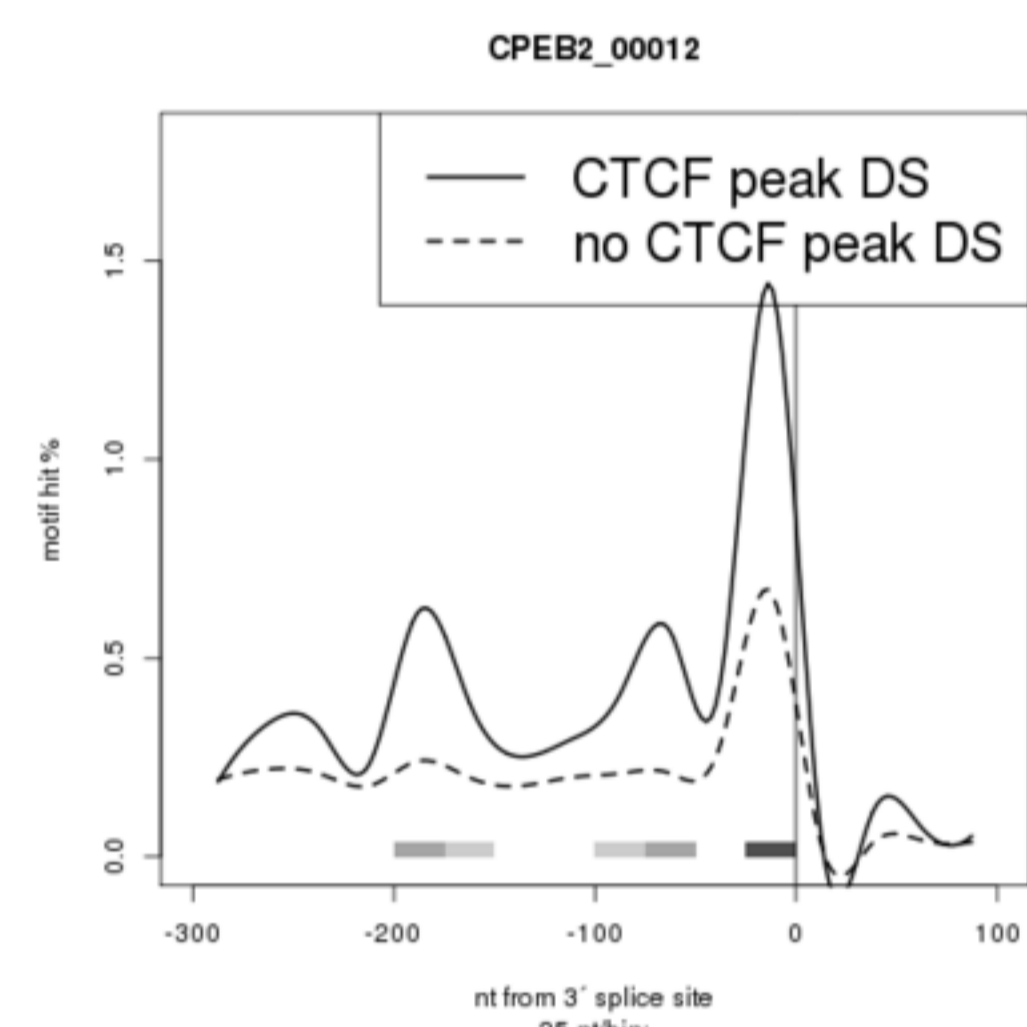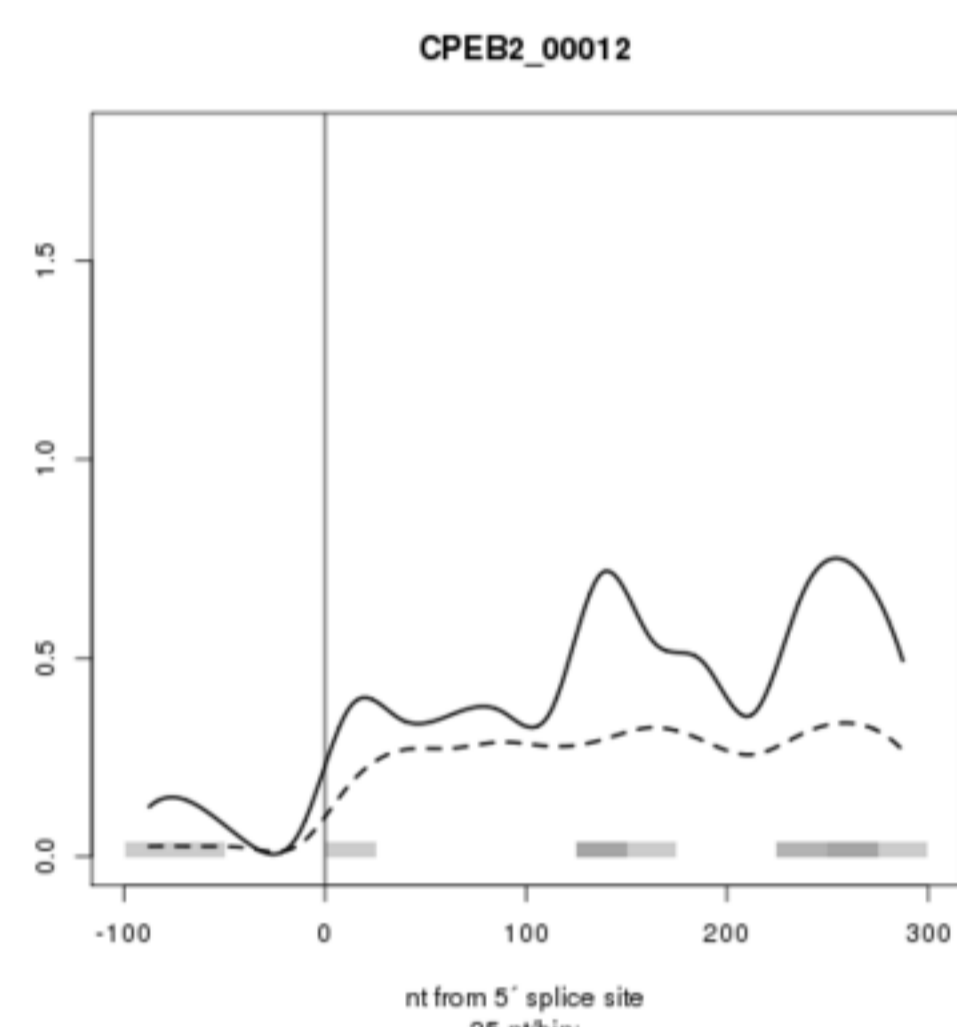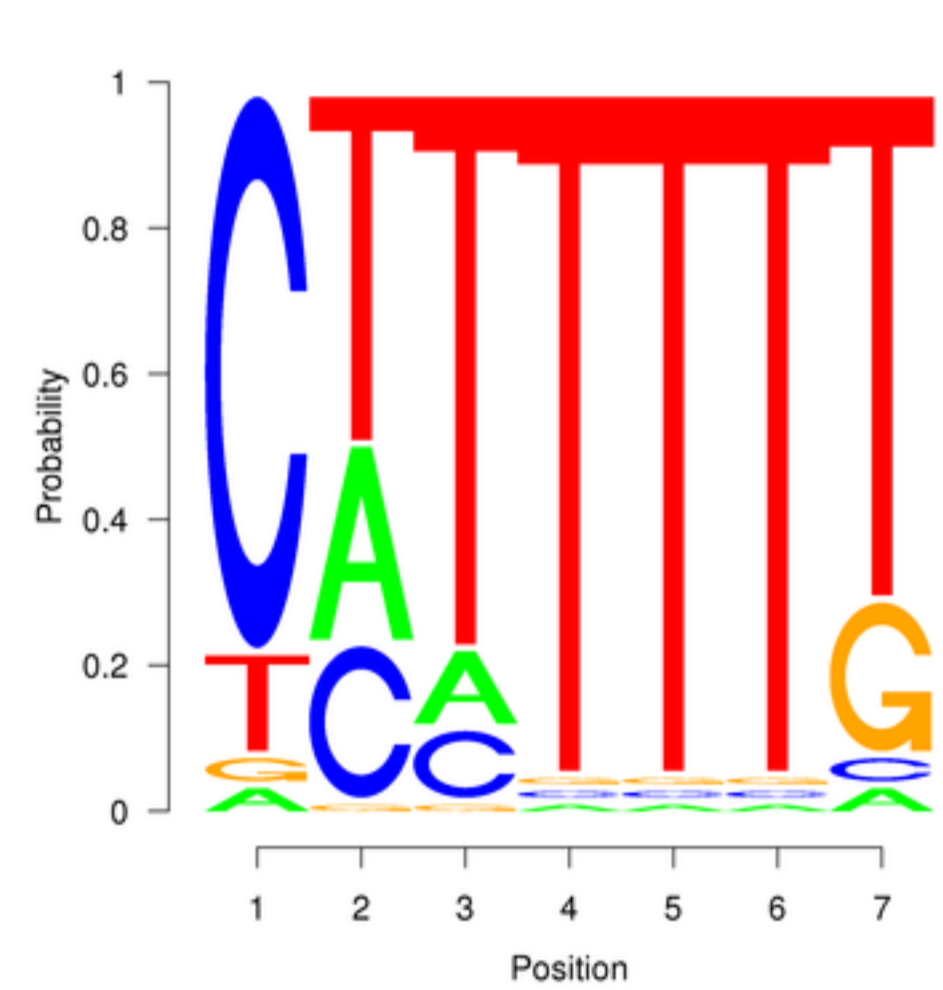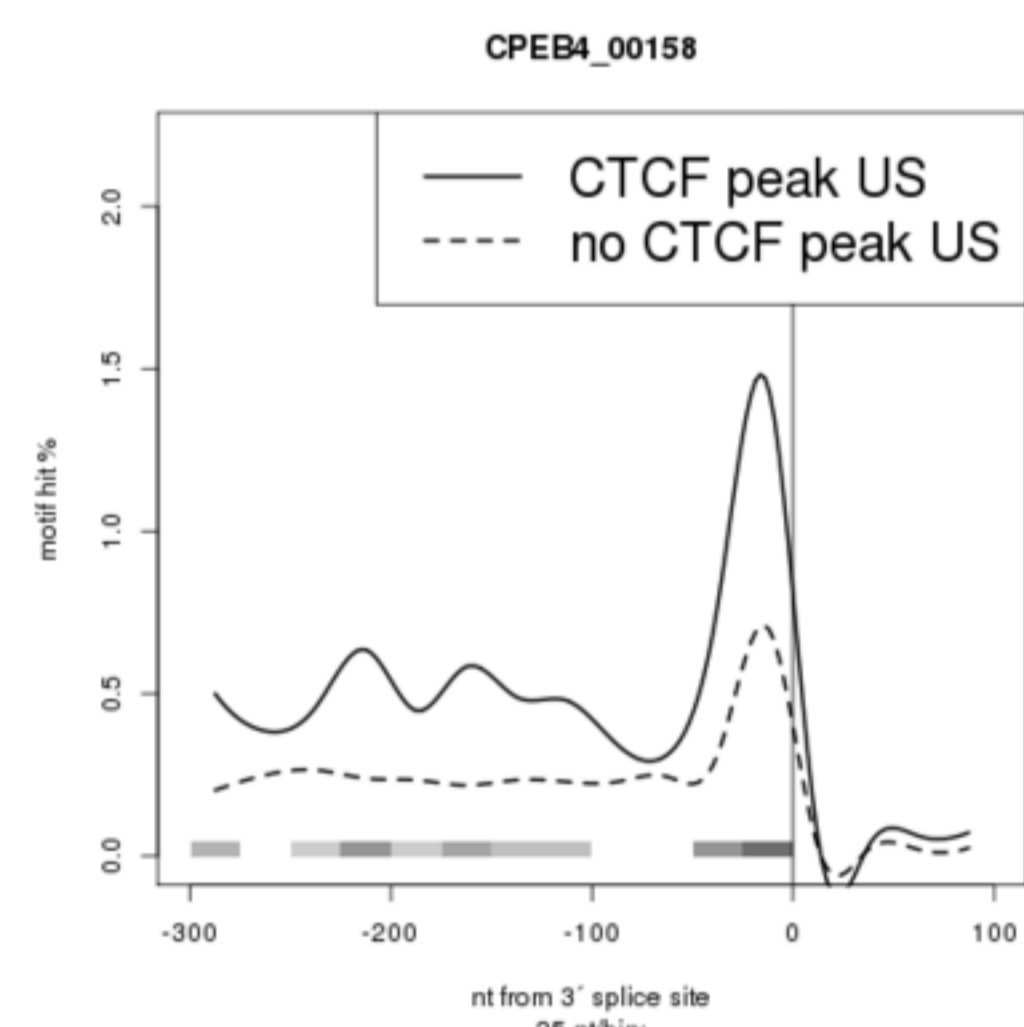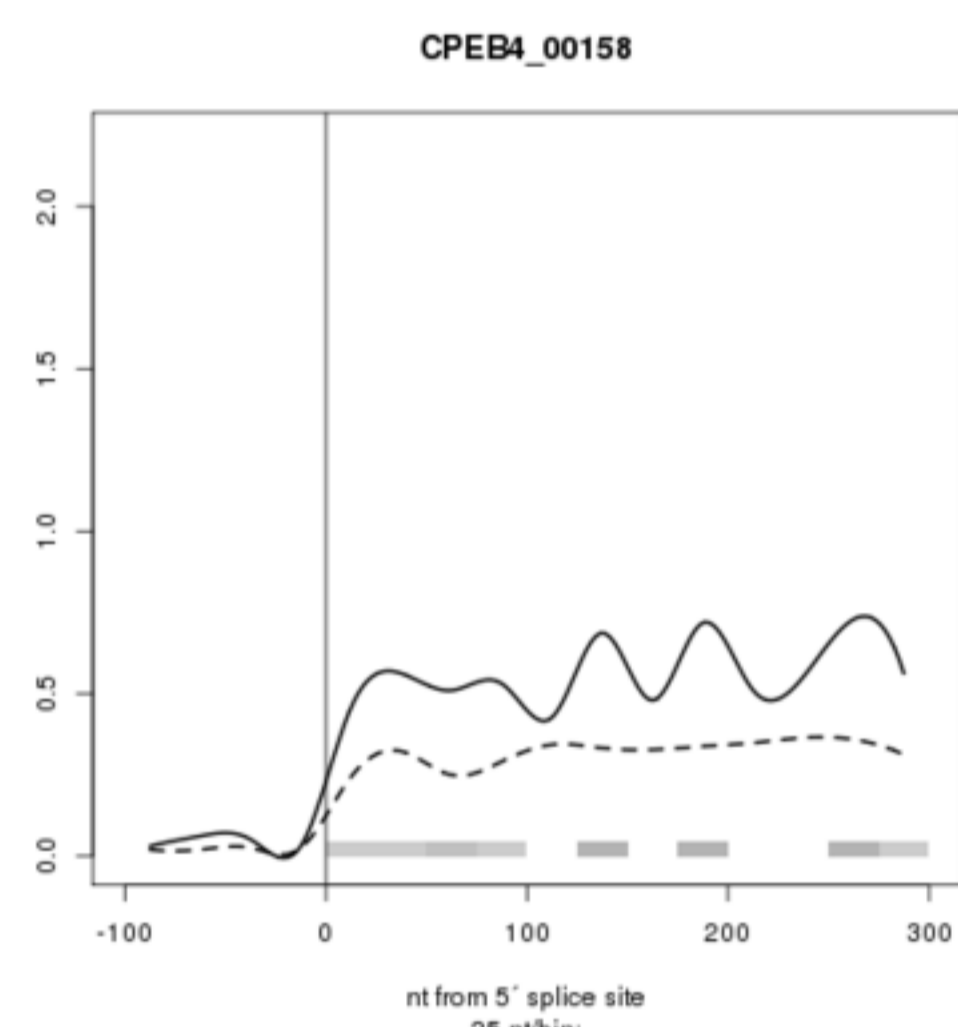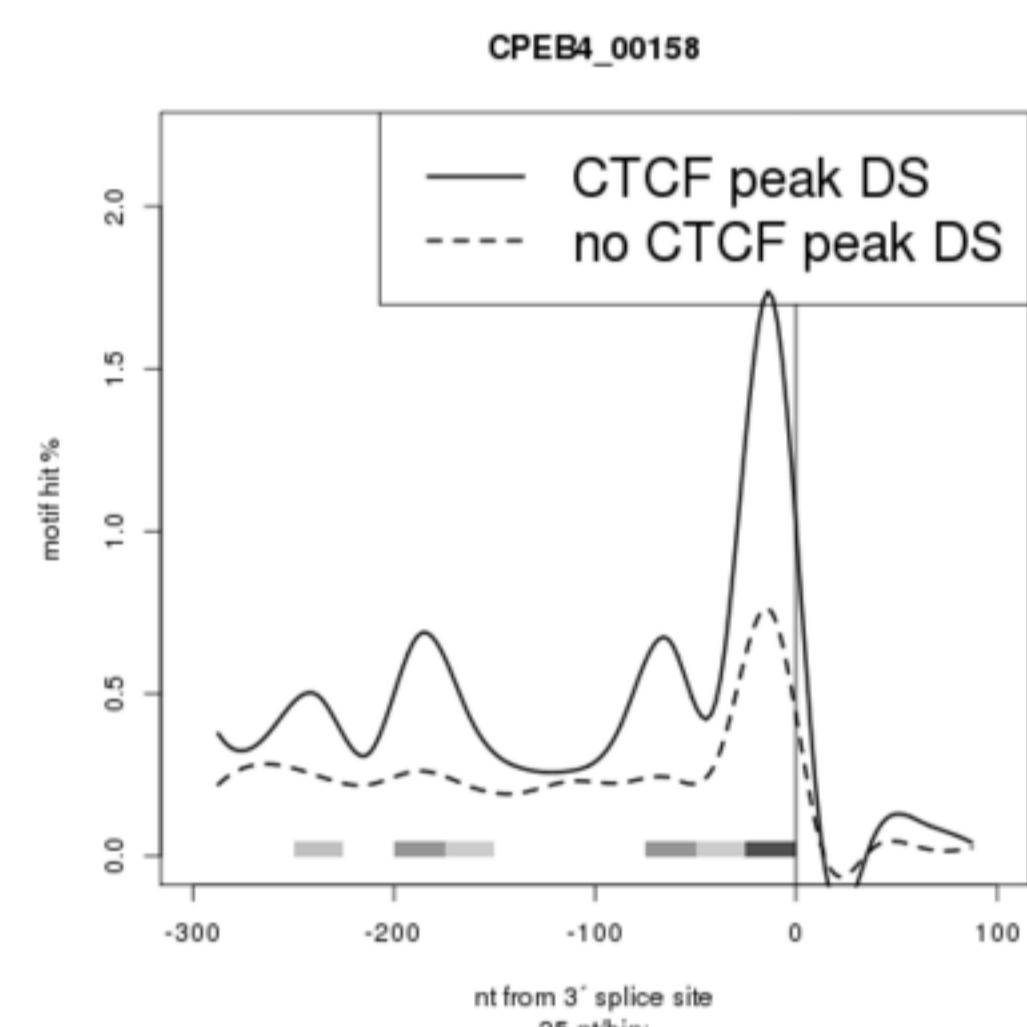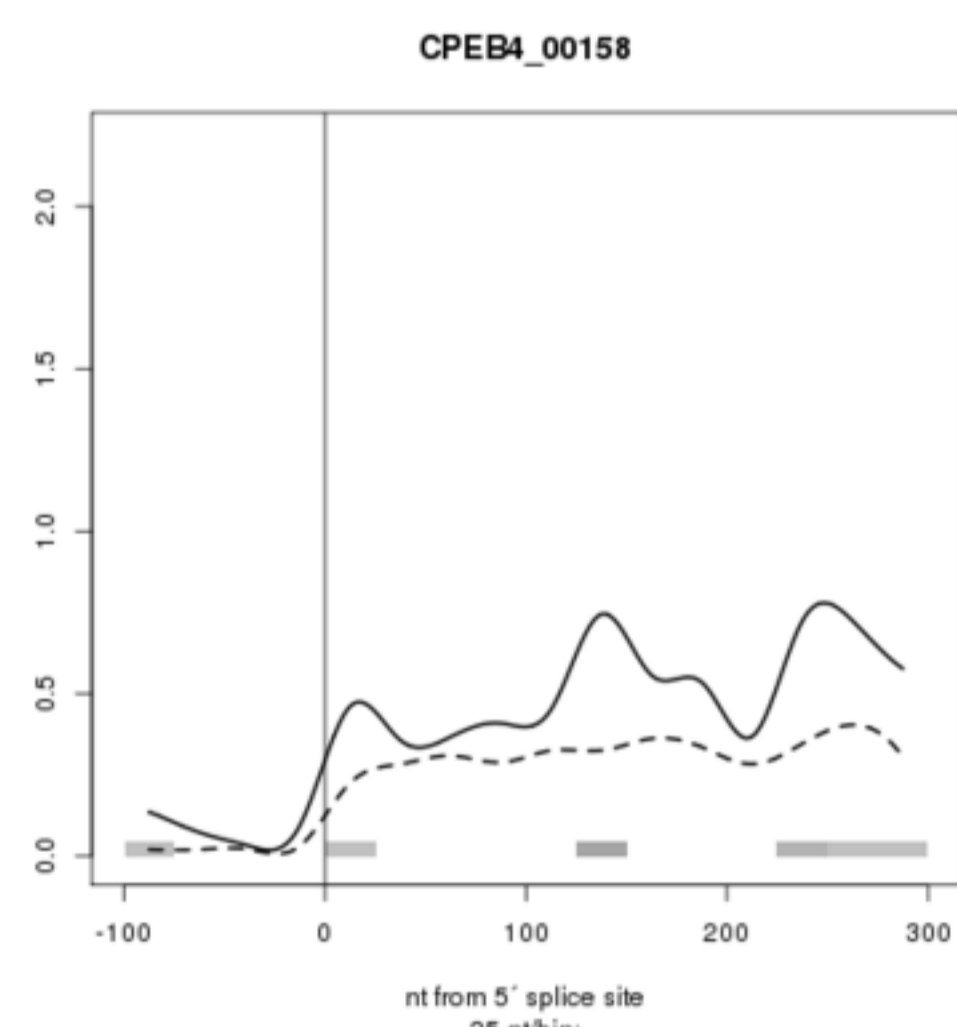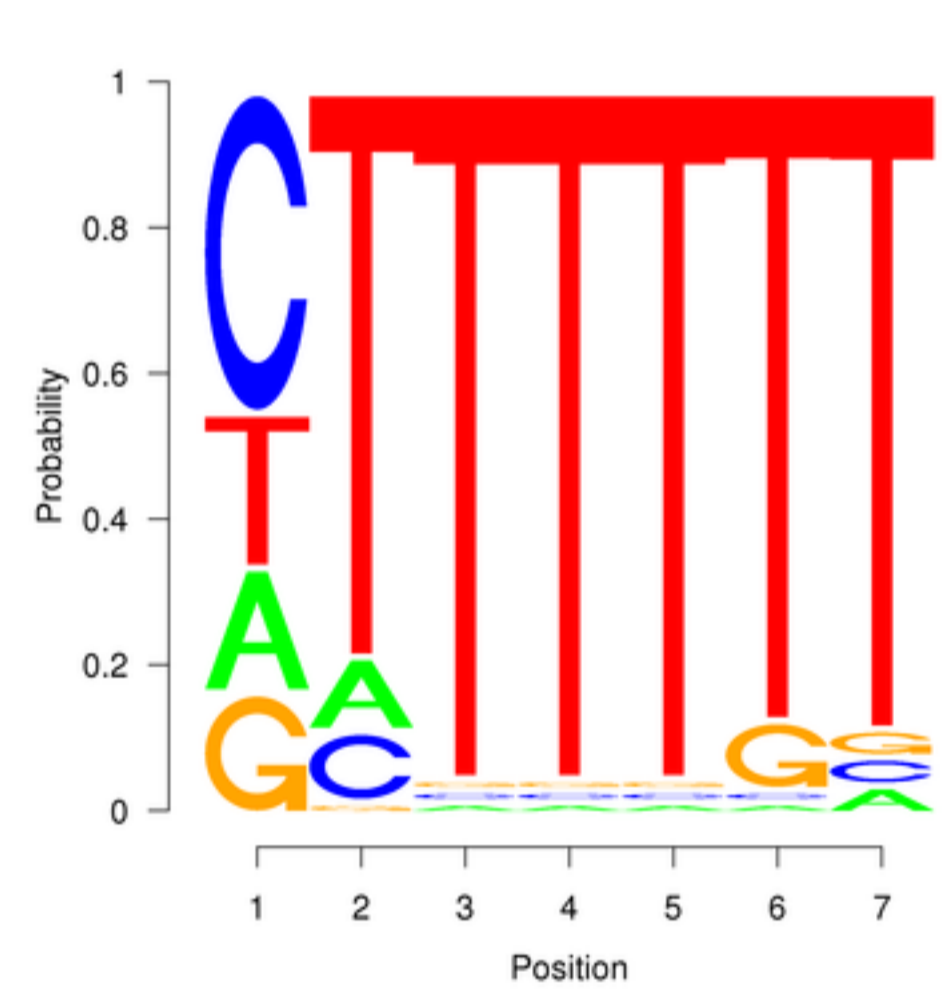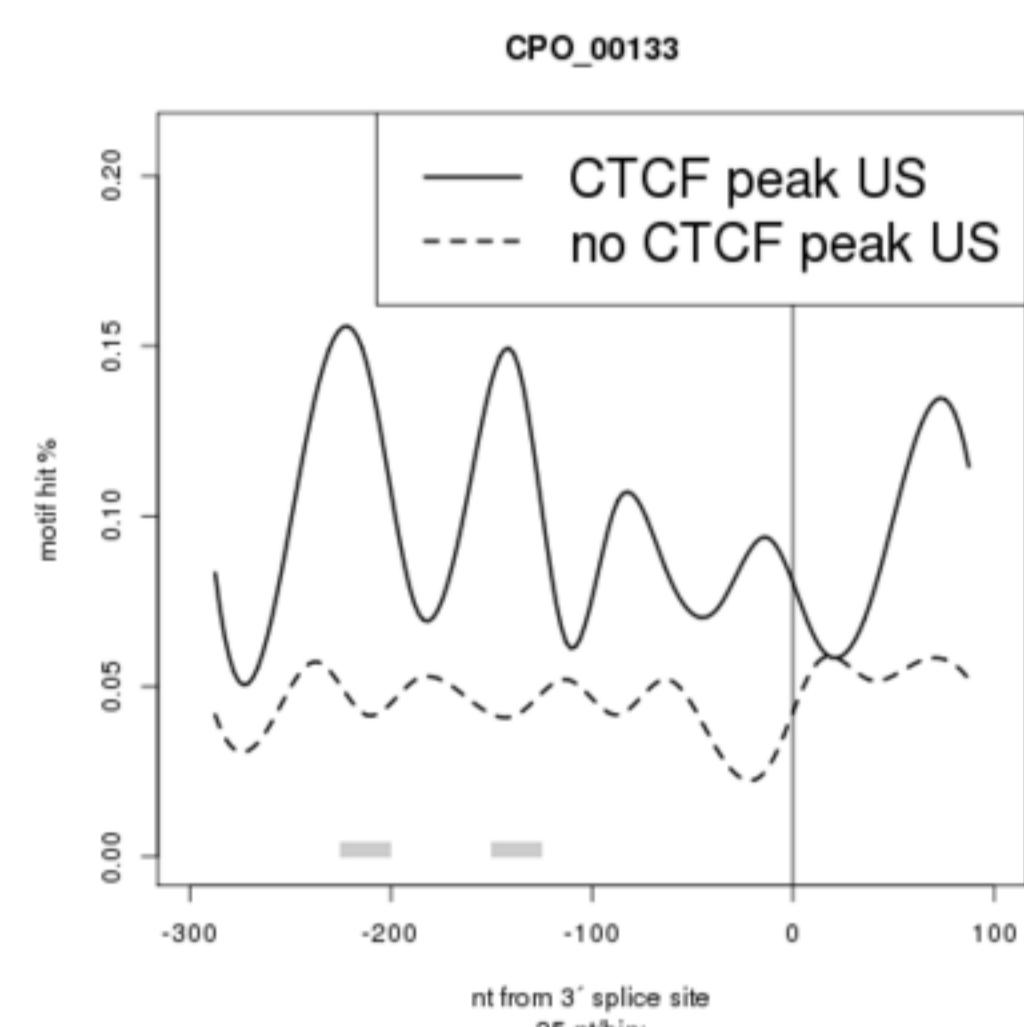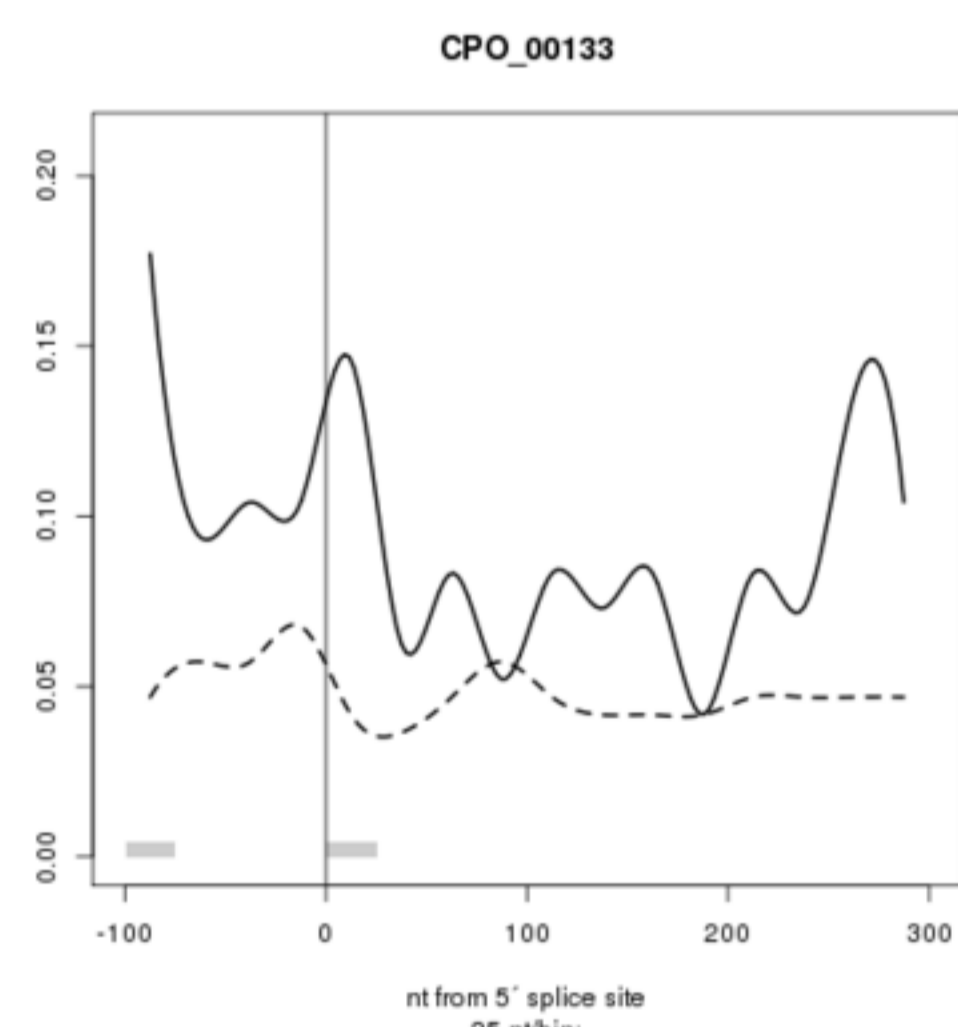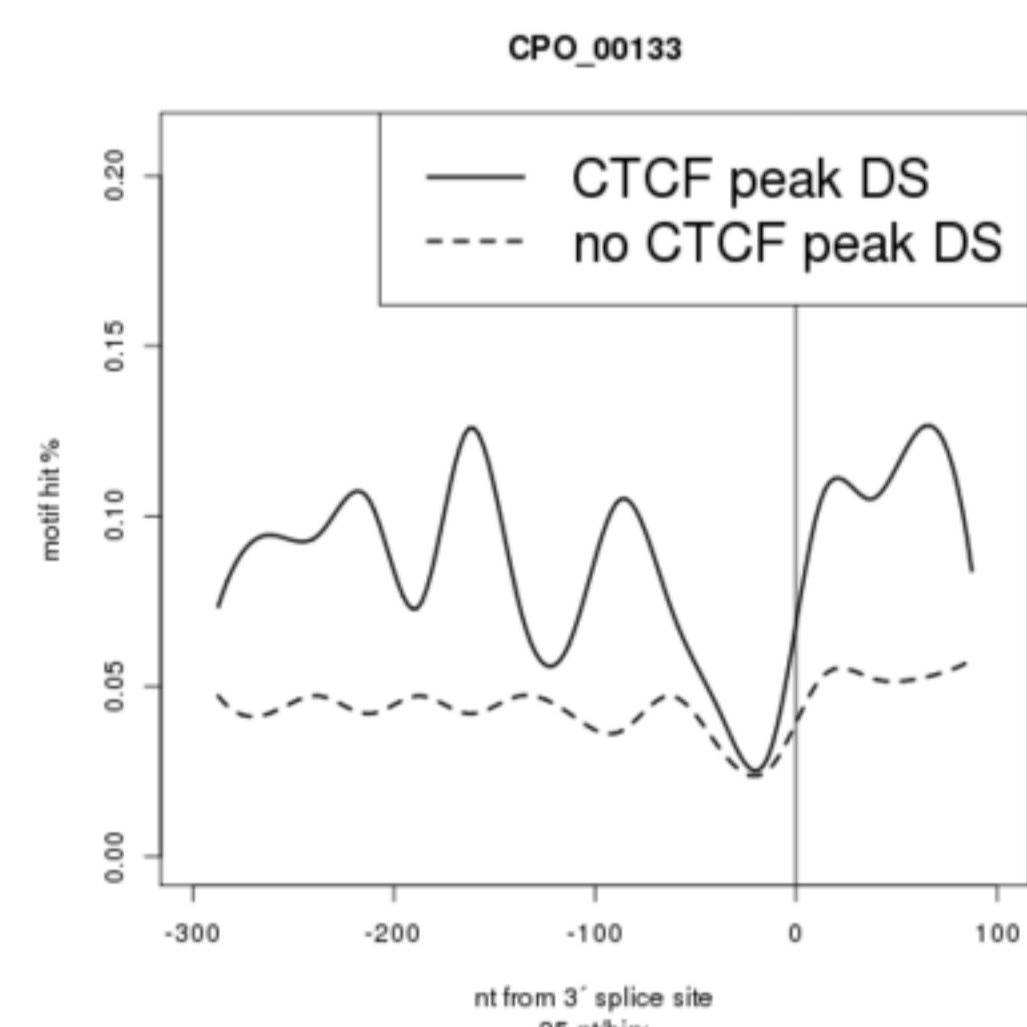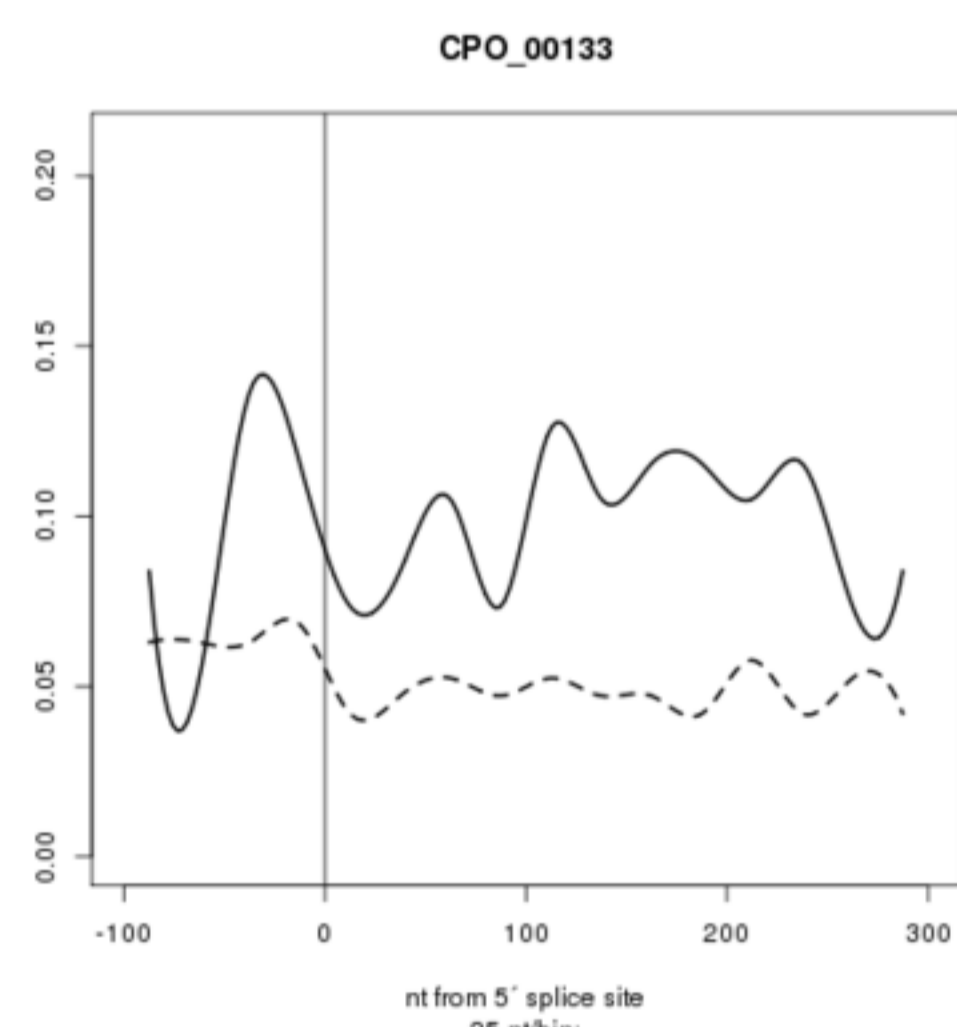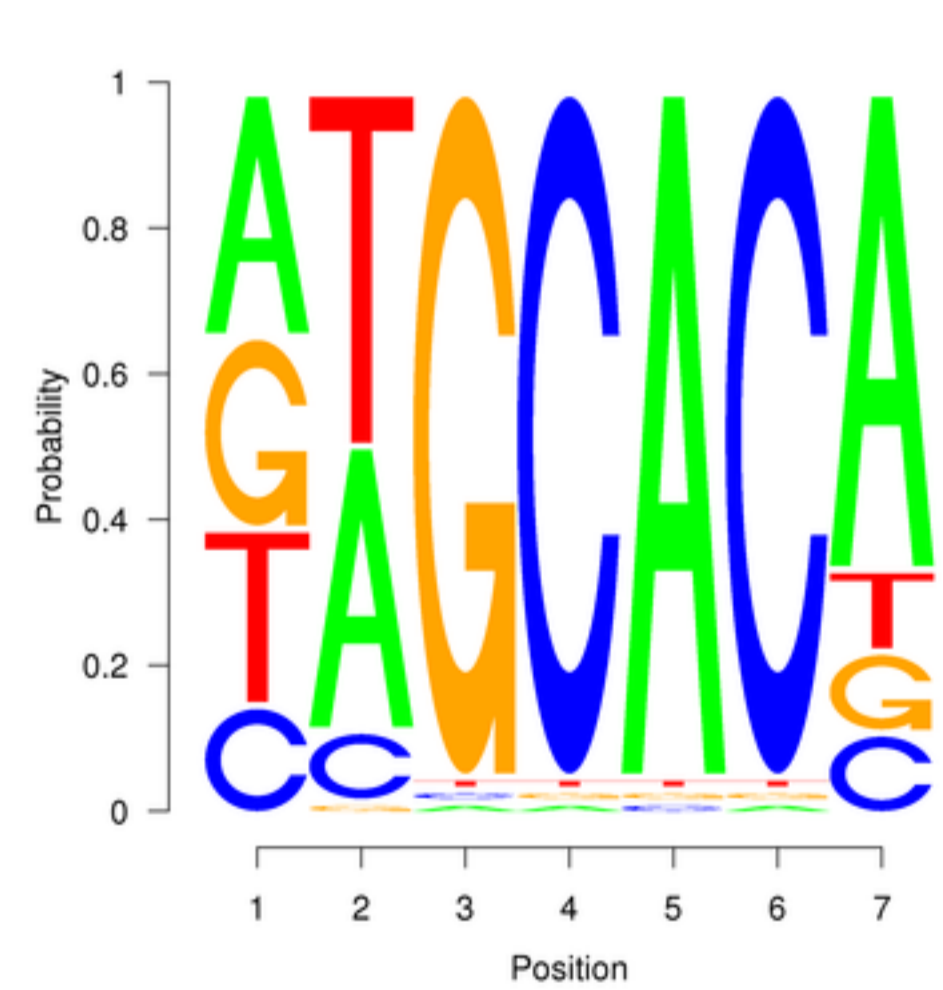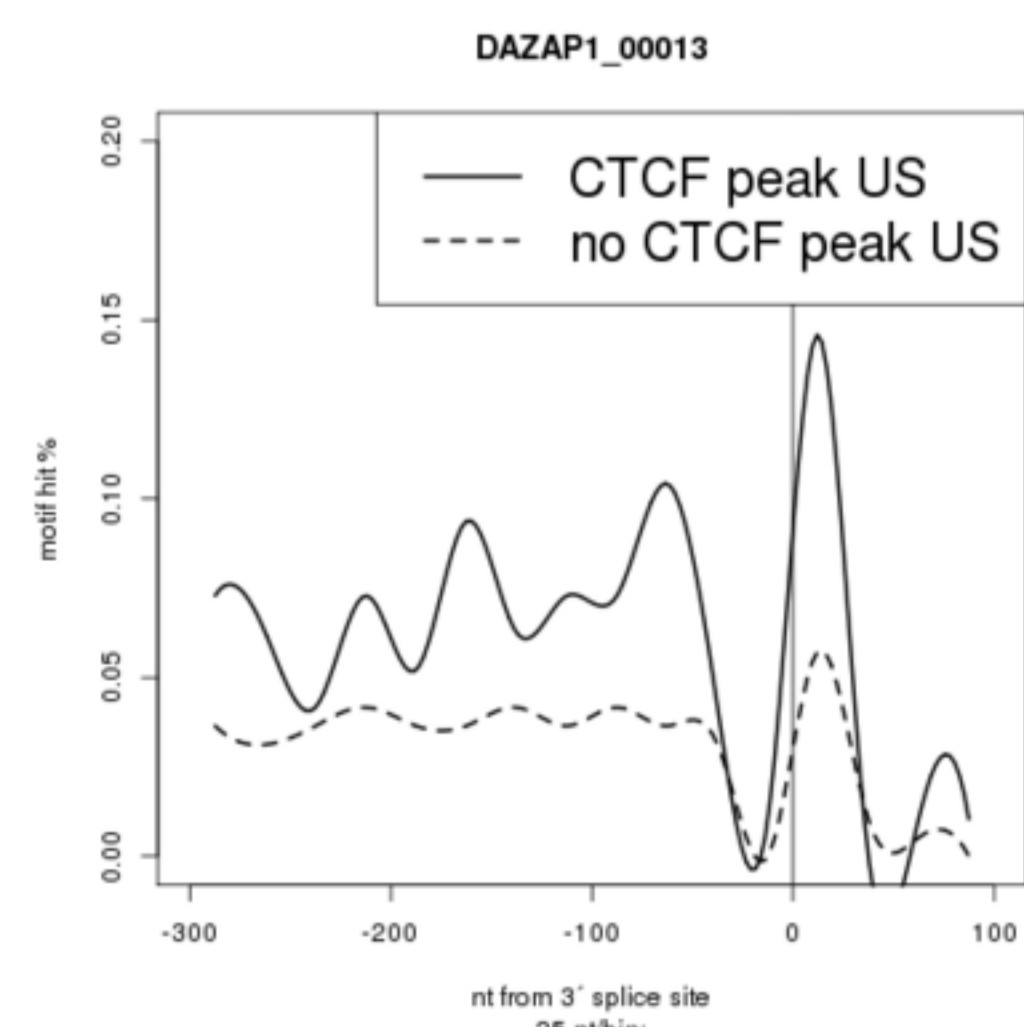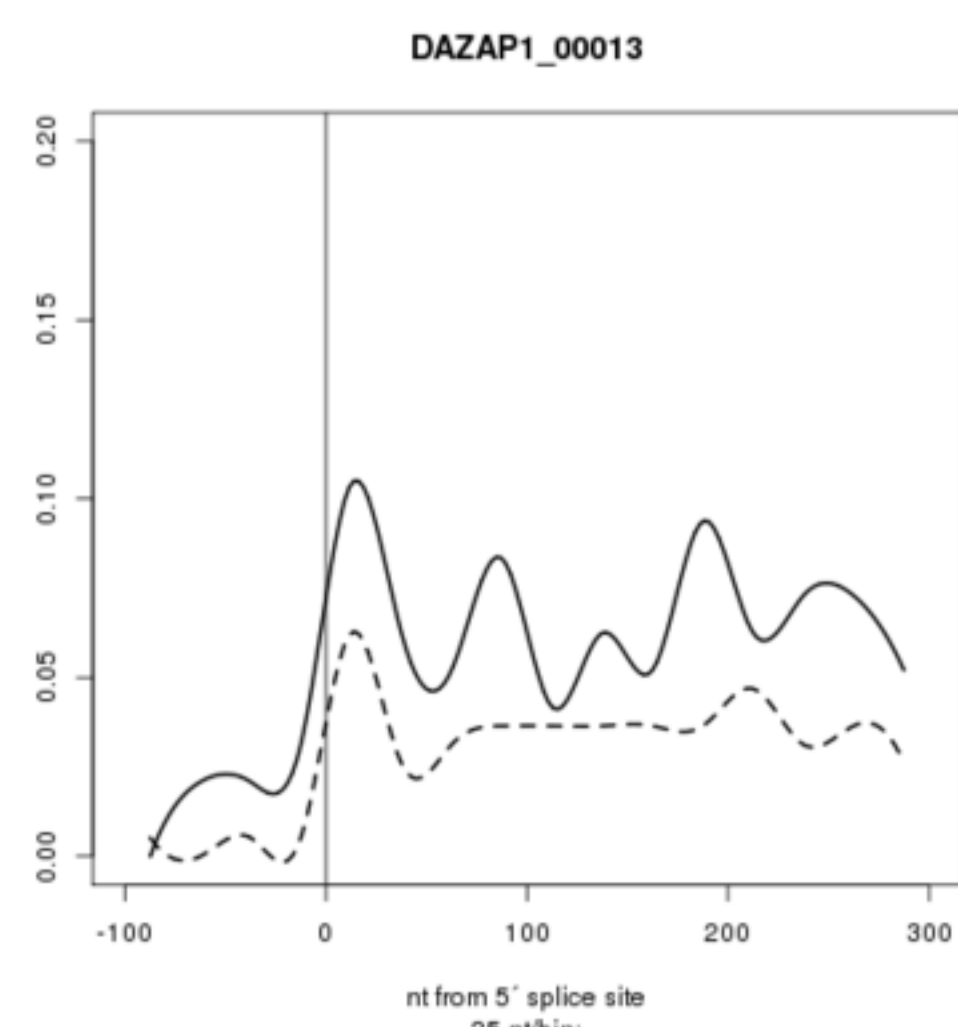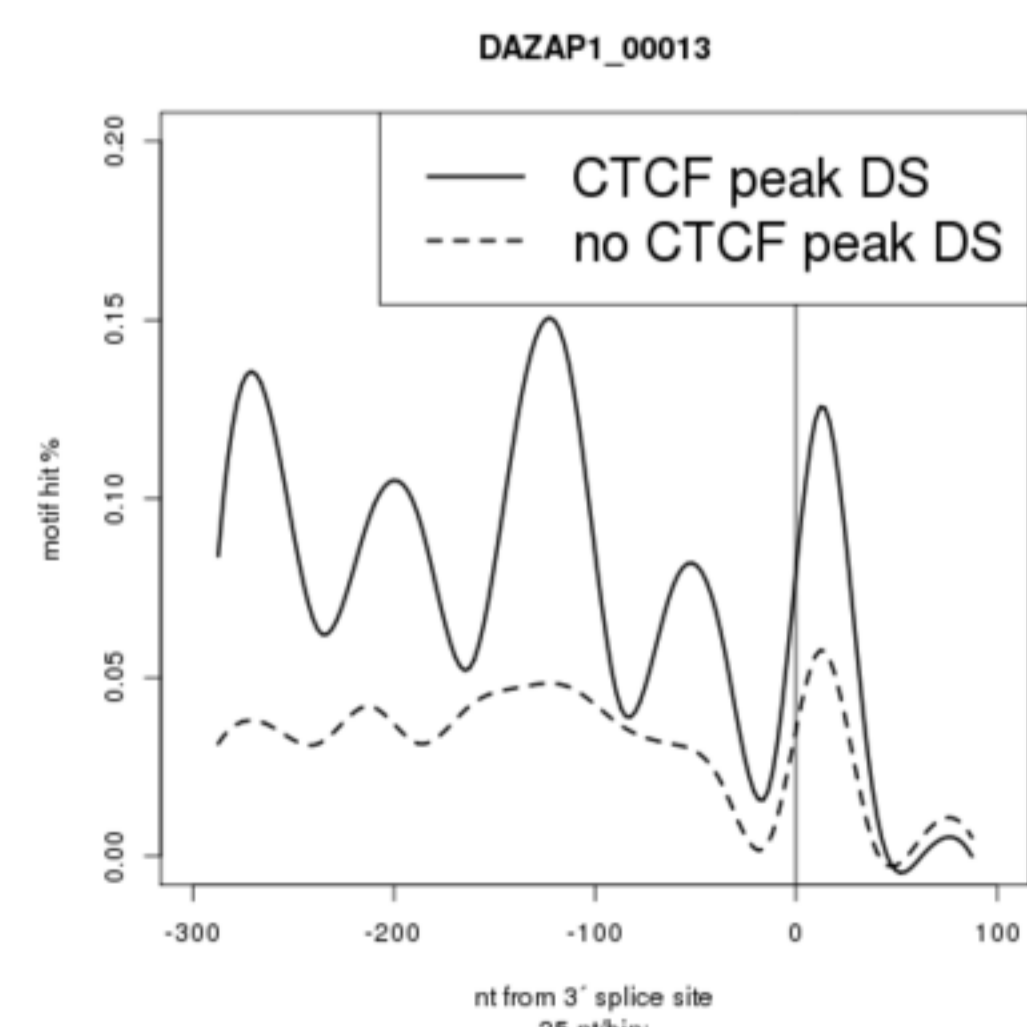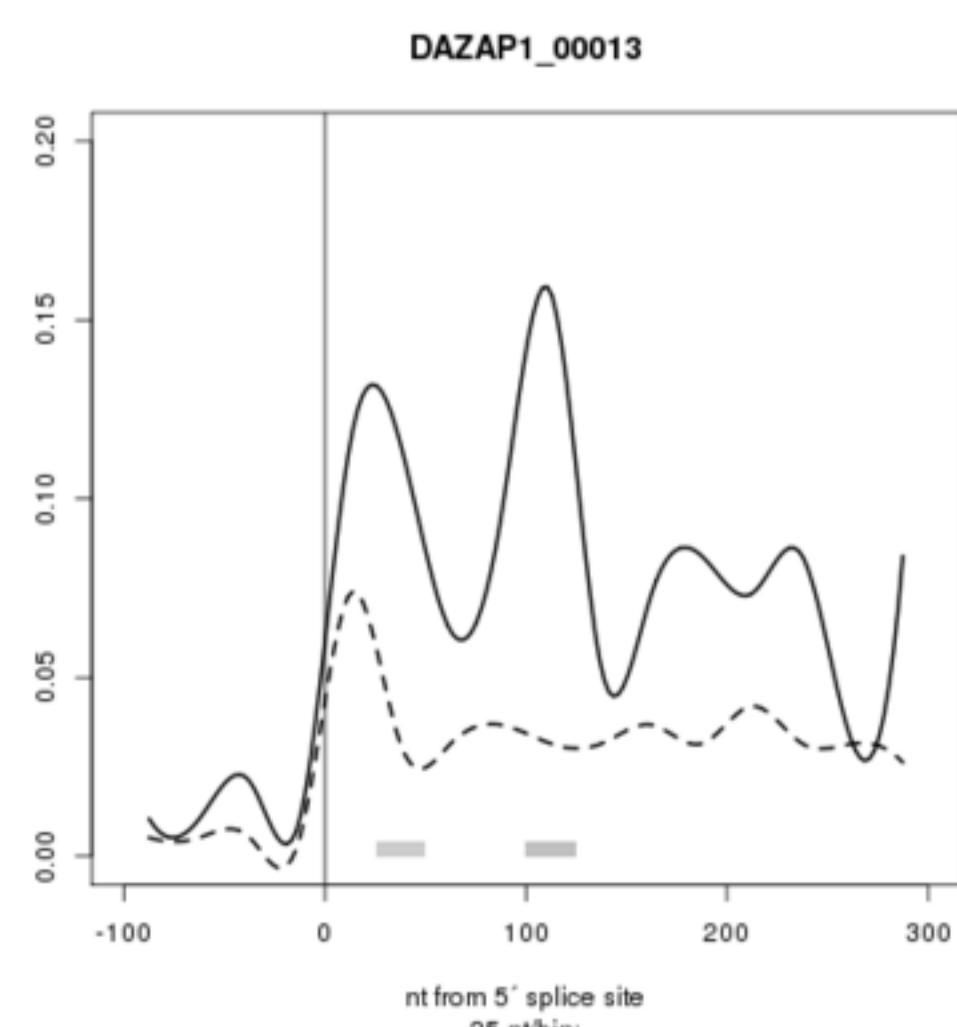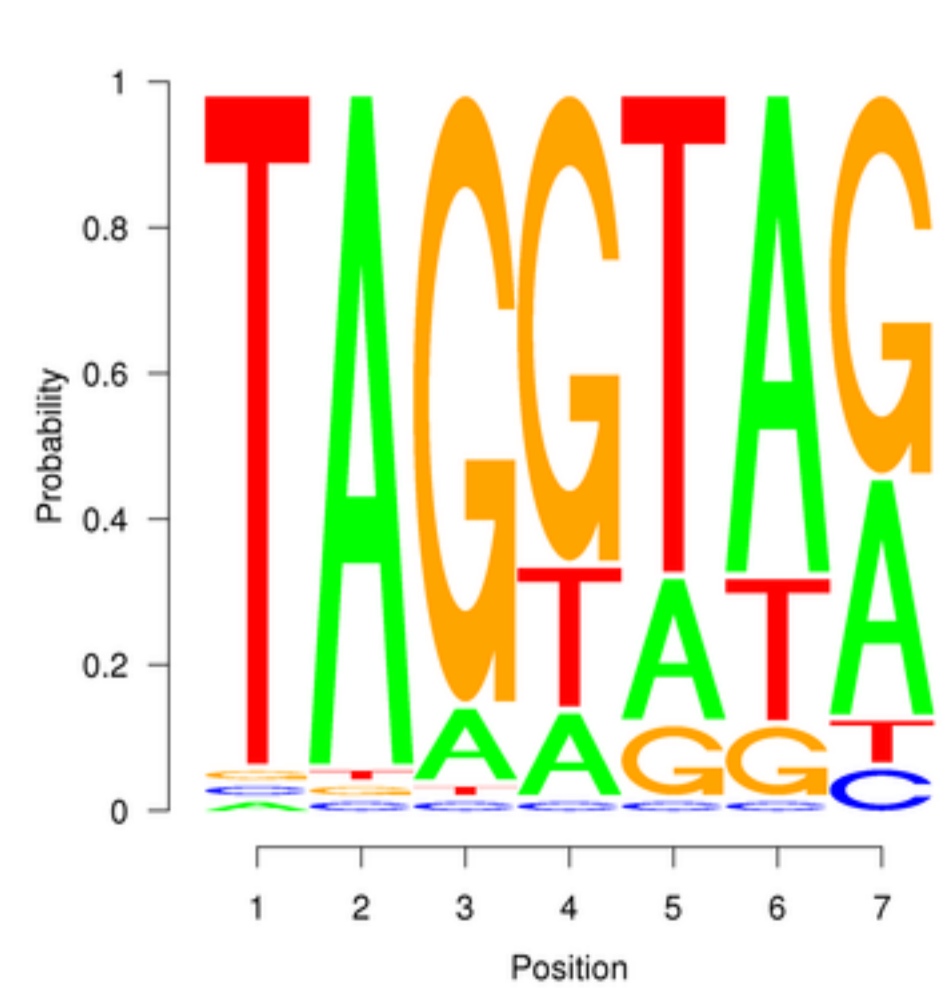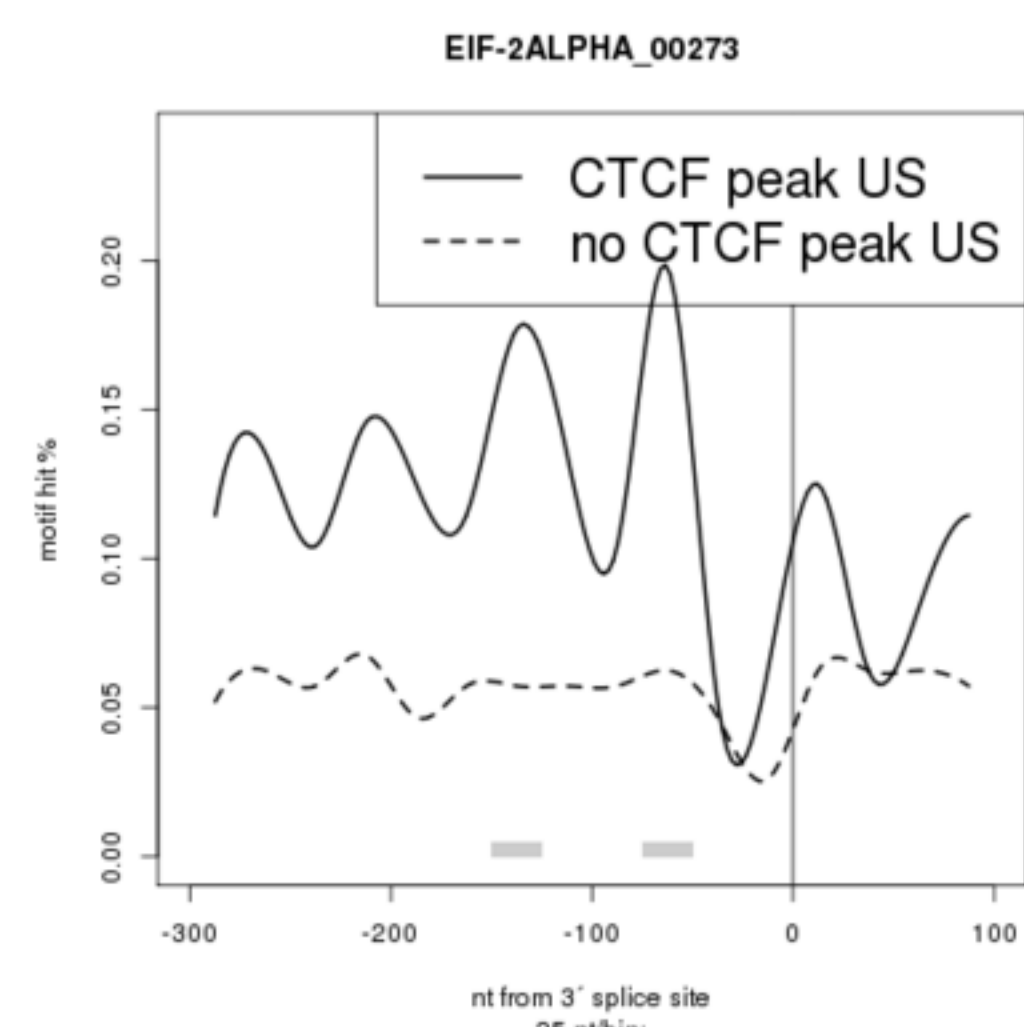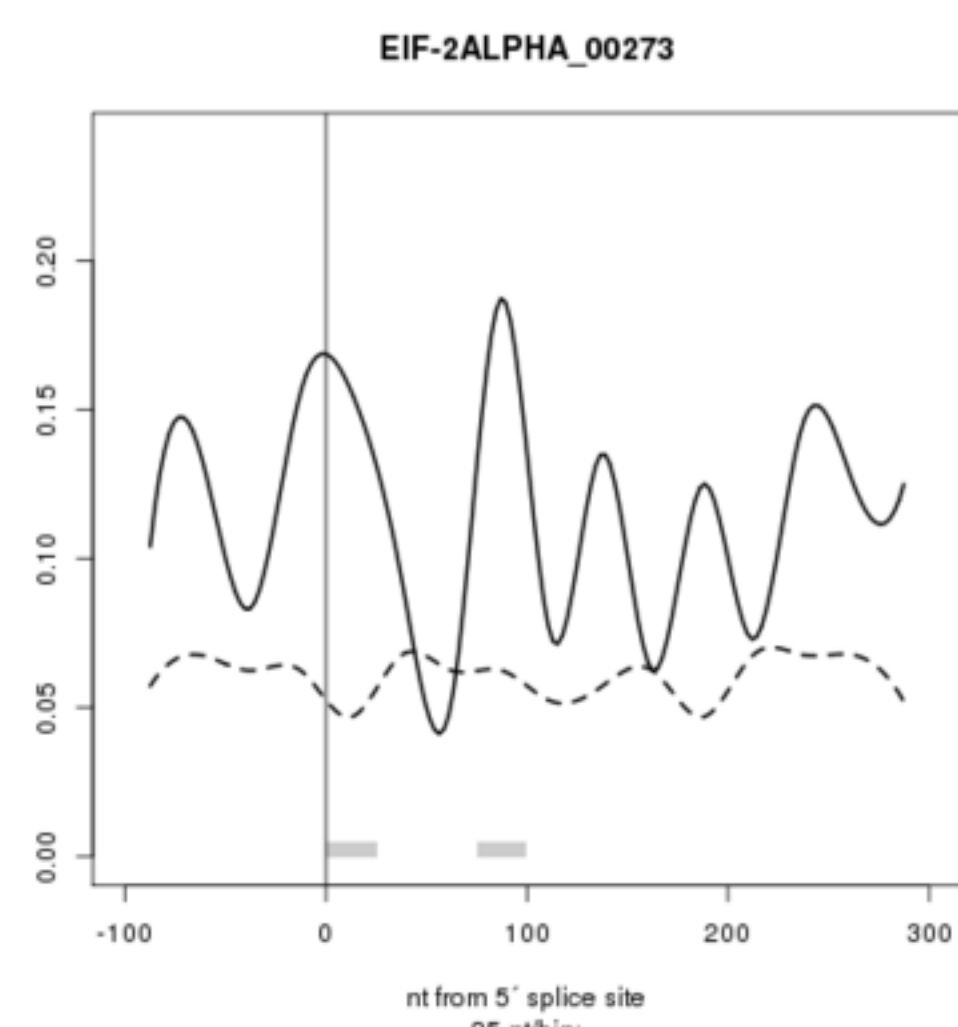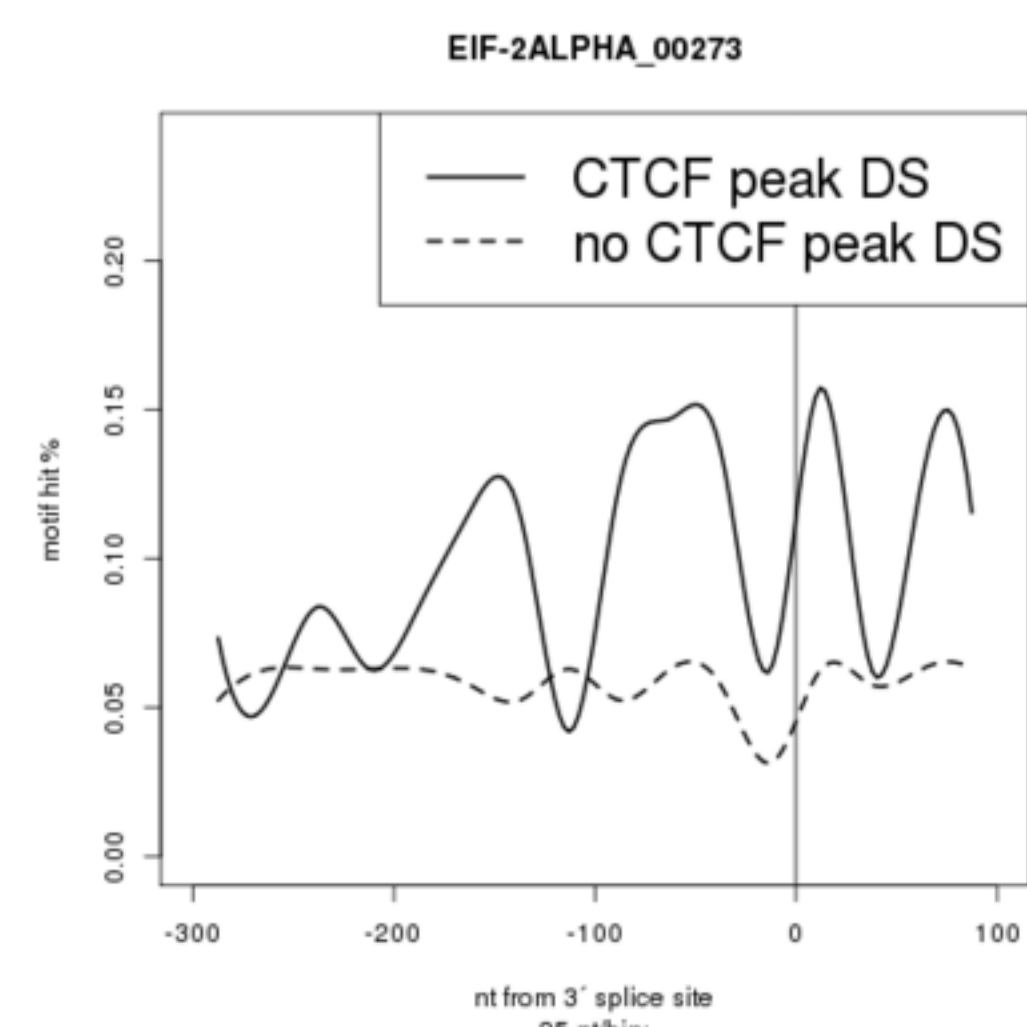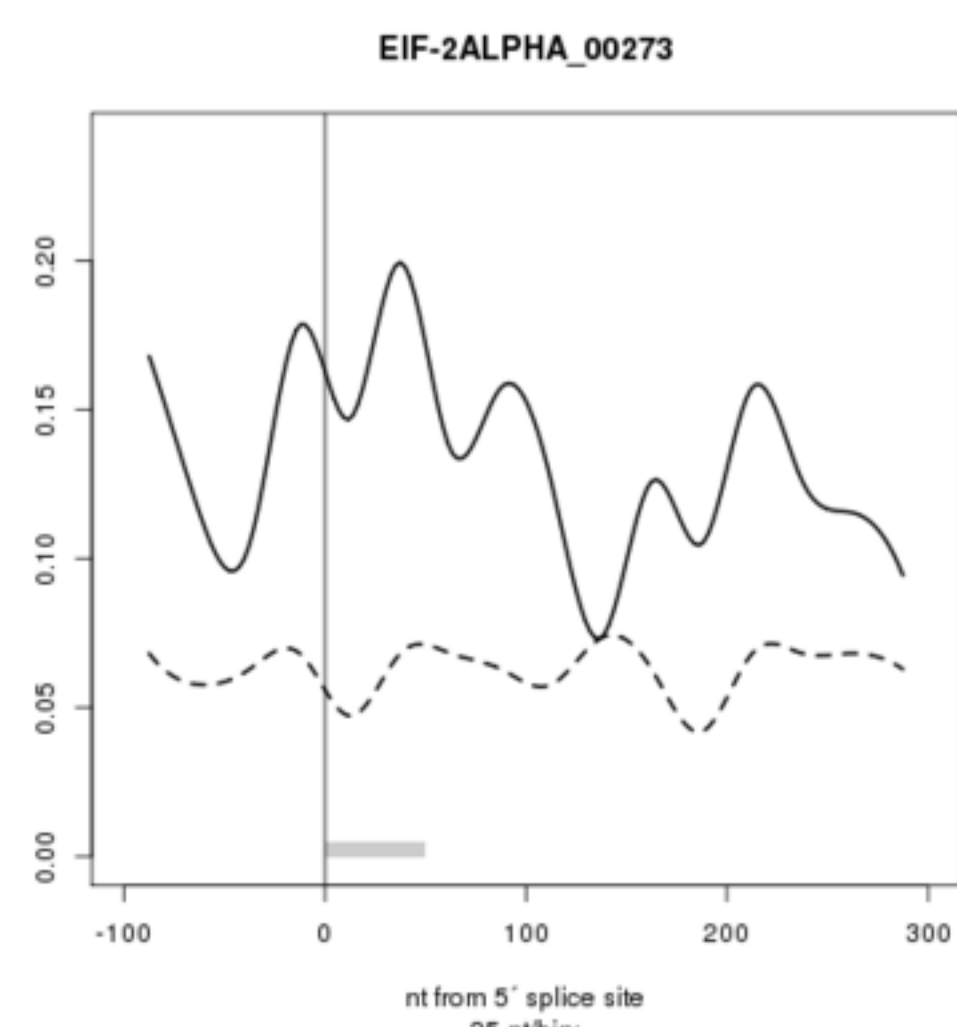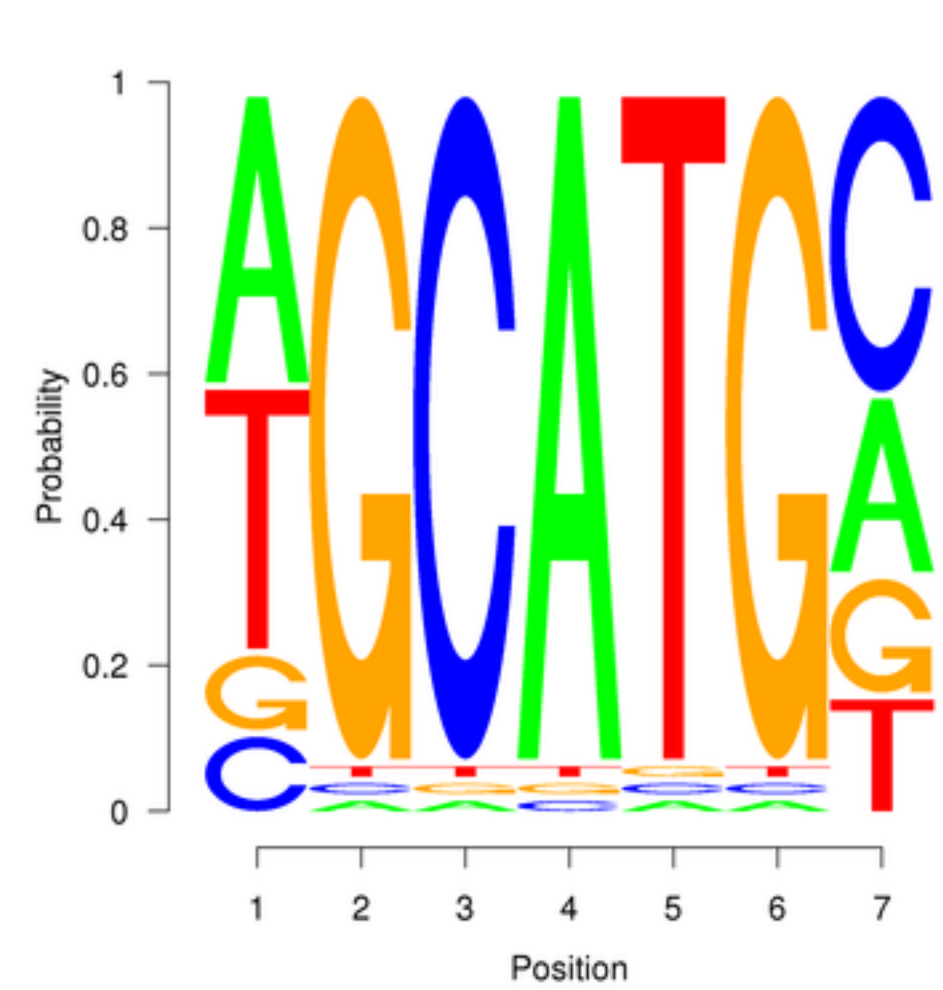

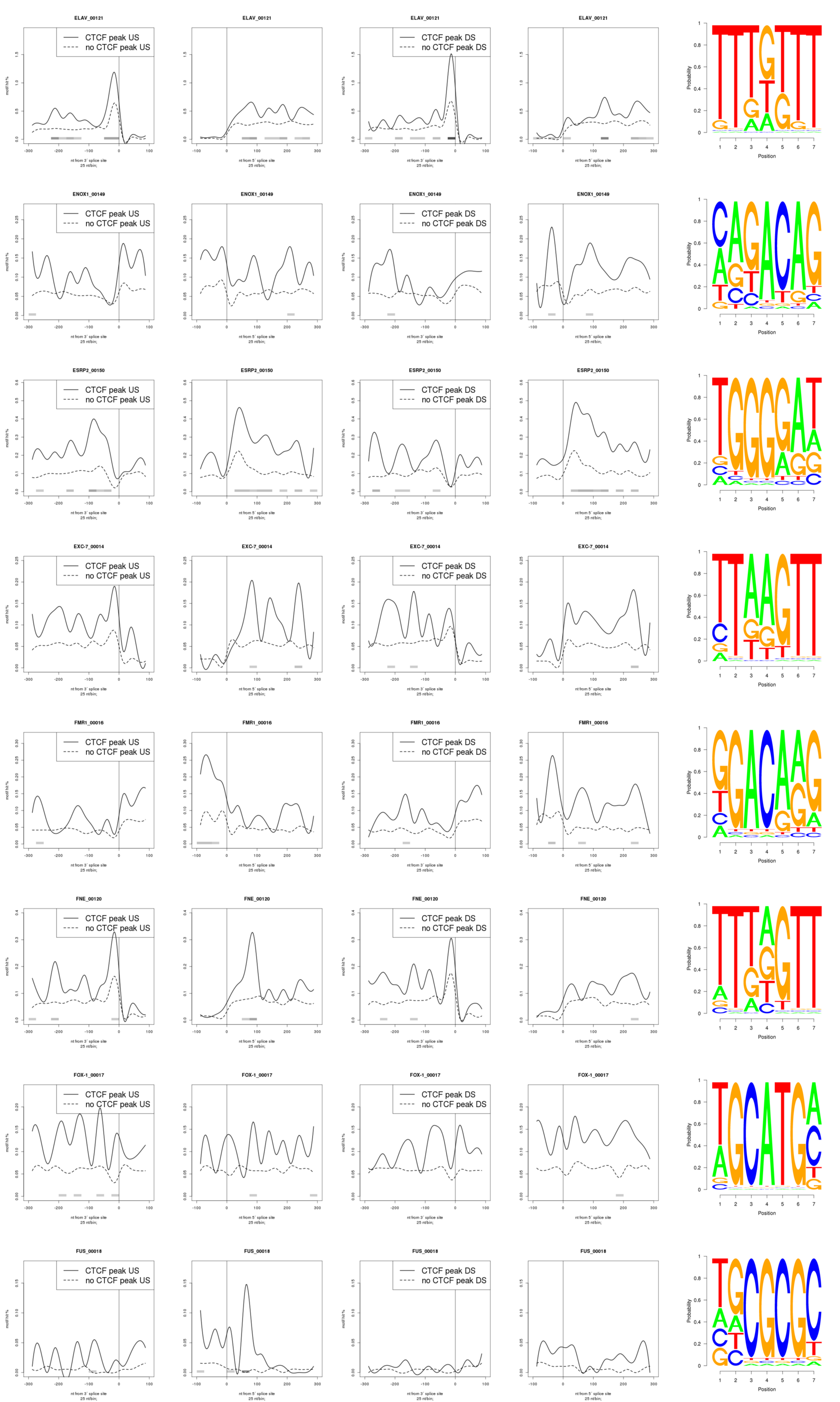

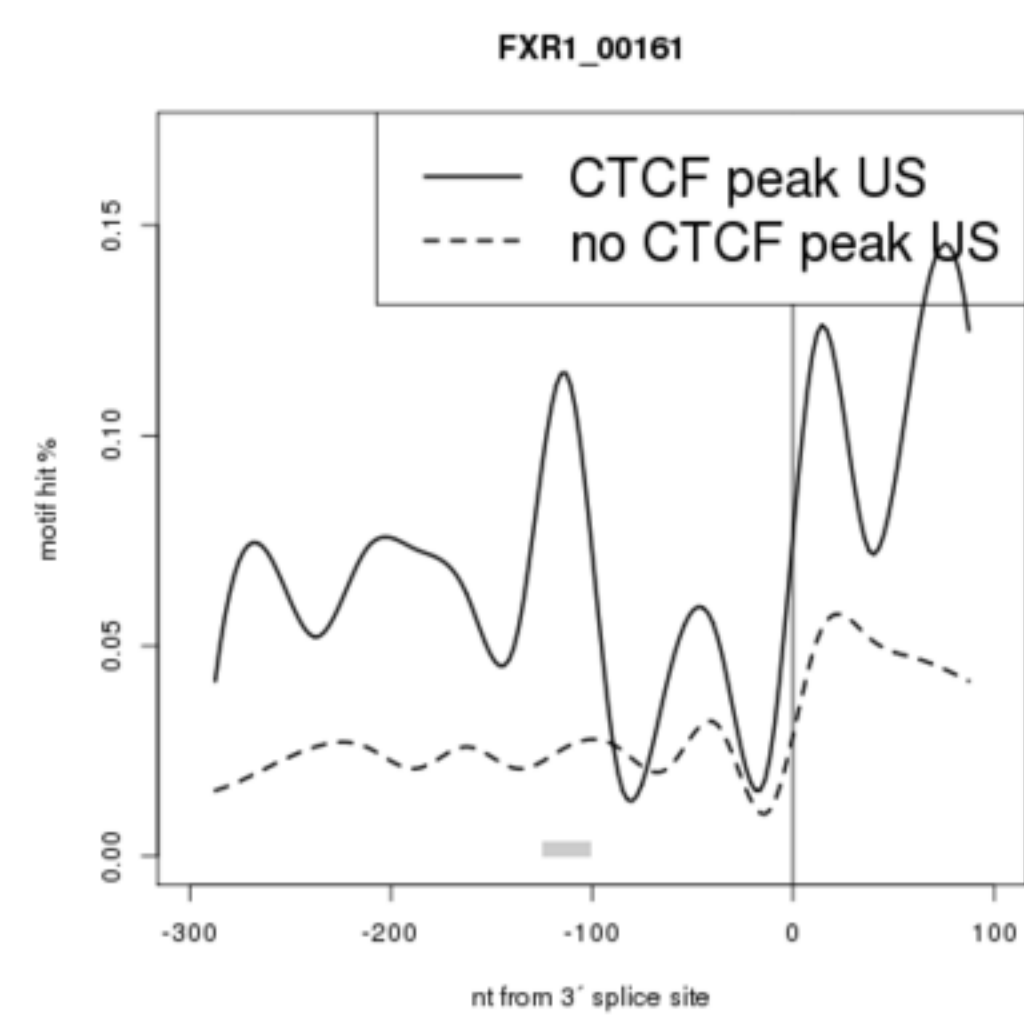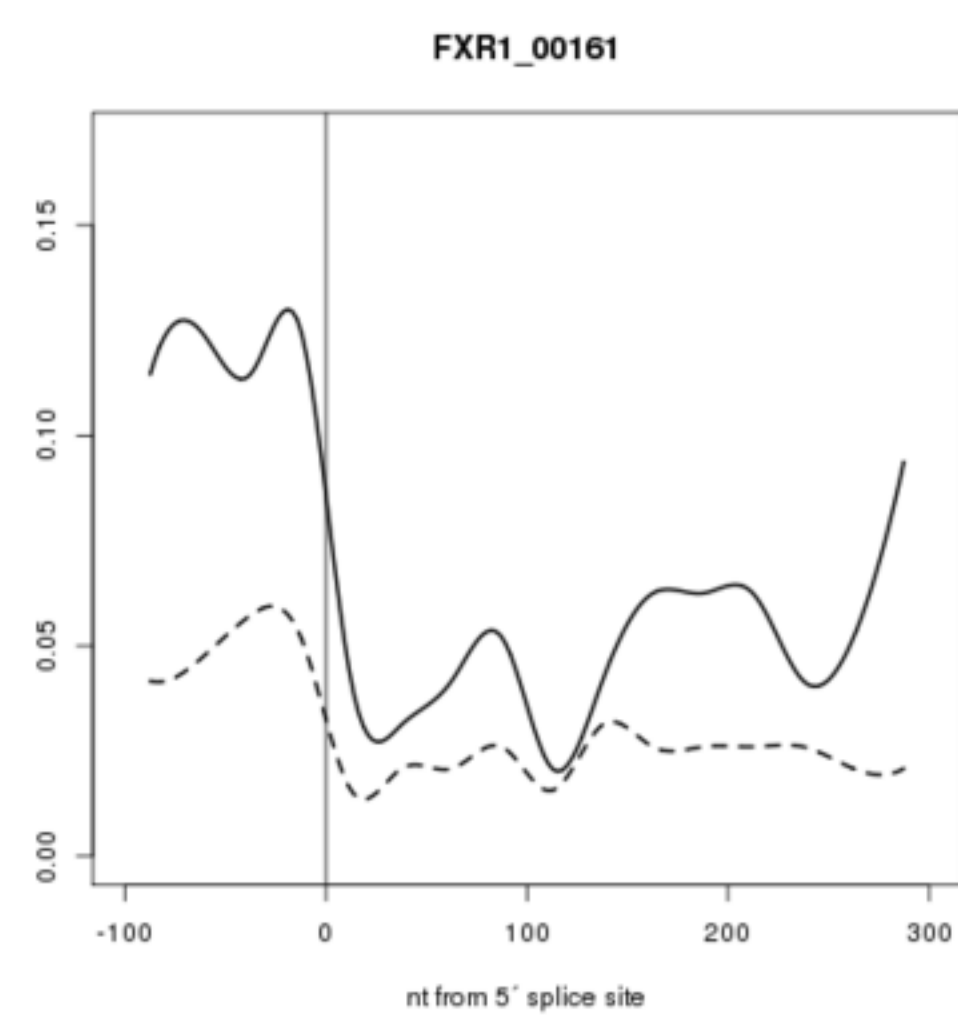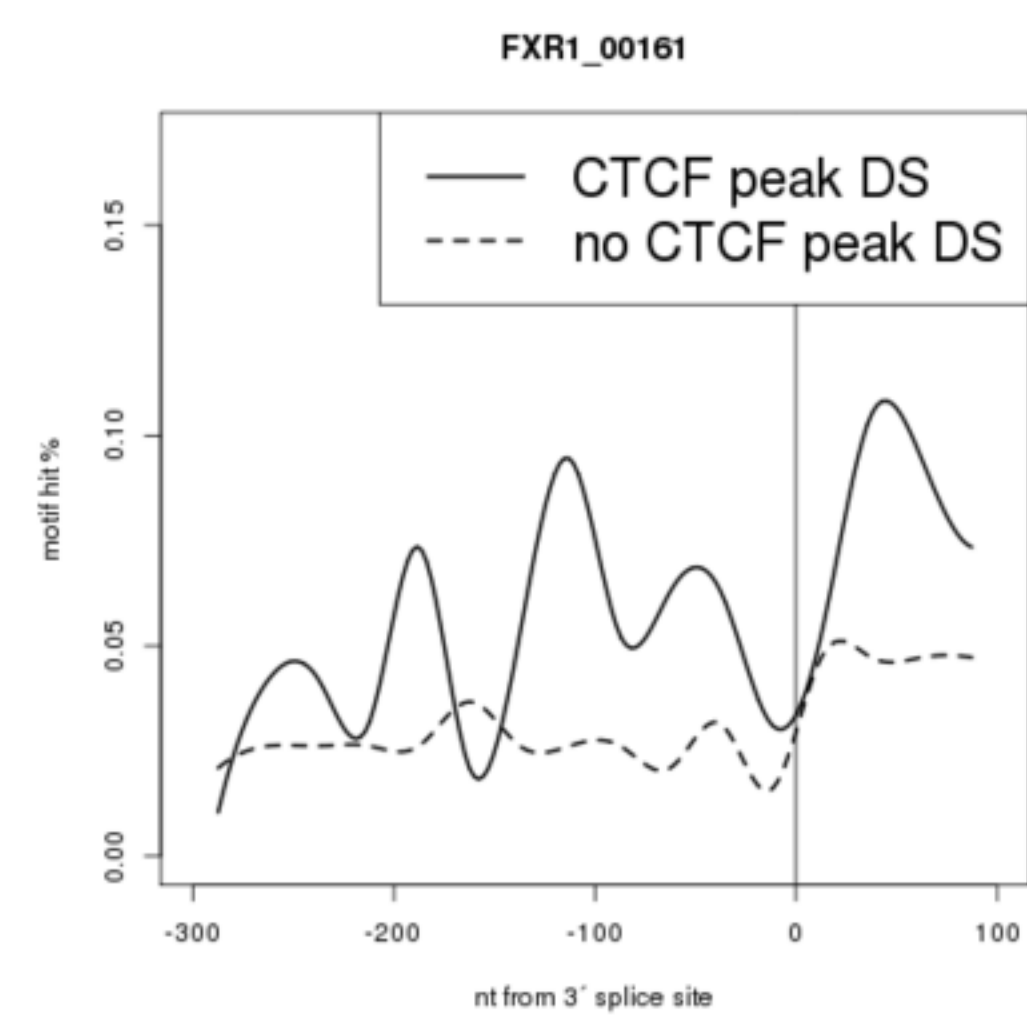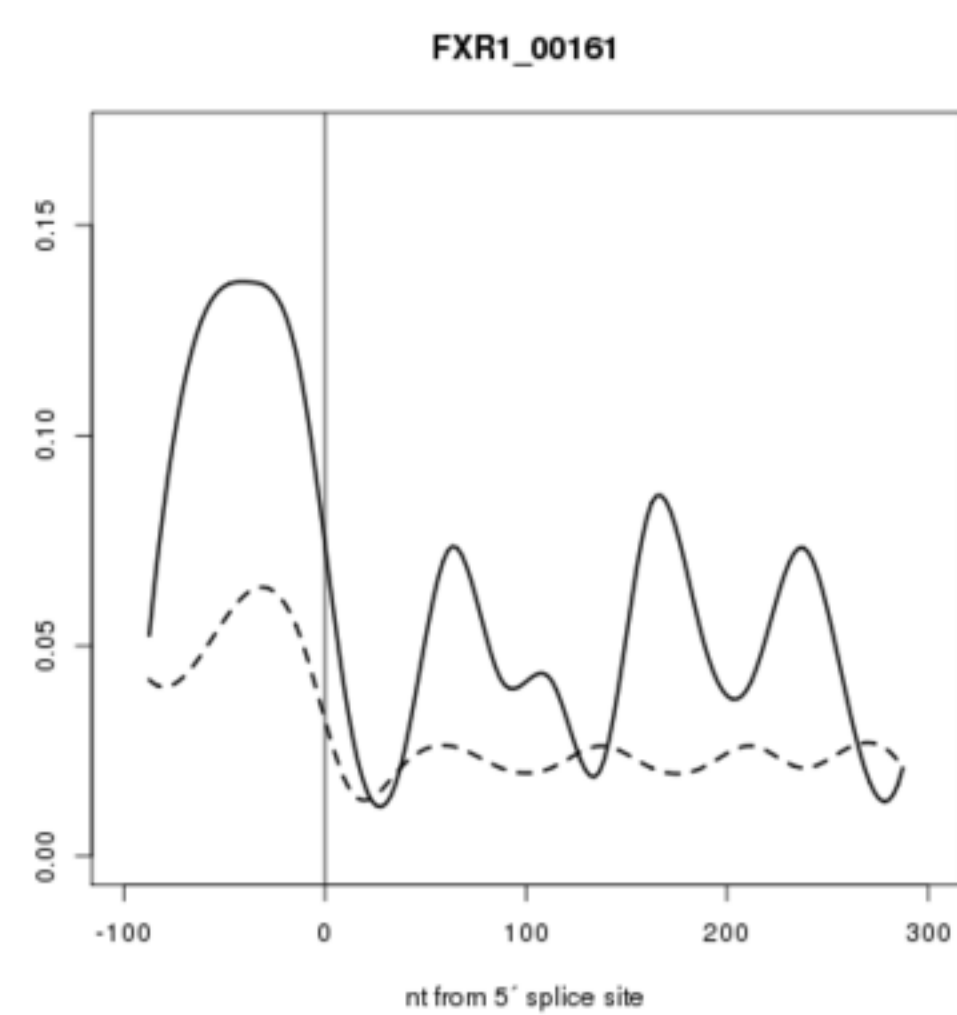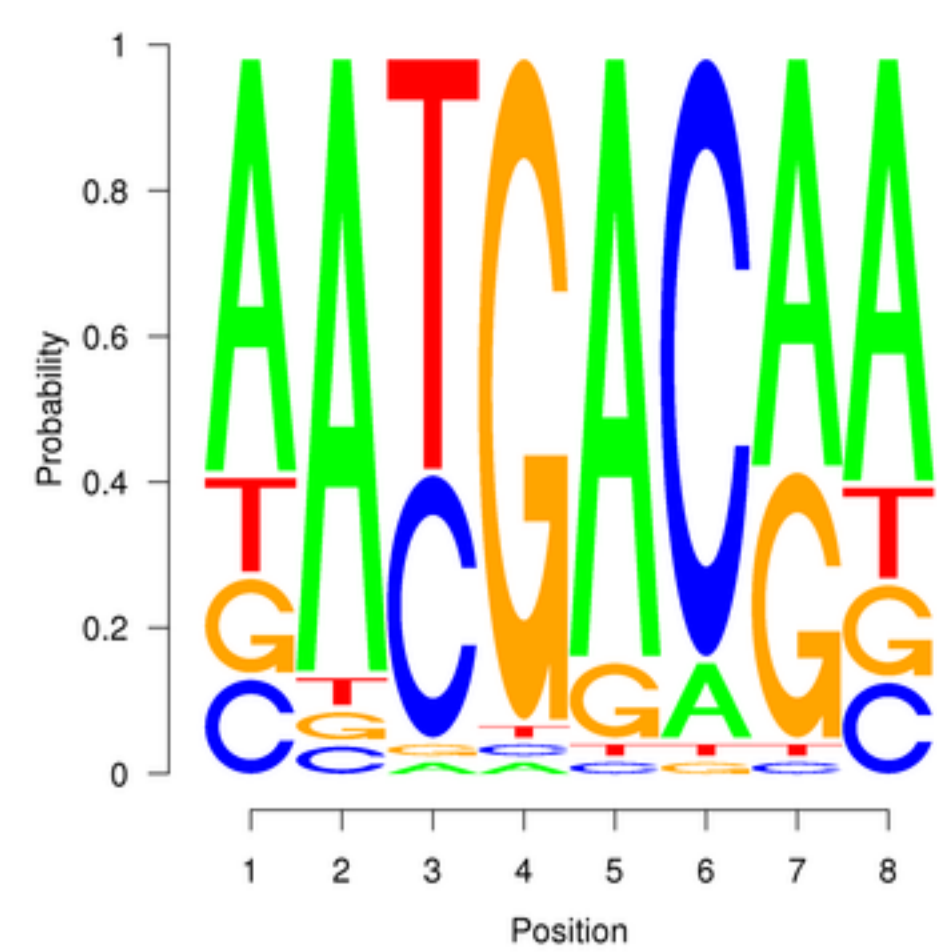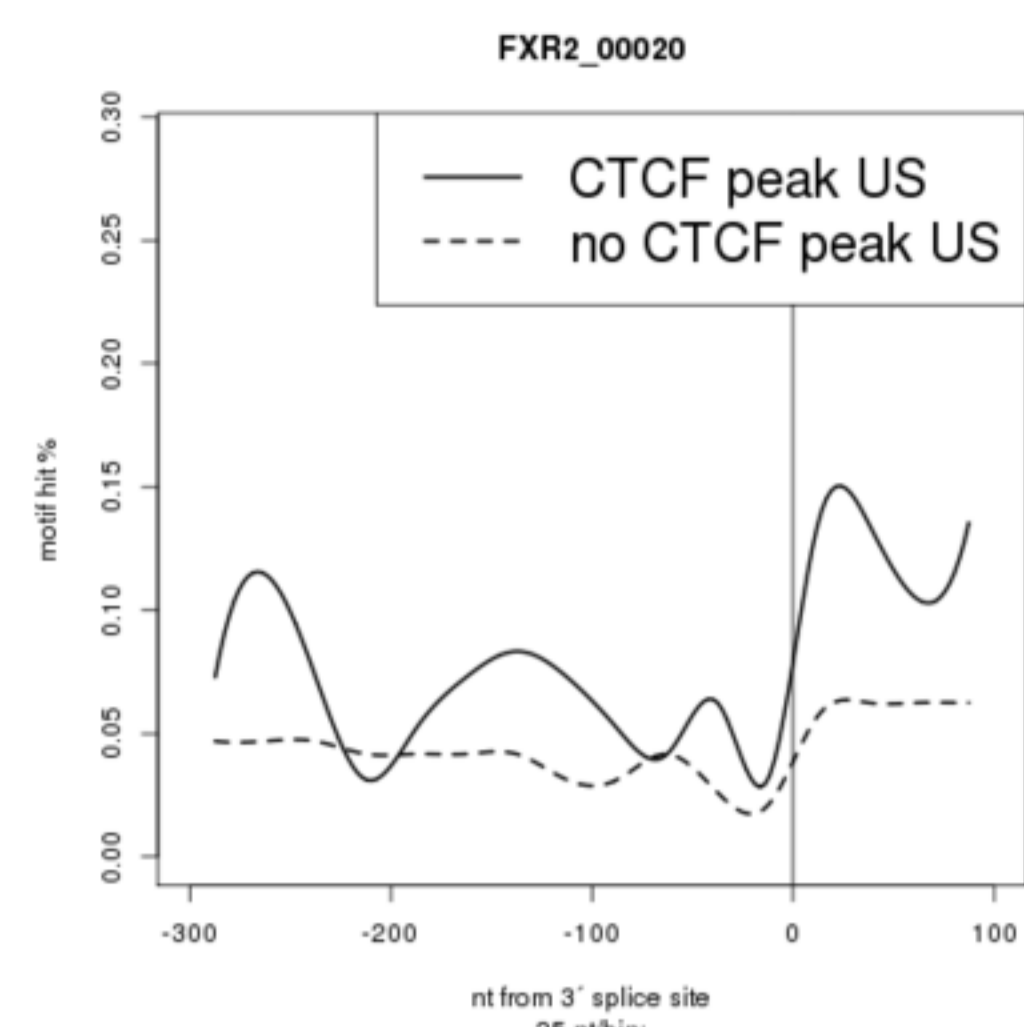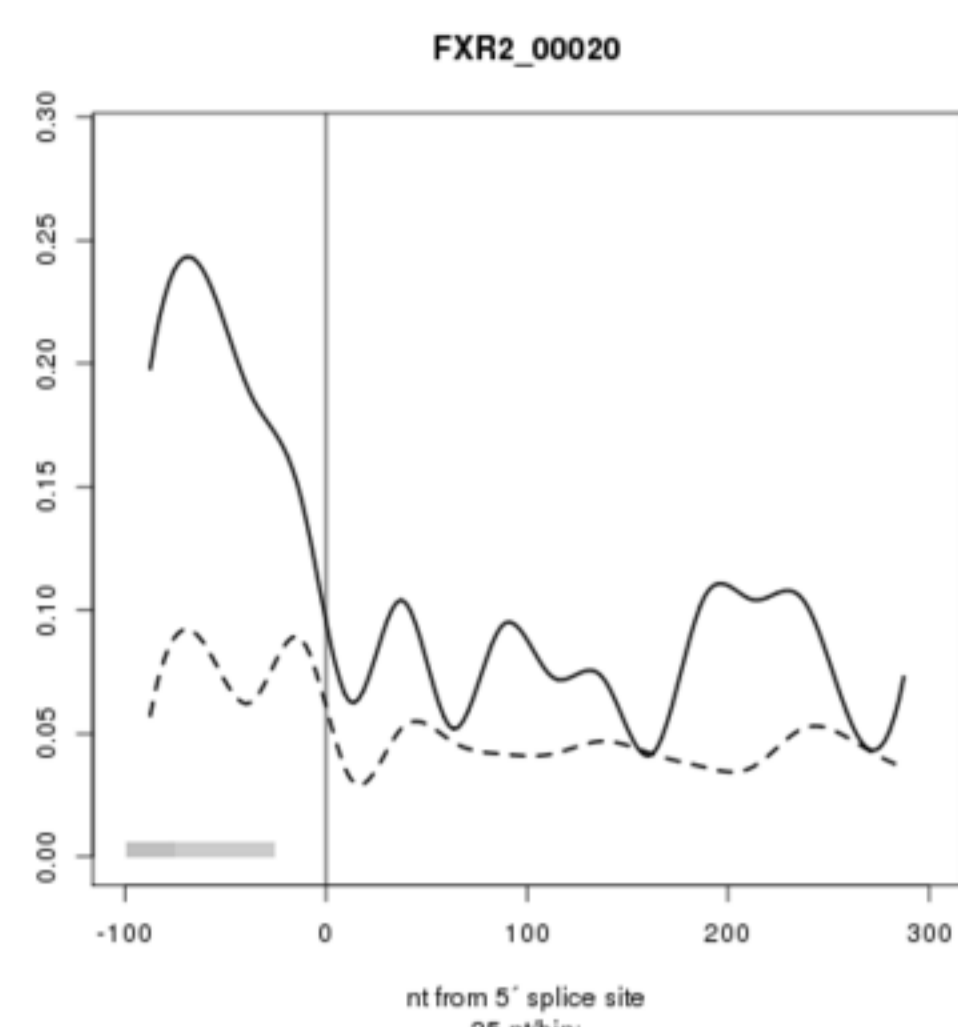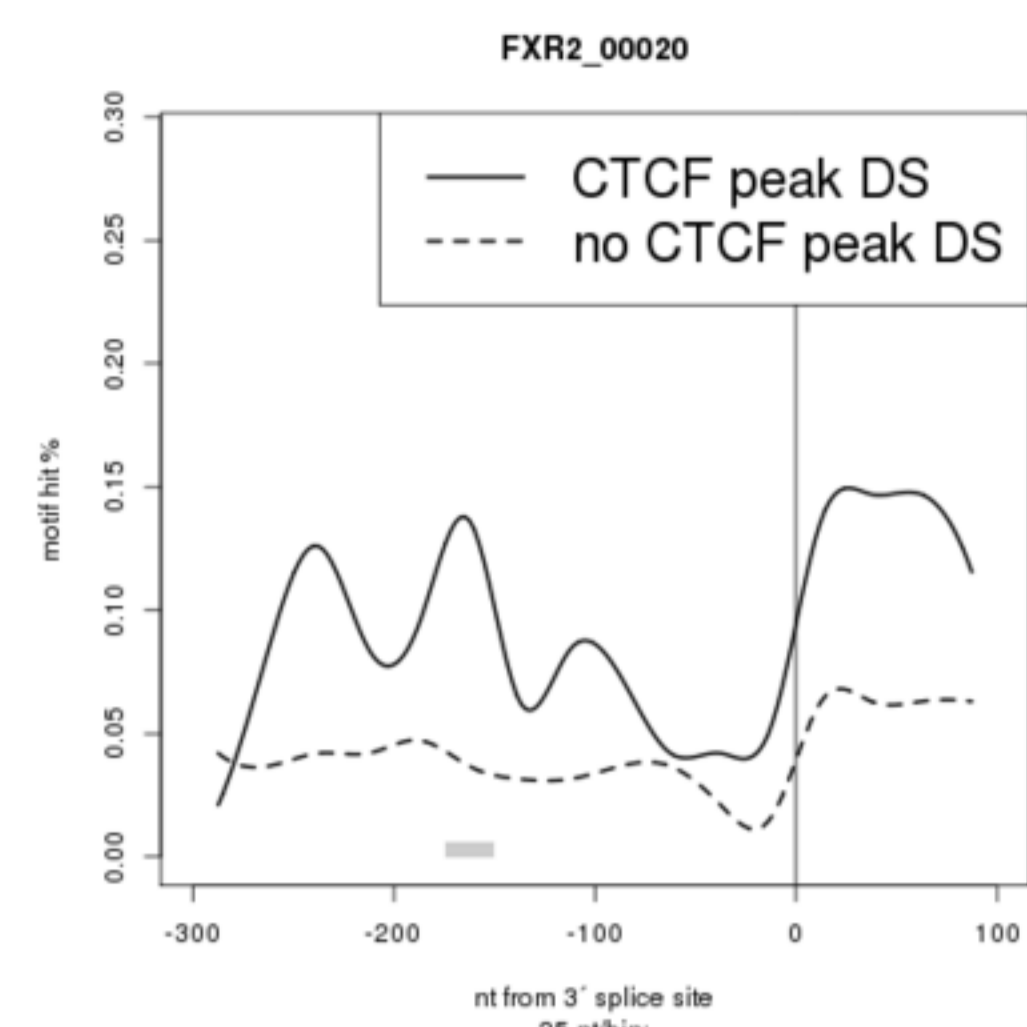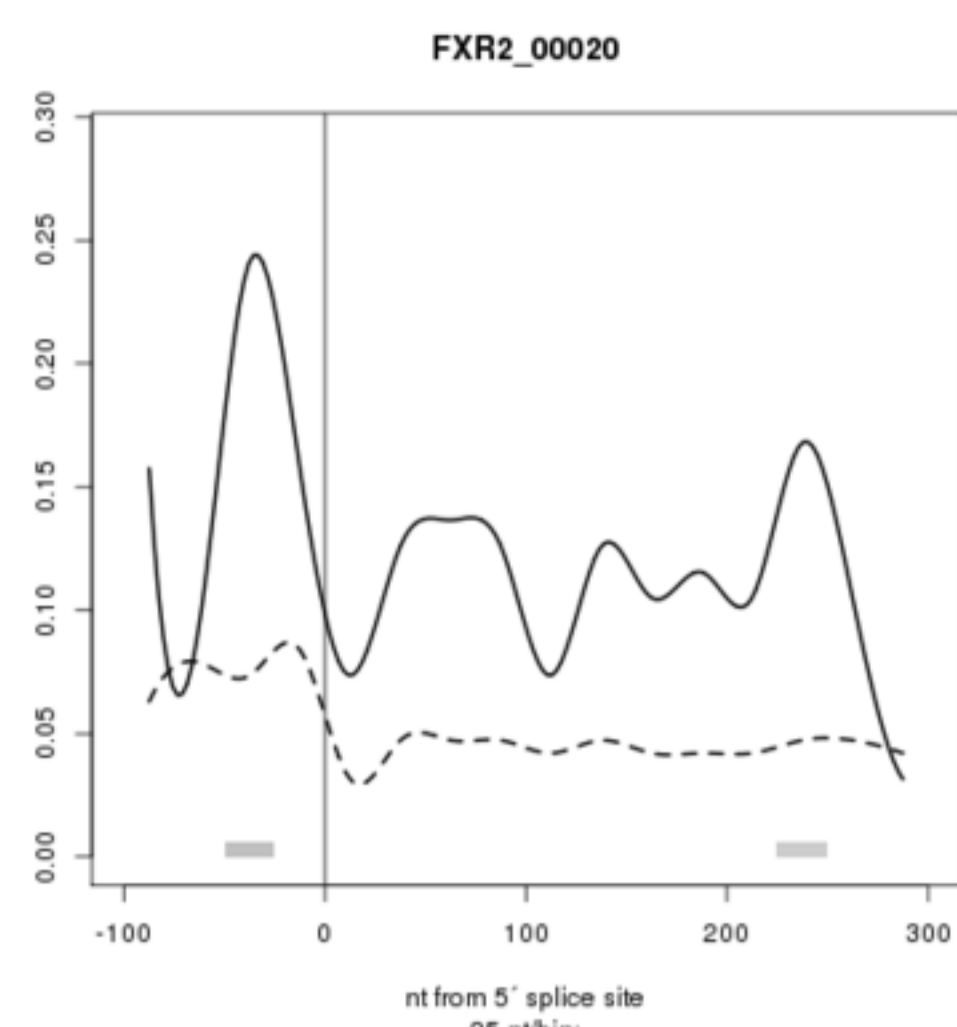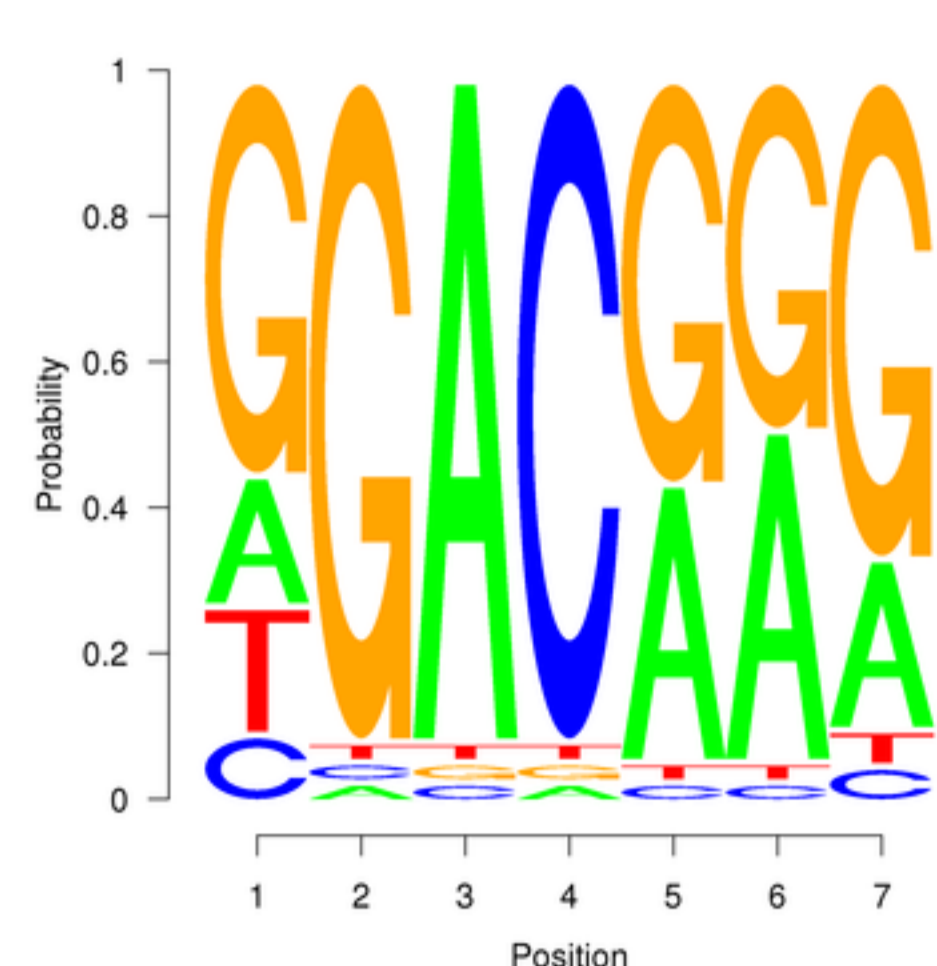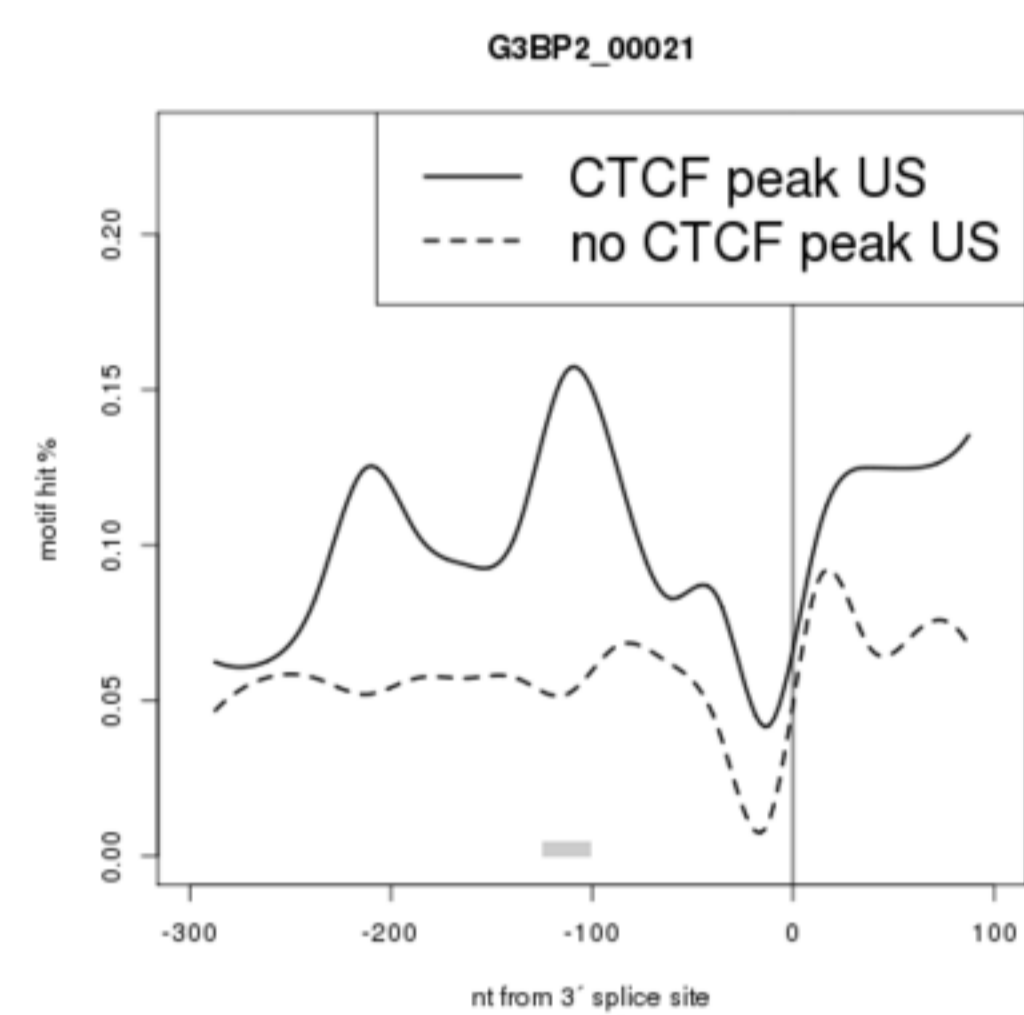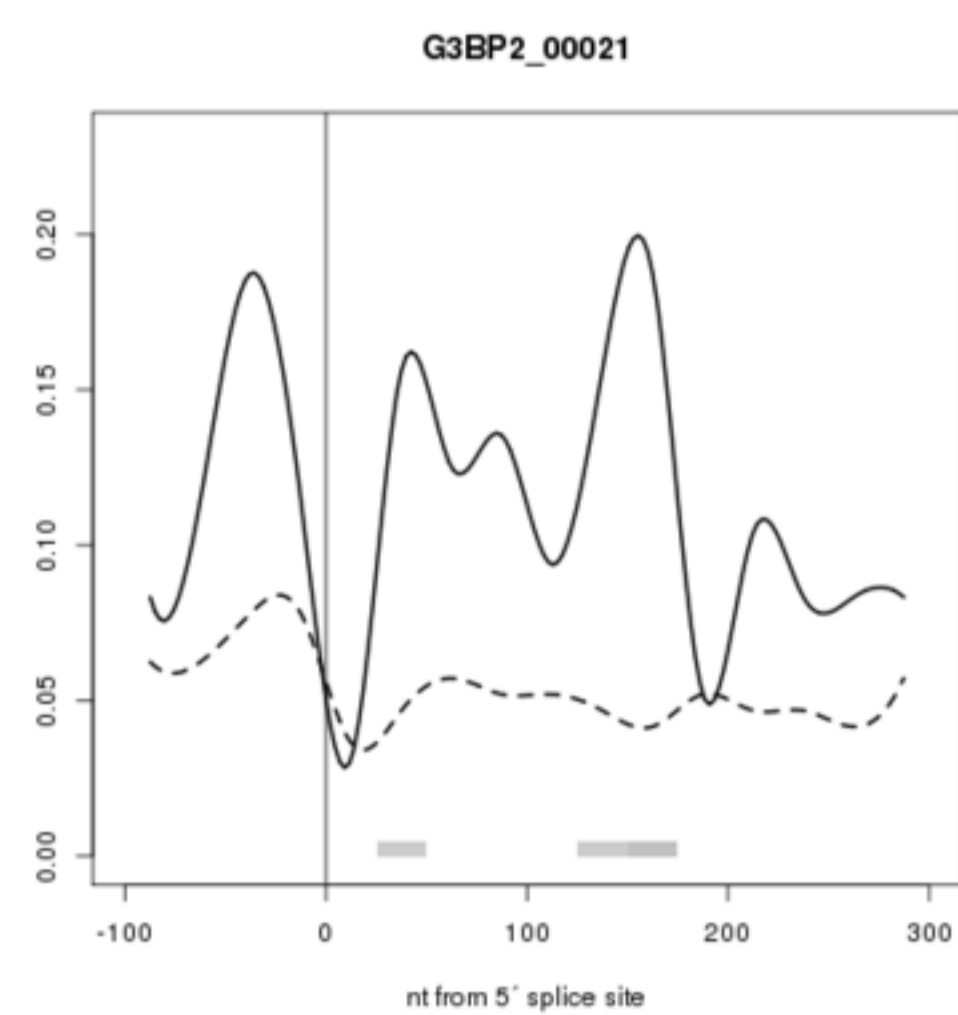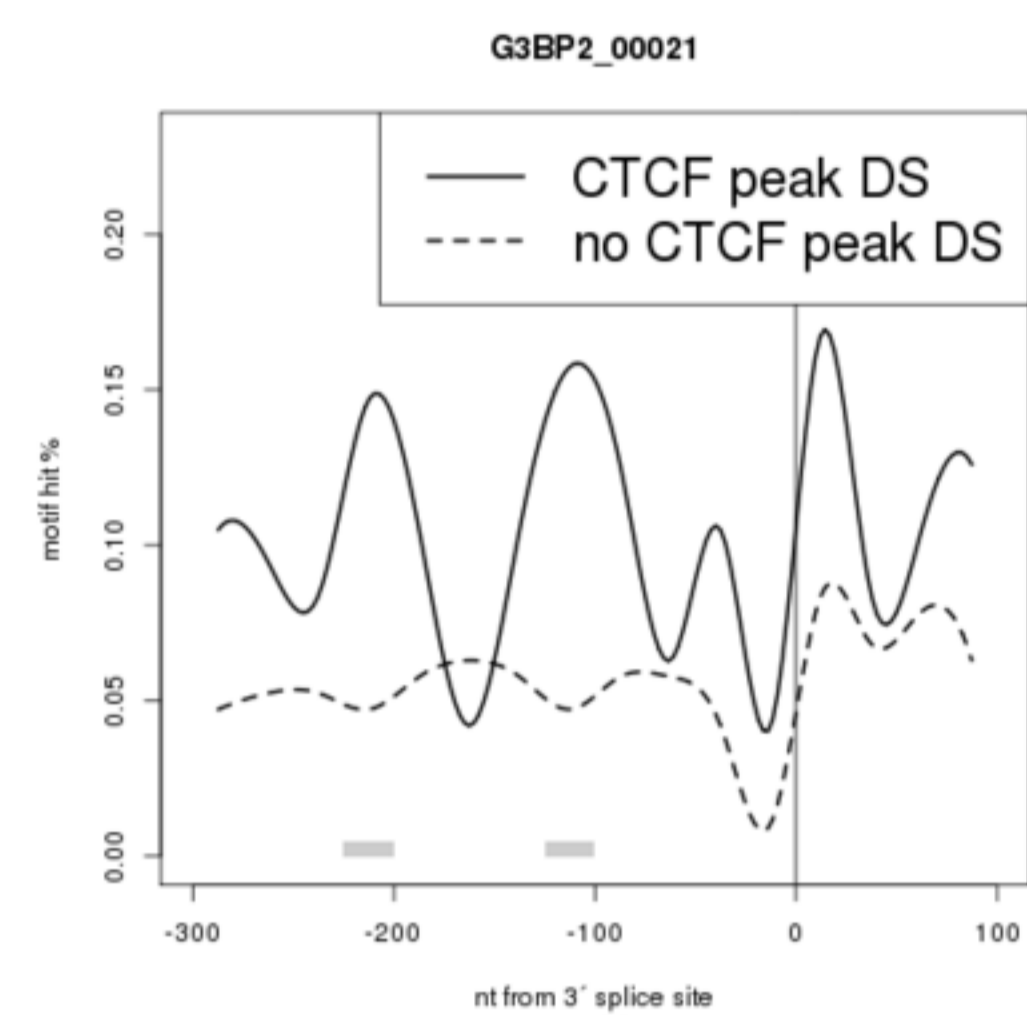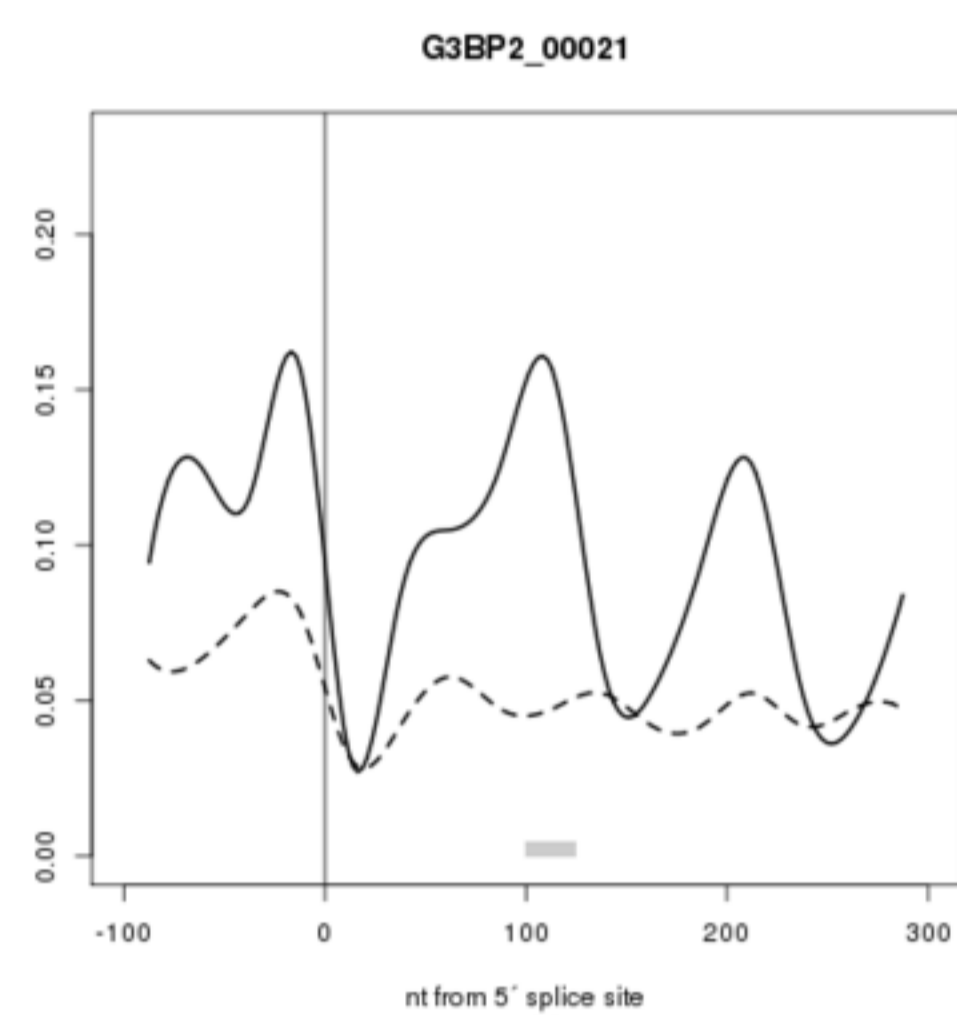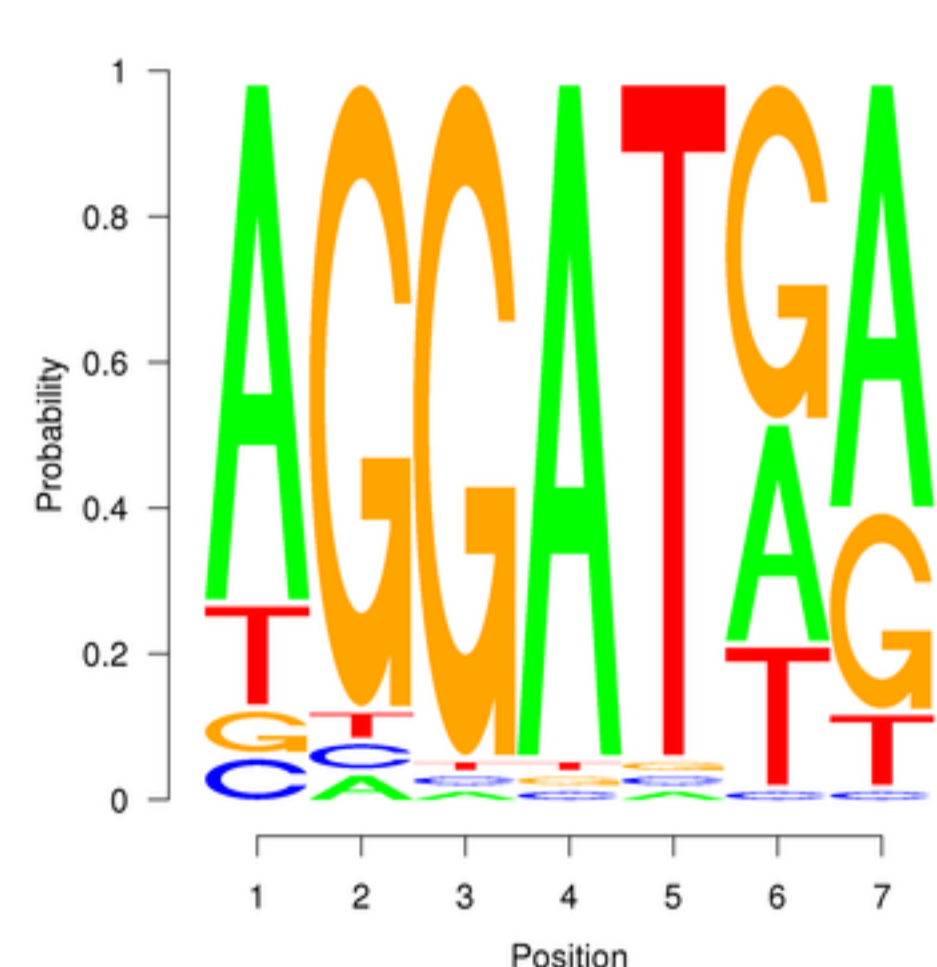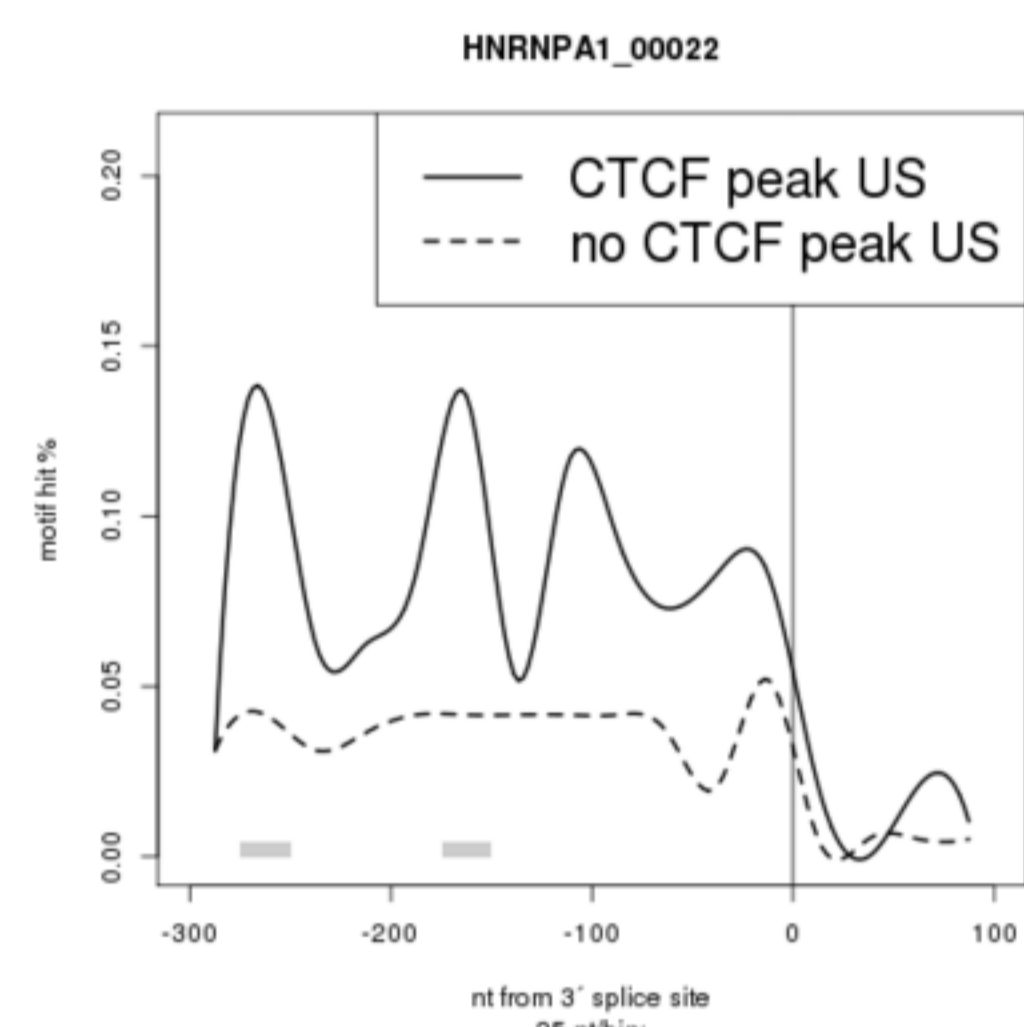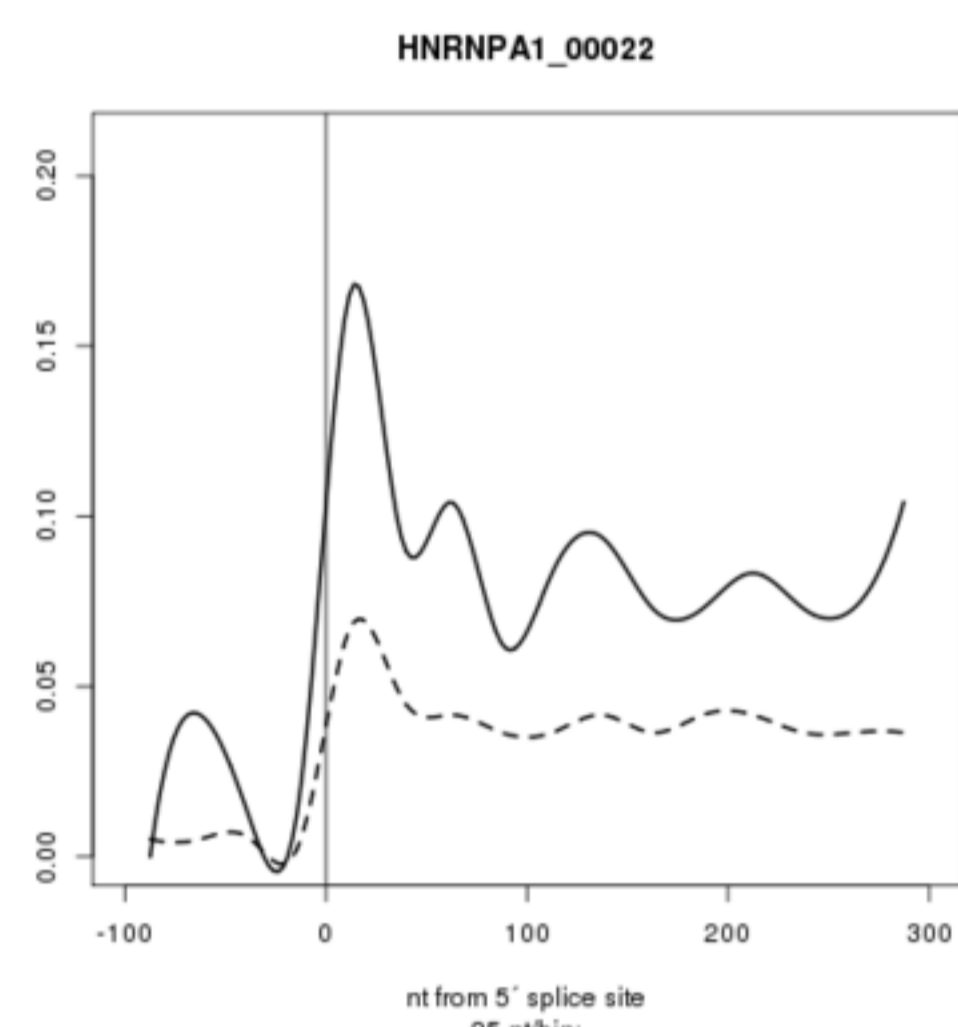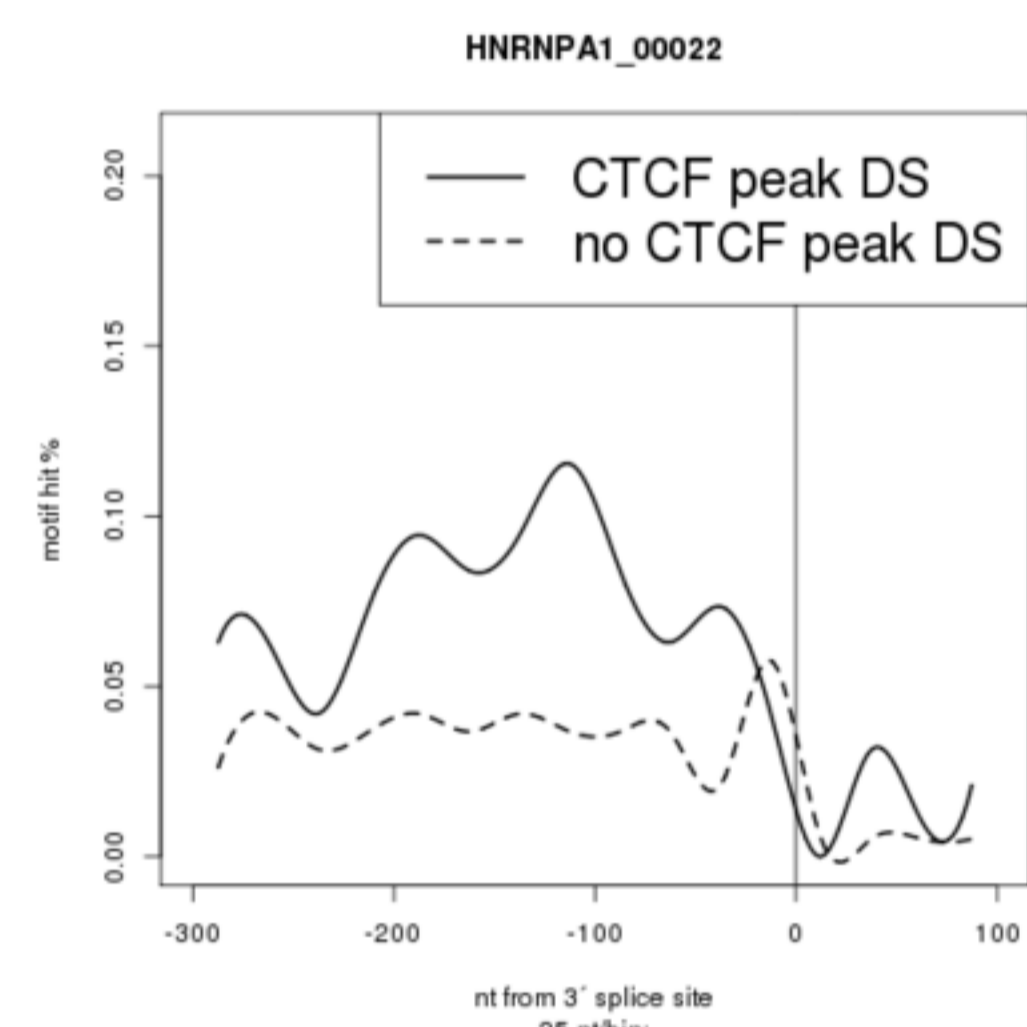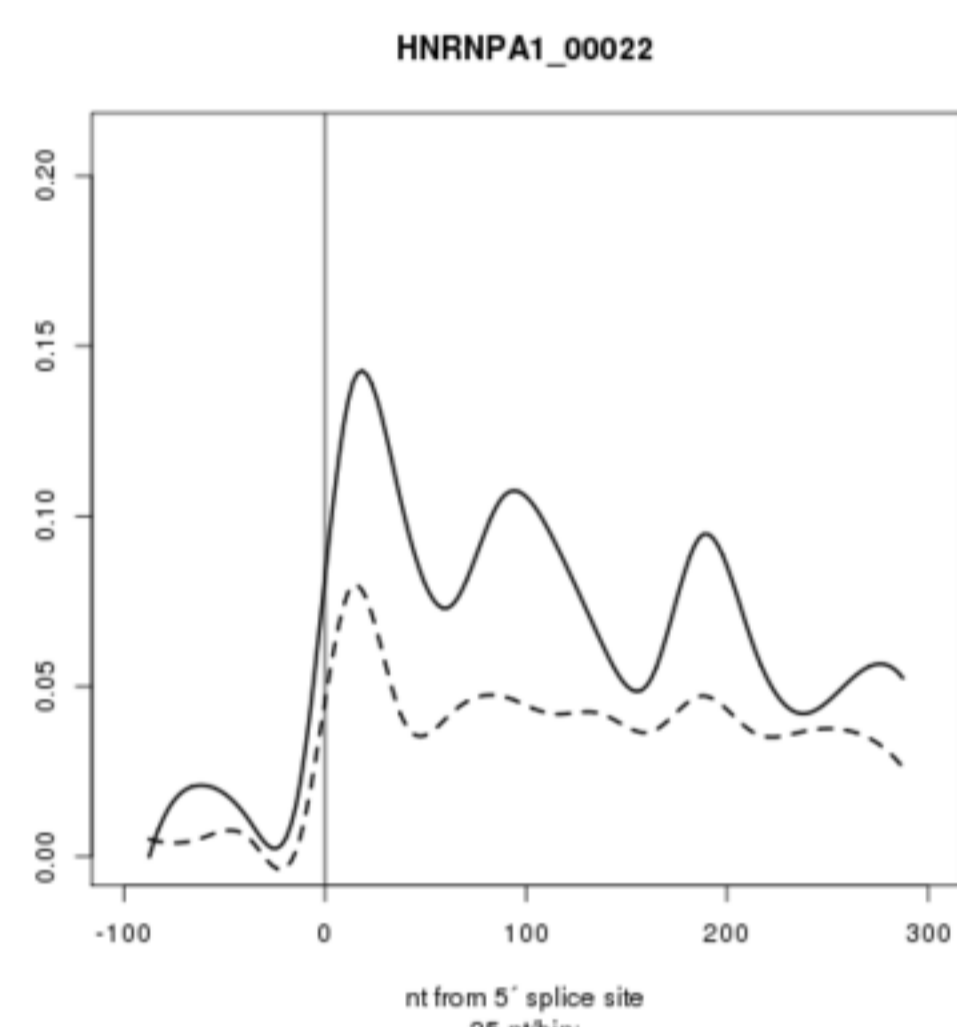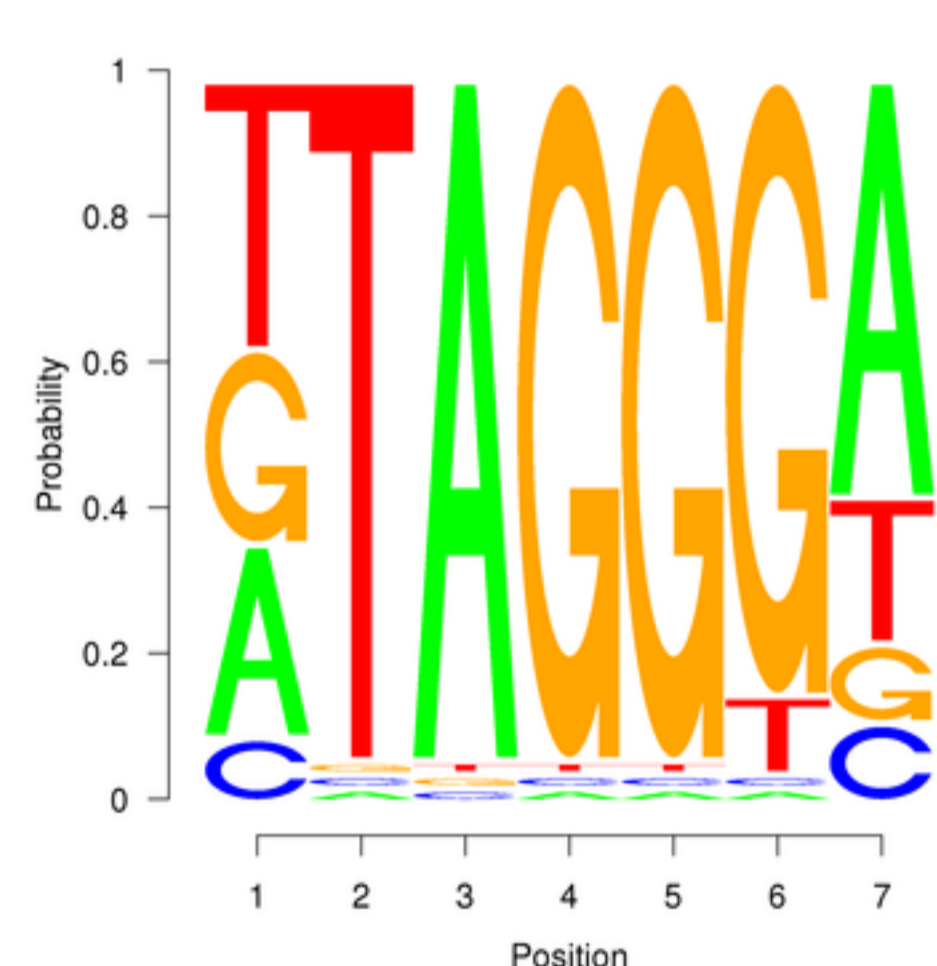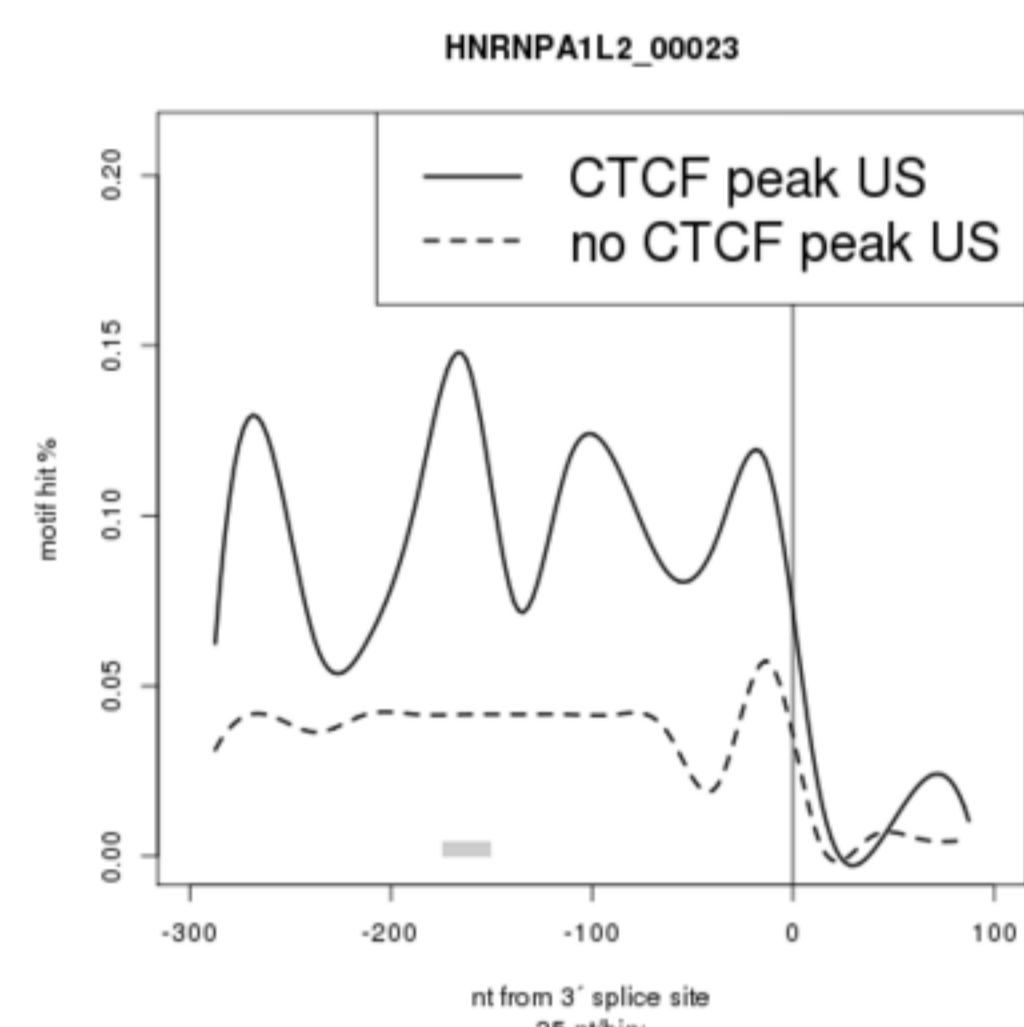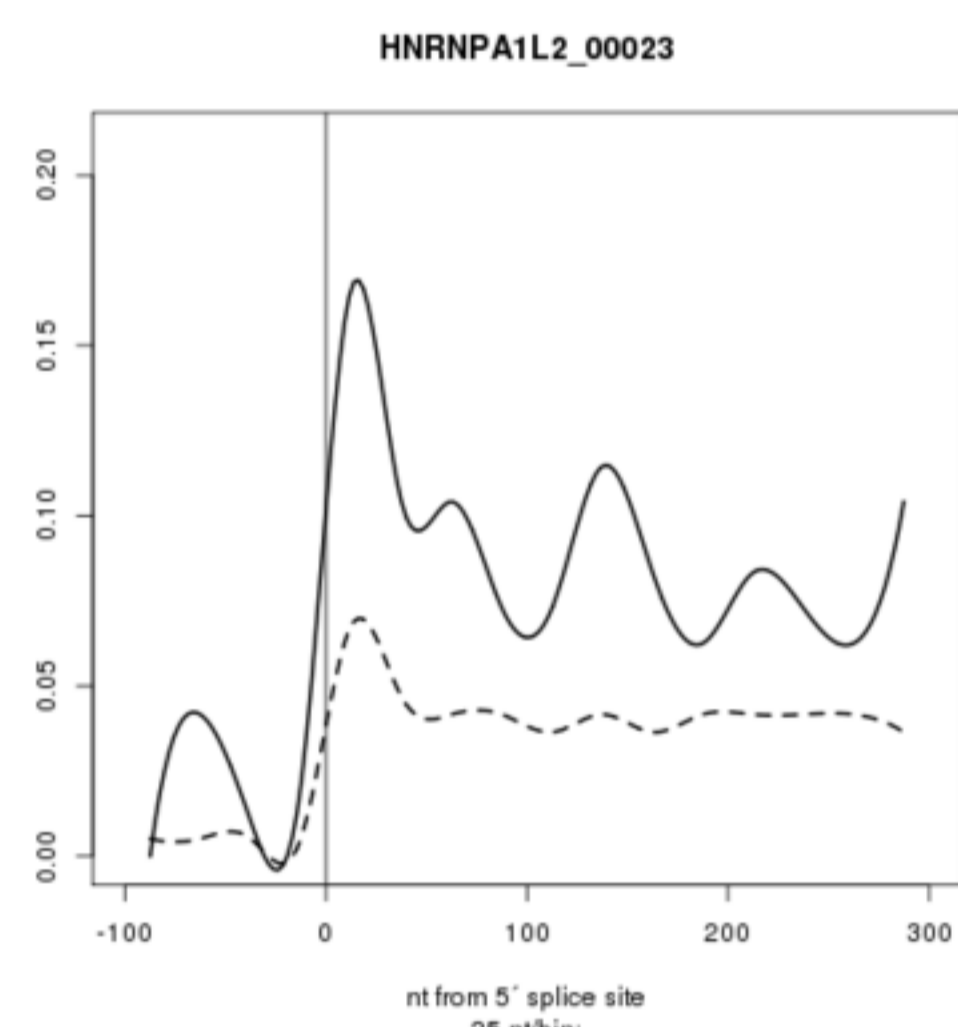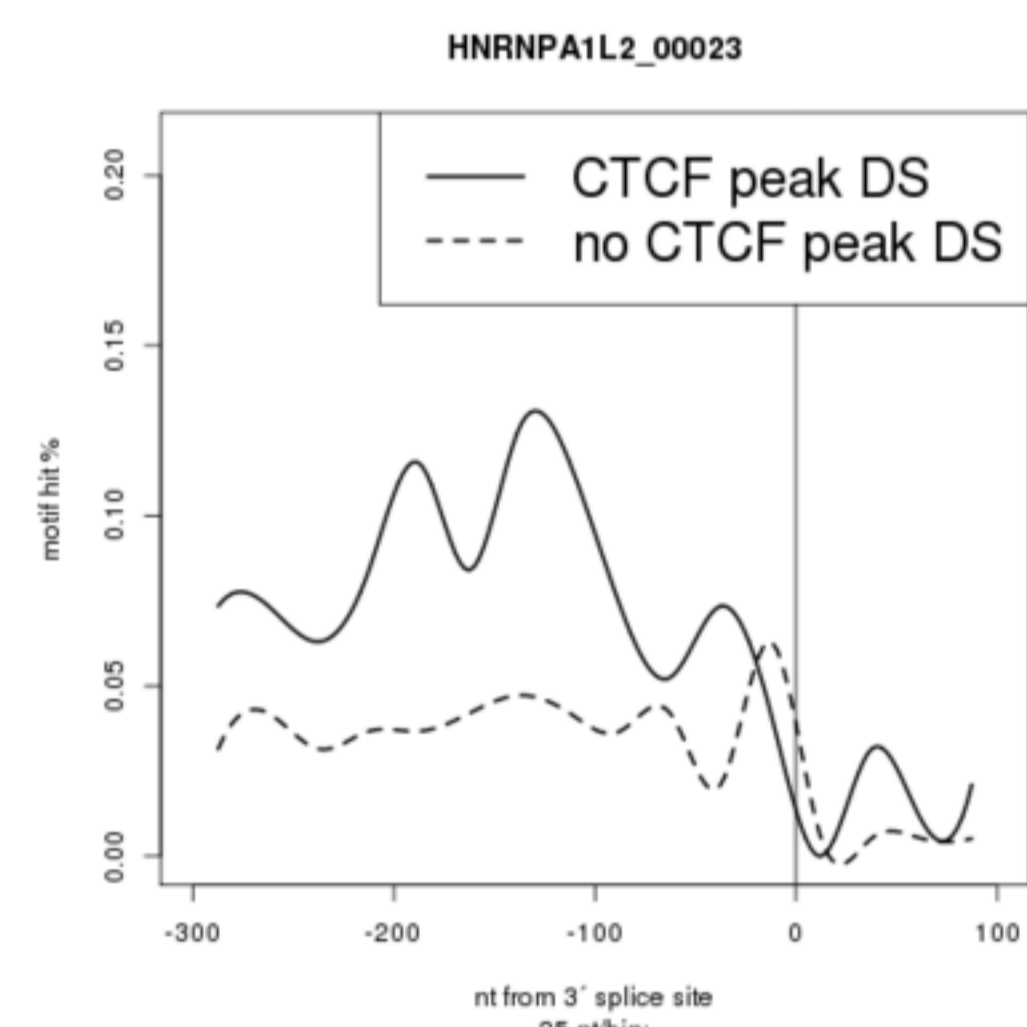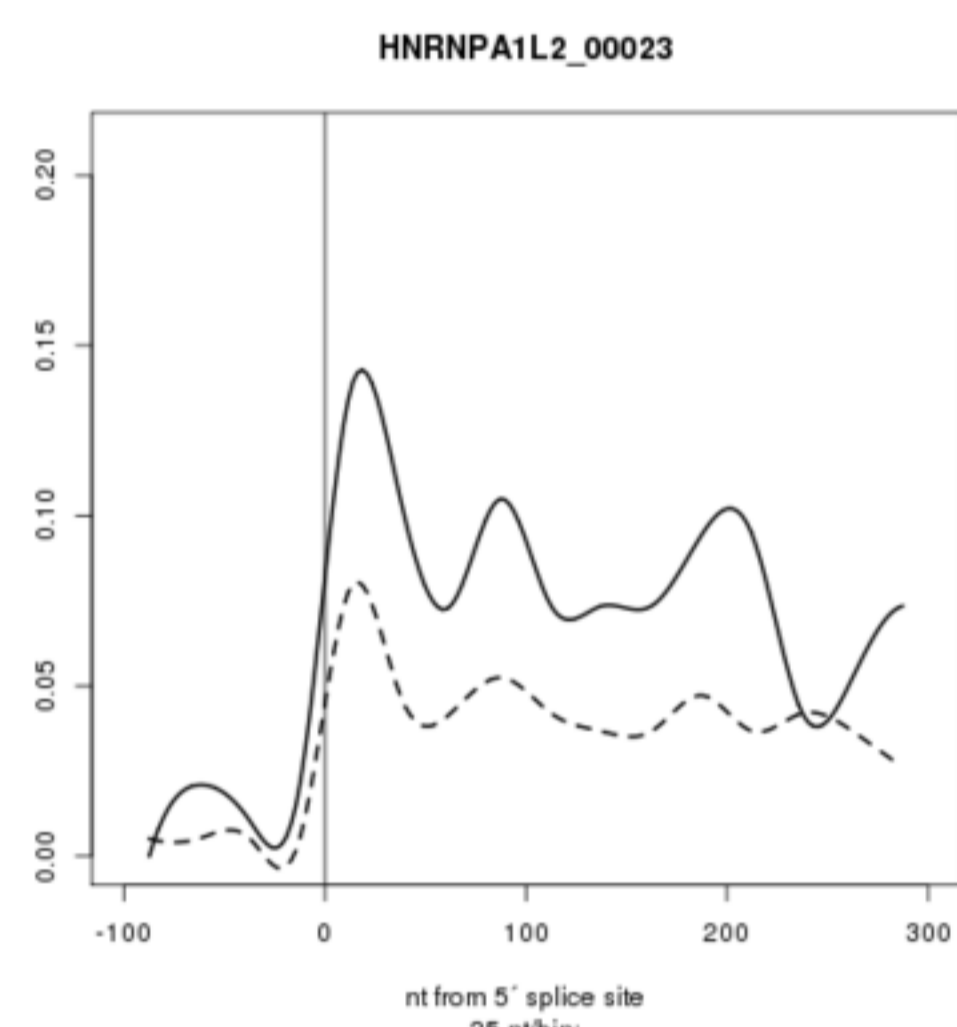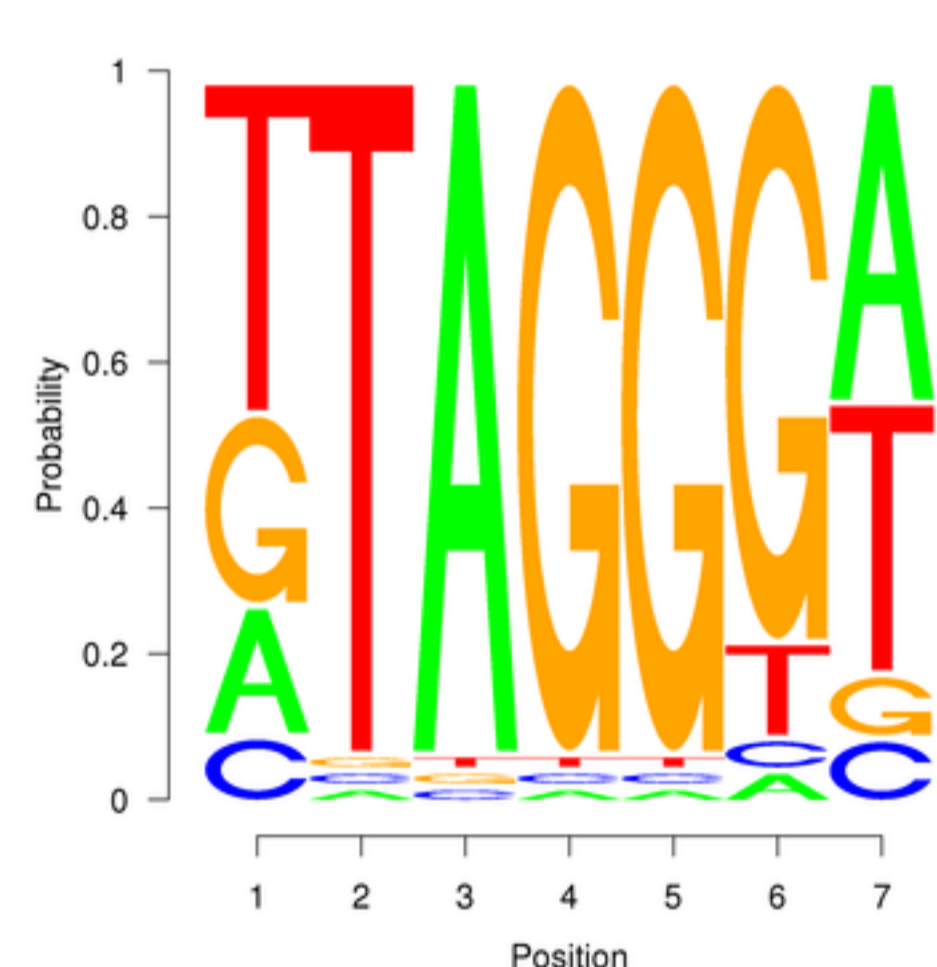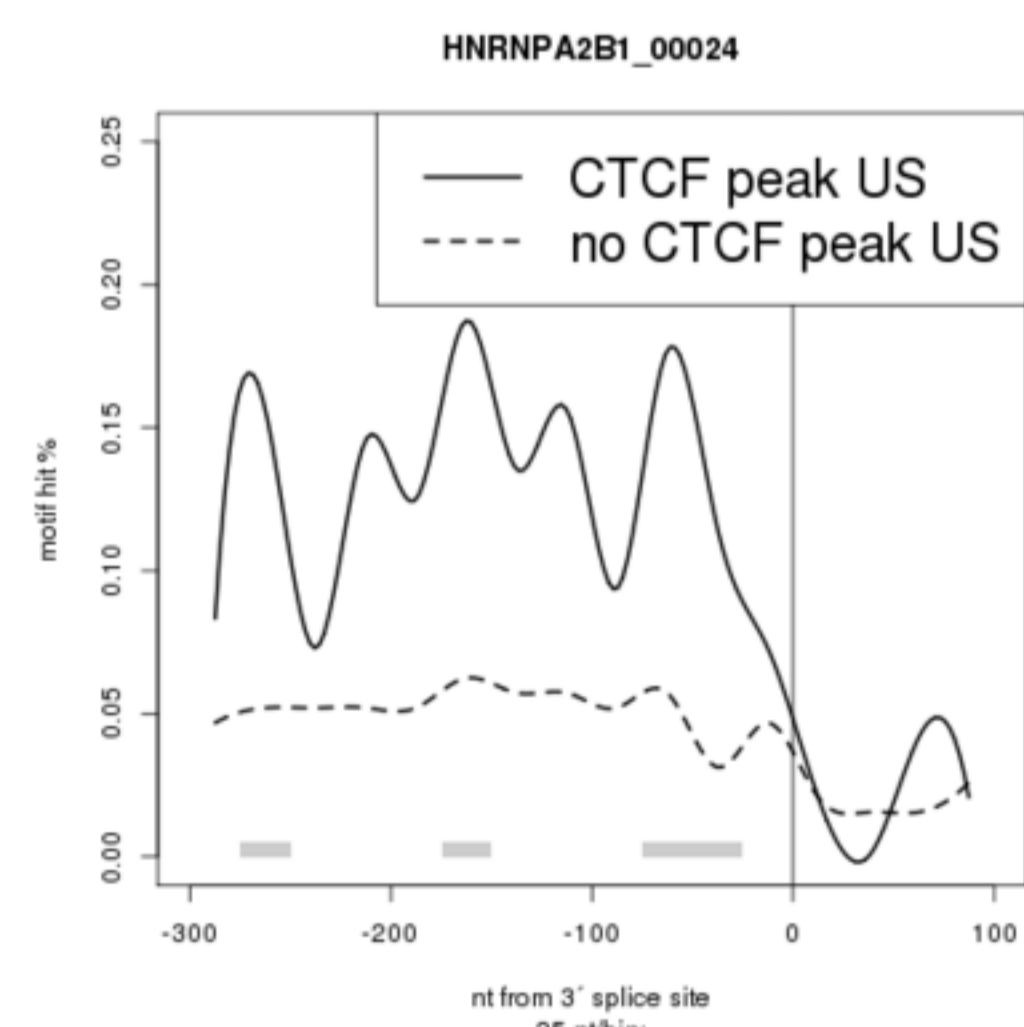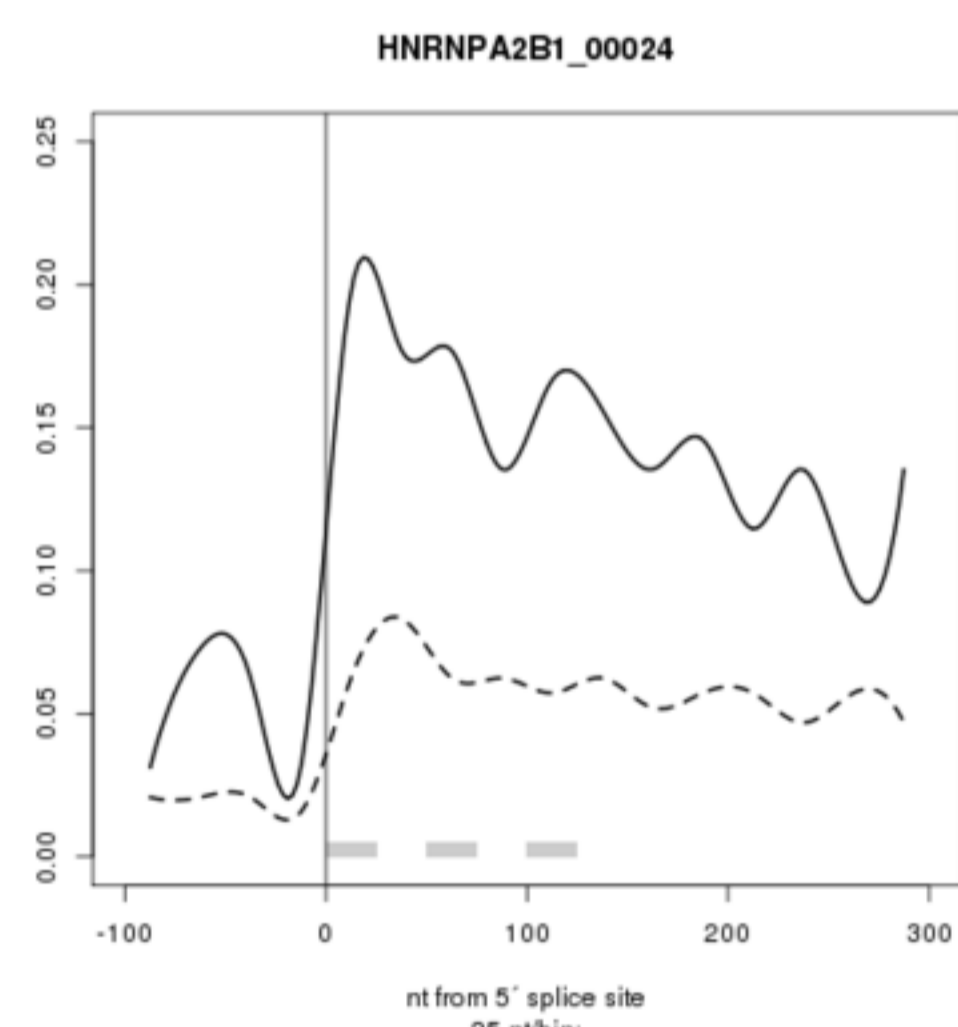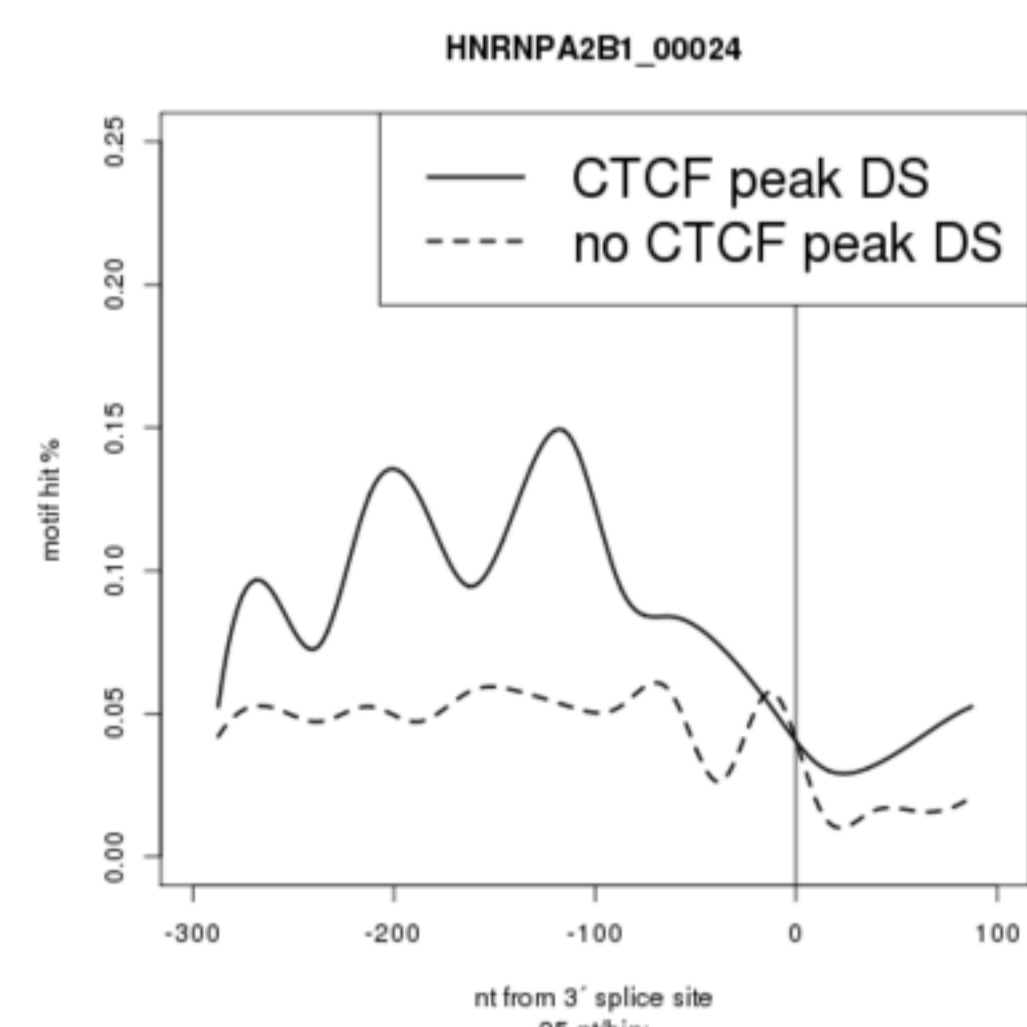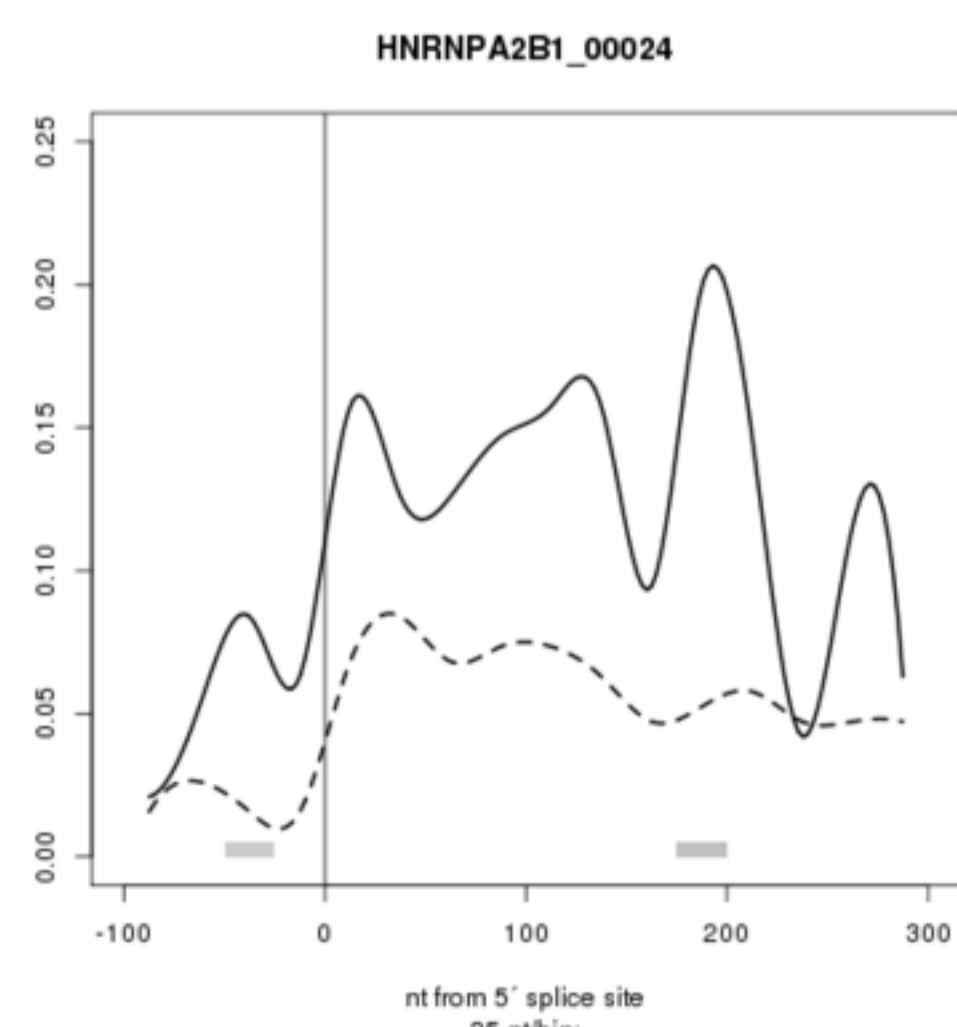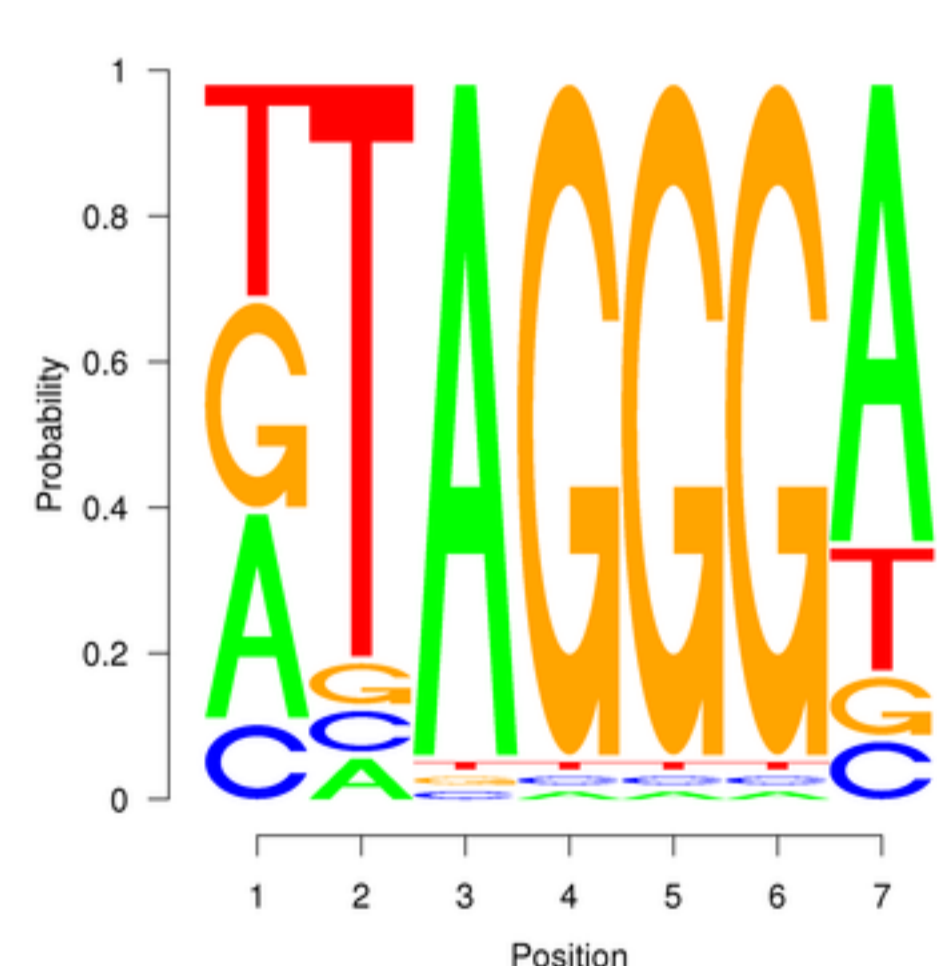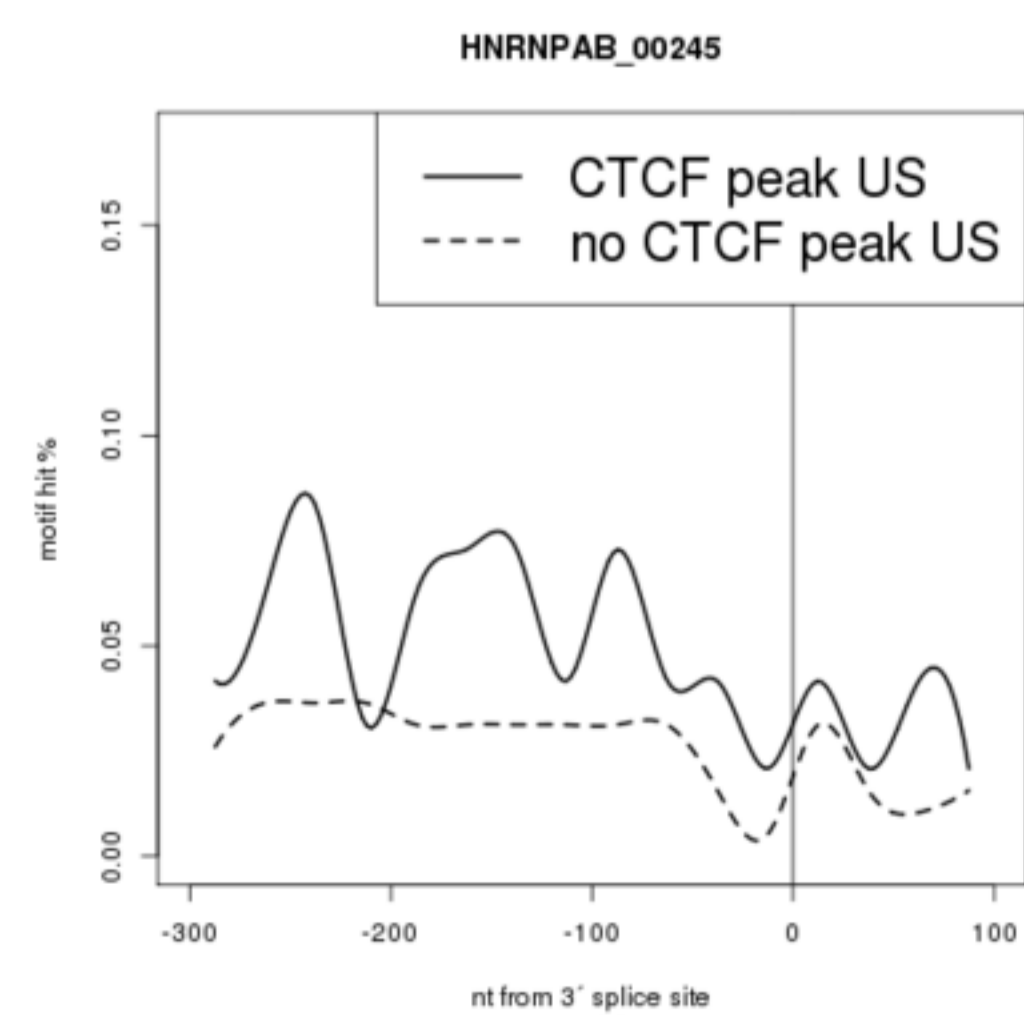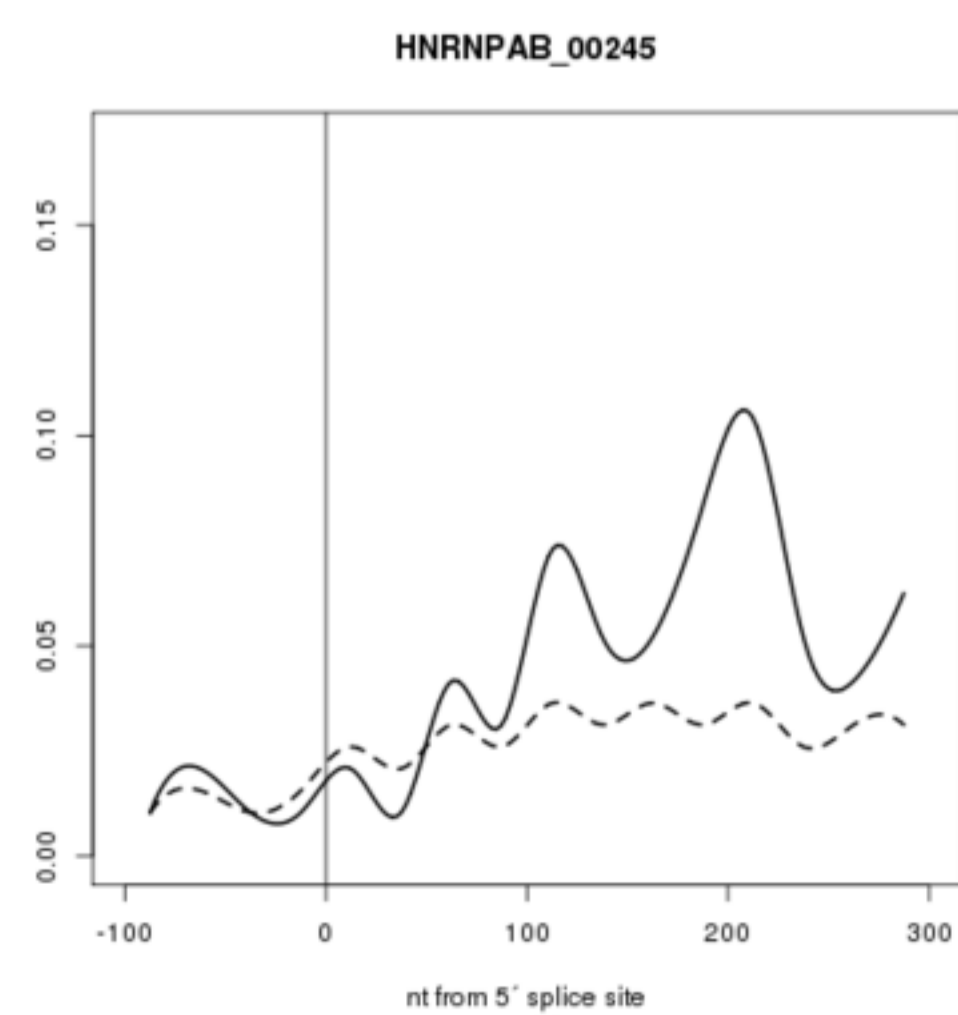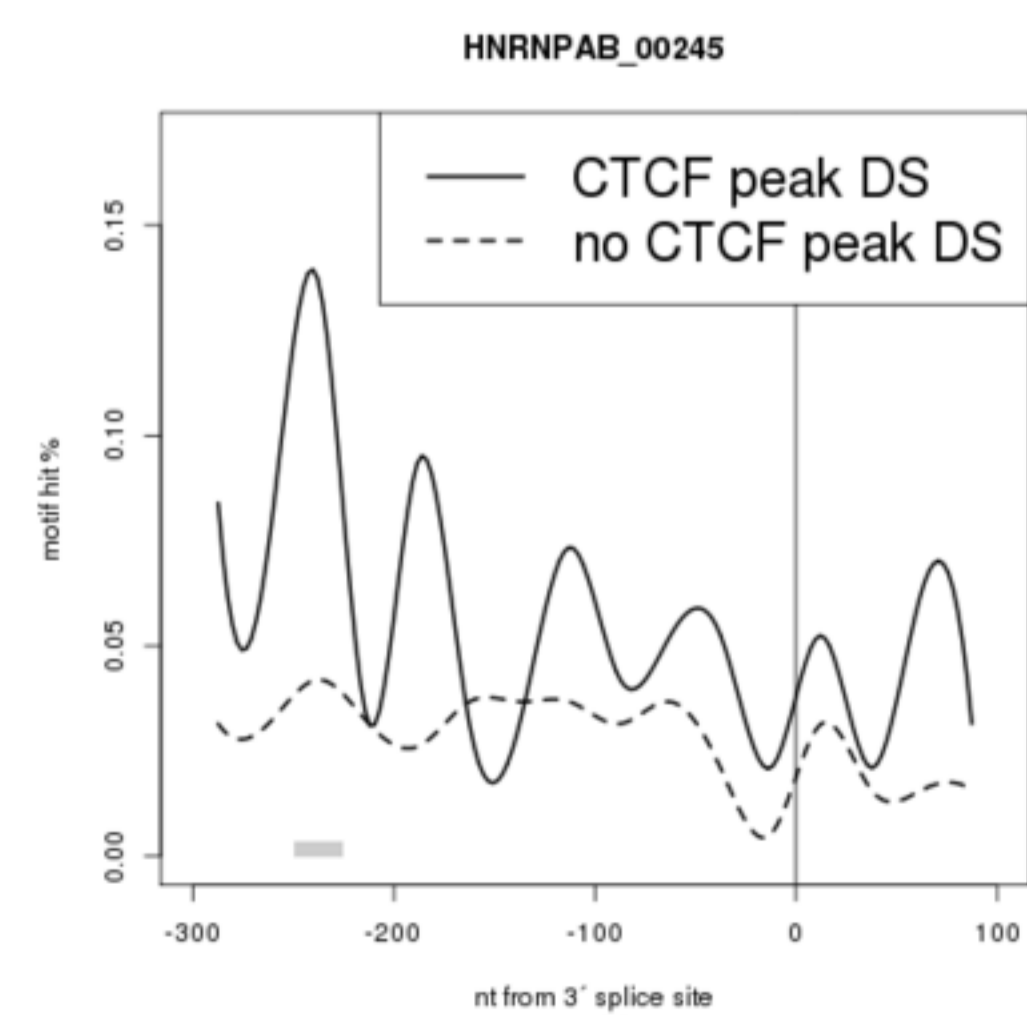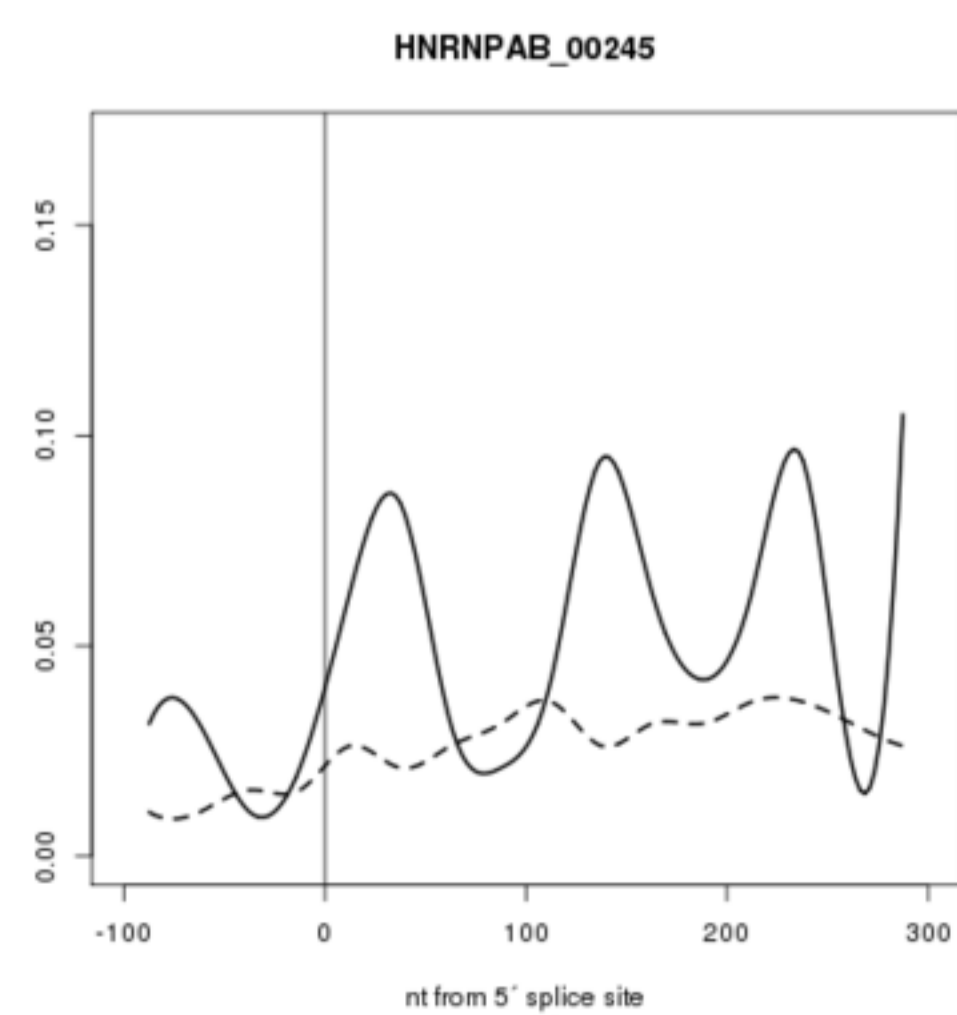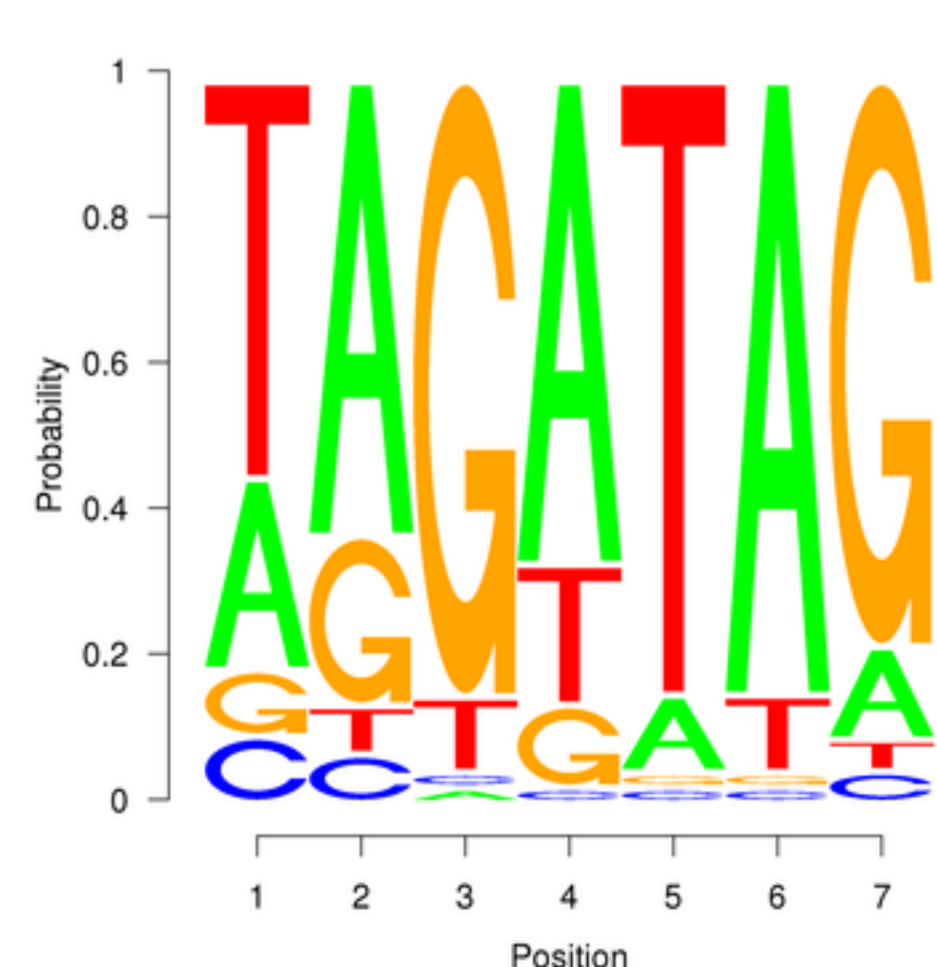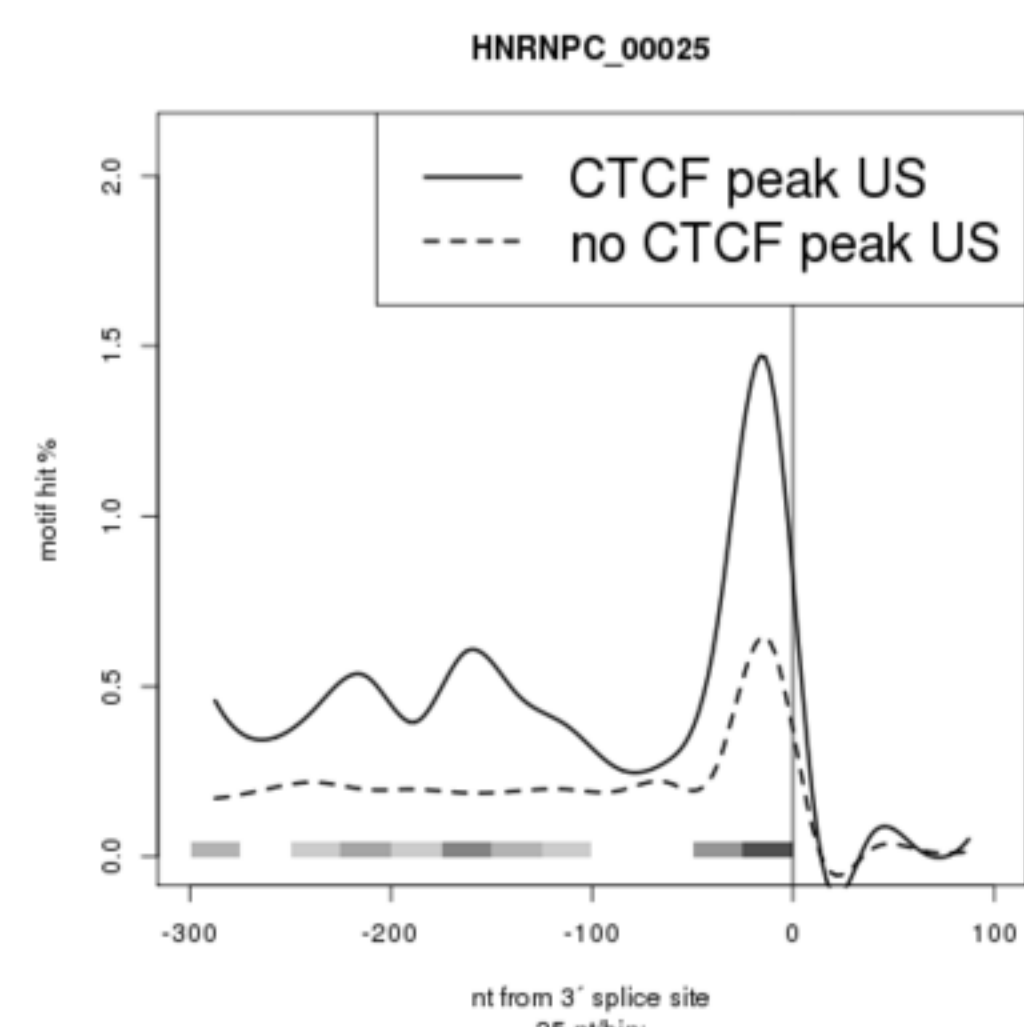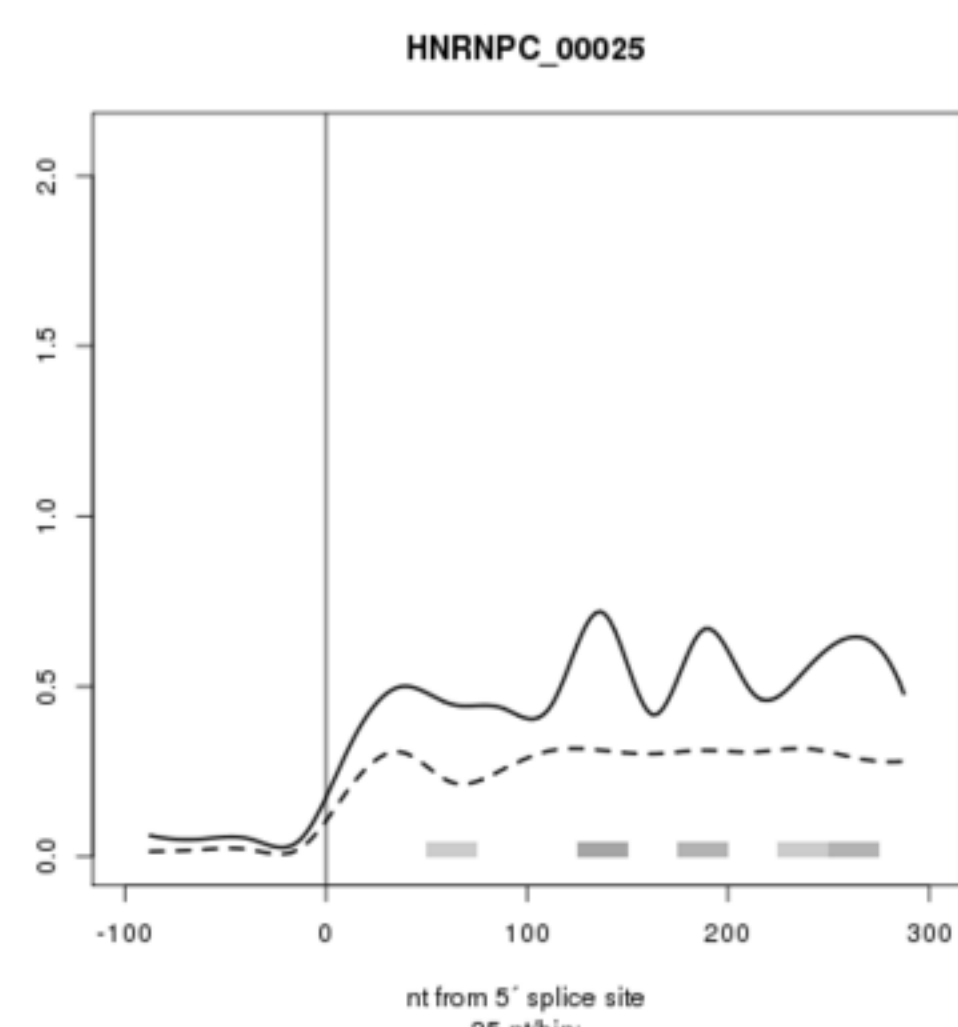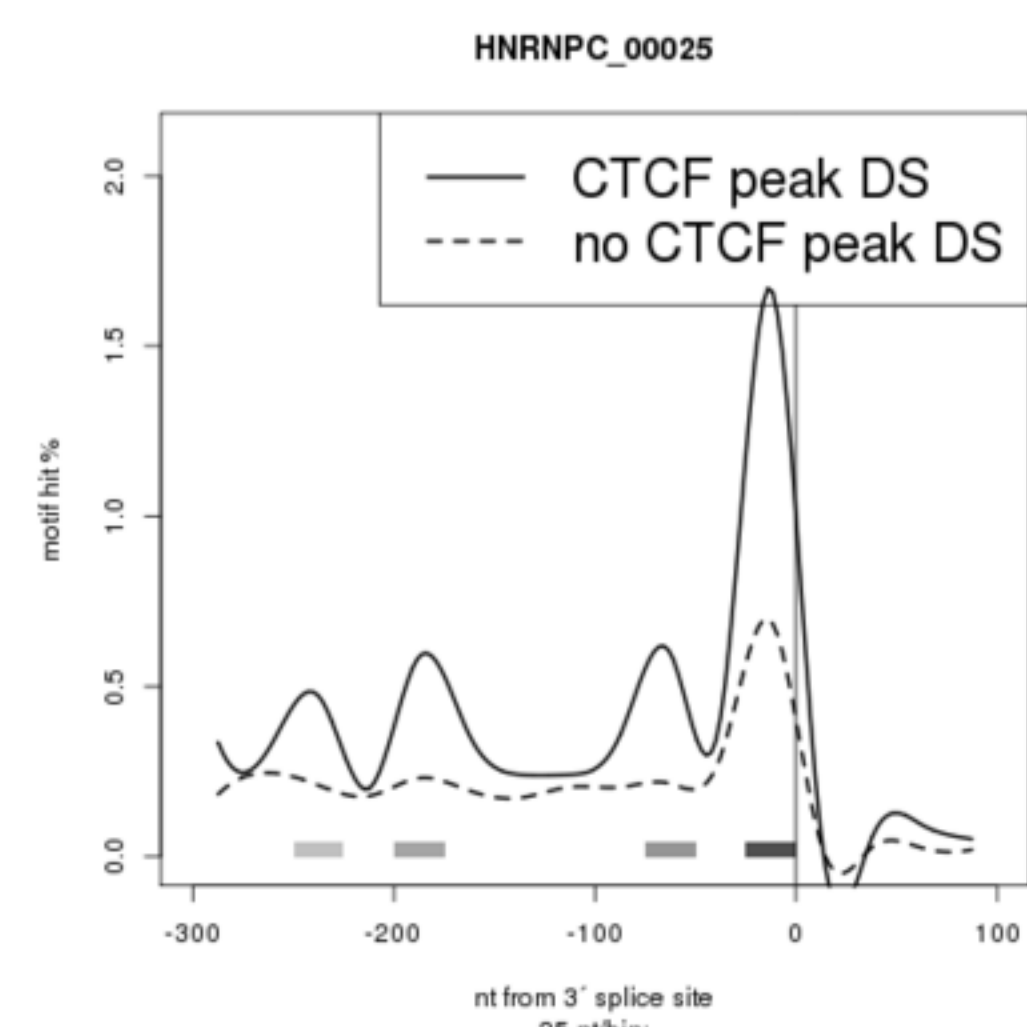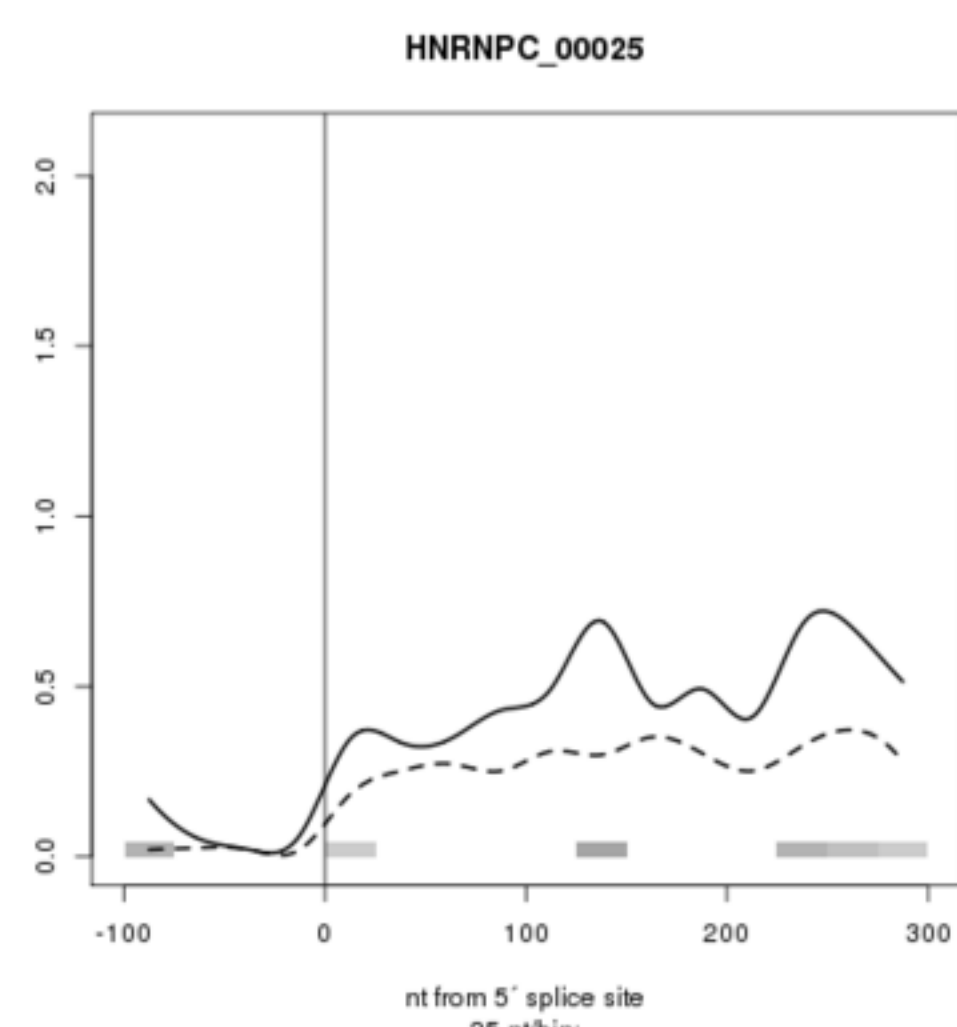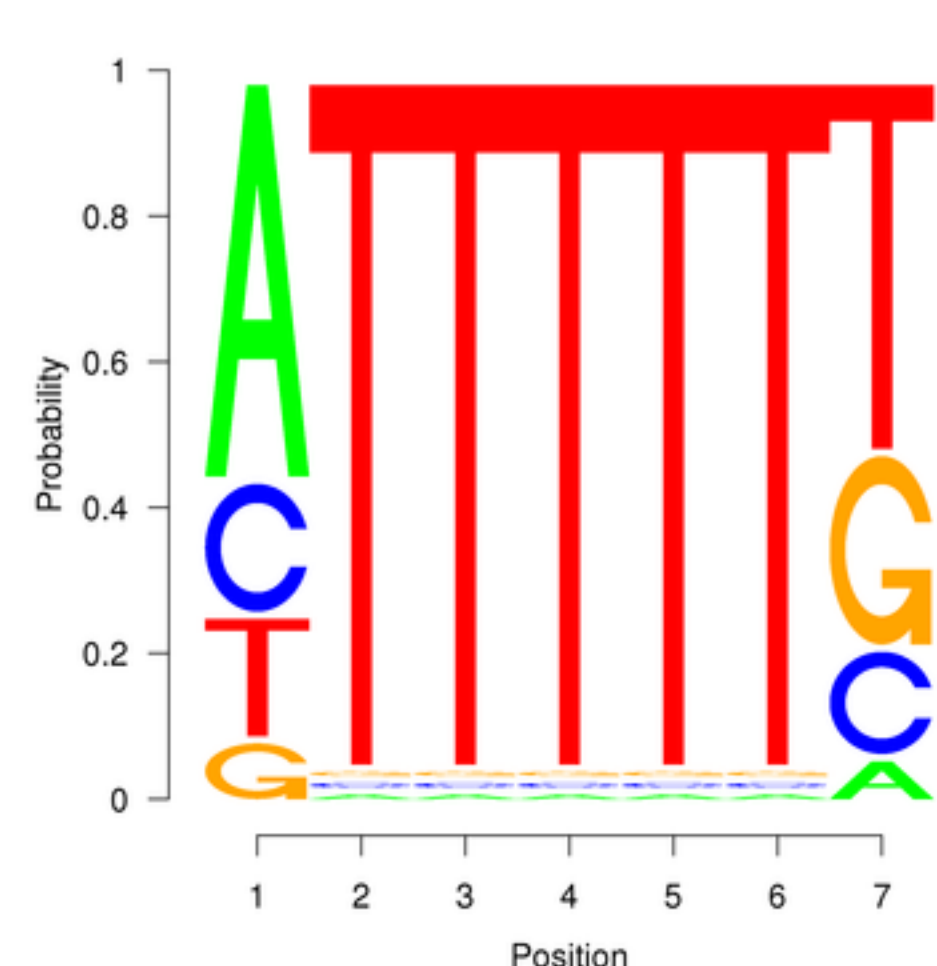

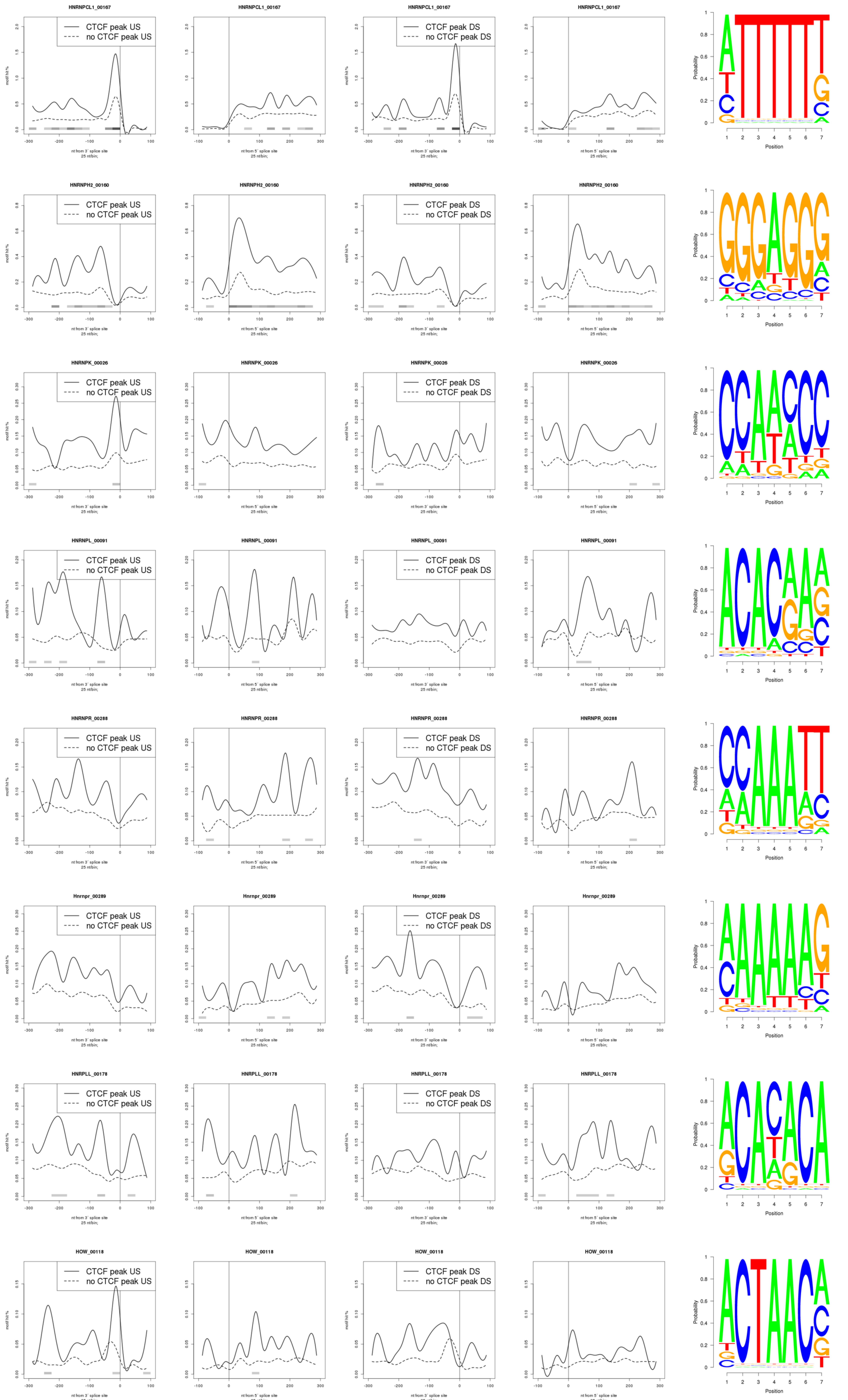

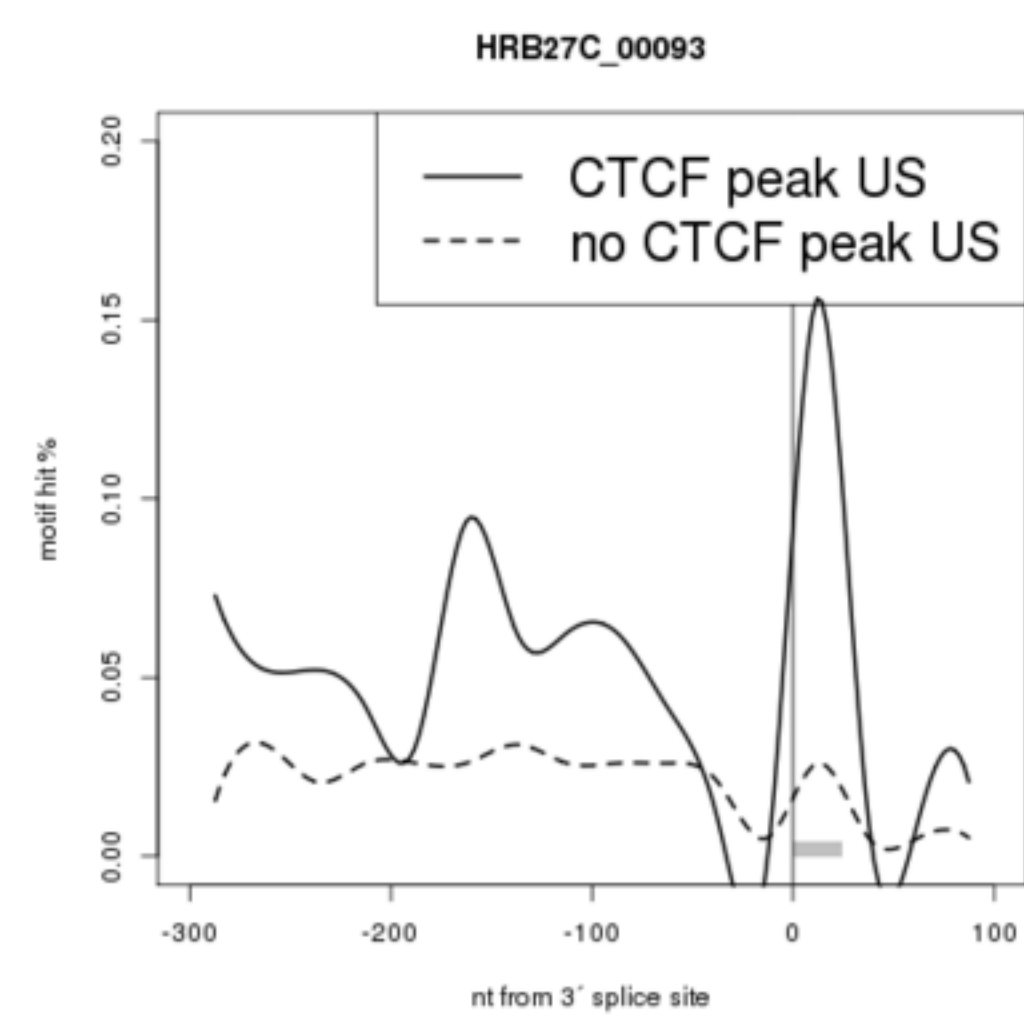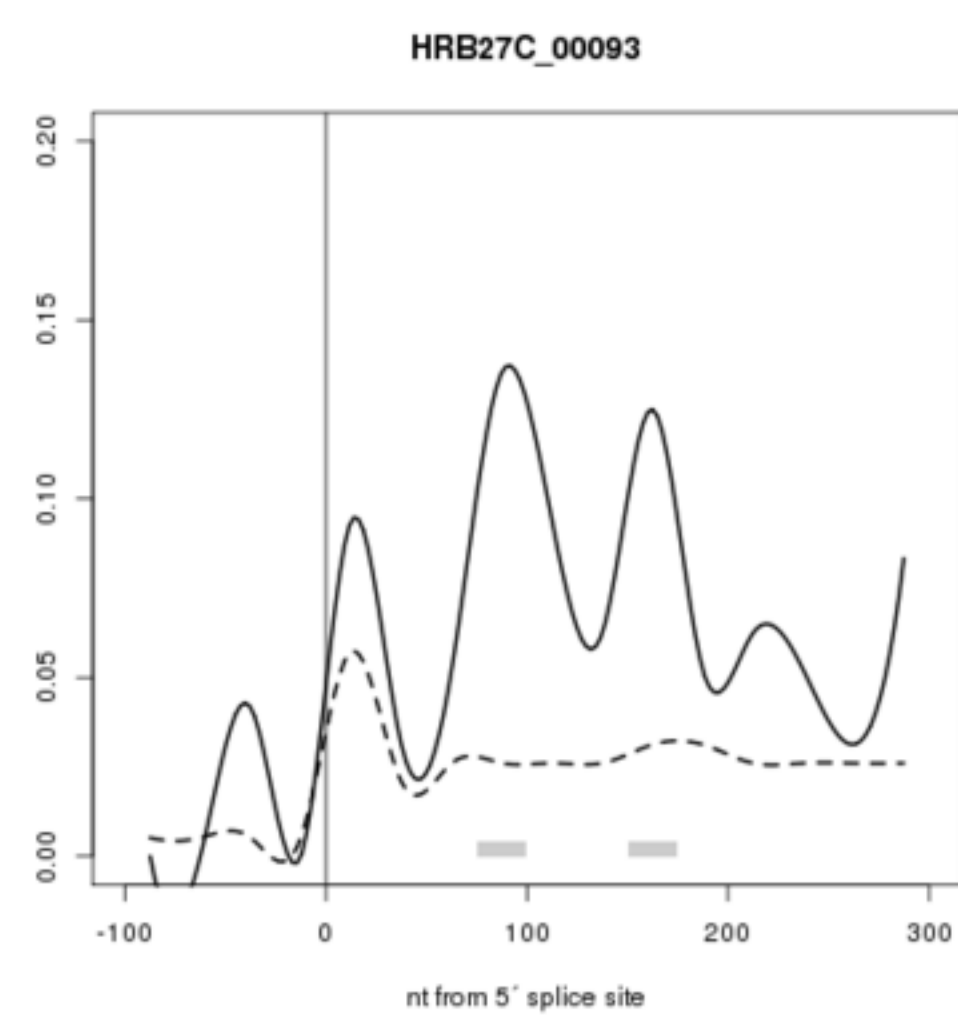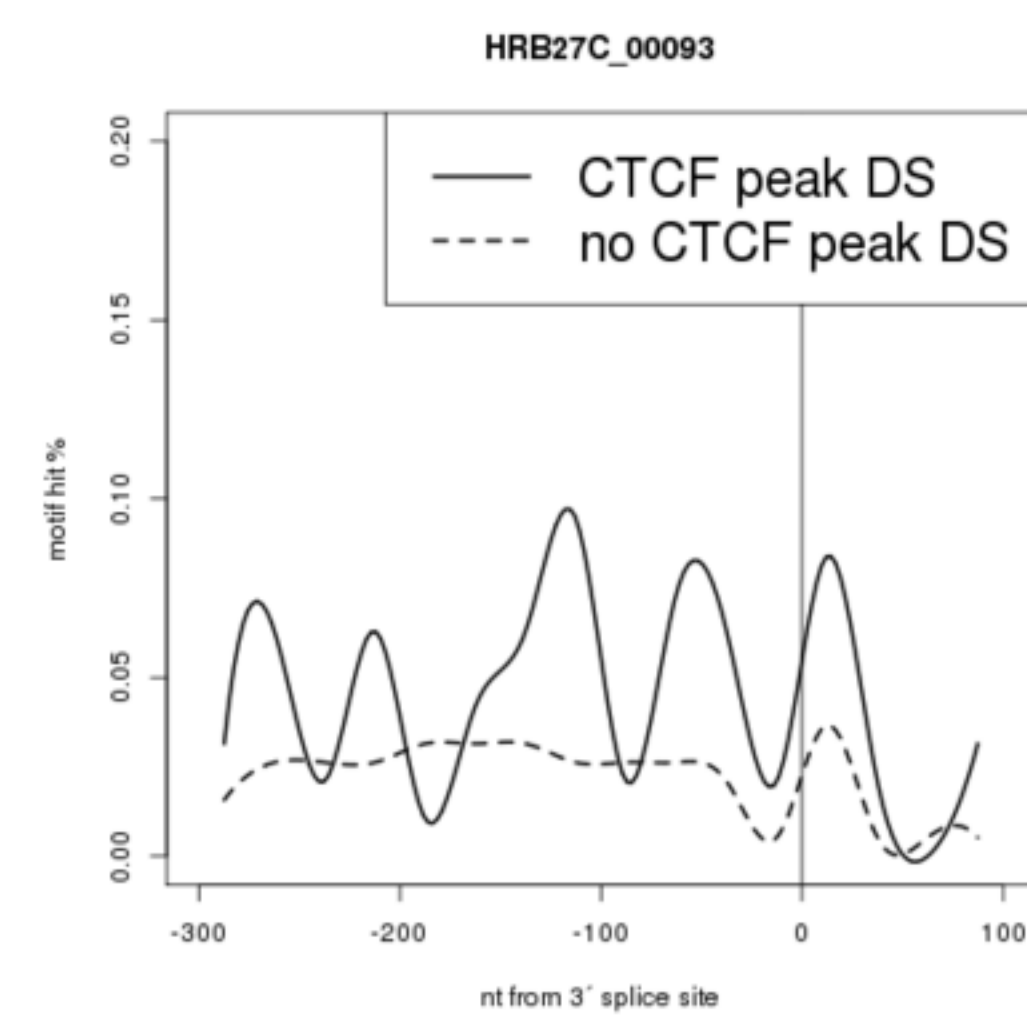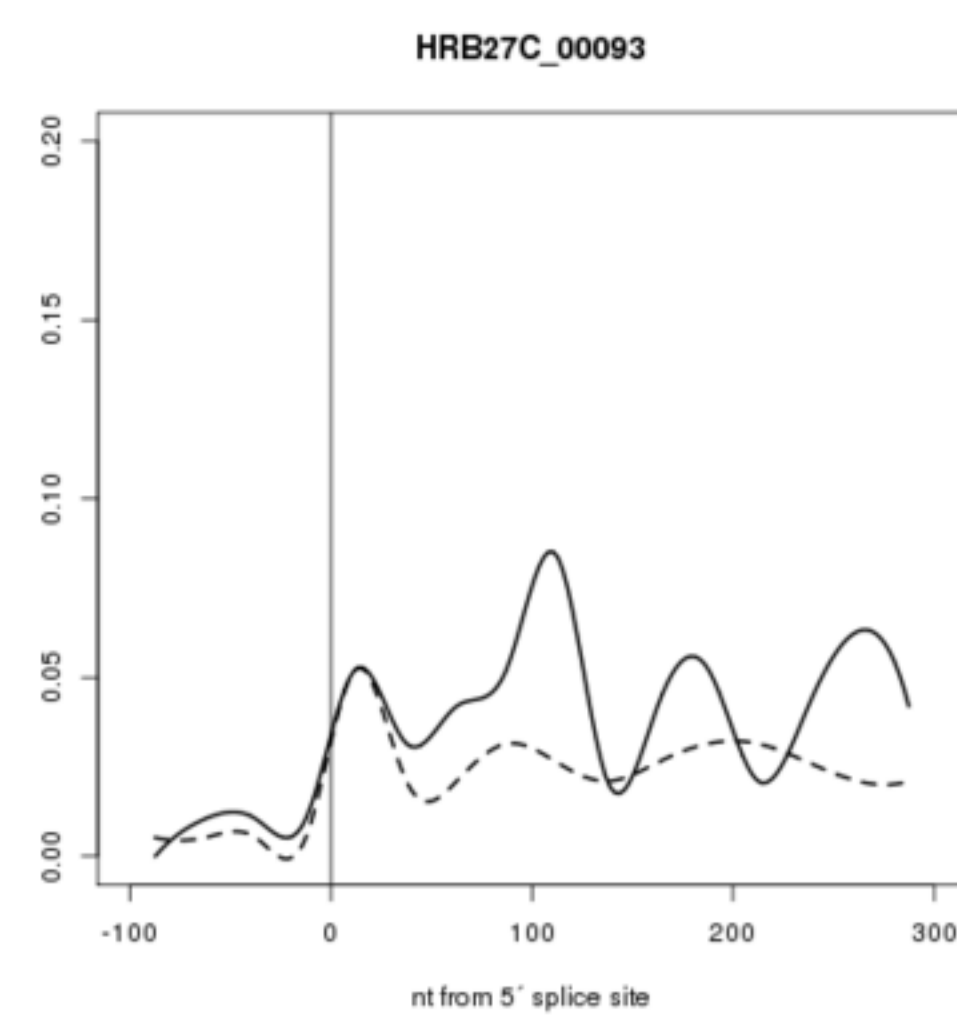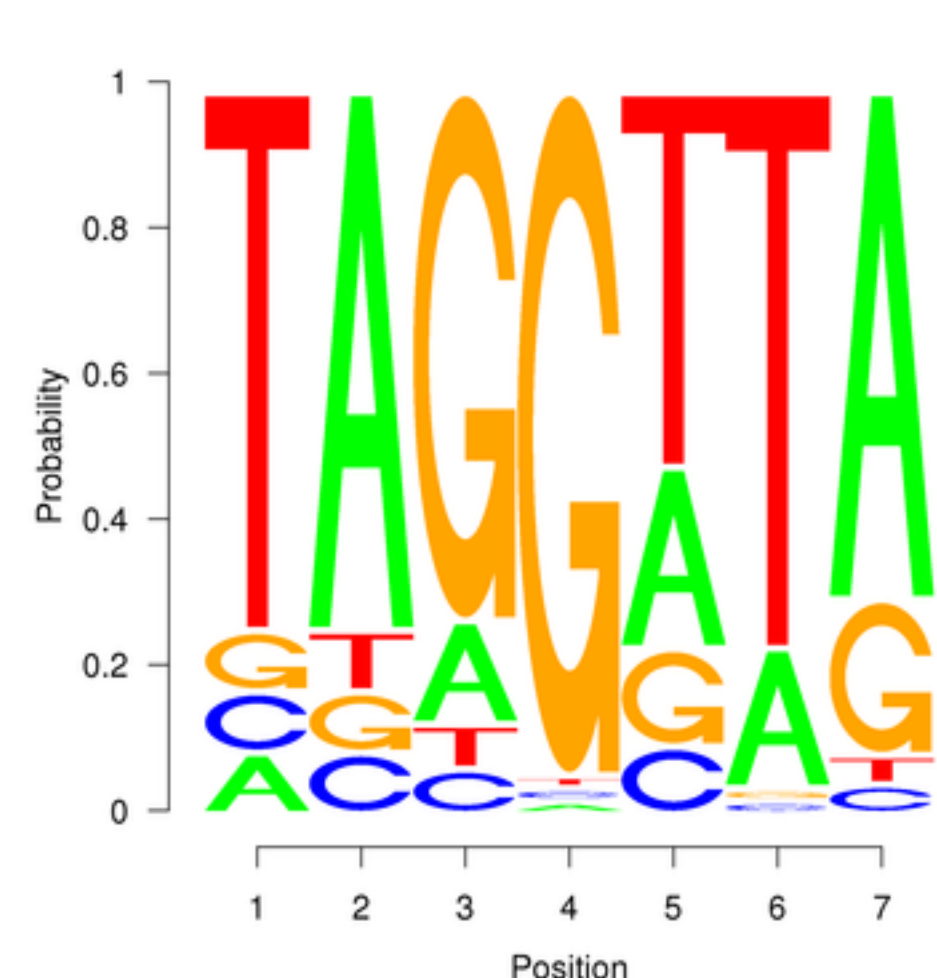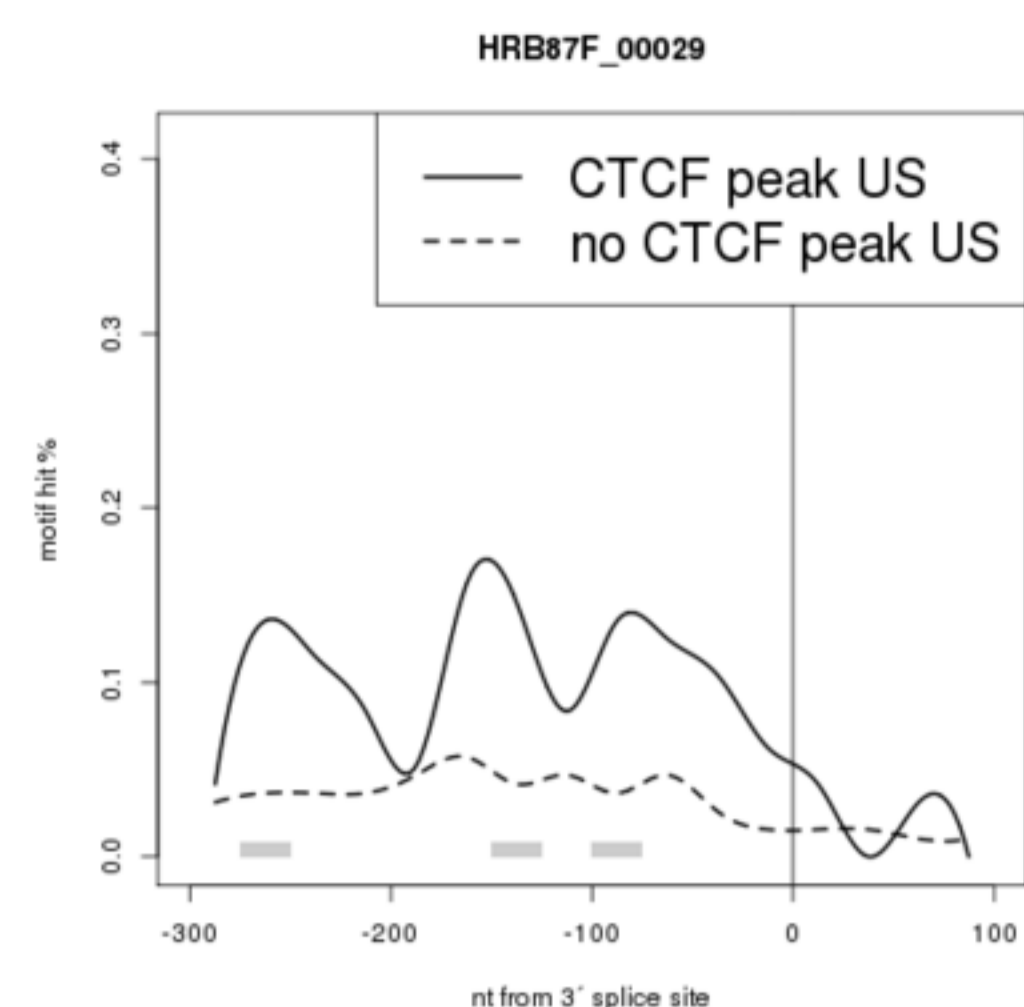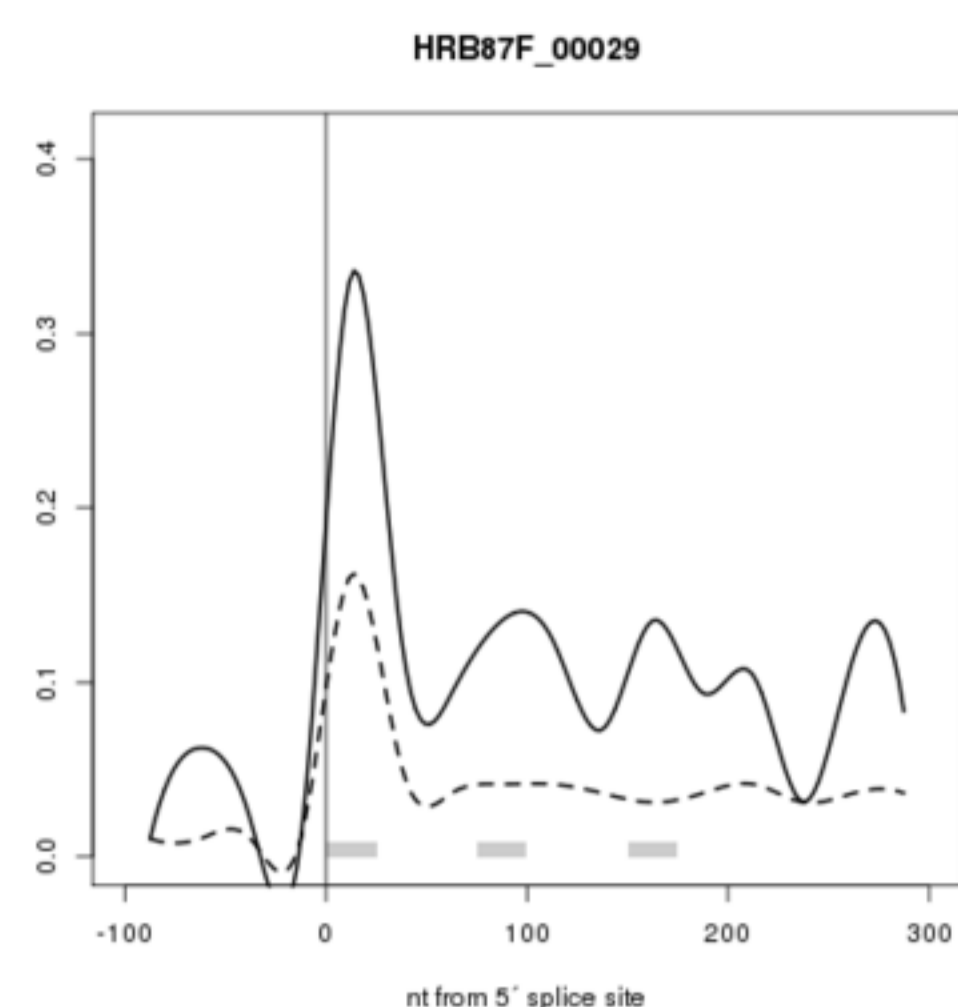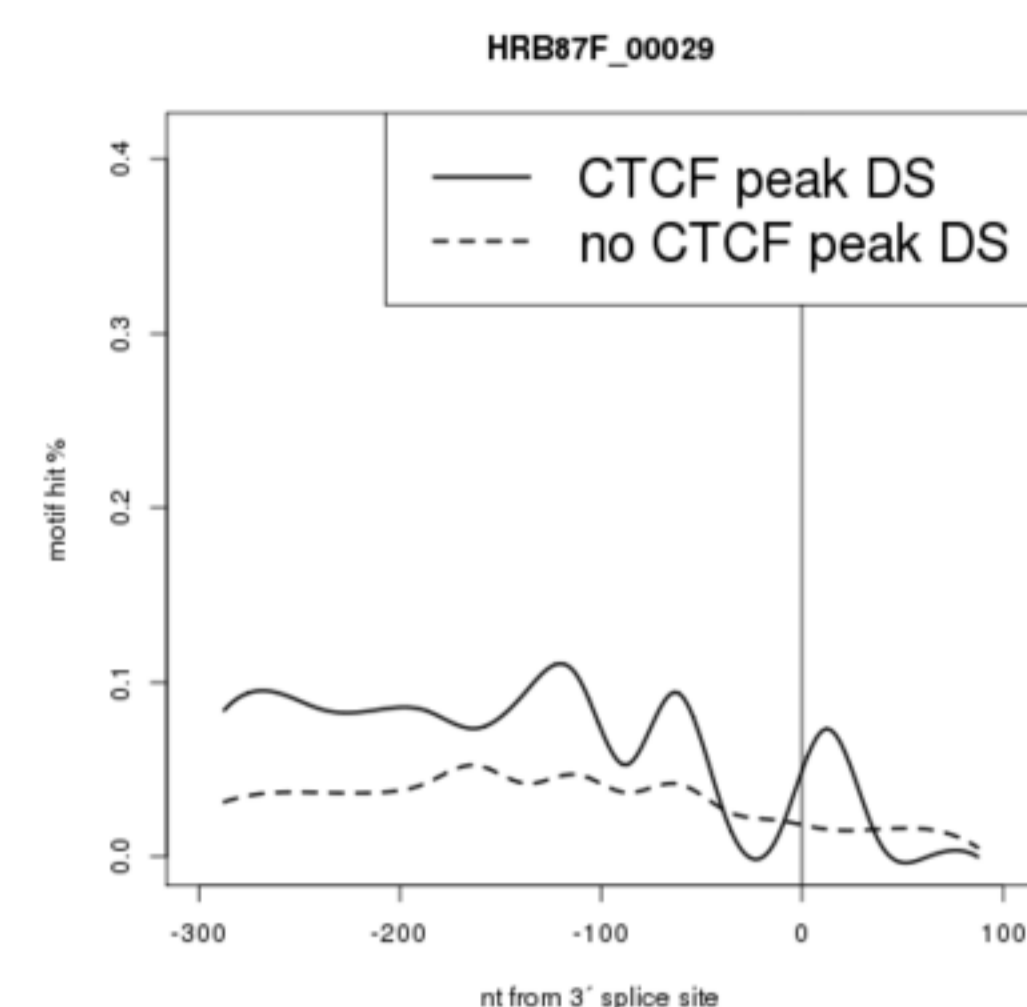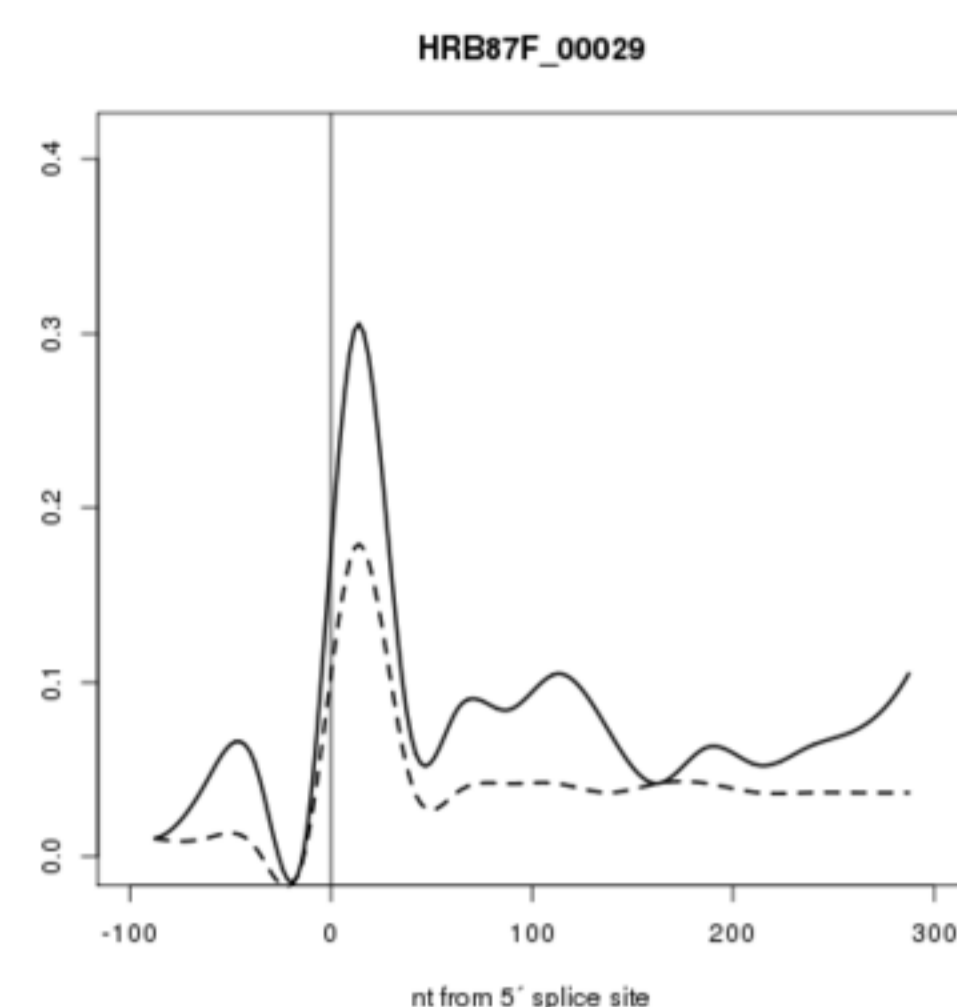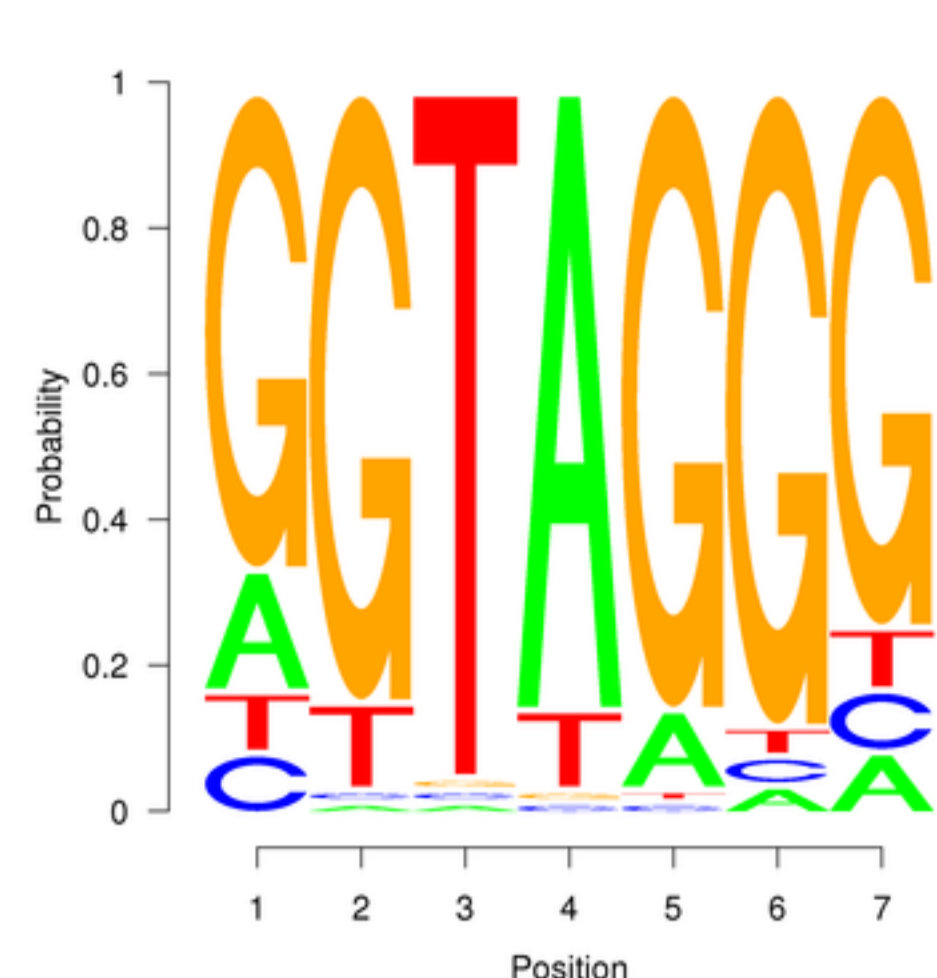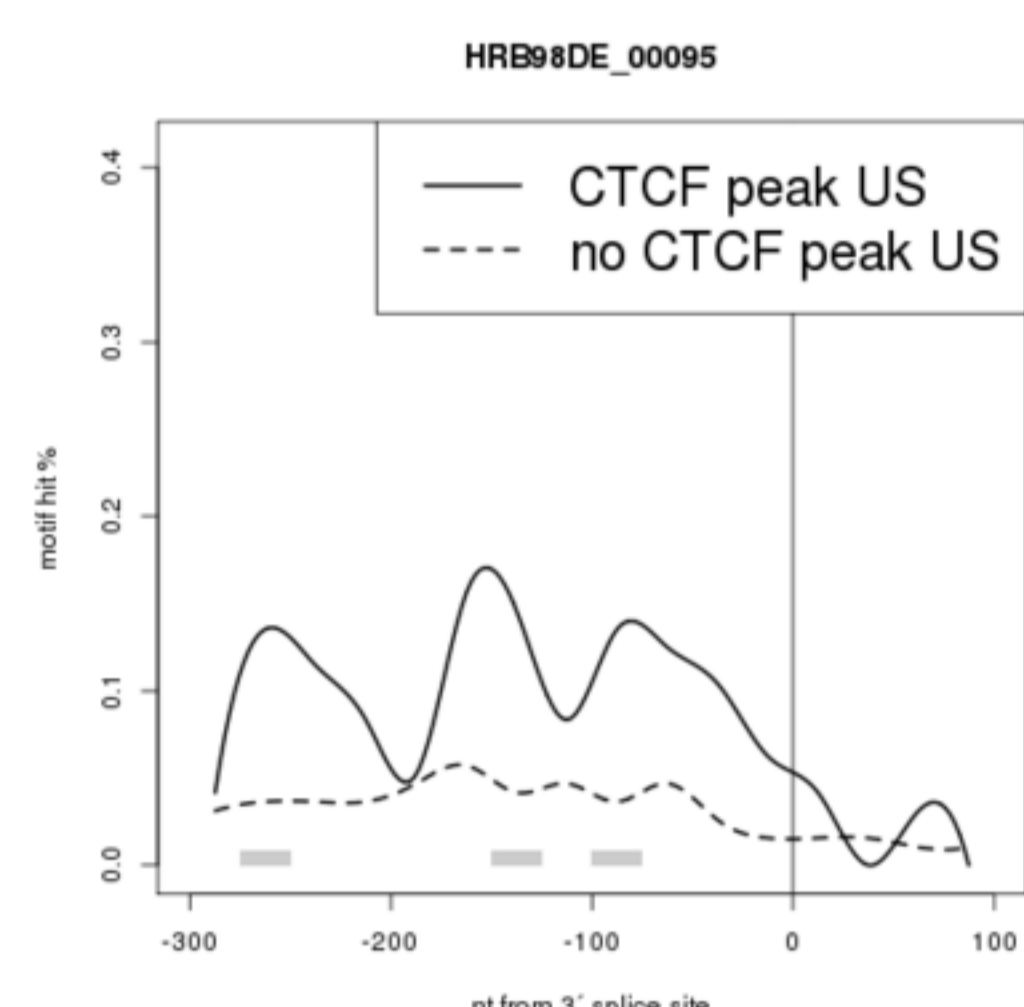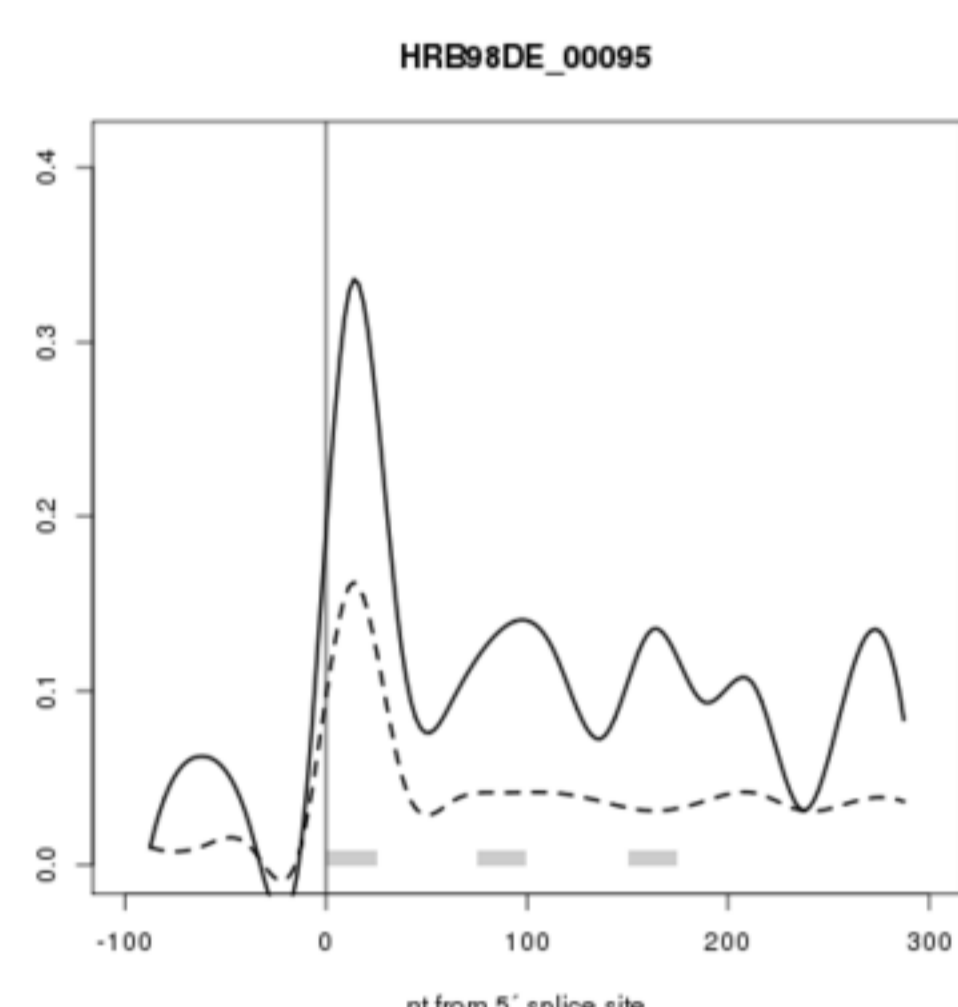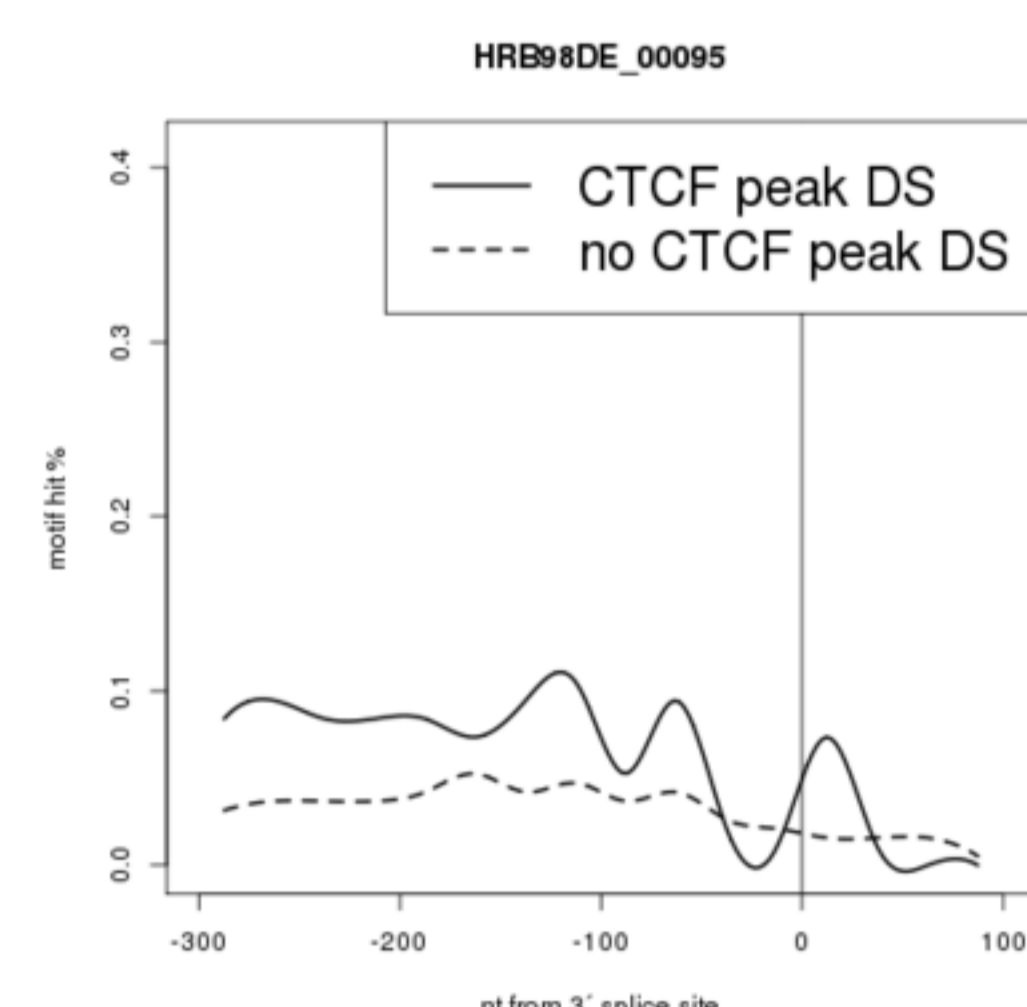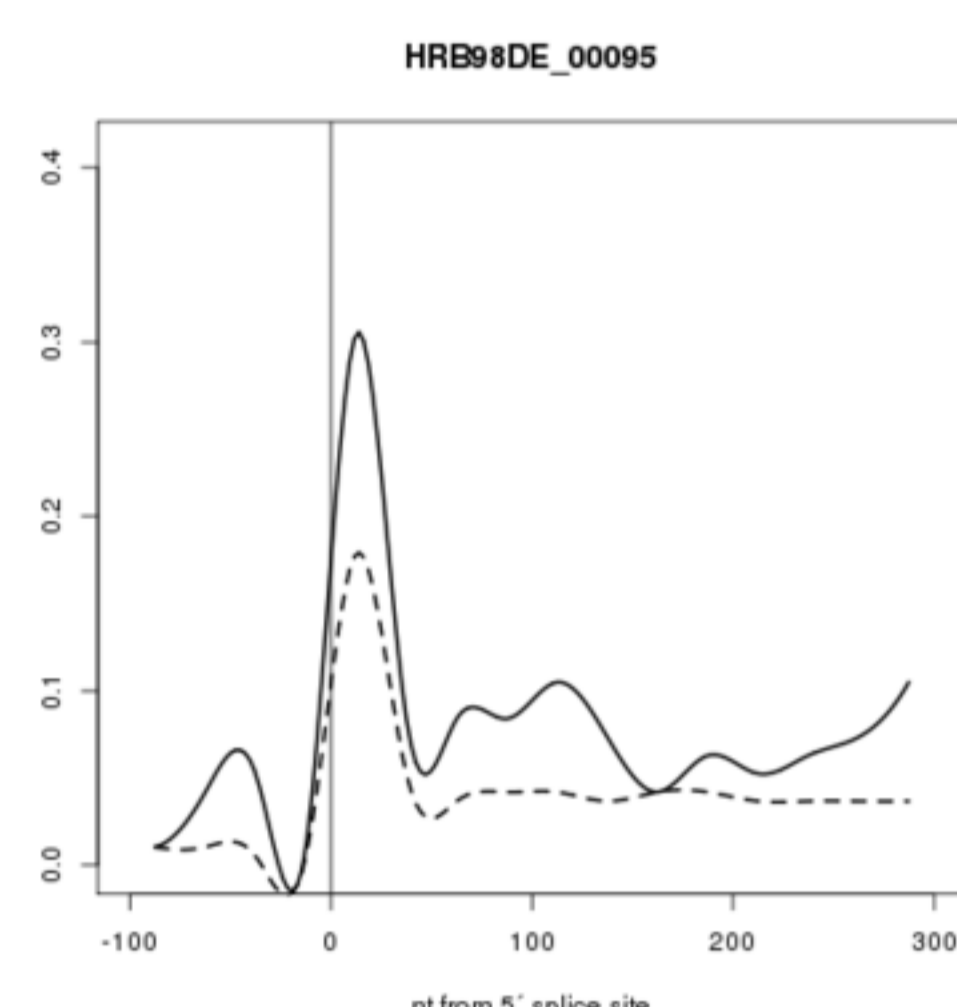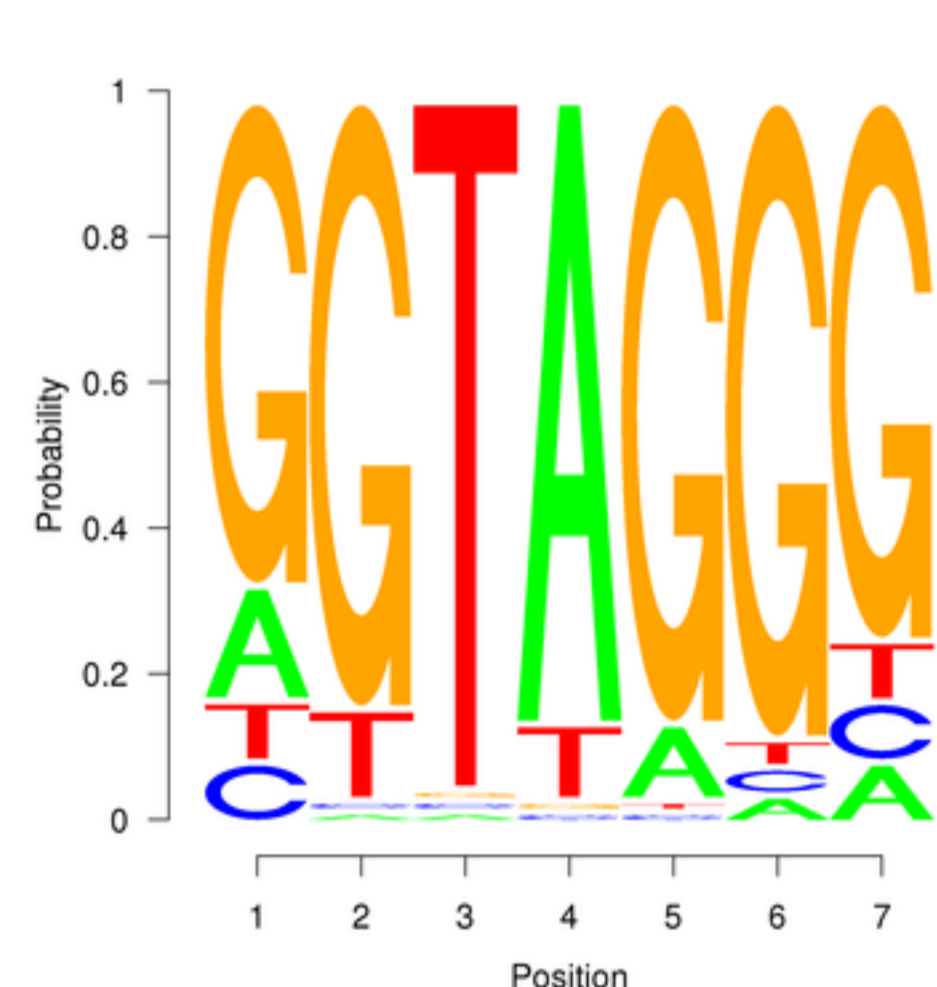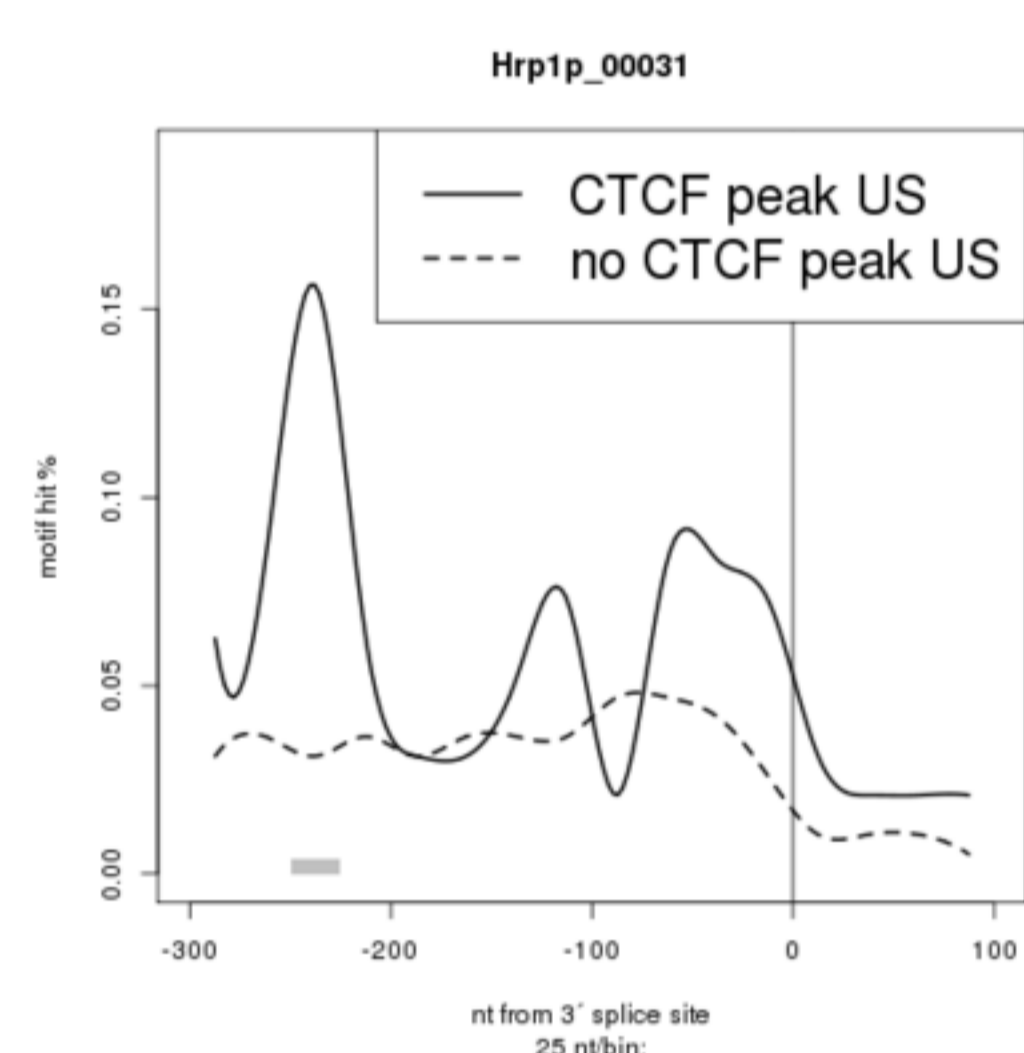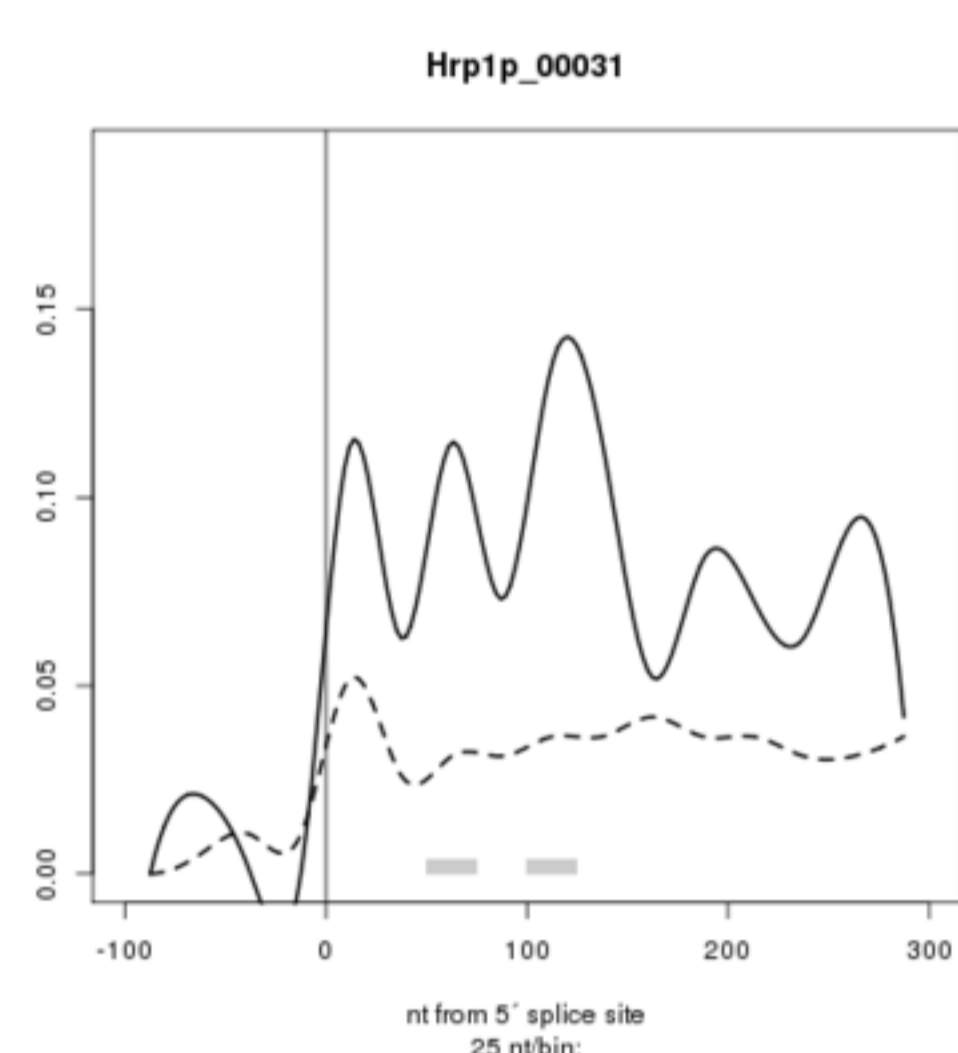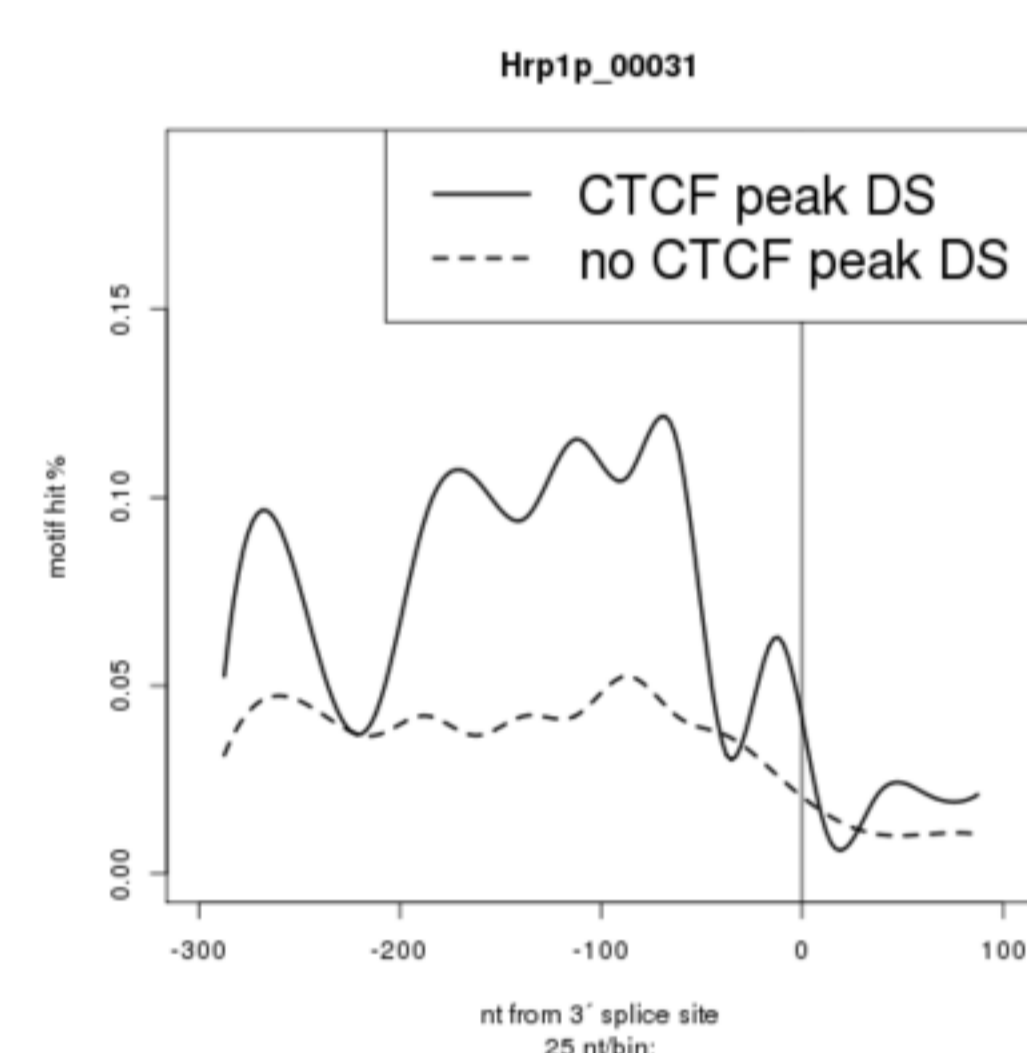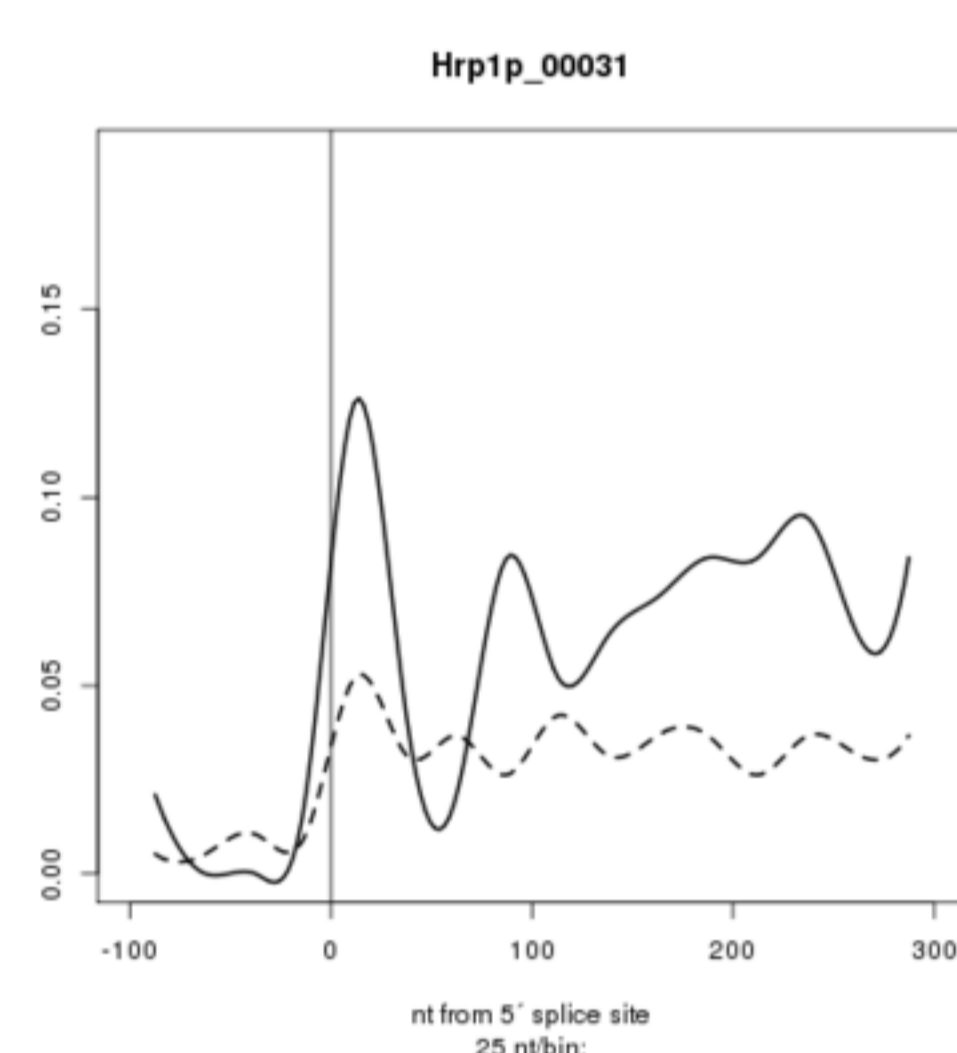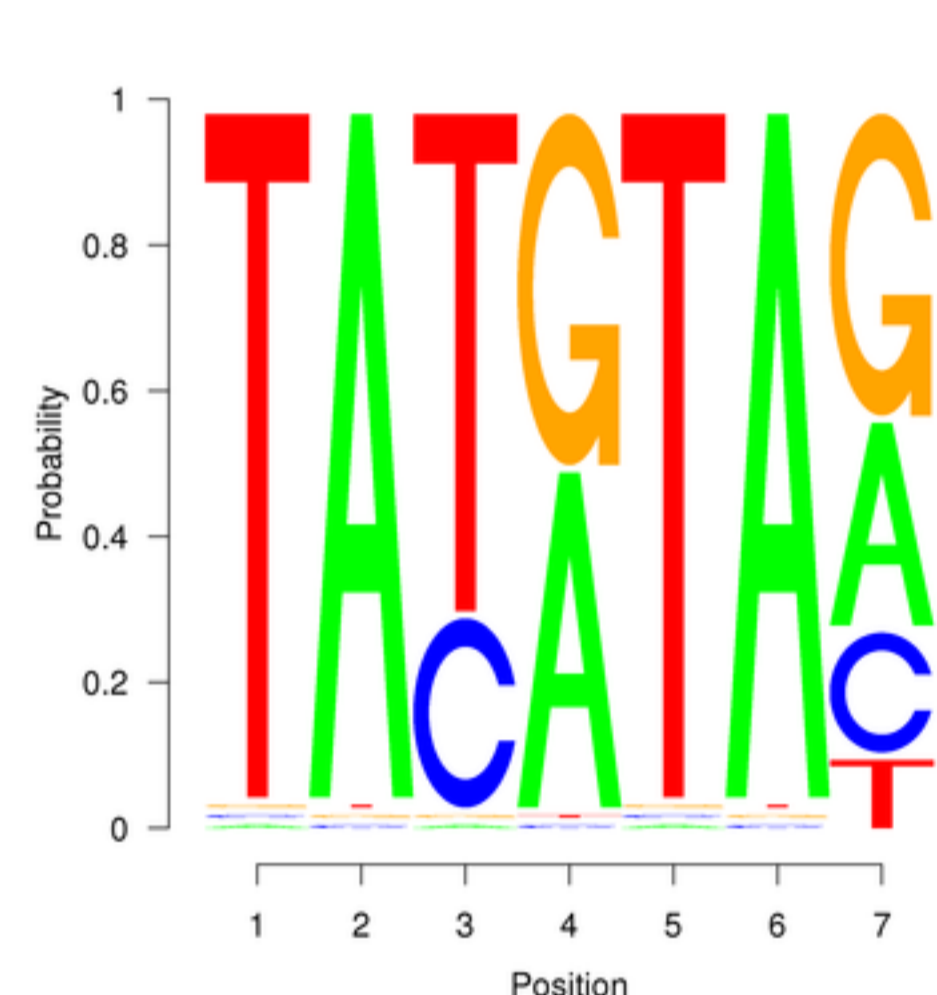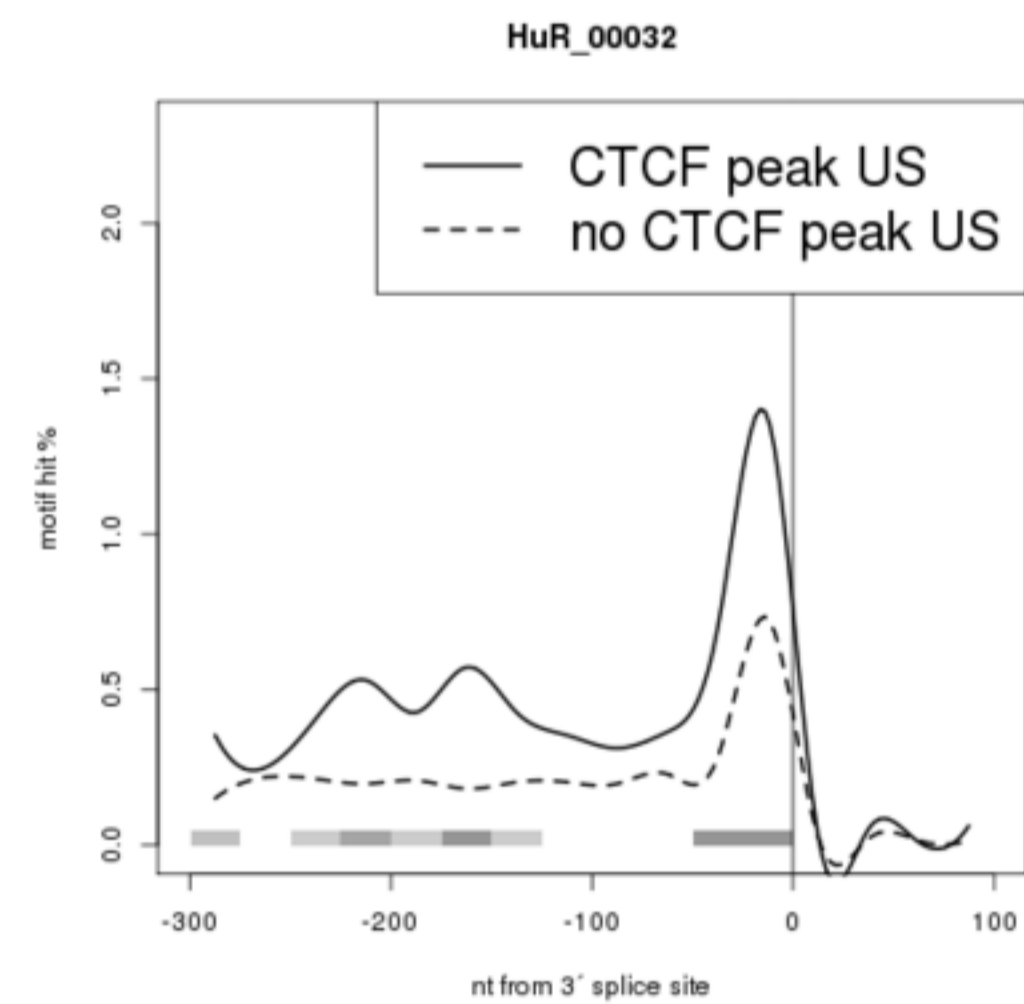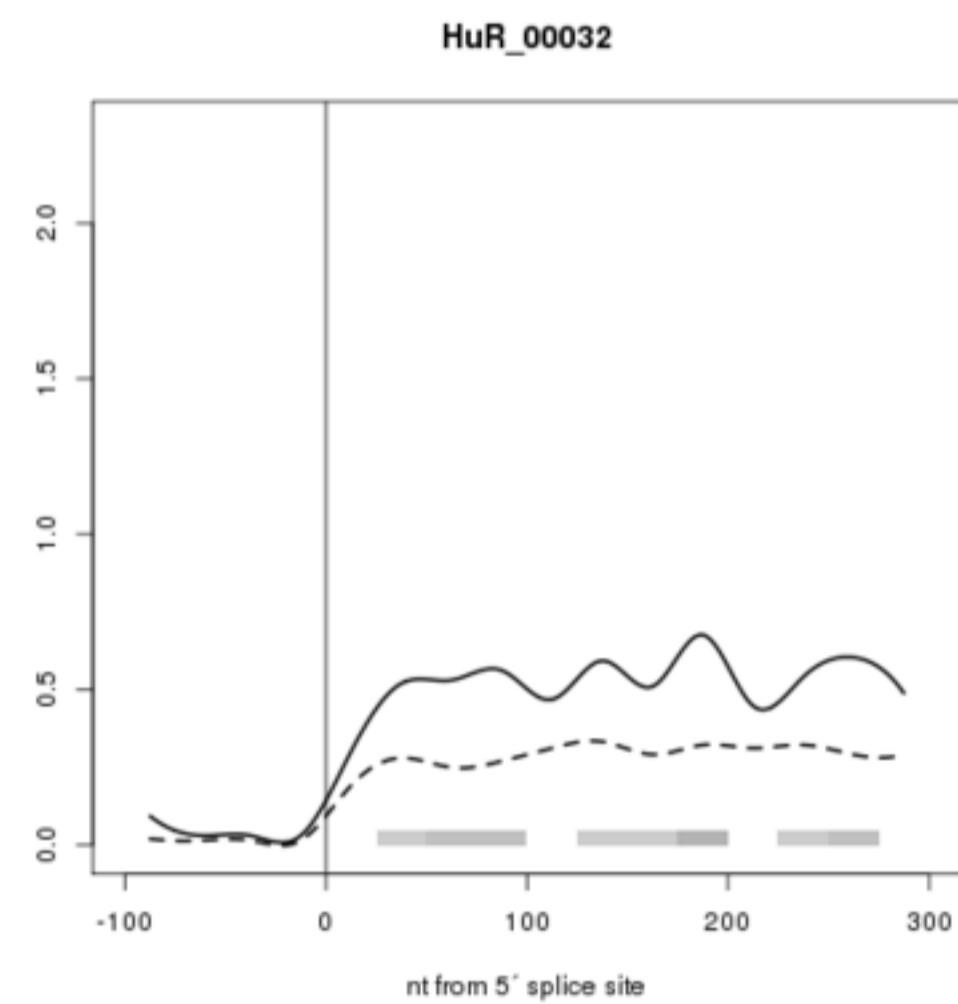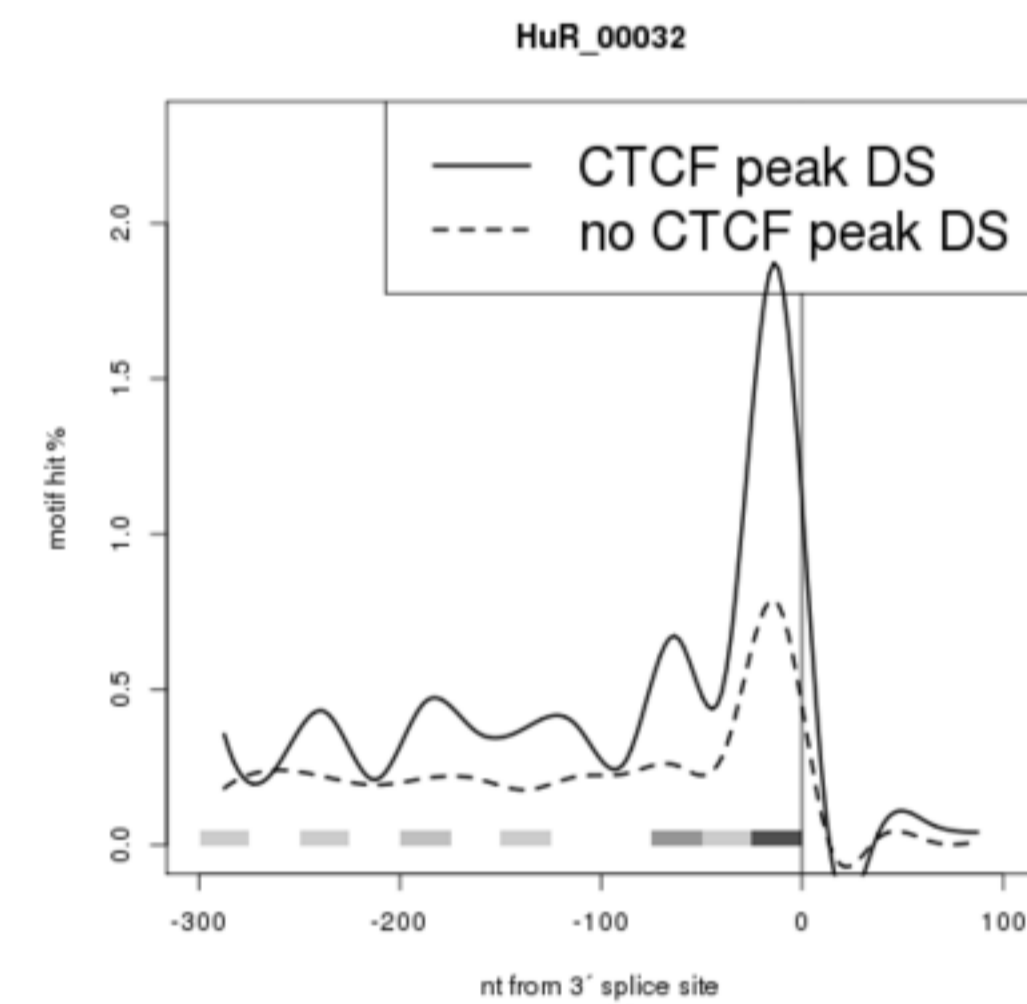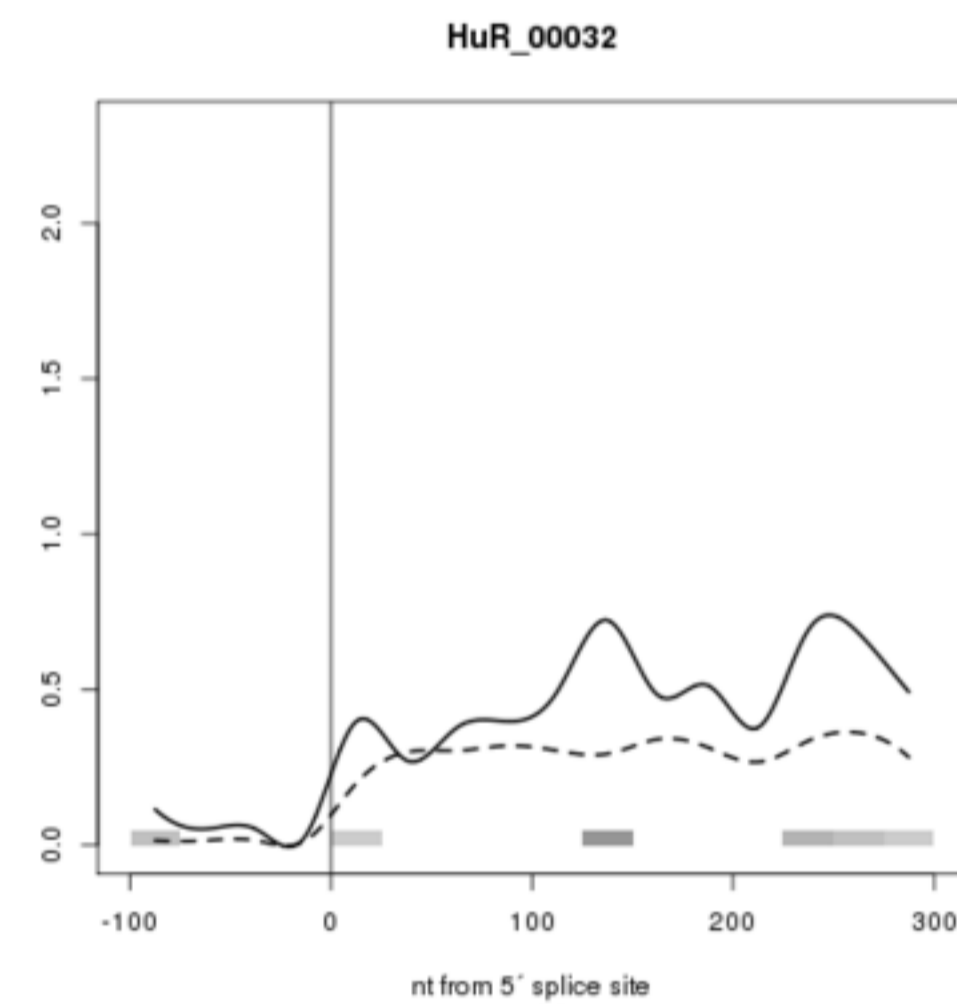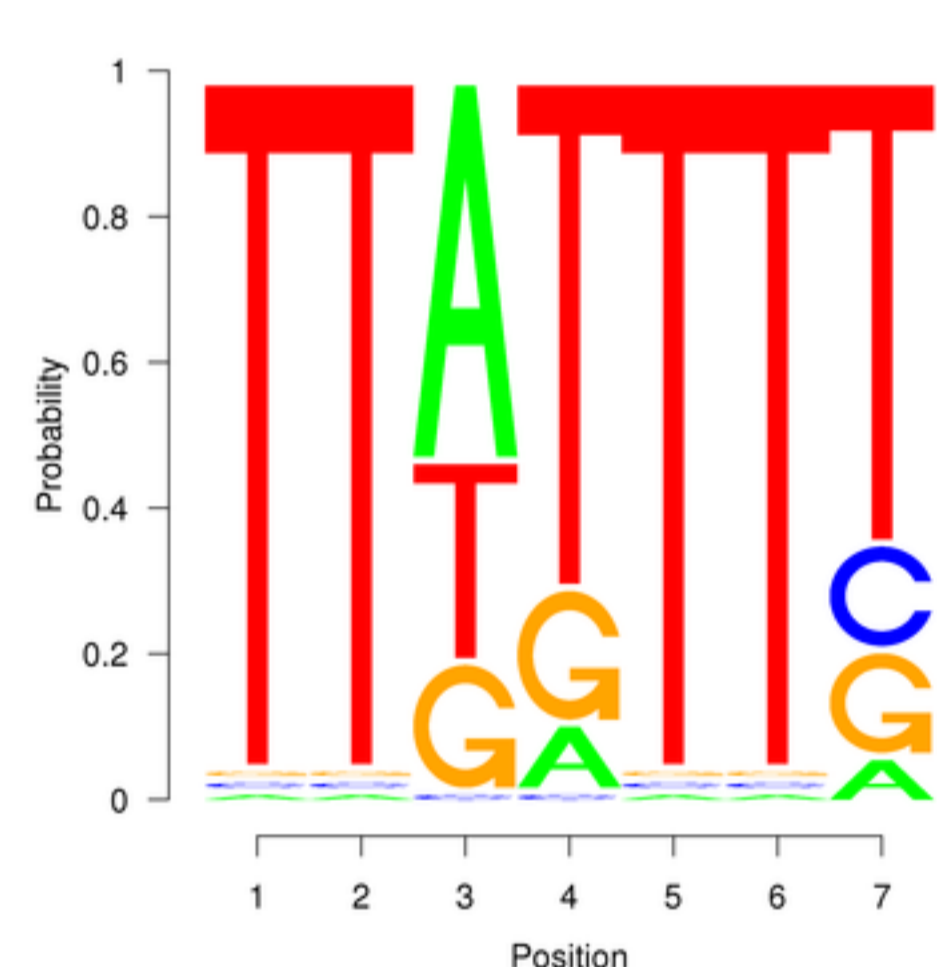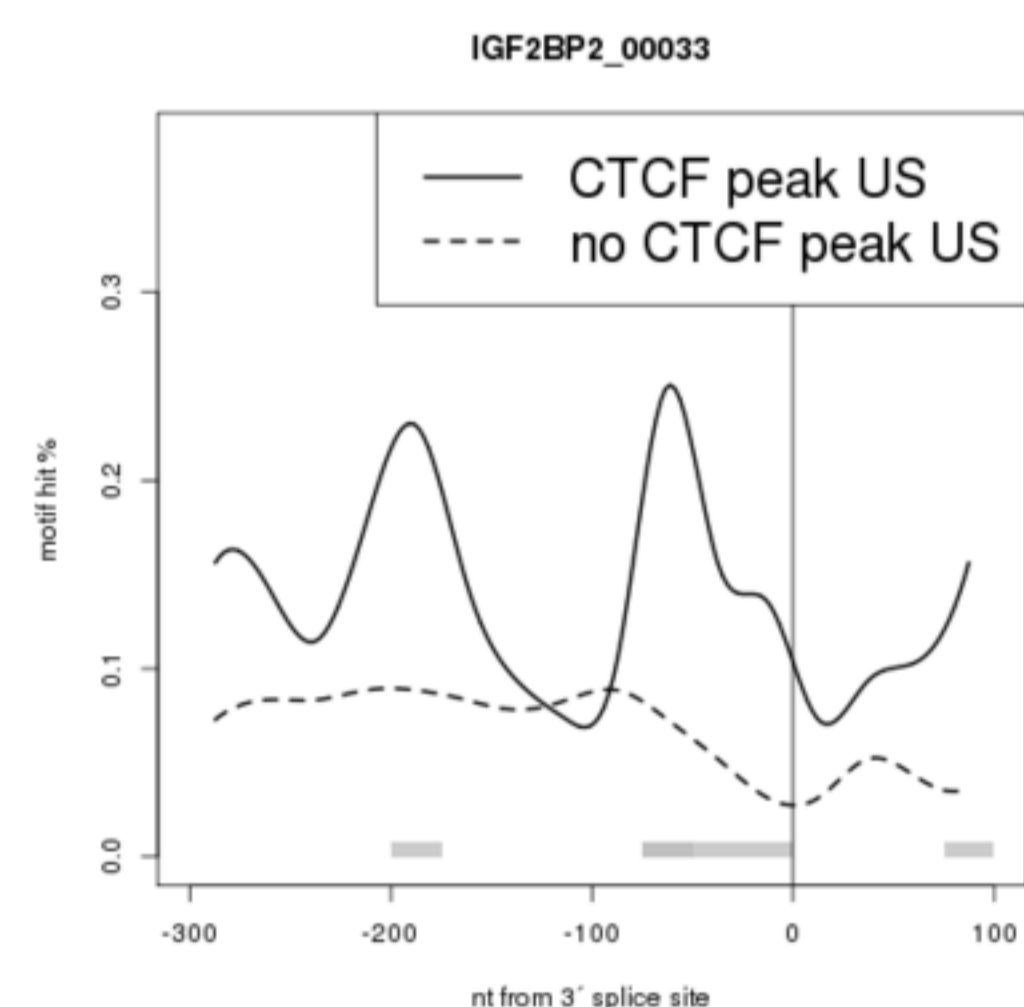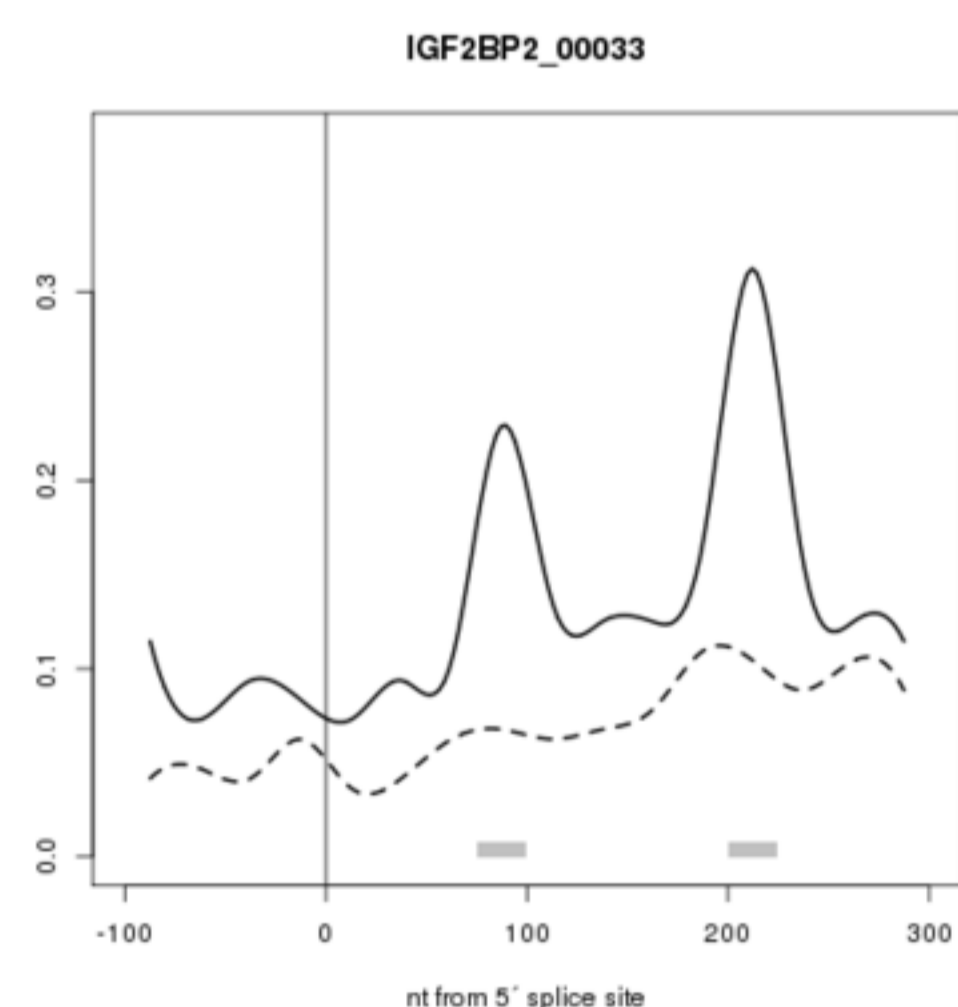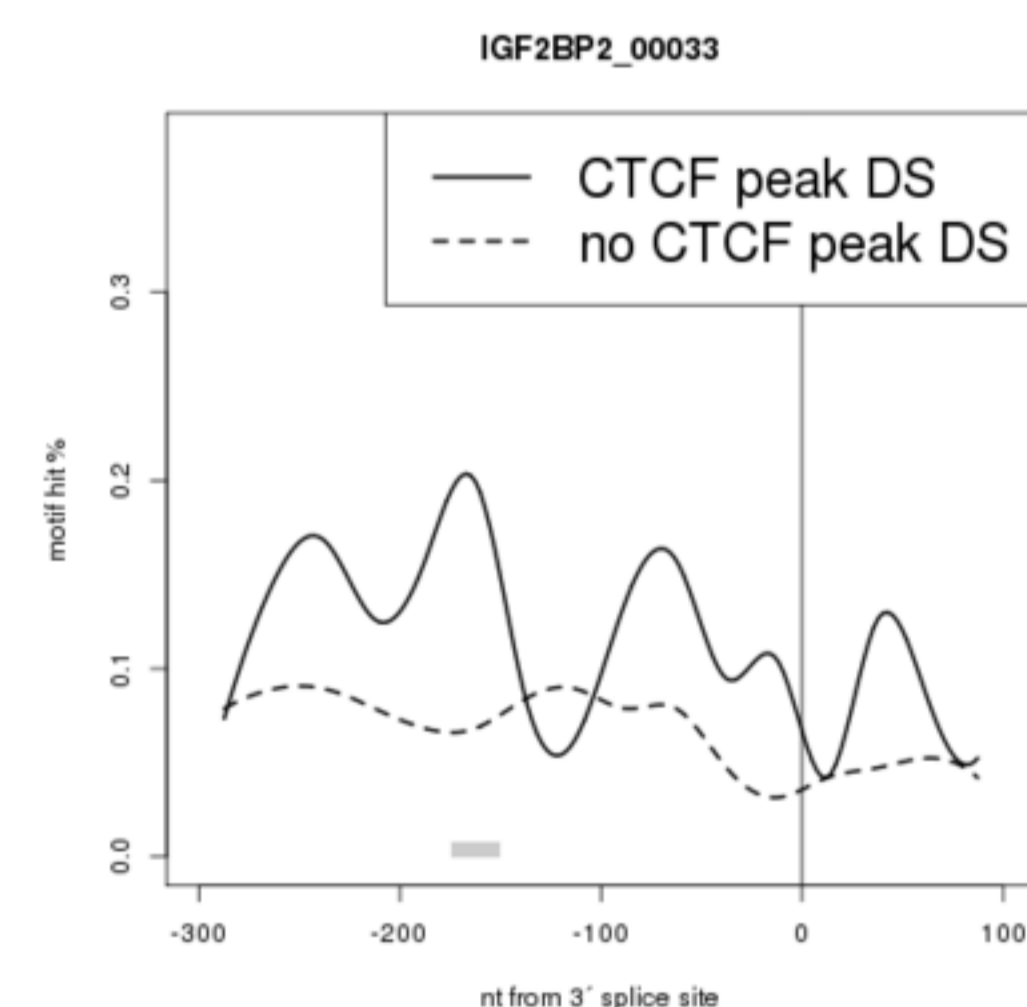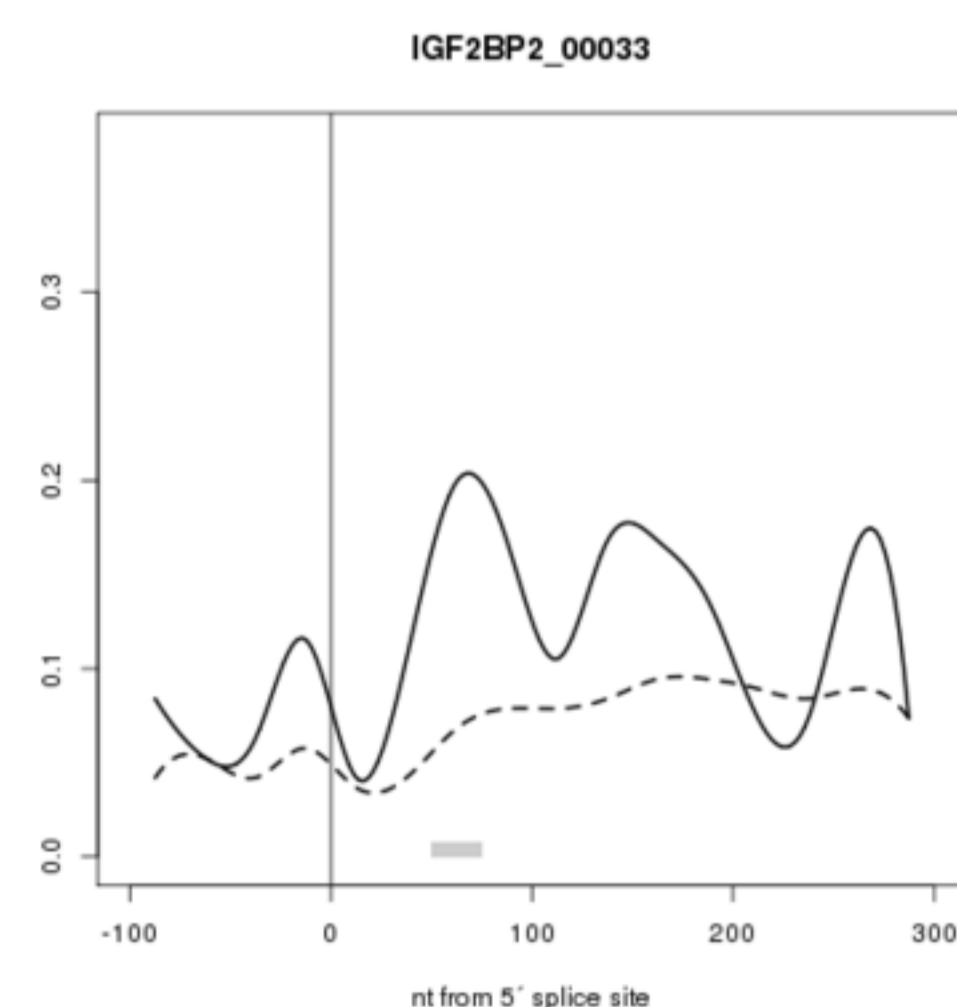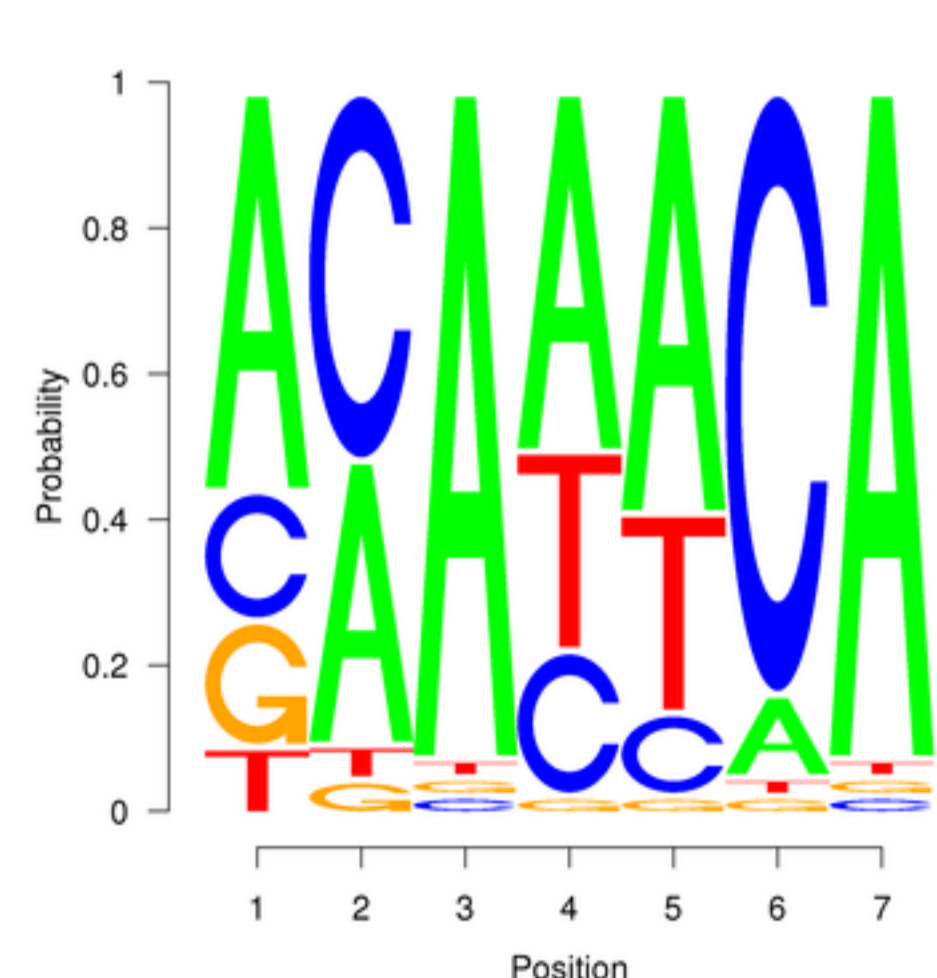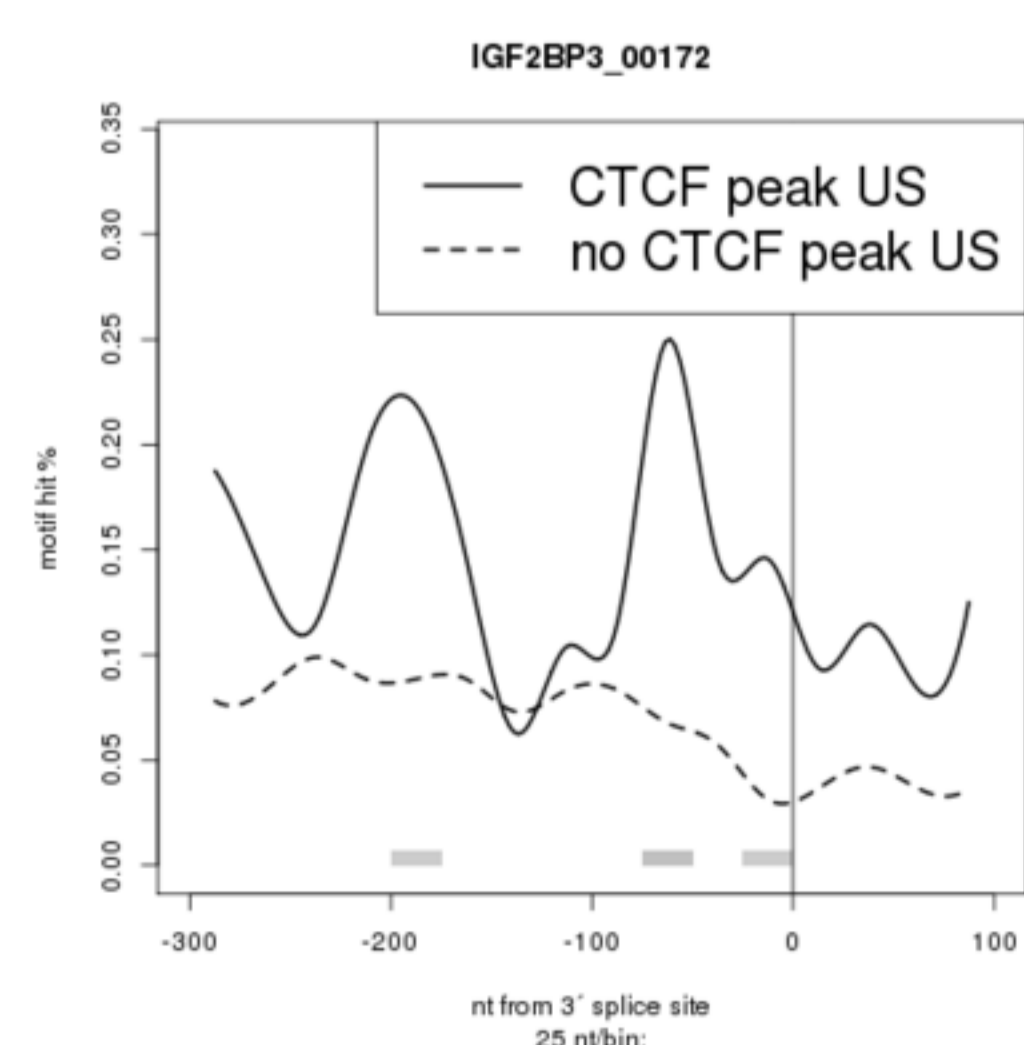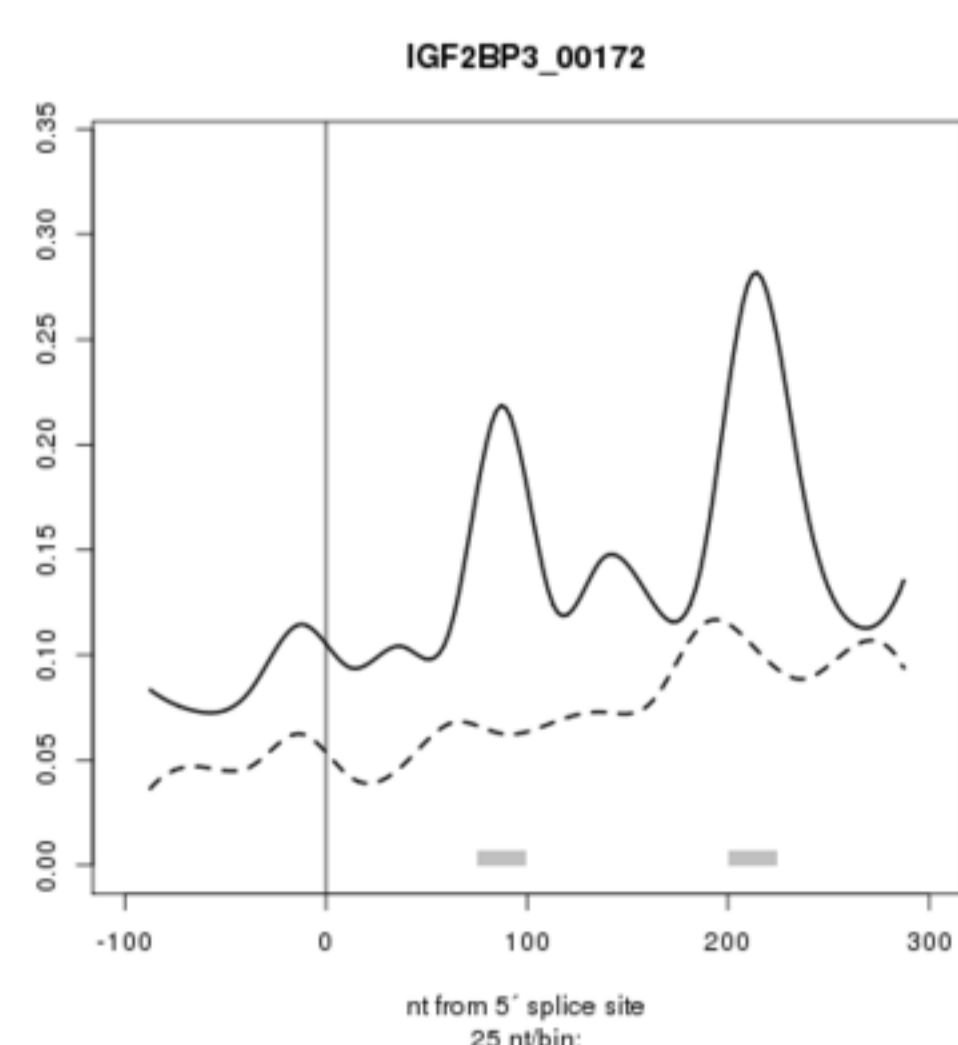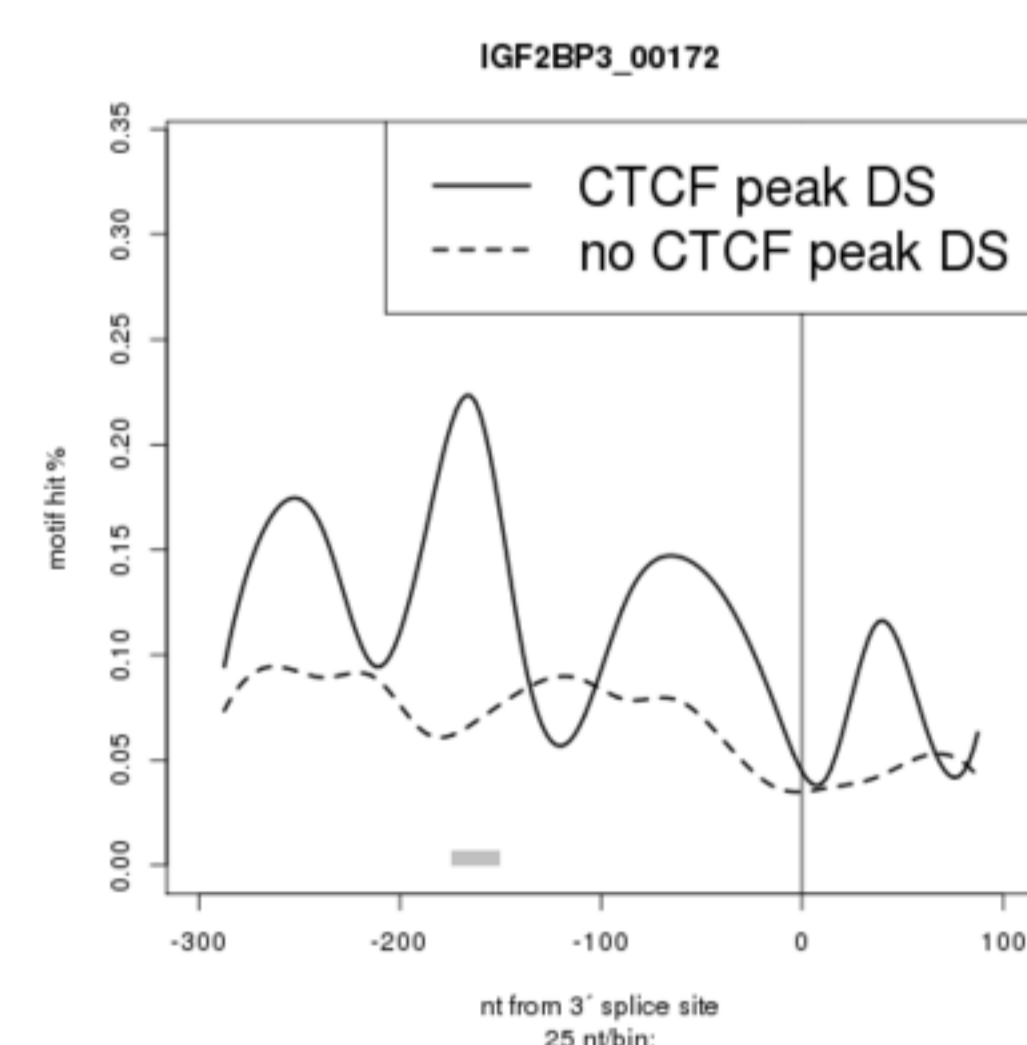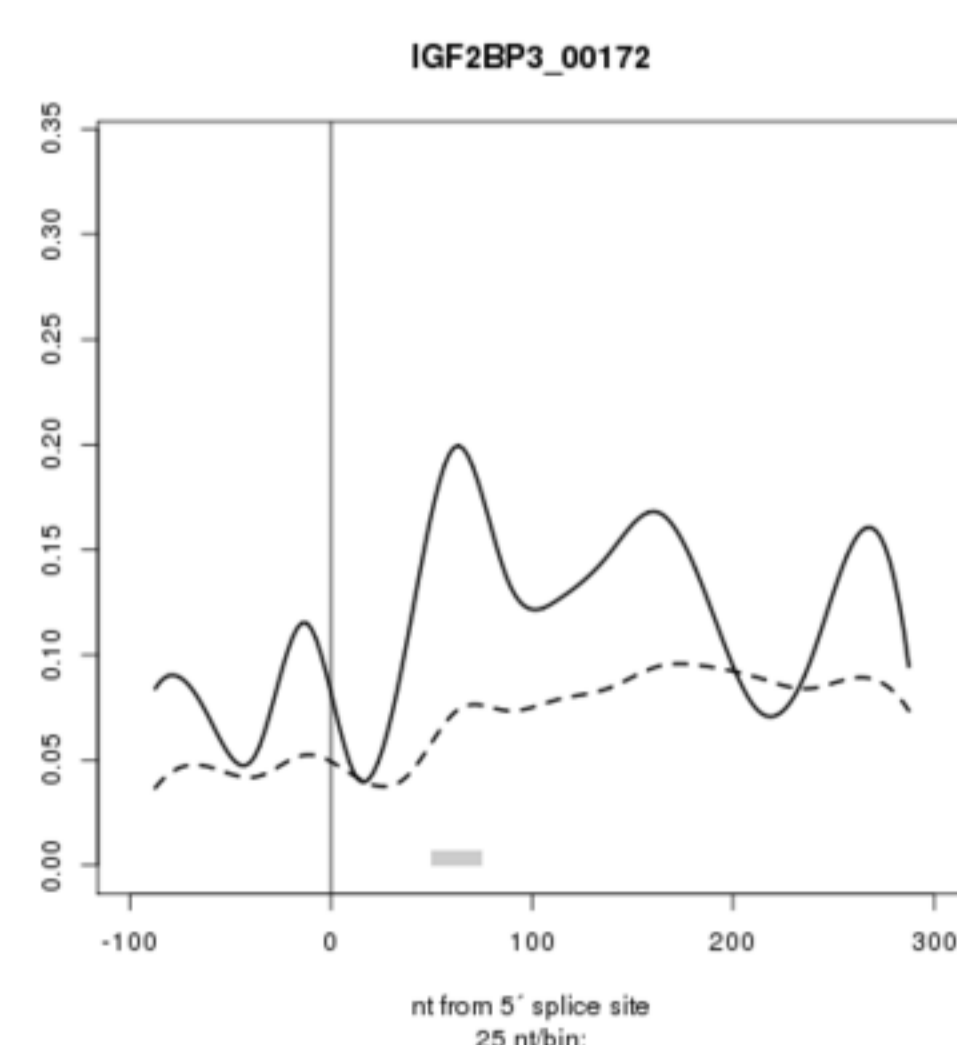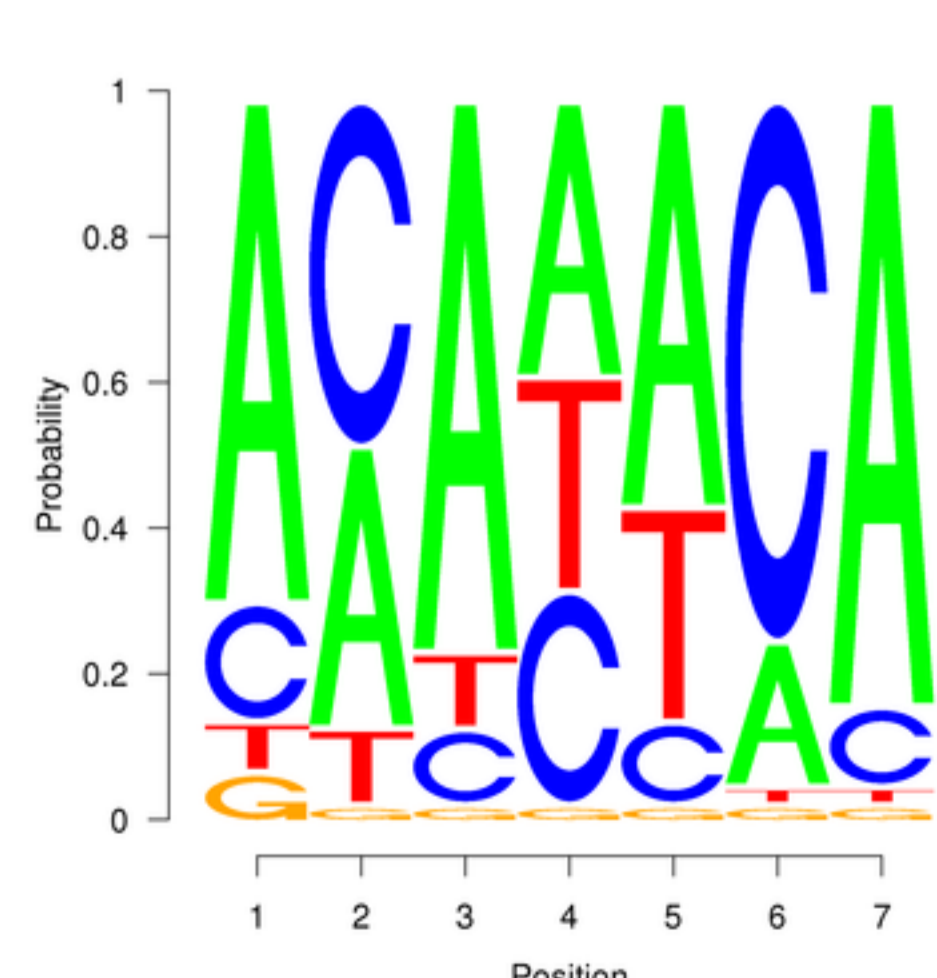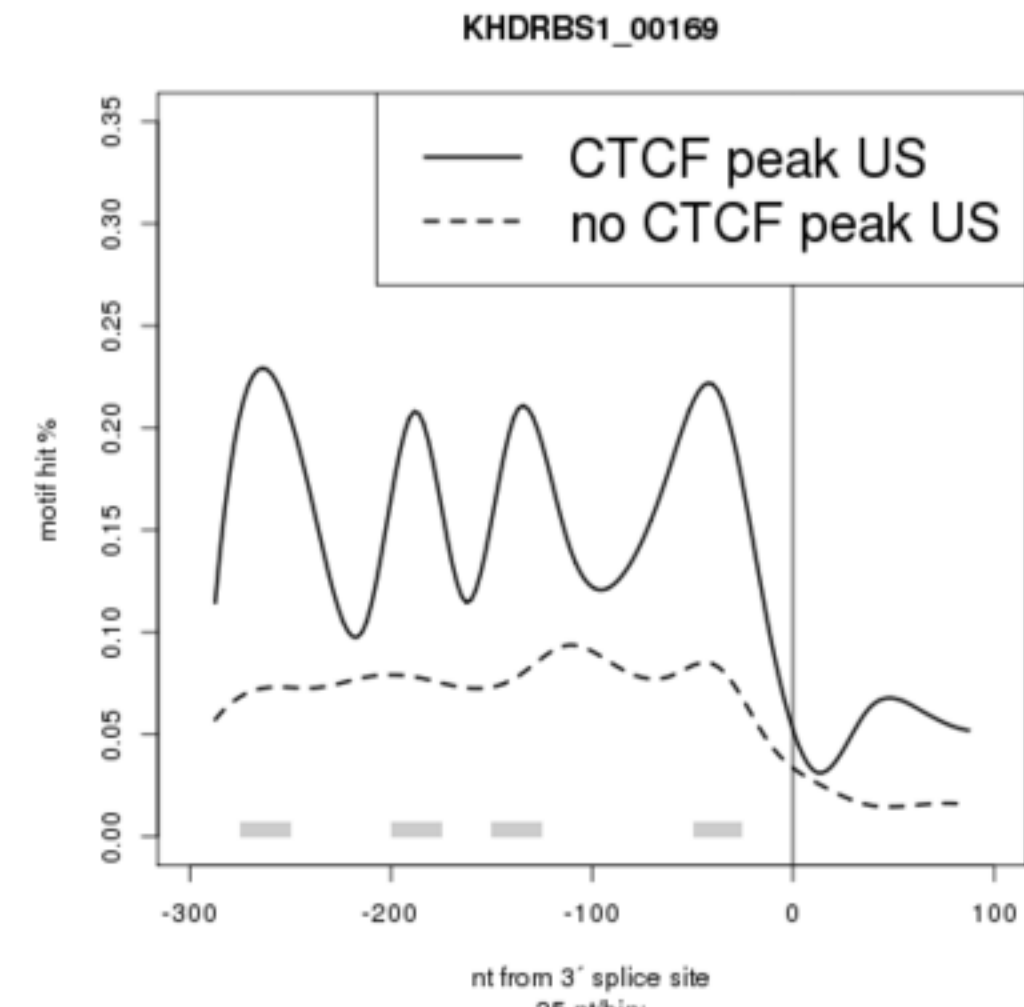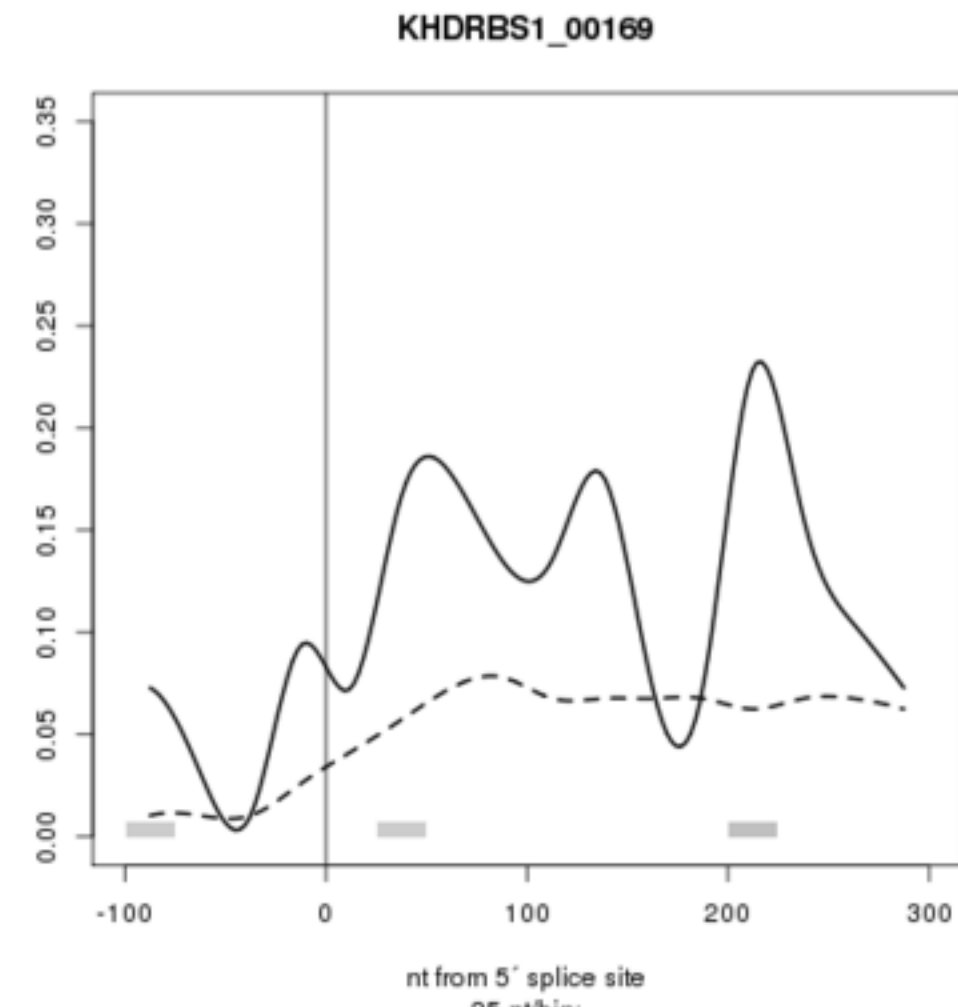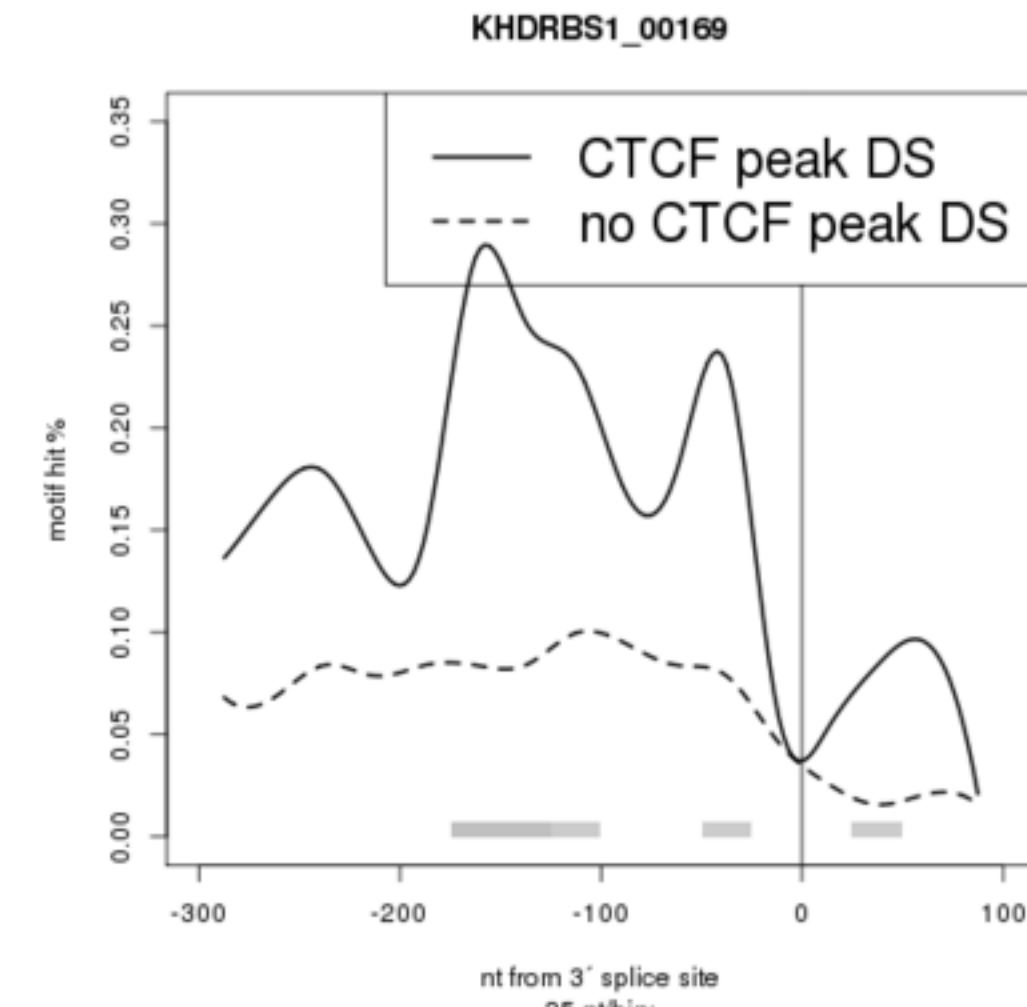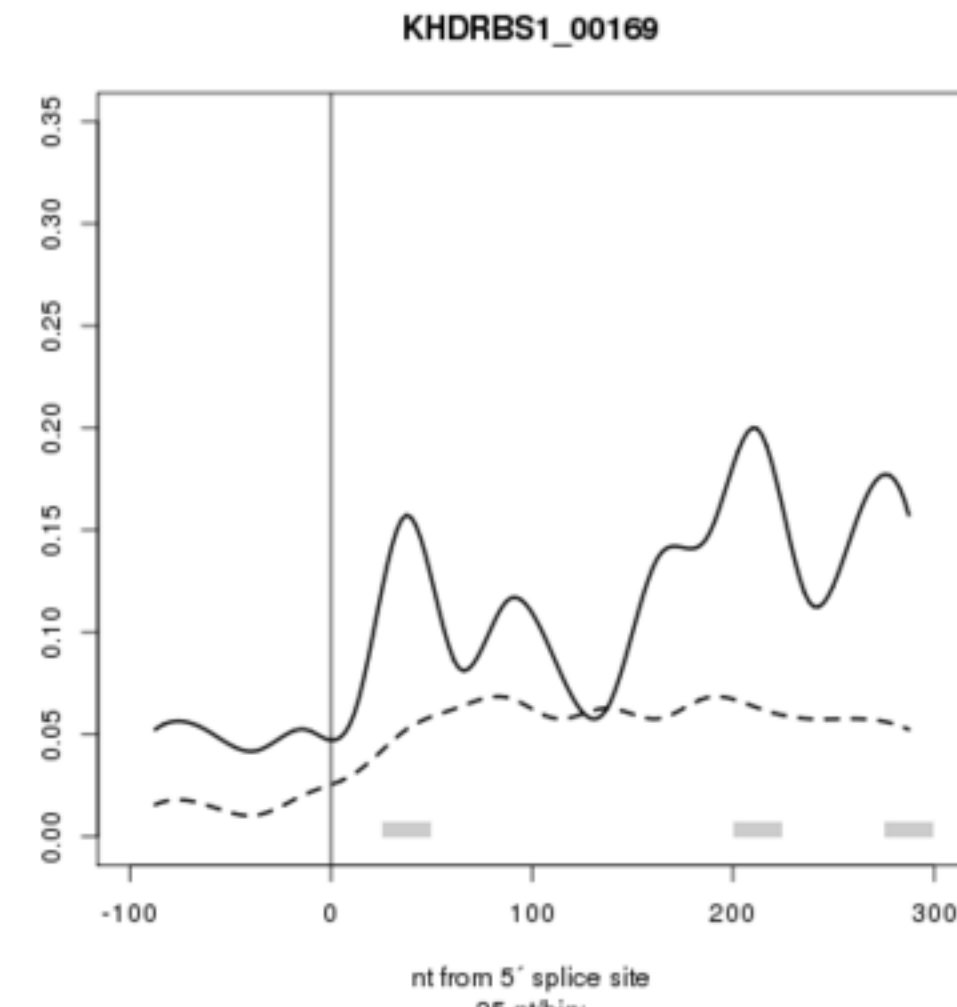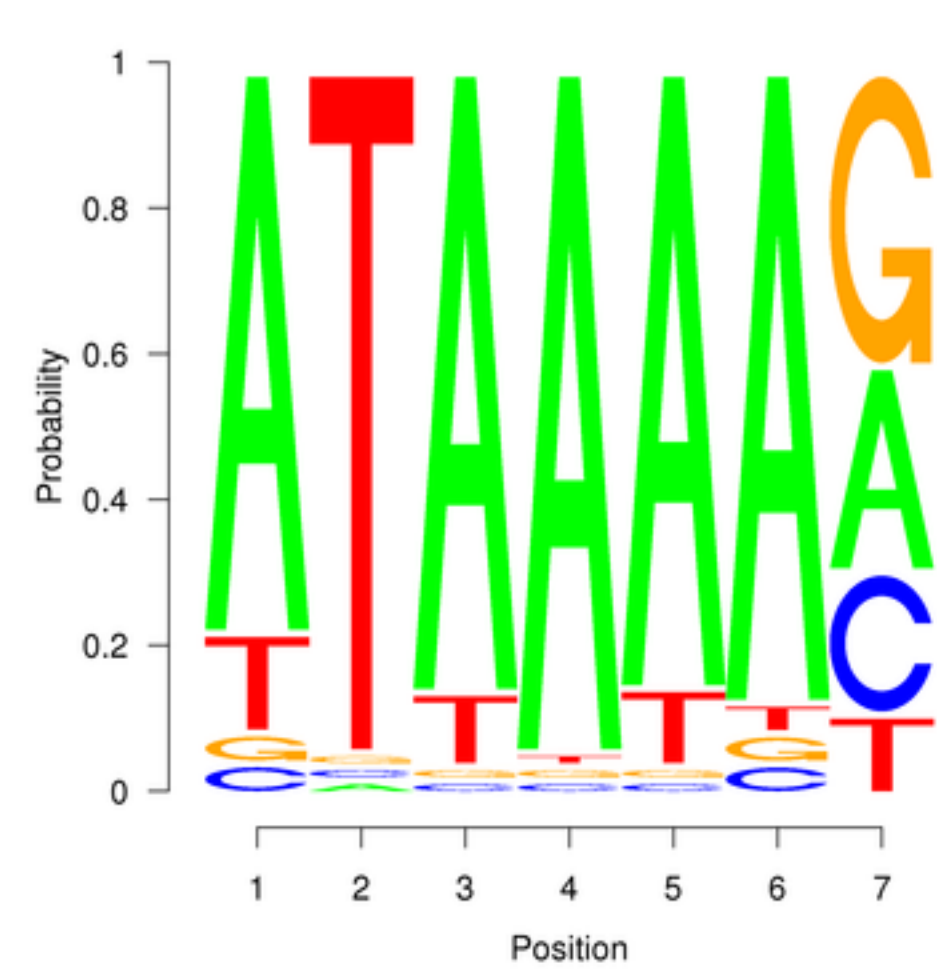

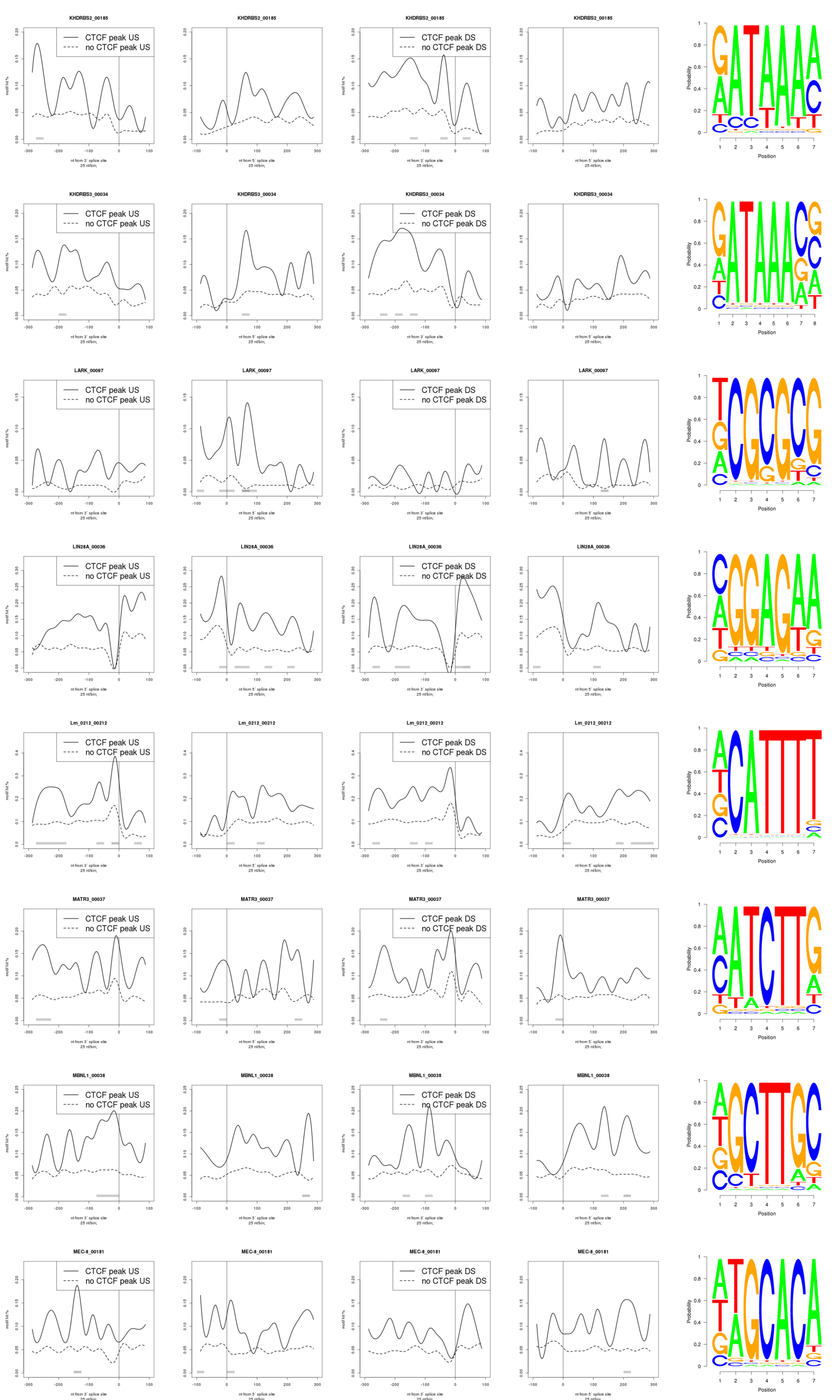

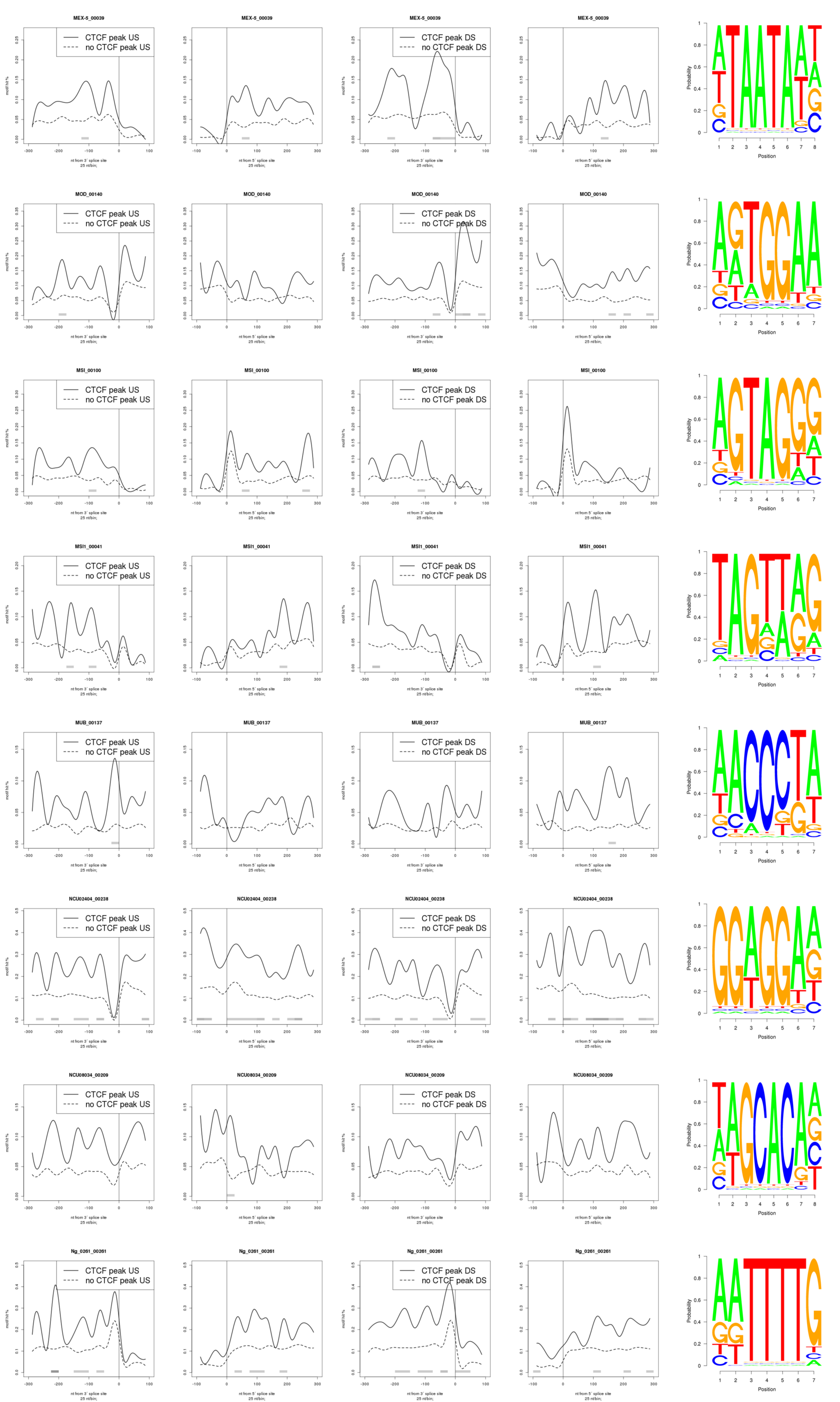

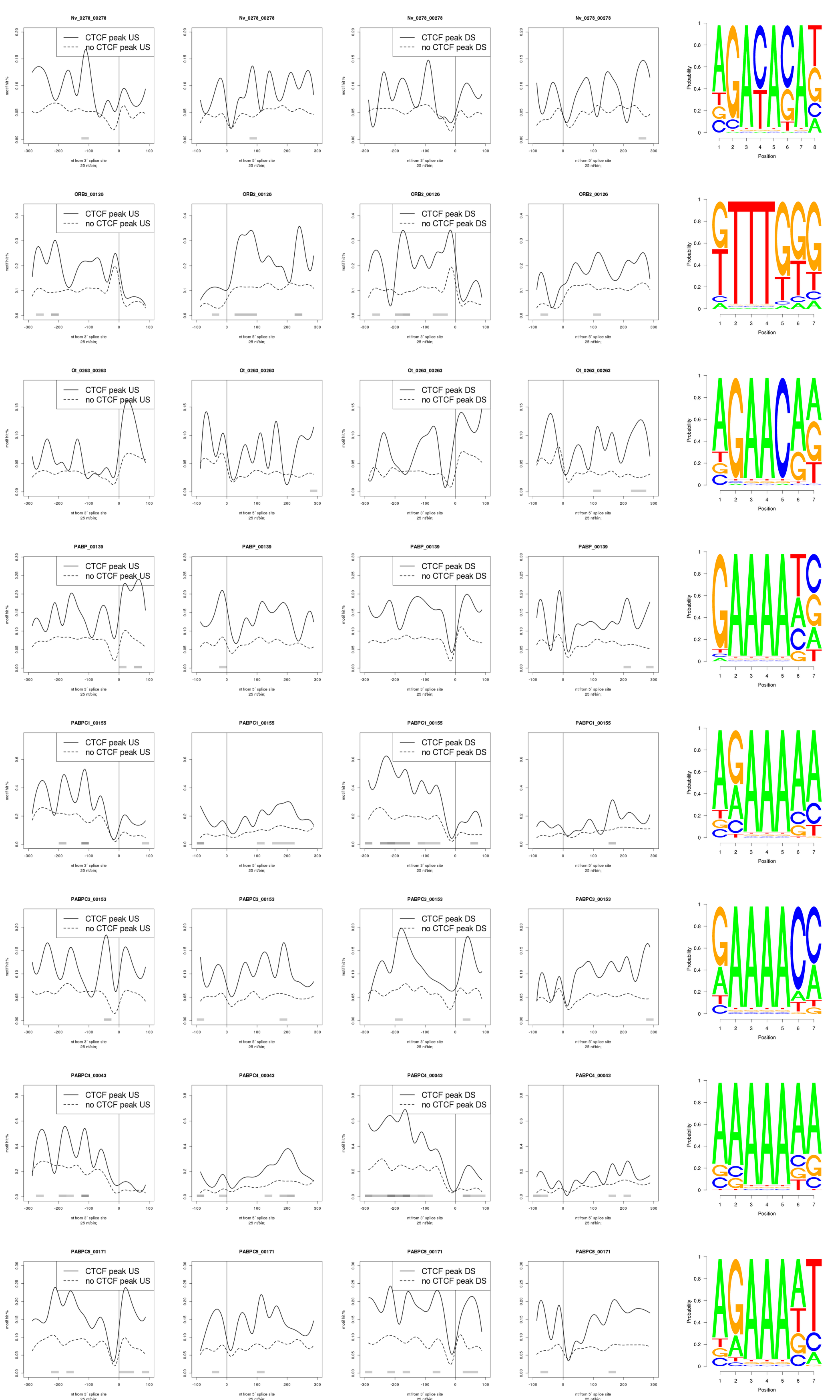

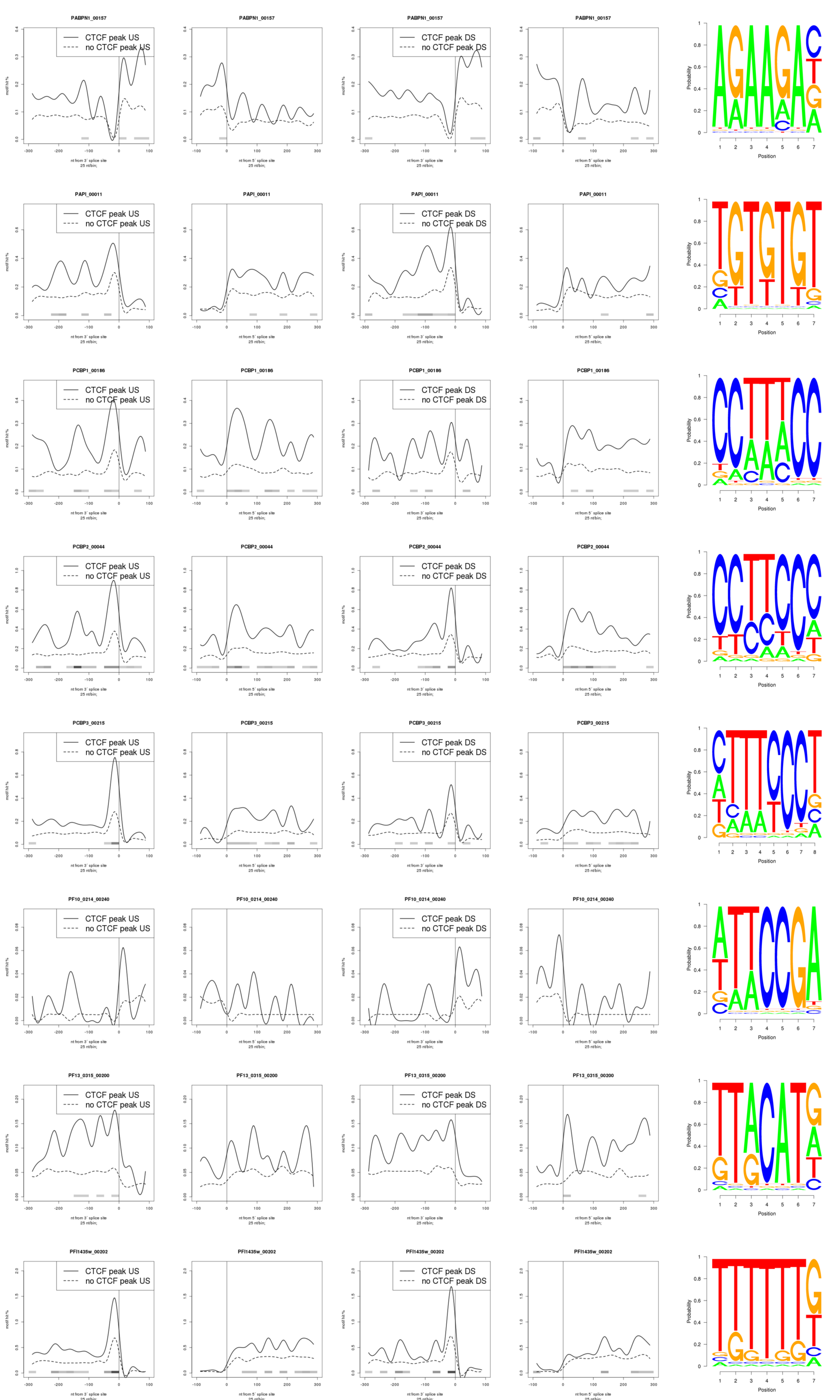

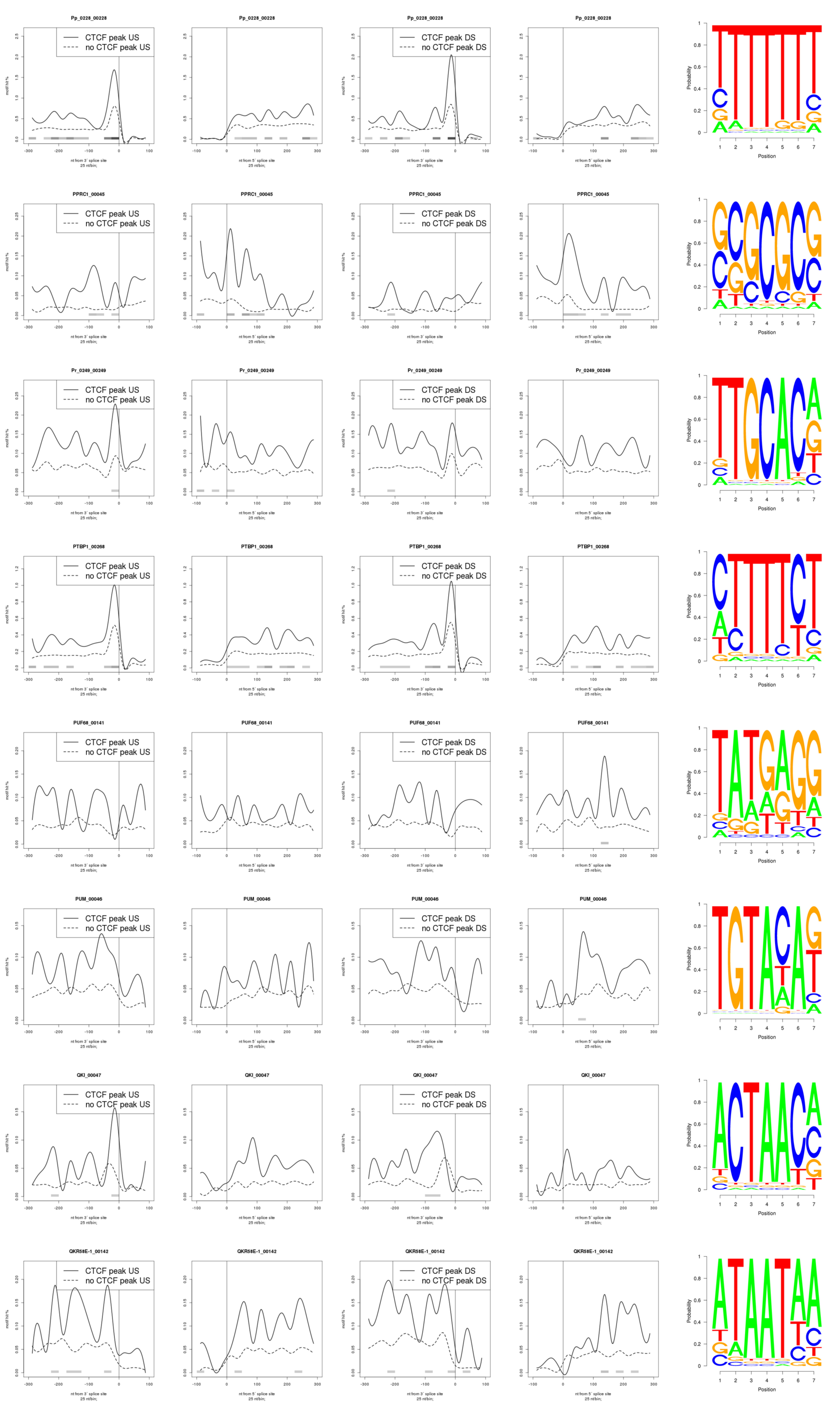

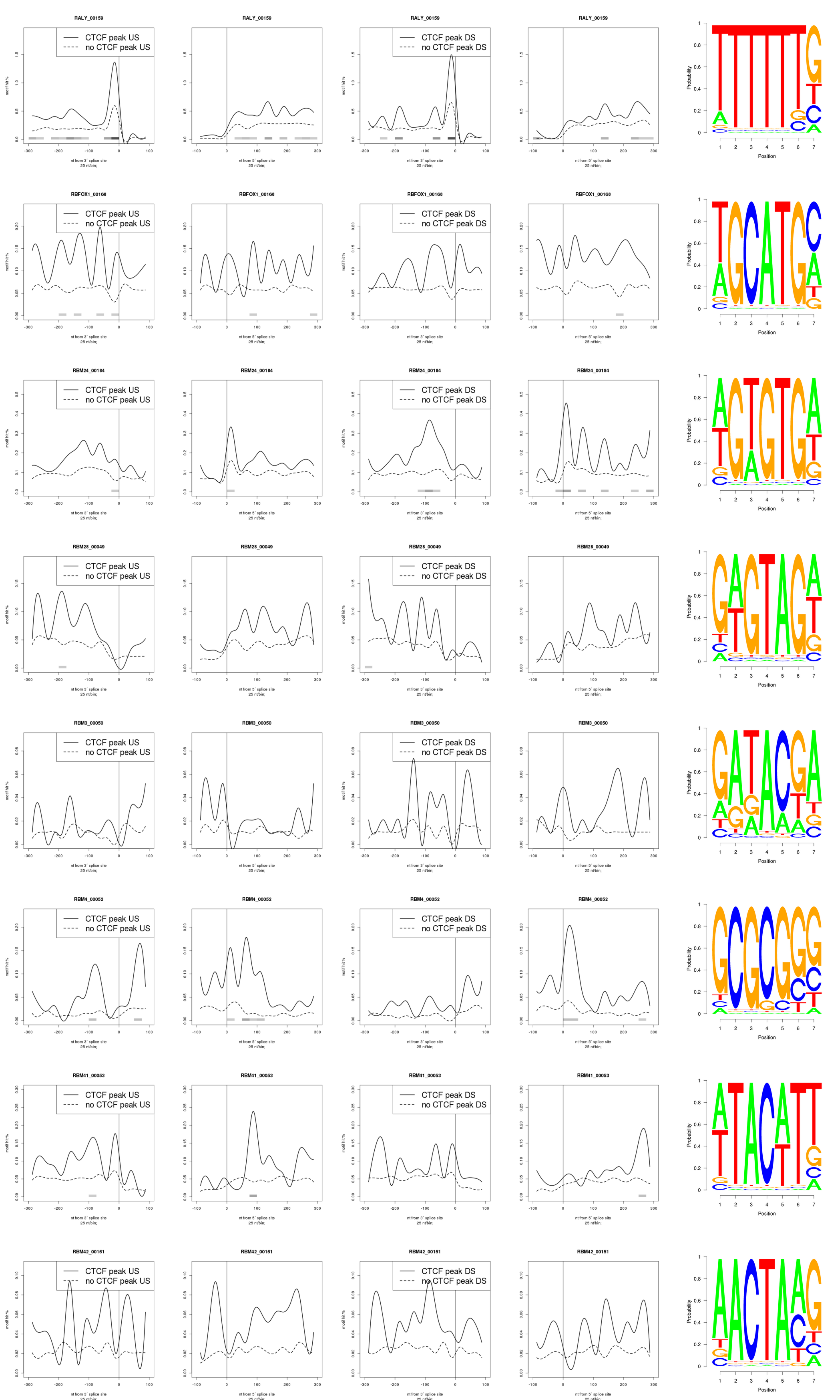

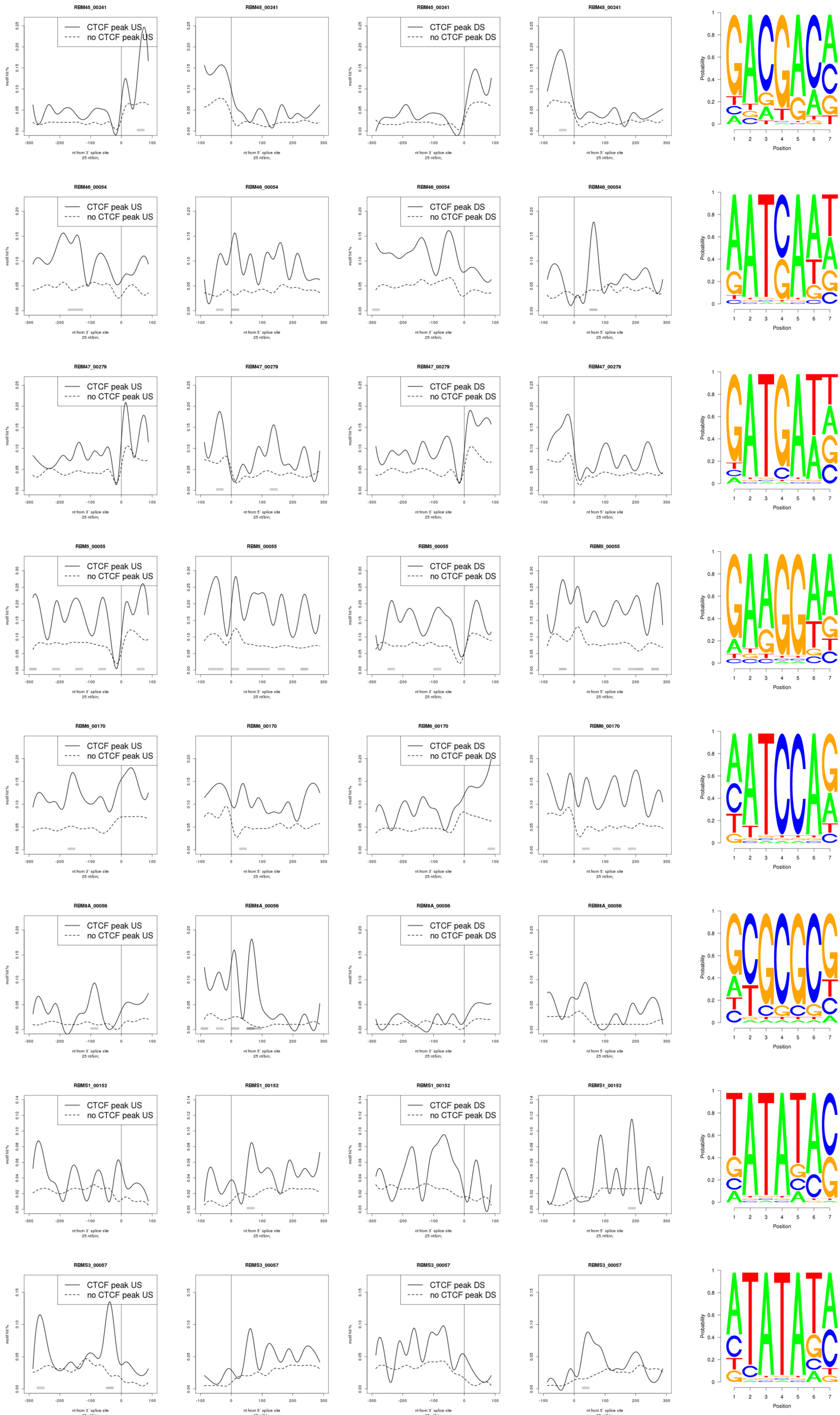

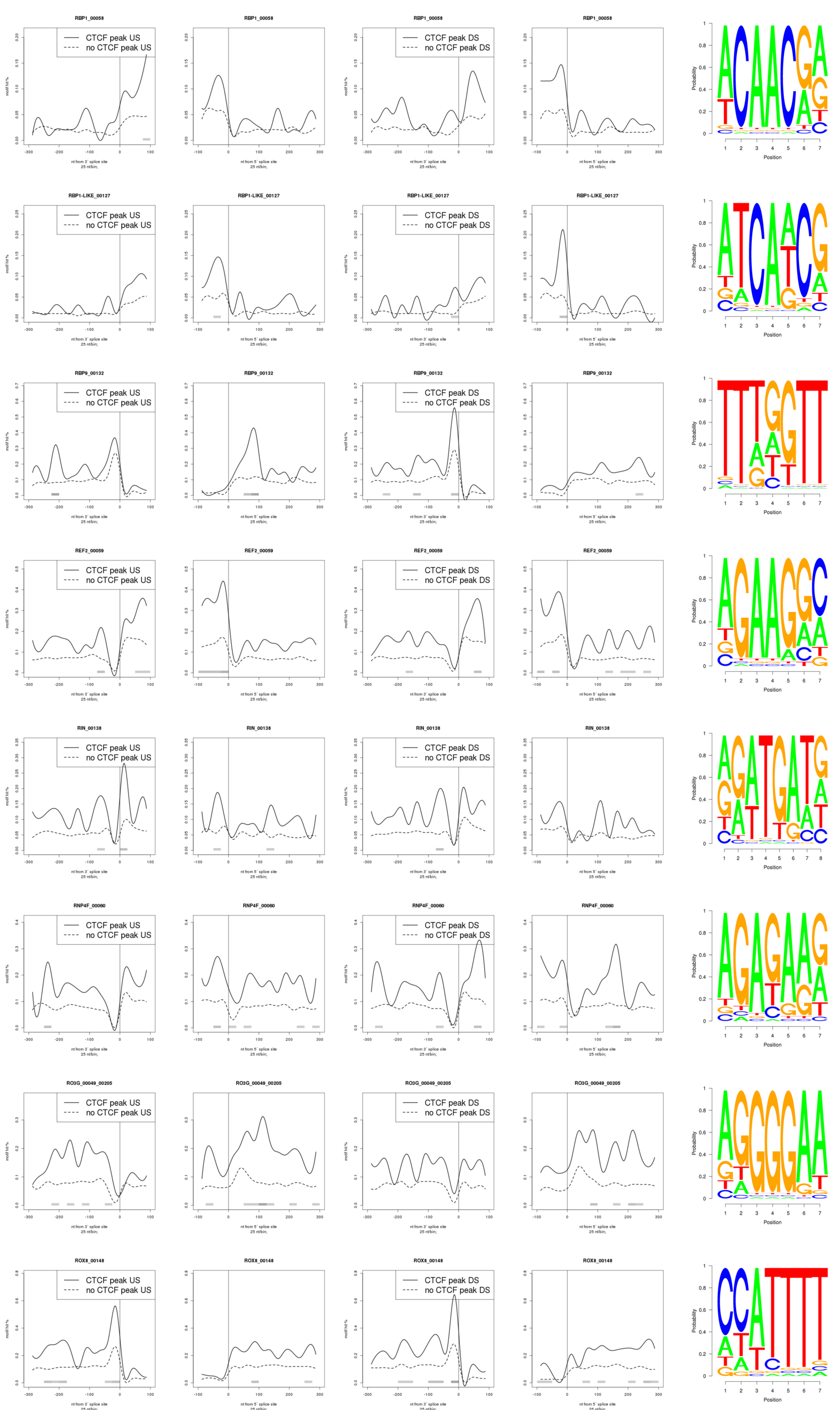

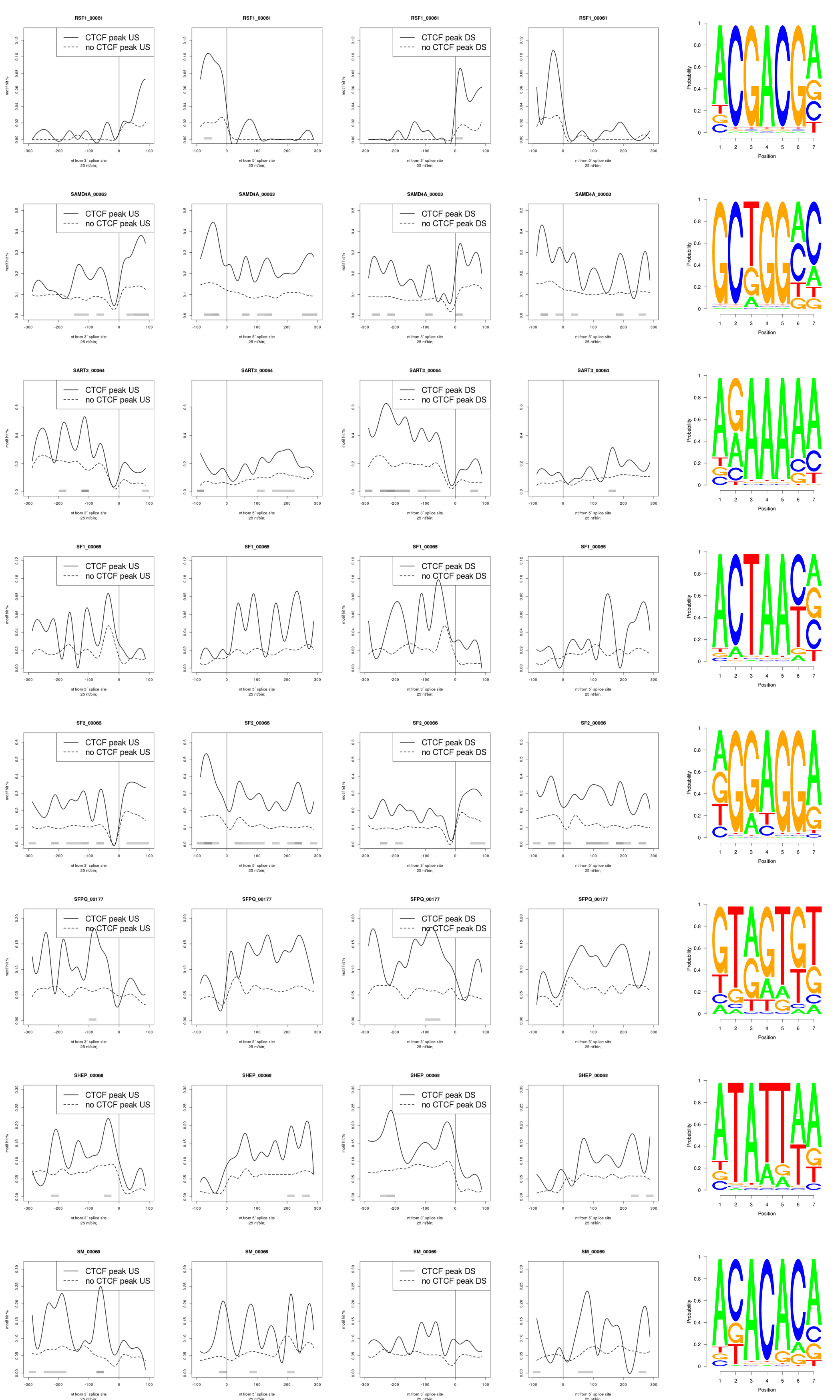

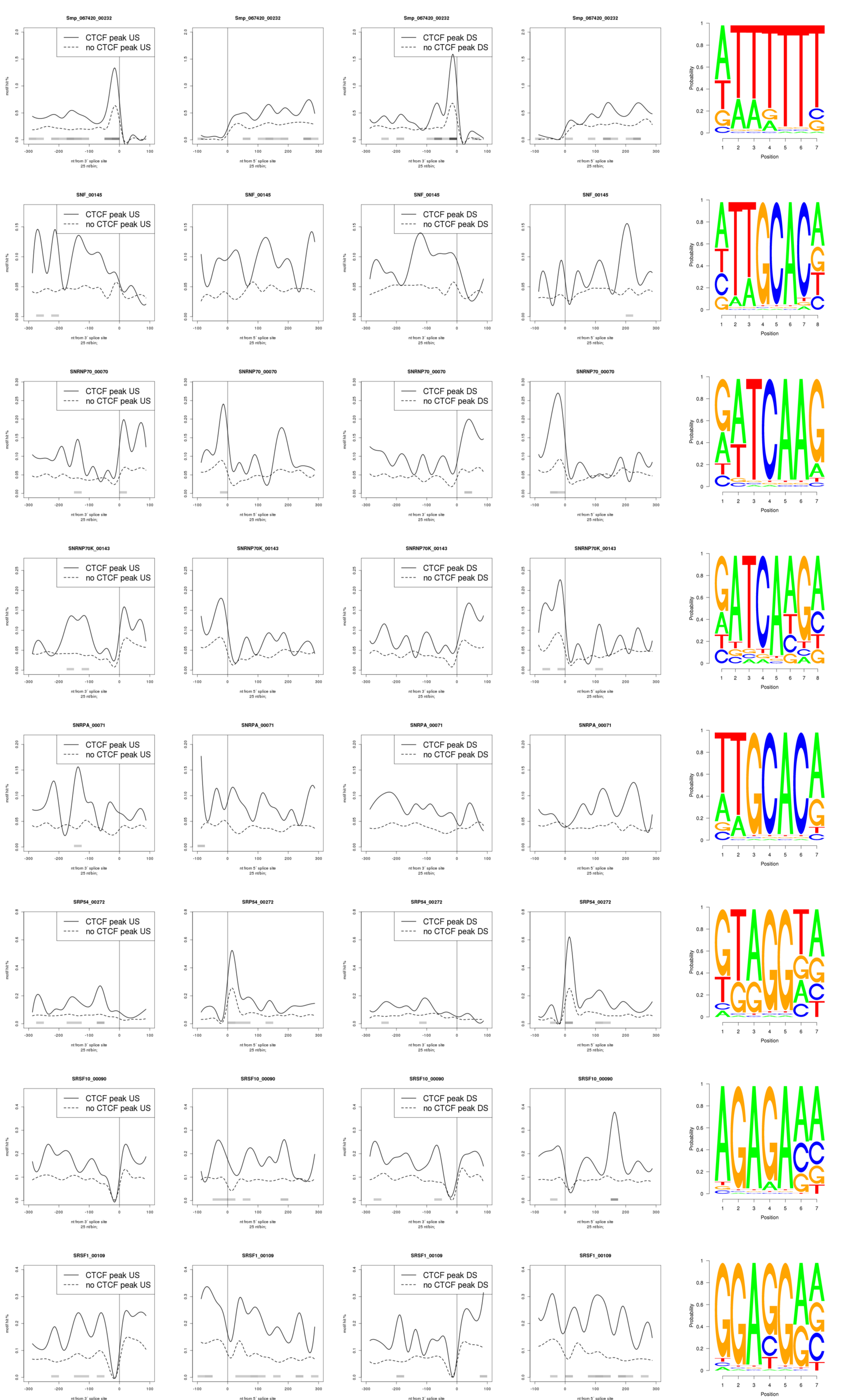

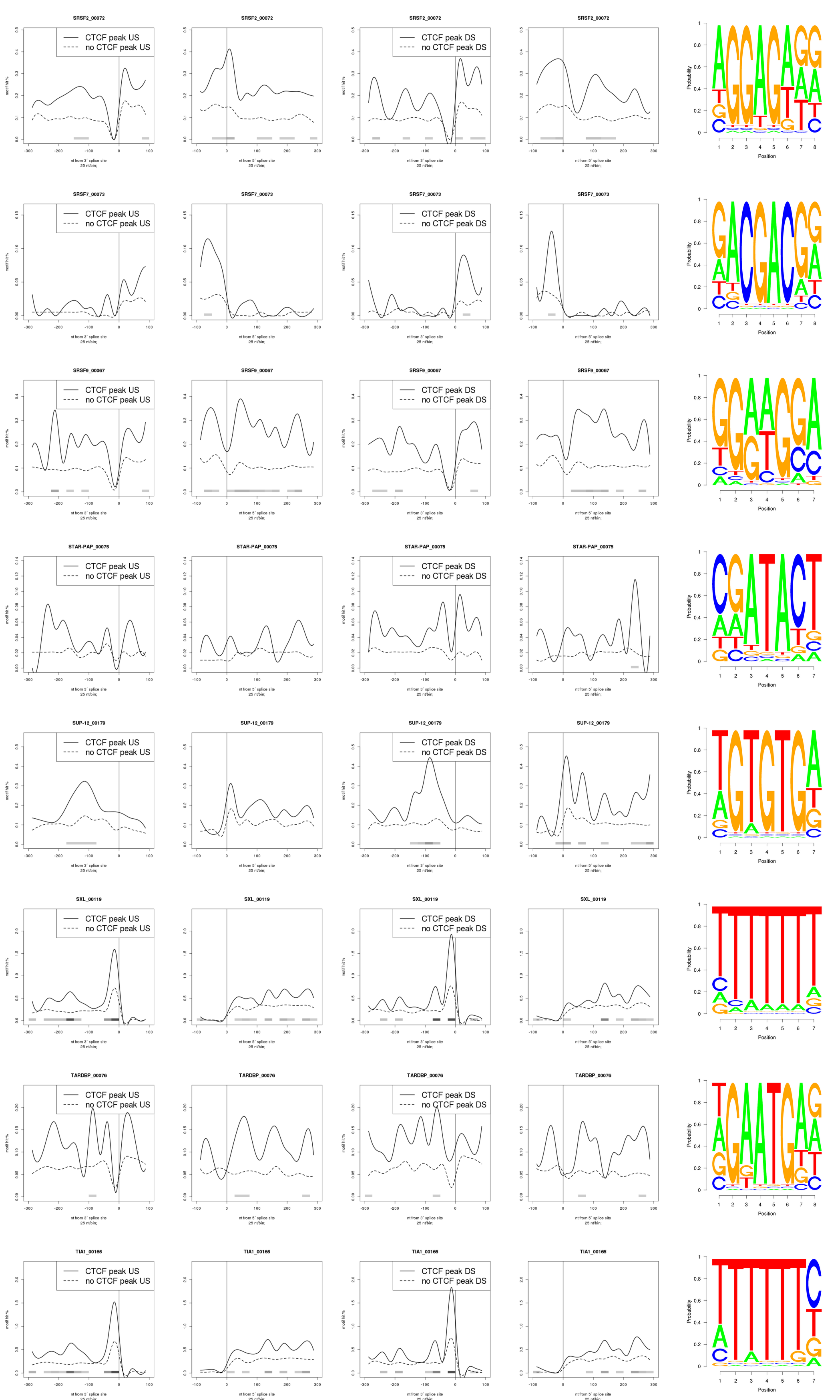

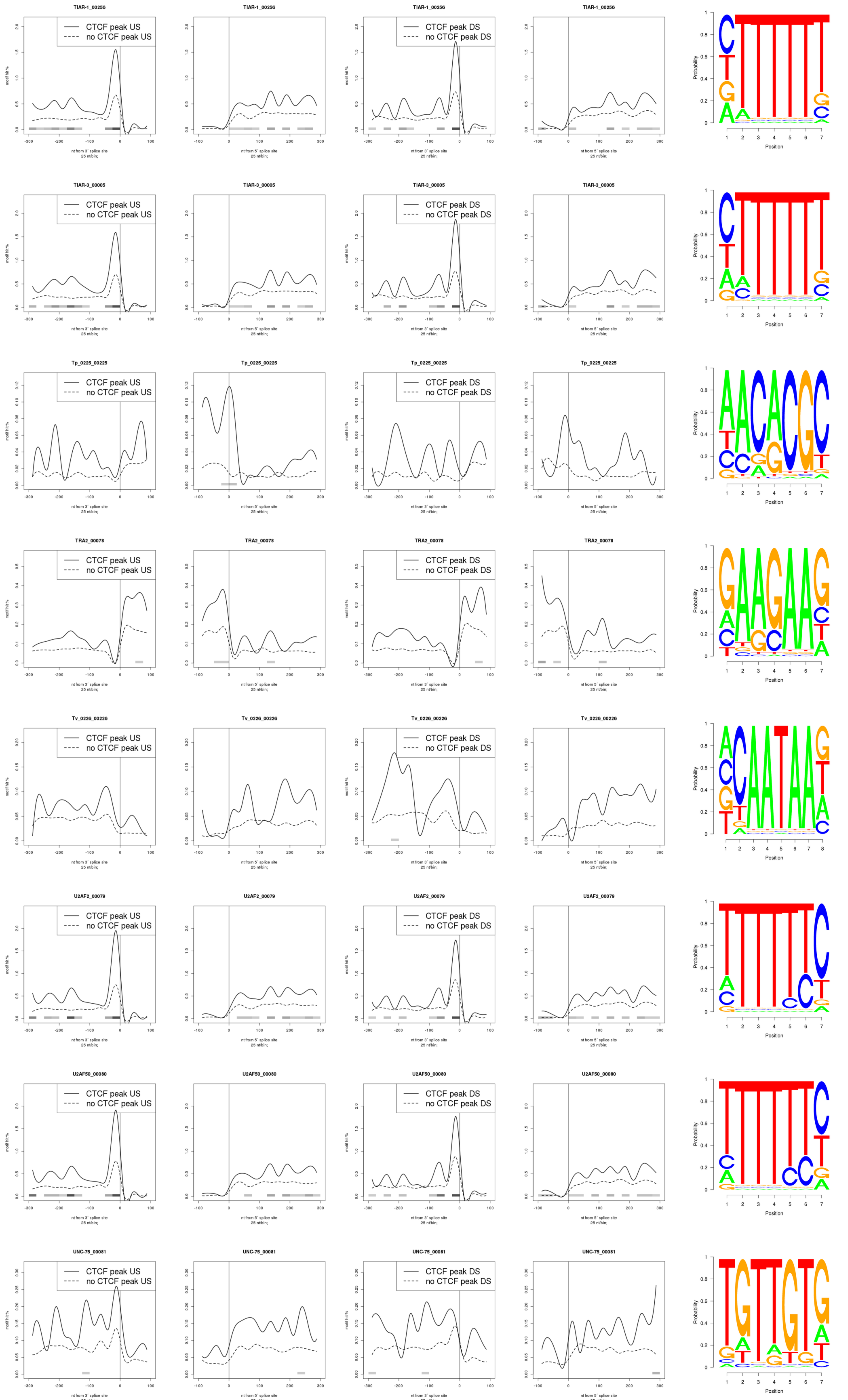

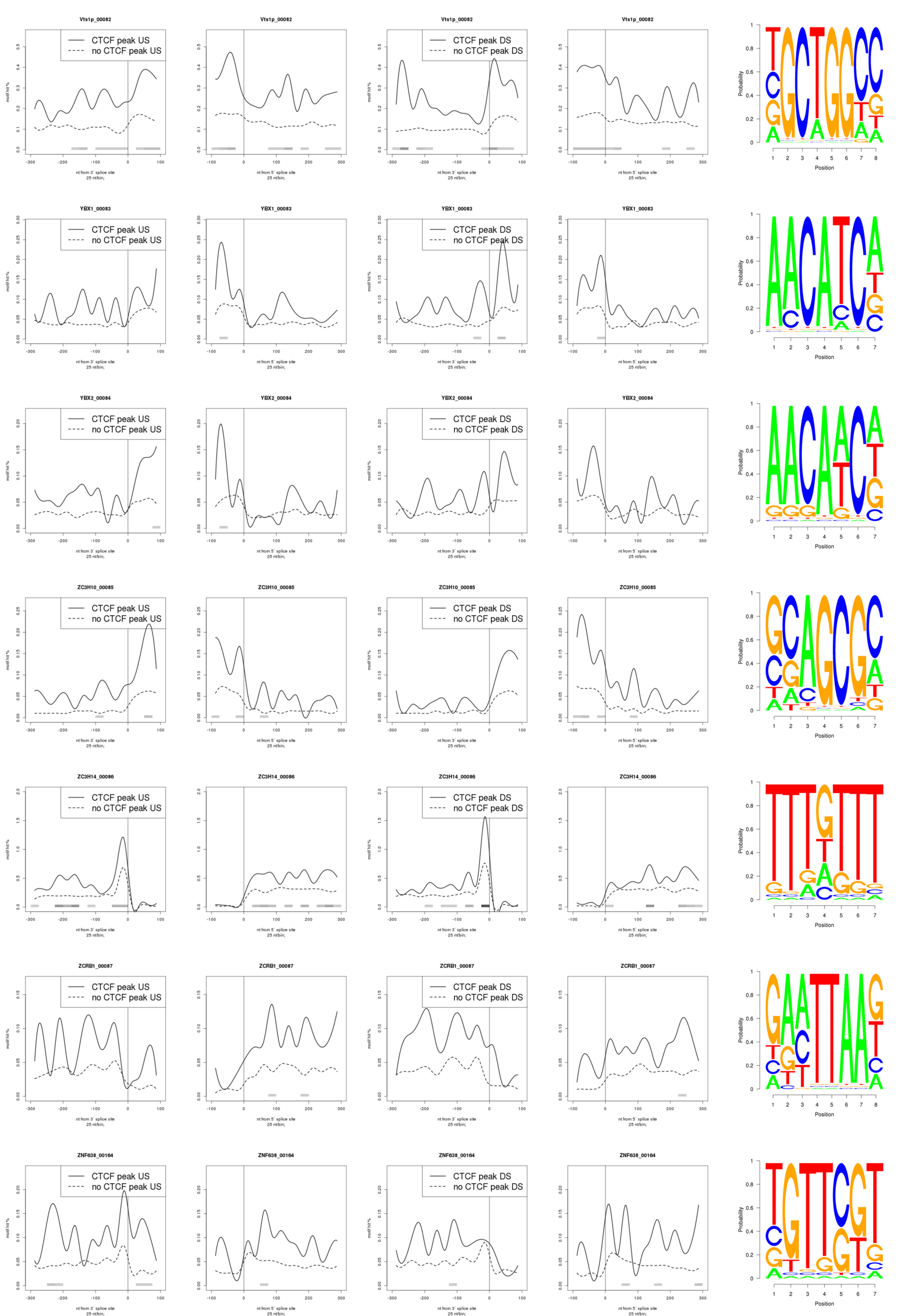

Supplement: S3 File — US = peak center within 1kb upstream of 3' ss. DS = peak center within 1kb downstream of 5'SS, “weak” means the lowest half of 3'SS strengths for profiles centered at the 3' ss, and the lowest half of 5'SS strengths for profiles centered at the 5' ss. Test vs. control P-values/bin are as shown in Fig 1B, with the lightest shade of grey corresponding to P-value < 0.01. (PDF) [file pone.0132448.s012.pdf]

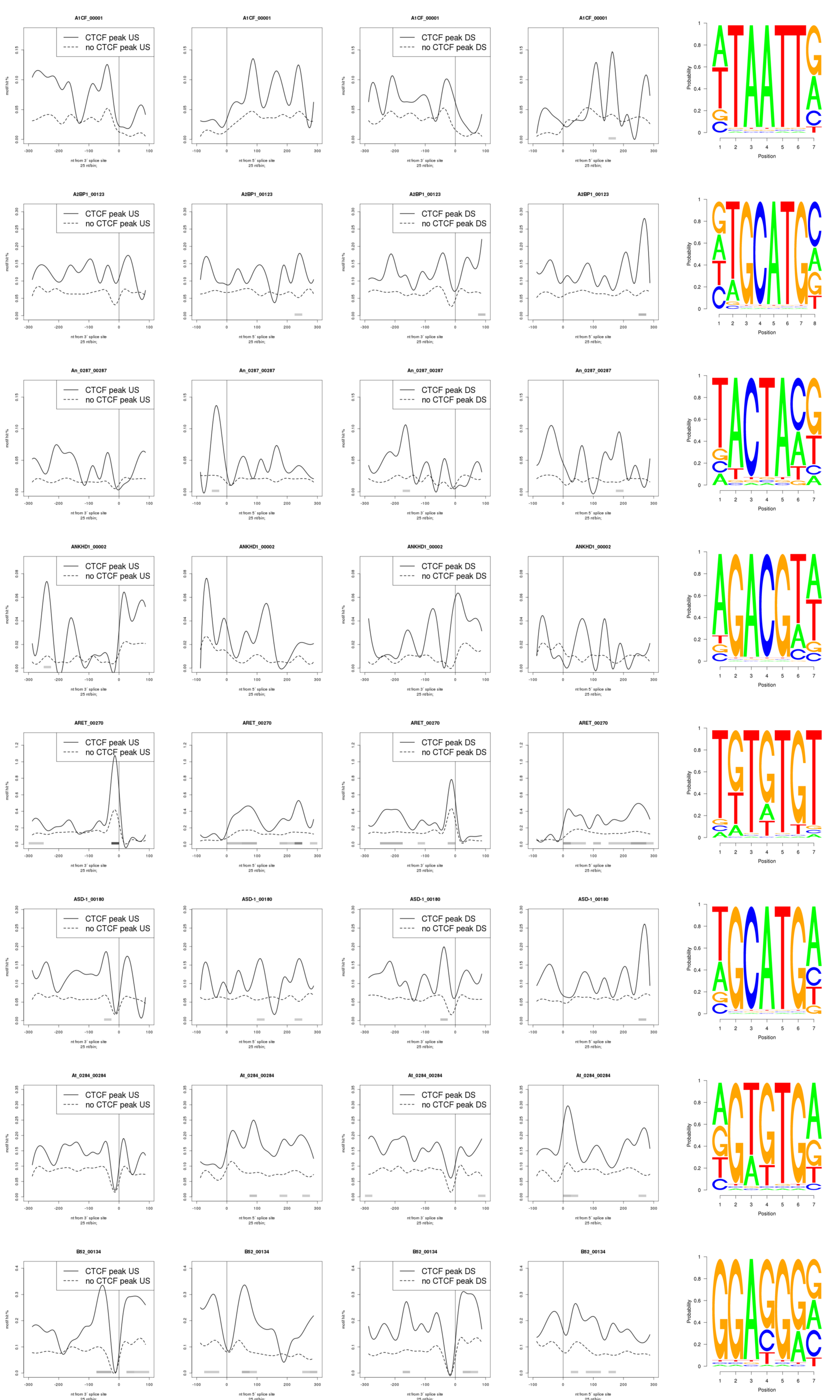

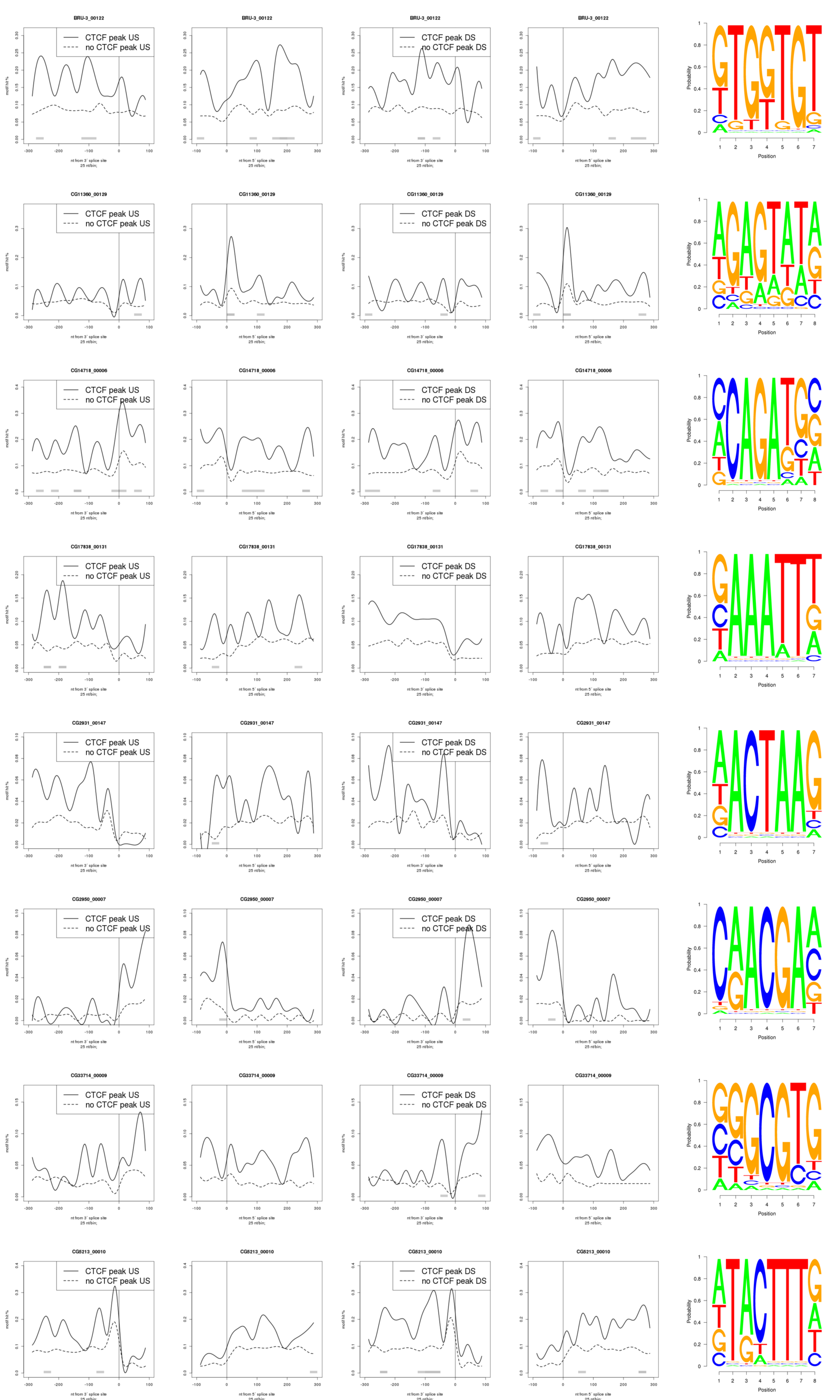

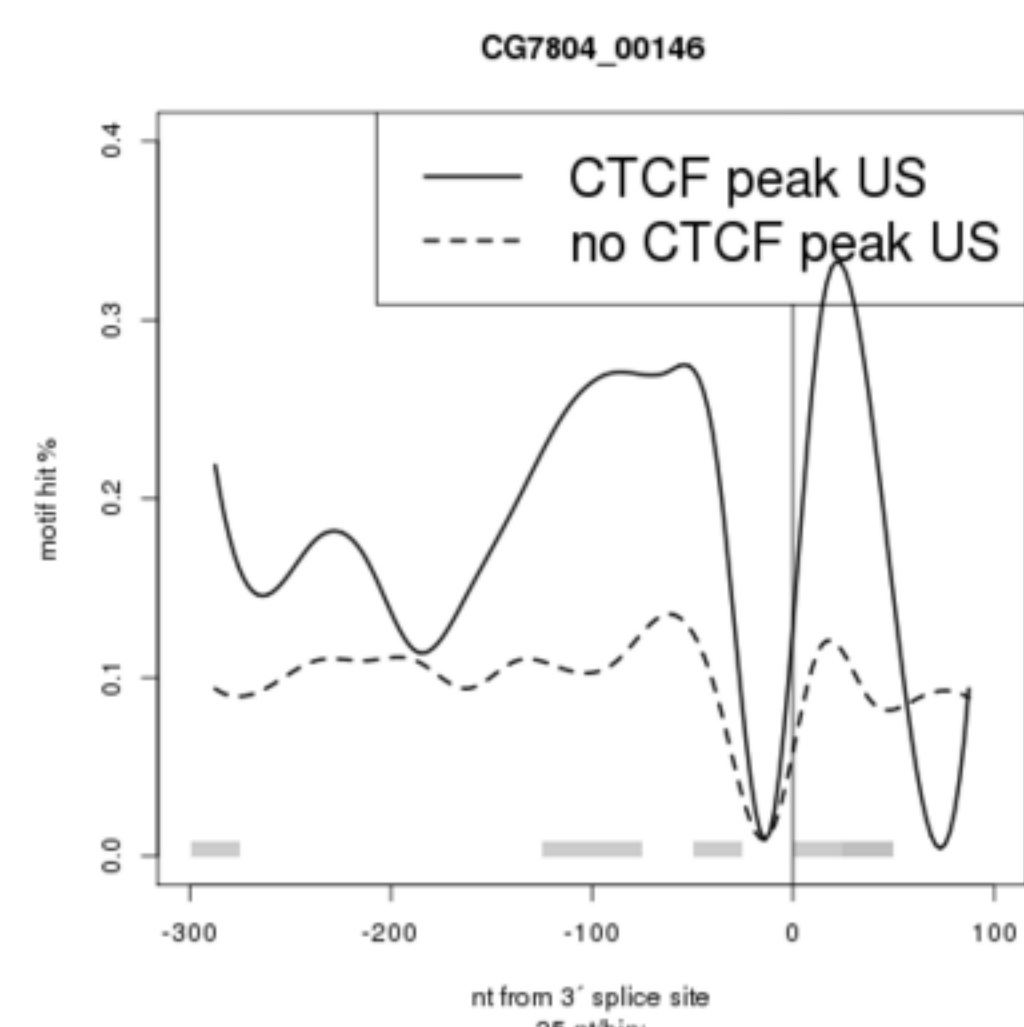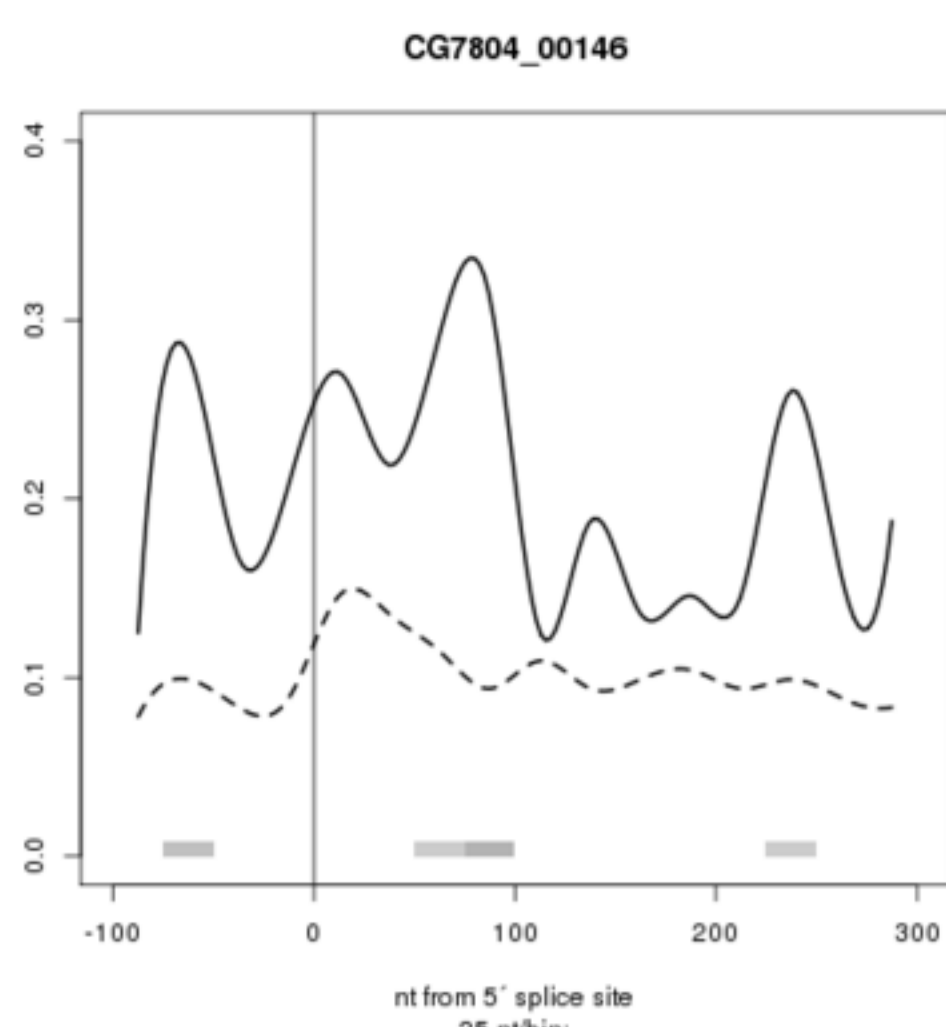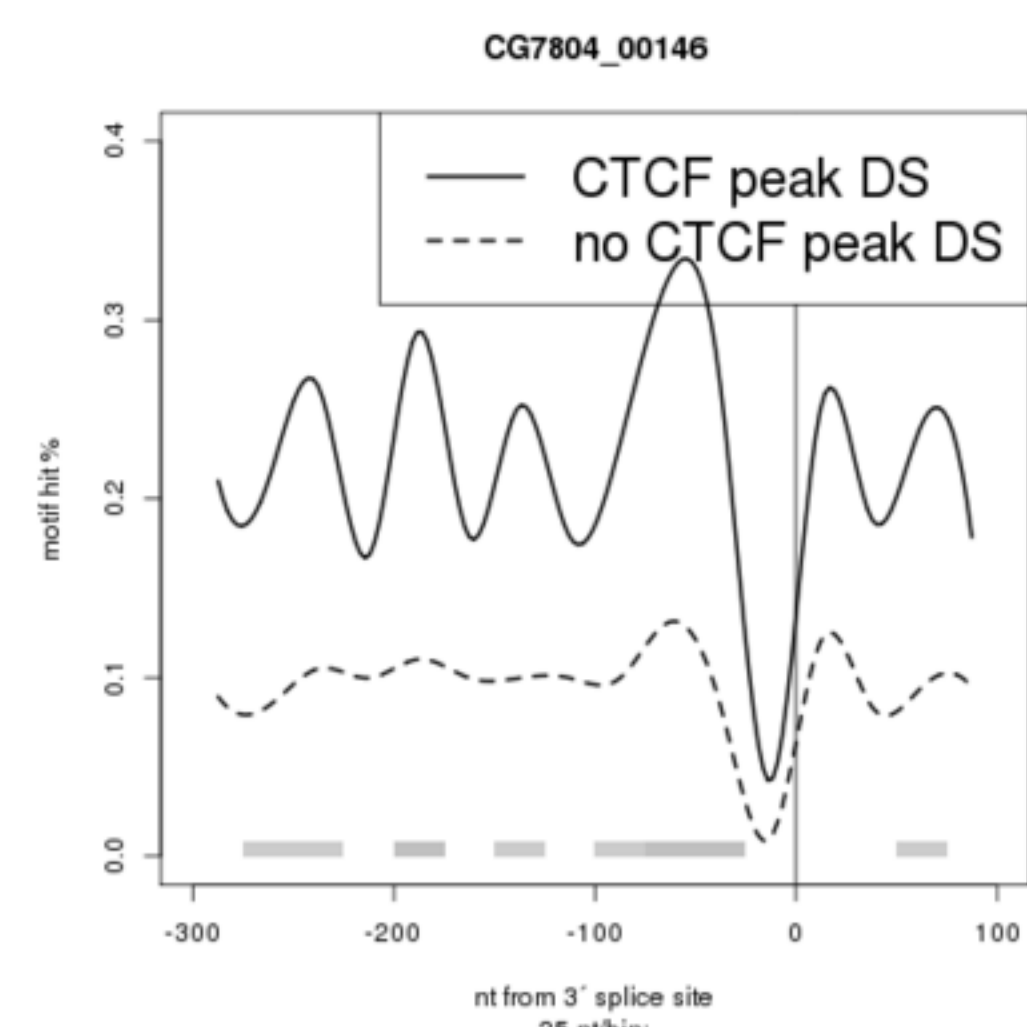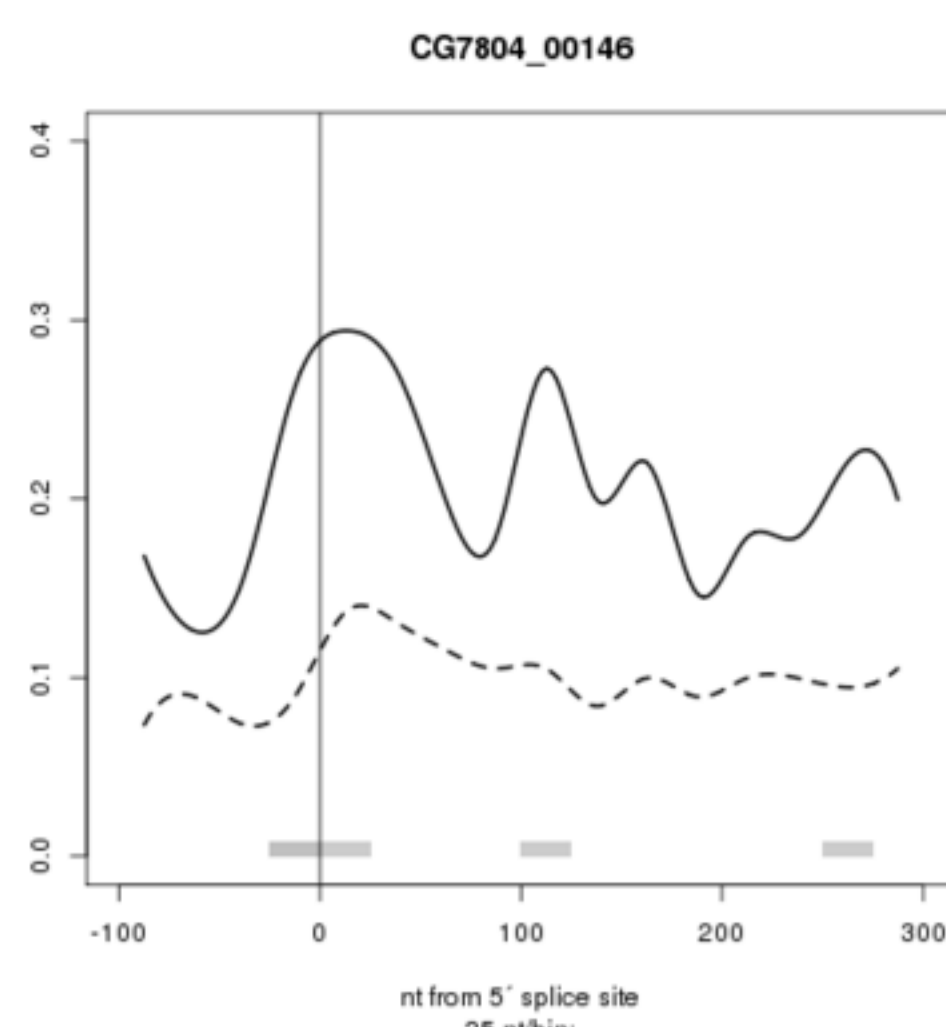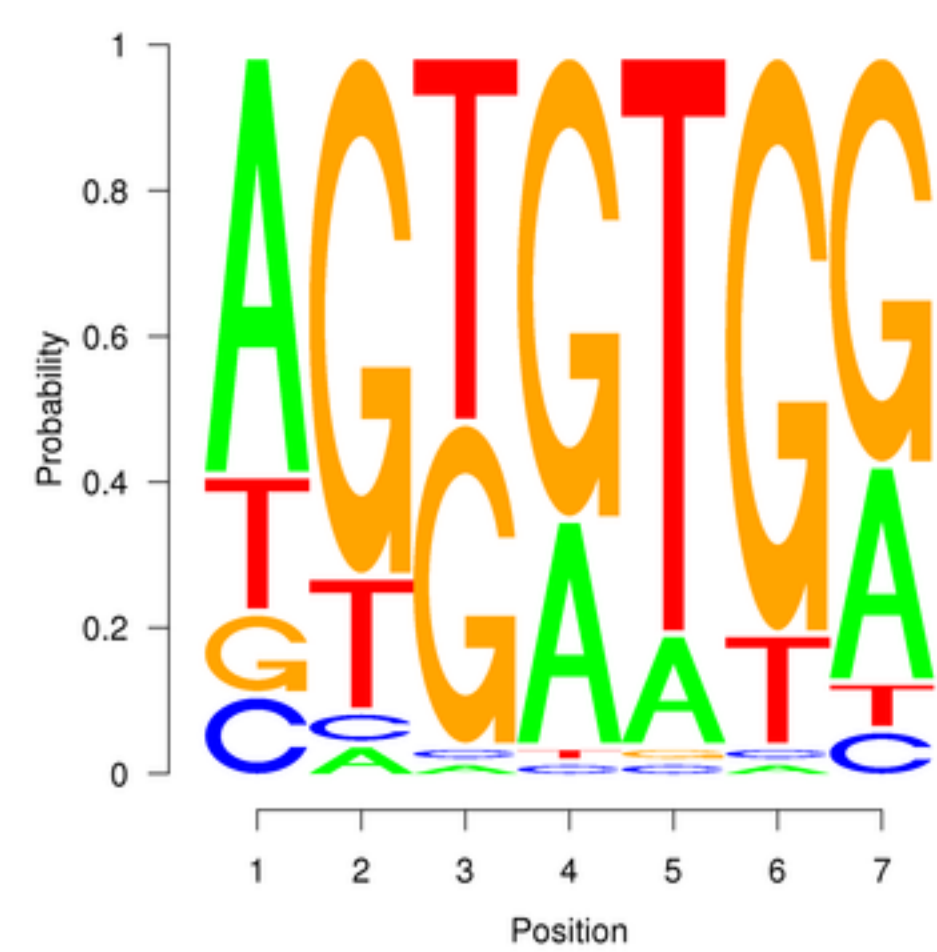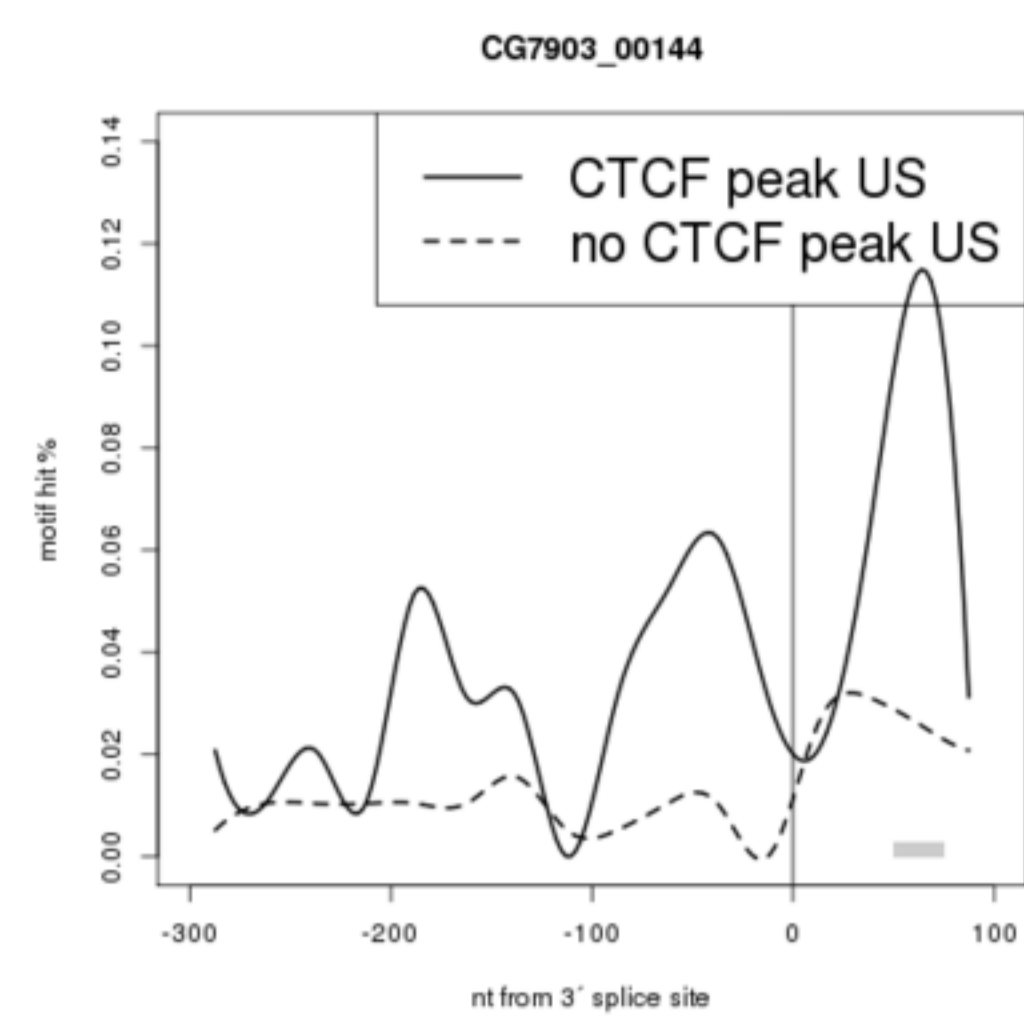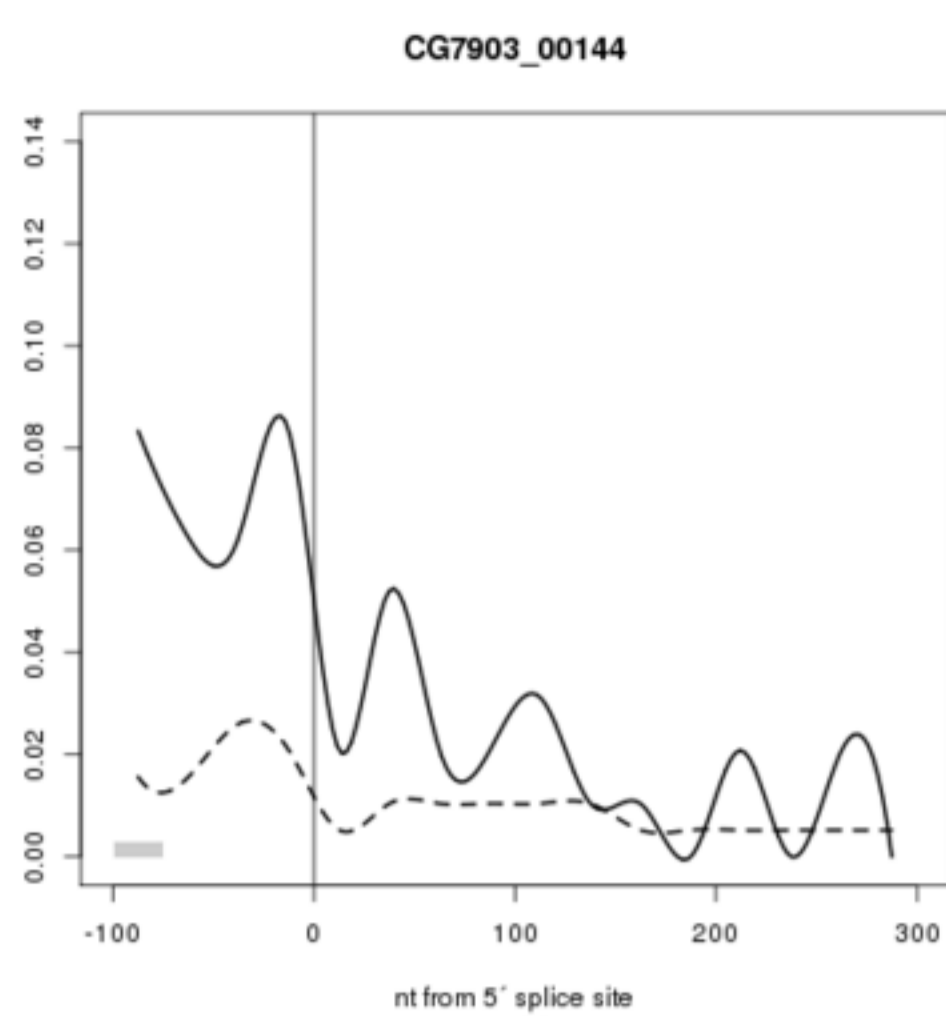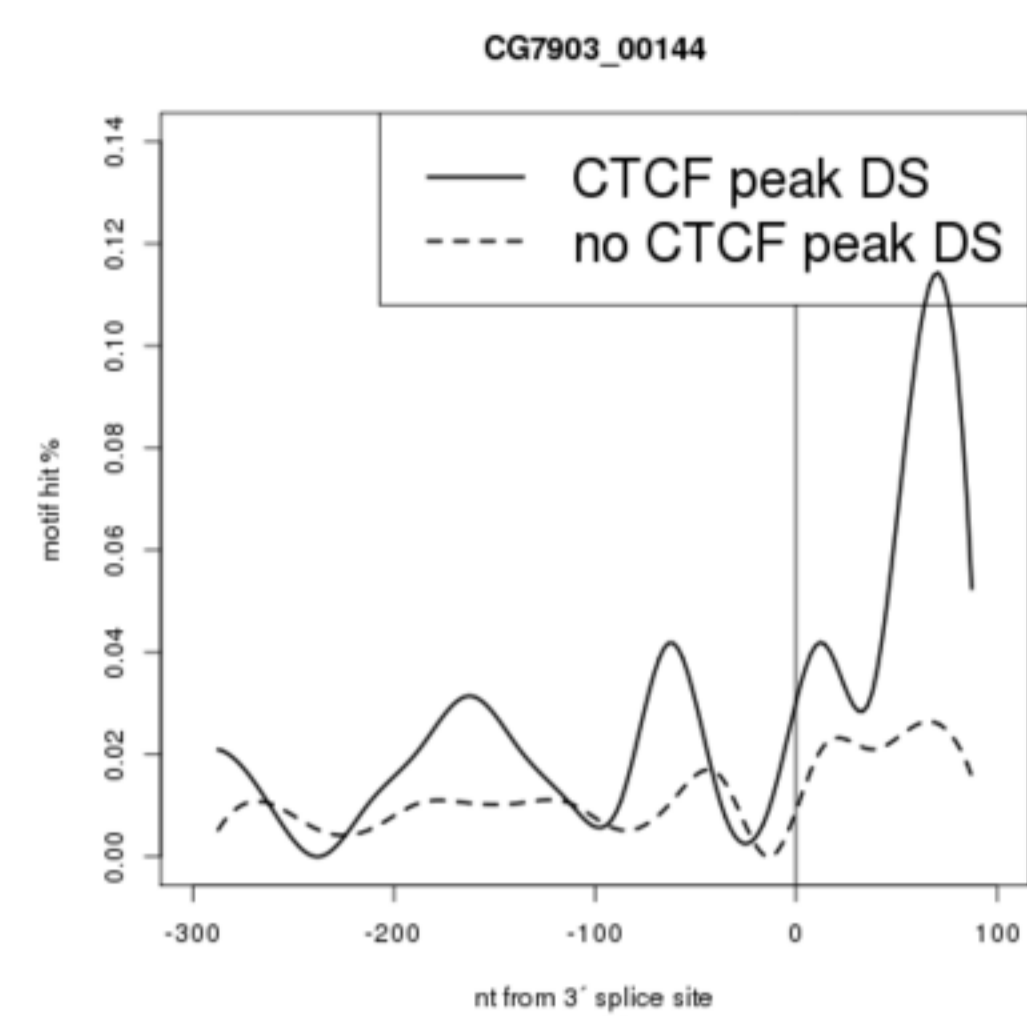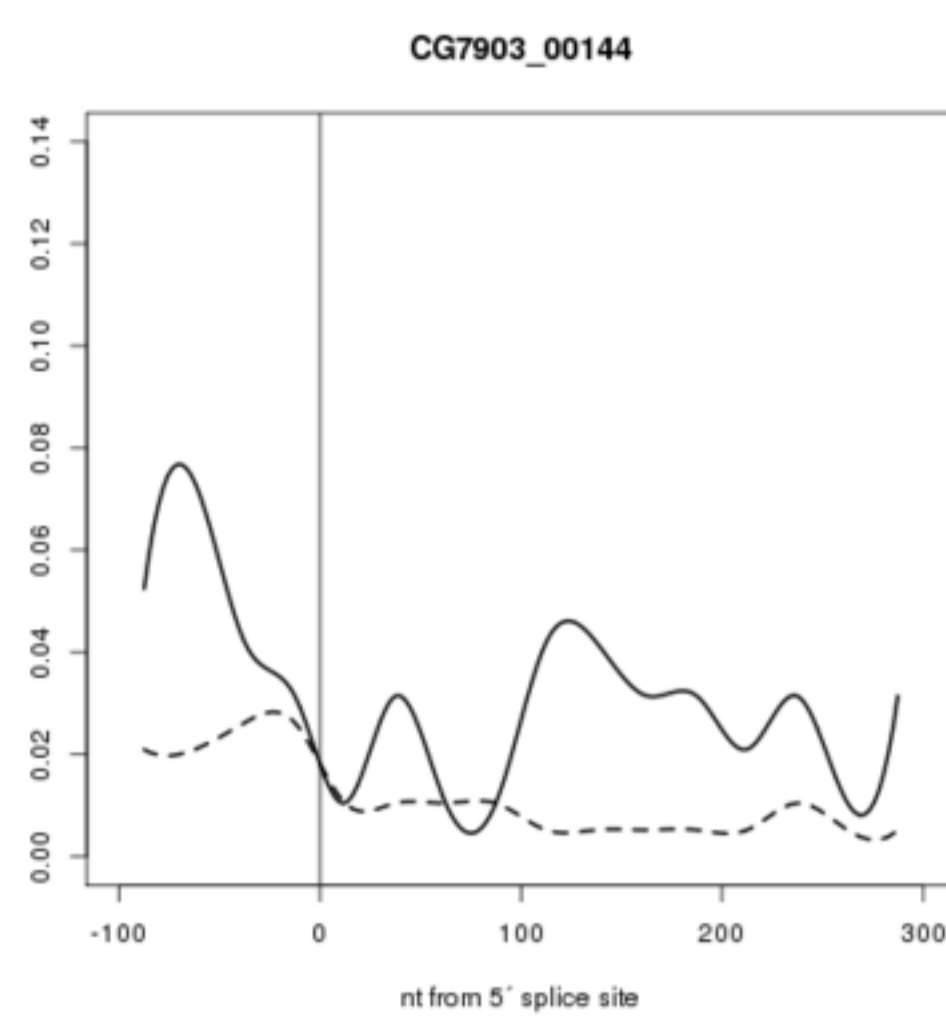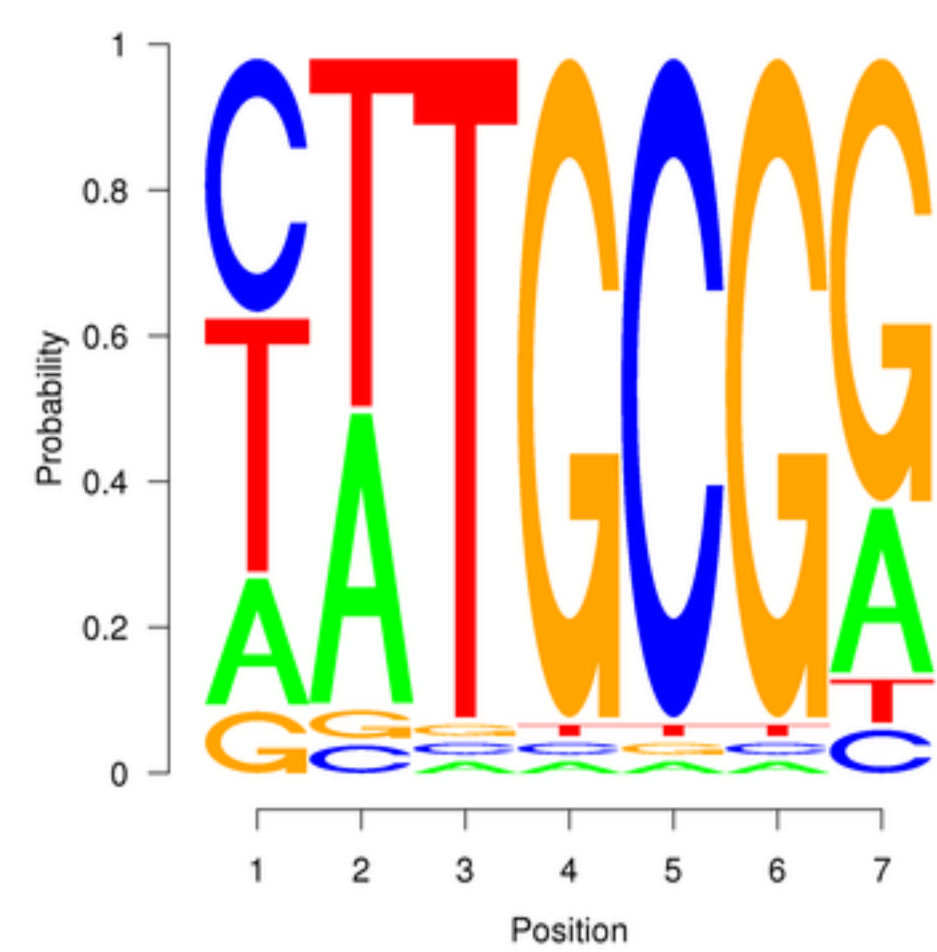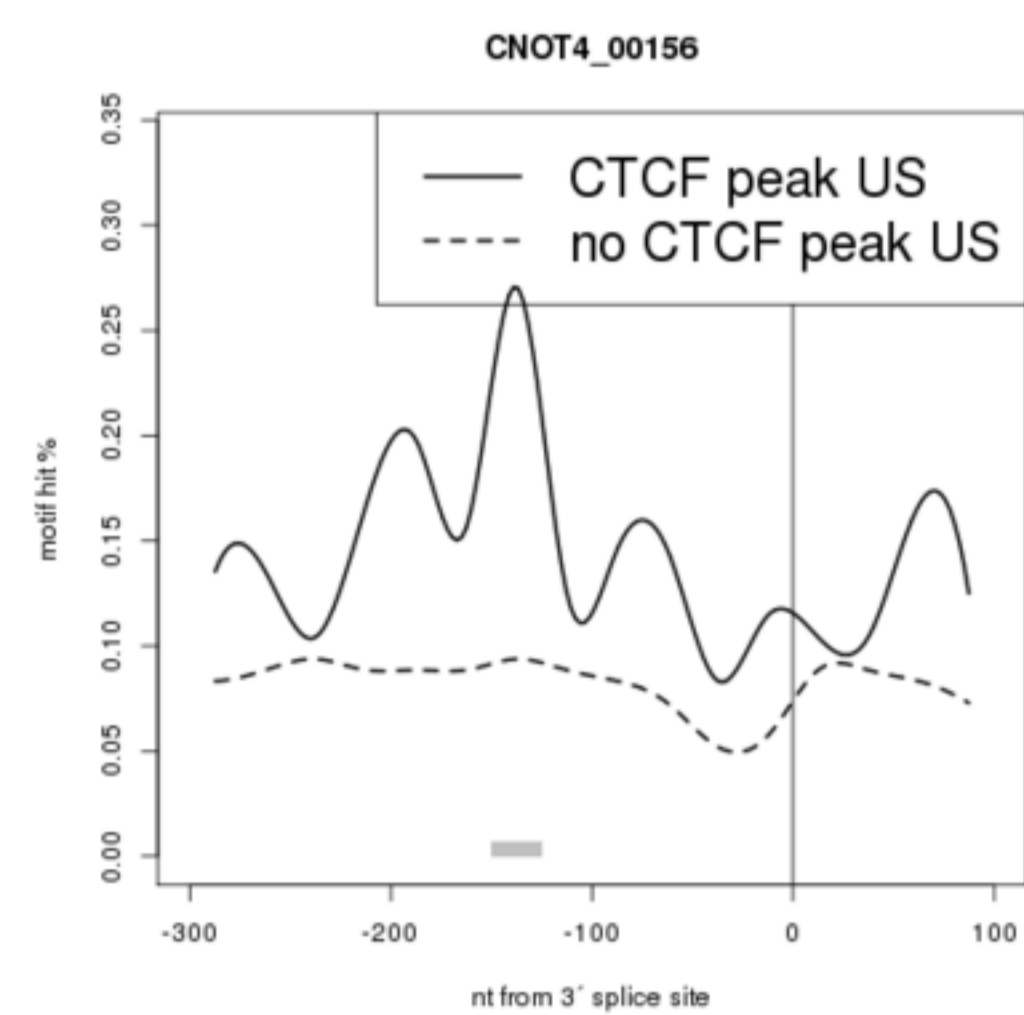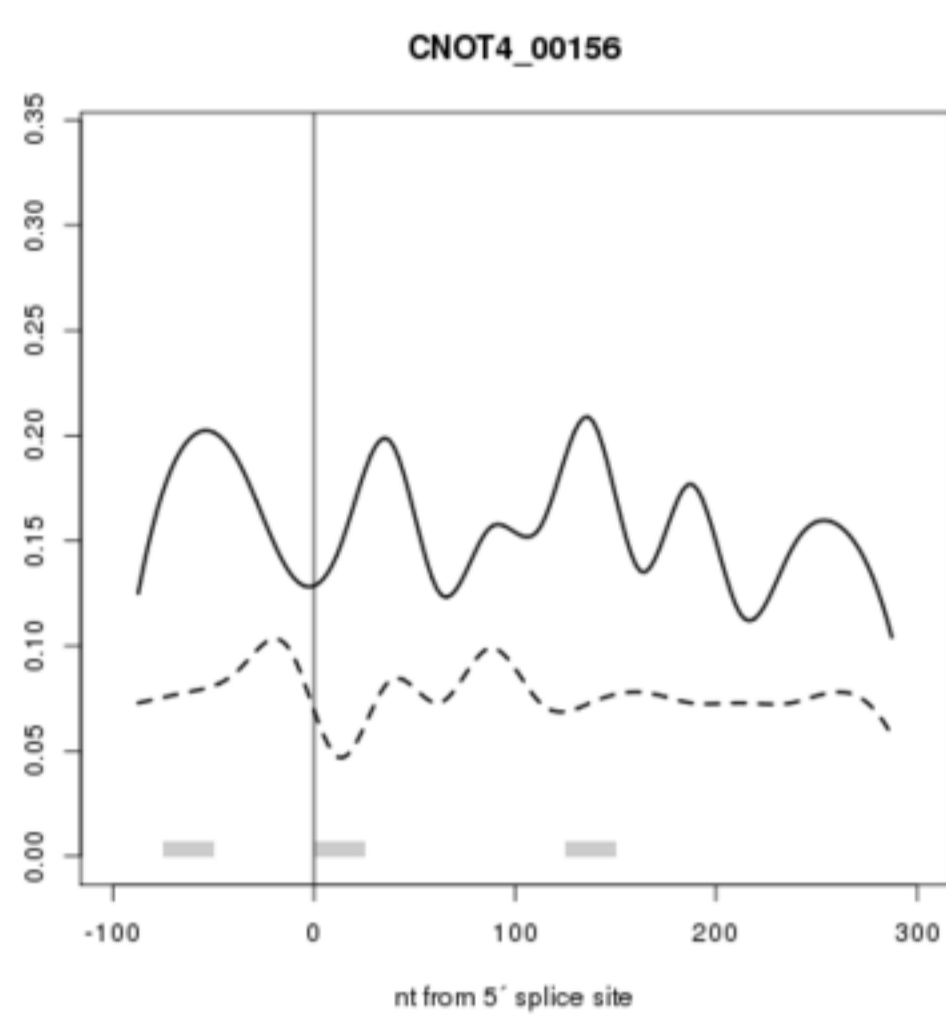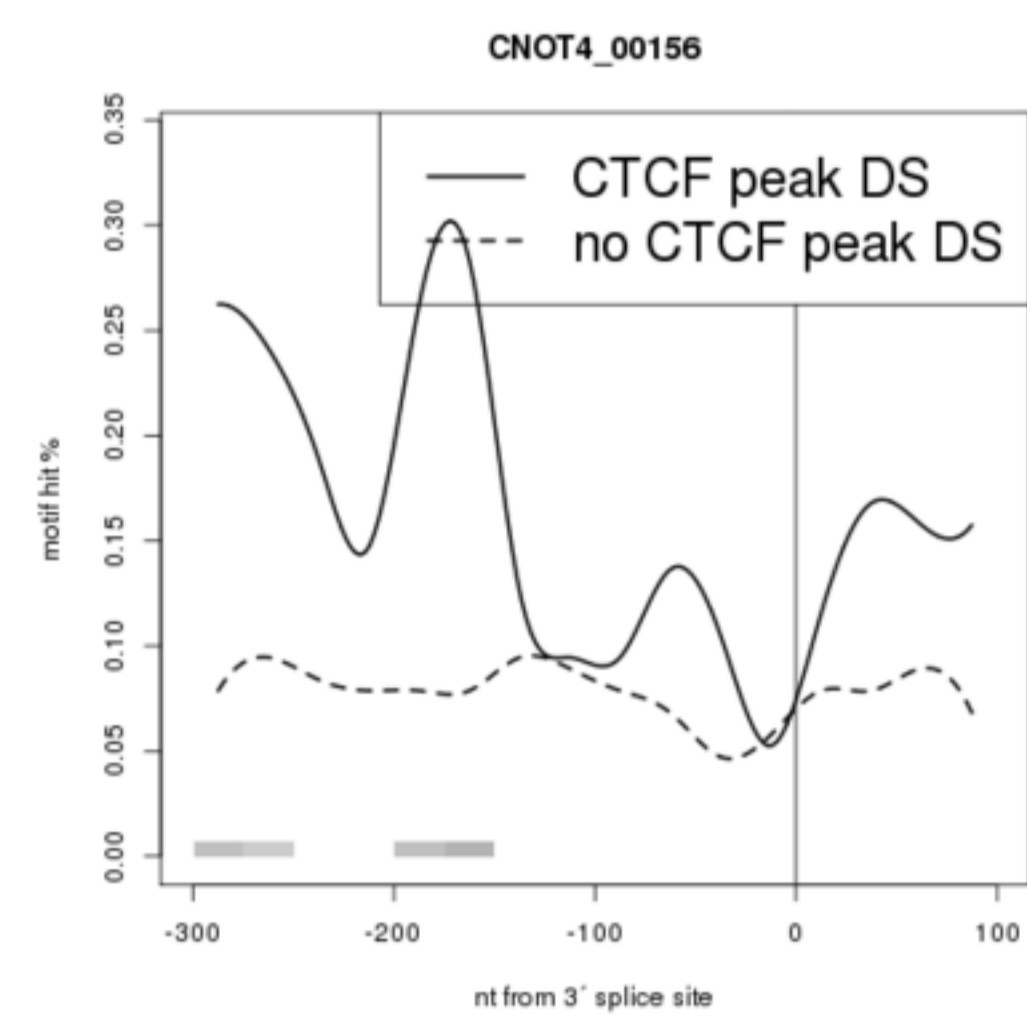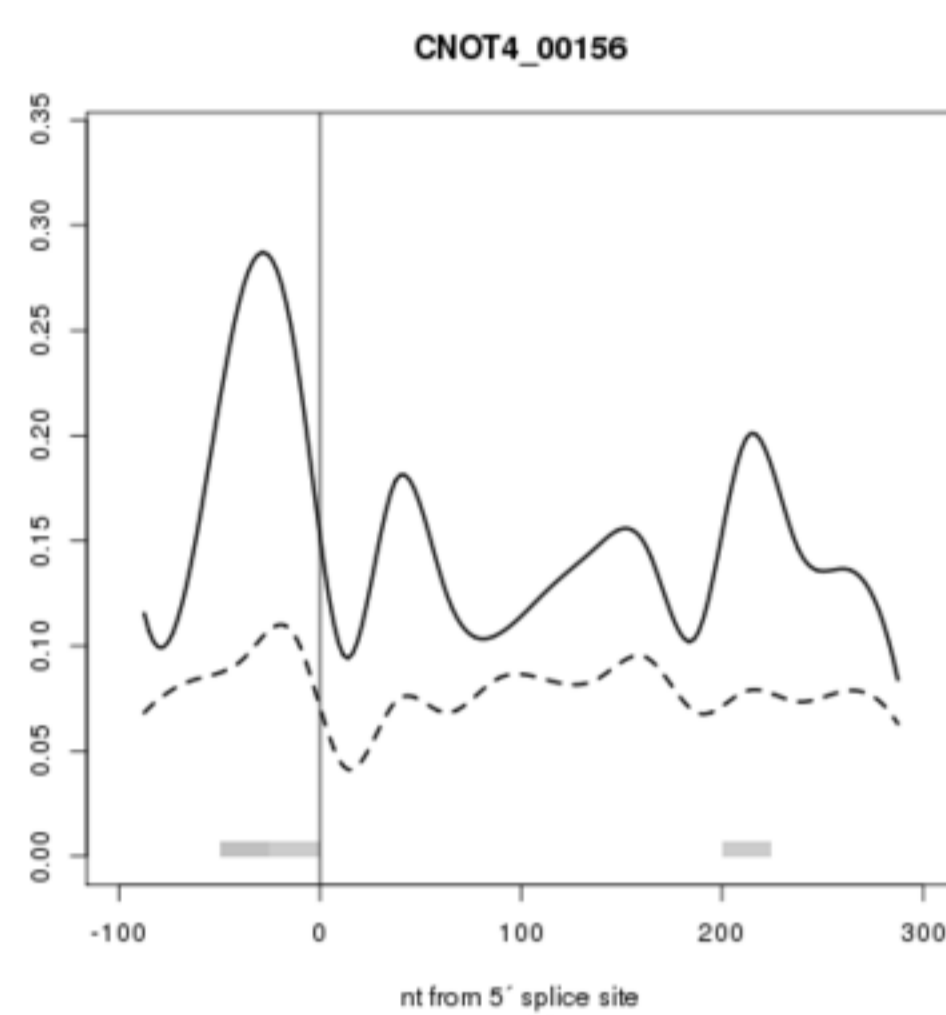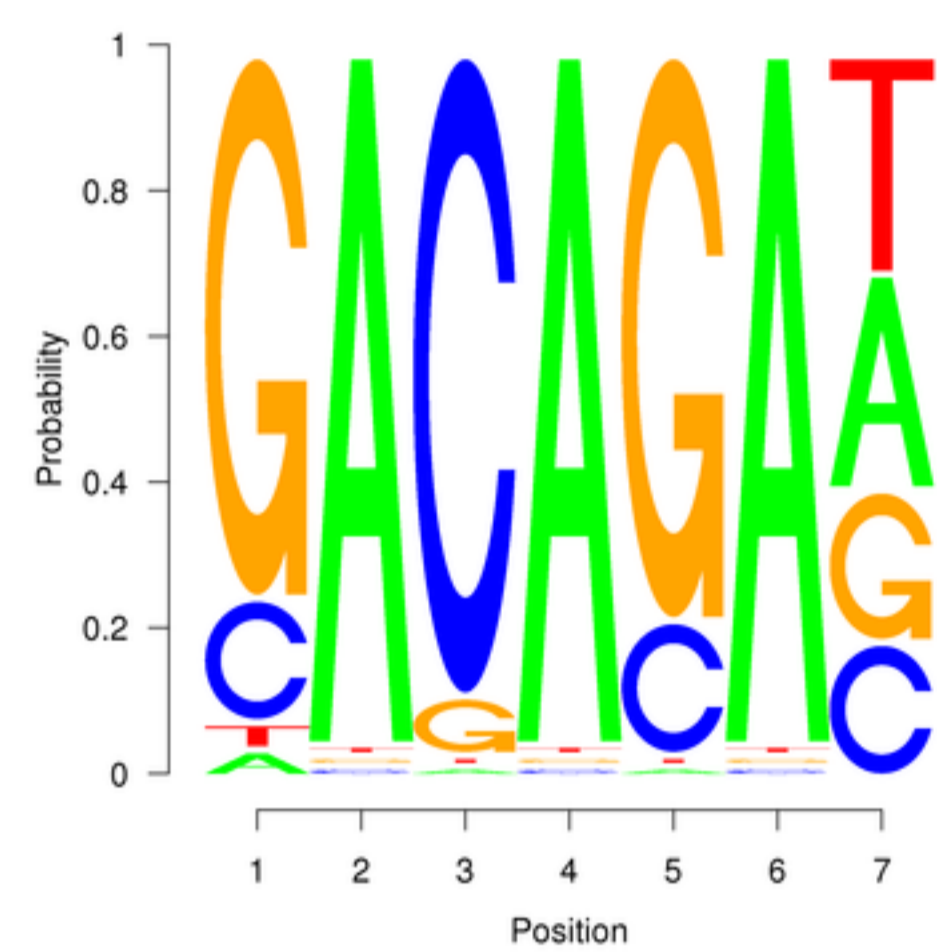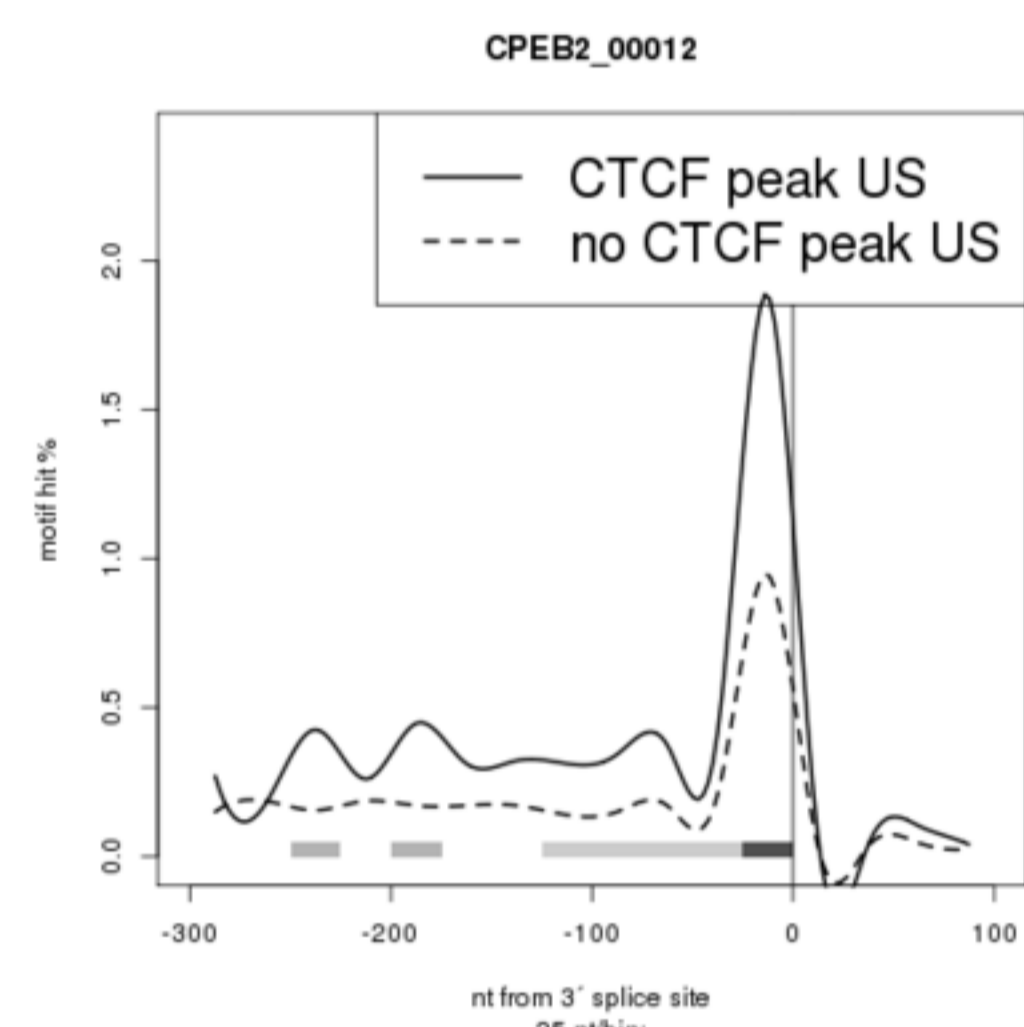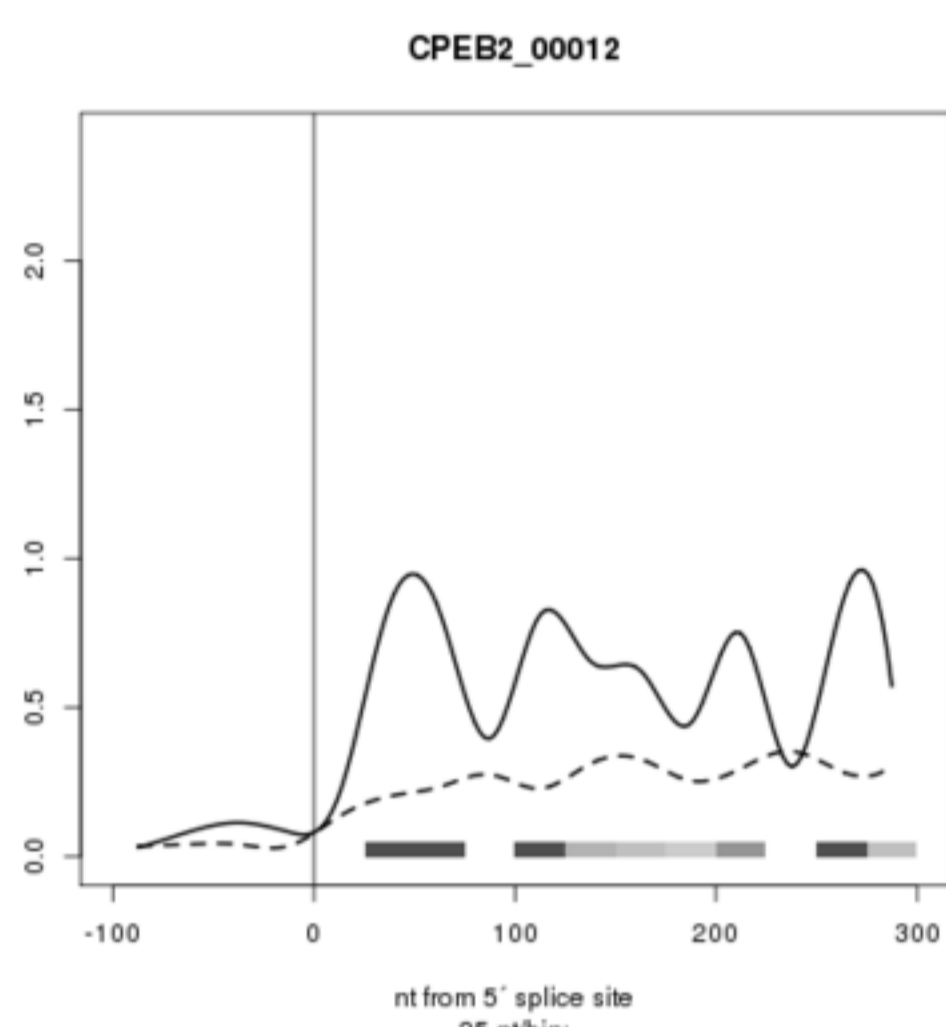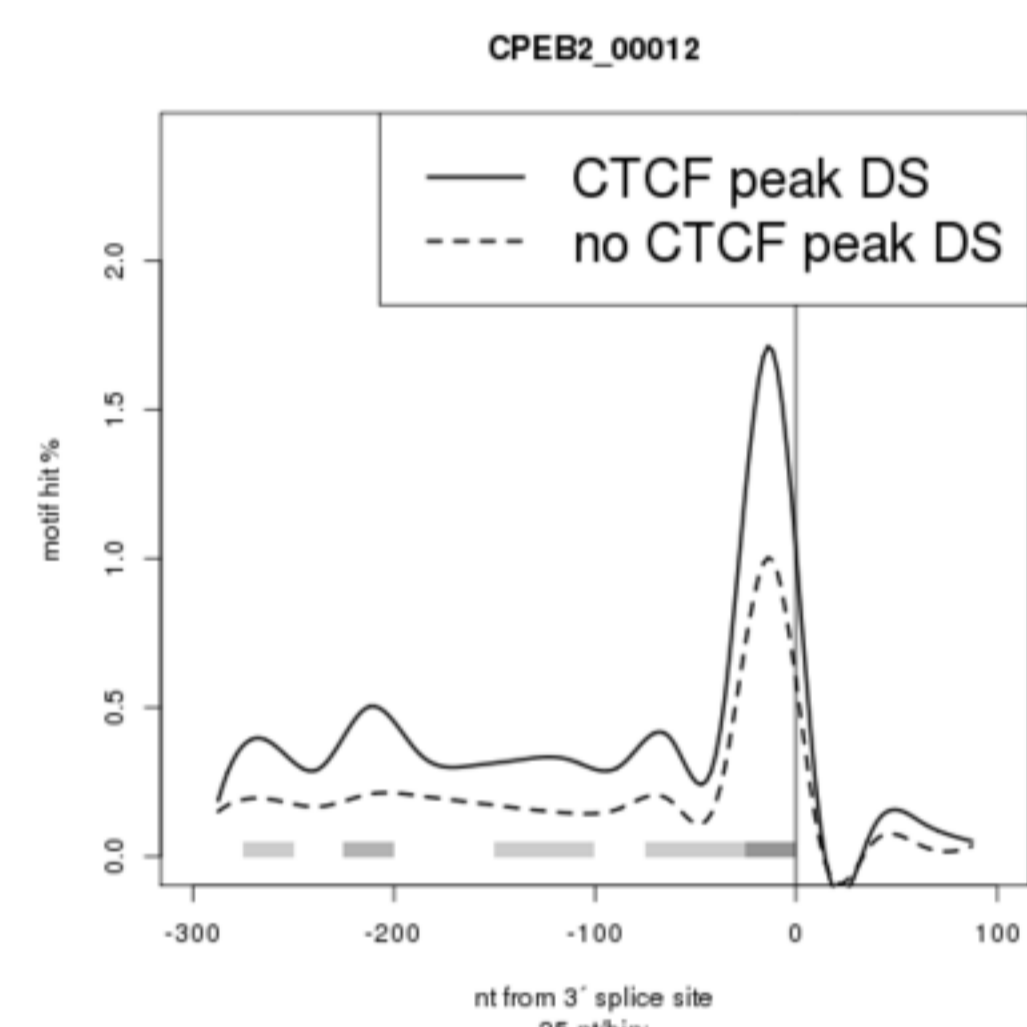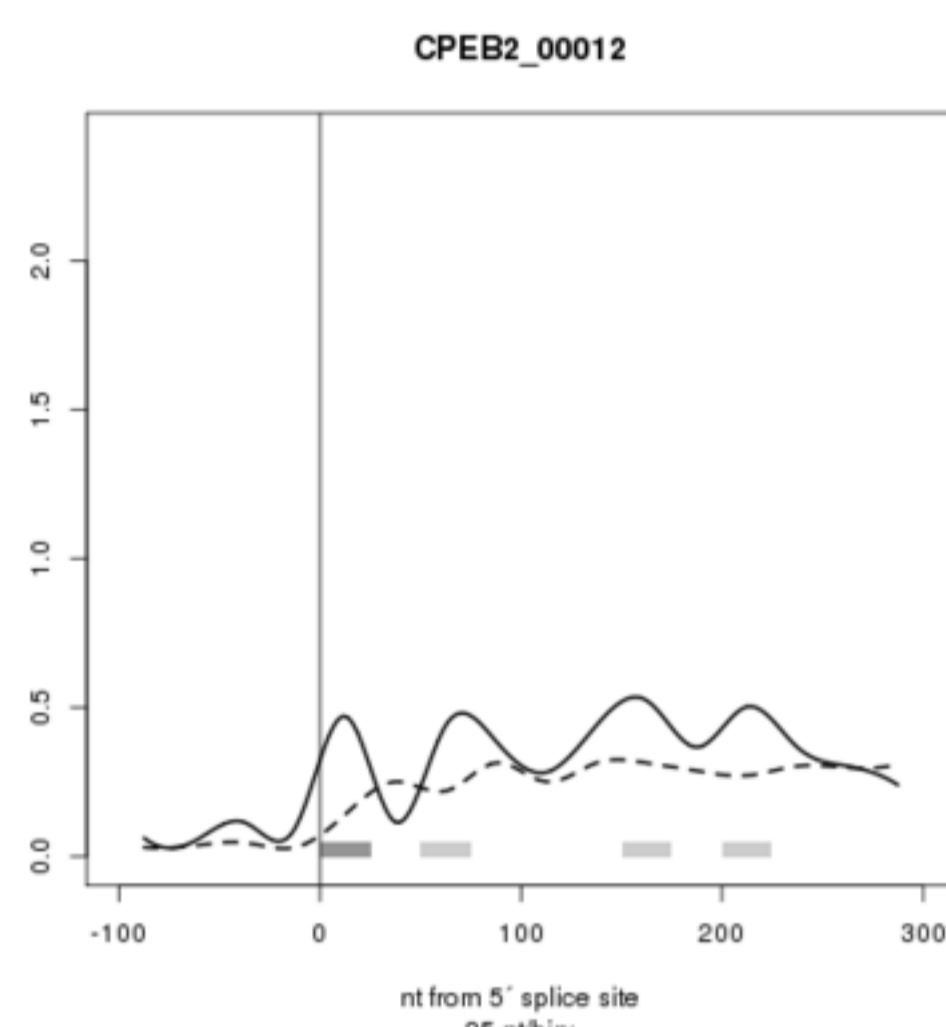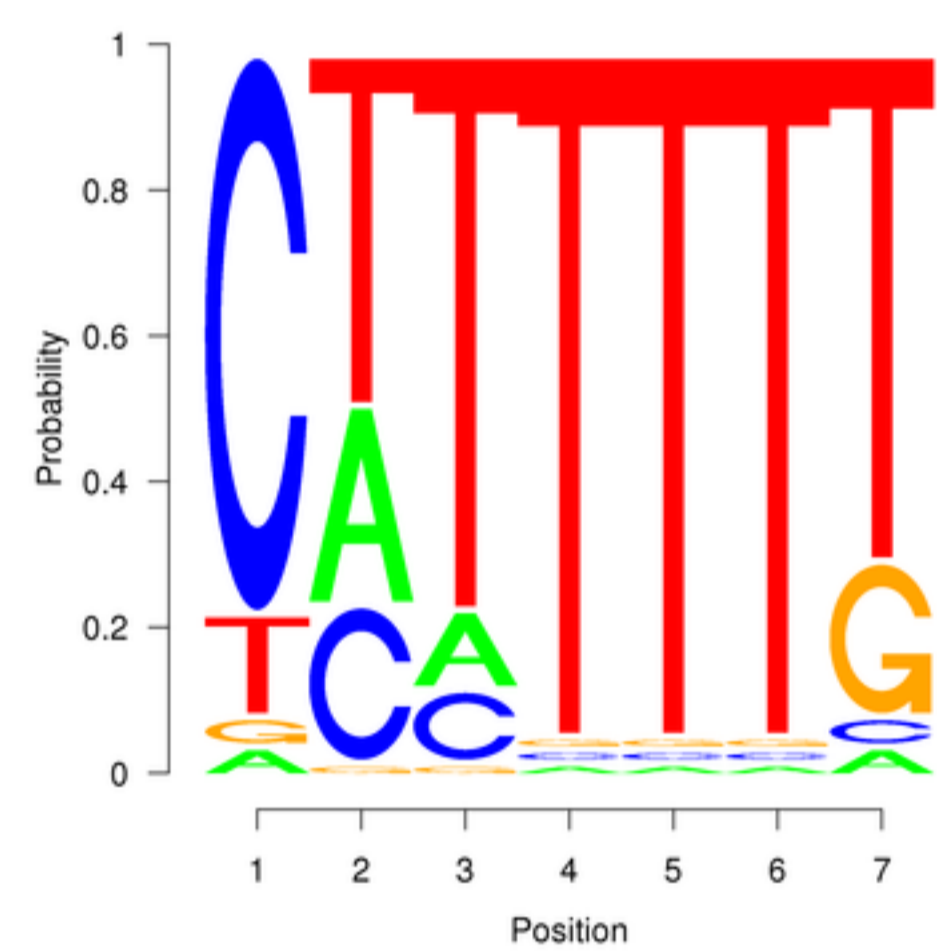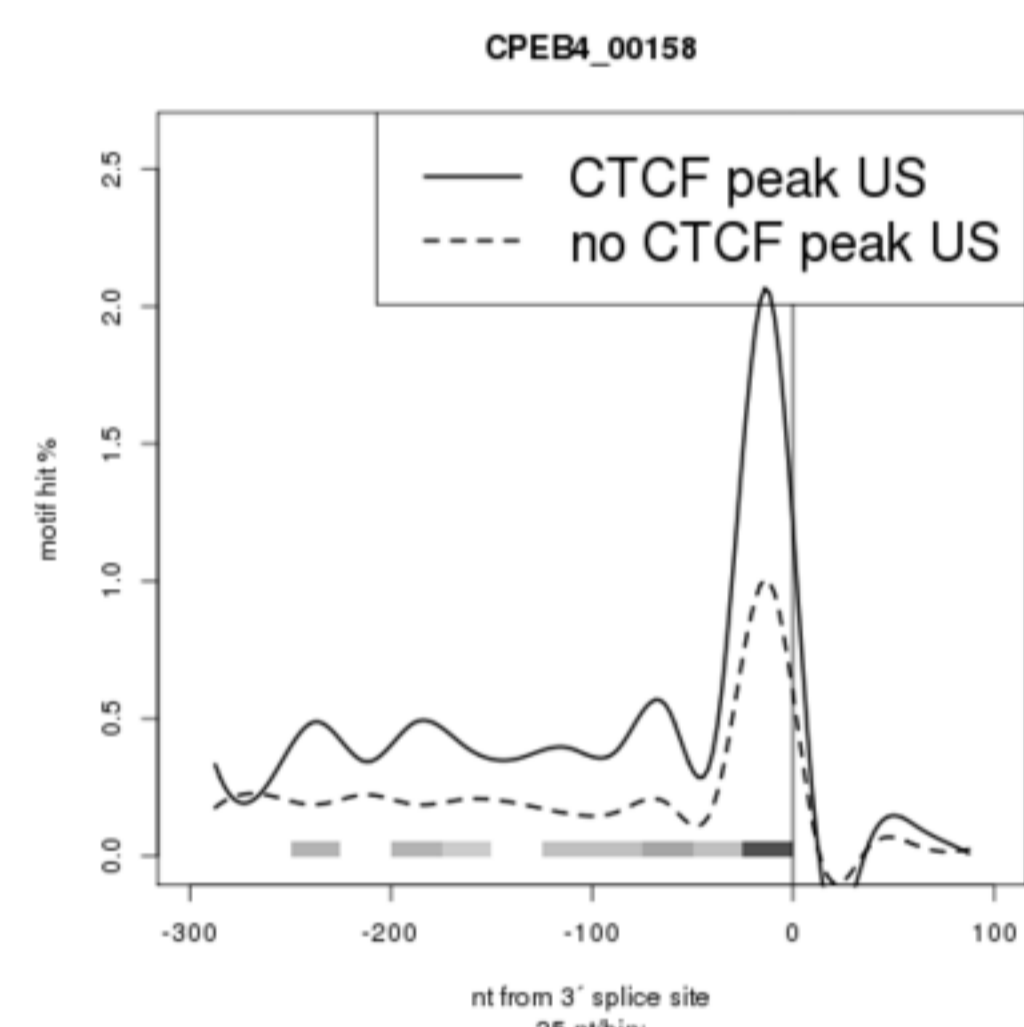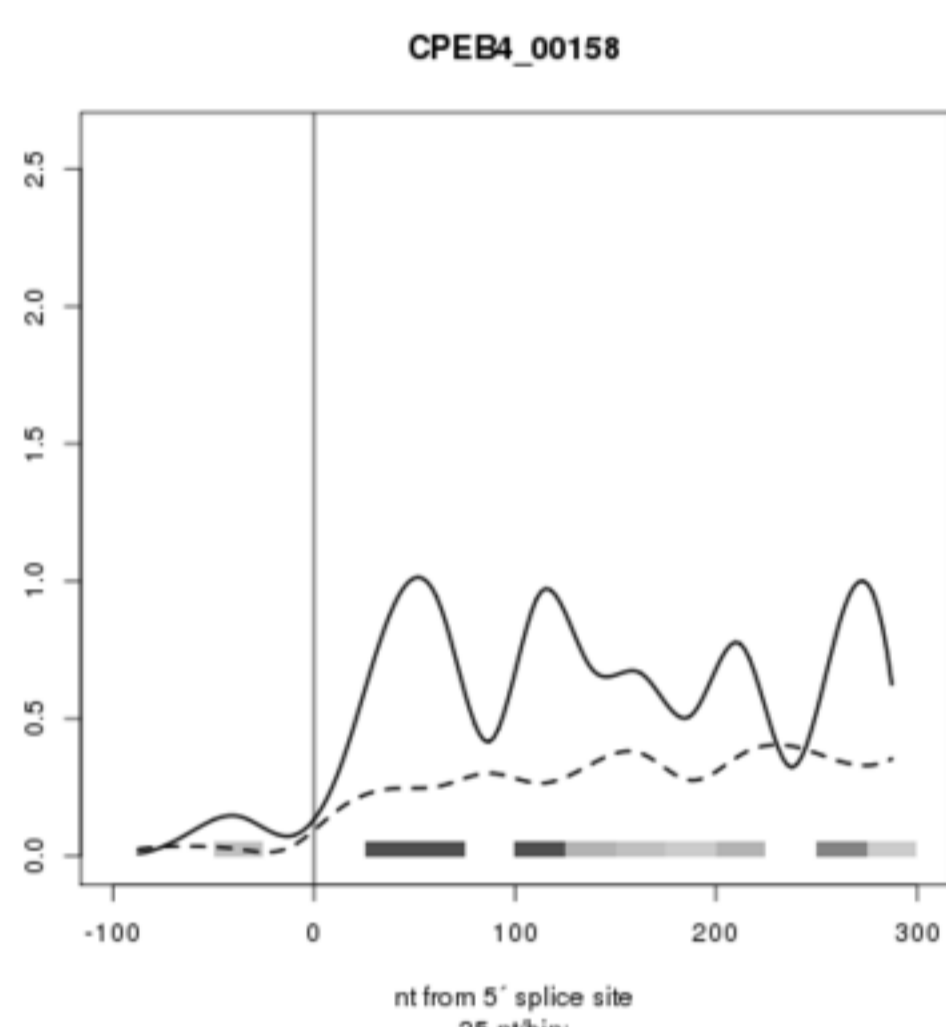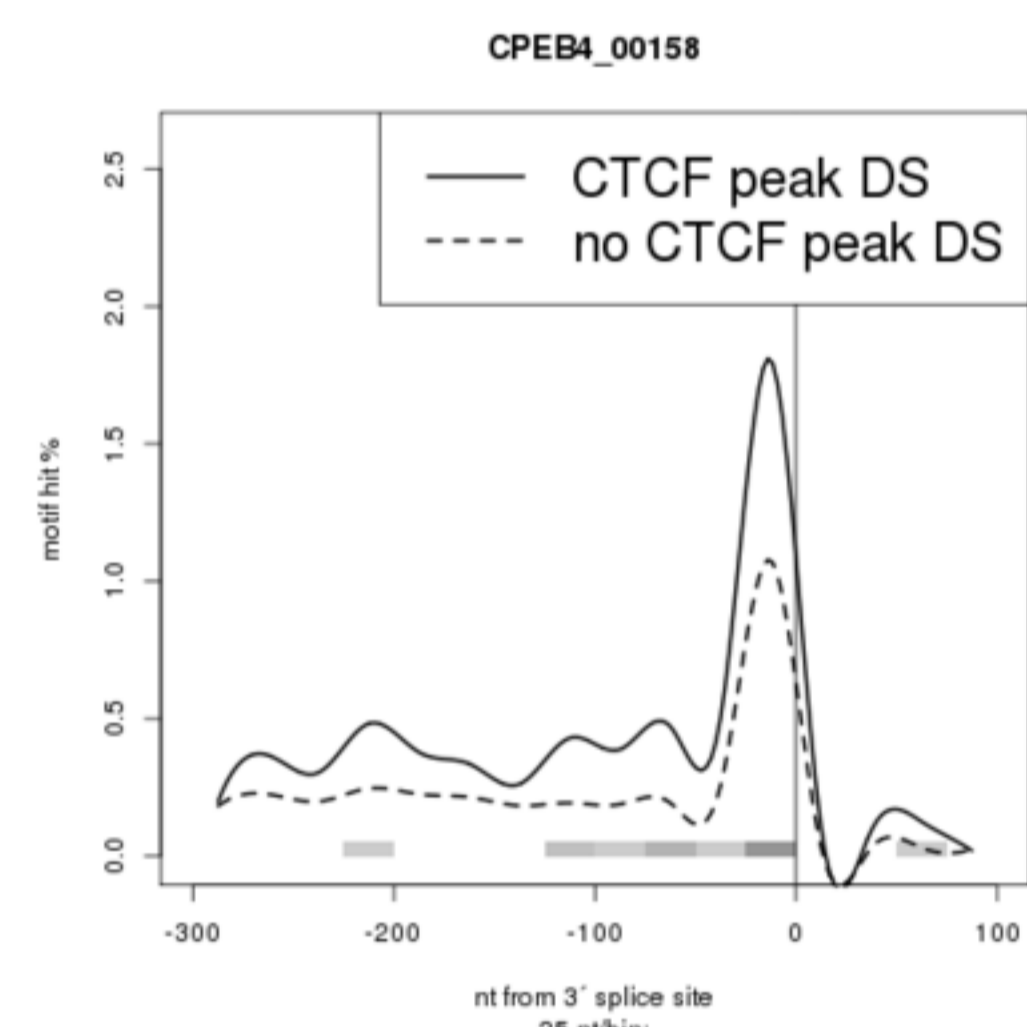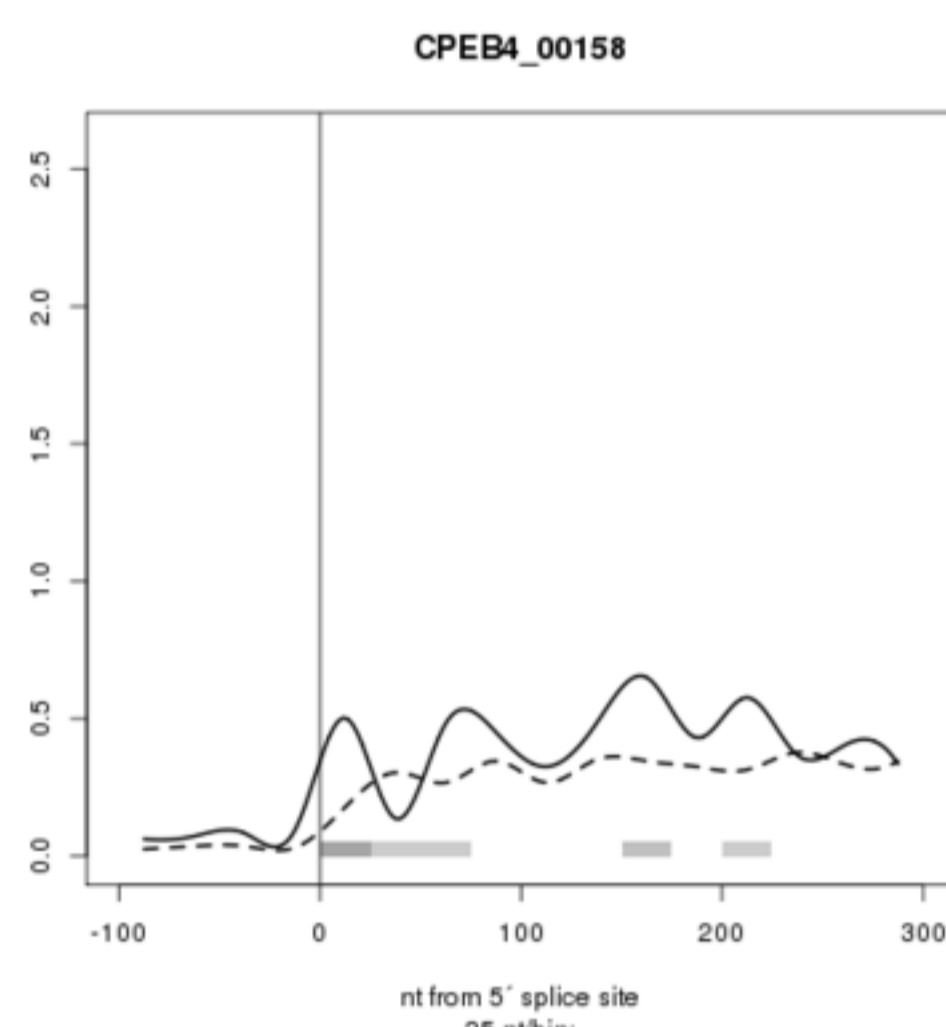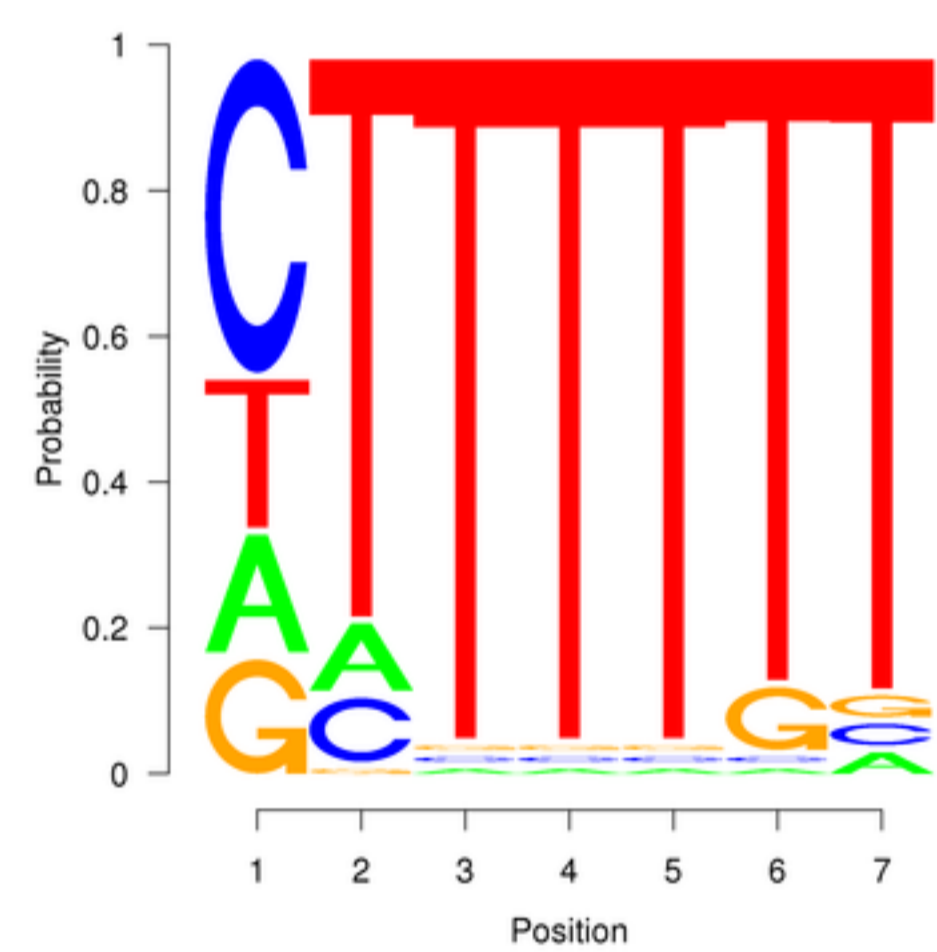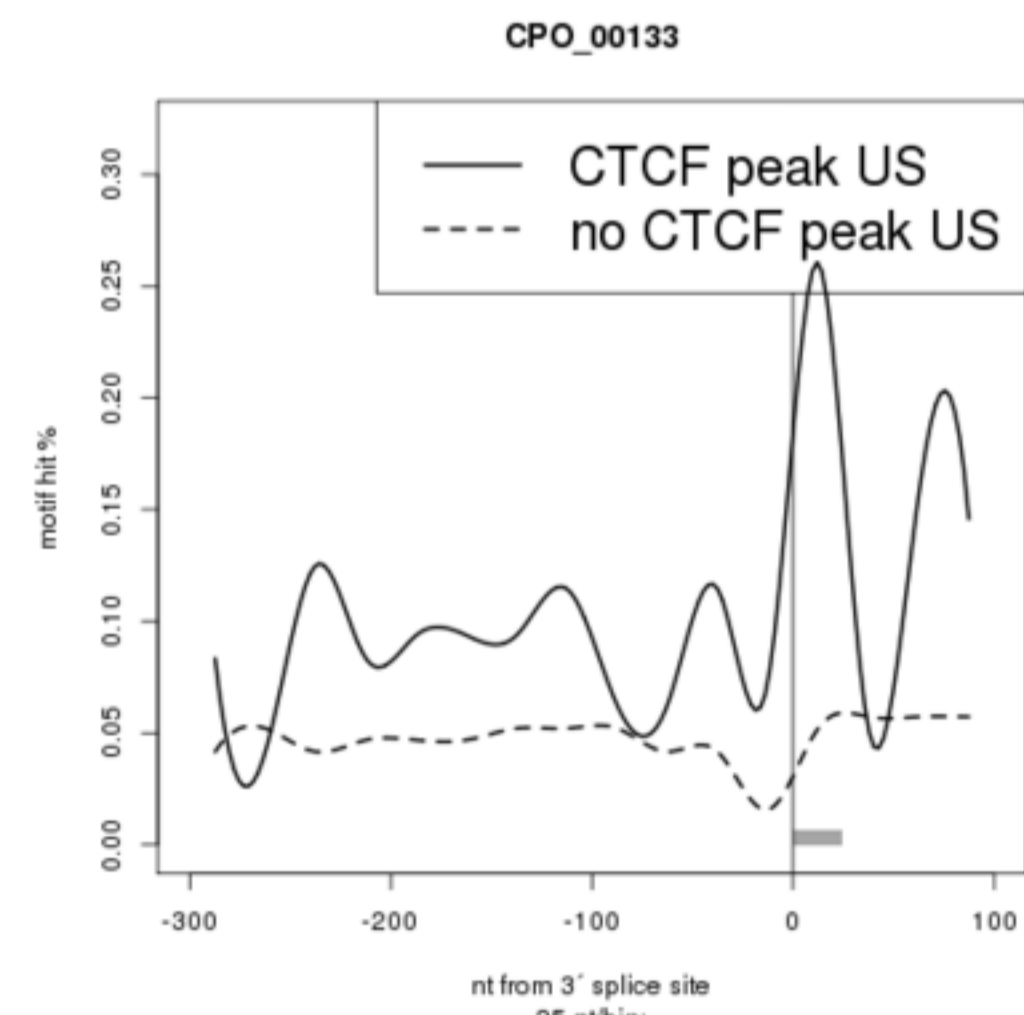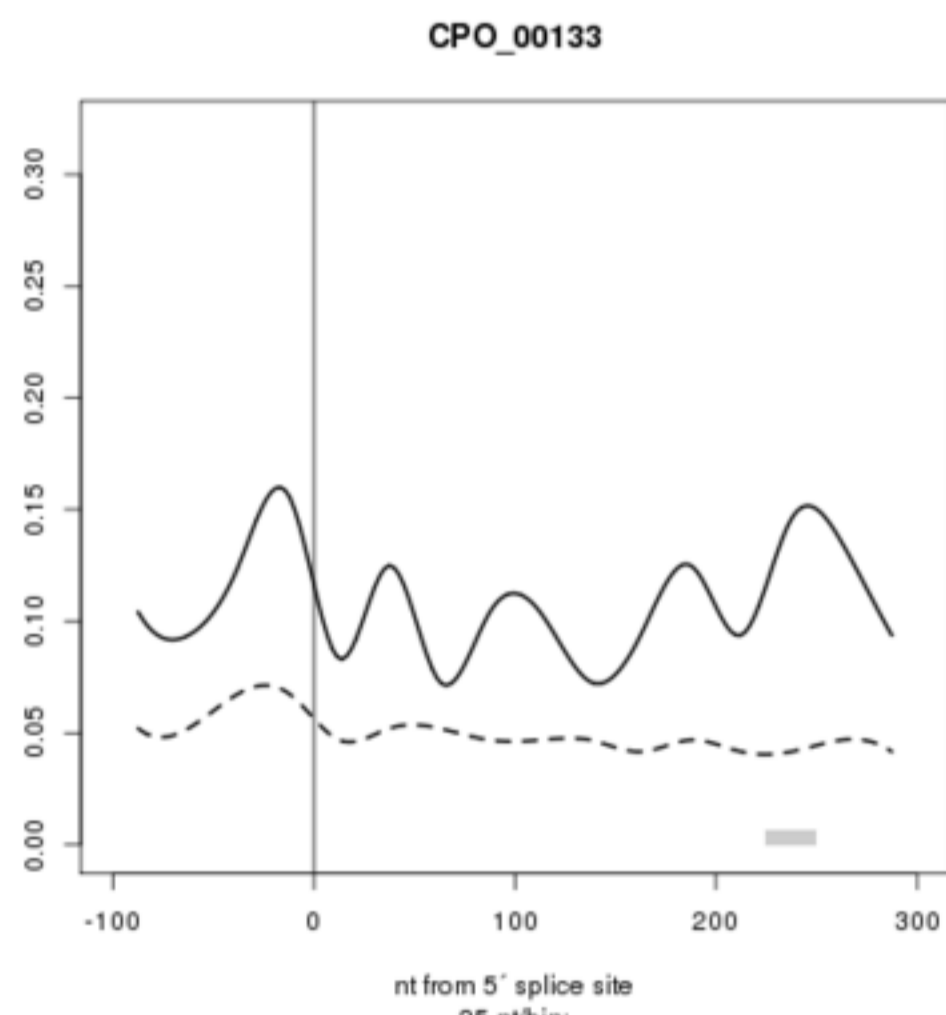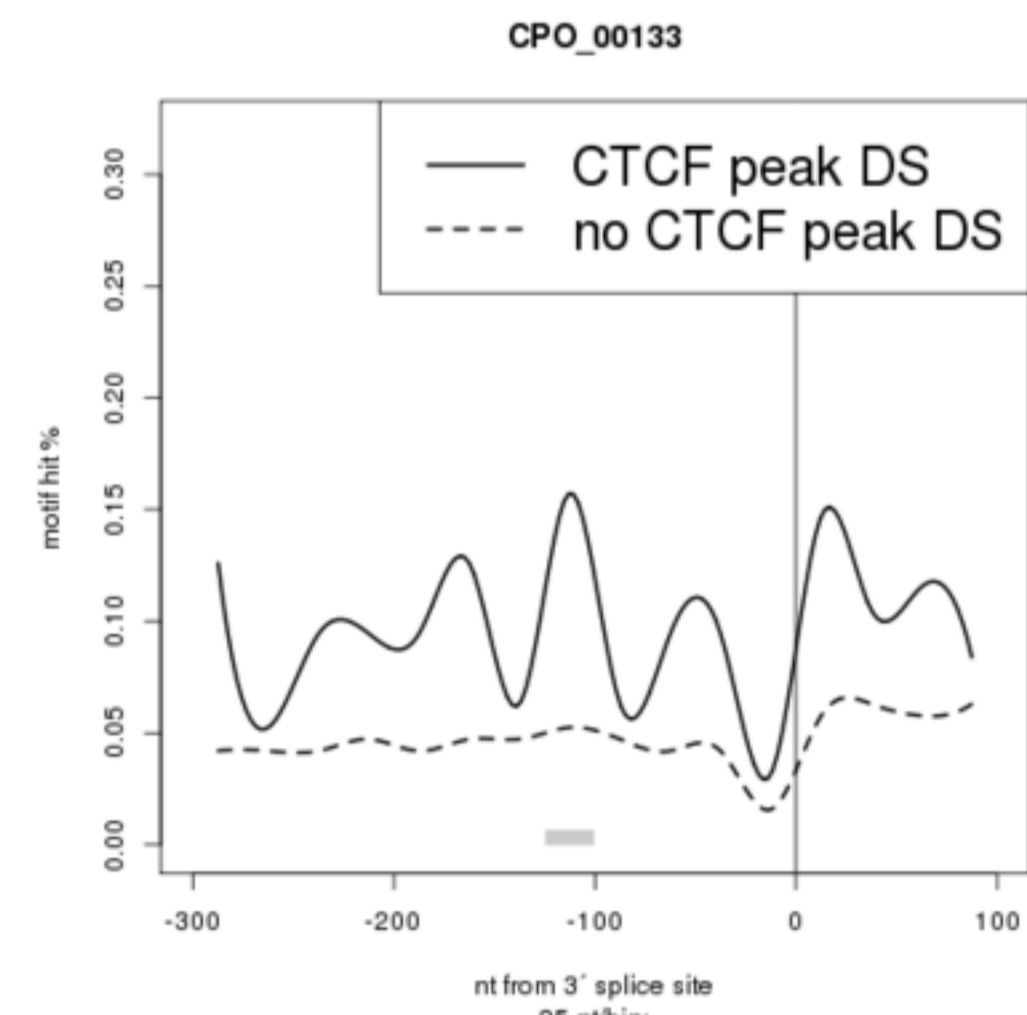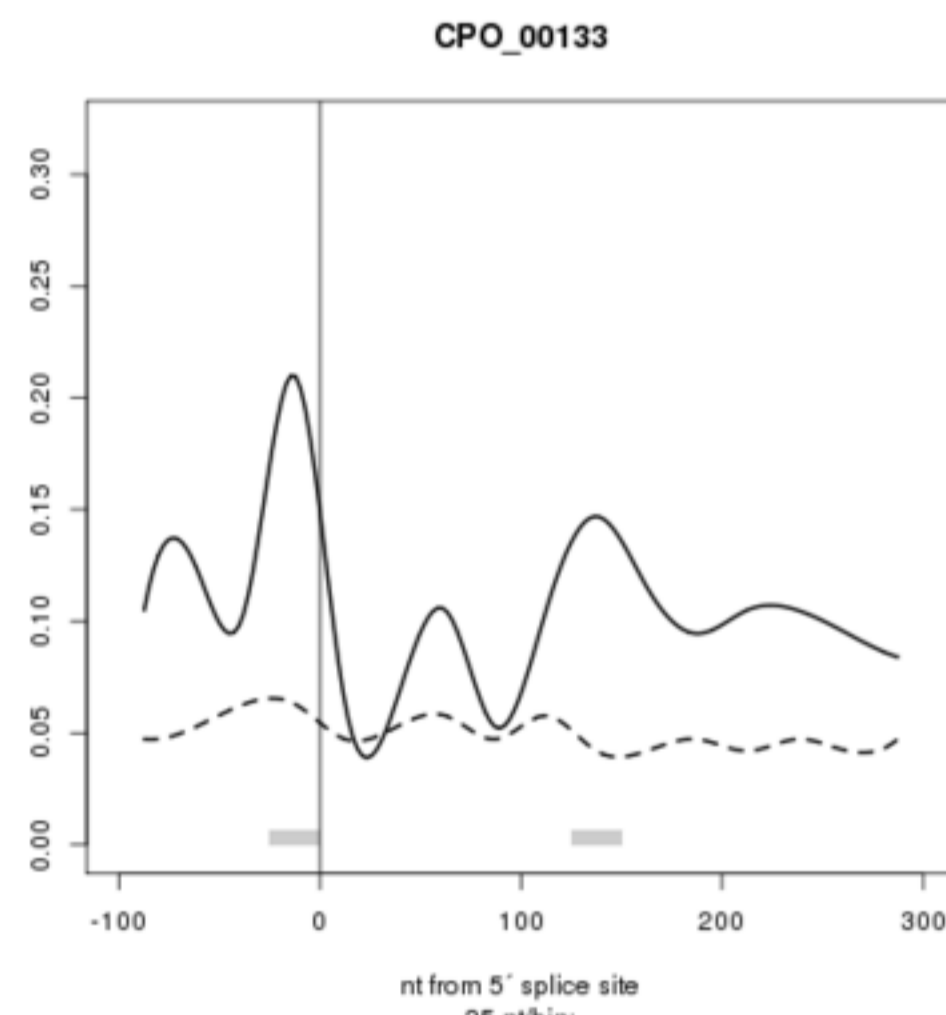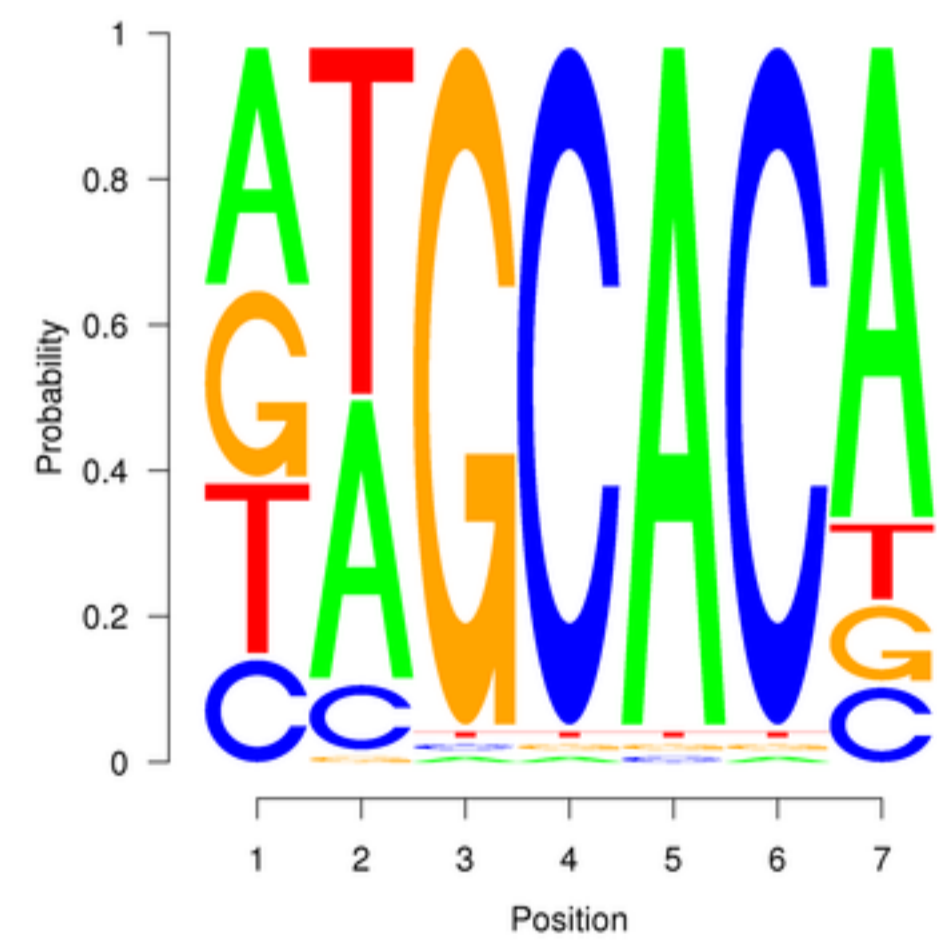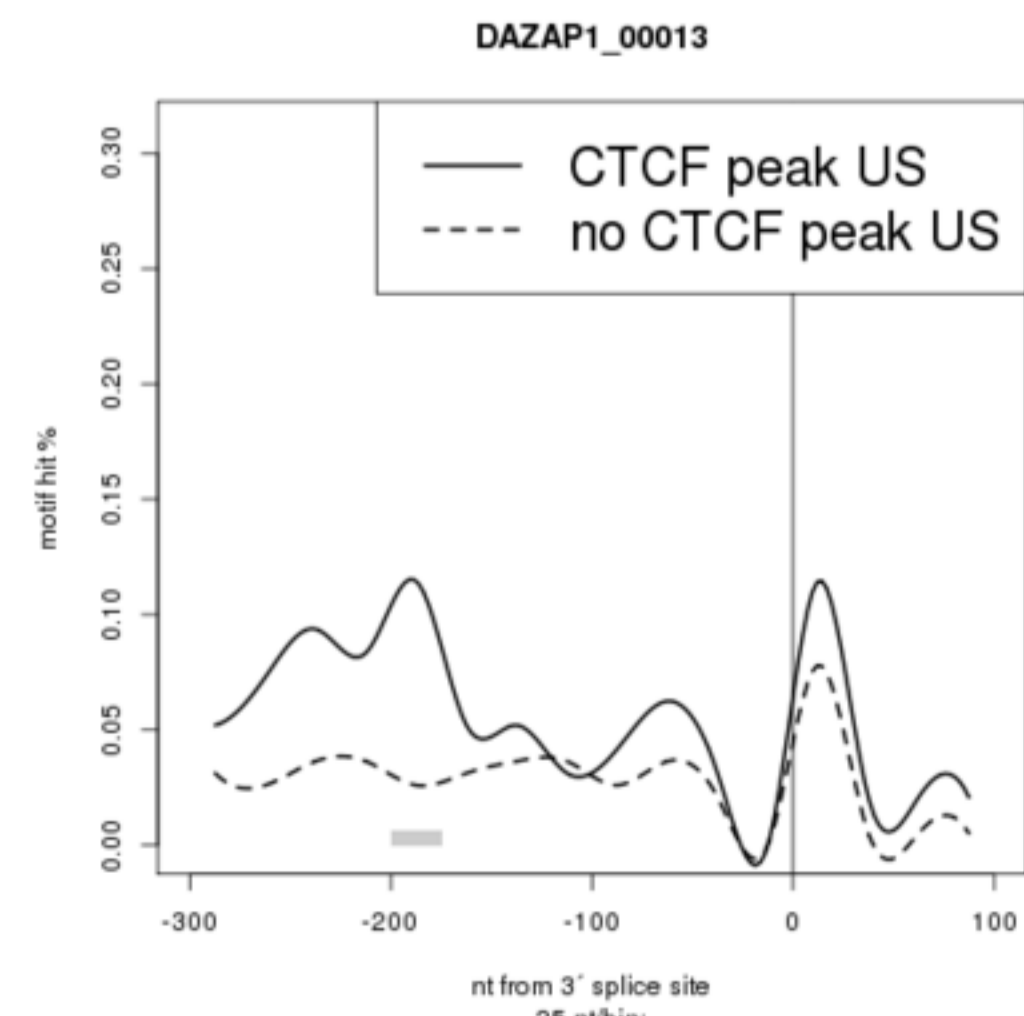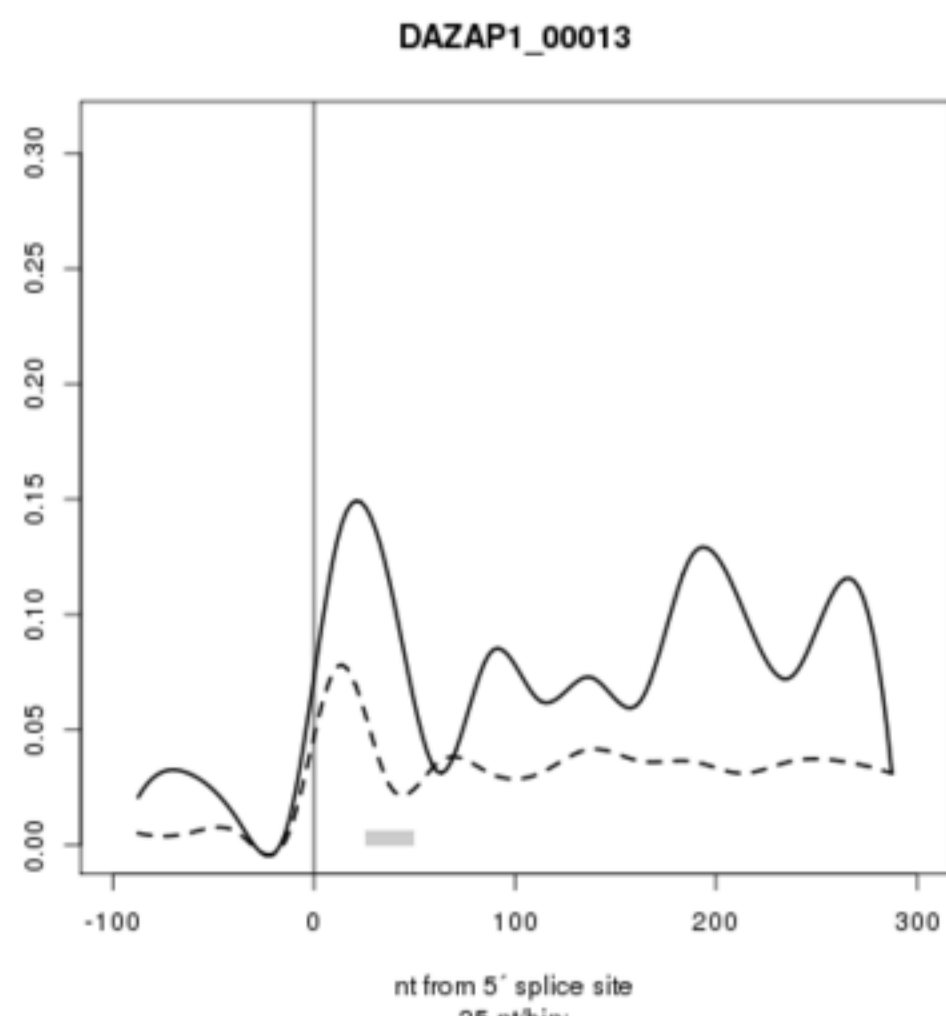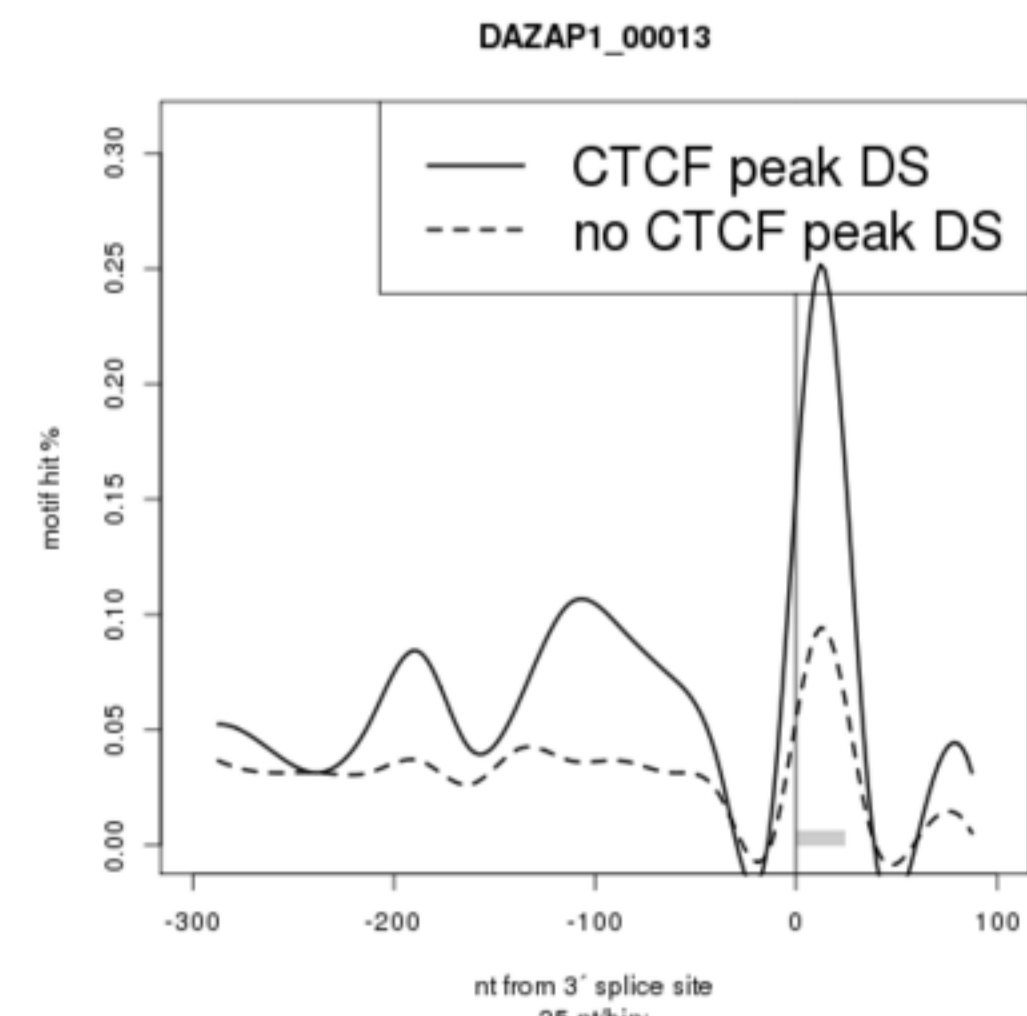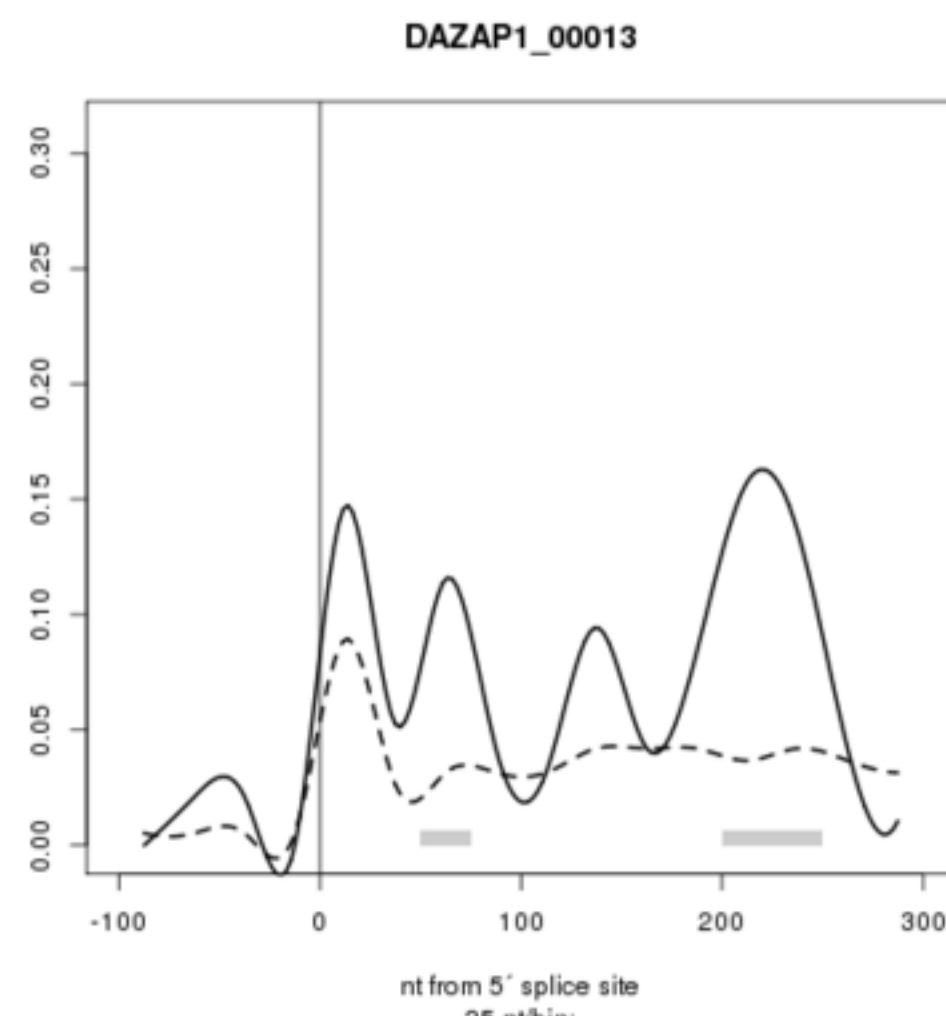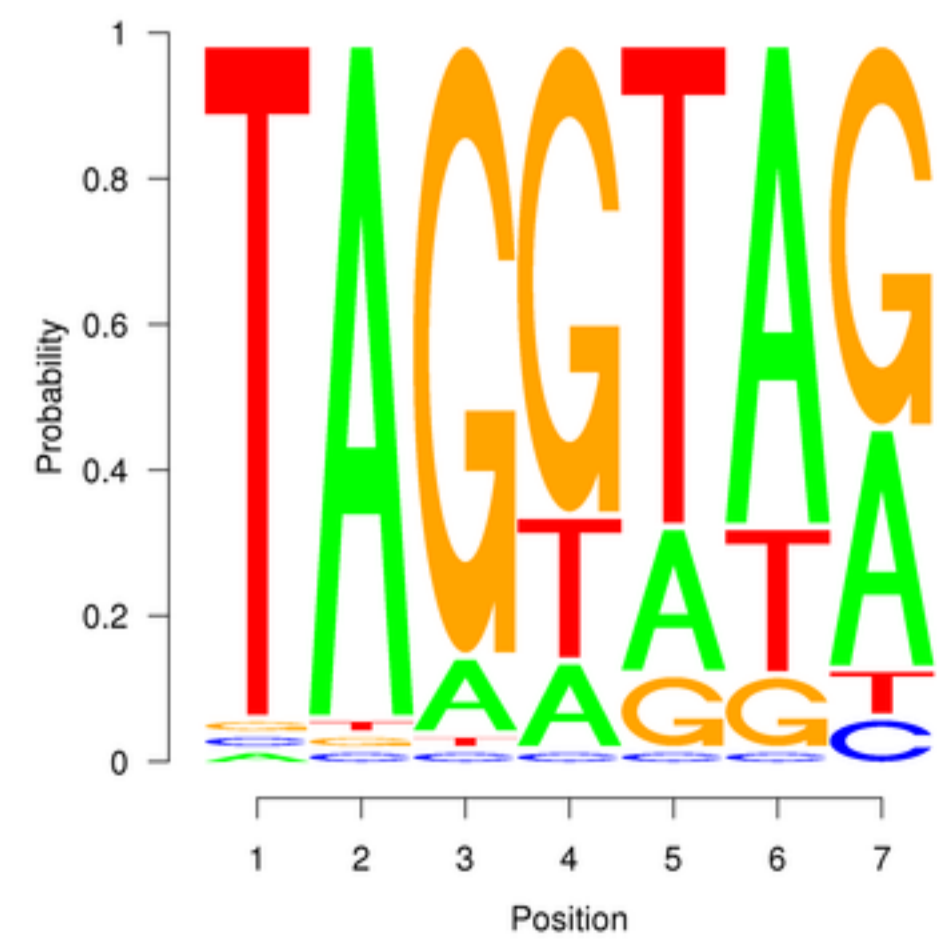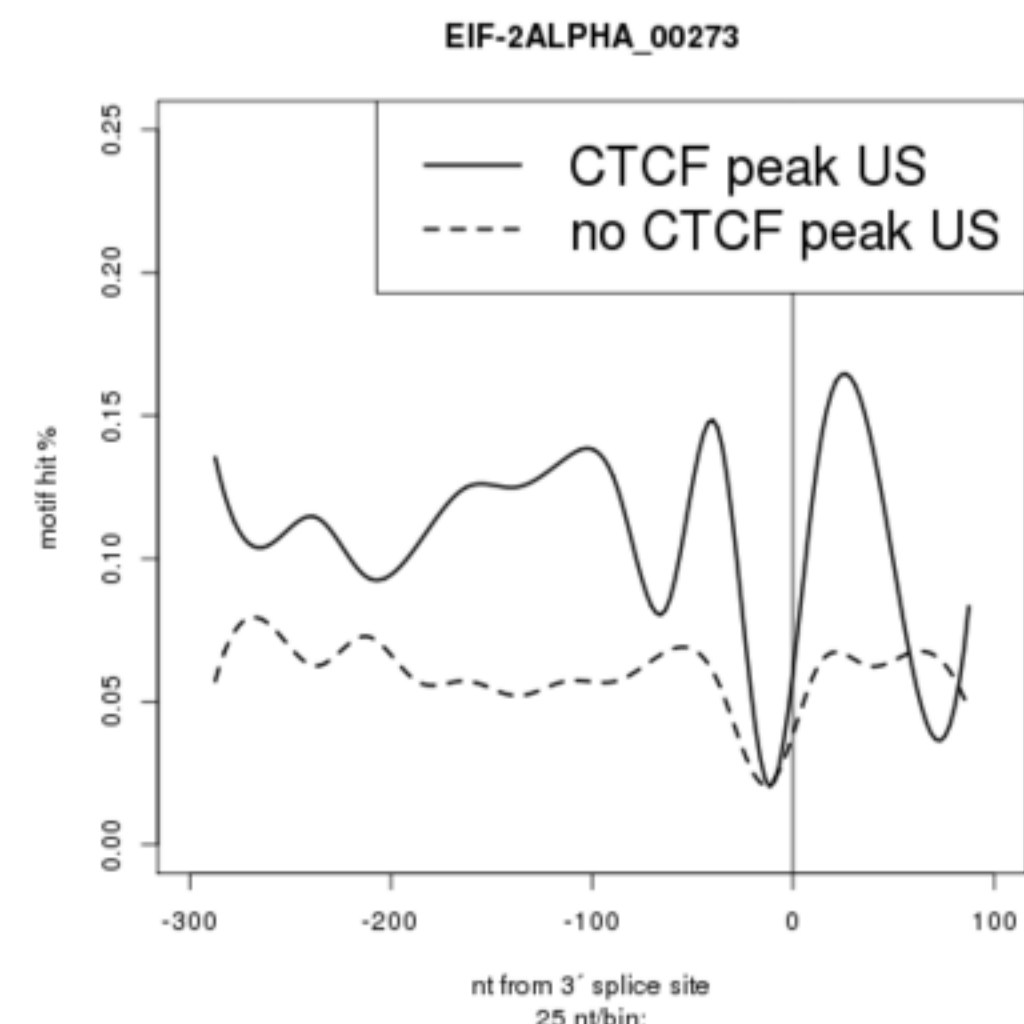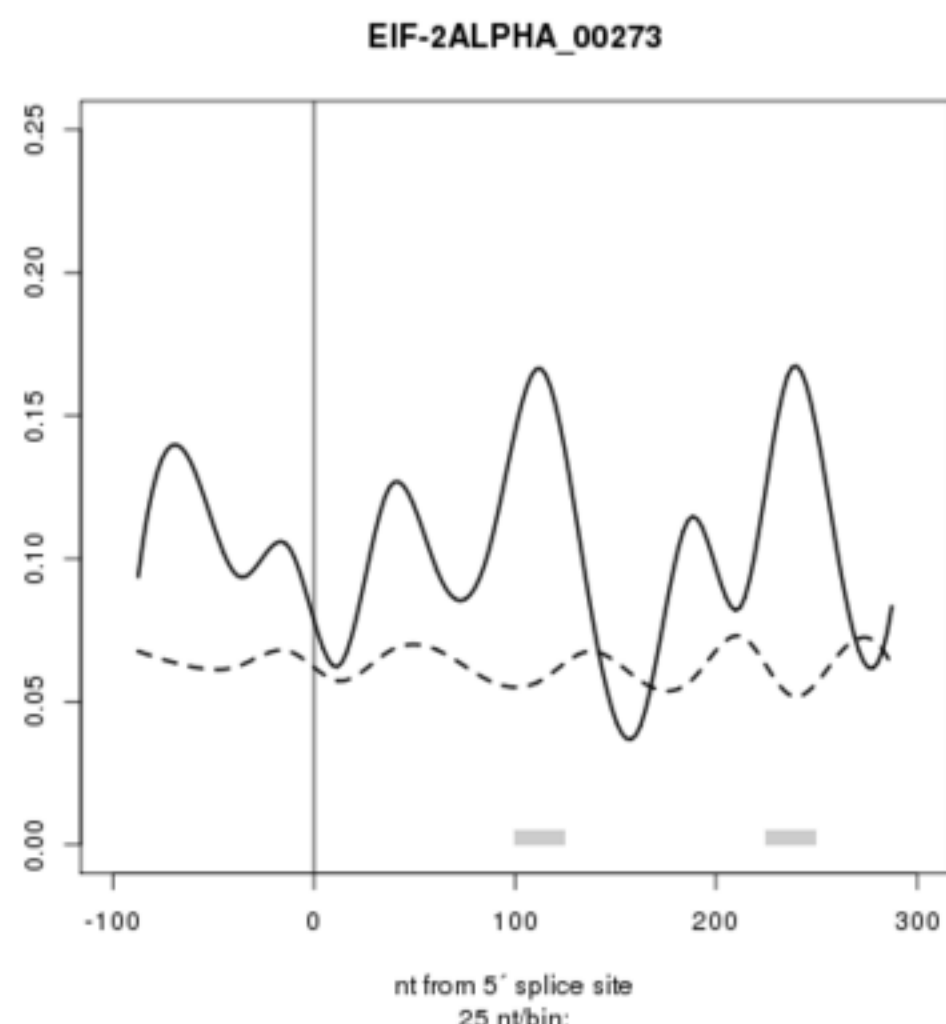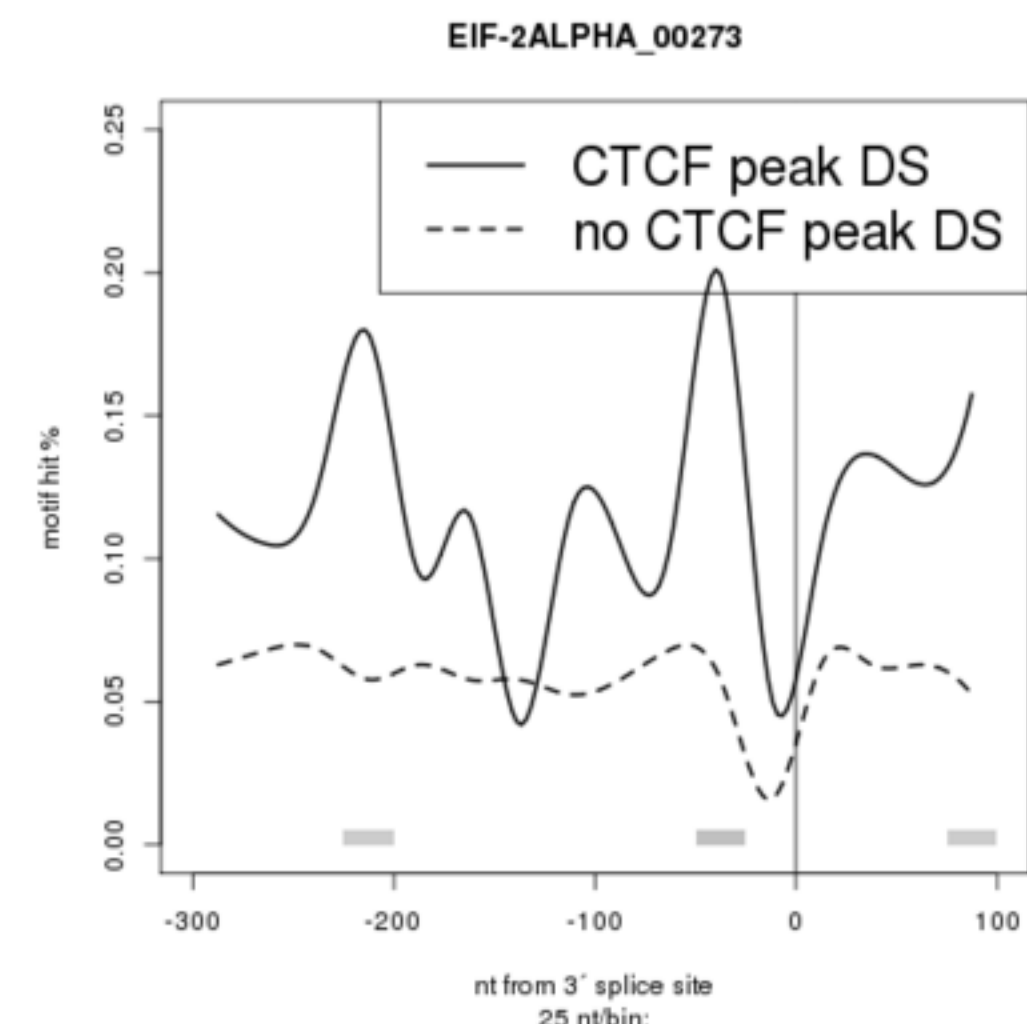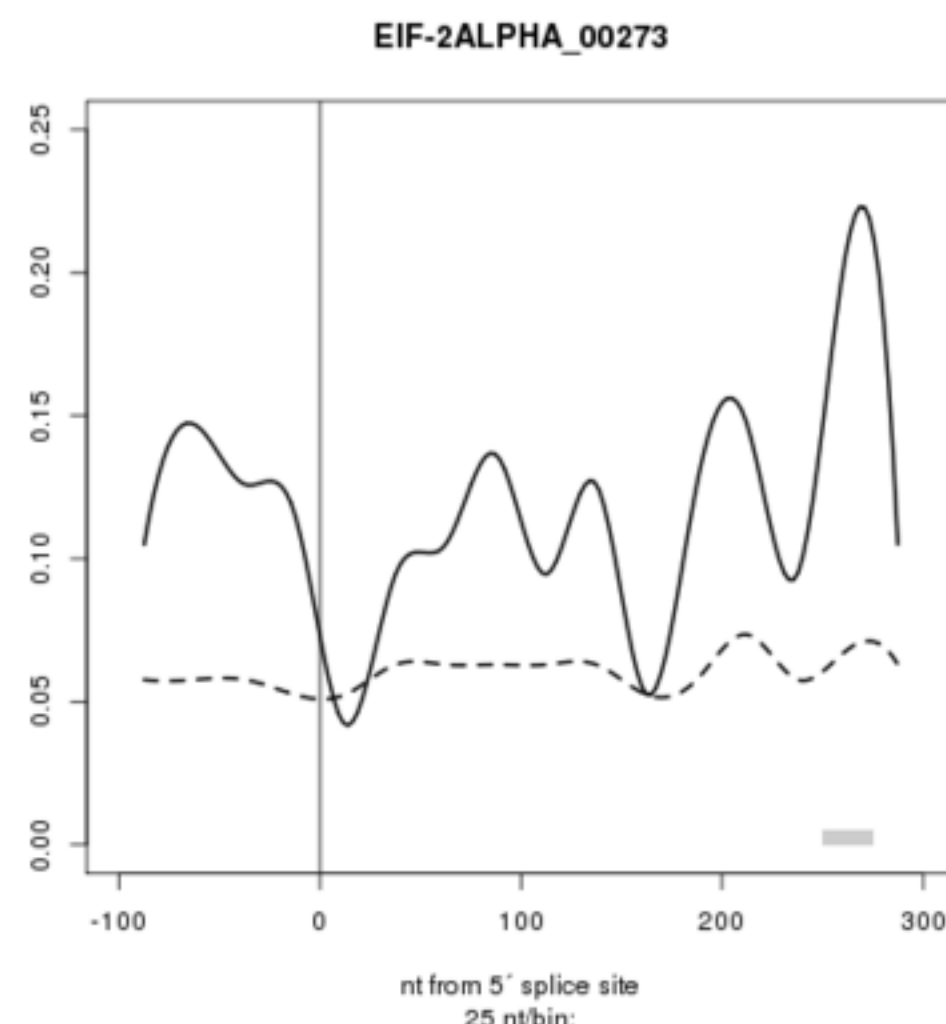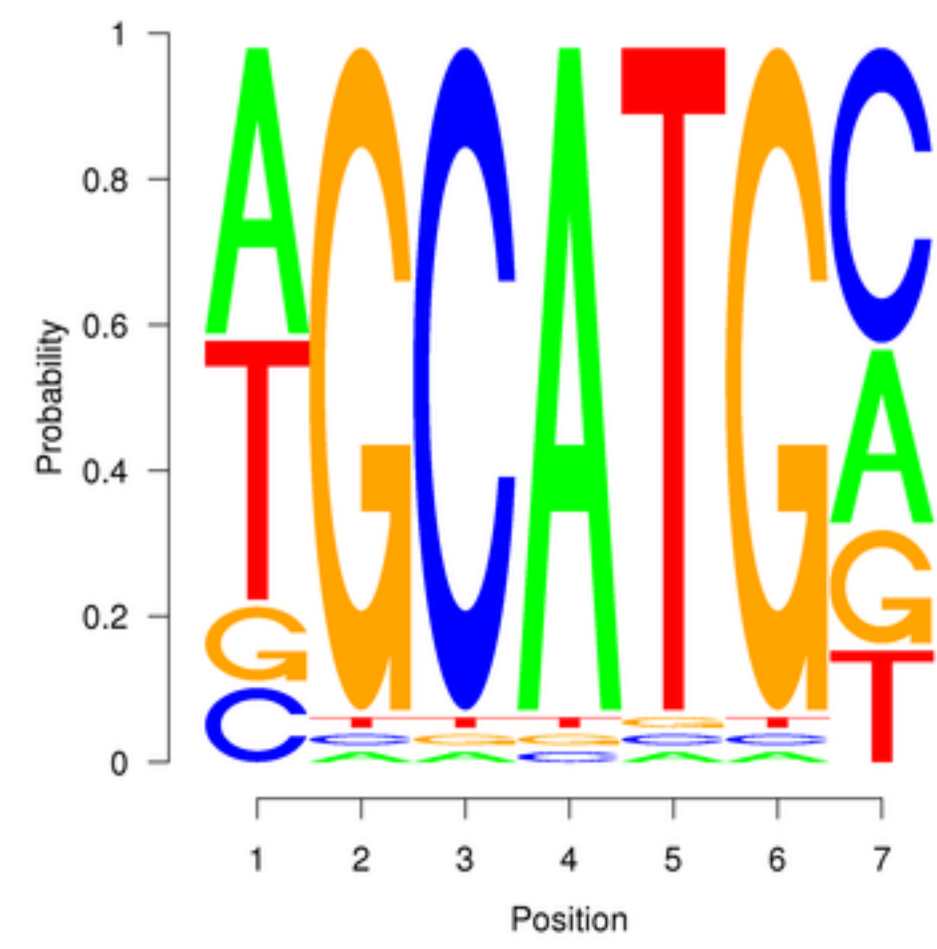

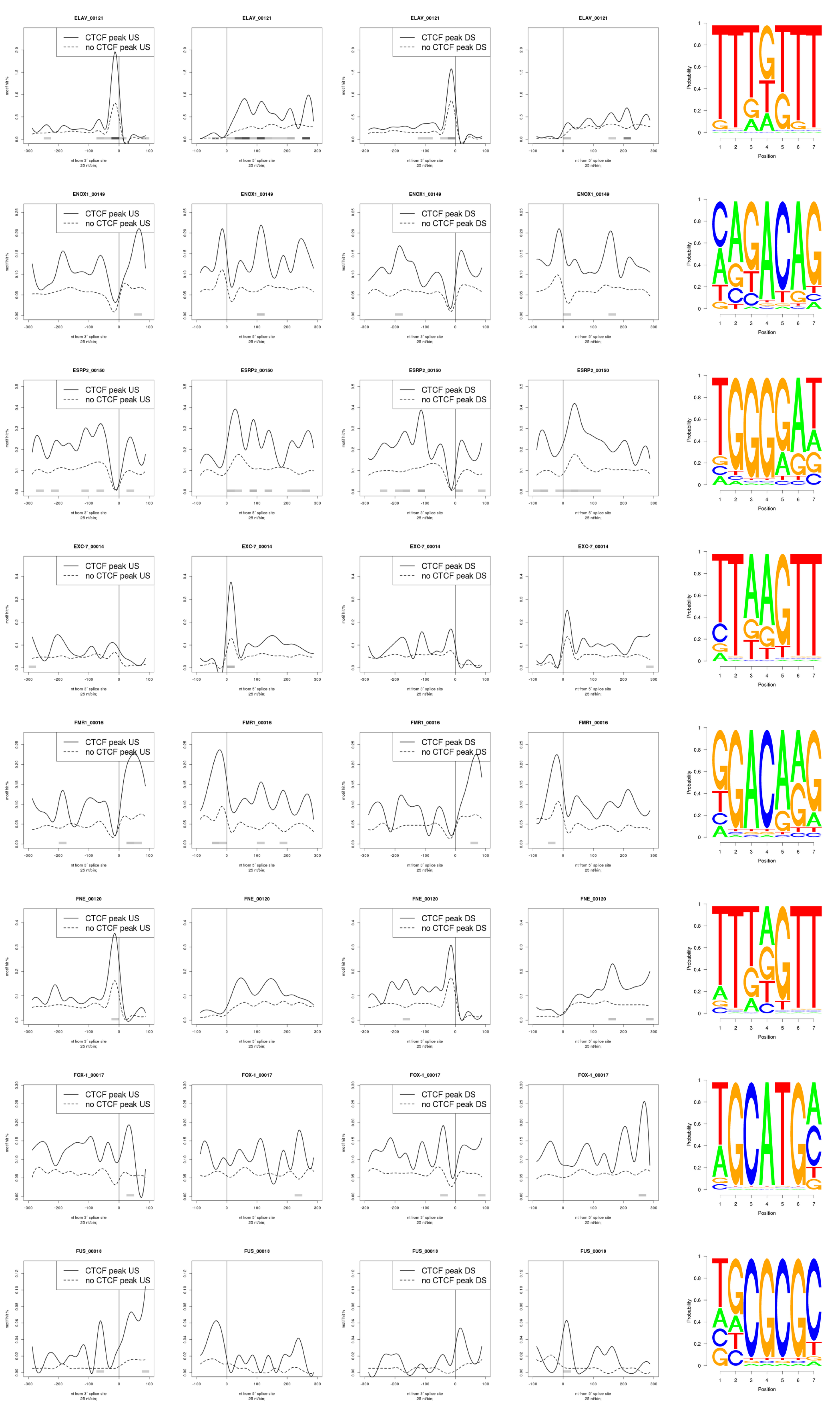

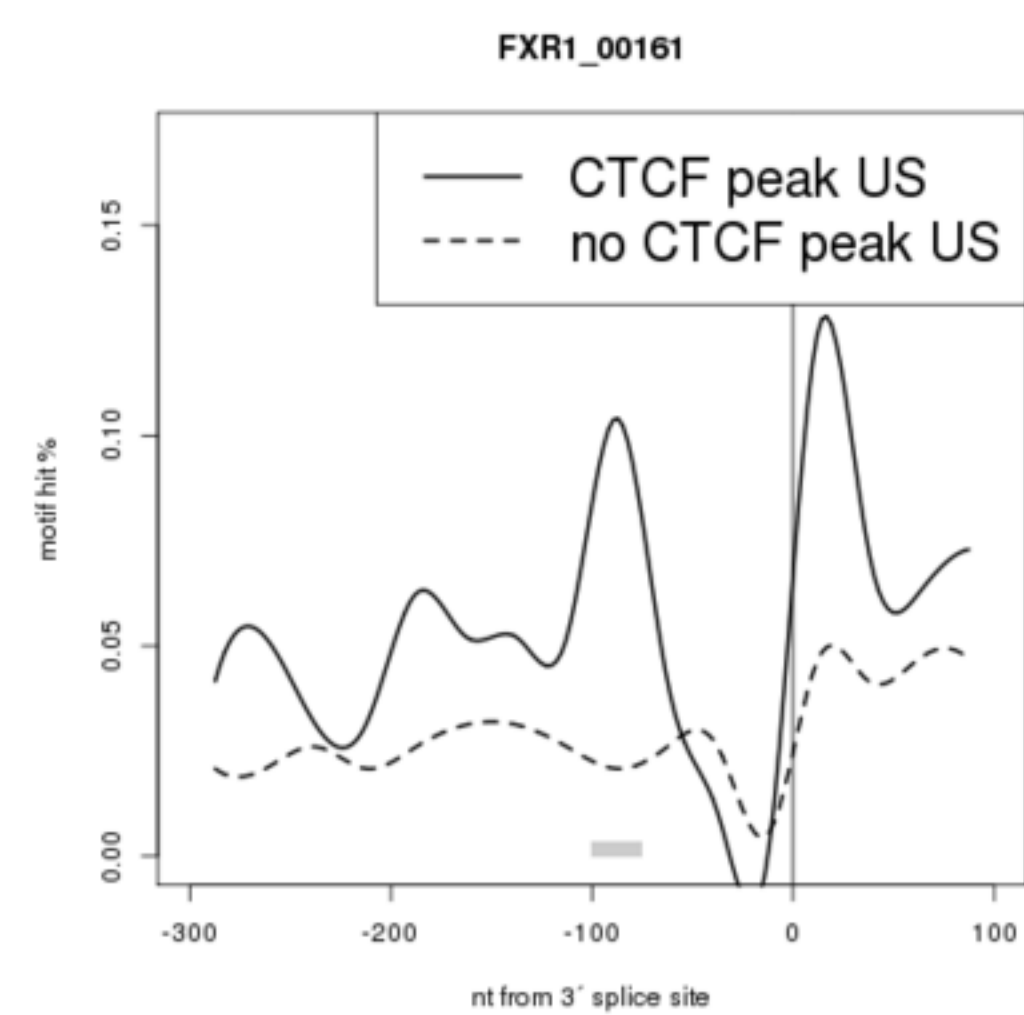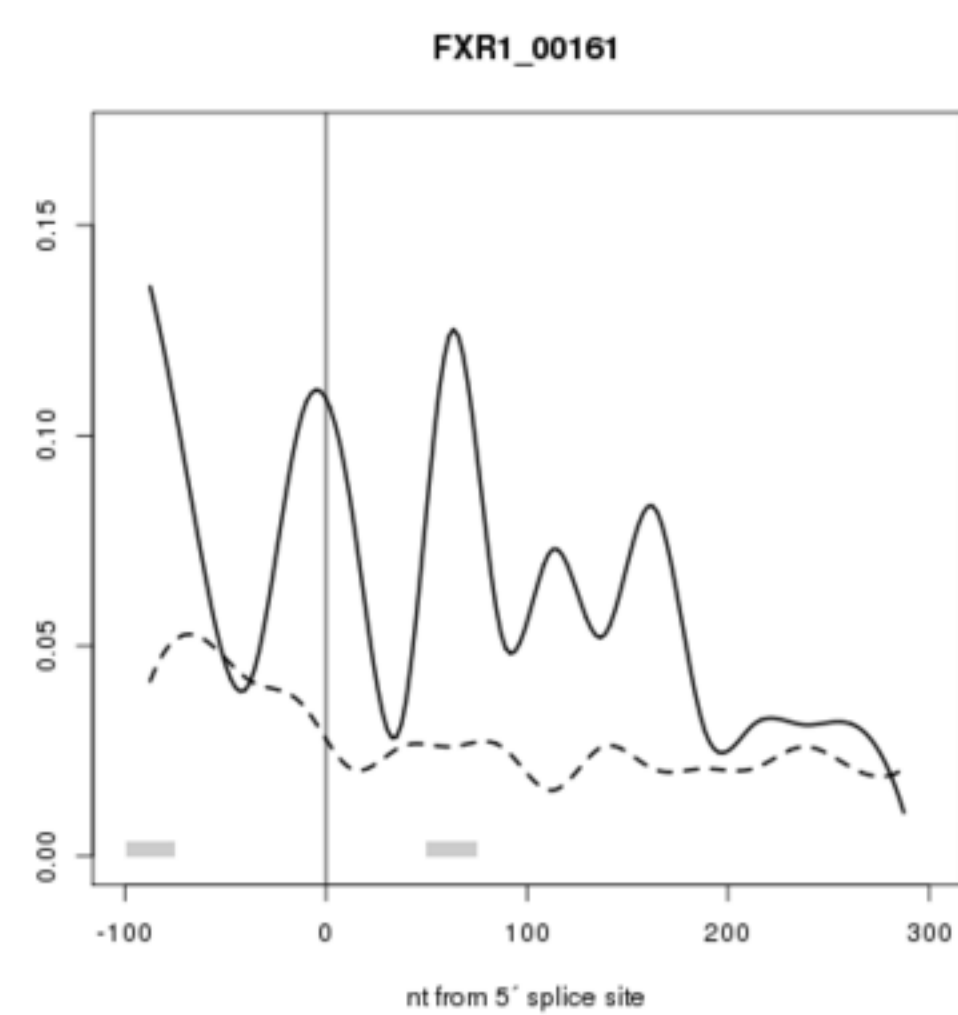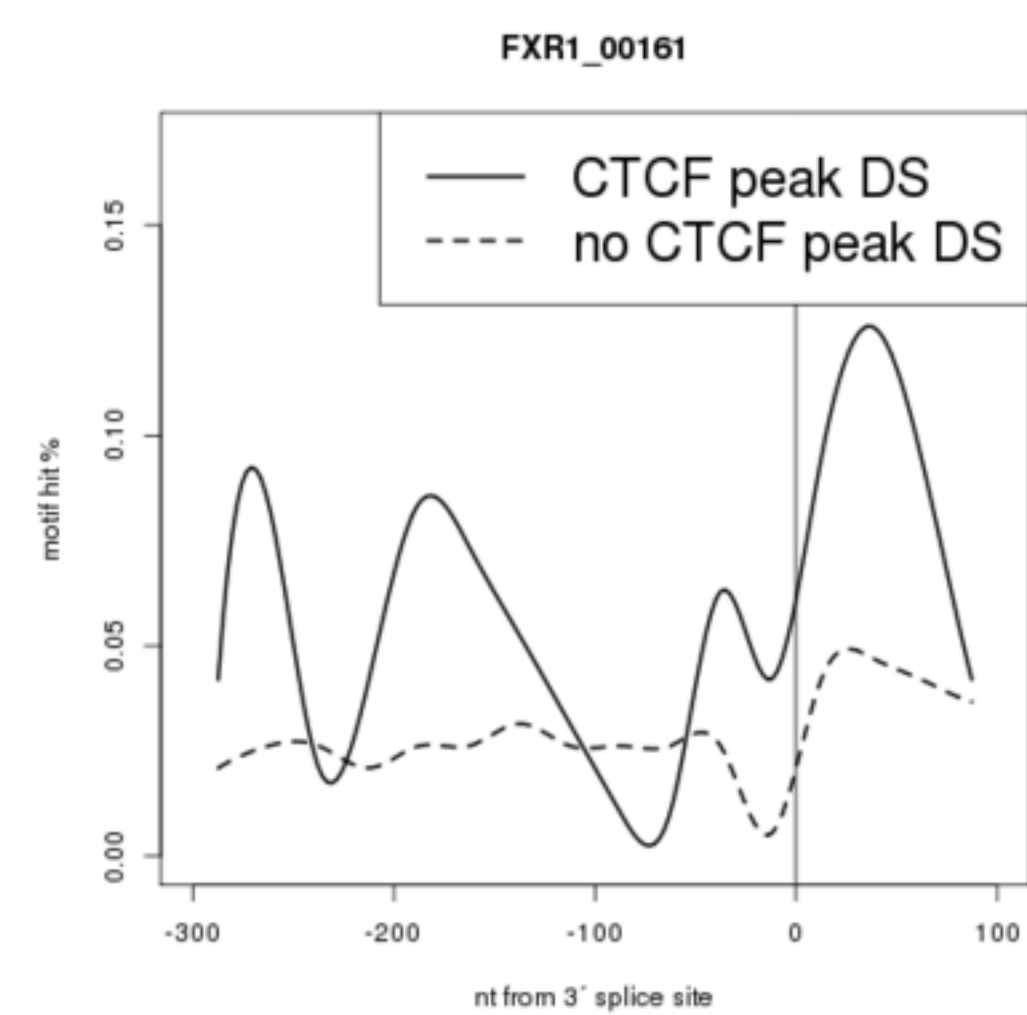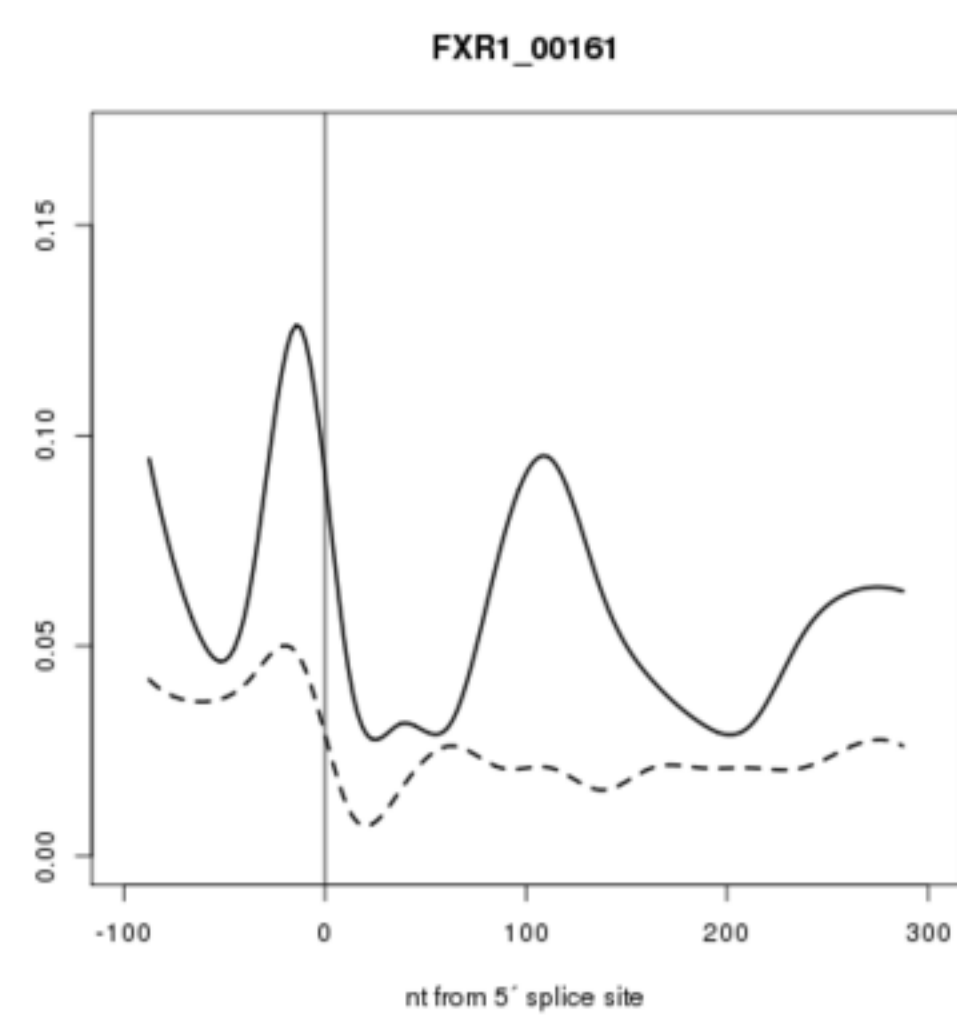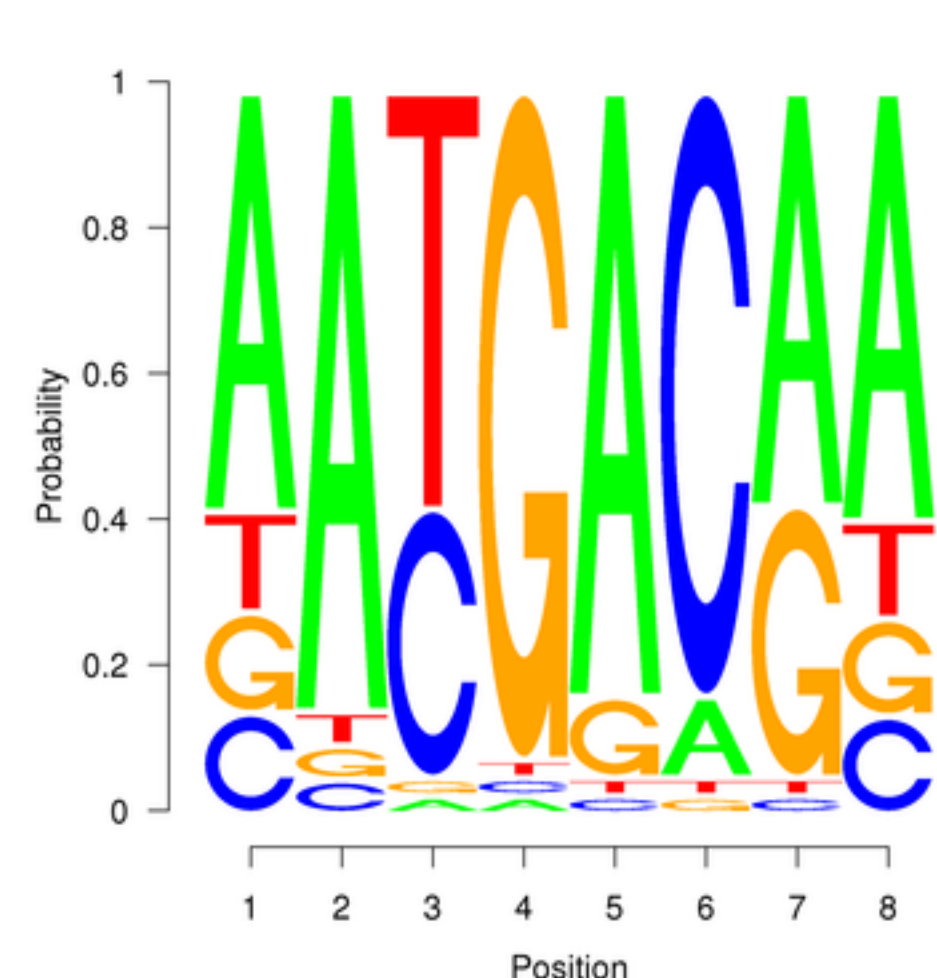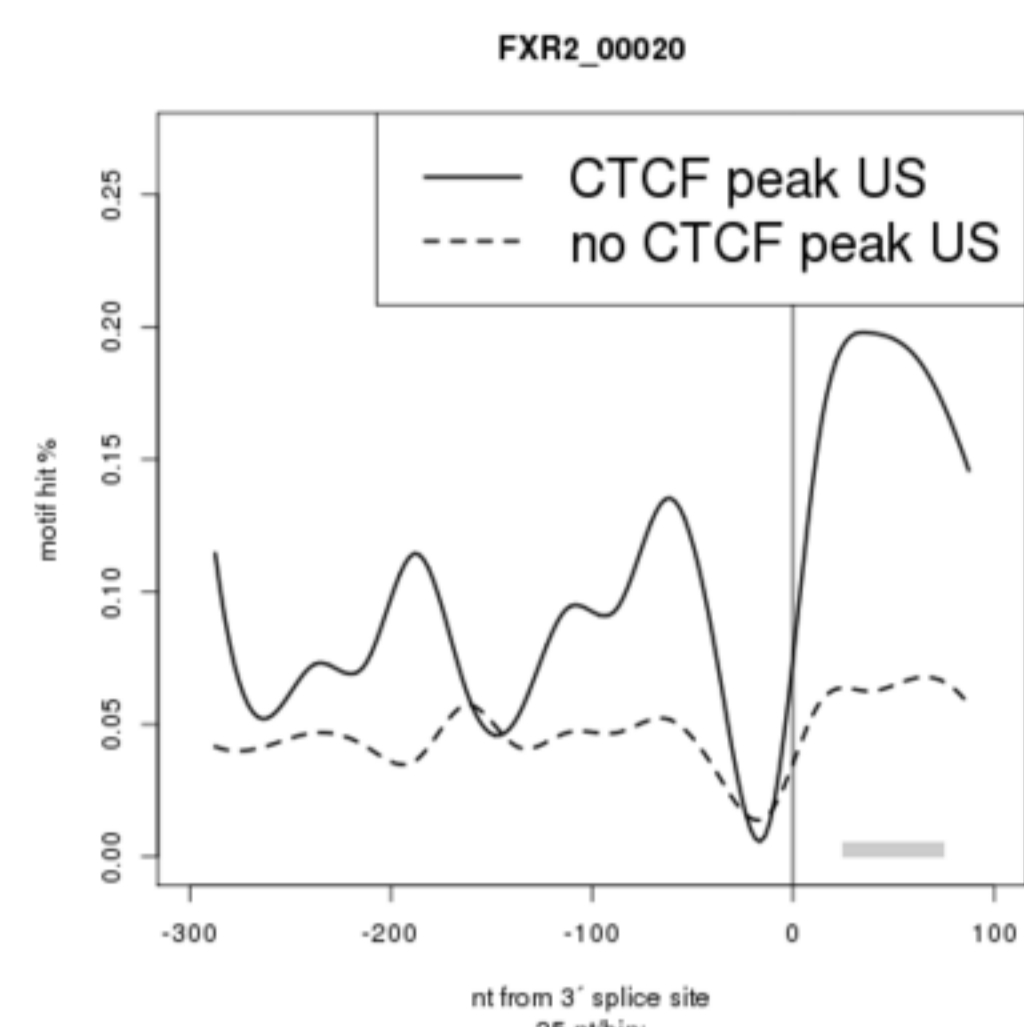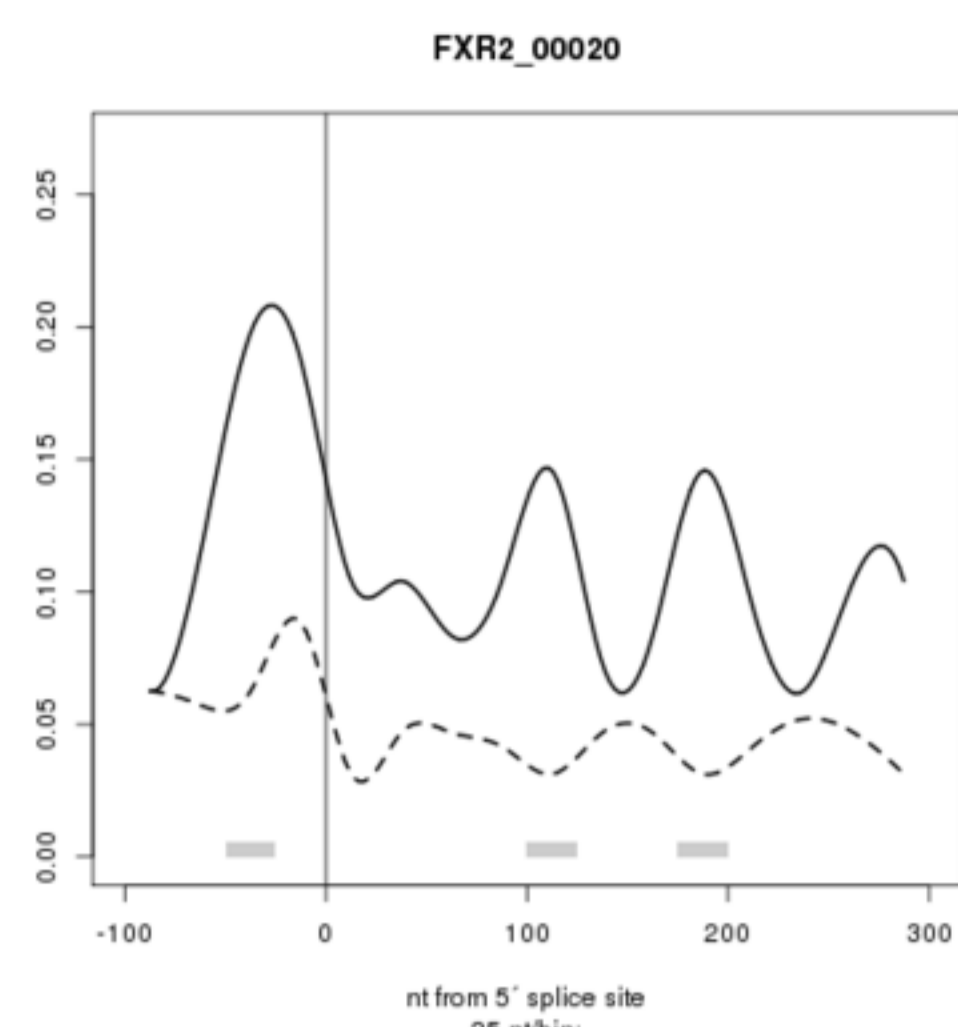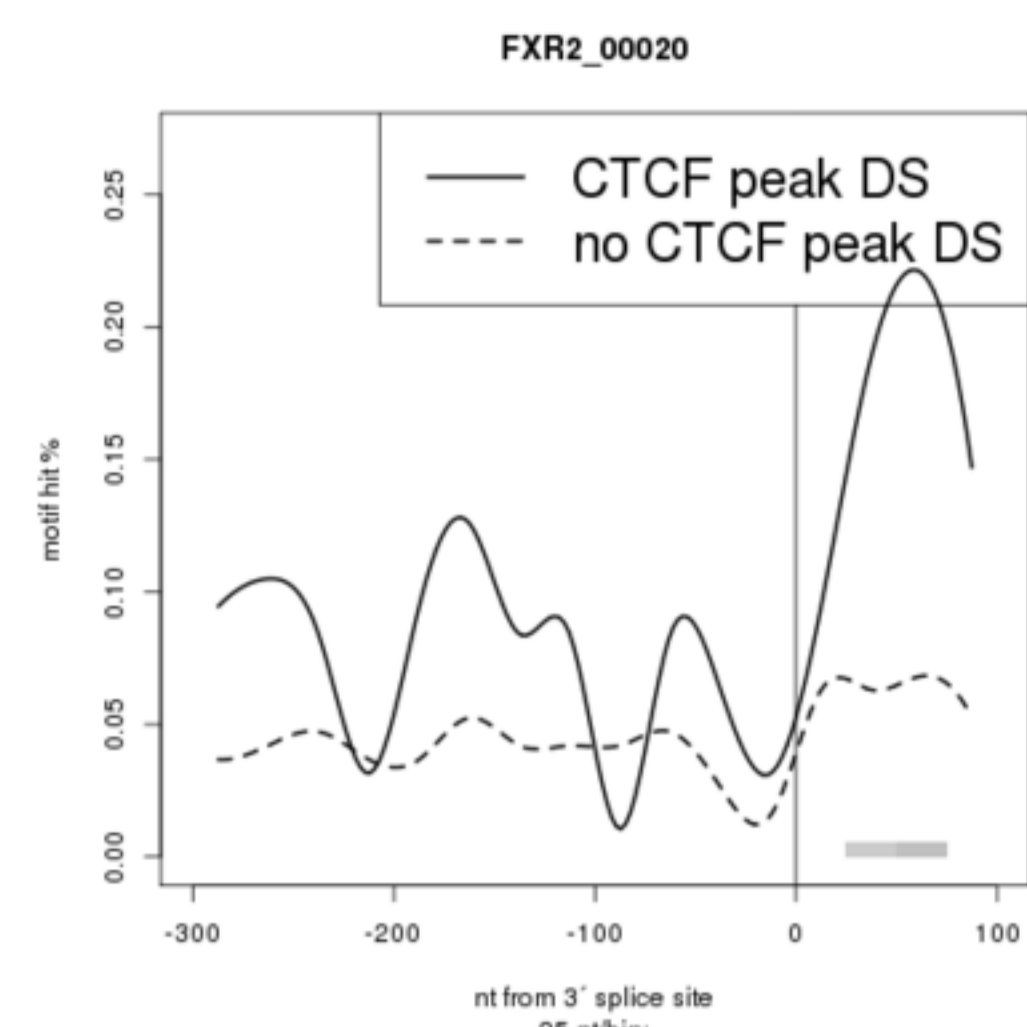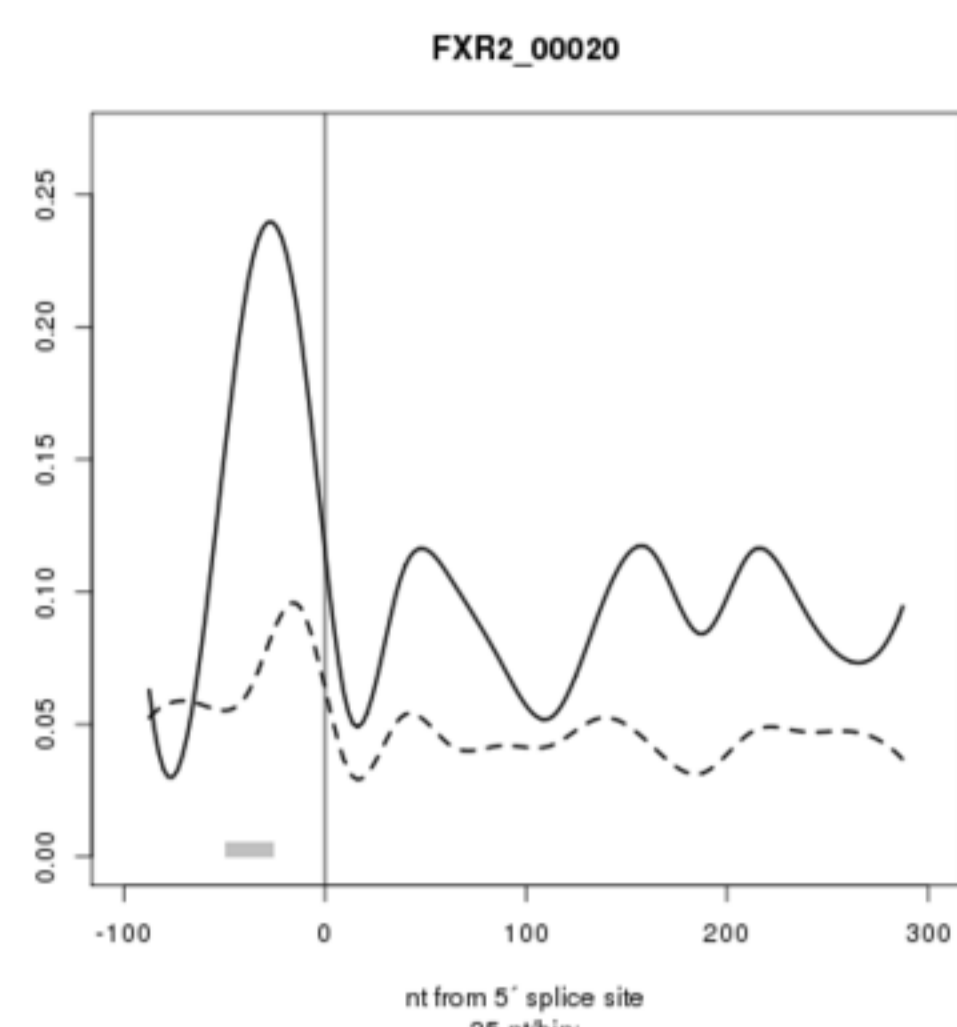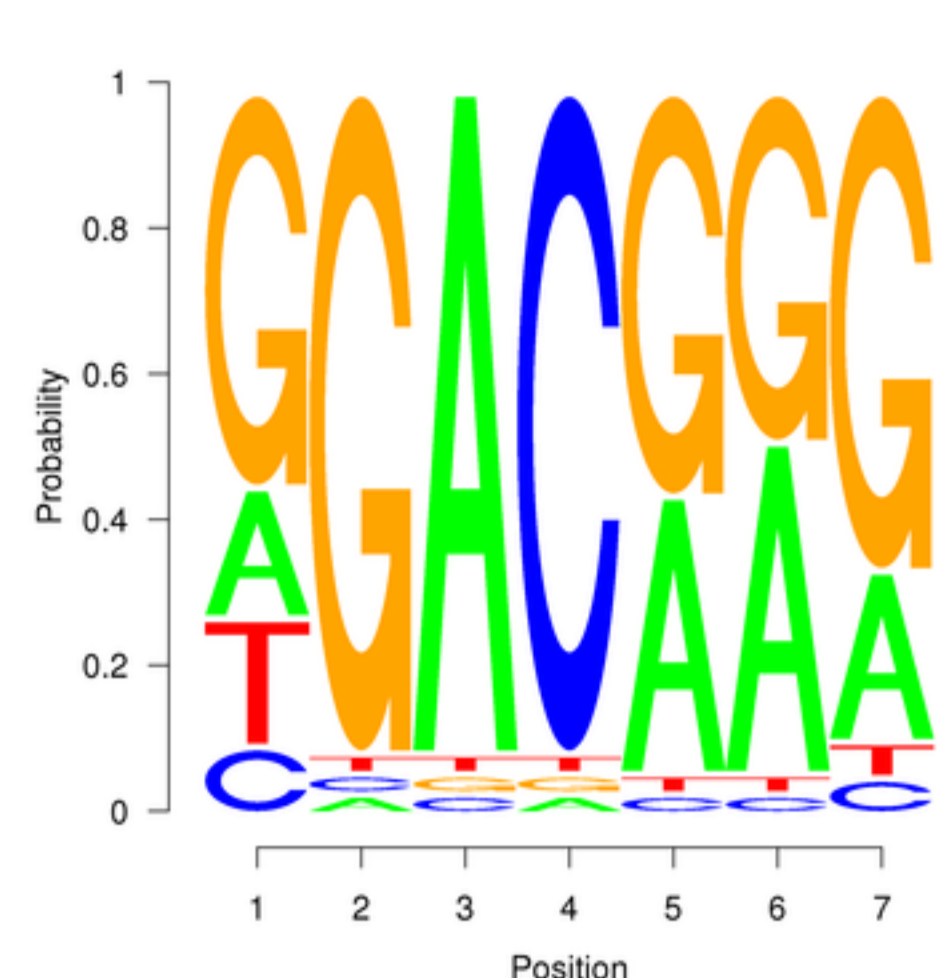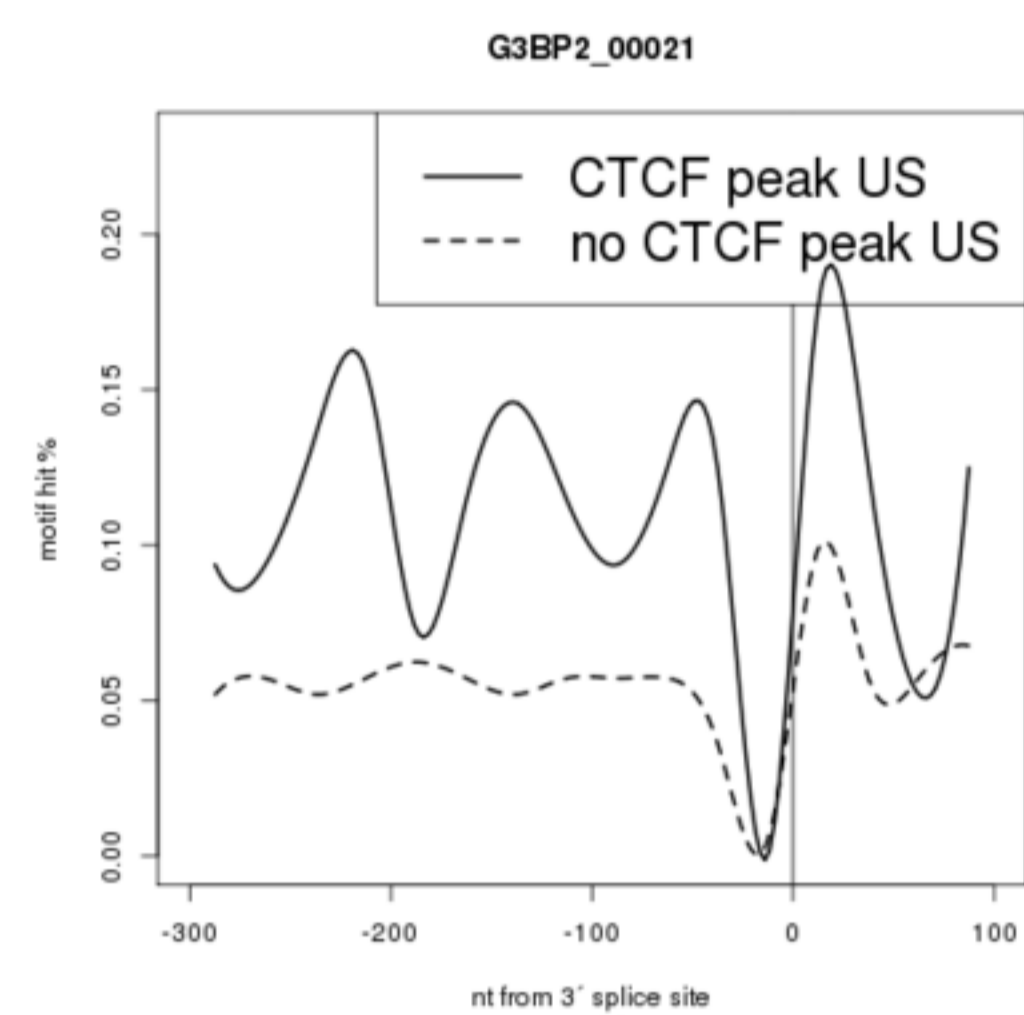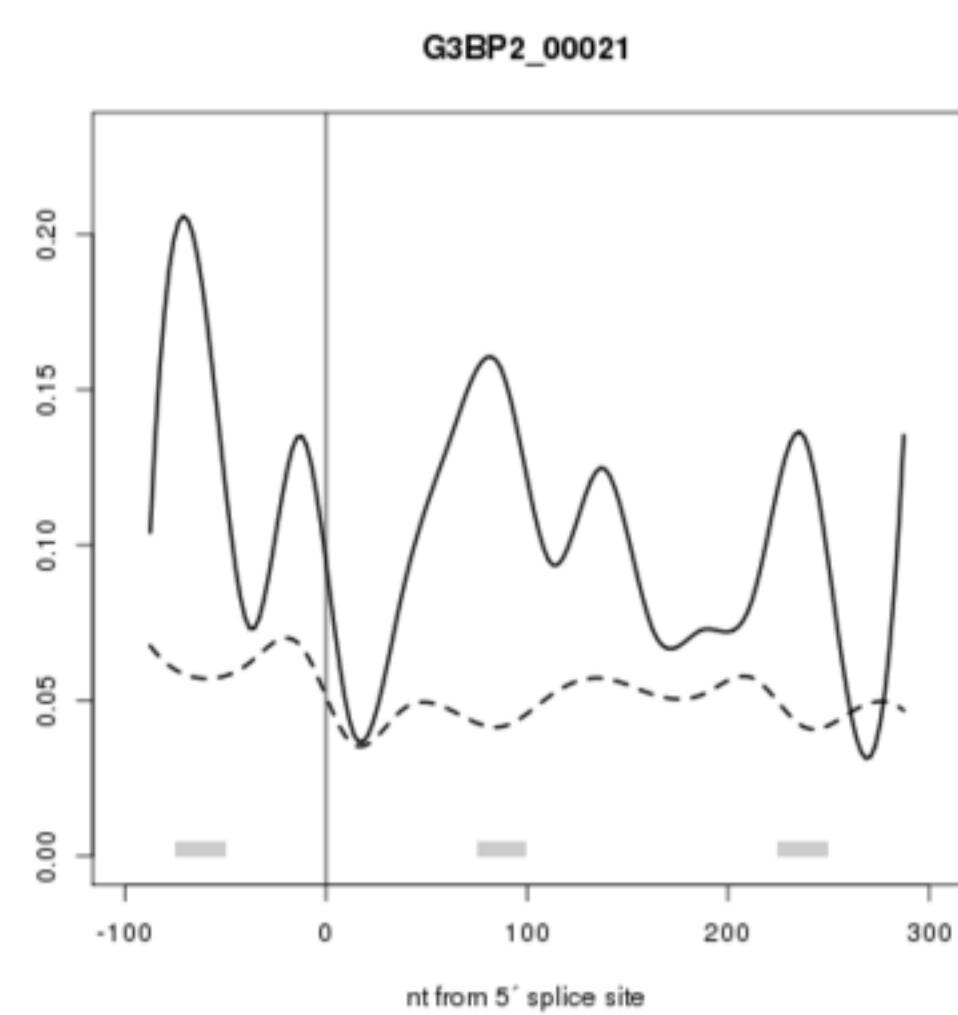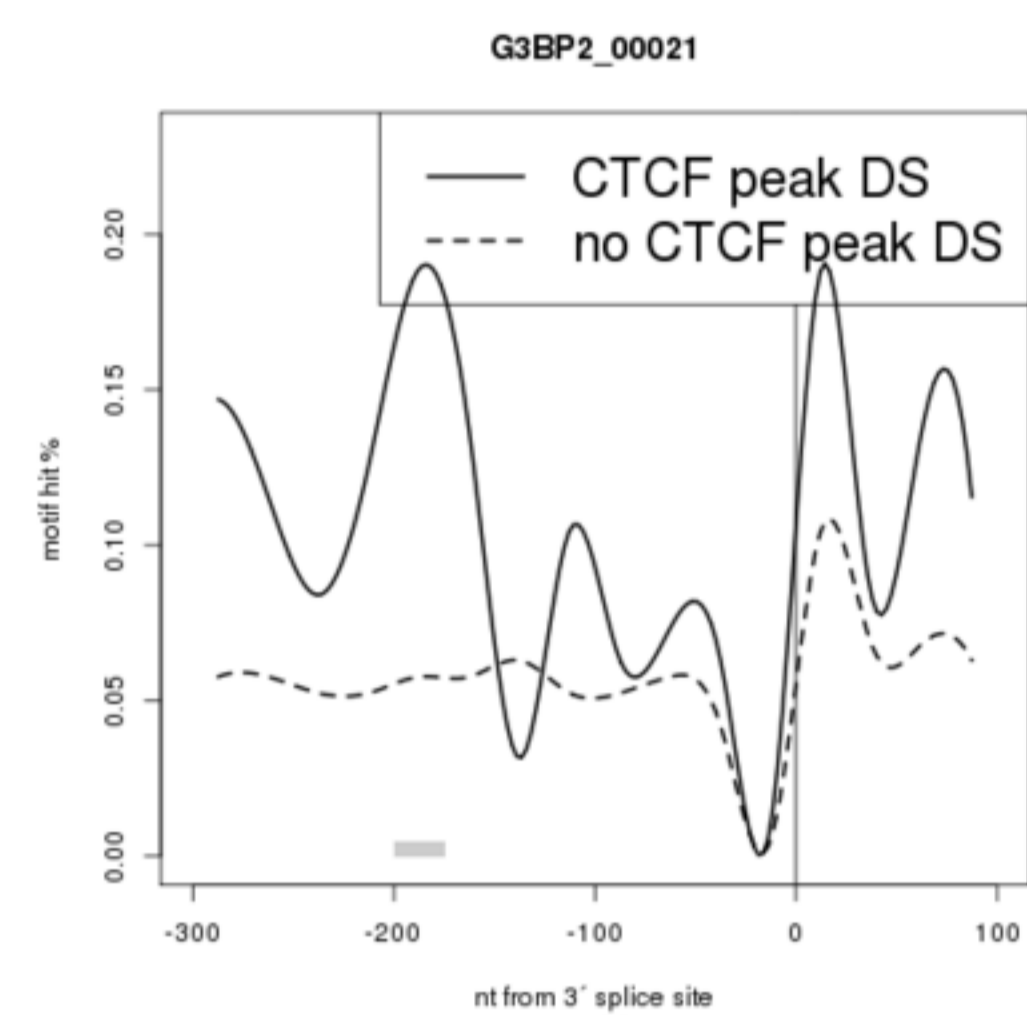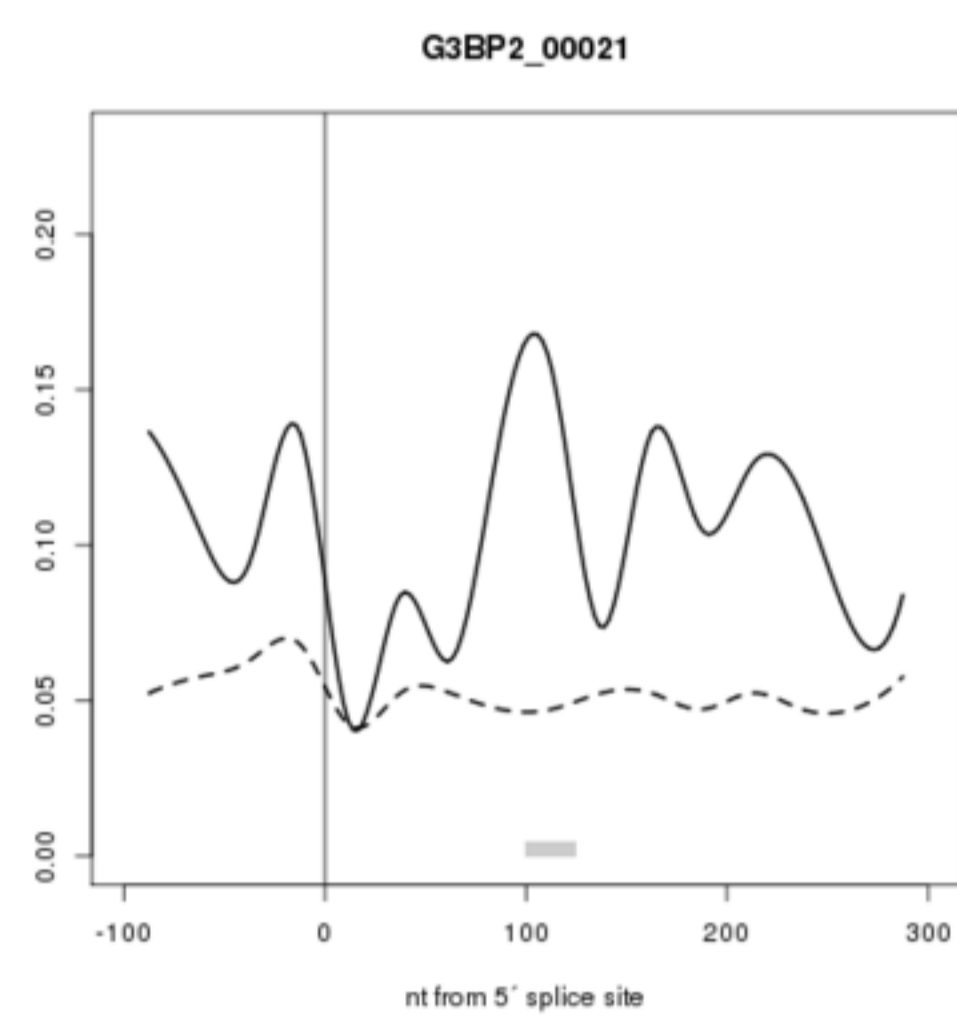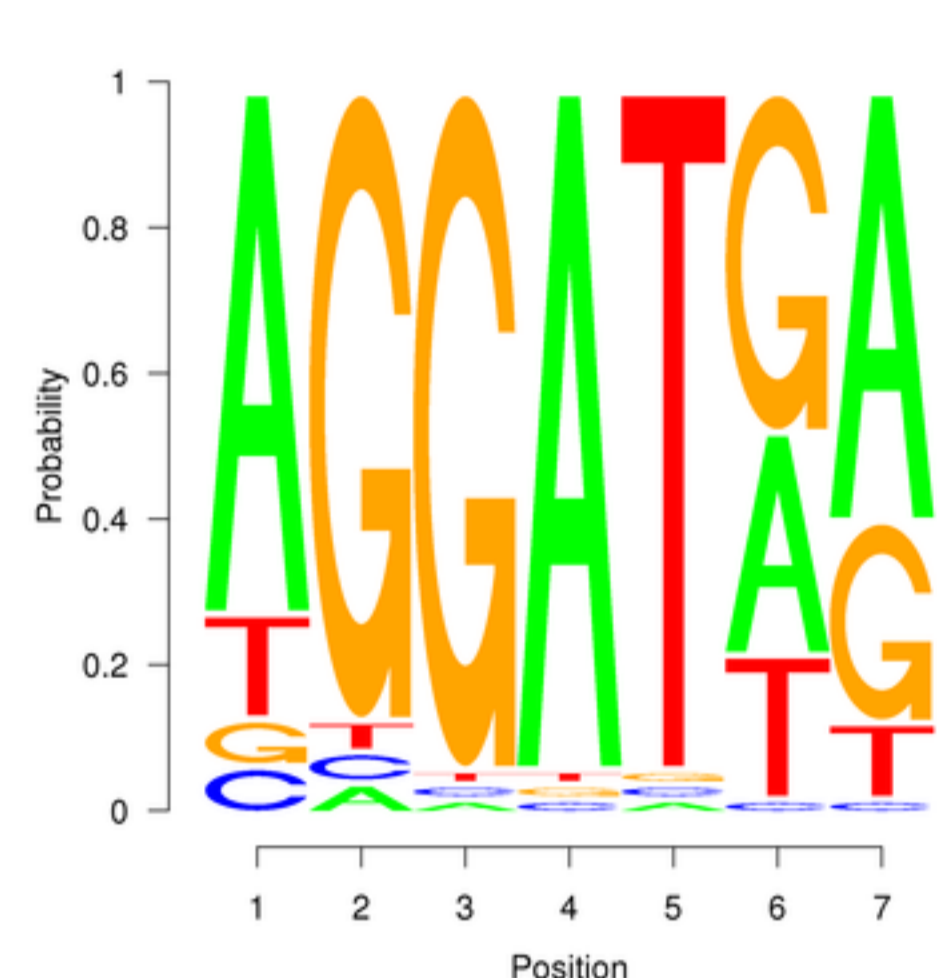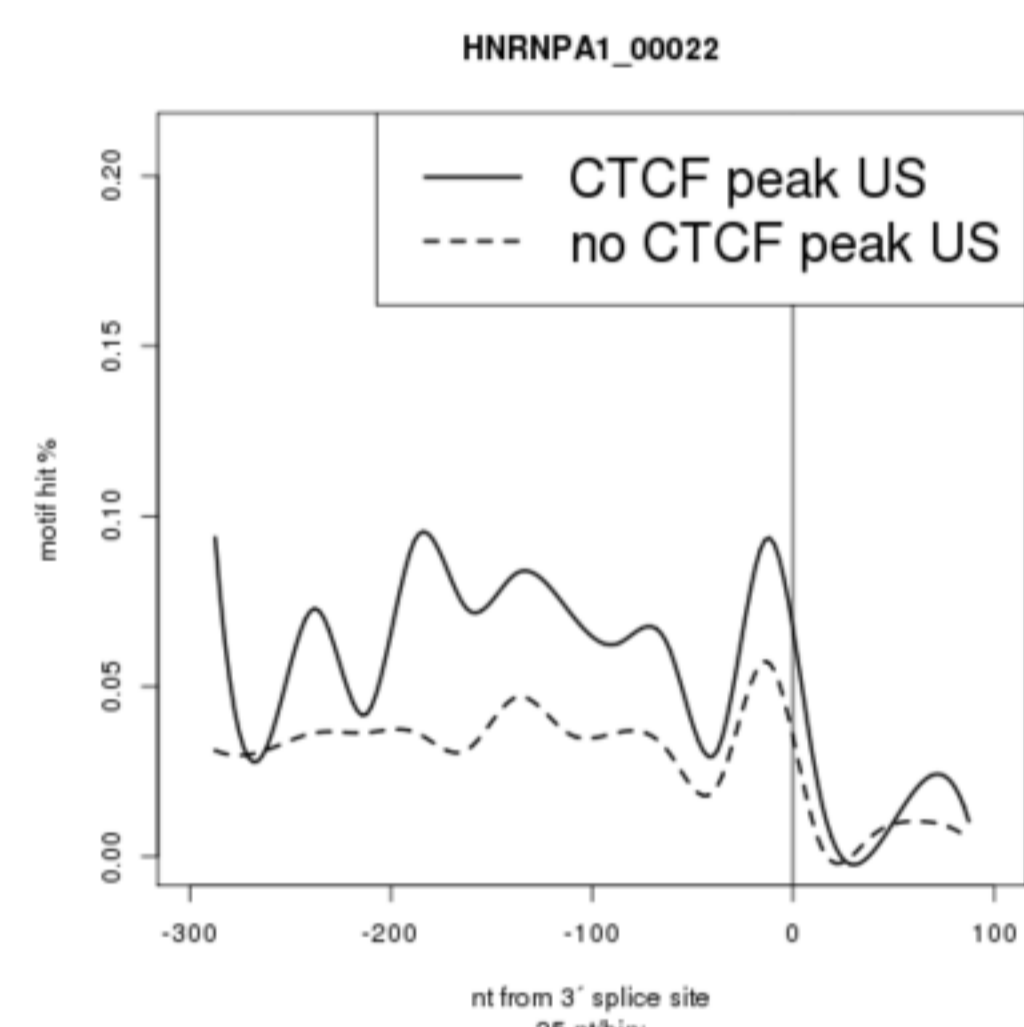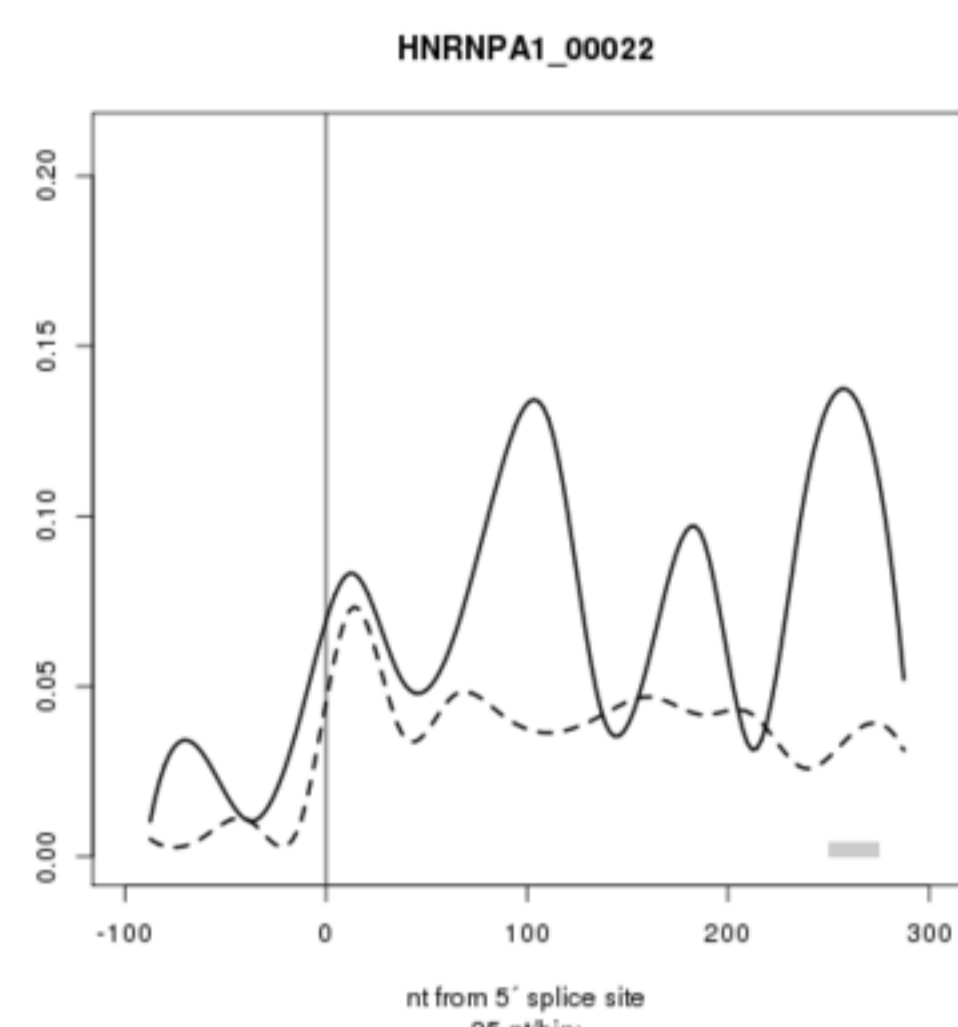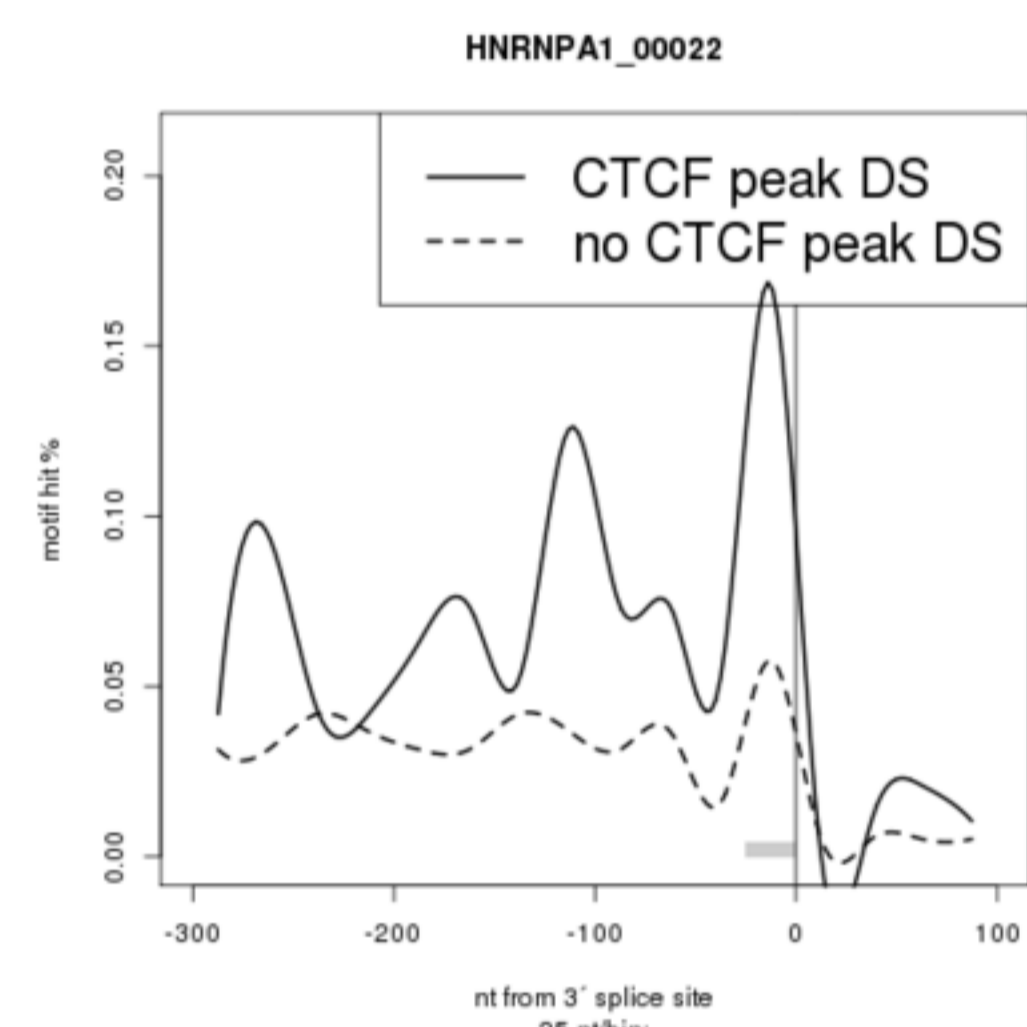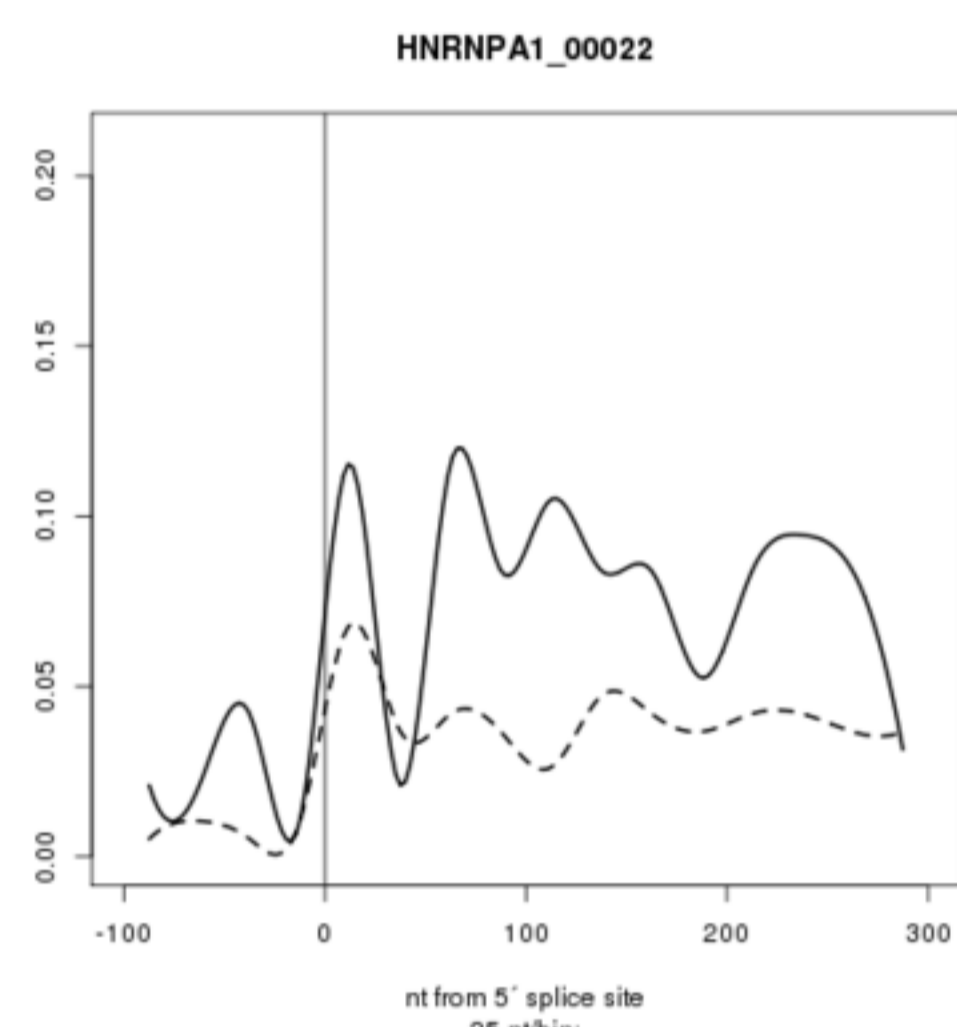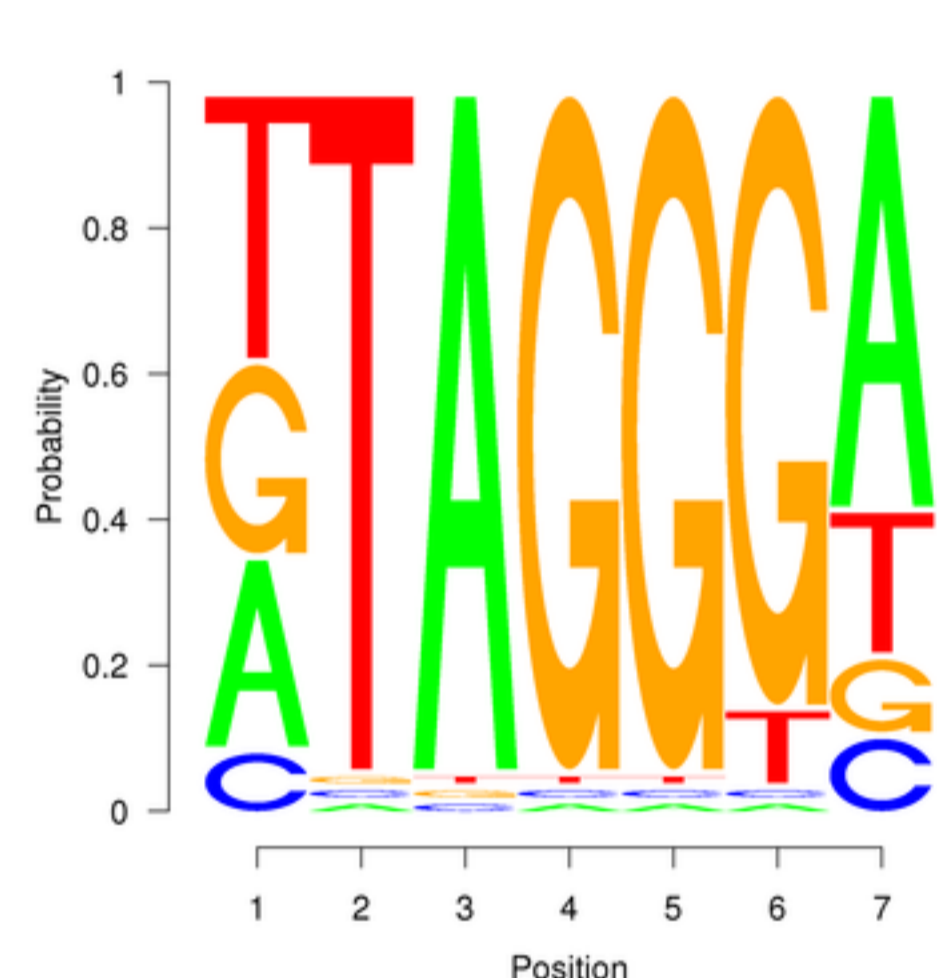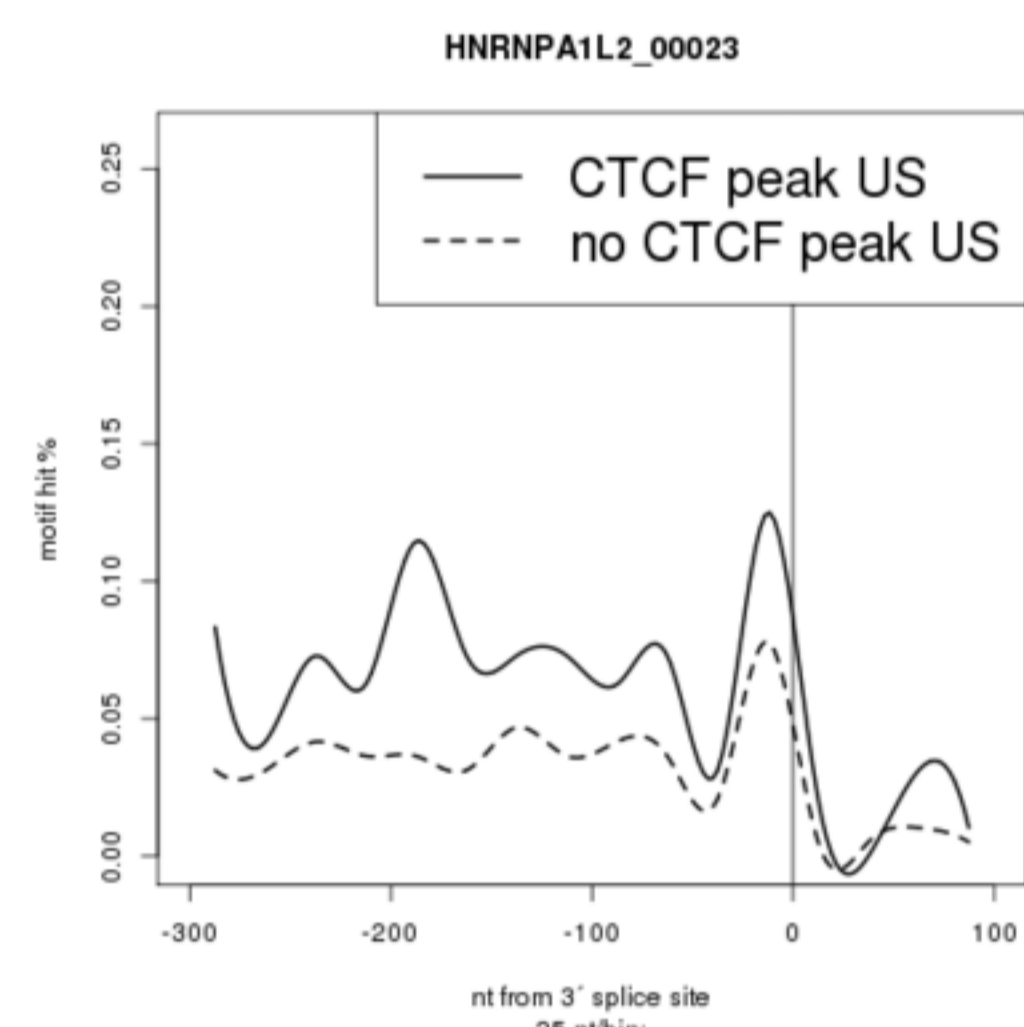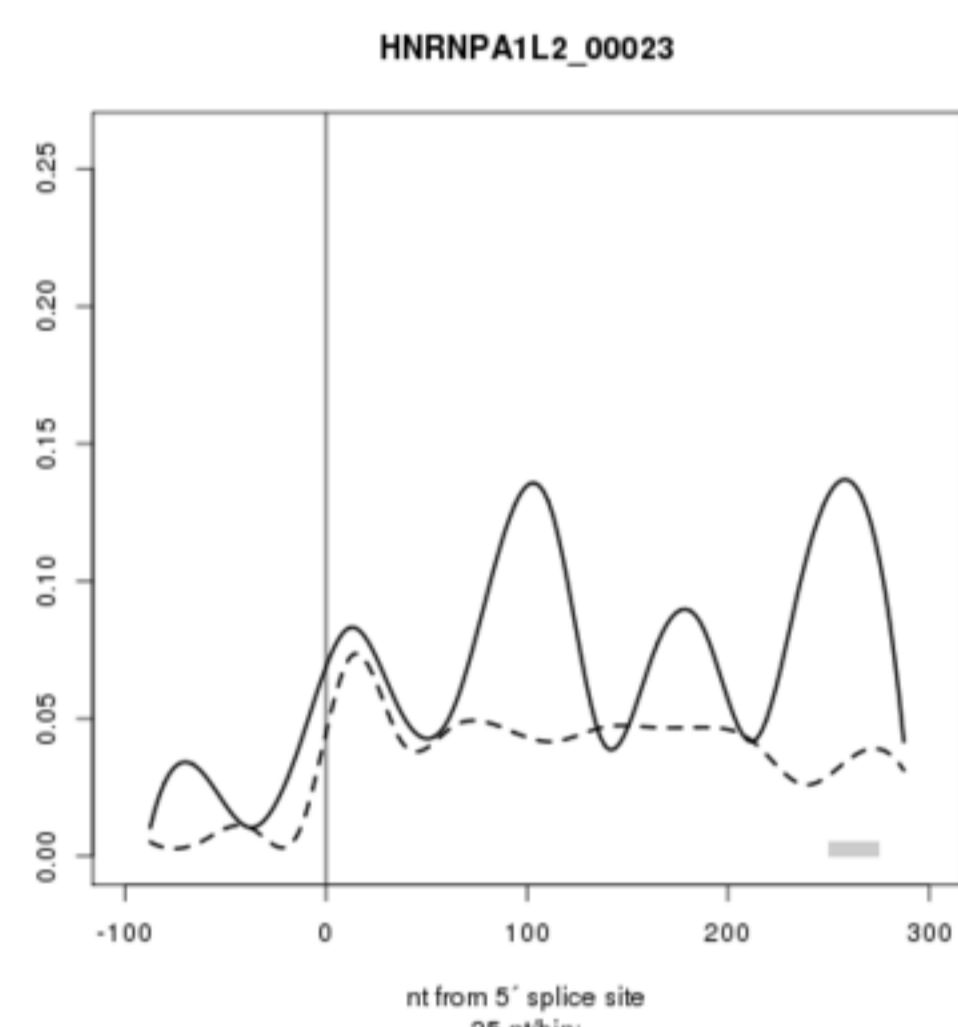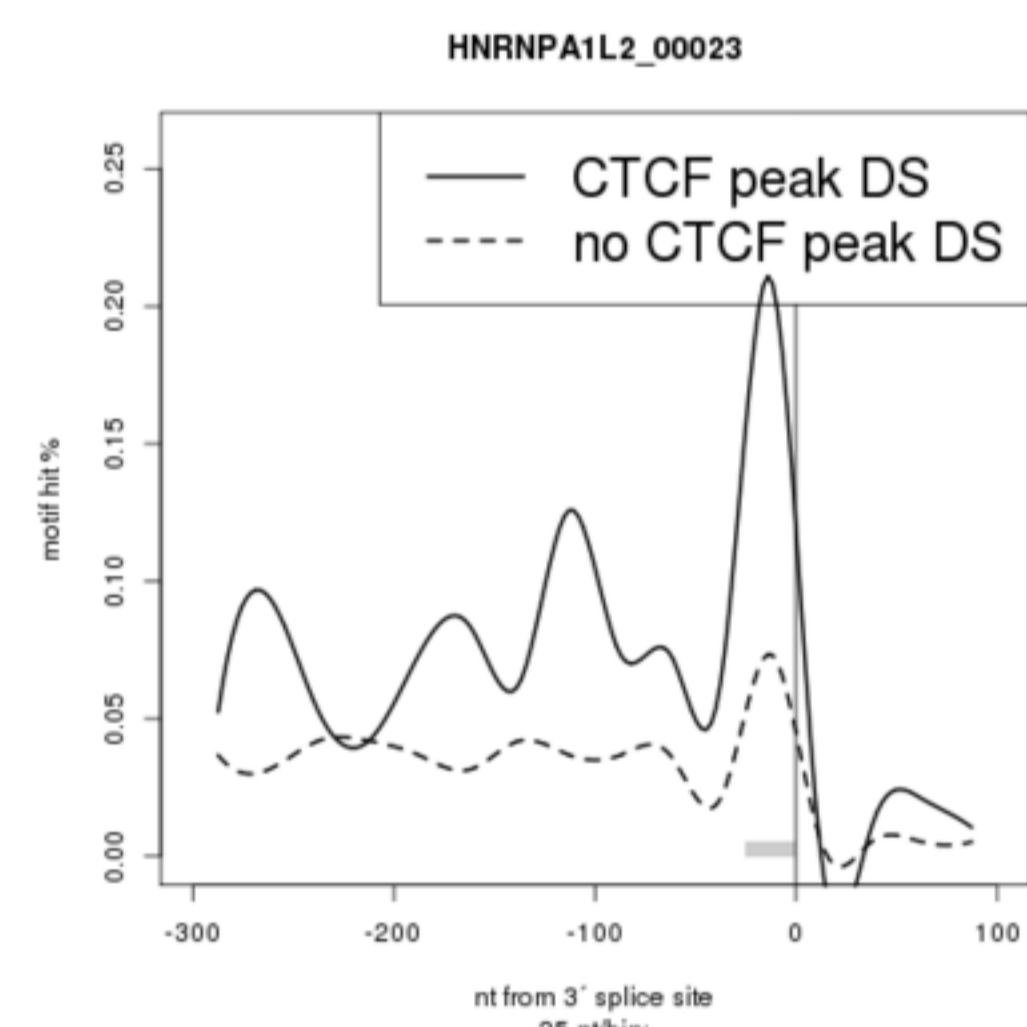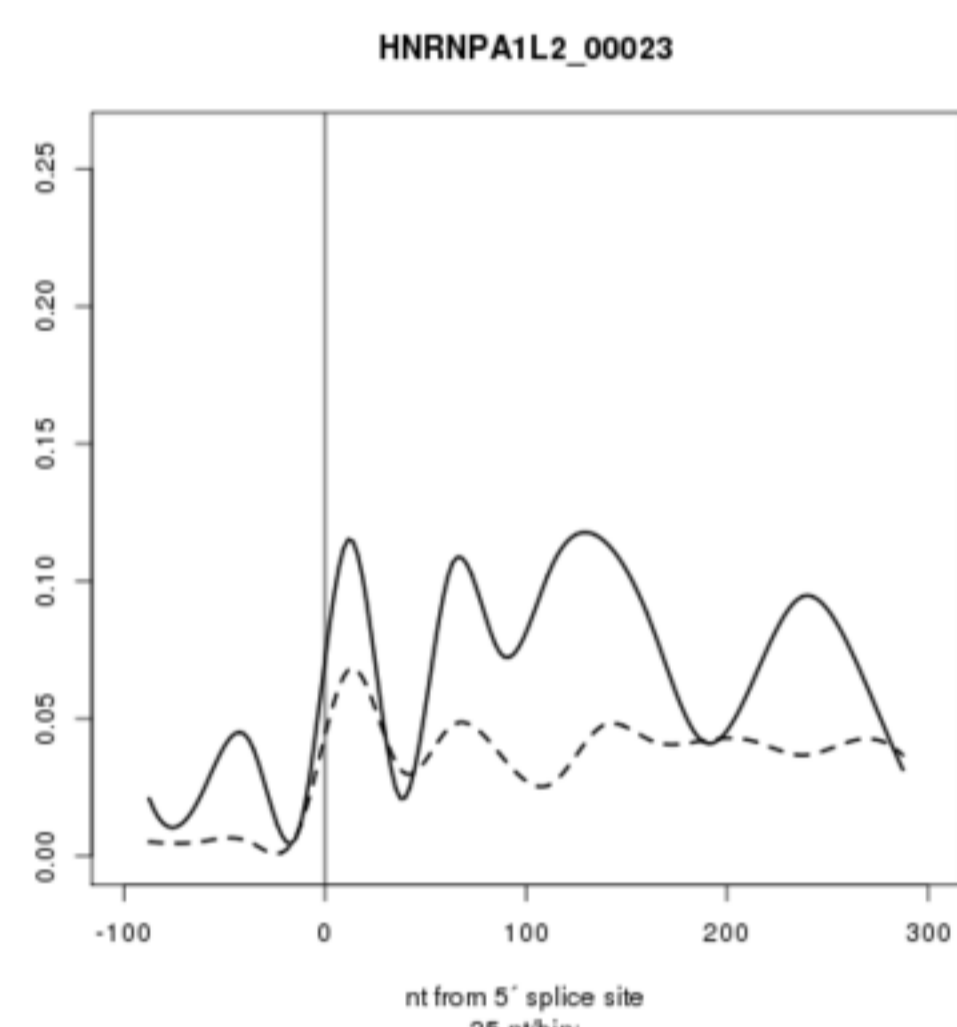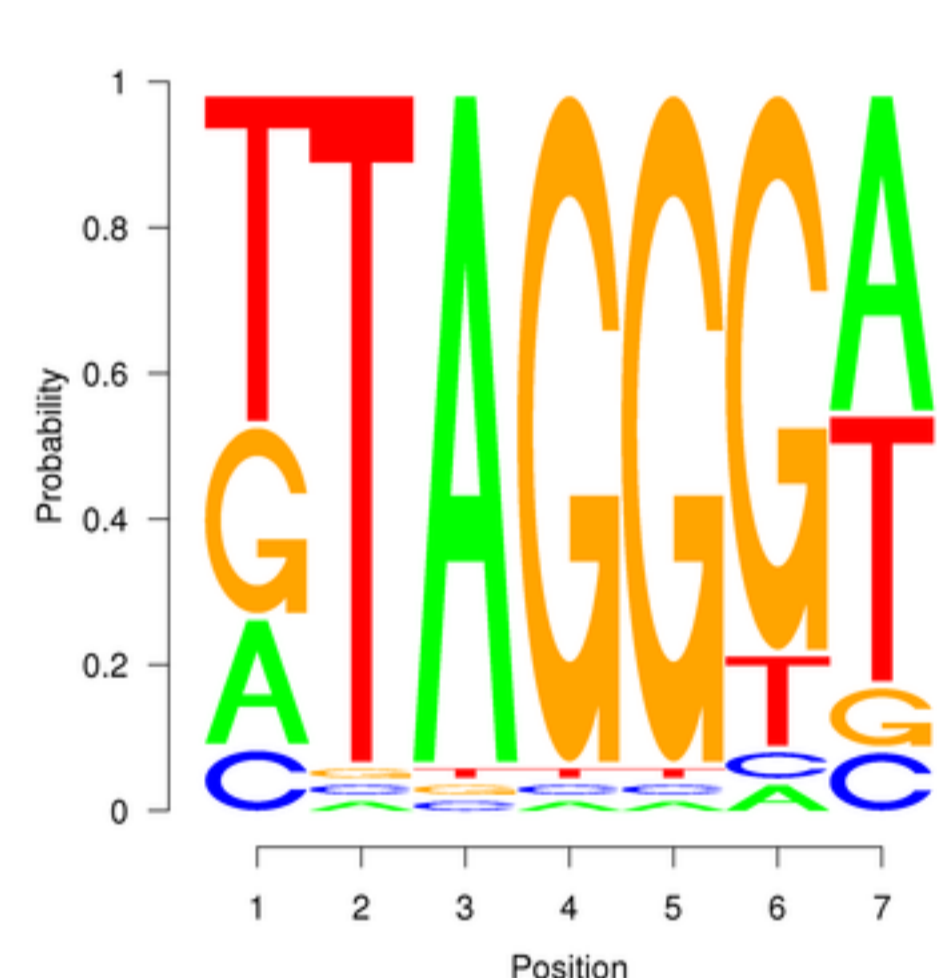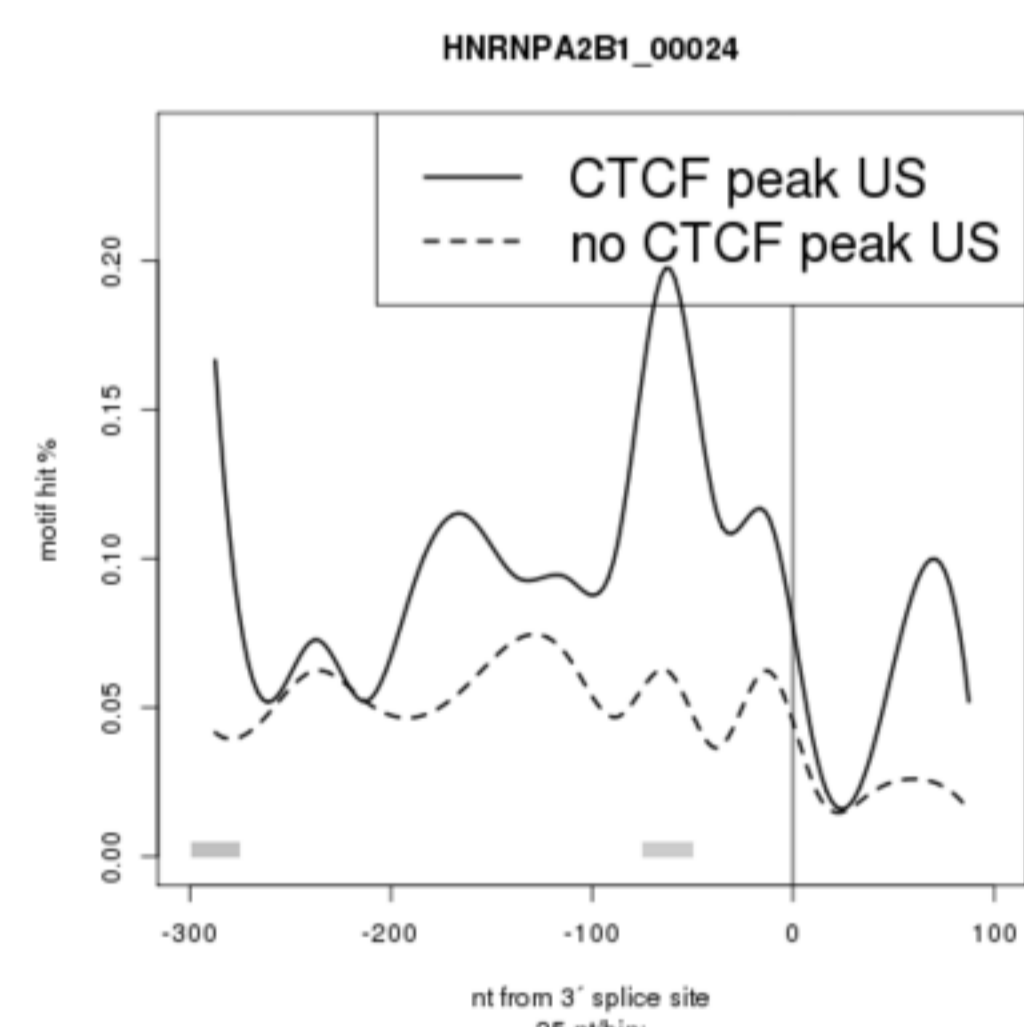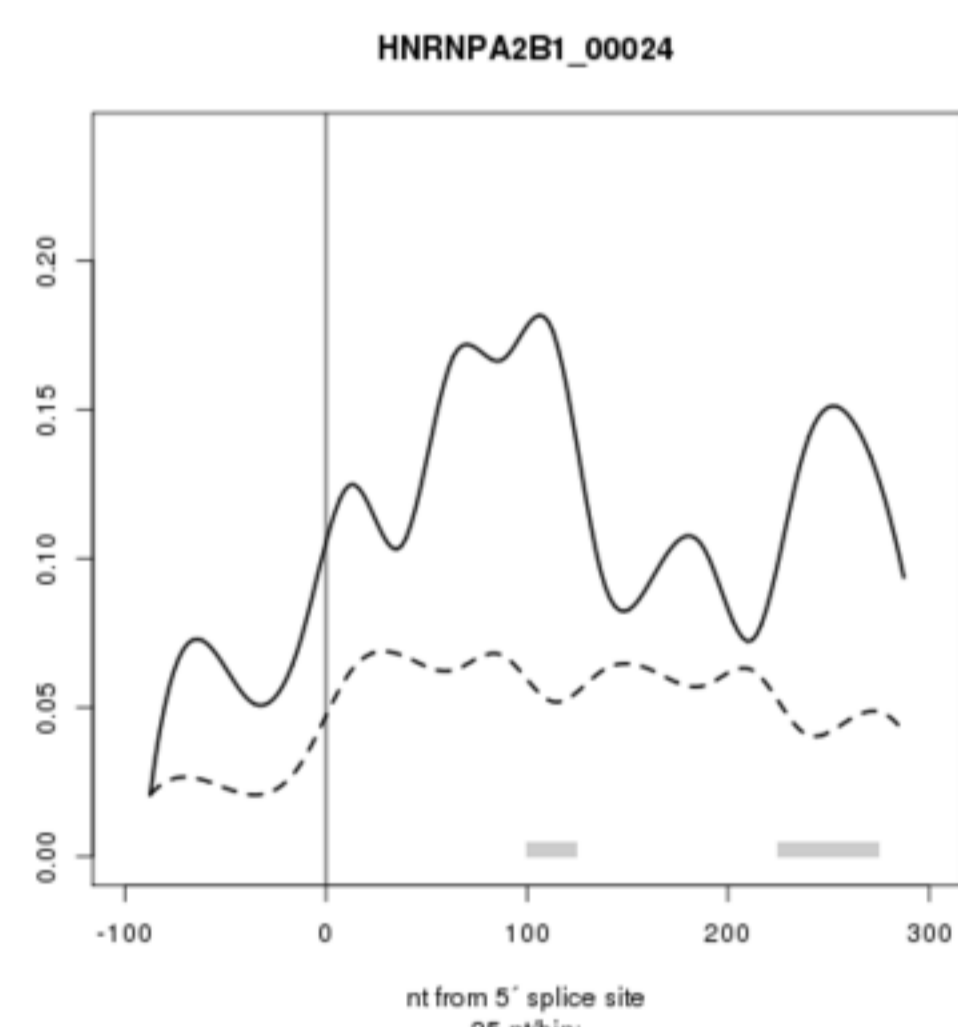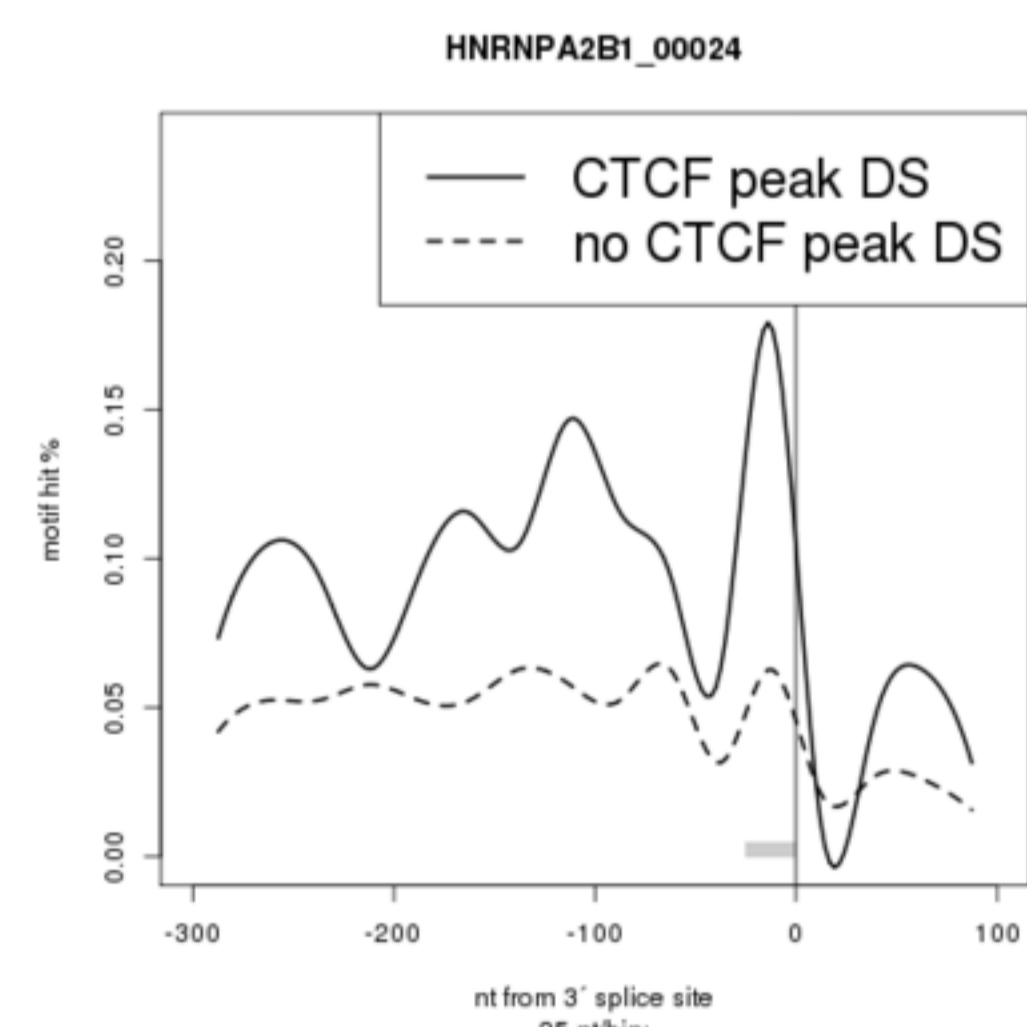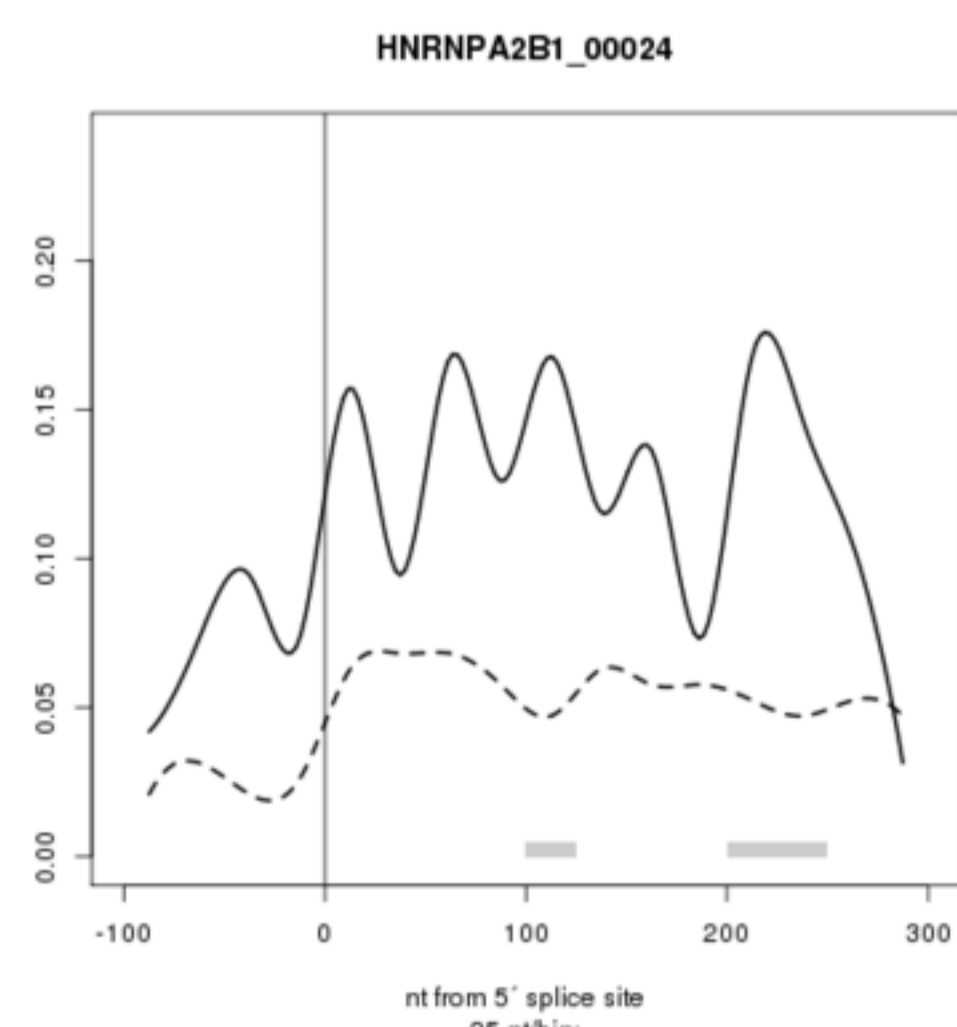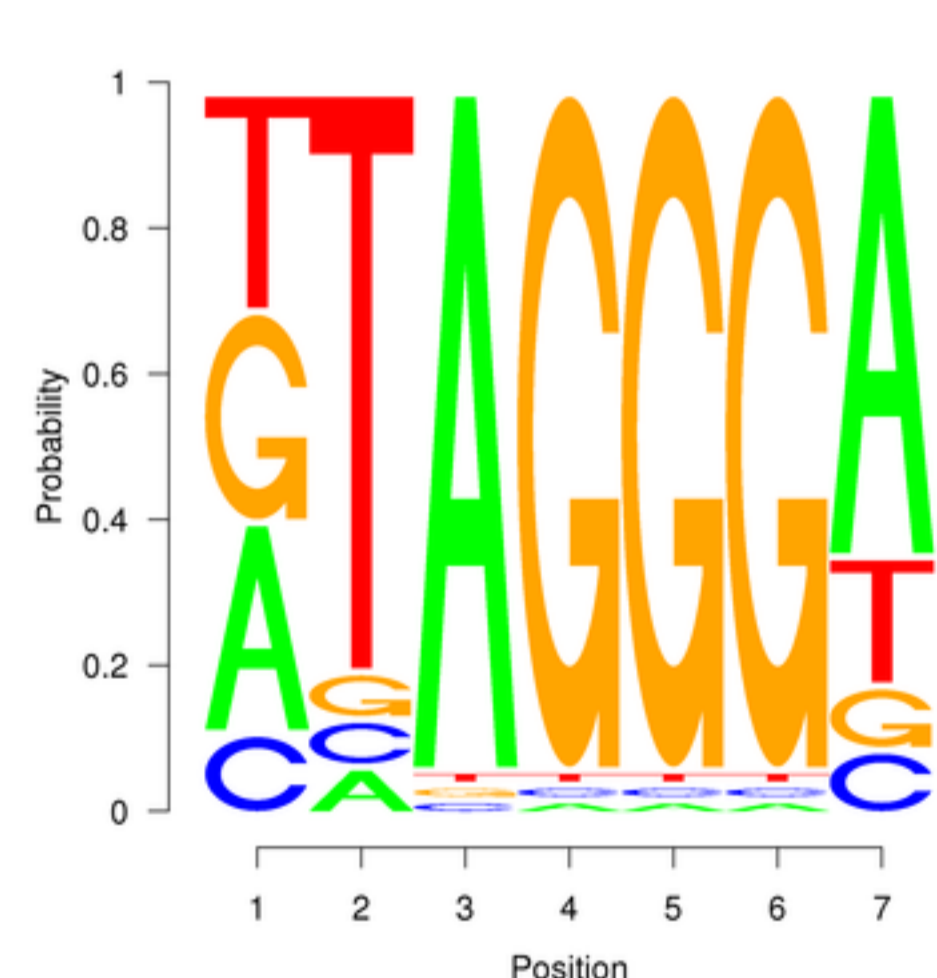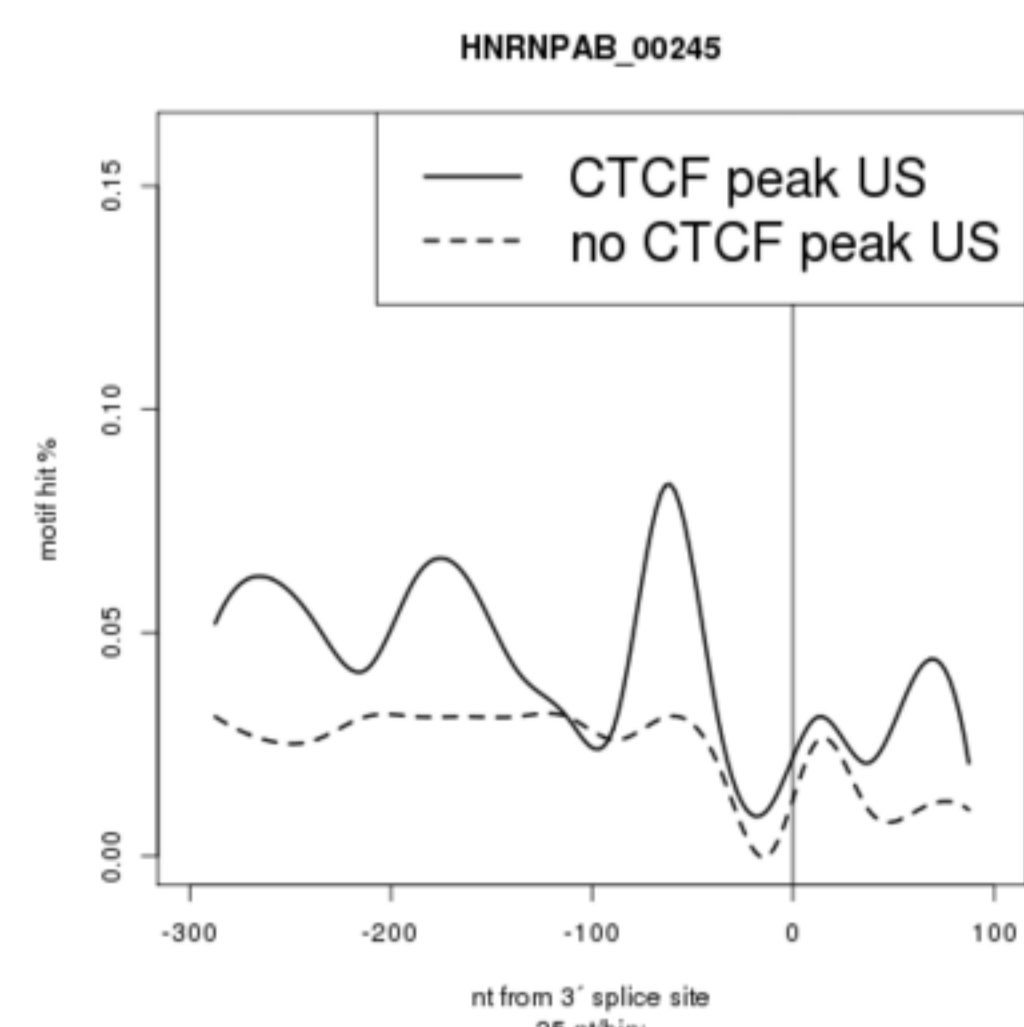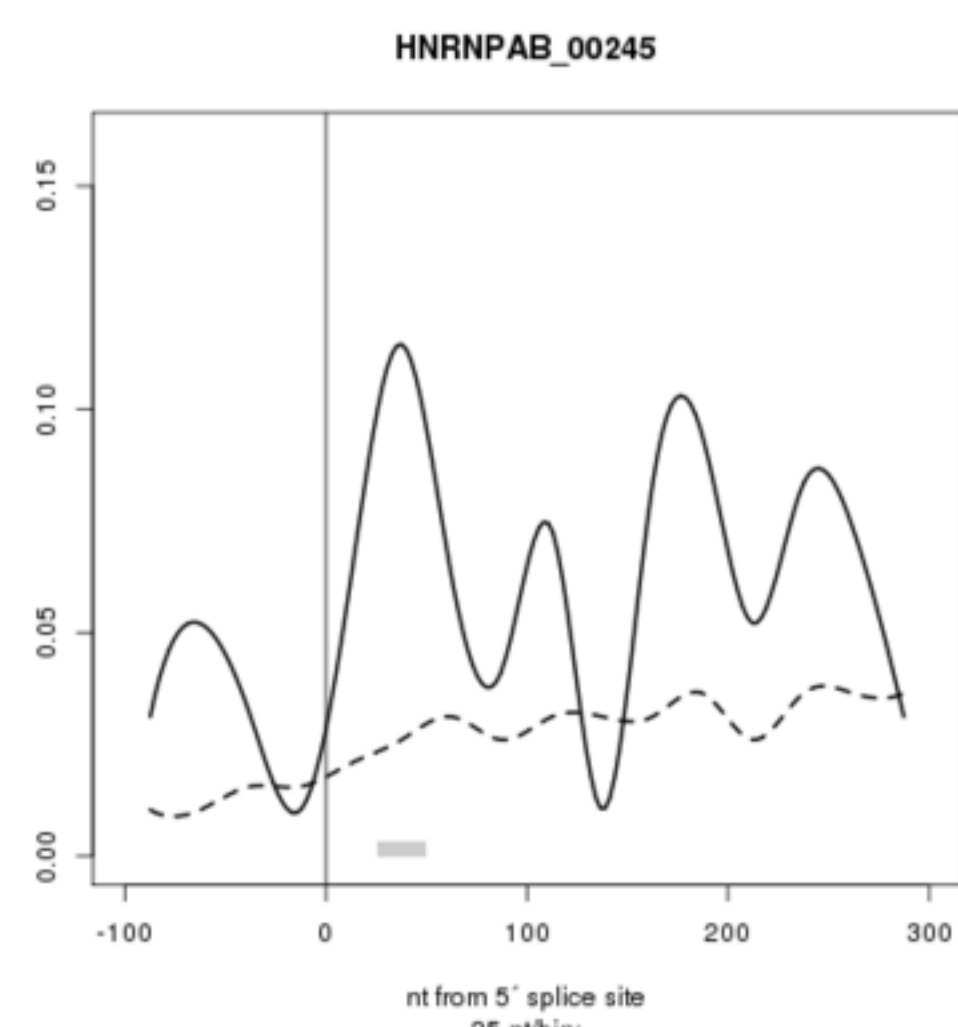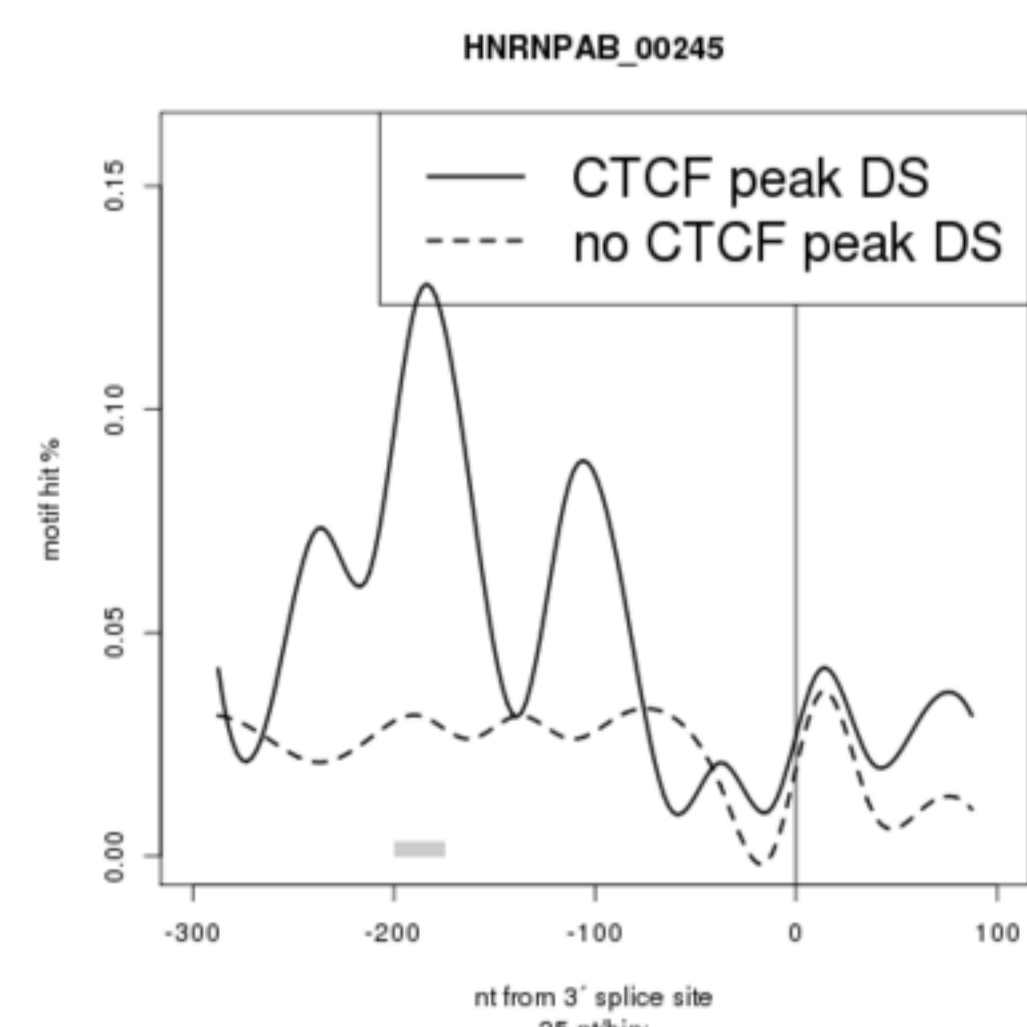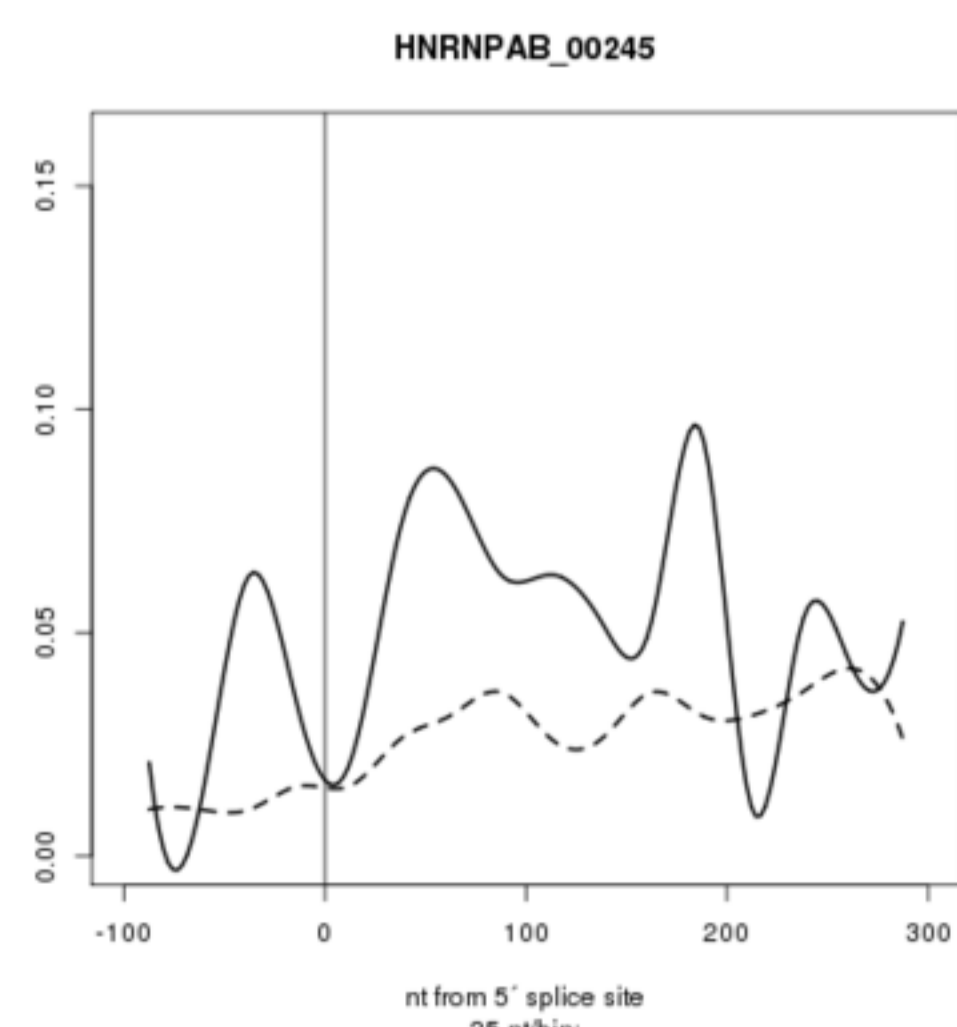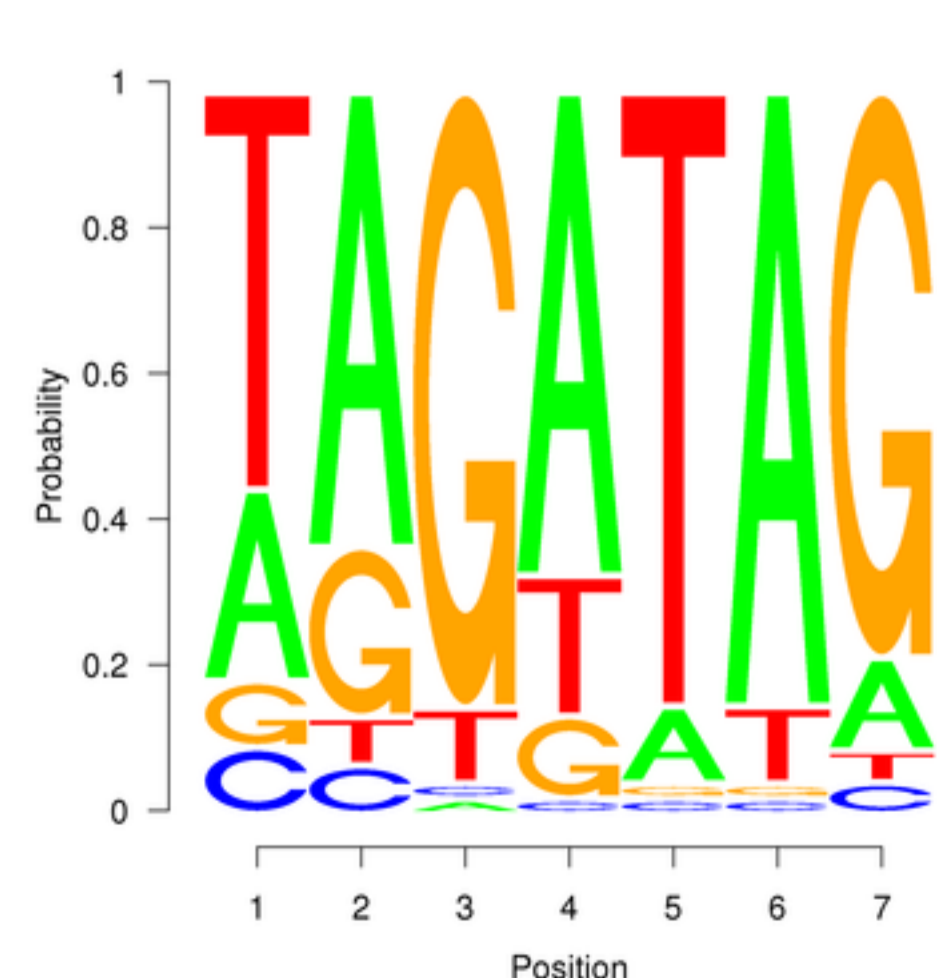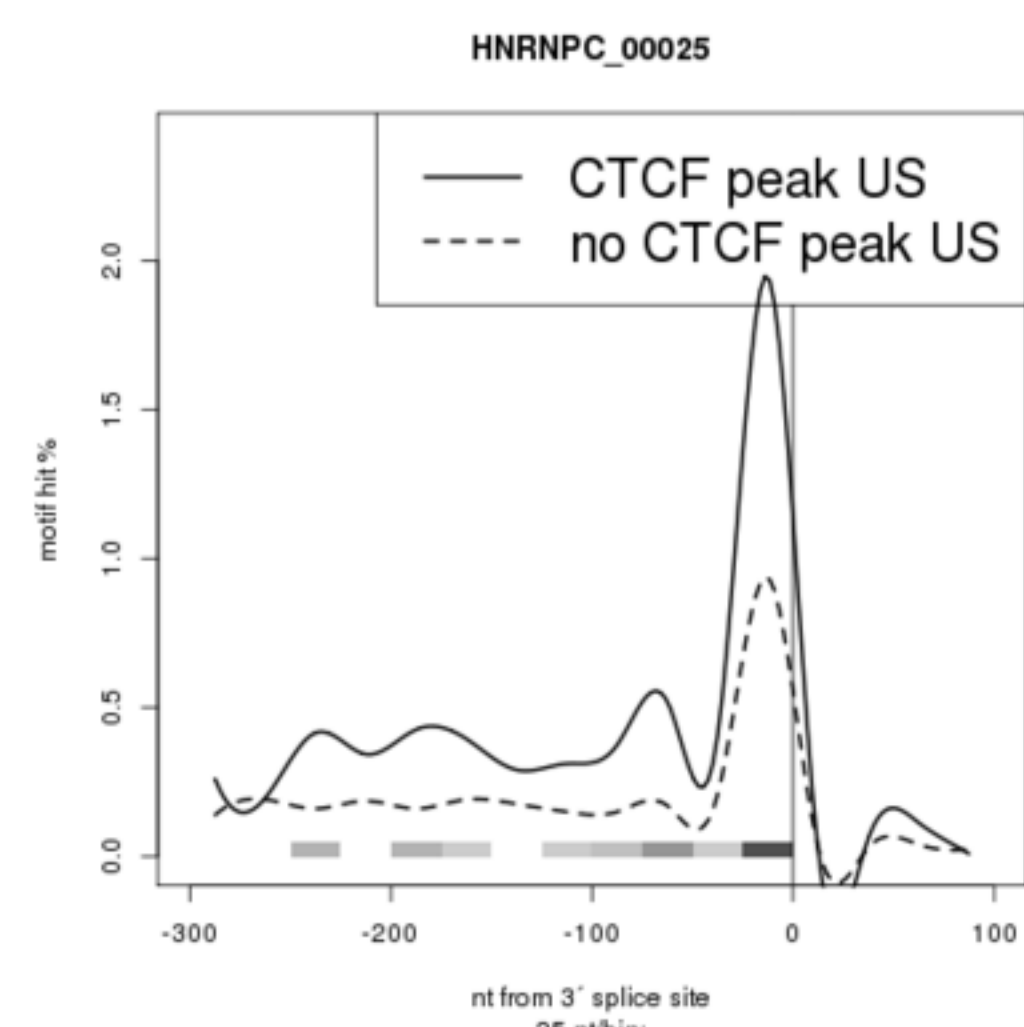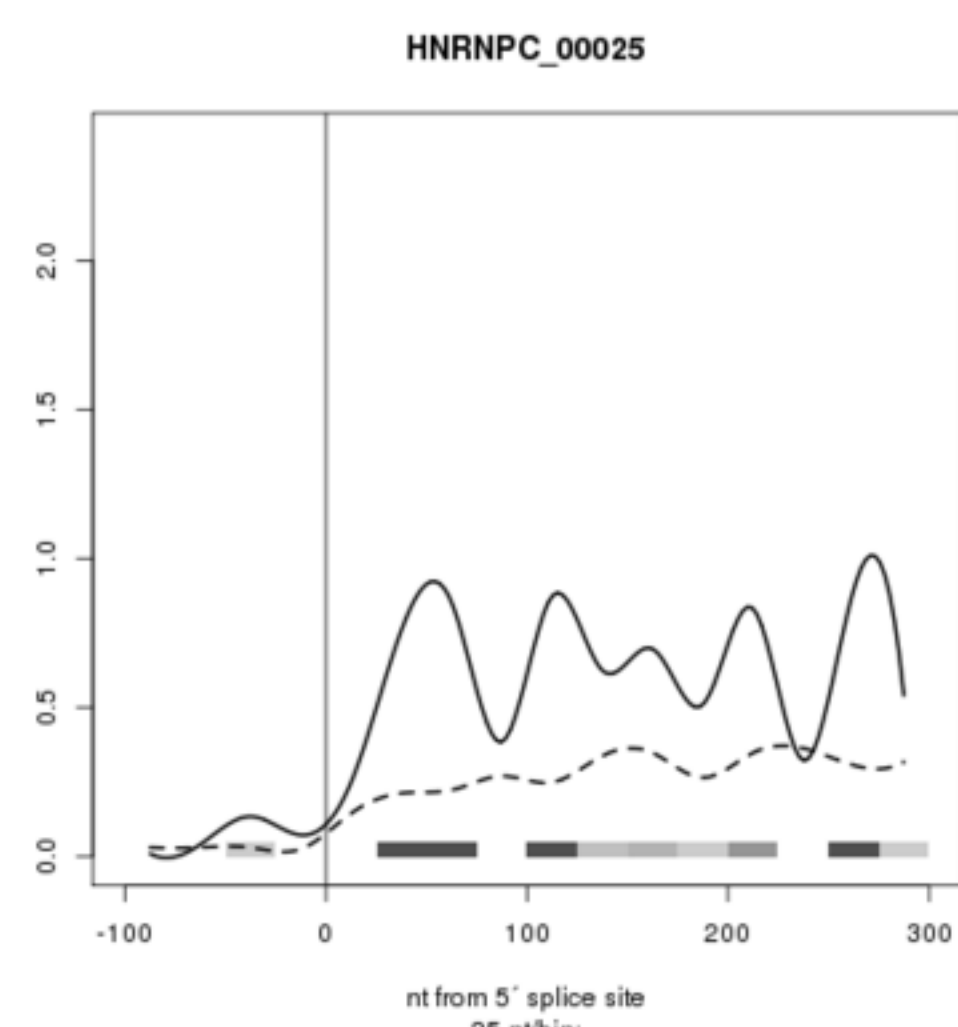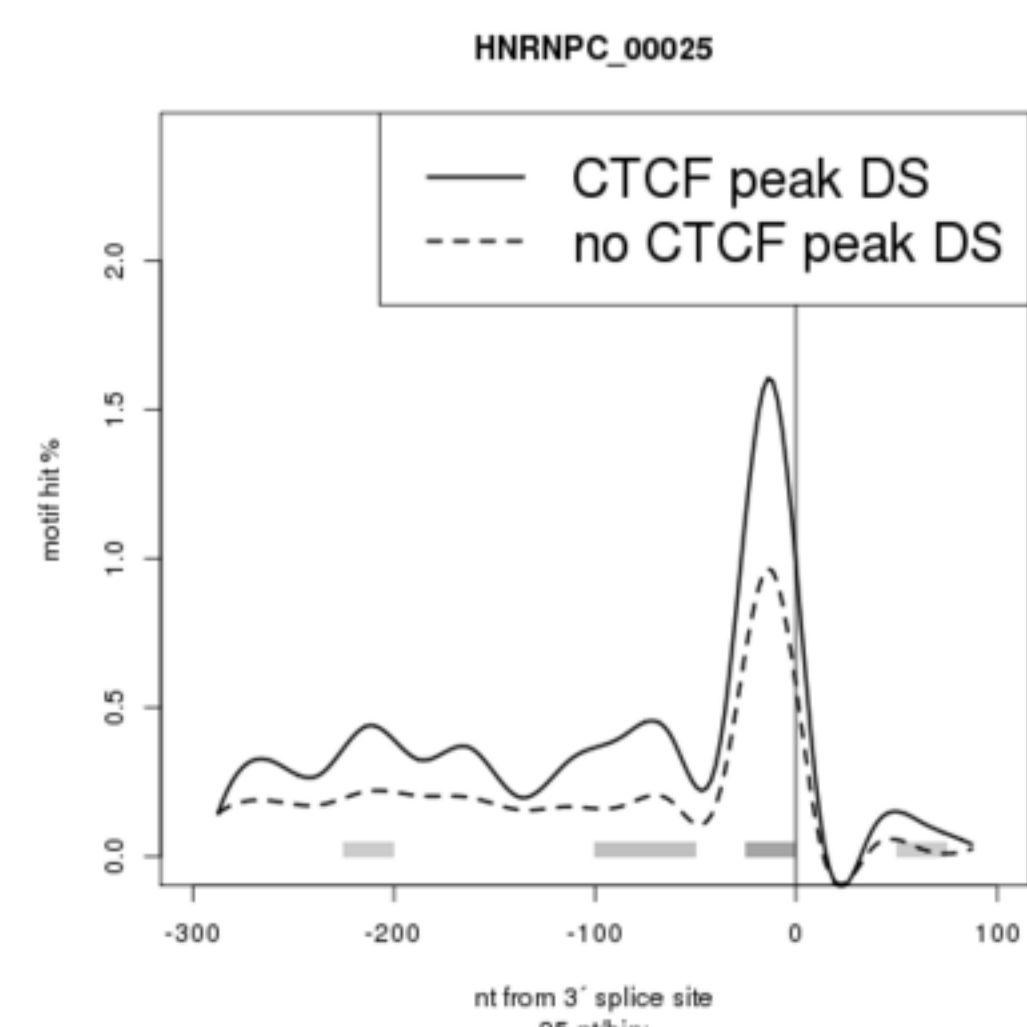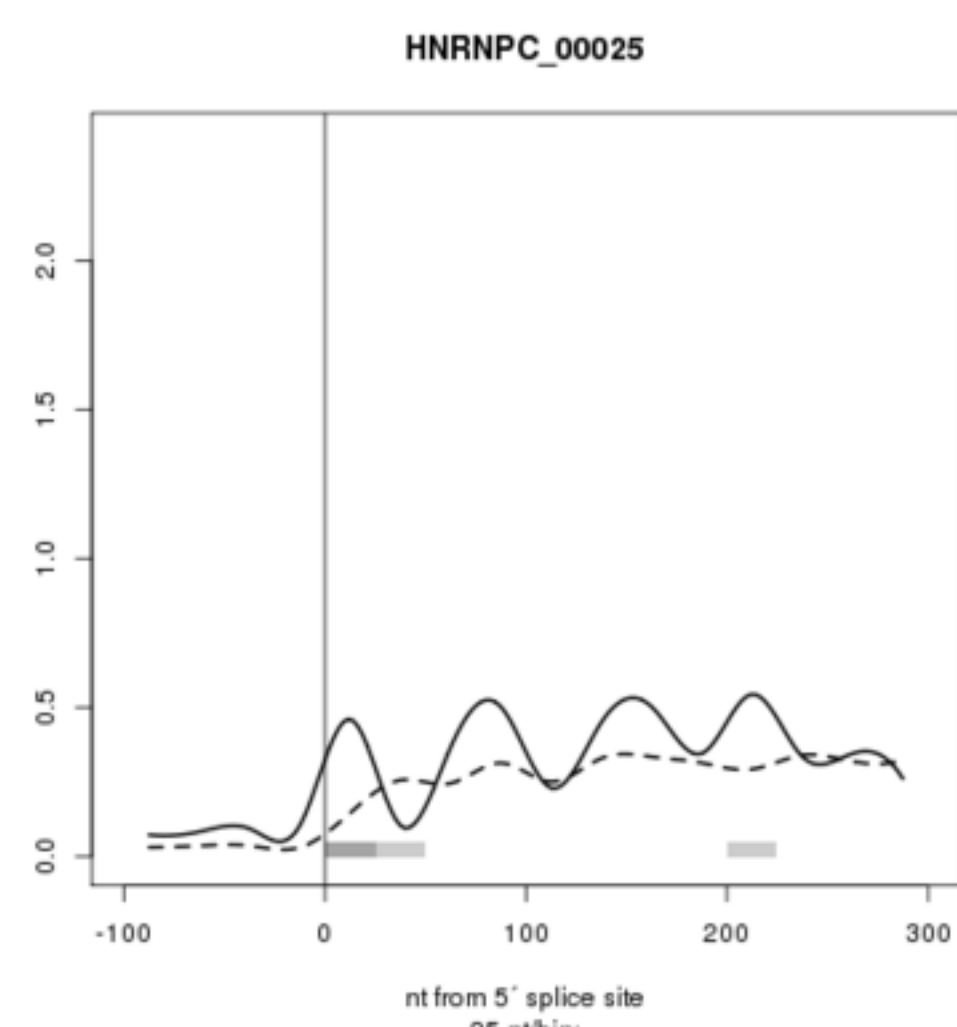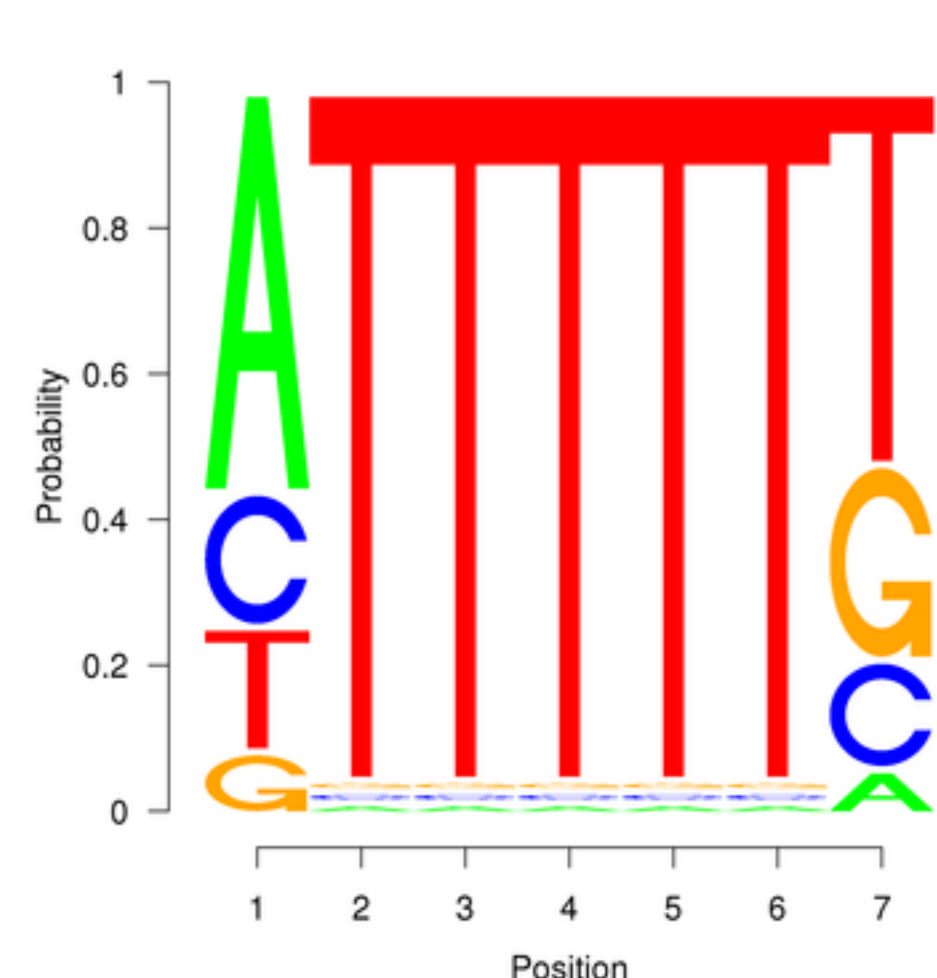

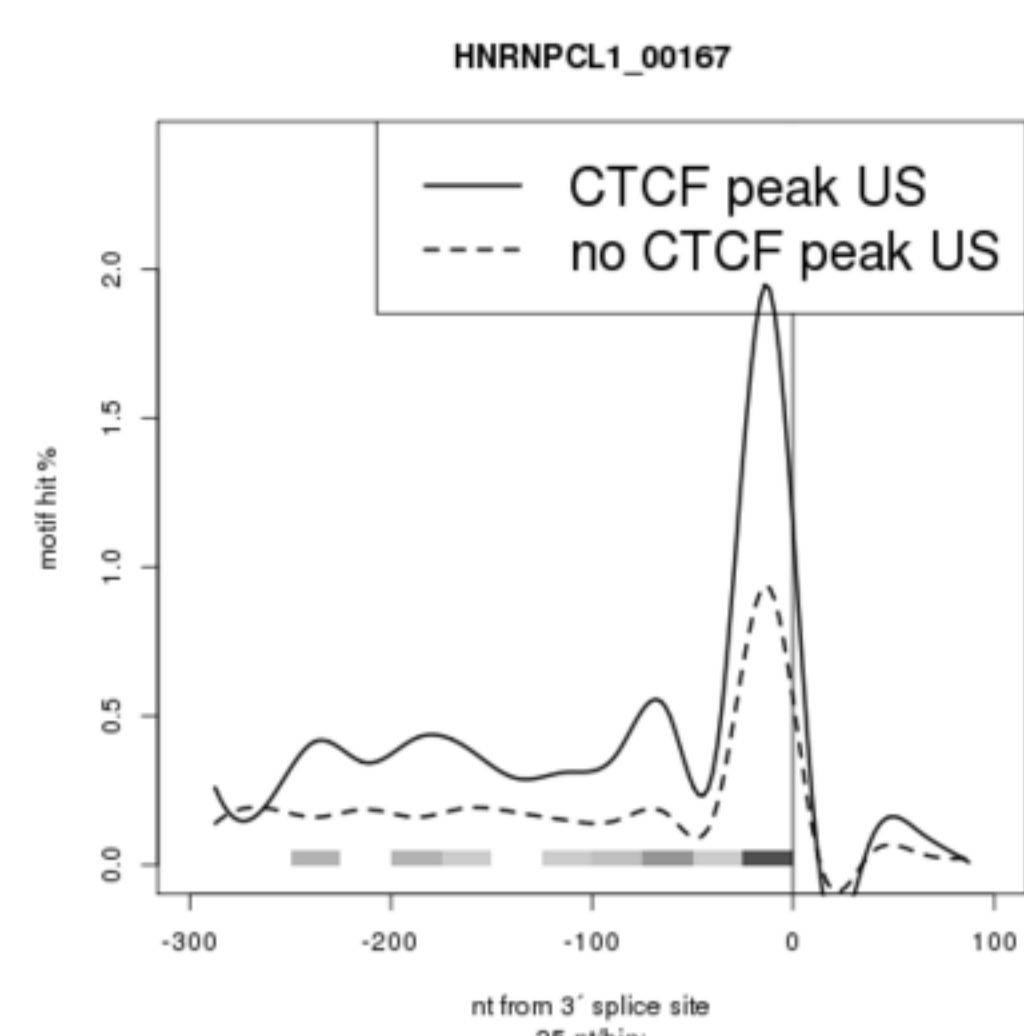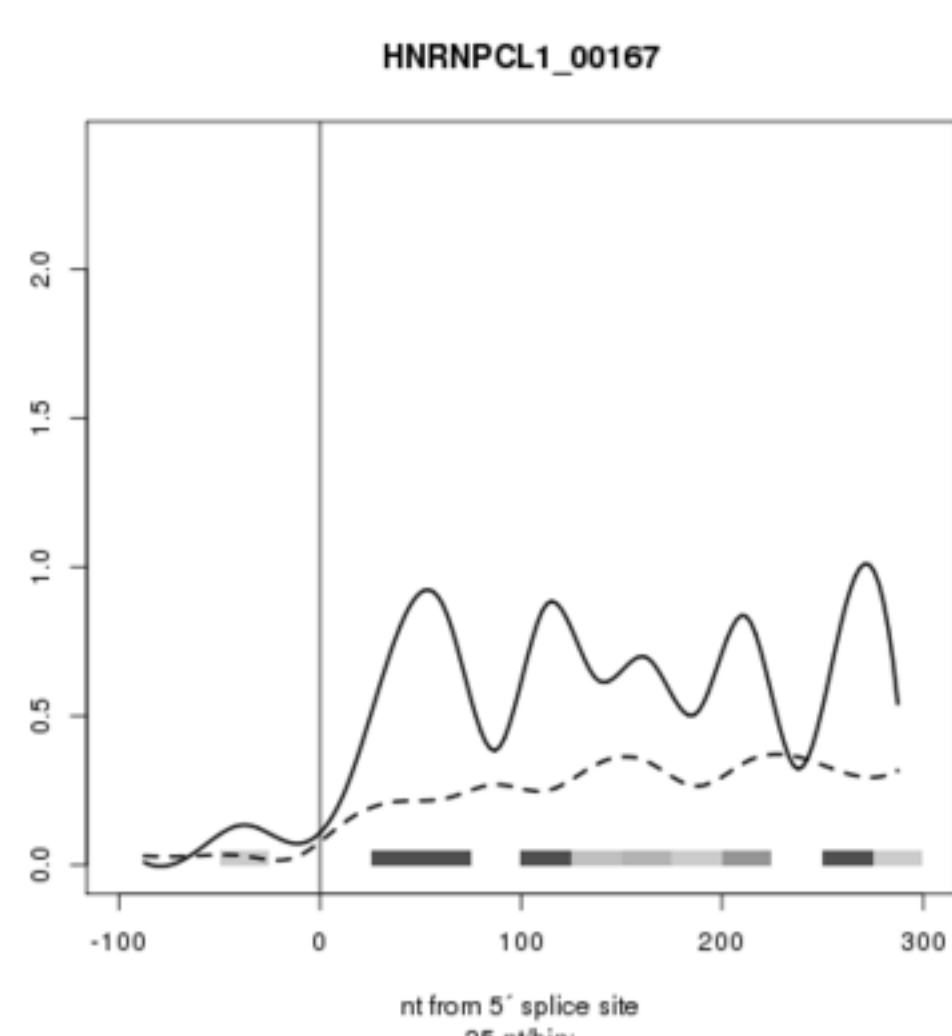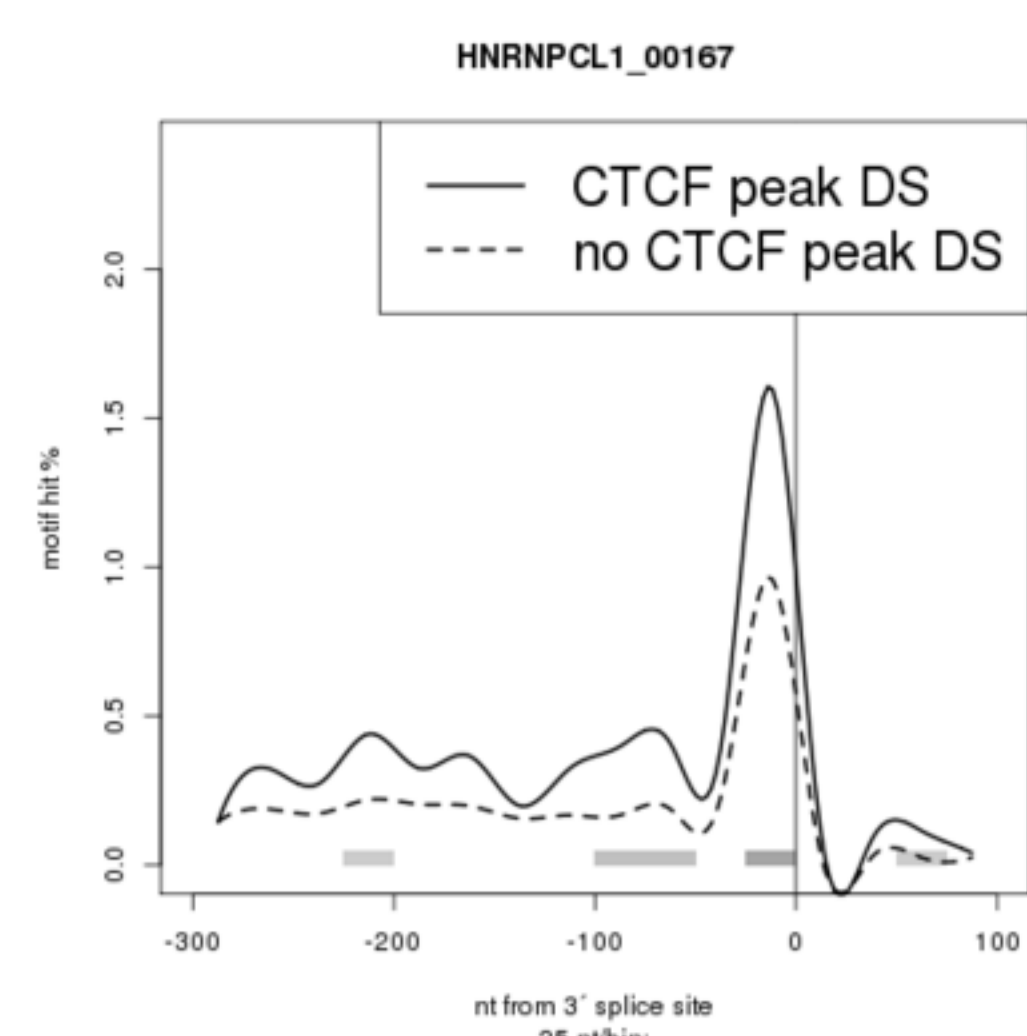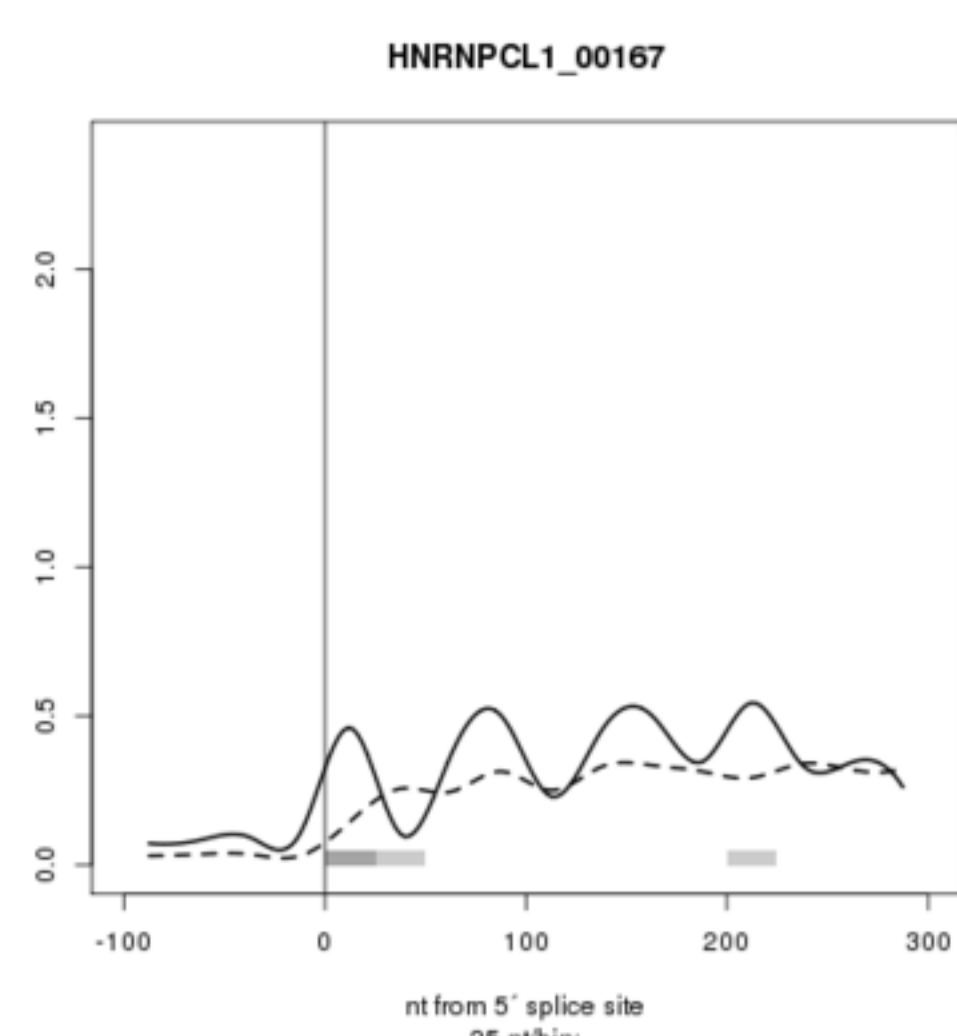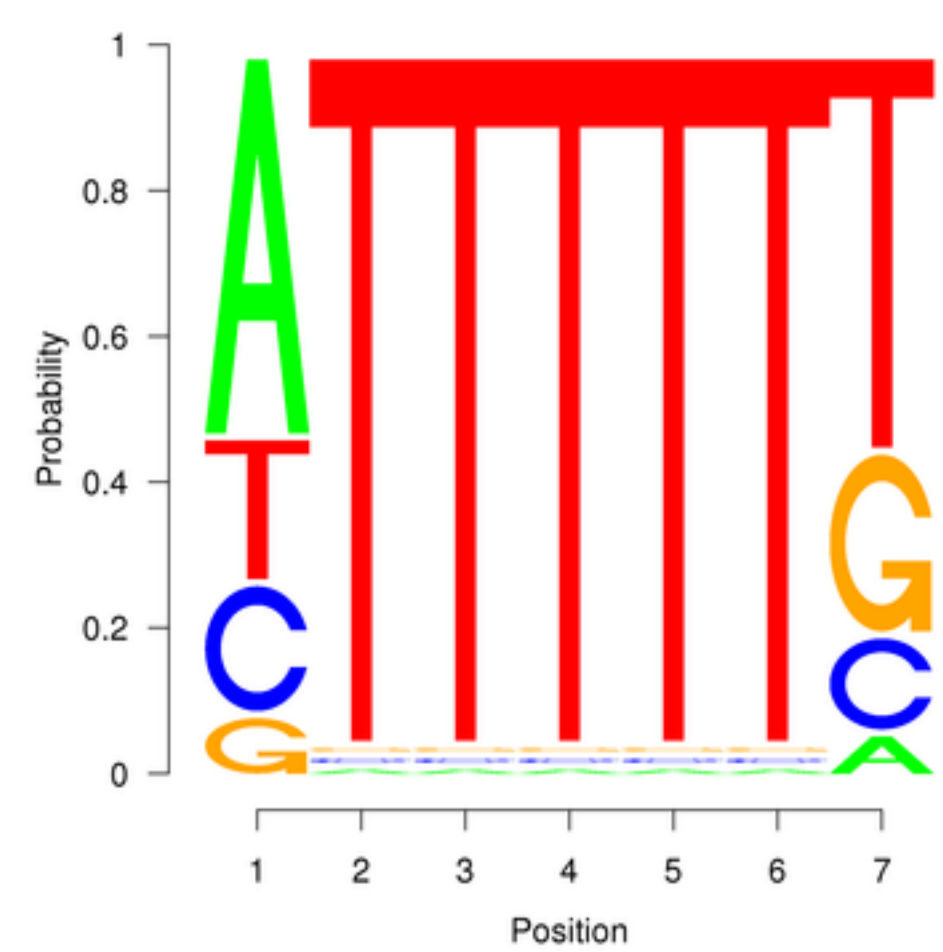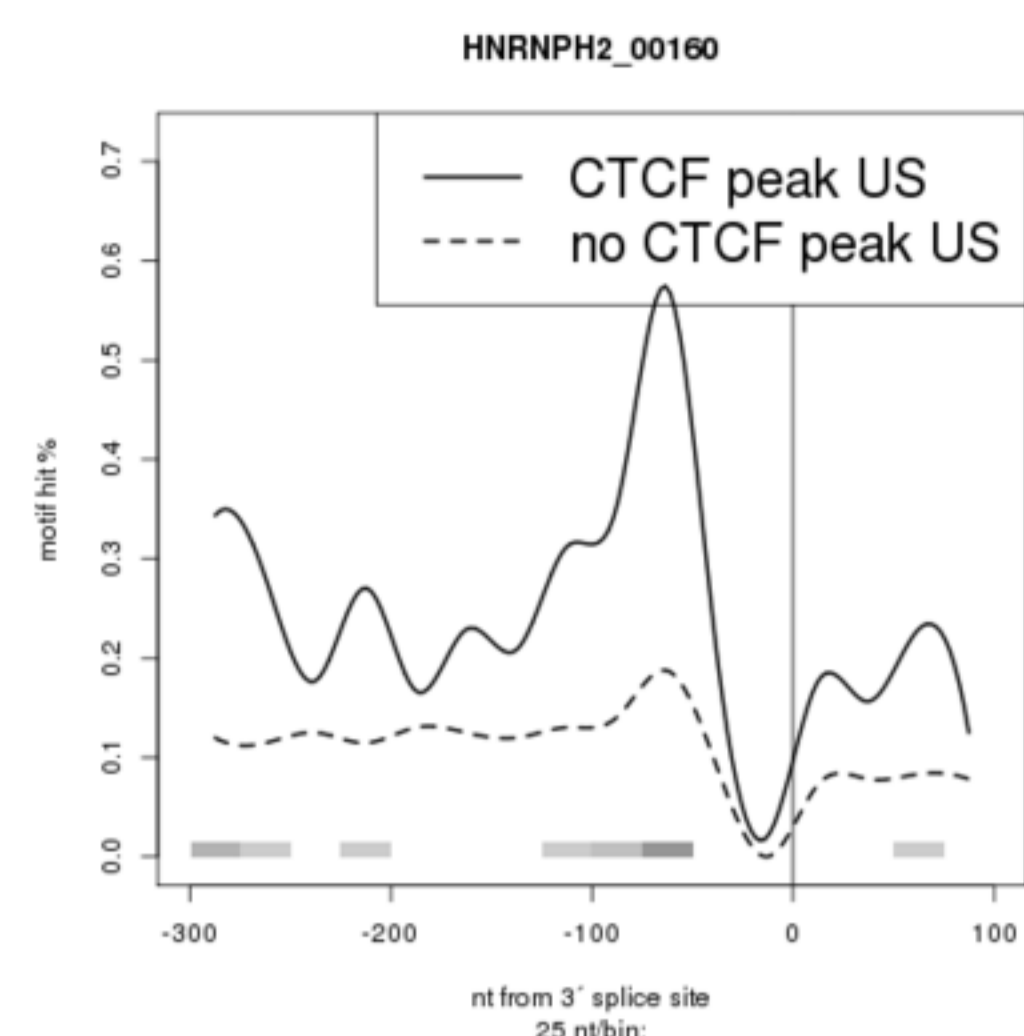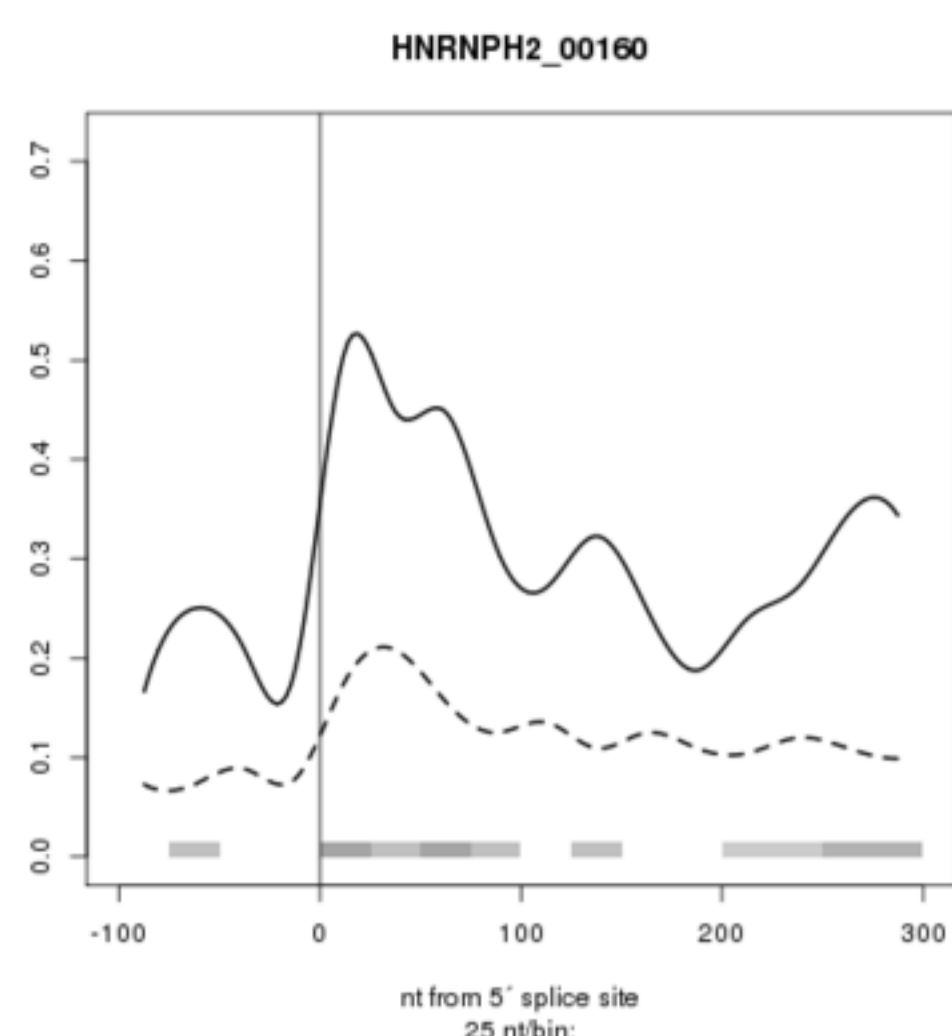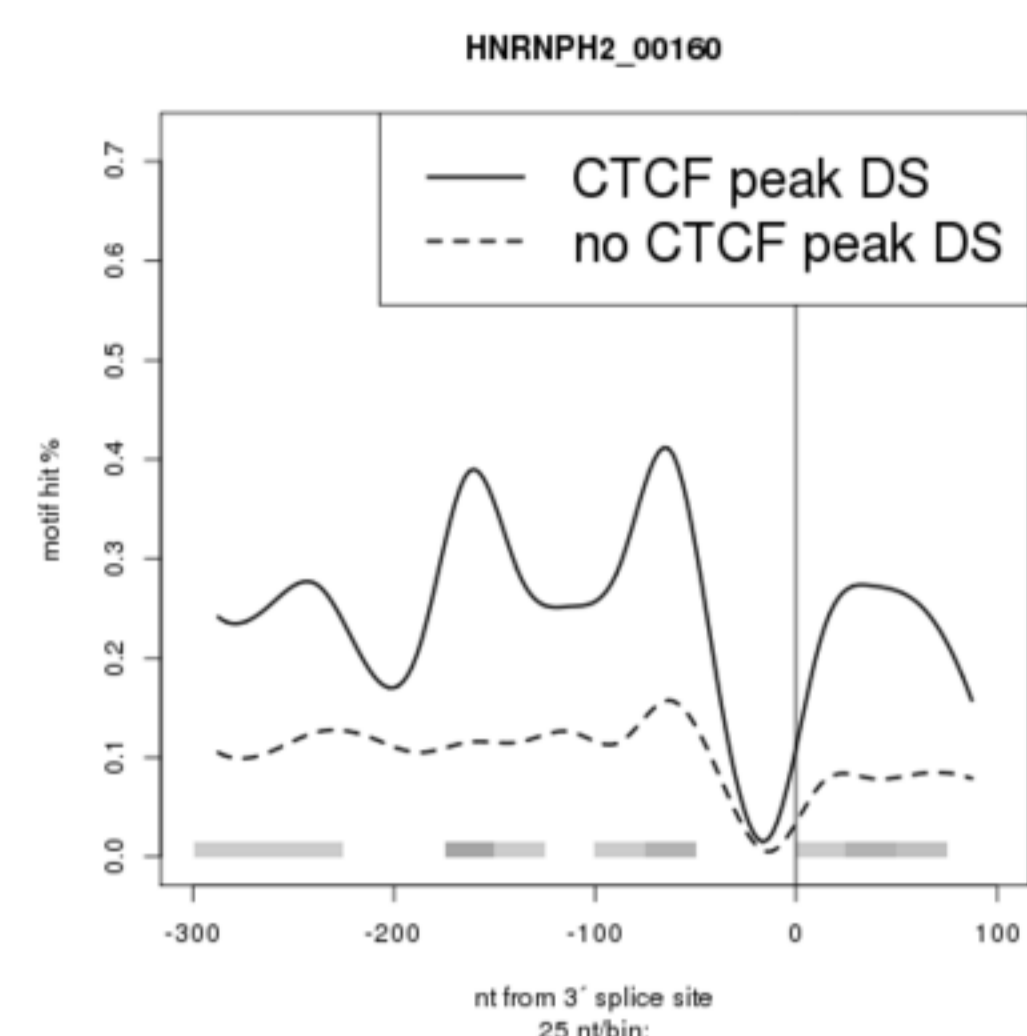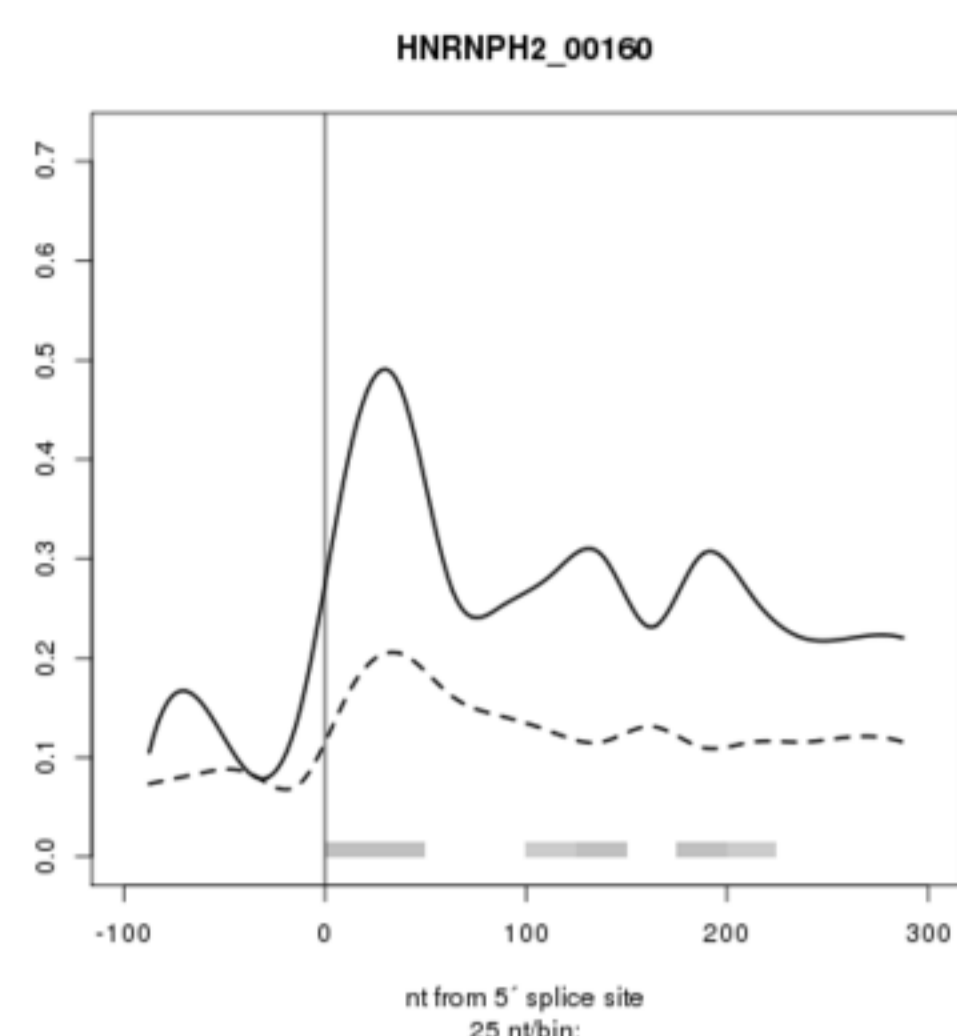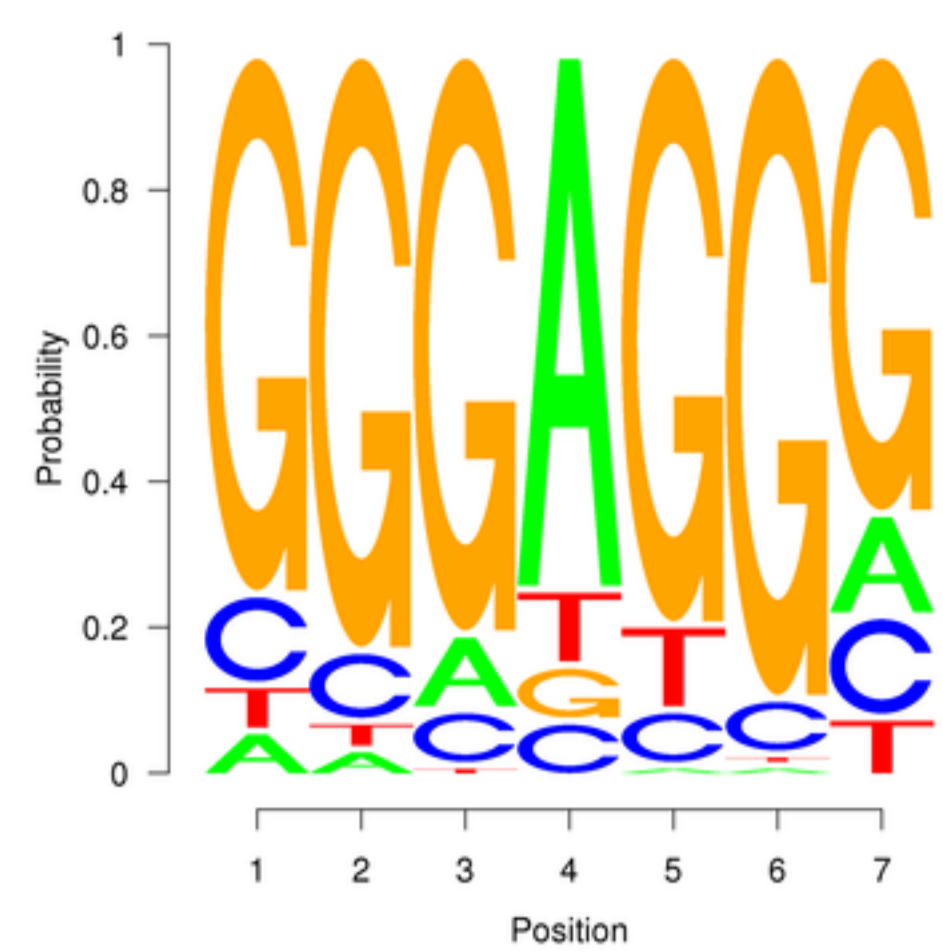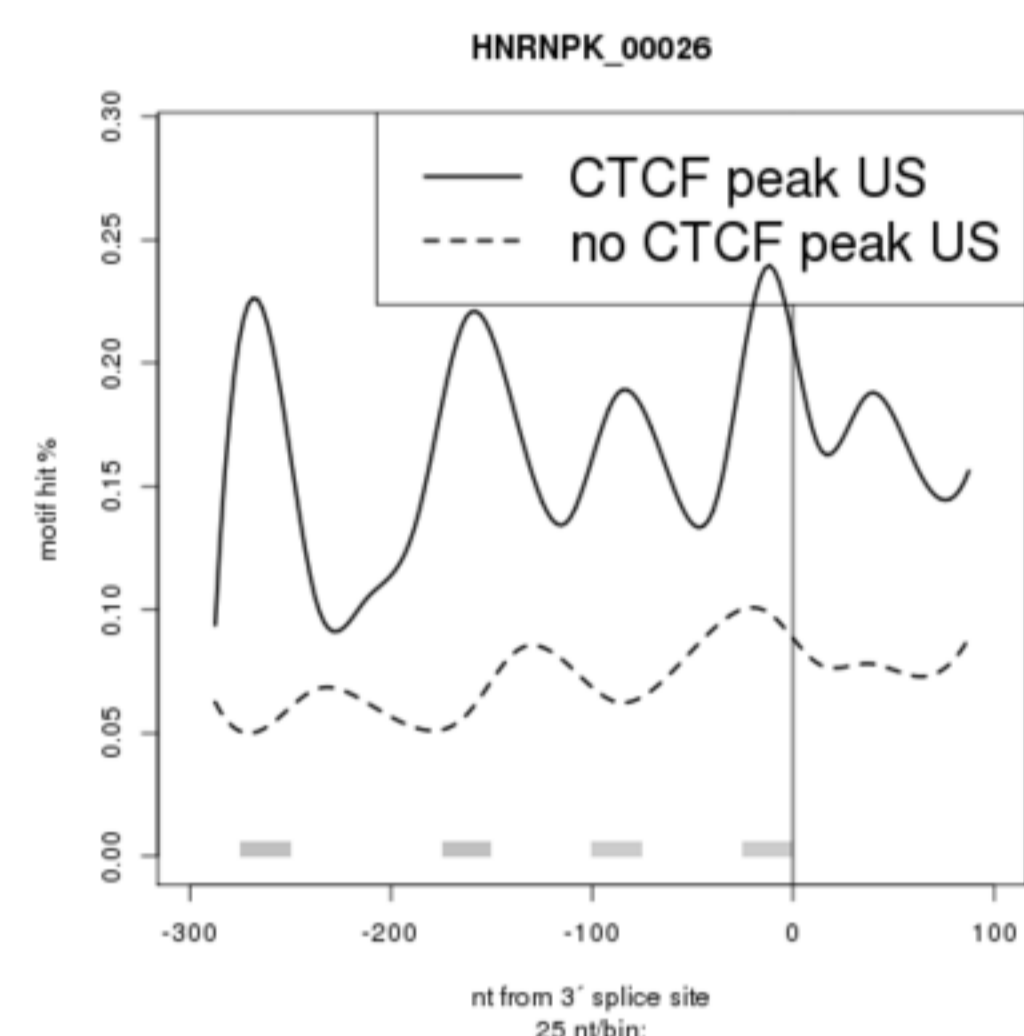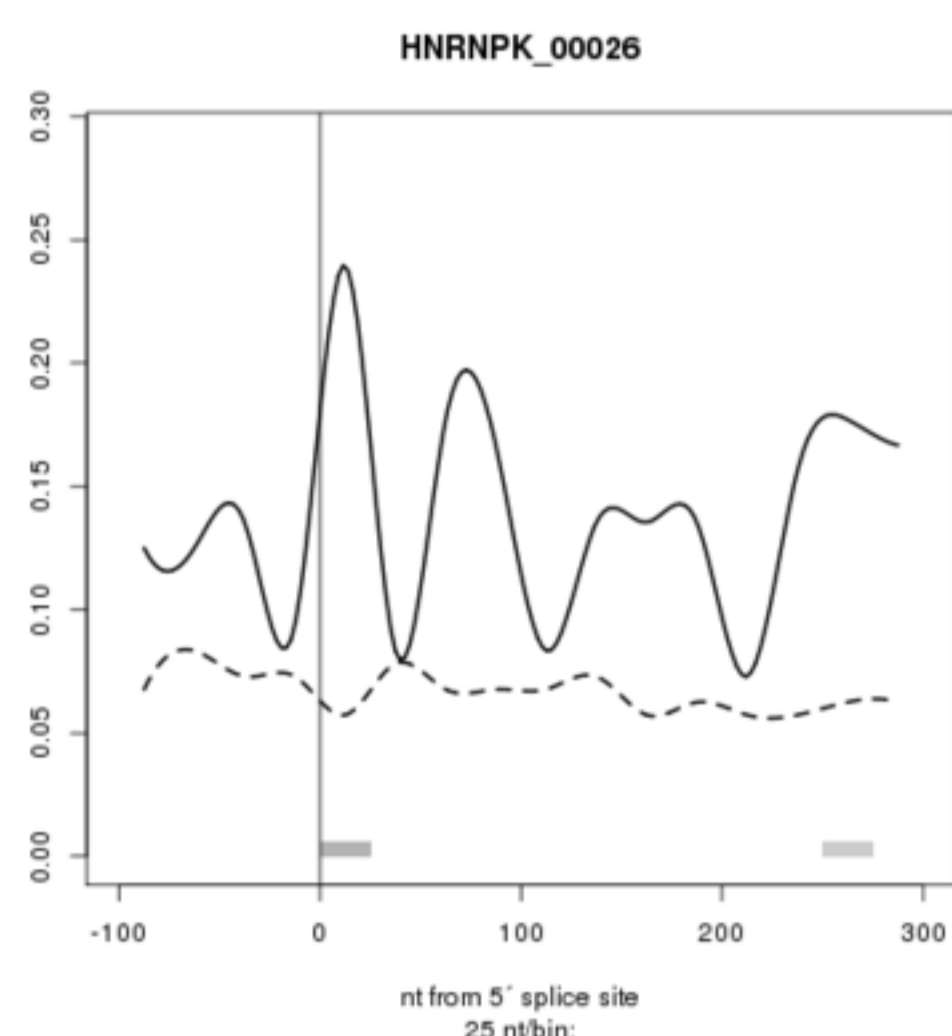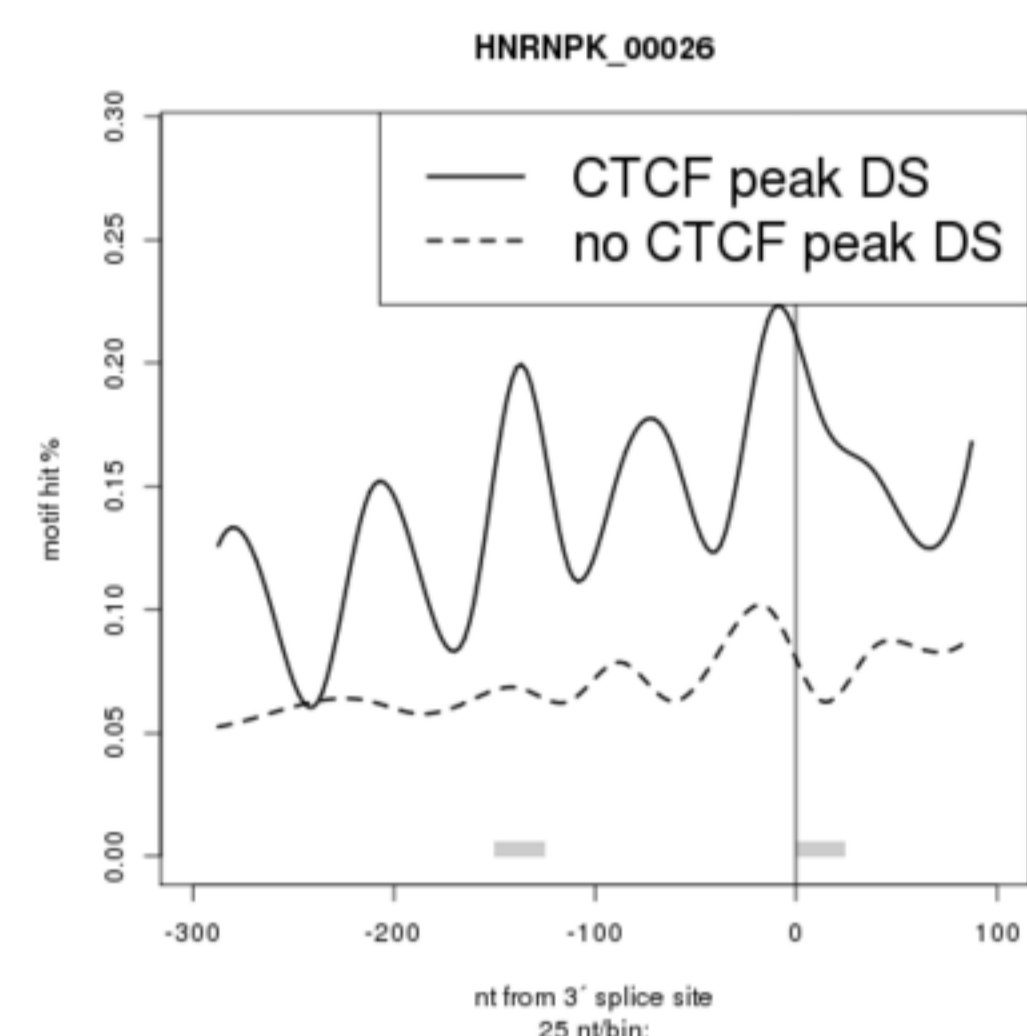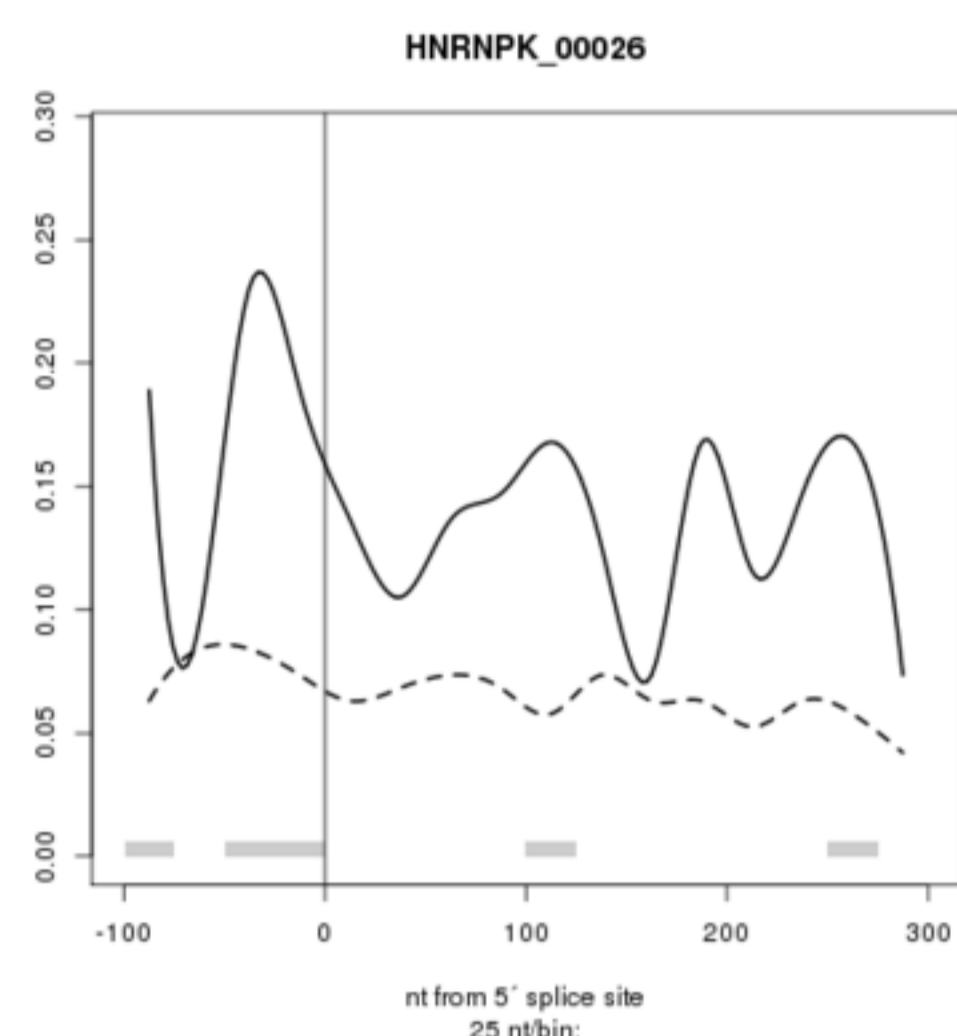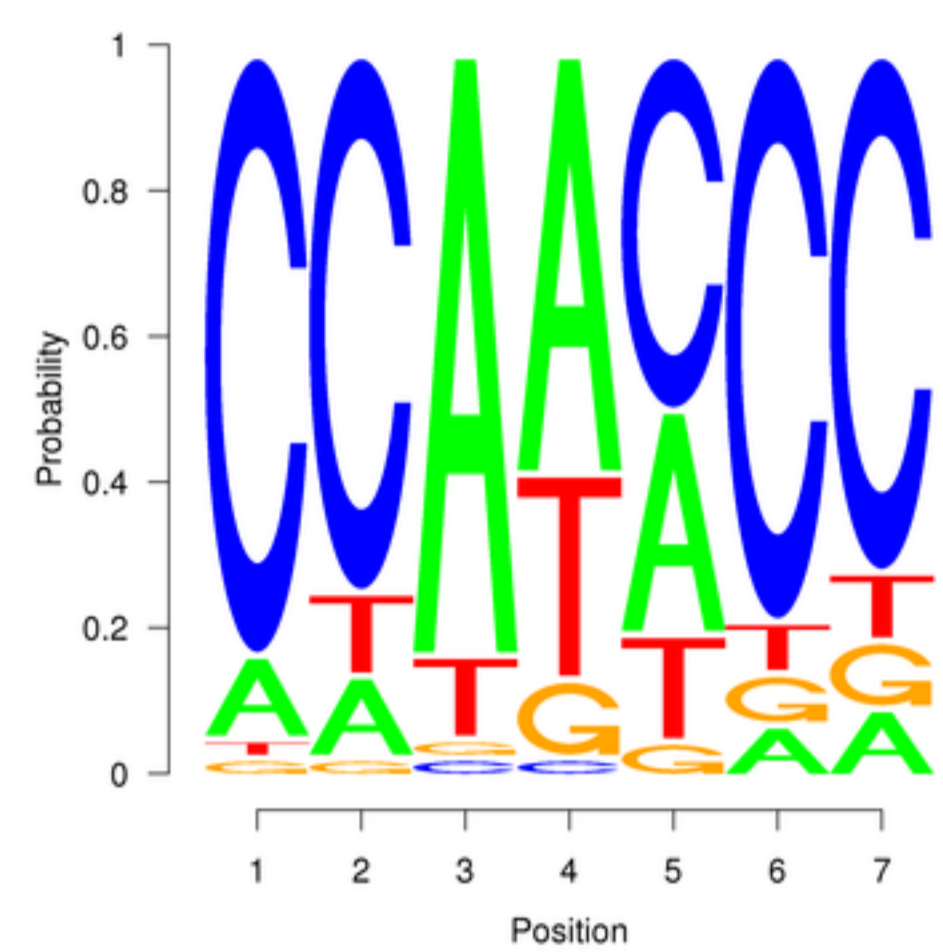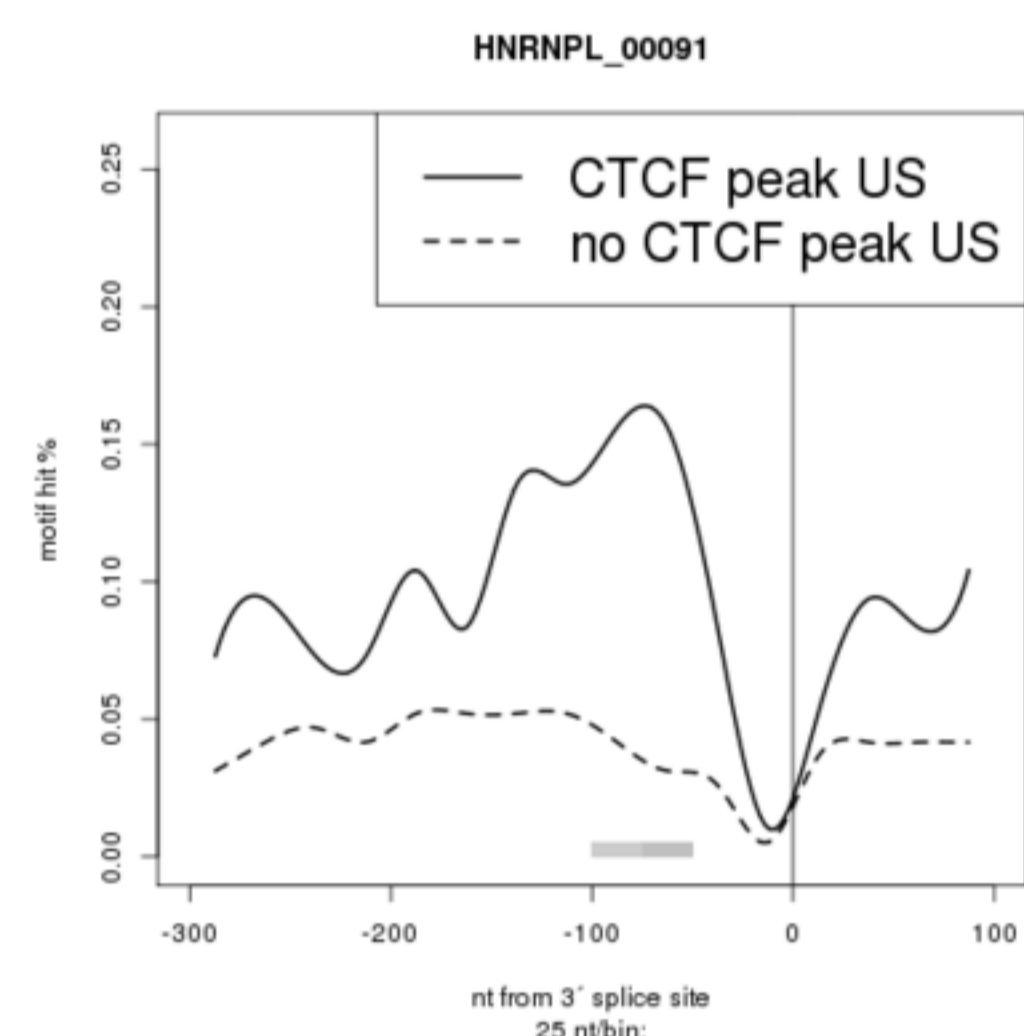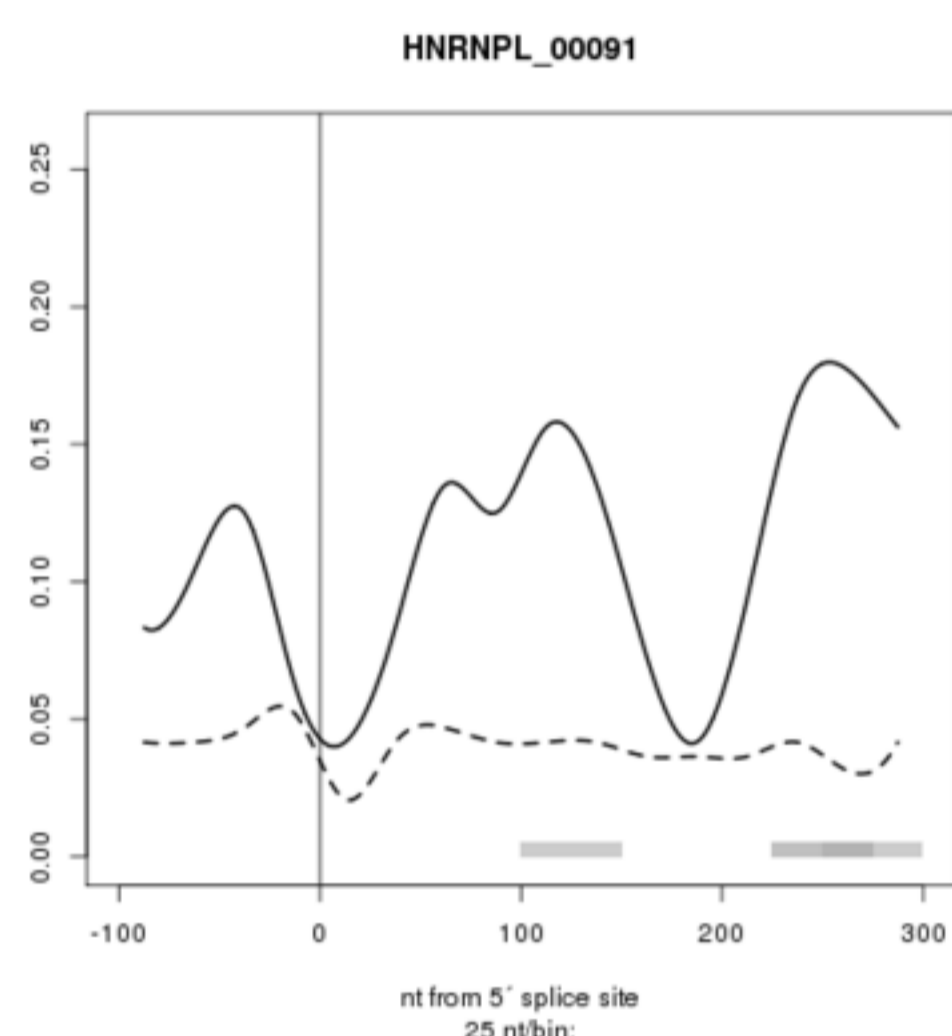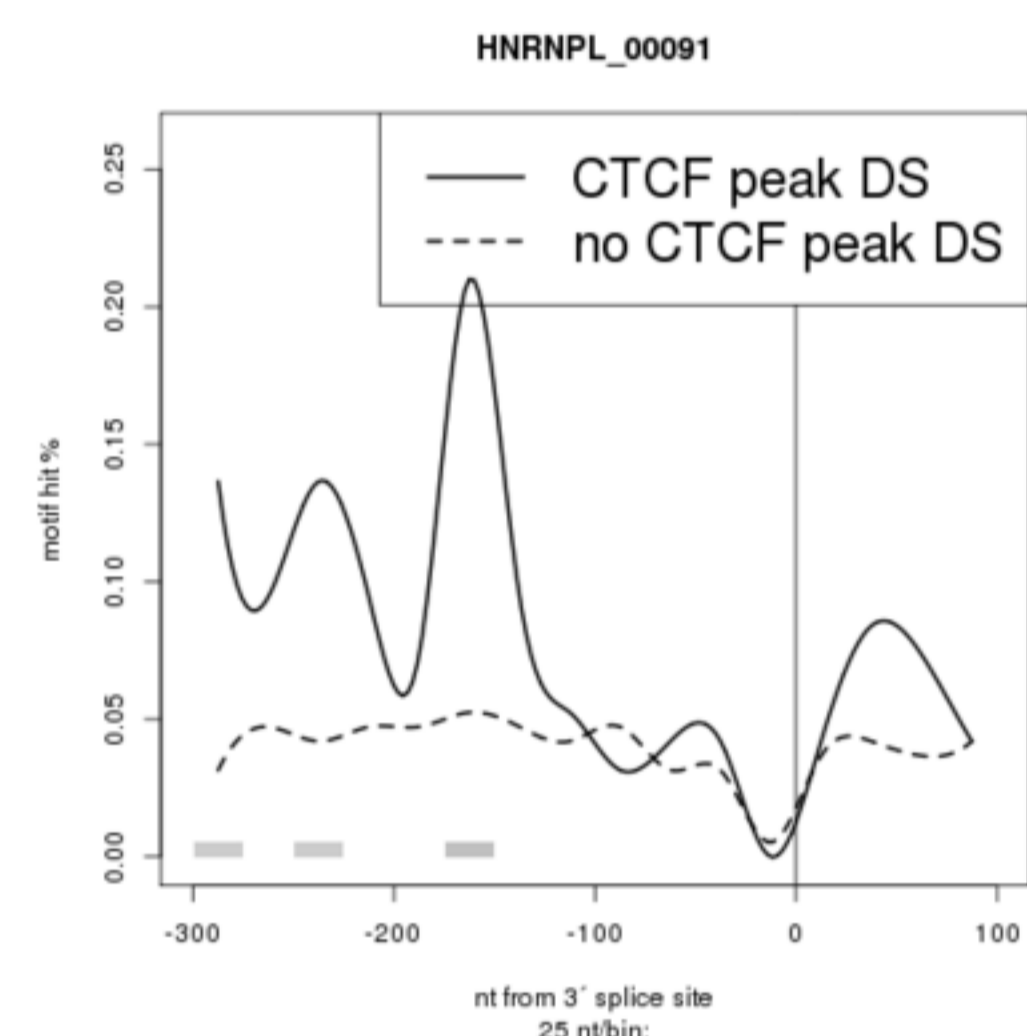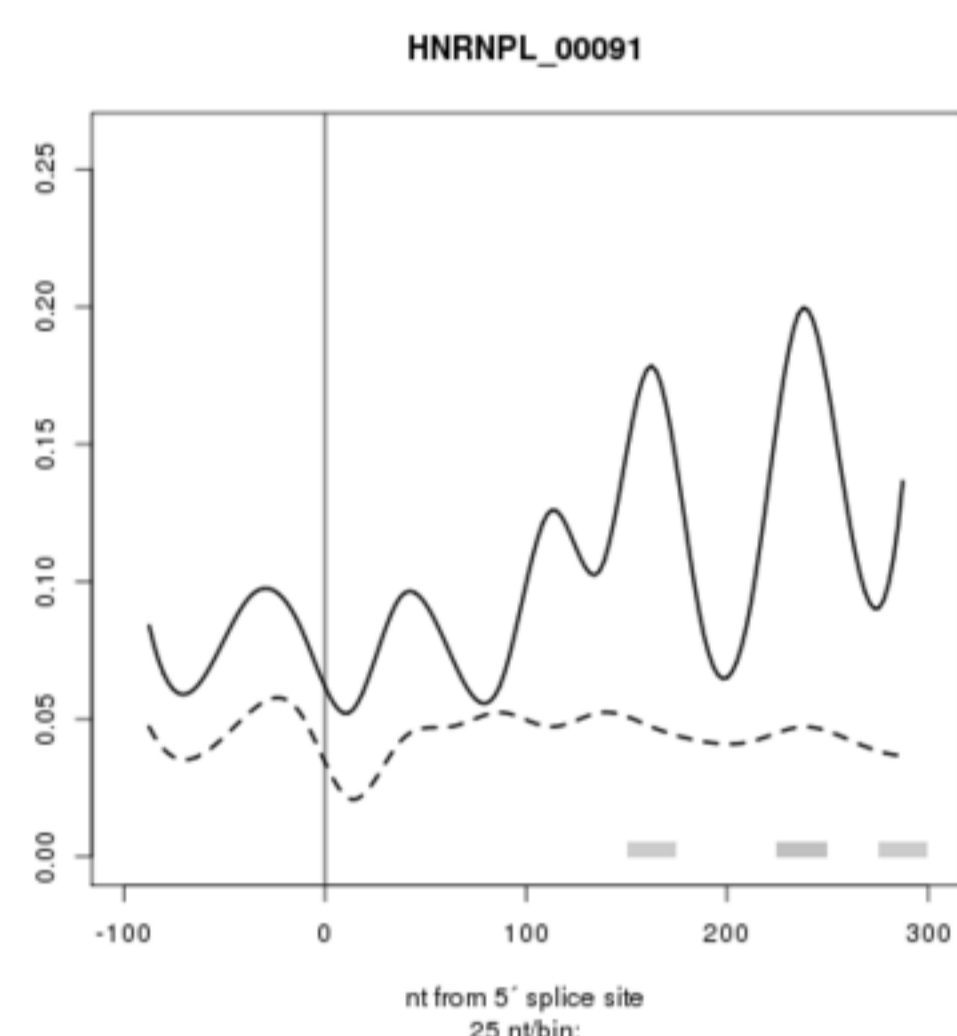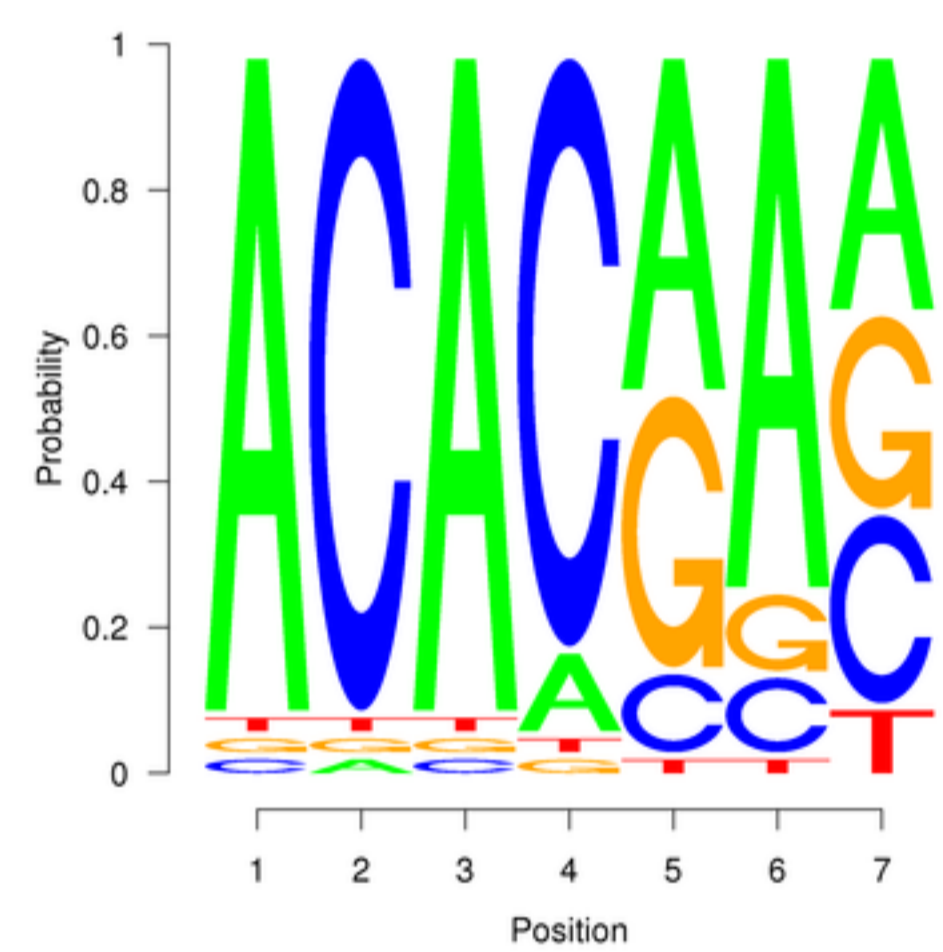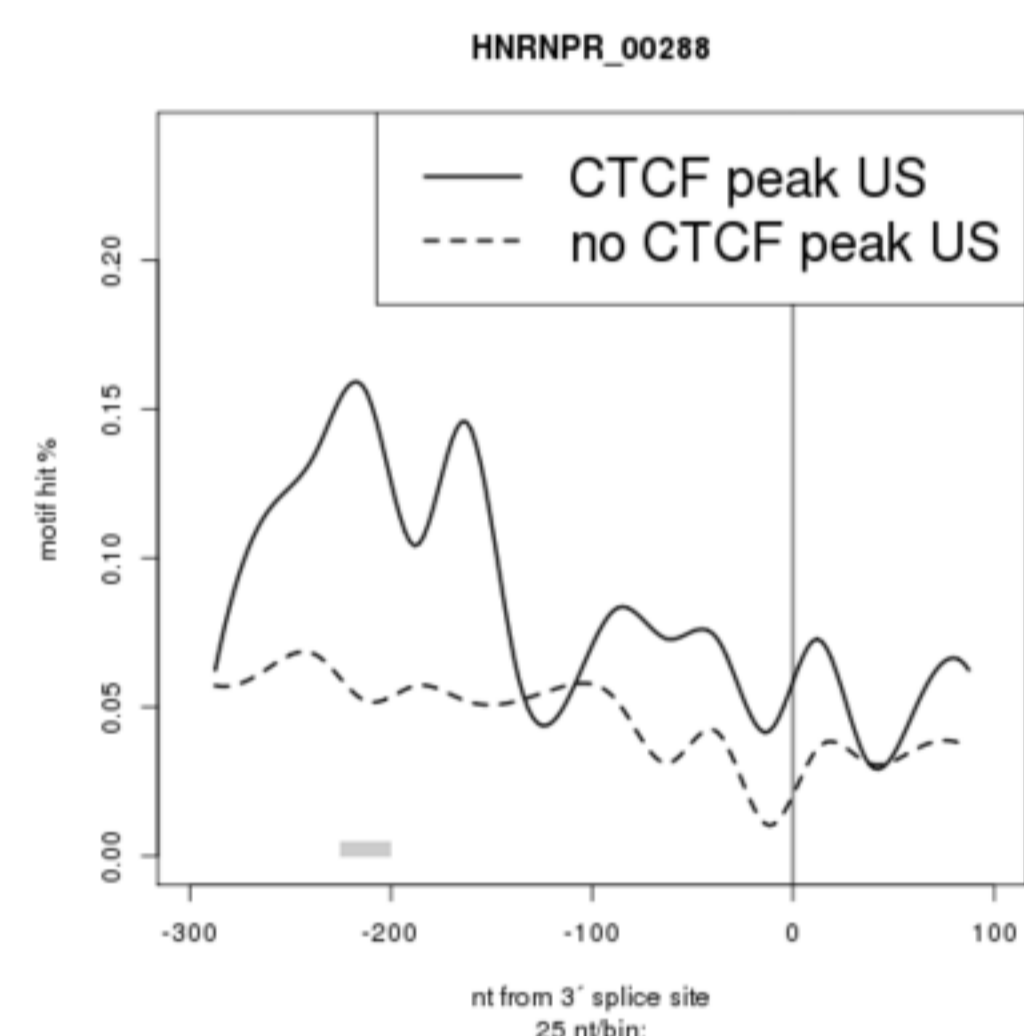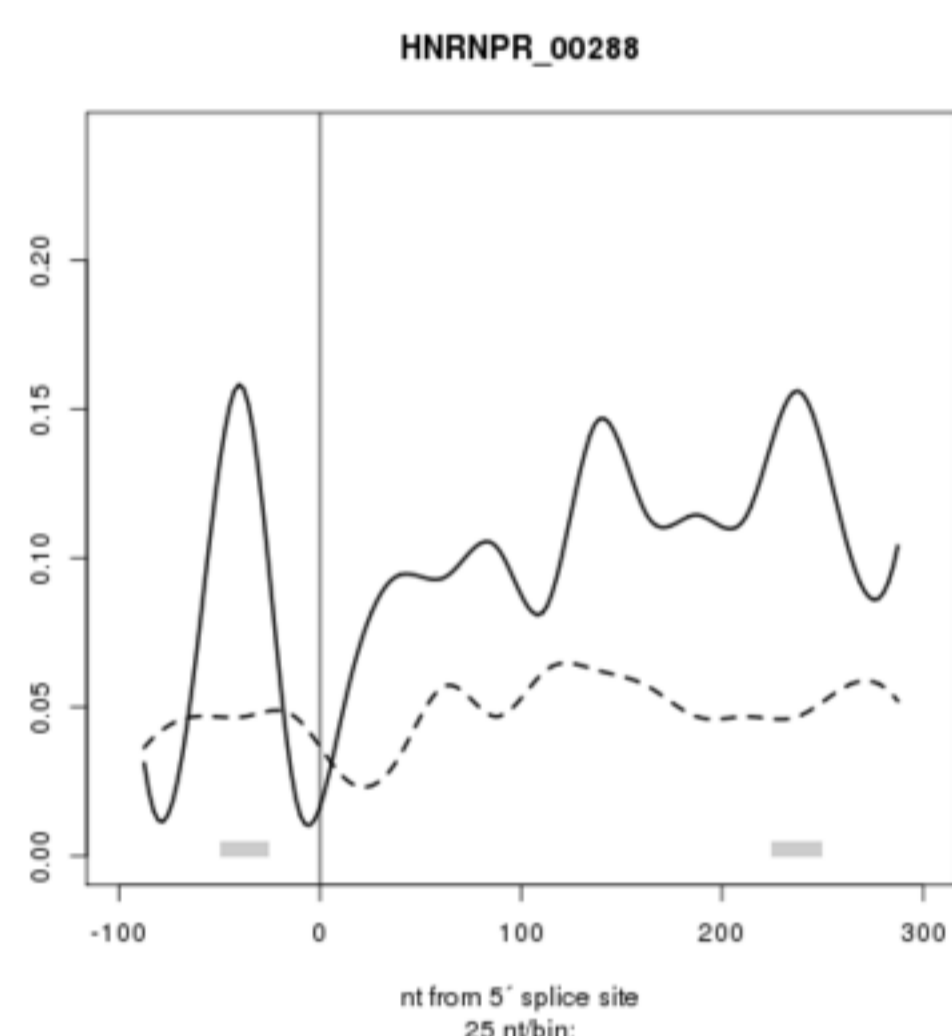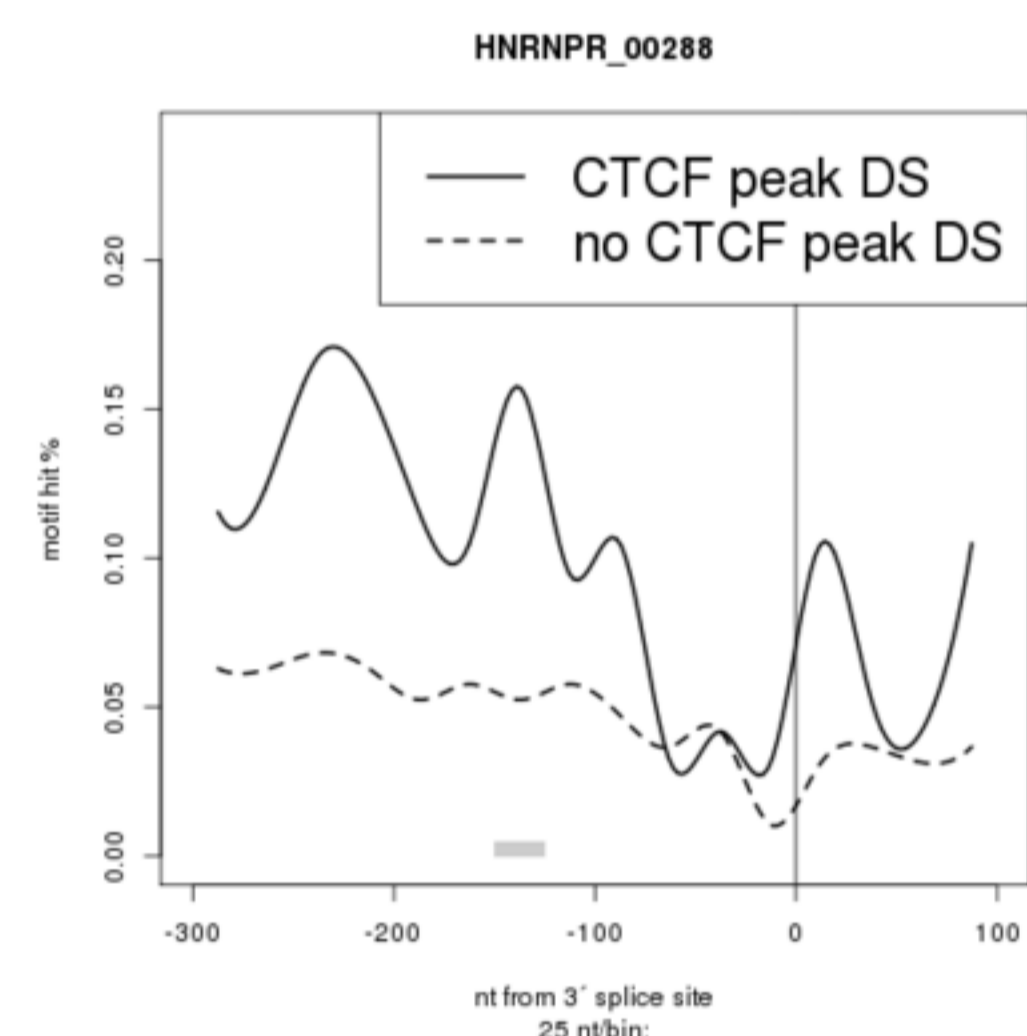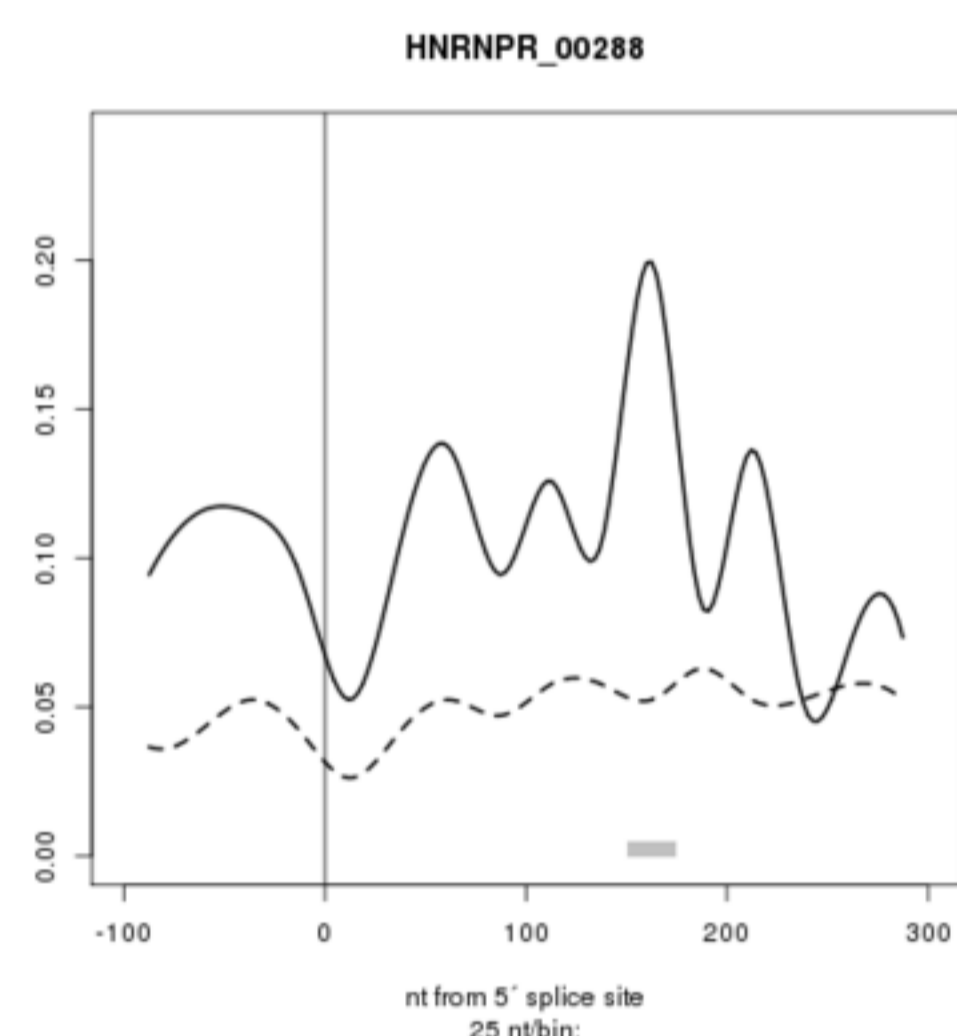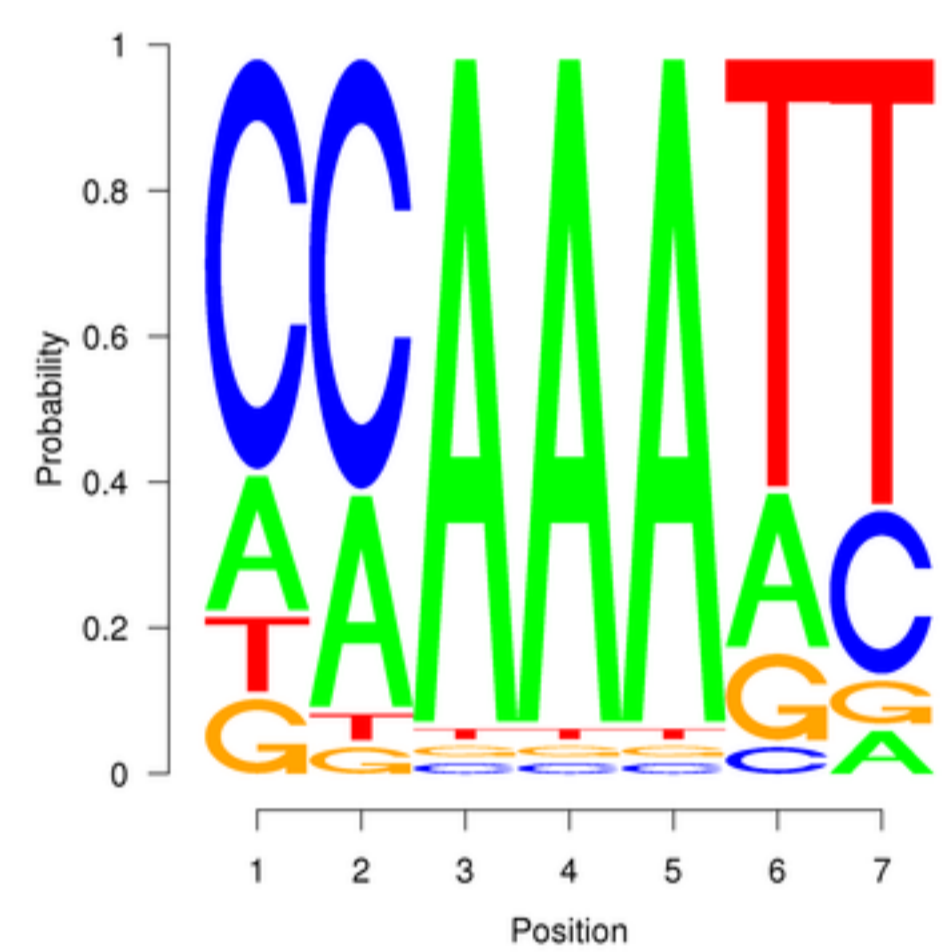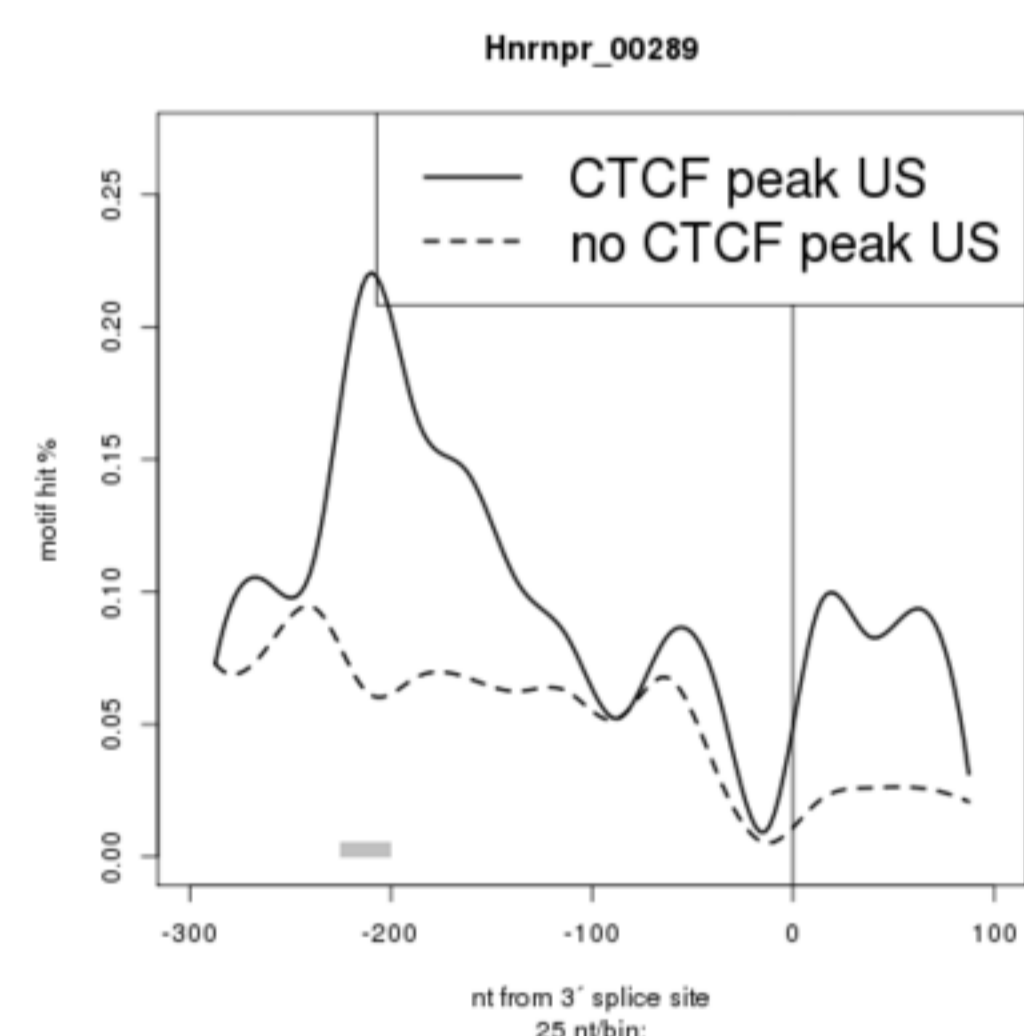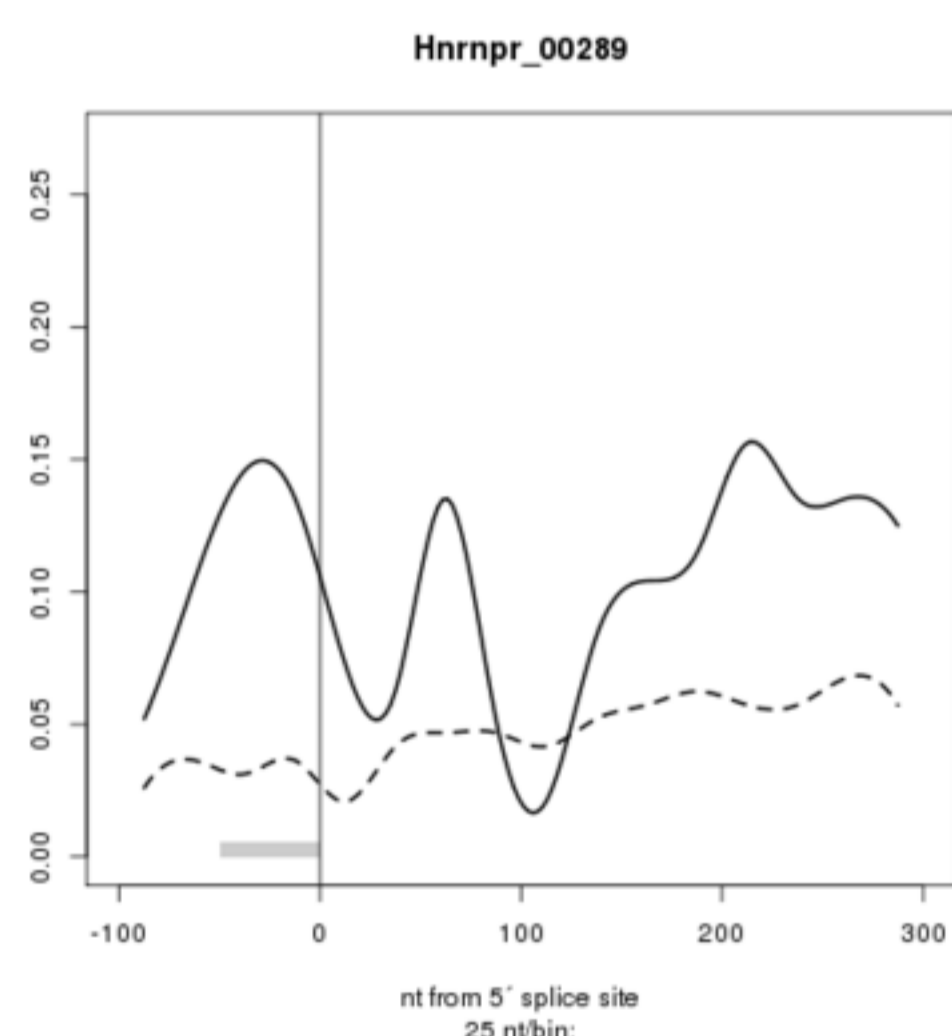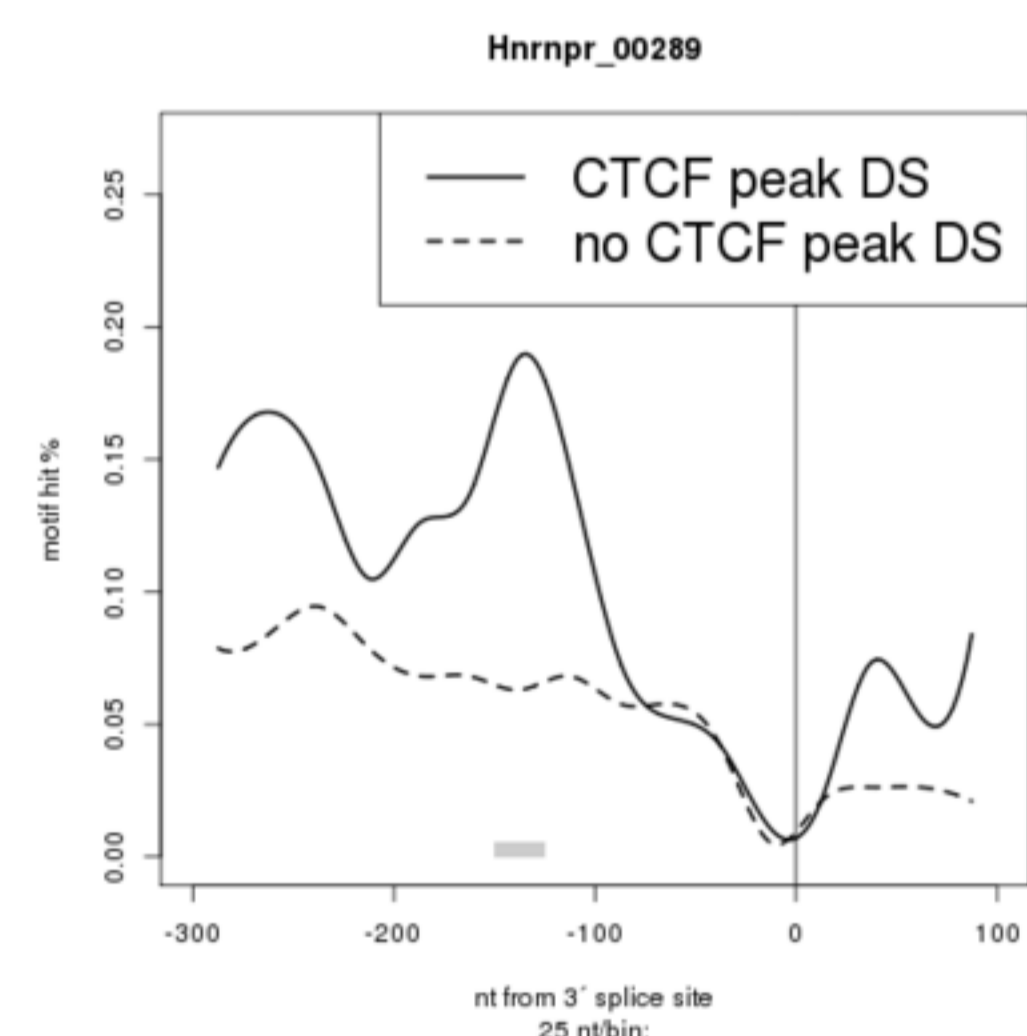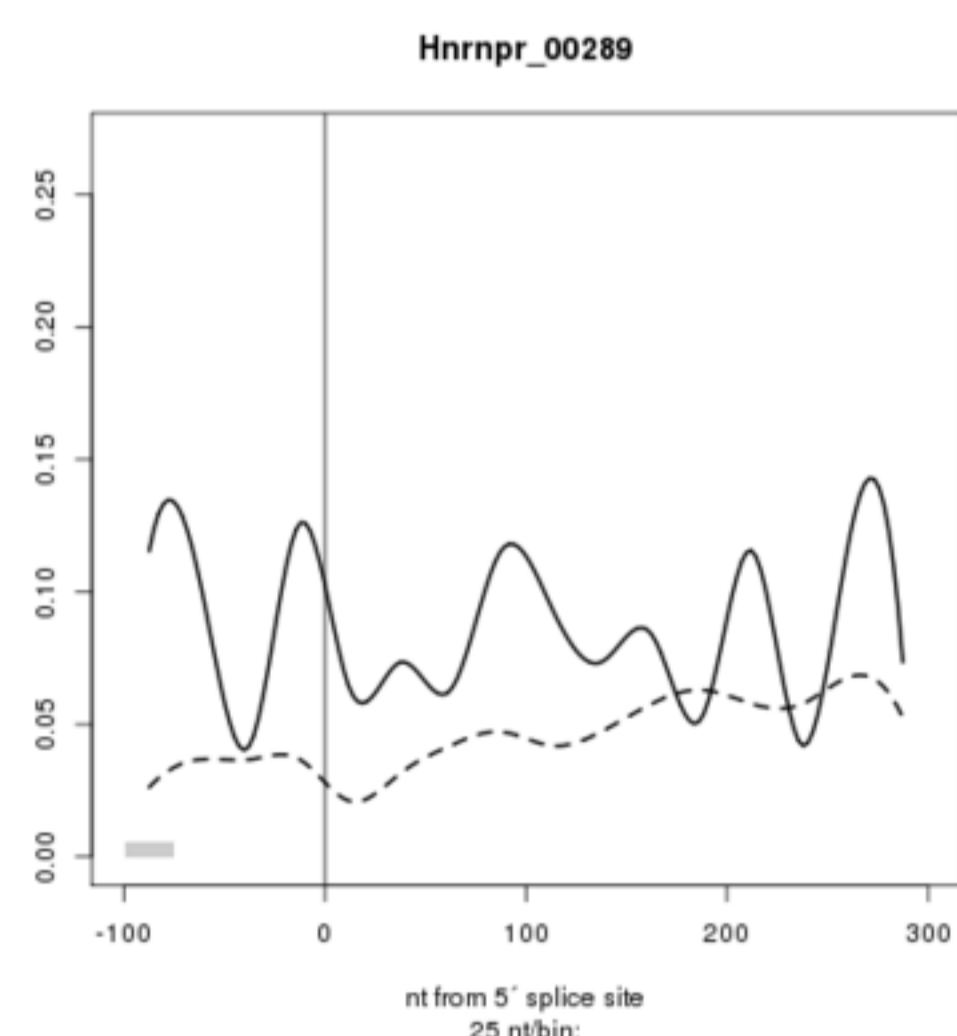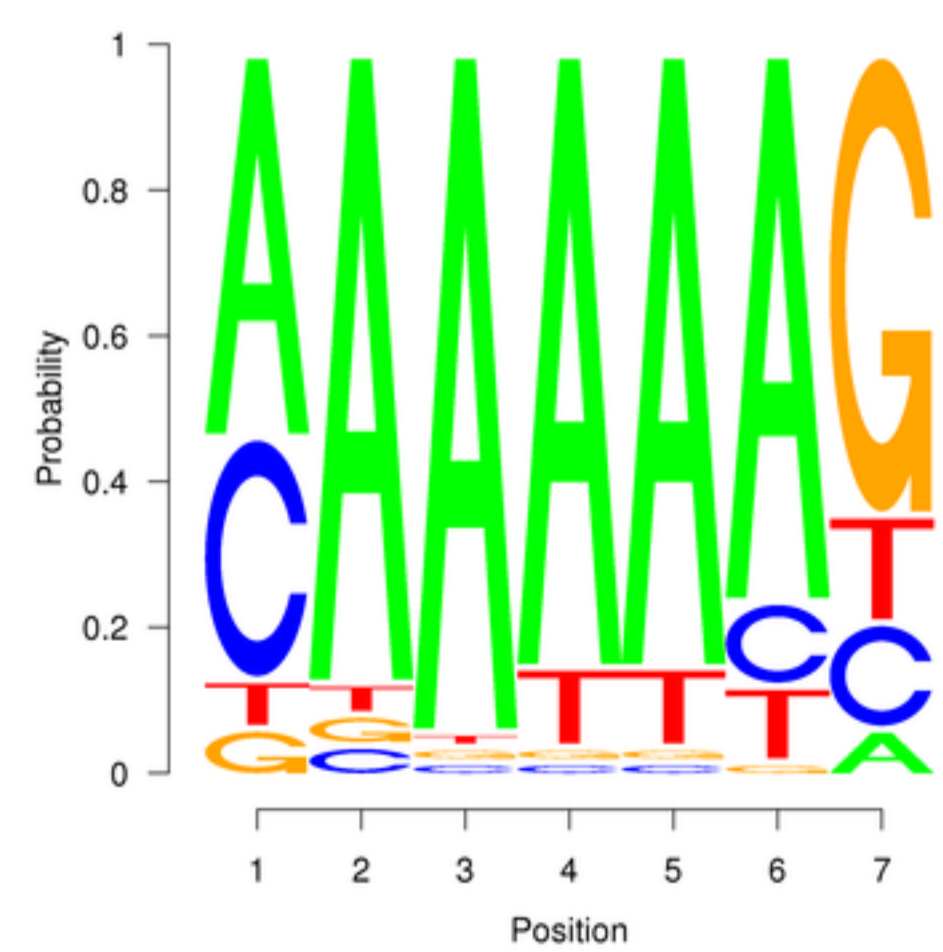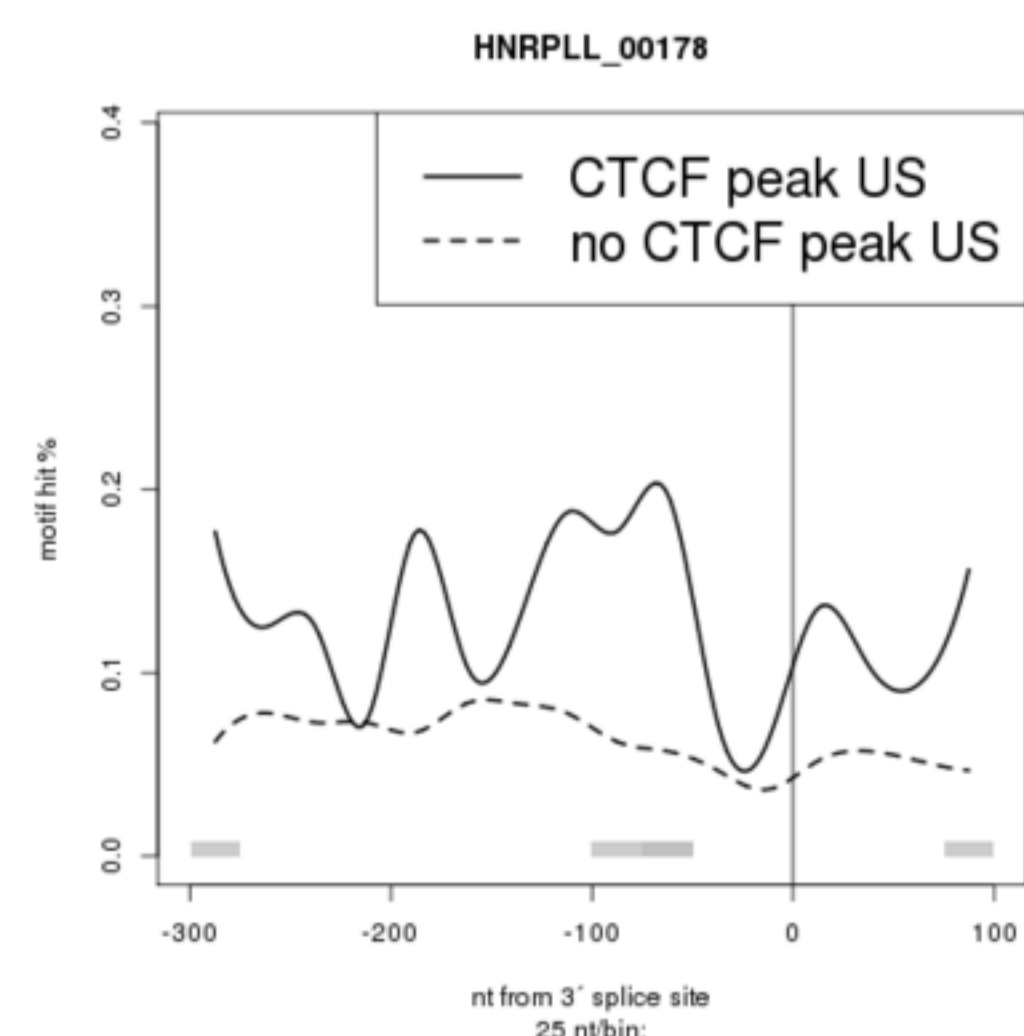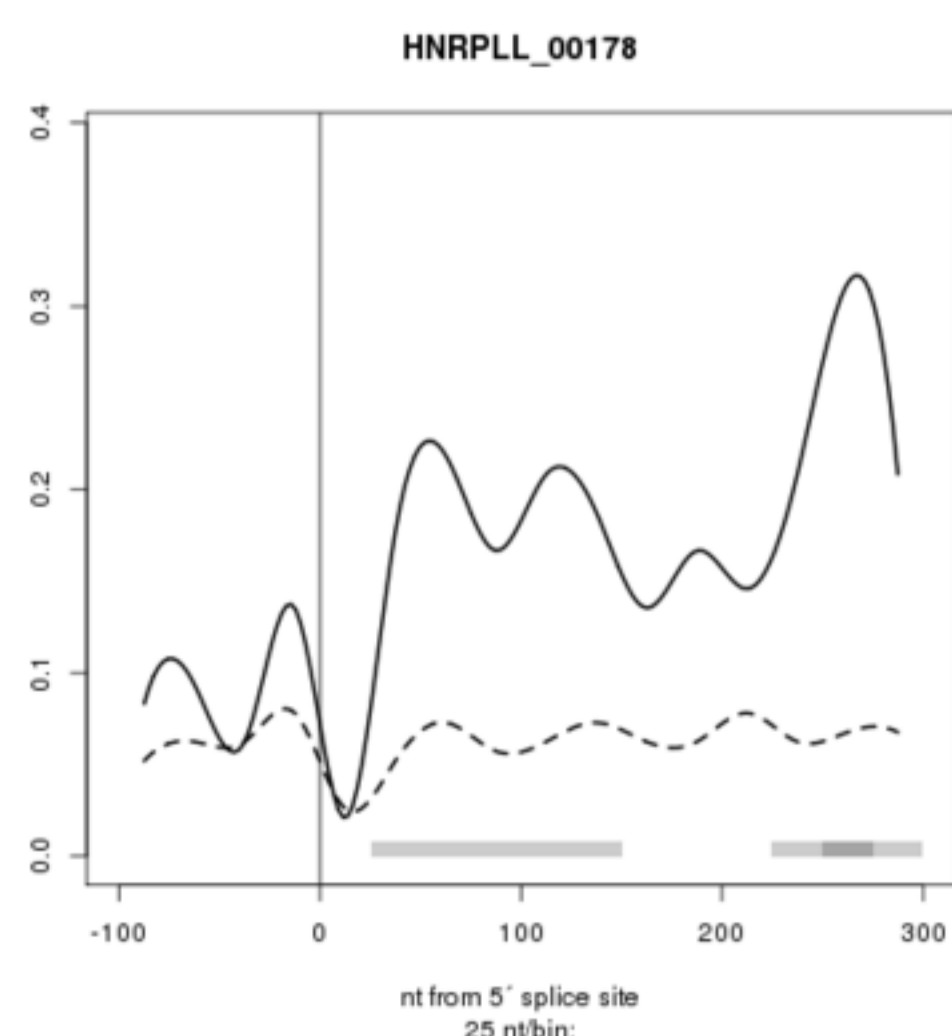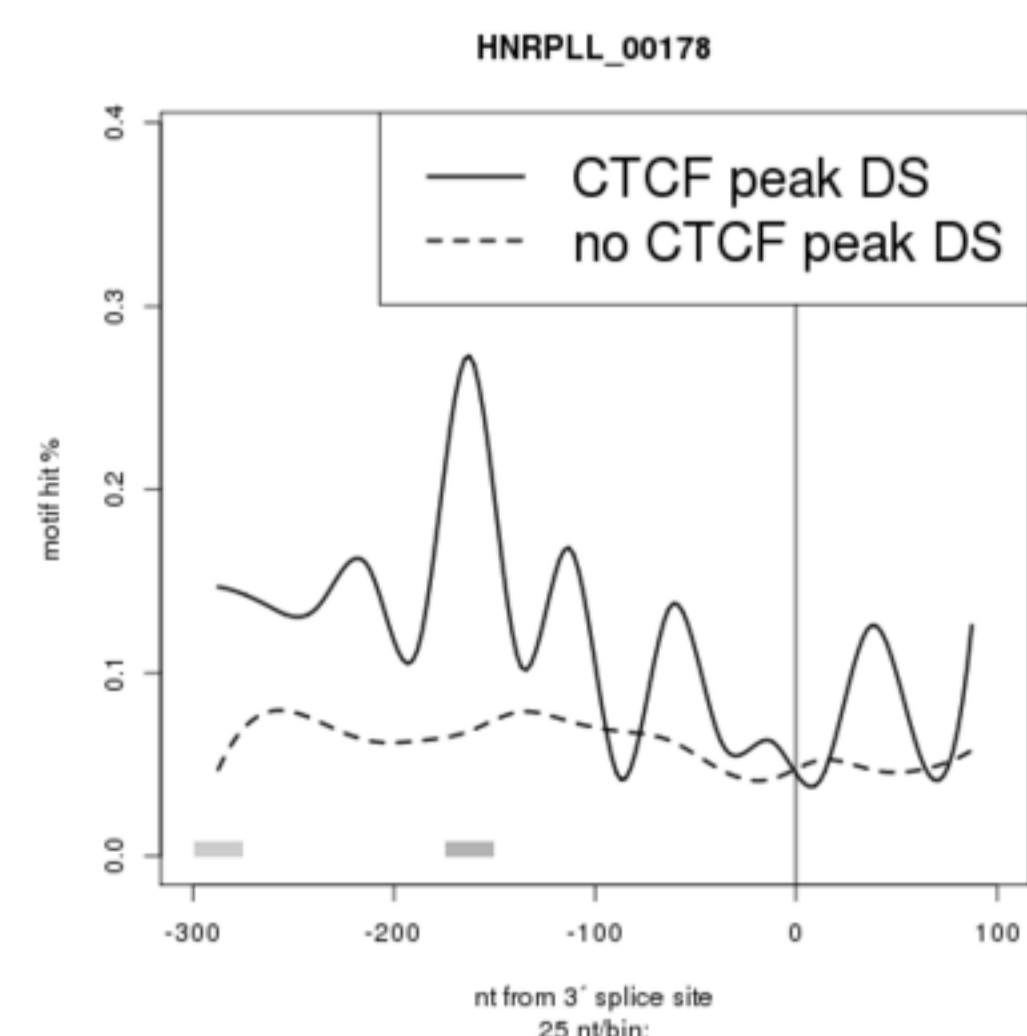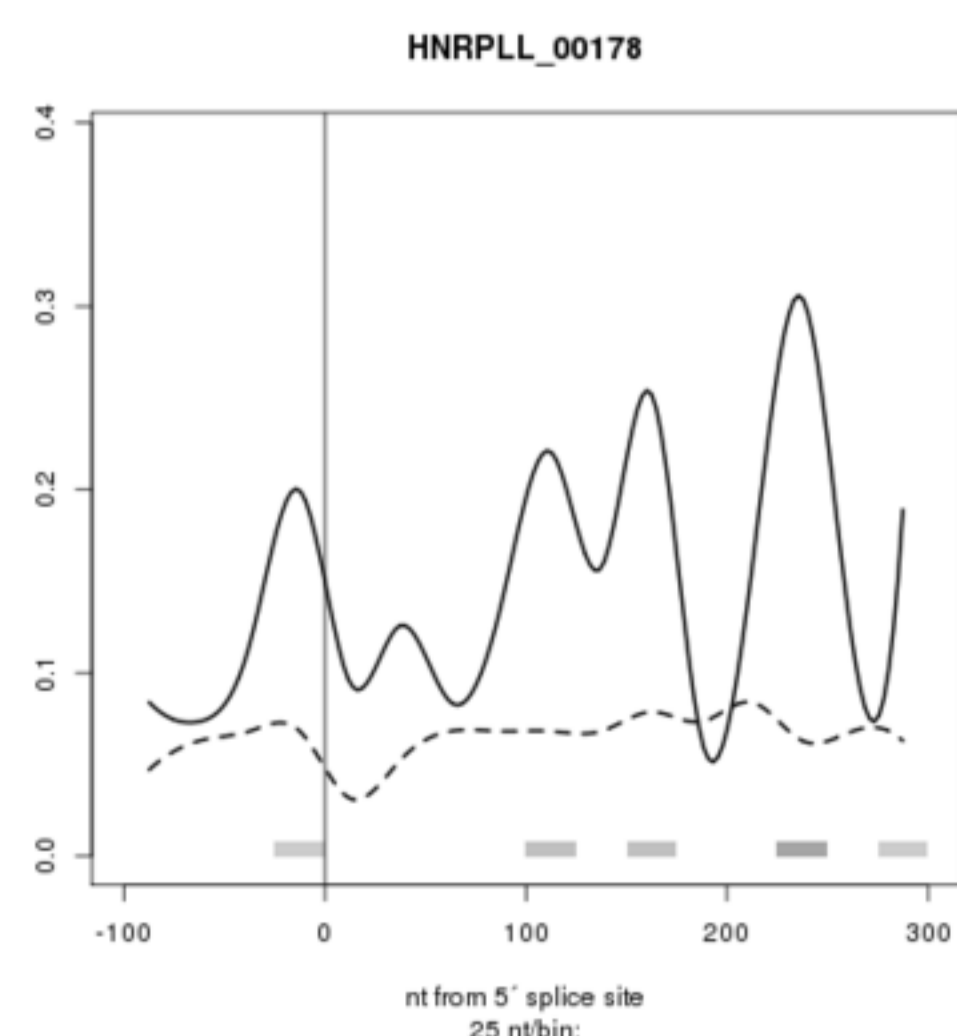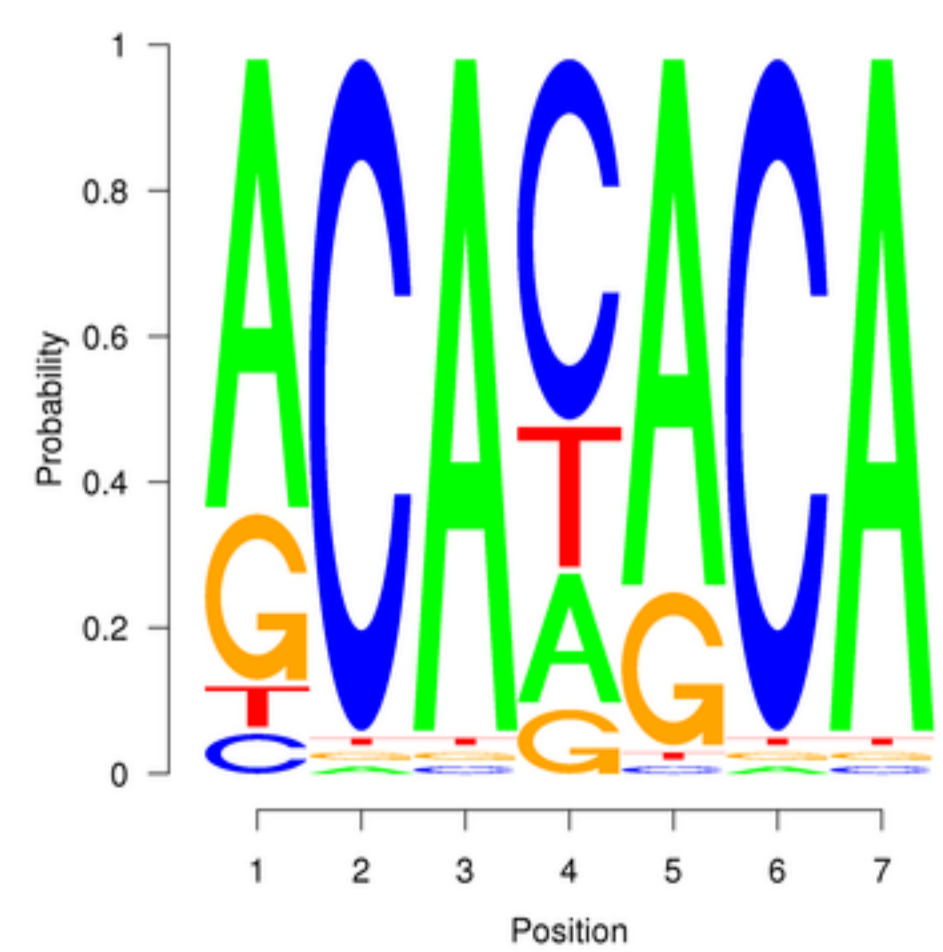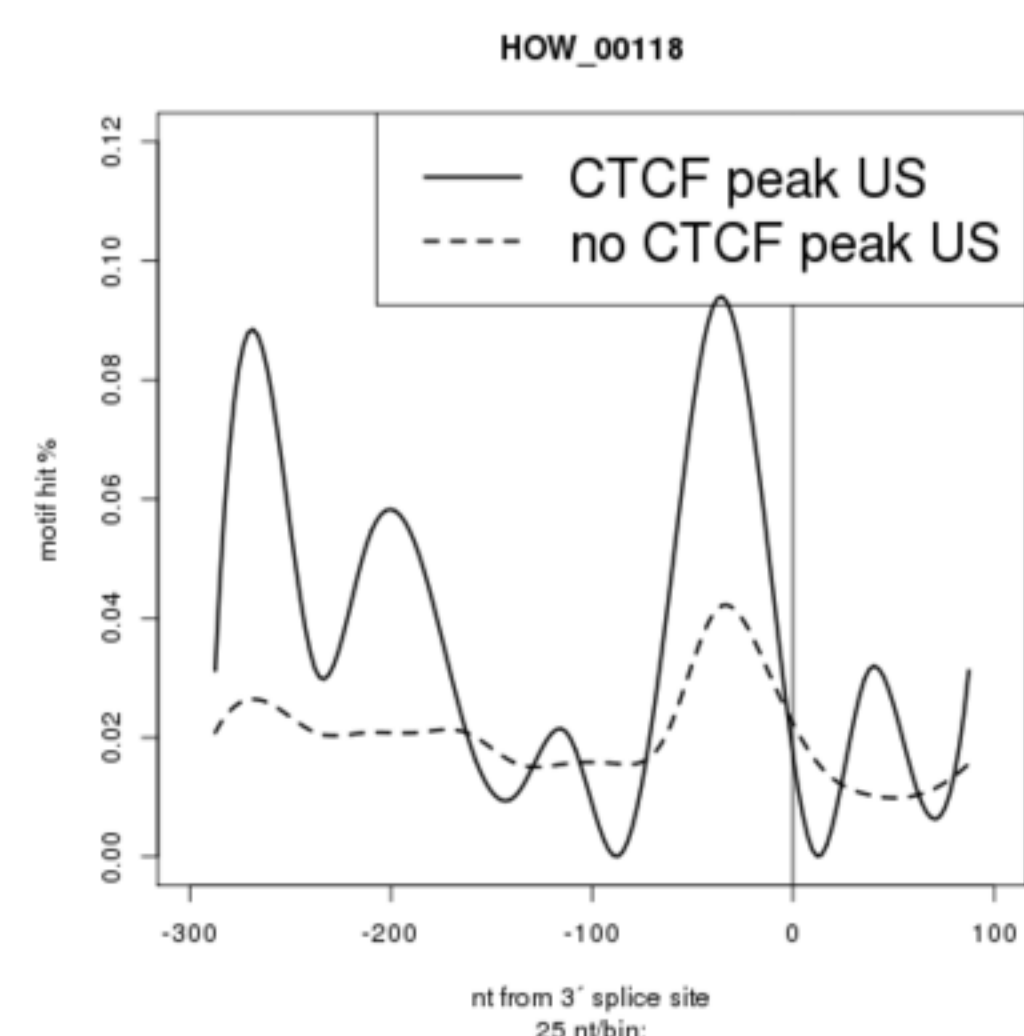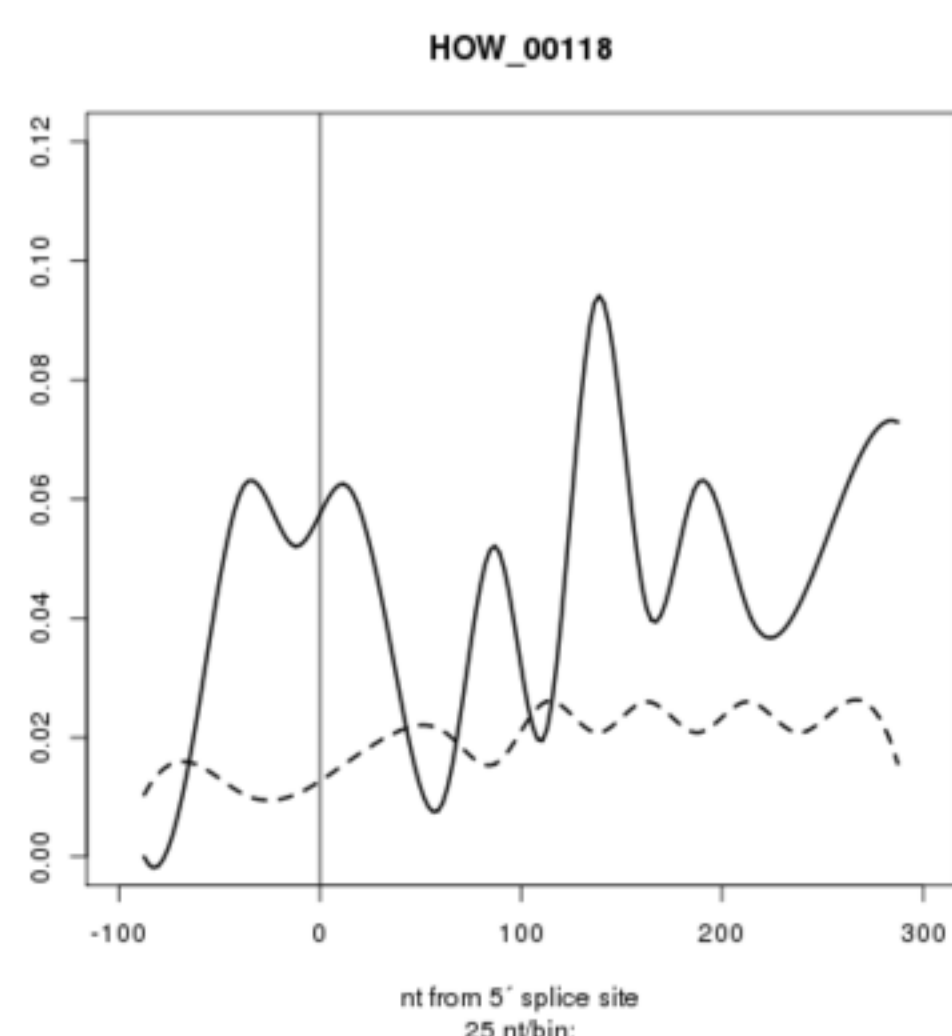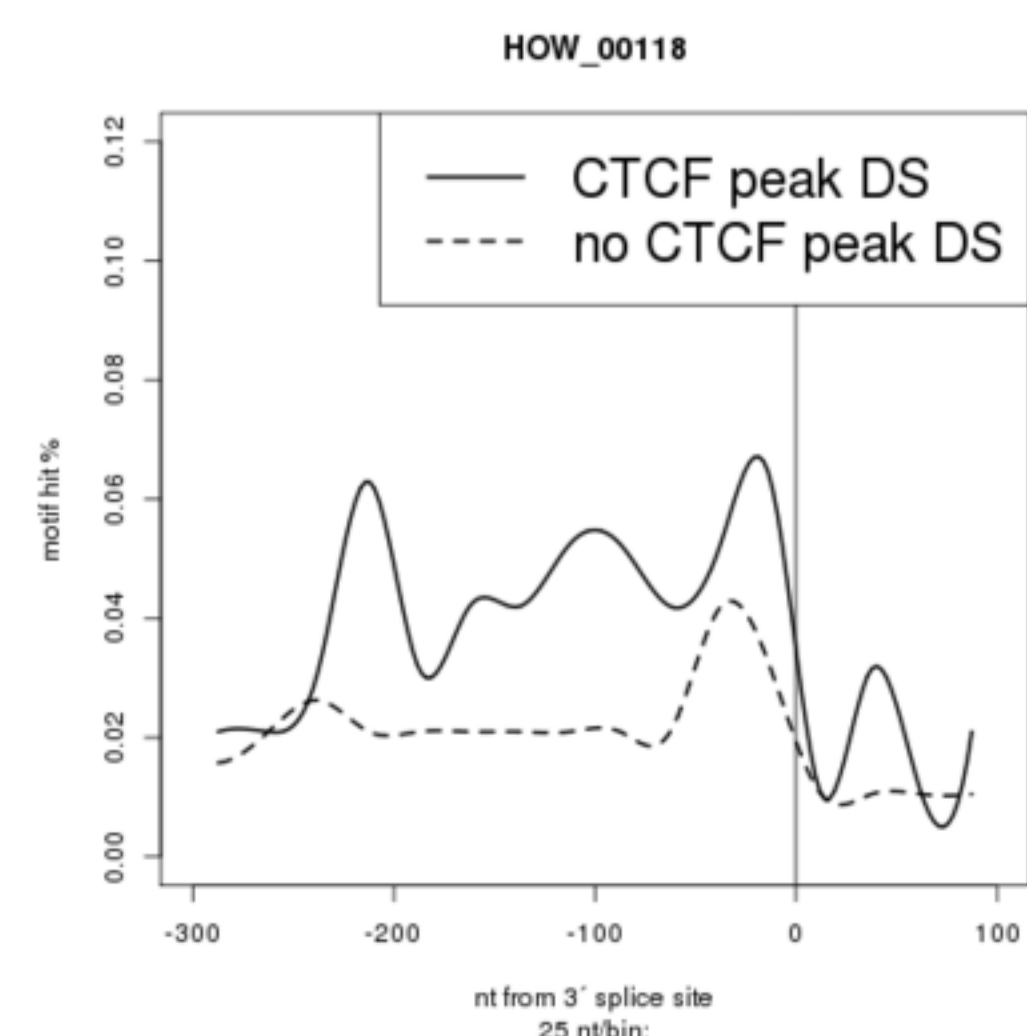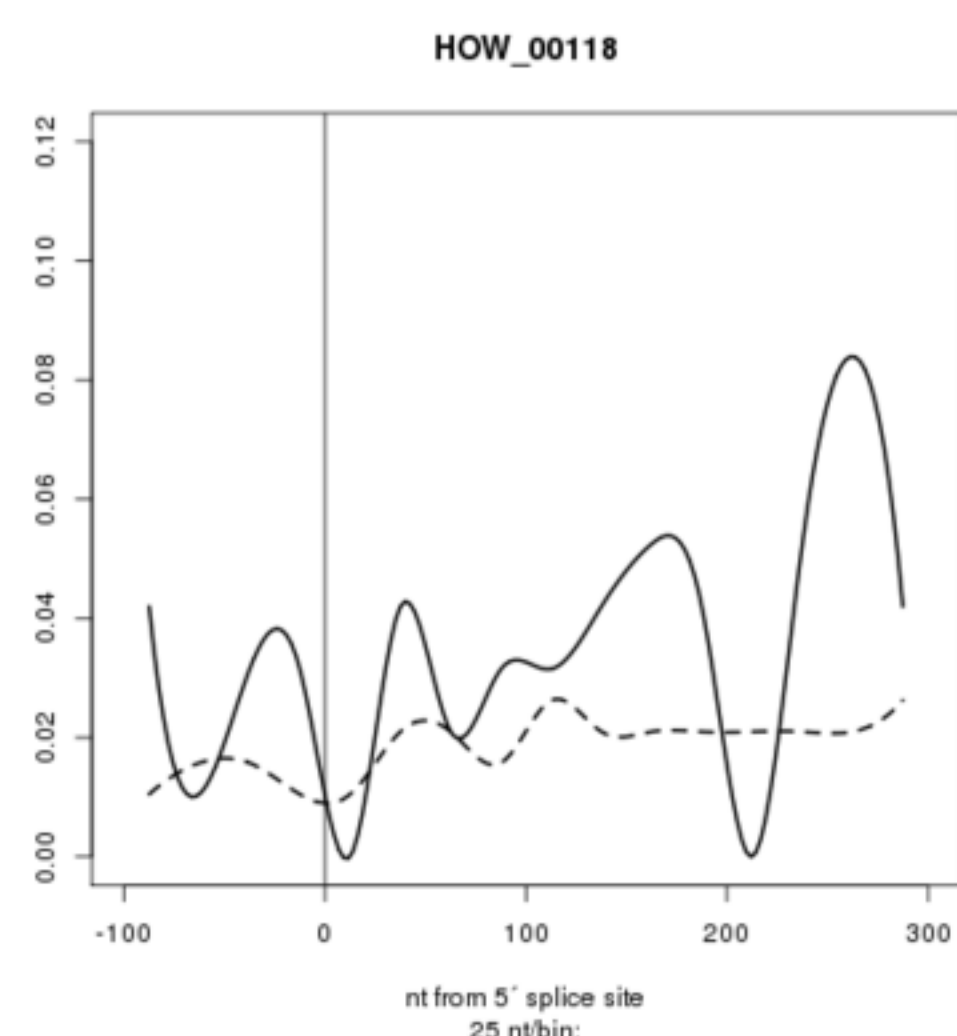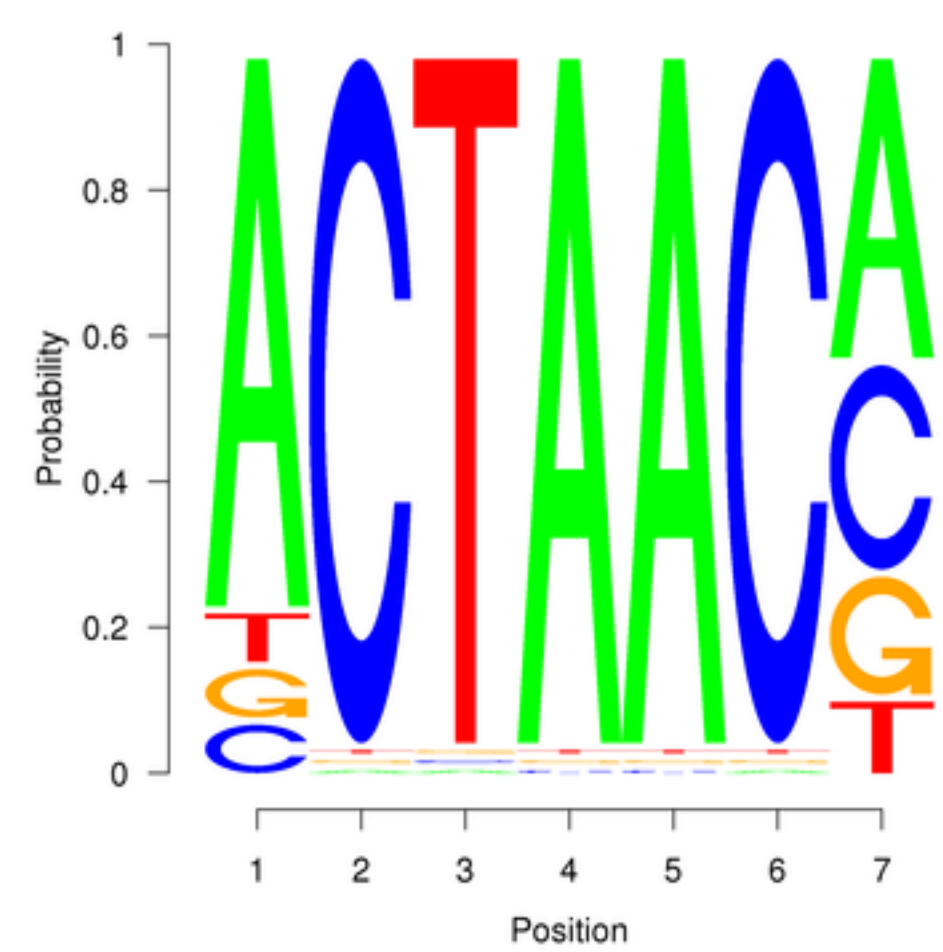

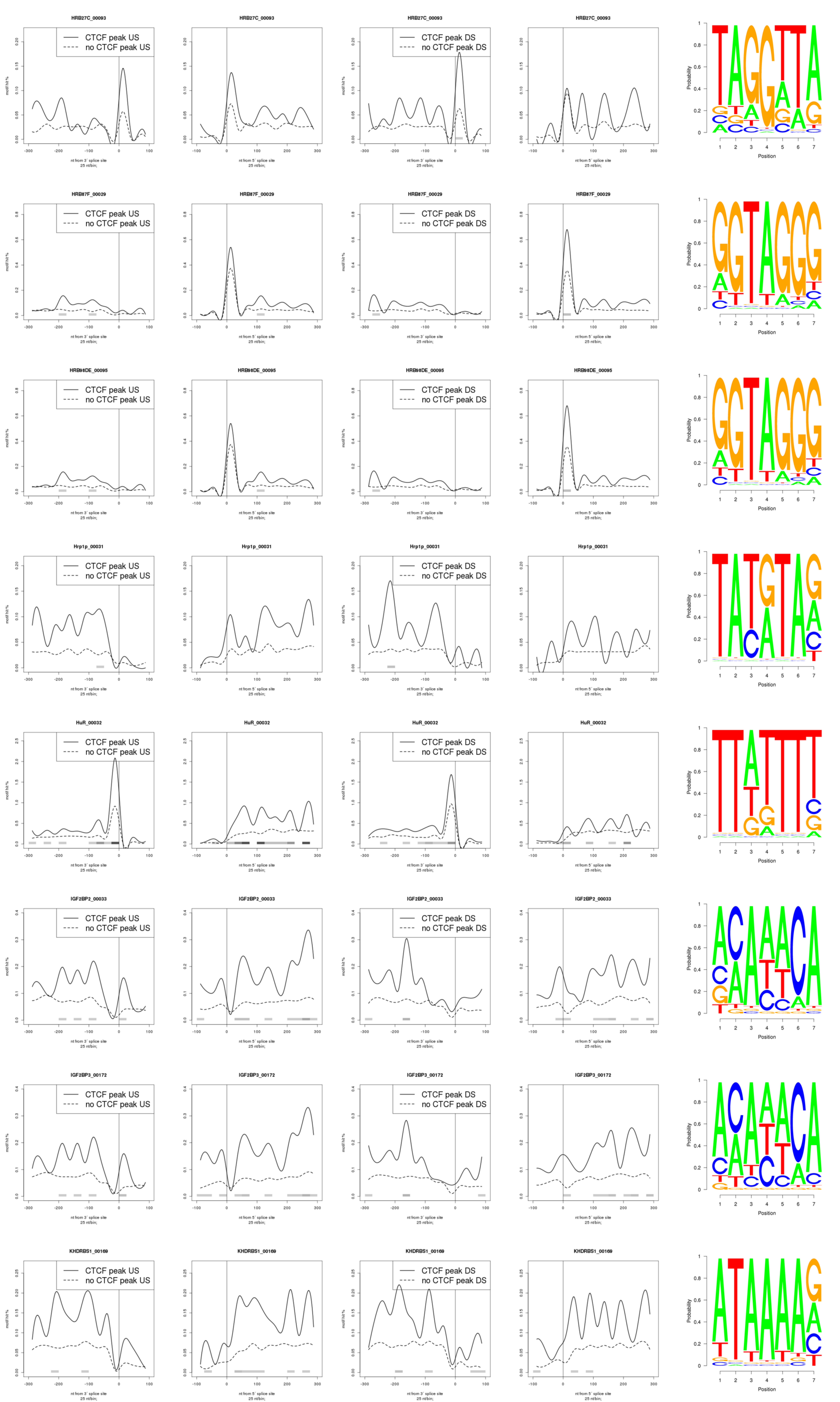

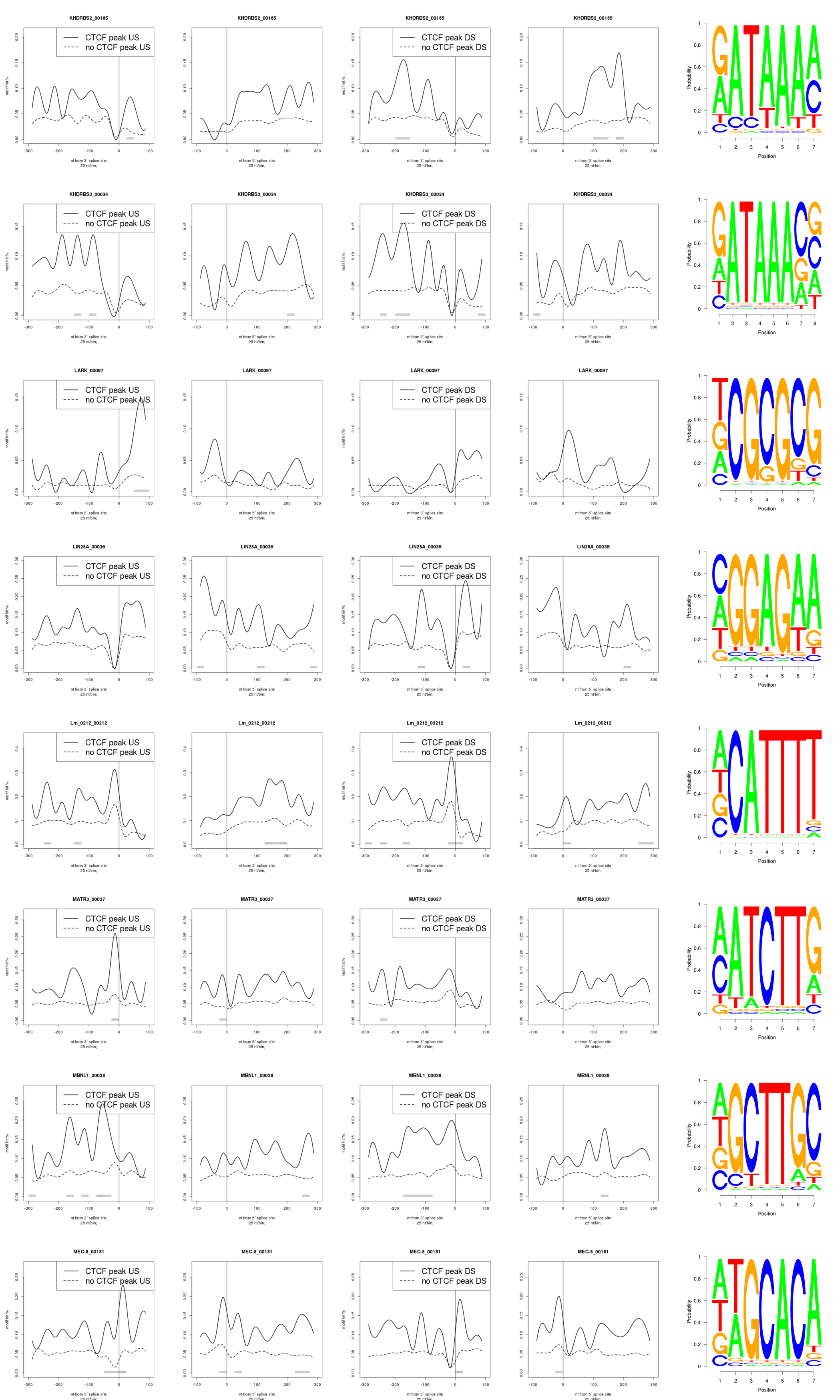

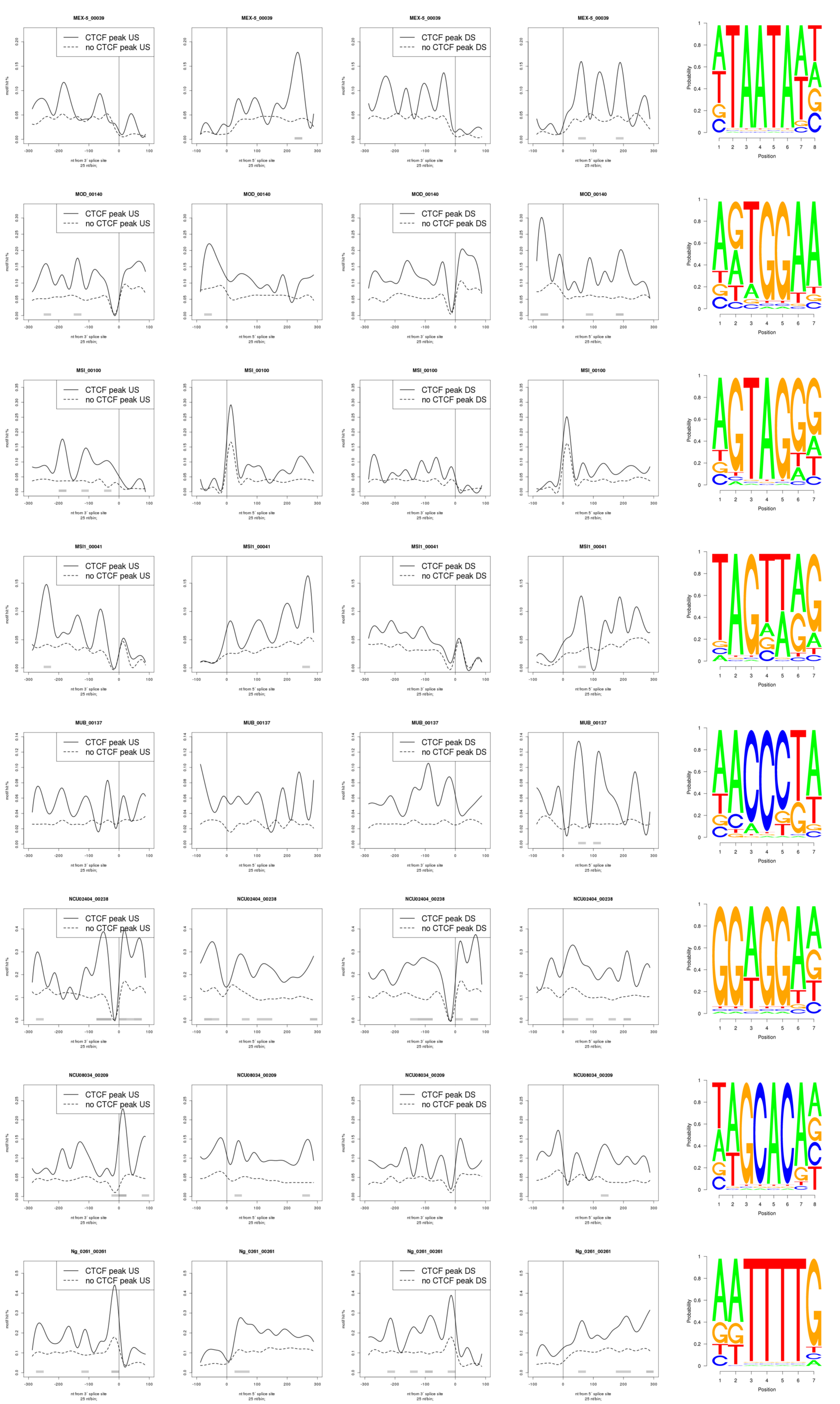

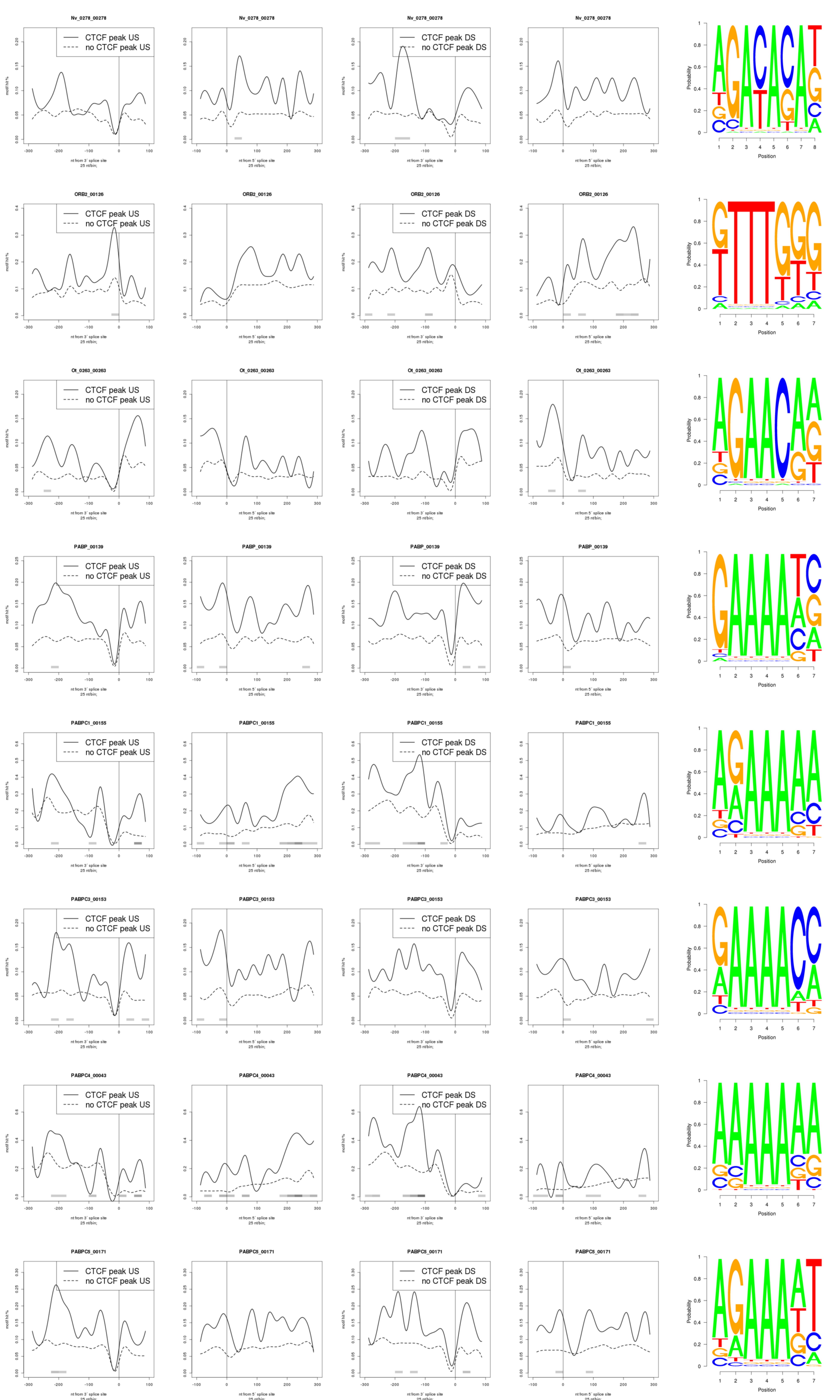

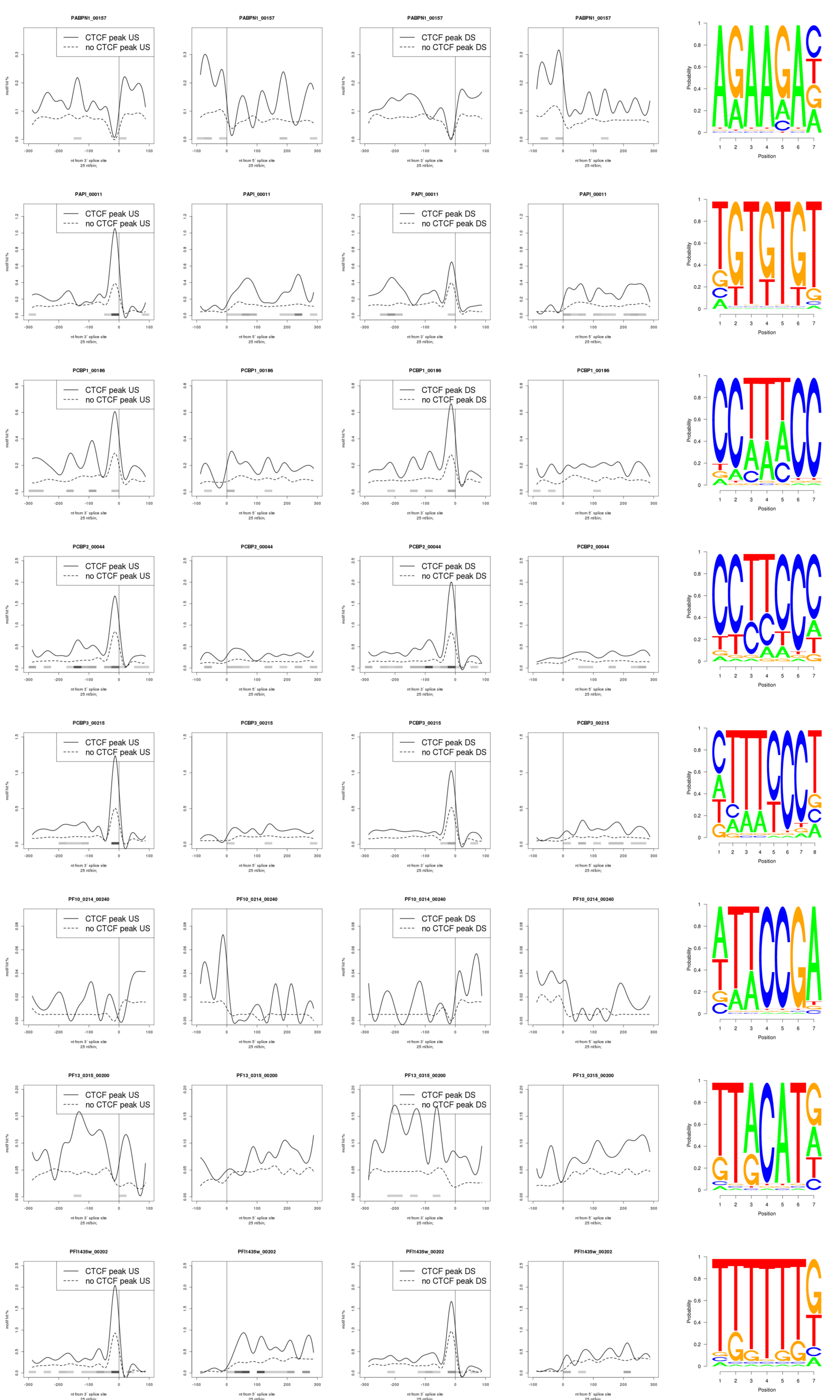

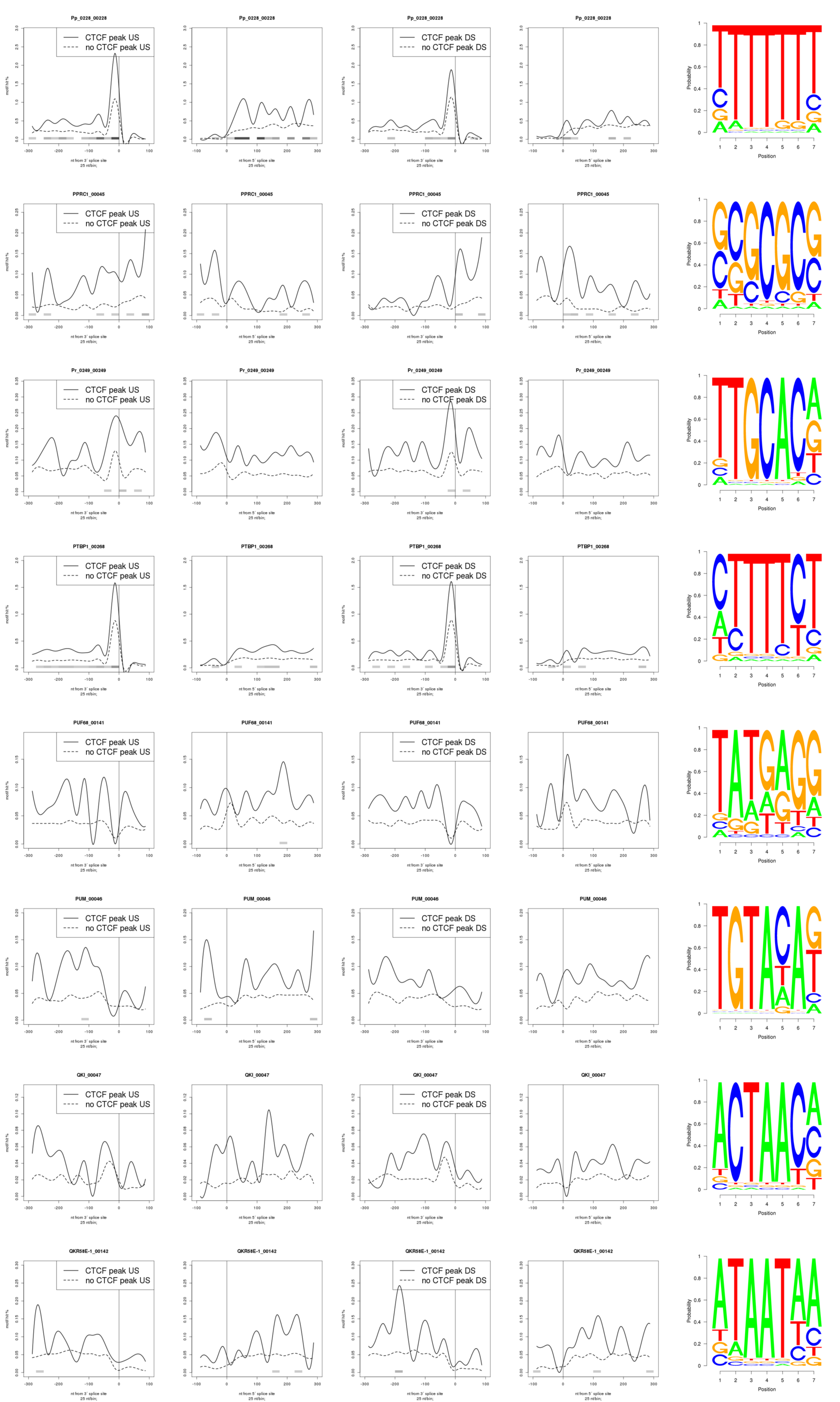

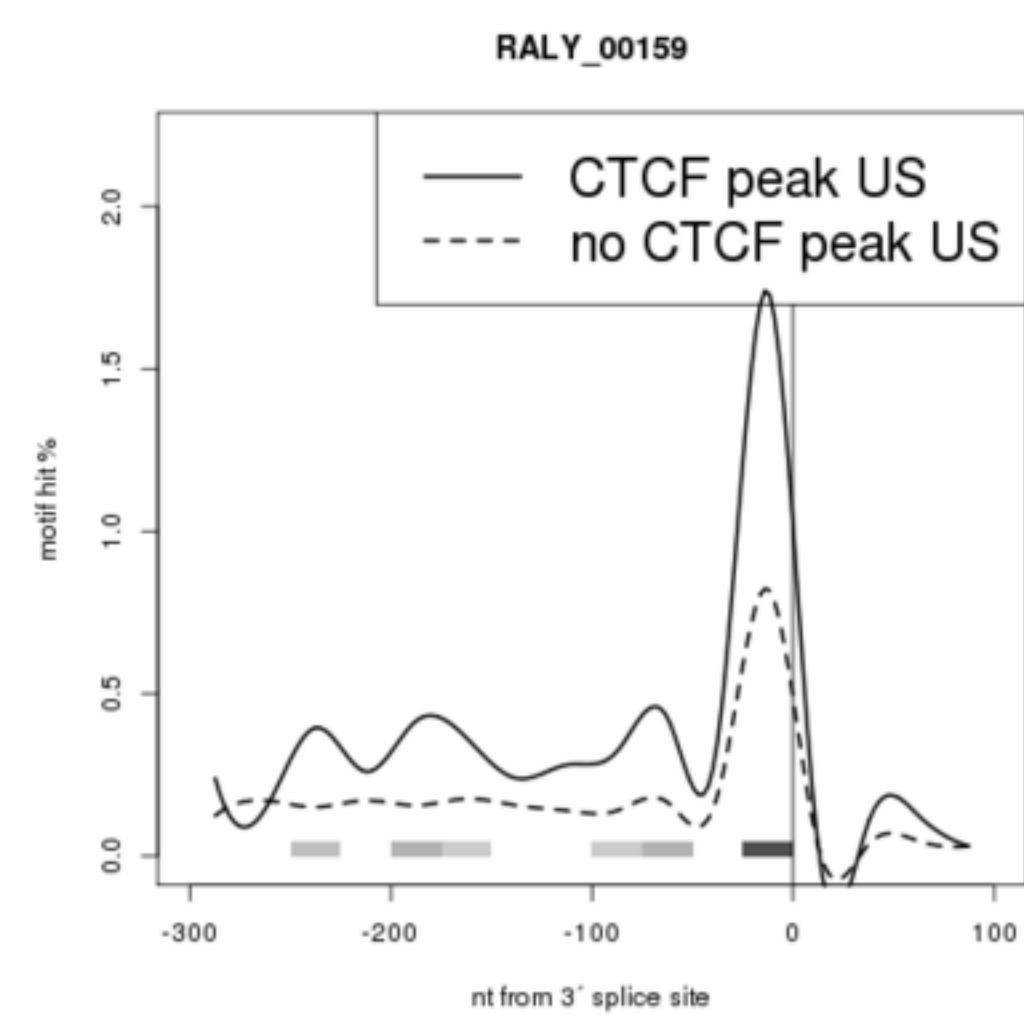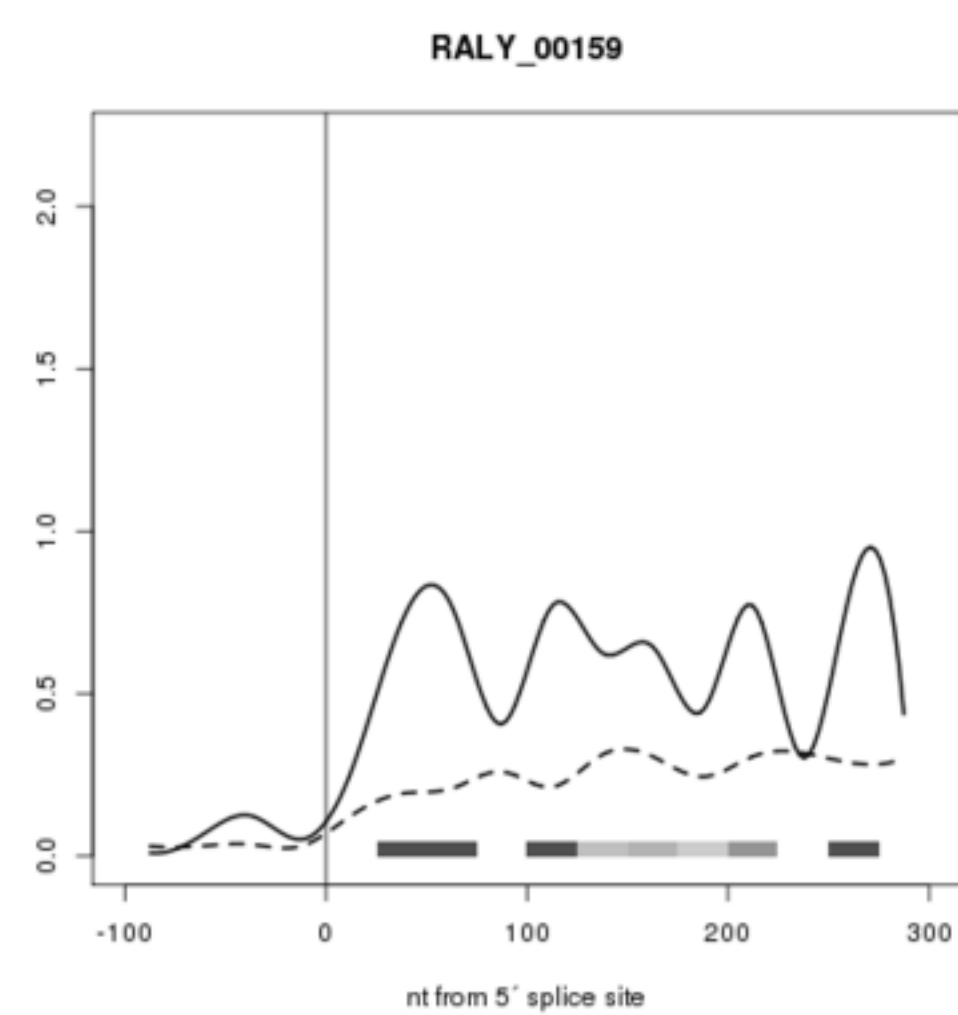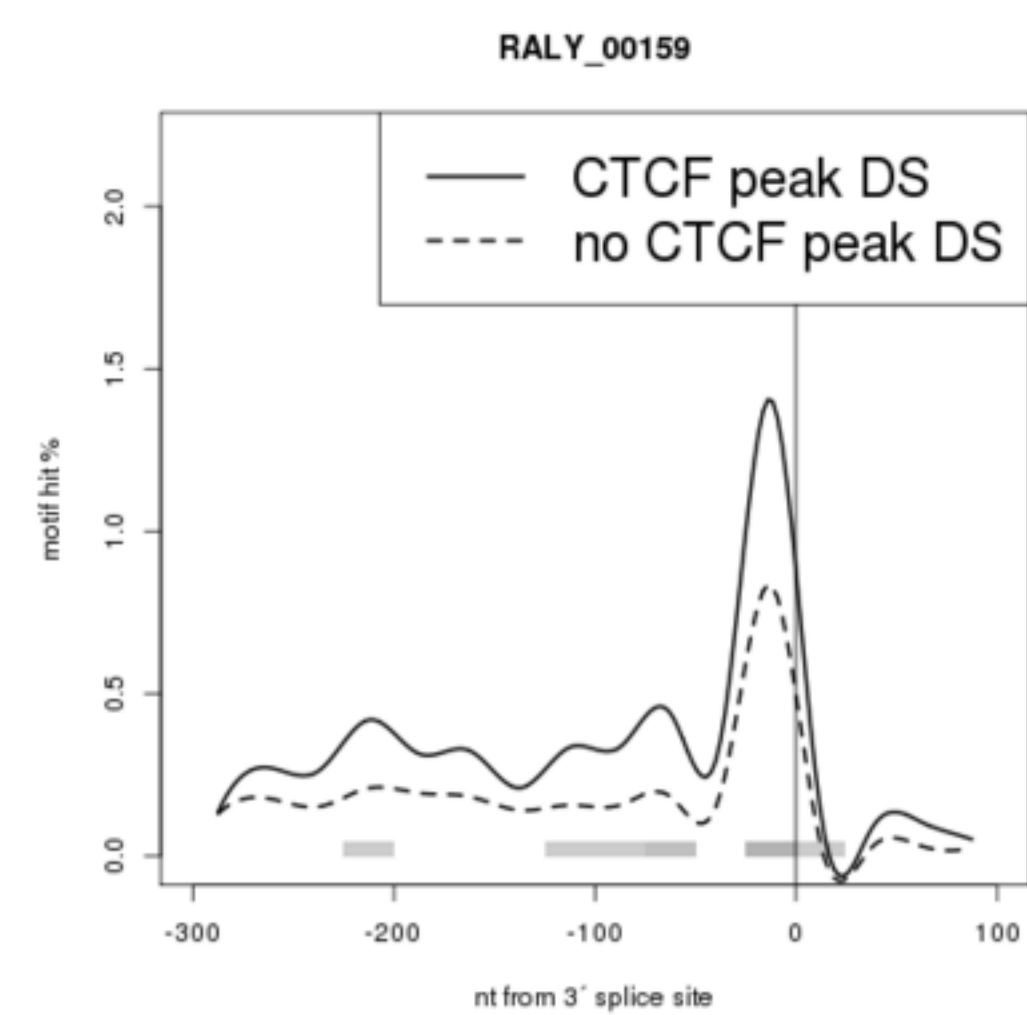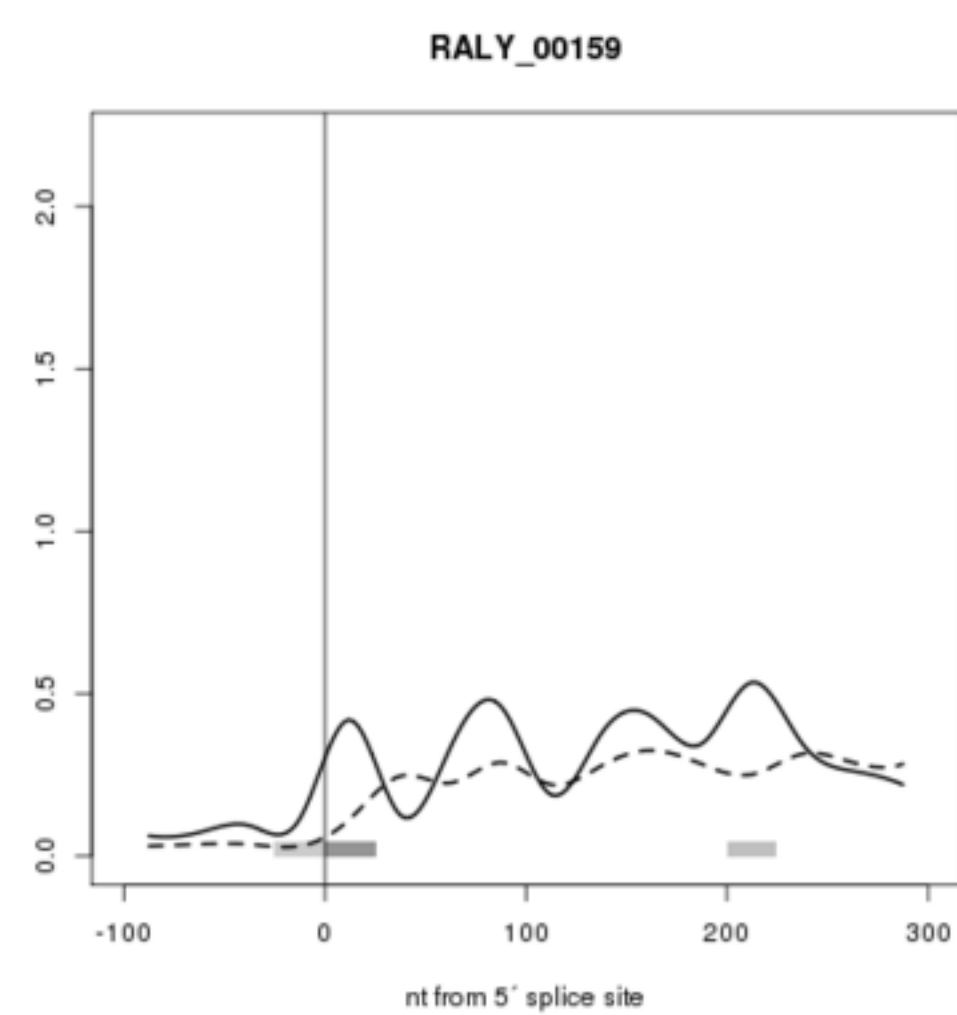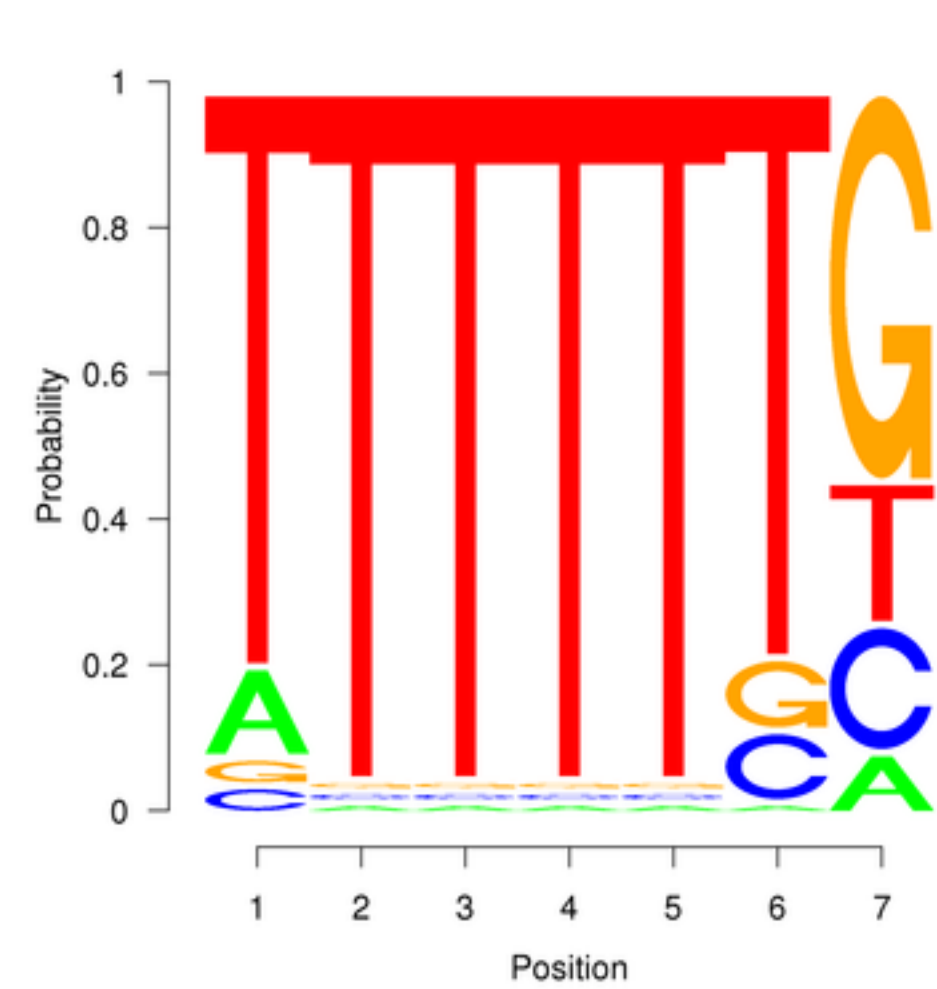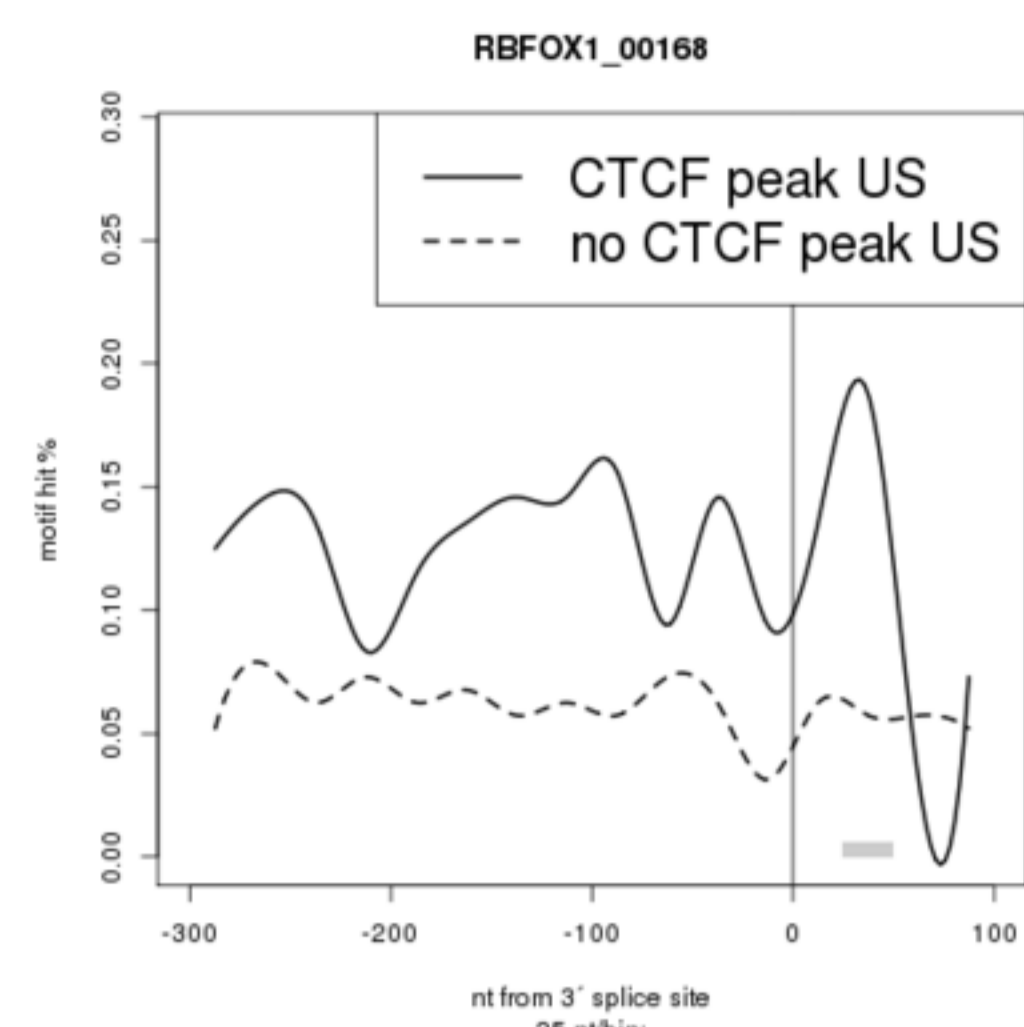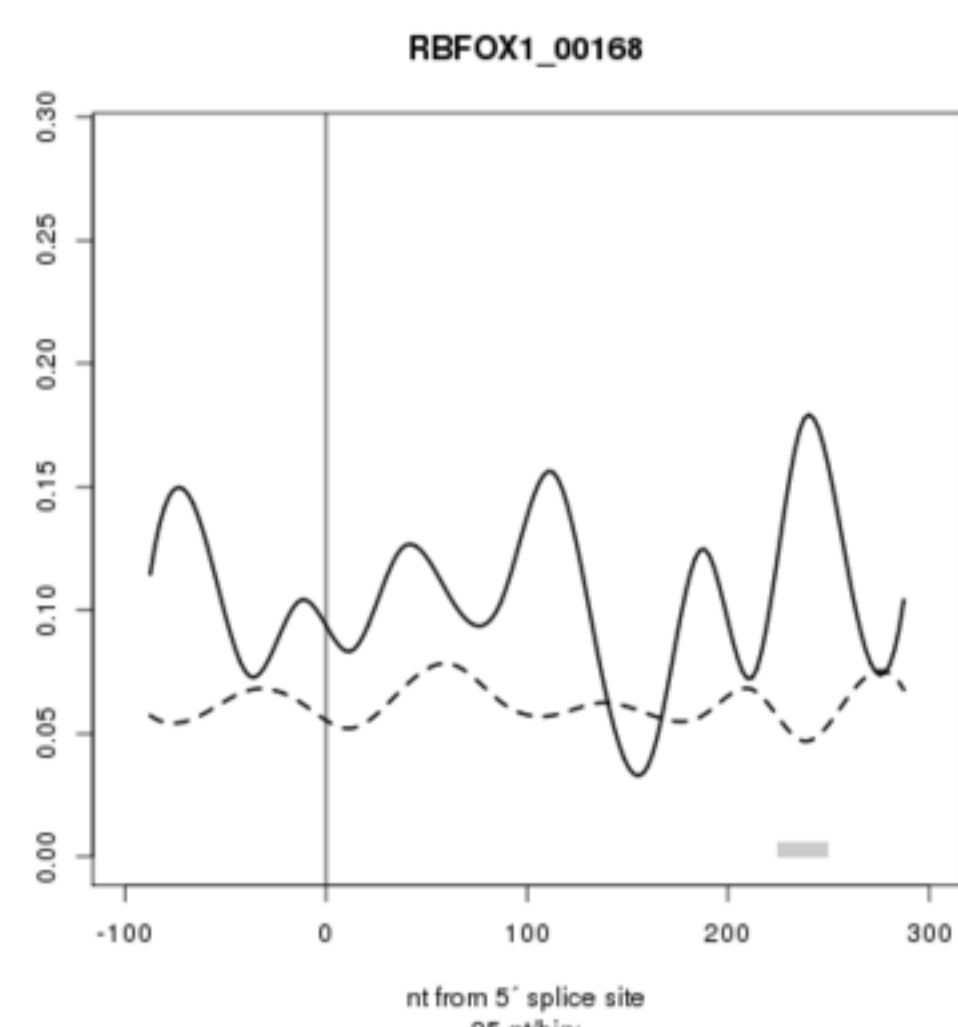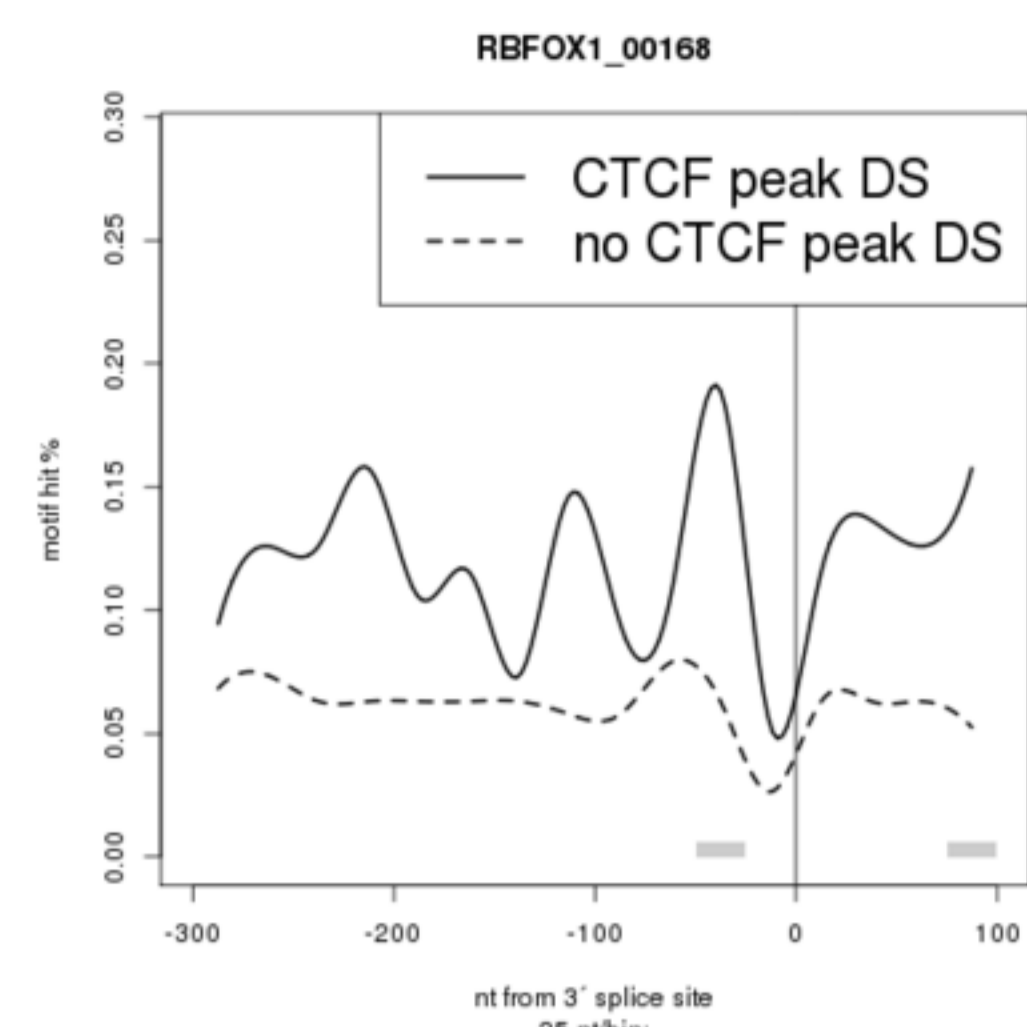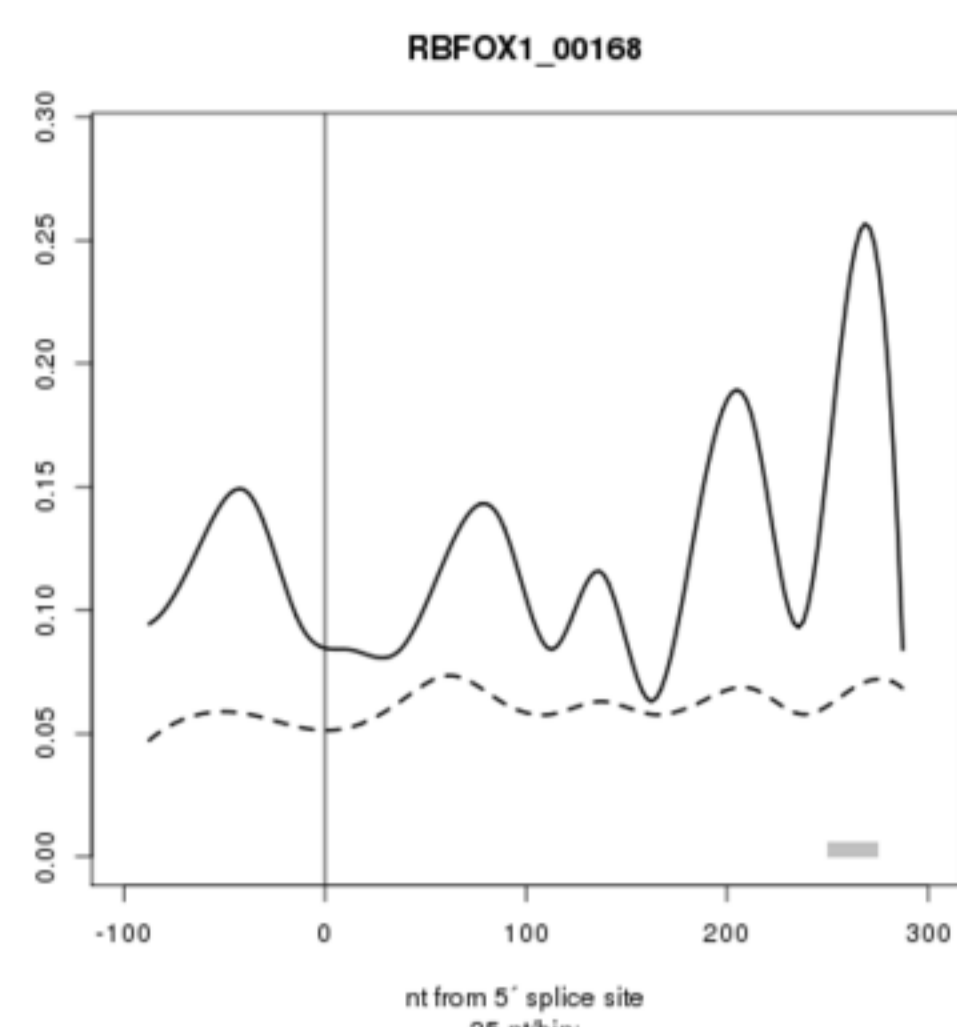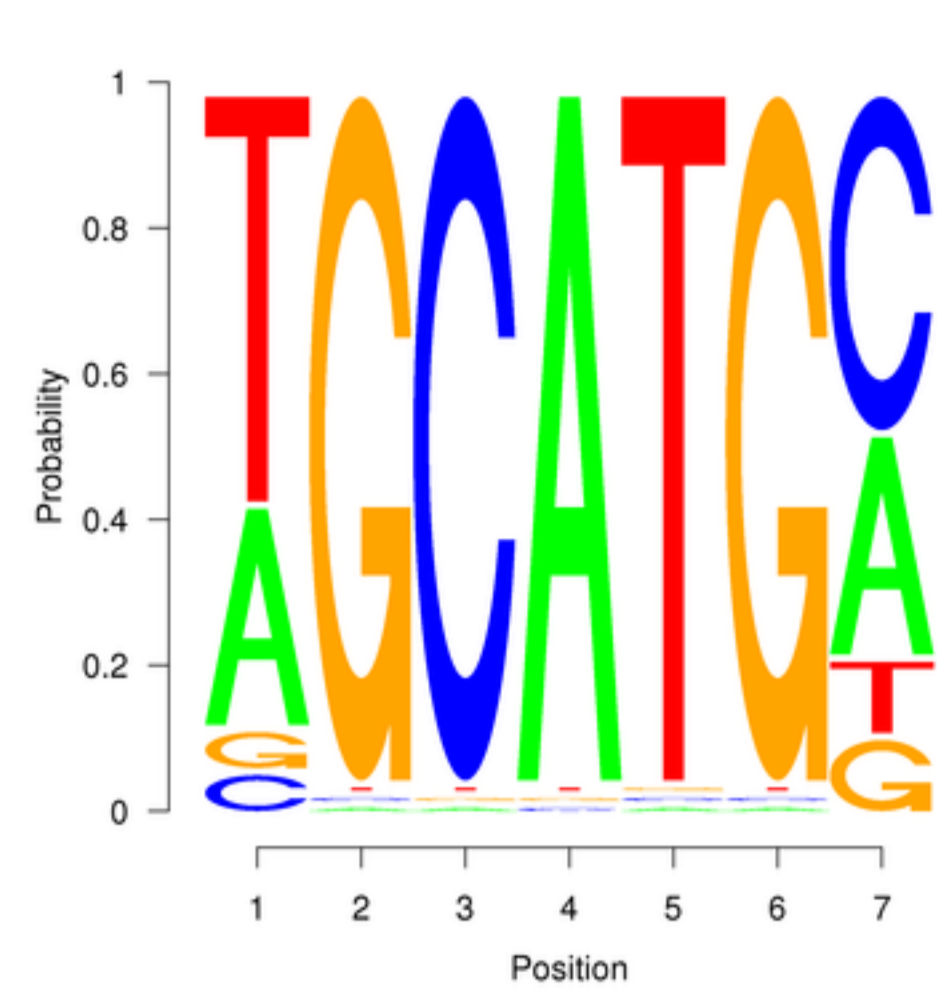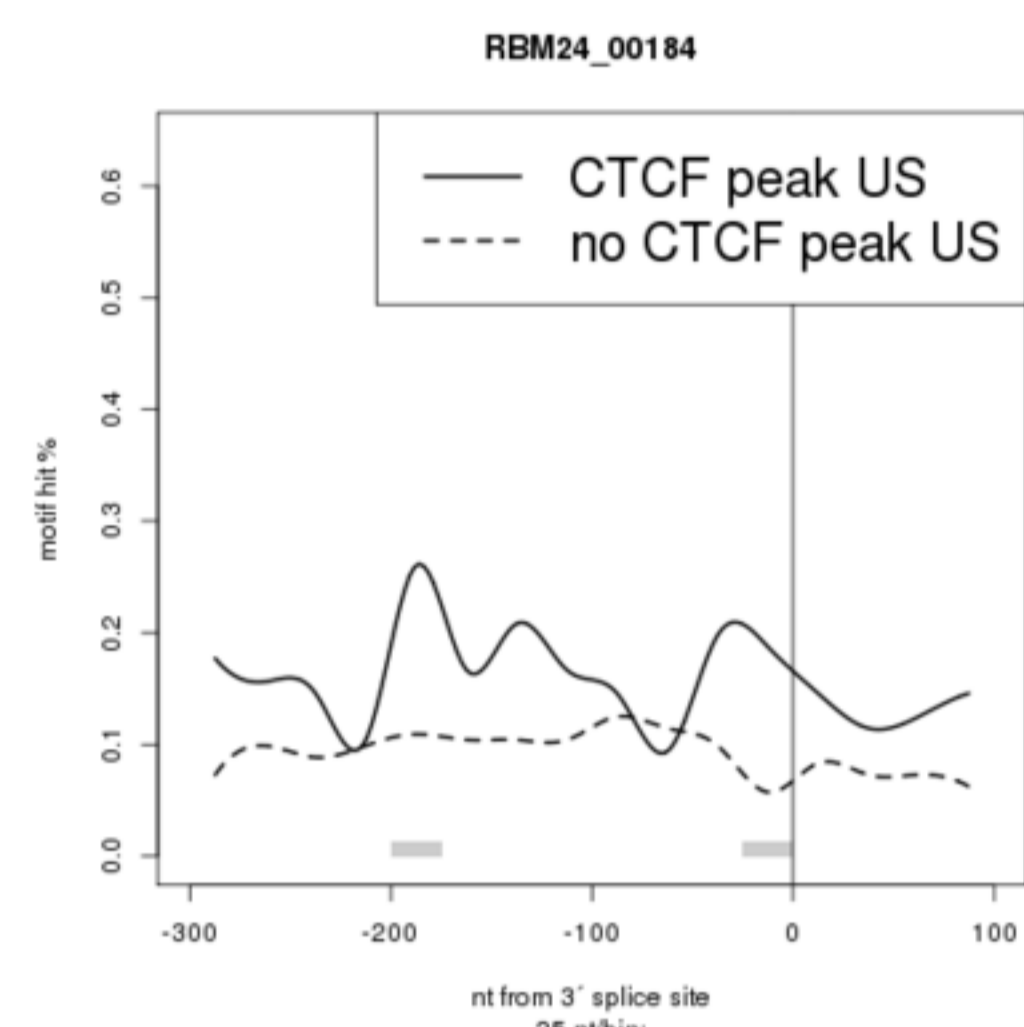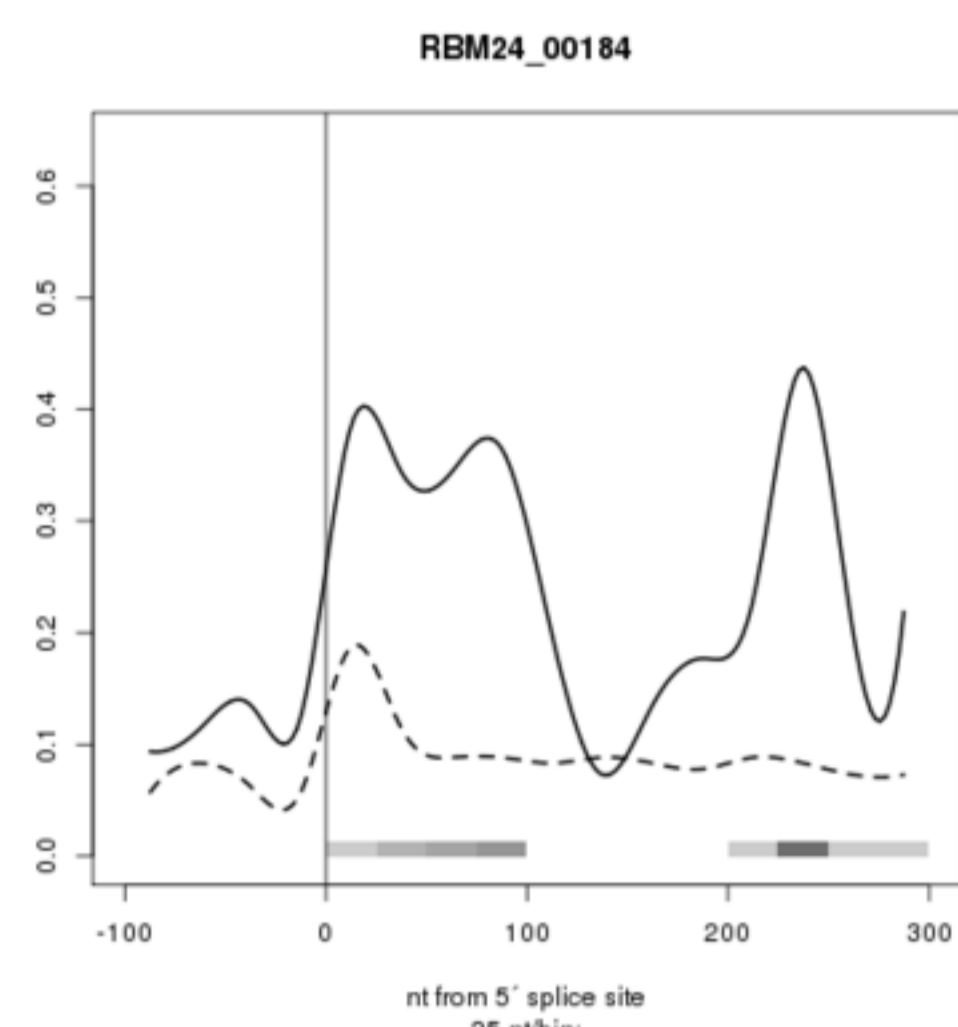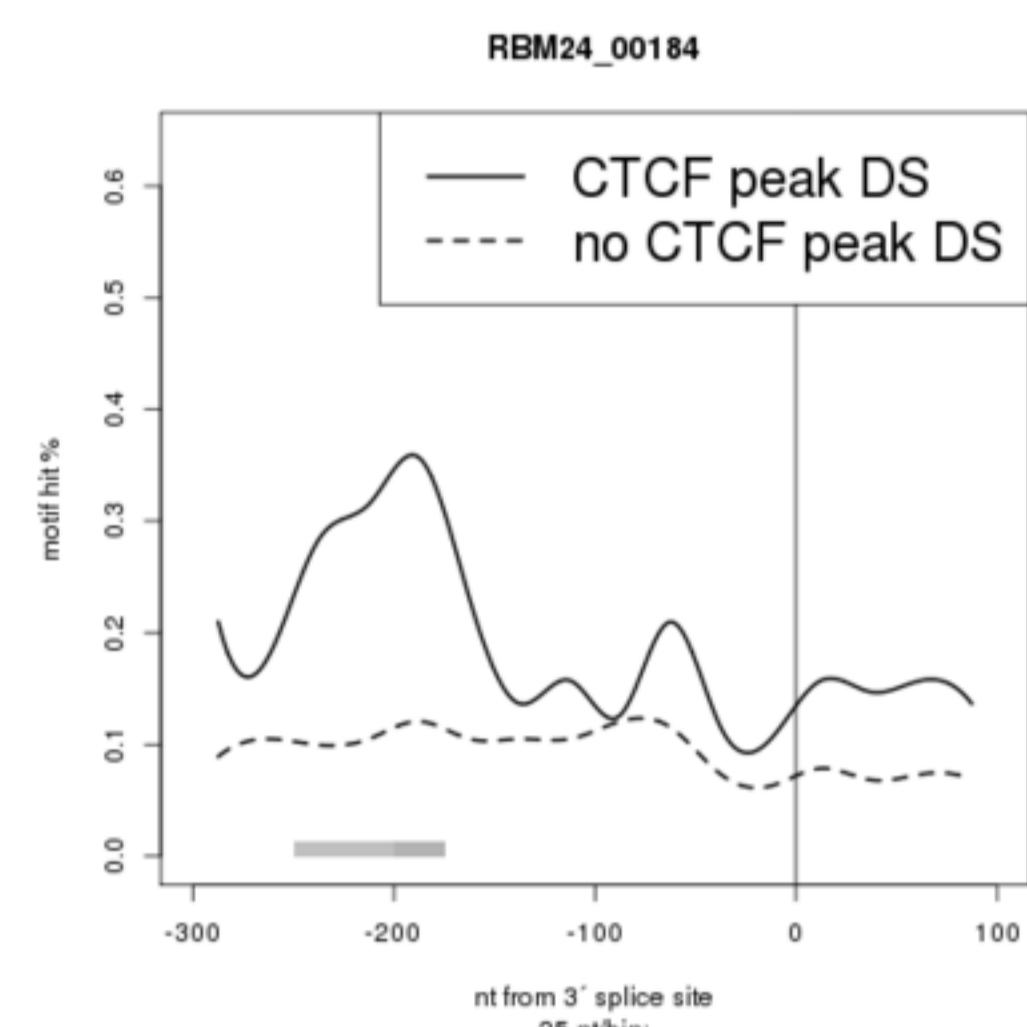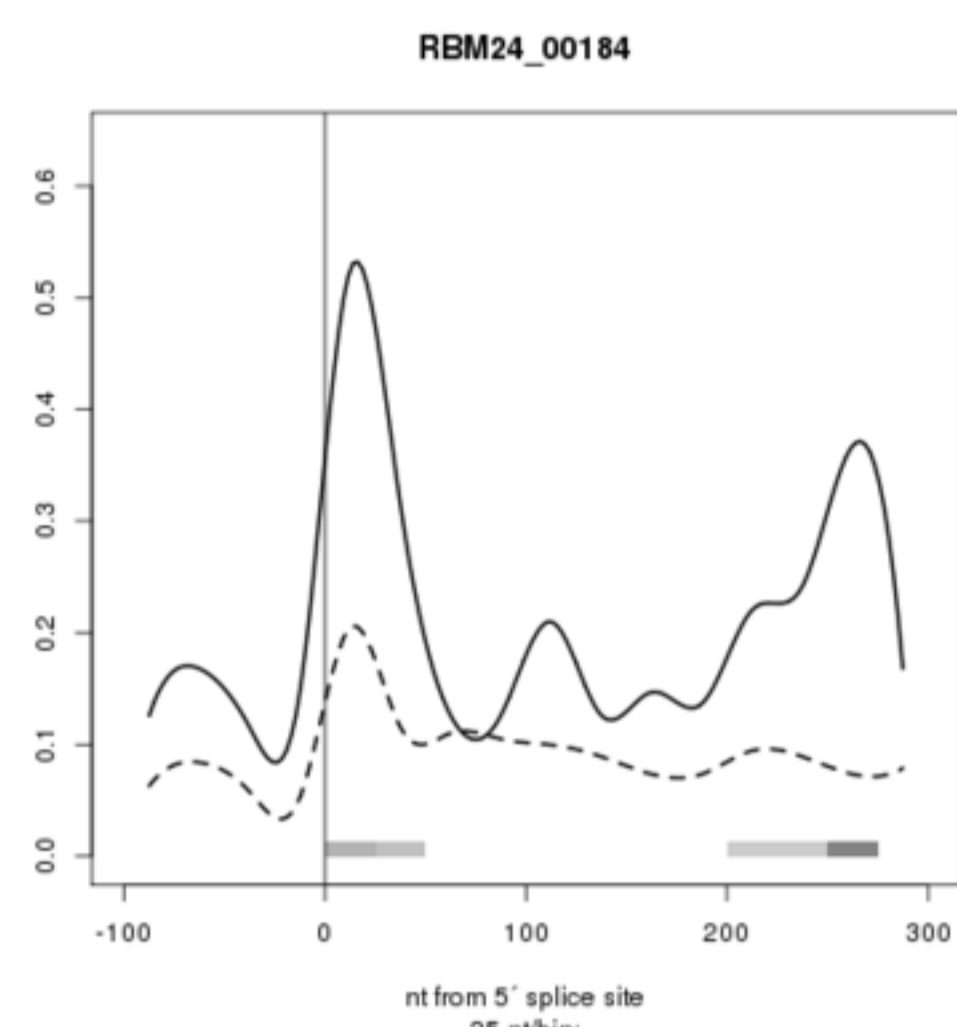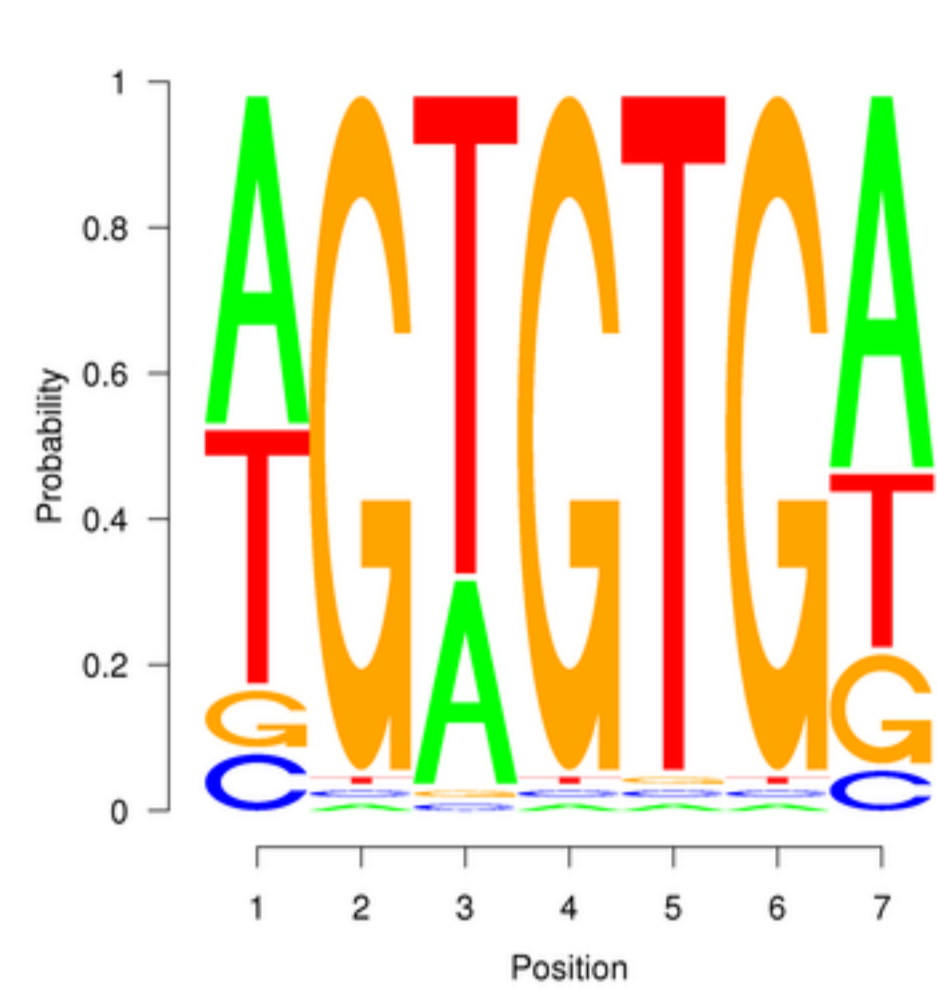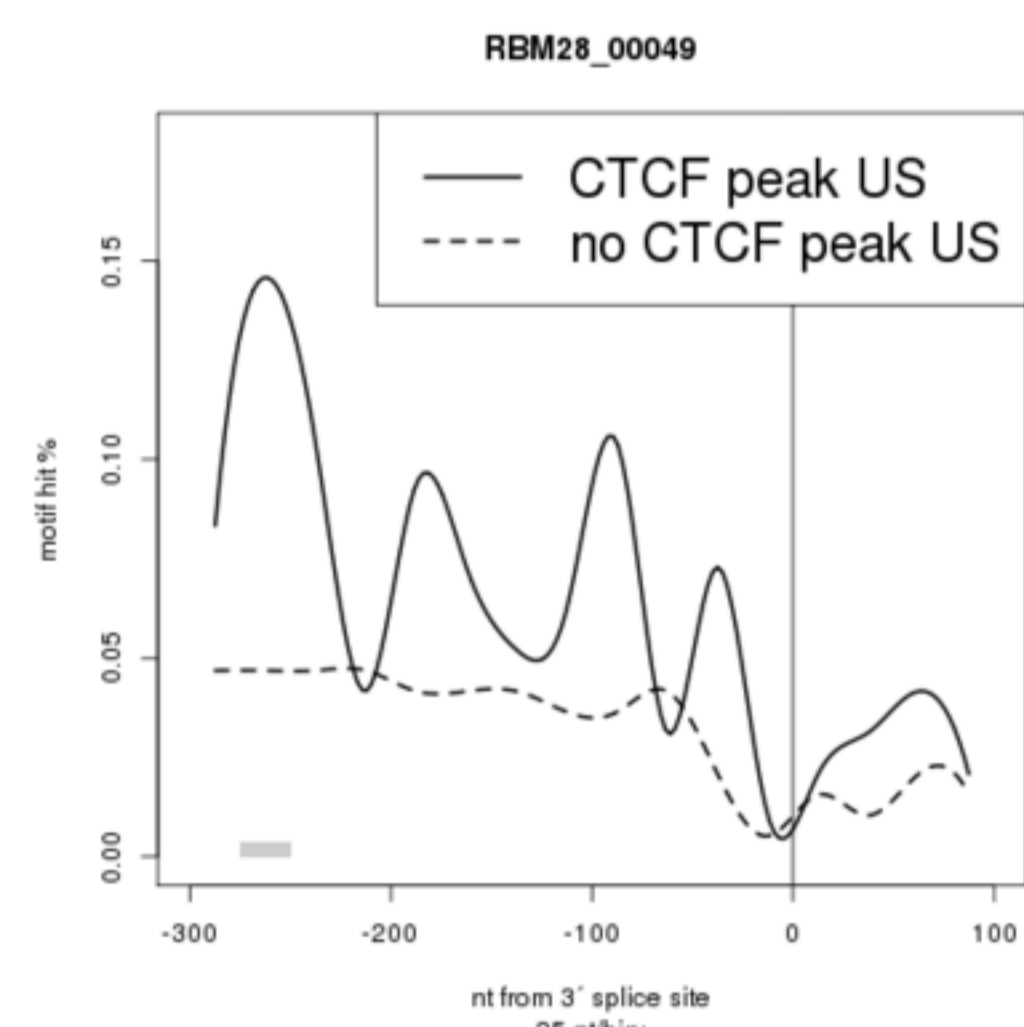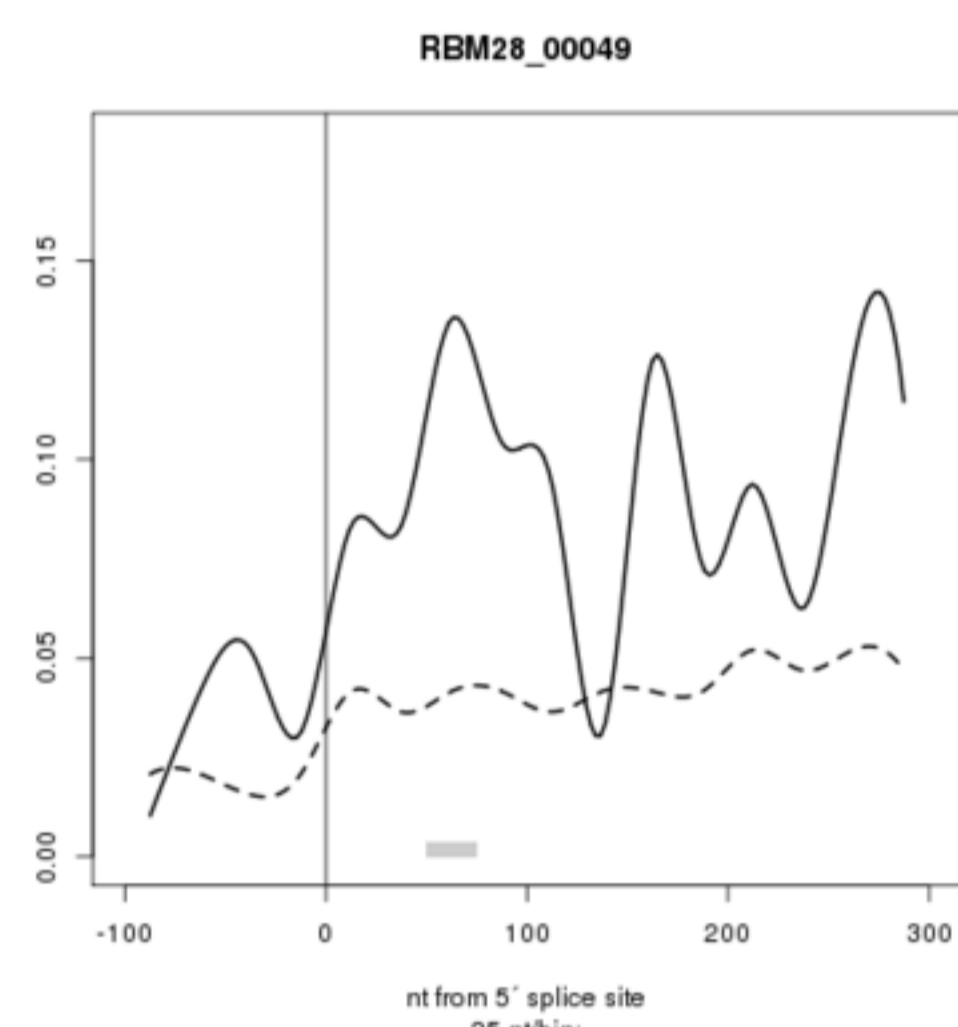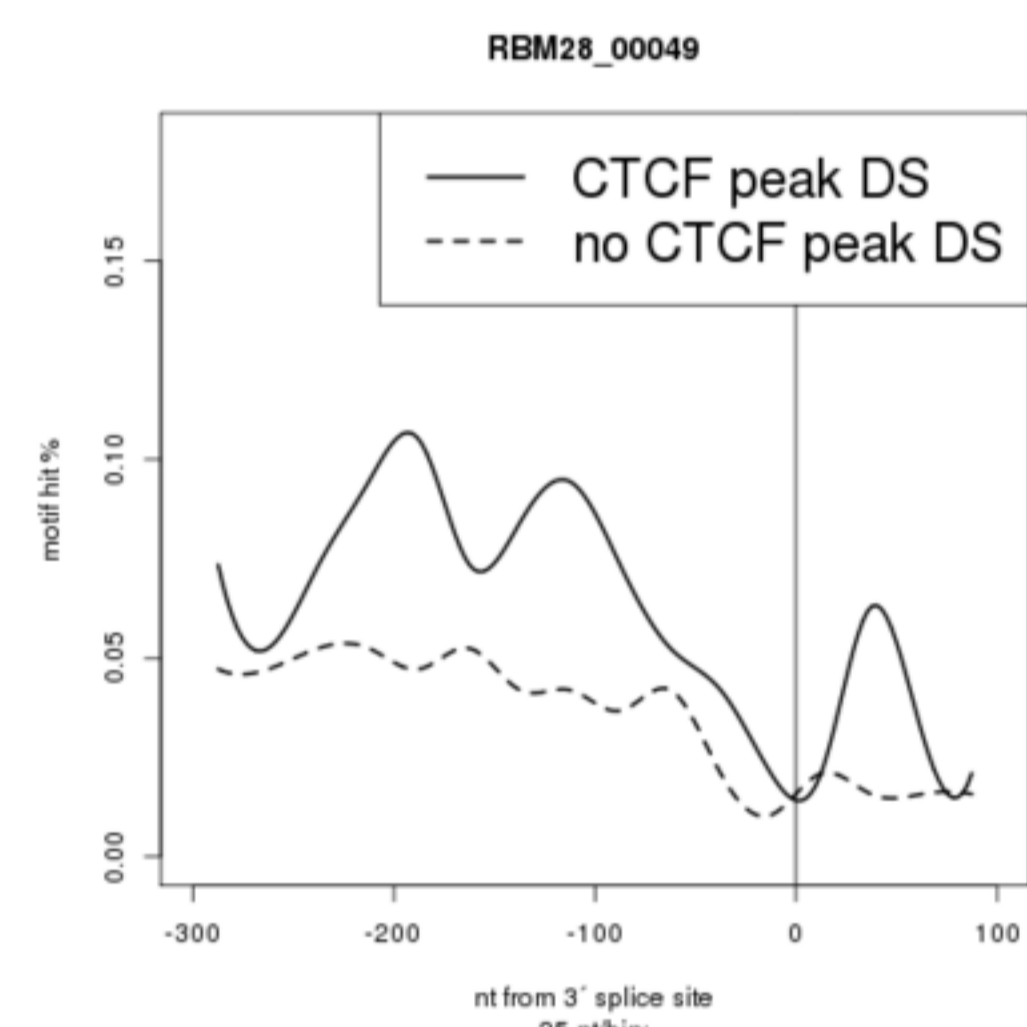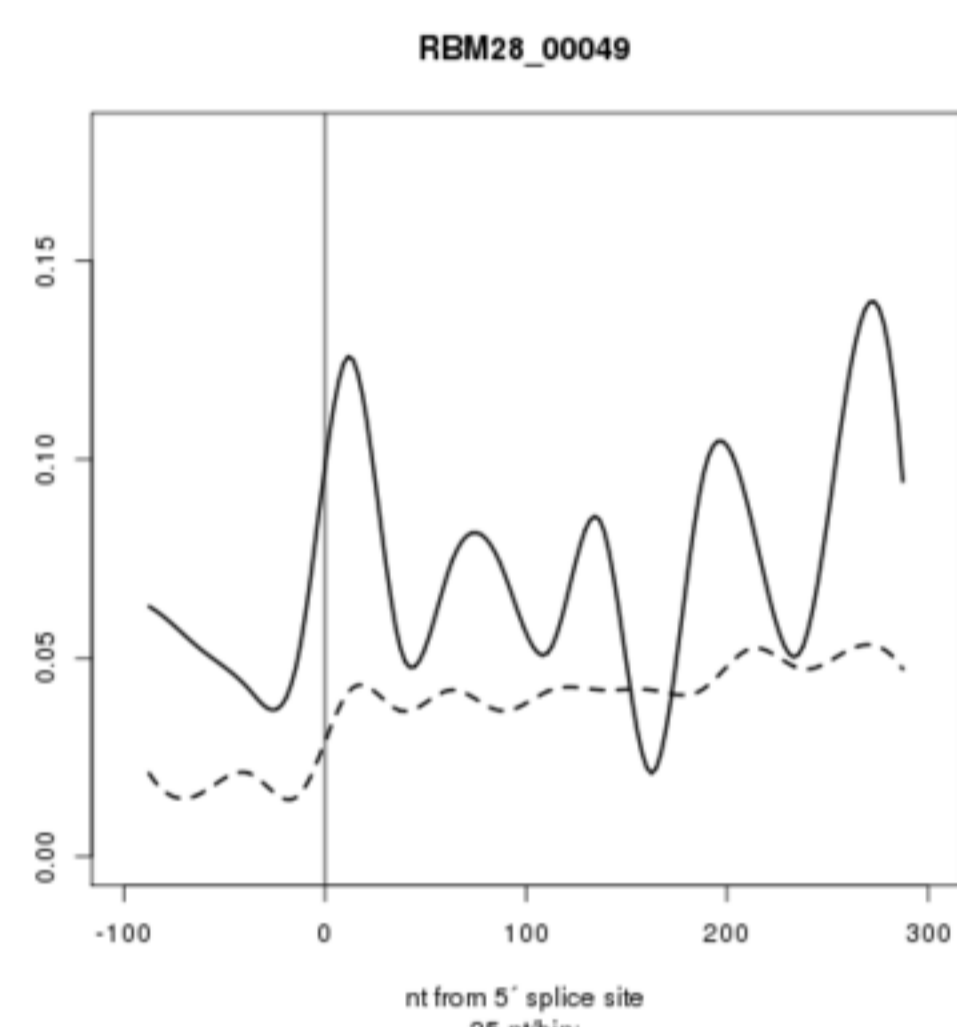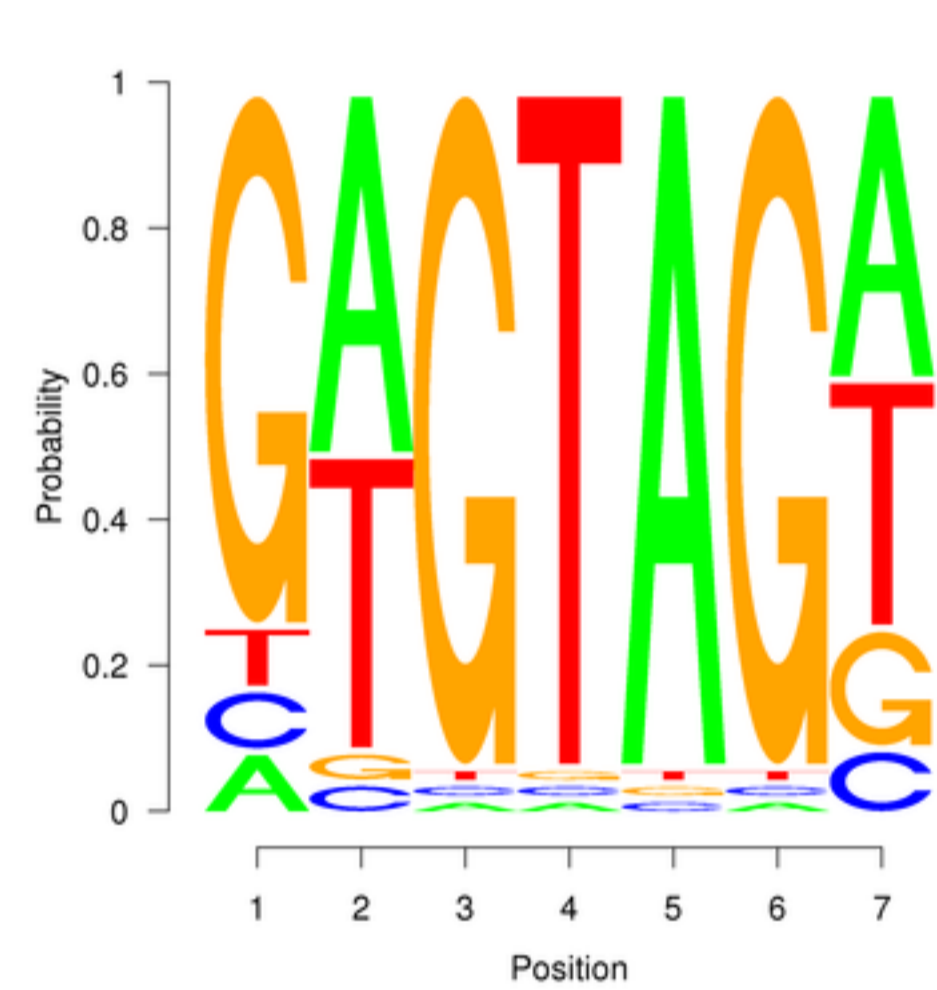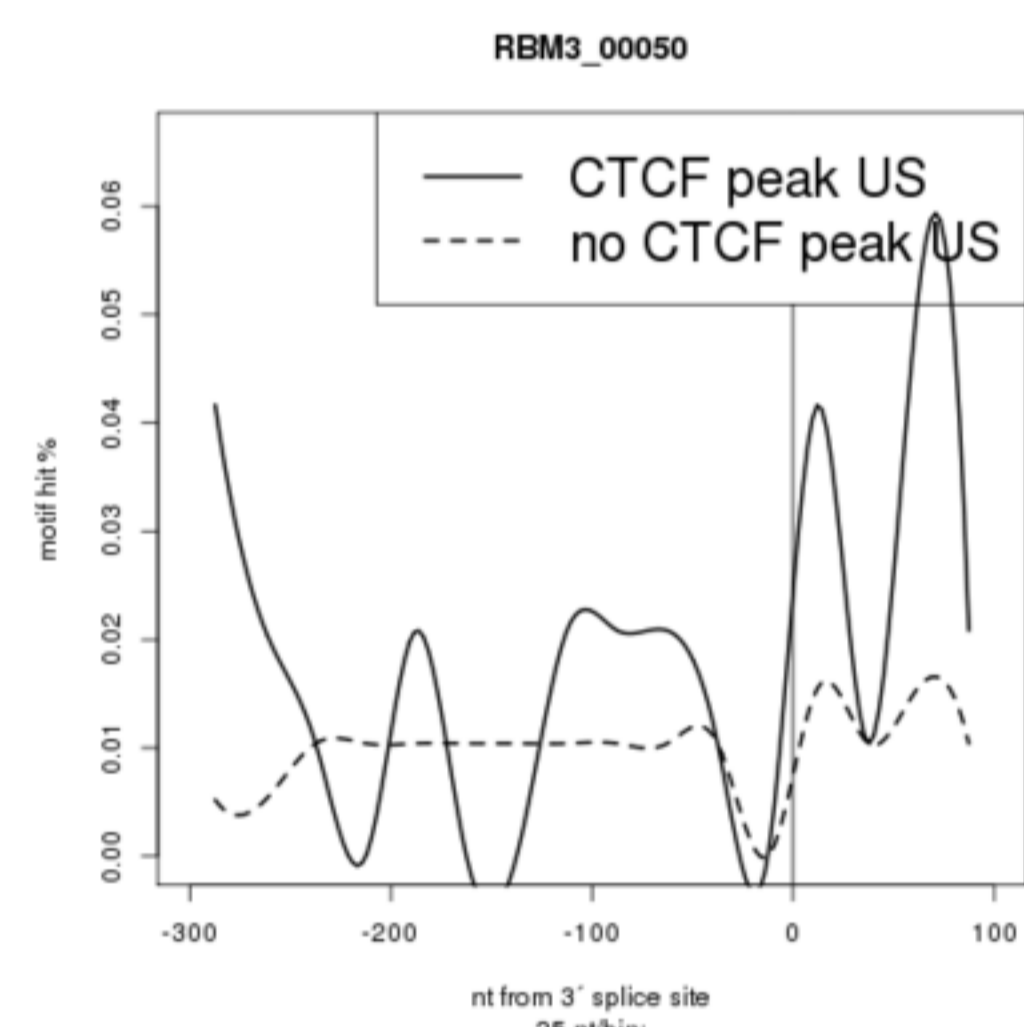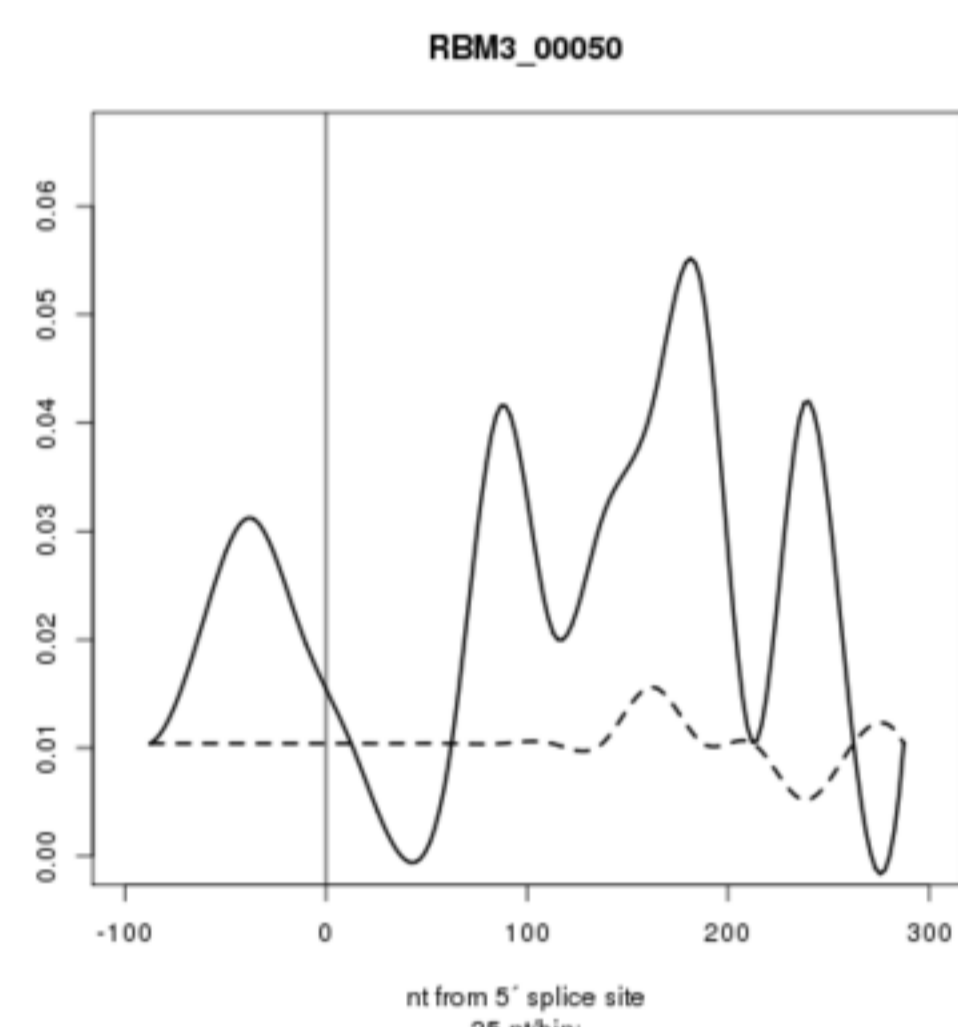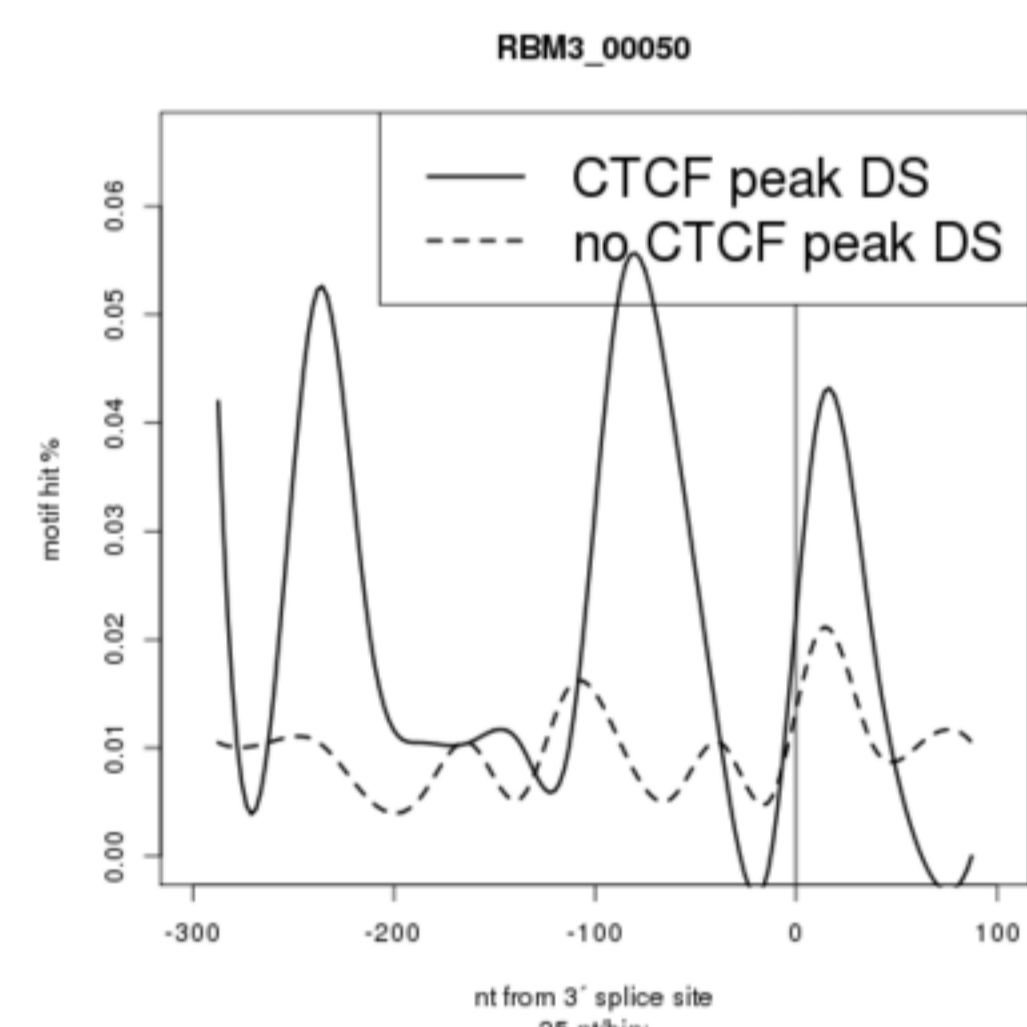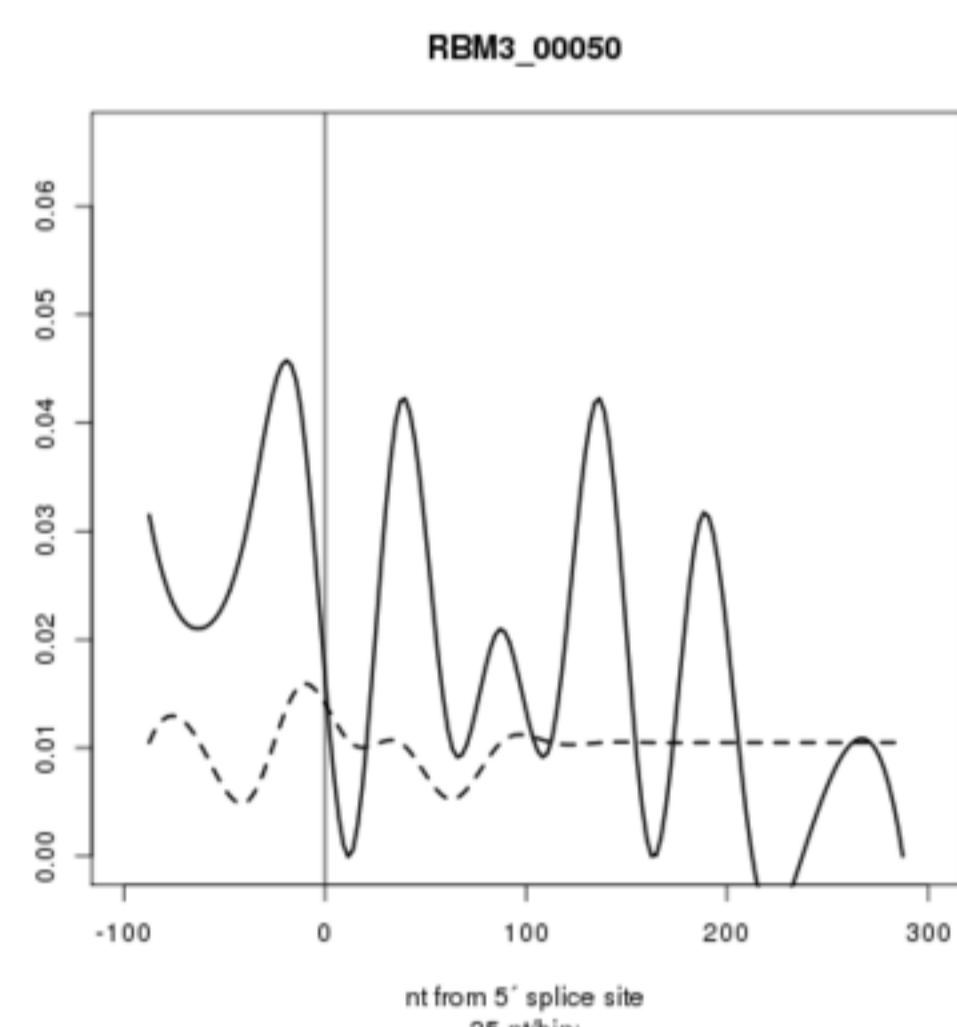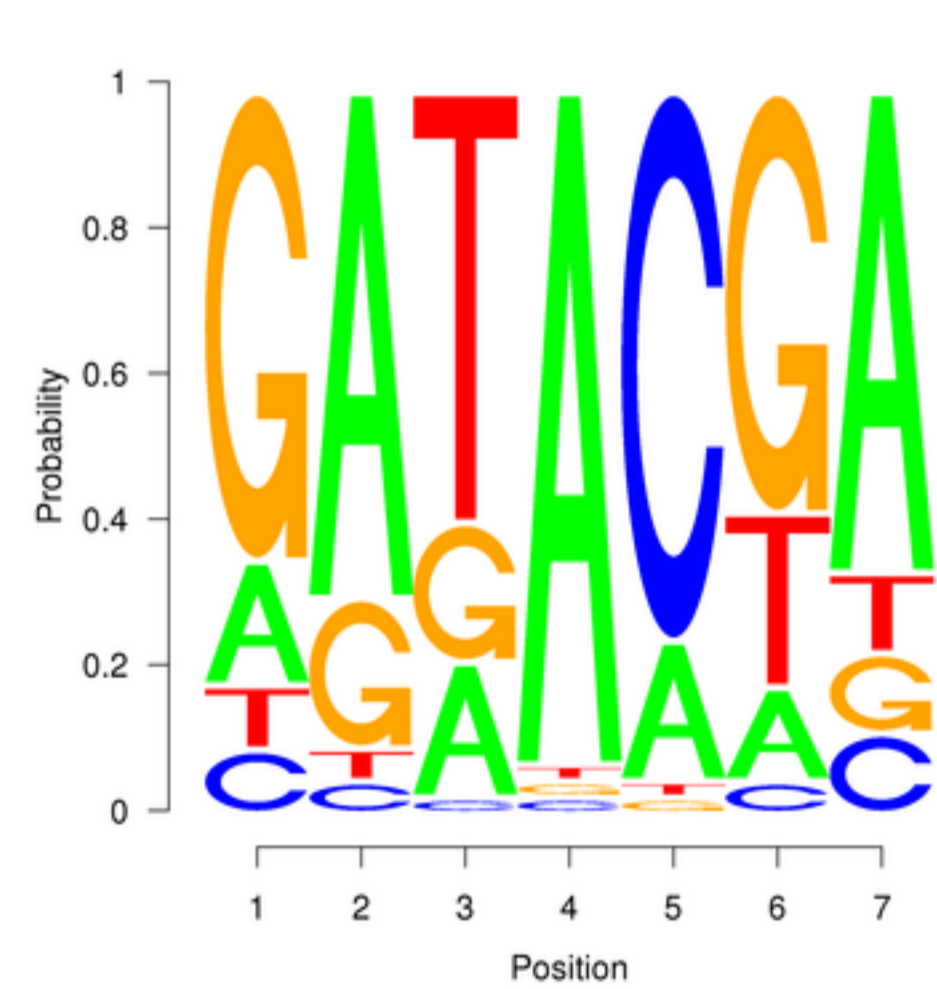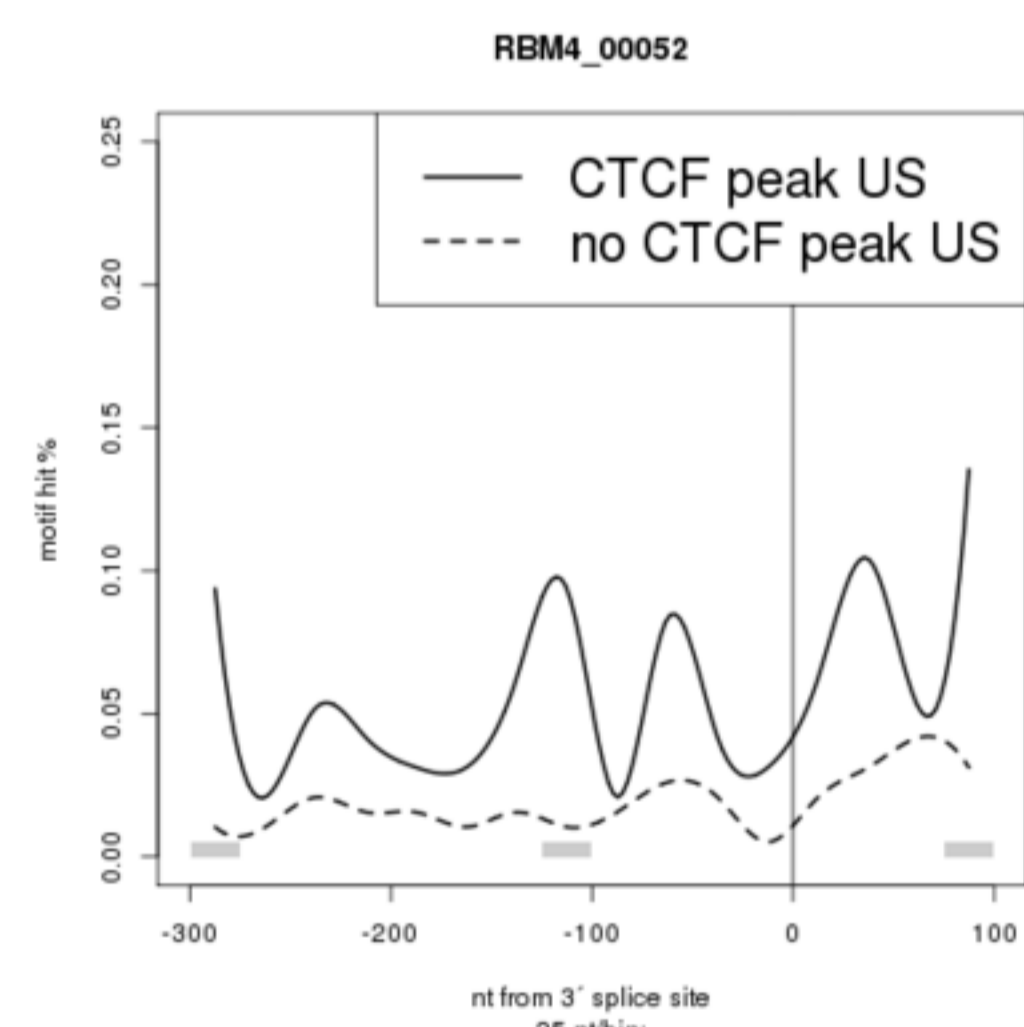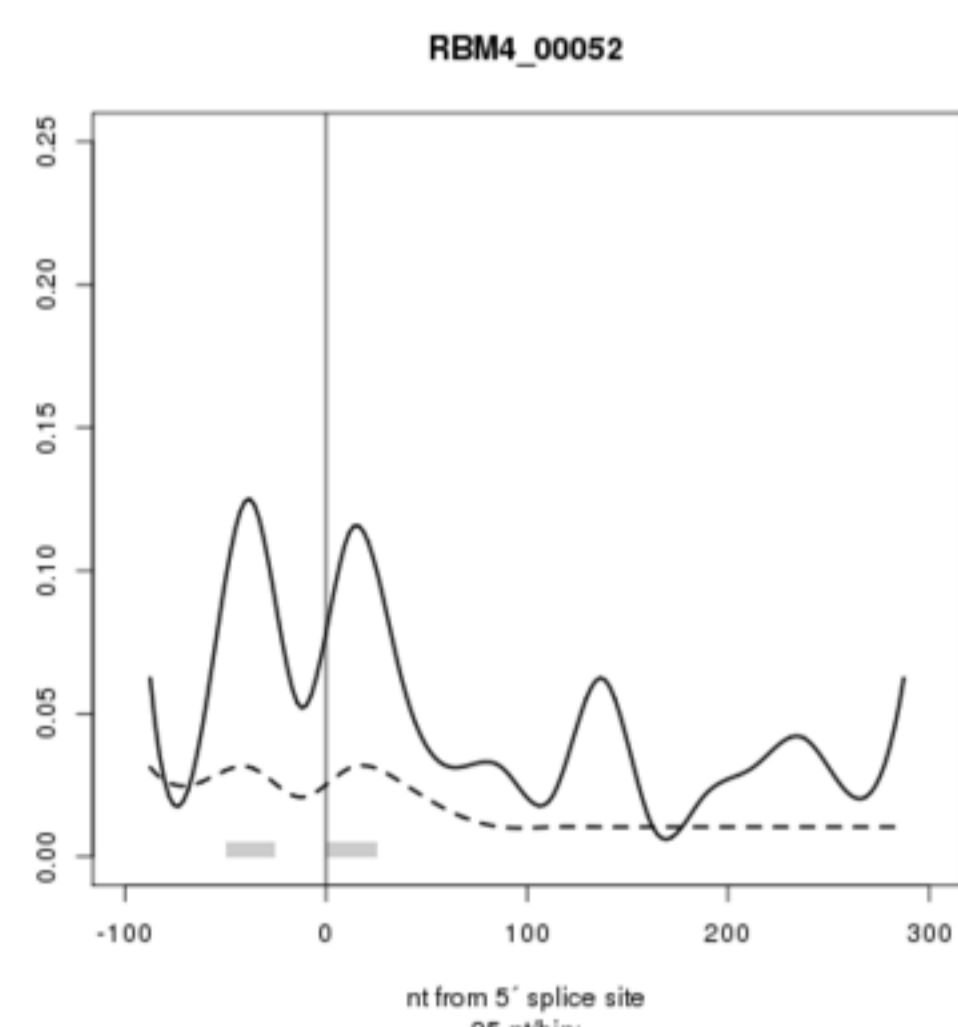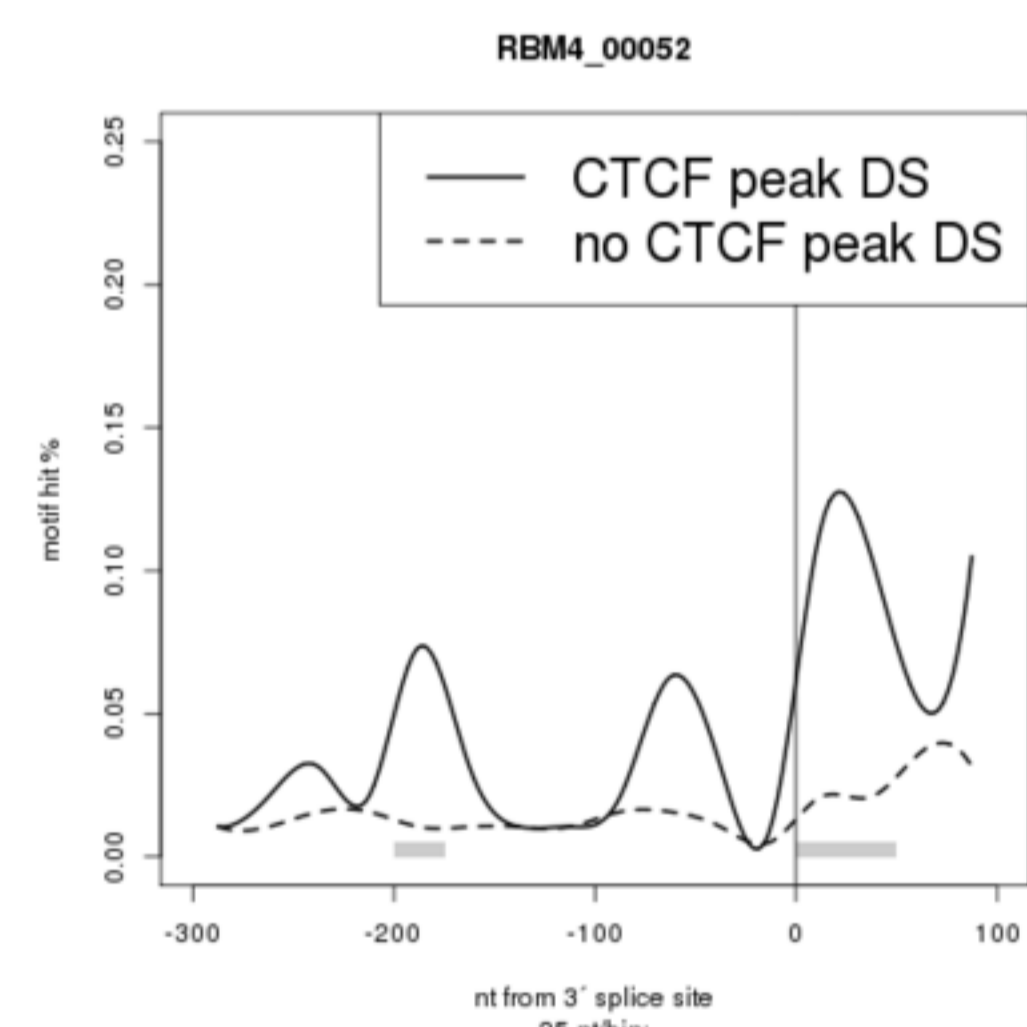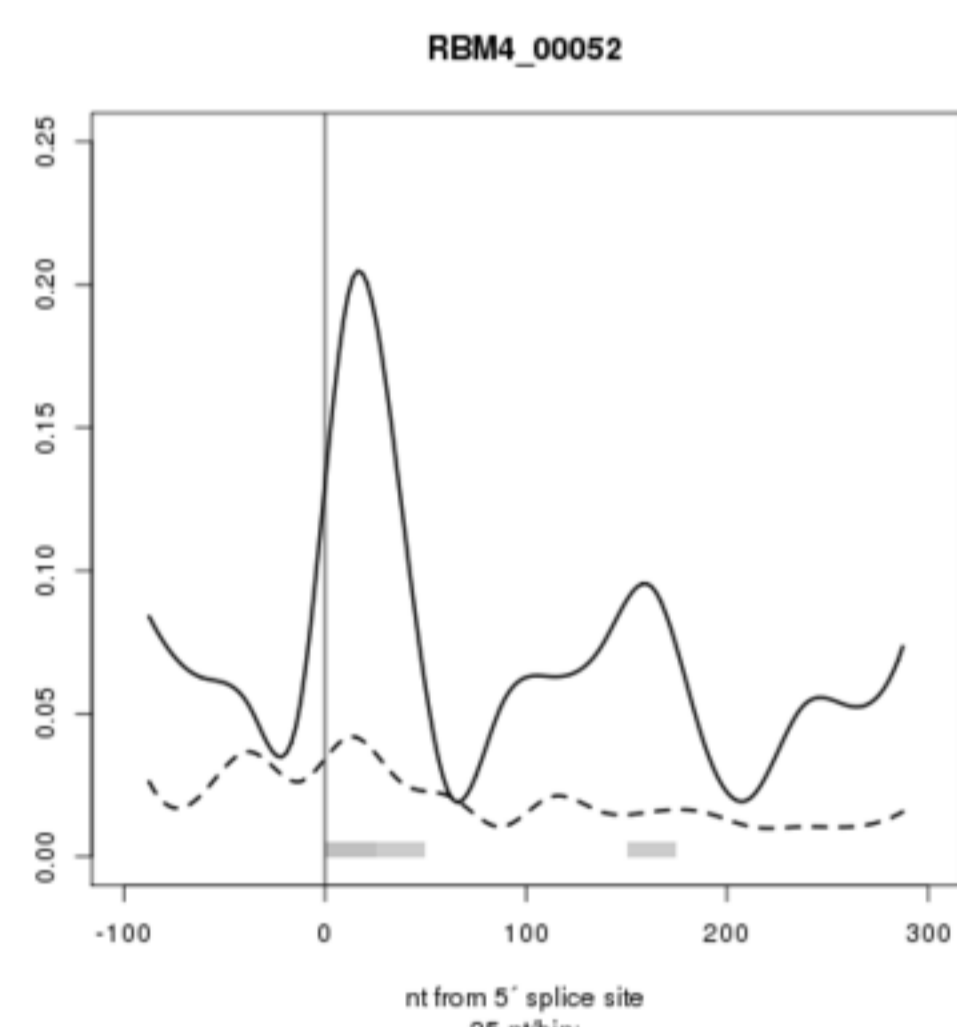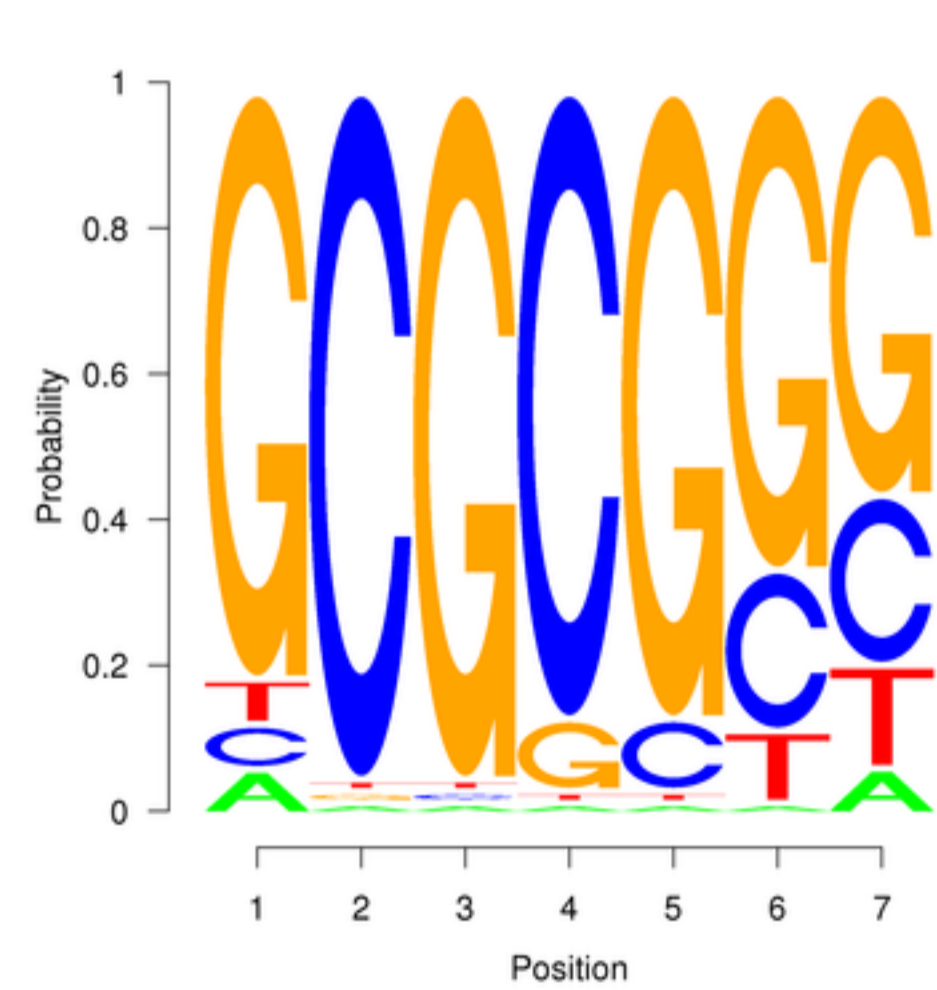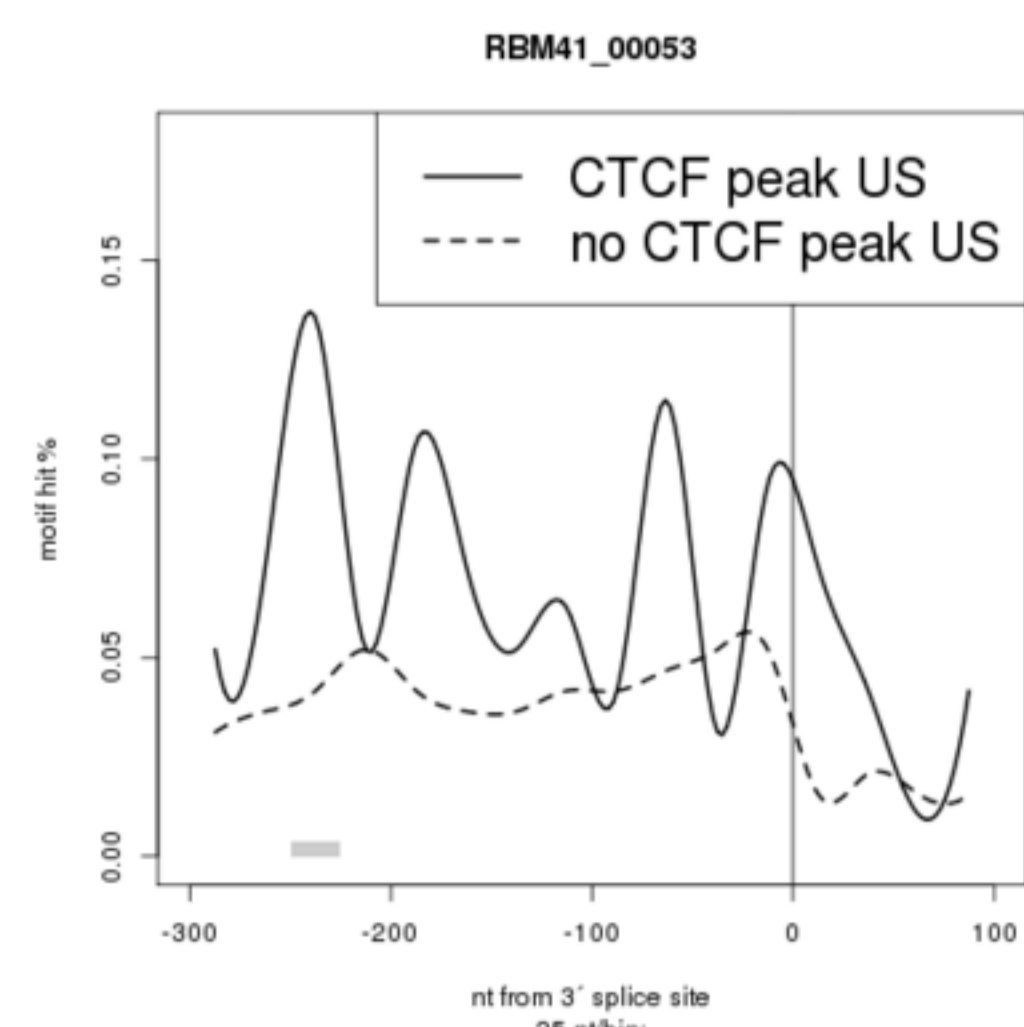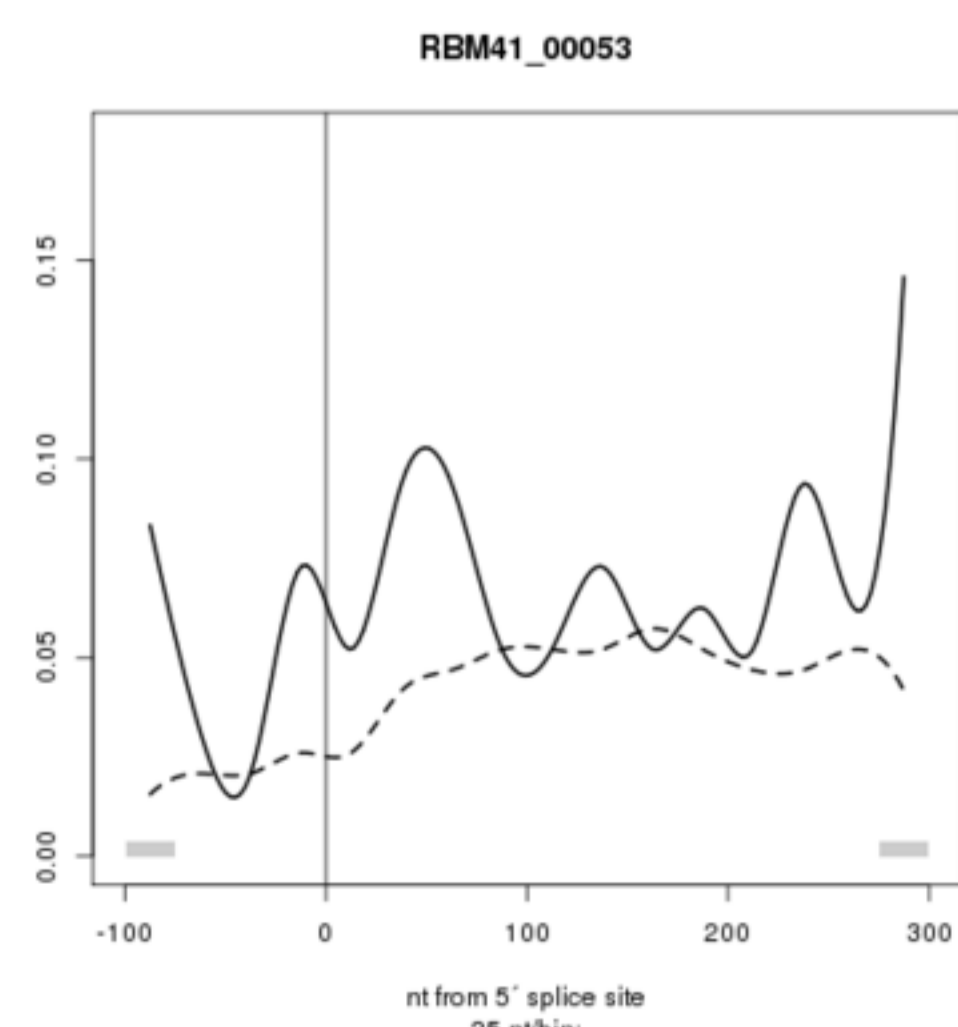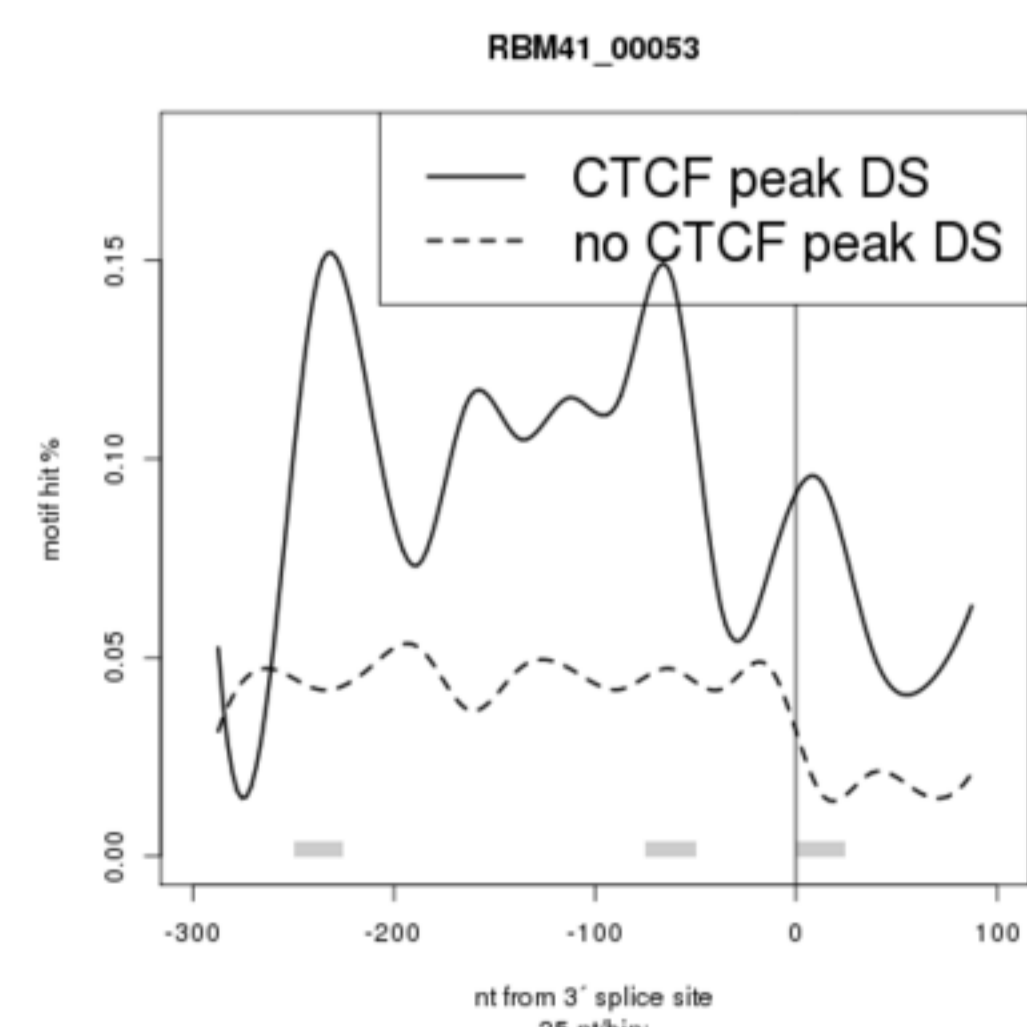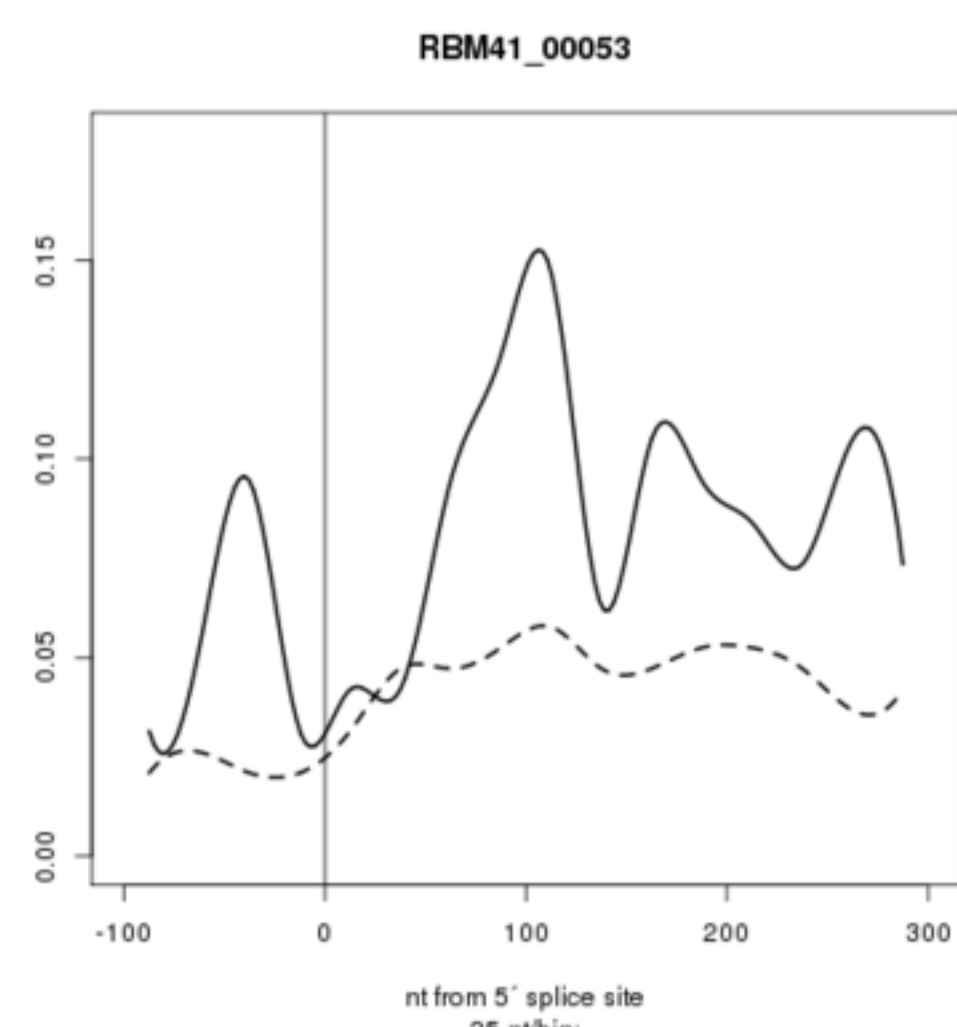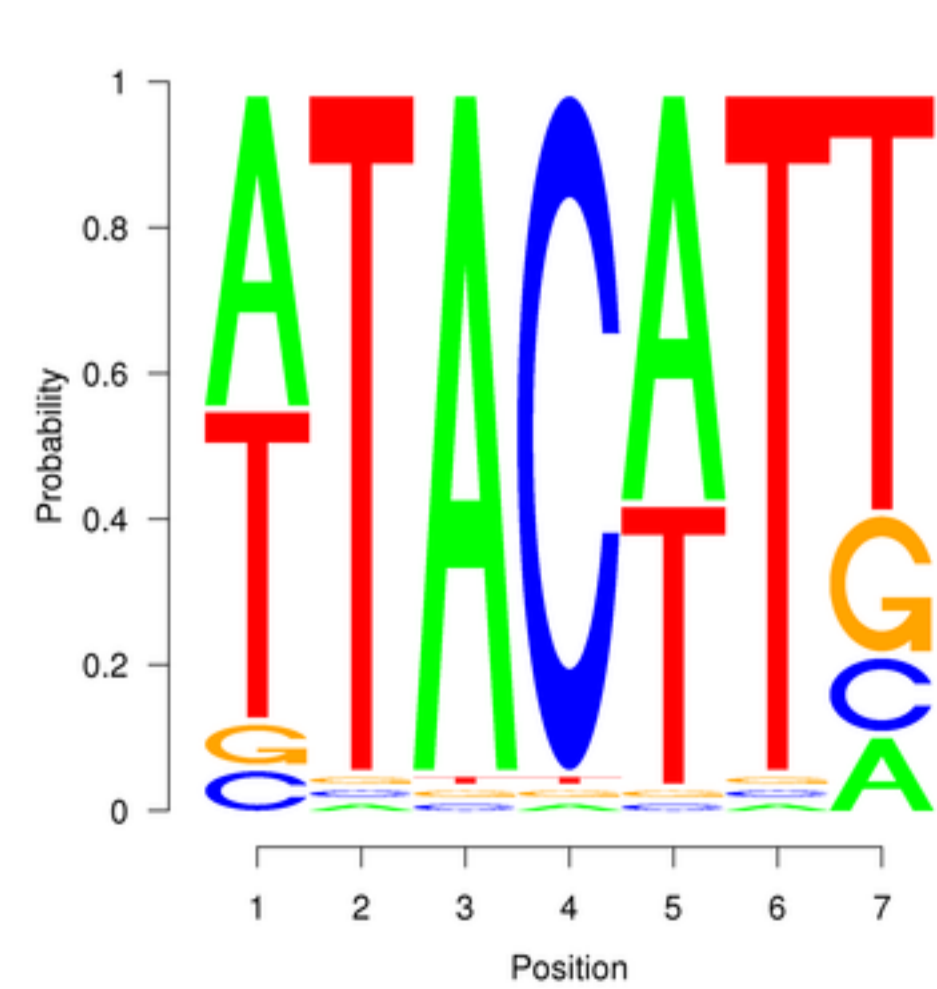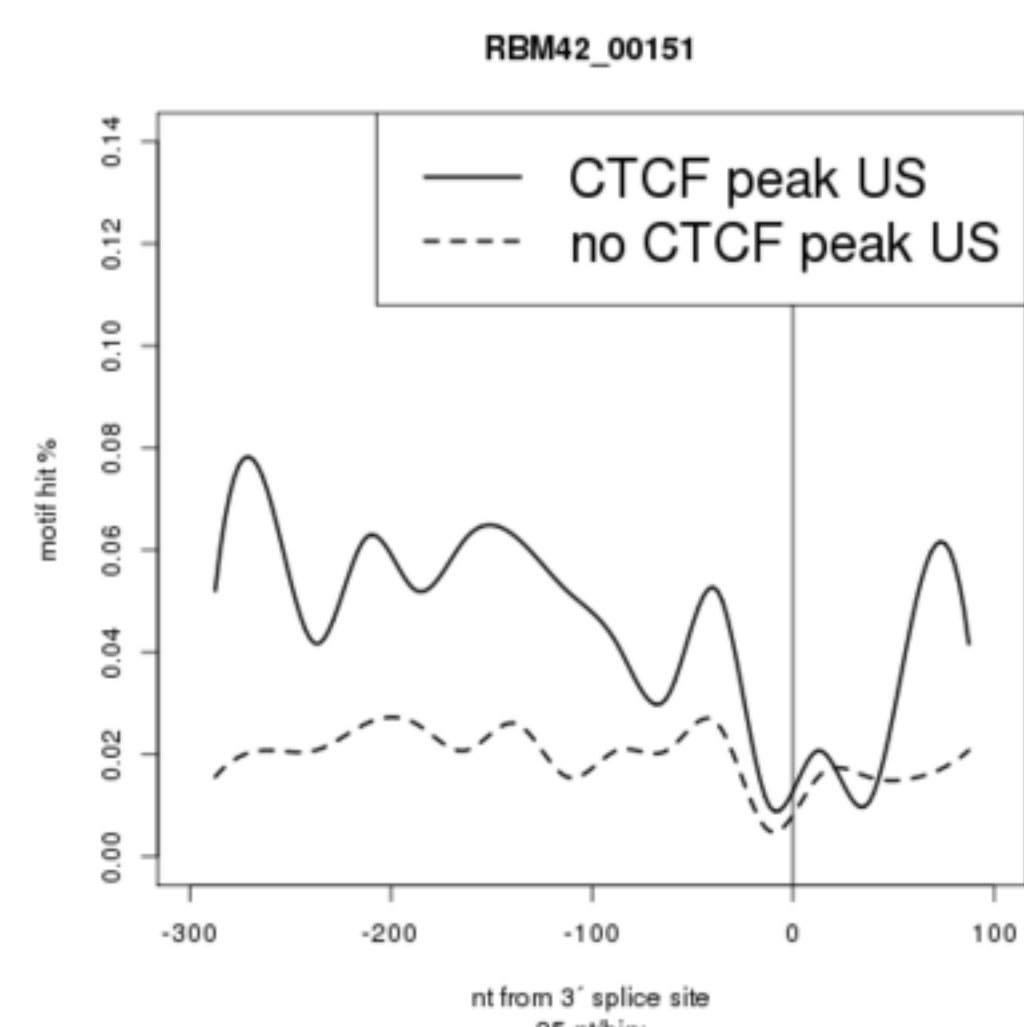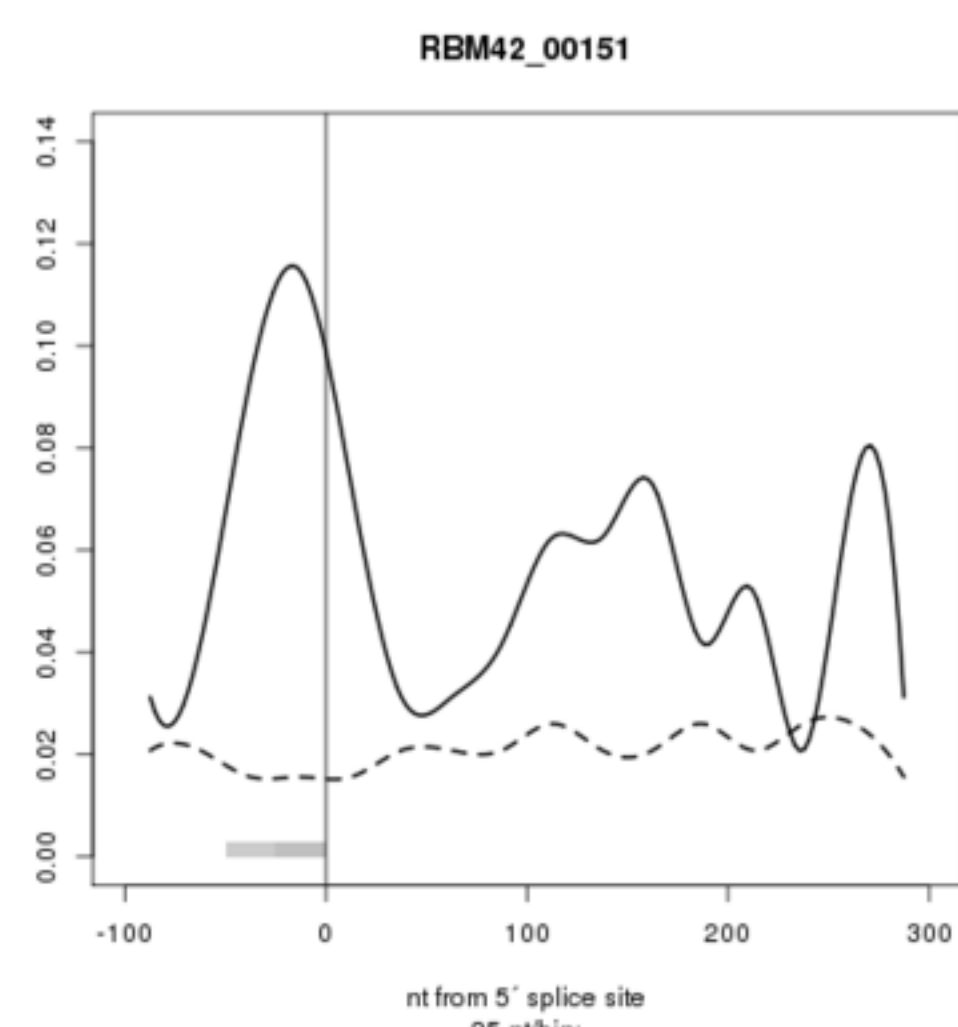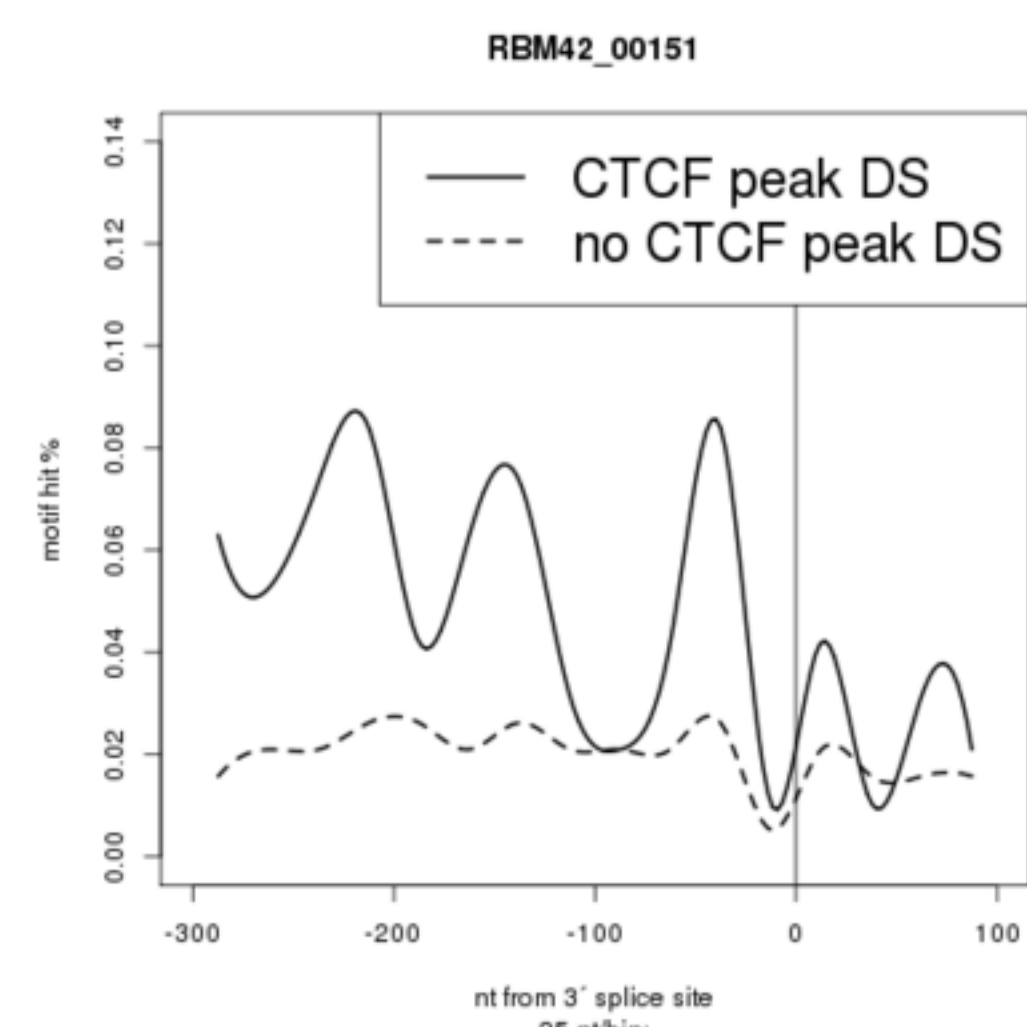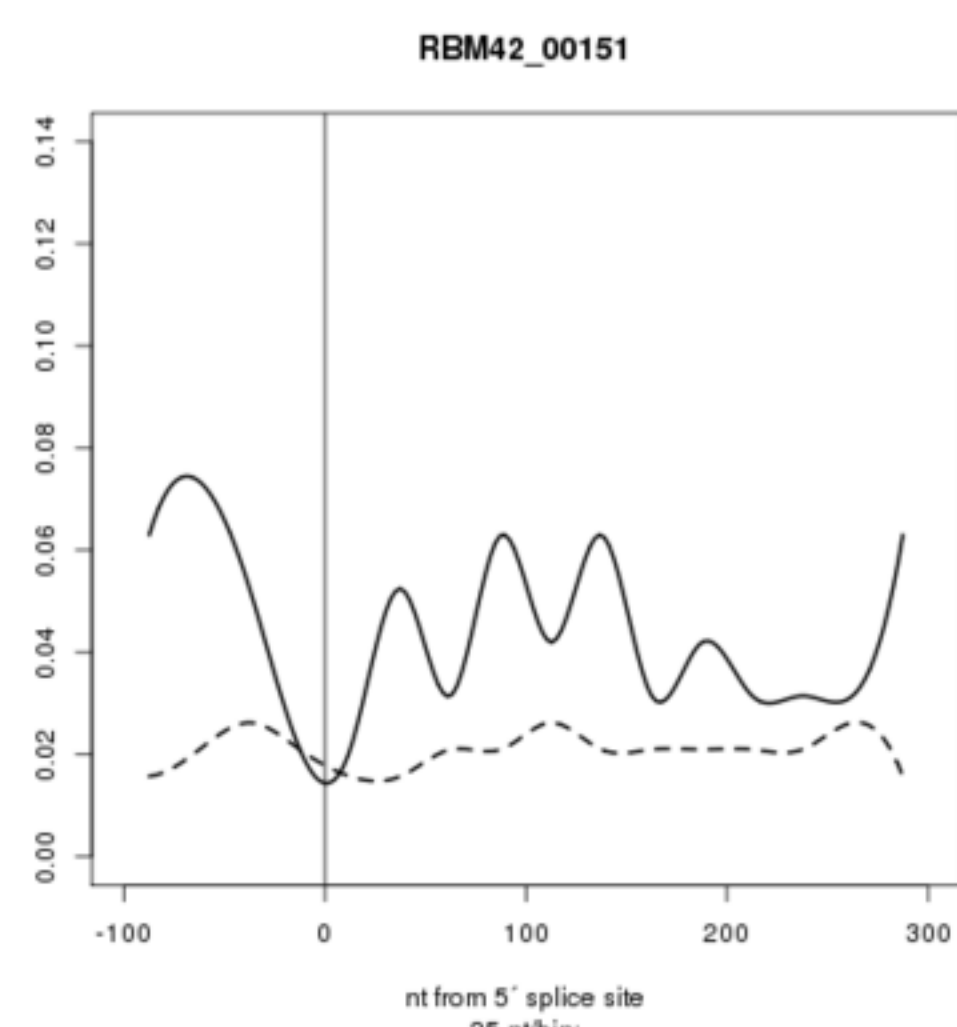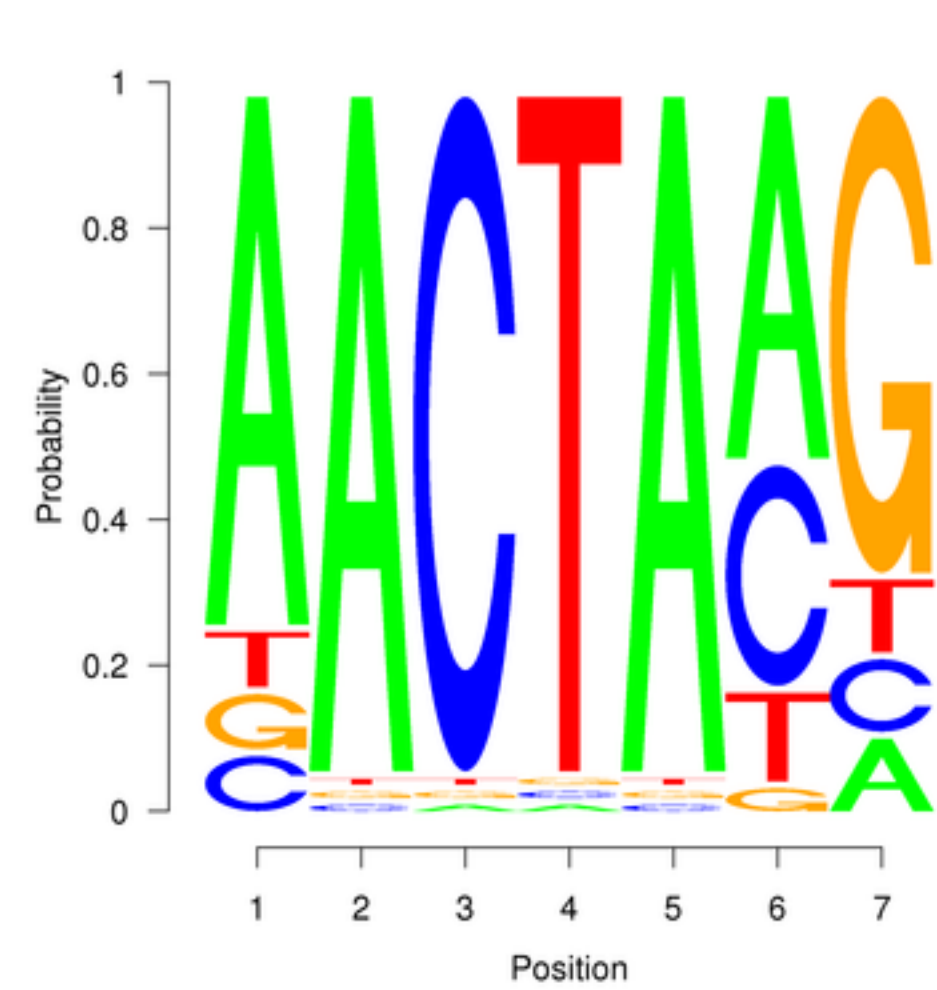

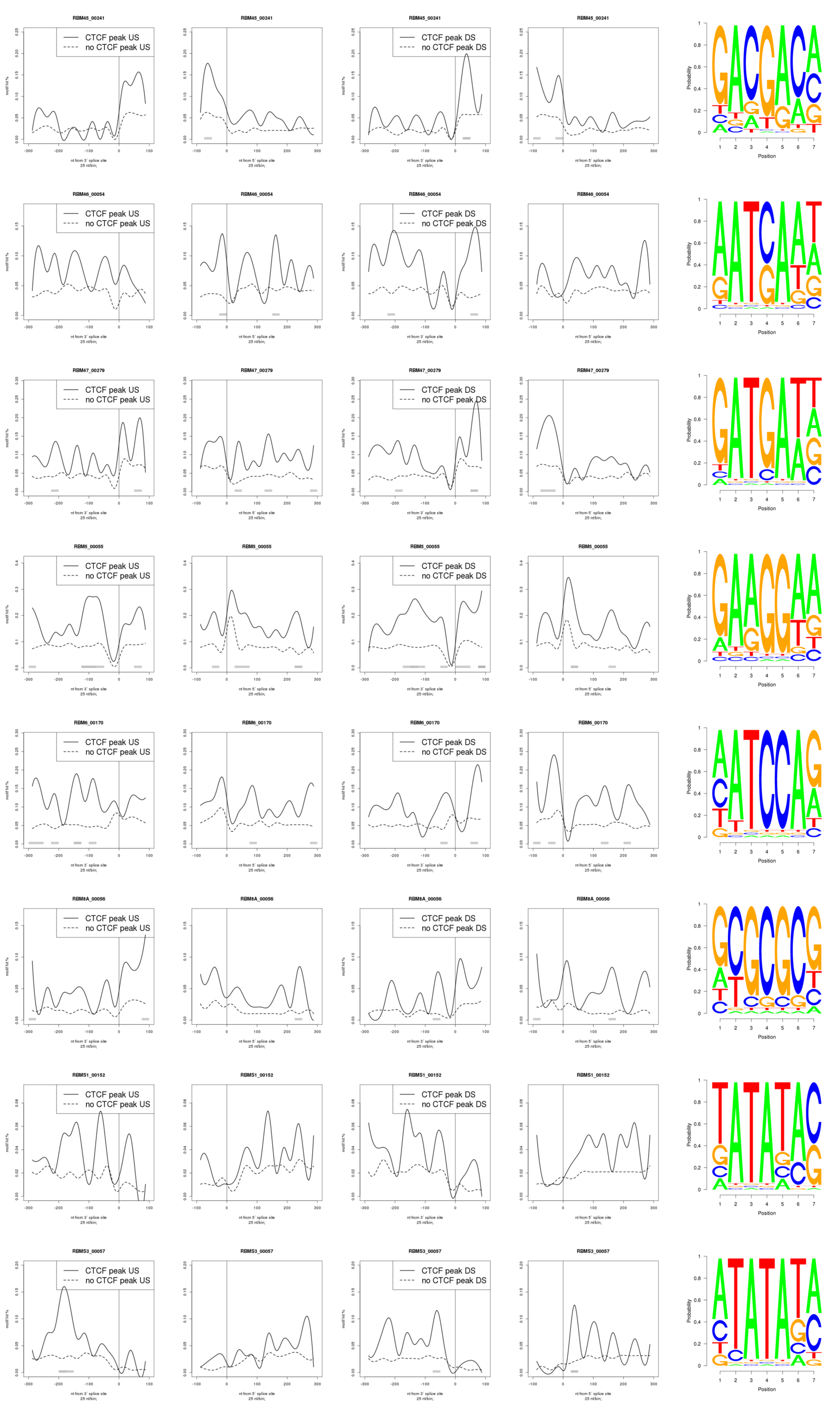

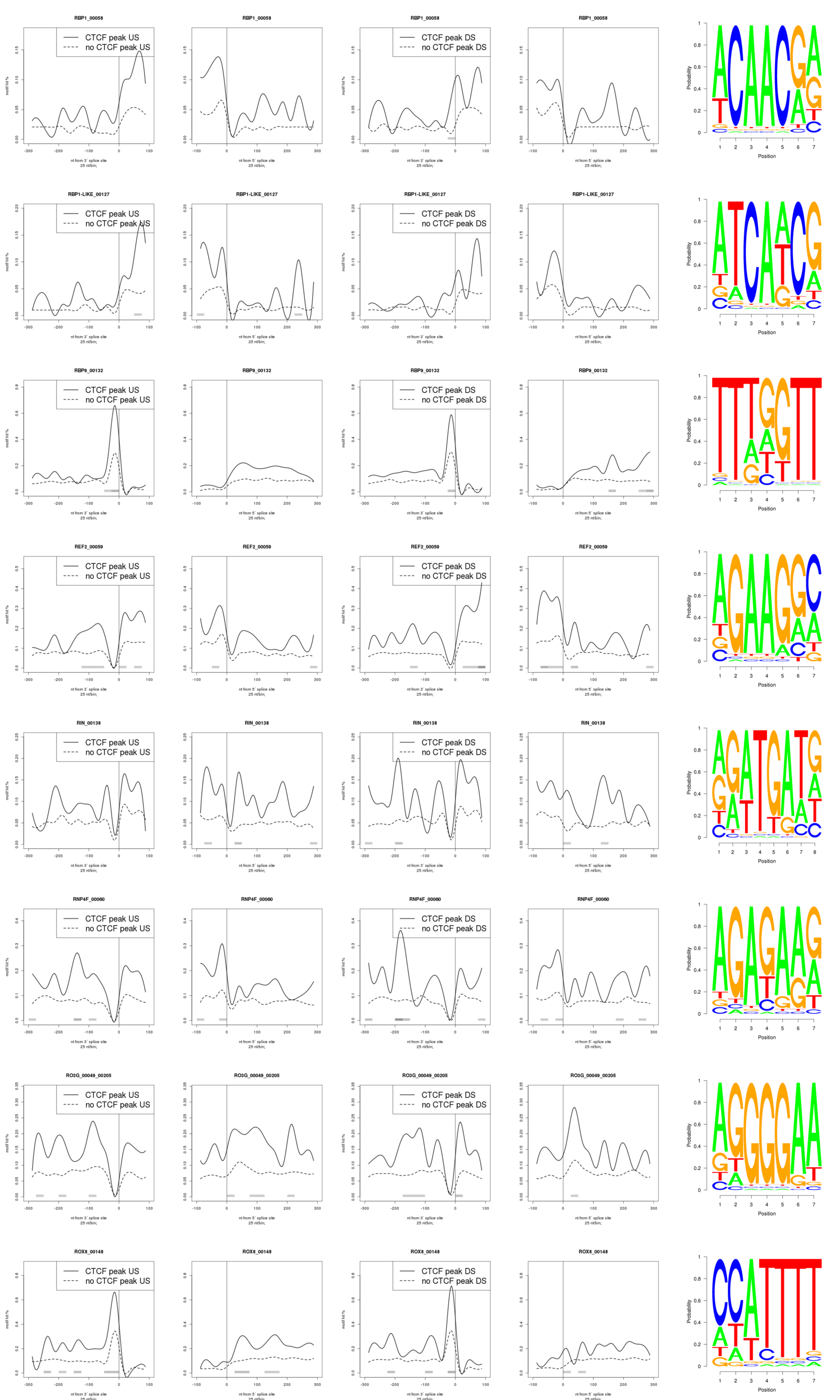

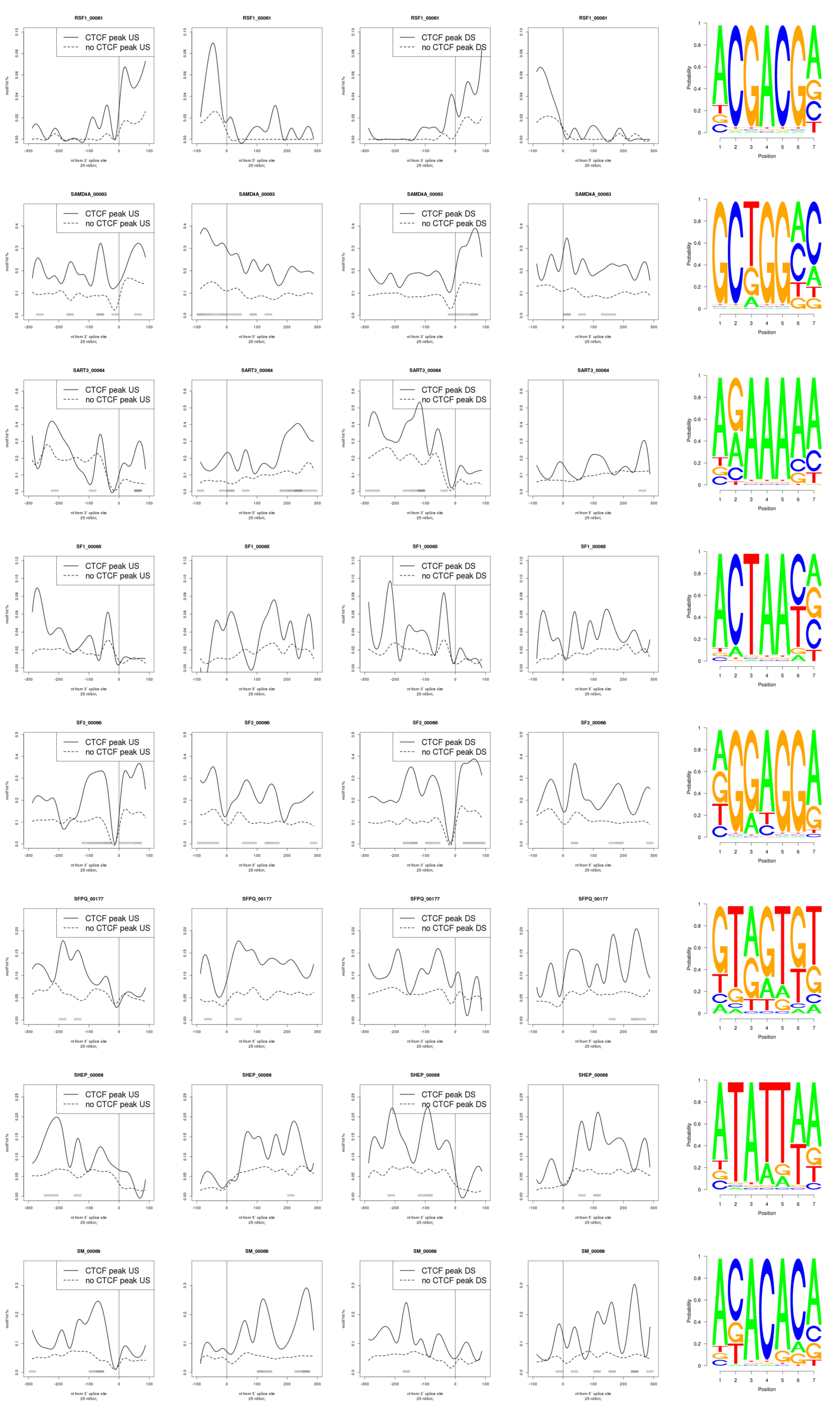

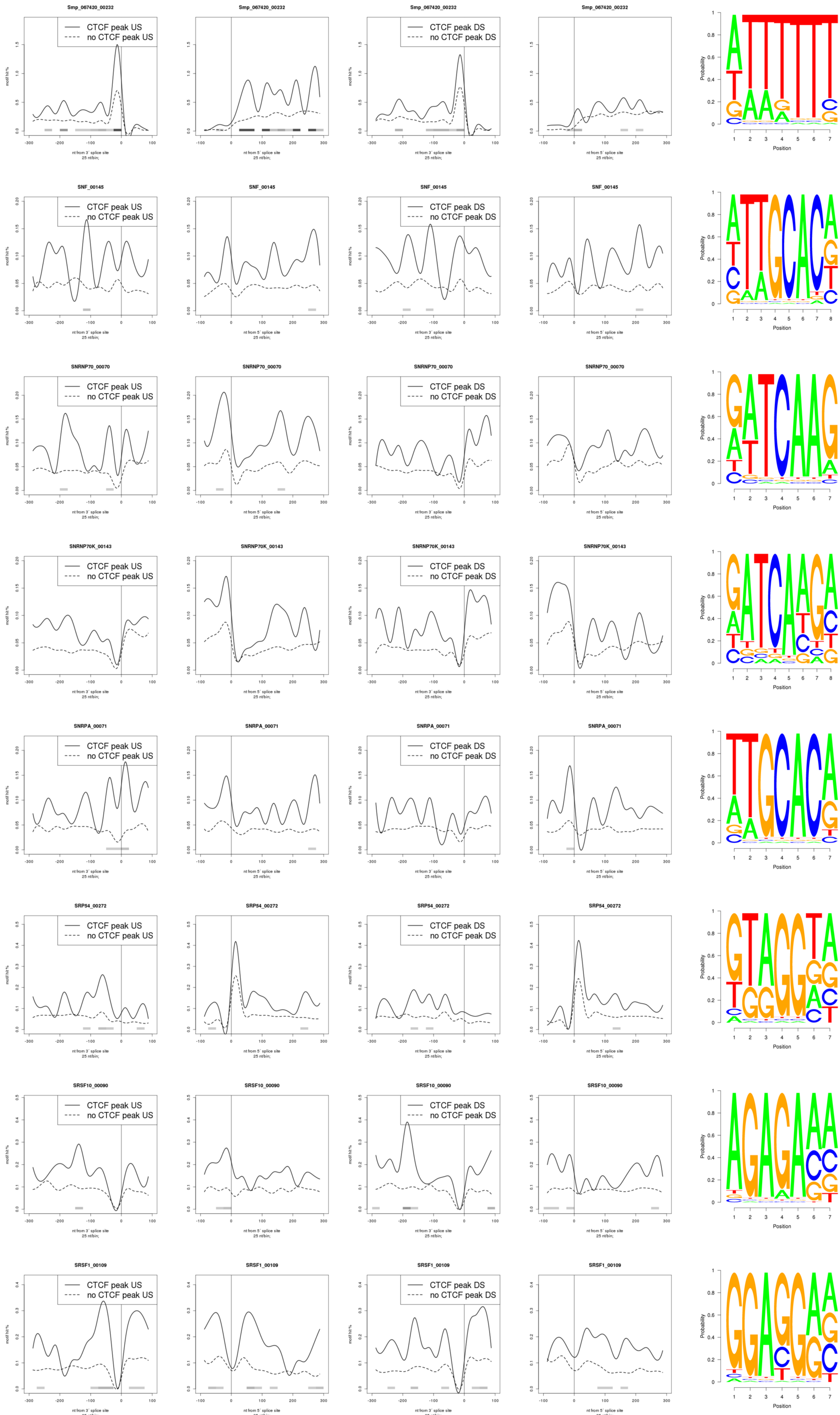

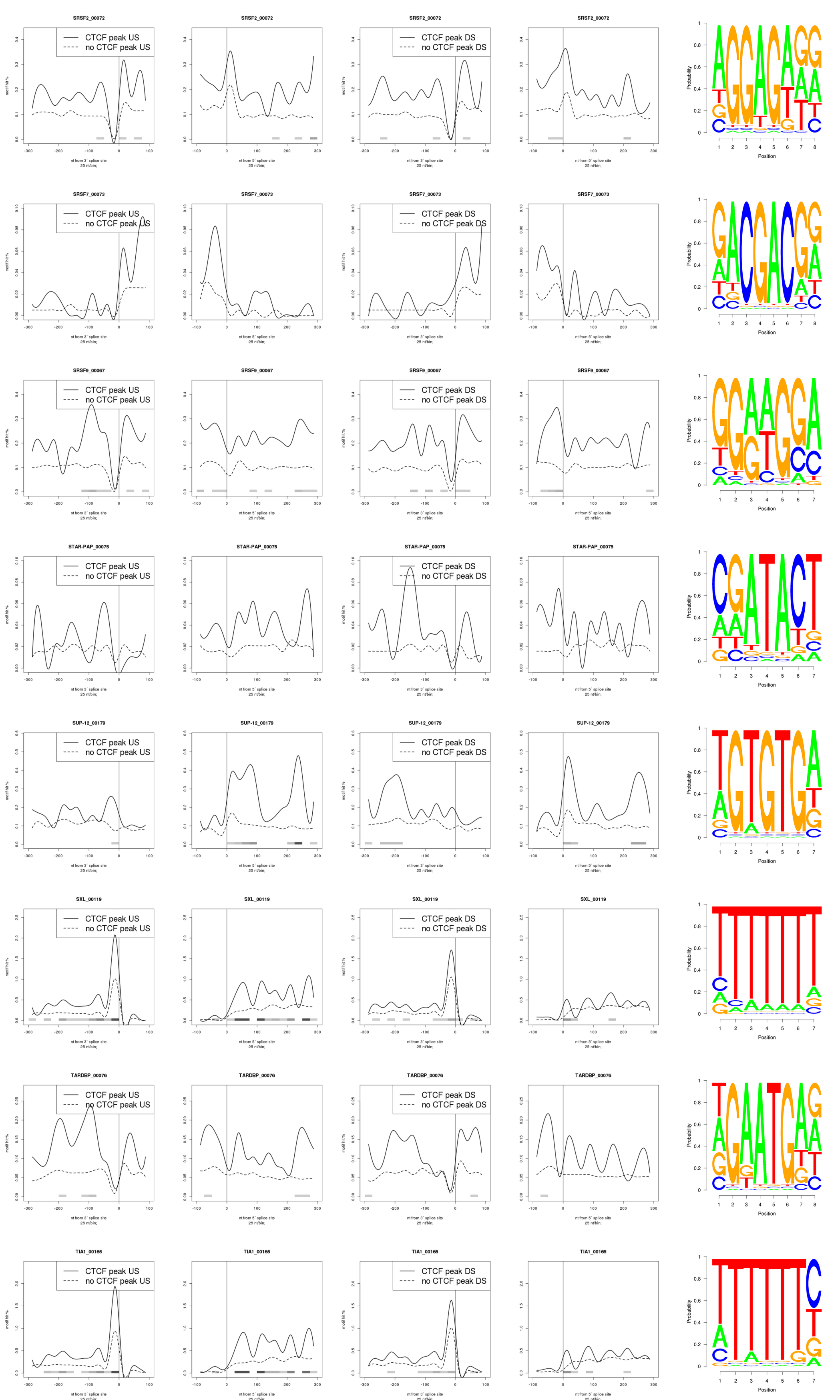

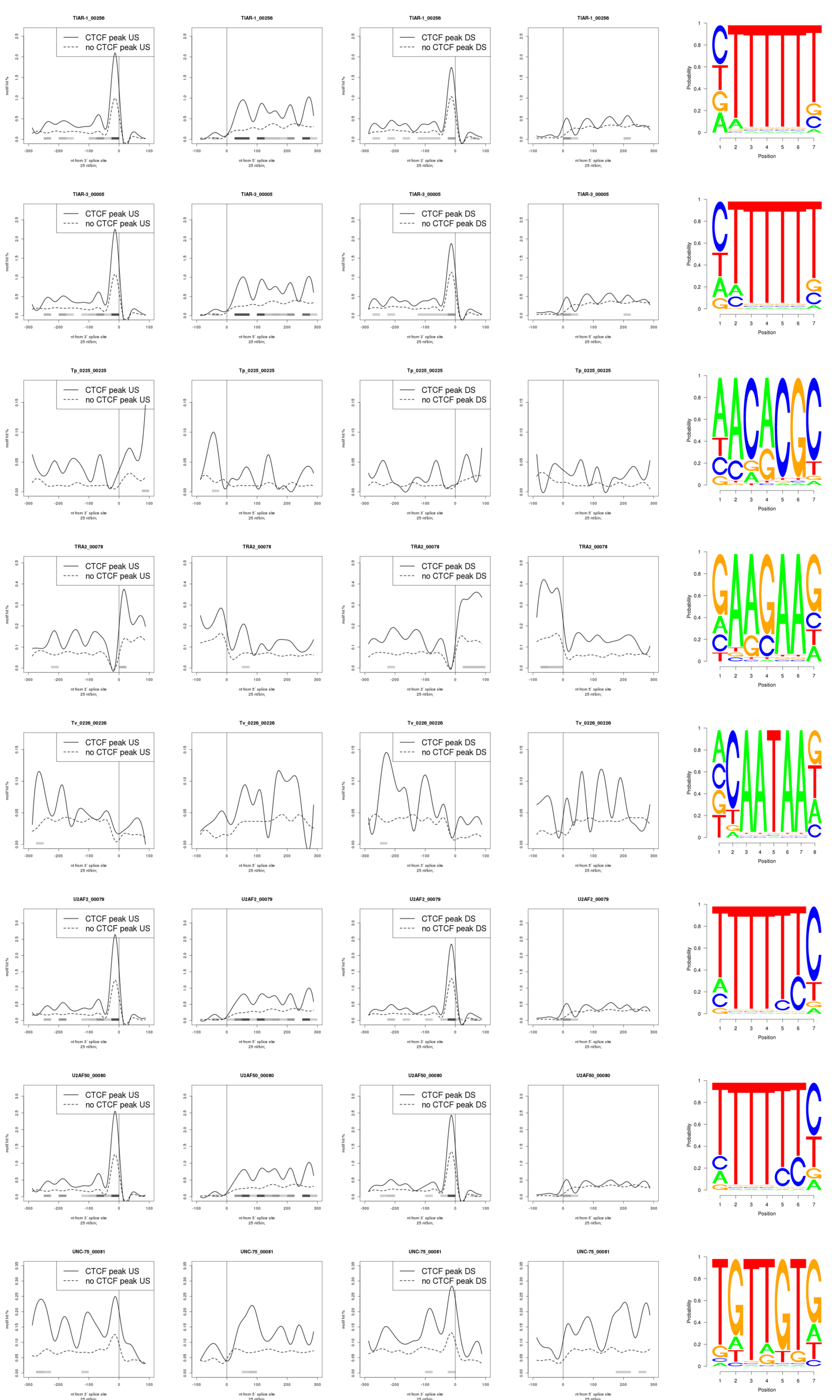

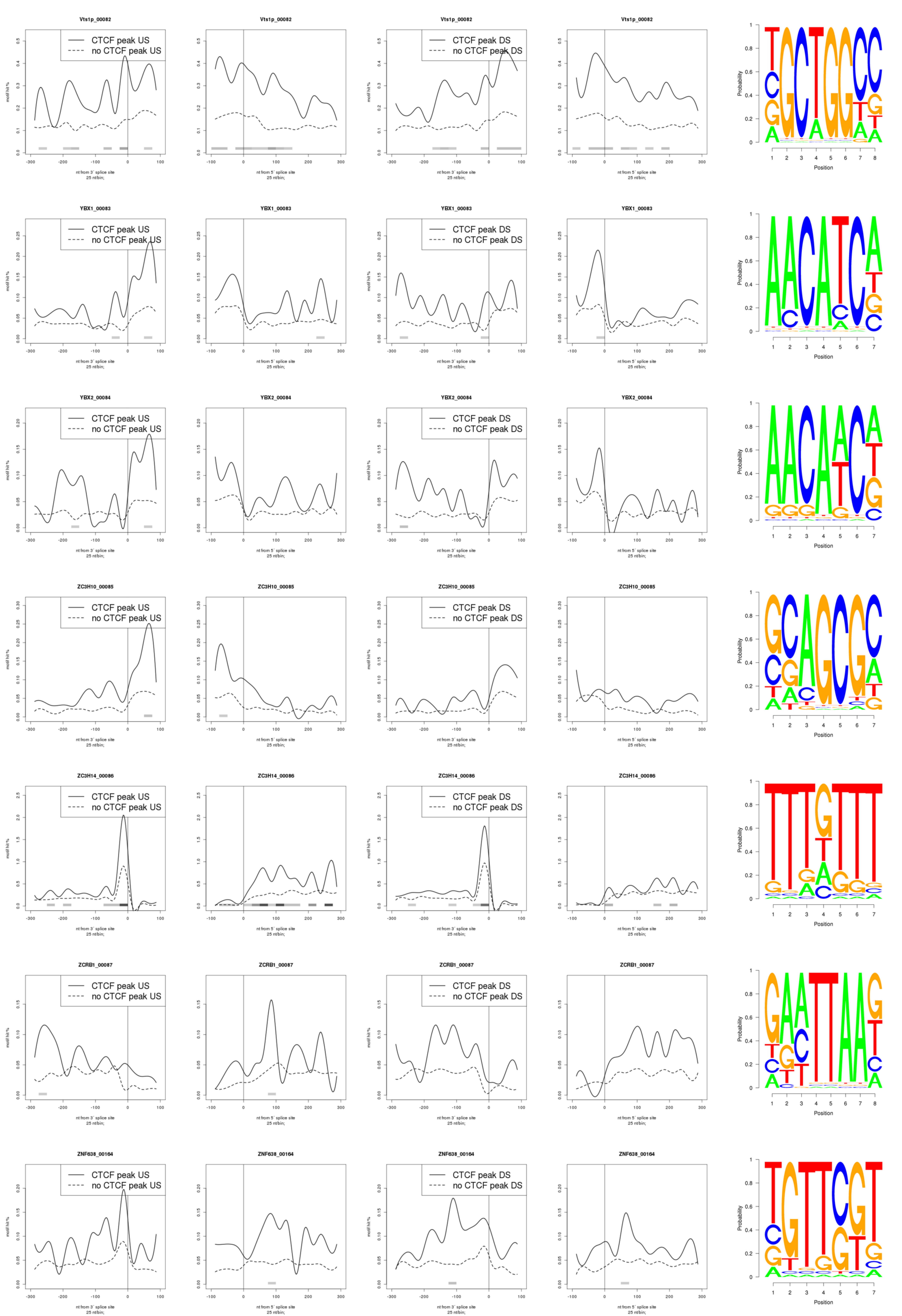

Supplement: S4 File — Same format as S3 File; “strong” is defined in the same way as “weak” from S3 File, but for the largest half of splice site strengths. Test vs. control P-values/bin are as shown in Fig 1B, with the lightest shade of grey corresponding to P-value < 0.01. (PDF) [file pone.0132448.s013.pdf]

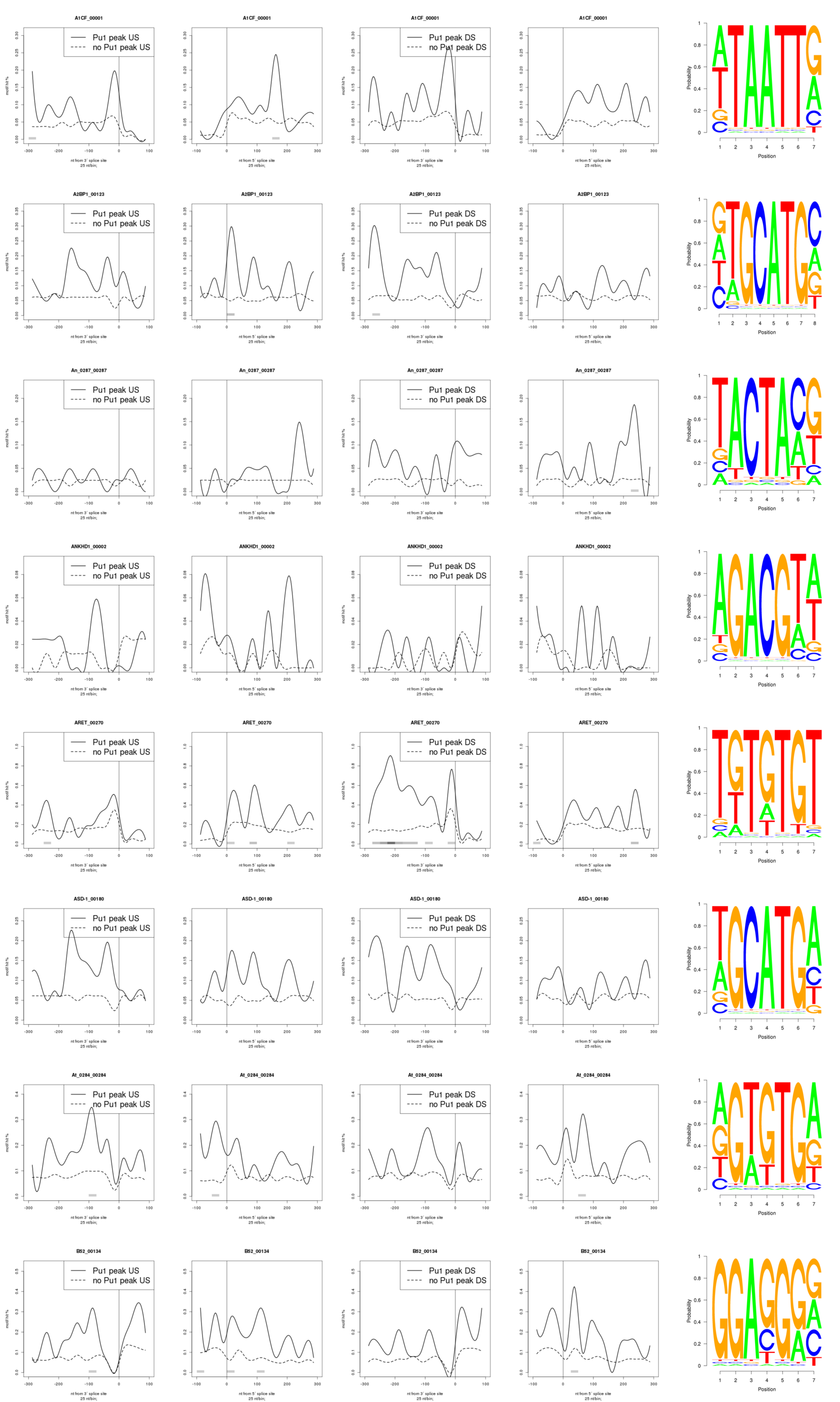

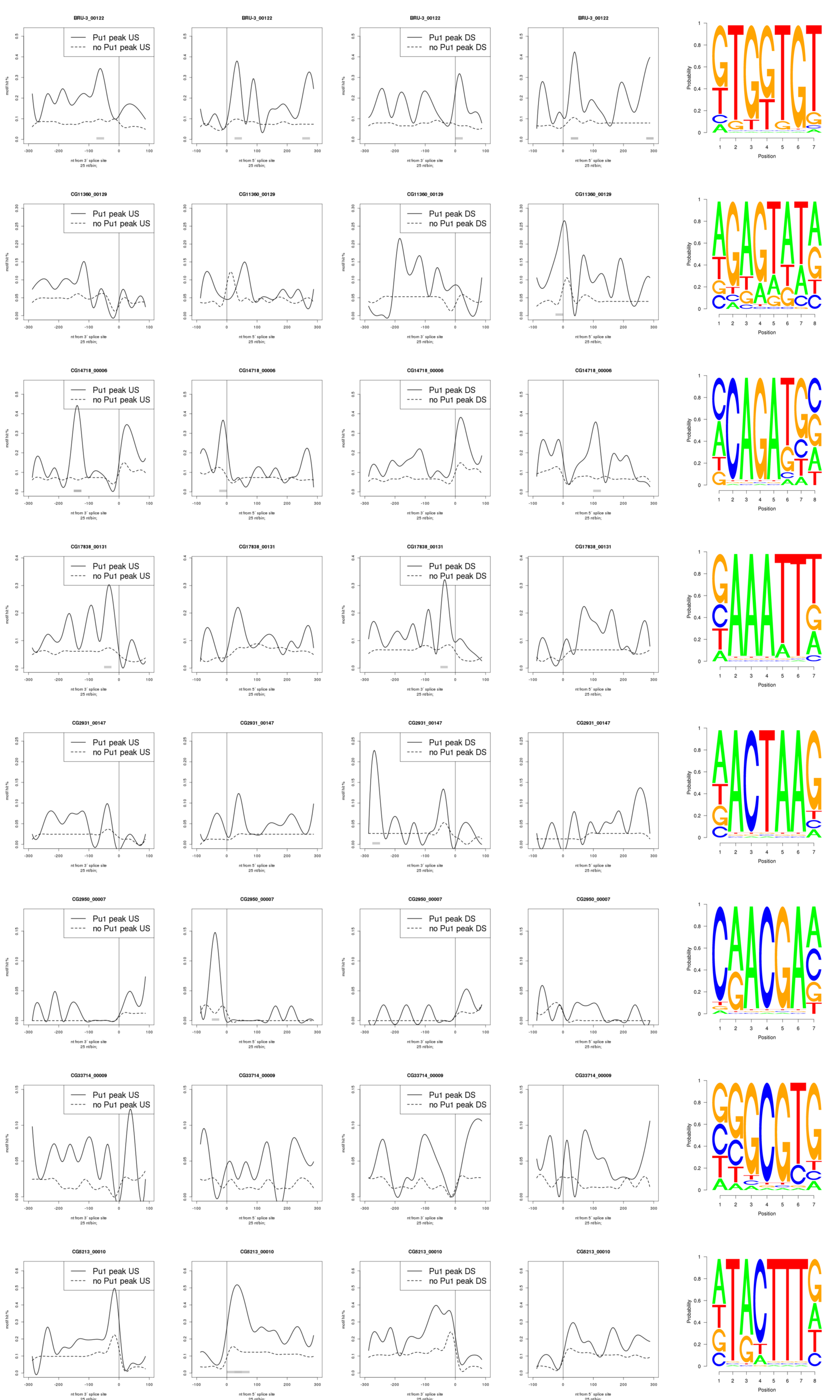

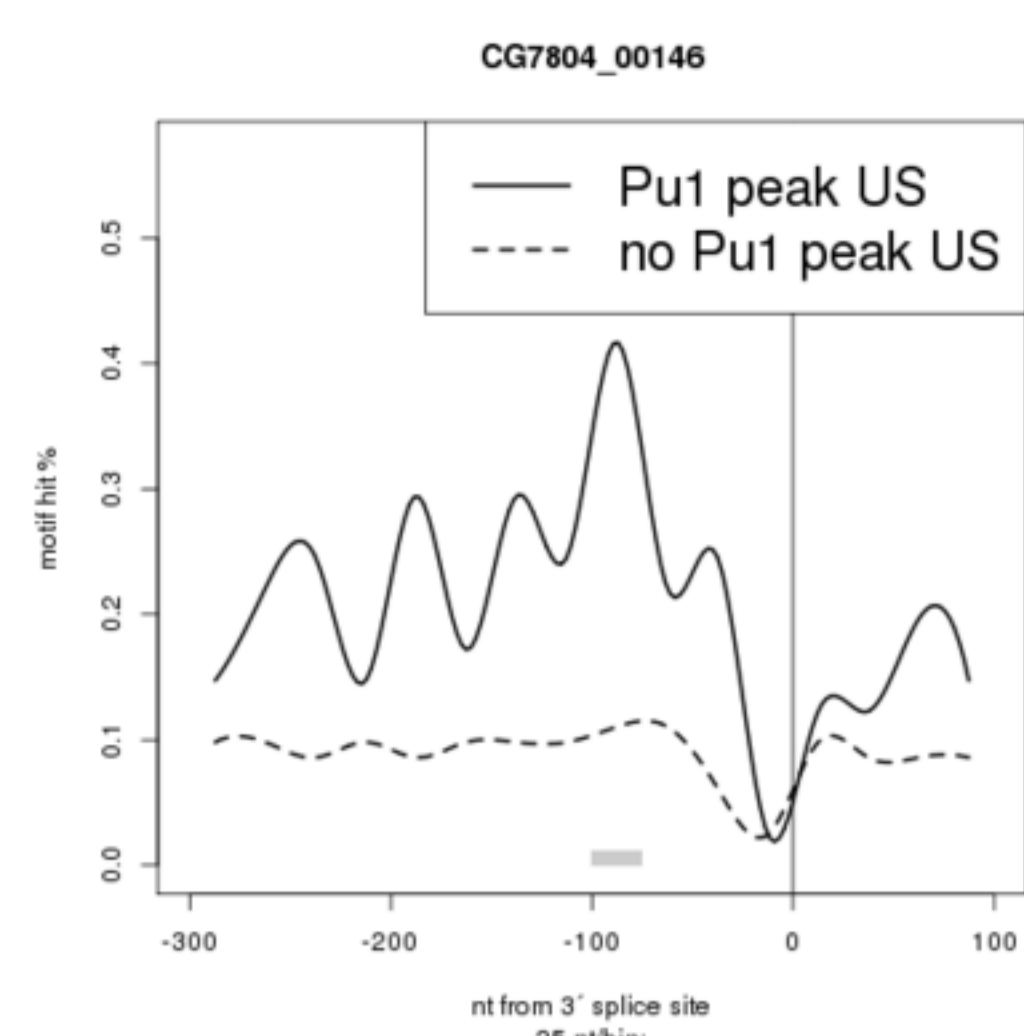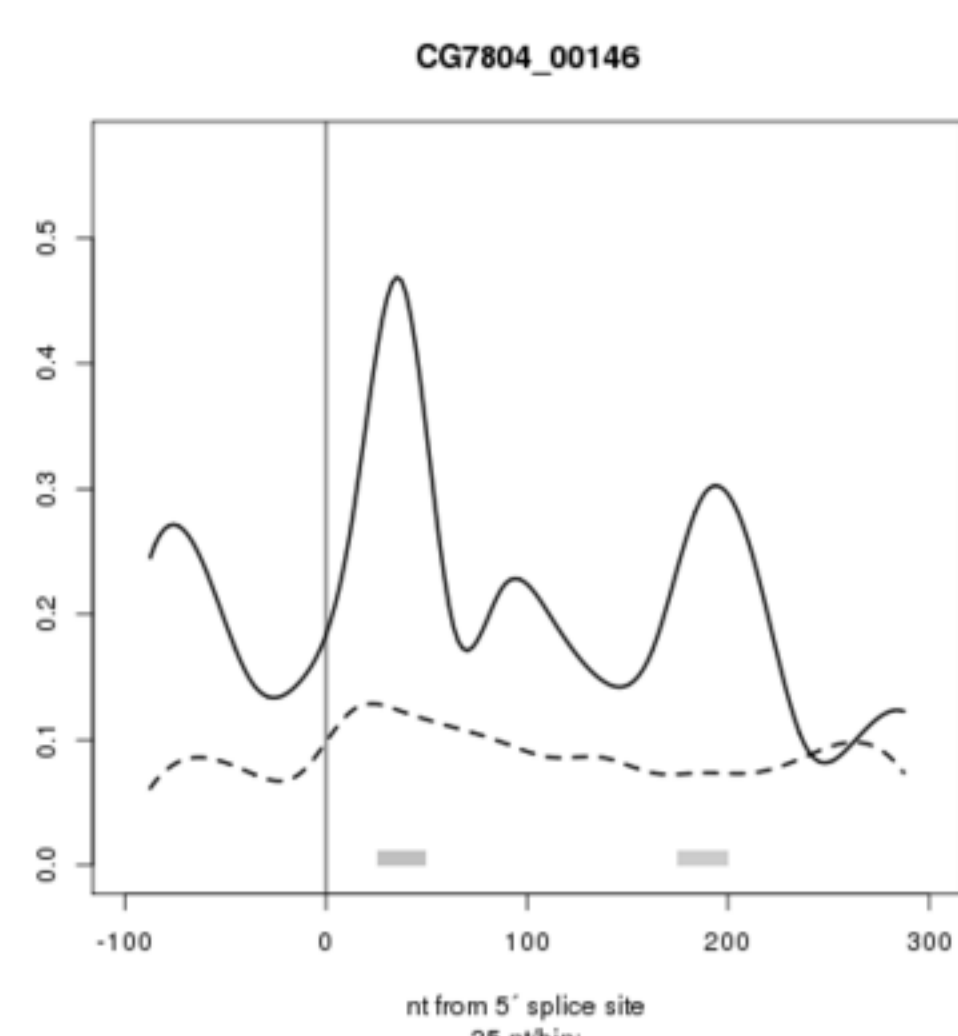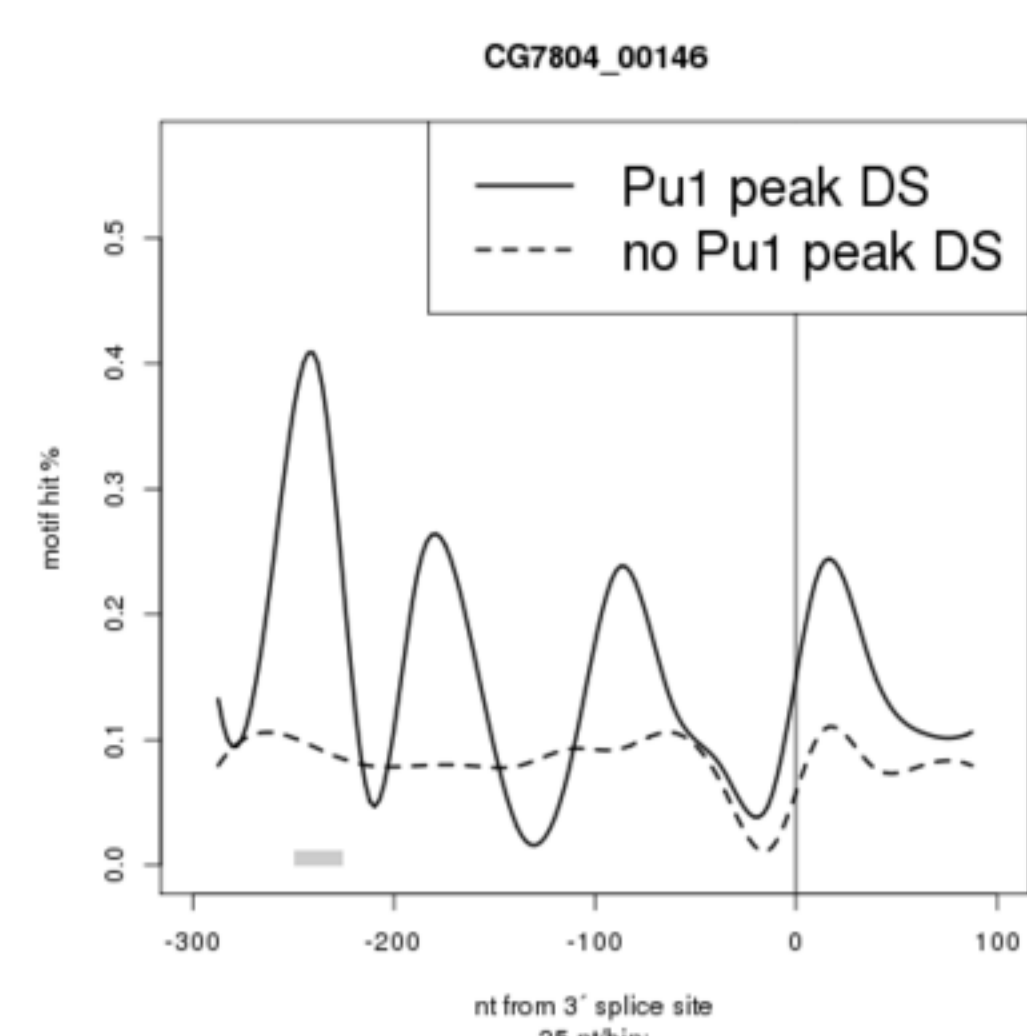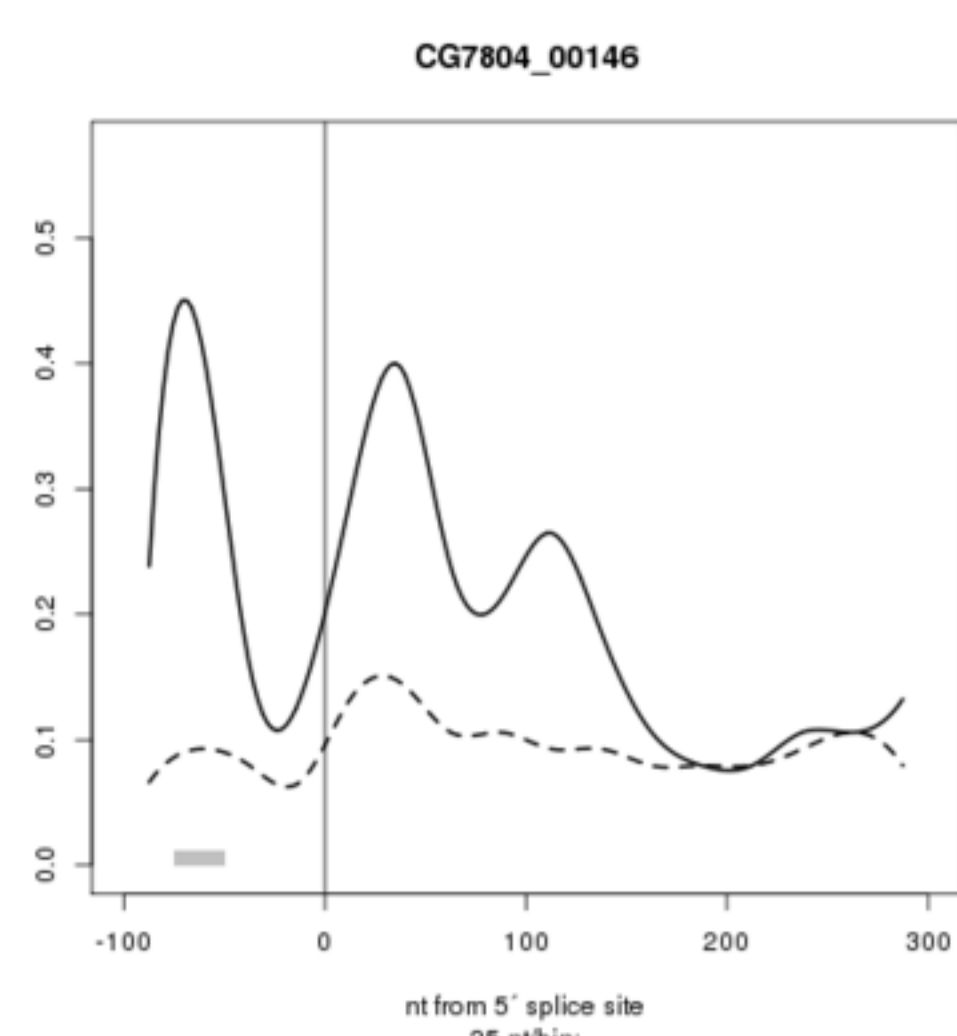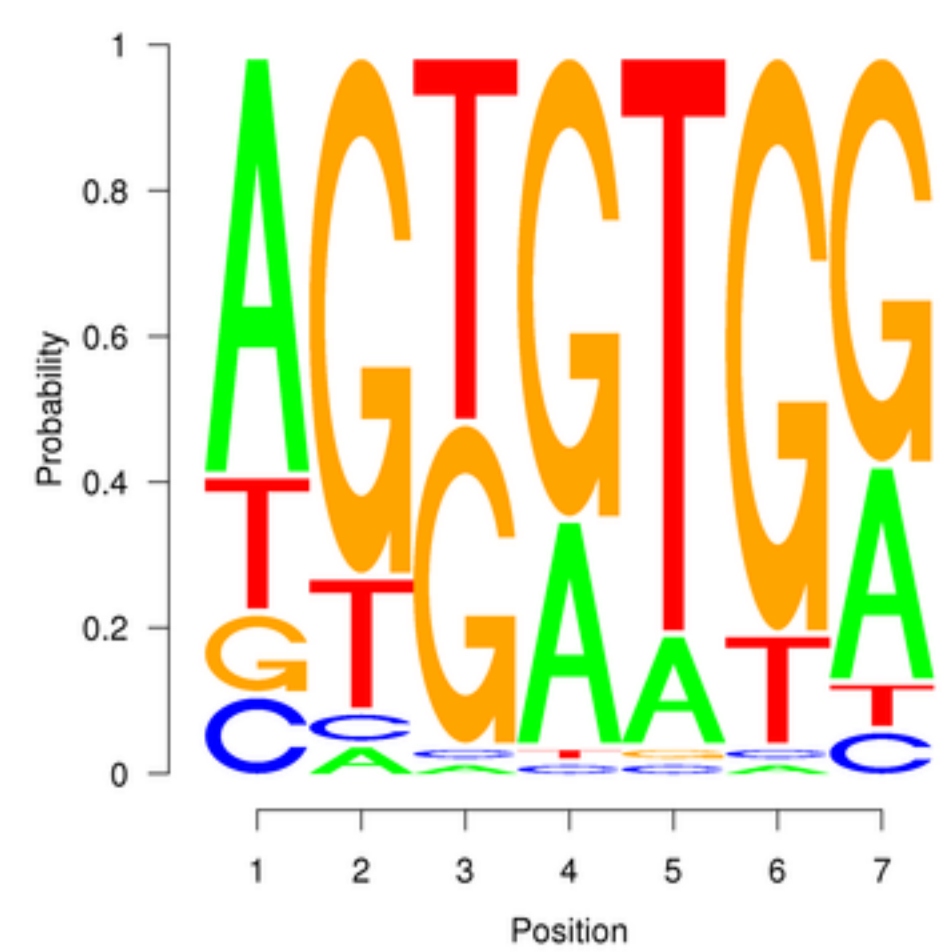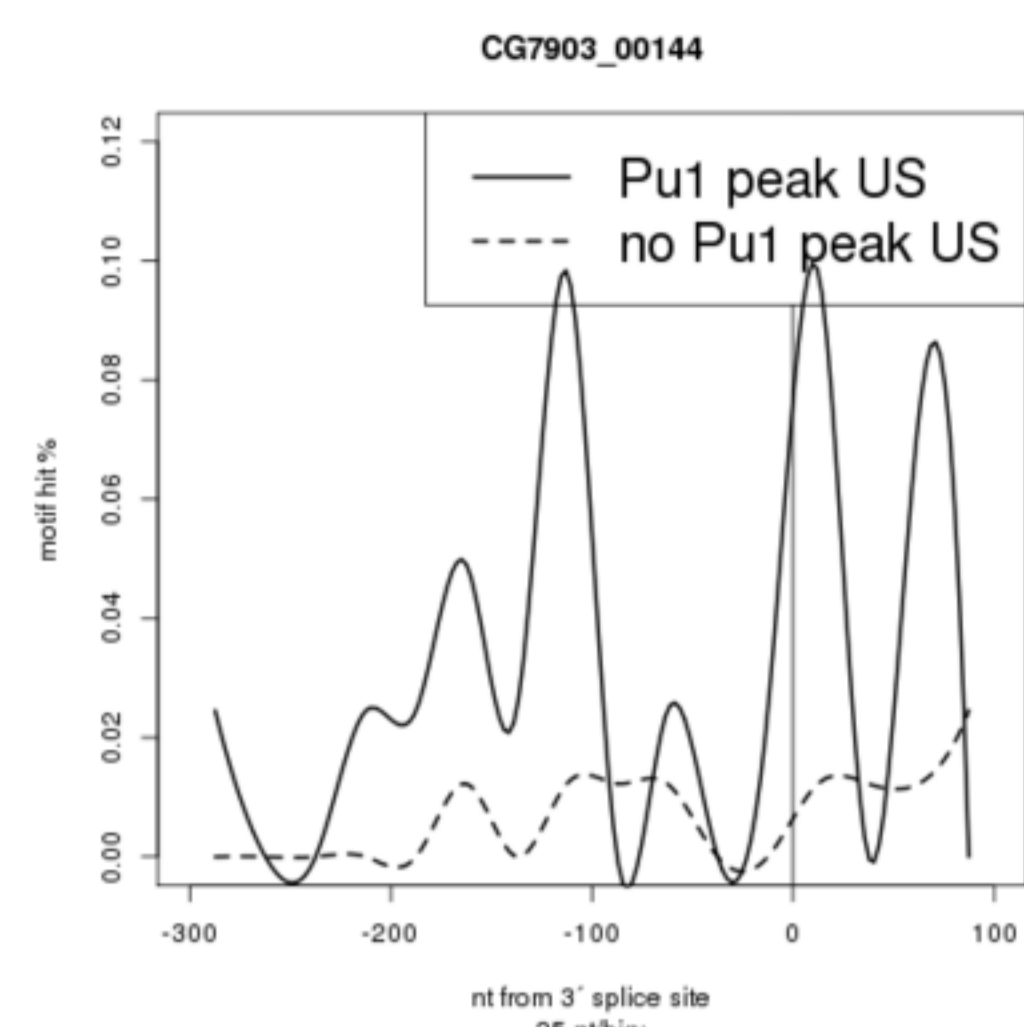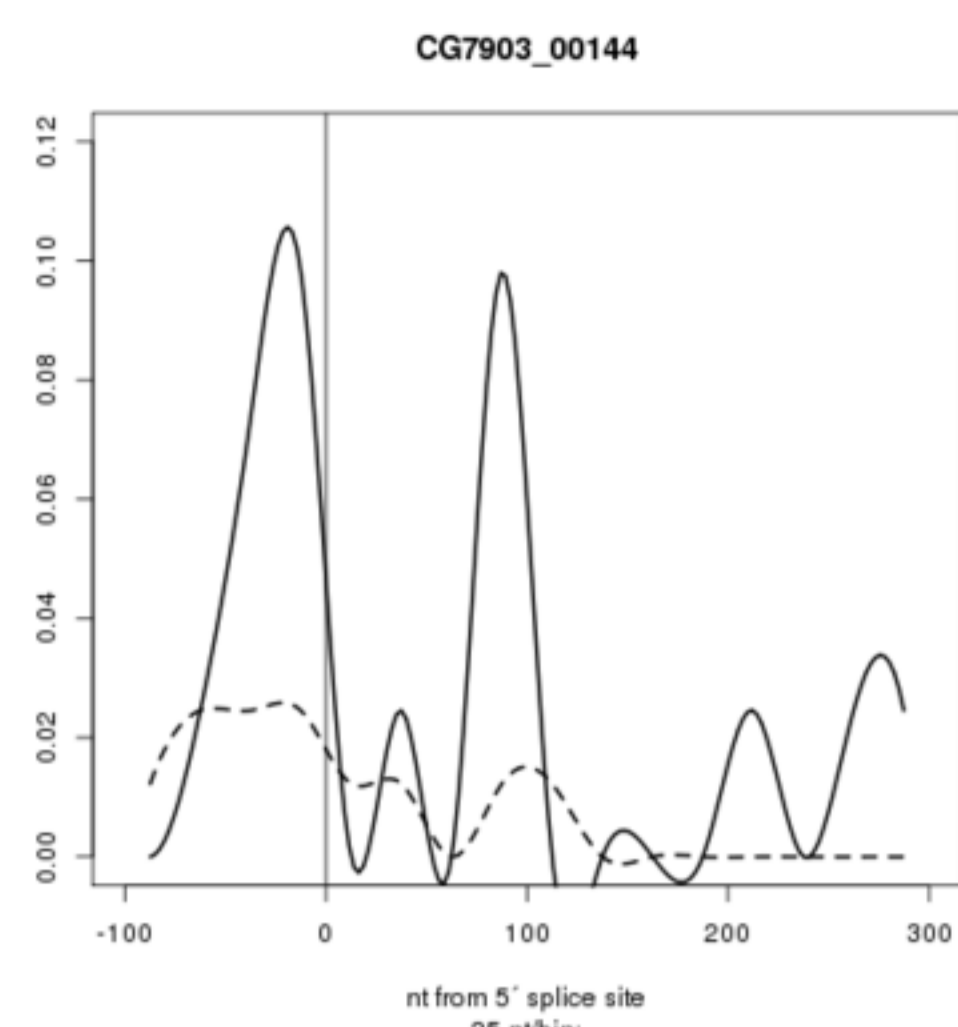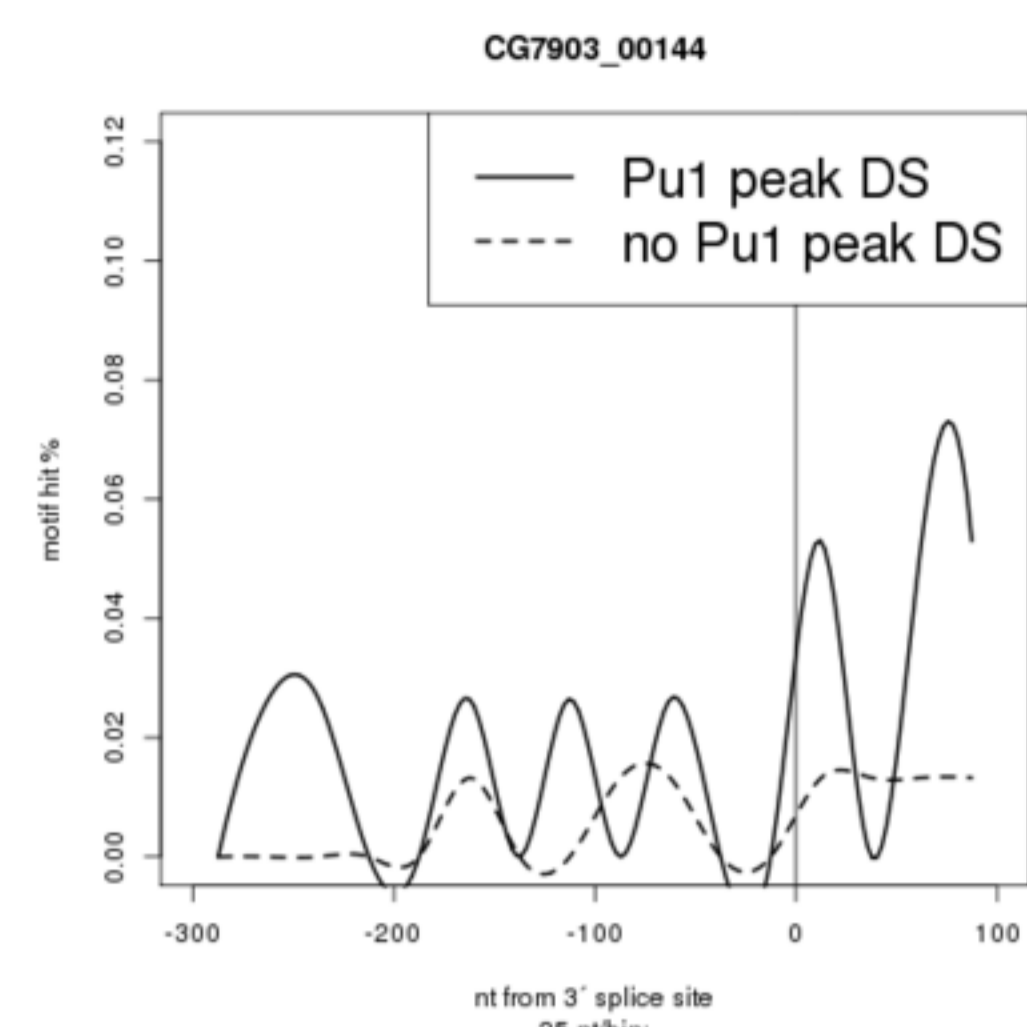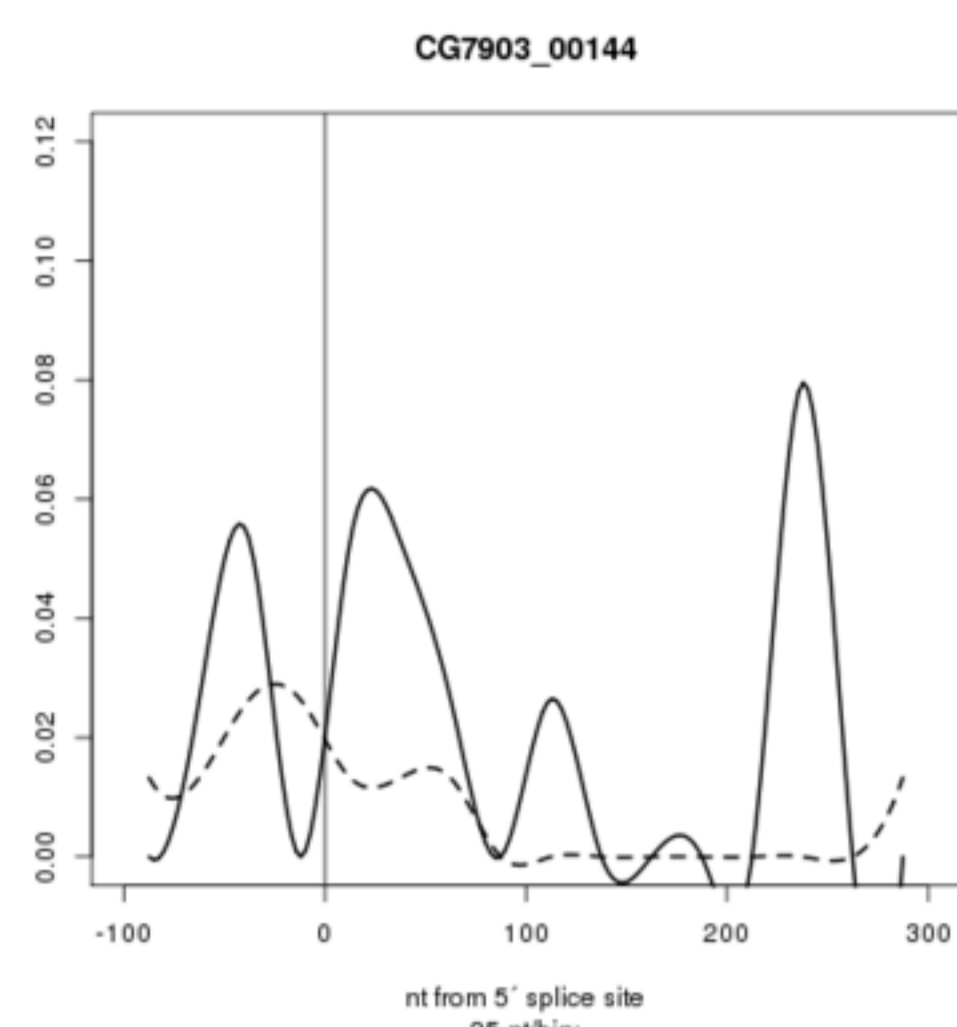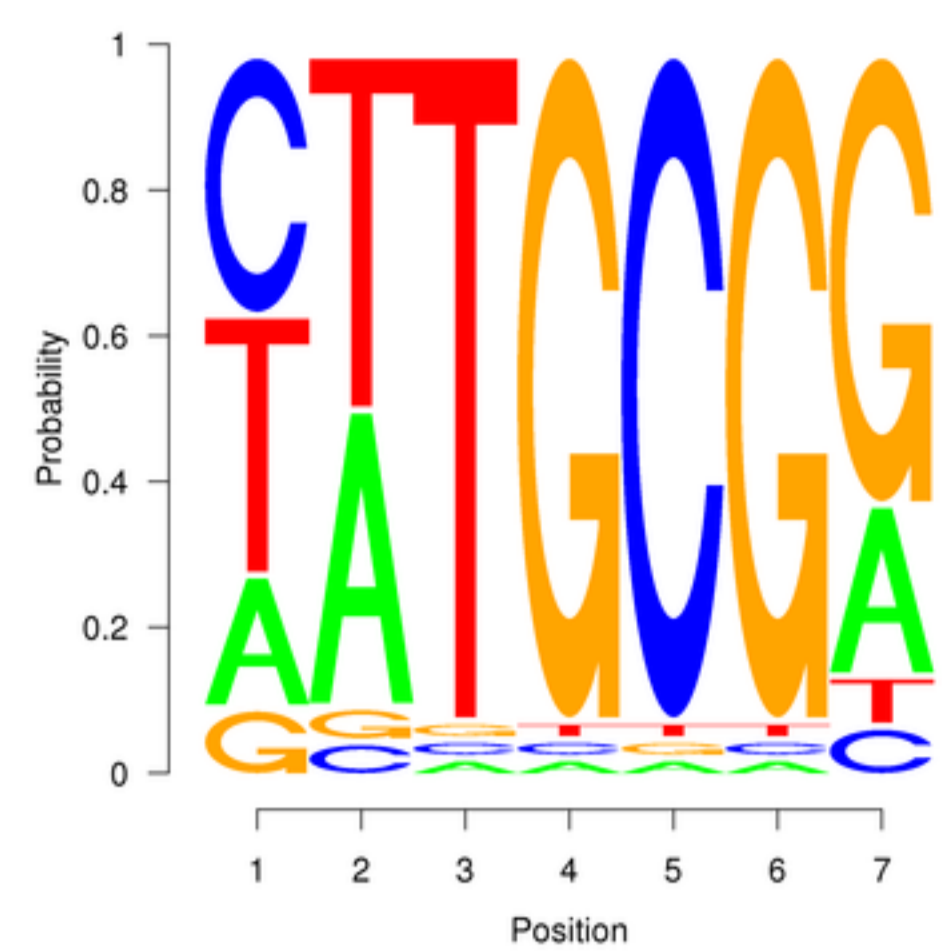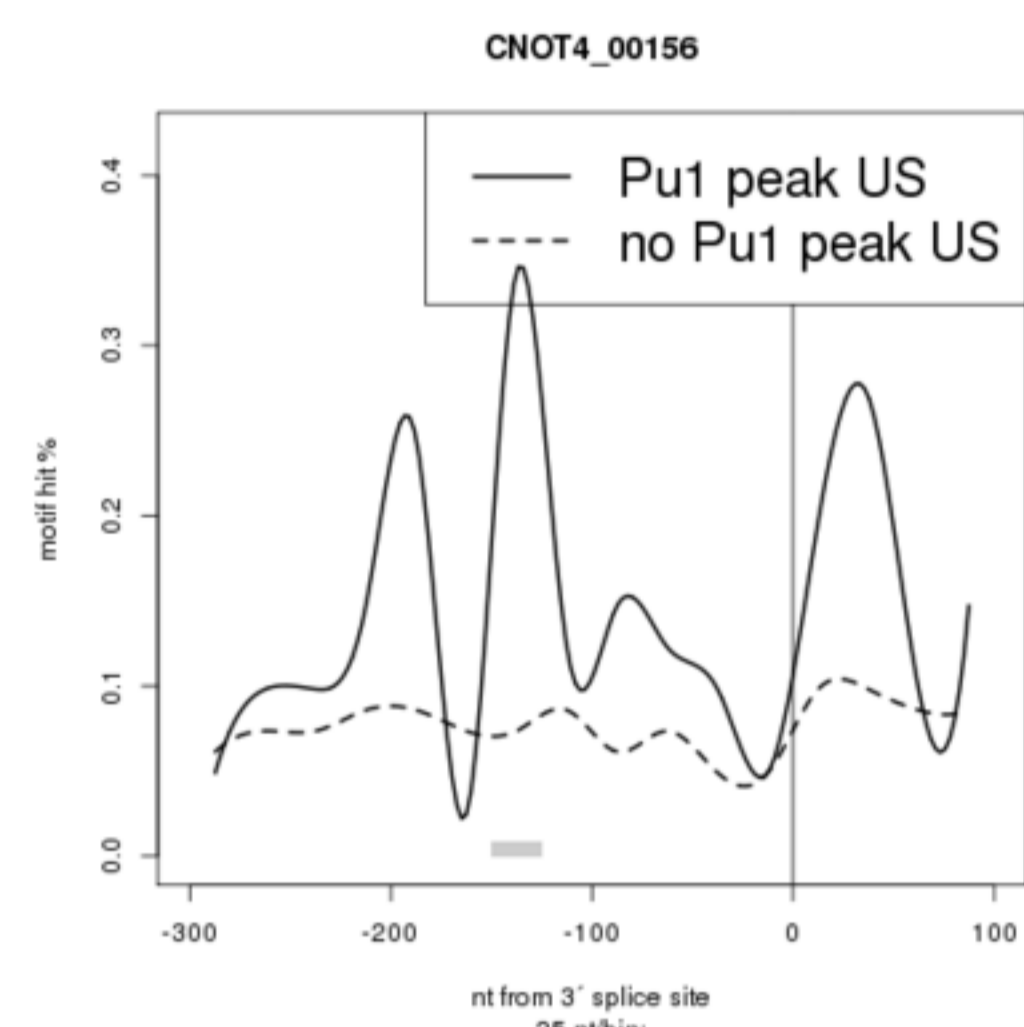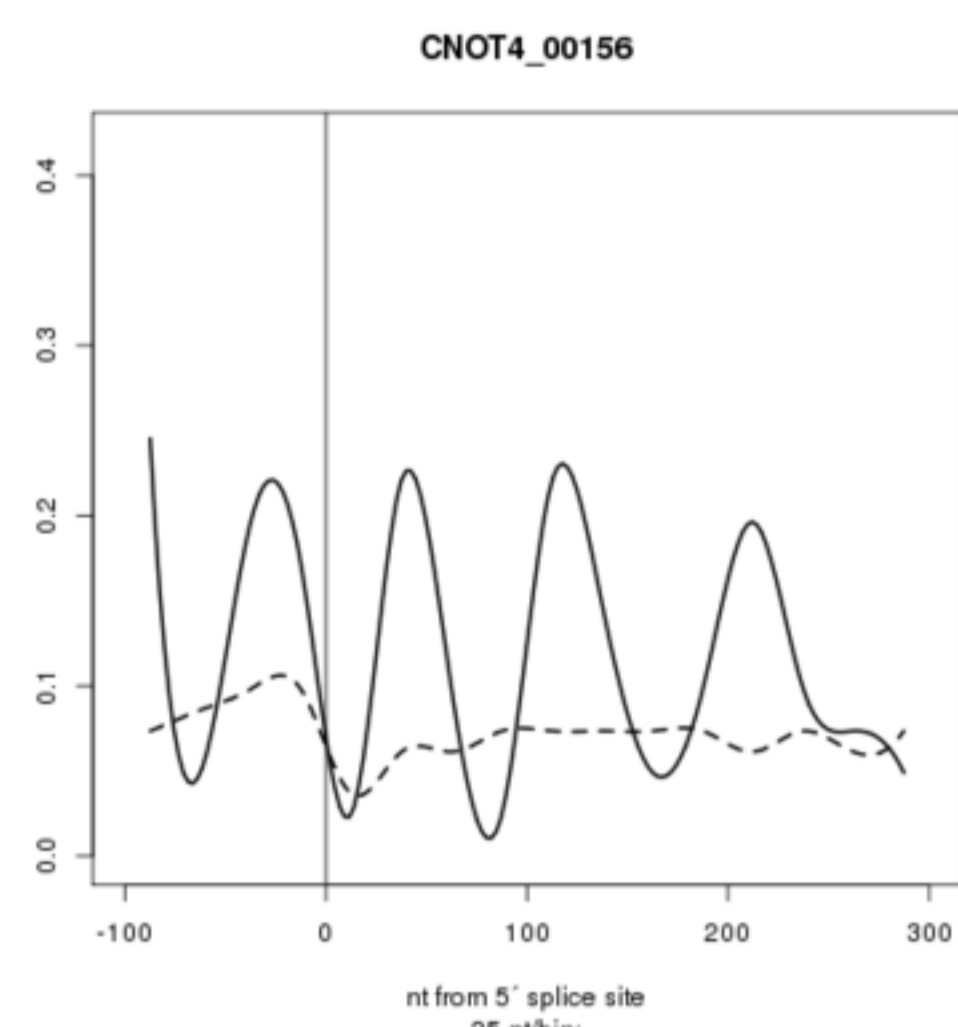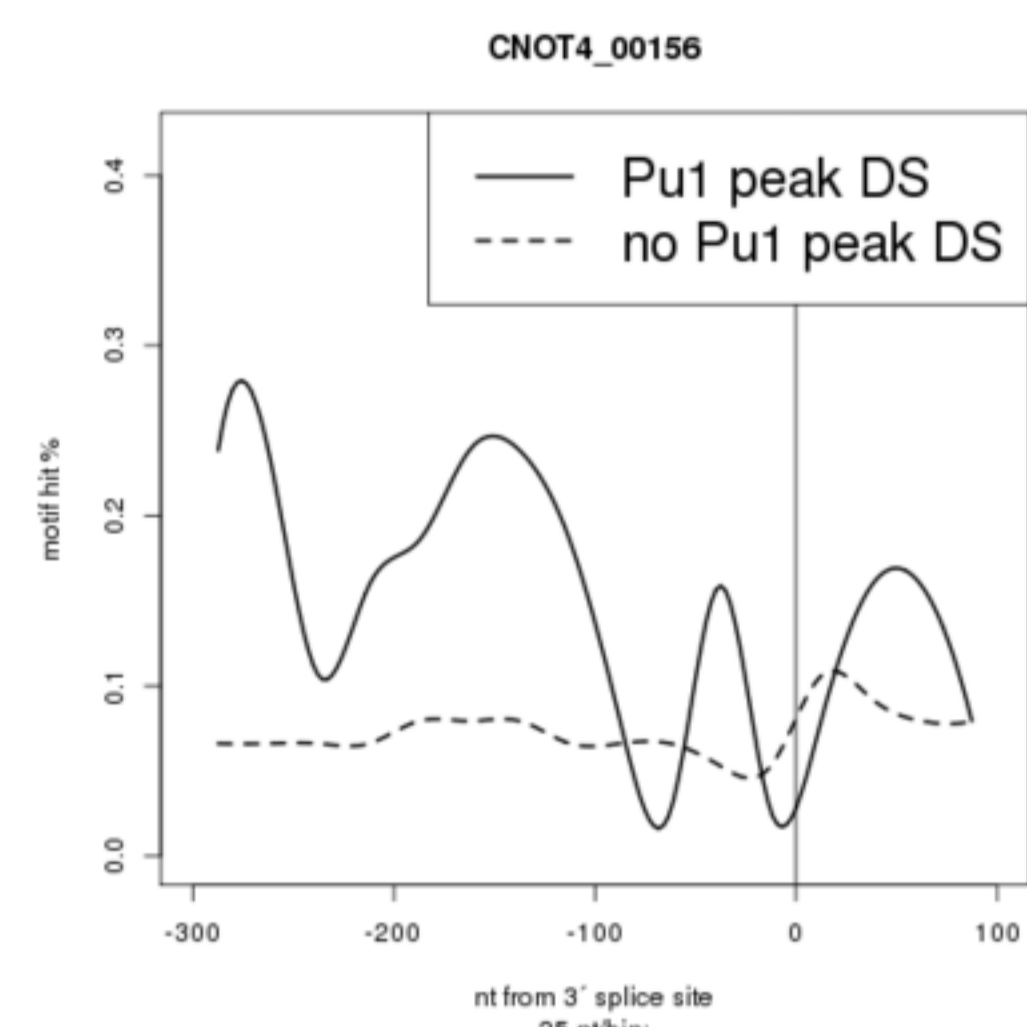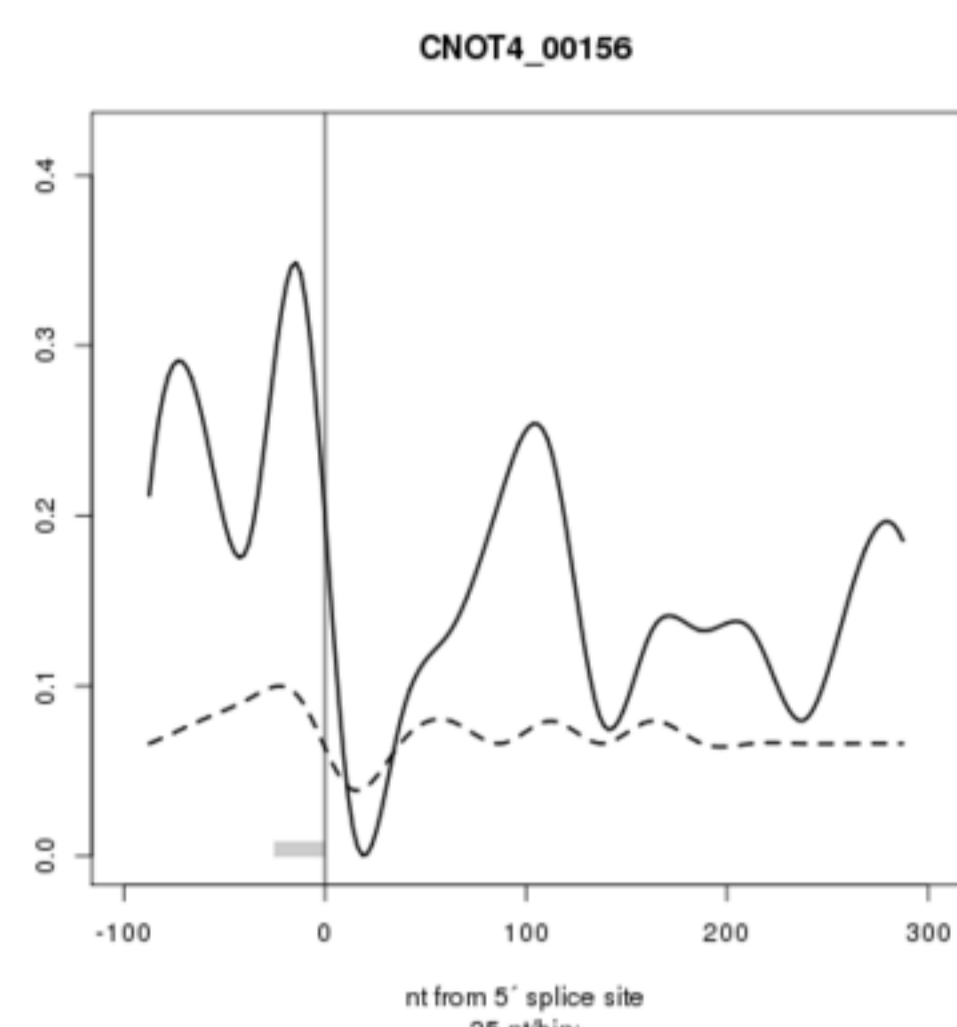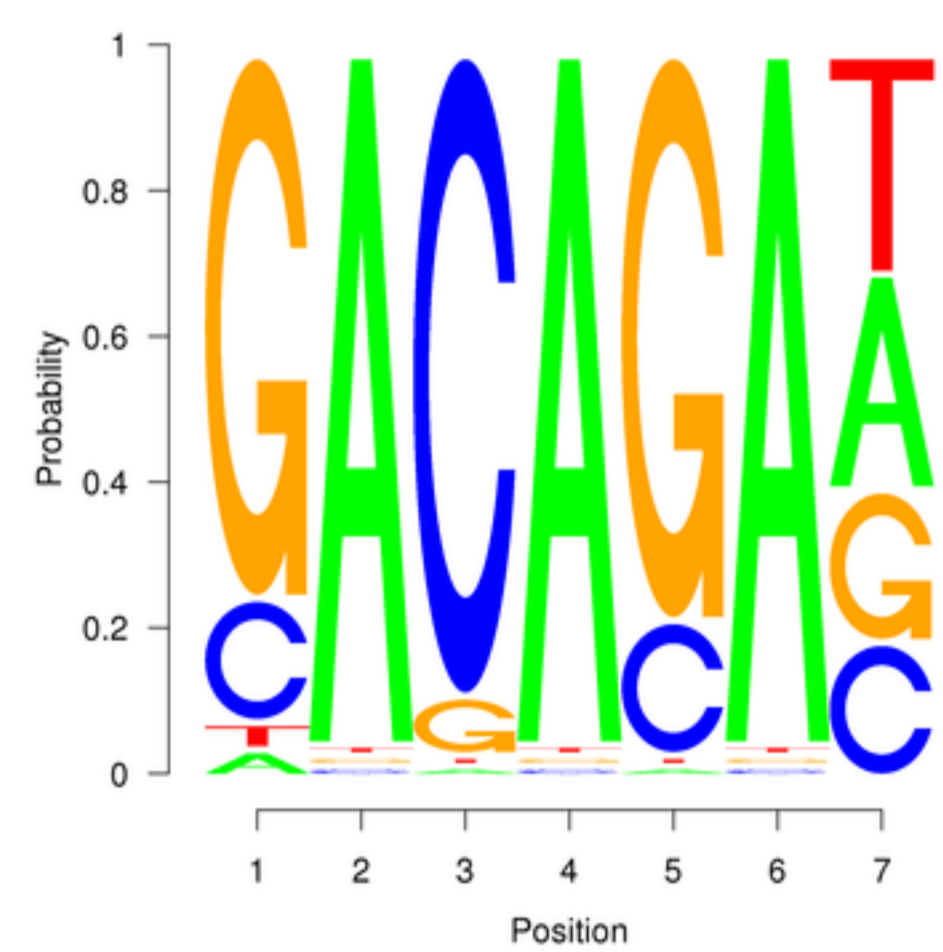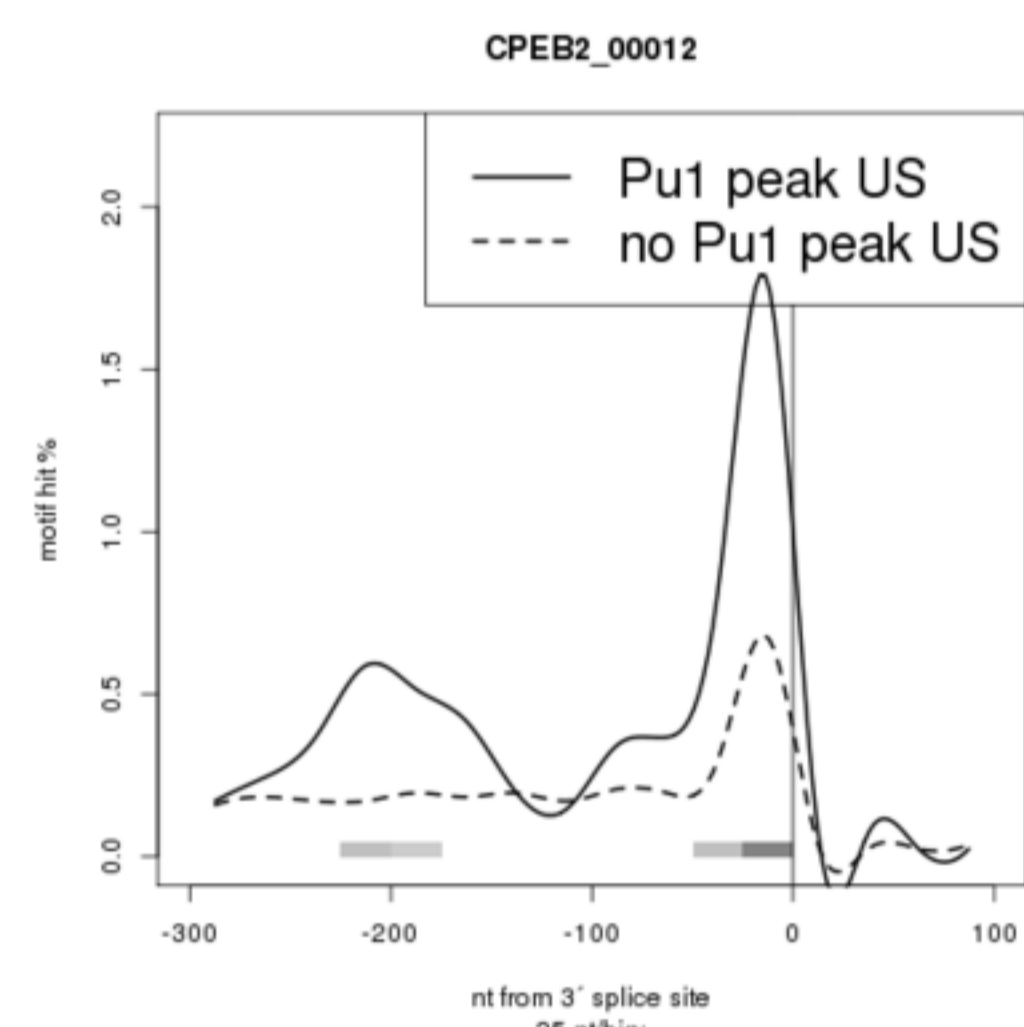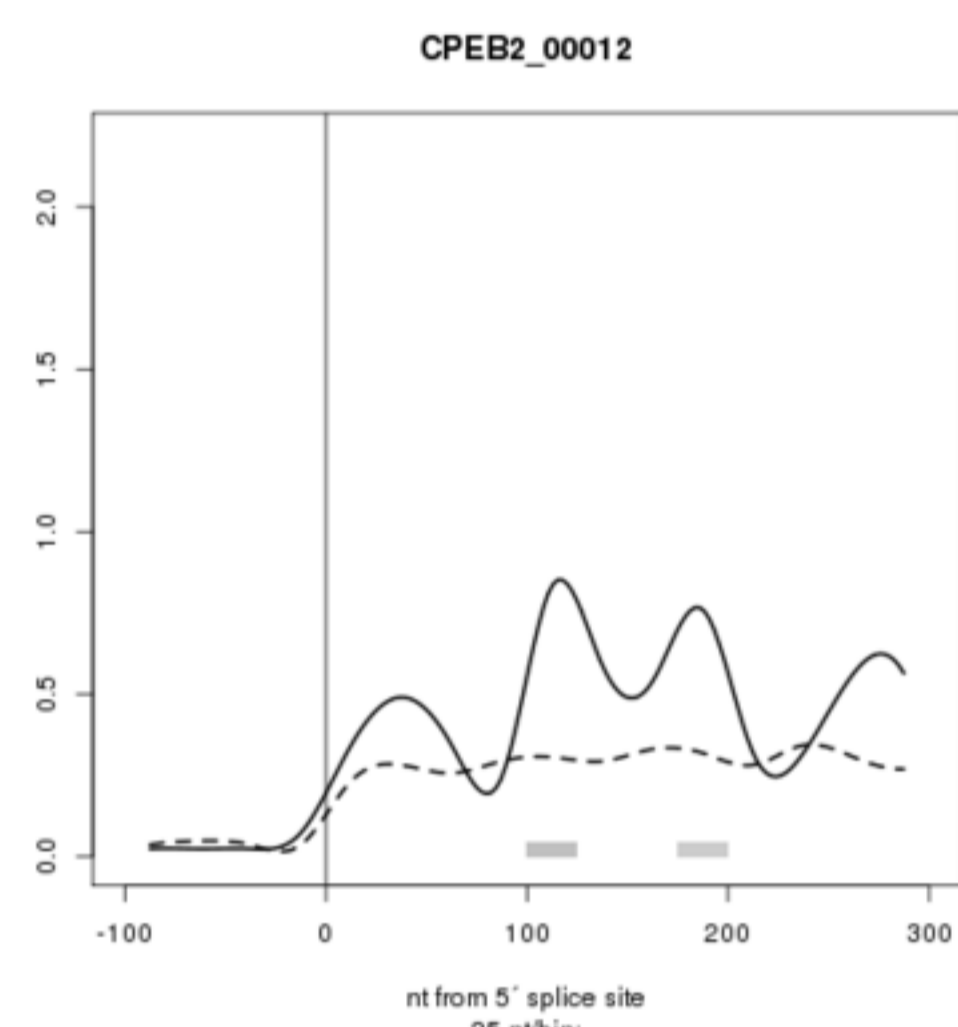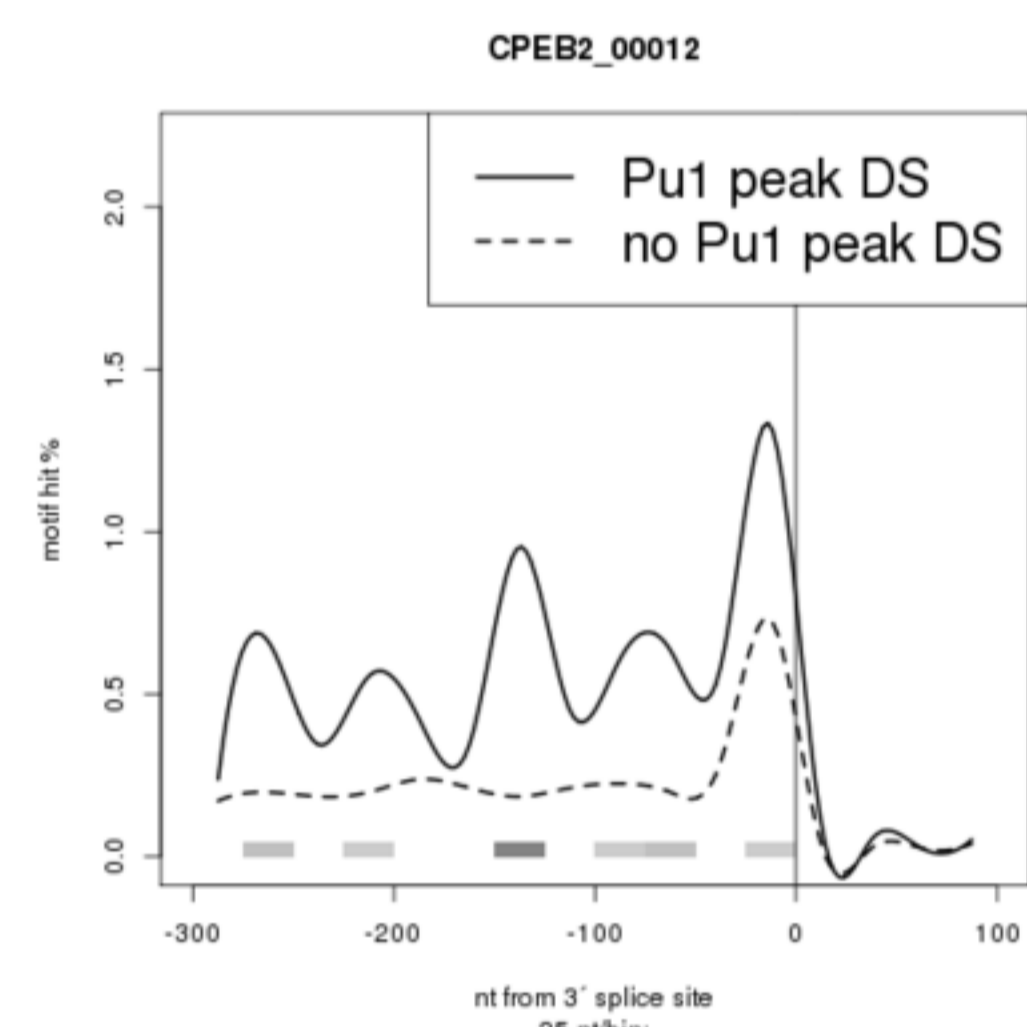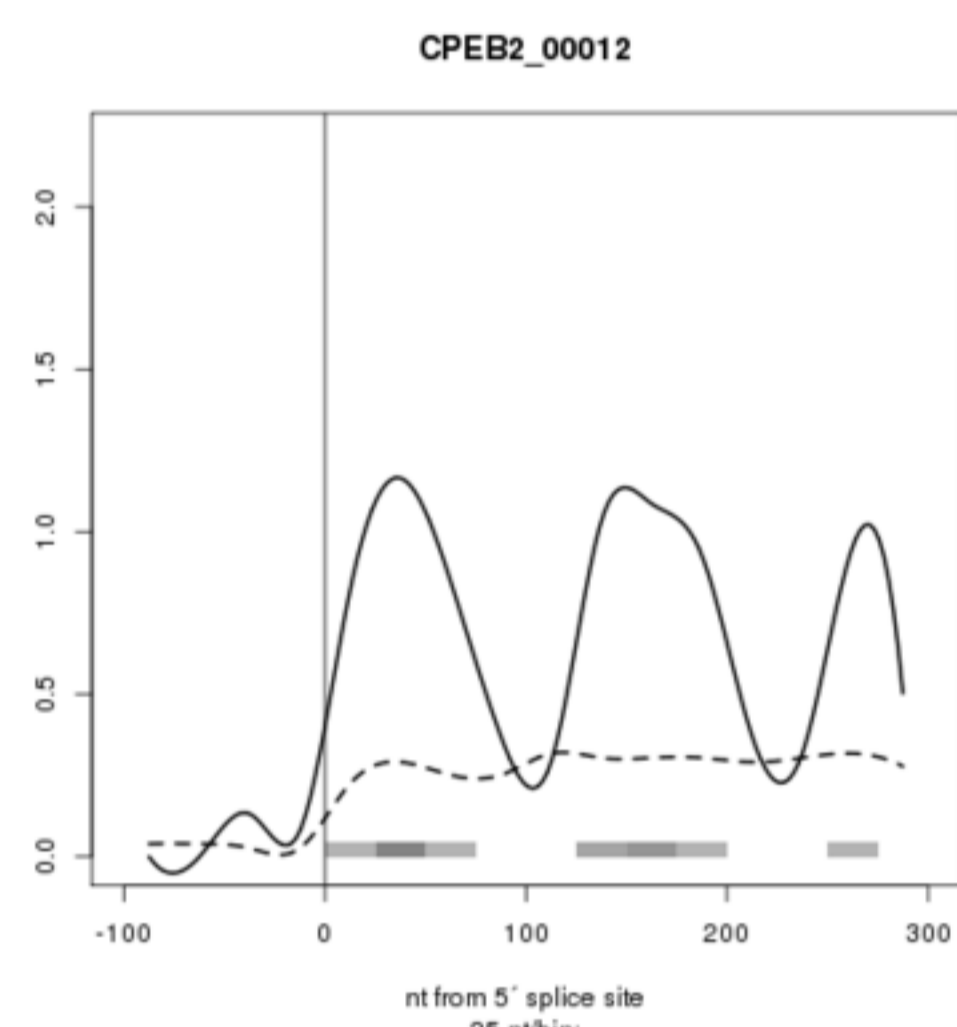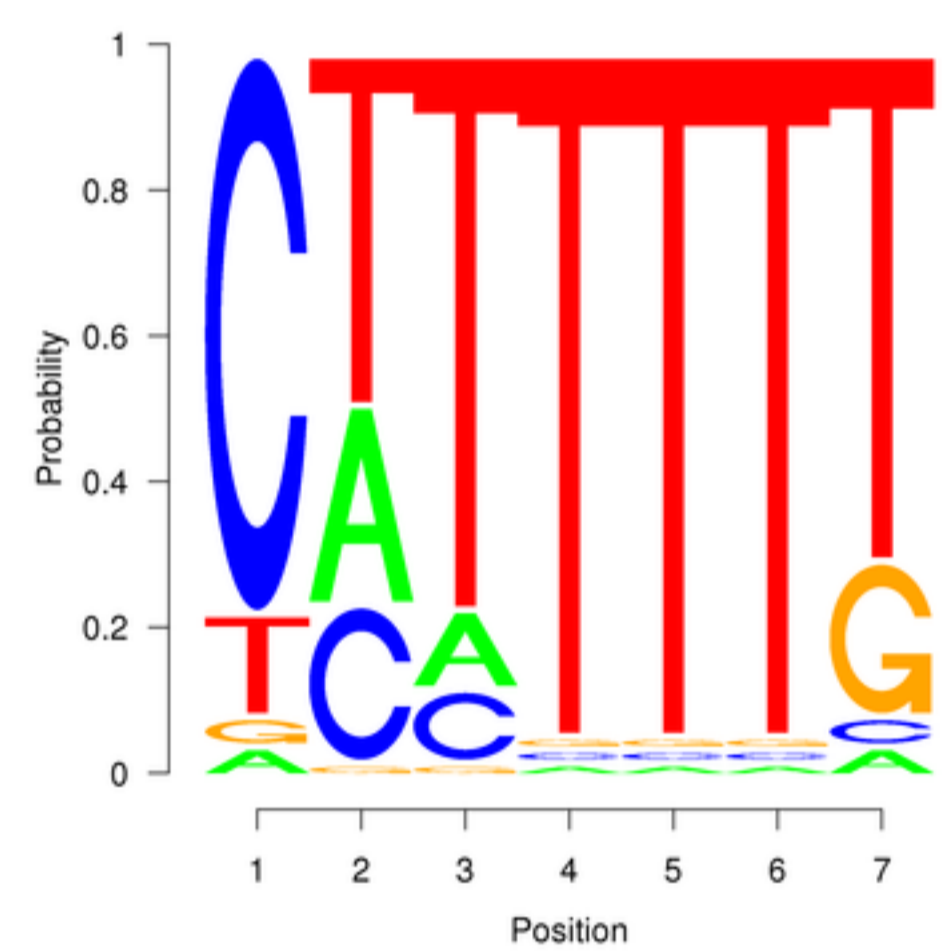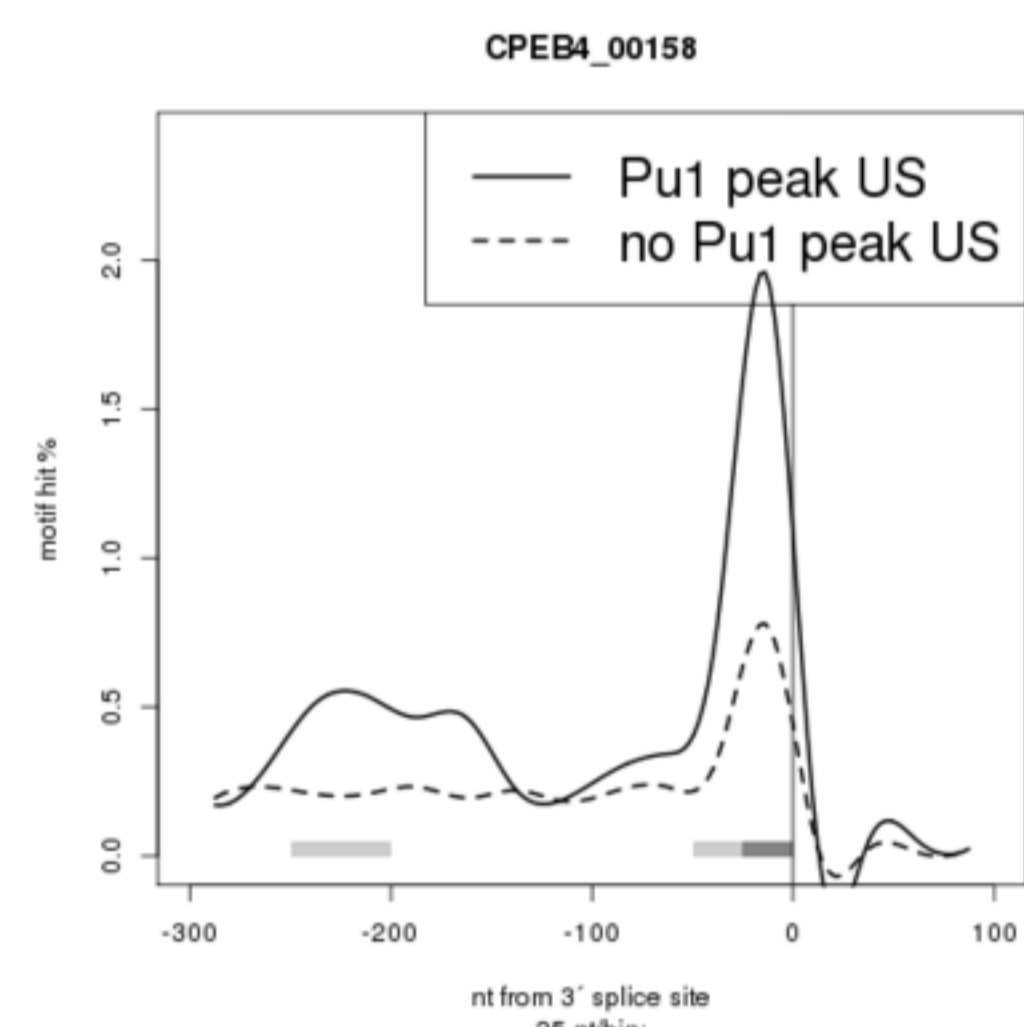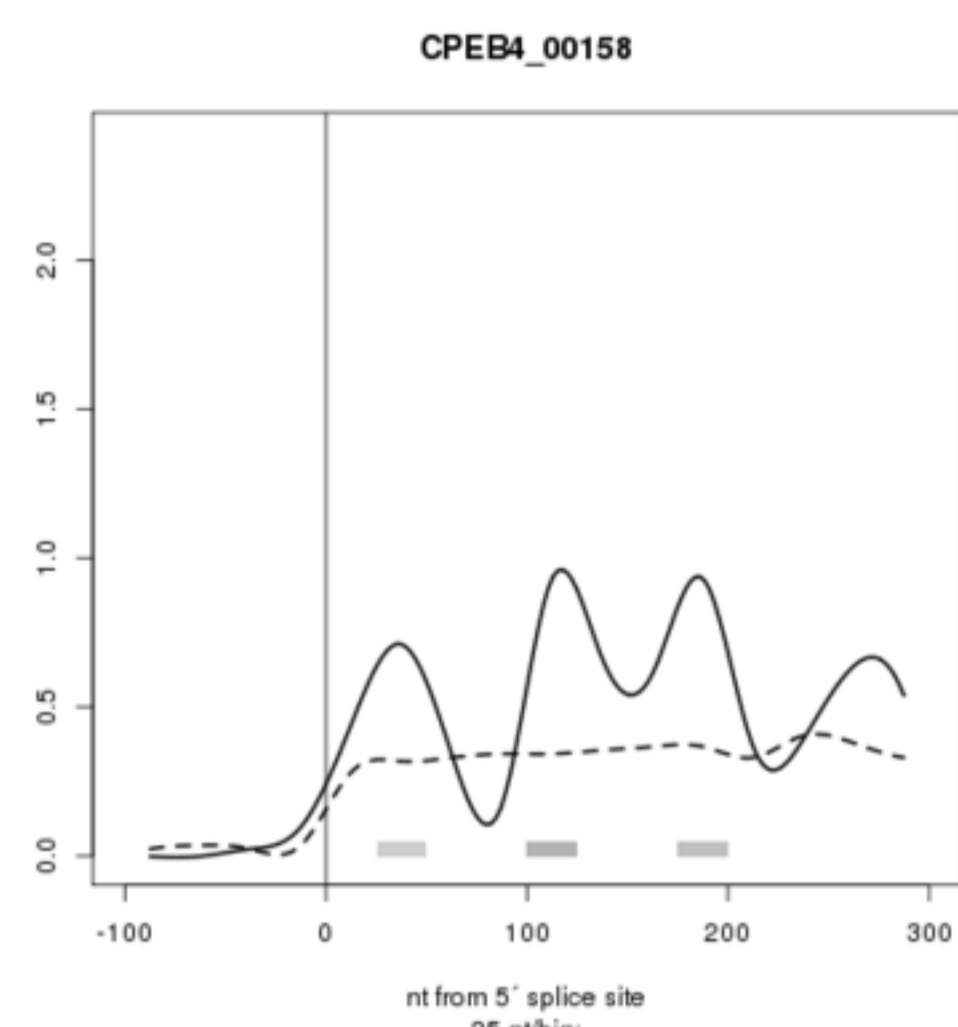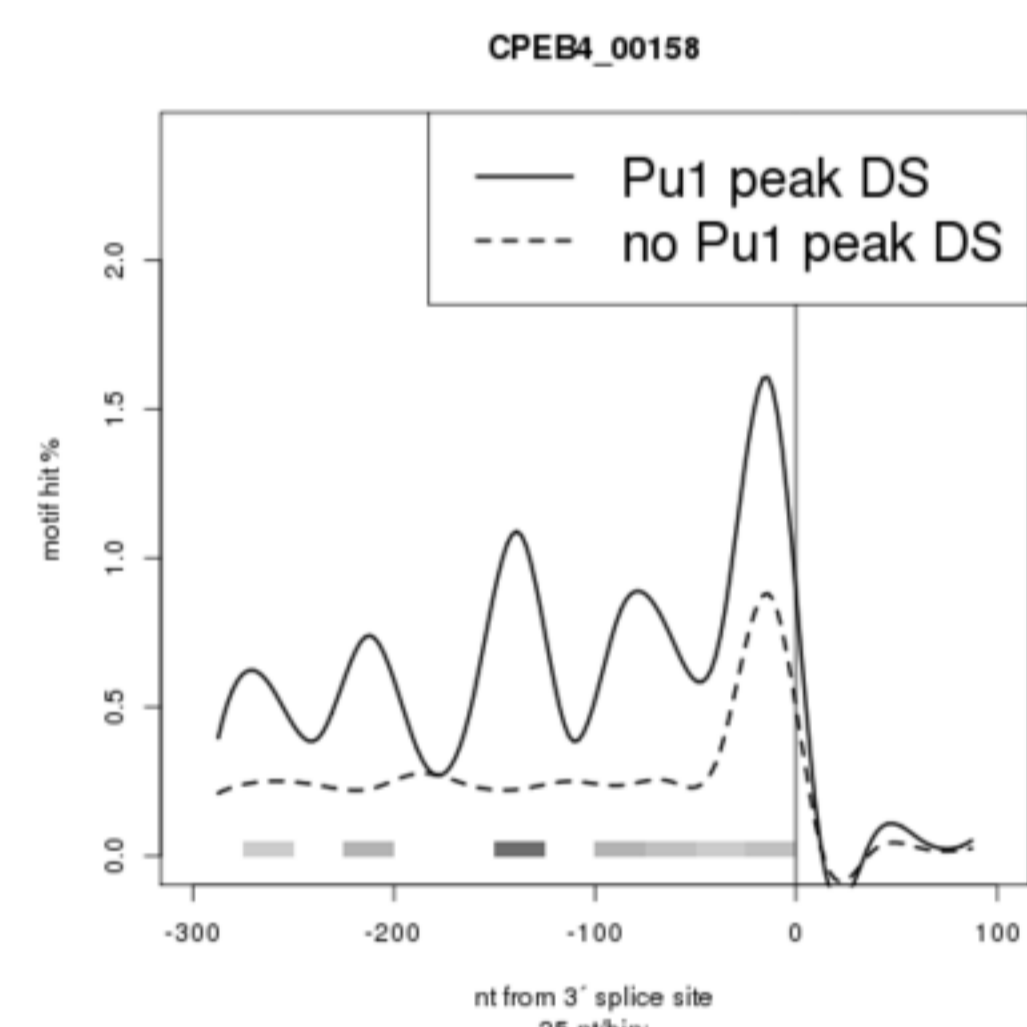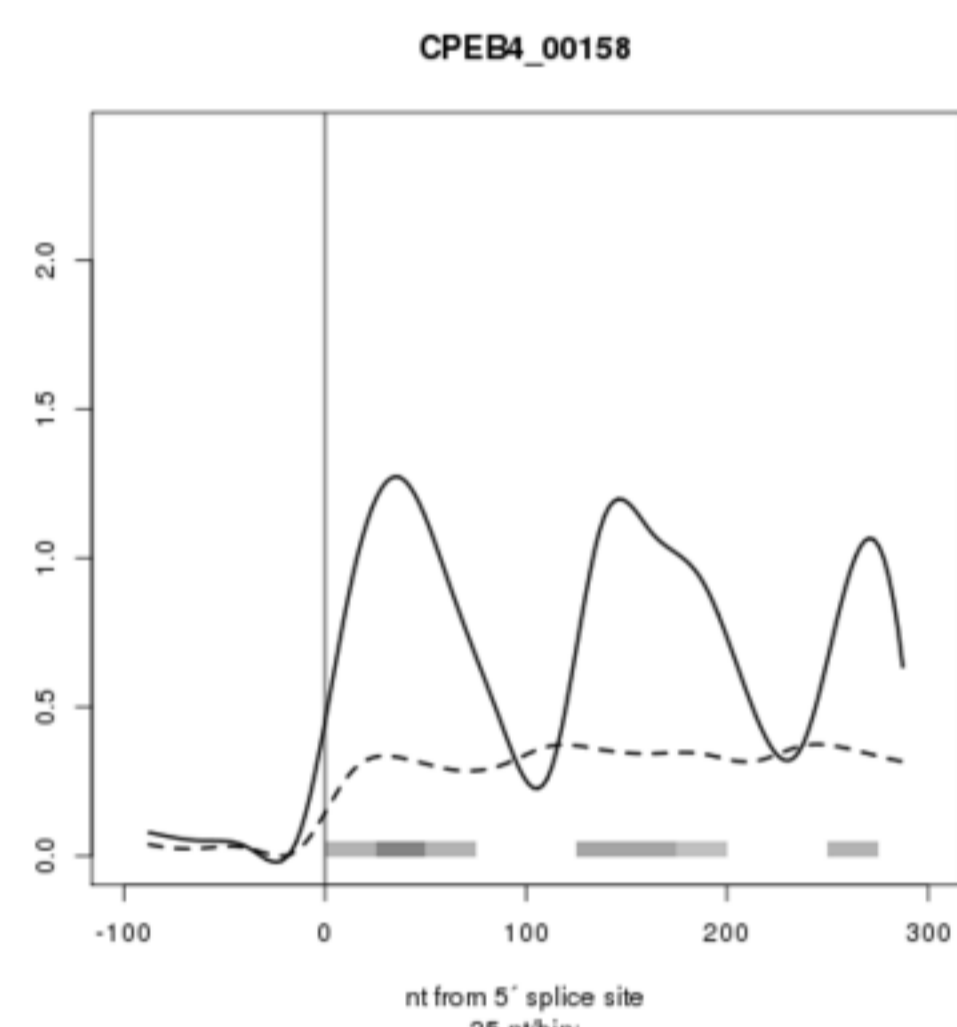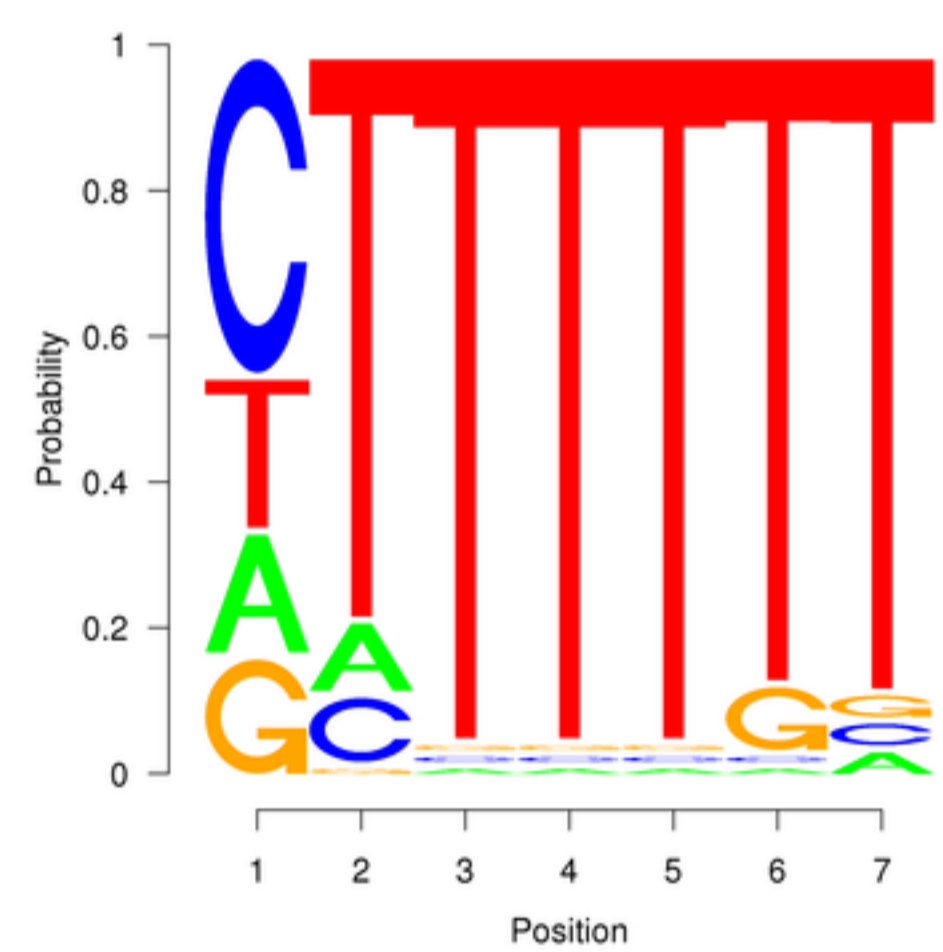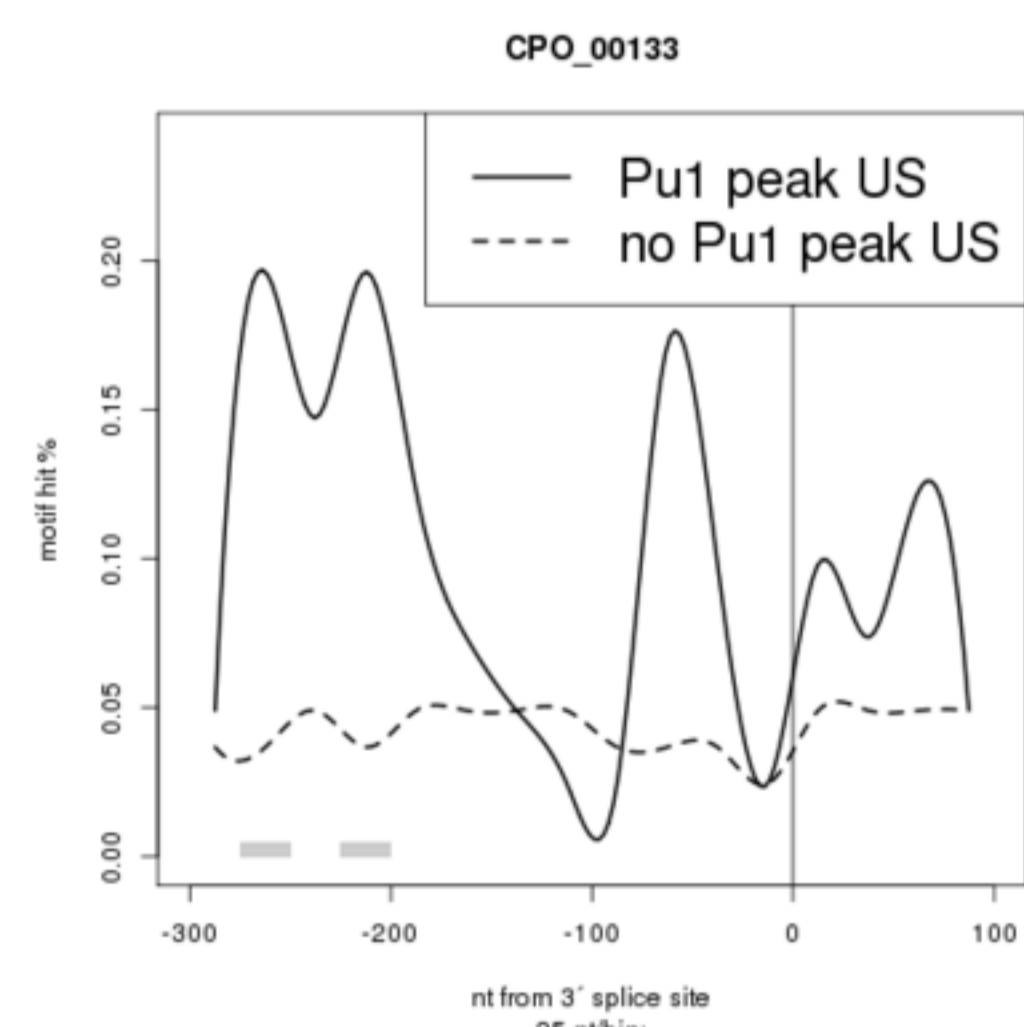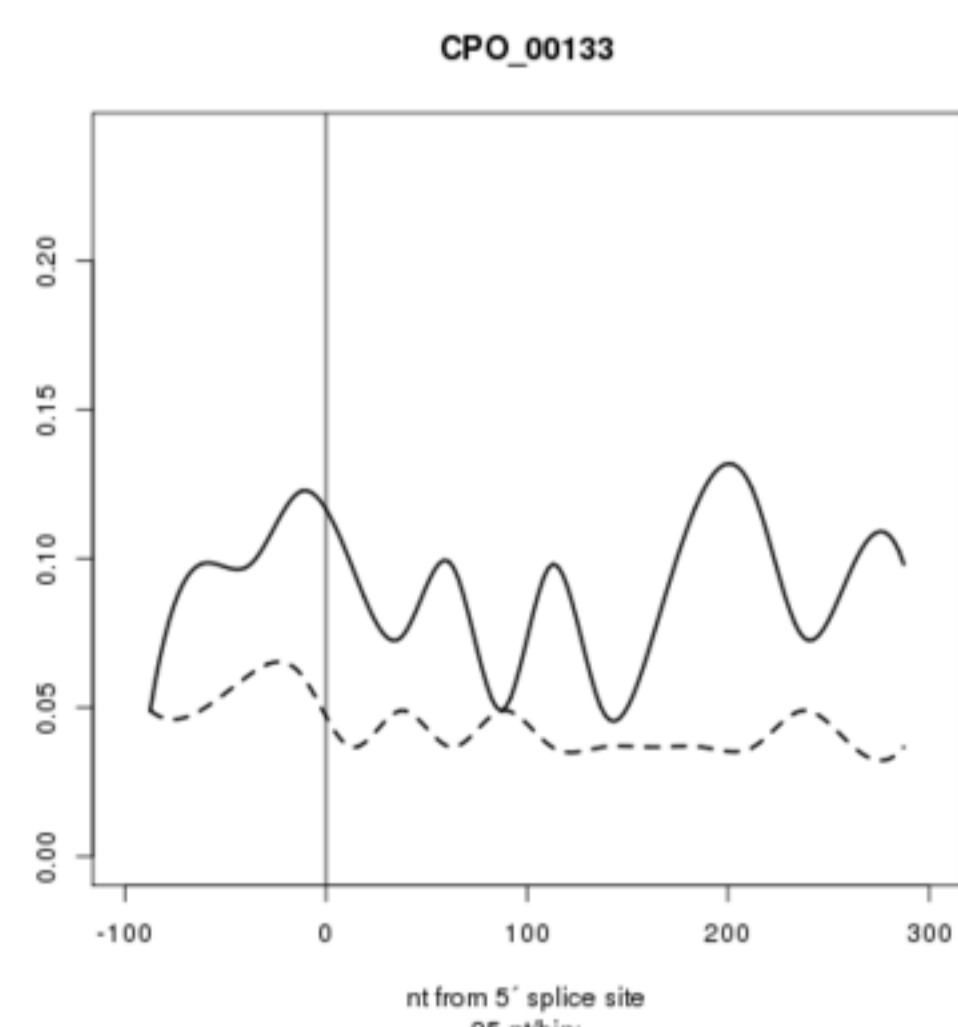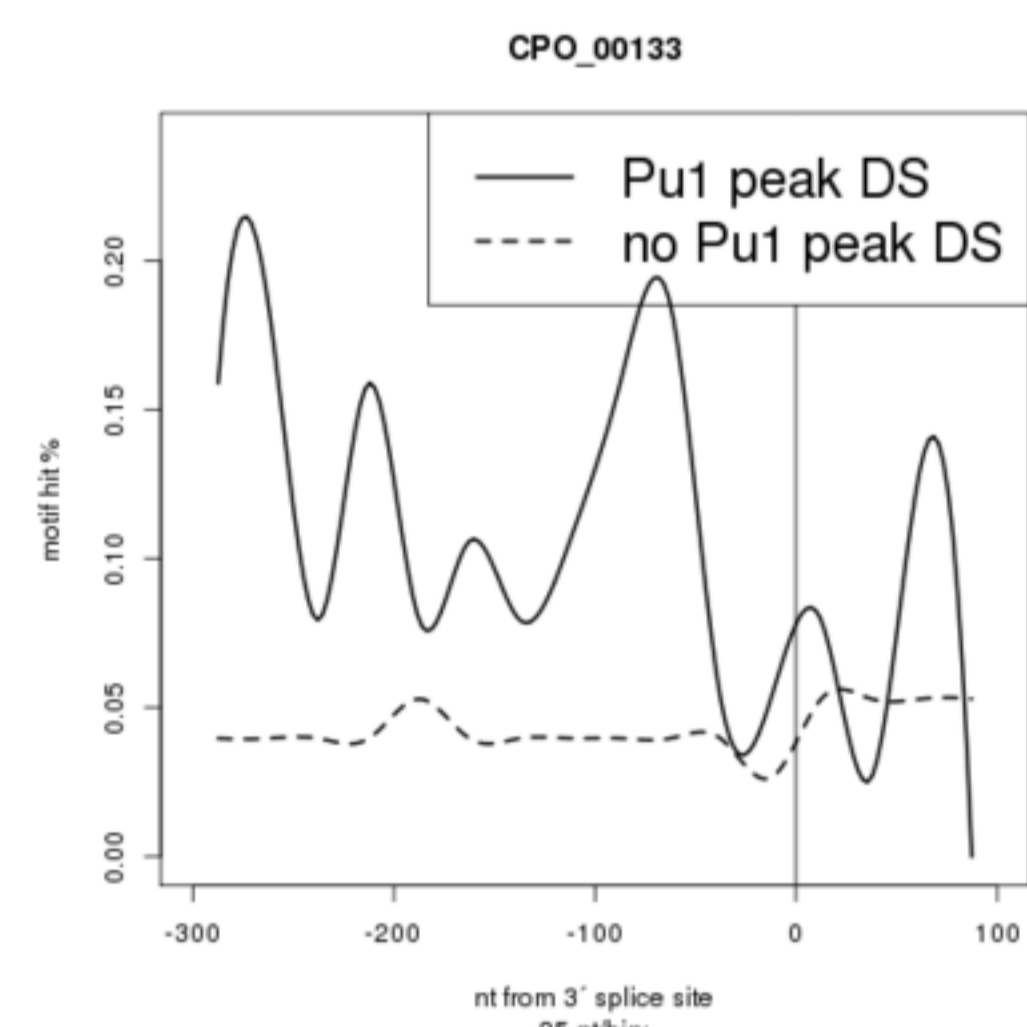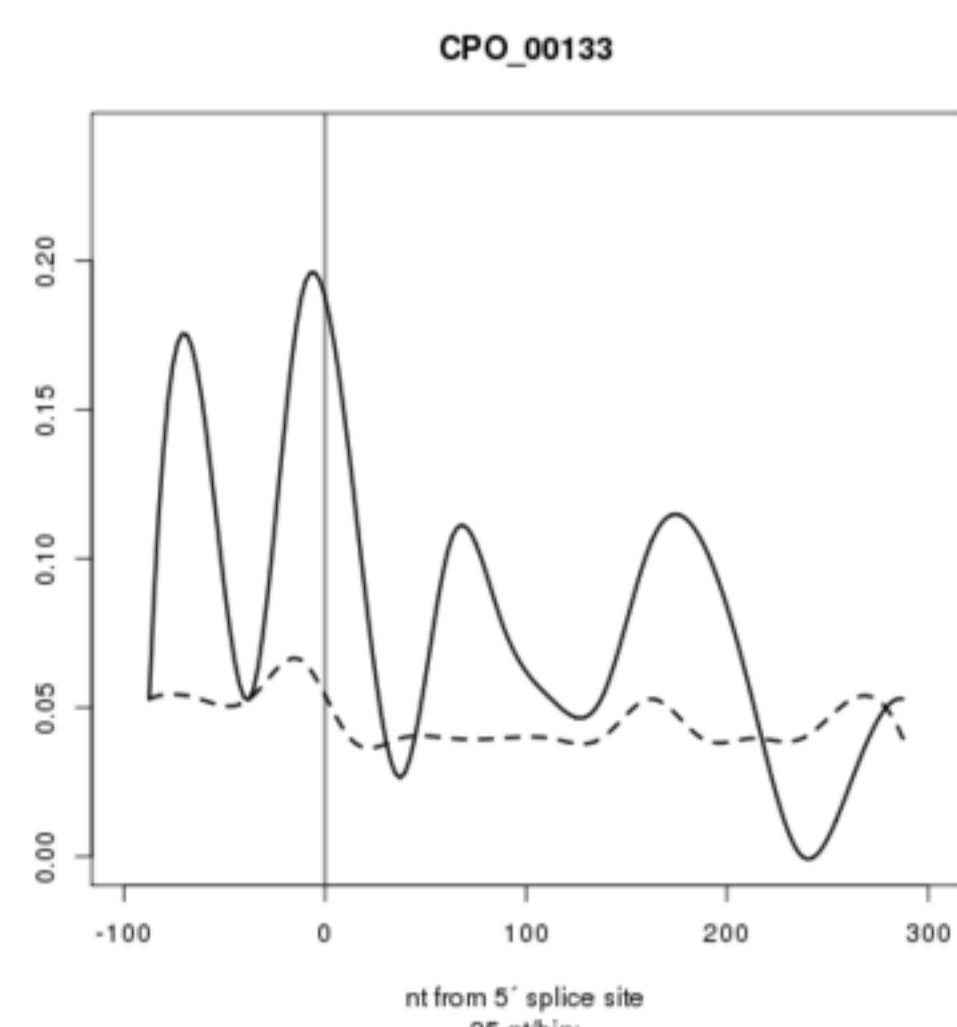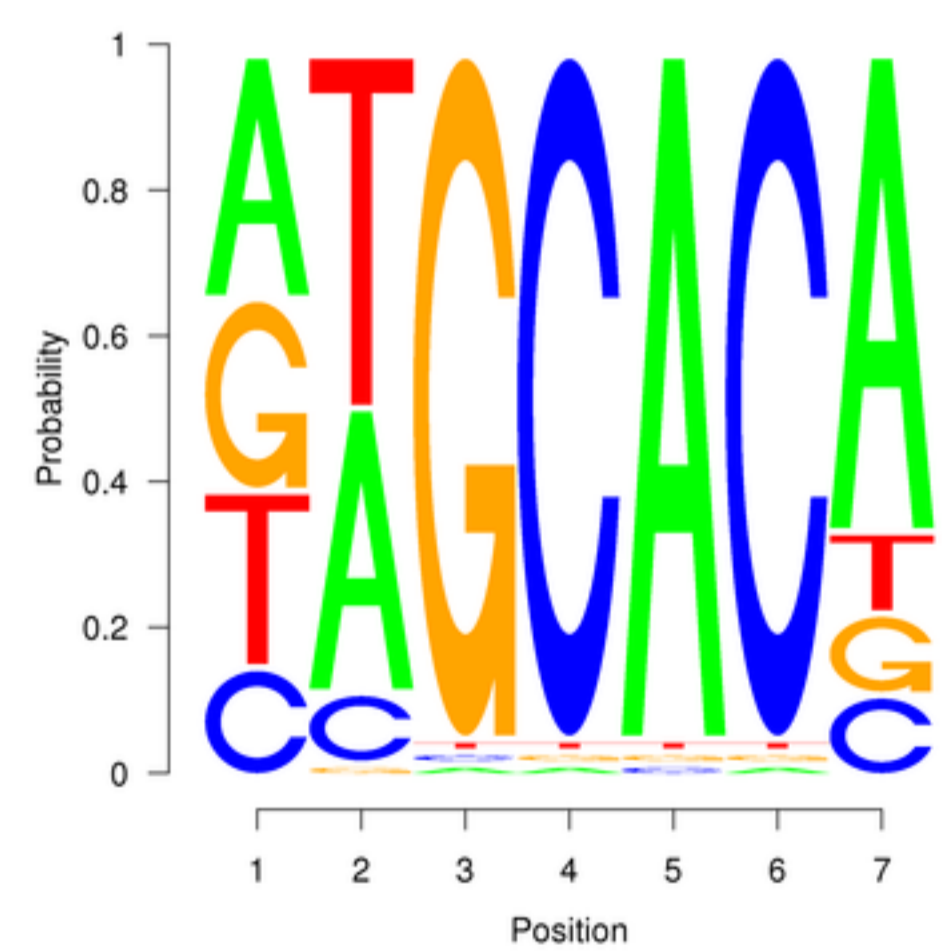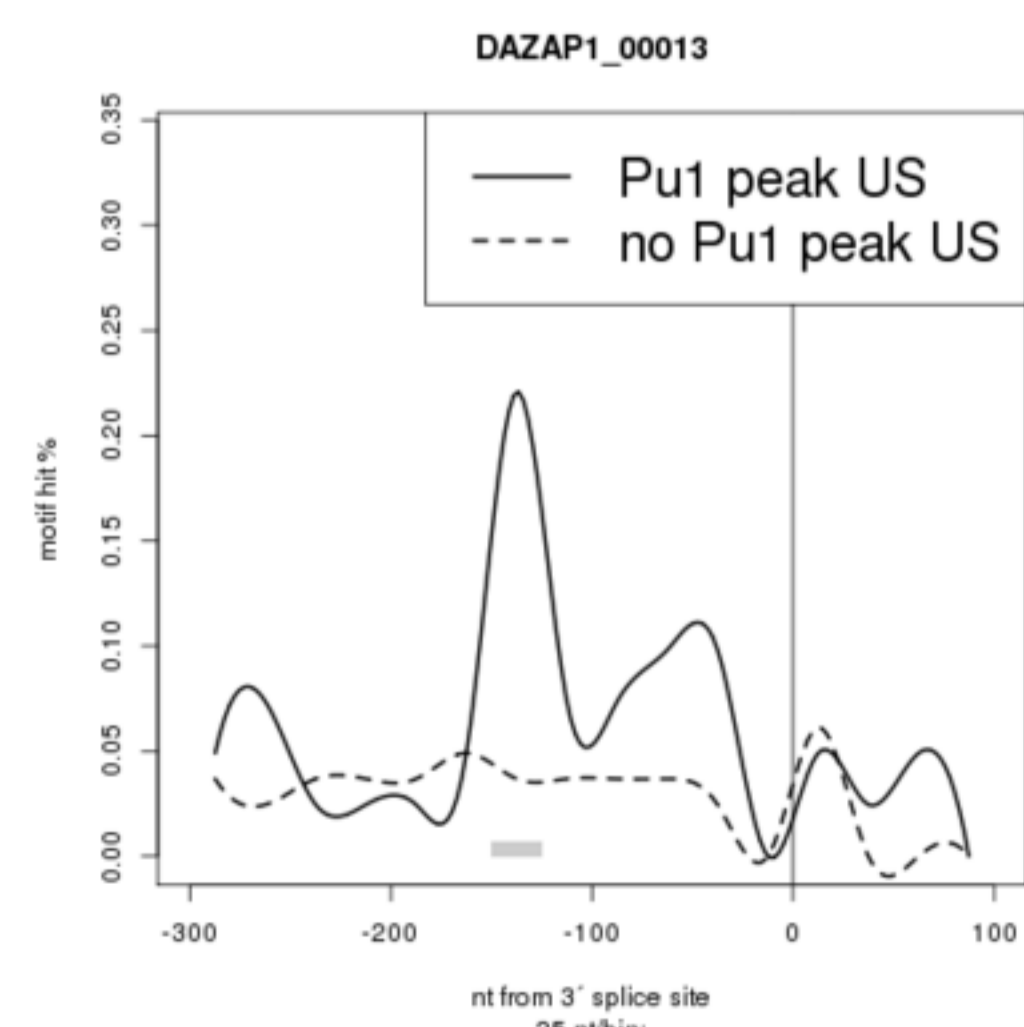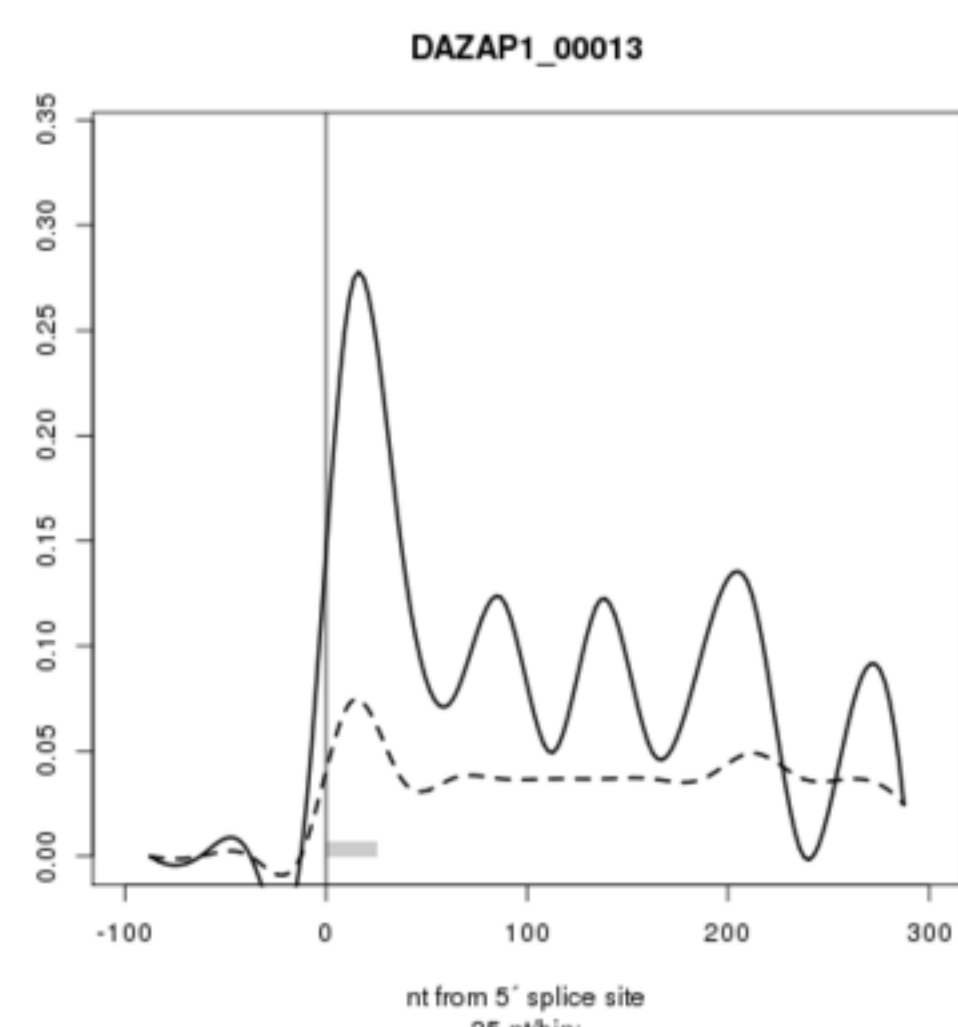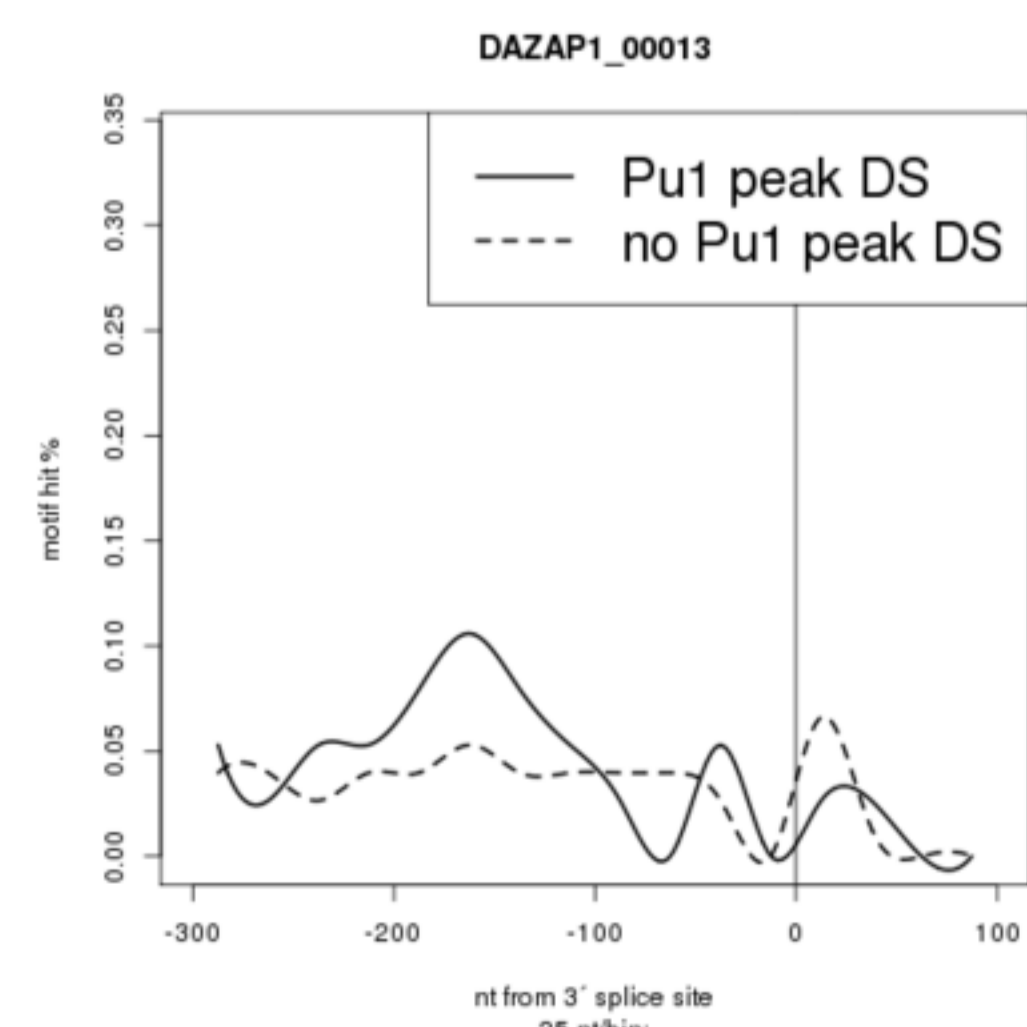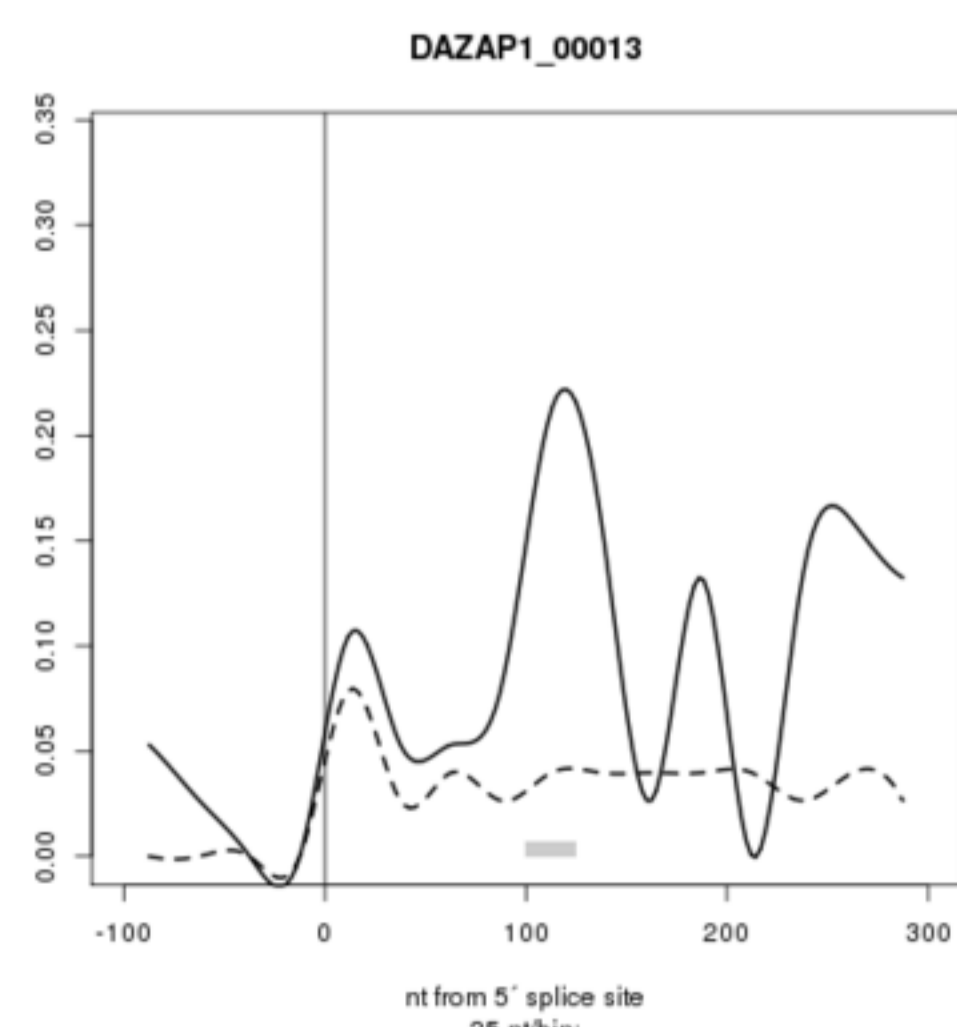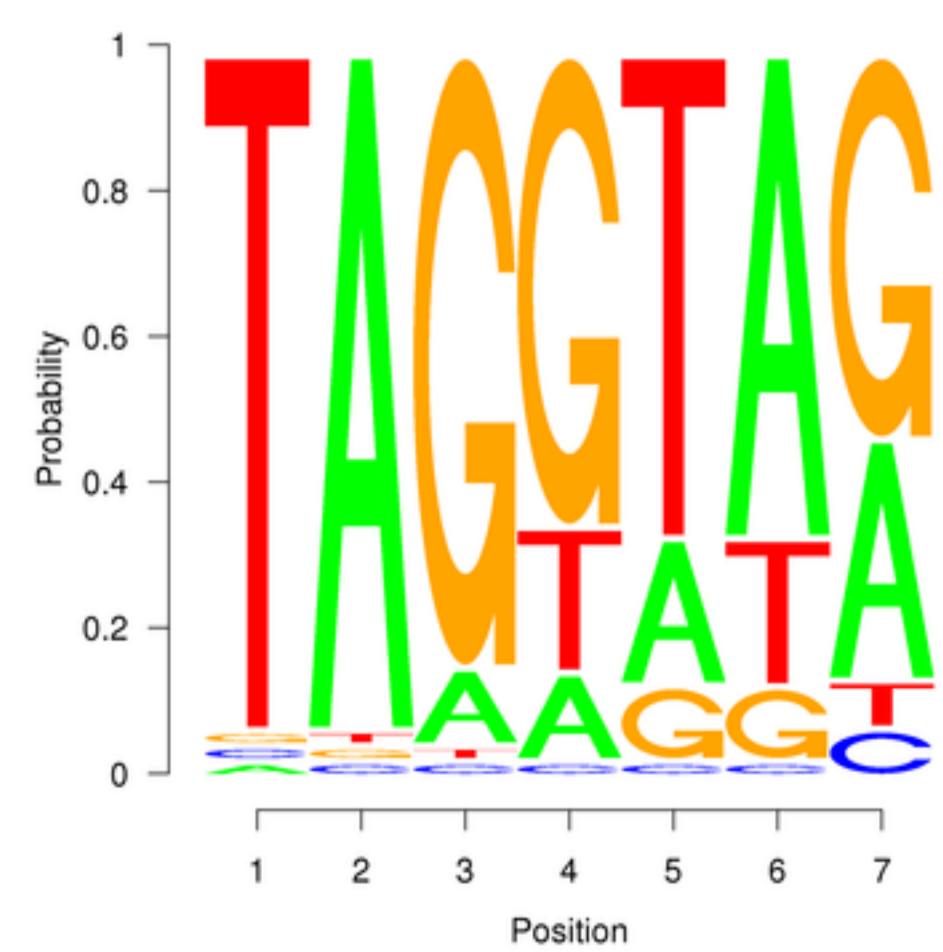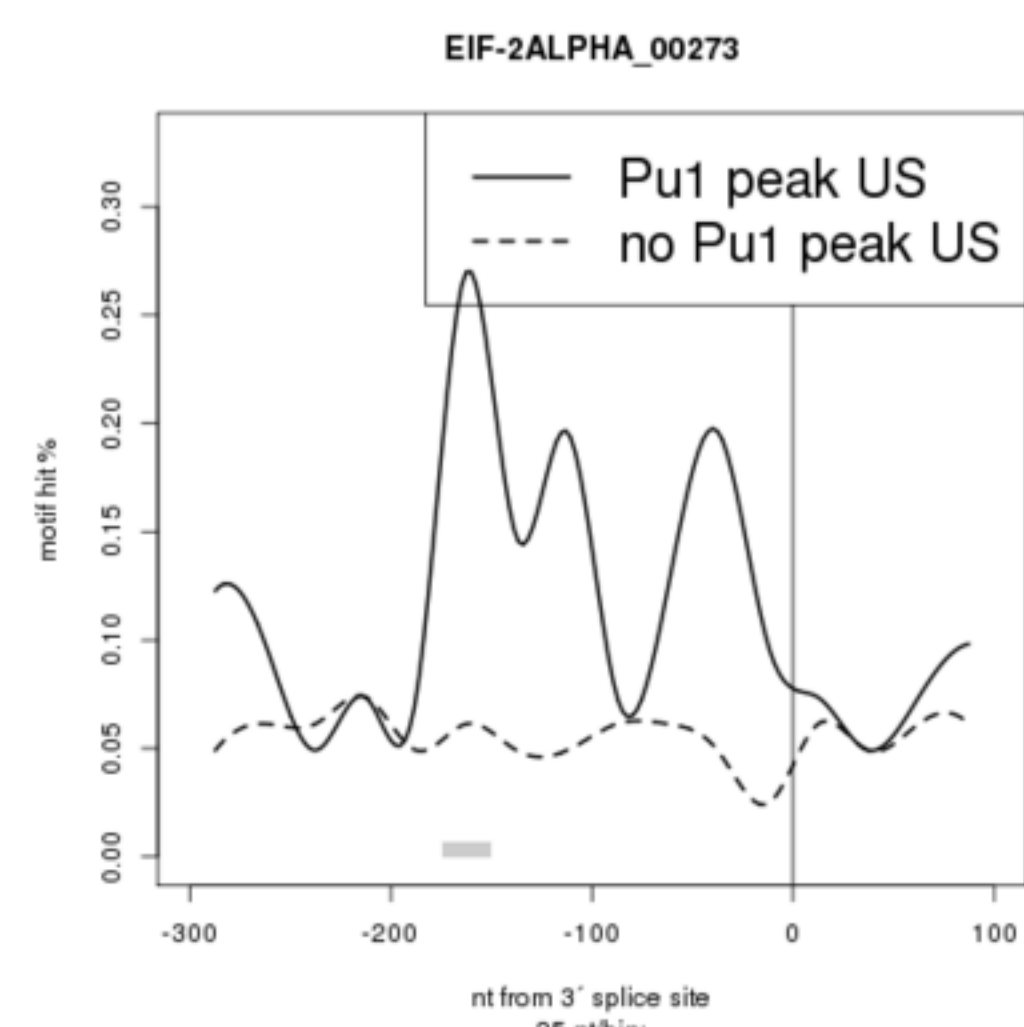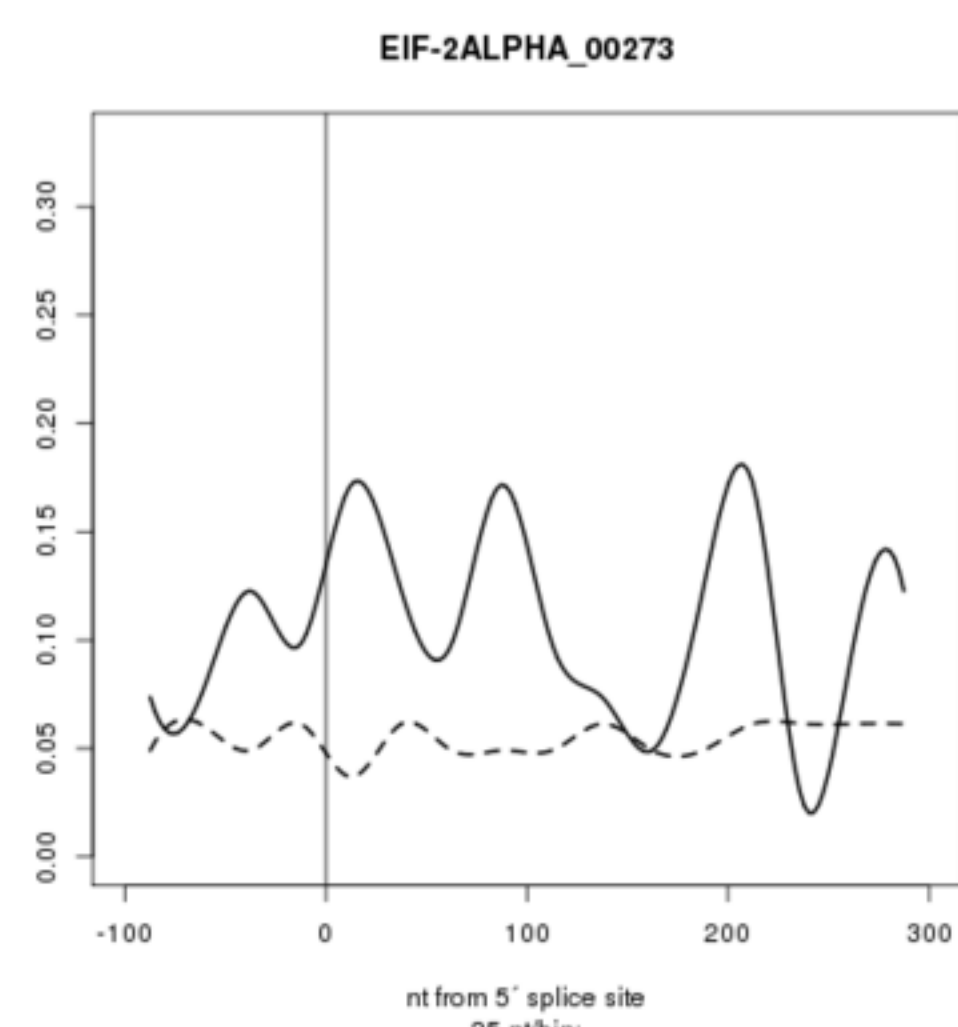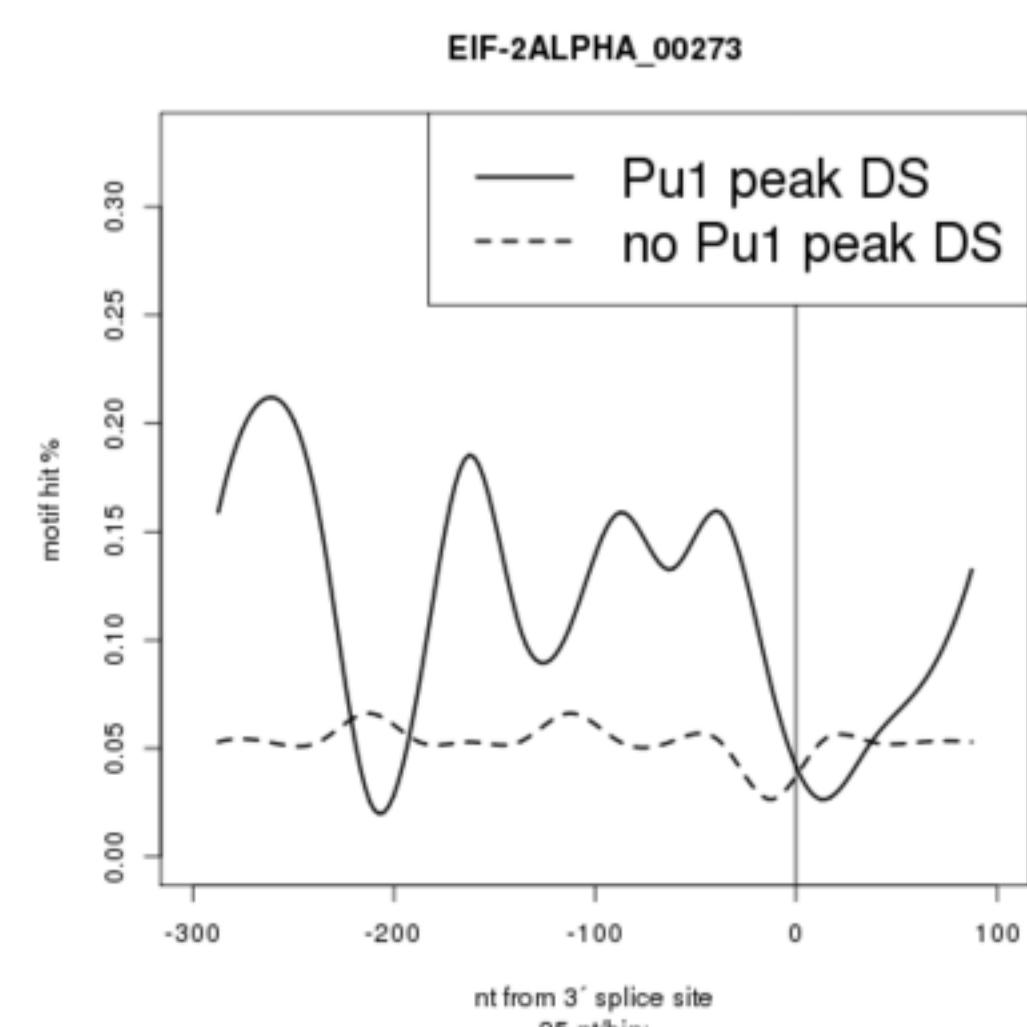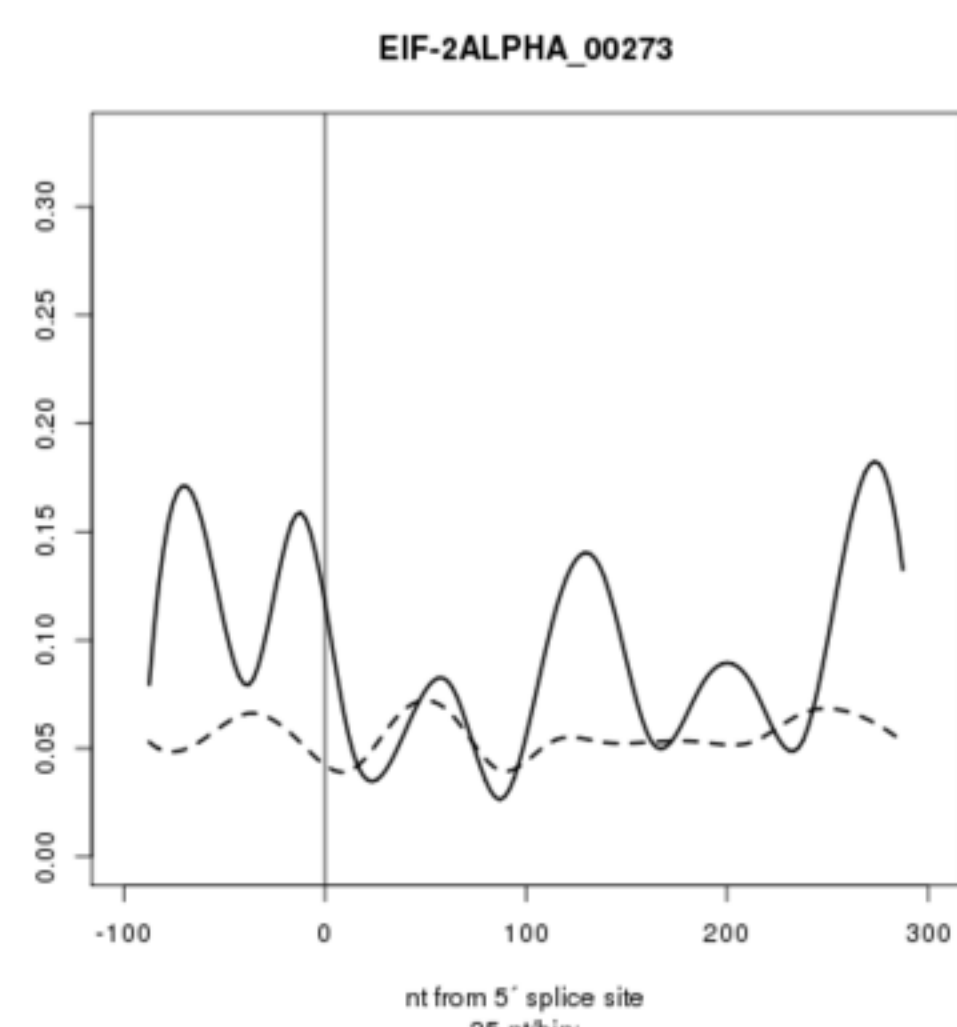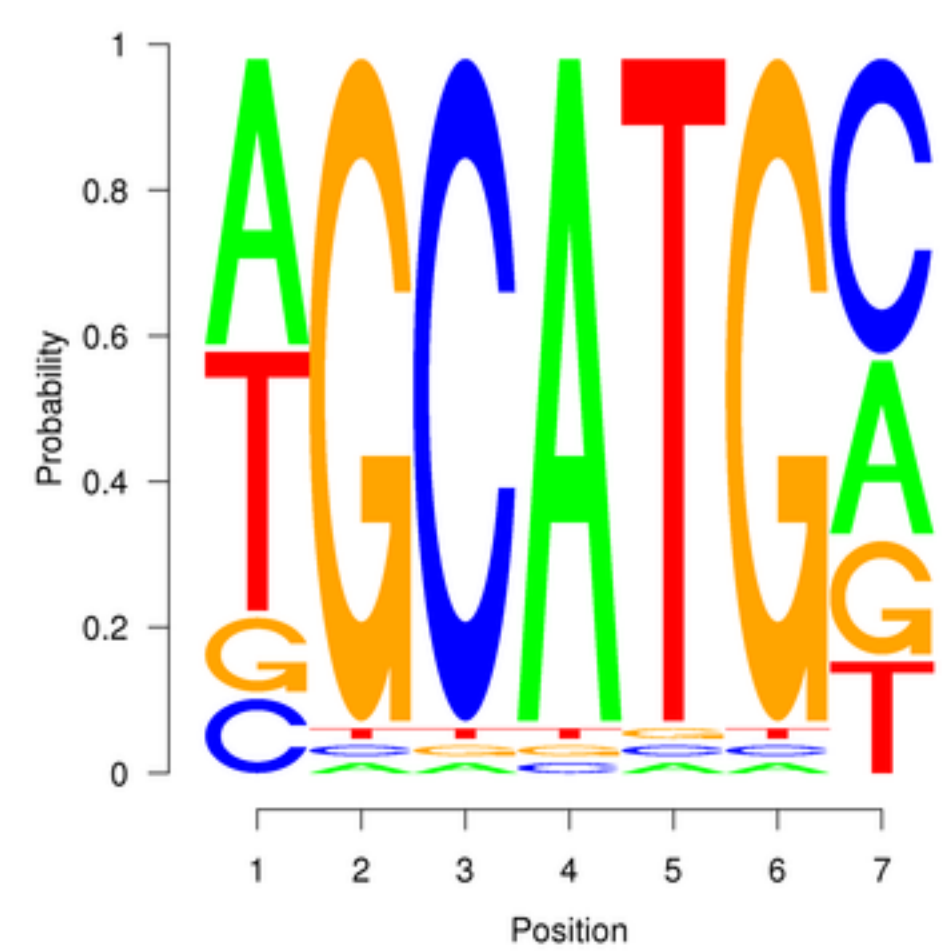

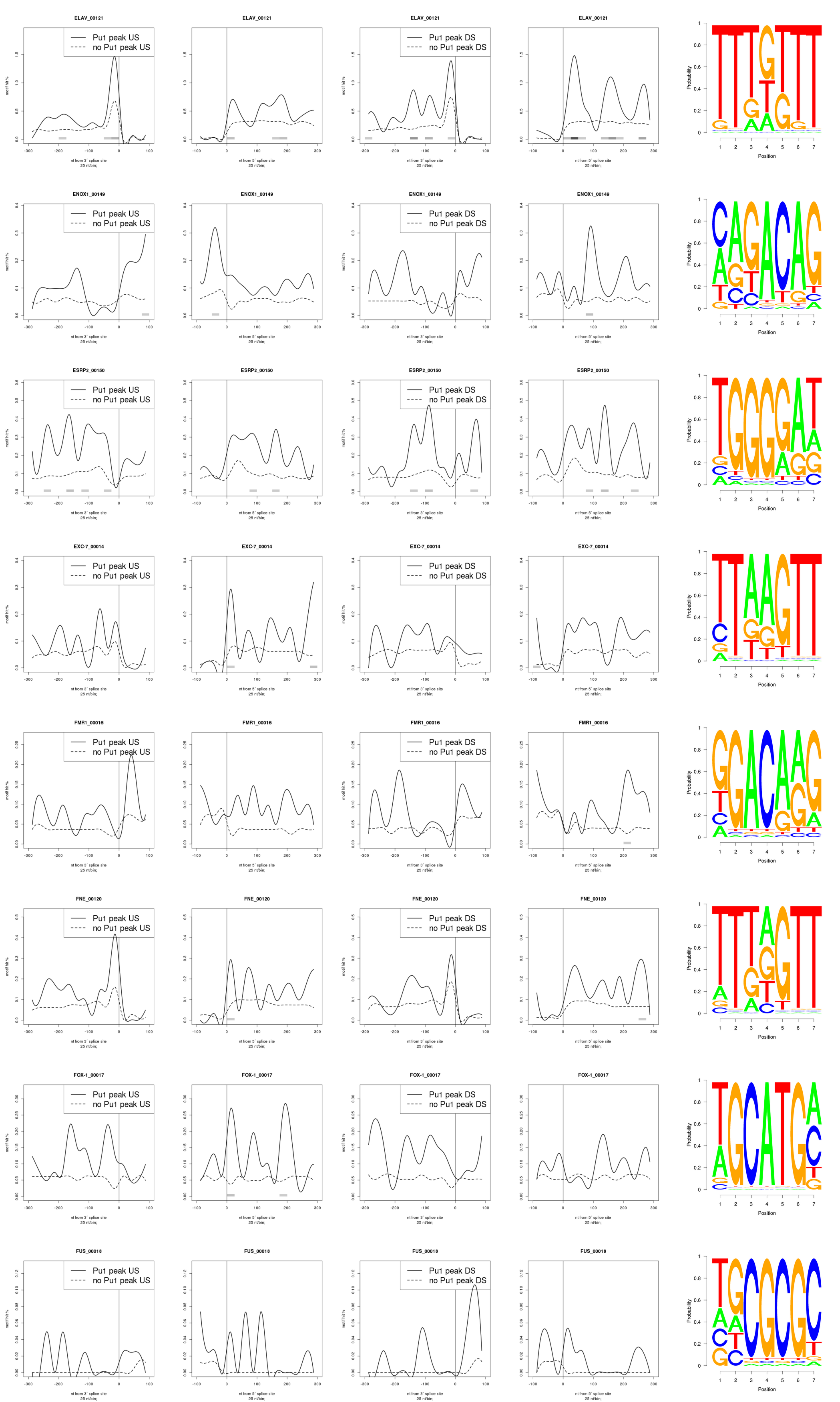

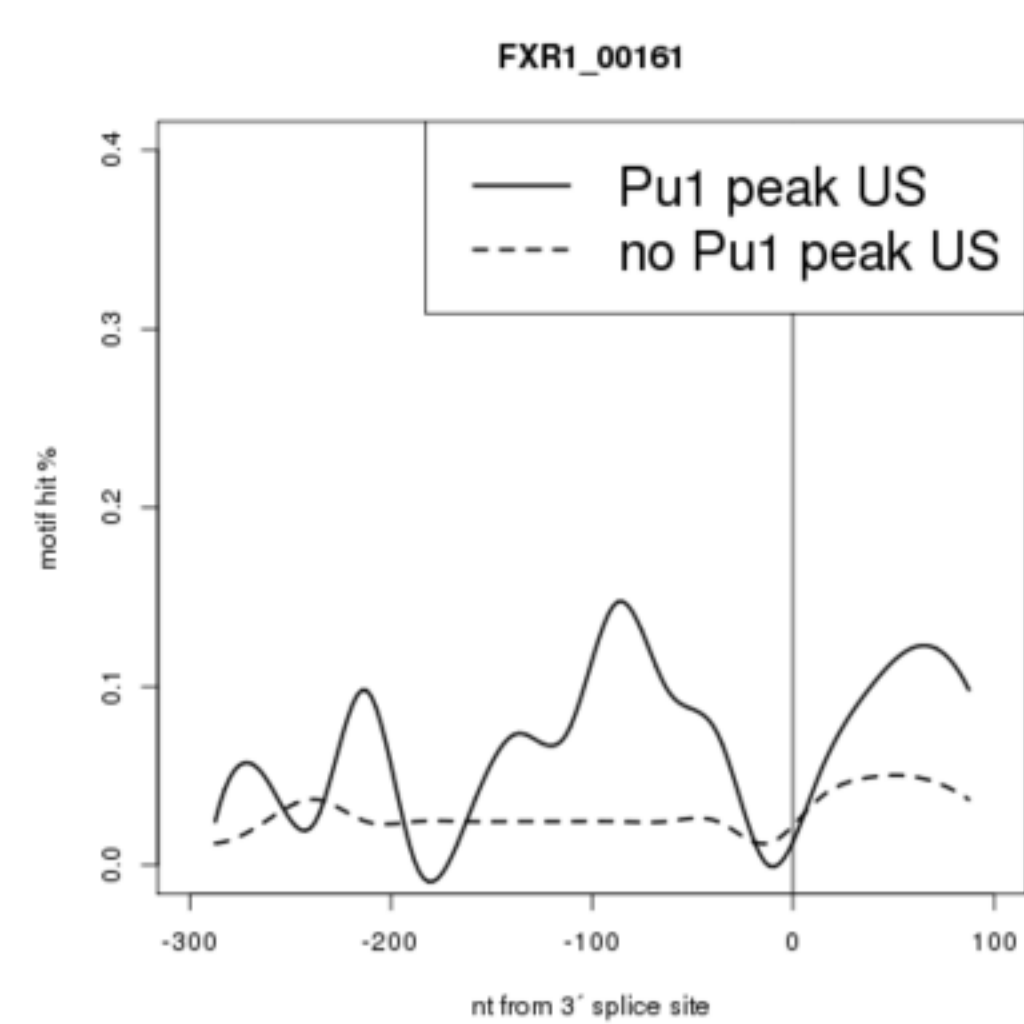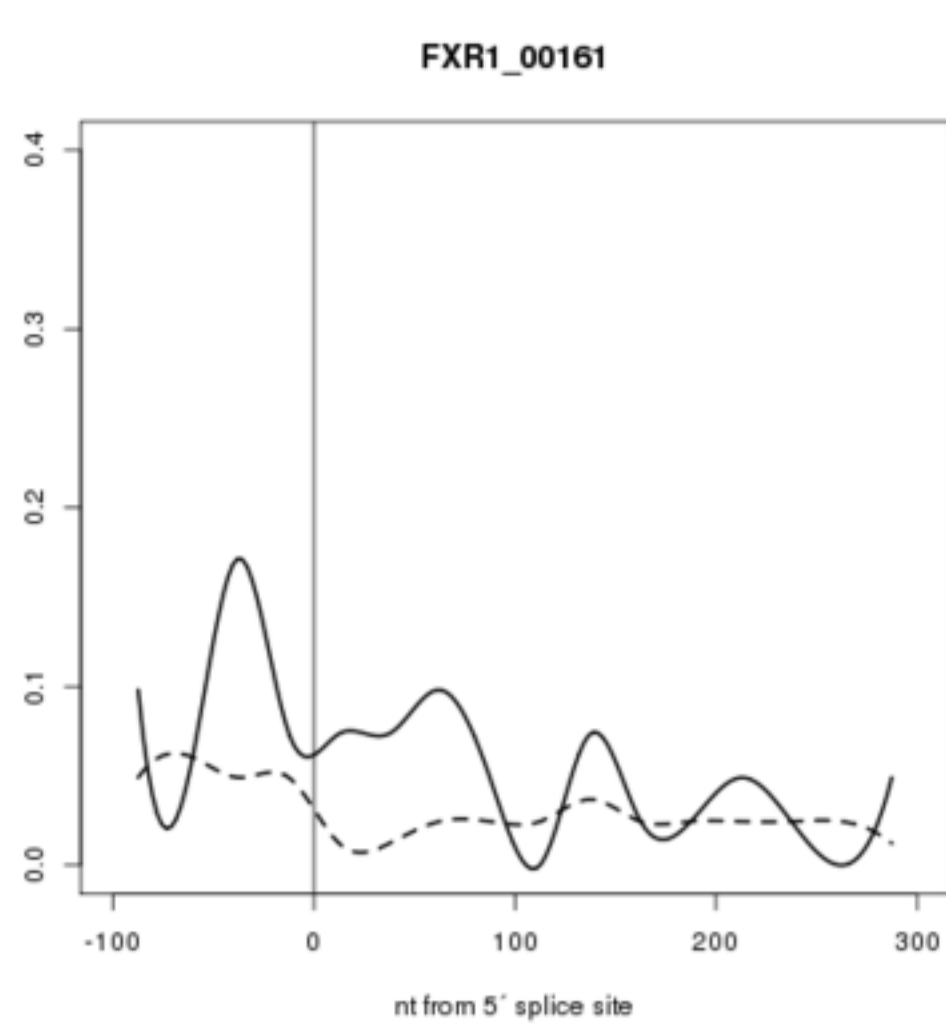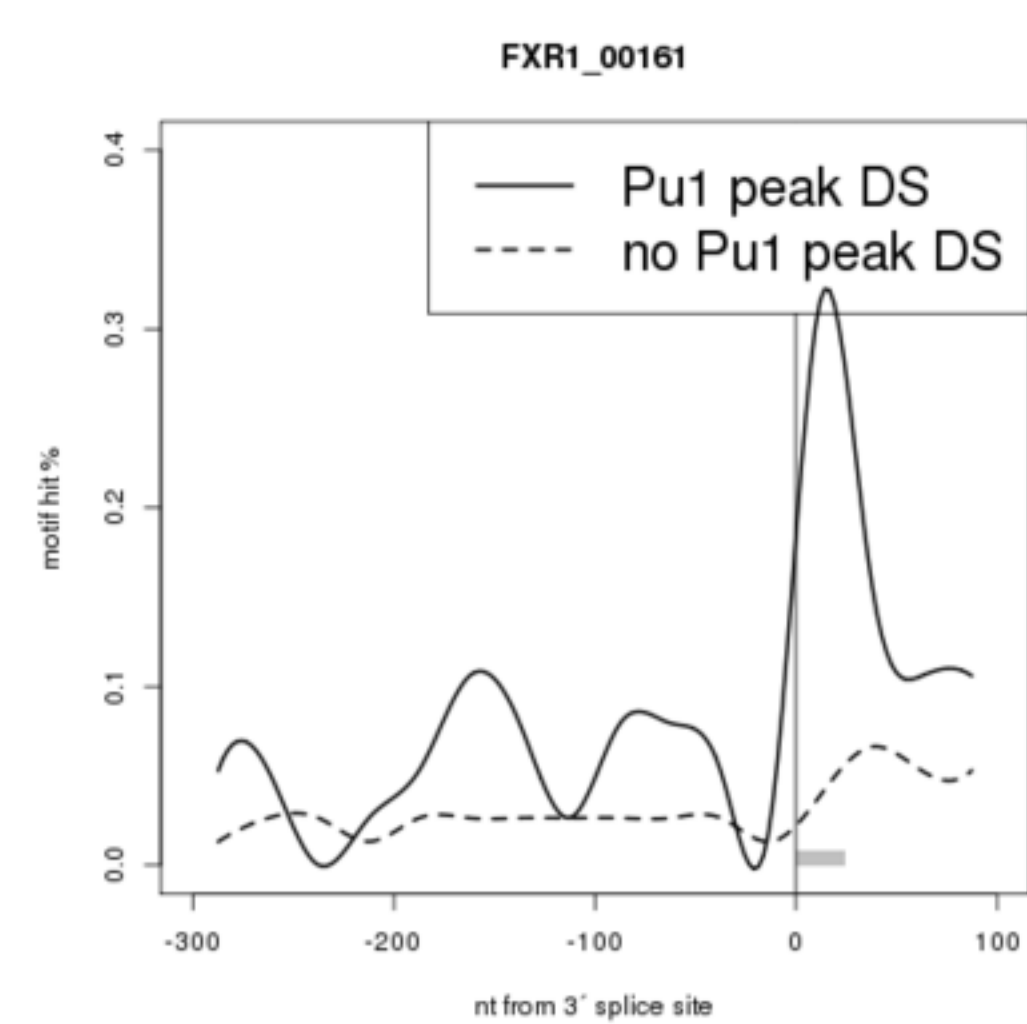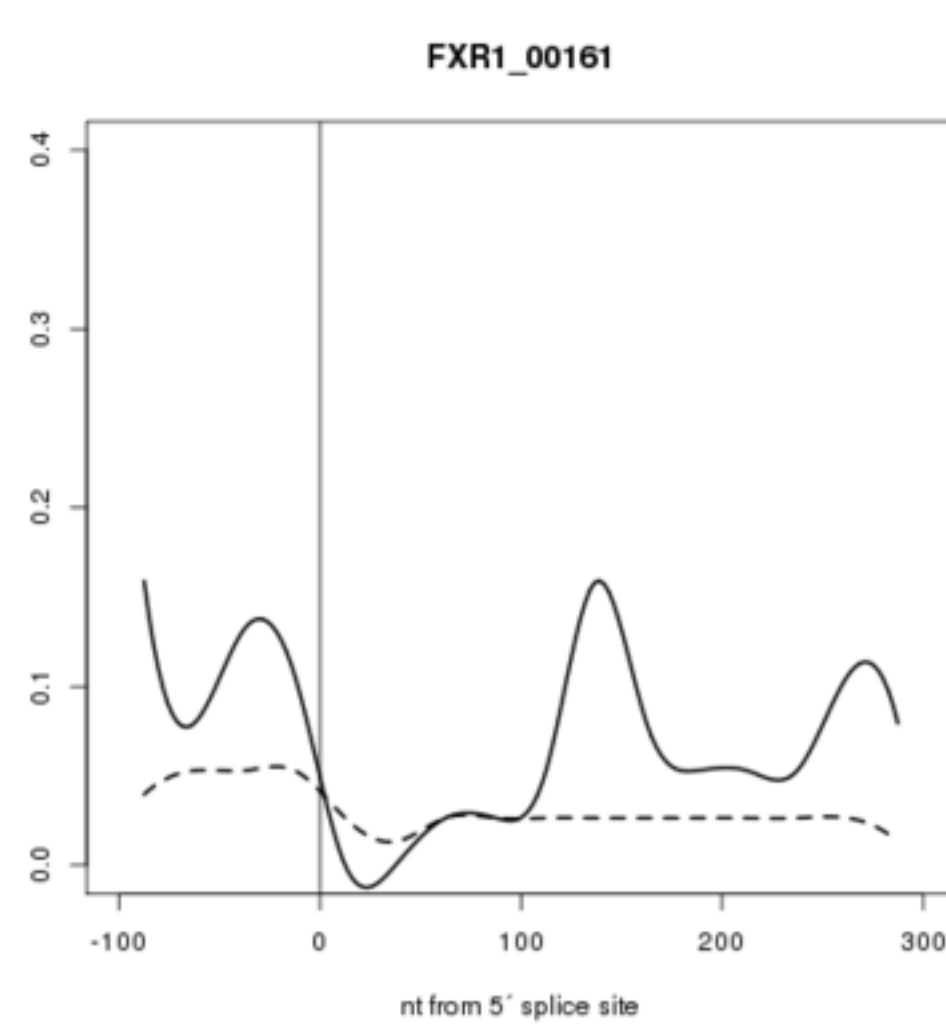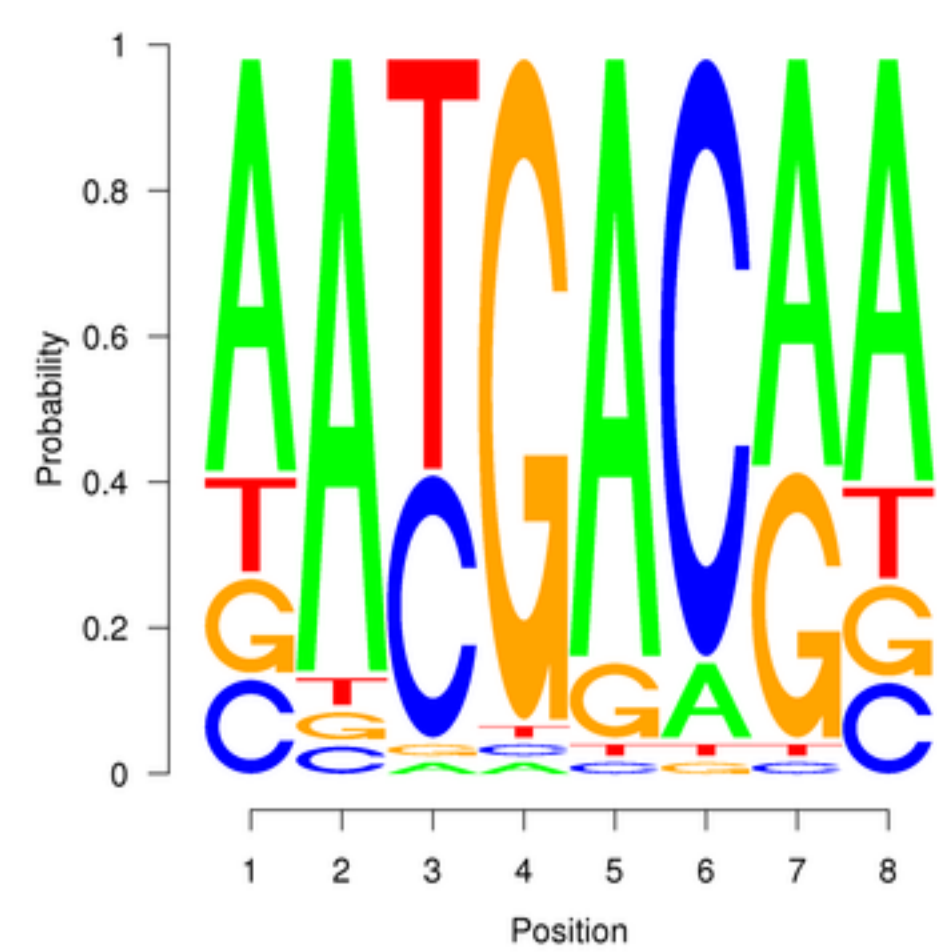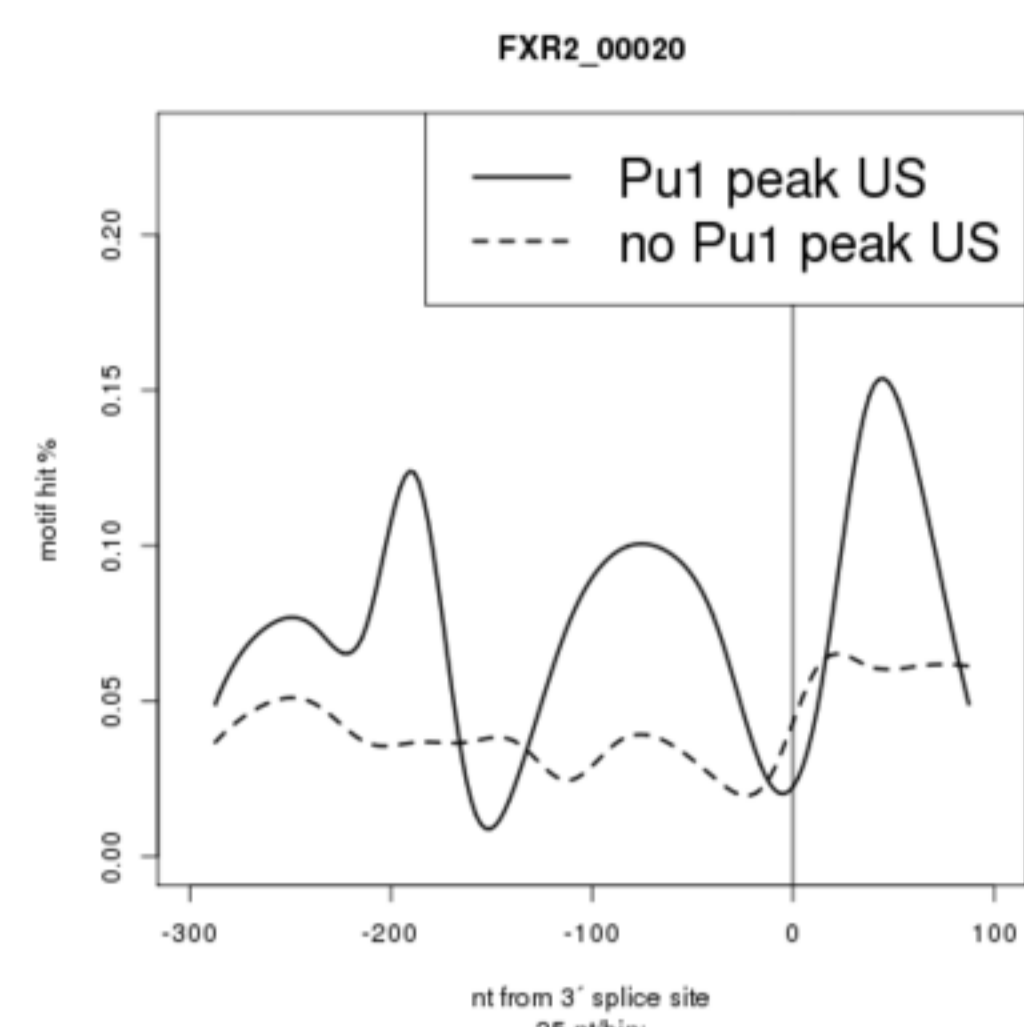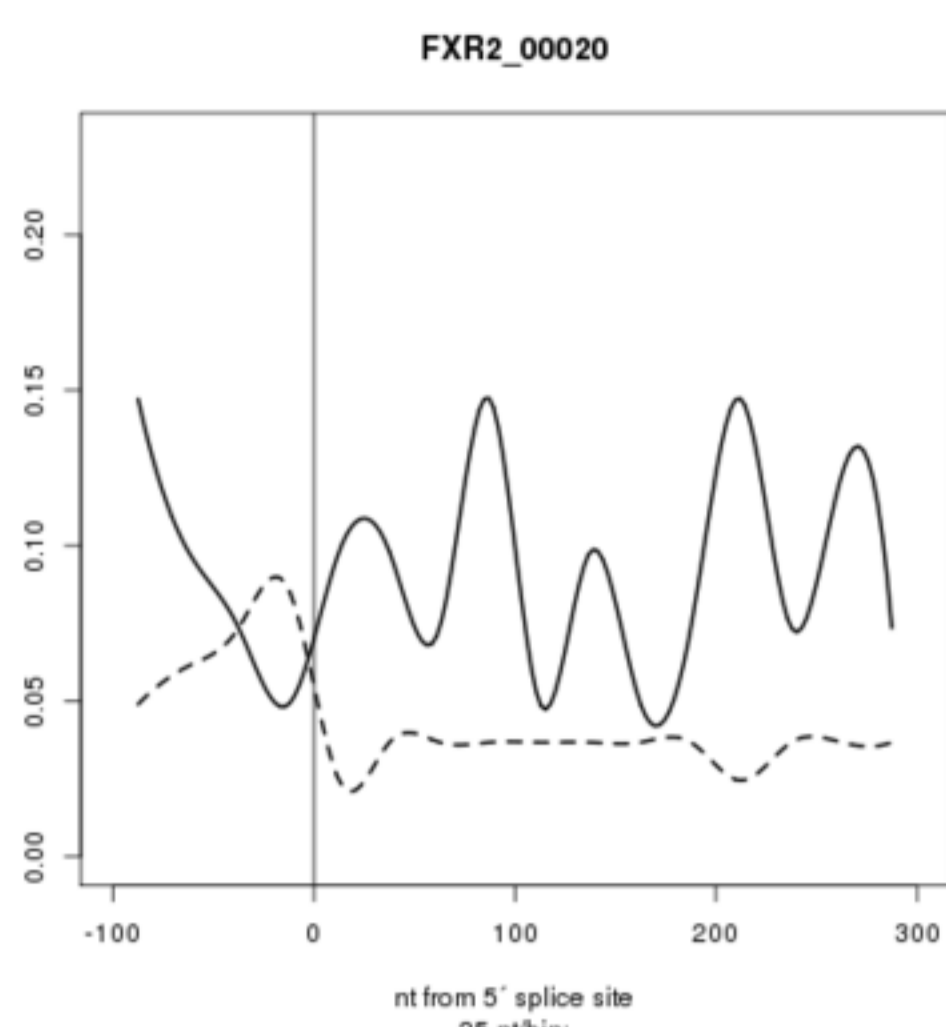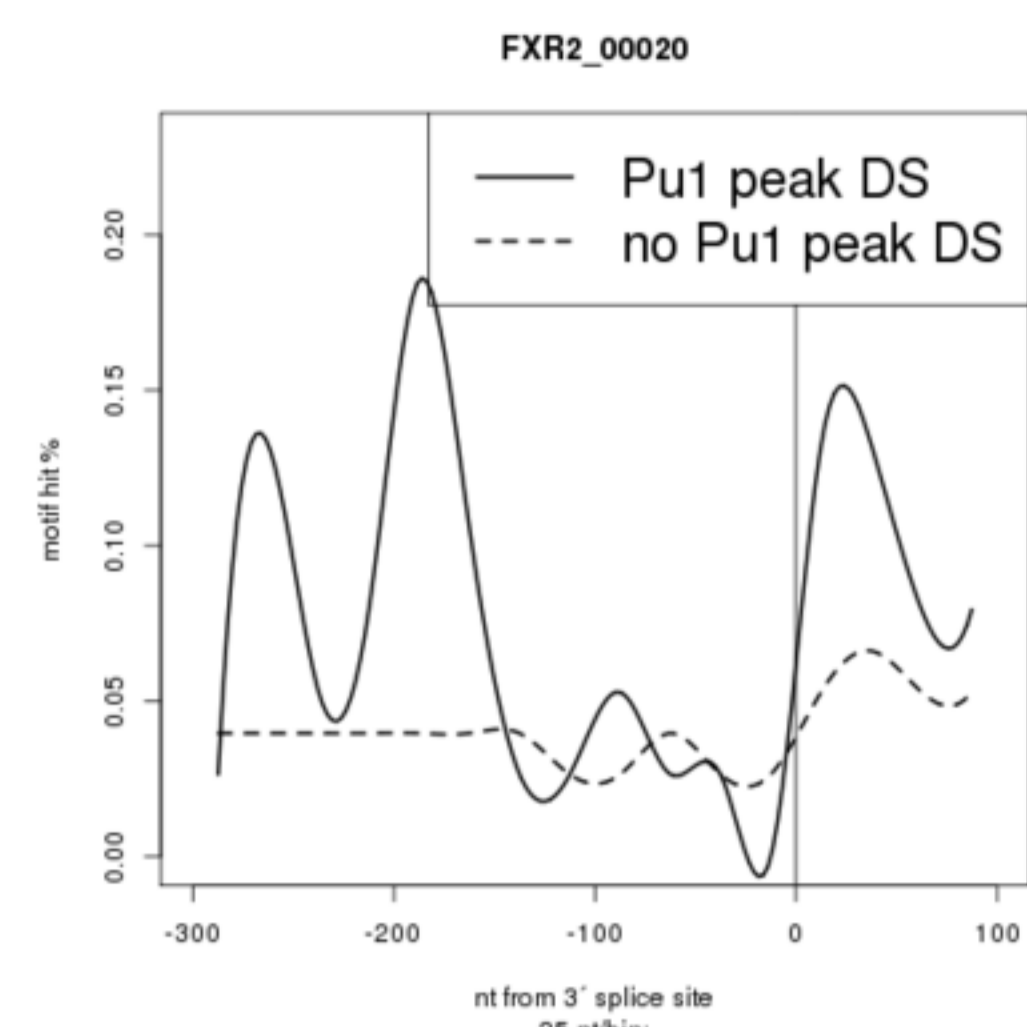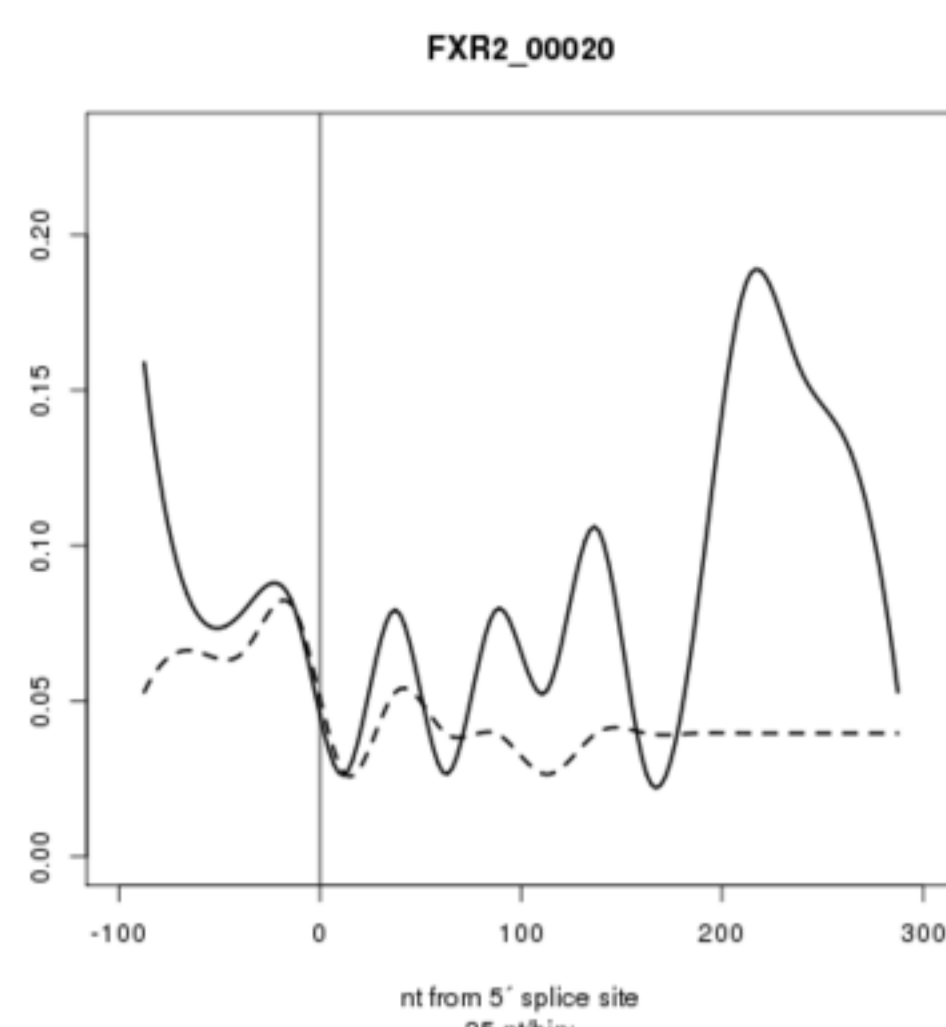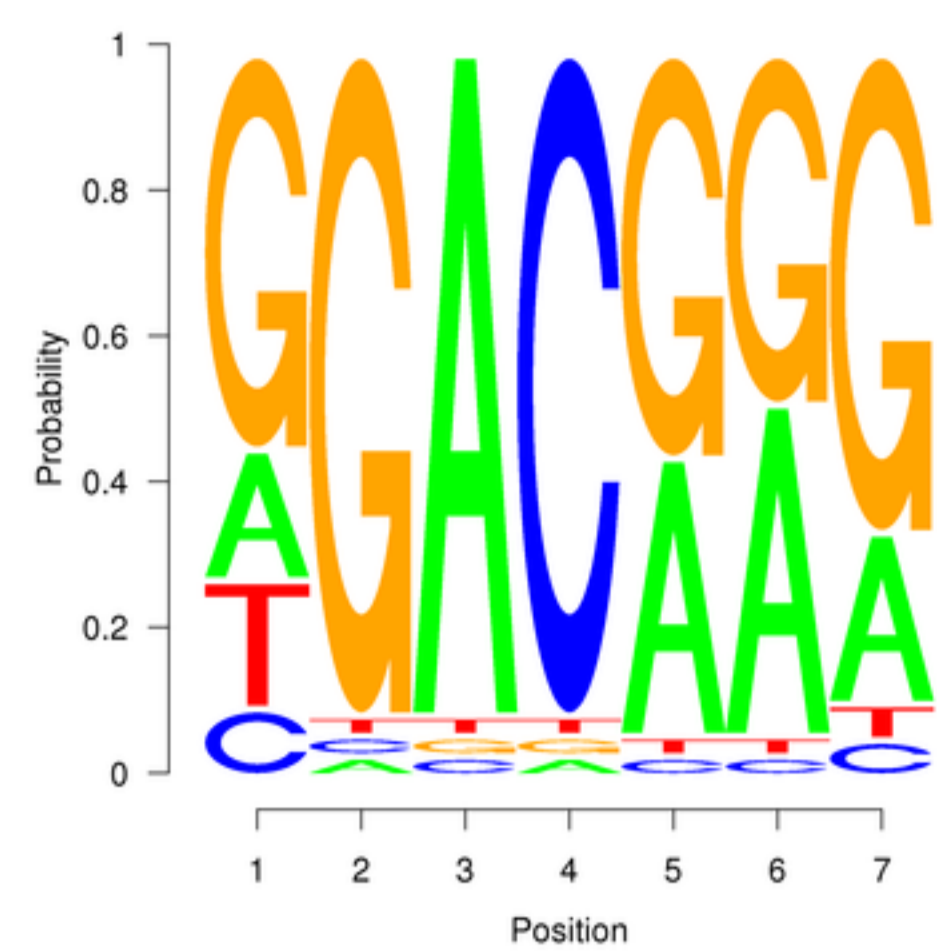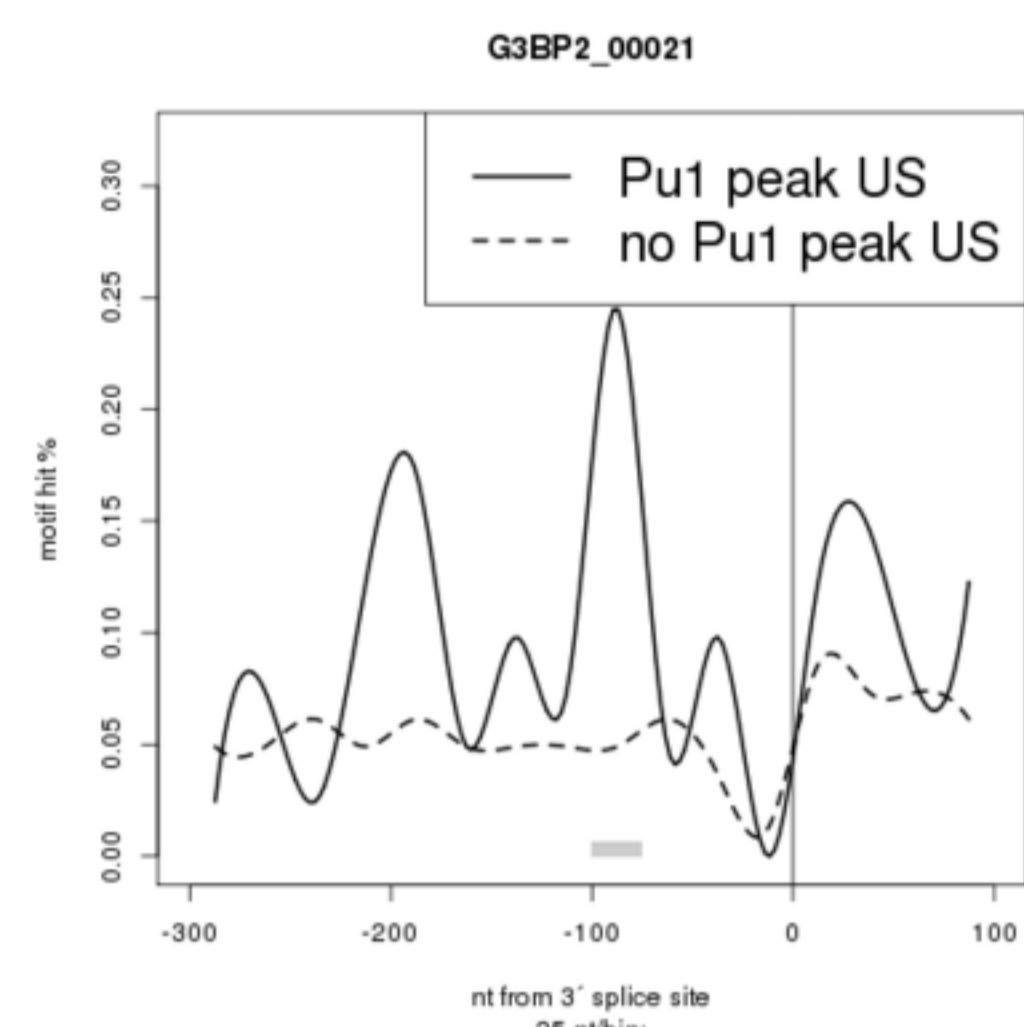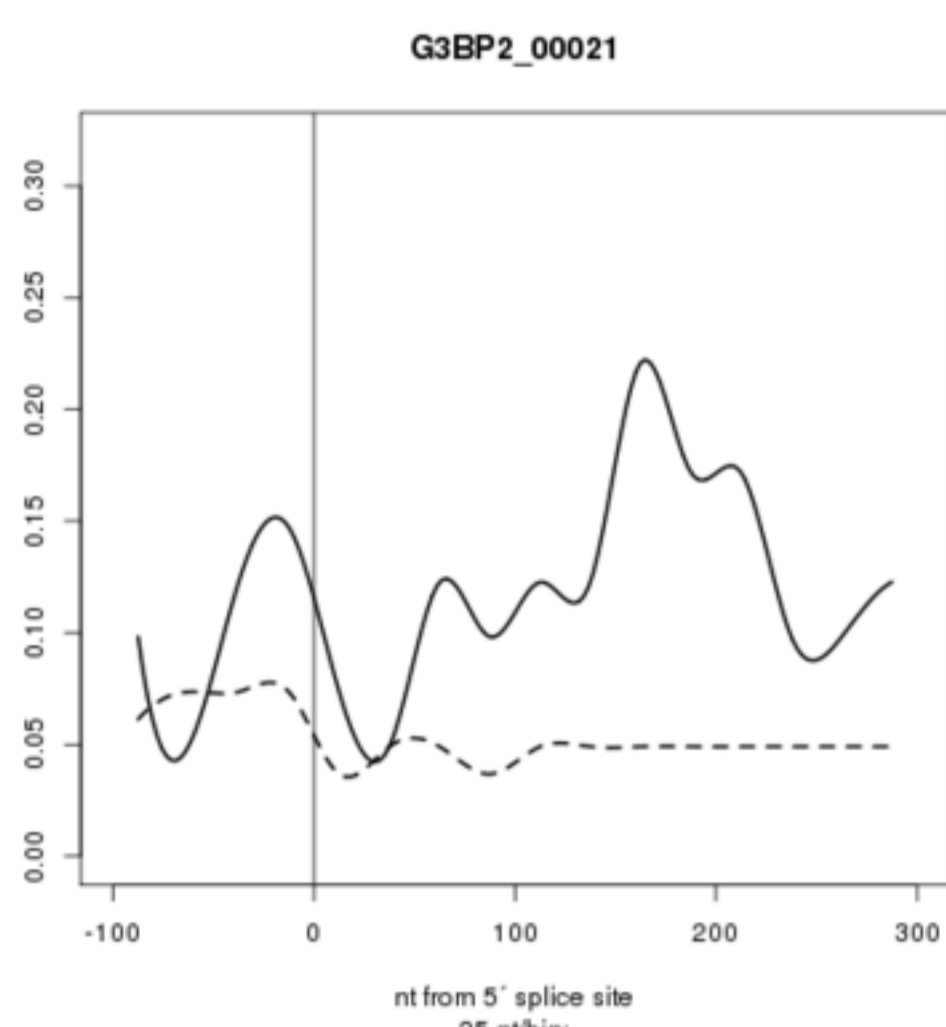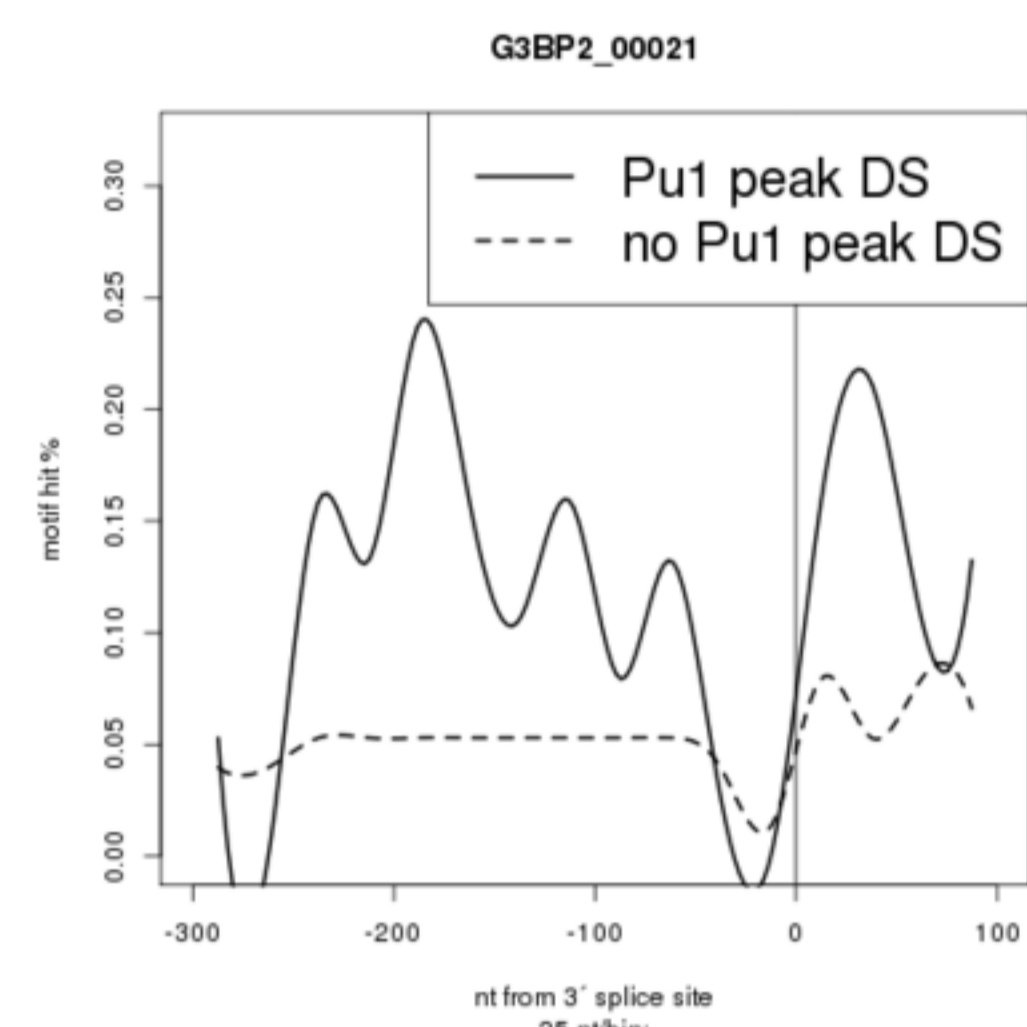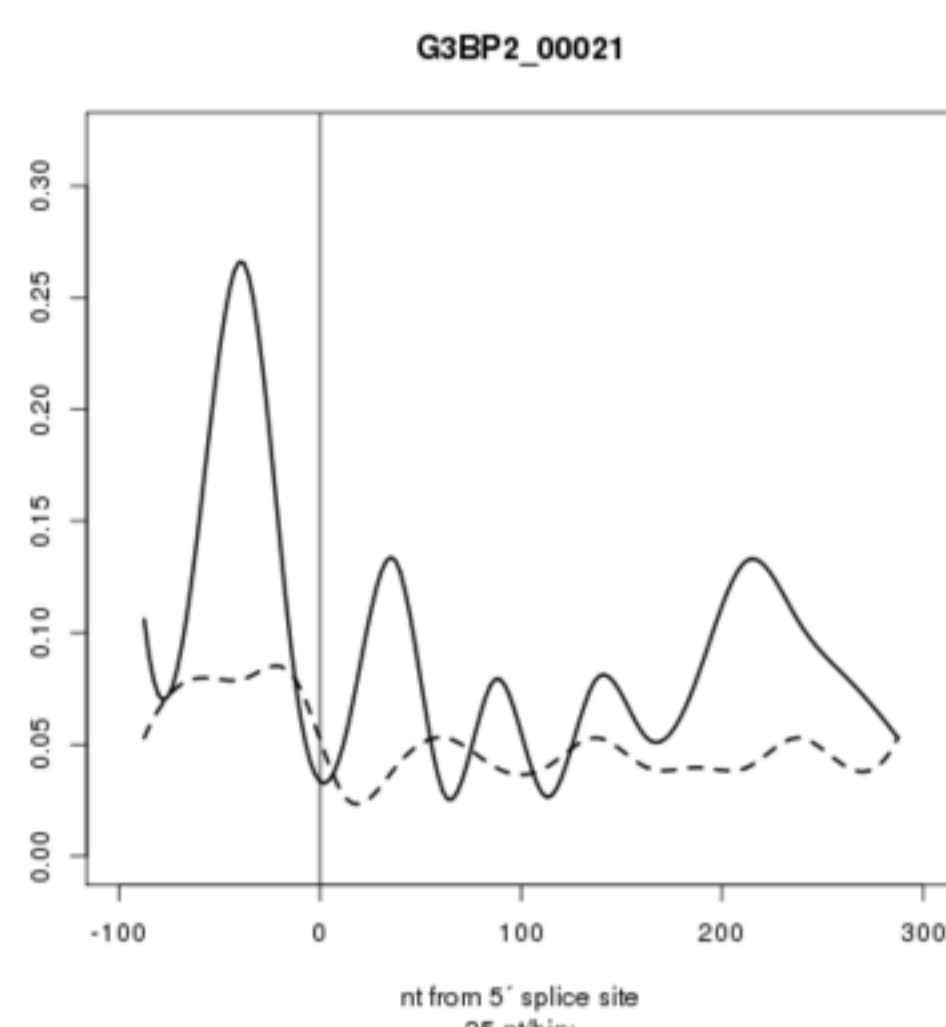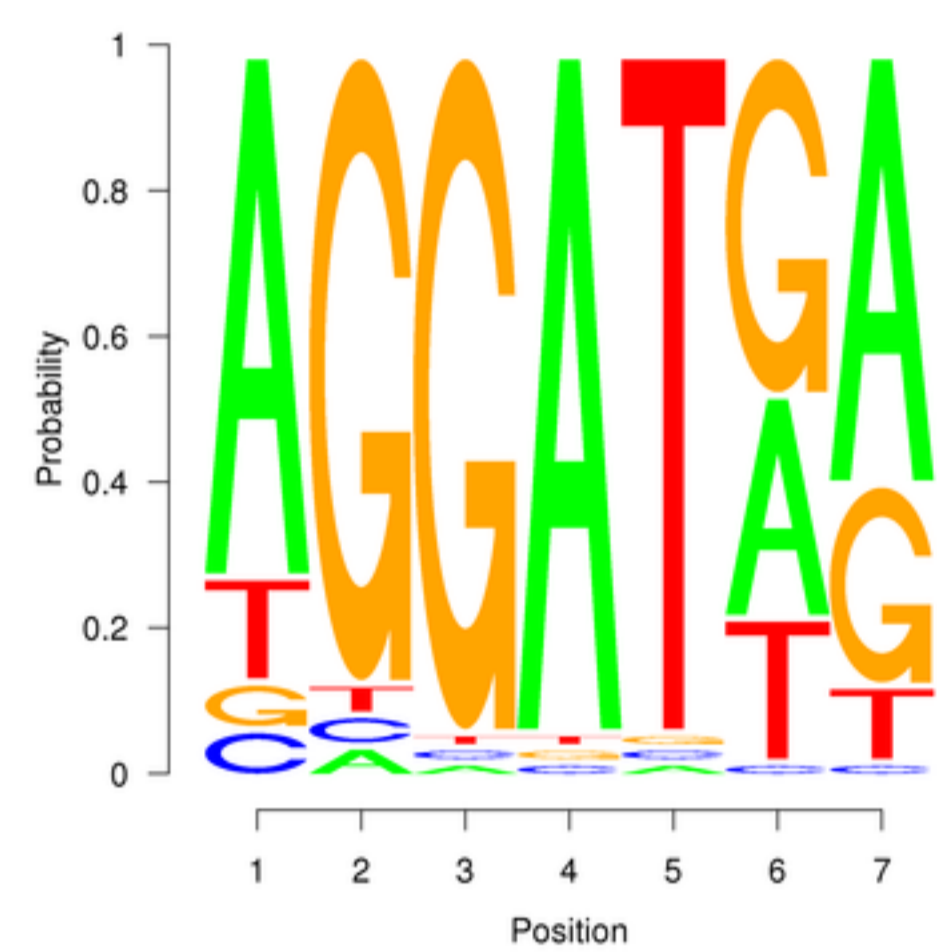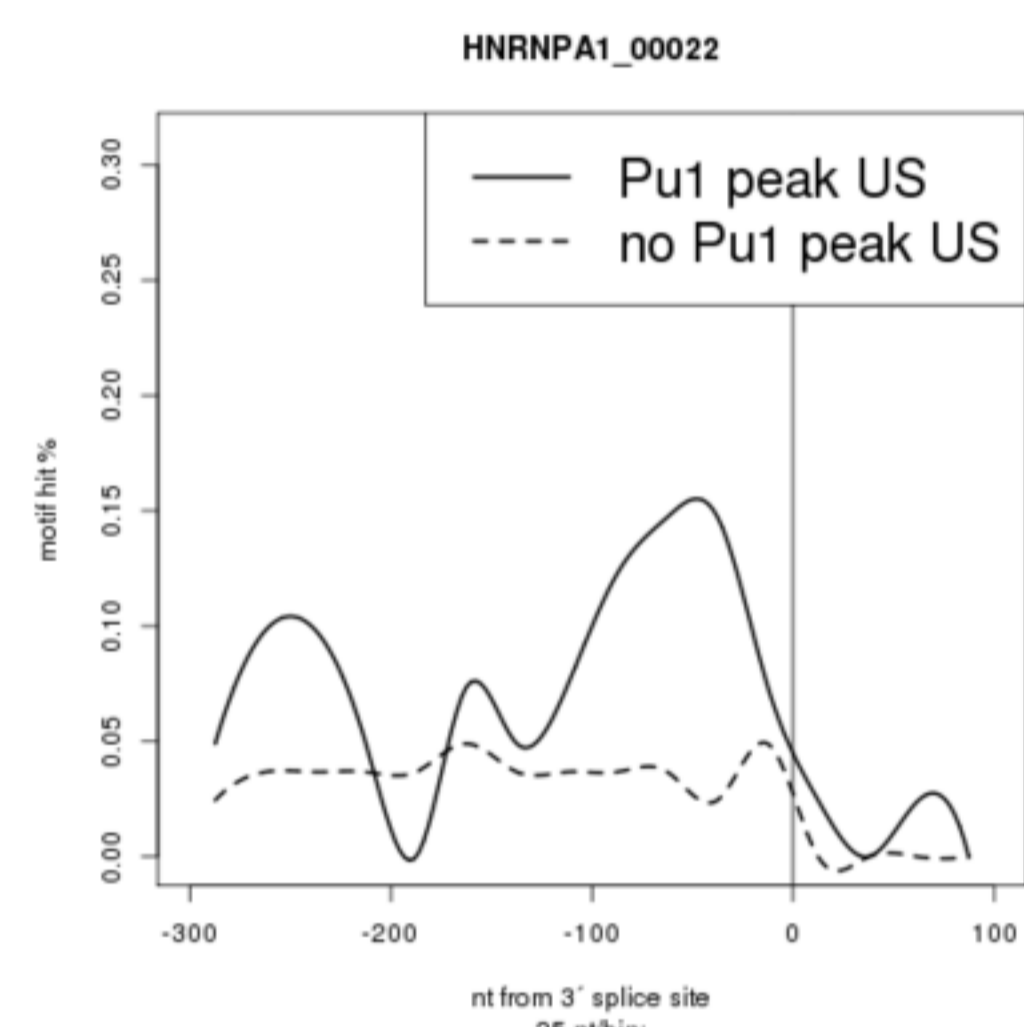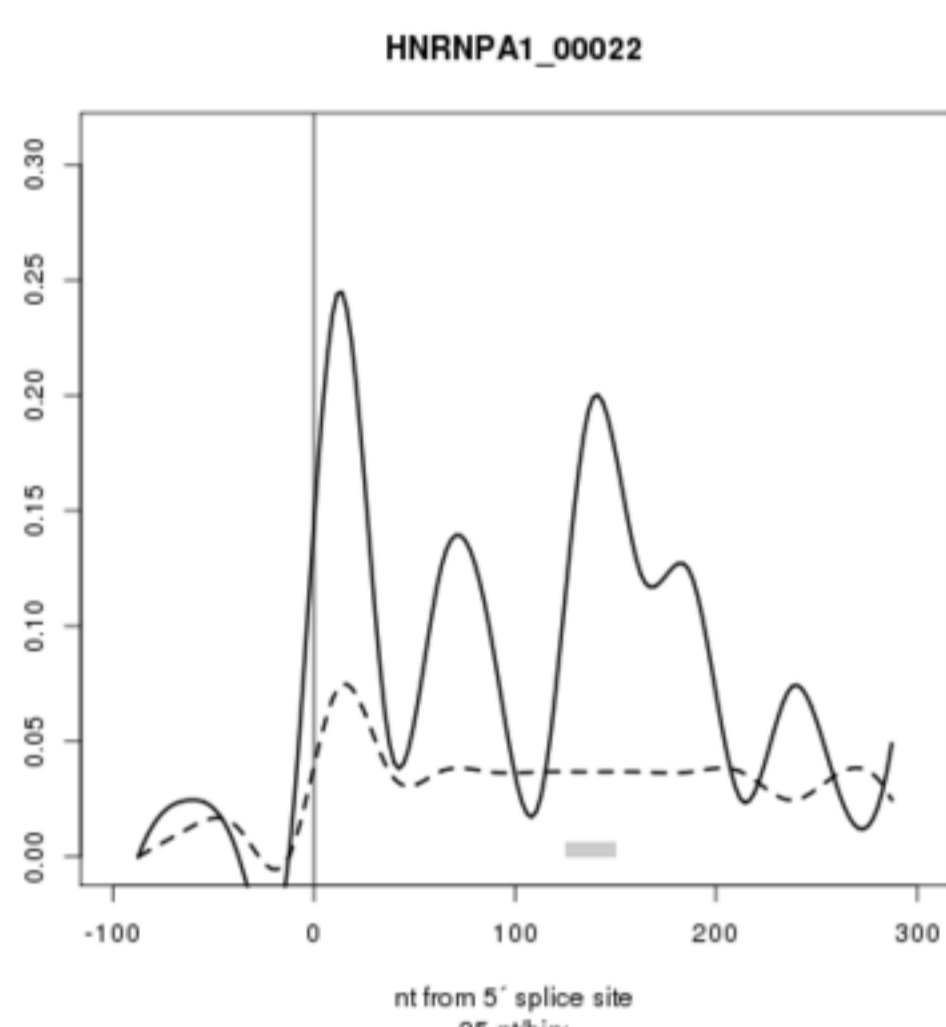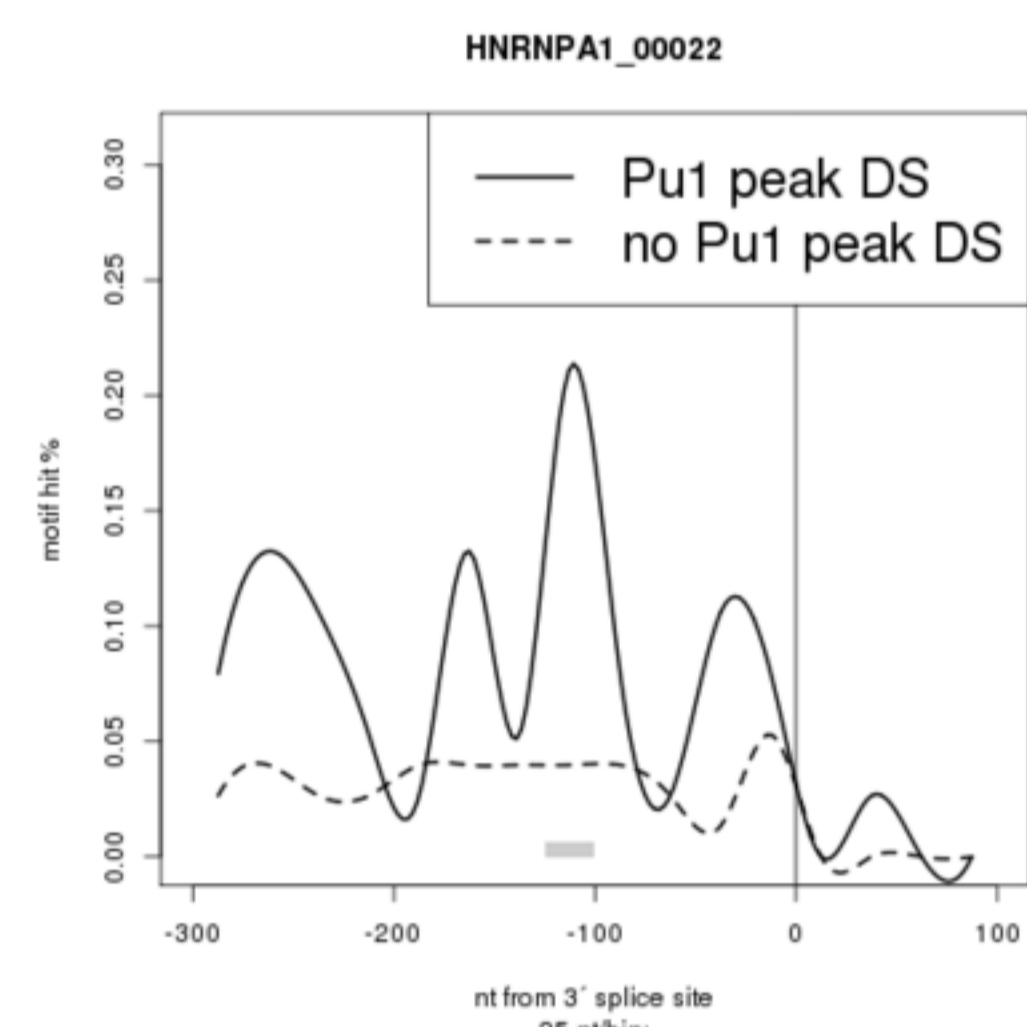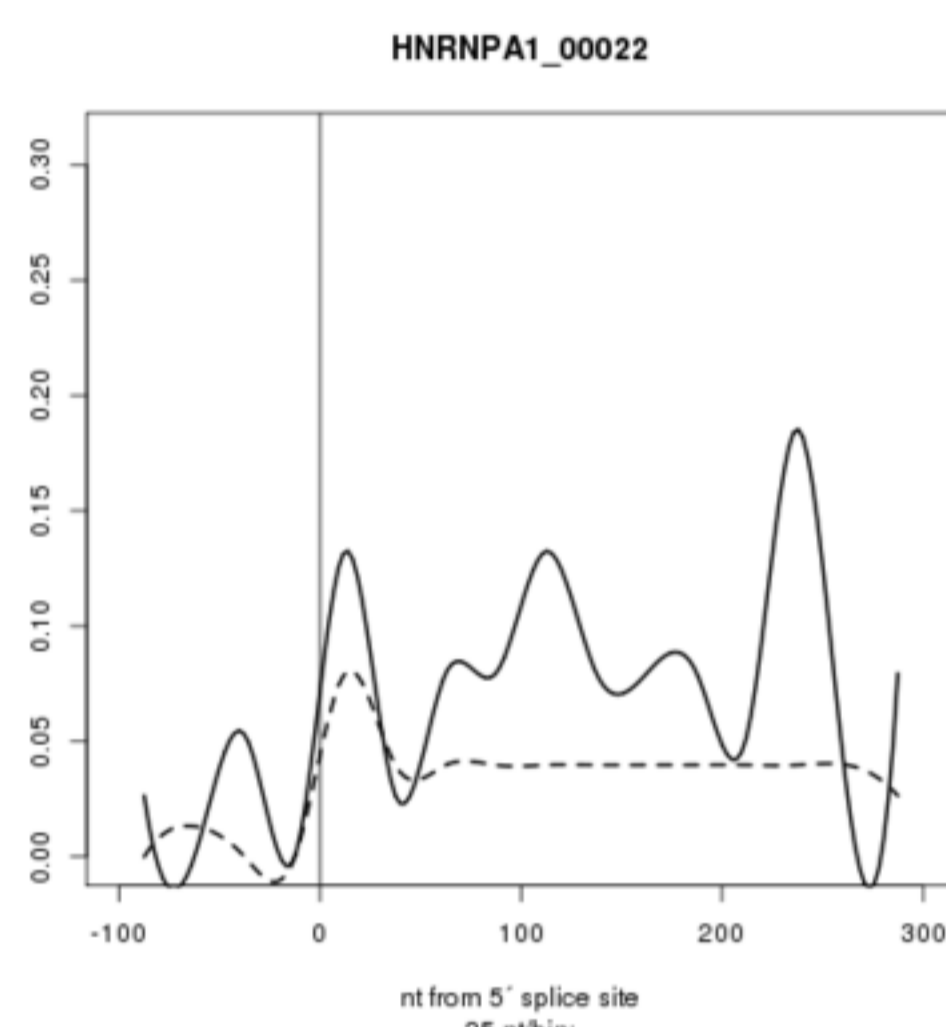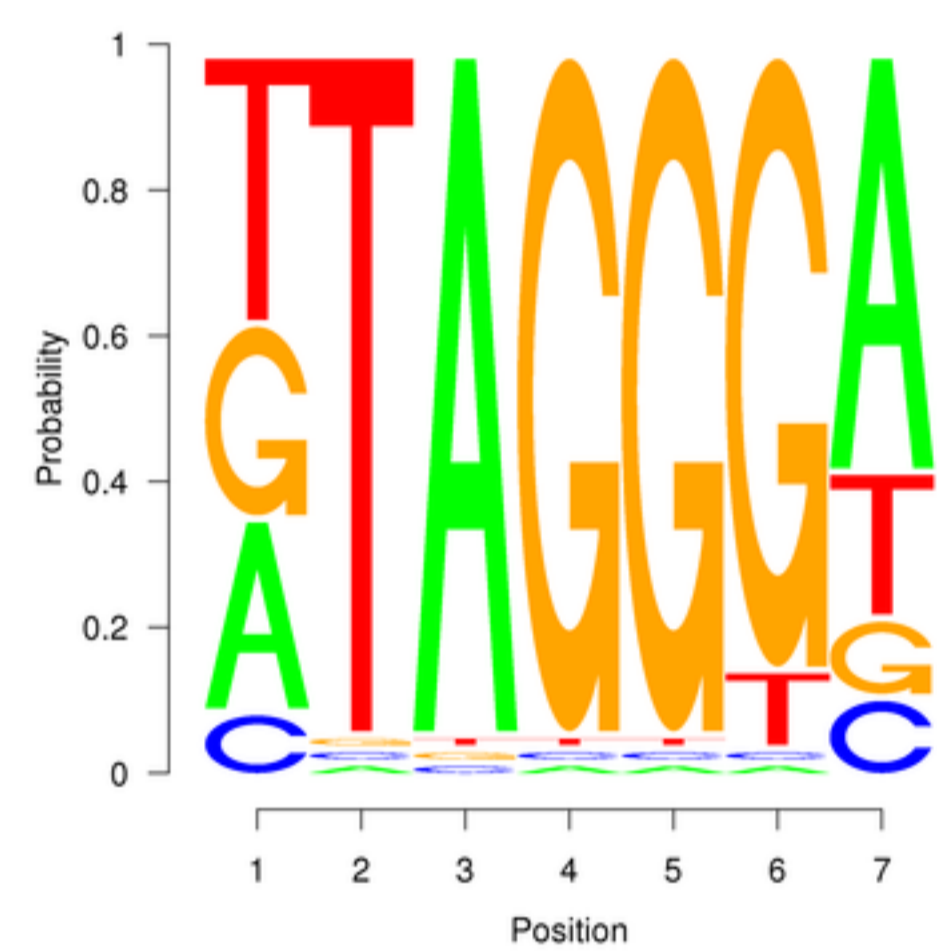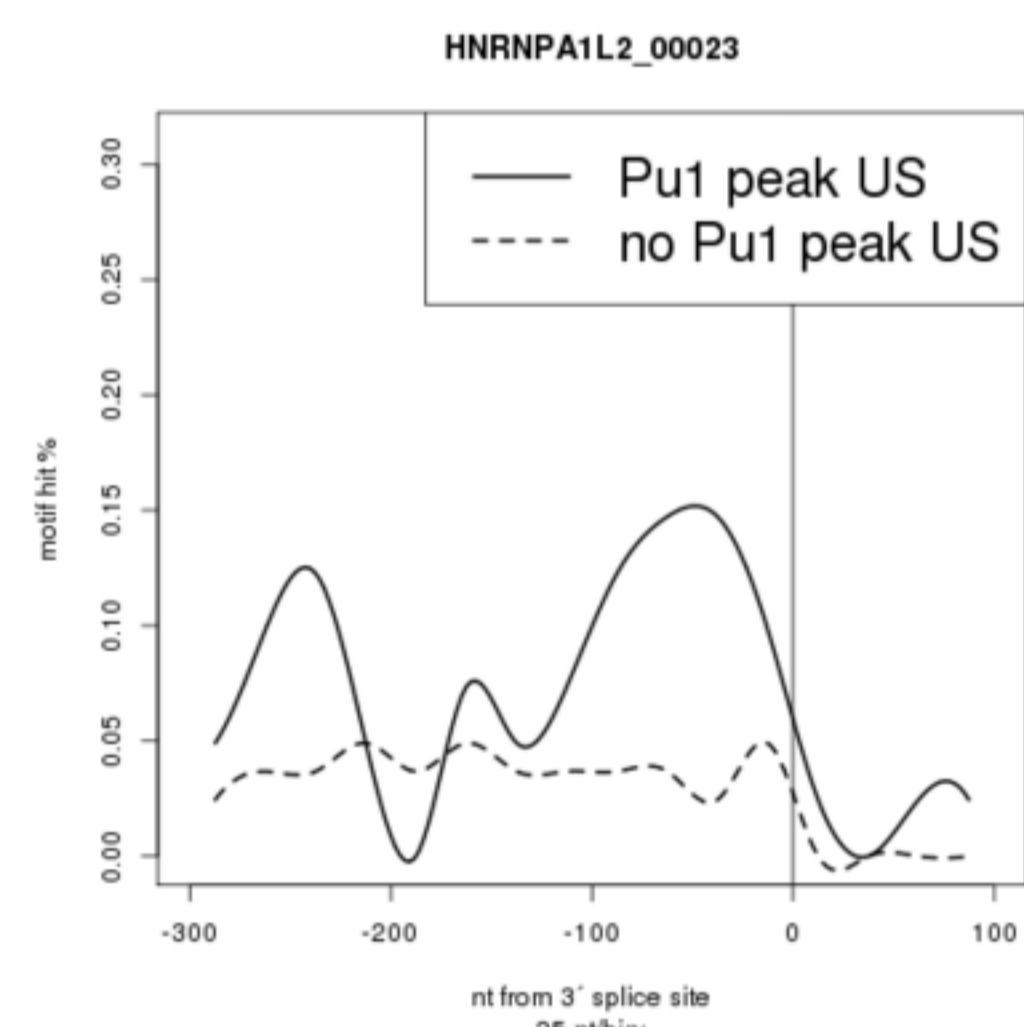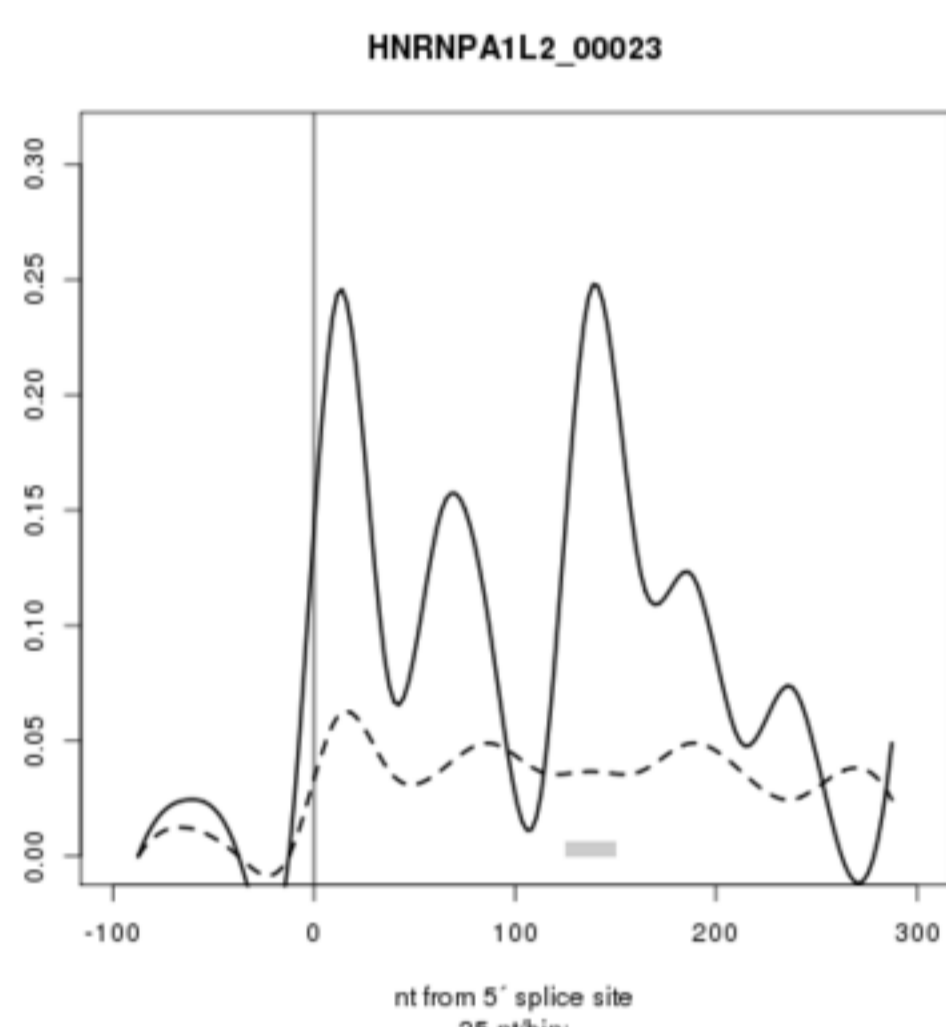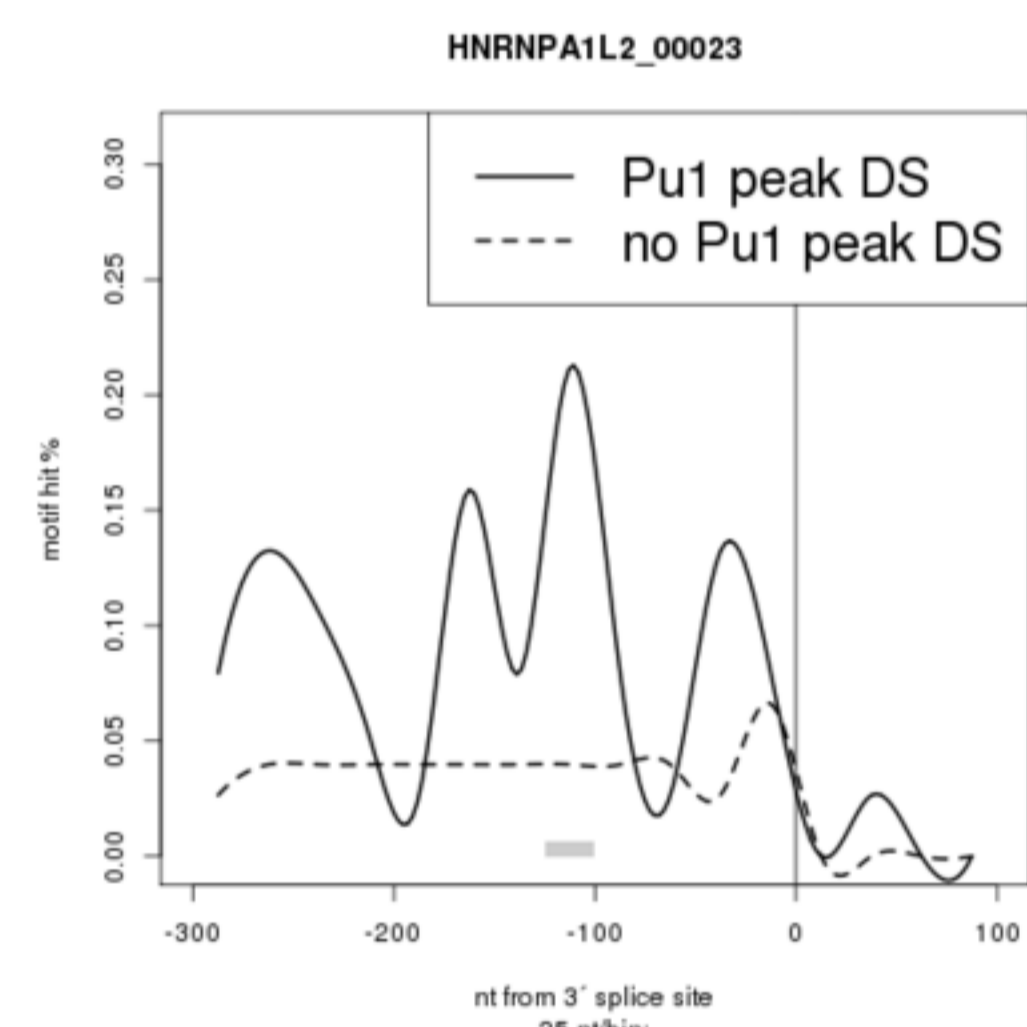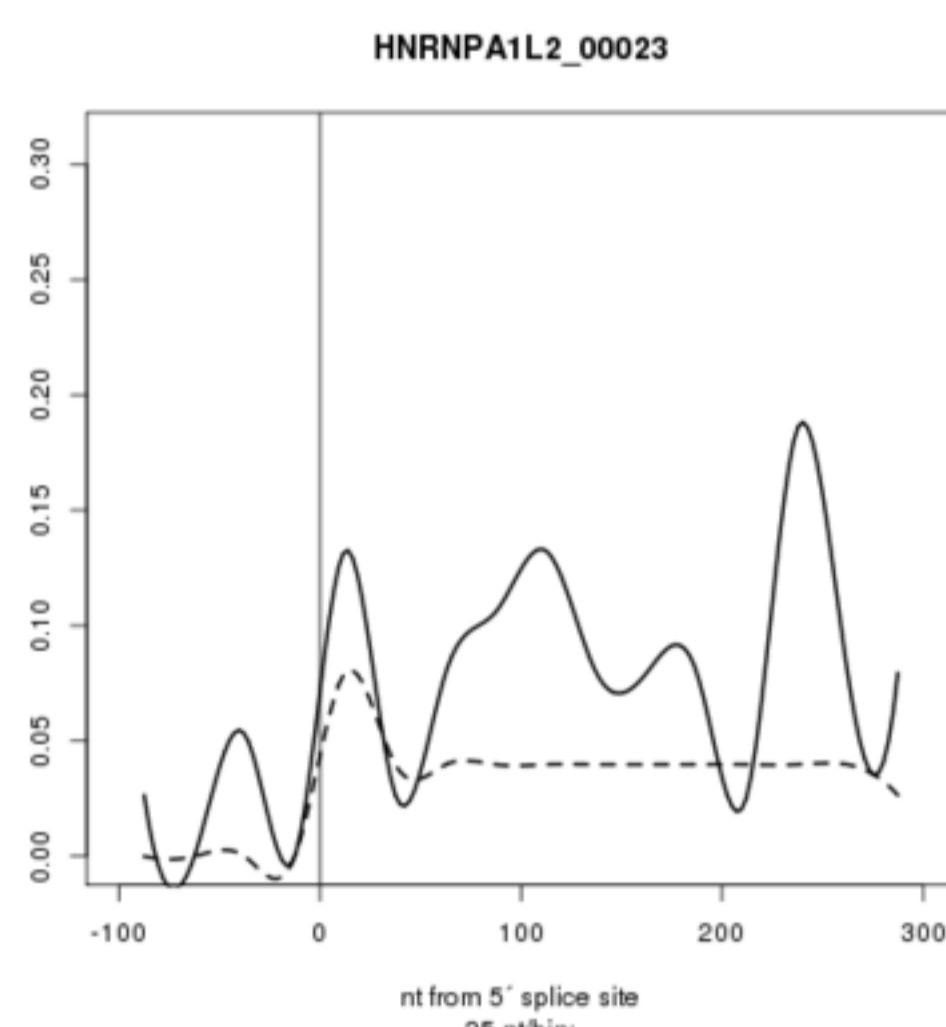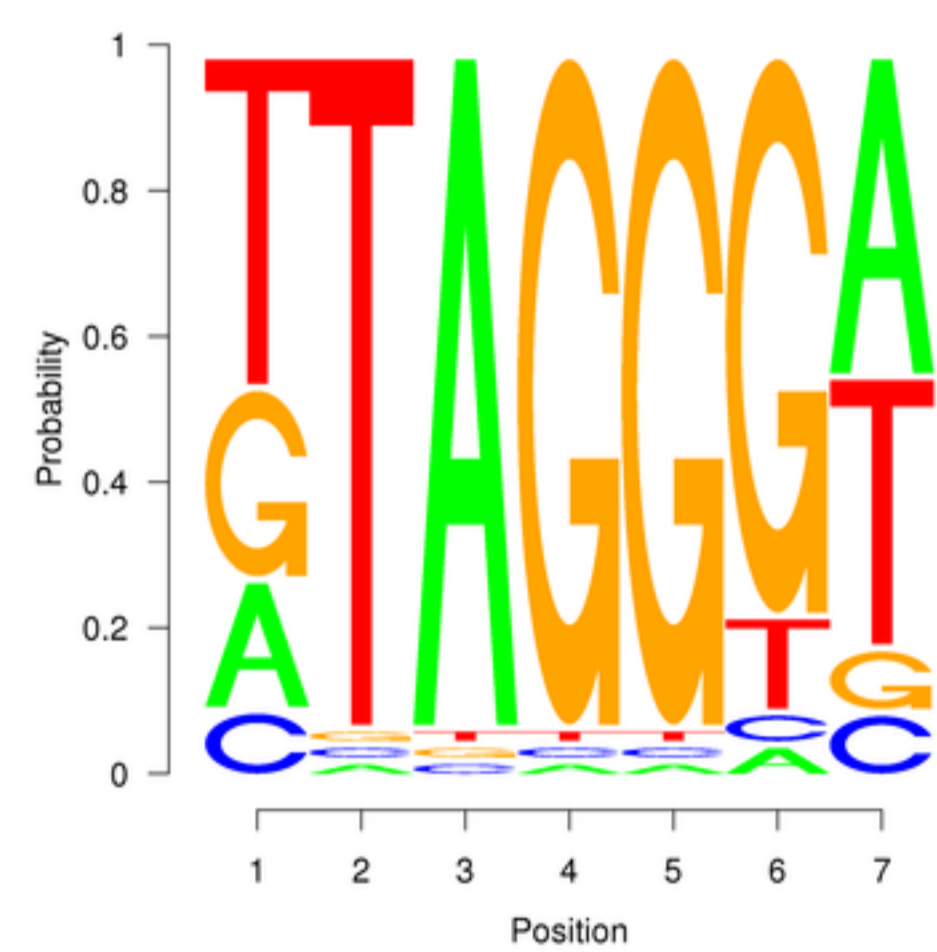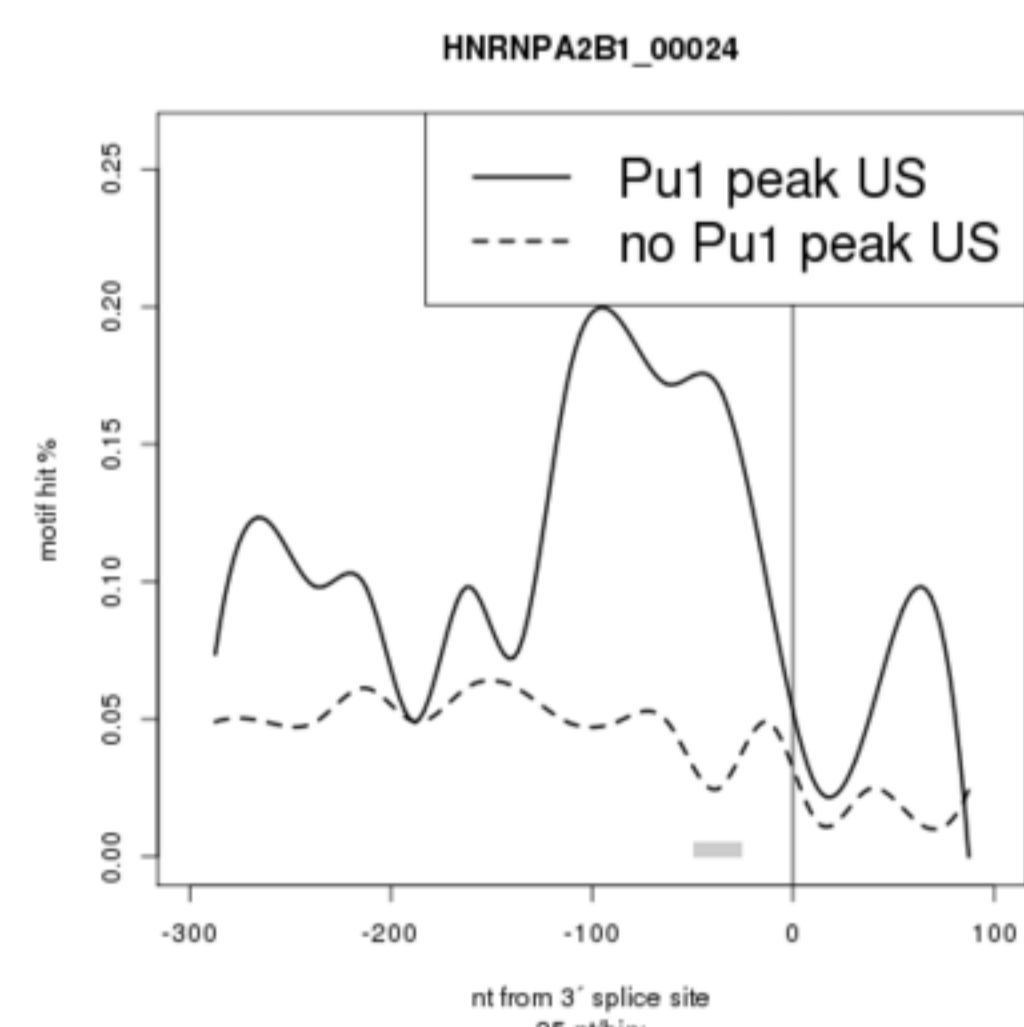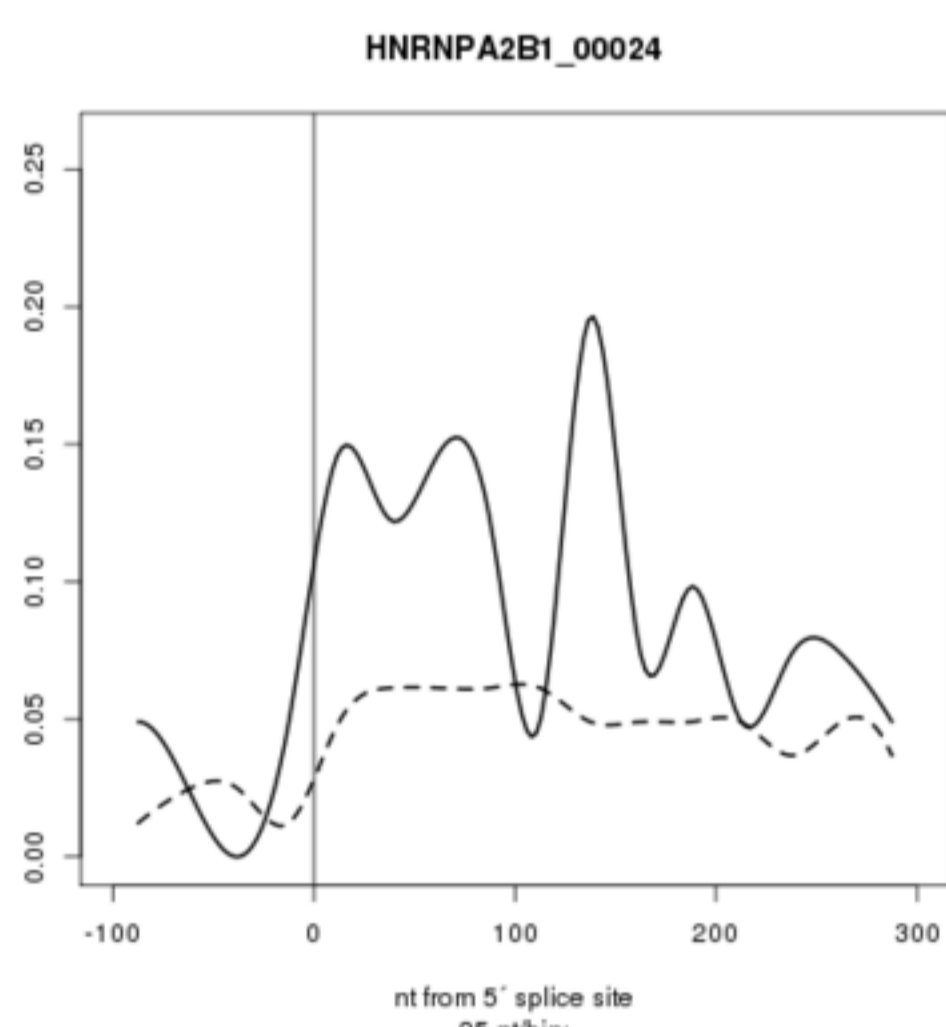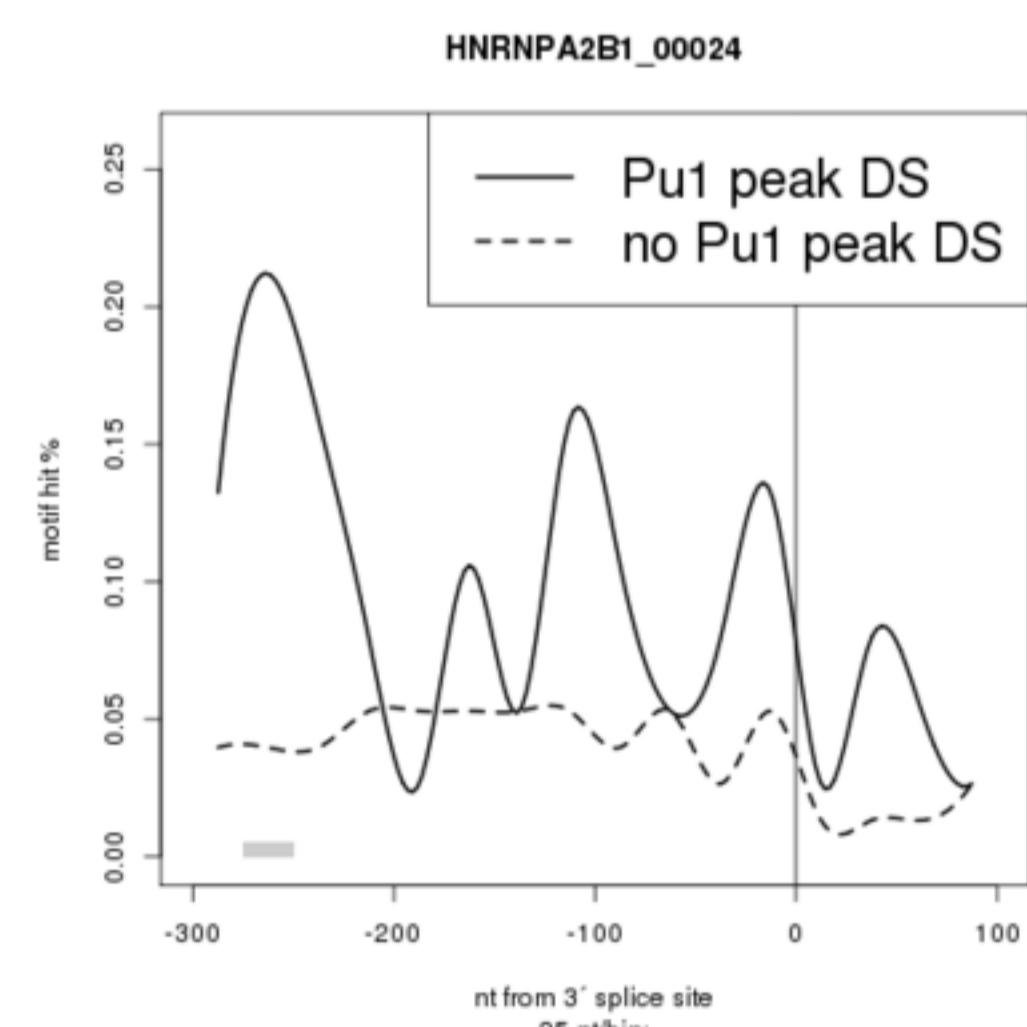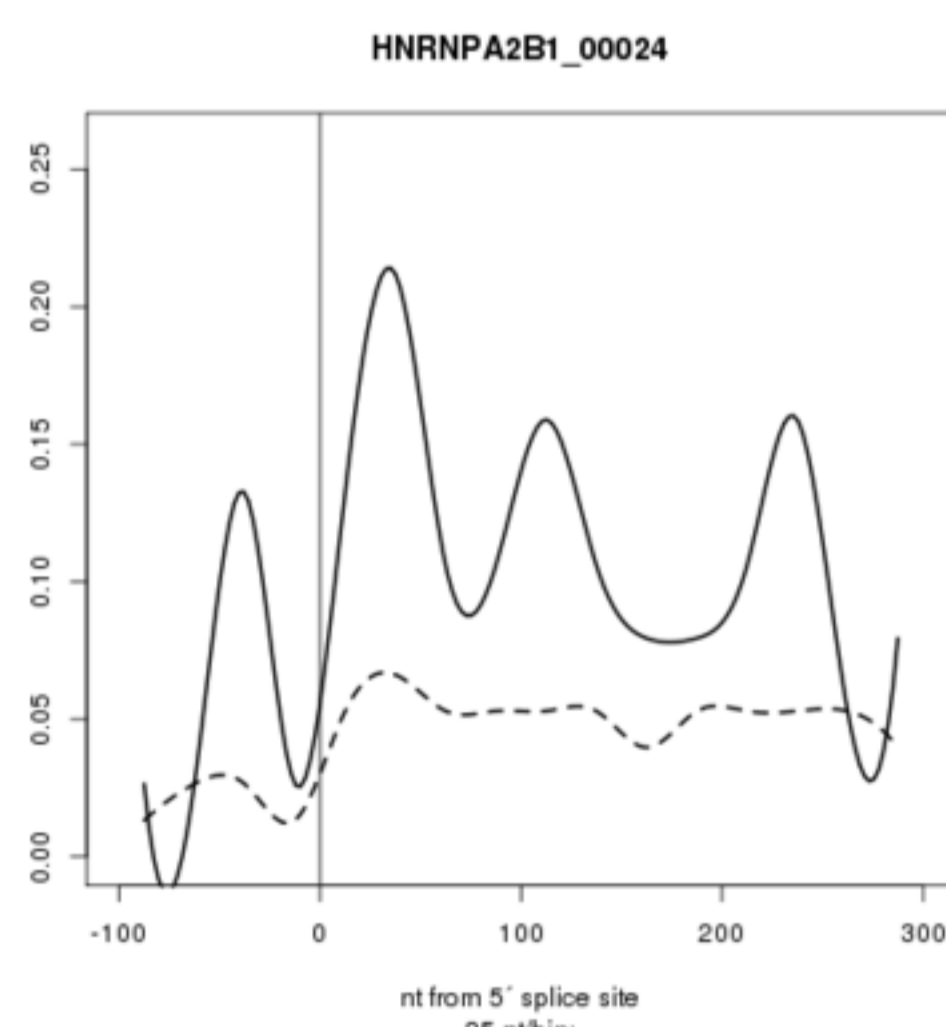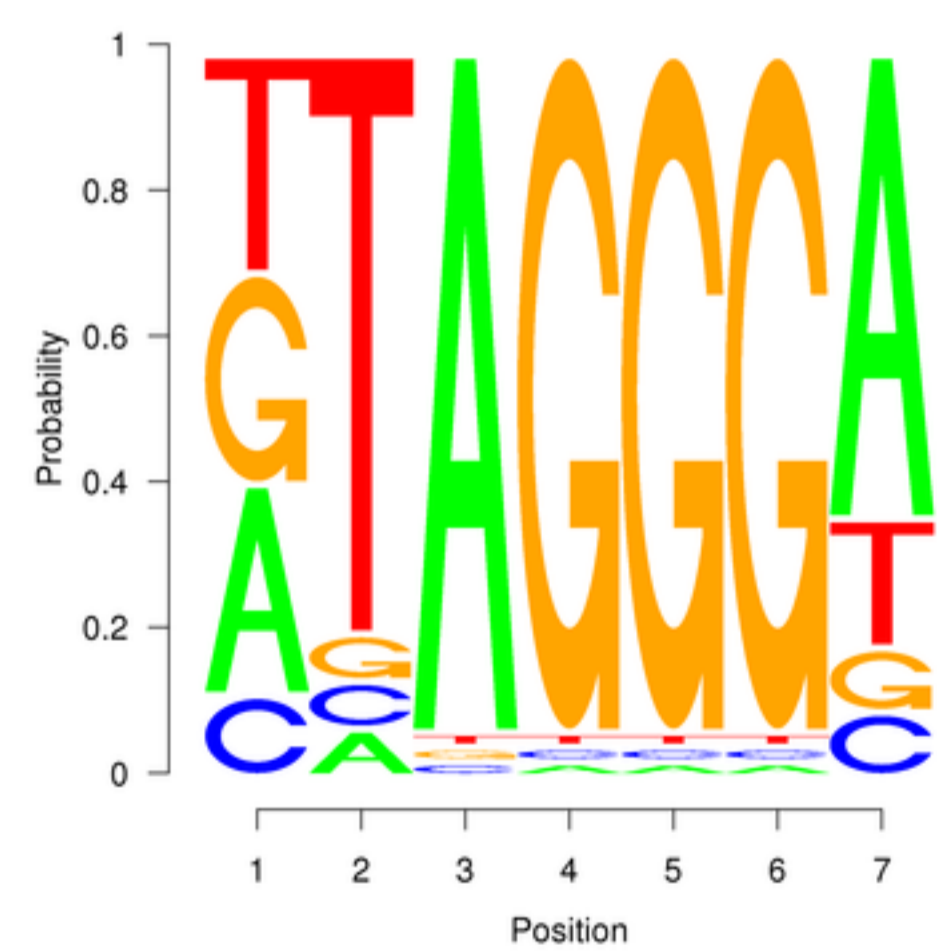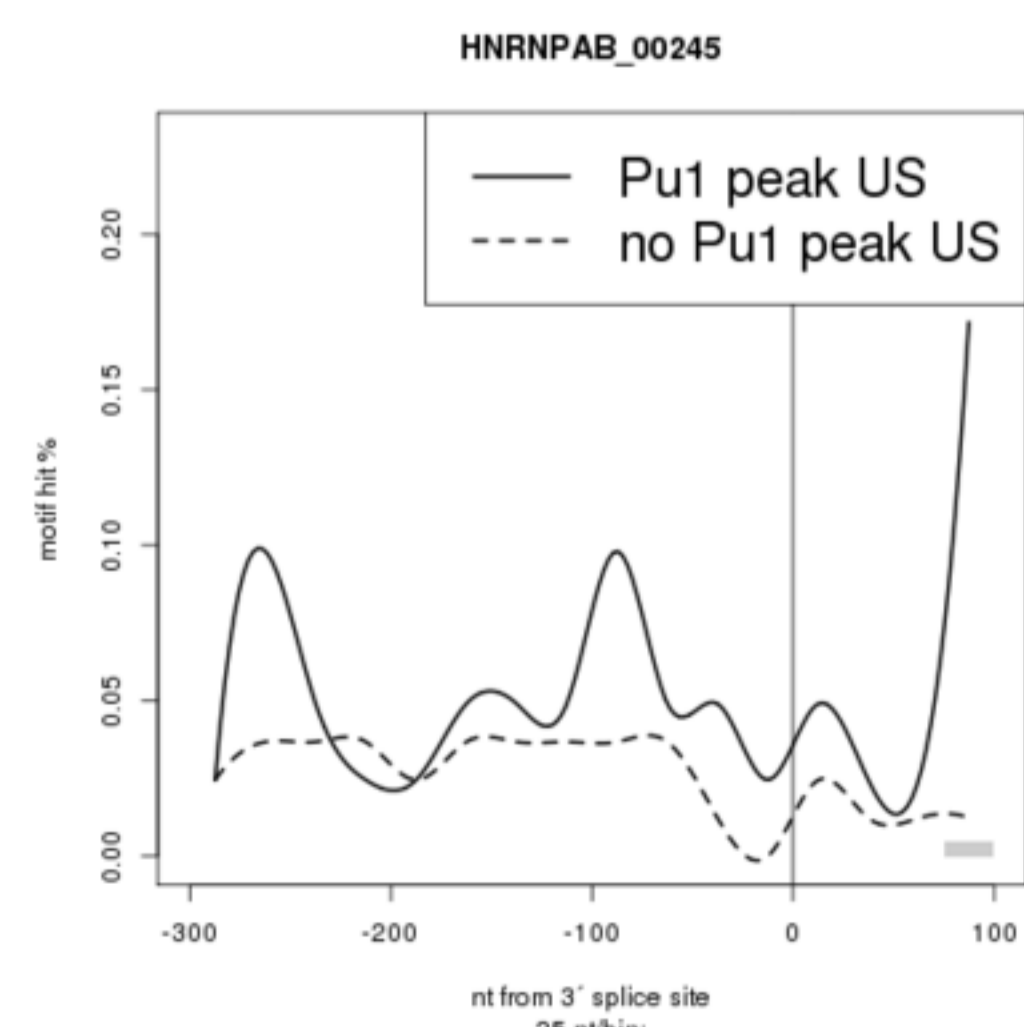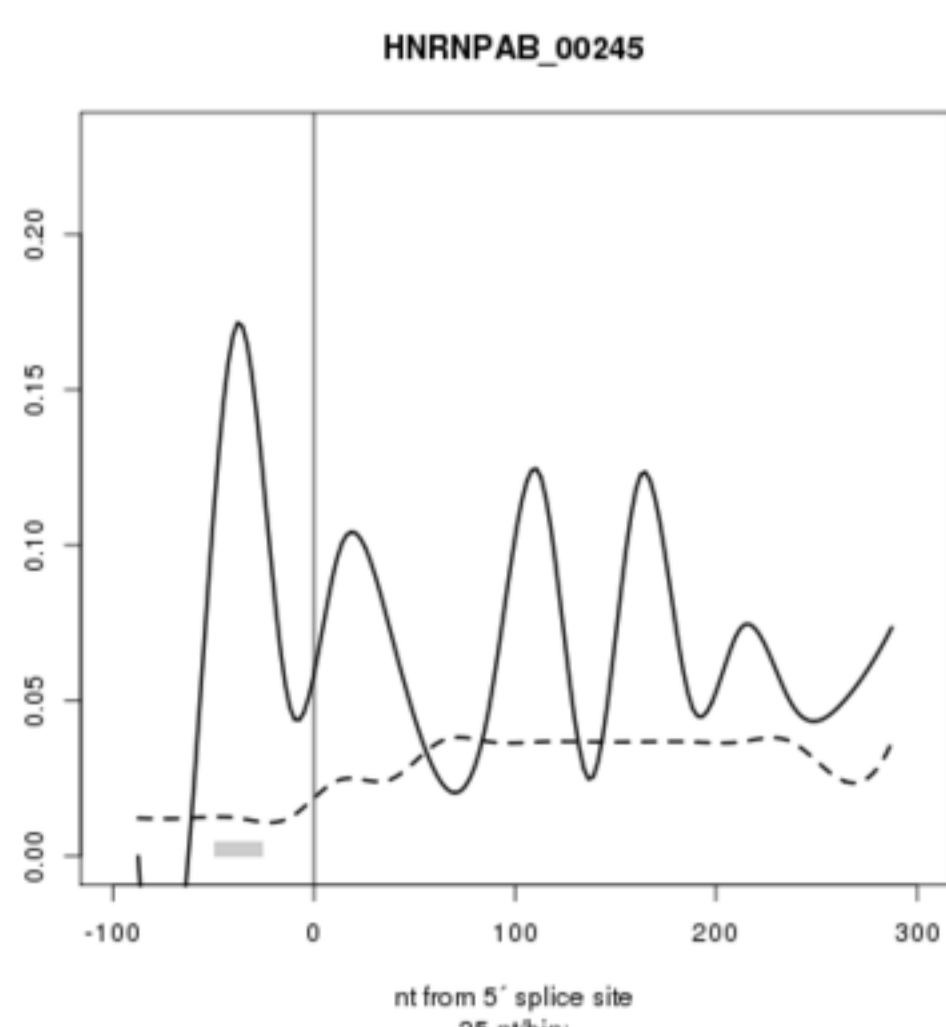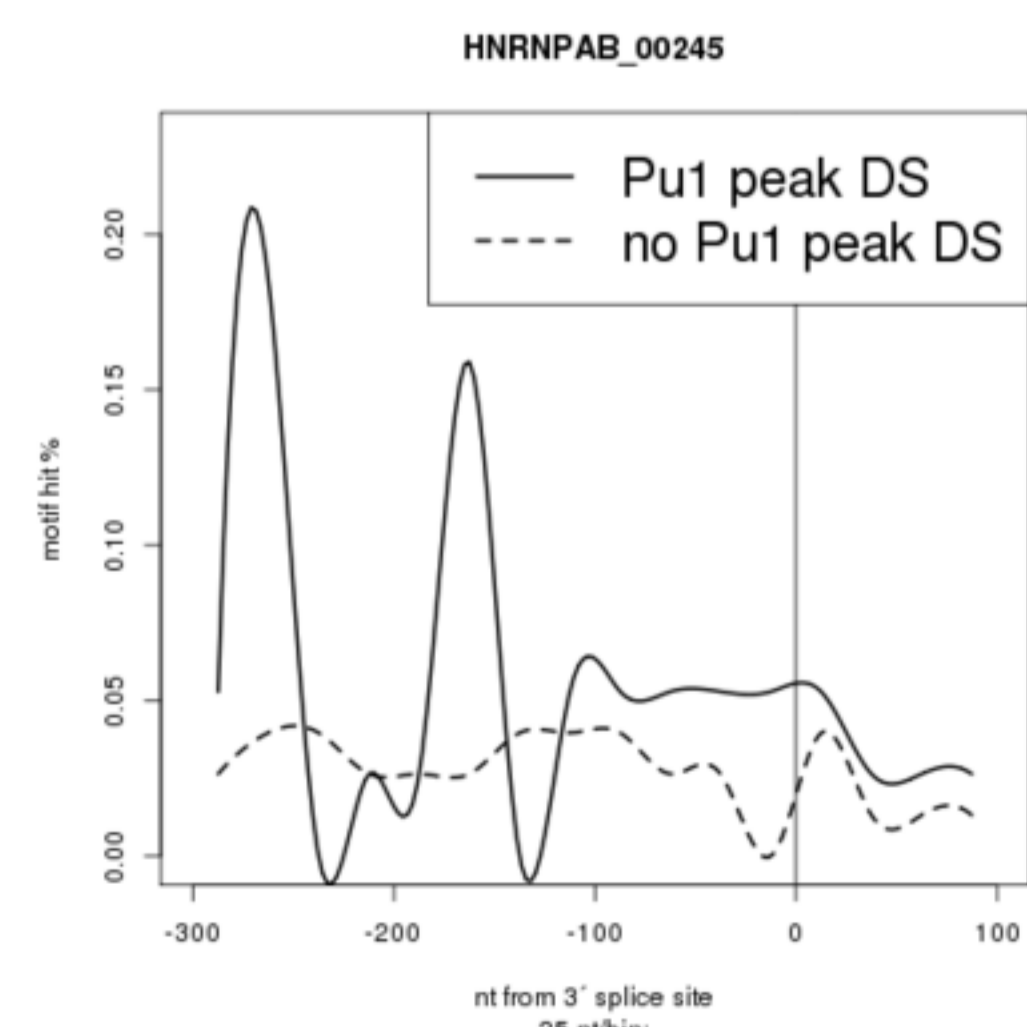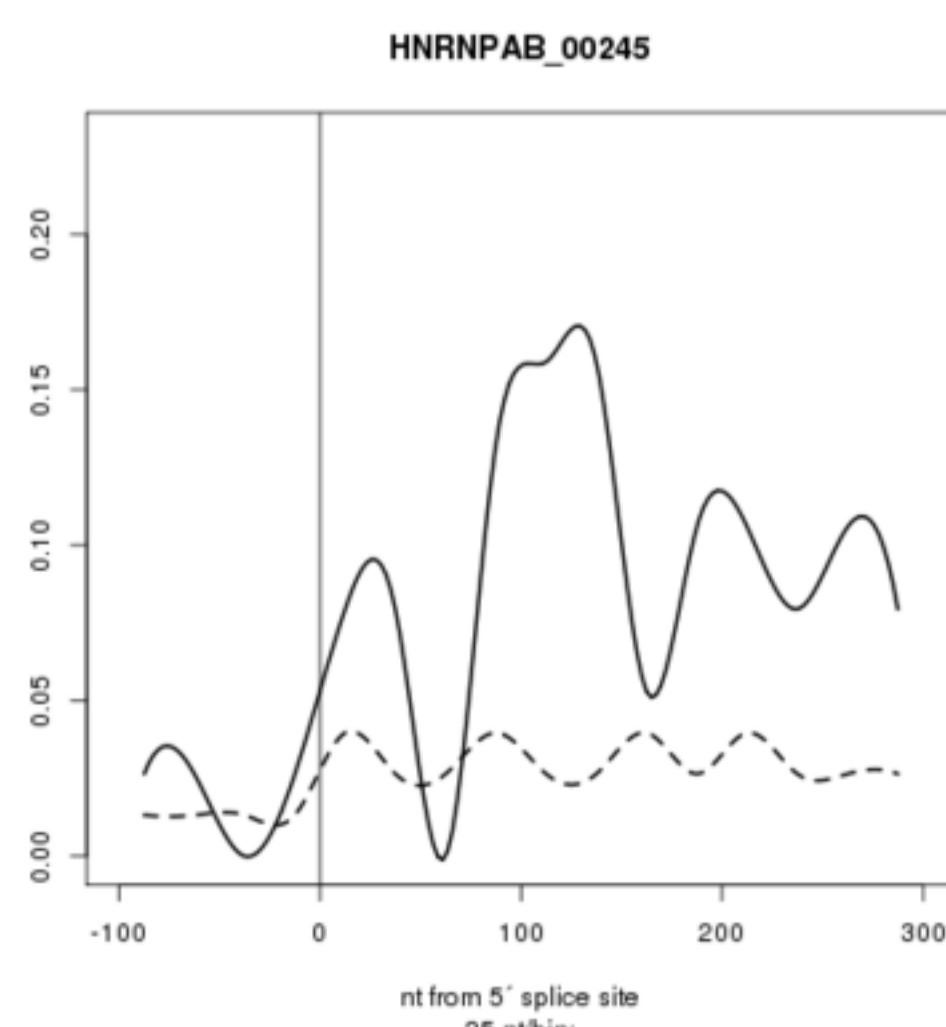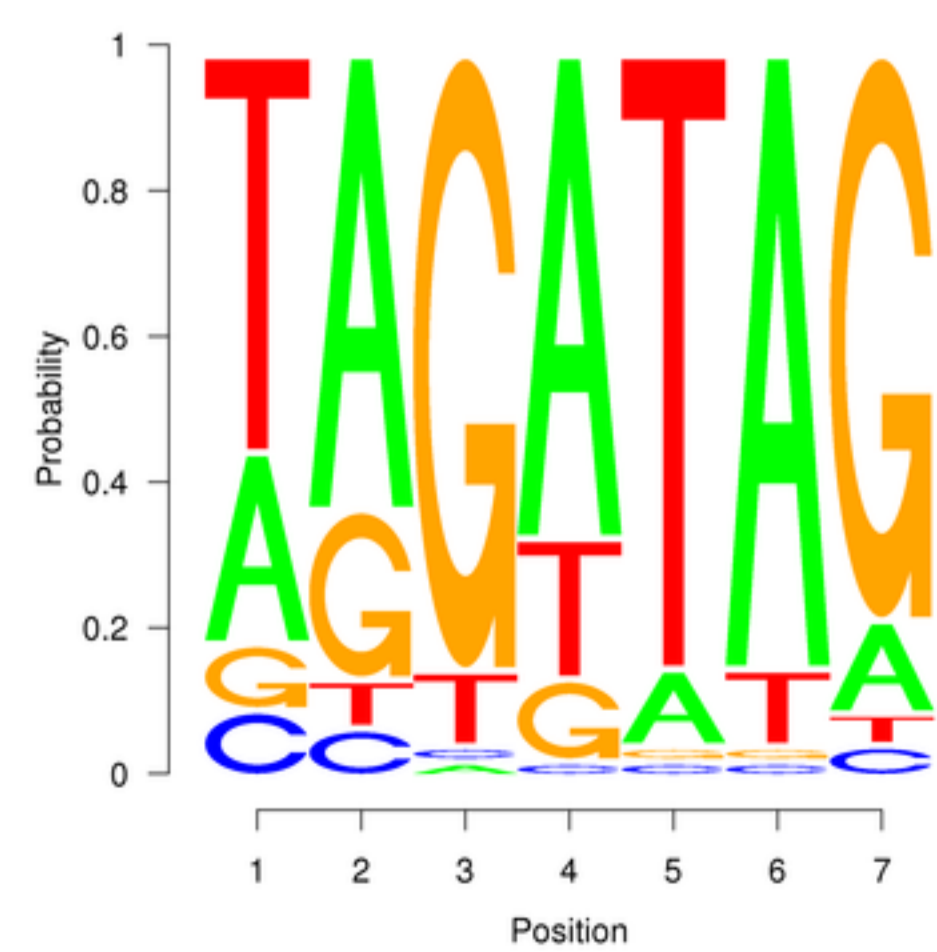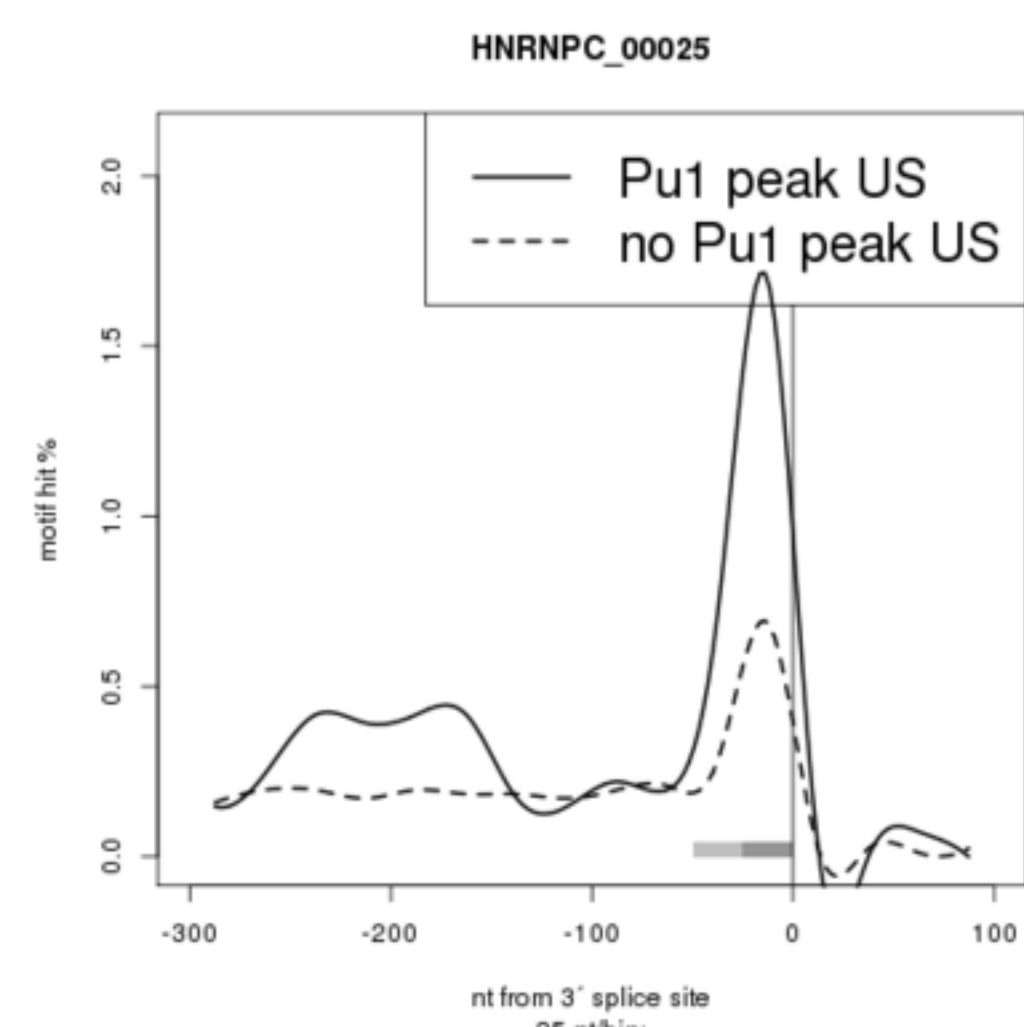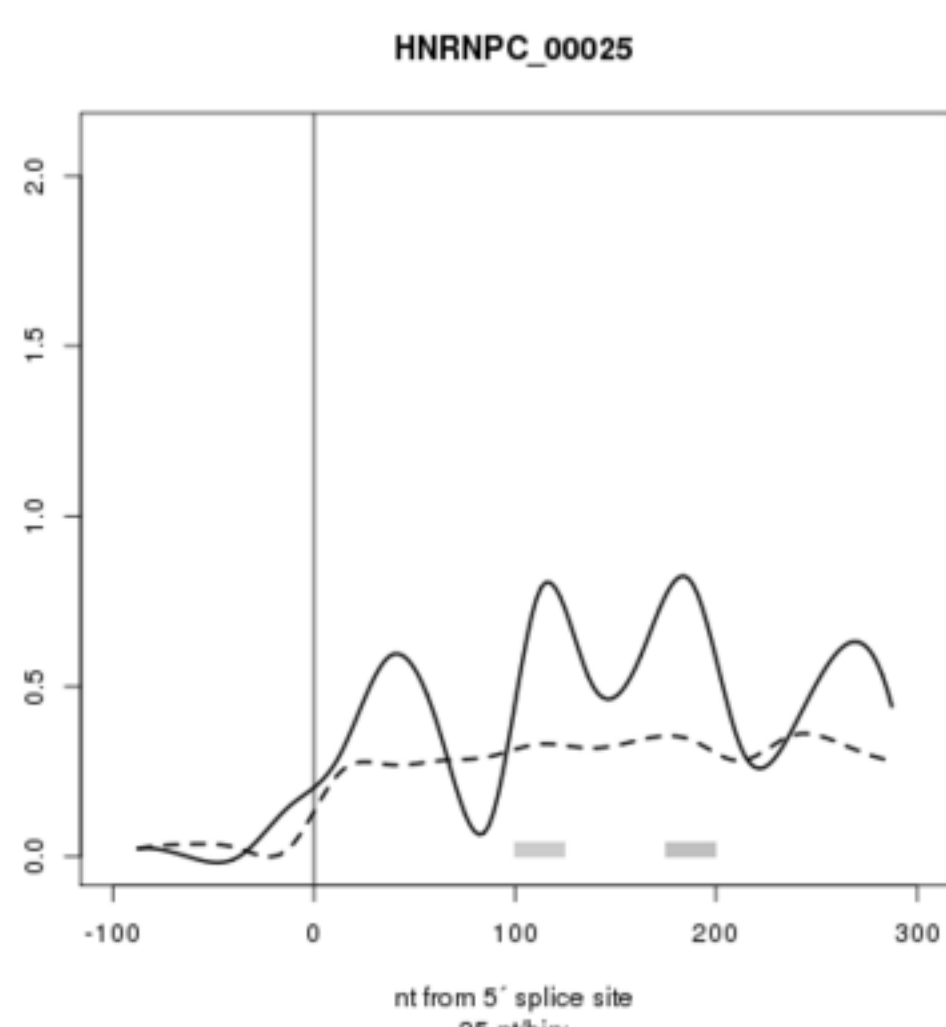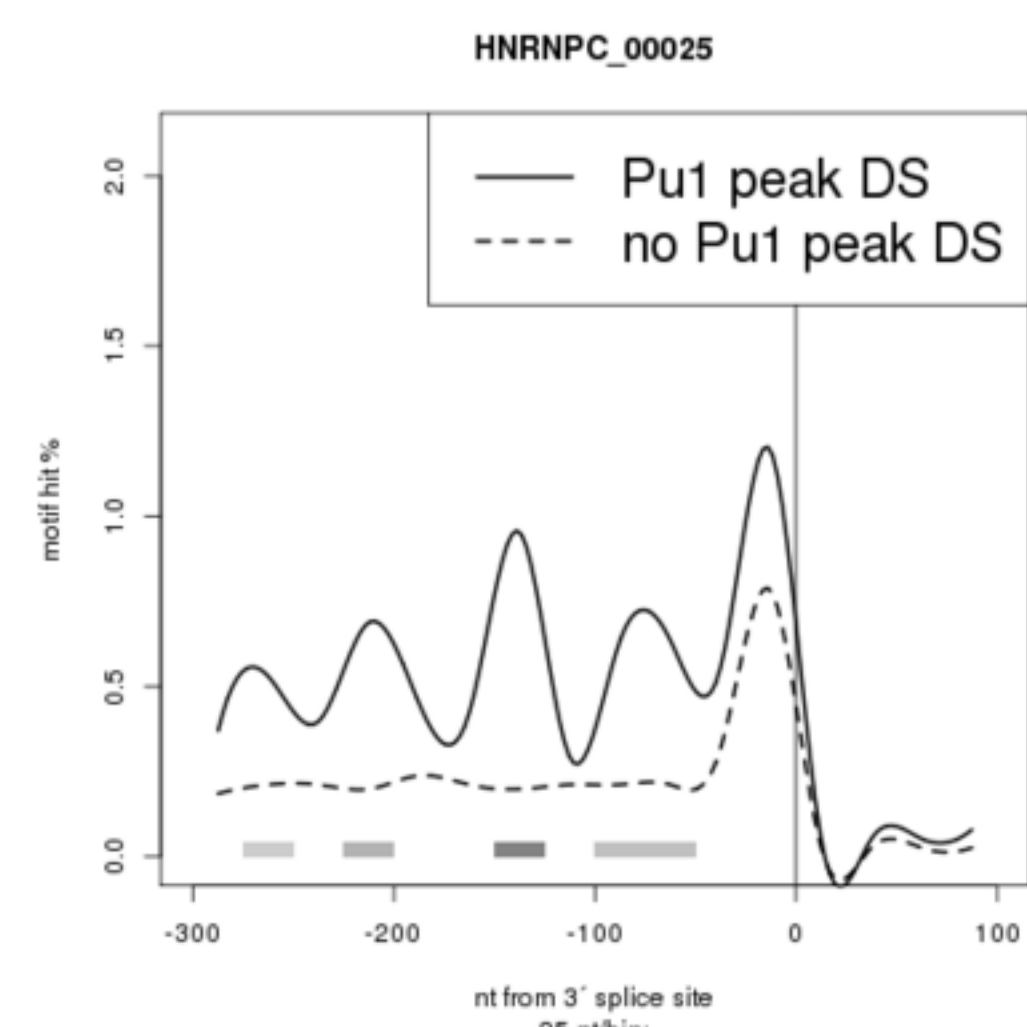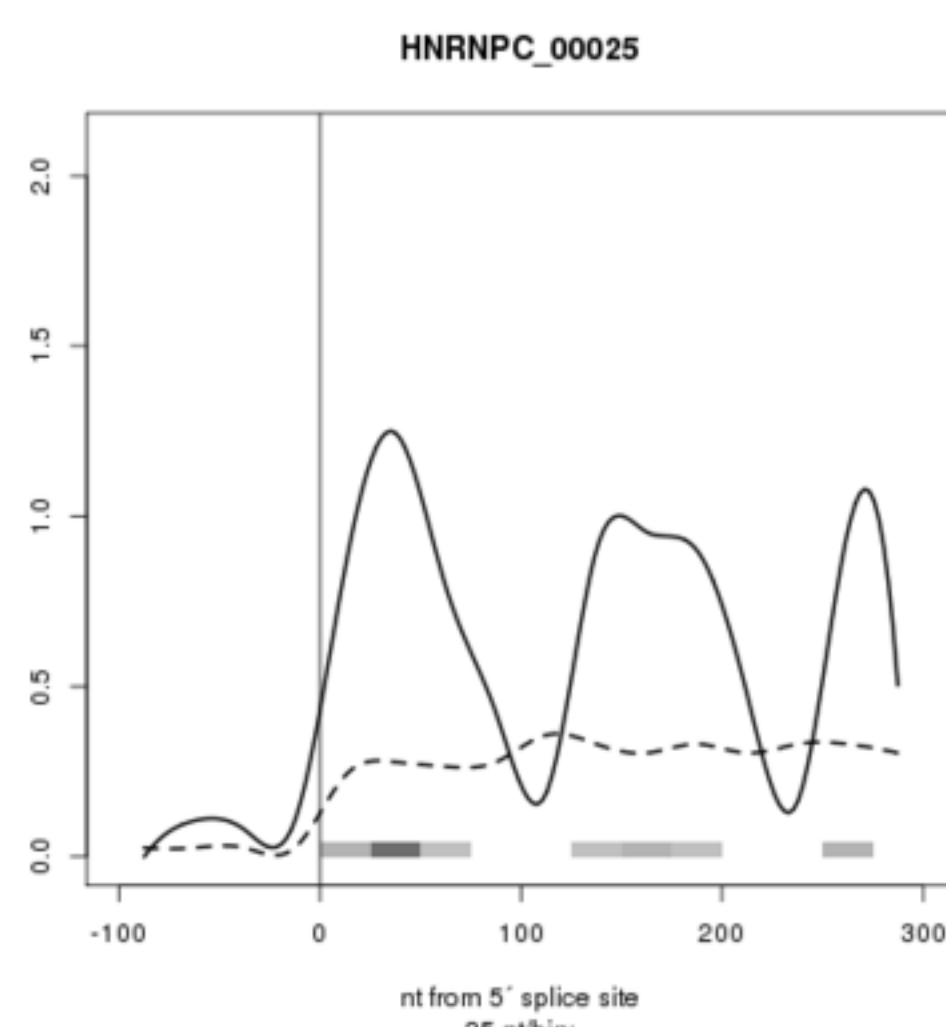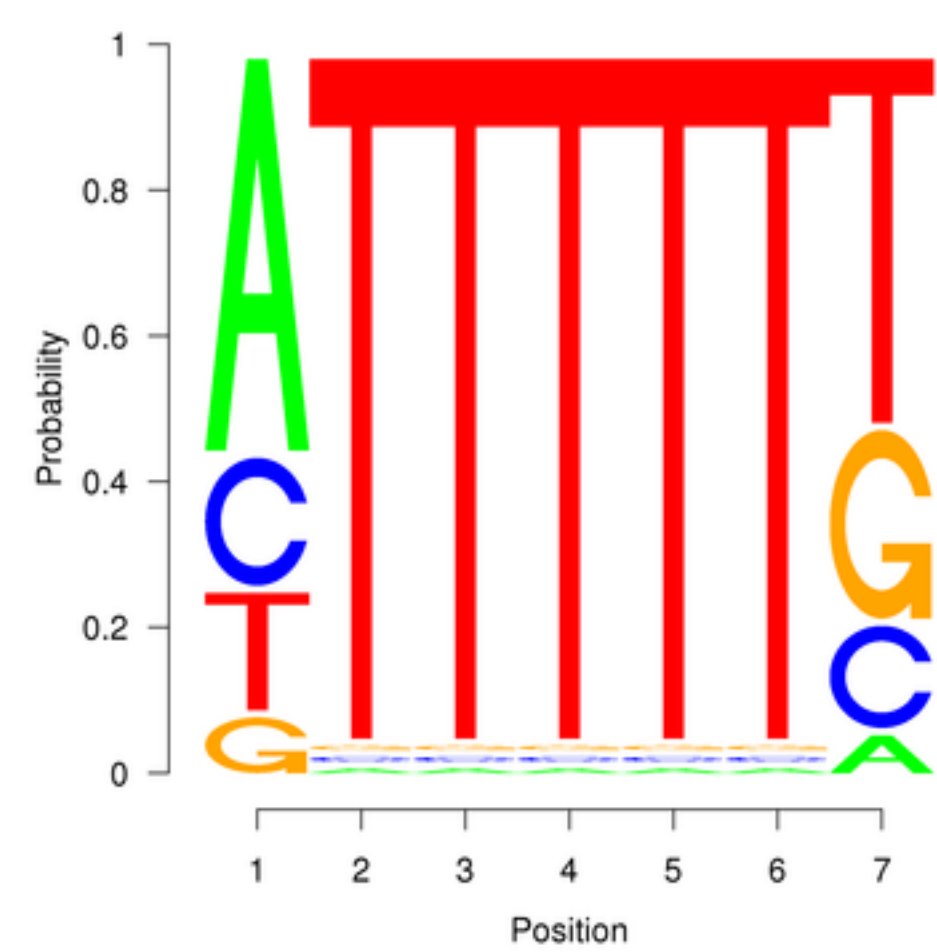

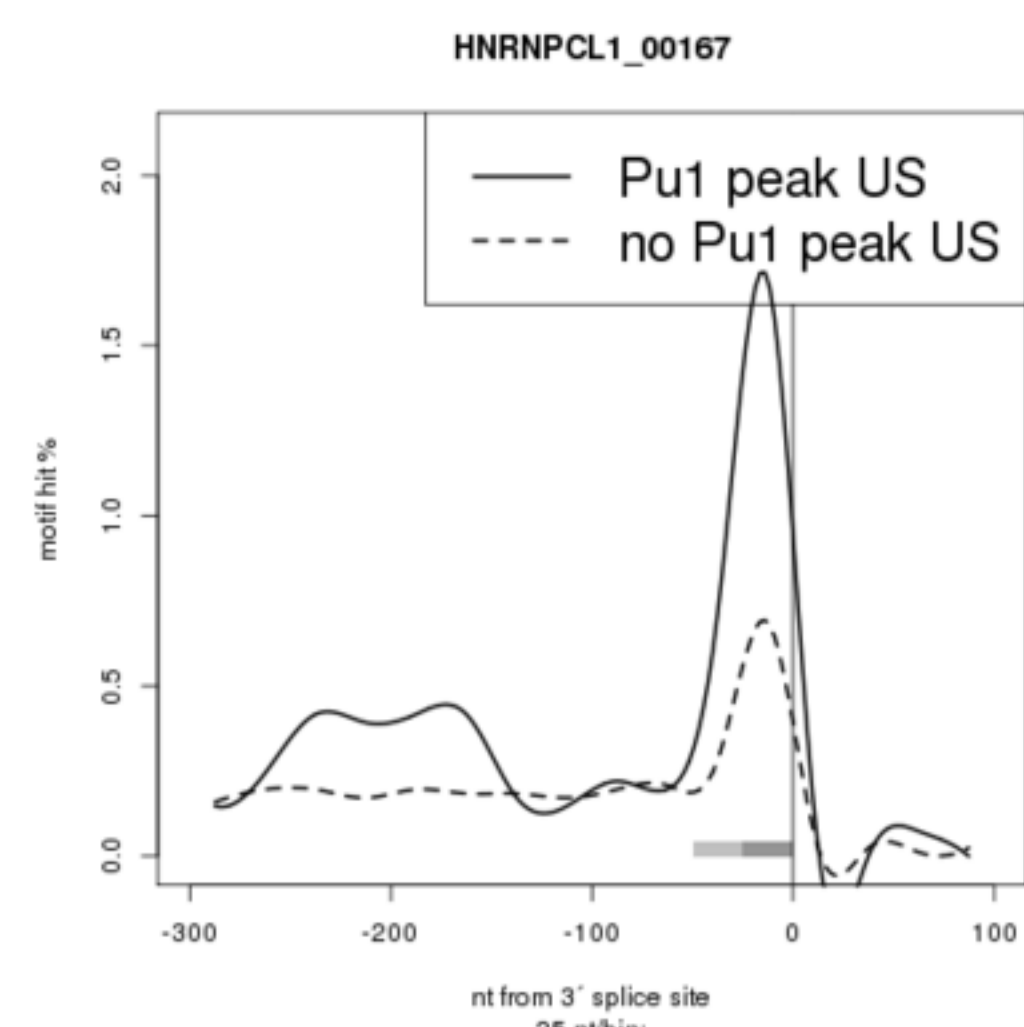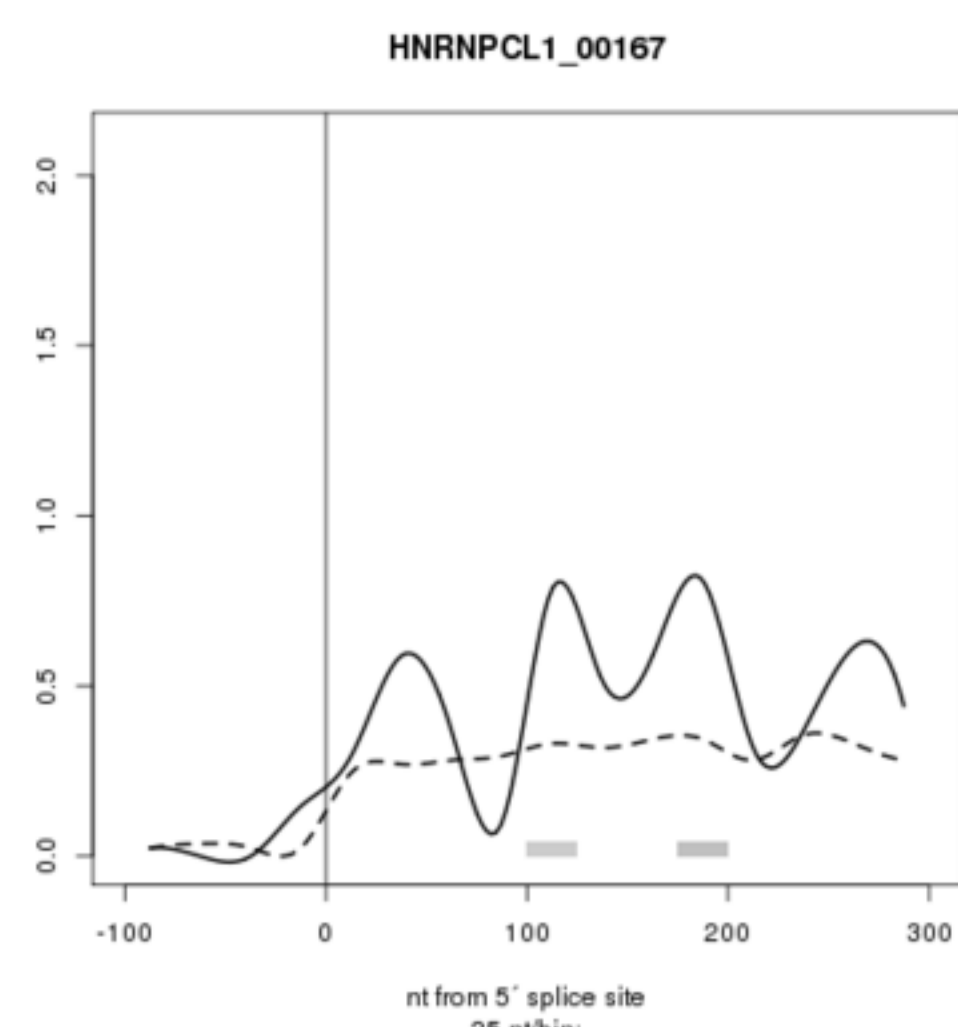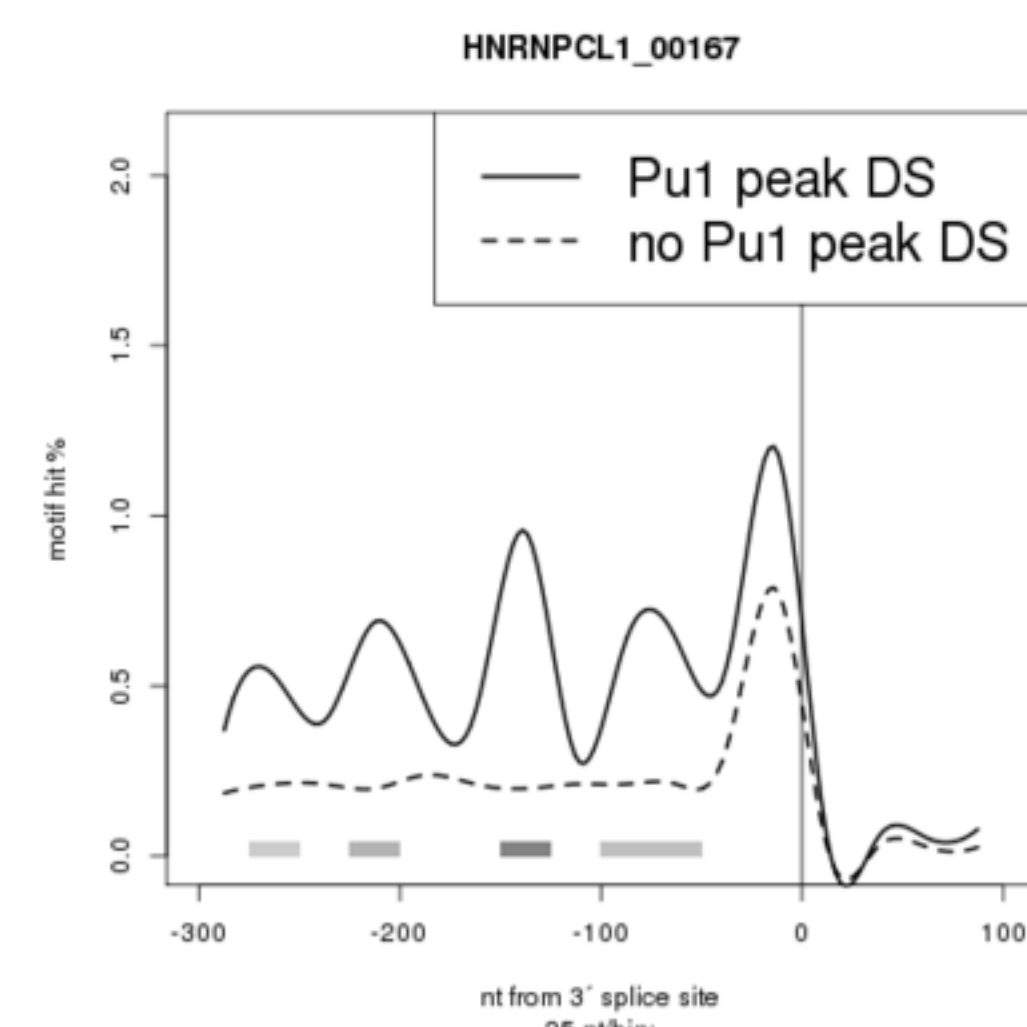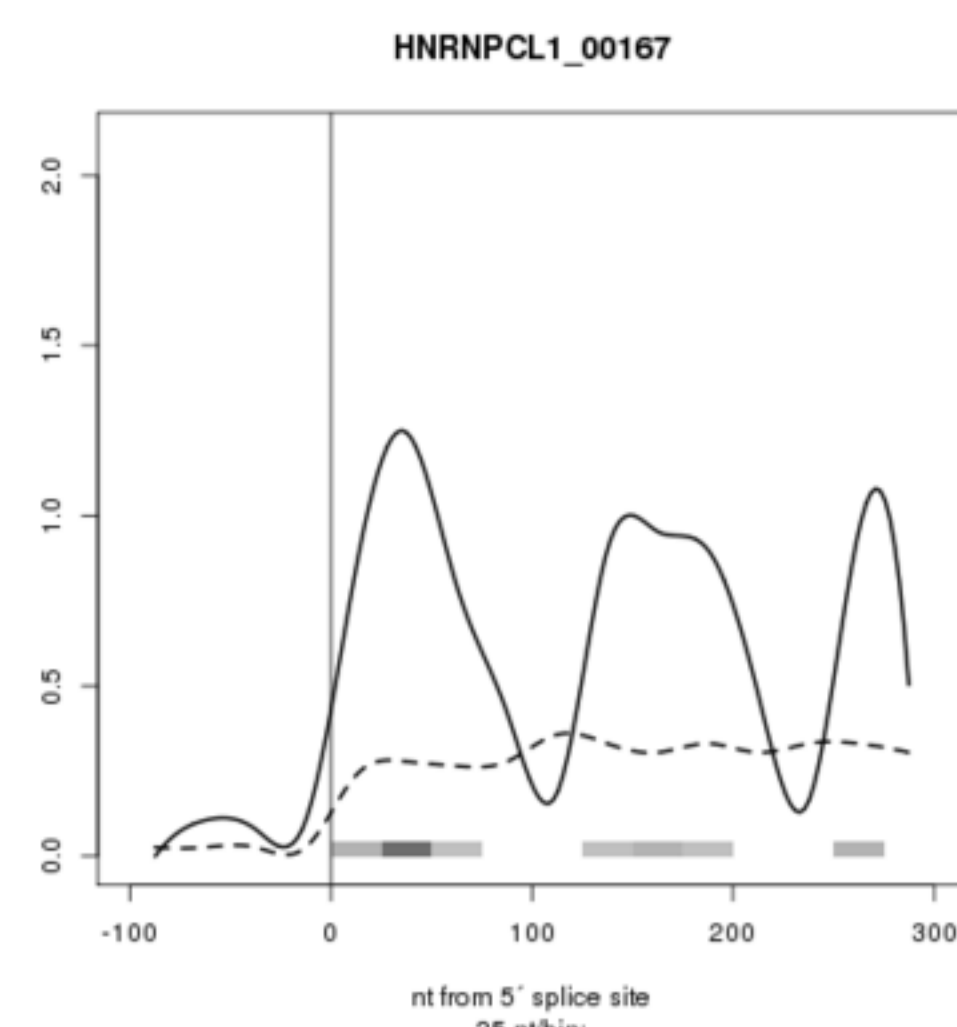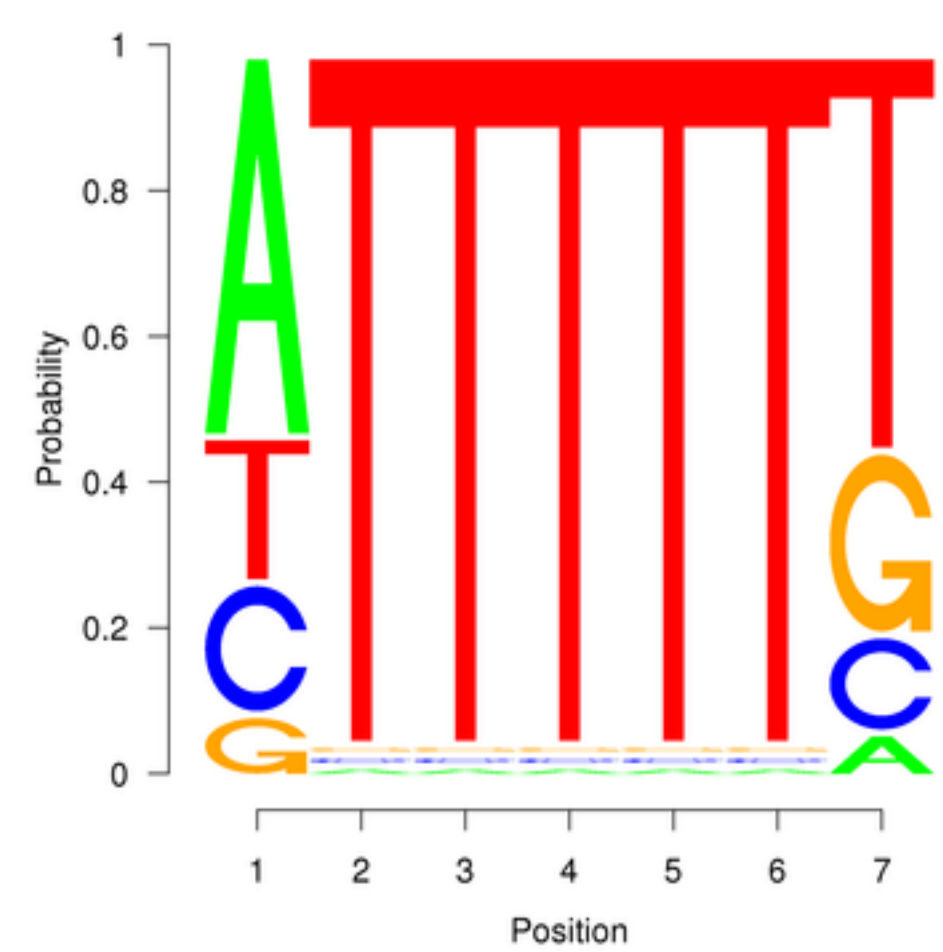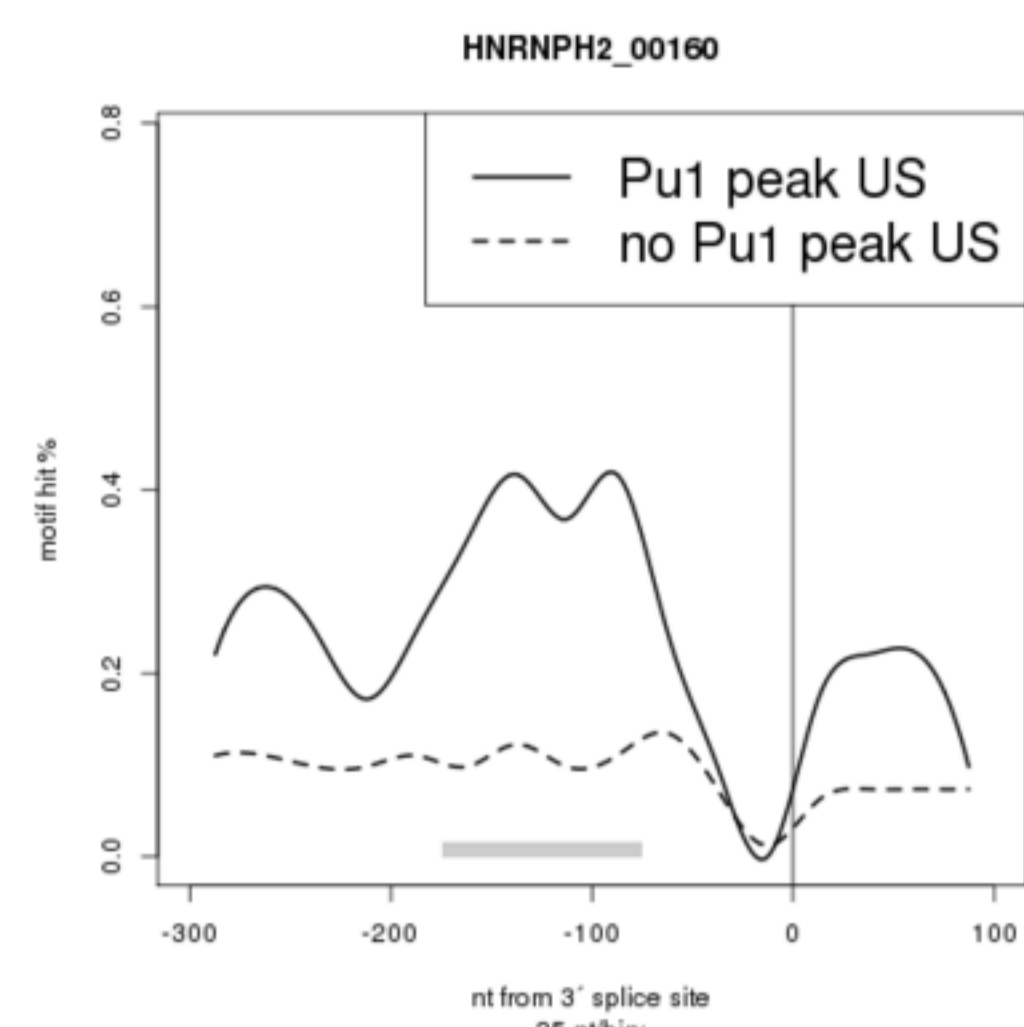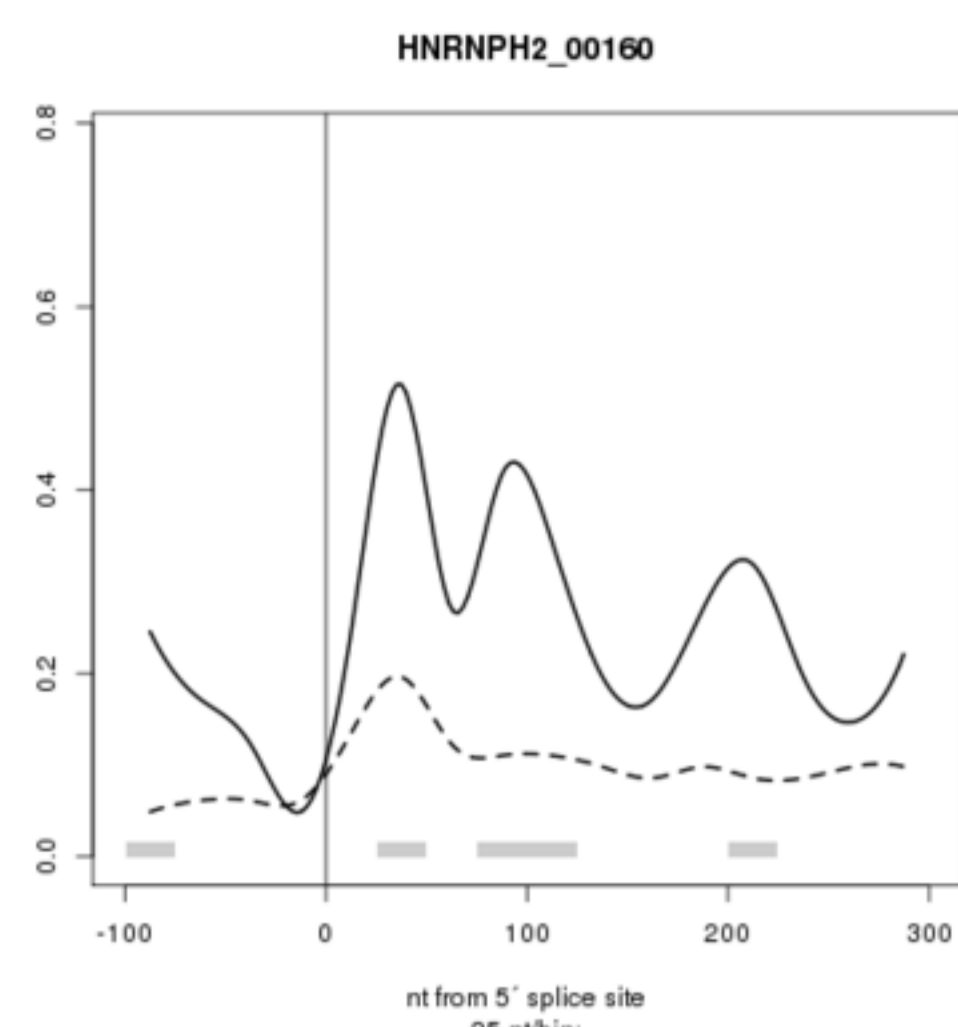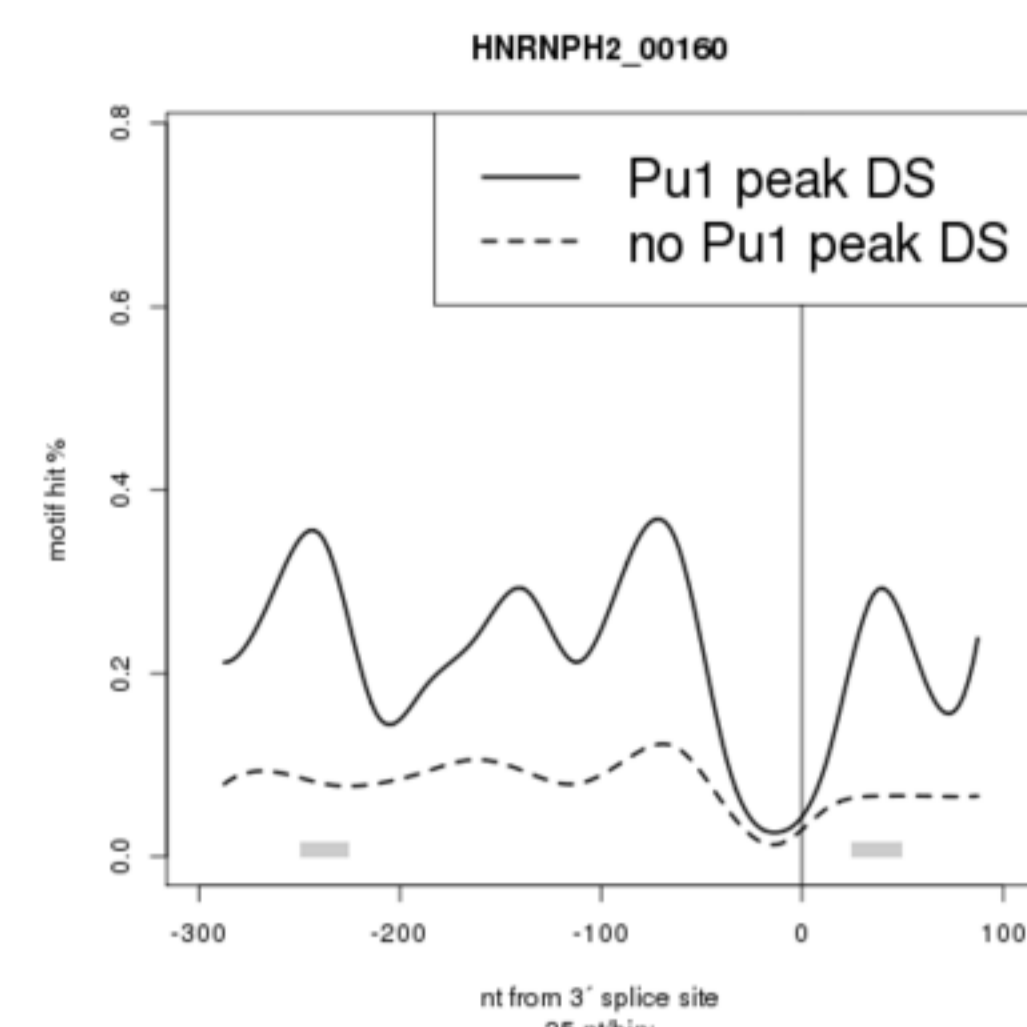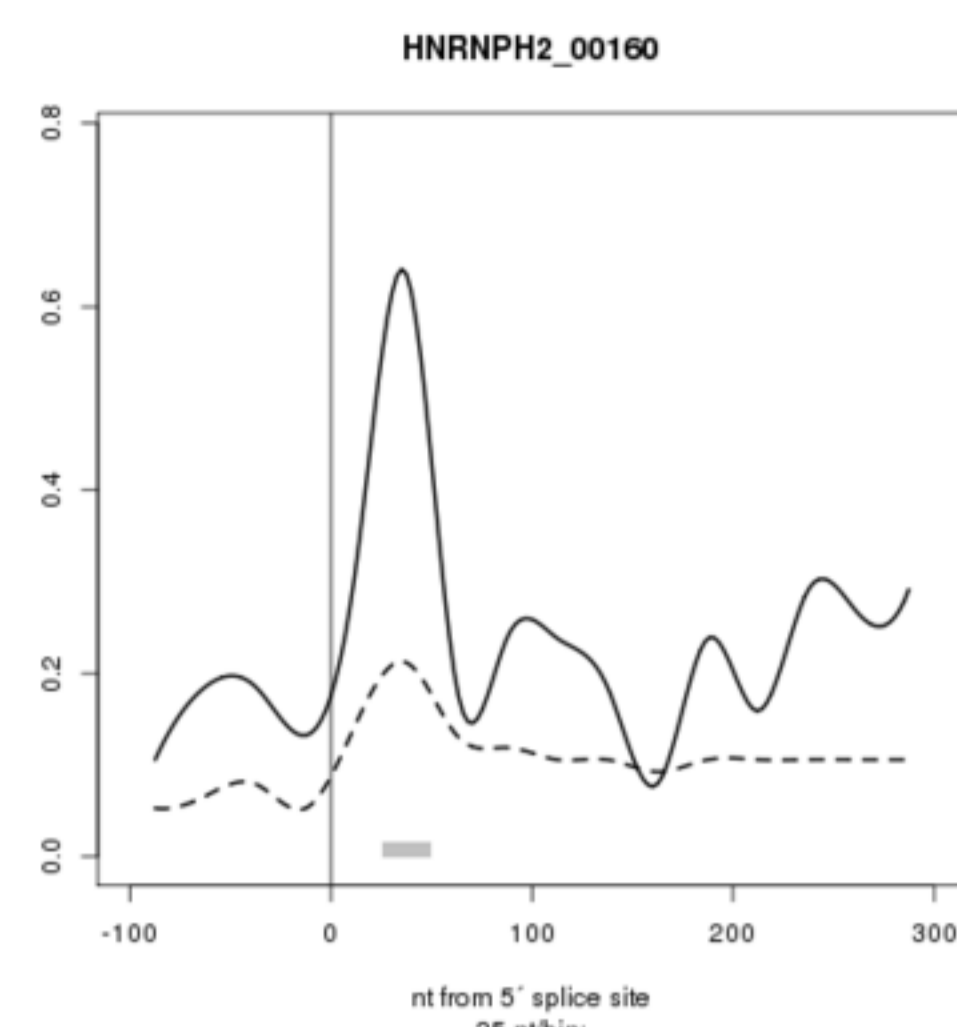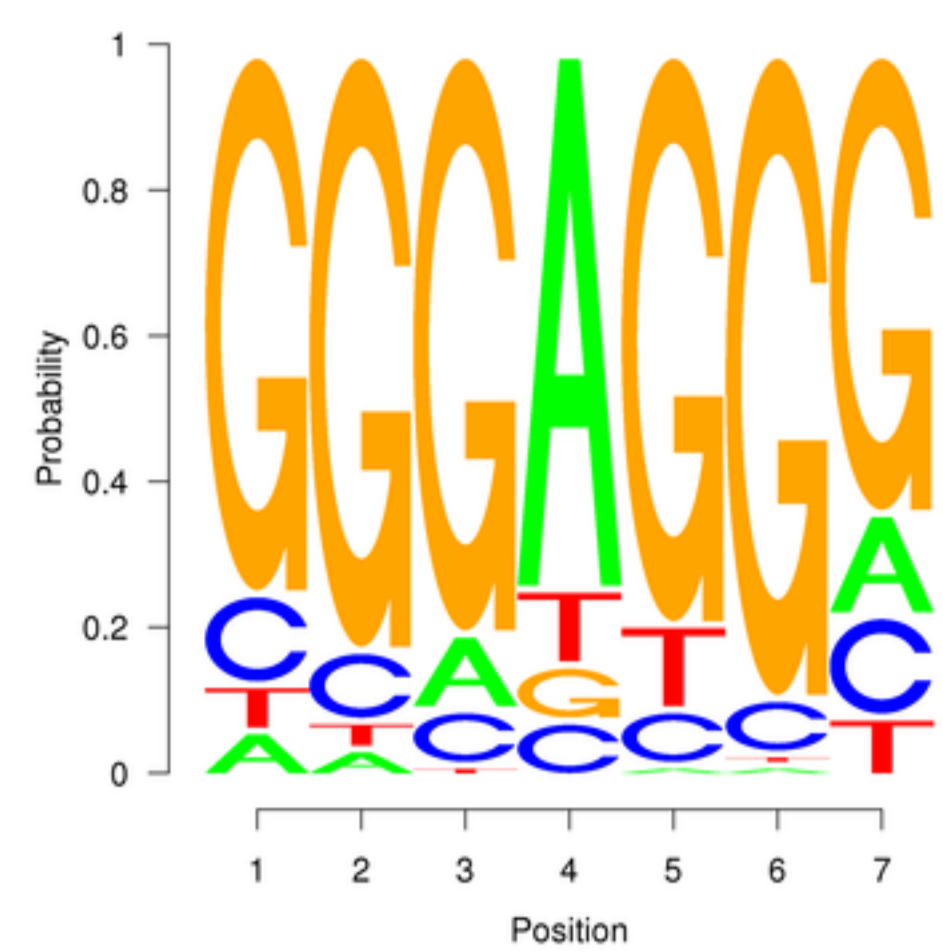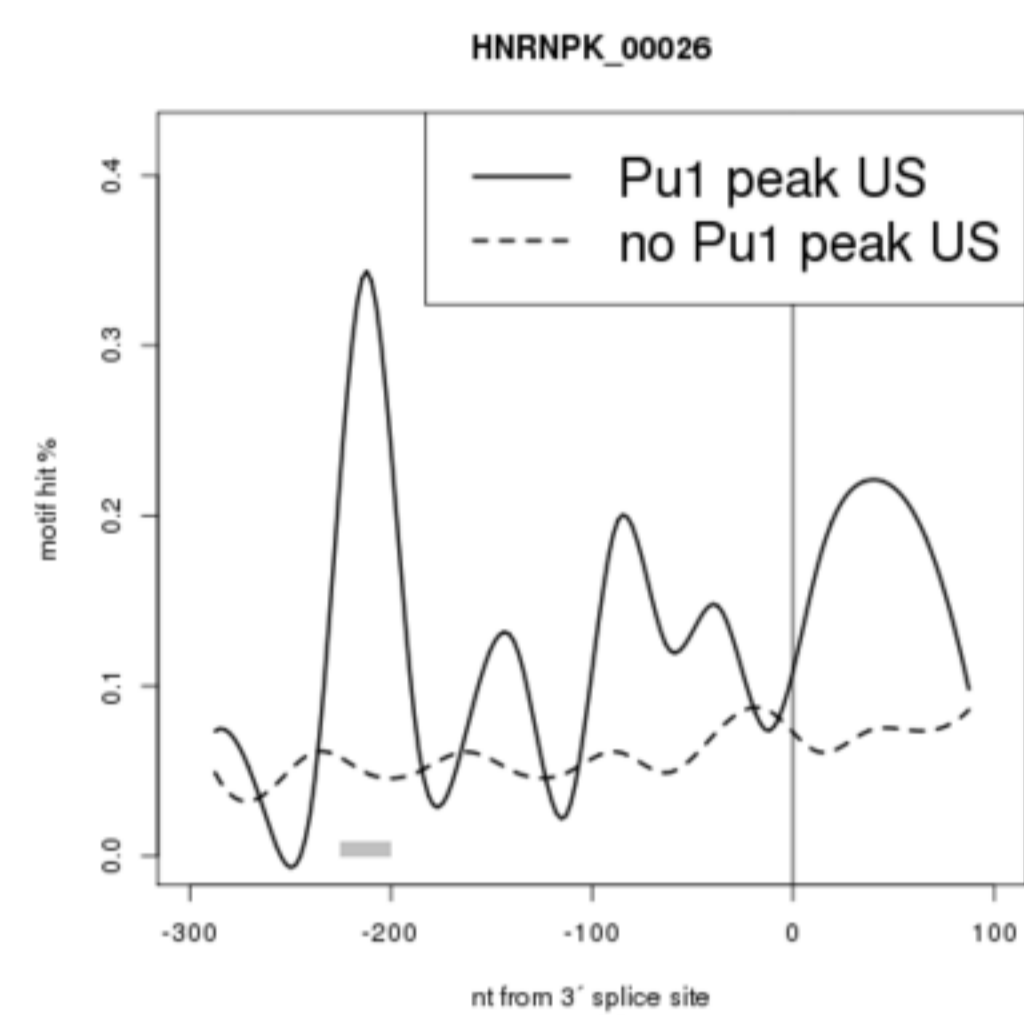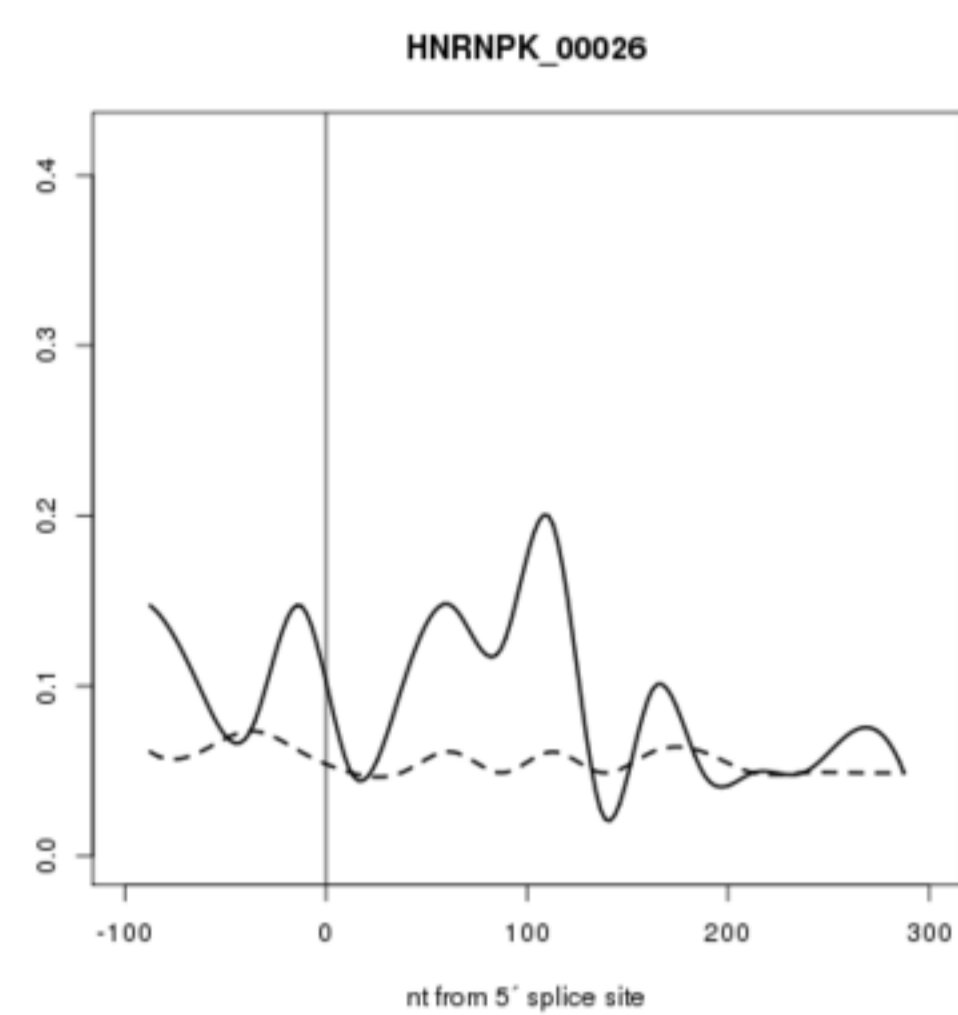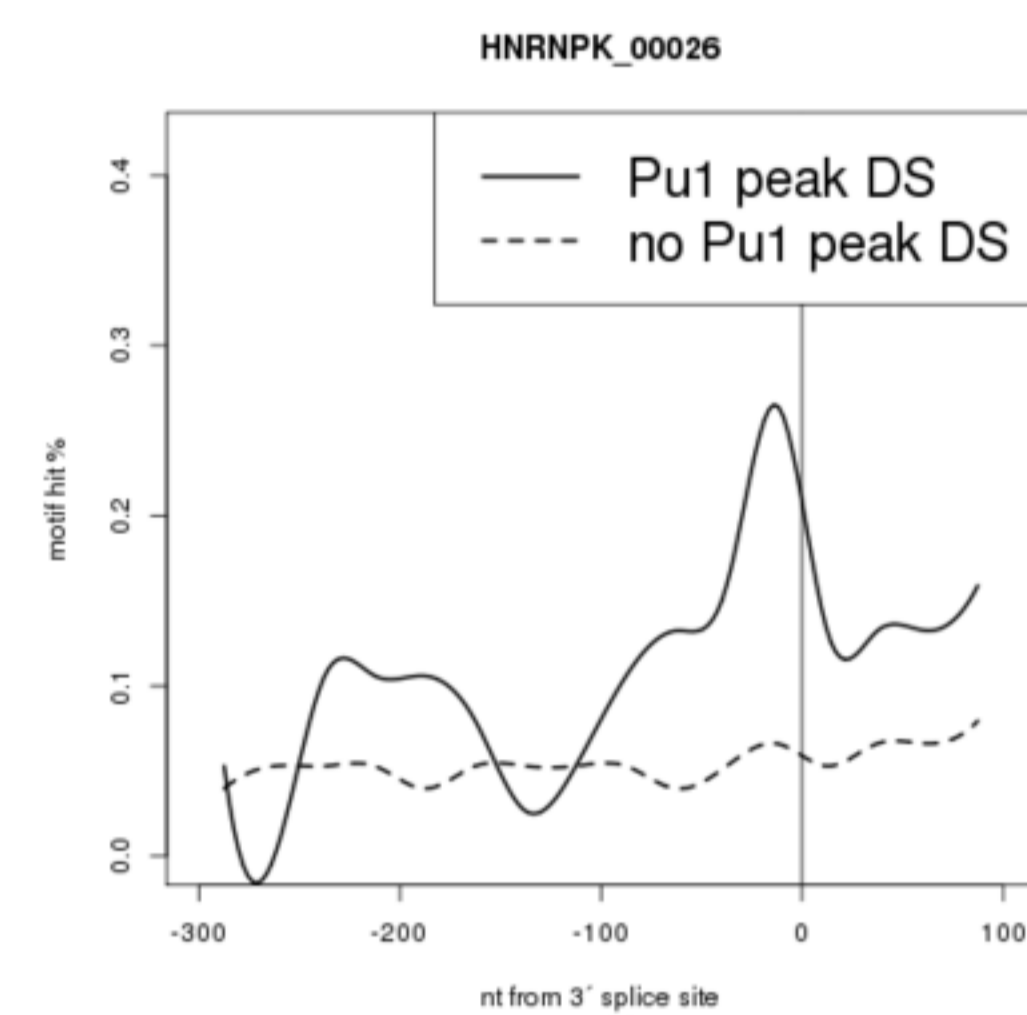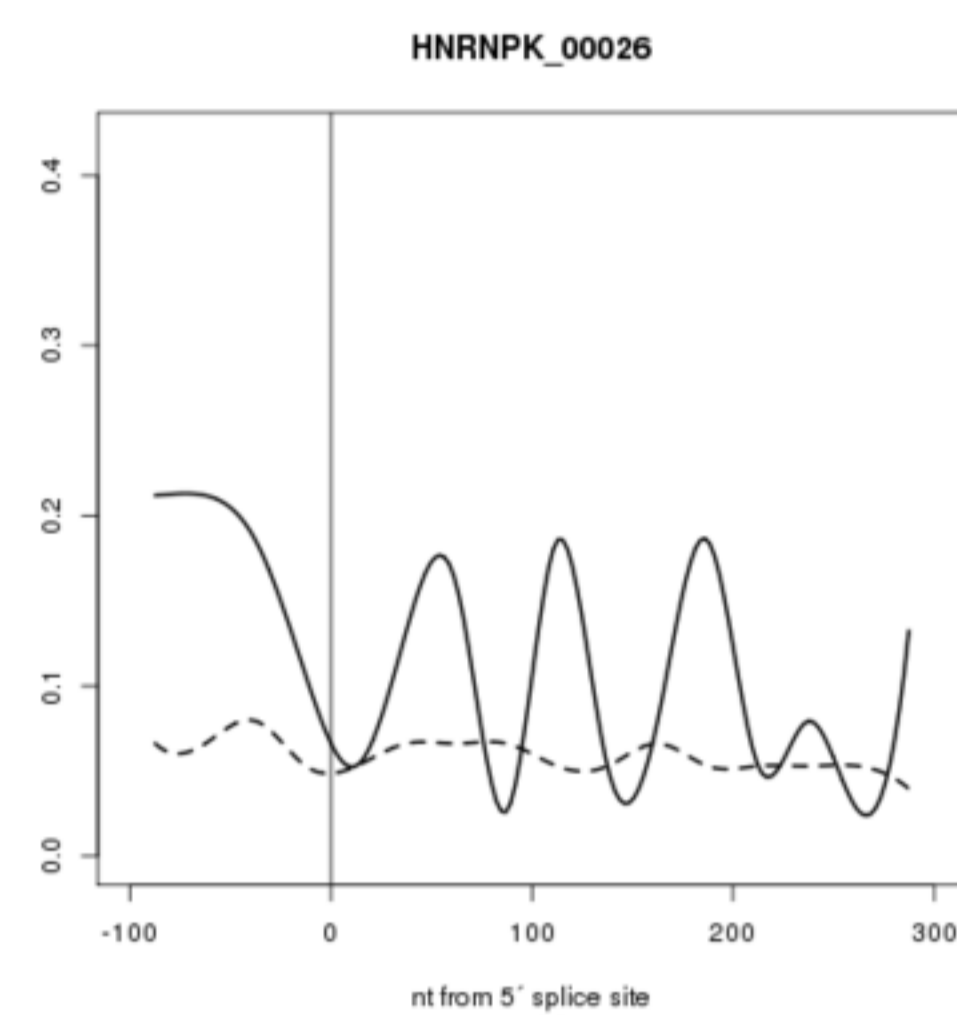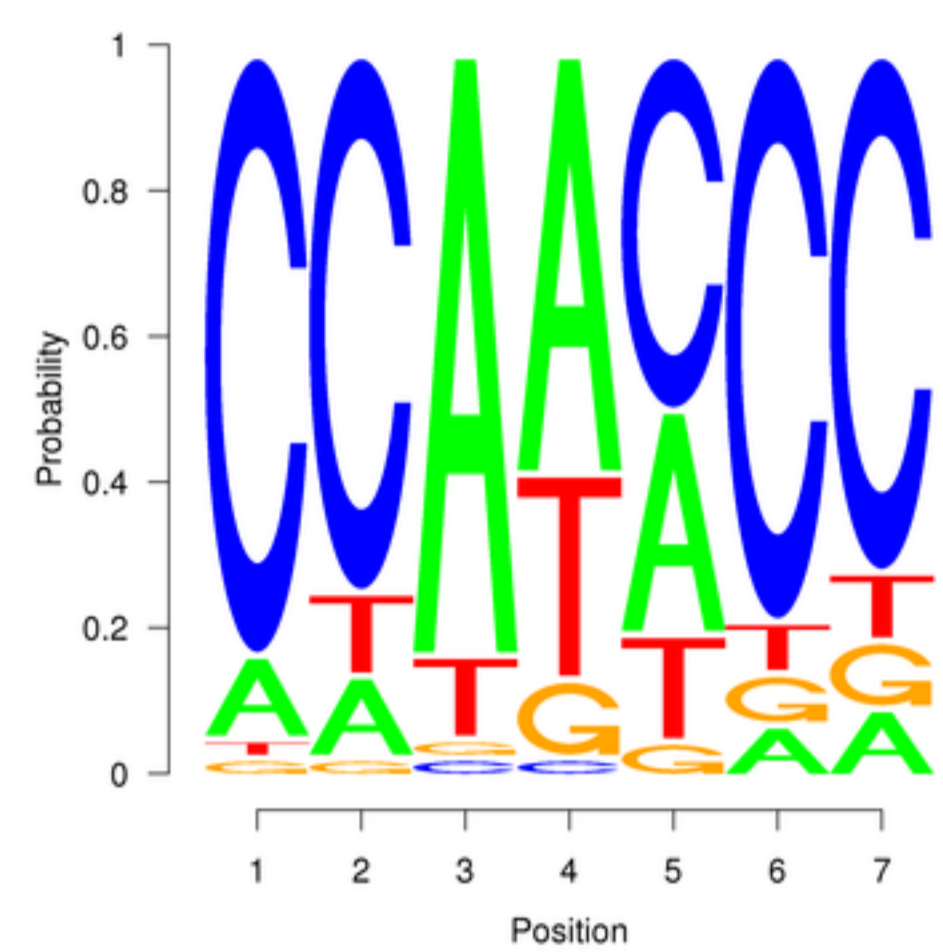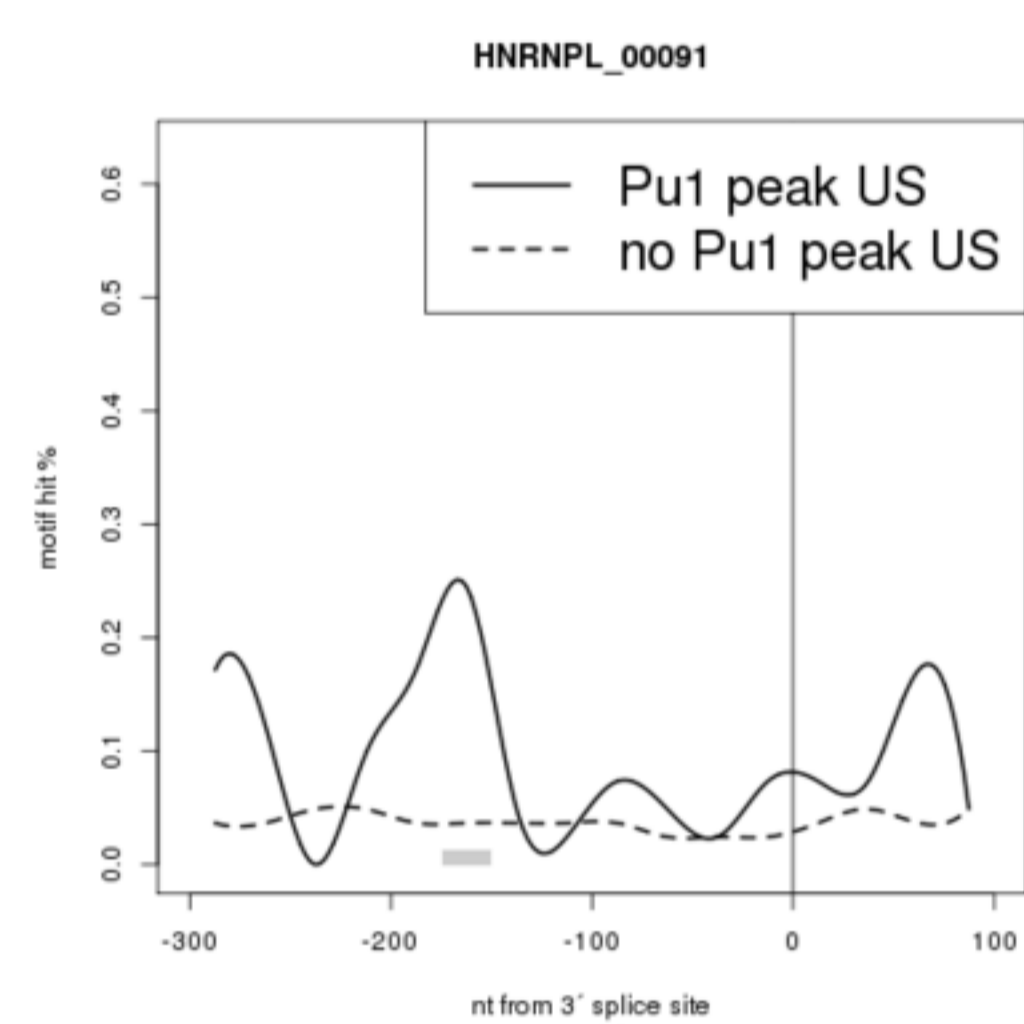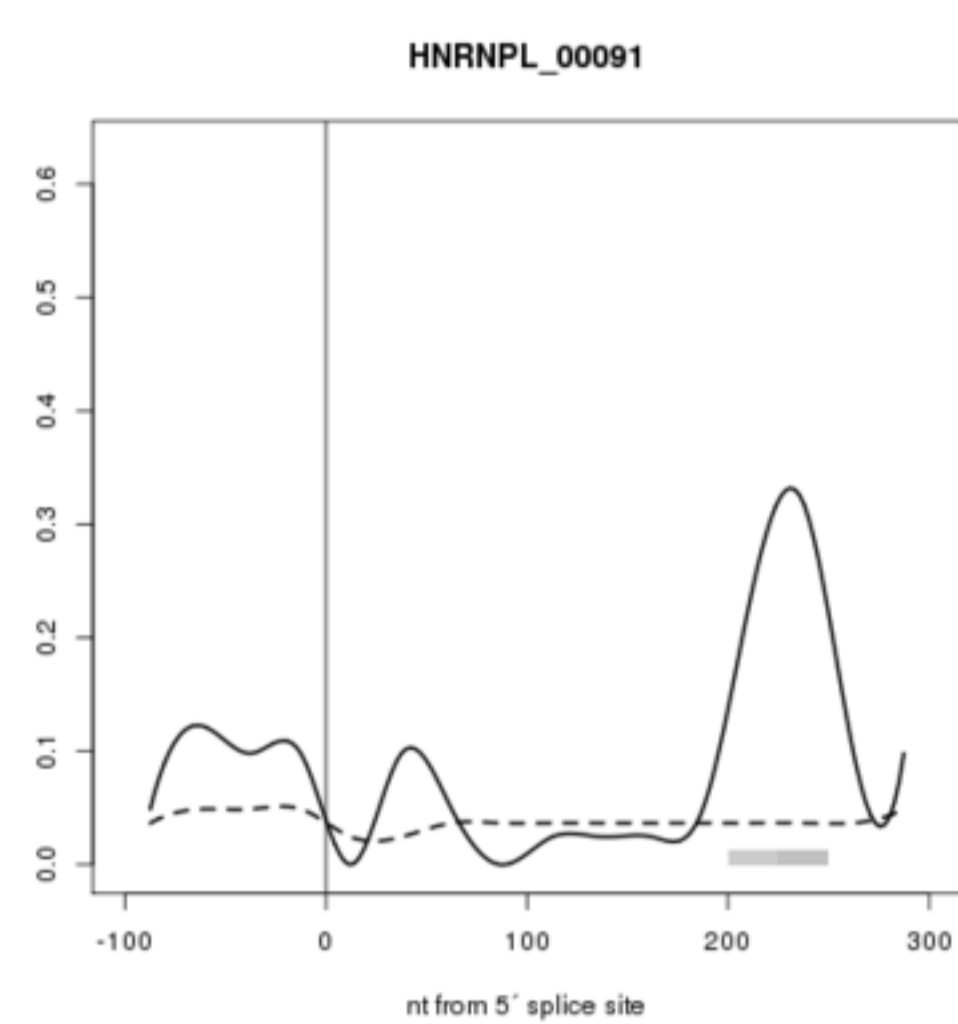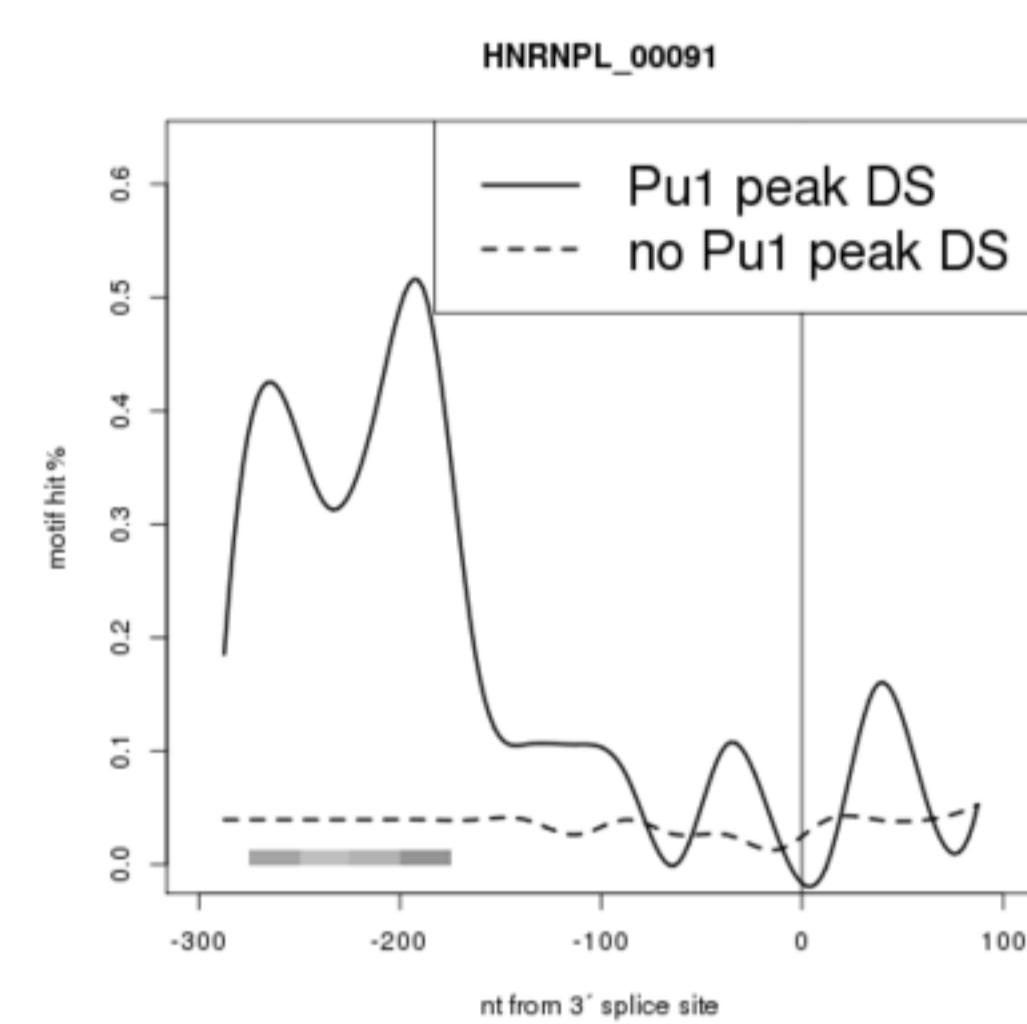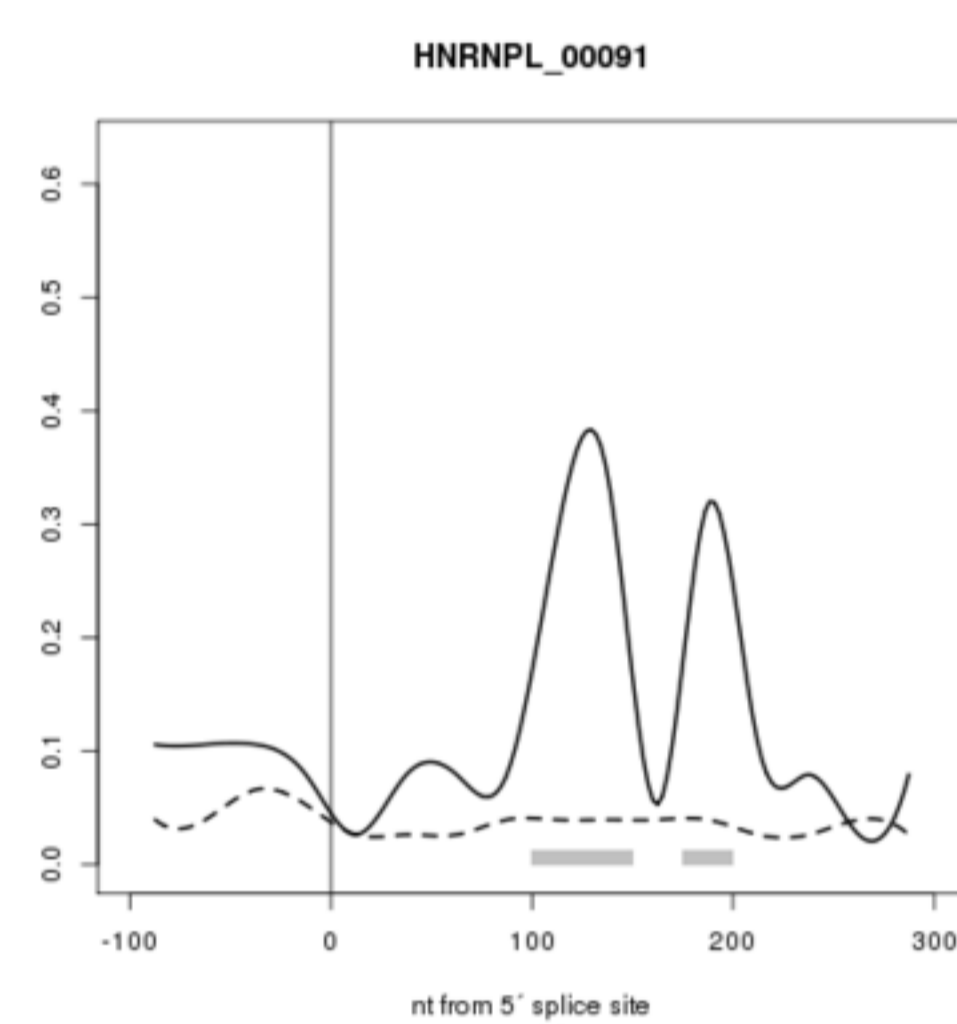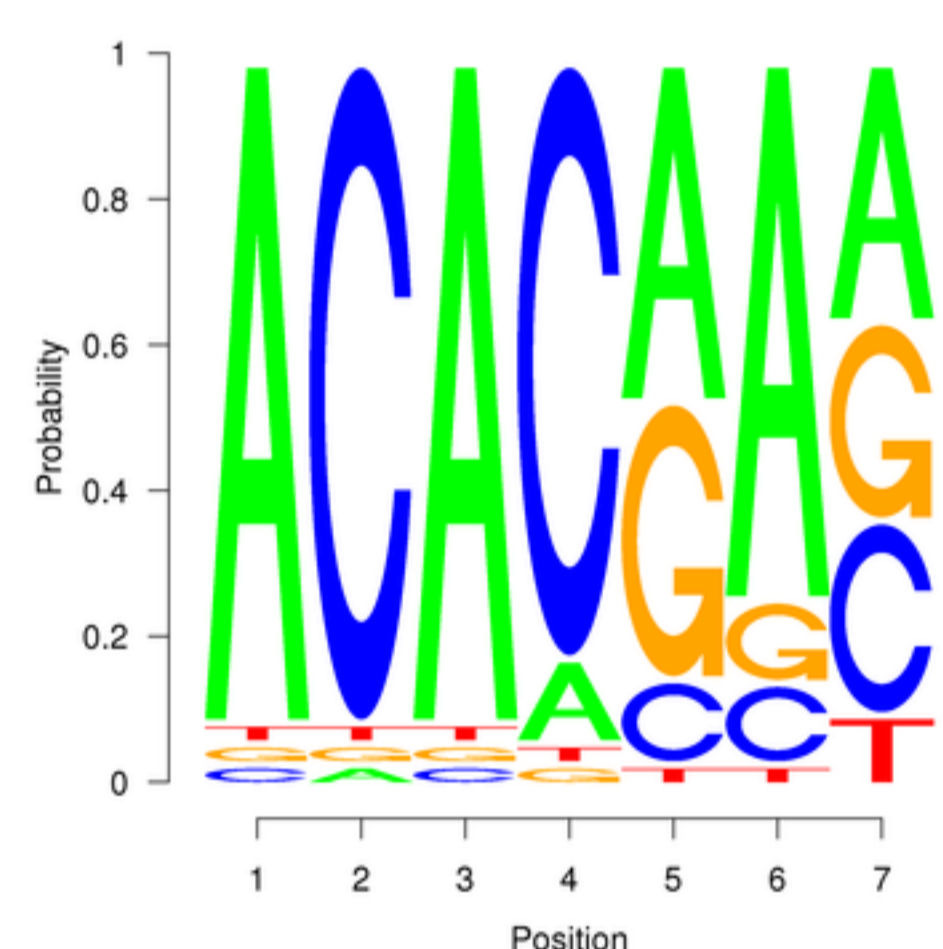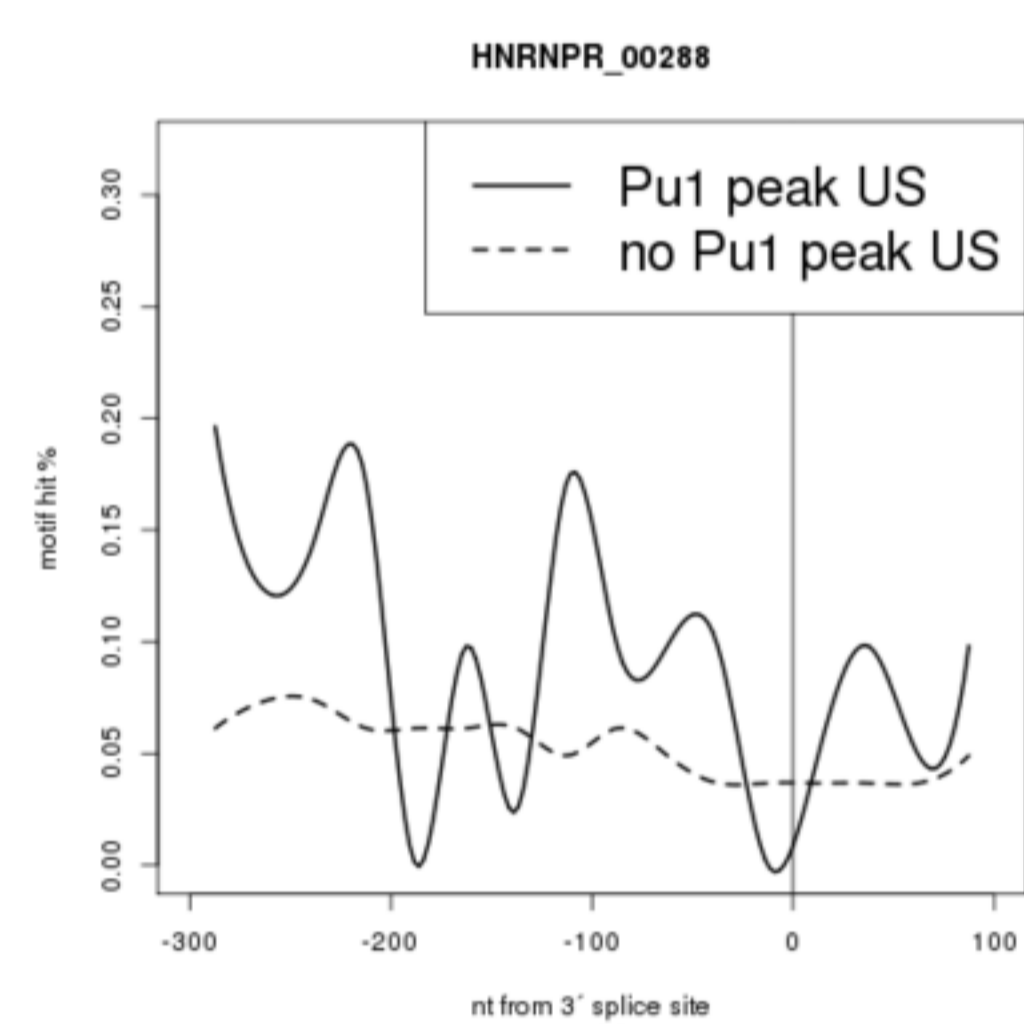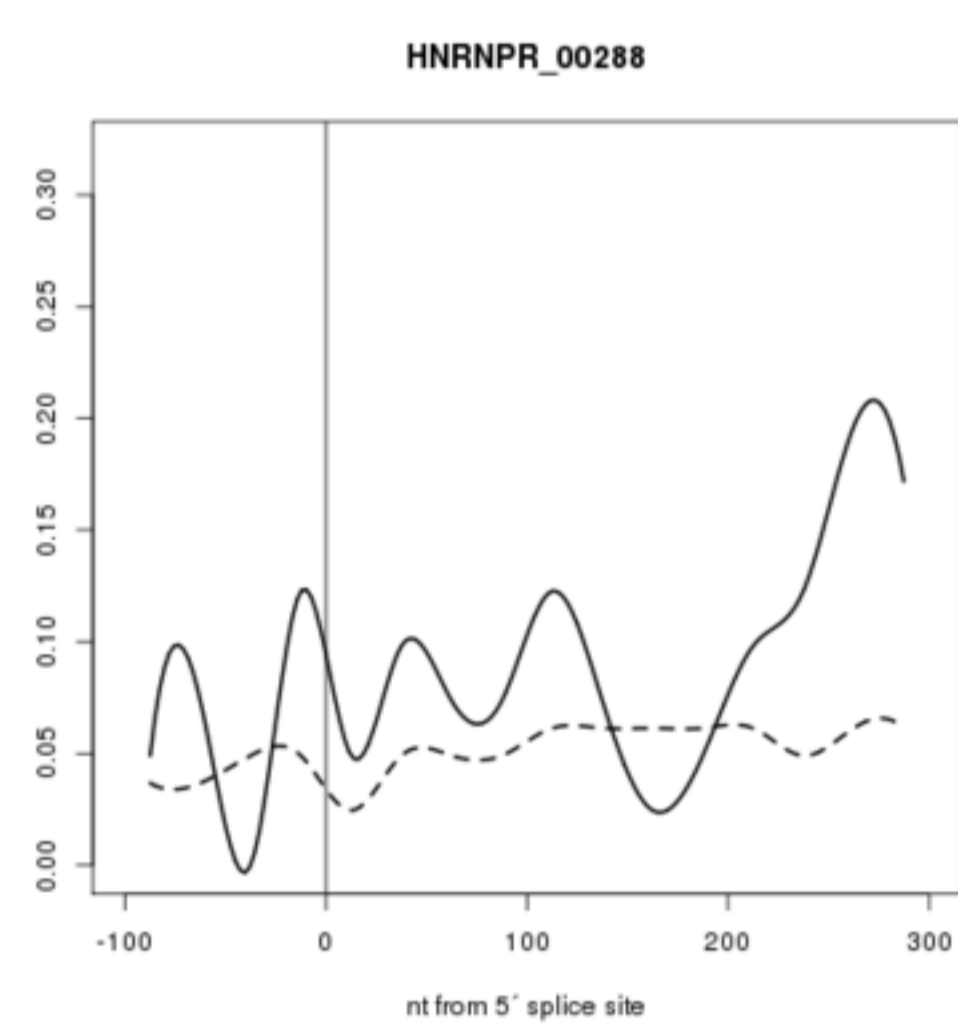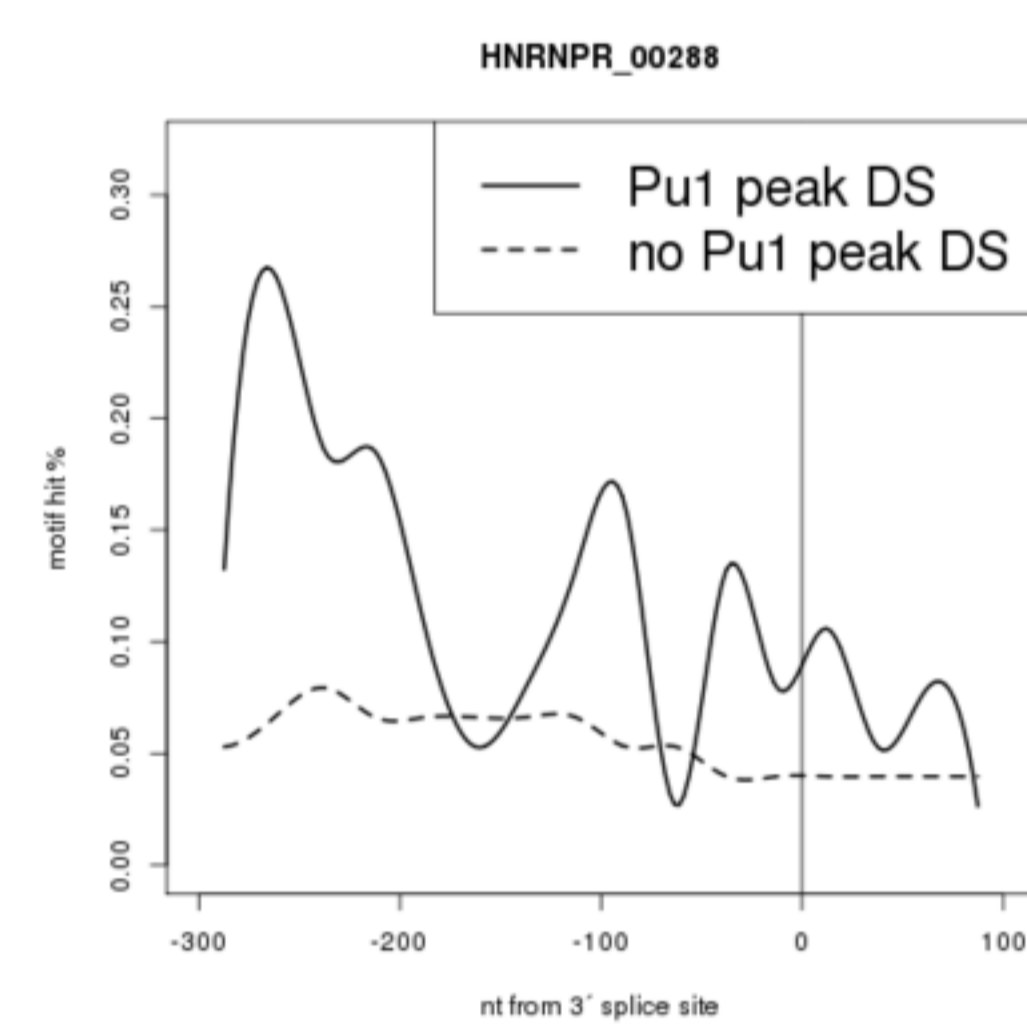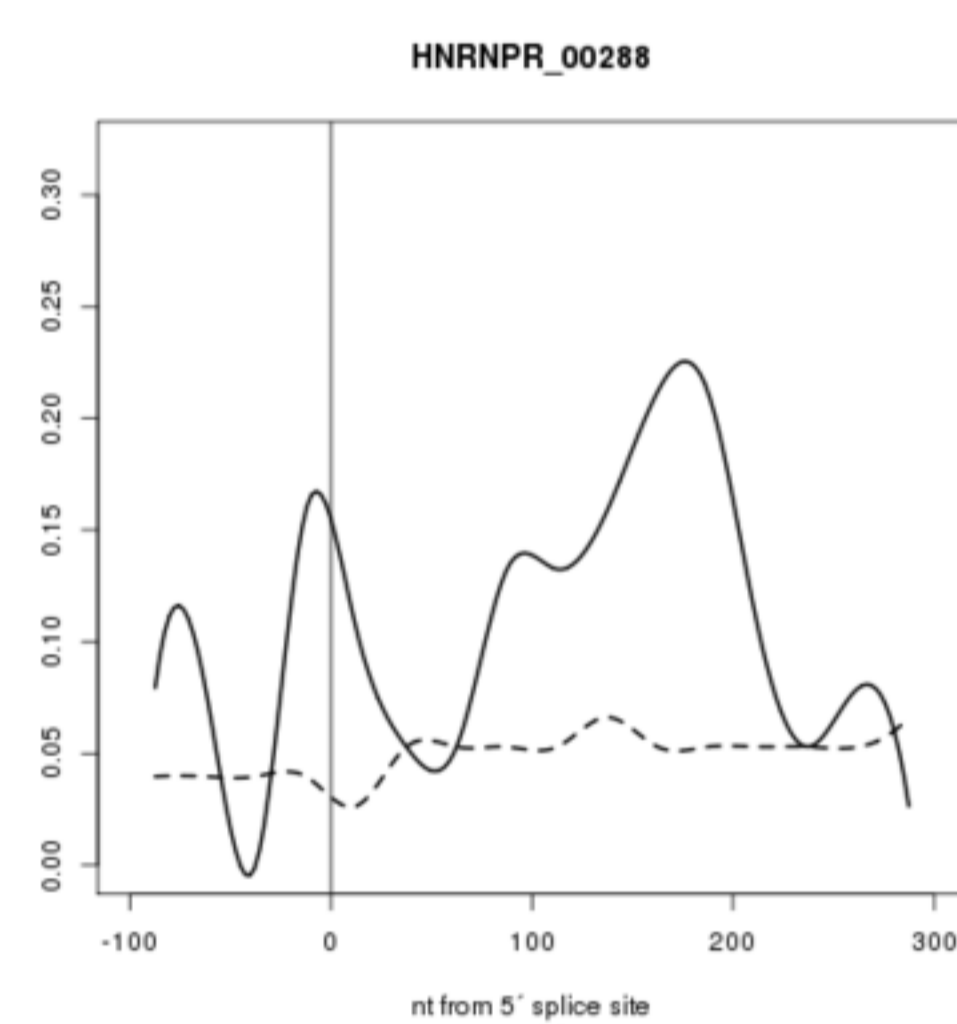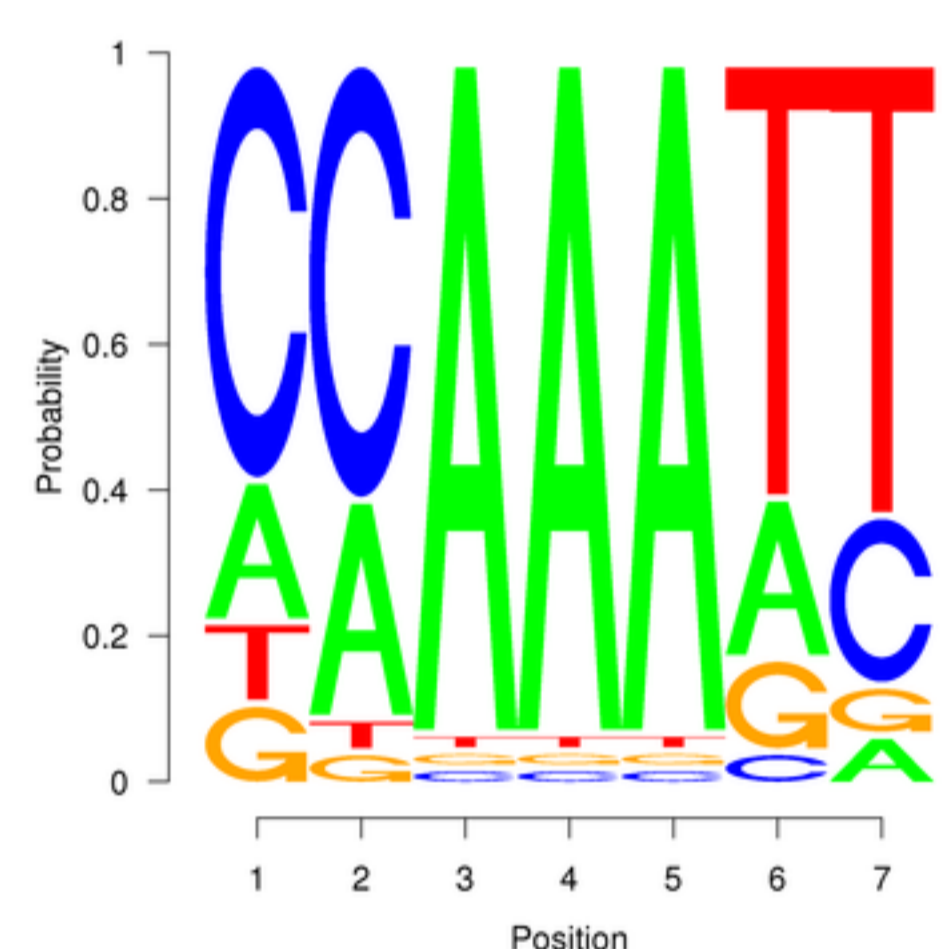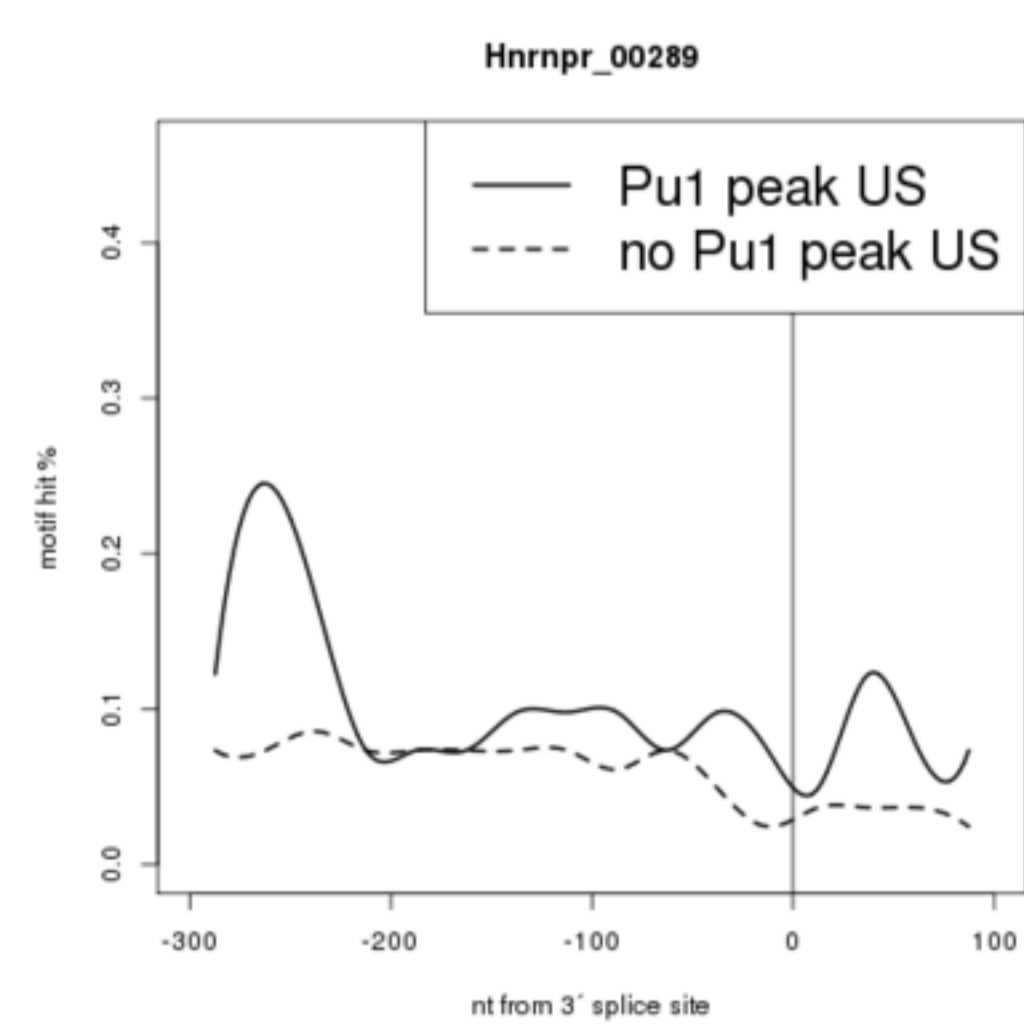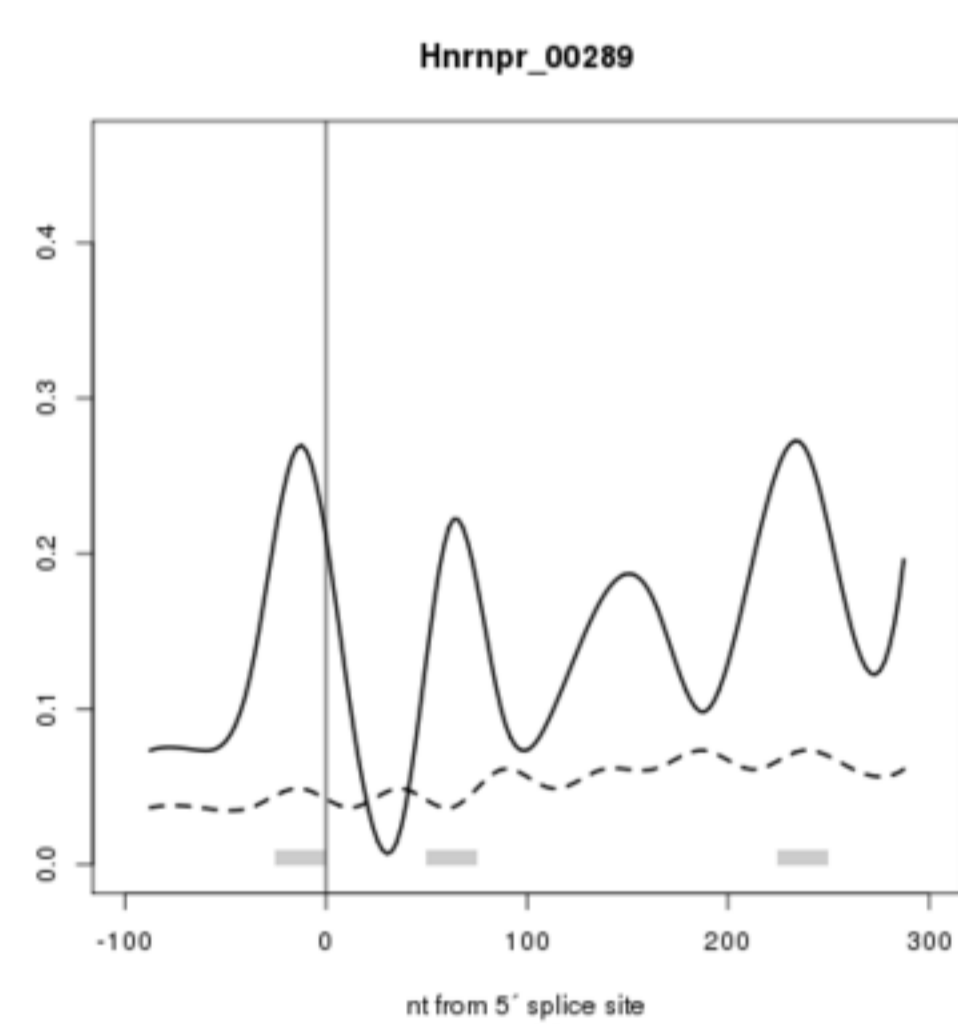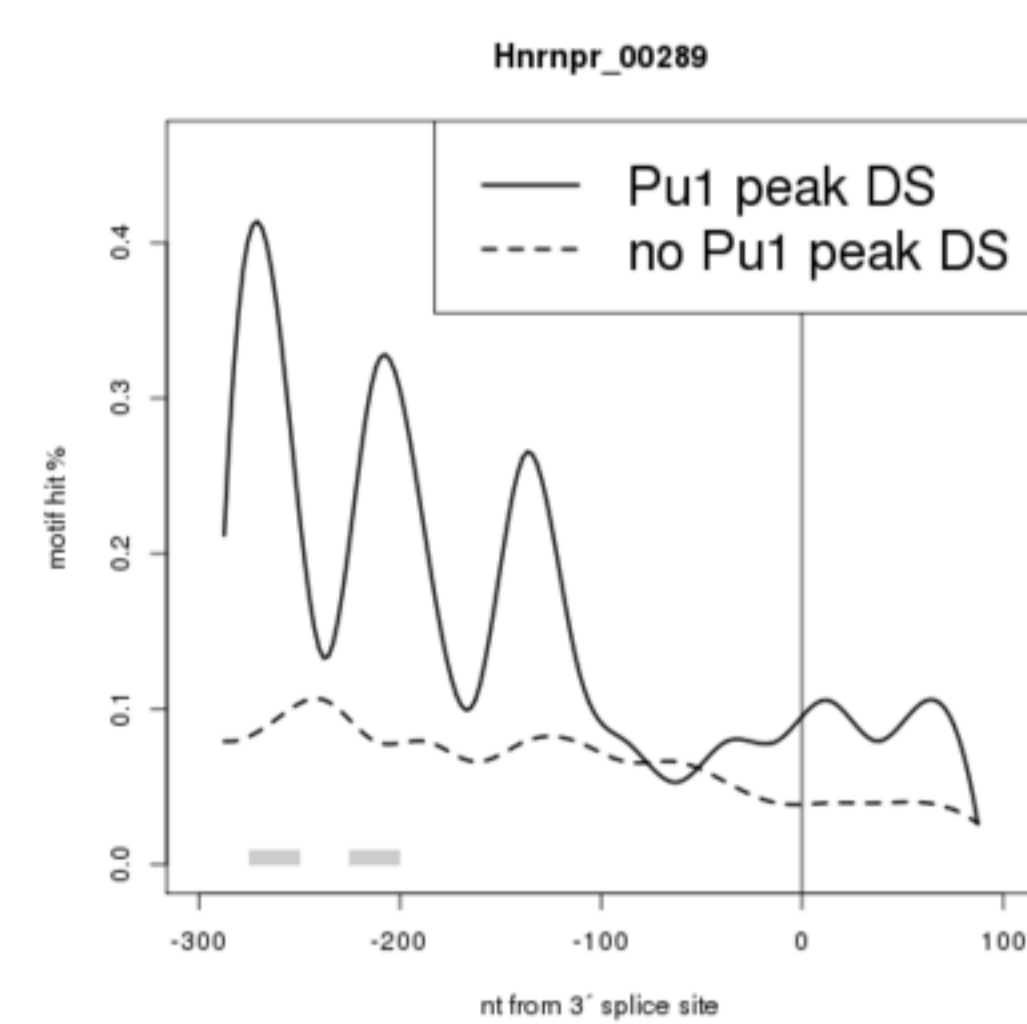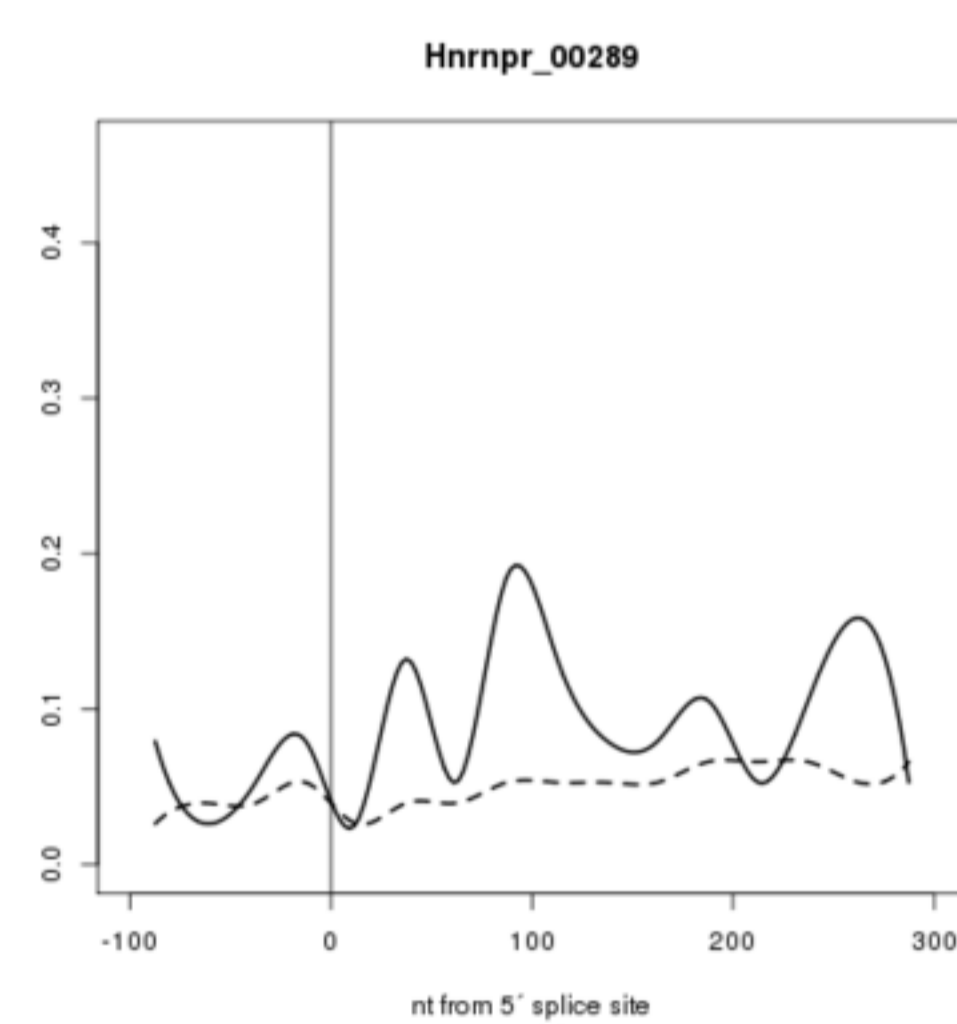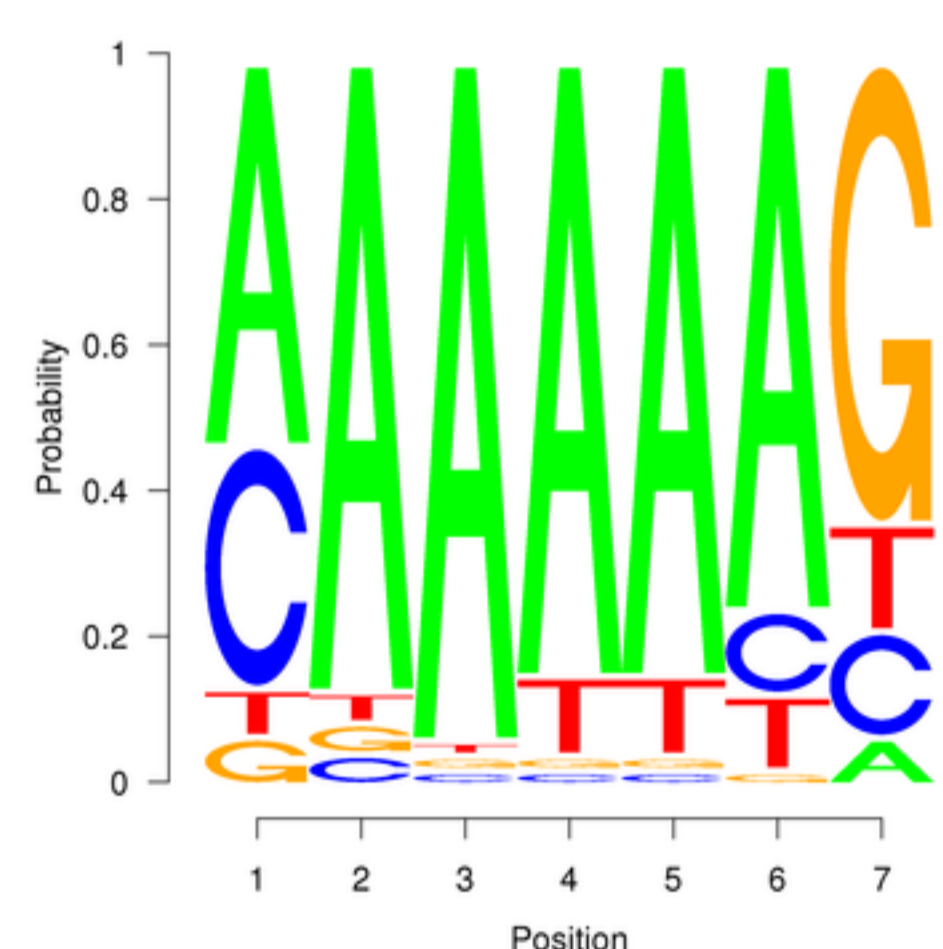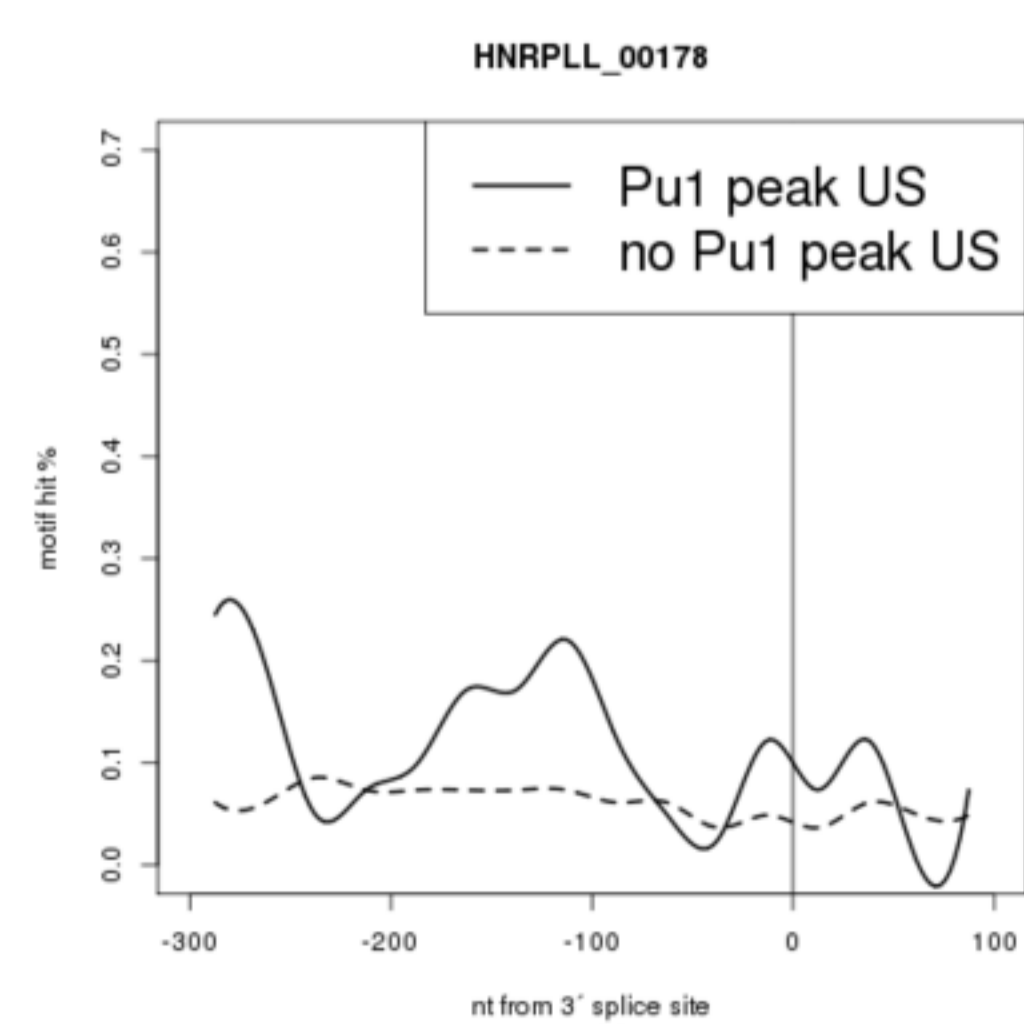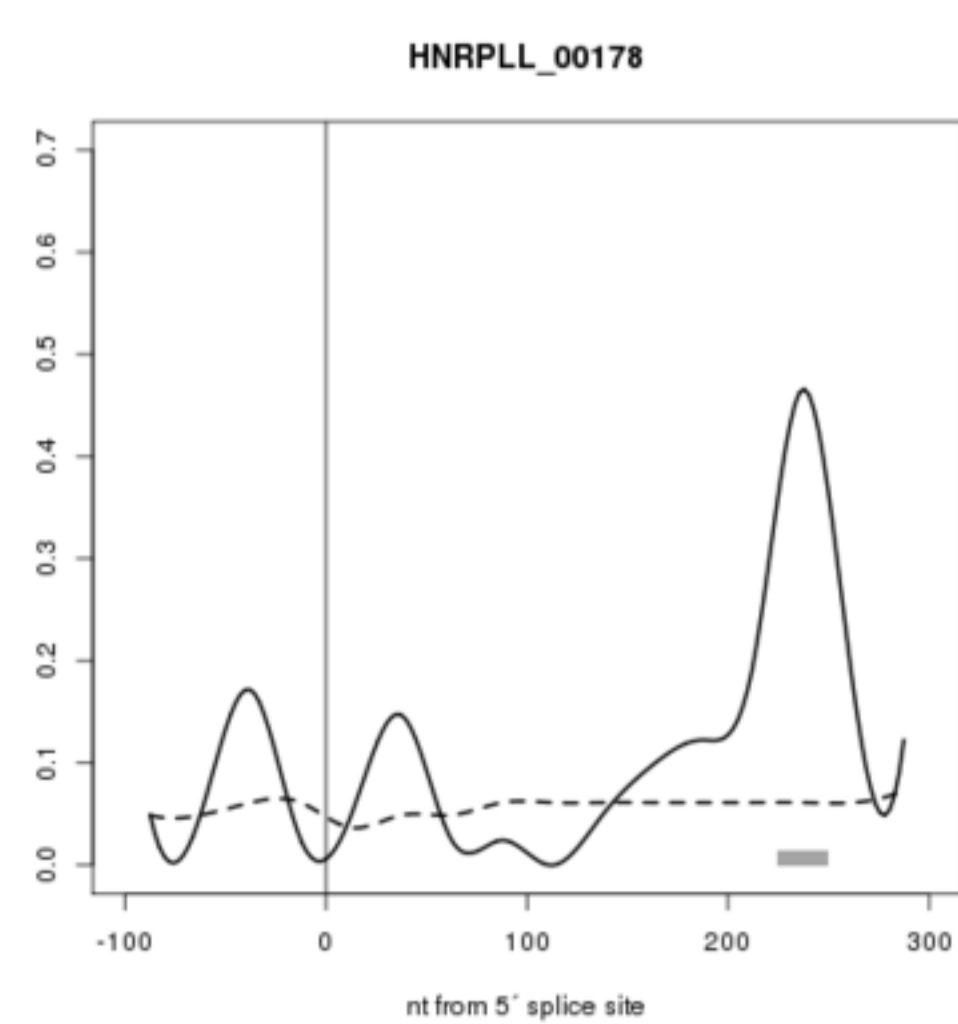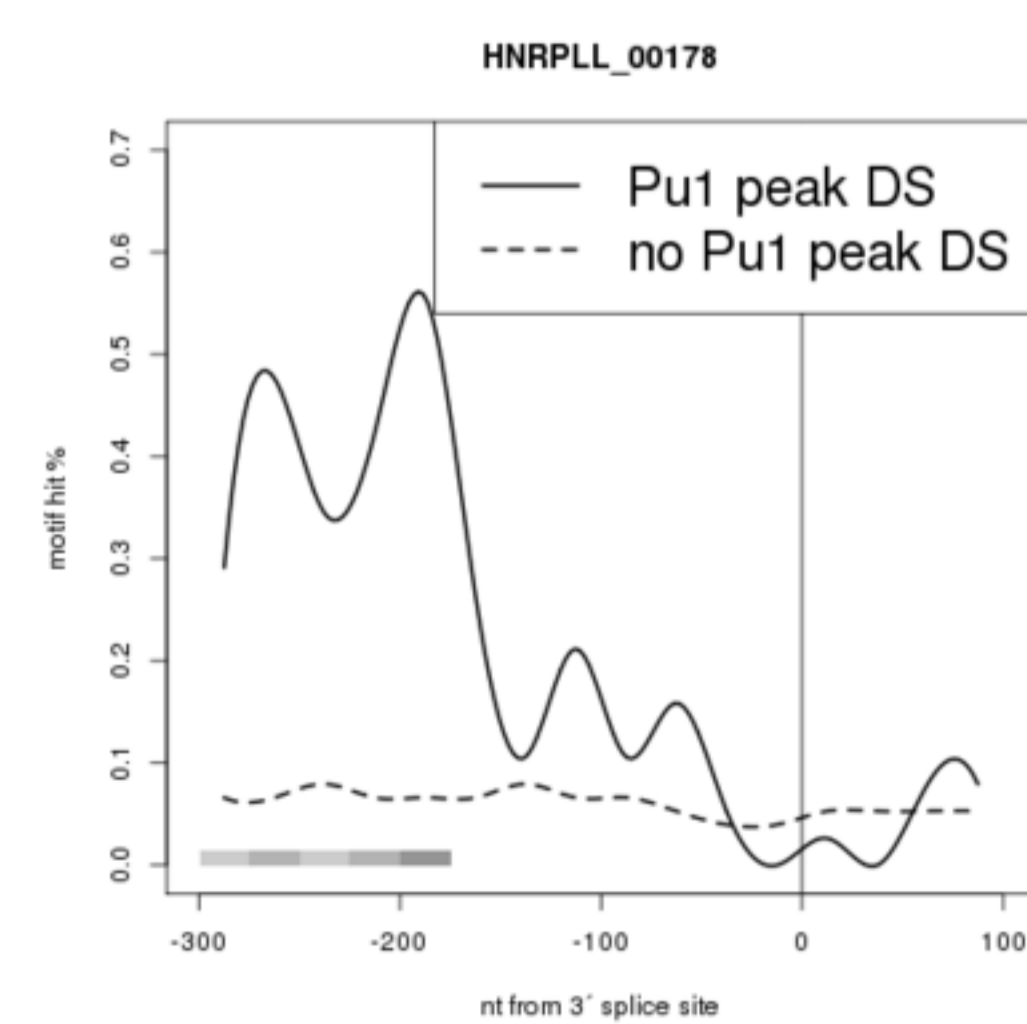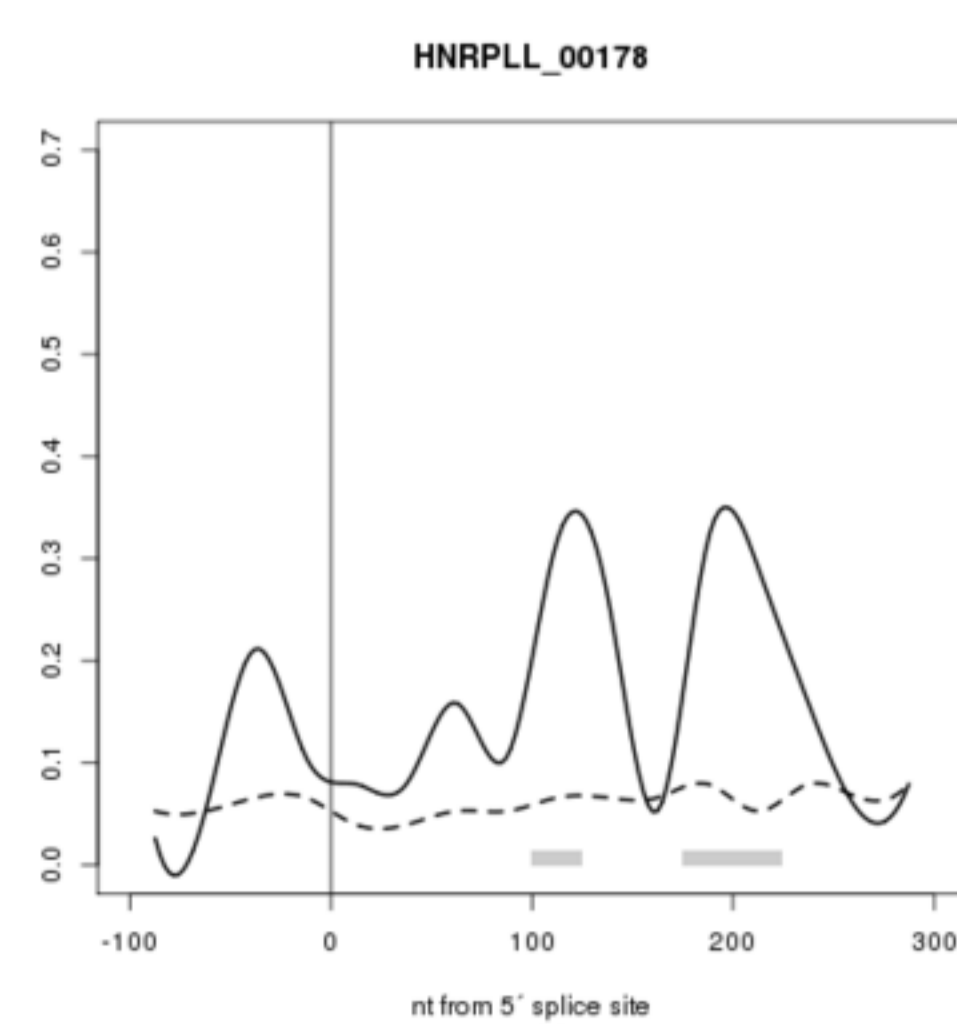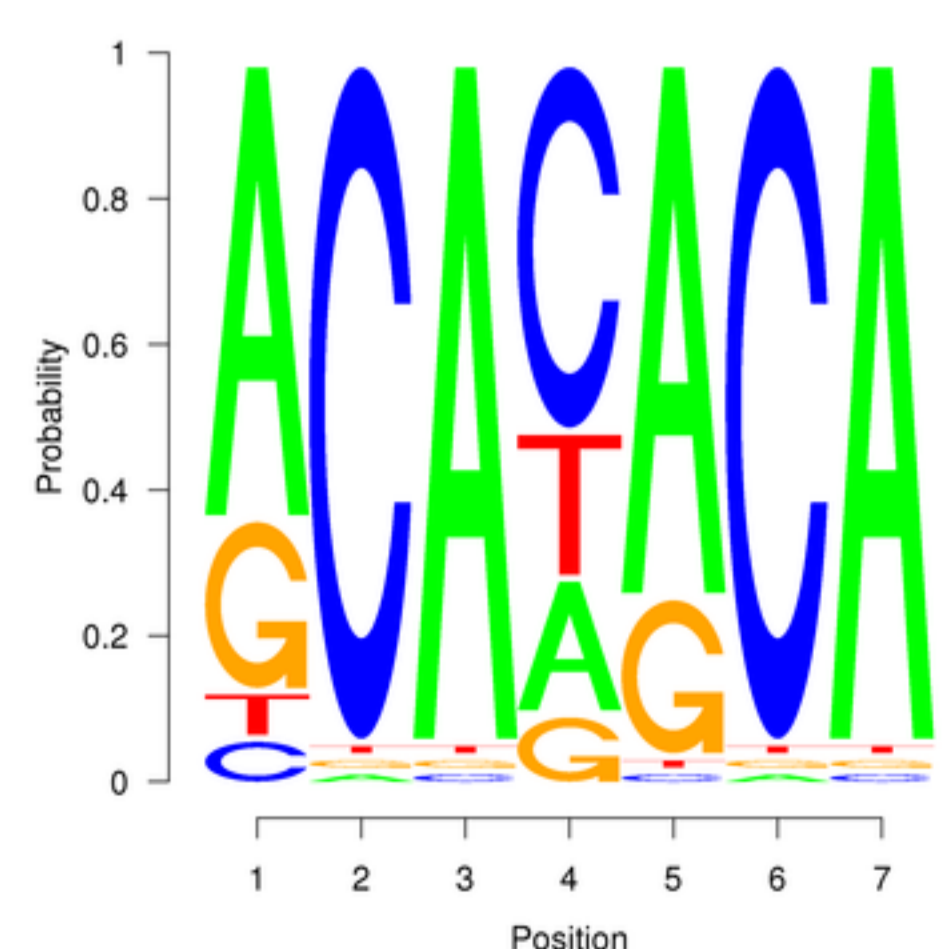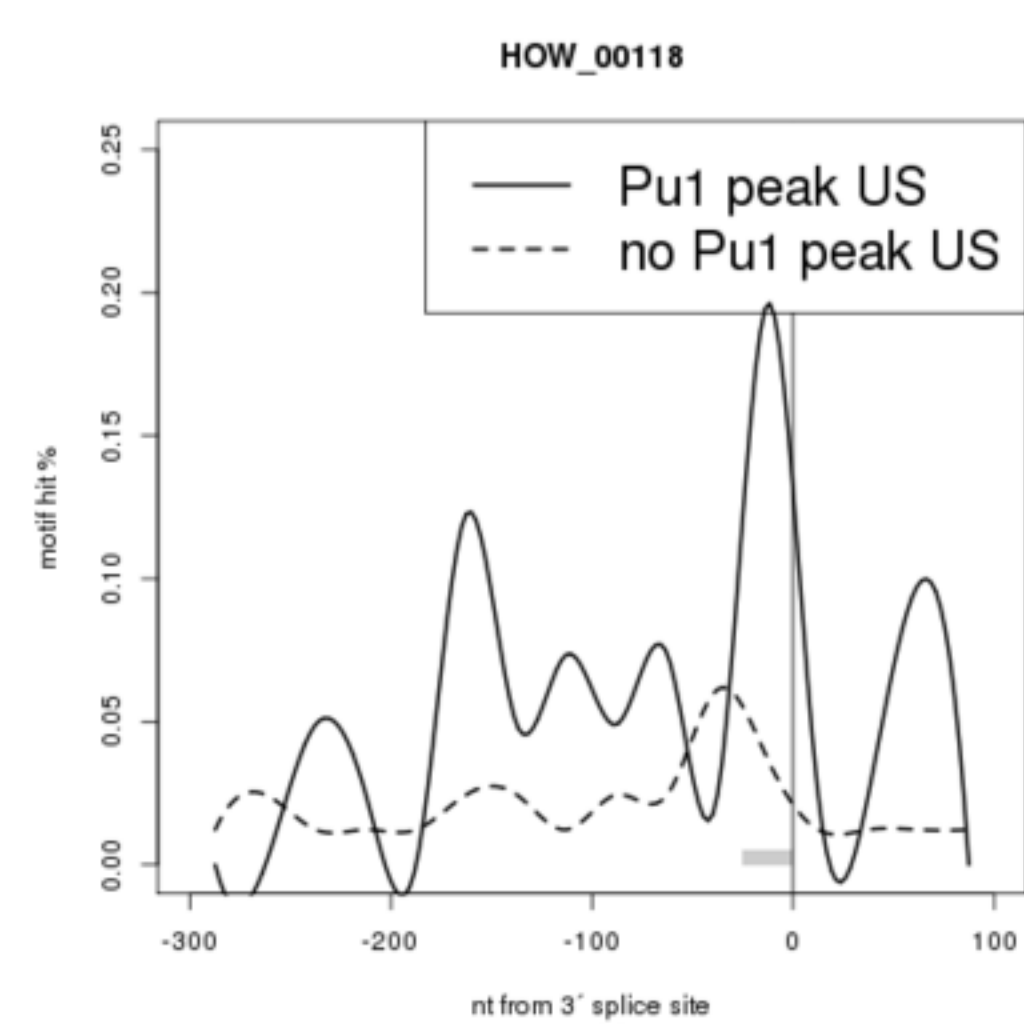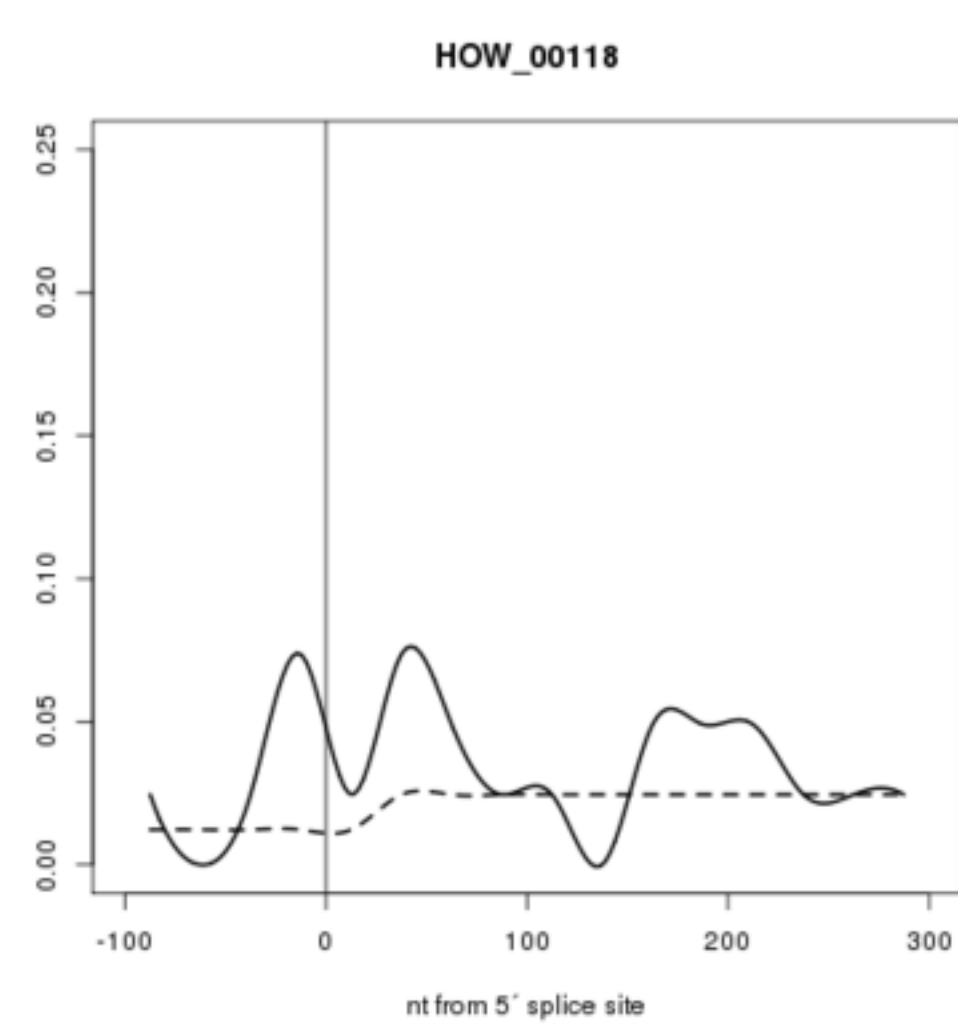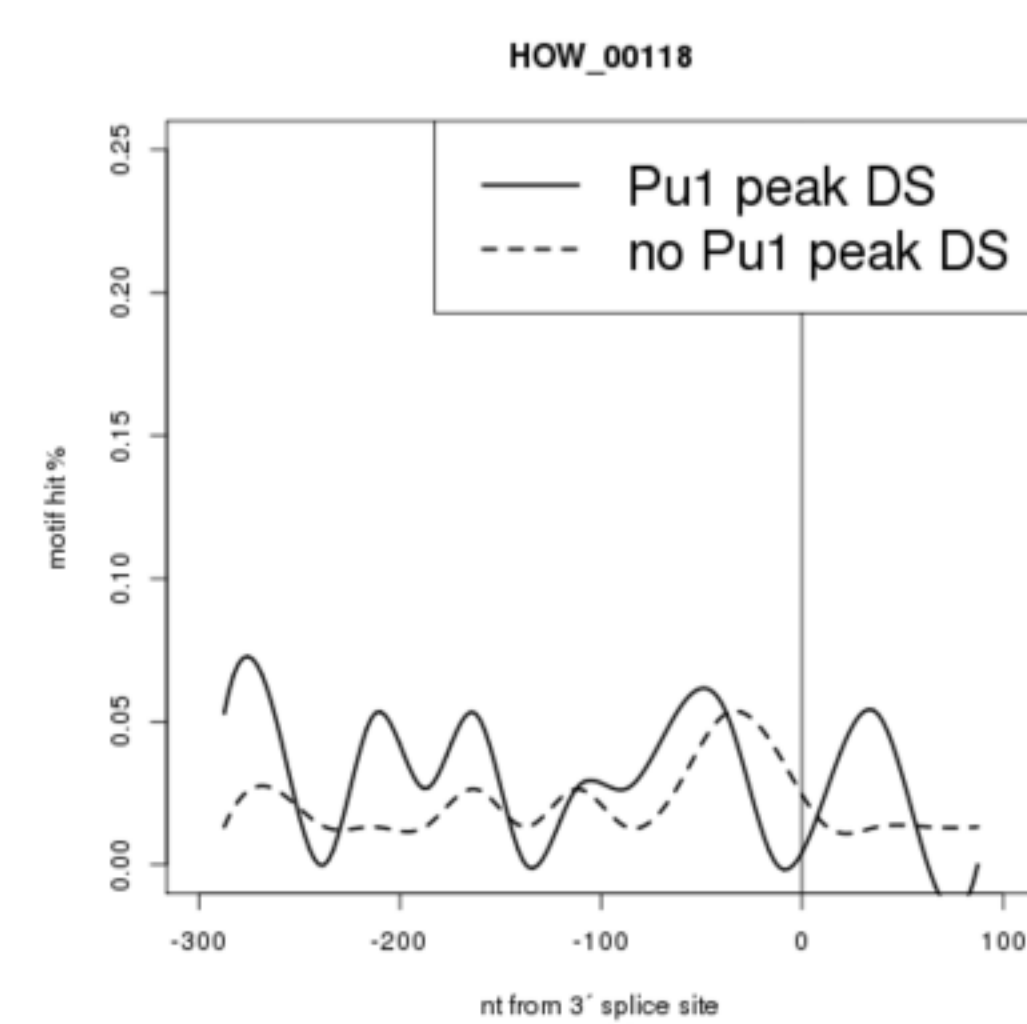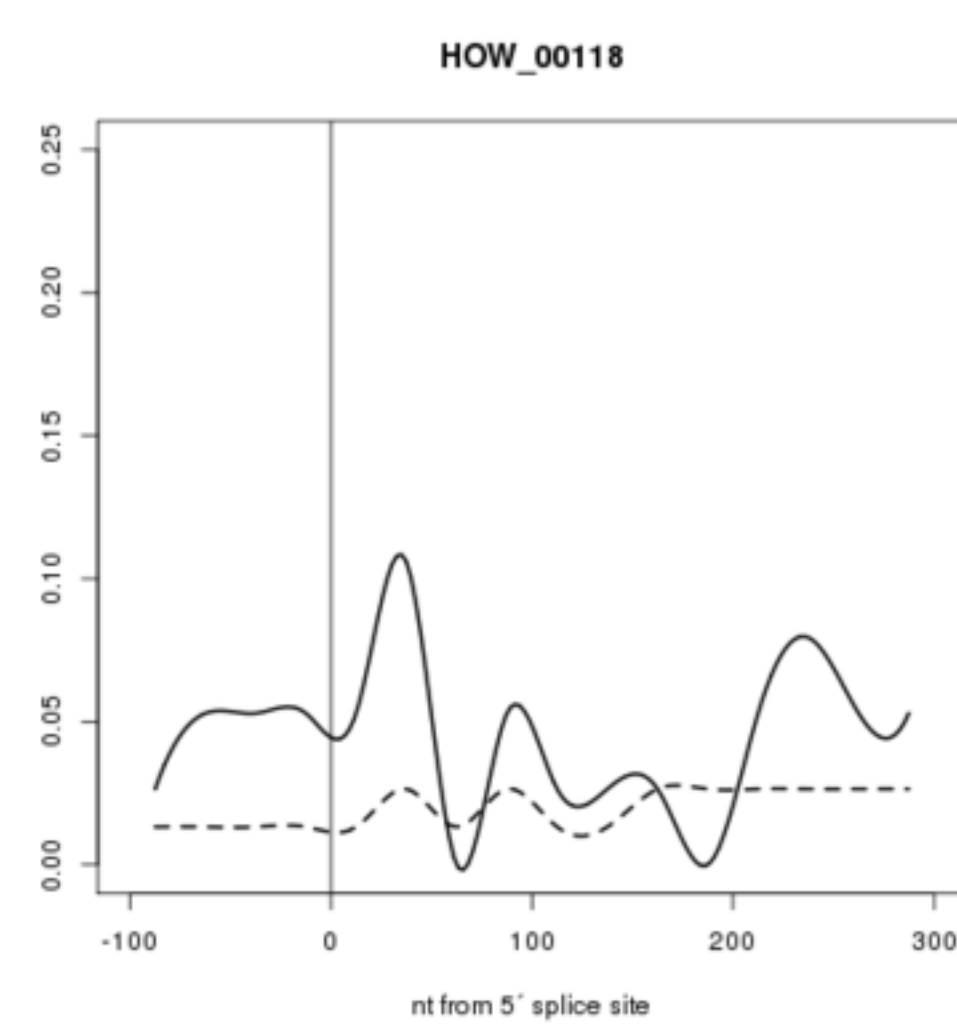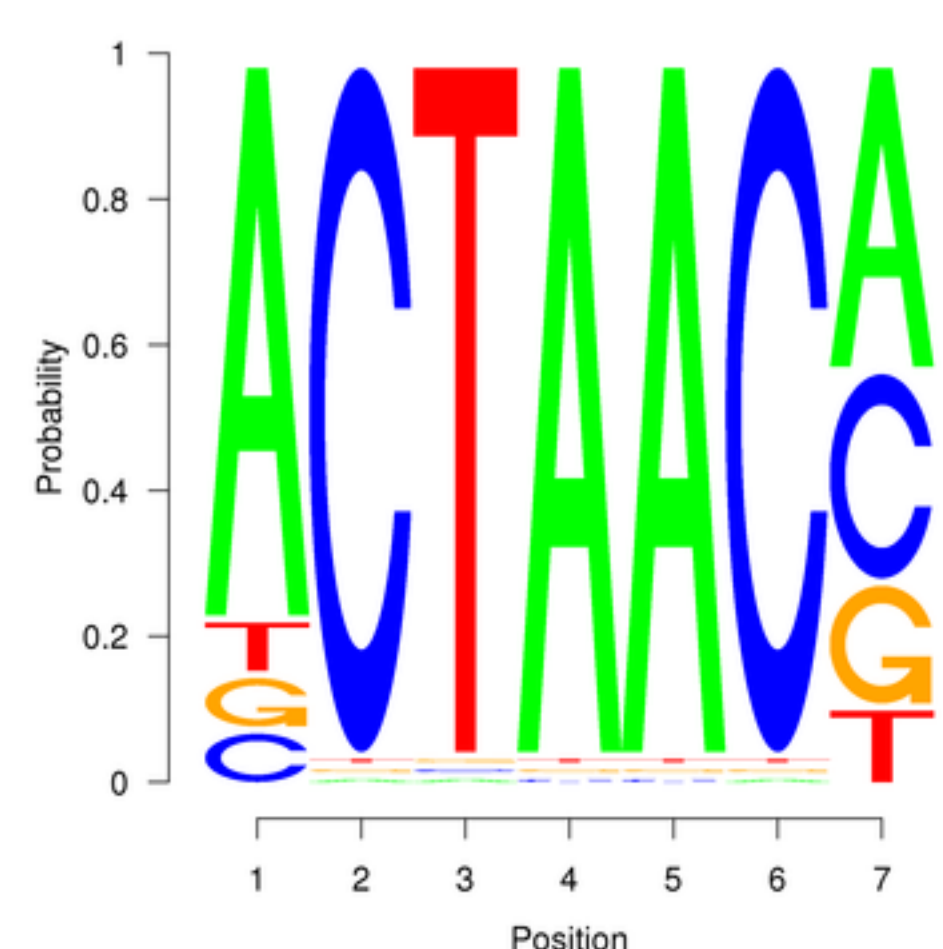

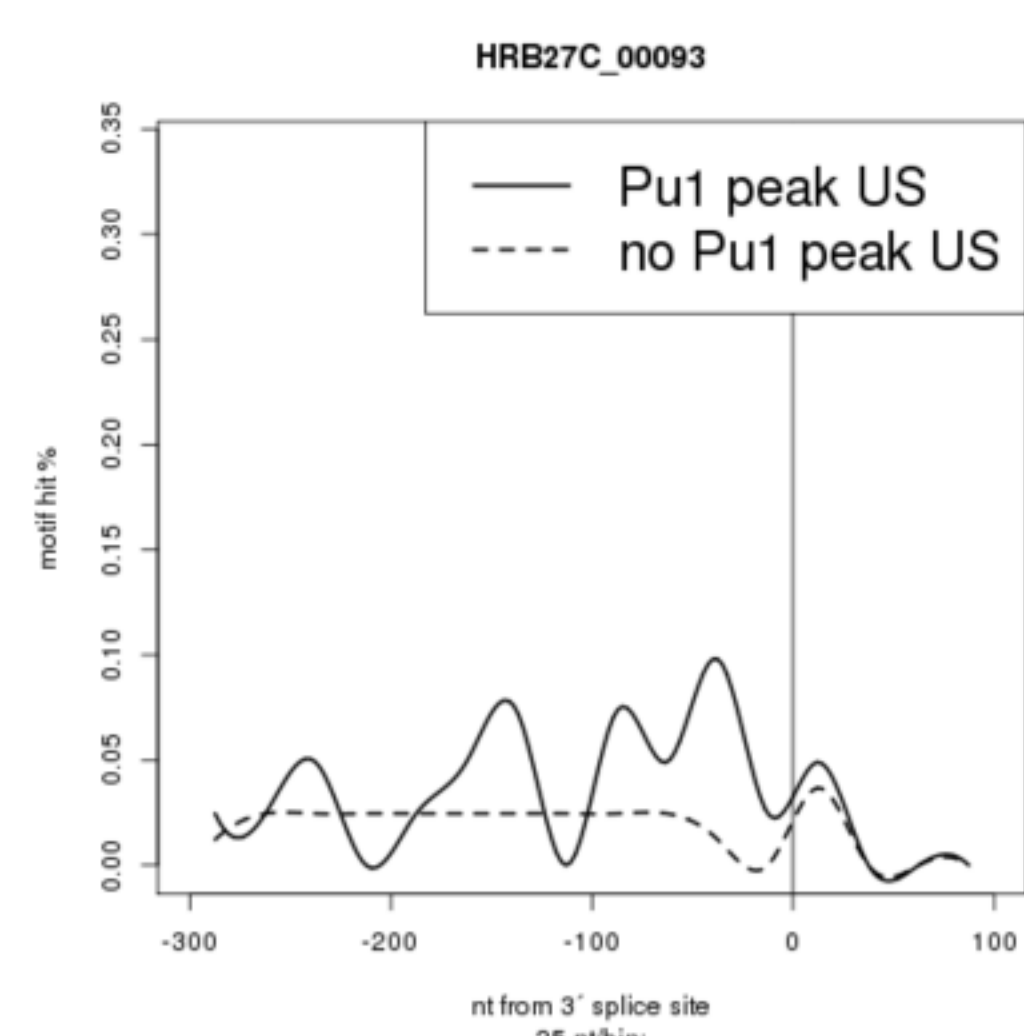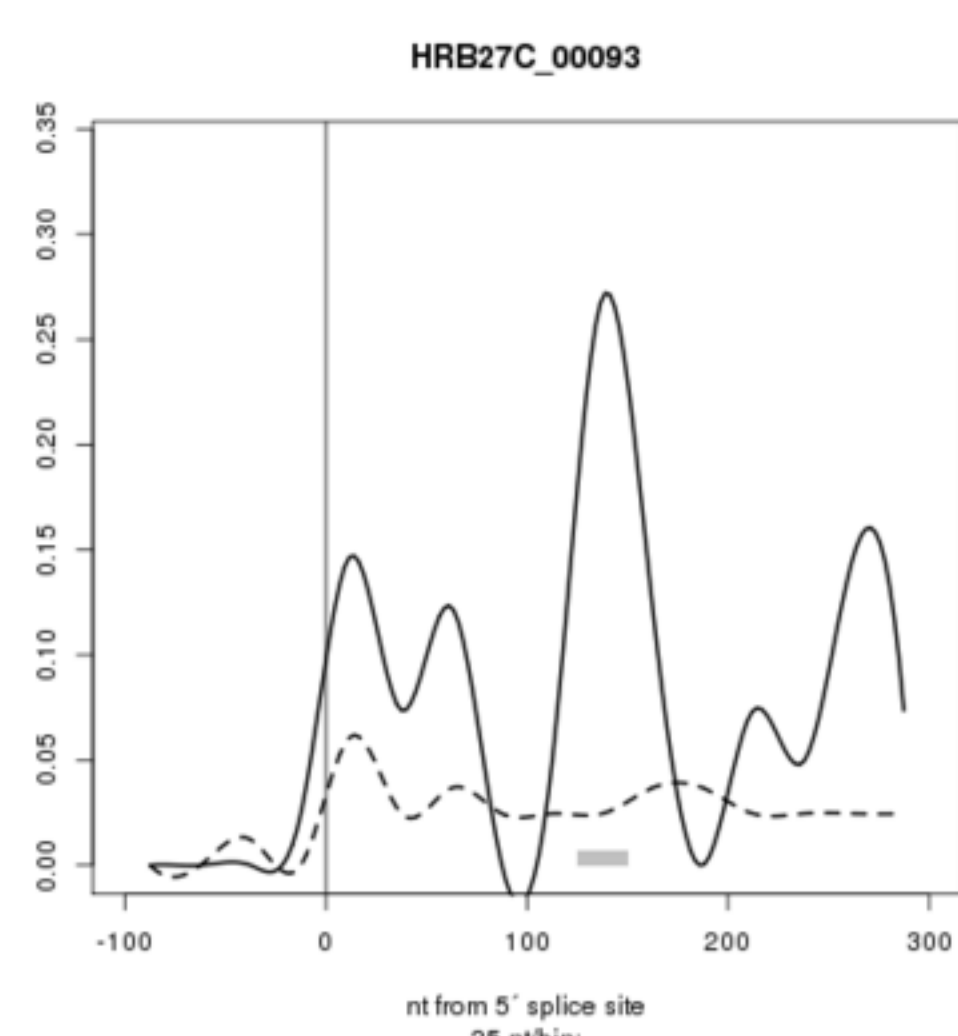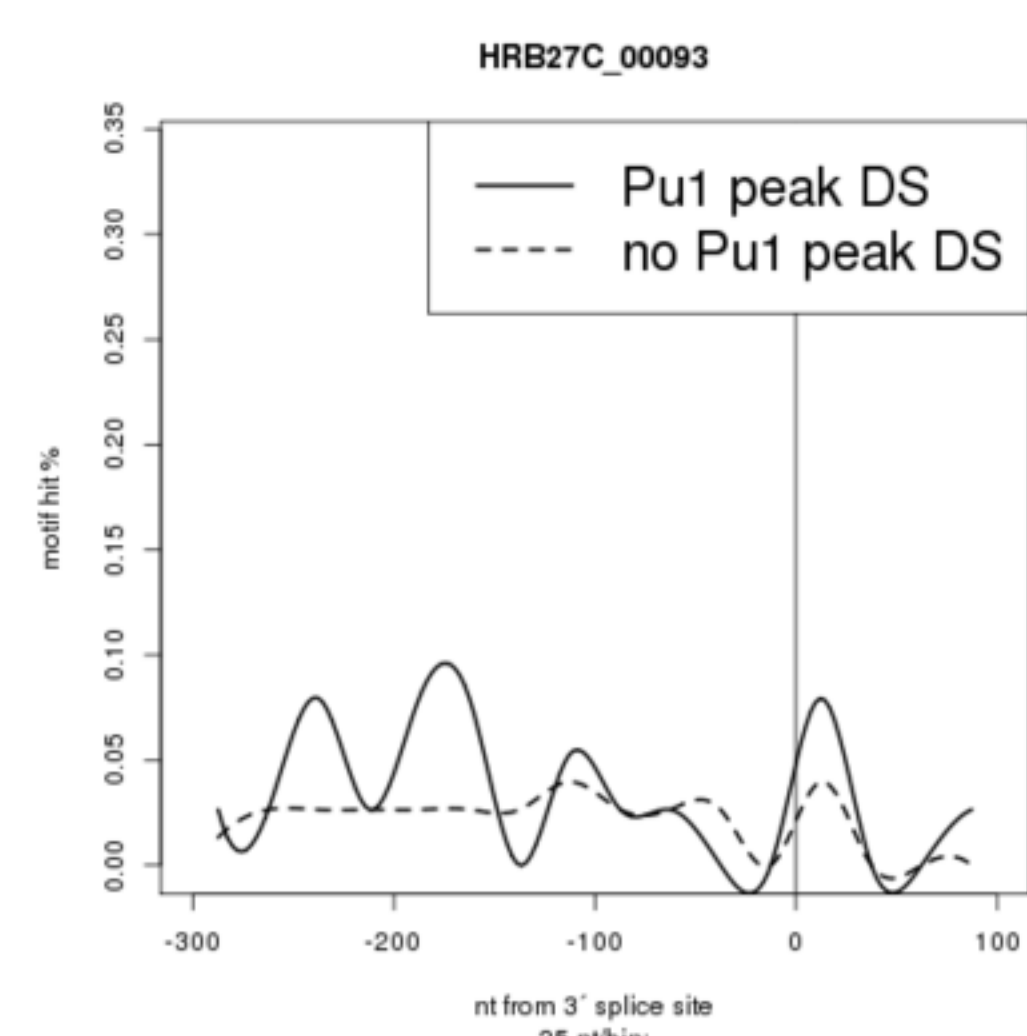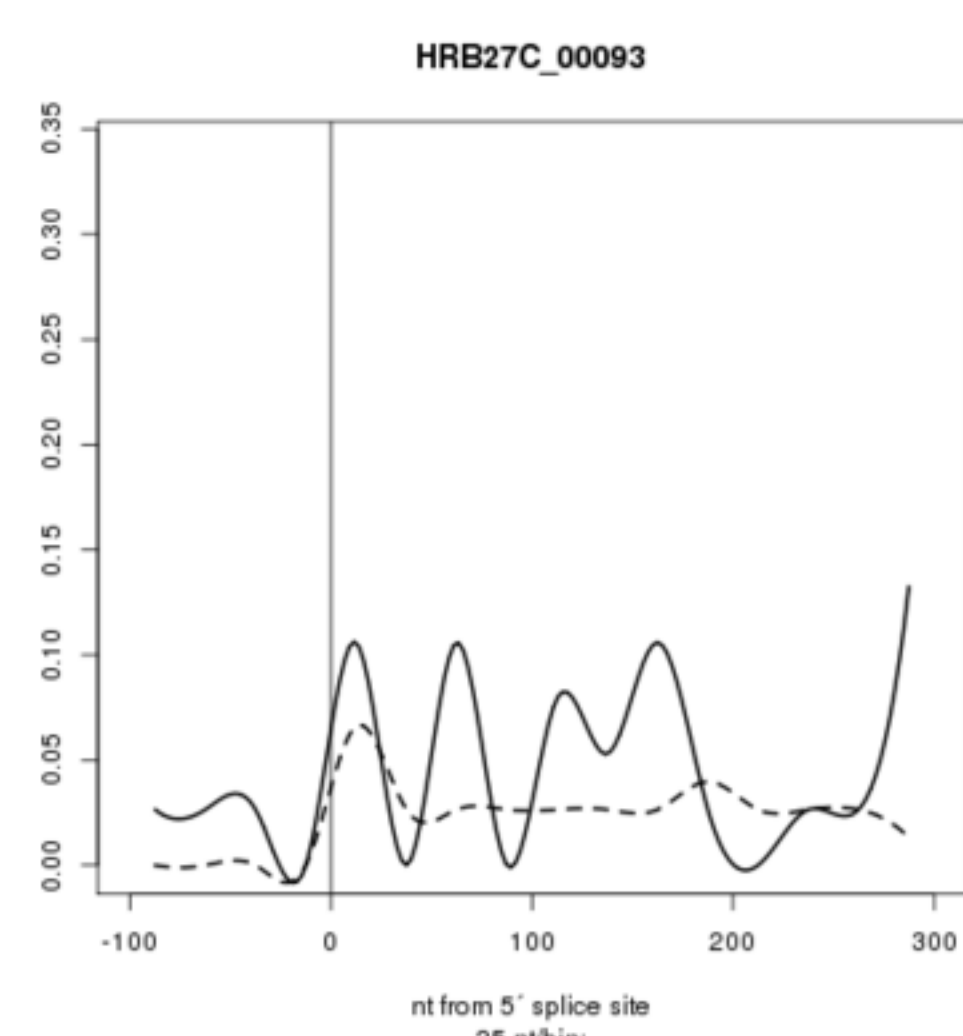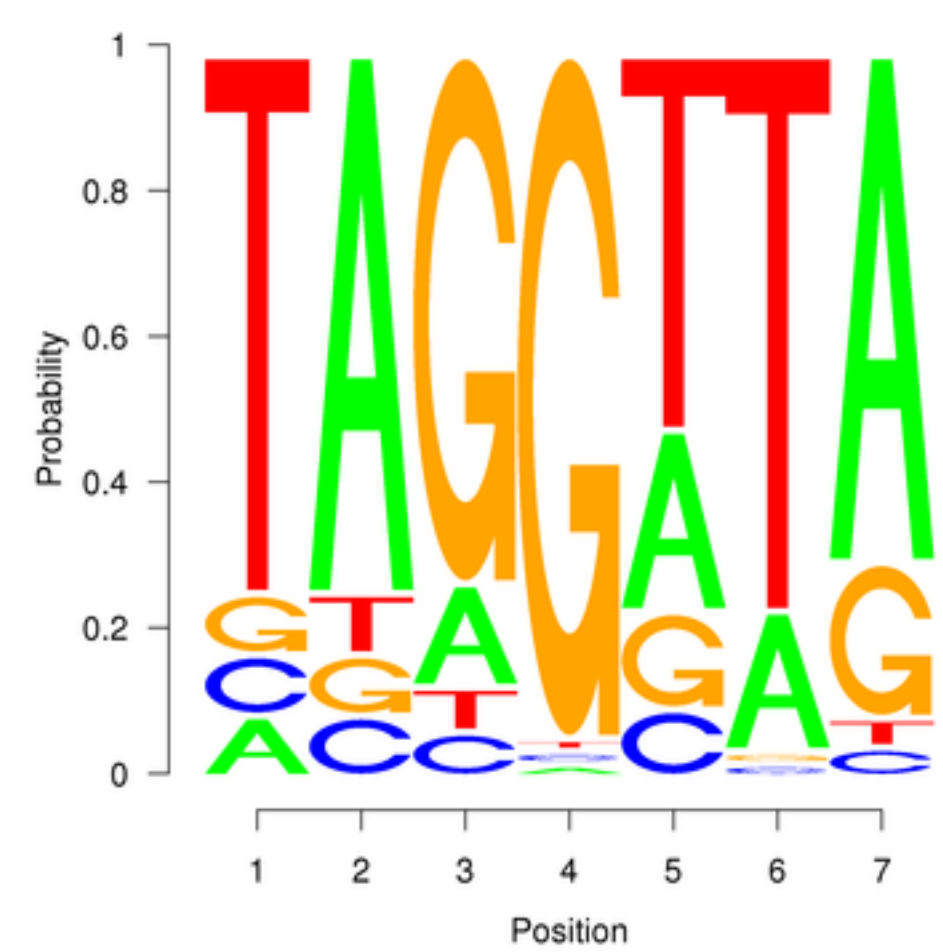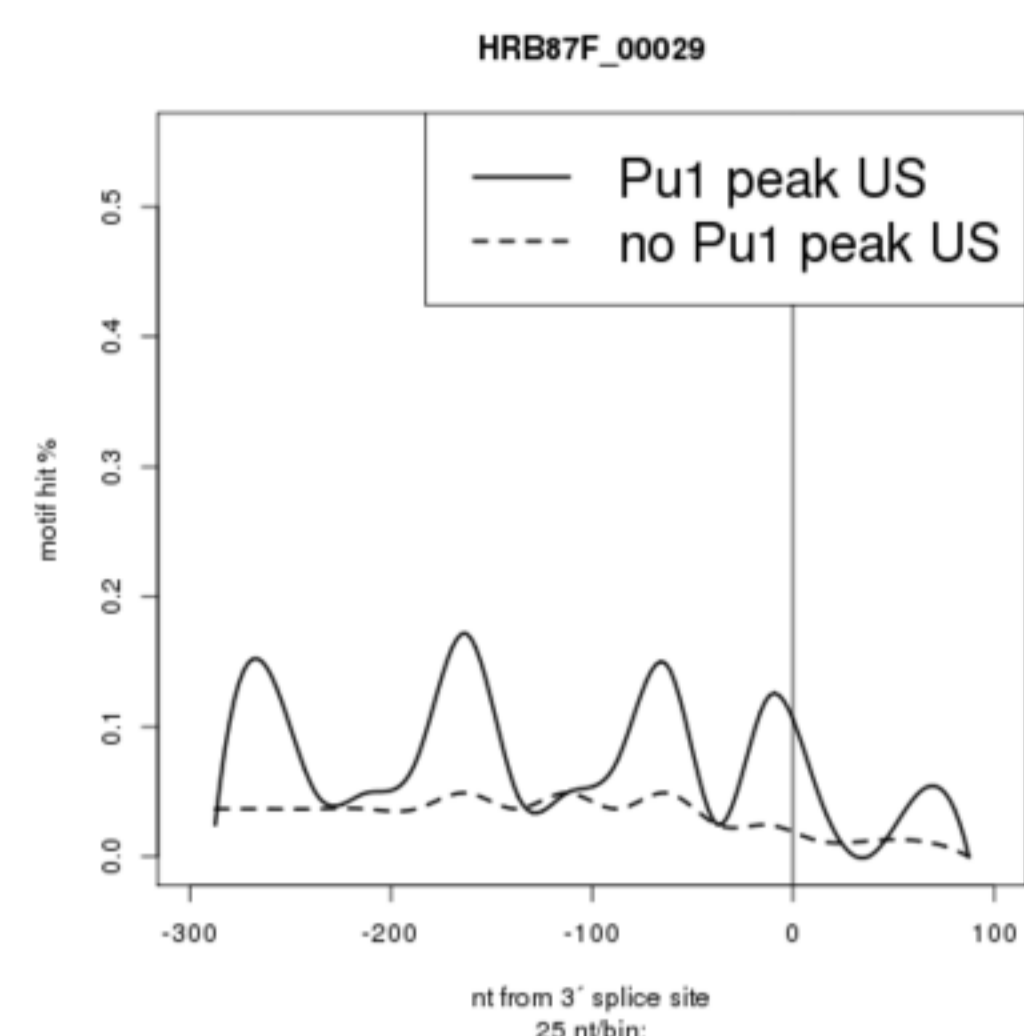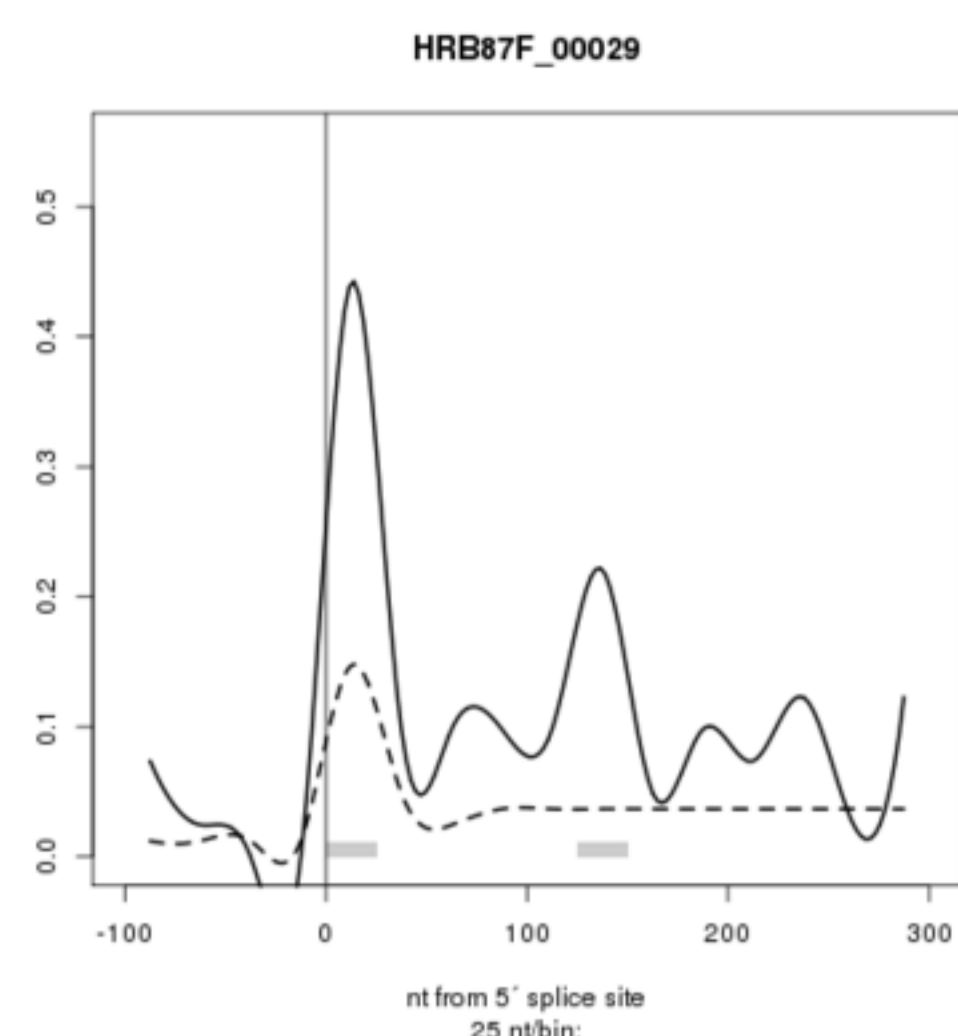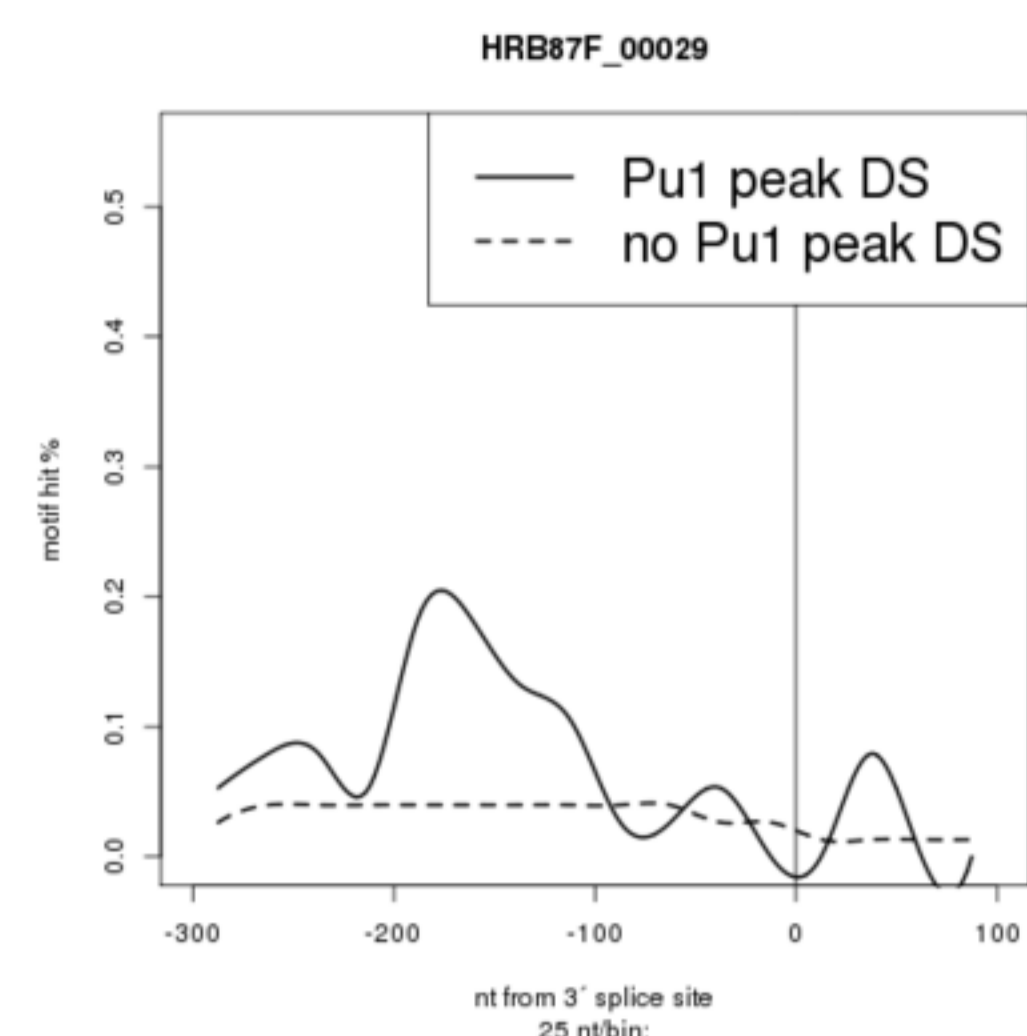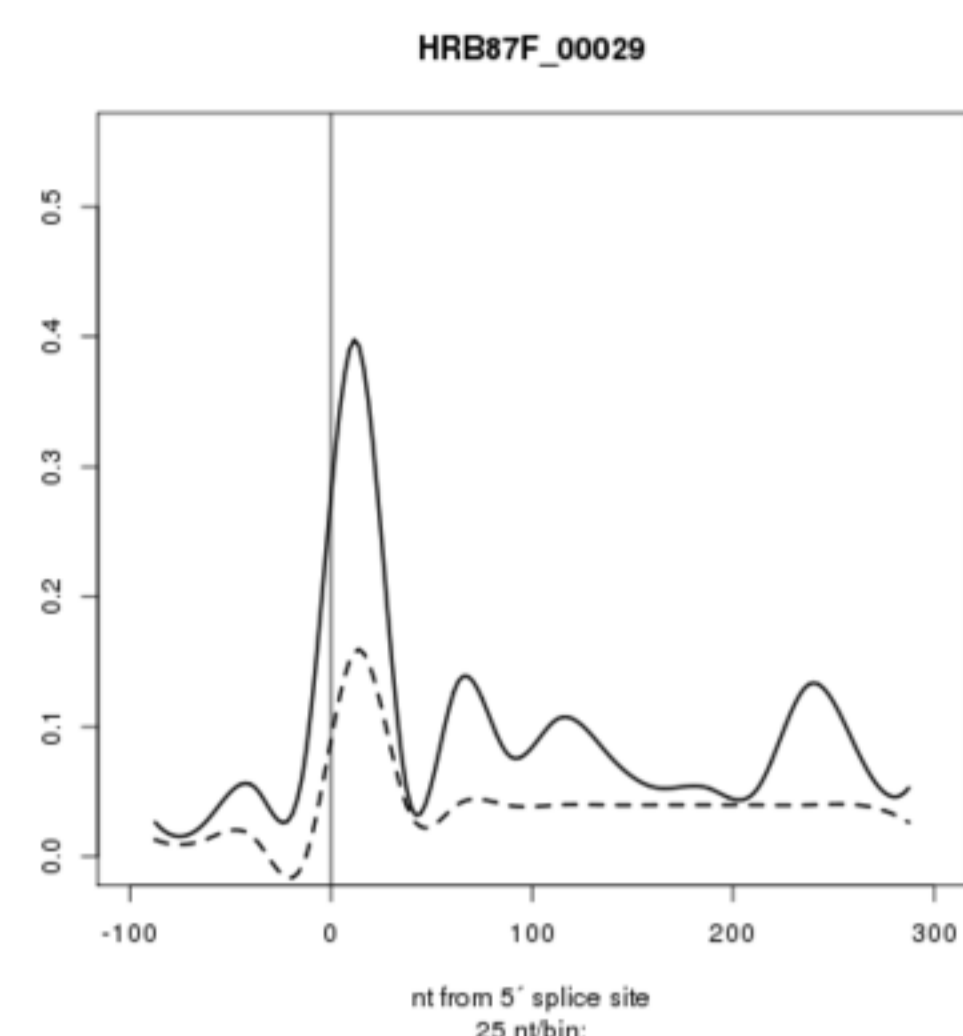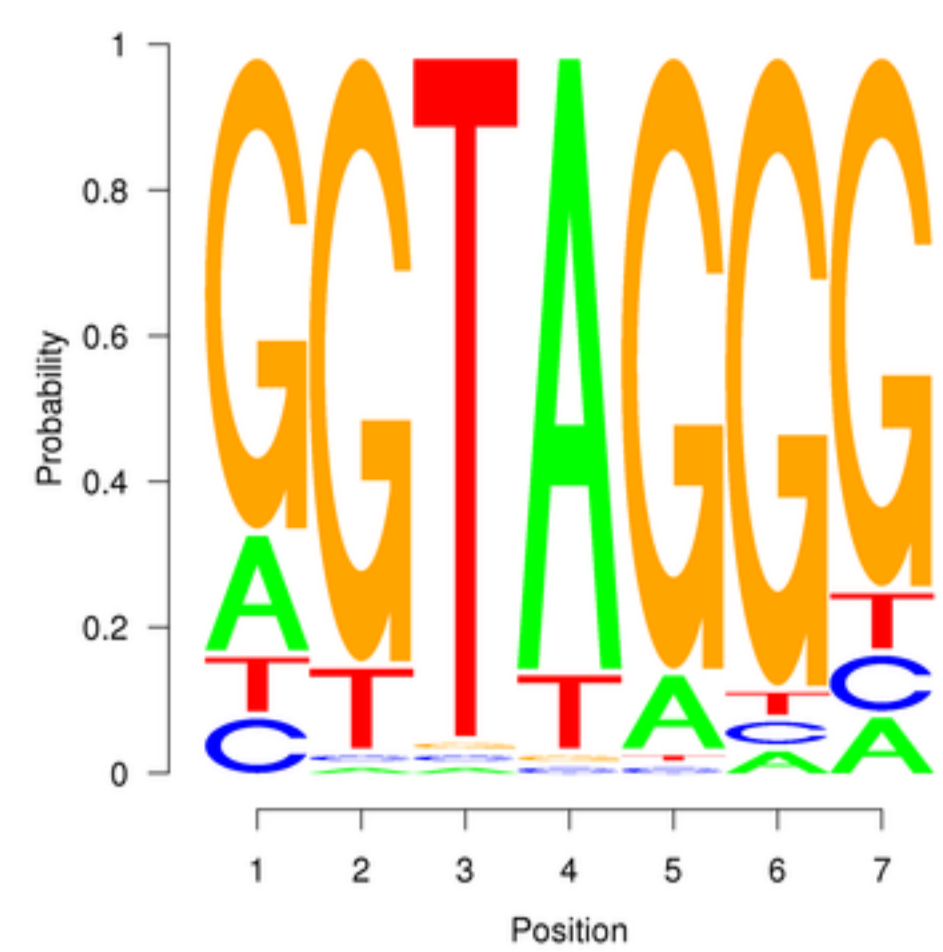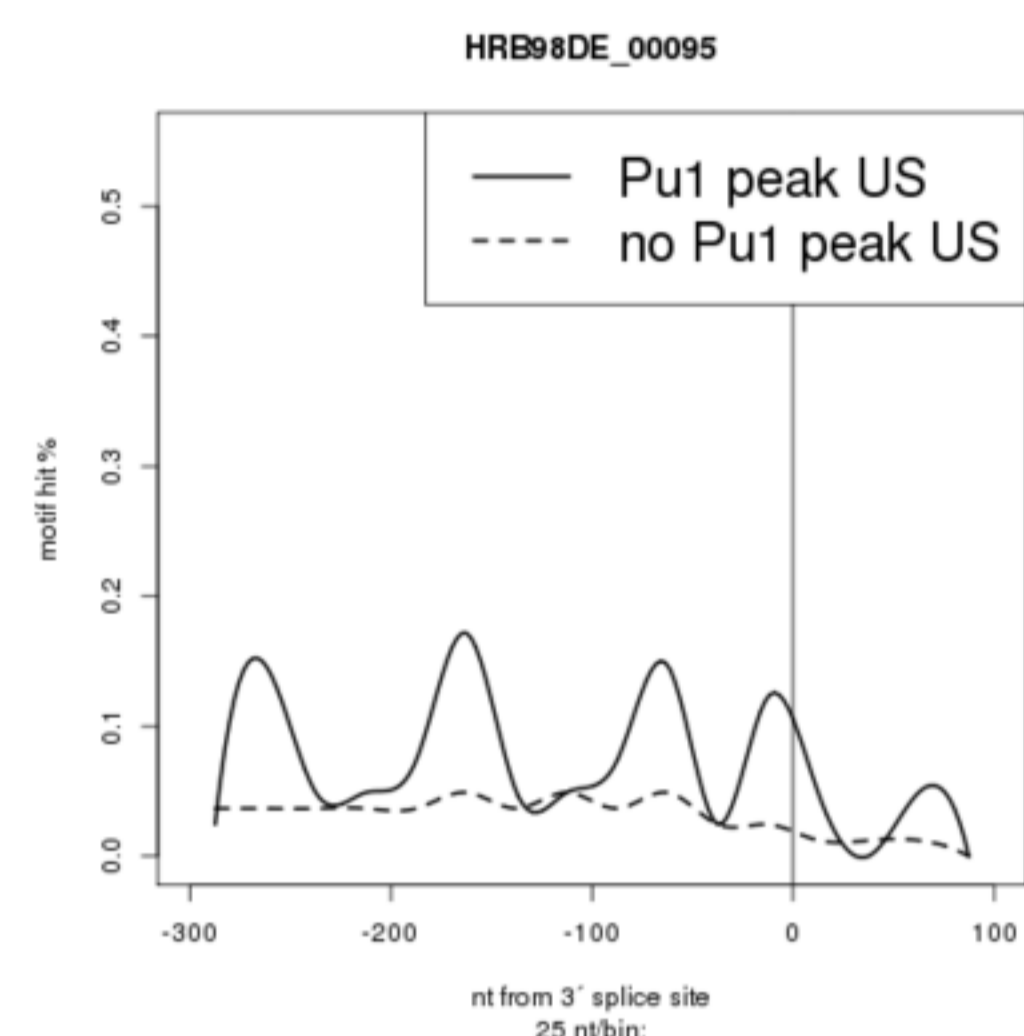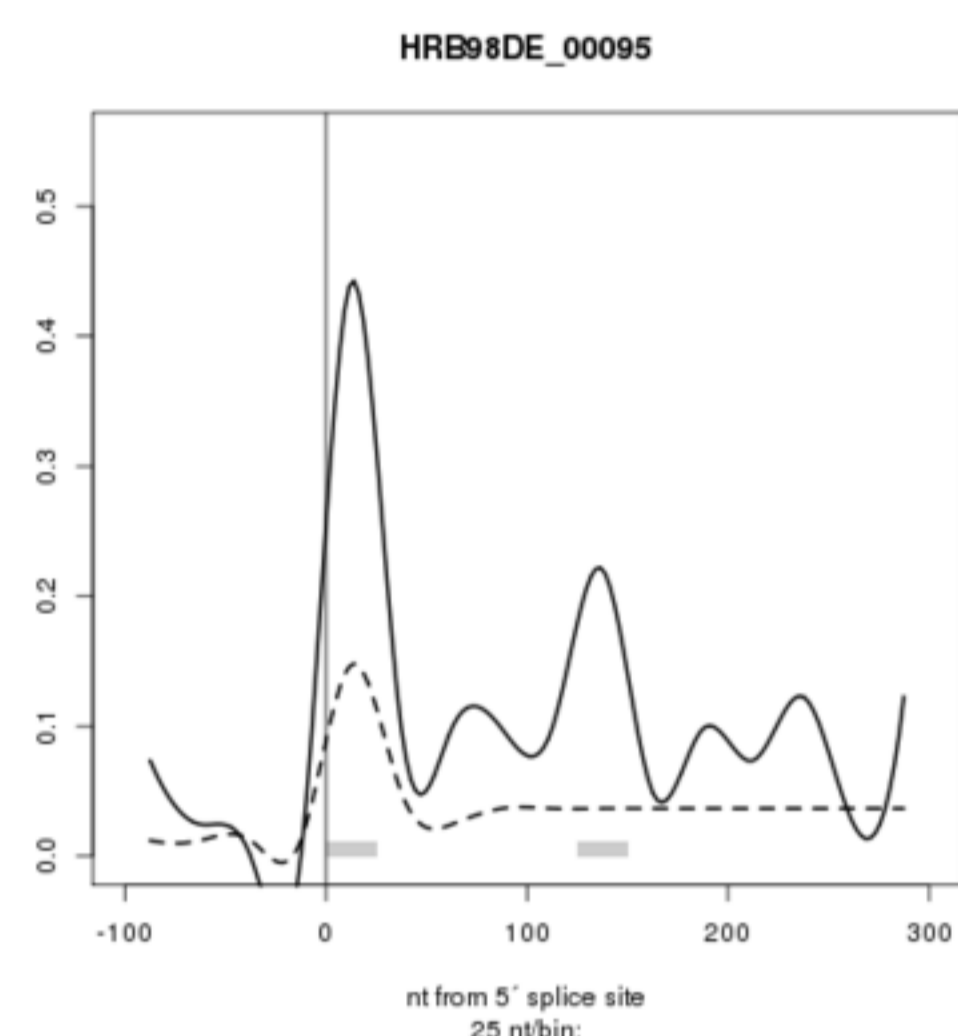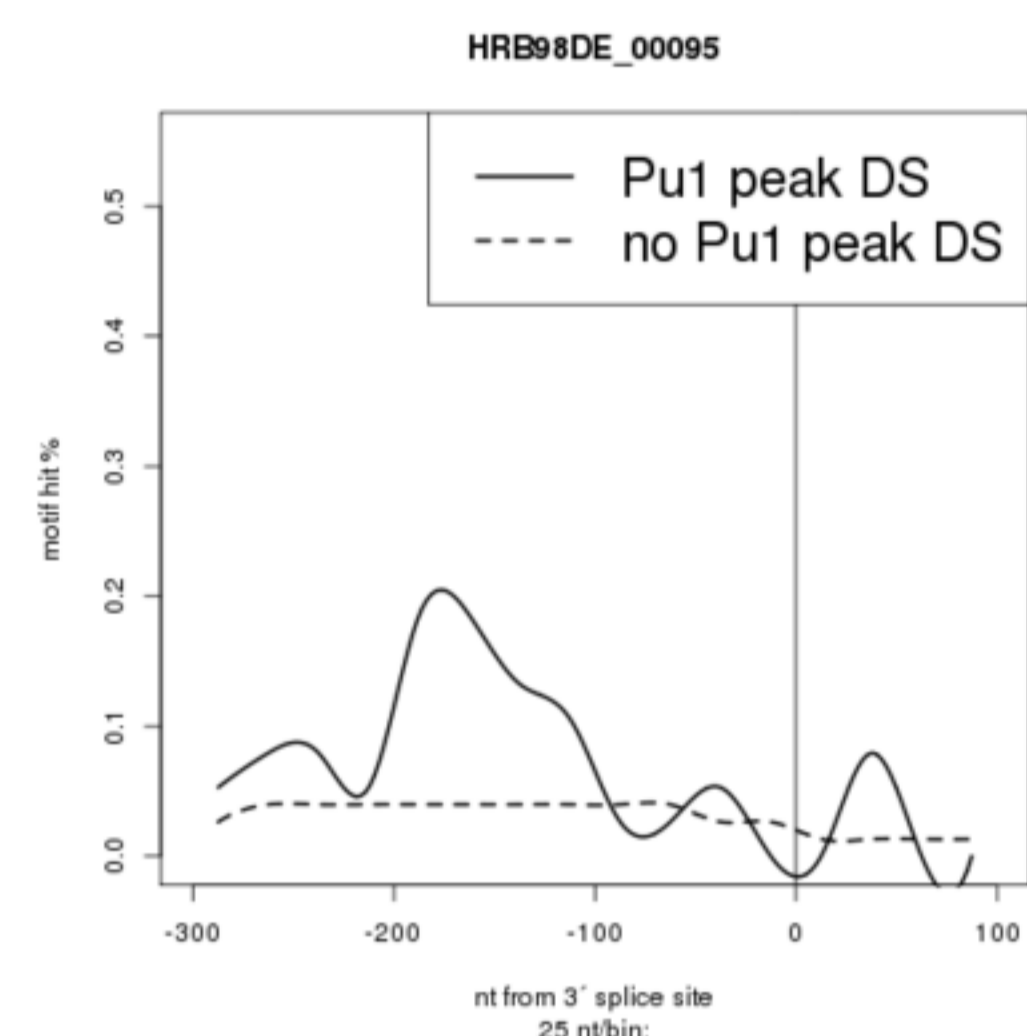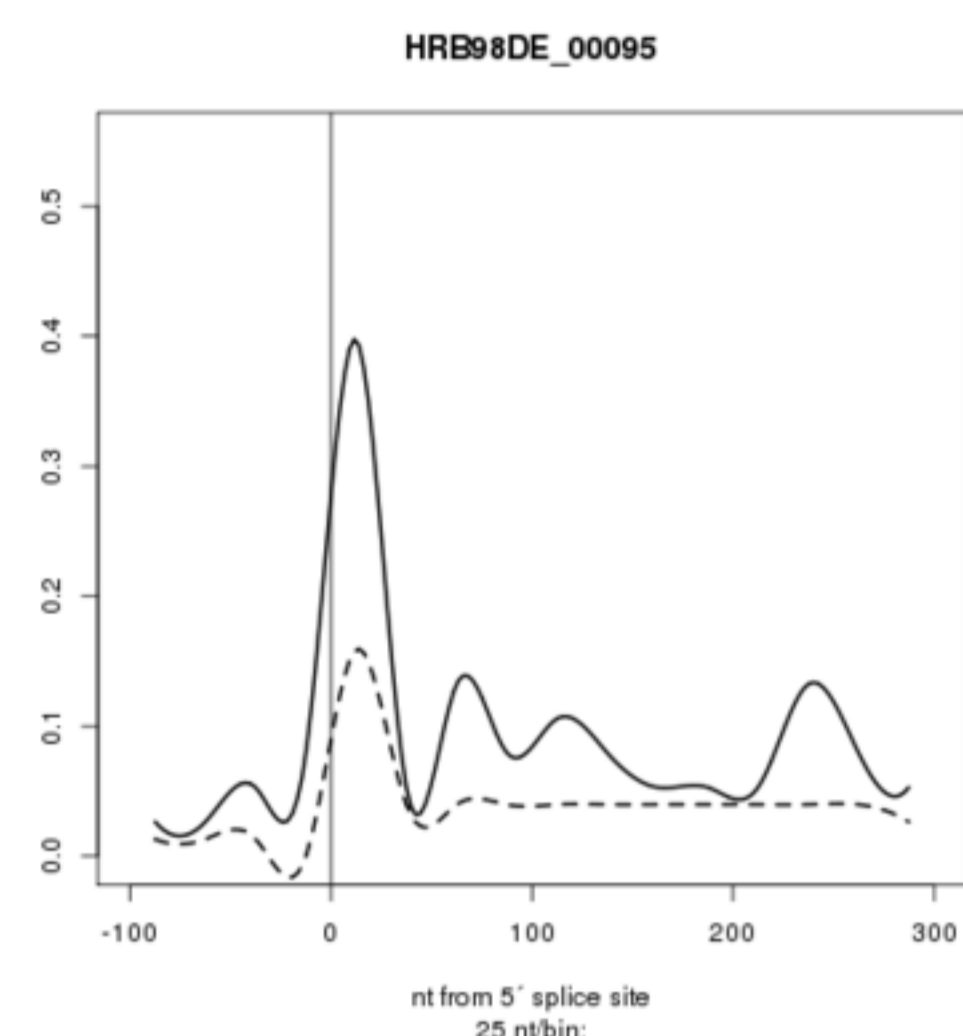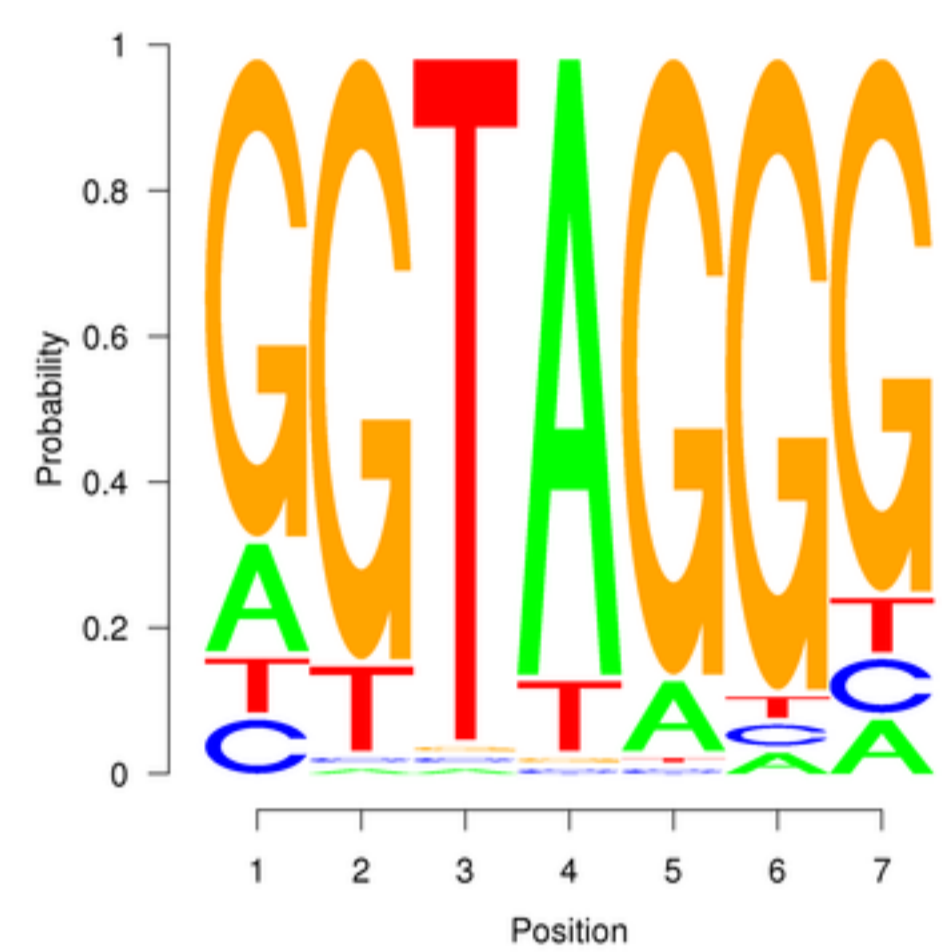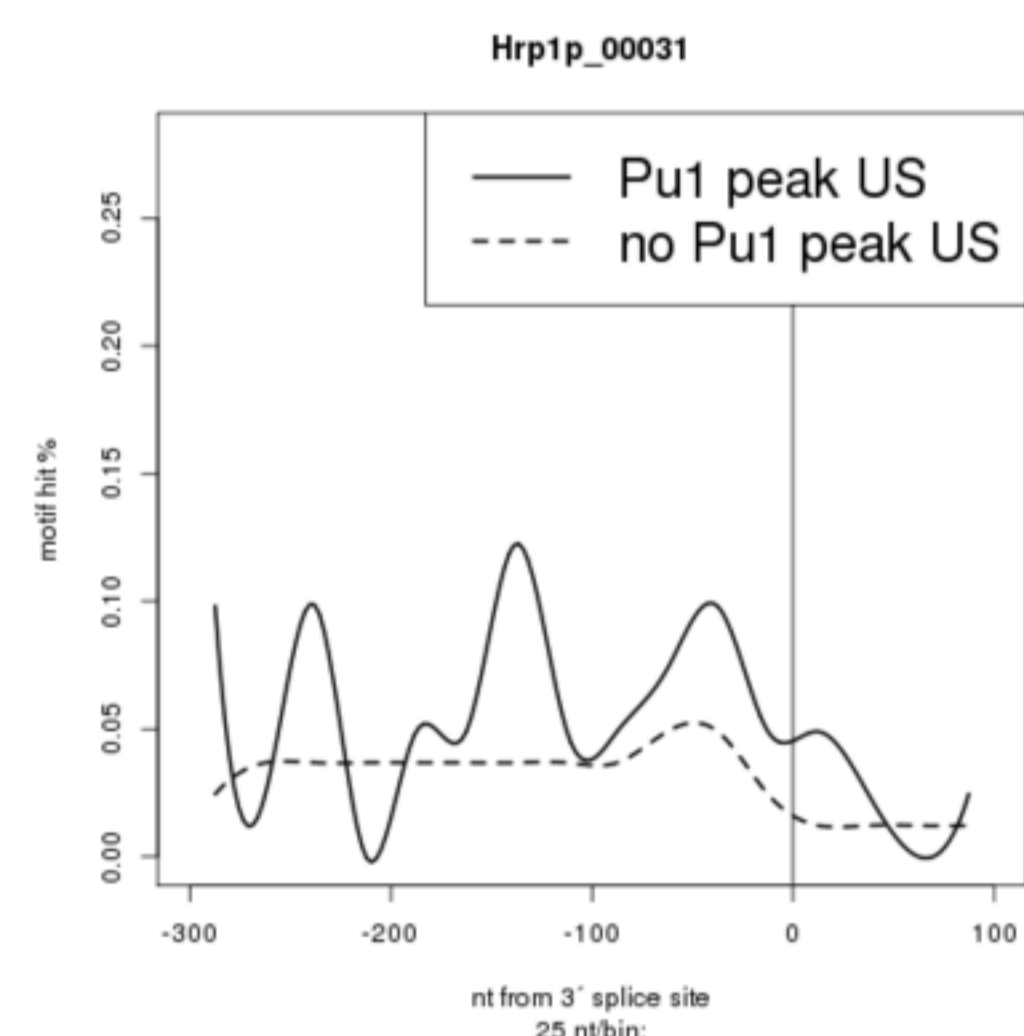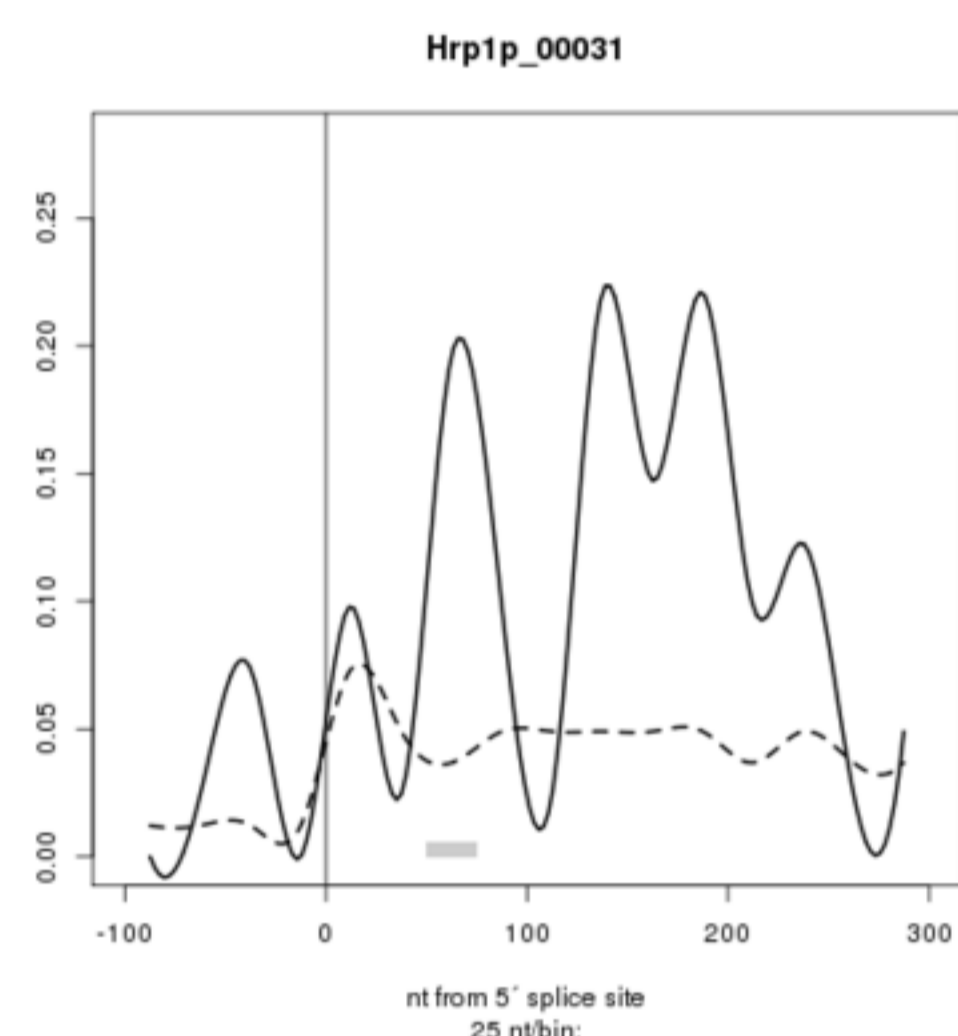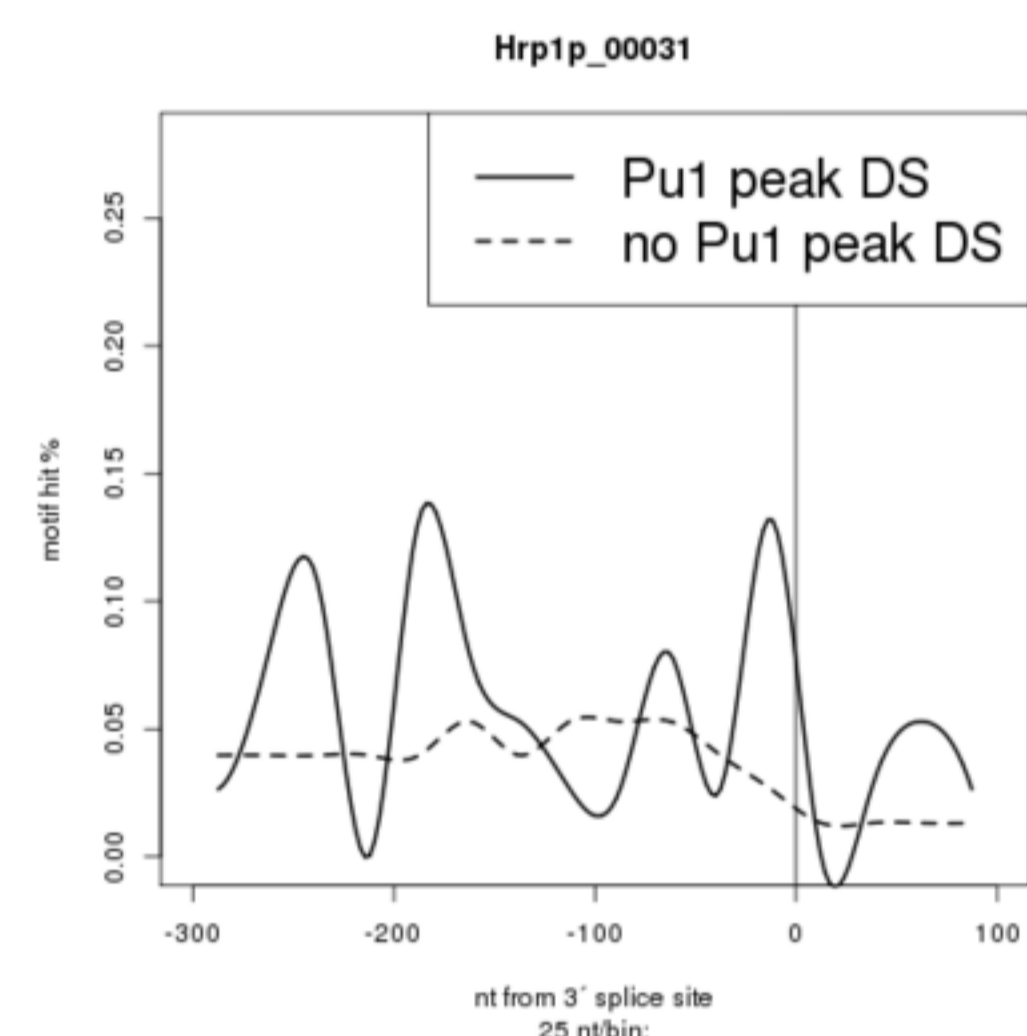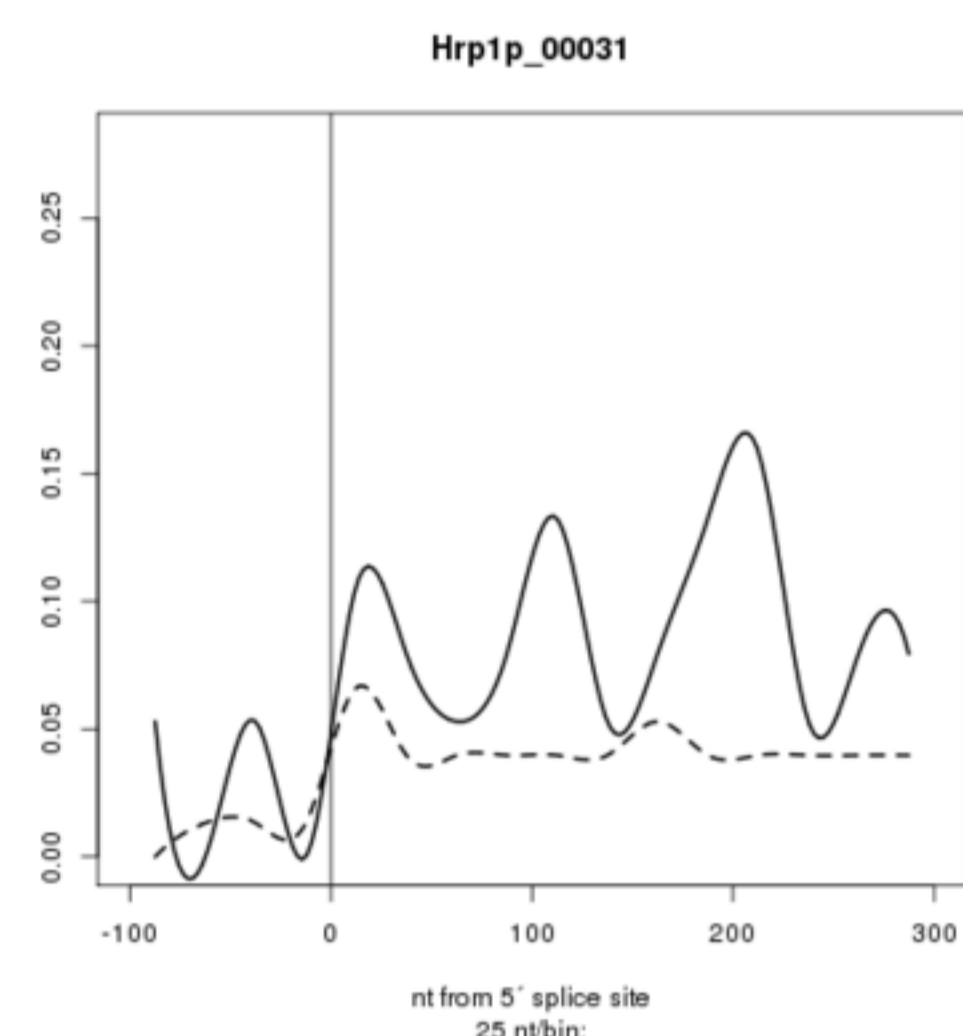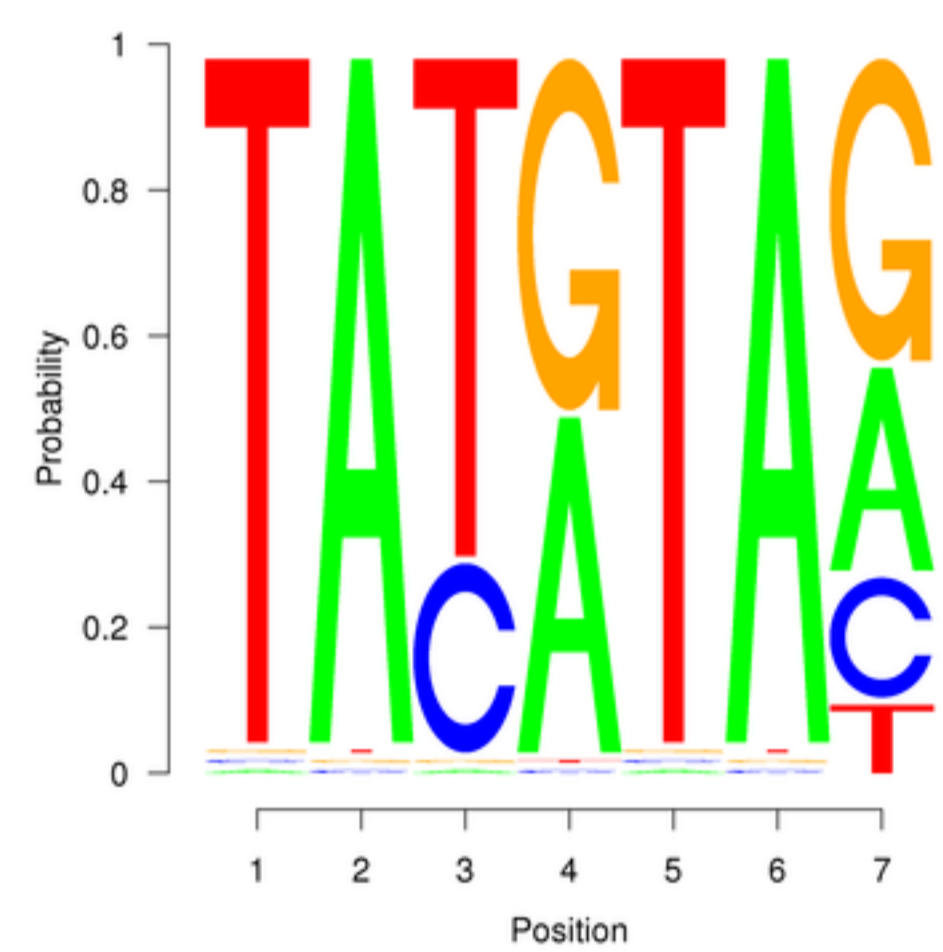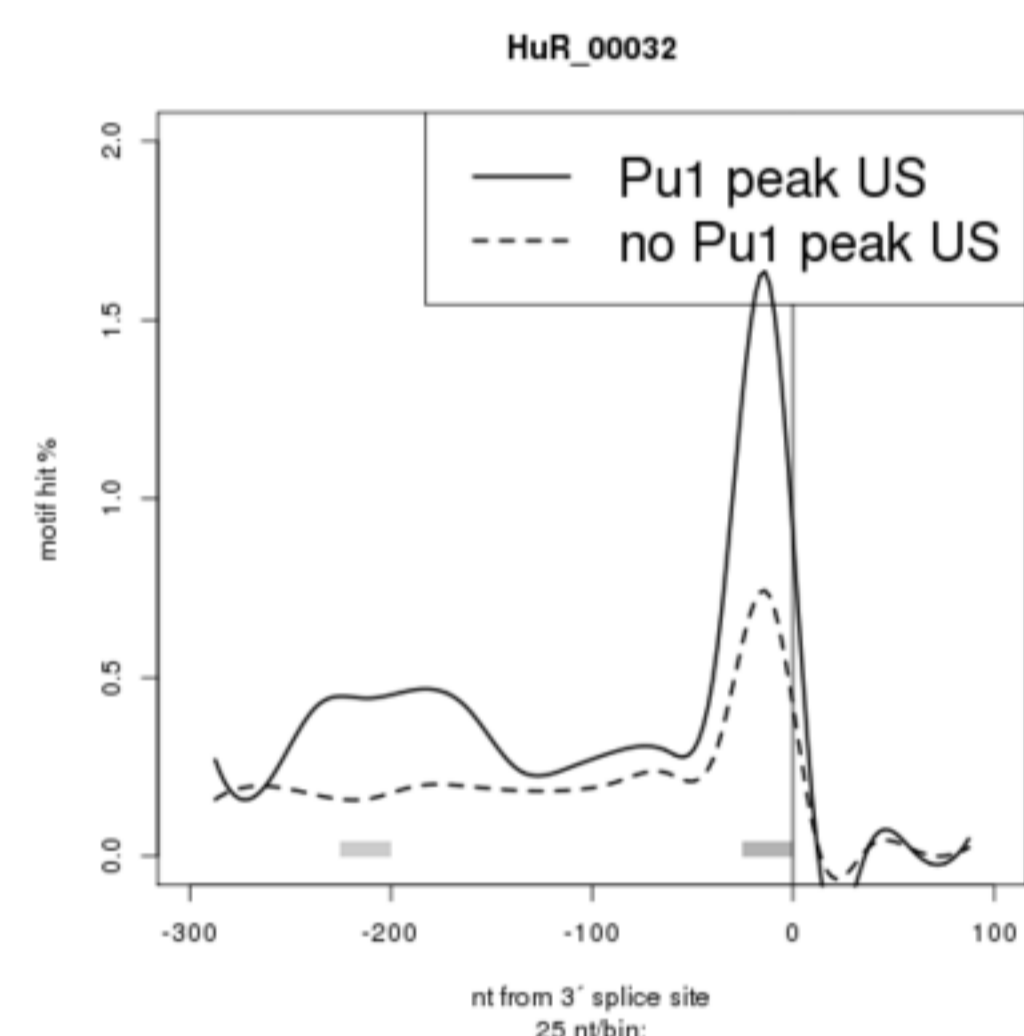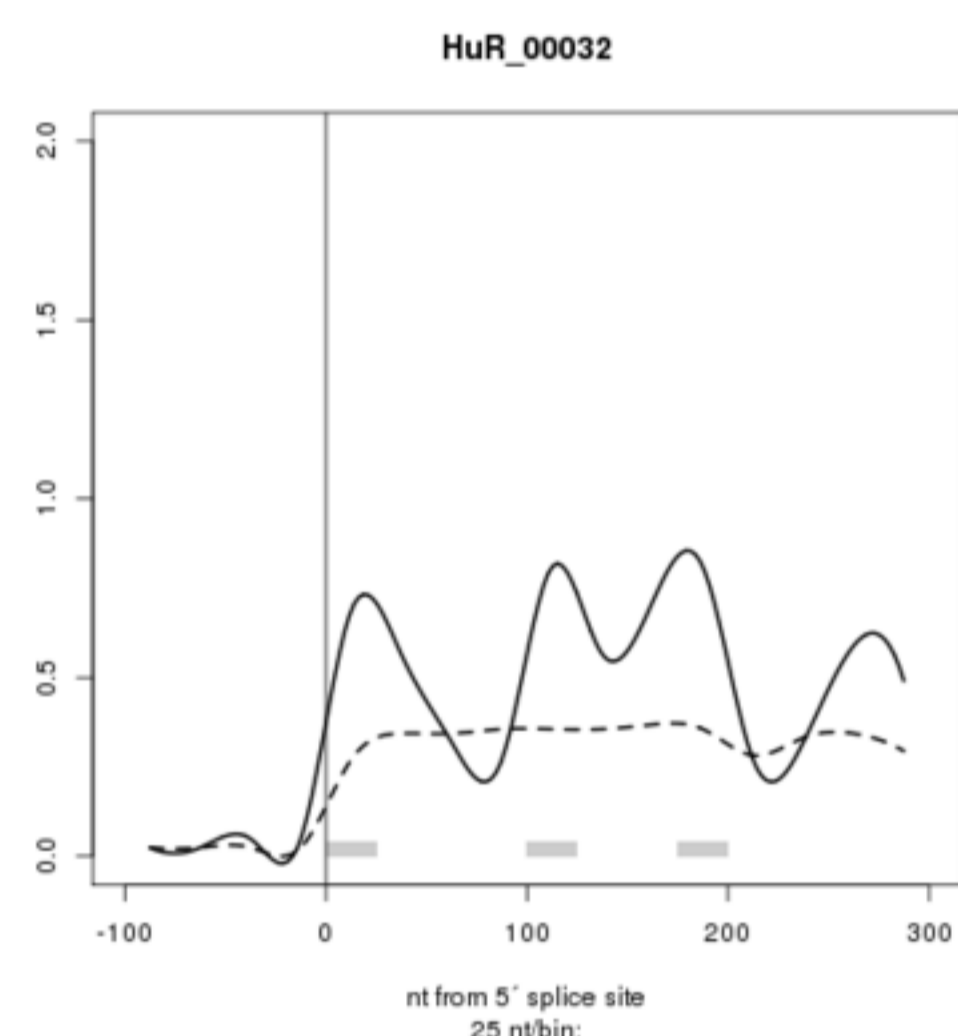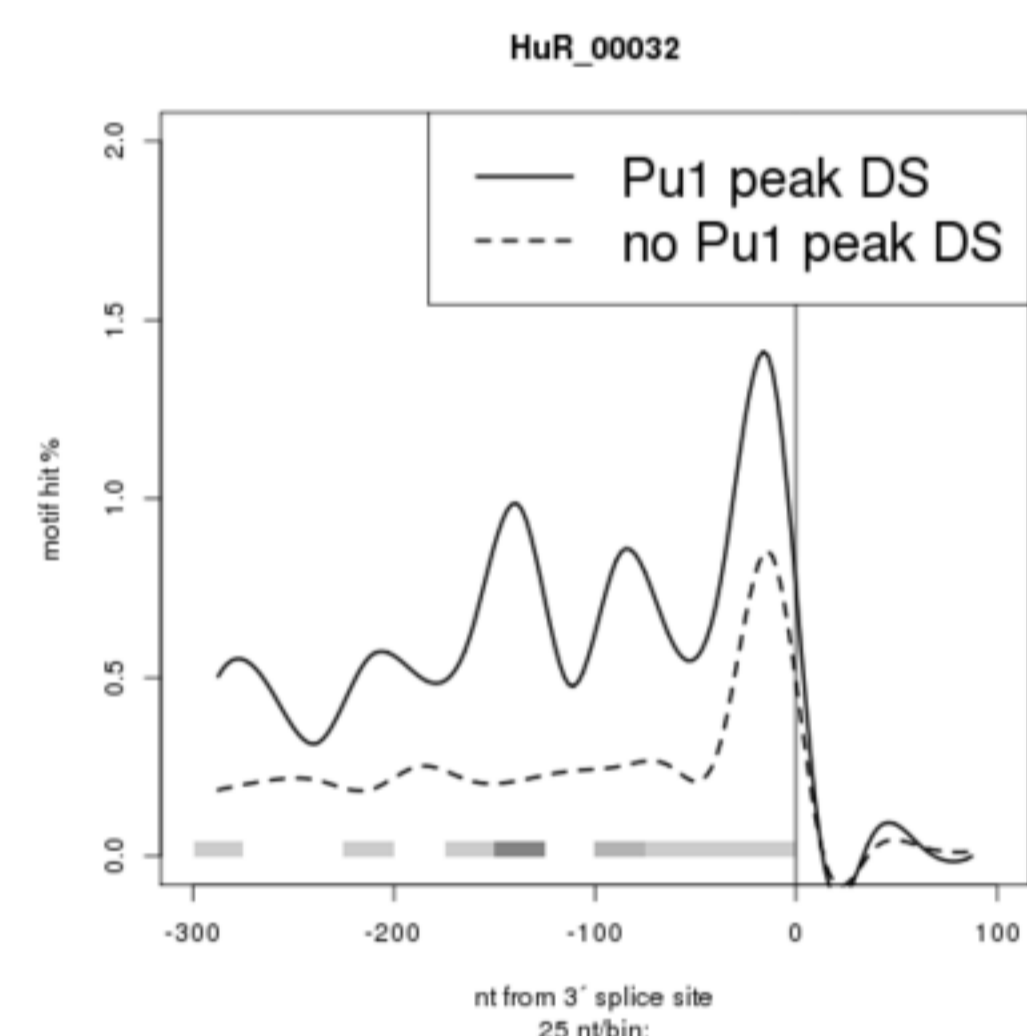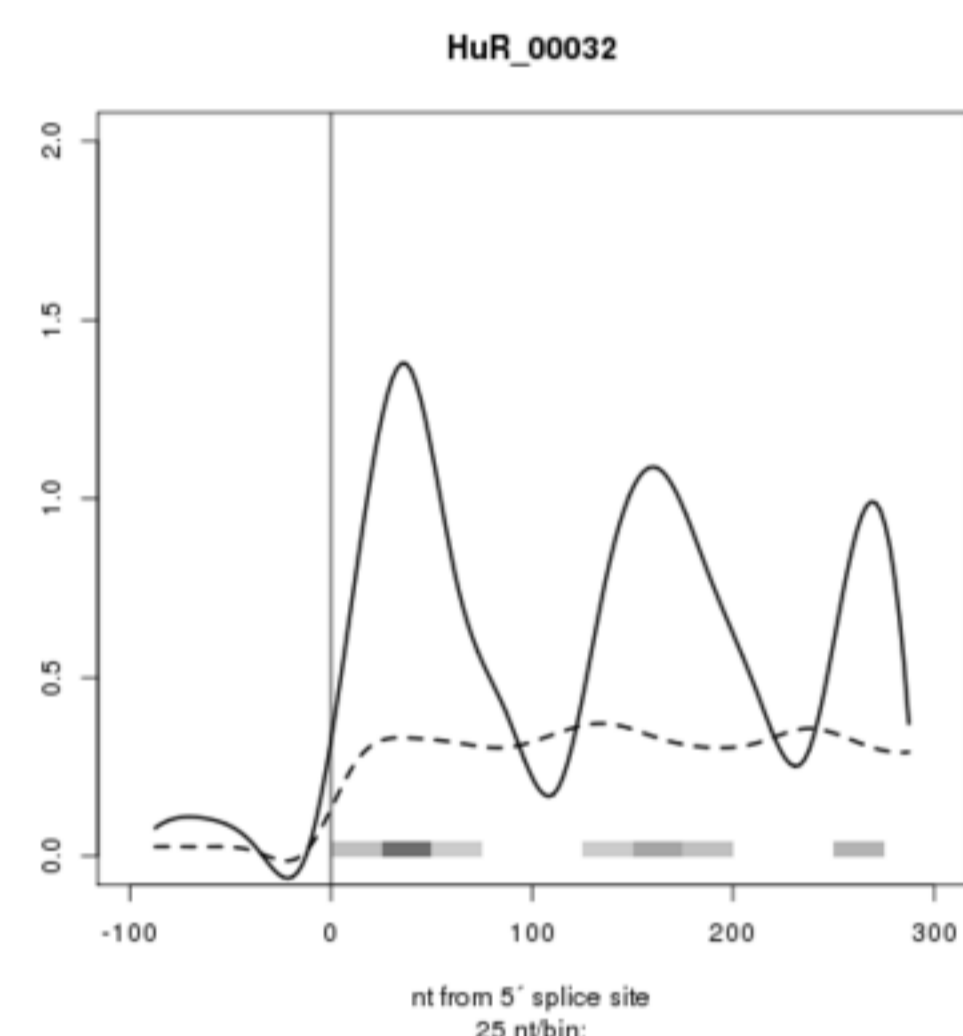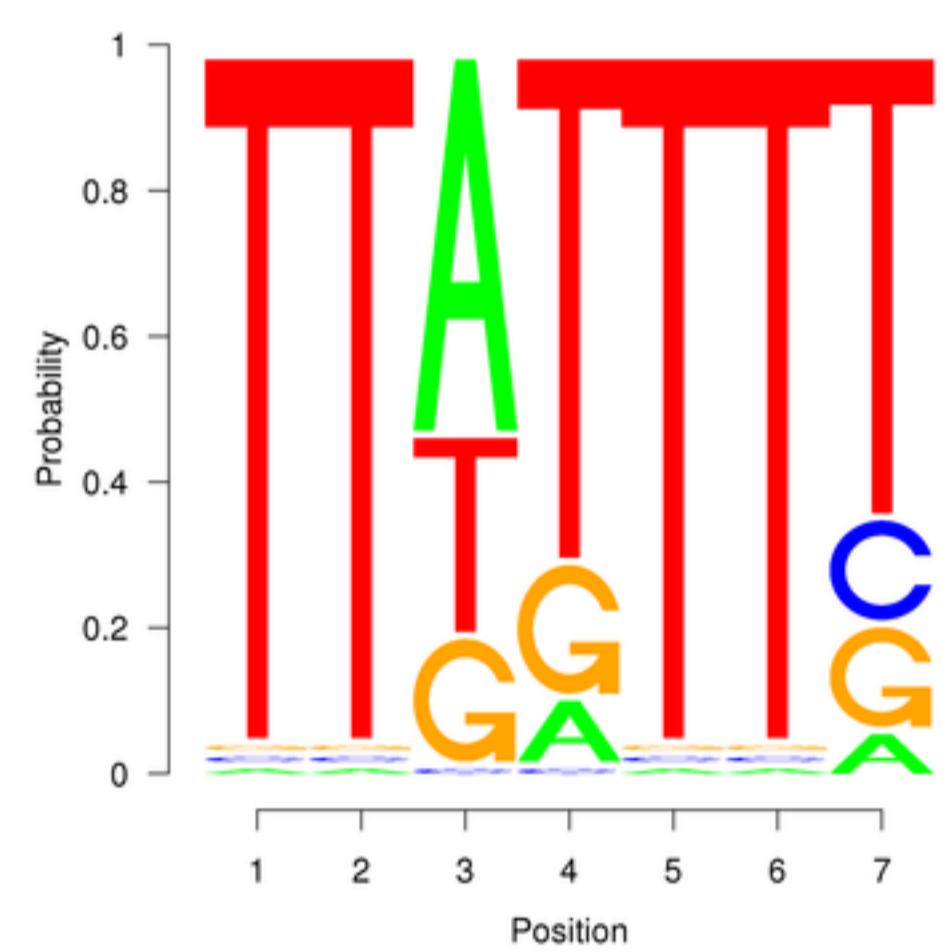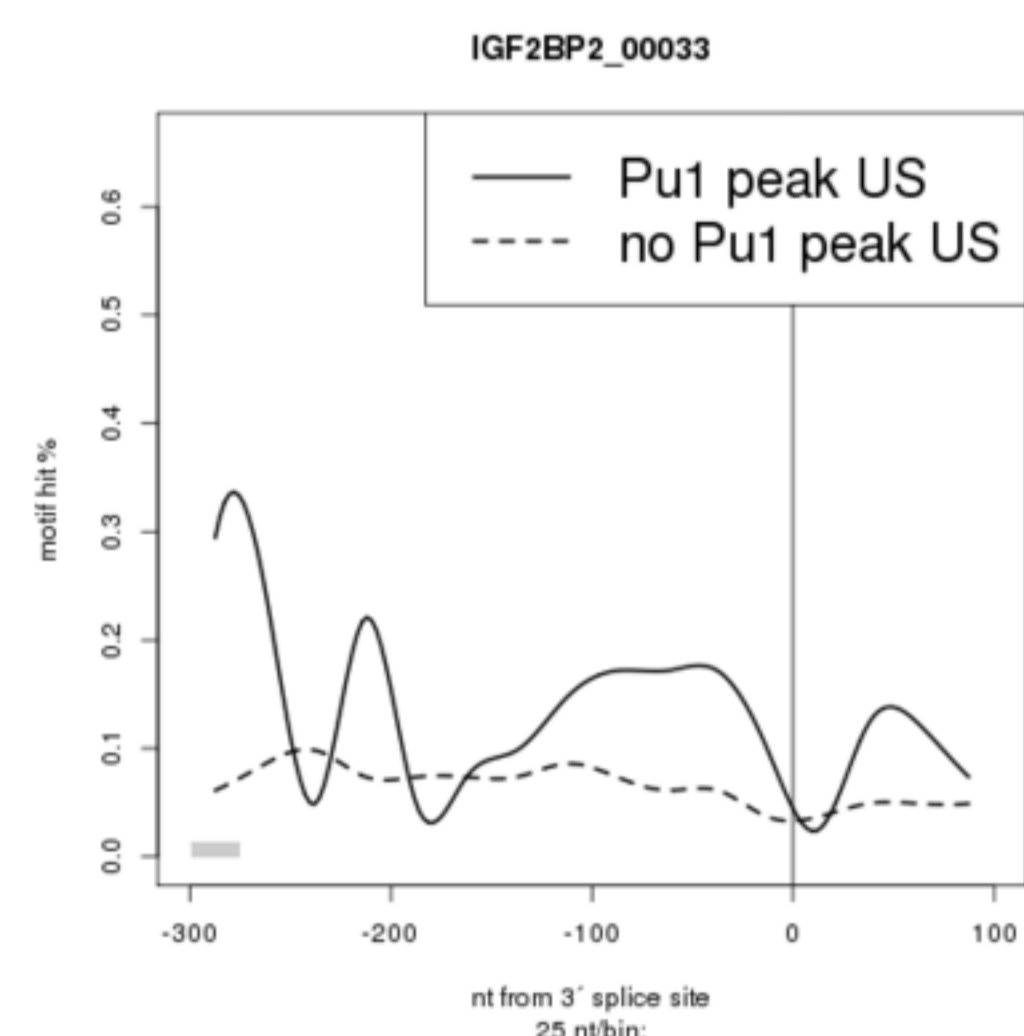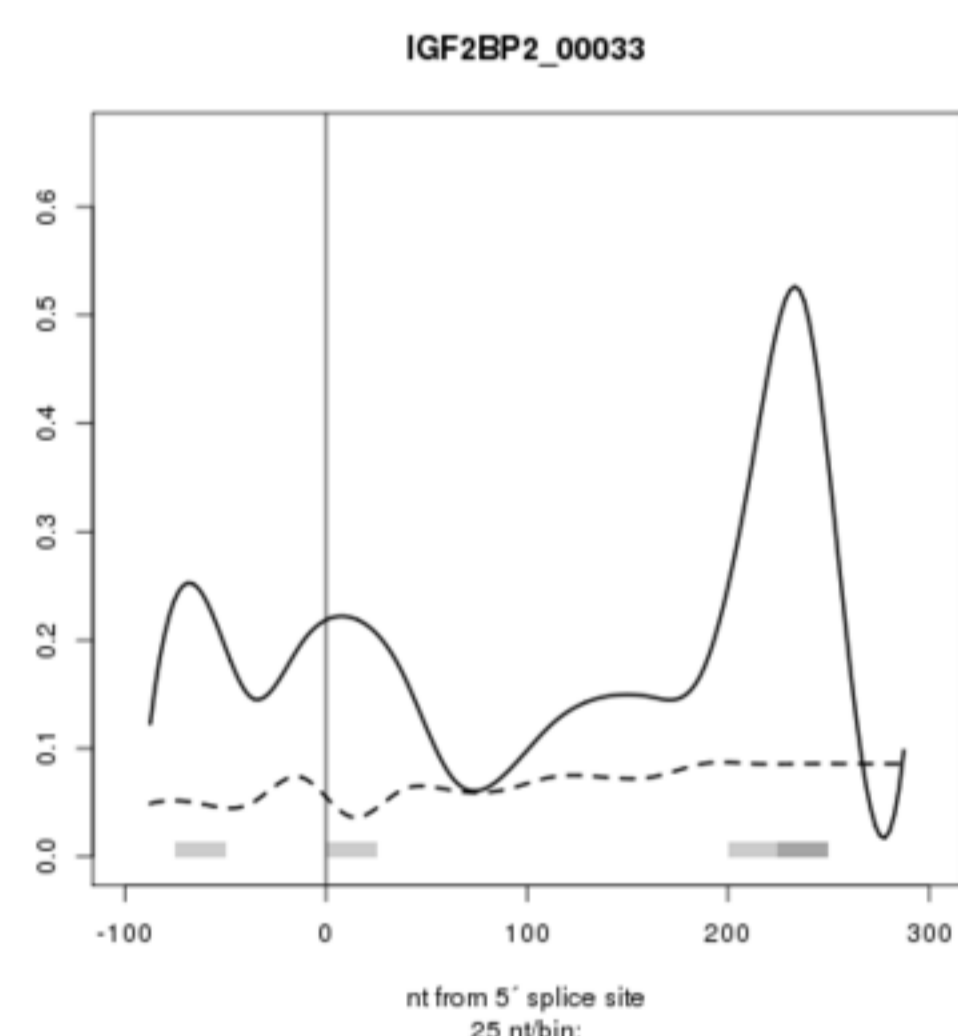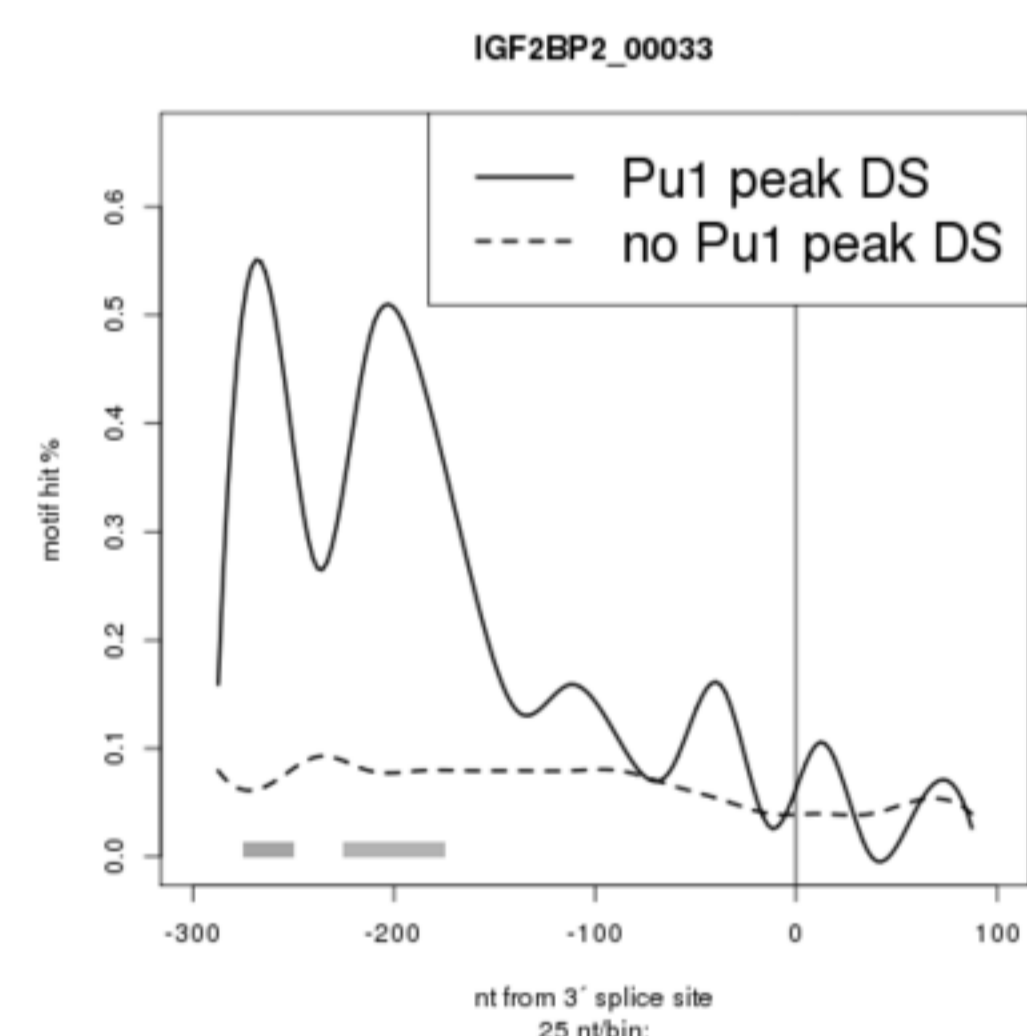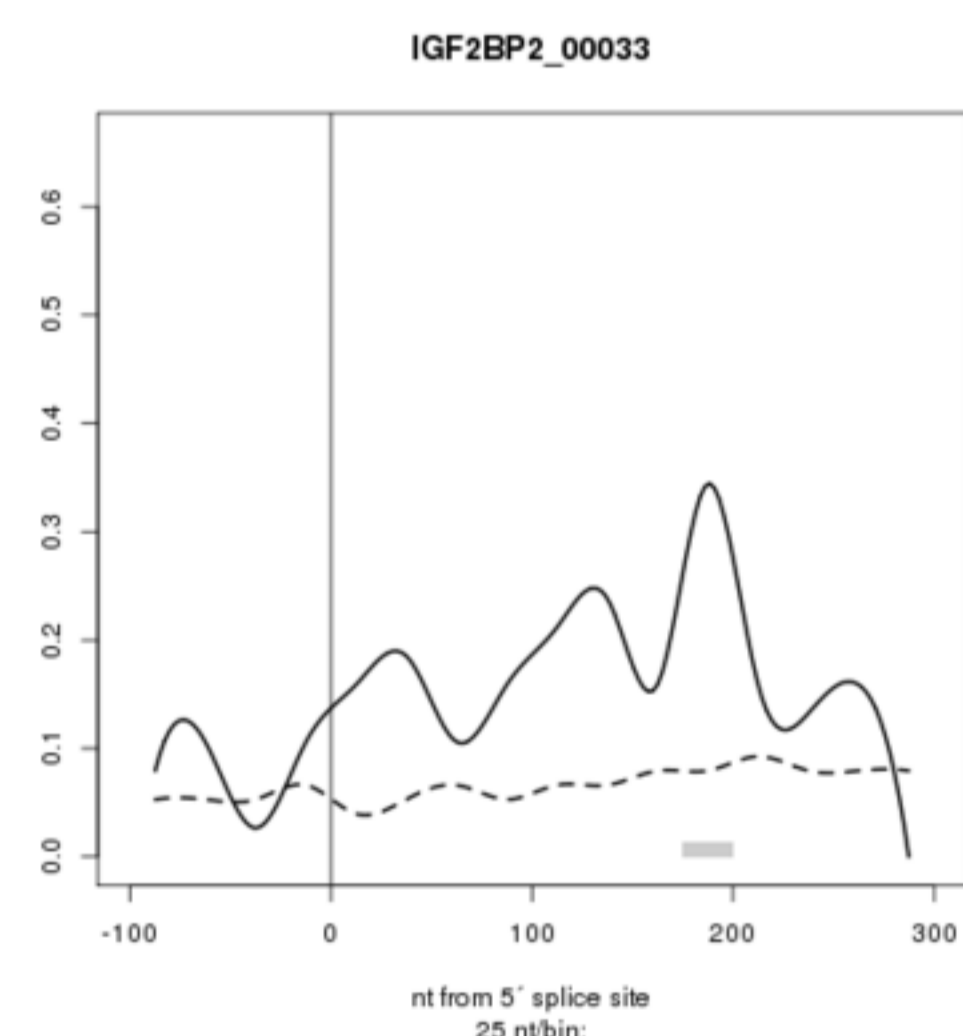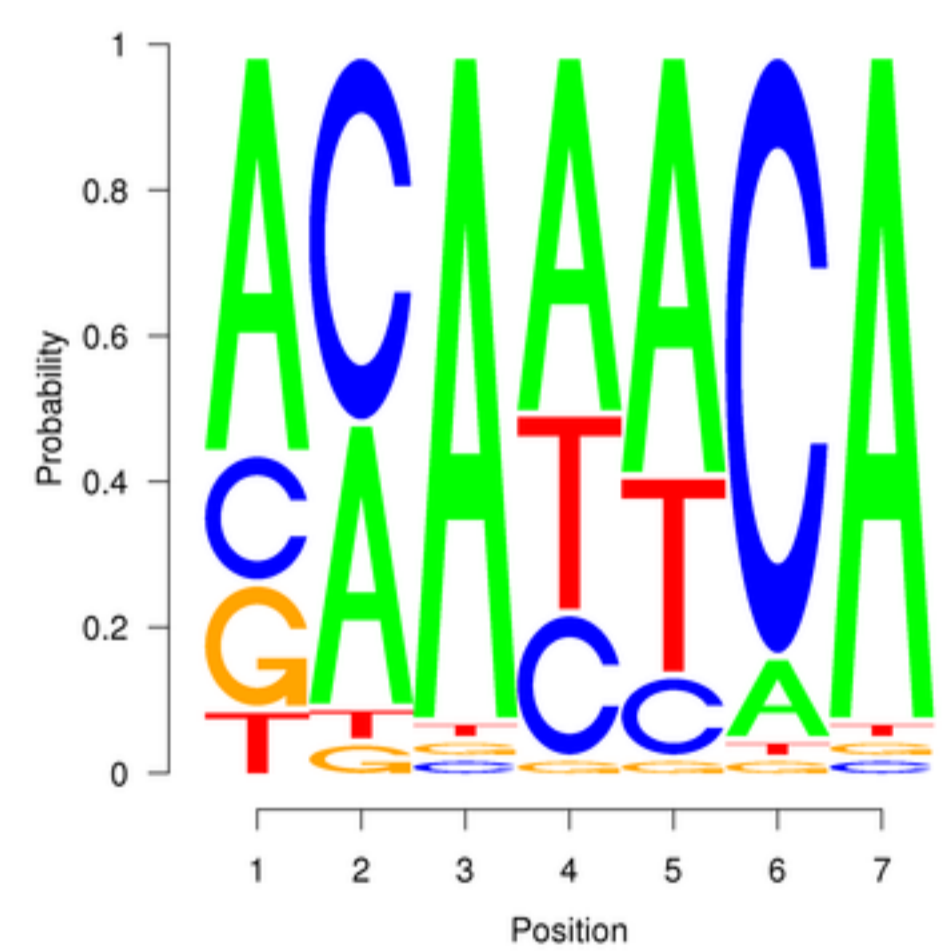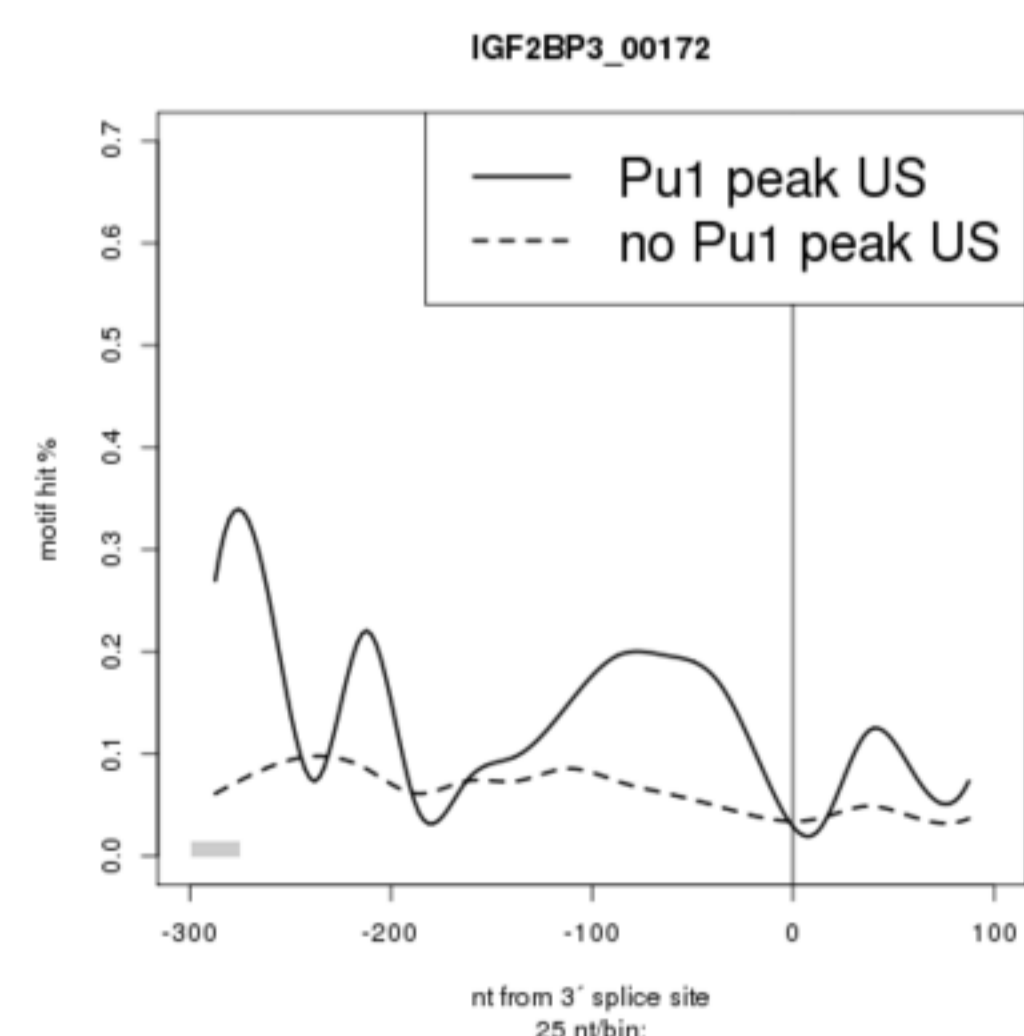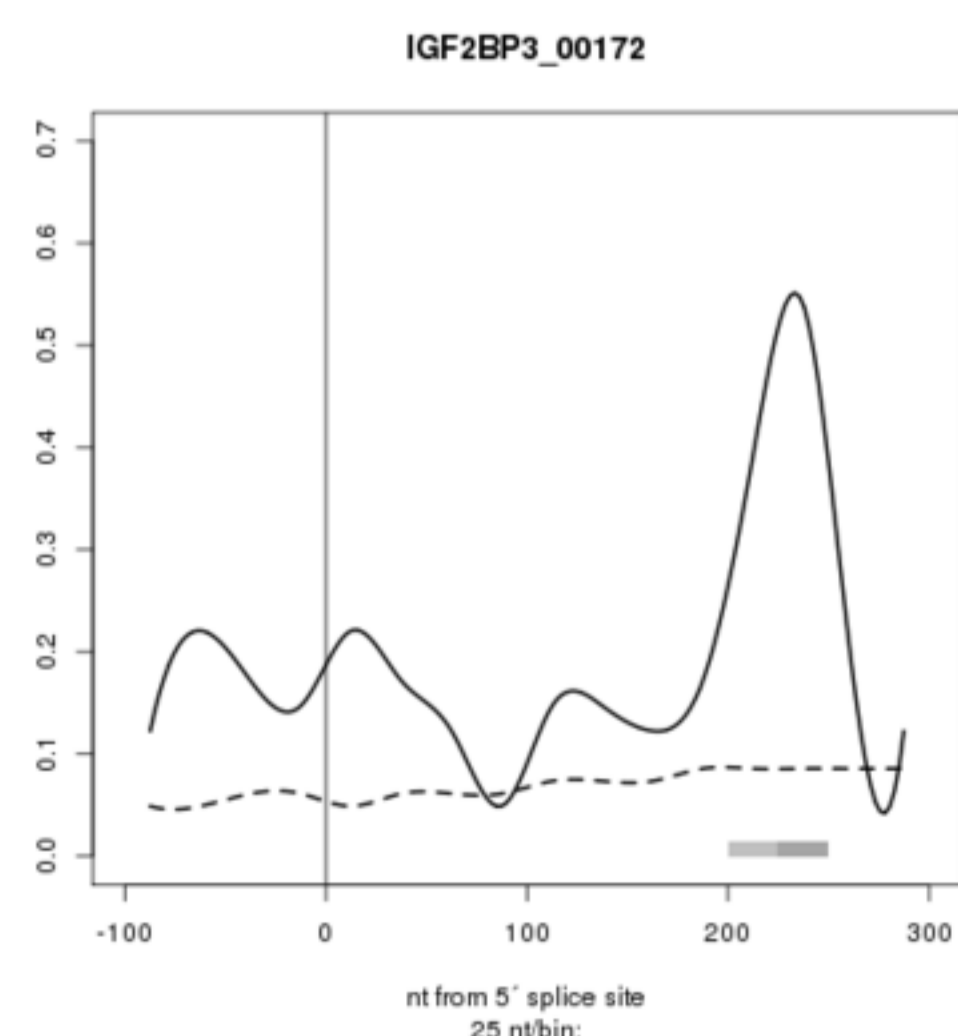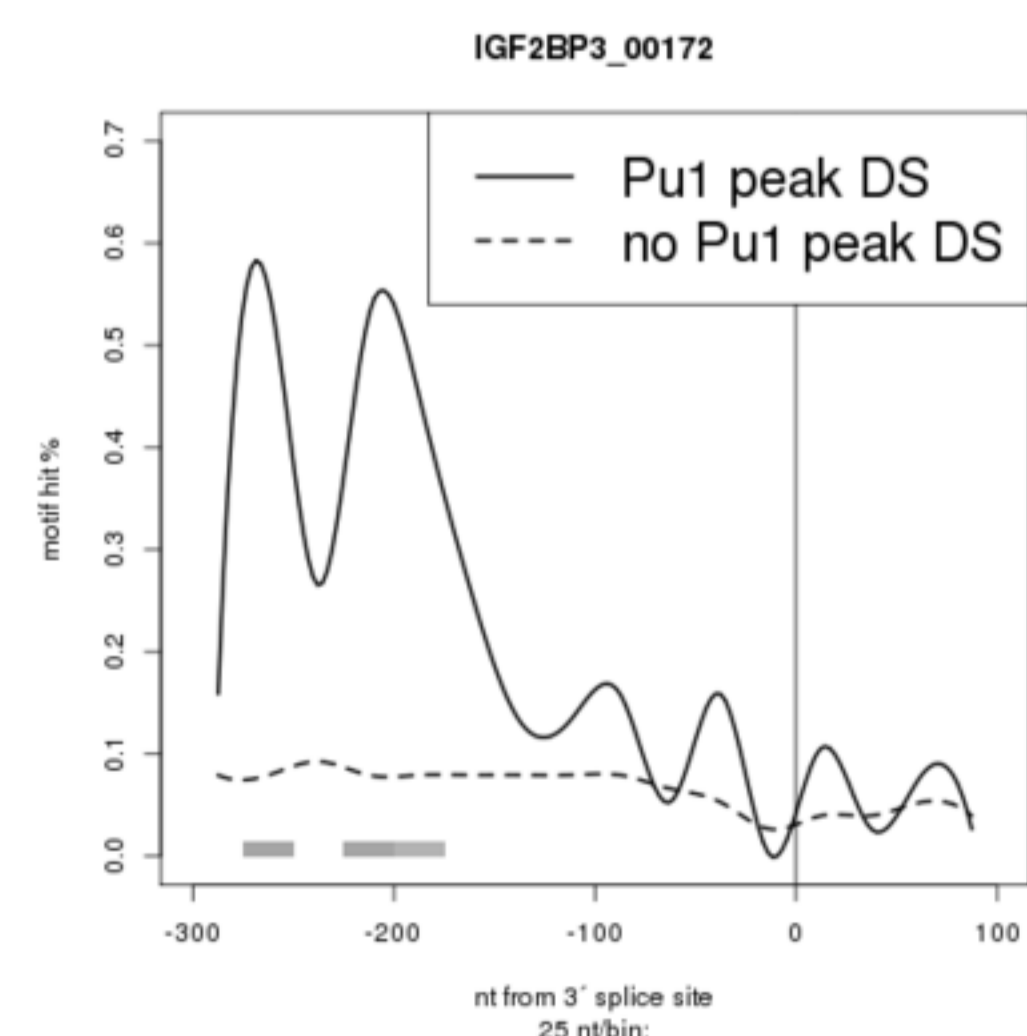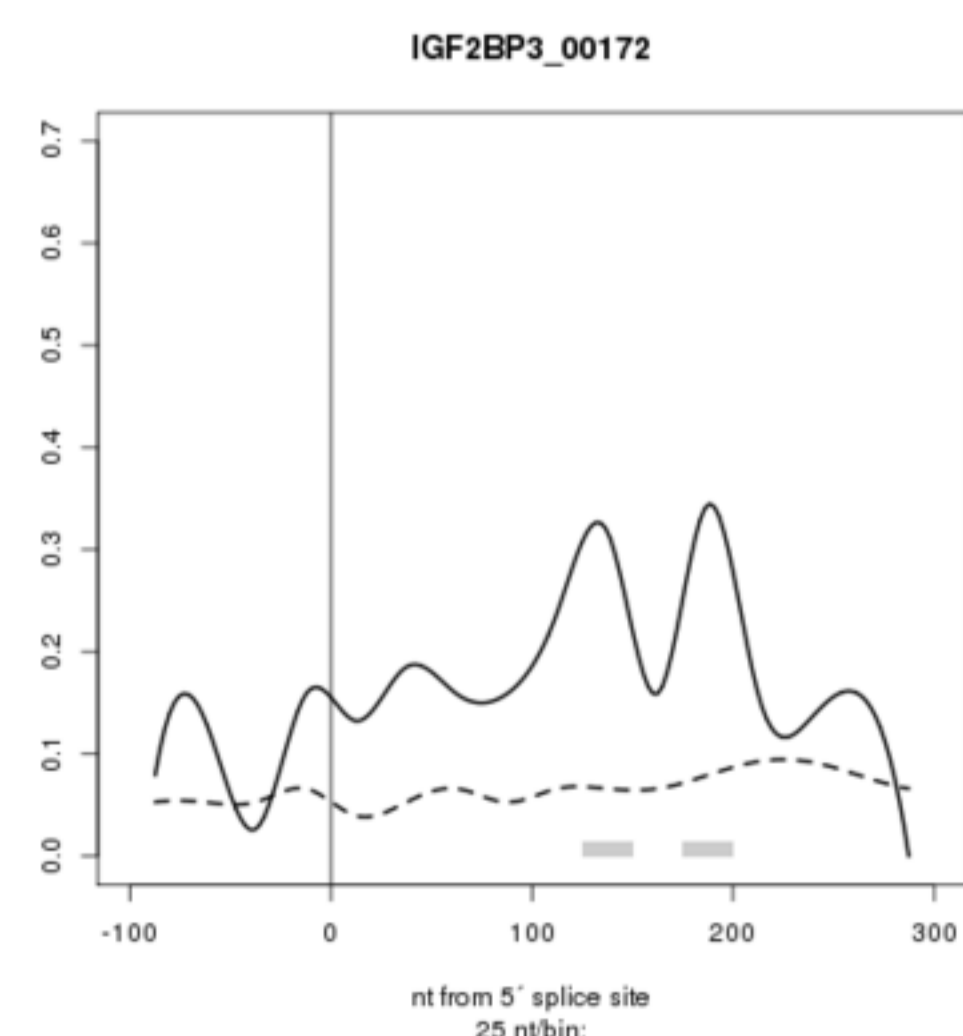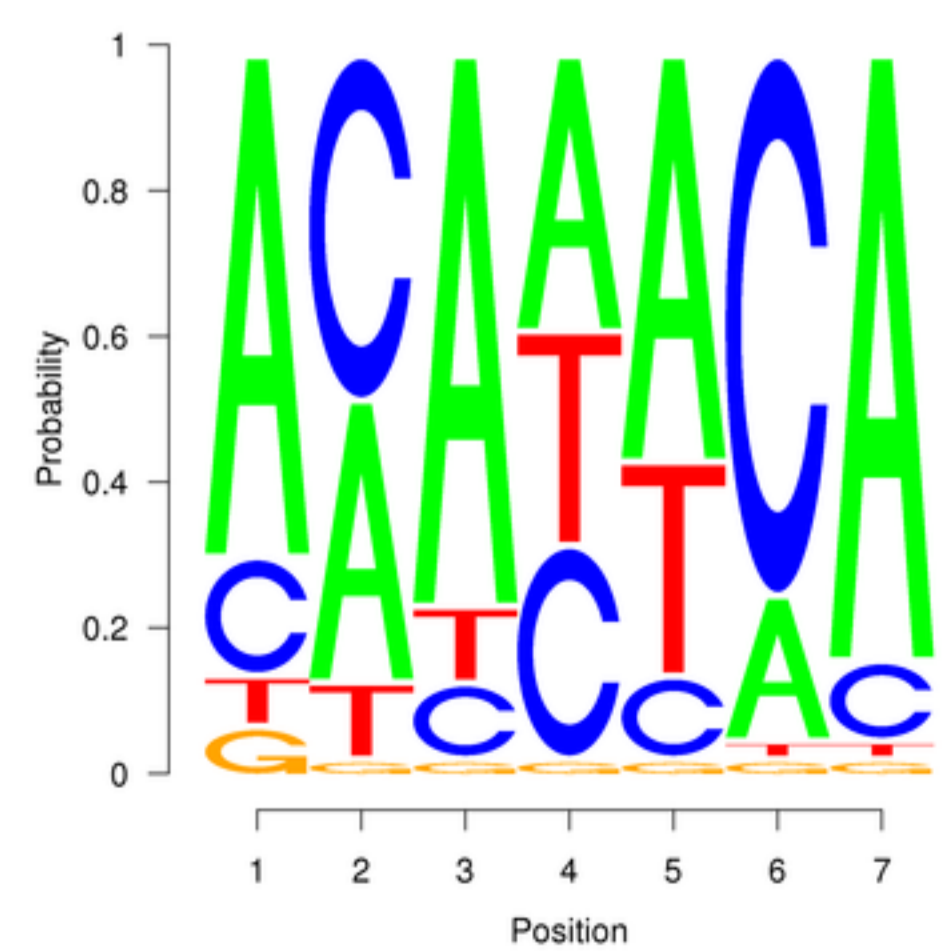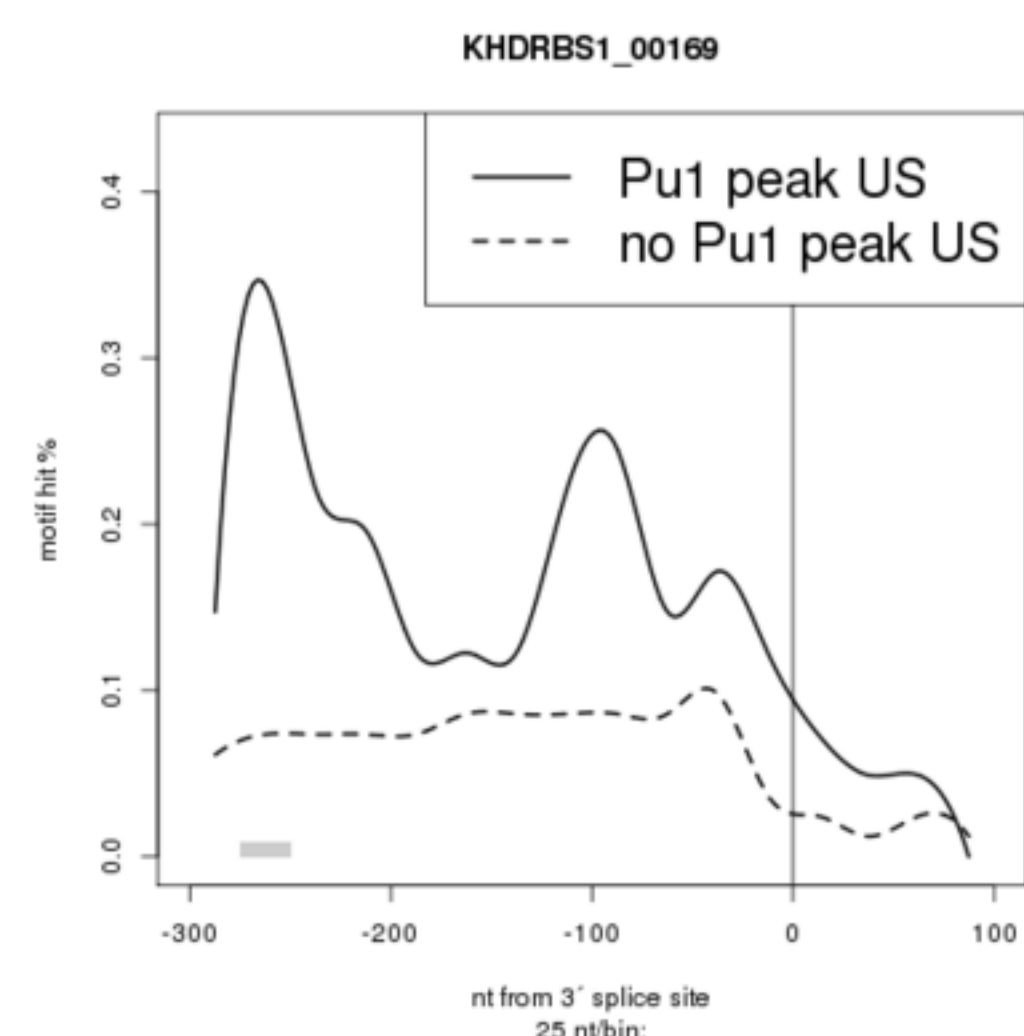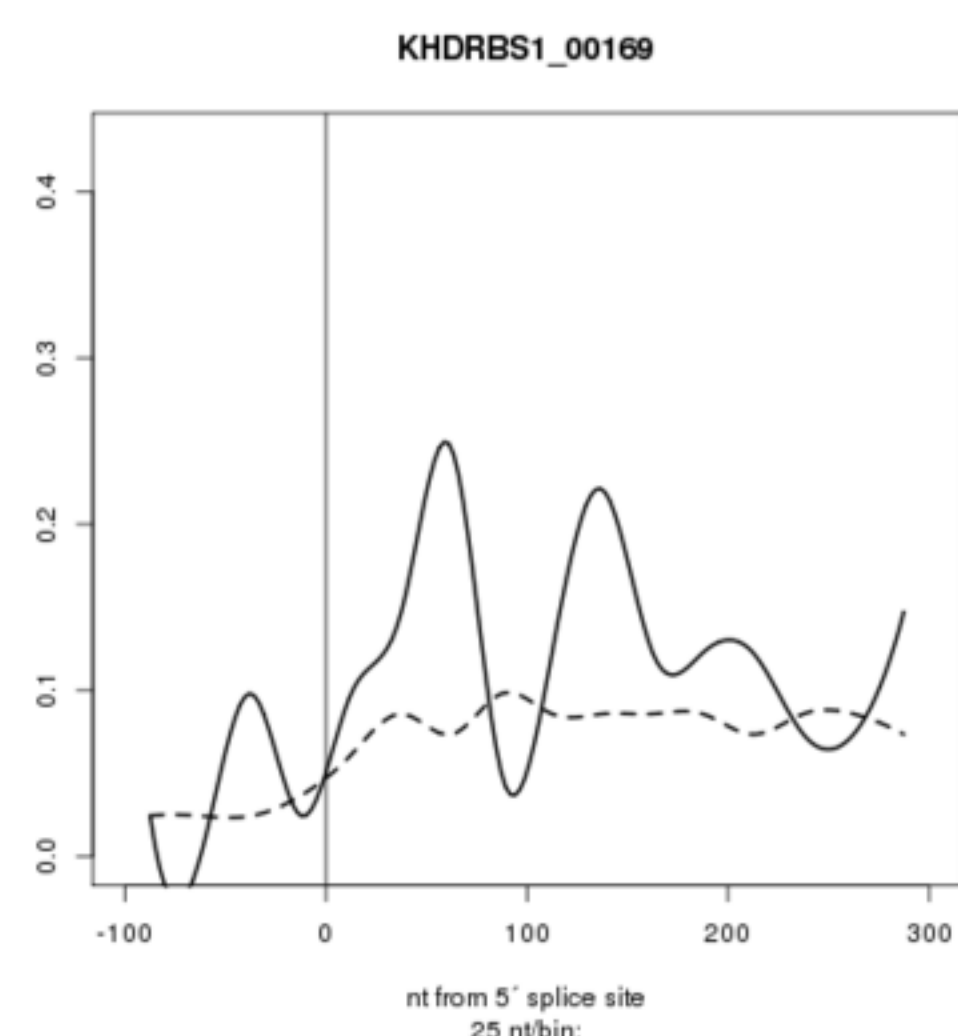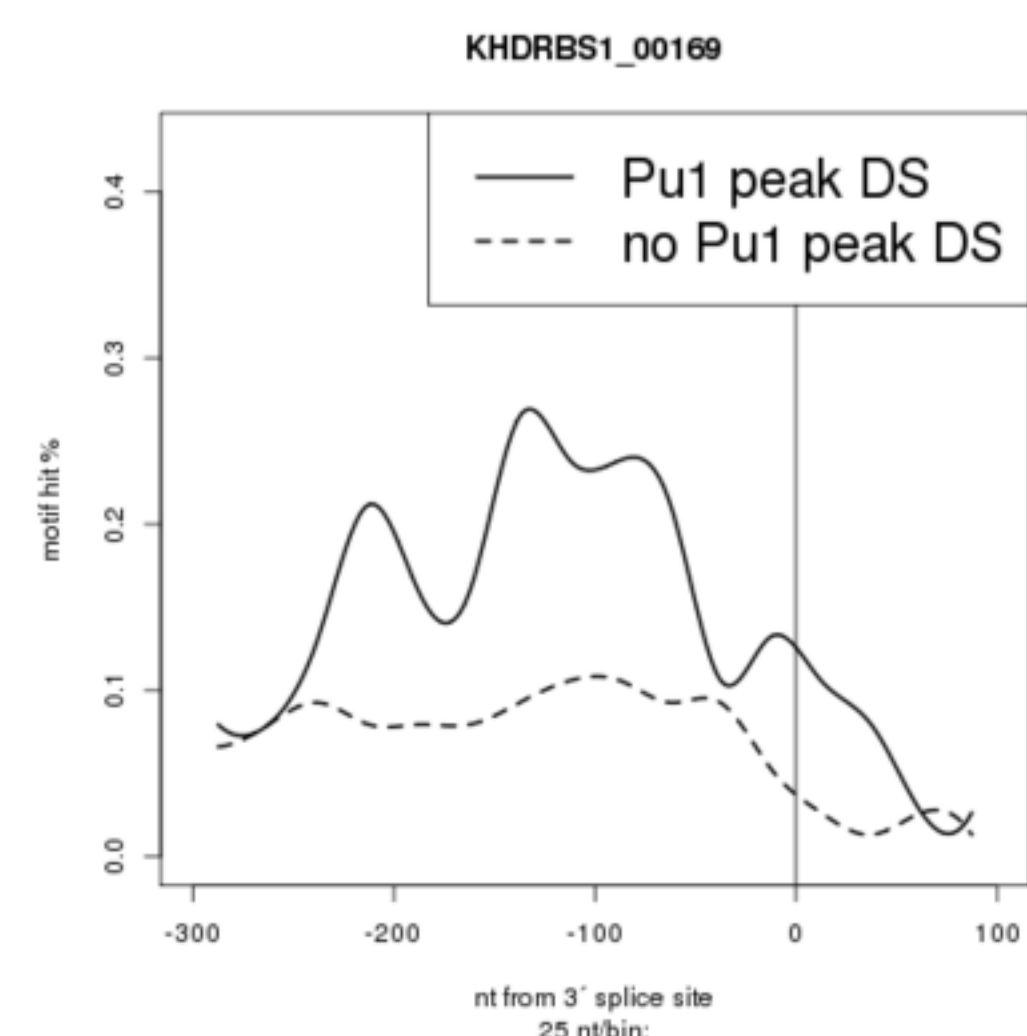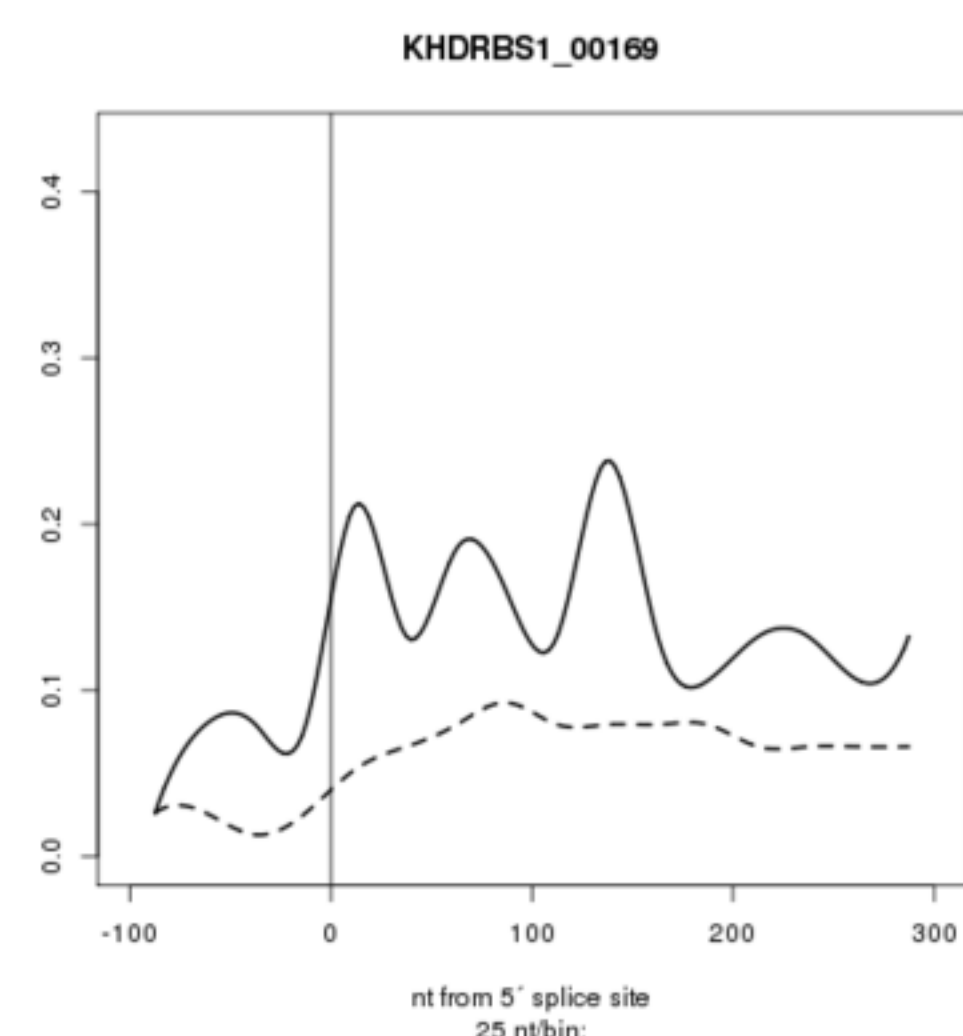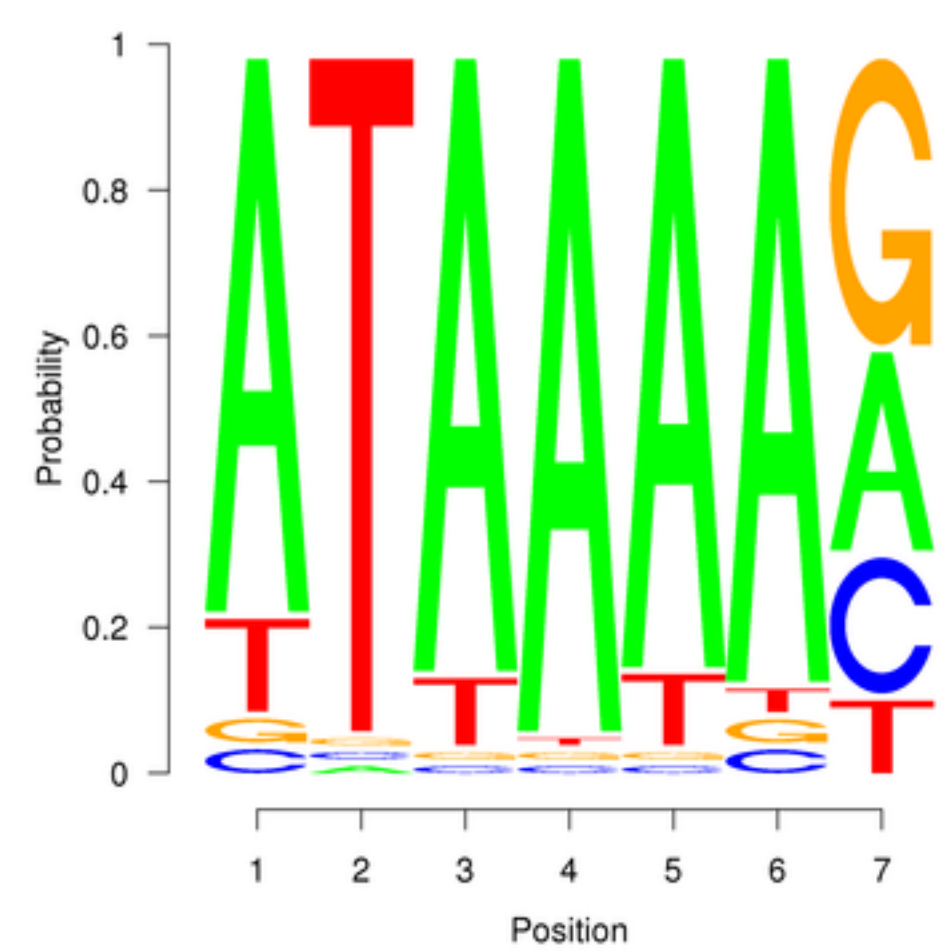

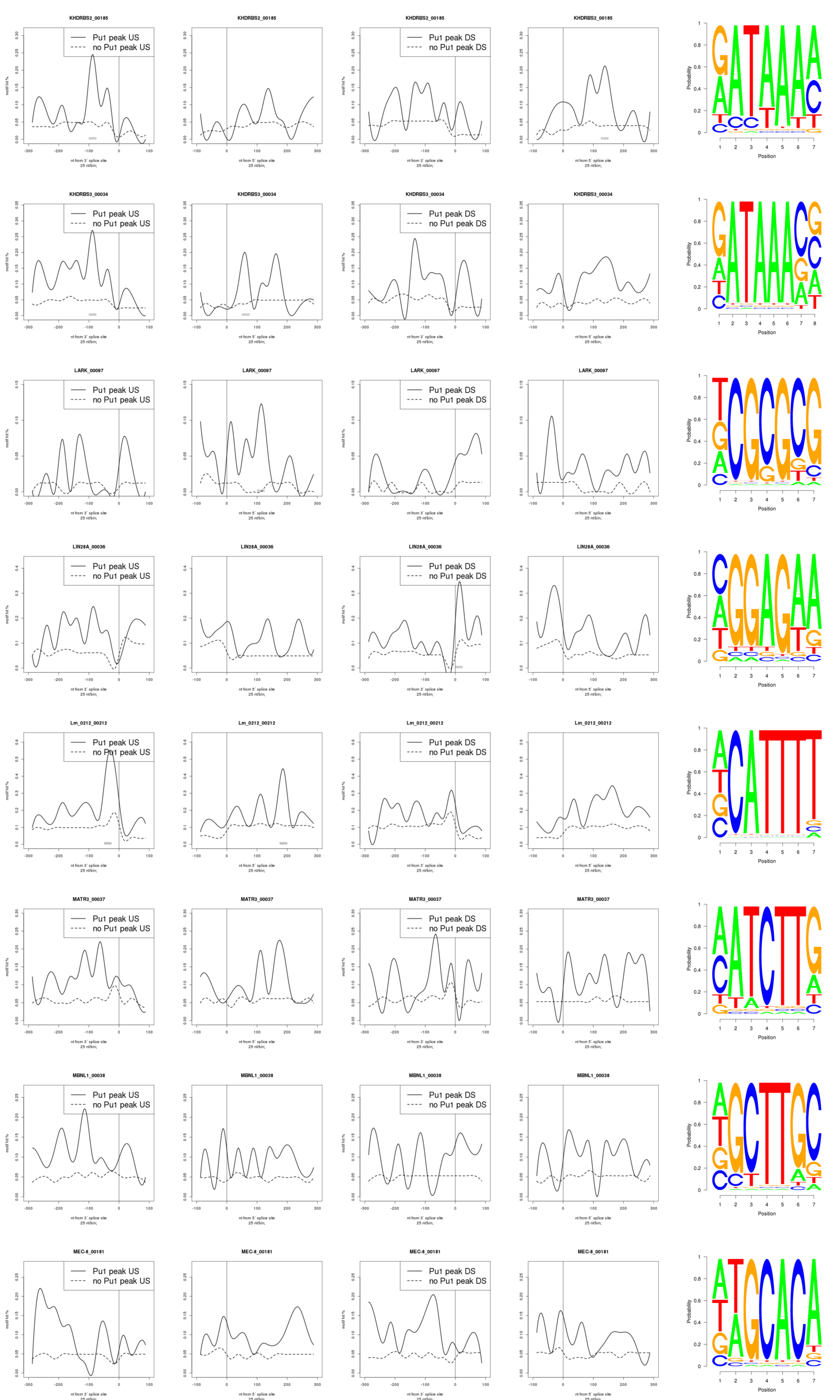

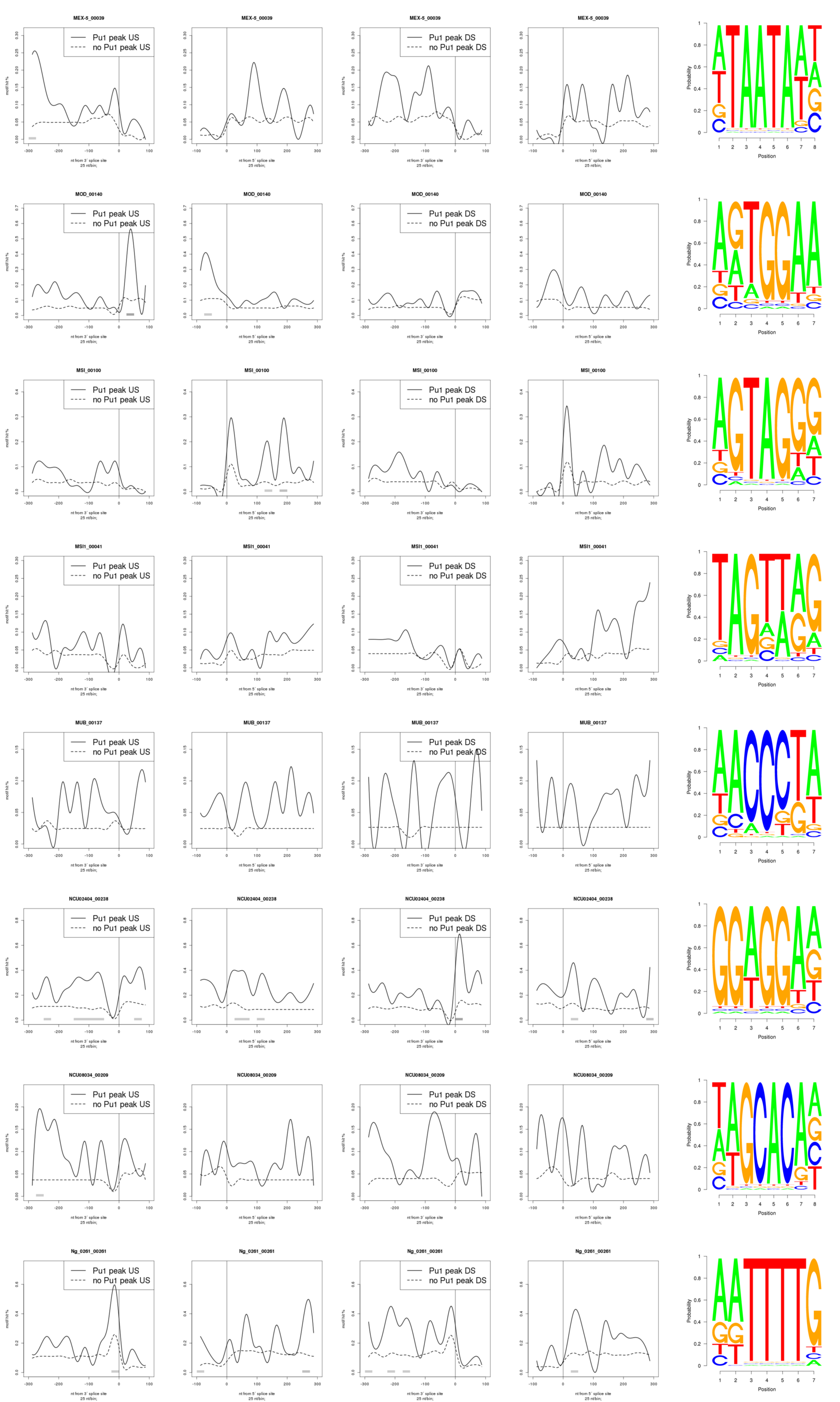

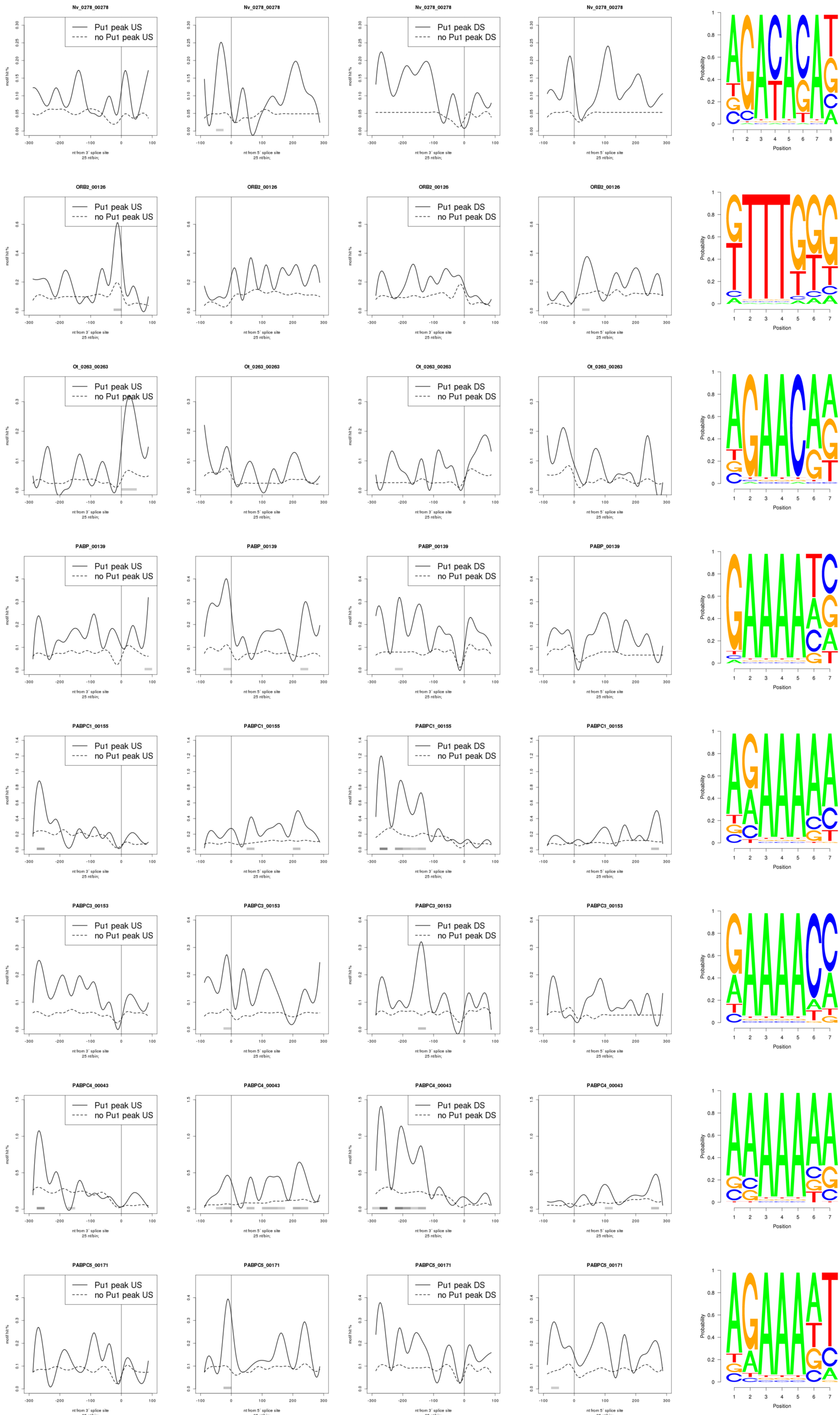

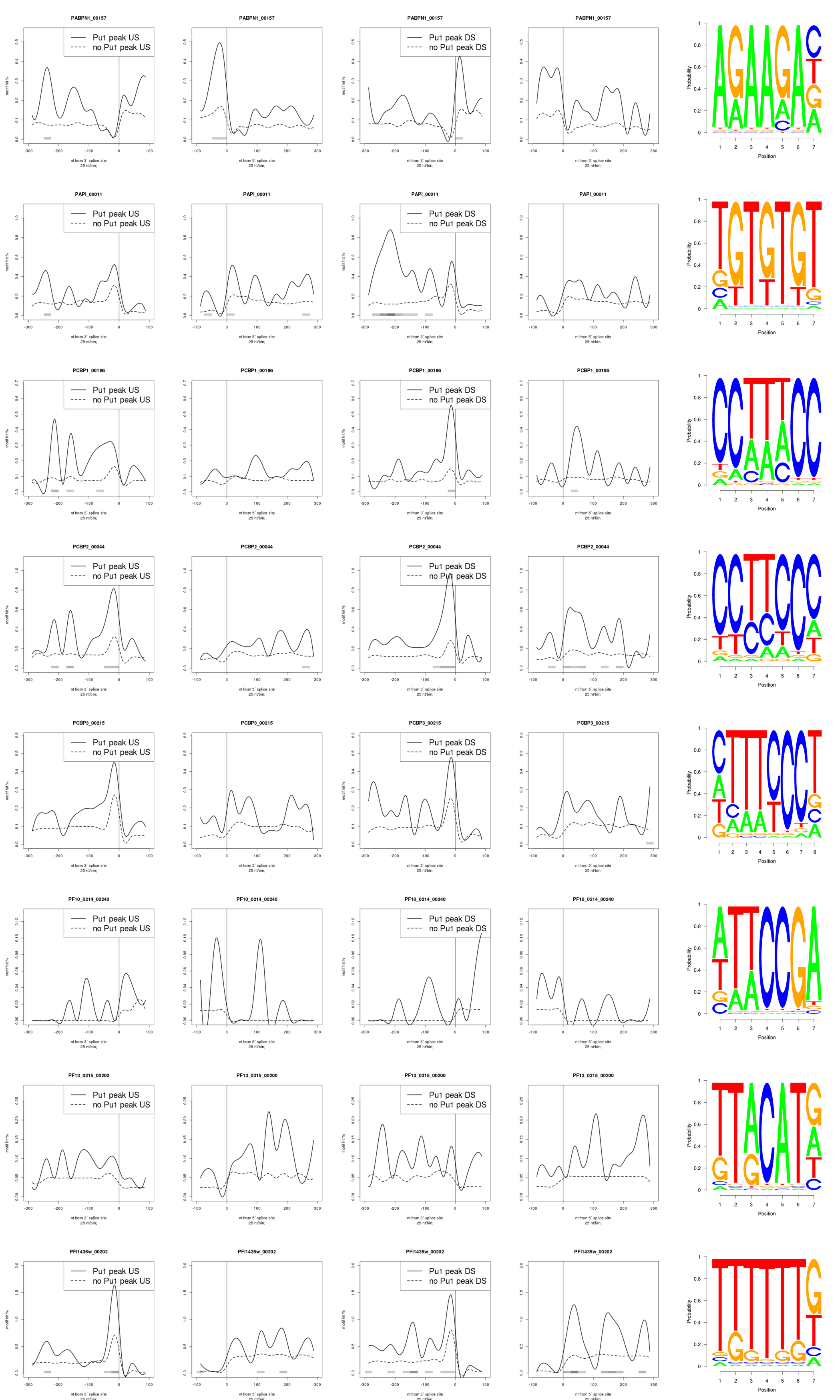

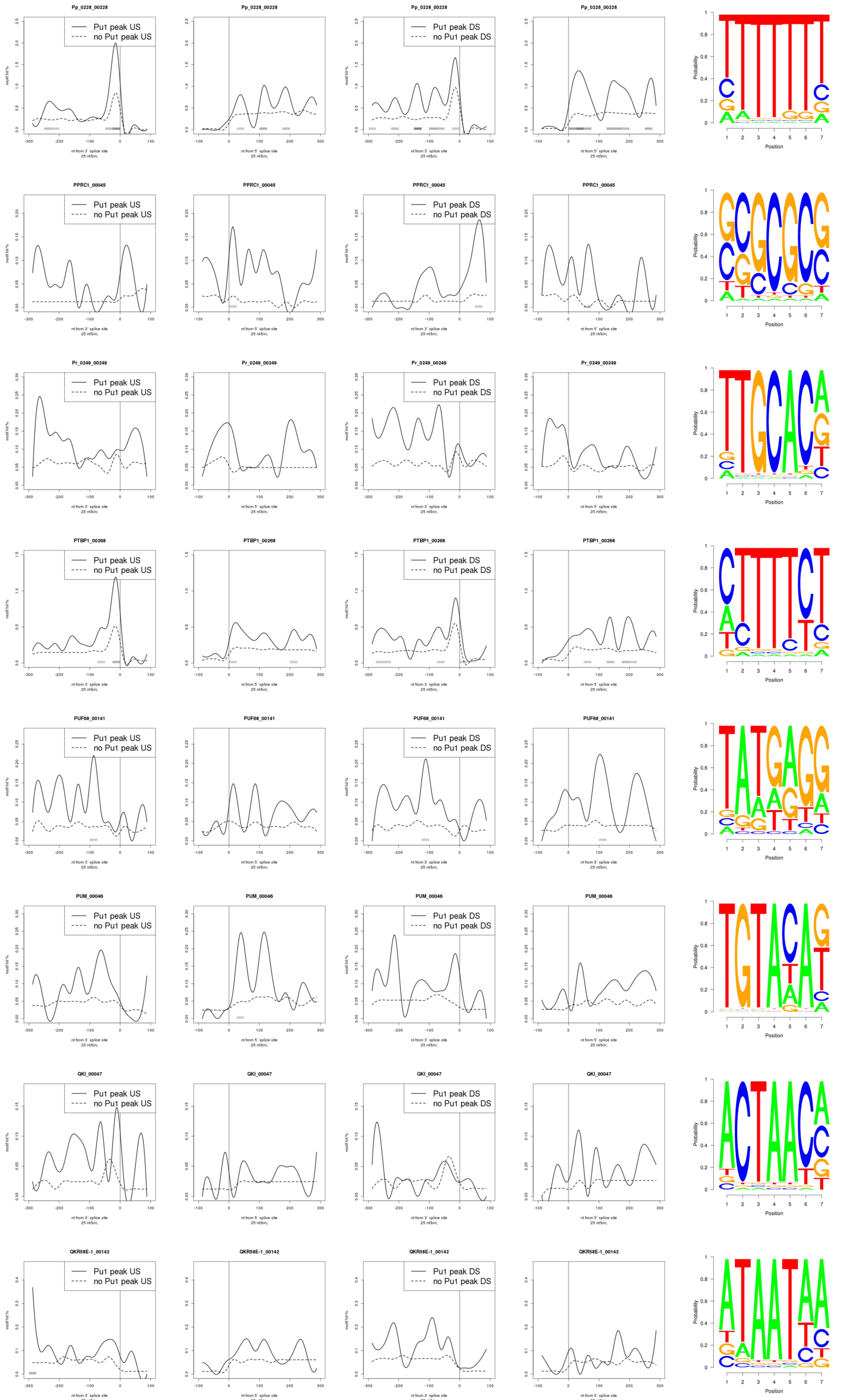

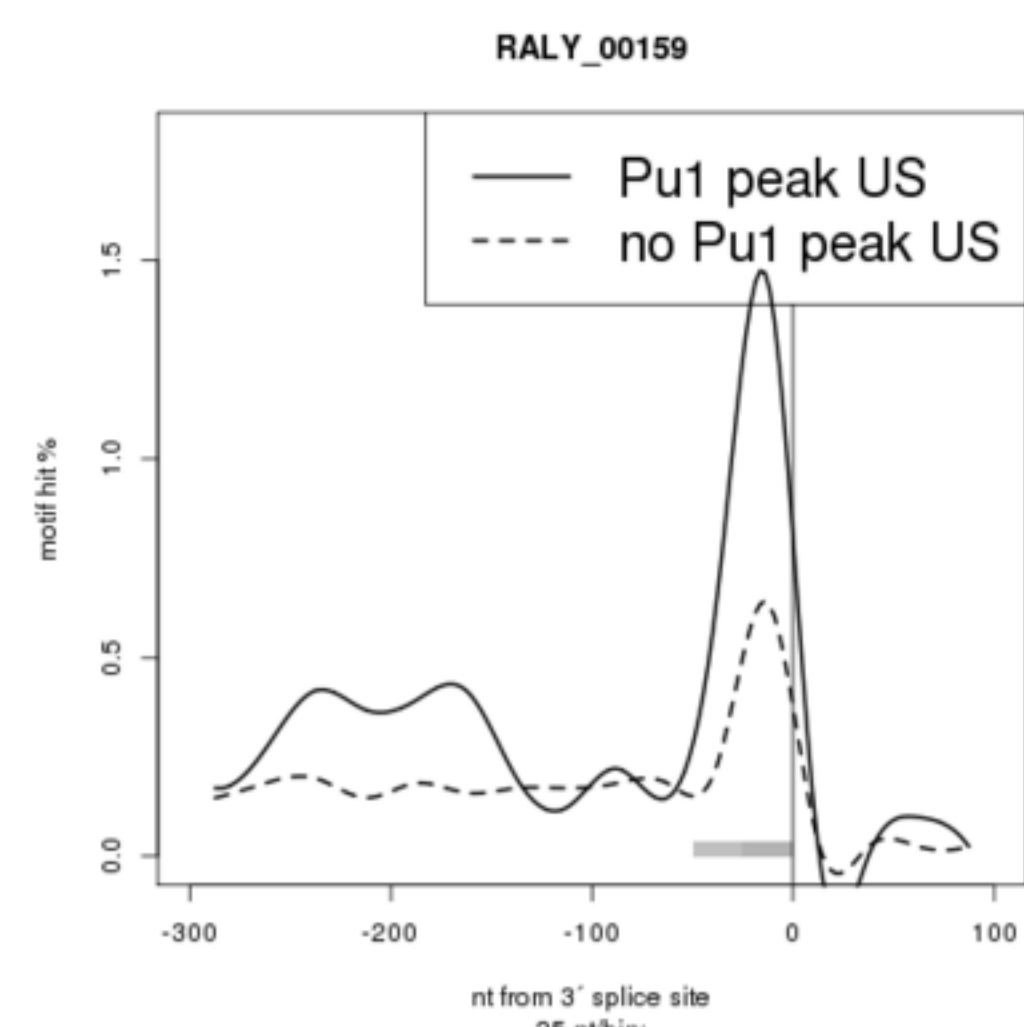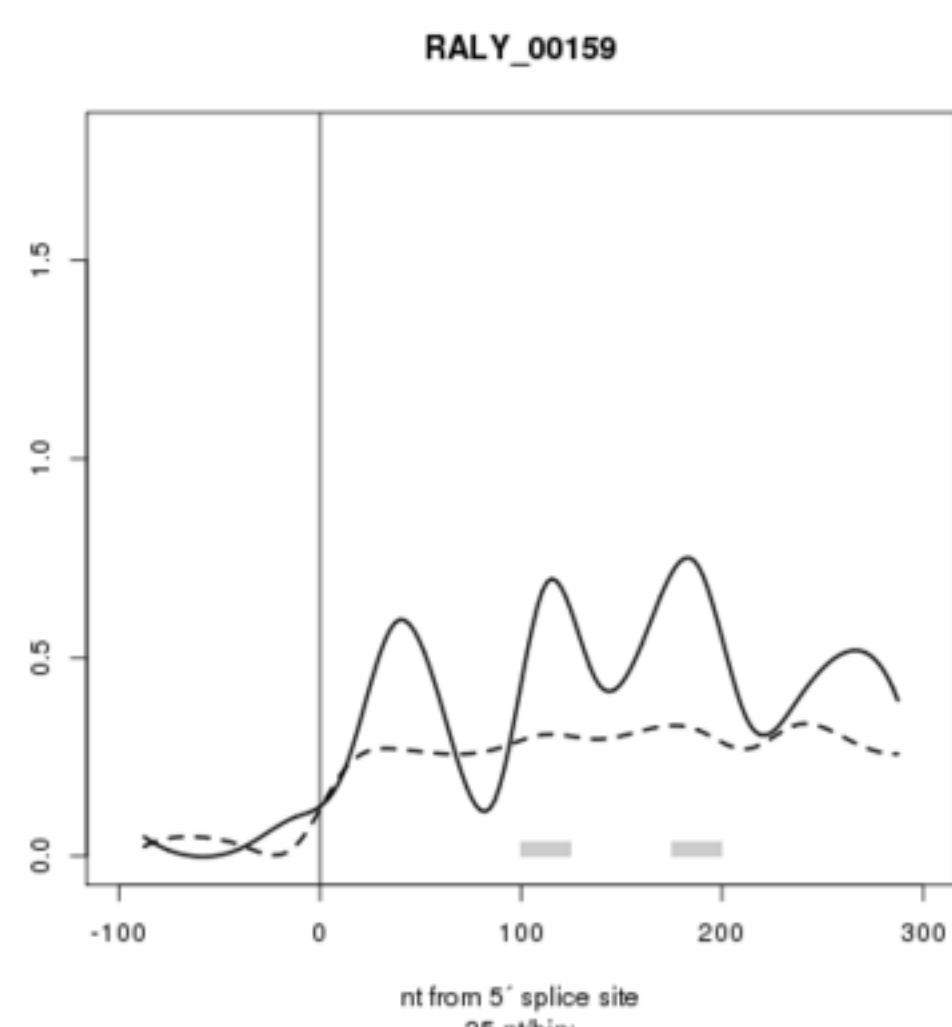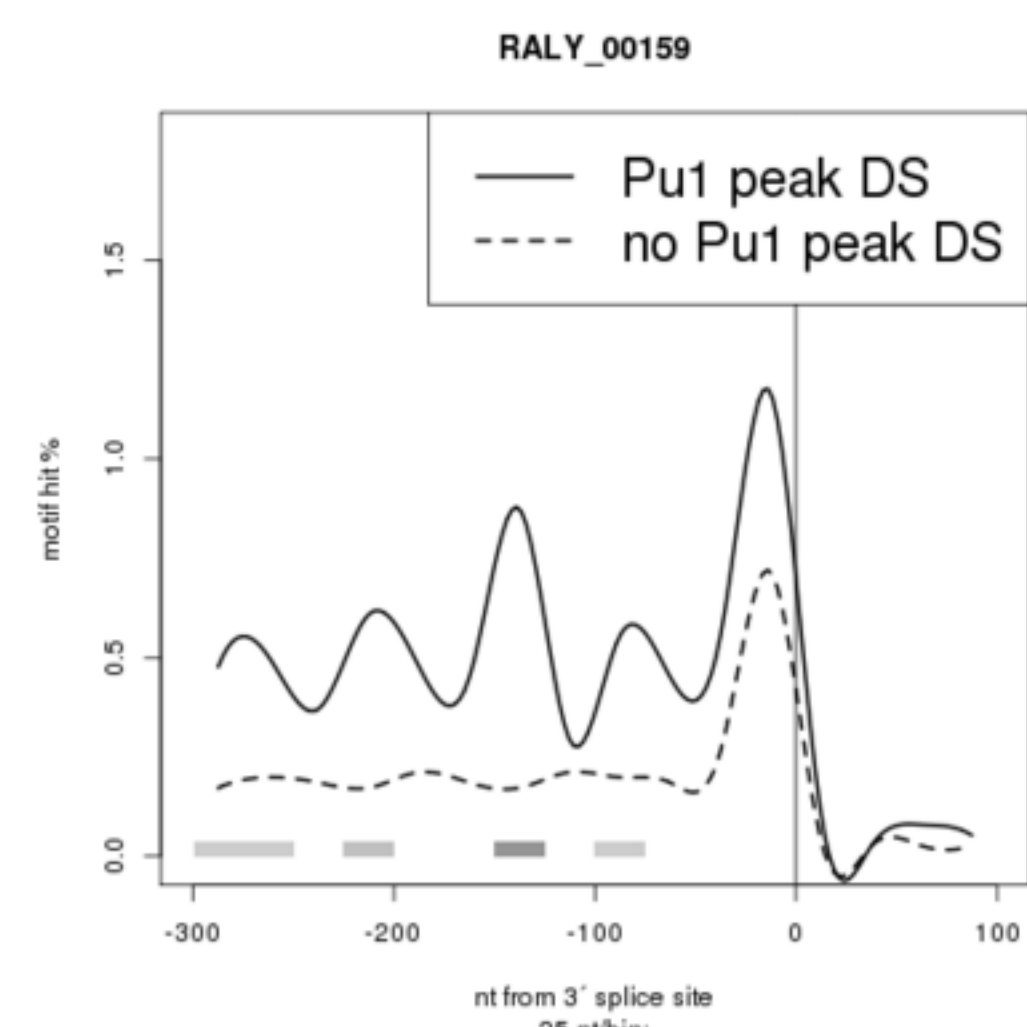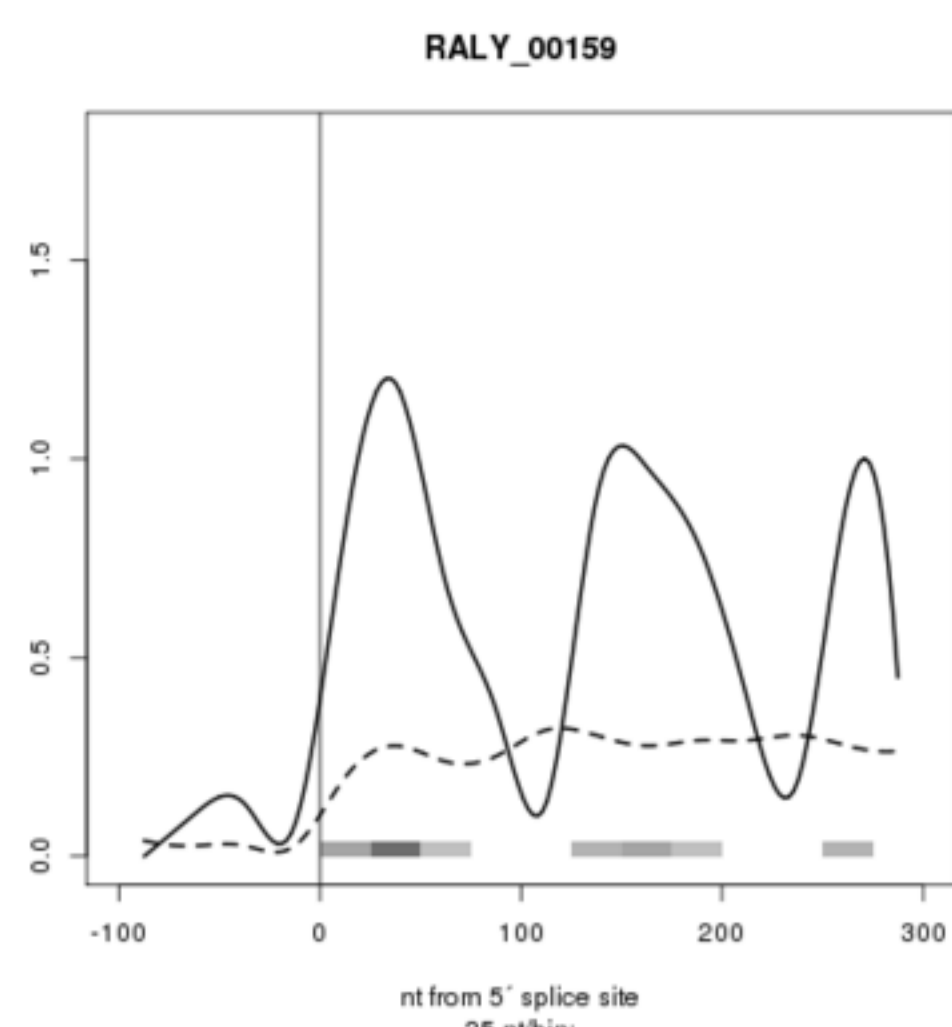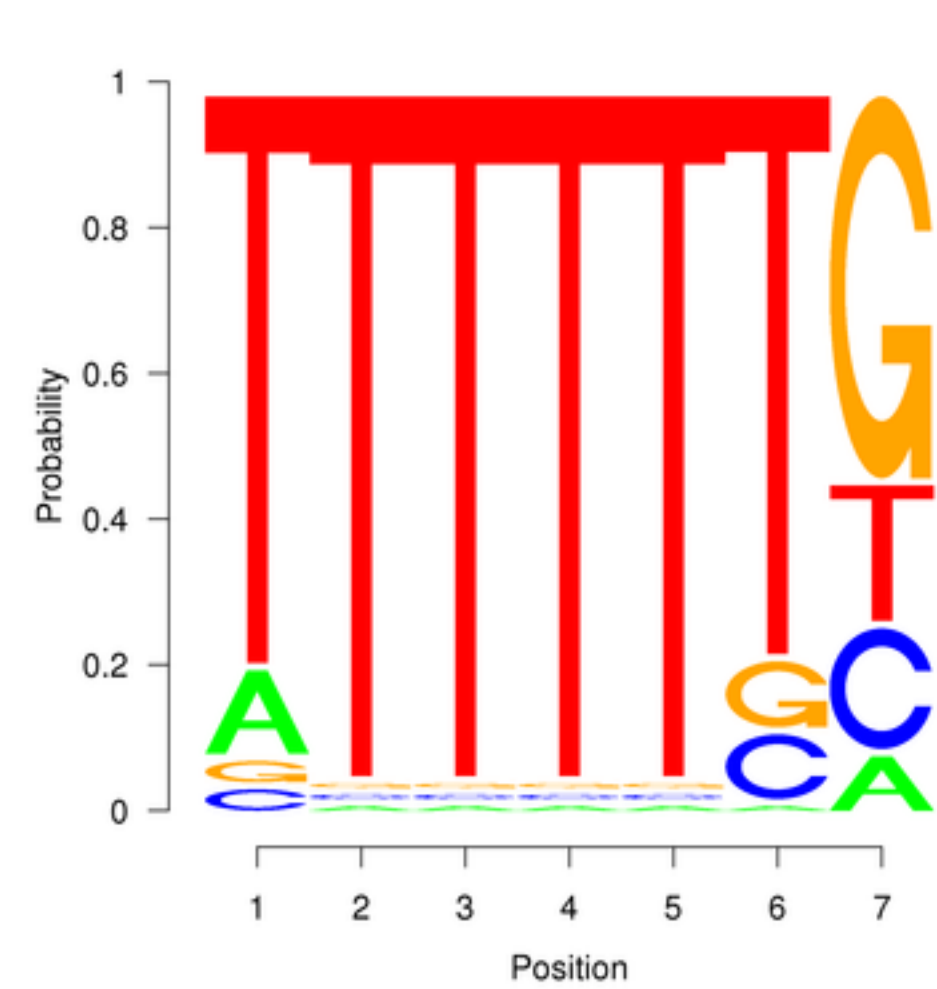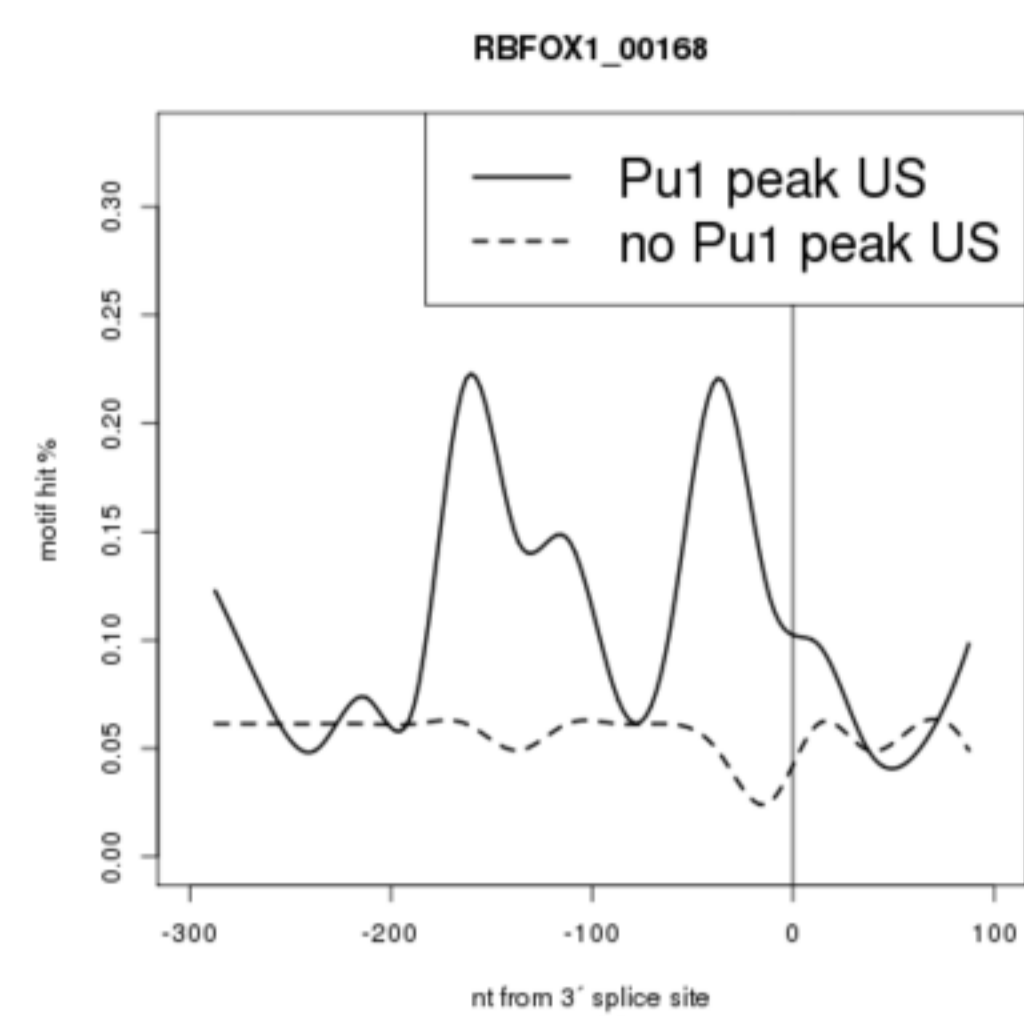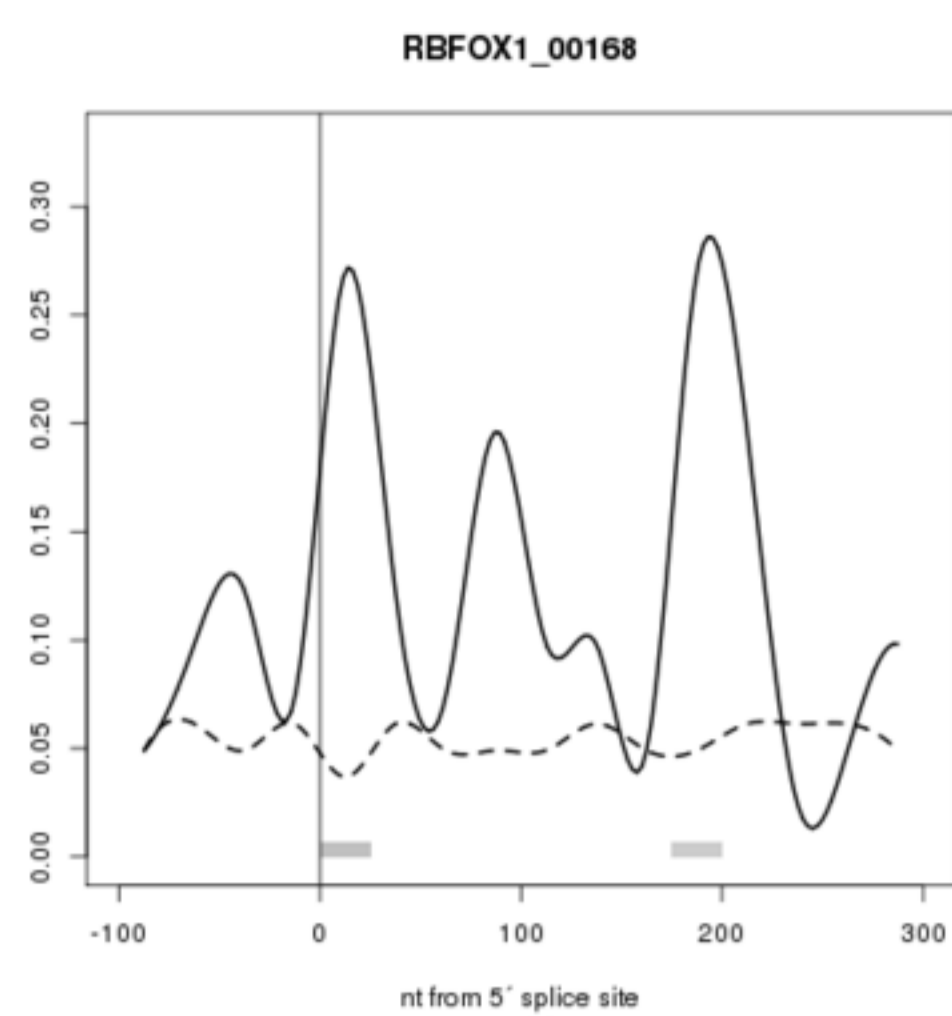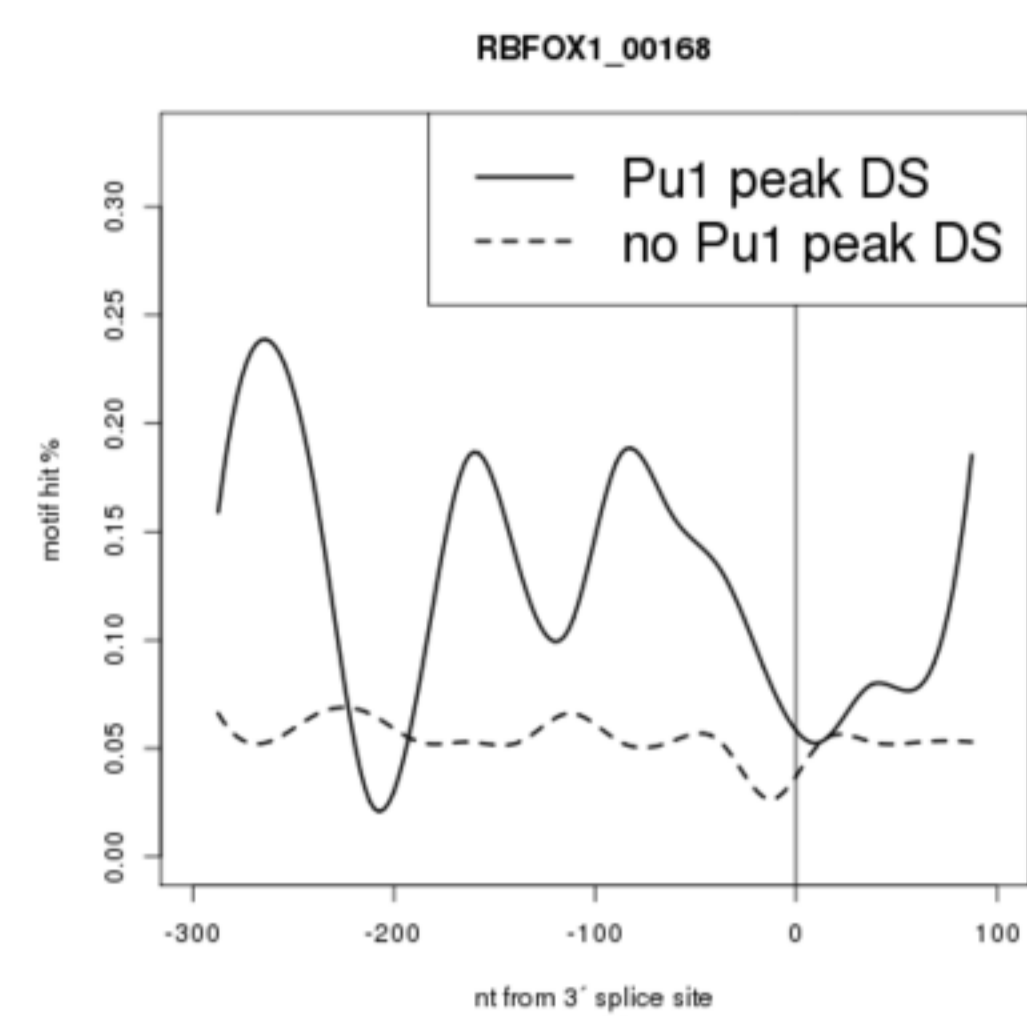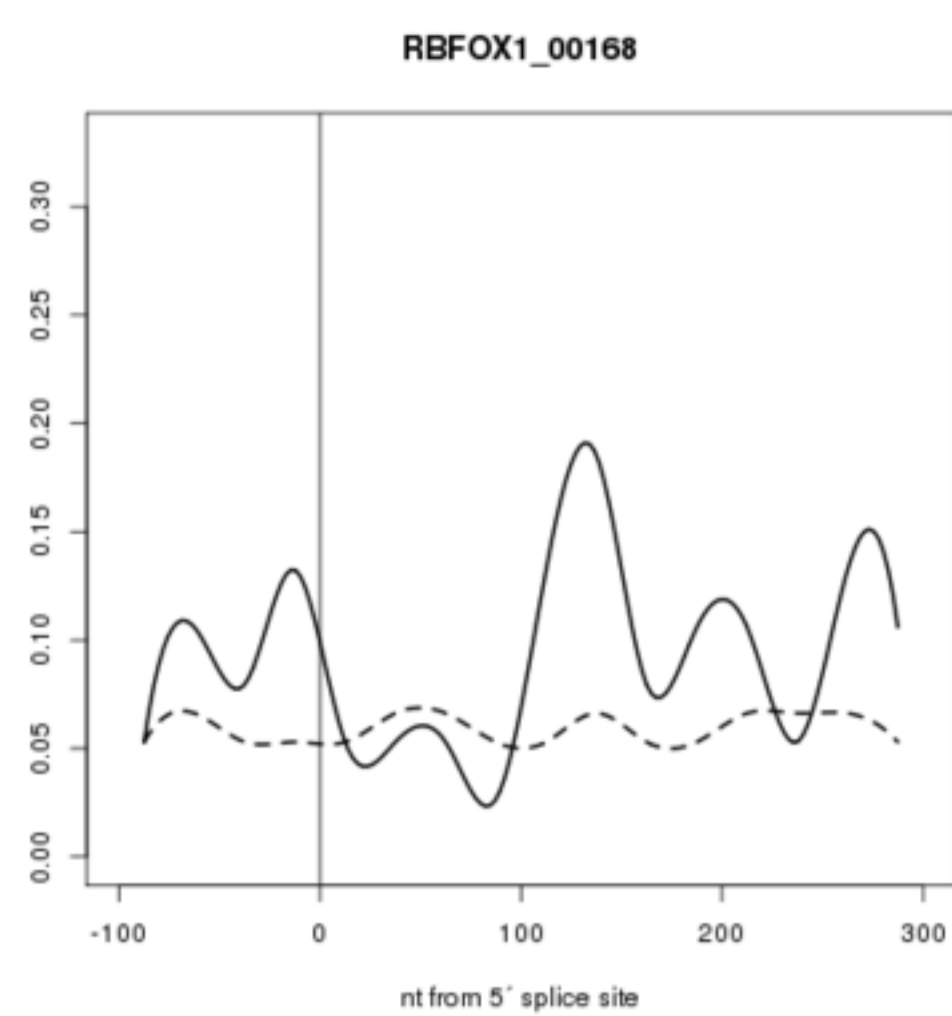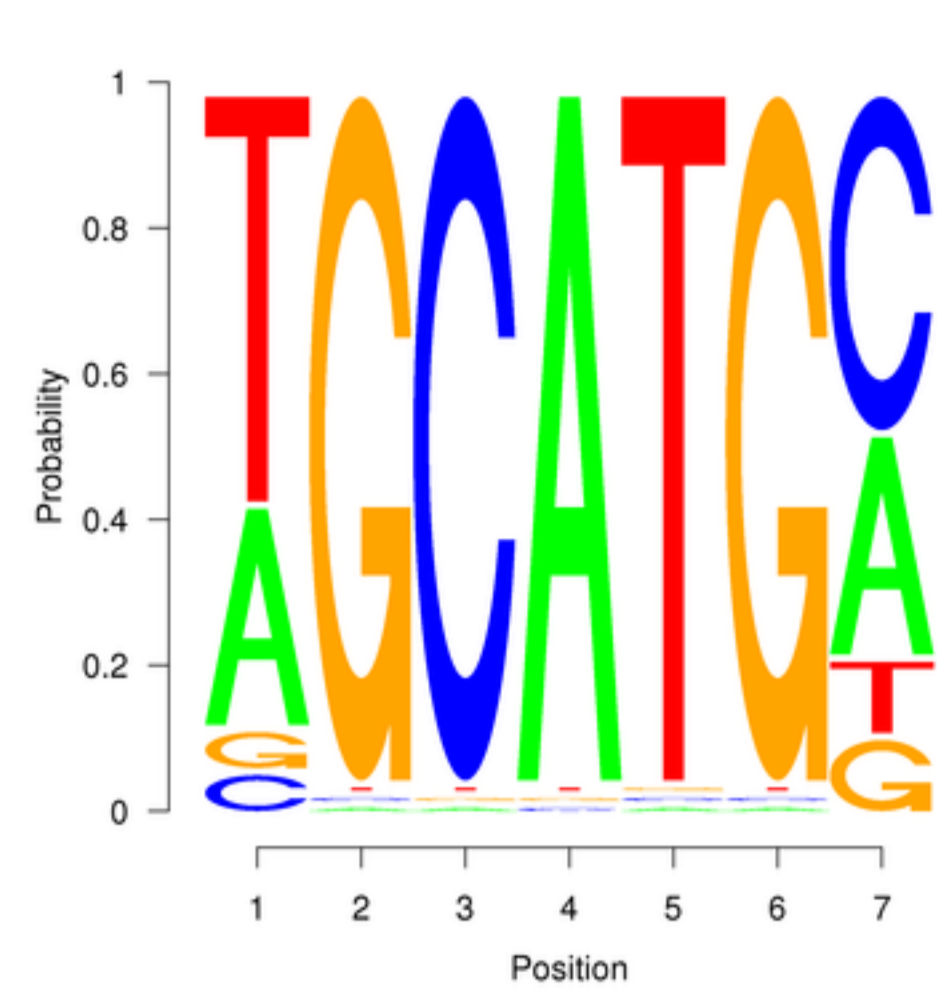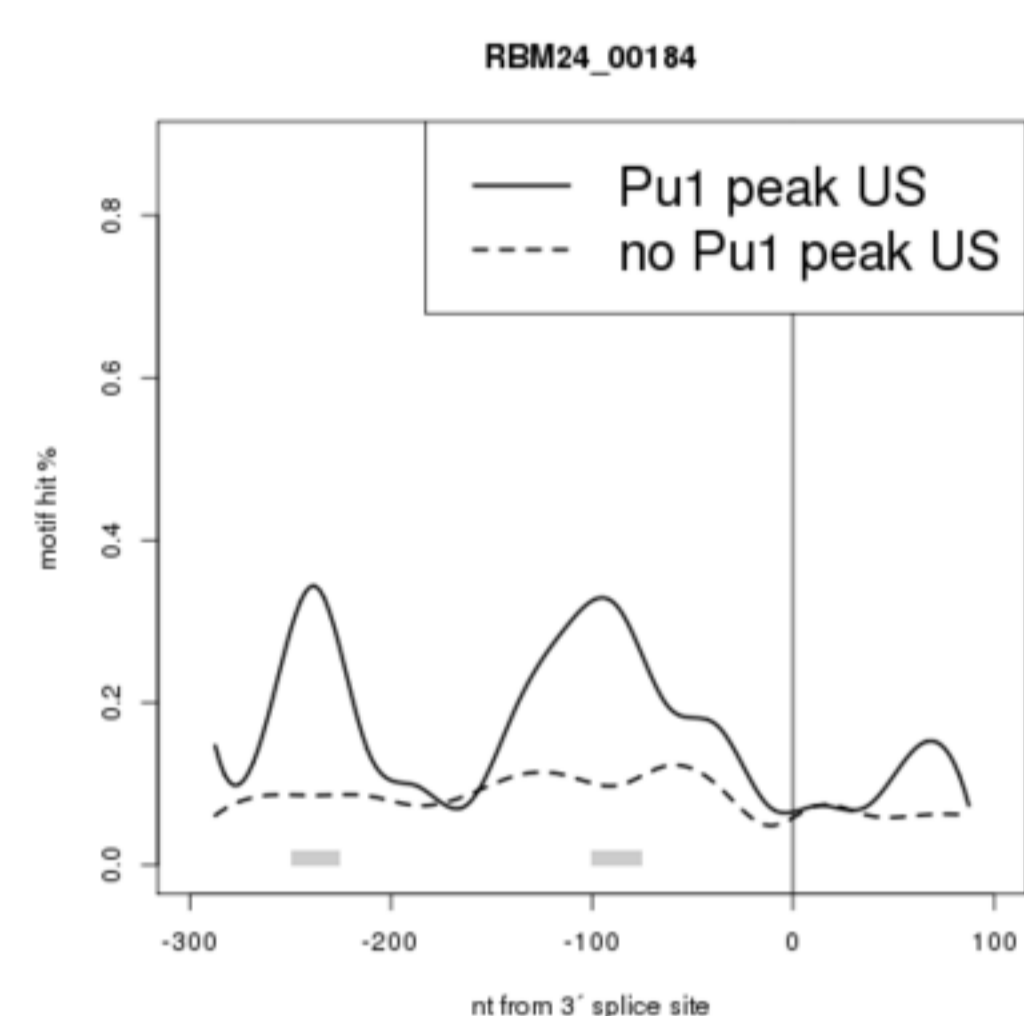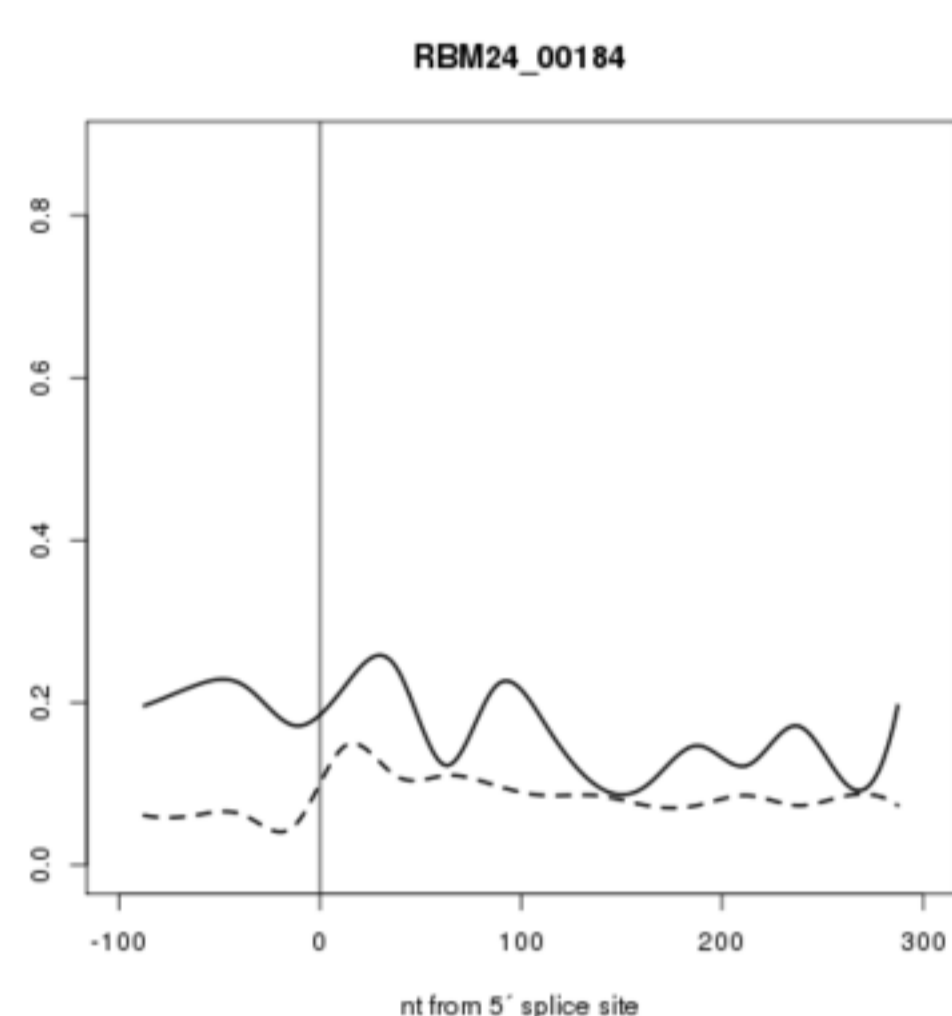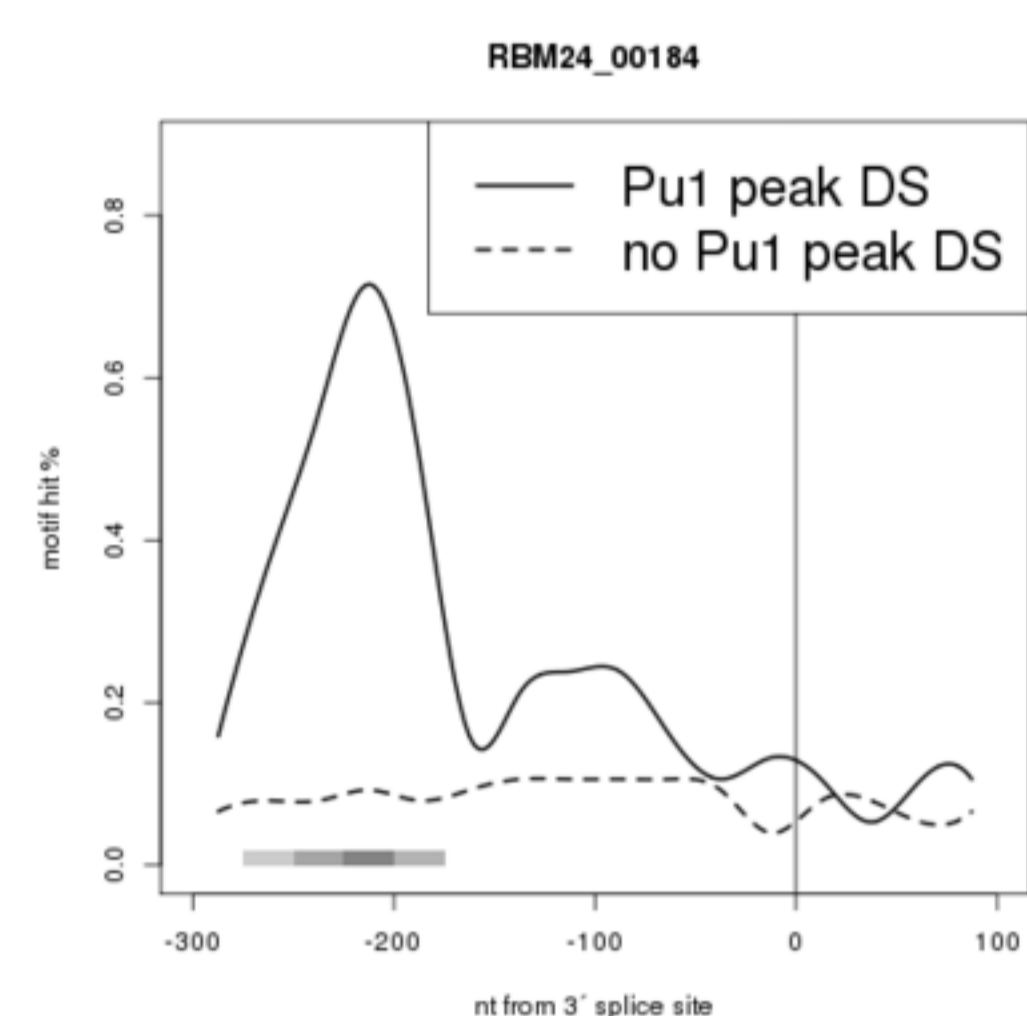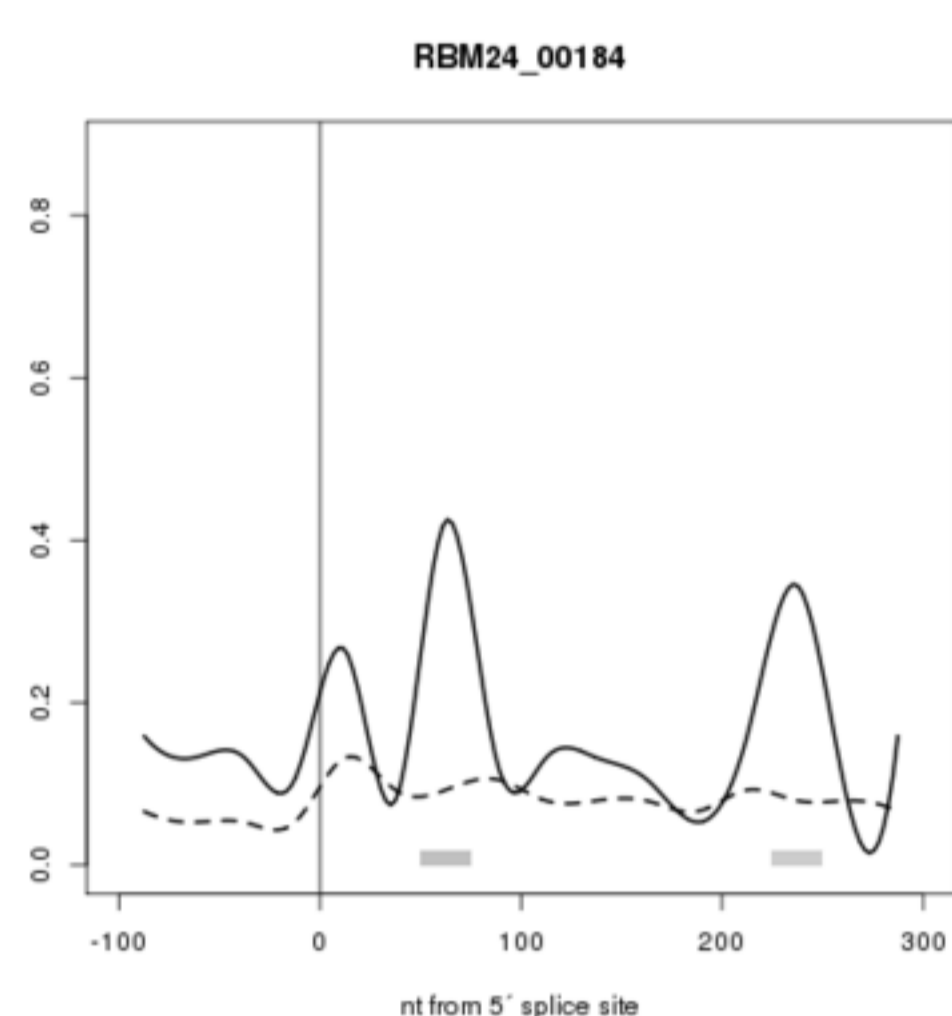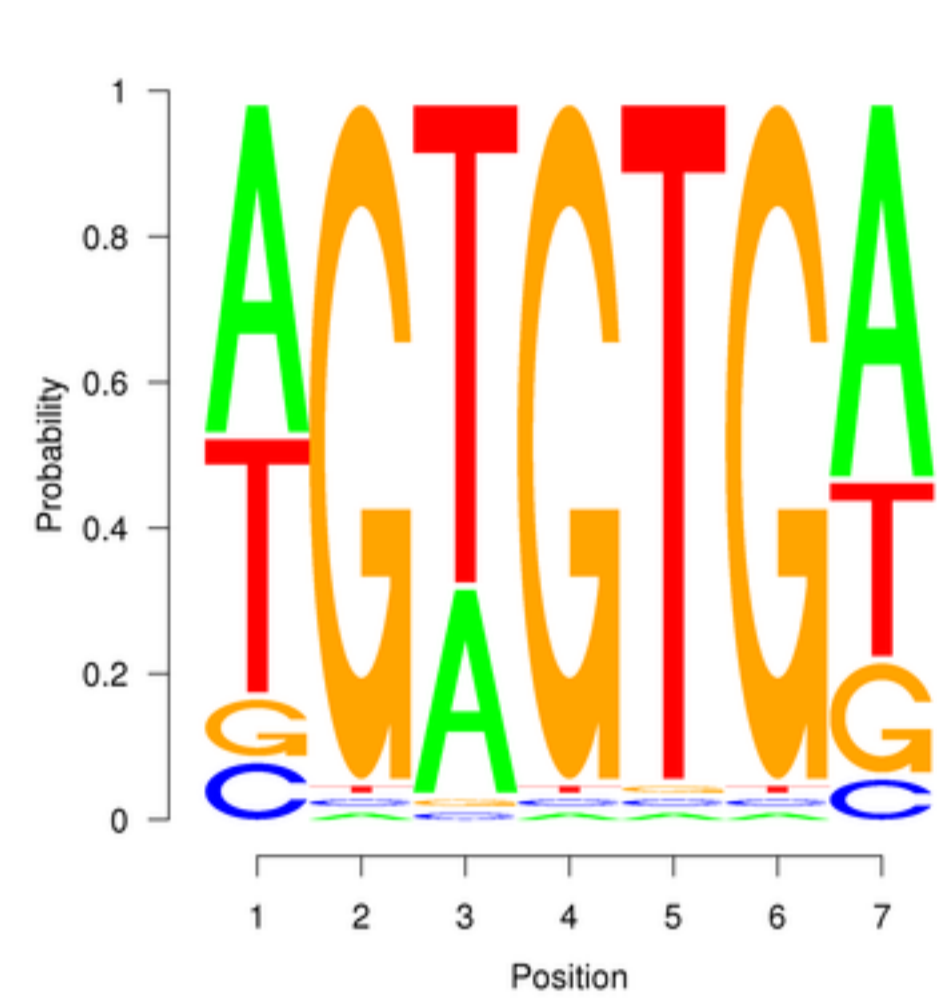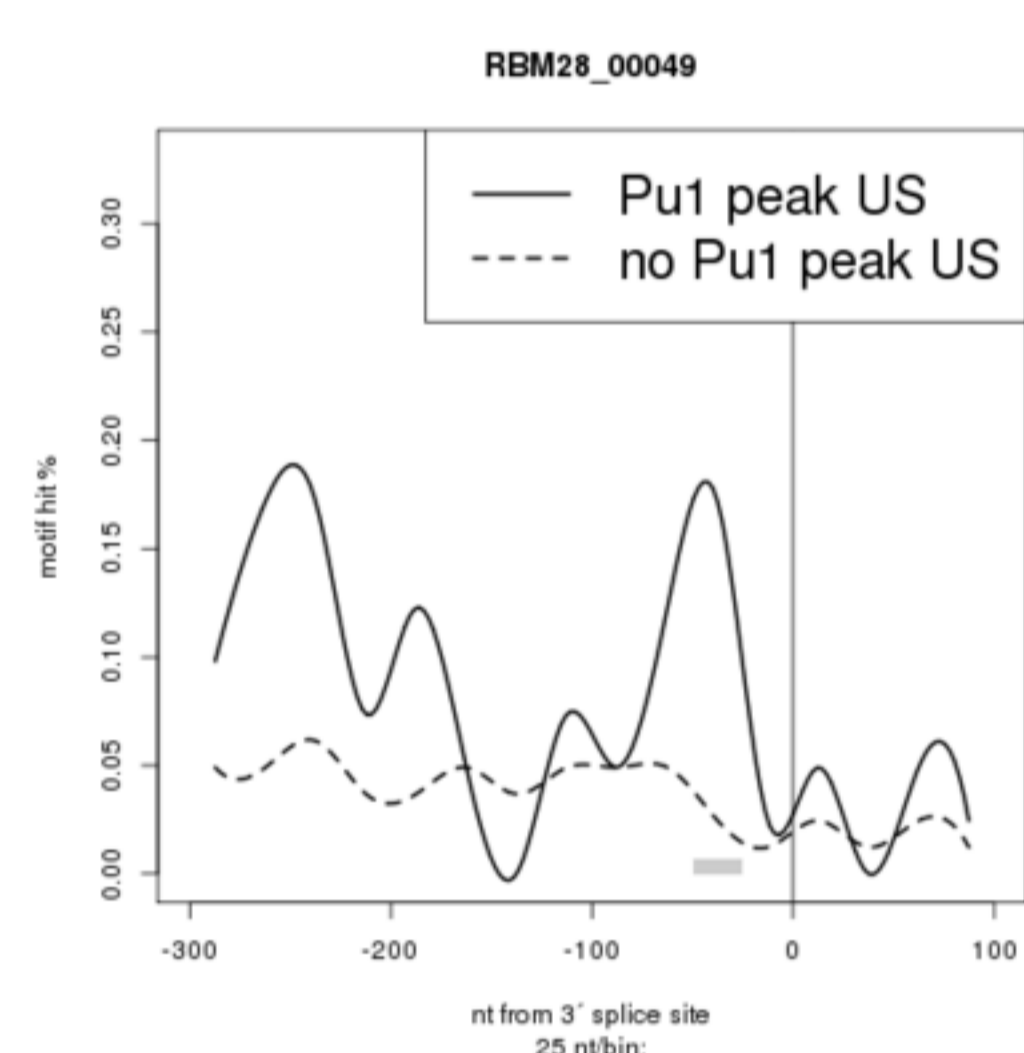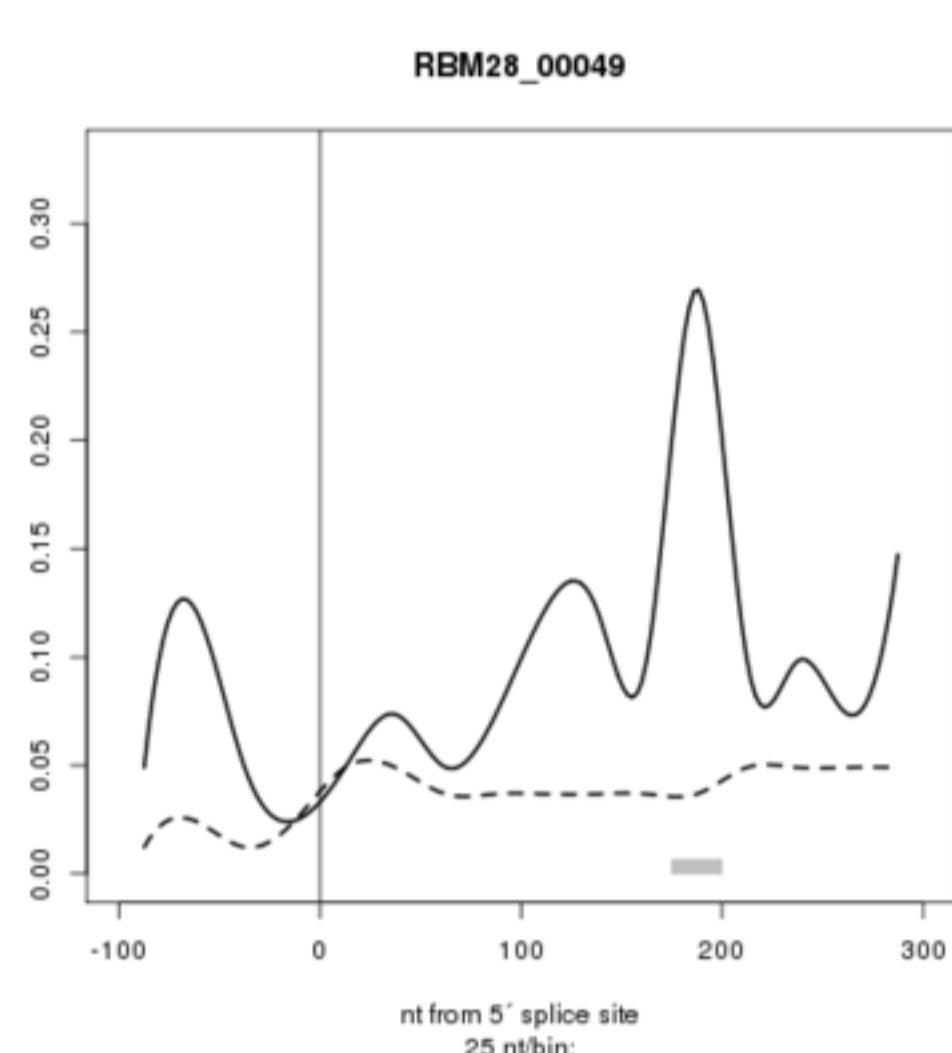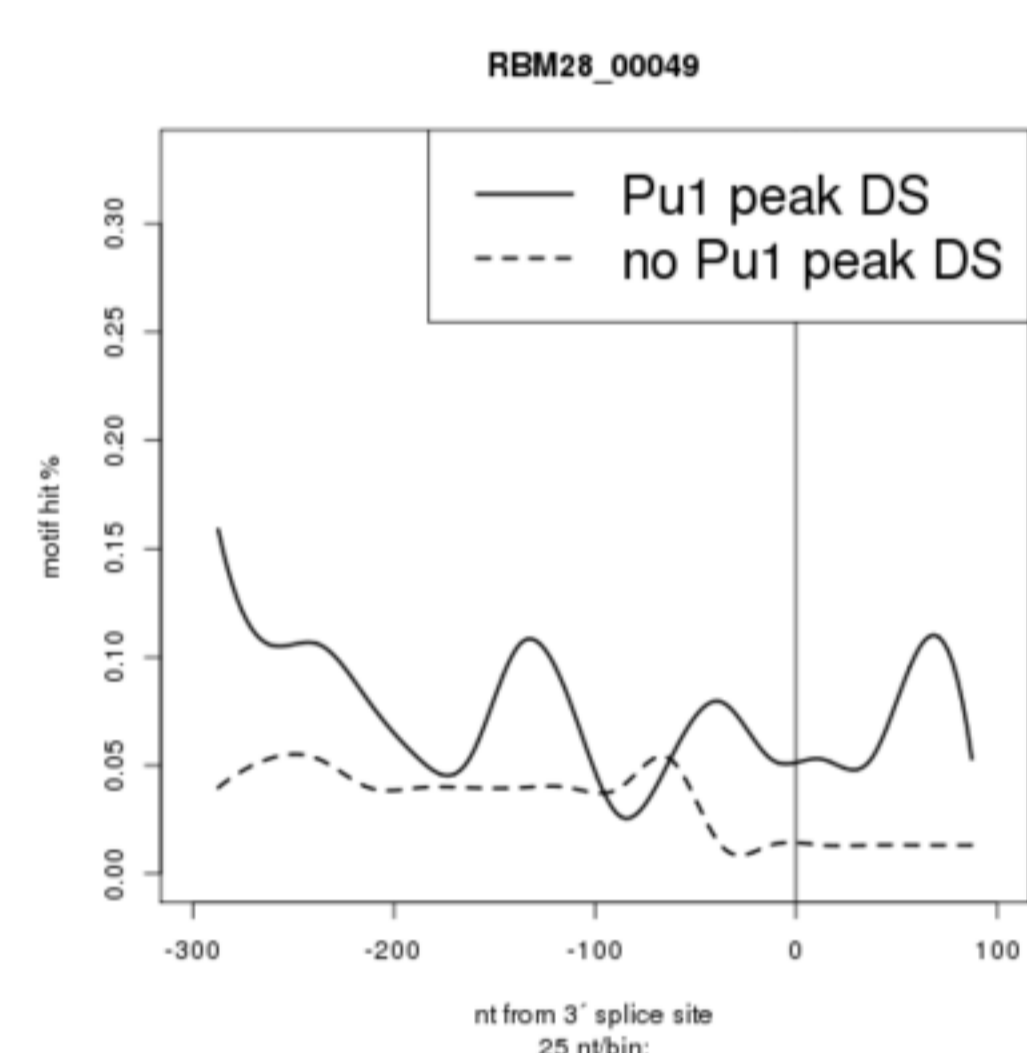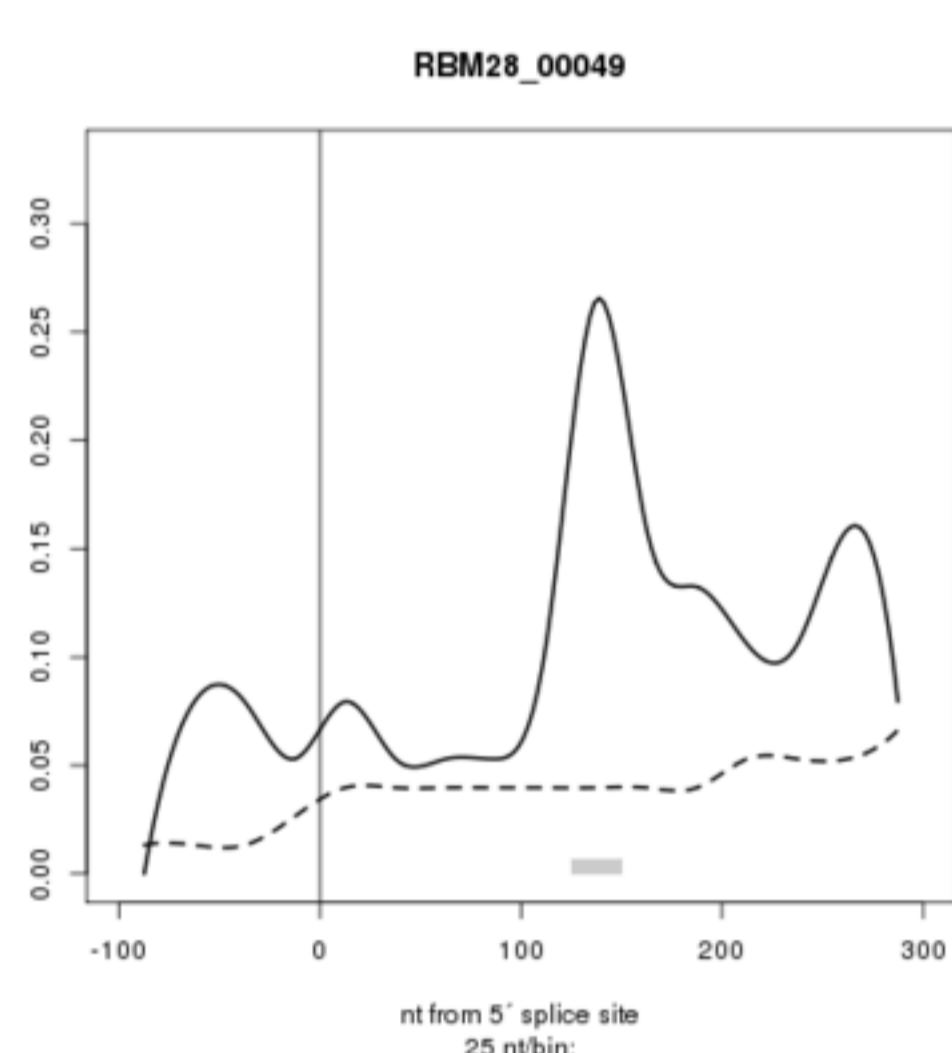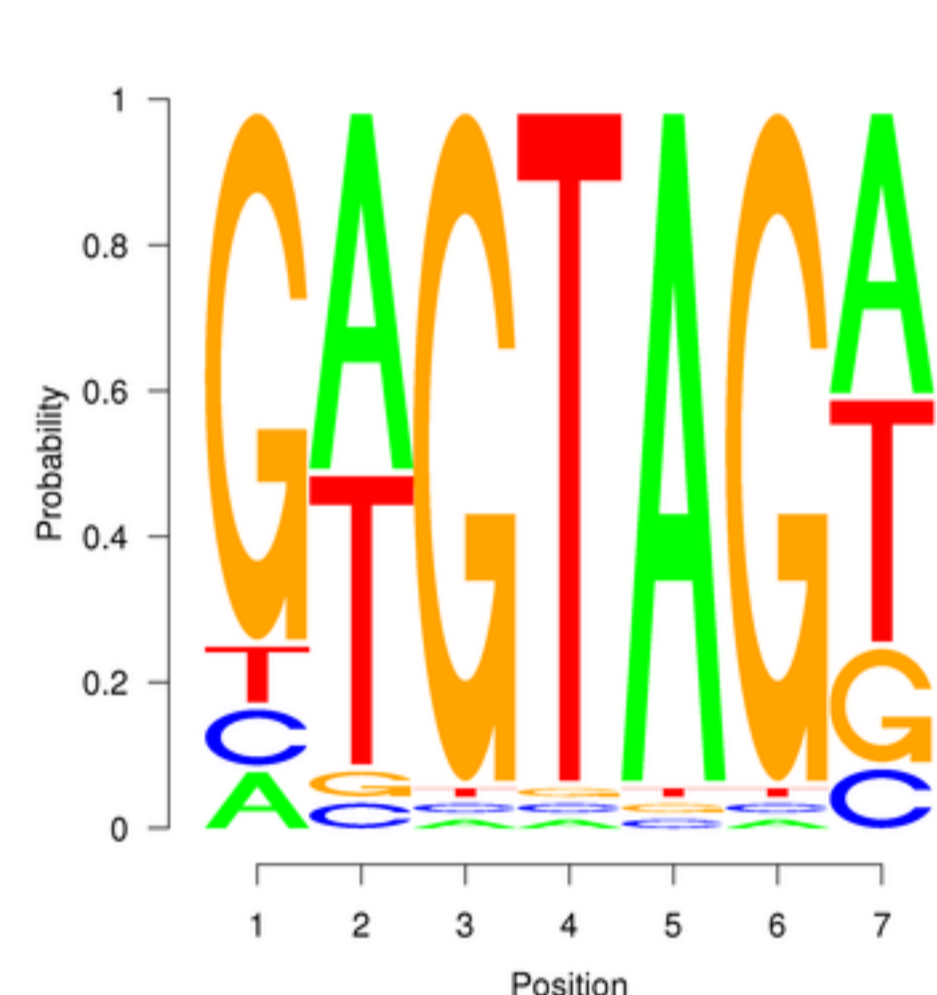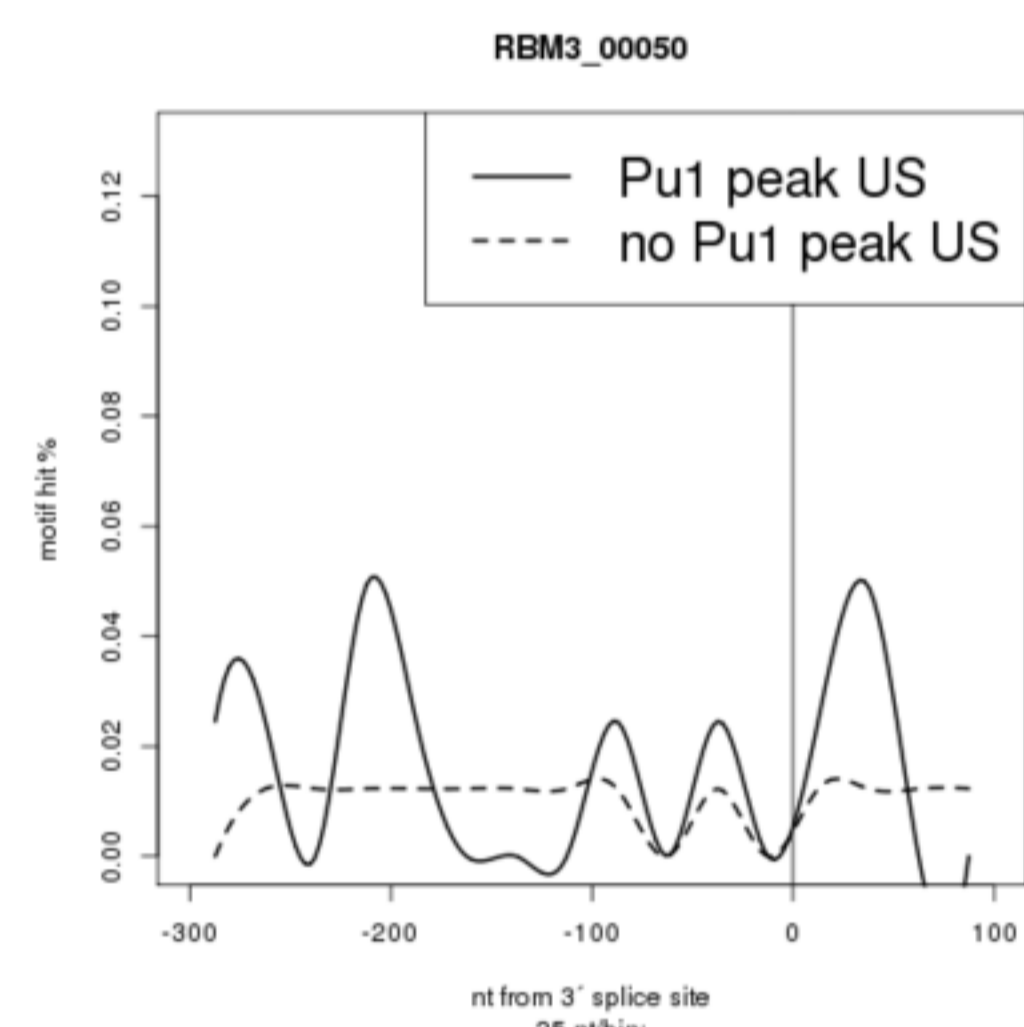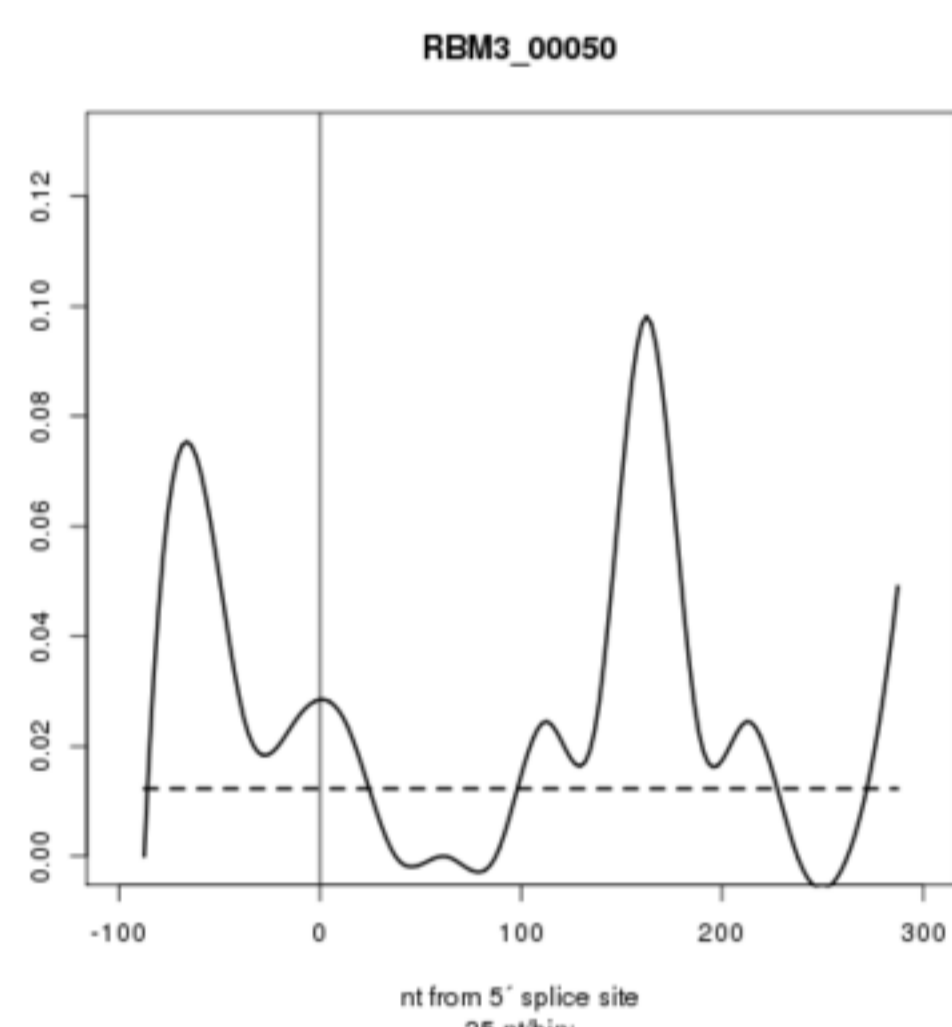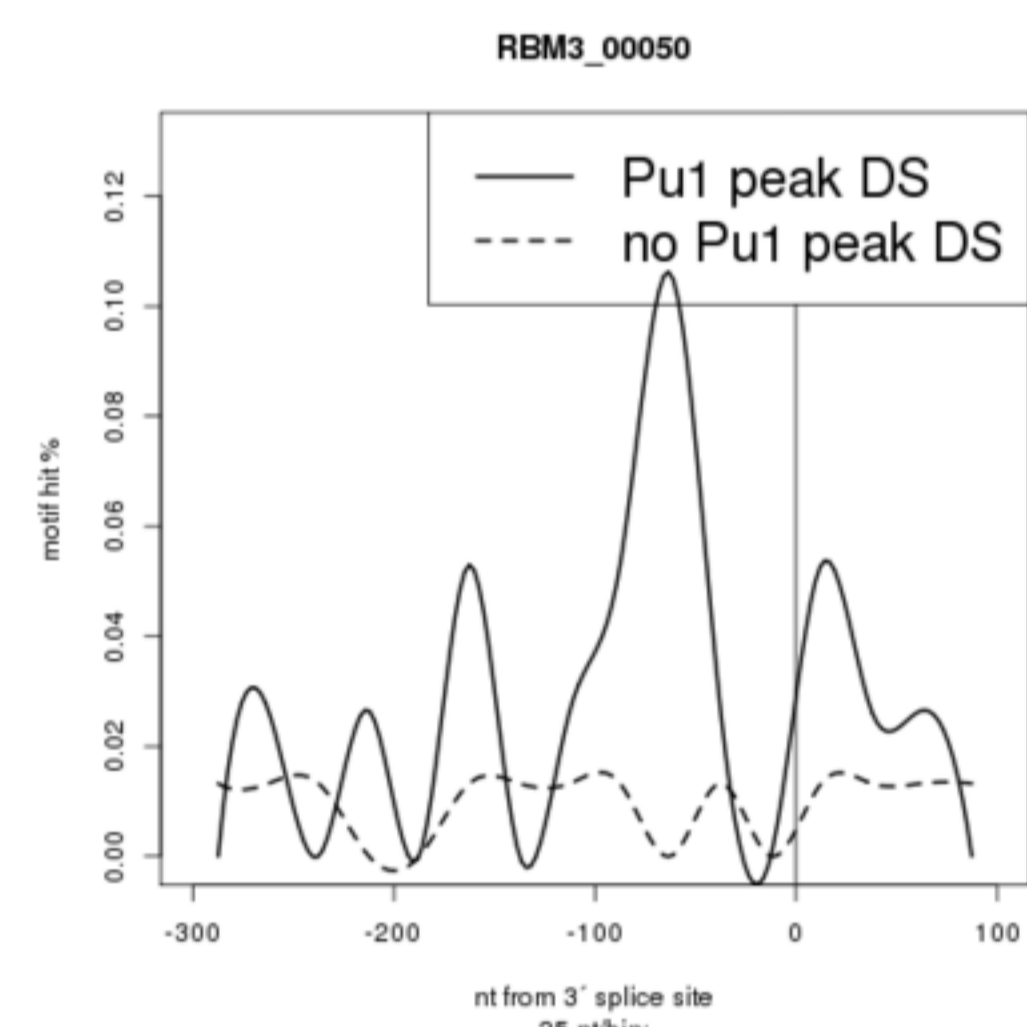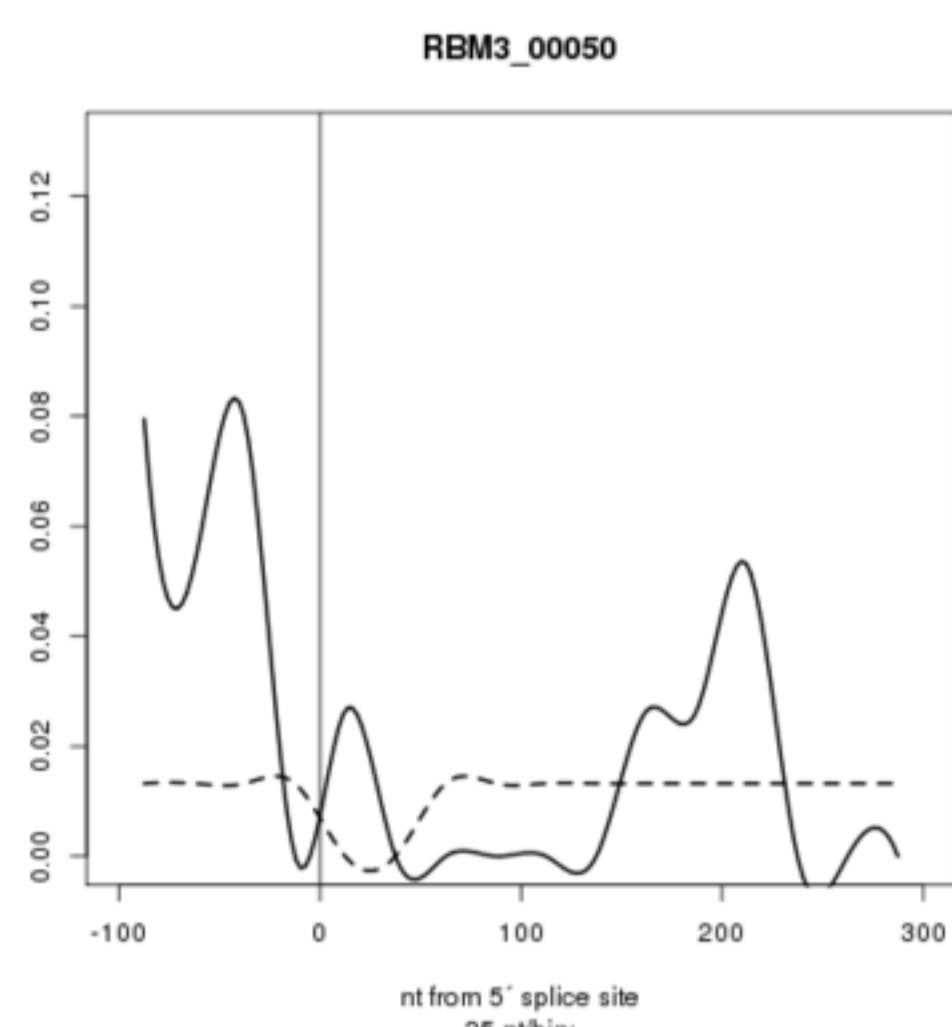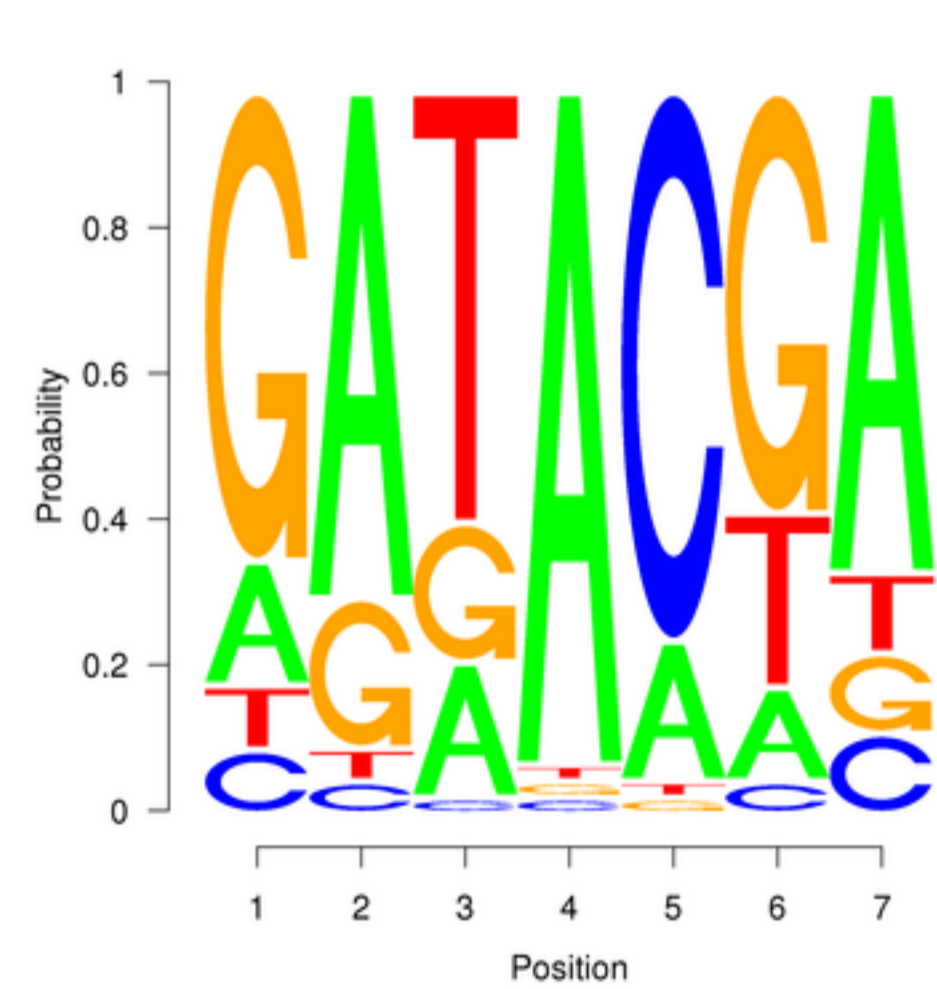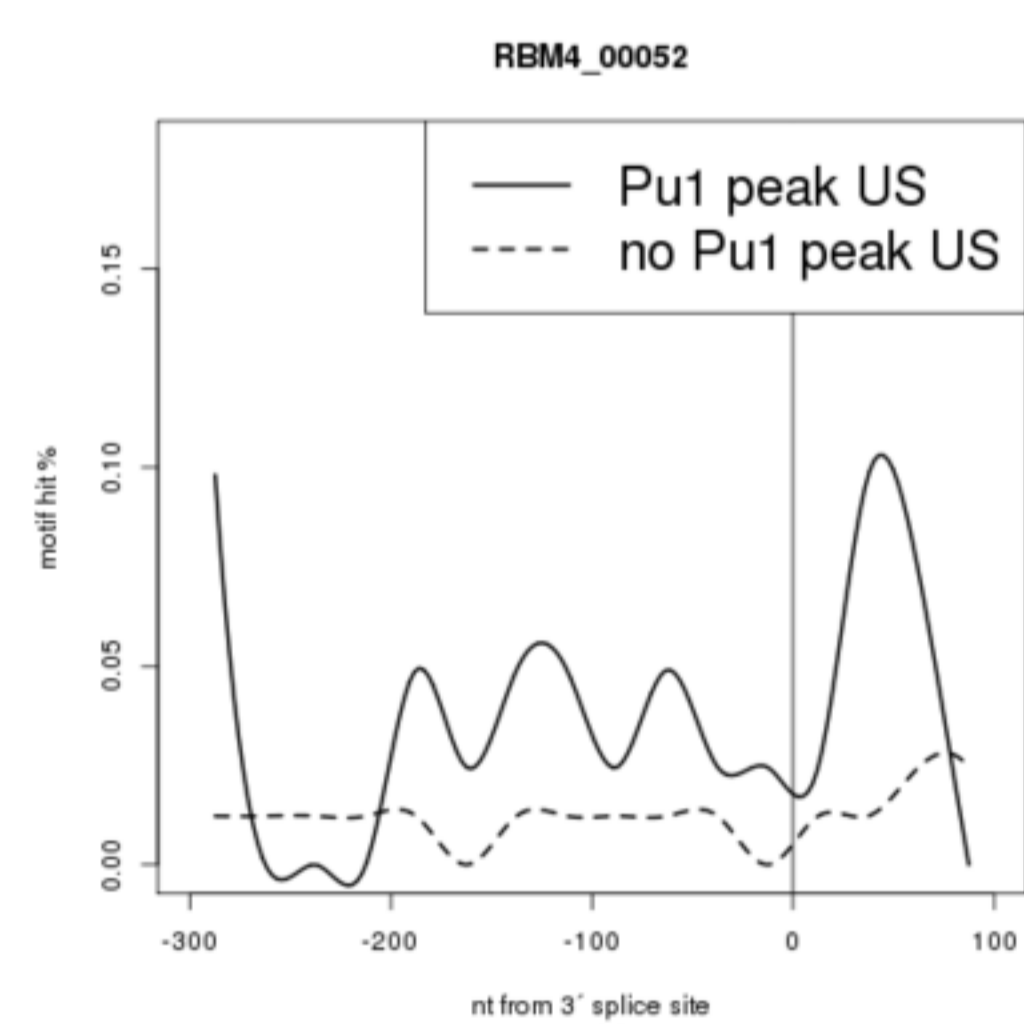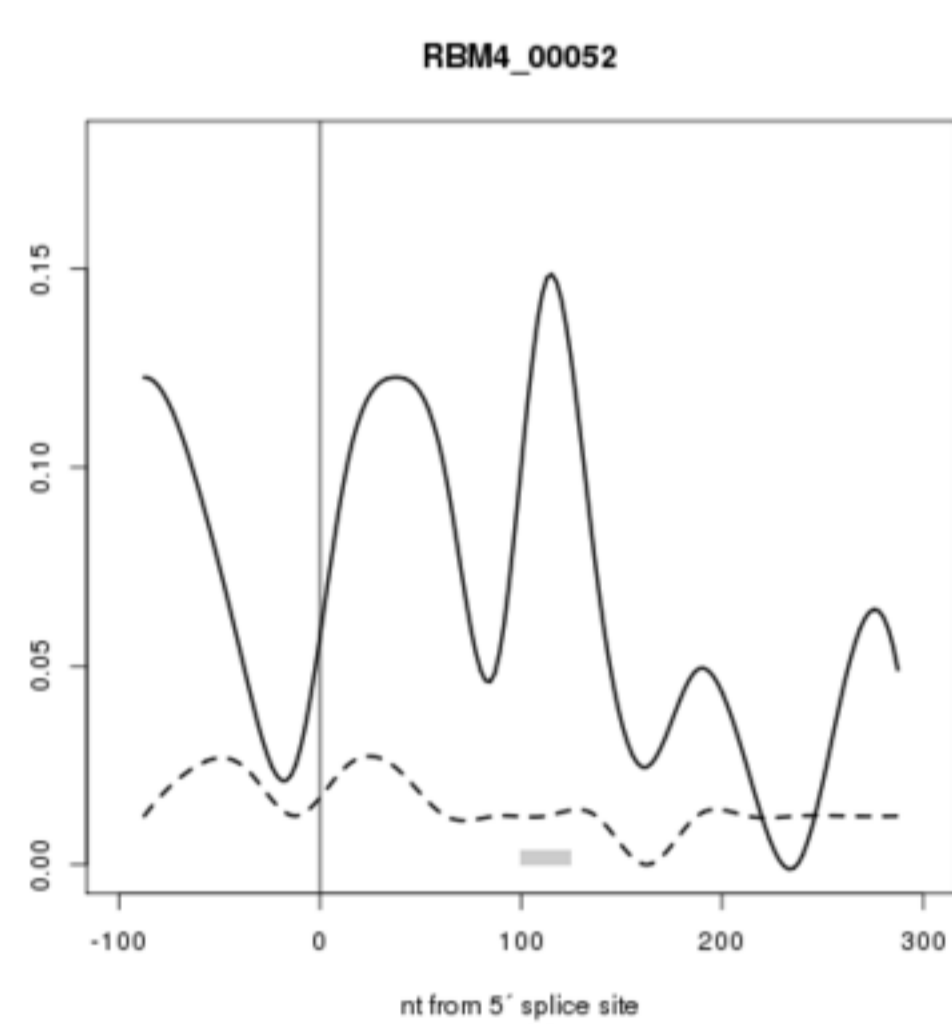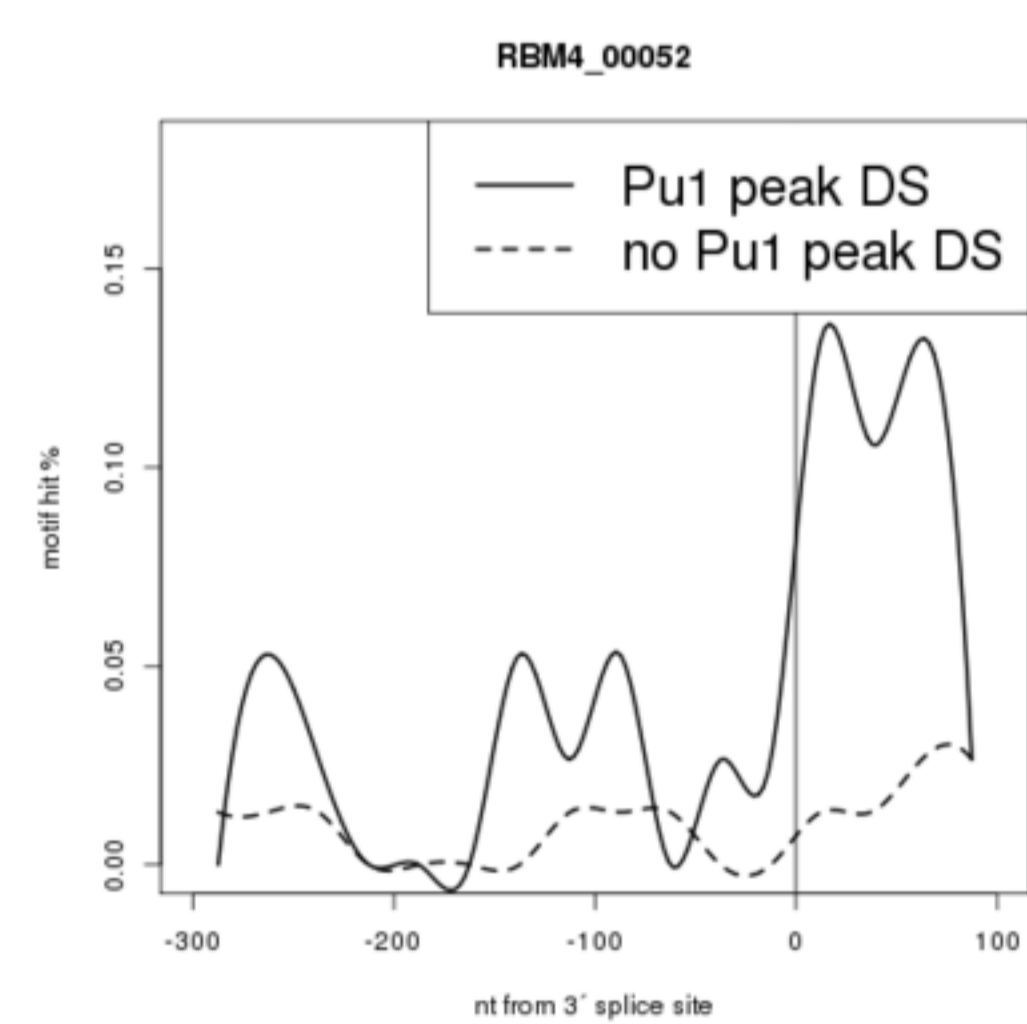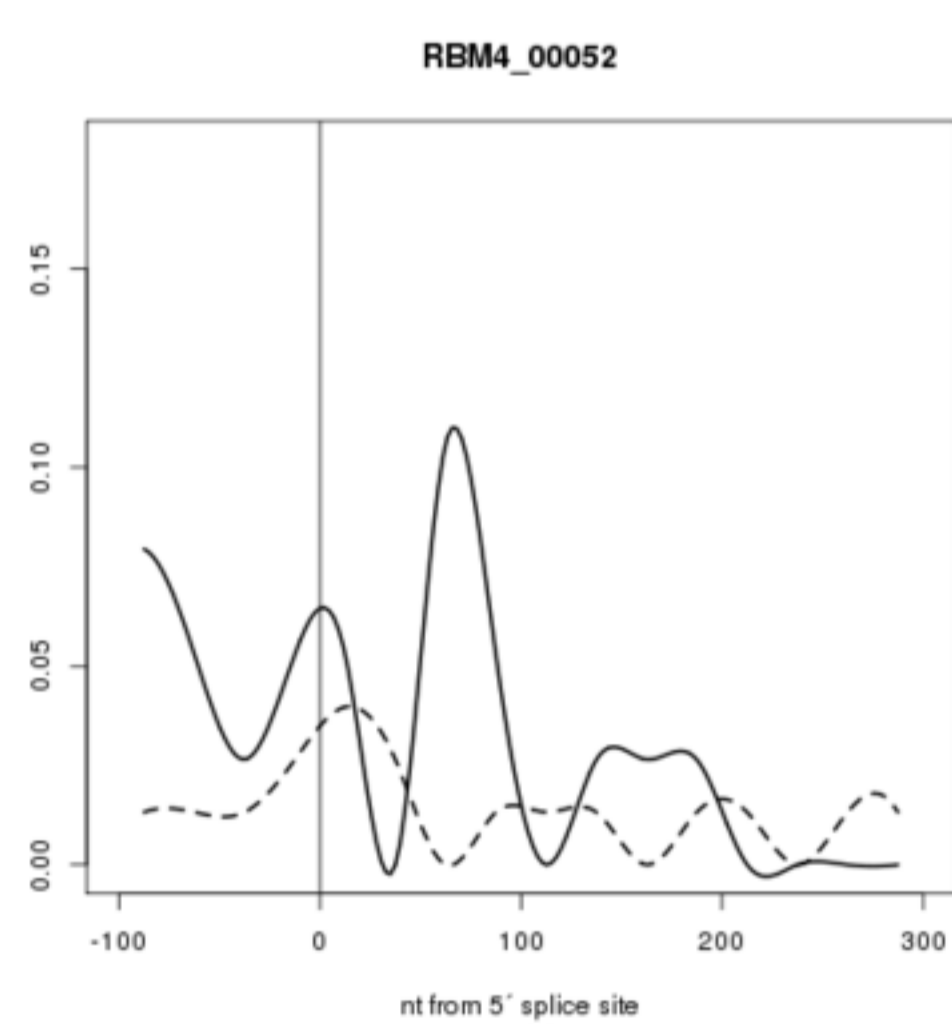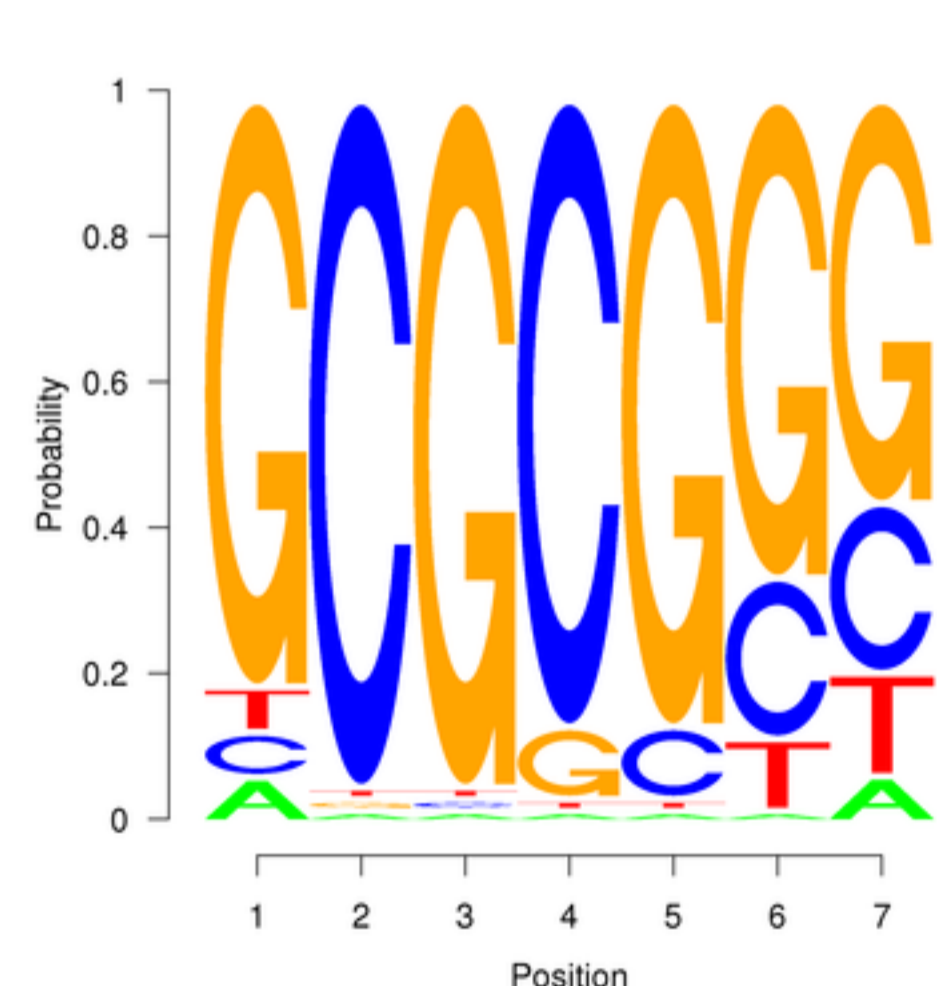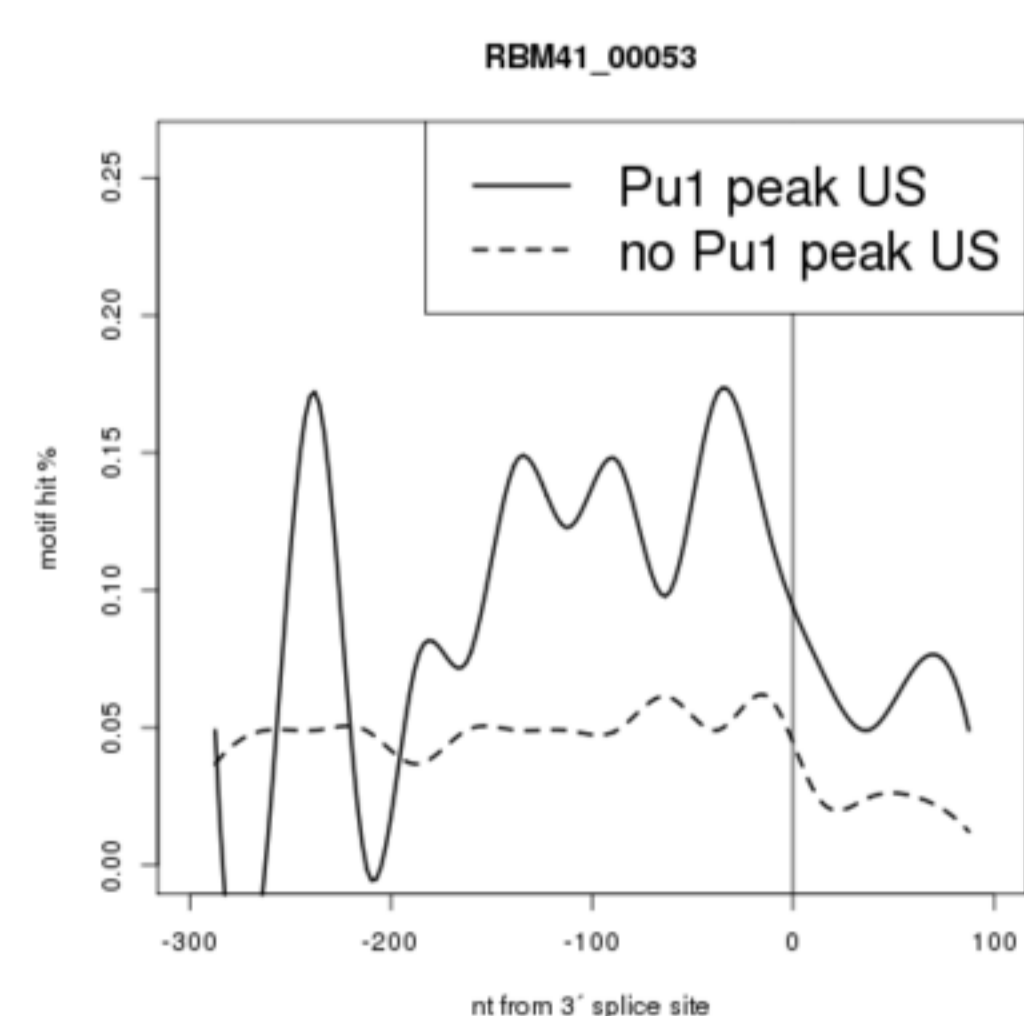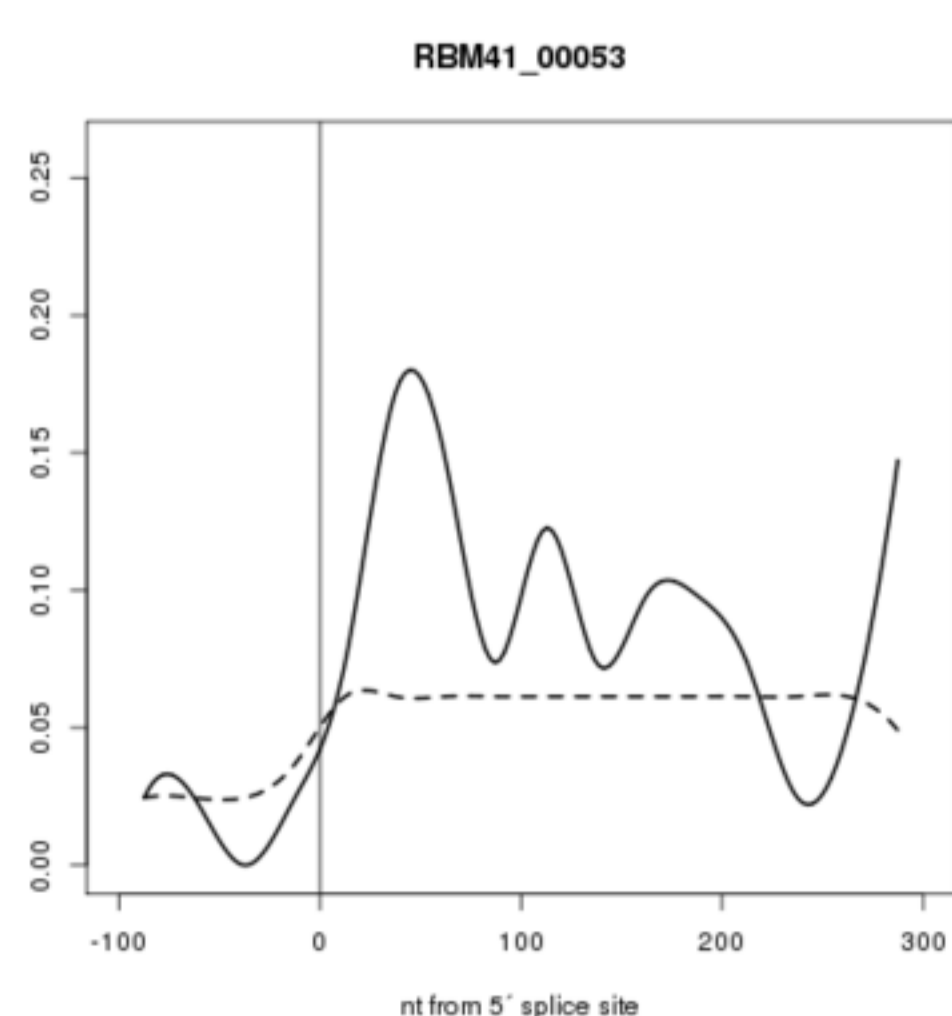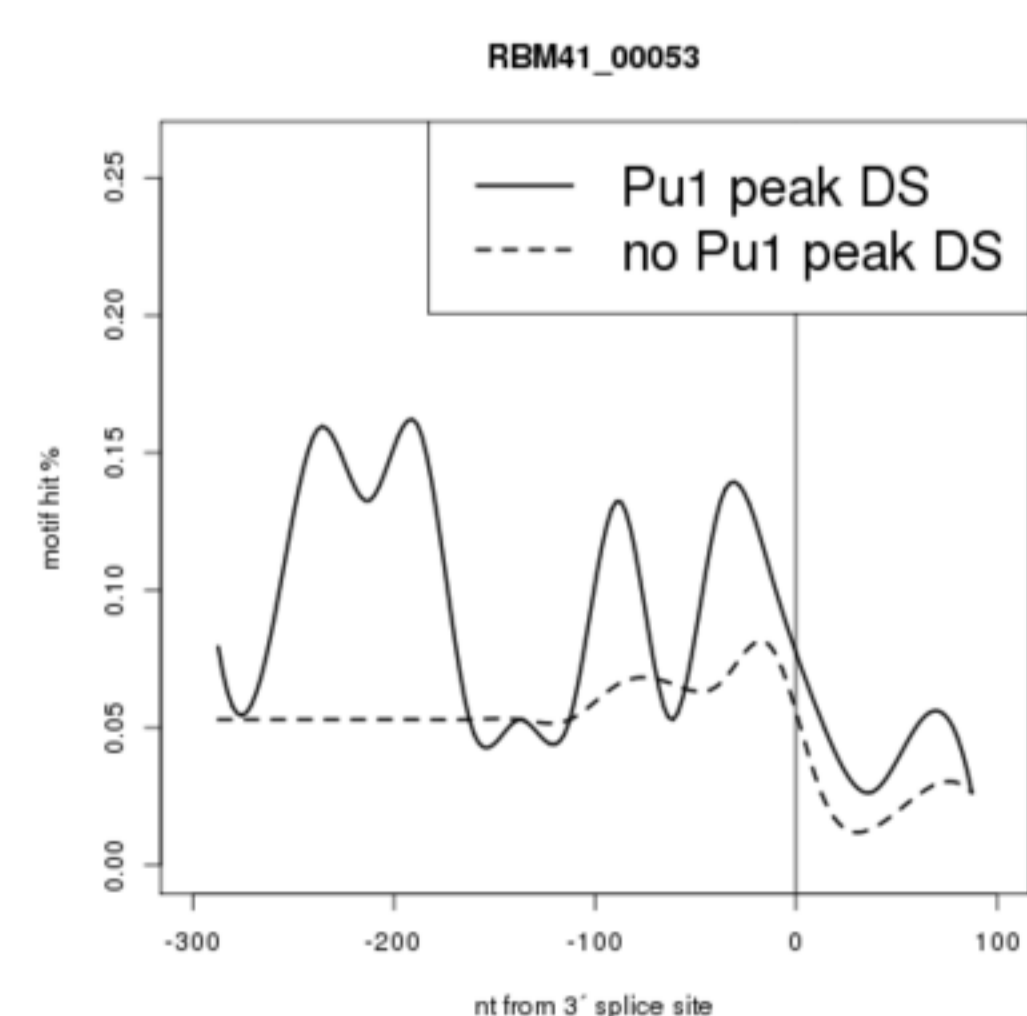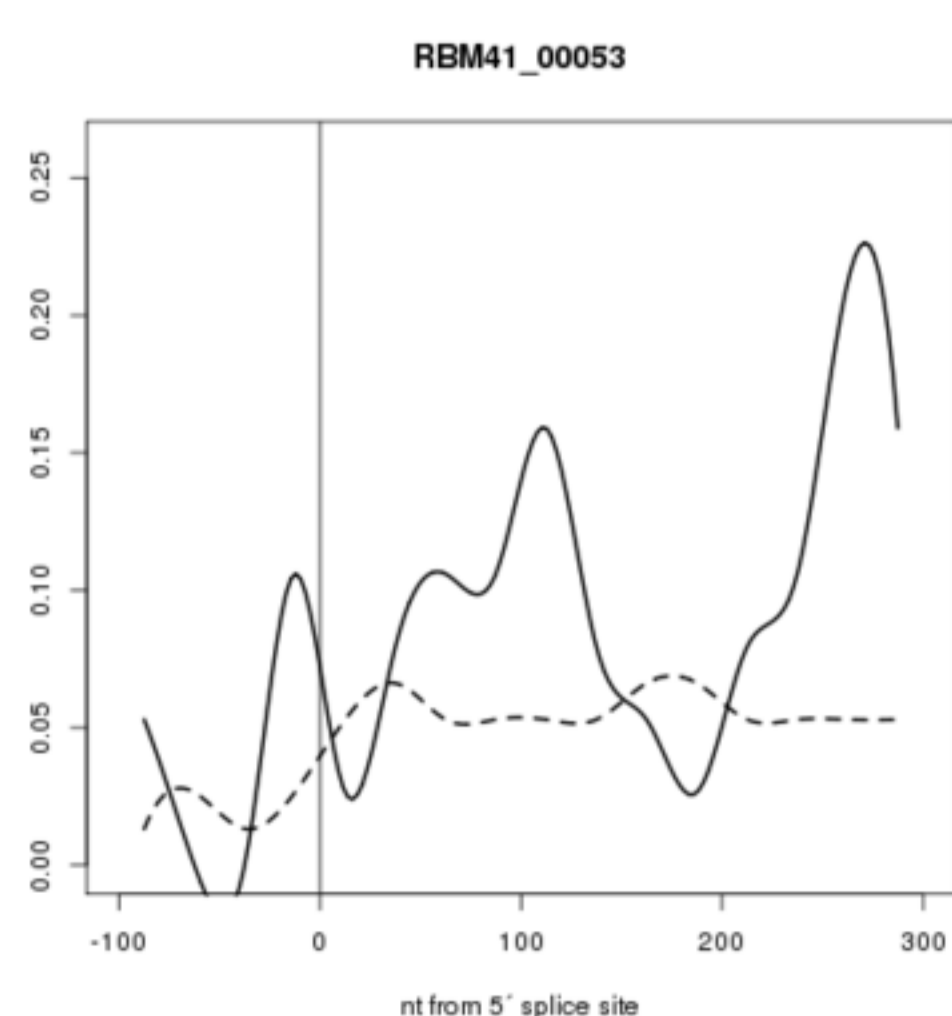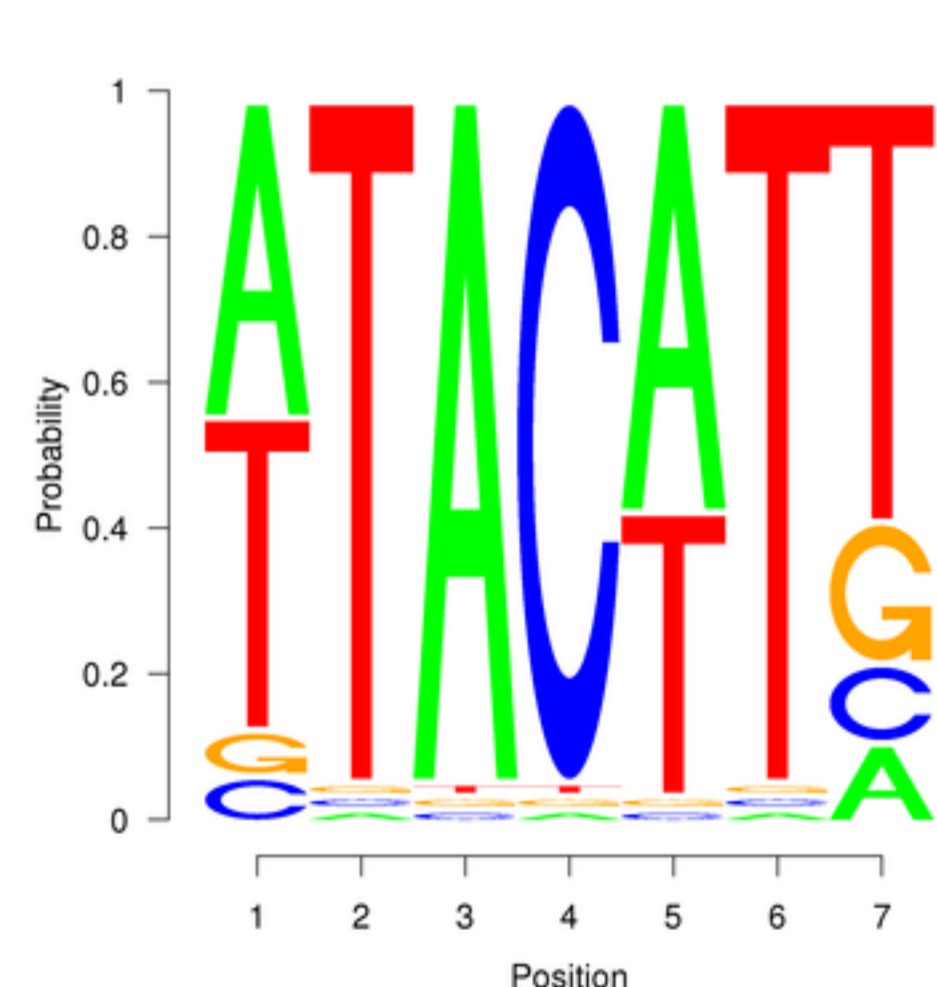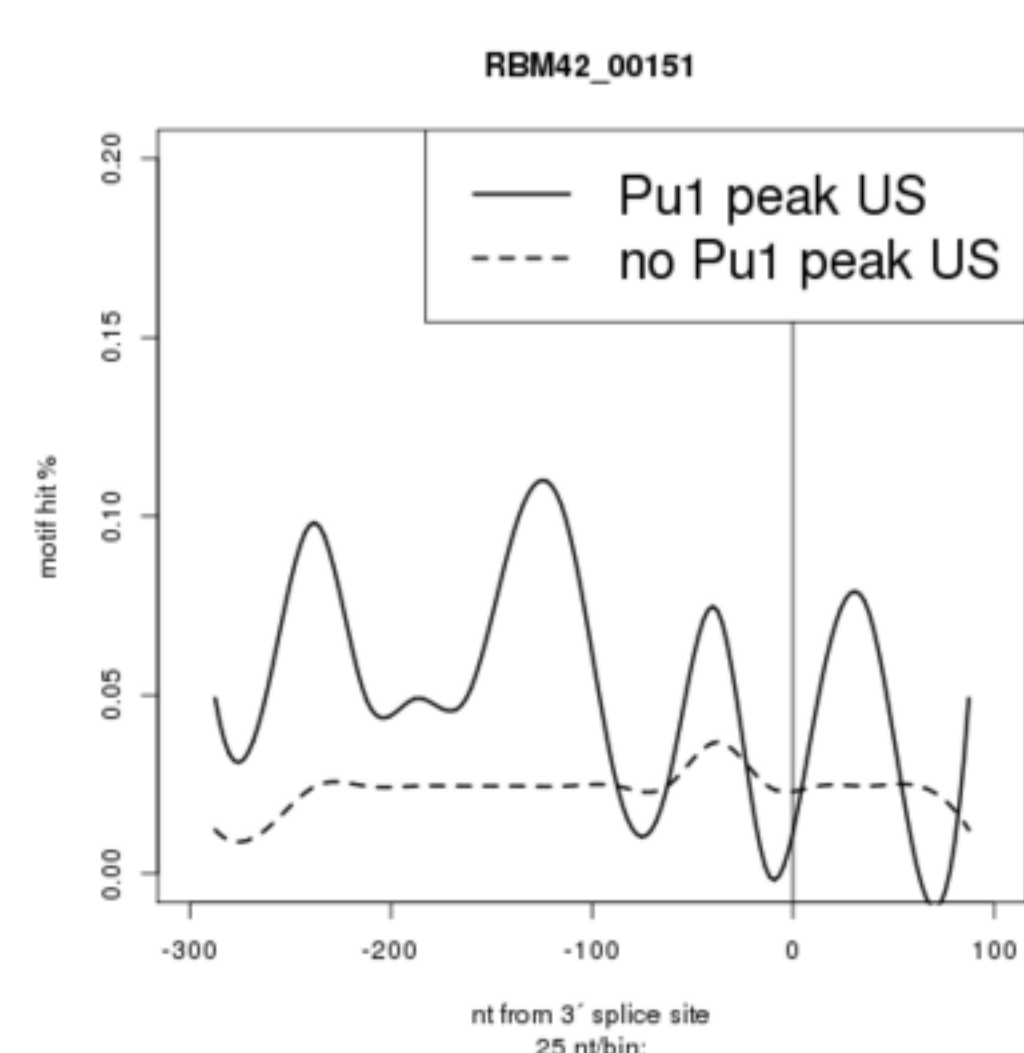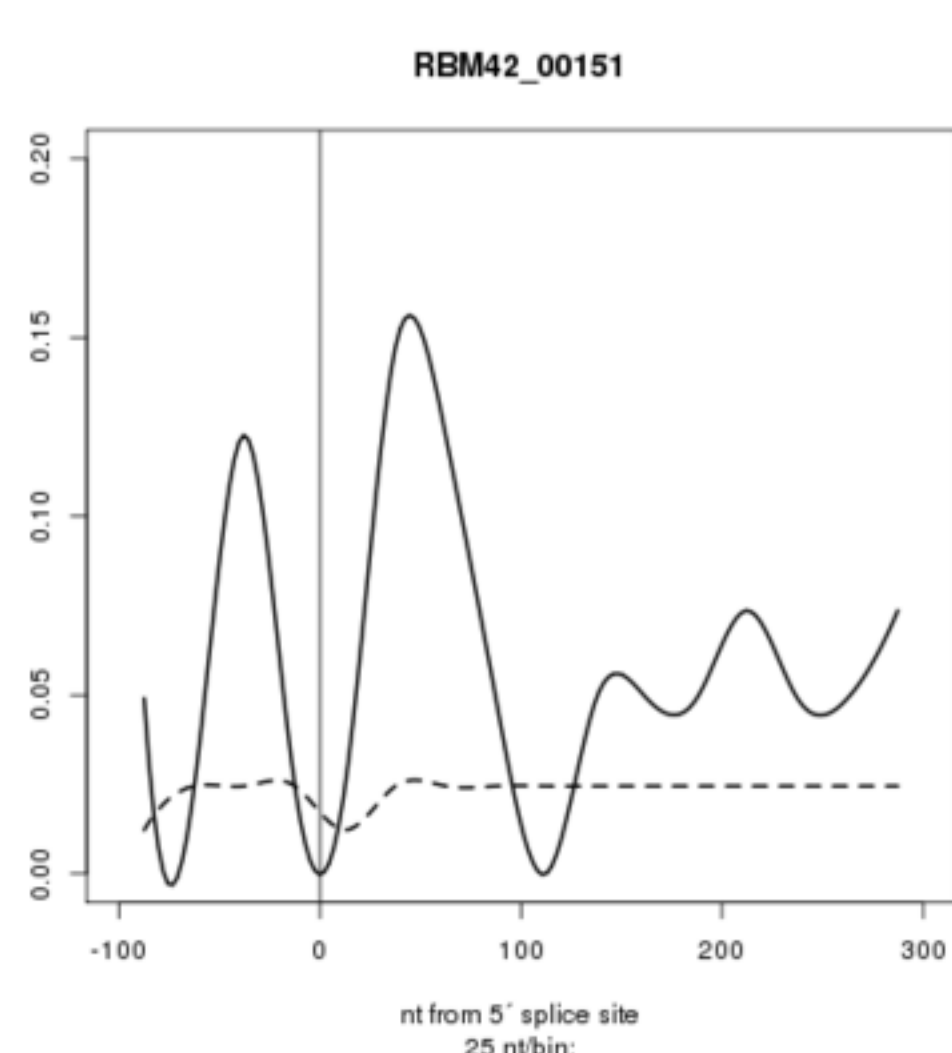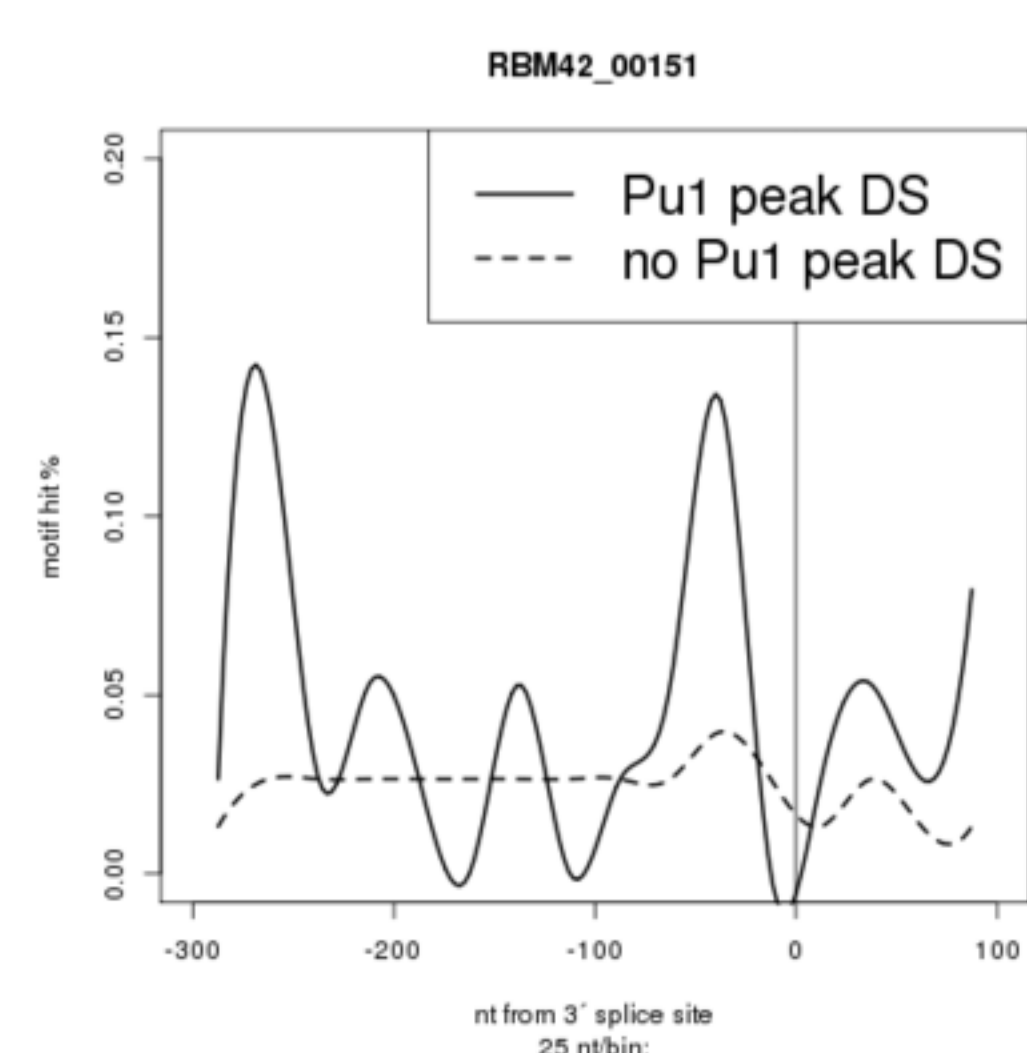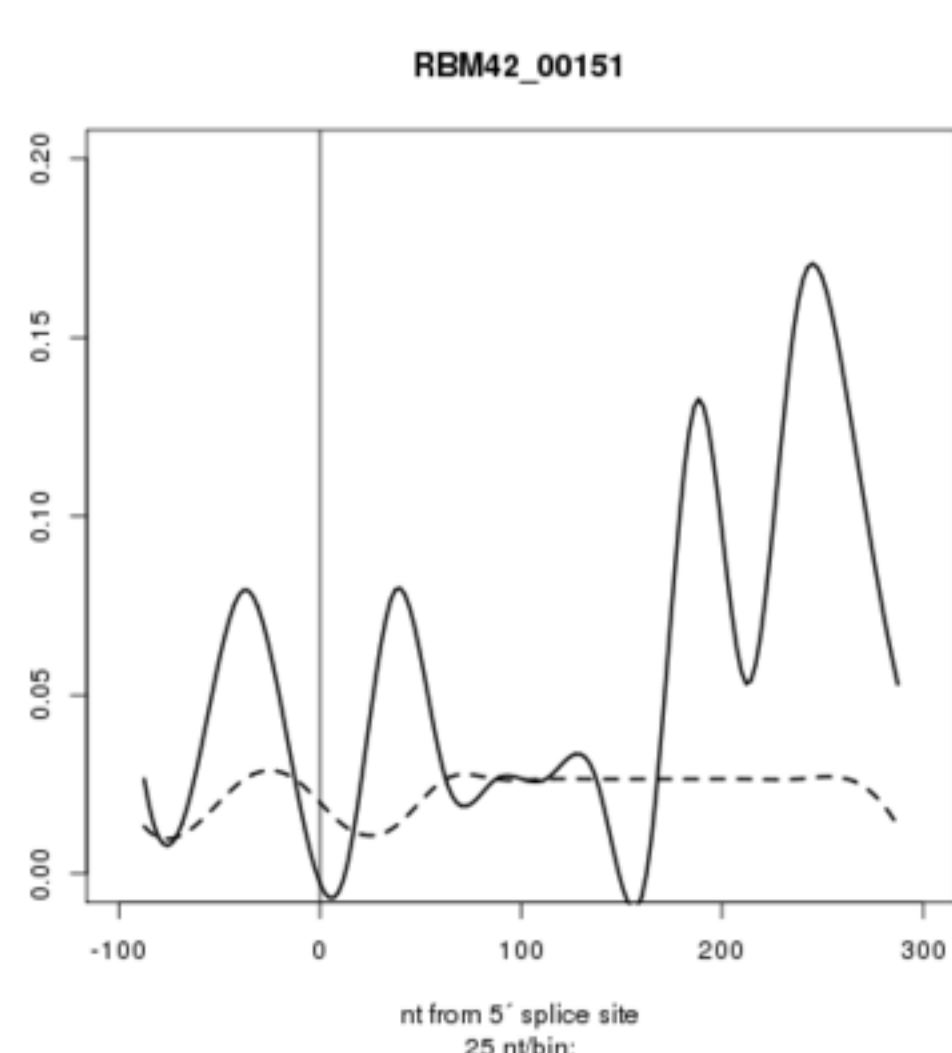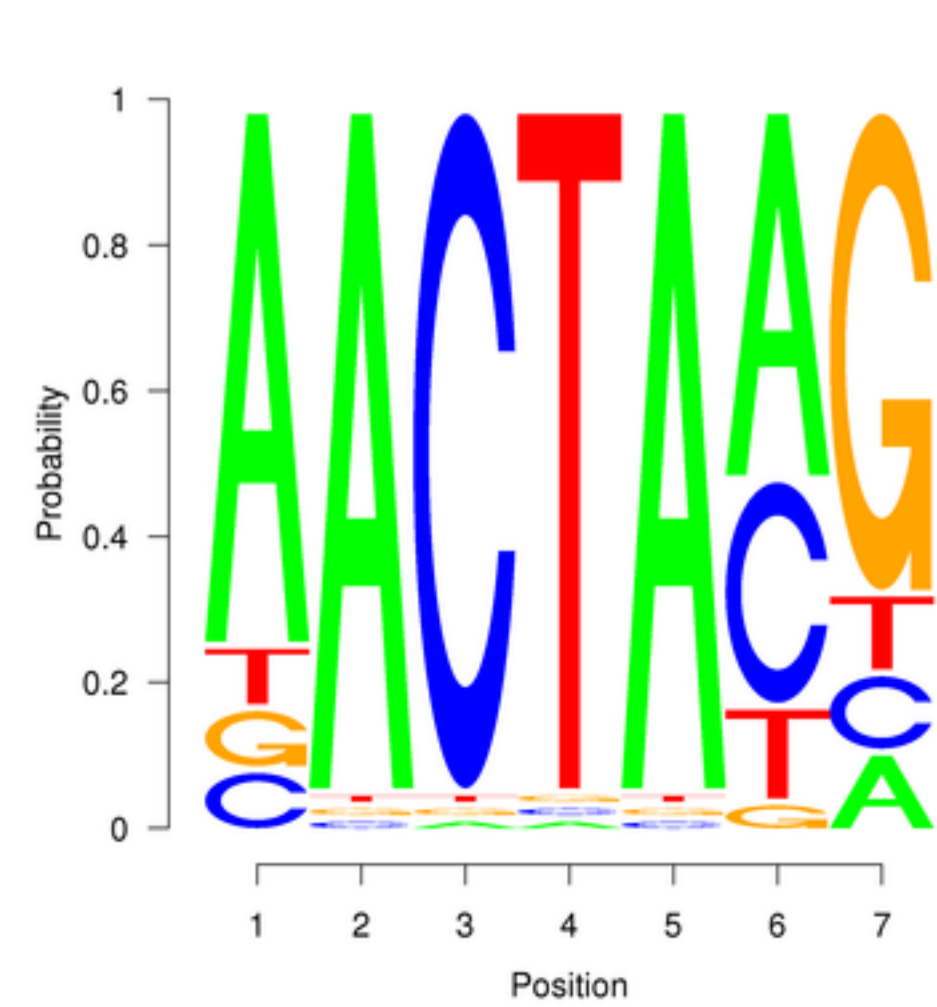

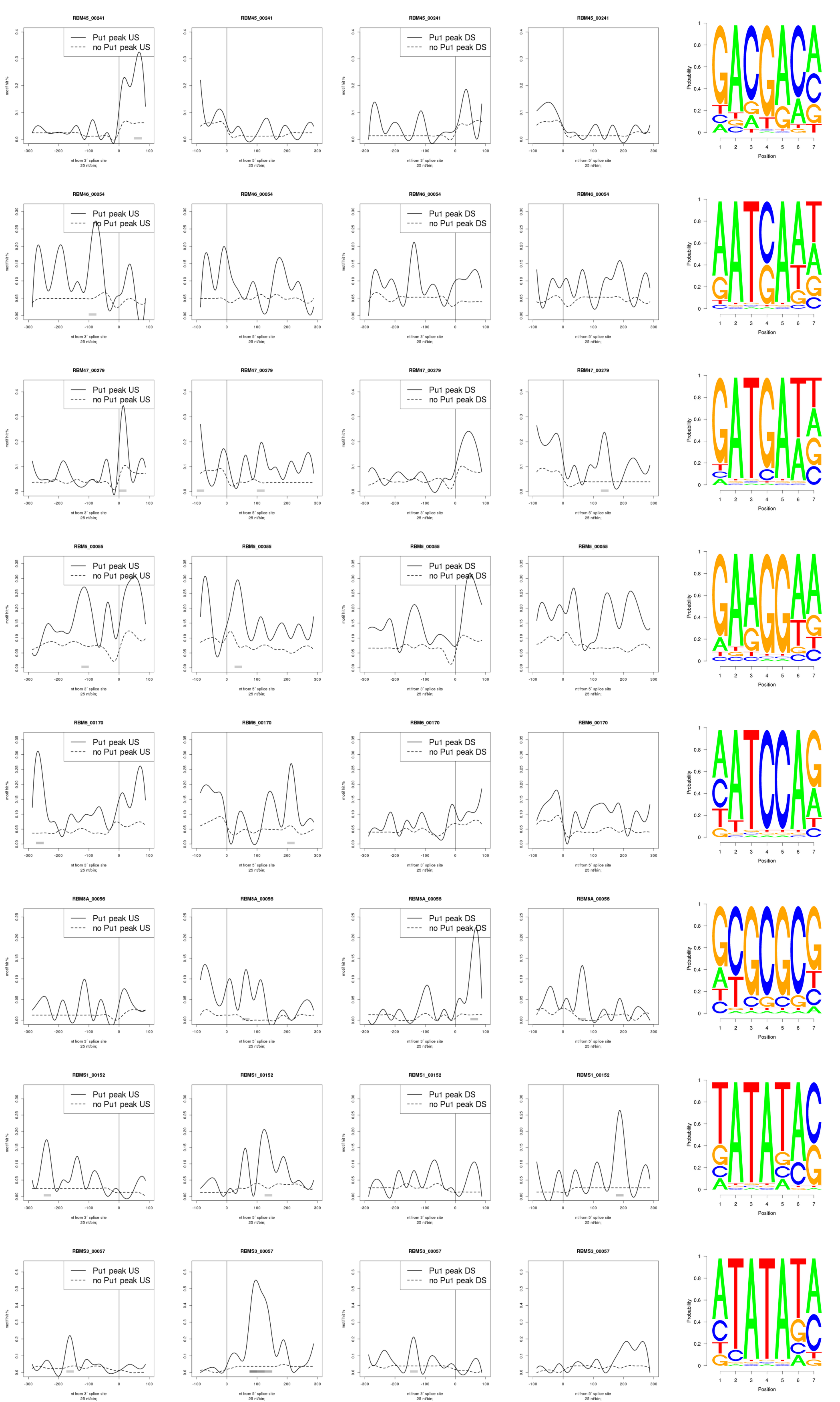

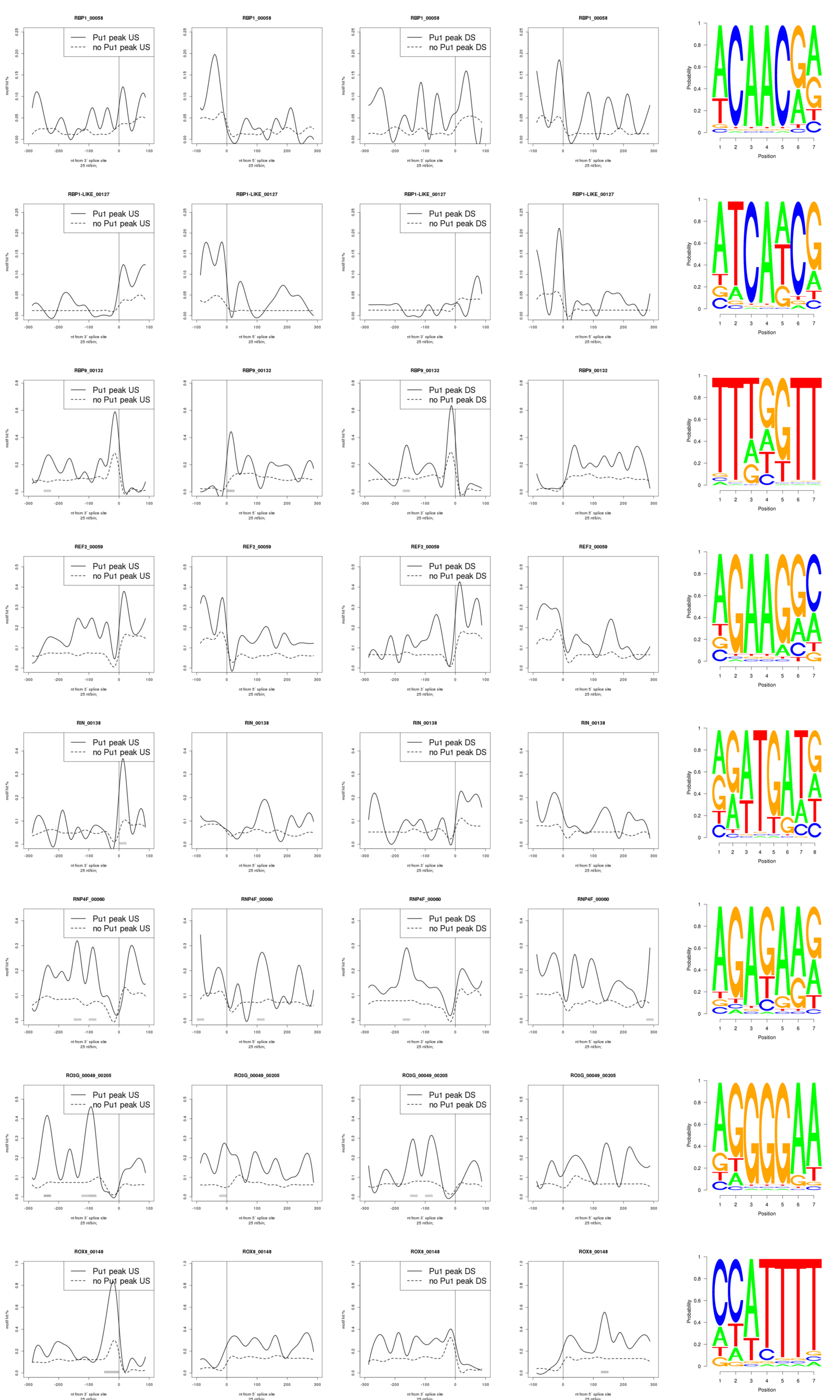

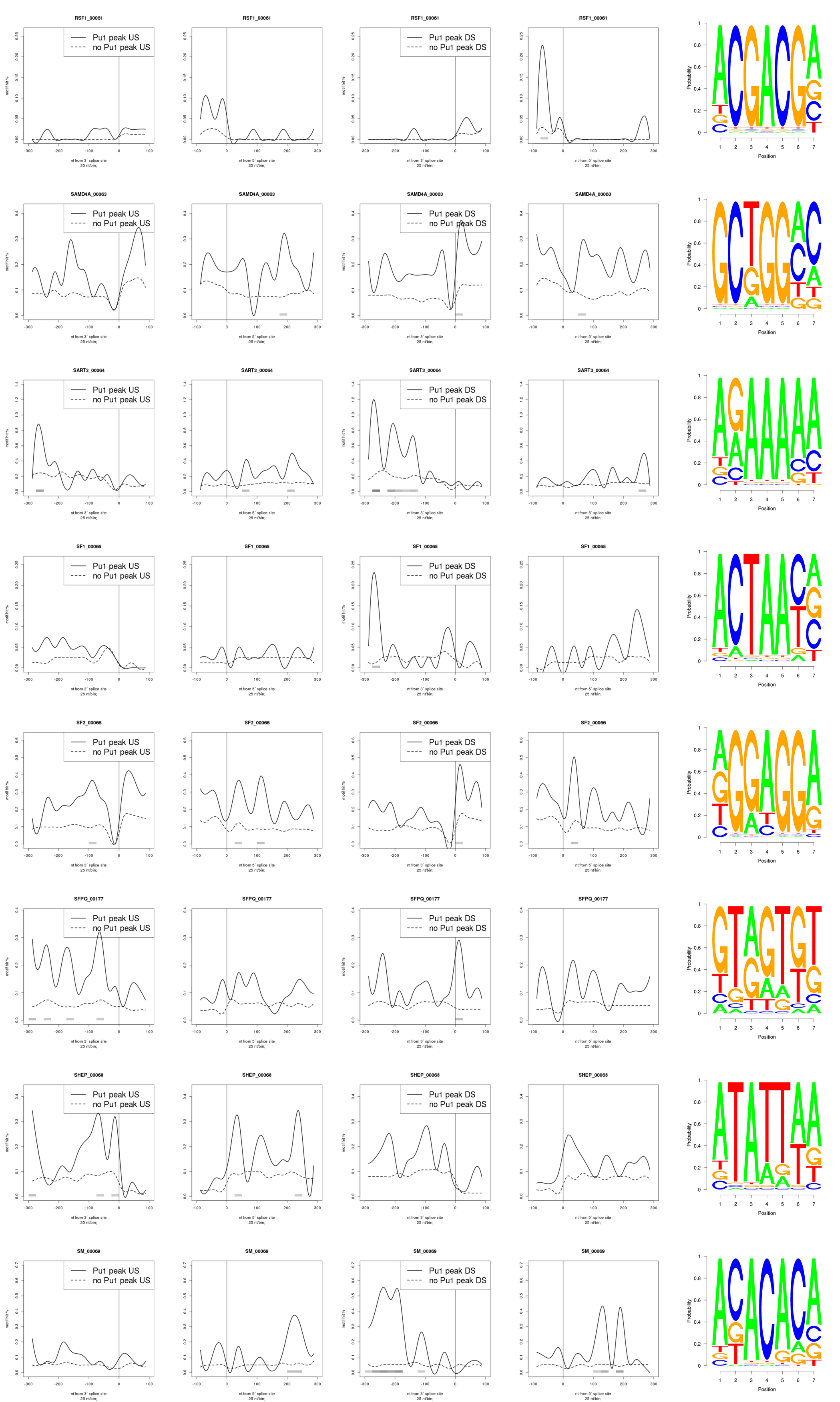

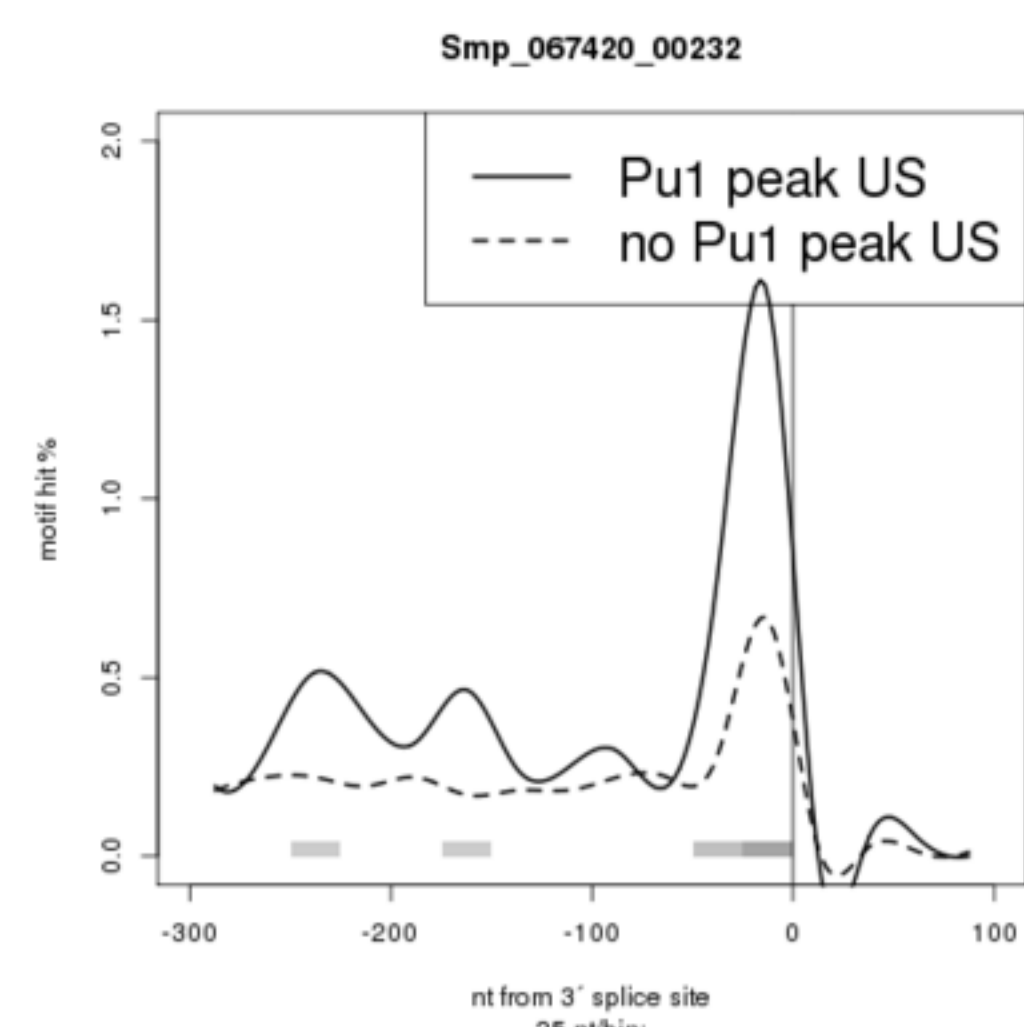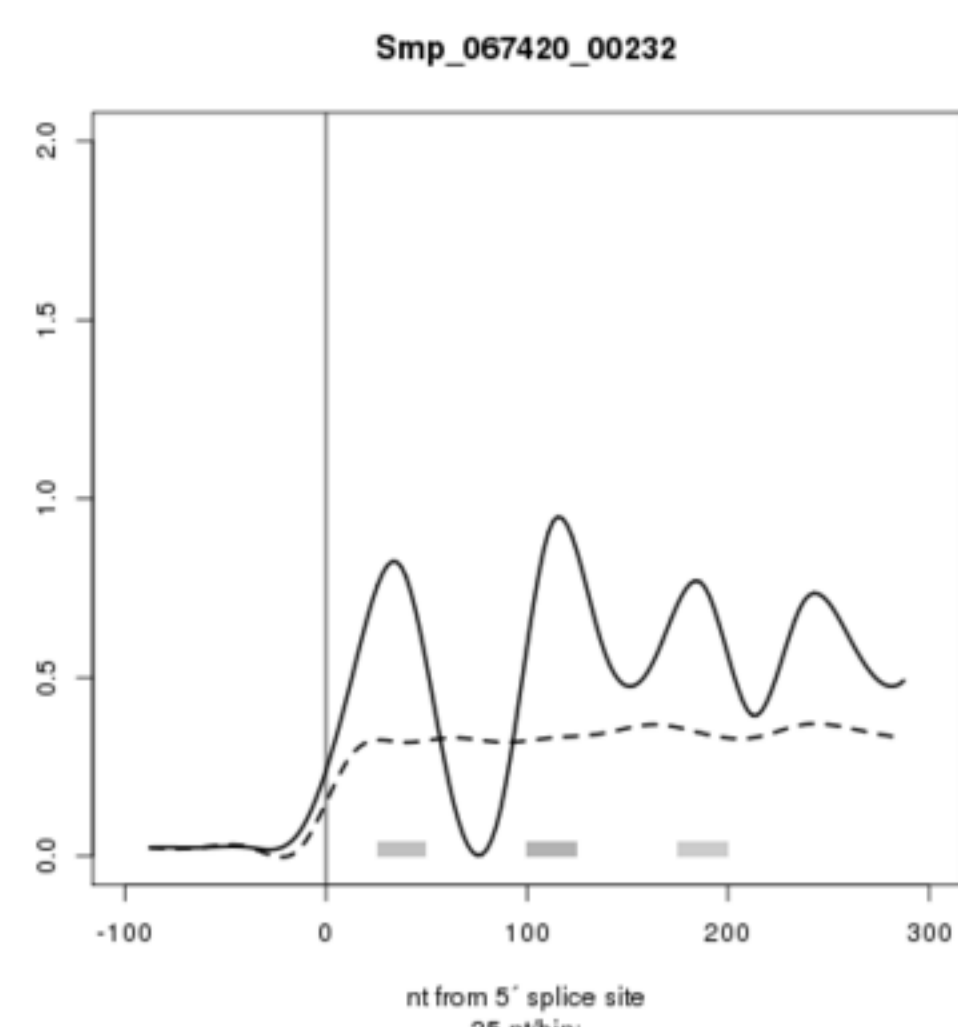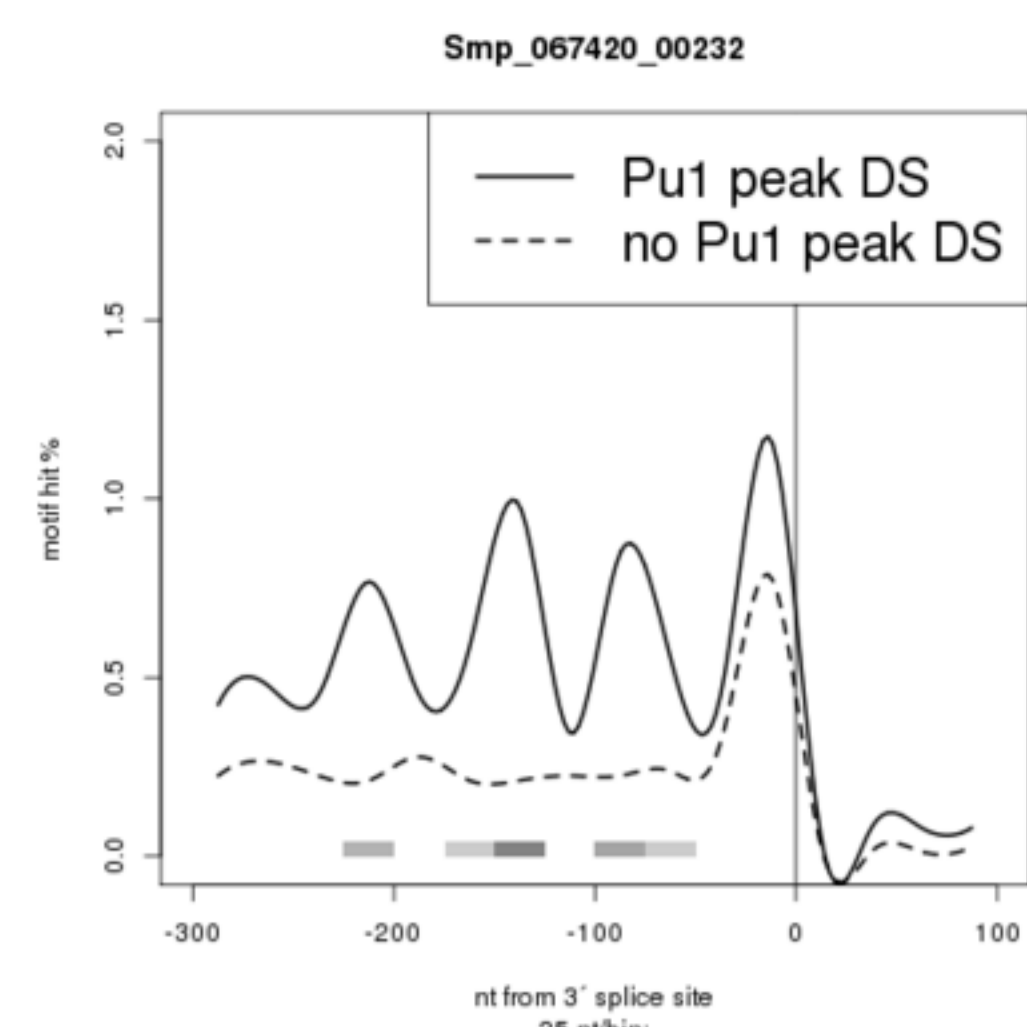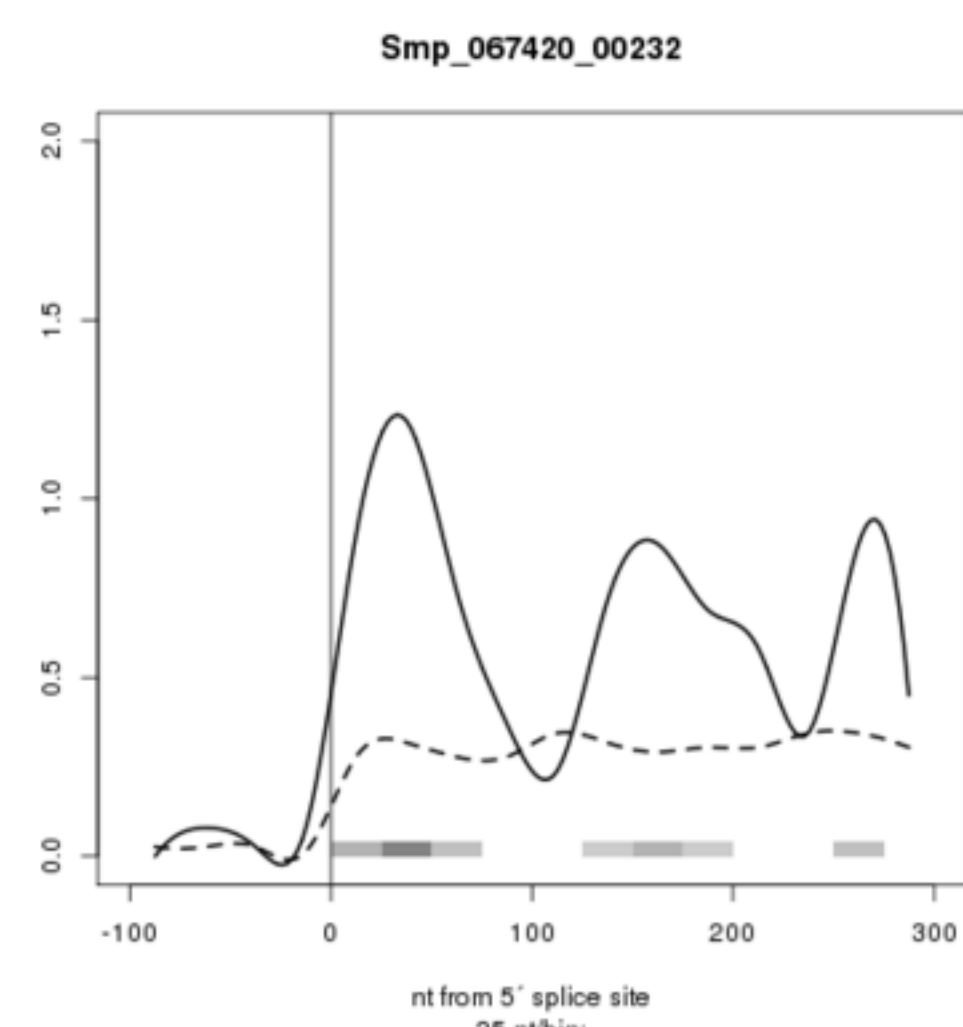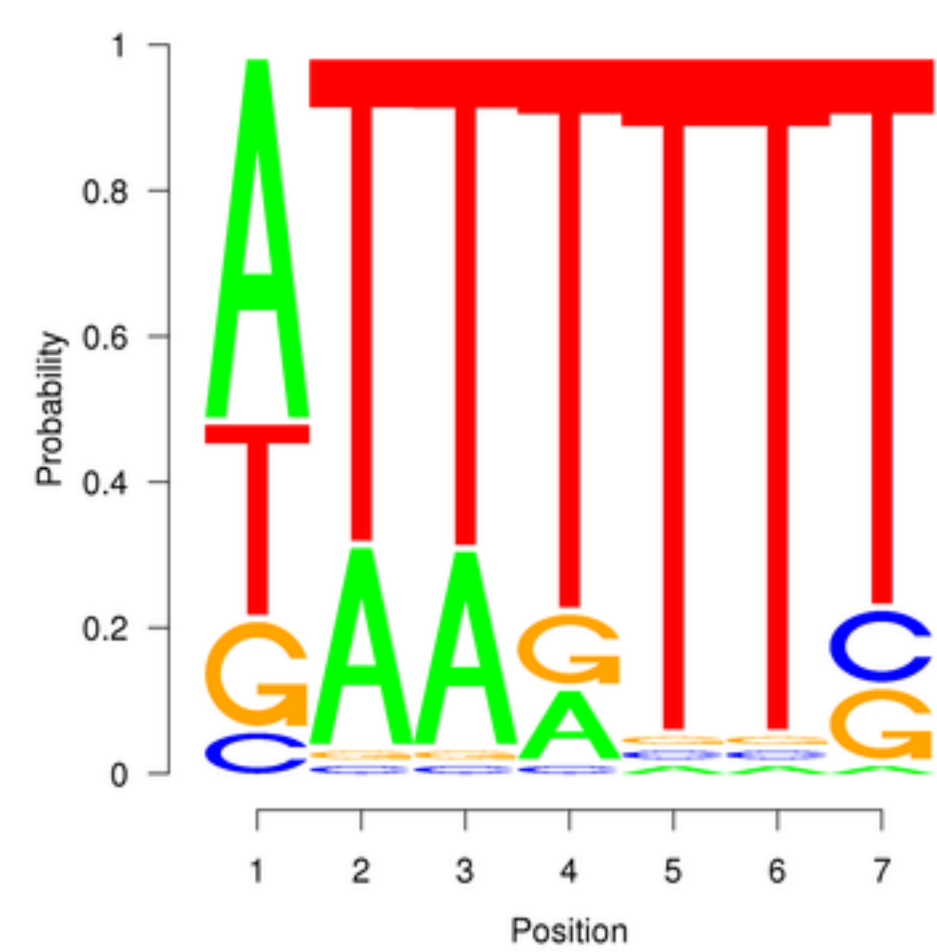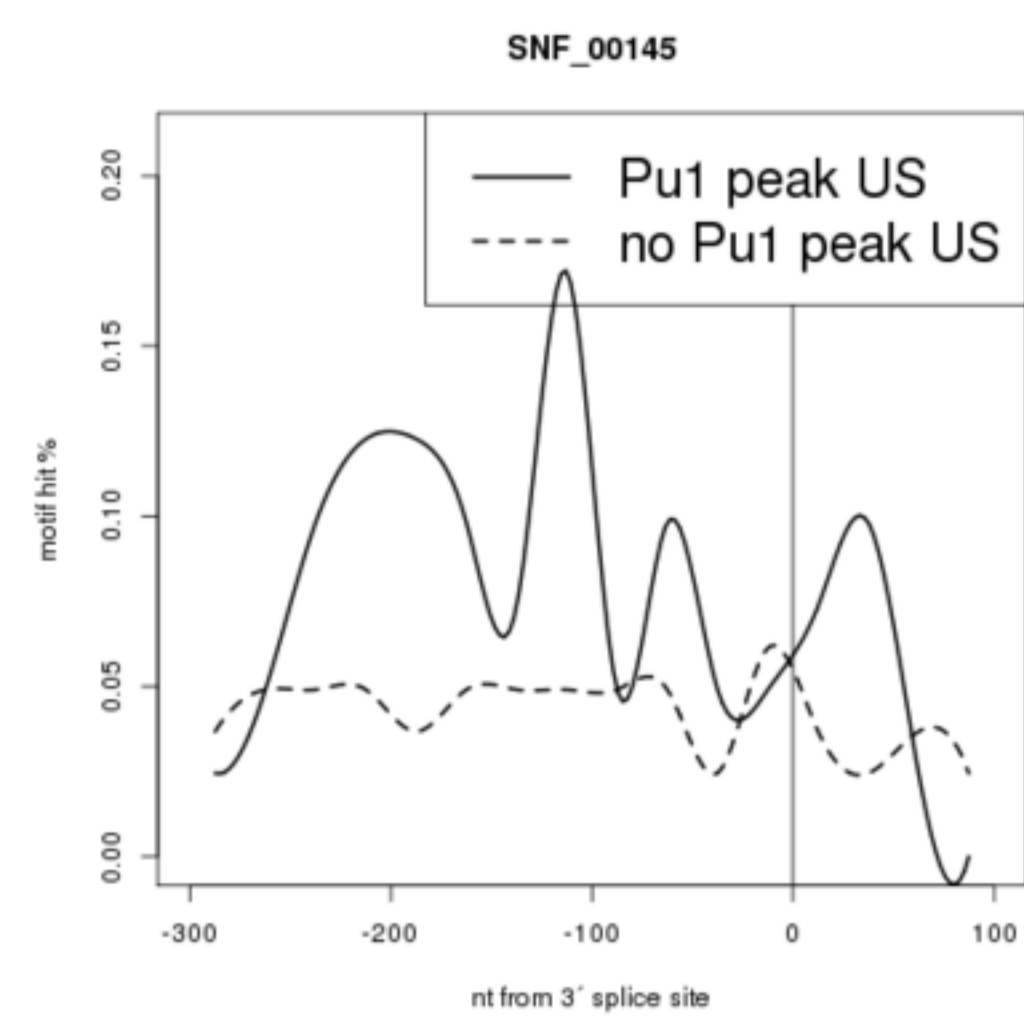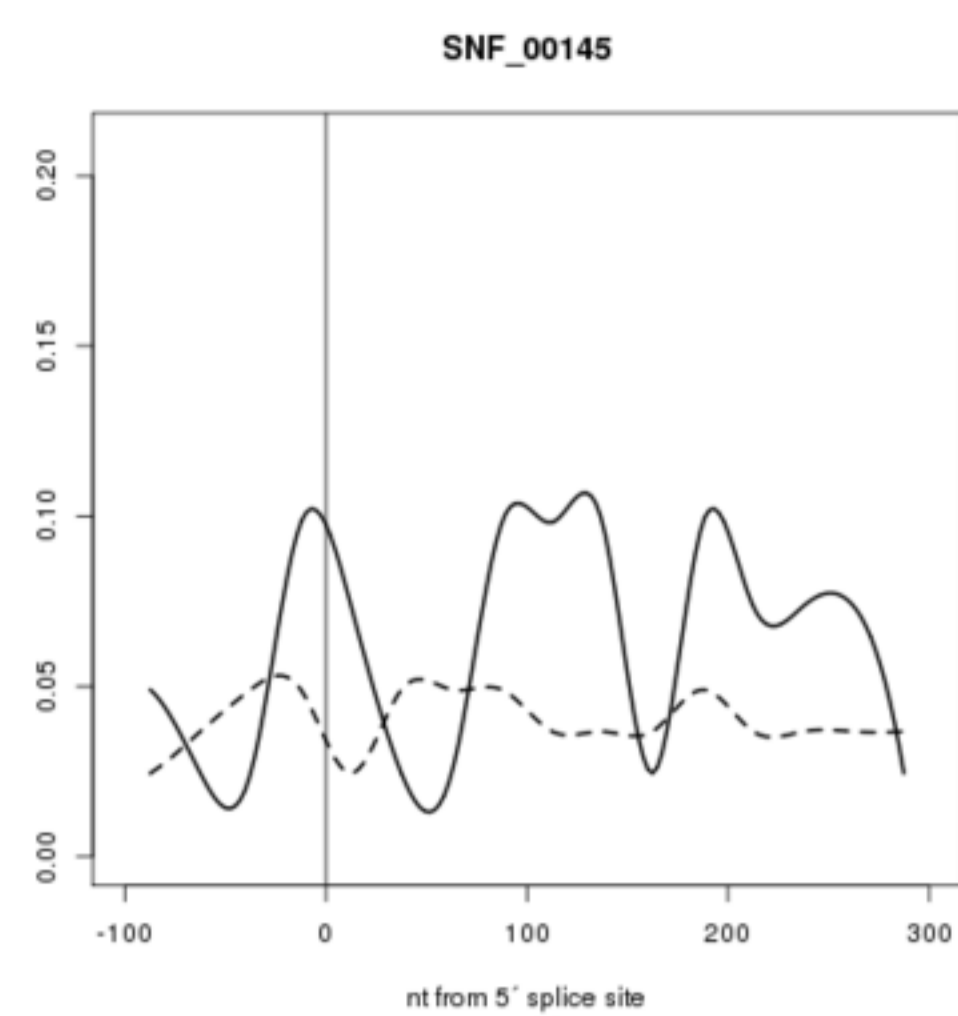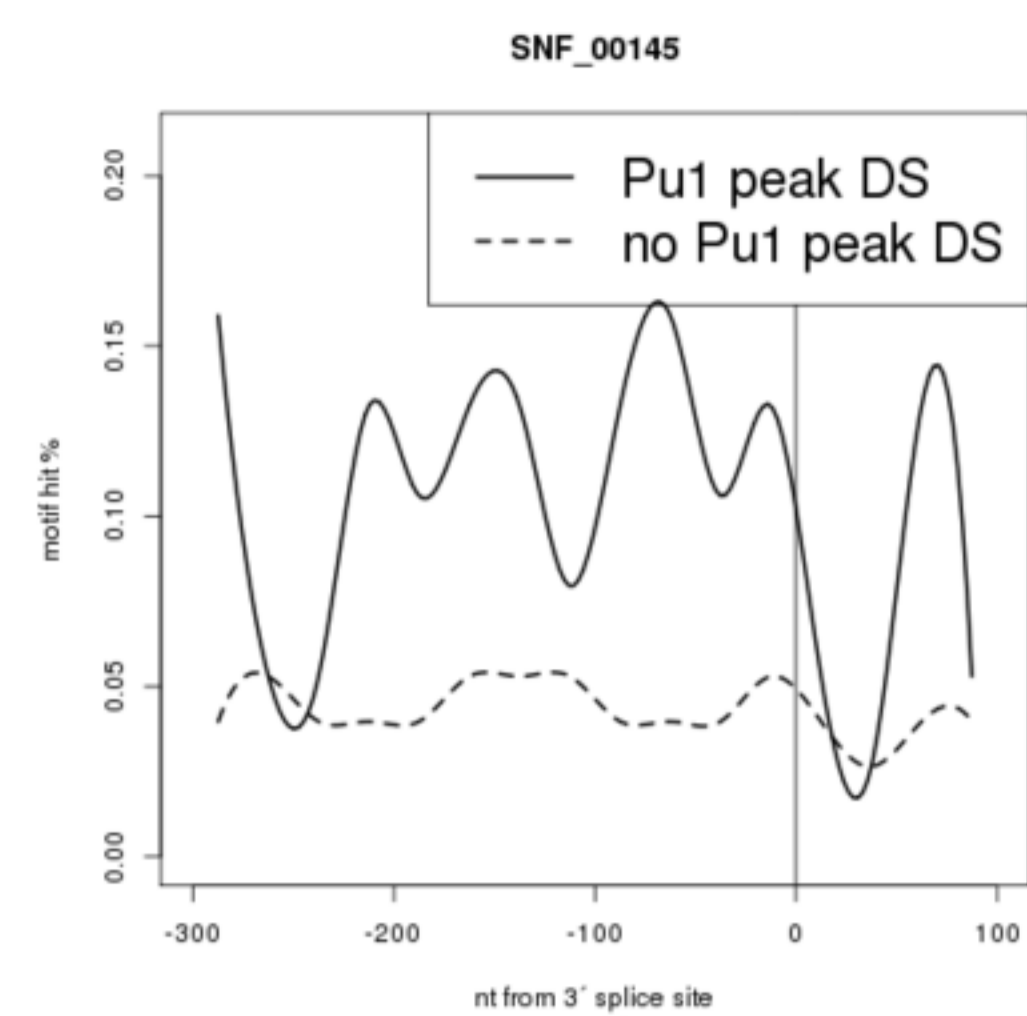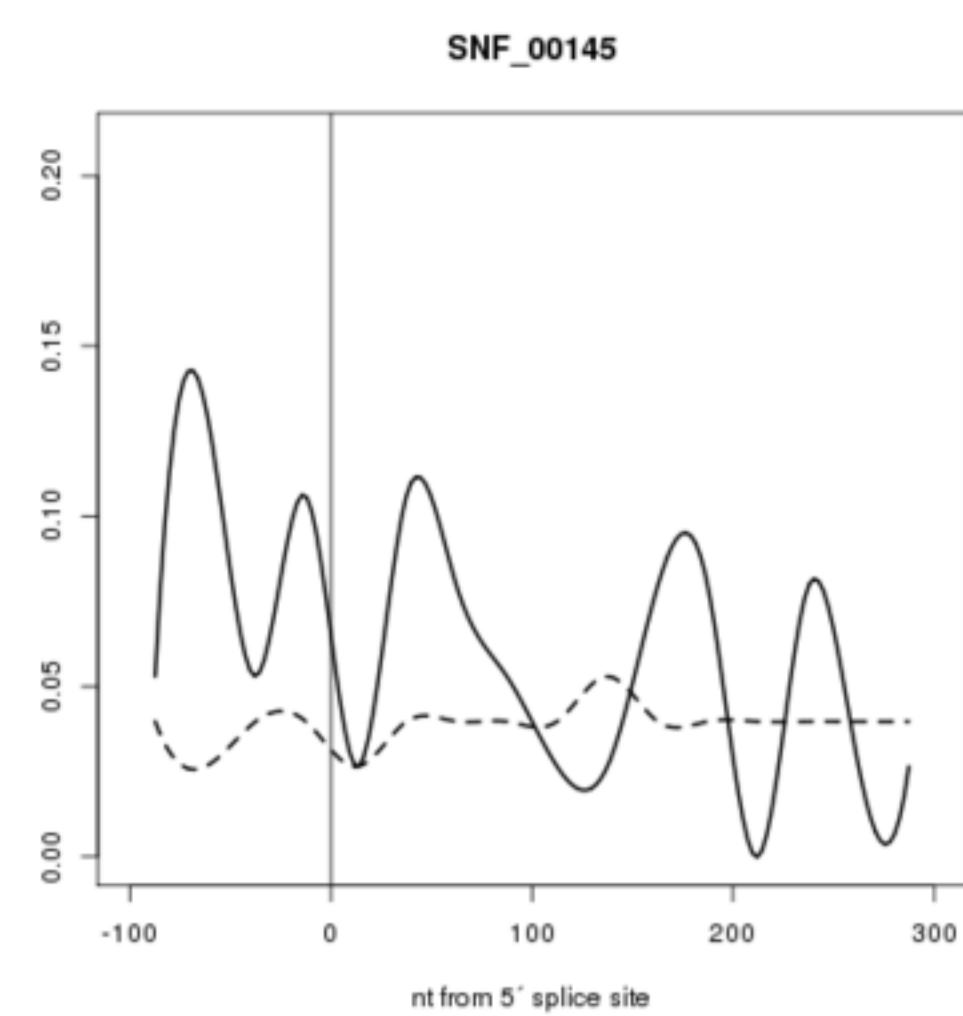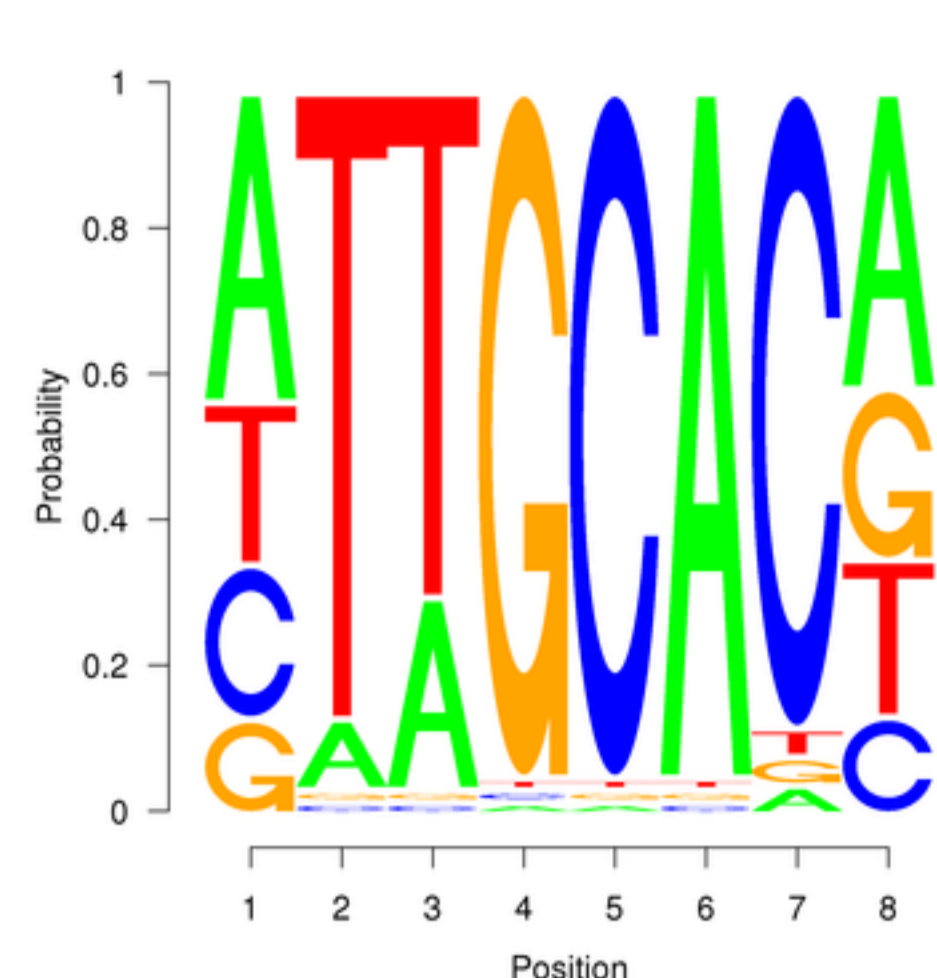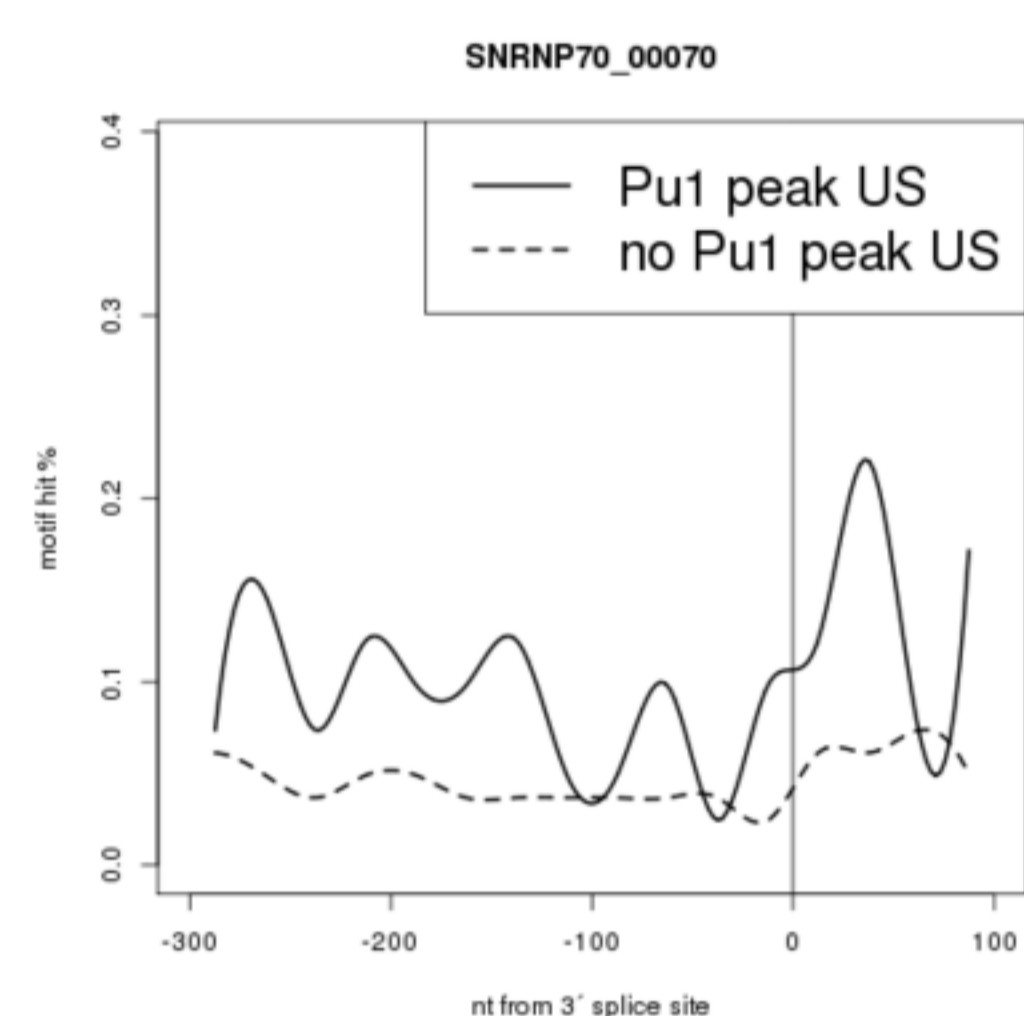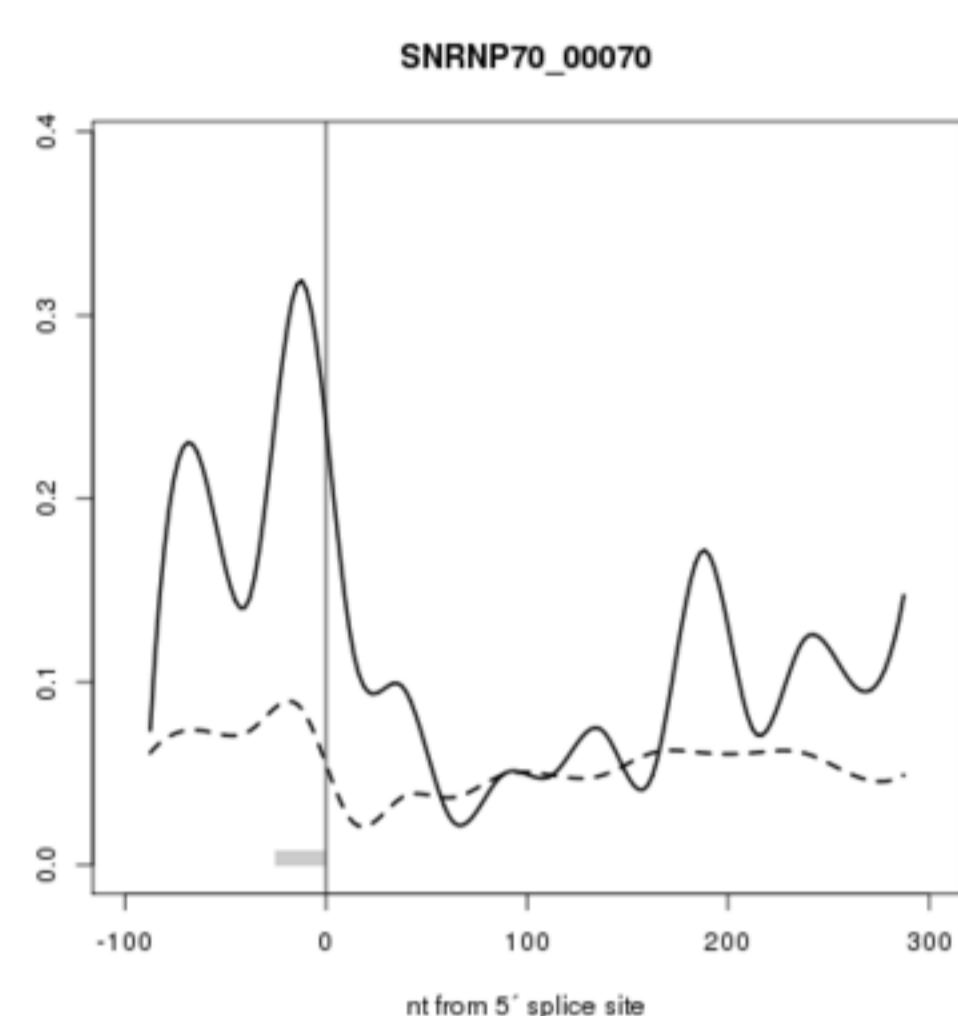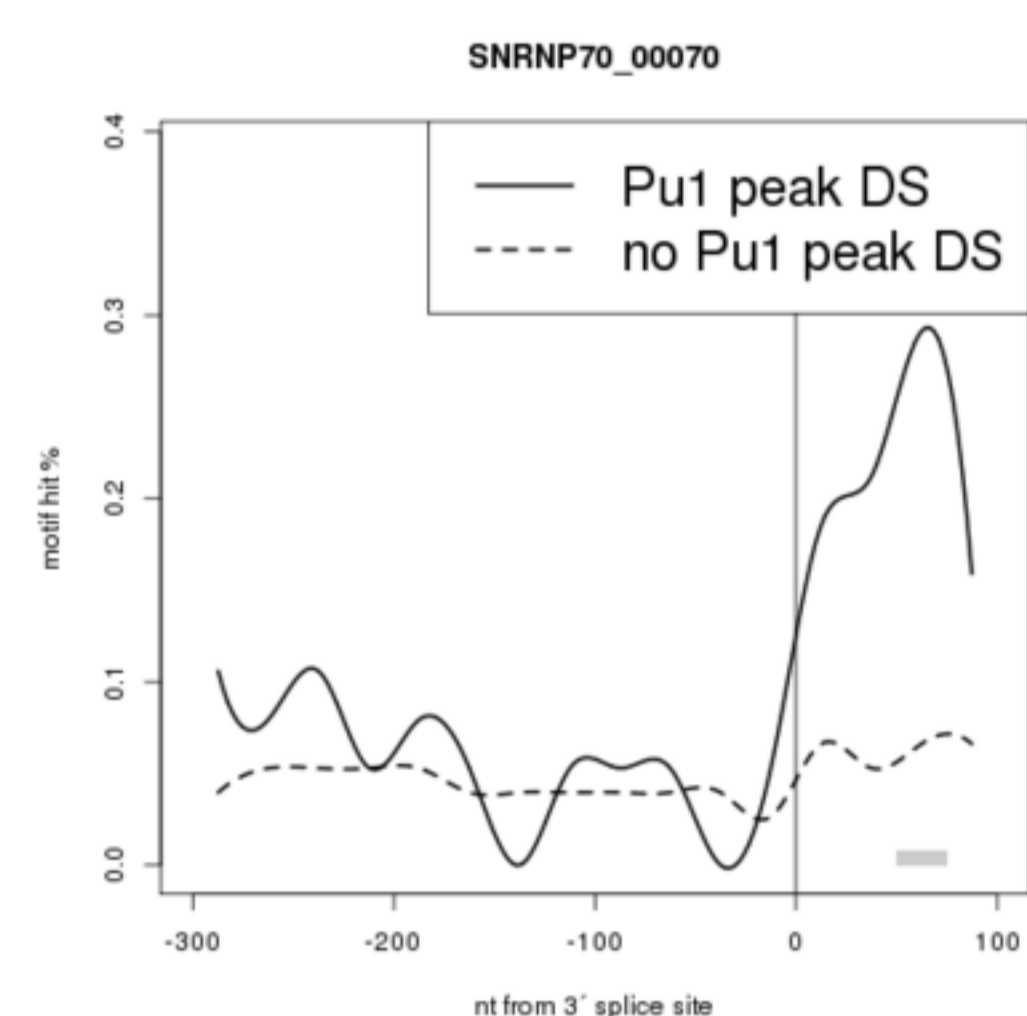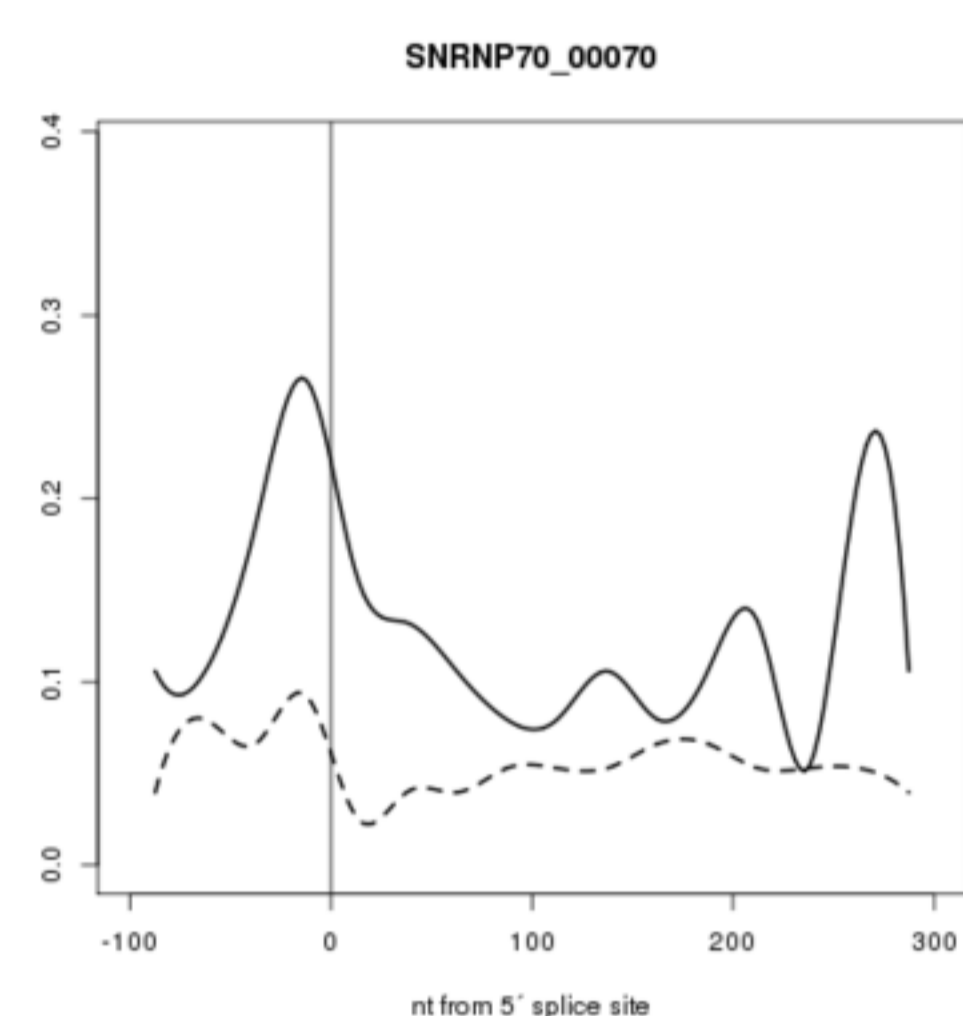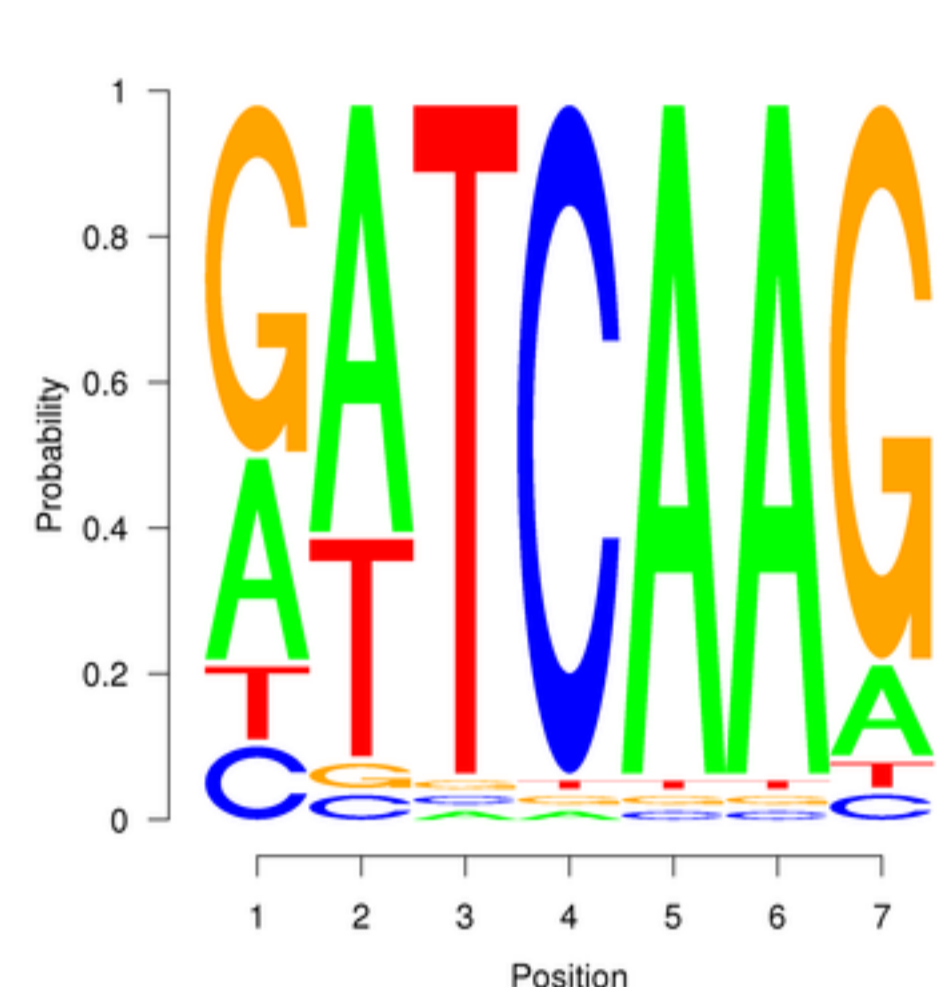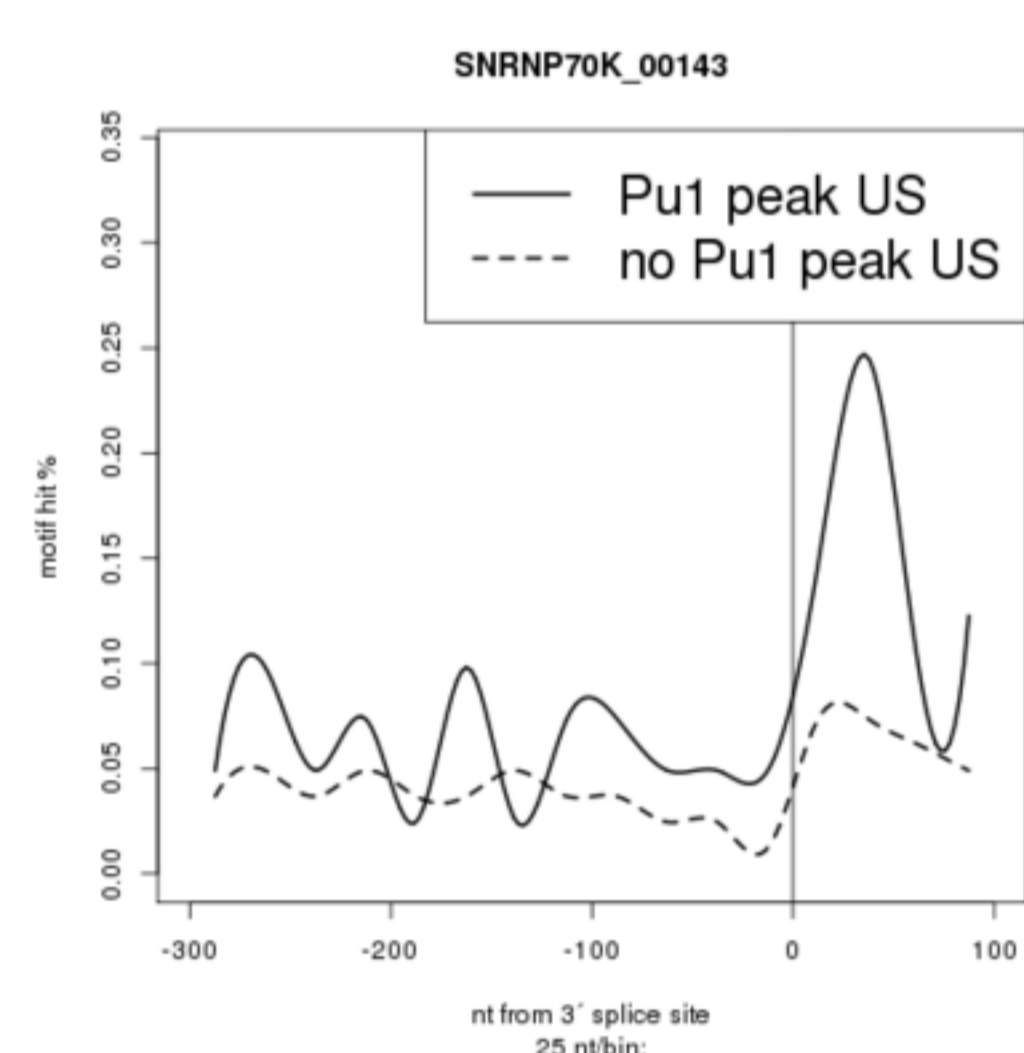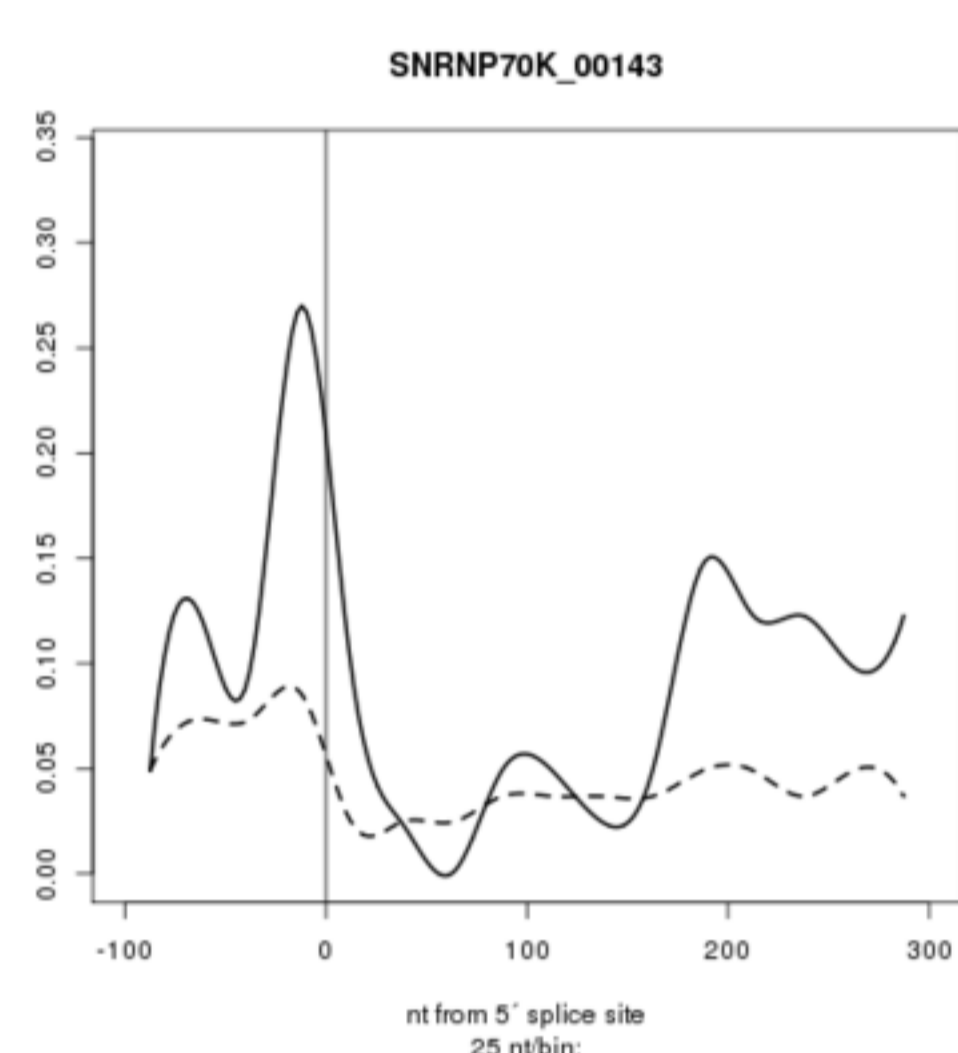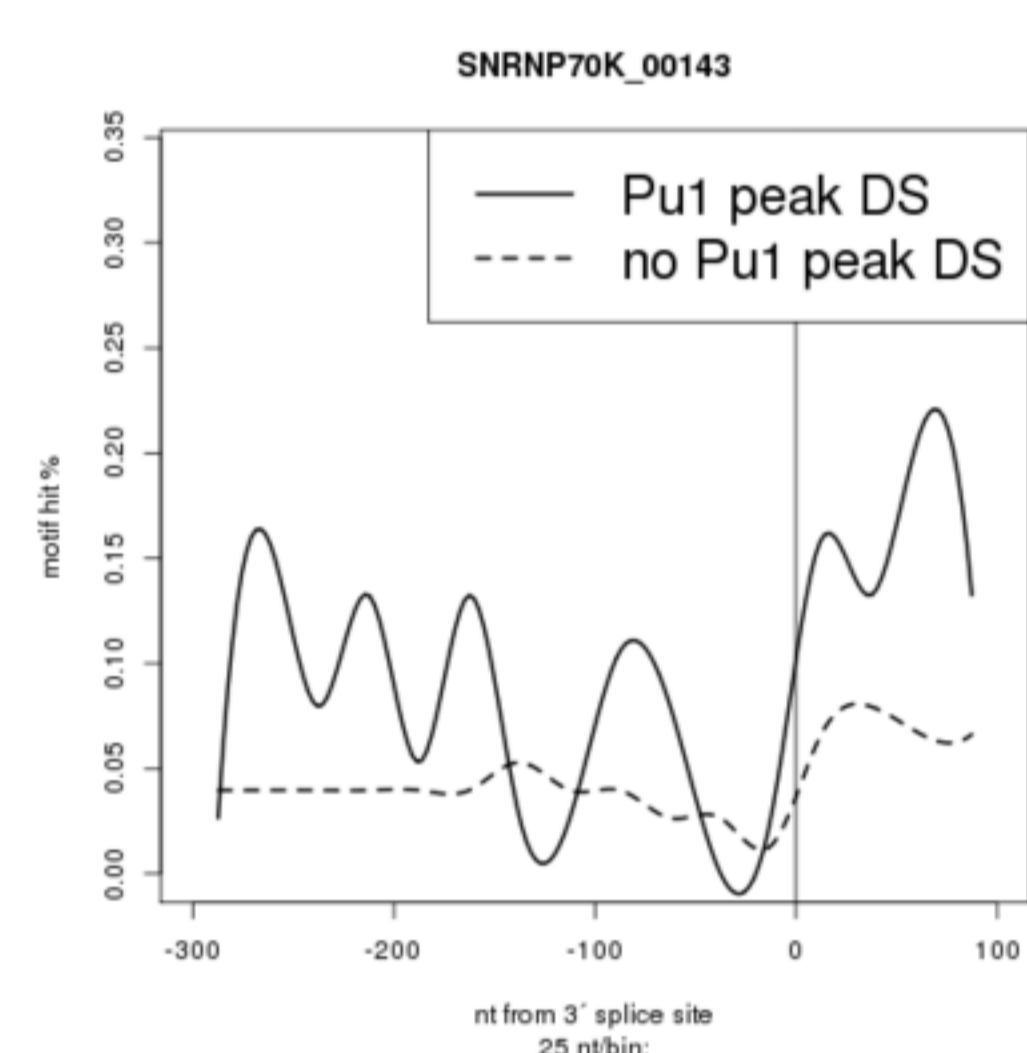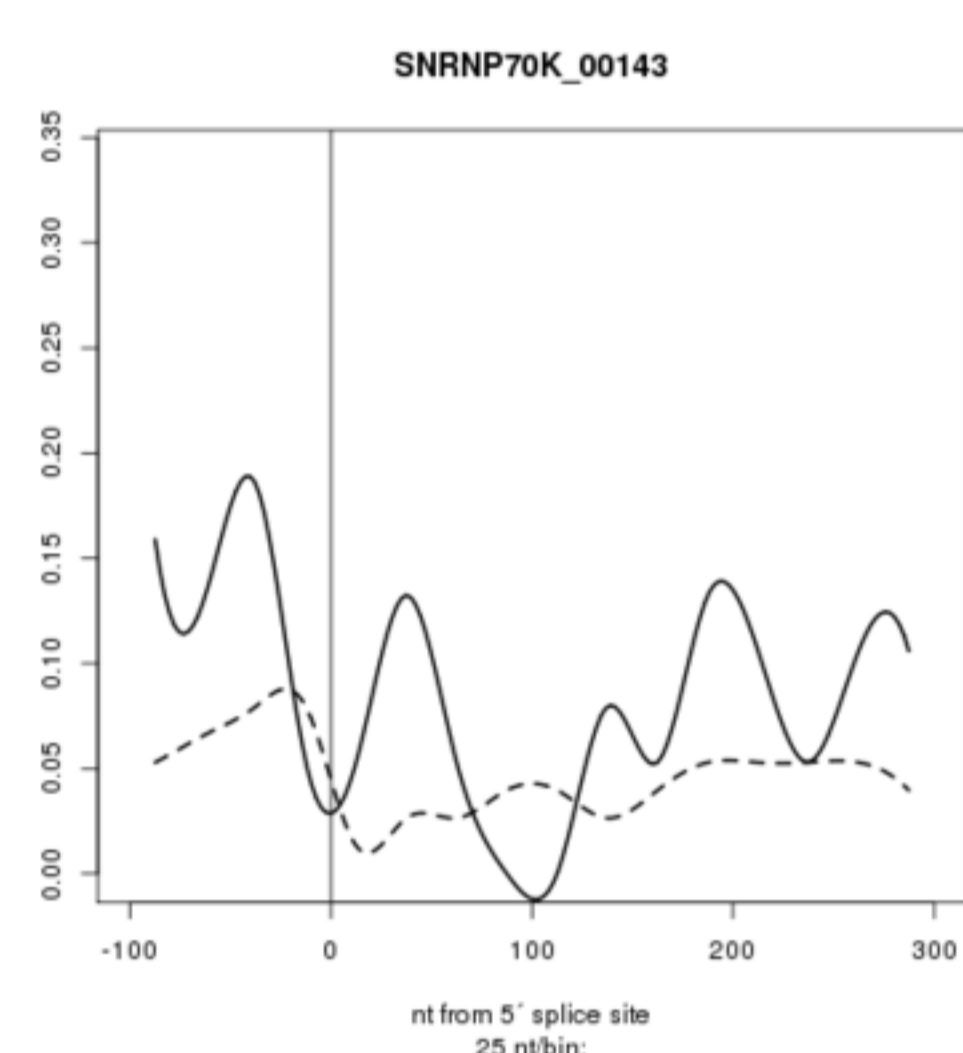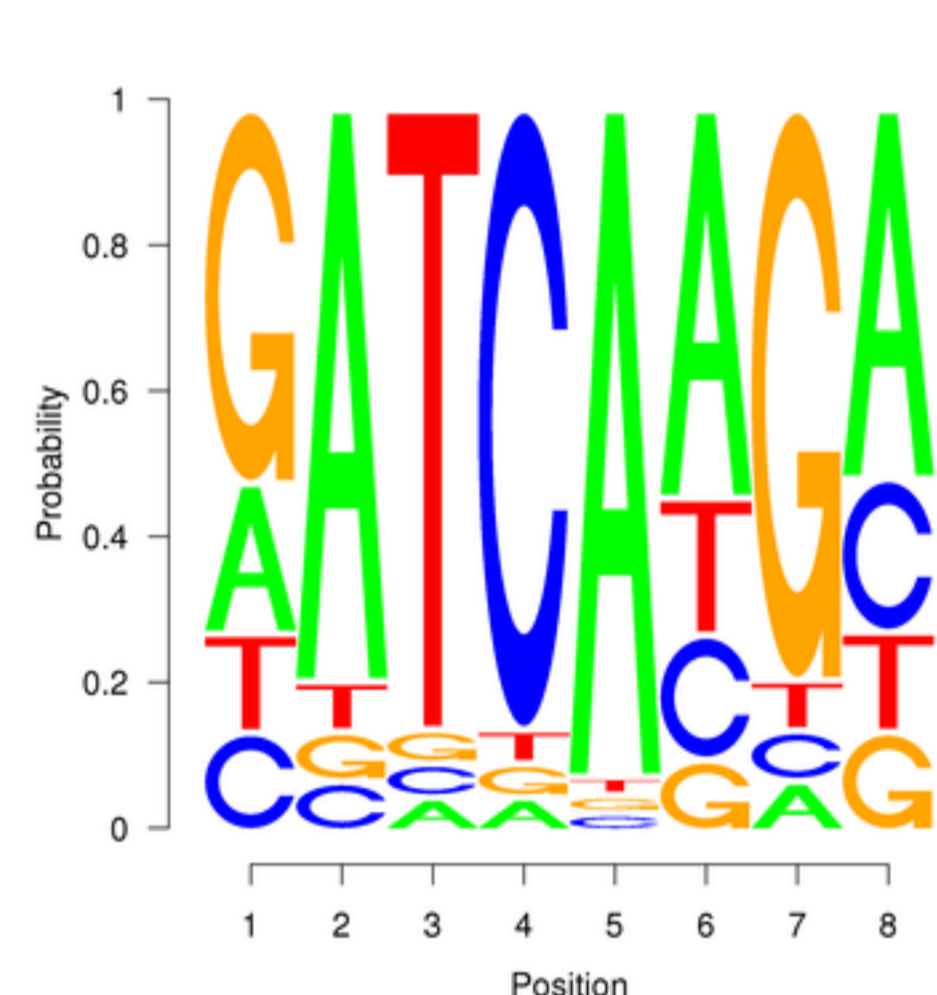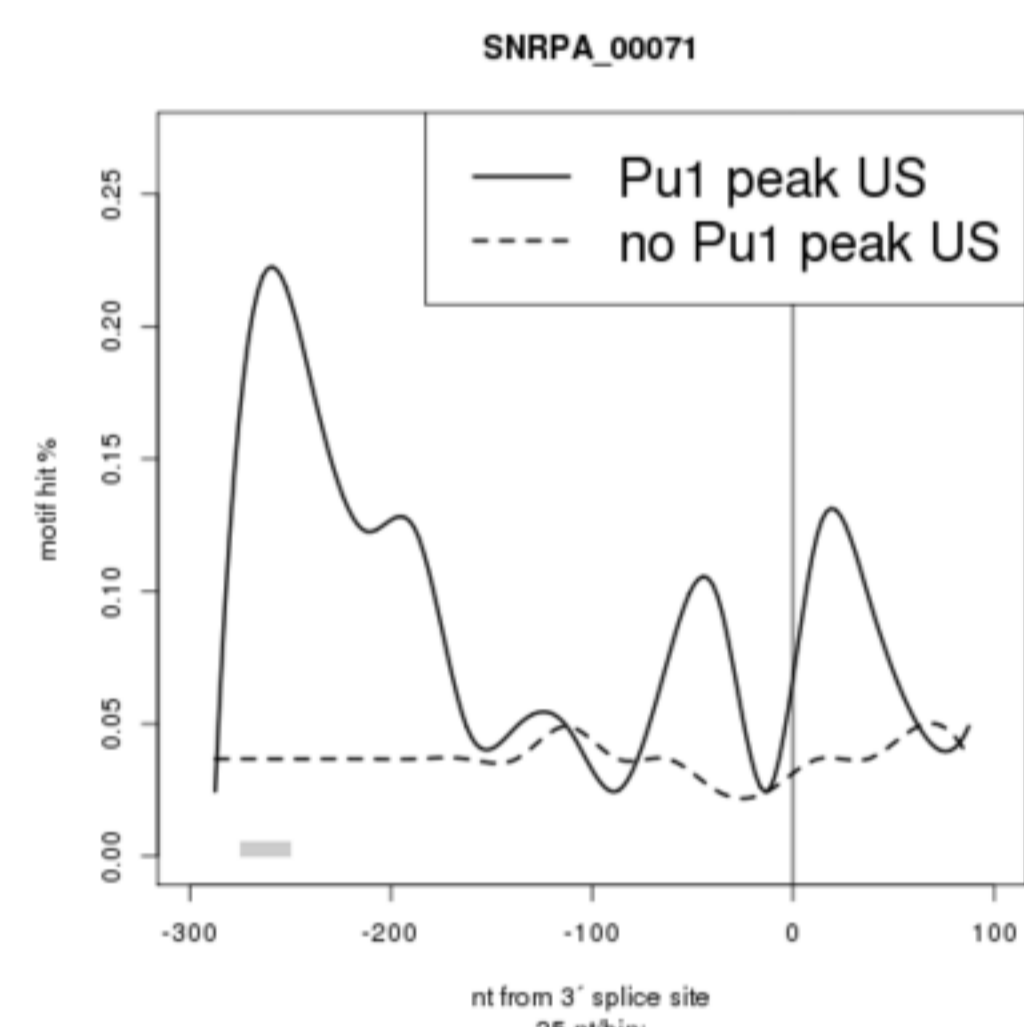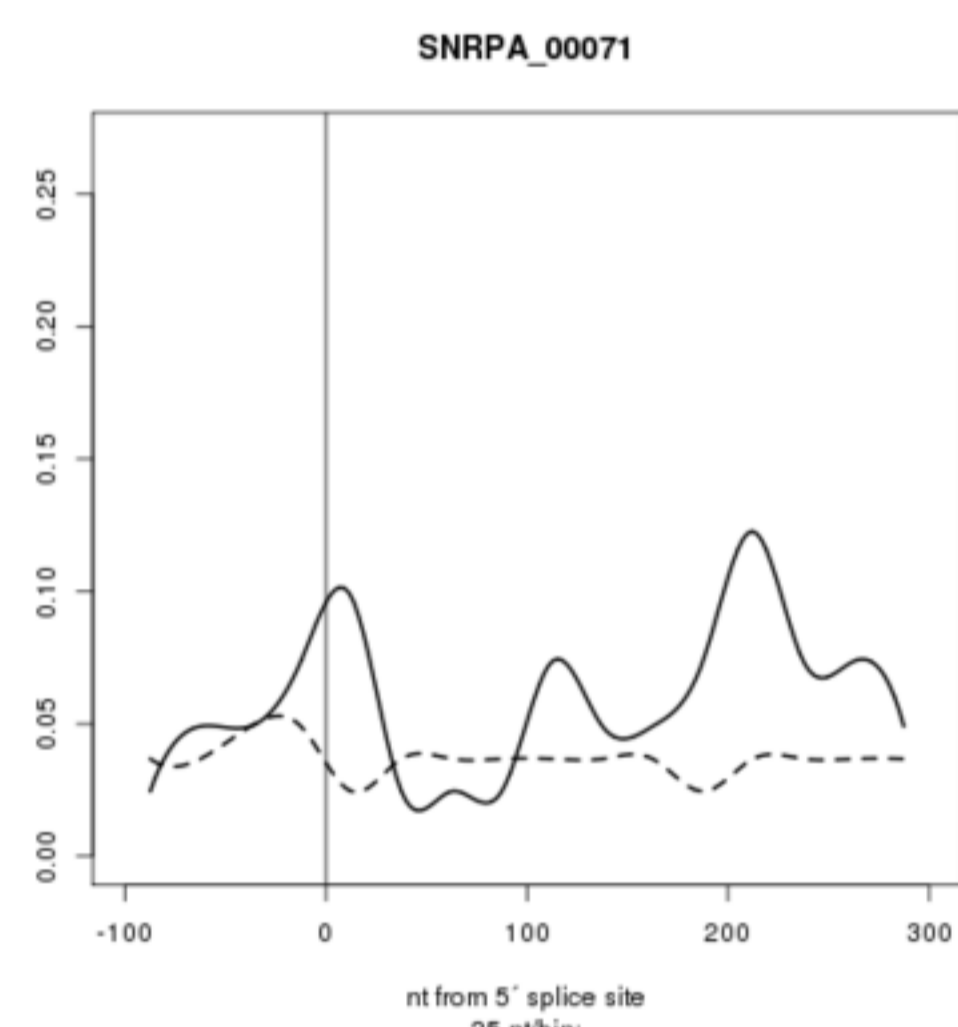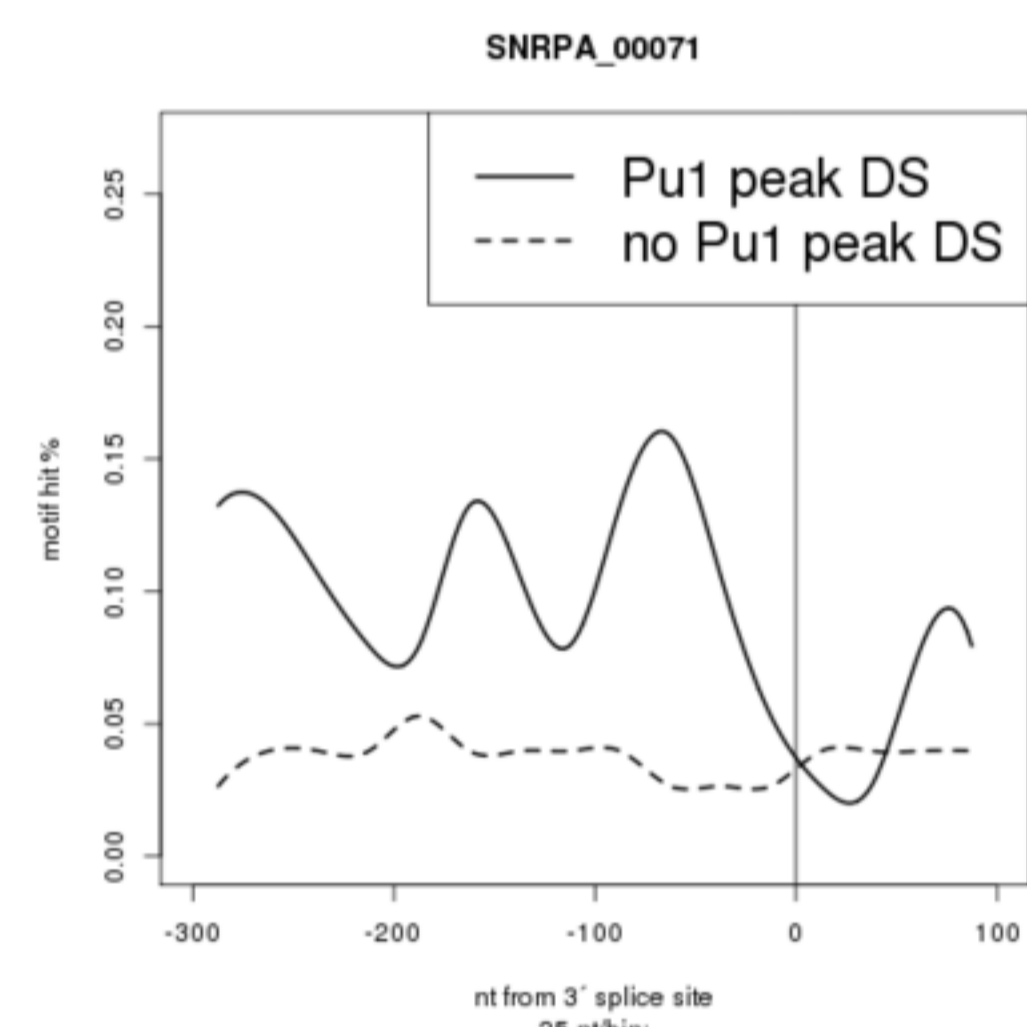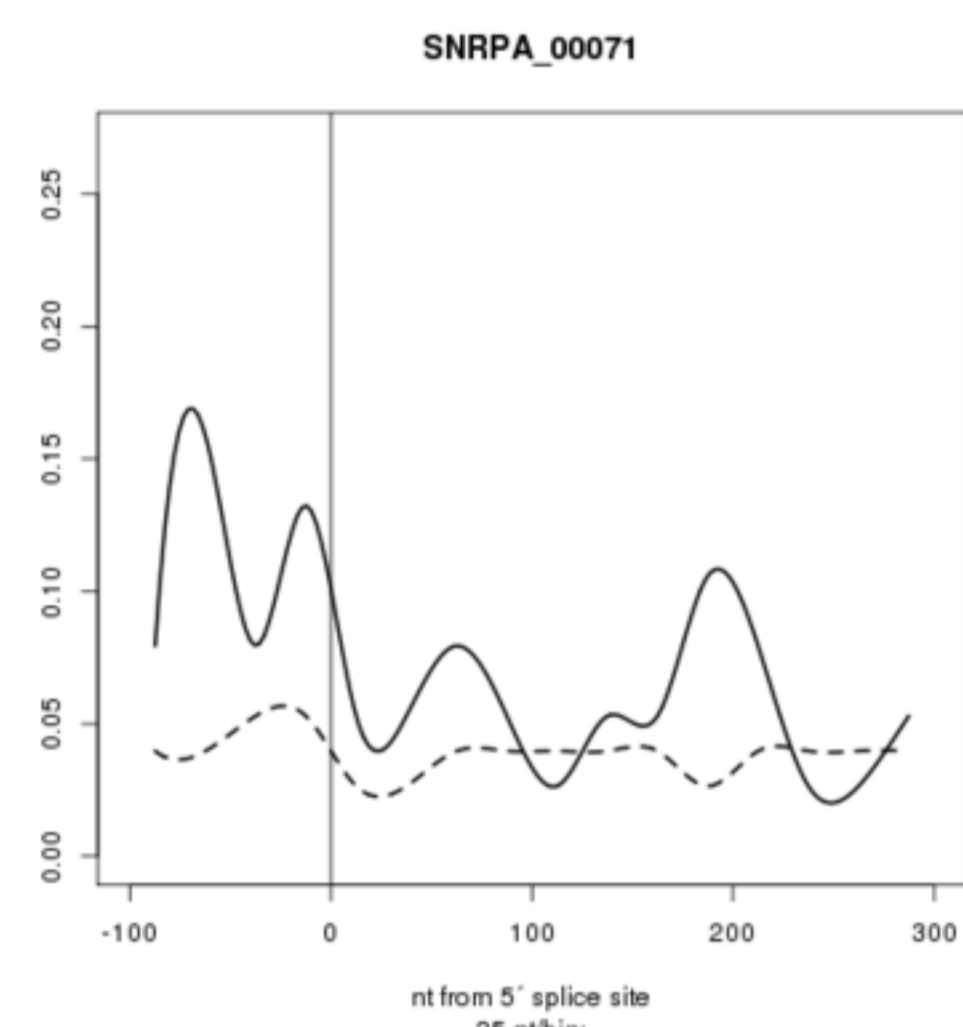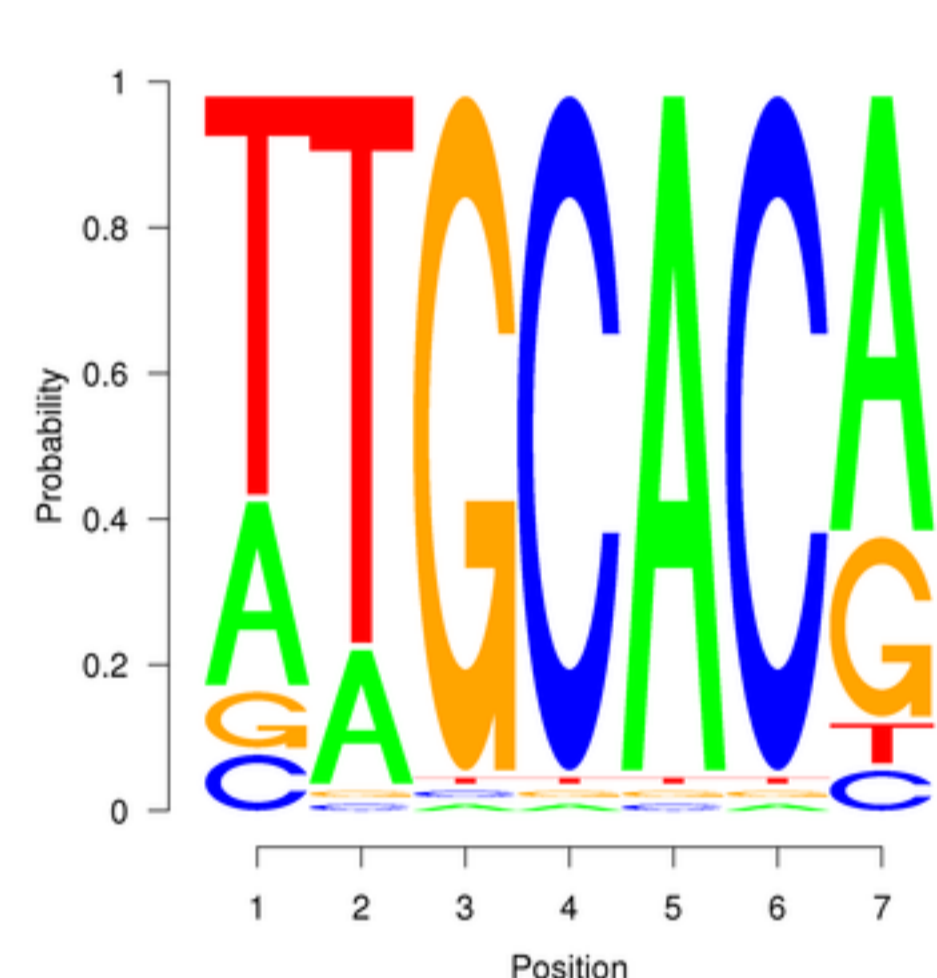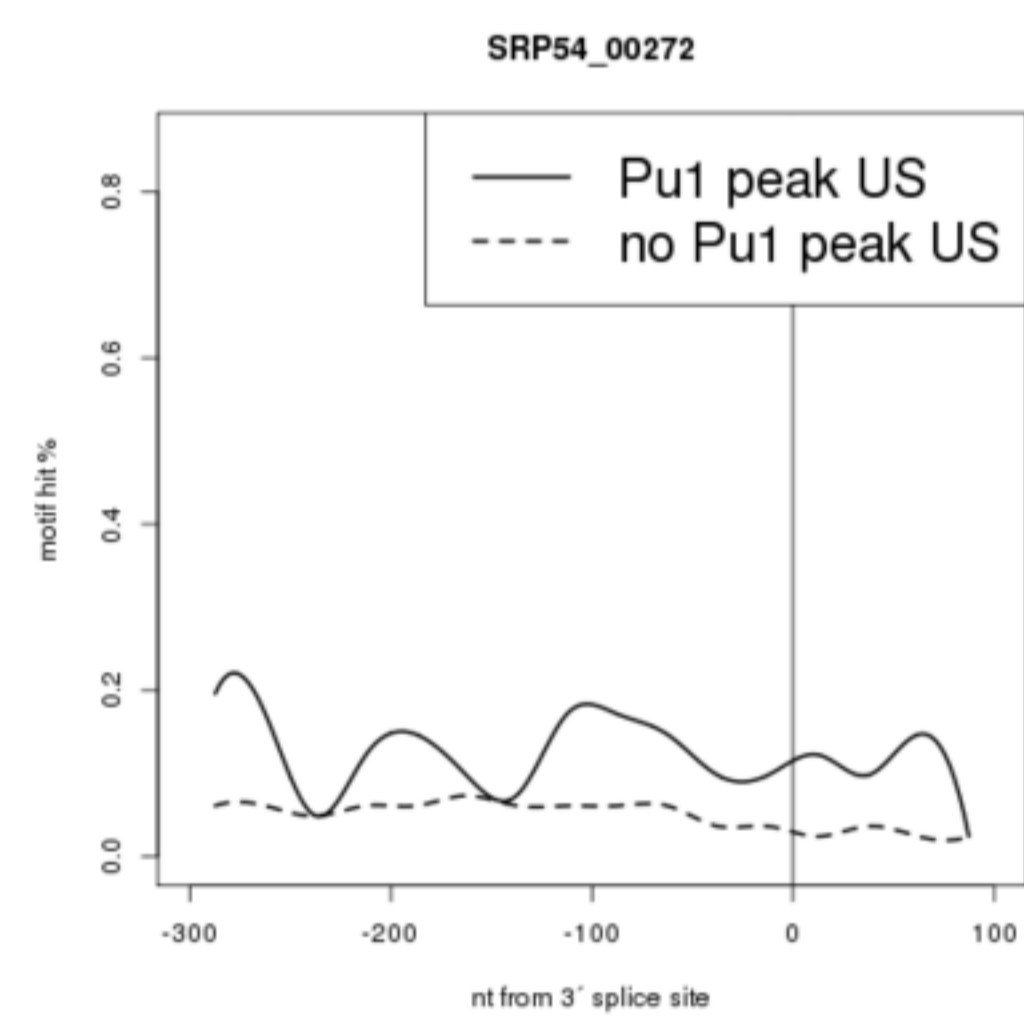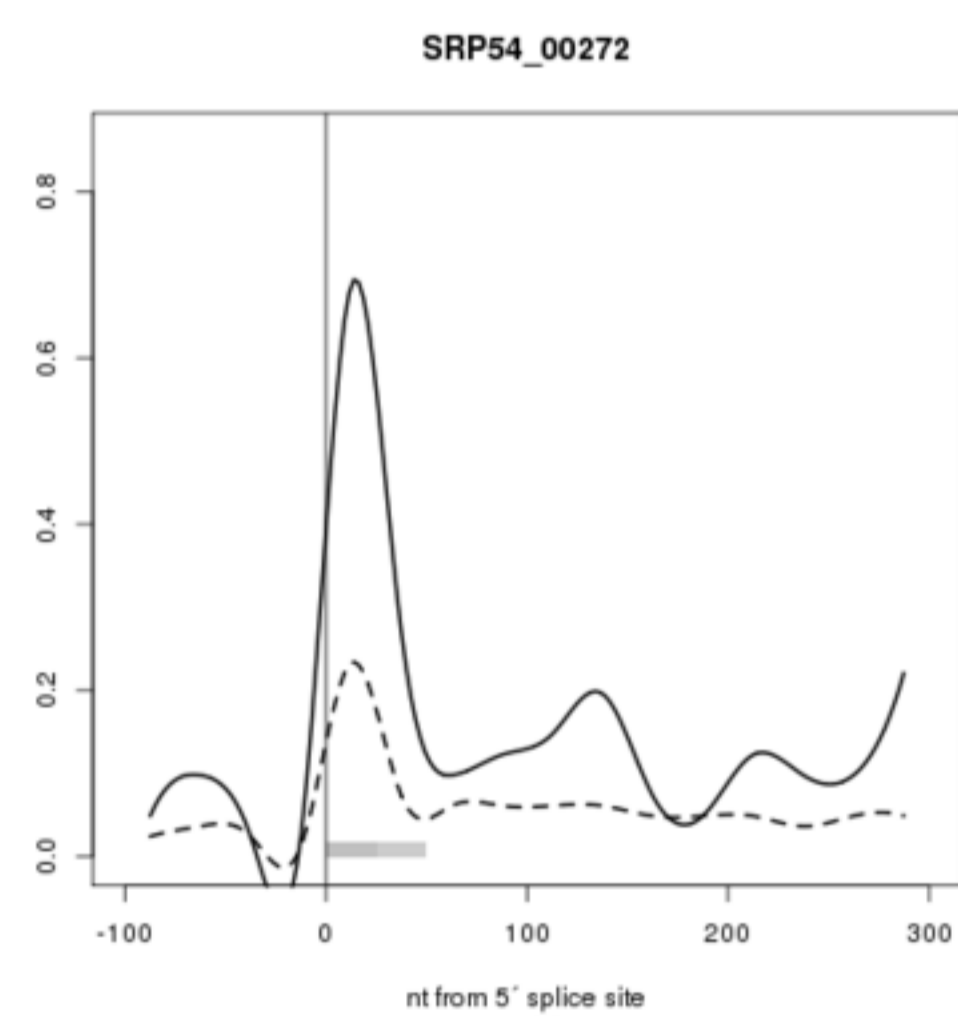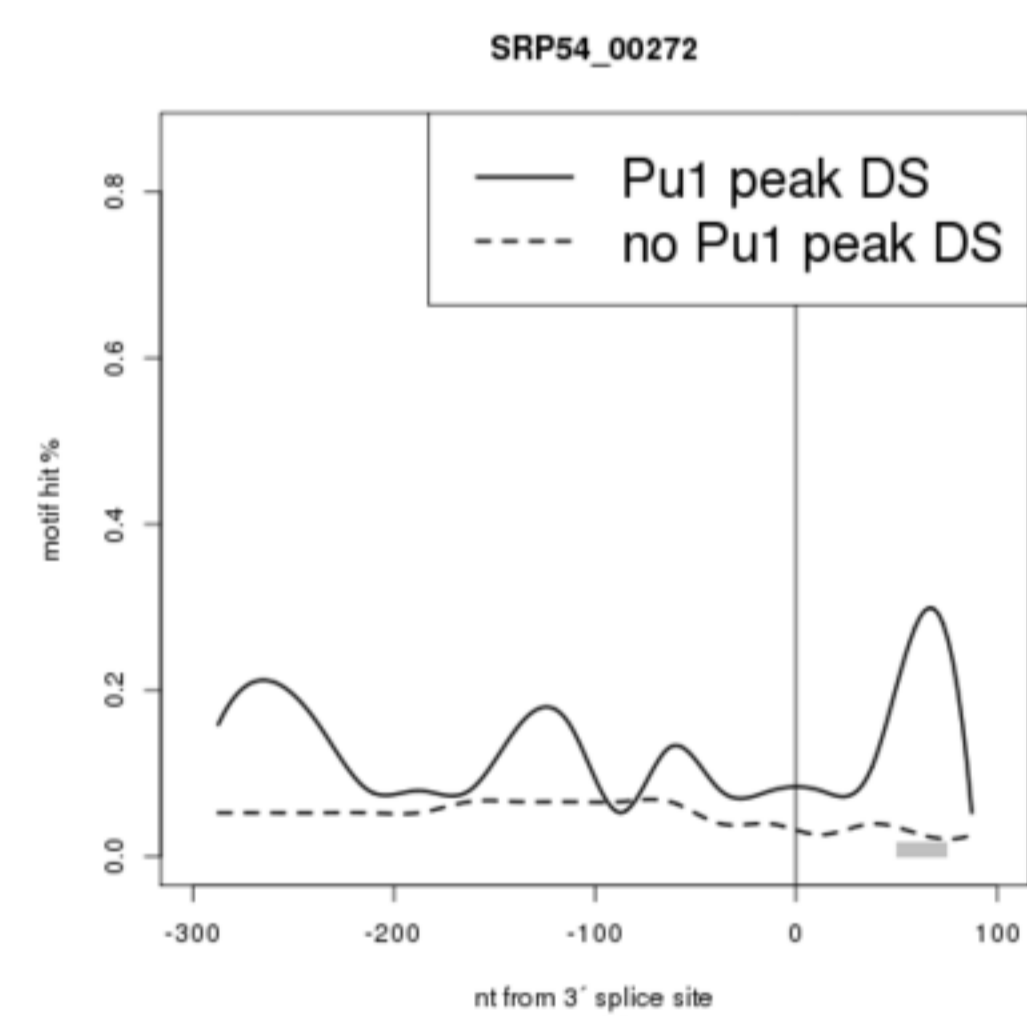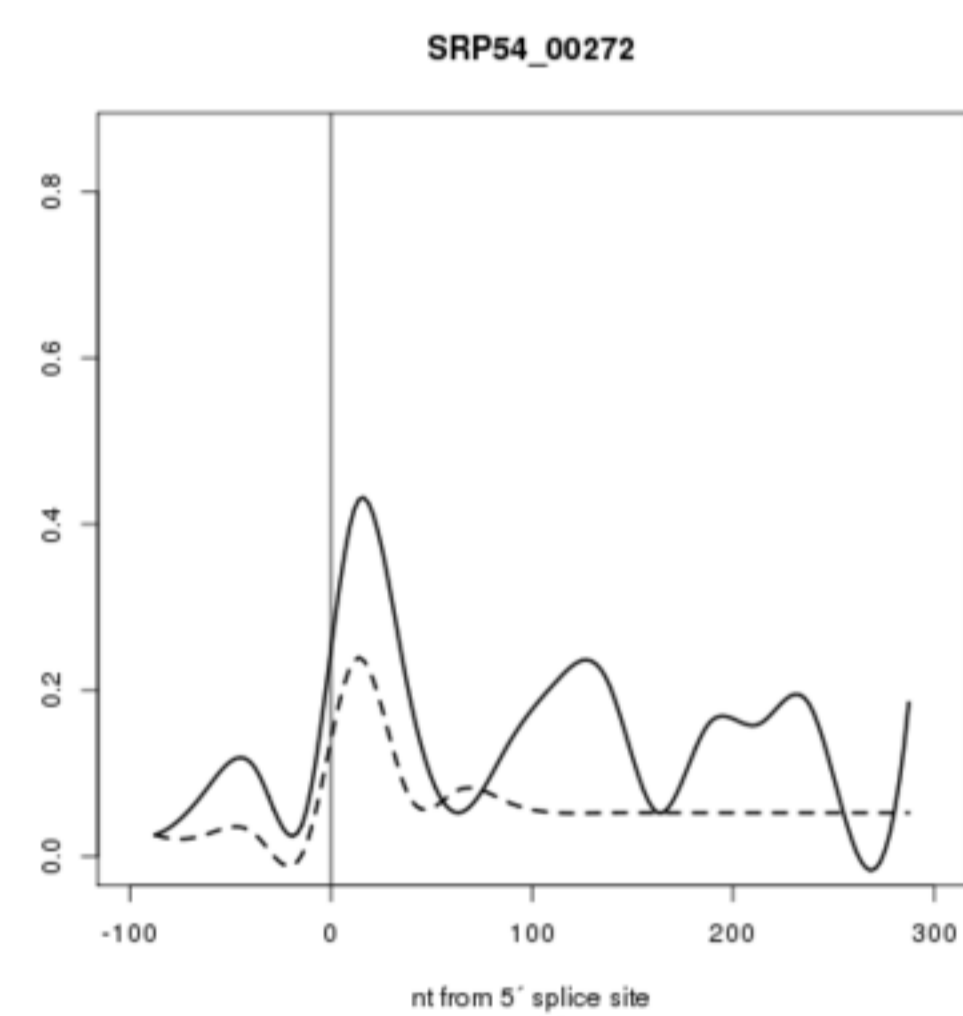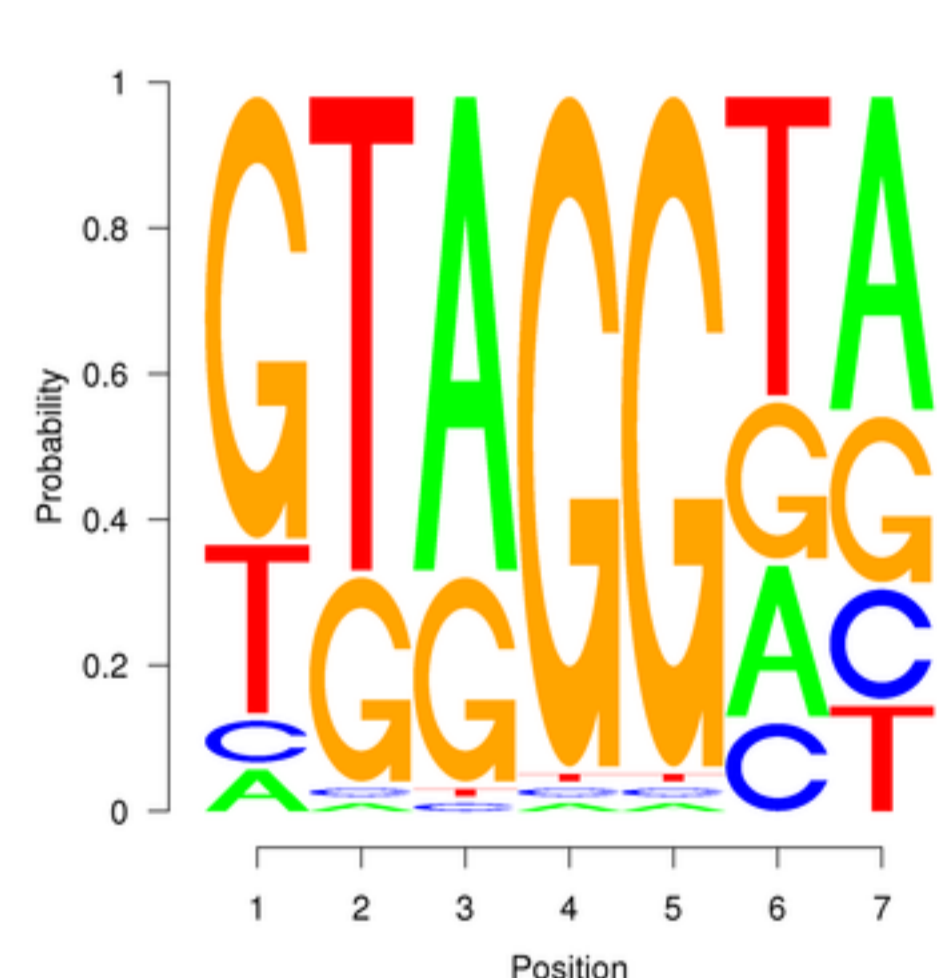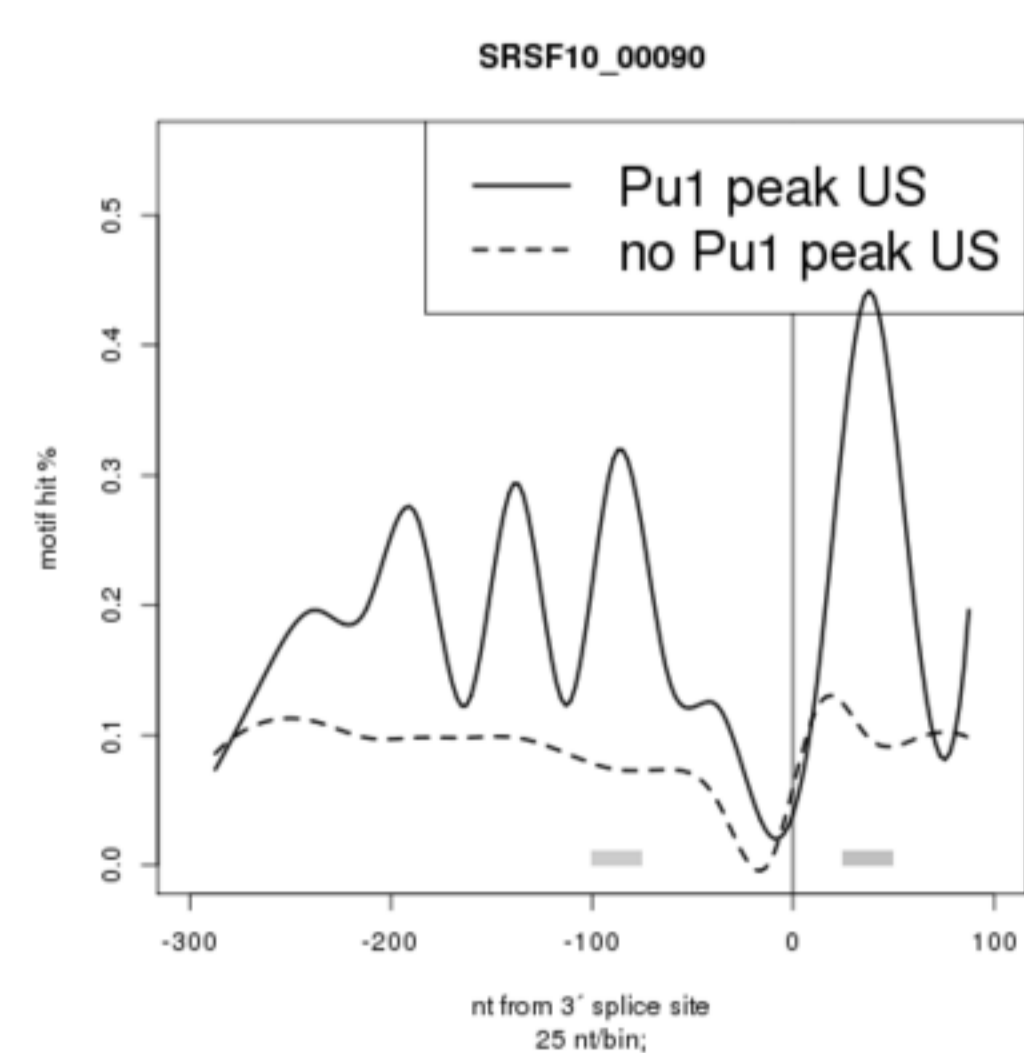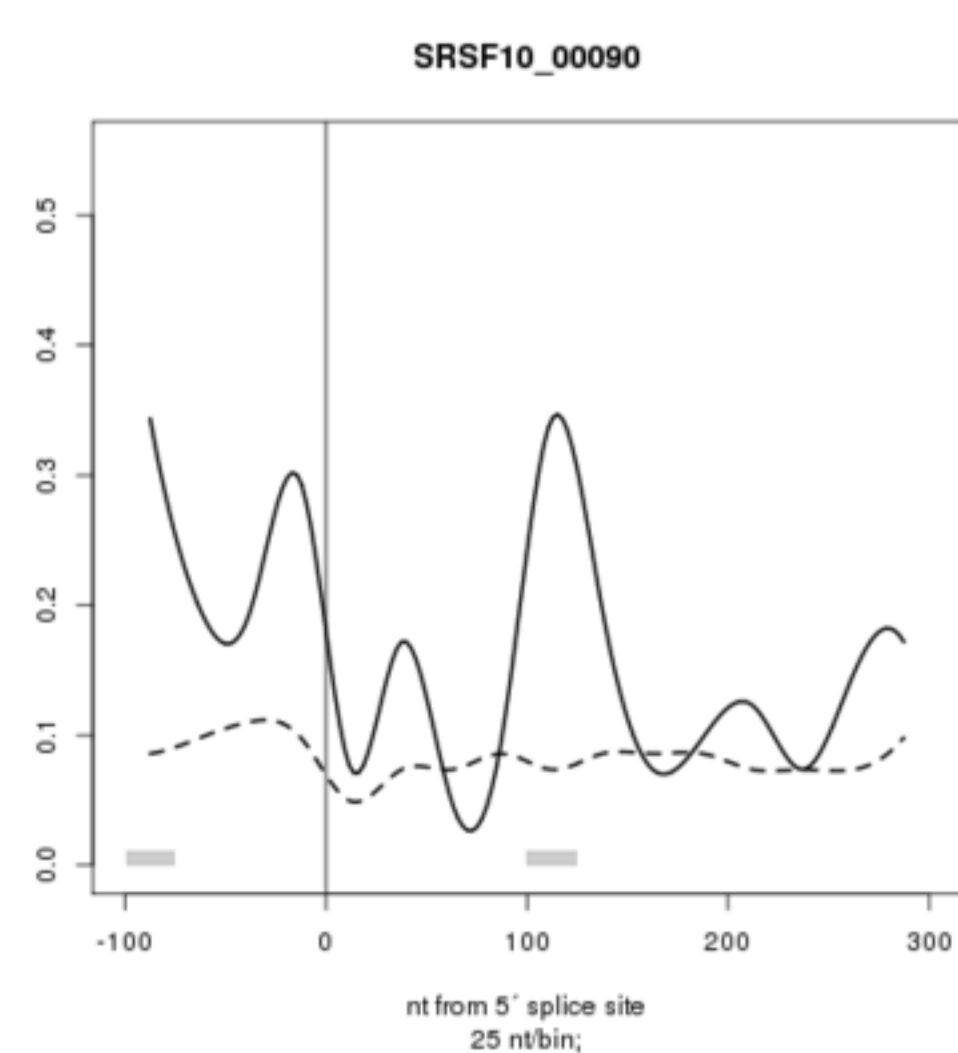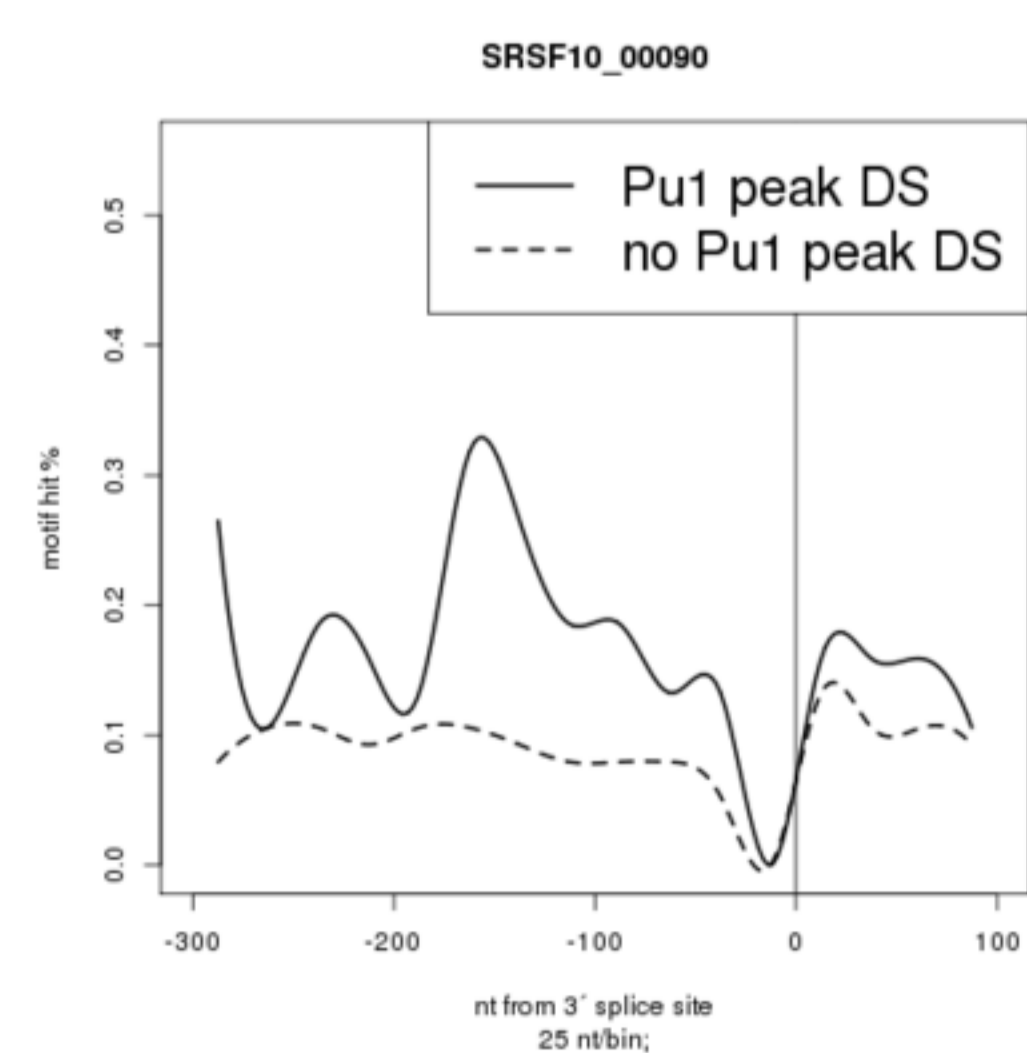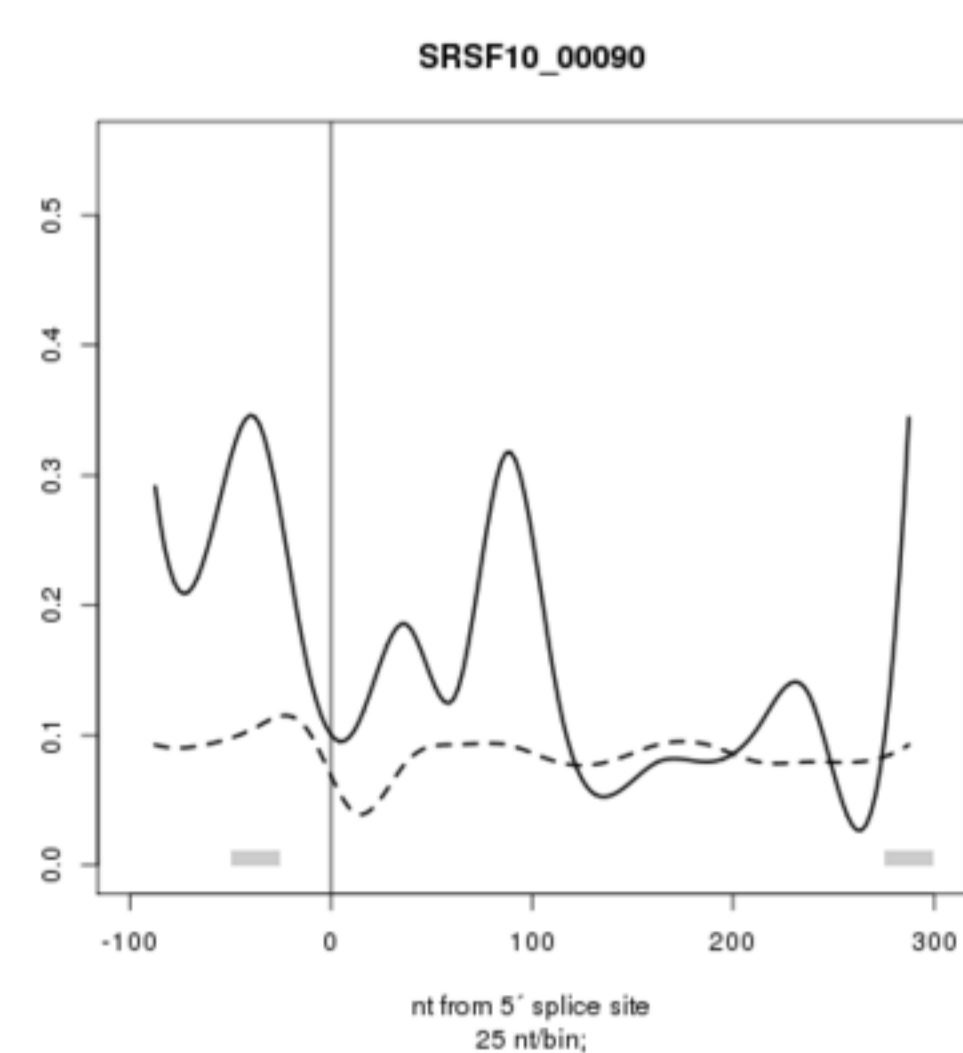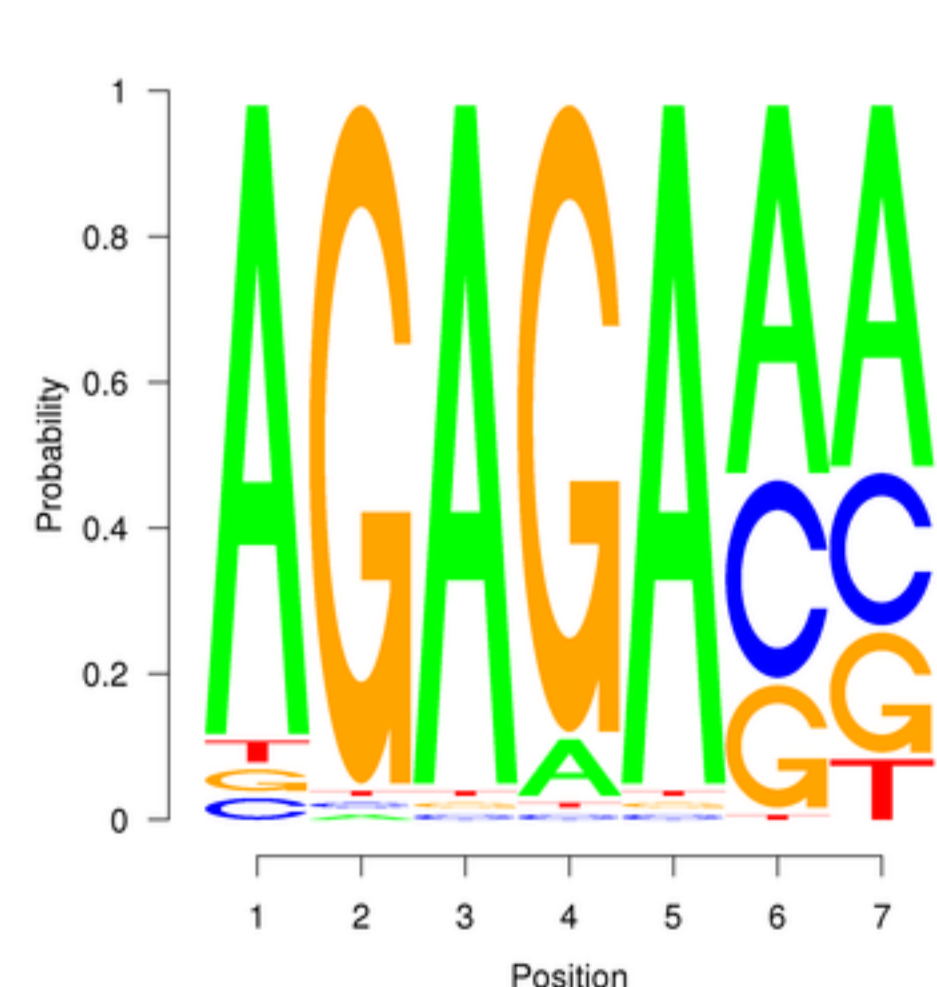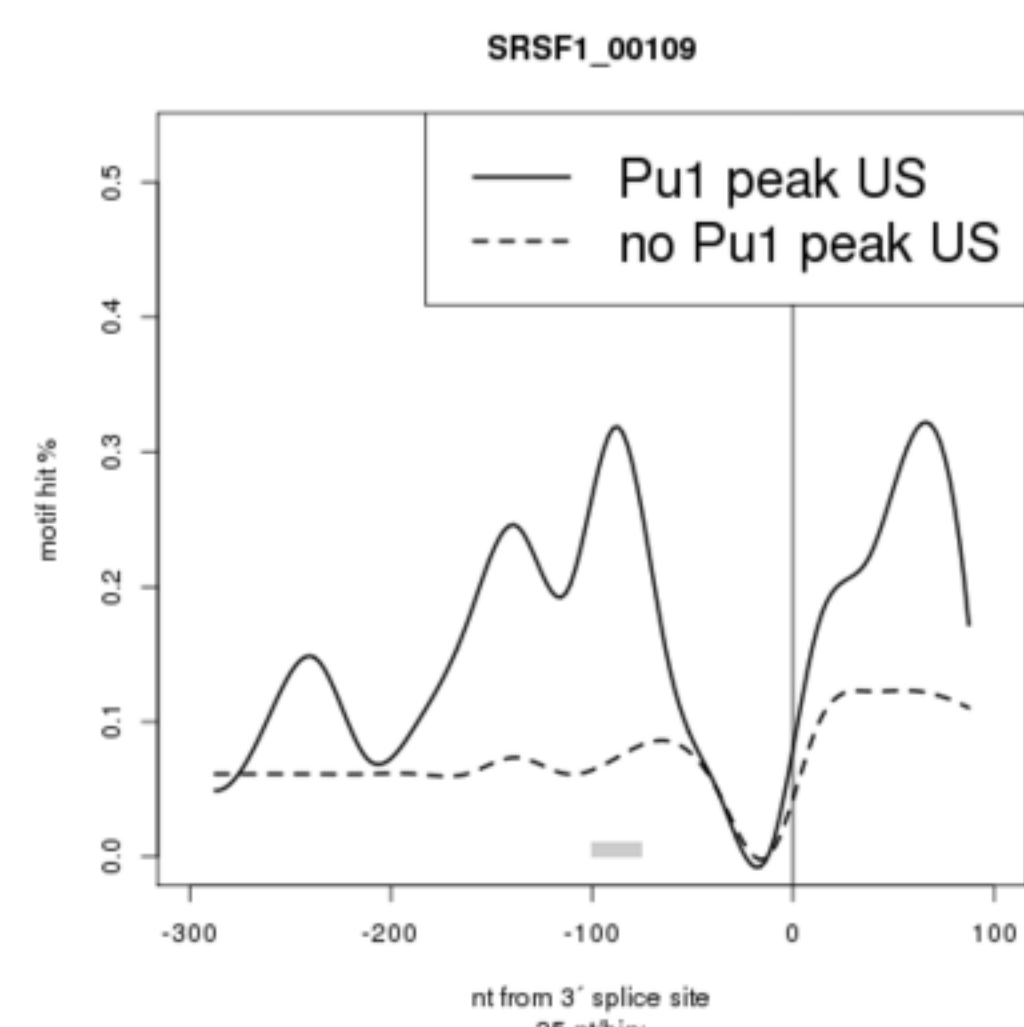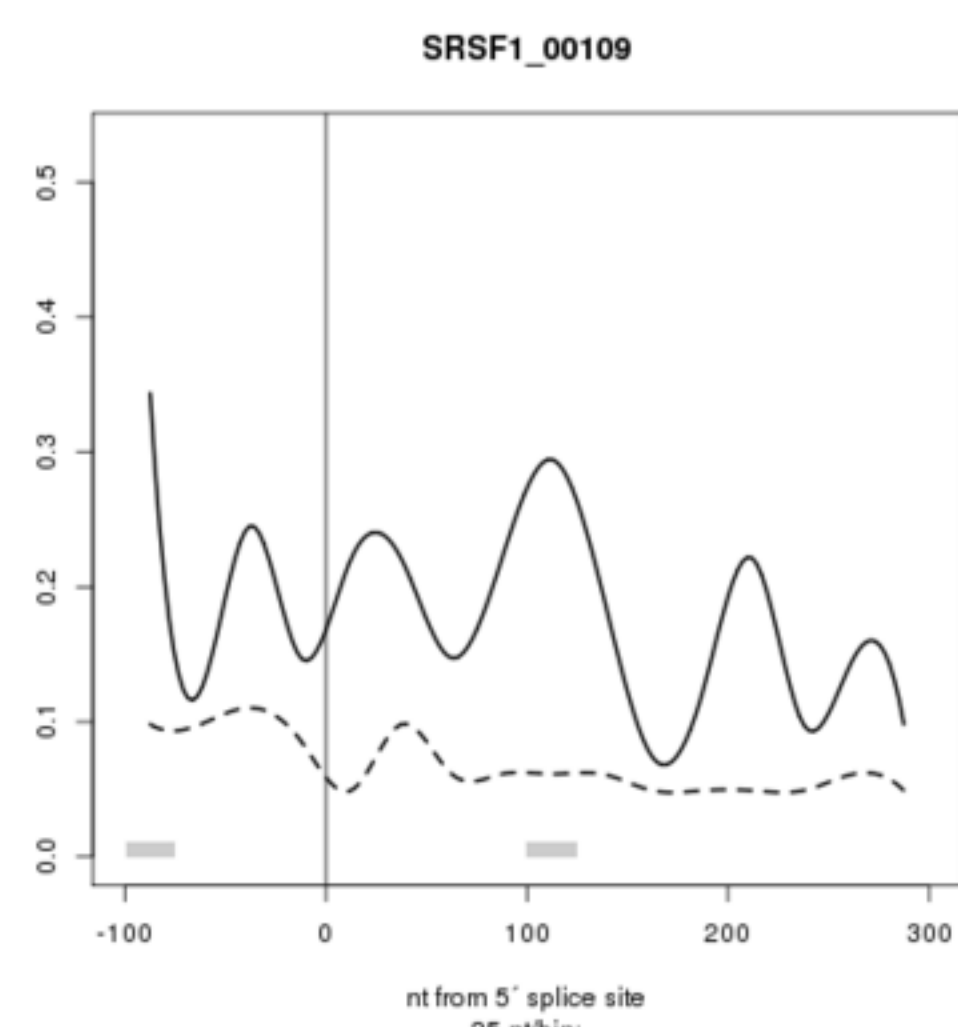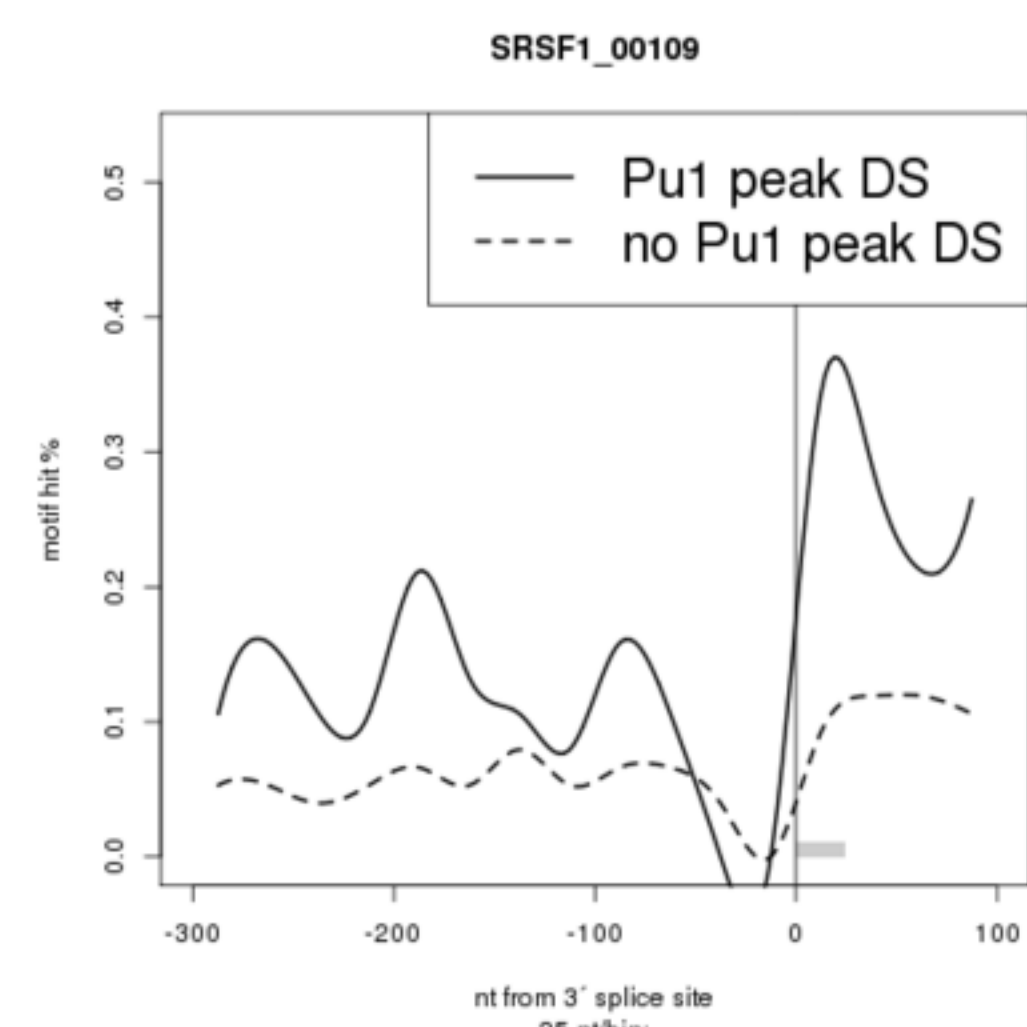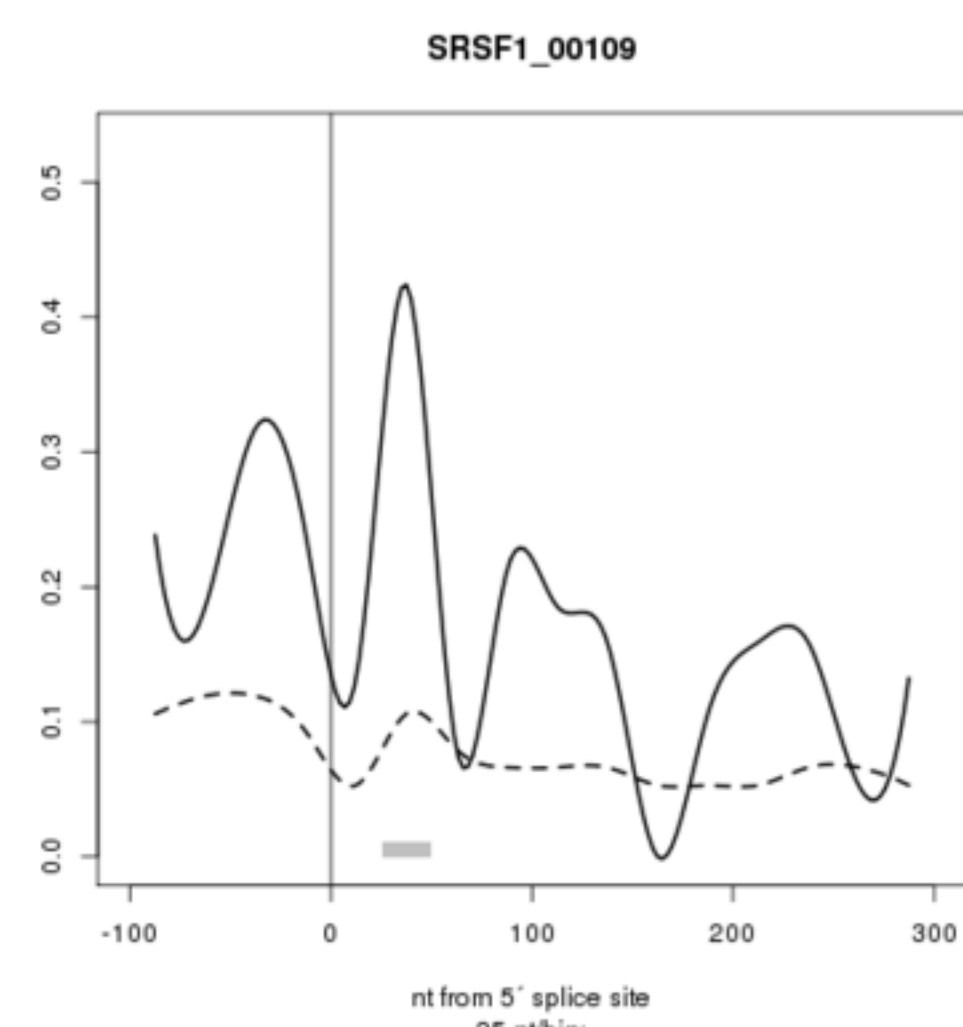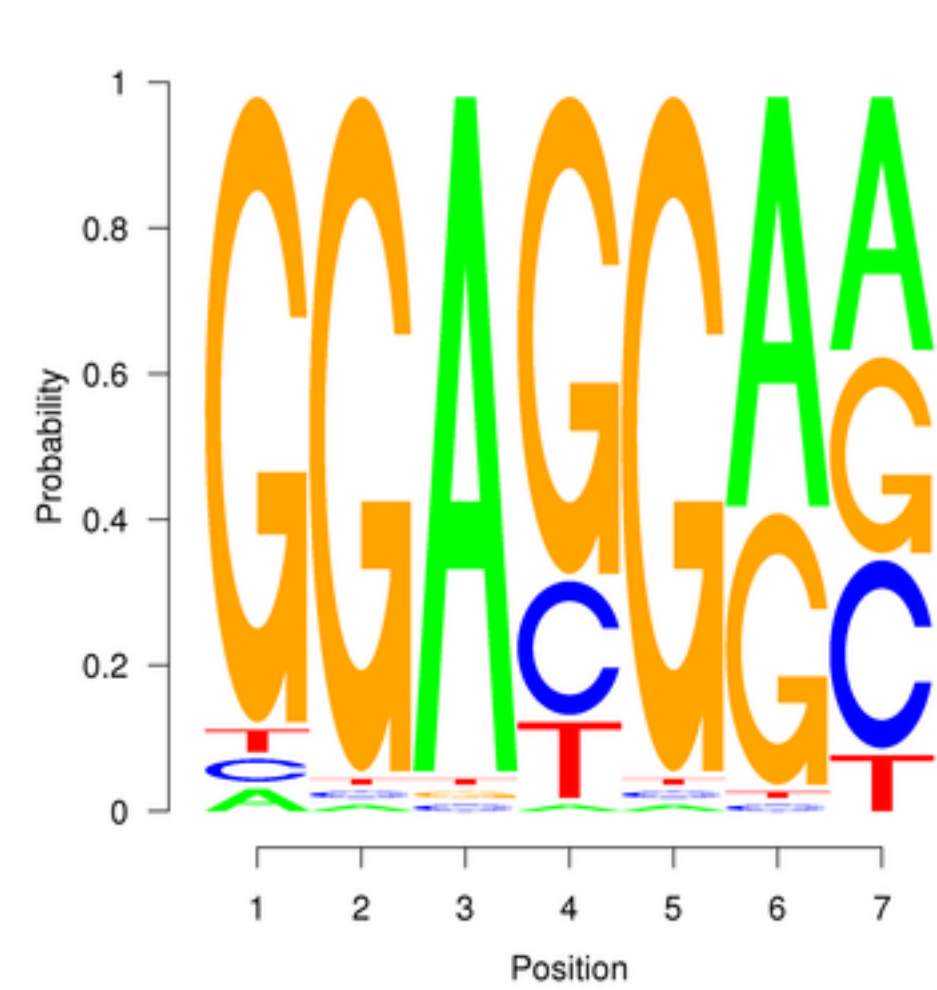

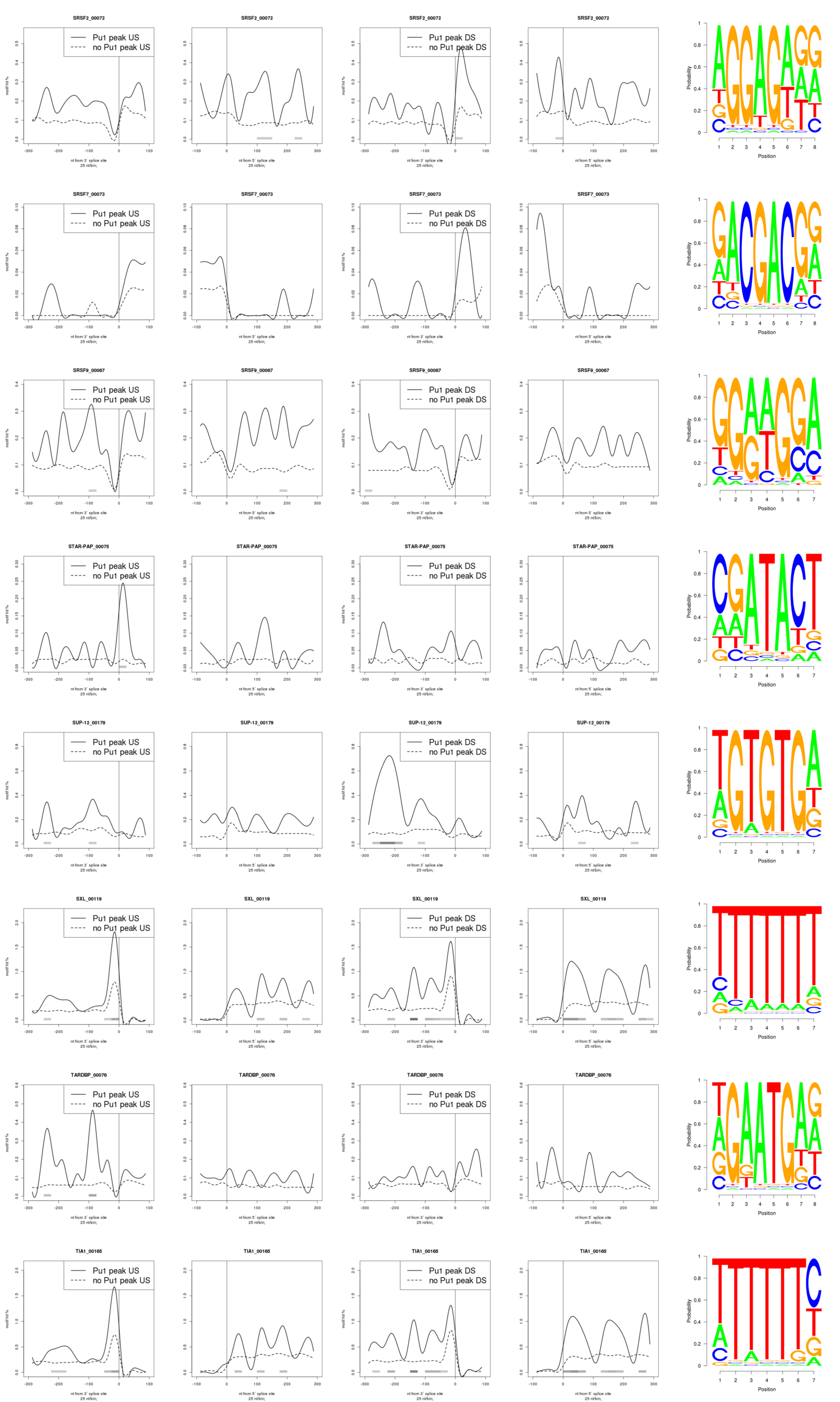

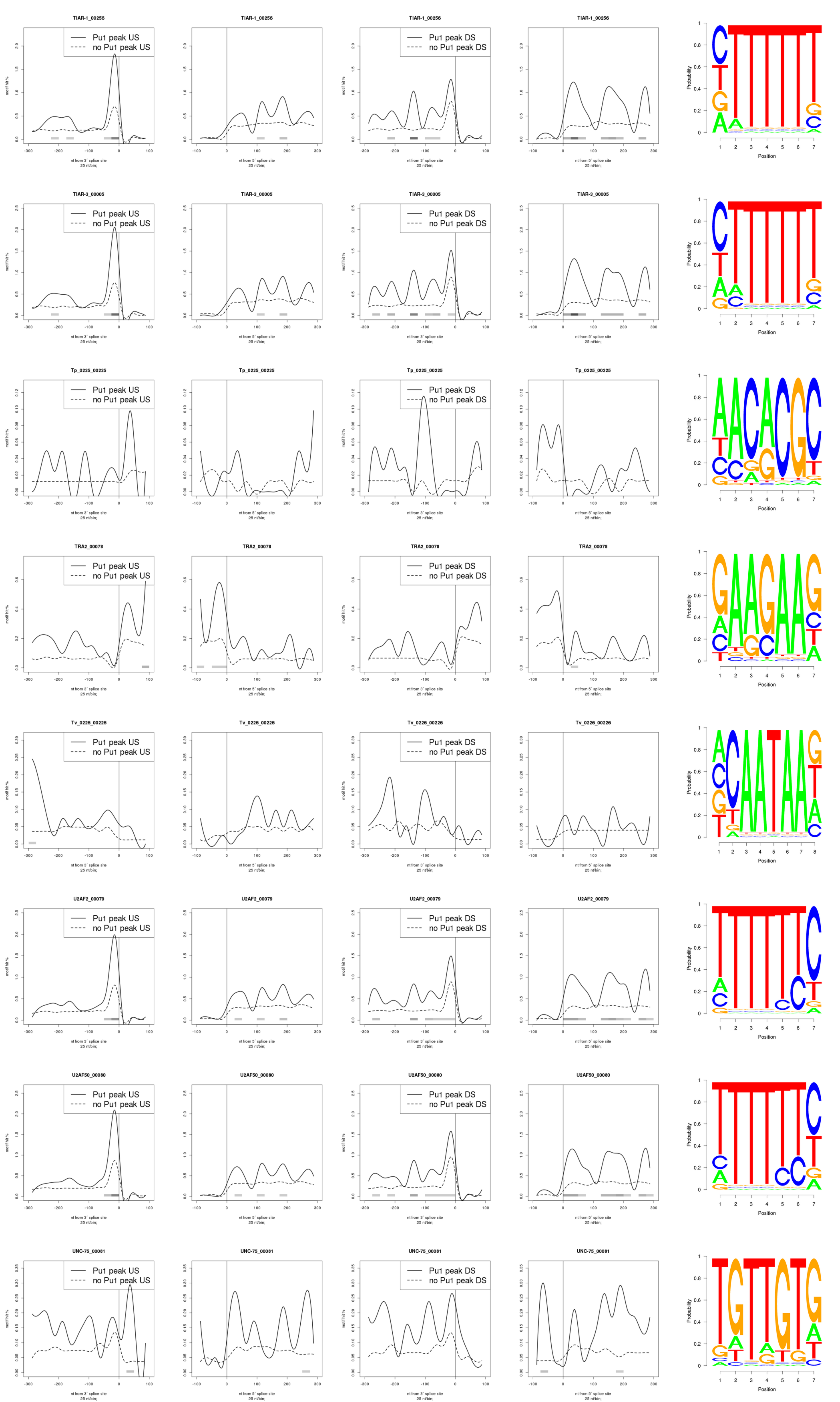

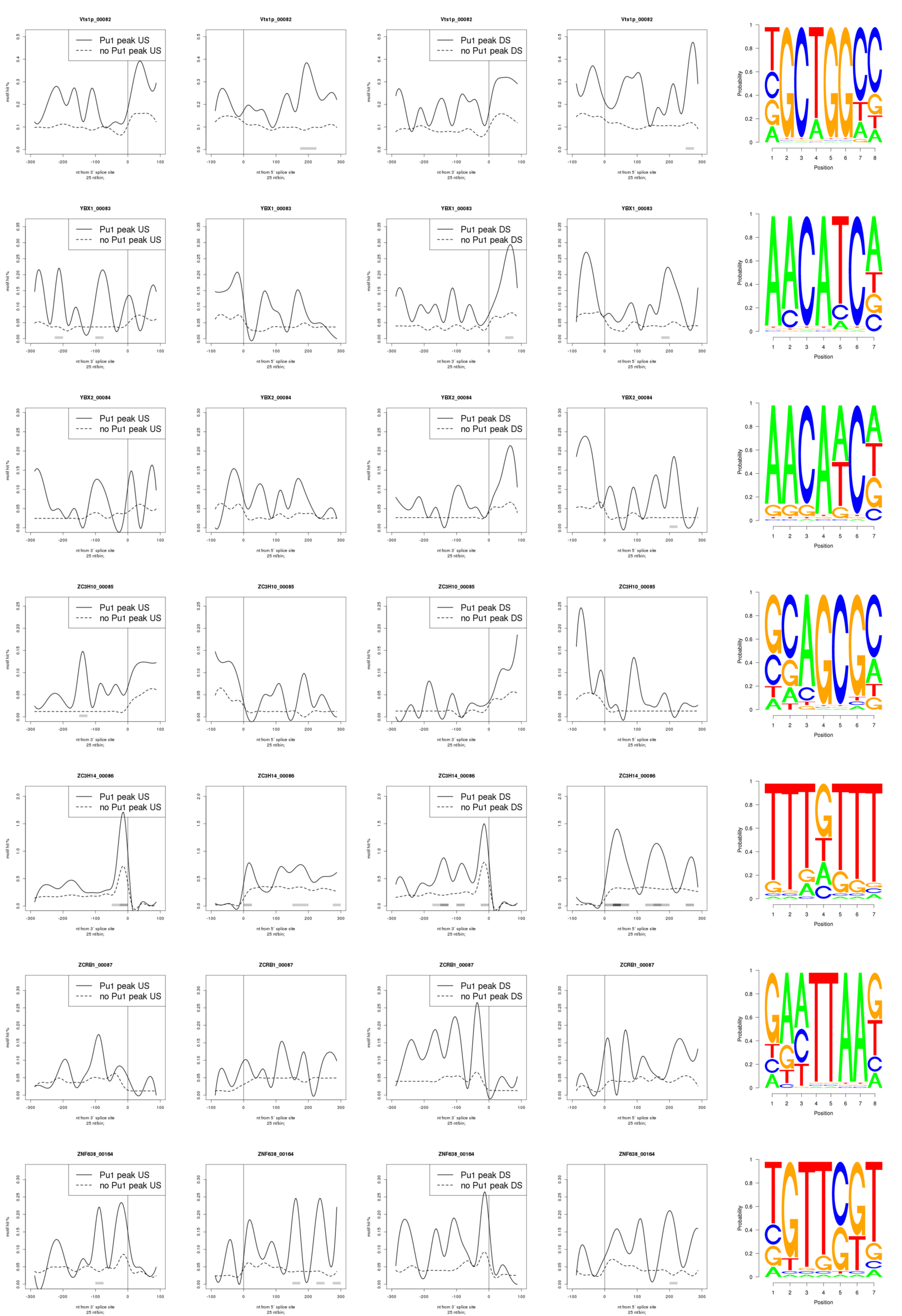

Supplement: S5 File — Same format as S3 File, Pu1 = SPI1/PU.1 gene. (PDF) [file pone.0132448.s014.pdf]

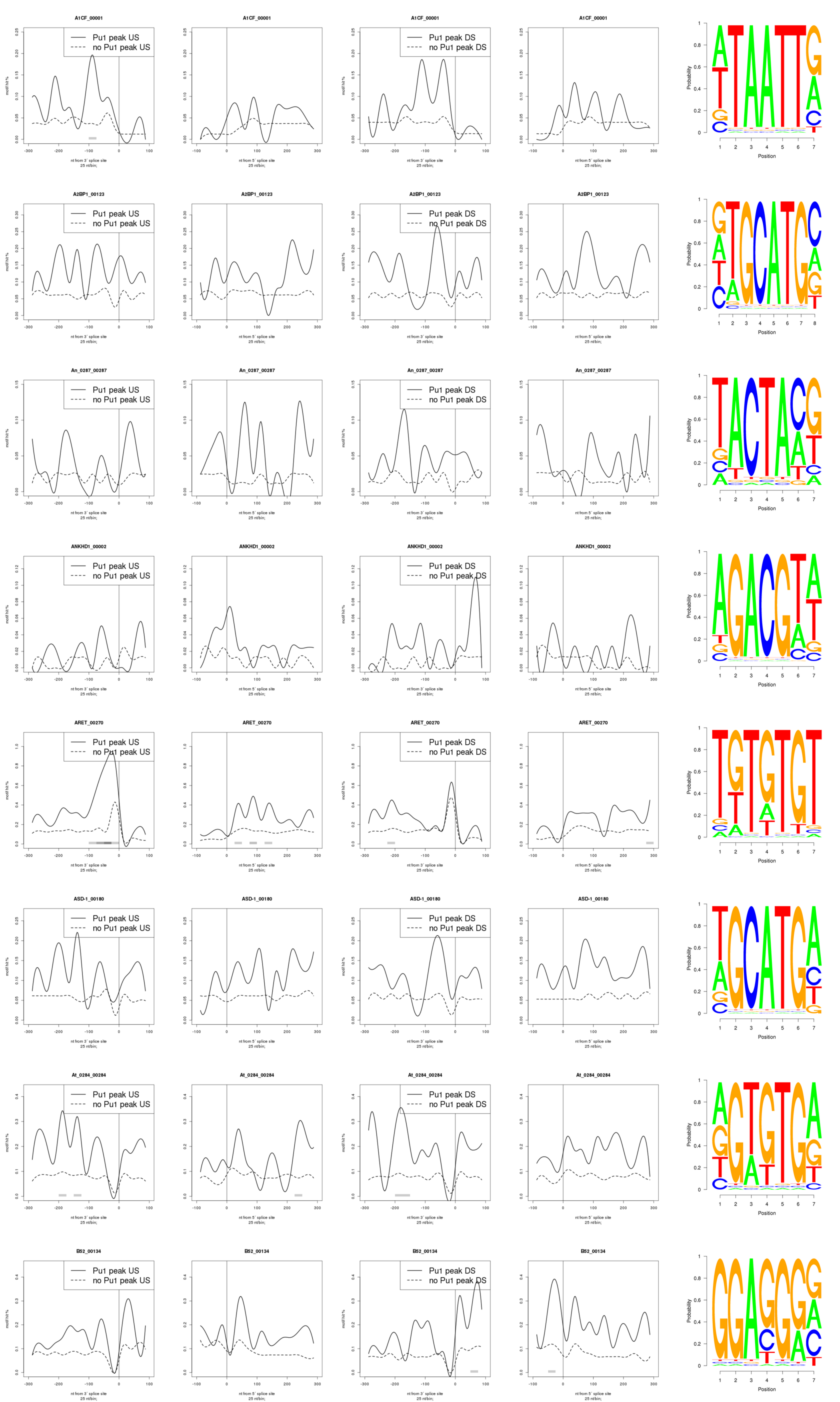

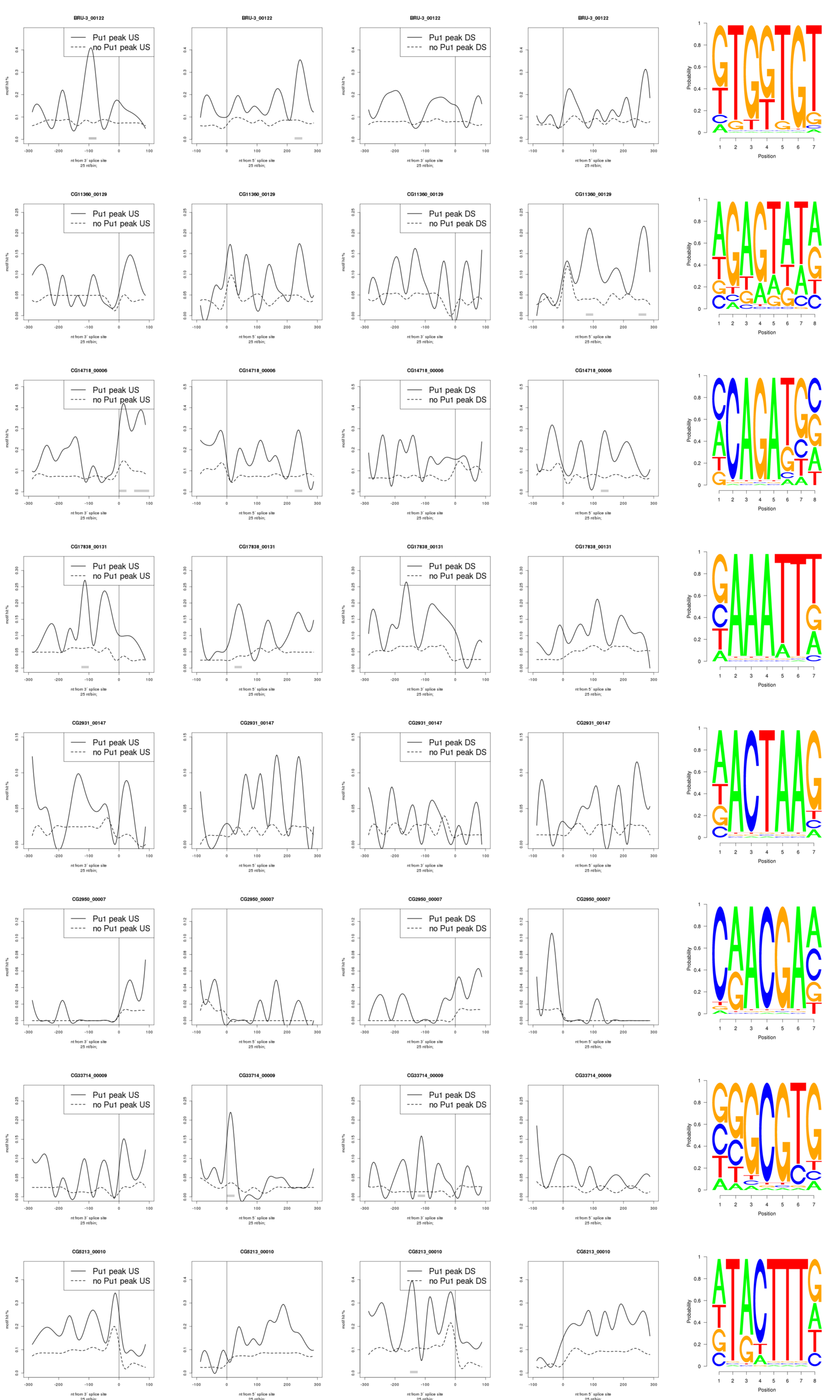

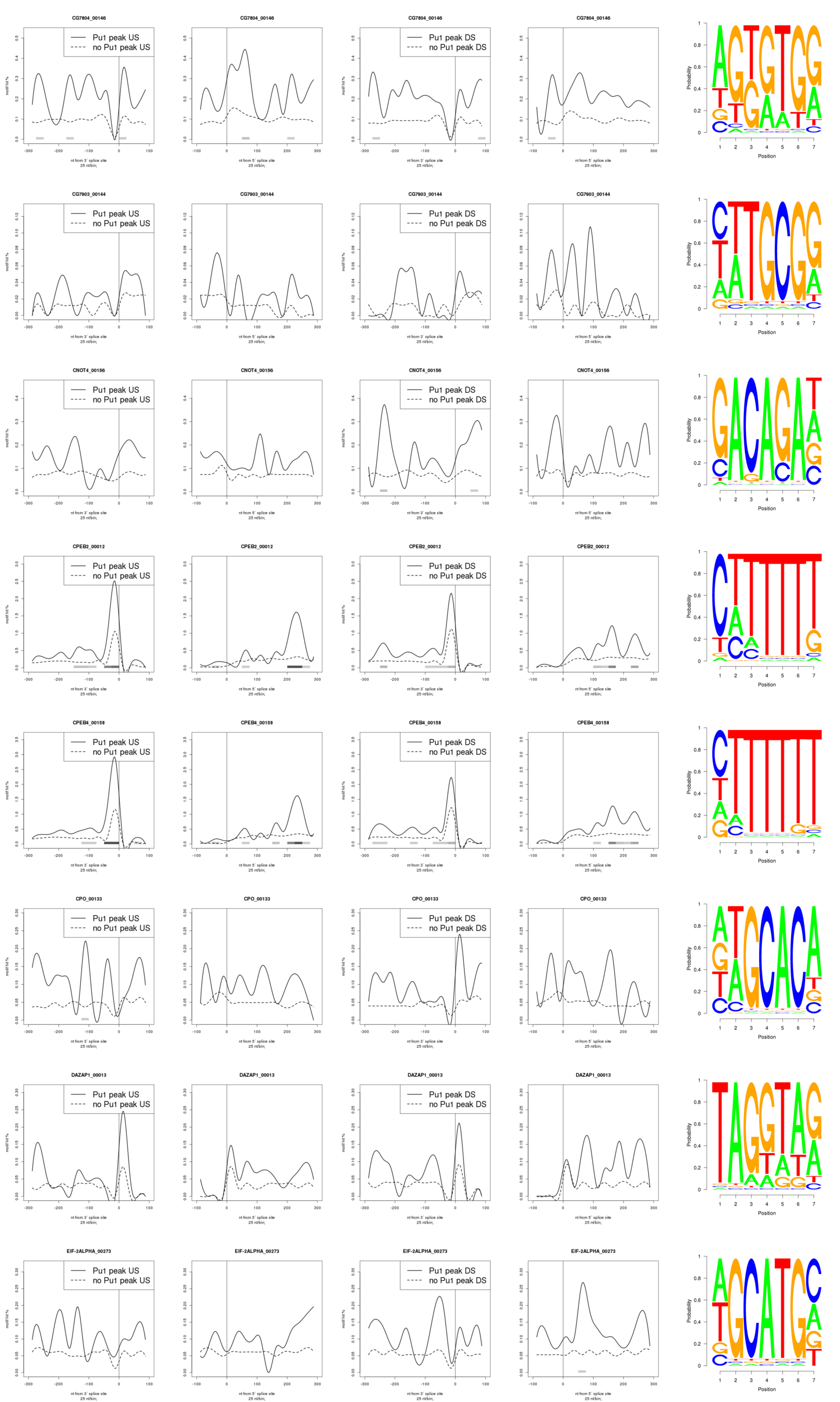

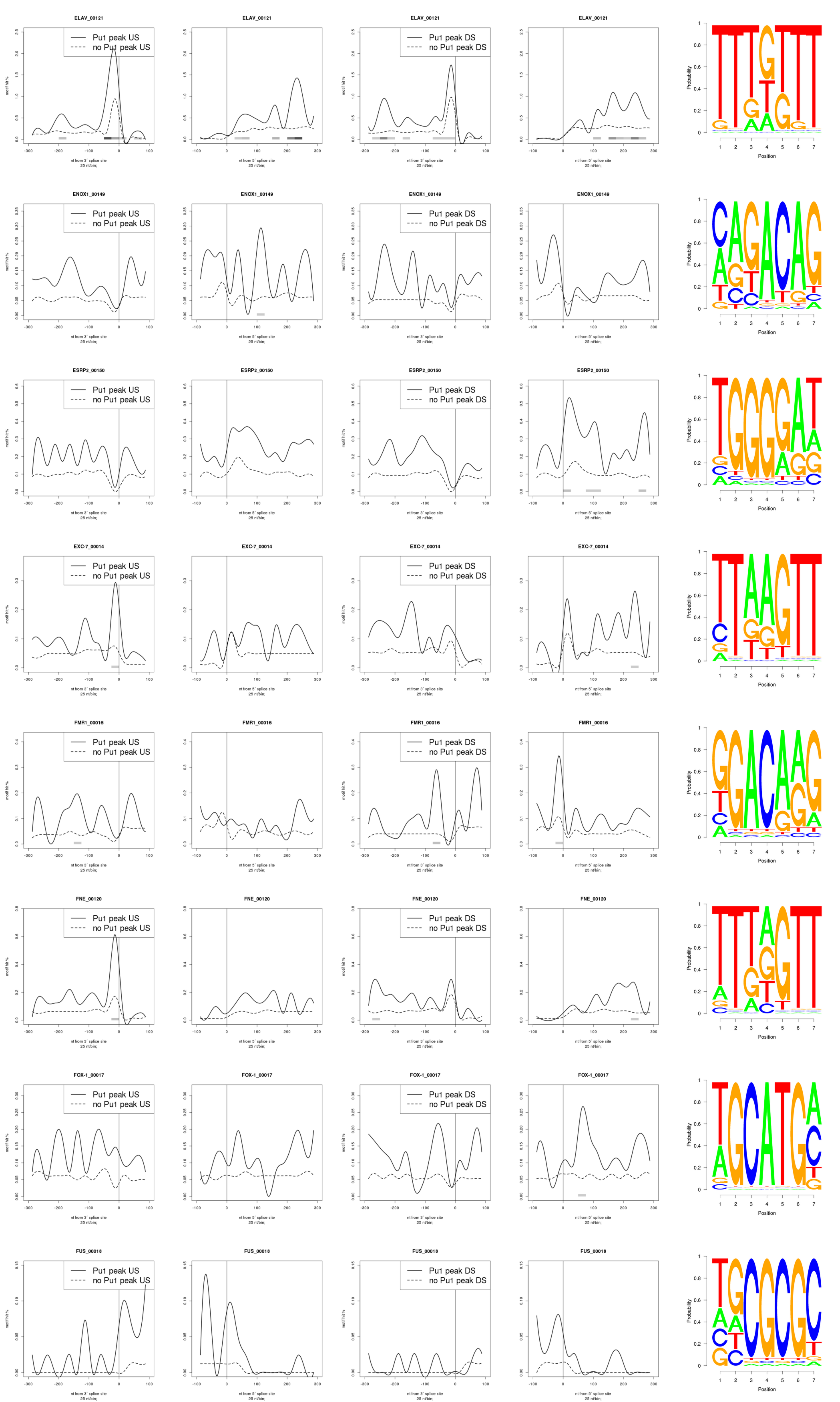

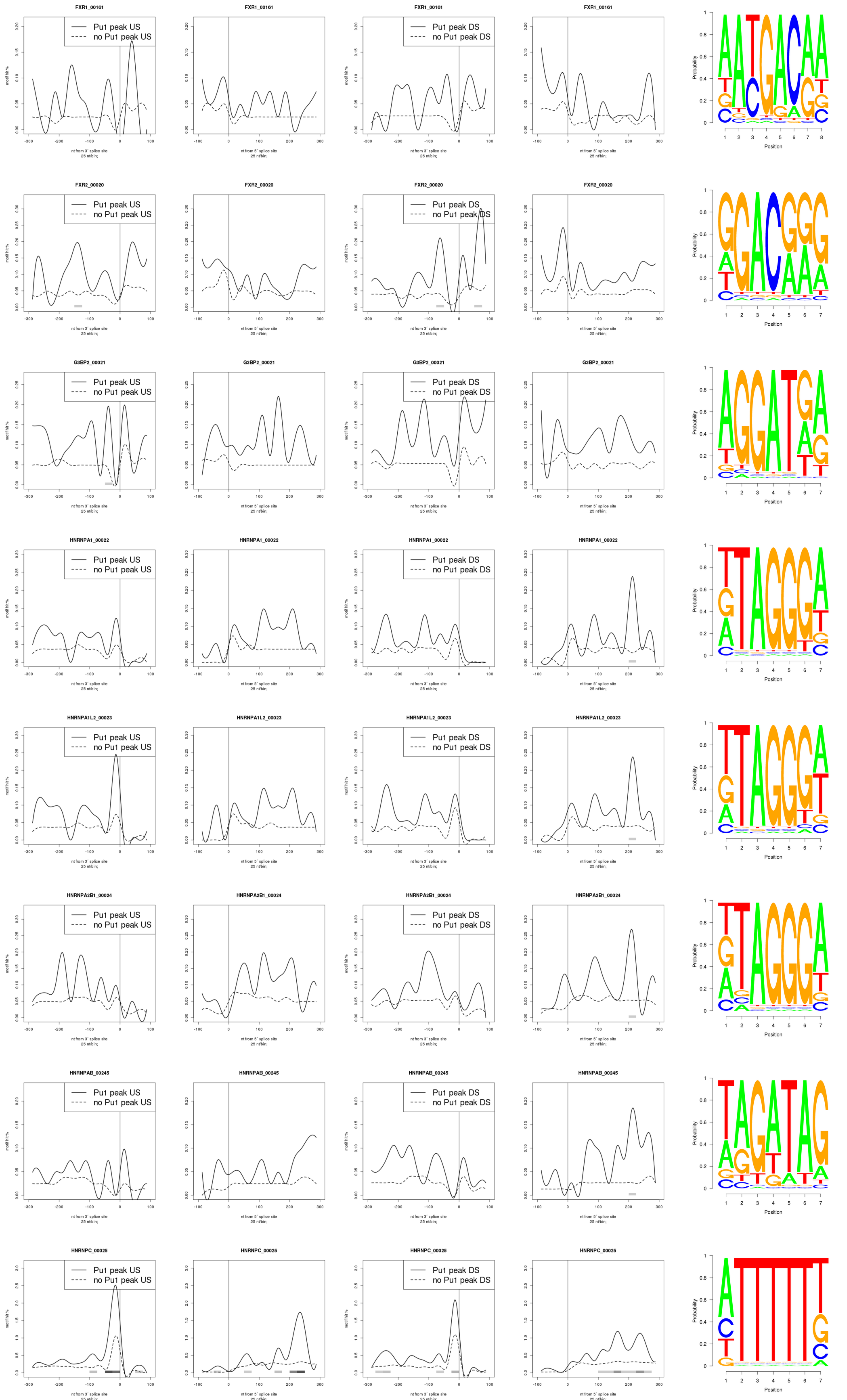

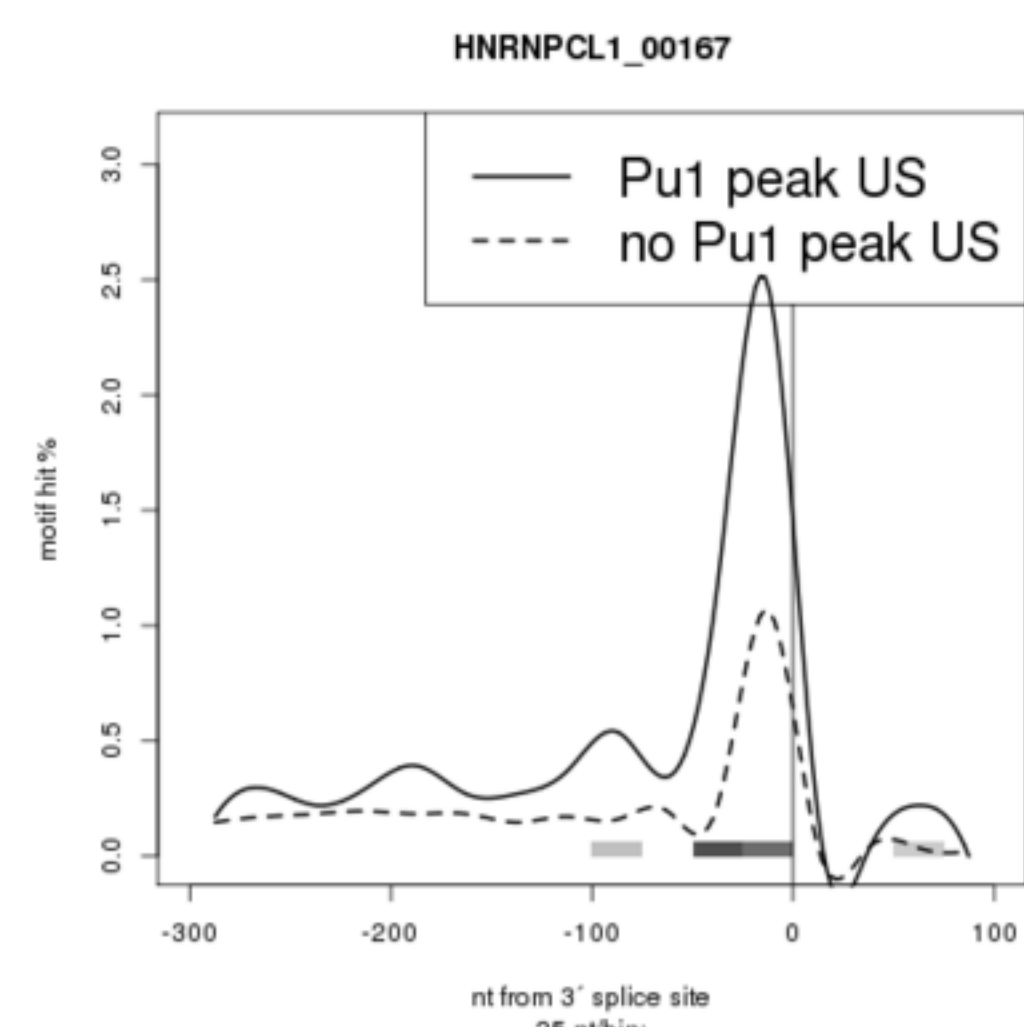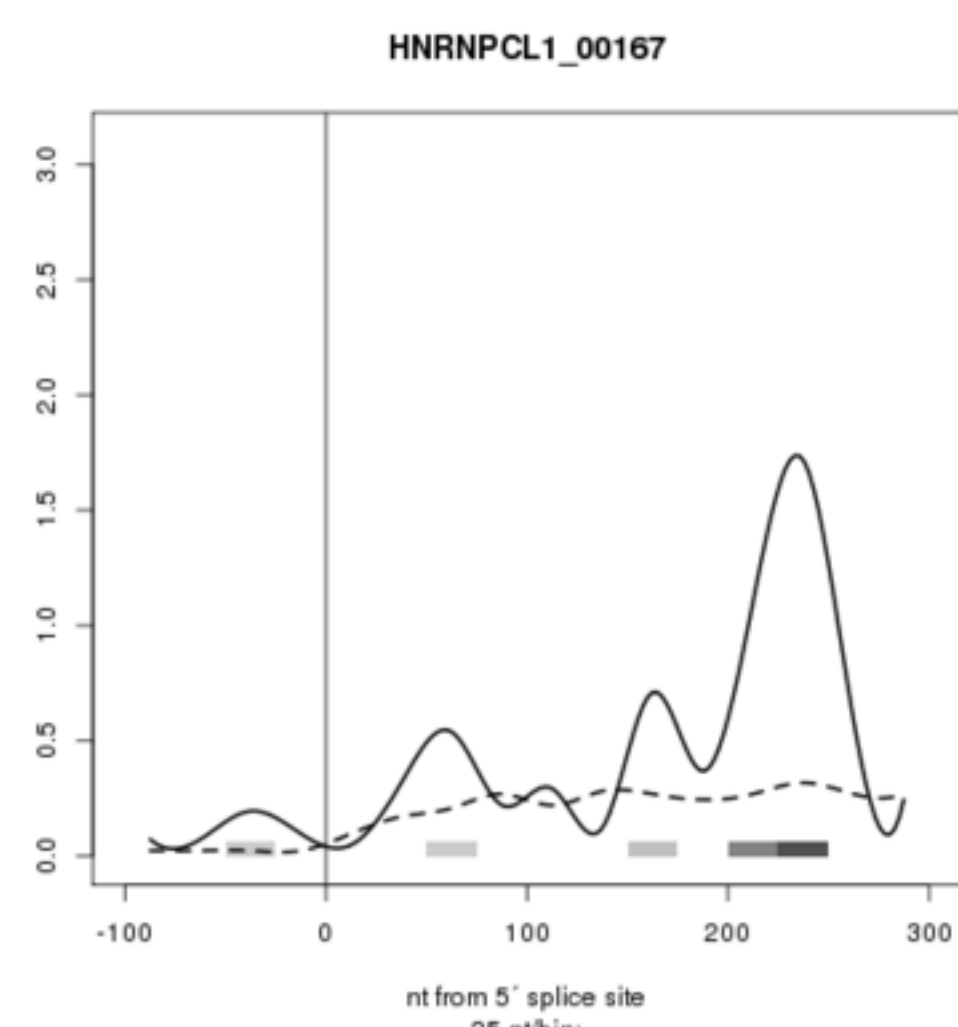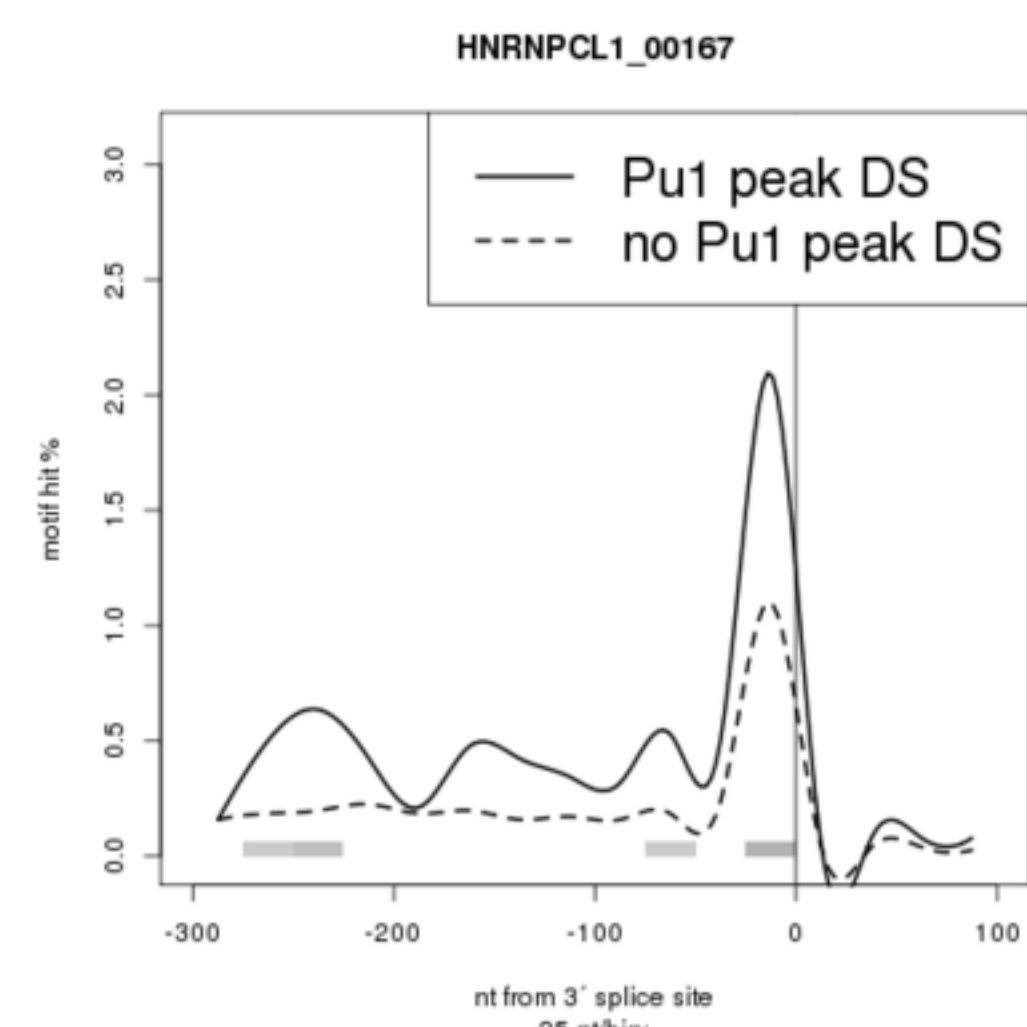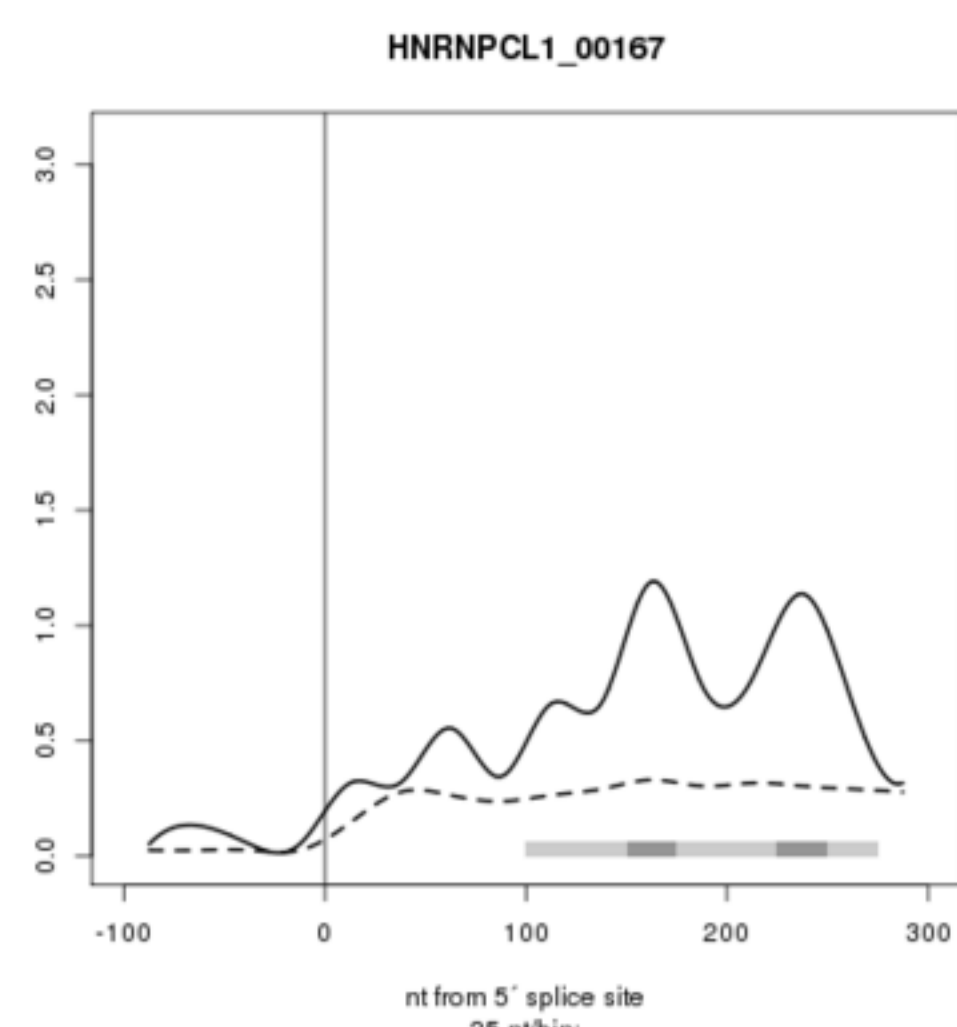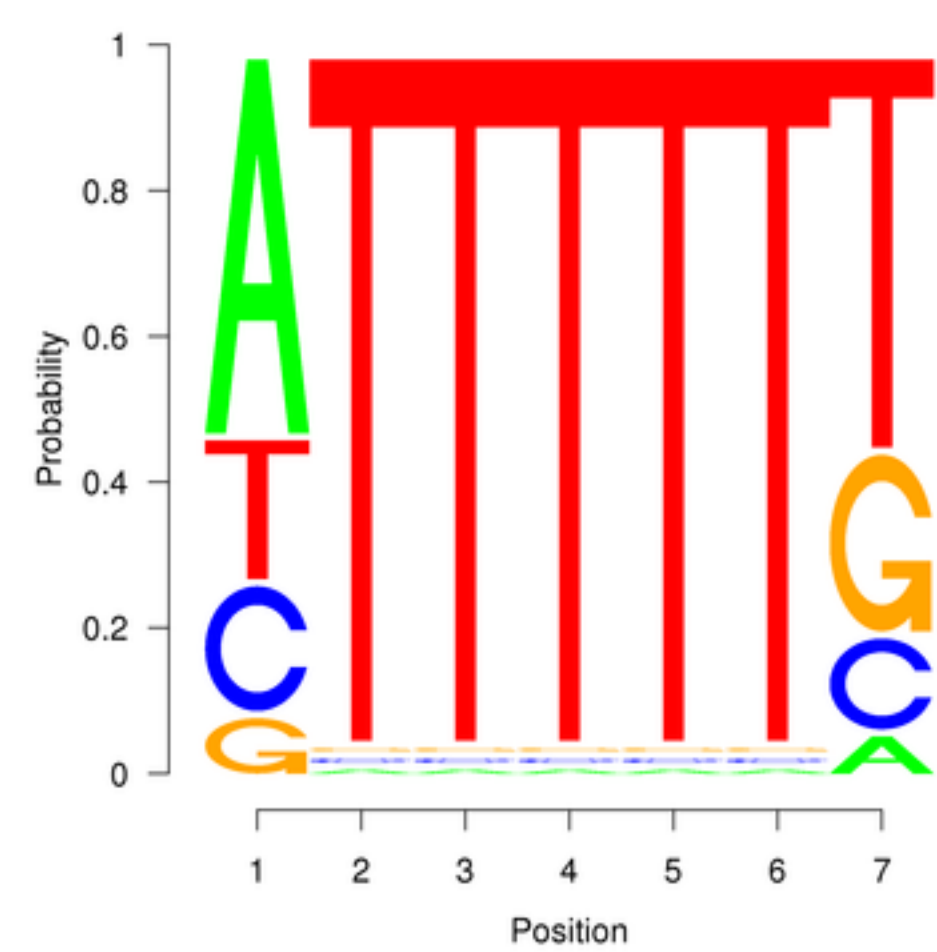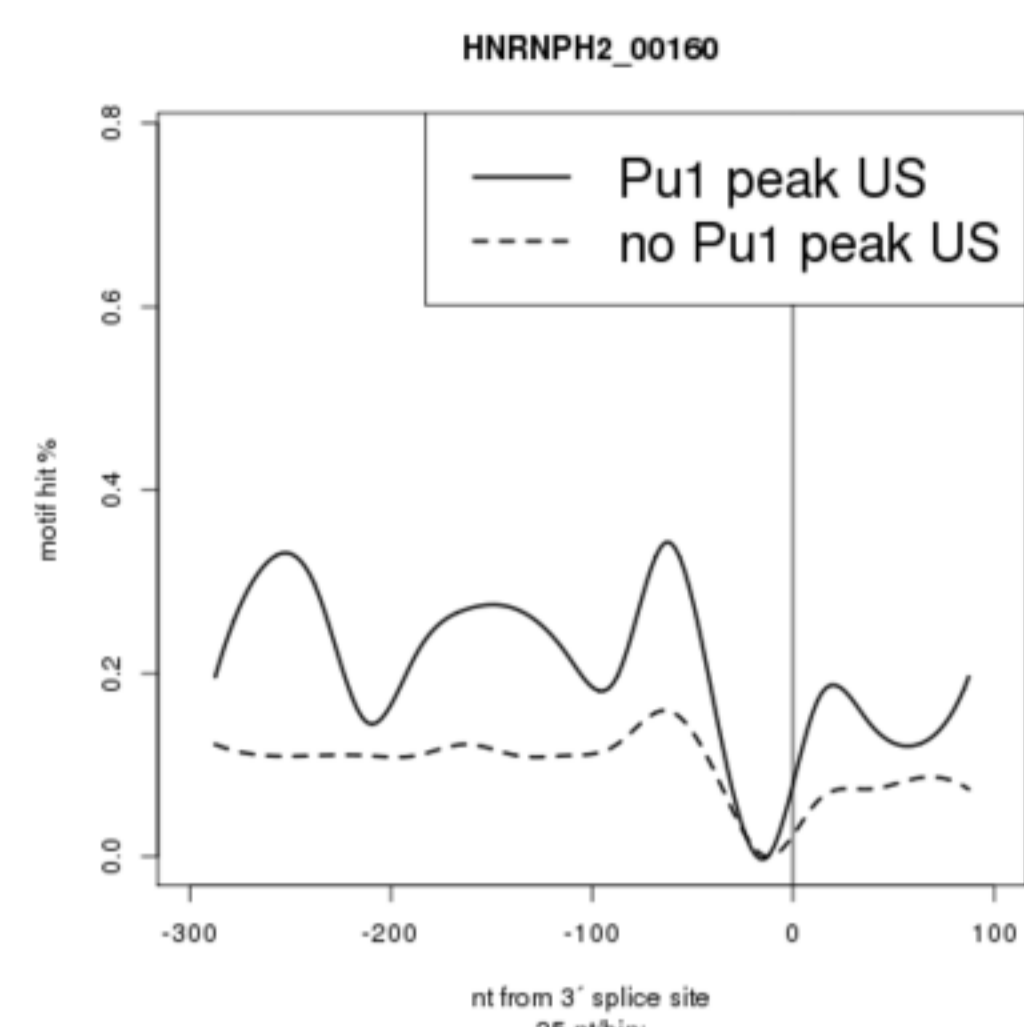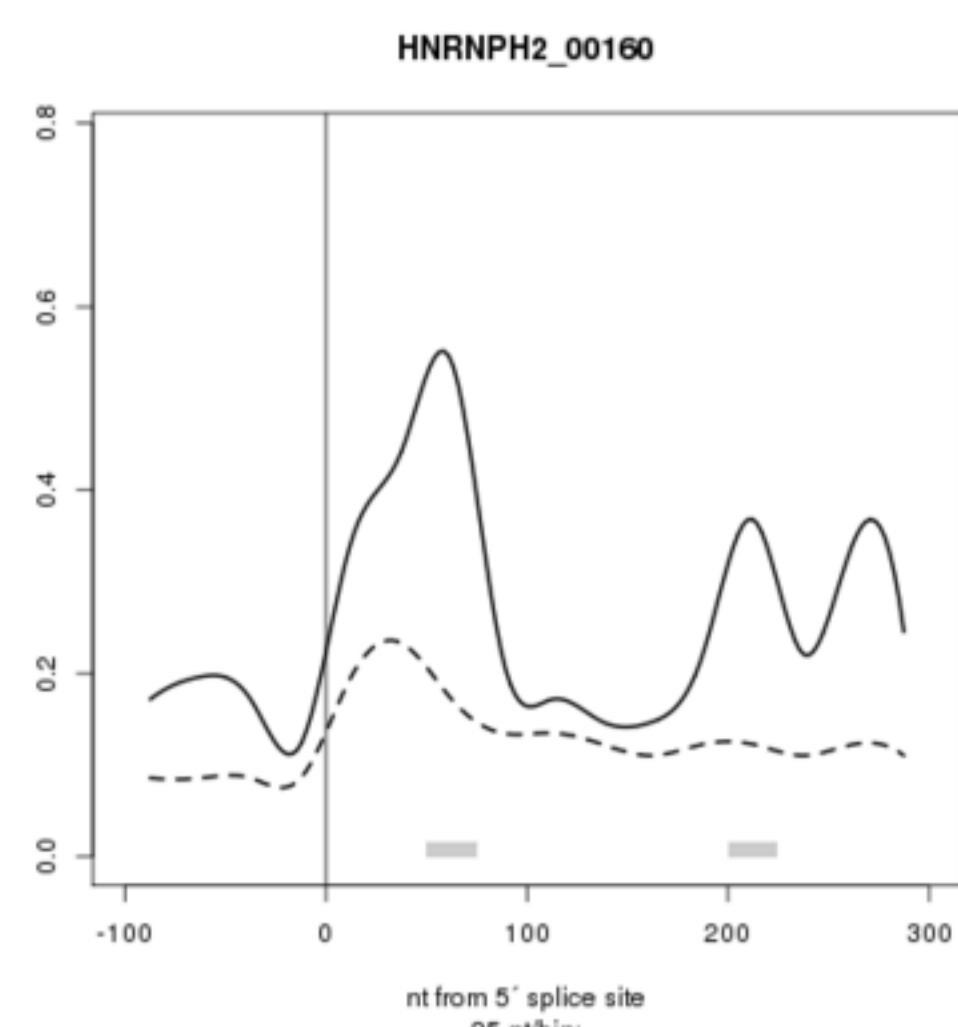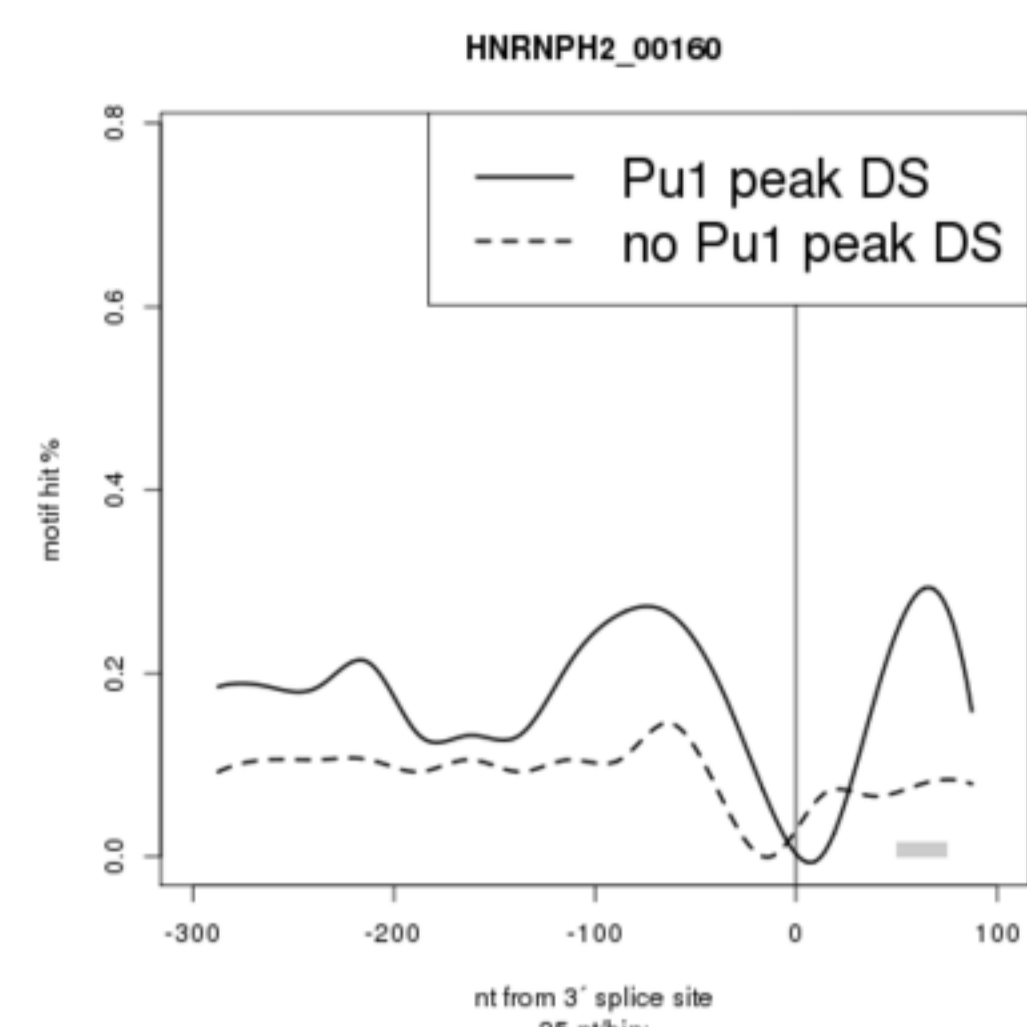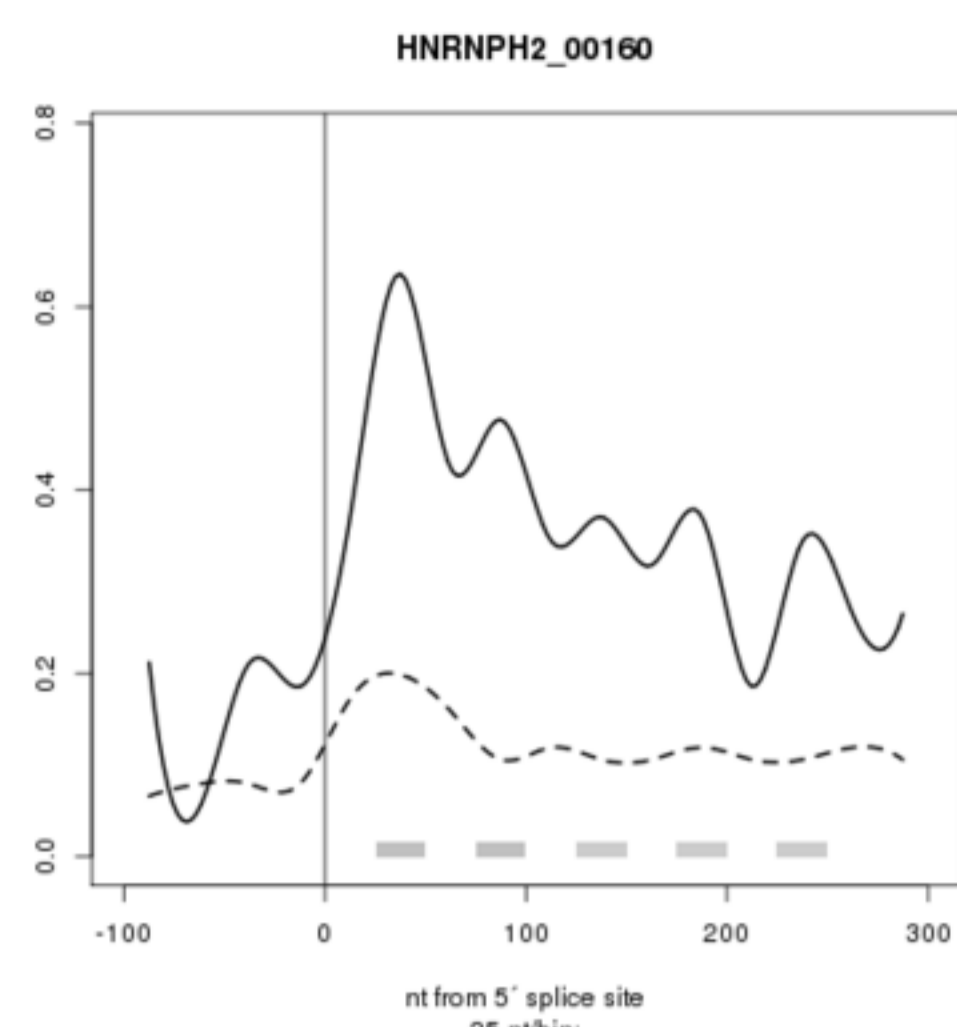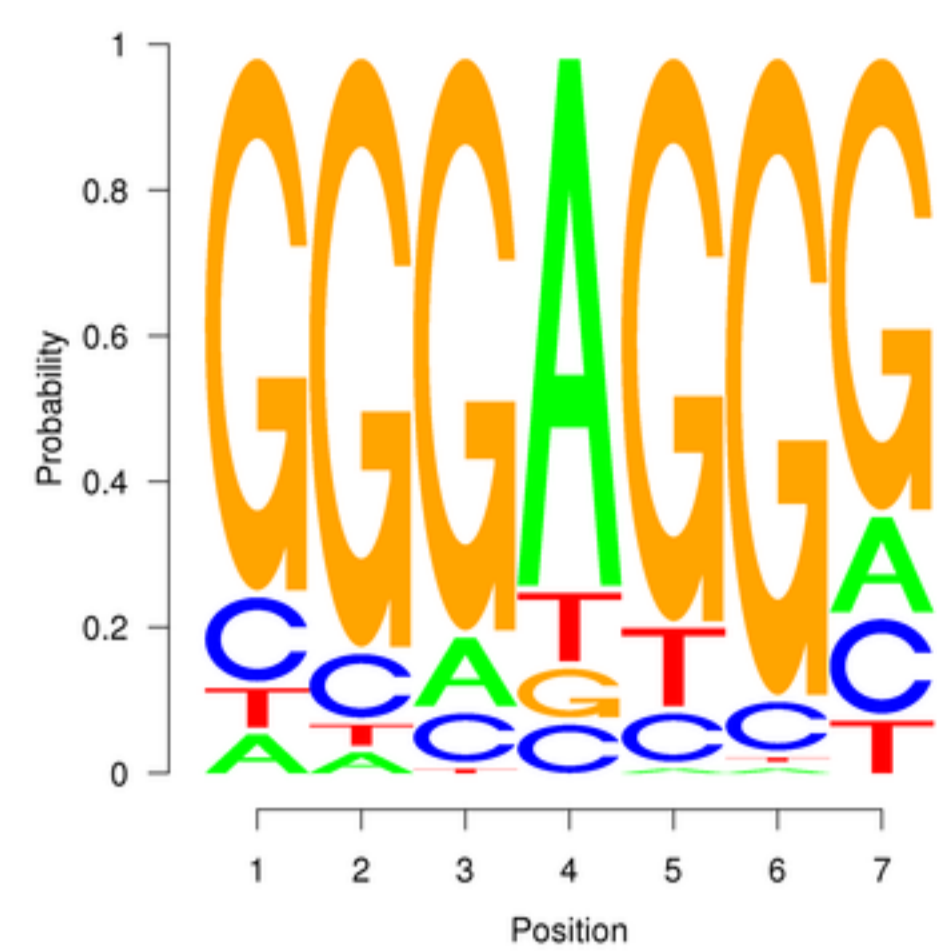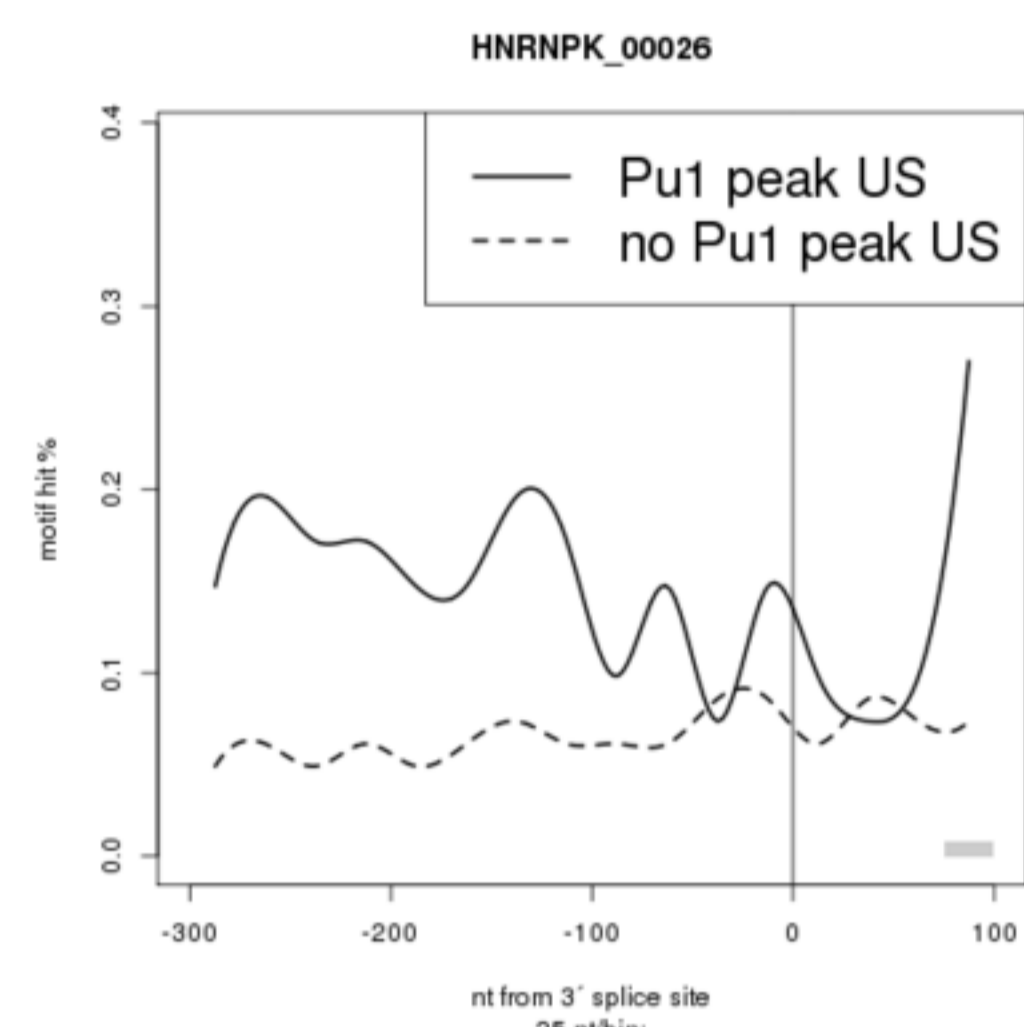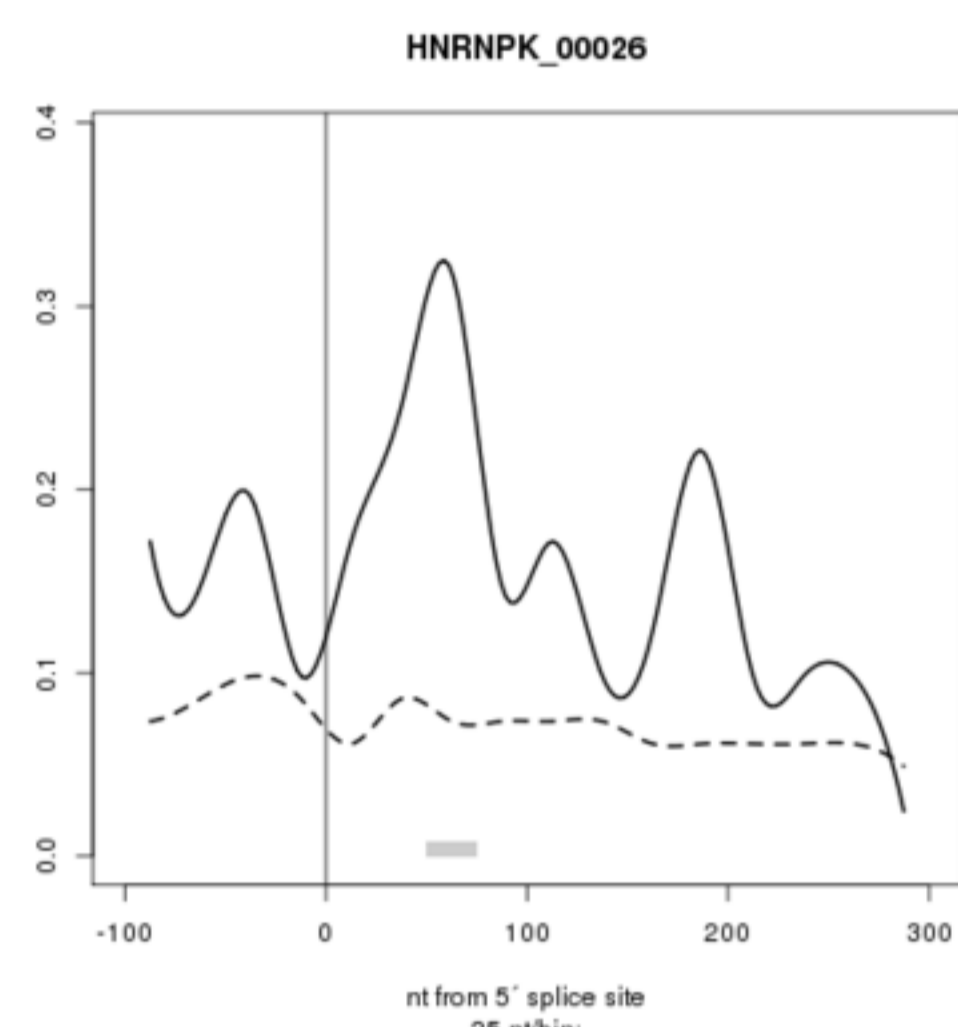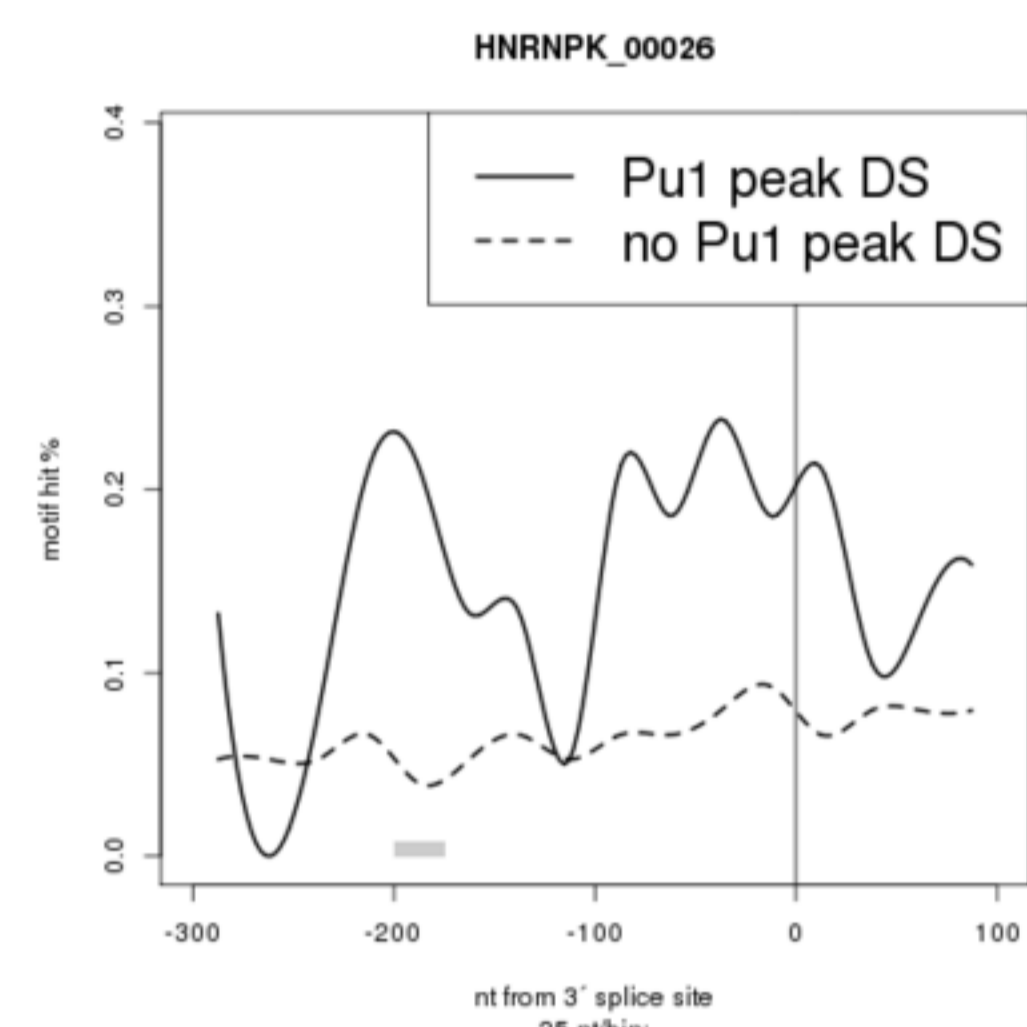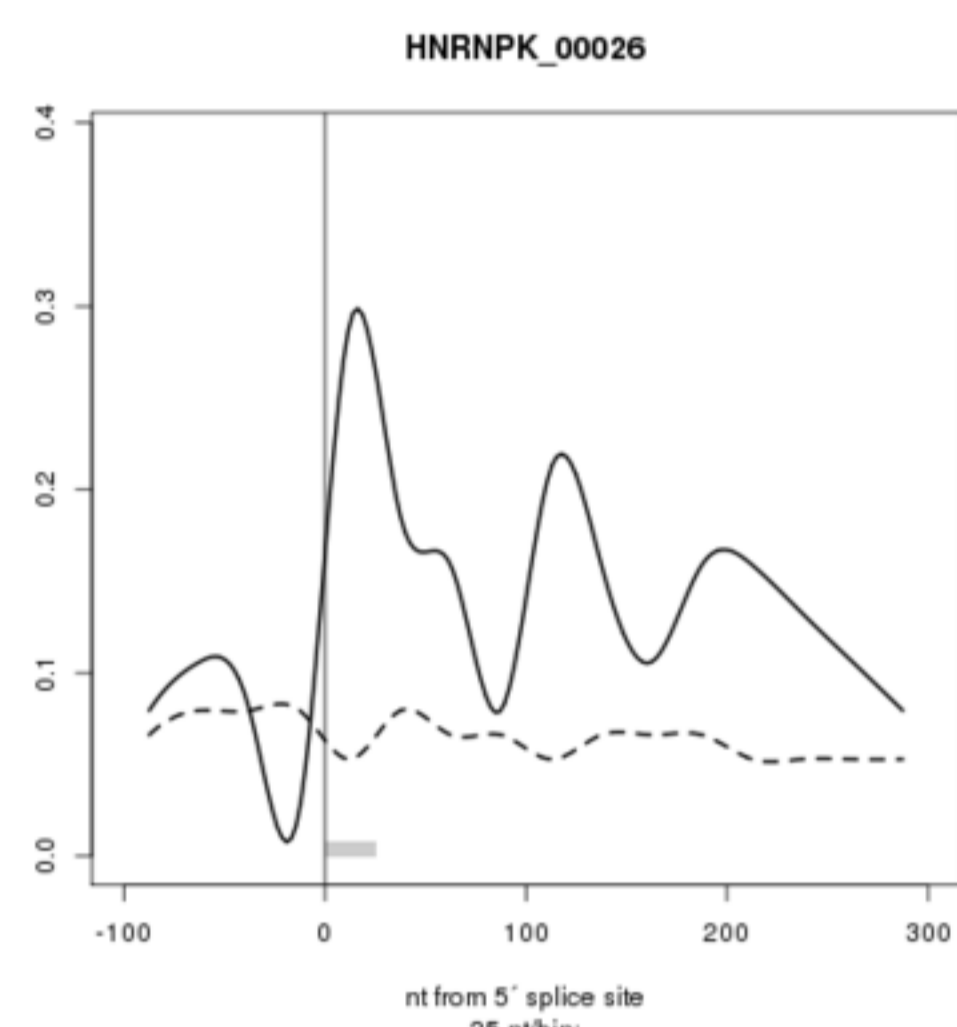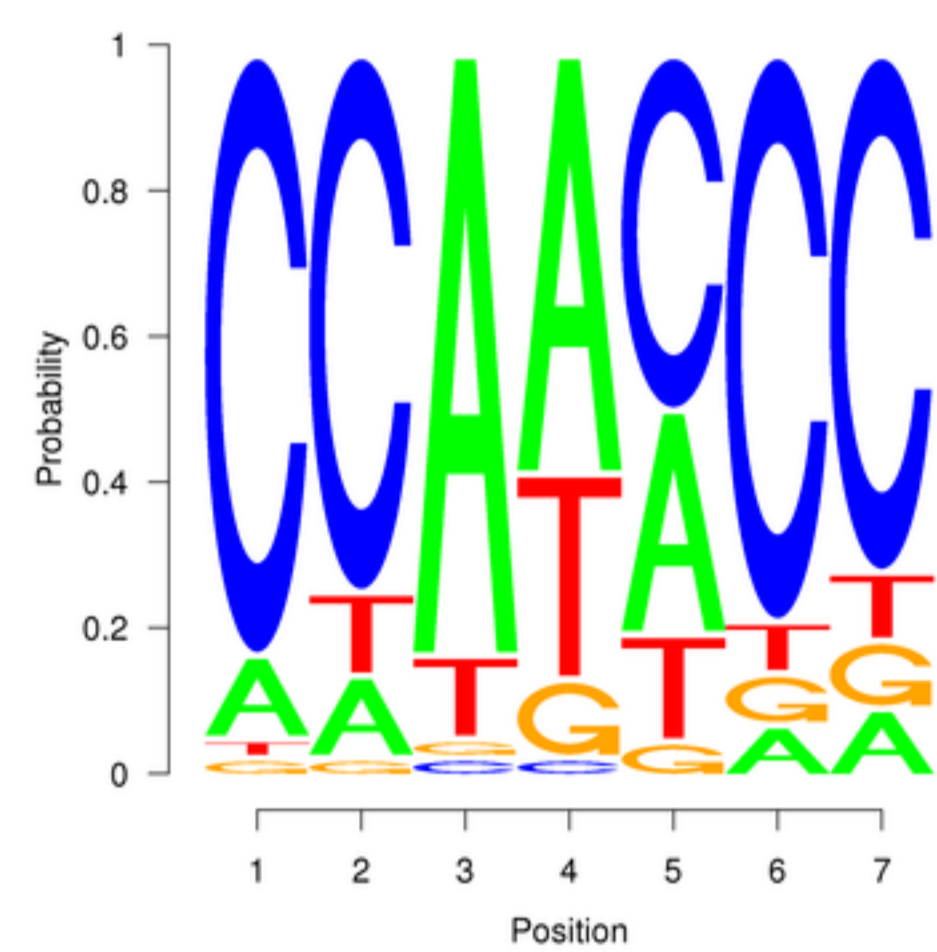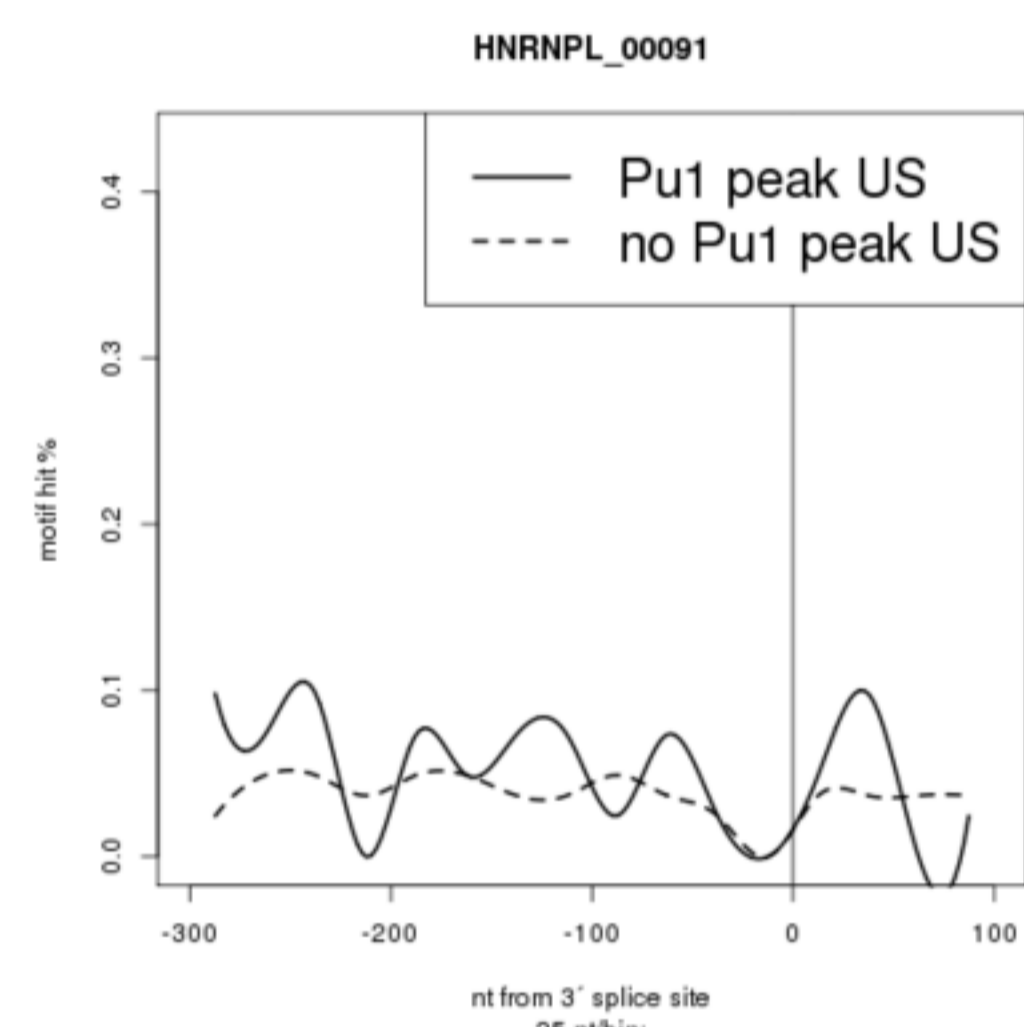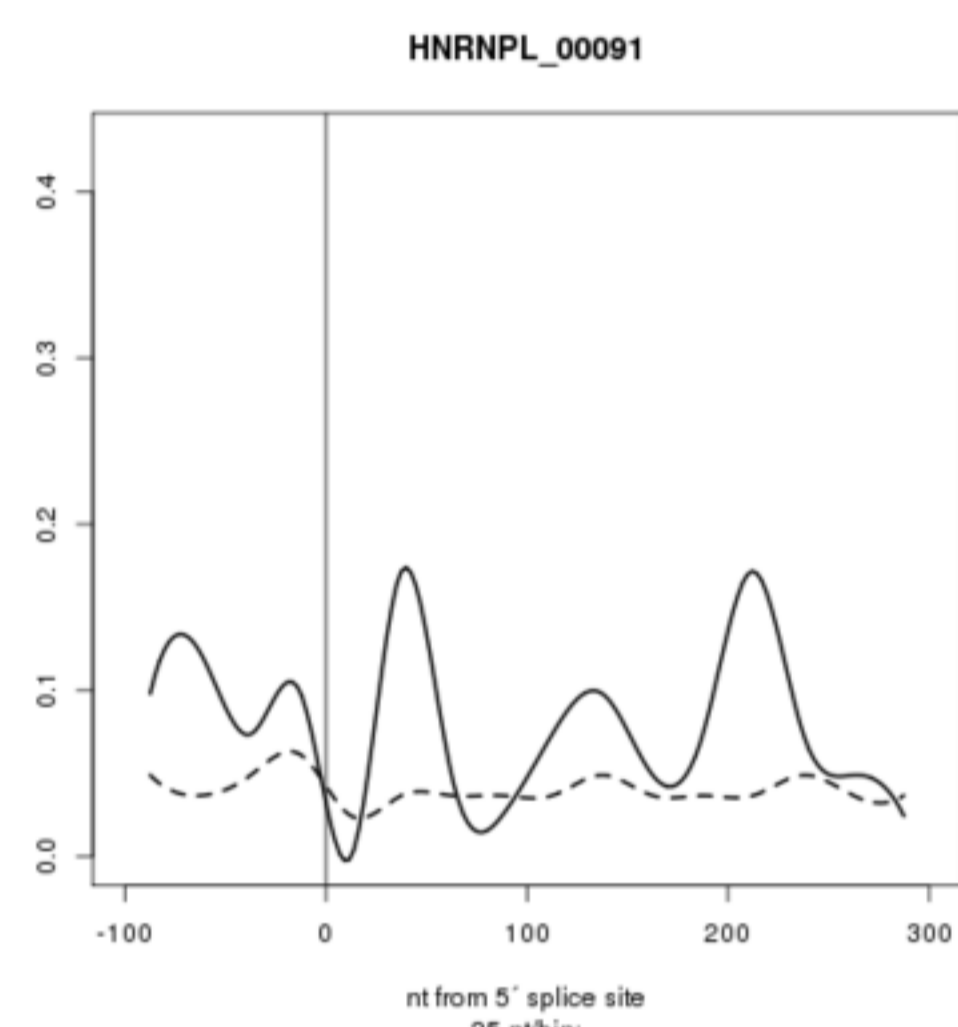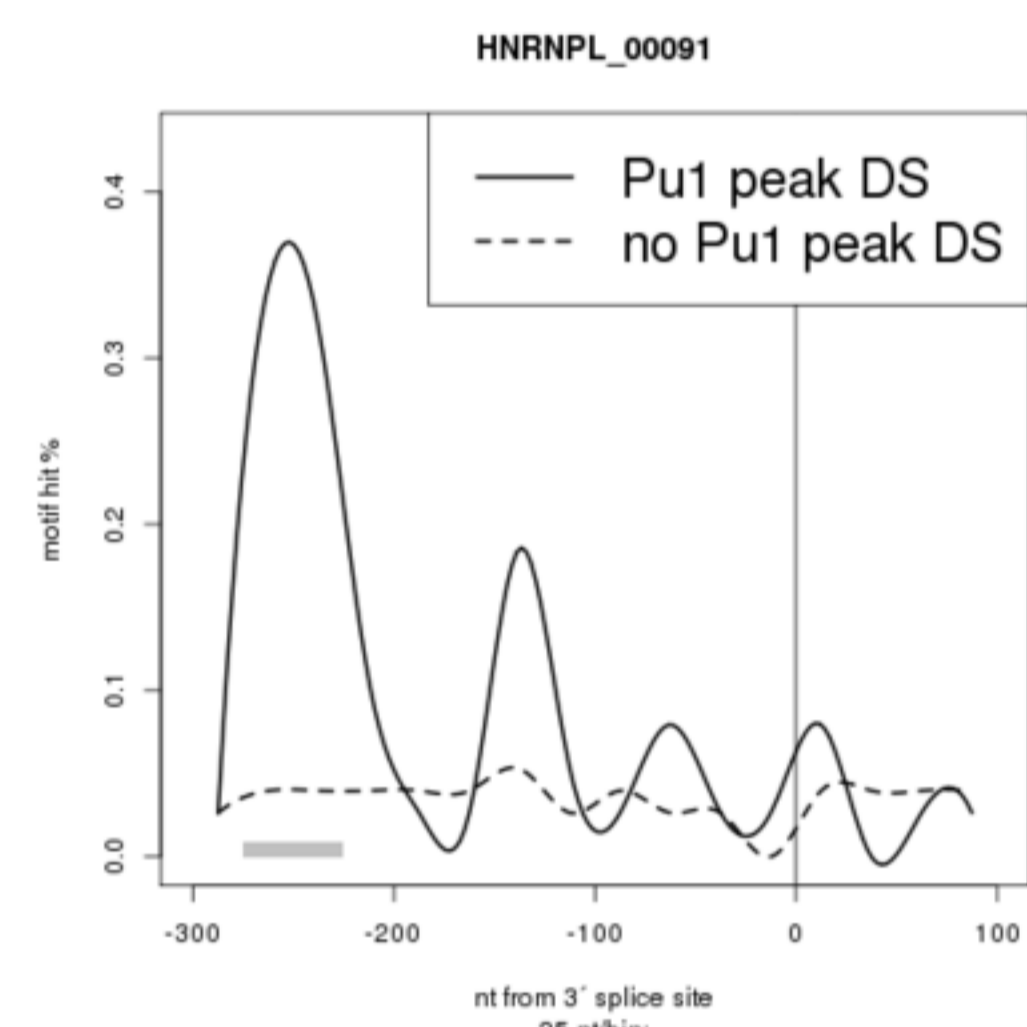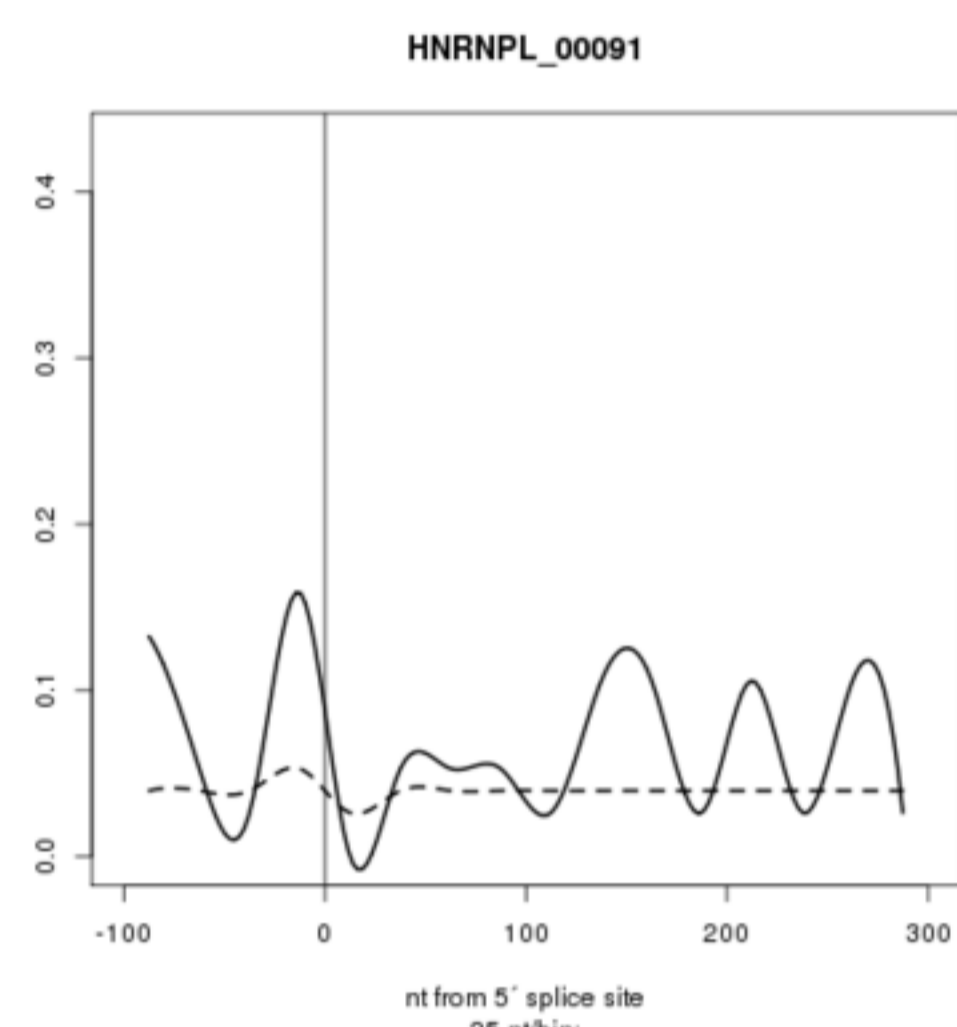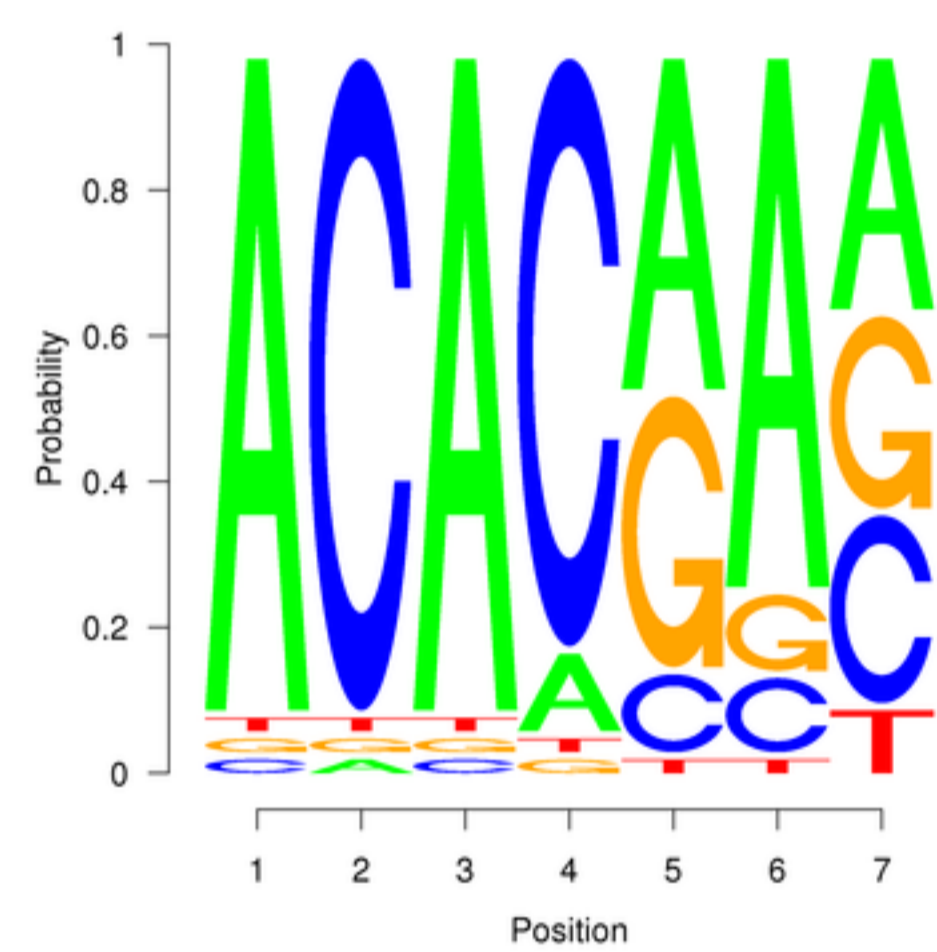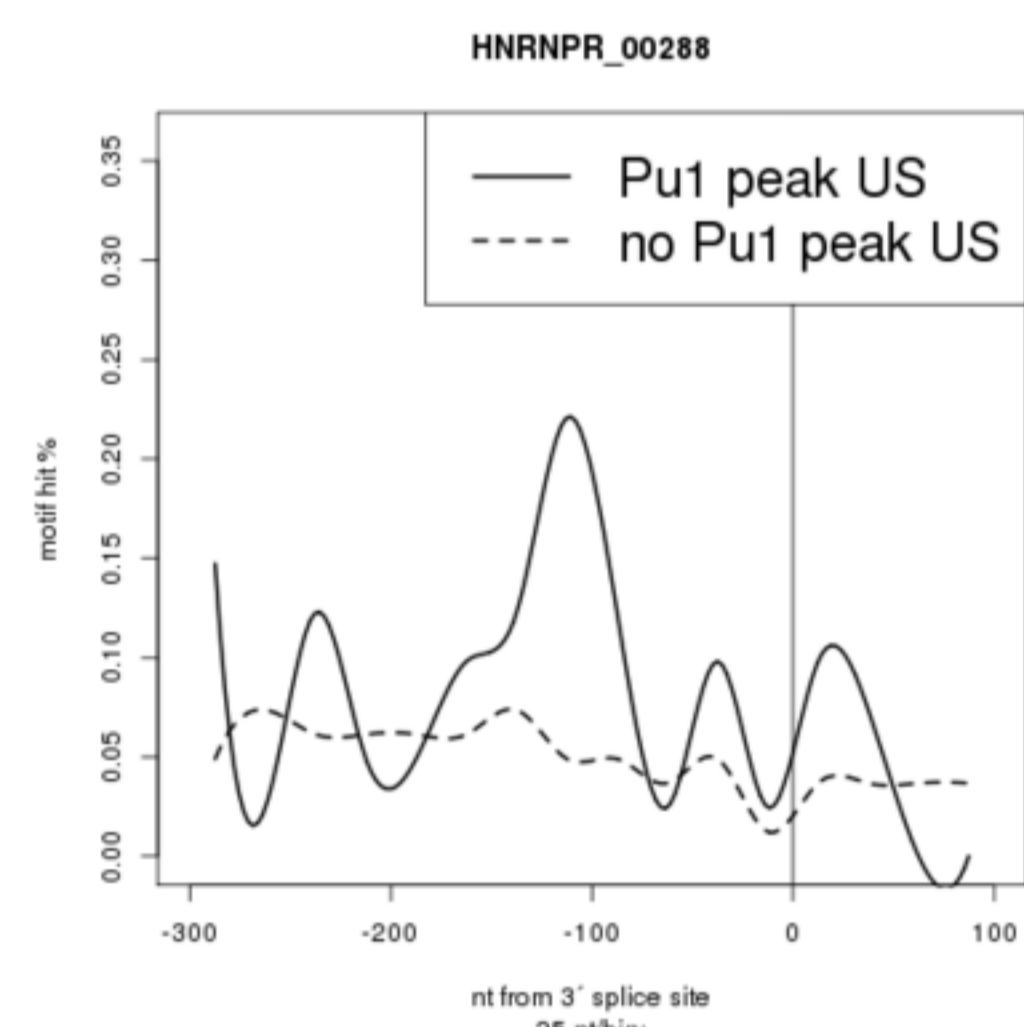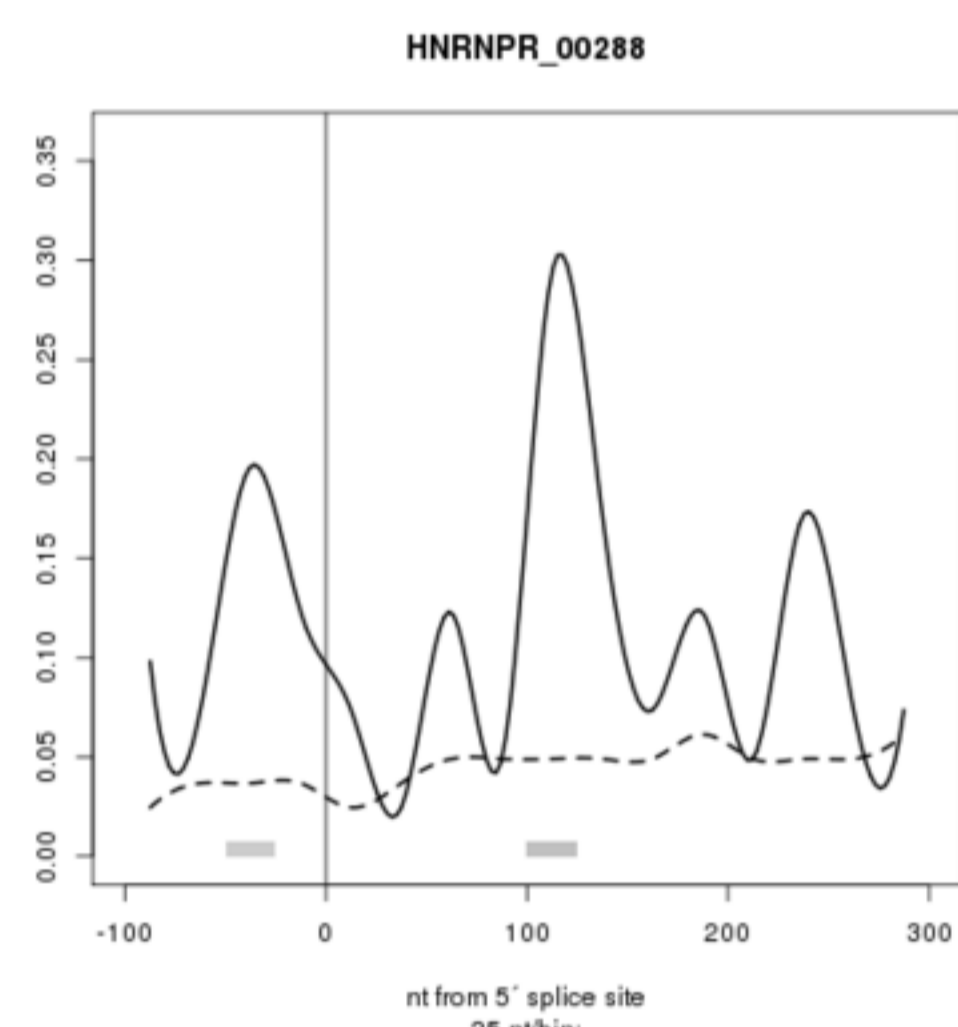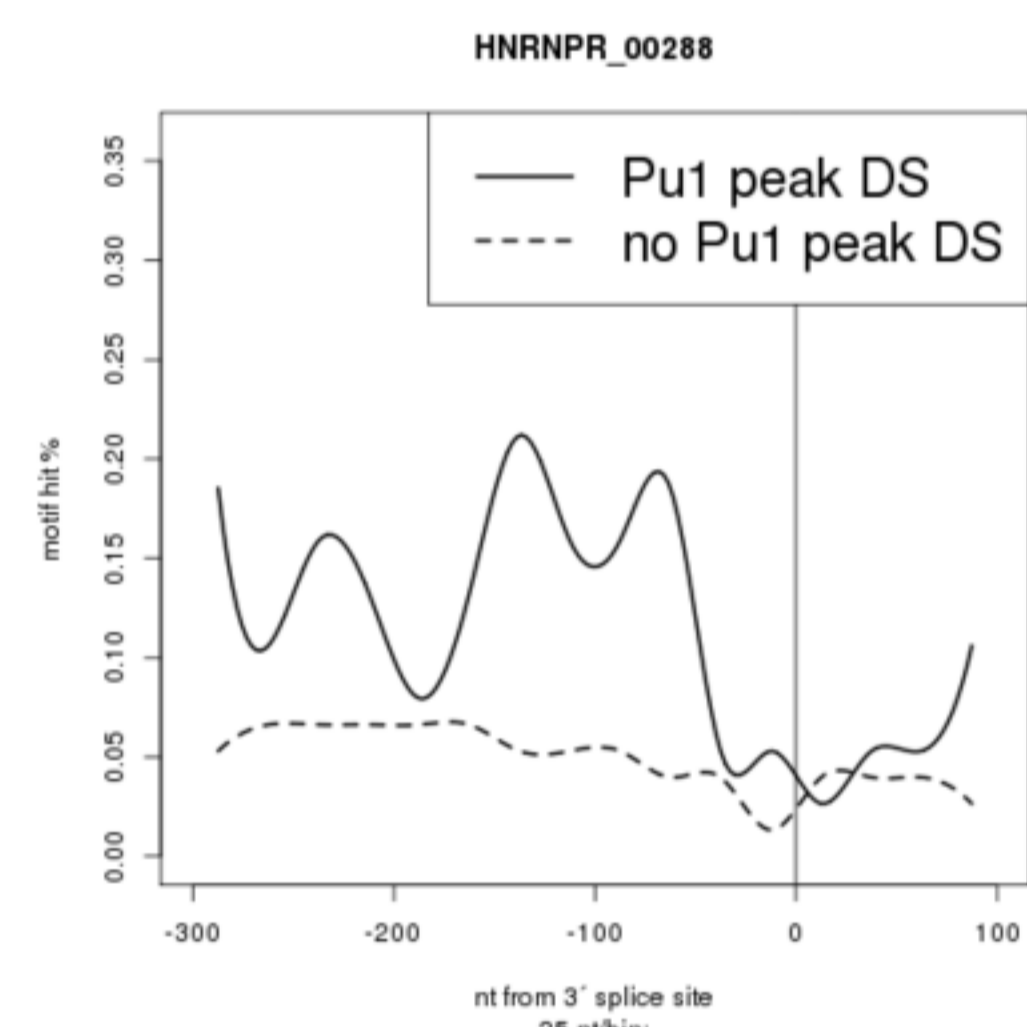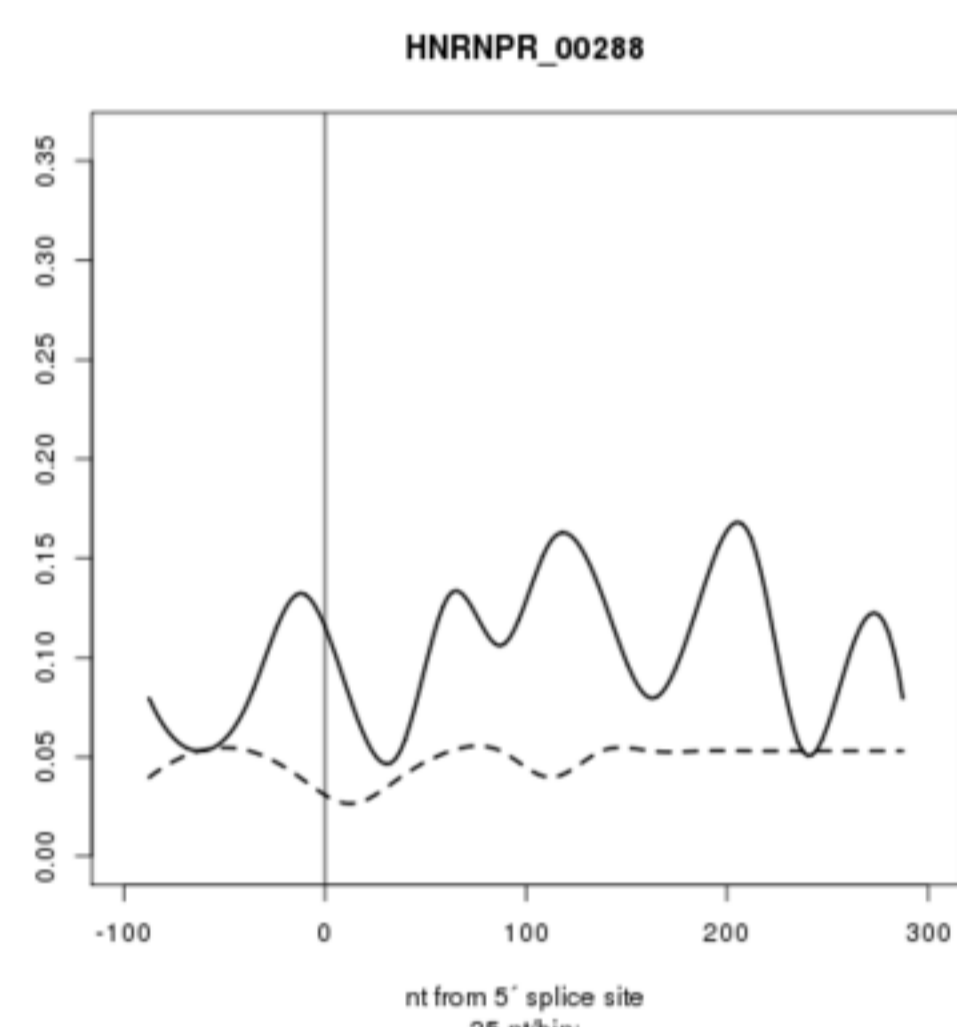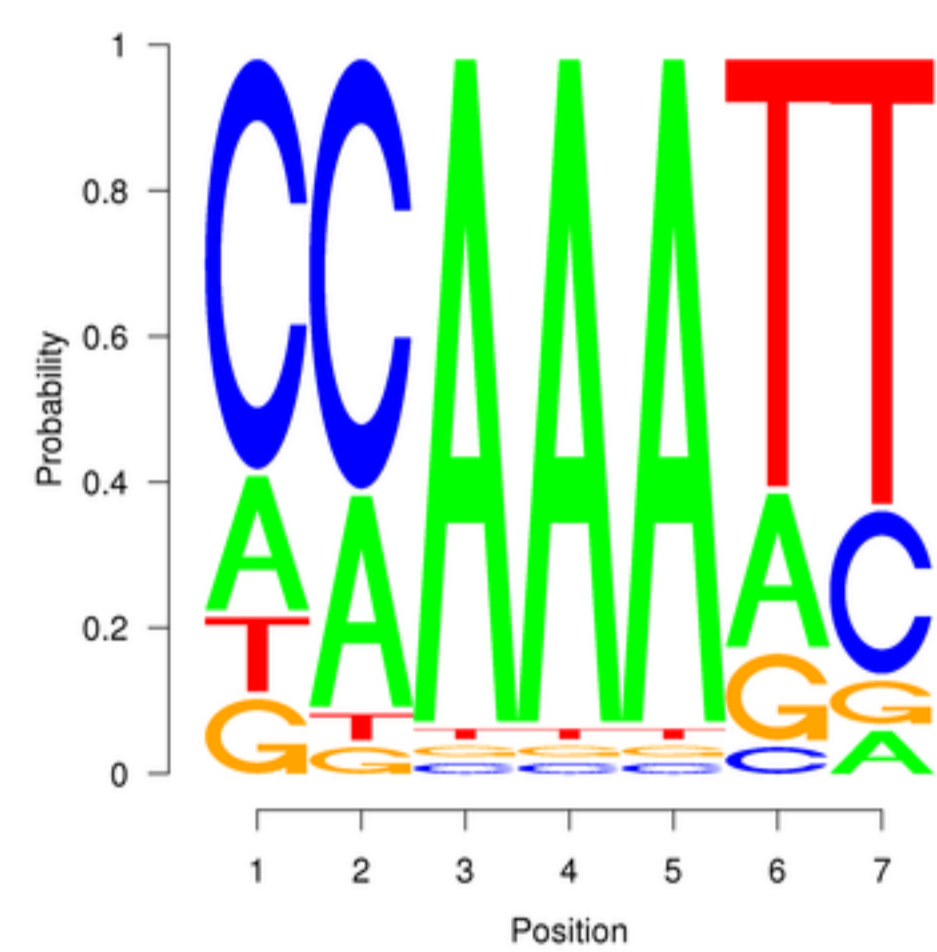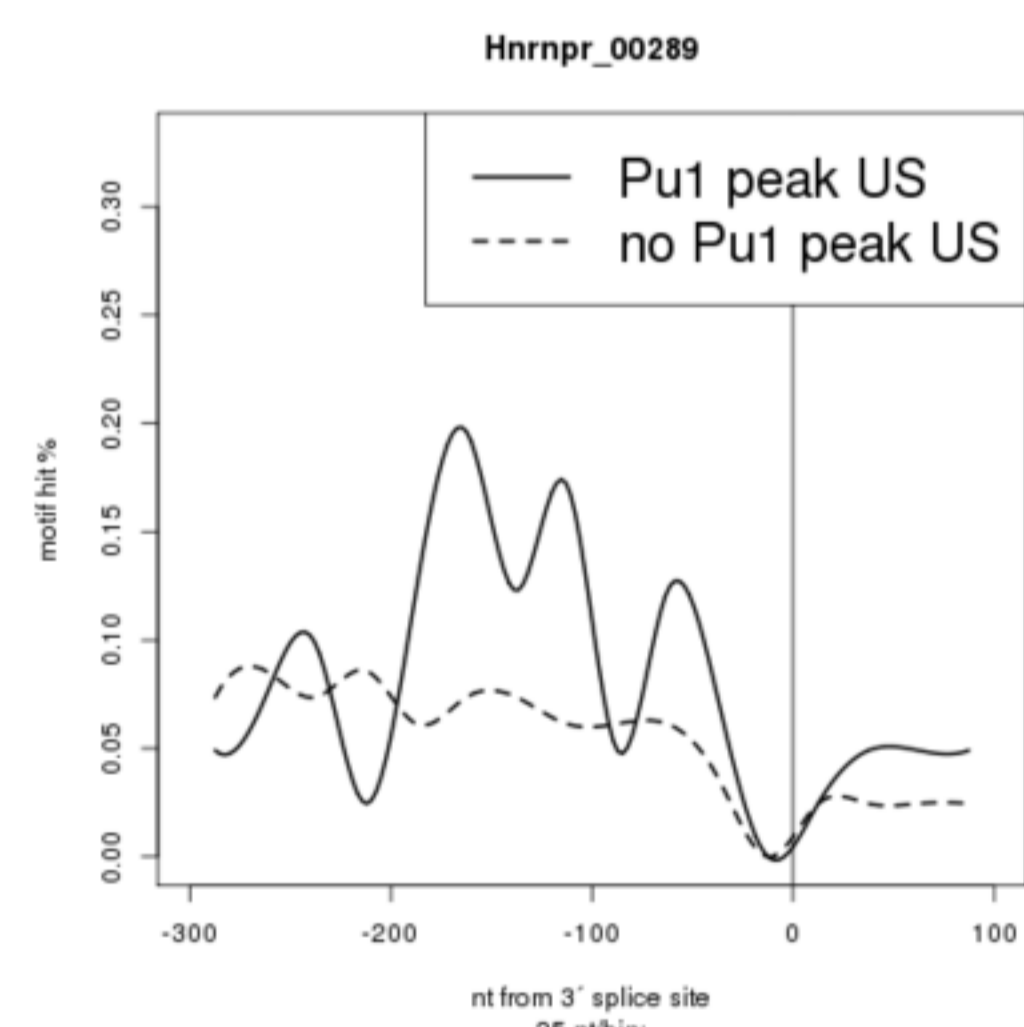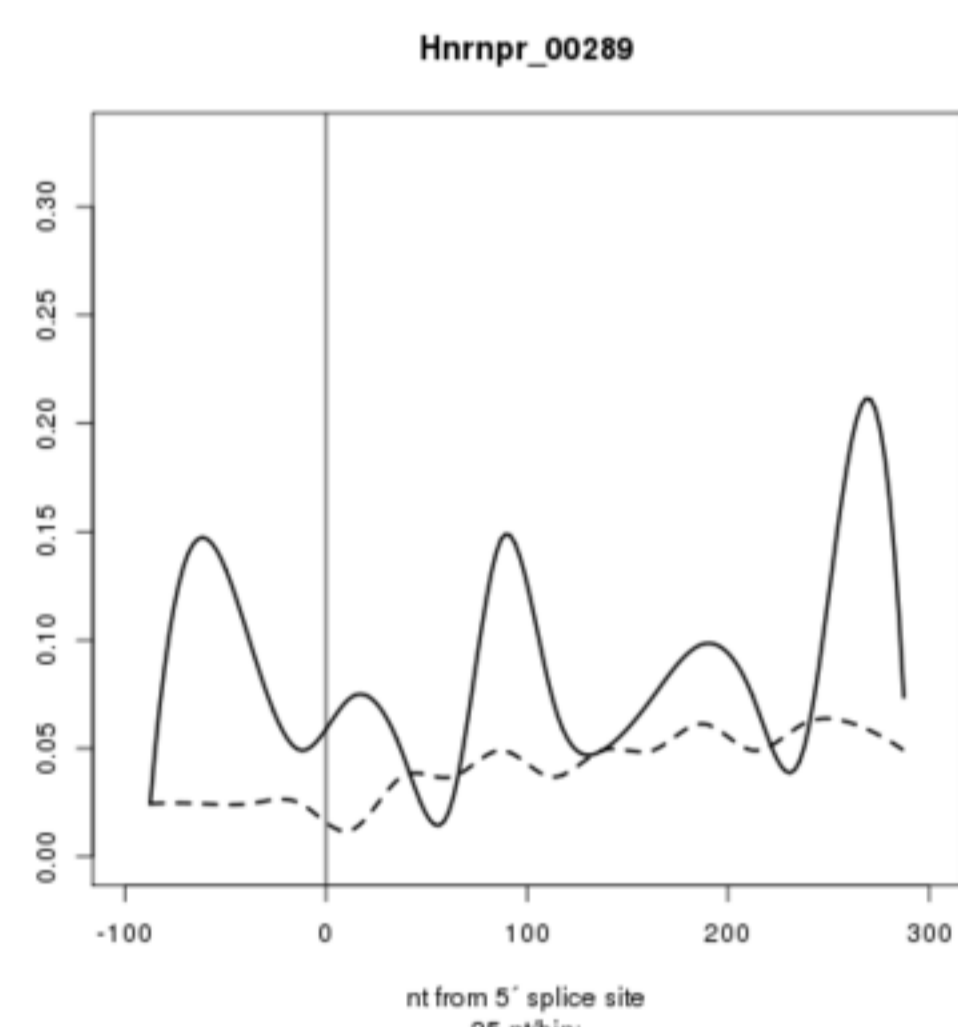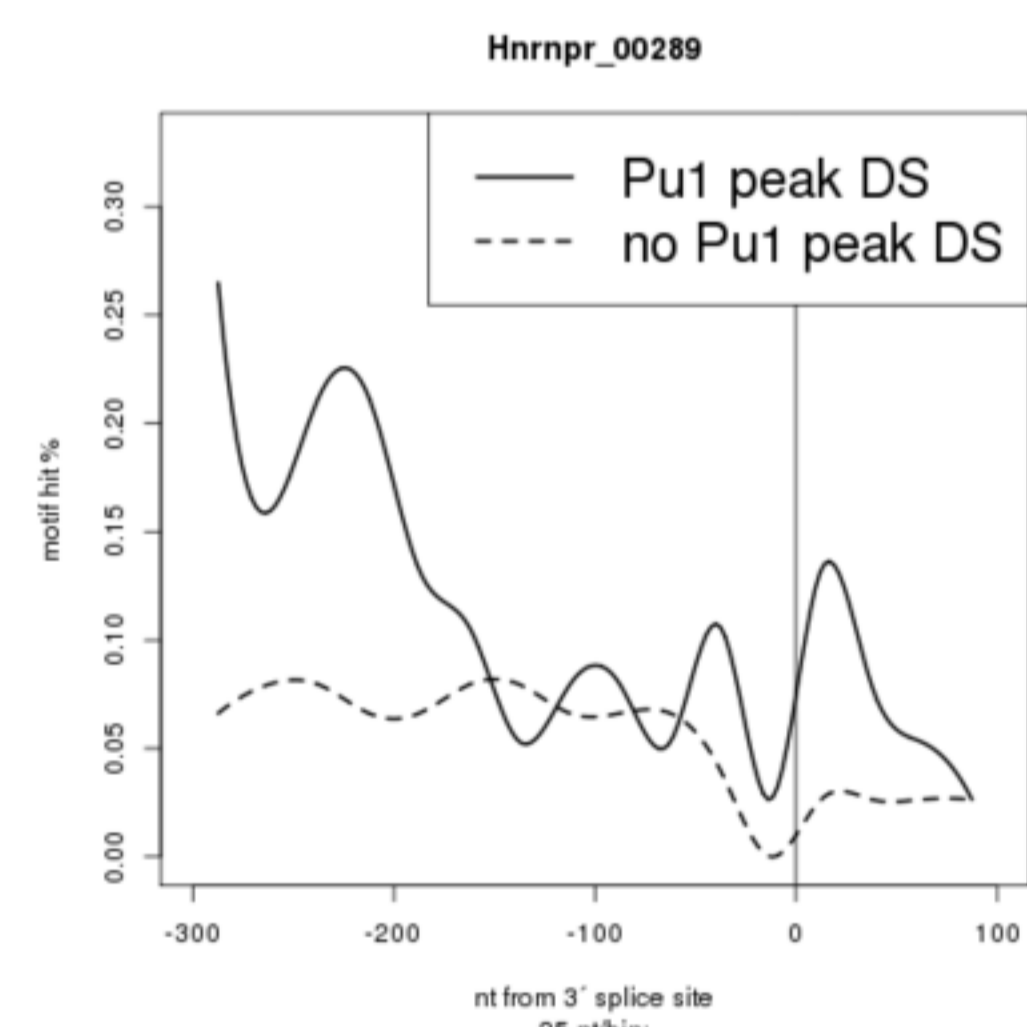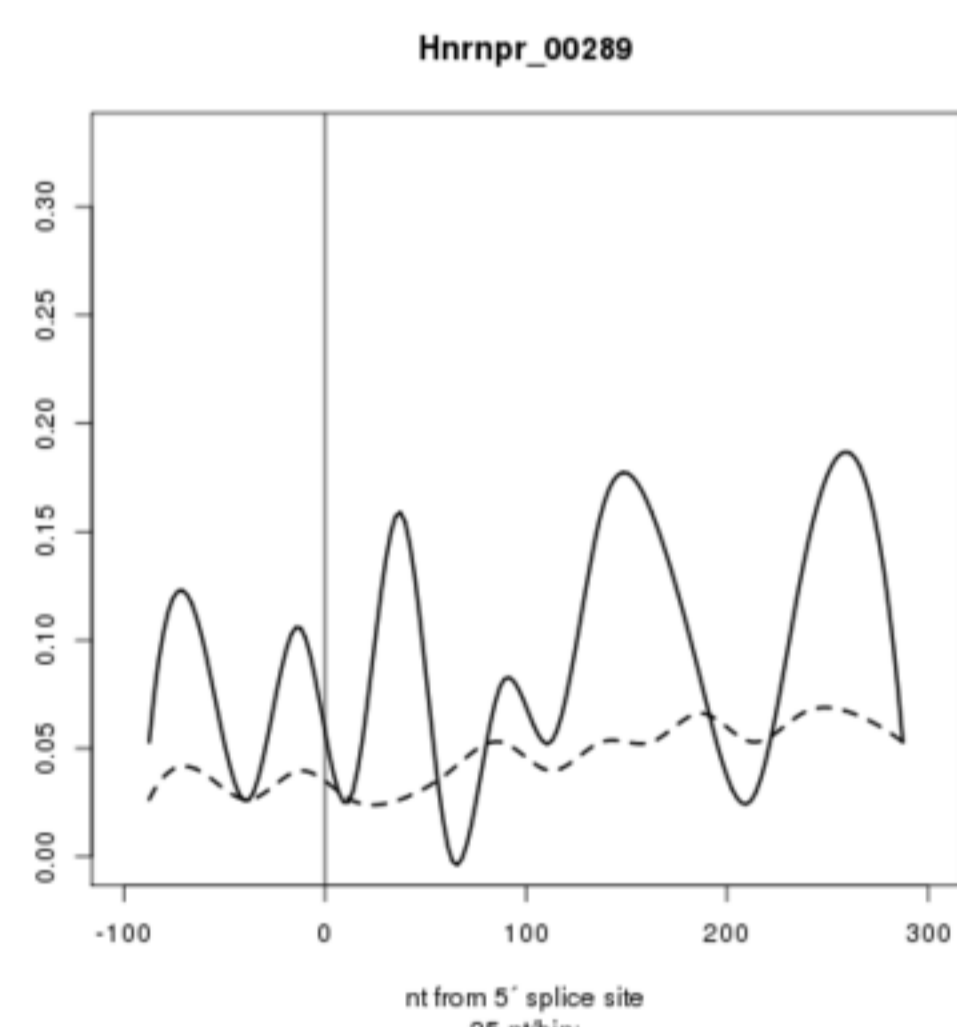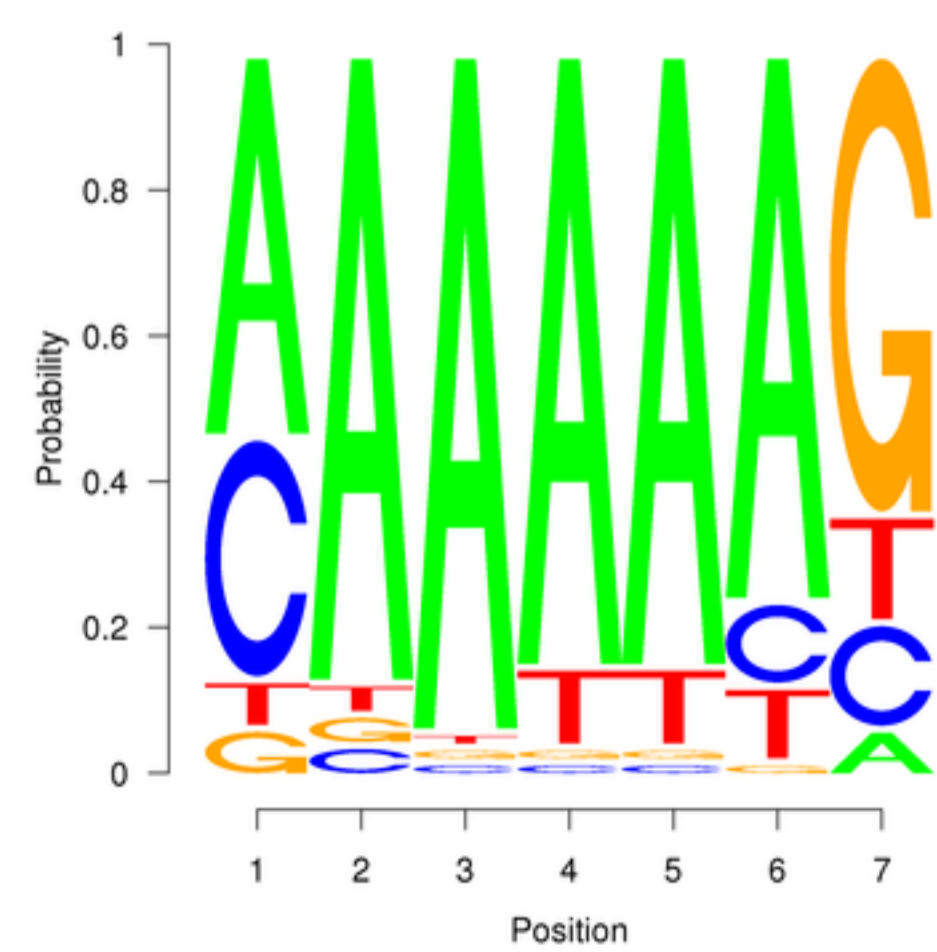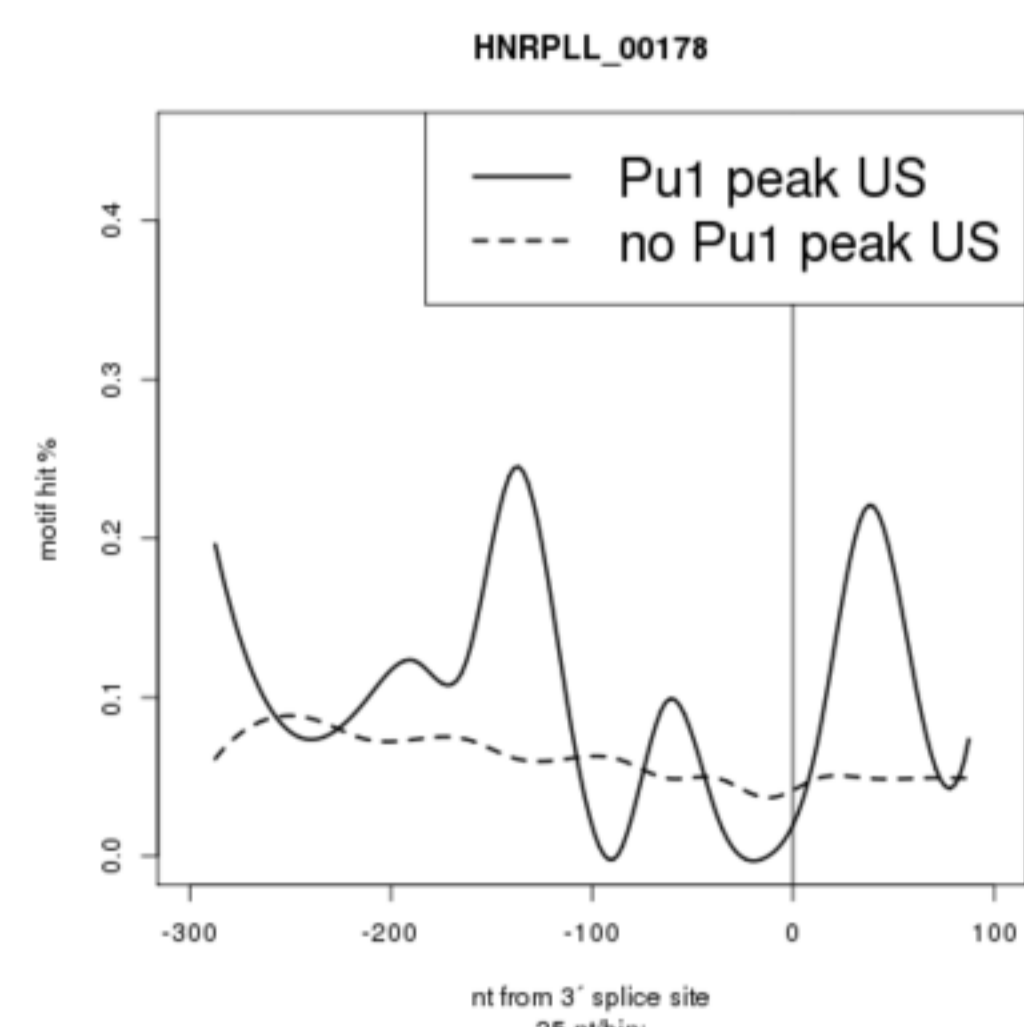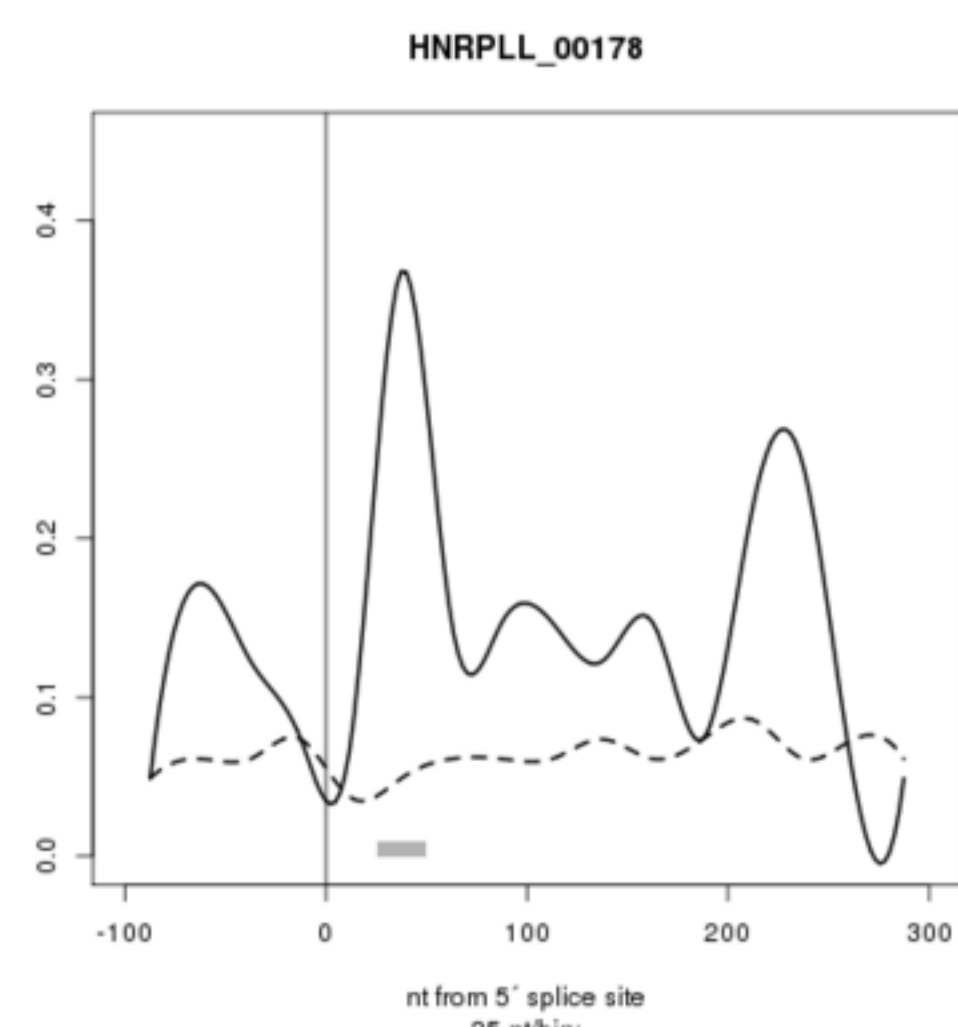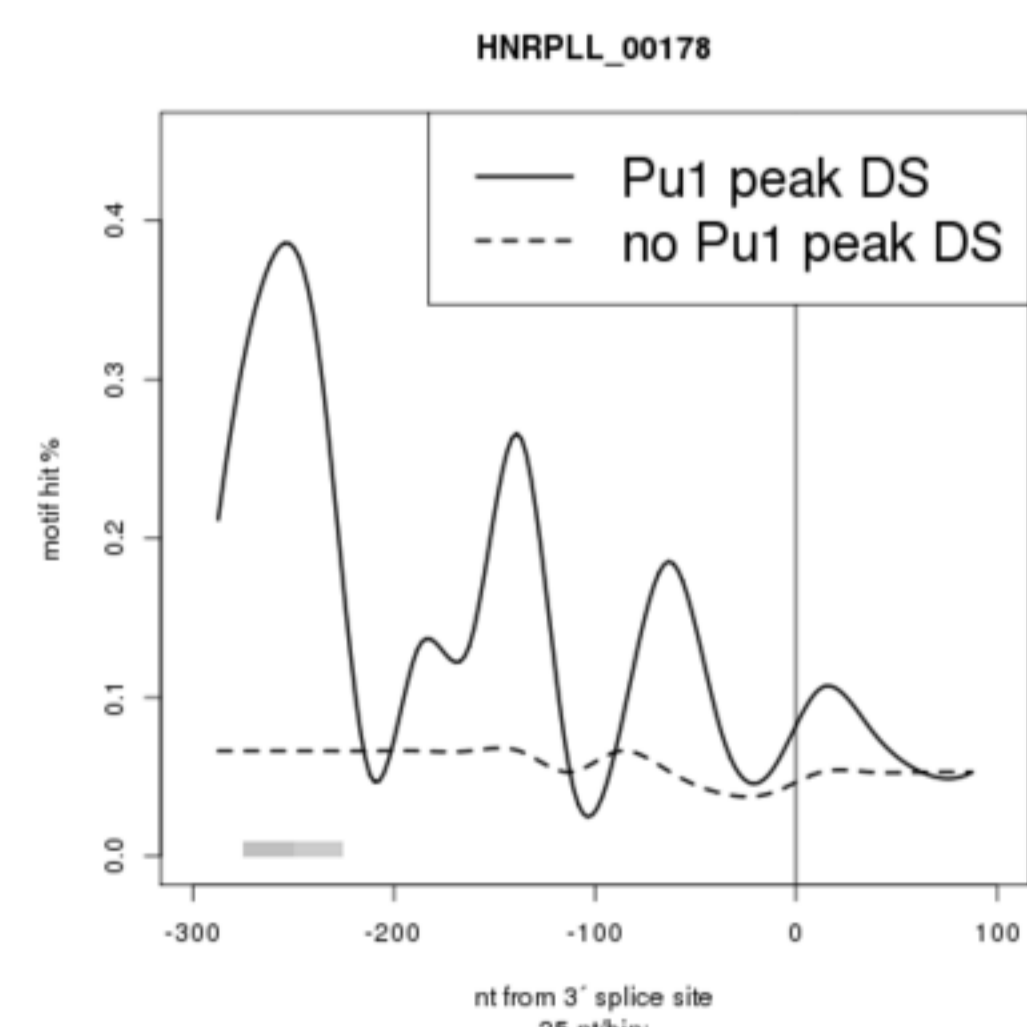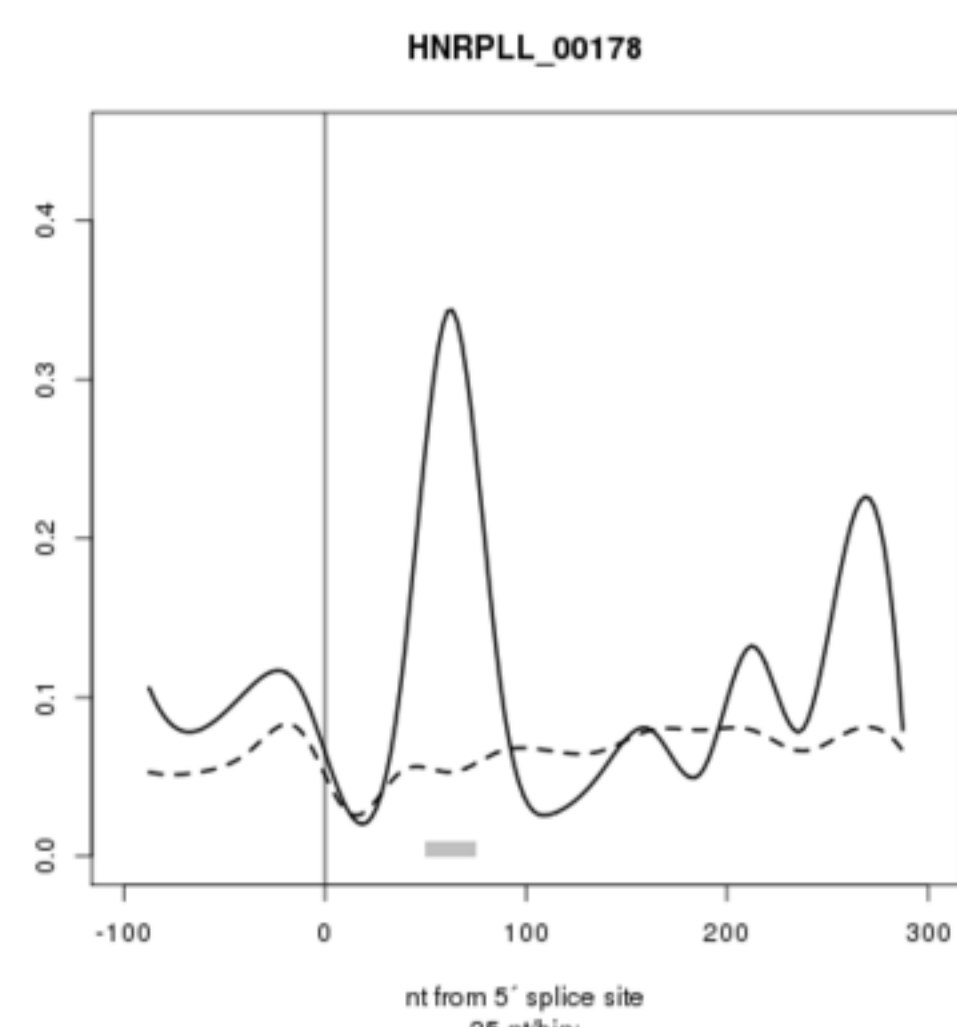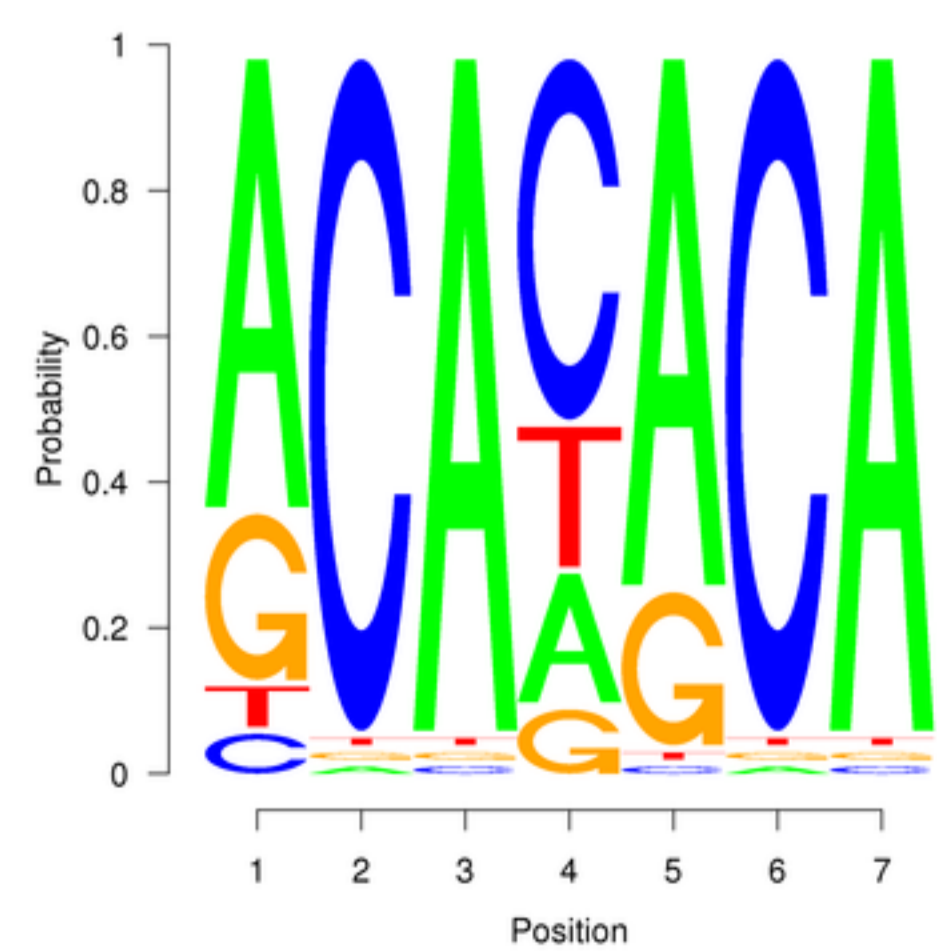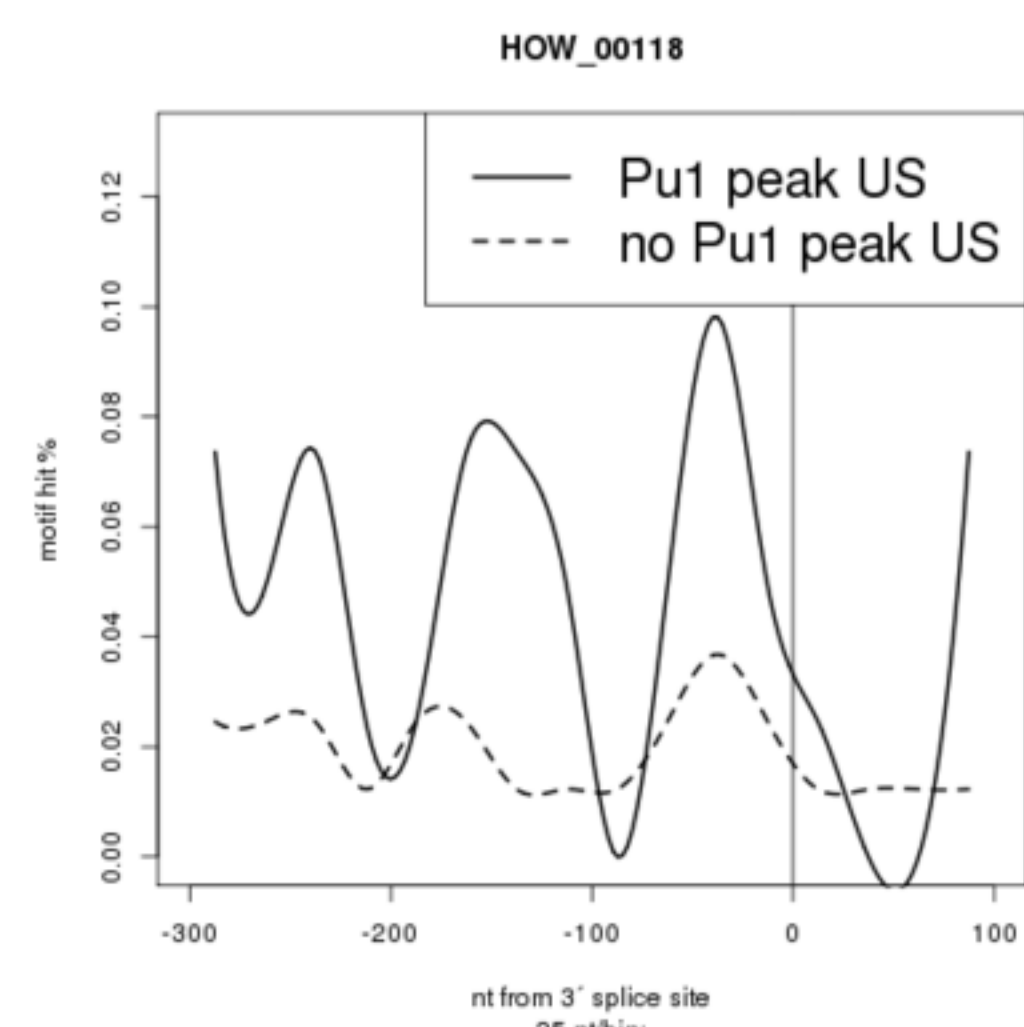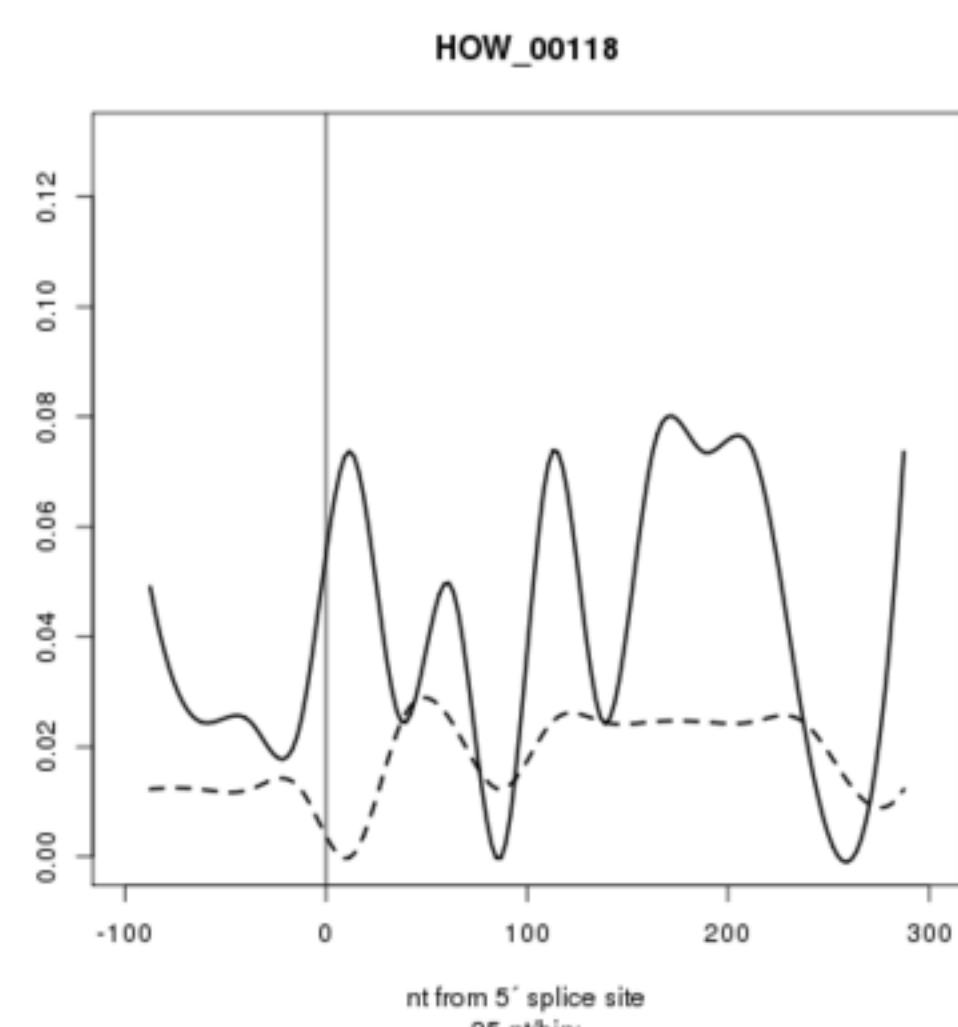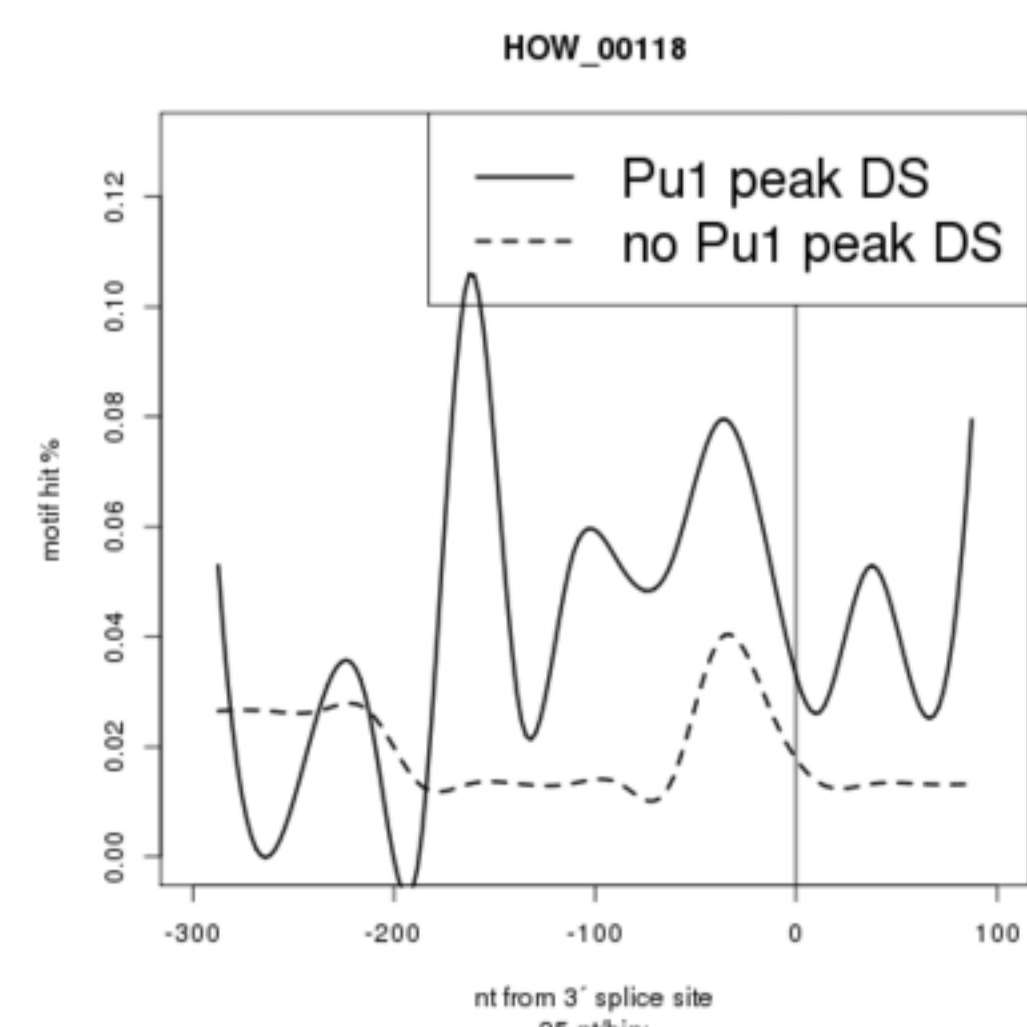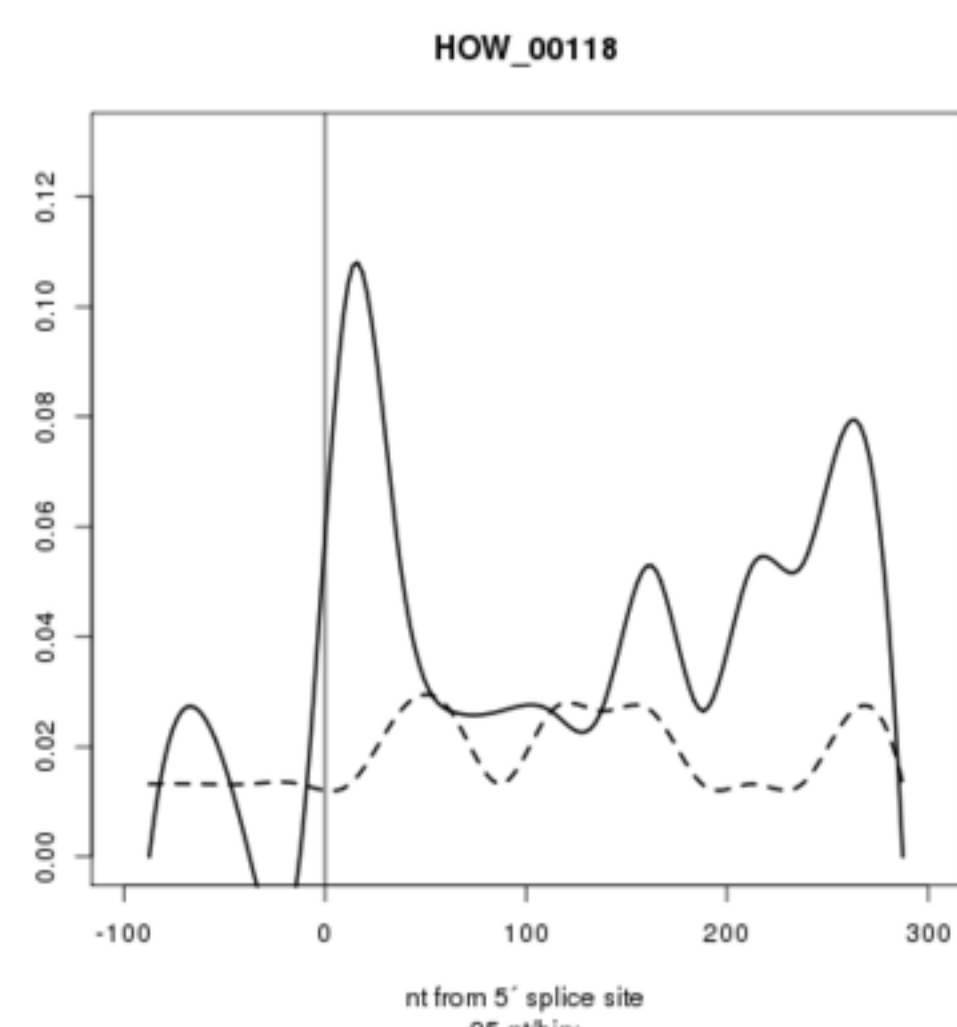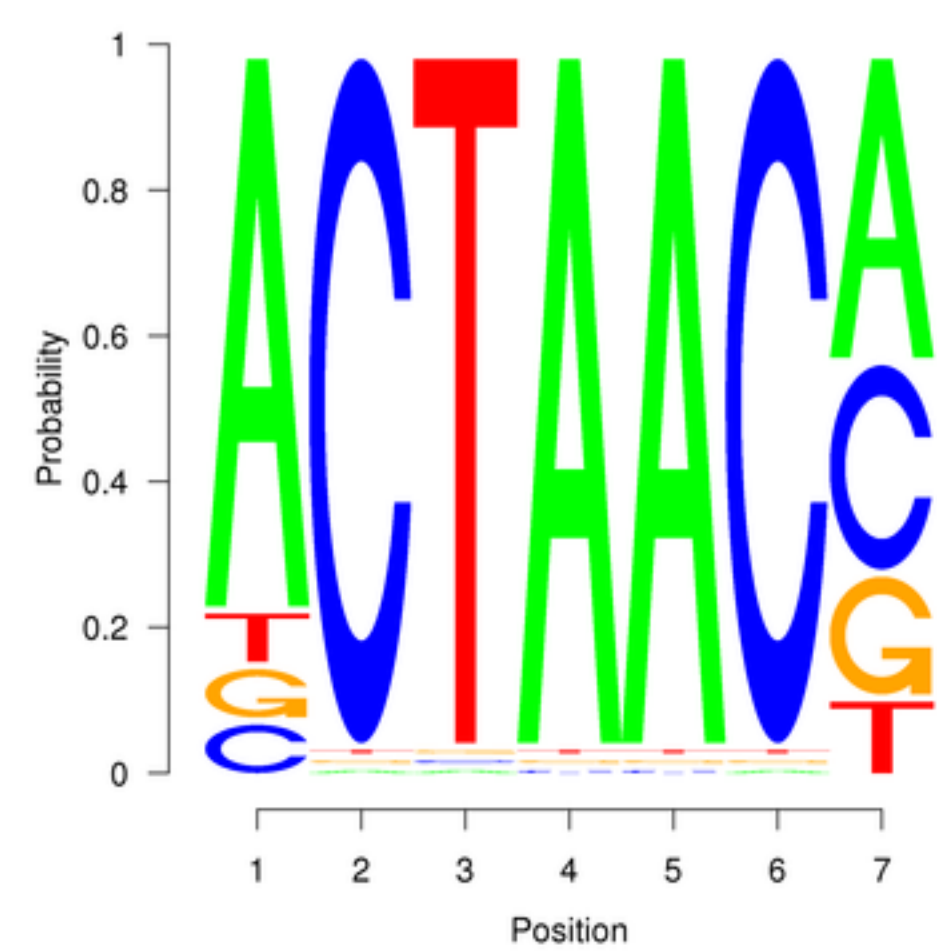

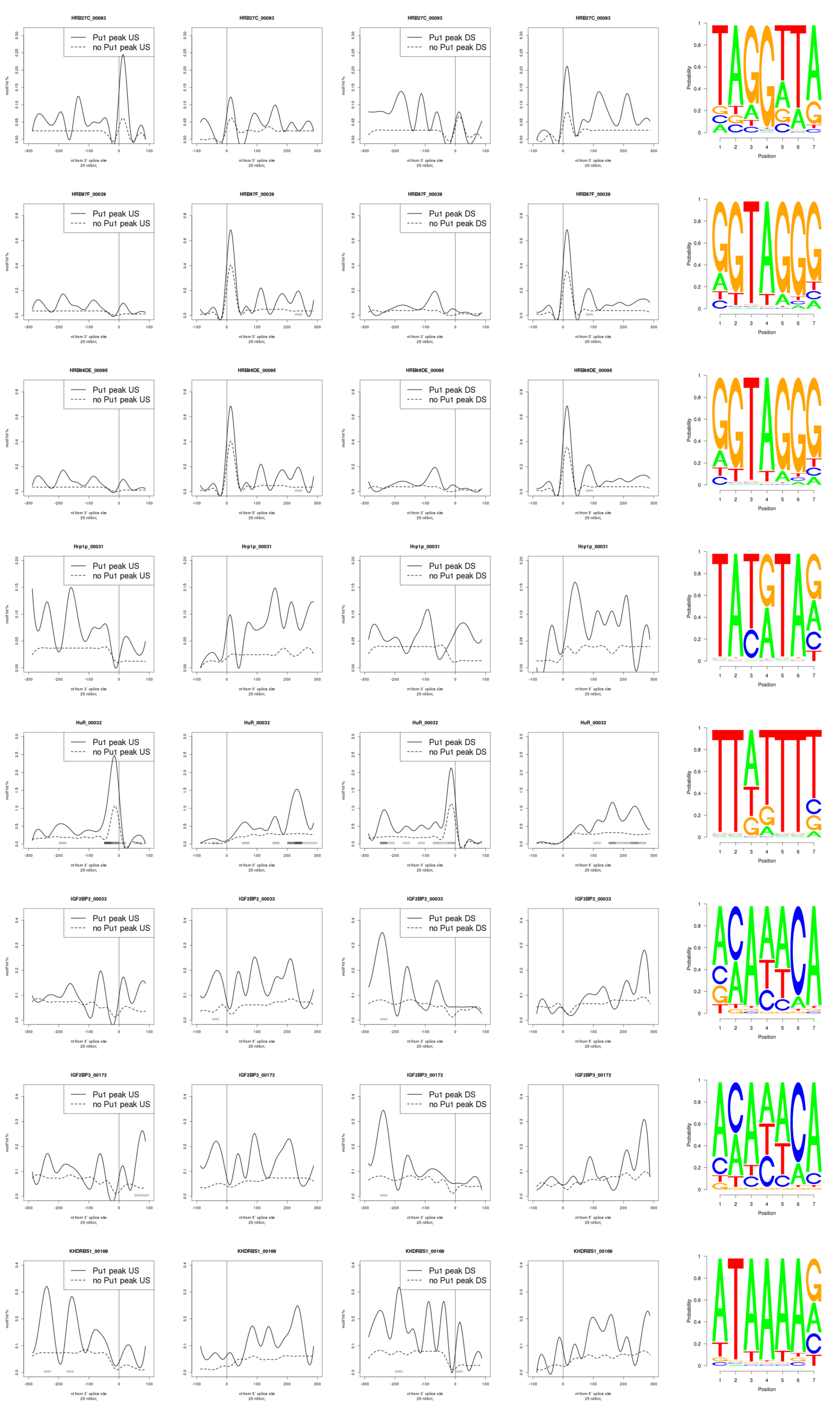

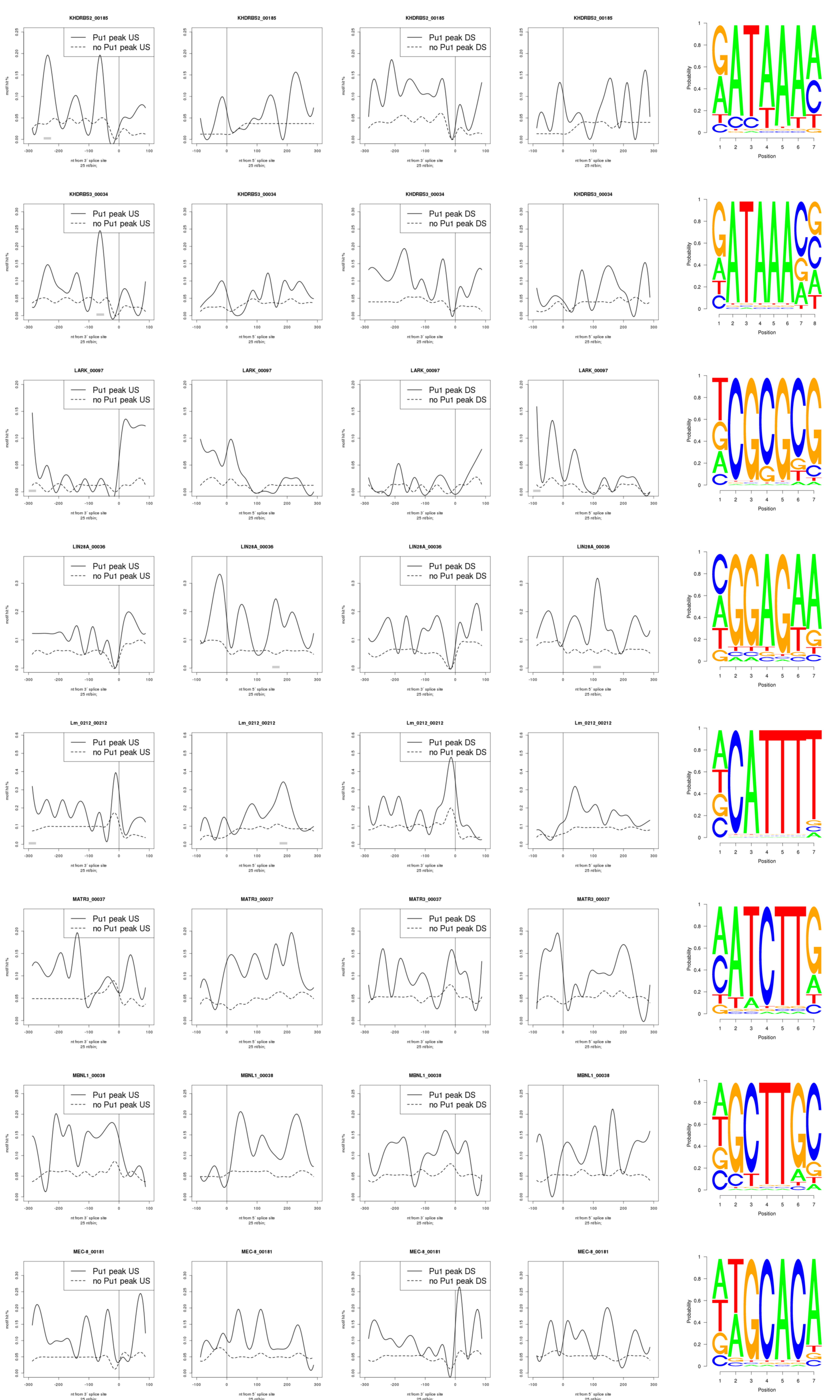

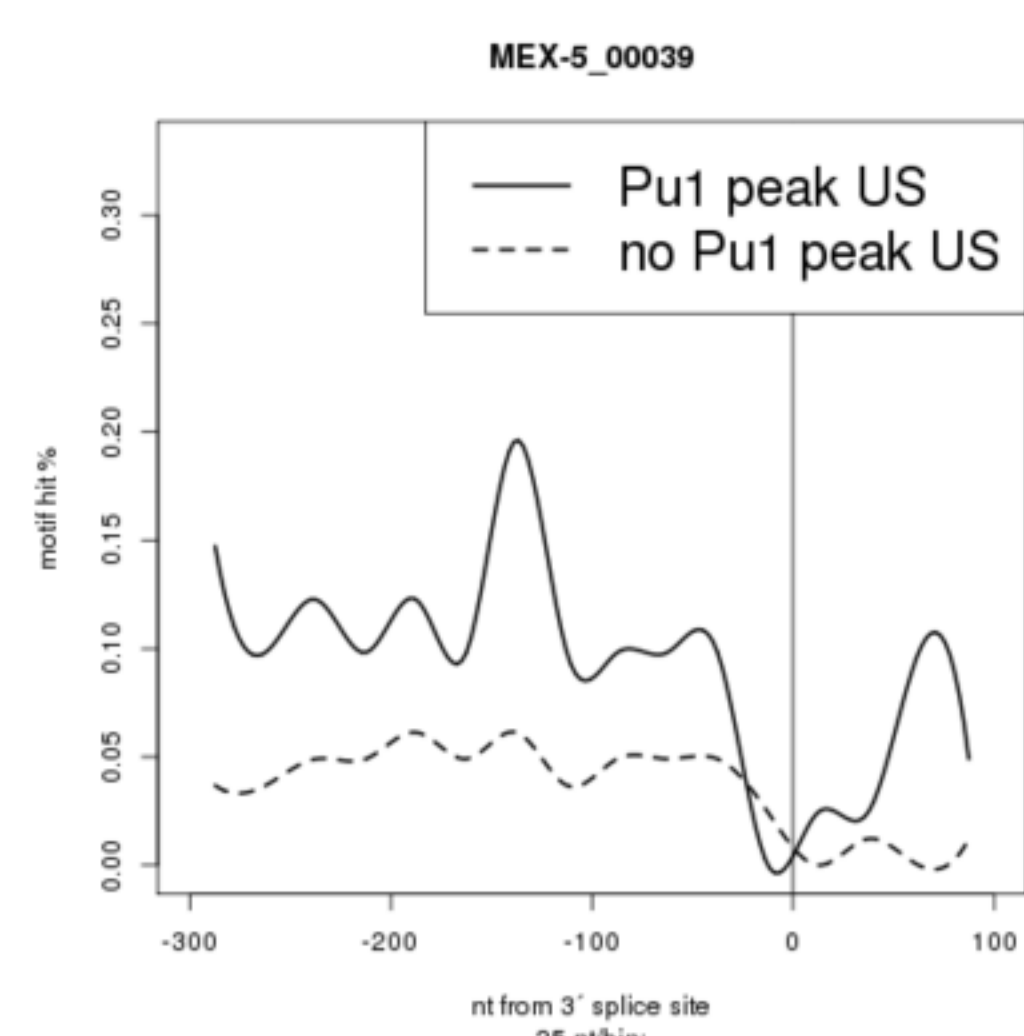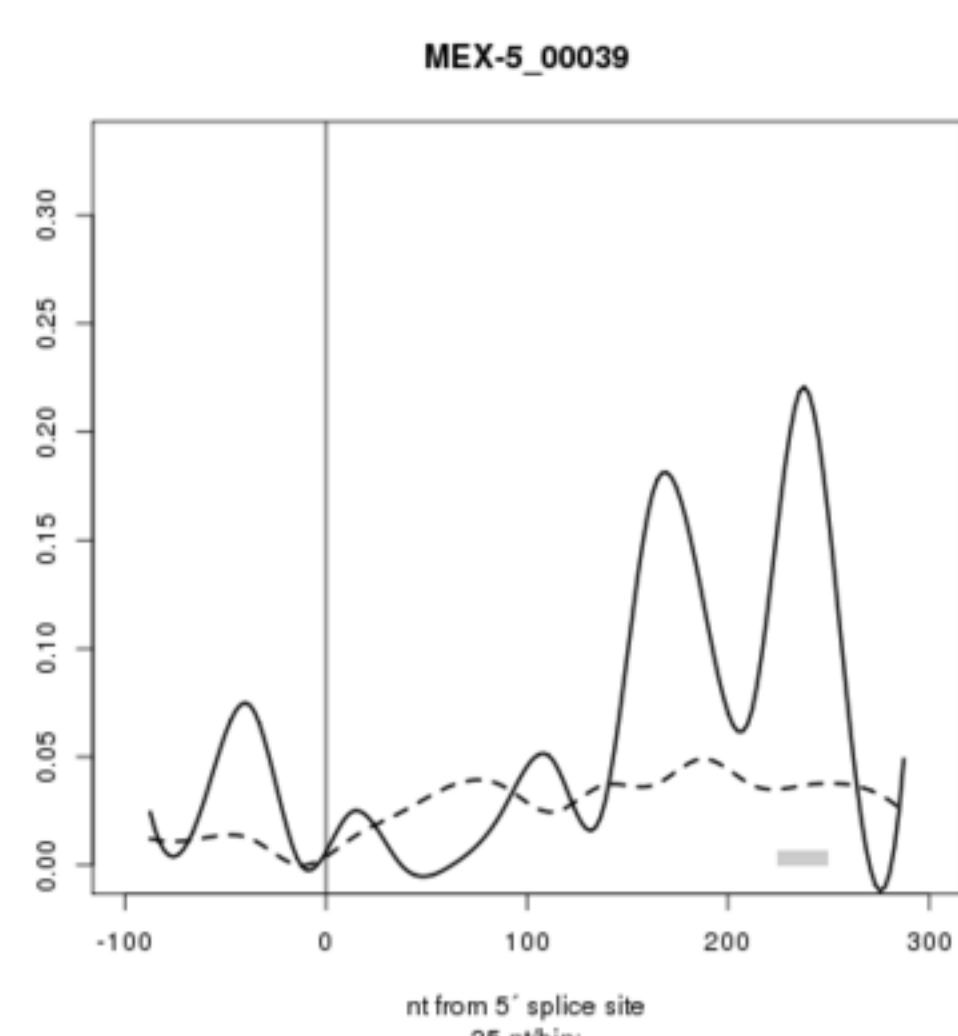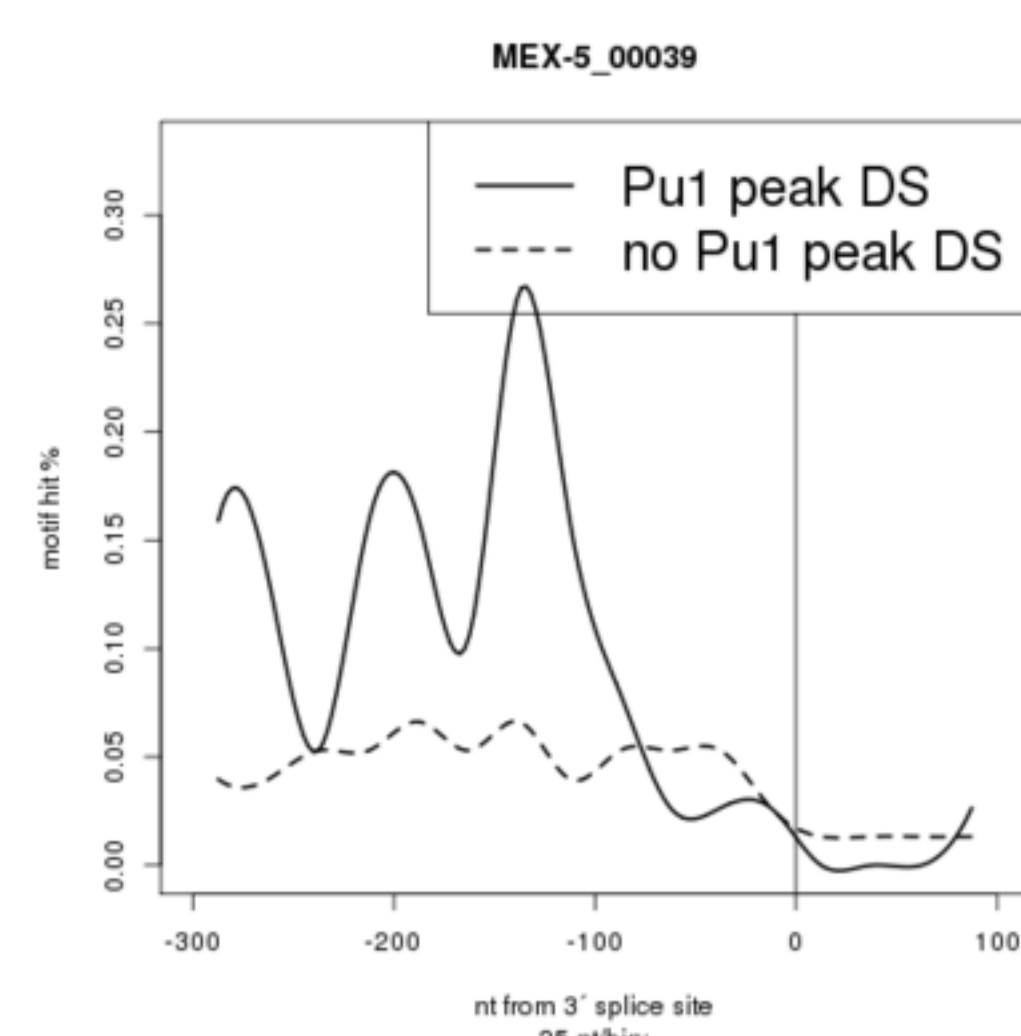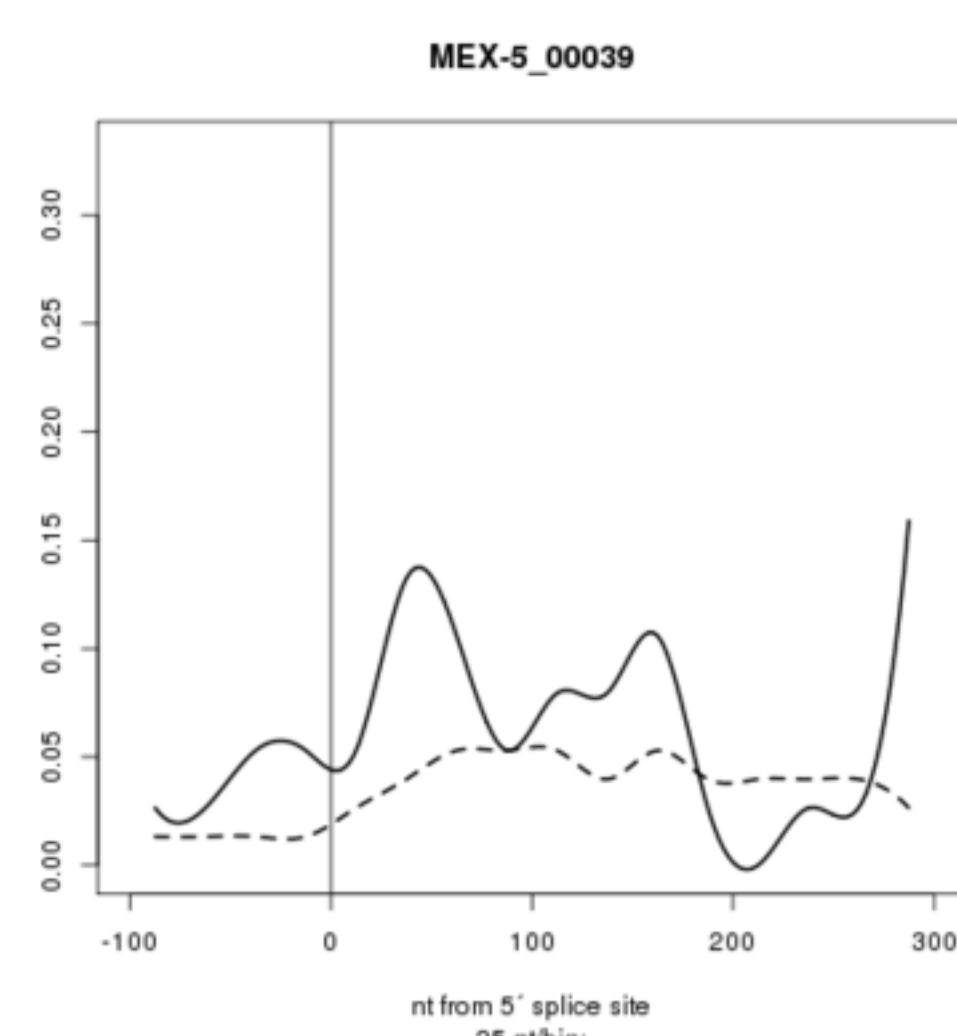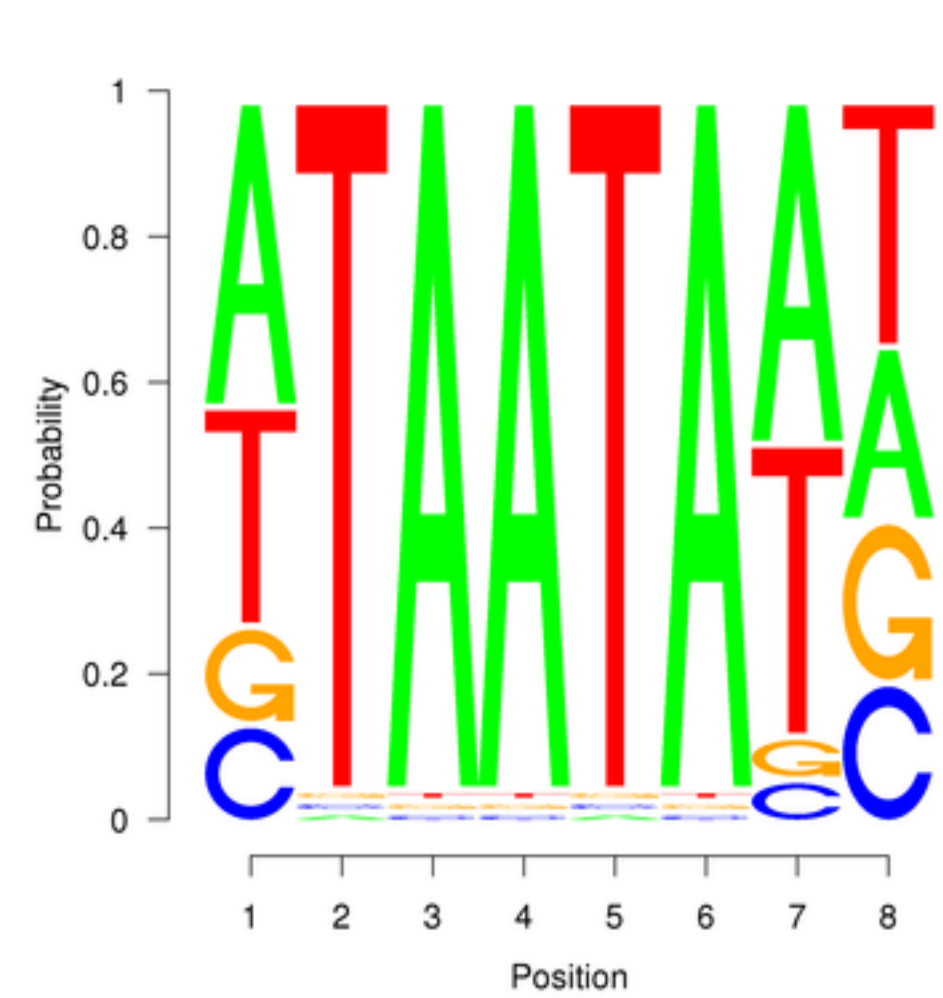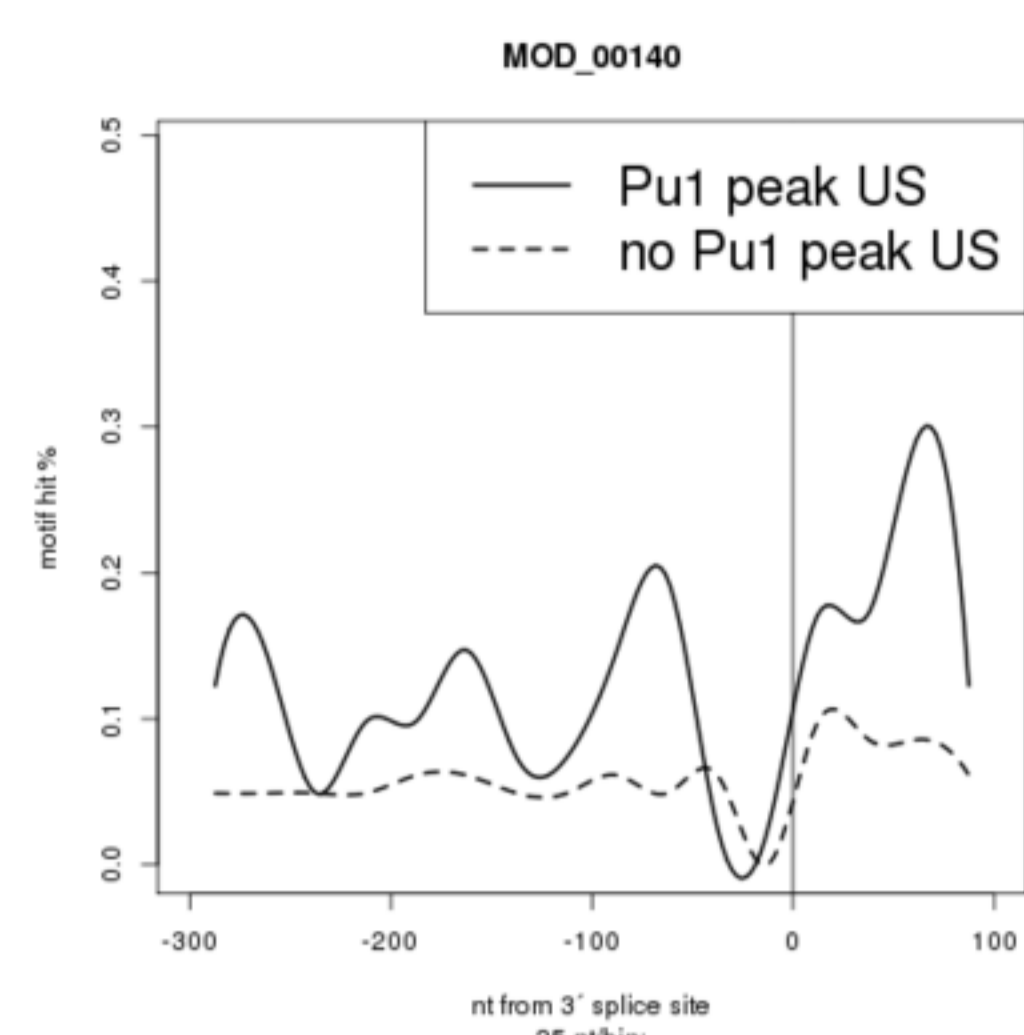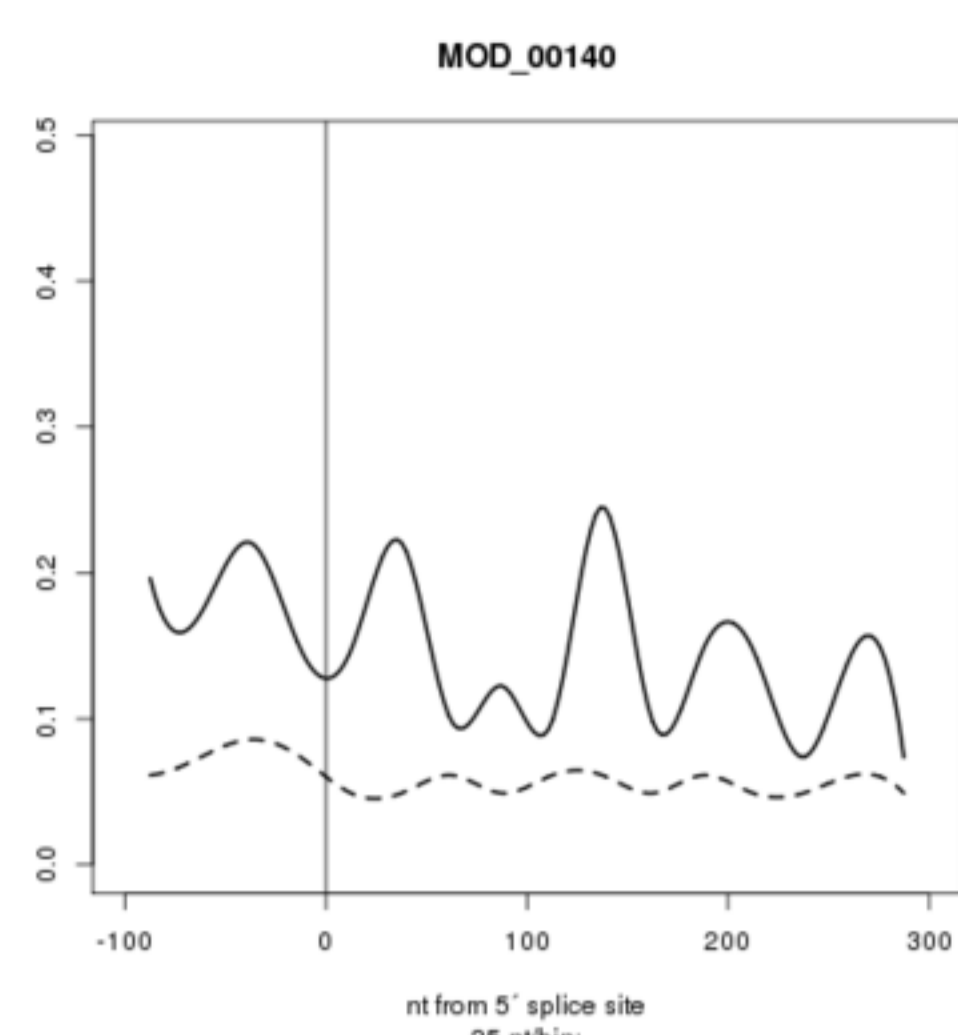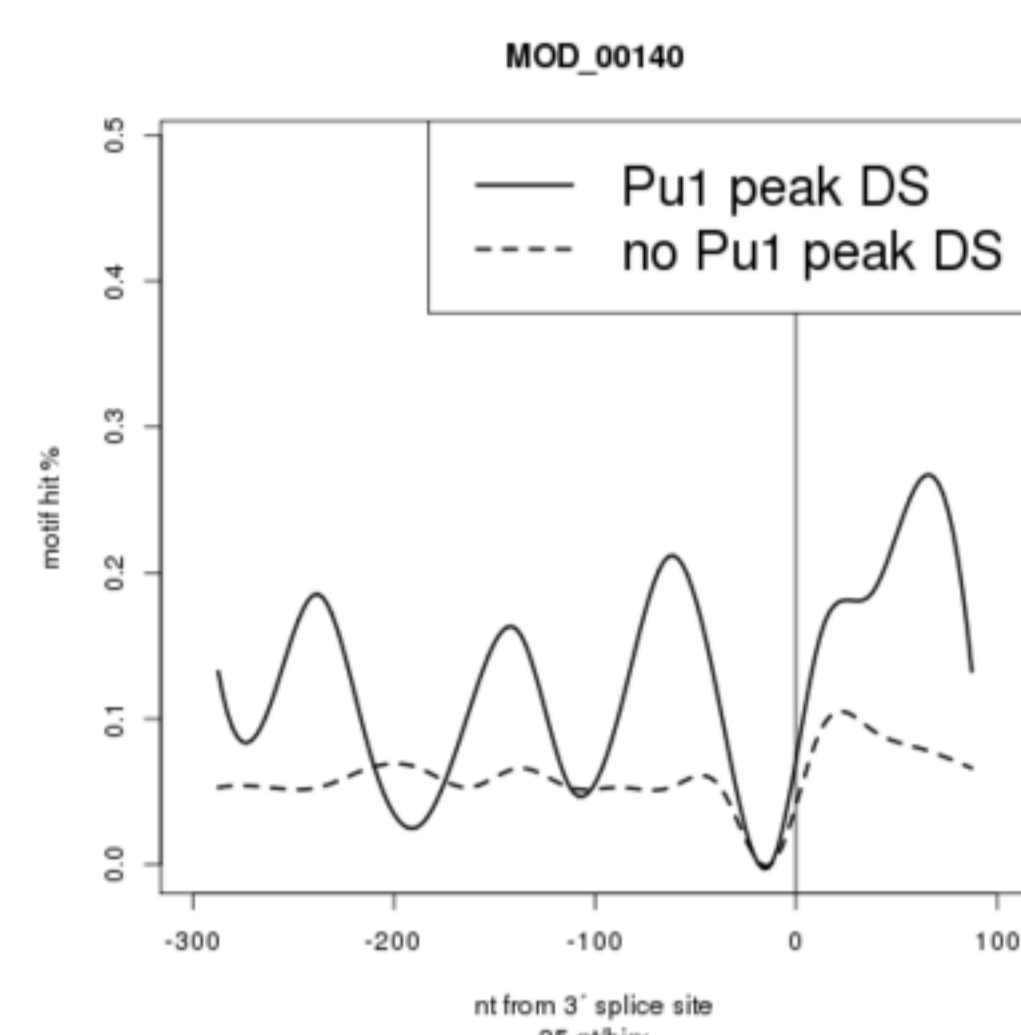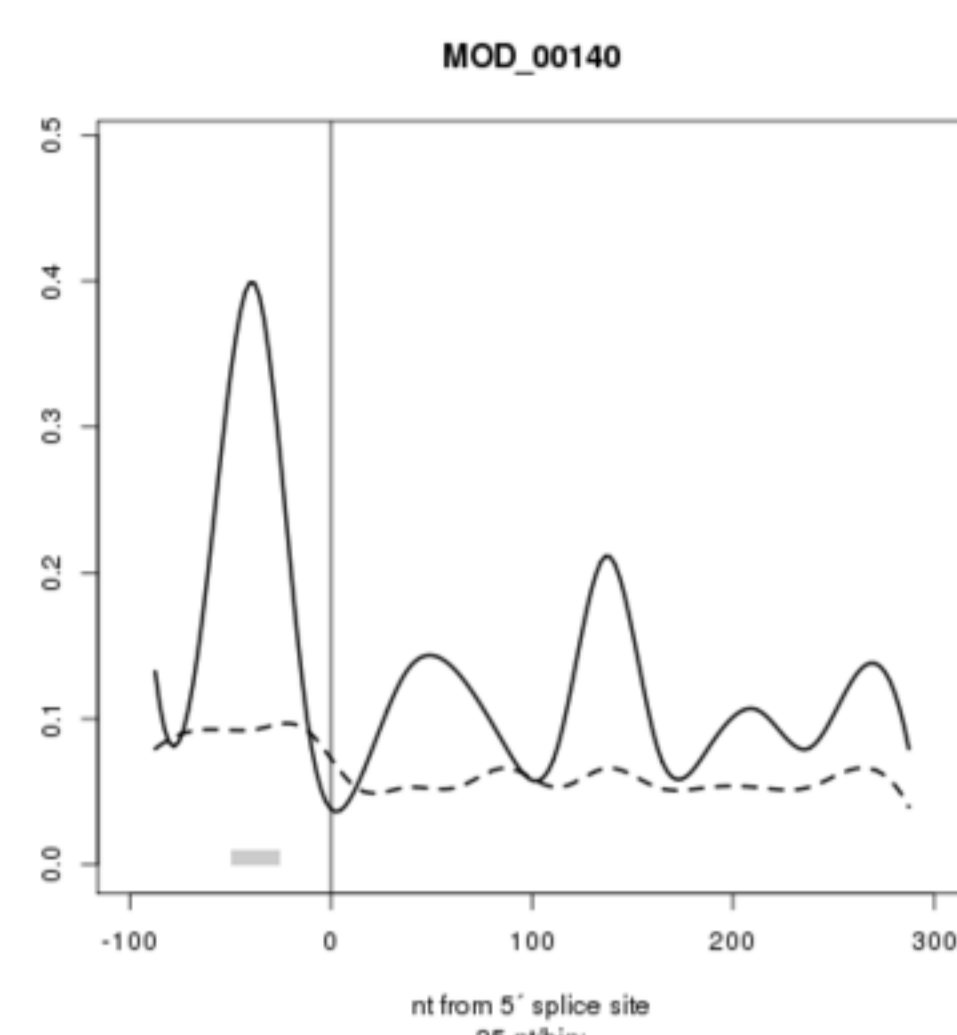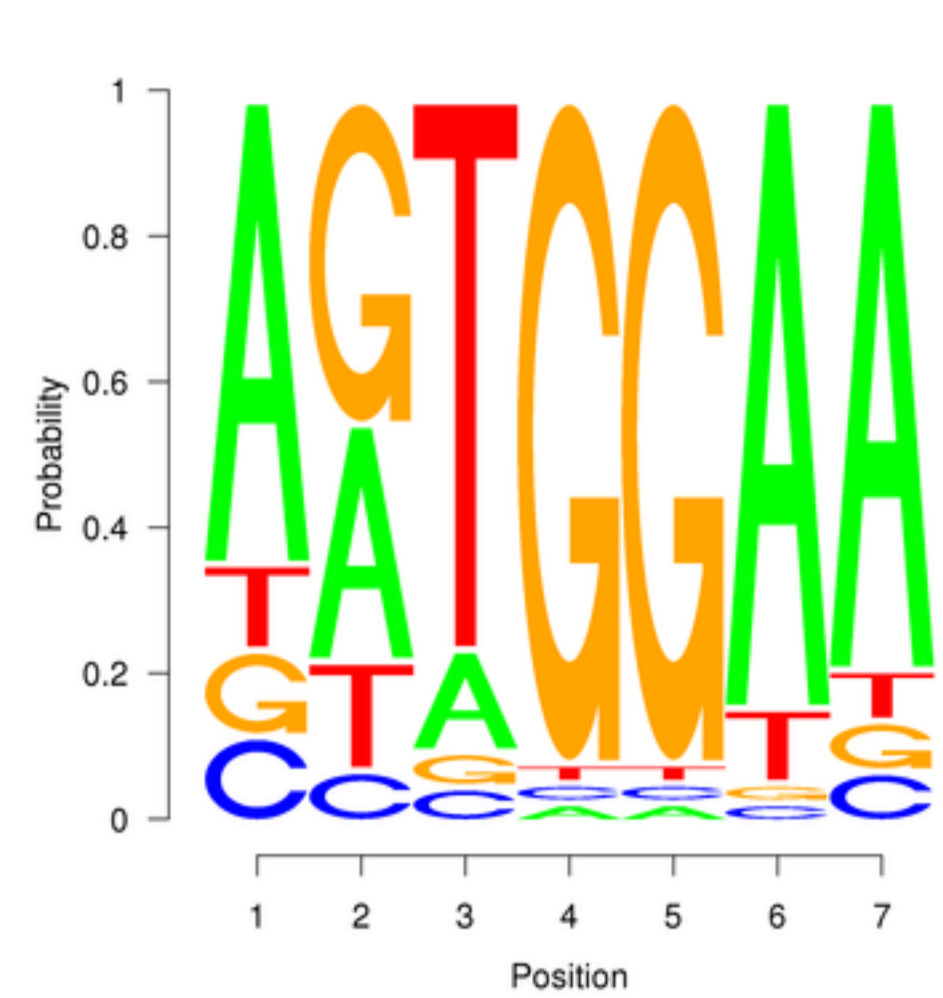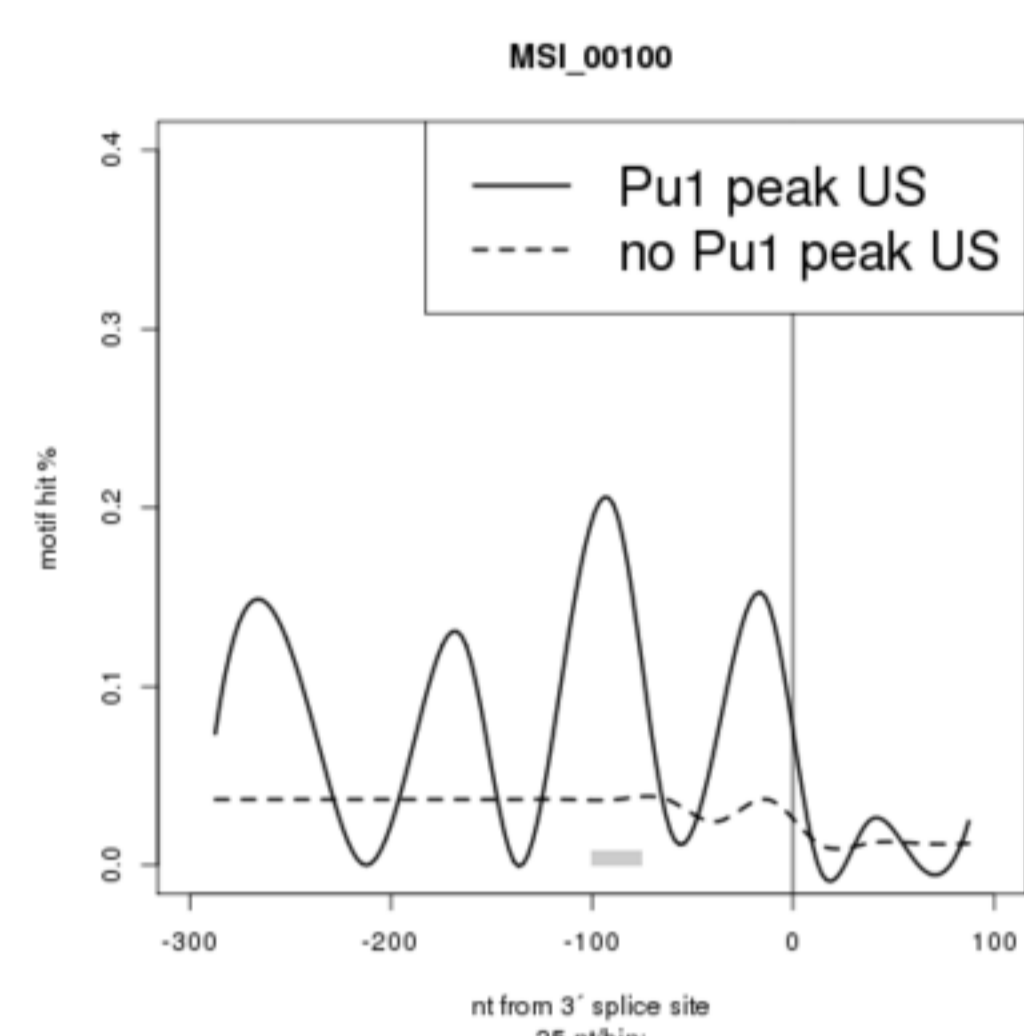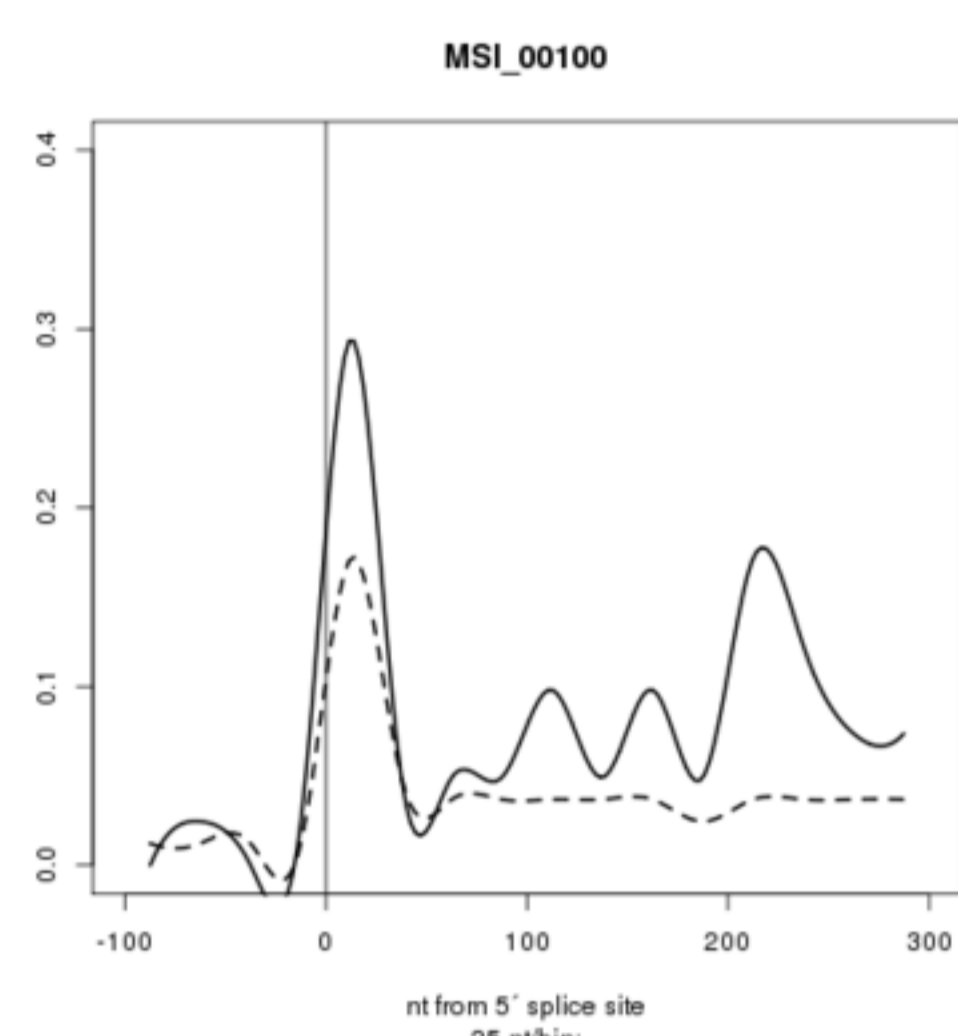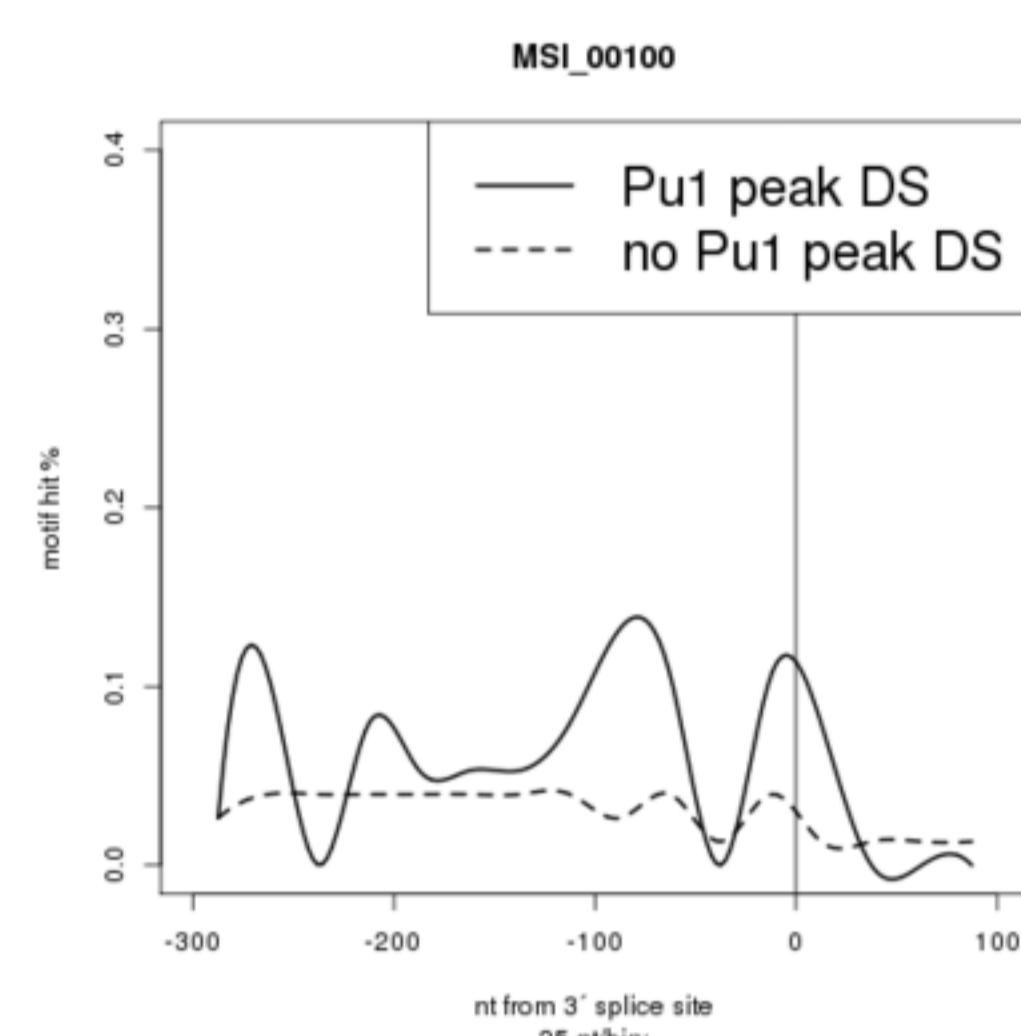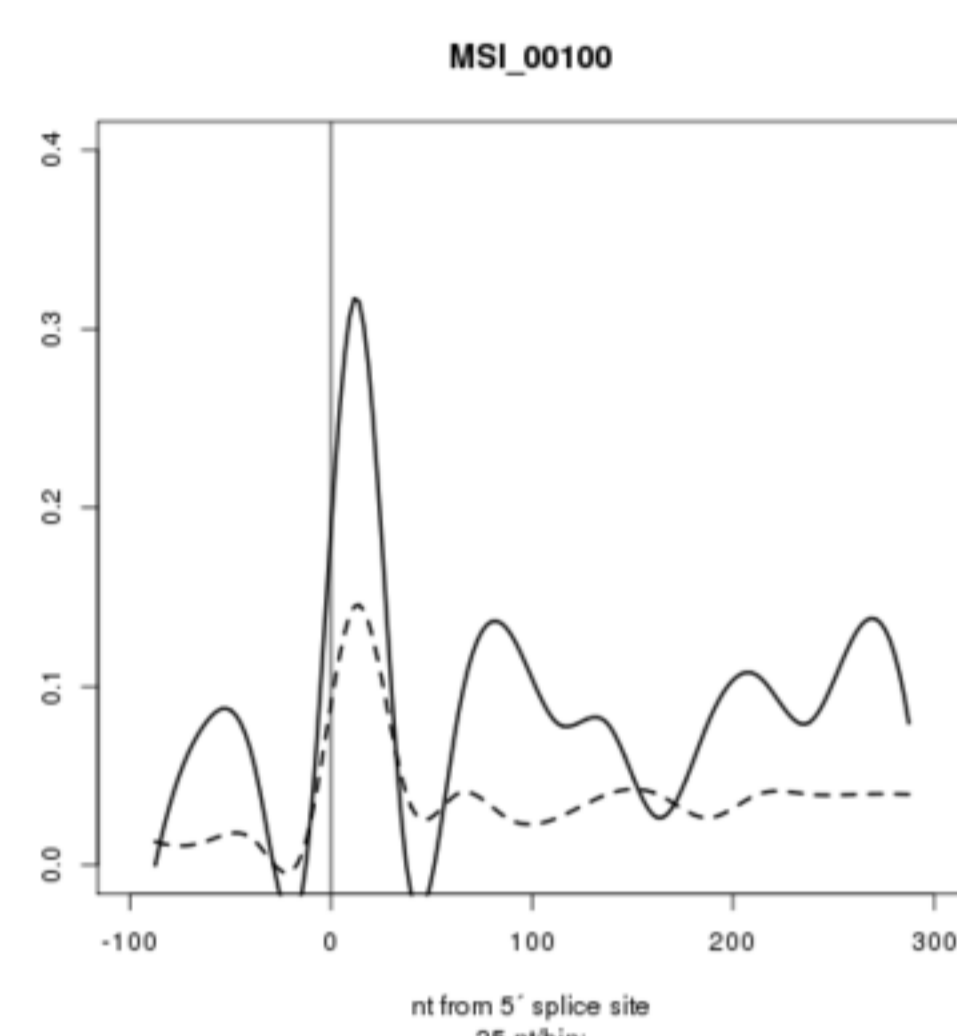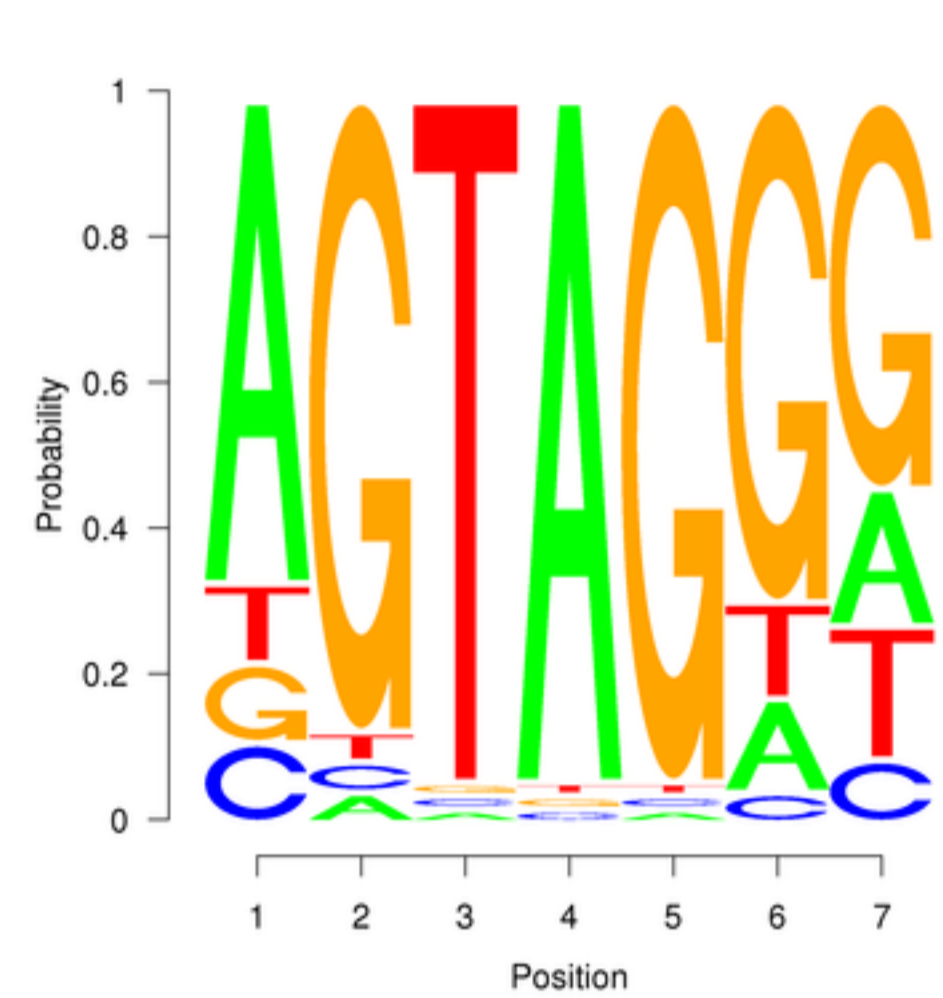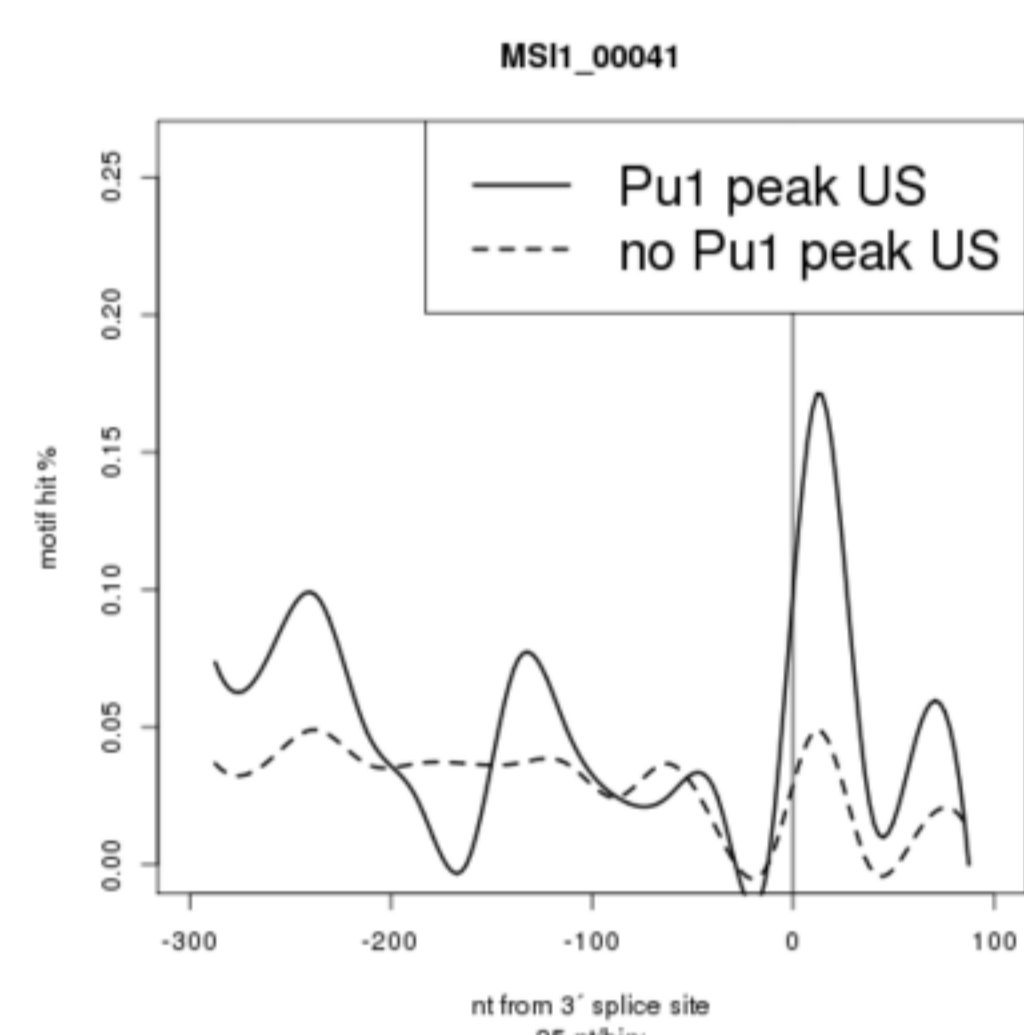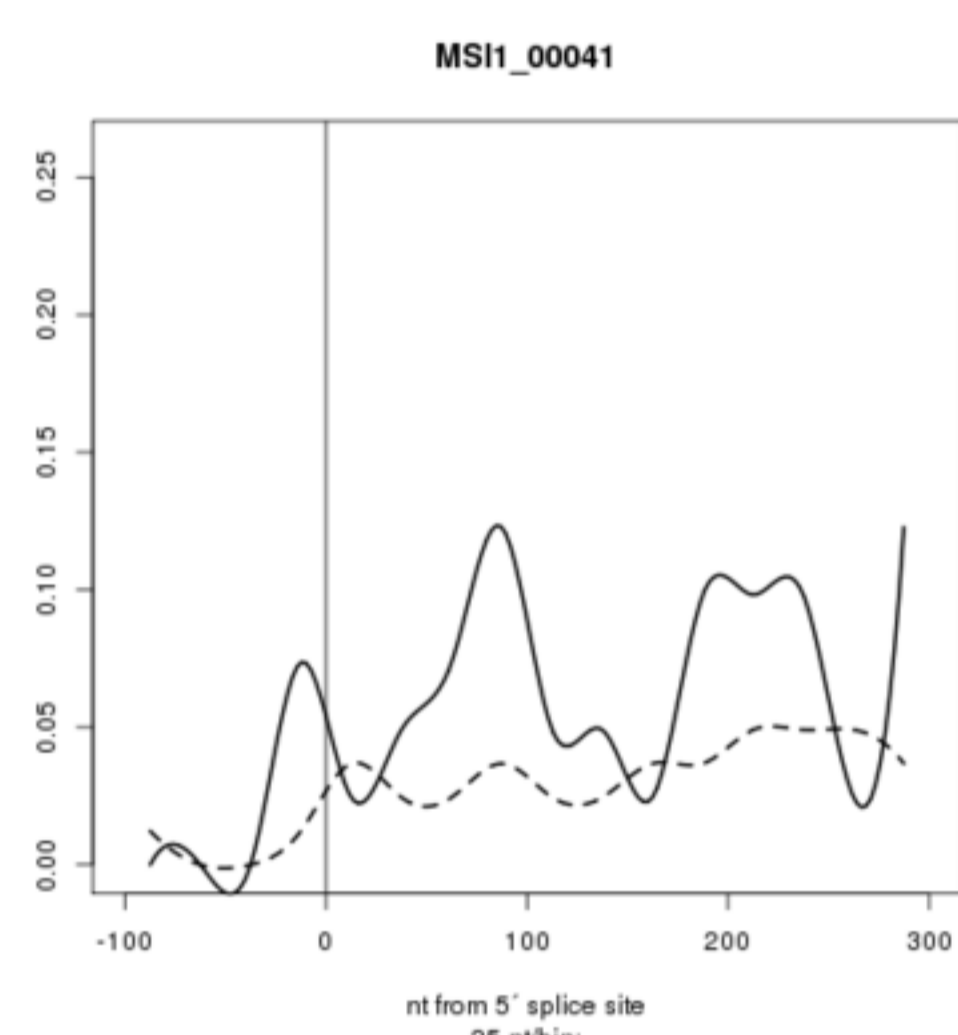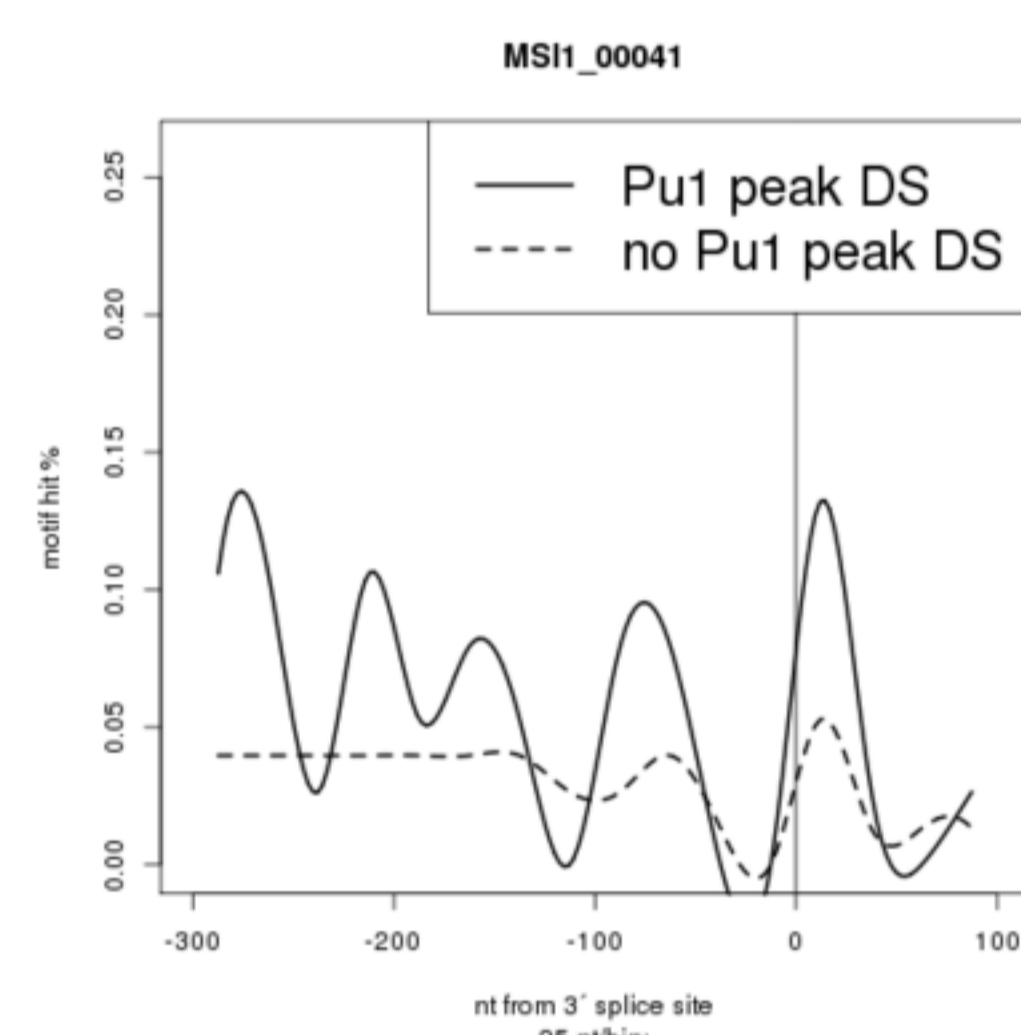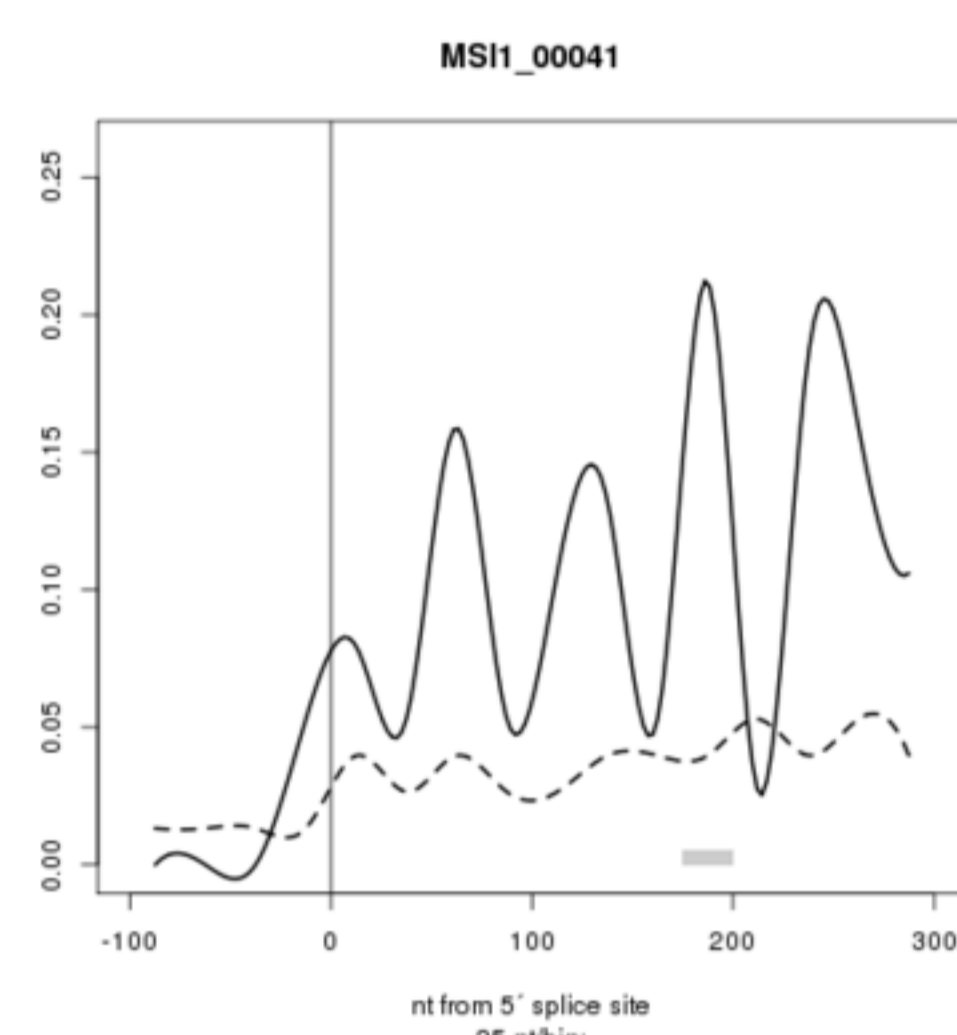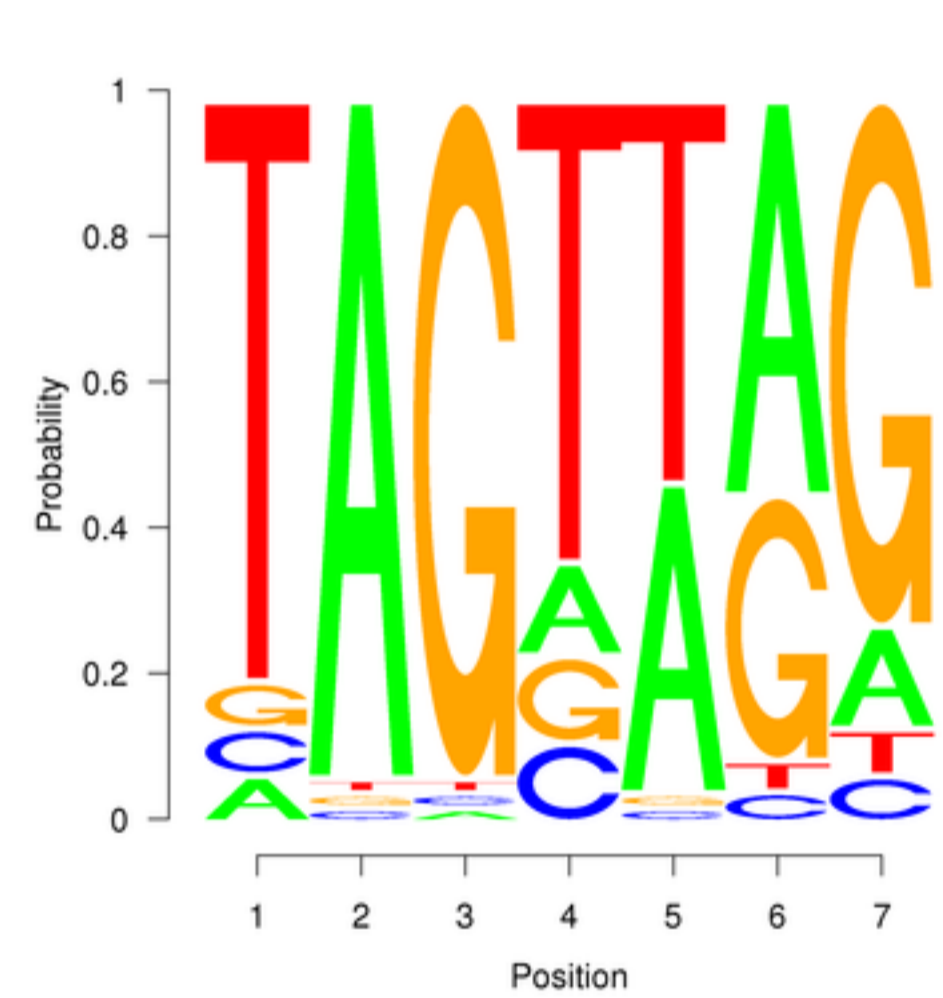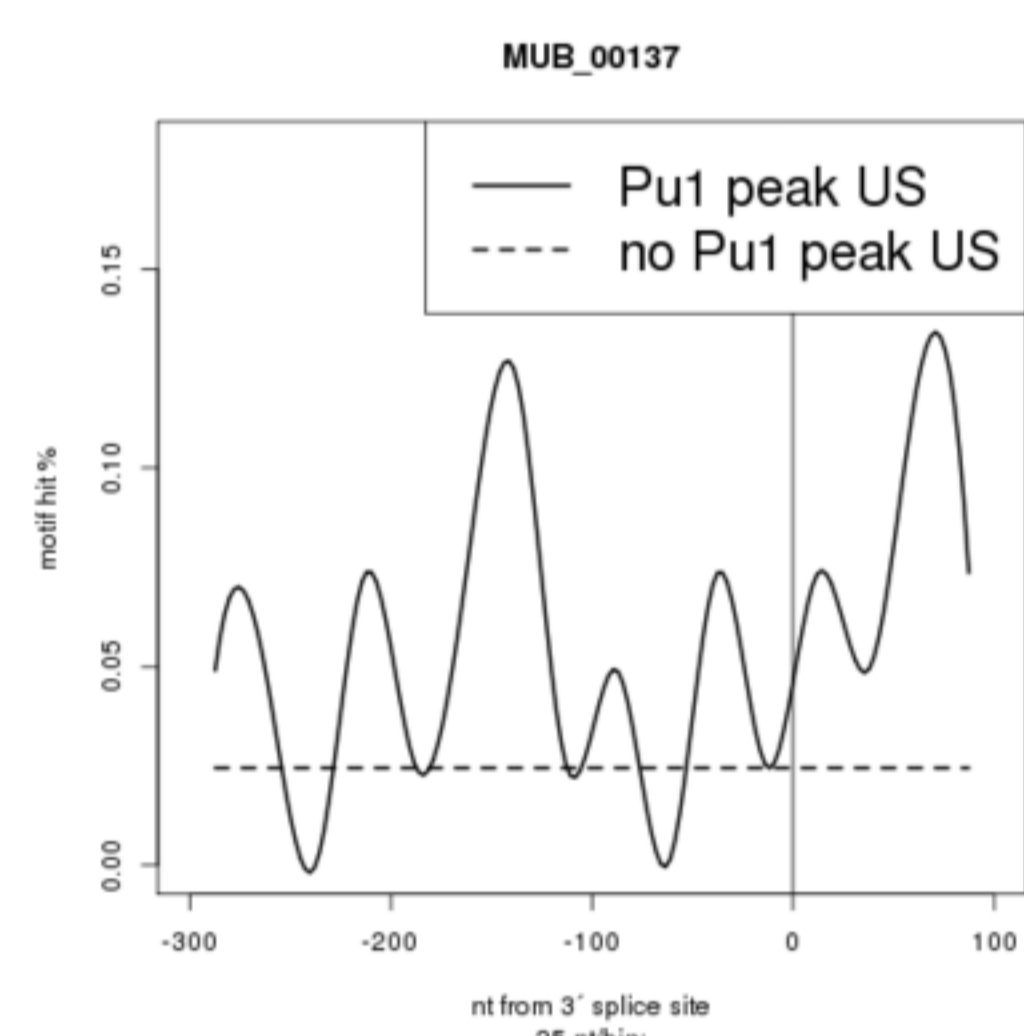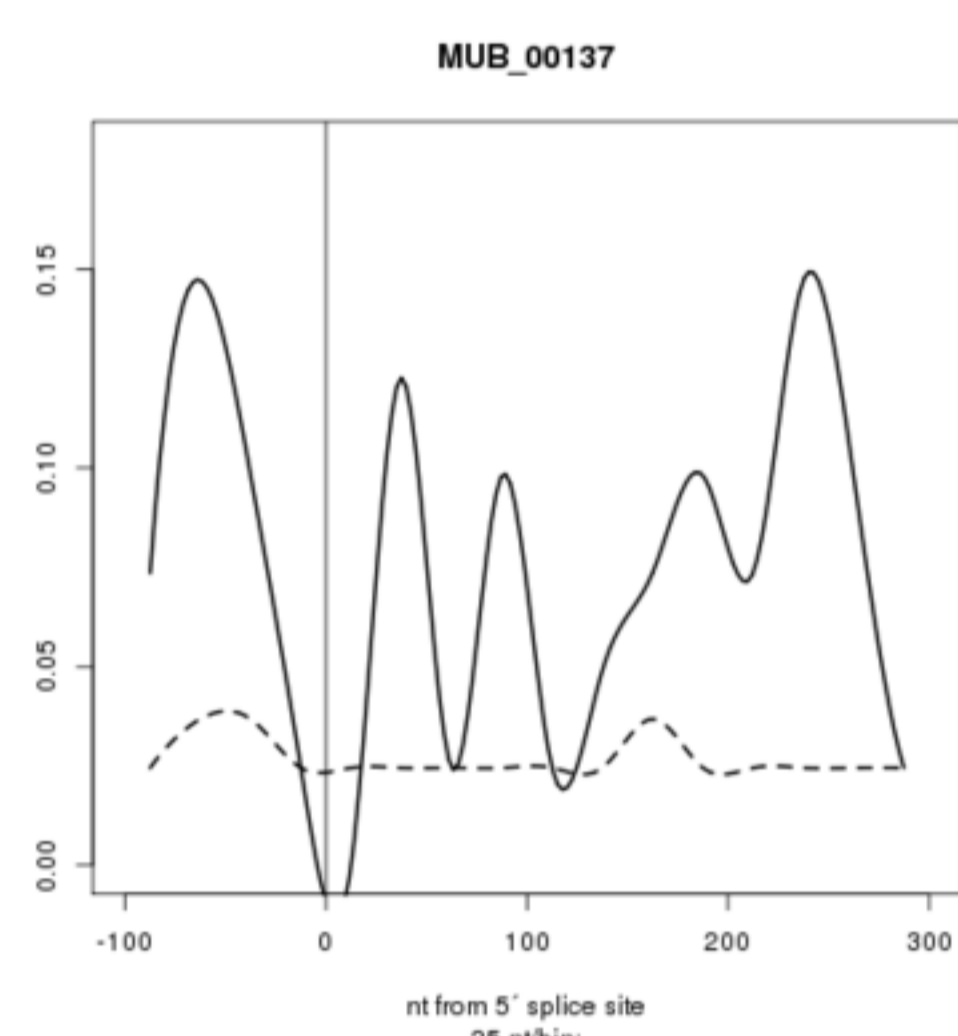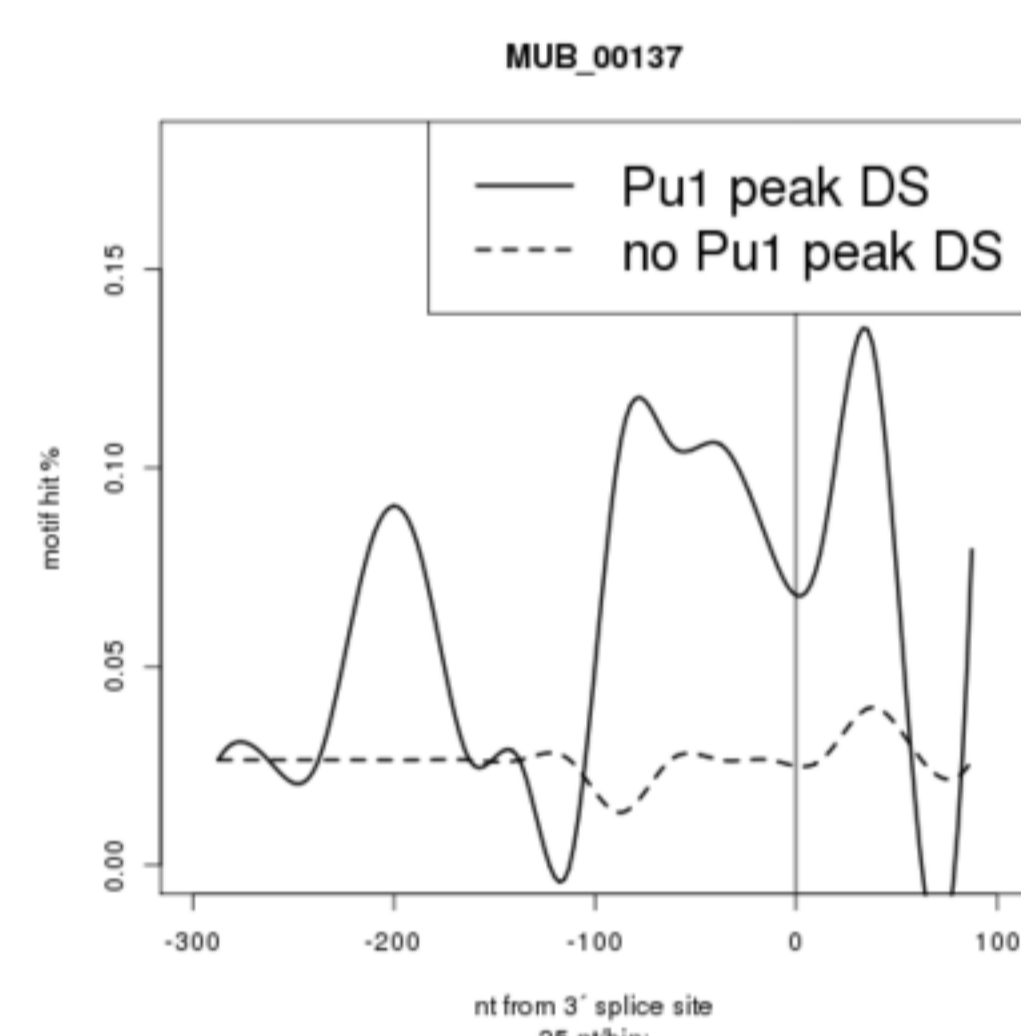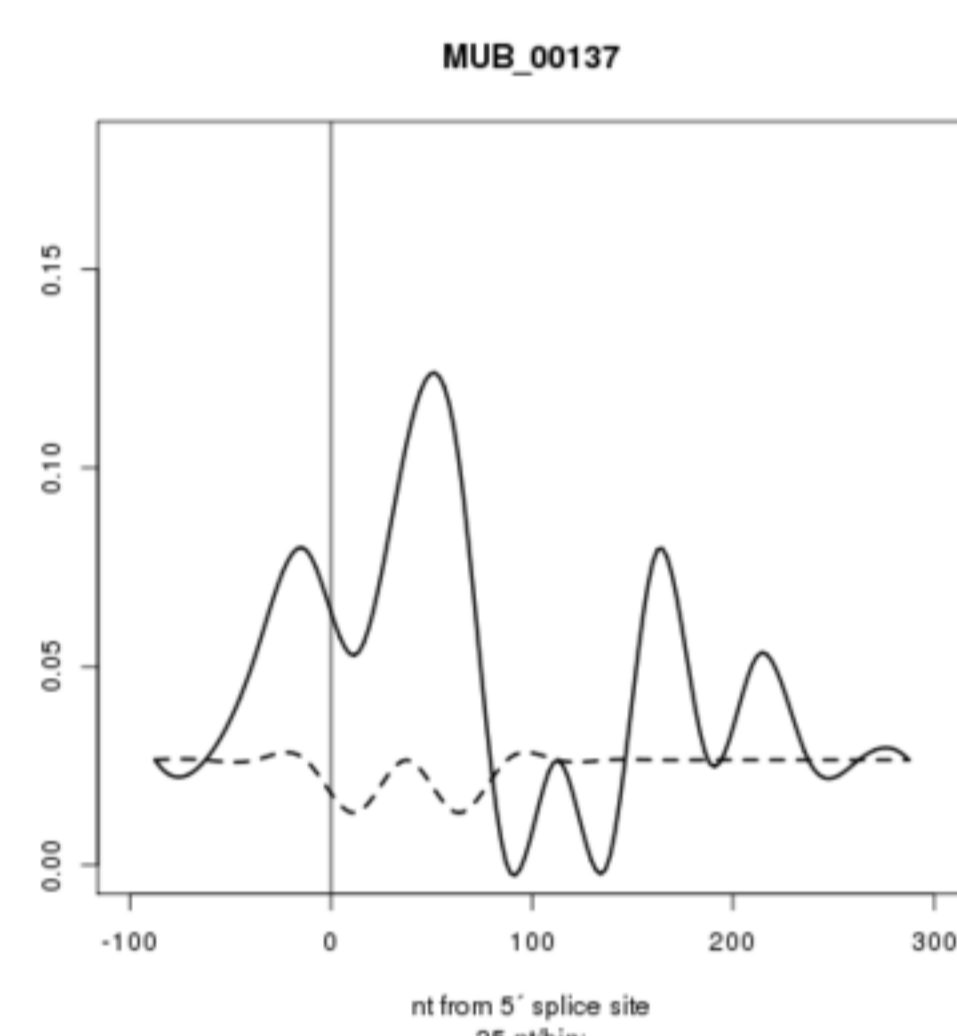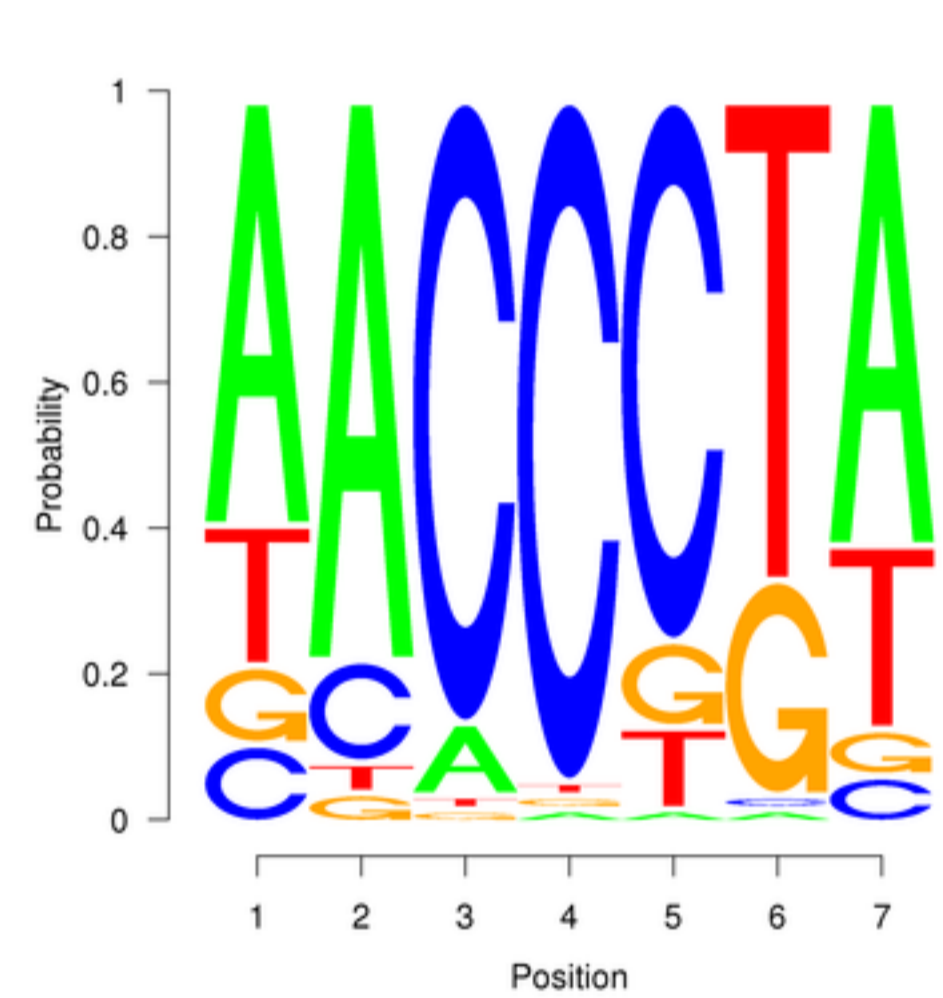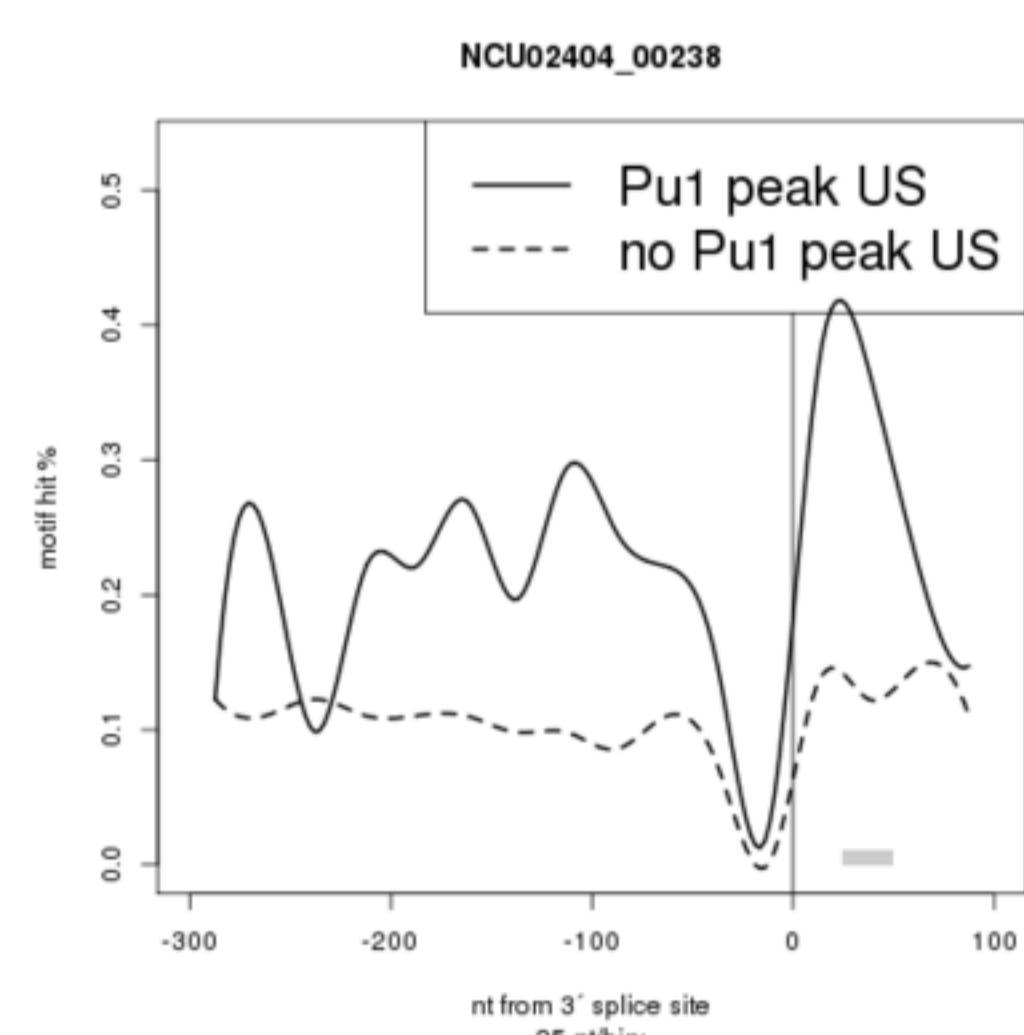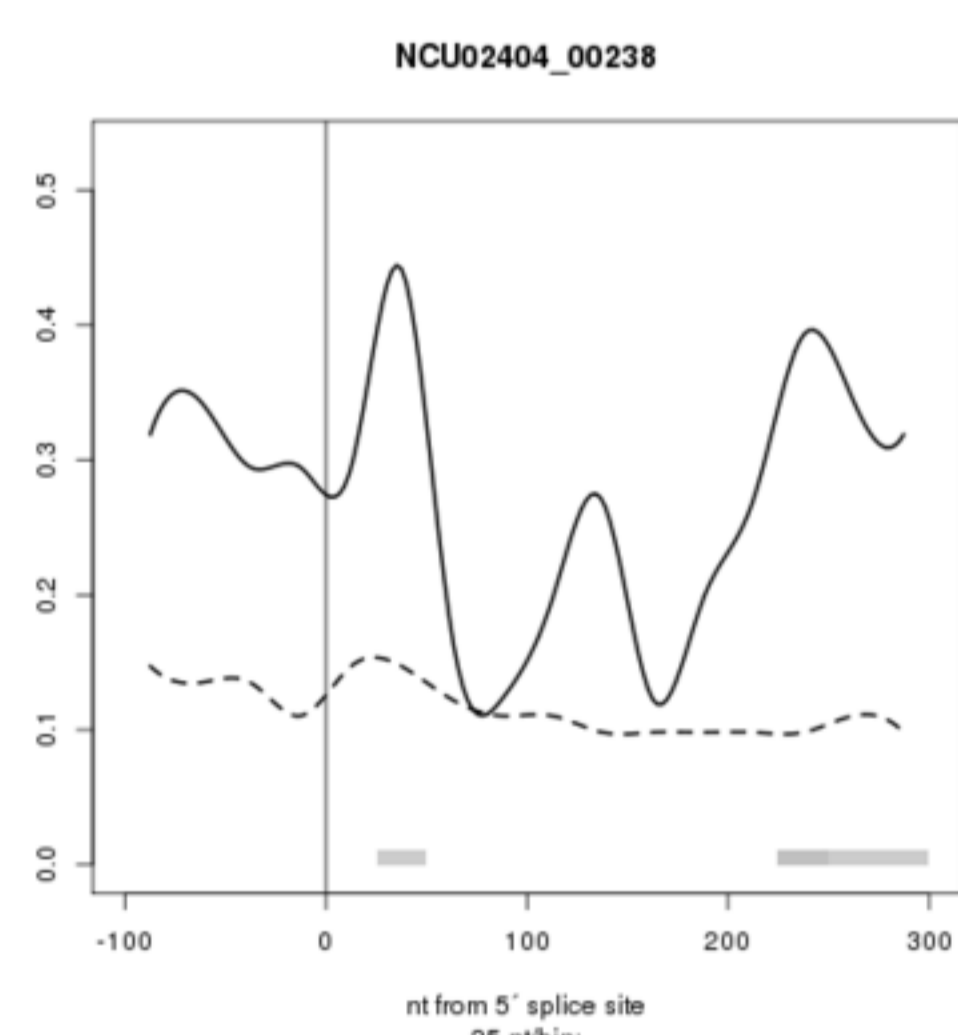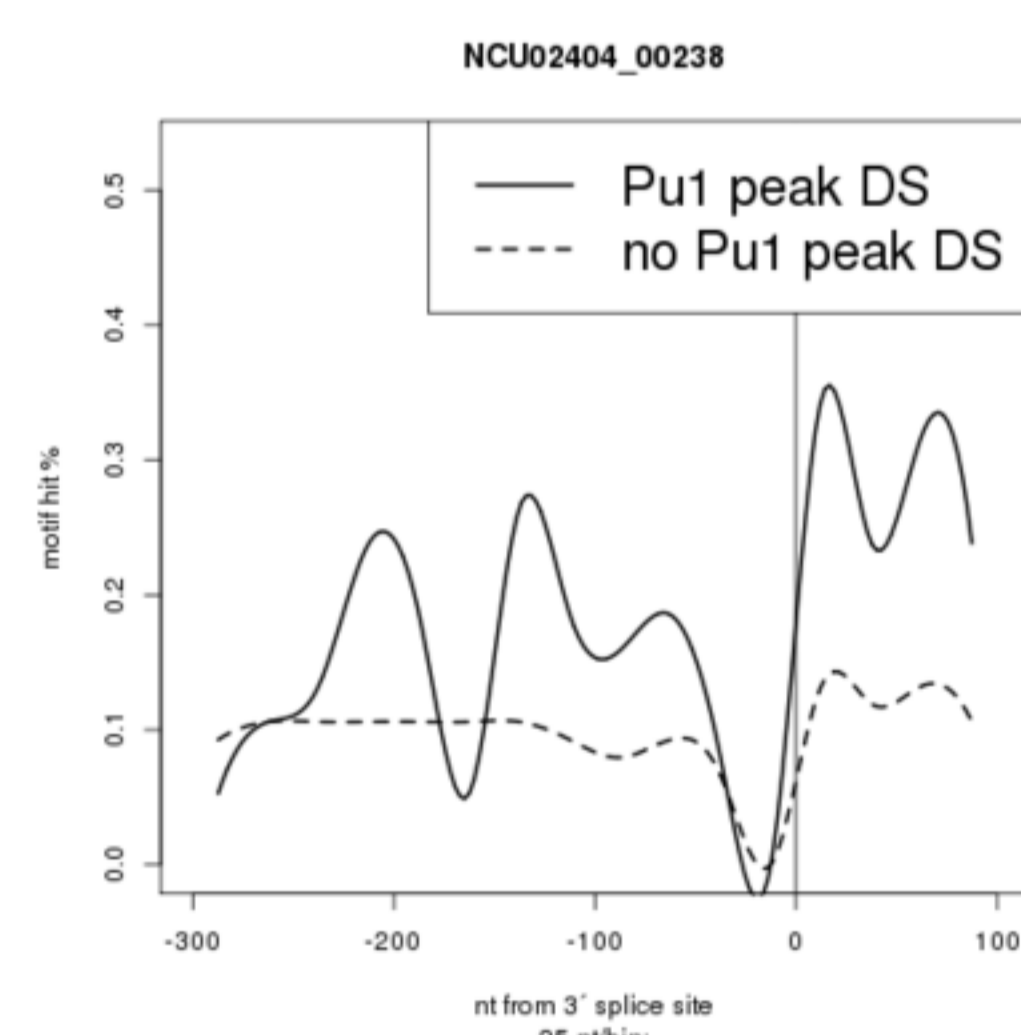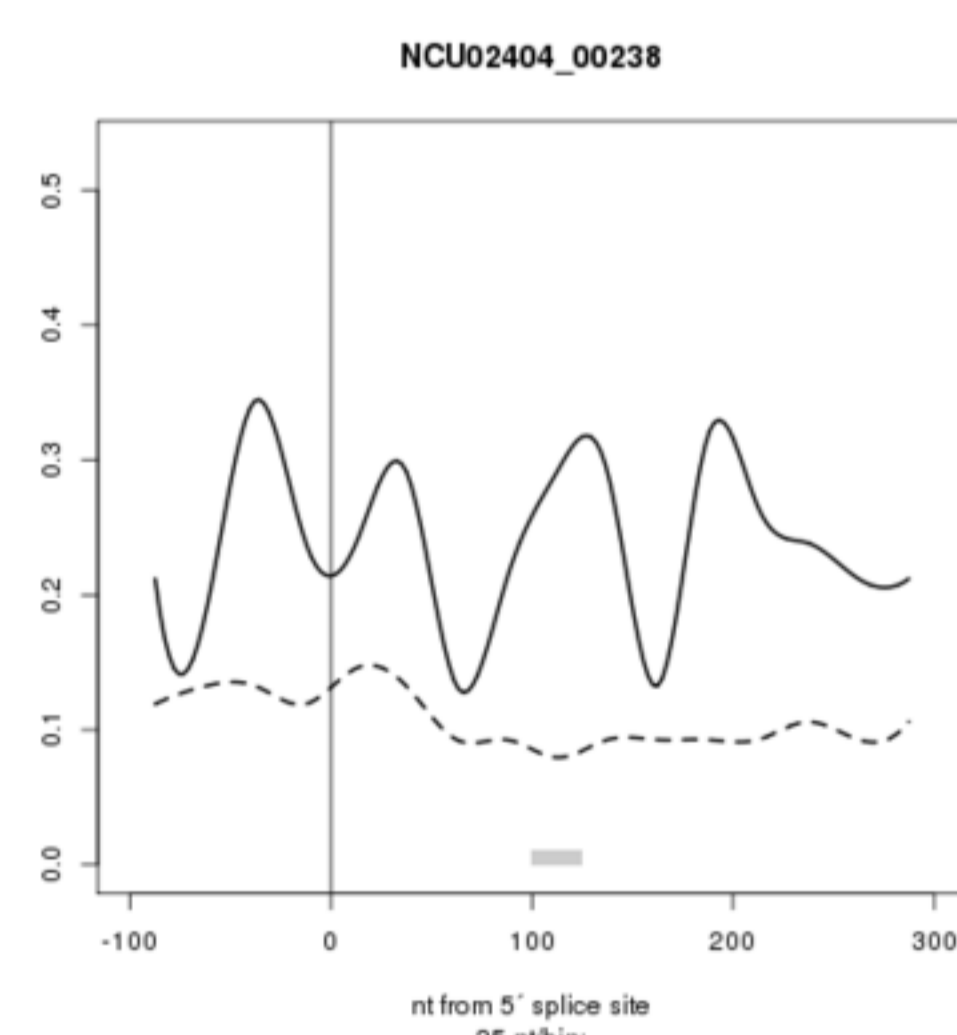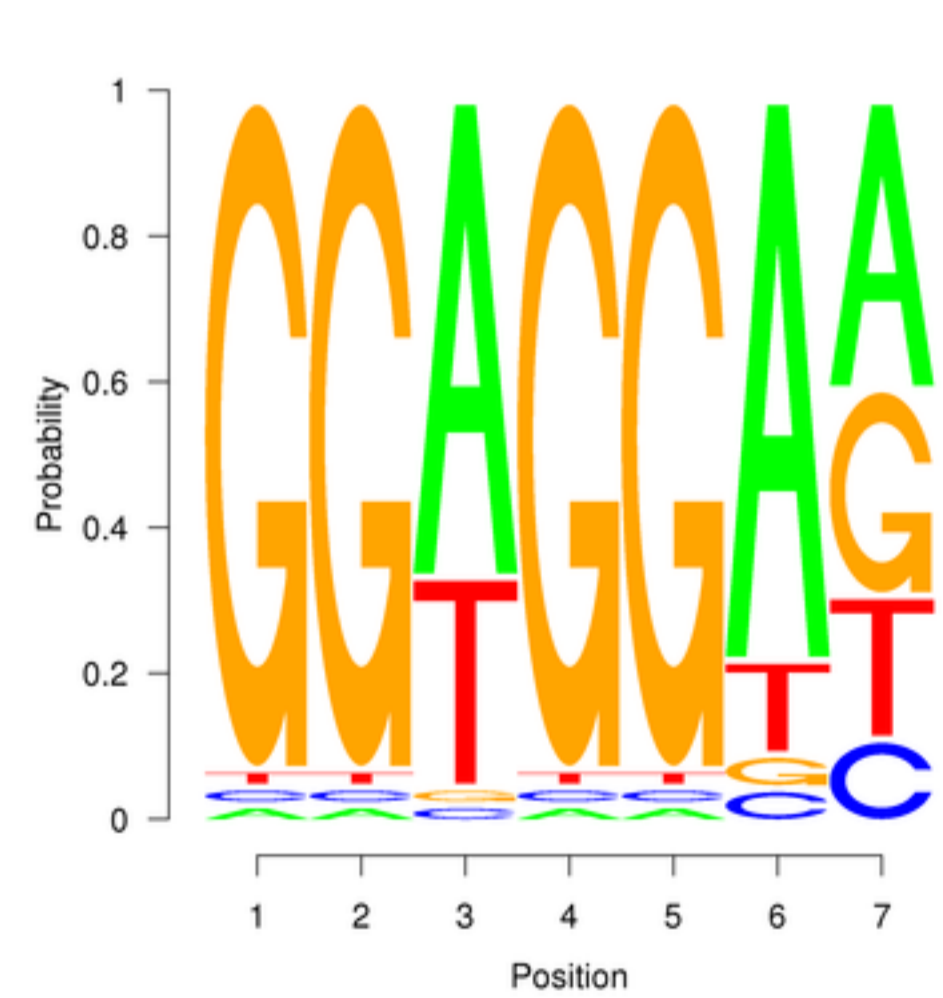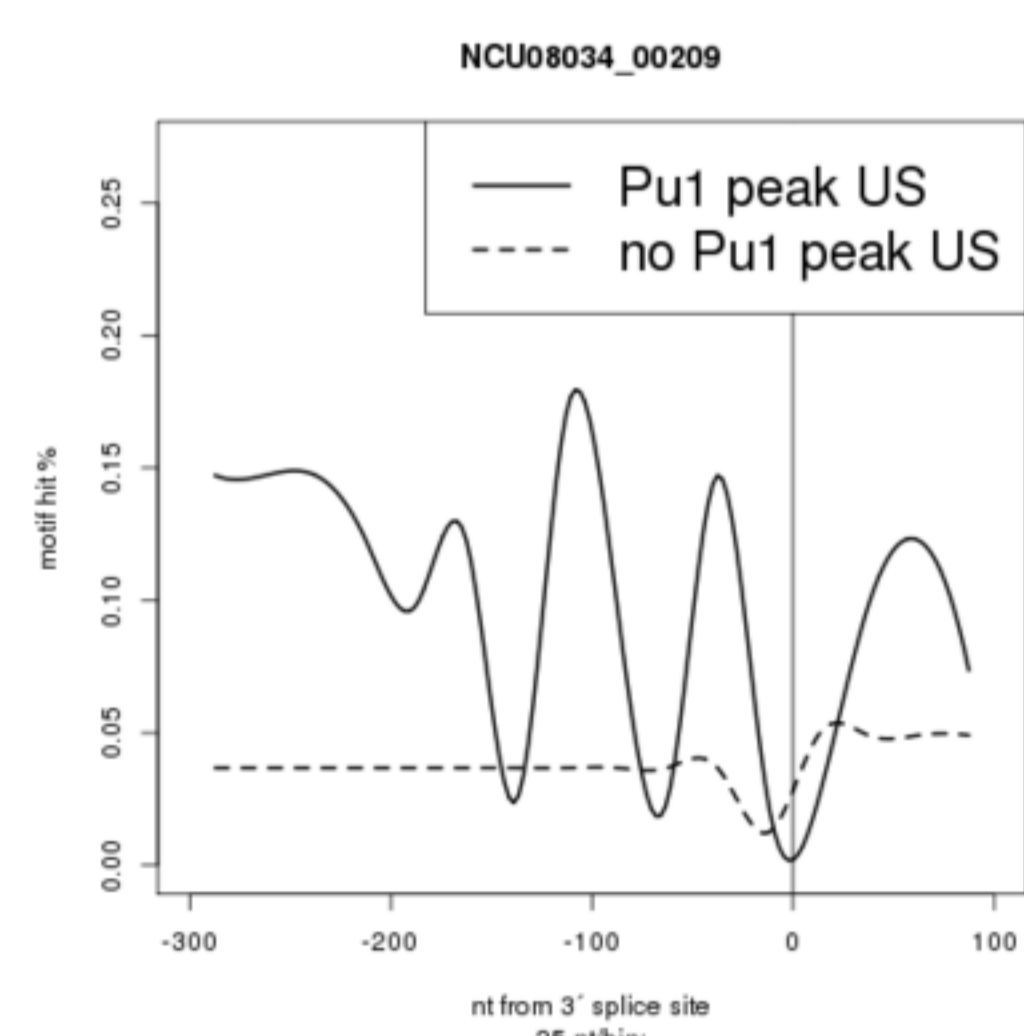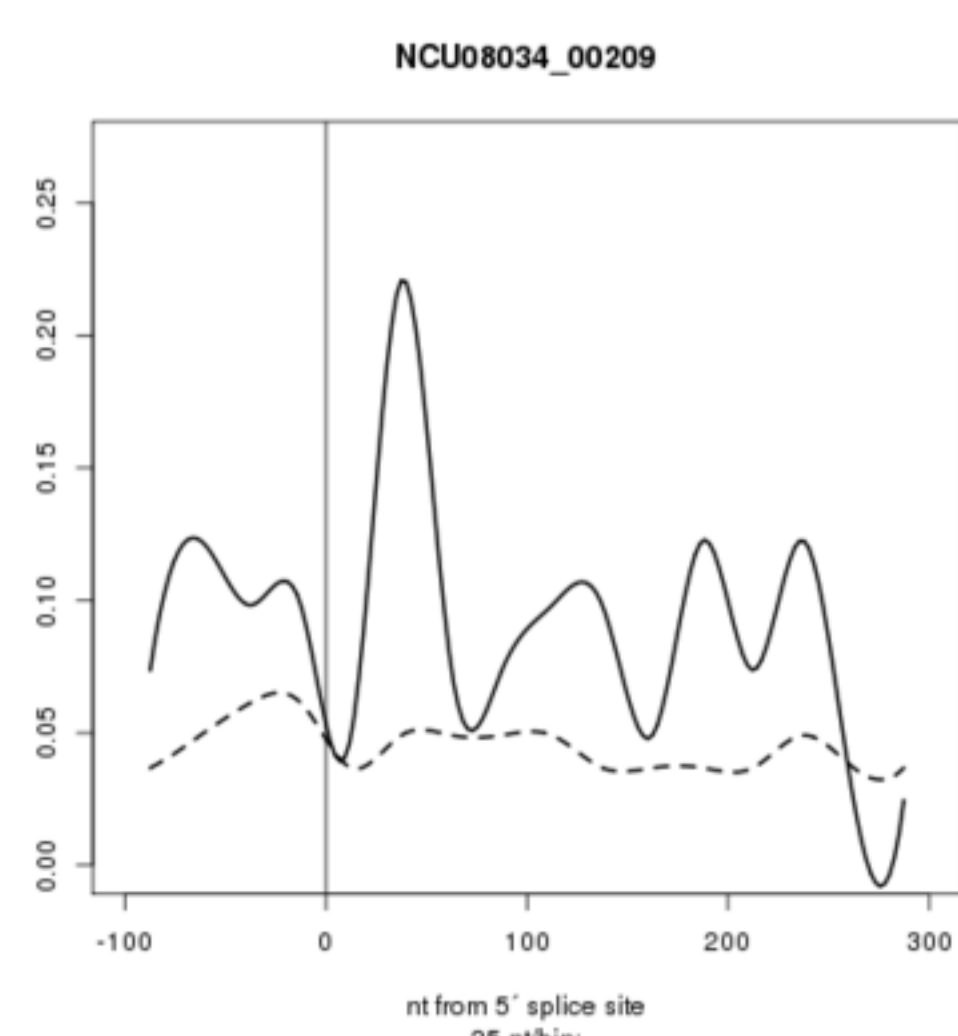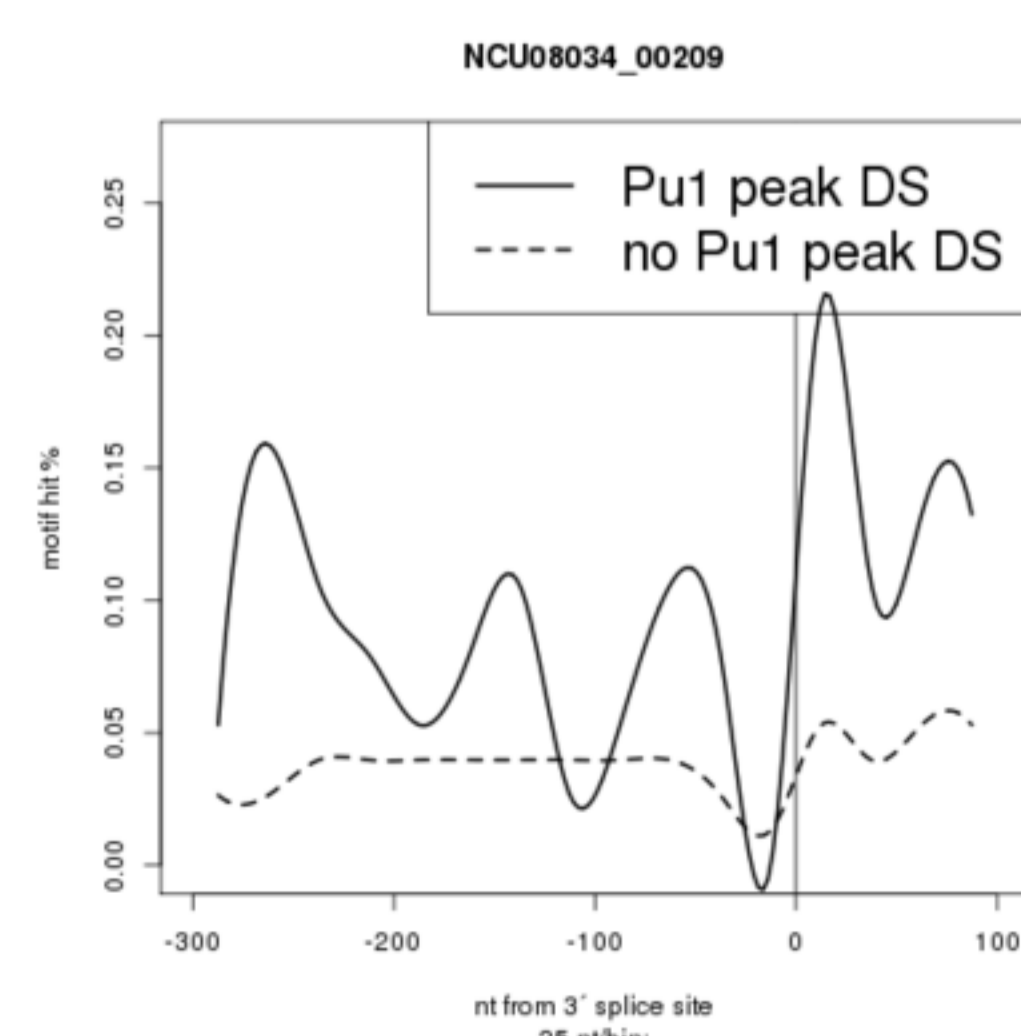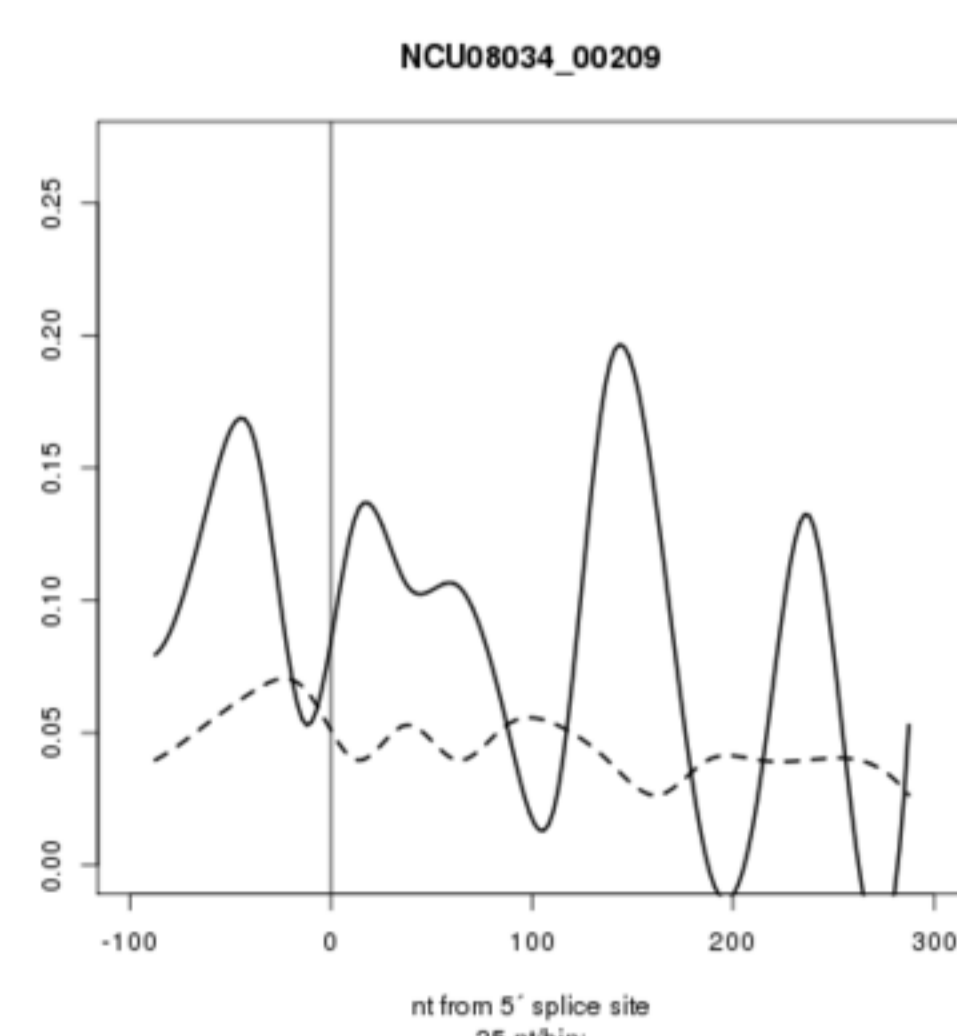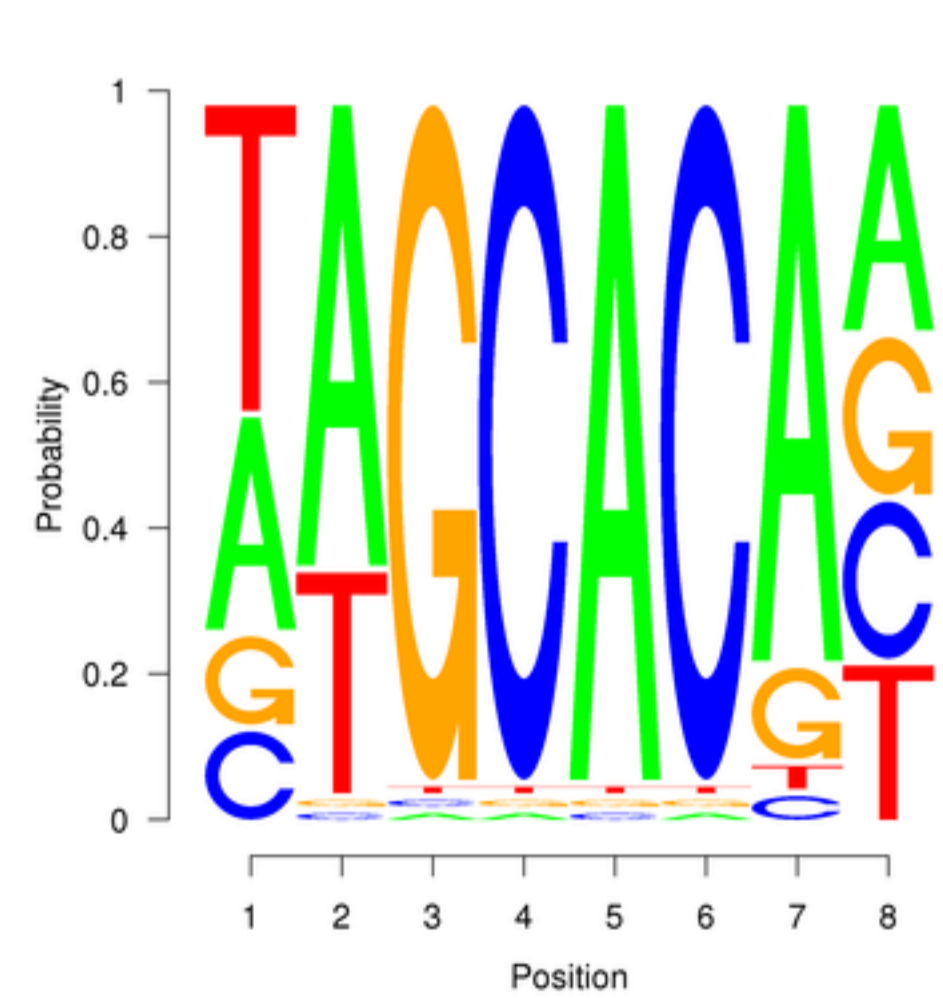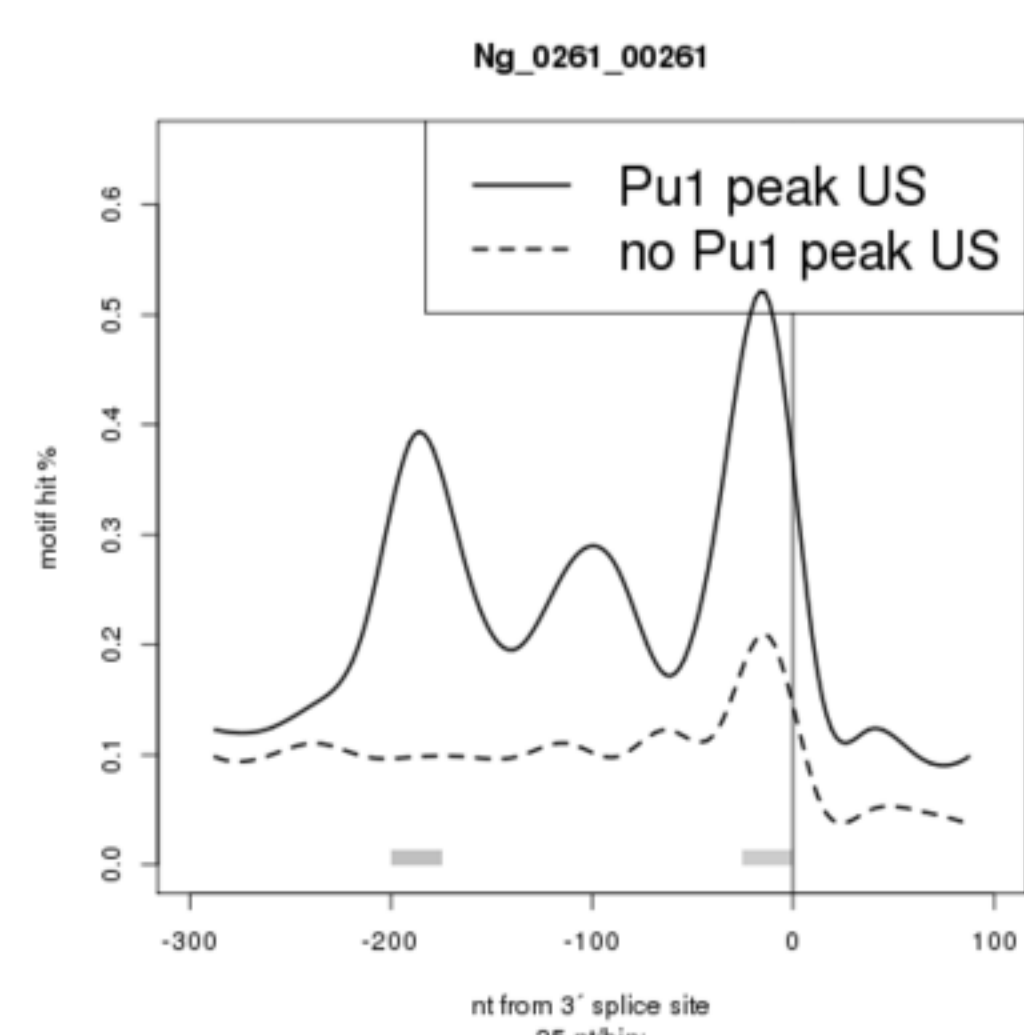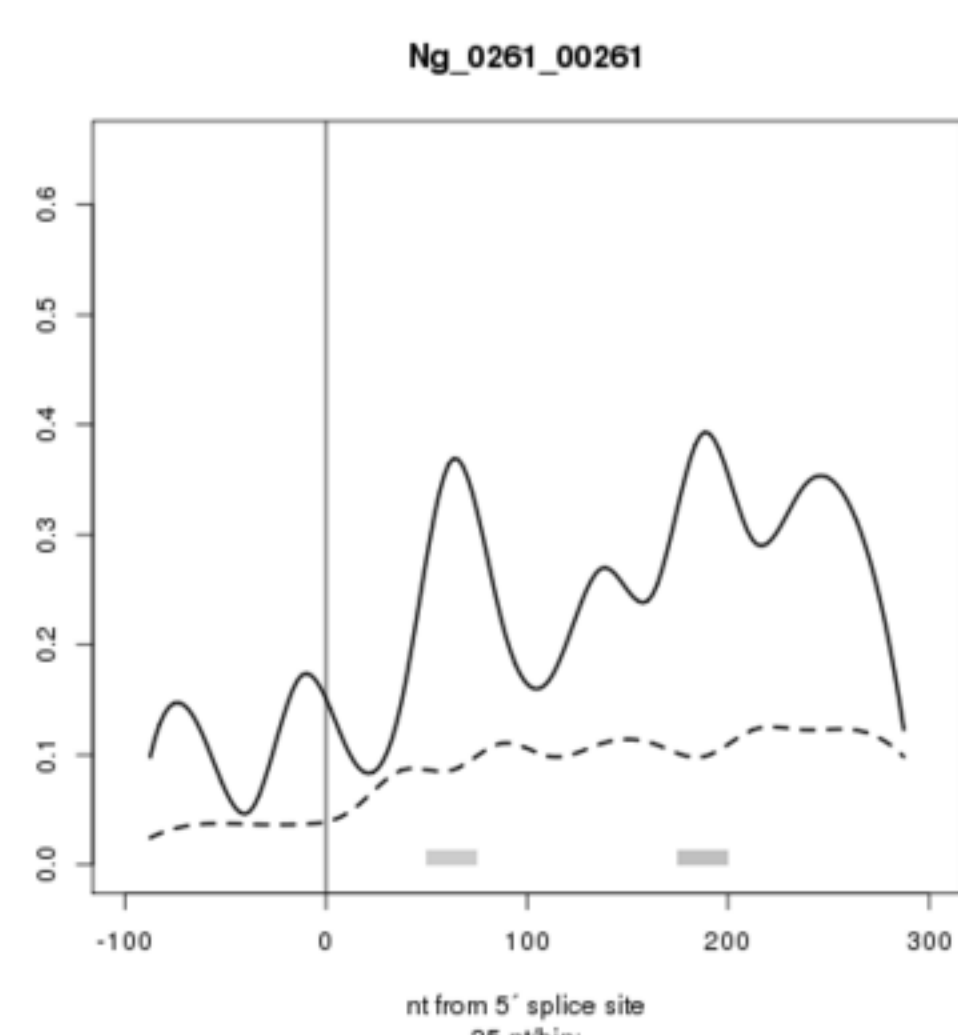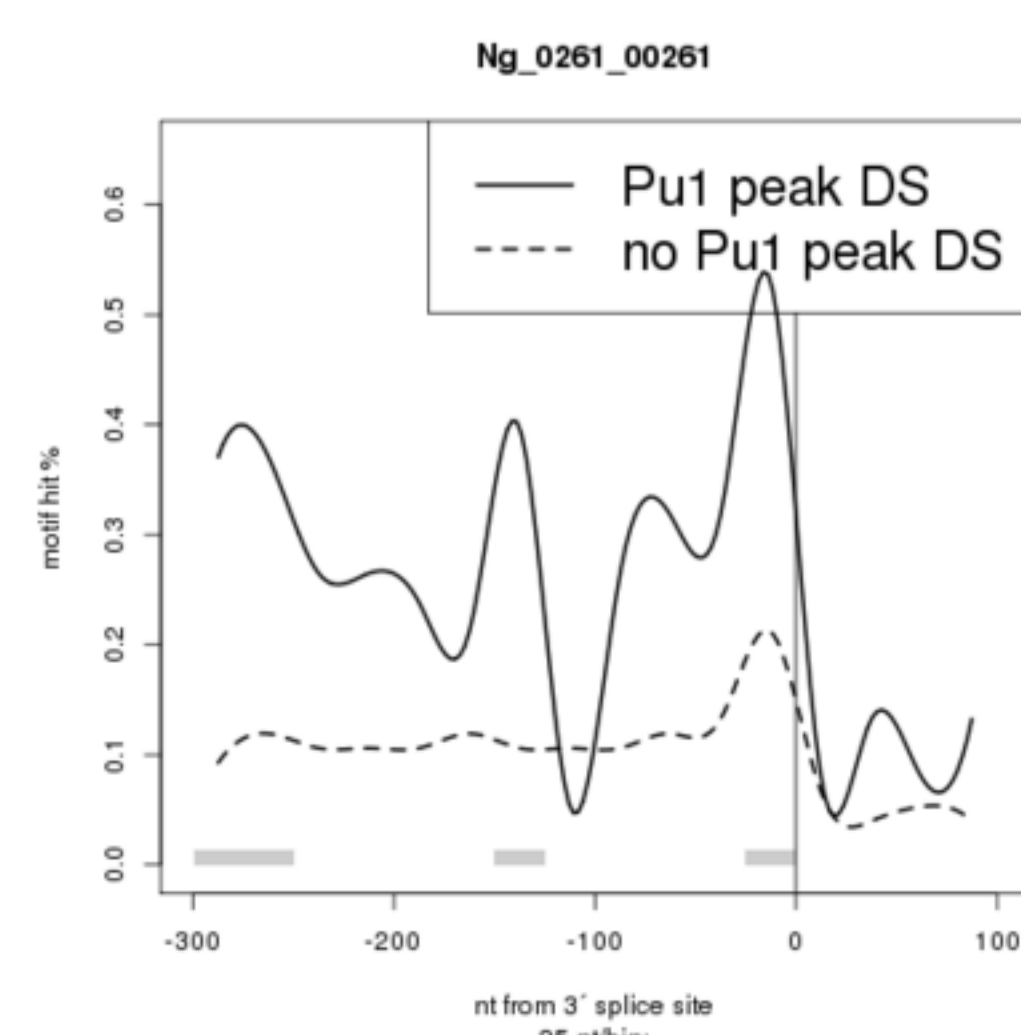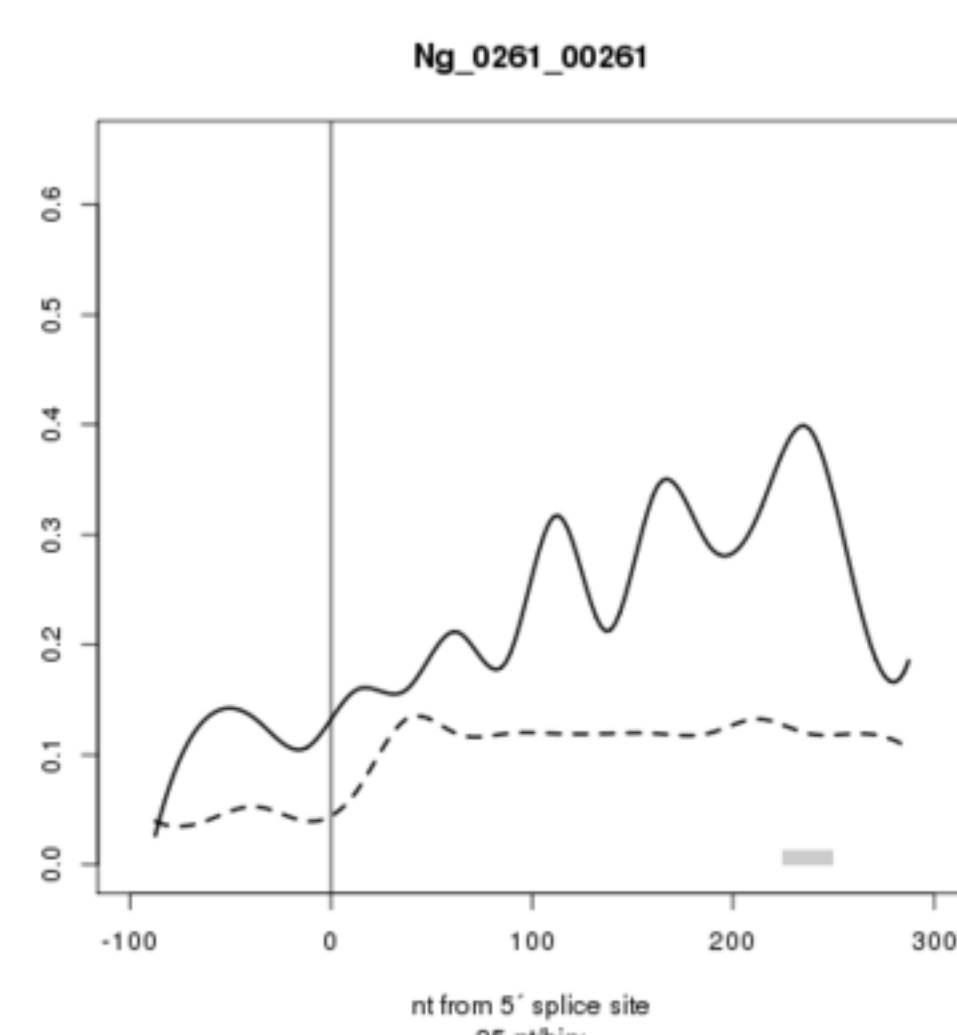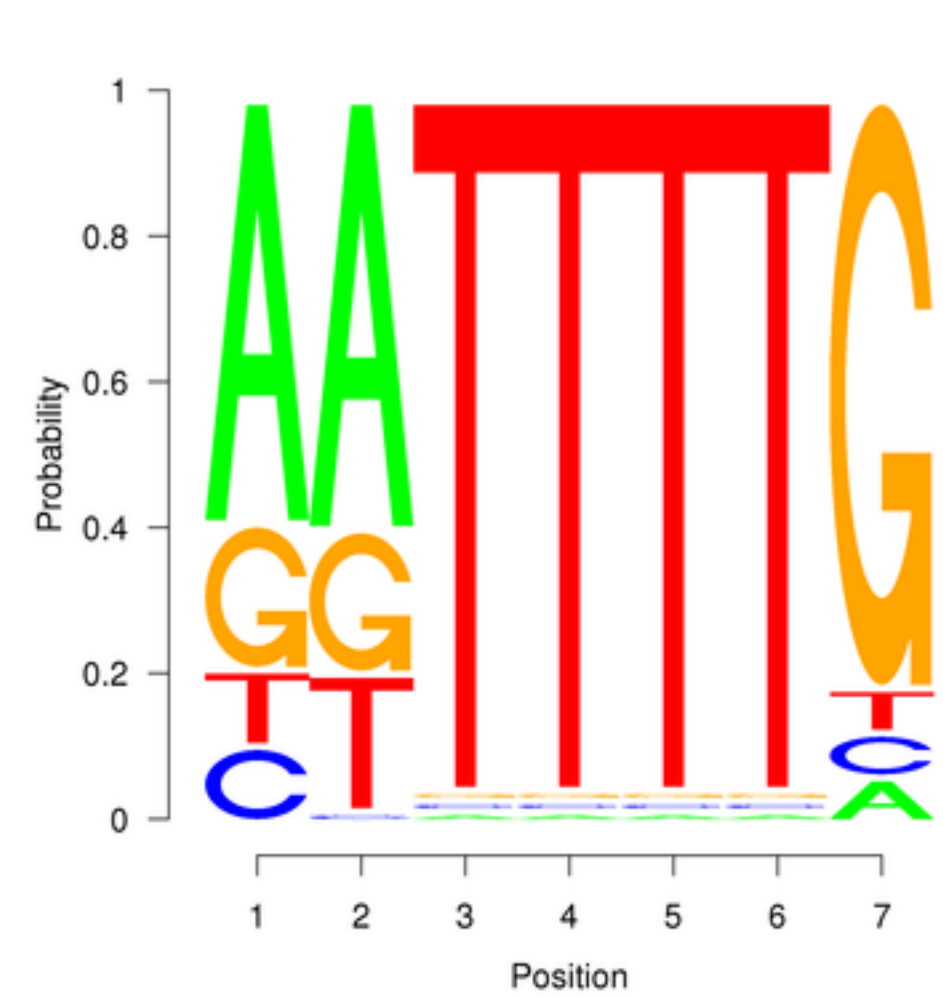

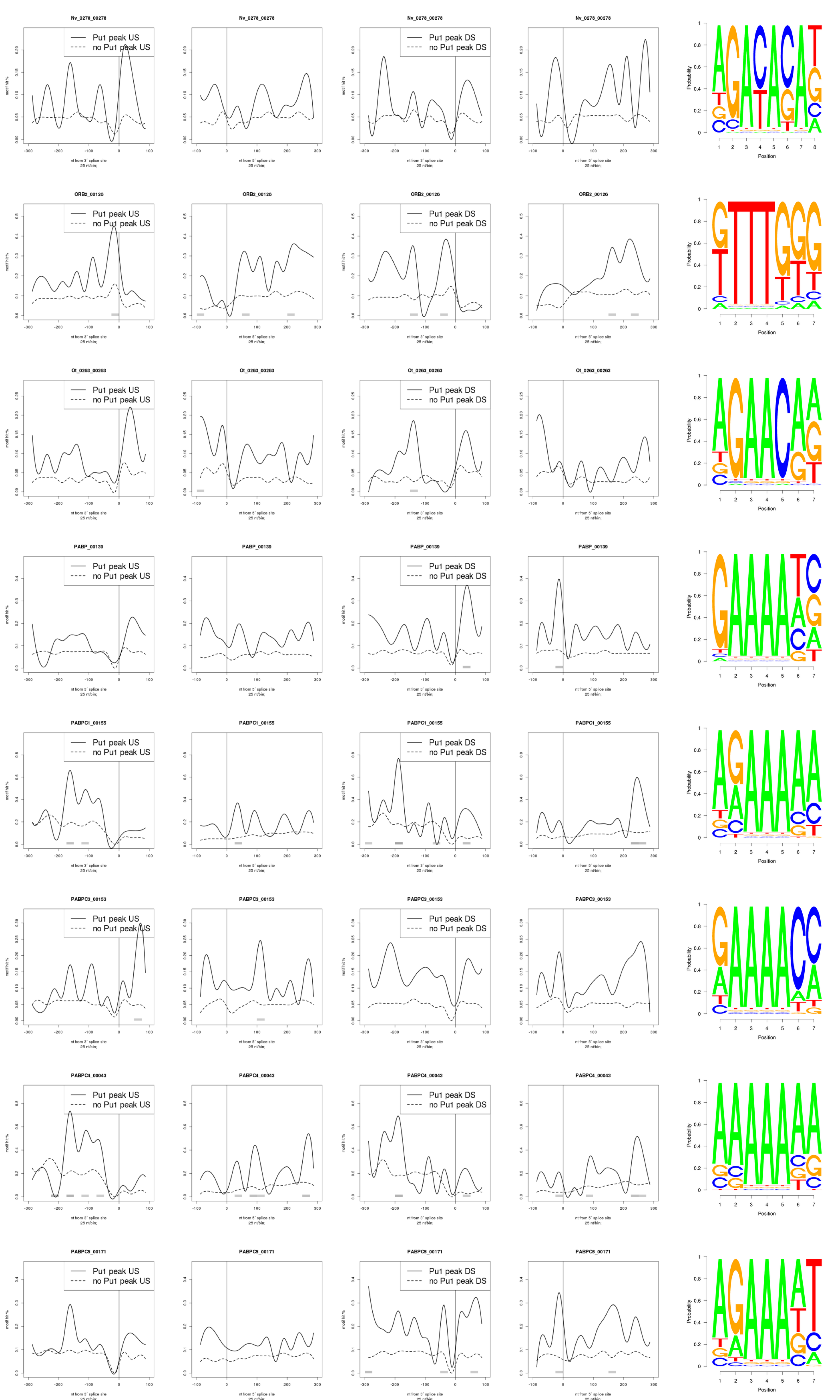

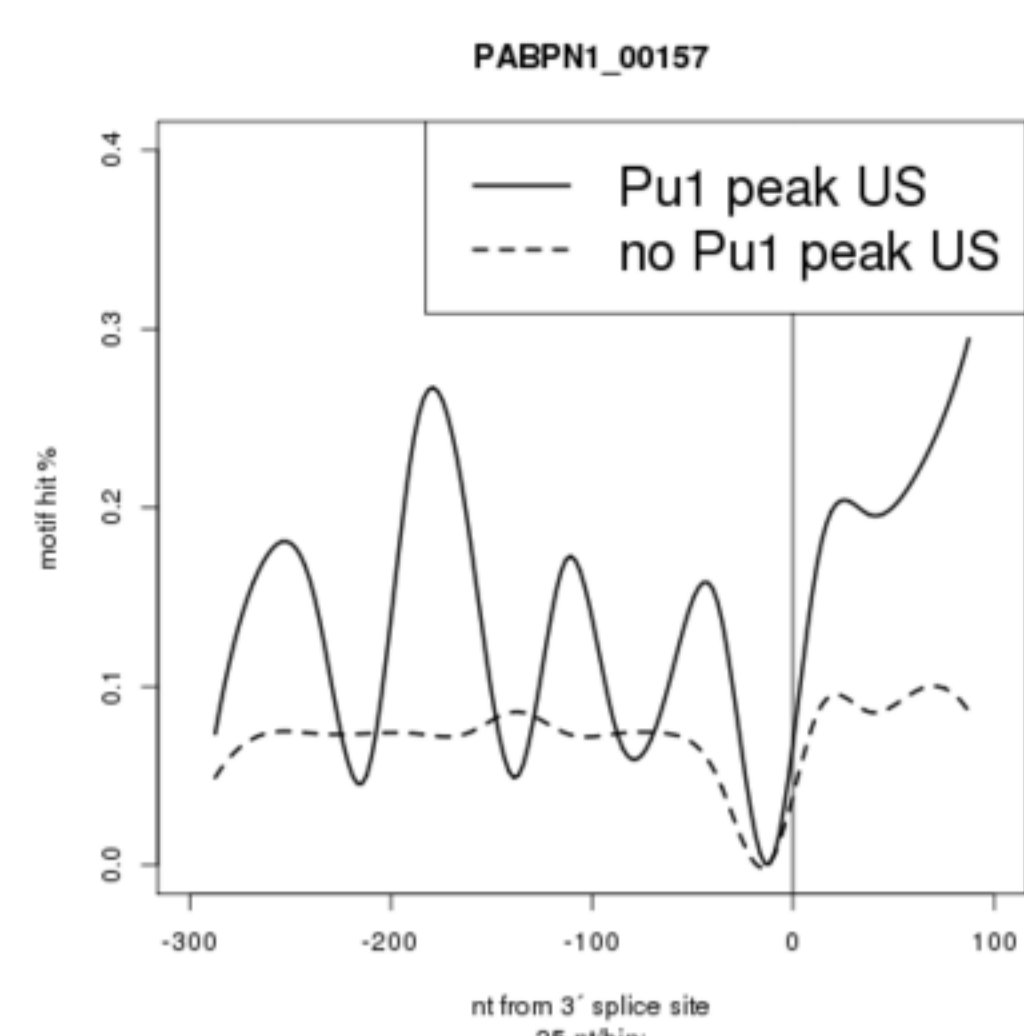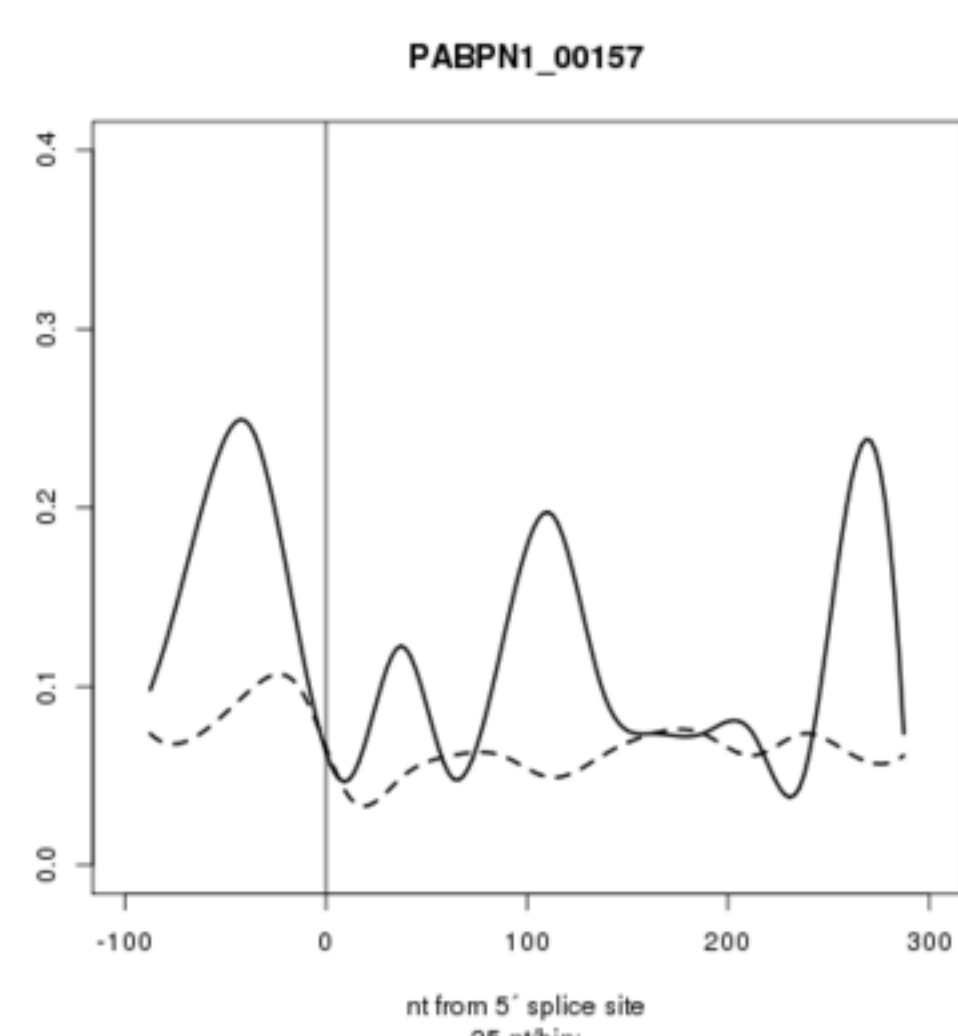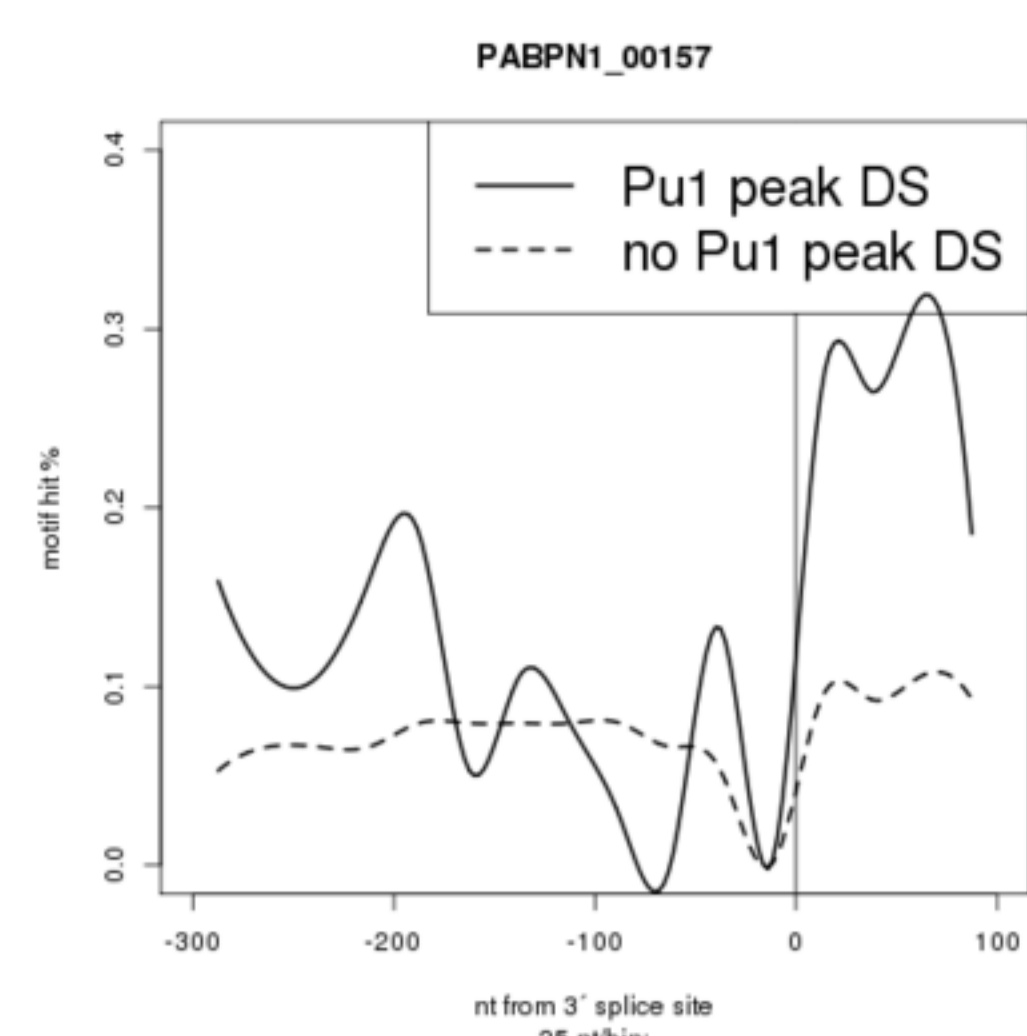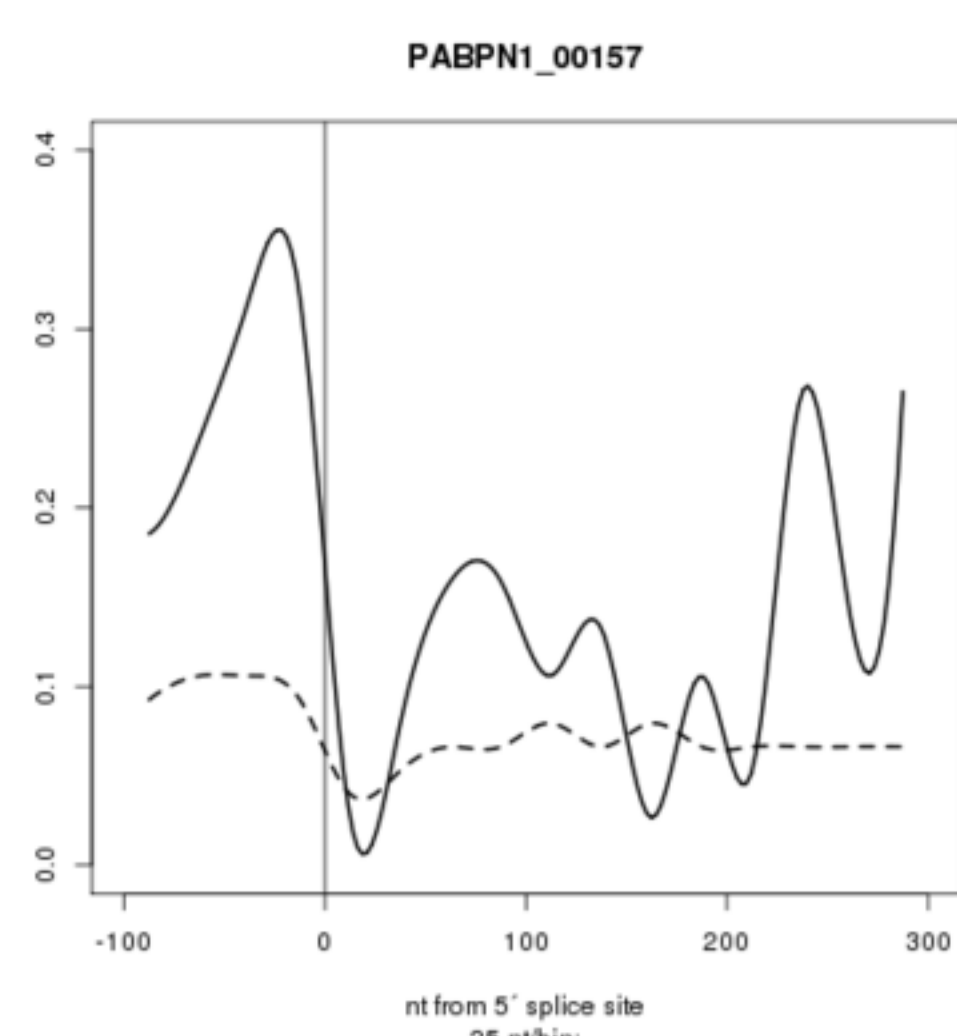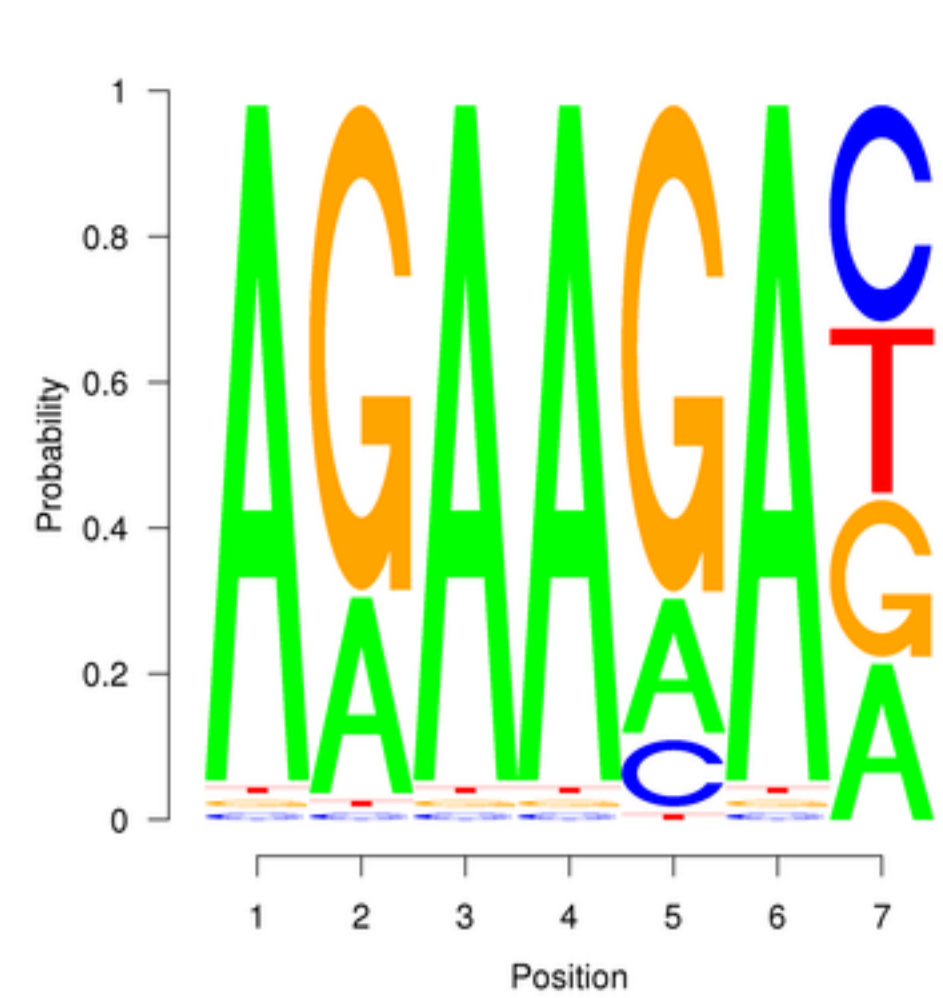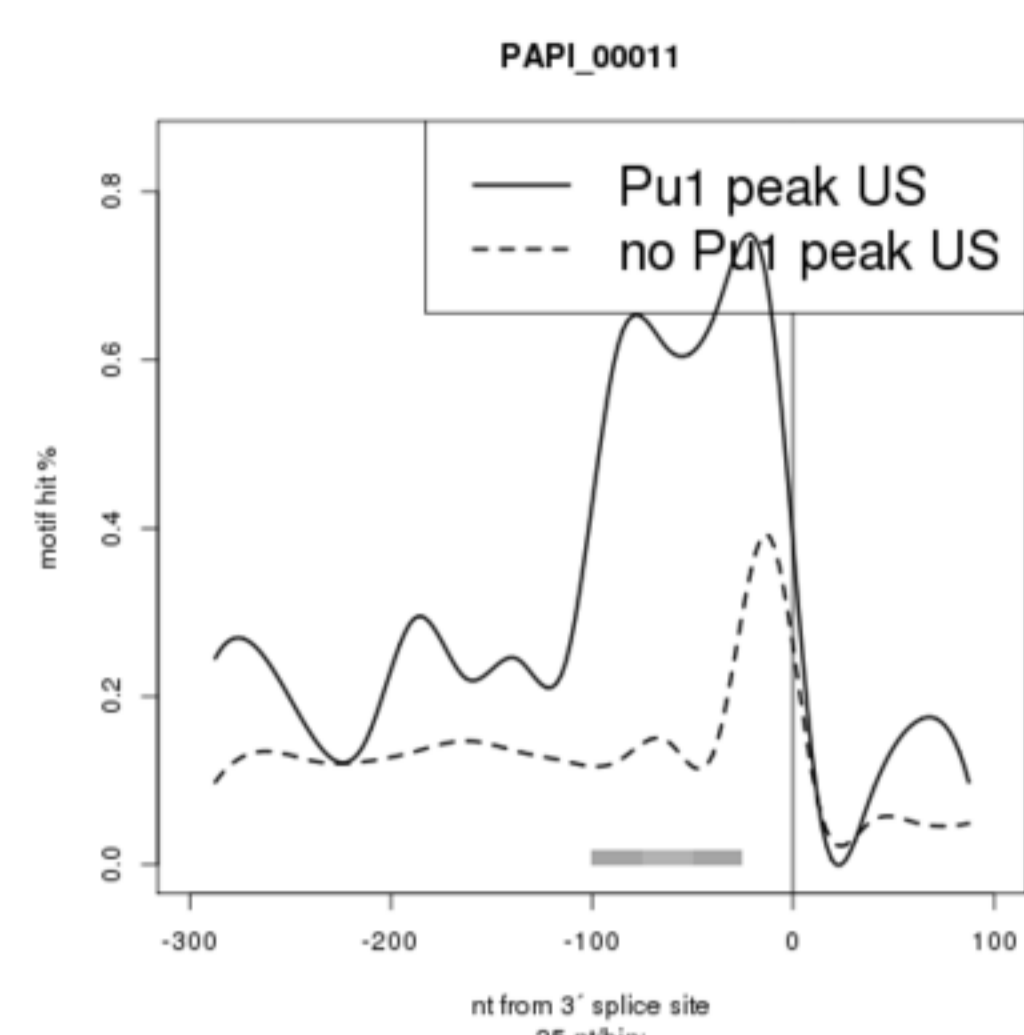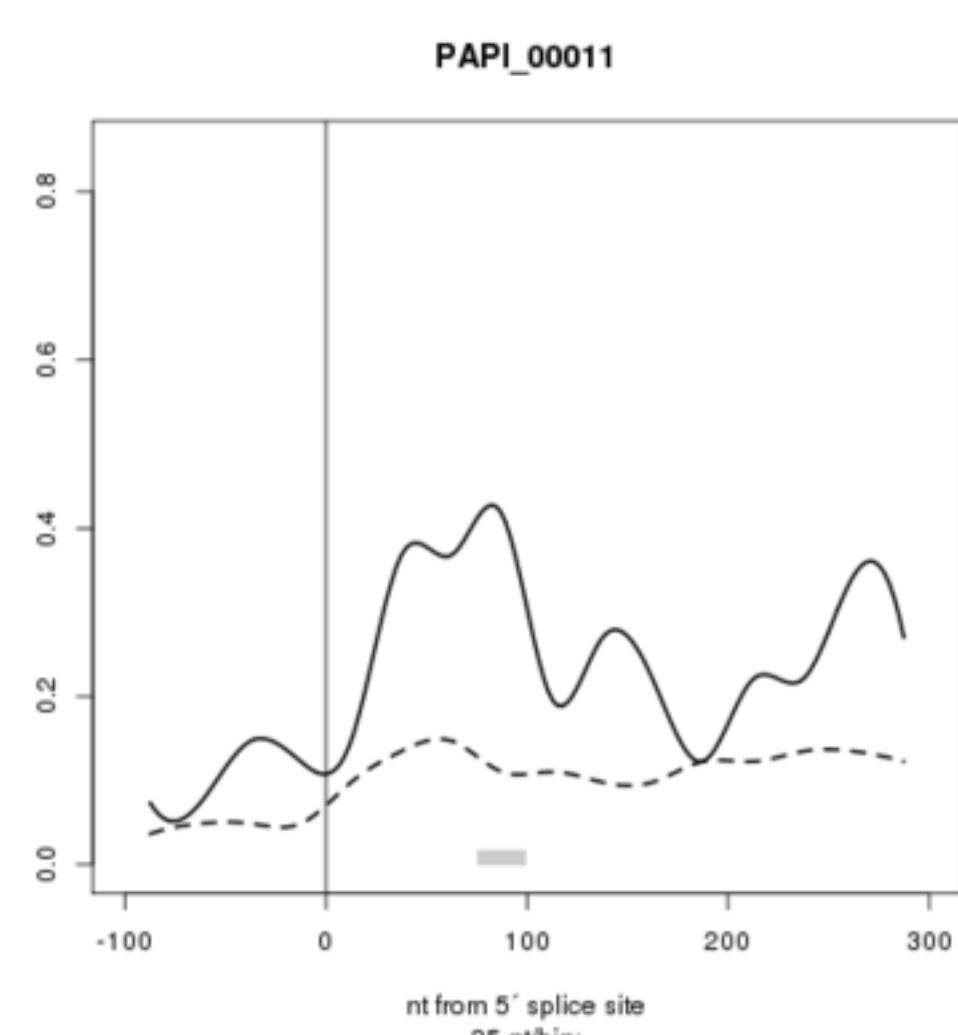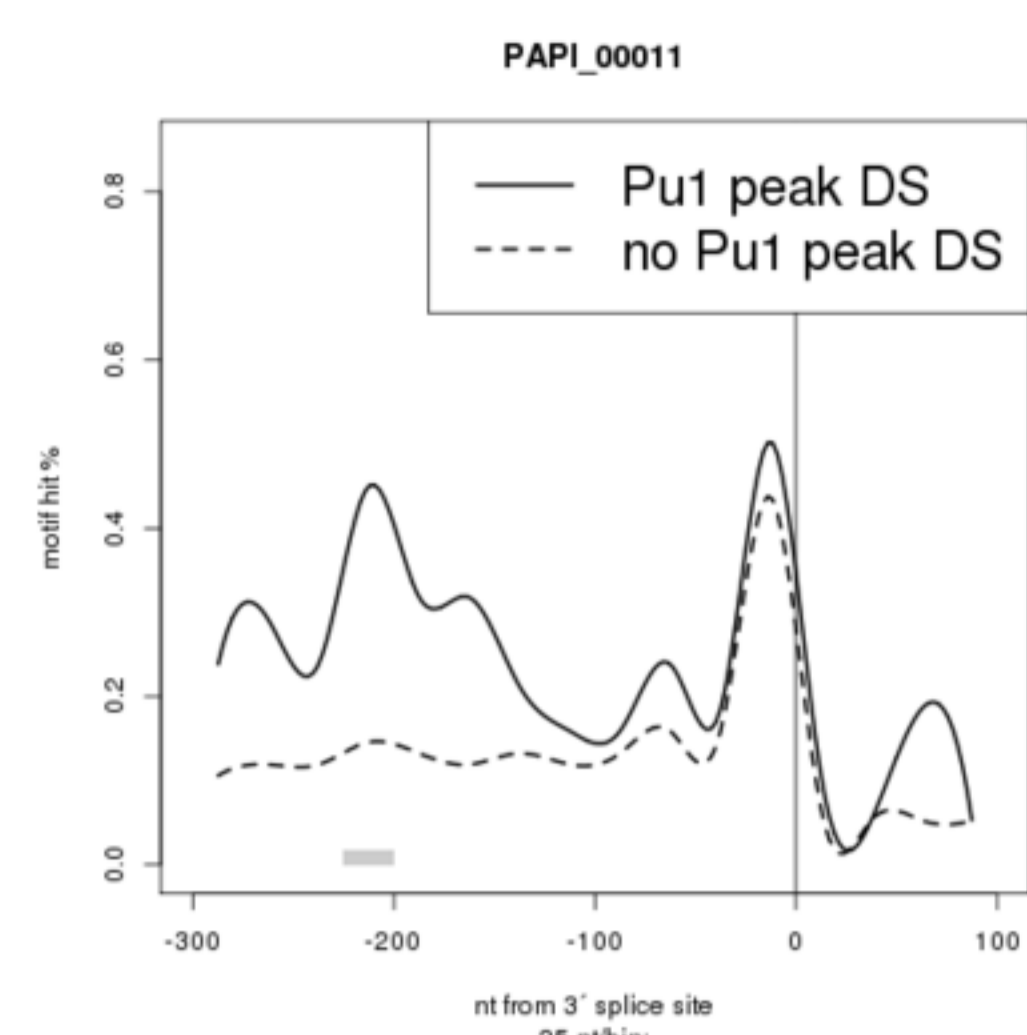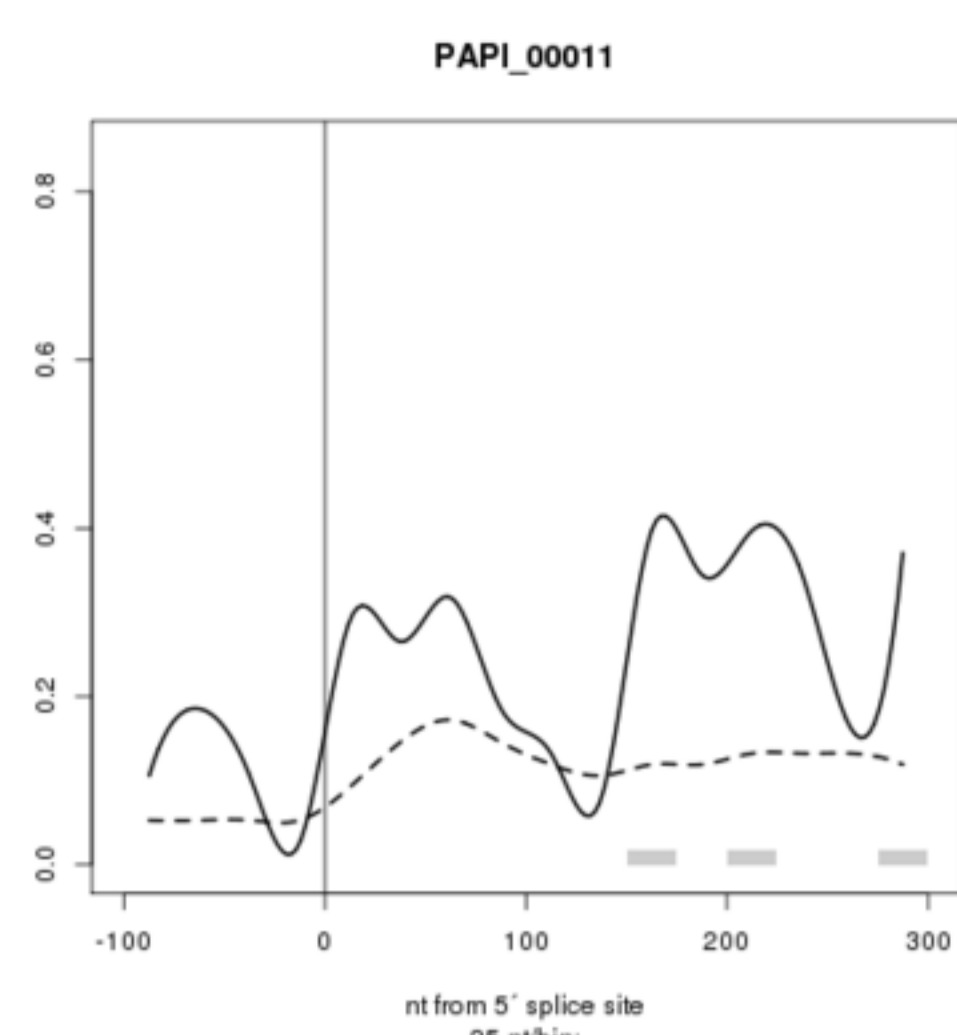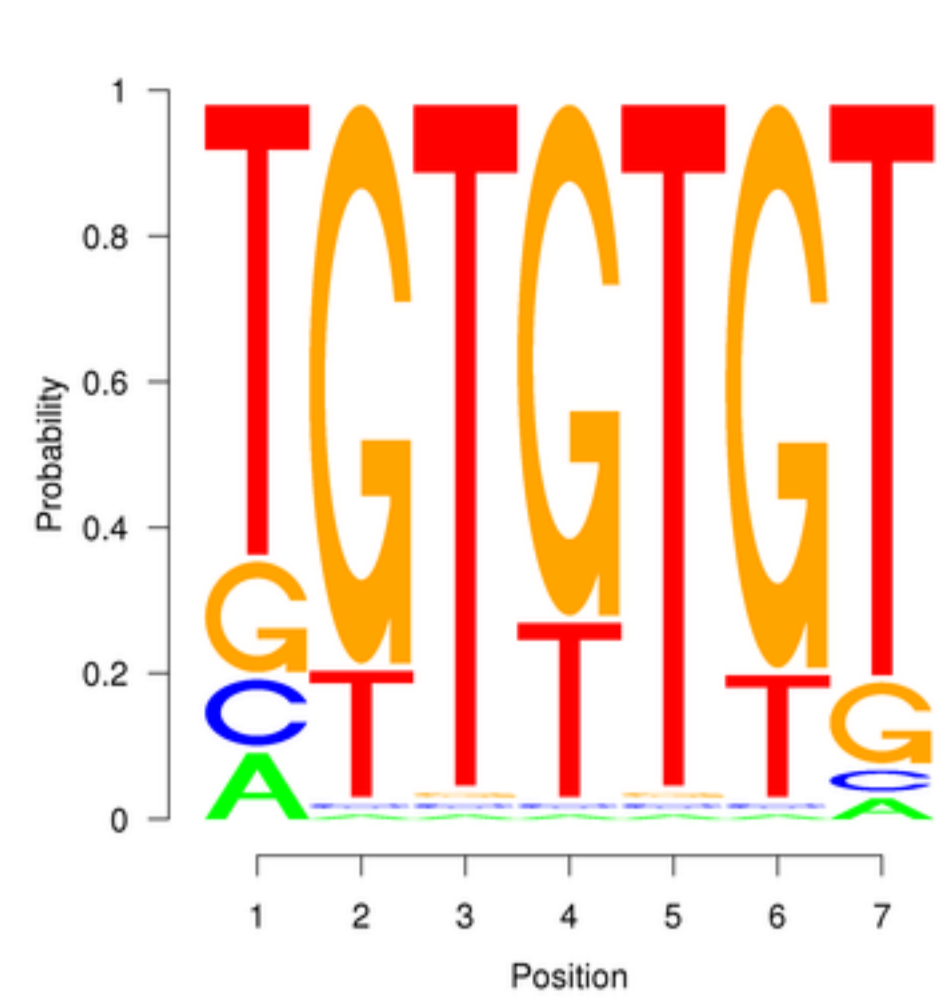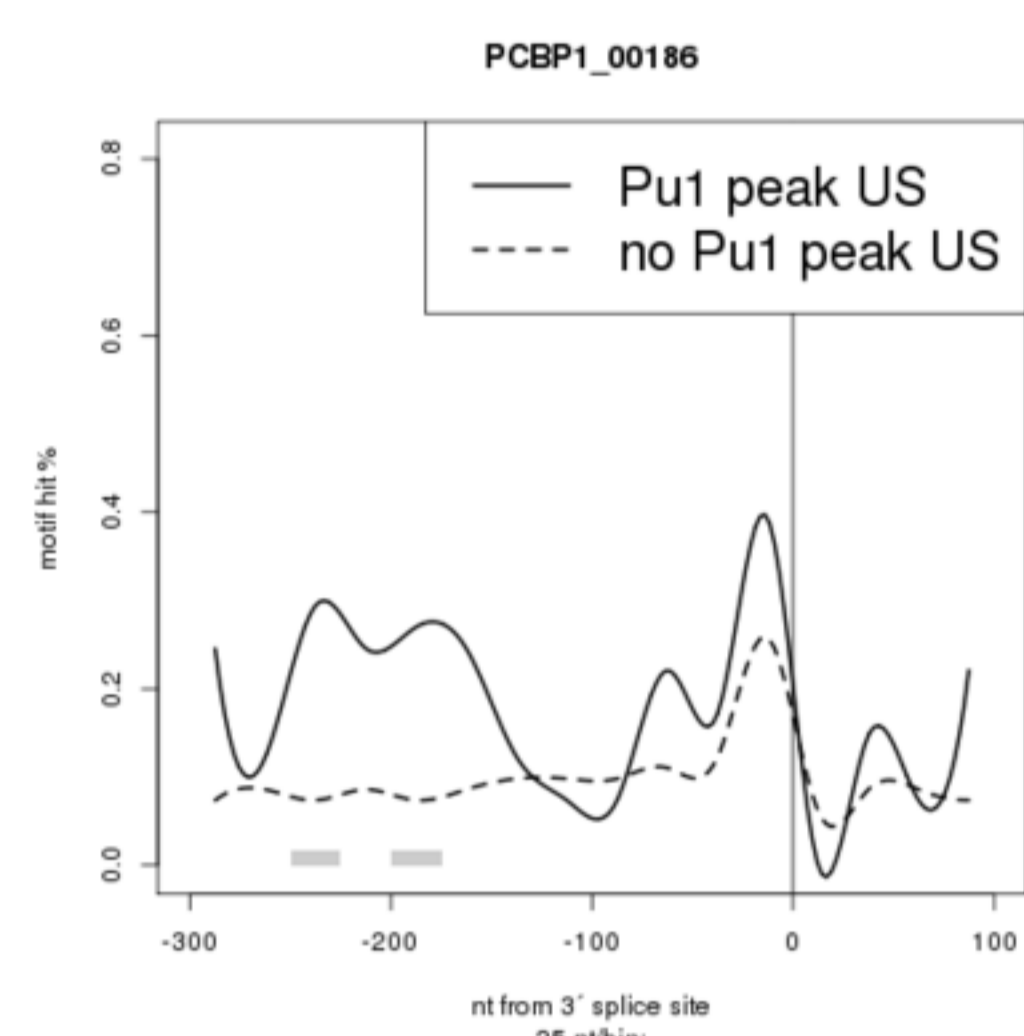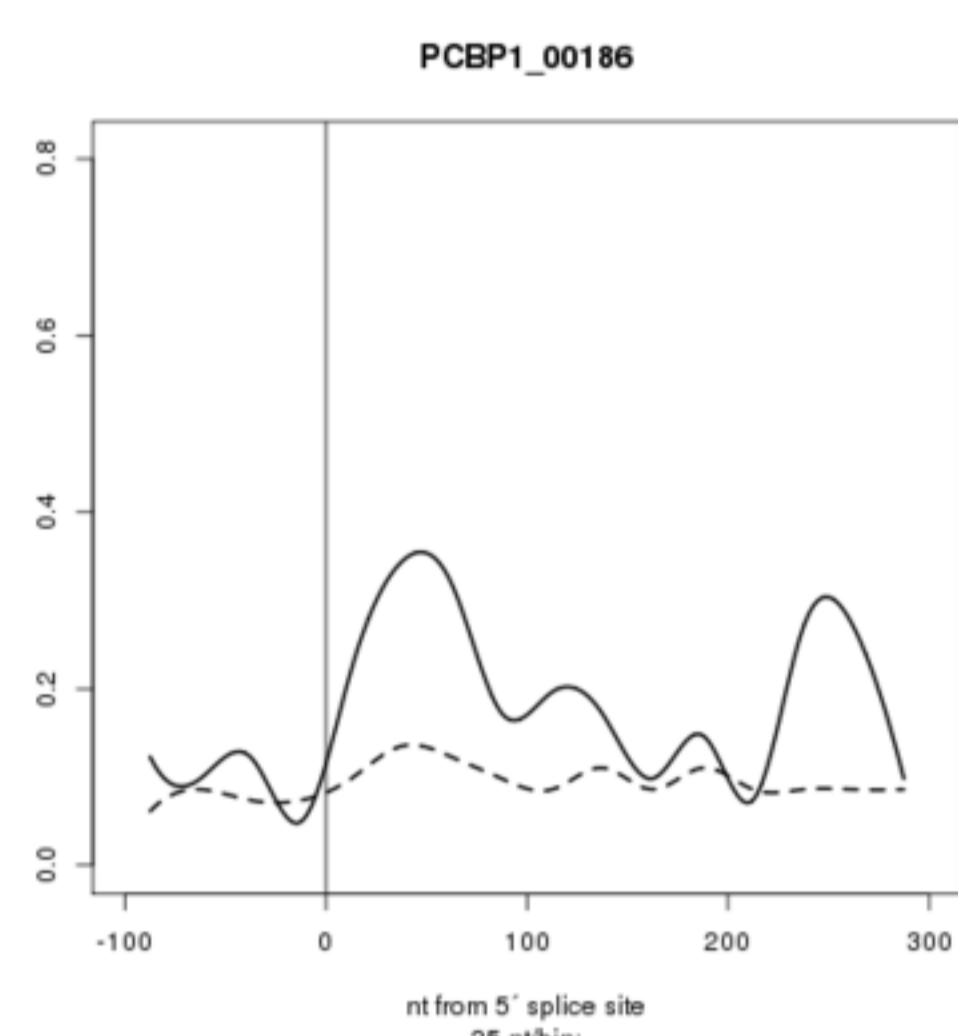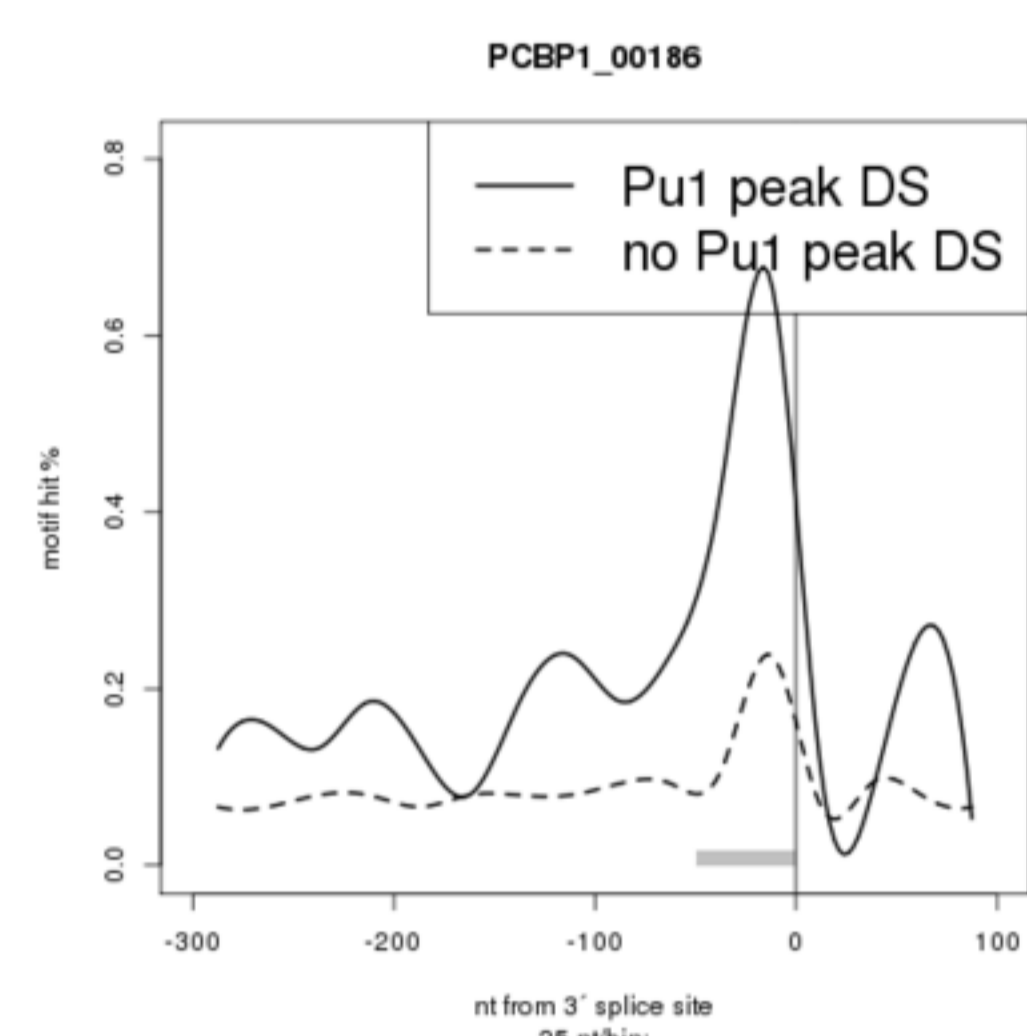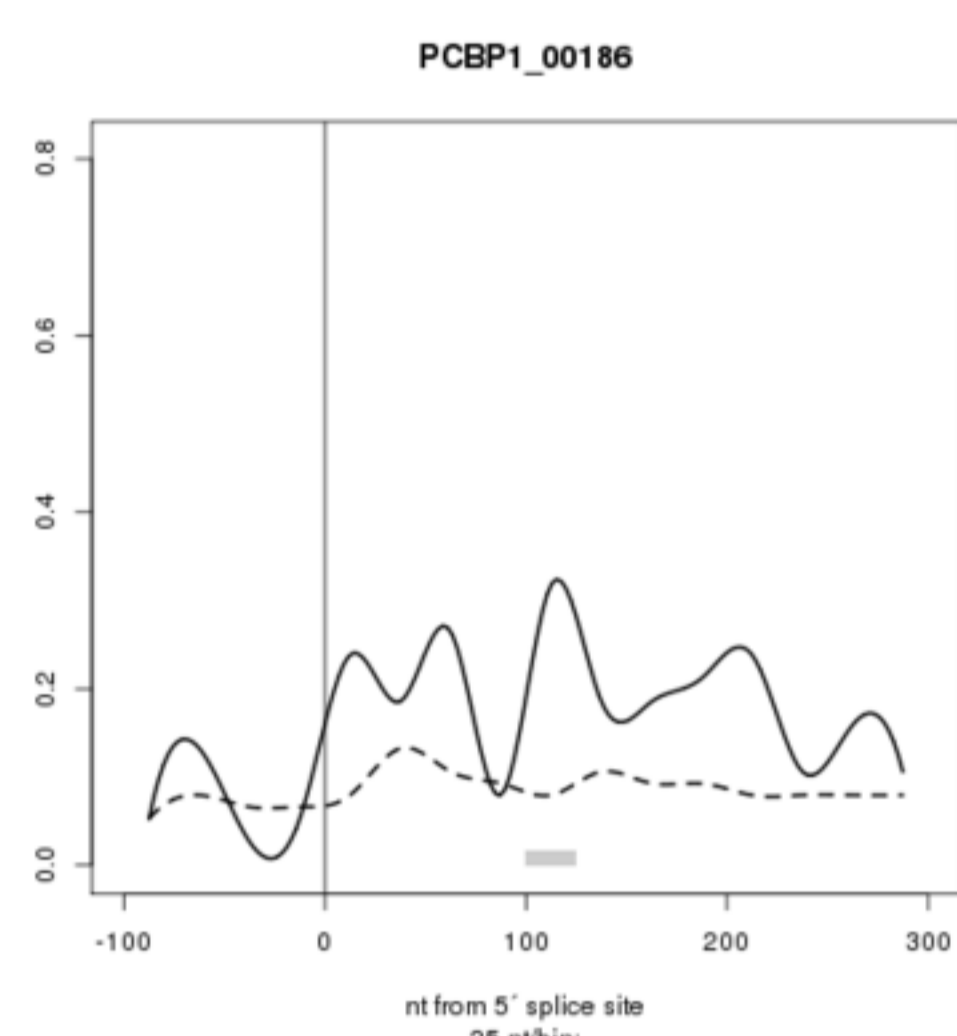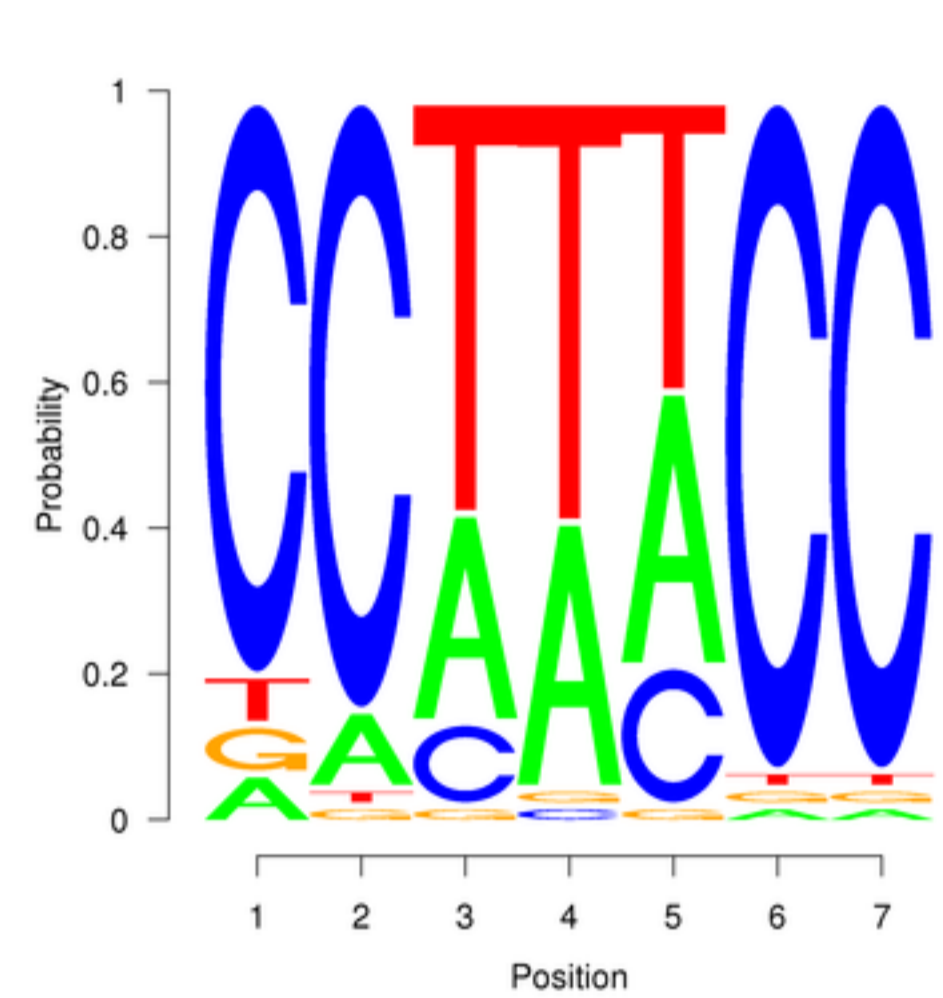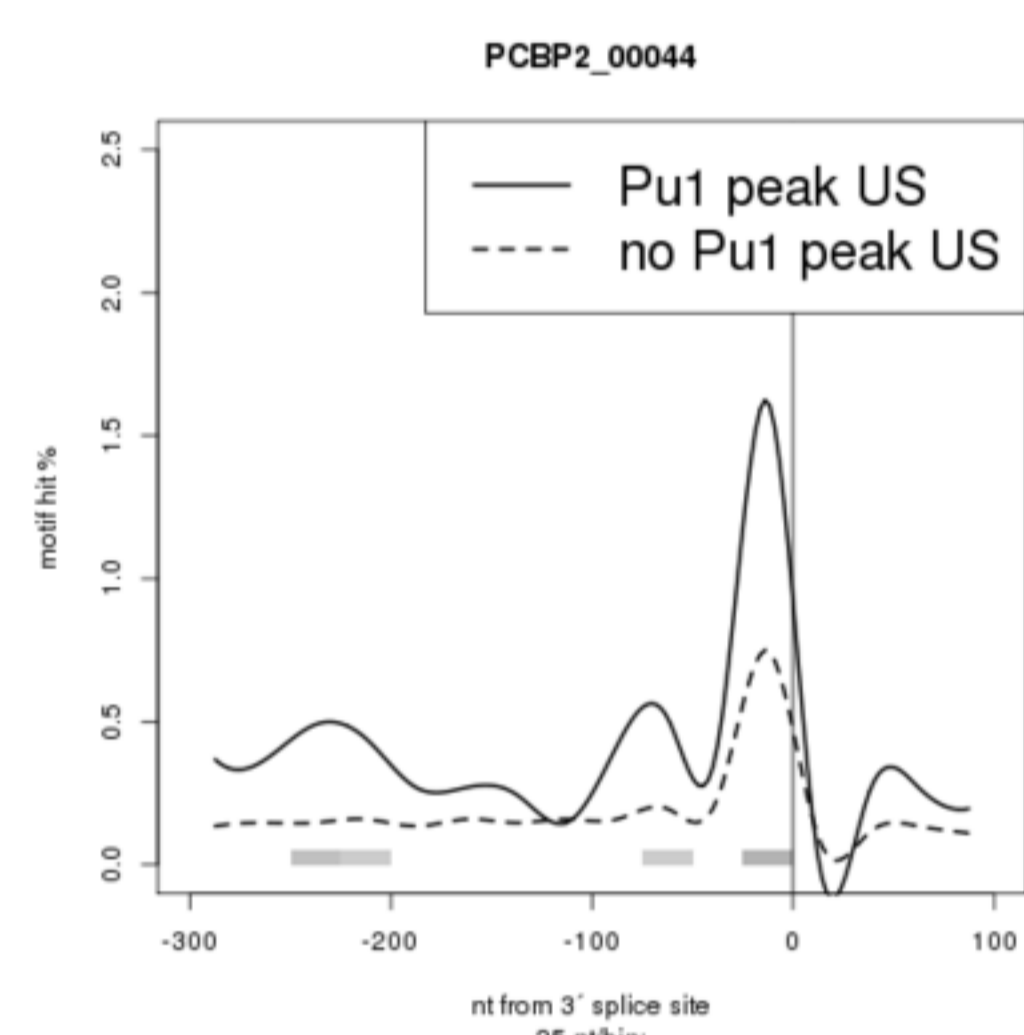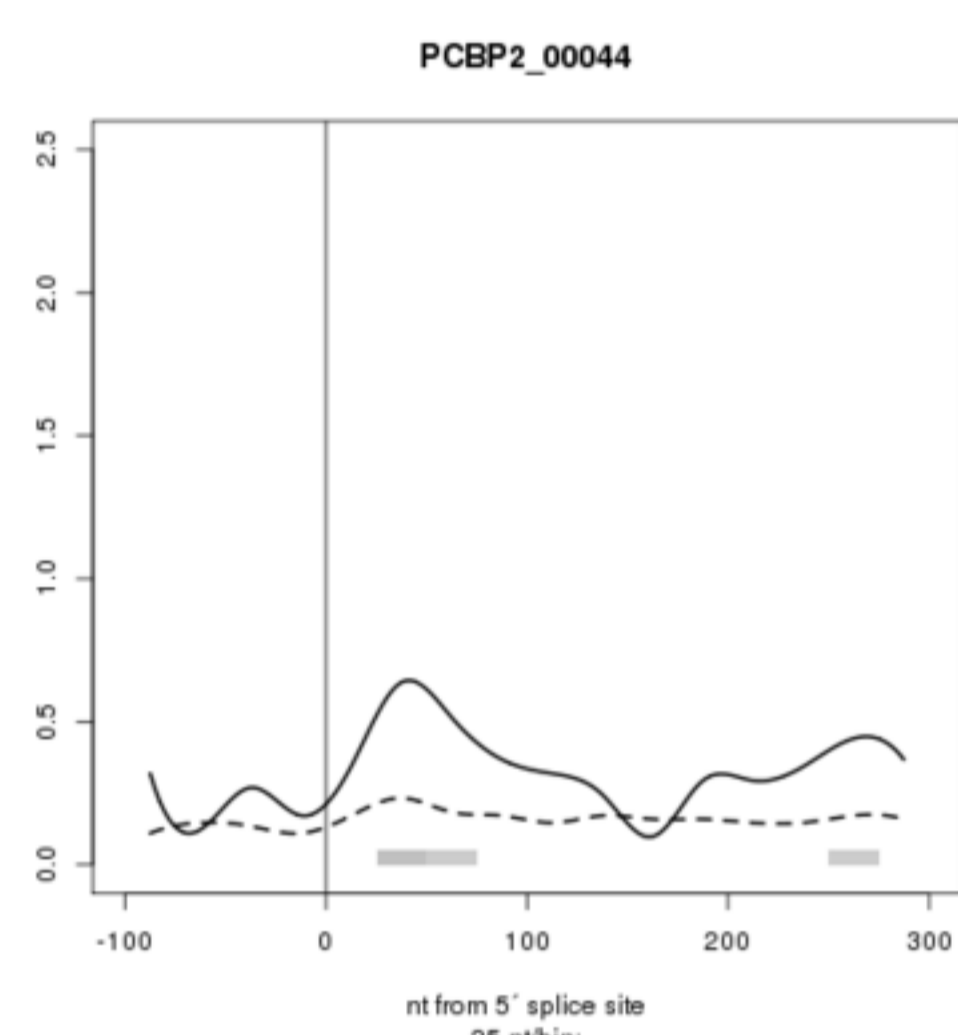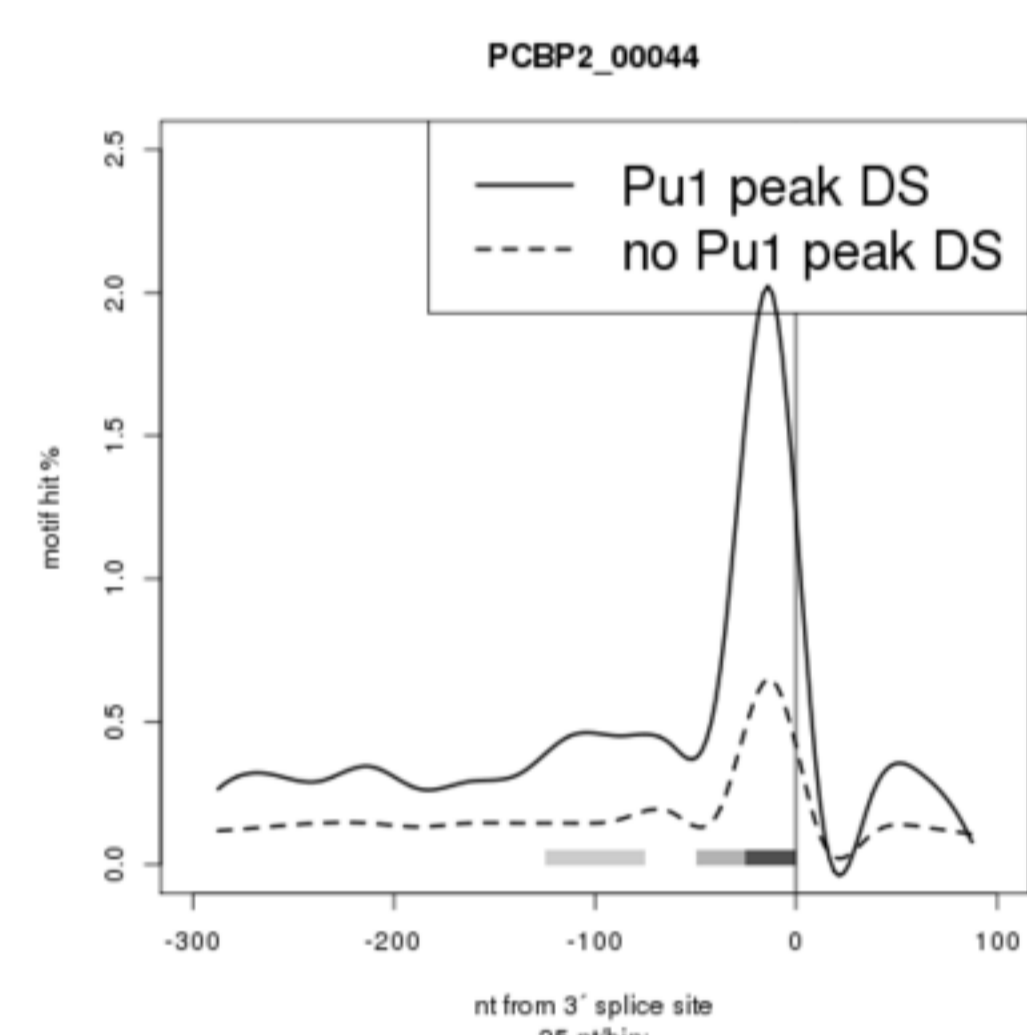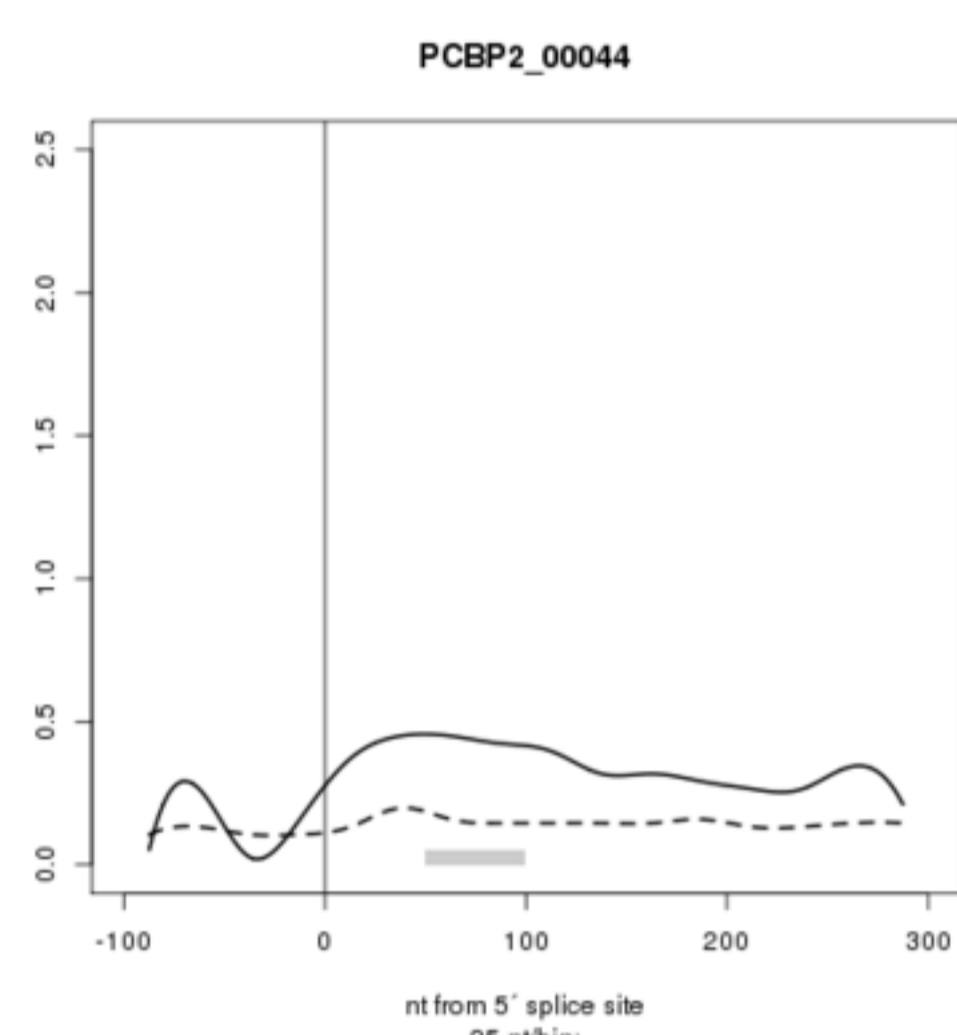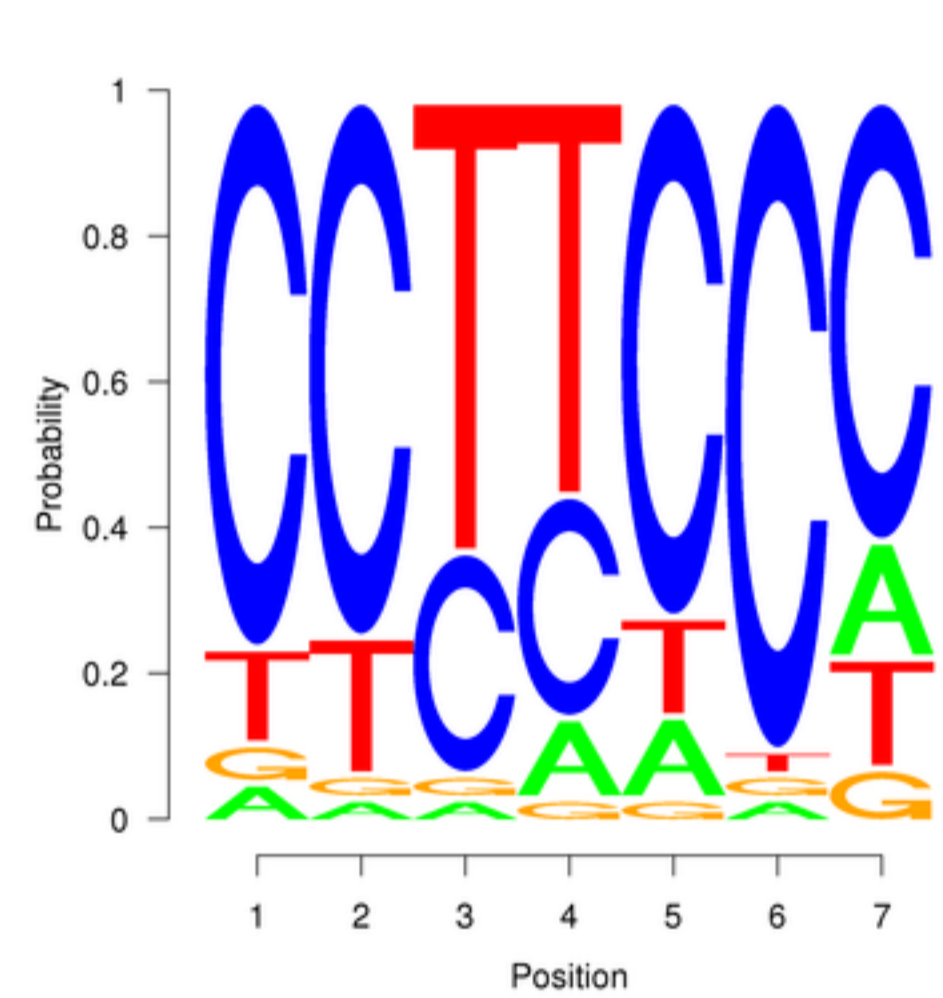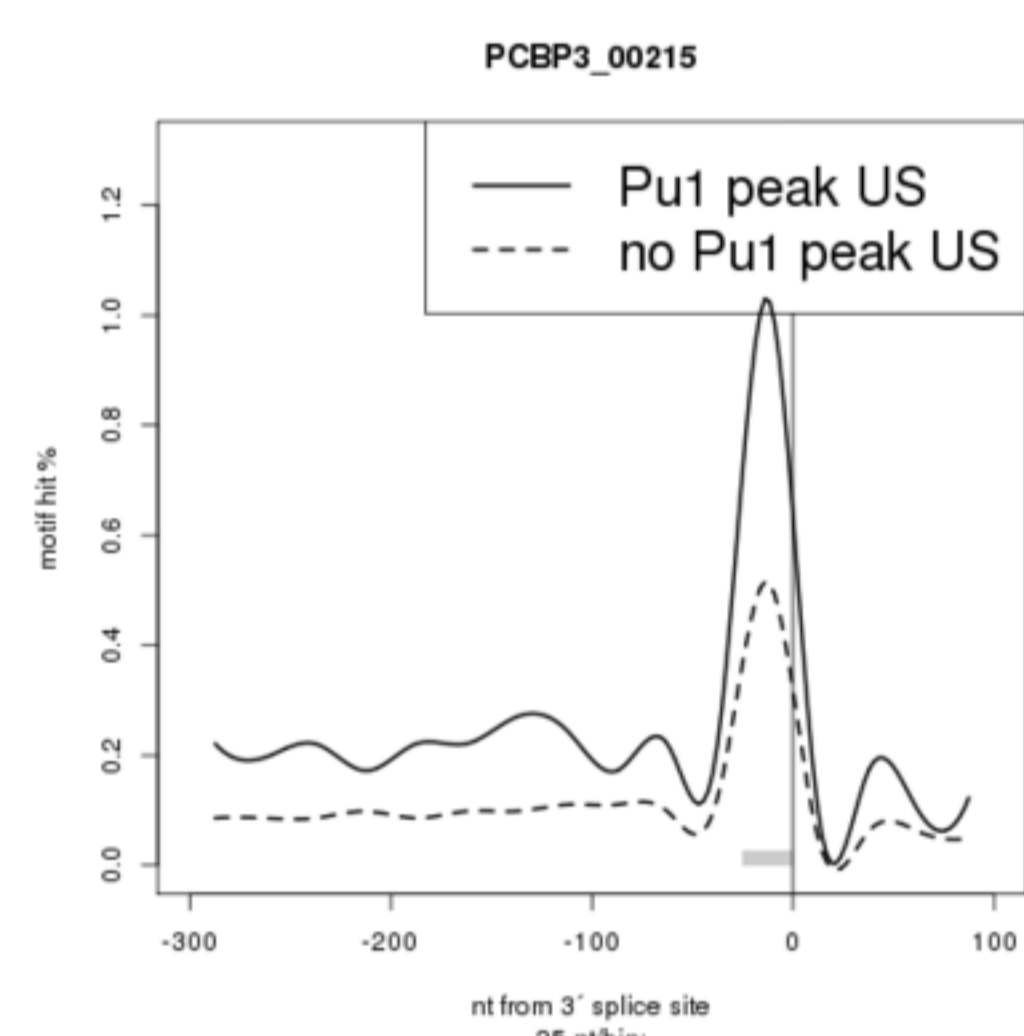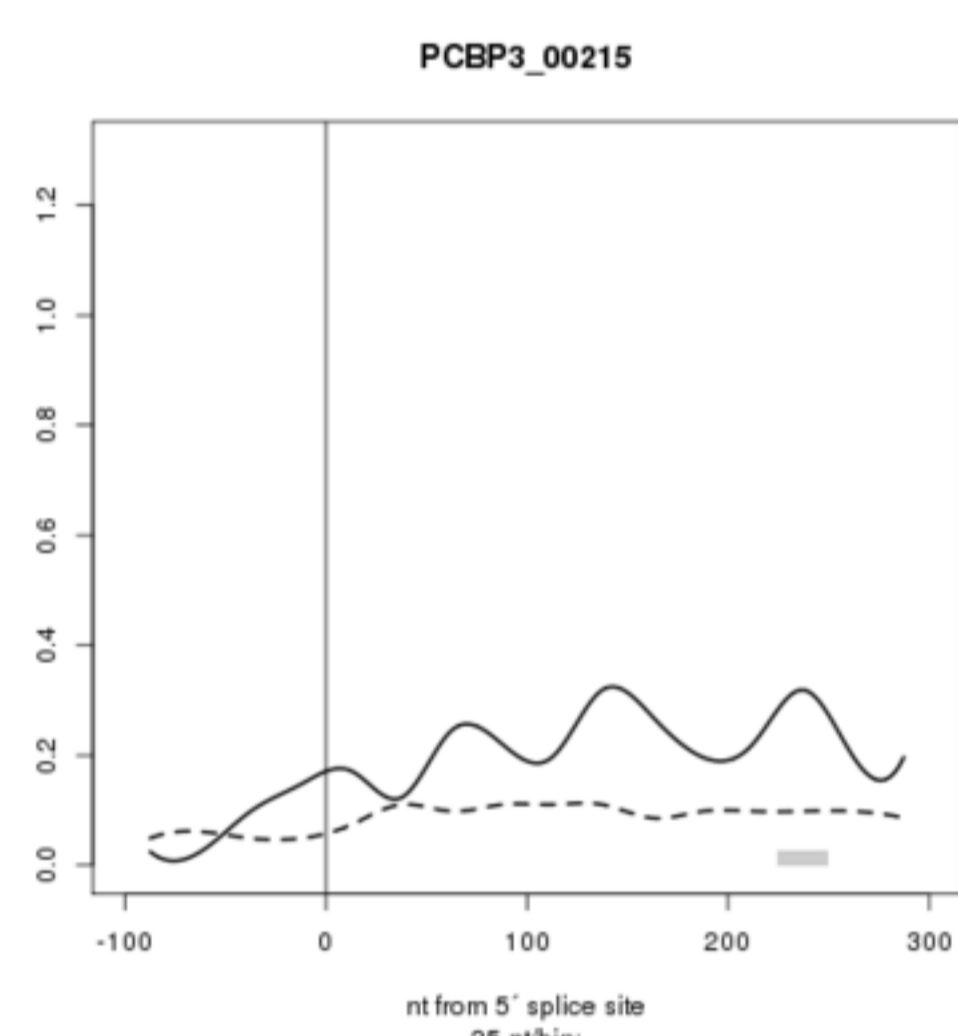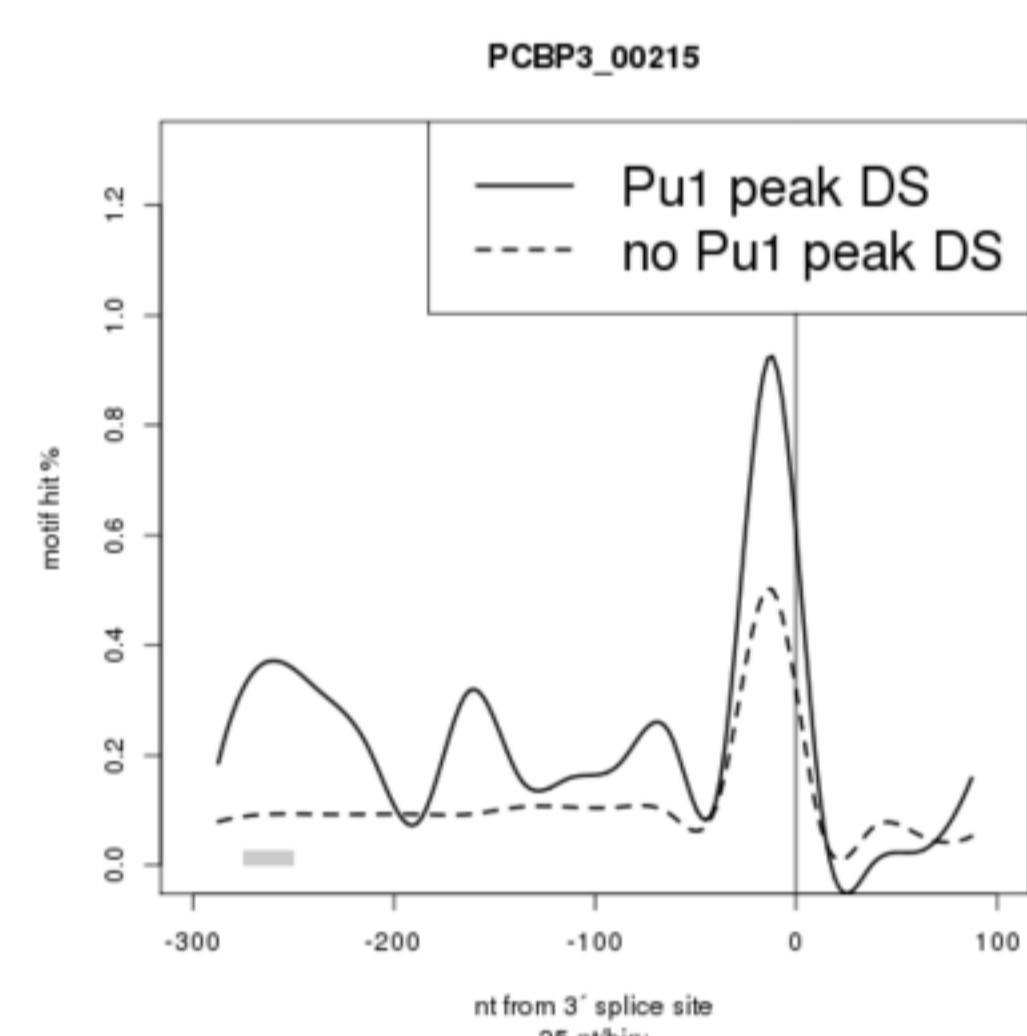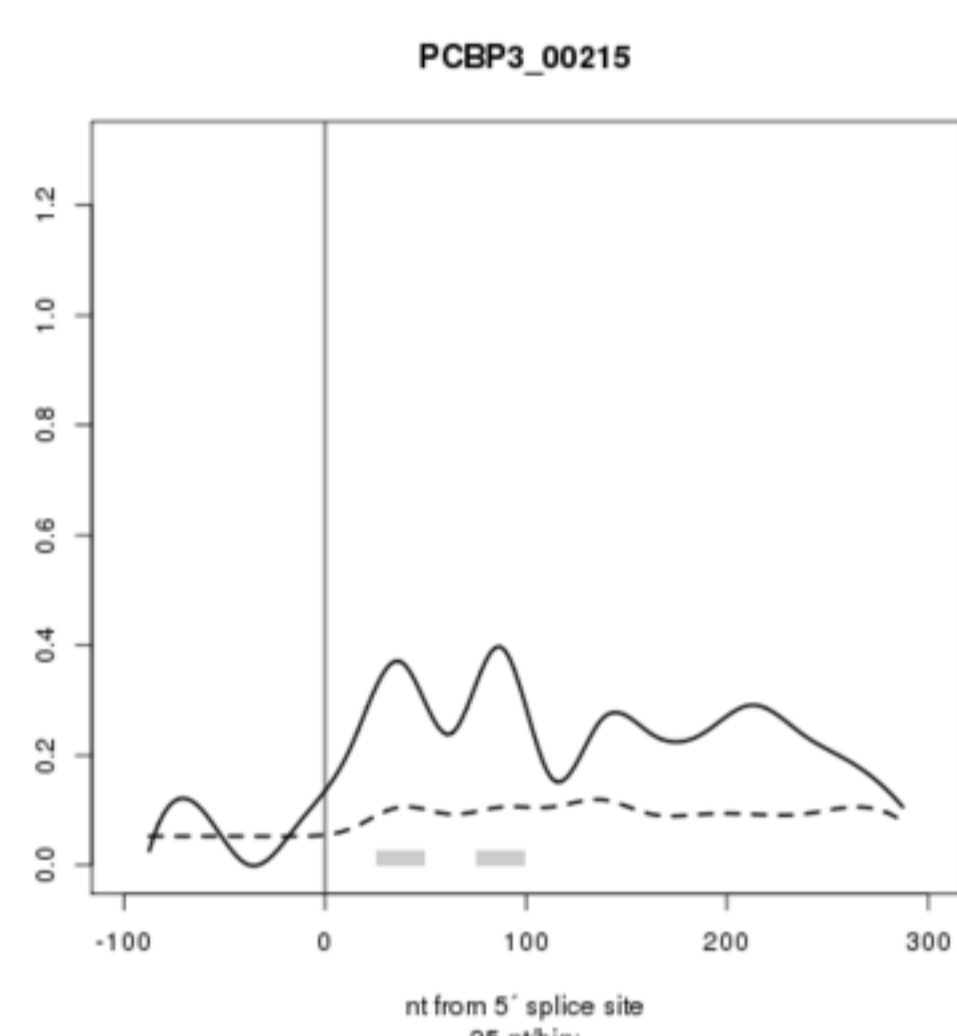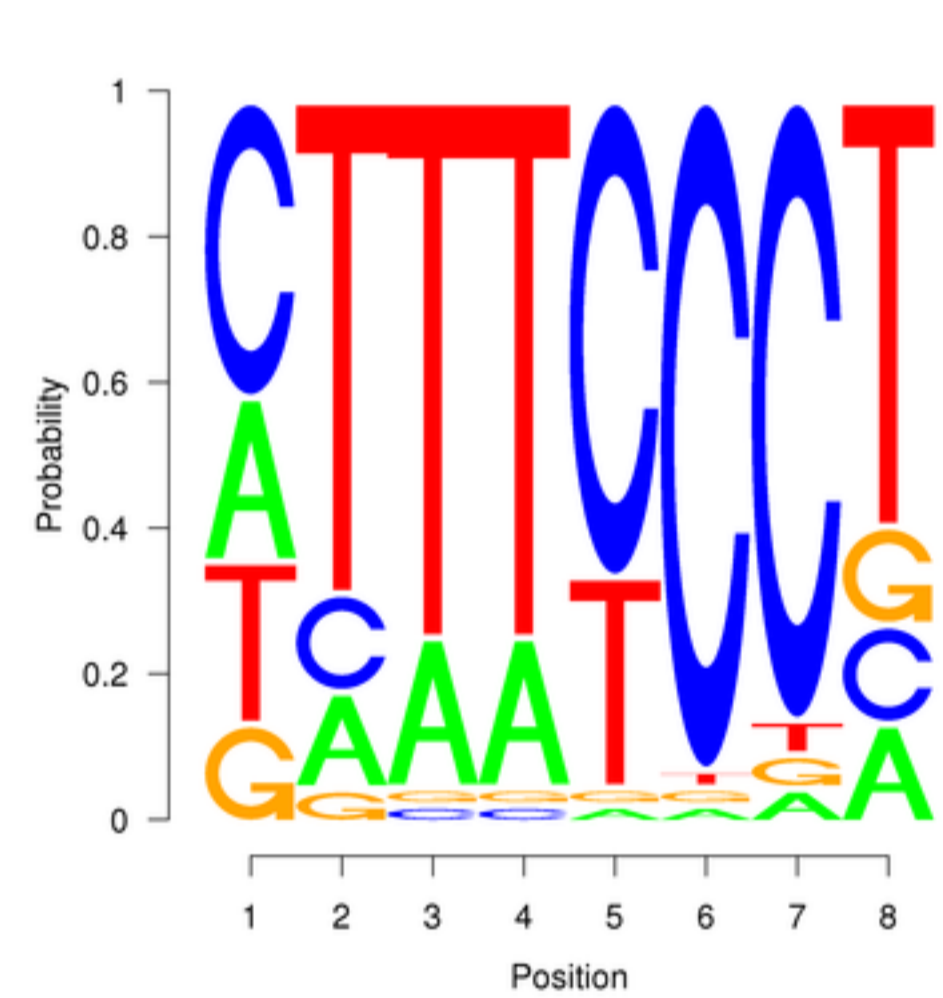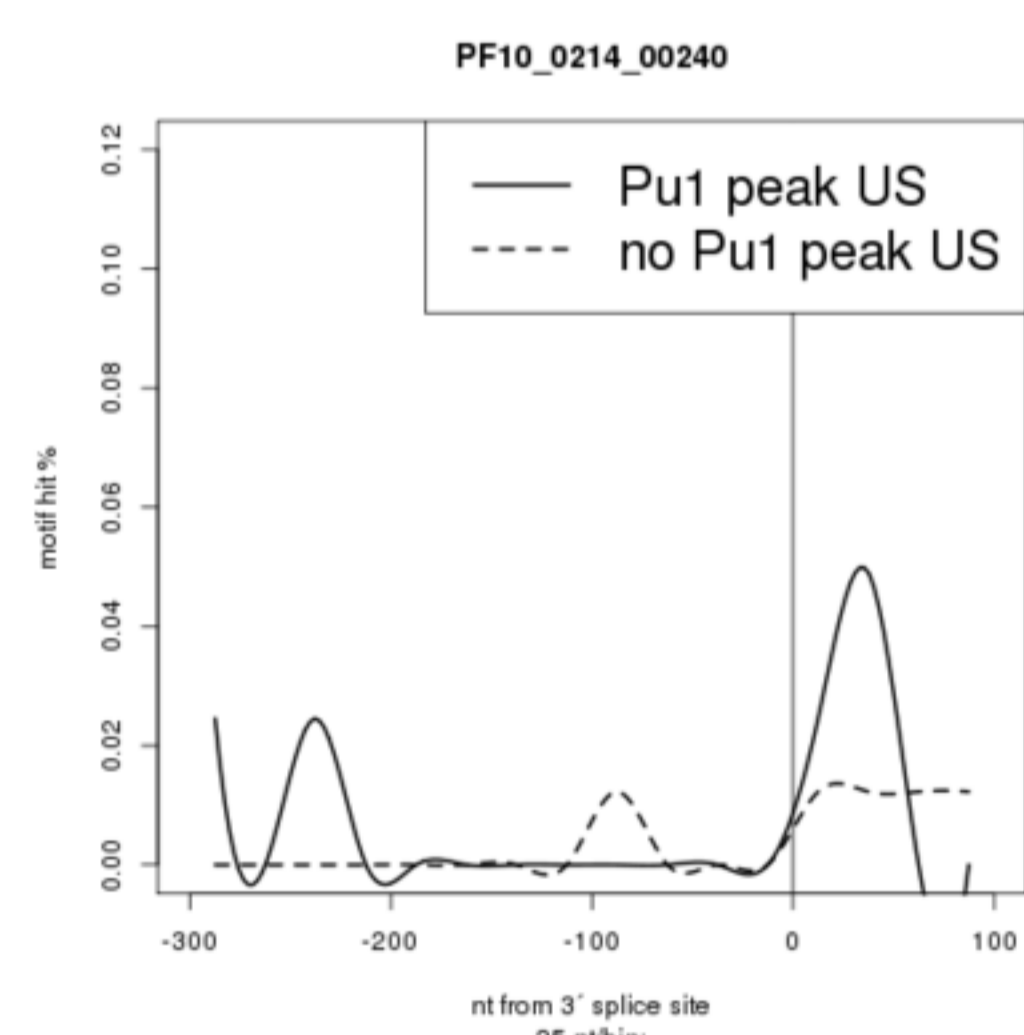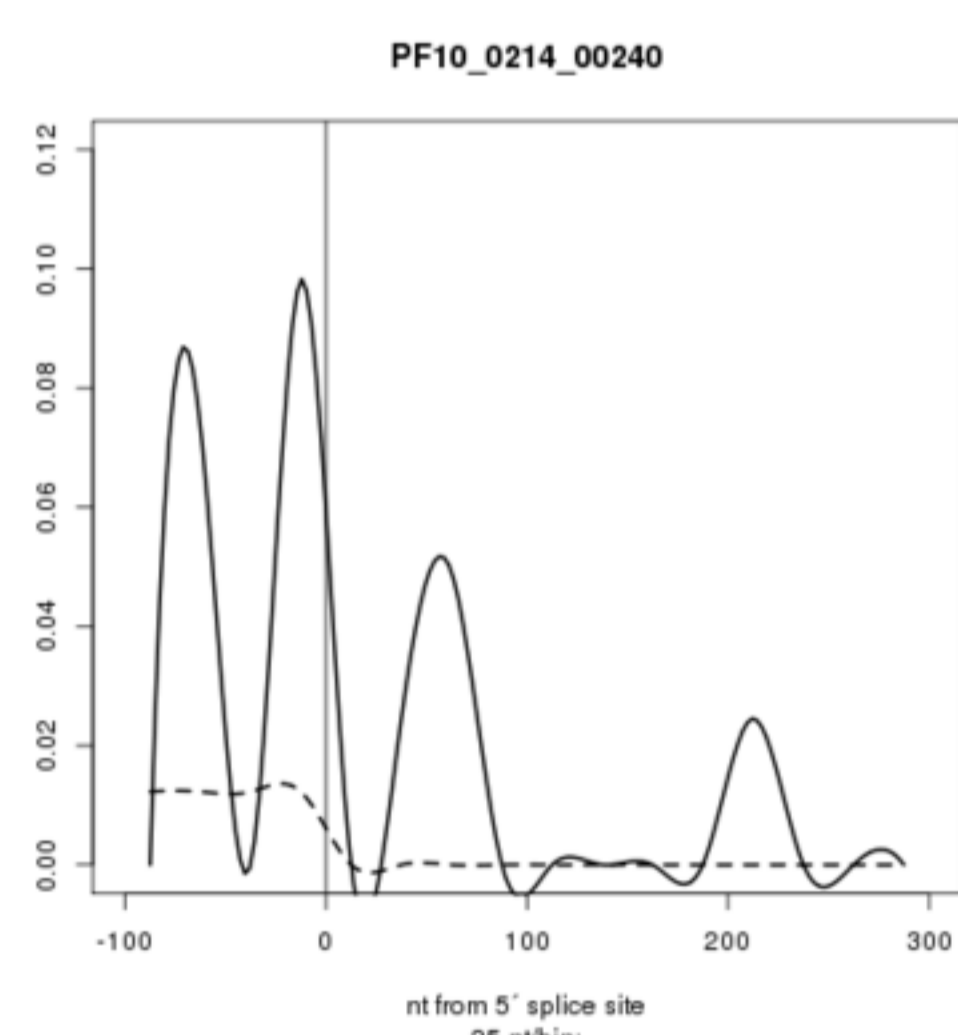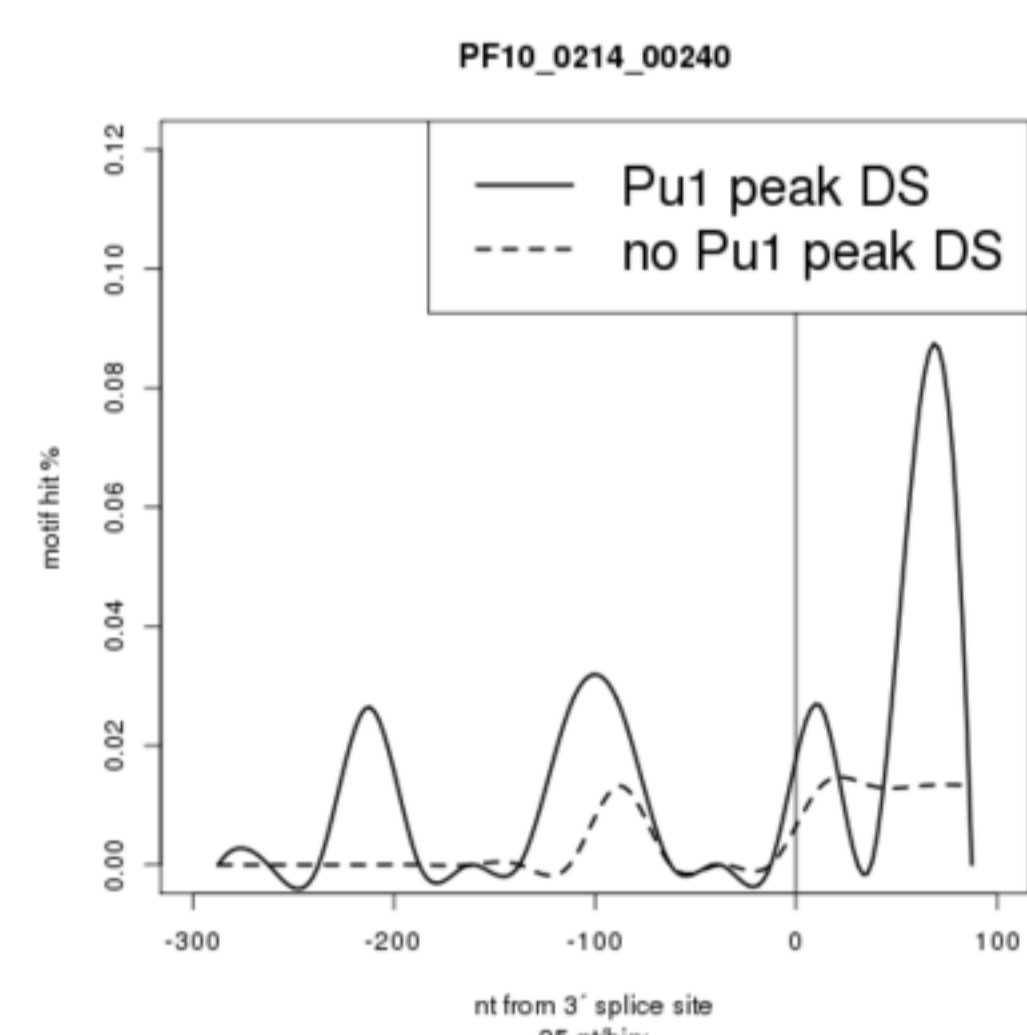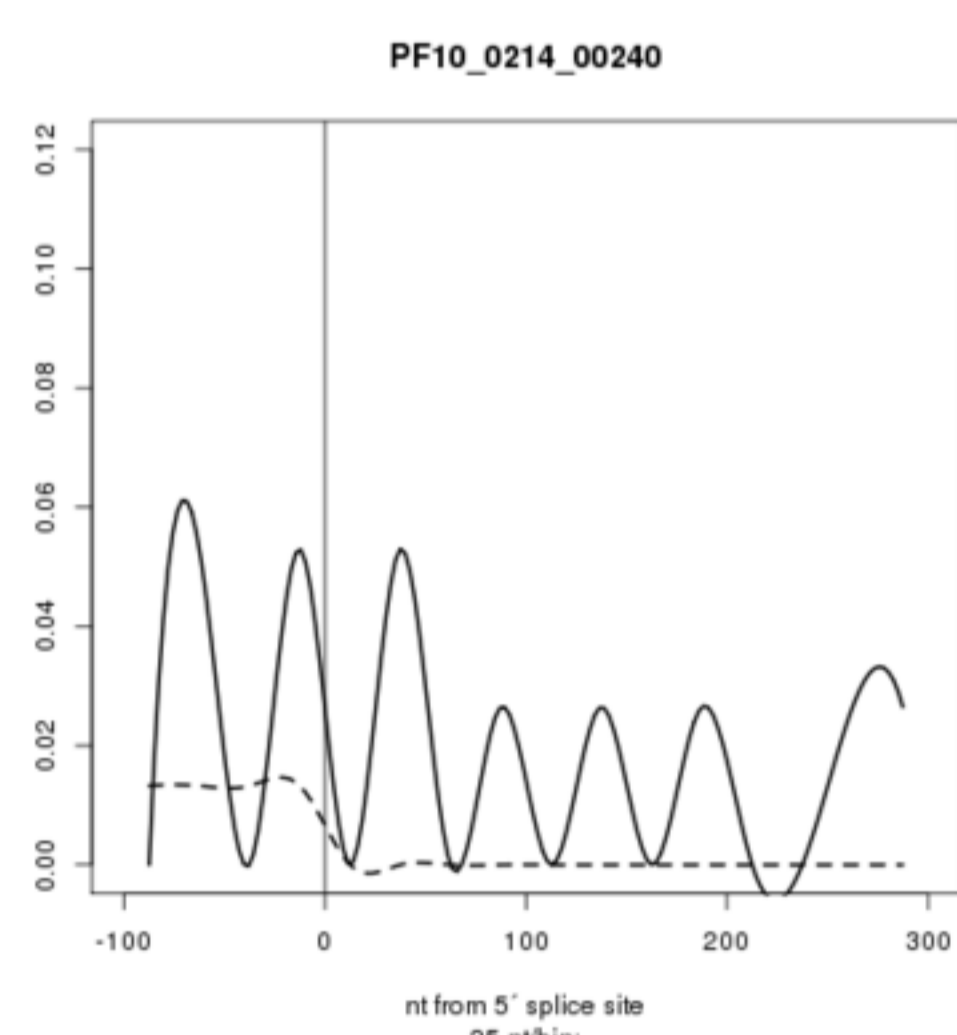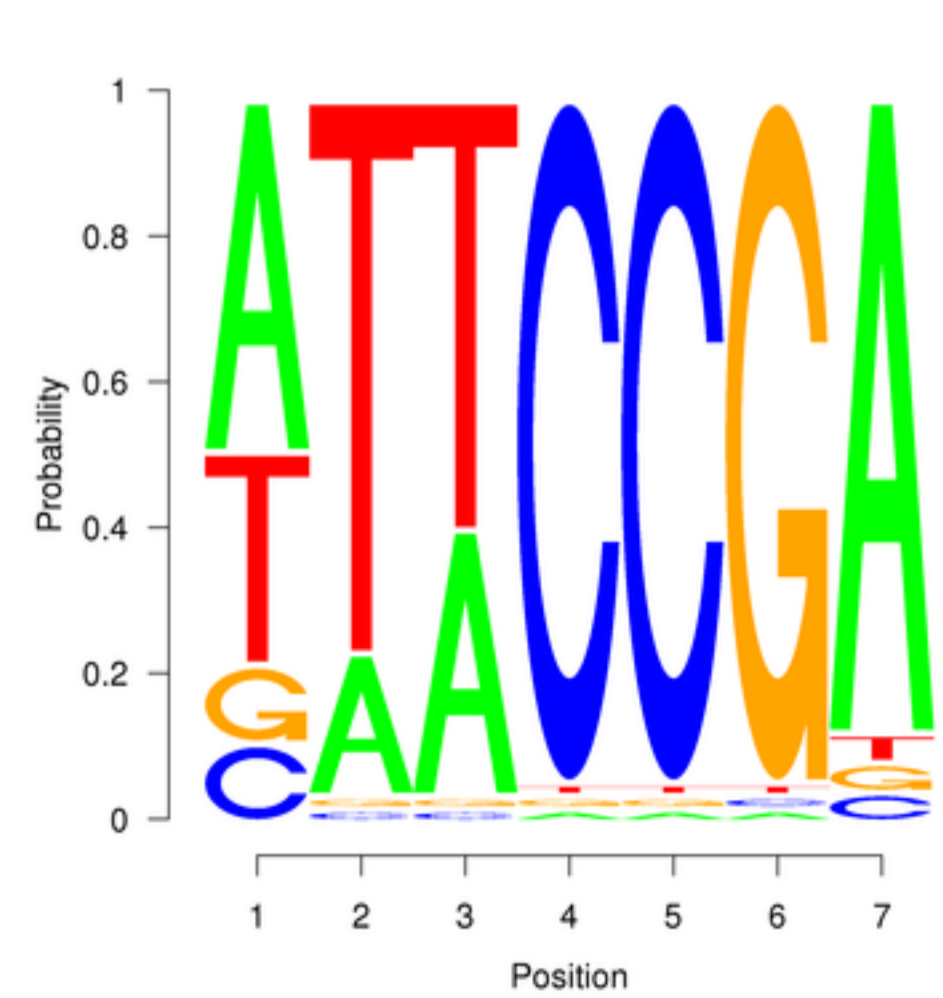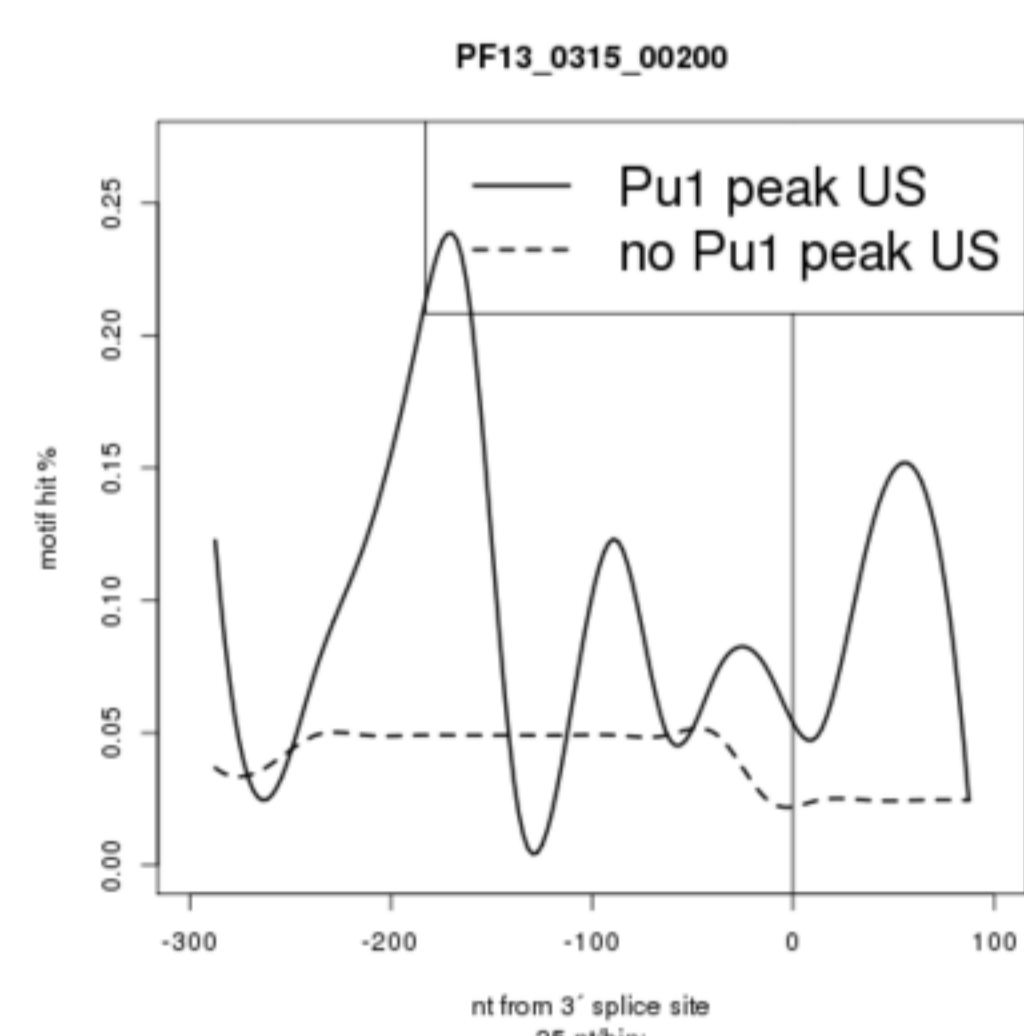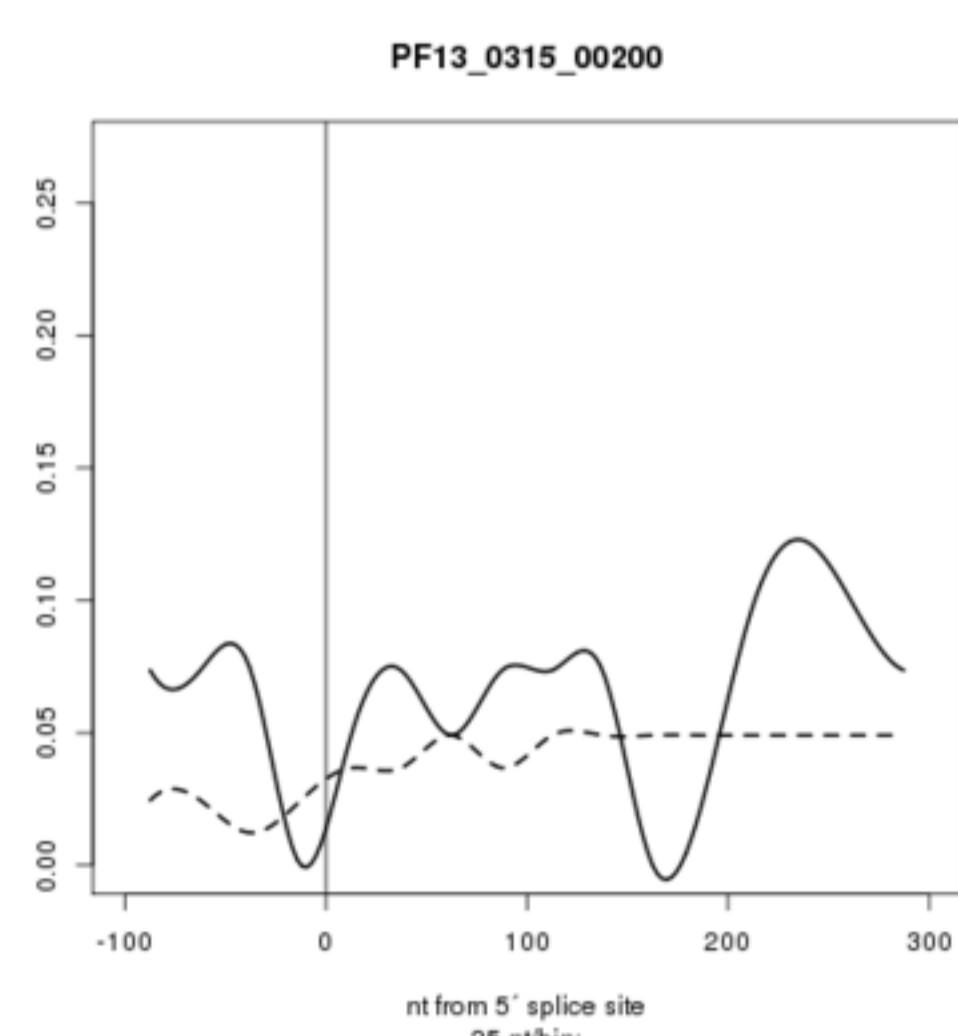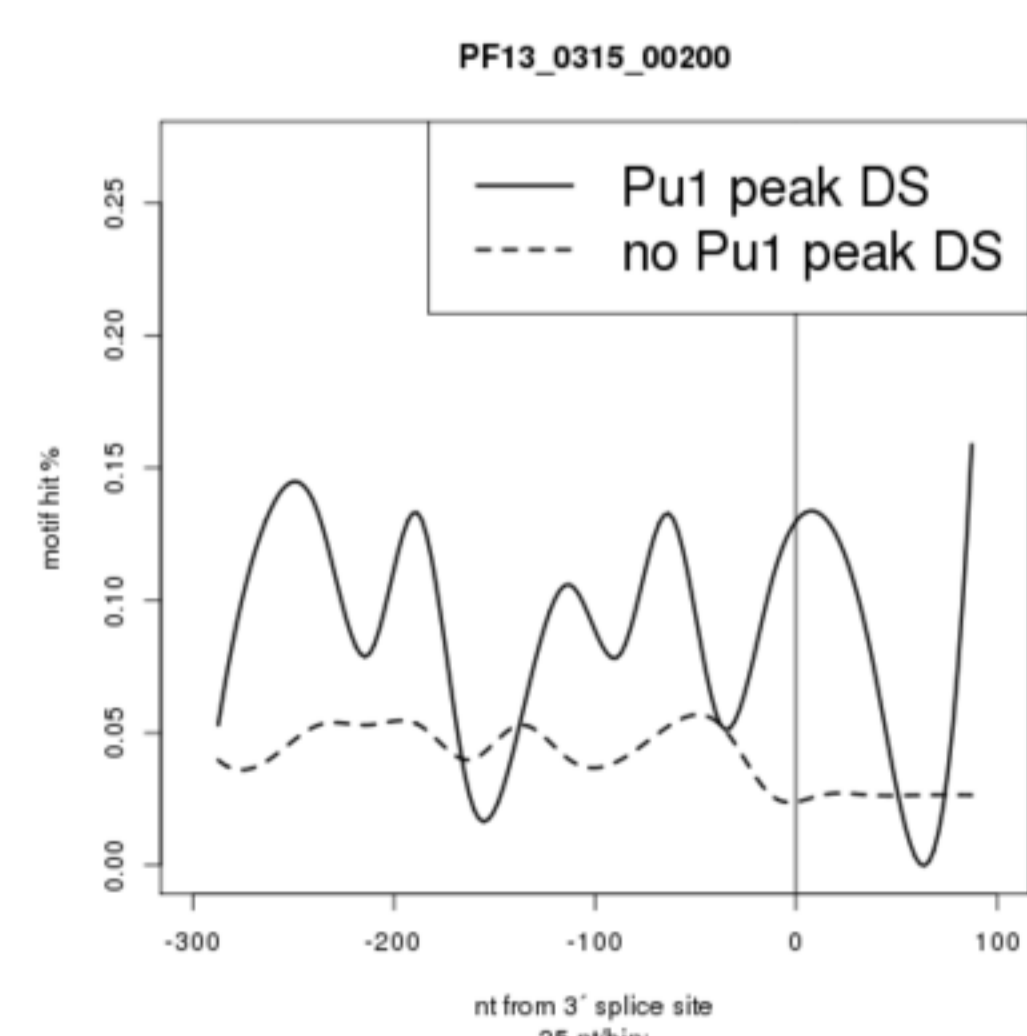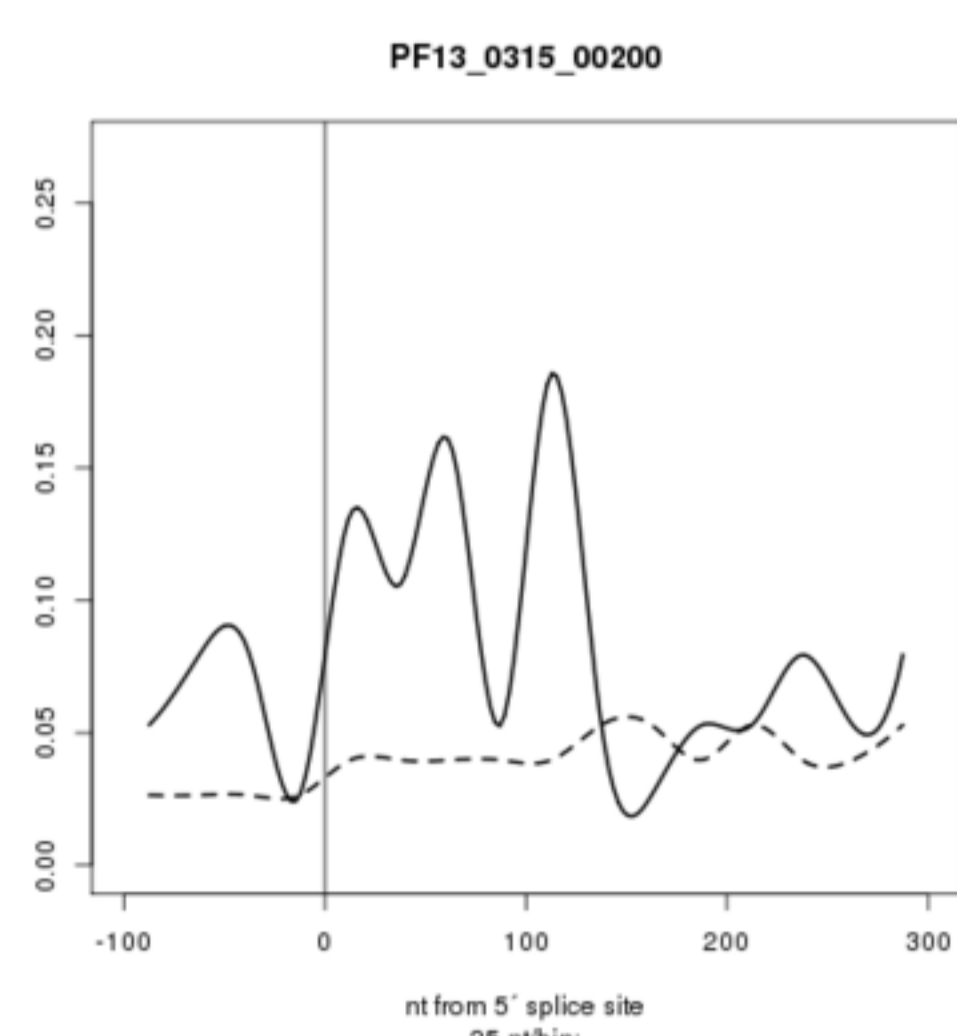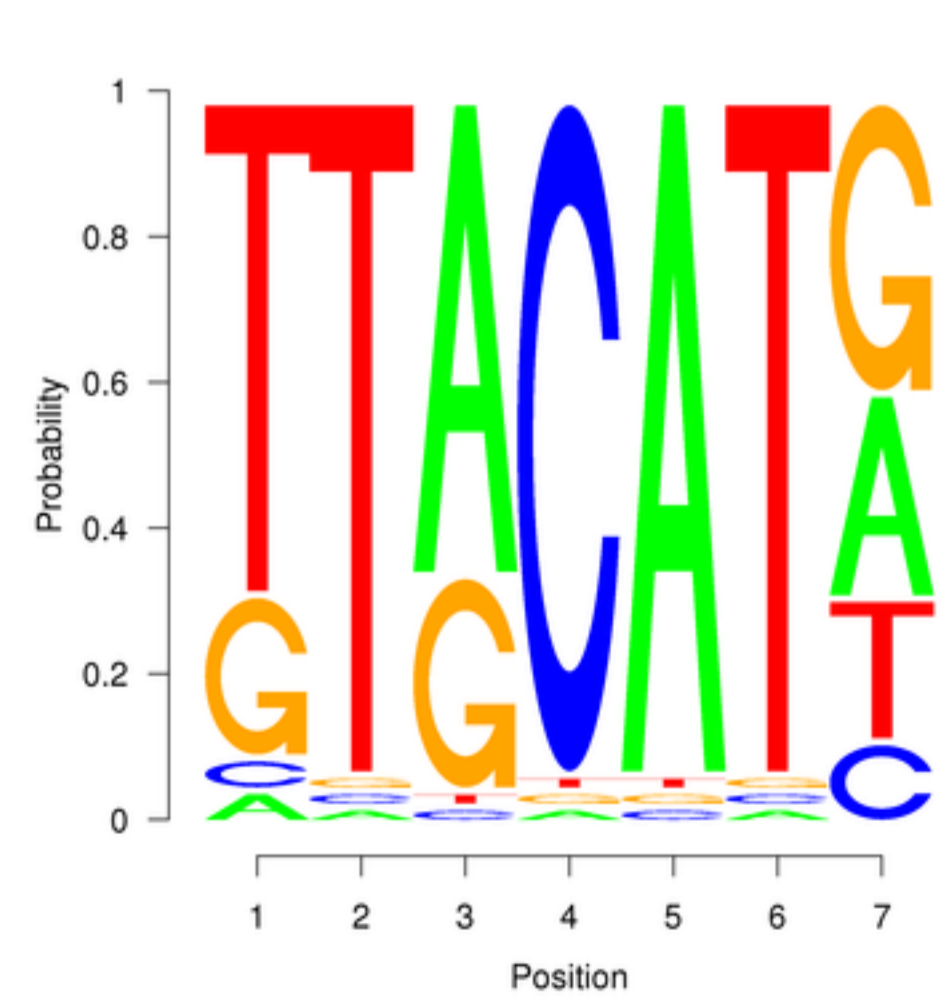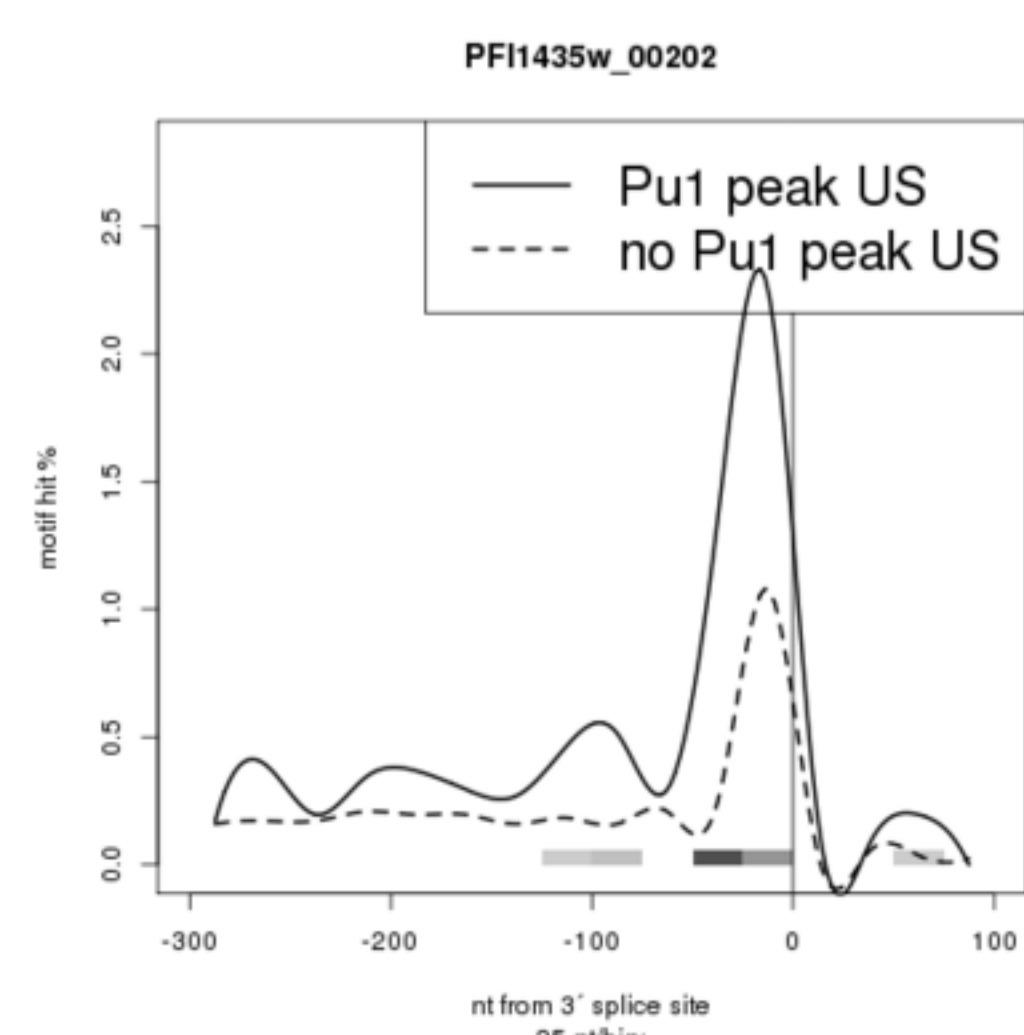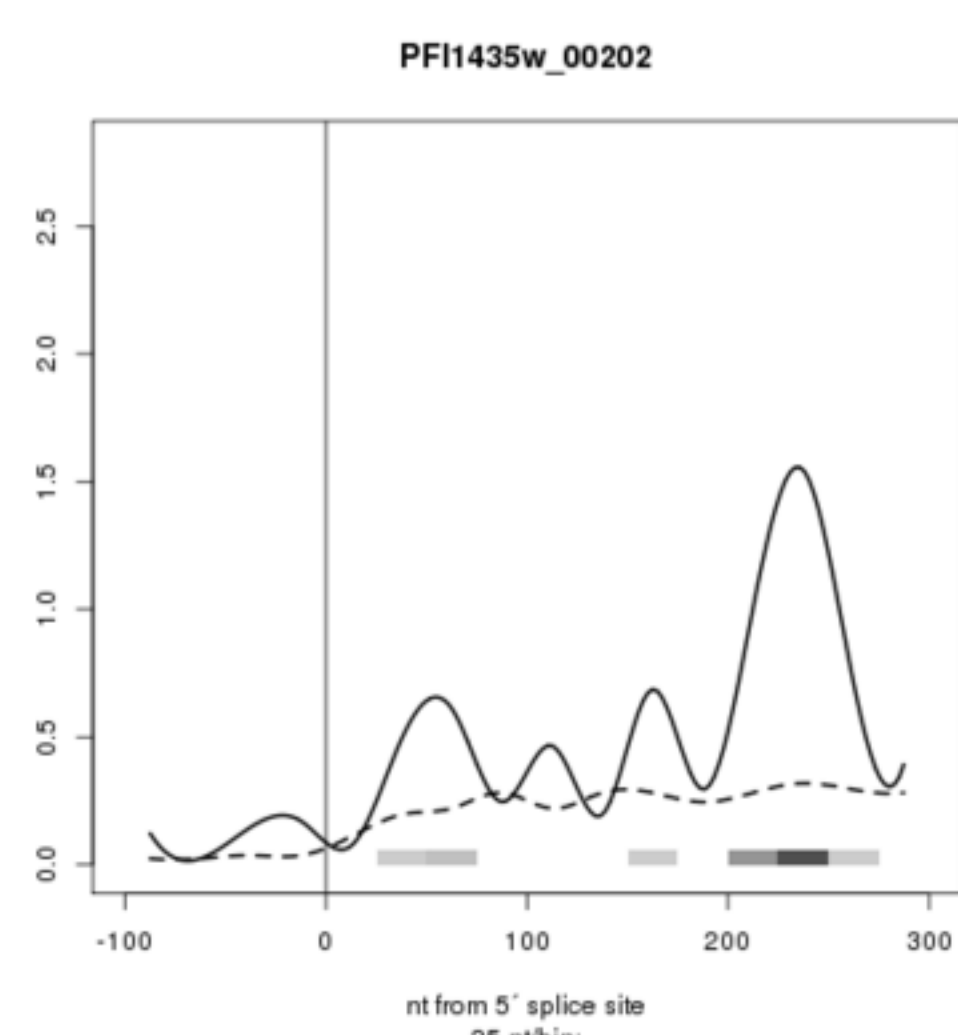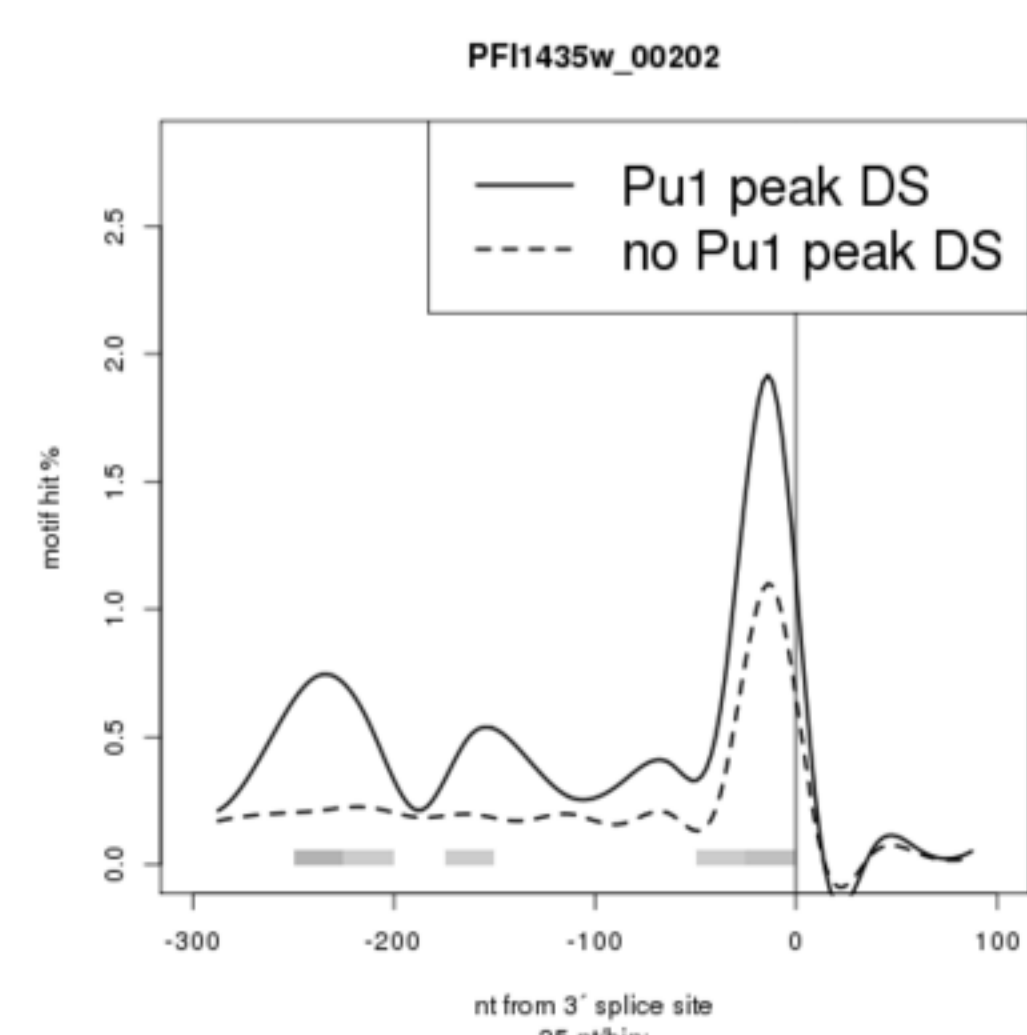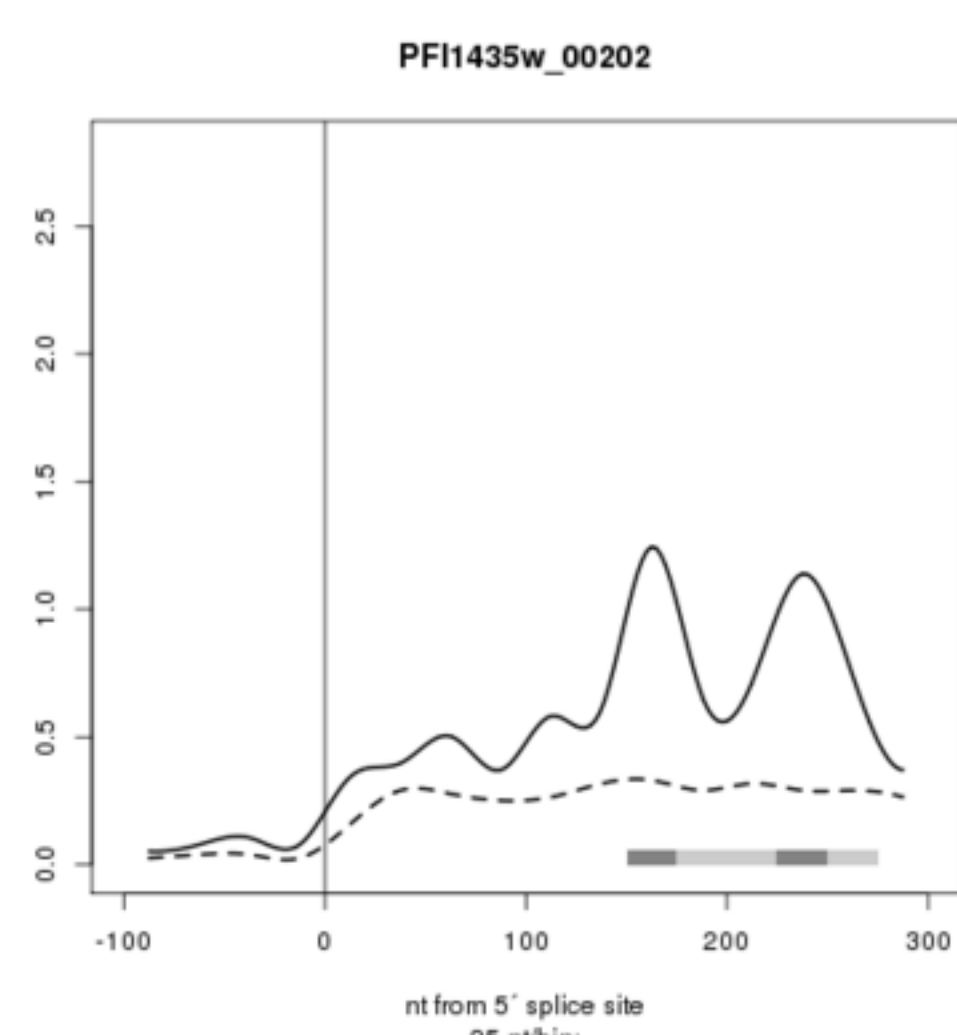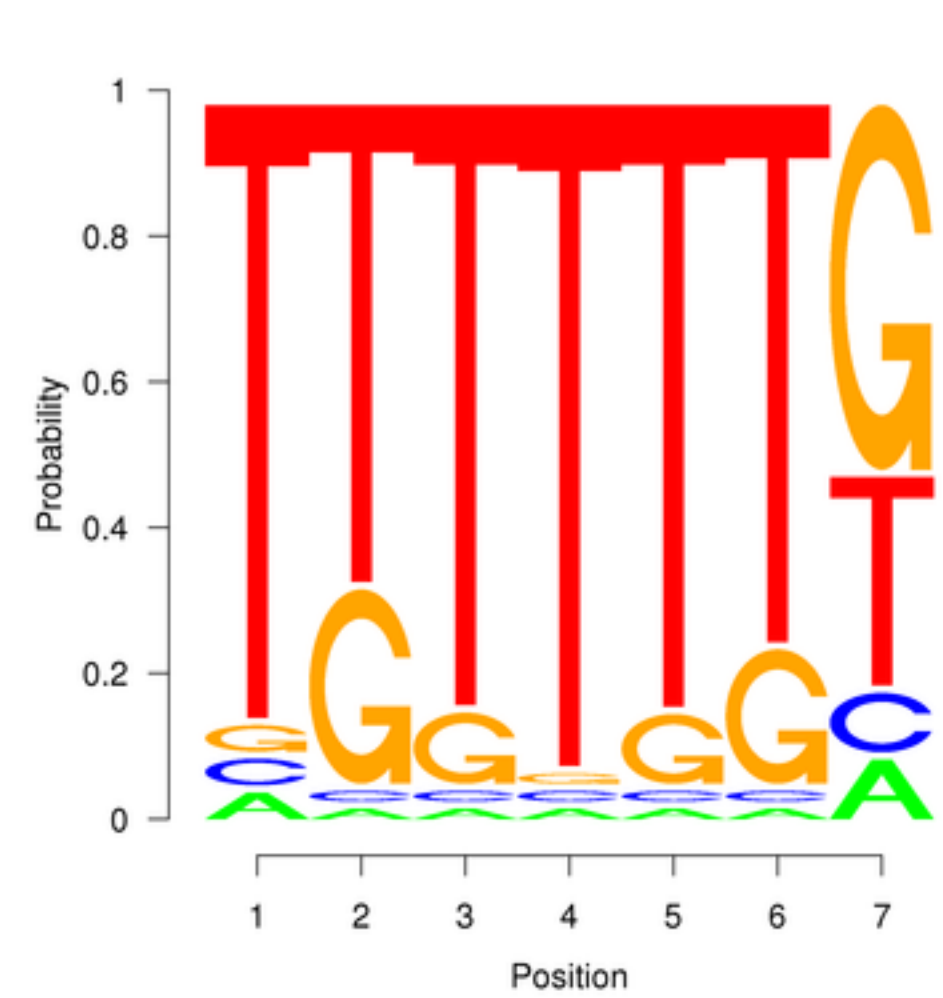

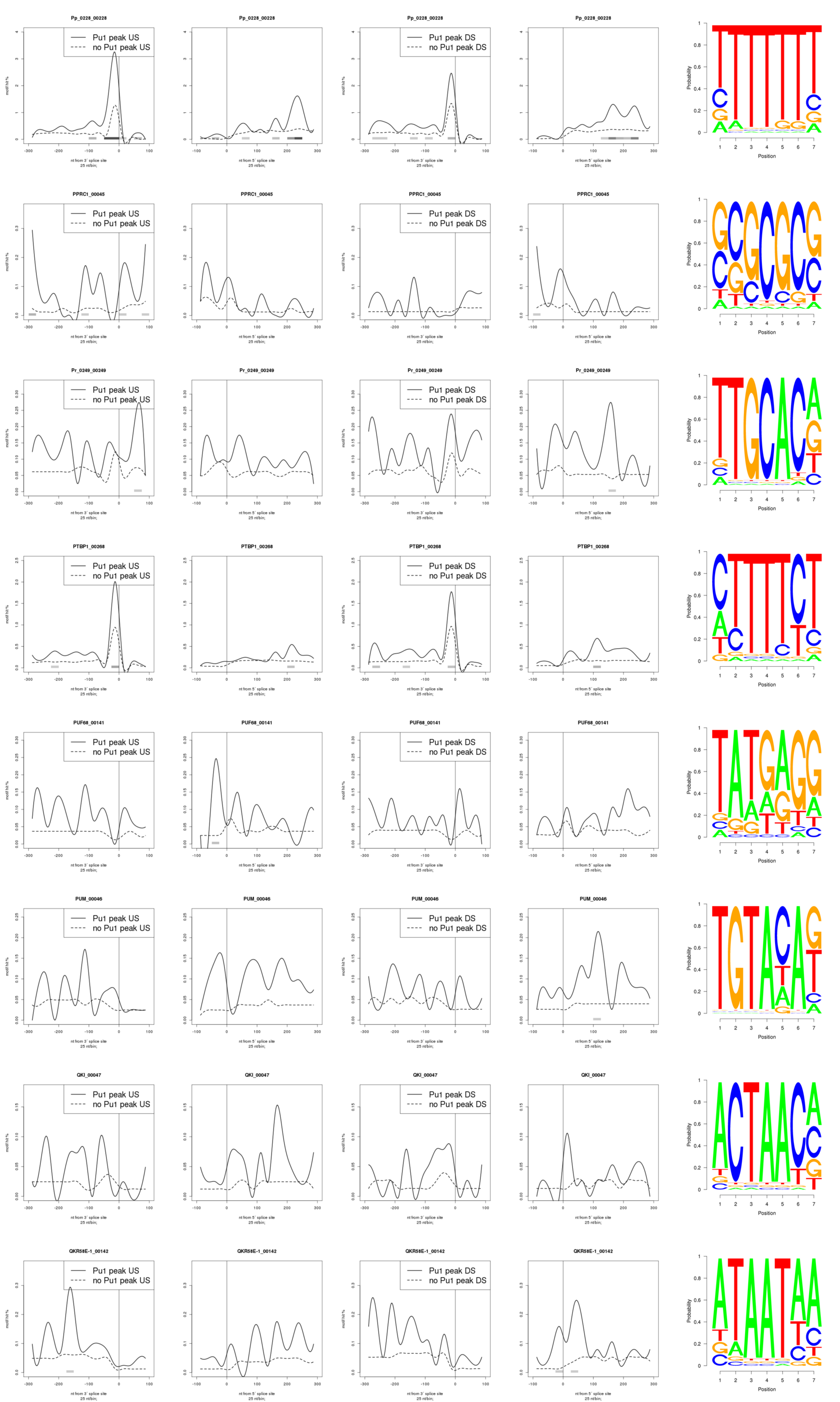

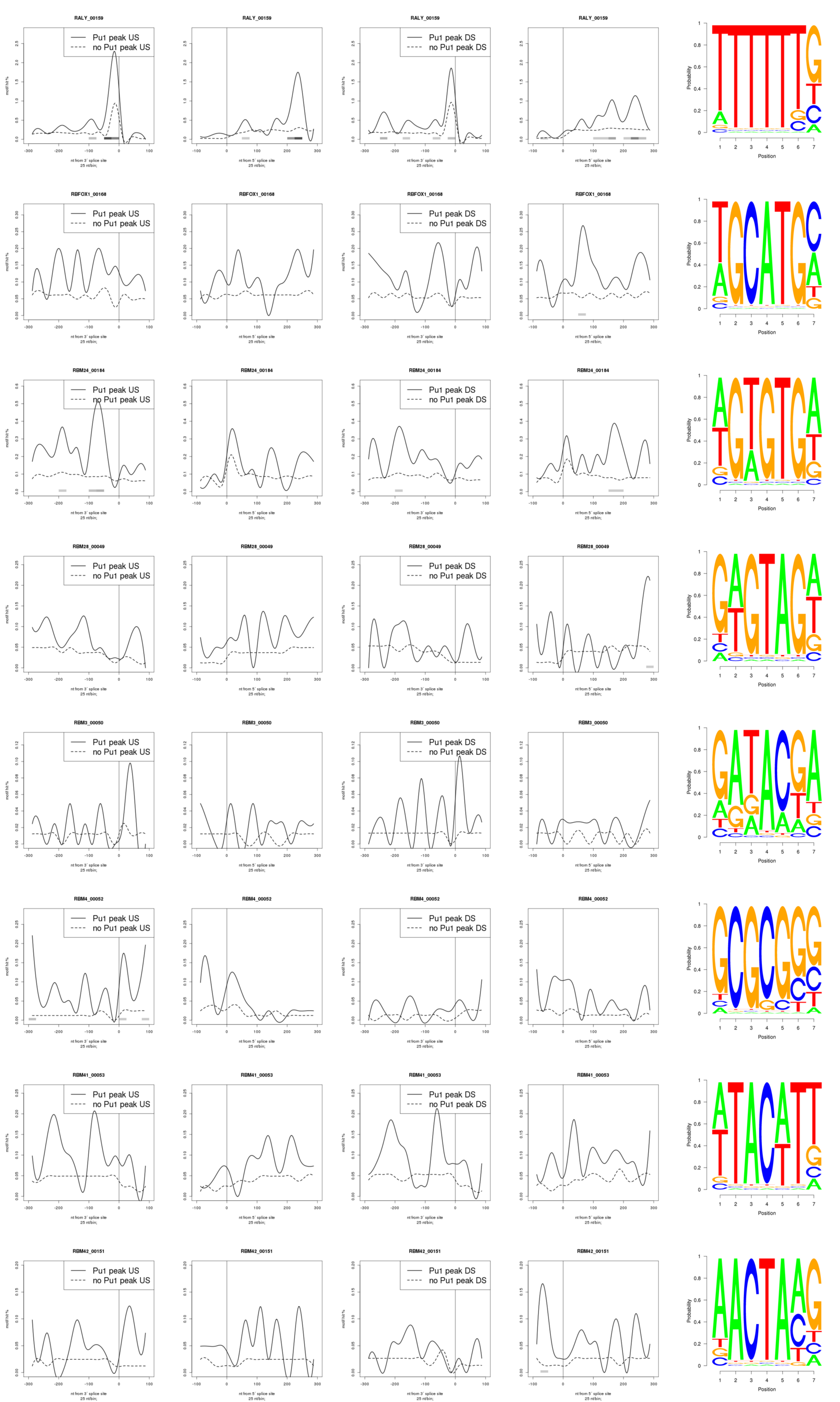

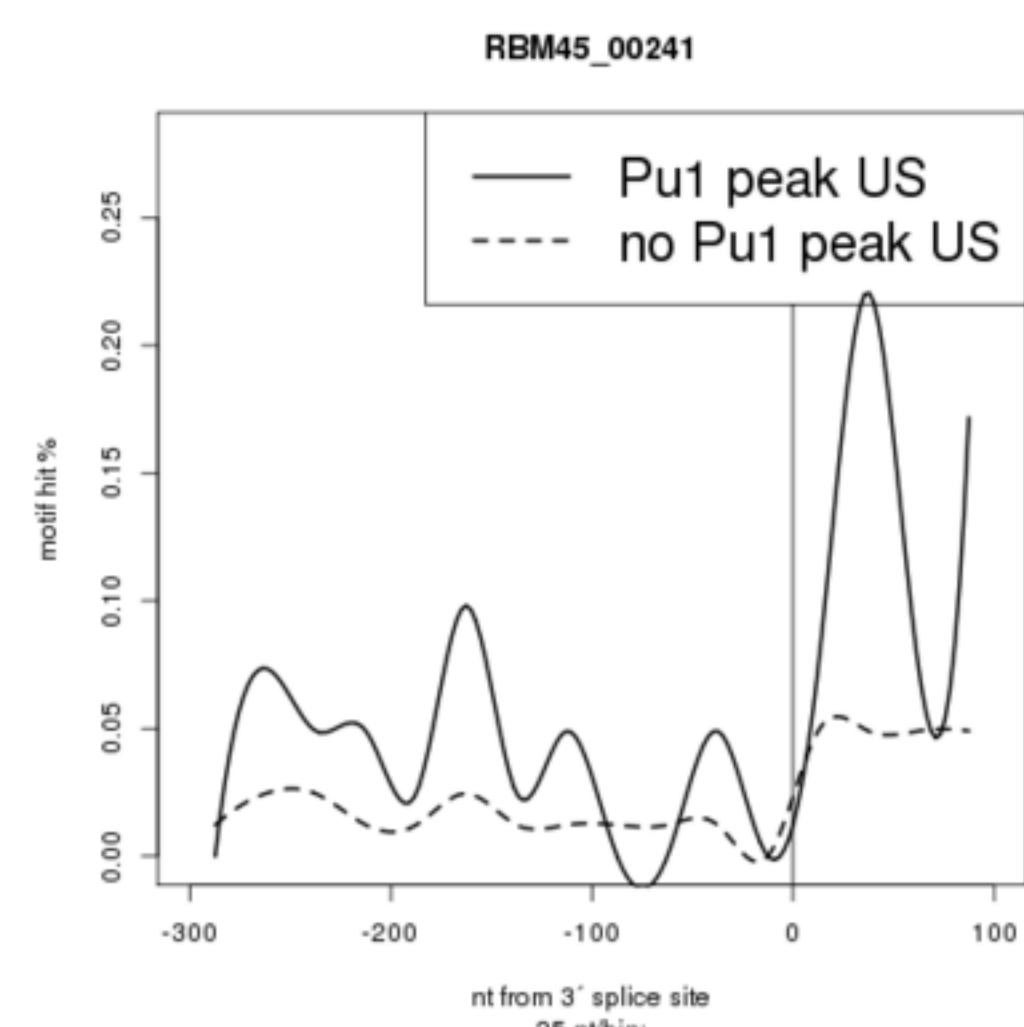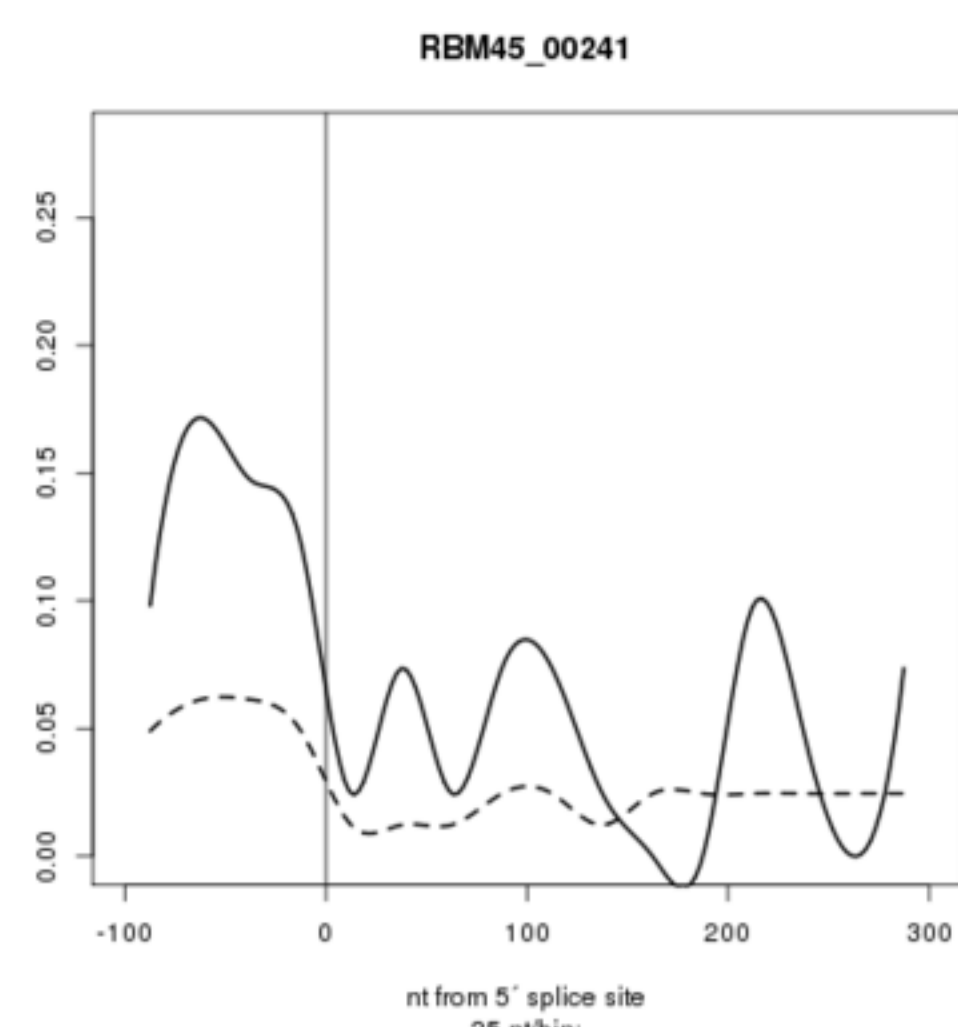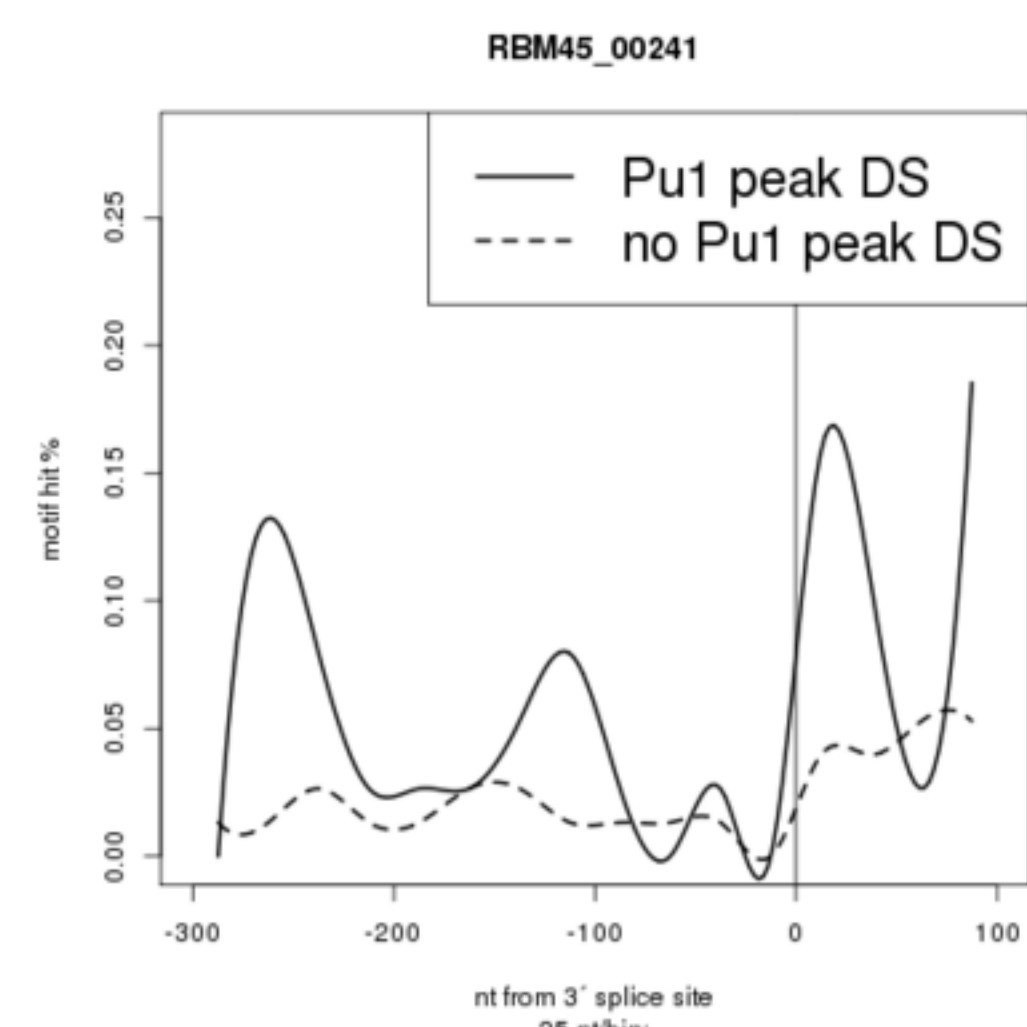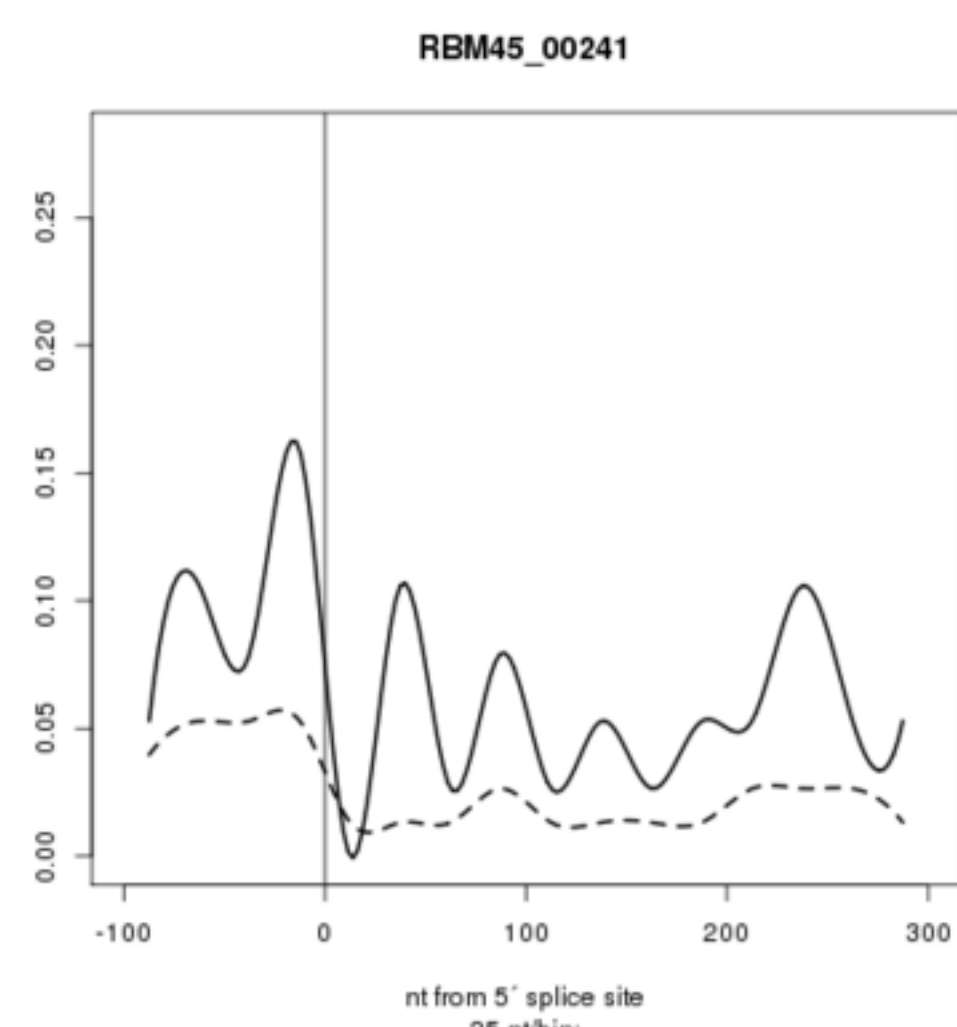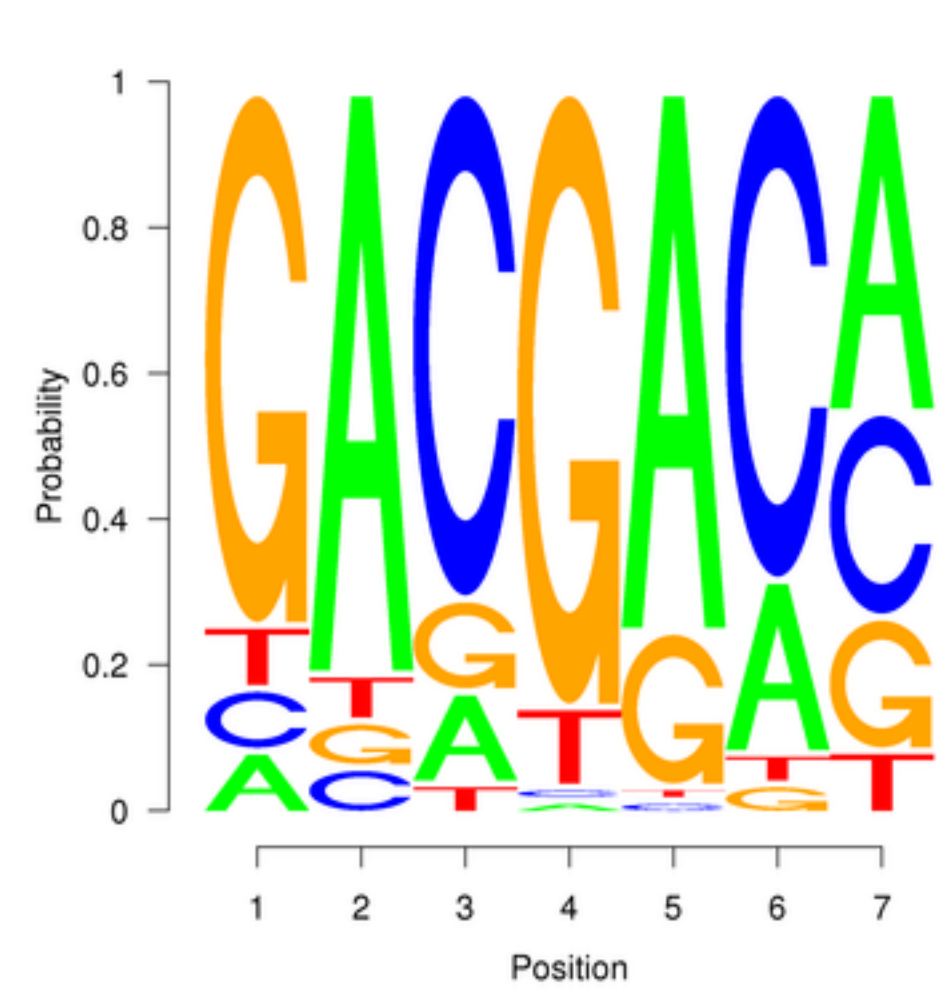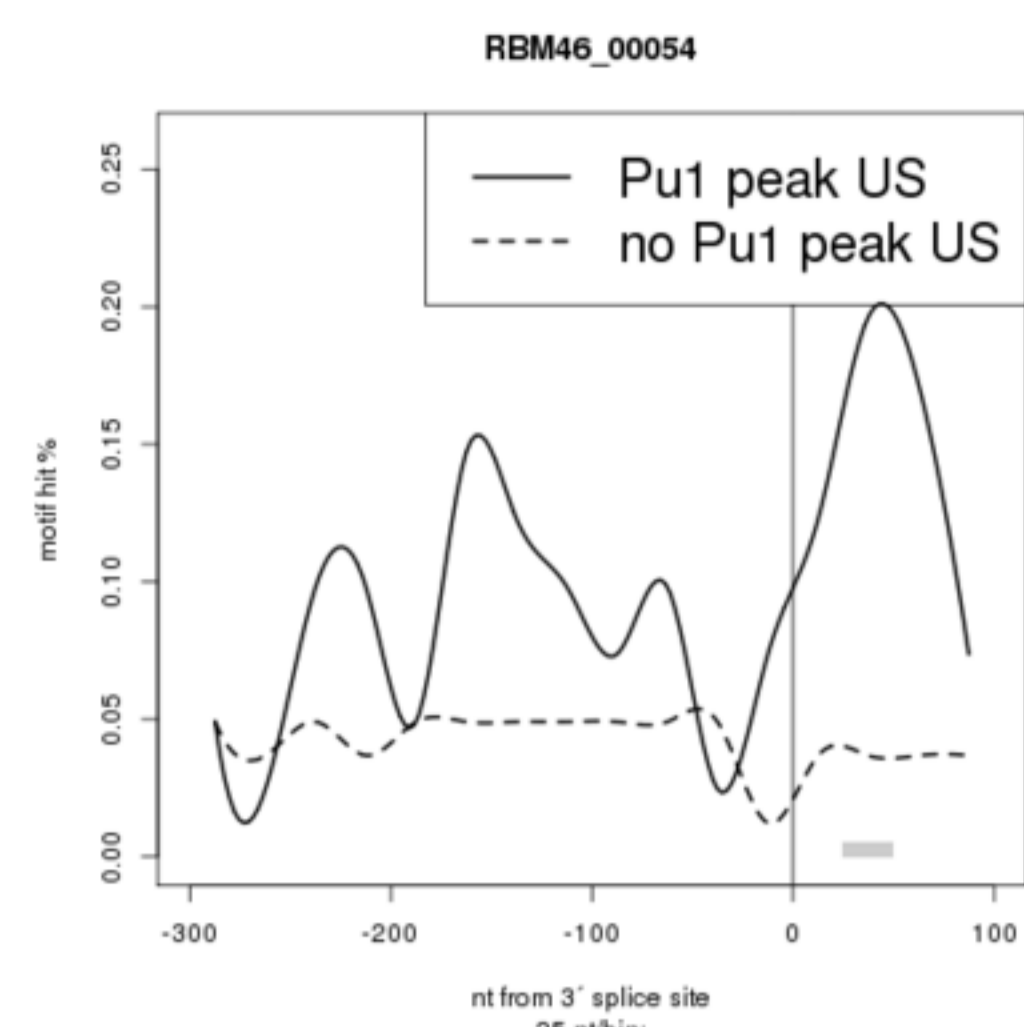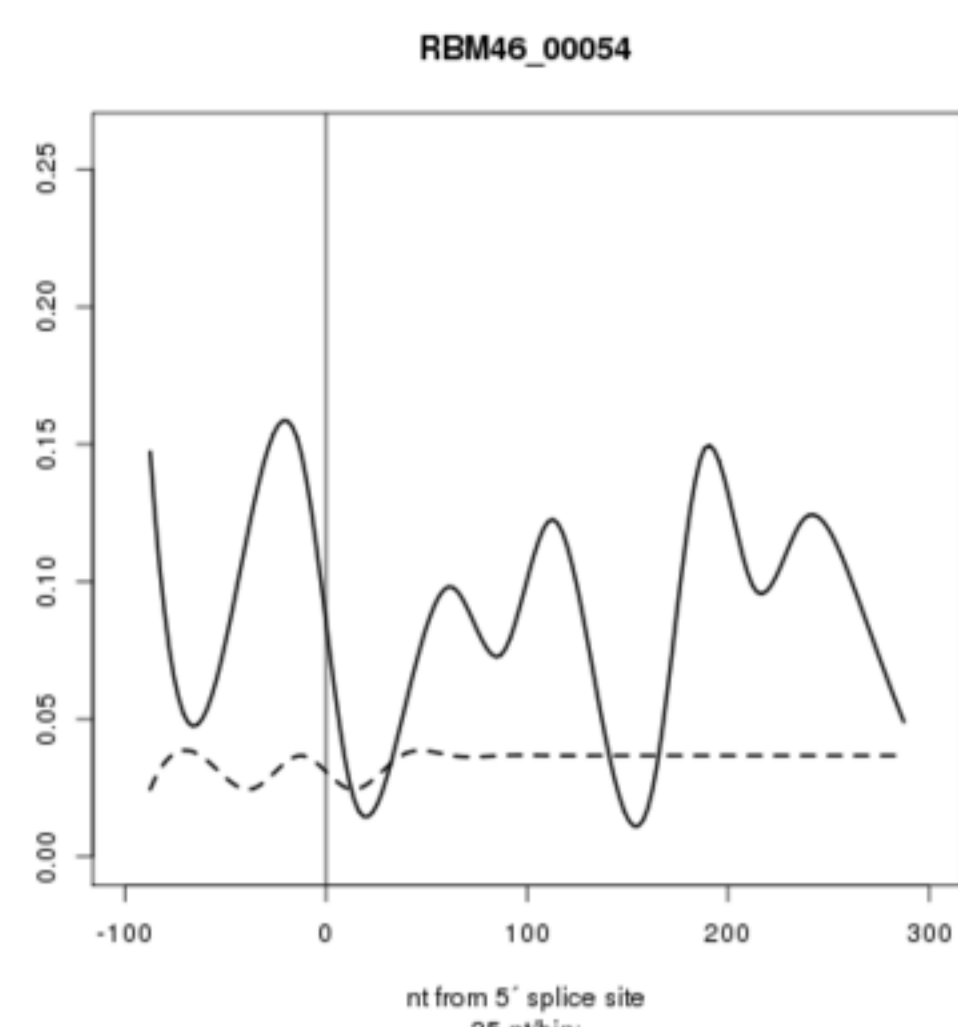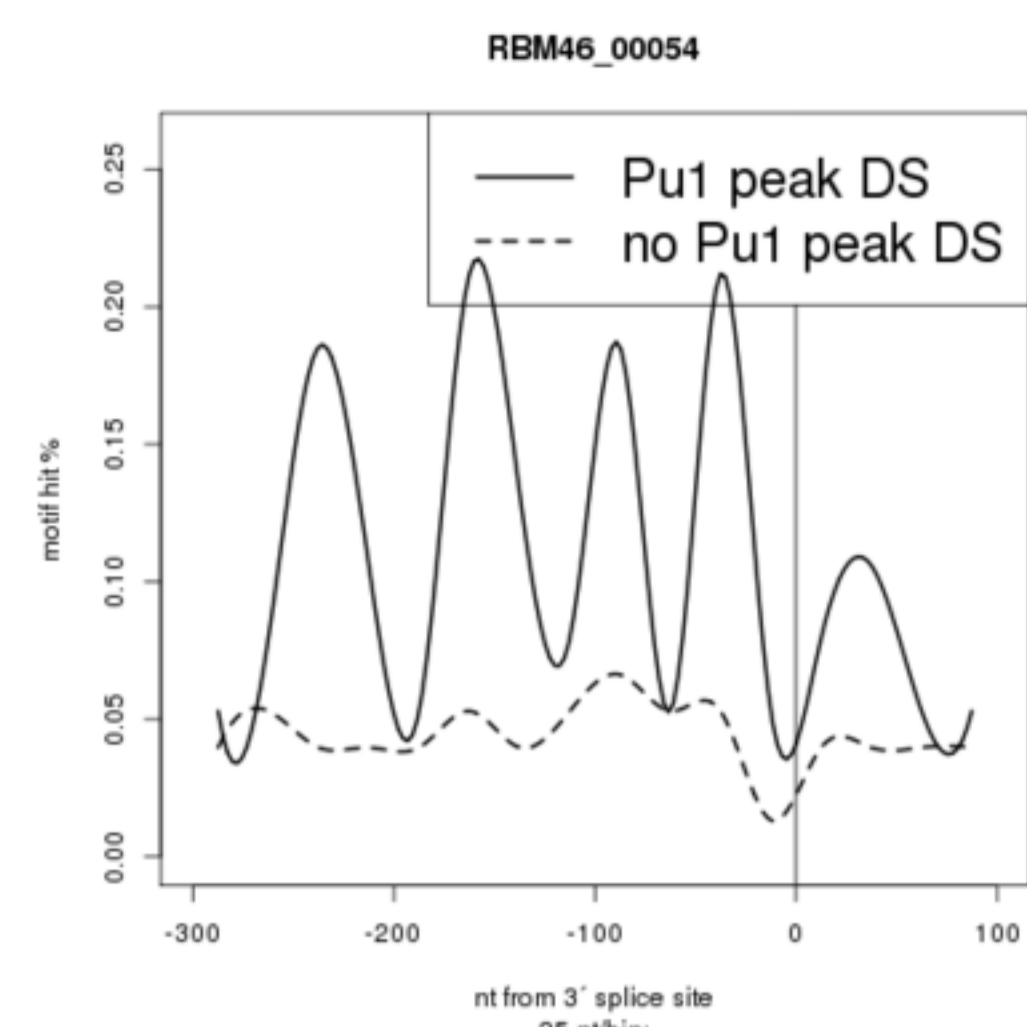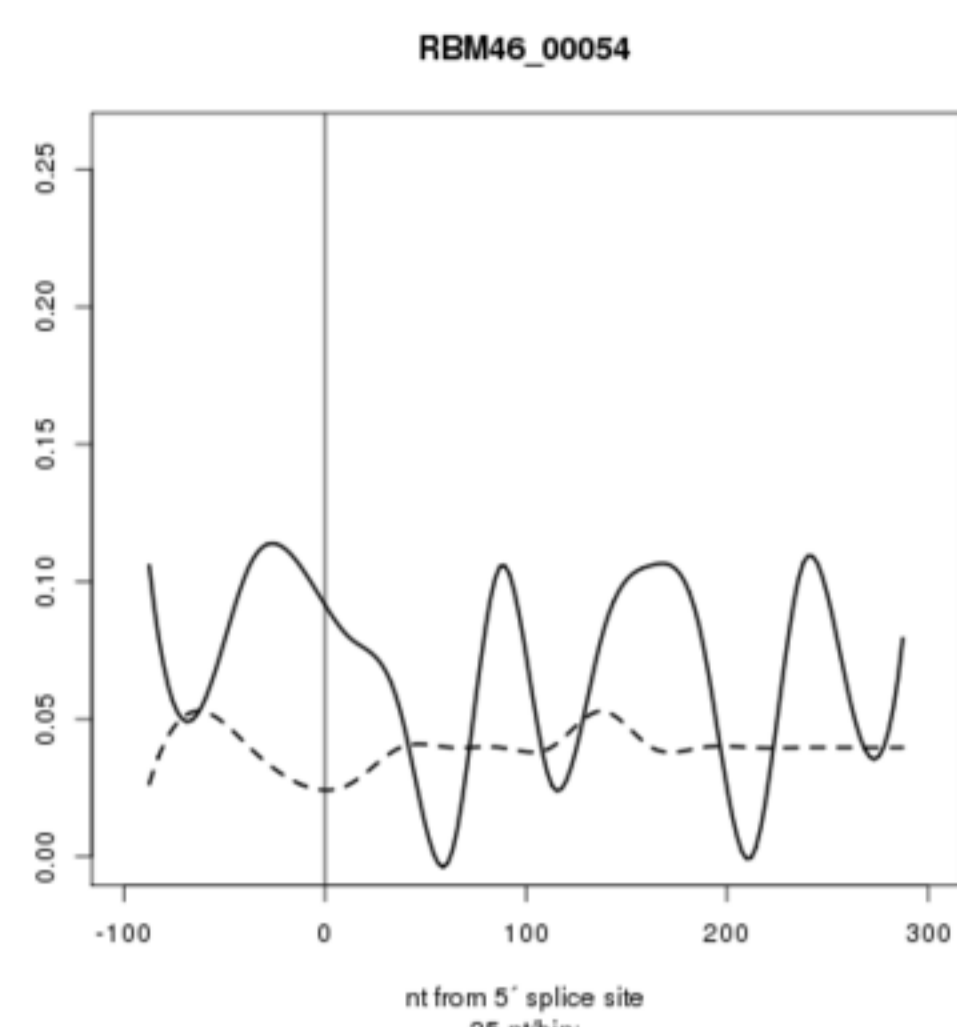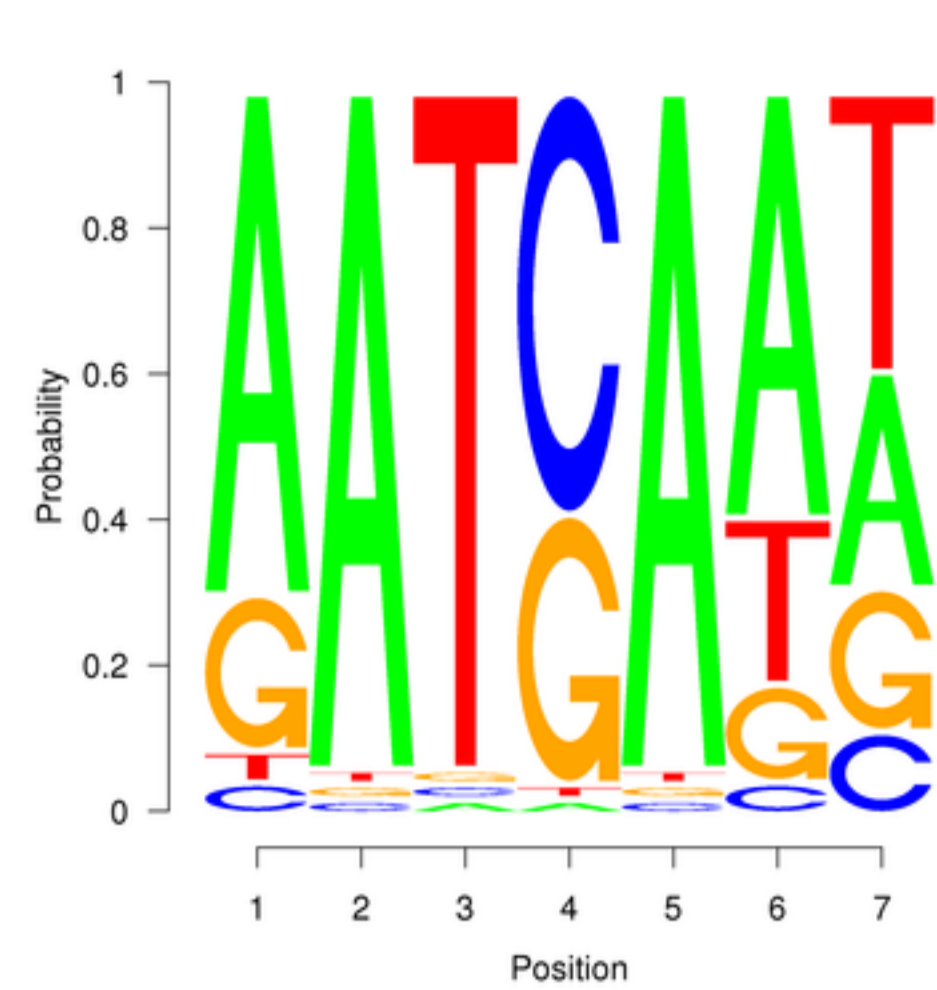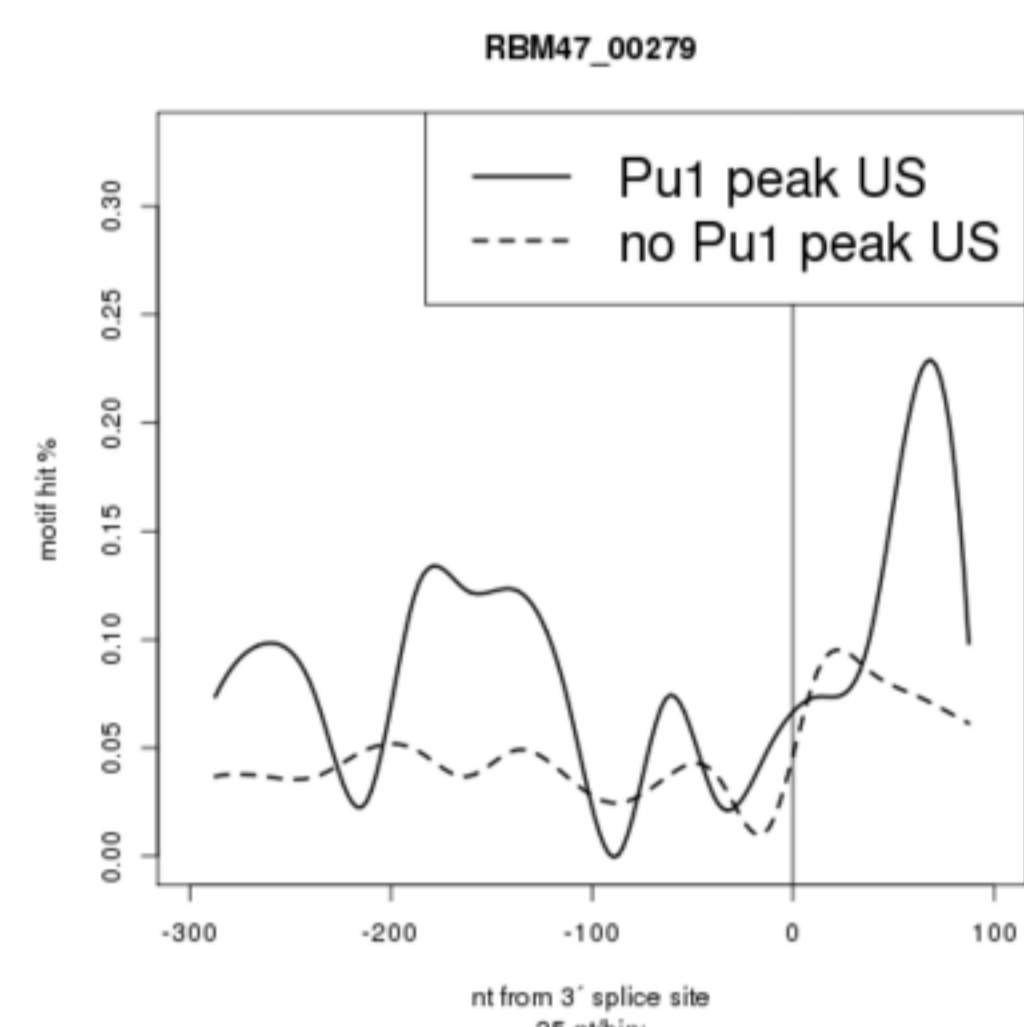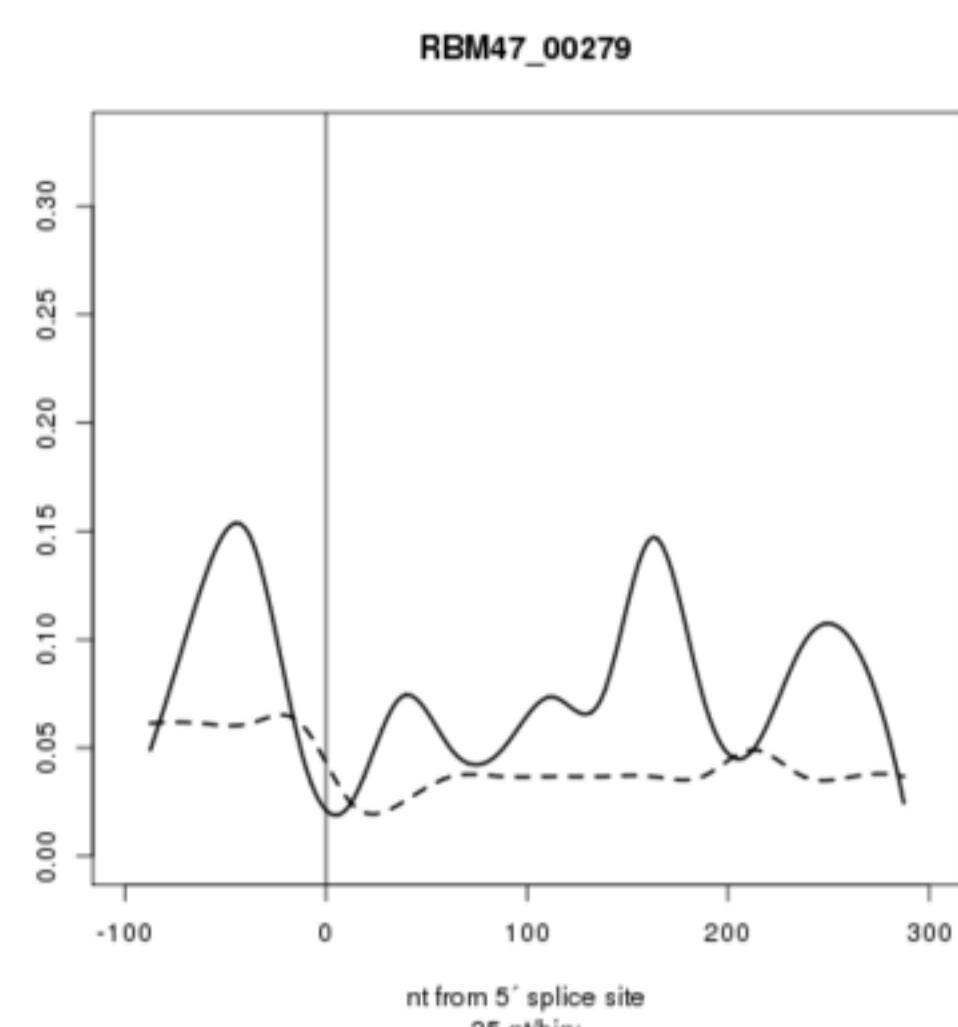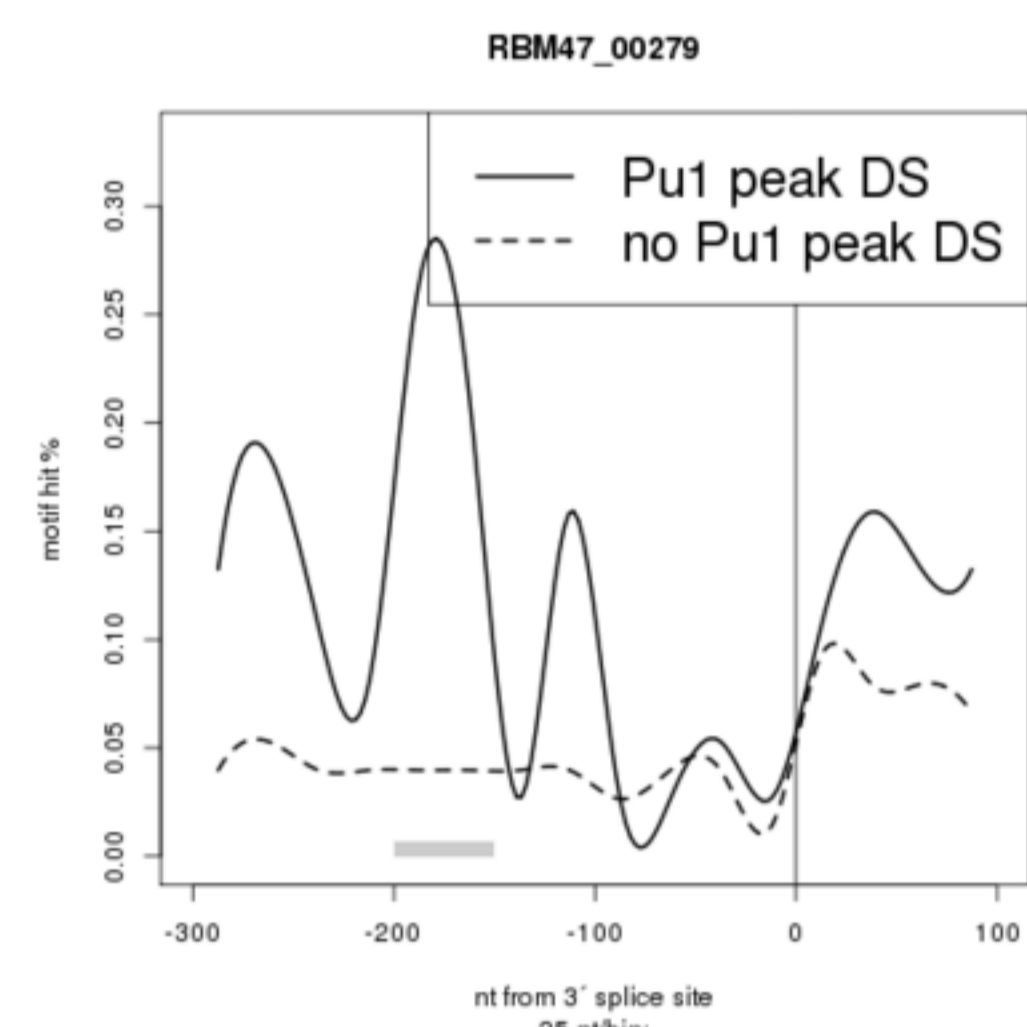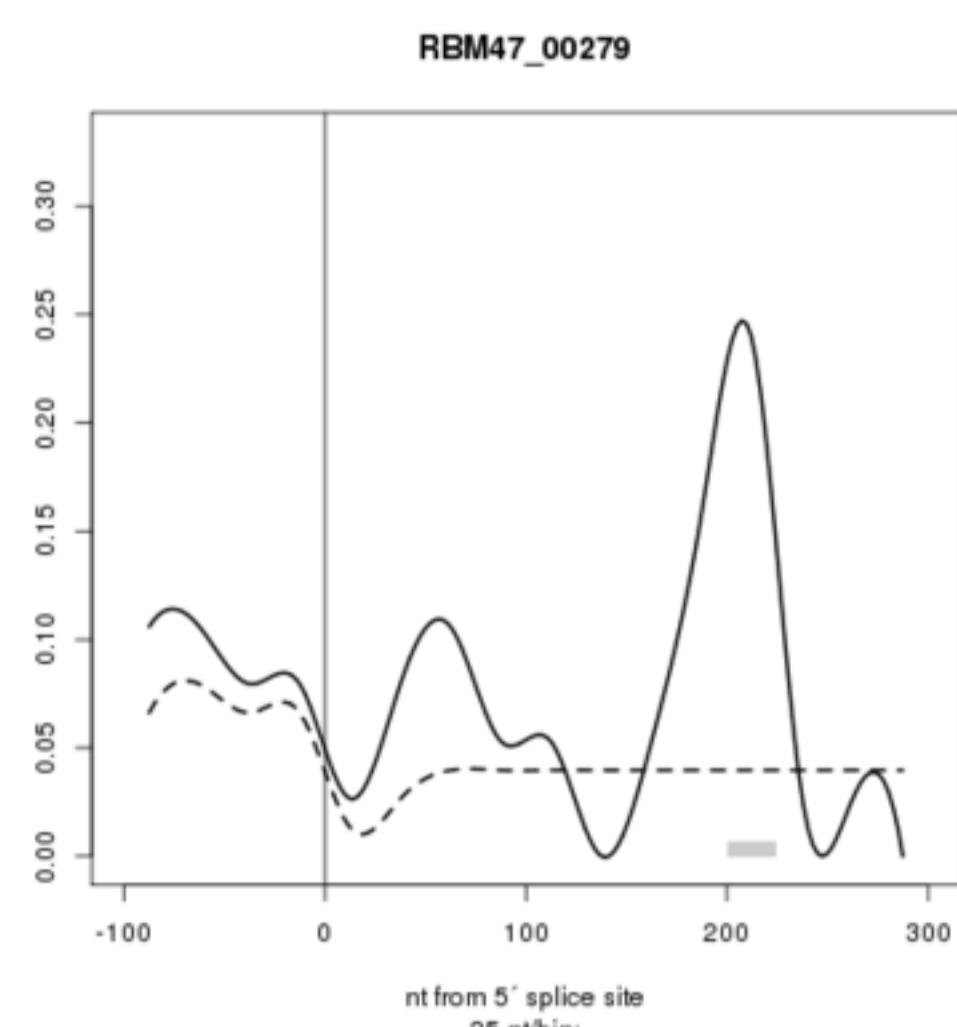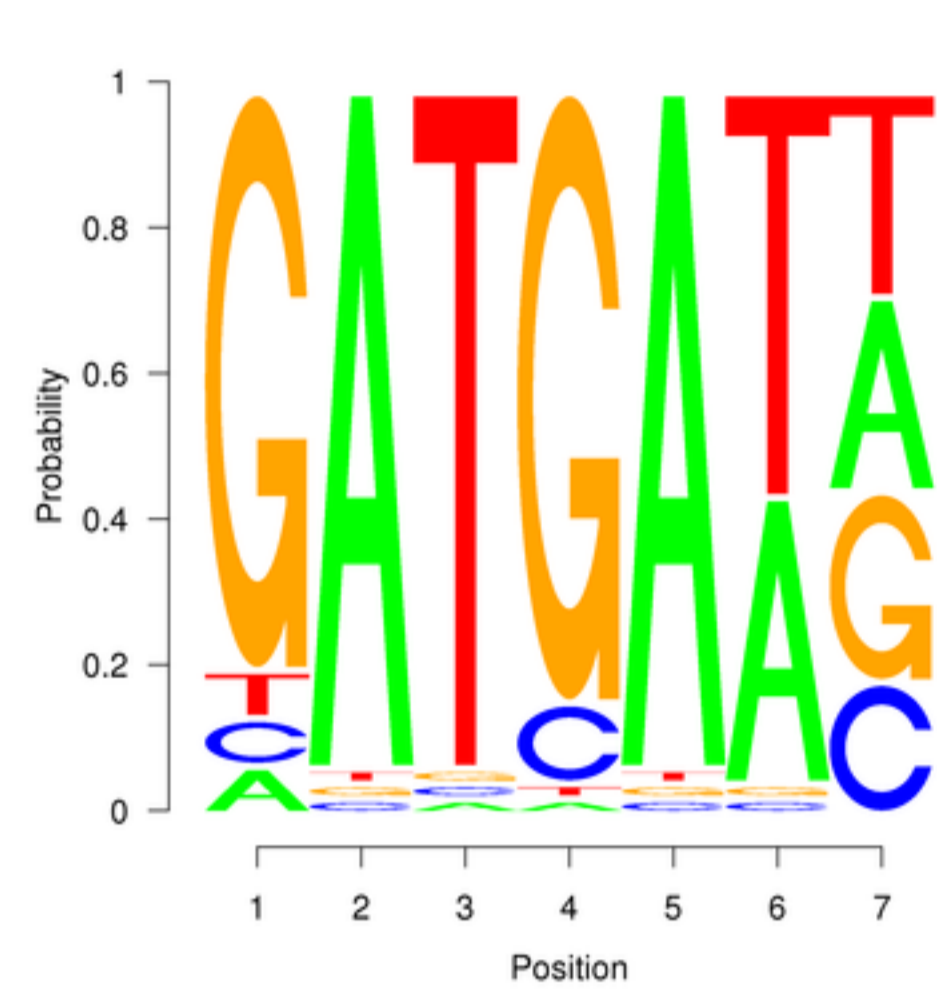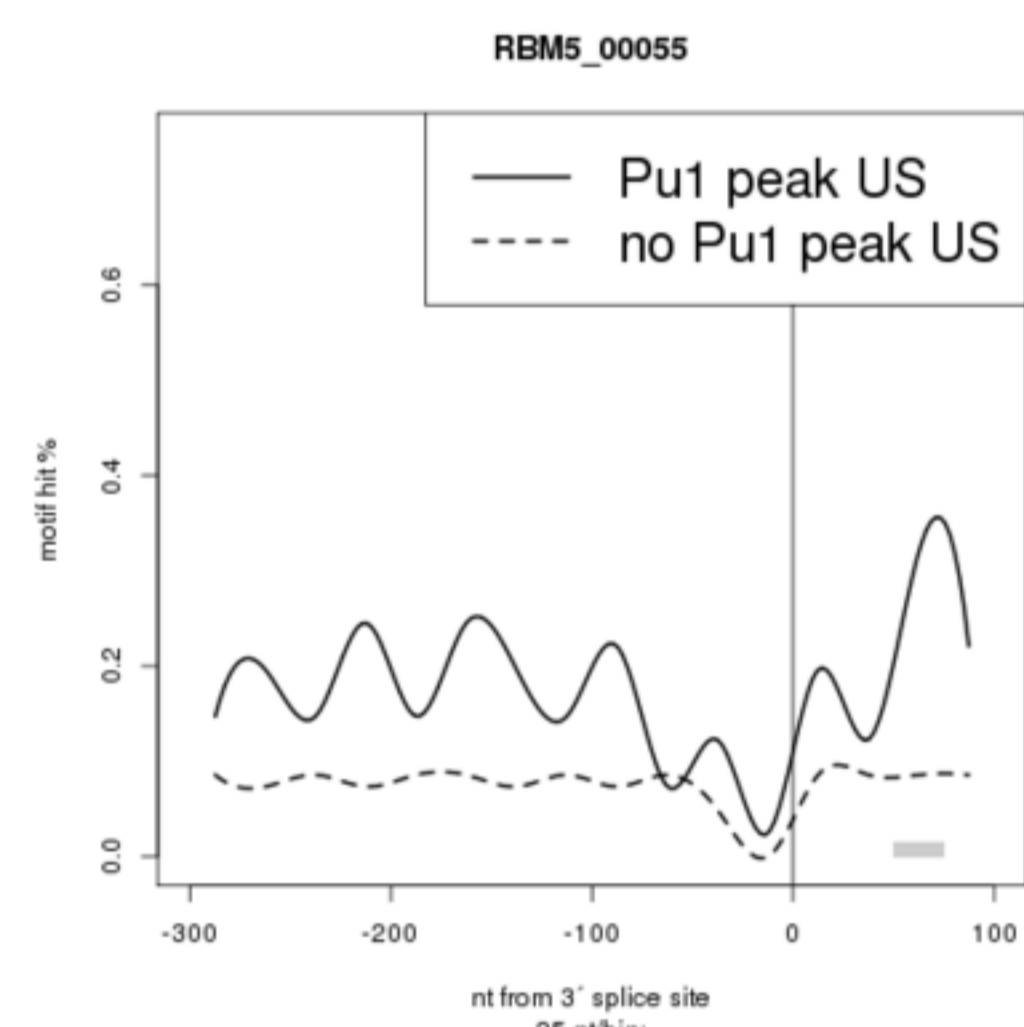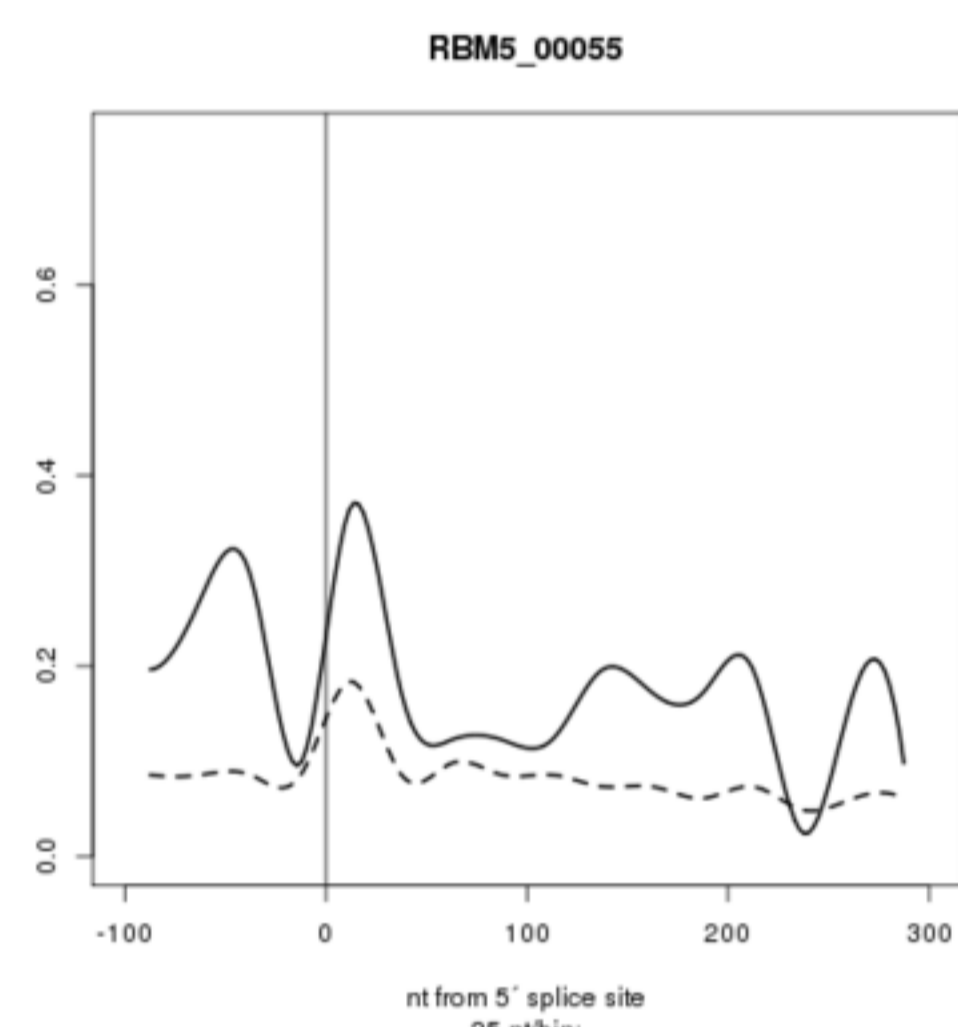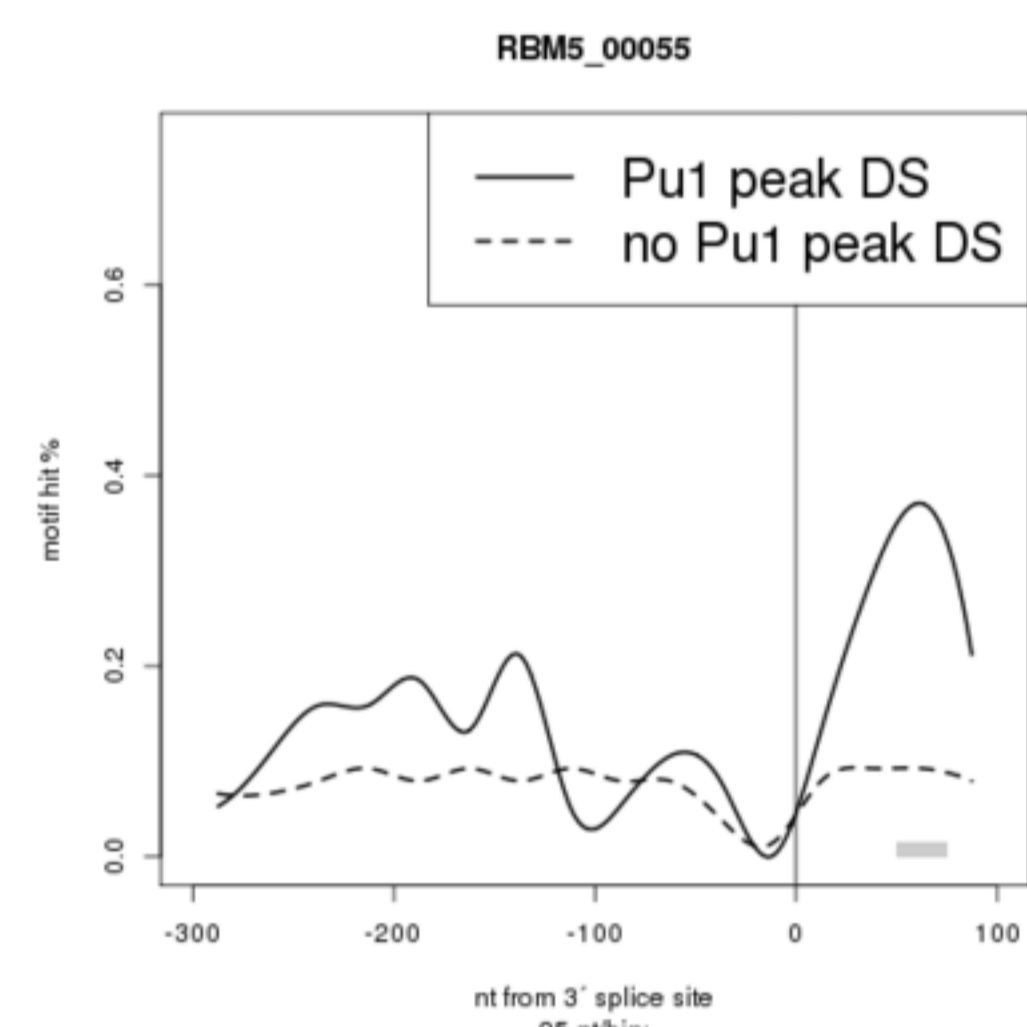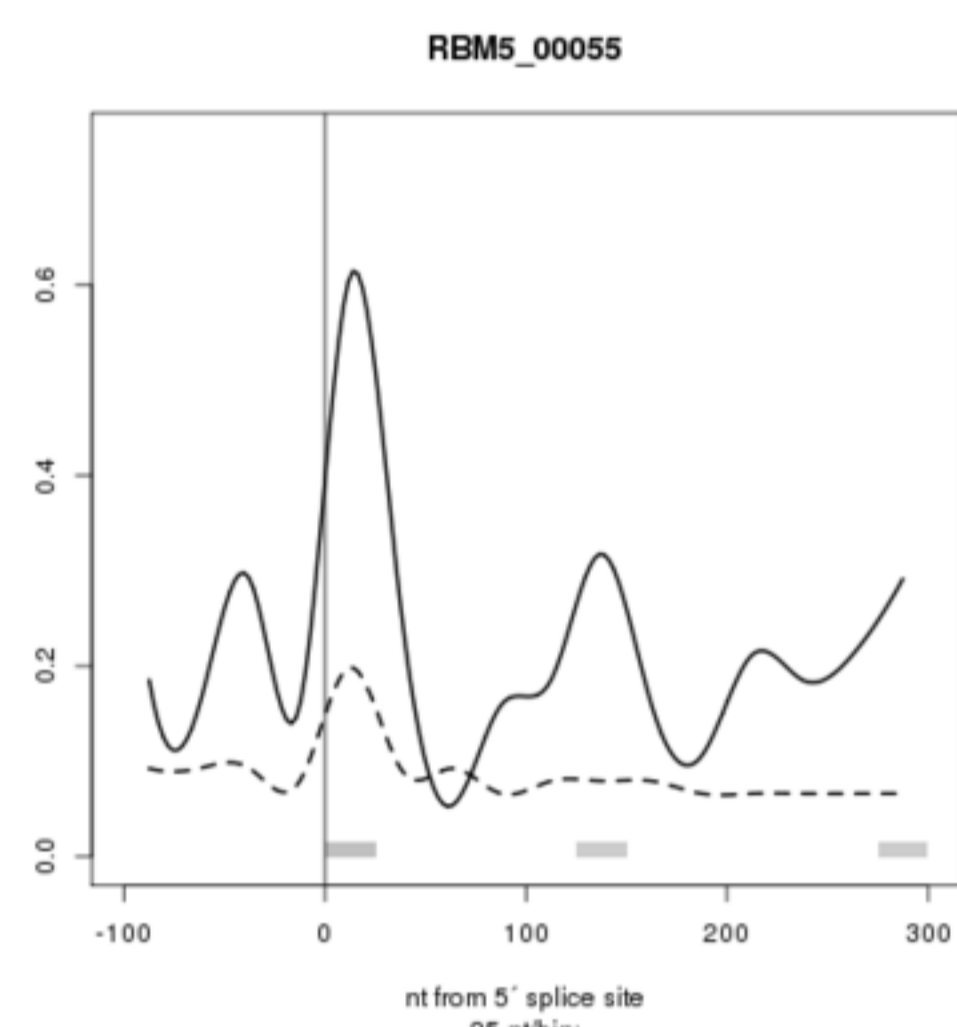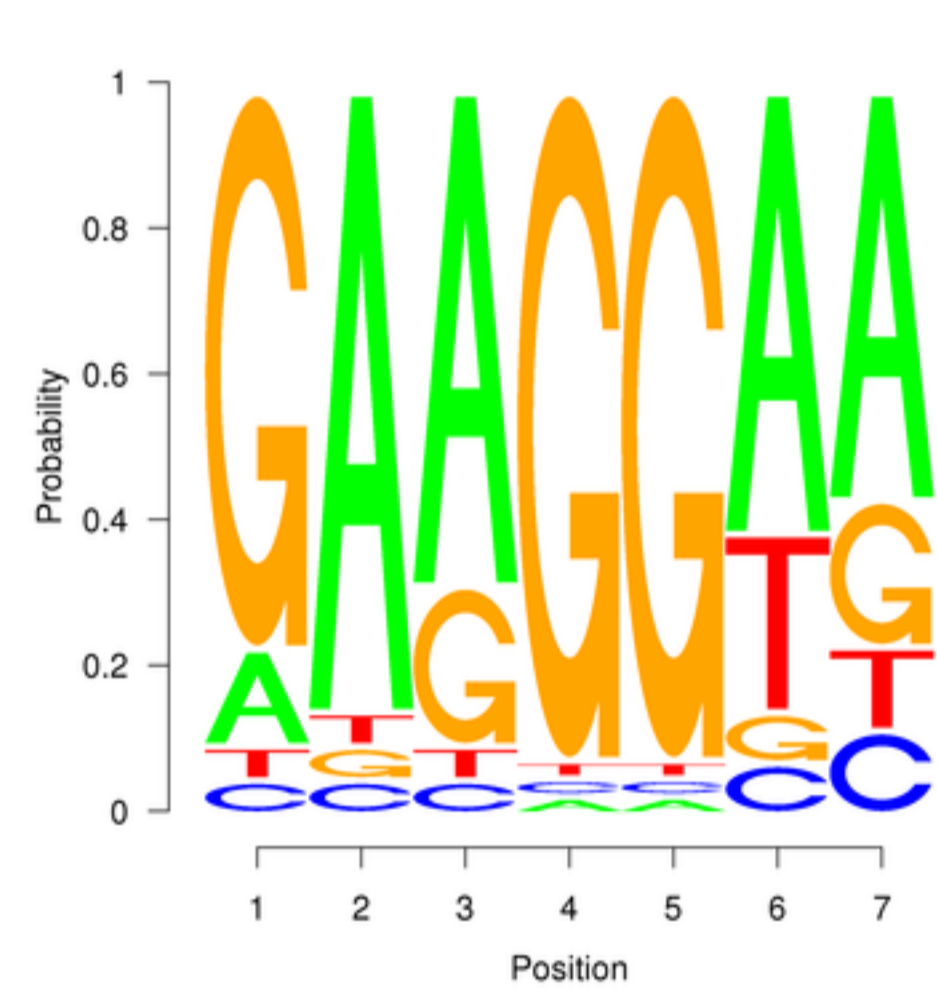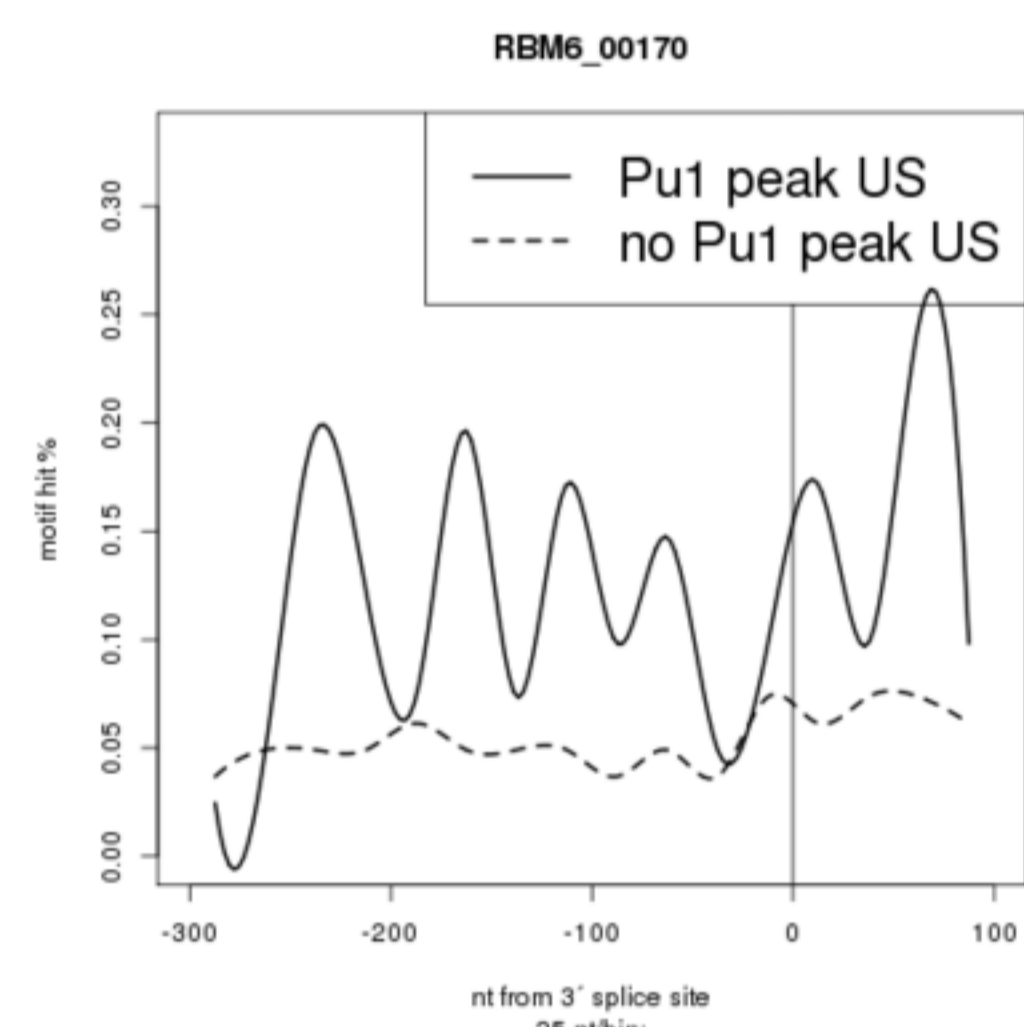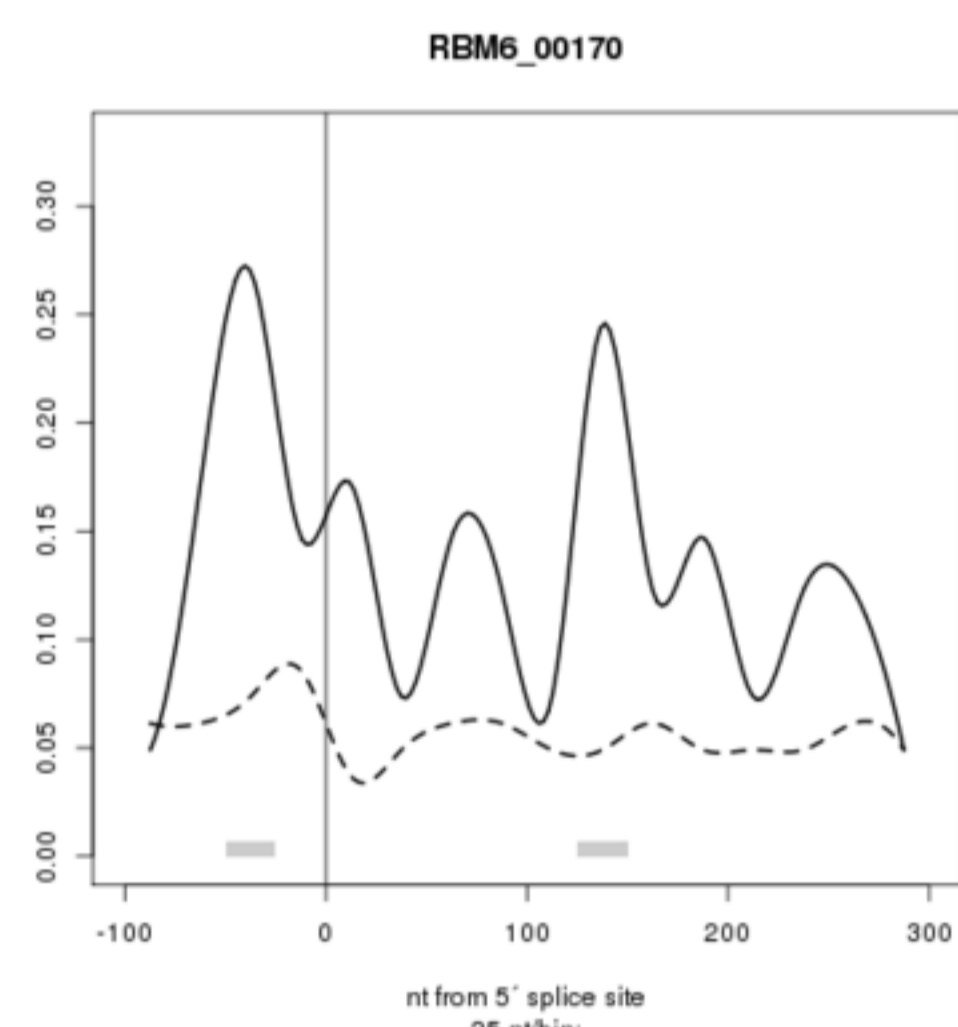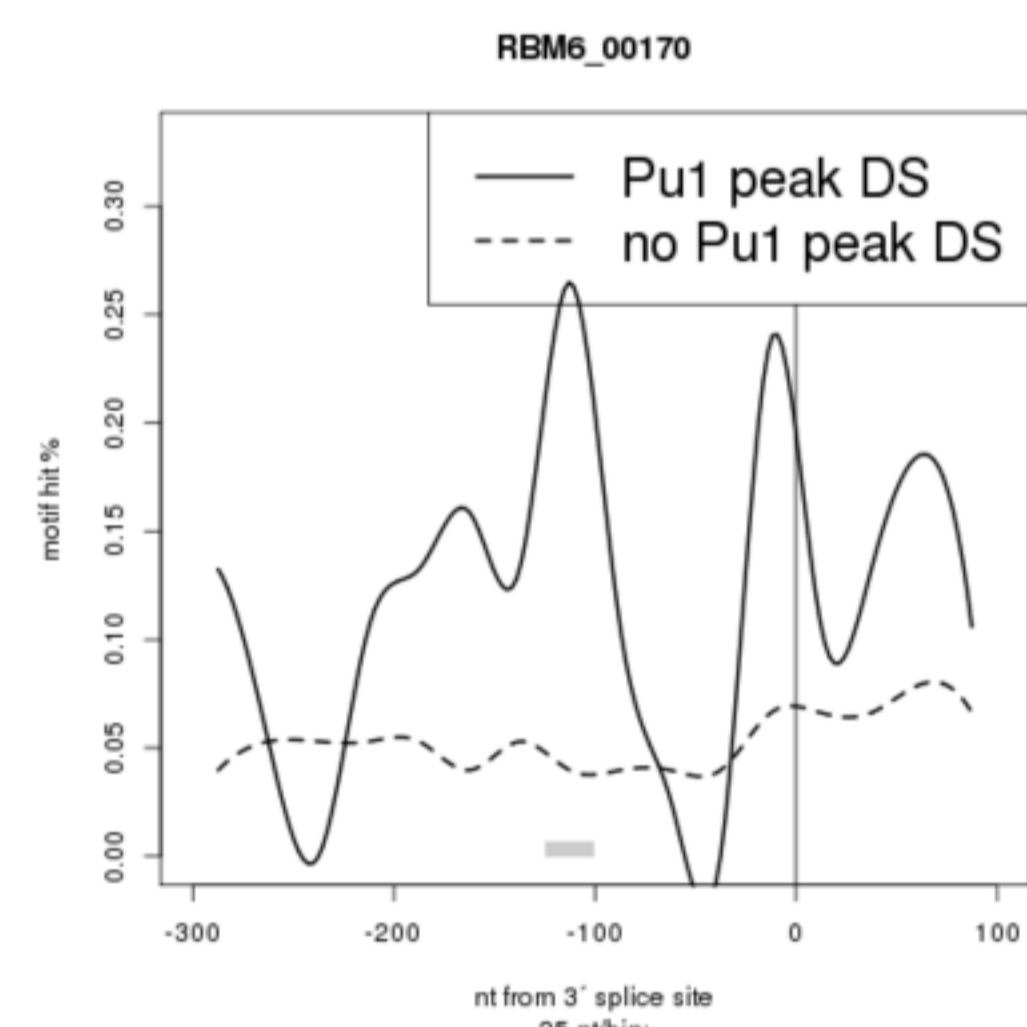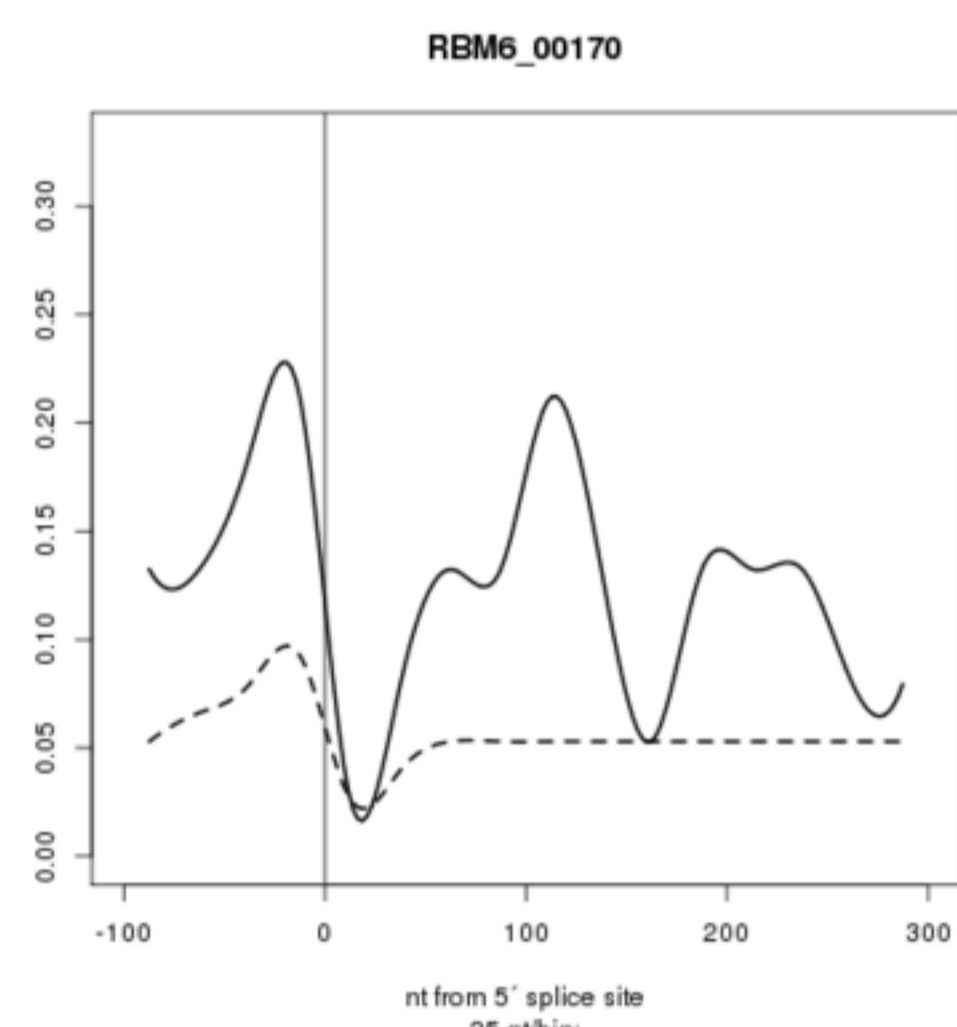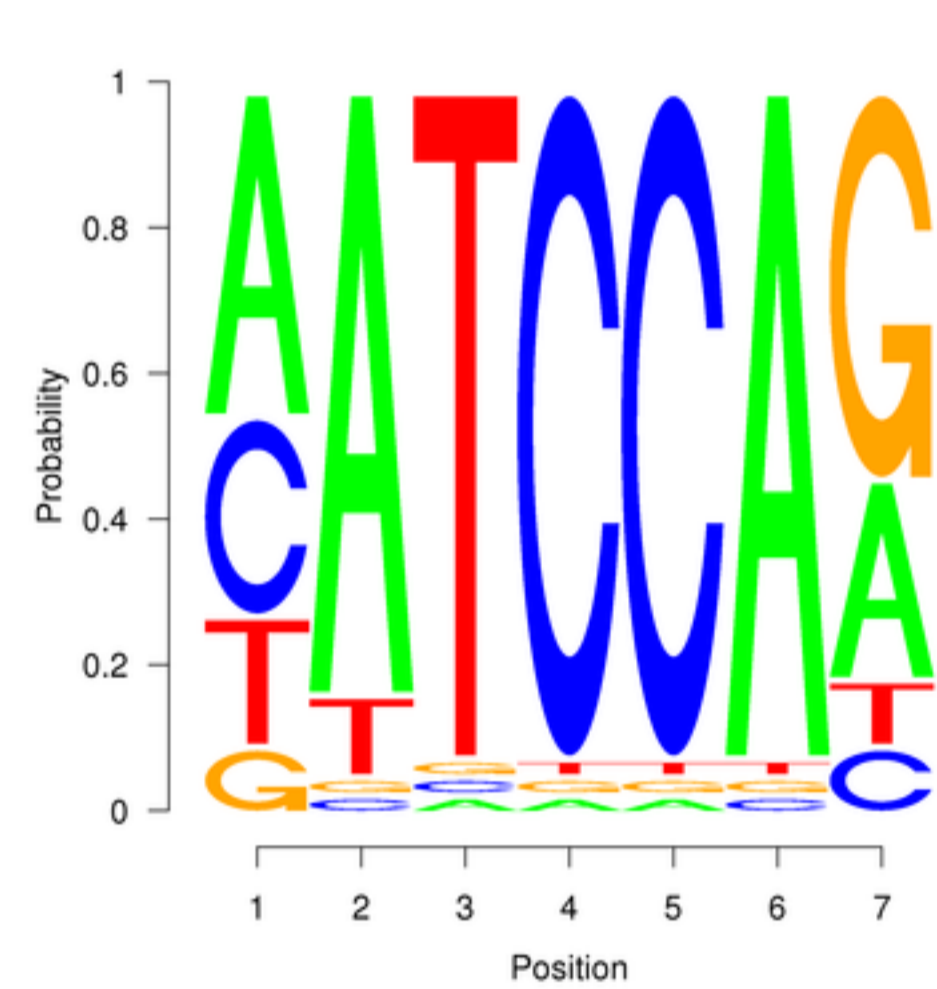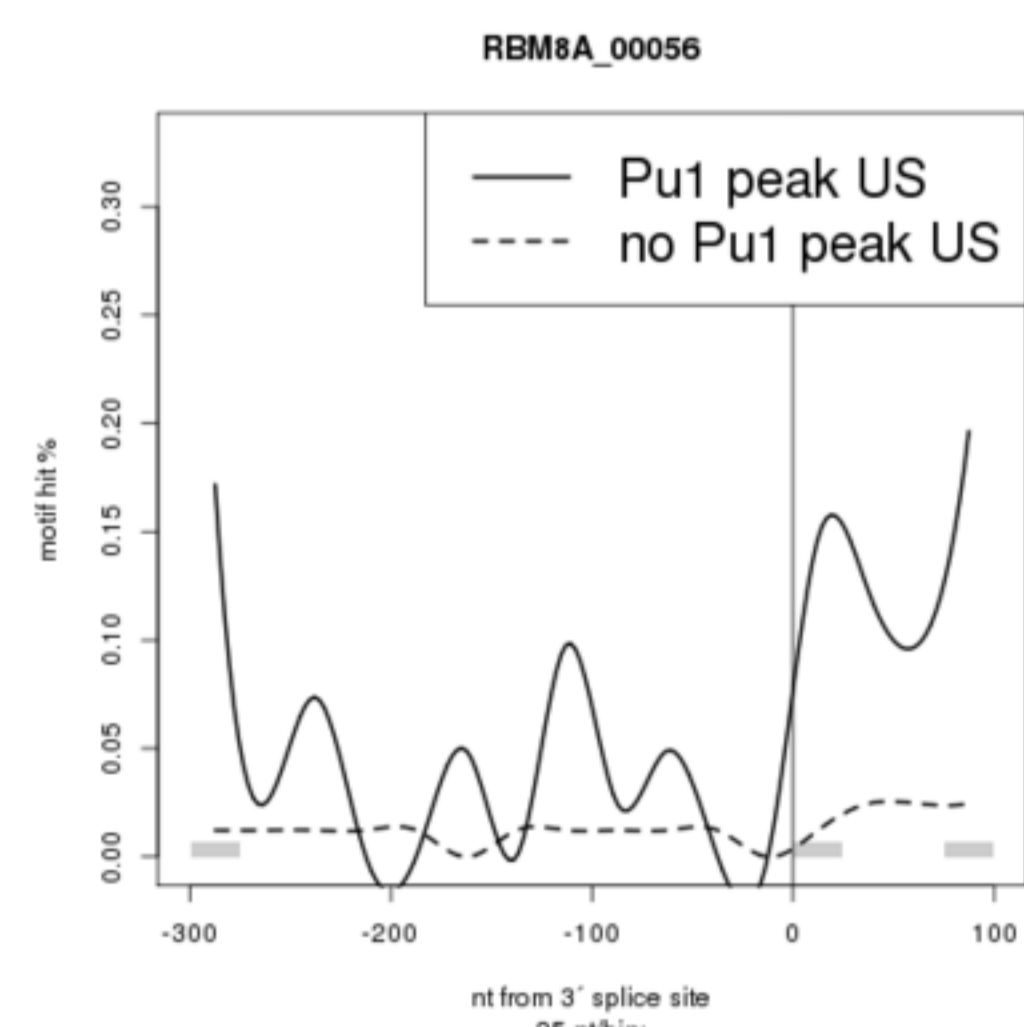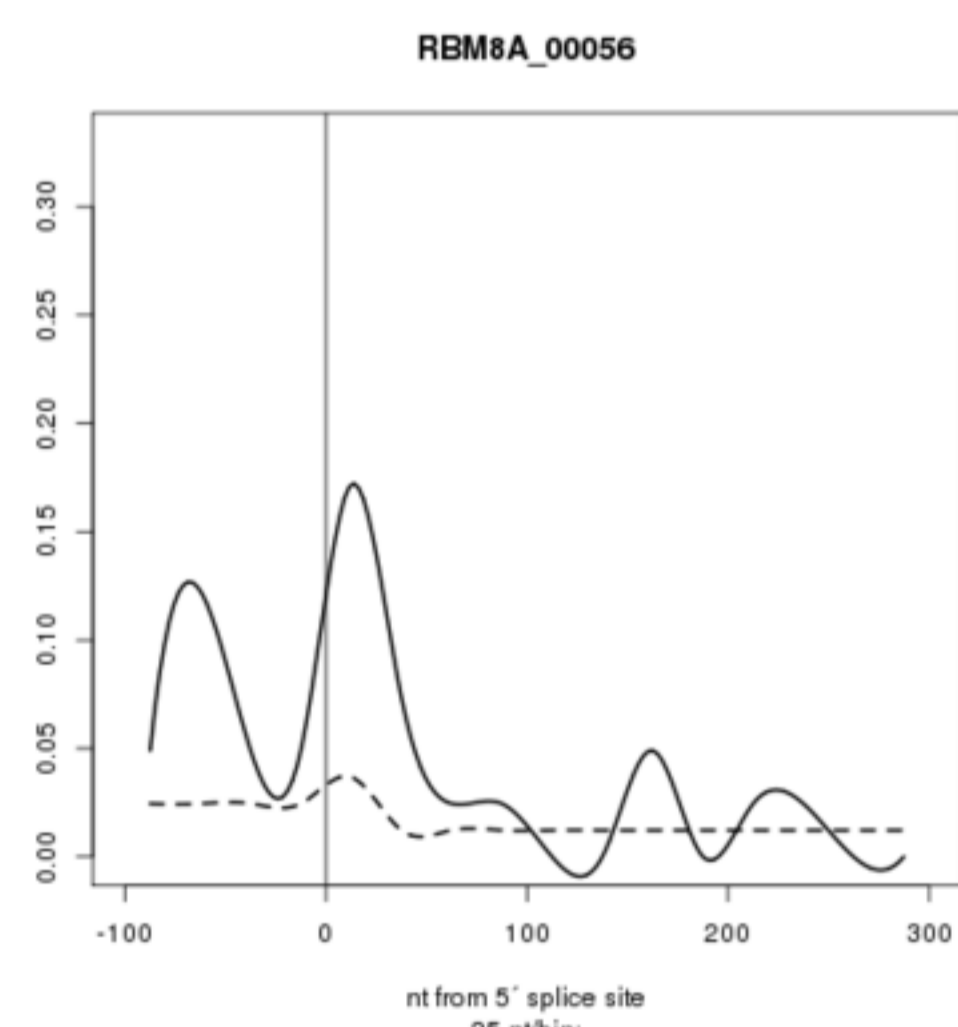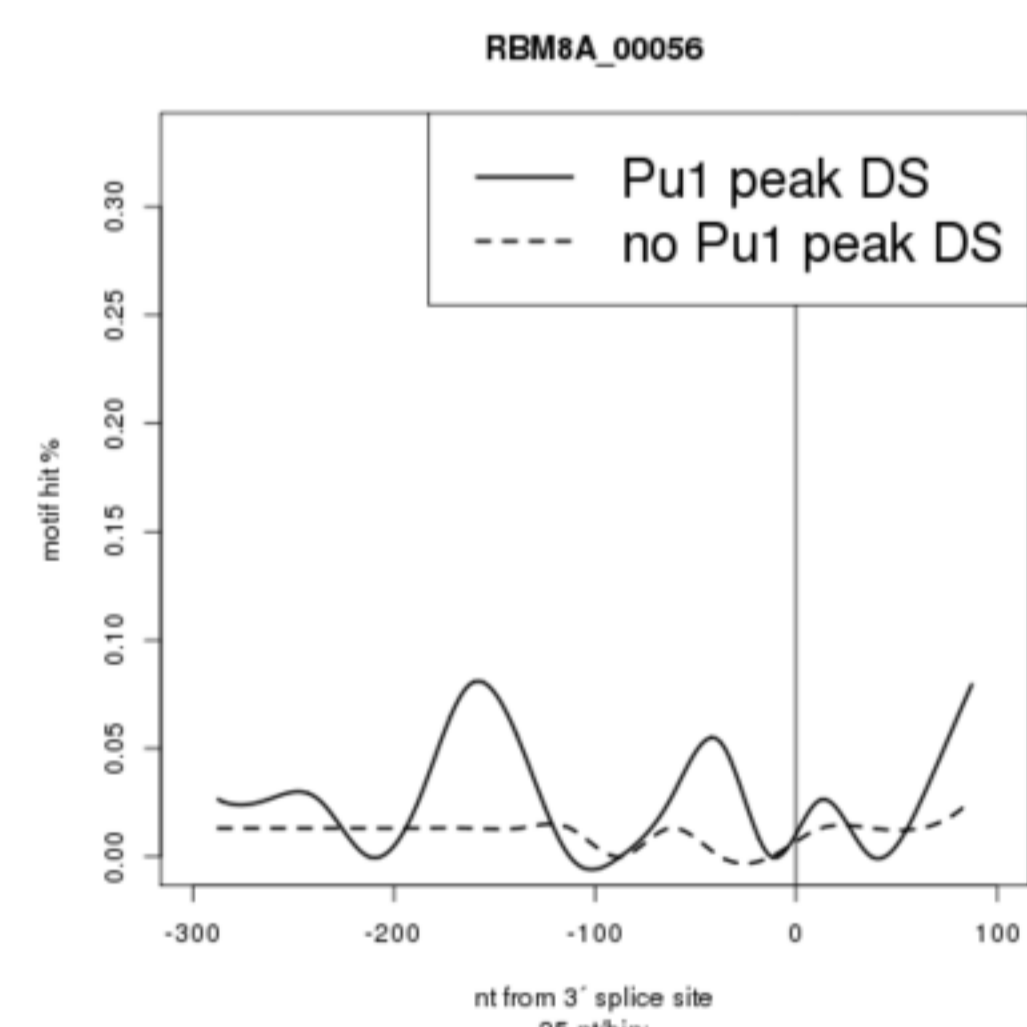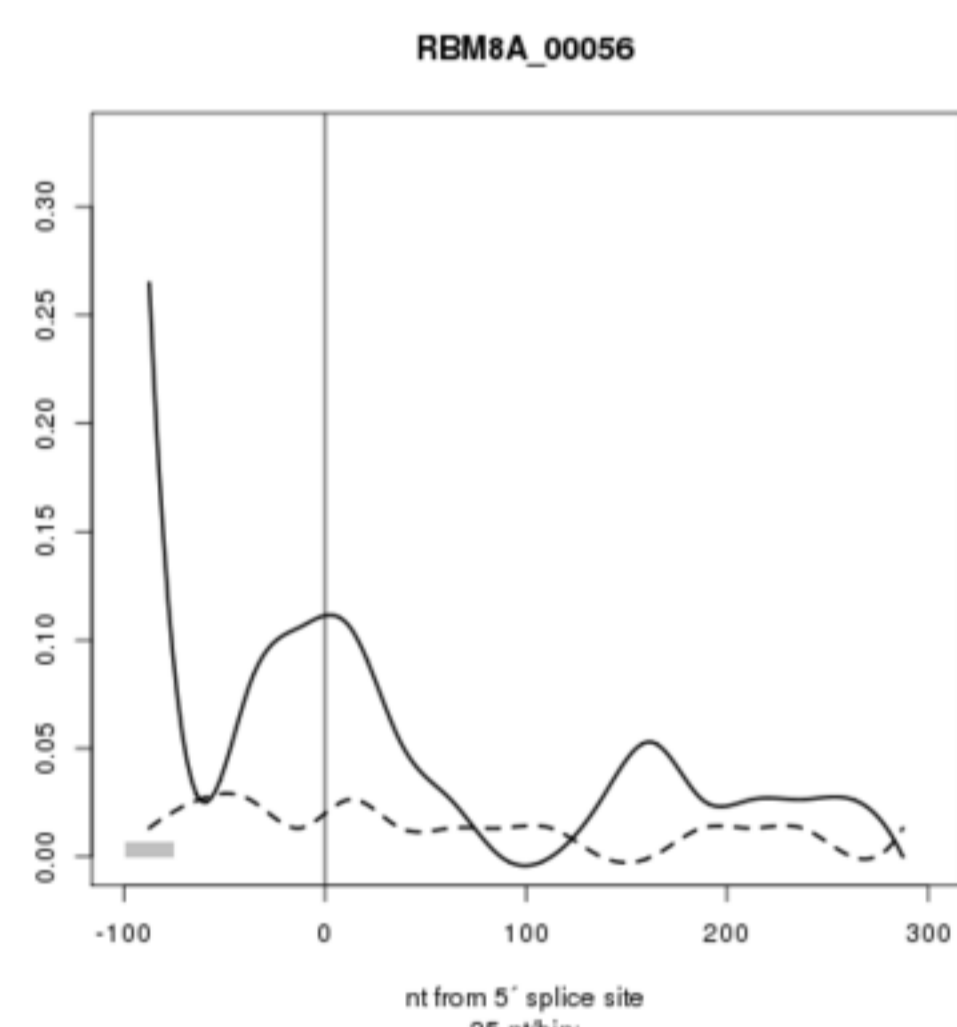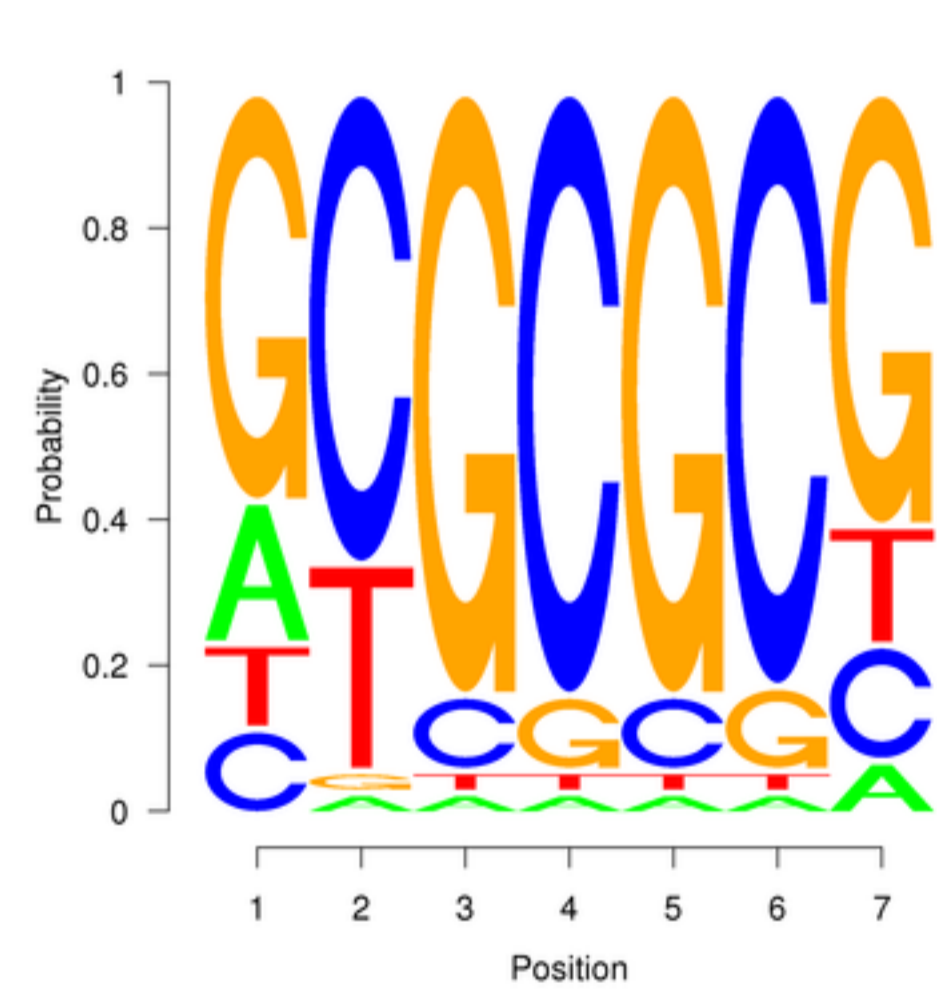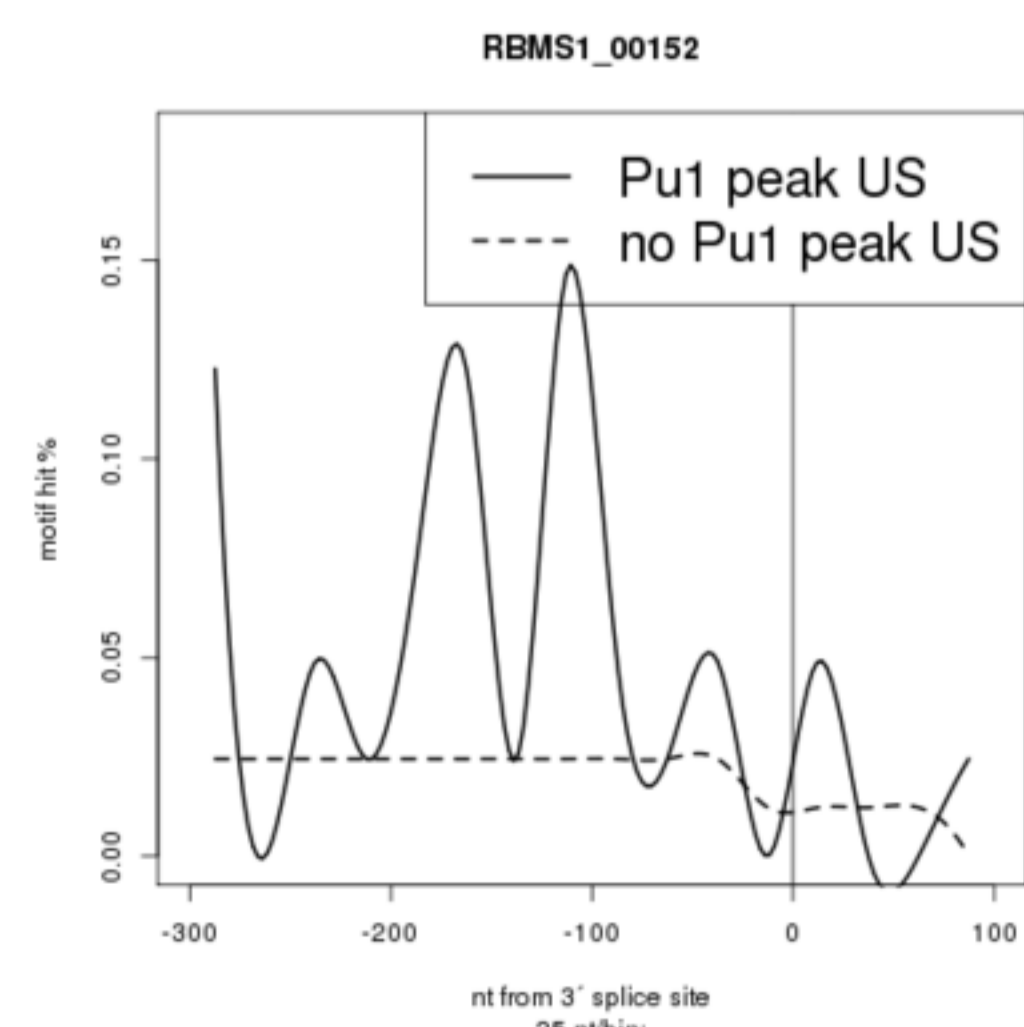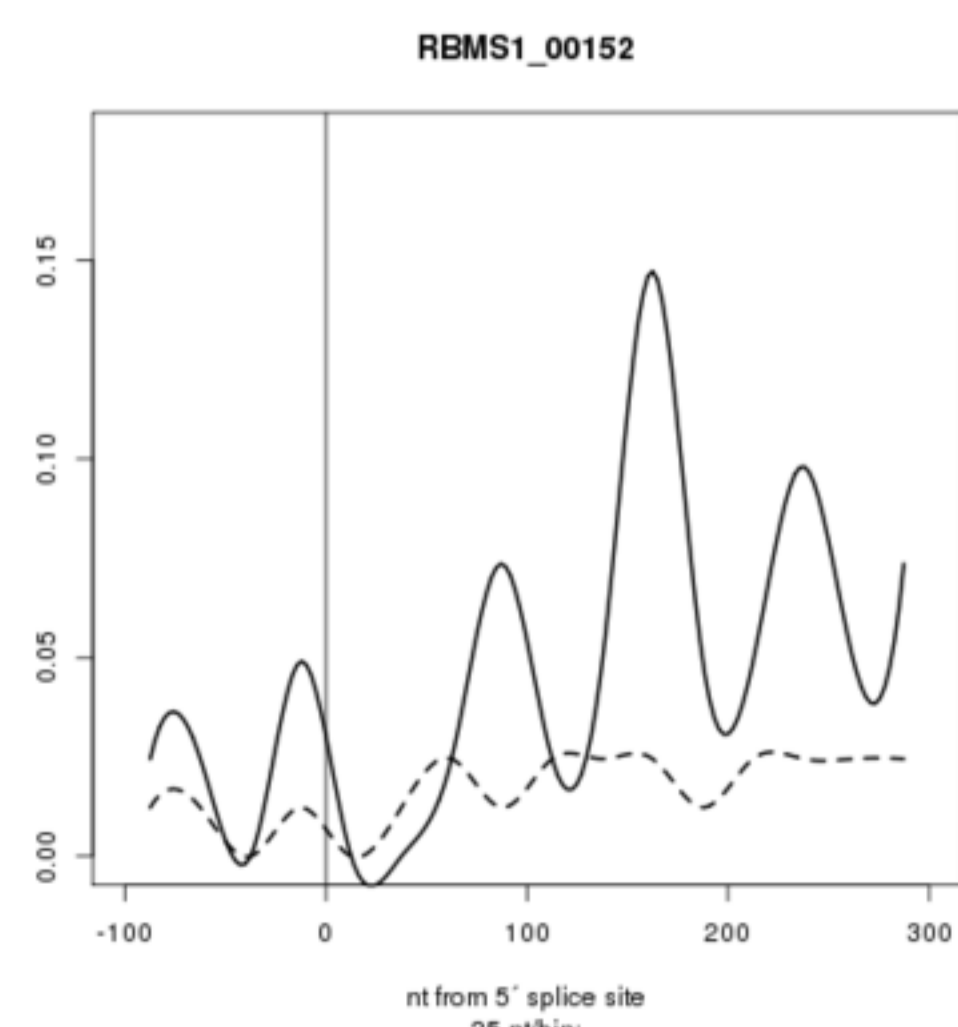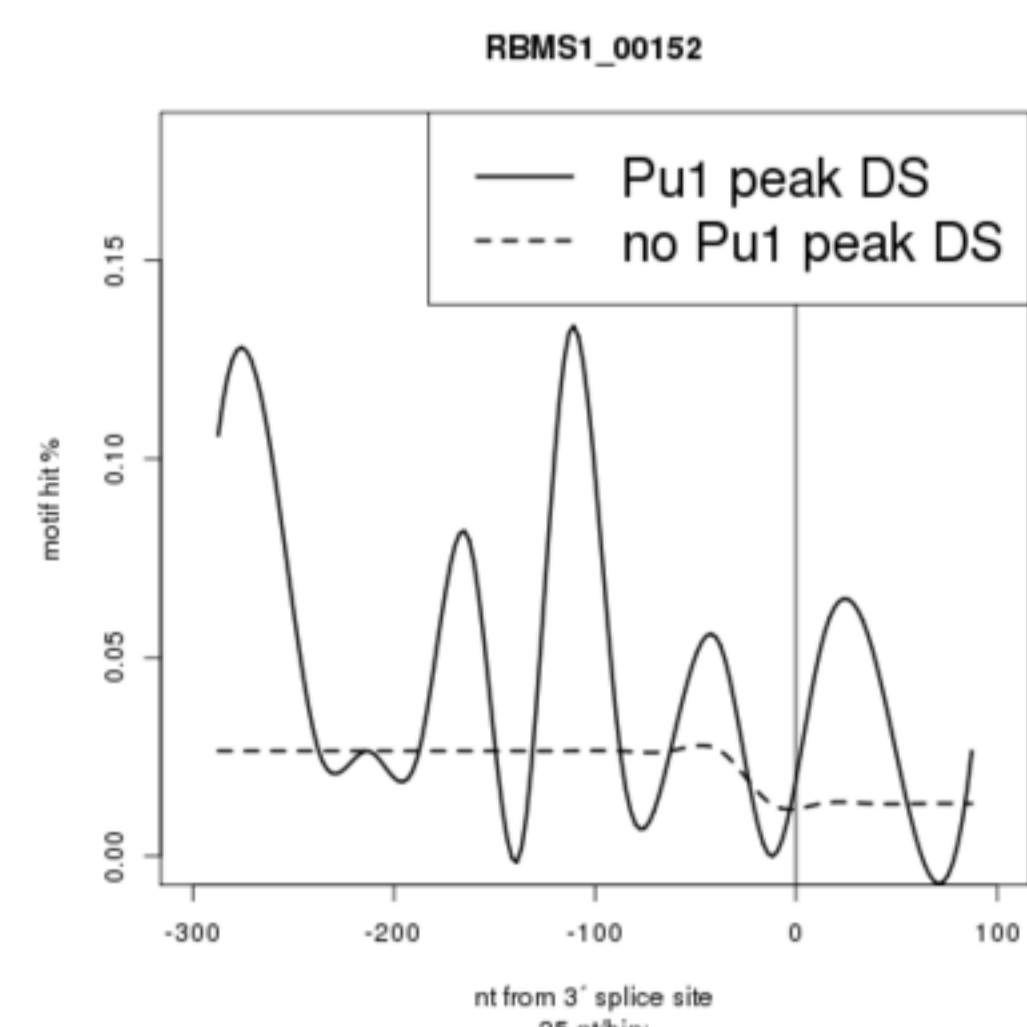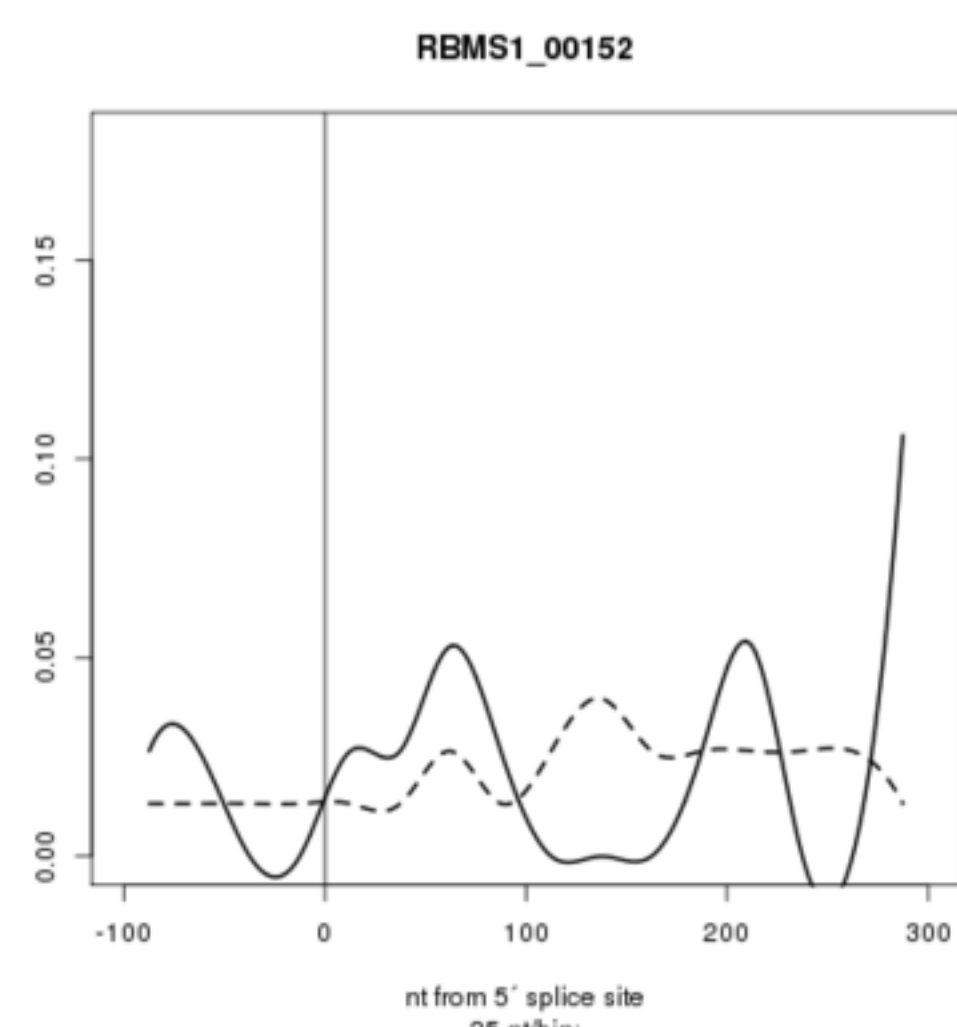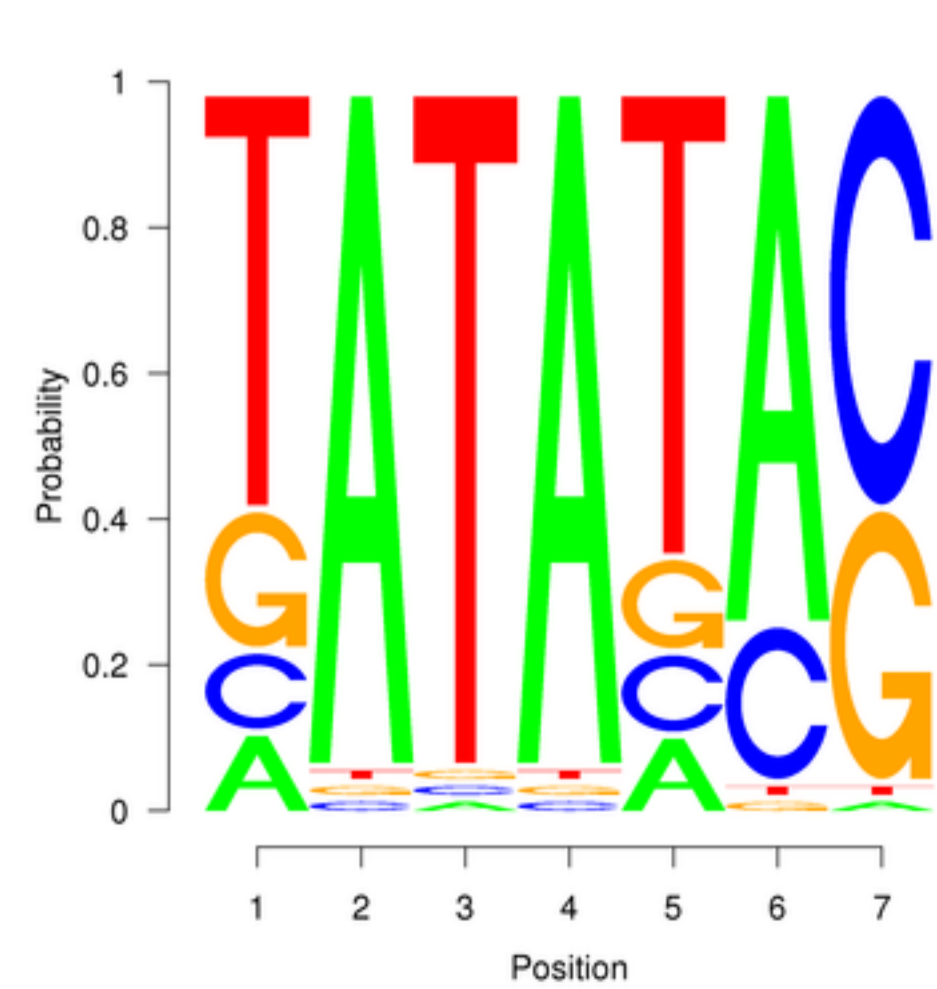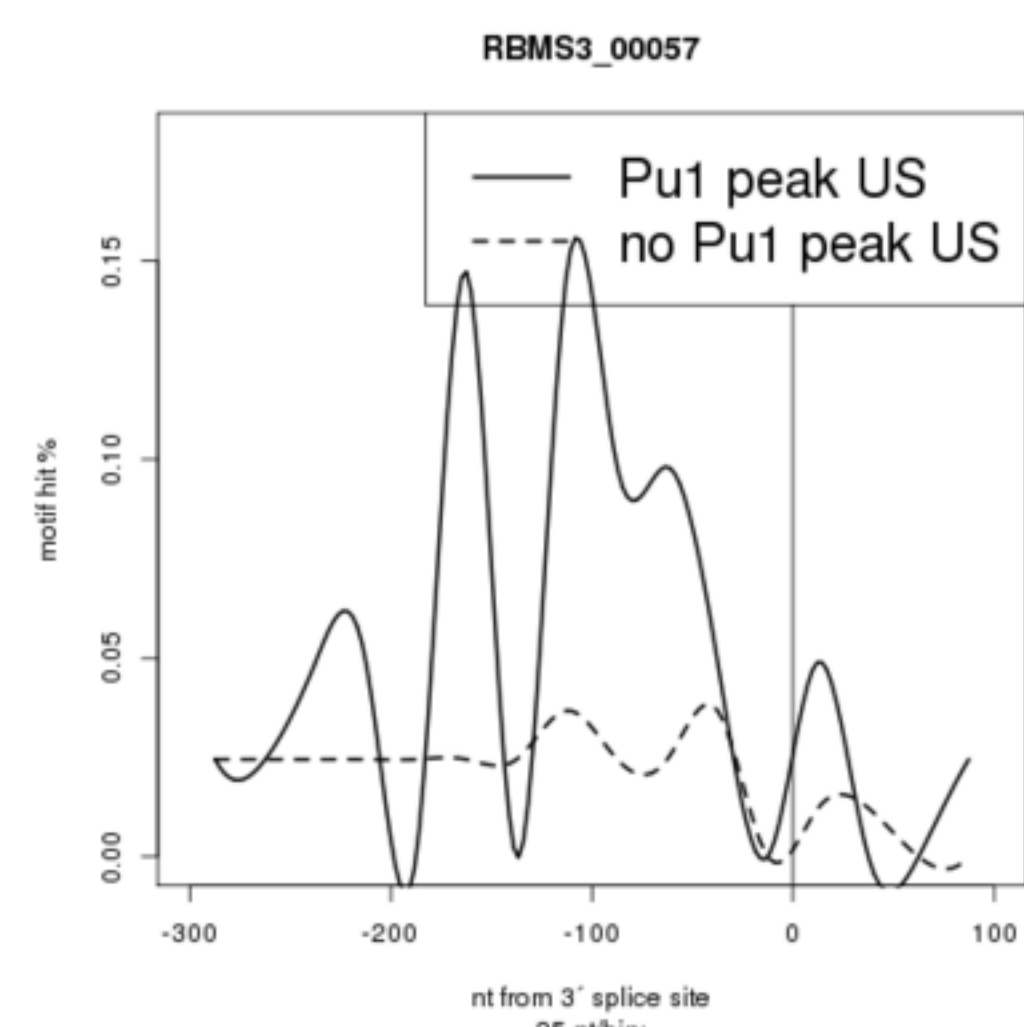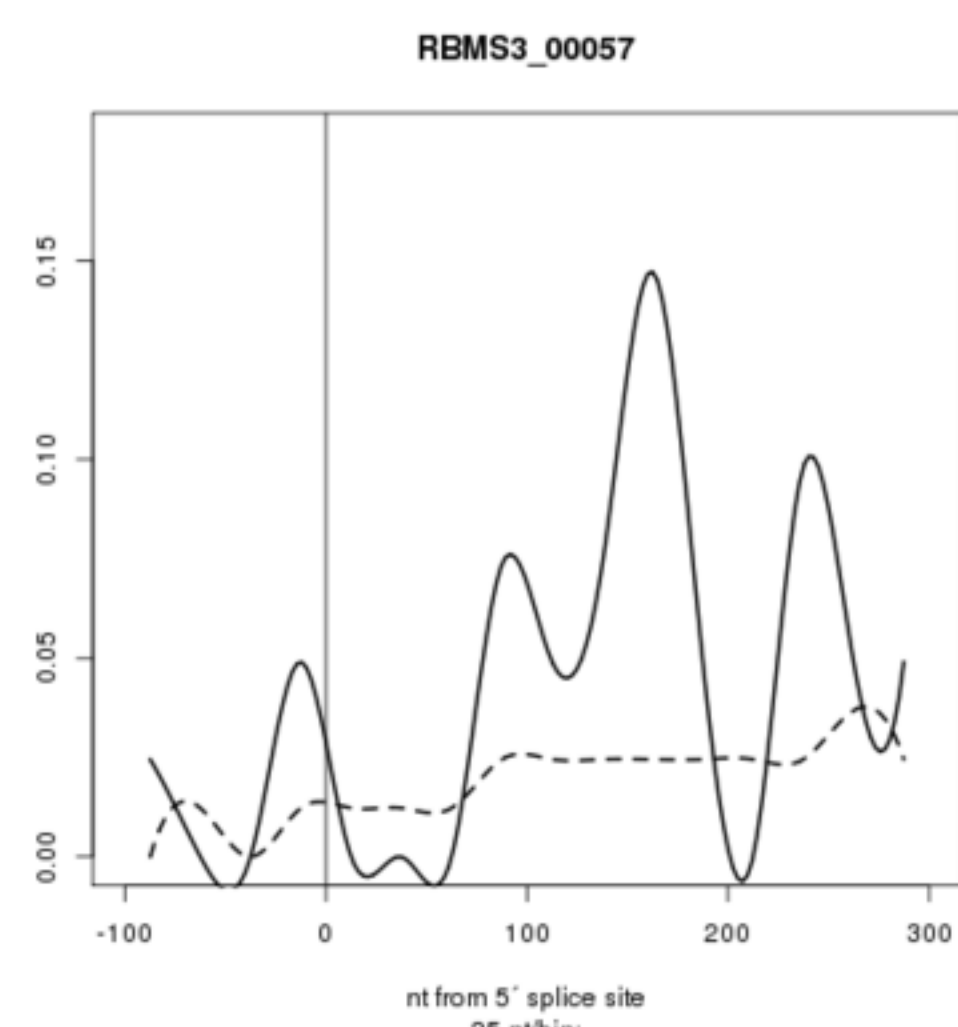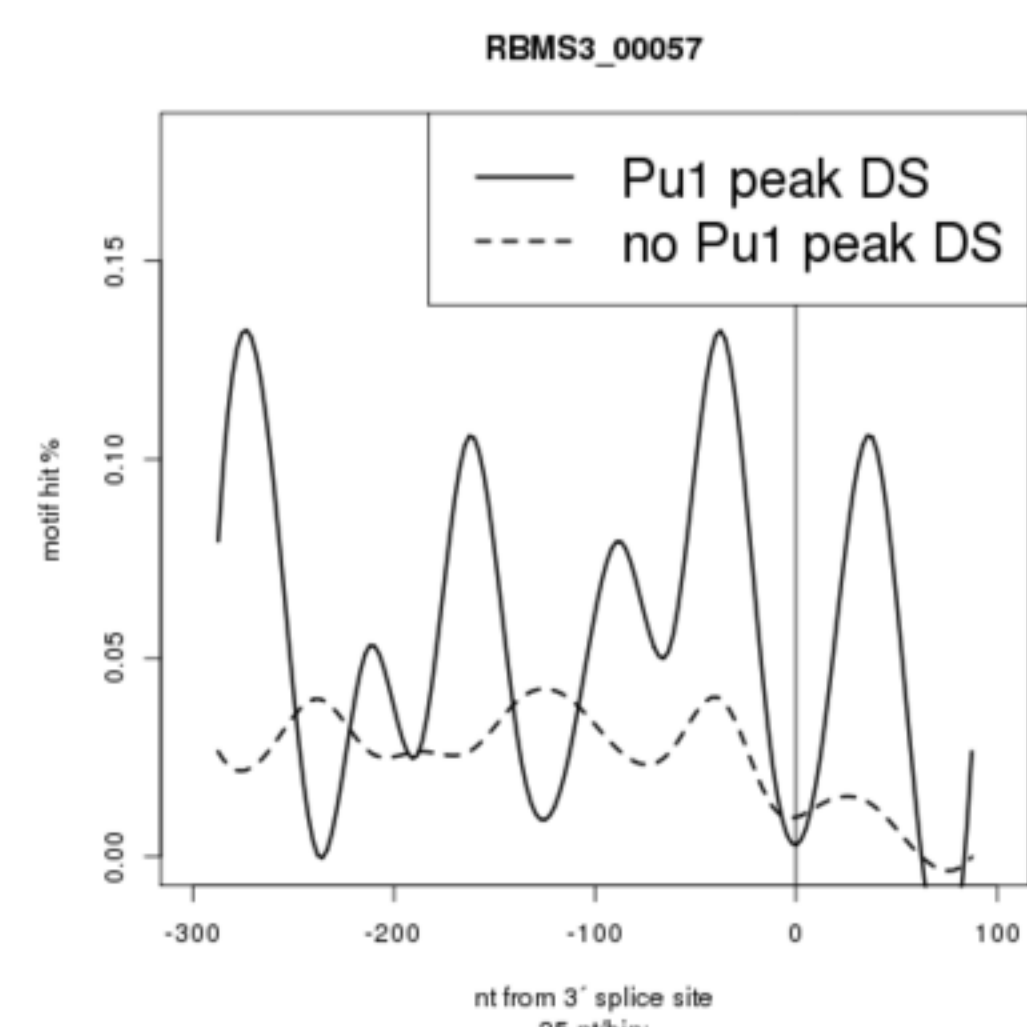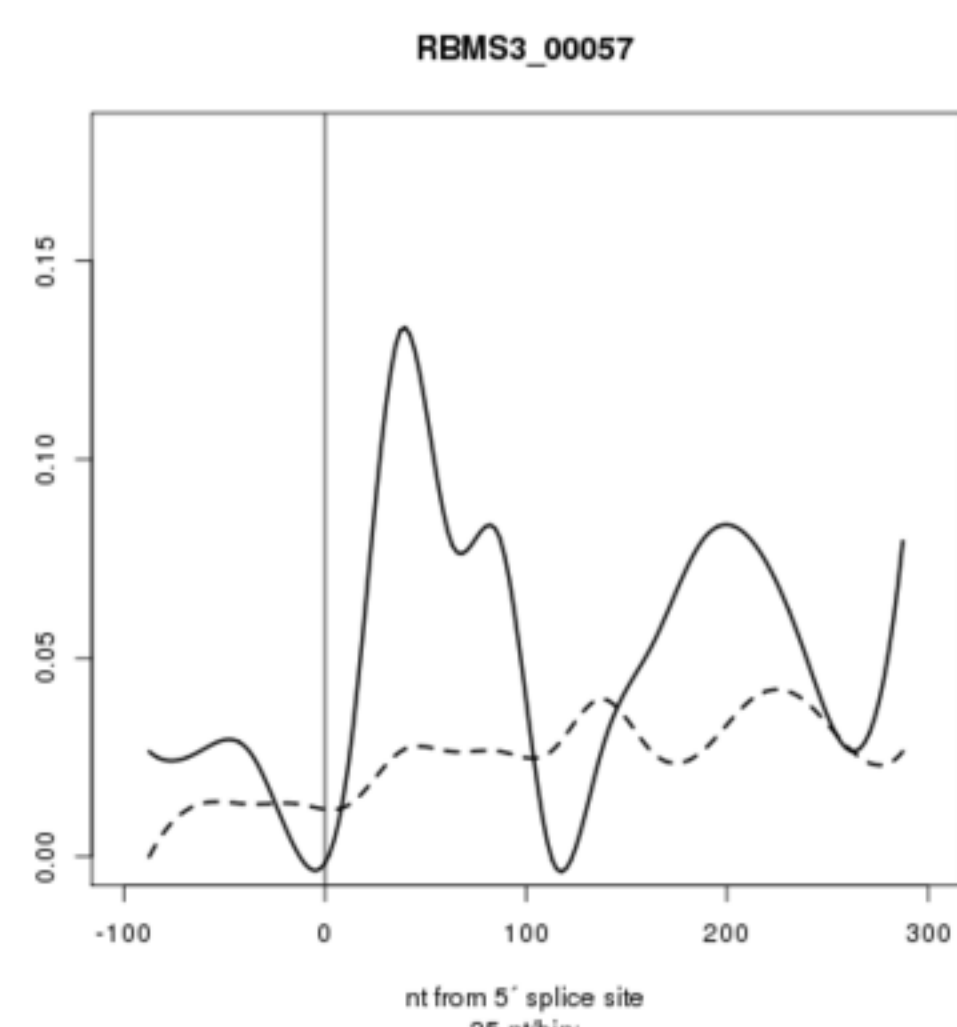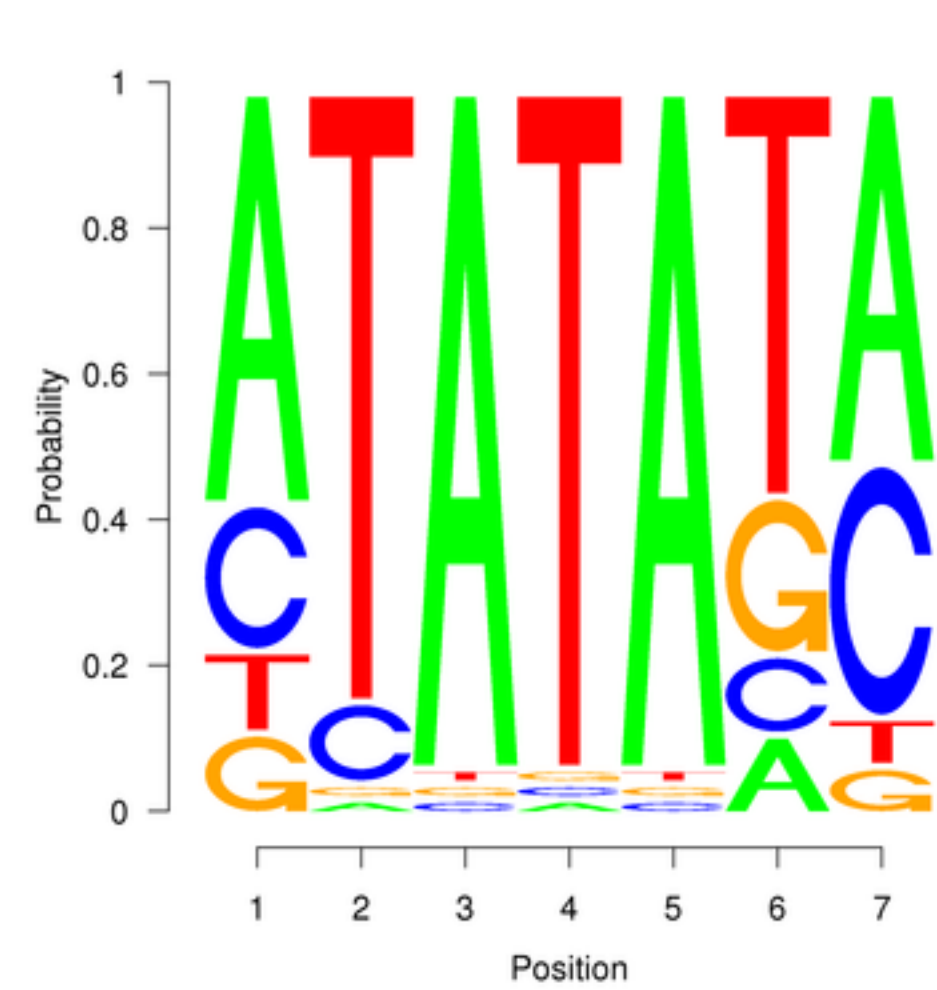

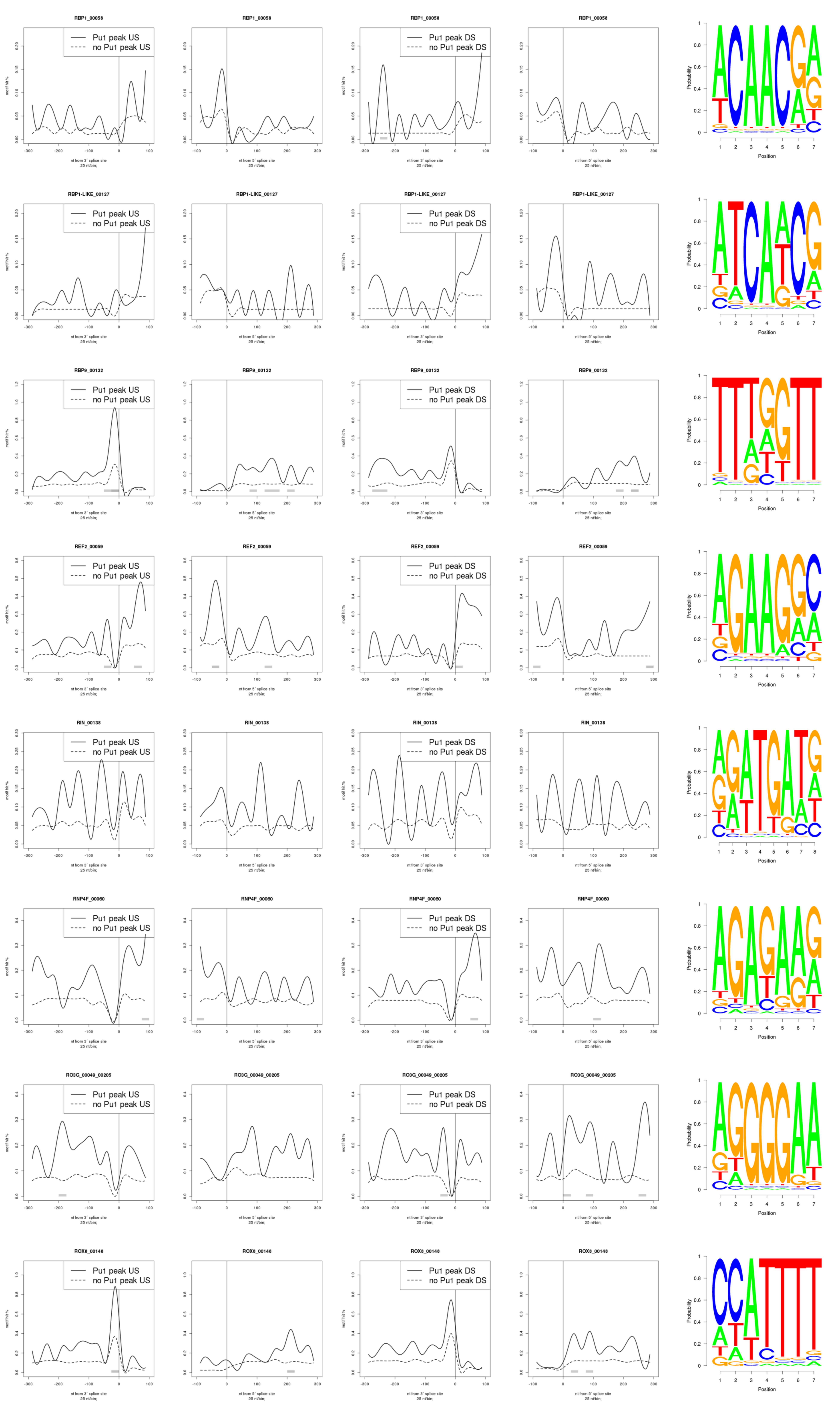

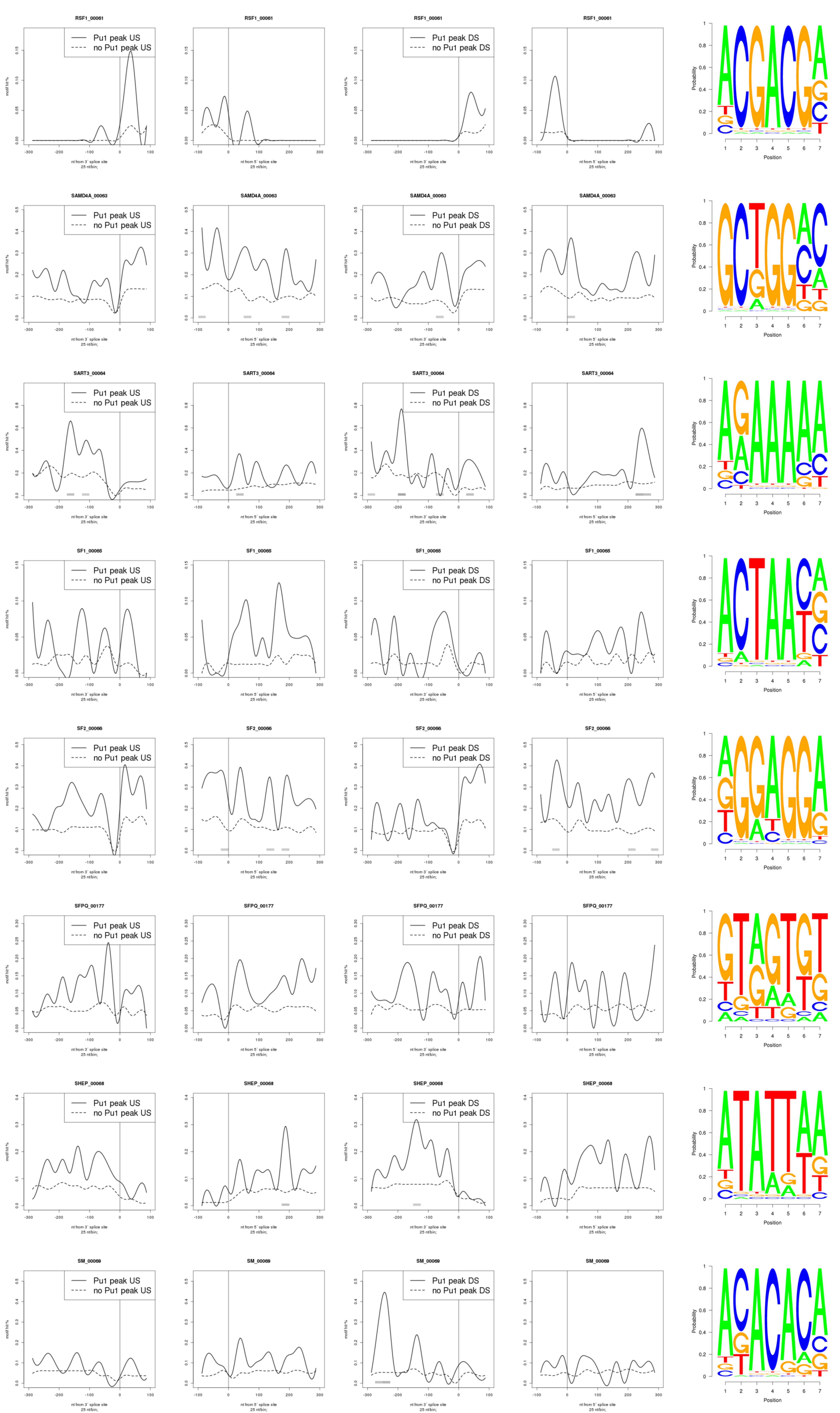

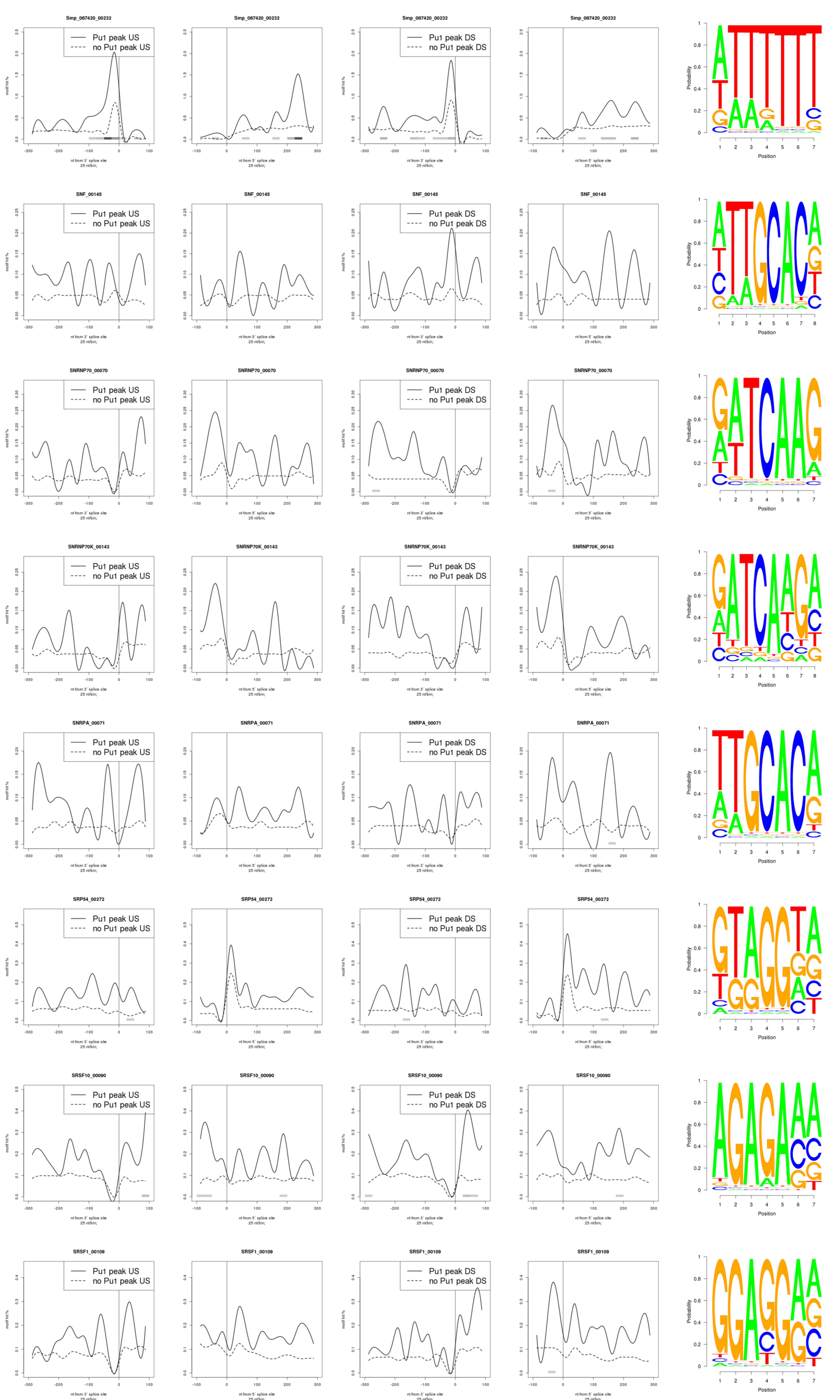

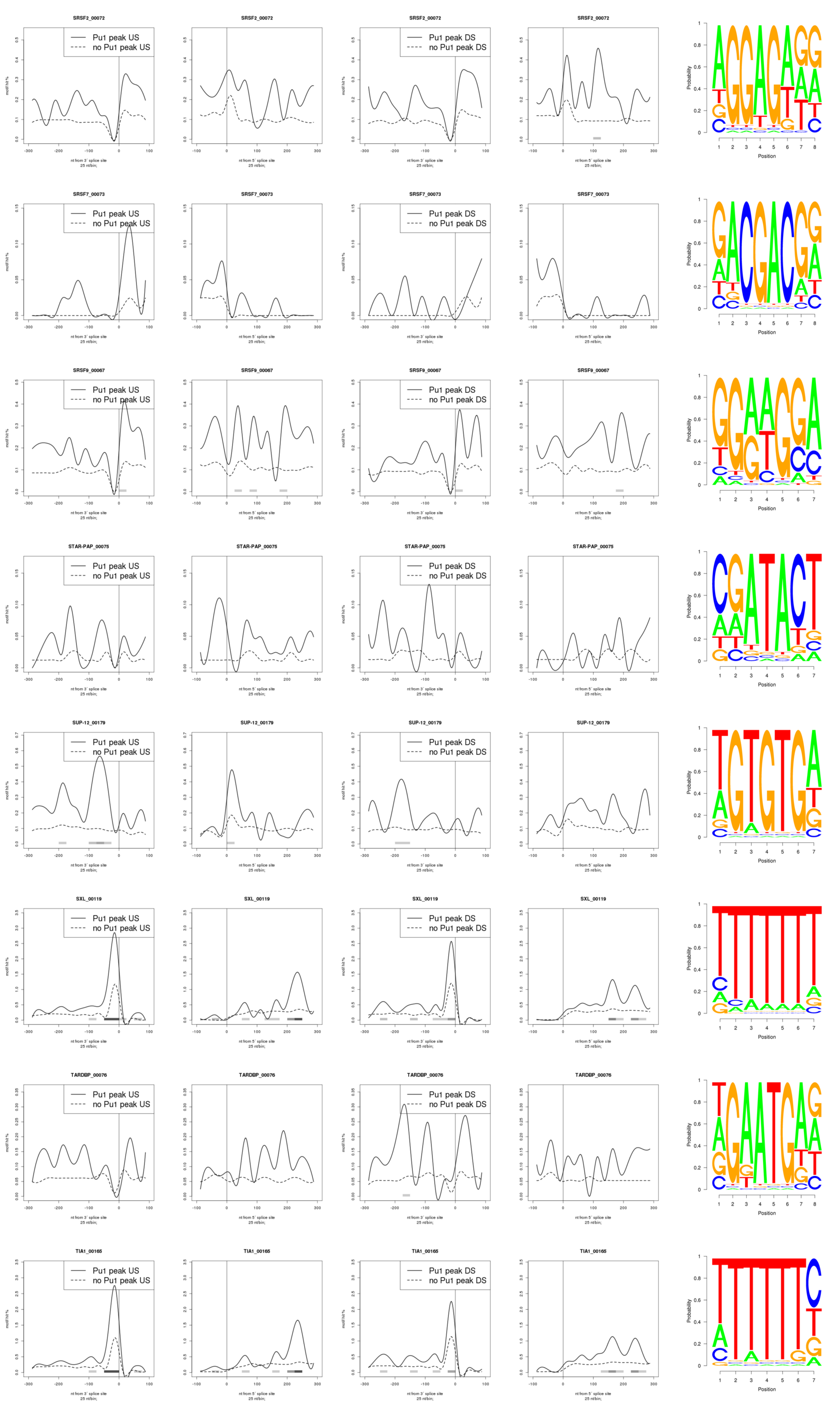

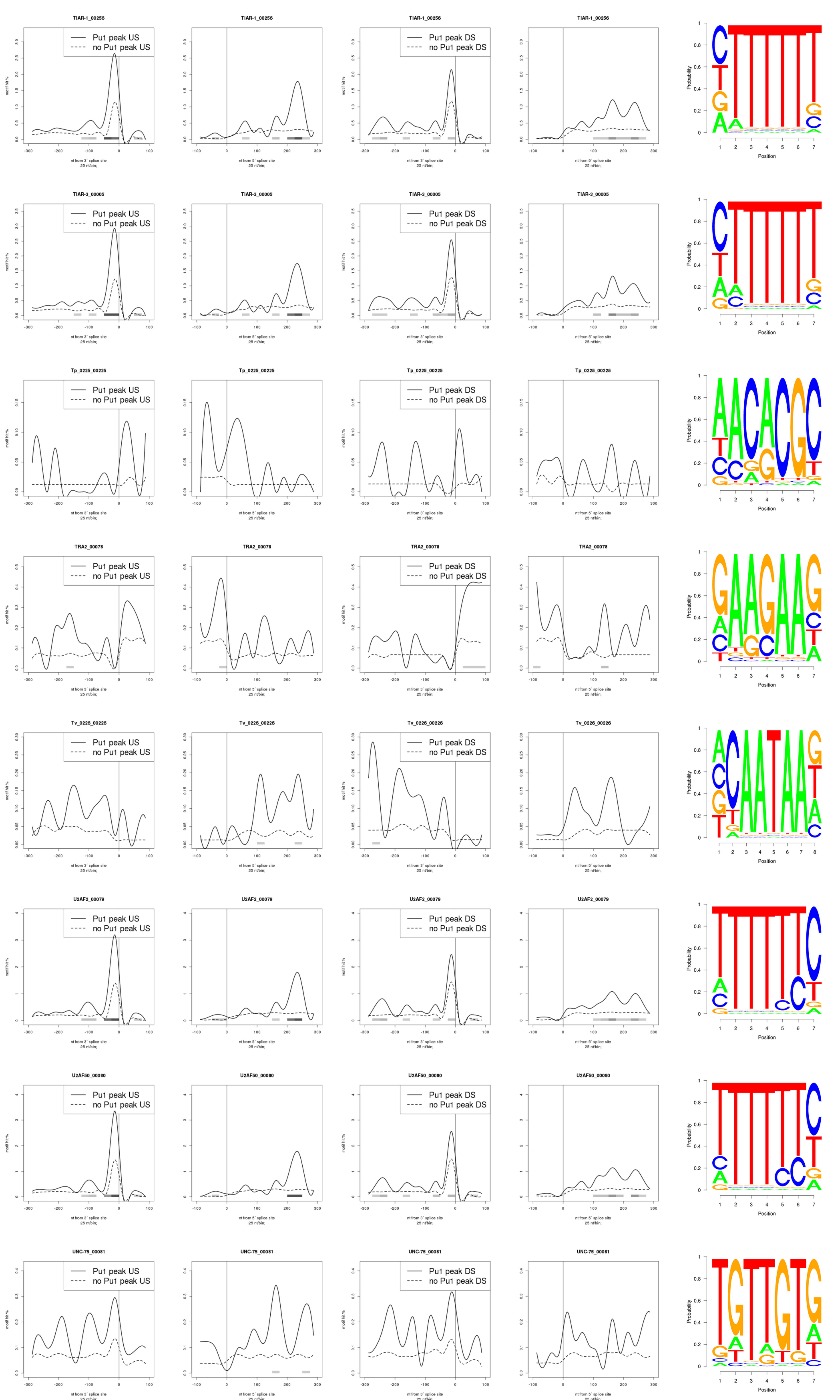

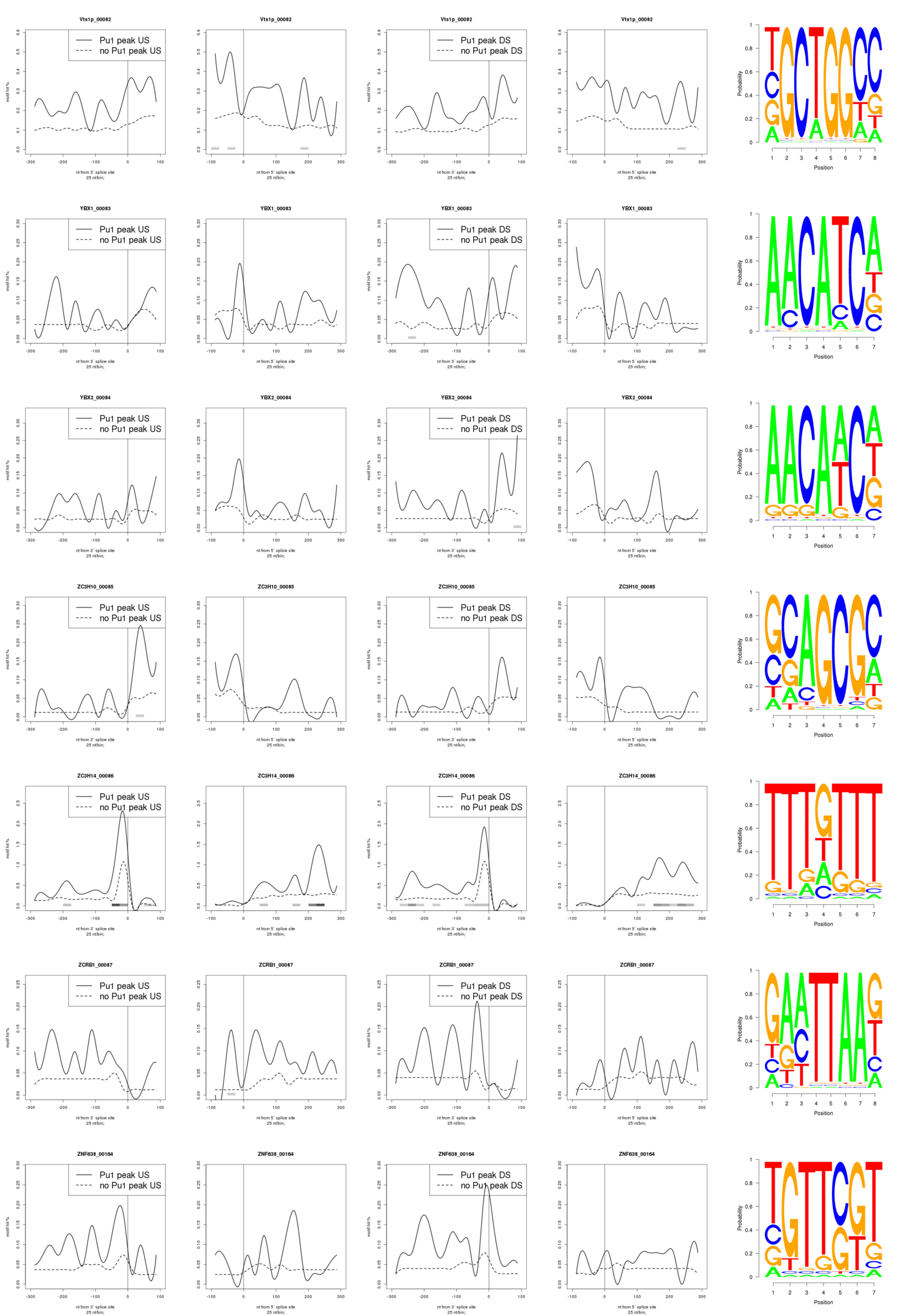

Supplement: S6 File — Same format as S5 File. (PDF) [file pone.0132448.s015.pdf]

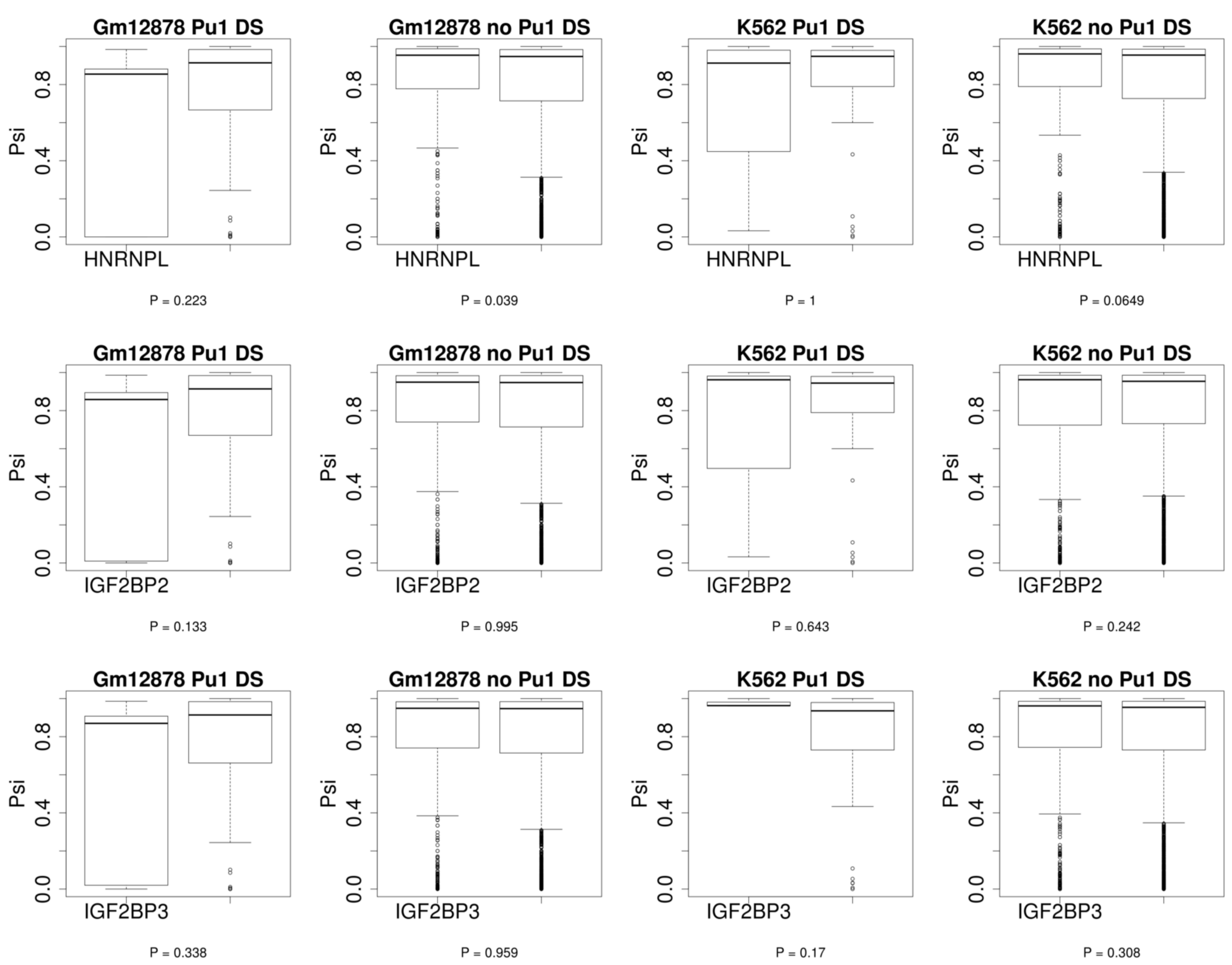

Supplement: S7 File — PSI values are from RNA-Seq from 1 of 2 cell lines–either Gm12878 or K562 – as indicated above each plot. Pu1 DS = a reproducible SPI1/PU.1 peak is found within 1 kb downstream of all internal exons considered in the given cell line. No Pu1 DS = only exons without a reproducible DS SPI1/PU.1 peak are considered. The left boxplot in each set of axes is the set of exons with an RBP motif hit from -275 to -175nt upstream of the 3' SS for the RBP indicated below the plot. The right boxplot corresponds to the exons without a motif hit in the same region (unlabeled). The P-value underneath each pair of axes is based on a Wilcoxon test. Only exons with PSI < 1 from RNA-Seq, or annotated as a skipped exon, are used in all boxplots. (PDF) [file pone.0132448.s016.pdf]

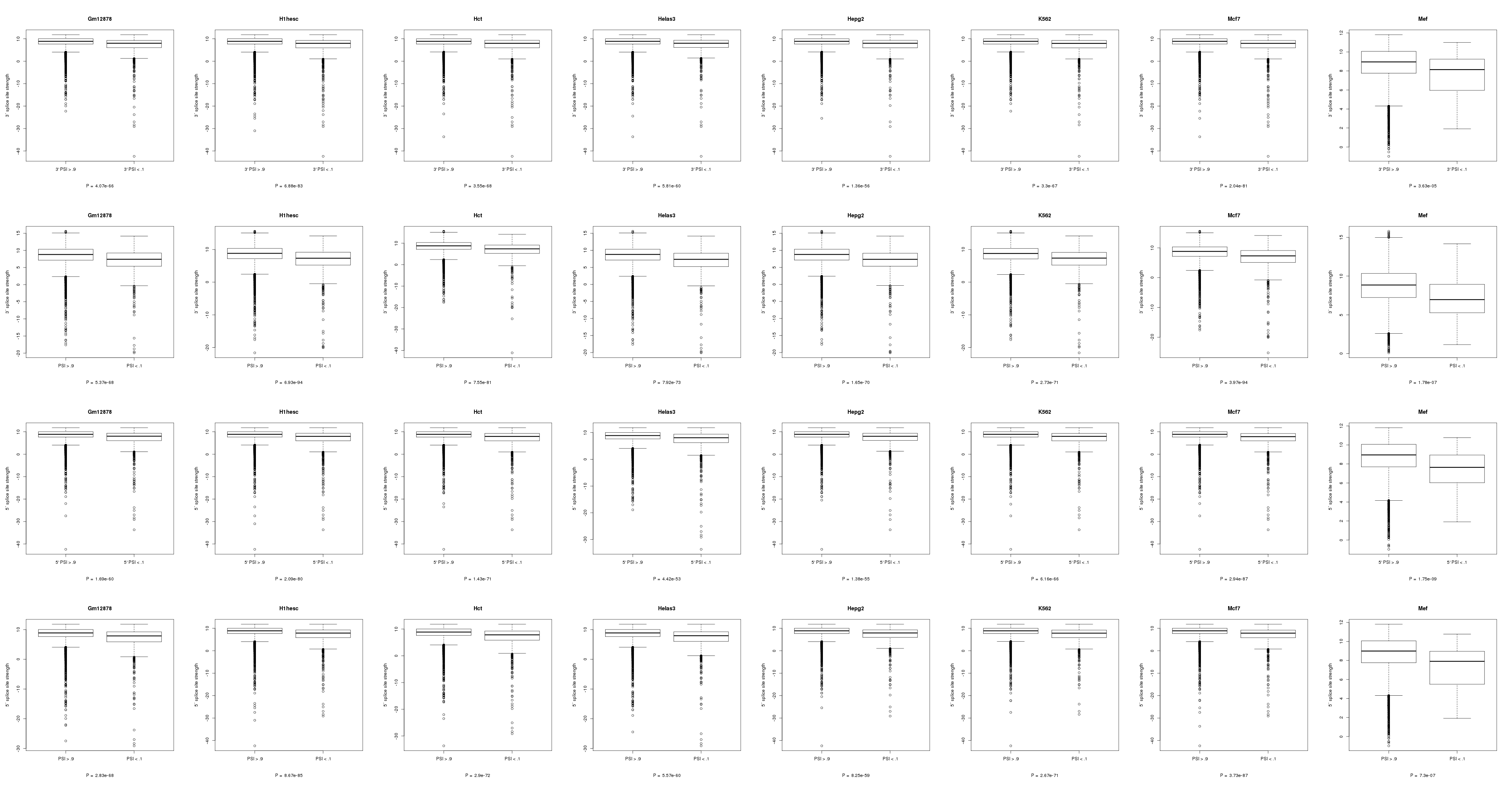

Supplement: S8 File — “3' PSI”, was calculated using only 3' junction reads (see Methods); “5' PSI” was similarly calculated for 5' junction reads, and “PSI” uses the average of 5' and 3' junction reads. The ENCODE cell line from which RNA-Seq data was derived is listed at the top of each plot. (PNG) [file pone.0132448.s017.png]
