# Supplementary material for: A Quantitative Profiling Tool for Diverse Genomic Data Types Reveals Potential Associations between Chromatin and Pre-mRNA Processing
Source: PLoS One. 2015 Jul 24;10(7):e0132448. doi: 10.1371/journal.pone.0132448 (PMC4514851; doi:10.1371/journal.pone.0132448)
Supplement: S3 Fig — All profiles shown centered at the polyA-site have been inverted such that positive x-axis values indicate distance into transcript body and negative values indicate distance outside of transcript body. (Figure C) SRSF1 wt is a conditional SRSF1-knock cell line in which SRSF1 is not knocked out; SRSF2 is defined similarly. Test vs. control P-values/bin are as shown in Fig 1B, with the lightest shade of grey corresponding to P-value < 0.01. (PDF) [file pone.0132448.s003.pdf]

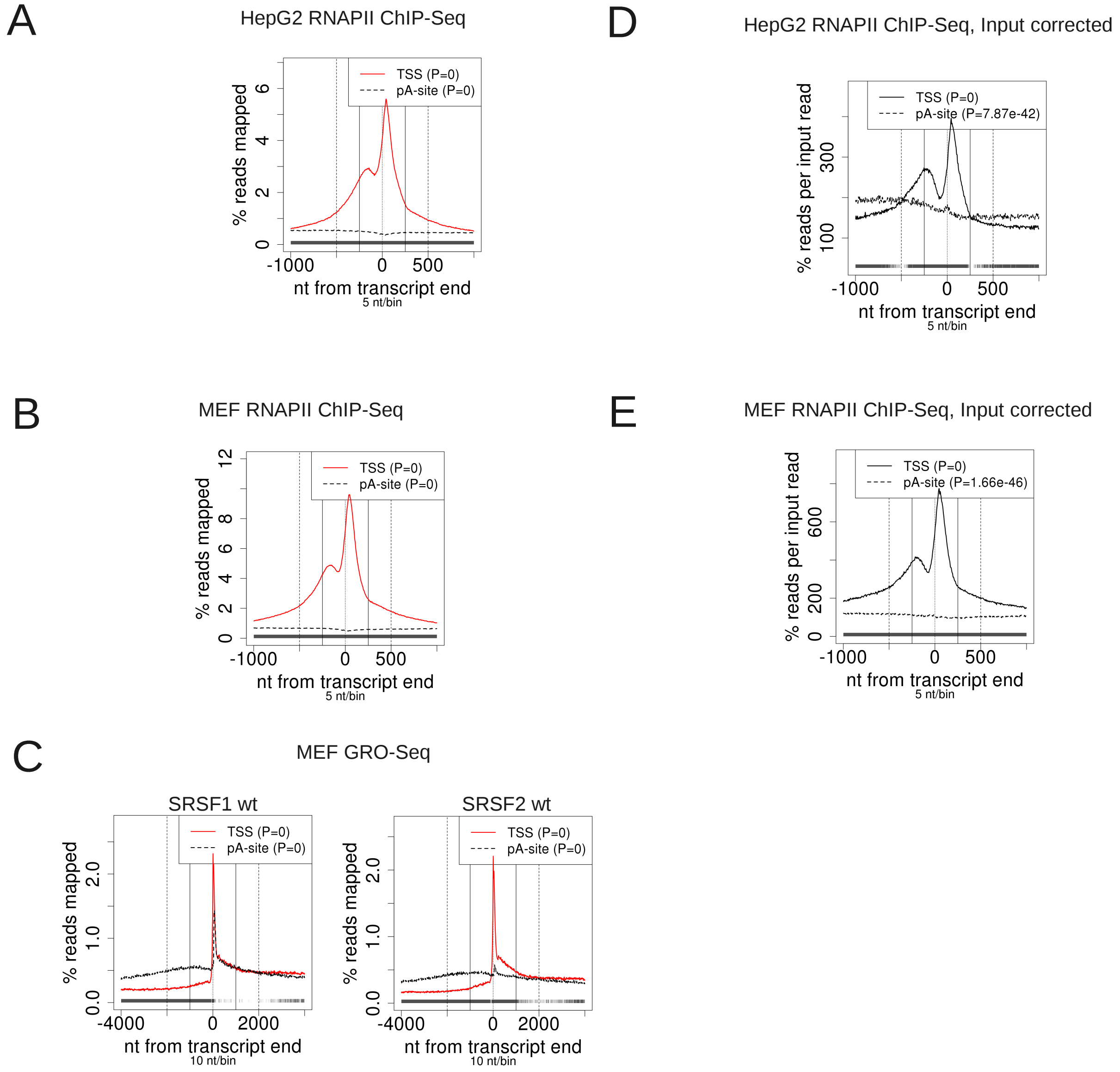
A
HepG2 RNAPII ChIP-Seq
TSS (P=0)
pA-site (P=0)
% reads mapped
0
2
4
6
-1000
0
500
nt from transcript end
5 nt/bin
B
MEF RNAPII ChIP-Seq
TSS (P=0)
pA-site (P=0)
% reads mapped
0
2
4
6
8
12
-1000
0
500
nt from transcript end
5 nt/bin
C
MEF GRO-Seq
SRSF1 wt
TSS (P=0)
pA-site (P=0)
% reads mapped
0.0
1.0
2.0
-4000
0
2000
nt from transcript end
10 nt/bin
SRSF2 wt
TSS (P=0)
pA-site (P=0)
% reads mapped
0.0
1.0
2.0
-4000
0
2000
nt from transcript end
10 nt/bin
D
HepG2 RNAPII ChIP-Seq, Input corrected
TSS (P=0)
pA-site (P=7.87e-42)
% reads per input read
100
300
-1000
0
500
nt from transcript end
5 nt/bin
E
MEF RNAPII ChIP-Seq, Input corrected
TSS (P=0)
pA-site (P=1.66e-46)
% reads per input read
0
200
600
-1000
0
500
nt from transcript end
5 nt/bin
