# Supplementary material for: A Quantitative Profiling Tool for Diverse Genomic Data Types Reveals Potential Associations between Chromatin and Pre-mRNA Processing
Source: PLoS One. 2015 Jul 24;10(7):e0132448. doi: 10.1371/journal.pone.0132448 (PMC4514851; doi:10.1371/journal.pone.0132448)

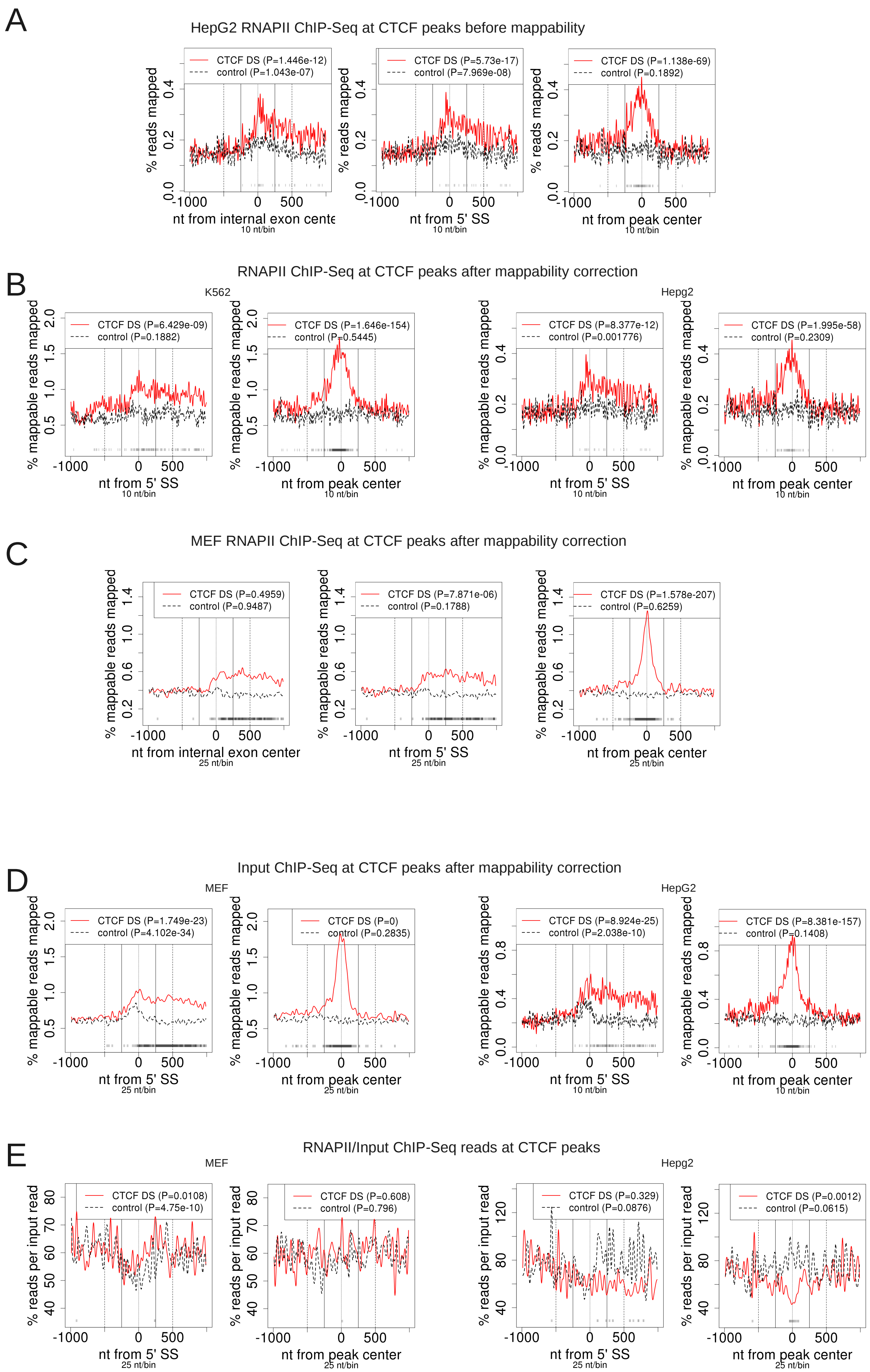

A
HepG2 RNAPII ChIP-Seq at CTCF peaks before mappability
CTCF DS (P=1.446e-12)
control (P=1.043e-07)
CTCF DS (P=5.73e-17)
control (P=7.969e-08)
CTCF DS (P=1.138e-69)
control (P=0.1892)
% reads mapped
nt from internal exon cente
nt from 5' SS
nt from peak center
10 nt/bin
B
RNAPII ChIP-Seq at CTCF peaks after mappability correction
K562
Hepg2
CTCF DS (P=6.429e-09)
control (P=0.1882)
CTCF DS (P=1.646e-154)
control (P=0.5445)
CTCF DS (P=8.377e-12)
control (P=0.001776)
CTCF DS (P=1.995e-58)
control (P=0.2309)
% mappable reads mapped
nt from 5' SS
nt from peak center
10 nt/bin
C
MEF RNAPII ChIP-Seq at CTCF peaks after mappability correction
CTCF DS (P=0.4959)
control (P=0.9487)
CTCF DS (P=7.871e-06)
control (P=0.1788)
CTCF DS (P=1.578e-207)
control (P=0.6259)
% mappable reads mapped
nt from internal exon center
nt from 5' SS
nt from peak center
25 nt/bin
D
Input ChIP-Seq at CTCF peaks after mappability correction
MEF
HepG2
CTCF DS (P=1.749e-23)
control (P=4.102e-34)
CTCF DS (P=0)
control (P=0.2835)
CTCF DS (P=8.924e-25)
control (P=2.038e-10)
CTCF DS (P=8.381e-157)
control (P=0.1408)
% mappable reads mapped
nt from 5' SS
nt from peak center
25 nt/bin
10 nt/bin
E
RNAPII/Input ChIP-Seq reads at CTCF peaks
MEF
Hepg2
CTCF DS (P=0.0108)
control (P=4.75e-10)
CTCF DS (P=0.608)
control (P=0.796)
CTCF DS (P=0.329)
control (P=0.0876)
CTCF DS (P=0.0012)
control (P=0.0615)
% reads per input read
nt from 5' SS
nt from peak center
25 nt/bin

F

IgG ChIP-Seq reads at CTCF peaks, Mef cell line

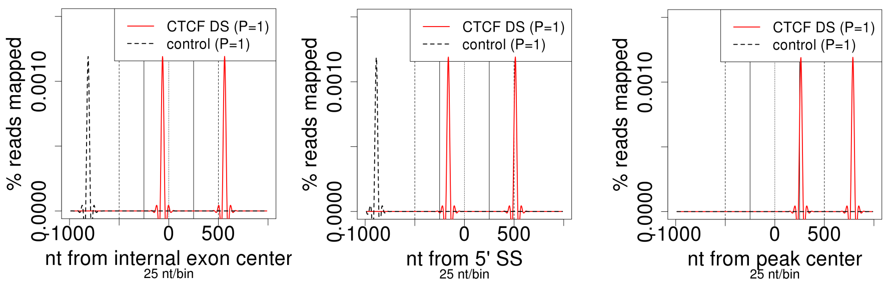

G

HepG2 ChIP-Seq reads at CTCF peaks downstream
of exons whose PSI changes with CTCF knockdown

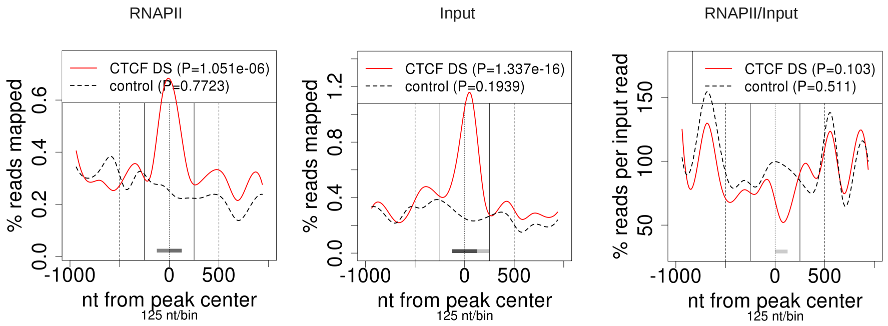

Supplement: S5 Fig — DS = peak center within 1kb downstream of 5'ss. Test vs. control P-values/bin are as shown in Fig 1B, with the lightest shade of grey corresponding to P-value < 0.01. (PDF) [file pone.0132448.s005.pdf]
