# Supplementary material for: A Quantitative Profiling Tool for Diverse Genomic Data Types Reveals Potential Associations between Chromatin and Pre-mRNA Processing
Source: PLoS One. 2015 Jul 24;10(7):e0132448. doi: 10.1371/journal.pone.0132448 (PMC4514851; doi:10.1371/journal.pone.0132448)

Full ChIA-PET interaction set: Interaction involving TSS's:

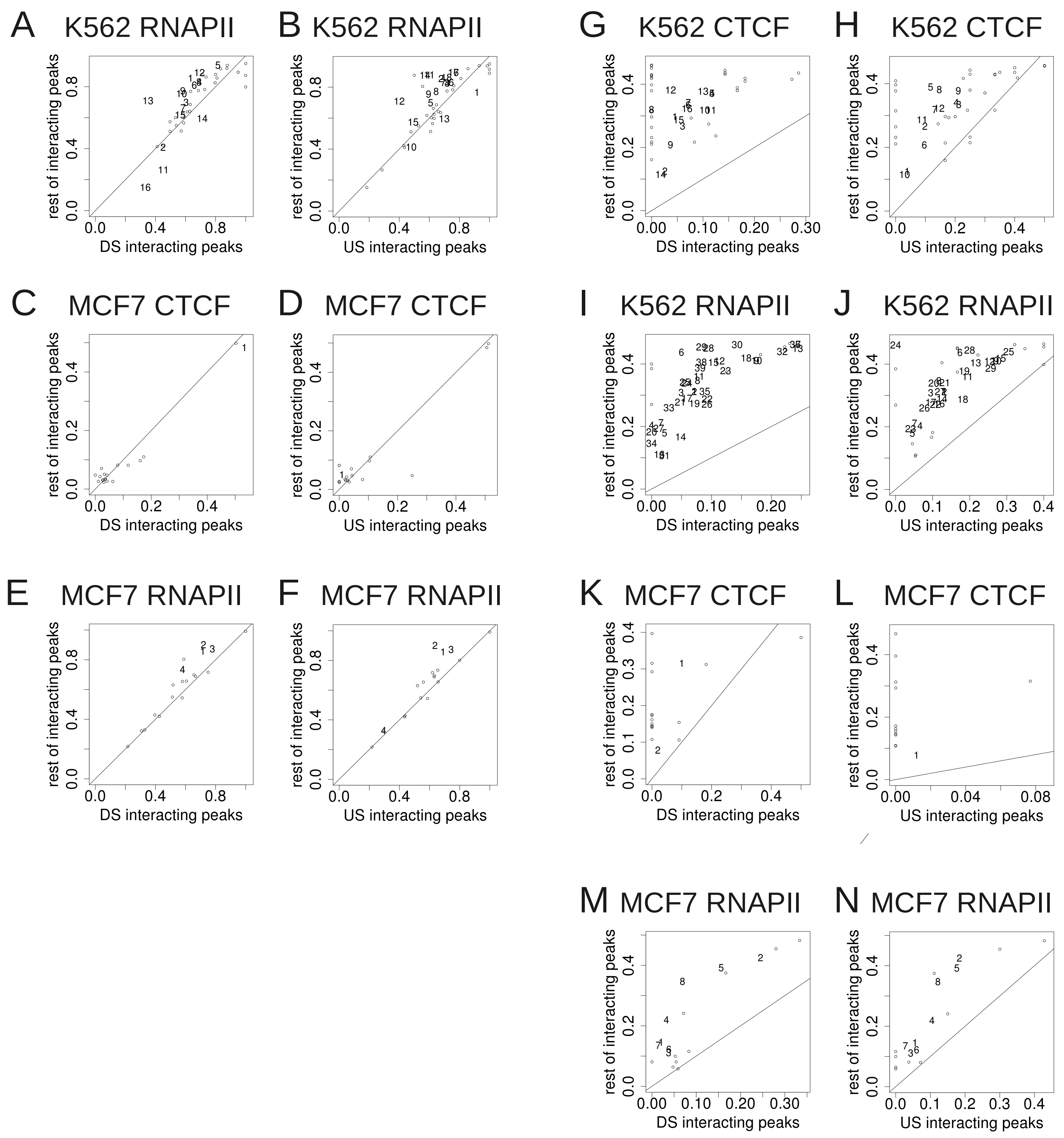

Supplement: S6 Fig — Diagonal line is of y = x. Statistically significant data points (Fisher's Exact Test P-value < 0.05) are shown with a number, whereas nonsignificant points are shown with an empty circle. Each point represents a set of peaks. The first 5 letters of the symbol for the numered points are listed here. (Figures A-F) Same format as in Fig 5. (Figure A) 1. Creb1 2. Ctcfc 3. Egr1V 4. Elf1s 5. Ets1V 6. GabpV 7. MaxV0 8. Pmlsc 9. Pol24 10. Pol2V 11. Six5P 12. Sp1Pc 13. SrfV0 14. Stat5 15. Tead4 16. Zbtb3 (Figure B) 1. Atf3V 2. Creb1 3. E2f6s 4. E2f6V 5. Egr1V 6. Elf1s 7. Pmlsc 8. Pol24 9. Pol2V 10. Pu1Pc 11. Sp2sc 12. SrfV0 13. Stat5 14. Taf7s 15. Usf1V 16. Yy1sc 17. Yy1V0 18. Yy1V0 (Figure C) 1. Rad21 (Figure D) 1. Sin3a (Figure E) 1. Elf1V 2. GabpV 3. MaxV0 4. Sin3a (Figure F) 1. Elf1V 2. GabpV 3. MaxV0 4. Rad21 (Figure G) 1. Cbx3s 2. Ctcfc 3. Ctcfl 4. E2f6s 5. E2f6V 6. Egr1V 7. Elf1s 8. Hdac2 9. MaxV0 10. Nr2f2 11. NrsfV 12. Pol24 13. Pol2V 14. Rad21 15. Tead4 16. Zbtb7 (Figure H) 1. Ctcfc 2. Ctcfl 3. Egr1V 4. Elf1s 5. GabpV 6. MaxV0 7. Nr2f2 8. Pol24 9. Pol2V 10. Rad21 11. Tead4 12. Zbtb7 (Figure I) 1. Atf3V 2. Atf3V 3. Cbx3s 4. Cebpb 5. Cebpd 6. Creb1 7. Ctcfc 8. Ctcfl 9. E2f6s 10. E2f6V 11. Egr1V 12. Elf1s 13. Ets1V 14. Fosl1 15. GabpV 16. Gata2 17. Hdac2 18. Hey1P 19. MaxV0 20. Mef2a 21. Nr2f2 22. NrsfV 23. Pmlsc 24. Pol24 25. Pol2V 26. Pu1Pc 27. Rad21 28. Six5P 29. Six5V 30. Sp2sc 31. Stat5 32. Taf1V 33. Tead4 34. Trim2 35. Usf1V 36. Yy1sc 37. Yy1V0 38. Zbtb3 39. Zbtb7 (Figure J) 1. Atf3V 2. Atf3V 3. Cbx3s 4. Cebpb 5. Cebpd 6. Creb1 7. Ctcfc 8. Ctcfl 9. E2f6s 10. E2f6V 11. Egr1V 12. Elf1s 13. GabpV 14. Hdac2 15. Hey1P 16. MaxV0 17. Nr2f2 18. NrsfV 19. Pmlsc 20. Pol24 21. Pol2V 22. Pu1Pc 23. Rad21 24. Sp2sc 25. Taf1V 26. Tead4 27. Usf1V 28. Yy1V0 29. Zbtb7 (Figure K) 1. MaxV0 2. Rad21 (Figure L) 1. Rad21 (Figure M) 1. Cebpb 2. Elf1V 3. Gata3 4. Hdac2 5. MaxV0 6. Nr2f2 7. Rad21 8. Sin3a (Figure N) 1. Cebpb 2. Elf1V 3. Gata3 4. Hdac2 5. MaxV0 6. Nr2f2 7. Rad21 8. Sin3a [file pone.0132448.s006.pdf]
