# Supplementary material for: A Quantitative Profiling Tool for Diverse Genomic Data Types Reveals Potential Associations between Chromatin and Pre-mRNA Processing
Source: PLoS One. 2015 Jul 24;10(7):e0132448. doi: 10.1371/journal.pone.0132448 (PMC4514851; doi:10.1371/journal.pone.0132448)
Supplement: S10 Table — (PDF) [file pone.0132448.s030.pdf]

**Experiment type (cell line)**	**source**	**link**
**GRO-Seq**		
(MEF)	[2]	http://www.ncbi.nlm.nih.gov/geo/query/acc.cgi?acc=GSE45517
(Imr90)	[3]	http://www.ncbi.nlm.nih.gov/geo/query/acc.cgi?acc=GSE13518
**ChIP-Seq[1]**		
RNAPII, input (K562[2], HepG2[3])	[34]	http://hgdownload.cse.ucsc.edu/goldenPath/hg19/encodeDCC/wgEncodeHaibTfbs/
RNAPII, input (MEF)	[34]	http://hgdownload.cse.ucsc.edu/goldenPath/mm9/encodeDCC/wgEncodeLicrTfbs/
IgG (MEF)	[2]	http://www.ncbi.nlm.nih.gov/geo/query/acc.cgi?acc=GSE45517
H3k4me1, H3k4me3, input (MEF)	[34]	http://hgdownload.cse.ucsc.edu/goldenPath/mm9/encodeDCC/wgEncodeLicrHistone/
Peaks (all human[4])	[34]	http://hgdownload.cse.ucsc.edu/goldenPath/hg19/encodeDCC/wgEncodeHaibTfbs/
Peaks (MEF)	[34]	http://hgdownload.cse.ucsc.edu/goldenPath/mm9/encodeDCC/wgEncodeLicrTfbs/
**CLIP-Seq**		
SRSF1, SRSF2 (MEF)	[2]	http://www.ncbi.nlm.nih.gov/geo/query/acc.cgi?acc=GSE45517
PTB[5] (Hela)	[13]	http://www.ncbi.nlm.nih.gov/geo/query/acc.cgi?acc=GSE19323
**CLIP-Seq putative binding sites**		
hnRNPL (CD4(+), Jurkat)	[29]	http://dorina.mdc-berlin.de/regulators
LIN28A[6] (hESC, HEK293)	[29]	http://dorina.mdc-berlin.de/regulators
**ChIA-PET**		
RNAPII, CTCF (K562, MCF7)	[34]	http://genome.ucsc.edu/cgi-bin/hgFileUi?db=hg19&g=wgEncodeGisChiaPet
**RNA-Seq**		
IGF2BP2 knockdown (K562)	[34]	https://www.encodeproject.org/search/?type=experiment&assay_term_name=shRNA%20knockdown%20followed%20by%20RNA-seq&limit=all
All other human samples	[34]	http://hgdownload.cse.ucsc.edu/goldenPath/hg19/encodeDCC/wgEncodeCaltechRnaSeq/
Mouse samples	[34]	http://hgdownload.cse.ucsc.edu/goldenPath/mm9/encodeDCC/wgEncodeLicrRnaSeq/

Notes:
1. Inputs were only used to compare data from corresponding cell line and lab samples.
2. “Pol2V0416101” samples.
3. “Pol2Pcr2x” samples.
4. Profiles at K562 CTCF peaks use “CtcfcPcr1x” samples.
5. Monomer samples.
6. Wilbert et al. (2012). LIN28 binds messenger RNAs at GGAGA motifs and regulates splicing factor abundance. Mol Cell. 48(2):195-206.
